# The Effect of Elevated Ozone Concentrations with Varying Shading on Dry Matter Loss in a Winter Wheat-Producing Region in China

**DOI:** 10.1371/journal.pone.0145446

**Published:** 2016-01-13

**Authors:** Jingxin Xu, Youfei Zheng, Yuhong He, Rongjun Wu, Boru Mai, Hanqing Kang

**Affiliations:** 1 Key Laboratory for Aerosol-Cloud-Precipitation of China Meteorological Administration, Nanjing University of Information Science & Technology, Nan Jing, Jiang Su, China; 2 Department of Geography, University of Toronto Mississauga, Mississauga, Toronto, Canada; Universidade de Vigo, SPAIN

## Abstract

Surface-level ozone pollution causes crop production loss by directly reducing healthy green leaf area available for carbon fixation. Ozone and its precursors also affect crop photosynthesis indirectly by decreasing solar irradiance. Pollutants are reported to have become even more severe in Eastern China over the last ten years. In this study, we investigated the effect of a combination of elevated ozone concentrations and reduced solar irradiance on a popular winter wheat Yangmai13 (*Triticum aestivum* L.) at field and regional levels in China. Winter wheat was grown in artificial shading and open-top-chamber environments. Treatment 1 (T1, i.e., 60% shading with an enhanced ozone of 100±9 ppb), Treatment 2 (T2, i.e., 20% shading with an enhanced ozone of 100±9 ppb), and Control Check Treatment (CK, i.e., no shading with an enhanced ozone of 100±9 ppb), with two plots under each, were established to investigate the response of winter wheat under elevated ozone concentrations and varying solar irradiance. At the field level, linear temporal relationships between dry matter loss and cumulative stomatal ozone uptake were first established through a parameterized stomatal-flux model. At the regional level, ozone concentrations and meteorological variables, including solar irradiance, were simulated using the WRF-CMAQ model (i.e., a meteorology and air quality modeling system). These variables were then used to estimate cumulative stomatal ozone uptake for the four major winter wheat-growing provinces. The regional-level cumulative ozone uptake was then used as the independent variable in field data-based regression models to predict dry matter loss over space and time. Field-level results showed that over 85% (T1: R^2^ = 0.85 & T2: R^2^ = 0.89) of variation in dry matter loss was explained by cumulative ozone uptake. Dry matter was reduced by 3.8% in T1 and 2.2% in T2 for each mmol O_3_·m^-2^ of cumulative ozone uptake. At the regional level, dry matter loss in winter wheat would reach 50% under elevated ozone concentrations and reduced solar irradiance as determined in T1, and 30% under conditions as determined in T2. Results from this study suggest that a combination of elevated ozone concentrations and reduced solar irradiance could result in substantial dry matter loss in the Chinese wheat-growing regions.

## Introduction

Man-made emissions of high levels of pollutants, especially nitrogen oxides (NO_x_) and volatile organic compounds (VOC), contribute to the chemical formation of surface-level ozone. Annual average background ozone concentrations over the mid-latitudes of the Northern Hemisphere range between approximately 20–45 ppb [1–2]. Background surface ozone will rise by 8 ppb, on average, by the year 2100 [3]. High surface-level ozone concentrations negatively affect plant photosynthesis, cause visible injuries to leaf, and result eventually in yield loss [4–9].

As part of ozone risk assessment, ozone exposure-response relationships have been characterized through open-top chamber (OTCs) experiments and simulated by several models [10–21]. Among these models, the ozone-dose exposure indices, AOT40 and SUM06, use ozone concentrations and exposure time as their main parameters to simulate the effects of ozone on plants. However, the two indices both overestimate loss in crop yield as a result of ignoring stomatal ozone uptake. To address this overestimation issue, the ozone-flux index (cumulative ozone uptake) has been proposed [18]. Specifically, the ozone-flux index is expressed through the stomatal-flux model, and this index has been used successfully to investigate relationships between yield loss and ozone uptake for wheat at field level [18, 22–25].

An increase in the concentrations of ozone and its precursors, such as NO_x_ and VOC, could also lead to atmospheric turbidity and hazy days, which are evident in many cities in Southwestern, Eastern, and Southern China [26–27]. Solar irradiance has decreased by more than 6% per decade in the Yangtze River region of China in the last five decades; this is much higher than the mean global decrease of 1.3% a^–1^ in photosynthetic active radiation [28–30]. Numerous studies have reported that reduced solar irradiance could alter plant chloroplast structure, hinder photosynthesis and stomatal conductance [31–32], and decrease light-use efficiency [33–35]. Thus, it is important to estimate accurately the combined effect of elevated ozone concentrations and reduced solar irradiance on crops. To the best of our knowledge, however, few studies have investigated this combined impact.

Winter wheat (*Triticum aestivum* L.) is a grass species that is planted widely in China, particularly in the Yellow and Huai River Valleys, which occupy about 43% of the country’s wheat growing area [36]. This region, however, has been experiencing high-level emissions of pollutants in recent years as a result of urbanization and industrialization [37–38]. Using field experiments and modeling approaches, the aim of this study was to quantify the effect of elevated ozone concentrations with reduced solar irradiance on winter wheat in four winter wheat-producing provinces in China. We estimate dry matter loss of wheat using the stomatal-flux model, which is strongly dependent on phenology and environmental variables such as solar irradiance, temperature, vapor pressure deficit, and soil moisture. Our specific objectives were 1) to establish relationships between dry matter loss and cumulative ozone uptake for winter wheat under elevated ozone concentration with varying solar irradiance at the field level, and 2) to estimate dry matter loss at the regional level by using dry matter loss-cumulative ozone uptake relationships established in 1). The study region for the second objective is the four provinces (i.e., Jiangsu, Anhui, Shandong, and Henan) within the major winter wheat-growing region in China.

## Materials and Methods

### Overview of methodology

**Fig 1:**
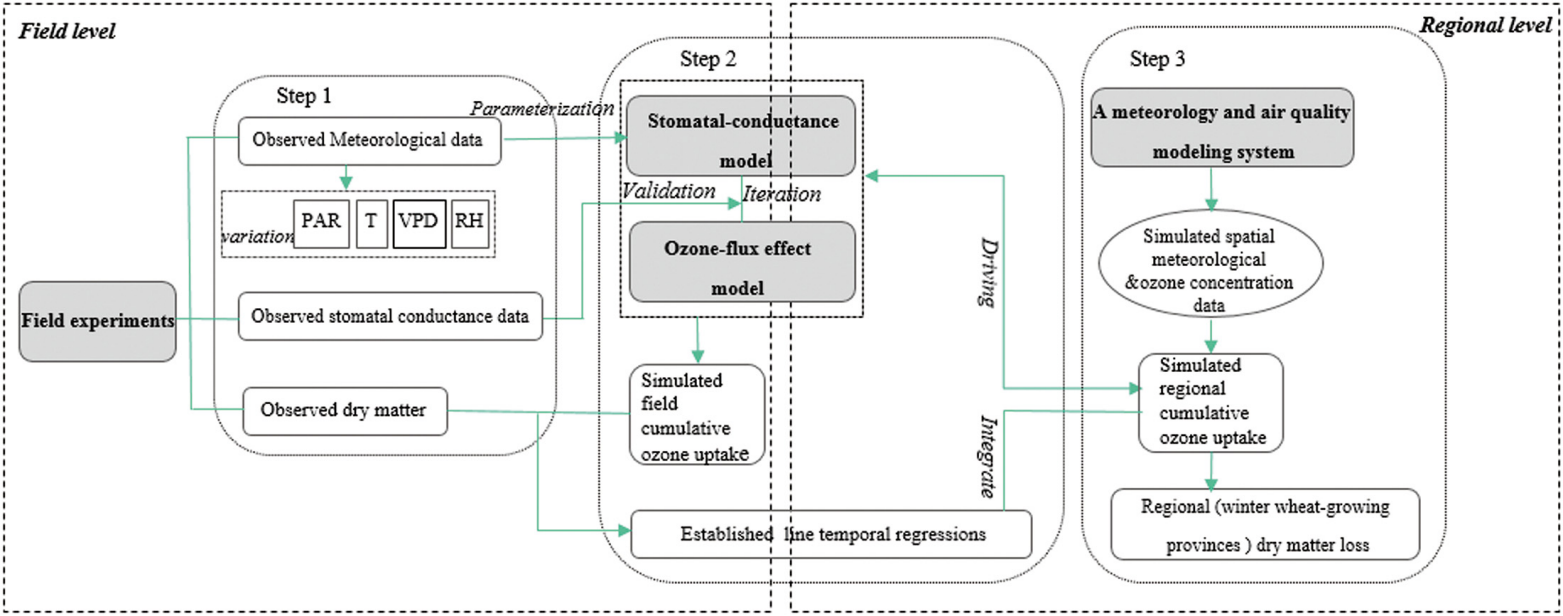
The overview of the methodology used in this study.

An overview of the methodology used in this study is shown in Fig 1. Specifically, we first collected data on stomatal conductance, dry matter, and environmental variables from field experiments (i.e., open-top-chambers and artificial shading). Second, cumulative ozone uptake was calculated through the parameterized and validated stomatal-flux model for the field site. Linear temporal relationships between cumulative ozone uptake and dry matter loss were established. Third, cumulative ozone uptake in winter wheat-growing provinces was simulated through the combination of the stomatal-flux model and an air quality modeling system. The uptake of regional cumulative ozone was used then as the independent variable in the regression models established in the second step to estimate dry matter loss at the regional level under elevated ozone concentrations with varying shading conditions.

### Field experiment and data collection

The experimental site is located at an agro-meteorological experimental station (32°14′N, 118°42′E), in Nanjing City, Jiangsu Province. The area has a subtropical monsoon climate with long, hot, wet summers and short, dry winters. Mean annual temperature is 16.5°C and accumulated precipitation is 1,107 mm. No specific permits were required for the described field experiment. The location is not owned privately or protected, and field work did not involve endangered or protected species.

Winter wheat Yangmai13 (*Triticum aestivum* L.), obtained from Lixiahe Agricultural Research Institute (Jiangsu, China) was planted in the field on November 18^th^, 2010. Prior to sowing, 692.3 kg ha^−2^ of nitrogen complex fertilizer (N8-P_2_O_5_6K_2_O6, *Tianbu Co*. *Ltd*., Shanghai, China) was applied. Winter wheat was harvested on May 31^th^, 2011.

**Fig 2:**
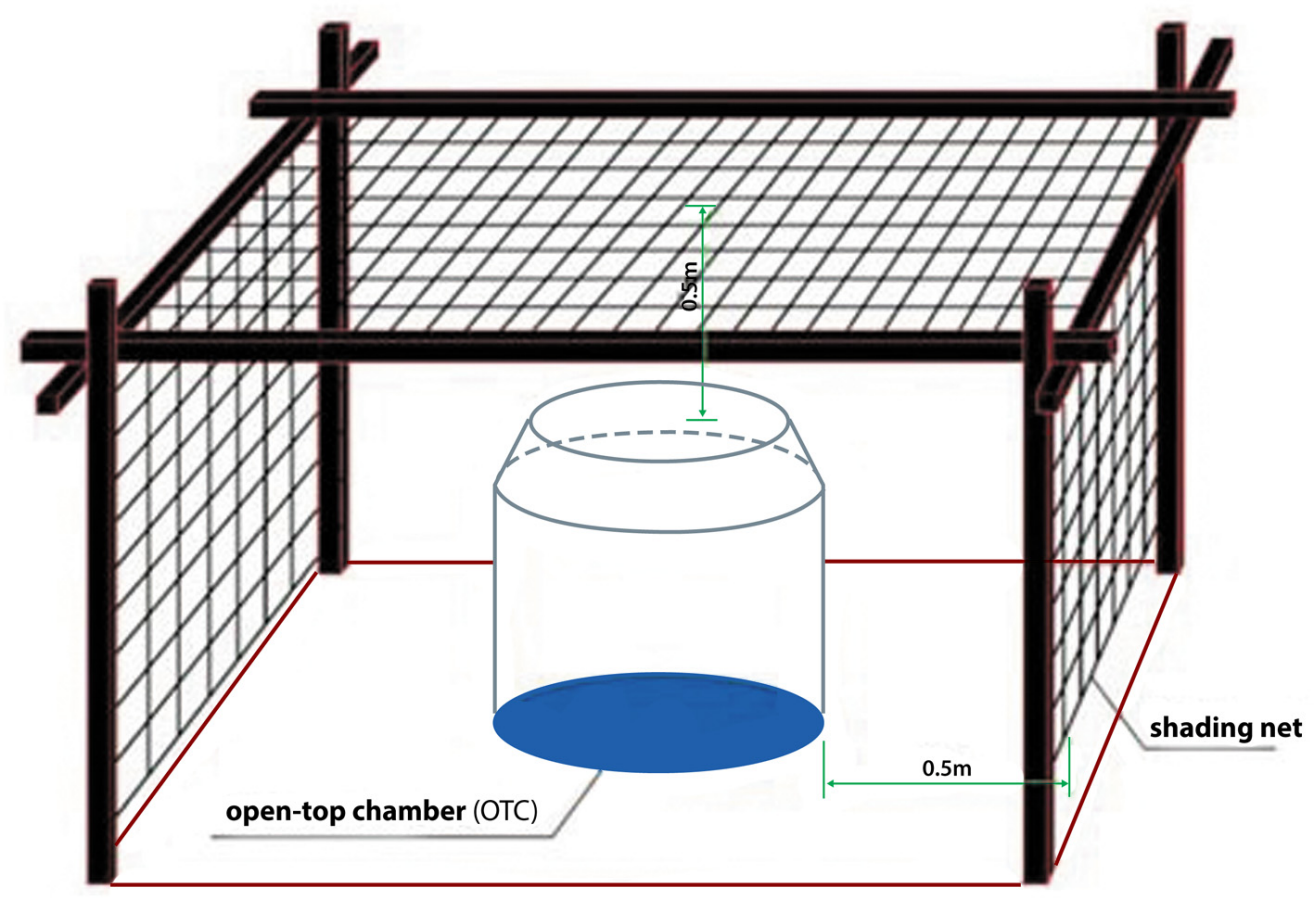
The experimental design of artificial shading and an open-top chamber.

The winter wheat was grown in a 3 × 3 m field plot. A 1.5 m buffer zone from the plot edge was used to prevent mutual interference. Three treatments, with two plots under each, were established to study the response of wheat to elevated surface-level ozone concentrations with varying levels of solar irradiance. Open-top-chambers (OTC, 2 m diameter and 1.9 m height) were used to elevate ozone concentrations. Polyethylene neutral shade cloths were used to reduce solar irradiance. Specifically, one polyethylene neutral shade cloth was placed 0.5 m above the open-top-chamber, and two additional shade cloths for eastwards and westwards were placed 0.5 m away from the OTC to cover each plot (Fig 2). The three treatments were in the uniform OTCs with an enhanced ozone of 100±9 ppb, and the shading levels were set as follows: T1: high-level shading (i.e., 40% of total solar irradiance); T2: low-level shading (i.e., 80% of total solar irradiance); and CK: no shading. We decided to set the enhanced ozone concentration at a level of 100 ppb in this experiment for the following reasons: 1) the daily background ozone concentrations of this region have nearly reached 50 ppb and it is therefore meaningful to investigate the impact of the doubled ozone concentration on plants; and 2) many pervious experiments adopted the level of 100ppb to study the effect of elevated ozone concentrations on plants [5] and the ozone concentration of 100ppb used in this work could enable a direct comparison between our results and those from previous work. The experiments were started on March 24^th^, 2011, when the crop was at the jointing stage. During the experimental period, artificial shading was applied throughout each day, and ozone exposure was set between 08:00 and 16:00 local time (a total of eight hours each day).

Photosynthetically active radiation (PAR) and the elevated ozone concentrations were monitored by a TBQ-5A portable spectroradiometer (JWF Ltd. Co., Shanghai, China) and a HK90 ozone detector (Hengaode Co Ltd., Beijing, China), respectively. Air temperature and relative humidity were measured using a HOBO U23-001 data-logger (Onset Computer Corp., Bourne, MA, USA), which was placed 10 cm above the wheat canopy in the chamber. Stomatal conductance of winter wheat was measured in each treatment between 09:00 and 16:00, using an SC-1 steady stomatal conductance meter that was placed over 5–8 randomly selected winter wheat plants. Ten plants for each plot were chosen randomly to determine the dry matter nondestructively (Table 1).

**Table 1:** The dates when the stomatal conductance and dry matter measurements were taken in 2011.

Growth Stage	Stomatal Conductance	Dry Matter
	Measurements	Measurements
**Jointing stage**	April 10	Mar 24 and April 02
**Heading stage**	April 20	April 12 and April 22
**Filling stage**	April 28	May 2
**Full ripe**	May 6	May 12 and May 22

### Simulation of field-level cumulative ozone uptake

Temporal uptake of stomatal cumulative ozone was calculated by the stomatal-flux model for the entire experimental period. The model included two modules: a multiplicative Javis stomatal conductance model and an ozone flux-effect model. Stomatal conductance was simulated from the multiplicative Javis stomatal conductance model and then used as input in the ozone flux-effect model to estimate stomatal cumulative ozone uptake. The details of the stomatal-flux model were fully described in a previous study [19].

The multiplicative Javis stomatal conductance model simulates stomatal conductance (G_ST_) by multiplying the maximum stomatal conductance value (G_max_) by six limiting driving parameter functions. These limiting driving parameters are PAR, air temperature (Temp), vapor pressure deficit (VPD), phenology (Phen), ozone, and soil water potential (SWP), and their functions (i.e. *f*_*PAR*_, *f*_*Temp*_, *f*_*VPD*_, *f*_*phen*_, *f*_*O3*_, *f*_SWP)_ were adopted from the earlier work [19] (Table 2). No limiting function of soil water content was considered in this model, because soil moisture was sufficient at the experimental station [39]. The constants in the limiting functions PAR, Temp, VPD, and Phen were obtained using the boundary-line analysis.

**Table 2:** The parameters used in the stomatal conductance model [19].

	Description/functions
***f***_***PAR***_	1-e^(-L^*^PAR)^
***f***_***T***_	If *T*_min_≤ *Temp* ≤*T*_*max*,_ *f*_*T*_ = 1- (*Temp* -*T*_*opt*_)^2^/(*T*_*opt*_*-T*_*min*_)^2^
	If *T*_*min*_<*Temp* or *Temp*>*T*_*max*_, *f*_*T*_ = *G*_*min*_
***f***_***VPD***_	min{1,((1-*G*_*min*_)*(*VPD*_*min*_-*VPD)*/(*VPD*_*min*_*-VPD*_*max*_))+*G*_*min*_}
***f***_***phen***_	Before anthesis, *f*_*phen*_ = 1-(1-*f*_*a*_)(*TT*_*max*_-*TT*)/*TT*_*max*_
	After anthesis, *f*_*phen*_ = 1-(1-*f*_*b*_*)(TT-TT*_*max*_*)/(TT*_*end*_*-TT*_*max*_)
***f***_***O3***_	(1+(AF_sto_0/11.5)^10^)^-1^

Cumulative ozone uptake was simulated using the ozone flux-effect model by dividing ozone concentrations (i.e., set to 100 ppb in this study) by two resistance factors (i.e., stomatal resistance (1/G_ST_) and leaf boundary layer resistance (a constant)). Specifically, the diffusivity ratio between ozone and water vapor is 0.663 [24].

### Relationship between field-level cumulative ozone uptake and dry matter

Regression analysis was performed to investigate relationships between dry matter loss and cumulative ozone uptake in T1 and T2. The dry matter loss (i.e., relative dry matter) was obtained by dividing dry matter from T1 or T2 by dry matter from CK [19]. Leave-One-Out Cross-Validation (LOOCV) was used to evaluate the established regression models, since this method is used commonly for studies with a small sample size. In this study, only seven sets of data were measured over time for each treatment, and thus the LOOCV was adopted for model evaluation. Specifically, each single observation (relative dry matter of winter wheat) from the original sample set was selected as validation datum, and the remaining observations (*n*–1 out of *n* relative dry matter) were used as training data to establish a regression model. This step was repeated n times. The prediction accuracy was computed using the absolute root mean square error (RMSE), relative root mean square error, absolute bias and relative bias [40].

### Simulation of regional-level cumulative ozone uptake and loss of dry matter

In short, a meteorology and air quality modeling system (WRF-CMAQ model) [41] was used to simulate regional meteorological variables (solar irradiation, T and VPD) and ozone concentrations. The stomatal-flux model was then used to simulate the uptake of cumulative ozone that was distributed spatially. Finally, the regional uptake of cumulative ozone was used as the independent variable in the regression models established in Sec. 2.4 to estimate regional dry matter loss under elevated ozone concentrations with varying shading conditions.

To be specific, the air quality model we used is the third-generation US Environmental Protection Agency (EPA) Community Multiscale Air Quality (CMAQ, version 4.7.1) model. In this study, CMAQ was driven by the outputs of the Weather Research and Forecasting (WRF, version 3.4) model. We chose the WRF-CMAQ coupled modelling system because previous studies have demonstrated that it performs well in simulating tropospheric ozone concentrations [41–45]. We set the WRF model with two one-way domains: 107 by 107 grids with a spatial resolution of 27 km (domain 1), and 151 by 133 grids with a spatial resolution of 9 km (domain 2). The latter domain covers the winter wheat-growing provinces. The center of latitude and longitude of the domains was located at 108°E, 32°30’N. Both domains had a total of 31 sigma levels vertically, with the top fixed at 100 hPa. The physical options used in this study were the WRF Single Moment 6-class microphysics scheme, RRTM (rapid radiative transfer mode) longwave radiation scheme, Dudhia shortwave radiation scheme, NOAH land surface model, Yonsei University Planetary Boundary Layer scheme, Kain-Fritsch cumulus parameterization scheme, and none urban physics [46]. The 6-hourly Final Analyses (FNL) data with 1°×1° resolution from the National Center for Environmental Prediction (NCEP) were used as initial and boundary conditions of the WRF model.

**Fig 3:**
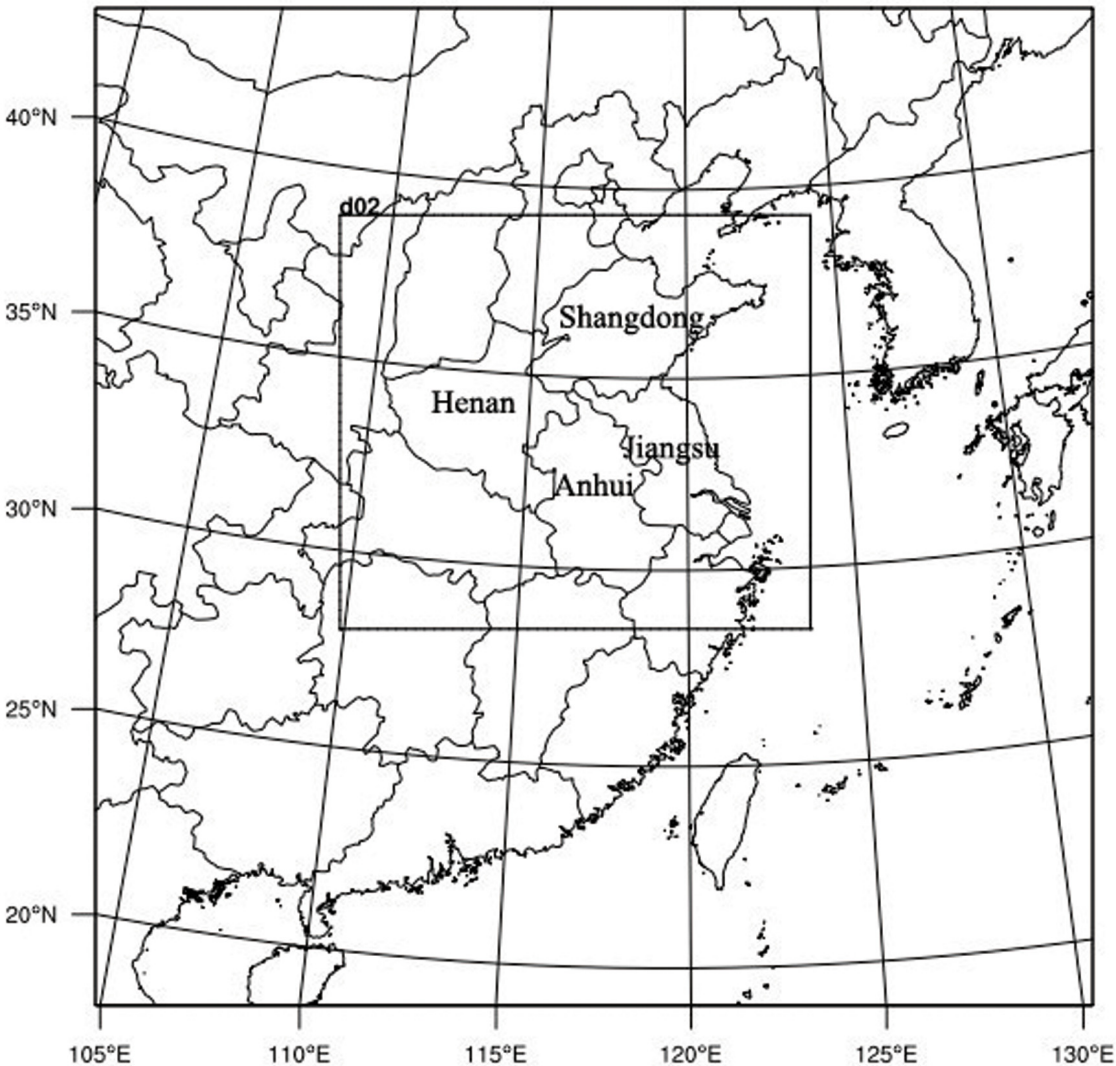
The two modelling domains for CMAQ simulations: the nested domain with a resolution of 9 × 9 km and the mother domain with a resolution of 27 × 27 km. This visualization was created by the NCAR Command Language (Version 6.3.0) [Software], 2015 under a CC BY license [63], with permission from University Corporation for Atmospheric Research, original copyright 2015.

Similarly to WRF, two domains were set in CMAQ with each domain slightly smaller than those in WRF, but the domain resolutions the same: an 80 ×80 grid system of 27 km resolution for Domain 1, and a 130 ×110 grid system of 9 km resolution for Domain 2 (Fig 3). CMAQ was set with a total of 23 vertical layers. Layer collapsing was used in the Meteorology-Chemistry Interface Processor (MCIP, version 3.6). CB05 and AERO4 were chosen as the chemistry and aerosol options of CMAQ [46–48]. Initial and boundary conditions (i.e., IC and BC) for Domain 1 were obtained from the default files prepared for CMAQ. For Domain 2, the IC and BC were interpolated from the previous day’s simulation in Domain 1 using initial conditions processor (ICON) and boundary conditions processor (BCON) modules. Emission data that included anthropogenic and biogenic volatile organic compounds (BVOC), biomass burning, and volcanic SO_2_ emissions were obtained from an emission inventory for Asia in 2006 [49]. The data have a horizontal resolution of 0.1°×0.1°.

The simulation period was from 00:00 local standard time (LST), March 1^st^, 2006 to 23:00 LST, May 31^th^, 2006. The period was chosen because the emission inventory data were collected from 2006, and the most important growing period of winter wheat is between March and May. The WRF and CMAQ model outputs had an hourly temporal resolution.

The WRF-CMAQ model outputs were compared with the observed ozone concentrations data from the Nanjing station between March and May in 2006. The modeling performance was determined using three measures, including Normalized Mean Bias (NMB), Normalized Mean Error (NME), and RMSE [50]. The closer the values of these measures approach zero, the better the simulation. The correlation coefficient (*r*) was calculated to show the correlation between the simulated and observational datasets.

## Results and Discussion

### Field data

#### Meteorological factors under varying conditions of reduced solar irradiance

**Fig 4:**
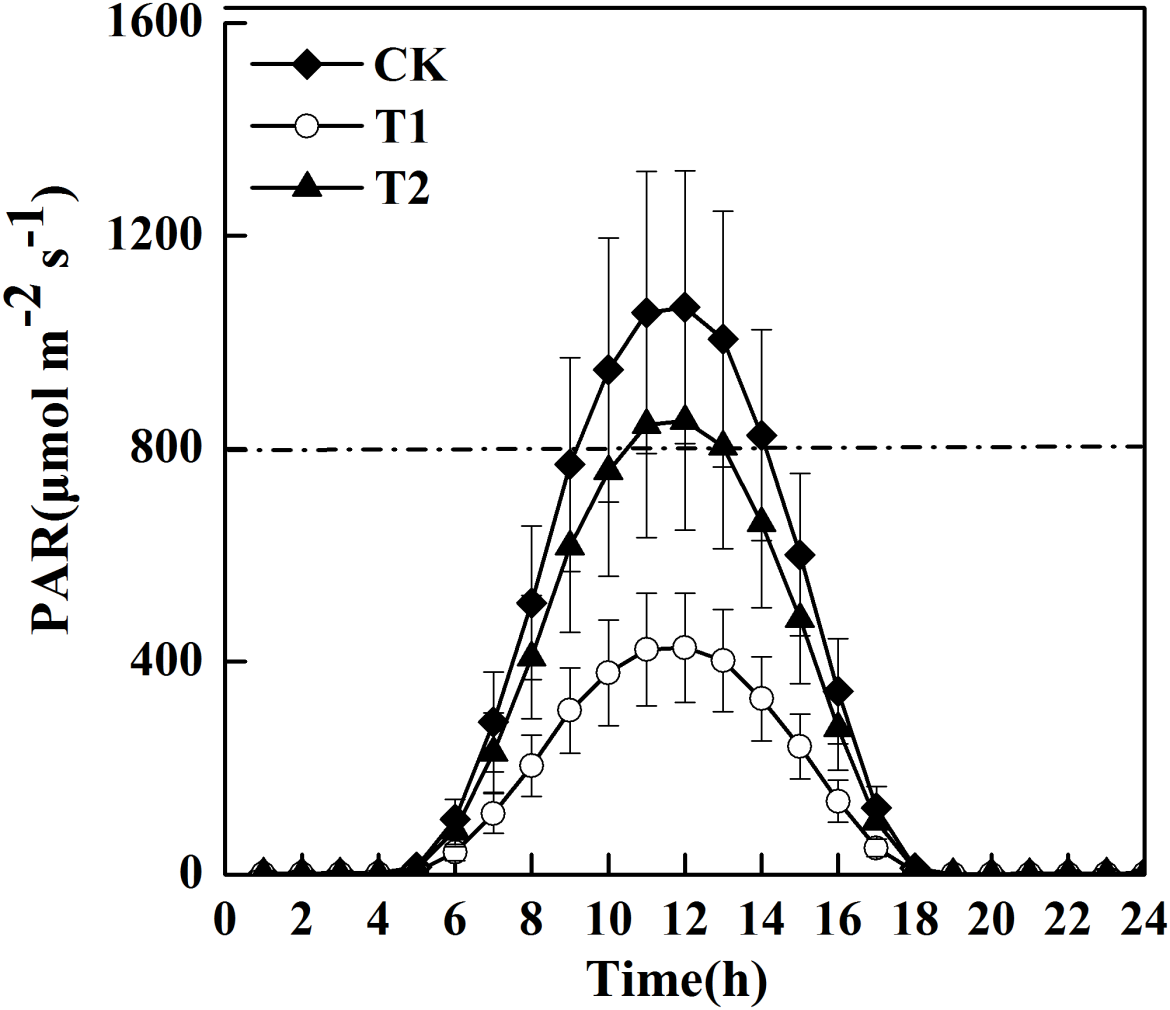
The diurnal variation of mean (x SD) PAR in each treatment (n = 68).

The mean observed diurnal PAR differed in the three treatments over the 24 h period, and the maximum difference occurred at noon (Fig 4). The maximum PAR at 12:00 was 426.26 μmol m^-2^·s^-1^ for T1, 852.52 μmol m^-2^·s^-1^ for T2, and 1065.65 μmol m^-2^·s^-1^ for CK. In comparison with CK, average temperatures (Temp) decreased 5.6°C in T1 and 4.1°C in T2, and vapor pressure deficit (VPD) decreased 0.84 kPa in T1 and 0.74 kPa in T2. However, relative humility (RH) increased 16.0% in T1 and 14.5% in T2 (Table 3). These results indicated that reduced solar irradiance led to substantial changes in field environments. The differences in these variables between CK and T1/T2 were used later to estimate regional meteorological conditions under shading treatments.

**Table 3:** Mean meteorological data for three variables (Temp, VPD, and RH) in each treatment.

Environmental parameters	CK	T1	T2
**Ave Temp (°C)**	26.8	21.3	22.7
**Ave VPD (kPa)**	1.46	0.62	0.72
**Ave RH (%)**	51.2	67.2	65.7

### Simulation of field-level uptake of cumulative ozone

#### The parameterization of the multiplicative Javis stomatal conductance model

**Fig 5:**
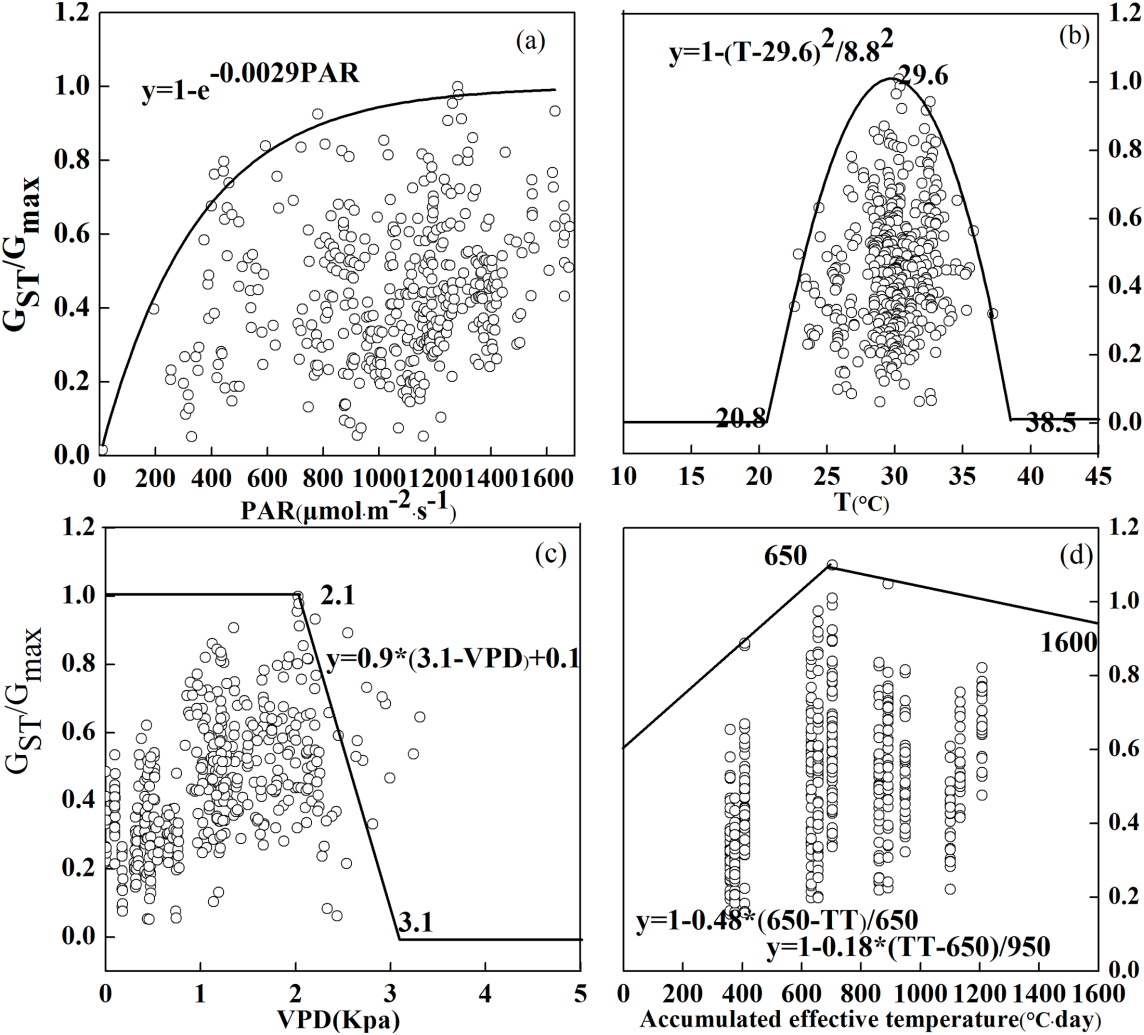
Boundary-line analysis of the relationships between relative stomatal conductance and environmental variables under elevated ozone concentrations with varying shading conditions. (a)PAR represents pohtosynthetically active radiation; (b) Temp represents temperature; (c) VPD represents vapor pressure deficit; (d) Phen represents accumulated effective temperature.

The multiplicative Javis stomatal conductance model was parameterized, given that the field environments changed under reduced solar irradiance conditions. Stomatal conductance as a function of PAR, Temp, VPD, and Phen is shown in Fig 5, and the parameterization for each function is summarized in Table 4.

**Table 4:** The unit and value of each parameter used in the stomatal conductance model.

Parameter	Units	Value
***G***_***max***_	**mmolH**_**2**_**O·m**^**-2**^**·s**^**-1**^	427(observed data)
***G***_***min***_	**Fraction**	0.1(observed data)
***L***	**Fraction**	-0.0029 (Fig 5a)
***T***_***min***_	**°C**	20.8(Fig 5b)
***T***_***opt***_	**°C**	29.6(Fig 5b)
***T***_***max***_	**°C**	38.5(Fig 5b)
***VPD***_***max***_	**kPa**	2.1(Fig 5c)
***VPD***_***min***_	**kPa**	3.1(Fig 5c)
***f***_***a***_	**Fraction**	0.52(Fig 5d)
***f***_***b***_	**Fraction**	0.82(Fig 5d)
***TT***_***max***_	**°C·d**	650(Fig 5d)
***TT***_***end***_	**°C·d**	1600(Fig 5d)

The parameter *G*_*max*_ was the maximum stomatal conductance; *G*_*min*_ was the lowest stomatal conductance expressed as a fraction of *G*_max_; *L* defined the rate of saturation of the *f*_*PAR*_ function; *T*_*opt*_ denoted the temperature where there was no limitation of stomatal conductance; *T*_*max*_ and *T*_*min*_ gave the temperature above and below which there was limited stomatal conductance, respectively; *VPD*_*max*_ defined the level where started to limit stomatal conductance and *VPD*_*min*_ indicated where there was limitation of stomatal conductance; *f*_*a*_ and *f*_*b*_ denoted the maximum fraction of *G*_*max*_ that stomatal conductance took at the beginning and end of the ozone exposure. The beginning and end of the ozone exposure period are expressed as temperature sums before (*TT*_*max*_) and after (*TT*_*end*_) anthesis in wheat.

The boundary line displayed a typical light saturation curve, and the light saturation was obtained at the PAR of 1000 μmol·m^-2^·s^-1^ (Fig 5a). The curve in Fig 5a provides the constant value (i.e., L) in the PAR function, which was -0.0029. These results are different from a previous study, which concluded that the light saturation was around 500 μmol·m^-2^·s^-1^ PAR and the constant value was -0.0105 [19]. The possible explanations for these differences are 1) the crop that the other study did the experiment on was spring wheat [19], while we studied winter wheat, and 2) different planting zones may result in differences in stomatal movements.

The effect of Temp on stomatal conductance is complicated, because it depends not only on plant species but also on vapor pressure. Relative stomatal conductance value (i.e., G_st_/G_max_), along with the change in Temp, was often characterized by a single-peak. In Fig 5b, the optimum temperature for stomatal conductance was approximately 29.6°C. The lack of measurements below 20.8°C and above 38.5°C posed a limitation for the calibration of the temperature function. Both minimum and optimum Temp were higher than the past results of 12°C and 26°C [51]. We speculate that our species has a stronger tolerance to high temperature, or has adapted to excessive ozone uptake and environmental changes under shading conditions.

We found that a VPD higher than 3.0 kPa induced stomatal closure (Fig 5c). Relative stomatal conductance had a marginal change when VPD was low, but declined rapidly when VPD was higher than 2.1 kPa. The VPD_min_ threshold (i.e., 3.1 kPa) in this study was lower than other experimental results (3.2~3.6 kPa) [19, 22, 51]. Constant exposure to high relative humidity and a wet environment may have affected the drought tolerance of winter wheat, thus leading to the lower VPD_min_ threshold.

Along with an increase in the accumulated effective temperature, winter wheat showed a high degree of senescence (Fig 5d). Relative stomatal conductance decreased especially rapidly when accumulated effective temperature exceeded 650°C·d. We thus used the phenology functions as introduced previously [52], because our two studies shared a similar response of relative stomatal conductance to the accumulated effective temperature.

#### The results and validation of the multiplicative Javis stomatal conductance model

**Fig 6:**
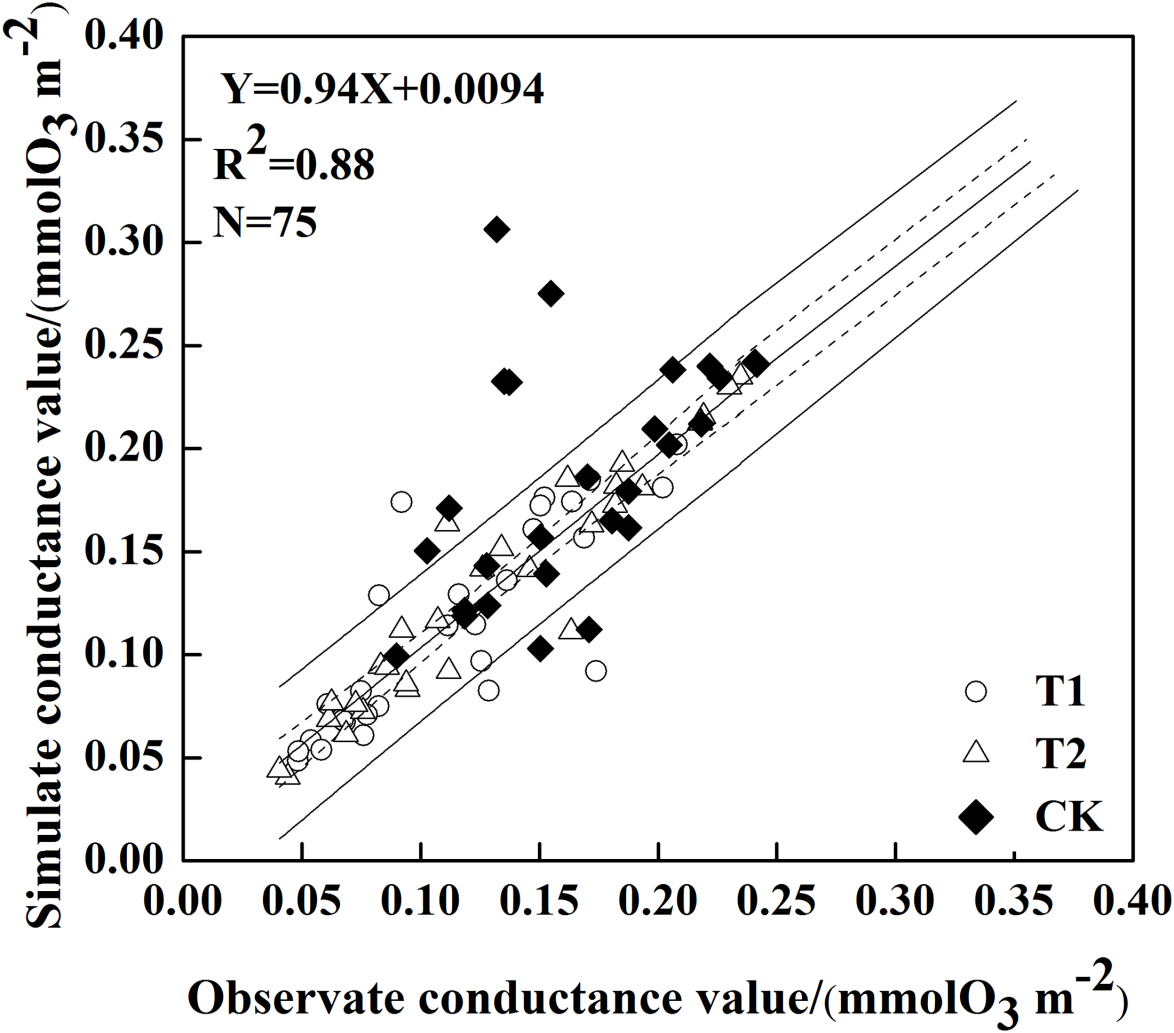
Simulated stomatal conductance plotted against measured stomatal conductance in all treatments. (“---” and “—” represent 95% confidence level and the overall mean confidence region, respectively).

The simulated stomatal conductance data ranged from 0.04 to 0.31 molO_3_·m^-2^·s^-1^, with a standard deviation of 0.062 molO_3_·m^-2^·s^-1^ for all three treatments. In comparison, mean stomatal conductance and its variation in CK were greater than those of T1 and T2. The model results were validated using observed stomatal conductance data for the three treatments in jointing, heading, flowering, and grain-filling stages of winter wheat (Fig 6). The regression line, with a slope of 0.94, was not significantly different from the 1:1 line at the 0.05 significance level. The R^2^ of 0.88 and RMSE of 0.035 indicated good agreement between the observed field data and modeling results.

#### Simulation of cumulative ozone uptake for the field site

**Fig 7:**
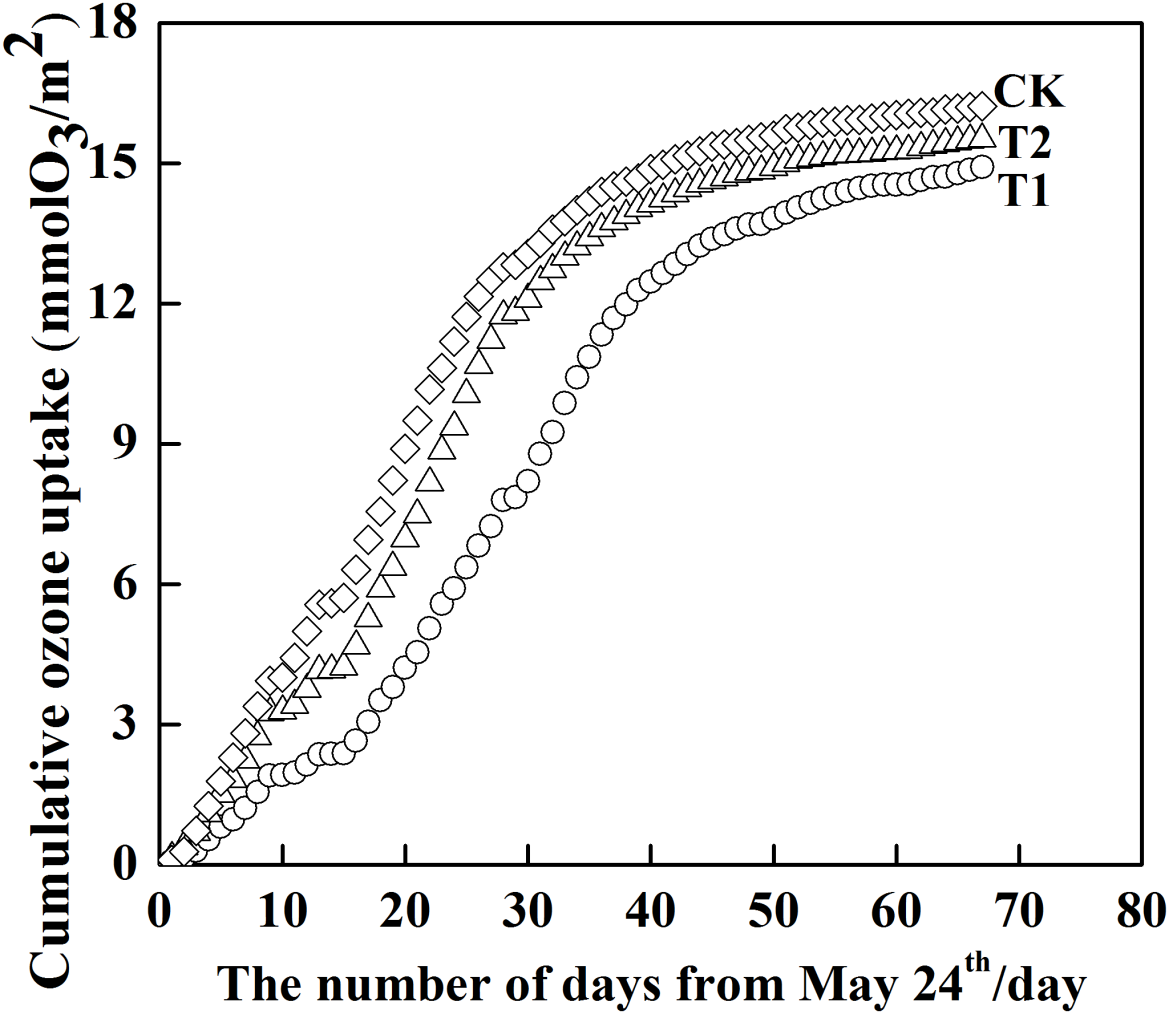
Cumulative ozone uptake in each treatment during the whole experiments period.

Simulated stomatal conductance was then used to drive the ozone flux-effect model to obtain daily cumulative ozone uptake in T1, T2, and CK over time (Fig 7). A similar trend was found in all treatments, with only a marginal difference in the magnitude of values. At the end of the experimental period, cumulative ozone uptake in T1, T2, and CK reached a maximum of 14.92, 15.52, and 16.23 mmolO_3_·m^-2^, respectively, suggesting that a decrease in solar irradiance reduced cumulative ozone uptake. It is well known that the uptake in cumulative ozone is dependent on both ozone concentrations and stomatal conductance [53]. Reduced solar irradiance is thus solely responsible for the decrease in stomatal conductance and stomatal ozone uptake, since the three treatments were all set under the same ozone exposures, which suggested that shading could alleviate ozone damage to the plant.

**Fig 8:**
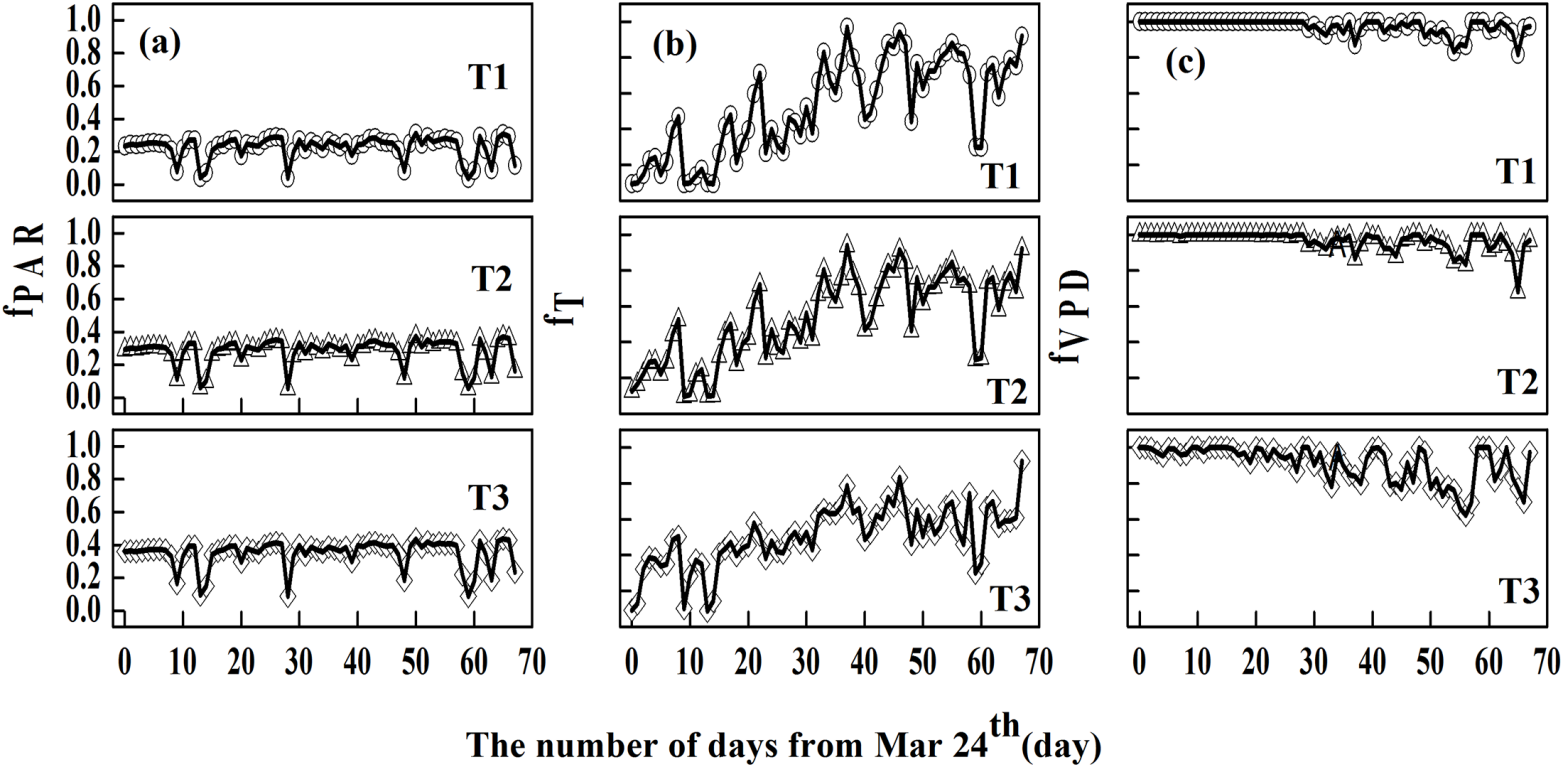
The daily variation of mean *f*_*PAR*_, *f*_*T*_, and *f*_*VPD*_ in each treatment (the black line represents the slide average of the five days).

**Fig 9:**
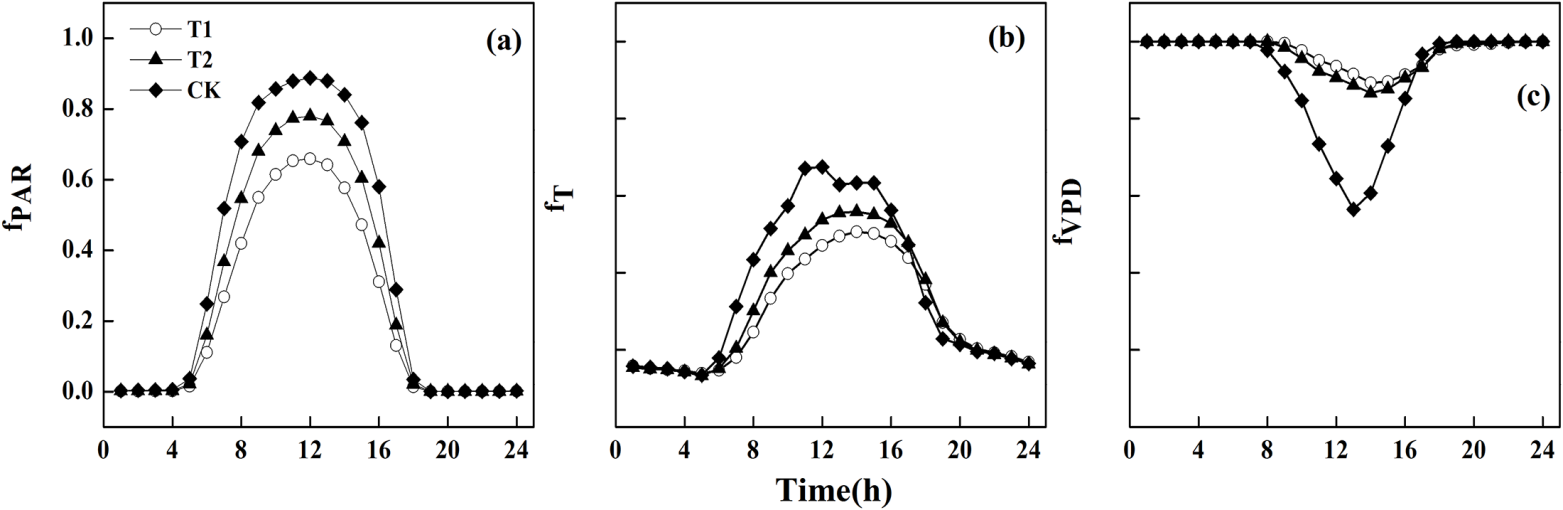
The hourly variation of mean *f*_*PAR*_, *f*_*T*_, and *f*_*VPD*_ in each treatment.

To further investigate the relative importance of environmental factors that influence the uptake in cumulative ozone under the reduced solar irradiance conditions, daily and hourly curves of environmental variables (PAR, Temp, and VPD) involved in the modelling of stomatal conductance were plotted for each treatment (Figs 8 and 9). The limiting effect of PAR on stomatal conductance was consistent over the entire season (around 0.2 in T1, 0.3 in T2, and 0.4 in CK in Fig 8a), except for several fluctuations that likely resulted from rainfall. The daily variation in Temp showed similar trends in all treatments, with a variation in the magnitude. The limiting effect of Temp on stomatal conductance was stronger at the beginning, but its impact became weaker with stronger diurnal fluctuations (Fig 8b). The daily variations in VPD in T1 and T2 showed a similar trend, but they were different from CK. VPD had no impact on stomatal conductance at the beginning in T1 and T2 (i.e., *f*_*VPD*_ = 1 before day 30 in Fig 8c), and had a relatively low effect in CK (i.e., *f*_*VPD*_ had a very small variation around 0.9). The hourly variations in PAR, Temp, and VPD had similar trends in all three treatments, except for an obvious difference in Temp at noon (Fig 9). Between 08:00 and 16:00, PAR and Temp was at its lowest in T1 and VPD was at its highest; PAR, Temp, and VPD were all moderate in T2; and PAR, Temp, and VPD in CK showed a trend opposite to that of T1. Temp in T1 and T2 reached its maximum value, but we observed a decrease in CK. The daily and hourly variations suggested that 1) PAR was the most limiting factor on stomatal conductance over the entire experimental period, and this effect became more negative with increasing shading. This was expected, because lower solar irradiance resulting from shading would lead to insufficient radiation needed for crop photosynthesis, and would lead also to a weaker stomatal conductance. This is also consistent with a past finding [54], 2) the limiting effect of Temp was stronger at the beginning, and the limiting effect of VPD became stronger over time, 3) the limiting impact of PAR combined with Temp was stronger than that of VPD, leading to the highest stomatal conductance in CK and the lowest in T1, and 4) the effect of each factor on stomatal conductance in T2 was intermediate as a result of moderate stomatal conductance in T2.

An interesting observation was that the wheat in CK showed an obvious "noon-nap" phenomenon (i.e., stomatal closure), but this phenomenon was not observed in T1 and T2 (Fig 9b). At noon, higher Temp and VPD in CK induced instantaneous stomata closure (i.e., “noon-nap” phenomenon), to decrease transpiration rate and moisture loss from the leaves [55]. Without a “noon-nap”, one would expect to see an increase in stomatal conductance in T1 and T2, which did not happen. A possible explanation is that the lower temperature in T1 and T2 influenced the crop’s enzyme activity [56], and resulted further in lower stomatal conductance. Therefore, both temperature and enzyme activity can affect stomatal conductance [57].

**Fig 10:**
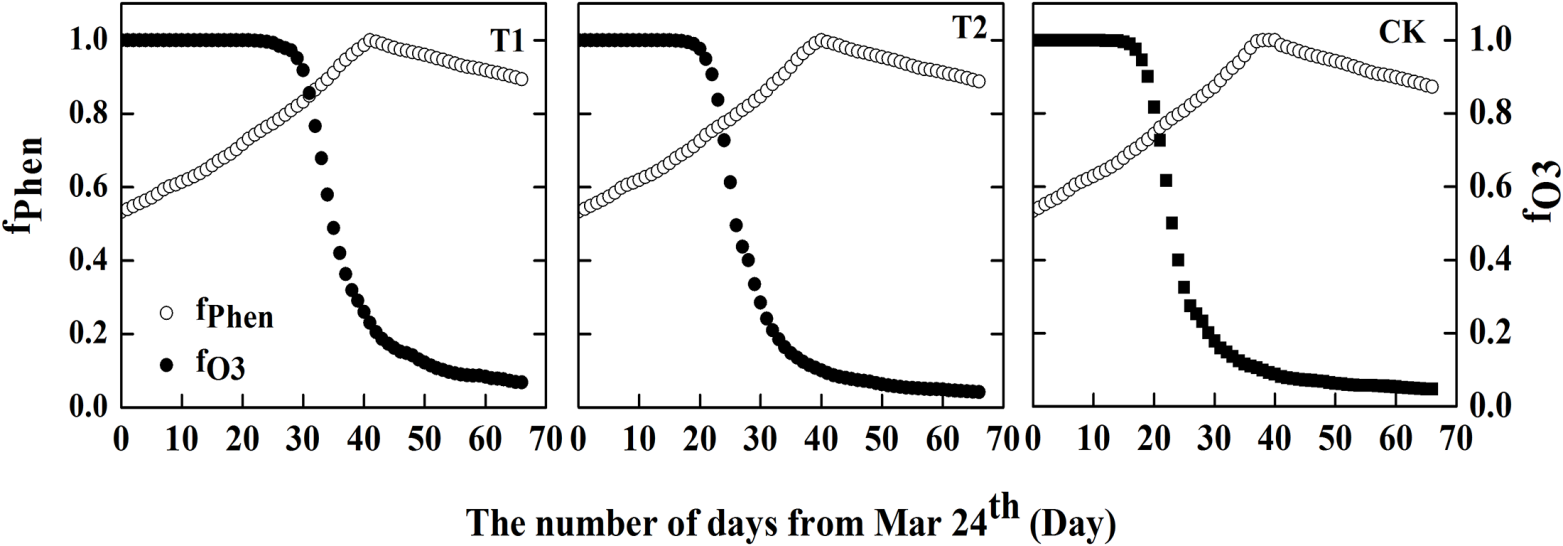
The daily variation of mean *f*_*phen*_ and *f*_*O3*_ in each treatment.

The relative importance of *f*_*Phen*_ and *f*_*O3*_ in the modeling of stomatal conductance is also expressed as daily variation curves for each treatment (Fig 10). Phen increased at first and decreased towards the end of the day in each treatment, while *O*_*3*_ decreased with a very different trend among the three treatments. The *f*_*O3*_ in each treatment had no impact on stomatal conductance at first (around 30 days in T1, 25 days in T2, and 20 days in CK), but it had a strong and increasing limiting effect towards the end of the growing season. Owing to the different trends for *f*_*O3*_ over time, the effect of phenology on stomatal conductance was dominant for the first 35 days in T1, for 27 days in T2, and for 23 days in CK. These results suggested that 1) the limiting effect of *f*_*O3*_ lasted longer under reduced solar irradiance, 2) phenology is the dominant limiting factor on stomatal conductance in the early growth period of winter wheat, and 3) ozone exposure became a stronger limiting factor in CK, which suggested that shading may have alleviated leaf senescence and ozone damage.

#### Regression models for dry matter loss for the field site

According to field measurements, dry matter losses were 3.8% (T1) and 2.2% (T2) for the increase in ozone cumulative uptake for each 1 mmol O_3_·m^-2^. It is interesting to note that reduced solar irradiance resulted in a decrease in cumulative ozone uptake, but an increase in dry matter loss. Shading hindered stomatal conductance and further affected CO_2_ uptake negatively, which led to reduced photosynthesis and a reduction in biomass products.

**Fig 11:**
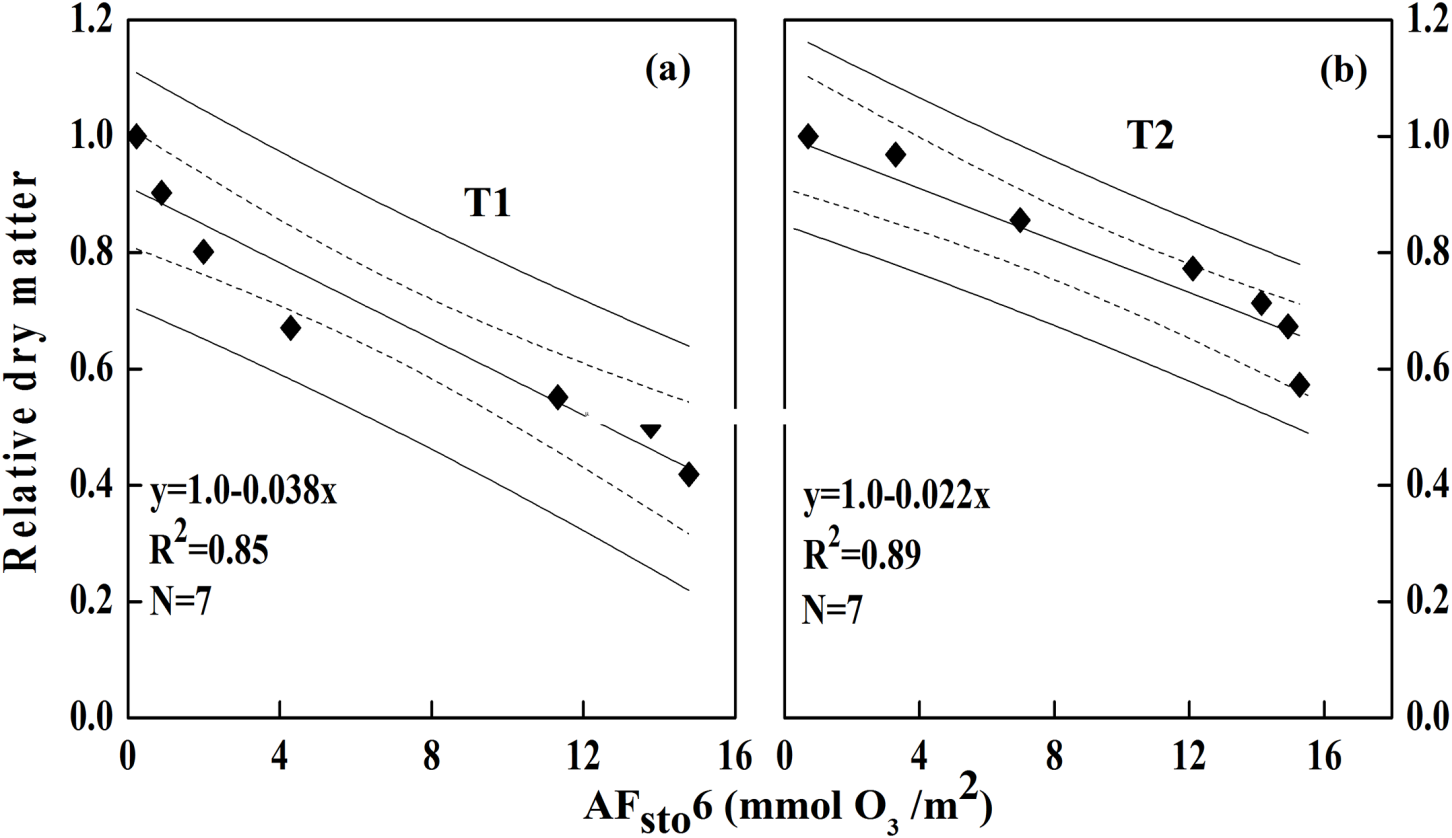
Temporal relationships between dry matter loss of winter wheat in T1 and AF_sto_6 (a) and in T2 and AF_sto_6 (b). (“---”and“—” represent 95% confidence level and the overall mean confidence region, respectively).

The strongest correlations for winter wheat were obtained using an ozone flux threshold of 6 nmol O_3_ ·m^-2^ (AF_sto_6) [19]. Therefore, cumulative ozone uptake (AF_sto_6) was used to establish regressions with dry matter loss for T1 and T2. A relatively stable modelling error calculated through the LOOCV validation approach (Tables 5 and 6) indicated that no outliers were included in the dataset. Given that model 4 for T1 and model 3 for T2 produced the lowest absolute RMSE, relative RMSE (i.e., RS_1_ and RS_2_), absolute bias and relative bias (i.e., B_ias1_ and B_ias2_), the two models (Fig 11) with R^2^ greater than 0.85 were then adopted to predict dry matter loss over time with T1 and T2 treatments.

**Table 5:** Prediction accuracy of the different regression models for T1.

MODEL(T1)	RS_1_	RS_2_ (%)	B_ias1_	B_ias2_ (%)
**1**	0.0875	12.65%	0.0077	1.11%
**2**	0.0839	12.12%	0.007	1.02%
**3**	0.0728	10.52%	0.0053	0.77%
**4**	0.0582	8.41%	0.0034	0.49%
**5**	0.0876	12.66%	0.0077	1.11%
**6**	0.0833	12.04%	0.0069	1.00%
**7**	0.0949	13.72%	0.009	1.30%

**Table 6:** Prediction accuracy of the different regression models for T2.

MODEL(T2)	RESE_1_	RESE (%)	Bias_1_	Bias (%)
**1**	0.042	5.30%	0.00177	0.22%
**2**	0.0421	5.31%	0.00177	0.22%
**3**	0.0347	4.38%	0.00121	0.15%
**4**	0.0388	4.89%	0.00151	0.19%
**5**	0.0357	4.50%	0.00127	0.16%
**6**	0.0379	4.78%	0.00143	0.18%
**7**	0.0354	4.46%	0.00125	0.16%

### Simulation of cumulative ozone uptake and dry matter loss at the regional level

#### Simulation of regional ozone concentrations

**Fig 12:**
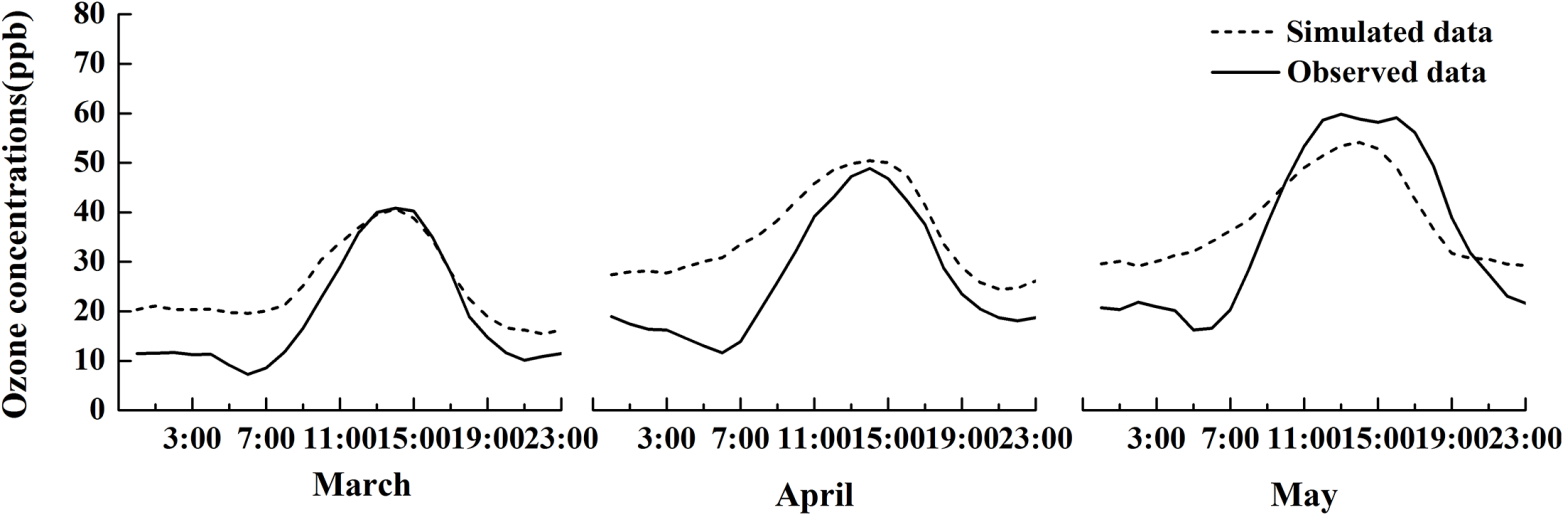
The hourly simulated and observed mean ozone concentrations in Nanjing station from March to May of 2006.

**Fig 13:**
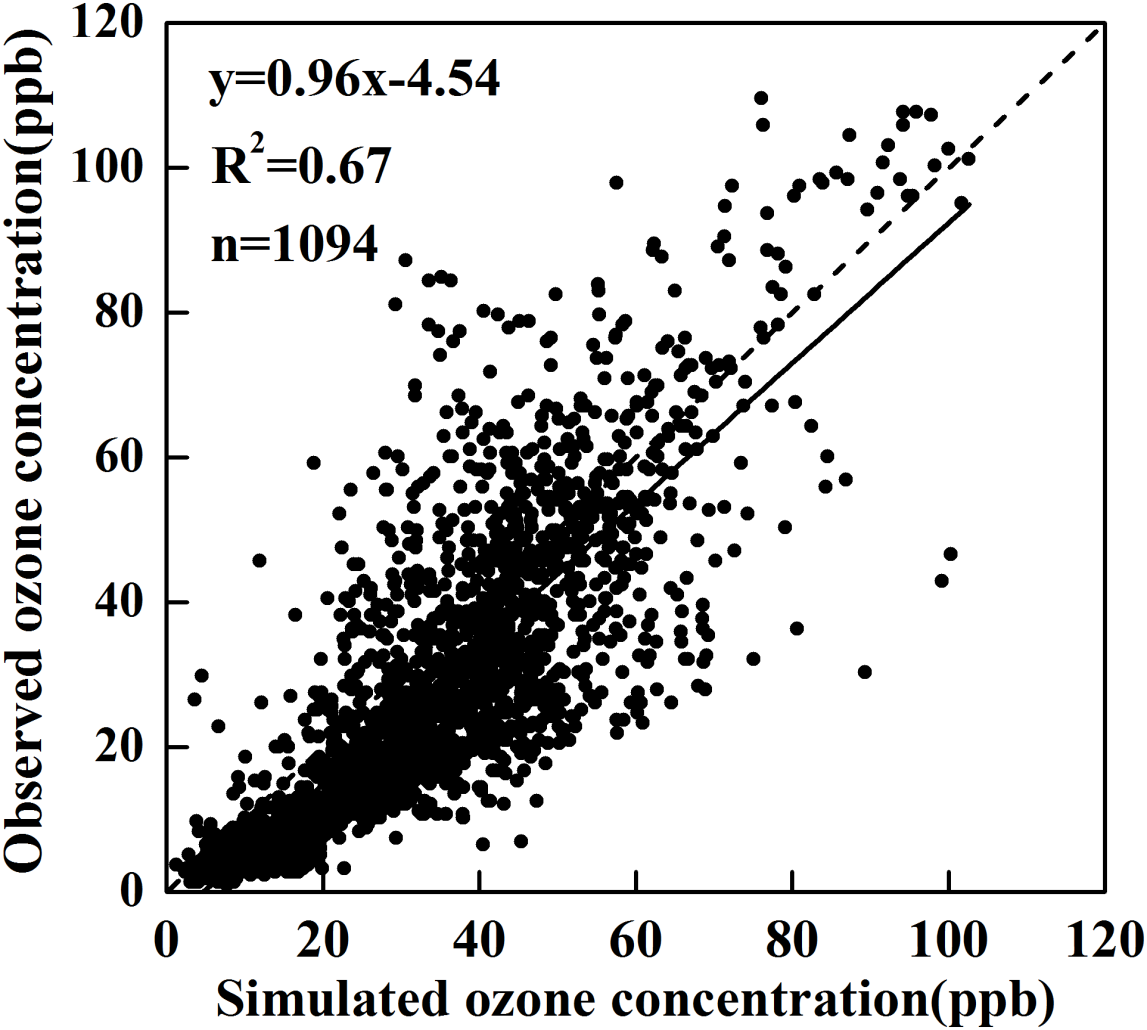
Simulated ozone concentrations plotted against observed ozone concentrations in Nanjing station from March to May of 2006.

The simulated regional ozone concentrations from Nanjing station using the WRF-CMAQ model were compared with observed hourly mean ozone concentrations in March, April, and May of 2006 (Table 7 and Fig 12). The simulated ozone concentrations have a mean of 33.25 ppb with a range of 24.94–38.31 ppb, while the observations have a mean of 27.7 ppb with a range of 19.28–36.31 ppb (Table 7). The daily variations in simulated and observed ozone concentrations are consistent as a function of solar irradiation: increasing with an increased solar irradiation in the morning, reaching the maximum value around 15:00, and decreasing with a decreased solar irradiation in the afternoon (Fig 12). However, the simulated data were higher in general, with a smaller difference around noon in March and April. In contrast, the simulated ozone concentrations were lower than the observations around noon in May. Overall, the model simulated diurnal ozone concentrations variations well (Table 7). The correlations between simulations and observations were all higher than 0.67, and significant (p<0.05) (Fig 13). The ranges of NMB, NNE, and RMSE were 6.08–33.85, 31.16–43.9, and 9.96–14.58, respectively, which were similar to the ranges of published studies [50, 58–62]. Therefore, the model demonstrated that the model used in this study is able to provide acceptable ozone concentration data in the absence of ground truth.

**Table 7:** The statistics of simulations and observations in Nanjing station from March to May of 2006.

	Total (from March to May)	March	April	May
**Average simulated concentrations (ppb)**	33.25	24.94	34.32	38.31
**Average observed concentrations(ppb)**	27.7	19.28	26.38	36.11
**Correlation Coefficient *r***	0.67(Fig 13)	0.72	0.69	0.68
**NMB (%)**	20.05	29.35	33.85	6.08
**NME (%)**	37.97	43.83	43.9	31.16
**RMSE(ppb)**	13.44	9.96	14.58	14.54

#### Simulation of regional cumulative ozone uptake

**Fig 14:**
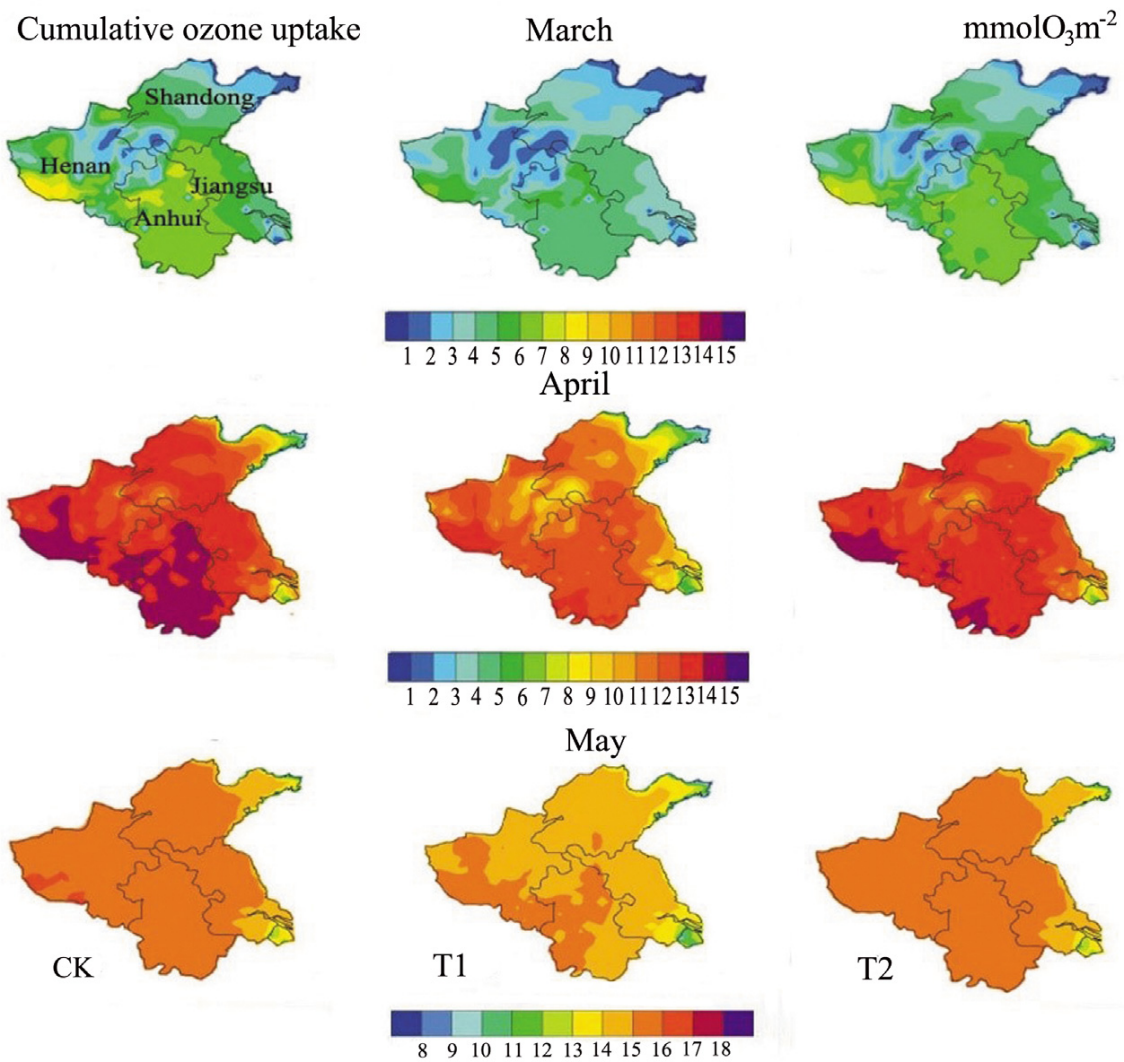
The temporal and spatial variations of simulated cumulative ozone uptake under elevated ozone concentrations and reduced solar radiation conditions in the major areas for production of winter wheat (from March to May of 2006). This visualization is created by the NCAR Command Language (Version 6.3.0) [Software], 2015 under a CC BY license [63], with permission from University Corporation for Atmospheric Research, original copyright 2015.

Spatial uptake of cumulative ozone was then simulated by the stomatal-flux model. The uptake in cumulative ozone in winter wheat-growing provinces under conditions of decreased solar irradiance (i.e., CK, T1, and T2 conditions) is shown in Fig 14. In March, the ozone uptake values were at their lowest, ranging from 1–7 mmol O_3_·m^-2^. In April, the ozone uptake values increased substantially, particularly in Southwest Henan Province and in most areas of Anhui Province under CK conditions, with a maximum of 15 mmol O_3_·m^-2^. In May, ozone uptake reached a maximum of 16 mmol O_3_·m^-2^ in most of the winter wheat-growing provinces under CK and T2 conditions, although the values under T1 were 15 mmol O_3_·m^-2^.

#### Estimation of regional dry matter loss under elevated ozone concentrations with different shading conditions

**Fig 15:**
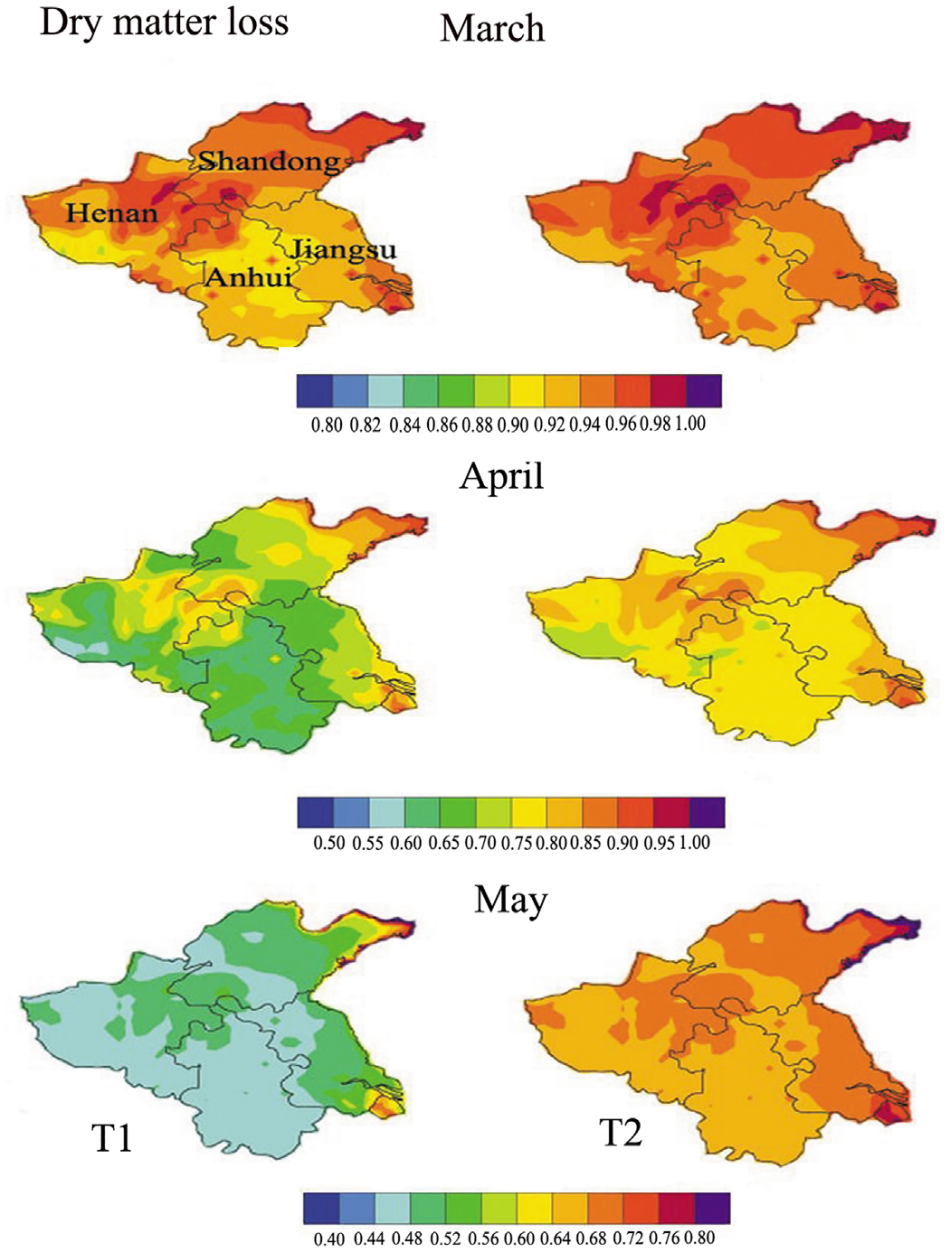
Regional loss of dry matter for winter wheat, simulated under elevated ozone concentrations and reduced solar radiation (from March to May of 2006). This visualization is created by the NCAR Command Language (Version 6.3.0) [Software], 2015 under a CC BY license [63], with permission from University Corporation for Atmospheric Research, original copyright 2015.

Regional dry matter loss (Fig 15) was estimated by the regression models (Fig 11), using regional cumulative ozone uptake as the independent variable (Fig 14). Dry matter loss under T1 was greater than under T2 from March to May. In March, the loss under T1 was between 0.92 and 0.96, and under T2 was between 0.94 and 0.96. In April, the dry matter loss in T1 and T2 decreased to 0.7 and 0.8, respectively. In May, the dry matter loss in T1 and T2 continued to decrease to a minimum of 0.5 and 0.7, respectively. In total, dry matter loss in winter wheat-growing provinces was 50% under high-level shading conditions (T1), and 30% under low-level shading conditions (T2). Our results suggested that the impact of shading and ozone pollution on winter wheat could be substantial. What we have to keep in mind is that our results are limited by an experiment that lasted only one season; the stomatal models and dry matter loss model would have benefited from data collected over multiple years. Furthermore, some other studies will be needed to do in the next step, such as the factors affecting simulated ozone concentrations, a sensitivity analysis of the WRF-CMAQ model and validation using more ground observations.

## Conclusions

A decrease in solar irradiance resulted in a decrease in average temperature of 5.6°C and 4.1°C, a decrease in vapor pressure deficit of 0.84 kPa and 0.74 kPa, and an increase in relative humidity of 16.0% and 14.5% under a high (T1) and low (T2) level of shading, respectively, in comparison with those in CK, in the field environment. The parameterization of the conductance model was obtained through boundary line technology, and a typical light response curve was formed with light saturation at approximately 1000 μmolm^-2^·s^-1^ photosynthetically active radiation (PAR). Optimum leaf temperature for stomatal conductance was 29.6°C. The stomatal conductance declined greatly when the vapor pressure deficit was greater than 2.1 kPa. PAR was the most limiting factor on stomatal conductance over the entire experimental period, and this negative effect became stronger with an increase in shading. The limiting effect of Temp was only strong at the early stage of growth, but the limiting effect of VPD became stronger over time. The limiting effects of PAR in combination with Temp were stronger than VPD. The combined effect of shading on stomatal conductance and dry matter could thus be negative; however, reduced solar irradiance was found to be responsible for the decrease in stomatal ozone uptake, which suggested that shading levels set in this experiment could alleviate ozone damage to the plant.

Over 85% (T1: R^2^ = 0.85 & T2: R^2^ = 0.89) of the dry matter loss can be explained by cumulative ozone uptake. Dry matter losses were 3.8% (T1) and 2.2% (T2) compared to CK for each 1 mmol O_3_·m^-2^ increase in ozone cumulative uptake. At the regional level, dry matter loss in winter wheat could be 50% under high-level shading (T1), and 30% under low-level shading (T2).

## Supporting Information

S1 Table The diurnal variation of mean (± SD) PAR in each treatment (n = 68). (PDF)

S2 Table Boundary-line analysis of the relationships between relative stomatal conductance and environmental variables under elevated ozone concentrations with varying shading conditions. (PDF)

S3 Table Simulated stomatal conductance plotted against measured stomatal conductance in all treatments. (PDF)

S4 Table Cumulative ozone uptake in each treatment during the whole experiments period. (PDF)

S5 Table The daily variation of mean *f*_*PAR*_, *f*_*T*_, and *f*_*VPD*_ in each treatment. (PDF)

S6 Table The hourly variation of mean *f*_*PAR*_, *f*_*T*_, and *f*_*VPD*_ in each treatment. (PDF)

S7 Table The daily variation of mean *f*_*phen*_ and *f*_*O3*_ in each treatment. (PDF)

S8 Table Temporal relationships between dry matter loss of winter wheat in T1 and AF_sto_6 and in T2 and AF_sto_6. (PDF)

S9 Table The hourly simulated and observed mean ozone concentrations in Nanjing station from March to May of 2006. (PDF)

S10 Table Simulated ozone concentrations plotted against observed ozone concentrations in Nanjing station from March to May of 2006. (PDF)

S1 File The temporal and spatial variations of simulated cumulative ozone uptake under elevated ozone concentrations and reduced solar radiation conditions in the major areas for production of winter wheat (from March to May of 2006). (PDF)

S2 File Regional loss of dry matter for winter wheat, simulated under elevated ozone concentrations and reduced solar radiation (from March to May of 2006). (PDF)
