# Supplementary material for: The Effect of Elevated Ozone Concentrations with Varying Shading on Dry Matter Loss in a Winter Wheat-Producing Region in China
Source: PLoS One. 2016 Jan 13;11(1):e0145446. doi: 10.1371/journal.pone.0145446 (PMC4711948; doi:10.1371/journal.pone.0145446)
Supplement: S1 Table — (PDF) [file pone.0145446.s001.pdf]

S1 Table.The diurnal variation of mean (±SD) PAR in each treatment (n = 68) unit: μmol m^{-2} s^{-1}

Time	CK	error	T1	error	T2	error
**1**	2.37	5.55	0.95	2.22	1.89	4.44
**2**	2.41	4.94	0.96	1.98	1.93	3.95
**3**	2.48	4.59	0.99	1.84	1.98	3.67
**4**	2.59	4.36	1.04	1.74	2.07	3.49
**5**	13.54	8.02	5.41	3.21	10.83	6.42
**6**	103.67	38.05	41.47	15.22	82.94	30.44
**7**	286.65	93.41	114.66	37.36	229.32	74.73
**8**	510.46	144.70	204.19	57.88	408.37	115.76
**9**	770.41	200.92	308.16	80.37	616.32	160.74
**10**	948.06	247.85	379.22	99.14	758.45	198.28
**11**	1055.90	264.73	422.36	105.89	844.72	211.78
**12**	1065.66	256.47	426.26	102.59	852.52	205.18
**13**	1005.75	240.30	402.30	96.12	804.60	192.24
**14**	825.48	198.52	330.19	79.41	660.39	158.82
**15**	601.01	152.95	240.40	61.18	480.81	122.36
**16**	344.03	98.95	137.61	39.58	275.22	79.16
**17**	124.83	40.95	49.93	16.38	99.86	32.76
**18**	12.24	6.26	4.90	2.50	9.79	5.01
**19**	0.59	1.57	0.24	0.63	0.47	1.26
**20**	0.89	2.49	0.35	1.00	0.71	1.99
**21**	1.48	4.16	0.59	1.66	1.18	3.33
**22**	1.96	6.33	0.78	2.53	1.57	5.06
**23**	2.43	8.54	0.97	3.41	1.95	6.83
**24**	2.62	7.29	1.05	5.83	2.09	5.83
