# Supplementary material for: The Effect of Elevated Ozone Concentrations with Varying Shading on Dry Matter Loss in a Winter Wheat-Producing Region in China
Source: PLoS One. 2016 Jan 13;11(1):e0145446. doi: 10.1371/journal.pone.0145446 (PMC4711948; doi:10.1371/journal.pone.0145446)
Supplement: S2 Table — (PDF) [file pone.0145446.s002.pdf]

S2 Table. Boundary-line analysis of the relationships between relative stomatal conductance and environmental variables under elevated ozone concentrations with varying shading conditions.

**PAR (μmol m^{-2} s^{-1})**	**G_{ST}/G_{max}**	**T (℃)**	**G_{ST}/G_{max}**	**VPD (kPa)**	**G_{ST}/G_{max}**	**Phen (°C•d)**	**G_{ST}/G_{max}**
254.21	0.21	30.30	1.00	0.18	0.09	303.56	0.09
255.89	0.23	30.40	0.98	0.18	0.14	303.56	0.14
299.66	0.20	30.10	0.95	0.18	0.14	303.56	0.14
304.71	0.27	32.60	0.93	0.18	0.14	303.56	0.14
308.08	0.11	30.50	0.91	0.18	0.10	303.56	0.10
316.50	0.16	32.30	0.91	0.18	0.24	303.56	0.24
319.87	0.13	29.20	0.86	0.18	0.25	303.56	0.25
328.28	0.05	32.20	0.85	0.18	0.24	303.56	0.24
345.12	0.27	28.50	0.84	0.18	0.27	303.56	0.27
351.85	0.23	29.60	0.84	0.18	0.20	303.56	0.20
355.22	0.29	29.80	0.83	0.18	0.08	303.56	0.08
373.74	0.58	32.70	0.82	0.18	0.17	303.56	0.17
387.21	0.46	28.80	0.82	0.30	0.22	303.56	0.22
390.57	0.49	31.40	0.82	0.31	0.20	303.56	0.20
390.57	0.37	33.00	0.81	0.32	0.20	303.56	0.20
398.99	0.68	29.60	0.81	0.32	0.15	303.56	0.15
410.77	0.76	29.90	0.81	0.32	0.28	303.56	0.28
410.77	0.39	30.20	0.80	0.32	0.25	303.56	0.25
419.19	0.21	32.30	0.80	0.32	0.31	303.56	0.31
424.24	0.25	30.50	0.80	0.32	0.30	303.56	0.30
434.34	0.28	32.80	0.78	0.32	0.35	303.56	0.35
437.71	0.28	26.80	0.77	0.31	0.26	303.56	0.26
442.76	0.77	32.70	0.77	0.31	0.26	303.56	0.26
444.44	0.80	30.30	0.76	0.31	0.20	303.56	0.20
447.81	0.64	31.90	0.76	0.31	0.20	303.56	0.20
449.49	0.18	32.40	0.75	0.31	0.15	303.56	0.15
456.23	0.67	32.50	0.75	0.32	0.17	303.56	0.17
456.23	0.54	28.90	0.75	0.32	0.15	303.56	0.15
462.96	0.74	31.00	0.74	0.33	0.21	303.56	0.21
473.06	0.15	26.90	0.74	0.33	0.20	303.56	0.20
473.06	0.65	32.50	0.73	0.38	0.58	303.56	0.58
483.16	0.19	29.50	0.73	0.37	0.51	303.56	0.51
498.32	0.63	27.70	0.72	0.35	0.53	303.56	0.53
498.32	0.46	32.50	0.72	0.35	0.44	303.56	0.44

500.00	0.19	30.60	0.72	0.35	0.37	303.56	0.37
510.10	0.51	29.60	0.71	0.34	0.38	303.56	0.38
531.99	0.54	27.70	0.71	0.34	0.30	303.56	0.30
538.72	0.45	28.50	0.70	0.34	0.38	303.56	0.38
540.40	0.40	33.00	0.70	0.34	0.28	303.56	0.28
542.09	0.35	32.00	0.69	0.34	0.24	303.56	0.24
547.14	0.54	29.60	0.69	0.35	0.22	303.56	0.22
558.92	0.51	33.10	0.69	0.35	0.21	303.56	0.21
567.34	0.45	28.40	0.68	0.35	0.31	303.56	0.31
580.81	0.34	30.40	0.68	0.42	0.29	303.56	0.29
584.17	0.25	30.30	0.68	0.42	0.32	303.56	0.32
587.54	0.49	29.80	0.68	0.42	0.27	303.56	0.27
621.21	0.35	32.90	0.67	0.42	0.35	303.56	0.35
627.95	0.30	26.60	0.67	0.42	0.36	303.56	0.37
634.68	0.76	28.40	0.67	0.42	0.37	303.56	0.37
641.41	0.67	29.70	0.66	0.42	0.29	303.56	0.29
693.60	0.69	28.20	0.66	0.42	0.46	303.56	0.46
710.44	0.36	33.40	0.66	0.42	0.41	303.56	0.41
713.80	0.26	32.20	0.66	0.42	0.31	303.56	0.31
720.54	0.34	26.90	0.65	0.42	0.33	303.56	0.33
720.54	0.84	28.70	0.65	0.42	0.19	303.56	0.19
725.59	0.40	32.90	0.65	0.42	0.18	303.56	0.18
742.42	0.30	30.40	0.65	0.43	0.18	303.56	0.18
747.47	0.61	33.00	0.64	0.44	0.19	303.56	0.19
747.47	0.13	34.60	0.64	0.44	0.29	303.56	0.29
749.16	0.53	29.00	0.64	0.44	0.05	303.56	0.05
759.26	0.35	30.20	0.64	0.44	0.29	303.56	0.29
767.68	0.22	30.10	0.64	0.44	0.40	303.56	0.40
769.36	0.43	28.80	0.64	0.45	0.39	303.56	0.39
772.73	0.25	30.40	0.63	0.44	0.52	303.56	0.52
776.09	0.33	29.80	0.63	0.44	0.35	303.56	0.35
777.78	0.30	31.60	0.63	0.44	0.47	303.56	0.47
781.14	0.23	24.40	0.62	0.70	0.38	303.56	0.38
781.14	0.25	32.10	0.62	0.71	0.33	303.56	0.33
787.88	0.58	28.00	0.62	0.71	0.34	303.56	0.34
796.30	0.29	32.50	0.62	0.70	0.30	303.56	0.30
804.71	0.52	29.80	0.62	0.70	0.32	303.56	0.32
806.40	0.84	32.60	0.62	0.71	0.26	303.56	0.26
808.08	0.37	32.60	0.62	0.72	0.22	303.56	0.22
808.08	0.59	32.50	0.62	0.73	0.38	303.56	0.38
809.76	0.40	28.40	0.61	0.73	0.34	303.56	0.34
816.50	0.39	28.90	0.61	0.74	0.08	303.56	0.08
818.18	0.23	30.20	0.61	0.74	0.06	303.56	0.06

818.18	0.30	31.50	0.61	0.75	0.20	303.56	0.20
823.23	0.57	33.00	0.61	0.75	0.48	303.56	0.48
823.23	0.43	30.20	0.61	0.63	0.25	303.56	0.25
824.92	0.54	29.60	0.60	0.64	0.33	303.56	0.33
828.28	0.65	30.20	0.60	0.64	0.35	303.56	0.35
831.65	0.51	33.60	0.59	0.65	0.23	303.56	0.23
835.02	0.43	29.60	0.59	0.65	0.30	303.56	0.30
836.70	0.56	29.90	0.59	0.65	0.37	303.56	0.37
843.43	0.55	32.60	0.59	0.65	0.30	303.56	0.30
848.48	0.50	28.50	0.59	0.66	0.23	303.56	0.23
853.53	0.68	33.20	0.59	0.66	0.25	303.56	0.25
860.27	0.54	30.00	0.59	0.67	0.22	303.56	0.22
867.00	0.83	30.20	0.58	0.67	0.31	303.56	0.31
872.05	0.22	29.50	0.58	0.67	0.36	303.56	0.36
872.05	0.33	32.30	0.58	0.67	0.26	303.56	0.26
872.05	0.62	29.60	0.58	0.47	0.19	303.56	0.19
872.05	0.63	32.60	0.58	0.47	0.19	303.56	0.19
875.42	0.14	33.00	0.58	0.47	0.15	303.56	0.15
875.42	0.10	29.00	0.58	0.47	0.18	303.56	0.18
875.42	0.49	33.00	0.57	0.48	0.25	303.56	0.25
875.42	0.60	28.80	0.57	0.48	0.21	303.56	0.21
877.10	0.14	33.80	0.44	1.36	0.75	303.56	0.13
878.79	0.41	30.80	0.57	0.48	0.13	303.56	0.16
880.47	0.14	28.70	0.56	0.48	0.16	303.56	0.23
888.89	0.22	27.10	0.56	0.48	0.23	303.56	0.29
890.57	0.52	30.90	0.56	0.48	0.29	303.56	0.27
890.57	0.44	29.20	0.56	0.47	0.27	303.56	0.27
892.26	0.81	29.40	0.56	0.47	0.27	303.56	0.11
893.94	0.56	35.80	0.55	0.46	0.11	303.56	0.05
893.94	0.68	32.60	0.55	0.46	0.05	303.56	0.20
895.62	0.09	32.00	0.55	0.46	0.20	303.56	0.23
897.31	0.53	28.60	0.55	0.46	0.23	303.56	0.21
900.67	0.59	31.50	0.54	0.46	0.21	526.35	0.53
902.36	0.25	29.70	0.54	0.01	0.22	526.35	0.56
902.36	0.49	26.70	0.54	0.01	0.22	526.35	0.59
905.72	0.41	32.90	0.54	0.01	0.33	526.35	0.44
905.72	0.25	29.20	0.54	0.01	0.25	526.35	0.68
905.72	0.26	32.70	0.54	0.01	0.49	526.35	0.53
907.41	0.48	33.60	0.54	0.01	0.41	526.35	0.52
910.77	0.52	30.90	0.54	0.01	0.25	526.35	0.45
910.77	0.66	29.50	0.54	0.01	0.27	526.35	0.37
912.46	0.26	31.20	0.54	0.01	0.26	526.35	0.41
912.46	0.37	24.50	0.54	0.01	0.37	526.35	0.69

920.87	0.34	30.50	0.53	0.01	0.34	526.35	0.41
922.56	0.06	29.30	0.53	0.09	0.39	526.35	0.62
929.29	0.20	30.70	0.53	0.09	0.39	526.35	0.61
929.29	0.51	30.60	0.53	0.10	0.54	526.35	0.43
936.03	0.08	27.20	0.53	0.10	0.32	526.35	0.76
949.49	0.59	29.00	0.53	0.10	0.44	526.35	0.59
949.49	0.54	29.20	0.53	0.10	0.48	526.35	0.33
956.23	0.24	30.70	0.53	0.10	0.41	526.35	0.64
956.23	0.38	28.80	0.52	0.10	0.35	526.35	0.67
956.23	0.30	31.10	0.52	0.10	0.41	526.35	0.78
957.91	0.42	28.40	0.52	0.10	0.45	526.35	0.27
959.60	0.33	29.60	0.52	0.10	0.43	526.35	0.33
964.65	0.24	29.90	0.52	0.10	0.43	526.35	0.70
964.65	0.38	29.50	0.52	0.10	0.38	526.35	0.69
966.33	0.26	28.70	0.52	0.10	0.31	526.35	0.63
969.70	0.34	28.20	0.52	0.10	0.30	526.35	0.62
971.38	0.32	32.00	0.52	0.10	0.41	526.35	0.45
973.06	0.26	32.80	0.52	0.10	0.39	526.35	0.35
974.75	0.34	30.60	0.52	0.10	0.22	526.35	0.52
978.11	0.38	30.90	0.51	0.10	0.32	526.35	0.58
979.80	0.56	29.80	0.51	0.52	0.49	526.35	0.80
984.85	0.24	29.50	0.51	0.43	0.62	526.35	0.82
986.53	0.22	28.40	0.51	0.52	0.53	526.35	0.61
993.27	0.25	26.90	0.51	0.44	0.48	526.35	0.59
993.27	0.32	32.50	0.51	0.51	0.48	526.35	0.66
993.27	0.65	28.70	0.51	0.45	0.50	526.35	0.60
1000.00	0.34	28.50	0.51	0.51	0.54	526.35	0.76
1001.68	0.20	31.40	0.51	0.46	0.44	526.35	0.86
1003.37	0.28	32.40	0.50	0.52	0.47	526.35	0.38
1005.05	0.19	28.50	0.50	0.47	0.51	526.35	0.34
1005.05	0.26	30.00	0.50	0.52	0.40	526.35	0.54
1006.73	0.23	28.60	0.50	0.48	0.53	526.35	0.82
1006.73	0.25	31.80	0.50	0.51	0.35	526.35	0.59
1008.42	0.28	25.70	0.49	0.51	0.41	526.35	0.47
1010.10	0.33	29.60	0.49	0.50	0.24	526.35	0.68
1016.83	0.85	29.70	0.49	0.84	0.56	526.35	0.76
1025.25	0.22	28.50	0.49	0.86	0.71	526.35	0.39
1031.99	0.81	29.30	0.49	0.87	0.66	526.35	0.28
1037.04	0.19	30.60	0.49	0.89	0.65	526.35	0.64
1038.72	0.51	30.20	0.49	0.90	0.75	526.35	0.63
1038.72	0.45	22.90	0.49	0.91	0.43	526.35	0.46
1040.40	0.37	33.00	0.48	0.92	0.71	526.35	0.80
1040.40	0.69	30.90	0.48	1.03	0.47	526.35	0.49

1042.09	0.41	29.20	0.48	1.04	0.30	526.35	0.37
1042.09	0.54	26.10	0.48	1.05	0.64	703.16	0.24
1045.45	0.41	25.00	0.48	1.08	0.73	703.16	0.25
1048.82	0.52	28.20	0.47	1.08	0.31	703.16	0.28
1048.82	0.61	29.40	0.47	1.09	0.59	703.16	0.27
1050.50	0.62	32.10	0.47	1.09	0.59	703.16	0.29
1052.19	0.34	31.50	0.47	1.09	0.38	703.16	0.45
1055.55	0.29	28.90	0.47	1.09	0.39	703.16	0.45
1058.92	0.61	30.20	0.46	1.01	0.72	703.16	0.49
1060.61	0.57	33.20	0.46	1.02	0.62	703.16	0.47
1062.29	0.28	31.50	0.46	1.04	0.61	703.16	0.48
1062.29	0.39	31.60	0.46	1.05	0.82	703.16	0.42
1069.02	0.08	30.40	0.46	1.06	0.26	703.16	0.34
1070.71	0.41	30.00	0.46	1.08	0.43	703.16	0.33
1075.76	0.25	30.40	0.46	1.16	0.49	703.16	0.45
1075.76	0.44	34.50	0.46	1.17	0.41	703.16	0.42
1079.12	0.64	30.80	0.45	1.18	0.72	703.16	0.24
1080.81	0.20	31.20	0.45	1.18	0.53	703.16	0.35
1080.81	0.35	32.30	0.45	1.18	0.45	703.16	0.54
1085.86	0.17	30.30	0.45	1.19	0.48	703.16	0.58
1095.96	0.44	30.80	0.45	1.19	0.45	703.16	0.53
1097.64	0.18	31.00	0.45	1.20	0.34	703.16	0.60
1101.01	0.15	28.50	0.45	1.20	0.45	703.16	0.52
1101.01	0.31	31.90	0.45	1.10	0.70	703.16	0.44
1101.01	0.32	31.30	0.45	1.10	0.52	703.16	0.39
1102.69	0.22	25.70	0.45	1.11	0.47	703.16	0.45
1102.69	0.44	30.10	0.45	1.11	0.53	703.16	0.27
1106.06	0.47	35.50	0.45	1.12	0.86	703.16	0.52
1107.74	0.61	30.00	0.44	1.13	0.56	703.16	0.33
1109.43	0.34	26.30	0.44	1.28	0.42	703.16	0.70
1111.11	0.20	25.60	0.44	1.30	0.40	703.16	0.80
1111.11	0.15	30.10	0.44	1.31	0.46	703.16	0.34
1111.11	0.51	30.10	0.44	1.33	0.45	703.16	0.65
1112.79	0.20	34.30	0.44	1.34	0.47	703.16	0.64
1114.48	0.26	34.80	0.44	1.35	0.91	703.16	0.41
1119.53	0.73	33.10	0.43	1.36	0.62	703.16	0.42
1121.21	0.44	29.20	0.43	1.36	0.72	703.16	0.54
1122.89	0.20	29.10	0.43	1.23	0.58	703.16	0.45
1122.89	0.45	30.20	0.43	1.23	0.52	703.16	0.79
1124.58	0.26	29.70	0.43	1.24	0.54	703.16	0.58
1127.95	0.30	32.10	0.43	1.25	0.81	703.16	0.50
1127.95	0.21	25.40	0.43	1.25	0.44	703.16	0.53
1129.63	0.20	28.50	0.43	1.26	0.30	703.16	0.50

1129.63	0.53	35.30	0.43	1.22	0.41	703.16	0.38
1136.36	0.40	29.50	0.43	1.22	0.49	703.16	0.50
1138.05	0.64	32.80	0.43	1.22	0.60	703.16	0.47
1139.73	0.29	28.00	0.43	1.21	0.54	703.16	0.44
1141.41	0.30	35.20	0.43	1.22	0.81	703.16	0.51
1141.41	0.18	25.50	0.43	1.22	0.52	703.16	0.49
1143.10	0.58	30.80	0.42	1.22	0.67	703.16	0.52
1146.46	0.15	32.30	0.42	1.22	0.83	703.16	1.00
1146.46	0.21	30.50	0.42	1.22	0.62	703.16	0.82
1146.46	0.38	30.00	0.41	1.22	0.63	703.16	0.68
1148.15	0.17	23.40	0.41	1.17	0.84	703.16	0.80
1151.51	0.31	32.30	0.41	1.18	0.40	703.16	0.45
1153.20	0.82	25.60	0.41	1.19	0.61	703.16	0.54
1154.88	0.35	30.50	0.41	1.19	0.13	703.16	0.66
1154.88	0.44	25.90	0.41	1.20	0.40	703.16	0.59
1158.25	0.37	31.60	0.41	1.21	0.34	703.16	0.89
1158.25	0.05	29.80	0.41	1.22	0.84	703.16	0.58
1158.25	0.39	25.80	0.41	1.22	0.69	703.16	0.73
1159.93	0.51	28.80	0.41	1.17	0.35	703.16	0.91
1161.62	0.19	28.70	0.40	1.17	0.35	703.16	0.68
1161.62	0.44	32.00	0.40	1.02	0.51	703.16	0.70
1161.62	0.30	29.50	0.40	1.02	0.67	703.16	0.39
1161.62	0.40	31.40	0.40	1.02	0.25	703.16	0.38
1161.62	0.74	31.10	0.40	1.02	0.49	703.16	0.56
1166.67	0.58	30.30	0.40	1.02	0.51	703.16	0.74
1166.67	0.52	32.40	0.40	1.02	0.34	703.16	0.27
1168.35	0.31	29.20	0.40	1.02	0.45	703.16	0.54
1168.35	0.31	24.00	0.39	0.96	0.67	703.16	0.56
1170.03	0.39	23.50	0.39	0.96	0.54	703.16	0.37
1170.03	0.39	32.00	0.39	0.97	0.77	703.16	0.50
1171.72	0.32	28.60	0.39	0.97	0.74	890.96	0.91
1171.72	0.34	30.30	0.39	0.97	0.65	890.96	0.86
1173.40	0.29	32.10	0.39	0.97	0.28	890.96	0.85
1175.08	0.29	25.70	0.38	1.32	0.53	890.96	0.81
1175.08	0.61	29.90	0.38	1.34	0.56	890.96	0.71
1175.08	0.50	32.50	0.38	1.36	0.59	890.96	0.71
1176.77	0.54	25.60	0.38	1.38	0.44	890.96	0.67
1176.77	0.81	29.40	0.38	1.39	0.68	890.96	0.67
1180.13	0.32	29.10	0.38	1.40	0.53	890.96	0.65
1180.13	0.31	32.10	0.38	1.36	0.51	890.96	0.64
1181.82	0.54	31.10	0.38	1.39	0.45	890.96	0.63
1181.82	0.31	29.90	0.38	1.40	0.37	890.96	0.63
1185.18	0.43	29.70	0.37	1.44	0.41	890.96	0.62

1185.18	0.29	33.20	0.37	1.46	0.69	890.96	0.61
1186.87	0.38	30.70	0.37	1.64	0.41	890.96	0.61
1186.87	0.75	30.70	0.37	1.66	0.62	890.96	0.59
1186.87	0.52	24.10	0.37	1.60	0.61	890.96	0.58
1188.55	0.44	30.60	0.37	1.63	0.43	890.96	0.58
1188.55	0.48	32.80	0.37	1.65	0.75	890.96	0.58
1188.55	0.41	30.20	0.37	1.66	0.59	890.96	0.55
1188.55	0.78	30.20	0.36	1.67	0.33	890.96	0.53
1188.55	0.27	29.00	0.36	1.68	0.64	890.96	0.52
1190.23	0.35	31.70	0.36	1.68	0.67	890.96	0.51
1190.23	0.59	32.30	0.36	1.67	0.78	890.96	0.50
1190.23	0.33	30.40	0.35	1.66	0.27	890.96	0.47
1190.23	0.64	28.50	0.35	1.66	0.33	890.96	0.47
1190.23	0.67	31.90	0.35	1.66	0.70	890.96	0.47
1191.92	0.52	26.80	0.35	1.67	0.69	890.96	0.46
1193.60	0.27	29.10	0.35	1.53	0.63	890.96	0.43
1193.60	0.33	29.10	0.35	1.55	0.62	890.96	0.43
1193.60	0.70	32.60	0.35	1.56	0.45	890.96	0.40
1193.60	0.66	30.20	0.35	1.58	0.35	890.96	0.39
1193.60	0.59	32.60	0.35	1.59	0.52	890.96	0.37
1195.29	0.69	28.50	0.35	1.60	0.58	890.96	0.36
1198.65	0.28	33.80	0.34	1.91	0.80	890.96	0.35
1203.70	0.36	24.50	0.34	1.93	0.82	890.96	0.30
1205.39	0.61	30.50	0.34	1.95	0.61	890.96	0.29
1208.75	0.29	29.60	0.34	1.96	0.59	890.96	0.24
1210.44	0.35	31.10	0.34	1.98	0.66	890.96	0.14
1212.12	0.45	33.60	0.34	1.99	0.59	1208.65	0.54
1217.17	0.24	29.40	0.34	2.06	0.76	1208.65	0.57
1217.17	0.32	26.70	0.34	2.08	0.85	1208.65	0.38
1218.85	0.37	31.20	0.34	2.10	0.38	1208.65	0.48
1218.85	0.31	32.30	0.34	2.12	0.34	1208.65	0.56
1220.54	0.10	32.70	0.34	2.12	0.54	1208.65	0.43
1222.22	0.57	28.50	0.34	2.14	0.82	1208.65	0.65
1223.90	0.62	29.70	0.33	1.87	0.58	1208.65	0.58
1228.96	0.58	30.10	0.33	1.87	0.47	1208.65	0.55
1230.64	0.43	32.80	0.33	1.87	0.68	1208.65	0.60
1232.32	0.46	22.60	0.33	1.87	0.76	1208.65	0.72
1232.32	0.30	32.70	0.33	1.87	0.39	1208.65	0.55
1234.01	0.75	30.30	0.33	1.88	0.28	1208.65	0.44
1239.06	0.41	31.20	0.33	1.86	0.64	1208.65	0.68
1239.06	0.45	25.10	0.32	1.81	0.63	1208.65	0.60
1240.74	0.33	25.70	0.32	1.81	0.46	1208.65	0.64
1240.74	0.72	30.40	0.32	1.81	0.80	1208.65	0.68

1240.74	0.52	35.30	0.32	1.82	0.49	1208.65	0.56
1242.42	0.62	34.10	0.32	1.82	0.37	1208.65	0.66
1242.42	0.45	25.00	0.32	0.53	0.32	1208.65	0.48
1245.79	0.35	31.10	0.32	0.55	0.23	1208.65	0.67
1245.79	0.91	26.80	0.31	0.55	0.25	1208.65	0.67
1249.16	0.41	29.20	0.31	0.57	0.28		
1250.84	0.38	30.20	0.31	0.71	0.29		
1254.21	0.43	27.70	0.31	0.72	0.32		
1254.21	0.52	30.10	0.31	0.74	0.31		
1255.89	0.63	28.50	0.31	0.75	0.29		
1257.57	0.71	25.70	0.31	0.76	0.31		
1259.26	0.38	29.70	0.31	0.94	0.43		
1260.94	0.22	31.60	0.30	0.96	0.43		
1260.94	0.46	30.80	0.30	0.97	0.36		
1262.63	0.47	30.20	0.30	0.99	0.43		
1262.63	0.95	29.40	0.30	1.01	0.50		
1264.31	0.40	28.50	0.30	1.13	0.46		
1264.31	0.45	34.20	0.30	1.14	0.53		
1269.36	0.42	30.60	0.30	1.14	0.29		
1281.14	0.80	29.40	0.30	1.14	0.52		
1281.14	1.00	31.10	0.30	1.14	0.10		
1284.51	0.98	29.20	0.30	1.14	0.57		
1289.56	0.72	25.60	0.30	1.24	0.45		
1289.56	0.62	28.00	0.29	1.25	0.40		
1291.24	0.47	30.10	0.29	1.26	0.34		
1294.61	0.91	28.90	0.29	1.27	0.45		
1297.98	0.40	26.10	0.29	1.28	0.74		
1303.03	0.50	30.20	0.29	1.28	0.53		
1308.08	0.66	31.70	0.29	1.25	0.65		
1311.45	0.55	32.00	0.29	1.26	0.42		
1316.50	0.45	34.00	0.29	1.27	0.54		
1318.18	0.80	30.60	0.29	1.27	0.52		
1318.18	0.82	30.20	0.29	1.27	0.56		
1319.86	0.55	29.60	0.28	1.21	0.29		
1326.60	0.42	30.40	0.28	1.21	0.58		
1335.02	0.86	28.80	0.28	1.21	0.43		
1336.70	0.70	29.90	0.28	1.22	0.53		
1338.38	0.35	27.00	0.28	1.23	0.30		
1341.75	0.45	27.00	0.28	0.76	0.26		
1343.43	0.26	30.40	0.27	0.77	0.24		
1345.12	0.72	32.60	0.27	0.77	0.33		
1345.12	0.47	29.90	0.27	0.77	0.28		
1346.80	0.53	29.70	0.27	0.77	0.20		

1346.80	0.43	26.20	0.26	1.17	0.42		
1351.85	0.56	23.70	0.26	1.18	0.35		
1355.22	0.52	29.20	0.26	1.20	0.25		
1356.90	0.25	24.30	0.26	1.21	0.26		
1356.90	0.43	31.70	0.26	1.22	0.54		
1358.58	0.36	33.40	0.26	1.34	0.39		
1360.27	0.43	28.90	0.26	1.35	0.36		
1361.95	0.46	29.60	0.26	1.37	0.37		
1363.64	0.49	31.40	0.26	1.39	0.46		
1365.32	0.50	28.70	0.26	1.43	0.29		
1367.00	0.43	25.80	0.25	1.39	0.37		
1368.69	0.54	23.90	0.25	1.60	0.34		
1370.37	0.36	28.40	0.25	1.61	0.35		
1370.37	0.43	30.00	0.25	1.63	0.30		
1373.74	0.37	26.20	0.25	1.65	0.46		
1373.74	0.34	33.00	0.25	1.78	0.43		
1375.42	0.32	24.10	0.25	1.79	0.32		
1377.10	0.53	29.60	0.25	1.79	0.43		
1382.15	0.62	29.90	0.25	1.80	0.45		
1382.15	0.53	31.10	0.25	1.82	0.55		
1385.52	0.29	25.80	0.24	2.00	0.44		
1390.57	0.48	29.20	0.24	2.02	0.32		
1390.57	0.48	28.60	0.24	2.03	0.44		
1390.57	0.35	29.70	0.24	2.05	0.64		
1393.94	0.64	25.80	0.24	2.06	0.44		
1393.94	0.53	30.50	0.23	2.11	0.43		
1393.94	0.45	29.00	0.23	2.12	0.65		
1393.94	0.46	30.10	0.23	2.12	0.54		
1395.62	0.41	31.00	0.23	2.12	0.57		
1395.62	0.24	25.80	0.23	2.13	0.44		
1404.04	0.51	29.80	0.22	1.94	0.55		
1404.04	0.40	23.70	0.22	1.93	0.40		
1404.04	0.53	31.80	0.22	1.92	0.45		
1404.04	0.34	23.60	0.22	1.92	0.54		
1404.04	0.30	31.40	0.22	2.02	0.96		
1404.04	0.46	27.80	0.22	2.02	0.80		
1407.41	0.29	25.60	0.22	2.03	1.00		
1410.77	0.47	30.20	0.21	2.04	0.98		
1410.77	0.37	30.10	0.21	2.04	0.91		
1412.46	0.48	30.00	0.21	2.13	0.50		
1414.14	0.34	29.00	0.21	2.14	0.55		
1415.82	0.45	28.10	0.20	2.16	0.66		
1417.51	0.49	28.50	0.20	2.17	0.50		

1419.19	0.41	30.00	0.20	2.17	0.40		
1420.87	0.26	29.20	0.20	2.13	0.82		
1420.87	0.43	30.40	0.20	2.14	0.46		
1422.56	0.46	33.00	0.20	2.20	0.73		
1441.08	0.49	29.00	0.20	2.21	0.93		
1441.08	0.54	28.20	0.20	2.22	0.77		
1441.08	0.44	26.20	0.19	2.20	0.62		
1451.18	0.82	30.10	0.19	2.21	0.55		
1466.33	0.59	29.60	0.19	2.00	0.58		
1491.58	0.58	29.80	0.19	2.00	0.62		
1493.26	0.30	31.40	0.19	2.00	0.51		
1496.63	0.38	29.90	0.18	1.72	0.58		
1503.37	0.31	30.60	0.18	1.73	0.68		
1506.73	0.38	31.10	0.18	1.73	0.52		
1511.78	0.55	29.40	0.17	1.75	0.64		
1537.04	0.59	27.30	0.17	1.75	0.60		
1543.77	0.43	30.10	0.16	1.75	0.43		
1547.14	0.75	29.40	0.15	1.75	0.61		
1548.82	0.66	29.80	0.15	1.18	0.52		
1548.82	0.65	28.90	0.15	1.20	0.50		
1553.87	0.56	26.20	0.14	1.20	0.57		
1607.74	0.50	26.00	0.14	1.21	0.64		
1621.21	0.77	26.30	0.14	1.22	0.49		
1622.89	0.73	29.00	0.13	1.23	0.51		
1629.63	0.93	30.10	0.13	1.24	0.34		
1629.63	0.62	29.20	0.11	1.25	0.44		
1658.25	0.58	30.70	0.10	1.49	0.68		
1661.61	0.68	26.40	0.10	1.49	0.50		
1663.30	0.52	25.80	0.09	1.50	0.55		
1663.30	0.60	26.80	0.08	1.50	0.56		
1663.30	0.43	32.60	0.08	1.50	0.51		
1664.98	0.64	32.70	0.06	1.50	0.39		
1678.45	0.51	31.80	0.05	1.49	0.59		
1681.82	0.62	28.90	0.05	1.49	0.52		
10.10	0.02	37.20	0.31	3.31	0.65		
193.60	0.40	29.80	0.44	3.24	0.54		
592.59	0.84	29.80	0.15	2.99	0.47		
781.14	0.92			2.95	0.68		
1116.16	0.49			2.91	0.71		
1548.82	0.71			2.81	0.33		
				2.75	0.73		
				2.71	0.52		
				2.65	0.58		

				2.64	0.53		
				2.55	0.89		
				2.54	0.22		
				2.45	0.59		
				2.44	0.06		
				2.43	0.37		
				2.38	0.36		
				2.35	0.66		
				2.34	0.08		
				2.33	0.34		
				2.30	0.27		
				2.28	0.24		
				2.26	0.48		
				2.25	0.38		
				2.25	0.54		
				2.24	0.42		
				2.24	0.37		
				2.23	0.46		
				2.23	0.52		
				0.56	0.34		
				1.75	0.61		
