# Supplementary material for: The Effect of Elevated Ozone Concentrations with Varying Shading on Dry Matter Loss in a Winter Wheat-Producing Region in China
Source: PLoS One. 2016 Jan 13;11(1):e0145446. doi: 10.1371/journal.pone.0145446 (PMC4711948; doi:10.1371/journal.pone.0145446)
Supplement: S3 Table — (PDF) [file pone.0145446.s003.pdf]

S3 Table. Simulated stomatal conductance plotted against measured stomatal conductance in all treatments. (unit: $molO_3$ m^{-2} s^{-1})

T1 measured value	T1 simulated value	T2 measured value	T2 simulated value	CK measured value	CK simulated value
0.048	0.048	0.069	0.061	0.103	0.150
0.054	0.058	0.076	0.073	0.112	0.171
0.068	0.067	0.094	0.083	0.135	0.233
0.082	0.075	0.112	0.092	0.155	0.275
0.129	0.083	0.163	0.111	0.137	0.232
0.174	0.092	0.044	0.041	0.132	0.306
0.061	0.076	0.094	0.086	0.241	0.242
0.048	0.048	0.061	0.069	0.150	0.103
0.058	0.054	0.073	0.076	0.171	0.112
0.067	0.068	0.083	0.094	0.119	0.119
0.075	0.082	0.092	0.112	0.188	0.179
0.083	0.129	0.111	0.163	0.150	0.157
0.092	0.174	0.041	0.044	0.112	0.171
0.076	0.061	0.086	0.094	0.242	0.241
0.049	0.053	0.193	0.181	0.226	0.234
0.116	0.129	0.235	0.235	0.170	0.186
0.171	0.185	0.219	0.216	0.153	0.139
0.152	0.176	0.182	0.173	0.222	0.240
0.148	0.161	0.172	0.163	0.218	0.212
0.164	0.174	0.126	0.142	0.181	0.165
0.202	0.181	0.182	0.182	0.119	0.121
0.111	0.114	0.162	0.185	0.128	0.143
0.151	0.172	0.134	0.152	0.090	0.099
0.208	0.202	0.218	0.213	0.188	0.162
0.169	0.157	0.218	0.213	0.205	0.202
0.136	0.136	0.230	0.230	0.206	0.238
0.123	0.114	0.185	0.193	0.199	0.209
0.126	0.097	0.146	0.141	0.128	0.124
