# Supplementary material for: The Effect of Elevated Ozone Concentrations with Varying Shading on Dry Matter Loss in a Winter Wheat-Producing Region in China
Source: PLoS One. 2016 Jan 13;11(1):e0145446. doi: 10.1371/journal.pone.0145446 (PMC4711948; doi:10.1371/journal.pone.0145446)
Supplement: S4 Table — (PDF) [file pone.0145446.s004.pdf]

S4 Table. Cumulative ozone uptake in each treatment during the whole experiments period.

Unit: mmolO_3 m^{-2}

DATE	T1	T2	CK
**1**	0.08	0.16	0.11
**2**	0.16	0.39	0.29
**3**	0.31	0.70	0.73
**4**	0.55	1.10	1.26
**5**	0.81	1.51	1.79
**6**	0.97	1.83	2.30
**7**	1.21	2.24	2.82
**8**	1.56	2.75	3.39
**9**	1.91	3.26	3.94
**10**	1.92	3.30	4.00
**11**	1.97	3.42	4.43
**12**	2.14	3.76	5.00
**13**	2.36	4.16	5.57
**14**	2.37	4.18	5.59
**15**	2.39	4.23	5.71
**16**	2.66	4.68	6.32
**17**	3.06	5.27	6.95
**18**	3.52	5.91	7.55
**19**	3.80	6.37	8.22
**20**	4.22	6.98	8.90
**21**	4.55	7.49	9.50
**22**	5.06	8.18	10.17
**23**	5.58	8.86	10.63
**24**	5.92	9.37	11.19
**25**	6.37	10.06	11.72
**26**	6.82	10.69	12.16
**27**	7.24	11.22	12.52
**28**	7.80	11.74	12.78
**29**	7.86	11.82	12.84
**30**	8.21	12.10	13.07
**31**	8.80	12.47	13.30
**32**	9.25	12.73	13.59
**33**	9.88	13.00	13.77
**34**	10.43	13.22	14.00
**35**	10.86	13.41	14.19
**36**	11.33	13.61	14.39
**37**	11.70	13.76	14.51
**38**	11.99	13.89	14.61
**39**	12.29	14.03	14.68

**40**	12.47	14.13	14.88
**41**	12.66	14.25	14.97
**42**	12.85	14.36	15.09
**43**	13.06	14.47	15.17
**44**	13.24	14.57	15.26
**45**	13.38	14.64	15.37
**46**	13.49	14.70	15.40
**47**	13.61	14.77	15.43
**48**	13.69	14.82	15.50
**49**	13.71	14.84	15.56
**50**	13.82	14.92	15.59
**51**	13.95	15.01	15.73
**52**	14.05	15.08	15.77
**53**	14.16	15.11	15.82
**54**	14.25	15.14	15.88
**55**	14.34	15.18	15.90
**56**	14.41	15.20	15.92
**57**	14.47	15.21	15.93
**58**	14.52	15.22	15.96
**59**	14.55	15.26	16.00
**60**	14.55	15.27	16.03
**61**	14.56	15.29	16.06
**62**	14.64	15.34	16.08
**63**	14.70	15.40	16.09
**64**	14.72	15.43	16.15
**65**	14.79	15.47	16.18
**66**	14.86	15.50	16.20
**67**	14.93	15.53	16.23
