# Supplementary material for: The Effect of Elevated Ozone Concentrations with Varying Shading on Dry Matter Loss in a Winter Wheat-Producing Region in China
Source: PLoS One. 2016 Jan 13;11(1):e0145446. doi: 10.1371/journal.pone.0145446 (PMC4711948; doi:10.1371/journal.pone.0145446)
Supplement: S5 Table — (PDF) [file pone.0145446.s005.pdf]

S5 Table. The daily variation of mean f_{PAR}, f_T, and f_{VPD} in each treatment.

The daily variation of mean of f_{PAR}

DATE	T1	T2	CK
**0**	0.23	0.29	0.36
**1**	0.24	0.30	0.36
**2**	0.24	0.30	0.36
**3**	0.24	0.30	0.36
**4**	0.25	0.31	0.37
**5**	0.26	0.31	0.37
**6**	0.25	0.31	0.37
**7**	0.25	0.30	0.37
**8**	0.21	0.26	0.33
**9**	0.08	0.11	0.16
**10**	0.22	0.27	0.33
**11**	0.27	0.33	0.39
**12**	0.27	0.33	0.39
**13**	0.04	0.06	0.09
**14**	0.07	0.10	0.15
**15**	0.21	0.27	0.34
**16**	0.23	0.29	0.36
**17**	0.24	0.30	0.37
**18**	0.27	0.33	0.39
**19**	0.27	0.33	0.39
**20**	0.17	0.23	0.29
**21**	0.25	0.31	0.38
**22**	0.24	0.30	0.36
**23**	0.23	0.29	0.35
**24**	0.27	0.33	0.39
**25**	0.28	0.34	0.40
**26**	0.29	0.35	0.41
**27**	0.28	0.34	0.40
**28**	0.04	0.05	0.09
**29**	0.20	0.26	0.33
**30**	0.27	0.33	0.39
**31**	0.21	0.27	0.34
**32**	0.26	0.32	0.38
**33**	0.24	0.30	0.37
**34**	0.22	0.28	0.36
**35**	0.27	0.32	0.39
**36**	0.25	0.31	0.38
**37**	0.23	0.29	0.36

**38**	0.25	0.31	0.38
**39**	0.18	0.23	0.30
**40**	0.24	0.31	0.40
**41**	0.25	0.31	0.39
**42**	0.28	0.34	0.41
**43**	0.28	0.35	0.41
**44**	0.26	0.33	0.40
**45**	0.26	0.32	0.39
**46**	0.25	0.32	0.40
**47**	0.21	0.27	0.34
**48**	0.08	0.12	0.18
**49**	0.25	0.31	0.39
**50**	0.31	0.37	0.43
**51**	0.24	0.31	0.39
**52**	0.29	0.35	0.42
**53**	0.26	0.32	0.40
**54**	0.27	0.34	0.41
**55**	0.28	0.34	0.40
**56**	0.27	0.34	0.41
**57**	0.26	0.33	0.40
**58**	0.10	0.15	0.22
**59**	0.04	0.05	0.09
**60**	0.08	0.12	0.18
**61**	0.29	0.36	0.42
**62**	0.21	0.27	0.34
**63**	0.09	0.13	0.19
**64**	0.28	0.35	0.42
**65**	0.31	0.37	0.44
**66**	0.29	0.36	0.43
**67**	0.12	0.16	0.23

The daily variation of mean of f_T

DATE	T1	T2	CK
**0**	0.10	0.10	0.10
**1**	0.10	0.10	0.22
**2**	0.10	0.10	0.15
**3**	0.10	0.10	0.10
**4**	0.10	0.10	0.20
**5**	0.10	0.10	0.15
**6**	0.10	0.10	0.10
**7**	0.11	0.20	0.19
**8**	0.17	0.23	0.15
**9**	0.10	0.10	0.10

**10**	0.10	0.10	0.22
**11**	0.10	0.10	0.11
**12**	0.10	0.10	0.12
**13**	0.10	0.10	0.17
**14**	0.10	0.10	0.10
**15**	0.10	0.10	0.13
**16**	0.14	0.20	0.29
**17**	0.21	0.29	0.12
**18**	0.10	0.10	0.34
**19**	0.10	0.16	0.45
**20**	0.12	0.17	0.17
**21**	0.28	0.37	0.57
**22**	0.35	0.43	0.51
**23**	0.10	0.10	0.30
**24**	0.13	0.23	0.83
**25**	0.10	0.11	0.42
**26**	0.10	0.12	0.56
**27**	0.18	0.26	0.67
**28**	0.10	0.10	0.61
**29**	0.10	0.10	0.85
**30**	0.25	0.34	0.56
**31**	0.15	0.22	0.72
**32**	0.53	0.55	0.77
**33**	0.47	0.50	0.56
**34**	0.23	0.29	0.70
**35**	0.31	0.39	0.72
**36**	0.64	0.64	0.45
**37**	0.84	0.82	0.73
**38**	0.40	0.44	0.70
**39**	0.25	0.30	0.40
**40**	0.16	0.22	0.71
**41**	0.21	0.25	0.66
**42**	0.32	0.38	0.33
**43**	0.47	0.49	0.65
**44**	0.65	0.65	0.69
**45**	0.81	0.78	0.27
**46**	0.88	0.87	0.49
**47**	0.75	0.73	0.70
**48**	0.10	0.10	0.13
**49**	0.39	0.42	0.40
**50**	0.39	0.44	0.39
**51**	0.39	0.41	0.10
**52**	0.47	0.50	0.33

**53**	0.46	0.50	0.23
**54**	0.58	0.58	0.13
**55**	0.67	0.64	0.38
**56**	0.74	0.65	0.11
**57**	0.65	0.58	0.18
**58**	0.09	0.11	0.29
**59**	0.10	0.10	0.10
**60**	0.10	0.10	0.20
**61**	0.32	0.39	0.25
**62**	0.30	0.35	0.10
**63**	0.13	0.14	0.24
**64**	0.38	0.43	0.17
**65**	0.46	0.49	0.10
**66**	0.55	0.55	0.24
**67**	0.51	0.55	0.18

The daily variation of mean of f_{VPD}

DATE	T1	T2	CK
**0**	1.00	1.00	1.00
**1**	1.00	1.00	1.00
**2**	1.00	1.00	0.99
**3**	1.00	1.00	0.97
**4**	1.00	1.00	0.95
**5**	1.00	1.00	1.00
**6**	1.00	1.00	0.99
**7**	1.00	0.99	0.96
**8**	1.00	1.00	0.96
**9**	1.00	1.00	1.00
**10**	1.00	1.00	1.00
**11**	1.00	1.00	0.97
**12**	1.00	1.00	1.00
**13**	1.00	1.00	1.00
**14**	1.00	1.00	1.00
**15**	1.00	1.00	1.00
**16**	1.00	1.00	0.99
**17**	1.00	1.00	0.96
**18**	1.00	1.00	0.97
**19**	1.00	1.00	0.92
**20**	1.00	1.00	1.00
**21**	1.00	0.99	0.99
**22**	1.00	1.00	0.93
**23**	1.00	1.00	0.99
**24**	1.00	0.99	0.95

**25**	1.00	1.00	0.94
**26**	1.00	0.99	0.96
**27**	1.00	1.00	0.87
**28**	1.00	1.00	1.00
**29**	0.96	0.94	1.00
**30**	0.98	0.96	0.90
**31**	0.95	0.94	0.97
**32**	0.92	0.92	0.86
**33**	0.97	0.97	0.78
**34**	0.98	0.98	0.97
**35**	0.94	0.97	0.90
**36**	1.00	0.99	0.85
**37**	0.87	0.87	0.84
**38**	0.97	0.94	0.80
**39**	1.00	1.00	0.94
**40**	1.00	0.98	0.99
**41**	1.00	0.98	1.00
**42**	0.94	0.92	0.96
**43**	0.97	0.92	0.79
**44**	0.96	0.88	0.80
**45**	0.99	0.97	0.77
**46**	0.97	0.98	0.92
**47**	1.00	1.00	0.81
**48**	1.00	1.00	1.00
**49**	0.91	0.95	0.97
**50**	0.95	0.99	0.77
**51**	0.93	0.97	0.83
**52**	0.95	0.95	0.73
**53**	0.92	0.93	0.78
**54**	0.83	0.85	0.76
**55**	0.87	0.87	0.66
**56**	0.87	0.83	0.62
**57**	1.00	1.00	0.69
**58**	1.00	1.00	1.00
**59**	1.00	1.00	1.00
**60**	0.95	0.91	1.00
**61**	0.96	0.94	0.82
**62**	1.00	1.00	0.88
**63**	0.98	0.95	1.00
**64**	0.94	0.89	0.83
**65**	0.82	0.68	0.77
**66**	0.96	0.94	0.70
**67**	0.97	0.97	0.97
