# Supplementary material for: The Effect of Elevated Ozone Concentrations with Varying Shading on Dry Matter Loss in a Winter Wheat-Producing Region in China
Source: PLoS One. 2016 Jan 13;11(1):e0145446. doi: 10.1371/journal.pone.0145446 (PMC4711948; doi:10.1371/journal.pone.0145446)
Supplement: S6 Table — (PDF) [file pone.0145446.s006.pdf]

S6 Table. The hourly variation of mean f_{PAR}, f_T, and f_{VPD} in each treatment.

The hourly variation of mean f_{PAR}.

Time	T1	T2	CK
**1**	0.001	0.002	0.003
**2**	0.002	0.002	0.004
**3**	0.002	0.003	0.004
**4**	0.002	0.003	0.005
**5**	0.015	0.022	0.036
**6**	0.111	0.160	0.248
**7**	0.268	0.368	0.518
**8**	0.419	0.546	0.707
**9**	0.549	0.680	0.817
**10**	0.615	0.739	0.857
**11**	0.653	0.773	0.879
**12**	0.659	0.780	0.888
**13**	0.641	0.766	0.879
**14**	0.576	0.707	0.840
**15**	0.472	0.604	0.761
**16**	0.310	0.420	0.580
**17**	0.131	0.188	0.289
**18**	0.014	0.021	0.034
**19**	0.000	0.000	0.001
**20**	0.000	0.000	0.000
**21**	0.000	0.001	0.001
**22**	0.000	0.001	0.001
**23**	0.000	0.001	0.001
**24**	0.001	0.001	0.002

The hourly variation of mean f_T.

Time	T1	T2	CK
**1**	0.16	0.15	0.16
**2**	0.15	0.15	0.15
**3**	0.15	0.15	0.15
**4**	0.15	0.14	0.14
**5**	0.14	0.13	0.13
**6**	0.15	0.15	0.18
**7**	0.18	0.20	0.31
**8**	0.25	0.30	0.43
**9**	0.33	0.40	0.51
**10**	0.40	0.46	0.57
**11**	0.44	0.50	0.67
**12**	0.47	0.54	0.67
**13**	0.49	0.56	0.63
**14**	0.51	0.56	0.63

**15**	0.50	0.55	0.63
**16**	0.48	0.53	0.56
**17**	0.44	0.48	0.47
**18**	0.37	0.38	0.32
**19**	0.27	0.27	0.23
**20**	0.23	0.22	0.21
**21**	0.20	0.20	0.20
**22**	0.19	0.19	0.19
**23**	0.18	0.18	0.18
**24**	0.17	0.16	0.16

The hourly variation of mean f_{VPD}.

Time	T1	T2	CK
**1**	1.00	1.00	1.00
**2**	1.00	1.00	1.00
**3**	1.00	1.00	1.00
**4**	1.00	1.00	1.00
**5**	1.00	1.00	1.00
**6**	1.00	1.00	1.00
**7**	1.00	1.00	1.00
**8**	1.00	1.00	0.98
**9**	1.00	0.98	0.92
**10**	0.98	0.96	0.85
**11**	0.95	0.92	0.73
**12**	0.94	0.91	0.64
**13**	0.92	0.89	0.56
**14**	0.89	0.87	0.61
**15**	0.90	0.88	0.73
**16**	0.91	0.91	0.85
**17**	0.94	0.93	0.97
**18**	0.98	0.98	0.99
**19**	0.99	0.99	1.00
**20**	0.99	1.00	1.00
**21**	0.99	1.00	1.00
**22**	1.00	1.00	1.00
**23**	1.00	1.00	1.00
**24**	1.00	1.00	1.00
