# Supplementary material for: The Effect of Elevated Ozone Concentrations with Varying Shading on Dry Matter Loss in a Winter Wheat-Producing Region in China
Source: PLoS One. 2016 Jan 13;11(1):e0145446. doi: 10.1371/journal.pone.0145446 (PMC4711948; doi:10.1371/journal.pone.0145446)
Supplement: S7 Table — (PDF) [file pone.0145446.s007.pdf]

S7 Table. The daily variation of mean fphen and f_{O3} in each treatment.

Date	T1 f_{Phen}	T1 F_{o3}	T2 f_{Phen}	T2 F_{o3}	CK f_{Phen}	CK F_{o3}
**0**	0.53	1.00	0.53	1.00	0.53	1.00
**1**	0.53	1.00	0.53	1.00	0.53	1.00
**2**	0.54	1.00	0.54	1.00	0.54	1.00
**3**	0.55	1.00	0.55	1.00	0.55	1.00
**4**	0.56	1.00	0.56	1.00	0.56	1.00
**5**	0.56	1.00	0.57	1.00	0.57	1.00
**6**	0.57	1.00	0.57	1.00	0.58	1.00
**7**	0.58	1.00	0.59	1.00	0.59	1.00
**8**	0.59	1.00	0.60	1.00	0.61	1.00
**9**	0.60	1.00	0.61	1.00	0.61	1.00
**10**	0.61	1.00	0.61	1.00	0.62	1.00
**11**	0.61	1.00	0.62	1.00	0.63	1.00
**12**	0.62	1.00	0.63	1.00	0.64	1.00
**13**	0.63	1.00	0.64	1.00	0.65	1.00
**14**	0.64	1.00	0.64	1.00	0.65	1.00
**15**	0.65	1.00	0.65	1.00	0.67	1.00
**16**	0.66	1.00	0.67	1.00	0.68	1.00
**17**	0.67	1.00	0.68	1.00	0.69	0.99
**18**	0.68	1.00	0.69	1.00	0.70	0.98
**19**	0.69	1.00	0.70	1.00	0.72	0.95
**20**	0.70	1.00	0.71	0.99	0.73	0.90
**21**	0.72	1.00	0.73	0.98	0.74	0.82
**22**	0.73	1.00	0.74	0.95	0.76	0.73
**23**	0.74	1.00	0.75	0.91	0.77	0.62
**24**	0.75	1.00	0.76	0.84	0.79	0.50
**25**	0.76	1.00	0.78	0.73	0.80	0.40
**26**	0.77	0.99	0.79	0.61	0.81	0.33
**27**	0.79	0.99	0.80	0.50	0.82	0.27
**28**	0.80	0.98	0.81	0.44	0.83	0.25
**29**	0.81	0.97	0.82	0.40	0.85	0.23
**30**	0.82	0.95	0.83	0.34	0.86	0.20
**31**	0.83	0.92	0.85	0.29	0.87	0.18
**32**	0.85	0.86	0.86	0.24	0.89	0.16
**33**	0.87	0.77	0.88	0.21	0.91	0.15
**34**	0.88	0.68	0.89	0.19	0.92	0.14
**35**	0.89	0.58	0.91	0.16	0.94	0.12
**36**	0.91	0.49	0.93	0.15	0.96	0.12
**37**	0.93	0.42	0.95	0.14	0.98	0.11
**38**	0.95	0.36	0.96	0.12	1.00	0.11
**39**	0.96	0.32	0.98	0.12	1.00	0.10
**40**	0.97	0.29	0.99	0.11	1.00	0.09
**41**	0.99	0.26	1.00	0.10	1.00	0.09

**42**	1.00	0.23	1.00	0.09	0.99	0.08
**43**	1.00	0.21	0.99	0.09	0.98	0.08
**44**	0.99	0.19	0.99	0.08	0.98	0.08
**45**	0.99	0.17	0.98	0.08	0.97	0.07
**46**	0.98	0.16	0.97	0.08	0.96	0.07
**47**	0.97	0.15	0.97	0.07	0.96	0.07
**48**	0.97	0.15	0.97	0.07	0.96	0.07
**49**	0.97	0.14	0.96	0.07	0.95	0.07
**50**	0.96	0.13	0.96	0.07	0.95	0.07
**51**	0.96	0.12	0.95	0.06	0.94	0.06
**52**	0.96	0.11	0.95	0.06	0.94	0.06
**53**	0.95	0.11	0.95	0.06	0.93	0.06
**54**	0.95	0.10	0.94	0.06	0.93	0.06
**55**	0.94	0.10	0.94	0.05	0.92	0.06
**56**	0.94	0.09	0.93	0.05	0.92	0.06
**57**	0.93	0.09	0.93	0.05	0.91	0.06
**58**	0.93	0.09	0.92	0.05	0.91	0.06
**59**	0.93	0.09	0.92	0.05	0.91	0.06
**60**	0.92	0.09	0.92	0.05	0.90	0.06
**61**	0.92	0.08	0.91	0.05	0.90	0.05
**62**	0.91	0.08	0.91	0.05	0.89	0.05
**63**	0.91	0.08	0.90	0.05	0.89	0.05
**64**	0.91	0.08	0.90	0.05	0.89	0.05
**65**	0.90	0.07	0.90	0.04	0.88	0.05
**66**	0.90	0.07	0.89	0.04	0.88	0.05
**67**	0.89	0.07	0.89	0.04	0.87	0.05
