# Supplementary material for: The Effect of Elevated Ozone Concentrations with Varying Shading on Dry Matter Loss in a Winter Wheat-Producing Region in China
Source: PLoS One. 2016 Jan 13;11(1):e0145446. doi: 10.1371/journal.pone.0145446 (PMC4711948; doi:10.1371/journal.pone.0145446)
Supplement: S8 Table — (PDF) [file pone.0145446.s008.pdf]

S8 Table. Temporal relationships between dry matter loss of winter wheat in T1 and AFsto6 and in T2 and AFsto6.

**T1 $AF_{sto}06$ (mmol O_3 m^{-2})**	**Dry matter loss**
0.23	1.00
0.88	0.90
1.99	0.80
4.29	0.67
11.33	0.55
13.78	0.50
14.78	0.42

**T2 $AF_{sto}06$ (mmol O_3 m^{-2})**	**Dry matter loss**
0.70	1.00
3.30	0.97
6.98	0.86
12.10	0.77
14.13	0.71
14.92	0.67
15.27	0.57
