# Supplementary material for: The Effect of Elevated Ozone Concentrations with Varying Shading on Dry Matter Loss in a Winter Wheat-Producing Region in China
Source: PLoS One. 2016 Jan 13;11(1):e0145446. doi: 10.1371/journal.pone.0145446 (PMC4711948; doi:10.1371/journal.pone.0145446)
Supplement: S9 Table — (PDF) [file pone.0145446.s009.pdf]

S9 Table. The hourly simulated and observed mean ozone concentrations in Nanjing station from March to May of 2006.
(unit: ppb)

March

**Time**	**simulated data**	**observed data**
0:00	20.37	11.50
1:00	21.09	11.52
2:00	20.35	11.70
3:00	20.38	11.28
4:00	20.46	11.34
5:00	19.77	9.11
6:00	19.60	7.28
7:00	20.08	8.53
8:00	21.26	11.84
9:00	25.15	16.56
10:00	30.52	22.94
11:00	33.83	28.94
12:00	36.87	35.82
13:00	39.56	39.95
14:00	40.69	40.82
15:00	38.84	40.23
16:00	34.57	35.03
17:00	27.87	27.92
18:00	22.56	18.90
19:00	18.92	14.75
20:00	16.66	11.59
21:00	16.22	10.08
22:00	15.42	10.93
23:00	16.22	11.46

April

**Time**	**simulated data**	**observed data**
0:00	27.43	18.95
1:00	27.95	17.48
2:00	28.11	16.37
3:00	27.75	16.23
4:00	28.99	14.60
5:00	30.06	13.00
6:00	30.84	11.63
7:00	33.48	13.87
8:00	35.41	19.90
9:00	38.41	25.89
10:00	42.21	32.20

11:00	45.82	39.16
12:00	48.47	42.88
13:00	49.82	47.26
14:00	50.42	48.90
15:00	50.02	46.84
16:00	47.58	42.37
17:00	41.41	37.56
18:00	33.69	28.76
19:00	28.81	23.48
20:00	25.81	20.44
21:00	24.45	18.73
22:00	24.70	18.11
23:00	26.14	18.73

May

Time	simulated data	observed data
0:00	29.59	20.69
1:00	30.12	20.37
2:00	29.17	21.88
3:00	30.09	20.90
4:00	31.30	20.18
5:00	32.09	16.22
6:00	34.08	16.57
7:00	36.27	20.35
8:00	38.48	28.56
9:00	41.85	37.69
10:00	45.59	46.28
11:00	49.02	53.34
12:00	51.42	58.63
13:00	53.45	59.79
14:00	54.12	58.86
15:00	52.83	58.21
16:00	49.10	59.10
17:00	42.64	56.09
18:00	36.68	49.37
19:00	31.66	38.91
20:00	30.77	31.78
21:00	30.49	27.50
22:00	29.52	23.07
23:00	29.30	21.65
