# Supplementary material for: The Effect of Elevated Ozone Concentrations with Varying Shading on Dry Matter Loss in a Winter Wheat-Producing Region in China
Source: PLoS One. 2016 Jan 13;11(1):e0145446. doi: 10.1371/journal.pone.0145446 (PMC4711948; doi:10.1371/journal.pone.0145446)
Supplement: S10 Table — (PDF) [file pone.0145446.s010.pdf]

S10 Table. Simulated ozone concentrations plotted against observed ozone concentrations in Nanjing station from March to May of 2006.
(unit: ppb)

Simulated data	Observed data
28.21	19.59
14.96	4.20
15.41	2.80
31.92	16.79
11.06	9.80
17.39	3.73
8.33	3.27
11.21	3.27
34.96	25.19
35.41	24.25
38.92	25.19
11.06	6.53
27.39	17.72
8.33	5.13
11.19	7.93
21.07	14.93
28.99	19.59
8.54	2.80
12.51	3.73
14.20	8.86
16.08	5.60
16.61	3.27
42.13	32.65
16.93	7.46
28.99	15.39
17.70	9.33
32.48	20.52
13.94	3.73
16.04	2.80
24.62	11.66
24.49	10.73
16.82	3.27
10.28	3.27
12.48	3.27
35.94	25.19
26.04	17.26
31.62	24.72

14.49	8.40
26.82	15.86
10.28	12.13
10.37	7.46
26.38	14.93
36.12	21.46
10.11	2.80
10.46	2.80
14.13	8.40
17.33	3.73
15.66	3.73
42.11	35.91
26.32	16.33
26.14	11.19
16.74	7.93
31.43	20.52
9.88	3.73
5.93	2.80
19.85	9.33
15.79	8.86
14.99	3.27
12.34	6.53
11.43	2.80
29.88	27.52
25.93	18.19
29.85	21.46
35.79	14.93
24.99	15.39
9.34	14.46
29.38	7.46
33.07	19.59
25.37	21.92
10.56	2.80
8.56	2.33
11.23	3.27
16.76	3.27
14.24	3.73
37.62	37.31
24.49	14.93
25.93	11.19
14.52	6.53
29.88	17.72
4.77	3.73

2.37	2.80
11.24	6.53
18.04	9.80
16.60	7.93
7.64	7.93
9.88	2.80
34.77	32.18
22.37	9.33
31.24	22.39
18.04	14.46
26.60	13.99
27.64	14.46
18.65	8.40
46.88	23.32
37.95	19.59
10.88	2.80
10.73	2.33
12.12	3.27
17.45	3.27
15.83	3.27
43.37	34.52
23.56	16.33
17.35	4.20
13.93	6.06
28.91	16.79
10.52	3.27
16.87	2.80
13.46	5.13
19.10	8.86
11.16	8.40
26.43	19.12
8.91	3.27
45.52	31.72
28.87	23.79
33.46	22.85
27.10	14.46
31.16	12.59
16.43	9.80
18.00	8.40
29.17	16.33
31.13	15.86
12.01	2.80
8.24	2.80

12.27	3.73
7.52	3.73
6.50	3.73
39.04	30.32
23.25	11.19
13.34	4.66
13.56	8.40
28.44	13.06
8.28	3.27
12.13	2.80
13.95	3.27
18.55	5.60
26.61	11.19
30.77	18.19
8.44	3.27
48.28	27.99
32.13	20.52
33.95	18.66
18.55	8.40
26.61	13.53
12.77	7.00
7.09	2.80
15.72	8.40
22.06	11.66
12.94	3.27
15.47	3.27
11.49	3.27
16.40	3.27
17.62	3.73
36.15	22.85
14.62	7.46
11.97	3.73
12.91	6.53
19.02	7.93
12.20	3.73
13.91	2.80
14.30	3.73
15.17	4.20
19.13	7.93
38.03	17.72
12.02	3.27
45.20	21.92
28.91	11.66

14.30	7.46
15.17	5.13
29.13	12.13
14.03	4.20
16.41	7.00
19.39	9.80
14.03	5.60
16.71	2.80
18.08	3.73
16.88	4.20
16.57	4.66
17.46	3.73
35.51	16.79
16.23	7.00
19.41	4.20
12.34	6.06
20.33	7.93
11.43	3.73
14.14	3.27
17.36	5.60
12.16	3.73
17.96	5.13
16.44	6.06
12.33	3.27
45.43	23.32
27.14	17.72
24.36	11.66
12.16	4.66
27.96	14.93
12.44	5.13
26.25	13.99
49.54	25.65
12.90	6.53
11.81	2.80
17.22	7.00
19.83	7.46
15.74	6.53
17.74	3.73
26.33	11.19
17.28	5.13
15.81	7.00
23.66	17.26
21.34	10.73

7.72	5.13
7.34	3.73
15.77	7.46
8.18	6.06
14.27	5.13
13.66	5.13
11.34	3.27
17.72	23.79
37.34	23.32
35.77	21.92
18.18	5.60
30.27	16.33
7.39	4.66
25.10	16.33
42.26	29.38
27.46	11.19
16.74	3.27
24.41	13.06
27.20	17.72
18.19	8.40
18.35	5.13
41.42	22.85
15.11	8.86
26.61	12.13
26.42	18.66
33.32	15.39
21.94	10.73
17.23	14.46
16.29	10.26
12.78	7.93
18.38	8.86
16.42	8.40
13.32	3.27
21.94	16.33
47.23	30.78
36.29	29.38
12.78	8.40
28.38	23.79
8.83	5.60
25.70	21.46
40.36	32.18
29.20	16.33
16.54	5.13

27.13	22.85
40.37	31.72
19.25	12.13
19.75	9.80
29.80	29.38
35.11	17.72
39.19	19.59
30.59	26.12
36.63	20.99
28.49	22.85
37.61	29.38
37.10	17.72
21.10	12.13
27.25	13.06
30.59	12.13
6.63	2.80
28.49	18.19
37.61	31.25
37.10	32.65
21.10	12.13
27.25	31.72
10.93	4.66
29.48	23.79
29.11	38.71
40.44	23.32
15.82	12.59
50.30	30.78
36.59	36.85
20.74	12.13
41.51	30.78
33.49	34.98
36.39	34.52
41.19	30.32
35.32	30.78
28.95	27.99
38.16	33.12
58.52	43.38
38.74	25.65
21.93	18.66
28.65	13.06
35.32	17.26
8.95	3.27
38.16	20.06

38.52	35.91
38.74	37.78
21.93	14.93
32.65	36.38
14.14	5.13
34.98	27.99
30.93	43.84
41.82	21.92
5.67	4.66
51.83	41.51
43.18	42.44
42.54	24.72
43.13	48.51
36.42	34.52
22.86	40.58
38.60	50.37
42.78	36.85
38.88	41.51
40.80	35.45
44.90	42.44
50.77	49.44
40.45	33.12
28.20	27.99
30.23	16.79
38.88	20.52
4.80	4.66
46.90	30.32
33.77	36.85
40.45	45.71
28.20	21.92
31.23	42.44
18.78	7.93
30.01	32.18
33.61	43.84
32.22	25.19
49.83	47.58
50.07	49.44
43.75	36.38
43.89	53.17
42.24	45.71
33.81	42.91
37.57	55.97
44.48	41.98

40.71	47.11
41.49	31.72
48.69	54.57
36.19	60.17
42.29	39.65
28.19	34.98
31.89	17.72
40.71	19.12
11.49	8.86
48.69	34.52
26.19	38.25
42.29	47.58
28.19	23.32
31.89	47.58
16.99	10.73
43.72	41.05
45.89	44.31
42.18	28.45
46.69	50.37
57.08	56.44
45.16	46.18
44.19	57.84
43.65	47.58
40.30	44.78
58.00	60.17
45.91	45.71
41.57	47.11
40.60	39.65
41.36	60.63
42.15	51.31
43.68	53.17
26.15	41.98
31.91	16.79
41.57	19.59
10.60	7.00
41.36	36.85
32.15	36.85
43.68	49.44
26.15	23.79
31.91	48.51
37.26	14.93
45.16	44.31
36.15	47.58

41.69	27.05
58.09	35.45
43.82	45.71
61.35	51.31
45.98	53.17
53.70	61.57
42.13	49.44
43.68	44.78
48.13	54.10
46.70	40.11
41.13	44.31
38.37	36.38
57.84	62.97
33.70	41.51
62.34	89.55
24.74	12.59
30.90	17.26
41.13	28.92
8.37	7.93
27.84	35.45
33.70	41.51
42.34	49.91
24.74	26.12
30.90	50.37
39.09	28.45
44.66	30.78
31.35	41.05
31.07	21.46
57.64	44.78
39.37	34.05
52.01	47.58
45.70	55.04
42.64	63.43
39.29	48.51
44.65	41.98
36.61	51.31
46.71	33.12
37.26	38.71
32.34	27.52
53.45	55.04
29.51	33.12
38.44	31.72
13.62	7.00

27.76	18.66
37.26	27.05
12.34	8.40
43.45	37.78
29.51	41.05
38.44	48.51
23.62	22.85
27.76	50.37
5.11	3.73
31.04	29.85
26.99	39.65
39.91	14.46
55.60	46.18
31.89	27.52
59.37	45.24
44.55	52.24
39.61	58.30
35.84	49.91
41.92	46.64
31.66	53.17
45.02	31.25
28.38	29.38
23.87	21.92
45.62	39.65
23.32	23.32
31.71	18.66
12.38	4.66
19.33	25.19
28.38	18.19
13.87	7.46
35.62	28.45
23.32	40.11
31.71	40.11
22.38	20.06
39.33	41.98
7.55	2.80
31.82	13.53
28.11	39.65
15.87	8.86
45.83	39.18
23.95	45.24
42.66	32.18
29.53	15.39

31.99	49.91
31.04	48.04
32.48	43.38
24.34	36.85
28.07	19.59
23.56	18.66
21.34	8.40
29.60	29.85
23.60	16.33
28.14	18.66
11.94	3.27
10.07	18.66
13.56	5.60
21.34	10.73
21.60	21.46
23.60	29.85
28.14	25.65
21.94	14.93
10.07	18.66
3.07	3.27
14.60	5.60
26.61	38.25
16.24	4.20
36.51	30.32
32.16	29.85
28.42	16.33
13.16	4.66
48.01	39.65
29.25	42.91
22.95	24.72
18.75	17.26
31.07	12.59
22.65	16.79
12.92	7.00
25.39	20.06
15.70	12.13
27.62	21.92
2.68	2.80
5.13	4.20
12.65	3.27
12.92	11.66
25.39	20.06
25.70	19.59

17.62	11.19
22.68	14.46
5.13	3.27
4.75	2.80
20.67	25.19
23.66	27.99
19.71	6.06
31.56	27.52
23.61	14.46
18.91	9.80
4.71	4.66
19.26	15.86
37.96	37.31
15.65	10.73
37.16	37.31
19.08	10.26
21.41	14.46
14.04	4.20
12.44	7.46
11.90	8.86
18.25	9.80
4.76	3.27
2.85	5.13
11.41	3.27
24.04	22.39
12.44	14.93
31.90	26.59
18.25	6.06
24.76	17.72
2.85	2.80
8.84	3.27
28.02	22.39
33.44	26.59
5.91	4.20
21.10	14.93
24.29	11.66
19.15	6.53
16.31	4.20
10.48	8.86
25.53	27.52
6.34	4.66
21.16	25.65
17.98	5.60

21.25	20.52
4.47	4.20
10.14	6.06
8.71	7.46
7.24	3.27
2.60	3.27
11.25	3.27
24.47	24.25
19.53	13.06
31.14	23.32
18.71	6.06
27.24	16.33
2.60	3.27
12.69	3.27
20.54	17.72
26.91	24.72
12.30	2.80
13.19	9.33
14.40	7.00
13.58	4.20
15.70	4.20
20.88	9.33
20.26	11.19
10.57	4.20
24.43	17.72
26.83	12.13
25.16	24.72
13.93	4.20
14.93	11.19
18.19	7.46
8.06	2.80
3.72	3.27
3.16	3.27
23.93	22.85
23.63	24.25
24.93	20.99
8.19	2.80
28.06	16.33
3.72	3.73
12.80	2.80
29.17	19.12
27.10	23.79
9.40	2.80

14.25	4.20
13.71	9.33
10.37	4.20
5.19	3.73
10.36	9.80
15.12	7.46
4.11	8.40
19.70	13.06
29.95	27.52
27.23	23.79
13.75	4.20
17.07	12.13
18.69	8.40
7.78	3.27
5.68	3.27
7.23	3.27
23.75	28.45
24.99	27.05
27.07	24.25
8.69	3.73
27.78	16.79
5.68	4.66
12.09	4.66
12.49	9.80
22.81	19.59
7.99	2.80
3.63	3.27
21.36	10.26
12.81	5.13
5.99	3.27
22.92	19.59
16.24	8.86
19.46	10.73
18.61	10.26
29.98	26.59
34.16	26.59
45.32	39.65
37.91	19.59
23.08	18.19
54.88	48.51
25.54	17.72
9.92	3.73
9.61	3.73

34.39	20.06
20.92	13.99
22.98	11.66
5.22	5.60
35.78	23.79
30.44	15.39
44.16	25.19
8.42	3.27
39.19	22.39
42.74	18.66
21.20	13.53
37.37	32.18
19.82	27.52
23.41	12.13
13.66	5.60
49.95	30.32
32.63	27.05
7.57	6.53
15.51	7.93
5.03	6.53
43.24	38.25
28.79	23.32
30.73	26.59
32.62	33.12
28.84	23.79
14.39	13.06
52.58	46.18
14.43	5.60
10.26	3.73
14.69	3.73
39.74	28.45
25.60	12.13
13.90	3.73
13.22	7.93
39.72	27.05
33.45	11.19
38.38	26.59
10.26	3.27
35.19	19.59
32.90	10.73
32.35	21.92
31.91	27.05
36.78	26.12

24.13	15.86
18.04	4.20
40.11	26.12
33.24	16.79
30.73	14.46
14.41	7.00
18.48	6.06
43.00	27.99
34.45	24.25
40.85	25.65
43.43	26.12
38.15	24.72
22.38	13.99
58.15	45.71
11.87	4.20
10.12	4.20
11.29	3.73
53.50	34.98
17.98	9.33
5.65	3.27
22.10	7.46
34.06	21.92
23.97	10.73
39.22	21.46
9.40	3.27
33.28	20.52
9.10	6.06
32.46	16.79
30.02	19.59
35.86	24.72
31.02	17.72
19.87	3.27
40.05	22.85
33.37	18.19
30.21	13.99
13.92	7.93
18.43	5.60
43.02	32.18
30.56	20.99
31.41	25.19
23.89	13.06
37.26	23.79
31.35	21.46

52.66	43.38
19.62	6.53
21.00	11.66
13.86	3.27
43.70	37.31
17.12	5.60
17.38	3.73
12.64	5.60
35.27	19.12
28.14	13.53
42.86	26.59
9.09	3.27
31.23	16.79
20.87	8.40
23.41	11.19
22.97	10.26
40.64	24.25
29.59	19.12
12.50	3.27
41.88	19.59
35.46	20.99
35.96	23.79
14.80	6.06
8.47	4.66
49.49	37.31
27.89	18.19
32.01	21.92
15.73	6.53
36.20	19.12
18.55	7.46
52.48	38.71
18.11	8.86
20.96	10.73
14.19	3.73
62.02	38.25
17.35	7.46
17.30	6.53
15.04	6.06
39.67	21.92
30.64	13.99
47.12	26.59
16.86	3.27
35.45	20.52

14.88	3.73
21.77	10.26
16.04	6.53
42.95	24.72
31.19	18.66
16.40	3.27
43.19	18.19
37.37	17.72
36.13	19.12
18.09	4.66
18.37	4.20
53.61	28.45
29.96	16.79
32.45	15.86
19.14	9.80
17.91	7.46
25.62	10.73
65.74	35.91
17.81	3.73
19.73	7.46
12.95	3.73
58.30	30.32
19.42	7.00
20.27	9.33
20.03	9.33
44.80	20.06
19.19	8.86
59.38	26.12
11.94	3.73
55.61	27.52
14.12	3.73
17.66	8.40
14.08	4.20
46.52	22.85
43.45	20.99
10.50	4.66
18.62	8.86
37.99	10.26
58.43	23.79
22.69	3.27
10.59	4.66
53.02	25.19
33.78	12.13

24.61	8.40
25.27	9.80
14.42	5.13
25.80	9.33
60.30	27.52
14.41	4.20
18.53	6.06
14.77	4.66
48.45	17.72
12.56	4.66
25.96	9.80
15.15	7.93
50.86	22.39
42.62	16.79
29.27	12.59
15.73	3.73
60.24	24.72
15.54	5.60
15.32	6.06
19.34	5.60
45.76	16.79
60.90	23.32
34.59	10.73
18.27	6.53
37.88	10.73
60.67	26.12
17.41	4.66
18.21	6.53
40.22	13.99
42.16	16.79
14.71	7.00
40.25	14.46
13.41	6.53
11.77	4.66
50.14	22.85
12.26	4.66
37.86	10.26
20.39	9.33
41.67	16.79
12.48	5.13
25.95	9.80
54.83	26.12
50.20	20.52

41.82	16.79
57.60	21.92
19.66	5.13
47.82	22.85
14.73	8.86
23.95	11.19
30.28	12.13
45.35	19.59
50.70	21.92
35.71	10.73
43.39	16.33
26.93	13.06
52.31	22.85
13.92	5.60
32.64	10.73
36.71	18.19
45.08	20.06
22.11	9.33
34.30	21.46
20.94	13.06
22.58	10.73
44.11	31.25
24.97	10.73
45.39	19.12
35.54	20.99
32.98	12.59
12.43	5.60
37.00	16.79
44.56	33.58
26.87	19.12
44.07	22.85
50.27	24.72
34.64	15.86
36.91	28.92
28.28	14.93
37.91	26.12
40.59	36.85
35.87	29.85
31.65	20.52
33.69	14.93
45.68	20.52
39.69	27.99
57.57	23.79

19.08	9.80
43.11	12.13
37.08	20.06
42.51	22.85
10.10	7.93
42.25	33.58
32.28	25.65
43.53	24.72
56.00	38.71
24.60	18.19
46.36	30.78
40.55	33.12
31.45	14.93
6.65	5.60
10.03	3.27
44.95	32.65
41.99	23.32
28.16	17.26
60.45	32.65
32.57	26.12
54.92	42.44
43.13	24.72
33.54	27.52
52.20	38.25
47.90	34.05
33.02	19.59
40.69	23.79
47.67	28.45
51.97	40.11
43.87	29.38
28.87	16.33
32.51	20.06
43.66	33.12
46.52	30.32
10.01	10.26
52.88	38.25
40.02	29.38
44.23	40.11
58.84	45.24
56.00	43.38
47.29	40.58
40.14	26.59
10.09	9.80

5.29	5.60
24.86	15.39
26.44	20.06
30.69	23.32
11.96	9.33
59.22	53.17
43.67	34.98
56.06	40.58
60.52	41.05
43.45	30.78
43.93	40.11
38.39	38.71
34.75	21.46
45.67	38.71
60.04	46.64
63.19	48.97
68.89	27.99
44.10	30.32
43.04	28.92
52.64	39.65
50.09	46.64
49.52	49.44
61.77	54.57
22.98	13.53
46.14	27.05
51.01	52.71
64.45	41.98
49.20	47.58
14.44	7.00
11.22	10.26
59.20	50.84
49.47	29.85
48.25	45.71
22.78	34.05
35.70	20.52
58.47	47.58
33.76	22.85
56.85	48.04
67.89	48.51
36.04	44.31
45.66	44.31
38.32	35.45
37.10	26.12

52.91	42.44
52.59	56.44
43.52	55.04
75.07	32.18
47.59	45.71
42.29	40.58
45.78	48.51
54.48	51.77
56.80	56.90
64.15	62.97
22.68	22.85
48.31	25.65
61.70	58.30
72.66	47.11
52.69	49.44
30.19	58.30
15.81	8.86
32.97	19.12
52.87	45.71
48.76	24.72
22.28	38.25
29.29	18.66
52.83	52.24
35.29	50.84
46.28	43.84
73.50	59.24
55.37	53.17
43.48	43.38
39.19	33.12
40.01	31.72
58.23	42.44
62.76	60.17
44.73	51.31
80.63	36.38
48.83	46.18
41.60	36.38
61.20	54.57
59.01	54.57
50.81	26.59
62.09	65.77
18.85	59.24
49.23	24.25
45.19	57.84

79.11	50.37
56.37	52.71
61.15	71.36
8.00	6.53
45.76	25.65
56.05	59.70
48.67	28.92
20.57	40.58
62.74	27.99
53.86	54.10
55.12	55.04
43.97	53.64
77.45	67.16
32.20	55.97
34.88	52.71
30.31	35.45
42.40	36.38
59.88	48.97
56.38	59.70
44.54	54.10
89.30	30.32
51.67	51.77
50.21	55.97
45.45	55.04
62.44	54.10
53.02	25.19
48.60	67.16
64.17	63.90
18.05	10.73
56.39	53.64
84.29	55.97
58.83	58.30
46.13	60.63
9.74	5.13
66.72	32.18
57.42	76.49
49.88	27.52
24.58	45.24
66.33	32.18
51.09	53.17
36.52	60.17
40.42	55.97
80.38	67.63

28.02	55.50
33.66	57.37
30.86	24.25
44.03	38.71
58.35	54.57
56.65	57.37
42.25	48.04
100.28	46.64
52.83	53.17
37.38	68.56
51.12	56.44
64.45	55.04
54.11	27.05
65.74	71.36
61.51	67.63
14.98	6.06
46.61	51.31
86.86	56.90
59.28	54.10
39.45	45.24
6.87	6.06
65.92	38.71
56.85	52.24
50.16	27.99
28.86	43.84
68.70	31.72
48.83	52.71
38.84	58.77
41.01	57.84
82.43	64.37
26.46	57.84
31.49	55.04
30.66	24.72
44.18	38.25
56.16	53.64
54.76	56.44
41.74	45.71
99.11	42.91
52.82	52.71
35.79	66.23
51.98	50.37
58.32	47.58
42.84	25.65

62.52	69.96
69.91	62.97
11.24	7.93
45.37	44.78
84.52	60.17
55.63	48.97
47.03	41.05
6.19	7.93
59.26	37.31
54.29	27.05
48.87	27.52
29.13	42.44
67.98	28.45
45.94	45.71
40.25	58.30
39.50	53.17
60.71	44.78
28.21	55.50
28.43	49.91
28.79	21.46
42.51	40.11
53.19	50.37
51.41	55.04
39.45	39.18
64.52	26.12
50.06	36.85
59.04	65.77
49.28	49.91
61.34	46.64
35.01	20.52
65.46	74.63
57.98	60.17
34.43	20.06
37.95	41.05
74.30	52.24
45.82	36.38
49.55	44.31
5.23	5.13
48.84	34.05
29.17	16.79
45.29	26.59
24.88	36.38
17.62	11.66

42.04	40.11
41.35	58.30
37.85	41.98
69.29	35.45
28.82	48.51
43.43	41.05
24.25	20.52
36.83	38.71
47.49	43.38
43.84	46.18
34.17	37.78
30.92	22.39
40.80	29.85
45.04	54.10
52.37	50.37
52.20	38.25
18.85	11.19
60.83	52.24
64.60	57.84
23.49	18.19
32.32	36.38
71.33	53.17
18.99	11.19
40.02	36.85
10.13	3.73
33.28	31.72
15.92	7.93
21.23	19.59
35.10	34.98
6.07	4.66
40.55	34.05
31.91	28.92
36.13	22.39
38.81	32.65
33.18	34.52
36.08	34.05
16.76	13.06
46.00	40.58
40.14	30.78
37.14	34.98
37.57	31.72
9.10	8.86
32.72	22.85

42.53	42.44
42.34	40.11
37.62	31.25
12.76	4.66
8.66	6.53
52.87	47.11
36.59	29.85
31.69	35.45
46.66	34.52
13.14	5.60
43.21	37.78
3.21	3.73
28.41	22.85
3.93	3.73
40.64	24.25
37.40	31.25
7.74	4.20
35.38	34.05
41.77	34.98
21.27	19.59
40.37	30.32
33.35	34.98
22.70	19.59
21.87	17.26
47.69	38.71
36.88	28.92
35.01	35.45
31.96	31.72
6.01	3.27
24.93	12.13
33.67	22.85
37.41	32.18
27.05	16.79
8.33	5.60
16.76	8.86
51.17	42.44
40.12	34.98
33.10	28.92
28.11	20.06
8.12	4.20
26.14	32.65
10.76	3.73
40.10	22.39

11.15	5.60
30.31	26.59
30.11	27.52
10.58	3.73
39.35	30.78
41.93	28.92
11.92	6.06
36.03	21.92
33.20	32.18
15.60	20.06
21.14	15.39
41.50	32.65
35.38	22.39
34.50	37.78
24.23	28.45
8.70	3.73
12.35	9.80
14.83	9.80
37.68	31.72
20.95	14.46
27.38	24.72
25.54	8.86
31.61	27.05
33.75	36.38
33.53	24.25
24.41	14.46
6.48	3.73
28.46	19.12
6.75	3.73
21.82	20.52
29.77	13.99
39.49	27.52
3.63	26.59
8.37	3.27
28.82	13.99
32.42	25.65
9.06	4.20
22.12	13.53
33.37	24.72
19.49	18.66
13.55	11.66
27.43	27.99
36.65	14.93

32.55	34.98
18.13	21.92
7.26	3.73
24.14	10.73
45.70	25.65
42.36	34.05
19.44	21.46
28.71	37.31
25.01	10.26
22.01	15.86
42.70	36.38
34.83	24.25
25.75	15.39
11.59	3.73
31.41	13.06
3.91	3.27
19.45	9.80
28.75	16.33
26.66	22.39
33.18	33.12
3.54	3.27
8.56	6.53
41.47	27.05
5.81	5.13
28.29	12.13
34.19	26.59
21.15	24.72
18.89	12.59
36.50	24.72
38.57	27.05
30.67	30.78
16.82	10.73
1.26	3.73
17.70	8.40
35.18	26.12
47.52	30.78
20.99	21.92
40.15	39.18
26.46	12.13
20.60	16.79
53.18	48.97
36.06	27.52
9.12	3.73

8.60	3.73
32.81	21.92
12.20	6.06
22.22	18.19
7.13	6.06
26.29	11.66
34.31	26.59
29.09	15.86
3.15	3.27
41.46	24.72
18.90	7.93
8.98	3.73
42.98	34.52
41.25	29.38
20.45	10.73
9.14	15.86
39.61	28.45
35.84	26.59
21.15	9.33
17.97	4.66
11.12	5.60
53.79	50.37
33.46	27.52
26.71	20.99
36.09	20.99
28.08	12.59
54.08	40.11
42.65	29.38
32.23	23.32
18.25	11.19
35.44	27.52
33.36	21.92
13.93	6.53
5.12	5.13
8.83	4.66
44.85	31.72
48.33	40.11
37.35	24.72
47.93	32.18
29.13	15.86
36.35	33.12
18.84	16.33
22.08	23.32

9.74	5.60
23.01	14.93
25.25	13.06
26.93	10.26
26.81	13.06
60.21	52.71
36.20	25.65
28.49	26.12
49.08	39.65
4.07	1.40
4.97	1.87
29.53	16.33
30.59	16.79
48.12	23.79
52.77	39.65
46.74	31.25
30.37	15.86
22.47	13.06
38.84	23.32
32.03	22.85
11.49	6.06
23.79	11.19
10.41	6.53
36.44	27.99
43.12	32.18
33.57	26.12
28.34	19.12
28.97	13.99
4.50	29.85
6.67	22.85
22.45	13.06
7.64	2.80
35.74	10.73
46.59	27.99
15.96	8.40
37.90	20.52
69.38	52.71
35.49	24.25
30.46	20.06
48.31	35.45
3.77	1.40
29.13	16.79
21.66	14.93

26.18	19.12
46.43	45.24
43.08	35.45
34.97	30.32
27.94	17.72
27.33	14.46
33.18	21.46
30.84	18.19
6.35	6.53
31.73	15.39
9.78	5.13
38.79	32.65
41.41	38.71
33.62	29.85
26.34	23.32
28.66	12.59
30.65	22.39
28.19	27.52
21.70	9.33
11.27	6.06
14.36	13.06
46.02	26.12
21.37	17.26
28.51	27.05
56.98	49.44
53.34	34.05
30.77	23.79
38.02	31.25
3.06	1.40
26.41	19.59
6.90	3.73
13.32	8.86
55.30	37.78
44.20	33.58
29.59	21.92
16.78	11.19
32.88	17.26
34.50	16.79
30.80	15.86
9.04	5.60
40.49	22.85
20.35	11.66
40.73	27.05

49.26	35.45
44.95	31.72
34.89	28.45
19.82	9.33
20.19	14.93
39.61	30.78
11.82	7.00
8.12	4.66
12.22	6.06
47.10	28.45
29.04	12.59
49.31	37.31
35.59	42.44
40.55	35.91
33.95	31.25
38.08	30.78
6.58	1.40
38.65	25.19
5.13	3.73
26.22	16.79
65.33	41.05
43.76	21.92
36.83	18.19
28.04	13.53
46.08	22.85
16.92	7.93
20.60	11.66
10.49	4.66
40.87	20.99
32.40	16.33
40.20	34.52
57.58	38.71
29.36	21.92
36.04	28.92
11.60	5.13
14.27	10.73
41.44	29.85
4.13	4.20
11.70	3.73
10.65	5.13
47.87	27.05
26.75	13.06
39.30	30.32

41.18	39.18
57.55	41.98
48.37	38.71
38.32	28.45
3.46	1.40
39.07	24.25
3.90	2.33
25.28	11.66
56.80	34.52
31.13	14.46
15.34	7.00
32.21	11.19
45.27	21.92
7.26	4.66
10.34	6.06
10.09	4.66
50.17	26.12
43.27	21.46
61.78	32.65
62.66	34.52
16.03	5.60
52.11	24.25
11.65	4.66
16.34	6.53
38.94	22.85
6.33	2.80
12.50	3.27
12.61	6.06
49.33	26.59
6.13	5.60
37.94	14.46
61.56	36.85
68.63	39.65
68.52	37.78
48.79	20.52
4.74	1.87
18.18	7.93
12.95	4.66
28.01	11.66
69.08	32.65
25.88	13.53
35.73	15.86
26.80	10.73

47.00	19.59
16.68	7.46
13.94	6.53
12.24	5.60
47.62	20.99
41.58	24.25
50.78	26.59
65.88	34.52
10.11	7.00
51.56	20.99
14.88	6.53
13.46	6.06
37.91	19.12
16.67	7.00
12.67	6.06
25.41	11.19
55.80	32.18
10.69	5.60
46.67	19.12
61.51	31.72
68.57	36.38
61.19	34.98
41.42	21.92
12.54	2.33
16.80	7.93
17.47	7.46
35.24	16.33
50.86	30.32
30.25	17.72
38.41	19.12
36.82	13.53
41.74	19.12
46.65	20.06
25.14	10.73
11.08	5.13
35.89	16.33
41.06	28.45
40.17	20.52
53.63	30.78
23.95	13.99
32.04	23.32
27.29	10.26
13.96	8.86

19.38	11.66
24.82	15.39
32.51	16.33
36.03	18.66
52.68	30.32
36.09	14.93
24.57	30.78
51.83	24.25
54.60	36.85
53.22	35.91
52.05	33.12
33.40	16.33
32.74	21.46
43.20	20.99
53.31	29.85
33.39	26.12
39.14	30.78
41.12	12.59
55.33	34.98
49.17	27.99
44.80	15.39
45.32	7.00
46.58	18.66
34.27	34.98
31.84	20.52
30.10	29.85
38.41	35.91
23.99	38.25
25.86	14.93
14.93	14.93
20.95	12.13
30.65	27.52
18.96	27.52
43.09	31.72
41.24	30.78
39.46	37.78
22.13	52.24
42.40	20.99
36.22	38.25
46.81	40.11
44.10	41.98
28.41	28.45
57.50	36.38

70.22	45.71
30.03	23.79
46.17	37.31
49.21	38.25
42.58	41.98
25.68	16.33
41.06	38.25
56.27	40.58
12.30	8.86
11.87	7.93
36.20	18.19
38.65	44.31
35.30	27.52
39.09	36.38
41.83	41.51
56.58	47.58
30.27	16.33
15.62	17.72
24.70	17.26
51.78	43.38
35.19	38.71
48.08	43.84
45.85	34.05
61.15	58.77
73.98	70.43
23.18	13.53
53.45	45.71
56.64	50.37
31.78	68.56
70.25	70.43
70.64	72.76
34.87	37.78
48.06	48.97
41.20	42.44
45.41	48.97
38.99	23.79
66.52	43.38
66.93	53.64
48.33	32.18
9.82	9.80
36.89	18.66
46.57	47.11
38.48	37.31

40.49	40.58
45.27	56.44
58.61	62.03
35.41	20.52
13.25	6.06
30.30	17.72
32.87	56.44
45.26	48.97
50.39	54.10
48.22	38.25
43.08	64.37
82.87	82.56
23.50	22.85
43.74	36.85
64.41	53.64
55.45	57.84
35.13	84.89
70.53	89.09
72.36	97.48
57.40	44.78
58.44	50.84
53.87	52.71
48.82	58.77
42.33	34.98
51.38	44.78
73.78	67.16
41.01	44.78
8.50	8.86
29.03	24.72
46.75	52.24
31.88	27.99
42.85	46.18
47.93	64.37
31.82	69.96
22.70	28.45
12.16	26.12
32.87	20.52
35.46	62.97
65.76	64.37
52.08	61.57
49.91	41.51
44.99	67.63
71.33	90.49

43.37	30.32
40.96	36.85
54.93	57.37
60.12	67.16
90.91	96.55
99.99	102.61
76.25	105.88
40.08	48.51
57.92	58.30
50.13	65.30
53.00	68.10
35.88	40.11
44.43	43.84
76.33	76.49
53.66	50.84
10.88	9.33
31.35	18.19
50.30	61.10
44.96	29.38
38.14	52.71
49.17	72.76
34.76	77.43
39.43	36.85
47.82	47.11
13.97	20.06
37.81	66.70
75.98	77.89
53.20	63.43
50.36	48.04
45.10	78.83
89.60	94.22
32.84	32.65
56.49	48.51
62.19	60.63
62.82	69.96
87.08	98.41
87.33	104.48
80.92	97.48
63.38	58.30
56.97	65.77
56.26	73.69
55.34	79.76
29.24	17.26

45.92	41.05
77.49	83.49
61.38	54.10
9.11	7.00
42.04	32.18
52.17	65.30
37.56	29.85
44.65	59.24
49.14	76.49
36.69	76.03
51.76	48.04
41.15	36.38
24.68	15.86
39.57	66.23
66.46	64.37
53.33	62.50
49.74	49.91
43.79	77.89
71.38	94.68
42.10	32.18
40.90	47.11
67.12	66.23
69.77	72.30
102.60	101.21
91.62	100.75
83.53	98.41
66.37	61.10
65.19	66.23
78.22	78.36
76.85	88.62
12.11	11.66
56.38	48.97
78.20	88.15
60.16	52.24
9.88	5.13
39.29	32.18
51.41	64.83
28.99	20.06
51.33	62.50
48.64	76.03
37.49	77.43
49.99	54.10
8.65	1.40

13.97	9.80
40.55	62.50
71.96	73.23
62.85	62.03
57.78	53.64
41.38	71.83
83.88	97.95
21.56	12.13
51.21	47.58
60.11	67.63
67.12	72.76
98.26	100.28
94.18	105.88
93.81	98.41
67.82	61.10
72.15	72.30
58.34	78.36
55.18	83.96
13.17	7.93
56.07	45.71
79.21	86.29
55.29	53.64
10.32	2.80
44.52	30.32
48.29	62.03
27.79	15.86
52.23	60.17
46.34	78.83
36.37	84.42
47.94	58.77
7.68	0.93
11.48	9.80
38.82	61.10
62.03	69.03
51.86	59.24
44.80	50.37
67.49	69.03
85.69	99.35
32.15	16.79
49.47	45.24
68.45	68.56
68.94	73.69
101.63	95.15

95.90	107.74
80.28	96.08
56.58	51.77
48.01	65.77
57.44	76.96
49.80	82.56
17.70	8.40
53.90	52.24
76.79	93.75
46.05	48.97
9.95	4.66
19.55	27.05
41.51	53.17
35.01	21.92
51.37	61.57
42.35	79.76
33.56	84.42
46.84	61.10
36.63	35.45
19.25	17.26
34.14	57.84
66.74	72.76
48.32	59.70
44.19	51.77
33.54	78.36
97.79	107.28
12.61	15.86
46.31	46.64
67.71	63.43
66.39	72.30
95.44	96.08
94.82	96.08
71.94	87.22
40.38	45.71
44.34	58.77
53.57	67.16
40.53	80.22
5.62	9.33
45.92	48.97
65.02	83.02
35.73	39.18
7.62	3.73
22.93	28.45

32.15	41.98
23.34	22.85
48.20	56.90
35.02	74.16
78.58	82.56
43.50	63.43
34.35	31.72
18.27	21.46
27.95	60.63
66.31	76.49
39.04	64.83
41.73	50.84
30.59	87.22
92.27	103.08
22.43	18.66
39.72	42.91
60.12	63.43
54.81	66.23
62.18	88.62
46.24	68.56
63.33	87.69
32.15	43.38
42.80	52.24
50.17	57.84
29.27	81.16
16.63	12.59
44.90	50.37
55.28	83.02
27.28	37.31
5.03	3.27
15.12	20.99
26.01	35.91
33.33	20.52
42.43	49.91
23.60	55.50
63.38	75.09
37.88	63.43
21.37	23.79
26.02	15.39
22.45	47.58
16.45	38.25
29.73	46.18
38.66	46.64

29.55	60.17
94.20	107.74
11.78	10.73
38.45	38.71
44.18	60.63
41.33	63.90
54.60	75.56
49.75	54.57
55.05	73.69
55.05	73.69
29.56	38.71
44.65	47.58
47.51	55.50
64.06	76.03
12.27	12.13
43.31	44.78
58.96	70.90
25.41	31.72
5.57	5.60
14.38	20.06
24.20	36.38
24.02	18.19
32.13	44.31
37.71	45.24
25.25	42.91
49.82	66.70
20.19	18.19
14.24	12.13
21.16	26.59
15.84	27.05
19.57	25.65
35.88	46.18
29.43	31.25
76.06	109.61
11.33	15.39
19.73	32.18
46.64	56.90
44.42	50.37
17.55	13.99
17.69	16.79
53.01	67.16
47.66	35.45
46.93	38.25

34.94	48.97
56.01	70.90
8.38	7.93
31.25	26.59
43.44	60.63
22.77	32.18
18.20	12.59
25.33	13.06
23.76	35.91
24.42	19.12
48.25	39.65
27.78	32.65
27.62	35.45
43.62	59.24
3.81	9.80
14.19	8.86
20.40	25.65
26.05	41.05
36.55	19.59
34.28	41.51
29.07	11.19
57.52	97.95
40.44	6.53
23.72	36.38
34.96	38.25
19.70	10.26
14.40	7.46
8.77	8.40
59.61	53.64
47.19	26.59
46.70	44.31
48.05	43.38
48.55	56.90
25.11	17.26
27.56	16.33
40.24	44.78
19.79	15.86
21.34	16.33
25.08	10.73
24.88	19.59
44.84	24.72
48.56	26.59
29.12	25.65

28.33	30.78
51.07	55.50
7.92	8.40
25.82	14.93
18.82	25.19
24.17	41.51
41.38	12.59
37.88	41.05
9.08	7.00
58.69	78.83
19.15	16.33
22.65	34.98
36.11	39.18
7.85	6.53
5.45	7.93
7.67	3.73
46.25	39.18
27.07	13.53
46.16	34.05
42.57	38.25
42.01	40.58
22.09	12.13
41.98	20.99
37.79	32.65
9.58	7.46
23.22	13.53
14.98	6.53
25.09	20.99
44.16	33.58
45.41	28.45
28.62	25.19
29.12	26.59
58.96	50.37
8.58	8.86
24.76	18.19
28.26	22.85
25.80	16.79
4.52	2.33
40.31	26.12
8.94	1.87
58.81	65.30
37.70	26.12
24.41	30.32

48.99	46.64
8.86	4.66
3.29	1.87
7.99	3.27
44.96	34.98
47.36	12.59
45.32	27.52
39.93	32.18
43.61	31.25
18.55	9.80
46.65	29.38
35.34	24.25
10.77	5.13
23.60	13.99
6.39	3.73
45.92	31.72
43.69	35.91
41.53	29.38
37.82	29.38
29.31	22.39
11.94	45.71
8.54	13.53
42.82	29.85
28.87	19.12
29.43	12.59
23.41	14.46
4.25	4.20
7.85	4.20
48.98	54.57
36.27	20.06
36.24	31.72
49.94	45.24
3.92	1.40
8.66	1.40
7.57	5.60
43.66	28.92
