# Supplementary material for: The Effect of Elevated Ozone Concentrations with Varying Shading on Dry Matter Loss in a Winter Wheat-Producing Region in China
Source: PLoS One. 2016 Jan 13;11(1):e0145446. doi: 10.1371/journal.pone.0145446 (PMC4711948; doi:10.1371/journal.pone.0145446)
Supplement: S1 File — (PDF) [file pone.0145446.s011.pdf]

S1 File. The temporal and spatial variations of simulated cumulative ozone uptake under elevated ozone concentrations and reduced solar radiation conditions in the major areas for production of winter wheat (from March to May of 2006). (unit: mmol O_3 m^{-2})

The temporal and spatial variations of simulated cumulative ozone uptake in CK (March)

```
%%Pages: (atend)
%%PageOrder: Ascend
%%Creator: NCAR GKS
%%DocumentFonts: (atend)
%%EndComments
%%BeginProlog

/M3
{
  2 copy lt {pop} {exch pop} ifelse
  2 copy lt {pop} {exch pop} ifelse
} bind def
/Sc
{
  1. exch sub /ylo exch def
  1. exch sub /mag exch def
  1. exch sub /cyn exch def
  ylo mag cyn M3 /b_cmy exch def
  cyn b_cmy sub /c_cmy exch def
  mag b_cmy sub /m_cmy exch def
  ylo b_cmy sub /y_cmy exch def
  c_cmy m_cmy y_cmy b_cmy setcmykcolor
} bind def
/P
{/n exch def
  newpath
  moveto
  2 1 n {pop lineto} for
  stroke
 } bind def

/F
{/n exch def
  newpath
  moveto
  2 1 n {pop lineto} for
```

```
  closepath
  eofill
} bind def

/B
{ /s exch def
  /n exch def
  1 1 n {newpath pop s 0 360 arc stroke} for
} bind def

/Q
{ /s exch def
  /n exch def
  /ms 0 s sub def
  /ts s s add def
  1 1 n {gsave newpath pop translate
  0 0 moveto ms 0 rmoveto ts 0 rlineto
  0 0 moveto 90 rotate ms  0 rmoveto ts 0 rlineto
  stroke grestore} for
} bind def

/Fs
{ /s exch def
  findfont
  s scalefont
  setfont
} bind def

/V
{ /s exch def
  /n exch def
  /ms 0 s sub def
  /ts s s add def
  1 1 n {gsave newpath pop translate
  0 0 moveto 30 rotate ms 0 rmoveto  ts 0 rlineto
  0 0 moveto 60 rotate ms 0 rmoveto  ts 0 rlineto
  0 0 moveto 60 rotate ms 0 rmoveto  ts 0 rlineto
  stroke grestore} for
} bind def

/X
{ /s exch def
  /n exch def
```

```
  /s s 1.25 mul def
  /ms 0 s sub def
  /ts s s add def
  1 1 n {gsave newpath pop translate
  0 0 moveto 45 rotate ms 0 rmoveto  ts 0 rlineto
  0 0 moveto 90 rotate ms 0 rmoveto  ts 0 rlineto
  stroke grestore} for
} bind def

/Ls
{matrix currentmatrix
 currentrgbcolor
 currentlinewidth
 currentlinecap
 currentlinejoin
 currentdash
} bind def

/Lr
{setdash
 setlinejoin
 setlinecap
 setlinewidth
 setrgbcolor
 setmatrix
} bind def

/M {stroke newpath moveto} bind def
/L {lineto} bind def
/E {eofill} def
/J {setlinejoin} def
/C {setlinecap} def
/W {setlinewidth} def
/S {stroke showpage} def
/A {scale} def
/U {put} def
/D {setdash} def
/I {currentdash} def
/H {currentlinewidth} def
/G {currentrgbcolor} def
/R {setrgbcolor} def
/N {closepath} def
/K {stroke} def
```

```
/Z {rotate} def
/As {ashow} def
/Ch {clippath} def
/Ci {colorimage} def
/Co {concat} def
/Cm {currentmatrix} def
/Cp {clip} def
/Ct {currentpoint} def
/Cu {currentfile} def
/Ex {exch} def
/Im {image} def
/In {initgraphics} def
/Ml {setmiterlimit} def
/Mx {matrix} def
/Np {newpath} def
/Ng {neg} def
/Rh {readhexstring} def
/Rm {rmoveto} def
/Sm {setmatrix} def
/Tr {translate} def
/Gs {gsave} def
/Gr {grestore} def

/Ov
{/strn exch def
 /ispc exch def
 /str1 1 string def
  strn
  {/code exch def
   /chr str1 0 code put str1 def
   0 ispc neg rmoveto
   gsave
   chr stringwidth pop 2 div neg
   0 rmoveto
   chr false charpath stroke
   grestore
  } forall
} bind def

/Oh
{/strn exch def
 /ispc exch def
 /tstr 1 string def
```

```
 strn
   {
    /code exch def
    /chr tstr 0 code put tstr def
    chr false charpath
    currentpoint
    /yt exch def /xt exch def
    stroke
    xt ispc add yt moveto
   } forall
 } bind def

/T 256 array def
/O
{ T
  exch
  get
  aload
  pop
  setrgbcolor
} bind def

/Y
{/strn exch def
 /ispc exch def
 /str1 1 string def
  strn
  {/code exch def
   /chr str1 0 code put str1 def
    0 ispc neg rmoveto
    gsave
      chr stringwidth pop 2 div neg 0 rmoveto
      chr show
    grestore
  } forall
} def

/Bg
{/ury Ex def /urx Ex def /lly Ex def /llx Ex def
 Np Ls Gr Gs 0 O Ch E Lr
 llx lly M urx lly L urx ury L
 llx ury L llx lly L
 Cp Np
```

```
} def

/Oc
{/ury exch def
 /urx exch def
 /lly exch def
 /llx exch def
 Np
 llx lly M urx lly L urx ury L  llx ury L llx lly L
 Cp Np
} def

/Cc
{/CellWidth exch def
 /strn CellWidth 1 add string def
 /strn1 CellWidth 1 add 3 mul string def
 systemdict /colorimage known
 { { Cu strn1 Rh pop } false 3 Ci}
 { { Cu strn1 Rh pop
     /ColorStr exch def
     0 1 CellWidth {
       /YIndx exch def
       /GrayVal YIndx 3 mul def
       strn YIndx
         ColorStr GrayVal       get 0.30 mul
         ColorStr GrayVal 1 add get 0.59 mul
         ColorStr GrayVal 2 add get 0.11 mul
         add add cvi
       put
     } for
     strn
   } Im
 } ifelse
} def

/Ld
{/ury exch def /urx exch def  /lly exch def /llx exch def
 -90 rotate
 lly urx add neg llx lly sub translate
} bind def
/Pt
{/ury exch def /urx exch def  /lly exch def /llx exch def
 90 rotate
```

```
 lly urx add lly llx sub translate
} bind def
%%EndProlog

%%BeginSetup
<</PageSize [00612 00792]>> setpagedevice
%%EndSetup

[1. 1. 1.] [0. 0. 0.] [1. 0. 0.] [0. 1. 0.] [0. 0. 1.] [0. 1. 1.] [1. 0. .8]
[1. 1. 0.] [1. .5 0.] [.6 .8 0.] [0. 1. .6] [0. .5 1.] [.5 0. .8] [1. 0. .6]
[.3 .3 .3] [.7 .7 .7] [1. 1. .3] [.7 1. .5] [.5 1. .6] [.2 1. .7] [.3 .8 .8]
[.5 .7 .8] [.7 .5 .8] [1. .3 .9] [.7 .9 .5] [.4 .9 .5] [.2 .9 .7] [.2 .7 .9]
[.2 .5 1.] [.5 .3 1.] [.7 .2 1.] [.9 .1 1.] [.9 1. .2] [.7 1. .3] [.5 1. .3]
[.2 1. .5] [.2 .8 .6] [.2 .7 .7] [.2 .5 .8] [.2 .4 .9] [.4 .3 .9] [.7 .2 .9]
[.8 .2 .8] [.9 .3 .7] [.8 .4 .6] [.8 .6 .5] [.9 .7 .4] [.9 .7 .3] [1. .9 .9]
[.8 1. .1] [.6 1. .2] [.5 1. .2] [.2 .9 .5] [.2 .8 .5] [.2 .7 .7] [.2 .5 .7]
[.2 .4 .9] [.4 .3 .9] [.5 .2 .9] [.8 .2 .7] [1. .2 .7] [1. .3 .6] [1. .4 .5]
[1. .5 .4] [1. .8 .1] [.7 1. .0] [.6 1. .1] [.4 1. .2] [.1 1. .3] [.1 .8 .4]
[.2 .7 .5] [.1 .6 .7] [.1 .5 .7] [.1 .4 .8] [.2 .3 .9] [.2 .2 1.] [.3 .1 1.]
[.5 .1 .9] [.7 .0 .8] [.9 .0 .7] [.9 .7 .1] [.7 .9 .1] [.5 .9 .1] [.3 .9 .2]
[.1 .9 .3] [.2 .7 .4] [.1 .7 .5] [.1 .5 .6] [.1 .4 .7] [.2 .3 .7] [.2 .2 .8]
[.3 .2 .8] [.5 .1 .7] [.7 .1 .7] [.9 .2 .5] [.8 .2 .5] [.8 .7 .0] [.6 .8 .1]
[.4 .9 .1] [.3 .9 .1] [.1 .9 .2] [.2 .8 .2] [.2 .7 .3] [.2 .6 .5] [.1 .5 .5]
[.1 .4 .6] [.2 .3 .7] [.3 .2 .7] [.4 .2 .6] [.7 .2 .5] [.8 .2 .4] [1. .2 .3]
[.7 .7 .0] [.5 .7 .1] [.4 .8 .1] [.2 .9 .1] [.2 .9 .1] [.2 .8 .2] [.1 .7 .2]
[.1 .7 .3] [.1 .6 .4] [.1 .4 .5] [.1 .4 .5] [.2 .3 .5] [.4 .3 .4] [.5 .3 .4]
[.7 .3 .3] [.8 .3 .2] [.7 .6 .0] [.5 .7 .1] [.4 .7 .1] [.2 .7 .2] [.1 .7 .2]
[.1 .6 .3] [.1 .5 .4] [.1 .4 .4] [.1 .3 .5] [.1 .2 .7] [.1 .1 .7] [.4 .1 .5]
[.5 .1 .5] [.6 .1 .4] [.7 .1 .4] [.9 .1 .3] [.5 .5 .1] [.4 .6 .1] [.3 .6 .1]
[.2 .6 .2] [.1 .5 .2] [.1 .4 .3] [.1 .4 .4] [.2 .3 .4] [.2 .2 .4] [.2 .2 .5]
[.2 .1 .5] [.3 .1 .5] [.4 .1 .4] [.5 .1 .4] [.7 .1 .3] [.8 .1 .2] [.5 .4 .1]
[.4 .5 .1] [.3 .6 .0] [.2 .6 .1] [.1 .7 .1] [.1 .6 .1] [.1 .5 .2] [.1 .4 .2]
[.1 .4 .2] [.1 .3 .3] [.1 .2 .4] [.1 .2 .5] [.1 .1 .5] [.1 .0 .6] [.3 .0 .5]
[.5 .0 .4] [.7 .3 .0] [.7 .4 .0] [.5 .4 .1] [.3 .4 .2] [.2 .3 .2] [.2 .2 .3]
[.2 .2 .4] [.3 .1 .4] [.5 .1 .3] [.6 .1 .2] [.7 .1 .2] [.8 .0 .2] [1. .0 .2]
[1. .1 .1] [.9 .2 .1] [.8 .2 .1] [.6 .2 .0] [.4 .4 .0] [.2 .4 .1] [.2 .3 .2]
[.2 .2 .2] [.2 .2 .3] [.2 .1 .3] [.3 .1 .3] [.5 .1 .2] [.7 .1 .2] [.8 .0 .2]
[.9 .0 .1] [1. .0 .1] [1. .1 .1] [.8 .2 .0] [.7 .2 .0] [.5 .2 .0] [.4 .3 .0]
[.1 .4 .1] [.1 .4 .1] [.1 .3 .2] [.1 .2 .2] [.1 .2 .3] [.2 .1 .4] [.2 .0 .3]
[.4 .0 .2] [.6 .0 .2] [.7 .0 .1] [.8 .0 .1] [.7 .1 .1] [.7 .1 .1] [.6 .2 .0]
[.4 .2 .0] [.2 .3 .0] [.1 .3 .0] [.1 .2 .1] [.1 .2 .1] [.1 .1 .2] [.1 .1 .3]
[.1 .0 .3] [.1 .0 .2] [.2 .0 .2] [.3 .0 .2] [.3 .0 .1] [.4 .0 .1] [.4 .1 .1]
[.4 .1 .0] [.2 .1 .0] [.0 .0 .0] [.0 .0 .0] [.0 .0 .0] [.0 .0 .0] [.0 .0 .0]
```

```
[.0 .0 .0] [.0 .0 .0] [.0 .0 .0] [.0 .0 .0] [.0 .0 .0] [.0 .0 .0] [.0 .0 .0]
[.0 .0 .0] [.0 .0 .0] [.0 .0 .0] [.0 .0 .0]  T astore pop
%%Page: 1 1
0.04000 0.04000 A 1 J 1 C 12 W [] 0 D
T   0 [1.000 1.000 1.000] U
T   1 [0.000 0.000 0.000] U
T   2 [0.000 0.000 1.000] U
T   3 [0.000 0.031 1.000] U
T   4 [0.000 0.059 1.000] U
T   5 [0.000 0.090 1.000] U
T   6 [0.000 0.122 1.000] U
T   7 [0.000 0.153 1.000] U
T   8 [0.000 0.180 1.000] U
T   9 [0.000 0.212 1.000] U
T  10 [0.000 0.243 1.000] U
T  11 [0.000 0.275 1.000] U
T  12 [0.000 0.302 1.000] U
T  13 [0.000 0.333 1.000] U
T  14 [0.000 0.365 1.000] U
T  15 [0.000 0.392 1.000] U
T  16 [0.000 0.424 1.000] U
T  17 [0.000 0.455 1.000] U
T  18 [0.000 0.486 1.000] U
T  19 [0.000 0.514 1.000] U
T  20 [0.000 0.545 1.000] U
T  21 [0.000 0.576 1.000] U
T  22 [0.000 0.608 1.000] U
T  23 [0.000 0.635 1.000] U
T  24 [0.000 0.667 1.000] U
T  25 [0.000 0.698 1.000] U
T  26 [0.000 0.725 1.000] U
T  27 [0.000 0.757 1.000] U
T  28 [0.000 0.788 1.000] U
T  29 [0.000 0.820 1.000] U
T  30 [0.000 0.847 1.000] U
T  31 [0.000 0.878 1.000] U
T  32 [0.000 0.910 1.000] U
T  33 [0.000 0.941 1.000] U
T  34 [0.000 0.969 1.000] U
T  35 [0.000 1.000 1.000] U
T  36 [0.000 0.992 0.973] U
T  37 [0.000 0.980 0.941] U
T  38 [0.004 0.973 0.914] U
```

T 39 [0.004 0.965 0.886] U
T 40 [0.004 0.953 0.859] U
T 41 [0.004 0.945 0.827] U
T 42 [0.008 0.937 0.800] U
T 43 [0.008 0.929 0.773] U
T 44 [0.008 0.918 0.745] U
T 45 [0.008 0.910 0.714] U
T 46 [0.012 0.902 0.686] U
T 47 [0.012 0.890 0.659] U
T 48 [0.012 0.882 0.627] U
T 49 [0.012 0.875 0.600] U
T 50 [0.016 0.863 0.573] U
T 51 [0.016 0.855 0.545] U
T 52 [0.016 0.847 0.514] U
T 53 [0.016 0.839 0.486] U
T 54 [0.020 0.827 0.459] U
T 55 [0.020 0.820 0.431] U
T 56 [0.020 0.812 0.400] U
T 57 [0.020 0.800 0.373] U
T 58 [0.024 0.792 0.345] U
T 59 [0.024 0.784 0.314] U
T 60 [0.024 0.773 0.286] U
T 61 [0.024 0.765 0.259] U
T 62 [0.027 0.757 0.231] U
T 63 [0.027 0.749 0.200] U
T 64 [0.027 0.737 0.173] U
T 65 [0.027 0.729 0.145] U
T 66 [0.031 0.722 0.118] U
T 67 [0.031 0.710 0.086] U
T 68 [0.031 0.702 0.059] U
T 69 [0.059 0.710 0.059] U
T 70 [0.090 0.718 0.055] U
T 71 [0.118 0.729 0.055] U
T 72 [0.145 0.737 0.051] U
T 73 [0.173 0.745 0.051] U
T 74 [0.204 0.753 0.047] U
T 75 [0.231 0.765 0.047] U
T 76 [0.259 0.773 0.043] U
T 77 [0.286 0.780 0.043] U
T 78 [0.318 0.788 0.043] U
T 79 [0.345 0.800 0.039] U
T 80 [0.373 0.808 0.039] U
T 81 [0.400 0.816 0.035] U

T 82 [0.431 0.824 0.035] U
T 83 [0.459 0.835 0.031] U
T 84 [0.486 0.843 0.031] U
T 85 [0.518 0.851 0.031] U
T 86 [0.545 0.859 0.027] U
T 87 [0.573 0.867 0.027] U
T 88 [0.600 0.878 0.024] U
T 89 [0.631 0.886 0.024] U
T 90 [0.659 0.894 0.020] U
T 91 [0.686 0.902 0.020] U
T 92 [0.714 0.914 0.016] U
T 93 [0.745 0.922 0.016] U
T 94 [0.773 0.929 0.016] U
T 95 [0.800 0.937 0.012] U
T 96 [0.827 0.949 0.012] U
T 97 [0.859 0.957 0.008] U
T 98 [0.886 0.965 0.008] U
T 99 [0.914 0.973 0.004] U
T 100 [0.941 0.984 0.004] U
T 101 [0.973 0.992 0.000] U
T 102 [1.000 1.000 0.000] U
T 103 [1.000 0.988 0.000] U
T 104 [1.000 0.980 0.000] U
T 105 [1.000 0.969 0.000] U
T 106 [1.000 0.957 0.000] U
T 107 [1.000 0.945 0.000] U
T 108 [1.000 0.937 0.000] U
T 109 [1.000 0.925 0.000] U
T 110 [1.000 0.914 0.000] U
T 111 [1.000 0.902 0.000] U
T 112 [1.000 0.894 0.000] U
T 113 [1.000 0.882 0.000] U
T 114 [1.000 0.871 0.000] U
T 115 [1.000 0.863 0.000] U
T 116 [1.000 0.851 0.000] U
T 117 [1.000 0.839 0.000] U
T 118 [1.000 0.827 0.000] U
T 119 [1.000 0.820 0.000] U
T 120 [1.000 0.808 0.000] U
T 121 [1.000 0.796 0.000] U
T 122 [1.000 0.784 0.000] U
T 123 [1.000 0.776 0.000] U
T 124 [1.000 0.765 0.000] U

T 125 [1.000 0.753 0.000] U
T 126 [1.000 0.745 0.000] U
T 127 [1.000 0.733 0.000] U
T 128 [1.000 0.722 0.000] U
T 129 [1.000 0.710 0.000] U
T 130 [1.000 0.702 0.000] U
T 131 [1.000 0.690 0.000] U
T 132 [1.000 0.678 0.000] U
T 133 [1.000 0.667 0.000] U
T 134 [1.000 0.659 0.000] U
T 135 [1.000 0.647 0.000] U
T 136 [1.000 0.627 0.000] U
T 137 [1.000 0.608 0.000] U
T 138 [1.000 0.588 0.000] U
T 139 [1.000 0.569 0.000] U
T 140 [1.000 0.549 0.000] U
T 141 [1.000 0.529 0.000] U
T 142 [1.000 0.510 0.000] U
T 143 [1.000 0.490 0.000] U
T 144 [1.000 0.471 0.000] U
T 145 [1.000 0.451 0.000] U
T 146 [1.000 0.431 0.000] U
T 147 [1.000 0.412 0.000] U
T 148 [1.000 0.392 0.000] U
T 149 [1.000 0.373 0.000] U
T 150 [1.000 0.353 0.000] U
T 151 [1.000 0.333 0.000] U
T 152 [1.000 0.314 0.000] U
T 153 [1.000 0.294 0.000] U
T 154 [1.000 0.275 0.000] U
T 155 [1.000 0.255 0.000] U
T 156 [1.000 0.235 0.000] U
T 157 [1.000 0.216 0.000] U
T 158 [1.000 0.196 0.000] U
T 159 [1.000 0.176 0.000] U
T 160 [1.000 0.157 0.000] U
T 161 [1.000 0.137 0.000] U
T 162 [1.000 0.118 0.000] U
T 163 [1.000 0.098 0.000] U
T 164 [1.000 0.078 0.000] U
T 165 [1.000 0.059 0.000] U
T 166 [1.000 0.039 0.000] U
T 167 [1.000 0.020 0.000] U

T 168 [1.000 0.000 0.000] U
T 169 [0.980 0.000 0.016] U
T 170 [0.961 0.000 0.031] U
T 171 [0.941 0.000 0.047] U
T 172 [0.922 0.000 0.063] U
T 173 [0.902 0.000 0.082] U
T 174 [0.878 0.000 0.098] U
T 175 [0.859 0.000 0.114] U
T 176 [0.839 0.000 0.129] U
T 177 [0.820 0.000 0.145] U
T 178 [0.800 0.000 0.161] U
T 179 [0.780 0.000 0.176] U
T 180 [0.761 0.000 0.192] U
T 181 [0.741 0.000 0.212] U
T 182 [0.722 0.000 0.227] U
T 183 [0.702 0.000 0.243] U
T 184 [0.682 0.000 0.259] U
T 185 [0.659 0.000 0.275] U
T 186 [0.639 0.000 0.290] U
T 187 [0.620 0.000 0.306] U
T 188 [0.600 0.000 0.322] U
T 189 [0.580 0.000 0.341] U
T 190 [0.561 0.000 0.357] U
T 191 [0.541 0.000 0.373] U
T 192 [0.522 0.000 0.388] U
T 193 [0.502 0.000 0.404] U
T 194 [0.482 0.000 0.420] U
T 195 [0.463 0.000 0.435] U
T 196 [0.439 0.000 0.451] U
T 197 [0.420 0.000 0.471] U
T 198 [0.400 0.000 0.486] U
T 199 [0.380 0.000 0.502] U
T 200 [0.361 0.000 0.518] U
T 201 [0.341 0.000 0.533] U
-1150 1075 16450 18700 Oc
Gs
1 O
0 O
2303 16210 M 2303 5501 L 2336 5501 L 2369 5501 L 2402 5501 L
2435 5501 L 2468 5501 L 2501 5501 L 2534 5501 L
2568 5501 L 2601 5501 L 2634 5501 L 2667 5501 L
2700 5501 L 2733 5501 L 2766 5501 L 2799 5501 L
2832 5501 L 2865 5501 L 2899 5501 L 2930 5501 L

2929 5504 L 2919 5532 L 2941 5551 L 2970 5550 L
2988 5528 L 2970 5505 L 2970 5501 L 2998 5501 L
3031 5501 L 3064 5501 L 3097 5501 L 3130 5501 L
3163 5501 L 3197 5501 L 3230 5501 L 3263 5501 L
3296 5501 L 3329 5501 L 3362 5501 L 3395 5501 L
3428 5501 L 3461 5501 L 3495 5501 L 3528 5501 L
3561 5501 L 3594 5501 L 3627 5501 L 3660 5501 L
3693 5501 L 3726 5501 L 3759 5501 L 3792 5501 L
3826 5501 L 3859 5501 L 3892 5501 L 3925 5501 L
3958 5501 L 3991 5501 L 4024 5501 L 4057 5501 L
4090 5501 L 4124 5501 L 4157 5501 L 4190 5501 L
4223 5501 L 4256 5501 L 4289 5501 L 4322 5501 L
4355 5501 L 4388 5501 L 4422 5501 L 4455 5501 L
4488 5501 L 4521 5501 L 4554 5501 L 4587 5501 L
4620 5501 L 4653 5501 L 4686 5501 L 4719 5501 L
4753 5501 L 4786 5501 L 4819 5501 L 4852 5501 L
4885 5501 L 4918 5501 L 4951 5501 L 4984 5501 L
5017 5501 L 5051 5501 L 5084 5501 L 5117 5501 L
5150 5501 L 5183 5501 L 5216 5501 L 5249 5501 L
5282 5501 L 5315 5501 L 5349 5501 L 5382 5501 L
5396 5501 L 5409 5511 L 5407 5540 L 5404 5569 L
5405 5598 L 5405 5628 L 5395 5654 L 5371 5671 L
5353 5694 L 5382 5690 L 5388 5661 L 5417 5660 L
5417 5631 L 5412 5602 L 5411 5573 L 5413 5544 L
5432 5522 L 5431 5501 L 5448 5501 L 5481 5501 L
5514 5501 L 5533 5501 L 5540 5514 L 5541 5521 L
5545 5528 L 5552 5533 L 5538 5570 L 5530 5598 L
5548 5576 L 5557 5548 L 5574 5525 L 5593 5503 L
5595 5501 L 5613 5501 L 5645 5501 L 5646 5518 L
5650 5501 L 5658 5501 L 5666 5516 L 5676 5543 L
5690 5568 L 5684 5597 L 5660 5613 L 5668 5641 L
5682 5666 L 5683 5695 L 5667 5720 L 5691 5736 L
5675 5761 L 5655 5782 L 5677 5762 L 5704 5772 L
5687 5795 L 5671 5819 L 5651 5840 L 5675 5824 L
5703 5831 L 5689 5857 L 5665 5873 L 5655 5900 L
5628 5911 L 5599 5912 L 5628 5917 L 5656 5922 L
5662 5950 L 5664 5979 L 5646 6002 L 5618 6006 L
5601 6030 L 5629 6021 L 5655 6010 L 5681 5996 L
5678 5967 L 5685 5938 L 5706 5919 L 5724 5942 L
5712 5915 L 5707 5886 L 5714 5875 L 5722 5862 L
5740 5839 L 5746 5811 L 5773 5821 L 5791 5799 L
5798 5771 L 5800 5767 L 5812 5746 L 5824 5719 L
5843 5697 L 5860 5674 L 5854 5645 L 5872 5667 L

5899 5678 L 5927 5671 L 5956 5668 L 5929 5658 L
5903 5644 L 5881 5625 L 5861 5605 L 5881 5584 L
5910 5580 L 5939 5580 L 5968 5581 L 5986 5559 L
6014 5551 L 6044 5553 L 6070 5541 L 6051 5562 L
6059 5590 L 6086 5580 L 6114 5588 L 6119 5616 L
6091 5610 L 6074 5634 L 6052 5615 L 6024 5607 L
6009 5631 L 6037 5636 L 6066 5637 L 6088 5657 L
6062 5670 L 6036 5683 L 6058 5702 L 6086 5709 L
6114 5715 L 6142 5708 L 6160 5686 L 6169 5658 L
6173 5629 L 6201 5636 L 6229 5643 L 6235 5671 L
6208 5681 L 6182 5695 L 6209 5706 L 6210 5735 L
6181 5735 L 6203 5754 L 6216 5779 L 6243 5792 L
6247 5793 L 6257 5796 L 6270 5801 L 6296 5813 L
6325 5807 L 6346 5827 L 6338 5799 L 6315 5781 L
6318 5753 L 6315 5724 L 6325 5697 L 6354 5697 L
6383 5694 L 6403 5673 L 6400 5702 L 6416 5726 L
6431 5750 L 6409 5768 L 6393 5793 L 6418 5778 L
6430 5751 L 6459 5755 L 6473 5781 L 6487 5806 L
6516 5813 L 6545 5812 L 6569 5828 L 6599 5816 L
6578 5808 L 6576 5799 L 6595 5800 L 6615 5801 L
6641 5787 L 6666 5774 L 6637 5772 L 6649 5746 L
6677 5739 L 6706 5744 L 6731 5731 L 6758 5718 L
6754 5747 L 6768 5772 L 6747 5753 L 6730 5776 L
6748 5799 L 6765 5823 L 6786 5842 L 6815 5844 L
6839 5828 L 6867 5820 L 6882 5796 L 6900 5773 L
6921 5792 L 6949 5800 L 6977 5809 L 7003 5820 L
7027 5836 L 7030 5865 L 7046 5842 L 7031 5817 L
7060 5817 L 7082 5835 L 7102 5856 L 7125 5874 L
7150 5889 L 7173 5872 L 7201 5866 L 7229 5874 L
7258 5872 L 7267 5900 L 7288 5920 L 7284 5948 L
7278 5977 L 7291 6002 L 7296 6031 L 7271 6016 L
7268 6045 L 7297 6047 L 7320 6029 L 7341 6010 L
7358 6033 L 7379 6044 L 7361 6060 L 7343 6076 L
7315 6082 L 7292 6100 L 7279 6126 L 7274 6154 L
7245 6159 L 7274 6158 L 7294 6137 L 7317 6120 L
7343 6108 L 7349 6108 L 7372 6108 L 7401 6107 L
7423 6131 L 7434 6144 L 7455 6164 L 7454 6175 L
7455 6201 L 7469 6227 L 7461 6250 L 7487 6261 L
7507 6266 L 7515 6269 L 7527 6263 L 7541 6255 L
7532 6283 L 7559 6273 L 7564 6244 L 7572 6216 L
7590 6232 L 7594 6235 L 7617 6252 L 7641 6268 L
7641 6297 L 7659 6274 L 7651 6247 L 7669 6269 L
7671 6298 L 7676 6326 L 7687 6353 L 7711 6337 L

7723 6353 L 7732 6352 L 7736 6352 L 7759 6336 L
7776 6359 L 7786 6386 L 7785 6415 L 7757 6421 L
7733 6438 L 7762 6435 L 7790 6429 L 7818 6421 L
7840 6402 L 7848 6375 L 7840 6347 L 7838 6319 L
7855 6342 L 7857 6370 L 7859 6399 L 7871 6426 L
7894 6442 L 7891 6471 L 7909 6493 L 7936 6502 L
7946 6475 L 7929 6452 L 7925 6423 L 7937 6449 L
7954 6473 L 7977 6490 L 8001 6505 L 8005 6533 L
8024 6556 L 8013 6582 L 8033 6603 L 8054 6583 L
8077 6601 L 8098 6620 L 8126 6626 L 8120 6654 L
8106 6679 L 8077 6679 L 8074 6708 L 8045 6705 L
8017 6702 L 7999 6706 L 7974 6719 L 7952 6737 L
7940 6749 L 7942 6764 L 7944 6761 L 7953 6749 L
7968 6752 L 7980 6753 L 7996 6754 L 8023 6744 L
8034 6741 L 8051 6736 L 8074 6753 L 8079 6781 L
8053 6794 L 8082 6795 L 8092 6822 L 8109 6799 L
8110 6802 L 8123 6824 L 8125 6853 L 8154 6853 L
8164 6827 L 8157 6799 L 8184 6792 L 8185 6792 L
8197 6818 L 8225 6810 L 8225 6810 L 8253 6815 L
8273 6827 L 8278 6829 L 8272 6857 L 8295 6875 L
8285 6848 L 8310 6835 L 8336 6822 L 8360 6807 L
8359 6779 L 8384 6792 L 8405 6813 L 8393 6839 L
8395 6868 L 8423 6865 L 8437 6890 L 8440 6919 L
8425 6943 L 8402 6925 L 8382 6945 L 8378 6974 L
8354 6990 L 8380 6978 L 8408 6984 L 8401 7012 L
8395 7040 L 8415 7020 L 8416 6992 L 8429 7017 L
8441 6992 L 8436 6963 L 8464 6971 L 8482 6993 L
8510 6989 L 8537 6978 L 8561 6993 L 8534 7000 L
8529 7028 L 8558 7030 L 8586 7028 L 8573 7054 L
8574 7082 L 8561 7057 L 8534 7065 L 8509 7080 L
8501 7107 L 8528 7114 L 8545 7138 L 8561 7161 L
8541 7181 L 8521 7202 L 8548 7212 L 8576 7215 L
8604 7208 L 8587 7185 L 8613 7197 L 8611 7168 L
8601 7142 L 8629 7138 L 8654 7126 L 8655 7126 L
8641 7151 L 8632 7178 L 8647 7202 L 8649 7173 L
8672 7157 L 8701 7159 L 8729 7163 L 8735 7191 L
8753 7196 L 8763 7198 L 8743 7219 L 8726 7242 L
8703 7225 L 8676 7216 L 8686 7242 L 8662 7258 L
8639 7275 L 8618 7294 L 8646 7302 L 8668 7319 L
8693 7334 L 8713 7354 L 8729 7378 L 8740 7404 L
8746 7400 L 8764 7389 L 8754 7362 L 8779 7349 L
8807 7346 L 8812 7318 L 8818 7309 L 8829 7295 L
8827 7266 L 8841 7291 L 8851 7317 L 8879 7309 L

8879 7280 L 8880 7252 L 8889 7259 L 8901 7270 L
8902 7271 L 8907 7299 L 8900 7327 L 8873 7336 L
8867 7364 L 8853 7389 L 8828 7377 L 8820 7404 L
8806 7429 L 8823 7452 L 8850 7463 L 8878 7468 L
8882 7476 L 8891 7493 L 8883 7520 L 8881 7549 L
8890 7576 L 8908 7577 L 8918 7578 L 8927 7605 L
8934 7632 L 8906 7635 L 8880 7648 L 8853 7656 L
8833 7677 L 8849 7701 L 8869 7720 L 8870 7720 L
8886 7743 L 8897 7769 L 8869 7765 L 8893 7781 L
8920 7787 L 8937 7791 L 8948 7795 L 8972 7810 L
8975 7810 L 9000 7815 L 9028 7819 L 9035 7828 L
9045 7842 L 9019 7831 L 8995 7846 L 8978 7868 L
8961 7846 L 8952 7819 L 8926 7806 L 8904 7824 L
8898 7852 L 8885 7877 L 8858 7869 L 8872 7894 L
8894 7911 L 8916 7892 L 8943 7886 L 8966 7868 L
8981 7892 L 8970 7918 L 8966 7946 L 8946 7967 L
8919 7972 L 8890 7972 L 8864 7962 L 8842 7980 L
8840 8008 L 8867 8018 L 8843 8034 L 8848 8062 L
8845 8090 L 8871 8079 L 8893 8061 L 8918 8048 L
8908 8075 L 8901 8102 L 8899 8130 L 8909 8104 L
8926 8081 L 8941 8068 L 8947 8062 L 8952 8090 L
8978 8102 L 8962 8079 L 8989 8071 L 8964 8058 L
8962 8030 L 8967 8029 L 8989 8023 L 9011 8005 L
9017 8033 L 9033 8056 L 9042 8083 L 9070 8080 L
9094 8064 L 9081 8039 L 9056 8026 L 9058 7998 L
9037 7979 L 9008 7978 L 8993 7954 L 8985 7927 L
9013 7926 L 9034 7945 L 9043 7972 L 9071 7974 L
9074 7976 L 9095 7989 L 9099 7991 L 9121 8000 L
9120 8029 L 9141 8048 L 9115 8060 L 9126 8086 L
9102 8100 L 9077 8115 L 9090 8140 L 9117 8135 L
9124 8162 L 9146 8161 L 9153 8160 L 9180 8155 L
9178 8183 L 9151 8190 L 9179 8190 L 9198 8211 L
9203 8239 L 9194 8266 L 9222 8262 L 9237 8266 L
9249 8270 L 9264 8293 L 9250 8318 L 9245 8346 L
9255 8319 L 9279 8305 L 9290 8331 L 9306 8308 L
9312 8318 L 9321 8332 L 9322 8360 L 9325 8365 L
9338 8383 L 9325 8408 L 9331 8435 L 9356 8423 L
9384 8427 L 9383 8455 L 9397 8480 L 9371 8490 L
9358 8515 L 9339 8537 L 9367 8544 L 9386 8564 L
9386 8593 L 9389 8620 L 9417 8624 L 9429 8649 L
9444 8673 L 9462 8694 L 9478 8718 L 9476 8746 L
9451 8760 L 9423 8765 L 9404 8785 L 9376 8789 L
9348 8786 L 9346 8814 L 9368 8796 L 9396 8798 L

9416 8793 L 9423 8791 L 9428 8792 L 9445 8774 L
9473 8775 L 9495 8774 L 9501 8773 L 9528 8771 L
9527 8784 L 9541 8808 L 9539 8837 L 9552 8861 L
9566 8868 L 9577 8874 L 9592 8898 L 9602 8924 L
9580 8942 L 9603 8952 L 9606 8953 L 9615 8979 L
9641 8990 L 9668 8982 L 9651 8959 L 9660 8945 L
9666 8935 L 9674 8908 L 9692 8886 L 9712 8867 L
9733 8885 L 9727 8913 L 9748 8931 L 9761 8956 L
9776 8980 L 9804 8975 L 9804 8975 L 9827 8960 L
9845 8939 L 9857 8964 L 9844 8989 L 9835 9015 L
9821 9040 L 9823 9068 L 9807 9091 L 9794 9116 L
9786 9143 L 9770 9166 L 9743 9173 L 9716 9181 L
9744 9181 L 9772 9181 L 9800 9182 L 9824 9197 L
9836 9209 L 9843 9217 L 9864 9236 L 9855 9263 L
9842 9288 L 9815 9294 L 9800 9318 L 9826 9308 L
9854 9305 L 9873 9326 L 9852 9345 L 9863 9371 L
9837 9382 L 9810 9376 L 9784 9365 L 9805 9384 L
9833 9387 L 9830 9415 L 9803 9420 L 9776 9413 L
9748 9409 L 9749 9437 L 9773 9451 L 9781 9478 L
9785 9506 L 9809 9502 L 9813 9501 L 9831 9480 L
9832 9508 L 9843 9533 L 9834 9507 L 9836 9479 L
9845 9452 L 9872 9460 L 9893 9479 L 9900 9506 L
9898 9534 L 9905 9561 L 9926 9542 L 9917 9515 L
9920 9499 L 9922 9488 L 9923 9460 L 9951 9455 L
9974 9470 L 9979 9476 L 9992 9491 L 9987 9519 L
9962 9531 L 9965 9559 L 9962 9587 L 9988 9597 L
9989 9625 L 9973 9649 L 9976 9676 L 9993 9698 L
9974 9719 L 9947 9711 L 9920 9705 L 9896 9692 L
9895 9664 L 9911 9641 L 9887 9627 L 9880 9654 L
9852 9654 L 9833 9633 L 9806 9625 L 9783 9610 L
9765 9589 L 9773 9615 L 9746 9608 L 9731 9584 L
9723 9611 L 9734 9637 L 9752 9658 L 9779 9653 L
9806 9658 L 9834 9665 L 9855 9682 L 9873 9704 L
9890 9726 L 9913 9742 L 9931 9764 L 9953 9781 L
9967 9805 L 9989 9822 L 10002 9827 L 10008 9830 L
10015 9833 L 10038 9848 L 10045 9875 L 10021 9861 L
9993 9861 L 9965 9860 L 9961 9861 L 9954 9867 L
9943 9877 L 9916 9885 L 9890 9895 L 9863 9900 L
9835 9904 L 9811 9919 L 9797 9942 L 9781 9965 L
9762 9986 L 9745 10008 L 9726 10029 L 9707 10049 L
9684 10065 L 9660 10079 L 9633 10083 L 9605 10082 L
9577 10078 L 9553 10065 L 9530 10048 L 9511 10028 L
9489 10011 L 9466 9996 L 9444 9979 L 9418 9968 L

9390 9968 L 9364 9978 L 9345 9958 L 9370 9945 L
9343 9953 L 9334 9980 L 9340 10007 L 9323 10029 L
9313 10055 L 9325 10081 L 9301 10095 L 9273 10097 L
9245 10094 L 9223 10078 L 9212 10052 L 9196 10030 L
9168 10028 L 9146 10044 L 9127 10024 L 9104 10009 L
9123 10029 L 9140 10051 L 9168 10052 L 9194 10043 L
9193 10071 L 9201 10097 L 9226 10110 L 9253 10116 L
9281 10117 L 9308 10119 L 9336 10119 L 9363 10111 L
9382 10090 L 9410 10093 L 9435 10105 L 9451 10128 L
9467 10150 L 9468 10178 L 9483 10202 L 9503 10221 L
9527 10235 L 9554 10239 L 9570 10262 L 9592 10279 L
9615 10295 L 9642 10299 L 9666 10314 L 9681 10337 L
9701 10356 L 9726 10369 L 9752 10377 L 9779 10384 L
9806 10389 L 9834 10392 L 9862 10395 L 9889 10399 L
9912 10414 L 9919 10442 L 9909 10468 L 9895 10492 L
9882 10516 L 9869 10541 L 9856 10565 L 9843 10590 L
9828 10613 L 9810 10635 L 9788 10651 L 9765 10666 L
9740 10680 L 9713 10680 L 9697 10703 L 9675 10720 L
9650 10731 L 9626 10745 L 9608 10766 L 9594 10790 L
9576 10811 L 9554 10828 L 9530 10842 L 9509 10859 L
9483 10869 L 9457 10879 L 9429 10878 L 9402 10879 L
9377 10891 L 9358 10912 L 9380 10929 L 9367 10953 L
9354 10978 L 9339 11001 L 9311 11004 L 9284 11001 L
9256 11004 L 9230 11013 L 9203 11010 L 9191 10985 L
9164 10980 L 9137 10973 L 9113 10961 L 9086 10953 L
9058 10953 L 9044 10977 L 9071 10971 L 9099 10969 L
9125 10979 L 9146 10996 L 9169 11013 L 9190 11031 L
9214 11044 L 9239 11055 L 9265 11044 L 9292 11043 L
9319 11035 L 9345 11045 L 9360 11022 L 9383 11007 L
9400 10985 L 9419 10964 L 9429 10939 L 9435 10912 L
9461 10904 L 9489 10907 L 9516 10903 L 9531 10927 L
9558 10933 L 9583 10943 L 9609 10953 L 9611 10951 L
9634 10941 L 9661 10935 L 9688 10926 L 9705 10905 L
9731 10894 L 9756 10882 L 9782 10872 L 9809 10869 L
9835 10860 L 9862 10853 L 9888 10863 L 9894 10890 L
9883 10915 L 9869 10939 L 9857 10964 L 9847 10990 L
9828 11010 L 9810 11031 L 9788 11049 L 9764 11062 L
9738 11071 L 9712 11081 L 9685 11080 L 9659 11090 L
9648 11116 L 9628 11135 L 9642 11158 L 9637 11185 L
9637 11213 L 9624 11238 L 9602 11254 L 9576 11264 L
9551 11275 L 9526 11287 L 9501 11297 L 9475 11307 L
9447 11308 L 9428 11327 L 9410 11348 L 9382 11352 L
9398 11375 L 9416 11395 L 9403 11420 L 9377 11429 L

9398 11447 L 9406 11473 L 9405 11501 L 9394 11526 L
9388 11553 L 9378 11579 L 9351 11584 L 9336 11607 L
9331 11634 L 9315 11657 L 9296 11677 L 9285 11702 L
9275 11728 L 9256 11748 L 9250 11774 L 9252 11802 L
9261 11828 L 9239 11844 L 9222 11866 L 9215 11893 L
9219 11920 L 9208 11945 L 9200 11972 L 9174 11981 L
9180 12008 L 9154 12019 L 9154 12046 L 9145 12072 L
9144 12100 L 9135 12126 L 9125 12152 L 9117 12178 L
9109 12204 L 9096 12228 L 9091 12255 L 9078 12279 L
9053 12290 L 9028 12301 L 9001 12308 L 8982 12328 L
8957 12339 L 8931 12348 L 8904 12353 L 8878 12359 L
8865 12334 L 8864 12362 L 8846 12383 L 8820 12389 L
8798 12405 L 8774 12418 L 8750 12433 L 8729 12450 L
8715 12474 L 8711 12501 L 8684 12503 L 8659 12515 L
8635 12503 L 8613 12519 L 8594 12499 L 8601 12526 L
8587 12549 L 8586 12577 L 8595 12603 L 8595 12630 L
8599 12657 L 8613 12680 L 8634 12698 L 8661 12693 L
8672 12718 L 8675 12746 L 8683 12772 L 8691 12798 L
8704 12815 L 8708 12820 L 8732 12832 L 8751 12852 L
8755 12879 L 8769 12902 L 8778 12928 L 8786 12954 L
8780 12981 L 8807 12978 L 8830 12994 L 8839 12968 L
8852 12993 L 8873 13010 L 8889 12989 L 8901 13014 L
8915 13037 L 8926 13062 L 8949 13046 L 8957 13073 L
8954 13100 L 8965 13125 L 8984 13144 L 9009 13155 L
9016 13158 L 9033 13169 L 9025 13143 L 9039 13166 L
9061 13183 L 9073 13207 L 9046 13204 L 9019 13208 L
9035 13230 L 9009 13239 L 8985 13252 L 8977 13278 L
8987 13304 L 9012 13314 L 9038 13305 L 9059 13322 L
9063 13349 L 9082 13329 L 9097 13307 L 9092 13280 L
9082 13255 L 9071 13230 L 9097 13225 L 9123 13233 L
9144 13251 L 9171 13256 L 9198 13260 L 9222 13272 L
9244 13289 L 9239 13316 L 9235 13343 L 9219 13365 L
9225 13392 L 9251 13400 L 9242 13426 L 9257 13449 L
9282 13461 L 9303 13444 L 9327 13431 L 9328 13458 L
9346 13479 L 9345 13506 L 9320 13517 L 9304 13540 L
9278 13533 L 9295 13554 L 9318 13568 L 9344 13561 L
9358 13537 L 9385 13538 L 9385 13566 L 9410 13555 L
9429 13574 L 9452 13589 L 9478 13595 L 9504 13605 L
9529 13615 L 9552 13630 L 9569 13651 L 9565 13678 L
9578 13655 L 9598 13674 L 9625 13677 L 9604 13659 L
9589 13636 L 9615 13643 L 9642 13636 L 9641 13663 L
9667 13673 L 9690 13686 L 9703 13710 L 9728 13721 L
9753 13733 L 9777 13745 L 9766 13770 L 9793 13775 L

9807 13751 L 9816 13725 L 9836 13744 L 9859 13759 L
9858 13786 L 9875 13807 L 9883 13781 L 9866 13759 L
9882 13737 L 9907 13727 L 9880 13723 L 9884 13696 L
9911 13695 L 9929 13692 L 9938 13690 L 9960 13706 L
9968 13712 L 9982 13723 L 9995 13747 L 9997 13747 L
10007 13750 L 10022 13753 L 10011 13728 L 10024 13752 L
10028 13779 L 10027 13806 L 10000 13802 L 9973 13805 L
9993 13823 L 9994 13850 L 10009 13873 L 10033 13887 L
10055 13902 L 10039 13924 L 10031 13950 L 10036 13977 L
10054 13998 L 10065 14009 L 10073 14017 L 10046 14019 L
10022 14005 L 9998 13994 L 9980 14015 L 9954 14007 L
9927 14006 L 9900 14014 L 9877 14027 L 9853 14015 L
9839 14038 L 9848 14064 L 9830 14084 L 9809 14067 L
9786 14053 L 9766 14034 L 9768 14007 L 9753 14029 L
9726 14025 L 9699 14020 L 9683 13999 L 9663 14017 L
9638 14007 L 9611 14002 L 9584 14004 L 9560 14016 L
9541 14036 L 9524 14057 L 9506 14077 L 9531 14089 L
9504 14085 L 9479 14075 L 9452 14072 L 9425 14077 L
9405 14096 L 9401 14123 L 9385 14146 L 9358 14144 L
9341 14165 L 9317 14178 L 9305 14202 L 9278 14199 L
9251 14202 L 9224 14201 L 9203 14183 L 9178 14174 L
9154 14160 L 9127 14156 L 9101 14149 L 9082 14130 L
9056 14122 L 9030 14114 L 9054 14102 L 9050 14075 L
9033 14054 L 9014 14035 L 8995 14015 L 8973 13999 L
8951 13984 L 8925 13977 L 8899 13966 L 8878 13949 L
8866 13925 L 8882 13904 L 8875 13877 L 8856 13858 L
8835 13841 L 8816 13821 L 8790 13814 L 8762 13814 L
8735 13812 L 8710 13823 L 8683 13820 L 8656 13818 L
8629 13819 L 8602 13826 L 8575 13829 L 8555 13847 L
8532 13861 L 8505 13860 L 8502 13887 L 8485 13908 L
8467 13929 L 8472 13956 L 8469 13983 L 8467 14010 L
8470 14037 L 8476 14064 L 8494 14085 L 8510 14106 L
8522 14130 L 8505 14151 L 8480 14162 L 8492 14186 L
8498 14213 L 8490 14239 L 8482 14265 L 8463 14285 L
8459 14311 L 8432 14312 L 8414 14291 L 8424 14316 L
8418 14343 L 8422 14370 L 8407 14393 L 8402 14367 L
8396 14393 L 8372 14381 L 8351 14365 L 8326 14354 L
8301 14342 L 8279 14326 L 8258 14343 L 8232 14347 L
8206 14355 L 8179 14357 L 8152 14354 L 8125 14358 L
8098 14354 L 8074 14367 L 8052 14383 L 8033 14402 L
8010 14417 L 7984 14424 L 7964 14443 L 7945 14462 L
7928 14483 L 7915 14507 L 7903 14531 L 7890 14555 L
7880 14580 L 7870 14606 L 7867 14633 L 7867 14660 L

7872 14686 L 7881 14712 L 7893 14736 L 7906 14760 L
7924 14781 L 7939 14803 L 7964 14814 L 7958 14840 L
7948 14866 L 7967 14885 L 7986 14904 L 8008 14919 L
8034 14928 L 8061 14933 L 8087 14928 L 8111 14915 L
8126 14892 L 8132 14866 L 8151 14846 L 8174 14832 L
8201 14827 L 8222 14843 L 8242 14862 L 8264 14878 L
8284 14896 L 8301 14904 L 8309 14908 L 8326 14907 L
8336 14907 L 8357 14890 L 8370 14891 L 8384 14891 L
8405 14907 L 8406 14907 L 8422 14909 L 8433 14910 L
8458 14920 L 8475 14929 L 8482 14933 L 8503 14950 L
8521 14970 L 8536 14993 L 8553 15014 L 8575 15029 L
8600 15040 L 8582 15061 L 8578 15088 L 8582 15114 L
8574 15140 L 8597 15154 L 8605 15180 L 8612 15206 L
8625 15230 L 8644 15249 L 8666 15266 L 8691 15276 L
8689 15303 L 8710 15321 L 8735 15330 L 8761 15337 L
8787 15345 L 8809 15361 L 8835 15368 L 8851 15390 L
8874 15405 L 8899 15415 L 8924 15423 L 8950 15433 L
8973 15446 L 8998 15458 L 9018 15475 L 9044 15482 L
9070 15489 L 9086 15511 L 9085 15538 L 9098 15561 L
9122 15575 L 9099 15591 L 9121 15607 L 9126 15633 L
9150 15647 L 9163 15670 L 9173 15677 L 9185 15686 L
9203 15706 L 9210 15721 L 9215 15731 L 9227 15745 L
9233 15751 L 9259 15757 L 9261 15760 L 9277 15777 L
9255 15792 L 9266 15817 L 9285 15836 L 9294 15835 L
9310 15832 L 9312 15832 L 9322 15857 L 9341 15876 L
9357 15898 L 9384 15896 L 9406 15880 L 9429 15893 L
9453 15880 L 9480 15881 L 9493 15872 L 9502 15865 L
9522 15846 L 9548 15849 L 9575 15851 L 9597 15867 L
9607 15893 L 9608 15920 L 9628 15938 L 9638 15963 L
9634 15936 L 9621 15912 L 9632 15887 L 9624 15861 L
9637 15838 L 9664 15840 L 9681 15818 L 9704 15804 L
9726 15788 L 9741 15773 L 9753 15772 L 9753 15785 L
9758 15790 L 9767 15786 L 9783 15778 L 9802 15784 L
9800 15790 L 9777 15784 L 9771 15795 L 9765 15802 L
9765 15808 L 9753 15815 L 9730 15831 L 9730 15833 L
9755 15818 L 9776 15806 L 9777 15793 L 9800 15794 L
9805 15786 L 9801 15780 L 9783 15775 L 9759 15788 L
9757 15781 L 9762 15755 L 9753 15738 L 9759 15728 L
9782 15712 L 9802 15695 L 9815 15671 L 9803 15647 L
9785 15626 L 9783 15599 L 9765 15579 L 9750 15556 L
9747 15529 L 9727 15511 L 9713 15487 L 9699 15465 L
9682 15444 L 9666 15422 L 9643 15408 L 9669 15401 L
9649 15382 L 9631 15362 L 9607 15349 L 9580 15350 L

9563 15329 L 9539 15315 L 9517 15301 L 9505 15276 L
9494 15251 L 9516 15235 L 9533 15213 L 9535 15186 L
9533 15165 L 9528 15153 L 9516 15136 L 9493 15127 L
9506 15095 L 9516 15065 L 9540 15051 L 9567 15054 L
9594 15056 L 9618 15068 L 9645 15068 L 9671 15075 L
9698 15073 L 9675 15061 L 9649 15053 L 9626 15039 L
9611 15016 L 9588 15002 L 9562 14995 L 9580 14975 L
9563 14954 L 9586 14939 L 9607 14921 L 9593 14898 L
9567 14889 L 9542 14878 L 9520 14862 L 9493 14859 L
9474 14878 L 9464 14852 L 9450 14829 L 9425 14819 L
9397 14819 L 9371 14812 L 9364 14786 L 9390 14779 L
9368 14762 L 9381 14738 L 9380 14711 L 9401 14694 L
9419 14715 L 9430 14740 L 9457 14739 L 9482 14751 L
9509 14750 L 9534 14759 L 9553 14778 L 9580 14779 L
9607 14781 L 9620 14806 L 9596 14820 L 9570 14825 L
9594 14837 L 9606 14862 L 9627 14879 L 9652 14868 L
9664 14843 L 9671 14840 L 9688 14831 L 9689 14858 L
9678 14882 L 9703 14874 L 9695 14900 L 9715 14882 L
9736 14899 L 9762 14905 L 9754 14931 L 9781 14936 L
9805 14948 L 9781 14960 L 9791 14985 L 9810 15004 L
9833 15018 L 9844 15043 L 9855 15068 L 9878 15082 L
9897 15099 L 9898 15100 L 9913 15099 L 9925 15098 L
9945 15116 L 9948 15143 L 9955 15139 L 9972 15129 L
9990 15150 L 10014 15162 L 10033 15181 L 10041 15186 L
10055 15197 L 10055 15197 L 10069 15203 L 10076 15206 L
10080 15208 L 10086 15210 L 10095 15214 L 10105 15217 L
10130 15229 L 10142 15241 L 10149 15248 L 10169 15256 L
10174 15258 L 10199 15249 L 10225 15259 L 10234 15266 L
10246 15275 L 10221 15286 L 10245 15300 L 10253 15326 L
10255 15325 L 10271 15323 L 10280 15321 L 10283 15321 L
10306 15316 L 10307 15315 L 10320 15308 L 10330 15302 L
10338 15320 L 10341 15326 L 10355 15323 L 10368 15321 L
10381 15344 L 10392 15369 L 10400 15395 L 10401 15368 L
10424 15353 L 10440 15354 L 10451 15354 L 10471 15372 L
10496 15360 L 10523 15358 L 10550 15357 L 10576 15363 L
10598 15378 L 10625 15384 L 10637 15408 L 10642 15381 L
10649 15355 L 10654 15382 L 10659 15408 L 10665 15435 L
10670 15435 L 10692 15437 L 10686 15410 L 10707 15394 L
10731 15382 L 10758 15375 L 10784 15373 L 10794 15347 L
10803 15322 L 10822 15302 L 10811 15277 L 10824 15253 L
10838 15271 L 10841 15275 L 10853 15290 L 10857 15296 L
10854 15323 L 10865 15348 L 10886 15331 L 10912 15323 L
10927 15303 L 10928 15302 L 10952 15315 L 10969 15294 L

10996 15295 L 11013 15283 L 11019 15279 L 11042 15266 L
11068 15274 L 11087 15255 L 11114 15256 L 11137 15270 L
11158 15287 L 11170 15311 L 11175 15323 L 11187 15320 L
11195 15323 L 11200 15329 L 11201 15328 L 11196 15320 L
11187 15317 L 11175 15322 L 11171 15309 L 11161 15281 L
11182 15282 L 11194 15292 L 11223 15309 L 11224 15307 L
11219 15303 L 11195 15291 L 11185 15277 L 11172 15276 L
11148 15263 L 11144 15236 L 11125 15217 L 11126 15190 L
11139 15166 L 11165 15173 L 11151 15149 L 11155 15122 L
11149 15095 L 11142 15069 L 11119 15055 L 11114 15028 L
11123 15002 L 11102 14984 L 11089 14961 L 11080 14935 L
11071 14910 L 11060 14885 L 11068 14859 L 11083 14836 L
11108 14827 L 11101 14853 L 11116 14831 L 11119 14804 L
11145 14812 L 11172 14809 L 11188 14831 L 11212 14819 L
11239 14819 L 11255 14796 L 11228 14792 L 11206 14807 L
11182 14795 L 11155 14791 L 11133 14775 L 11106 14769 L
11083 14755 L 11066 14777 L 11073 14750 L 11069 14724 L
11045 14711 L 11018 14710 L 11029 14685 L 11019 14660 L
10999 14642 L 10989 14616 L 10975 14593 L 10981 14567 L
10982 14539 L 11001 14521 L 11022 14532 L 11032 14527 L
11003 14508 L 11000 14511 L 11002 14519 L 10980 14532 L
10961 14506 L 10941 14490 L 10922 14471 L 10896 14463 L
10922 14456 L 10926 14455 L 10949 14452 L 10958 14455 L
10971 14458 L 10975 14459 L 11000 14469 L 11024 14456 L
11050 14448 L 11077 14452 L 11102 14462 L 11129 14465 L
11154 14455 L 11152 14428 L 11131 14444 L 11104 14440 L
11097 14413 L 11121 14401 L 11096 14391 L 11069 14395 L
11046 14381 L 11061 14359 L 11077 14352 L 11086 14348 L
11112 14341 L 11138 14349 L 11139 14349 L 11158 14363 L
11160 14365 L 11184 14351 L 11179 14324 L 11205 14325 L
11207 14325 L 11215 14350 L 11242 14355 L 11219 14370 L
11246 14371 L 11273 14369 L 11251 14353 L 11266 14331 L
11239 14329 L 11237 14302 L 11210 14295 L 11199 14270 L
11215 14249 L 11227 14273 L 11253 14278 L 11270 14299 L
11284 14308 L 11293 14314 L 11296 14313 L 11320 14311 L
11328 14337 L 11324 14364 L 11321 14391 L 11341 14391 L
11347 14390 L 11348 14390 L 11355 14417 L 11327 14416 L
11302 14427 L 11298 14454 L 11323 14443 L 11350 14444 L
11367 14440 L 11374 14438 L 11377 14438 L 11388 14416 L
11389 14413 L 11398 14407 L 11403 14403 L 11410 14398 L
11411 14398 L 11420 14388 L 11430 14378 L 11447 14357 L
11454 14357 L 11474 14355 L 11476 14383 L 11502 14376 L
11503 14376 L 11507 14350 L 11513 14343 L 11518 14339 L

11526 14330 L 11547 14315 L 11548 14314 L 11555 14340 L
11553 14368 L 11558 14369 L 11579 14376 L 11596 14379 L
11606 14380 L 11627 14397 L 11654 14400 L 11665 14392 L
11677 14384 L 11700 14370 L 11724 14358 L 11751 14363 L
11768 14384 L 11794 14398 L 11776 14374 L 11781 14348 L
11783 14321 L 11795 14296 L 11820 14285 L 11842 14269 L
11865 14255 L 11885 14243 L 11885 14242 L 11863 14251 L
11841 14267 L 11816 14279 L 11792 14291 L 11768 14304 L
11776 14331 L 11763 14355 L 11739 14341 L 11714 14329 L
11719 14303 L 11726 14276 L 11746 14258 L 11772 14250 L
11782 14225 L 11785 14198 L 11760 14186 L 11761 14185 L
11779 14166 L 11800 14149 L 11826 14140 L 11810 14118 L
11834 14106 L 11859 14094 L 11837 14077 L 11810 14073 L
11817 14046 L 11804 14023 L 11821 14001 L 11839 14021 L
11861 14038 L 11866 14011 L 11854 13986 L 11848 13960 L
11874 13954 L 11893 13934 L 11911 13914 L 11938 13909 L
11957 13928 L 11948 13902 L 11926 13886 L 11906 13867 L
11907 13840 L 11892 13863 L 11887 13890 L 11871 13912 L
11845 13917 L 11824 13935 L 11802 13920 L 11777 13932 L
11753 13945 L 11764 13920 L 11739 13931 L 11758 13912 L
11769 13887 L 11748 13904 L 11744 13877 L 11738 13850 L
11737 13878 L 11730 13904 L 11721 13930 L 11695 13921 L
11674 13903 L 11697 13888 L 11715 13867 L 11699 13845 L
11675 13832 L 11691 13810 L 11669 13826 L 11670 13853 L
11664 13879 L 11658 13906 L 11658 13879 L 11634 13866 L
11627 13840 L 11628 13813 L 11602 13807 L 11592 13781 L
11598 13758 L 11598 13755 L 11608 13780 L 11610 13779 L
11619 13775 L 11628 13771 L 11633 13769 L 11632 13741 L
11637 13743 L 11640 13744 L 11650 13747 L 11654 13748 L
11658 13749 L 11676 13729 L 11676 13702 L 11681 13694 L
11686 13688 L 11692 13682 L 11693 13680 L 11695 13670 L
11699 13654 L 11703 13627 L 11718 13607 L 11719 13605 L
11740 13622 L 11730 13647 L 11718 13671 L 11714 13698 L
11689 13709 L 11694 13736 L 11680 13760 L 11700 13779 L
11708 13753 L 11713 13726 L 11731 13746 L 11747 13758 L
11753 13762 L 11759 13735 L 11756 13708 L 11755 13681 L
11779 13668 L 11766 13645 L 11770 13637 L 11776 13626 L
11779 13621 L 11805 13649 L 11804 13634 L 11781 13619 L
11782 13592 L 11785 13589 L 11789 13586 L 11804 13575 L
11791 13551 L 11805 13528 L 11807 13503 L 11807 13501 L
11829 13484 L 11807 13468 L 11824 13462 L 11828 13460 L
11833 13459 L 11844 13449 L 11854 13441 L 11867 13417 L
11886 13398 L 11910 13411 L 11935 13422 L 11957 13438 L

11963 13465 L 11962 13437 L 11941 13420 L 11916 13409 L
11895 13391 L 11871 13379 L 11856 13356 L 11864 13330 L
11875 13332 L 11891 13336 L 11918 13338 L 11945 13344 L
11926 13324 L 11907 13305 L 11922 13282 L 11897 13271 L
11892 13244 L 11865 13243 L 11847 13222 L 11826 13204 L
11808 13184 L 11821 13161 L 11849 13162 L 11876 13168 L
11903 13167 L 11904 13140 L 11877 13142 L 11851 13133 L
11825 13125 L 11814 13100 L 11809 13073 L 11796 13049 L
11823 13044 L 11814 13018 L 11792 13034 L 11786 13008 L
11781 12981 L 11771 12955 L 11798 12948 L 11794 12921 L
11816 12905 L 11843 12899 L 11833 12873 L 11815 12852 L
11797 12873 L 11778 12893 L 11780 12920 L 11768 12896 L
11741 12902 L 11750 12876 L 11762 12864 L 11769 12857 L
11767 12884 L 11787 12865 L 11809 12850 L 11797 12826 L
11771 12815 L 11774 12812 L 11792 12797 L 11808 12819 L
11835 12824 L 11820 12801 L 11830 12776 L 11824 12749 L
11833 12723 L 11857 12737 L 11885 12736 L 11885 12764 L
11876 12789 L 11877 12817 L 11879 12789 L 11903 12776 L
11907 12749 L 11934 12747 L 11909 12735 L 11882 12731 L
11875 12704 L 11848 12708 L 11827 12690 L 11854 12692 L
11882 12689 L 11903 12672 L 11913 12646 L 11885 12644 L
11859 12650 L 11853 12677 L 11825 12676 L 11824 12648 L
11850 12640 L 11871 12621 L 11843 12619 L 11818 12630 L
11804 12653 L 11802 12681 L 11778 12695 L 11770 12669 L
11770 12666 L 11772 12641 L 11785 12617 L 11812 12612 L
11833 12595 L 11845 12590 L 11859 12584 L 11880 12602 L
11907 12601 L 11887 12581 L 11869 12561 L 11877 12534 L
11901 12522 L 11884 12500 L 11907 12485 L 11909 12463 L
11909 12458 L 11931 12474 L 11931 12474 L 11949 12495 L
11953 12522 L 11955 12524 L 11975 12538 L 11979 12552 L
11982 12565 L 12006 12578 L 12006 12606 L 12008 12633 L
12019 12608 L 12021 12580 L 12035 12556 L 12043 12558 L
12062 12563 L 12089 12562 L 12096 12571 L 12105 12584 L
12094 12609 L 12103 12635 L 12107 12662 L 12130 12676 L
12137 12682 L 12151 12694 L 12176 12705 L 12194 12726 L
12220 12735 L 12245 12745 L 12249 12718 L 12231 12697 L
12211 12715 L 12212 12688 L 12213 12660 L 12189 12646 L
12185 12673 L 12174 12648 L 12165 12622 L 12191 12613 L
12206 12636 L 12214 12629 L 12226 12617 L 12242 12595 L
12247 12591 L 12263 12577 L 12274 12602 L 12275 12603 L
12285 12611 L 12296 12619 L 12298 12646 L 12271 12641 L
12274 12669 L 12301 12663 L 12316 12663 L 12328 12662 L
12329 12690 L 12307 12706 L 12284 12722 L 12267 12743 L

12259 12769 L 12282 12785 L 12257 12794 L 12284 12795 L
12310 12803 L 12322 12827 L 12335 12803 L 12343 12789 L
12349 12780 L 12361 12755 L 12351 12729 L 12363 12705 L
12387 12718 L 12386 12746 L 12405 12751 L 12412 12752 L
12439 12747 L 12434 12720 L 12435 12692 L 12444 12695 L
12461 12701 L 12460 12728 L 12451 12754 L 12436 12777 L
12439 12804 L 12434 12831 L 12407 12824 L 12386 12807 L
12362 12819 L 12348 12843 L 12355 12870 L 12372 12848 L
12396 12861 L 12412 12883 L 12439 12889 L 12465 12881 L
12485 12895 L 12488 12897 L 12511 12911 L 12536 12921 L
12544 12947 L 12546 12920 L 12548 12893 L 12548 12893 L
12573 12882 L 12599 12873 L 12626 12874 L 12628 12880 L
12635 12900 L 12643 12896 L 12660 12888 L 12661 12888 L
12685 12898 L 12682 12870 L 12709 12871 L 12716 12844 L
12689 12850 L 12697 12840 L 12705 12828 L 12724 12814 L
12727 12812 L 12739 12837 L 12741 12864 L 12722 12885 L
12723 12912 L 12726 12939 L 12745 12959 L 12727 12980 L
12740 13004 L 12764 13003 L 12768 13003 L 12794 12995 L
12786 13021 L 12780 13048 L 12804 13035 L 12826 13019 L
12830 13018 L 12853 13015 L 12880 13014 L 12892 13017 L
12906 13021 L 12906 13022 L 12908 13022 L 12922 13023 L
12934 13024 L 12949 13047 L 12948 13019 L 12956 12993 L
12977 13010 L 12996 13020 L 12996 13042 L 12995 13049 L
12996 13048 L 12996 14120 L 12996 14126 L 12977 14146 L
12964 14170 L 12958 14196 L 12948 14222 L 12931 14243 L
12912 14262 L 12897 14285 L 12875 14302 L 12861 14325 L
12841 14343 L 12835 14369 L 12815 14389 L 12795 14407 L
12790 14433 L 12769 14451 L 12749 14469 L 12728 14486 L
12709 14506 L 12689 14524 L 12672 14546 L 12653 14565 L
12637 14587 L 12619 14607 L 12603 14629 L 12584 14648 L
12572 14672 L 12560 14697 L 12544 14718 L 12531 14743 L
12516 14765 L 12500 14787 L 12491 14813 L 12473 14833 L
12460 14857 L 12446 14880 L 12435 14905 L 12430 14931 L
12407 14945 L 12381 14954 L 12355 14944 L 12344 14969 L
12325 14989 L 12303 15004 L 12276 15007 L 12253 15021 L
12232 15039 L 12210 15054 L 12194 15076 L 12176 15096 L
12160 15118 L 12144 15140 L 12121 15126 L 12096 15136 L
12070 15142 L 12042 15143 L 12015 15145 L 11992 15159 L
11978 15181 L 11981 15208 L 12004 15223 L 12010 15249 L
11984 15259 L 12010 15266 L 12037 15270 L 12051 15246 L
12042 15272 L 12031 15297 L 12023 15323 L 12026 15350 L
12039 15374 L 12032 15400 L 12006 15408 L 12000 15434 L
12007 15461 L 12020 15484 L 12038 15504 L 12065 15503 L

12079 15527 L 12105 15534 L 12126 15551 L 12146 15568 L
12144 15595 L 12166 15611 L 12180 15634 L 12202 15650 L
12228 15644 L 12255 15641 L 12280 15651 L 12296 15673 L
12321 15664 L 12331 15689 L 12357 15697 L 12375 15717 L
12399 15729 L 12417 15750 L 12442 15759 L 12446 15786 L
12423 15800 L 12431 15826 L 12455 15839 L 12479 15851 L
12504 15863 L 12525 15879 L 12542 15900 L 12558 15922 L
12581 15936 L 12606 15948 L 12618 15972 L 12623 15998 L
12630 16025 L 12635 16051 L 12653 16071 L 12679 16079 L
12695 16101 L 12716 16118 L 12740 16130 L 12764 16142 L
12782 16162 L 12809 16167 L 12833 16179 L 12839 16205 L
12838 16210 L 12831 16210 L 12798 16210 L 12765 16210 L
12731 16210 L 12698 16210 L 12665 16210 L 12632 16210 L
12599 16210 L 12566 16210 L 12533 16210 L 12500 16210 L
12467 16210 L 12434 16210 L 12400 16210 L 12367 16210 L
12334 16210 L 12301 16210 L 12268 16210 L 12235 16210 L
12202 16210 L 12169 16210 L 12136 16210 L 12102 16210 L
12069 16210 L 12036 16210 L 12003 16210 L 11970 16210 L
11937 16210 L 11904 16210 L 11871 16210 L 11838 16210 L
11804 16210 L 11771 16210 L 11738 16210 L 11705 16210 L
11672 16210 L 11639 16210 L 11606 16210 L 11573 16210 L
11540 16210 L 11507 16210 L 11473 16210 L 11440 16210 L
11407 16210 L 11374 16210 L 11341 16210 L 11308 16210 L
11275 16210 L 11242 16210 L 11209 16210 L 11175 16210 L
11142 16210 L 11109 16210 L 11076 16210 L 11043 16210 L
11010 16210 L 10977 16210 L 10944 16210 L 10911 16210 L
10877 16210 L 10844 16210 L 10811 16210 L 10778 16210 L
10745 16210 L 10712 16210 L 10679 16210 L 10646 16210 L
10613 16210 L 10580 16210 L 10546 16210 L 10513 16210 L
10480 16210 L 10447 16210 L 10414 16210 L 10381 16210 L
10348 16210 L 10315 16210 L 10282 16210 L 10248 16210 L
10215 16210 L 10182 16210 L 10149 16210 L 10116 16210 L
10083 16210 L 10050 16210 L 10017 16210 L 9984 16210 L
9950 16210 L 9917 16210 L 9884 16210 L 9851 16210 L
9818 16210 L 9785 16210 L 9752 16210 L 9719 16210 L
9686 16210 L 9653 16210 L 9619 16210 L 9586 16210 L
9553 16210 L 9520 16210 L 9487 16210 L 9454 16210 L
9421 16210 L 9388 16210 L 9355 16210 L 9321 16210 L
9288 16210 L 9255 16210 L 9222 16210 L 9189 16210 L
9156 16210 L 9123 16210 L 9090 16210 L 9057 16210 L
9023 16210 L 8990 16210 L 8957 16210 L 8924 16210 L
8891 16210 L 8858 16210 L 8825 16210 L 8792 16210 L
8759 16210 L 8725 16210 L 8692 16210 L 8659 16210 L

8626 16210 L 8593 16210 L 8560 16210 L 8527 16210 L
8494 16210 L 8461 16210 L 8428 16210 L 8394 16210 L
8361 16210 L 8328 16210 L 8295 16210 L 8262 16210 L
8229 16210 L 8196 16210 L 8163 16210 L 8130 16210 L
8096 16210 L 8063 16210 L 8030 16210 L 7997 16210 L
7964 16210 L 7931 16210 L 7898 16210 L 7865 16210 L
7832 16210 L 7798 16210 L 7765 16210 L 7732 16210 L
7699 16210 L 7666 16210 L 7633 16210 L 7600 16210 L
7567 16210 L 7534 16210 L 7501 16210 L 7467 16210 L
7434 16210 L 7401 16210 L 7368 16210 L 7335 16210 L
7302 16210 L 7269 16210 L 7236 16210 L 7203 16210 L
7169 16210 L 7136 16210 L 7103 16210 L 7070 16210 L
7037 16210 L 7004 16210 L 6971 16210 L 6938 16210 L
6905 16210 L 6871 16210 L 6838 16210 L 6805 16210 L
6772 16210 L 6739 16210 L 6706 16210 L 6673 16210 L
6640 16210 L 6607 16210 L 6574 16210 L 6540 16210 L
6507 16210 L 6474 16210 L 6441 16210 L 6408 16210 L
6375 16210 L 6342 16210 L 6309 16210 L 6276 16210 L
6242 16210 L 6209 16210 L 6176 16210 L 6143 16210 L
6110 16210 L 6077 16210 L 6044 16210 L 6011 16210 L
5978 16210 L 5944 16210 L 5911 16210 L 5878 16210 L
5845 16210 L 5812 16210 L 5779 16210 L 5746 16210 L
5713 16210 L 5680 16210 L 5646 16210 L 5613 16210 L
5580 16210 L 5547 16210 L 5514 16210 L 5481 16210 L
5448 16210 L 5415 16210 L 5382 16210 L 5349 16210 L
5315 16210 L 5282 16210 L 5249 16210 L 5216 16210 L
5183 16210 L 5150 16210 L 5117 16210 L 5084 16210 L
5051 16210 L 5017 16210 L 4984 16210 L 4951 16210 L
4918 16210 L 4885 16210 L 4852 16210 L 4819 16210 L
4786 16210 L 4753 16210 L 4719 16210 L 4686 16210 L
4653 16210 L 4620 16210 L 4587 16210 L 4554 16210 L
4521 16210 L 4488 16210 L 4455 16210 L 4422 16210 L
4388 16210 L 4355 16210 L 4322 16210 L 4289 16210 L
4256 16210 L 4223 16210 L 4190 16210 L 4157 16210 L
4124 16210 L 4090 16210 L 4057 16210 L 4024 16210 L
3991 16210 L 3958 16210 L 3925 16210 L 3892 16210 L
3859 16210 L 3826 16210 L 3792 16210 L 3759 16210 L
3726 16210 L 3693 16210 L 3660 16210 L 3627 16210 L
3594 16210 L 3561 16210 L 3528 16210 L 3495 16210 L
3461 16210 L 3428 16210 L 3395 16210 L 3362 16210 L
3329 16210 L 3296 16210 L 3263 16210 L 3230 16210 L
3197 16210 L 3163 16210 L 3130 16210 L 3097 16210 L
3064 16210 L 3031 16210 L 2998 16210 L 2965 16210 L

2932 16210 L 2899 16210 L 2865 16210 L 2832 16210 L
2799 16210 L 2766 16210 L 2733 16210 L 2700 16210 L
2667 16210 L 2634 16210 L 2601 16210 L 2568 16210 L
2534 16210 L 2501 16210 L 2468 16210 L 2435 16210 L
2402 16210 L 2369 16210 L 2336 16210 L 2303 16210 L
N E
5544 5522 5542 5515 5544 5511 5546 5507 5549 5503 5549 5501
5576 5501 5576 5504 5574 5508 5572 5512 5570 5516 5568 5520
5564 5523 5561 5526 5557 5529 5553 5532 5551 5528 5546 5526
5544 5522 19 N F
5703 5619 5699 5613 5701 5609 5705 5606 5709 5609 5713 5610
5718 5612 5718 5616 5722 5618 5725 5622 5725 5626 5727 5631
5722 5631 5718 5633 5714 5634 5712 5629 5708 5627 5705 5623
5703 5619 19 N F
5797 5618 5791 5617 5795 5615 5799 5612 5802 5609 5806 5607
5811 5607 5809 5612 5807 5616 5804 5619 5800 5621 5797 5618
12 N F
5818 5507 5817 5501 5845 5501 5866 5501 5872 5504 5880 5504
5886 5507 5891 5502 5891 5501 5911 5501 5904 5506 5905 5514
5909 5520 5903 5523 5910 5526 5916 5523 5918 5530 5917 5537
5917 5545 5922 5550 5925 5557 5931 5553 5937 5557 5938 5564
5934 5570 5928 5565 5922 5562 5915 5561 5909 5556 5904 5551
5898 5548 5894 5542 5887 5542 5880 5540 5873 5537 5867 5533
5863 5539 5856 5540 5848 5540 5844 5533 5839 5529 5833 5525
5826 5523 5828 5516 5824 5511 5818 5507 46 N F
5965 5504 5963 5501 5970 5501 5971 5505 5965 5504 5 N F
5978 5533 5968 5526 5973 5520 5976 5514 5982 5510 5989 5509
5993 5503 6000 5504 6007 5501 6007 5501 6011 5501 6025 5501
6025 5505 6029 5501 6040 5501 6039 5504 6043 5510 6039 5517
6035 5523 6032 5529 6025 5530 6019 5534 6011 5535 6006 5530
5999 5529 5992 5531 5985 5534 5978 5533 28 N F
6056 5509 6055 5505 6057 5503 6059 5502 6059 5501 6060 5501
6061 5501 6063 5502 6064 5504 6065 5506 6065 5508 6065 5510
6064 5512 6062 5512 6060 5510 6058 5510 6056 5509 17 N F
6244 5628 6241 5626 6242 5623 6243 5620 6245 5617 6248 5619
6251 5620 6255 5620 6258 5622 6260 5624 6260 5624 6257 5627
6254 5627 6251 5628 6247 5628 6244 5628 16 N F
6253 5608 6252 5605 6254 5606 6256 5605 6257 5606 6259 5608
6259 5610 6261 5612 6260 5614 6258 5613 6256 5612 6254 5610
6253 5608 13 N F
6257 5696 6253 5693 6254 5690 6258 5689 6260 5687 6261 5683
6264 5685 6263 5689 6260 5691 6257 5693 6257 5696 11 N F
7343 6097 7335 6089 7339 6083 7343 6077 7349 6074 7356 6070

7360 6064 7367 6062 7374 6064 7378 6058 7381 6052 7388 6049
7395 6047 7389 6043 7393 6037 7398 6033 7405 6032 7408 6025
7415 6025 7417 6032 7422 6037 7415 6040 7409 6037 7404 6043
7402 6050 7403 6057 7404 6064 7402 6071 7398 6077 7397 6084
7392 6089 7385 6092 7380 6087 7372 6087 7365 6089 7361 6094
7356 6100 7349 6100 7343 6097 39 N F
7490 6225 7483 6221 7488 6216 7496 6215 7503 6214 7508 6219
7506 6225 7506 6232 7511 6238 7514 6244 7507 6246 7501 6242
7500 6235 7497 6228 7490 6225 15 N F
7499 6211 7491 6210 7493 6203 7499 6199 7506 6198 7513 6196
7520 6194 7522 6201 7523 6208 7527 6214 7533 6217 7527 6226
7520 6224 7513 6225 7510 6218 7506 6212 7499 6211 17 N F
7498 6156 7493 6150 7497 6144 7504 6143 7511 6141 7519 6142
7526 6142 7533 6140 7538 6135 7545 6132 7552 6132 7559 6135
7566 6136 7573 6134 7575 6127 7581 6130 7586 6125 7593 6128
7597 6134 7595 6142 7594 6149 7600 6152 7598 6159 7592 6163
7589 6170 7590 6177 7583 6177 7579 6171 7576 6164 7569 6166
7565 6171 7559 6175 7552 6173 7548 6167 7545 6160 7539 6156
7535 6150 7526 6158 7523 6164 7516 6167 7509 6165 7503 6161
7498 6156 43 N F
7698 6241 7691 6229 7698 6229 7705 6231 7712 6233 7718 6231
7717 6224 7723 6228 7729 6231 7736 6233 7743 6234 7750 6236
7758 6236 7754 6242 7747 6243 7745 6250 7745 6257 7746 6264
7748 6271 7753 6277 7759 6279 7766 6277 7772 6274 7780 6274
7781 6281 7775 6286 7775 6293 7777 6300 7784 6304 7782 6311
7788 6315 7794 6311 7801 6312 7802 6319 7809 6321 7801 6321
7797 6326 7791 6330 7786 6335 7780 6330 7773 6332 7770 6326
7762 6327 7756 6323 7753 6330 7749 6336 7744 6341 7737 6344
7732 6349 7725 6346 7724 6339 7727 6332 7727 6325 7722 6319
7717 6314 7715 6307 7711 6301 7706 6296 7708 6289 7714 6285
7719 6291 7726 6293 7732 6289 7739 6289 7746 6287 7740 6283
7739 6276 7734 6270 7728 6266 7722 6262 7718 6256 7713 6250
7714 6243 7710 6249 7704 6245 7698 6241 76 N F
7944 6758 7942 6753 7943 6750 7945 6746 7948 6745 7950 6744
7951 6744 7952 6747 7950 6749 7947 6752 7946 6755 7944 6758
12 N F
7955 6742 7950 6742 7954 6740 7958 6737 7961 6734 7964 6730
7967 6727 7971 6724 7975 6723 7979 6723 7984 6724 7988 6723
7989 6723 7993 6725 7991 6729 7990 6733 7986 6735 7982 6733
7977 6732 7973 6734 7971 6738 7968 6741 7963 6741 7959 6739
7955 6742 25 N F
7957 6748 7953 6744 7957 6742 7961 6741 7965 6743 7970 6741
7973 6738 7975 6734 7980 6734 7984 6736 7988 6738 7992 6735

7994 6731 7998 6729 8002 6732 8007 6732 8006 6736 8003 6740
7999 6743 7996 6746 7993 6749 7988 6750 7984 6751 7980 6753
7975 6752 7970 6751 7966 6750 7962 6749 7957 6748 29 N F
7984 6721 7979 6720 7983 6718 7987 6716 7991 6714 7996 6714
7997 6718 7993 6720 7988 6722 7984 6721 10 N F
8030 6713 8022 6709 8026 6706 8031 6708 8035 6705 8039 6708
8043 6710 8039 6713 8034 6714 8030 6713 10 N F
8100 6744 8089 6739 8093 6733 8096 6727 8103 6723 8109 6720
8116 6718 8123 6717 8129 6721 8134 6726 8140 6730 8145 6736
8146 6743 8149 6749 8156 6751 8156 6758 8156 6765 8156 6772
8152 6777 8145 6774 8141 6780 8136 6785 8129 6783 8124 6787
8117 6790 8110 6792 8110 6784 8105 6780 8104 6773 8100 6767
8095 6762 8091 6756 8093 6749 8100 6750 8105 6755 8112 6758
8113 6751 8107 6747 8100 6744 39 N F
8168 6713 8164 6711 8167 6709 8171 6709 8174 6708 8176 6711
8179 6712 8182 6711 8184 6713 8185 6716 8187 6718 8191 6717
8193 6720 8193 6723 8194 6727 8193 6730 8190 6732 8187 6734
8184 6735 8181 6733 8177 6733 8174 6732 8174 6728 8173 6725
8173 6722 8169 6720 8168 6717 8168 6713 28 N F
8211 6716 8200 6712 8205 6708 8209 6702 8216 6699 8223 6696
8229 6700 8231 6707 8235 6712 8241 6717 8247 6720 8254 6723
8261 6724 8268 6722 8274 6718 8280 6715 8286 6711 8292 6714
8297 6720 8297 6727 8302 6732 8298 6738 8297 6746 8294 6752
8292 6759 8289 6766 8282 6767 8278 6773 8273 6778 8269 6773
8263 6769 8265 6762 8260 6757 8254 6753 8253 6746 8249 6740
8242 6741 8235 6744 8229 6747 8224 6752 8219 6757 8212 6753
8207 6749 8208 6742 8215 6745 8212 6738 8214 6732 8216 6725
8218 6718 8211 6716 50 N F
8212 6799 8209 6796 8212 6795 8215 6794 8218 6792 8221 6789
8223 6787 8227 6787 8230 6787 8231 6791 8231 6794 8234 6797
8232 6800 8229 6801 8225 6802 8222 6801 8219 6801 8215 6800
8212 6799 19 N F
8275 14779 8272 14774 8276 14774 8279 14777 8282 14780 8285 14784
8287 14787 8290 14791 8292 14794 8294 14798 8295 14803 8293 14807
8291 14803 8289 14799 8285 14797 8282 14794 8282 14789 8281 14785
8278 14781 8275 14779 20 N F
8292 14820 8291 14817 8293 14817 8294 14816 8297 14818 8299 14821
8301 14823 8301 14827 8298 14825 8295 14823 8292 14820 11 N F
8313 14837 8310 14835 8313 14833 8315 14835 8318 14837 8320 14839
8320 14842 8322 14845 8325 14847 8326 14850 8323 14849 8321 14847
8318 14844 8318 14841 8315 14840 8313 14837 16 N F
8361 14829 8356 14826 8360 14827 8364 14829 8368 14830 8371 14834
8375 14836 8378 14839 8379 14844 8376 14847 8373 14850 8370 14853

8368 14849 8370 14845 8374 14844 8374 14839 8371 14837 8368 14834
8365 14831 8361 14829 20 N F
8396 14878 8392 14874 8396 14874 8400 14876 8405 14877 8409 14878
8413 14879 8415 14882 8417 14886 8419 14890 8423 14892 8424 14896
8422 14900 8417 14899 8415 14895 8412 14892 8410 14888 8408 14885
8404 14882 8400 14880 8396 14878 21 N F
8398 14892 8396 14889 8399 14887 8402 14888 8404 14890 8405 14893
8406 14896 8407 14900 8405 14903 8404 14900 8402 14897 8400 14894
8398 14892 13 N F
8430 14891 8426 14890 8430 14887 8434 14889 8438 14890 8442 14892
8446 14893 8449 14896 8452 14899 8455 14902 8458 14906 8460 14909
8464 14912 8467 14915 8470 14918 8473 14921 8475 14926 8471 14925
8467 14922 8463 14921 8459 14919 8455 14917 8455 14913 8452 14910
8448 14908 8444 14905 8441 14902 8437 14901 8437 14896 8434 14893
8430 14891 31 N F
8627 7082 8622 7080 8626 7079 8631 7078 8633 7074 8634 7069
8638 7068 8637 7072 8639 7076 8643 7079 8646 7083 8644 7087
8647 7091 8644 7094 8644 7099 8647 7102 8651 7103 8655 7106
8654 7111 8650 7108 8646 7107 8641 7106 8638 7104 8635 7100
8630 7099 8631 7095 8633 7091 8636 7087 8635 7082 8631 7080
8627 7082 31 N F
8709 12522 8704 12523 8705 12519 8708 12515 8712 12513 8714 12509
8718 12508 8722 12507 8726 12509 8730 12511 8728 12514 8724 12515
8719 12516 8715 12517 8711 12519 8709 12522 16 N F
8716 7332 8712 7321 8716 7315 8720 7309 8720 7302 8724 7296
8730 7299 8736 7303 8743 7303 8743 7310 8741 7317 8741 7324
8740 7331 8742 7338 8745 7344 8748 7351 8746 7358 8749 7364
8750 7371 8749 7378 8746 7385 8740 7382 8736 7376 8733 7370
8727 7366 8726 7358 8724 7352 8720 7345 8719 7338 8716 7332
30 N F
8750 6137 8748 6134 8750 6133 8752 6133 8755 6134 8756 6136
8757 6138 8755 6138 8753 6140 8751 6139 8750 6137 11 N F
8809 7190 8799 7188 8805 7183 8802 7177 8807 7172 8814 7171
8817 7169 8820 7168 8826 7172 8833 7174 8839 7170 8836 7163
8836 7156 8842 7153 8849 7151 8853 7151 8856 7151 8858 7157
8853 7162 8856 7169 8864 7169 8868 7175 8861 7173 8856 7178
8852 7173 8845 7173 8839 7177 8832 7180 8828 7186 8828 7193
8821 7194 8818 7201 8814 7196 8809 7190 34 N F
8808 6031 8801 6027 8804 6024 8806 6020 8807 6015 8811 6013
8812 6017 8815 6020 8818 6024 8819 6028 8817 6032 8813 6032
8808 6031 13 N F
8836 6236 8827 6228 8835 6230 8841 6232 8843 6231 8849 6231
8851 6238 8851 6245 8856 6251 8855 6258 8854 6265 8860 6269

8865 6273 8866 6274 8858 6275 8853 6279 8848 6274 8845 6268
8846 6260 8845 6253 8840 6248 8846 6243 8844 6237 8836 6236
24 N F
8843 6116 8840 6110 8844 6109 8849 6110 8853 6110 8853 6109
8851 6113 8854 6117 8851 6121 8851 6125 8851 6130 8846 6130
8843 6134 8843 6129 8842 6125 8843 6121 8843 6116 17 N F
8847 7685 8841 7674 8848 7673 8853 7673 8855 7672 8862 7670
8869 7670 8875 7666 8881 7662 8888 7662 8895 7665 8895 7672
8893 7679 8890 7685 8887 7692 8881 7695 8875 7698 8869 7702
8863 7698 8856 7696 8851 7691 8847 7685 22 N F
8853 7671 8846 7669 8850 7666 8853 7663 8857 7661 8861 7661
8866 7662 8870 7663 8874 7665 8871 7668 8866 7669 8862 7670
8857 7670 8853 7671 14 N F
8850 6190 8849 6186 8850 6184 8853 6184 8855 6185 8857 6186
8859 6186 8861 6187 8863 6188 8865 6190 8865 6190 8867 6190
8867 6192 8865 6193 8863 6192 8861 6192 8859 6190 8857 6190
8855 6188 8853 6187 8852 6188 8850 6190 22 N F
8853 6063 8850 6060 8851 6059 8854 6059 8854 6061 8853 6063
6 N F
8855 6055 8854 6052 8856 6052 8858 6054 8860 6056 8859 6058
8857 6057 8855 6055 8 N F
8861 7426 8856 7423 8859 7420 8864 7418 8868 7418 8870 7422
8874 7423 8877 7427 8880 7431 8882 7435 8882 7439 8878 7439
8875 7436 8871 7433 8868 7430 8866 7426 8864 7422 8861 7426
18 N F
8858 6117 8856 6114 8858 6113 8859 6113 8860 6113 8861 6115
8863 6116 8866 6116 8868 6116 8868 6119 8868 6121 8865 6121
8863 6121 8861 6120 8860 6118 8858 6117 16 N F
8871 6223 8866 6216 8872 6212 8878 6208 8882 6202 8889 6200
8896 6202 8902 6205 8901 6212 8906 6217 8911 6221 8912 6222
8918 6225 8923 6230 8930 6230 8938 6230 8939 6229 8944 6227
8941 6234 8940 6241 8938 6248 8934 6254 8929 6249 8927 6256
8921 6253 8919 6246 8918 6253 8914 6247 8910 6240 8910 6248
8911 6255 8904 6255 8900 6249 8896 6243 8895 6235 8891 6241
8888 6248 8882 6244 8879 6237 8875 6231 8882 6234 8884 6227
8877 6228 8883 6224 8890 6224 8898 6224 8895 6217 8889 6214
8885 6207 8881 6213 8875 6217 8871 6223 52 N F
8875 6293 8869 6287 8875 6282 8880 6288 8885 6283 8890 6277
8892 6270 8896 6264 8899 6271 8897 6278 8902 6283 8899 6289
8896 6296 8889 6297 8882 6294 8875 6293 16 N F
8896 8008 8893 8003 8895 7999 8897 7995 8897 7990 8902 7990
8904 7994 8908 7992 8912 7990 8916 7991 8921 7993 8925 7993
8927 7997 8930 8001 8933 8003 8938 8004 8942 8006 8945 8009

8945 8013 8941 8015 8936 8015 8933 8012 8932 8007 8929 8003
8925 8005 8927 8009 8922 8009 8918 8008 8914 8009 8909 8010
8905 8009 8900 8009 8896 8008 33 N F
8894 6335 8893 6332 8896 6329 8896 6326 8897 6329 8899 6332
8902 6334 8905 6335 8906 6338 8904 6340 8901 6343 8899 6340
8897 6337 8894 6335 14 N F
8902 7437 8901 7433 8904 7433 8907 7433 8910 7434 8912 7435
8912 7438 8910 7440 8908 7442 8906 7441 8903 7440 8902 7437
12 N F
8907 6052 8905 6050 8907 6049 8909 6048 8911 6049 8912 6051
8911 6053 8909 6053 8907 6052 9 N F
8913 7431 8910 7425 8913 7423 8918 7422 8922 7422 8927 7423
8931 7424 8935 7422 8939 7425 8940 7430 8938 7434 8937 7438
8933 7435 8929 7433 8925 7432 8921 7429 8917 7432 8913 7431
18 N F
8920 7337 8917 7329 8924 7332 8930 7336 8937 7335 8943 7339
8941 7332 8941 7325 8934 7327 8931 7320 8936 7315 8933 7309
8938 7304 8943 7299 8945 7299 8949 7297 8954 7292 8960 7287
8967 7285 8970 7292 8967 7298 8961 7302 8963 7308 8969 7312
8975 7315 8981 7320 8988 7322 8993 7327 9000 7328 9005 7323
9011 7326 9012 7333 9005 7333 8998 7335 8992 7339 8986 7344
8984 7351 8984 7358 8986 7364 8992 7369 8998 7372 9005 7374
9009 7369 9016 7371 9020 7377 9024 7382 9017 7382 9010 7382
9003 7384 8998 7389 8992 7386 8995 7393 8999 7399 8996 7405
8990 7408 8984 7413 8977 7411 8971 7413 8967 7419 8967 7427
8972 7432 8975 7438 8968 7438 8962 7434 8957 7429 8954 7423
8947 7426 8943 7421 8940 7414 8933 7414 8929 7408 8925 7403
8926 7396 8929 7389 8936 7391 8941 7397 8948 7397 8950 7391
8949 7384 8945 7377 8946 7370 8945 7363 8944 7356 8950 7352
8955 7357 8958 7364 8963 7359 8970 7358 8971 7351 8965 7347
8959 7343 8952 7343 8947 7348 8940 7350 8933 7351 8928 7346
8921 7344 8920 7337 98 N F
8928 7249 8925 7246 8925 7242 8928 7240 8926 7237 8927 7234
8927 7235 8929 7236 8932 7238 8934 7240 8938 7242 8934 7242
8932 7244 8931 7248 8933 7250 8932 7253 8929 7252 8928 7249
18 N F
8936 7269 8930 7267 8931 7264 8932 7263 8933 7262 8936 7263
8940 7263 8943 7264 8946 7266 8949 7267 8946 7269 8944 7272
8945 7275 8942 7274 8939 7272 8936 7273 8936 7269 17 N F
8937 6058 8935 6056 8936 6054 8935 6052 8937 6050 8938 6048
8939 6050 8940 6053 8941 6054 8942 6056 8942 6059 8939 6059
8937 6058 13 N F
8951 7984 8947 7981 8948 7978 8950 7975 8954 7975 8956 7973

8959 7975 8962 7976 8964 7979 8968 7978 8971 7978 8974 7980
8975 7983 8973 7985 8970 7986 8967 7988 8964 7987 8960 7986
8957 7985 8954 7985 8951 7984 21 N F
9010 13068 9010 13062 9012 13059 9016 13058 9020 13061 9023 13063
9024 13068 9021 13071 9020 13075 9020 13080 9016 13080 9015 13076
9013 13072 9010 13068 14 N F
9032 7717 9030 7712 9035 7711 9039 7709 9042 7712 9047 7712
9050 7715 9052 7716 9055 7717 9057 7721 9058 7725 9054 7726
9049 7726 9047 7722 9043 7719 9041 7723 9036 7722 9038 7727
9035 7730 9034 7725 9031 7722 9032 7717 22 N F
9045 7620 9042 7617 9043 7614 9046 7612 9049 7613 9051 7615
9054 7617 9052 7620 9049 7620 9045 7620 10 N F
9059 7748 9058 7743 9061 7740 9061 7745 9061 7749 9064 7753
9066 7754 9068 7755 9072 7755 9077 7754 9080 7751 9083 7754
9082 7759 9078 7761 9074 7765 9070 7765 9067 7761 9063 7759
9059 7757 9059 7752 9059 7748 21 N F
9064 7608 9061 7602 9065 7604 9069 7605 9072 7609 9075 7612
9071 7612 9066 7613 9064 7608 9 N F
9087 7921 9084 7914 9088 7913 9092 7913 9094 7917 9098 7916
9103 7917 9103 7922 9104 7926 9104 7931 9099 7931 9098 7927
9094 7930 9090 7928 9086 7925 9087 7921 16 N F
9121 6192 9120 6187 9121 6182 9121 6178 9122 6173 9123 6169
9125 6164 9125 6169 9124 6173 9123 6178 9123 6182 9125 6187
9126 6191 9127 6196 9128 6200 9129 6205 9132 6208 9135 6212
9137 6216 9136 6221 9134 6217 9131 6213 9129 6209 9127 6205
9124 6201 9123 6197 9121 6192 27 N F
9133 7960 9128 7956 9131 7953 9135 7950 9136 7950 9139 7951
9143 7953 9143 7958 9147 7959 9151 7960 9152 7964 9154 7969
9153 7973 9151 7977 9146 7978 9144 7974 9141 7971 9139 7967
9137 7963 9133 7960 20 N F
9139 5973 M 9135 5959 L 9146 5949 L 9160 5952 L 9152 5940 L
9163 5931 L 9175 5923 L 9189 5927 L 9203 5931 L
9216 5935 L 9220 5921 L 9207 5915 L 9192 5912 L
9199 5899 L 9207 5887 L 9211 5873 L 9211 5858 L
9215 5845 L 9220 5831 L 9235 5828 L 9226 5816 L
9231 5803 L 9236 5789 L 9242 5776 L 9248 5763 L
9254 5750 L 9258 5736 L 9258 5721 L 9254 5707 L
9262 5695 L 9275 5689 L 9279 5675 L 9290 5665 L
9301 5656 L 9288 5661 L 9279 5672 L 9271 5685 L
9276 5671 L 9285 5660 L 9295 5649 L 9305 5639 L
9315 5628 L 9327 5621 L 9327 5620 L 9340 5612 L
9346 5625 L 9352 5612 L 9362 5601 L 9372 5591 L
9385 5585 L 9398 5577 L 9411 5571 L 9423 5564 L

9435 5556 L 9446 5546 L 9453 5533 L 9464 5524 L
9471 5511 L 9474 5501 L 9487 5501 L 9520 5501 L
9553 5501 L 9586 5501 L 9607 5501 L 9606 5510 L
9605 5525 L 9607 5539 L 9613 5553 L 9615 5567 L
9621 5581 L 9627 5594 L 9631 5608 L 9634 5622 L
9637 5636 L 9642 5650 L 9645 5664 L 9651 5677 L
9661 5688 L 9663 5702 L 9666 5716 L 9672 5730 L
9683 5738 L 9694 5748 L 9706 5756 L 9719 5763 L
9729 5773 L 9739 5784 L 9749 5794 L 9753 5808 L
9757 5822 L 9752 5836 L 9759 5849 L 9772 5854 L
9783 5863 L 9789 5876 L 9796 5889 L 9800 5903 L
9811 5913 L 9818 5926 L 9823 5939 L 9829 5952 L
9831 5967 L 9836 5980 L 9847 5990 L 9856 6001 L
9858 6016 L 9861 6030 L 9856 6043 L 9858 6057 L
9864 6070 L 9867 6085 L 9871 6098 L 9876 6112 L
9888 6120 L 9890 6135 L 9891 6149 L 9893 6163 L
9898 6177 L 9902 6191 L 9905 6205 L 9910 6218 L
9904 6232 L 9906 6246 L 9909 6260 L 9915 6274 L
9916 6288 L 9916 6302 L 9917 6305 L 9922 6316 L
9923 6330 L 9924 6344 L 9927 6359 L 9932 6372 L
9933 6386 L 9933 6401 L 9938 6414 L 9943 6428 L
9946 6442 L 9948 6456 L 9948 6470 L 9948 6485 L
9950 6499 L 9959 6510 L 9967 6522 L 9959 6534 L
9952 6547 L 9949 6561 L 9954 6575 L 9964 6585 L
9973 6596 L 9971 6610 L 9969 6624 L 9976 6637 L
9987 6646 L 9996 6657 L 10007 6666 L 10012 6679 L
10020 6691 L 10025 6705 L 10025 6719 L 10029 6733 L
10031 6747 L 10033 6761 L 10039 6774 L 10046 6787 L
10057 6796 L 10054 6810 L 10059 6824 L 10071 6831 L
10069 6845 L 10069 6860 L 10067 6874 L 10057 6885 L
10052 6898 L 10049 6912 L 10047 6926 L 10042 6940 L
10038 6953 L 10036 6968 L 10036 6982 L 10037 6996 L
10040 7010 L 10045 7024 L 10053 7036 L 10063 7046 L
10068 7059 L 10079 7069 L 10087 7080 L 10100 7087 L
10103 7089 L 10112 7094 L 10124 7103 L 10116 7114 L
10101 7113 L 10089 7120 L 10087 7134 L 10079 7146 L
10082 7160 L 10072 7170 L 10057 7169 L 10043 7170 L
10029 7167 L 10018 7176 L 10005 7182 L 9992 7175 L
9983 7186 L 9970 7192 L 9958 7200 L 9963 7214 L
9949 7209 L 9939 7219 L 9930 7230 L 9926 7244 L
9915 7252 L 9902 7259 L 9888 7258 L 9874 7254 L
9860 7249 L 9848 7242 L 9839 7231 L 9831 7219 L
9823 7207 L 9816 7195 L 9827 7186 L 9814 7181 L

9804 7171 L 9792 7164 L 9779 7158 L 9765 7155 L
9751 7152 L 9737 7153 L 9723 7148 L 9710 7142 L
9698 7135 L 9686 7127 L 9672 7124 L 9659 7119 L
9648 7110 L 9635 7103 L 9625 7093 L 9617 7077 L
9614 7063 L 9606 7052 L 9597 7040 L 9591 7027 L
9585 7015 L 9581 7001 L 9572 6990 L 9563 6978 L
9570 6965 L 9566 6952 L 9562 6938 L 9552 6927 L
9542 6918 L 9539 6904 L 9528 6893 L 9539 6883 L
9524 6884 L 9510 6882 L 9497 6877 L 9489 6865 L
9495 6852 L 9481 6849 L 9468 6844 L 9463 6830 L
9458 6817 L 9455 6803 L 9451 6789 L 9444 6776 L
9437 6764 L 9426 6754 L 9418 6743 L 9408 6732 L
9402 6719 L 9396 6706 L 9391 6692 L 9385 6679 L
9383 6665 L 9374 6653 L 9370 6640 L 9363 6627 L
9355 6615 L 9367 6607 L 9353 6607 L 9346 6595 L
9339 6582 L 9331 6570 L 9328 6556 L 9324 6542 L
9315 6531 L 9305 6521 L 9295 6510 L 9288 6497 L
9279 6486 L 9273 6474 L 9273 6459 L 9266 6446 L
9261 6433 L 9256 6420 L 9246 6409 L 9255 6398 L
9270 6396 L 9284 6394 L 9298 6397 L 9287 6388 L
9273 6385 L 9258 6385 L 9244 6387 L 9230 6386 L
9222 6374 L 9213 6363 L 9211 6349 L 9209 6335 L
9206 6320 L 9194 6312 L 9188 6299 L 9182 6286 L
9180 6272 L 9179 6257 L 9186 6245 L 9186 6230 L
9192 6217 L 9180 6209 L 9190 6198 L 9187 6184 L
9198 6175 L 9187 6165 L 9191 6151 L 9195 6138 L
9194 6123 L 9192 6109 L 9198 6096 L 9184 6099 L
9183 6084 L 9175 6072 L 9176 6058 L 9165 6049 L
9178 6044 L 9164 6042 L 9163 6027 L 9164 6013 L
9154 6003 L 9168 5999 L 9173 5985 L 9168 5972 L
9157 5963 L 9143 5959 L 9139 5973 L N E
9169 14283 9167 14277 9168 14273 9172 14270 9175 14268 9179 14265
9181 14269 9181 14273 9180 14278 9181 14282 9177 14284 9173 14285
9169 14283 13 N F
9197 15646 9195 15639 9198 15636 9202 15638 9205 15641 9208 15643
9209 15644 9213 15644 9217 15645 9219 15649 9218 15653 9218 15657
9214 15660 9210 15662 9208 15658 9204 15658 9203 15654 9204 15650
9200 15650 9197 15646 20 N F
9208 14292 9204 14290 9206 14287 9209 14283 9211 14279 9215 14277
9219 14277 9222 14274 9225 14277 9224 14281 9223 14285 9220 14289
9216 14289 9213 14291 9208 14292 15 N F
9225 14252 9220 14249 9224 14246 9228 14247 9231 14243 9234 14240
9237 14237 9239 14241 9238 14245 9237 14250 9239 14254 9237 14257

9235 14261 9233 14265 9231 14269 9227 14271 9224 14269 9223 14265
9223 14260 9224 14256 9225 14252 21 N F
9224 14395 9223 14391 9224 14384 9227 14382 9227 14382 9231 14384
9235 14383 9240 14383 9242 14386 9239 14389 9235 14390 9230 14390
9231 14394 9230 14399 9227 14402 9225 14399 9224 14395 17 N F
9226 8181 9224 8176 9226 8172 9230 8169 9233 8166 9237 8164
9241 8161 9245 8161 9249 8163 9253 8166 9258 8167 9262 8167
9258 8169 9254 8171 9253 8175 9251 8179 9249 8183 9245 8184
9242 8187 9237 8188 9233 8187 9229 8184 9226 8181 23 N F
9250 14460 9246 14455 9250 14452 9253 14455 9255 14459 9259 14460
9263 14460 9265 14464 9265 14469 9261 14470 9257 14469 9253 14468
9251 14464 9250 14460 14 N F
9290 14499 9286 14496 9288 14494 9290 14491 9293 14489 9295 14486
9295 14489 9294 14492 9296 14494 9293 14495 9293 14498 9290 14499
12 N F
9290 14511 9288 14507 9290 14507 9291 14507 9294 14507 9297 14509
9300 14507 9301 14510 9298 14512 9297 14516 9294 14516 9293 14513
9290 14511 13 N F
9311 7860 9308 7856 9311 7857 9312 7854 9315 7856 9317 7859
9315 7862 9312 7863 9311 7860 9 N F
9310 14318 9308 14314 9311 14312 9314 14310 9316 14312 9316 14316
9313 14317 9310 14318 8 N F
9317 5528 9315 5523 9319 5521 9323 5522 9326 5526 9329 5530
9333 5532 9334 5536 9331 5540 9327 5541 9323 5539 9319 5536
9320 5531 9317 5528 14 N F
9317 7856 9315 7849 9319 7847 9323 7850 9326 7853 9330 7852
9328 7856 9325 7859 9321 7858 9317 7856 10 N F
9412 15142 9402 15141 9404 15134 9403 15128 9409 15125 9415 15123
9422 15125 9428 15125 9433 15119 9435 15113 9438 15119 9444 15123
9448 15128 9453 15132 9456 15133 9460 15133 9467 15133 9472 15129
9478 15126 9485 15126 9491 15128 9495 15133 9500 15138 9507 15139
9510 15145 9514 15151 9520 15149 9526 15153 9529 15159 9531 15166
9530 15172 9530 15179 9526 15185 9525 15191 9518 15193 9512 15196
9506 15199 9500 15202 9493 15203 9498 15197 9498 15191 9494 15185
9488 15182 9482 15182 9477 15177 9471 15174 9464 15174 9457 15175
9451 15173 9444 15171 9438 15171 9432 15174 9430 15168 9430 15161
9425 15157 9419 15153 9416 15147 9412 15142 58 N F
9420 15062 9415 15057 9421 15054 9428 15052 9432 15057 9434 15064
9439 15068 9444 15073 9448 15079 9449 15085 9455 15089 9461 15089
9468 15089 9470 15082 9465 15078 9461 15072 9457 15067 9454 15061
9451 15055 9458 15053 9464 15051 9471 15050 9476 15046 9483 15047
9489 15050 9489 15057 9493 15063 9496 15069 9496 15076 9493 15082
9487 15086 9481 15089 9488 15088 9495 15088 9501 15090 9499 15099

9496 15105 9490 15107 9484 15110 9477 15112 9471 15110 9465 15106
9461 15111 9456 15115 9449 15114 9445 15108 9442 15102 9442 15096
9438 15090 9431 15089 9424 15089 9422 15082 9424 15076 9418 15075
9421 15069 9420 15062 56 N F
9416 8788 9416 8785 9417 8782 9419 8779 9421 8775 9424 8774
9427 8771 9428 8769 9431 8767 9435 8766 9438 8765 9441 8767
9441 8771 9441 8774 9438 8776 9437 8779 9435 8782 9432 8784
9429 8785 9426 8786 9423 8787 9419 8788 9416 8788 23 N F
9475 8763 9469 8762 9472 8760 9475 8758 9478 8757 9481 8755
9484 8753 9486 8751 9489 8749 9493 8748 9496 8747 9499 8748
9502 8749 9505 8751 9507 8753 9509 8756 9509 8759 9507 8762
9505 8764 9501 8765 9498 8765 9495 8766 9491 8766 9488 8765
9485 8765 9481 8765 9478 8765 9475 8763 28 N F
9544 10905 9537 10896 9541 10890 9546 10886 9552 10881 9556 10876
9561 10871 9565 10865 9570 10861 9576 10864 9582 10860 9589 10858
9594 10853 9599 10848 9605 10844 9610 10840 9615 10835 9619 10830
9625 10826 9631 10822 9637 10819 9644 10816 9647 10811 9653 10807
9660 10807 9667 10804 9672 10800 9678 10797 9685 10795 9691 10791
9698 10790 9704 10787 9710 10783 9716 10780 9722 10776 9727 10772
9734 10770 9740 10768 9746 10764 9752 10761 9755 10760 9759 10759
9765 10758 9772 10759 9779 10757 9786 10755 9792 10753 9799 10751
9805 10748 9811 10745 9818 10742 9824 10739 9831 10737 9837 10735
9844 10734 9851 10732 9857 10731 9858 10731 9864 10730 9871 10733
9876 10737 9879 10743 9883 10749 9889 10752 9891 10759 9892 10766
9893 10773 9890 10779 9885 10783 9879 10787 9873 10790 9866 10790
9859 10790 9852 10792 9846 10794 9839 10796 9833 10800 9827 10803
9822 10807 9816 10810 9810 10814 9803 10817 9797 10819 9790 10821
9784 10824 9777 10826 9771 10828 9764 10830 9757 10831 9751 10835
9745 10838 9741 10843 9738 10849 9733 10854 9727 10859 9722 10863
9716 10866 9709 10868 9703 10871 9697 10874 9690 10876 9684 10878
9677 10881 9671 10884 9667 10890 9664 10896 9661 10902 9657 10908
9653 10914 9649 10919 9643 10923 9638 10927 9632 10931 9625 10933
9618 10934 9611 10935 9605 10935 9598 10935 9591 10933 9584 10931
9578 10929 9571 10926 9566 10922 9561 10917 9556 10913 9550 10909
9544 10905 127 N F
9560 8697 9557 8693 9560 8689 9563 8693 9567 8695 9571 8697
9575 8699 9578 8696 9582 8694 9582 8699 9583 8699 9586 8701
9589 8705 9592 8708 9592 8708 9597 8708 9593 8710 9589 8708
9584 8707 9581 8704 9576 8706 9573 8709 9570 8713 9566 8713
9566 8708 9562 8706 9561 8702 9560 8697 28 N F
9567 8757 9566 8752 9570 8754 9575 8755 9579 8755 9583 8757
9588 8758 9592 8757 9590 8753 9592 8748 9596 8751 9600 8751
9605 8752 9609 8754 9613 8756 9616 8759 9620 8761 9617 8763

9613 8765 9610 8769 9607 8772 9603 8774 9599 8772 9595 8772
9591 8773 9587 8770 9583 8769 9584 8773 9579 8773 9578 8768
9575 8766 9570 8764 9567 8761 9567 8757 34 N F
9584 8482 9579 8479 9582 8475 9586 8473 9589 8470 9589 8474
9589 8478 9593 8476 9597 8475 9601 8473 9605 8473 9609 8470
9609 8474 9606 8478 9602 8479 9599 8483 9595 8486 9591 8487
9588 8491 9583 8491 9587 8488 9590 8485 9586 8486 9584 8482
24 N F
9588 8660 9585 8658 9588 8657 9591 8656 9593 8657 9595 8658
9597 8660 9596 8663 9595 8665 9592 8667 9590 8665 9589 8663
9588 8660 13 N F
9595 8686 9592 8681 9597 8680 9601 8679 9605 8680 9610 8679
9614 8681 9618 8681 9621 8684 9625 8682 9629 8681 9633 8678
9637 8680 9638 8684 9638 8688 9638 8693 9634 8695 9638 8697
9639 8693 9643 8696 9646 8700 9643 8703 9644 8708 9640 8709
9637 8705 9633 8703 9629 8704 9625 8702 9628 8698 9626 8694
9622 8694 9617 8693 9615 8696 9612 8700 9610 8696 9606 8693
9603 8690 9599 8688 9595 8686 39 N F
9616 8860 9607 8858 9609 8851 9610 8844 9613 8838 9617 8832
9621 8827 9621 8820 9614 8816 9620 8813 9619 8806 9626 8808
9633 8808 9639 8811 9640 8811 9646 8814 9653 8816 9652 8823
9659 8820 9664 8816 9667 8823 9661 8827 9663 8833 9669 8830
9675 8833 9677 8826 9684 8828 9680 8834 9686 8837 9687 8844
9680 8844 9674 8848 9670 8853 9671 8860 9669 8867 9666 8874
9669 8880 9673 8886 9680 8887 9678 8893 9672 8897 9666 8901
9660 8904 9653 8904 9646 8902 9642 8896 9641 8889 9642 8882
9636 8879 9632 8873 9627 8869 9621 8865 9616 8860 53 N F
9632 8778 9630 8775 9633 8771 9635 8767 9639 8766 9640 8767
9642 8769 9646 8772 9650 8775 9654 8776 9656 8777 9658 8778
9662 8780 9657 8780 9653 8779 9649 8781 9644 8782 9640 8783
9636 8781 9632 8778 20 N F
9651 8574 9647 8571 9650 8571 9650 8569 9653 8568 9655 8569
9656 8571 9658 8572 9659 8574 9660 8575 9661 8576 9662 8578
9661 8579 9659 8579 9656 8579 9655 8578 9655 8575 9653 8574
9651 8574 19 N F
9655 8568 9654 8565 9656 8564 9658 8565 9659 8567 9660 8569
9660 8572 9658 8572 9656 8570 9655 8568 10 N F
9670 14795 9667 14792 9668 14789 9668 14786 9670 14783 9673 14784
9675 14787 9674 14790 9673 14793 9673 14796 9674 14799 9671 14800
9669 14798 9670 14795 14 N F
9755 10719 9746 10714 9752 10711 9758 10708 9764 10705 9770 10702
9775 10697 9782 10694 9788 10691 9794 10687 9799 10683 9805 10679
9812 10677 9817 10673 9823 10670 9827 10664 9831 10658 9835 10653

9841 10649 9847 10651 9848 10658 9847 10665 9844 10671 9839 10676
9835 10682 9831 10687 9825 10691 9820 10696 9813 10698 9808 10702
9802 10705 9795 10707 9789 10710 9782 10711 9775 10714 9769 10715
9762 10717 9755 10719 38 N F
9791 8840 9790 8837 9790 8833 9793 8833 9796 8835 9797 8839
9799 8841 9802 8842 9805 8843 9806 8847 9804 8850 9801 8849
9799 8846 9797 8844 9794 8842 9791 8840 16 N F
9794 9440 9792 9437 9795 9436 9798 9434 9800 9431 9804 9432
9807 9432 9810 9433 9813 9434 9814 9437 9814 9440 9812 9443
9809 9443 9806 9442 9807 9438 9804 9437 9800 9437 9798 9439
9794 9440 19 N F
9830 9087 9828 9081 9830 9079 9833 9078 9836 9075 9839 9074
9841 9076 9839 9079 9841 9081 9842 9082 9845 9083 9845 9086
9842 9087 9842 9091 9840 9094 9839 9097 9836 9097 9833 9097
9831 9093 9831 9090 9830 9087 21 N F
9841 9071 9837 9068 9838 9065 9841 9063 9844 9062 9847 9063
9849 9066 9847 9069 9844 9069 9841 9071 10 N F
9858 10684 9850 10683 9853 10677 9856 10671 9857 10664 9859 10657
9861 10650 9866 10646 9872 10643 9878 10638 9884 10641 9887 10647
9891 10653 9894 10659 9893 10666 9890 10672 9884 10676 9878 10679
9872 10682 9865 10682 9858 10684 21 N F
9876 14972 9865 14967 9869 14962 9868 14955 9873 14951 9878 14946
9884 14950 9887 14956 9888 14962 9894 14958 9900 14957 9899 14963
9897 14970 9895 14976 9897 14983 9903 14985 9897 14985 9892 14980
9885 14979 9879 14978 9876 14972 21 N F
9912 5749 9903 5747 9907 5742 9909 5737 9912 5732 9917 5730
9921 5725 9926 5725 9925 5731 9927 5737 9928 5742 9928 5748
9923 5750 9917 5751 9912 5749 15 N F
9907 9436 9904 9429 9906 9425 9906 9425 9910 9425 9915 9425
9919 9424 9921 9423 9923 9422 9927 9420 9929 9416 9932 9413
9937 9414 9941 9412 9945 9415 9949 9416 9946 9420 9951 9421
9952 9422 9953 9424 9956 9427 9953 9431 9949 9431 9945 9434
9947 9438 9945 9441 9940 9440 9936 9439 9932 9438 9927 9438
9923 9440 9920 9443 9916 9442 9912 9439 9909 9443 9905 9440
9907 9436 37 N F
9907 9191 9904 9187 9908 9186 9911 9185 9913 9182 9915 9180
9919 9180 9921 9182 9922 9185 9922 9188 9925 9190 9927 9193
9923 9194 9922 9191 9919 9189 9916 9187 9913 9186 9911 9188
9912 9192 9909 9193 9906 9194 9907 9191 22 N F
9910 15016 9906 15014 9909 15014 9912 15013 9915 15011 9915 15011
9917 15008 9920 15009 9923 15009 9921 15012 9918 15013 9915 15014
9913 15016 9910 15016 14 N F
9910 14994 9907 14991 9910 14991 9913 14989 9915 14991 9919 14992

9919 14995 9918 14998 9915 14998 9912 14997 9910 14994 11 N F
9914 9408 9907 9403 9912 9402 9916 9402 9915 9397 9920 9397
9922 9393 9926 9391 9930 9393 9930 9398 9931 9402 9929 9405
9932 9408 9929 9412 9926 9414 9921 9416 9917 9415 9914 9412
9914 9408 19 N F
9912 9925 9908 9918 9913 9913 9915 9906 9920 9902 9927 9903
9932 9908 9938 9912 9944 9916 9949 9920 9952 9927 9950 9933
9947 9940 9946 9947 9942 9952 9936 9955 9932 9961 9929 9967
9922 9967 9915 9965 9914 9959 9911 9952 9908 9946 9909 9939
9911 9932 9912 9925 26 N F
9943 15000 9938 14998 9941 14996 9942 14993 9945 14994 9948 14993
9951 14994 9955 14993 9958 14994 9960 14996 9962 14999 9959 15000
9956 14999 9953 15000 9949 15000 9946 15000 9943 15000 17 N F
9945 15034 9938 15033 9941 15030 9943 15029 9945 15028 9949 15027
9954 15027 9957 15024 9961 15022 9965 15021 9969 15021 9973 15019
9973 15014 9976 15011 9978 15015 9978 15019 9982 15020 9986 15020
9988 15020 9990 15019 9995 15019 9999 15019 10003 15018 10007 15017
10011 15014 10014 15017 10017 15020 10020 15023 10016 15023 10012 15024
10008 15025 10004 15027 10001 15030 9997 15032 9995 15028 9991 15027
9986 15025 9982 15025 9978 15026 9974 15027 9971 15030 9968 15033
9968 15037 9965 15034 9963 15031 9959 15033 9959 15037 9955 15037
9952 15034 9948 15037 9945 15034 51 N F
9942 9235 9938 9233 9941 9231 9943 9233 9945 9235 9948 9237
9951 9239 9951 9243 9951 9246 9953 9249 9956 9248 9955 9251
9954 9254 9952 9256 9949 9256 9946 9254 9945 9251 9946 9248
9945 9244 9942 9244 9943 9241 9944 9238 9942 9235 23 N F
9942 9978 9939 9972 9942 9969 9947 9968 9950 9966 9949 9961
9953 9960 9958 9960 9958 9964 9959 9969 9962 9973 9961 9977
9963 9981 9960 9983 9956 9986 9952 9985 9948 9983 9944 9981
9942 9978 19 N F
9948 9215 9945 9209 9949 9209 9951 9207 9954 9207 9957 9208
9957 9211 9954 9214 9957 9216 9959 9218 9961 9221 9965 9222
9968 9222 9968 9225 9972 9226 9975 9227 9972 9228 9968 9228
9966 9226 9963 9224 9961 9222 9957 9222 9955 9220 9951 9220
9948 9220 9946 9217 9948 9215 27 N F
9950 9440 9947 9433 9952 9433 9956 9431 9958 9427 9961 9424
9965 9423 9968 9420 9969 9416 9969 9411 9967 9407 9964 9404
9968 9405 9968 9405 9973 9405 9977 9405 9977 9405 9981 9403
9980 9399 9979 9395 9983 9394 9987 9392 9989 9394 9990 9395
9995 9396 9992 9400 9993 9404 9994 9409 9998 9410 10001 9413
9999 9416 9994 9416 9997 9420 10001 9422 10004 9426 10002 9430
10001 9434 10001 9438 9998 9442 10001 9446 10000 9450 9996 9453
9993 9456 9989 9458 9986 9461 9983 9465 9979 9466 9975 9463

9974 9459 9971 9456 9967 9454 9963 9451 9960 9449 9956 9447
9952 9444 9950 9440 56 N F
9957 10018 9950 10015 9954 10009 9956 10006 9959 10004 9964 10000
9967 9994 9970 9987 9971 9986 9975 9982 9976 9975 9977 9968
9976 9962 9973 9955 9977 9949 9982 9945 9987 9940 9991 9934
9995 9928 9997 9921 10003 9918 10007 9921 10009 9922 10014 9927
10020 9929 10020 9929 10027 9929 10034 9930 10040 9926 10042 9923
10044 9920 10051 9921 10058 9921 10065 9922 10065 9922 10071 9920
10077 9917 10084 9914 10090 9912 10097 9910 10104 9908 10110 9904
10115 9900 10116 9900 10122 9897 10129 9898 10135 9899 10142 9902
10139 9908 10137 9915 10140 9922 10143 9928 10145 9935 10151 9939
10144 9942 10141 9948 10135 9952 10130 9957 10126 9962 10120 9966
10113 9965 10106 9966 10102 9972 10096 9975 10095 9981 10088 9981
10081 9981 10074 9984 10071 9990 10065 9989 10058 9990 10051 9991
10047 9997 10042 10002 10038 10008 10033 10013 10026 10014 10020 10010
10014 10007 10007 10005 10001 10003 9994 10005 9989 10010 9982 10004
9976 10008 9970 10009 9970 10016 9965 10021 9959 10024 9957 10018
90 N F
9956 10096 9955 10093 9955 10090 9956 10086 9959 10089 9959 10089
9962 10091 9964 10093 9965 10096 9963 10099 9966 10101 9964 10104
9967 10106 9969 10109 9970 10112 9968 10114 9965 10113 9965 10109
9963 10107 9960 10109 9959 10106 9959 10102 9958 10099 9956 10096
24 N F
9957 9877 9955 9869 9958 9866 9961 9863 9965 9862 9970 9863
9974 9865 9978 9865 9983 9867 9987 9869 9987 9873 9987 9878
9984 9881 9980 9883 9977 9886 9976 9890 9972 9892 9971 9896
9967 9895 9965 9891 9961 9889 9958 9886 9957 9882 9957 9877
24 N F
9966 9081 9963 9078 9967 9078 9970 9079 9968 9076 9971 9074
9969 9071 9966 9069 9968 9066 9971 9067 9973 9064 9976 9067
9978 9069 9981 9067 9981 9071 9982 9074 9984 9077 9985 9078
9986 9079 9983 9080 9979 9080 9976 9081 9976 9085 9977 9088
9973 9087 9970 9087 9969 9083 9966 9081 28 N F
9967 9043 9964 9040 9967 9040 9970 9041 9972 9044 9974 9042
9977 9044 9979 9046 9982 9048 9985 9050 9987 9053 9985 9055
9981 9055 9978 9054 9975 9052 9972 9051 9970 9049 9970 9045
9967 9043 19 N F
9978 9809 9976 9805 9978 9801 9977 9796 9980 9794 9985 9793
9989 9793 9994 9794 9996 9797 9998 9801 10000 9805 10003 9808
10005 9812 10008 9816 10008 9820 10006 9824 10002 9825 9997 9823
9992 9820 9988 9818 9984 9815 9981 9812 9978 9809 23 N F
9982 9205 9978 9202 9981 9199 9981 9196 9984 9198 9986 9200
9989 9201 9991 9204 9989 9206 9986 9205 9982 9205 11 N F

9983 14991 9979 14988 9982 14986 9984 14988 9987 14989 9989 14991
9989 14994 9988 14997 9986 14994 9983 14995 9983 14991 11 N F
9989 10029 9986 10025 9989 10026 9992 10025 9994 10022 9996 10020
9994 10018 9992 10015 9995 10015 9998 10017 10001 10019 10003 10021
10005 10023 10007 10026 10010 10028 10007 10031 10007 10034 10005 10037
10002 10036 9999 10034 9999 10031 9997 10034 9994 10035 9991 10033
9993 10030 9989 10029 26 N F
9993 10282 9990 10278 9993 10276 9995 10274 9998 10275 9999 10278
10001 10281 10003 10283 10000 10282 9997 10284 9996 10281 9993 10282
12 N F
9999 10254 9994 10252 9997 10250 10000 10250 10002 10247 10003 10244
10005 10245 10006 10245 10009 10248 10009 10251 10011 10254 10014 10255
10011 10256 10007 10257 10004 10257 10003 10254 9999 10254 17 N F
10002 15006 9995 15001 9998 14998 10003 14998 10006 15001 10010 15001
10014 15002 10019 15002 10023 15003 10027 15002 10026 14997 10025 14993
10023 14989 10023 14985 10026 14988 10029 14991 10032 14994 10037 14994
10037 14998 10041 14999 10044 15000 10045 15001 10048 15003 10045 15006
10043 15009 10039 15011 10041 15015 10037 15012 10035 15008 10031 15010
10027 15010 10022 15009 10018 15008 10014 15006 10010 15006 10006 15007
10002 15006 37 N F
10001 9784 9998 9782 9999 9779 9999 9775 10000 9772 9999 9769
10002 9770 10004 9773 10007 9775 10008 9778 10010 9781 10013 9782
10013 9786 10012 9789 10008 9790 10005 9789 10002 9787 10001 9784
18 N F
10001 9900 10000 9896 10002 9893 10002 9890 10005 9888 10008 9888
10010 9890 10012 9893 10014 9896 10014 9899 10013 9902 10010 9904
10007 9904 10004 9902 10001 9900 15 N F
10017 9458 10015 9451 10019 9452 10024 9452 10026 9457 10029 9460
10032 9457 10036 9457 10040 9460 10043 9464 10038 9465 10034 9464
10030 9466 10030 9471 10035 9472 10032 9476 10035 9479 10030 9480
10026 9482 10024 9478 10026 9474 10023 9471 10027 9468 10023 9466
10021 9463 10016 9462 10017 9458 27 N F
10021 9779 10016 9767 10023 9767 10026 9772 10027 9773 10034 9773
10039 9768 10046 9769 10047 9762 10054 9759 10060 9755 10060 9748
10060 9741 10067 9741 10069 9748 10072 9742 10078 9745 10080 9738
10086 9737 10090 9743 10091 9750 10095 9756 10090 9761 10088 9768
10082 9770 10077 9775 10070 9777 10064 9781 10060 9786 10055 9792
10057 9799 10055 9805 10050 9811 10045 9806 10039 9804 10032 9804
10026 9799 10026 9792 10024 9785 10021 9779 40 N F
10022 10094 10017 10085 10023 10082 10029 10079 10035 10075 10041 10072
10046 10068 10053 10066 10060 10066 10062 10066 10067 10066 10071 10065
10074 10064 10080 10061 10087 10059 10084 10066 10082 10072 10087 10077
10088 10084 10084 10090 10089 10095 10094 10100 10087 10100 10082 10105

10084 10112 10078 10115 10071 10113 10065 10116 10063 10109 10057 10106
10050 10105 10045 10100 10039 10102 10032 10102 10028 10097 10022 10094
36 N F
10020 9923 10018 9920 10020 9917 10019 9914 10020 9911 10024 9912
10026 9914 10029 9915 10028 9918 10026 9921 10023 9923 10020 9923
12 N F
10039 14903 10035 14900 10038 14897 10041 14894 10037 14892 10041 14890
10044 14887 10048 14886 10053 14885 10057 14883 10060 14886 10059 14890
10055 14891 10054 14895 10051 14898 10047 14896 10044 14900 10044 14904
10039 14903 19 N F
10039 9911 10038 9907 10039 9904 10042 9904 10046 9905 10046 9908
10047 9911 10046 9914 10042 9915 10039 9914 10039 9911 11 N F
10052 15005 10049 15004 10052 15003 10055 15003 10058 15005 10055 15007
10052 15005 7 N F
10055 9905 10050 9901 10050 9901 10052 9899 10055 9898 10059 9898
10062 9897 10065 9899 10067 9901 10065 9904 10064 9907 10061 9908
10057 9907 10055 9905 14 N F
10052 10030 10050 10020 10053 10013 10058 10008 10065 10010 10069 10016
10070 10023 10077 10025 10081 10030 10075 10034 10069 10037 10062 10039
10056 10035 10052 10030 14 N F
10057 9914 10053 9910 10056 9909 10059 9911 10061 9910 10062 9910
10065 9911 10068 9909 10071 9907 10073 9904 10076 9906 10077 9909
10075 9912 10072 9914 10070 9916 10068 9919 10065 9920 10062 9919
10060 9916 10057 9914 20 N F
10060 15003 10055 15000 10058 14999 10061 14997 10063 14999 10066 15000
10069 15000 10072 15001 10069 15003 10066 15003 10063 15002 10060 15003
12 N F
10068 10057 10064 10053 10065 10051 10068 10050 10070 10048 10072 10047
10075 10048 10076 10051 10075 10054 10073 10055 10071 10057 10068 10057
12 N F
10071 14922 10065 14921 10067 14918 10070 14917 10073 14918 10076 14918
10079 14917 10081 14919 10079 14921 10076 14923 10074 14921 10071 14922
12 N F
10082 14908 10081 14904 10084 14901 10087 14903 10089 14905 10091 14907
10089 14910 10086 14911 10085 14908 10082 14908 10 N F
10089 9607 10088 9604 10089 9601 10092 9602 10095 9601 10097 9604
10100 9606 10100 9609 10103 9611 10106 9613 10109 9613 10109 9616
10105 9616 10102 9617 10100 9615 10097 9614 10097 9610 10095 9608
10092 9606 10089 9607 20 N F
10095 9768 10093 9764 10096 9763 10099 9761 10102 9760 10102 9760
10105 9759 10107 9756 10110 9754 10113 9752 10114 9755 10117 9756
10120 9755 10124 9755 10126 9753 10128 9751 10129 9754 10130 9757
10127 9758 10124 9758 10120 9757 10118 9759 10114 9760 10111 9761

10109 9763 10105 9764 10105 9767 10102 9769 10099 9769 10097 9771
10094 9771 10095 9768 32 N F
10094 10220 10093 10216 10096 10215 10100 10216 10101 10219 10102 10222
10099 10224 10097 10222 10094 10220 9 N F
10098 7010 10094 7010 10096 7009 10098 7007 10100 7006 10102 7005
10104 7005 10106 7006 10108 7005 10110 7006 10111 7008 10109 7009
10108 7011 10107 7013 10105 7014 10103 7015 10101 7014 10100 7011
10098 7010 19 N F
10101 14915 10095 14917 10098 14915 10100 14912 10101 14909 10103 14908
10104 14908 10105 14911 10107 14913 10105 14916 10101 14915 11 N F
10100 9806 10095 9801 10097 9800 10100 9800 10102 9796 10105 9792
10107 9789 10111 9786 10115 9784 10118 9781 10122 9783 10126 9781
10130 9779 10134 9782 10138 9783 10143 9782 10143 9786 10139 9786
10138 9790 10134 9791 10131 9788 10126 9790 10122 9790 10118 9791
10114 9794 10111 9797 10114 9800 10116 9804 10112 9806 10108 9809
10104 9809 10100 9806 32 N F
10103 9843 10097 9836 10103 9832 10108 9828 10109 9828 10112 9822
10118 9817 10123 9814 10128 9809 10135 9807 10141 9803 10147 9806
10143 9812 10140 9818 10140 9825 10134 9829 10140 9833 10136 9839
10129 9841 10123 9843 10116 9845 10110 9843 10103 9843 23 N F
10101 10288 10099 10284 10099 10284 10102 10284 10104 10285 10105 10286
10108 10288 10106 10291 10103 10292 10105 10289 10101 10288 11 N F
10104 10081 10101 10071 10102 10064 10108 10067 10114 10070 10121 10072
10128 10072 10124 10078 10118 10080 10111 10080 10104 10081 11 N F
10115 10191 10107 10192 10110 10186 10108 10179 10113 10174 10112 10167
10118 10164 10124 10161 10131 10160 10130 10167 10136 10168 10142 10172
10148 10168 10155 10169 10161 10167 10167 10167 10168 10167 10174 10163
10179 10163 10181 10163 10179 10170 10173 10173 10178 10177 10174 10183
10167 10185 10160 10185 10154 10186 10152 10193 10145 10192 10148 10186
10141 10187 10135 10189 10128 10191 10121 10189 10115 10191 35 N F
10120 10068 10111 10063 10116 10058 10121 10064 10128 10065 10134 10063
10141 10065 10147 10067 10152 10073 10155 10074 10159 10075 10164 10079
10161 10073 10164 10066 10169 10062 10175 10065 10179 10071 10177 10078
10174 10084 10167 10086 10160 10085 10153 10085 10146 10083 10139 10083
10133 10082 10132 10075 10127 10070 10120 10068 28 N F
10120 15169 10118 15165 10123 15164 10127 15164 10129 15160 10131 15157
10133 15153 10135 15153 10138 15154 10142 15154 10144 15157 10147 15161
10150 15164 10152 15168 10152 15168 10154 15171 10155 15176 10152 15179
10148 15182 10142 15185 10139 15182 10141 15179 10140 15174 10137 15178
10133 15178 10133 15182 10129 15183 10127 15180 10125 15176 10123 15172
10120 15169 31 N F
10126 10221 10123 10218 10126 10218 10129 10216 10131 10214 10135 10214
10136 10217 10135 10220 10136 10223 10135 10227 10133 10224 10132 10221

10129 10220 10126 10221 14 N F
10130 9859 10126 9853 10130 9851 10134 9849 10139 9849 10143 9850
10147 9853 10148 9853 10151 9854 10151 9859 10155 9861 10153 9865
10148 9864 10144 9863 10140 9865 10135 9864 10131 9863 10130 9859
18 N F
10144 7464 10144 7461 10146 7460 10148 7459 10149 7460 10151 7462
10151 7464 10148 7465 10146 7465 10144 7464 10 N F
10150 9902 10145 9896 10151 9891 10153 9888 10154 9885 10160 9881
10166 9877 10166 9876 10168 9871 10173 9866 10168 9861 10169 9854
10171 9848 10176 9842 10183 9841 10188 9845 10184 9851 10182 9857
10185 9864 10190 9867 10197 9870 10197 9877 10195 9884 10188 9885
10182 9888 10189 9888 10196 9890 10191 9895 10191 9902 10185 9906
10179 9902 10172 9901 10166 9903 10159 9901 10155 9907 10150 9902
36 N F
10149 15026 10147 15024 10148 15021 10151 15020 10152 15021 10154 15022
10155 15025 10152 15028 10149 15026 9 N F
10166 7382 10163 7381 10164 7379 10167 7379 10166 7382 5 N F
10169 15133 10166 15128 10169 15125 10171 15121 10174 15120 10176 15120
10179 15118 10180 15122 10180 15126 10184 15127 10182 15130 10177 15131
10173 15131 10169 15133 14 N F
10169 9928 10166 9924 10167 9920 10171 9917 10176 9917 10176 9921
10175 9926 10175 9930 10178 9933 10182 9933 10187 9934 10185 9938
10181 9940 10179 9944 10181 9948 10179 9952 10177 9948 10172 9947
10168 9947 10169 9942 10169 9938 10167 9931 10169 9928 23 N F
10174 10347 10168 10346 10174 10342 10175 10335 10174 10329 10169 10321
10176 10322 10182 10322 10189 10323 10196 10326 10199 10326 10203 10326
10209 10327 10212 10326 10216 10326 10223 10328 10217 10333 10222 10338
10215 10338 10208 10337 10202 10333 10196 10331 10190 10336 10184 10338
10180 10344 10174 10347 26 N F
10194 10176 10190 10171 10193 10171 10196 10173 10199 10172 10200 10169
10202 10166 10204 10167 10206 10167 10207 10170 10204 10172 10202 10174
10201 10178 10199 10180 10196 10179 10194 10176 16 N F
10204 9905 10203 9901 10204 9898 10206 9895 10209 9896 10209 9899
10208 9902 10207 9905 10204 9905 9 N F
10209 10065 10206 10061 10209 10060 10212 10059 10215 10058 10218 10057
10220 10060 10219 10063 10215 10065 10212 10065 10209 10065 11 N F
10222 14935 10216 14930 10222 14930 10227 14928 10229 14923 10224 14925
10221 14921 10221 14916 10220 14910 10222 14905 10227 14902 10230 14907
10234 14911 10235 14911 10239 14913 10242 14917 10243 14922 10239 14927
10236 14931 10234 14936 10234 14941 10232 14936 10227 14934 10222 14935
24 N F
10229 10307 10228 10300 10233 10299 10237 10297 10241 10297 10239 10300
10242 10303 10246 10306 10242 10308 10239 10312 10235 10314 10233 10310

10229 10307 13 N F
10255 10398 10251 10395 10255 10395 10258 10395 10261 10393 10264 10393
10265 10396 10262 10397 10258 10398 10255 10398 10 N F
10269 10397 10267 10394 10270 10392 10273 10394 10276 10395 10274 10398
10271 10400 10269 10397 8 N F
10284 10414 10279 10412 10282 10411 10285 10409 10288 10409 10288 10409
10292 10409 10293 10412 10297 10412 10297 10415 10294 10416 10291 10414
10288 10416 10286 10418 10283 10420 10282 10416 10284 10414 17 N F
10288 10058 10284 10054 10287 10052 10288 10049 10290 10046 10292 10045
10293 10048 10293 10051 10292 10054 10291 10058 10288 10058 11 N F
10307 10056 10302 10054 10304 10052 10306 10049 10309 10048 10310 10045
10311 10042 10313 10045 10312 10048 10312 10052 10309 10054 10307 10056
12 N F
10320 10058 10316 10054 10317 10051 10319 10048 10322 10047 10322 10050
10320 10053 10322 10056 10320 10058 9 N F
10327 10044 10324 10039 10325 10037 10328 10037 10330 10039 10328 10041
10327 10044 7 N F
10328 10341 10325 10337 10327 10334 10328 10333 10330 10337 10335 10337
10337 10334 10341 10332 10341 10336 10340 10341 10342 10345 10343 10349
10338 10349 10335 10347 10331 10345 10328 10341 16 N F
10338 10384 10333 10381 10335 10379 10338 10381 10338 10381 10341 10382
10338 10384 7 N F
10349 10023 10346 10021 10347 10017 10348 10015 10352 10014 10355 10014
10358 10014 10358 10017 10356 10020 10353 10022 10349 10023 11 N F
10355 10352 10349 10346 10349 10345 10351 10342 10355 10342 10360 10343
10362 10338 10365 10335 10365 10340 10364 10344 10362 10348 10358 10349
10355 10352 13 N F
10429 15312 10428 15308 10431 15308 10434 15310 10437 15310 10440 15312
10443 15311 10446 15312 10444 15314 10443 15317 10440 15318 10437 15317
10434 15315 10431 15314 10429 15312 15 N F
10635 6811 10631 6809 10634 6806 10639 6806 10643 6805 10647 6803
10652 6803 10656 6804 10661 6806 10665 6805 10669 6804 10674 6806
10677 6809 10681 6811 10683 6815 10687 6818 10683 6820 10679 6822
10675 6823 10670 6825 10664 6822 10660 6824 10656 6822 10651 6821
10647 6823 10643 6821 10641 6817 10637 6815 10635 6811 29 N F
10809 15230 10806 15224 10810 15225 10812 15228 10815 15231 10819 15230
10820 15229 10823 15227 10825 15230 10825 15235 10830 15233 10830 15229
10834 15227 10838 15227 10842 15228 10845 15232 10846 15236 10849 15239
10850 15243 10846 15245 10842 15247 10838 15248 10837 15244 10833 15244
10828 15244 10826 15241 10822 15243 10818 15241 10819 15237 10816 15234
10811 15233 10809 15230 32 N F
10810 15176 10809 15171 10812 15171 10811 15168 10814 15166 10817 15166
10820 15167 10822 15169 10822 15173 10820 15175 10818 15178 10818 15181

10815 15179 10813 15177 10810 15176 15 N F
10819 15202 10818 15198 10818 15198 10820 15197 10822 15198 10822 15199
10824 15201 10822 15203 10820 15204 10819 15202 10 N F
10819 15191 10818 15186 10821 15185 10824 15185 10823 15188 10824 15191
10822 15193 10819 15191 8 N F
10846 15223 10844 15219 10846 15215 10845 15210 10848 15207 10851 15210
10855 15211 10855 15216 10857 15219 10854 15223 10856 15226 10853 15228
10849 15226 10846 15223 14 N F
10880 7584 10878 7580 10880 7578 10882 7578 10884 7579 10886 7580
10888 7582 10890 7581 10892 7581 10895 7582 10897 7582 10897 7584
10895 7586 10894 7588 10891 7588 10889 7588 10887 7588 10885 7588
10883 7587 10881 7585 10880 7584 21 N F
10890 14381 10885 14376 10886 14372 10889 14370 10890 14370 10893 14367
10893 14363 10895 14359 10897 14355 10900 14352 10904 14353 10909 14352
10913 14351 10917 14353 10921 14350 10925 14351 10925 14356 10927 14359
10925 14363 10921 14364 10919 14368 10921 14372 10925 14374 10929 14372
10927 14368 10930 14368 10931 14368 10935 14371 10939 14371 10936 14374
10941 14374 10938 14378 10936 14382 10935 14386 10931 14387 10926 14387
10922 14387 10918 14385 10914 14385 10910 14384 10906 14382 10902 14380
10898 14377 10894 14379 10890 14381 45 N F
10906 15215 10901 15209 10906 15210 10909 15213 10912 15215 10917 15215
10920 15217 10923 15220 10925 15224 10929 15226 10932 15230 10933 15234
10937 15234 10936 15238 10940 15239 10944 15238 10946 15240 10947 15241
10950 15244 10949 15249 10944 15248 10940 15247 10943 15251 10939 15252
10942 15256 10938 15256 10940 15260 10937 15262 10936 15266 10939 15270
10943 15269 10943 15273 10939 15275 10936 15278 10931 15278 10927 15279
10924 15276 10920 15274 10924 15271 10923 15267 10919 15265 10922 15262
10923 15258 10919 15255 10915 15253 10914 15249 10909 15250 10906 15247
10904 15244 10902 15239 10906 15241 10910 15242 10914 15244 10918 15242
10916 15238 10912 15237 10913 15233 10917 15232 10918 15227 10916 15223
10912 15224 10908 15224 10907 15219 10906 15215 64 N F
10918 14298 10913 14298 10916 14297 10919 14295 10922 14295 10923 14292
10922 14289 10922 14289 10925 14290 10927 14292 10931 14291 10934 14292
10936 14294 10938 14297 10938 14300 10936 14303 10936 14306 10938 14308
10941 14310 10939 14313 10936 14314 10933 14313 10930 14315 10930 14312
10928 14310 10925 14309 10923 14306 10921 14303 10921 14300 10918 14298
30 N F
10922 7579 10920 7577 10921 7575 10923 7574 10925 7575 10923 7577
10922 7579 7 N F
10932 14683 10926 14681 10929 14678 10933 14677 10937 14678 10935 14681
10936 14685 10940 14682 10944 14683 10947 14686 10950 14682 10954 14680
10958 14678 10961 14676 10965 14674 10970 14674 10970 14678 10973 14675
10977 14675 10980 14678 10977 14682 10975 14686 10973 14689 10969 14692

10970 14696 10965 14696 10961 14696 10958 14699 10953 14700 10949 14701
10946 14704 10945 14700 10942 14697 10940 14693 10936 14692 10931 14692
10932 14687 10932 14683 38 N F
10946 14273 10943 14267 10946 14268 10948 14271 10951 14272 10954 14274
10957 14273 10958 14273 10960 14272 10960 14276 10962 14279 10958 14279
10955 14278 10952 14277 10949 14278 10948 14275 10946 14273 17 N F
10956 7681 10955 7678 10957 7676 10959 7676 10961 7676 10962 7678
10961 7680 10960 7682 10958 7682 10956 7681 10 N F
10967 6683 10964 6681 10967 6681 10969 6682 10971 6683 10973 6684
10971 6685 10968 6685 10967 6683 9 N F
11009 14751 11009 14747 11012 14743 11014 14747 11018 14749 11022 14751
11023 14755 11024 14760 11026 14763 11026 14768 11024 14764 11017 14764
11016 14769 11013 14772 11012 14768 11013 14763 11010 14760 11013 14756
11017 14756 11017 14752 11013 14754 11009 14751 22 N F
11028 6755 11016 6752 11020 6746 11024 6741 11031 6738 11038 6738
11045 6737 11047 6743 11053 6748 11059 6743 11065 6739 11072 6738
11079 6738 11086 6737 11092 6736 11093 6736 11100 6737 11107 6738
11114 6735 11120 6732 11127 6730 11133 6733 11133 6740 11129 6746
11122 6749 11129 6747 11136 6745 11142 6741 11143 6748 11140 6755
11143 6762 11146 6768 11153 6766 11155 6773 11153 6780 11157 6786
11159 6793 11152 6795 11146 6800 11141 6804 11134 6806 11127 6807
11125 6814 11119 6810 11112 6814 11105 6813 11099 6810 11094 6805
11089 6810 11087 6817 11083 6823 11077 6827 11070 6830 11064 6826
11070 6821 11072 6814 11075 6808 11069 6811 11070 6818 11063 6816
11059 6810 11054 6804 11057 6797 11056 6790 11060 6784 11065 6779
11066 6772 11063 6778 11061 6772 11060 6764 11063 6758 11062 6751
11061 6758 11056 6763 11050 6767 11049 6774 11043 6770 11037 6773
11038 6766 11041 6760 11035 6764 11029 6767 11025 6761 11028 6755
84 N F
11057 14306 11056 14303 11059 14302 11062 14303 11065 14301 11068 14302
11069 14305 11070 14306 11071 14307 11072 14310 11075 14309 11076 14312
11076 14315 11077 14318 11077 14321 11074 14321 11071 14321 11067 14321
11064 14320 11067 14318 11070 14316 11069 14313 11069 14310 11066 14310
11063 14311 11060 14312 11060 14309 11057 14306 28 N F
11075 6616 11074 6611 11076 6607 11081 6607 11086 6606 11090 6607
11095 6606 11099 6608 11102 6612 11104 6616 11101 6619 11097 6621
11092 6621 11088 6621 11083 6619 11079 6617 11075 6616 17 N F
11120 14306 11115 14302 11119 14302 11122 14301 11122 14298 11121 14295
11124 14297 11125 14300 11128 14302 11131 14303 11133 14305 11136 14307
11135 14311 11138 14310 11141 14311 11139 14314 11139 14317 11136 14316
11132 14315 11129 14314 11126 14312 11125 14309 11122 14308 11120 14306
24 N F
11154 14284 11150 14282 11154 14281 11156 14280 11159 14278 11161 14275

11164 14277 11161 14279 11163 14281 11161 14284 11158 14285 11154 14284
12 N F
11155 14257 11153 14253 11155 14251 11158 14249 11160 14250 11161 14250
11163 14252 11167 14252 11169 14254 11172 14256 11174 14258 11176 14261
11178 14263 11179 14265 11180 14266 11182 14268 11185 14269 11188 14271
11191 14273 11192 14276 11194 14279 11194 14279 11196 14281 11198 14283
11199 14286 11201 14289 11203 14292 11205 14295 11202 14293 11199 14294
11196 14292 11193 14290 11191 14288 11188 14286 11185 14287 11183 14284
11180 14283 11178 14281 11179 14278 11180 14275 11181 14272 11181 14269
11178 14271 11178 14274 11176 14276 11173 14278 11170 14277 11167 14276
11169 14273 11169 14270 11166 14268 11165 14265 11163 14262 11161 14260
11158 14259 11155 14257 56 N F
11157 6713 11154 6710 11157 6707 11160 6706 11163 6707 11163 6711
11160 6713 11157 6713 8 N F
11163 6721 11162 6715 11166 6716 11168 6719 11170 6721 11171 6725
11168 6726 11166 6724 11163 6721 9 N F
11170 6779 11166 6779 11168 6778 11170 6778 11173 6777 11176 6777
11180 6777 11183 6778 11186 6775 11189 6774 11192 6775 11194 6778
11190 6781 11187 6782 11186 6785 11185 6789 11182 6790 11179 6791
11176 6791 11173 6789 11171 6786 11174 6784 11173 6781 11170 6779
24 N F
11191 6732 11190 6729 11190 6726 11192 6723 11194 6720 11196 6717
11199 6715 11202 6715 11205 6717 11208 6719 11210 6722 11211 6725
11211 6728 11209 6731 11205 6731 11202 6731 11199 6732 11194 6734
11191 6732 19 N F
11227 6846 11223 6838 11229 6834 11234 6836 11235 6836 11241 6841
11248 6842 11253 6837 11257 6831 11262 6826 11261 6818 11258 6812
11251 6810 11247 6804 11252 6799 11259 6797 11266 6794 11271 6790
11277 6786 11284 6785 11291 6788 11296 6793 11297 6800 11304 6798
11310 6795 11317 6797 11320 6804 11321 6811 11321 6818 11320 6825
11322 6832 11321 6839 11317 6846 11321 6852 11317 6858 11317 6865
11321 6871 11326 6876 11330 6882 11331 6889 11336 6894 11340 6900
11343 6906 11341 6913 11344 6919 11350 6923 11355 6929 11356 6936
11355 6943 11356 6950 11348 6950 11341 6951 11341 6944 11340 6937
11336 6930 11332 6925 11329 6918 11334 6914 11331 6907 11329 6900
11328 6893 11321 6895 11314 6892 11311 6886 11304 6884 11304 6877
11301 6870 11303 6863 11297 6858 11290 6859 11283 6857 11277 6853
11270 6854 11263 6852 11261 6859 11257 6865 11250 6868 11250 6860
11248 6854 11244 6847 11238 6852 11231 6851 11227 6846 83 N F
11233 6780 11232 6776 11233 6772 11236 6770 11239 6770 11242 6773
11244 6775 11244 6779 11243 6782 11241 6784 11238 6786 11234 6787
11233 6784 11233 6780 14 N F
11277 12274 11271 12274 11275 12272 11279 12272 11283 12270 11287 12267

11288 12263 11291 12259 11295 12261 11299 12261 11301 12266 11298 12268
11294 12271 11291 12274 11290 12278 11291 12283 11286 12283 11284 12287
11280 12286 11277 12283 11277 12279 11277 12274 22 N F
11288 12612 11286 12608 11287 12604 11289 12601 11291 12605 11293 12608
11298 12609 11299 12613 11301 12617 11302 12621 11300 12625 11296 12627
11295 12623 11294 12619 11292 12615 11288 12612 16 N F
11347 12475 11343 12475 11345 12472 11346 12470 11347 12469 11348 12472
11347 12475 7 N F
11346 12483 11343 12479 11347 12479 11347 12479 11349 12481 11351 12483
11350 12486 11347 12486 11346 12483 9 N F
11351 12459 11346 12461 11348 12459 11350 12456 11352 12453 11353 12450
11356 12452 11359 12451 11361 12452 11361 12456 11358 12457 11355 12458
11353 12456 11351 12459 14 N F
11354 14261 11353 14257 11356 14255 11357 14252 11356 14249 11357 14246
11360 14245 11363 14247 11365 14250 11367 14252 11368 14255 11370 14258
11370 14261 11367 14262 11364 14261 11360 14260 11357 14260 11354 14261
18 N F
11371 12616 11369 12612 11371 12610 11374 12611 11377 12613 11377 12616
11374 12617 11371 12616 8 N F
11381 12622 11377 12618 11380 12618 11382 12621 11384 12623 11381 12622
6 N F
11384 12601 11380 12597 11383 12593 11386 12590 11389 12587 11393 12587
11392 12591 11393 12596 11396 12598 11399 12602 11398 12606 11402 12607
11405 12610 11407 12614 11406 12618 11409 12622 11414 12623 11412 12627
11408 12625 11405 12622 11401 12621 11400 12626 11404 12628 11399 12629
11395 12628 11391 12627 11388 12623 11389 12619 11386 12615 11385 12611
11384 12607 11381 12604 11384 12601 33 N F
11384 14332 11381 14329 11384 14328 11384 14328 11387 14328 11389 14331
11388 14334 11388 14337 11385 14335 11384 14332 10 N F
11398 12659 11394 12658 11396 12656 11399 12656 11400 12656 11398 12659
6 N F
11403 12653 11400 12652 11400 12649 11403 12647 11405 12650 11403 12653
6 N F
11408 12647 11406 12644 11408 12642 11411 12641 11412 12637 11415 12640
11414 12643 11415 12646 11419 12645 11419 12649 11416 12649 11413 12651
11410 12653 11411 12649 11408 12647 15 N F
11413 12602 11412 12598 11415 12598 11418 12599 11420 12601 11423 12604
11424 12607 11420 12607 11417 12607 11416 12604 11413 12602 11 N F
11440 12364 11436 12364 11439 12362 11441 12359 11443 12361 11446 12360
11448 12362 11445 12363 11443 12365 11440 12364 10 N F
11441 14322 11437 14319 11439 14317 11442 14318 11443 14318 11445 14318
11449 14319 11451 14321 11452 14324 11454 14327 11451 14325 11448 14324
11446 14321 11443 14323 11441 14322 15 N F

11500 13949 11498 13945 11500 13942 11503 13940 11503 13943 11504 13946
11503 13949 11507 13948 11510 13949 11508 13952 11505 13953 11502 13954
11500 13952 11500 13949 14 N F
11504 14005 11502 14002 11506 14001 11508 14003 11510 14005 11514 14005
11512 14008 11515 14009 11517 14012 11513 14013 11512 14010 11508 14009
11507 14006 11504 14005 14 N F
11518 13927 11517 13924 11518 13921 11521 13919 11524 13917 11525 13915
11528 13916 11531 13918 11531 13918 11534 13920 11530 13920 11527 13920
11524 13921 11524 13924 11522 13926 11518 13927 16 N F
11528 7052 11525 7050 11529 7048 11532 7047 11535 7045 11537 7045
11538 7044 11539 7048 11537 7050 11534 7052 11531 7054 11528 7052
12 N F
11529 7000 11526 6997 11526 6990 11528 6986 11532 6983 11536 6983
11541 6982 11545 6982 11549 6985 11552 6988 11556 6991 11556 6996
11554 7000 11551 7003 11546 7004 11542 7005 11537 7005 11533 7003
11529 7000 19 N F
11547 14286 11544 14283 11545 14281 11548 14281 11551 14280 11553 14282
11552 14284 11549 14285 11547 14286 9 N F
11551 13963 11549 13960 11550 13960 11552 13961 11555 13959 11552 13959
11551 13956 11554 13954 11557 13955 11559 13957 11561 13960 11562 13963
11561 13966 11558 13968 11555 13967 11552 13967 11551 13963 17 N F
11552 13490 11550 13492 11551 13489 11552 13487 11554 13485 11556 13484
11557 13487 11558 13489 11556 13491 11554 13489 11552 13490 11 N F
11555 14033 11553 14029 11557 14031 11558 14036 11560 14033 11560 14032
11564 14030 11565 14026 11570 14025 11574 14026 11578 14028 11579 14032
11580 14032 11583 14034 11586 14037 11584 14040 11579 14042 11575 14044
11572 14046 11567 14045 11566 14049 11562 14051 11561 14056 11558 14058
11558 14054 11556 14050 11555 14046 11554 14042 11555 14038 11555 14033
30 N F
11557 14010 11555 14005 11558 14003 11558 14002 11559 14000 11562 14000
11565 14000 11566 14000 11568 14002 11566 14005 11568 14008 11565 14008
11563 14010 11560 14012 11557 14010 15 N F
11562 14281 11558 14278 11561 14276 11563 14274 11566 14275 11569 14276
11572 14274 11574 14272 11577 14270 11580 14270 11582 14272 11583 14276
11580 14277 11578 14280 11576 14283 11575 14286 11572 14285 11571 14282
11568 14281 11565 14280 11562 14281 21 N F
11565 13430 11561 13429 11563 13427 11564 13424 11565 13422 11568 13421
11570 13422 11570 13425 11572 13425 11573 13422 11575 13424 11575 13427
11573 13428 11570 13429 11568 13430 11565 13430 16 N F
11570 13732 11567 13729 11569 13727 11571 13729 11573 13730 11576 13730
11578 13730 11581 13731 11581 13732 11581 13734 11583 13736 11582 13738
11583 13741 11580 13742 11580 13739 11579 13737 11577 13735 11574 13735
11572 13734 11570 13732 20 N F

11575 12601 11569 12602 11573 12600 11575 12597 11580 12597 11577 12594
11573 12592 11576 12588 11580 12590 11584 12592 11588 12591 11591 12594
11596 12595 11596 12599 11594 12603 11594 12608 11589 12609 11587 12606
11583 12604 11579 12601 11575 12601 21 N F
11578 14337 11574 14331 11575 14330 11578 14329 11581 14327 11585 14325
11586 14325 11590 14326 11591 14330 11595 14331 11599 14332 11601 14333
11603 14334 11607 14335 11610 14332 11614 14334 11618 14336 11622 14339
11624 14342 11626 14346 11627 14350 11627 14350 11626 14355 11624 14358
11620 14355 11616 14354 11612 14354 11608 14354 11604 14356 11600 14358
11596 14360 11592 14359 11588 14358 11584 14356 11581 14353 11581 14349
11579 14345 11581 14341 11578 14337 39 N F
11585 13483 11581 13483 11582 13481 11585 13479 11587 13478 11587 13481
11585 13483 7 N F
11586 14308 11583 14305 11585 14304 11588 14304 11591 14304 11593 14305
11596 14305 11594 14307 11591 14307 11588 14307 11586 14308 11 N F
11589 14036 11585 14033 11586 14031 11587 14028 11589 14027 11589 14027
11592 14027 11594 14026 11595 14023 11595 14021 11598 14022 11599 14025
11600 14027 11598 14028 11596 14028 11595 14030 11593 14032 11591 14034
11589 14036 19 N F
11593 14270 11589 14268 11589 14267 11591 14266 11592 14263 11595 14261
11598 14259 11600 14258 11604 14257 11607 14256 11605 14259 11602 14261
11602 14264 11602 14267 11603 14270 11600 14270 11596 14269 11593 14270
18 N F
11601 12598 11598 12602 11599 12599 11599 12596 11601 12598 5 N F
11601 13489 11599 13485 11600 13485 11602 13485 11605 13486 11607 13488
11609 13488 11612 13489 11612 13492 11610 13494 11607 13493 11605 13493
11602 13492 11601 13489 14 N F
11601 14316 11599 14314 11599 14310 11602 14310 11603 14311 11604 14312
11603 14315 11601 14316 8 N F
11605 12600 11600 12602 11602 12599 11605 12598 11607 12596 11610 12593
11609 12596 11608 12600 11605 12600 9 N F
11602 12674 11601 12670 11604 12667 11607 12663 11610 12660 11614 12659
11618 12660 11622 12662 11626 12664 11624 12668 11629 12669 11626 12672
11625 12677 11621 12679 11620 12683 11624 12685 11628 12686 11633 12687
11636 12688 11637 12689 11638 12685 11642 12685 11646 12687 11650 12688
11649 12694 11645 12693 11647 12697 11649 12701 11650 12706 11648 12710
11645 12712 11640 12710 11636 12708 11636 12704 11633 12700 11629 12701
11628 12697 11624 12694 11620 12693 11616 12692 11611 12692 11608 12695
11604 12694 11604 12690 11601 12687 11603 12683 11602 12678 11602 12674
48 N F
11613 14303 11605 14302 11608 14300 11612 14298 11615 14294 11619 14292
11622 14292 11623 14291 11627 14289 11630 14287 11630 14287 11630 14287
11634 14284 11634 14280 11632 14276 11635 14273 11638 14270 11641 14273

11644 14276 11647 14279 11651 14282 11653 14285 11653 14290 11652 14294
11649 14297 11645 14298 11640 14299 11636 14299 11633 14302 11629 14304
11626 14307 11626 14312 11626 14316 11627 14320 11629 14324 11627 14327
11623 14327 11619 14324 11615 14323 11612 14320 11612 14316 11613 14312
11612 14307 11613 14303 44 N F
11616 14016 11611 14016 11613 14013 11616 14012 11619 14013 11622 14012
11624 14015 11622 14018 11619 14017 11616 14016 10 N F
11616 12384 11611 12383 11614 12382 11616 12380 11619 12379 11619 12382
11616 12384 7 N F
11617 12656 11615 12652 11615 12648 11616 12644 11616 12641 11617 12639
11619 12636 11623 12639 11625 12635 11628 12632 11632 12633 11635 12630
11639 12628 11639 12629 11643 12630 11646 12633 11646 12637 11650 12639
11653 12642 11654 12647 11657 12646 11658 12646 11659 12650 11661 12654
11653 12656 11650 12658 11646 12661 11642 12661 11640 12665 11638 12669
11636 12672 11633 12669 11632 12664 11629 12662 11632 12659 11630 12655
11629 12659 11625 12660 11621 12658 11617 12656 40 N F
11617 12996 11616 12993 11619 12995 11622 12997 11619 12999 11617 12996
6 N F
11625 13007 11621 13006 11624 13004 11625 13001 11628 13001 11631 13001
11634 13004 11636 13006 11635 13009 11633 13012 11630 13013 11628 13015
11626 13014 11622 13014 11622 13011 11626 13010 11628 13008 11625 13007
18 N F
11628 14013 11624 14011 11626 14009 11629 14009 11632 14008 11635 14007
11637 14004 11640 14003 11641 14006 11638 14007 11636 14010 11636 14013
11633 14014 11630 14016 11628 14013 15 N F
11635 14221 11630 14222 11632 14219 11635 14217 11637 14215 11638 14214
11640 14213 11641 14210 11640 14206 11643 14205 11647 14206 11650 14207
11653 14208 11656 14208 11658 14208 11659 14209 11662 14208 11664 14211
11664 14214 11661 14213 11658 14212 11655 14211 11651 14211 11648 14212
11646 14215 11644 14217 11641 14219 11638 14220 11635 14221 29 N F
11635 12749 11631 12749 11635 12747 11633 12743 11637 12742 11641 12742
11645 12744 11645 12744 11649 12742 11652 12745 11655 12748 11655 12752
11655 12748 11655 12744 11659 12742 11661 12738 11663 12737 11664 12736
11667 12739 11669 12740 11671 12742 11675 12743 11677 12747 11681 12747
11680 12752 11680 12756 11684 12754 11688 12756 11689 12760 11686 12763
11681 12764 11682 12768 11686 12771 11682 12774 11683 12778 11678 12778
11677 12782 11673 12785 11669 12786 11668 12782 11669 12778 11668 12774
11664 12772 11661 12775 11658 12772 11654 12770 11650 12772 11648 12768
11647 12763 11643 12757 11642 12753 11639 12749 11635 12749 53 N F
11633 12406 11632 12402 11636 12402 11638 12400 11638 12403 11641 12405
11643 12408 11644 12405 11643 12402 11646 12404 11645 12407 11644 12410
11641 12411 11638 12410 11635 12408 11633 12406 16 N F
11634 12619 11633 12615 11635 12613 11638 12611 11641 12612 11644 12611

11645 12608 11648 12610 11648 12613 11647 12616 11644 12618 11643 12621
11639 12622 11637 12620 11634 12619 15 N F
11637 13024 11634 13021 11637 13019 11639 13022 11637 13024 5 N F
11638 14065 11634 14060 11638 14058 11642 14056 11646 14055 11649 14054
11651 14054 11655 14054 11659 14054 11663 14057 11664 14061 11661 14063
11658 14059 11654 14060 11650 14062 11646 14062 11642 14063 11638 14065
18 N F
11641 14354 11635 14349 11638 14343 11640 14336 11640 14330 11640 14323
11641 14316 11640 14309 11643 14303 11650 14302 11656 14301 11658 14295
11663 14290 11666 14284 11668 14277 11665 14272 11659 14269 11652 14267
11654 14261 11654 14254 11660 14252 11667 14249 11673 14250 11677 14249
11680 14249 11684 14248 11687 14247 11693 14245 11693 14252 11694 14259
11697 14257 11700 14256 11704 14253 11705 14252 11712 14251 11717 14256
11719 14258 11722 14261 11723 14268 11721 14274 11719 14280 11721 14287
11715 14291 11715 14298 11715 14305 11713 14311 11710 14317 11710 14324
11713 14330 11710 14337 11706 14342 11705 14349 11700 14354 11694 14356
11688 14359 11682 14363 11677 14367 11672 14372 11665 14373 11659 14370
11652 14370 11651 14363 11647 14358 11641 14354 64 N F
11637 12872 11636 12867 11639 12865 11641 12862 11644 12861 11646 12864
11646 12867 11644 12870 11642 12872 11640 12875 11637 12872 11 N F
11642 12597 11640 12594 11641 12594 11643 12595 11643 12595 11647 12595
11647 12592 11646 12589 11646 12585 11650 12586 11650 12590 11650 12593
11649 12596 11652 12598 11652 12598 11648 12598 11645 12597 11642 12597
18 N F
11642 12473 11641 12470 11645 12470 11646 12473 11647 12476 11643 12477
11642 12473 7 N F
11649 14013 11644 14012 11646 14009 11648 14007 11652 14007 11655 14006
11658 14006 11657 14009 11655 14011 11652 14012 11649 14013 11 N F
11647 13185 11645 13182 11648 13181 11649 13183 11650 13186 11647 13185
6 N F
11648 12402 11646 12398 11647 12395 11650 12395 11653 12395 11656 12398
11656 12398 11658 12400 11659 12403 11656 12404 11653 12402 11651 12404
11648 12402 13 N F
11650 12460 11648 12457 11651 12458 11655 12458 11654 12461 11652 12463
11650 12460 7 N F
11656 12857 11650 12855 11654 12854 11658 12855 11659 12850 11662 12854
11665 12857 11667 12853 11669 12852 11671 12851 11675 12850 11679 12848
11680 12852 11684 12854 11682 12858 11685 12862 11687 12866 11683 12867
11679 12869 11679 12873 11679 12877 11683 12879 11684 12884 11681 12887
11684 12891 11683 12895 11687 12897 11692 12898 11695 12901 11699 12902
11695 12905 11692 12901 11688 12901 11684 12900 11679 12900 11679 12896
11676 12893 11673 12889 11671 12885 11669 12882 11665 12880 11661 12879
11656 12877 11654 12874 11653 12869 11651 12866 11655 12863 11658 12861

11656 12857 49 N F
11651 13175 11651 13172 11653 13171 11656 13171 11656 13169 11659 13170
11658 13173 11656 13175 11654 13176 11651 13175 10 N F
11654 12608 11653 12605 11655 12603 11657 12606 11657 12606 11657 12603
11660 12605 11663 12606 11663 12609 11662 12612 11660 12615 11657 12617
11654 12616 11654 12612 11657 12610 11654 12608 16 N F
11655 12442 11653 12438 11656 12437 11656 12437 11659 12440 11658 12443
11655 12442 7 N F
11655 12476 11654 12470 11657 12470 11658 12472 11659 12473 11659 12476
11657 12479 11655 12476 8 N F
11656 12359 11654 12356 11656 12353 11657 12350 11658 12347 11660 12350
11658 12352 11657 12356 11656 12359 9 N F
11656 14175 11656 14172 11658 14169 11661 14169 11663 14166 11663 14162
11665 14160 11668 14158 11671 14160 11672 14163 11675 14164 11677 14162
11677 14158 11679 14160 11680 14160 11682 14158 11685 14156 11686 14153
11687 14156 11690 14158 11690 14161 11690 14165 11688 14168 11685 14169
11683 14171 11681 14173 11677 14174 11674 14175 11674 14171 11671 14170
11668 14171 11666 14173 11664 14176 11661 14178 11658 14178 11656 14175
36 N F
11663 12680 11657 12680 11658 12677 11658 12674 11660 12671 11662 12668
11665 12668 11666 12671 11665 12674 11663 12677 11663 12680 11 N F
11664 12445 11660 12444 11661 12441 11662 12438 11664 12439 11665 12439
11666 12442 11664 12445 8 N F
11664 12437 11660 12435 11662 12433 11665 12433 11669 12433 11667 12436
11664 12437 7 N F
11663 12619 11662 12615 11664 12613 11666 12610 11665 12607 11664 12604
11664 12603 11665 12600 11668 12600 11671 12598 11669 12595 11667 12593
11667 12590 11669 12587 11669 12584 11671 12581 11674 12581 11675 12585
11676 12588 11679 12585 11682 12585 11685 12585 11688 12587 11689 12590
11689 12593 11688 12596 11685 12597 11684 12600 11684 12604 11681 12603
11678 12603 11676 12606 11674 12608 11676 12611 11677 12614 11676 12618
11673 12619 11670 12620 11666 12620 11663 12619 40 N F
11669 12726 11666 12725 11667 12722 11669 12719 11672 12719 11674 12716
11676 12717 11677 12717 11677 12721 11675 12722 11672 12724 11669 12726
12 N F
11668 12422 11667 12419 11668 12416 11671 12417 11673 12414 11675 12411
11678 12411 11678 12414 11680 12417 11680 12420 11677 12417 11676 12420
11674 12423 11671 12424 11668 12422 15 N F
11671 12690 11668 12687 11670 12687 11671 12688 11674 12689 11676 12692
11676 12695 11673 12693 11671 12690 9 N F
11673 12459 11670 12457 11671 12454 11671 12451 11674 12449 11677 12449
11679 12449 11681 12449 11684 12449 11685 12453 11688 12452 11691 12454
11691 12454 11694 12452 11697 12451 11700 12450 11701 12453 11699 12455

11696 12456 11693 12458 11690 12459 11687 12461 11684 12459 11681 12459
11680 12456 11677 12456 11673 12459 27 N F
11675 14215 11672 14211 11674 14209 11675 14212 11677 14214 11677 14216
11675 14215 7 N F
11679 12740 11672 12739 11675 12735 11677 12731 11680 12728 11681 12724
11681 12724 11685 12721 11689 12720 11691 12716 11690 12712 11694 12712
11698 12714 11701 12717 11703 12721 11700 12725 11699 12729 11701 12733
11705 12732 11709 12730 11709 12734 11713 12736 11713 12741 11710 12743
11706 12746 11710 12748 11713 12748 11714 12748 11714 12753 11710 12752
11710 12757 11707 12760 11704 12757 11700 12759 11696 12760 11692 12757
11694 12753 11696 12749 11699 12745 11697 12741 11692 12742 11688 12743
11685 12746 11681 12744 11679 12740 45 N F
11677 12579 11673 12577 11677 12575 11680 12578 11684 12577 11688 12578
11687 12574 11683 12572 11681 12568 11677 12570 11674 12565 11678 12564
11683 12564 11687 12564 11690 12565 11691 12566 11694 12569 11698 12571
11701 12574 11700 12578 11704 12579 11706 12583 11704 12587 11707 12590
11707 12595 11710 12598 11707 12601 11703 12603 11705 12606 11701 12608
11697 12610 11693 12611 11693 12616 11689 12618 11685 12618 11680 12618
11680 12614 11683 12611 11687 12609 11690 12606 11692 12602 11692 12597
11696 12595 11700 12594 11696 12592 11694 12588 11695 12584 11692 12581
11688 12580 11684 12583 11680 12582 11677 12579 52 N F
11679 12446 11674 12446 11675 12443 11677 12440 11680 12442 11683 12443
11683 12446 11679 12446 8 N F
11678 12639 11675 12636 11676 12632 11677 12629 11678 12626 11679 12629
11679 12632 11679 12635 11678 12639 9 N F
11678 13534 11676 13534 11677 13531 11679 13529 11680 13531 11682 13533
11682 13536 11684 13538 11681 13539 11679 13539 11678 13537 11678 13534
12 N F
11681 12717 11677 12715 11678 12714 11679 12712 11681 12710 11684 12711
11682 12714 11681 12717 8 N F
11679 14140 11678 14136 11681 14135 11683 14132 11683 14129 11683 14126
11685 14123 11688 14122 11691 14123 11691 14123 11695 14124 11695 14127
11695 14131 11698 14133 11695 14135 11692 14136 11690 14139 11688 14141
11685 14143 11684 14145 11680 14146 11680 14143 11679 14140 23 N F
11681 12470 11678 12467 11681 12465 11679 12463 11681 12461 11684 12461
11684 12464 11683 12467 11681 12470 9 N F
11685 12696 11678 12694 11682 12692 11686 12689 11687 12688 11688 12685
11690 12682 11691 12682 11694 12682 11698 12680 11697 12675 11697 12671
11702 12671 11702 12671 11705 12668 11708 12671 11711 12675 11715 12675
11716 12671 11720 12669 11719 12664 11723 12663 11725 12662 11727 12662
11732 12662 11733 12667 11733 12671 11733 12675 11731 12679 11728 12682
11731 12686 11727 12688 11725 12691 11721 12693 11717 12690 11713 12691
11709 12693 11707 12697 11703 12698 11699 12696 11695 12697 11691 12699

11690 12695 11685 12696 44 N F
11680 14213 11679 14208 11682 14207 11684 14210 11684 14213 11684 14217
11686 14219 11684 14221 11681 14220 11681 14216 11680 14213 11 N F
11682 12984 11679 12979 11681 12977 11683 12975 11684 12975 11684 12971
11688 12972 11690 12975 11689 12978 11689 12981 11687 12984 11684 12986
11682 12984 13 N F
11683 12536 11679 12536 11681 12535 11682 12534 11684 12531 11683 12528
11685 12525 11687 12526 11688 12527 11690 12529 11691 12533 11693 12534
11696 12536 11696 12540 11693 12538 11692 12541 11695 12542 11692 12544
11690 12547 11687 12545 11685 12543 11685 12539 11683 12536 23 N F
11683 12933 11680 12932 11680 12929 11680 12925 11683 12927 11684 12930
11683 12933 7 N F
11687 12827 11680 12822 11684 12820 11688 12822 11692 12823 11697 12822
11701 12821 11705 12823 11704 12819 11700 12820 11699 12815 11700 12811
11700 12807 11699 12803 11697 12799 11696 12795 11700 12796 11704 12797
11705 12802 11704 12806 11707 12807 11708 12807 11711 12809 11715 12807
11715 12812 11719 12813 11718 12817 11718 12822 11715 12825 11711 12826
11709 12829 11708 12834 11705 12837 11701 12834 11697 12837 11693 12837
11693 12833 11689 12831 11687 12827 39 N F
11681 13524 11681 13522 11682 13519 11684 13519 11685 13519 11685 13521
11686 13524 11686 13526 11684 13526 11681 13524 10 N F
11685 12650 11683 12645 11685 12642 11687 12644 11688 12647 11689 12648
11690 12649 11691 12652 11687 12652 11685 12650 10 N F
11685 12438 11683 12433 11687 12435 11687 12430 11691 12429 11695 12429
11700 12428 11701 12427 11702 12425 11702 12429 11704 12433 11705 12429
11708 12425 11710 12429 11714 12430 11718 12428 11717 12432 11716 12437
11715 12441 11715 12445 11711 12444 11707 12444 11703 12442 11698 12443
11695 12446 11691 12448 11689 12444 11685 12444 11688 12441 11685 12438
30 N F
11685 12681 11683 12677 11686 12676 11688 12679 11689 12682 11687 12684
11685 12681 7 N F
11687 12936 11684 12934 11688 12934 11691 12934 11693 12936 11695 12939
11692 12940 11690 12937 11687 12936 9 N F
11686 14210 11685 14205 11688 14204 11690 14206 11694 14207 11697 14206
11699 14204 11702 14203 11702 14207 11702 14210 11703 14213 11702 14216
11700 14219 11697 14220 11694 14219 11694 14216 11691 14214 11688 14212
11686 14210 19 N F
11693 12709 11685 12707 11689 12705 11691 12705 11693 12704 11697 12702
11701 12703 11705 12704 11708 12701 11711 12697 11713 12694 11717 12696
11722 12697 11723 12701 11723 12705 11721 12709 11724 12712 11722 12716
11718 12716 11717 12720 11713 12722 11711 12718 11714 12715 11709 12715
11705 12713 11702 12711 11697 12710 11693 12709 28 N F
11688 13985 11686 13980 11689 13978 11692 13976 11693 13979 11693 13982

11691 13985 11688 13985 8 N F
11690 12404 11690 12400 11693 12400 11695 12398 11698 12400 11701 12400
11704 12398 11706 12401 11707 12404 11710 12405 11708 12408 11707 12411
11704 12412 11701 12414 11702 12411 11702 12408 11700 12405 11696 12405
11693 12404 11690 12404 20 N F
11696 14195 11691 14194 11694 14191 11698 14189 11700 14185 11699 14181
11703 14180 11702 14185 11705 14188 11707 14192 11704 14195 11700 14195
11696 14195 13 N F
11697 12854 11691 12850 11693 12850 11695 12849 11696 12845 11700 12844
11704 12845 11707 12842 11710 12839 11711 12843 11711 12847 11713 12843
11714 12839 11717 12842 11717 12846 11716 12851 11714 12855 11709 12855
11705 12854 11701 12855 11697 12854 21 N F
11699 12665 11696 12661 11698 12658 11701 12658 11703 12661 11704 12664
11702 12666 11699 12665 8 N F
11701 14073 11699 14067 11701 14063 11702 14059 11703 14055 11704 14050
11708 14053 11709 14054 11711 14056 11713 14052 11712 14048 11716 14050
11719 14054 11721 14057 11723 14061 11725 14065 11729 14066 11729 14070
11727 14074 11727 14079 11722 14080 11718 14080 11714 14080 11709 14080
11709 14075 11705 14073 11701 14073 27 N F
11704 12879 11702 12873 11706 12873 11709 12869 11707 12865 11709 12865
11712 12864 11713 12861 11718 12860 11722 12861 11725 12858 11729 12858
11733 12860 11736 12857 11736 12857 11740 12859 11742 12855 11738 12852
11738 12848 11742 12848 11743 12847 11744 12843 11746 12842 11747 12840
11747 12845 11751 12843 11753 12846 11756 12850 11752 12852 11748 12854
11749 12858 11748 12862 11744 12865 11741 12862 11737 12862 11735 12866
11731 12868 11730 12873 11733 12876 11737 12874 11740 12877 11739 12882
11735 12882 11732 12878 11731 12882 11728 12885 11725 12883 11723 12878
11719 12879 11715 12882 11714 12886 11710 12883 11706 12883 11704 12879
54 N F
11709 13960 11705 13957 11707 13955 11708 13951 11708 13948 11711 13947
11714 13948 11717 13949 11720 13951 11719 13954 11718 13957 11715 13959
11713 13957 11710 13957 11709 13960 15 N F
11709 14175 11707 14171 11709 14169 11712 14168 11714 14171 11714 14174
11712 14177 11709 14175 8 N F
11709 12634 11708 12630 11713 12629 11715 12626 11714 12622 11715 12618
11714 12614 11718 12613 11721 12616 11726 12617 11728 12621 11732 12622
11735 12619 11739 12622 11742 12625 11746 12626 11748 12630 11744 12631
11739 12632 11740 12636 11738 12640 11735 12642 11732 12639 11729 12636
11725 12636 11728 12639 11729 12644 11725 12646 11721 12644 11719 12641
11715 12639 11713 12635 11709 12634 33 N F
11711 12512 11709 12505 11713 12499 11718 12494 11721 12488 11727 12485
11726 12491 11724 12498 11726 12504 11728 12497 11733 12493 11740 12492
11736 12486 11733 12480 11735 12474 11739 12479 11743 12473 11750 12473

11757 12473 11761 12475 11763 12477 11769 12479 11764 12484 11771 12483
11772 12483 11778 12485 11781 12490 11788 12491 11788 12498 11794 12501
11801 12501 11802 12508 11807 12509 11809 12509 11810 12515 11815 12511
11817 12505 11822 12509 11820 12516 11824 12518 11826 12519 11828 12526
11832 12531 11829 12537 11833 12543 11828 12547 11832 12553 11833 12560
11834 12566 11829 12570 11825 12576 11819 12580 11814 12584 11808 12581
11806 12575 11801 12579 11798 12585 11800 12592 11796 12598 11790 12601
11783 12603 11777 12604 11770 12604 11768 12598 11771 12591 11778 12591
11785 12593 11782 12586 11784 12580 11781 12574 11776 12569 11773 12563
11767 12564 11766 12571 11764 12578 11760 12572 11763 12566 11757 12563
11760 12557 11761 12550 11765 12544 11772 12547 11769 12541 11762 12542
11766 12536 11764 12530 11763 12523 11762 12516 11758 12510 11759 12517
11757 12524 11758 12531 11761 12537 11757 12543 11757 12550 11751 12551
11750 12558 11744 12554 11743 12547 11746 12541 11744 12535 11739 12540
11732 12540 11728 12534 11722 12532 11720 12525 11725 12520 11719 12522
11713 12518 11711 12512 110 N F
11711 14191 11709 14186 11711 14182 11712 14181 11714 14179 11719 14180
11721 14184 11725 14184 11728 14187 11732 14189 11736 14188 11740 14189
11744 14191 11745 14195 11748 14198 11748 14203 11745 14206 11743 14210
11739 14212 11735 14213 11730 14212 11726 14214 11722 14216 11719 14218
11715 14216 11713 14212 11713 14208 11713 14203 11711 14199 11712 14195
11711 14191 31 N F
11713 13562 11713 13560 11713 13556 11714 13556 11716 13556 11718 13558
11720 13556 11722 13558 11724 13560 11724 13562 11722 13564 11721 13567
11722 13569 11720 13571 11718 13572 11717 13569 11717 13566 11715 13564
11713 13562 19 N F
11720 13151 11714 13148 11717 13145 11719 13141 11723 13144 11727 13145
11730 13149 11732 13152 11735 13156 11739 13157 11741 13161 11742 13165
11740 13169 11737 13171 11737 13167 11734 13164 11729 13165 11725 13166
11728 13163 11723 13162 11724 13158 11720 13155 11720 13151 23 N F
11728 12657 11726 12651 11730 12651 11733 12650 11736 12652 11737 12654
11739 12652 11742 12654 11743 12657 11740 12660 11743 12662 11744 12665
11741 12666 11738 12665 11735 12663 11734 12660 11734 12657 11731 12657
11728 12657 19 N F
11729 12813 11728 12809 11730 12807 11731 12804 11734 12802 11736 12805
11735 12808 11735 12812 11735 12815 11736 12818 11733 12817 11729 12816
11729 12813 13 N F
11730 13587 11729 13582 11730 13579 11733 13578 11735 13580 11737 13580
11738 13580 11740 13579 11743 13579 11744 13579 11745 13578 11748 13579
11751 13578 11754 13578 11756 13576 11757 13576 11758 13576 11761 13576
11764 13578 11763 13580 11760 13580 11757 13579 11755 13581 11753 13582
11754 13584 11753 13587 11751 13589 11750 13591 11747 13591 11745 13591
11743 13590 11742 13587 11743 13585 11740 13585 11738 13586 11735 13587

11733 13588 11730 13587 38 N F
11744 13298 11739 13296 11742 13295 11746 13295 11744 13298 5 N F
11740 12790 11740 12783 11741 12782 11743 12781 11747 12779 11750 12783
11754 12783 11754 12787 11755 12792 11751 12793 11746 12793 11742 12793
11740 12790 13 N F
11750 14084 11745 14085 11748 14081 11751 14078 11754 14075 11755 14071
11757 14067 11757 14063 11758 14058 11754 14057 11750 14054 11752 14050
11756 14051 11754 14047 11752 14043 11749 14040 11747 14035 11750 14037
11753 14041 11757 14040 11759 14036 11763 14038 11763 14043 11765 14047
11766 14051 11770 14050 11774 14050 11774 14054 11778 14057 11782 14058
11783 14062 11782 14066 11780 14069 11779 14074 11775 14075 11771 14075
11767 14076 11764 14079 11759 14078 11759 14083 11761 14086 11761 14091
11759 14087 11757 14083 11753 14081 11750 14084 46 N F
11749 12832 11746 12833 11748 12829 11750 12826 11750 12823 11750 12819
11748 12817 11751 12816 11754 12817 11757 12816 11760 12818 11759 12822
11759 12825 11756 12827 11754 12830 11752 12832 11749 12832 17 N F
11747 12751 11747 12745 11751 12745 11755 12744 11760 12743 11764 12743
11766 12746 11768 12750 11772 12752 11776 12751 11781 12752 11779 12756
11784 12755 11786 12751 11790 12751 11794 12750 11796 12746 11800 12745
11798 12741 11795 12738 11797 12734 11801 12734 11803 12737 11807 12736
11810 12739 11809 12743 11811 12748 11811 12752 11810 12756 11806 12758
11803 12760 11799 12760 11794 12759 11790 12758 11786 12759 11788 12763
11784 12765 11788 12768 11789 12772 11793 12770 11794 12775 11798 12775
11800 12779 11796 12779 11793 12783 11789 12784 11785 12783 11781 12783
11779 12787 11774 12787 11770 12786 11767 12783 11770 12779 11774 12777
11778 12774 11778 12770 11775 12767 11775 12763 11771 12764 11771 12759
11768 12757 11765 12760 11764 12757 11762 12753 11757 12753 11753 12752
11750 12755 11747 12751 68 N F
11752 13297 11748 13296 11751 13295 11755 13295 11756 13295 11757 13296
11760 13298 11757 13300 11754 13299 11752 13297 10 N F
11756 13279 11754 13275 11756 13273 11758 13270 11761 13272 11760 13275
11758 13278 11756 13279 8 N F
11757 12415 11755 12411 11758 12409 11761 12409 11759 12412 11760 12415
11761 12418 11762 12421 11761 12424 11758 12421 11757 12418 11757 12415
12 N F
11762 12814 11756 12811 11759 12808 11762 12805 11764 12801 11766 12797
11770 12795 11774 12797 11773 12801 11771 12805 11769 12808 11765 12811
11762 12814 13 N F
11760 7118 11759 7109 11761 7105 11764 7102 11768 7100 11773 7099
11777 7100 11780 7103 11785 7104 11789 7102 11793 7102 11798 7102
11802 7104 11804 7109 11803 7113 11801 7117 11799 7121 11797 7125
11793 7128 11789 7129 11785 7131 11781 7133 11776 7134 11772 7135
11768 7132 11768 7127 11769 7123 11768 7118 11764 7116 11760 7118

30 N F
11767 13281 11763 13281 11766 13280 11766 13276 11769 13277 11769 13274
11772 13276 11775 13275 11776 13278 11774 13281 11777 13282 11776 13285
11774 13288 11771 13289 11768 13288 11768 13285 11771 13284 11773 13282
11772 13279 11769 13278 11767 13281 21 N F
11768 13429 11765 13427 11767 13425 11769 13423 11772 13423 11775 13423
11774 13425 11772 13427 11770 13429 11768 13429 10 N F
11773 13304 11769 13303 11771 13302 11772 13302 11775 13302 11778 13302
11776 13304 11773 13304 8 N F
11777 13274 11776 13271 11778 13269 11782 13269 11784 13272 11787 13273
11787 13276 11785 13279 11784 13276 11783 13273 11779 13273 11781 13276
11778 13277 11777 13274 14 N F
11789 13291 11786 13289 11788 13286 11790 13283 11790 13280 11793 13279
11793 13282 11795 13283 11796 13284 11798 13281 11801 13281 11804 13282
11807 13284 11804 13286 11801 13287 11798 13288 11795 13288 11793 13291
11789 13291 19 N F
11795 13246 11791 13242 11794 13241 11795 13238 11797 13235 11798 13238
11799 13241 11797 13244 11795 13246 9 N F
11799 12493 11797 12487 11800 12486 11803 12485 11802 12488 11805 12488
11808 12489 11811 12492 11811 12495 11808 12497 11806 12495 11808 12498
11807 12501 11804 12500 11801 12498 11798 12496 11799 12493 17 N F
11810 11923 11804 11919 11804 11912 11806 11906 11805 11899 11805 11890
11809 11885 11813 11880 11817 11874 11823 11870 11824 11870 11829 11867
11836 11866 11842 11862 11846 11857 11851 11852 11856 11847 11862 11844
11868 11844 11869 11851 11870 11858 11874 11864 11878 11869 11884 11872
11891 11870 11898 11870 11905 11869 11911 11872 11917 11876 11924 11877
11930 11873 11936 11875 11943 11877 11948 11872 11955 11871 11961 11875
11967 11875 11972 11880 11978 11881 11985 11882 11992 11882 11999 11885
12005 11882 12011 11884 12016 11889 12021 11893 12025 11899 12031 11902
12038 11904 12045 11904 12051 11906 12058 11909 12064 11911 12071 11912
12075 11917 12080 11922 12084 11928 12091 11928 12098 11929 12105 11931
12111 11933 12115 11939 12114 11946 12119 11950 12122 11956 12124 11963
12125 11970 12128 11976 12134 11979 12139 11985 12138 11991 12140 11998
12141 12005 12146 12001 12144 12007 12143 12014 12146 12020 12153 12019
12148 12024 12143 12019 12142 12026 12136 12028 12132 12034 12135 12040
12134 12047 12130 12053 12124 12056 12117 12057 12111 12054 12105 12057
12099 12060 12094 12064 12090 12070 12083 12068 12076 12070 12069 12070
12063 12069 12057 12066 12050 12066 12043 12063 12037 12061 12031 12058
12024 12058 12018 12054 12011 12056 12005 12052 11999 12049 11993 12046
11987 12044 11980 12041 11974 12039 11968 12036 11961 12033 11956 12029
11949 12031 11942 12032 11939 12027 11932 12026 11927 12021 11921 12017
11914 12016 11909 12012 11903 12008 11896 12010 11891 12006 11885 12004
11879 11999 11873 11996 11866 11996 11863 11990 11859 11984 11853 11981

11848 11977 11843 11972 11843 11965 11839 11959 11835 11953 11830 11949
11824 11945 11821 11939 11816 11934 11810 11930 11810 11923 143 N F
11807 13301 11804 13300 11806 13298 11809 13299 11810 13301 11807 13301
6 N F
11817 13360 11814 13357 11817 13356 11820 13356 11821 13358 11820 13360
11817 13360 7 N F
11819 13410 11816 13407 11819 13406 11820 13406 11822 13406 11824 13405
11825 13407 11825 13410 11824 13413 11821 13412 11819 13410 11 N F
11824 7166 11817 7165 11820 7159 11825 7153 11828 7147 11832 7141
11837 7135 11839 7129 11833 7126 11832 7119 11834 7112 11832 7105
11825 7102 11823 7096 11829 7092 11834 7087 11838 7081 11834 7076
11823 7076 11826 7069 11831 7064 11837 7060 11844 7058 11845 7065
11847 7058 11853 7054 11860 7057 11866 7059 11873 7060 11880 7064
11887 7066 11894 7067 11901 7068 11908 7068 11915 7068 11922 7070
11929 7071 11934 7066 11930 7072 11925 7078 11921 7083 11916 7088
11909 7089 11902 7091 11895 7093 11892 7099 11885 7102 11878 7101
11871 7103 11864 7105 11861 7111 11862 7118 11857 7123 11850 7125
11847 7131 11845 7138 11842 7145 11836 7149 11831 7154 11827 7160
11824 7166 61 N F
11824 12268 11822 12263 11826 12261 11830 12259 11831 12263 11828 12267
11824 12268 7 N F
11828 13372 11825 13369 11828 13369 11830 13367 11832 13365 11835 13366
11837 13367 11839 13369 11836 13370 11834 13371 11831 13371 11828 13372
12 N F
11837 12262 11832 12258 11836 12256 11839 12253 11840 12249 11845 12249
11843 12253 11844 12257 11848 12258 11849 12262 11845 12264 11842 12261
11837 12262 13 N F
11844 13365 11840 13363 11843 13363 11846 13362 11847 13360 11850 13360
11847 13362 11847 13364 11844 13365 9 N F
11869 13280 11867 13275 11869 13272 11873 13272 11875 13274 11876 13277
11877 13280 11875 13283 11871 13283 11869 13280 10 N F
11903 12396 11901 12393 11904 12392 11906 12395 11908 12398 11911 12397
11912 12401 11914 12403 11911 12406 11909 12408 11907 12405 11905 12402
11905 12399 11903 12396 14 N F
11914 12387 11911 12384 11913 12380 11911 12372 11914 12369 11916 12365
11920 12364 11925 12364 11928 12366 11932 12368 11936 12371 11939 12374
11940 12378 11944 12378 11945 12383 11949 12384 11949 12389 11953 12386
11953 12386 11955 12382 11955 12387 11959 12385 11964 12386 11963 12390
11959 12390 11955 12390 11951 12393 11946 12393 11942 12391 11938 12392
11934 12390 11932 12395 11928 12397 11924 12396 11920 12393 11917 12390
11914 12387 37 N F
11931 12407 11928 12403 11928 12403 11932 12402 11935 12398 11938 12395
11942 12395 11947 12394 11951 12395 11947 12397 11946 12401 11950 12404

11952 12400 11956 12400 11959 12404 11956 12407 11959 12410 11963 12412
11960 12415 11957 12419 11954 12422 11950 12424 11946 12423 11942 12424
11938 12422 11935 12425 11931 12427 11932 12423 11935 12420 11931 12418
11930 12414 11934 12415 11932 12411 11931 12407 34 N F
11947 12434 11946 12428 11950 12427 11952 12430 11955 12428 11957 12431
11960 12431 11960 12434 11958 12437 11955 12438 11952 12437 11949 12435
11947 12434 13 N F
11953 12363 11950 12361 11952 12359 11950 12354 11953 12355 11956 12354
11959 12353 11959 12357 11957 12359 11955 12362 11953 12363 11 N F
11961 12515 11958 12510 11960 12506 11960 12502 11961 12497 11963 12493
11962 12489 11965 12486 11964 12482 11968 12483 11972 12481 11975 12479
11976 12479 11979 12477 11982 12480 11986 12482 11989 12479 11992 12476
11989 12473 11985 12473 11985 12468 11988 12465 11992 12467 11996 12468
12001 12467 12004 12465 12008 12463 12008 12468 12006 12472 12010 12470
12013 12467 12017 12467 12021 12467 12023 12466 12026 12466 12023 12470
12019 12471 12021 12475 12023 12479 12019 12481 12014 12481 12010 12481
12011 12485 12010 12489 12005 12490 12005 12495 12003 12499 12002 12503
12002 12507 12001 12512 11998 12515 11995 12517 11991 12520 11987 12522
11983 12524 11979 12525 11975 12523 11972 12520 11967 12520 11963 12519
11961 12515 61 N F
11971 12386 11967 12383 11969 12379 11972 12375 11972 12371 11976 12373
11979 12370 11980 12366 11984 12364 11987 12368 11987 12372 11989 12376
11988 12380 11991 12383 11991 12387 11987 12385 11986 12389 11984 12393
11980 12394 11980 12398 11984 12399 11989 12400 11991 12404 11987 12407
11984 12409 11979 12410 11975 12412 11972 12410 11972 12405 11967 12405
11968 12401 11973 12402 11975 12398 11975 12394 11972 12390 11971 12386
36 N F
12010 12520 12008 12515 12012 12517 12016 12517 12020 12518 12019 12514
12017 12509 12017 12505 12020 12503 12023 12506 12024 12510 12026 12514
12028 12518 12032 12520 12036 12520 12040 12521 12040 12526 12043 12529
12047 12528 12048 12528 12051 12526 12055 12526 12060 12526 12059 12530
12056 12533 12052 12535 12052 12540 12048 12538 12044 12539 12047 12542
12048 12546 12045 12548 12043 12552 12039 12552 12035 12550 12031 12547
12027 12546 12023 12546 12020 12542 12017 12539 12016 12535 12013 12532
12015 12528 12012 12524 12010 12520 45 N F
12015 12493 12012 12490 12016 12490 12020 12488 12025 12488 12028 12486
12031 12482 12034 12486 12037 12489 12042 12489 12046 12490 12050 12487
12052 12483 12052 12479 12054 12476 12059 12477 12060 12481 12064 12484
12063 12488 12067 12487 12067 12483 12072 12483 12073 12487 12071 12492
12072 12496 12076 12495 12078 12499 12076 12503 12073 12501 12068 12501
12066 12505 12061 12504 12057 12504 12054 12501 12051 12505 12048 12507
12044 12505 12041 12501 12042 12497 12038 12494 12034 12493 12031 12496
12028 12499 12026 12495 12024 12491 12020 12492 12015 12493 47 N F

12020 12422 12019 12419 12020 12415 12020 12409 12022 12408 12025 12405
12026 12408 12026 12411 12029 12414 12029 12417 12028 12420 12026 12423
12023 12423 12020 12422 14 N F
12063 12536 12057 12535 12061 12532 12063 12529 12060 12525 12060 12521
12061 12516 12064 12519 12066 12524 12070 12525 12069 12521 12069 12517
12071 12512 12075 12512 12079 12509 12079 12509 12083 12510 12087 12512
12088 12516 12092 12517 12097 12517 12098 12521 12096 12525 12095 12529
12095 12534 12097 12538 12096 12542 12094 12538 12091 12534 12090 12530
12086 12531 12082 12530 12079 12533 12074 12533 12070 12534 12067 12536
12065 12532 12063 12536 38 N F
12068 12417 12064 12407 12068 12401 12075 12403 12080 12399 12087 12399
12090 12398 12094 12398 12098 12403 12099 12410 12098 12417 12093 12421
12092 12428 12086 12431 12079 12433 12074 12429 12070 12424 12068 12417
18 N F
12107 12310 12104 12304 12106 12300 12111 12299 12115 12301 12117 12304
12115 12308 12111 12310 12107 12310 9 N F
12108 12501 12105 12497 12106 12493 12106 12489 12110 12488 12111 12485
12112 12484 12116 12486 12120 12487 12123 12490 12127 12491 12128 12495
12132 12497 12130 12501 12124 12505 12119 12504 12115 12505 12111 12504
12108 12501 19 N F
12122 12516 12118 12517 12120 12513 12124 12512 12124 12512 12128 12513
12132 12512 12136 12509 12133 12507 12137 12506 12140 12503 12141 12507
12145 12508 12148 12505 12145 12502 12141 12499 12142 12495 12143 12491
12145 12495 12147 12498 12150 12502 12153 12505 12158 12504 12159 12508
12154 12508 12153 12512 12157 12511 12162 12511 12165 12508 12169 12508
12167 12512 12163 12515 12163 12520 12158 12520 12154 12521 12151 12518
12147 12516 12143 12514 12144 12518 12142 12522 12138 12524 12136 12528
12133 12531 12135 12527 12130 12527 12126 12525 12129 12521 12126 12518
12122 12516 49 N F
12133 12571 12127 12569 12130 12567 12133 12563 12133 12563 12135 12559
12135 12555 12137 12551 12141 12548 12142 12553 12141 12557 12145 12555
12145 12551 12146 12547 12148 12543 12150 12547 12151 12551 12152 12555
12151 12559 12149 12563 12148 12567 12145 12571 12141 12573 12137 12573
12133 12571 25 N F
12163 12582 12157 12581 12160 12578 12165 12579 12167 12575 12164 12573
12163 12569 12167 12569 12169 12566 12173 12564 12177 12561 12181 12559
12184 12562 12189 12561 12191 12565 12196 12565 12200 12565 12204 12565
12209 12565 12213 12566 12216 12569 12217 12574 12216 12578 12218 12582
12219 12586 12221 12590 12220 12594 12217 12598 12214 12601 12210 12600
12206 12597 12202 12597 12197 12598 12194 12596 12190 12593 12188 12589
12184 12590 12181 12586 12177 12588 12173 12589 12171 12593 12167 12593
12167 12589 12165 12585 12163 12582 45 N F
12233 12453 12231 12446 12235 12448 12239 12448 12243 12449 12248 12449

12248 12453 12250 12457 12248 12461 12247 12465 12247 12470 12243 12469
12239 12467 12239 12463 12235 12462 12234 12457 12233 12453 17 N F
12271 12366 12266 12363 12268 12359 12269 12355 12273 12352 12274 12348
12277 12344 12280 12342 12284 12342 12289 12341 12291 12344 12287 12344
12283 12346 12281 12350 12278 12353 12276 12357 12274 12361 12276 12365
12275 12369 12271 12366 20 N F
12285 12367 12279 12366 12282 12363 12284 12359 12288 12357 12292 12355
12293 12360 12289 12362 12290 12366 12285 12367 10 N F
12310 12630 12307 12626 12311 12624 12315 12624 12316 12619 12320 12617
12315 12616 12313 12612 12315 12609 12318 12605 12322 12605 12325 12608
12329 12609 12333 12611 12337 12610 12339 12614 12339 12618 12336 12621
12332 12623 12328 12623 12323 12623 12327 12625 12324 12628 12320 12630
12324 12633 12326 12636 12325 12640 12321 12641 12316 12642 12314 12638
12311 12635 12310 12630 32 N F
12320 12594 12318 12588 12323 12589 12327 12590 12331 12591 12334 12587
12329 12587 12325 12587 12327 12583 12331 12581 12335 12583 12339 12581
12339 12576 12337 12572 12341 12570 12344 12573 12348 12575 12351 12578
12356 12579 12359 12582 12360 12586 12359 12590 12357 12594 12353 12595
12350 12598 12346 12601 12343 12604 12342 12600 12338 12599 12334 12596
12331 12599 12327 12600 12323 12602 12319 12600 12322 12598 12320 12594
36 N F
12397 12670 12395 12666 12396 12660 12397 12664 12400 12661 12403 12657
12407 12657 12410 12660 12414 12662 12415 12666 12418 12663 12422 12664
12426 12666 12423 12669 12419 12672 12416 12669 12411 12669 12409 12673
12405 12674 12402 12671 12397 12670 21 N F
12430 12651 12424 12650 12427 12647 12431 12646 12435 12643 12438 12645
12438 12641 12441 12638 12443 12634 12447 12633 12451 12634 12454 12632
12459 12631 12460 12627 12464 12627 12469 12627 12471 12631 12470 12635
12467 12638 12463 12638 12458 12639 12462 12640 12462 12645 12459 12648
12456 12651 12454 12655 12452 12659 12448 12660 12444 12661 12440 12660
12436 12658 12433 12655 12430 12651 33 N F
12456 12835 12454 12829 12455 12822 12458 12816 12462 12810 12468 12806
12469 12799 12474 12794 12477 12788 12474 12782 12472 12775 12475 12775
12479 12773 12481 12767 12487 12763 12493 12762 12500 12762 12506 12766
12506 12767 12508 12772 12504 12778 12502 12785 12508 12788 12513 12792
12520 12794 12521 12787 12521 12780 12522 12773 12523 12772 12525 12767
12527 12760 12533 12759 12536 12760 12540 12760 12542 12761 12546 12763
12553 12764 12557 12759 12564 12761 12569 12762 12570 12762 12566 12768
12563 12774 12567 12779 12571 12785 12569 12791 12568 12798 12567 12805
12566 12812 12570 12817 12569 12824 12563 12828 12557 12826 12552 12831
12540 12828 12533 12826 12530 12819 12524 12816 12520 12811 12515 12816
12513 12822 12506 12824 12499 12825 12500 12832 12495 12836 12492 12842
12493 12849 12498 12853 12501 12860 12502 12866 12501 12873 12497 12879

12491 12882 12485 12883 12478 12881 12473 12876 12469 12871 12469 12864
12473 12859 12467 12856 12460 12854 12460 12847 12460 12840 12456 12835
84 N F
12474 12601 12470 12597 12475 12597 12472 12593 12472 12589 12477 12589
12480 12586 12482 12590 12482 12594 12481 12598 12482 12603 12482 12607
12478 12609 12479 12613 12475 12613 12473 12609 12474 12605 12474 12601
18 N F
12482 8073 12474 8065 12479 8060 12486 8058 12493 8058 12500 8057
12505 8052 12508 8046 12512 8040 12518 8036 12524 8033 12532 8032
12529 8039 12527 8046 12526 8053 12530 8058 12533 8065 12526 8069
12523 8075 12516 8077 12512 8083 12506 8087 12499 8085 12493 8082
12489 8076 12482 8073 26 N F
12522 12848 12520 12844 12521 12840 12523 12836 12525 12832 12529 12830
12533 12829 12538 12829 12542 12830 12546 12832 12547 12836 12551 12837
12555 12835 12559 12833 12563 12834 12567 12832 12572 12833 12575 12830
12579 12830 12578 12835 12574 12837 12570 12839 12567 12842 12566 12846
12568 12850 12567 12854 12563 12857 12559 12858 12555 12861 12557 12865
12553 12865 12550 12862 12548 12858 12547 12854 12547 12850 12545 12846
12542 12848 12542 12853 12543 12857 12543 12861 12546 12865 12547 12869
12549 12873 12548 12878 12544 12876 12540 12875 12534 12871 12532 12867
12531 12862 12529 12859 12527 12855 12525 12851 12522 12848 53 N F
12522 8205 12521 8204 12521 8203 12521 8203 12522 8203 12523 8203
12523 8203 12523 8203 12523 8204 12524 8205 12523 8205 12522 8205
12 N F
12533 8060 12531 8057 12533 8058 12535 8058 12537 8059 12537 8061
12536 8063 12534 8062 12533 8060 9 N F
12540 8063 12540 8060 12542 8060 12543 8062 12542 8064 12540 8063
6 N F
12546 8060 12545 8057 12546 8059 12548 8060 12550 8061 12552 8061
12554 8063 12556 8064 12557 8066 12559 8067 12561 8068 12563 8068
12565 8069 12566 8071 12569 8071 12571 8072 12569 8073 12566 8072
12565 8070 12563 8069 12561 8069 12559 8069 12556 8068 12555 8066
12553 8065 12552 8063 12549 8063 12547 8062 12546 8060 29 N F
12616 12850 12614 12842 12618 12841 12622 12839 12621 12835 12625 12834
12628 12837 12629 12842 12632 12845 12637 12845 12641 12845 12640 12850
12636 12851 12632 12852 12628 12854 12624 12853 12620 12851 12616 12850
18 N F
12636 12834 12630 12830 12634 12830 12635 12826 12639 12826 12642 12823
12647 12822 12651 12820 12651 12825 12648 12828 12653 12829 12657 12829
12660 12833 12657 12836 12653 12837 12650 12841 12647 12843 12643 12845
12639 12844 12636 12841 12633 12837 12636 12834 22 N F
12656 12738 12653 12733 12656 12730 12660 12728 12660 12724 12662 12720
12665 12723 12669 12725 12671 12728 12675 12728 12680 12727 12684 12728

12683 12732 12686 12735 12684 12739 12680 12738 12678 12734 12677 12730
12673 12733 12676 12736 12673 12739 12669 12739 12665 12742 12661 12743
12657 12742 12656 12738 26 N F
12693 8098 12690 8092 12693 8089 12695 8085 12699 8087 12703 8090
12699 8091 12696 8095 12697 8100 12693 8098 10 N F
12718 8227 12712 8215 12719 8217 12726 8218 12732 8221 12730 8228
12724 8231 12718 8227 8 N F
12750 12884 12748 12878 12749 12871 12752 12865 12755 12858 12757 12858
12761 12856 12764 12850 12768 12844 12774 12847 12773 12854 12780 12856
12786 12859 12790 12864 12793 12870 12800 12869 12801 12862 12803 12856
12802 12849 12802 12842 12796 12838 12790 12837 12784 12834 12785 12827
12786 12828 12790 12832 12790 12825 12797 12826 12803 12829 12809 12826
12804 12822 12803 12816 12805 12809 12812 12808 12809 12802 12806 12795
12813 12793 12814 12793 12819 12791 12825 12795 12831 12796 12829 12803
12824 12807 12830 12810 12837 12811 12843 12813 12850 12814 12844 12817
12838 12816 12833 12821 12829 12827 12831 12833 12838 12833 12842 12838
12840 12844 12840 12851 12846 12854 12849 12848 12853 12854 12860 12853
12861 12852 12864 12847 12870 12845 12871 12852 12866 12856 12861 12862
12863 12868 12857 12867 12854 12873 12860 12877 12867 12875 12864 12881
12860 12886 12866 12888 12866 12894 12869 12901 12862 12901 12857 12896
12856 12890 12849 12891 12845 12897 12850 12902 12853 12908 12856 12914
12854 12920 12852 12927 12851 12934 12849 12940 12843 12944 12840 12950
12838 12957 12841 12963 12847 12965 12848 12972 12844 12977 12837 12980
12830 12980 12826 12974 12826 12968 12830 12958 12823 12956 12820 12949
12819 12943 12819 12936 12812 12937 12806 12934 12800 12937 12798 12930
12793 12925 12797 12919 12801 12914 12805 12908 12810 12903 12815 12899
12808 12900 12801 12899 12795 12902 12790 12896 12784 12898 12778 12901
12772 12905 12766 12904 12763 12897 12757 12896 12753 12890 12750 12884
126 N F
12749 12834 12748 12829 12751 12826 12754 12823 12753 12819 12754 12815
12754 12815 12758 12814 12762 12814 12766 12816 12769 12819 12772 12822
12772 12822 12772 12826 12769 12829 12765 12828 12762 12832 12762 12836
12759 12840 12759 12844 12756 12842 12757 12846 12753 12846 12749 12843
12751 12839 12755 12840 12758 12837 12755 12839 12754 12834 12751 12831
12751 12835 12748 12838 12749 12834 33 N F
12754 7994 12751 7989 12753 7985 12757 7983 12757 7988 12758 7992
12754 7994 7 N F
12754 8016 12753 8011 12754 8011 12757 8011 12758 8015 12754 8016
6 N F
12760 12933 12759 12929 12761 12925 12762 12921 12760 12917 12763 12913
12765 12909 12770 12910 12767 12914 12770 12916 12767 12919 12765 12923
12768 12926 12770 12930 12768 12934 12764 12935 12760 12933 17 N F
12769 8010 12766 8006 12770 8003 12772 7999 12777 8001 12780 8003

12778 8007 12776 8011 12772 8013 12769 8010 10 N F
12779 8028 12773 8023 12777 8023 12782 8022 12783 8022 12784 8021
12785 8019 12788 8022 12790 8027 12790 8031 12793 8028 12797 8027
12797 8031 12795 8035 12790 8036 12786 8036 12785 8032 12781 8032
12779 8028 19 N F
12783 7999 12776 7997 12779 7993 12783 7995 12783 7999 5 N F
12784 7993 12780 7988 12783 7985 12784 7989 12784 7993 5 N F
12809 8003 12805 8000 12807 7996 12810 7993 12809 7988 12809 7984
12812 7980 12813 7984 12817 7986 12818 7991 12817 7995 12816 8000
12819 8003 12822 8006 12821 8011 12821 8015 12820 8020 12818 8024
12814 8026 12810 8025 12810 8020 12809 8016 12807 8012 12808 8007
12809 8003 25 N F
12856 8023 12854 8017 12858 8018 12859 8022 12861 8026 12861 8031
12857 8028 12856 8023 8 N F
12882 12996 12879 12992 12883 12991 12886 12988 12886 12984 12889 12981
12885 12978 12889 12976 12893 12975 12893 12971 12893 12966 12896 12964
12898 12964 12901 12964 12902 12969 12900 12973 12900 12977 12901 12981
12903 12986 12904 12990 12903 12994 12902 12998 12897 12997 12895 13001
12892 13004 12889 13000 12885 12999 12882 12996 28 N F
12893 11300 12892 11300 12893 11299 12893 11299 12893 11299 12893 11299
12894 11298 12895 11298 12895 11299 12895 11299 12895 11300 12894 11301
12893 11300 13 N F
12894 11303 12893 11302 12894 11301 12895 11301 12896 11299 12896 11298
12895 11296 12895 11294 12895 11293 12894 11291 12896 11291 12896 11292
12897 11294 12898 11294 12900 11294 12900 11296 12898 11297 12898 11299
12898 11300 12899 11301 12900 11303 12900 11304 12898 11305 12897 11304
12896 11303 12894 11303 26 N F
12901 11309 12900 11308 12900 11307 12901 11306 12902 11306 12903 11307
12903 11308 12903 11309 12903 11310 12903 11310 12902 11310 12901 11309
12901 11309 13 N F
12902 11315 12902 11314 12902 11313 12902 11313 12903 11313 12903 11313
12903 11314 12903 11315 12903 11315 12902 11315 10 N F
12904 11317 12903 11316 12903 11314 12903 11314 12904 11314 12904 11315
12905 11314 12906 11314 12906 11315 12906 11316 12906 11317 12906 11317
12906 11318 12904 11317 12904 11317 12904 11317 16 N F
12906 11321 12906 11321 12906 11320 12907 11320 12907 11319 12907 11319
12907 11318 12908 11318 12908 11318 12909 11319 12909 11320 12908 11320
12908 11321 12908 11322 12906 11321 15 N F
12908 11325 12908 11323 12910 11323 12911 11324 12913 11324 12914 11324
12916 11325 12917 11326 12918 11325 12919 11324 12920 11325 12921 11327
12922 11328 12923 11329 12923 11331 12923 11333 12922 11333 12921 11332
12919 11332 12918 11332 12916 11332 12915 11332 12913 11332 12912 11331
12912 11329 12912 11328 12911 11326 12910 11326 12908 11325 29 N F

12960 8037 12957 8030 12958 8023 12963 8018 12966 8011 12971 8007
12976 8002 12977 7995 12976 7988 12976 7980 12974 7974 12974 7967
12979 7962 12986 7960 12990 7965 12996 7969 12996 7969 12996 8077
12992 8075 12985 8072 12981 8066 12978 8060 12975 8053 12975 8046
12970 8041 12974 8035 12979 8030 12973 8034 12967 8037 12960 8037
30 N F
12971 11664 12967 11651 12974 11649 12981 11649 12985 11644 12992 11643
12996 11649 12996 11649 12996 11679 12990 11679 12995 11674 12988 11676
12991 11669 12985 11665 12984 11658 12991 11661 12996 11665 12992 11659
12992 11652 12985 11654 12980 11658 12976 11664 12973 11670 12971 11664
24 N F
12979 8339 12976 8328 12980 8323 12987 8323 12994 8325 12996 8325
12996 8340 12993 8339 12986 8339 12979 8339 10 N F
12981 11744 12978 11737 12979 11731 12984 11725 12981 11719 12983 11712
12988 11707 12984 11701 12987 11695 12989 11689 12996 11686 12996 11680
12996 11742 12992 11747 12985 11749 12981 11744 16 N F
12980 8163 12980 8156 12982 8149 12985 8143 12989 8137 12993 8131
12996 8128 12996 8164 12993 8161 12988 8165 12982 8170 12980 8163
12 N F
1 O
12997 16211 2301 16211 2301 5500 12997 5500 12997 16211 5 P
Ls Gr Gs Lr
2301 5499 12998 16212 Oc
95 O
2301 5634 2301 5600 2301 5600 2301 6188 2301 6188 2301 6177
2301 6041 2301 5906 2301 5770 2301 5634 10 N F
82 O
2301 6313 2301 6188 2301 6188 2301 6437 2301 6437 2301 6313
6 N F
95 O
2301 6448 2301 6437 2301 6437 2301 6918 2301 6918 2301 6855
2301 6719 2301 6584 2301 6448 9 N F
82 O
2301 6991 2301 6918 2301 6918 2301 7455 2301 7455 2301 7397
2301 7262 2301 7126 2301 6991 9 N F
95 O
2301 7533 2301 7455 2301 7455 2301 7681 2301 7681 2301 7669
2301 7533 7 N F
82 O
2301 7804 2301 7681 2301 7681 2301 7825 2301 7825 2301 7804
6 N F
68 O
2301 7940 2301 7825 2301 7825 2301 7988 2301 7988 2301 7940

6 N F
55 O
2301 8075 2301 7988 2301 7988 2301 8413 2301 8413 2301 8347
2301 8211 2301 8075 8 N F
42 O
2301 8482 2301 8413 2301 8413 2301 8619 2301 8619 2301 8618
2301 8482 7 N F
55 O
2301 8754 2301 8619 2301 8619 2301 9570 2301 9570 2301 9567
2301 9432 2301 9296 2301 9160 2301 9025 2301 8889 2301 8754
12 N F
68 O
2301 9687 2301 9570 2301 9570 2301 9687 2301 9687 5 N F
82 O
2301 9703 2301 9687 2301 9687 2301 10179 2301 10179 2301 10110
2301 9974 2301 9838 2301 9703 9 N F
95 O
2301 10245 2301 10179 2301 10179 2301 10521 2301 10521 2301 10516
2301 10381 2301 10245 8 N F
108 O
2301 10652 2301 10521 2301 10521 2301 10917 2301 10917 2301 10788
2301 10652 7 N F
95 O
2301 10923 2301 10917 2301 10917 2301 11347 2301 11347 2301 11330
2301 11194 2301 11059 2301 10923 9 N F
82 O
2301 11466 2301 11347 2301 11347 2301 11559 2301 11559 2301 11466
6 N F
68 O
2301 11601 2301 11559 2301 11559 2301 11776 2301 11776 2301 11737
2301 11601 7 N F
55 O
2301 11852 2301 11776 2301 11776 2301 11852 2301 11852 5 N F
42 O
2301 11873 2301 11852 2301 11852 2301 11932 2301 11932 2301 11873
6 N F
29 O
2301 12008 2301 11932 2301 11932 2301 12027 2301 12027 2301 12008
6 N F
15 O
2301 12144 2301 12027 2301 12027 2301 12353 2301 12353 2301 12279
2301 12144 7 N F
29 O

2301 12415 2301 12353 2301 12353 2301 12431 2301 12431 2301 12415
6 N F
15 O
2301 12551 2301 12431 2301 12431 2301 15624 2301 15624 2301 15534
2301 15398 2301 15263 2301 15127 2301 14992 2301 14856 2301 14720
2301 14585 2301 14449 2301 14314 2301 14178 2301 14042 2301 13907
2301 13771 2301 13635 2301 13500 2301 13364 2301 13229 2301 13093
2301 12957 2301 12822 2301 12686 2301 12551 28 N F
82 O
2301 5600 2301 5499 2301 5499 2301 5499 2301 5600 2301 5600
6 N F
95 O
2301 6188 2301 5600 2301 5600 2301 6188 2301 6188 5 N F
82 O
2301 6437 2301 6188 2301 6188 2301 6437 2301 6437 5 N F
95 O
2301 6918 2301 6437 2301 6437 2301 6918 2301 6918 5 N F
82 O
2301 7455 2301 6918 2301 6918 2301 7455 2301 7455 5 N F
95 O
2301 7681 2301 7455 2301 7455 2301 7681 2301 7681 5 N F
82 O
2301 7825 2301 7681 2301 7681 2301 7825 2301 7825 5 N F
68 O
2301 7988 2301 7825 2301 7825 2301 7988 2301 7988 5 N F
55 O
2301 8413 2301 7988 2301 7988 2301 8413 2301 8413 5 N F
42 O
2301 8619 2301 8413 2301 8413 2301 8619 2301 8619 5 N F
55 O
2301 9570 2301 8619 2301 8619 2301 9570 2301 9570 5 N F
68 O
2301 9687 2301 9570 2301 9570 2301 9687 2301 9687 5 N F
82 O
2301 10179 2301 9687 2301 9687 2301 10179 2301 10179 5 N F
95 O
2301 10521 2301 10179 2301 10179 2301 10521 2301 10521 5 N F
108 O
2301 10917 2301 10521 2301 10521 2301 10917 2301 10917 5 N F
95 O
2301 11347 2301 10917 2301 10917 2301 11347 2301 11347 5 N F
82 O
2301 11559 2301 11347 2301 11347 2301 11559 2301 11559 5 N F

68 O
2301 11776 2301 11559 2301 11559 2301 11776 2301 11776 5 N F
55 O
2301 11852 2301 11776 2301 11776 2301 11852 2301 11852 5 N F
42 O
2301 11932 2301 11852 2301 11852 2301 11932 2301 11932 5 N F
29 O
2301 12027 2301 11932 2301 11932 2301 12027 2301 12027 5 N F
15 O
2301 12353 2301 12027 2301 12027 2301 12352 2301 12353 5 N F
29 O
2301 12431 2301 12353 2301 12352 2301 12431 2301 12431 5 N F
15 O
2301 15624 2301 12431 2301 12431 2301 15624 2301 15624 5 N F
2 O
2301 16212 2301 15624 2301 15624 2301 16212 2301 16212 2301 16212
6 N F
82 O
2468 5499 2301 5499 2301 5600 2436 5611 2481 5634 2571 5704
2691 5634 2661 5499 2635 5499 2468 5499 10 N F
95 O
2436 5611 M 2301 5600 L 2301 6188 L 2323 6177 L 2436 6127 L
2502 6177 L 2461 6313 L 2436 6331 L 2301 6437 L
2301 6918 L 2436 6943 L 2563 6991 L 2571 6997 L
2574 6991 L 2582 6855 L 2601 6719 L 2707 6614 L
2707 5777 L 2699 5770 L 2707 5731 L 2714 5770 L
2707 5777 L 2707 6614 L 2842 6635 L 2906 6584 L
2903 6448 L 2978 6315 L 2982 6313 L 3113 6286 L
3113 5688 L 3094 5634 L 3113 5622 L 3140 5634 L
3113 5688 L 3113 6286 L 3148 6313 L 3210 6448 L
3248 6510 L 3384 6525 L 3426 6584 L 3515 6719 L
3519 6730 L 3600 6855 L 3655 6908 L 3790 6885 L
3841 6855 L 3837 6719 L 3813 6584 L 3858 6448 L
3926 6427 L 4002 6448 L 4061 6468 L 4196 6525 L
4309 6584 L 4332 6598 L 4350 6584 L 4467 6487 L
4603 6503 L 4688 6584 L 4696 6719 L 4603 6844 L
4598 6855 L 4603 6857 L 4738 6877 L 4772 6855 L
4777 6719 L 4873 6637 L 5009 6588 L 5144 6627 L
5254 6719 L 5271 6855 L 5144 6948 L 5106 6991 L
5144 7036 L 5248 7126 L 5280 7161 L 5354 7262 L
5415 7310 L 5495 7262 L 5415 7212 L 5306 7126 L
5280 7003 L 5272 6991 L 5280 6951 L 5298 6855 L
5415 6814 L 5551 6799 L 5622 6855 L 5621 6991 L

5660 7126 L 5686 7164 L 5821 7158 L 5957 7175 L
6014 7126 L 6024 6991 L 6092 6910 L 6183 6855 L
6228 6833 L 6323 6855 L 6363 6904 L 6387 6991 L
6363 7043 L 6228 7106 L 6201 7126 L 6228 7147 L
6355 7262 L 6363 7272 L 6498 7387 L 6531 7397 L
6634 7406 L 6634 7288 L 6626 7262 L 6634 7258 L
6644 7262 L 6634 7288 L 6634 7406 L 6769 7440 L
6905 7483 L 6905 7313 L 6849 7262 L 6905 7234 L
6966 7262 L 6905 7313 L 6905 7483 L 6960 7533 L
7040 7579 L 7103 7533 L 7141 7397 L 7176 7325 L
7259 7397 L 7311 7419 L 7357 7397 L 7391 7262 L
7446 7216 L 7582 7164 L 7667 7262 L 7717 7328 L
7836 7397 L 7853 7402 L 7864 7397 L 7988 7306 L
8123 7320 L 8259 7352 L 8394 7354 L 8530 7331 L
8639 7262 L 8665 7253 L 8673 7262 L 8743 7397 L
8801 7436 L 8915 7533 L 8923 7669 L 8936 7705 L
9071 7801 L 9075 7804 L 9071 7818 L 9018 7940 L
9071 8010 L 9135 8075 L 9207 8092 L 9342 8094 L
9357 8075 L 9342 8033 L 9258 7940 L 9243 7804 L
9325 7669 L 9342 7657 L 9478 7660 L 9613 7649 L
9748 7576 L 9884 7544 L 9917 7533 L 10019 7518 L
10155 7507 L 10290 7513 L 10417 7533 L 10426 7536 L
10561 7577 L 10696 7584 L 10832 7553 L 10849 7533 L
10967 7467 L 11103 7454 L 11238 7447 L 11373 7456 L
11509 7466 L 11644 7477 L 11780 7489 L 11915 7506 L
12041 7533 L 12051 7542 L 12079 7669 L 12099 7804 L
12114 7940 L 12123 8075 L 12132 8211 L 12141 8347 L
12146 8482 L 12152 8618 L 12159 8754 L 12165 8889 L
12172 9025 L 12181 9160 L 12186 9217 L 12229 9296 L
12284 9432 L 12321 9555 L 12328 9567 L 12405 9703 L
12457 9787 L 12508 9838 L 12592 9939 L 12625 9974 L
12728 10084 L 12751 10110 L 12863 10221 L 12909 10245 L
12998 10298 L 12998 9519 L 12863 9468 L 12820 9432 L
12728 9354 L 12592 9336 L 12553 9296 L 12457 9237 L
12371 9160 L 12321 9092 L 12302 9025 L 12236 8889 L
12186 8768 L 12185 8754 L 12180 8618 L 12174 8482 L
12170 8347 L 12164 8211 L 12158 8075 L 12152 7940 L
12142 7804 L 12129 7669 L 12108 7533 L 12051 7474 L
11915 7454 L 11780 7444 L 11644 7435 L 11509 7426 L
11373 7417 L 11238 7410 L 11103 7411 L 10967 7420 L
10832 7463 L 10696 7488 L 10561 7491 L 10426 7458 L
10290 7438 L 10155 7424 L 10019 7431 L 9884 7442 L
9748 7455 L 9613 7485 L 9478 7483 L 9342 7484 L

9207 7486 L 9071 7449 L 8972 7397 L 8936 7375 L
8801 7272 L 8794 7262 L 8673 7126 L 8665 7119 L
8534 6991 L 8530 6987 L 8523 6991 L 8394 7123 L
8389 7126 L 8259 7262 L 8181 7126 L 8123 7064 L
7988 7034 L 7853 7068 L 7737 6991 L 7717 6973 L
7602 6855 L 7582 6830 L 7572 6855 L 7534 6991 L
7446 7069 L 7311 7101 L 7176 7039 L 7040 6992 L
7035 6991 L 6905 6874 L 6769 6926 L 6634 6917 L
6502 6855 L 6498 6852 L 6432 6719 L 6363 6622 L
6284 6584 L 6228 6562 L 6092 6543 L 6043 6584 L
5997 6719 L 5957 6753 L 5821 6735 L 5773 6719 L
5686 6672 L 5583 6584 L 5567 6448 L 5627 6313 L
5645 6177 L 5686 6082 L 5720 6041 L 5821 5932 L
5833 5906 L 5957 5775 L 5962 5770 L 6092 5705 L
6136 5634 L 6097 5499 L 6029 5499 L 6064 5634 L
5957 5680 L 5821 5720 L 5779 5770 L 5686 5883 L
5555 5770 L 5551 5765 L 5516 5770 L 5418 5906 L
5476 6041 L 5415 6080 L 5388 6041 L 5407 5906 L
5280 5836 L 5241 5770 L 5144 5672 L 5009 5669 L
4985 5634 L 5009 5519 L 5023 5499 L 4975 5499 L
4808 5499 L 4641 5499 L 4474 5499 L 4370 5499 L
4413 5634 L 4409 5770 L 4338 5906 L 4332 5916 L
4288 6041 L 4332 6131 L 4370 6177 L 4332 6204 L
4196 6295 L 4061 6268 L 3926 6264 L 3790 6196 L
3754 6177 L 3655 6107 L 3519 6042 L 3519 6041 L
3393 5906 L 3519 5829 L 3647 5906 L 3655 5910 L
3790 5949 L 3887 5906 L 3867 5770 L 3926 5689 L
3968 5634 L 4061 5589 L 4188 5499 L 4139 5499 L
3972 5499 L 3805 5499 L 3638 5499 L 3471 5499 L
3304 5499 L 3136 5499 L 2969 5499 L 2802 5499 L
2661 5499 L 2691 5634 L 2571 5704 L 2481 5634 L
2436 5611 L N E
82 O
2301 6188 2301 6437 2436 6331 2461 6313 2502 6177 2436 6127
2323 6177 2301 6188 8 N F
2301 6918 M 2301 7455 L 2397 7533 L 2330 7669 L 2301 7681 L
2301 7825 L 2357 7804 L 2436 7774 L 2564 7669 L
2571 7655 L 2624 7533 L 2692 7397 L 2707 7381 L
2842 7349 L 2962 7397 L 2978 7406 L 3009 7397 L
3113 7317 L 3127 7262 L 3248 7142 L 3248 6895 L
3202 6855 L 3248 6803 L 3338 6855 L 3248 6895 L
3248 7142 L 3384 7176 L 3485 7262 L 3519 7283 L
3615 7262 L 3655 7234 L 3790 7128 L 3799 7126 L

3926 7099 L 4061 7105 L 4061 6791 L 4009 6719 L
4061 6654 L 4123 6719 L 4061 6791 L 4061 7105 L
4196 7122 L 4332 7089 L 4422 6991 L 4467 6944 L
4603 6990 L 4605 6991 L 4654 7126 L 4636 7262 L
4731 7397 L 4738 7411 L 4869 7533 L 4873 7536 L
5009 7582 L 5009 7356 L 4873 7273 L 4857 7262 L
4795 7126 L 4859 6991 L 4873 6982 L 4887 6991 L
5009 7047 L 5077 7126 L 5144 7212 L 5191 7262 L
5144 7330 L 5009 7356 L 5009 7582 L 5144 7616 L
5280 7662 L 5301 7669 L 5415 7711 L 5473 7804 L
5551 7895 L 5686 7881 L 5718 7804 L 5735 7669 L
5712 7533 L 5686 7475 L 5645 7397 L 5686 7359 L
5821 7301 L 5957 7377 L 6092 7389 L 6096 7397 L
6228 7480 L 6302 7533 L 6363 7567 L 6498 7587 L
6602 7669 L 6623 7804 L 6629 7940 L 6634 7954 L
6672 8075 L 6769 8164 L 6850 8211 L 6905 8254 L
7000 8347 L 7040 8460 L 7042 8482 L 7176 8613 L
7197 8618 L 7186 8754 L 7176 8784 L 7162 8889 L
7153 9025 L 7176 9062 L 7276 9025 L 7311 9010 L
7311 7814 L 7220 7804 L 7311 7795 L 7330 7804 L
7311 7814 L 7311 9010 L 7329 9025 L 7405 9160 L
7428 9296 L 7429 9432 L 7446 9436 L 7582 9493 L
7582 9174 L 7541 9160 L 7582 9143 L 7639 9160 L
7582 9174 L 7582 9493 L 7626 9567 L 7582 9617 L
7509 9703 L 7446 9758 L 7339 9838 L 7311 9880 L
7176 9936 L 7040 9844 L 7015 9838 L 6905 9823 L
6889 9838 L 6889 9974 L 6905 10012 L 6961 10110 L
6905 10222 L 6824 10245 L 6769 10361 L 6634 10290 L
6559 10381 L 6634 10406 L 6733 10516 L 6769 10549 L
6905 10645 L 6910 10652 L 6925 10788 L 6905 10812 L
6769 10868 L 6681 10923 L 6634 11047 L 6498 11032 L
6461 11059 L 6498 11121 L 6593 11194 L 6634 11229 L
6708 11330 L 6634 11405 L 6605 11466 L 6634 11493 L
6722 11466 L 6769 11419 L 6905 11414 L 7001 11466 L
7040 11487 L 7176 11518 L 7311 11521 L 7421 11601 L
7446 11620 L 7446 11512 L 7311 11483 L 7176 11468 L
7168 11466 L 7040 11378 L 6905 11336 L 6875 11330 L
6806 11194 L 6905 11072 L 7040 11171 L 7086 11194 L
7176 11264 L 7176 11116 L 7040 11074 L 7020 11059 L
7040 11031 L 7040 10717 L 7029 10652 L 7040 10639 L
7043 10652 L 7040 10717 L 7040 11031 L 7145 10923 L
7176 10899 L 7176 10763 L 7070 10652 L 7176 10530 L
7295 10652 L 7176 10763 L 7176 10899 L 7214 10923 L

7243 11059 L 7176 11116 L 7176 11264 L 7311 11221 L
7325 11194 L 7446 11122 L 7550 11194 L 7446 11324 L
7434 11330 L 7446 11339 L 7531 11466 L 7446 11512 L
7446 11620 L 7582 11698 L 7614 11737 L 7582 11763 L
7551 11873 L 7582 11996 L 7592 12008 L 7659 12144 L
7698 12279 L 7717 12319 L 7782 12415 L 7827 12551 L
7853 12623 L 7853 12088 L 7717 12059 L 7690 12008 L
7582 11879 L 7580 11873 L 7582 11867 L 7717 11791 L
7717 11214 L 7710 11194 L 7717 11192 L 7717 9440 L
7698 9432 L 7717 9407 L 7717 9139 L 7646 9025 L
7624 8889 L 7582 8839 L 7502 8754 L 7459 8618 L
7582 8498 L 7704 8618 L 7717 8629 L 7717 8286 L
7582 8247 L 7572 8211 L 7446 8111 L 7379 8075 L
7446 8046 L 7582 8038 L 7624 8075 L 7717 8136 L
7853 8166 L 7942 8211 L 7853 8256 L 7717 8286 L
7717 8629 L 7853 8696 L 7979 8618 L 7988 8606 L
7988 8116 L 7975 8075 L 7853 7984 L 7813 7940 L
7738 7804 L 7717 7768 L 7582 7756 L 7509 7669 L
7582 7575 L 7582 7470 L 7557 7397 L 7582 7363 L
7610 7397 L 7582 7470 L 7582 7575 L 7684 7533 L
7717 7524 L 7853 7515 L 7988 7465 L 8123 7443 L
8259 7460 L 8394 7465 L 8530 7481 L 8665 7495 L
8723 7533 L 8795 7669 L 8665 7786 L 8530 7768 L
8394 7707 L 8259 7706 L 8123 7704 L 7988 7737 L
7936 7804 L 7988 7848 L 8086 7940 L 8123 8048 L
8177 8075 L 8123 8093 L 7988 8116 L 7988 8606 L
8005 8618 L 8113 8754 L 8097 8889 L 8064 9025 L
7988 9053 L 7925 9025 L 7853 9018 L 7781 9025 L
7717 9139 L 7717 9407 L 7748 9432 L 7717 9440 L
7717 11192 L 7724 11194 L 7717 11214 L 7717 11791 L
7853 11770 L 7853 9675 L 7734 9567 L 7853 9436 L
7956 9567 L 7853 9675 L 7853 11770 L 7970 11873 L
7917 12008 L 7853 12088 L 7853 12623 L 7988 12620 L
7988 11646 L 7975 11601 L 7947 11466 L 7988 11465 L
7988 11436 L 7881 11330 L 7988 11221 L 8094 11330 L
7988 11436 L 7988 11465 L 8122 11330 L 8123 11269 L
8259 11324 L 8265 11330 L 8259 11349 L 8132 11466 L
8123 11469 L 7997 11601 L 7988 11646 L 7988 12620 L
8123 12572 L 8196 12551 L 8259 12518 L 8394 12436 L
8427 12415 L 8426 12279 L 8530 12160 L 8559 12144 L
8624 12008 L 8577 11873 L 8665 11779 L 8712 11737 L
8754 11601 L 8749 11466 L 8667 11330 L 8665 11328 L
8561 11194 L 8530 11140 L 8442 11059 L 8530 10923 L

8530 10923 L 8647 10788 L 8665 10769 L 8801 10676 L
8829 10652 L 8936 10542 L 8969 10516 L 8952 10381 L
8936 10363 L 8854 10245 L 8830 10110 L 8801 9993 L
8795 9974 L 8665 9962 L 8545 9838 L 8665 9718 L
8801 9789 L 8913 9703 L 8895 9567 L 8819 9432 L
8917 9296 L 8801 9259 L 8665 9270 L 8530 9295 L
8528 9296 L 8513 9432 L 8394 9537 L 8265 9432 L
8259 9357 L 8123 9308 L 8107 9296 L 8123 9252 L
8123 8444 L 8097 8347 L 8123 8317 L 8228 8347 L
8123 8444 L 8123 9252 L 8146 9160 L 8218 9025 L
8159 8889 L 8259 8839 L 8304 8754 L 8289 8618 L
8259 8581 L 8138 8482 L 8259 8358 L 8261 8347 L
8323 8211 L 8283 8075 L 8394 8005 L 8526 7940 L
8530 7934 L 8665 7814 L 8735 7940 L 8733 8075 L
8665 8160 L 8567 8211 L 8661 8347 L 8665 8356 L
8801 8399 L 8912 8347 L 8936 8328 L 8973 8347 L
9071 8380 L 9124 8347 L 9207 8279 L 9342 8229 L
9355 8211 L 9457 8075 L 9427 7940 L 9478 7870 L
9560 7940 L 9613 8048 L 9748 7945 L 9884 7962 L
9999 7940 L 9884 7924 L 9760 7804 L 9884 7701 L
9950 7669 L 10019 7627 L 10121 7669 L 10155 7679 L
10290 7698 L 10426 7748 L 10500 7669 L 10561 7652 L
10642 7669 L 10696 7693 L 10760 7669 L 10832 7652 L
10935 7533 L 10967 7515 L 11103 7498 L 11238 7484 L
11373 7494 L 11509 7505 L 11644 7518 L 11775 7533 L
11780 7535 L 11915 7617 L 11970 7669 L 12051 7770 L
12057 7804 L 12077 7940 L 12089 8075 L 12100 8211 L
12111 8347 L 12118 8482 L 12125 8618 L 12132 8754 L
12139 8889 L 12146 9025 L 12155 9160 L 12166 9296 L
12175 9432 L 12183 9567 L 12186 9611 L 12219 9703 L
12260 9838 L 12310 9974 L 12321 10001 L 12400 10110 L
12457 10223 L 12474 10245 L 12591 10381 L 12592 10382 L
12672 10516 L 12728 10635 L 12738 10652 L 12863 10779 L
12875 10788 L 12998 10876 L 12998 10298 L 12909 10245 L
12863 10221 L 12751 10110 L 12728 10084 L 12625 9974 L
12592 9939 L 12508 9838 L 12457 9787 L 12405 9703 L
12328 9567 L 12321 9555 L 12284 9432 L 12229 9296 L
12186 9217 L 12181 9160 L 12172 9025 L 12165 8889 L
12159 8754 L 12152 8618 L 12146 8482 L 12141 8347 L
12132 8211 L 12123 8075 L 12114 7940 L 12099 7804 L
12079 7669 L 12051 7542 L 12041 7533 L 11915 7506 L
11780 7489 L 11644 7477 L 11509 7466 L 11373 7456 L
11238 7447 L 11103 7454 L 10967 7467 L 10849 7533 L

10832 7553 L 10696 7584 L 10561 7577 L 10426 7536 L
10417 7533 L 10290 7513 L 10155 7507 L 10019 7518 L
9917 7533 L 9884 7544 L 9748 7576 L 9613 7649 L
9478 7660 L 9342 7657 L 9325 7669 L 9243 7804 L
9258 7940 L 9342 8033 L 9357 8075 L 9342 8094 L
9207 8092 L 9135 8075 L 9071 8010 L 9018 7940 L
9071 7818 L 9075 7804 L 9071 7801 L 8936 7705 L
8923 7669 L 8915 7533 L 8801 7436 L 8743 7397 L
8673 7262 L 8665 7253 L 8639 7262 L 8530 7331 L
8394 7354 L 8259 7352 L 8123 7320 L 7988 7306 L
7864 7397 L 7853 7402 L 7836 7397 L 7717 7328 L
7667 7262 L 7582 7164 L 7446 7216 L 7391 7262 L
7357 7397 L 7311 7419 L 7259 7397 L 7176 7325 L
7141 7397 L 7103 7533 L 7040 7579 L 6960 7533 L
6905 7483 L 6769 7440 L 6634 7406 L 6531 7397 L
6498 7387 L 6363 7272 L 6355 7262 L 6228 7147 L
6201 7126 L 6228 7106 L 6363 7043 L 6387 6991 L
6363 6904 L 6323 6855 L 6228 6833 L 6183 6855 L
6092 6910 L 6024 6991 L 6014 7126 L 5957 7175 L
5821 7158 L 5686 7164 L 5660 7126 L 5621 6991 L
5622 6855 L 5551 6799 L 5415 6814 L 5298 6855 L
5280 6951 L 5272 6991 L 5280 7003 L 5306 7126 L
5415 7212 L 5415 7055 L 5381 6991 L 5415 6949 L
5507 6991 L 5415 7055 L 5415 7212 L 5495 7262 L
5415 7310 L 5354 7262 L 5280 7161 L 5248 7126 L
5144 7036 L 5106 6991 L 5144 6948 L 5271 6855 L
5254 6719 L 5144 6627 L 5009 6588 L 4873 6637 L
4777 6719 L 4772 6855 L 4738 6877 L 4603 6857 L
4598 6855 L 4603 6844 L 4696 6719 L 4688 6584 L
4603 6503 L 4467 6487 L 4350 6584 L 4332 6598 L
4309 6584 L 4196 6525 L 4061 6468 L 4002 6448 L
3926 6427 L 3858 6448 L 3813 6584 L 3837 6719 L
3841 6855 L 3790 6885 L 3655 6908 L 3600 6855 L
3519 6730 L 3515 6719 L 3426 6584 L 3384 6525 L
3248 6510 L 3210 6448 L 3148 6313 L 3113 6286 L
2982 6313 L 2978 6315 L 2903 6448 L 2906 6584 L
2842 6635 L 2707 6614 L 2601 6719 L 2582 6855 L
2574 6991 L 2571 6997 L 2563 6991 L 2436 6943 L
2301 6918 L N E
95 O
2397 7533 2301 7455 2301 7681 2330 7669 2397 7533 5 N F
68 O
2301 7825 M 2301 7988 L 2436 7945 L 2457 7940 L 2571 7912 L

2683 7804 L 2707 7675 L 2791 7804 L 2836 7940 L
2842 7948 L 2978 7942 L 2981 7940 L 3113 7870 L
3248 7826 L 3287 7804 L 3248 7773 L 3113 7720 L
3021 7669 L 3113 7598 L 3149 7533 L 3205 7397 L
3248 7315 L 3384 7356 L 3443 7397 L 3519 7484 L
3560 7533 L 3596 7669 L 3655 7691 L 3762 7804 L
3790 7852 L 3926 7904 L 3945 7804 L 3926 7745 L
3872 7669 L 3852 7533 L 3926 7399 L 4061 7471 L
4194 7533 L 4196 7534 L 4196 7398 L 4195 7397 L
4061 7288 L 4022 7262 L 4061 7194 L 4093 7262 L
4196 7396 L 4197 7397 L 4196 7398 L 4196 7534 L
4332 7563 L 4451 7669 L 4467 7689 L 4602 7804 L
4603 7806 L 4603 7753 L 4597 7669 L 4467 7643 L
4375 7533 L 4381 7397 L 4467 7330 L 4565 7397 L
4603 7438 L 4656 7533 L 4738 7659 L 4759 7669 L
4738 7685 L 4603 7753 L 4603 7806 L 4659 7940 L
4738 8047 L 4776 8075 L 4873 8164 L 4969 8211 L
5009 8240 L 5009 7831 L 4978 7804 L 5009 7778 L
5110 7804 L 5009 7831 L 5009 8240 L 5075 8211 L
5144 8188 L 5171 8211 L 5204 8347 L 5144 8418 L
5099 8482 L 5009 8555 L 4980 8618 L 5009 8678 L
5085 8754 L 5144 8796 L 5280 8763 L 5285 8754 L
5398 8618 L 5415 8593 L 5422 8618 L 5502 8754 L
5551 8799 L 5569 8889 L 5551 8944 L 5461 9025 L
5415 9084 L 5280 9146 L 5263 9160 L 5144 9213 L
5074 9160 L 5009 9134 L 4923 9160 L 4903 9296 L
4873 9323 L 4764 9432 L 4738 9447 L 4710 9567 L
4715 9703 L 4680 9838 L 4738 9963 L 4744 9974 L
4796 10110 L 4738 10225 L 4735 10245 L 4681 10381 L
4661 10516 L 4603 10622 L 4548 10652 L 4603 10659 L
4616 10652 L 4738 10562 L 4873 10618 L 4896 10652 L
4927 10788 L 4873 10918 L 4870 10923 L 4738 11007 L
4695 11059 L 4603 11123 L 4467 11129 L 4332 11112 L
4196 11193 L 4196 11194 L 4127 11330 L 4061 11377 L
3926 11421 L 3790 11449 L 3716 11466 L 3655 11482 L
3519 11542 L 3493 11601 L 3384 11734 L 3380 11737 L
3248 11784 L 3113 11803 L 2978 11825 L 2856 11873 L
2878 12008 L 2978 12034 L 3113 12077 L 3113 12032 L
3041 12008 L 2978 11873 L 3113 11844 L 3248 11829 L
3384 11806 L 3501 11737 L 3519 11712 L 3591 11601 L
3655 11557 L 3790 11519 L 3926 11479 L 3955 11466 L
4061 11439 L 4166 11466 L 4178 11601 L 4061 11670 L
3981 11737 L 3926 11770 L 3790 11764 L 3655 11809 L

3585 11873 L 3519 11911 L 3399 12008 L 3384 12014 L
3248 12022 L 3113 12032 L 3113 12077 L 3248 12061 L
3384 12049 L 3493 12008 L 3519 11986 L 3655 11896 L
3713 11873 L 3790 11832 L 3926 11856 L 4061 11794 L
4196 11811 L 4262 11873 L 4332 11916 L 4467 11931 L
4603 11953 L 4649 12008 L 4738 12064 L 4873 12043 L
4943 12008 L 5009 11995 L 5144 11971 L 5280 11936 L
5415 11991 L 5427 12008 L 5415 12049 L 5301 12144 L
5280 12157 L 5175 12279 L 5144 12316 L 5060 12415 L
5059 12551 L 5009 12657 L 4962 12551 L 4873 12517 L
4738 12481 L 4603 12543 L 4583 12551 L 4467 12581 L
4332 12565 L 4311 12551 L 4196 12482 L 4082 12415 L
4061 12405 L 3926 12403 L 3790 12412 L 3769 12415 L
3655 12445 L 3519 12527 L 3469 12551 L 3519 12606 L
3655 12672 L 3709 12686 L 3790 12702 L 3926 12748 L
4061 12795 L 4154 12822 L 4196 12835 L 4332 12905 L
4467 12924 L 4467 12846 L 4332 12823 L 4330 12822 L
4196 12738 L 4061 12714 L 3950 12686 L 3926 12675 L
3790 12593 L 3655 12557 L 3644 12551 L 3655 12545 L
3790 12513 L 3926 12478 L 4061 12480 L 4196 12550 L
4197 12551 L 4332 12644 L 4467 12651 L 4603 12679 L
4621 12686 L 4603 12728 L 4567 12822 L 4467 12846 L
4467 12924 L 4603 12935 L 4727 12957 L 4738 12961 L
4873 12980 L 4913 12957 L 4947 12822 L 5009 12704 L
5020 12686 L 5144 12599 L 5258 12686 L 5280 12695 L
5415 12743 L 5551 12761 L 5686 12766 L 5686 12665 L
5551 12647 L 5425 12551 L 5415 12533 L 5322 12415 L
5415 12318 L 5442 12279 L 5513 12144 L 5512 12008 L
5417 11873 L 5415 11871 L 5280 11846 L 5233 11873 L
5144 11910 L 5009 11945 L 4873 11945 L 4738 11924 L
4695 11873 L 4603 11808 L 4467 11792 L 4332 11750 L
4317 11737 L 4332 11700 L 4365 11601 L 4332 11562 L
4231 11466 L 4225 11330 L 4275 11194 L 4332 11160 L
4467 11168 L 4603 11173 L 4738 11121 L 4873 11060 L
4880 11059 L 5009 11020 L 5063 10923 L 5044 10788 L
5009 10747 L 4981 10652 L 4977 10516 L 4873 10445 L
4812 10381 L 4858 10245 L 4873 10226 L 5009 10216 L
5071 10245 L 5090 10381 L 5032 10516 L 5144 10578 L
5280 10625 L 5415 10604 L 5469 10516 L 5551 10420 L
5597 10381 L 5686 10309 L 5686 10195 L 5554 10110 L
5614 9974 L 5680 9838 L 5686 9831 L 5686 8638 L
5672 8618 L 5686 8573 L 5691 8618 L 5686 8638 L
5686 9831 L 5690 9838 L 5712 9974 L 5717 10110 L

5686 10195 L 5686 10309 L 5714 10381 L 5741 10516 L
5727 10652 L 5768 10788 L 5810 10923 L 5779 11059 L
5821 11166 L 5901 11194 L 5957 11218 L 5982 11194 L
6092 11167 L 6228 11167 L 6264 11194 L 6228 11289 L
6092 11249 L 6015 11330 L 6092 11395 L 6228 11356 L
6345 11466 L 6228 11567 L 6159 11601 L 6228 11651 L
6363 11617 L 6363 11252 L 6327 11194 L 6228 11121 L
6092 11125 L 5957 11159 L 5832 11059 L 5882 10923 L
5853 10788 L 5842 10652 L 5845 10516 L 5849 10381 L
5833 10245 L 5848 10110 L 5899 9974 L 5912 9838 L
5821 9755 L 5774 9703 L 5720 9567 L 5749 9432 L
5821 9338 L 5880 9296 L 5821 9160 L 5821 9160 L
5788 9025 L 5821 8934 L 5830 8889 L 5821 8862 L
5769 8754 L 5787 8618 L 5793 8482 L 5808 8347 L
5821 8307 L 5847 8211 L 5821 8083 L 5818 8075 L
5821 8058 L 5838 7940 L 5828 7804 L 5894 7669 L
5957 7626 L 6092 7566 L 6228 7654 L 6259 7669 L
6228 7798 L 6226 7804 L 6218 7940 L 6228 7963 L
6279 8075 L 6363 8139 L 6451 8211 L 6498 8308 L
6532 8347 L 6498 8351 L 6364 8482 L 6363 8490 L
6228 8542 L 6209 8618 L 6228 8657 L 6297 8754 L
6228 8795 L 6167 8889 L 6105 9025 L 6228 9146 L
6250 9160 L 6291 9296 L 6363 9405 L 6401 9432 L
6458 9567 L 6477 9703 L 6440 9838 L 6498 9939 L
6498 9314 L 6363 9298 L 6362 9296 L 6363 9292 L
6471 9160 L 6498 9114 L 6562 9160 L 6520 9296 L
6498 9314 L 6498 9939 L 6559 9974 L 6551 10110 L
6498 10152 L 6462 10245 L 6403 10381 L 6363 10453 L
6286 10516 L 6363 10545 L 6498 10527 L 6524 10516 L
6634 10472 L 6674 10516 L 6769 10602 L 6836 10652 L
6797 10788 L 6769 10802 L 6731 10788 L 6634 10749 L
6578 10788 L 6594 10923 L 6498 10985 L 6397 11059 L
6452 11194 L 6363 11252 L 6363 11617 L 6498 11716 L
6517 11737 L 6508 11873 L 6498 11887 L 6490 11873 L
6363 11766 L 6228 11762 L 6092 11777 L 6066 11737 L
6009 11601 L 5957 11578 L 5922 11601 L 5821 11646 L
5762 11737 L 5739 11873 L 5729 12008 L 5702 12144 L
5686 12212 L 5639 12279 L 5686 12385 L 5691 12415 L
5717 12551 L 5686 12665 L 5686 12766 L 5762 12686 L
5785 12551 L 5769 12415 L 5756 12279 L 5771 12144 L
5812 12008 L 5789 11873 L 5808 11737 L 5821 11716 L
5957 11650 L 6021 11737 L 6092 11847 L 6228 11801 L
6363 11821 L 6425 11873 L 6498 11997 L 6516 12008 L

6634 12123 L 6675 12008 L 6634 11944 L 6579 11873 L
6584 11737 L 6498 11637 L 6454 11601 L 6407 11466 L
6498 11361 L 6566 11466 L 6634 11530 L 6769 11490 L
6905 11519 L 7040 11569 L 7176 11568 L 7311 11558 L
7370 11601 L 7446 11658 L 7546 11737 L 7521 11873 L
7543 12008 L 7533 12144 L 7582 12241 L 7594 12279 L
7688 12415 L 7714 12551 L 7717 12557 L 7807 12686 L
7816 12822 L 7717 12934 L 7625 12957 L 7582 12974 L
7446 13001 L 7311 13009 L 7176 13009 L 7040 12975 L
6905 13018 L 6769 13035 L 6634 13039 L 6498 12996 L
6367 12957 L 6363 12956 L 6354 12957 L 6228 13024 L
6212 13093 L 6228 13207 L 6234 13229 L 6280 13364 L
6301 13500 L 6363 13632 L 6381 13635 L 6498 13666 L
6498 13279 L 6363 13241 L 6357 13229 L 6339 13093 L
6363 13074 L 6498 13064 L 6634 13086 L 6725 13093 L
6715 13229 L 6634 13261 L 6498 13279 L 6498 13666 L
6581 13635 L 6634 13608 L 6769 13554 L 6905 13634 L
6909 13635 L 7040 13667 L 7176 13676 L 7311 13661 L
7410 13635 L 7446 13618 L 7493 13500 L 7485 13364 L
7565 13229 L 7582 13218 L 7717 13138 L 7756 13093 L
7815 12957 L 7853 12933 L 7988 12901 L 8123 12899 L
8259 12907 L 8394 12898 L 8530 12890 L 8665 12868 L
8801 12946 L 8831 12957 L 8936 12980 L 9071 12966 L
9116 12957 L 9207 12922 L 9342 12867 L 9342 12440 L
9207 12435 L 9149 12415 L 9071 12394 L 8936 12326 L
8856 12279 L 8936 12248 L 9071 12240 L 9207 12252 L
9342 12242 L 9478 12270 L 9613 12247 L 9695 12279 L
9613 12325 L 9478 12341 L 9400 12415 L 9342 12440 L
9342 12867 L 9449 12822 L 9478 12804 L 9613 12718 L
9631 12686 L 9680 12551 L 9748 12490 L 9833 12415 L
9884 12353 L 9978 12279 L 9963 12144 L 10003 12008 L
9884 11932 L 9748 11949 L 9613 11987 L 9607 12008 L
9500 12144 L 9478 12157 L 9424 12144 L 9342 12109 L
9276 12144 L 9207 12160 L 9088 12144 L 9071 12139 L
8936 12017 L 8926 12008 L 8936 11977 L 9069 11873 L
9071 11872 L 9075 11873 L 9207 11940 L 9241 11873 L
9342 11811 L 9342 11619 L 9326 11601 L 9342 11556 L
9365 11601 L 9342 11619 L 9342 11811 L 9427 11737 L
9478 11678 L 9585 11601 L 9478 11523 L 9391 11466 L
9342 11434 L 9207 11432 L 9156 11330 L 9148 11194 L
9071 11191 L 8952 11059 L 9071 10929 L 9071 9554 L
8961 9432 L 9071 9314 L 9178 9296 L 9207 9266 L
9272 9296 L 9304 9432 L 9207 9513 L 9071 9554 L

9071 10929 L 9176 10923 L 9145 10788 L 9207 10733 L
9265 10652 L 9342 10548 L 9349 10516 L 9420 10381 L
9475 10245 L 9478 10244 L 9613 10168 L 9748 10204 L
9799 10245 L 9884 10363 L 9905 10381 L 9884 10433 L
9874 10516 L 9884 10550 L 10014 10652 L 10019 10662 L
10019 10236 L 9893 10110 L 10019 9986 L 10139 10110 L
10019 10236 L 10019 10662 L 10155 10683 L 10192 10652 L
10254 10516 L 10290 10458 L 10319 10381 L 10351 10245 L
10396 10110 L 10378 9974 L 10296 9838 L 10290 9838 L
10163 9703 L 10155 9574 L 10148 9567 L 10025 9432 L
10155 9298 L 10162 9296 L 10155 9253 L 10101 9160 L
10155 9113 L 10266 9025 L 10230 8889 L 10155 8857 L
10019 8836 L 9884 8762 L 9868 8754 L 9748 8623 L
9613 8736 L 9534 8754 L 9543 8889 L 9613 8953 L
9699 8889 L 9748 8851 L 9770 8889 L 9782 9025 L
9884 9038 L 10010 9160 L 9884 9296 L 9884 9296 L
9748 9421 L 9748 9432 L 9736 9567 L 9748 9659 L
9761 9703 L 9748 9735 L 9613 9789 L 9478 9703 L
9477 9567 L 9478 9564 L 9481 9432 L 9478 9400 L
9388 9296 L 9342 9180 L 9336 9160 L 9239 9025 L
9207 8990 L 9098 8889 L 9139 8754 L 9207 8631 L
9216 8618 L 9342 8566 L 9478 8534 L 9613 8568 L
9748 8614 L 9748 8124 L 9712 8075 L 9748 8042 L
9831 8075 L 9748 8124 L 9748 8614 L 9808 8482 L
9884 8380 L 10019 8393 L 10153 8482 L 10155 8492 L
10155 7863 L 10028 7804 L 10155 7763 L 10261 7804 L
10155 7863 L 10155 8492 L 10290 8552 L 10290 8446 L
10209 8347 L 10290 8267 L 10375 8347 L 10290 8446 L
10290 8552 L 10348 8482 L 10426 8422 L 10561 8474 L
10561 8101 L 10504 8075 L 10561 8049 L 10573 8075 L
10561 8101 L 10561 8474 L 10674 8347 L 10695 8211 L
10649 8075 L 10625 7940 L 10696 7866 L 10790 7940 L
10820 8075 L 10700 8211 L 10722 8347 L 10710 8482 L
10832 8534 L 10832 7878 L 10779 7804 L 10832 7778 L
10871 7804 L 10832 7878 L 10832 8534 L 10885 8482 L
10887 8347 L 10849 8211 L 10877 8075 L 10967 8045 L
11103 7945 L 11106 7940 L 11107 7804 L 11118 7669 L
11154 7533 L 11238 7521 L 11373 7532 L 11383 7533 L
11509 7572 L 11644 7630 L 11728 7669 L 11780 7699 L
11904 7804 L 11915 7814 L 12003 7940 L 12051 8042 L
12054 8075 L 12068 8211 L 12081 8347 L 12089 8482 L
12097 8618 L 12105 8754 L 12113 8889 L 12120 9025 L
12129 9160 L 12139 9296 L 12147 9432 L 12154 9567 L

12162 9703 L 12170 9838 L 12181 9974 L 12186 10028 L
12219 10110 L 12269 10245 L 12311 10381 L 12321 10419 L
12364 10516 L 12421 10652 L 12457 10731 L 12491 10788 L
12585 10923 L 12592 10933 L 12707 11059 L 12728 11081 L
12846 11194 L 12863 11212 L 12998 11327 L 12998 10876 L
12875 10788 L 12863 10779 L 12738 10652 L 12728 10635 L
12672 10516 L 12592 10382 L 12591 10381 L 12474 10245 L
12457 10223 L 12400 10110 L 12321 10001 L 12310 9974 L
12260 9838 L 12219 9703 L 12186 9611 L 12183 9567 L
12175 9432 L 12166 9296 L 12155 9160 L 12146 9025 L
12139 8889 L 12132 8754 L 12125 8618 L 12118 8482 L
12111 8347 L 12100 8211 L 12089 8075 L 12077 7940 L
12057 7804 L 12051 7770 L 11970 7669 L 11915 7617 L
11780 7535 L 11775 7533 L 11644 7518 L 11509 7505 L
11373 7494 L 11238 7484 L 11103 7498 L 10967 7515 L
10935 7533 L 10832 7652 L 10760 7669 L 10696 7693 L
10642 7669 L 10561 7652 L 10500 7669 L 10426 7748 L
10290 7698 L 10155 7679 L 10121 7669 L 10019 7627 L
9950 7669 L 9884 7701 L 9760 7804 L 9884 7924 L
9884 7817 L 9871 7804 L 9884 7793 L 9990 7804 L
9884 7817 L 9884 7924 L 9999 7940 L 9884 7962 L
9748 7945 L 9613 8048 L 9560 7940 L 9478 7870 L
9427 7940 L 9457 8075 L 9355 8211 L 9342 8229 L
9207 8279 L 9124 8347 L 9071 8380 L 8973 8347 L
8936 8328 L 8912 8347 L 8801 8399 L 8665 8356 L
8661 8347 L 8567 8211 L 8665 8160 L 8733 8075 L
8735 7940 L 8665 7814 L 8530 7934 L 8526 7940 L
8394 8005 L 8283 8075 L 8323 8211 L 8261 8347 L
8259 8358 L 8138 8482 L 8259 8581 L 8289 8618 L
8304 8754 L 8259 8839 L 8159 8889 L 8218 9025 L
8146 9160 L 8123 9252 L 8107 9296 L 8123 9308 L
8259 9357 L 8265 9432 L 8394 9537 L 8394 9506 L
8303 9432 L 8394 9309 L 8394 9253 L 8296 9160 L
8394 9102 L 8459 9025 L 8530 8916 L 8665 8938 L
8665 8734 L 8545 8618 L 8530 8604 L 8422 8482 L
8394 8445 L 8311 8347 L 8394 8251 L 8530 8253 L
8530 8101 L 8496 8075 L 8530 8053 L 8583 8075 L
8530 8101 L 8530 8253 L 8615 8347 L 8665 8458 L
8719 8482 L 8790 8618 L 8665 8734 L 8665 8938 L
8748 9025 L 8665 9121 L 8626 9160 L 8530 9215 L
8394 9253 L 8394 9309 L 8479 9432 L 8394 9506 L
8394 9537 L 8513 9432 L 8528 9296 L 8530 9295 L
8665 9270 L 8801 9259 L 8917 9296 L 8819 9432 L

8895 9567 L 8913 9703 L 8801 9789 L 8665 9718 L
8545 9838 L 8665 9962 L 8665 9926 L 8580 9838 L
8665 9753 L 8764 9838 L 8665 9926 L 8665 9962 L
8795 9974 L 8801 9993 L 8830 10110 L 8854 10245 L
8936 10363 L 8952 10381 L 8969 10516 L 8936 10542 L
8829 10652 L 8801 10676 L 8665 10769 L 8647 10788 L
8530 10923 L 8530 10923 L 8442 11059 L 8530 11140 L
8561 11194 L 8665 11328 L 8667 11330 L 8749 11466 L
8754 11601 L 8712 11737 L 8665 11779 L 8577 11873 L
8624 12008 L 8559 12144 L 8530 12160 L 8426 12279 L
8427 12415 L 8394 12436 L 8259 12518 L 8196 12551 L
8123 12572 L 7988 12620 L 7853 12623 L 7827 12551 L
7782 12415 L 7717 12319 L 7698 12279 L 7659 12144 L
7592 12008 L 7582 11996 L 7551 11873 L 7582 11763 L
7614 11737 L 7582 11698 L 7446 11620 L 7421 11601 L
7311 11521 L 7176 11518 L 7040 11487 L 7001 11466 L
6905 11414 L 6769 11419 L 6722 11466 L 6634 11493 L
6605 11466 L 6634 11405 L 6708 11330 L 6634 11229 L
6593 11194 L 6498 11121 L 6461 11059 L 6498 11032 L
6634 11047 L 6681 10923 L 6769 10868 L 6905 10812 L
6925 10788 L 6910 10652 L 6905 10645 L 6769 10549 L
6733 10516 L 6634 10406 L 6559 10381 L 6634 10290 L
6769 10361 L 6824 10245 L 6905 10222 L 6961 10110 L
6905 10012 L 6889 9974 L 6889 9838 L 6905 9823 L
7015 9838 L 7040 9844 L 7176 9936 L 7176 9375 L
7140 9296 L 7176 9249 L 7237 9296 L 7176 9375 L
7176 9936 L 7311 9880 L 7339 9838 L 7446 9758 L
7509 9703 L 7582 9617 L 7626 9567 L 7582 9493 L
7446 9436 L 7429 9432 L 7428 9296 L 7405 9160 L
7329 9025 L 7311 9010 L 7276 9025 L 7176 9062 L
7153 9025 L 7162 8889 L 7176 8784 L 7186 8754 L
7197 8618 L 7176 8613 L 7042 8482 L 7040 8460 L
7000 8347 L 6905 8254 L 6850 8211 L 6769 8164 L
6672 8075 L 6634 7954 L 6629 7940 L 6623 7804 L
6602 7669 L 6498 7587 L 6363 7567 L 6302 7533 L
6228 7480 L 6096 7397 L 6092 7389 L 5957 7377 L
5821 7301 L 5686 7359 L 5645 7397 L 5686 7475 L
5712 7533 L 5735 7669 L 5718 7804 L 5686 7881 L
5551 7895 L 5473 7804 L 5415 7711 L 5301 7669 L
5280 7662 L 5144 7616 L 5009 7582 L 4873 7536 L
4869 7533 L 4738 7411 L 4731 7397 L 4636 7262 L
4654 7126 L 4605 6991 L 4603 6990 L 4467 6944 L
4422 6991 L 4332 7089 L 4196 7122 L 4061 7105 L

3926 7099 L 3799 7126 L 3790 7128 L 3655 7234 L
3615 7262 L 3519 7283 L 3485 7262 L 3384 7176 L
3248 7142 L 3127 7262 L 3113 7317 L 3009 7397 L
2978 7406 L 2962 7397 L 2842 7349 L 2707 7381 L
2692 7397 L 2624 7533 L 2571 7655 L 2564 7669 L
2436 7774 L 2357 7804 L 2301 7825 L N E
55 O
2301 7988 M 2301 8413 L 2360 8482 L 2302 8618 L 2301 8619 L
2301 9570 L 2310 9567 L 2436 9535 L 2571 9563 L
2575 9567 L 2664 9703 L 2707 9766 L 2756 9838 L
2842 9903 L 2945 9974 L 2978 9996 L 3113 10069 L
3168 10110 L 3248 10169 L 3329 10245 L 3384 10304 L
3471 10381 L 3519 10424 L 3655 10465 L 3790 10506 L
3811 10516 L 3926 10577 L 4061 10616 L 4190 10652 L
4147 10788 L 4061 10837 L 3926 10822 L 3790 10836 L
3677 10923 L 3655 10945 L 3532 11059 L 3519 11089 L
3444 11194 L 3410 11330 L 3384 11353 L 3248 11402 L
3113 11451 L 3068 11466 L 2978 11490 L 2842 11526 L
2707 11531 L 2571 11571 L 2534 11601 L 2481 11737 L
2436 11795 L 2301 11776 L 2301 11852 L 2366 11873 L
2436 11909 L 2524 12008 L 2571 12102 L 2707 12079 L
2707 11838 L 2571 11751 L 2566 11737 L 2571 11725 L
2657 11601 L 2707 11583 L 2842 11586 L 2978 11559 L
3113 11523 L 3248 11501 L 3384 11519 L 3415 11601 L
3384 11639 L 3254 11737 L 3248 11739 L 3113 11762 L
2978 11777 L 2842 11811 L 2707 11838 L 2707 12079 L
2842 12052 L 2978 12082 L 3113 12122 L 3248 12101 L
3384 12085 L 3519 12043 L 3570 12008 L 3655 11947 L
3790 11905 L 3926 12002 L 4061 11997 L 4196 11954 L
4332 12004 L 4342 12008 L 4416 12144 L 4467 12191 L
4603 12199 L 4738 12182 L 4873 12229 L 4917 12144 L
5009 12096 L 5144 12106 L 5163 12144 L 5144 12165 L
5031 12279 L 5009 12304 L 4873 12315 L 4738 12356 L
4642 12415 L 4603 12437 L 4467 12500 L 4332 12492 L
4197 12415 L 4196 12414 L 4061 12348 L 3926 12350 L
3790 12359 L 3655 12378 L 3590 12415 L 3519 12463 L
3384 12531 L 3342 12551 L 3248 12655 L 3180 12686 L
3248 12721 L 3384 12707 L 3519 12728 L 3655 12769 L
3790 12784 L 3926 12816 L 3942 12822 L 4061 12875 L
4196 12923 L 4266 12957 L 4332 12998 L 4467 13000 L
4603 13032 L 4694 13093 L 4738 13139 L 4873 13153 L
5009 13192 L 5077 13229 L 5144 13278 L 5214 13229 L
5183 13093 L 5144 13002 L 5117 12957 L 5144 12855 L

5239 12822 L 5280 12818 L 5314 12822 L 5415 12829 L
5551 12858 L 5686 12855 L 5730 12822 L 5821 12724 L
5842 12686 L 5867 12551 L 5855 12415 L 5855 12279 L
5851 12144 L 5879 12008 L 5844 11873 L 5907 11737 L
5957 11712 L 5975 11737 L 6057 11873 L 6052 12008 L
6092 12127 L 6133 12008 L 6152 11873 L 6228 11841 L
6355 11873 L 6363 11877 L 6452 12008 L 6498 12057 L
6586 12144 L 6633 12279 L 6634 12283 L 6635 12279 L
6680 12144 L 6736 12008 L 6652 11873 L 6657 11737 L
6634 11707 L 6549 11601 L 6634 11568 L 6769 11530 L
6877 11601 L 6877 11737 L 6905 11825 L 7040 11752 L
7176 11792 L 7195 11737 L 7268 11601 L 7311 11595 L
7319 11601 L 7446 11696 L 7498 11737 L 7492 11873 L
7499 12008 L 7446 12107 L 7363 12144 L 7444 12279 L
7446 12281 L 7582 12342 L 7635 12415 L 7666 12551 L
7717 12637 L 7751 12686 L 7730 12822 L 7717 12836 L
7582 12863 L 7446 12922 L 7311 12925 L 7176 12884 L
7040 12825 L 6905 12932 L 6829 12957 L 6769 12973 L
6634 12992 L 6548 12957 L 6498 12932 L 6363 12890 L
6228 12894 L 6129 12957 L 6154 13093 L 6181 13229 L
6186 13364 L 6160 13500 L 6214 13635 L 6228 13649 L
6316 13771 L 6363 13876 L 6392 13907 L 6482 14042 L
6498 14090 L 6594 14178 L 6634 14226 L 6769 14188 L
6905 14187 L 7040 14182 L 7176 14233 L 7270 14178 L
7311 14153 L 7446 14152 L 7582 14063 L 7627 14042 L
7717 14012 L 7853 13995 L 7988 13953 L 8123 13929 L
8123 13579 L 7988 13525 L 7913 13500 L 7853 13456 L
7717 13373 L 7712 13364 L 7717 13359 L 7853 13305 L
7914 13229 L 7988 13101 L 8012 13093 L 8123 13081 L
8176 13093 L 8259 13155 L 8318 13229 L 8351 13364 L
8333 13500 L 8259 13545 L 8123 13579 L 8123 13929 L
8162 13907 L 8259 13822 L 8367 13771 L 8394 13732 L
8418 13635 L 8494 13500 L 8530 13488 L 8665 13471 L
8801 13413 L 8861 13364 L 8936 13259 L 8937 13229 L
9071 13113 L 9207 13106 L 9221 13093 L 9342 12984 L
9424 12957 L 9478 12916 L 9613 12891 L 9748 12900 L
9765 12822 L 9768 12686 L 9772 12551 L 9884 12442 L
10019 12416 L 10155 12418 L 10170 12415 L 10252 12279 L
10290 12189 L 10307 12144 L 10290 12113 L 10248 12008 L
10176 11873 L 10155 11841 L 10084 11737 L 10019 11609 L
10008 11601 L 9913 11466 L 9886 11330 L 9887 11194 L
10019 11067 L 10028 11059 L 10155 10942 L 10260 10923 L
10290 10901 L 10379 10788 L 10426 10716 L 10438 10652 L

10447 10516 L 10518 10381 L 10561 10320 L 10578 10245 L
10622 10110 L 10696 9997 L 10704 9974 L 10832 9844 L
10838 9838 L 10910 9703 L 10832 9627 L 10760 9567 L
10832 9511 L 10832 9262 L 10726 9160 L 10832 9052 L
10935 9160 L 10832 9262 L 10832 9511 L 10850 9432 L
10967 9319 L 11034 9296 L 11103 9215 L 11119 9160 L
11103 9144 L 10968 9025 L 10967 9024 L 10832 8949 L
10781 8889 L 10696 8856 L 10561 8754 L 10592 8618 L
10696 8529 L 10805 8618 L 10832 8640 L 10967 8650 L
11058 8618 L 11103 8573 L 11238 8492 L 11259 8482 L
11238 8469 L 11150 8347 L 11238 8250 L 11273 8211 L
11238 8153 L 11103 8116 L 11065 8075 L 11103 8042 L
11174 7940 L 11184 7804 L 11222 7669 L 11238 7644 L
11373 7659 L 11399 7669 L 11509 7723 L 11644 7803 L
11646 7804 L 11780 7904 L 11818 7940 L 11915 8059 L
11923 8075 L 11988 8211 L 12051 8344 L 12051 8347 L
12061 8482 L 12070 8618 L 12078 8754 L 12086 8889 L
12094 9025 L 12103 9160 L 12112 9296 L 12119 9432 L
12126 9567 L 12132 9703 L 12138 9838 L 12147 9974 L
12157 10110 L 12169 10245 L 12182 10381 L 12186 10429 L
12208 10516 L 12239 10652 L 12275 10788 L 12315 10923 L
12321 10942 L 12385 11059 L 12457 11186 L 12463 11194 L
12563 11330 L 12592 11368 L 12675 11466 L 12728 11526 L
12822 11601 L 12863 11639 L 12998 11692 L 12998 11327 L
12863 11212 L 12846 11194 L 12728 11081 L 12707 11059 L
12592 10933 L 12585 10923 L 12491 10788 L 12457 10731 L
12421 10652 L 12364 10516 L 12321 10419 L 12311 10381 L
12269 10245 L 12219 10110 L 12186 10028 L 12181 9974 L
12170 9838 L 12162 9703 L 12154 9567 L 12147 9432 L
12139 9296 L 12129 9160 L 12120 9025 L 12113 8889 L
12105 8754 L 12097 8618 L 12089 8482 L 12081 8347 L
12068 8211 L 12054 8075 L 12051 8042 L 12003 7940 L
11915 7814 L 11904 7804 L 11780 7699 L 11728 7669 L
11644 7630 L 11509 7572 L 11383 7533 L 11373 7532 L
11238 7521 L 11154 7533 L 11118 7669 L 11107 7804 L
11106 7940 L 11103 7945 L 10967 8045 L 10877 8075 L
10849 8211 L 10887 8347 L 10885 8482 L 10832 8534 L
10710 8482 L 10722 8347 L 10700 8211 L 10820 8075 L
10790 7940 L 10696 7866 L 10625 7940 L 10649 8075 L
10695 8211 L 10674 8347 L 10561 8474 L 10426 8422 L
10348 8482 L 10290 8552 L 10155 8492 L 10153 8482 L
10019 8393 L 9884 8380 L 9808 8482 L 9748 8614 L
9613 8568 L 9478 8534 L 9342 8566 L 9216 8618 L

9207 8631 L 9139 8754 L 9098 8889 L 9207 8990 L
9239 9025 L 9336 9160 L 9342 9180 L 9388 9296 L
9478 9400 L 9481 9432 L 9478 9564 L 9477 9567 L
9478 9703 L 9613 9789 L 9613 9552 L 9519 9432 L
9613 9299 L 9709 9432 L 9613 9552 L 9613 9789 L
9748 9735 L 9761 9703 L 9748 9659 L 9736 9567 L
9748 9432 L 9748 9421 L 9884 9296 L 9884 9296 L
9884 9243 L 9794 9160 L 9884 9086 L 9960 9160 L
9884 9243 L 9884 9296 L 10010 9160 L 9884 9038 L
9782 9025 L 9770 8889 L 9748 8851 L 9699 8889 L
9613 8953 L 9543 8889 L 9534 8754 L 9613 8736 L
9748 8623 L 9868 8754 L 9884 8762 L 9884 8651 L
9851 8618 L 9884 8553 L 9919 8618 L 9884 8651 L
9884 8762 L 10019 8836 L 10155 8857 L 10155 8782 L
10097 8754 L 10155 8715 L 10203 8754 L 10155 8782 L
10155 8857 L 10230 8889 L 10266 9025 L 10155 9113 L
10101 9160 L 10155 9253 L 10162 9296 L 10155 9298 L
10025 9432 L 10148 9567 L 10155 9574 L 10163 9703 L
10290 9838 L 10290 9794 L 10204 9703 L 10290 9606 L
10290 9531 L 10155 9524 L 10067 9432 L 10155 9341 L
10278 9296 L 10290 9193 L 10292 9160 L 10426 9041 L
10561 9052 L 10670 9160 L 10561 9295 L 10560 9296 L
10426 9414 L 10405 9432 L 10290 9531 L 10290 9606 L
10386 9703 L 10290 9794 L 10290 9838 L 10296 9838 L
10378 9974 L 10396 10110 L 10351 10245 L 10319 10381 L
10290 10458 L 10254 10516 L 10192 10652 L 10155 10683 L
10019 10662 L 10014 10652 L 9884 10550 L 9874 10516 L
9884 10433 L 9905 10381 L 9884 10363 L 9799 10245 L
9748 10204 L 9613 10168 L 9478 10244 L 9475 10245 L
9420 10381 L 9349 10516 L 9342 10548 L 9265 10652 L
9207 10733 L 9145 10788 L 9176 10923 L 9071 10929 L
8952 11059 L 9071 11191 L 9071 11154 L 8985 11059 L
9071 10965 L 9169 11059 L 9071 11154 L 9071 11191 L
9148 11194 L 9156 11330 L 9207 11432 L 9342 11434 L
9391 11466 L 9478 11523 L 9478 10903 L 9370 10788 L
9433 10652 L 9471 10516 L 9478 10507 L 9506 10381 L
9613 10252 L 9748 10272 L 9852 10381 L 9779 10516 L
9772 10652 L 9748 10692 L 9619 10788 L 9613 10792 L
9478 10903 L 9478 11523 L 9585 11601 L 9478 11678 L
9427 11737 L 9342 11811 L 9241 11873 L 9207 11940 L
9075 11873 L 9071 11872 L 9069 11873 L 8936 11977 L
8926 12008 L 8936 12017 L 9071 12139 L 9088 12144 L
9207 12160 L 9276 12144 L 9342 12109 L 9424 12144 L

9478 12157 L 9478 12021 L 9456 12008 L 9478 11990 L
9488 12008 L 9478 12021 L 9478 12157 L 9500 12144 L
9607 12008 L 9613 11987 L 9748 11949 L 9884 11932 L
10003 12008 L 9963 12144 L 9978 12279 L 9884 12353 L
9833 12415 L 9748 12490 L 9680 12551 L 9631 12686 L
9613 12718 L 9478 12804 L 9449 12822 L 9342 12867 L
9207 12922 L 9116 12957 L 9071 12966 L 8936 12980 L
8831 12957 L 8801 12946 L 8665 12868 L 8530 12890 L
8394 12898 L 8259 12907 L 8123 12899 L 7988 12901 L
7853 12933 L 7815 12957 L 7756 13093 L 7717 13138 L
7582 13218 L 7565 13229 L 7485 13364 L 7493 13500 L
7446 13618 L 7410 13635 L 7311 13661 L 7176 13676 L
7040 13667 L 6909 13635 L 6905 13634 L 6769 13554 L
6634 13608 L 6581 13635 L 6498 13666 L 6381 13635 L
6363 13632 L 6301 13500 L 6280 13364 L 6234 13229 L
6228 13207 L 6212 13093 L 6228 13024 L 6354 12957 L
6363 12956 L 6367 12957 L 6498 12996 L 6634 13039 L
6769 13035 L 6905 13018 L 7040 12975 L 7176 13009 L
7311 13009 L 7446 13001 L 7582 12974 L 7625 12957 L
7717 12934 L 7816 12822 L 7807 12686 L 7717 12557 L
7714 12551 L 7688 12415 L 7594 12279 L 7582 12241 L
7533 12144 L 7543 12008 L 7521 11873 L 7546 11737 L
7446 11658 L 7370 11601 L 7311 11558 L 7176 11568 L
7040 11569 L 6905 11519 L 6769 11490 L 6634 11530 L
6566 11466 L 6498 11361 L 6407 11466 L 6454 11601 L
6498 11637 L 6498 11542 L 6461 11466 L 6498 11422 L
6526 11466 L 6498 11542 L 6498 11637 L 6584 11737 L
6579 11873 L 6634 11944 L 6675 12008 L 6634 12123 L
6516 12008 L 6498 11997 L 6425 11873 L 6363 11821 L
6228 11801 L 6092 11847 L 6021 11737 L 5957 11650 L
5821 11716 L 5808 11737 L 5789 11873 L 5812 12008 L
5771 12144 L 5756 12279 L 5769 12415 L 5785 12551 L
5762 12686 L 5686 12766 L 5551 12761 L 5415 12743 L
5280 12695 L 5258 12686 L 5144 12599 L 5020 12686 L
5009 12704 L 4947 12822 L 4913 12957 L 4873 12980 L
4738 12961 L 4727 12957 L 4603 12935 L 4467 12924 L
4332 12905 L 4196 12835 L 4154 12822 L 4061 12795 L
3926 12748 L 3790 12702 L 3709 12686 L 3655 12672 L
3519 12606 L 3469 12551 L 3519 12527 L 3655 12445 L
3769 12415 L 3790 12412 L 3926 12403 L 4061 12405 L
4082 12415 L 4196 12482 L 4311 12551 L 4332 12565 L
4467 12581 L 4583 12551 L 4603 12543 L 4738 12481 L
4873 12517 L 4962 12551 L 5009 12657 L 5059 12551 L

5060 12415 L 5144 12316 L 5175 12279 L 5280 12157 L
5301 12144 L 5415 12049 L 5427 12008 L 5415 11991 L
5280 11936 L 5144 11971 L 5009 11995 L 4943 12008 L
4873 12043 L 4738 12064 L 4649 12008 L 4603 11953 L
4467 11931 L 4332 11916 L 4262 11873 L 4196 11811 L
4061 11794 L 3926 11856 L 3790 11832 L 3713 11873 L
3655 11896 L 3519 11986 L 3493 12008 L 3384 12049 L
3248 12061 L 3113 12077 L 2978 12034 L 2878 12008 L
2856 11873 L 2978 11825 L 3113 11803 L 3248 11784 L
3380 11737 L 3384 11734 L 3493 11601 L 3519 11542 L
3655 11482 L 3655 11395 L 3566 11330 L 3551 11194 L
3601 11059 L 3655 11009 L 3743 10923 L 3790 10887 L
3926 10885 L 4061 10910 L 4196 10842 L 4248 10788 L
4332 10684 L 4332 10575 L 4196 10563 L 4061 10548 L
3965 10516 L 3926 10492 L 3790 10437 L 3655 10418 L
3554 10381 L 3519 10364 L 3385 10245 L 3384 10244 L
3248 10117 L 3239 10110 L 3113 10018 L 3038 9974 L
2978 9921 L 2855 9838 L 2842 9824 L 2755 9703 L
2707 9614 L 2677 9567 L 2581 9432 L 2571 9422 L
2436 9345 L 2417 9296 L 2436 9204 L 2444 9160 L
2486 9025 L 2571 8998 L 2670 9025 L 2707 9035 L
2707 8514 L 2674 8482 L 2627 8347 L 2707 8323 L
2719 8347 L 2785 8482 L 2707 8514 L 2707 9035 L
2748 9025 L 2842 9000 L 2978 8997 L 3055 9025 L
3113 9043 L 3243 9160 L 3248 9188 L 3261 9160 L
3274 9025 L 3280 8889 L 3248 8806 L 3206 8754 L
3113 8648 L 3085 8618 L 3113 8587 L 3208 8482 L
3248 8434 L 3307 8347 L 3248 8281 L 3113 8242 L
3080 8211 L 3059 8075 L 3113 8029 L 3248 8036 L
3371 7940 L 3384 7933 L 3519 7906 L 3566 7940 L
3605 8075 L 3651 8211 L 3655 8215 L 3715 8347 L
3721 8482 L 3790 8567 L 3846 8618 L 3885 8754 L
3907 8889 L 3926 8941 L 3952 9025 L 3972 9160 L
4037 9296 L 3926 9357 L 3853 9432 L 3926 9500 L
3995 9567 L 4061 9627 L 4061 8148 L 3926 8093 L
3920 8075 L 3926 8052 L 4061 8044 L 4121 8075 L
4061 8148 L 4061 9627 L 4091 9703 L 4150 9838 L
4196 9867 L 4196 9482 L 4154 9432 L 4196 9361 L
4196 8767 L 4190 8754 L 4196 8729 L 4229 8754 L
4196 8767 L 4196 9361 L 4332 9420 L 4343 9432 L
4332 9457 L 4196 9482 L 4196 9867 L 4332 9866 L
4467 9867 L 4467 8771 L 4454 8754 L 4343 8618 L
4332 8606 L 4225 8482 L 4196 8425 L 4155 8347 L

4196 8297 L 4332 8340 L 4332 7992 L 4196 7972 L
4129 7940 L 4061 7882 L 4034 7804 L 4019 7669 L
4061 7613 L 4196 7640 L 4255 7669 L 4332 7738 L
4386 7804 L 4467 7897 L 4498 7940 L 4467 7990 L
4332 7992 L 4332 8340 L 4340 8347 L 4467 8451 L
4509 8482 L 4603 8602 L 4618 8618 L 4613 8754 L
4603 8768 L 4467 8771 L 4467 9867 L 4585 9974 L
4603 9996 L 4667 10110 L 4646 10245 L 4609 10381 L
4603 10397 L 4467 10474 L 4392 10516 L 4332 10575 L
4332 10684 L 4467 10705 L 4603 10741 L 4738 10684 L
4819 10788 L 4738 10917 L 4736 10923 L 4623 11059 L
4603 11073 L 4467 11090 L 4332 11064 L 4196 11111 L
4148 11194 L 4061 11313 L 4028 11330 L 3926 11371 L
3790 11390 L 3655 11395 L 3655 11482 L 3716 11466 L
3790 11449 L 3926 11421 L 4061 11377 L 4127 11330 L
4196 11194 L 4196 11193 L 4332 11112 L 4467 11129 L
4603 11123 L 4695 11059 L 4738 11007 L 4870 10923 L
4873 10918 L 4927 10788 L 4896 10652 L 4873 10618 L
4738 10562 L 4616 10652 L 4603 10659 L 4548 10652 L
4603 10622 L 4661 10516 L 4681 10381 L 4735 10245 L
4738 10225 L 4796 10110 L 4744 9974 L 4738 9963 L
4680 9838 L 4715 9703 L 4710 9567 L 4738 9447 L
4764 9432 L 4873 9323 L 4903 9296 L 4923 9160 L
5009 9134 L 5074 9160 L 5144 9213 L 5263 9160 L
5280 9146 L 5415 9084 L 5461 9025 L 5551 8944 L
5569 8889 L 5551 8799 L 5502 8754 L 5422 8618 L
5415 8593 L 5398 8618 L 5285 8754 L 5280 8763 L
5144 8796 L 5085 8754 L 5009 8678 L 4980 8618 L
5009 8555 L 5099 8482 L 5144 8418 L 5204 8347 L
5171 8211 L 5144 8188 L 5075 8211 L 5009 8240 L
4969 8211 L 4873 8164 L 4776 8075 L 4738 8047 L
4659 7940 L 4603 7806 L 4602 7804 L 4467 7689 L
4451 7669 L 4332 7563 L 4196 7534 L 4194 7533 L
4061 7471 L 3926 7399 L 3852 7533 L 3872 7669 L
3926 7745 L 3945 7804 L 3926 7904 L 3790 7852 L
3762 7804 L 3655 7691 L 3596 7669 L 3560 7533 L
3519 7484 L 3443 7397 L 3384 7356 L 3248 7315 L
3205 7397 L 3149 7533 L 3113 7598 L 3021 7669 L
3113 7720 L 3248 7773 L 3287 7804 L 3248 7826 L
3113 7870 L 2981 7940 L 2978 7942 L 2842 7948 L
2836 7940 L 2791 7804 L 2707 7675 L 2683 7804 L
2571 7912 L 2457 7940 L 2436 7945 L 2301 7988 L
N E

42 O
2301 8413 2301 8619 2302 8618 2360 8482 2301 8413 5 N F
68 O
2310 9567 2301 9570 2301 9687 2436 9663 2560 9703 2571 9711
2635 9838 2707 9906 2839 9974 2842 9976 2978 10066 3078 10110
3113 10128 3248 10221 3274 10245 3384 10363 3404 10381 3519 10484
3655 10513 3664 10516 3790 10583 3925 10652 3790 10783 3787 10788
3655 10878 3594 10923 3519 10990 3418 11059 3384 11108 3251 11194
3248 11198 3190 11330 3113 11368 2978 11414 2842 11465 2837 11466
2707 11478 2571 11495 2441 11601 2436 11616 2402 11601 2301 11559
2301 11776 2436 11795 2481 11737 2534 11601 2571 11571 2707 11531
2842 11526 2978 11490 3068 11466 3113 11451 3248 11402 3384 11353
3410 11330 3444 11194 3519 11089 3532 11059 3655 10945 3677 10923
3790 10836 3926 10822 4061 10837 4147 10788 4190 10652 4061 10616
3926 10577 3811 10516 3790 10506 3655 10465 3519 10424 3471 10381
3384 10304 3329 10245 3248 10169 3168 10110 3113 10069 2978 9996
2945 9974 2842 9903 2756 9838 2707 9766 2664 9703 2575 9567
2571 9563 2436 9535 2310 9567 87 N F
82 O
2436 9663 2301 9687 2301 10179 2436 10209 2473 10245 2571 10339
2597 10381 2693 10516 2707 10528 2842 10627 2905 10652 2978 10676
3113 10680 3248 10783 3248 10455 3165 10381 3113 10357 2978 10301
2909 10245 2978 10203 3113 10215 3166 10245 3248 10356 3268 10381
3248 10455 3248 10783 3339 10788 3316 10923 3248 11026 3231 11059
3113 11191 3111 11194 2984 11330 2978 11332 2842 11356 2707 11387
2571 11387 2436 11391 2301 11347 2301 11559 2402 11601 2436 11616
2441 11601 2571 11495 2707 11478 2837 11466 2842 11465 2978 11414
3113 11368 3190 11330 3248 11198 3251 11194 3384 11108 3418 11059
3519 10990 3519 10892 3385 10788 3440 10652 3519 10595 3655 10594
3771 10652 3684 10788 3655 10808 3519 10892 3519 10990 3594 10923
3655 10878 3787 10788 3790 10783 3925 10652 3790 10583 3664 10516
3655 10513 3519 10484 3404 10381 3384 10363 3274 10245 3248 10221
3113 10128 3078 10110 2978 10066 2842 9976 2839 9974 2707 9906
2635 9838 2571 9711 2560 9703 2436 9663 88 N F
95 O
2436 10209 2301 10179 2301 10521 2436 10630 2451 10652 2571 10744
2630 10788 2670 10923 2707 11002 2842 11046 2862 11059 2950 11194
2842 11208 2731 11194 2707 11164 2571 11154 2436 11149 2369 11059
2307 10923 2301 10917 2301 11347 2436 11391 2571 11387 2707 11387
2842 11356 2978 11332 2984 11330 3111 11194 3113 11191 3231 11059
3248 11026 3316 10923 3339 10788 3248 10783 3113 10680 2978 10676
2905 10652 2842 10627 2707 10528 2693 10516 2597 10381 2571 10339
2473 10245 2436 10209 44 N F

108 O
2436 10630 2301 10521 2301 10917 2307 10923 2369 11059 2436 11149
2571 11154 2707 11164 2731 11194 2842 11208 2950 11194 2862 11059
2842 11046 2707 11002 2670 10923 2630 10788 2571 10744 2451 10652
2436 10630 19 N F
42 O
2301 11852 M 2301 11932 L 2414 12008 L 2436 12052 L 2479 12144 L
2468 12279 L 2571 12388 L 2707 12375 L 2734 12279 L
2748 12144 L 2842 12106 L 2978 12131 L 3025 12144 L
3113 12189 L 3232 12144 L 3248 12141 L 3384 12120 L
3519 12091 L 3640 12008 L 3655 11998 L 3790 11989 L
3816 12008 L 3926 12099 L 4061 12110 L 4153 12144 L
4196 12210 L 4332 12249 L 4365 12279 L 4417 12415 L
4332 12420 L 4324 12415 L 4196 12298 L 4061 12291 L
3926 12297 L 3790 12306 L 3655 12325 L 3519 12394 L
3495 12415 L 3384 12477 L 3248 12536 L 3216 12551 L
3113 12609 L 3021 12686 L 3041 12822 L 3113 12951 L
3142 12957 L 3248 13051 L 3290 13093 L 3384 13192 L
3384 13024 L 3317 12957 L 3317 12822 L 3384 12796 L
3519 12820 L 3525 12822 L 3519 12834 L 3429 12957 L
3384 13024 L 3384 13192 L 3519 13212 L 3626 13093 L
3655 13016 L 3668 12957 L 3790 12894 L 3926 12900 L
4061 12953 L 4072 12957 L 4119 13093 L 4061 13228 L
4061 13229 L 4061 13230 L 4196 13322 L 4245 13229 L
4332 13125 L 4379 13093 L 4467 13074 L 4526 13093 L
4603 13146 L 4682 13229 L 4701 13364 L 4603 13443 L
4570 13500 L 4493 13635 L 4467 13698 L 4403 13771 L
4467 13813 L 4553 13907 L 4603 13963 L 4738 13988 L
4781 13907 L 4773 13771 L 4786 13635 L 4794 13500 L
4753 13364 L 4873 13257 L 5009 13296 L 5090 13364 L
5144 13442 L 5280 13486 L 5381 13364 L 5370 13229 L
5280 13111 L 5270 13093 L 5249 12957 L 5280 12932 L
5415 12915 L 5548 12957 L 5551 12962 L 5566 12957 L
5686 12952 L 5821 12864 L 5871 12822 L 5918 12686 L
5957 12572 L 5957 12444 L 5954 12415 L 5957 12396 L
5957 12124 L 5943 12008 L 5905 11873 L 5957 11794 L
6002 11873 L 5973 12008 L 5957 12124 L 5957 12396 L
5962 12415 L 5957 12444 L 5957 12572 L 6092 12681 L
6099 12686 L 6092 12706 L 6065 12822 L 5974 12957 L
6092 13082 L 6096 13093 L 6131 13229 L 6122 13364 L
6092 13440 L 6013 13500 L 6092 13587 L 6113 13635 L
6228 13750 L 6243 13771 L 6301 13907 L 6363 14031 L
6371 14042 L 6363 14082 L 6330 14178 L 6289 14314 L

6285 14449 L 6351 14585 L 6363 14595 L 6494 14720 L
6498 14724 L 6634 14765 L 6769 14780 L 6905 14856 L
6905 14856 L 7040 14978 L 7176 14893 L 7210 14856 L
7311 14764 L 7446 14765 L 7506 14720 L 7582 14670 L
7717 14599 L 7739 14585 L 7853 14500 L 7861 14449 L
7951 14314 L 7988 14282 L 8123 14244 L 8259 14194 L
8295 14178 L 8394 14042 L 8394 14041 L 8410 13907 L
8530 13781 L 8607 13771 L 8665 13747 L 8801 13722 L
8936 13670 L 9065 13635 L 9071 13633 L 9207 13629 L
9218 13500 L 9221 13364 L 9207 13350 L 9071 13341 L
8983 13229 L 9071 13152 L 9207 13148 L 9267 13093 L
9342 13025 L 9478 12990 L 9613 12975 L 9748 12994 L
9785 12957 L 9807 12822 L 9809 12686 L 9814 12551 L
9884 12483 L 10019 12463 L 10155 12463 L 10250 12551 L
10290 12590 L 10419 12686 L 10426 12693 L 10561 12738 L
10696 12706 L 10832 12688 L 10834 12686 L 10832 12636 L
10696 12588 L 10617 12551 L 10696 12440 L 10704 12415 L
10696 12410 L 10600 12279 L 10561 12194 L 10520 12144 L
10426 12097 L 10357 12008 L 10293 11873 L 10290 11867 L
10191 11737 L 10155 11711 L 10065 11601 L 10019 11488 L
10003 11466 L 9933 11330 L 9935 11194 L 10019 11113 L
10075 11059 L 10155 10986 L 10290 10966 L 10367 10923 L
10426 10855 L 10460 10788 L 10481 10652 L 10486 10516 L
10561 10416 L 10595 10381 L 10618 10245 L 10696 10120 L
10707 10110 L 10748 9974 L 10832 9889 L 10883 9838 L
10967 9741 L 10967 9518 L 10897 9432 L 10967 9364 L
11094 9432 L 10967 9518 L 10967 9741 L 11064 9703 L
11103 9657 L 11134 9567 L 11109 9432 L 11130 9296 L
11163 9160 L 11103 9097 L 11021 9025 L 10967 8975 L
10866 8889 L 10832 8847 L 10696 8806 L 10627 8754 L
10648 8618 L 10696 8577 L 10746 8618 L 10832 8688 L
10967 8695 L 11103 8655 L 11147 8618 L 11238 8546 L
11340 8618 L 11373 8646 L 11404 8618 L 11377 8482 L
11373 8478 L 11238 8395 L 11203 8347 L 11238 8309 L
11325 8211 L 11264 8075 L 11256 7940 L 11373 7858 L
11509 7924 L 11531 7940 L 11644 8038 L 11684 8075 L
11780 8177 L 11806 8211 L 11901 8347 L 11915 8375 L
11948 8482 L 12001 8618 L 12051 8738 L 12052 8754 L
12060 8889 L 12068 9025 L 12077 9160 L 12085 9296 L
12091 9432 L 12097 9567 L 12102 9703 L 12106 9838 L
12112 9974 L 12119 10110 L 12127 10245 L 12136 10381 L
12144 10516 L 12152 10652 L 12162 10788 L 12174 10923 L
12186 11025 L 12197 11059 L 12240 11194 L 12288 11330 L

12321 11425 L 12342 11466 L 12411 11601 L 12457 11709 L
12472 11737 L 12520 11873 L 12590 12008 L 12592 12011 L
12715 12144 L 12728 12159 L 12863 12262 L 12922 12279 L
12998 12298 L 12998 11692 L 12863 11639 L 12822 11601 L
12728 11526 L 12675 11466 L 12592 11368 L 12563 11330 L
12463 11194 L 12457 11186 L 12385 11059 L 12321 10942 L
12315 10923 L 12275 10788 L 12239 10652 L 12208 10516 L
12186 10429 L 12182 10381 L 12169 10245 L 12157 10110 L
12147 9974 L 12138 9838 L 12132 9703 L 12126 9567 L
12119 9432 L 12112 9296 L 12103 9160 L 12094 9025 L
12086 8889 L 12078 8754 L 12070 8618 L 12061 8482 L
12051 8347 L 12051 8344 L 11988 8211 L 11923 8075 L
11915 8059 L 11818 7940 L 11780 7904 L 11646 7804 L
11644 7803 L 11509 7723 L 11399 7669 L 11373 7659 L
11238 7644 L 11222 7669 L 11184 7804 L 11174 7940 L
11103 8042 L 11065 8075 L 11103 8116 L 11238 8153 L
11273 8211 L 11238 8250 L 11150 8347 L 11238 8469 L
11259 8482 L 11238 8492 L 11103 8573 L 11058 8618 L
10967 8650 L 10832 8640 L 10805 8618 L 10696 8529 L
10592 8618 L 10561 8754 L 10696 8856 L 10781 8889 L
10832 8949 L 10967 9024 L 10968 9025 L 11103 9144 L
11119 9160 L 11103 9215 L 11034 9296 L 10967 9319 L
10850 9432 L 10832 9511 L 10760 9567 L 10832 9627 L
10910 9703 L 10838 9838 L 10832 9844 L 10704 9974 L
10696 9997 L 10622 10110 L 10578 10245 L 10561 10320 L
10518 10381 L 10447 10516 L 10438 10652 L 10426 10716 L
10379 10788 L 10290 10901 L 10260 10923 L 10155 10942 L
10028 11059 L 10019 11067 L 9887 11194 L 9886 11330 L
9913 11466 L 10008 11601 L 10019 11609 L 10084 11737 L
10155 11841 L 10176 11873 L 10248 12008 L 10290 12113 L
10307 12144 L 10290 12189 L 10252 12279 L 10170 12415 L
10155 12418 L 10019 12416 L 9884 12442 L 9772 12551 L
9768 12686 L 9765 12822 L 9748 12900 L 9613 12891 L
9478 12916 L 9424 12957 L 9342 12984 L 9221 13093 L
9207 13106 L 9071 13113 L 8937 13229 L 8936 13259 L
8861 13364 L 8801 13413 L 8665 13471 L 8530 13488 L
8494 13500 L 8418 13635 L 8394 13732 L 8367 13771 L
8259 13822 L 8162 13907 L 8123 13929 L 7988 13953 L
7853 13995 L 7717 14012 L 7627 14042 L 7582 14063 L
7446 14152 L 7311 14153 L 7270 14178 L 7176 14233 L
7040 14182 L 6905 14187 L 6769 14188 L 6634 14226 L
6594 14178 L 6498 14090 L 6482 14042 L 6392 13907 L
6363 13876 L 6316 13771 L 6228 13649 L 6214 13635 L

6160 13500 L 6186 13364 L 6181 13229 L 6154 13093 L
6129 12957 L 6228 12894 L 6363 12890 L 6498 12932 L
6548 12957 L 6634 12992 L 6634 12940 L 6498 12874 L
6363 12824 L 6344 12822 L 6302 12686 L 6228 12590 L
6194 12551 L 6159 12415 L 6228 12307 L 6263 12279 L
6228 12199 L 6211 12144 L 6212 12008 L 6228 11940 L
6363 11953 L 6401 12008 L 6498 12111 L 6531 12144 L
6566 12279 L 6577 12415 L 6588 12551 L 6634 12600 L
6735 12686 L 6769 12732 L 6905 12798 L 6942 12822 L
6905 12852 L 6769 12894 L 6634 12940 L 6634 12992 L
6769 12973 L 6829 12957 L 6905 12932 L 7040 12825 L
7040 12812 L 6905 12736 L 6826 12686 L 6769 12643 L
6659 12551 L 6769 12499 L 6905 12541 L 6916 12551 L
7040 12668 L 7067 12686 L 7040 12812 L 7040 12825 L
7176 12884 L 7311 12925 L 7446 12922 L 7446 12857 L
7311 12849 L 7263 12822 L 7176 12723 L 7154 12686 L
7040 12607 L 6981 12551 L 6905 12490 L 6769 12457 L
6682 12415 L 6688 12279 L 6732 12144 L 6769 12061 L
6769 11947 L 6728 11873 L 6746 11737 L 6649 11601 L
6769 11569 L 6817 11601 L 6792 11737 L 6823 11873 L
6769 11947 L 6769 12061 L 6881 12008 L 6905 11994 L
7040 11914 L 7067 12008 L 7040 12118 L 6990 12144 L
7006 12279 L 7040 12312 L 7158 12415 L 7176 12433 L
7176 12057 L 7162 12008 L 7176 11966 L 7202 12008 L
7176 12057 L 7176 12433 L 7190 12415 L 7225 12279 L
7311 12217 L 7361 12279 L 7446 12358 L 7582 12414 L
7583 12415 L 7619 12551 L 7636 12686 L 7582 12726 L
7510 12822 L 7446 12857 L 7446 12922 L 7582 12863 L
7717 12836 L 7730 12822 L 7751 12686 L 7717 12637 L
7666 12551 L 7635 12415 L 7582 12342 L 7446 12281 L
7444 12279 L 7363 12144 L 7446 12107 L 7446 12026 L
7399 12008 L 7311 11934 L 7266 11873 L 7281 11737 L
7311 11698 L 7446 11735 L 7449 11737 L 7463 11873 L
7456 12008 L 7446 12026 L 7446 12107 L 7499 12008 L
7492 11873 L 7498 11737 L 7446 11696 L 7319 11601 L
7311 11595 L 7268 11601 L 7195 11737 L 7176 11792 L
7040 11752 L 6905 11825 L 6877 11737 L 6877 11601 L
6769 11530 L 6634 11568 L 6549 11601 L 6634 11707 L
6657 11737 L 6652 11873 L 6736 12008 L 6680 12144 L
6635 12279 L 6634 12283 L 6633 12279 L 6586 12144 L
6498 12057 L 6452 12008 L 6363 11877 L 6355 11873 L
6228 11841 L 6152 11873 L 6133 12008 L 6092 12127 L
6052 12008 L 6057 11873 L 5975 11737 L 5957 11712 L

5907 11737 L 5844 11873 L 5879 12008 L 5851 12144 L
5855 12279 L 5855 12415 L 5867 12551 L 5842 12686 L
5821 12724 L 5730 12822 L 5686 12855 L 5551 12858 L
5415 12829 L 5314 12822 L 5280 12818 L 5239 12822 L
5144 12855 L 5117 12957 L 5144 13002 L 5183 13093 L
5214 13229 L 5144 13278 L 5077 13229 L 5009 13192 L
4873 13153 L 4738 13139 L 4694 13093 L 4603 13032 L
4467 13000 L 4332 12998 L 4266 12957 L 4196 12923 L
4061 12875 L 3942 12822 L 3926 12816 L 3790 12784 L
3655 12769 L 3519 12728 L 3384 12707 L 3248 12721 L
3180 12686 L 3248 12655 L 3342 12551 L 3384 12531 L
3519 12463 L 3590 12415 L 3655 12378 L 3790 12359 L
3926 12350 L 4061 12348 L 4196 12414 L 4197 12415 L
4332 12492 L 4467 12500 L 4603 12437 L 4642 12415 L
4738 12356 L 4873 12315 L 5009 12304 L 5031 12279 L
5144 12165 L 5163 12144 L 5144 12106 L 5009 12096 L
4917 12144 L 4873 12229 L 4738 12182 L 4603 12199 L
4467 12191 L 4416 12144 L 4342 12008 L 4332 12004 L
4196 11954 L 4061 11997 L 3926 12002 L 3790 11905 L
3655 11947 L 3570 12008 L 3519 12043 L 3384 12085 L
3248 12101 L 3113 12122 L 2978 12082 L 2842 12052 L
2707 12079 L 2571 12102 L 2524 12008 L 2436 11909 L
2366 11873 L 2301 11852 L N E
29 O
2301 11932 M 2301 12027 L 2357 12144 L 2306 12279 L 2301 12352 L
2301 12431 L 2436 12502 L 2525 12551 L 2571 12661 L
2707 12644 L 2842 12580 L 2862 12686 L 2876 12822 L
2948 12957 L 2978 13017 L 3003 13093 L 3035 13229 L
3037 13364 L 3063 13500 L 3113 13576 L 3167 13635 L
3227 13771 L 3248 13823 L 3322 13907 L 3384 13934 L
3446 13907 L 3519 13872 L 3655 13828 L 3790 13808 L
3896 13907 L 3926 13909 L 4061 13984 L 4127 14042 L
4196 14132 L 4232 14178 L 4196 14277 L 4061 14284 L
3986 14314 L 3926 14393 L 3907 14449 L 3926 14466 L
4061 14490 L 4196 14492 L 4332 14487 L 4467 14485 L
4603 14553 L 4738 14558 L 4786 14585 L 4873 14689 L
4973 14585 L 4958 14449 L 5009 14363 L 5028 14314 L
5069 14178 L 5046 14042 L 5009 13974 L 4982 13907 L
4969 13771 L 5009 13641 L 5009 13610 L 5000 13500 L
5009 13483 L 5017 13500 L 5009 13610 L 5009 13641 L
5116 13771 L 5144 13796 L 5190 13907 L 5280 14001 L
5311 14042 L 5415 14136 L 5457 14178 L 5534 14314 L
5551 14334 L 5686 14415 L 5686 14193 L 5649 14178 L

5551 14139 L 5455 14042 L 5415 13996 L 5348 13907 L
5415 13856 L 5526 13907 L 5551 13927 L 5686 14009 L
5686 13808 L 5551 13805 L 5472 13771 L 5415 13700 L
5365 13635 L 5415 13589 L 5515 13500 L 5551 13420 L
5580 13500 L 5622 13635 L 5686 13745 L 5725 13771 L
5686 13808 L 5686 14009 L 5722 14042 L 5704 14178 L
5686 14193 L 5686 14415 L 5772 14449 L 5733 14585 L
5800 14720 L 5821 14751 L 5904 14720 L 5957 14600 L
5964 14585 L 5957 14579 L 5844 14449 L 5821 14350 L
5812 14314 L 5821 14308 L 5927 14178 L 5888 14042 L
5827 13907 L 5931 13771 L 5957 13754 L 5957 13273 L
5911 13229 L 5957 13123 L 6015 13229 L 5957 13273 L
5957 13754 L 6092 13742 L 6122 13771 L 6216 13907 L
6188 14042 L 6127 14178 L 6092 14271 L 6052 14314 L
6092 14354 L 6119 14449 L 6168 14585 L 6228 14605 L
6363 14713 L 6371 14720 L 6498 14850 L 6516 14856 L
6634 14897 L 6769 14959 L 6801 14992 L 6905 15112 L
6927 15127 L 7040 15198 L 7176 15244 L 7207 15263 L
7264 15398 L 7311 15474 L 7434 15534 L 7446 15539 L
7465 15534 L 7582 15483 L 7661 15398 L 7717 15349 L
7853 15334 L 7988 15334 L 8054 15263 L 8121 15127 L
8044 14992 L 7988 14983 L 7866 14856 L 7892 14720 L
7908 14585 L 7922 14449 L 7988 14372 L 8123 14361 L
8259 14344 L 8394 14321 L 8402 14314 L 8450 14178 L
8460 14042 L 8470 13907 L 8530 13844 L 8665 13833 L
8801 13829 L 8911 13907 L 8936 13932 L 9066 14042 L
9071 14048 L 9207 14143 L 9342 14074 L 9478 14078 L
9514 14042 L 9613 13925 L 9682 13907 L 9613 13788 L
9604 13771 L 9513 13635 L 9478 13599 L 9342 13576 L
9282 13500 L 9286 13364 L 9207 13286 L 9071 13283 L
9029 13229 L 9071 13192 L 9207 13190 L 9312 13093 L
9342 13066 L 9478 13047 L 9613 13038 L 9748 13046 L
9837 12957 L 9848 12822 L 9851 12686 L 9855 12551 L
9884 12523 L 10019 12509 L 10155 12509 L 10201 12551 L
10290 12640 L 10352 12686 L 10426 12759 L 10510 12822 L
10561 12872 L 10612 12822 L 10696 12765 L 10832 12756 L
10902 12686 L 10967 12594 L 11103 12553 L 11111 12551 L
11103 12547 L 10967 12429 L 10955 12415 L 10832 12350 L
10744 12279 L 10696 12245 L 10625 12144 L 10579 12008 L
10561 11990 L 10426 11976 L 10361 11873 L 10290 11757 L
10275 11737 L 10155 11653 L 10113 11601 L 10082 11466 L
10019 11399 L 9980 11330 L 9982 11194 L 10019 11158 L
10123 11059 L 10155 11029 L 10290 11015 L 10426 10960 L

10462 10923 L 10513 10788 L 10523 10652 L 10526 10516 L
10561 10470 L 10647 10381 L 10658 10245 L 10696 10184 L
10770 10110 L 10792 9974 L 10832 9933 L 10928 9838 L
10967 9793 L 11103 9758 L 11157 9703 L 11186 9567 L
11175 9432 L 11187 9296 L 11207 9160 L 11103 9051 L
11073 9025 L 10967 8926 L 10924 8889 L 10832 8778 L
10696 8757 L 10692 8754 L 10696 8711 L 10832 8736 L
10967 8740 L 11103 8714 L 11216 8618 L 11238 8600 L
11263 8618 L 11373 8710 L 11474 8618 L 11478 8482 L
11373 8359 L 11343 8347 L 11373 8275 L 11509 8278 L
11574 8347 L 11644 8420 L 11688 8482 L 11780 8613 L
11782 8618 L 11853 8754 L 11915 8848 L 11931 8889 L
11987 9025 L 12050 9160 L 12051 9161 L 12057 9296 L
12064 9432 L 12069 9567 L 12072 9703 L 12075 9838 L
12078 9974 L 12082 10110 L 12086 10245 L 12090 10381 L
12095 10516 L 12098 10652 L 12103 10788 L 12108 10923 L
12116 11059 L 12126 11194 L 12137 11330 L 12148 11466 L
12165 11601 L 12186 11714 L 12193 11737 L 12208 11873 L
12221 12008 L 12217 12144 L 12249 12279 L 12290 12415 L
12321 12495 L 12353 12551 L 12400 12686 L 12457 12764 L
12592 12807 L 12728 12820 L 12734 12822 L 12728 12834 L
12592 12892 L 12566 12957 L 12552 13093 L 12558 13229 L
12592 13332 L 12728 13233 L 12730 13229 L 12797 13093 L
12766 12957 L 12863 12883 L 12998 12909 L 12998 12298 L
12922 12279 L 12863 12262 L 12728 12159 L 12715 12144 L
12592 12011 L 12590 12008 L 12520 11873 L 12472 11737 L
12457 11709 L 12411 11601 L 12342 11466 L 12321 11425 L
12288 11330 L 12240 11194 L 12197 11059 L 12186 11025 L
12174 10923 L 12162 10788 L 12152 10652 L 12144 10516 L
12136 10381 L 12127 10245 L 12119 10110 L 12112 9974 L
12106 9838 L 12102 9703 L 12097 9567 L 12091 9432 L
12085 9296 L 12077 9160 L 12068 9025 L 12060 8889 L
12052 8754 L 12051 8738 L 12001 8618 L 11948 8482 L
11915 8375 L 11901 8347 L 11806 8211 L 11780 8177 L
11684 8075 L 11644 8038 L 11531 7940 L 11509 7924 L
11373 7858 L 11256 7940 L 11264 8075 L 11325 8211 L
11238 8309 L 11203 8347 L 11238 8395 L 11373 8478 L
11377 8482 L 11404 8618 L 11373 8646 L 11340 8618 L
11238 8546 L 11147 8618 L 11103 8655 L 10967 8695 L
10832 8688 L 10746 8618 L 10696 8577 L 10648 8618 L
10627 8754 L 10696 8806 L 10832 8847 L 10866 8889 L
10967 8975 L 11021 9025 L 11103 9097 L 11163 9160 L
11130 9296 L 11109 9432 L 11134 9567 L 11103 9657 L

11064 9703 L 10967 9741 L 10883 9838 L 10832 9889 L
10748 9974 L 10707 10110 L 10696 10120 L 10618 10245 L
10595 10381 L 10561 10416 L 10486 10516 L 10481 10652 L
10460 10788 L 10426 10855 L 10367 10923 L 10290 10966 L
10155 10986 L 10075 11059 L 10019 11113 L 9935 11194 L
9933 11330 L 10003 11466 L 10019 11488 L 10065 11601 L
10155 11711 L 10191 11737 L 10290 11867 L 10293 11873 L
10357 12008 L 10426 12097 L 10520 12144 L 10561 12194 L
10600 12279 L 10696 12410 L 10704 12415 L 10696 12440 L
10617 12551 L 10696 12588 L 10832 12636 L 10834 12686 L
10832 12688 L 10696 12706 L 10561 12738 L 10426 12693 L
10419 12686 L 10290 12590 L 10250 12551 L 10155 12463 L
10019 12463 L 9884 12483 L 9814 12551 L 9809 12686 L
9807 12822 L 9785 12957 L 9748 12994 L 9613 12975 L
9478 12990 L 9342 13025 L 9267 13093 L 9207 13148 L
9071 13152 L 8983 13229 L 9071 13341 L 9207 13350 L
9221 13364 L 9218 13500 L 9207 13629 L 9071 13633 L
9065 13635 L 8936 13670 L 8801 13722 L 8665 13747 L
8607 13771 L 8530 13781 L 8410 13907 L 8394 14041 L
8394 14042 L 8295 14178 L 8259 14194 L 8123 14244 L
7988 14282 L 7951 14314 L 7861 14449 L 7853 14500 L
7739 14585 L 7717 14599 L 7582 14670 L 7506 14720 L
7446 14765 L 7311 14764 L 7210 14856 L 7176 14893 L
7040 14978 L 6905 14856 L 6905 14856 L 6769 14780 L
6634 14765 L 6498 14724 L 6494 14720 L 6363 14595 L
6351 14585 L 6285 14449 L 6289 14314 L 6330 14178 L
6363 14082 L 6371 14042 L 6363 14031 L 6301 13907 L
6243 13771 L 6228 13750 L 6113 13635 L 6092 13587 L
6013 13500 L 6092 13440 L 6122 13364 L 6131 13229 L
6096 13093 L 6092 13082 L 5974 12957 L 6065 12822 L
6092 12706 L 6099 12686 L 6092 12681 L 5957 12572 L
5918 12686 L 5871 12822 L 5821 12864 L 5686 12952 L
5566 12957 L 5551 12962 L 5548 12957 L 5415 12915 L
5280 12932 L 5249 12957 L 5270 13093 L 5280 13111 L
5370 13229 L 5381 13364 L 5280 13486 L 5144 13442 L
5090 13364 L 5009 13296 L 4873 13257 L 4753 13364 L
4794 13500 L 4786 13635 L 4773 13771 L 4781 13907 L
4738 13988 L 4603 13963 L 4553 13907 L 4467 13813 L
4403 13771 L 4467 13698 L 4467 13253 L 4457 13229 L
4467 13221 L 4479 13229 L 4467 13253 L 4467 13698 L
4493 13635 L 4570 13500 L 4603 13443 L 4701 13364 L
4682 13229 L 4603 13146 L 4526 13093 L 4467 13074 L
4379 13093 L 4332 13125 L 4245 13229 L 4196 13322 L

4061 13230 L 4061 13229 L 4061 13228 L 4119 13093 L
4072 12957 L 4061 12953 L 3926 12900 L 3790 12894 L
3668 12957 L 3655 13016 L 3626 13093 L 3519 13212 L
3384 13192 L 3290 13093 L 3248 13051 L 3142 12957 L
3113 12951 L 3041 12822 L 3021 12686 L 3113 12609 L
3113 12481 L 2978 12464 L 2962 12415 L 2978 12323 L
3058 12279 L 3113 12278 L 3248 12224 L 3384 12182 L
3489 12144 L 3519 12139 L 3655 12103 L 3782 12144 L
3655 12243 L 3619 12279 L 3519 12309 L 3398 12415 L
3384 12423 L 3248 12458 L 3113 12481 L 3113 12609 L
3216 12551 L 3248 12536 L 3384 12477 L 3495 12415 L
3519 12394 L 3655 12325 L 3790 12306 L 3926 12297 L
4061 12291 L 4196 12298 L 4324 12415 L 4332 12420 L
4417 12415 L 4365 12279 L 4332 12249 L 4196 12210 L
4153 12144 L 4061 12110 L 3926 12099 L 3816 12008 L
3790 11989 L 3655 11998 L 3640 12008 L 3519 12091 L
3384 12120 L 3248 12141 L 3232 12144 L 3113 12189 L
3025 12144 L 2978 12131 L 2842 12106 L 2748 12144 L
2734 12279 L 2707 12375 L 2571 12388 L 2468 12279 L
2479 12144 L 2436 12052 L 2414 12008 L 2301 11932 L
N E
15 O
2301 12027 2301 12352 2306 12279 2357 12144 2301 12027 5 N F
2301 12431 M 2301 15624 L 2436 15604 L 2436 14194 L 2418 14178 L
2397 14042 L 2436 13936 L 2516 14042 L 2450 14178 L
2436 14194 L 2436 15604 L 2571 15633 L 2571 13998 L
2458 13907 L 2478 13771 L 2514 13635 L 2571 13581 L
2571 13128 L 2510 13093 L 2436 13057 L 2318 12957 L
2397 12822 L 2436 12753 L 2571 12692 L 2707 12731 L
2782 12822 L 2707 12950 L 2704 12957 L 2602 13093 L
2571 13128 L 2571 13581 L 2671 13500 L 2707 13458 L
2752 13364 L 2777 13229 L 2842 13152 L 2894 13229 L
2897 13364 L 2851 13500 L 2842 13514 L 2761 13635 L
2707 13736 L 2682 13771 L 2614 13907 L 2571 13998 L
2571 15633 L 2641 15670 L 2707 15706 L 2807 15805 L
2842 15825 L 2978 15882 L 3045 15941 L 3113 15978 L
3113 15363 L 2978 15351 L 2863 15263 L 2842 15238 L
2765 15127 L 2777 14992 L 2842 14916 L 2945 14992 L
2978 15009 L 3069 15127 L 3113 15198 L 3175 15263 L
3113 15363 L 3113 15978 L 3248 16009 L 3248 15154 L
3223 15127 L 3113 15017 L 3095 14992 L 2989 14856 L
2978 14843 L 2914 14720 L 2871 14585 L 2842 14452 L
2831 14449 L 2842 14448 L 2850 14449 L 2978 14463 L

3102 14585 L 3113 14610 L 3151 14720 L 3223 14856 L
3248 14882 L 3324 14992 L 3295 15127 L 3248 15154 L
3248 16009 L 3331 16076 L 3384 16109 L 3519 16158 L
3519 15732 L 3436 15670 L 3428 15534 L 3450 15398 L
3519 15330 L 3655 15307 L 3784 15398 L 3750 15534 L
3702 15670 L 3655 15716 L 3519 15732 L 3519 16158 L
3585 16212 L 3638 16212 L 3790 16212 L 3790 15364 L
3762 15263 L 3790 15239 L 3926 15218 L 4061 15190 L
4141 15263 L 4061 15339 L 3926 15285 L 3790 15364 L
3790 16212 L 3805 16212 L 3972 16212 L 4139 16212 L
4304 16212 L 4332 16178 L 4332 15752 L 4221 15670 L
4196 15647 L 4121 15534 L 4107 15398 L 4196 15305 L
4243 15263 L 4332 15214 L 4394 15263 L 4467 15366 L
4603 15340 L 4738 15303 L 4840 15398 L 4738 15528 L
4734 15534 L 4603 15592 L 4467 15638 L 4420 15670 L
4332 15752 L 4332 16178 L 4363 16076 L 4370 15941 L
4467 15857 L 4603 15837 L 4738 15820 L 4793 15805 L
4873 15741 L 4978 15805 L 5009 15809 L 5144 15828 L
5280 15814 L 5331 15805 L 5415 15748 L 5449 15670 L
5446 15534 L 5415 15493 L 5336 15398 L 5337 15263 L
5355 15127 L 5355 14992 L 5292 14856 L 5280 14835 L
5219 14720 L 5226 14585 L 5280 14466 L 5280 14438 L
5208 14314 L 5262 14178 L 5280 14164 L 5295 14178 L
5350 14314 L 5280 14438 L 5280 14466 L 5415 14552 L
5484 14585 L 5551 14603 L 5647 14720 L 5686 14842 L
5694 14856 L 5686 14878 L 5655 14992 L 5681 15127 L
5686 15135 L 5780 15263 L 5821 15301 L 5922 15398 L
5957 15492 L 6027 15534 L 6092 15550 L 6127 15534 L
6228 15471 L 6332 15398 L 6228 15339 L 6173 15263 L
6126 15127 L 6092 15080 L 6007 14992 L 6002 14856 L
6092 14754 L 6228 14744 L 6307 14856 L 6330 14992 L
6363 15021 L 6498 15065 L 6634 15034 L 6759 15127 L
6769 15135 L 6901 15263 L 6905 15275 L 7002 15398 L
6905 15455 L 6769 15485 L 6672 15398 L 6634 15357 L
6532 15263 L 6498 15203 L 6478 15263 L 6405 15398 L
6498 15492 L 6553 15534 L 6545 15670 L 6607 15805 L
6634 15815 L 6769 15810 L 6777 15805 L 6905 15716 L
6984 15670 L 7040 15590 L 7176 15581 L 7274 15670 L
7311 15712 L 7446 15785 L 7476 15805 L 7582 15884 L
7699 15805 L 7717 15788 L 7787 15670 L 7853 15596 L
7988 15664 L 8000 15670 L 7988 15716 L 7939 15805 L
7988 15854 L 8123 15807 L 8125 15805 L 8230 15670 L
8259 15656 L 8394 15629 L 8530 15616 L 8613 15534 L

8651 15398 L 8606 15263 L 8592 15127 L 8530 15065 L
8489 14992 L 8394 14895 L 8259 14883 L 8123 14880 L
7988 14880 L 7965 14856 L 7974 14720 L 7979 14585 L
7983 14449 L 7988 14443 L 8123 14440 L 8259 14438 L
8394 14435 L 8518 14314 L 8524 14178 L 8526 14042 L
8530 13937 L 8557 13907 L 8665 13902 L 8801 13900 L
8810 13907 L 8936 14032 L 8948 14042 L 9071 14166 L
9085 14178 L 9207 14300 L 9335 14178 L 9342 14173 L
9478 14170 L 9608 14042 L 9613 14037 L 9748 14034 L
9884 14031 L 10011 13907 L 10016 13771 L 10019 13719 L
10139 13771 L 10155 13779 L 10290 13820 L 10426 13880 L
10471 13907 L 10561 13998 L 10601 14042 L 10696 14117 L
10832 14155 L 10904 14178 L 10967 14188 L 10994 14178 L
11103 14125 L 11238 14082 L 11373 14065 L 11509 14045 L
11519 14042 L 11644 13917 L 11780 14011 L 11780 13511 L
11762 13500 L 11765 13364 L 11733 13229 L 11644 13177 L
11509 13121 L 11373 13117 L 11238 13105 L 11103 13094 L
11088 13093 L 11060 12957 L 11103 12870 L 11131 12822 L
11238 12689 L 11239 12686 L 11373 12681 L 11509 12626 L
11644 12657 L 11724 12551 L 11780 12511 L 11915 12512 L
11958 12551 L 12051 12641 L 12053 12686 L 12064 12822 L
12081 12957 L 12090 13093 L 12051 13126 L 11915 13172 L
11796 13229 L 11781 13364 L 11782 13500 L 11780 13511 L
11780 14011 L 11818 14042 L 11915 14145 L 11978 14178 L
12051 14305 L 12066 14314 L 12186 14371 L 12268 14314 L
12321 14291 L 12427 14178 L 12457 14150 L 12592 14067 L
12592 13722 L 12460 13635 L 12520 13500 L 12592 13400 L
12728 13458 L 12753 13500 L 12728 13590 L 12711 13635 L
12592 13722 L 12592 14067 L 12656 14042 L 12728 14019 L
12767 14042 L 12811 14178 L 12728 14308 L 12725 14314 L
12655 14449 L 12592 14524 L 12535 14585 L 12580 14720 L
12584 14856 L 12592 14890 L 12625 14856 L 12686 14720 L
12728 14690 L 12819 14720 L 12863 14753 L 12904 14720 L
12998 14632 L 12998 13532 L 12978 13500 L 12998 13438 L
12998 13186 L 12888 13093 L 12998 13015 L 12998 12909 L
12863 12883 L 12766 12957 L 12797 13093 L 12730 13229 L
12728 13233 L 12592 13332 L 12558 13229 L 12552 13093 L
12566 12957 L 12592 12892 L 12728 12834 L 12734 12822 L
12728 12820 L 12592 12807 L 12457 12764 L 12400 12686 L
12353 12551 L 12321 12495 L 12290 12415 L 12249 12279 L
12217 12144 L 12221 12008 L 12208 11873 L 12193 11737 L
12186 11714 L 12165 11601 L 12148 11466 L 12137 11330 L
12126 11194 L 12116 11059 L 12108 10923 L 12103 10788 L

12098 10652 L 12095 10516 L 12090 10381 L 12086 10245 L
12082 10110 L 12078 9974 L 12075 9838 L 12072 9703 L
12069 9567 L 12064 9432 L 12057 9296 L 12051 9161 L
12050 9160 L 11987 9025 L 11931 8889 L 11915 8848 L
11853 8754 L 11782 8618 L 11780 8613 L 11688 8482 L
11644 8420 L 11574 8347 L 11509 8278 L 11373 8275 L
11343 8347 L 11373 8359 L 11478 8482 L 11474 8618 L
11373 8710 L 11263 8618 L 11238 8600 L 11216 8618 L
11103 8714 L 10967 8740 L 10832 8736 L 10696 8711 L
10692 8754 L 10696 8757 L 10832 8778 L 10924 8889 L
10967 8926 L 11073 9025 L 11103 9051 L 11207 9160 L
11187 9296 L 11175 9432 L 11186 9567 L 11157 9703 L
11103 9758 L 10967 9793 L 10928 9838 L 10832 9933 L
10792 9974 L 10770 10110 L 10696 10184 L 10658 10245 L
10647 10381 L 10561 10470 L 10526 10516 L 10523 10652 L
10513 10788 L 10462 10923 L 10426 10960 L 10290 11015 L
10155 11029 L 10123 11059 L 10019 11158 L 9982 11194 L
9980 11330 L 10019 11399 L 10082 11466 L 10113 11601 L
10155 11653 L 10275 11737 L 10290 11757 L 10361 11873 L
10426 11976 L 10561 11990 L 10579 12008 L 10625 12144 L
10696 12245 L 10744 12279 L 10832 12350 L 10955 12415 L
10967 12429 L 11103 12547 L 11103 10166 L 10967 10150 L
10907 10110 L 10960 9974 L 10967 9969 L 11089 9838 L
11103 9837 L 11236 9703 L 11238 9612 L 11298 9567 L
11353 9432 L 11373 9401 L 11469 9432 L 11509 9491 L
11566 9567 L 11644 9685 L 11650 9703 L 11644 9724 L
11566 9838 L 11509 9914 L 11442 9974 L 11373 9993 L
11238 10092 L 11219 10110 L 11103 10166 L 11103 12547 L
11111 12551 L 11103 12553 L 10967 12594 L 10902 12686 L
10832 12756 L 10696 12765 L 10612 12822 L 10561 12872 L
10510 12822 L 10426 12759 L 10352 12686 L 10290 12640 L
10201 12551 L 10155 12509 L 10019 12509 L 9884 12523 L
9855 12551 L 9851 12686 L 9848 12822 L 9837 12957 L
9748 13046 L 9613 13038 L 9478 13047 L 9342 13066 L
9312 13093 L 9207 13190 L 9071 13192 L 9029 13229 L
9071 13283 L 9207 13286 L 9286 13364 L 9282 13500 L
9342 13576 L 9478 13599 L 9513 13635 L 9604 13771 L
9613 13788 L 9613 13636 L 9613 13635 L 9613 13626 L
9632 13635 L 9613 13636 L 9613 13788 L 9682 13907 L
9613 13925 L 9514 14042 L 9478 14078 L 9342 14074 L
9207 14143 L 9071 14048 L 9066 14042 L 8936 13932 L
8911 13907 L 8801 13829 L 8665 13833 L 8530 13844 L
8470 13907 L 8460 14042 L 8450 14178 L 8402 14314 L

8394 14321 L 8259 14344 L 8123 14361 L 7988 14372 L
7922 14449 L 7908 14585 L 7892 14720 L 7866 14856 L
7988 14983 L 8044 14992 L 8121 15127 L 8054 15263 L
7988 15334 L 7853 15334 L 7717 15349 L 7661 15398 L
7582 15483 L 7465 15534 L 7446 15539 L 7434 15534 L
7311 15474 L 7264 15398 L 7207 15263 L 7176 15244 L
7040 15198 L 6927 15127 L 6905 15112 L 6801 14992 L
6769 14959 L 6634 14897 L 6516 14856 L 6498 14850 L
6371 14720 L 6363 14713 L 6228 14605 L 6168 14585 L
6119 14449 L 6092 14354 L 6052 14314 L 6092 14271 L
6127 14178 L 6188 14042 L 6216 13907 L 6122 13771 L
6092 13742 L 5957 13754 L 5931 13771 L 5827 13907 L
5888 14042 L 5927 14178 L 5821 14308 L 5812 14314 L
5821 14350 L 5844 14449 L 5957 14579 L 5964 14585 L
5957 14600 L 5904 14720 L 5821 14751 L 5800 14720 L
5733 14585 L 5772 14449 L 5686 14415 L 5551 14334 L
5534 14314 L 5457 14178 L 5415 14136 L 5311 14042 L
5280 14001 L 5190 13907 L 5144 13796 L 5116 13771 L
5009 13641 L 4969 13771 L 4982 13907 L 5009 13974 L
5046 14042 L 5069 14178 L 5028 14314 L 5009 14363 L
4958 14449 L 4973 14585 L 4873 14689 L 4786 14585 L
4738 14558 L 4603 14553 L 4467 14485 L 4332 14487 L
4196 14492 L 4061 14490 L 3926 14466 L 3907 14449 L
3926 14393 L 3986 14314 L 4061 14284 L 4196 14277 L
4232 14178 L 4196 14132 L 4127 14042 L 4061 13984 L
3926 13909 L 3896 13907 L 3790 13808 L 3655 13828 L
3519 13872 L 3446 13907 L 3384 13934 L 3322 13907 L
3248 13823 L 3227 13771 L 3167 13635 L 3113 13576 L
3063 13500 L 3037 13364 L 3035 13229 L 3003 13093 L
2978 13017 L 2948 12957 L 2876 12822 L 2862 12686 L
2842 12580 L 2707 12644 L 2571 12661 L 2525 12551 L
2436 12502 L 2301 12431 L N E
2 O
2301 15624 2301 16212 2468 16212 2571 16212 2571 16088 2556 16076
2571 16054 2640 16076 2571 16088 2571 16212 2635 16212 2802 16212
2969 16212 3136 16212 3304 16212 3471 16212 3585 16212 3519 16158
3384 16109 3331 16076 3248 16009 3113 15978 3045 15941 2978 15882
2842 15825 2807 15805 2707 15706 2641 15670 2571 15633 2436 15604
2301 15624 31 N F
29 O
2397 12822 2318 12957 2436 13057 2510 13093 2571 13128 2602 13093
2704 12957 2707 12950 2782 12822 2707 12731 2571 12692 2436 12753
2397 12822 13 N F

2 O
2418 14178 2397 14042 2436 13936 2516 14042 2450 14178 2436 14194
2418 14178 7 N F
42 O
2436 9345 2417 9296 2436 9204 2444 9160 2486 9025 2571 8998
2670 9025 2707 9035 2748 9025 2842 9000 2978 8997 3055 9025
3113 9043 3243 9160 3248 9188 3261 9160 3274 9025 3280 8889
3248 8806 3206 8754 3113 8648 3085 8618 3113 8587 3208 8482
3248 8434 3307 8347 3248 8281 3113 8242 3080 8211 3059 8075
3113 8029 3248 8036 3371 7940 3384 7933 3519 7906 3566 7940
3605 8075 3651 8211 3655 8215 3715 8347 3721 8482 3790 8567
3846 8618 3885 8754 3907 8889 3926 8941 3952 9025 3972 9160
4037 9296 3926 9357 3853 9432 3926 9500 3995 9567 4061 9627
4091 9703 4150 9838 4196 9867 4332 9866 4467 9867 4585 9974
4603 9996 4667 10110 4646 10245 4609 10381 4603 10397 4467 10474
4467 10219 4559 10110 4467 9987 4403 9974 4332 9967 4196 9954
4061 9905 4003 9974 4061 10058 4090 10110 4196 10173 4332 10195
4467 10219 4467 10474 4392 10516 4332 10575 4196 10563 4196 10395
4218 10381 4196 10266 4191 10245 4061 10213 3961 10110 3926 10061
3867 10110 3918 10245 3926 10252 4061 10293 4180 10381 4196 10395
4196 10563 4061 10548 3965 10516 3926 10492 3790 10437 3655 10418
3655 10352 3766 10245 3707 10110 3655 9999 3655 9891 3671 9838
3683 9703 3655 9615 3554 9703 3636 9838 3655 9891 3655 9999
3519 9978 3519 9666 3605 9567 3519 9488 3488 9567 3519 9666
3519 9978 3511 9974 3384 9905 3248 9851 3142 9974 3248 10025
3323 10110 3384 10166 3457 10245 3519 10300 3655 10352 3655 10418
3554 10381 3519 10364 3385 10245 3384 10244 3248 10117 3239 10110
3113 10018 3113 9940 3234 9838 3152 9703 3196 9567 3138 9432
3113 9373 3028 9432 2978 9459 2842 9529 2830 9567 2842 9696
2843 9703 2971 9838 2978 9843 3113 9940 3113 10018 3038 9974
2978 9921 2855 9838 2842 9824 2755 9703 2707 9614 2677 9567
2581 9432 2571 9422 2436 9345 165 N F
2 O
2571 13998 2458 13907 2478 13771 2514 13635 2571 13581 2671 13500
2707 13458 2752 13364 2777 13229 2842 13152 2894 13229 2897 13364
2851 13500 2842 13514 2761 13635 2707 13736 2682 13771 2614 13907
2571 13998 19 N F
15 O
2571 16054 2556 16076 2571 16088 2640 16076 2571 16054 5 N F
42 O
2571 11751 2566 11737 2571 11725 2657 11601 2707 11583 2842 11586
2978 11559 3113 11523 3248 11501 3384 11519 3415 11601 3384 11639
3254 11737 3248 11739 3113 11762 3113 11634 3248 11624 3281 11601

3248 11585 3113 11593 3081 11601 3113 11634 3113 11762 2978 11777
2842 11811 2707 11838 2707 11746 2842 11741 2877 11737 2842 11728
2707 11719 2694 11737 2707 11746 2707 11838 2571 11751 35 N F
2674 8482 2627 8347 2707 8323 2719 8347 2785 8482 2707 8514
2674 8482 7 N F
29 O
2707 11746 2694 11737 2707 11719 2842 11728 2877 11737 2842 11741
2707 11746 7 N F
82 O
2707 5777 2699 5770 2707 5731 2714 5770 2707 5777 5 N F
2 O
2842 15238 2765 15127 2777 14992 2842 14916 2945 14992 2978 15009
3069 15127 3113 15198 3175 15263 3113 15363 2978 15351 2863 15263
2842 15238 13 N F
29 O
2842 9696 2830 9567 2842 9529 2978 9459 3028 9432 3113 9373
3138 9432 3196 9567 3152 9703 3234 9838 3113 9940 2978 9843
2971 9838 2843 9703 2842 9696 15 N F
2842 14448 2831 14449 2842 14452 2871 14585 2914 14720 2978 14843
2989 14856 3095 14992 3113 15017 3223 15127 3248 15154 3295 15127
3324 14992 3248 14882 3223 14856 3151 14720 3113 14610 3102 14585
2978 14463 2850 14449 2842 14448 21 N F
95 O
2978 10203 2909 10245 2978 10301 3113 10357 3165 10381 3248 10455
3268 10381 3248 10356 3166 10245 3113 10215 2978 10203 11 N F
15 O
2978 12464 2962 12415 2978 12323 3058 12279 3113 12278 3248 12224
3384 12182 3489 12144 3519 12139 3655 12103 3782 12144 3655 12243
3619 12279 3519 12309 3398 12415 3384 12423 3248 12458 3113 12481
2978 12464 19 N F
82 O
3113 11844 2978 11873 3041 12008 3113 12032 3248 12022 3384 12014
3399 12008 3519 11911 3585 11873 3655 11809 3790 11764 3790 11626
3757 11601 3790 11592 3926 11589 3954 11601 3926 11622 3790 11626
3790 11764 3926 11770 3981 11737 4061 11670 4178 11601 4166 11466
4061 11439 3955 11466 3926 11479 3790 11519 3655 11557 3591 11601
3519 11712 3501 11737 3384 11806 3248 11829 3113 11844 35 N F
29 O
3113 11634 3081 11601 3113 11593 3248 11585 3281 11601 3248 11624
3113 11634 7 N F
108 O
3113 5622 3094 5634 3113 5688 3140 5634 3113 5622 5 N F
29 O

3248 10025 3142 9974 3248 9851 3384 9905 3511 9974 3519 9978
3655 9999 3707 10110 3766 10245 3655 10352 3519 10300 3457 10245
3384 10166 3323 10110 3248 10025 15 N F
68 O
3248 6895 3202 6855 3248 6803 3338 6855 3248 6895 5 N F
29 O
3384 13024 3317 12957 3317 12822 3384 12796 3519 12820 3525 12822
3519 12834 3429 12957 3384 13024 9 N F
95 O
3440 10652 3385 10788 3519 10892 3655 10808 3684 10788 3771 10652
3655 10594 3519 10595 3440 10652 9 N F
108 O
3519 5829 3393 5906 3519 6041 3519 6042 3655 6107 3754 6177
3790 6196 3926 6264 4061 6268 4196 6295 4332 6204 4370 6177
4332 6131 4288 6041 4332 5916 4338 5906 4409 5770 4413 5634
4370 5499 4306 5499 4188 5499 4061 5589 3968 5634 3926 5689
3867 5770 3887 5906 3790 5949 3655 5910 3647 5906 3519 5829
30 N F
2 O
3436 15670 3428 15534 3450 15398 3519 15330 3655 15307 3784 15398
3750 15534 3702 15670 3655 15716 3519 15732 3436 15670 11 N F
29 O
3519 9666 3488 9567 3519 9488 3605 9567 3519 9666 5 N F
42 O
3566 11330 3551 11194 3601 11059 3655 11009 3743 10923 3790 10887
3926 10885 4061 10910 4196 10842 4248 10788 4332 10684 4467 10705
4603 10741 4738 10684 4819 10788 4738 10917 4736 10923 4623 11059
4603 11073 4467 11090 4467 11043 4603 11006 4673 10923 4603 10826
4526 10788 4467 10767 4342 10788 4332 10791 4206 10923 4332 11001
4467 11043 4467 11090 4332 11064 4196 11111 4148 11194 4061 11313
4028 11330 3926 11371 3790 11390 3790 11330 3791 11330 3926 11316
4061 11249 4101 11194 4159 11059 4061 10987 3926 10950 3790 10946
3674 11059 3655 11131 3638 11194 3655 11249 3790 11330 3790 11330
3790 11390 3655 11395 3566 11330 57 N F
29 O
3636 9838 3554 9703 3655 9615 3683 9703 3671 9838 3655 9891
3636 9838 7 N F
3655 11249 3638 11194 3655 11131 3674 11059 3790 10946 3926 10950
4061 10987 4159 11059 4101 11194 4061 11249 3926 11316 3926 11238
4033 11194 4048 11059 3926 11017 3790 11028 3758 11059 3774 11194
3790 11208 3926 11238 3926 11316 3791 11330 3790 11330 3790 11330
3655 11249 25 N F
82 O

3655 12545 3644 12551 3655 12557 3790 12593 3926 12675 3950 12686
4061 12714 4196 12738 4330 12822 4332 12823 4467 12846 4567 12822
4603 12728 4621 12686 4603 12679 4467 12651 4332 12644 4197 12551
4196 12550 4061 12480 3926 12478 3790 12513 3655 12545 23 N F
95 O
3790 11592 3757 11601 3790 11626 3926 11622 3954 11601 3926 11589
3790 11592 7 N F
15 O
3774 11194 3758 11059 3790 11028 3926 11017 4048 11059 4033 11194
3926 11238 3790 11208 3774 11194 9 N F
2 O
3790 15364 3762 15263 3790 15239 3926 15218 4061 15190 4141 15263
4061 15339 3926 15285 3790 15364 9 N F
55 O
3926 10061 3867 10110 3918 10245 3926 10252 4061 10293 4180 10381
4196 10395 4218 10381 4196 10266 4191 10245 4061 10213 3961 10110
3926 10061 13 N F
68 O
3926 8052 3920 8075 3926 8093 4061 8148 4121 8075 4061 8044
3926 8052 7 N F
29 O
4061 10058 4003 9974 4061 9905 4196 9954 4332 9967 4403 9974
4467 9987 4559 10110 4467 10219 4332 10195 4196 10173 4090 10110
4061 10058 13 N F
68 O
4061 6791 4009 6719 4061 6654 4123 6719 4061 6791 5 N F
42 O
4034 7804 4019 7669 4061 7613 4196 7640 4255 7669 4332 7738
4386 7804 4467 7897 4498 7940 4467 7990 4332 7992 4196 7972
4129 7940 4061 7882 4034 7804 15 N F
82 O
4061 7194 4022 7262 4061 7288 4195 7397 4196 7398 4197 7397
4196 7396 4093 7262 4061 7194 9 N F
2 O
4121 15534 4107 15398 4196 15305 4243 15263 4332 15214 4394 15263
4467 15366 4603 15340 4738 15303 4840 15398 4738 15528 4734 15534
4603 15592 4467 15638 4420 15670 4332 15752 4221 15670 4196 15647
4121 15534 19 N F
42 O
4196 9482 4154 9432 4196 9361 4332 9420 4343 9432 4332 9457
4196 9482 7 N F
4196 8425 4155 8347 4196 8297 4332 8340 4340 8347 4467 8451
4509 8482 4603 8602 4618 8618 4613 8754 4603 8768 4467 8771

4454 8754 4343 8618 4332 8606 4225 8482 4196 8425 17 N F
108 O
4306 5499 4188 5499 4188 5499 4306 5499 4370 5499 4370 5499
4306 5499 7 N F
68 O
4196 8729 4190 8754 4196 8767 4229 8754 4196 8729 5 N F
29 O
4332 11001 4206 10923 4332 10791 4342 10788 4467 10767 4526 10788
4603 10826 4673 10923 4603 11006 4467 11043 4467 10971 4603 10931
4609 10923 4603 10914 4467 10855 4332 10901 4311 10923 4332 10936
4467 10971 4467 11043 4332 11001 21 N F
82 O
4275 11194 4225 11330 4231 11466 4332 11562 4365 11601 4332 11700
4317 11737 4332 11750 4467 11792 4467 11364 4384 11330 4467 11277
4535 11330 4467 11364 4467 11792 4603 11808 4695 11873 4738 11924
4873 11945 5009 11945 5009 11895 4912 11873 4873 11835 4818 11737
4738 11673 4603 11676 4546 11601 4603 11477 4613 11466 4738 11361
4756 11330 4873 11200 4878 11194 5009 11116 5085 11059 5144 10952
5280 10961 5359 11059 5415 11095 5443 11059 5463 10923 5486 10788
5551 10733 5628 10788 5686 10831 5733 10923 5726 11059 5755 11194
5821 11260 5923 11330 5957 11431 6092 11462 6228 11457 6237 11466
6228 11474 6092 11475 5957 11476 5821 11547 5791 11601 5717 11737
5689 11873 5686 11889 5574 11873 5551 11870 5415 11766 5280 11764
5144 11833 5082 11873 5009 11895 5009 11945 5144 11910 5233 11873
5280 11846 5415 11871 5417 11873 5512 12008 5513 12144 5442 12279
5415 12318 5322 12415 5415 12533 5425 12551 5551 12647 5686 12665
5717 12551 5691 12415 5686 12385 5639 12279 5686 12212 5702 12144
5729 12008 5739 11873 5762 11737 5821 11646 5922 11601 5957 11578
6009 11601 6066 11737 6092 11777 6228 11762 6363 11766 6490 11873
6498 11887 6508 11873 6517 11737 6498 11716 6363 11617 6228 11651
6159 11601 6228 11567 6345 11466 6228 11356 6092 11395 6015 11330
6092 11249 6228 11289 6264 11194 6228 11167 6092 11167 5982 11194
5957 11218 5901 11194 5821 11166 5779 11059 5810 10923 5768 10788
5727 10652 5741 10516 5714 10381 5686 10309 5597 10381 5551 10420
5469 10516 5415 10604 5280 10625 5144 10578 5032 10516 5090 10381
5071 10245 5009 10216 4873 10226 4858 10245 4812 10381 4873 10445
4977 10516 4981 10652 5009 10747 5044 10788 5063 10923 5009 11020
4880 11059 4873 11060 4738 11121 4603 11173 4467 11168 4332 11160
4275 11194 157 N F
2 O
4304 16212 M 4306 16212 L 4474 16212 L 4641 16212 L 4808 16212 L
4975 16212 L 5142 16212 L 5309 16212 L 5477 16212 L
5644 16212 L 5811 16212 L 5978 16212 L 6145 16212 L

6312 16212 L 6479 16212 L 6647 16212 L 6814 16212 L
6981 16212 L 7148 16212 L 7315 16212 L 7482 16212 L
7650 16212 L 7817 16212 L 7984 16212 L 8151 16212 L
8318 16212 L 8485 16212 L 8652 16212 L 8820 16212 L
8987 16212 L 9053 16212 L 8956 16076 L 8943 15941 L
8936 15938 L 8927 15941 L 8801 15973 L 8665 15968 L
8642 15941 L 8642 15805 L 8665 15751 L 8693 15670 L
8713 15534 L 8801 15432 L 8936 15522 L 8957 15534 L
9071 15645 L 9081 15670 L 9115 15805 L 9195 15941 L
9207 15965 L 9223 16076 L 9207 16118 L 9111 16212 L
9154 16212 L 9321 16212 L 9488 16212 L 9655 16212 L
9822 16212 L 9884 16212 L 9884 15952 L 9879 15941 L
9877 15805 L 9884 15795 L 9891 15805 L 9895 15941 L
9884 15952 L 9884 16212 L 9990 16212 L 10157 16212 L
10324 16212 L 10491 16212 L 10658 16212 L 10825 16212 L
10993 16212 L 11160 16212 L 11327 16212 L 11494 16212 L
11661 16212 L 11828 16212 L 11995 16212 L 12163 16212 L
12330 16212 L 12497 16212 L 12664 16212 L 12831 16212 L
12998 16212 L 12998 14632 L 12904 14720 L 12863 14753 L
12819 14720 L 12728 14690 L 12686 14720 L 12625 14856 L
12592 14890 L 12584 14856 L 12580 14720 L 12535 14585 L
12592 14524 L 12655 14449 L 12725 14314 L 12728 14308 L
12811 14178 L 12767 14042 L 12728 14019 L 12656 14042 L
12592 14067 L 12457 14150 L 12427 14178 L 12321 14291 L
12268 14314 L 12186 14371 L 12066 14314 L 12051 14305 L
11978 14178 L 11915 14145 L 11818 14042 L 11780 14011 L
11644 13917 L 11519 14042 L 11509 14045 L 11373 14065 L
11238 14082 L 11103 14125 L 10994 14178 L 10967 14188 L
10904 14178 L 10832 14155 L 10696 14117 L 10601 14042 L
10561 13998 L 10471 13907 L 10426 13880 L 10290 13820 L
10155 13779 L 10139 13771 L 10019 13719 L 10016 13771 L
10011 13907 L 9884 14031 L 9748 14034 L 9613 14037 L
9608 14042 L 9478 14170 L 9342 14173 L 9335 14178 L
9207 14300 L 9085 14178 L 9071 14166 L 8948 14042 L
8936 14032 L 8810 13907 L 8801 13900 L 8665 13902 L
8557 13907 L 8530 13937 L 8526 14042 L 8524 14178 L
8518 14314 L 8394 14435 L 8259 14438 L 8123 14440 L
7988 14443 L 7983 14449 L 7979 14585 L 7974 14720 L
7965 14856 L 7988 14880 L 8123 14880 L 8259 14883 L
8394 14895 L 8489 14992 L 8530 15065 L 8592 15127 L
8606 15263 L 8651 15398 L 8613 15534 L 8530 15616 L
8394 15629 L 8259 15656 L 8230 15670 L 8125 15805 L
8123 15807 L 7988 15854 L 7939 15805 L 7988 15716 L

8000 15670 L 7988 15664 L 7853 15596 L 7787 15670 L
7717 15788 L 7699 15805 L 7582 15884 L 7476 15805 L
7446 15785 L 7311 15712 L 7274 15670 L 7176 15581 L
7040 15590 L 6984 15670 L 6905 15716 L 6777 15805 L
6769 15810 L 6634 15815 L 6607 15805 L 6545 15670 L
6553 15534 L 6498 15492 L 6405 15398 L 6478 15263 L
6498 15203 L 6532 15263 L 6634 15357 L 6672 15398 L
6769 15485 L 6905 15455 L 7002 15398 L 6905 15275 L
6901 15263 L 6769 15135 L 6759 15127 L 6634 15034 L
6498 15065 L 6363 15021 L 6330 14992 L 6307 14856 L
6228 14744 L 6092 14754 L 6002 14856 L 6007 14992 L
6092 15080 L 6126 15127 L 6173 15263 L 6228 15339 L
6332 15398 L 6228 15471 L 6127 15534 L 6092 15550 L
6027 15534 L 5957 15492 L 5922 15398 L 5821 15301 L
5780 15263 L 5686 15135 L 5681 15127 L 5655 14992 L
5686 14878 L 5694 14856 L 5686 14842 L 5647 14720 L
5551 14603 L 5484 14585 L 5415 14552 L 5280 14466 L
5226 14585 L 5219 14720 L 5280 14835 L 5292 14856 L
5355 14992 L 5355 15127 L 5337 15263 L 5336 15398 L
5415 15493 L 5446 15534 L 5449 15670 L 5415 15748 L
5331 15805 L 5280 15814 L 5144 15828 L 5009 15809 L
4978 15805 L 4873 15741 L 4793 15805 L 4738 15820 L
4603 15837 L 4467 15857 L 4370 15941 L 4363 16076 L
4332 16178 L 4304 16212 L N E
15 O
4332 10936 4311 10923 4332 10901 4467 10855 4603 10914 4609 10923
4603 10931 4467 10971 4332 10936 9 N F
55 O
4467 7643 4375 7533 4381 7397 4467 7330 4565 7397 4603 7438
4656 7533 4738 7659 4759 7669 4738 7685 4603 7753 4597 7669
4467 7643 13 N F
95 O
4467 11277 4384 11330 4467 11364 4535 11330 4467 11277 5 N F
15 O
4467 13253 4457 13229 4467 13221 4479 13229 4467 13253 5 N F
95 O
4603 11477 4546 11601 4603 11676 4738 11673 4818 11737 4873 11835
4912 11873 5009 11895 5009 11772 4972 11737 4883 11601 4873 11556
4864 11466 4873 11444 4903 11330 5000 11194 5009 11189 5144 11140
5213 11194 5280 11288 5356 11330 5415 11400 5529 11330 5551 11312
5578 11330 5686 11361 5731 11466 5704 11601 5686 11655 5551 11612
5527 11601 5415 11523 5375 11601 5280 11642 5144 11724 5114 11737
5009 11772 5009 11895 5082 11873 5144 11833 5280 11764 5415 11766

5551 11870 5574 11873 5686 11889 5689 11873 5717 11737 5791 11601
5821 11547 5957 11476 6092 11475 6228 11474 6237 11466 6228 11457
6092 11462 5957 11431 5923 11330 5821 11260 5755 11194 5726 11059
5733 10923 5686 10831 5628 10788 5551 10733 5486 10788 5463 10923
5443 11059 5415 11095 5359 11059 5280 10961 5144 10952 5085 11059
5009 11116 4878 11194 4873 11200 4756 11330 4738 11361 4613 11466
4603 11477 79 N F
68 O
4857 7262 4795 7126 4859 6991 4873 6982 4887 6991 5009 7047
5077 7126 5144 7212 5191 7262 5144 7330 5009 7356 4873 7273
4857 7262 13 N F
108 O
4873 11444 4864 11466 4873 11556 4883 11601 4972 11737 5009 11772
5114 11737 5144 11724 5280 11642 5375 11601 5415 11523 5527 11601
5551 11612 5686 11655 5704 11601 5731 11466 5686 11361 5578 11330
5551 11312 5529 11330 5415 11400 5356 11330 5280 11288 5213 11194
5144 11140 5009 11189 5000 11194 4903 11330 4873 11444 29 N F
55 O
5009 7831 4978 7804 5009 7778 5110 7804 5009 7831 5 N F
82 O
5009 5669 4985 5634 5009 5519 5023 5499 5142 5499 5309 5499
5319 5499 5415 5618 5454 5499 5477 5499 5644 5499 5811 5499
5949 5499 5957 5511 5960 5499 5978 5499 6029 5499 6064 5634
5957 5680 5821 5720 5779 5770 5686 5883 5686 5773 5689 5770
5791 5634 5686 5538 5572 5634 5682 5770 5686 5773 5686 5883
5555 5770 5551 5765 5516 5770 5418 5906 5476 6041 5415 6080
5388 6041 5407 5906 5280 5836 5241 5770 5144 5672 5009 5669
42 N F
15 O
5009 13610 5000 13500 5009 13483 5017 13500 5009 13610 5 N F
2 O
5280 14438 5208 14314 5262 14178 5280 14164 5295 14178 5350 14314
5280 14438 7 N F
95 O
5319 5499 5319 5499 5454 5499 5454 5499 5415 5499 5319 5499
6 N F
5319 5499 5319 5499 5454 5499 5454 5499 5319 5499 5 N F
5454 5499 5319 5499 5415 5618 5454 5499 4 N F
42 O
5415 13856 5348 13907 5415 13996 5455 14042 5551 14139 5649 14178
5686 14193 5704 14178 5722 14042 5686 14009 5551 13927 5526 13907
5415 13856 13 N F
15 O

5415 13700 5365 13635 5415 13589 5515 13500 5551 13420 5580 13500
5622 13635 5686 13745 5725 13771 5686 13808 5551 13805 5472 13771
5415 13700 13 N F
68 O
5415 7055 5381 6991 5415 6949 5507 6991 5415 7055 5 N F
82 O
5614 9974 5554 10110 5686 10195 5717 10110 5712 9974 5690 9838
5686 9831 5680 9838 5614 9974 9 N F
108 O
5627 6313 M 5567 6448 L 5583 6584 L 5686 6672 L 5773 6719 L
5821 6735 L 5957 6753 L 5997 6719 L 6043 6584 L
6092 6543 L 6228 6562 L 6284 6584 L 6363 6622 L
6432 6719 L 6498 6852 L 6502 6855 L 6634 6917 L
6769 6926 L 6905 6874 L 7035 6991 L 7040 6992 L
7176 7039 L 7311 7101 L 7446 7069 L 7534 6991 L
7572 6855 L 7582 6830 L 7602 6855 L 7717 6973 L
7737 6991 L 7853 7068 L 7988 7034 L 7988 6881 L
7922 6855 L 7988 6800 L 8052 6855 L 7988 6881 L
7988 7034 L 8123 7064 L 8181 7126 L 8259 7262 L
8389 7126 L 8394 7123 L 8523 6991 L 8530 6987 L
8534 6991 L 8665 7119 L 8673 7126 L 8794 7262 L
8801 7272 L 8936 7375 L 8972 7397 L 9071 7449 L
9207 7486 L 9342 7484 L 9478 7483 L 9613 7485 L
9748 7455 L 9884 7442 L 10019 7431 L 10019 7109 L
9884 7066 L 9843 6991 L 9748 6945 L 9665 6855 L
9613 6736 L 9602 6719 L 9613 6668 L 9620 6584 L
9628 6448 L 9613 6423 L 9546 6313 L 9505 6177 L
9508 6041 L 9527 5906 L 9603 5770 L 9613 5761 L
9623 5770 L 9748 5880 L 9772 5906 L 9830 6041 L
9873 6177 L 9884 6223 L 9920 6313 L 9956 6448 L
9991 6584 L 10019 6660 L 10065 6719 L 10088 6855 L
10054 6991 L 10019 7109 L 10019 7431 L 10155 7424 L
10290 7438 L 10426 7458 L 10561 7491 L 10696 7488 L
10832 7463 L 10967 7420 L 11103 7411 L 11238 7410 L
11373 7417 L 11509 7426 L 11644 7435 L 11780 7444 L
11915 7454 L 12051 7474 L 12108 7533 L 12129 7669 L
12142 7804 L 12152 7940 L 12158 8075 L 12164 8211 L
12170 8347 L 12174 8482 L 12180 8618 L 12185 8754 L
12186 8768 L 12236 8889 L 12302 9025 L 12321 9092 L
12371 9160 L 12457 9237 L 12553 9296 L 12592 9336 L
12728 9354 L 12820 9432 L 12863 9468 L 12998 9519 L
12998 5499 L 12864 5499 L 12903 5634 L 12922 5770 L
12907 5906 L 12915 6041 L 12925 6177 L 12923 6313 L

12912 6448 L 12894 6584 L 12880 6719 L 12884 6855 L
12892 6991 L 12883 7126 L 12875 7262 L 12863 7346 L
12813 7397 L 12728 7452 L 12684 7533 L 12708 7669 L
12728 7689 L 12861 7804 L 12841 7940 L 12863 8075 L
12774 8211 L 12728 8283 L 12642 8347 L 12592 8426 L
12458 8347 L 12457 8346 L 12321 8242 L 12310 8211 L
12272 8075 L 12245 7940 L 12186 7844 L 12184 7804 L
12180 7669 L 12168 7533 L 12051 7413 L 11915 7401 L
11780 7400 L 11729 7397 L 11644 7376 L 11509 7345 L
11373 7316 L 11238 7287 L 11103 7277 L 10967 7309 L
10832 7340 L 10696 7391 L 10561 7382 L 10426 7354 L
10290 7291 L 10235 7262 L 10164 7126 L 10155 7115 L
10129 6991 L 10133 6855 L 10114 6719 L 10019 6598 L
10014 6584 L 9981 6448 L 9962 6313 L 9932 6177 L
9884 6131 L 9856 6041 L 9817 5906 L 9748 5831 L
9679 5770 L 9613 5706 L 9548 5770 L 9501 5906 L
9488 6041 L 9486 6177 L 9522 6313 L 9592 6448 L
9567 6584 L 9545 6719 L 9613 6822 L 9627 6855 L
9748 6986 L 9757 6991 L 9757 7126 L 9748 7137 L
9743 7126 L 9613 7042 L 9550 6991 L 9566 6855 L
9478 6803 L 9412 6855 L 9396 6991 L 9431 7126 L
9478 7200 L 9547 7262 L 9478 7302 L 9342 7302 L
9207 7292 L 9109 7262 L 9071 7242 L 8936 7149 L
8897 7126 L 8801 7057 L 8730 6991 L 8665 6948 L
8559 6855 L 8530 6838 L 8394 6755 L 8282 6719 L
8259 6711 L 8129 6584 L 8123 6579 L 8093 6584 L
7988 6630 L 7919 6584 L 7853 6557 L 7717 6520 L
7582 6545 L 7524 6584 L 7517 6719 L 7468 6855 L
7446 6900 L 7311 6908 L 7231 6855 L 7176 6806 L
7095 6719 L 7040 6656 L 6917 6584 L 6905 6521 L
6769 6529 L 6726 6448 L 6769 6388 L 6905 6429 L
6965 6313 L 7040 6270 L 7176 6234 L 7214 6177 L
7176 6095 L 7154 6041 L 7040 5982 L 6905 5998 L
6857 6041 L 6769 6113 L 6698 6177 L 6634 6269 L
6498 6309 L 6363 6247 L 6258 6177 L 6228 6152 L
6092 6072 L 5957 6047 L 5956 6041 L 5957 6034 L
5987 5906 L 6092 5819 L 6121 5770 L 6187 5634 L
6167 5499 L 6145 5499 L 6097 5499 L 6136 5634 L
6092 5705 L 5962 5770 L 5957 5775 L 5833 5906 L
5821 5932 L 5720 6041 L 5686 6082 L 5645 6177 L
5627 6313 L N E
68 O
5682 5770 5572 5634 5686 5538 5791 5634 5689 5770 5686 5773

5682 5770 7 N F
82 O
5686 8573 5672 8618 5686 8638 5691 8618 5686 8573 5 N F
55 O
5774 9703 5720 9567 5749 9432 5821 9338 5880 9296 5821 9160
5821 9160 5788 9025 5821 8934 5830 8889 5821 8862 5769 8754
5787 8618 5793 8482 5808 8347 5821 8307 5847 8211 5821 8083
5818 8075 5821 8058 5838 7940 5828 7804 5894 7669 5957 7626
6092 7566 6228 7654 6259 7669 6228 7798 6226 7804 6218 7940
6228 7963 6279 8075 6363 8139 6451 8211 6498 8308 6532 8347
6498 8351 6364 8482 6363 8490 6228 8542 6209 8618 6228 8657
6297 8754 6228 8795 6167 8889 6105 9025 6228 9146 6250 9160
6291 9296 6363 9405 6401 9432 6458 9567 6477 9703 6440 9838
6498 9939 6559 9974 6551 10110 6498 10152 6462 10245 6403 10381
6363 10453 6286 10516 6363 10545 6498 10527 6524 10516 6634 10472
6674 10516 6769 10602 6836 10652 6797 10788 6769 10802 6731 10788
6634 10749 6578 10788 6594 10923 6498 10985 6397 11059 6452 11194
6363 11252 6327 11194 6228 11121 6092 11125 5957 11159 5957 11117
6092 11083 6228 11074 6284 11059 6228 10979 6188 10923 6228 10877
6305 10923 6363 10960 6498 10939 6523 10923 6498 10842 6498 10760
6634 10677 6747 10652 6634 10583 6498 10594 6368 10652 6498 10760
6498 10842 6484 10788 6363 10655 6299 10652 6228 10646 6228 10307
6293 10245 6340 10110 6228 9984 6111 10110 6158 10245 6228 10307
6228 10646 6215 10652 6092 10683 5957 10695 5957 10614 6070 10516
6092 10489 6182 10381 6092 10284 6092 10012 6152 9974 6092 9903
6048 9974 6092 10012 6092 10284 5957 10311 5957 9796 6092 9770
6135 9703 6228 9630 6303 9703 6363 9739 6386 9703 6387 9567
6363 9525 6235 9432 6228 9390 6215 9296 6092 9227 5993 9296
5957 9371 5957 9146 6041 9025 6058 8889 6075 8754 5957 8651
5861 8754 5905 8889 5868 9025 5957 9146 5957 9371 5821 9422
5814 9432 5775 9567 5821 9650 5859 9703 5957 9796 5957 10311
5928 10381 5904 10516 5957 10614 5957 10695 5912 10788 5952 10923
5884 11059 5957 11117 5957 11159 5832 11059 5882 10923 5853 10788
5842 10652 5845 10516 5849 10381 5833 10245 5848 10110 5899 9974
5912 9838 5821 9755 5774 9703 183 N F
42 O
5821 9650 5775 9567 5814 9432 5821 9422 5957 9371 5993 9296
6092 9227 6215 9296 6228 9390 6235 9432 6363 9525 6387 9567
6386 9703 6363 9739 6303 9703 6228 9630 6135 9703 6092 9770
5957 9796 5957 9729 5999 9703 6092 9600 6129 9567 6155 9432
6092 9302 6019 9432 5957 9484 5837 9567 5930 9703 5957 9729
5957 9796 5859 9703 5821 9650 33 N F
29 O

5930 9703 5837 9567 5957 9484 6019 9432 6092 9302 6155 9432
6129 9567 6092 9600 5999 9703 5957 9729 5957 9590 5976 9567
5957 9558 5944 9567 5957 9590 5957 9729 5930 9703 17 N F
42 O
5905 8889 5861 8754 5957 8651 6075 8754 6058 8889 6041 9025
5957 9146 5957 9064 5984 9025 5957 8942 5957 8828 5990 8754
5957 8725 5930 8754 5957 8828 5957 8942 5928 9025 5957 9064
5957 9146 5868 9025 5905 8889 21 N F
5957 11117 5884 11059 5952 10923 5912 10788 5957 10695 6092 10683
6215 10652 6228 10646 6299 10652 6363 10655 6484 10788 6498 10842
6523 10923 6498 10939 6363 10960 6363 10855 6417 10788 6363 10728
6282 10788 6363 10855 6363 10960 6305 10923 6228 10877 6188 10923
6228 10979 6284 11059 6228 11074 6092 11083 6092 10836 6133 10788
6092 10768 6026 10788 6092 10836 6092 11083 5957 11117 5957 11075
6023 11059 5957 11018 5936 11059 5957 11075 5957 11117 41 N F
5957 10614 5904 10516 5928 10381 5957 10311 6092 10284 6182 10381
6092 10489 6070 10516 5957 10614 9 N F
29 O
5943 12008 5905 11873 5957 11794 6002 11873 5973 12008 5957 12124
5943 12008 7 N F
15 O
5957 13273 5911 13229 5957 13123 6015 13229 5957 13273 5 N F
29 O
5957 9064 5928 9025 5957 8942 5984 9025 5957 9064 5 N F
5957 8828 5930 8754 5957 8725 5990 8754 5957 8828 5 N F
5957 11075 5936 11059 5957 11018 6023 11059 5957 11075 5 N F
15 O
5957 9590 5944 9567 5957 9558 5976 9567 5957 9590 5 N F
68 O
5949 5499 5949 5499 5957 5499 5960 5499 5960 5499 5949 5499
6 N F
5949 5499 5949 5499 5960 5499 5960 5499 5949 5499 5 N F
5957 5511 5949 5499 5960 5499 5957 5511 4 N F
29 O
5957 12444 5954 12415 5957 12396 5962 12415 5957 12444 5 N F
121 O
5957 6034 M 5956 6041 L 5957 6047 L 6092 6072 L 6228 6152 L
6258 6177 L 6363 6247 L 6498 6309 L 6634 6269 L
6698 6177 L 6769 6113 L 6857 6041 L 6905 5998 L
7040 5982 L 7154 6041 L 7176 6095 L 7214 6177 L
7176 6234 L 7040 6270 L 6965 6313 L 6905 6429 L
6769 6388 L 6726 6448 L 6769 6529 L 6905 6521 L
6917 6584 L 7040 6656 L 7095 6719 L 7176 6806 L

7231 6855 L 7311 6908 L 7446 6900 L 7468 6855 L
7517 6719 L 7524 6584 L 7582 6545 L 7717 6520 L
7853 6557 L 7919 6584 L 7988 6630 L 8093 6584 L
8123 6579 L 8129 6584 L 8259 6711 L 8282 6719 L
8394 6755 L 8530 6838 L 8559 6855 L 8665 6948 L
8730 6991 L 8801 7057 L 8897 7126 L 8936 7149 L
9071 7242 L 9109 7262 L 9207 7292 L 9342 7302 L
9342 6694 L 9294 6584 L 9342 6504 L 9401 6584 L
9342 6694 L 9342 7302 L 9478 7302 L 9547 7262 L
9478 7200 L 9431 7126 L 9396 6991 L 9412 6855 L
9478 6803 L 9566 6855 L 9550 6991 L 9613 7042 L
9743 7126 L 9748 7137 L 9757 7126 L 9757 6991 L
9748 6986 L 9627 6855 L 9613 6822 L 9545 6719 L
9567 6584 L 9592 6448 L 9522 6313 L 9486 6177 L
9488 6041 L 9501 5906 L 9548 5770 L 9613 5706 L
9679 5770 L 9748 5831 L 9817 5906 L 9856 6041 L
9884 6131 L 9932 6177 L 9962 6313 L 9981 6448 L
10014 6584 L 10019 6598 L 10114 6719 L 10133 6855 L
10129 6991 L 10155 7115 L 10164 7126 L 10235 7262 L
10290 7291 L 10426 7354 L 10561 7382 L 10696 7391 L
10832 7340 L 10967 7309 L 11103 7277 L 11238 7287 L
11373 7316 L 11509 7345 L 11644 7376 L 11729 7397 L
11780 7400 L 11915 7401 L 12051 7413 L 12168 7533 L
12180 7669 L 12184 7804 L 12186 7844 L 12245 7940 L
12272 8075 L 12310 8211 L 12321 8242 L 12457 8346 L
12458 8347 L 12592 8426 L 12642 8347 L 12728 8283 L
12774 8211 L 12863 8075 L 12841 7940 L 12861 7804 L
12728 7689 L 12708 7669 L 12684 7533 L 12728 7452 L
12813 7397 L 12863 7346 L 12875 7262 L 12883 7126 L
12892 6991 L 12884 6855 L 12880 6719 L 12894 6584 L
12912 6448 L 12923 6313 L 12925 6177 L 12915 6041 L
12907 5906 L 12922 5770 L 12903 5634 L 12864 5499 L
12831 5499 L 12664 5499 L 12497 5499 L 12330 5499 L
12163 5499 L 11995 5499 L 11828 5499 L 11661 5499 L
11494 5499 L 11371 5499 L 11373 5500 L 11509 5544 L
11644 5550 L 11780 5619 L 11797 5634 L 11780 5711 L
11766 5770 L 11780 5782 L 11898 5906 L 11915 5948 L
11978 6041 L 11929 6177 L 12031 6313 L 11974 6448 L
11915 6541 L 11879 6584 L 11854 6719 L 11803 6855 L
11780 6868 L 11644 6904 L 11509 6933 L 11402 6991 L
11373 7011 L 11238 7022 L 11103 7048 L 10967 7059 L
10832 7043 L 10696 7054 L 10561 7052 L 10426 7055 L
10360 6991 L 10290 6910 L 10267 6855 L 10203 6719 L

10188 6584 L 10189 6448 L 10155 6380 L 10121 6448 L
10019 6502 L 10006 6448 L 10004 6313 L 10016 6177 L
9884 6050 L 9881 6041 L 9861 5906 L 9748 5782 L
9735 5770 L 9613 5652 L 9492 5770 L 9478 5871 L
9469 5906 L 9449 6041 L 9437 6177 L 9478 6228 L
9499 6313 L 9533 6448 L 9499 6584 L 9488 6719 L
9478 6732 L 9452 6719 L 9459 6584 L 9375 6448 L
9342 6403 L 9300 6448 L 9247 6584 L 9269 6719 L
9274 6855 L 9207 6910 L 9071 6913 L 8936 6885 L
8821 6855 L 8801 6850 L 8665 6763 L 8578 6719 L
8530 6687 L 8394 6593 L 8367 6584 L 8259 6519 L
8188 6448 L 8123 6401 L 7988 6315 L 7965 6313 L
7853 6300 L 7717 6299 L 7582 6289 L 7446 6244 L
7415 6177 L 7315 6041 L 7311 6038 L 7176 5929 L
7135 5906 L 7040 5841 L 6905 5824 L 6769 5815 L
6712 5770 L 6634 5730 L 6498 5726 L 6363 5756 L
6338 5634 L 6291 5499 L 6167 5499 L 6187 5634 L
6121 5770 L 6092 5819 L 5987 5906 L 5957 6034 L
N E
82 O
5978 5499 5960 5499 5960 5499 5978 5499 6029 5499 6029 5499
5978 5499 7 N F
29 O
6092 10836 6026 10788 6092 10768 6133 10788 6092 10836 5 N F
95 O
6029 5499 6029 5499 6097 5499 6097 5499 6092 5499 6029 5499
6 N F
6029 5499 6029 5499 6097 5499 6097 5499 6029 5499 5 N F
42 O
6092 10012 6048 9974 6092 9903 6152 9974 6092 10012 5 N F
108 O
6097 5499 6097 5499 6145 5499 6167 5499 6167 5499 6145 5499
6097 5499 7 N F
42 O
6158 10245 6111 10110 6228 9984 6340 10110 6293 10245 6228 10307
6228 10205 6297 10110 6228 10032 6156 10110 6228 10205 6228 10307
6158 10245 13 N F
29 O
6228 10205 6156 10110 6228 10032 6297 10110 6228 10205 6228 10146
6254 10110 6228 10080 6200 10110 6228 10146 6228 10205 11 N F
6194 12551 6159 12415 6228 12307 6263 12279 6228 12199 6211 12144
6212 12008 6228 11940 6363 11953 6401 12008 6498 12111 6531 12144
6566 12279 6577 12415 6588 12551 6634 12600 6735 12686 6769 12732

6905 12798 6942 12822 6905 12852 6769 12894 6634 12940 6634 12872
6747 12822 6656 12686 6634 12667 6525 12551 6498 12465 6498 12288
6500 12279 6498 12274 6494 12279 6498 12288 6498 12465 6429 12415
6363 12369 6300 12415 6343 12551 6363 12575 6449 12686 6498 12791
6516 12822 6634 12872 6634 12940 6498 12874 6363 12824 6344 12822
6302 12686 6228 12590 6194 12551 51 N F
121 O
6167 5499 6167 5499 6291 5499 6291 5499 6228 5499 6167 5499
6 N F
6167 5499 6167 5499 6291 5499 6291 5499 6167 5499 5 N F
15 O
6228 10146 6200 10110 6228 10080 6254 10110 6228 10146 5 N F
29 O
6363 10855 6282 10788 6363 10728 6417 10788 6363 10855 5 N F
135 O
6312 5499 6291 5499 6338 5634 6363 5756 6498 5726 6634 5730
6712 5770 6769 5815 6905 5824 7040 5841 7135 5906 7176 5929
7311 6038 7315 6041 7415 6177 7446 6244 7582 6289 7717 6299
7853 6300 7965 6313 7988 6315 8123 6401 8188 6448 8259 6519
8367 6584 8394 6593 8530 6687 8578 6719 8665 6763 8801 6850
8821 6855 8936 6885 9071 6913 9207 6910 9274 6855 9269 6719
9247 6584 9300 6448 9342 6403 9375 6448 9459 6584 9452 6719
9478 6732 9488 6719 9499 6584 9533 6448 9499 6313 9478 6228
9437 6177 9449 6041 9469 5906 9478 5871 9492 5770 9613 5652
9735 5770 9748 5782 9861 5906 9881 6041 9884 6050 10016 6177
10004 6313 10006 6448 10019 6502 10019 5995 9999 5906 10019 5855
10042 5906 10019 5995 10019 6502 10121 6448 10155 6380 10189 6448
10188 6584 10203 6719 10267 6855 10290 6910 10360 6991 10426 7055
10561 7052 10696 7054 10832 7043 10967 7059 11103 7048 11238 7022
11373 7011 11402 6991 11509 6933 11644 6904 11780 6868 11803 6855
11854 6719 11879 6584 11915 6541 11974 6448 12031 6313 11929 6177
11978 6041 11915 5948 11898 5906 11780 5782 11766 5770 11780 5711
11797 5634 11780 5619 11644 5550 11509 5544 11373 5500 11371 5499
11327 5499 11160 5499 10993 5499 10828 5499 10696 5523 10561 5532
10493 5499 10491 5499 10324 5499 10157 5499 9990 5499 9822 5499
9666 5499 9613 5524 9478 5571 9342 5548 9234 5499 9185 5499
9108 5634 9101 5770 9207 5901 9207 5858 9136 5770 9167 5634
9207 5577 9299 5634 9342 5758 9357 5770 9342 5773 9207 5858
9207 5901 9342 5874 9373 5906 9390 6041 9361 6177 9342 6230
9207 6311 9206 6313 9152 6448 9138 6584 9071 6639 8936 6623
8801 6590 8791 6584 8665 6496 8589 6448 8530 6396 8394 6318
8385 6313 8259 6214 8132 6177 8123 6174 7988 6122 7853 6108
7717 6130 7582 6147 7458 6041 7446 6025 7322 5906 7446 5798

7537 5770 7582 5753 7717 5699 7853 5667 7930 5634 7988 5622
8123 5605 8259 5573 8394 5507 8427 5499 8318 5499 8151 5499
7984 5499 7817 5499 7650 5499 7482 5499 7315 5499 7148 5499
6981 5499 6814 5499 6647 5499 6479 5499 6312 5499 191 N F
15 O
6343 12551 6300 12415 6363 12369 6429 12415 6498 12465 6525 12551
6634 12667 6656 12686 6747 12822 6634 12872 6516 12822 6498 12791
6449 12686 6363 12575 6343 12551 15 N F
82 O
6363 13074 6339 13093 6357 13229 6363 13241 6498 13279 6634 13261
6715 13229 6725 13093 6634 13086 6498 13064 6363 13074 11 N F
6363 9292 6362 9296 6363 9298 6498 9314 6520 9296 6562 9160
6498 9114 6471 9160 6363 9292 9 N F
42 O
6498 10760 6368 10652 6498 10594 6634 10583 6747 10652 6634 10677
6498 10760 7 N F
6498 11542 6461 11466 6498 11422 6526 11466 6498 11542 5 N F
15 O
6498 12288 6494 12279 6498 12274 6500 12279 6498 12288 5 N F
82 O
6634 7288 6626 7262 6634 7258 6644 7262 6634 7288 5 N F
29 O
6746 11737 6649 11601 6769 11569 6817 11601 6792 11737 6823 11873
6769 11947 6728 11873 6746 11737 9 N F
55 O
6769 12499 6659 12551 6769 12643 6769 12567 6750 12551 6769 12542
6797 12551 6769 12567 6769 12643 6826 12686 6905 12736 7040 12812
7067 12686 7040 12668 6916 12551 6905 12541 6769 12499 17 N F
29 O
6769 12457 6682 12415 6688 12279 6732 12144 6769 12061 6881 12008
6905 11994 7040 11914 7067 12008 7040 12118 6990 12144 7006 12279
7040 12312 7158 12415 7176 12433 7190 12415 7225 12279 7311 12217
7361 12279 7446 12358 7582 12414 7583 12415 7619 12551 7636 12686
7582 12726 7510 12822 7446 12857 7446 12750 7494 12686 7536 12551
7446 12465 7311 12470 7268 12551 7265 12686 7311 12737 7446 12750
7446 12857 7311 12849 7263 12822 7176 12723 7154 12686 7040 12607
6981 12551 6905 12490 6905 12439 6993 12415 6905 12332 6776 12415
6905 12439 6905 12490 6769 12457 6769 12409 6855 12279 6769 12194
6742 12279 6769 12409 6769 12457 57 N F
15 O
6769 12409 6742 12279 6769 12194 6855 12279 6769 12409 5 N F
68 O
6769 12542 6750 12551 6769 12567 6797 12551 6769 12542 5 N F

15 O
6905 12439 6776 12415 6905 12332 6993 12415 6905 12439 5 N F
95 O
6905 11072 6806 11194 6875 11330 6905 11336 7040 11378 7168 11466
7176 11468 7311 11483 7446 11512 7531 11466 7446 11339 7434 11330
7446 11324 7550 11194 7446 11122 7325 11194 7311 11221 7176 11264
7086 11194 7040 11171 6905 11072 21 N F
82 O
6905 7313 6849 7262 6905 7234 6966 7262 6905 7313 5 N F
68 O
7040 11074 7020 11059 7040 11031 7145 10923 7176 10899 7214 10923
7243 11059 7176 11116 7040 11074 9 N F
95 O
7040 10639 7029 10652 7040 10717 7043 10652 7040 10639 5 N F
68 O
7176 10763 7070 10652 7176 10530 7295 10652 7176 10763 7176 10735
7264 10652 7176 10561 7097 10652 7176 10735 7176 10763 11 N F
55 O
7176 10735 7097 10652 7176 10561 7264 10652 7176 10735 7176 10706
7233 10652 7176 10593 7125 10652 7176 10706 7176 10735 11 N F
42 O
7176 10706 7125 10652 7176 10593 7233 10652 7176 10706 7176 10677
7202 10652 7176 10625 7152 10652 7176 10677 7176 10706 11 N F
82 O
7176 9249 7140 9296 7176 9375 7237 9296 7176 9249 5 N F
29 O
7176 10677 7152 10652 7176 10625 7202 10652 7176 10677 5 N F
55 O
7176 11966 7162 12008 7176 12057 7202 12008 7176 11966 5 N F
95 O
7311 7795 7220 7804 7311 7814 7330 7804 7311 7795 5 N F
15 O
7311 12737 7265 12686 7268 12551 7311 12470 7446 12465 7536 12551
7494 12686 7446 12750 7311 12737 9 N F
29 O
7311 11934 7266 11873 7281 11737 7311 11698 7446 11735 7449 11737
7463 11873 7456 12008 7446 12026 7399 12008 7311 11934 11 N F
148 O
7446 5798 7322 5906 7446 6025 7458 6041 7582 6147 7717 6130
7853 6108 7988 6122 8123 6174 8132 6177 8259 6214 8385 6313
8394 6318 8530 6396 8589 6448 8665 6496 8791 6584 8801 6590
8936 6623 8936 6169 8801 6124 8668 6041 8665 6038 8591 5906
8601 5770 8665 5738 8801 5702 8936 5704 9000 5770 9071 5830

9097 5906 9114 6041 9071 6121 8936 6169 8936 6623 9071 6639
9138 6584 9152 6448 9206 6313 9207 6311 9342 6230 9361 6177
9390 6041 9373 5906 9342 5874 9207 5901 9101 5770 9108 5634
9185 5499 9154 5499 8987 5499 8820 5499 8652 5499 8485 5499
8427 5499 8394 5507 8259 5573 8123 5605 7988 5622 7930 5634
7853 5667 7717 5699 7582 5753 7537 5770 7446 5798 65 N F
68 O
7446 8111 7379 8075 7446 8046 7582 8038 7624 8075 7717 8136
7853 8166 7942 8211 7853 8256 7717 8286 7582 8247 7572 8211
7446 8111 13 N F
7502 8754 7459 8618 7582 8498 7704 8618 7717 8629 7853 8696
7979 8618 7988 8606 8005 8618 8113 8754 8097 8889 8064 9025
7988 9053 7988 8841 8066 8754 7988 8668 7895 8754 7988 8841
7988 9053 7925 9025 7853 9018 7853 8980 7950 8889 7853 8787
7751 8889 7853 8980 7853 9018 7781 9025 7717 9139 7717 8851
7816 8754 7717 8670 7621 8754 7717 8851 7717 9139 7646 9025
7624 8889 7582 8839 7582 8705 7655 8618 7582 8546 7508 8618
7582 8705 7582 8839 7502 8754 45 N F
55 O
7582 8705 7508 8618 7582 8546 7655 8618 7582 8705 7582 8646
7605 8618 7582 8595 7558 8618 7582 8646 7582 8705 11 N F
68 O
7582 7756 7509 7669 7582 7575 7684 7533 7717 7524 7853 7515
7988 7465 8123 7443 8259 7460 8394 7465 8530 7481 8665 7495
8723 7533 8795 7669 8665 7786 8530 7768 8394 7707 8259 7706
8123 7704 7988 7737 7936 7804 7988 7848 8086 7940 8123 8048
8177 8075 8123 8093 7988 8116 7975 8075 7853 7984 7813 7940
7738 7804 7717 7768 7582 7756 33 N F
7582 9174 7541 9160 7582 9143 7639 9160 7582 9174 5 N F
7582 7470 7557 7397 7582 7363 7610 7397 7582 7470 5 N F
42 O
7582 8646 7558 8618 7582 8595 7605 8618 7582 8646 5 N F
95 O
7582 11867 7580 11873 7582 11879 7690 12008 7717 12059 7853 12088
7917 12008 7970 11873 7853 11770 7717 11791 7582 11867 11 N F
55 O
7717 8851 7621 8754 7717 8670 7816 8754 7717 8851 7717 8802
7766 8754 7717 8712 7669 8754 7717 8802 7717 8851 11 N F
42 O
7717 8802 7669 8754 7717 8712 7766 8754 7717 8802 7717 8754
7717 8754 7717 8753 7717 8754 7717 8754 7717 8802 11 N F
68 O
7717 9440 7698 9432 7717 9407 7748 9432 7717 9440 5 N F

95 O
7717 11192 7710 11194 7717 11214 7724 11194 7717 11192 5 N F
42 O
7717 13373 7712 13364 7717 13359 7853 13305 7914 13229 7988 13101
8012 13093 8123 13081 8176 13093 8259 13155 8318 13229 8351 13364
8333 13500 8259 13545 8123 13579 7988 13525 7913 13500 7853 13456
7717 13373 19 N F
29 O
7717 8754 7717 8754 7717 8753 7717 8754 7717 8754 5 N F
68 O
7853 9675 7734 9567 7853 9436 7956 9567 7853 9675 7853 9641
7924 9567 7853 9476 7771 9567 7853 9641 7853 9675 11 N F
55 O
7853 8980 7751 8889 7853 8787 7950 8889 7853 8980 7853 8941
7908 8889 7853 8831 7795 8889 7853 8941 7853 8980 11 N F
7853 9641 7771 9567 7853 9476 7924 9567 7853 9641 7853 9608
7892 9567 7853 9517 7807 9567 7853 9608 7853 9641 11 N F
42 O
7853 8941 7795 8889 7853 8831 7908 8889 7853 8941 7853 8902
7866 8889 7853 8875 7838 8889 7853 8902 7853 8941 11 N F
7853 9608 7807 9567 7853 9517 7892 9567 7853 9608 7853 9575
7860 9567 7853 9558 7844 9567 7853 9575 7853 9608 11 N F
29 O
7853 8902 7838 8889 7853 8875 7866 8889 7853 8902 5 N F
7853 9575 7844 9567 7853 9558 7860 9567 7853 9575 5 N F
68 O
7988 11436 7881 11330 7988 11221 8094 11330 7988 11436 7988 11408
8066 11330 7988 11250 7909 11330 7988 11408 7988 11436 11 N F
55 O
7988 8841 7895 8754 7988 8668 8066 8754 7988 8841 7988 8788
8019 8754 7988 8720 7951 8754 7988 8788 7988 8841 11 N F
7988 11408 7909 11330 7988 11250 8066 11330 7988 11408 7988 11380
8037 11330 7988 11279 7938 11330 7988 11380 7988 11408 11 N F
121 O
7988 6800 7922 6855 7988 6881 8052 6855 7988 6800 5 N F
42 O
7988 11380 7938 11330 7988 11279 8037 11330 7988 11380 7988 11351
8009 11330 7988 11308 7967 11330 7988 11351 7988 11380 11 N F
95 O
7988 11465 7947 11466 7975 11601 7988 11646 7997 11601 8123 11469
8132 11466 8259 11349 8265 11330 8259 11324 8123 11269 8122 11330
7988 11465 13 N F
42 O

7988 8788 7951 8754 7988 8720 8019 8754 7988 8788 5 N F
29 O
7988 11351 7967 11330 7988 11308 8009 11330 7988 11351 5 N F
68 O
8123 8444 8097 8347 8123 8317 8228 8347 8123 8444 5 N F
55 O
8394 9253 8296 9160 8394 9102 8459 9025 8530 8916 8665 8938
8748 9025 8665 9121 8626 9160 8530 9215 8394 9253 11 N F
8394 9506 8303 9432 8394 9309 8479 9432 8394 9506 8394 9475
8444 9432 8394 9359 8341 9432 8394 9475 8394 9506 11 N F
8394 8445 8311 8347 8394 8251 8530 8253 8615 8347 8665 8458
8719 8482 8790 8618 8665 8734 8665 8681 8733 8618 8665 8536
8600 8618 8665 8681 8665 8734 8545 8618 8530 8604 8530 8553
8617 8482 8570 8347 8530 8303 8394 8309 8362 8347 8394 8385
8467 8482 8530 8553 8530 8604 8422 8482 8394 8445 29 N F
42 O
8394 9475 8341 9432 8394 9359 8444 9432 8394 9475 8394 9444
8409 9432 8394 9410 8378 9432 8394 9444 8394 9475 11 N F
8394 8385 8362 8347 8394 8309 8530 8303 8570 8347 8617 8482
8530 8553 8530 8502 8554 8482 8530 8377 8512 8482 8530 8502
8530 8553 8467 8482 8394 8385 15 N F
29 O
8394 9444 8378 9432 8394 9410 8409 9432 8394 9444 5 N F
55 O
8530 8101 8496 8075 8530 8053 8583 8075 8530 8101 5 N F
29 O
8530 8502 8512 8482 8530 8377 8554 8482 8530 8502 5 N F
55 O
8665 9926 8580 9838 8665 9753 8764 9838 8665 9926 8665 9889
8723 9838 8665 9789 8615 9838 8665 9889 8665 9926 11 N F
161 O
8601 5770 8591 5906 8665 6038 8668 6041 8801 6124 8936 6169
9071 6121 9114 6041 9097 5906 9071 5830 9000 5770 8936 5704
8801 5702 8665 5738 8601 5770 15 N F
42 O
8665 8681 8600 8618 8665 8536 8733 8618 8665 8681 8665 8627
8675 8618 8665 8605 8655 8618 8665 8627 8665 8681 11 N F
8665 9889 8615 9838 8665 9789 8723 9838 8665 9889 8665 9853
8682 9838 8665 9824 8651 9838 8665 9853 8665 9889 11 N F
15 O
8642 15805 8642 15941 8665 15968 8801 15973 8927 15941 8936 15938
8943 15941 8956 16076 9053 16212 9111 16212 9207 16118 9223 16076
9207 15965 9195 15941 9115 15805 9081 15670 9071 15645 8957 15534

8936 15522 8801 15432 8713 15534 8693 15670 8665 15751 8642 15805
24 N F
29 O
8665 9853 8651 9838 8665 9824 8682 9838 8665 9853 5 N F
8665 8627 8655 8618 8665 8605 8675 8618 8665 8627 5 N F
82 O
8936 12248 8856 12279 8936 12326 9071 12394 9149 12415 9207 12435
9342 12440 9400 12415 9478 12341 9613 12325 9695 12279 9613 12247
9478 12270 9342 12242 9207 12252 9071 12240 8936 12248 17 N F
55 O
9071 9554 8961 9432 9071 9314 9178 9296 9207 9266 9272 9296
9304 9432 9207 9513 9071 9554 9071 9516 9188 9432 9071 9350
8994 9432 9071 9516 9071 9554 15 N F
42 O
9071 11154 8985 11059 9071 10965 9169 11059 9071 11154 9071 11118
9132 11059 9071 11001 9018 11059 9071 11118 9071 11154 11 N F
9071 9516 8994 9432 9071 9350 9188 9432 9071 9516 9071 9479
9137 9432 9071 9385 9028 9432 9071 9479 9071 9516 11 N F
29 O
9071 11118 9018 11059 9071 11001 9132 11059 9071 11118 9071 11081
9094 11059 9071 11037 9051 11059 9071 11081 9071 11118 11 N F
9071 9479 9028 9432 9071 9385 9137 9432 9071 9479 9071 9442
9086 9432 9071 9421 9062 9432 9071 9442 9071 9479 11 N F
15 O
9071 11081 9051 11059 9071 11037 9094 11059 9071 11081 5 N F
9053 16212 9053 16212 9111 16212 9111 16212 9053 16212 5 N F
9111 16212 9053 16212 9053 16212 9071 16212 9111 16212 9111 16212
6 N F
9071 9442 9062 9432 9071 9421 9086 9432 9071 9442 5 N F
121 O
9207 5858 9136 5770 9167 5634 9207 5577 9299 5634 9342 5758
9357 5770 9342 5773 9207 5858 9207 5815 9277 5770 9207 5667
9171 5770 9207 5815 9207 5858 15 N F
108 O
9207 5815 9171 5770 9207 5667 9277 5770 9207 5815 9207 5772
9209 5770 9207 5766 9205 5770 9207 5772 9207 5815 11 N F
135 O
9234 5499 9185 5499 9185 5499 9233 5499 9234 5499 5 N F
9185 5499 9185 5499 9207 5499 9233 5499 9233 5499 9185 5499
6 N F
95 O
9207 5772 9205 5770 9207 5766 9209 5770 9207 5772 5 N F
148 O

9321 5499 9234 5499 9342 5548 9478 5571 9613 5524 9666 5499
9655 5499 9488 5499 9321 5499 9 N F
108 O
9342 6694 9294 6584 9342 6504 9401 6584 9342 6694 9342 6584
9342 6584 9342 6583 9342 6584 9342 6584 9342 6694 11 N F
55 O
9342 11619 9326 11601 9342 11556 9365 11601 9342 11619 5 N F
95 O
9342 6584 9342 6584 9342 6583 9342 6584 9342 6584 5 N F
42 O
9478 10903 9370 10788 9433 10652 9471 10516 9478 10507 9506 10381
9613 10252 9748 10272 9852 10381 9779 10516 9772 10652 9748 10692
9619 10788 9613 10792 9613 10668 9631 10652 9656 10516 9748 10482
9818 10381 9748 10307 9613 10294 9541 10381 9579 10516 9589 10652
9613 10668 9613 10792 9478 10903 9478 10863 9567 10788 9478 10685
9407 10788 9478 10863 9478 10903 33 N F
29 O
9478 10863 9407 10788 9478 10685 9567 10788 9478 10863 9478 10823
9519 10788 9478 10740 9444 10788 9478 10823 9478 10863 11 N F
15 O
9478 10823 9444 10788 9478 10740 9519 10788 9478 10823 5 N F
42 O
9478 12021 9456 12008 9478 11990 9488 12008 9478 12021 5 N F
95 O
9546 6313 9505 6177 9508 6041 9527 5906 9603 5770 9613 5761
9623 5770 9748 5880 9772 5906 9830 6041 9873 6177 9884 6223
9920 6313 9956 6448 9991 6584 10019 6660 10065 6719 10088 6855
10054 6991 10019 7109 9884 7066 9884 6967 10019 6921 10042 6855
10019 6733 10017 6719 9968 6584 9931 6448 9884 6323 9879 6313
9846 6177 9805 6041 9748 5929 9726 5906 9613 5806 9553 5906
9528 6041 9524 6177 9569 6313 9613 6384 9651 6448 9641 6584
9632 6719 9703 6855 9748 6905 9884 6967 9884 7066 9843 6991
9748 6945 9665 6855 9613 6736 9602 6719 9613 6668 9620 6584
9628 6448 9613 6423 9546 6313 57 N F
42 O
9613 9552 9519 9432 9613 9299 9709 9432 9613 9552 9613 9503
9669 9432 9613 9353 9558 9432 9613 9503 9613 9552 11 N F
82 O
9569 6313 9524 6177 9528 6041 9553 5906 9613 5806 9726 5906
9748 5929 9805 6041 9846 6177 9879 6313 9884 6323 9931 6448
9968 6584 10017 6719 10019 6733 10042 6855 10019 6921 9884 6967
9884 6923 9986 6855 9985 6719 9945 6584 9906 6448 9884 6389
9846 6313 9820 6177 9780 6041 9748 5980 9677 5906 9613 5848

9579 5906 9548 6041 9543 6177 9593 6313 9613 6346 9674 6448
9663 6584 9656 6719 9740 6855 9748 6864 9884 6923 9884 6967
9748 6905 9703 6855 9632 6719 9641 6584 9651 6448 9613 6384
9569 6313 49 N F
29 O
9579 10516 9541 10381 9613 10294 9748 10307 9818 10381 9748 10482
9656 10516 9631 10652 9613 10668 9613 10464 9748 10433 9784 10381
9748 10343 9613 10336 9575 10381 9613 10464 9613 10668 9589 10652
9579 10516 19 N F
68 O
9593 6313 9543 6177 9548 6041 9579 5906 9613 5848 9677 5906
9748 5980 9780 6041 9820 6177 9846 6313 9884 6389 9906 6448
9945 6584 9985 6719 9986 6855 9884 6923 9884 6878 9918 6855
9953 6719 9923 6584 9884 6457 9877 6448 9813 6313 9793 6177
9754 6041 9748 6030 9629 5906 9613 5891 9604 5906 9568 6041
9562 6177 9613 6306 9623 6313 9697 6448 9685 6584 9680 6719
9748 6826 9831 6855 9884 6878 9884 6923 9748 6864 9740 6855
9656 6719 9663 6584 9674 6448 9613 6346 9593 6313 47 N F
29 O
9613 9503 9558 9432 9613 9353 9669 9432 9613 9503 9613 9453
9630 9432 9613 9408 9596 9432 9613 9453 9613 9503 11 N F
55 O
9613 6306 9562 6177 9568 6041 9604 5906 9613 5891 9629 5906
9748 6030 9754 6041 9793 6177 9813 6313 9877 6448 9884 6457
9923 6584 9953 6719 9918 6855 9884 6878 9884 6815 9921 6719
9900 6584 9884 6532 9819 6448 9780 6313 9767 6177 9748 6113
9696 6041 9613 5954 9589 6041 9580 6177 9613 6258 9687 6313
9721 6448 9706 6584 9704 6719 9748 6788 9884 6815 9884 6878
9831 6855 9748 6826 9680 6719 9685 6584 9697 6448 9623 6313
9613 6306 43 N F
15 O
9613 10464 9575 10381 9613 10336 9748 10343 9784 10381 9748 10433
9613 10464 9613 10387 9748 10383 9750 10381 9748 10379 9613 10377
9610 10381 9613 10387 9613 10464 15 N F
42 O
9613 6258 9580 6177 9589 6041 9613 5954 9696 6041 9748 6113
9767 6177 9780 6313 9819 6448 9884 6532 9900 6584 9921 6719
9884 6815 9748 6788 9748 6751 9884 6733 9889 6719 9884 6672
9851 6584 9760 6448 9748 6334 9744 6448 9728 6584 9728 6719
9748 6751 9748 6788 9704 6719 9706 6584 9721 6448 9687 6313
9613 6258 9613 6211 9709 6177 9628 6041 9613 6026 9609 6041
9599 6177 9613 6211 9613 6258 39 N F
15 O

9613 9453 9596 9432 9613 9408 9630 9432 9613 9453 5 N F
29 O
9613 6211 9599 6177 9609 6041 9613 6026 9628 6041 9709 6177
9613 6211 7 N F
2 O
9613 10387 9610 10381 9613 10377 9748 10379 9750 10381 9748 10383
9613 10387 7 N F
9613 13636 9613 13635 9613 13626 9632 13635 9613 13636 5 N F
55 O
9748 8124 9712 8075 9748 8042 9831 8075 9748 8124 5 N F
29 O
9728 6719 9728 6584 9744 6448 9748 6334 9760 6448 9851 6584
9884 6672 9889 6719 9884 6733 9748 6751 9728 6719 11 N F
42 O
9884 9243 9794 9160 9884 9086 9960 9160 9884 9243 9884 9189
9911 9160 9884 9134 9852 9160 9884 9189 9884 9243 11 N F
9884 8651 9851 8618 9884 8553 9919 8618 9884 8651 5 N F
29 O
9884 9189 9852 9160 9884 9134 9911 9160 9884 9189 5 N F
55 O
9884 7817 9871 7804 9884 7793 9990 7804 9884 7817 5 N F
15 O
9884 15795 9877 15805 9879 15941 9884 15952 9895 15941 9891 15805
9884 15795 7 N F
55 O
10019 10236 9893 10110 10019 9986 10139 10110 10019 10236 10019 10195
10101 10110 10019 10026 9934 10110 10019 10195 10019 10236 11 N F
42 O
10019 10195 9934 10110 10019 10026 10101 10110 10019 10195 10019 10155
10062 10110 10019 10066 9974 10110 10019 10155 10019 10195 11 N F
29 O
10019 10155 9974 10110 10019 10066 10062 10110 10019 10155 10019 10114
10023 10110 10019 10106 10015 10110 10019 10114 10019 10155 11 N F
121 O
10019 5995 9999 5906 10019 5855 10042 5906 10019 5995 5 N F
15 O
10019 10114 10015 10110 10019 10106 10023 10110 10019 10114 5 N F
55 O
10155 7863 10028 7804 10155 7763 10261 7804 10155 7863 5 N F
42 O
10155 9524 10067 9432 10155 9341 10278 9296 10290 9193 10292 9160
10426 9041 10561 9052 10670 9160 10561 9295 10560 9296 10426 9414
10426 9373 10513 9296 10561 9235 10621 9160 10561 9100 10426 9090

10347 9160 10334 9296 10426 9373 10426 9414 10405 9432 10290 9531
10290 9489 10356 9432 10290 9350 10155 9384 10108 9432 10155 9480
10290 9489 10290 9531 10155 9524 33 N F
10155 8782 10097 8754 10155 8715 10203 8754 10155 8782 5 N F
29 O
10155 9480 10108 9432 10155 9384 10290 9350 10356 9432 10290 9489
10290 9446 10307 9432 10290 9410 10155 9426 10150 9432 10155 9437
10290 9446 10290 9489 10155 9480 15 N F
15 O
10155 9437 10150 9432 10155 9426 10290 9410 10307 9432 10290 9446
10155 9437 7 N F
42 O
10290 9794 10204 9703 10290 9606 10386 9703 10290 9794 10290 9751
10340 9703 10290 9652 10245 9703 10290 9751 10290 9794 11 N F
55 O
10290 8446 10209 8347 10290 8267 10375 8347 10290 8446 5 N F
29 O
10290 9751 10245 9703 10290 9652 10340 9703 10290 9751 10290 9707
10295 9703 10290 9698 10286 9703 10290 9707 10290 9751 11 N F
15 O
10290 9707 10286 9703 10290 9698 10295 9703 10290 9707 5 N F
29 O
10426 9373 10334 9296 10347 9160 10426 9090 10561 9100 10621 9160
10561 9235 10513 9296 10426 9373 10426 9331 10465 9296 10561 9175
10572 9160 10561 9149 10426 9139 10402 9160 10384 9296 10426 9331
10426 9373 19 N F
15 O
10426 9331 10384 9296 10402 9160 10426 9139 10561 9149 10572 9160
10561 9175 10465 9296 10426 9331 9 N F
121 O
10561 5532 10493 5499 10658 5499 10825 5499 10828 5499 10696 5523
10561 5532 7 N F
82 O
10561 8049 10504 8075 10561 8101 10573 8075 10561 8049 5 N F
42 O
10832 9262 10726 9160 10832 9052 10935 9160 10832 9262 10832 9210
10882 9160 10832 9108 10781 9160 10832 9210 10832 9262 11 N F
55 O
10832 7878 10779 7804 10832 7778 10871 7804 10832 7878 5 N F
29 O
10832 9210 10781 9160 10832 9108 10882 9160 10832 9210 5 N F
10967 9518 10897 9432 10967 9364 11094 9432 10967 9518 10967 9461
11011 9432 10967 9408 10943 9432 10967 9461 10967 9518 11 N F

2 O
10967 10150 10907 10110 10960 9974 10967 9969 11089 9838 11103 9837
11236 9703 11238 9612 11298 9567 11353 9432 11373 9401 11469 9432
11509 9491 11566 9567 11644 9685 11650 9703 11644 9724 11566 9838
11509 9914 11442 9974 11373 9993 11238 10092 11219 10110 11103 10166
10967 10150 25 N F
15 O
10967 9461 10943 9432 10967 9408 11011 9432 10967 9461 5 N F
2 O
11088 13093 11060 12957 11103 12870 11131 12822 11238 12689 11239 12686
11373 12681 11509 12626 11644 12657 11724 12551 11780 12511 11915 12512
11958 12551 12051 12641 12053 12686 12064 12822 12081 12957 12090 13093
12051 13126 11915 13172 11796 13229 11781 13364 11782 13500 11780 13511
11762 13500 11765 13364 11733 13229 11644 13177 11509 13121 11373 13117
11238 13105 11103 13094 11088 13093 33 N F
29 O
12520 13500 12460 13635 12592 13722 12711 13635 12728 13590 12753 13500
12728 13458 12592 13400 12520 13500 9 N F
108 O
12864 5499 12864 5499 12998 5499 12998 5499 12864 5499 5 N F
29 O
12998 13015 12888 13093 12998 13186 12998 13015 4 N F
12998 13438 12978 13500 12998 13532 12998 13438 4 N F
108 O
12998 5499 12998 5499 12998 9519 12998 9519 12998 5499 12998 5499
6 N F
95 O
12998 10298 12998 9519 12998 9519 12998 10298 12998 10298 5 N F
82 O
12998 10876 12998 10298 12998 10298 12998 10876 12998 10876 5 N F
68 O
12998 11327 12998 10876 12998 10876 12998 11327 12998 11327 5 N F
55 O
12998 11692 12998 11327 12998 11327 12998 11692 12998 11692 5 N F
42 O
12998 12298 12998 11692 12998 11692 12998 12298 12998 12298 5 N F
29 O
12998 12909 12998 12298 12998 12298 12998 12909 12998 12909 5 N F
15 O
12998 13015 12998 12909 12998 12909 12998 13015 12998 13015 5 N F
29 O
12998 13186 12998 13015 12998 13015 12998 13186 12998 13186 5 N F
15 O

12998 13438 12998 13186 12998 13186 12998 13438 12998 13438 5 N F
29 O
12998 13532 12998 13438 12998 13438 12998 13532 12998 13532 5 N F
15 O
12998 14632 12998 13532 12998 13532 12998 14632 12998 14632 5 N F
2 O
12998 16212 12998 14632 12998 14632 12998 16212 12998 16212 12998 16212
6 N F
95 O
12998 9519 12998 10298 12998 10298 12998 10245 12998 10110 12998 9974
12998 9838 12998 9703 12998 9567 12998 9519 12998 9519 11 N F
82 O
12998 10298 12998 10876 12998 10876 12998 10788 12998 10652 12998 10516
12998 10381 12998 10298 12998 10298 9 N F
68 O
12998 10876 12998 11327 12998 11327 12998 11194 12998 11059 12998 10923
12998 10876 12998 10876 8 N F
55 O
12998 11327 12998 11692 12998 11692 12998 11601 12998 11466 12998 11330
12998 11327 12998 11327 8 N F
42 O
12998 11692 12998 12298 12998 12298 12998 12279 12998 12144 12998 12008
12998 11873 12998 11737 12998 11692 12998 11692 10 N F
29 O
12998 12298 12998 12909 12998 12909 12998 12822 12998 12686 12998 12551
12998 12415 12998 12298 12998 12298 9 N F
15 O
12998 12909 12998 13015 12998 13015 12998 12957 12998 12909 12998 12909
6 N F
29 O
12998 13015 12998 13015 12998 13186 12998 13186 12998 13093 12998 13015
6 N F
15 O
12998 13186 12998 13438 12998 13438 12998 13364 12998 13229 12998 13186
12998 13186 7 N F
29 O
12998 13438 12998 13438 12998 13532 12998 13532 12998 13500 12998 13438
6 N F
15 O
12998 13532 12998 14632 12998 14632 12998 14585 12998 14449 12998 14314
12998 14178 12998 14042 12998 13907 12998 13771 12998 13635 12998 13532
12998 13532 13 N F
Ls Gr Gs Lr

-1150 1075 16450 18700 Oc
1 O
10637 15408 10638 15449 10677 15486 10674 15526 10709 15568 10751 15601
10790 15639 10825 15680 10870 15710 10918 15735 10944 15783 10983 15820
11028 15850 11078 15870 11120 15905 11158 15942 11194 15973 11245 16000
18 P
11245 16000 11297 16011 11327 16055 11353 16103 11387 16145 11417 16190
11426 16212 7 P
12837 16212 12838 16205 12833 16178 12809 16167 12782 16162 12764 16142
12740 16129 12715 16118 12694 16100 12678 16079 12653 16071 12634 16051
12630 16024 12623 15998 12618 15971 12605 15948 12581 15936 12558 15921
12542 15900 12525 15879 12503 15863 12479 15851 12455 15838 12431 15826
12423 15800 12446 15786 12441 15759 12416 15750 12398 15729 12374 15717
12357 15697 12331 15689 12321 15664 12295 15673 12280 15650 12255 15641
12228 15644 12201 15650 12179 15634 12166 15610 12143 15595 12146 15568
12125 15551 12105 15533 12078 15526 12065 15503 12038 15504 12020 15484
12007 15460 12000 15434 50 P
12000 15434 12006 15408 12032 15400 12038 15374 12026 15350 12023 15323
12031 15296 12042 15272 12050 15246 12037 15269 12010 15266 11 P
12010 15266 11984 15258 12010 15249 12004 15223 11981 15208 11977 15181
11992 15159 12015 15145 12042 15142 12069 15142 12096 15135 12121 15126
12144 15140 12159 15118 12176 15096 12193 15075 12210 15054 12232 15039
12253 15021 12276 15006 12303 15004 12325 14988 12344 14969 12355 14944
12381 14953 12407 14944 12430 14931 12435 14905 28 P
12435 14905 12445 14879 12460 14857 12473 14833 12491 14812 12500 14787
12516 14765 12531 14742 12544 14718 12560 14697 12571 14672 12583 14648
12603 14628 12619 14606 12637 14587 12653 14565 12672 14545 12689 14523
12708 14505 12728 14486 12749 14468 12769 14451 12790 14433 12795 14407
12815 14388 12835 14369 12841 14343 12861 14324 12875 14301 12896 14285
12911 14262 12931 14243 12947 14222 12957 14196 12964 14169 12976 14145
12995 14126 12998 14101 38 P
12998 13048 12994 13048 12998 13033 3 P
12998 13020 12976 13010 12955 12993 12948 13019 12949 13046 12933 13024
12906 13022 12880 13013 12852 13015 9 P
12852 13015 12826 13018 12803 13034 12779 13047 12786 13021 12794 12995
6:00 PM
12794 12995 12768 13002 12740 13004 12727 12980 12744 12959 12726 12939
12723 12912 12722 12884 12740 12864 12738 12837 12727 12812 12705 12828
12689 12850 12715 12844 12709 12870 12682 12870 12685 12897 12660 12888
12635 12899 12626 12874 12598 12873 12573 12882 12547 12892 12546 12920
12544 12947 12536 12920 12511 12911 12487 12897 12465 12881 12439 12889
12412 12883 12395 12861 12372 12848 12354 12869 12348 12843 12362 12819
12386 12806 12407 12824 12433 12830 12439 12804 12435 12776 12451 12754

12460 12728 12461 12700 12435 12692 12433 12720 12439 12746 12412 12752
12386 12746 12387 12718 50 P
12387 12718 12363 12704 12351 12729 12360 12755 12349 12779 12334 12803
12322 12827 12310 12803 12284 12795 12256 12794 12282 12784 12259 12769
12266 12743 12284 12722 12307 12706 12329 12690 12328 12662 12301 12662
12274 12668 12271 12641 12298 12646 12295 12619 12274 12602 12263 12577
12242 12595 12225 12617 12206 12636 12191 12613 12165 12621 12173 12648
12185 12672 12189 12646 12212 12660 12212 12688 12211 12715 12231 12696
12249 12718 12245 12745 12219 12734 12193 12726 12176 12705 12150 12694
12130 12676 12106 12662 12103 12635 12094 12609 12105 12584 12089 12561
12061 12563 12035 12556 50 P
12035 12556 12021 12580 2 P
12021 12580 12019 12607 12008 12633 12006 12605 12006 12578 11982 12565
11975 12538 11953 12522 11948 12495 11931 12474 11909 12458 11907 12485
11884 12500 11901 12522 11876 12534 11869 12561 11887 12581 11907 12600
11880 12601 11858 12584 11833 12595 11811 12612 11785 12617 11772 12641
11770 12668 11778 12695 11801 12680 11804 12653 11818 12629 11843 12619
11871 12621 11850 12639 11824 12648 11825 12676 11852 12677 11858 12650
11885 12643 11912 12646 11903 12671 11881 12689 11854 12692 11827 12690
11848 12708 11875 12704 11882 12731 11909 12735 11934 12747 11906 12749
11903 12776 11879 12789 50 P
11879 12789 11876 12817 11875 12789 11884 12763 11884 12736 11857 12736
11833 12723 11823 12749 11830 12775 11819 12800 11835 12823 11808 12819
11792 12797 11771 12815 11796 12825 11809 12849 11786 12864 11766 12884
11769 12856 11749 12875 11741 12901 11767 12895 11780 12920 11778 12892
11796 12873 11815 12852 11832 12873 11843 12898 11816 12905 11794 12920
11797 12948 11771 12955 11780 12981 11786 13008 11792 13034 11814 13018
11823 13044 11796 13049 11809 13073 11813 13100 11825 13124 11851 13133
11877 13142 11904 13140 11903 13167 11875 13167 11848 13162 11821 13160
11807 13184 11826 13204 50 P
11826 13204 11846 13222 2 P
11846 13222 11865 13243 11892 13244 11897 13271 11922 13282 11906 13304
11926 13324 11945 13344 11918 13338 11890 13336 11864 13330 11855 13356
11871 13379 11895 13391 11916 13408 11941 13420 11962 13437 11963 13464
11957 13438 11935 13422 11910 13411 11886 13398 11866 13417 11854 13441
11833 13458 11807 13467 11829 13484 11807 13500 11804 13528 11790 13551
11803 13575 11782 13592 11780 13619 11803 13633 11804 13649 11779 13621
11765 13644 11779 13668 11755 13680 11756 13708 11758 13735 11753 13762
11730 13746 11713 13726 11708 13753 11700 13778 11680 13760 11694 13736
11689 13709 11714 13698 50 P
11714 13698 11717 13671 11729 13647 11740 13621 11719 13605 11703 13627
11699 13654 11692 13680 11676 13701 11676 13729 11658 13749 11632 13741
11633 13768 11608 13780 11598 13754 11592 13781 11601 13806 11628 13812

11627 13840 11634 13866 11658 13878 11658 13905 11664 13879 11670 13853
11669 13825 11691 13810 11675 13832 11699 13845 11715 13867 11696 13888
11674 13903 11695 13921 11721 13930 11729 13904 11737 13877 11738 13850
11744 13877 11748 13904 11768 13886 11758 13912 11739 13931 11764 13920
11753 13945 11777 13932 11801 13920 11824 13935 11844 13917 11871 13911
11887 13890 11892 13863 50 P
11892 13863 11906 13840 2 P
11906 13840 11906 13867 11926 13886 11947 13902 11957 13927 11938 13909
11911 13914 11893 13934 11874 13954 11847 13960 11854 13986 11866 14011
11861 14038 11839 14021 11821 14001 11804 14022 11817 14046 11810 14073
11837 14077 11858 14094 11834 14105 11810 14118 11825 14140 11800 14149
11779 14166 11760 14186 11784 14197 11782 14225 11772 14250 11745 14258
11725 14276 11719 14302 11714 14329 11738 14341 11762 14354 11775 14330
11768 14304 11792 14291 11816 14279 11840 14267 11862 14251 11885 14241
11885 14243 11865 14254 11842 14268 11819 14285 11795 14296 11783 14320
11781 14347 11775 14374 50 P
11775 14374 11793 14397 11767 14384 3 P
11767 14384 11751 14363 11724 14358 11699 14369 11677 14384 11654 14400
11627 14397 11606 14380 11579 14376 11553 14367 11554 14340 11548 14314
11526 14330 11507 14349 11503 14376 11476 14382 11474 14355 11447 14357
11430 14378 11411 14397 11389 14413 11376 14437 11350 14444 11323 14443
11298 14453 11302 14427 11327 14416 11354 14416 11348 14390 11320 14391
11324 14364 11328 14337 11319 14311 11293 14314 11269 14299 11253 14278
11226 14273 11215 14248 11199 14270 11210 14295 11236 14301 11239 14329
11266 14331 11251 14353 11273 14368 11246 14371 11218 14369 11242 14355
11215 14350 11206 14324 50 P
11206 14324 11179 14324 11184 14351 11160 14365 11138 14349 11112 14340
11086 14348 11061 14359 11045 14381 11069 14394 11096 14390 11121 14401
11097 14413 11103 14439 11130 14444 11152 14428 11153 14454 11128 14464
11101 14462 11077 14451 11049 14448 11023 14456 11000 14469 10975 14459
10948 14452 10921 14456 10896 14463 10921 14471 10941 14489 10961 14506
10979 14532 11001 14518 11000 14511 11003 14507 11032 14527 11021 14531
11001 14521 10982 14539 10981 14566 10975 14593 10989 14616 10999 14641
11019 14659 11029 14685 11018 14709 11045 14711 11069 14723 11073 14750
11065 14776 11083 14755 50 P
11083 14755 11106 14769 2 P
11106 14769 11132 14775 11155 14791 11181 14794 11206 14807 11228 14792
11255 14796 11239 14818 11212 14819 11188 14831 11172 14809 11145 14812
11119 14803 11116 14830 11101 14853 11108 14827 11083 14835 11068 14858
11060 14884 11071 14909 11079 14935 11089 14960 11102 14984 11122 15002
11114 15028 11119 15055 11142 15069 11149 15095 11155 15121 11151 15148
11165 15173 11138 15166 11126 15190 11124 15217 11144 15235 11148 15262
11172 15276 11185 15277 11194 15291 11218 15303 11224 15307 11223 15309

11194 15292 11182 15281 11161 15281 11171 15308 11174 15322 11186 15317
11196 15319 11201 15327 50 P
11201 15327 11200 15329 11194 15323 11187 15319 11174 15323 11170 15311
11158 15287 11137 15270 11114 15255 11087 15255 11067 15274 11042 15266
11018 15279 10996 15295 10969 15294 10951 15315 10928 15302 10912 15323
10885 15331 10864 15348 10854 15323 10857 15296 10841 15274 10824 15253
10811 15277 10821 15302 10803 15321 10794 15347 10784 15372 10757 15375
10731 15381 10707 15394 10685 15410 10692 15437 10665 15435 10659 15408
10654 15381 10649 15355 10642 15381 10637 15408 40 P
10637 15408 10625 15383 10598 15378 10576 15363 10549 15357 10522 15358
10495 15360 10471 15372 10451 15353 10424 15353 10401 15368 10399 15395
10391 15369 10381 15344 10368 15320 10341 15326 10329 15302 10306 15316
10280 15321 10253 15325 10245 15300 10221 15286 10246 15275 10225 15259
10199 15249 10174 15258 10148 15248 10129 15229 10105 15217 10080 15208
10054 15197 10033 15180 10014 15161 9989 15150 9972 15129 9948 15142
9945 15116 9925 15097 9898 15100 9878 15082 9855 15068 9844 15042
9833 15018 9810 15004 9791 14984 9781 14960 9805 14948 9781 14936
9754 14931 9762 14905 50 P
9762 14905 9735 14899 9715 14882 9695 14899 9703 14873 9677 14882
9689 14857 9688 14830 9663 14843 9652 14868 9627 14878 9606 14861
9594 14837 9569 14825 9596 14819 9619 14806 9607 14781 9580 14779
9553 14778 9534 14758 9508 14750 9481 14750 9457 14738 9430 14739
9419 14715 9401 14694 9380 14711 9380 14738 9368 14762 9390 14779
9364 14786 9371 14812 9397 14819 9424 14819 9450 14829 9464 14852
9473 14877 9493 14859 9520 14862 9542 14878 9567 14889 9593 14898
9607 14921 9586 14938 9563 14954 9580 14975 9562 14995 9588 15002
9611 15016 9626 15038 50 P
9626 15038 9648 15053 2 P
9648 15053 9674 15060 9698 15073 9671 15075 9645 15068 9618 15068
9594 15056 9566 15054 9539 15051 9516 15064 9506 15095 9493 15127
9516 15136 9528 15153 9532 15164 9535 15186 9532 15213 9516 15234
9494 15251 9505 15276 9516 15301 9539 15315 9562 15329 9580 15350
9607 15349 9631 15362 9649 15382 9669 15400 9643 15408 9666 15422
9682 15443 9698 15465 9713 15487 9727 15510 9747 15529 9750 15556
9764 15578 9782 15599 9785 15626 9803 15646 9814 15671 9801 15694
9781 15712 9759 15728 9753 15737 9761 15755 9756 15781 9759 15787
9783 15775 9800 15779 50 P
9800 15779 9805 15785 9799 15794 9777 15793 9776 15806 9755 15817
9730 15833 9730 15831 9753 15814 9764 15808 9764 15802 9771 15794
9777 15784 9800 15790 9801 15784 9783 15778 9767 15786 9758 15790
9753 15785 9753 15772 9741 15772 9725 15787 9704 15804 9681 15818
9664 15840 9637 15838 9624 15861 9632 15887 9621 15912 9633 15936
9638 15962 9627 15937 9608 15919 9607 15892 9597 15867 9575 15851

9548 15849 9521 15846 9502 15865 9480 15881 9453 15880 9429 15892
9405 15880 9383 15895 9357 15898 9341 15876 9322 15857 9312 15831
9285 15836 9266 15816 50 P
9266 15816 9254 15792 2 P
9254 15792 9277 15777 9259 15757 9233 15751 9214 15730 9203 15706
9184 15686 9163 15670 9149 15646 9126 15633 9121 15607 9099 15591
9121 15575 9098 15561 9085 15537 9086 15510 9070 15488 9044 15482
9018 15475 8997 15458 8973 15446 8950 15432 8924 15423 8898 15415
8873 15404 8851 15390 8835 15368 27 P
8835 15368 8809 15361 8787 15344 8761 15337 8735 15330 8710 15320
8689 15303 8691 15276 8665 15265 8644 15249 8625 15230 8612 15206
8604 15180 8597 15154 8574 15140 8582 15114 8578 15088 8582 15061
8599 15040 8574 15029 8552 15014 8535 14992 8521 14969 8503 14949
8481 14933 8458 14920 8432 14910 8406 14907 8383 14891 8357 14890
8335 14906 8308 14907 8284 14896 8264 14878 8242 14861 8222 14843
8200 14827 8173 14832 8150 14846 8132 14866 8126 14892 8111 14915
8087 14928 8060 14933 44 P
8060 14933 8034 14928 8008 14919 7985 14904 7966 14885 7947 14865
7957 14840 7963 14814 7939 14803 7923 14780 7906 14760 7893 14736
7881 14712 7872 14686 7867 14659 7866 14632 7869 14605 17 P
7869 14605 7879 14580 7890 14555 7902 14531 7915 14507 7927 14483
7945 14462 7964 14443 7983 14424 8010 14417 8032 14402 11 P
8032 14402 8052 14382 8074 14367 8098 14354 8125 14357 8151 14354
8178 14357 8205 14354 8231 14346 8258 14343 8279 14325 8301 14342
8325 14353 8350 14364 8372 14380 8396 14393 8402 14366 8407 14393
8421 14370 8418 14343 8423 14316 8414 14290 8431 14311 8458 14311
8463 14285 8482 14265 8490 14239 8498 14212 8491 14186 8480 14162
8504 14151 8522 14130 8510 14106 8493 14084 8476 14063 8469 14037
8467 14010 8469 13983 8472 13955 8467 13929 8484 13908 8502 13887
8504 13860 8532 13861 8554 13847 8575 13828 8602 13825 8628 13819
8656 13818 8683 13819 50 P
8683 13819 8710 13822 8735 13812 8762 13814 8790 13814 8816 13821
8835 13840 8855 13858 8875 13877 8882 13903 8865 13925 8878 13949
8899 13966 8924 13976 8951 13984 8973 13999 8995 14015 9014 14034
9033 14054 9050 14075 9054 14102 9030 14113 9055 14122 9081 14130
9101 14149 9127 14155 9154 14160 9177 14174 9203 14182 9224 14201
9251 14201 9278 14198 9305 14202 9317 14177 9341 14165 9358 14144
9385 14145 9400 14123 9405 14096 9424 14077 9451 14072 9478 14075
9503 14085 9530 14089 9506 14077 9524 14056 9541 14035 9559 14016
9584 14003 9611 14002 50 P
9611 14002 9638 14007 2 P
9638 14007 9663 14017 9683 13998 9699 14020 9726 14025 9753 14029
9768 14006 9766 14034 9786 14053 9808 14067 9830 14084 9848 14063

9839 14038 9853 14014 9877 14027 9900 14013 9926 14006 9954 14007
9980 14014 9998 13994 10022 14005 10046 14018 10073 14017 10054 13997
10036 13977 10031 13950 10039 13924 10054 13902 10032 13886 10009 13872
9993 13850 9993 13823 9973 13805 10000 13802 10027 13806 10028 13779
10024 13752 10010 13728 10021 13753 9995 13747 9981 13723 9960 13706
9938 13690 9911 13695 9884 13696 9879 13723 9907 13726 9882 13737
9866 13759 9882 13781 50 P
9882 13781 9875 13807 9857 13786 9859 13758 9836 13744 9816 13725
9807 13751 9793 13775 9766 13770 9777 13744 9753 13733 9728 13721
9703 13710 9690 13686 9666 13673 9641 13663 9641 13636 9615 13643
9589 13636 9604 13659 9624 13676 9597 13674 9578 13655 9564 13678
9568 13651 9552 13629 9529 13615 9503 13605 9478 13595 9452 13588
9429 13574 9409 13555 9384 13565 9385 13538 9358 13537 9344 13561
9318 13568 9294 13554 9278 13533 9304 13539 9320 13516 9345 13506
9345 13479 9328 13458 9327 13430 9303 13444 9281 13460 9257 13449
9242 13426 9250 13400 50 P
9250 13400 9225 13392 2 P
9225 13392 9219 13365 9234 13343 9239 13316 9243 13289 9222 13272
9197 13260 9170 13256 9144 13251 9123 13233 9097 13224 9070 13230
9082 13254 9092 13280 9097 13307 9081 13329 9063 13349 9059 13322
9038 13305 9012 13314 8987 13304 8977 13278 8985 13252 9009 13238
9034 13230 9019 13207 9046 13203 9073 13207 9061 13182 9039 13166
9025 13143 9032 13169 9009 13154 8984 13144 8964 13125 8954 13100
8957 13073 8949 13046 8926 13061 8915 13037 8900 13013 8889 12988
8873 13010 8852 12993 8839 12968 8829 12994 8807 12977 8780 12981
8786 12954 8778 12928 50 P
8778 12928 8769 12902 8755 12878 8751 12852 8732 12832 8707 12819
8691 12798 8682 12771 8675 12745 8672 12718 8661 12693 8634 12698

12:00 PM

8634 12698 8613 12680 8598 12657 8595 12630 8595 12603 8586 12576
8587 12549 8600 12525 8593 12499 8612 12519 8634 12502 8659 12515
8684 12503 8711 12501 8715 12473 8729 12450 8750 12433 8773 12418
8798 12405 8820 12388 8846 12382 8864 12362 8865 12334 8877 12358
8904 12352 8931 12348 8957 12339 8982 12327 9001 12307 9027 12301
9052 12290 9077 12279 9091 12255 9096 12228 9109 12203 9117 12178
9125 12151 9135 12125 9144 12100 9145 12072 9153 12046 9154 12018
9180 12008 9174 11981 9200 11972 9208 11945 9219 11920 9215 11893
9222 11866 9239 11844 50 P
9239 11844 9261 11827 9251 11802 9249 11774 9256 11747 9275 11728
9285 11702 9296 11677 9315 11656 9330 11634 9336 11607 9350 11583
9378 11578 9387 11553 9393 11526 9405 11500 9406 11473 9398 11447
9377 11428 9402 11419 9416 11395 9398 11375 9382 11351 9409 11348
9428 11327 9446 11307 9474 11306 9500 11297 9526 11287 9551 11275

9576 11264 9602 11254 9624 11237 9637 11213 9636 11185 9642 11158
9627 11135 9647 11115 9659 11090 9684 11080 9712 11080 9738 11071
9764 11061 9788 11048 9810 11030 9828 11010 9847 10990 9856 10964
9869 10939 9883 10915 50 P
9883 10915 9894 10890 2 P
9894 10890 9888 10863 9862 10853 9835 10860 9809 10868 9781 10872
9755 10882 9731 10894 9705 10905 9687 10926 9661 10935 9634 10940
9609 10952 9583 10943 9557 10932 9530 10926 9516 10903 9488 10907
9461 10903 9435 10911 9429 10938 9419 10964 9400 10985 9383 11006
9360 11022 9344 11045 9319 11035 9292 11043 9264 11043 9239 11054
9213 11044 9189 11030 9168 11013 9146 10996 9125 10979 9098 10969
9071 10971 9044 10976 9058 10953 9086 10953 9112 10960 9137 10973
9164 10980 9191 10985 9203 11010 9230 11013 9256 11003 9284 11001
9311 11003 9338 11001 50 P
9338 11001 9354 10977 9366 10953 9380 10929 9358 10911 9377 10891
9401 10879 9429 10878 9457 10879 9482 10869 9509 10859 9530 10841
9554 10828 9576 10811 9594 10790 9608 10766 9626 10745 17 P
9626 10745 9650 10731 9675 10720 9697 10703 9712 10680 9740 10679
9764 10666 9788 10651 9810 10634 9828 10613 9842 10589 9856 10565
9869 10541 9882 10516 9895 10491 9909 10468 9919 10441 9912 10414
9889 10399 9862 10394 9834 10391 9806 10389 9779 10384 9752 10377
9725 10369 9701 10356 9681 10336 9666 10313 9642 10299 9615 10294
30 P
9615 10294 9591 10279 9569 10262 9554 10239 9526 10234 9502 10221
9482 10202 9467 10178 9467 10150 9451 10127 9435 10105 9409 10093
9382 10090 9363 10110 9336 10119 9308 10119 9280 10116 9253 10116
9225 10110 9200 10097 9193 10070 9194 10043 9168 10052 9140 10051
9123 10028 9103 10009 9127 10024 9146 10044 9168 10027 9196 10029
9212 10052 9222 10078 9245 10094 9273 10097 9300 10094 9325 10080
9313 10055 9322 10029 9340 10007 9334 9979 9343 9953 9370 9945
9345 9958 9364 9978 9390 9968 9417 9968 9444 9978 9465 9996
9489 10010 9511 10028 50 P
9511 10028 9530 10048 9552 10065 9577 10078 9604 10082 9632 10083
9660 10079 9684 10064 9706 10049 9726 10029 9745 10008 9762 9986
9781 9965 9796 9942 9811 9918 9835 9904 9862 9900 9890 9895
9915 9884 9943 9877 9954 9867 9960 9861 9965 9860 9993 9860
10021 9861 10045 9875 10037 9848 10014 9832 9988 9822 9967 9804
9952 9781 9930 9764 9913 9742 9890 9725 9873 9703 9855 9682
9833 9665 9806 9658 9779 9652 9752 9658 9734 9636 9723 9610
9731 9584 9746 9608 9773 9615 9764 9588 9783 9609 9806 9625
9833 9633 9851 9654 50 P
9851 9654 9879 9654 2 P
9879 9654 9886 9627 9911 9640 9895 9664 9895 9691 9920 9705

9947 9711 9974 9718 9993 9698 9976 9676 9973 9648 9988 9625
9987 9597 9962 9587 9964 9559 9962 9531 9987 9519 9992 9491
9974 9470 9950 9455 9923 9460 9922 9488 9916 9515 9926 9541
9905 9560 9898 9533 9900 9505 9893 9478 9872 9460 9845 9452
9836 9478 9834 9506 9842 9533 9831 9508 9830 9480 9813 9501
9785 9505 9781 9478 9773 9451 9749 9437 9748 9409 9776 9412
9803 9420 9830 9414 9833 9386 9805 9384 9784 9365 9810 9376
9837 9382 9863 9370 50 P
9863 9370 9852 9345 9872 9326 9854 9304 9826 9307 9800 9317
9815 9293 9842 9288 9855 9263 9863 9236 9843 9217 9824 9197
9800 9182 9771 9180 9743 9180 9716 9180 9742 9173 9770 9165
9786 9143 9794 9116 9806 9091 9822 9068 9821 9040 9835 9015
9844 8988 9857 8964 9845 8938 9827 8960 9804 8975 9776 8979
9761 8956 9748 8931 9726 8913 9733 8885 9712 8866 9691 8886
9674 8908 9666 8935 9651 8959 9668 8981 9641 8990 9615 8979
9605 8953 9580 8942 9602 8924 9592 8898 9577 8874 9552 8861
9538 8836 9540 8808 50 P
9540 8808 9527 8784 2 P
9527 8784 9528 8771 9501 8773 9473 8774 9445 8774 9428 8792
9423 8791 9395 8798 9367 8795 9346 8814 9348 8786 9376 8789
9403 8785 9423 8764 9451 8759 9475 8746 9478 8718 9462 8694
9444 8673 9429 8649 9417 8623 9389 8620 9385 8592 9386 8564
9366 8544 9339 8537 9357 8515 9370 8490 9397 8480 9383 8455
9384 8427 9356 8422 9330 8435 9325 8408 9338 8382 9321 8360
9321 8331 9306 8307 9290 8331 9279 8305 9255 8319 9244 8345
42 P
9244 8345 9250 8318 9264 8293 9249 8269 9221 8262 9193 8266
9203 8239 9198 8211 9179 8190 9151 8190 9178 8183 9180 8155
9152 8160 9124 8162 9117 8135 9089 8140 9077 8114 9101 8100
9126 8086 9115 8060 9141 8048 9120 8028 9121 8000 9095 7989
9071 7974 9043 7971 9034 7944 9013 7926 8985 7927 8992 7954
9008 7977 9037 7978 9057 7998 9056 8026 9081 8039 9094 8064
9070 8080 9041 8083 9032 8056 9016 8033 9011 8005 8989 8023
8961 8029 8964 8058 8989 8071 8961 8078 8978 8101 8952 8090
8947 8062 8926 8081 50 P
8926 8081 8909 8104 8899 8130 8900 8101 8908 8075 8917 8048
8893 8061 8871 8079 8844 8090 8848 8061 8843 8034 8866 8018
8840 8008 8842 7979 8864 7961 8890 7972 8918 7972 8946 7966
8965 7946 8969 7918 8981 7892 8966 7868 8943 7885 8915 7892
8894 7911 8871 7894 8858 7869 8885 7877 8898 7852 8904 7824
8926 7806 8952 7819 8960 7845 8978 7868 8995 7845 9019 7830
9045 7841 9027 7819 9000 7815 8972 7809 8947 7794 8920 7787
8892 7781 8869 7765 8897 7769 8886 7743 8869 7720 8849 7700

8833 7677 8852 7656 50 P
8852 7656 8880 7648 2 P
8880 7648 8906 7635 8933 7632 8927 7604 8918 7578 8890 7575
8881 7549 8883 7520 8891 7493 8878 7467 8849 7462 8823 7452
8806 7429 8820 7404 8828 7376 8853 7389 8867 7364 8873 7336
8900 7326 8907 7299 8901 7270 8880 7252 8879 7280 8878 7309
8851 7317 8841 7290 8826 7266 8829 7294 8812 7317 8807 7345
8779 7349 8754 7361 8763 7388 8740 7404 8728 7378 8713 7354
8693 7334 8668 7319 8646 7302 8618 7294 8639 7275 8662 7258
8686 7242 8676 7216 8703 7225 8726 7241 8743 7218 8763 7198
8735 7191 8729 7163 50 P
8729 7163 8700 7159 8672 7157 8648 7173 8647 7202 8631 7177
8641 7150 8655 7126 8628 7138 8600 7141 8611 7168 8613 7197
8587 7185 8604 7208 8576 7215 8547 7212 8521 7202 8540 7181
8561 7161 8544 7137 8528 7114 8500 7106 8509 7079 8533 7064
8561 7057 8574 7082 8573 7054 8586 7028 8558 7030 8529 7028
8533 6999 8561 6993 8537 6978 8510 6989 8482 6993 8464 6971
8436 6963 8441 6991 8429 7017 8416 6991 8415 7020 8395 7040
8401 7012 8408 6984 8380 6978 8354 6990 8378 6974 8382 6945
8402 6925 8425 6942 50 P
8425 6942 8440 6918 2 P
8440 6918 8437 6890 8423 6864 8395 6868 8393 6839 8404 6813
8384 6792 8359 6778 8360 6807 8335 6822 8309 6834 8284 6848
8294 6875 8272 6857 8278 6829 8253 6815 8224 6810 8197 6818
8185 6792 8157 6799 8164 6826 8154 6853 8125 6853 8122 6824
8108 6799 8091 6822 8082 6795 8053 6794 8079 6781 8074 6752
8050 6736 8023 6743 7996 6753 7968 6752 7953 6749 7941 6764
7940 6748 7951 6737 7974 6719 7999 6706 8017 6701 8045 6705
8074 6707 8077 6679 8105 6679 8119 6654 8126 6626 8098 6620
8076 6601 8054 6583 50 P
8054 6583 8033 6603 8013 6582 8024 6556 8005 6533 8001 6504
7976 6490 7953 6473 7937 6449 7925 6423 7929 6451 7946 6475
7936 6502 7909 6493 7890 6471 7894 6442 7871 6425 7859 6399
7857 6370 7855 6342 7838 6318 7839 6347 7848 6375 7839 6402
7817 6421 7790 6429 7762 6435 7733 6438 7757 6421 7785 6414
7786 6386 7776 6358 7758 6336 7735 6352 7723 6352 7711 6336
7687 6352 7676 6326 7671 6297 7669 6269 7651 6246 7659 6274
7641 6296 7641 6268 7616 6252 45 P
7616 6252 7594 6234 7572 6216 7564 6244 7558 6272 7532 6283
7540 6255 7515 6268 7487 6261 7461 6250 7469 6226 7455 6201
7454 6175 7455 6164 7434 6144 7423 6130 7401 6107 7372 6108
7343 6108 7317 6119 7294 6137 7274 6158 7245 6159 7273 6154
7279 6125 7292 6099 7315 6082 7343 6075 7379 6044 7358 6033

7341 6009 7319 6029 7297 6047 7268 6045 7271 6016 7296 6030
7291 6002 7278 5976 7284 5948 7287 5919 7266 5899 7258 5872
7229 5874 7201 5866 7172 5871 7149 5889 7125 5874 7102 5856
7082 5835 7060 5817 50 P
7060 5817 7031 5817 7046 5841 7029 5865 7027 5836 7003 5820
6976 5809 6949 5800 6921 5792 6899 5772 6882 5796 6866 5820
6839 5828 6815 5844 6786 5842 6765 5822 6747 5799 6730 5776
6747 5753 6768 5772 6754 5747 6757 5718 6731 5730 6706 5744
6677 5739 6649 5746 6637 5772 6666 5773 6641 5787 6615 5800
6576 5799 6577 5808 6599 5816 6569 5827 6544 5812 6515 5812
6487 5806 6472 5780 6459 5755 6430 5751 6417 5777 6392 5792
6408 5768 6431 5750 6415 5726 6399 5701 6403 5673 6383 5694
6354 5697 6325 5697 50 P
6325 5697 6314 5723 2 P
6314 5723 6317 5752 6315 5781 6338 5799 6346 5827 6325 5807
6296 5813 6270 5800 6242 5791 6216 5779 6202 5754 6180 5735
6209 5734 6209 5705 6182 5695 6208 5681 6234 5671 6229 5642
6201 5635 6173 5629 6169 5658 6160 5685 6142 5708 6114 5715
6086 5708 6057 5701 6036 5682 27 P
6036 5682 6061 5669 6088 5657 6066 5637 6037 5636 6008 5631
6024 5607 6052 5615 6074 5634 6091 5610 6119 5616 6114 5587
6086 5580 6058 5590 6050 5562 6070 5541 6043 5552 6014 5551
5986 5559 5967 5581 5938 5580 5909 5580 5880 5584 5861 5604
5881 5625 5903 5644 5928 5657 5956 5668 28 P
5956 5668 5927 5670 5899 5678 5872 5667 5853 5645 5860 5673
5843 5697 5824 5719 5812 5746 5797 5770 5791 5799 5773 5821
5746 5811 5740 5839 5721 5861 5706 5886 5712 5914 5724 5941
5706 5918 5684 5938 5678 5967 5680 5995 5655 6010 5629 6020
5601 6030 5617 6006 5646 6002 5663 5979 5662 5950 5656 5921
5627 5917 5599 5912 5628 5911 5655 5900 5665 5872 5689 5856
5703 5831 5675 5824 5651 5840 5671 5819 5687 5795 5704 5771
5676 5762 5655 5782 5675 5761 5690 5736 5667 5720 5682 5695
5682 5666 5668 5641 50 P
5668 5641 5660 5613 5684 5597 5690 5568 5676 5543 5666 5515
5657 5499 7 P
5650 5499 5645 5518 5645 5499 3 P
5597 5499 5593 5502 5574 5524 5557 5548 5548 5576 5530 5598
5538 5570 5552 5533 5545 5527 5540 5520 5540 5514 5532 5499

12:00 PM

5430 5499 5431 5521 5413 5544 5411 5573 5412 5602 5416 5630
5416 5659 5387 5661 5381 5690 5353 5694 5370 5670 5394 5654
5405 5627 5405 5598 5404 5569 5407 5540 5408 5511 5393 5499
18 P

2969 5499 2970 5505 2988 5527 2970 5550 2941 5551 2919 5531
2928 5504 2931 5499 8 P
12998 8165 12993 8160 12988 8165 12982 8169 12980 8163 12980 8156
12982 8149 12985 8142 12989 8136 12992 8131 12998 8126 12998 8125
12:00 PM
12998 8078 12998 8077 12991 8075 12985 8072 12981 8066 12978 8060
12975 8053 12975 8046 12970 8041 12974 8035 12979 8030 12973 8034
12967 8037 12959 8036 12957 8030 12958 8023 12962 8018 12965 8011
12971 8007 12976 8001 12976 7994 12976 7987 12976 7980 12974 7973
12973 7966 12979 7962 12986 7959 12990 7965 12996 7969 12998 7969
30 P
11823 7075 11826 7069 11831 7064 11837 7060 11844 7058 11845 7065
11847 7058 11853 7054 11859 7056 11866 7059 11873 7060 11880 7063
11886 7066 11894 7066 11901 7068 11908 7068 11915 7068 11922 7070
11929 7071 11934 7066 11930 7072 11925 7077 11921 7083 11916 7088
11909 7089 11902 7090 11895 7093 11891 7099 11884 7102 11877 7100
11871 7103 11864 7104 11861 7111 11862 7118 11857 7123 11850 7125
11846 7131 11845 7138 11841 7145 11836 7149 11831 7154 11826 7160
11824 7166 11817 7164 11820 7159 11825 7153 11828 7147 11832 7141
11836 7135 11839 7128 50 P
11839 7128 11832 7126 11832 7119 11834 7112 11831 7105 11825 7102
11823 7095 11829 7092 11834 7087 11838 7081 11833 7075 11823 7075
12:00 PM
11223 6838 11228 6834 11235 6836 11240 6841 11247 6842 11253 6837
11257 6831 11261 6825 11261 6818 11257 6812 11251 6809 11247 6804
11252 6798 11259 6797 11266 6794 11271 6790 11277 6786 11284 6785
11291 6787 11296 6793 11297 6800 11304 6798 11310 6794 11317 6797
11320 6804 11321 6811 11320 6818 11320 6825 11322 6832 11320 6839
11317 6845 11320 6851 11317 6858 11317 6865 11320 6871 11326 6876
11330 6882 11331 6889 11336 6894 11340 6899 11343 6906 11340 6912
11344 6918 11349 6923 11354 6928 11355 6935 11355 6942 11355 6950
11348 6949 11341 6951 50 P
11341 6951 11340 6943 11339 6936 11336 6930 11331 6925 11329 6918
11334 6913 11331 6907 11329 6900 11327 6893 11320 6895 11314 6892
11310 6886 11304 6884 11303 6876 11301 6870 11302 6863 11297 6858
11290 6858 11283 6857 11277 6853 11270 6854 11263 6852 11260 6859
11257 6865 11250 6868 11250 6860 11247 6854 11244 6847 11238 6852
11231 6851 11226 6846 11223 6838 33 P
11015 6752 11020 6746 11024 6741 11030 6737 11038 6738 11045 6736
11047 6743 11053 6748 11058 6743 11064 6739 11071 6738 11079 6738
11086 6737 11093 6736 11100 6736 11107 6737 11114 6735 11120 6731
11127 6729 11133 6733 11132 6740 11128 6746 11122 6749 11129 6747
11136 6745 11142 6741 11142 6748 11140 6755 11142 6762 11146 6768

11153 6766 11155 6773 11152 6779 11156 6786 11158 6792 11152 6795
11146 6799 11141 6804 11134 6806 11127 6807 11124 6814 11118 6810
11112 6814 11105 6813 11098 6810 11093 6805 11089 6810 11087 6817
11083 6822 11077 6827 50 P
11077 6827 11070 6829 11064 6825 11070 6821 11071 6814 11075 6808
11069 6811 11069 6818 11063 6815 11058 6809 11054 6804 11057 6797
11056 6790 11060 6784 11065 6779 11066 6772 11063 6778 11061 6771
11059 6764 11063 6758 11062 6751 11061 6758 11056 6763 11050 6767
11049 6774 11043 6769 11037 6773 11037 6766 11041 6759 11035 6764
11029 6767 11025 6761 11028 6755 11015 6752 34 P
9617 7077 9614 7063 9605 7052 9597 7040 9591 7027 9584 7014
9581 7000 9572 6990 9563 6978 9569 6965 9566 6951 9561 6937
9552 6927 9542 6917 9538 6903 9528 6893 9538 6883 9524 6884
9510 6882 9497 6877 9488 6865 9495 6852 9481 6849 9467 6843
9463 6830 9458 6816 9455 6802 9450 6788 9444 6776 9436 6764
9426 6754 9417 6742 9408 6731 9402 6719 9396 6705 9391 6692
9385 6679 9383 6665 9374 6653 9370 6639 9363 6627 9355 6615
9367 6607 9353 6607 9345 6594 9338 6582 9331 6570 9328 6556
9323 6542 9314 6531 50 P
9314 6531 9304 6521 9294 6510 9288 6497 9279 6486 9272 6473
9272 6459 9266 6446 9261 6433 9256 6420 9246 6409 9255 6397
9269 6396 9284 6394 9298 6396 9287 6387 9272 6385 9258 6384
9244 6387 9229 6386 9221 6374 9212 6363 9211 6348 9209 6335
9206 6320 9194 6312 9188 6299 9182 6286 9180 6272 9179 6257
9185 6244 9186 6230 9191 6217 9180 6209 9190 6198 9186 6184
9198 6175 9187 6165 9191 6151 9195 6137 9193 6123 9191 6109
9198 6096 9184 6098 9182 6084 9174 6072 9176 6058 9164 6049
9178 6044 9164 6041 50 P
9164 6041 9162 6027 2 P
9162 6027 9163 6013 9154 6003 9168 5999 9173 5985 9168 5971
9157 5962 9143 5959 9139 5973 9135 5959 9145 5949 9160 5952
9152 5940 9163 5931 9175 5922 9189 5927 9202 5931 9216 5935
9219 5921 9206 5914 9192 5912 9198 5899 9206 5886 9210 5872
9210 5858 9215 5844 9220 5831 9234 5827 9225 5816 9231 5803
9236 5789 9242 5776 9248 5763 9254 5749 9258 5735 9257 5721
9254 5707 9262 5694 9275 5688 9279 5675 9290 5665 9301 5656
9287 5661 9278 5672 9270 5684 9276 5671 9285 5659 9294 5649
9305 5638 9315 5628 50 P
9315 5628 9327 5620 9339 5612 9346 5625 9352 5612 9361 5601
9372 5591 9385 5584 9397 5577 9410 5571 9423 5564 9435 5556
9445 5545 9453 5533 9464 5523 9471 5511 9475 5499 17 P
9607 5499 9605 5510 9605 5525 9607 5539 9613 5552 9615 5567
9620 5580 9626 5594 9631 5607 9634 5622 9637 5636 9641 5650

9645 5664 9651 5677 9661 5687 9663 5702 9666 5716 9672 5729
9683 5738 9694 5748 9706 5756 9719 5763 9729 5773 9739 5783
9749 5794 9753 5808 9757 5821 9752 5835 9758 5848 9772 5854
9783 5863 9789 5876 9796 5889 9800 5903 9810 5913 9818 5925
9823 5939 9828 5952 9831 5967 9836 5980 9847 5990 9856 6001
9858 6016 9861 6030 9856 6043 9857 6057 9864 6070 9866 6084
9871 6098 9876 6111 50 P
9876 6111 9888 6120 9890 6134 9891 6149 9893 6163 9897 6177
9902 6190 9905 6205 9909 6218 9904 6232 9905 6246 9909 6260
9914 6273 9916 6288 9916 6302 9921 6315 9922 6330 9923 6344
9926 6358 9932 6372 9933 6386 9933 6400 9938 6414 9943 6428
9945 6442 9948 6456 9948 6470 9948 6485 9950 6499 9958 6510
9966 6522 9959 6534 9951 6546 9949 6560 9954 6574 9964 6584
9972 6596 9971 6610 9969 6624 9976 6637 9987 6645 9995 6657
10007 6665 10012 6679 10020 6691 10025 6705 10024 6719 10029 6733
10031 6747 49 P
10031 6747 10032 6761 10038 6774 10045 6786 10056 6796 10054 6810
10059 6823 10071 6831 10068 6845 10069 6859 10066 6873 10057 6884
10052 6898 10049 6912 10046 6926 10042 6940 10037 6953 10036 6967
10036 6982 10037 6996 10040 7010 10045 7024 10053 7035 10063 7046
10067 7059 10078 7068 10087 7080 10100 7087 10112 7093 10123 7102
10115 7114 10101 7113 10089 7120 10087 7134 10079 7146 10082 7160
10072 7169 10057 7169 10043 7170 10029 7167 10018 7176 10005 7182
9992 7175 9983 7185 9969 7191 9958 7200 9963 7213 9949 7209
9938 7218 9930 7230 50 P
9930 7230 9926 7244 9914 7252 9902 7259 9887 7258 9873 7253
9860 7249 9848 7242 9839 7231 9830 7219 9823 7207 9816 7195
9827 7185 9814 7181 9804 7170 9791 7164 9778 7157 9764 7155
9750 7152 9737 7153 9723 7148 9710 7142 9698 7135 9685 7127
9672 7124 9659 7118 9647 7110 9634 7103 9624 7093 9617 7077
30 P
12967 11650 12974 11649 12981 11649 12985 11643 12991 11642 12995 11648
12998 11649 7 P
12998 11741 12997 11742 12992 11747 12985 11748 12980 11744 12978 11737
12979 11730 12983 11725 12981 11718 12982 11712 12988 11707 12984 11701
12987 11695 12989 11689 12995 11685 12996 11678 12989 11678 12994 11674
12988 11675 12990 11669 12985 11665 12984 11658 12990 11660 12996 11664
12992 11659 12991 11652 12985 11654 12979 11658 12976 11664 12972 11670
12970 11663 12967 11650 32 P
11804 11890 11809 11885 11813 11879 11817 11874 11823 11870 11829 11867
11836 11866 11841 11862 11845 11856 11850 11852 11855 11847 11861 11844
11868 11844 11868 11851 11869 11858 11873 11863 11878 11868 11884 11872
11891 11869 11898 11870 11904 11869 11911 11872 11917 11875 11924 11876

11930 11873 11936 11875 11943 11876 11947 11872 11954 11871 11960 11874
11967 11874 11971 11880 11978 11881 11985 11882 11992 11882 11998 11884
12004 11881 12011 11883 12016 11888 12021 11893 12025 11898 12031 11902
12038 11904 12045 11904 12051 11905 12057 11908 12064 11911 12070 11912
12075 11917 12080 11922 50 P
12080 11922 12084 11927 12091 11927 12098 11929 12104 11931 12111 11933
12114 11939 12113 11946 12119 11950 12122 11956 12123 11963 12125 11969
12128 11976 12134 11979 12139 11984 12138 11991 12140 11998 12141 12005
12146 12001 12144 12007 12143 12014 12146 12020 12153 12019 12148 12024
12143 12019 12142 12026 12136 12028 12132 12034 12135 12040 12133 12047
12130 12053 12123 12055 12117 12056 12111 12053 12105 12057 12098 12059
12093 12064 12090 12069 12083 12068 12076 12069 12069 12069 12062 12069
12056 12065 12049 12065 12043 12063 12036 12060 12030 12058 12024 12058
12018 12054 12011 12055 50 P
12011 12055 12005 12052 2 P
12005 12052 11999 12049 11992 12046 11986 12043 11980 12040 11973 12039
11967 12036 11961 12033 11955 12029 11949 12031 11942 12032 11938 12026
11932 12025 11926 12021 11920 12017 11914 12015 11908 12011 11903 12008
11896 12010 11891 12006 11884 12003 11879 11999 11873 11996 11866 11995
11862 11990 11859 11983 11853 11981 11848 11976 11843 11972 11843 11965
11839 11959 11835 11953 11829 11949 11824 11945 11821 11939 11816 11933
11810 11930 11809 11923 11803 11919 11804 11912 11806 11905 11804 11899
11804 11890 43 P
12975 8328 12980 8323 12987 8323 12994 8325 12998 8325 5 P
12998 8341 12993 8339 12986 8339 12979 8339 12975 8328 5 P
12712 8214 12719 8217 12726 8218 12732 8221 12730 8228 12723 8231
12718 8227 12712 8214 8 P
12474 8065 12479 8060 12486 8058 12492 8057 12499 8057 12505 8052
12508 8046 12512 8040 12518 8036 12524 8033 12531 8032 12529 8039
12526 8046 12526 8053 12530 8058 12532 8065 12526 8068 12523 8075
12516 8077 12512 8083 12506 8086 12499 8085 12493 8082 12488 8076
12482 8073 12474 8065 26 P
12690 8092 12693 8089 12695 8085 12699 8087 12703 8090 12699 8091
12696 8095 12697 8099 12692 8098 12690 8092 10 P
12531 8056 12533 8057 12535 8058 12537 8059 12537 8061 12536 8063
12534 8062 12532 8060 12531 8056 9 P
12539 8060 12541 8060 12543 8062 12542 8064 12540 8063 12539 8060

6:00 PM

12544 8057 12546 8059 12548 8060 12550 8061 12552 8061 12554 8063
12555 8064 12556 8066 12559 8067 12561 8067 12563 8068 12565 8068
12566 8070 12568 8071 12570 8071 12568 8072 12566 8072 12564 8070
12563 8069 12561 8069 12559 8068 12556 8068 12555 8066 12553 8064
12552 8063 12549 8062 12547 8062 12545 8060 12544 8057 29 P

12805 7999 12806 7996 12810 7992 12809 7988 12809 7983 12812 7979
12813 7984 12817 7986 12818 7990 12817 7995 12816 7999 12818 8003
12822 8006 12821 8011 12821 8015 12820 8020 12817 8023 12814 8026
12809 8024 12809 8020 12809 8015 12807 8011 12807 8007 12808 8003
12805 7999 25 P
12773 8023 12777 8022 12782 8022 12785 8019 12787 8022 12790 8026
12789 8031 12793 8028 12797 8027 12797 8031 12794 8035 12790 8036
12785 8036 12785 8032 12780 8032 12778 8028 12773 8023 17 P
12766 8006 12770 8003 12772 7999 12776 8001 12780 8003 12778 8007
12776 8011 12772 8013 12769 8010 12766 8006 10 P
12776 7997 12778 7993 12783 7994 12783 7999 12776 7997 5 P
12780 7988 12783 7984 12784 7989 12783 7993 12780 7988 5 P
12751 7989 12752 7985 12757 7983 12757 7987 12757 7992 12754 7994
12751 7989 7 P
12752 8011 12757 8011 12758 8015 12754 8016 12752 8011 5 P
12853 8016 12858 8018 12858 8022 12860 8026 12860 8030 12857 8027
12856 8023 12853 8016 8 P
12829 12958 12823 12955 12820 12949 12819 12942 12819 12935 12812 12937
12806 12934 12799 12937 12798 12930 12793 12925 12797 12919 12801 12913
12805 12908 12809 12903 12814 12898 12808 12899 12801 12898 12795 12901
12790 12896 12784 12897 12777 12901 12772 12905 12765 12903 12763 12897
12756 12896 12753 12890 12750 12884 12747 12877 12749 12871 12751 12864
12755 12858 12761 12856 12764 12850 12768 12844 12774 12847 12773 12854
12779 12855 12786 12859 12790 12864 12793 12870 12799 12869 12800 12862
12803 12855 12801 12849 12802 12842 12796 12838 12790 12837 12783 12834
12785 12827 12790 12832 50 P
12790 12832 12790 12825 12797 12826 12803 12829 12809 12826 12803 12822
12802 12816 12805 12809 12812 12807 12808 12802 12806 12795 12813 12793
12819 12790 12824 12795 12831 12796 12829 12803 12823 12807 12830 12810
12837 12811 12843 12813 12850 12814 12844 12817 12837 12816 12833 12821
12829 12826 12831 12833 12838 12832 12842 12838 12839 12844 12839 12851
12845 12854 12849 12848 12853 12853 12859 12852 12864 12847 12870 12845
12870 12852 12865 12856 12861 12862 12863 12868 12856 12867 12854 12873
12860 12876 12866 12875 12864 12881 12859 12886 12866 12887 12866 12894
12869 12901 12862 12901 50 P
12862 12901 12857 12896 2 P
12857 12896 12856 12889 12849 12891 12845 12897 12850 12902 12853 12908
12856 12914 12853 12920 12852 12927 12851 12933 12849 12940 12843 12944
12840 12950 12837 12956 12841 12962 12846 12965 12848 12972 12843 12977
12837 12979 12830 12980 12826 12974 12826 12967 12829 12958 23 P
12539 12828 12533 12825 12530 12819 12524 12816 12519 12811 12515 12816
12512 12822 12506 12824 12499 12825 12500 12831 12495 12836 12491 12842
12492 12849 12498 12853 12501 12859 12502 12866 12501 12873 12497 12878

12491 12882 12484 12883 12478 12880 12473 12876 12468 12870 12469 12863
12473 12858 12467 12856 12460 12854 12460 12847 12460 12840 12455 12835
12453 12828 12455 12821 12458 12816 12462 12810 12467 12805 12469 12799
12474 12794 12476 12788 12474 12782 12472 12775 12479 12773 12481 12767
12487 12762 12493 12761 12500 12762 12506 12766 12508 12772 12504 12778
12502 12784 12508 12788 50 P
12508 12788 12513 12792 12520 12793 12521 12786 12520 12780 12522 12773
12525 12767 12526 12760 12533 12759 12540 12760 12546 12763 12553 12764
12557 12759 12563 12761 12570 12762 12566 12768 12563 12774 12567 12778
12571 12784 12569 12791 12568 12798 12567 12805 12566 12811 12569 12817
12569 12824 12563 12828 12556 12826 12552 12831 12539 12828 29 P
12064 12407 12068 12401 12074 12403 12080 12399 12086 12399 12093 12398
12098 12402 12099 12409 12098 12416 12093 12421 12091 12427 12086 12431
12079 12433 12074 12429 12070 12423 12068 12417 12064 12407 17 P
12230 12446 12235 12448 12239 12448 12243 12449 12248 12449 12248 12453
12250 12457 12248 12461 12246 12465 12246 12470 12242 12469 12238 12467
12239 12463 12235 12462 12234 12457 12233 12453 12230 12446 17 P
12157 12581 12160 12578 12164 12578 12167 12575 12163 12572 12162 12568
12166 12569 12169 12565 12173 12564 12177 12561 12180 12559 12184 12562
12188 12561 12191 12565 12195 12565 12200 12564 12204 12564 12208 12564
12213 12566 12215 12569 12216 12573 12216 12578 12218 12582 12219 12586
12221 12590 12220 12594 12217 12598 12214 12600 12209 12599 12206 12597
12201 12597 12197 12598 12193 12596 12190 12593 12187 12589 12183 12590
12181 12586 12177 12587 12172 12589 12171 12593 12166 12593 12167 12589
12165 12585 12162 12582 12157 12581 45 P
12318 12588 12323 12589 12327 12590 12331 12590 12334 12586 12329 12586
12325 12587 12327 12583 12330 12581 12335 12583 12338 12580 12338 12576
12337 12572 12340 12570 12344 12573 12347 12575 12351 12577 12356 12578
12359 12582 12360 12585 12358 12590 12357 12594 12352 12594 12350 12598
12346 12600 12343 12604 12342 12600 12337 12598 12334 12596 12330 12598
12326 12599 12323 12601 12318 12600 12322 12598 12320 12593 12318 12588
36 P
12423 12650 12426 12647 12431 12646 12434 12643 12438 12645 12438 12641
12441 12638 12443 12634 12446 12632 12451 12634 12454 12632 12458 12631
12460 12627 12464 12627 12468 12627 12471 12631 12469 12634 12467 12638
12462 12638 12458 12639 12462 12640 12462 12644 12459 12648 12455 12650
12454 12654 12452 12658 12448 12660 12444 12661 12439 12660 12436 12657
12433 12654 12430 12651 12423 12650 33 P
12470 12597 12474 12597 12472 12593 12472 12589 12476 12589 12480 12586
12482 12590 12481 12594 12481 12598 12482 12603 12482 12607 12478 12608
12479 12613 12474 12613 12473 12609 12474 12605 12474 12600 12470 12597
18 P
11958 12510 11960 12506 11960 12501 11961 12497 11962 12493 11962 12488

11965 12485 11964 12482 11968 12483 11971 12480 11975 12479 11979 12477
11982 12479 11985 12482 11989 12478 11992 12476 11989 12472 11984 12472
11984 12468 11988 12465 11992 12467 11996 12468 12000 12467 12004 12464
12008 12463 12007 12468 12006 12471 12010 12470 12012 12466 12017 12466
12021 12466 12025 12466 12023 12470 12019 12471 12020 12475 12023 12479
12019 12481 12014 12480 12010 12480 12011 12485 12009 12488 12005 12490
12004 12494 12003 12498 12002 12502 12002 12507 12000 12512 11998 12515
11994 12517 11990 12520 50 P
11990 12520 11987 12522 11983 12523 11978 12525 11975 12523 11971 12520
11967 12520 11963 12518 11961 12515 11958 12510 10 P
11709 12504 11713 12499 11717 12494 11721 12488 11727 12484 11726 12491
11723 12498 11726 12504 11728 12497 11732 12493 11739 12491 11736 12485
11732 12480 11735 12473 11739 12479 11743 12473 11750 12472 11757 12473
11763 12477 11769 12479 11764 12484 11771 12483 11777 12484 11781 12490
11788 12491 11788 12498 11794 12501 11801 12501 11802 12508 11808 12508
11809 12515 11815 12511 11816 12504 11822 12509 11819 12515 11825 12519
11828 12526 11832 12531 11828 12537 11832 12542 11828 12547 11832 12553
11832 12560 11834 12566 11828 12570 11825 12576 11819 12579 11814 12584
11808 12581 11806 12575 50 P
11806 12575 11801 12579 11798 12585 11800 12592 11796 12598 11790 12601
11783 12603 11777 12603 11770 12604 11768 12598 11771 12591 11778 12591
11784 12593 11782 12586 11784 12580 11780 12574 11776 12569 11773 12563
11766 12564 11766 12571 11764 12577 11760 12572 11763 12565 11756 12563
11759 12556 11761 12550 11765 12544 11771 12547 11768 12541 11762 12542
11765 12536 11764 12529 11763 12523 11761 12516 11758 12510 11758 12517
11757 12523 11758 12530 11761 12537 11757 12542 11757 12549 11750 12551
11749 12558 11744 12554 11743 12547 11746 12541 11743 12535 11738 12540
11732 12540 11728 12534 50 P
11728 12534 11722 12532 2 P
11722 12532 11720 12525 11725 12520 11718 12522 11713 12518 11711 12511
11709 12504 7 P
11911 12372 11914 12369 11916 12365 11920 12364 11924 12364 11928 12366
11932 12367 11935 12370 11939 12373 11939 12378 11944 12378 11945 12383
11948 12384 11949 12388 11952 12386 11955 12382 11955 12386 11959 12385
11963 12386 11963 12390 11959 12390 11954 12390 11951 12392 11946 12392
11942 12391 11938 12391 11933 12390 11932 12394 11928 12397 11924 12395
11920 12393 11917 12390 11914 12386 11911 12384 11913 12380 11911 12372
36 P
11927 12402 11932 12401 11934 12398 11938 12395 11942 12394 11946 12394
11951 12394 11947 12397 11946 12401 11949 12404 11952 12400 11956 12400
11958 12404 11955 12407 11959 12409 11962 12412 11959 12415 11957 12419
11954 12422 11950 12423 11946 12422 11941 12423 11938 12422 11935 12425
11931 12427 11932 12423 11934 12420 11931 12417 11930 12413 11934 12415

11932 12411 11931 12407 11927 12402 33 P
11967 12382 11969 12379 11971 12375 11972 12371 11976 12373 11978 12370
11980 12365 11984 12364 11987 12367 11987 12372 11988 12376 11988 12380
11991 12383 11991 12387 11987 12385 11986 12389 11984 12393 11980 12393
11980 12398 11984 12399 11989 12400 11990 12404 11987 12407 11983 12409
11979 12410 11975 12412 11971 12409 11971 12405 11967 12405 11968 12401
11973 12401 11975 12398 11975 12393 11971 12390 11971 12386 11967 12382
36 P
12012 12490 12016 12490 12020 12488 12024 12488 12028 12485 12031 12482
12034 12485 12037 12488 12041 12489 12046 12489 12049 12487 12052 12483
12052 12479 12054 12476 12058 12477 12060 12481 12063 12484 12062 12488
12067 12487 12067 12483 12071 12483 12072 12487 12071 12491 12072 12495
12076 12495 12077 12499 12076 12503 12072 12500 12068 12501 12065 12504
12061 12504 12056 12504 12053 12501 12051 12505 12047 12507 12044 12505
12041 12501 12042 12497 12038 12494 12034 12493 12031 12496 12027 12498
12025 12494 12024 12491 12019 12492 12015 12493 12012 12490 47 P
12008 12515 12011 12517 12016 12516 12020 12518 12018 12513 12017 12509
12017 12505 12020 12502 12023 12506 12024 12510 12026 12514 12027 12518
12032 12520 12036 12520 12040 12521 12040 12525 12043 12528 12047 12528
12050 12526 12055 12526 12060 12526 12059 12530 12056 12533 12052 12535
12052 12540 12048 12538 12043 12539 12047 12541 12048 12546 12045 12548
12043 12552 12039 12551 12034 12549 12031 12547 12027 12546 12023 12546
12020 12542 12017 12539 12016 12535 12013 12532 12014 12527 12012 12523
12010 12520 12008 12515 44 P
12057 12534 12061 12532 12063 12528 12060 12525 12060 12520 12061 12516
12064 12519 12065 12523 12069 12525 12069 12521 12069 12516 12070 12512
12075 12512 12078 12509 12083 12510 12086 12512 12088 12516 12092 12517
12096 12517 12098 12521 12095 12525 12094 12529 12095 12533 12097 12537
12095 12541 12093 12537 12091 12534 12090 12529 12085 12531 12082 12529
12078 12533 12074 12533 12070 12534 12066 12536 12064 12532 12062 12536
12057 12534 37 P
12122 12516 12118 12517 12120 12513 12124 12511 12128 12513 12132 12512
12136 12509 12133 12506 12137 12506 12140 12502 12141 12507 12145 12508
12148 12505 12144 12501 12141 12499 12142 12495 12143 12491 12145 12494
12147 12498 12150 12502 12153 12505 12157 12504 12158 12508 12154 12508
12153 12512 12157 12511 12162 12511 12164 12508 12169 12508 12166 12512
12163 12515 12163 12519 12158 12519 12154 12521 12151 12518 12147 12516
12143 12514 12144 12518 12142 12522 12138 12524 12136 12528 12133 12531
12134 12527 12130 12526 12126 12525 12129 12521 12126 12518 12122 12516
48 P
12127 12569 12130 12566 12133 12563 12134 12559 12135 12555 12137 12551
12140 12548 12142 12553 12141 12557 12145 12555 12144 12551 12146 12547
12148 12542 12149 12546 12151 12550 12152 12555 12151 12559 12149 12563

12147 12567 12145 12570 12141 12573 12137 12573 12133 12571 12127 12569
24 P
12893 12966 12896 12963 12901 12964 12902 12968 12900 12973 12900 12977
12901 12981 12902 12986 12903 12989 12903 12994 12901 12998 12897 12997
12895 13001 12891 13003 12889 12999 12885 12998 12881 12996 12879 12992
12883 12991 12886 12988 12886 12983 12888 12980 12885 12978 12888 12976
12893 12975 12893 12970 12893 12966 27 P
12747 12828 12751 12826 12754 12823 12752 12819 12753 12814 12758 12814
12762 12813 12765 12816 12769 12819 12772 12821 12771 12826 12769 12829
12764 12828 12762 12832 12762 12836 12758 12840 12759 12844 12756 12841
12757 12846 12752 12846 12749 12842 12751 12838 12755 12839 12758 12837
12754 12838 12754 12834 12751 12831 12751 12835 12748 12838 12749 12834
12747 12828 31 P
12760 12916 12763 12913 12765 12909 12769 12910 12766 12913 12770 12916
12767 12919 12765 12923 12768 12926 12770 12930 12768 12933 12764 12934
12759 12933 12759 12928 12761 12925 12762 12920 12760 12916 17 P
12613 12842 12618 12840 12621 12839 12621 12834 12625 12834 12628 12837
12629 12841 12632 12845 12636 12845 12641 12845 12640 12849 12636 12851
12632 12851 12628 12854 12624 12853 12620 12851 12615 12849 12613 12842
18 P
12630 12830 12634 12830 12635 12825 12639 12826 12642 12823 12646 12822
12650 12820 12651 12825 12648 12828 12653 12828 12657 12829 12660 12832
12657 12835 12653 12837 12650 12840 12647 12843 12642 12845 12639 12844
12635 12840 12633 12837 12635 12833 12630 12830 22 P
12653 12733 12656 12729 12660 12728 12660 12724 12662 12720 12664 12723
12669 12724 12671 12728 12675 12727 12679 12727 12684 12728 12683 12732
12686 12735 12684 12739 12680 12738 12678 12734 12677 12729 12673 12732
12676 12735 12673 12739 12669 12739 12665 12742 12661 12743 12657 12742
12655 12738 12653 12733 26 P
12534 12870 12532 12867 12531 12862 12529 12859 12526 12855 12525 12850
12522 12848 12520 12844 12521 12839 12523 12835 12525 12831 12529 12830
12533 12829 12538 12829 12542 12830 12546 12831 12547 12835 12551 12837
12555 12835 12559 12833 12563 12834 12567 12832 12571 12833 12575 12830
12579 12830 12577 12834 12574 12837 12570 12839 12567 12841 12566 12846
12567 12849 12567 12854 12563 12856 12559 12858 12555 12861 12557 12864
12553 12865 12549 12862 12548 12858 12547 12854 12547 12849 12545 12845
12542 12848 12542 12853 12542 12857 12543 12861 12546 12865 12547 12869
12548 12873 12548 12877 50 P
12548 12877 12544 12876 12539 12875 12534 12870 4 P
12395 12660 12397 12664 12400 12661 12402 12657 12407 12657 12410 12660
12414 12662 12415 12666 12418 12663 12422 12663 12425 12666 12422 12669
12419 12671 12416 12668 12411 12669 12409 12672 12405 12674 12401 12671
12397 12670 12395 12666 12395 12660 21 P

12307 12626 12311 12624 12315 12624 12316 12619 12320 12617 12315 12615
12312 12612 12315 12608 12317 12605 12322 12605 12324 12608 12329 12608
12333 12611 12337 12610 12339 12613 12339 12618 12336 12620 12332 12622
12327 12622 12323 12622 12327 12625 12324 12628 12320 12629 12323 12633
12326 12636 12325 12640 12320 12641 12316 12641 12313 12638 12311 12634
12310 12630 12307 12626 32 P
12123 12505 12119 12504 12115 12505 12111 12504 12107 12501 12105 12497
12105 12493 12106 12488 12110 12487 12112 12484 12115 12485 12120 12487
12122 12490 12127 12491 12128 12495 12132 12497 12130 12501 12123 12505
18 P
12266 12363 12268 12358 12269 12355 12273 12352 12274 12348 12277 12344
12280 12342 12284 12342 12288 12341 12291 12344 12287 12344 12282 12345
12281 12350 12278 12352 12276 12357 12274 12360 12275 12365 12274 12369
12271 12366 12266 12363 20 P
12278 12366 12282 12363 12284 12359 12287 12357 12291 12355 12292 12359
12288 12362 12289 12366 12285 12367 12278 12366 10 P
12104 12303 12106 12299 12110 12299 12114 12301 12117 12304 12115 12308
12111 12310 12107 12310 12104 12303 9 P
11831 12258 11836 12256 11838 12252 11840 12249 11845 12249 11843 12252
11844 12257 11848 12258 11849 12262 11845 12264 11841 12261 11837 12262
11831 12258 13 P
11822 12263 11826 12261 11829 12259 11831 12263 11828 12266 11824 12267
11822 12263 7 P
11901 12393 11904 12392 11905 12395 11908 12397 11911 12397 11911 12400
11913 12403 11911 12405 11909 12407 11907 12405 11905 12402 11904 12399
11903 12395 11901 12393 14 P
11946 12428 11949 12427 11952 12429 11955 12428 11957 12430 11960 12430
11960 12434 11958 12437 11955 12438 11952 12437 11949 12435 11946 12434
11946 12428 13 P
11949 12353 11953 12354 11956 12354 11959 12353 11959 12356 11956 12358
11955 12362 11952 12363 11949 12361 11952 12359 11949 12353 11 P
12019 12409 12022 12407 12024 12405 12026 12408 12026 12411 12028 12413
12029 12416 12027 12420 12026 12422 12023 12423 12020 12421 12019 12419
12020 12415 12019 12409 14 P
11271 12274 11275 12272 11279 12271 11283 12270 11287 12267 11288 12263
11290 12259 11295 12260 11299 12261 11301 12265 11297 12268 11294 12271
11291 12274 11289 12278 11290 12282 11286 12282 11284 12287 11280 12286
11276 12283 11277 12279 11276 12274 11271 12274 22 P
11436 12364 11439 12362 11440 12359 11443 12361 11446 12360 11448 12362
11445 12363 11442 12365 11439 12364 11436 12364 10 P
11683 12433 11687 12434 11686 12430 11691 12429 11695 12429 11699 12428
11702 12424 11701 12429 11704 12433 11705 12428 11707 12425 11709 12429
11714 12430 11717 12428 11717 12432 11716 12436 11715 12441 11715 12445

11710 12444 11706 12443 11702 12442 11698 12443 11695 12445 11691 12448
11689 12444 11685 12444 11688 12441 11685 12438 11683 12433 29 P
11689 12400 11693 12400 11695 12398 11698 12400 11701 12399 11704 12398
11706 12401 11707 12404 11710 12405 11708 12407 11707 12411 11703 12412
11701 12414 11701 12411 11702 12407 11699 12405 11696 12405 11693 12404
11690 12404 11689 12400 20 P
11645 12398 11647 12395 11650 12394 11653 12395 11655 12398 11658 12400
11658 12403 11655 12404 11653 12401 11651 12404 11648 12402 11645 12398

12:00 PM

11632 12401 11635 12401 11638 12400 11638 12403 11640 12405 11642 12408
11644 12405 11643 12402 11646 12404 11645 12407 11644 12410 11641 12411
11637 12409 11635 12408 11633 12406 11632 12401 16 P
11673 12458 11670 12457 11670 12454 11671 12450 11674 12449 11677 12449
11680 12449 11684 12449 11685 12452 11688 12452 11691 12454 11693 12451
11696 12451 11699 12449 11701 12452 11699 12455 11695 12456 11693 12457
11689 12458 11687 12461 11684 12458 11681 12459 11680 12456 11677 12456
11673 12458 25 P
11677 12417 11676 12420 11673 12423 11671 12423 11667 12422 11666 12419
11667 12416 11671 12416 11673 12414 11674 12411 11678 12411 11678 12414
11680 12417 11679 12420 11677 12417 15 P
11755 12411 11758 12409 11761 12409 11759 12412 11760 12415 11761 12418
11762 12421 11760 12423 11758 12421 11757 12418 11756 12415 11755 12411

12:00 PM

11380 12597 11383 12593 11385 12590 11389 12587 11393 12587 11392 12591
11392 12596 11396 12598 11398 12601 11397 12606 11402 12607 11405 12610
11406 12614 11406 12618 11409 12621 11413 12623 11412 12627 11408 12625
11405 12621 11401 12621 11399 12625 11403 12627 11399 12628 11395 12627
11391 12627 11388 12623 11389 12619 11385 12615 11385 12611 11384 12607
11381 12604 11384 12600 11380 12597 33 P
11405 12644 11408 12641 11411 12640 11412 12637 11414 12639 11414 12643
11415 12646 11418 12645 11419 12648 11416 12649 11413 12651 11410 12652
11411 12649 11408 12647 11405 12644 15 P
11286 12604 11289 12600 11291 12604 11293 12608 11297 12608 11299 12612
11301 12617 11302 12621 11300 12625 11296 12627 11295 12622 11293 12619
11292 12614 11288 12612 11286 12608 11286 12604 16 P
11343 12475 11345 12472 11346 12469 11348 12472 11347 12475 11343 12475

6:00 PM

11343 12478 11346 12478 11349 12480 11351 12483 11350 12486 11347 12486
11346 12483 11343 12478 8 P
11346 12461 11348 12458 11349 12456 11351 12453 11353 12450 11355 12451
11359 12450 11361 12452 11360 12455 11357 12456 11354 12458 11352 12456
11351 12458 11346 12461 14 P
11368 12612 11371 12610 11374 12611 11376 12613 11376 12616 11373 12617

11370 12615 11368 12612 8 P
11377 12618 11380 12618 11381 12621 11384 12622 11381 12622 11377 12618
6:00 PM
11400 12648 11403 12647 11404 12650 11403 12653 11399 12652 11400 12648
6:00 PM
11569 12601 11573 12600 11575 12597 11579 12597 11577 12593 11573 12592
11576 12588 11580 12590 11584 12592 11588 12591 11591 12594 11595 12595
11595 12599 11593 12603 11593 12607 11589 12609 11586 12605 11583 12604
11579 12600 11575 12601 11569 12601 21 P
11653 12656 11649 12658 11646 12661 11642 12661 11640 12664 11638 12669
11635 12672 11633 12669 11632 12664 11629 12662 11631 12658 11630 12654
11629 12658 11625 12660 11620 12658 11616 12656 11615 12652 11615 12648
11615 12643 11616 12639 11619 12636 11622 12639 11625 12634 11628 12632
11631 12633 11635 12629 11638 12628 11643 12629 11646 12632 11646 12636
11650 12639 11652 12642 11654 12646 11658 12646 11659 12650 11660 12654
11653 12656 37 P
11649 12694 11645 12693 11647 12697 11649 12701 11650 12705 11648 12710
11644 12711 11640 12710 11636 12708 11635 12704 11633 12700 11628 12700
11628 12696 11623 12694 11620 12692 11615 12692 11611 12691 11608 12695
11604 12694 11604 12690 11601 12686 11603 12682 11601 12678 11601 12674
11600 12670 11604 12667 11607 12663 11609 12660 11614 12658 11618 12660
11622 12661 11626 12664 11624 12668 11628 12669 11626 12672 11625 12676
11621 12678 11620 12683 11624 12684 11628 12686 11633 12686 11636 12689
11637 12684 11642 12685 11645 12686 11650 12688 11649 12694 47 P
11412 12598 11415 12598 11418 12598 11420 12601 11423 12603 11423 12606
11420 12607 11417 12607 11416 12604 11413 12602 11412 12598 11 P
11394 12658 11396 12656 11399 12656 11398 12658 11394 12658 5 P
11600 12602 11601 12599 11605 12598 11607 12596 11609 12593 11608 12596
11607 12599 11604 12600 11600 12602 9 P
11598 12602 11599 12599 11598 12596 11601 12598 11598 12602 5 P
11633 12615 11635 12613 11637 12611 11641 12612 11643 12611 11645 12608
11648 12610 11648 12613 11647 12616 11643 12618 11642 12621 11639 12622
11637 12619 11634 12618 11633 12615 15 P
11657 12617 11654 12615 11654 12612 11657 12610 11654 12608 11652 12605
11655 12603 11657 12606 11657 12603 11659 12605 11663 12606 11663 12609
11662 12612 11660 12615 11657 12617 15 P
11663 12603 11664 12600 11667 12599 11671 12598 11669 12595 11667 12593
11667 12590 11669 12586 11669 12583 11671 12580 11674 12581 11674 12584
11676 12587 11679 12585 11682 12585 11685 12584 11687 12586 11688 12590
11689 12593 11687 12596 11685 12597 11684 12600 11684 12604 11681 12603
11678 12603 11676 12605 11674 12608 11676 12611 11676 12614 11676 12618
11672 12619 11670 12620 11666 12619 11663 12618 11662 12615 11664 12612
11665 12610 11664 12606 11663 12603 39 P

11674 12565 11678 12564 11683 12564 11687 12564 11691 12565 11693 12569
11698 12570 11701 12573 11700 12578 11704 12579 11706 12583 11704 12586
11706 12590 11707 12594 11710 12598 11706 12600 11702 12603 11705 12606
11701 12608 11697 12610 11693 12611 11692 12615 11689 12618 11685 12618
11680 12618 11680 12614 11683 12611 11687 12609 11689 12605 11692 12601
11692 12597 11695 12595 11700 12594 11696 12592 11694 12588 11695 12584
11692 12580 11688 12580 11684 12583 11680 12582 11677 12579 11673 12577
11677 12575 11679 12578 11684 12577 11688 12577 11687 12573 11683 12572
11680 12568 11677 12570 50 P
11677 12570 11674 12565 2 P
11640 12594 11643 12594 11647 12595 11647 12592 11646 12589 11646 12585
11649 12586 11649 12589 11649 12592 11649 12596 11651 12598 11648 12598
11645 12597 11642 12597 11640 12594 15 P
11708 12629 11713 12629 11715 12626 11714 12621 11715 12618 11714 12613
11718 12613 11721 12616 11725 12617 11727 12620 11731 12621 11735 12619
11738 12622 11741 12625 11746 12626 11748 12629 11743 12631 11739 12632
11740 12635 11738 12639 11735 12642 11731 12639 11729 12635 11725 12635
11728 12639 11728 12643 11725 12646 11721 12644 11718 12641 11714 12639
11713 12635 11709 12634 11708 12629 33 P
11678 12693 11682 12692 11686 12689 11688 12685 11690 12681 11694 12682
11698 12679 11697 12675 11697 12671 11701 12671 11705 12668 11708 12671
11710 12675 11715 12675 11716 12671 11720 12668 11719 12664 11723 12662
11727 12662 11731 12662 11733 12667 11732 12671 11733 12675 11731 12679
11728 12682 11730 12686 11727 12688 11724 12691 11720 12693 11717 12690
11713 12691 11709 12693 11707 12697 11703 12698 11699 12696 11695 12697
11691 12699 11689 12695 11685 12695 11678 12693 40 P
11685 12706 11689 12705 11693 12704 11696 12702 11701 12703 11705 12704
11708 12700 11710 12697 11713 12693 11717 12695 11721 12697 11723 12700
11722 12705 11720 12709 11724 12712 11722 12715 11717 12715 11717 12720
11713 12722 11711 12718 11713 12714 11709 12714 11705 12712 11701 12711
11697 12710 11693 12708 11685 12706 27 P
11672 12738 11674 12735 11677 12731 11680 12728 11681 12724 11685 12721
11688 12720 11691 12716 11690 12711 11694 12711 11698 12713 11701 12717
11702 12721 11700 12725 11699 12729 11701 12733 11705 12732 11709 12729
11709 12734 11713 12736 11713 12741 11709 12743 11706 12746 11709 12747
11714 12748 11714 12753 11709 12752 11710 12756 11707 12760 11703 12756
11700 12759 11695 12759 11692 12756 11694 12753 11696 12749 11699 12745
11696 12741 11692 12741 11688 12743 11685 12746 11681 12744 11679 12740
11672 12738 43 P
11643 12757 11641 12753 11639 12749 11635 12748 11630 12748 11634 12747
11633 12743 11637 12742 11641 12741 11644 12744 11648 12742 11652 12744
11655 12748 11655 12752 11655 12748 11655 12743 11659 12742 11660 12738
11664 12735 11667 12738 11670 12741 11674 12743 11676 12747 11681 12747

11680 12752 11679 12756 11684 12754 11687 12756 11688 12760 11685 12763
11681 12764 11682 12768 11686 12771 11682 12774 11683 12778 11678 12778
11676 12782 11673 12785 11669 12786 11667 12782 11669 12777 11668 12774
11664 12771 11660 12775 11657 12771 11654 12769 11650 12771 11648 12768
11647 12763 11643 12757 50 P
11677 12715 11679 12712 11681 12710 11684 12711 11682 12713 11681 12717
11677 12715 7 P
11665 12724 11667 12721 11669 12719 11672 12719 11674 12716 11677 12717
11677 12720 11674 12722 11671 12724 11669 12726 11665 12724 11 P
11657 12680 11658 12677 11658 12674 11659 12670 11662 12668 11665 12668
11666 12671 11665 12674 11663 12677 11663 12680 11657 12680 11 P
11726 12651 11729 12650 11732 12650 11736 12651 11737 12654 11739 12651
11742 12654 11743 12657 11740 12659 11742 12662 11744 12664 11741 12665
11738 12664 11735 12662 11734 12660 11734 12656 11730 12657 11727 12656
11726 12651 19 P
11695 12660 11698 12658 11701 12658 11703 12661 11704 12664 11702 12665
11699 12665 11695 12660 8 P
11683 12676 11686 12676 11688 12678 11688 12682 11686 12684 11685 12681
11683 12676 7 P
11668 12686 11671 12688 11674 12689 11676 12691 11676 12695 11673 12693
11671 12690 11668 12686 8 P
11683 12644 11684 12642 11687 12643 11687 12647 11690 12649 11691 12652
11687 12651 11685 12649 11683 12644 9 P
11676 12632 11677 12629 11677 12626 11679 12628 11679 12632 11678 12635
11678 12638 11675 12636 11676 12632 9 P
11679 12536 11681 12534 11684 12531 11683 12528 11685 12525 11687 12527
11690 12529 11690 12533 11693 12534 11695 12536 11695 12540 11693 12537
11692 12541 11694 12542 11692 12544 11690 12547 11687 12545 11685 12542
11684 12539 11683 12536 11679 12536 21 P
11797 12487 11800 12485 11803 12485 11802 12488 11805 12487 11808 12489
11810 12491 11810 12494 11808 12497 11805 12495 11808 12498 11807 12501
11803 12500 11801 12498 11798 12496 11799 12493 11797 12487 17 P
11641 12470 11644 12470 11646 12473 11647 12476 11643 12476 11642 12473
11641 12470 7 P
11654 12470 11657 12470 11658 12473 11659 12476 11657 12478 11654 12476
11654 12470 7 P
11652 12437 11656 12437 11658 12439 11658 12442 11655 12441 11652 12437

6:00 PM

11659 12435 11662 12433 11665 12432 11668 12433 11666 12435 11664 12437
11659 12435 7 P
11659 12444 11660 12441 11662 12438 11664 12439 11665 12442 11664 12445
11659 12444 7 P
11648 12457 11651 12457 11654 12457 11654 12461 11651 12463 11649 12460

11648 12457 7 P
11678 12466 11681 12465 11679 12463 11681 12461 11684 12461 11684 12464
11683 12467 11681 12470 11678 12466 9 P
11674 12446 11675 12443 11677 12440 11679 12442 11683 12442 11683 12446
11679 12446 11674 12446 8 P
11611 12383 11613 12381 11616 12379 11619 12378 11619 12381 11616 12384
11611 12383 7 P
11654 12356 11656 12353 11657 12350 11658 12346 11659 12349 11658 12352
11657 12355 11656 12358 11654 12356 9 P
11746 12745 11751 12745 11755 12744 11759 12742 11764 12742 11766 12746
11768 12750 11772 12751 11776 12750 11780 12752 11779 12756 11783 12754
11785 12750 11790 12750 11794 12750 11796 12746 11800 12745 11797 12741
11795 12738 11796 12734 11801 12733 11803 12737 11807 12735 11810 12739
11809 12743 11811 12747 11811 12752 11810 12756 11806 12757 11803 12760
11799 12759 11794 12759 11790 12758 11786 12759 11788 12763 11784 12765
11788 12768 11789 12772 11793 12770 11794 12775 11798 12775 11800 12778
11795 12779 11793 12783 11789 12784 11785 12783 11780 12783 11779 12787
11774 12787 11770 12786 50 P
11770 12786 11767 12782 11770 12779 11774 12776 11777 12774 11778 12770
11775 12767 11774 12763 11771 12763 11771 12759 11767 12756 11765 12760
11763 12756 11761 12752 11757 12752 11753 12752 11749 12754 11747 12750
11746 12745 19 P
11756 12811 11759 12808 11762 12805 11764 12801 11766 12797 11770 12795
11774 12796 11773 12800 11771 12805 11768 12808 11765 12811 11761 12813
11756 12811 13 P
11739 12783 11743 12781 11747 12779 11750 12782 11754 12783 11753 12787
11754 12791 11750 12793 11746 12793 11742 12793 11740 12789 11739 12783
12:00 PM
11649 12854 11654 12854 11658 12854 11659 12850 11662 12854 11665 12856
11666 12853 11670 12851 11674 12850 11678 12848 11680 12852 11684 12854
11682 12858 11685 12861 11687 12866 11683 12867 11678 12868 11679 12873
11679 12877 11683 12879 11684 12883 11681 12887 11684 12890 11683 12895
11687 12897 11691 12897 11695 12901 11699 12902 11695 12904 11692 12901
11688 12901 11684 12900 11679 12900 11679 12896 11676 12892 11673 12889
11671 12885 11669 12882 11664 12880 11660 12878 11656 12877 11654 12873
11653 12869 11651 12866 11655 12863 11658 12861 11656 12857 11649 12854
48 P
11701 12873 11706 12873 11708 12869 11707 12865 11712 12864 11713 12860
11717 12860 11722 12861 11724 12857 11729 12857 11732 12860 11736 12856
11740 12859 11742 12855 11738 12852 11738 12848 11742 12847 11744 12843
11747 12840 11747 12844 11751 12842 11753 12846 11756 12849 11752 12852
11748 12854 11749 12858 11748 12862 11744 12865 11741 12862 11737 12862
11734 12866 11731 12868 11730 12873 11733 12876 11737 12874 11739 12877

11739 12881 11734 12881 11732 12877 11730 12882 11728 12885 11724 12882
11723 12878 11718 12878 11715 12881 11714 12885 11710 12883 11706 12882
11703 12878 11701 12873 50 P
11691 12849 11695 12849 11695 12845 11700 12844 11703 12845 11707 12842
11709 12839 11711 12842 11711 12847 11712 12843 11714 12839 11717 12842
11717 12846 11715 12850 11714 12854 11709 12855 11705 12854 11701 12855
11696 12854 11691 12849 20 P
11680 12822 11684 12819 11688 12821 11692 12823 11696 12822 11700 12821
11705 12823 11704 12818 11700 12819 11699 12815 11699 12811 11700 12806
11699 12802 11696 12798 11695 12794 11700 12796 11704 12797 11705 12802
11703 12805 11708 12806 11711 12809 11715 12807 11714 12811 11718 12813
11718 12817 11718 12821 11715 12825 11711 12826 11708 12829 11708 12833
11705 12837 11701 12834 11697 12837 11693 12837 11693 12833 11688 12831
11687 12827 11680 12822 38 P
11748 12829 11749 12826 11750 12823 11750 12819 11748 12817 11751 12816
11754 12816 11757 12816 11759 12818 11759 12821 11758 12825 11756 12827
11754 12830 11752 12832 11749 12832 11745 12833 11748 12829 17 P
11728 12809 11730 12807 11731 12804 11734 12802 11736 12805 11735 12808
11735 12811 11735 12814 11736 12818 11732 12817 11729 12816 11729 12812
11728 12809 13 P
11714 13148 11717 13145 11719 13141 11723 13144 11727 13145 11730 13148
11732 13152 11735 13155 11739 13157 11741 13161 11742 13165 11740 13168
11736 13171 11737 13167 11733 13164 11729 13164 11725 13166 11728 13162
11723 13162 11723 13158 11720 13155 11720 13151 11714 13148 23 P
11679 12979 11681 12976 11684 12974 11684 12971 11687 12972 11689 12974
11689 12977 11688 12981 11687 12984 11684 12986 11681 12984 11679 12979
12:00 PM
11621 13005 11623 13003 11625 13001 11628 13001 11631 13001 11633 13003
11636 13006 11635 13009 11633 13012 11630 13012 11628 13015 11626 13013
11622 13014 11622 13010 11626 13010 11628 13008 11625 13006 11621 13005
18 P
11619 12999 11617 12996 11616 12993 11619 12995 11621 12997 11619 12999
6:00 PM
11634 13021 11637 13019 11639 13022 11636 13024 11634 13021 5 P
11684 12933 11687 12933 11691 12933 11692 12936 11694 12939 11691 12939
11689 12937 11687 12935 11684 12933 9 P
11680 12925 11683 12927 11684 12930 11683 12933 11680 12932 11680 12928
11680 12925 7 P
11636 12867 11639 12865 11640 12862 11643 12861 11645 12863 11645 12867
11644 12869 11642 12872 11640 12874 11637 12872 11636 12867 11 P
11650 13172 11653 13171 11656 13171 11656 13168 11659 13169 11658 13172
11656 13174 11654 13175 11651 13174 11650 13172 10 P
11645 13181 11648 13181 11649 13183 11649 13186 11647 13185 11645 13181

6:00 PM

11790 13242 11794 13240 11795 13237 11796 13235 11798 13237 11799 13240
11797 13244 11794 13245 11790 13242 9 P
11867 13274 11869 13272 11872 13272 11875 13274 11876 13277 11876 13280
11874 13282 11871 13282 11869 13280 11867 13274 10 P
11786 13288 11788 13286 11789 13283 11790 13280 11793 13279 11793 13282
11795 13283 11797 13281 11801 13280 11804 13281 11807 13283 11804 13285
11801 13287 11798 13288 11795 13288 11793 13290 11789 13291 11786 13288
18 P
11763 13280 11766 13279 11766 13276 11769 13277 11768 13274 11772 13275
11775 13275 11776 13278 11774 13280 11777 13282 11776 13285 11774 13288
11771 13289 11768 13288 11768 13285 11771 13284 11773 13282 11772 13279
11768 13278 11767 13281 11763 13280 21 P
11775 13271 11778 13269 11781 13269 11783 13271 11786 13273 11787 13276
11785 13279 11783 13276 11782 13273 11779 13273 11781 13275 11778 13277
11777 13274 11775 13271 14 P
11753 13275 11756 13273 11758 13270 11760 13272 11760 13275 11758 13278
11756 13279 11753 13275 8 P
11748 13295 11751 13294 11754 13295 11757 13296 11760 13298 11757 13300
11753 13299 11751 13296 11748 13295 9 P
11768 13302 11772 13302 11775 13301 11778 13301 11776 13304 11773 13303
11768 13302 7 P
11739 13296 11742 13295 11745 13295 11743 13297 11739 13296 5 P
11804 13300 11806 13297 11808 13299 11809 13301 11807 13301 11804 13300

6:00 PM

11814 13357 11817 13356 11819 13355 11821 13357 11819 13360 11817 13359
11814 13357 7 P
11840 13363 11843 13363 11845 13362 11847 13359 11850 13360 11847 13361
11846 13364 11844 13365 11840 13363 9 P
11825 13369 11828 13368 11830 13367 11832 13365 11835 13366 11837 13367
11838 13369 11836 13370 11833 13371 11831 13371 11828 13371 11825 13369

12:00 PM

11816 13407 11818 13406 11821 13406 11824 13405 11825 13407 11824 13410
11824 13412 11821 13412 11819 13410 11816 13407 10 P
11765 13427 11767 13424 11769 13423 11772 13423 11774 13422 11773 13425
11772 13427 11770 13429 11767 13429 11765 13427 10 P
11561 13429 11562 13427 11563 13424 11565 13422 11568 13421 11570 13422
11569 13424 11572 13425 11573 13422 11575 13424 11575 13427 11572 13428
11570 13429 11567 13430 11564 13430 11561 13429 16 P
11580 13483 11582 13481 11584 13479 11586 13478 11586 13480 11585 13483
11580 13483 7 P
11599 13485 11602 13485 11604 13486 11607 13487 11609 13488 11612 13488
11612 13491 11609 13493 11607 13492 11605 13493 11602 13492 11601 13489

11599 13485 13 P
11550 13492 11550 13489 11551 13486 11554 13485 11556 13484 11556 13486
11557 13489 11555 13491 11554 13488 11551 13490 11550 13492 11 P
11713 13556 11715 13556 11717 13557 11720 13556 11722 13558 11724 13559
11724 13562 11722 13564 11721 13566 11722 13569 11720 13571 11717 13572
11717 13569 11716 13566 11715 13564 11713 13562 11713 13559 11713 13556
18 P
11729 13581 11730 13579 11732 13578 11735 13580 11737 13580 11740 13578
11743 13579 11745 13578 11748 13578 11751 13578 11753 13578 11756 13576
11758 13576 11761 13576 11763 13578 11763 13580 11760 13579 11757 13579
11755 13580 11752 13581 11753 13584 11752 13587 11751 13588 11750 13591
11747 13591 11744 13591 11742 13589 11741 13587 11743 13584 11740 13584
11738 13586 11735 13587 11732 13587 11730 13587 11729 13581 35 P
11676 13534 11677 13531 11678 13529 11680 13531 11682 13533 11682 13536
11684 13538 11681 13539 11679 13539 11678 13536 11678 13534 11676 13534

12:00 PM

11681 13521 11681 13519 11684 13519 11685 13521 11686 13523 11686 13526
11683 13526 11681 13524 11681 13521 9 P
11567 13729 11569 13727 11571 13728 11572 13730 11575 13730 11578 13730
11580 13731 11581 13734 11583 13735 11582 13738 11582 13741 11580 13742
11580 13739 11579 13736 11577 13735 11574 13735 11571 13734 11570 13732
11567 13729 19 P
11553 14028 11557 14031 11558 14035 11560 14032 11564 14030 11565 14026
11569 14025 11573 14026 11578 14027 11579 14032 11583 14034 11586 14037
11583 14040 11579 14041 11575 14044 11571 14046 11567 14045 11565 14049
11562 14051 11561 14055 11557 14058 11558 14054 11556 14050 11555 14046
11554 14041 11555 14038 11555 14033 11553 14028 28 P
11585 14033 11586 14030 11586 14027 11589 14027 11591 14027 11594 14026
11595 14023 11595 14020 11597 14022 11598 14024 11600 14026 11598 14028
11595 14027 11594 14030 11593 14032 11591 14034 11589 14036 11585 14033
18 P
11699 14067 11701 14063 11702 14059 11703 14054 11704 14050 11707 14053
11710 14056 11713 14052 11712 14048 11716 14049 11718 14053 11721 14057
11723 14061 11724 14065 11729 14066 11728 14070 11727 14074 11726 14078
11722 14080 11718 14080 11714 14080 11709 14080 11708 14075 11705 14073
11700 14073 11699 14067 26 P
11747 14034 11750 14037 11753 14041 11757 14039 11759 14035 11763 14038
11763 14042 11765 14046 11765 14051 11770 14049 11774 14049 11774 14054
11778 14056 11782 14058 11783 14061 11782 14066 11779 14069 11779 14074
11775 14075 11771 14075 11766 14076 11763 14079 11759 14078 11759 14082
11761 14086 11761 14090 11759 14087 11757 14083 11753 14081 11750 14084
11745 14085 11748 14081 11751 14078 11754 14075 11755 14071 11757 14067
11757 14062 11758 14058 11753 14056 11750 14054 11751 14050 11756 14051

11753 14047 11752 14042 11749 14039 11747 14034 46 P
11634 14059 11638 14058 11642 14055 11646 14055 11650 14054 11655 14053
11659 14054 11662 14057 11664 14061 11660 14063 11658 14059 11654 14059
11650 14061 11645 14062 11641 14063 11637 14065 11634 14059 17 P
11644 14012 11645 14009 11648 14007 11651 14006 11655 14005 11658 14005
11657 14009 11655 14011 11651 14012 11648 14013 11644 14012 11 P
11623 14011 11626 14008 11629 14009 11632 14008 11635 14006 11637 14004
11640 14003 11641 14006 11638 14007 11636 14009 11636 14012 11633 14014
11630 14016 11628 14013 11623 14011 15 P
11611 14016 11613 14013 11616 14012 11619 14012 11622 14011 11623 14014
11622 14017 11619 14017 11616 14016 11611 14016 10 P
11555 14005 11557 14003 11559 14000 11562 13999 11565 13999 11568 14002
11566 14005 11568 14007 11564 14007 11563 14010 11560 14011 11557 14010
11555 14005 13 P
11549 13960 11552 13961 11555 13959 11551 13959 11551 13955 11554 13954
11557 13954 11559 13957 11561 13960 11562 13963 11561 13966 11558 13968
11555 13967 11551 13966 11550 13963 11549 13960 16 P
11498 13944 11500 13941 11503 13939 11503 13942 11503 13946 11503 13949
11506 13948 11510 13949 11508 13952 11505 13953 11502 13954 11499 13952
11500 13949 11498 13944 14 P
11527 13920 11524 13920 11524 13924 11521 13926 11518 13927 11516 13924
11518 13921 11521 13919 11523 13917 11525 13914 11528 13916 11531 13918
11533 13919 11530 13919 11527 13920 15 P
11502 14002 11505 14001 11508 14003 11510 14005 11513 14005 11512 14007
11514 14009 11516 14012 11513 14012 11511 14010 11508 14009 11507 14005
11504 14005 11502 14002 14 P
11686 13980 11689 13978 11692 13976 11693 13979 11692 13982 11691 13985
11687 13985 11686 13980 8 P
11705 13957 11707 13954 11708 13951 11708 13948 11711 13947 11714 13948
11717 13949 11720 13950 11718 13954 11717 13957 11715 13959 11713 13956
11710 13957 11708 13960 11705 13957 15 P
11677 14136 11680 14135 11683 14132 11683 14129 11683 14126 11685 14123
11688 14122 11691 14123 11694 14124 11695 14127 11694 14131 11697 14132
11695 14134 11692 14136 11690 14139 11688 14141 11685 14143 11683 14145
11680 14146 11679 14143 11678 14139 11677 14136 22 P
11656 14172 11658 14169 11661 14168 11662 14165 11663 14162 11665 14160
11667 14158 11670 14160 11672 14163 11674 14164 11677 14161 11677 14158
11679 14160 11682 14158 11684 14156 11686 14153 11687 14156 11689 14158
11690 14161 11689 14165 11688 14167 11685 14168 11683 14170 11680 14173
11677 14174 11674 14174 11673 14171 11671 14169 11667 14170 11665 14173
11663 14175 11661 14177 11658 14177 11656 14175 11656 14172 35 P
11709 14186 11710 14181 11714 14179 11718 14180 11720 14184 11725 14184
11728 14187 11731 14189 11736 14188 11740 14189 11744 14190 11745 14195

11748 14198 11748 14202 11745 14205 11743 14209 11738 14211 11735 14212
11730 14212 11726 14214 11722 14215 11718 14218 11715 14216 11713 14212
11713 14208 11713 14203 11711 14199 11712 14195 11710 14191 11709 14186
30 P
11691 14194 11694 14191 11698 14189 11699 14185 11699 14181 11703 14180
11702 14184 11705 14188 11707 14192 11704 14195 11700 14195 11695 14195
11691 14194 13 P
11685 14205 11687 14204 11690 14206 11693 14207 11696 14205 11699 14203
11702 14203 11702 14207 11701 14210 11702 14213 11702 14216 11700 14218
11697 14220 11694 14219 11693 14216 11691 14214 11688 14212 11686 14210
11685 14205 19 P
11707 14170 11709 14168 11712 14168 11714 14171 11714 14174 11712 14176
11708 14175 11707 14170 8 P
11630 14222 11632 14219 11634 14217 11637 14215 11639 14212 11640 14209
11640 14206 11643 14205 11647 14205 11650 14207 11652 14208 11656 14208
11659 14208 11662 14208 11664 14210 11664 14214 11660 14213 11657 14212
11654 14211 11651 14211 11648 14212 11645 14214 11644 14217 11641 14218
11638 14219 11635 14221 11630 14222 27 P
11678 14208 11681 14207 11684 14210 11684 14213 11684 14216 11686 14218
11684 14221 11680 14219 11680 14216 11680 14213 11678 14208 11 P
11672 14211 11673 14209 11675 14211 11677 14214 11677 14216 11674 14215
11672 14211 7 P
11635 14349 11638 14343 11640 14336 11640 14329 11640 14323 11641 14316
11640 14309 11643 14303 11649 14302 11656 14301 11658 14294 11663 14290
11666 14283 11668 14277 11665 14272 11658 14269 11652 14267 11654 14260
11654 14253 11660 14251 11666 14248 11673 14250 11680 14248 11686 14247
11693 14245 11693 14252 11694 14259 11700 14256 11705 14252 11712 14251
11717 14256 11722 14261 11723 14267 11721 14274 11718 14280 11720 14287
11715 14291 11714 14298 11715 14304 11713 14311 11710 14317 11710 14324
11713 14330 11710 14336 11706 14342 11705 14349 11700 14353 11693 14356
11687 14359 11682 14363 50 P
11682 14363 11676 14367 11671 14372 11665 14373 11659 14369 11652 14369
11650 14363 11647 14357 11641 14353 11635 14349 10 P
11605 14302 11608 14299 11612 14297 11615 14294 11619 14292 11623 14291
11627 14289 11630 14287 11634 14283 11634 14280 11632 14275 11635 14273
11638 14269 11641 14273 11644 14275 11647 14279 11650 14281 11653 14285
11653 14289 11652 14294 11648 14296 11644 14298 11640 14298 11636 14299
11633 14302 11629 14304 11626 14307 11626 14311 11626 14316 11627 14320
11629 14324 11627 14327 11622 14326 11619 14324 11615 14323 11612 14320
11612 14316 11612 14311 11612 14307 11613 14303 11605 14302 41 P
11573 14331 11577 14329 11581 14326 11585 14325 11589 14325 11591 14330
11595 14331 11599 14331 11602 14334 11607 14335 11610 14332 11614 14334
11618 14336 11621 14338 11623 14342 11626 14346 11627 14350 11626 14354

11623 14358 11620 14355 11616 14354 11612 14353 11607 14353 11604 14356
11600 14358 11596 14360 11592 14359 11587 14358 11584 14356 11581 14353
11580 14349 11579 14344 11580 14340 11578 14337 11573 14331 35 P
11589 14268 11591 14266 11592 14263 11594 14261 11597 14259 11600 14257
11604 14257 11606 14256 11605 14259 11602 14260 11601 14264 11602 14267
11602 14270 11599 14270 11596 14269 11593 14270 11589 14268 17 P
11558 14278 11561 14276 11563 14274 11566 14275 11569 14276 11572 14274
11574 14272 11577 14270 11580 14269 11582 14272 11582 14275 11579 14277
11577 14280 11576 14282 11575 14286 11572 14285 11570 14282 11568 14280
11564 14280 11561 14281 11558 14278 21 P
11543 14283 11545 14280 11548 14281 11550 14280 11553 14281 11551 14284
11549 14285 11547 14286 11543 14283 9 P
11599 14310 11602 14309 11604 14312 11602 14315 11601 14316 11599 14314
11599 14310 7 P
11583 14305 11585 14304 11587 14303 11590 14304 11593 14304 11595 14305
11593 14307 11591 14307 11588 14307 11585 14307 11583 14305 11 P
10884 14376 10886 14372 10889 14370 10892 14367 10893 14363 10895 14359
10897 14355 10900 14352 10904 14352 10909 14352 10913 14351 10917 14352
10920 14350 10924 14351 10925 14356 10927 14359 10925 14363 10920 14364
10919 14368 10920 14372 10925 14373 10928 14372 10927 14367 10931 14368
10934 14371 10939 14371 10936 14374 10940 14374 10938 14378 10936 14381
10934 14385 10931 14387 10926 14387 10922 14387 10918 14385 10913 14385
10909 14383 10905 14381 10902 14380 10898 14377 10894 14379 10890 14381
10884 14376 43 P
10913 14298 10915 14296 10919 14295 10922 14295 10922 14292 10921 14288
10924 14290 10927 14292 10930 14290 10933 14292 10935 14294 10938 14296
10938 14300 10935 14302 10935 14305 10938 14308 10940 14310 10939 14312
10936 14314 10933 14313 10930 14315 10929 14312 10927 14309 10924 14308
10923 14305 10921 14303 10920 14300 10918 14298 10913 14298 29 P
10943 14267 10946 14268 10948 14271 10951 14272 10954 14274 10957 14273
10960 14272 10960 14275 10961 14278 10958 14279 10955 14278 10952 14277
10949 14278 10948 14274 10945 14273 10943 14267 16 P
11056 14306 11056 14303 11058 14302 11062 14302 11064 14301 11068 14301
11069 14304 11071 14307 11072 14310 11074 14309 11076 14311 11076 14315
11077 14318 11077 14321 11073 14320 11070 14321 11067 14321 11064 14319
11066 14317 11070 14316 11069 14313 11069 14310 11066 14310 11063 14311
11059 14312 11059 14309 11056 14306 27 P
11115 14302 11119 14302 11122 14301 11122 14297 11121 14295 11123 14296
11125 14299 11128 14302 11130 14303 11133 14305 11135 14307 11135 14310
11138 14309 11141 14311 11139 14314 11138 14317 11135 14315 11132 14315
11129 14314 11126 14312 11125 14309 11122 14308 11120 14305 11115 14302
24 P
11152 14253 11155 14251 11157 14249 11160 14250 11163 14252 11166 14252

11169 14253 11172 14255 11174 14258 11175 14261 11178 14263 11180 14265
11182 14268 11185 14269 11188 14271 11191 14273 11192 14275 11194 14279
11196 14281 11198 14283 11199 14286 11201 14289 11203 14292 11204 14294
11201 14293 11199 14294 11195 14292 11193 14290 11191 14288 11188 14286
11185 14287 11183 14284 11180 14283 11177 14281 11178 14278 11180 14275
11180 14272 11180 14268 11178 14271 11178 14274 11175 14276 11173 14278
11170 14276 11167 14275 11169 14273 11168 14269 11166 14268 11164 14265
11163 14262 11160 14260 50 P
11160 14260 11158 14258 11155 14257 11152 14253 4 P
11353 14257 11355 14255 11357 14252 11356 14249 11357 14246 11360 14245
11362 14247 11365 14250 11367 14252 11368 14255 11370 14258 11370 14261
11367 14261 11363 14261 11360 14260 11357 14260 11354 14260 11353 14257
18 P
11437 14319 11439 14316 11442 14317 11445 14318 11448 14318 11450 14321
11452 14324 11454 14326 11450 14325 11448 14324 11446 14321 11443 14323
11441 14321 11437 14319 14 P
11381 14329 11384 14328 11387 14328 11389 14331 11388 14334 11388 14337
11385 14335 11384 14332 11381 14329 9 P
11150 14281 11153 14281 11156 14280 11159 14278 11160 14275 11163 14276
11161 14279 11163 14281 11160 14283 11157 14285 11154 14283 11150 14281

12:00 PM

10926 14680 10929 14678 10933 14677 10937 14677 10934 14681 10936 14685
10940 14682 10944 14683 10947 14685 10949 14682 10954 14680 10957 14678
10961 14676 10965 14674 10969 14673 10969 14678 10972 14675 10977 14674
10979 14678 10977 14681 10975 14686 10972 14689 10969 14691 10969 14695
10965 14695 10961 14695 10957 14698 10953 14700 10949 14701 10946 14704
10944 14700 10941 14697 10940 14693 10935 14692 10931 14691 10931 14687
10932 14683 10926 14680 38 P
11016 14764 11015 14768 11013 14772 11012 14768 11012 14763 11010 14759
11012 14756 11016 14756 11016 14751 11012 14754 11009 14751 11008 14747
11011 14743 11014 14747 11018 14749 11021 14751 11023 14755 11023 14759
11026 14763 11026 14768 11023 14763 11016 14764 22 P
10901 15209 10905 15209 10909 15212 10912 15215 10916 15215 10920 15217
10923 15220 10925 15224 10929 15226 10932 15229 10933 15233 10937 15234
10936 15238 10940 15239 10944 15237 10947 15240 10949 15244 10948 15248
10944 15248 10940 15247 10942 15251 10939 15252 10942 15255 10938 15256
10940 15260 10936 15262 10935 15266 10939 15269 10943 15268 10942 15273
10939 15275 10935 15277 10931 15277 10927 15279 10924 15275 10920 15274
10924 15271 10922 15267 10919 15265 10921 15262 10922 15258 10919 15255
10915 15253 10913 15249 10909 15249 10906 15247 10903 15243 10902 15239
10906 15241 10910 15242 50 P
10910 15242 10914 15243 10918 15241 10915 15238 10911 15237 10913 15232
10917 15232 10918 15227 10916 15223 10912 15223 10907 15224 10907 15219

10906 15215 10901 15209 14 P
10806 15224 10810 15224 10812 15228 10815 15231 10819 15230 10823 15226
10825 15230 10825 15234 10829 15233 10830 15229 10834 15227 10838 15226
10842 15228 10845 15231 10846 15235 10849 15239 10850 15243 10846 15245
10842 15246 10838 15247 10837 15244 10832 15244 10828 15244 10825 15241
10821 15243 10818 15241 10818 15237 10816 15233 10811 15233 10809 15230
10806 15224 31 P
10845 15210 10847 15207 10851 15210 10855 15211 10855 15216 10857 15219
10854 15222 10856 15226 10852 15228 10848 15226 10846 15223 10844 15219
10846 15215 10845 15210 14 P
10809 15171 10812 15170 10811 15167 10814 15165 10817 15166 10820 15166
10821 15169 10822 15173 10819 15175 10818 15177 10818 15181 10815 15179
10813 15177 10810 15175 10809 15171 15 P
10818 15186 10821 15184 10824 15184 10823 15187 10824 15190 10821 15192
10819 15190 10818 15186 8 P
10817 15198 10820 15197 10822 15199 10824 15201 10822 15203 10820 15204
10818 15202 10817 15198 8 P
9401 15141 9403 15134 9403 15127 9409 15125 9415 15123 9422 15125
9428 15124 9432 15119 9435 15113 9438 15118 9444 15122 9448 15128
9453 15132 9460 15132 9466 15133 9472 15129 9478 15126 9485 15125
9491 15127 9495 15133 9500 15138 9507 15139 9510 15145 9514 15151
9520 15149 9526 15153 9529 15159 9530 15166 9529 15172 9530 15179
9526 15184 9524 15191 9518 15193 9512 15196 9506 15199 9500 15202
9493 15202 9497 15197 9498 15190 9494 15184 9488 15182 9481 15182
9477 15176 9471 15174 9464 15174 9457 15174 9451 15173 9444 15170
9437 15170 9431 15174 50 P
9431 15174 9429 15167 9430 15161 9424 15157 9419 15153 9416 15147
9412 15142 9401 15141 8 P
9499 15099 9496 15105 9489 15107 9484 15109 9477 15111 9470 15110
9465 15105 9461 15111 9456 15115 9449 15113 9445 15108 9442 15102
9442 15095 9437 15090 9431 15089 9424 15089 9422 15082 9424 15076
9417 15075 9421 15069 9420 15062 9415 15057 9421 15054 9428 15052
9432 15057 9434 15063 9439 15068 9443 15073 9448 15078 9449 15085
9455 15089 9461 15089 9468 15089 9470 15082 9465 15077 9461 15072
9457 15067 9454 15061 9451 15055 9457 15053 9464 15051 9471 15050
9475 15046 9482 15047 9488 15050 9489 15057 9493 15062 9495 15069
9495 15075 9492 15082 50 P
9492 15082 9487 15085 9481 15089 9488 15088 9494 15088 9501 15090
9499 15099 7 P
9865 14967 9869 14961 9868 14955 9873 14950 9878 14946 9884 14949
9886 14956 9888 14962 9893 14958 9900 14956 9899 14963 9897 14970
9894 14976 9897 14982 9903 14984 9897 14985 9892 14980 9885 14978
9878 14978 9876 14971 9865 14967 21 P

10216 14929 10222 14929 10226 14928 10229 14922 10224 14925 10220 14921
10220 14915 10220 14910 10222 14905 10226 14902 10230 14906 10233 14911
10238 14913 10242 14917 10242 14922 10239 14926 10236 14931 10233 14936
10233 14941 10231 14936 10226 14933 10221 14934 10216 14929 23 P
10035 14900 10037 14897 10041 14894 10037 14892 10041 14889 10044 14886
10048 14886 10052 14885 10057 14883 10060 14885 10059 14890 10055 14891
10054 14895 10051 14898 10047 14896 10044 14899 10043 14904 10039 14903
10035 14900 19 P
10141 15185 10138 15182 10141 15178 10140 15174 10137 15177 10133 15177
10133 15182 10129 15183 10126 15180 10125 15176 10123 15172 10119 15169
10118 15165 10122 15164 10126 15164 10129 15160 10131 15156 10133 15153
10137 15153 10141 15153 10144 15157 10147 15160 10150 15164 10151 15168
10154 15171 10154 15175 10152 15178 10148 15181 10141 15185 29 P
9938 15033 9941 15030 9945 15028 9949 15027 9954 15026 9957 15024
9960 15022 9965 15021 9969 15020 9973 15018 9973 15014 9976 15011
9978 15015 9977 15019 9981 15020 9986 15020 9990 15019 9994 15018
9999 15019 10003 15018 10007 15017 10010 15014 10014 15017 10016 15020
10020 15022 10016 15022 10012 15024 10007 15025 10003 15026 10001 15030
9997 15031 9994 15028 9991 15026 9986 15025 9982 15025 9978 15026
9973 15026 9970 15029 9967 15033 9967 15037 9964 15034 9962 15031
9959 15033 9959 15037 9955 15037 9951 15034 9948 15037 9945 15034
9938 15033 49 P
9995 15001 9998 14998 10002 14998 10006 15001 10010 15000 10014 15002
10018 15002 10022 15003 10027 15002 10026 14997 10024 14993 10023 14989
10023 14985 10025 14988 10029 14991 10032 14993 10036 14993 10036 14998
10041 14999 10044 15001 10048 15003 10045 15005 10042 15009 10038 15011
10041 15014 10037 15012 10035 15008 10031 15010 10027 15010 10022 15009
10018 15007 10014 15006 10010 15006 10006 15006 10001 15005 9995 15001
36 P
10049 15004 10052 15003 10055 15003 10058 15005 10055 15006 10052 15005
10049 15004 7 P
10055 15000 10058 14999 10060 14997 10063 14999 10066 15000 10069 14999
10072 15001 10069 15003 10066 15003 10063 15002 10060 15003 10055 15000

12:00 PM

10166 15128 10168 15124 10171 15121 10175 15120 10179 15118 10180 15122
10180 15126 10184 15126 10181 15130 10177 15131 10173 15131 10169 15133
10166 15128 13 P
10151 15028 10149 15025 10146 15024 10147 15021 10151 15020 10153 15021
10155 15025 10151 15028 8 P
9938 14997 9941 14995 9942 14992 9945 14993 9948 14992 9951 14993
9955 14993 9958 14993 9960 14996 9962 14998 9959 14999 9956 14999
9952 14999 9949 15000 9946 15000 9943 15000 9938 14997 17 P
9979 14988 9981 14985 9984 14988 9987 14988 9989 14991 9989 14994

9987 14997 9986 14994 9983 14995 9983 14991 9979 14988 11 P
10065 14920 10067 14918 10070 14917 10073 14918 10076 14918 10079 14917
10081 14919 10079 14921 10076 14922 10073 14921 10070 14921 10065 14920

12:00 PM

10095 14917 10097 14914 10100 14912 10101 14909 10103 14907 10105 14911
10107 14913 10104 14915 10101 14915 10095 14917 10 P
10081 14903 10083 14901 10086 14903 10089 14905 10091 14907 10089 14910
10086 14911 10085 14908 10082 14907 10081 14903 10 P
10428 15308 10431 15308 10433 15310 10436 15310 10440 15311 10443 15311
10446 15311 10444 15313 10442 15317 10440 15318 10436 15317 10434 15315
10431 15314 10428 15312 10428 15308 15 P
9907 14991 9910 14991 9913 14989 9915 14991 9919 14991 9919 14995
9918 14998 9915 14998 9912 14996 9910 14994 9907 14991 11 P
9906 15014 9909 15013 9912 15012 9915 15011 9916 15008 9920 15009
9923 15009 9921 15011 9918 15013 9915 15013 9913 15016 9910 15016
9906 15014 13 P
9667 14792 9668 14789 9668 14785 9670 14783 9673 14784 9674 14787
9674 14790 9672 14793 9673 14796 9674 14799 9670 14800 9668 14798
9670 14794 9667 14792 14 P
9195 15639 9198 15636 9202 15638 9205 15641 9209 15643 9213 15644
9217 15644 9218 15649 9218 15653 9218 15657 9214 15659 9210 15662
9208 15658 9204 15658 9203 15653 9204 15649 9200 15649 9197 15646
9195 15639 19 P
8356 14826 8360 14827 8364 14828 8368 14830 8371 14833 8374 14836
8378 14839 8379 14843 8376 14847 8373 14850 8370 14853 8368 14849
8370 14845 8374 14843 8374 14839 8371 14837 8367 14834 8365 14830
8361 14828 8356 14826 20 P
8392 14873 8396 14874 8400 14876 8404 14876 8409 14877 8412 14878
8415 14882 8416 14886 8418 14890 8423 14892 8424 14896 8422 14900
8417 14899 8415 14895 8412 14892 8410 14888 8408 14884 8404 14882
8400 14879 8396 14878 8392 14873 21 P
8474 14926 8470 14925 8467 14922 8462 14920 8459 14918 8455 14917
8454 14913 8451 14910 8447 14907 8444 14905 8440 14902 8437 14900
8436 14896 8433 14892 8430 14890 8426 14889 8430 14887 8433 14889
8438 14890 8441 14891 8446 14893 8449 14896 8452 14899 8455 14902
8457 14906 8460 14909 8464 14912 8466 14915 8469 14918 8473 14921
8474 14926 31 P
8396 14889 8399 14887 8402 14888 8404 14890 8405 14893 8406 14896
8407 14899 8405 14903 8403 14899 8402 14897 8400 14894 8398 14892
8396 14889 13 P
8271 14773 8276 14774 8279 14777 8282 14780 8285 14783 8287 14787
8289 14791 8292 14794 8294 14798 8294 14802 8293 14806 8290 14802
8289 14799 8285 14796 8282 14793 8281 14789 8280 14785 8278 14781

8275 14778 8271 14773 20 P
8292 14820 8291 14817 8294 14816 8297 14818 8299 14820 8300 14823
8301 14826 8298 14825 8295 14823 8292 14820 10 P
8310 14835 8313 14833 8315 14835 8318 14836 8320 14839 8320 14842
8322 14845 8324 14847 8325 14850 8323 14849 8321 14846 8318 14844
8317 14841 8315 14839 8313 14837 8310 14835 16 P
9246 14455 9249 14452 9253 14454 9255 14459 9259 14459 9263 14460
9265 14464 9265 14468 9261 14470 9257 14469 9253 14468 9250 14464
9250 14460 9246 14455 14 P
9224 14384 9227 14381 9231 14383 9235 14382 9239 14382 9242 14386
9239 14389 9234 14390 9230 14390 9231 14394 9229 14399 9227 14402
9224 14399 9224 14395 9223 14390 9224 14384 16 P
9166 14277 9168 14273 9171 14270 9175 14268 9178 14265 9181 14269
9181 14273 9180 14278 9181 14282 9177 14284 9173 14285 9169 14282
9166 14277 13 P
9203 14290 9206 14287 9209 14283 9211 14279 9214 14276 9219 14276
9222 14274 9225 14277 9224 14281 9222 14285 9220 14289 9216 14289
9212 14291 9208 14292 9203 14290 15 P
9220 14248 9224 14246 9228 14246 9231 14243 9233 14240 9237 14237
9239 14241 9238 14245 9237 14250 9239 14253 9237 14257 9234 14261
9232 14265 9231 14268 9227 14271 9224 14268 9222 14264 9222 14260
9224 14256 9224 14252 9220 14248 21 P
9308 14314 9310 14311 9313 14310 9316 14312 9315 14315 9313 14317
9309 14317 9308 14314 8 P
9287 14507 9291 14507 9294 14507 9297 14509 9299 14507 9300 14510
9298 14512 9297 14515 9294 14516 9293 14513 9290 14511 9287 14507
12:00 PM
9286 14496 9288 14494 9290 14491 9292 14489 9294 14486 9295 14489
9293 14492 9296 14494 9293 14494 9293 14498 9290 14499 9286 14496
12:00 PM
9009 13062 9012 13059 9016 13058 9019 13060 9023 13063 9023 13067
9021 13070 9020 13075 9019 13079 9015 13080 9015 13075 9012 13072
9010 13068 9009 13062 14 P
8704 12522 8705 12518 8708 12515 8712 12513 8714 12509 8718 12508
8722 12506 8726 12509 8730 12510 8727 12514 8723 12515 8719 12516
8715 12517 8711 12519 8708 12522 8704 12522 16 P
9537 10895 9541 10890 9546 10886 9551 10881 9556 10876 9561 10871
9565 10865 9570 10860 9576 10864 9582 10860 9589 10858 9594 10853
9599 10848 9604 10844 9610 10839 9615 10834 9619 10829 9625 10825
9631 10822 9637 10819 9644 10816 9647 10810 9653 10807 9660 10807
9667 10804 9672 10800 9678 10797 9685 10795 9691 10791 9697 10790
9704 10787 9709 10783 9716 10780 9721 10776 9727 10772 9734 10770
9740 10768 9746 10764 9752 10761 9759 10758 9765 10758 9772 10759

9779 10757 9785 10755 9792 10753 9799 10751 9805 10748 9811 10745
9817 10741 9824 10739 50 P
9824 10739 9830 10737 9837 10734 9844 10733 9850 10732 9857 10731
9864 10730 9870 10733 9876 10737 9879 10743 9883 10748 9889 10752
9890 10759 9892 10766 9893 10773 9890 10779 9884 10783 9879 10787
9872 10789 9865 10790 9858 10790 9852 10791 9846 10794 9839 10795
9833 10800 9827 10803 9821 10807 9815 10810 9810 10814 9803 10817
9797 10819 9790 10821 9784 10824 9777 10826 9770 10827 9764 10830
9757 10831 9751 10834 9745 10838 9740 10843 9738 10849 9732 10854
9727 10858 9722 10863 9716 10866 9709 10868 9703 10871 9697 10874
9690 10876 9683 10878 50 P
9683 10878 9677 10881 2 P
9677 10881 9671 10884 9667 10889 9663 10896 9660 10902 9656 10908
9653 10914 9648 10919 9643 10923 9637 10927 9631 10930 9625 10932
9618 10933 9611 10935 9604 10935 9597 10935 9590 10933 9584 10931
9578 10929 9571 10926 9566 10922 9561 10917 9555 10912 9550 10909
9544 10904 9537 10895 26 P
9745 10713 9752 10710 9758 10708 9764 10704 9770 10702 9775 10697
9781 10694 9788 10690 9793 10687 9799 10682 9805 10679 9811 10677
9817 10673 9823 10670 9826 10663 9830 10658 9835 10653 9841 10649
9847 10651 9847 10658 9847 10665 9843 10671 9839 10676 9835 10681
9830 10687 9825 10690 9820 10695 9813 10697 9808 10702 9801 10704
9795 10707 9788 10709 9782 10711 9775 10713 9768 10715 9762 10716
9755 10718 9745 10713 38 P
9849 10683 9853 10676 9855 10670 9857 10663 9859 10657 9861 10650
9866 10646 9872 10642 9877 10638 9884 10641 9887 10647 9891 10652
9894 10659 9893 10666 9890 10672 9884 10675 9878 10679 9871 10682
9864 10682 9858 10684 9849 10683 21 P
9982 10004 9976 10008 9969 10009 9969 10015 9965 10021 9959 10024
9956 10017 9950 10015 9954 10009 9959 10004 9964 10000 9967 9994
9970 9987 9974 9982 9976 9975 9977 9968 9976 9961 9973 9955
9977 9949 9982 9944 9987 9939 9991 9934 9995 9928 9996 9921
10003 9918 10008 9922 10013 9927 10020 9929 10027 9929 10034 9929
10040 9926 10043 9920 10050 9920 10057 9921 10064 9922 10071 9920
10077 9917 10083 9914 10090 9912 10097 9910 10103 9908 10109 9904
10115 9900 10122 9897 10128 9898 10135 9899 10141 9902 10138 9908
10137 9915 10139 9921 50 P
10139 9921 10143 9928 10145 9934 10151 9939 10144 9942 10140 9948
10135 9951 10130 9957 10126 9962 10120 9966 10113 9965 10106 9966
10102 9972 10096 9974 10094 9981 10087 9980 10080 9980 10074 9984
10071 9990 10064 9989 10057 9989 10051 9991 10046 9997 10042 10002
10038 10008 10032 10012 10026 10014 10020 10009 10014 10007 10007 10005
10001 10002 9994 10005 9989 10009 9982 10004 34 P

10016 10085 10022 10081 10029 10079 10035 10075 10041 10072 10046 10067
10053 10066 10060 10065 10067 10065 10074 10064 10080 10061 10087 10059
10084 10066 10082 10072 10087 10077 10088 10084 10084 10090 10089 10095
10094 10100 10087 10100 10082 10105 10083 10112 10078 10114 10071 10113
10065 10116 10063 10109 10057 10106 10050 10104 10045 10099 10039 10102
10032 10102 10028 10097 10021 10093 10016 10085 34 P
10107 10192 10109 10186 10108 10179 10113 10174 10111 10167 10118 10164
10124 10161 10131 10160 10129 10167 10136 10168 10141 10172 10147 10168
10154 10169 10161 10167 10168 10166 10174 10163 10181 10163 10179 10170
10173 10173 10178 10177 10174 10183 10167 10185 10160 10185 10153 10186
10152 10192 10145 10192 10148 10185 10141 10187 10135 10189 10128 10191
10121 10188 10115 10191 10107 10192 33 P
10168 10321 10175 10321 10182 10321 10189 10323 10195 10326 10202 10326
10209 10326 10216 10326 10223 10328 10217 10332 10221 10338 10215 10337
10208 10337 10202 10332 10195 10331 10190 10336 10183 10338 10180 10344
10174 10347 10167 10346 10173 10342 10175 10335 10174 10328 10168 10321
24 P
10016 9767 10023 9767 10027 9773 10034 9773 10039 9768 10046 9768
10047 9761 10053 9758 10059 9755 10059 9748 10059 9741 10066 9741
10068 9748 10072 9742 10078 9745 10079 9738 10086 9737 10089 9743
10090 9750 10094 9755 10090 9761 10088 9767 10081 9770 10077 9775
10070 9776 10064 9781 10059 9786 10055 9792 10057 9798 10054 9805
10050 9810 10045 9806 10038 9803 10031 9803 10026 9799 10025 9792
10024 9785 10021 9779 10016 9767 39 P
10097 9836 10103 9831 10109 9828 10112 9822 10117 9817 10123 9814
10128 9808 10135 9807 10141 9803 10147 9806 10143 9812 10139 9818
10140 9825 10134 9829 10140 9833 10136 9838 10129 9840 10122 9843
10116 9845 10109 9843 10102 9843 10097 9836 22 P
10145 9895 10151 9891 10154 9885 10160 9881 10166 9877 10168 9871
10173 9865 10168 9861 10169 9854 10170 9847 10175 9842 10182 9841
10188 9845 10184 9850 10182 9857 10184 9864 10190 9867 10196 9870
10196 9877 10195 9884 10188 9885 10182 9888 10189 9888 10196 9889
10191 9895 10191 9902 10185 9906 10179 9902 10172 9900 10166 9902
10159 9901 10154 9907 10149 9902 10145 9895 34 P
9908 9917 9913 9913 9915 9906 9920 9902 9927 9903 9932 9908
9938 9911 9944 9915 9949 9920 9951 9926 9949 9933 9947 9939
9946 9946 9942 9952 9936 9955 9932 9961 9929 9967 9922 9966
9915 9965 9913 9958 9911 9952 9908 9945 9908 9938 9911 9932
9912 9925 9908 9917 26 P
10050 10019 10053 10013 10058 10008 10064 10010 10069 10015 10070 10022
10077 10025 10081 10030 10075 10034 10068 10036 10062 10039 10056 10035
10052 10030 10050 10019 14 P
10111 10063 10116 10058 10121 10063 10128 10065 10134 10063 10141 10064

10147 10067 10152 10072 10158 10075 10164 10079 10161 10073 10164 10066
10169 10062 10175 10065 10179 10071 10176 10077 10173 10084 10167 10086
10160 10085 10153 10084 10146 10083 10139 10083 10132 10082 10131 10075
10126 10070 10120 10067 10111 10063 27 P
10100 10070 10102 10064 10108 10066 10114 10070 10121 10071 10128 10072
10124 10078 10117 10080 10110 10080 10103 10081 10100 10070 11 P
10325 10336 10327 10333 10330 10336 10334 10337 10336 10333 10341 10332
10341 10336 10340 10340 10341 10344 10343 10349 10338 10349 10334 10347
10331 10344 10328 10341 10325 10336 15 P
10348 10346 10350 10342 10355 10342 10360 10342 10361 10338 10364 10335
10365 10339 10364 10343 10362 10348 10357 10349 10355 10352 10348 10346

12:00 PM

10228 10299 10232 10299 10237 10297 10241 10297 10238 10300 10242 10303
10246 10305 10242 10308 10239 10311 10235 10313 10232 10310 10229 10307
10228 10299 13 P
10167 9931 10169 9927 10166 9924 10167 9920 10171 9917 10175 9917
10176 9921 10174 9925 10175 9930 10177 9933 10182 9932 10186 9934
10184 9938 10180 9939 10179 9943 10181 9948 10179 9951 10176 9948
10172 9947 10168 9946 10169 9942 10168 9938 10167 9931 23 P
9955 9869 9958 9866 9960 9863 9965 9862 9970 9863 9974 9864
9978 9865 9983 9866 9986 9868 9987 9873 9987 9878 9984 9881
9980 9882 9977 9886 9976 9890 9972 9892 9971 9896 9966 9895
9965 9890 9961 9889 9957 9886 9957 9881 9956 9877 9955 9869
24 P
9991 9820 9988 9817 9984 9815 9980 9812 9977 9809 9976 9804
9978 9801 9977 9796 9980 9793 9985 9793 9989 9793 9993 9793
9996 9797 9998 9801 10000 9805 10003 9808 10005 9812 10007 9816
10008 9820 10006 9824 10001 9825 9997 9823 9991 9820 23 P
10095 9801 10100 9800 10102 9796 10104 9792 10107 9788 10111 9786
10115 9784 10118 9781 10122 9782 10126 9781 10130 9779 10133 9782
10138 9782 10143 9782 10143 9786 10139 9785 10138 9790 10134 9791
10130 9788 10126 9789 10122 9789 10117 9790 10114 9794 10111 9796
10113 9800 10116 9804 10111 9806 10108 9808 10104 9808 10100 9806
10095 9801 31 P
10126 9853 10130 9850 10134 9849 10138 9849 10143 9850 10146 9852
10151 9854 10151 9858 10154 9861 10152 9865 10148 9864 10144 9863
10139 9865 10135 9864 10131 9863 10130 9858 10126 9853 17 P
9938 9971 9942 9968 9947 9968 9950 9965 9949 9961 9953 9960
9957 9960 9958 9964 9959 9968 9962 9972 9960 9977 9963 9980
9959 9983 9956 9986 9952 9985 9948 9982 9944 9981 9941 9977
9938 9971 19 P
9998 9781 9999 9778 9999 9775 10000 9772 9999 9768 10002 9770
10004 9773 10007 9775 10008 9778 10009 9781 10013 9782 10013 9786

10012 9788 10008 9789 10005 9788 10002 9787 10000 9784 9998 9781
18 P
9986 10025 9989 10026 9992 10025 9993 10022 9996 10020 9993 10018
9992 10015 9995 10015 9998 10017 10000 10019 10003 10021 10005 10023
10007 10026 10009 10028 10007 10030 10007 10034 10005 10037 10002 10036
9999 10034 9999 10031 9997 10034 9994 10035 9991 10033 9992 10030
9989 10029 9986 10025 26 P
9954 10093 9955 10090 9956 10086 9959 10088 9962 10091 9964 10093
9965 10096 9963 10099 9965 10101 9964 10104 9967 10106 9969 10109
9970 10112 9967 10114 9965 10112 9965 10109 9963 10106 9960 10108
9959 10105 9959 10102 9958 10099 9956 10096 9954 10093 23 P
10346 10020 10346 10017 10348 10014 10352 10014 10355 10014 10358 10014
10357 10017 10355 10020 10353 10022 10349 10022 10346 10020 11 P
9947 9433 9952 9433 9956 9431 9958 9427 9960 9424 9965 9423
9968 9420 9969 9416 9969 9411 9966 9407 9964 9404 9968 9405
9972 9405 9977 9405 9981 9403 9980 9399 9979 9395 9983 9393
9987 9391 9990 9395 9994 9396 9992 9399 9993 9404 9993 9408
9998 9410 10001 9412 9999 9416 9994 9416 9997 9419 10001 9422
10003 9425 10002 9430 10000 9434 10000 9438 9998 9442 10000 9445
10000 9450 9996 9453 9992 9455 9988 9457 9986 9461 9983 9464
9979 9466 9975 9463 9974 9459 9970 9456 9967 9453 9963 9451
9959 9448 9955 9446 50 P
9955 9446 9951 9444 9949 9440 9947 9433 4 P
9904 9429 9906 9425 9910 9425 9914 9425 9919 9424 9923 9422
9927 9419 9929 9416 9932 9412 9936 9413 9941 9412 9944 9414
9949 9416 9946 9420 9950 9420 9953 9424 9956 9427 9953 9431
9949 9431 9945 9433 9947 9438 9944 9441 9940 9440 9936 9439
9931 9438 9927 9438 9923 9439 9920 9442 9915 9441 9911 9439
9908 9442 9905 9440 9906 9435 9904 9429 34 P
9907 9402 9912 9401 9916 9402 9915 9397 9920 9397 9922 9393
9926 9390 9930 9393 9929 9397 9931 9402 9928 9405 9932 9408
9929 9411 9926 9414 9921 9416 9917 9415 9913 9412 9914 9407
9907 9402 19 P
10015 9451 10019 9452 10024 9452 10025 9456 10028 9460 10032 9457
10036 9457 10039 9460 10042 9463 10038 9465 10034 9464 10030 9466
10030 9470 10035 9472 10032 9475 10034 9479 10030 9480 10026 9482
10024 9478 10025 9474 10023 9470 10027 9468 10023 9466 10020 9462
10016 9462 10016 9458 10015 9451 27 P
9607 8857 9609 8851 9609 8844 9613 8837 9617 8832 9621 8827
9620 8820 9614 8816 9620 8812 9619 8806 9626 8808 9633 8808
9639 8811 9646 8814 9652 8816 9652 8823 9659 8820 9664 8816
9667 8822 9661 8827 9662 8833 9668 8830 9675 8832 9677 8826
9684 8828 9680 8834 9686 8837 9687 8844 9680 8844 9673 8848

9669 8853 9670 8860 9668 8867 9666 8873 9668 8880 9673 8886
9680 8886 9677 8893 9672 8897 9666 8900 9660 8904 9653 8903
9646 8901 9642 8896 9640 8889 9642 8882 9636 8879 9632 8873
9626 8869 9620 8865 50 P
9620 8865 9616 8859 9607 8857 3 P
9566 8752 9570 8753 9575 8754 9579 8755 9583 8757 9588 8758
9591 8757 9590 8752 9592 8748 9595 8751 9600 8751 9604 8752
9608 8754 9612 8756 9616 8759 9620 8760 9617 8763 9612 8765
9610 8769 9607 8772 9603 8774 9599 8772 9595 8772 9590 8773
9587 8770 9582 8768 9583 8773 9579 8772 9578 8768 9574 8765
9570 8763 9566 8761 9567 8757 9566 8752 34 P
9557 8693 9560 8689 9562 8693 9567 8694 9571 8697 9574 8699
9578 8696 9582 8694 9582 8699 9586 8701 9588 8704 9592 8707
9596 8708 9593 8710 9589 8708 9584 8706 9580 8704 9576 8706
9573 8709 9570 8713 9566 8713 9565 8708 9561 8706 9561 8701
9560 8697 9557 8693 26 P
9592 8680 9596 8680 9601 8679 9605 8680 9609 8679 9614 8681
9618 8681 9620 8684 9624 8682 9629 8680 9632 8678 9637 8679
9638 8683 9638 8688 9637 8693 9633 8695 9638 8697 9639 8693
9642 8696 9645 8700 9643 8703 9644 8708 9639 8708 9637 8704
9633 8703 9629 8704 9625 8701 9628 8698 9626 8694 9622 8694
9617 8693 9615 8696 9611 8699 9609 8695 9605 8693 9603 8689
9599 8687 9595 8686 9592 8680 39 P
9640 8783 9636 8781 9632 8778 9630 8774 9633 8771 9634 8767
9639 8766 9642 8769 9646 8772 9649 8774 9654 8775 9658 8778
9662 8780 9657 8780 9653 8779 9648 8781 9644 8782 9640 8783
18 P
9579 8479 9582 8475 9586 8472 9589 8469 9589 8474 9588 8478
9593 8476 9597 8475 9601 8473 9605 8473 9609 8469 9608 8474
9605 8478 9601 8479 9598 8482 9595 8486 9591 8487 9588 8490
9583 8491 9587 8488 9590 8485 9586 8486 9584 8482 9579 8479
24 P
9903 5746 9907 5742 9909 5736 9912 5732 9917 5729 9920 5725
9926 5725 9925 5731 9927 5736 9928 5742 9928 5748 9922 5750
9917 5750 9912 5748 9903 5746 15 P
9314 5522 9319 5520 9323 5522 9325 5526 9328 5529 9333 5532
9334 5536 9331 5540 9327 5541 9322 5538 9319 5536 9319 5531
9317 5527 9314 5522 14 P
8866 6216 8872 6212 8878 6208 8881 6202 8888 6200 8895 6201
8902 6204 8901 6211 8906 6217 8911 6222 8918 6225 8923 6230
8930 6230 8937 6230 8944 6227 8941 6233 8939 6240 8938 6247
8934 6254 8929 6249 8927 6255 8921 6253 8919 6246 8917 6253
8914 6246 8910 6240 8910 6247 8911 6254 8904 6254 8900 6248

8896 6242 8895 6235 8891 6241 8888 6247 8882 6244 8879 6237
8875 6231 8882 6233 8884 6227 8877 6227 8883 6224 8890 6224
8898 6224 8895 6217 8889 6213 8885 6207 8880 6212 8874 6217
8871 6223 8866 6216 50 P
8869 6287 8875 6282 8880 6288 8885 6283 8889 6277 8892 6270
8896 6264 8899 6271 8897 6277 8902 6283 8899 6289 8895 6296
8888 6296 8881 6294 8874 6293 8869 6287 16 P
8827 6228 8834 6230 8841 6231 8849 6231 8850 6238 8851 6245
8856 6251 8855 6258 8853 6265 8859 6269 8865 6273 8858 6274
8852 6279 8847 6274 8845 6267 8845 6260 8845 6253 8840 6247
8845 6243 8843 6236 8836 6236 8827 6228 22 P
8840 6110 8844 6109 8849 6110 8853 6109 8851 6113 8854 6117
8851 6120 8851 6125 8851 6130 8846 6130 8843 6134 8842 6129
8842 6125 8843 6120 8843 6116 8840 6110 16 P
8801 6027 8804 6024 8806 6019 8807 6015 8810 6013 8812 6017
8815 6020 8818 6024 8819 6028 8817 6032 8813 6032 8808 6030
8801 6027 13 P
8893 6331 8895 6329 8896 6325 8897 6329 8899 6331 8902 6333
8905 6335 8906 6338 8903 6340 8901 6343 8899 6340 8897 6337
8894 6335 8893 6331 14 P
9120 6187 9120 6182 9121 6177 9122 6173 9123 6168 9124 6164
9125 6168 9124 6173 9123 6177 9123 6182 9125 6187 9126 6191
9126 6195 9128 6200 9129 6204 9132 6208 9134 6212 9137 6216
9136 6220 9133 6216 9131 6212 9128 6209 9127 6204 9124 6201
9122 6196 9121 6192 9120 6187 27 P
8917 7329 8923 7332 8930 7336 8937 7334 8943 7339 8941 7332
8941 7325 8934 7326 8931 7320 8936 7315 8932 7309 8937 7304
8943 7299 8949 7297 8954 7291 8959 7287 8966 7285 8970 7291
8966 7297 8960 7301 8963 7308 8969 7312 8975 7315 8980 7320
8987 7322 8993 7326 9000 7327 9004 7322 9010 7326 9011 7333
9004 7333 8997 7335 8992 7339 8986 7344 8984 7350 8984 7358
8986 7364 8992 7368 8998 7372 9004 7374 9009 7368 9016 7370
9020 7376 9024 7382 9017 7382 9010 7382 9003 7384 8998 7389
8992 7386 8994 7393 50 P
8994 7393 8999 7398 8995 7404 8989 7408 8984 7412 8977 7410
8971 7413 8967 7419 8967 7426 8971 7432 8975 7438 8968 7438
8961 7434 8957 7429 8953 7423 8947 7426 8943 7421 8940 7414
8933 7414 8929 7408 8925 7402 8925 7395 8929 7389 8936 7391
8940 7397 8947 7397 8950 7390 8949 7383 8945 7377 8945 7370
8945 7363 8943 7356 8949 7352 8954 7357 8958 7363 8963 7359
8970 7358 8971 7351 8965 7346 8959 7343 8952 7343 8947 7348
8939 7350 8932 7351 8928 7345 8921 7343 8920 7336 8917 7329
48 P

8799 7188 8804 7183 8801 7176 8806 7171 8813 7170 8820 7168
8826 7172 8833 7174 8838 7170 8836 7163 8835 7156 8842 7153
8848 7150 8856 7150 8858 7157 8853 7162 8856 7168 8863 7169
8868 7175 8861 7173 8856 7178 8852 7173 8844 7173 8838 7177
8832 7180 8828 7185 8828 7192 8821 7194 8818 7201 8813 7196
8809 7190 8799 7188 32 P
8199 6712 8205 6707 8209 6701 8215 6698 8222 6696 8228 6700
8231 6706 8235 6712 8241 6716 8247 6720 8253 6722 8260 6723
8267 6722 8274 6718 8280 6714 8286 6710 8292 6714 8296 6720
8297 6727 8301 6732 8298 6738 8297 6745 8294 6751 8292 6758
8289 6765 8282 6767 8278 6773 8273 6778 8269 6772 8263 6769
8265 6762 8260 6757 8254 6752 8253 6745 8249 6740 8242 6741
8235 6743 8229 6747 8223 6752 8218 6757 8212 6753 8207 6748
8208 6741 8214 6745 8212 6738 8214 6731 8215 6724 8218 6717
8211 6716 8199 6712 50 P
8088 6738 8092 6733 8096 6727 8103 6723 8109 6720 8116 6718
8123 6716 8128 6721 8134 6726 8140 6730 8144 6735 8146 6742
8149 6749 8156 6750 8156 6757 8156 6765 8156 6772 8151 6777
8145 6774 8140 6779 8136 6785 8129 6783 8124 6787 8117 6790
8110 6791 8110 6784 8105 6780 8104 6773 8100 6766 8095 6762
8091 6756 8092 6749 8099 6750 8105 6755 8111 6758 8113 6751
8106 6747 8100 6744 8088 6738 39 P
7690 6229 7698 6229 7705 6231 7712 6233 7718 6231 7716 6224
7722 6228 7729 6230 7736 6233 7743 6233 7750 6236 7757 6236
7754 6242 7746 6243 7745 6250 7745 6257 7745 6264 7748 6271
7752 6276 7759 6279 7766 6276 7772 6273 7779 6274 7780 6281
7775 6286 7775 6293 7777 6300 7784 6303 7781 6310 7787 6315
7793 6310 7801 6311 7802 6318 7808 6321 7801 6321 7797 6326
7790 6330 7786 6335 7780 6330 7773 6332 7770 6325 7762 6326
7756 6323 7753 6330 7749 6336 7743 6340 7737 6344 7732 6348
7725 6346 7724 6339 50 P
7724 6339 7726 6332 7727 6324 7722 6319 7717 6314 7714 6307
7710 6301 7705 6296 7707 6289 7714 6285 7719 6290 7726 6293
7731 6288 7738 6288 7745 6287 7740 6282 7738 6275 7734 6270
7728 6266 7722 6262 7717 6256 7713 6250 7714 6243 7709 6248
7703 6245 7698 6240 7690 6229 27 P
7526 6158 7522 6164 7515 6167 7509 6165 7503 6160 7498 6156
7493 6150 7497 6144 7504 6142 7511 6141 7518 6142 7526 6141
7533 6139 7538 6135 7545 6132 7552 6132 7558 6135 7566 6135
7573 6134 7574 6126 7581 6130 7586 6125 7592 6128 7596 6134
7595 6141 7593 6148 7599 6152 7598 6159 7591 6163 7589 6170
7590 6177 7583 6177 7578 6171 7576 6164 7569 6166 7564 6171
7558 6175 7551 6173 7547 6167 7544 6160 7539 6156 7535 6149

7526 6158 43 P
7527 6225 7520 6224 7513 6224 7510 6218 7505 6212 7498 6211
7491 6210 7493 6203 7499 6199 7506 6198 7513 6196 7520 6194
7522 6201 7523 6208 7527 6214 7533 6217 7527 6225 17 P
7483 6220 7488 6216 7495 6215 7503 6213 7508 6218 7505 6225
7506 6232 7511 6237 7513 6244 7506 6246 7500 6241 7500 6234
7497 6228 7490 6225 7483 6220 15 P
7334 6089 7339 6083 7343 6077 7349 6073 7355 6070 7360 6064
7367 6062 7374 6063 7378 6058 7381 6052 7388 6049 7395 6047
7388 6043 7392 6037 7398 6032 7405 6032 7408 6025 7415 6025
7417 6032 7422 6037 7415 6040 7409 6037 7404 6042 7402 6049
7403 6056 7404 6064 7402 6070 7398 6077 7397 6084 7392 6089
7385 6091 7380 6087 7372 6087 7365 6089 7360 6094 7356 6099
7348 6100 7343 6097 7334 6089 39 P
8712 7320 8715 7315 8720 7309 8720 7302 8723 7296 8730 7298
8735 7303 8742 7303 8743 7310 8740 7317 8741 7324 8740 7331
8742 7338 8745 7344 8748 7351 8746 7358 8749 7364 8749 7371
8749 7378 8746 7384 8740 7382 8736 7375 8732 7369 8726 7365
8726 7358 8723 7352 8720 7345 8719 7338 8716 7332 8712 7320
30 P
8841 7673 8848 7673 8855 7672 8862 7670 8869 7670 8874 7665
8881 7662 8888 7662 8895 7665 8894 7672 8893 7679 8889 7685
8887 7692 8881 7695 8874 7698 8869 7702 8863 7698 8856 7696
8851 7691 8847 7685 8841 7673 21 P
8845 7668 8850 7666 8852 7663 8857 7660 8861 7660 8865 7662
8870 7663 8874 7664 8871 7667 8866 7668 8862 7670 8857 7670
8853 7671 8845 7668 14 P
8892 8003 8894 7998 8897 7994 8897 7990 8902 7990 8903 7994
8907 7992 8911 7990 8916 7991 8921 7992 8925 7993 8927 7997
8930 8000 8933 8003 8938 8004 8942 8005 8945 8008 8944 8013
8940 8015 8936 8014 8932 8011 8931 8007 8929 8003 8925 8005
8927 8008 8922 8009 8917 8007 8913 8009 8909 8010 8904 8009
8900 8009 8896 8008 8892 8003 33 P
9224 8176 9226 8171 9229 8168 9233 8165 9237 8163 9241 8161
9245 8161 9249 8163 9253 8165 9257 8166 9262 8167 9258 8169
9254 8170 9253 8175 9251 8179 9249 8183 9244 8184 9241 8187
9237 8188 9233 8187 9229 8184 9226 8181 9224 8176 23 P
10664 6822 10659 6824 10656 6822 10651 6821 10646 6822 10642 6821
10640 6816 10636 6815 10635 6811 10630 6809 10634 6806 10638 6806
10643 6805 10647 6803 10652 6803 10656 6804 10660 6806 10665 6805
10669 6804 10673 6805 10676 6808 10680 6811 10683 6815 10687 6818
10683 6820 10679 6822 10674 6823 10670 6825 10664 6822 29 P
11073 6610 11076 6607 11081 6606 11085 6606 11090 6606 11094 6606

11098 6608 11101 6612 11103 6615 11101 6619 11097 6621 11092 6621
11087 6621 11083 6619 11079 6617 11074 6615 11073 6610 17 P
11526 6990 11528 6986 11531 6983 11536 6982 11540 6982 11545 6982
11549 6984 11552 6988 11555 6991 11556 6996 11554 7000 11550 7003
11546 7004 11541 7005 11537 7005 11533 7003 11529 7000 11526 6997
11526 6990 19 P
11758 7109 11760 7105 11764 7102 11768 7100 11772 7098 11777 7099
11780 7103 11785 7104 11788 7102 11793 7101 11797 7102 11802 7104
11803 7109 11803 7113 11801 7117 11799 7121 11797 7125 11793 7127
11789 7128 11785 7130 11780 7133 11776 7134 11772 7135 11768 7132
11768 7127 11768 7123 11768 7118 11764 7116 11759 7117 11758 7109
30 P
11525 7049 11528 7048 11532 7047 11535 7045 11538 7044 11539 7047
11537 7050 11534 7052 11531 7054 11528 7052 11525 7049 11 P
11194 6734 11191 6732 11190 6729 11190 6725 11192 6722 11194 6720
11196 6716 11199 6715 11202 6715 11205 6717 11208 6719 11210 6721
11211 6724 11210 6728 11209 6731 11205 6731 11202 6731 11199 6732
11194 6734 19 P
11190 6781 11187 6782 11186 6785 11185 6788 11182 6790 11179 6791
11175 6791 11172 6789 11171 6786 11174 6784 11172 6781 11169 6779
11166 6778 11169 6778 11173 6777 11176 6777 11179 6777 11183 6777
11186 6775 11188 6773 11192 6775 11194 6778 11190 6781 23 P
11231 6776 11232 6772 11235 6770 11239 6770 11242 6772 11244 6775
11244 6778 11243 6781 11240 6784 11238 6786 11234 6786 11232 6783
11232 6780 11231 6776 14 P
11154 6709 11156 6707 11159 6705 11163 6707 11163 6710 11160 6713
11156 6712 11154 6709 8 P
11162 6715 11165 6716 11167 6719 11170 6721 11171 6724 11168 6726
11165 6723 11163 6721 11162 6715 9 P
10964 6681 10967 6680 10968 6681 10970 6683 10972 6684 10970 6685
10968 6684 10967 6683 10964 6681 9 P
10878 7580 10880 7578 10882 7578 10884 7579 10886 7580 10888 7581
10890 7581 10892 7581 10895 7582 10897 7582 10896 7584 10895 7586
10893 7588 10891 7587 10889 7588 10887 7588 10884 7588 10883 7586
10881 7585 10880 7583 10878 7580 21 P
10919 7576 10921 7575 10922 7573 10924 7575 10923 7576 10922 7579
10919 7576 7 P
10955 7677 10956 7676 10959 7675 10961 7676 10962 7678 10961 7680
10960 7681 10957 7682 10956 7680 10955 7677 10 P
10144 7461 10146 7460 10147 7458 10149 7460 10151 7462 10150 7464
10148 7465 10146 7465 10144 7464 10144 7461 10 P
10163 7380 10164 7379 10166 7379 10166 7381 10163 7380 5 P
10094 7010 10096 7008 10097 7007 10099 7006 10101 7005 10103 7005

10106 7005 10108 7005 10110 7005 10111 7008 10109 7009 10108 7011
10107 7013 10104 7014 10102 7014 10100 7013 10099 7011 10098 7010
10094 7010 19 P
8935 6055 8936 6054 8935 6052 8937 6050 8938 6048 8939 6050
8939 6053 8941 6054 8942 6056 8942 6059 8939 6059 8937 6058
8935 6055 13 P
8748 6134 8750 6132 8752 6133 8755 6134 8756 6135 8756 6138
8754 6138 8752 6139 8750 6139 8749 6137 8748 6134 11 P
8904 6050 8907 6049 8909 6048 8911 6048 8912 6051 8911 6053
8909 6053 8907 6052 8904 6050 9 P
8856 6114 8858 6113 8860 6113 8861 6115 8863 6116 8865 6116
8867 6116 8868 6118 8867 6120 8865 6121 8863 6120 8860 6120
8860 6118 8858 6117 8856 6114 15 P
8849 6186 8850 6184 8852 6184 8855 6184 8857 6186 8859 6186
8861 6187 8863 6188 8865 6190 8867 6190 8867 6192 8865 6193
8863 6192 8860 6191 8859 6190 8857 6189 8855 6188 8853 6187
8852 6188 8850 6189 8849 6186 21 P
8849 6060 8851 6059 8853 6059 8854 6061 8852 6062 8849 6060

6:00 PM

8854 6052 8856 6052 8858 6054 8859 6055 8858 6058 8856 6057
8855 6055 8854 6052 8 P
8164 6711 8167 6709 8170 6709 8173 6708 8176 6710 8178 6712
8182 6710 8184 6712 8184 6715 8187 6717 8191 6717 8192 6720
8193 6723 8194 6727 8192 6729 8190 6731 8187 6734 8184 6735
8180 6733 8177 6733 8174 6731 8173 6728 8173 6724 8172 6721
8169 6720 8168 6716 8168 6713 8164 6711 28 P
7950 6742 7954 6739 7958 6737 7961 6734 7964 6730 7967 6726
7970 6724 7975 6722 7979 6723 7984 6723 7988 6723 7992 6724
7990 6728 7990 6733 7985 6734 7981 6733 7977 6731 7973 6734
7970 6737 7967 6741 7963 6740 7959 6738 7955 6741 7950 6742
24 P
7953 6744 7956 6741 7961 6741 7965 6743 7969 6741 7973 6738
7975 6734 7980 6734 7983 6736 7988 6737 7991 6735 7994 6730
7998 6729 8002 6731 8006 6731 8005 6736 8002 6740 7999 6742
7996 6746 7992 6749 7988 6750 7983 6751 7979 6752 7975 6752
7970 6751 7966 6750 7961 6748 7957 6748 7953 6744 29 P
8022 6708 8026 6706 8031 6707 8034 6705 8038 6707 8042 6710
8039 6712 8034 6714 8030 6713 8022 6708 10 P
7978 6720 7983 6718 7987 6716 7991 6714 7996 6713 7997 6718
7992 6720 7988 6721 7983 6721 7978 6720 10 P
7942 6752 7943 6749 7945 6746 7947 6744 7951 6743 7952 6747
7949 6749 7947 6751 7946 6755 7944 6757 7942 6752 11 P
8622 7079 8626 7078 8631 7077 8633 7074 8634 7069 8638 7068

8637 7072 8639 7076 8643 7079 8646 7083 8644 7087 8647 7091
8643 7094 8643 7098 8647 7102 8651 7103 8654 7106 8654 7111
8650 7108 8646 7106 8641 7106 8638 7103 8634 7100 8630 7099
8631 7095 8633 7090 8635 7086 8635 7082 8631 7080 8627 7081
8622 7079 31 P
9030 7712 9034 7710 9038 7709 9042 7712 9046 7712 9050 7715
9054 7716 9057 7721 9058 7725 9053 7726 9049 7725 9047 7721
9043 7719 9040 7723 9036 7722 9038 7726 9034 7729 9033 7725
9031 7721 9032 7717 9030 7712 21 P
9074 7765 9069 7764 9067 7761 9062 7759 9059 7756 9059 7752
9058 7747 9058 7743 9061 7740 9061 7744 9061 7749 9063 7753
9068 7755 9072 7755 9076 7753 9080 7750 9082 7754 9081 7758
9078 7761 9074 7765 20 P
9041 7617 9043 7614 9045 7611 9048 7613 9051 7615 9054 7617
9052 7620 9048 7620 9045 7620 9041 7617 10 P
9061 7601 9065 7603 9069 7605 9072 7609 9075 7612 9070 7611
9066 7612 9064 7608 9061 7601 9 P
9315 7849 9319 7847 9323 7849 9326 7853 9330 7851 9328 7856
9325 7859 9320 7858 9317 7856 9315 7849 10 P
9308 7856 9311 7857 9312 7854 9315 7856 9317 7859 9315 7862
9312 7863 9311 7860 9308 7856 9 P
9127 7955 9131 7952 9134 7949 9139 7951 9142 7953 9142 7957
9147 7959 9151 7959 9152 7964 9153 7969 9153 7973 9150 7977
9146 7978 9144 7974 9141 7970 9139 7966 9137 7962 9133 7960
9127 7955 19 P
9083 7913 9088 7912 9092 7913 9094 7917 9098 7916 9103 7917
9103 7921 9103 7926 9104 7930 9099 7931 9097 7927 9094 7929
9090 7928 9086 7925 9087 7920 9083 7913 16 P
8947 7981 8948 7978 8950 7975 8953 7975 8956 7973 8959 7974
8962 7976 8964 7978 8967 7978 8971 7978 8973 7980 8975 7983
8973 7985 8969 7986 8967 7988 8964 7987 8960 7986 8957 7985
8953 7985 8951 7984 8947 7981 21 P
9469 8761 9472 8760 9475 8758 9478 8756 9481 8754 9484 8753
9486 8751 9489 8749 9492 8748 9495 8746 9499 8747 9502 8749
9504 8751 9507 8753 9509 8756 9509 8759 9507 8762 9504 8764
9501 8765 9497 8765 9494 8766 9491 8765 9488 8765 9485 8765
9481 8765 9478 8764 9475 8763 9469 8761 28 P
9421 8775 9424 8774 9426 8771 9428 8768 9431 8767 9435 8766
9438 8765 9440 8767 9440 8770 9441 8774 9438 8775 9436 8779
9435 8781 9432 8784 9429 8785 9426 8786 9422 8787 9419 8787
9416 8788 9416 8785 9416 8781 9419 8779 9421 8775 23 P
9585 8658 9588 8657 9590 8656 9593 8656 9595 8658 9597 8660
9596 8663 9594 8665 9592 8666 9589 8665 9589 8662 9587 8660

9585 8658 13 P
9654 8565 9655 8564 9657 8565 9659 8567 9659 8569 9660 8571
9658 8571 9656 8570 9655 8568 9654 8565 10 P
9647 8571 9649 8571 9650 8568 9652 8568 9654 8569 9656 8571
9658 8572 9659 8574 9661 8576 9662 8578 9661 8579 9659 8579
9656 8579 9655 8578 9654 8575 9653 8574 9651 8573 9647 8571
18 P
9828 9081 9829 9078 9833 9077 9835 9075 9838 9074 9841 9076
9839 9079 9841 9082 9844 9083 9844 9086 9841 9087 9842 9091
9840 9093 9839 9097 9835 9097 9832 9096 9831 9093 9830 9090
9829 9087 9828 9081 20 P
9836 9068 9838 9065 9840 9063 9843 9062 9847 9063 9849 9065
9847 9069 9844 9069 9841 9070 9836 9068 10 P
9963 9078 9966 9078 9970 9078 9968 9076 9970 9073 9969 9071
9966 9069 9968 9066 9971 9067 9973 9064 9976 9066 9978 9069
9981 9067 9980 9070 9981 9073 9984 9076 9986 9079 9983 9079
9979 9079 9976 9081 9976 9084 9976 9088 9973 9087 9970 9086
9969 9083 9966 9081 9963 9078 27 P
9963 9040 9967 9040 9970 9041 9971 9044 9974 9042 9977 9044
9979 9046 9982 9048 9985 9050 9987 9052 9985 9055 9981 9054
9978 9054 9975 9052 9972 9051 9970 9048 9969 9045 9967 9043
9963 9040 19 P
9938 9233 9941 9231 9943 9233 9945 9235 9948 9237 9950 9239
9950 9242 9950 9246 9952 9248 9956 9248 9955 9250 9954 9254
9952 9256 9948 9256 9945 9254 9945 9251 9945 9248 9945 9244
9942 9244 9943 9241 9943 9237 9941 9235 9938 9233 23 P
9945 9209 9948 9209 9951 9206 9954 9206 9957 9208 9956 9211
9954 9213 9957 9216 9959 9218 9961 9221 9964 9222 9967 9222
9968 9225 9971 9226 9974 9226 9972 9228 9968 9228 9965 9226
9963 9224 9960 9221 9957 9222 9955 9220 9951 9219 9948 9220
9945 9217 9947 9214 9945 9209 27 P
9904 9186 9907 9185 9911 9184 9913 9182 9915 9179 9919 9179
9921 9182 9922 9185 9922 9188 9925 9190 9927 9193 9923 9193
9921 9191 9919 9189 9916 9186 9913 9186 9911 9188 9912 9191
9908 9192 9905 9193 9907 9191 9904 9186 22 P
10087 9603 10089 9600 10092 9602 10095 9601 10097 9604 10100 9605
10100 9609 10103 9611 10106 9612 10109 9613 10108 9616 10105 9615
10102 9617 10100 9615 10096 9613 10097 9610 10095 9607 10092 9605
10089 9607 10087 9603 20 P
10093 9764 10095 9762 10099 9761 10102 9760 10104 9758 10107 9756
10110 9754 10112 9752 10114 9755 10117 9756 10120 9755 10123 9755
10125 9753 10128 9750 10129 9754 10130 9757 10127 9758 10123 9758
10120 9757 10117 9759 10114 9760 10111 9761 10109 9763 10105 9763

10104 9767 10102 9769 10099 9768 10097 9771 10094 9771 10094 9767
10093 9764 31 P
10050 9901 10052 9899 10055 9897 10058 9898 10061 9897 10065 9899
10067 9901 10065 9904 10064 9907 10060 9908 10057 9907 10054 9905
10050 9901 13 P
10052 9910 10056 9909 10059 9910 10062 9910 10065 9910 10068 9909
10070 9906 10073 9904 10075 9905 10076 9909 10075 9911 10072 9914
10070 9916 10068 9918 10065 9920 10062 9919 10060 9916 10057 9914
10052 9910 19 P
10038 9907 10039 9903 10042 9903 10045 9904 10045 9908 10046 9911
10045 9914 10042 9915 10039 9914 10038 9910 10038 9907 11 P
10020 9923 10018 9920 10020 9917 10018 9914 10020 9911 10023 9911
10026 9914 10029 9915 10028 9918 10025 9921 10023 9923 10020 9923
12:00 PM
10000 9896 10001 9893 10001 9889 10005 9888 10008 9888 10010 9890
10012 9893 10014 9896 10014 9899 10013 9902 10010 9904 10006 9904
10004 9902 10001 9900 10000 9896 15 P
10279 10412 10282 10411 10284 10409 10288 10408 10291 10408 10293 10411
10297 10411 10297 10414 10294 10415 10291 10413 10288 10415 10285 10418
10283 10419 10282 10416 10284 10413 10279 10412 16 P
10251 10394 10254 10394 10258 10395 10260 10393 10264 10392 10264 10396
10261 10397 10258 10397 10255 10398 10251 10394 10 P
10267 10393 10270 10392 10273 10394 10276 10395 10273 10398 10270 10399
10268 10397 10267 10393 8 P
10332 10381 10335 10379 10338 10381 10341 10382 10338 10384 10332 10381
6:00 PM
9994 10252 9996 10250 10000 10250 10001 10247 10003 10244 10006 10245
10008 10247 10008 10250 10010 10254 10014 10255 10010 10256 10007 10257
10004 10256 10002 10254 9999 10254 9994 10252 16 P
9990 10278 9992 10276 9994 10273 9998 10275 9999 10278 10000 10280
10003 10283 10000 10282 9996 10284 9996 10280 9993 10282 9990 10278
12:00 PM
10099 10284 10102 10284 10104 10286 10107 10287 10106 10291 10103 10291
10104 10289 10101 10288 10099 10284 9 P
10093 10215 10096 10215 10100 10215 10101 10219 10102 10222 10099 10223
10096 10222 10094 10220 10093 10215 9 P
10122 10218 10126 10218 10128 10216 10131 10214 10135 10213 10136 10216
10135 10220 10136 10223 10135 10226 10132 10224 10132 10221 10129 10220
10125 10221 10122 10218 14 P
10189 10171 10193 10171 10196 10172 10199 10172 10200 10169 10202 10166
10205 10167 10207 10170 10204 10172 10202 10174 10201 10177 10198 10179
10195 10178 10194 10176 10189 10171 15 P
10205 10061 10209 10060 10212 10058 10215 10058 10218 10057 10219 10060

10218 10063 10215 10064 10212 10065 10209 10065 10205 10061 11 P
10284 10054 10287 10052 10288 10049 10289 10046 10292 10044 10293 10048
10292 10051 10291 10054 10291 10057 10288 10058 10284 10054 11 P
10302 10054 10304 10051 10306 10049 10309 10048 10310 10045 10311 10042
10312 10045 10312 10048 10312 10051 10309 10053 10307 10056 10302 10054

12:00 PM

10316 10053 10317 10051 10319 10048 10322 10046 10321 10050 10320 10052
10321 10056 10319 10058 10316 10053 9 P
10324 10039 10325 10037 10327 10036 10329 10038 10328 10041 10327 10043
10324 10039 7 P
10202 9901 10204 9898 10205 9895 10209 9895 10209 9899 10208 9902
10207 9905 10204 9905 10202 9901 9 P
10064 10053 10065 10050 10067 10049 10070 10048 10072 10047 10075 10048
10075 10051 10075 10053 10073 10055 10071 10056 10068 10056 10064 10053

12:00 PM

9792 9437 9795 9435 9798 9434 9800 9431 9803 9431 9806 9432
9810 9433 9813 9433 9814 9437 9813 9440 9812 9442 9808 9443
9806 9441 9806 9438 9803 9437 9800 9437 9797 9439 9794 9439
9792 9437 19 P
9978 9201 9980 9199 9981 9196 9984 9198 9986 9200 9989 9201
9991 9204 9988 9206 9986 9205 9982 9205 9978 9201 11 P
9790 8833 9793 8833 9796 8835 9796 8838 9798 8841 9801 8842
9804 8843 9805 8846 9804 8849 9801 8849 9799 8846 9797 8843
9793 8842 9791 8839 9789 8837 9790 8833 16 P
8909 7425 8913 7423 8917 7422 8922 7422 8927 7423 8931 7424
8935 7422 8938 7425 8939 7430 8938 7433 8936 7438 8933 7434
8929 7433 8924 7431 8921 7429 8917 7431 8913 7431 8909 7425
18 P
8856 7423 8859 7419 8864 7418 8868 7417 8870 7422 8874 7423
8877 7426 8880 7431 8882 7434 8882 7439 8878 7439 8874 7435
8871 7433 8867 7430 8866 7425 8863 7422 8861 7425 8856 7423
18 P
8901 7432 8904 7433 8907 7433 8909 7434 8912 7435 8912 7438
8909 7439 8908 7442 8906 7441 8903 7439 8902 7437 8901 7432

12:00 PM

8925 7242 8928 7240 8926 7237 8927 7233 8929 7236 8931 7238
8934 7240 8937 7241 8934 7241 8932 7244 8930 7247 8933 7250
8932 7253 8929 7252 8927 7248 8925 7246 8925 7242 17 P
8929 7267 8931 7264 8933 7261 8936 7262 8939 7262 8943 7264
8946 7265 8949 7267 8946 7269 8944 7272 8945 7275 8942 7274
8939 7272 8935 7273 8936 7269 8929 7267 16 P
8208 6796 8212 6794 8215 6793 8218 6791 8220 6789 8223 6787
8226 6786 8229 6787 8230 6791 8231 6794 8233 6797 8232 6800

8228 6800 8225 6801 8222 6801 8219 6801 8215 6799 8212 6798
8208 6796 19 P
5904 5506 5905 5513 5909 5520 5902 5523 5909 5526 5916 5523
5918 5530 5917 5537 5917 5544 5922 5550 5925 5556 5931 5552
5936 5557 5938 5564 5934 5570 5928 5565 5922 5561 5915 5560
5909 5556 5904 5551 5897 5548 5893 5542 5886 5542 5879 5540
5873 5537 5867 5533 5863 5539 5855 5540 5848 5540 5844 5533
5839 5528 5832 5525 5826 5523 5828 5516 5824 5510 5818 5507
5817 5499 5817 5499 38 P
5840 5499 5847 5500 5848 5499 3 P
5862 5499 5865 5500 5872 5503 5879 5504 5886 5507 5891 5501
5891 5499 7 P
5911 5499 5913 5500 2 P
5913 5500 5904 5506 2 P
5968 5526 5972 5520 5975 5513 5981 5510 5988 5508 5993 5502
6000 5504 6007 5501 6007 5499 9 P
6024 5499 6024 5504 6030 5500 6030 5499 4 P
6041 5499 6038 5504 6043 5510 6039 5516 6035 5522 6032 5529
6024 5530 6019 5534 6011 5535 6006 5530 5999 5529 5991 5530
5985 5534 5978 5533 5968 5526 15 P
5970 5499 5970 5505 5964 5504 5963 5499 5962 5499 5 P
6055 5505 6057 5503 6059 5502 6059 5500 6060 5501 6063 5502
6064 5503 6065 5506 6065 5508 6065 5510 6064 5512 6061 5512
6060 5510 6058 5510 6056 5508 6055 5505 16 P
5542 5515 5544 5511 5546 5507 5548 5502 5550 5499 5 P
5576 5499 5575 5503 5574 5508 5572 5512 5570 5516 5568 5520
5564 5523 5560 5526 5557 5529 5553 5531 5550 5528 5546 5526
5543 5522 5542 5515 14 P
5699 5613 5701 5608 5705 5606 5709 5608 5713 5610 5718 5612
5717 5616 5721 5618 5725 5621 5725 5626 5726 5630 5722 5630
5718 5633 5713 5634 5711 5629 5708 5627 5705 5623 5703 5619
5699 5613 19 P
5791 5617 5795 5614 5798 5612 5802 5608 5806 5607 5811 5607
5809 5612 5807 5615 5803 5619 5799 5621 5797 5618 5791 5617

12:00 PM

6252 5605 6253 5606 6255 5605 6257 5606 6259 5608 6259 5610
6261 5612 6260 5614 6257 5613 6255 5612 6254 5610 6253 5608
6252 5605 13 P
6240 5626 6241 5622 6242 5619 6245 5616 6248 5618 6251 5620
6254 5620 6258 5621 6260 5624 6257 5626 6254 5627 6251 5628
6247 5628 6244 5628 6240 5626 15 P
6253 5693 6254 5690 6258 5689 6260 5686 6261 5683 6263 5685
6262 5689 6260 5691 6257 5693 6257 5696 6253 5693 11 P

12520 8203 12522 8203 12523 8203 12523 8203 12523 8204 12523 8204
12523 8205 12522 8205 12520 8204 12520 8203 10 P
12908 11323 12909 11323 12911 11323 12913 11324 12914 11324 12915 11325
12917 11326 12918 11325 12919 11324 12920 11325 12921 11326 12922 11328
12923 11329 12923 11330 12923 11332 12922 11333 12921 11332 12919 11332
12917 11332 12916 11332 12914 11332 12913 11332 12912 11330 12912 11329
12912 11327 12911 11326 12910 11325 12908 11325 12908 11323 29 P
12893 11299 12894 11298 12895 11298 12895 11299 12894 11300 12892 11300
12892 11299 12893 11299 12893 11299 9 P
12895 11299 12895 11297 12895 11296 12895 11294 12894 11293 12894 11291
12895 11291 12896 11292 12896 11293 12898 11294 12900 11294 12899 11295
12898 11297 12897 11298 12898 11300 12899 11301 12900 11302 12900 11304
12898 11305 12897 11304 12895 11303 12894 11303 12893 11301 12894 11300
12895 11299 25 P
12900 11307 12901 11306 12902 11307 12903 11308 12903 11309 12902 11310
12901 11309 12900 11309 12900 11308 12900 11307 10 P
12901 11313 12902 11313 12902 11314 12902 11314 12901 11313 5 P
12903 11314 12903 11314 12904 11314 12904 11314 12906 11314 12906 11316
12906 11316 12906 11318 12904 11317 12903 11316 12903 11316 12903 11314

12:00 PM

12906 11320 12907 11320 12907 11319 12907 11318 12908 11318 12909 11319
12908 11320 12908 11321 12906 11321 12906 11320 10 P
2300 14445 2332 14440 2324 14386 2300 14390 4 P
2300 14335 2328 14330 2376 14306 2383 14252 2415 14208 2462 14179
2493 14135 2525 14090 2511 14038 2492 13987 2510 13935 2528 13883
2545 13832 2558 13779 2566 13725 2533 13681 2489 13649 2470 13598
2483 13544 2510 13498 2550 13460 2604 13452 2632 13405 2655 13355
2673 13303 2725 13285 2776 13266 2825 13243 2839 13190 2871 13146
2915 13179 2943 13225 2930 13279 2938 13333 2951 13386 3004 13400
3055 13381 3110 13384 3148 13399 3138 13466 3181 13499 3189 13553
3191 13594 3153 13634 3106 13662 3059 13690 46 P
3059 13690 3019 13696 3016 13750 3029 13803 2997 13847 2974 13872
3023 13896 3051 13942 3105 13940 3113 13994 3118 14028 3087 14055
3124 14073 3133 14133 3187 14131 3242 14129 3296 14127 3350 14119
3396 14108 19 P
3396 14108 3399 14054 3418 14003 3468 13980 3515 13953 3562 13926
3612 13903 3653 13868 3686 13825 3720 13781 3772 13764 3822 13742
3863 13706 3908 13675 3959 13658 3974 13606 3948 13558 3952 13503
3940 13450 3932 13410 3865 13376 3864 13322 3852 13268 3861 13214
3881 13163 3875 13109 3868 13054 3814 13055 3759 13057 3705 13058
3650 13059 3596 13055 3542 13067 3492 13090 3452 13052 3467 12999
3486 12948 3454 12904 3399 12905 3345 12907 3290 12915 3236 12911
3188 12939 3156 12982 3122 13026 3068 13033 3014 13041 2946 13051

2910 13010 2907 12955 50 P
2907 12955 2921 12902 2880 12865 2837 12832 2803 12808 2795 12754
2849 12743 2898 12717 2933 12675 2933 12599 2871 12599 2871 12544
2841 12498 2811 12452 2790 12401 2796 12347 2817 12296 2822 12241
2816 12208 2775 12215 2759 12253 2704 12259 2651 12248 2602 12274
2548 12285 2506 12250 2475 12204 2426 12179 2442 12126 2484 12091
2510 12042 2489 11991 2463 11943 2410 11927 2355 11922 2302 11938
2300 11939 37 P
2300 10513 2316 10506 2360 10472 2384 10422 2439 10414 2494 10417
2550 10425 2604 10412 2607 10356 2621 10302 2666 10269 2721 10266
2776 10275 2824 10305 2868 10339 2901 10384 2933 10429 2966 10474
3003 10516 3040 10557 3020 10610 3048 10658 3102 10667 3157 10676
3187 10629 3241 10644 3273 10689 3295 10740 3328 10785 3335 10840
3366 10886 3403 10928 3442 10966 3487 11000 3530 11035 3577 11064
3594 11117 3555 11157 3521 11200 3499 11236 3535 11278 3562 11326
3567 11360 3607 11355 3611 11379 45 P
3611 11379 3613 11400 3558 11406 3503 11413 3450 11398 3398 11378
3360 11364 3318 11400 3280 11440 3256 11490 3222 11534 3167 11541
3143 11590 3127 11621 3084 11588 3045 11558 3012 11603 2983 11650
3003 11668 2970 11712 2919 11735 2865 11744 2810 11735 2755 11737
2701 11729 2649 11747 2601 11776 2564 11817 2537 11821 2540 11869
2485 11867 2422 11870 2412 11830 2357 11833 2300 11854 35 P
3396 14108 3434 14123 3420 14175 3412 14229 3430 14281 3452 14330
3480 14377 3519 14414 3562 14447 3616 14456 3621 14496 11 P
3621 14496 3570 14514 3533 14554 3513 14605 3476 14644 3430 14672
3375 14669 3321 14671 3281 14707 3237 14739 3196 14776 3168 14822
3215 14850 3255 14887 3268 14940 3275 14993 3311 15034 3339 15081
3379 15117 3411 15161 3397 15214 3389 15267 3365 15316 3322 15348
3271 15330 3218 15316 3167 15298 3115 15285 3065 15262 3026 15225
2993 15182 2965 15135 2947 15084 2928 15033 2920 14979 2906 14927
2883 14878 2880 14823 2847 14780 2839 14727 2847 14672 2833 14620
2810 14571 2757 14558 2710 14530 2657 14539 2603 14548 2549 14540
2502 14513 2455 14486 50 P
2455 14486 2400 14484 2346 14487 2300 14500 4 P
3621 14496 3671 14474 3720 14452 3758 14413 3760 14372 3808 14345
3861 14338 3916 14343 3970 14341 4024 14335 4085 14328 4097 14381
4097 14435 4109 14488 4138 14507 4167 14461 4224 14498 4214 14551
4185 14597 4206 14647 4233 14694 4267 14736 4306 14774 4349 14808
4391 14842 4426 14884 4468 14918 4513 14948 4548 14989 4587 15027
4628 15062 4674 15092 4724 15112 4774 15133 4763 15187 4759 15233
4804 15203 4838 15162 4876 15146 4879 15180 4933 15175 4946 15109
5000 15119 5025 15167 5062 15206 5108 15237 5160 15248 5188 15245
5169 15209 5156 15178 50 P

5156 15178 5187 15133 5210 15099 5185 15052 5175 14998 5130 14967
5096 14925 5087 14871 5071 14820 5046 14771 5041 14717 5021 14667
4976 14636 4935 14601 4889 14571 4855 14529 4850 14475 4844 14407
4879 14365 4905 14317 4925 14267 4936 14214 4931 14160 4927 14105
4872 14110 4867 14056 4878 14031 4848 13985 4814 13943 4762 13900
4754 13846 4746 13792 4741 13737 4741 13683 4751 13629 4747 13575
4741 13521 4736 13466 4731 13412 4736 13358 4742 13303 4748 13249
4754 13195 4759 13140 4743 13088 4723 13037 4693 12991 4658 12949
4638 12898 4621 12846 50 P
4621 12846 4596 12797 4585 12743 4580 12689 4559 12638 4582 12604
4616 12570 7 P
4616 12570 4605 12516 4636 12471 4626 12417 4656 12371 4695 12333
4690 12278 4706 12225 4669 12168 4719 12147 4758 12109 4770 12054
4816 12024 4842 11976 4843 11921 4838 11866 4815 11809 17 P
4815 11809 4746 11815 4730 11762 4676 11756 4641 11798 4610 11844
4567 11809 4513 11798 4461 11814 4408 11830 4356 11846 4301 11857
4248 11868 4193 11879 4139 11880 4138 11824 4191 11808 4241 11787
4262 11735 4241 11685 4296 11679 4346 11658 4389 11622 4410 11571
4431 11521 4380 11499 4328 11484 4277 11505 4222 11516 4168 11521
4133 11479 4102 11433 4085 11380 4100 11327 4116 11274 4121 11219
4115 11164 4115 11109 4095 11043 39 P
4095 11043 4039 11043 3984 11044 3945 11083 3910 11127 3876 11170
3837 11209 3787 11231 3744 11267 3702 11303 3664 11343 3640 11368
3611 11379 13 P
4095 11043 4149 11031 4143 10976 4140 10942 4194 10936 4243 10910
4283 10871 4331 10844 4337 10789 4331 10734 4325 10679 4320 10623
4315 10568 4272 10532 4221 10554 4165 10560 4114 10538 4071 10503
4027 10468 3979 10442 3939 10404 3897 10408 3901 10442 3850 10422
3795 10412 3801 10467 3745 10473 3702 10439 3655 10408 3603 10425
3583 10463 3538 10410 3483 10411 3451 10365 3485 10321 3515 10274
3503 10220 3486 10166 3479 10111 3475 10077 3405 10071 3426 10019
3464 9979 3471 10034 3509 10053 3534 10003 3559 9954 3608 9927
3623 9873 3591 9826 50 P
3591 9826 3637 9795 3686 9768 3717 9721 3721 9665 3756 9621
3783 9598 7 P
3783 9598 3789 9654 3821 9701 3843 9752 3869 9801 3905 9844
3941 9887 3993 9865 4026 9855 4042 9908 4092 9935 4147 9945
4202 9945 4256 9929 4299 9894 4349 9867 4403 9878 4454 9900
4460 9956 4433 10005 4454 10056 4506 10078 4557 10100 4626 10093
4623 10052 4678 10047 4734 10042 4790 10037 4826 9995 4872 9964
4924 9944 4979 9934 5035 9929 5088 9914 5129 9876 5169 9837
5205 9795 5249 9760 5304 9746 5358 9758 5414 9754 5469 9761
5511 9799 5557 9831 5633 9826 5630 9784 5602 9786 5583 9733

5579 9664 5631 9644 50 P
5631 9644 5687 9641 5720 9685 5759 9726 5796 9767 5838 9804
5886 9833 5883 9777 5928 9744 5972 9710 5943 9662 5980 9620
5939 9583 5906 9538 5883 9487 5903 9435 5946 9398 5988 9362
6043 9372 6104 9389 20 P
6104 9389 6118 9444 6166 9472 6207 9510 6245 9552 6297 9533
6353 9541 6403 9566 6459 9569 6513 9583 6550 9625 6602 9644
6657 9631 6670 9686 6725 9695 6779 9709 6816 9751 6817 9807
6873 9805 6929 9803 6971 9767 7022 9744 7078 9743 7143 9784
24 P
7143 9784 7128 9838 7118 9893 7120 9949 7105 10002 7059 10034
7044 10088 7046 10144 7047 10186 7008 10226 6980 10256 6982 10312
7022 10350 7062 10389 7106 10423 7077 10470 7034 10506 6986 10534
6934 10552 6883 10574 6841 10611 6819 10646 6804 10685 23 P
6804 10685 6736 10681 6732 10736 6712 10788 6676 10746 6629 10717
6575 10703 6520 10705 6472 10733 6443 10781 6435 10836 6428 10877
6374 10885 6304 10888 6267 10929 6234 10953 6197 10912 6159 10895
6136 10945 6091 10978 6058 11023 6045 11077 6059 11130 6061 11185
6070 11240 6029 11277 5992 11237 5944 11208 5889 11212 5857 11256
5813 11290 5759 11277 5704 11270 5649 11267 5594 11271 5539 11280
5485 11284 5430 11277 5375 11270 5326 11295 5282 11329 5239 11363
5192 11393 5145 11422 5096 11448 5053 11482 5017 11524 4981 11566
4942 11605 4930 11659 50 P
4930 11659 4904 11707 4873 11753 4843 11781 4815 11809 5 P
6804 10685 6817 10739 6840 10790 6881 10827 6924 10861 6978 10876
7061 10882 7068 10937 7069 10992 7070 11047 7072 11103 7068 11158
7069 11213 7076 11268 7082 11316 7055 11314 7054 11273 6998 11274
6964 11276 6924 11237 6878 11270 6845 11313 6790 11321 6743 11348
6745 11404 6746 11459 6709 11499 6659 11522 6611 11550 6613 11605
6615 11653 6657 11652 6667 11612 6721 11620 6797 11625 6804 11680
6822 11732 6818 11787 6841 11837 6888 11866 6941 11881 6948 11936
6949 11990 6941 12045 6942 12100 6982 12137 7037 12141 7085 12114
7133 12087 7147 12034 50 P
7147 12034 7162 11981 7209 11954 7264 11958 7316 11978 7359 12012
7405 12042 7401 12096 7381 12147 7381 12202 7382 12251 7329 12263
7279 12285 7240 12324 7221 12375 7222 12416 16 P
7222 12416 7170 12434 7116 12424 7061 12420 7006 12422 6952 12423
6907 12456 6888 12507 6868 12558 6827 12594 6790 12634 6738 12652
6683 12654 6636 12682 6638 12736 6670 12781 6707 12822 6724 12874
6775 12893 6827 12912 6867 12949 6894 12997 6934 13034 6981 13063
7023 13097 7072 13121 7108 13162 7155 13192 7208 13180 7247 13171
7238 13224 7210 13254 7167 13221 7114 13206 7062 13192 7013 13167
6960 13153 6917 13119 6868 13095 6870 13150 6897 13197 6925 13245

6931 13292 43 P
6931 13292 6888 13259 6860 13257 6836 13306 6782 13314 6739 13280
6695 13247 6654 13283 6600 13286 6545 13288 6498 13316 6451 13344
6400 13362 6350 13385 6301 13409 6247 13417 6207 13423 6166 13429
18 P
6166 13429 6158 13375 6150 13321 6142 13267 6128 13214 6104 13165
6091 13112 6082 13058 6069 13005 6050 12953 6022 12906 5985 12867
5937 12839 5883 12831 5829 12834 5775 12844 5720 12847 5666 12850
5611 12849 5557 12853 5503 12867 5450 12876 5395 12881 5386 12826
5381 12772 5329 12786 5275 12780 5229 12749 5188 12713 5143 12682
5091 12665 5037 12670 4983 12658 4932 12636 4879 12625 4834 12594
4779 12589 4731 12562 4676 12568 4636 12575 4616 12570 41 P
6166 13429 6134 13473 6111 13522 6088 13572 6065 13622 6109 13654
6150 13690 6194 13722 6202 13776 6205 13831 6233 13877 6266 13921
6294 13968 6322 14014 6350 14061 6368 14112 6350 14163 6332 14215
6313 14266 6290 14315 6272 14367 6244 14413 6198 14442 6151 14470
6170 14521 6173 14575 6187 14628 6195 14681 6198 14736 6241 14768
6278 14808 6287 14862 6323 14901 6363 14938 6399 14898 6454 14900
6505 14919 6541 14959 6543 15013 6571 15060 6589 15111 6606 15148
6570 15188 6556 15240 6558 15295 6515 15327 6464 15345 6415 15368
6375 15404 6403 15451 50 P
6403 15451 6435 15494 6487 15508 6540 15521 6589 15544 6621 15588
6567 15591 6513 15598 6521 15652 6524 15719 6484 15721 6486 15775
6441 15816 13 P
6441 15816 6433 15763 6420 15710 6377 15678 6323 15670 6270 15657
6231 15621 6184 15594 6130 15596 6087 15629 6033 15632 5979 15625
5941 15612 5915 15543 5861 15536 5818 15568 5773 15597 5719 15591
5678 15555 5645 15512 5616 15466 5592 15418 5562 15372 5538 15324
5518 15273 5474 15242 5420 15246 5375 15276 5311 15268 5301 15215
5270 15167 5216 15171 5156 15178 33 P
6441 15816 6433 15870 6415 15921 6383 15964 6347 16004 6390 16036
6418 16067 6390 16113 6396 16153 6372 16185 6407 16212 11 P
8148 16212 8163 16184 8184 16134 8200 16083 8251 16098 8305 16088
8358 16092 8412 16093 8460 16067 8513 16068 8545 16042 11 P
8545 16042 8570 16090 8595 16138 8604 16191 8598 16212 5 P
11245 16000 11191 15994 11137 15995 11158 16044 11179 16095 11184 16148
11158 16196 11146 16212 8 P
8545 16042 8508 16003 8474 15961 8426 15935 8465 15897 8414 15881
8383 15806 8425 15772 8468 15738 8520 15723 8551 15678 8598 15653
8653 15655 8693 15655 8705 15602 8711 15549 8728 15497 8754 15450
8785 15405 8835 15368 20 P
8060 14933 8055 14986 8030 15034 7995 15076 8003 15102 8023 15102
8019 15142 7966 15138 7926 15130 7892 15153 7908 15190 7860 15216

7855 15270 7855 15324 7905 15344 7936 15389 7894 15424 7868 15487
7821 15487 7787 15487 20 P
7787 15487 7750 15525 7729 15576 7730 15630 7768 15668 7822 15673
7817 15727 7763 15727 7709 15733 7656 15734 7606 15755 7581 15803
7547 15845 7518 15891 7489 15936 7444 15907 7398 15878 7366 15865
7376 15812 7380 15758 7353 15711 7302 15696 7247 15693 7195 15678
7153 15645 7122 15601 7086 15560 7059 15513 7057 15459 7026 15415
6987 15377 7011 15329 7009 15275 6997 15222 7026 15176 7072 15149
7116 15118 7165 15142 7219 15146 7273 15145 7314 15109 7366 15092
7411 15121 7446 15163 7504 15144 45 P
7504 15144 7555 15164 7593 15202 3 P
7593 15202 7643 15181 7693 15159 7688 15213 7719 15258 7714 15312
7709 15366 7730 15416 8 P
7730 15416 7770 15442 7787 15487 3 P
7504 15144 7514 15091 7558 15060 7568 15007 7573 14953 7593 14902
7567 14855 7532 14813 7505 14766 7494 14713 7496 14658 7507 14606
7534 14558 7583 14534 7637 14530 7691 14538 7744 14550 7796 14564
7832 14584 7869 14605 20 P
6931 13292 6932 13346 6895 13387 6862 13430 6864 13485 6881 13536
6917 13577 6961 13611 7007 13639 7050 13672 7077 13720 7104 13767
7126 13817 7143 13869 7170 13916 7223 13931 7276 13940 7325 13964
7364 14002 7403 14040 7438 14082 7474 14123 7513 14161 7555 14195
7609 14194 7664 14194 7718 14193 7772 14193 7815 14226 7845 14272
7876 14317 7926 14337 7974 14363 8005 14388 8032 14402 35 P
7222 12416 7276 12421 7314 12465 3 P
7314 12465 7321 12519 7332 12573 7381 12598 7434 12613 7489 12617
7539 12596 7577 12556 7615 12517 7630 12464 7651 12413 7676 12365
7725 12390 7746 12407 7788 12372 7822 12349 7875 12365 7925 12385
7960 12428 8009 12454 8063 12448 8098 12406 8114 12353 8114 12312
8169 12312 8220 12334 8235 12386 8235 12427 8273 12466 8308 12489
8356 12464 8381 12512 8402 12563 8423 12614 8438 12667 8493 12662
8547 12668 8585 12707 8626 12708 8634 12698 40 P
7314 12465 7356 12429 7390 12386 7435 12355 7485 12333 7538 12316
7578 12312 7577 12257 7593 12204 7635 12168 7680 12138 7735 12132
7788 12121 7842 12109 7888 12079 7931 12025 7935 11984 7909 11952
7962 11941 8017 11947 8071 11958 8117 11927 8096 11876 8091 11821
8064 11773 8039 11724 8013 11675 8043 11629 8098 11624 8141 11589
8157 11536 8157 11482 8183 11433 8218 11390 8273 11385 8327 11396
8381 11407 8411 11454 8441 11500 8494 11517 8530 11474 8573 11440
8608 11398 8609 11342 8589 11291 8550 11251 8497 11266 8458 11305
8403 11315 8349 11321 50 P
8349 11321 8318 11274 8353 11232 8365 11178 8344 11127 8298 11095
8268 11049 8226 11013 8211 10960 8218 10904 8240 10854 8280 10816

8327 10786 8377 10761 8329 10734 8314 10680 8321 10625 8343 10575
8390 10545 8444 10530 8499 10526 8622 10527 8677 10528 8733 10524
8774 10520 8829 10516 26 P
8829 10516 8880 10538 8936 10534 8972 10492 9008 10450 9055 10420
9107 10400 9161 10412 9208 10383 9215 10328 9261 10360 9313 10378
9337 10428 9390 10446 9445 10448 15 P
9445 10448 9478 10403 9522 10369 9563 10331 9598 10310 9615 10294

6:00 PM

8829 10516 8820 10461 8799 10409 8790 10355 8759 10307 8725 10264
8679 10232 8637 10196 8677 10157 8699 10106 8710 10081 8656 10069
8603 10052 8547 10051 8495 10072 8480 10019 8469 9964 8470 9908
8470 9852 8440 9805 8410 9758 8384 9709 8345 9669 8302 9633
8256 9602 8209 9570 8148 9538 27 P
8148 9538 8108 9577 8065 9612 8010 9623 7954 9629 7899 9634
7844 9646 7795 9673 7779 9726 7758 9778 7705 9795 7679 9827
7629 9801 7612 9748 7585 9699 7534 9679 7484 9653 7428 9648
7394 9692 7416 9743 7463 9773 7499 9816 7504 9858 7457 9889
7402 9896 7359 9861 7315 9826 7265 9801 7218 9771 7177 9776
7143 9784 31 P
8148 9538 8126 9485 8075 9464 8048 9414 8048 9359 8079 9312
8069 9257 8095 9207 8142 9176 8197 9165 8252 9155 8252 9099
8258 9043 8269 8988 8275 8932 8275 8910 16 P
8275 8910 8275 8889 8331 8895 8385 8912 8425 8873 8425 8816
8409 8763 8441 8716 8458 8662 8475 8608 8497 8557 8492 8500
8497 8458 8553 8464 8600 8433 8656 8429 8706 8456 8755 8483
8780 8534 8827 8566 8867 8527 8885 8473 8908 8422 8940 8375
8996 8371 9043 8403 9098 8416 9149 8439 9202 8419 9247 8384
9244 8345 31 P
8275 8910 8219 8909 8219 8853 8179 8813 8133 8782 8083 8755
8030 8739 7977 8717 7937 8678 7894 8642 7854 8682 7818 8726
7765 8710 7710 8694 7684 8644 7648 8601 7604 8566 7547 8566
7547 8510 7588 8501 7587 8445 7598 8389 7608 8334 7580 8284
7549 8238 7502 8206 7447 8191 7391 8181 7347 8146 7310 8103
7304 8046 7292 7991 7312 7938 7338 7887 7290 7857 7250 7817
7227 7765 7226 7722 7268 7722 7251 7667 7214 7624 7170 7589
7124 7554 7097 7504 7064 7458 7041 7405 7023 7351 7011 7296
7004 7239 6966 7196 50 P
6966 7196 6943 7143 6963 7090 6939 7046 6938 7004 5 P
6104 9389 6101 9333 6125 9282 6149 9232 6141 9176 6133 9120
6162 9072 6190 9024 6172 8971 6138 8926 6093 8892 6074 8839
6023 8815 5972 8791 5935 8750 5926 8694 5892 8649 5854 8607
5835 8554 5832 8497 5850 8444 5907 8447 5963 8438 5960 8382
5935 8331 5933 8274 5966 8228 5969 8172 5944 8121 5952 8065

5998 8031 6039 7993 6064 7941 6035 7893 6021 7837 5970 7769
6027 7772 6104 7783 6096 7727 6042 7708 6015 7701 6012 7644
5998 7589 5964 7543 5956 7487 5970 7431 5957 7367 47 P
3783 9598 3777 9543 3755 9491 3749 9435 3759 9381 3723 9338
3778 9326 3744 9256 8 P
3744 9256 3738 9200 3764 9150 3764 9094 3752 9039 3741 8984
3734 8928 3728 8865 3771 8829 3781 8773 3781 8731 3738 8731
3694 8696 3642 8675 3595 8644 3554 8604 3507 8574 3463 8538
3418 8503 3462 8448 3507 8483 3563 8477 3619 8482 3650 8511
3720 8510 3751 8463 3791 8423 3768 8370 3757 8315 3797 8275
3741 8275 3688 8254 3647 8242 3693 8171 3666 8121 3634 8074
3603 8027 3659 8021 3701 8016 3701 7959 3716 7920 3689 7855
42 P
3689 7855 3652 7811 3616 7767 3599 7713 3543 7724 3490 7746
3463 7696 3423 7709 3412 7735 3376 7712 3416 7671 3456 7621
3399 7622 3343 7628 3315 7631 3311 7673 3268 7678 3251 7623
3210 7548 3166 7585 3120 7617 3076 7654 3031 7618 2986 7582
2969 7528 2932 7485 2877 7469 2807 7457 2760 7489 2721 7530
2701 7568 2641 7530 2615 7581 2590 7632 2569 7686 2539 7734
2485 7752 2442 7757 2410 7711 2386 7659 2368 7604 2330 7561
2300 7559 43 P
3689 7855 3745 7854 3802 7848 3795 7777 3851 7771 3859 7842
3906 7873 3946 7914 4003 7914 4009 7857 4053 7821 4108 7810
4152 7846 4192 7886 4240 7918 4279 7958 4335 7970 4379 7934
4431 7956 4483 7979 4534 7952 4582 7921 4613 7874 4603 7819
4582 7766 4583 7709 4638 7693 4688 7689 4683 7632 4628 7615
4613 7560 4602 7504 4592 7448 4548 7411 4509 7370 4469 7329
4433 7284 4397 7240 4409 7184 4423 7159 4473 7186 4504 7234
4544 7275 4597 7254 4634 7210 4646 7154 4658 7098 4642 7043
4628 6995 4685 6990 50 P
4685 6990 4738 7013 4788 7041 4844 7031 4908 7021 5 P
4908 7021 4955 7039 5023 7062 5026 7104 5003 7157 5024 7210
5054 7259 5108 7238 5164 7234 5218 7213 5273 7199 5301 7149
5330 7099 5381 7074 5438 7082 5458 7135 5462 7192 5471 7249
5429 7288 5472 7326 5510 7368 5553 7405 5610 7402 5652 7363
5698 7329 5749 7304 5806 7301 5863 7303 5917 7323 5957 7367
30 P
5957 7367 5986 7318 6000 7263 6057 7266 6113 7274 6169 7283
6225 7297 6281 7305 6316 7260 6354 7218 6326 7185 6356 7155
6309 7124 6254 7106 6210 7070 6166 7033 6139 6983 6099 6942
6059 6901 6105 6868 6151 6834 6197 6800 6251 6819 6308 6822
6360 6848 6412 6823 6444 6879 6501 6883 6559 6880 6613 6901
6666 6921 6719 6941 6766 6908 6787 6854 6841 6836 6854 6802

6909 6818 6899 6874 6890 6930 6901 6979 6938 7004 41 P
4908 7021 4888 6967 4908 6908 4934 6857 4950 6802 4922 6752
4868 6731 4842 6720 4840 6663 4850 6606 4818 6559 4799 6504
4720 6498 4703 6444 4679 6391 4614 6393 4607 6335 4562 6300
4554 6243 4532 6222 4547 6185 4543 6127 4555 6071 4540 6015
4504 5969 4464 5927 4411 5904 4368 5899 4359 5849 4301 5854
4248 5831 4192 5813 4142 5785 4148 5727 4168 5680 4128 5639
4101 5587 4044 5598 3983 5622 3943 5581 3932 5523 3934 5499
42 P
6938 7004 6994 7002 7049 6984 7105 6971 7157 6948 7212 6953
7242 6882 7278 6837 7335 6842 7416 6857 7415 6799 7436 6746
7467 6698 7493 6646 7515 6593 7492 6541 7491 6483 7507 6428
7529 6374 7555 6323 7587 6274 7616 6252 22 P
2300 9487 2316 9477 2367 9454 2421 9440 2477 9432 2533 9441
2585 9460 2630 9494 2653 9545 2686 9590 2743 9588 2797 9575
2852 9584 2897 9618 2934 9660 2962 9709 3012 9734 3065 9716
3075 9661 3099 9611 3149 9636 3200 9661 3253 9643 3308 9636
3318 9581 3328 9526 3326 9470 3336 9414 3382 9382 3421 9341
3414 9286 3435 9233 3445 9207 3481 9250 3502 9287 3501 9231
3495 9175 3485 9134 3540 9122 3592 9099 3647 9109 3675 9159
3686 9214 3716 9243 3744 9256 45 P
5956 5668 5985 5672 6013 5679 6036 5682 4 P
9445 10448 9438 10503 9480 10540 9509 10587 9523 10641 9547 10691
9592 10723 9626 10745 8 P
7593 15202 7599 15229 7602 15255 7587 15278 7565 15294 7544 15311
7542 15338 7527 15361 7528 15387 7554 15383 7582 15382 7603 15382
7626 15395 7652 15402 7679 15402 7706 15409 7730 15416 17 P
3310 4924 3333 4924 3333 4651 3298 4651 3298 4846 3238 4846
3238 4870 3262 4873 3273 4875 3283 4879 3292 4885 3296 4889
3300 4894 3306 4907 3310 4924 15 N F
3525 4924 3547 4924 3547 4651 3513 4651 3513 4846 3453 4846
3453 4870 3477 4873 3488 4875 3498 4879 3507 4885 3511 4889
3514 4894 3520 4907 3525 4924 15 N F
3734 4924 3744 4923 3753 4921 3762 4917 3771 4912 3779 4905
3786 4898 3793 4889 3799 4879 3805 4869 3810 4858 3814 4846
3817 4834 3820 4822 3822 4809 3824 4796 3824 4784 3824 4771
3822 4758 3820 4745 3817 4732 3814 4720 3810 4708 3805 4697
3799 4687 3793 4677 3786 4668 3779 4660 3771 4654 3762 4648
3753 4644 3744 4642 3734 4641 3724 4642 3714 4644 3705 4648
3697 4654 3689 4660 3681 4668 3674 4677 3668 4687 3663 4697
3658 4708 3653 4720 3650 4732 3647 4745 3645 4758 3644 4771
3643 4784 3680 4784 3681 4759 3684 4737 3690 4718 3697 4702
3705 4689 3714 4679 3719 4676 3724 4673 3729 4672 3734 4671

3739 4672 3743 4673 3748 4676 3753 4679 3762 4689 3771 4702
3778 4718 3783 4737 3787 4759 3788 4784 3787 4808 3783 4830
3778 4849 3771 4864 3762 4877 3753 4886 3748 4890 3743 4892
3739 4894 3734 4894 3729 4894 3724 4892 3719 4890 3714 4886
3705 4877 3697 4864 3690 4849 3684 4830 3681 4808 3680 4784
3643 4784 3644 4796 3645 4809 3647 4822 3650 4834 3653 4846
3658 4858 3663 4869 3668 4879 3674 4889 3681 4898 3689 4905
3697 4912 3705 4917 3714 4921 3724 4923 3734 4924 107 N F
3991 4872 3990 4860 3986 4848 3980 4837 3973 4827 3963 4819
3952 4814 3940 4810 3928 4808 3928 4833 3920 4834 3912 4836
3906 4840 3900 4845 3895 4850 3891 4857 3889 4865 3888 4872
3889 4880 3891 4888 3895 4894 3900 4900 3906 4905 3912 4909
3920 4911 3928 4912 3935 4911 3943 4909 3950 4905 3955 4900
3960 4894 3964 4888 3966 4880 3967 4872 3966 4865 3964 4857
3960 4850 3955 4845 3950 4840 3943 4836 3935 4834 3928 4833
3928 4808 3915 4810 3903 4814 3892 4819 3882 4827 3875 4837
3869 4848 3865 4860 3864 4872 3865 4885 3869 4897 3875 4908
3882 4918 3892 4925 3903 4931 3915 4935 3928 4936 3940 4935
3952 4931 3963 4925 3973 4918 3980 4908 3986 4897 3990 4885
3991 4872 67 N F
4044 4932 4239 4932 4239 4900 4081 4900 4081 4810 4233 4810
4233 4778 4081 4778 4081 4683 4245 4683 4245 4651 4044 4651
4044 4932 13 N F
5473 4924 5495 4924 5495 4651 5461 4651 5461 4846 5401 4846
5401 4870 5425 4873 5436 4875 5446 4879 5455 4885 5459 4889
5462 4894 5468 4907 5473 4924 15 N F
5688 4924 5710 4924 5710 4651 5676 4651 5676 4846 5615 4846
5615 4870 5640 4873 5651 4875 5660 4879 5669 4885 5673 4889
5677 4894 5683 4907 5688 4924 15 N F
5926 4924 5950 4924 5950 4747 5992 4747 5992 4715 5950 4715
5950 4651 5916 4651 5916 4715 5802 4715 5802 4747 5832 4747
5916 4747 5916 4866 5832 4747 5802 4747 5802 4751 5926 4924
18 N F
6154 4872 6153 4860 6149 4848 6143 4837 6135 4827 6126 4819
6115 4814 6103 4810 6090 4808 6090 4833 6082 4834 6075 4836
6068 4840 6062 4845 6057 4850 6054 4857 6052 4865 6051 4872
6052 4880 6054 4888 6057 4894 6062 4900 6068 4905 6075 4909
6082 4911 6090 4912 6098 4911 6105 4909 6112 4905 6118 4900
6123 4894 6126 4888 6129 4880 6129 4872 6129 4865 6126 4857
6123 4850 6118 4845 6112 4840 6105 4836 6098 4834 6090 4833
6090 4808 6077 4810 6065 4814 6054 4819 6045 4827 6037 4837
6031 4848 6027 4860 6026 4872 6027 4885 6031 4897 6037 4908
6045 4918 6054 4925 6065 4931 6077 4935 6090 4936 6103 4935

6115 4931 6126 4925 6135 4918 6143 4908 6149 4897 6153 4885
6154 4872 67 N F
6207 4932 6402 4932 6402 4900 6243 4900 6243 4810 6396 4810
6396 4778 6243 4778 6243 4683 6408 4683 6408 4651 6207 4651
6207 4932 13 N F
7620 4924 7642 4924 7642 4651 7608 4651 7608 4846 7548 4846
7548 4870 7572 4873 7583 4875 7593 4879 7602 4885 7606 4889
7610 4894 7616 4907 7620 4924 15 N F
7835 4924 7857 4924 7857 4651 7823 4651 7823 4846 7763 4846
7763 4870 7787 4873 7798 4875 7808 4879 7817 4885 7821 4889
7824 4894 7830 4907 7835 4924 15 N F
8044 4924 8060 4923 8076 4919 8090 4913 8102 4905 8107 4900
8112 4895 8116 4889 8120 4883 8122 4876 8124 4868 8125 4860
8126 4852 8125 4840 8123 4830 8119 4821 8114 4814 8108 4807
8102 4802 8090 4794 8100 4788 8109 4782 8117 4775 8123 4766
8129 4757 8133 4747 8135 4736 8136 4725 8134 4712 8130 4698
8123 4684 8112 4670 8106 4664 8099 4659 8091 4654 8083 4649
8073 4646 8064 4643 8053 4642 8042 4641 8030 4642 8020 4643
8010 4646 8001 4649 7993 4654 7986 4659 7979 4664 7973 4670
7964 4684 7957 4698 7953 4712 7951 4725 7988 4725 7989 4714
7992 4704 7997 4695 8004 4687 8013 4680 8022 4675 8033 4672
8044 4671 8055 4672 8065 4675 8075 4680 8083 4687 8090 4695
8095 4704 8099 4714 8100 4725 8099 4736 8095 4747 8090 4756
8083 4764 8075 4770 8065 4775 8055 4778 8044 4780 8033 4778
8022 4775 8013 4770 8004 4764 7997 4756 7992 4747 7989 4736
7988 4725 7951 4725 7952 4736 7955 4747 7959 4756 7964 4765
7971 4774 7978 4781 7987 4788 7998 4794 7985 4802 7979 4807
7973 4814 7969 4821 7965 4830 7962 4840 7961 4852 7996 4852
7997 4843 7999 4834 8004 4827 8010 4821 8017 4816 8025 4813
8034 4810 8044 4810 8053 4810 8062 4813 8070 4816 8078 4821
8083 4827 8088 4834 8091 4843 8092 4852 8091 4861 8088 4869
8083 4877 8078 4883 8070 4888 8062 4891 8053 4893 8044 4894
8034 4893 8025 4891 8017 4888 8010 4883 8004 4877 7999 4869
7997 4861 7996 4852 7961 4852 7962 4860 7963 4868 7965 4876
7968 4883 7971 4889 7975 4895 7980 4900 7985 4905 7997 4913
8011 4919 8027 4923 8044 4924 147 N F
8301 4872 8300 4860 8296 4848 8290 4837 8283 4827 8273 4819
8262 4814 8250 4810 8238 4808 8238 4833 8230 4834 8222 4836
8216 4840 8210 4845 8205 4850 8201 4857 8199 4865 8198 4872
8199 4880 8201 4888 8205 4894 8210 4900 8216 4905 8222 4909
8230 4911 8238 4912 8245 4911 8253 4909 8260 4905 8265 4900
8270 4894 8274 4888 8276 4880 8277 4872 8276 4865 8274 4857
8270 4850 8265 4845 8260 4840 8253 4836 8245 4834 8238 4833

8238 4808 8225 4810 8213 4814 8202 4819 8192 4827 8185 4837
8179 4848 8175 4860 8174 4872 8175 4885 8179 4897 8185 4908
8192 4918 8202 4925 8213 4931 8225 4935 8238 4936 8250 4935
8262 4931 8273 4925 8283 4918 8290 4908 8296 4897 8300 4885
8301 4872 67 N F
8354 4932 8549 4932 8549 4900 8391 4900 8391 4810 8543 4810
8543 4778 8391 4778 8391 4683 8555 4683 8555 4651 8354 4651
8354 4932 13 N F
9781 4924 9804 4924 9804 4651 9769 4651 9769 4846 9709 4846
9709 4870 9734 4873 9744 4875 9754 4879 9763 4885 9767 4889
9771 4894 9777 4907 9781 4924 15 N F
9904 4830 9938 4830 9939 4843 9941 4856 9946 4867 9952 4876
9960 4884 9969 4889 9981 4893 9994 4894 10004 4893 10014 4890
10023 4886 10031 4880 10037 4872 10042 4864 10045 4853 10046 4842
10045 4830 10042 4820 10038 4812 10031 4805 10022 4798 10012 4790
9984 4774 9961 4760 9942 4746 9927 4732 9915 4718 9907 4702
9902 4686 9899 4669 9898 4651 10080 4651 10080 4683 9936 4683
9940 4695 9944 4706 9951 4714 9959 4722 9969 4730 9981 4737
10012 4755 10030 4764 10045 4774 10057 4785 10067 4797 10074 4809
10079 4821 10081 4834 10082 4846 10082 4854 10081 4862 10078 4870
10075 4878 10072 4884 10067 4891 10062 4897 10056 4902 10043 4912
10028 4918 10012 4923 9994 4924 9975 4923 9966 4921 9958 4918
9950 4915 9943 4911 9936 4907 9929 4901 9924 4895 9919 4888
9914 4880 9911 4872 9908 4862 9906 4852 9904 4841 9904 4830
78 N F
10118 4830 10153 4830 10153 4843 10156 4856 10160 4867 10166 4876
10174 4884 10184 4889 10195 4893 10209 4894 10219 4893 10229 4890
10238 4886 10245 4880 10252 4872 10257 4864 10260 4853 10261 4842
10260 4830 10257 4820 10252 4812 10246 4805 10237 4798 10226 4790
10199 4774 10175 4760 10156 4746 10141 4732 10130 4718 10122 4702
10116 4686 10113 4669 10112 4651 10295 4651 10295 4683 10151 4683
10154 4695 10159 4706 10166 4714 10174 4722 10184 4730 10196 4737
10227 4755 10245 4764 10260 4774 10272 4785 10281 4797 10289 4809
10293 4821 10296 4834 10297 4846 10297 4854 10295 4862 10293 4870
10290 4878 10286 4884 10282 4891 10277 4897 10271 4902 10258 4912
10243 4918 10226 4923 10209 4924 10190 4923 10181 4921 10173 4918
10165 4915 10157 4911 10150 4907 10144 4901 10138 4895 10133 4888
10129 4880 10125 4872 10122 4862 10120 4852 10119 4841 10118 4830
78 N F
10462 4872 10461 4860 10457 4848 10452 4837 10444 4827 10434 4819
10423 4814 10411 4810 10399 4808 10399 4833 10391 4834 10383 4836
10377 4840 10371 4845 10366 4850 10362 4857 10360 4865 10359 4872
10360 4880 10362 4888 10366 4894 10371 4900 10377 4905 10383 4909

10391 4911 10399 4912 10407 4911 10414 4909 10421 4905 10426 4900
10431 4894 10435 4888 10437 4880 10438 4872 10437 4865 10435 4857
10431 4850 10426 4845 10421 4840 10414 4836 10407 4834 10399 4833
10399 4808 10386 4810 10374 4814 10363 4819 10353 4827 10346 4837
10340 4848 10336 4860 10335 4872 10336 4885 10340 4897 10346 4908
10353 4918 10363 4925 10374 4931 10386 4935 10399 4936 10411 4935
10423 4931 10434 4925 10444 4918 10452 4908 10457 4897 10461 4885
10462 4872 67 N F
10516 4932 10710 4932 10710 4900 10552 4900 10552 4810 10704 4810
10704 4778 10552 4778 10552 4683 10716 4683 10716 4651 10516 4651
10516 4932 13 N F
11943 4924 11965 4924 11965 4651 11930 4651 11930 4846 11870 4846
11870 4870 11895 4873 11905 4875 11915 4879 11924 4885 11928 4889
11932 4894 11938 4907 11943 4924 15 N F
12065 4830 12099 4830 12100 4843 12102 4856 12107 4867 12113 4876
12121 4884 12130 4889 12142 4893 12155 4894 12165 4893 12175 4890
12184 4886 12192 4880 12198 4872 12203 4864 12206 4853 12207 4842
12206 4830 12203 4820 12199 4812 12192 4805 12183 4798 12173 4790
12145 4774 12122 4760 12103 4746 12088 4732 12076 4718 12068 4702
12063 4686 12060 4669 12059 4651 12241 4651 12241 4683 12097 4683
12101 4695 12105 4706 12112 4714 12120 4722 12130 4730 12142 4737
12173 4755 12191 4764 12206 4774 12218 4785 12228 4797 12235 4809
12240 4821 12243 4834 12243 4846 12243 4854 12242 4862 12239 4870
12236 4878 12233 4884 12228 4891 12223 4897 12217 4902 12204 4912
12189 4918 12173 4923 12155 4924 12136 4923 12127 4921 12119 4918
12111 4915 12104 4911 12097 4907 12090 4901 12085 4895 12080 4888
12075 4880 12072 4872 12069 4862 12067 4852 12065 4841 12065 4830
78 N F
12452 4854 12418 4854 12416 4863 12412 4871 12407 4878 12402 4884
12395 4888 12389 4891 12381 4893 12374 4894 12366 4894 12360 4892
12353 4890 12347 4887 12342 4883 12336 4879 12332 4873 12328 4867
12321 4852 12316 4835 12313 4814 12312 4792 12320 4800 12328 4807
12336 4813 12344 4816 12352 4819 12360 4821 12378 4822 12391 4820
12405 4816 12419 4810 12431 4800 12437 4794 12443 4788 12447 4781
12451 4773 12454 4764 12457 4754 12458 4744 12458 4733 12424 4731
12423 4743 12419 4753 12414 4764 12407 4773 12399 4780 12389 4786
12379 4790 12368 4792 12357 4790 12346 4786 12337 4780 12328 4773
12321 4764 12316 4753 12313 4743 12312 4731 12313 4720 12316 4709
12321 4699 12328 4690 12337 4682 12346 4676 12357 4672 12368 4671
12379 4672 12389 4676 12399 4682 12407 4690 12414 4699 12419 4709
12423 4720 12424 4731 12458 4733 12457 4717 12453 4701 12446 4686
12441 4678 12436 4671 12430 4665 12424 4659 12416 4654 12408 4649
12399 4646 12389 4643 12379 4642 12368 4641 12355 4642 12344 4644

12334 4647 12325 4652 12316 4657 12309 4664 12302 4672 12296 4681
12291 4690 12287 4701 12283 4712 12280 4724 12277 4750 12275 4778
12276 4794 12277 4810 12279 4825 12282 4839 12286 4852 12290 4864
12295 4875 12301 4885 12308 4894 12315 4902 12323 4908 12332 4914
12342 4918 12352 4922 12362 4924 12374 4924 12390 4923 12404 4919
12417 4912 12428 4903 12437 4893 12444 4881 12449 4868 12452 4854
126 N F
12624 4872 12622 4860 12618 4848 12613 4837 12605 4827 12595 4819
12584 4814 12572 4810 12560 4808 12560 4833 12552 4834 12544 4836
12538 4840 12532 4845 12527 4850 12523 4857 12521 4865 12520 4872
12521 4880 12523 4888 12527 4894 12532 4900 12538 4905 12544 4909
12552 4911 12560 4912 12568 4911 12575 4909 12582 4905 12587 4900
12592 4894 12596 4888 12598 4880 12599 4872 12598 4865 12596 4857
12592 4850 12587 4845 12582 4840 12575 4836 12568 4834 12560 4833
12560 4808 12547 4810 12535 4814 12524 4819 12515 4827 12507 4837
12501 4848 12497 4860 12496 4872 12497 4885 12501 4897 12507 4908
12515 4918 12524 4925 12535 4931 12547 4935 12560 4936 12572 4935
12584 4931 12595 4925 12605 4918 12613 4908 12618 4897 12622 4885
12624 4872 67 N F
12677 4932 12871 4932 12871 4900 12713 4900 12713 4810 12865 4810
12865 4778 12713 4778 12713 4683 12877 4683 12877 4651 12677 4651
12677 4932 13 N F
2319 17058 2341 17058 2341 16785 2307 16785 2307 16979 2247 16979
2247 17003 2271 17006 2282 17009 2292 17013 2301 17019 2305 17023
2308 17028 2314 17041 2319 17058 15 N F
2528 17058 2537 17057 2547 17054 2556 17051 2565 17045 2572 17039
2580 17031 2587 17023 2593 17013 2599 17003 2604 16992 2608 16980
2611 16968 2614 16955 2616 16943 2617 16930 2618 16917 2617 16904
2616 16891 2614 16879 2611 16866 2608 16854 2604 16842 2599 16831
2593 16820 2587 16810 2580 16802 2572 16794 2565 16787 2556 16782
2547 16778 2537 16775 2528 16774 2518 16775 2508 16778 2499 16782
2491 16787 2483 16794 2475 16802 2468 16810 2462 16820 2457 16831
2452 16842 2447 16854 2444 16866 2441 16879 2439 16891 2438 16904
2437 16917 2473 16917 2475 16893 2478 16871 2484 16851 2491 16835
2499 16822 2508 16813 2513 16809 2518 16807 2523 16805 2528 16805
2532 16805 2537 16807 2542 16809 2547 16813 2556 16822 2564 16835
2571 16851 2577 16871 2580 16893 2582 16917 2580 16941 2577 16963
2571 16982 2564 16998 2556 17011 2547 17020 2542 17023 2537 17026
2532 17027 2528 17028 2523 17027 2518 17026 2513 17023 2508 17020
2499 17011 2491 16998 2484 16982 2478 16963 2475 16941 2473 16917
2437 16917 2438 16930 2439 16943 2441 16955 2444 16968 2447 16980
2452 16992 2457 17003 2462 17013 2468 17023 2475 17031 2483 17039
2491 17045 2499 17051 2508 17054 2518 17057 2528 17058 107 N F

2678 17058 2820 17058 2820 17026 2706 17026 2696 16947 2706 16954
2718 16960 2732 16964 2748 16965 2762 16964 2777 16960 2791 16954
2798 16950 2805 16945 2811 16939 2817 16932 2822 16925 2826 16916
2830 16907 2832 16896 2834 16885 2834 16873 2833 16854 2831 16844
2828 16836 2825 16827 2821 16819 2816 16811 2811 16804 2804 16798
2797 16792 2790 16787 2781 16783 2772 16779 2762 16777 2751 16775
2740 16774 2730 16775 2720 16776 2711 16778 2703 16780 2695 16784
2688 16788 2682 16792 2676 16797 2667 16808 2659 16821 2654 16835
2650 16851 2684 16851 2687 16841 2692 16832 2697 16824 2704 16817
2711 16812 2719 16808 2728 16805 2738 16805 2752 16806 2764 16809
2775 16814 2784 16821 2791 16831 2796 16842 2799 16855 2800 16871
2799 16882 2797 16894 2792 16904 2786 16914 2778 16923 2767 16929
2755 16934 2748 16935 2740 16935 2732 16935 2726 16934 2719 16932
2713 16929 2702 16921 2688 16909 2658 16909 2678 17058 83 N F
3000 17006 2999 16993 2995 16981 2989 16970 2981 16961 2972 16953
2961 16947 2949 16943 2936 16942 2936 16967 2928 16967 2921 16970
2914 16973 2908 16978 2903 16984 2900 16991 2898 16998 2897 17006
2898 17014 2900 17021 2903 17028 2908 17034 2914 17039 2921 17042
2928 17045 2936 17045 2944 17045 2951 17042 2958 17039 2964 17034
2969 17028 2972 17021 2975 17014 2975 17006 2975 16998 2972 16991
2969 16984 2964 16978 2958 16973 2951 16970 2944 16967 2936 16967
2936 16942 2923 16943 2911 16947 2900 16953 2891 16961 2883 16970
2877 16981 2873 16993 2872 17006 2873 17019 2877 17031 2883 17042
2891 17051 2900 17059 2911 17065 2923 17069 2936 17070 2949 17069
2961 17065 2972 17059 2981 17051 2989 17042 2995 17031 2999 17019
3000 17006 67 N F
3053 17066 3248 17066 3248 17034 3089 17034 3089 16943 3242 16943
3242 16911 3089 16911 3089 16817 3254 16817 3254 16785 3053 16785
3053 17066 13 N F
4383 17058 4405 17058 4405 16785 4371 16785 4371 16979 4311 16979
4311 17003 4335 17006 4346 17009 4356 17013 4365 17019 4369 17023
4372 17028 4378 17041 4383 17058 15 N F
4597 17058 4620 17058 4620 16785 4585 16785 4585 16979 4525 16979
4525 17003 4550 17006 4560 17009 4570 17013 4579 17019 4583 17023
4587 17028 4593 17041 4597 17058 15 N F
4806 17058 4816 17057 4825 17054 4835 17051 4843 17045 4851 17039
4858 17031 4865 17023 4872 17013 4877 17003 4882 16992 4886 16980
4890 16968 4893 16955 4895 16943 4896 16930 4896 16917 4896 16904
4895 16891 4893 16879 4890 16866 4886 16854 4882 16842 4877 16831
4872 16820 4865 16810 4858 16802 4851 16794 4843 16787 4835 16782
4825 16778 4816 16775 4806 16774 4796 16775 4787 16778 4778 16782
4769 16787 4761 16794 4754 16802 4747 16810 4741 16820 4735 16831
4730 16842 4726 16854 4722 16866 4719 16879 4717 16891 4716 16904

4716 16917 4752 16917 4753 16893 4757 16871 4762 16851 4769 16835
4777 16822 4787 16813 4791 16809 4796 16807 4801 16805 4806 16805
4811 16805 4816 16807 4821 16809 4826 16813 4835 16822 4843 16835
4850 16851 4855 16871 4859 16893 4860 16917 4859 16941 4855 16963
4850 16982 4843 16998 4835 17011 4826 17020 4821 17023 4816 17026
4811 17027 4806 17028 4801 17027 4796 17026 4791 17023 4787 17020
4777 17011 4769 16998 4762 16982 4757 16963 4753 16941 4752 16917
4716 16917 4716 16930 4717 16943 4719 16955 4722 16968 4726 16980
4730 16992 4735 17003 4741 17013 4747 17023 4754 17031 4761 17039
4769 17045 4778 17051 4787 17054 4796 17057 4806 17058 107 N F
5064 17006 5063 16993 5059 16981 5053 16970 5045 16961 5036 16953
5025 16947 5013 16943 5000 16942 5000 16967 4992 16967 4985 16970
4978 16973 4972 16978 4967 16984 4964 16991 4961 16998 4961 17006
4961 17014 4964 17021 4967 17028 4972 17034 4978 17039 4985 17042
4992 17045 5000 17045 5008 17045 5015 17042 5022 17039 5028 17034
5033 17028 5036 17021 5038 17014 5039 17006 5038 16998 5036 16991
5033 16984 5028 16978 5022 16973 5015 16970 5008 16967 5000 16967
5000 16942 4987 16943 4975 16947 4964 16953 4955 16961 4947 16970
4941 16981 4937 16993 4936 17006 4937 17019 4941 17031 4947 17042
4955 17051 4964 17059 4975 17065 4987 17069 5000 17070 5013 17069
5025 17065 5036 17059 5045 17051 5053 17042 5059 17031 5063 17019
5064 17006 67 N F
5117 17066 5311 17066 5311 17034 5153 17034 5153 16943 5305 16943
5305 16911 5153 16911 5153 16817 5317 16817 5317 16785 5117 16785
5117 17066 13 N F
6413 17058 6435 17058 6435 16785 6400 16785 6400 16979 6340 16979
6340 17003 6365 17006 6375 17009 6385 17013 6394 17019 6398 17023
6402 17028 6408 17041 6413 17058 15 N F
6627 17058 6649 17058 6649 16785 6615 16785 6615 16979 6555 16979
6555 17003 6579 17006 6590 17009 6600 17013 6609 17019 6613 17023
6616 17028 6623 17041 6627 17058 15 N F
6772 17058 6914 17058 6914 17026 6800 17026 6790 16947 6799 16954
6811 16960 6825 16964 6842 16965 6856 16964 6870 16960 6885 16954
6892 16950 6898 16945 6905 16939 6910 16932 6915 16925 6920 16916
6923 16907 6926 16896 6927 16885 6928 16873 6927 16854 6925 16844
6922 16836 6919 16827 6915 16819 6910 16811 6904 16804 6898 16798
6891 16792 6883 16787 6875 16783 6866 16779 6856 16777 6845 16775
6834 16774 6823 16775 6813 16776 6804 16778 6796 16780 6789 16784
6782 16788 6775 16792 6770 16797 6760 16808 6753 16821 6747 16835
6743 16851 6778 16851 6781 16841 6785 16832 6791 16824 6797 16817
6804 16812 6813 16808 6822 16805 6832 16805 6845 16806 6858 16809
6868 16814 6877 16821 6884 16831 6890 16842 6893 16855 6894 16871
6893 16882 6890 16894 6886 16904 6880 16914 6871 16923 6861 16929

6848 16934 6841 16935 6834 16935 6826 16935 6819 16934 6813 16932
6807 16929 6795 16921 6782 16909 6751 16909 6772 17058 83 N F
7093 17006 7092 16993 7088 16981 7083 16970 7075 16961 7065 16953
7054 16947 7042 16943 7030 16942 7030 16967 7022 16967 7014 16970
7008 16973 7002 16978 6997 16984 6993 16991 6991 16998 6990 17006
6991 17014 6993 17021 6997 17028 7002 17034 7008 17039 7014 17042
7022 17045 7030 17045 7038 17045 7045 17042 7052 17039 7057 17034
7062 17028 7066 17021 7068 17014 7069 17006 7068 16998 7066 16991
7062 16984 7057 16978 7052 16973 7045 16970 7038 16967 7030 16967
7030 16942 7017 16943 7005 16947 6994 16953 6984 16961 6977 16970
6971 16981 6967 16993 6966 17006 6967 17019 6971 17031 6977 17042
6984 17051 6994 17059 7005 17065 7017 17069 7030 17070 7042 17069
7054 17065 7065 17059 7075 17051 7083 17042 7088 17031 7092 17019
7093 17006 67 N F
7147 17066 7341 17066 7341 17034 7183 17034 7183 16943 7335 16943
7335 16911 7183 16911 7183 16817 7347 16817 7347 16785 7147 16785
7147 17066 13 N F
8432 17058 8455 17058 8455 16785 8420 16785 8420 16979 8360 16979
8360 17003 8385 17006 8395 17009 8405 17013 8414 17019 8418 17023
8422 17028 8428 17041 8432 17058 15 N F
8555 16963 8589 16963 8590 16977 8592 16989 8597 17000 8603 17010
8611 17017 8620 17023 8632 17026 8645 17028 8655 17027 8665 17024
8674 17020 8682 17014 8688 17006 8693 16997 8696 16987 8697 16975
8696 16964 8693 16954 8689 16946 8682 16938 8673 16931 8663 16924
8635 16907 8612 16893 8593 16880 8578 16866 8566 16851 8558 16836
8553 16820 8550 16803 8549 16785 8731 16785 8731 16817 8587 16817
8591 16829 8595 16839 8602 16848 8610 16856 8620 16863 8632 16871
8663 16889 8681 16898 8696 16908 8708 16919 8718 16930 8725 16942
8730 16955 8732 16967 8733 16979 8733 16988 8732 16996 8729 17004
8726 17011 8723 17018 8718 17024 8713 17030 8707 17036 8694 17045
8679 17052 8663 17056 8645 17058 8626 17056 8617 17055 8609 17052
8601 17049 8594 17045 8587 17040 8580 17035 8575 17028 8570 17021
8565 17014 8562 17005 8559 16996 8557 16986 8555 16975 8555 16963
78 N F
8856 17058 8866 17057 8875 17054 8884 17051 8893 17045 8901 17039
8908 17031 8915 17023 8921 17013 8927 17003 8932 16992 8936 16980
8939 16968 8942 16955 8944 16943 8946 16930 8946 16917 8946 16904
8944 16891 8942 16879 8939 16866 8936 16854 8932 16842 8927 16831
8921 16820 8915 16810 8908 16802 8901 16794 8893 16787 8884 16782
8875 16778 8866 16775 8856 16774 8846 16775 8836 16778 8827 16782
8819 16787 8811 16794 8803 16802 8797 16810 8790 16820 8785 16831
8780 16842 8775 16854 8772 16866 8769 16879 8767 16891 8766 16904
8765 16917 8802 16917 8803 16893 8806 16871 8812 16851 8819 16835

8827 16822 8836 16813 8841 16809 8846 16807 8851 16805 8856 16805
8861 16805 8866 16807 8870 16809 8875 16813 8884 16822 8893 16835
8900 16851 8905 16871 8909 16893 8910 16917 8909 16941 8905 16963
8900 16982 8893 16998 8884 17011 8875 17020 8870 17023 8866 17026
8861 17027 8856 17028 8851 17027 8846 17026 8841 17023 8836 17020
8827 17011 8819 16998 8812 16982 8806 16963 8803 16941 8802 16917
8765 16917 8766 16930 8767 16943 8769 16955 8772 16968 8775 16980
8780 16992 8785 17003 8790 17013 8797 17023 8803 17031 8811 17039
8819 17045 8827 17051 8836 17054 8846 17057 8856 17058 107 N F
9113 17006 9112 16993 9108 16981 9103 16970 9095 16961 9085 16953
9074 16947 9062 16943 9050 16942 9050 16967 9042 16967 9034 16970
9028 16973 9022 16978 9017 16984 9013 16991 9011 16998 9010 17006
9011 17014 9013 17021 9017 17028 9022 17034 9028 17039 9034 17042
9042 17045 9050 17045 9058 17045 9065 17042 9072 17039 9077 17034
9082 17028 9086 17021 9088 17014 9089 17006 9088 16998 9086 16991
9082 16984 9077 16978 9072 16973 9065 16970 9058 16967 9050 16967
9050 16942 9037 16943 9025 16947 9014 16953 9004 16961 8997 16970
8991 16981 8987 16993 8986 17006 8987 17019 8991 17031 8997 17042
9004 17051 9014 17059 9025 17065 9037 17069 9050 17070 9062 17069
9074 17065 9085 17059 9095 17051 9103 17042 9108 17031 9112 17019
9113 17006 67 N F
9167 17066 9361 17066 9361 17034 9203 17034 9203 16943 9355 16943
9355 16911 9203 16911 9203 16817 9367 16817 9367 16785 9167 16785
9167 17066 13 N F
10459 17058 10481 17058 10481 16785 10447 16785 10447 16979 10386 16979
10386 17003 10411 17006 10422 17009 10432 17013 10440 17019 10444 17023
10448 17028 10454 17041 10459 17058 15 N F
10581 16963 10615 16963 10616 16977 10619 16989 10623 17000 10629 17010
10637 17017 10646 17023 10658 17026 10671 17028 10682 17027 10691 17024
10700 17020 10708 17014 10714 17006 10719 16997 10722 16987 10723 16975
10723 16964 10720 16954 10715 16946 10708 16938 10699 16931 10689 16924
10661 16907 10638 16893 10619 16880 10604 16866 10593 16851 10584 16836
10579 16820 10576 16803 10575 16785 10758 16785 10758 16817 10613 16817
10617 16829 10622 16839 10628 16848 10636 16856 10646 16863 10658 16871
10689 16889 10707 16898 10722 16908 10735 16919 10744 16930 10751 16942
10756 16955 10759 16967 10760 16979 10759 16988 10758 16996 10756 17004
10753 17011 10749 17018 10744 17024 10739 17030 10734 17036 10720 17045
10705 17052 10689 17056 10671 17058 10652 17056 10644 17055 10635 17052
10627 17049 10620 17045 10613 17040 10607 17035 10601 17028 10596 17021
10591 17014 10588 17005 10585 16996 10583 16986 10582 16975 10581 16963
78 N F
10818 17058 10960 17058 10960 17026 10846 17026 10836 16947 10846 16954
10857 16960 10871 16964 10888 16965 10902 16964 10917 16960 10931 16954

10938 16950 10945 16945 10951 16939 10956 16932 10961 16925 10966 16916
10969 16907 10972 16896 10974 16885 10974 16873 10973 16854 10971 16844
10968 16836 10965 16827 10961 16819 10956 16811 10950 16804 10944 16798
10937 16792 10929 16787 10921 16783 10912 16779 10902 16777 10891 16775
10880 16774 10869 16775 10860 16776 10851 16778 10842 16780 10835 16784
10828 16788 10822 16792 10816 16797 10806 16808 10799 16821 10793 16835
10790 16851 10824 16851 10827 16841 10832 16832 10837 16824 10843 16817
10851 16812 10859 16808 10868 16805 10878 16805 10892 16806 10904 16809
10914 16814 10923 16821 10930 16831 10936 16842 10939 16855 10940 16871
10939 16882 10937 16894 10932 16904 10926 16914 10917 16923 10907 16929
10895 16934 10888 16935 10880 16935 10872 16935 10865 16934 10859 16932
10853 16929 10841 16921 10828 16909 10798 16909 10818 17058 83 N F
11140 17006 11138 16993 11135 16981 11129 16970 11121 16961 11111 16953
11101 16947 11089 16943 11076 16942 11076 16967 11068 16967 11060 16970
11054 16973 11048 16978 11043 16984 11040 16991 11037 16998 11036 17006
11037 17014 11040 17021 11043 17028 11048 17034 11054 17039 11060 17042
11068 17045 11076 17045 11084 17045 11091 17042 11098 17039 11104 17034
11108 17028 11112 17021 11114 17014 11115 17006 11114 16998 11112 16991
11108 16984 11104 16978 11098 16973 11091 16970 11084 16967 11076 16967
11076 16942 11063 16943 11051 16947 11040 16953 11031 16961 11023 16970
11017 16981 11013 16993 11012 17006 11013 17019 11017 17031 11023 17042
11031 17051 11040 17059 11051 17065 11063 17069 11076 17070 11089 17069
11101 17065 11111 17059 11121 17051 11129 17042 11135 17031 11138 17019
11140 17006 67 N F
11193 17066 11387 17066 11387 17034 11229 17034 11229 16943 11381 16943
11381 16911 11229 16911 11229 16817 11393 16817 11393 16785 11193 16785
11193 17066 13 N F
12508 17058 12530 17058 12530 16785 12496 16785 12496 16979 12436 16979
12436 17003 12460 17006 12471 17009 12481 17013 12490 17019 12494 17023
12497 17028 12504 17041 12508 17058 15 N F
12663 16969 12629 16969 12631 16990 12633 16999 12636 17008 12639 17016
12643 17023 12648 17029 12653 17035 12659 17041 12665 17045 12672 17049
12680 17052 12688 17055 12696 17056 12715 17058 12732 17056 12748 17053
12763 17047 12775 17039 12780 17034 12785 17028 12789 17022 12793 17015
12795 17008 12797 17000 12799 16992 12799 16983 12799 16973 12797 16964
12794 16956 12790 16948 12785 16941 12778 16935 12769 16930 12759 16925
12771 16920 12781 16915 12789 16908 12796 16901 12801 16892 12804 16883
12806 16872 12807 16861 12806 16846 12802 16831 12796 16817 12791 16810
12786 16804 12780 16798 12773 16793 12766 16788 12757 16784 12748 16781
12737 16778 12725 16777 12713 16776 12702 16777 12692 16778 12682 16780
12674 16783 12666 16786 12658 16791 12652 16795 12646 16801 12641 16807
12637 16813 12633 16821 12630 16828 12626 16845 12625 16863 12659 16863
12659 16853 12661 16843 12664 16834 12669 16825 12676 16818 12686 16812

12699 16808 12715 16807 12729 16807 12740 16810 12750 16815 12758 16822
12764 16830 12768 16839 12770 16850 12771 16861 12770 16875 12768 16881
12766 16887 12763 16891 12759 16895 12750 16902 12739 16906 12726 16909
12711 16911 12695 16911 12695 16939 12711 16940 12725 16941 12737 16943
12746 16947 12753 16952 12759 16960 12762 16969 12763 16981 12762 16991
12760 17000 12757 17007 12752 17014 12745 17020 12737 17024 12727 17027
12715 17028 12704 17027 12694 17025 12686 17021 12678 17016 12672 17008
12667 16998 12664 16985 12663 16969 123 N F
12931 17058 12941 17057 12951 17054 12960 17051 12968 17045 12976 17039
12984 17031 12991 17023 12997 17013 13002 17003 13007 16992 13012 16980
13015 16968 13018 16955 13020 16943 13021 16930 13022 16917 13021 16904
13020 16891 13018 16879 13015 16866 13012 16854 13007 16842 13002 16831
12997 16820 12991 16810 12984 16802 12976 16794 12968 16787 12960 16782
12951 16778 12941 16775 12931 16774 12921 16775 12912 16778 12903 16782
12894 16787 12886 16794 12879 16802 12872 16810 12866 16820 12860 16831
12855 16842 12851 16854 12848 16866 12845 16879 12843 16891 12842 16904
12841 16917 12877 16917 12879 16893 12882 16871 12888 16851 12895 16835
12903 16822 12912 16813 12917 16809 12922 16807 12926 16805 12931 16805
12936 16805 12941 16807 12946 16809 12951 16813 12960 16822 12968 16835
12975 16851 12981 16871 12984 16893 12986 16917 12984 16941 12981 16963
12975 16982 12968 16998 12960 17011 12951 17020 12946 17023 12941 17026
12936 17027 12931 17028 12926 17027 12922 17026 12917 17023 12912 17020
12903 17011 12895 16998 12888 16982 12882 16963 12879 16941 12877 16917
12841 16917 12842 16930 12843 16943 12845 16955 12848 16968 12851 16980
12855 16992 12860 17003 12866 17013 12872 17023 12879 17031 12886 17039
12894 17045 12903 17051 12912 17054 12921 17057 12931 17058 107 N F
13189 17006 13188 16993 13184 16981 13178 16970 13170 16961 13161 16953
13150 16947 13138 16943 13125 16942 13125 16967 13117 16967 13110 16970
13103 16973 13097 16978 13093 16984 13089 16991 13087 16998 13086 17006
13087 17014 13089 17021 13093 17028 13097 17034 13103 17039 13110 17042
13117 17045 13125 17045 13133 17045 13141 17042 13147 17039 13153 17034
13158 17028 13162 17021 13164 17014 13165 17006 13164 16998 13162 16991
13158 16984 13153 16978 13147 16973 13141 16970 13133 16967 13125 16967
13125 16942 13112 16943 13100 16947 13090 16953 13080 16961 13072 16970
13066 16981 13063 16993 13061 17006 13063 17019 13066 17031 13072 17042
13080 17051 13090 17059 13100 17065 13112 17069 13125 17070 13138 17069
13150 17065 13161 17059 13170 17051 13178 17042 13184 17031 13188 17019
13189 17006 67 N F
13242 17066 13437 17066 13437 17034 13278 17034 13278 16943 13431 16943
13431 16911 13278 16911 13278 16817 13443 16817 13443 16785 13242 16785
13242 17066 13 N F
915 5728 949 5728 950 5742 952 5754 956 5765 962 5775
970 5782 980 5788 991 5791 1005 5793 1015 5792 1025 5789

1034 5784 1041 5778 1048 5771 1053 5762 1056 5752 1057 5740
1056 5729 1053 5719 1048 5711 1042 5703 1033 5696 1022 5689
995 5672 972 5658 953 5645 938 5631 926 5616 918 5601
912 5585 909 5568 909 5549 1091 5549 1091 5582 947 5582
950 5594 955 5604 962 5613 970 5621 980 5628 992 5636
1023 5654 1041 5663 1056 5673 1068 5684 1078 5695 1085 5707
1089 5720 1092 5732 1093 5744 1093 5753 1091 5761 1089 5769
1086 5776 1082 5783 1078 5789 1073 5795 1067 5801 1054 5810
1039 5817 1022 5821 1005 5823 986 5821 977 5819 969 5817
961 5814 953 5810 946 5805 940 5800 934 5793 929 5786
925 5779 921 5770 918 5761 916 5751 915 5740 915 5728
78 N F
1129 5728 1163 5728 1164 5742 1167 5754 1171 5765 1177 5775
1185 5782 1195 5788 1206 5791 1219 5793 1230 5792 1239 5789
1248 5784 1256 5778 1263 5771 1267 5762 1271 5752 1272 5740
1271 5729 1268 5719 1263 5711 1256 5703 1248 5696 1237 5689
1209 5672 1186 5658 1167 5645 1152 5631 1141 5616 1132 5601
1127 5585 1124 5568 1123 5549 1306 5549 1306 5582 1161 5582
1165 5594 1170 5604 1176 5613 1184 5621 1194 5628 1206 5636
1238 5654 1256 5663 1270 5673 1283 5684 1292 5695 1299 5707
1304 5720 1307 5732 1308 5744 1307 5753 1306 5761 1304 5769
1301 5776 1297 5783 1292 5789 1287 5795 1282 5801 1269 5810
1254 5817 1237 5821 1219 5823 1201 5821 1192 5819 1183 5817
1175 5814 1168 5810 1161 5805 1155 5800 1149 5793 1144 5786
1140 5779 1136 5770 1133 5761 1131 5751 1130 5740 1129 5728
78 N F
1473 5771 1472 5758 1468 5746 1462 5735 1454 5726 1445 5718
1434 5712 1422 5708 1409 5707 1409 5732 1401 5732 1394 5735
1387 5738 1381 5743 1377 5749 1373 5756 1371 5763 1370 5771
1371 5779 1373 5786 1377 5793 1381 5799 1387 5804 1394 5807
1401 5810 1409 5810 1417 5810 1425 5807 1431 5804 1437 5799
1442 5793 1446 5786 1448 5779 1449 5771 1448 5763 1446 5756
1442 5749 1437 5743 1431 5738 1425 5735 1417 5732 1409 5732
1409 5707 1396 5708 1385 5712 1374 5718 1364 5726 1356 5735
1350 5746 1347 5758 1345 5771 1347 5784 1350 5796 1356 5807
1364 5816 1374 5824 1385 5830 1396 5834 1409 5835 1422 5834
1434 5830 1445 5824 1454 5816 1462 5807 1468 5796 1472 5784
1473 5771 67 N F
1520 5831 1560 5831 1705 5602 1705 5831 1739 5831 1739 5549
1699 5549 1554 5778 1554 5549 1520 5549 1520 5831 11 N F
915 6904 949 6904 950 6918 952 6930 956 6941 962 6951
970 6958 980 6964 991 6967 1005 6969 1015 6968 1025 6965
1034 6961 1041 6955 1048 6947 1053 6938 1056 6928 1057 6916

1056 6905 1053 6895 1048 6887 1042 6879 1033 6872 1022 6865
995 6848 972 6834 953 6821 938 6807 926 6792 918 6777
912 6761 909 6744 909 6726 1091 6726 1091 6758 947 6758
950 6770 955 6780 962 6789 970 6797 980 6804 992 6812
1023 6830 1041 6839 1056 6849 1068 6860 1078 6871 1085 6883
1089 6896 1092 6908 1093 6920 1093 6929 1091 6937 1089 6945
1086 6952 1082 6959 1078 6965 1073 6971 1067 6977 1054 6986
1039 6993 1022 6997 1005 6999 986 6997 977 6995 969 6993
961 6990 953 6986 946 6981 940 6976 934 6969 929 6962
925 6955 921 6946 918 6937 916 6927 915 6916 915 6904
78 N F
1246 6999 1270 6999 1270 6822 1312 6822 1312 6790 1270 6790
1270 6726 1236 6726 1236 6790 1121 6790 1121 6822 1151 6822
1236 6822 1236 6940 1151 6822 1121 6822 1121 6826 1246 6999
18 N F
1473 6947 1472 6934 1468 6922 1462 6911 1454 6902 1445 6894
1434 6888 1422 6884 1409 6883 1409 6908 1401 6908 1394 6911
1387 6914 1381 6919 1377 6925 1373 6932 1371 6939 1370 6947
1371 6955 1373 6962 1377 6969 1381 6975 1387 6980 1394 6983
1401 6986 1409 6986 1417 6986 1425 6983 1431 6980 1437 6975
1442 6969 1446 6962 1448 6955 1449 6947 1448 6939 1446 6932
1442 6925 1437 6919 1431 6914 1425 6911 1417 6908 1409 6908
1409 6883 1396 6884 1385 6888 1374 6894 1364 6902 1356 6911
1350 6922 1347 6934 1345 6947 1347 6960 1350 6972 1356 6983
1364 6992 1374 7000 1385 7006 1396 7010 1409 7011 1422 7010
1434 7006 1445 7000 1454 6992 1462 6983 1468 6972 1472 6960
1473 6947 67 N F
1520 7007 1560 7007 1705 6778 1705 7007 1739 7007 1739 6726
1699 6726 1554 6955 1554 6726 1520 6726 1520 7007 11 N F
915 8065 949 8065 950 8079 952 8091 956 8102 962 8112
970 8119 980 8125 991 8128 1005 8129 1015 8129 1025 8126
1034 8121 1041 8115 1048 8108 1053 8099 1056 8089 1057 8077
1056 8066 1053 8056 1048 8047 1042 8040 1033 8033 1022 8026
995 8009 972 7995 953 7982 938 7968 926 7953 918 7938
912 7922 909 7905 909 7886 1091 7886 1091 7919 947 7919
950 7931 955 7941 962 7950 970 7958 980 7965 992 7973
1023 7991 1041 8000 1056 8010 1068 8021 1078 8032 1085 8044
1089 8057 1092 8069 1093 8081 1093 8090 1091 8098 1089 8106
1086 8113 1082 8120 1078 8126 1073 8132 1067 8138 1054 8147
1039 8154 1022 8158 1005 8160 986 8158 977 8156 969 8154
961 8151 953 8147 946 8142 940 8136 934 8130 929 8123
925 8116 921 8107 918 8098 916 8088 915 8077 915 8065
78 N F

1302 8089 1268 8089 1265 8098 1262 8107 1257 8113 1252 8119
1245 8124 1238 8127 1231 8129 1223 8129 1216 8129 1209 8128
1203 8126 1197 8122 1191 8119 1186 8114 1182 8109 1177 8102
1170 8088 1165 8070 1162 8050 1161 8027 1169 8036 1178 8043
1186 8048 1194 8052 1202 8054 1210 8056 1227 8057 1241 8056
1255 8052 1268 8045 1281 8036 1287 8030 1292 8023 1297 8016
1301 8008 1304 7999 1306 7990 1308 7980 1308 7969 1274 7967
1272 7978 1269 7989 1264 7999 1257 8008 1248 8016 1239 8022
1229 8026 1217 8027 1206 8026 1196 8022 1186 8016 1178 8008
1171 7999 1166 7989 1162 7978 1161 7967 1162 7956 1166 7945
1171 7934 1178 7925 1186 7918 1196 7912 1206 7908 1217 7906
1229 7908 1239 7912 1248 7918 1257 7925 1264 7934 1269 7945
1272 7956 1274 7967 1308 7969 1306 7953 1302 7937 1296 7921
1291 7914 1286 7907 1280 7900 1273 7894 1266 7889 1258 7885
1249 7881 1239 7879 1229 7877 1217 7876 1205 7877 1194 7879
1184 7882 1174 7887 1166 7893 1158 7899 1152 7907 1146 7916
1141 7926 1136 7936 1133 7947 1130 7959 1126 7985 1125 8013
1126 8029 1127 8045 1129 8060 1132 8074 1135 8087 1140 8099
1145 8110 1151 8120 1158 8129 1165 8137 1173 8144 1182 8149
1191 8154 1201 8157 1212 8159 1223 8160 1239 8158 1254 8154
1267 8147 1278 8139 1287 8128 1294 8116 1299 8103 1302 8089
126 N F
1473 8108 1472 8095 1468 8083 1462 8072 1454 8063 1445 8055
1434 8049 1422 8045 1409 8044 1409 8068 1401 8069 1394 8072
1387 8075 1381 8080 1377 8086 1373 8093 1371 8100 1370 8108
1371 8116 1373 8123 1377 8130 1381 8136 1387 8141 1394 8144
1401 8146 1409 8147 1417 8146 1425 8144 1431 8141 1437 8136
1442 8130 1446 8123 1448 8116 1449 8108 1448 8100 1446 8093
1442 8086 1437 8080 1431 8075 1425 8072 1417 8069 1409 8068
1409 8044 1396 8045 1385 8049 1374 8055 1364 8063 1356 8072
1350 8083 1347 8095 1345 8108 1347 8121 1350 8133 1356 8144
1364 8153 1374 8161 1385 8167 1396 8171 1409 8172 1422 8171
1434 8167 1445 8161 1454 8153 1462 8144 1468 8133 1472 8121
1473 8108 67 N F
1520 8168 1560 8168 1705 7939 1705 8168 1739 8168 1739 7886
1699 7886 1554 8115 1554 7886 1520 7886 1520 8168 11 N F
915 9210 949 9210 950 9224 952 9236 956 9247 962 9256
970 9264 980 9269 991 9273 1005 9274 1015 9273 1025 9270
1034 9266 1041 9260 1048 9253 1053 9244 1056 9233 1057 9222
1056 9210 1053 9200 1048 9192 1042 9185 1033 9178 1022 9170
995 9154 972 9140 953 9126 938 9112 926 9098 918 9082
912 9066 909 9049 909 9031 1091 9031 1091 9063 947 9063
950 9076 955 9086 962 9095 970 9102 980 9110 992 9117

1023 9135 1041 9144 1056 9154 1068 9165 1078 9177 1085 9189
1089 9201 1092 9214 1093 9226 1093 9234 1091 9243 1089 9250
1086 9258 1082 9265 1078 9271 1073 9277 1067 9282 1054 9292
1039 9298 1022 9303 1005 9304 986 9303 977 9301 969 9299
961 9295 953 9291 946 9287 940 9281 934 9275 929 9268
925 9260 921 9252 918 9242 916 9232 915 9221 915 9210
78 N F
1215 9304 1232 9303 1248 9299 1262 9294 1274 9285 1279 9281
1284 9275 1288 9269 1291 9263 1294 9256 1296 9248 1297 9240
1298 9232 1297 9220 1294 9210 1290 9201 1286 9194 1280 9187
1274 9182 1262 9174 1272 9168 1281 9162 1289 9155 1295 9146
1301 9137 1304 9127 1307 9116 1308 9105 1306 9092 1302 9078
1294 9064 1284 9050 1278 9044 1271 9039 1263 9034 1254 9029
1245 9026 1235 9023 1225 9022 1213 9021 1202 9022 1192 9023
1182 9026 1173 9029 1165 9034 1158 9039 1151 9044 1145 9050
1135 9064 1129 9078 1125 9092 1123 9105 1159 9105 1160 9094
1164 9084 1169 9075 1176 9067 1184 9060 1194 9055 1204 9052
1215 9051 1226 9052 1237 9055 1246 9060 1255 9067 1262 9075
1267 9084 1270 9094 1272 9105 1270 9116 1267 9127 1262 9136
1255 9144 1246 9150 1237 9155 1226 9159 1215 9160 1204 9159
1194 9155 1184 9150 1176 9144 1169 9136 1164 9127 1160 9116
1159 9105 1123 9105 1124 9116 1126 9127 1130 9137 1136 9146
1142 9154 1150 9161 1159 9168 1169 9174 1157 9182 1151 9187
1145 9194 1140 9201 1137 9210 1134 9220 1133 9232 1167 9232
1168 9223 1171 9214 1176 9207 1182 9201 1189 9196 1197 9193
1206 9190 1215 9190 1225 9190 1234 9193 1242 9196 1249 9201
1255 9207 1260 9214 1263 9223 1264 9232 1263 9241 1260 9250
1255 9257 1249 9263 1242 9268 1234 9271 1225 9273 1215 9274
1206 9273 1197 9271 1189 9268 1182 9263 1176 9257 1171 9250
1168 9241 1167 9232 1133 9232 1134 9240 1135 9248 1137 9256
1140 9263 1143 9269 1147 9275 1152 9281 1157 9285 1169 9294
1183 9299 1199 9303 1215 9304 147 N F
1473 9253 1472 9240 1468 9228 1462 9217 1454 9207 1445 9199
1434 9194 1422 9190 1409 9189 1409 9213 1401 9214 1394 9216
1387 9220 1381 9225 1377 9230 1373 9237 1371 9245 1370 9253
1371 9260 1373 9268 1377 9275 1381 9280 1387 9285 1394 9289
1401 9291 1409 9292 1417 9291 1425 9289 1431 9285 1437 9280
1442 9275 1446 9268 1448 9260 1449 9253 1448 9245 1446 9237
1442 9230 1437 9225 1431 9220 1425 9216 1417 9214 1409 9213
1409 9189 1396 9190 1385 9194 1374 9199 1364 9207 1356 9217
1350 9228 1347 9240 1345 9253 1347 9265 1350 9277 1356 9288
1364 9298 1374 9306 1385 9311 1396 9315 1409 9316 1422 9315
1434 9311 1445 9306 1454 9298 1462 9288 1468 9277 1472 9265

1473 9253 67 N F
1520 9312 1560 9312 1705 9083 1705 9312 1739 9312 1739 9031
1699 9031 1554 9260 1554 9031 1520 9031 1520 9312 11 N F
947 10350 913 10350 915 10371 917 10380 920 10389 923 10397
927 10404 932 10411 937 10416 943 10422 949 10426 956 10430
964 10433 972 10436 980 10437 999 10439 1016 10438 1032 10434
1047 10428 1059 10420 1064 10415 1069 10409 1073 10403 1077 10396
1079 10389 1081 10381 1083 10373 1083 10364 1083 10354 1081 10345
1078 10337 1074 10329 1069 10322 1062 10316 1053 10311 1043 10306
1055 10301 1065 10296 1073 10289 1080 10282 1085 10273 1088 10264
1090 10253 1091 10242 1090 10227 1086 10212 1080 10198 1075 10191
1070 10185 1064 10179 1057 10174 1050 10169 1041 10165 1032 10162
1021 10160 1009 10158 997 10158 986 10158 976 10159 966 10161
958 10164 950 10167 942 10172 936 10176 930 10182 925 10188
921 10195 917 10202 914 10209 910 10226 909 10244 943 10244
943 10234 945 10224 948 10215 953 10206 960 10199 970 10193
983 10189 999 10188 1013 10188 1025 10191 1034 10196 1042 10203
1048 10211 1052 10220 1054 10231 1055 10242 1054 10256 1052 10262
1050 10268 1047 10272 1043 10276 1034 10283 1023 10287 1010 10290
995 10292 979 10292 979 10320 995 10321 1009 10322 1021 10324
1030 10328 1038 10333 1043 10341 1046 10350 1047 10362 1046 10372
1044 10381 1041 10389 1036 10395 1029 10401 1021 10405 1011 10408
999 10409 988 10408 978 10406 970 10402 962 10397 956 10389
951 10379 948 10366 947 10350 123 N F
1215 10439 1225 10438 1235 10436 1244 10432 1252 10426 1260 10420
1268 10412 1275 10404 1281 10394 1286 10384 1291 10373 1296 10361
1299 10349 1302 10336 1304 10324 1305 10311 1306 10298 1305 10285
1304 10272 1302 10260 1299 10247 1296 10235 1291 10223 1286 10212
1281 10201 1275 10191 1268 10183 1260 10175 1252 10168 1244 10163
1235 10159 1225 10156 1215 10156 1206 10156 1196 10159 1187 10163
1178 10168 1171 10175 1163 10183 1156 10191 1150 10201 1144 10212
1139 10223 1135 10235 1132 10247 1129 10260 1127 10272 1126 10285
1125 10298 1161 10298 1163 10274 1166 10252 1172 10233 1179 10216
1187 10203 1196 10194 1201 10190 1206 10188 1211 10186 1215 10186
1220 10186 1225 10188 1230 10190 1235 10194 1244 10203 1252 10216
1259 10233 1265 10252 1268 10274 1270 10298 1268 10323 1265 10344
1259 10363 1252 10379 1244 10392 1235 10401 1230 10404 1225 10407
1220 10408 1215 10409 1211 10408 1206 10407 1201 10404 1196 10401
1187 10392 1179 10379 1172 10363 1166 10344 1163 10323 1161 10298
1125 10298 1126 10311 1127 10324 1129 10336 1132 10349 1135 10361
1139 10373 1144 10384 1150 10394 1156 10404 1163 10412 1171 10420
1178 10426 1187 10432 1196 10436 1206 10438 1215 10439 107 N F
1473 10387 1472 10374 1468 10362 1462 10351 1454 10342 1445 10334

1434 10328 1422 10324 1409 10323 1409 10348 1401 10348 1394 10351
1387 10354 1381 10359 1377 10365 1373 10372 1371 10379 1370 10387
1371 10395 1373 10402 1377 10409 1381 10415 1387 10420 1394 10423
1401 10426 1409 10426 1417 10426 1425 10423 1431 10420 1437 10415
1442 10409 1446 10402 1448 10395 1449 10387 1448 10379 1446 10372
1442 10365 1437 10359 1431 10354 1425 10351 1417 10348 1409 10348
1409 10323 1396 10324 1385 10328 1374 10334 1364 10342 1356 10351
1350 10362 1347 10374 1345 10387 1347 10400 1350 10412 1356 10423
1364 10432 1374 10440 1385 10446 1396 10450 1409 10451 1422 10450
1434 10446 1445 10440 1454 10432 1462 10423 1468 10412 1472 10400
1473 10387 67 N F
1520 10447 1560 10447 1705 10218 1705 10447 1739 10447 1739 10166
1699 10166 1554 10395 1554 10166 1520 10166 1520 10447 11 N F
947 11475 913 11475 915 11495 917 11505 920 11513 923 11521
927 11528 932 11535 937 11541 943 11546 949 11551 956 11555
964 11558 972 11560 980 11562 999 11563 1016 11562 1032 11558
1047 11552 1059 11544 1064 11539 1069 11534 1073 11528 1077 11521
1079 11514 1081 11506 1083 11498 1083 11489 1083 11479 1081 11470
1078 11461 1074 11454 1069 11447 1062 11441 1053 11436 1043 11431
1055 11426 1065 11420 1073 11414 1080 11406 1085 11398 1088 11388
1090 11378 1091 11366 1090 11351 1086 11336 1080 11322 1075 11315
1070 11309 1064 11303 1057 11298 1050 11294 1041 11290 1032 11286
1021 11284 1009 11283 997 11282 986 11282 976 11284 966 11286
958 11289 950 11292 942 11296 936 11301 930 11306 925 11312
921 11319 917 11326 914 11334 910 11350 909 11368 943 11368
943 11359 945 11349 948 11339 953 11331 960 11323 970 11317
983 11314 999 11312 1013 11313 1025 11316 1034 11321 1042 11327
1048 11336 1052 11345 1054 11355 1055 11366 1054 11381 1052 11387
1050 11392 1047 11397 1043 11401 1034 11407 1023 11412 1010 11415
995 11416 979 11417 979 11445 995 11445 1009 11446 1021 11449
1030 11452 1038 11458 1043 11465 1046 11475 1047 11487 1046 11496
1044 11505 1041 11513 1036 11520 1029 11525 1021 11530 1011 11532
999 11533 988 11533 978 11531 970 11527 962 11521 956 11514
951 11504 948 11491 947 11475 123 N F
1129 11469 1163 11469 1164 11483 1167 11495 1171 11506 1177 11515
1185 11523 1195 11528 1206 11532 1219 11533 1230 11532 1239 11530
1248 11525 1256 11519 1263 11512 1267 11503 1271 11492 1272 11481
1271 11469 1268 11460 1263 11451 1256 11444 1248 11437 1237 11430
1209 11413 1186 11399 1167 11385 1152 11371 1141 11357 1132 11341
1127 11325 1124 11308 1123 11290 1306 11290 1306 11322 1161 11322
1165 11335 1170 11345 1176 11354 1184 11361 1194 11369 1206 11376
1238 11395 1256 11403 1270 11413 1283 11424 1292 11436 1299 11448
1304 11460 1307 11473 1308 11485 1307 11493 1306 11502 1304 11509

1301 11517 1297 11524 1292 11530 1287 11536 1282 11541 1269 11551
1254 11558 1237 11562 1219 11563 1201 11562 1192 11560 1183 11558
1175 11554 1168 11550 1161 11546 1155 11540 1149 11534 1144 11527
1140 11519 1136 11511 1133 11501 1131 11491 1130 11481 1129 11469
78 N F
1473 11512 1472 11499 1468 11487 1462 11476 1454 11466 1445 11459
1434 11453 1422 11449 1409 11448 1409 11472 1401 11473 1394 11475
1387 11479 1381 11484 1377 11490 1373 11496 1371 11504 1370 11512
1371 11520 1373 11527 1377 11534 1381 11539 1387 11544 1394 11548
1401 11550 1409 11551 1417 11550 1425 11548 1431 11544 1437 11539
1442 11534 1446 11527 1448 11520 1449 11512 1448 11504 1446 11496
1442 11490 1437 11484 1431 11479 1425 11475 1417 11473 1409 11472
1409 11448 1396 11449 1385 11453 1374 11459 1364 11466 1356 11476
1350 11487 1347 11499 1345 11512 1347 11524 1350 11536 1356 11547
1364 11557 1374 11565 1385 11570 1396 11574 1409 11576 1422 11574
1434 11570 1445 11565 1454 11557 1462 11547 1468 11536 1472 11524
1473 11512 67 N F
1520 11571 1560 11571 1705 11342 1705 11571 1739 11571 1739 11290
1699 11290 1554 11519 1554 11290 1520 11290 1520 11571 11 N F
947 12593 913 12593 915 12613 917 12622 920 12631 923 12639
927 12646 932 12653 937 12659 943 12664 949 12668 956 12672
964 12675 972 12678 980 12680 999 12681 1016 12680 1032 12676
1047 12670 1059 12662 1064 12657 1069 12651 1073 12645 1077 12639
1079 12631 1081 12624 1083 12615 1083 12607 1083 12597 1081 12587
1078 12579 1074 12572 1069 12565 1062 12559 1053 12553 1043 12548
1055 12544 1065 12538 1073 12531 1080 12524 1085 12515 1088 12506
1090 12496 1091 12484 1090 12469 1086 12454 1080 12440 1075 12433
1070 12427 1064 12421 1057 12416 1050 12411 1041 12407 1032 12404
1021 12402 1009 12400 997 12400 986 12400 976 12401 966 12403
958 12406 950 12410 942 12414 936 12419 930 12424 925 12430
921 12437 917 12444 914 12451 910 12468 909 12486 943 12486
943 12476 945 12466 948 12457 953 12448 960 12441 970 12435
983 12431 999 12430 1013 12430 1025 12433 1034 12438 1042 12445
1048 12453 1052 12463 1054 12473 1055 12484 1054 12498 1052 12504
1050 12510 1047 12515 1043 12519 1034 12525 1023 12530 1010 12532
995 12534 979 12534 979 12563 995 12563 1009 12564 1021 12566
1030 12570 1038 12575 1043 12583 1046 12593 1047 12605 1046 12614
1044 12623 1041 12631 1036 12638 1029 12643 1021 12647 1011 12650
999 12651 988 12650 978 12648 970 12645 962 12639 956 12631
951 12621 948 12608 947 12593 123 N F
1246 12681 1270 12681 1270 12504 1312 12504 1312 12472 1270 12472
1270 12408 1236 12408 1236 12472 1121 12472 1121 12504 1151 12504
1236 12504 1236 12623 1151 12504 1121 12504 1121 12508 1246 12681

18 N F
1473 12629 1472 12616 1468 12604 1462 12594 1454 12584 1445 12576
1434 12570 1422 12567 1409 12565 1409 12590 1401 12591 1394 12593
1387 12597 1381 12601 1377 12607 1373 12614 1371 12621 1370 12629
1371 12637 1373 12645 1377 12651 1381 12657 1387 12662 1394 12666
1401 12668 1409 12669 1417 12668 1425 12666 1431 12662 1437 12657
1442 12651 1446 12645 1448 12637 1449 12629 1448 12621 1446 12614
1442 12607 1437 12601 1431 12597 1425 12593 1417 12591 1409 12590
1409 12565 1396 12567 1385 12570 1374 12576 1364 12584 1356 12594
1350 12604 1347 12616 1345 12629 1347 12642 1350 12654 1356 12665
1364 12674 1374 12682 1385 12688 1396 12692 1409 12693 1422 12692
1434 12688 1445 12682 1454 12674 1462 12665 1468 12654 1472 12642
1473 12629 67 N F
1520 12689 1560 12689 1705 12460 1705 12689 1739 12689 1739 12408
1699 12408 1554 12637 1554 12408 1520 12408 1520 12689 11 N F
947 13705 913 13705 915 13725 917 13735 920 13743 923 13751
927 13758 932 13765 937 13771 943 13776 949 13781 956 13784
964 13788 972 13790 980 13792 999 13793 1016 13792 1032 13788
1047 13782 1059 13774 1064 13769 1069 13764 1073 13757 1077 13751
1079 13744 1081 13736 1083 13728 1083 13719 1083 13709 1081 13700
1078 13691 1074 13684 1069 13677 1062 13671 1053 13665 1043 13661
1055 13656 1065 13650 1073 13643 1080 13636 1085 13628 1088 13618
1090 13608 1091 13596 1090 13581 1086 13566 1080 13552 1075 13545
1070 13539 1064 13533 1057 13528 1050 13523 1041 13519 1032 13516
1021 13514 1009 13512 997 13512 986 13512 976 13514 966 13516
958 13518 950 13522 942 13526 936 13531 930 13536 925 13542
921 13549 917 13556 914 13564 910 13580 909 13598 943 13598
943 13588 945 13579 948 13569 953 13560 960 13553 970 13547
983 13543 999 13542 1013 13543 1025 13545 1034 13550 1042 13557
1048 13565 1052 13575 1054 13585 1055 13596 1054 13611 1052 13617
1050 13622 1047 13627 1043 13631 1034 13637 1023 13642 1010 13645
995 13646 979 13647 979 13675 995 13675 1009 13676 1021 13678
1030 13682 1038 13688 1043 13695 1046 13705 1047 13717 1046 13726
1044 13735 1041 13743 1036 13750 1029 13755 1021 13759 1011 13762
999 13763 988 13762 978 13760 970 13757 962 13751 956 13744
951 13733 948 13721 947 13705 123 N F
1302 13723 1268 13723 1265 13732 1262 13740 1257 13747 1252 13753
1245 13757 1238 13760 1231 13762 1223 13763 1216 13763 1209 13761
1203 13759 1197 13756 1191 13752 1186 13748 1182 13742 1177 13736
1170 13721 1165 13704 1162 13683 1161 13661 1169 13669 1178 13676
1186 13682 1194 13685 1202 13688 1210 13690 1227 13691 1241 13689
1255 13685 1268 13679 1281 13669 1287 13663 1292 13657 1297 13650
1301 13642 1304 13633 1306 13623 1308 13613 1308 13602 1274 13600

1272 13612 1269 13622 1264 13633 1257 13642 1248 13649 1239 13655
1229 13659 1217 13661 1206 13659 1196 13655 1186 13649 1178 13642
1171 13633 1166 13622 1162 13612 1161 13600 1162 13589 1166 13578
1171 13568 1178 13559 1186 13551 1196 13545 1206 13541 1217 13540
1229 13541 1239 13545 1248 13551 1257 13559 1264 13568 1269 13578
1272 13589 1274 13600 1308 13602 1306 13586 1302 13570 1296 13555
1291 13547 1286 13540 1280 13534 1273 13528 1266 13523 1258 13518
1249 13515 1239 13512 1229 13511 1217 13510 1205 13511 1194 13513
1184 13516 1174 13521 1166 13526 1158 13533 1152 13541 1146 13550
1141 13559 1136 13570 1133 13581 1130 13593 1126 13619 1125 13647
1126 13663 1127 13679 1129 13694 1132 13708 1135 13721 1140 13733
1145 13744 1151 13754 1158 13763 1165 13771 1173 13777 1182 13783
1191 13787 1201 13791 1212 13793 1223 13793 1239 13792 1254 13788
1267 13781 1278 13772 1287 13762 1294 13750 1299 13737 1302 13723
126 N F
1473 13741 1472 13729 1468 13717 1462 13706 1454 13696 1445 13688
1434 13683 1422 13679 1409 13677 1409 13702 1401 13703 1394 13705
1387 13709 1381 13714 1377 13719 1373 13726 1371 13733 1370 13741
1371 13749 1373 13757 1377 13763 1381 13769 1387 13774 1394 13778
1401 13780 1409 13781 1417 13780 1425 13778 1431 13774 1437 13769
1442 13763 1446 13757 1448 13749 1449 13741 1448 13733 1446 13726
1442 13719 1437 13714 1431 13709 1425 13705 1417 13703 1409 13702
1409 13677 1396 13679 1385 13683 1374 13688 1364 13696 1356 13706
1350 13717 1347 13729 1345 13741 1347 13754 1350 13766 1356 13777
1364 13787 1374 13794 1385 13800 1396 13804 1409 13805 1422 13804
1434 13800 1445 13794 1454 13787 1462 13777 1468 13766 1472 13754
1473 13741 67 N F
1520 13801 1560 13801 1705 13572 1705 13801 1739 13801 1739 13520
1699 13520 1554 13749 1554 13520 1520 13520 1520 13801 11 N F
947 14811 913 14811 915 14831 917 14840 920 14849 923 14857
927 14864 932 14871 937 14877 943 14882 949 14886 956 14890
964 14893 972 14896 980 14898 999 14899 1016 14898 1032 14894
1047 14888 1059 14880 1064 14875 1069 14869 1073 14863 1077 14857
1079 14849 1081 14842 1083 14833 1083 14825 1083 14815 1081 14805
1078 14797 1074 14790 1069 14783 1062 14777 1053 14771 1043 14766
1055 14762 1065 14756 1073 14749 1080 14742 1085 14733 1088 14724
1090 14714 1091 14702 1090 14687 1086 14672 1080 14658 1075 14651
1070 14645 1064 14639 1057 14634 1050 14629 1041 14625 1032 14622
1021 14620 1009 14618 997 14618 986 14618 976 14619 966 14621
958 14624 950 14628 942 14632 936 14637 930 14642 925 14648
921 14655 917 14662 914 14669 910 14686 909 14704 943 14704
943 14694 945 14684 948 14675 953 14666 960 14659 970 14653
983 14649 999 14648 1013 14648 1025 14651 1034 14656 1042 14663

1048 14671 1052 14681 1054 14691 1055 14702 1054 14716 1052 14722
1050 14728 1047 14732 1043 14737 1034 14743 1023 14748 1010 14750
995 14752 979 14752 979 14780 995 14781 1009 14782 1021 14784
1030 14788 1038 14793 1043 14801 1046 14810 1047 14823 1046 14832
1044 14841 1041 14849 1036 14856 1029 14861 1021 14865 1011 14868
999 14869 988 14868 978 14866 970 14863 962 14857 956 14849
951 14839 948 14826 947 14811 123 N F
1215 14899 1232 14898 1248 14894 1262 14888 1274 14880 1279 14875
1284 14870 1288 14864 1291 14857 1294 14851 1296 14843 1297 14835
1298 14827 1297 14815 1294 14805 1290 14796 1286 14789 1280 14782
1274 14777 1262 14768 1272 14763 1281 14757 1289 14749 1295 14741
1301 14732 1304 14722 1307 14711 1308 14700 1306 14687 1302 14673
1294 14658 1284 14645 1278 14639 1271 14633 1263 14628 1254 14624
1245 14621 1235 14618 1225 14616 1213 14616 1202 14616 1192 14618
1182 14621 1173 14624 1165 14628 1158 14633 1151 14639 1145 14645
1135 14658 1129 14673 1125 14687 1123 14700 1159 14700 1160 14689
1164 14679 1169 14670 1176 14662 1184 14655 1194 14650 1204 14647
1215 14646 1226 14647 1237 14650 1246 14655 1255 14662 1262 14670
1267 14679 1270 14689 1272 14700 1270 14711 1267 14721 1262 14731
1255 14739 1246 14745 1237 14750 1226 14753 1215 14754 1204 14753
1194 14750 1184 14745 1176 14739 1169 14731 1164 14721 1160 14711
1159 14700 1123 14700 1124 14711 1126 14722 1130 14731 1136 14740
1142 14749 1150 14756 1159 14763 1169 14768 1157 14777 1151 14782
1145 14789 1140 14796 1137 14805 1134 14815 1133 14827 1167 14827
1168 14817 1171 14809 1176 14802 1182 14796 1189 14791 1197 14787
1206 14785 1215 14784 1225 14785 1234 14787 1242 14791 1249 14796
1255 14802 1260 14809 1263 14817 1264 14827 1263 14836 1260 14844
1255 14852 1249 14858 1242 14862 1234 14866 1225 14868 1215 14869
1206 14868 1197 14866 1189 14862 1182 14858 1176 14852 1171 14844
1168 14836 1167 14827 1133 14827 1134 14835 1135 14843 1137 14851
1140 14857 1143 14864 1147 14870 1152 14875 1157 14880 1169 14888
1183 14894 1199 14898 1215 14899 147 N F
1473 14847 1472 14834 1468 14822 1462 14812 1454 14802 1445 14794
1434 14788 1422 14785 1409 14783 1409 14808 1401 14809 1394 14811
1387 14815 1381 14819 1377 14825 1373 14832 1371 14839 1370 14847
1371 14855 1373 14863 1377 14869 1381 14875 1387 14880 1394 14884
1401 14886 1409 14887 1417 14886 1425 14884 1431 14880 1437 14875
1442 14869 1446 14863 1448 14855 1449 14847 1448 14839 1446 14832
1442 14825 1437 14819 1431 14815 1425 14811 1417 14809 1409 14808
1409 14783 1396 14785 1385 14788 1374 14794 1364 14802 1356 14812
1350 14822 1347 14834 1345 14847 1347 14860 1350 14872 1356 14883
1364 14892 1374 14900 1385 14906 1396 14910 1409 14911 1422 14910
1434 14906 1445 14900 1454 14892 1462 14883 1468 14872 1472 14860

1473 14847 67 N F
1520 14907 1560 14907 1705 14678 1705 14907 1739 14907 1739 14626
1699 14626 1554 14855 1554 14626 1520 14626 1520 14907 11 N F
1031 15992 1055 15992 1055 15815 1097 15815 1097 15783 1055 15783
1055 15718 1021 15718 1021 15783 907 15783 907 15815 937 15815
1021 15815 1021 15933 937 15815 907 15815 907 15819 1031 15992
18 N F
1215 15992 1225 15991 1235 15988 1244 15984 1252 15979 1260 15973
1268 15965 1275 15956 1281 15947 1286 15936 1291 15925 1296 15914
1299 15902 1302 15889 1304 15877 1305 15864 1306 15851 1305 15838
1304 15825 1302 15812 1299 15800 1296 15788 1291 15776 1286 15765
1281 15754 1275 15744 1268 15735 1260 15728 1252 15721 1244 15716
1235 15712 1225 15709 1215 15708 1206 15709 1196 15712 1187 15716
1178 15721 1171 15728 1163 15735 1156 15744 1150 15754 1144 15765
1139 15776 1135 15788 1132 15800 1129 15812 1127 15825 1126 15838
1125 15851 1161 15851 1163 15827 1166 15805 1172 15785 1179 15769
1187 15756 1196 15746 1201 15743 1206 15740 1211 15739 1215 15738
1220 15739 1225 15740 1230 15743 1235 15746 1244 15756 1252 15769
1259 15785 1265 15805 1268 15827 1270 15851 1268 15875 1265 15897
1259 15916 1252 15932 1244 15944 1235 15954 1230 15957 1225 15959
1220 15961 1215 15961 1211 15961 1206 15959 1201 15957 1196 15954
1187 15944 1179 15932 1172 15916 1166 15897 1163 15875 1161 15851
1125 15851 1126 15864 1127 15877 1129 15889 1132 15902 1135 15914
1139 15925 1144 15936 1150 15947 1156 15956 1163 15965 1171 15973
1178 15979 1187 15984 1196 15988 1206 15991 1215 15992 107 N F
1473 15940 1472 15927 1468 15915 1462 15904 1454 15895 1445 15887
1434 15881 1422 15877 1409 15876 1409 15900 1401 15901 1394 15904
1387 15907 1381 15912 1377 15918 1373 15924 1371 15932 1370 15940
1371 15948 1373 15955 1377 15962 1381 15968 1387 15972 1394 15976
1401 15978 1409 15979 1417 15978 1425 15976 1431 15972 1437 15968
1442 15962 1446 15955 1448 15948 1449 15940 1448 15932 1446 15924
1442 15918 1437 15912 1431 15907 1425 15904 1417 15901 1409 15900
1409 15876 1396 15877 1385 15881 1374 15887 1364 15895 1356 15904
1350 15915 1347 15927 1345 15940 1347 15953 1350 15965 1356 15976
1364 15985 1374 15993 1385 15999 1396 16003 1409 16004 1422 16003
1434 15999 1445 15993 1454 15985 1462 15976 1468 15965 1472 15953
1473 15940 67 N F
1520 16000 1560 16000 1705 15771 1705 16000 1739 16000 1739 15718
1699 15718 1554 15947 1554 15718 1520 15718 1520 16000 11 N F
13566 5631 13600 5631 13601 5645 13604 5657 13608 5668 13614 5677
13622 5685 13632 5690 13643 5694 13656 5695 13667 5694 13676 5691
13685 5687 13693 5681 13699 5674 13704 5665 13707 5654 13709 5643
13708 5631 13705 5621 13700 5613 13693 5606 13685 5599 13674 5591

13646 5575 13623 5561 13604 5547 13589 5533 13578 5519 13569 5503
13564 5487 13561 5470 13560 5452 13743 5452 13743 5484 13598 5484
13602 5497 13607 5507 13613 5516 13621 5523 13631 5531 13643 5538
13674 5557 13692 5565 13707 5575 13720 5586 13729 5598 13736 5610
13741 5622 13744 5635 13745 5647 13744 5655 13743 5664 13741 5671
13738 5679 13734 5686 13729 5692 13724 5698 13719 5703 13705 5713
13691 5720 13674 5724 13656 5725 13638 5724 13629 5722 13620 5720
13612 5716 13605 5712 13598 5708 13592 5702 13586 5696 13581 5689
13577 5681 13573 5673 13570 5663 13568 5653 13567 5643 13566 5631
78 N F
13781 5631 13815 5631 13816 5645 13818 5657 13823 5668 13829 5677
13837 5685 13846 5690 13858 5694 13871 5695 13881 5694 13891 5691
13900 5687 13908 5681 13914 5674 13919 5665 13922 5654 13923 5643
13922 5631 13919 5621 13915 5613 13908 5606 13899 5599 13888 5591
13861 5575 13838 5561 13819 5547 13804 5533 13792 5519 13784 5503
13779 5487 13776 5470 13775 5452 13957 5452 13957 5484 13813 5484
13816 5497 13821 5507 13828 5516 13836 5523 13846 5531 13858 5538
13889 5557 13907 5565 13922 5575 13934 5586 13944 5598 13951 5610
13956 5622 13958 5635 13959 5647 13959 5655 13957 5664 13955 5671
13952 5679 13949 5686 13944 5692 13939 5698 13933 5703 13920 5713
13905 5720 13889 5724 13871 5725 13852 5724 13843 5722 13835 5720
13827 5716 13820 5712 13813 5708 13806 5702 13801 5696 13796 5689
13791 5681 13788 5673 13785 5663 13783 5653 13781 5643 13781 5631
78 N F
14125 5674 14123 5661 14120 5649 14114 5638 14106 5628 14097 5621
14086 5615 14074 5611 14061 5610 14061 5634 14053 5635 14046 5637
14039 5641 14033 5646 14028 5651 14025 5658 14022 5666 14022 5674
14022 5681 14025 5689 14028 5696 14033 5701 14039 5706 14046 5710
14053 5712 14061 5713 14069 5712 14076 5710 14083 5706 14089 5701
14094 5696 14097 5689 14099 5681 14100 5674 14099 5666 14097 5658
14094 5651 14089 5646 14083 5641 14076 5637 14069 5635 14061 5634
14061 5610 14048 5611 14036 5615 14025 5621 14016 5628 14008 5638
14002 5649 13998 5661 13997 5674 13998 5686 14002 5698 14008 5709
14016 5719 14025 5727 14036 5732 14048 5736 14061 5738 14074 5736
14086 5732 14097 5727 14106 5719 14114 5709 14120 5698 14123 5686
14125 5674 67 N F
14172 5733 14212 5733 14356 5504 14356 5733 14390 5733 14390 5452
14350 5452 14206 5681 14206 5452 14172 5452 14172 5733 11 N F
13566 6800 13600 6800 13601 6814 13604 6826 13608 6837 13614 6846
13622 6854 13632 6860 13643 6863 13656 6864 13667 6863 13676 6861
13685 6856 13693 6850 13699 6843 13704 6834 13707 6824 13709 6812
13708 6800 13705 6791 13700 6782 13693 6775 13685 6768 13674 6761
13646 6744 13623 6730 13604 6716 13589 6702 13578 6688 13569 6673

13564 6656 13561 6639 13560 6621 13743 6621 13743 6653 13598 6653
13602 6666 13607 6676 13613 6685 13621 6693 13631 6700 13643 6708
13674 6726 13692 6735 13707 6745 13720 6756 13729 6767 13736 6779
13741 6792 13744 6804 13745 6816 13744 6825 13743 6833 13741 6840
13738 6848 13734 6855 13729 6861 13724 6867 13719 6873 13705 6882
13691 6889 13674 6893 13656 6894 13638 6893 13629 6891 13620 6889
13612 6885 13605 6881 13598 6877 13592 6871 13586 6865 13581 6858
13577 6850 13573 6842 13570 6833 13568 6823 13567 6812 13566 6800
78 N F
13897 6894 13921 6894 13921 6718 13963 6718 13963 6686 13921 6686
13921 6621 13887 6621 13887 6686 13773 6686 13773 6718 13803 6718
13887 6718 13887 6836 13803 6718 13773 6718 13773 6722 13897 6894
18 N F
14125 6843 14123 6830 14120 6818 14114 6807 14106 6798 14097 6790
14086 6784 14074 6780 14061 6779 14061 6803 14053 6804 14046 6806
14039 6810 14033 6815 14028 6821 14025 6827 14022 6835 14022 6843
14022 6851 14025 6858 14028 6865 14033 6871 14039 6875 14046 6879
14053 6881 14061 6882 14069 6881 14076 6879 14083 6875 14089 6871
14094 6865 14097 6858 14099 6851 14100 6843 14099 6835 14097 6827
14094 6821 14089 6815 14083 6810 14076 6806 14069 6804 14061 6803
14061 6779 14048 6780 14036 6784 14025 6790 14016 6798 14008 6807
14002 6818 13998 6830 13997 6843 13998 6856 14002 6868 14008 6878
14016 6888 14025 6896 14036 6902 14048 6905 14061 6907 14074 6905
14086 6902 14097 6896 14106 6888 14114 6878 14120 6868 14123 6856
14125 6843 67 N F
14172 6902 14212 6902 14356 6673 14356 6902 14390 6902 14390 6621
14350 6621 14206 6850 14206 6621 14172 6621 14172 6902 11 N F
13566 7958 13600 7958 13601 7972 13604 7984 13608 7995 13614 8004
13622 8012 13632 8017 13643 8021 13656 8022 13667 8021 13676 8018
13685 8014 13693 8008 13699 8001 13704 7992 13707 7981 13709 7970
13708 7958 13705 7948 13700 7940 13693 7933 13685 7926 13674 7918
13646 7902 13623 7888 13604 7874 13589 7860 13578 7846 13569 7830
13564 7814 13561 7797 13560 7779 13743 7779 13743 7811 13598 7811
13602 7824 13607 7834 13613 7843 13621 7850 13631 7858 13643 7865
13674 7884 13692 7892 13707 7902 13720 7913 13729 7925 13736 7937
13741 7949 13744 7962 13745 7974 13744 7982 13743 7991 13741 7998
13738 8006 13734 8013 13729 8019 13724 8025 13719 8030 13705 8040
13691 8047 13674 8051 13656 8052 13638 8051 13629 8049 13620 8047
13612 8043 13605 8039 13598 8035 13592 8029 13586 8023 13581 8016
13577 8008 13573 8000 13570 7990 13568 7980 13567 7969 13566 7958
78 N F
13953 7982 13919 7982 13917 7991 13913 7999 13909 8006 13903 8012
13897 8016 13890 8020 13883 8021 13875 8022 13868 8022 13861 8020

13854 8018 13848 8015 13843 8011 13838 8007 13833 8001 13829 7995
13822 7980 13817 7963 13814 7943 13813 7920 13821 7928 13829 7935
13837 7941 13845 7945 13853 7947 13862 7949 13879 7950 13892 7948
13906 7944 13920 7938 13933 7928 13939 7922 13944 7916 13948 7909
13952 7901 13956 7892 13958 7883 13959 7872 13959 7861 13925 7859
13924 7871 13921 7882 13915 7892 13908 7901 13900 7909 13890 7914
13880 7918 13869 7920 13858 7918 13848 7914 13838 7909 13830 7901
13823 7892 13817 7882 13814 7871 13813 7859 13814 7848 13817 7837
13823 7827 13830 7818 13838 7810 13848 7804 13858 7801 13869 7799
13880 7801 13890 7804 13900 7810 13908 7818 13915 7827 13921 7837
13924 7848 13925 7859 13959 7861 13958 7845 13954 7829 13947 7814
13943 7806 13937 7799 13932 7793 13925 7787 13918 7782 13909 7778
13900 7774 13891 7771 13880 7770 13869 7769 13857 7770 13845 7772
13835 7775 13826 7780 13817 7785 13810 7792 13803 7800 13797 7809
13792 7818 13788 7829 13784 7840 13782 7852 13778 7878 13777 7906
13777 7922 13778 7938 13781 7953 13783 7967 13787 7980 13791 7992
13797 8003 13803 8013 13809 8022 13817 8030 13825 8036 13833 8042
13843 8046 13853 8050 13864 8052 13875 8052 13891 8051 13905 8047
13918 8040 13929 8031 13938 8021 13946 8009 13951 7996 13953 7982
126 N F
14125 8001 14123 7988 14120 7976 14114 7965 14106 7955 14097 7948
14086 7942 14074 7938 14061 7937 14061 7961 14053 7962 14046 7964
14039 7968 14033 7973 14028 7978 14025 7985 14022 7993 14022 8001
14022 8008 14025 8016 14028 8023 14033 8028 14039 8033 14046 8037
14053 8039 14061 8040 14069 8039 14076 8037 14083 8033 14089 8028
14094 8023 14097 8016 14099 8008 14100 8001 14099 7993 14097 7985
14094 7978 14089 7973 14083 7968 14076 7964 14069 7962 14061 7961
14061 7937 14048 7938 14036 7942 14025 7948 14016 7955 14008 7965
14002 7976 13998 7988 13997 8001 13998 8013 14002 8025 14008 8036
14016 8046 14025 8054 14036 8059 14048 8063 14061 8065 14074 8063
14086 8059 14097 8054 14106 8046 14114 8036 14120 8025 14123 8013
14125 8001 67 N F
14172 8060 14212 8060 14356 7831 14356 8060 14390 8060 14390 7779
14350 7779 14206 8008 14206 7779 14172 7779 14172 8060 11 N F
13566 9099 13600 9099 13601 9113 13604 9125 13608 9136 13614 9146
13622 9153 13632 9159 13643 9162 13656 9164 13667 9163 13676 9160
13685 9156 13693 9150 13699 9142 13704 9133 13707 9123 13709 9111
13708 9100 13705 9090 13700 9082 13693 9074 13685 9067 13674 9060
13646 9043 13623 9030 13604 9016 13589 9002 13578 8987 13569 8972
13564 8956 13561 8939 13560 8921 13743 8921 13743 8953 13598 8953
13602 8965 13607 8975 13613 8984 13621 8992 13631 8999 13643 9007
13674 9025 13692 9034 13707 9044 13720 9055 13729 9066 13736 9079
13741 9091 13744 9103 13745 9115 13744 9124 13743 9132 13741 9140

13738 9147 13734 9154 13729 9160 13724 9166 13719 9172 13705 9181
13691 9188 13674 9192 13656 9194 13638 9192 13629 9191 13620 9188
13612 9185 13605 9181 13598 9176 13592 9171 13586 9164 13581 9157
13577 9150 13573 9141 13570 9132 13568 9122 13567 9111 13566 9099
78 N F
13867 9194 13884 9193 13899 9189 13913 9183 13925 9175 13931 9170
13935 9165 13939 9159 13943 9152 13946 9145 13948 9138 13949 9130
13949 9121 13948 9110 13946 9099 13942 9091 13937 9083 13932 9077
13926 9072 13913 9063 13923 9058 13932 9052 13940 9044 13947 9036
13952 9027 13956 9017 13958 9006 13959 8995 13958 8981 13953 8967
13946 8953 13935 8940 13929 8934 13922 8928 13914 8923 13906 8919
13897 8915 13887 8913 13876 8911 13865 8911 13854 8911 13843 8913
13834 8915 13825 8919 13817 8923 13809 8928 13802 8934 13797 8940
13787 8953 13780 8967 13776 8981 13775 8995 13811 8995 13812 8984
13815 8974 13821 8964 13828 8956 13836 8950 13846 8945 13856 8942
13867 8941 13878 8942 13888 8945 13898 8950 13906 8956 13913 8964
13919 8974 13922 8984 13923 8995 13922 9006 13919 9016 13913 9025
13906 9033 13898 9040 13888 9045 13878 9048 13867 9049 13856 9048
13846 9045 13836 9040 13828 9033 13821 9025 13815 9016 13812 9006
13811 8995 13775 8995 13776 9006 13778 9016 13782 9026 13787 9035
13794 9043 13802 9051 13811 9057 13821 9063 13808 9072 13802 9077
13797 9083 13792 9091 13788 9099 13786 9110 13785 9121 13819 9121
13820 9112 13823 9104 13827 9097 13833 9091 13840 9086 13849 9082
13857 9080 13867 9079 13877 9080 13886 9082 13894 9086 13901 9091
13907 9097 13911 9104 13914 9112 13915 9121 13914 9131 13911 9139
13907 9146 13901 9152 13894 9157 13886 9161 13877 9163 13867 9164
13857 9163 13849 9161 13840 9157 13833 9152 13827 9146 13823 9139
13820 9131 13819 9121 13785 9121 13785 9130 13786 9138 13788 9145
13791 9152 13795 9159 13799 9165 13803 9170 13809 9175 13821 9183
13835 9189 13850 9193 13867 9194 147 N F
14125 9142 14123 9129 14120 9117 14114 9106 14106 9097 14097 9089
14086 9083 14074 9079 14061 9078 14061 9103 14053 9103 14046 9106
14039 9109 14033 9114 14028 9120 14025 9127 14022 9134 14022 9142
14022 9150 14025 9157 14028 9164 14033 9170 14039 9175 14046 9178
14053 9181 14061 9181 14069 9181 14076 9178 14083 9175 14089 9170
14094 9164 14097 9157 14099 9150 14100 9142 14099 9134 14097 9127
14094 9120 14089 9114 14083 9109 14076 9106 14069 9103 14061 9103
14061 9078 14048 9079 14036 9083 14025 9089 14016 9097 14008 9106
14002 9117 13998 9129 13997 9142 13998 9155 14002 9167 14008 9178
14016 9187 14025 9195 14036 9201 14048 9205 14061 9206 14074 9205
14086 9201 14097 9195 14106 9187 14114 9178 14120 9167 14123 9155
14125 9142 67 N F
14172 9202 14212 9202 14356 8973 14356 9202 14390 9202 14390 8921

14350 8921 14206 9150 14206 8921 14172 8921 14172 9202 11 N F
13598 10237 13564 10237 13566 10257 13568 10266 13571 10275 13575 10283
13579 10290 13583 10297 13588 10303 13594 10308 13601 10312 13608 10316
13615 10319 13623 10322 13632 10324 13650 10325 13668 10324 13684 10320
13698 10314 13711 10306 13716 10301 13721 10295 13725 10289 13728 10283
13731 10275 13733 10268 13734 10259 13735 10251 13734 10241 13733 10231
13730 10223 13726 10216 13720 10209 13713 10203 13705 10197 13695 10192
13706 10188 13716 10182 13725 10175 13731 10168 13736 10159 13740 10150
13742 10140 13743 10128 13741 10113 13738 10098 13731 10084 13727 10077
13722 10071 13716 10065 13709 10060 13701 10055 13693 10051 13683 10048
13673 10046 13661 10044 13648 10044 13637 10044 13627 10045 13618 10047
13609 10050 13601 10054 13594 10058 13587 10063 13582 10068 13577 10074
13572 10081 13568 10088 13565 10095 13561 10112 13560 10130 13594 10130
13595 10120 13596 10110 13599 10101 13604 10092 13611 10085 13621 10079
13634 10075 13650 10074 13664 10074 13676 10077 13686 10082 13694 10089
13699 10097 13703 10107 13706 10117 13707 10128 13705 10142 13703 10148
13701 10154 13698 10158 13695 10163 13686 10169 13675 10174 13662 10176
13647 10178 13630 10178 13630 10206 13647 10207 13660 10208 13672 10210
13682 10214 13689 10219 13694 10227 13697 10236 13699 10249 13698 10258
13696 10267 13692 10275 13687 10282 13681 10287 13672 10291 13662 10294
13650 10295 13640 10294 13630 10292 13621 10289 13614 10283 13608 10275
13603 10265 13600 10252 13598 10237 123 N F
13867 10325 13877 10324 13886 10322 13895 10318 13904 10313 13912 10306
13919 10299 13926 10290 13932 10280 13938 10270 13943 10259 13947 10247
13951 10235 13954 10223 13956 10210 13957 10197 13957 10184 13957 10172
13956 10159 13954 10146 13951 10133 13947 10121 13943 10109 13938 10098
13932 10087 13926 10078 13919 10069 13912 10061 13904 10054 13895 10049
13886 10045 13877 10043 13867 10042 13857 10043 13848 10045 13839 10049
13830 10054 13822 10061 13815 10069 13808 10078 13802 10087 13796 10098
13791 10109 13787 10121 13783 10133 13780 10146 13778 10159 13777 10172
13777 10184 13813 10184 13814 10160 13818 10138 13823 10119 13830 10103
13838 10089 13848 10080 13852 10076 13857 10074 13862 10072 13867 10072
13872 10072 13877 10074 13882 10076 13886 10080 13896 10089 13904 10103
13911 10119 13916 10138 13920 10160 13921 10184 13920 10209 13916 10230
13911 10249 13904 10265 13896 10278 13886 10287 13882 10291 13877 10293
13872 10294 13867 10295 13862 10294 13857 10293 13852 10291 13848 10287
13838 10278 13830 10265 13823 10249 13818 10230 13814 10209 13813 10184
13777 10184 13777 10197 13778 10210 13780 10223 13783 10235 13787 10247
13791 10259 13796 10270 13802 10280 13808 10290 13815 10299 13822 10306
13830 10313 13839 10318 13848 10322 13857 10324 13867 10325 107 N F
14125 10273 14123 10260 14120 10248 14114 10238 14106 10228 14097 10220
14086 10214 14074 10211 14061 10209 14061 10234 14053 10235 14046 10237
14039 10241 14033 10245 14028 10251 14025 10258 14022 10265 14022 10273

14022 10281 14025 10289 14028 10295 14033 10301 14039 10306 14046 10310
14053 10312 14061 10313 14069 10312 14076 10310 14083 10306 14089 10301
14094 10295 14097 10289 14099 10281 14100 10273 14099 10265 14097 10258
14094 10251 14089 10245 14083 10241 14076 10237 14069 10235 14061 10234
14061 10209 14048 10211 14036 10214 14025 10220 14016 10228 14008 10238
14002 10248 13998 10260 13997 10273 13998 10286 14002 10298 14008 10309
14016 10318 14025 10326 14036 10332 14048 10336 14061 10337 14074 10336
14086 10332 14097 10326 14106 10318 14114 10309 14120 10298 14123 10286
14125 10273 67 N F
14172 10333 14212 10333 14356 10104 14356 10333 14390 10333 14390 10052
14350 10052 14206 10281 14206 10052 14172 10052 14172 10333 11 N F
13598 11358 13564 11358 13566 11378 13568 11388 13571 11396 13575 11404
13579 11411 13583 11418 13588 11424 13594 11429 13601 11434 13608 11437
13615 11441 13623 11443 13632 11445 13650 11446 13668 11445 13684 11441
13698 11435 13711 11427 13716 11422 13721 11416 13725 11410 13728 11404
13731 11397 13733 11389 13734 11381 13735 11372 13734 11362 13733 11353
13730 11344 13726 11337 13720 11330 13713 11324 13705 11318 13695 11314
13706 11309 13716 11303 13725 11296 13731 11289 13736 11281 13740 11271
13742 11261 13743 11249 13741 11234 13738 11219 13731 11205 13727 11198
13722 11192 13716 11186 13709 11181 13701 11176 13693 11172 13683 11169
13673 11167 13661 11165 13648 11165 13637 11165 13627 11167 13618 11169
13609 11171 13601 11175 13594 11179 13587 11184 13582 11189 13577 11195
13572 11202 13568 11209 13565 11217 13561 11233 13560 11251 13594 11251
13595 11241 13596 11231 13599 11222 13604 11213 13611 11206 13621 11200
13634 11196 13650 11195 13664 11196 13676 11198 13686 11203 13694 11210
13699 11218 13703 11228 13706 11238 13707 11249 13705 11264 13703 11270
13701 11275 13698 11280 13695 11284 13686 11290 13675 11295 13662 11298
13647 11299 13630 11299 13630 11328 13647 11328 13660 11329 13672 11331
13682 11335 13689 11341 13694 11348 13697 11358 13699 11370 13698 11379
13696 11388 13692 11396 13687 11403 13681 11408 13672 11412 13662 11415
13650 11416 13640 11415 13630 11413 13621 11410 13614 11404 13608 11396
13603 11386 13600 11374 13598 11358 123 N F
13781 11352 13815 11352 13816 11365 13818 11378 13823 11389 13829 11398
13837 11406 13846 11411 13858 11415 13871 11416 13881 11415 13891 11412
13900 11408 13908 11402 13914 11394 13919 11385 13922 11375 13923 11364
13922 11352 13919 11342 13915 11334 13908 11327 13899 11320 13888 11312
13861 11295 13838 11282 13819 11268 13804 11254 13792 11239 13784 11224
13779 11208 13776 11191 13775 11173 13957 11173 13957 11205 13813 11205
13816 11217 13821 11228 13828 11236 13836 11244 13846 11252 13858 11259
13889 11277 13907 11286 13922 11296 13934 11307 13944 11319 13951 11331
13956 11343 13958 11356 13959 11368 13959 11376 13957 11384 13955 11392
13952 11400 13949 11406 13944 11413 13939 11419 13933 11424 13920 11434
13905 11440 13889 11445 13871 11446 13852 11445 13843 11443 13835 11440

13827 11437 13820 11433 13813 11428 13806 11423 13801 11417 13796 11410
13791 11402 13788 11394 13785 11384 13783 11374 13781 11363 13781 11352
78 N F
14125 11394 14123 11382 14120 11370 14114 11359 14106 11349 14097 11341
14086 11335 14074 11332 14061 11330 14061 11355 14053 11356 14046 11358
14039 11362 14033 11367 14028 11372 14025 11379 14022 11386 14022 11394
14022 11402 14025 11410 14028 11416 14033 11422 14039 11427 14046 11431
14053 11433 14061 11434 14069 11433 14076 11431 14083 11427 14089 11422
14094 11416 14097 11410 14099 11402 14100 11394 14099 11386 14097 11379
14094 11372 14089 11367 14083 11362 14076 11358 14069 11356 14061 11355
14061 11330 14048 11332 14036 11335 14025 11341 14016 11349 14008 11359
14002 11370 13998 11382 13997 11394 13998 11407 14002 11419 14008 11430
14016 11440 14025 11447 14036 11453 14048 11457 14061 11458 14074 11457
14086 11453 14097 11447 14106 11440 14114 11430 14120 11419 14123 11407
14125 11394 67 N F
14172 11454 14212 11454 14356 11225 14356 11454 14390 11454 14390 11173
14350 11173 14206 11402 14206 11173 14172 11173 14172 11454 11 N F
13598 12472 13564 12472 13566 12492 13568 12502 13571 12510 13575 12518
13579 12525 13583 12532 13588 12538 13594 12543 13601 12548 13608 12551
13615 12555 13623 12557 13632 12559 13650 12560 13668 12559 13684 12555
13698 12549 13711 12541 13716 12536 13721 12531 13725 12525 13728 12518
13731 12511 13733 12503 13734 12495 13735 12486 13734 12476 13733 12467
13730 12458 13726 12451 13720 12444 13713 12438 13705 12432 13695 12428
13706 12423 13716 12417 13725 12411 13731 12403 13736 12395 13740 12385
13742 12375 13743 12363 13741 12348 13738 12333 13731 12319 13727 12312
13722 12306 13716 12300 13709 12295 13701 12291 13693 12287 13683 12283
13673 12281 13661 12280 13648 12279 13637 12279 13627 12281 13618 12283
13609 12285 13601 12289 13594 12293 13587 12298 13582 12303 13577 12309
13572 12316 13568 12323 13565 12331 13561 12347 13560 12365 13594 12365
13595 12355 13596 12346 13599 12336 13604 12327 13611 12320 13621 12314
13634 12311 13650 12309 13664 12310 13676 12313 13686 12317 13694 12324
13699 12332 13703 12342 13706 12352 13707 12363 13705 12378 13703 12384
13701 12389 13698 12394 13695 12398 13686 12404 13675 12409 13662 12412
13647 12413 13630 12414 13630 12442 13647 12442 13660 12443 13672 12446
13682 12449 13689 12455 13694 12462 13697 12472 13699 12484 13698 12493
13696 12502 13692 12510 13687 12517 13681 12522 13672 12527 13662 12529
13650 12530 13640 12530 13630 12528 13621 12524 13614 12518 13608 12511
13603 12500 13600 12488 13598 12472 123 N F
13897 12560 13921 12560 13921 12383 13963 12383 13963 12351 13921 12351
13921 12287 13887 12287 13887 12351 13773 12351 13773 12383 13803 12383
13887 12383 13887 12502 13803 12383 13773 12383 13773 12388 13897 12560
18 N F
14125 12509 14123 12496 14120 12484 14114 12473 14106 12463 14097 12456

14086 12450 14074 12446 14061 12445 14061 12469 14053 12470 14046 12472
14039 12476 14033 12481 14028 12486 14025 12493 14022 12501 14022 12509
14022 12516 14025 12524 14028 12531 14033 12536 14039 12541 14046 12545
14053 12547 14061 12548 14069 12547 14076 12545 14083 12541 14089 12536
14094 12531 14097 12524 14099 12516 14100 12509 14099 12501 14097 12493
14094 12486 14089 12481 14083 12476 14076 12472 14069 12470 14061 12469
14061 12445 14048 12446 14036 12450 14025 12456 14016 12463 14008 12473
14002 12484 13998 12496 13997 12509 13998 12521 14002 12533 14008 12544
14016 12554 14025 12562 14036 12567 14048 12571 14061 12573 14074 12571
14086 12567 14097 12562 14106 12554 14114 12544 14120 12533 14123 12521
14125 12509 67 N F
14172 12568 14212 12568 14356 12339 14356 12568 14390 12568 14390 12287
14350 12287 14206 12516 14206 12287 14172 12287 14172 12568 11 N F
13598 13580 13564 13580 13566 13601 13568 13610 13571 13619 13575 13627
13579 13634 13583 13640 13588 13646 13594 13651 13601 13656 13608 13660
13615 13663 13623 13665 13632 13667 13650 13669 13668 13667 13684 13664
13698 13658 13711 13650 13716 13645 13721 13639 13725 13633 13728 13626
13731 13619 13733 13611 13734 13603 13735 13594 13734 13584 13733 13575
13730 13567 13726 13559 13720 13552 13713 13546 13705 13541 13695 13536
13706 13531 13716 13525 13725 13519 13731 13511 13736 13503 13740 13494
13742 13483 13743 13472 13741 13457 13738 13442 13731 13427 13727 13421
13722 13414 13716 13409 13709 13403 13701 13399 13693 13395 13683 13392
13673 13389 13661 13388 13648 13387 13637 13388 13627 13389 13618 13391
13609 13394 13601 13397 13594 13401 13587 13406 13582 13412 13577 13418
13572 13424 13568 13431 13565 13439 13561 13456 13560 13474 13594 13474
13595 13464 13596 13454 13599 13444 13604 13436 13611 13428 13621 13423
13634 13419 13650 13418 13664 13418 13676 13421 13686 13426 13694 13433
13699 13441 13703 13450 13706 13461 13707 13472 13705 13486 13703 13492
13701 13497 13698 13502 13695 13506 13686 13513 13675 13517 13662 13520
13647 13522 13630 13522 13630 13550 13647 13550 13660 13552 13672 13554
13682 13558 13689 13563 13694 13570 13697 13580 13699 13592 13698 13602
13696 13610 13692 13618 13687 13625 13681 13631 13672 13635 13662 13638
13650 13638 13640 13638 13630 13636 13621 13632 13614 13627 13608 13619
13603 13609 13600 13596 13598 13580 123 N F
13953 13598 13919 13598 13917 13608 13913 13616 13909 13622 13903 13628
13897 13633 13890 13636 13883 13638 13875 13638 13868 13638 13861 13637
13854 13635 13848 13631 13843 13628 13838 13623 13833 13618 13829 13611
13822 13597 13817 13579 13814 13559 13813 13536 13821 13545 13829 13552
13837 13557 13845 13561 13853 13564 13862 13565 13879 13566 13892 13565
13906 13561 13920 13554 13933 13545 13939 13539 13944 13532 13948 13525
13952 13517 13956 13508 13958 13499 13959 13489 13959 13478 13925 13476
13924 13487 13921 13498 13915 13508 13908 13517 13900 13525 13890 13531
13880 13535 13869 13536 13858 13535 13848 13531 13838 13525 13830 13517

13823 13508 13817 13498 13814 13487 13813 13476 13814 13465 13817 13454
13823 13443 13830 13434 13838 13427 13848 13421 13858 13417 13869 13415
13880 13417 13890 13421 13900 13427 13908 13434 13915 13443 13921 13454
13924 13465 13925 13476 13959 13478 13958 13462 13954 13446 13947 13430
13943 13423 13937 13416 13932 13409 13925 13403 13918 13398 13909 13394
13900 13390 13891 13388 13880 13386 13869 13385 13857 13386 13845 13388
13835 13391 13826 13396 13817 13402 13810 13408 13803 13416 13797 13425
13792 13435 13788 13445 13784 13456 13782 13468 13778 13494 13777 13522
13777 13539 13778 13554 13781 13569 13783 13583 13787 13596 13791 13608
13797 13619 13803 13629 13809 13638 13817 13646 13825 13653 13833 13658
13843 13663 13853 13666 13864 13668 13875 13669 13891 13667 13905 13663
13918 13656 13929 13648 13938 13637 13946 13625 13951 13612 13953 13598
126 N F
14125 13617 14123 13604 14120 13592 14114 13581 14106 13572 14097 13564
14086 13558 14074 13554 14061 13553 14061 13578 14053 13578 14046 13581
14039 13584 14033 13589 14028 13595 14025 13602 14022 13609 14022 13617
14022 13625 14025 13632 14028 13639 14033 13645 14039 13650 14046 13653
14053 13655 14061 13656 14069 13655 14076 13653 14083 13650 14089 13645
14094 13639 14097 13632 14099 13625 14100 13617 14099 13609 14097 13602
14094 13595 14089 13589 14083 13584 14076 13581 14069 13578 14061 13578
14061 13553 14048 13554 14036 13558 14025 13564 14016 13572 14008 13581
14002 13592 13998 13604 13997 13617 13998 13630 14002 13642 14008 13653
14016 13662 14025 13670 14036 13676 14048 13680 14061 13681 14074 13680
14086 13676 14097 13670 14106 13662 14114 13653 14120 13642 14123 13630
14125 13617 67 N F
14172 13677 14212 13677 14356 13448 14356 13677 14390 13677 14390 13395
14350 13395 14206 13624 14206 13395 14172 13395 14172 13677 11 N F
13598 14682 13564 14682 13566 14703 13568 14712 13571 14720 13575 14728
13579 14736 13583 14742 13588 14748 13594 14753 13601 14758 13608 14762
13615 14765 13623 14767 13632 14769 13650 14770 13668 14769 13684 14766
13698 14760 13711 14751 13716 14746 13721 14741 13725 14735 13728 14728
13731 14721 13733 14713 13734 14705 13735 14696 13734 14686 13733 14677
13730 14668 13726 14661 13720 14654 13713 14648 13705 14643 13695 14638
13706 14633 13716 14627 13725 14621 13731 14613 13736 14605 13740 14595
13742 14585 13743 14574 13741 14558 13738 14543 13731 14529 13727 14523
13722 14516 13716 14511 13709 14505 13701 14501 13693 14497 13683 14494
13673 14491 13661 14490 13648 14489 13637 14490 13627 14491 13618 14493
13609 14496 13601 14499 13594 14503 13587 14508 13582 14514 13577 14520
13572 14526 13568 14533 13565 14541 13561 14557 13560 14576 13594 14576
13595 14566 13596 14556 13599 14546 13604 14538 13611 14530 13621 14524
13634 14521 13650 14519 13664 14520 13676 14523 13686 14528 13694 14534
13699 14543 13703 14552 13706 14562 13707 14574 13705 14588 13703 14594
13701 14599 13698 14604 13695 14608 13686 14614 13675 14619 13662 14622

13647 14623 13630 14624 13630 14652 13647 14652 13660 14653 13672 14656
13682 14659 13689 14665 13694 14672 13697 14682 13699 14694 13698 14704
13696 14712 13692 14720 13687 14727 13681 14733 13672 14737 13662 14739
13650 14740 13640 14740 13630 14738 13621 14734 13614 14728 13608 14721
13603 14711 13600 14698 13598 14682 123 N F
13867 14770 13884 14769 13899 14766 13913 14760 13925 14752 13931 14747
13935 14741 13939 14735 13943 14729 13946 14722 13948 14714 13949 14706
13949 14698 13948 14686 13946 14676 13942 14667 13937 14660 13932 14654
13926 14648 13913 14640 13923 14635 13932 14628 13940 14621 13947 14612
13952 14603 13956 14593 13958 14583 13959 14572 13958 14558 13953 14544
13946 14530 13935 14517 13929 14510 13922 14505 13914 14500 13906 14496
13897 14492 13887 14489 13876 14488 13865 14487 13854 14488 13843 14489
13834 14492 13825 14496 13817 14500 13809 14505 13802 14510 13797 14517
13787 14530 13780 14544 13776 14558 13775 14572 13811 14572 13812 14561
13815 14550 13821 14541 13828 14533 13836 14527 13846 14522 13856 14518
13867 14517 13878 14518 13888 14522 13898 14527 13906 14533 13913 14541
13919 14550 13922 14561 13923 14572 13922 14583 13919 14593 13913 14602
13906 14610 13898 14617 13888 14622 13878 14625 13867 14626 13856 14625
13846 14622 13836 14617 13828 14610 13821 14602 13815 14593 13812 14583
13811 14572 13775 14572 13776 14583 13778 14593 13782 14603 13787 14612
13794 14620 13802 14627 13811 14634 13821 14640 13808 14648 13802 14654
13797 14660 13792 14667 13788 14676 13786 14686 13785 14698 13819 14698
13820 14689 13823 14680 13827 14673 13833 14667 13840 14662 13849 14659
13857 14657 13867 14656 13877 14657 13886 14659 13894 14662 13901 14667
13907 14673 13911 14680 13914 14689 13915 14698 13914 14707 13911 14716
13907 14723 13901 14729 13894 14734 13886 14737 13877 14740 13867 14740
13857 14740 13849 14737 13840 14734 13833 14729 13827 14723 13823 14716
13820 14707 13819 14698 13785 14698 13785 14706 13786 14714 13788 14722
13791 14729 13795 14735 13799 14741 13803 14747 13809 14752 13821 14760
13835 14766 13850 14769 13867 14770 147 N F
14125 14719 14123 14706 14120 14694 14114 14683 14106 14673 14097 14666
14086 14660 14074 14656 14061 14655 14061 14679 14053 14680 14046 14682
14039 14686 14033 14691 14028 14697 14025 14703 14022 14711 14022 14719
14022 14727 14025 14734 14028 14741 14033 14747 14039 14751 14046 14755
14053 14757 14061 14758 14069 14757 14076 14755 14083 14751 14089 14747
14094 14741 14097 14734 14099 14727 14100 14719 14099 14711 14097 14703
14094 14697 14089 14691 14083 14686 14076 14682 14069 14680 14061 14679
14061 14655 14048 14656 14036 14660 14025 14666 14016 14673 14008 14683
14002 14694 13998 14706 13997 14719 13998 14732 14002 14743 14008 14754
14016 14764 14025 14772 14036 14778 14048 14781 14061 14783 14074 14781
14086 14778 14097 14772 14106 14764 14114 14754 14120 14743 14123 14732
14125 14719 67 N F
14172 14778 14212 14778 14356 14549 14356 14778 14390 14778 14390 14497

14350 14497 14206 14726 14206 14497 14172 14497 14172 14778 11 N F
13684 15868 13709 15868 13709 15691 13751 15691 13751 15659 13709 15659
13709 15595 13674 15595 13674 15659 13560 15659 13560 15691 13590 15691
13674 15691 13674 15810 13590 15691 13560 15691 13560 15695 13684 15868
18 N F
13869 15868 13879 15867 13888 15865 13897 15861 13906 15856 13914 15849
13921 15841 13928 15833 13934 15823 13940 15813 13945 15802 13949 15790
13953 15778 13956 15766 13958 15753 13959 15740 13959 15727 13959 15714
13958 15702 13956 15689 13953 15676 13949 15664 13945 15652 13940 15641
13934 15630 13928 15621 13921 15612 13914 15604 13906 15597 13897 15592
13888 15588 13879 15586 13869 15585 13859 15586 13850 15588 13841 15592
13832 15597 13824 15604 13817 15612 13810 15621 13804 15630 13798 15641
13793 15652 13789 15664 13785 15676 13782 15689 13780 15702 13779 15714
13779 15727 13815 15727 13816 15703 13820 15681 13825 15662 13832 15645
13840 15632 13850 15623 13854 15619 13859 15617 13864 15615 13869 15615
13874 15615 13879 15617 13884 15619 13888 15623 13898 15632 13906 15645
13913 15662 13918 15681 13922 15703 13923 15727 13922 15752 13918 15773
13913 15792 13906 15808 13898 15821 13888 15830 13884 15833 13879 15836
13874 15837 13869 15838 13864 15837 13859 15836 13854 15833 13850 15830
13840 15821 13832 15808 13825 15792 13820 15773 13816 15752 13815 15727
13779 15727 13779 15740 13780 15753 13782 15766 13785 15778 13789 15790
13793 15802 13798 15813 13804 15823 13810 15833 13817 15841 13824 15849
13832 15856 13841 15861 13850 15865 13859 15867 13869 15868 107 N F
14127 15816 14125 15803 14122 15791 14116 15781 14108 15771 14099 15763
14088 15757 14076 15754 14063 15752 14063 15777 14055 15778 14048 15780
14041 15784 14035 15788 14030 15794 14027 15801 14024 15808 14024 15816
14024 15824 14027 15832 14030 15838 14035 15844 14041 15849 14048 15852
14055 15855 14063 15856 14071 15855 14078 15852 14085 15849 14091 15844
14096 15838 14099 15832 14101 15824 14102 15816 14101 15808 14099 15801
14096 15794 14091 15788 14085 15784 14078 15780 14071 15778 14063 15777
14063 15752 14050 15754 14038 15757 14027 15763 14018 15771 14010 15781
14004 15791 14000 15803 13999 15816 14000 15829 14004 15841 14010 15852
14018 15861 14027 15869 14038 15875 14050 15879 14063 15880 14076 15879
14088 15875 14099 15869 14108 15861 14116 15852 14122 15841 14125 15829
14127 15816 67 N F
14174 15876 14214 15876 14358 15647 14358 15876 14392 15876 14392 15595
14352 15595 14208 15824 14208 15595 14174 15595 14174 15876 11 N F
25 W
2649 5218 2649 5499 2 P
3742 5218 3742 5499 2 P
4825 5218 4825 5499 2 P
5904 5218 5904 5499 2 P
6971 5218 6971 5499 2 P

8052 5218 8052 5499 2 P
9132 5218 9132 5499 2 P
10213 5218 10213 5499 2 P
11293 5218 11293 5499 2 P
12374 5218 12374 5499 2 P
2750 16493 2750 16212 2 P
4814 16493 4814 16212 2 P
6844 16493 6844 16212 2 P
8864 16493 8864 16212 2 P
10890 16493 10890 16212 2 P
12939 16493 12939 16212 2 P
2020 5692 2301 5692 2 P
2020 6868 2301 6868 2 P
2020 8024 2301 8024 2 P
2020 9169 2301 9169 2 P
2020 10303 2301 10303 2 P
2020 11429 2301 11429 2 P
2020 12547 2301 12547 2 P
2020 13658 2301 13658 2 P
2020 14764 2301 14764 2 P
2020 15856 2301 15856 2 P
13279 5595 12998 5595 2 P
13279 6764 12998 6764 2 P
13279 7917 12998 7917 2 P
13279 9058 12998 9058 2 P
13279 10190 12998 10190 2 P
13279 11312 12998 11312 2 P
13279 12426 12998 12426 2 P
13279 13533 12998 13533 2 P
13279 14635 12998 14635 2 P
13279 15732 12998 15732 2 P
12998 5499 2301 5499 2301 16212 12998 16212 12998 5499 2301 5499

6:00 PM

12633 17555 12632 17544 12629 17534 12624 17525 12618 17517 12610 17511
12601 17506 12591 17503 12580 17502 12569 17503 12559 17506 12550 17511
12542 17517 12536 17525 12531 17534 12527 17544 12526 17555 12548 17555
12548 17549 12550 17543 12553 17537 12557 17532 12562 17528 12567 17525
12573 17524 12580 17523 12586 17524 12592 17525 12598 17528 12603 17532
12607 17537 12609 17543 12611 17549 12612 17555 12611 17562 12609 17568
12607 17573 12603 17578 12598 17582 12592 17585 12586 17587 12580 17587
12573 17587 12567 17585 12562 17582 12557 17578 12553 17573 12550 17568
12548 17562 12548 17555 12526 17555 12527 17566 12531 17576 12536 17585
12542 17593 12550 17600 12559 17604 12569 17608 12580 17609 12591 17608

12601 17604 12610 17600 12618 17593 12624 17585 12629 17576 12632 17566
12633 17555 67 N F
12993 17486 12990 17501 12986 17515 12981 17528 12976 17540 12970 17550
12962 17560 12955 17568 12946 17576 12937 17583 12927 17588 12917 17593
12905 17597 12893 17599 12881 17601 12867 17603 12853 17603 12835 17602
12817 17599 12801 17595 12785 17589 12771 17581 12758 17572 12746 17562
12735 17550 12725 17537 12716 17523 12709 17508 12702 17491 12698 17474
12694 17456 12691 17437 12690 17417 12691 17395 12694 17374 12697 17354
12703 17336 12710 17319 12718 17304 12727 17290 12737 17278 12749 17267
12761 17257 12775 17249 12789 17243 12804 17237 12820 17234 12836 17231
12853 17231 12872 17231 12889 17234 12904 17237 12918 17242 12931 17248
12943 17255 12953 17263 12962 17272 12970 17282 12977 17293 12983 17305
12988 17317 12992 17330 12995 17343 12997 17357 12998 17371 12953 17371
12948 17346 12941 17325 12937 17316 12933 17308 12928 17301 12922 17294
12916 17289 12909 17284 12901 17280 12893 17277 12884 17275 12875 17273
12853 17271 12839 17272 12825 17274 12812 17278 12801 17283 12790 17289
12780 17296 12772 17304 12764 17314 12757 17324 12752 17335 12747 17347
12743 17360 12740 17373 12738 17387 12737 17402 12736 17417 12737 17434
12738 17449 12741 17464 12745 17478 12750 17491 12756 17503 12762 17514
12770 17524 12778 17533 12787 17540 12797 17547 12807 17552 12818 17557
12829 17560 12841 17562 12853 17562 12871 17561 12888 17557 12903 17551
12917 17542 12928 17531 12933 17525 12937 17518 12941 17511 12944 17503
12946 17495 12948 17486 12993 17486 123 N F
7006 17601 7070 17601 7174 17289 7279 17601 7343 17601 7343 17243
7299 17243 7299 17542 7197 17243 7149 17243 7050 17542 7050 17243
7006 17243 7006 17601 14 N F
7409 17425 7410 17440 7414 17455 7418 17462 7422 17469 7426 17476
7432 17482 7439 17488 7446 17493 7454 17497 7464 17501 7474 17504
7485 17507 7497 17508 7511 17509 7525 17508 7541 17506 7557 17502
7572 17496 7580 17492 7586 17488 7592 17482 7597 17476 7602 17470
7605 17462 7607 17454 7607 17445 7607 17282 7608 17277 7611 17273
7614 17270 7619 17267 7624 17266 7629 17265 7634 17265 7638 17266
7638 17236 7628 17234 7613 17233 7602 17233 7593 17234 7586 17236
7581 17239 7577 17244 7574 17249 7567 17266 7545 17250 7536 17245
7527 17240 7517 17237 7507 17235 7496 17234 7483 17233 7471 17234
7461 17235 7451 17237 7443 17239 7436 17243 7429 17246 7423 17251
7418 17256 7414 17261 7410 17266 7405 17278 7402 17291 7401 17305
7439 17305 7440 17297 7443 17290 7448 17284 7454 17278 7461 17273
7471 17269 7481 17267 7493 17266 7510 17269 7525 17273 7537 17279
7548 17286 7556 17295 7562 17303 7566 17312 7567 17320 7567 17371
7548 17364 7532 17360 7485 17353 7472 17349 7461 17344 7453 17339
7447 17333 7443 17326 7441 17319 7440 17312 7439 17305 7401 17305
7402 17324 7405 17339 7411 17352 7418 17361 7427 17369 7439 17375

7452 17380 7467 17384 7534 17394 7550 17398 7560 17403 7563 17406
7565 17409 7566 17411 7567 17414 7567 17437 7566 17442 7564 17447
7560 17452 7554 17458 7546 17463 7537 17467 7525 17469 7511 17470
7496 17469 7483 17466 7472 17461 7464 17455 7457 17448 7453 17440
7450 17432 7450 17425 7409 17425 129 N F
7684 17501 7720 17501 7720 17453 7735 17473 7749 17489 7762 17499
7773 17505 7783 17508 7792 17508 7800 17506 7806 17504 7806 17465
7786 17463 7769 17459 7761 17456 7755 17452 7749 17448 7743 17443
7739 17438 7735 17432 7729 17419 7726 17403 7725 17386 7725 17243
7684 17243 7684 17501 26 N F
8043 17414 8002 17414 8000 17425 7997 17435 7992 17444 7985 17453
7977 17460 7966 17466 7955 17469 7941 17470 7933 17470 7925 17468
7918 17466 7911 17463 7904 17459 7898 17453 7893 17448 7888 17441
7884 17434 7880 17426 7875 17408 7871 17388 7870 17366 7871 17344
7875 17325 7881 17308 7885 17301 7890 17294 7895 17289 7900 17283
7906 17279 7913 17275 7920 17273 7927 17271 7935 17269 7944 17269
7952 17269 7959 17271 7965 17273 7972 17275 7982 17282 7990 17291
7997 17301 8001 17312 8004 17323 8005 17333 8046 17333 8045 17323
8044 17313 8042 17303 8039 17294 8035 17285 8031 17277 8025 17269
8019 17262 8012 17255 8004 17249 7995 17244 7985 17239 7975 17235
7964 17233 7952 17231 7939 17231 7924 17231 7910 17234 7898 17238
7887 17243 7876 17249 7867 17257 7859 17266 7852 17275 7846 17286
7840 17297 7836 17309 7833 17321 7830 17334 7828 17347 7827 17374
7828 17392 7831 17409 7835 17424 7840 17438 7846 17450 7853 17461
7861 17471 7870 17479 7878 17487 7888 17493 7897 17498 7906 17502
7916 17505 7925 17507 7941 17509 7959 17507 7976 17504 7985 17501
7993 17497 8000 17493 8007 17487 8014 17481 8020 17474 8026 17467
8031 17458 8035 17449 8038 17438 8041 17427 8043 17414 107 N F
8091 17601 8132 17601 8132 17465 8132 17465 8144 17479 8156 17489
8168 17497 8179 17502 8189 17506 8198 17508 8214 17509 8229 17507
8243 17503 8257 17497 8268 17489 8278 17480 8286 17469 8291 17457
8292 17451 8293 17445 8293 17243 8252 17243 8252 17430 8251 17436
8249 17442 8244 17449 8239 17456 8231 17462 8222 17466 8211 17469
8198 17470 8191 17470 8185 17469 8178 17467 8172 17464 8161 17456
8151 17447 8143 17435 8137 17422 8133 17408 8132 17394 8132 17243
8091 17243 8091 17601 44 N F
2301 17501 2336 17501 2336 17463 2346 17475 2356 17485 2365 17493
2375 17499 2384 17504 2394 17507 2404 17508 2415 17509 2433 17506
2442 17504 2450 17501 2459 17496 2467 17489 2475 17480 2484 17468
2494 17479 2504 17488 2514 17495 2523 17501 2533 17505 2543 17507
2553 17508 2563 17509 2576 17508 2588 17505 2601 17500 2612 17493
2622 17484 2626 17478 2630 17472 2633 17466 2635 17458 2636 17451
2637 17442 2637 17243 2596 17243 2596 17427 2595 17436 2592 17445

2587 17453 2581 17460 2574 17465 2566 17469 2558 17472 2550 17473
2539 17472 2529 17468 2518 17462 2509 17455 2501 17446 2495 17435
2490 17424 2489 17412 2489 17243 2448 17243 2448 17427 2447 17436
2444 17445 2439 17453 2433 17460 2425 17465 2417 17469 2409 17472
2400 17473 2391 17472 2381 17468 2371 17463 2362 17456 2354 17447
2347 17436 2343 17425 2342 17418 2341 17412 2341 17243 2301 17243
2301 17501 79 N F
2708 17501 2744 17501 2744 17463 2754 17475 2764 17485 2773 17493
2782 17499 2792 17504 2801 17507 2812 17508 2823 17509 2841 17506
2850 17504 2858 17501 2866 17496 2875 17489 2883 17480 2891 17468
2902 17479 2912 17488 2921 17495 2931 17501 2941 17505 2950 17507
2960 17508 2970 17509 2983 17508 2996 17505 3008 17500 3020 17493
3030 17484 3034 17478 3037 17472 3040 17466 3042 17458 3044 17451
3044 17442 3044 17243 3004 17243 3004 17427 3002 17436 2999 17445
2994 17453 2988 17460 2981 17465 2973 17469 2965 17472 2958 17473
2947 17472 2936 17468 2926 17462 2917 17455 2909 17446 2902 17435
2898 17424 2897 17412 2897 17243 2856 17243 2856 17427 2855 17436
2851 17445 2847 17453 2840 17460 2833 17465 2825 17469 2816 17472
2807 17473 2798 17472 2789 17468 2779 17463 2770 17456 2761 17447
2755 17436 2750 17425 2749 17418 2749 17412 2749 17243 2708 17243
2708 17501 79 N F
3212 17509 3224 17508 3236 17506 3247 17502 3258 17498 3268 17492
3277 17484 3286 17476 3295 17467 3302 17457 3309 17446 3315 17435
3320 17423 3324 17410 3327 17396 3329 17383 3330 17368 3329 17354
3327 17341 3324 17327 3320 17315 3315 17303 3309 17291 3302 17281
3295 17271 3286 17262 3277 17254 3268 17247 3258 17241 3247 17237
3236 17233 3224 17231 3212 17231 3201 17231 3189 17233 3178 17237
3167 17241 3157 17247 3147 17254 3138 17262 3130 17271 3122 17281
3116 17291 3110 17303 3105 17315 3101 17327 3098 17341 3096 17354
3095 17368 3139 17368 3140 17349 3144 17330 3150 17313 3158 17299
3163 17292 3169 17286 3175 17281 3182 17277 3189 17274 3196 17271
3204 17269 3212 17269 3221 17269 3229 17271 3236 17274 3243 17277
3250 17281 3256 17286 3261 17292 3267 17299 3275 17313 3281 17330
3285 17349 3286 17368 3285 17388 3281 17407 3275 17424 3267 17440
3261 17446 3256 17452 3250 17458 3243 17462 3236 17466 3229 17468
3221 17470 3212 17470 3204 17470 3196 17468 3189 17466 3182 17462
3175 17458 3169 17452 3163 17446 3158 17440 3150 17424 3144 17407
3140 17388 3139 17368 3095 17368 3096 17383 3098 17396 3101 17410
3105 17423 3110 17435 3116 17446 3122 17457 3130 17467 3138 17476
3147 17484 3157 17492 3167 17498 3178 17502 3189 17506 3201 17508
3212 17509 115 N F
3386 17601 3426 17601 3426 17243 3386 17243 3386 17601 5 N F
3788 17606 3807 17605 3825 17601 3842 17597 3858 17590 3873 17581

3887 17572 3900 17560 3912 17548 3922 17534 3932 17520 3940 17504
3946 17488 3952 17472 3956 17455 3958 17437 3959 17419 3958 17402
3956 17384 3953 17367 3947 17350 3941 17334 3933 17318 3923 17303
3913 17290 3901 17277 3887 17266 3873 17255 3858 17247 3842 17240
3825 17235 3807 17232 3788 17231 3769 17232 3751 17235 3734 17240
3718 17247 3703 17255 3688 17266 3675 17277 3663 17290 3652 17303
3643 17318 3634 17334 3628 17350 3622 17367 3618 17384 3616 17402
3615 17419 3661 17419 3661 17404 3663 17390 3666 17375 3671 17362
3676 17349 3682 17337 3689 17325 3698 17315 3707 17305 3716 17297
3727 17289 3738 17283 3750 17278 3762 17274 3775 17272 3788 17271
3801 17272 3814 17274 3826 17278 3838 17283 3849 17289 3859 17297
3868 17305 3877 17315 3885 17325 3892 17337 3898 17349 3903 17362
3907 17375 3910 17390 3912 17404 3913 17419 3912 17435 3910 17449
3907 17463 3903 17477 3898 17489 3892 17501 3885 17512 3877 17523
3868 17532 3859 17540 3849 17548 3838 17554 3826 17558 3814 17562
3801 17564 3788 17565 3775 17564 3762 17562 3750 17558 3738 17554
3727 17548 3716 17540 3707 17532 3698 17523 3689 17512 3682 17501
3676 17489 3671 17477 3666 17463 3663 17449 3661 17435 3661 17419
3615 17419 3616 17437 3618 17455 3622 17472 3628 17488 3634 17504
3643 17520 3652 17534 3663 17548 3675 17560 3688 17572 3703 17581
3718 17590 3734 17597 3751 17601 3769 17605 3788 17606 131 N F
4040 17478 3997 17478 3998 17492 4000 17504 4002 17516 4006 17527
4010 17537 4015 17546 4021 17554 4028 17562 4035 17569 4043 17574
4052 17579 4062 17583 4072 17586 4083 17589 4094 17590 4106 17590
4118 17590 4129 17589 4139 17587 4149 17584 4158 17581 4167 17577
4175 17572 4183 17566 4190 17560 4196 17553 4201 17545 4205 17537
4209 17527 4211 17518 4213 17507 4213 17496 4213 17483 4211 17471
4207 17461 4202 17451 4195 17443 4187 17435 4176 17428 4162 17422
4178 17416 4190 17408 4201 17400 4209 17391 4216 17380 4220 17368
4223 17355 4224 17340 4222 17321 4220 17312 4217 17302 4214 17293
4209 17284 4204 17276 4197 17268 4190 17260 4181 17254 4171 17248
4160 17243 4148 17239 4135 17236 4120 17234 4104 17233 4090 17234
4077 17235 4065 17238 4054 17241 4044 17246 4035 17251 4027 17257
4019 17264 4013 17272 4007 17280 4002 17289 3999 17299 3996 17309
3993 17320 3992 17331 3992 17343 4035 17343 4036 17330 4038 17318
4042 17306 4048 17295 4052 17290 4057 17285 4063 17281 4069 17278
4077 17275 4086 17273 4096 17272 4106 17271 4116 17271 4124 17272
4132 17274 4139 17276 4146 17279 4151 17282 4157 17286 4161 17291
4169 17301 4174 17313 4177 17326 4178 17340 4177 17350 4176 17358
4174 17366 4171 17373 4167 17379 4163 17384 4157 17389 4151 17392
4145 17395 4137 17398 4121 17402 4102 17404 4081 17404 4081 17440
4102 17440 4119 17442 4134 17445 4146 17449 4151 17453 4156 17456
4159 17461 4162 17466 4165 17471 4166 17478 4168 17493 4167 17505

4164 17517 4160 17527 4153 17535 4145 17542 4134 17548 4121 17551
4106 17552 4093 17551 4080 17549 4069 17544 4060 17537 4056 17533
4052 17527 4046 17514 4042 17498 4040 17478 148 N F
4361 17601 4389 17601 4272 17233 4244 17233 4359 17601 5 N F
4422 17501 4458 17501 4458 17463 4468 17475 4478 17485 4487 17493
4496 17499 4506 17504 4516 17507 4526 17508 4537 17509 4555 17506
4564 17504 4572 17501 4581 17496 4589 17489 4597 17480 4606 17468
4616 17479 4626 17488 4636 17495 4645 17501 4655 17505 4664 17507
4674 17508 4685 17509 4697 17508 4710 17505 4723 17500 4734 17493
4744 17484 4748 17478 4752 17472 4755 17466 4757 17458 4758 17451
4758 17442 4758 17243 4718 17243 4718 17427 4717 17436 4713 17445
4709 17453 4702 17460 4695 17465 4688 17469 4680 17472 4672 17473
4661 17472 4651 17468 4640 17462 4631 17455 4623 17446 4616 17435
4612 17424 4611 17412 4611 17243 4570 17243 4570 17427 4569 17436
4566 17445 4561 17453 4554 17460 4547 17465 4539 17469 4530 17472
4522 17473 4513 17472 4503 17468 4493 17463 4484 17456 4476 17447
4469 17436 4465 17425 4463 17418 4463 17412 4463 17243 4422 17243
4422 17501 79 N F
4817 17470 4860 17470 4861 17488 4865 17504 4870 17518 4878 17529
4883 17535 4888 17539 4894 17543 4900 17546 4907 17549 4915 17551
4923 17552 4932 17552 4945 17551 4957 17547 4968 17542 4978 17534
4986 17525 4993 17513 4997 17500 4998 17486 4997 17471 4993 17459
4987 17448 4979 17439 4967 17430 4954 17420 4919 17399 4889 17382
4877 17373 4865 17364 4855 17355 4846 17346 4838 17337 4832 17328
4826 17318 4821 17309 4817 17299 4814 17288 4811 17267 4809 17243
5041 17243 5041 17284 4858 17284 4862 17300 4869 17313 4877 17324
4887 17334 4900 17343 4915 17353 4955 17376 4967 17382 4977 17387
4987 17394 4996 17400 5012 17414 5024 17429 5033 17444 5039 17460
5043 17475 5044 17491 5043 17502 5041 17512 5039 17522 5035 17531
5030 17540 5024 17548 5018 17556 5011 17563 5003 17569 4994 17574
4985 17579 4975 17583 4965 17586 4954 17588 4943 17590 4932 17590
4919 17590 4908 17589 4897 17586 4886 17583 4876 17579 4866 17574
4858 17568 4850 17561 4842 17553 4836 17544 4830 17534 4826 17524
4822 17512 4819 17499 4818 17485 4817 17470 94 N F
2 O
2461 2836 3110 2836 3110 3791 2461 3791 2461 2836 5 N F
1 O
12 W
2461 2836 3110 2836 3110 3791 2461 3791 2461 2836 5 P
15 O
3110 2836 3758 2836 3758 3791 3110 3791 3110 2836 5 N F
1 O
3110 2836 3758 2836 3758 3791 3110 3791 3110 2836 5 P

29 O
3758 2836 4407 2836 4407 3791 3758 3791 3758 2836 5 N F
1 O
3758 2836 4407 2836 4407 3791 3758 3791 3758 2836 5 P
42 O
4407 2836 5055 2836 5055 3791 4407 3791 4407 2836 5 N F
1 O
4407 2836 5055 2836 5055 3791 4407 3791 4407 2836 5 P
55 O
5055 2836 5704 2836 5704 3791 5055 3791 5055 2836 5 N F
1 O
5055 2836 5704 2836 5704 3791 5055 3791 5055 2836 5 P
68 O
5704 2836 6352 2836 6352 3791 5704 3791 5704 2836 5 N F
1 O
5704 2836 6352 2836 6352 3791 5704 3791 5704 2836 5 P
82 O
6352 2836 7001 2836 7001 3791 6352 3791 6352 2836 5 N F
1 O
6352 2836 7001 2836 7001 3791 6352 3791 6352 2836 5 P
95 O
7001 2836 7650 2836 7650 3791 7001 3791 7001 2836 5 N F
1 O
7001 2836 7650 2836 7650 3791 7001 3791 7001 2836 5 P
108 O
7650 2836 8298 2836 8298 3791 7650 3791 7650 2836 5 N F
1 O
7650 2836 8298 2836 8298 3791 7650 3791 7650 2836 5 P
121 O
8298 2836 8947 2836 8947 3791 8298 3791 8298 2836 5 N F
1 O
8298 2836 8947 2836 8947 3791 8298 3791 8298 2836 5 P
135 O
8947 2836 9595 2836 9595 3791 8947 3791 8947 2836 5 N F
1 O
8947 2836 9595 2836 9595 3791 8947 3791 8947 2836 5 P
148 O
9595 2836 10244 2836 10244 3791 9595 3791 9595 2836 5 N F
1 O
9595 2836 10244 2836 10244 3791 9595 3791 9595 2836 5 P
161 O
10244 2836 10892 2836 10892 3791 10244 3791 10244 2836 5 N F
1 O

10244 2836 10892 2836 10892 3791 10244 3791 10244 2836 5 P
174 O
10892 2836 11541 2836 11541 3791 10892 3791 10892 2836 5 N F
1 O
10892 2836 11541 2836 11541 3791 10892 3791 10892 2836 5 P
188 O
11541 2836 12189 2836 12189 3791 11541 3791 11541 2836 5 N F
1 O
11541 2836 12189 2836 12189 3791 11541 3791 11541 2836 5 P
201 O
12189 2836 12838 2836 12838 3791 12189 3791 12189 2836 5 N F
1 O
12189 2836 12838 2836 12838 3791 12189 3791 12189 2836 5 P
3133 2542 3154 2542 3154 2282 3122 2282 3122 2468 3065 2468
3065 2491 3088 2493 3098 2495 3108 2499 3116 2505 3123 2514
3129 2526 3133 2542 14 N F
3676 2452 3708 2452 3709 2465 3712 2477 3716 2488 3722 2496
3729 2504 3738 2509 3749 2512 3762 2513 3772 2513 3781 2510
3789 2506 3797 2500 3803 2493 3808 2484 3811 2475 3812 2464
3811 2453 3808 2443 3803 2435 3797 2428 3789 2422 3779 2415
3752 2399 3730 2386 3712 2373 3698 2359 3687 2345 3679 2331
3674 2316 3671 2299 3670 2282 3844 2282 3844 2313 3707 2313
3710 2324 3715 2334 3721 2342 3729 2350 3738 2357 3750 2364
3779 2381 3796 2390 3811 2399 3822 2410 3831 2421 3838 2432
3843 2444 3845 2456 3846 2468 3846 2476 3844 2483 3842 2491
3839 2498 3836 2504 3831 2510 3821 2521 3809 2530 3794 2537
3779 2541 3762 2542 3744 2541 3728 2537 3720 2534 3713 2530
3706 2525 3700 2520 3695 2514 3690 2508 3686 2500 3682 2492
3680 2483 3678 2474 3676 2463 3676 2452 76 N F
4356 2462 4324 2462 4326 2481 4330 2498 4333 2506 4337 2513
4342 2519 4347 2525 4352 2530 4358 2534 4365 2538 4372 2541
4388 2545 4406 2546 4422 2545 4438 2541 4451 2536 4463 2528
4473 2518 4477 2512 4480 2506 4482 2499 4484 2491 4486 2484
4486 2475 4485 2466 4484 2457 4481 2449 4478 2442 4472 2435
4466 2429 4458 2424 4448 2420 4459 2415 4469 2410 4477 2403
4483 2396 4488 2388 4491 2379 4493 2369 4494 2359 4492 2344
4489 2330 4483 2316 4479 2310 4474 2304 4468 2298 4462 2293
4454 2289 4446 2285 4437 2282 4427 2280 4416 2279 4404 2278
4393 2279 4384 2280 4375 2282 4367 2284 4359 2288 4352 2292
4346 2296 4340 2301 4335 2307 4331 2313 4328 2320 4325 2327
4321 2343 4320 2360 4352 2360 4353 2351 4354 2342 4357 2333
4362 2324 4369 2317 4378 2312 4390 2308 4406 2307 4419 2307
4430 2310 4439 2315 4447 2321 4452 2329 4456 2338 4458 2348

4459 2359 4458 2372 4456 2378 4454 2383 4451 2387 4448 2391
4439 2397 4429 2402 4417 2405 4402 2406 4387 2406 4387 2433
4402 2433 4415 2435 4426 2437 4436 2440 4443 2445 4448 2452
4451 2462 4452 2473 4451 2482 4449 2491 4446 2498 4441 2505
4435 2510 4427 2514 4417 2516 4406 2517 4395 2517 4386 2515
4378 2511 4371 2506 4365 2499 4361 2489 4358 2477 4356 2462
120 N F
5083 2542 5106 2542 5106 2374 5146 2374 5146 2343 5106 2343
5106 2282 5073 2282 5073 2343 4965 2343 4965 2374 4993 2374
5073 2374 5073 2487 4993 2374 4965 2374 4965 2378 5083 2542
18 N F
5643 2547 5778 2547 5778 2516 5669 2516 5660 2442 5669 2448
5680 2454 5694 2458 5710 2459 5723 2458 5737 2454 5751 2448
5757 2444 5764 2439 5769 2434 5775 2427 5780 2420 5784 2412
5787 2403 5790 2393 5791 2383 5792 2371 5790 2353 5786 2335
5783 2327 5779 2319 5774 2312 5769 2305 5763 2299 5756 2294
5749 2289 5741 2285 5732 2282 5723 2279 5713 2278 5702 2277
5683 2279 5674 2280 5666 2283 5659 2286 5652 2290 5641 2298
5632 2309 5625 2322 5620 2335 5616 2350 5648 2350 5652 2340
5656 2332 5661 2324 5667 2318 5674 2313 5682 2309 5690 2307
5700 2306 5713 2307 5725 2310 5735 2315 5743 2322 5750 2331
5755 2341 5758 2354 5759 2369 5758 2380 5756 2391 5752 2401
5746 2410 5738 2418 5728 2425 5716 2429 5702 2430 5695 2430
5688 2429 5682 2427 5676 2424 5665 2417 5652 2405 5624 2405
5643 2547 79 N F
6434 2480 6401 2480 6399 2489 6396 2496 6391 2503 6386 2508
6380 2513 6373 2516 6366 2518 6359 2518 6352 2518 6346 2517
6339 2514 6334 2512 6328 2508 6324 2504 6319 2498 6315 2492
6309 2478 6304 2462 6301 2442 6300 2421 6308 2429 6315 2436
6323 2441 6331 2444 6338 2447 6346 2448 6363 2449 6376 2448
6389 2444 6402 2438 6414 2429 6419 2423 6425 2417 6429 2410
6433 2403 6436 2394 6438 2385 6439 2376 6439 2365 6407 2363
6406 2374 6402 2384 6397 2394 6391 2403 6383 2410 6374 2416
6364 2419 6353 2421 6343 2419 6333 2416 6324 2410 6316 2403
6309 2394 6304 2384 6301 2374 6300 2363 6301 2353 6304 2342
6309 2333 6316 2324 6324 2316 6333 2311 6343 2307 6353 2306
6364 2307 6374 2311 6383 2316 6391 2324 6397 2333 6402 2342
6406 2353 6407 2363 6439 2365 6438 2350 6434 2335 6428 2320
6423 2313 6419 2306 6413 2300 6407 2294 6400 2290 6392 2285
6383 2282 6374 2279 6364 2278 6353 2277 6342 2278 6331 2280
6321 2283 6312 2287 6304 2293 6297 2299 6291 2307 6285 2315
6280 2324 6276 2334 6273 2345 6270 2356 6267 2381 6265 2407
6266 2423 6267 2438 6269 2452 6272 2465 6275 2478 6279 2489

6284 2500 6290 2509 6296 2518 6303 2525 6311 2532 6319 2537
6328 2541 6338 2544 6348 2546 6359 2547 6374 2546 6388 2542
6400 2535 6411 2527 6419 2517 6426 2506 6431 2493 6434 2480
126 N F
6914 2542 7088 2542 7088 2515 7063 2478 7043 2446 7027 2418
7015 2392 7004 2367 6996 2341 6989 2313 6983 2282 6948 2282
6958 2318 6968 2349 6977 2375 6988 2400 7001 2424 7016 2449
7055 2512 6914 2512 6914 2542 21 N F
7650 2547 7665 2546 7680 2542 7694 2537 7705 2529 7715 2519
7719 2514 7722 2507 7724 2501 7726 2494 7727 2486 7728 2478
7727 2467 7725 2457 7721 2449 7716 2442 7711 2436 7705 2431
7693 2423 7703 2418 7712 2412 7719 2405 7725 2397 7731 2388
7734 2378 7737 2368 7737 2358 7736 2345 7732 2331 7725 2318
7715 2305 7709 2299 7702 2294 7695 2289 7687 2285 7678 2282
7668 2279 7658 2278 7648 2277 7637 2278 7627 2279 7618 2282
7609 2285 7601 2289 7594 2294 7588 2299 7582 2305 7573 2318
7567 2331 7563 2345 7562 2358 7596 2358 7597 2347 7600 2337
7605 2329 7612 2321 7620 2315 7629 2310 7639 2307 7650 2306
7660 2307 7670 2310 7679 2315 7687 2321 7694 2329 7699 2337
7702 2347 7703 2358 7702 2368 7699 2378 7694 2387 7687 2394
7679 2400 7670 2405 7660 2408 7650 2409 7639 2408 7629 2405
7620 2400 7612 2394 7605 2387 7600 2378 7597 2368 7596 2358
7562 2358 7562 2368 7565 2378 7568 2387 7574 2396 7580 2404
7587 2411 7596 2417 7606 2423 7594 2431 7588 2436 7583 2442
7578 2449 7574 2457 7572 2467 7571 2478 7604 2478 7605 2469
7607 2461 7612 2454 7617 2449 7624 2444 7632 2441 7640 2439
7650 2438 7659 2439 7667 2441 7675 2444 7682 2449 7687 2454
7692 2461 7694 2469 7695 2478 7694 2487 7692 2495 7687 2502
7682 2507 7675 2512 7667 2515 7659 2518 7650 2518 7640 2518
7632 2515 7624 2512 7617 2507 7612 2502 7607 2495 7605 2487
7604 2478 7571 2478 7572 2486 7573 2494 7575 2501 7577 2507
7580 2514 7584 2519 7594 2529 7605 2537 7619 2542 7634 2546
7650 2547 145 N F
8297 2547 8307 2547 8316 2545 8324 2543 8332 2540 8340 2536
8347 2531 8354 2525 8360 2518 8365 2510 8370 2501 8374 2491
8378 2479 8381 2466 8383 2452 8384 2437 8385 2421 8384 2400
8383 2382 8380 2365 8377 2350 8372 2337 8367 2325 8361 2315
8354 2306 8347 2298 8340 2292 8332 2287 8324 2283 8315 2281
8307 2279 8289 2277 8276 2278 8264 2281 8252 2286 8242 2294
8233 2303 8226 2315 8219 2329 8215 2346 8247 2346 8251 2337
8254 2329 8259 2322 8264 2316 8269 2312 8276 2308 8283 2307
8291 2306 8299 2306 8305 2308 8312 2310 8318 2314 8323 2318
8328 2323 8336 2335 8342 2349 8347 2366 8350 2384 8351 2403

8342 2395 8333 2388 8325 2383 8317 2379 8310 2377 8304 2375
8291 2375 8281 2376 8272 2377 8263 2380 8255 2383 8248 2387
8241 2391 8235 2396 8230 2402 8225 2408 8221 2415 8215 2429
8212 2445 8211 2461 8244 2461 8245 2451 8247 2441 8252 2431
8258 2422 8266 2415 8275 2409 8285 2405 8297 2403 8309 2405
8319 2409 8328 2415 8336 2422 8342 2431 8347 2441 8350 2451
8351 2461 8350 2471 8347 2481 8342 2491 8336 2500 8328 2507
8319 2513 8309 2517 8297 2518 8285 2517 8275 2513 8266 2507
8258 2500 8252 2491 8247 2481 8245 2471 8244 2461 8211 2461
8211 2469 8212 2478 8214 2486 8216 2494 8219 2501 8223 2508
8228 2515 8233 2521 8239 2527 8246 2532 8253 2536 8261 2540
8269 2543 8278 2545 8287 2546 8297 2547 130 N F
8839 2547 8860 2547 8860 2287 8827 2287 8827 2472 8770 2472
8770 2495 8793 2498 8803 2500 8813 2504 8821 2510 8828 2518
8834 2531 8839 2547 14 N F
9037 2547 9047 2546 9056 2544 9064 2540 9073 2535 9080 2529
9087 2522 9094 2513 9100 2504 9105 2495 9110 2484 9117 2461
9122 2437 9123 2413 9122 2389 9117 2364 9110 2341 9105 2331
9100 2321 9094 2311 9087 2303 9080 2296 9073 2289 9064 2284
9056 2280 9047 2278 9037 2277 9028 2278 9019 2280 9010 2284
9002 2289 8995 2296 8988 2303 8981 2311 8975 2321 8970 2331
8965 2341 8958 2364 8953 2389 8951 2413 8986 2413 8987 2390
8990 2369 8996 2351 9002 2335 9010 2323 9019 2313 9023 2310
9028 2308 9033 2306 9037 2306 9042 2306 9047 2308 9051 2310
9056 2313 9065 2323 9072 2335 9079 2351 9084 2369 9088 2390
9089 2413 9088 2436 9084 2457 9079 2475 9072 2490 9065 2502
9056 2511 9051 2514 9047 2516 9042 2518 9037 2518 9033 2518
9028 2516 9023 2514 9019 2511 9010 2502 9002 2490 8996 2475
8990 2457 8987 2436 8986 2413 8951 2413 8953 2437 8958 2461
8965 2484 8970 2495 8975 2504 8981 2513 8988 2522 8995 2529
9002 2535 9010 2540 9019 2544 9028 2546 9037 2547 95 N F
9517 2542 9538 2542 9538 2282 9505 2282 9505 2468 9448 2468
9448 2491 9471 2493 9482 2495 9491 2499 9499 2505 9507 2514
9513 2526 9517 2542 14 N F
9721 2542 9742 2542 9742 2282 9710 2282 9710 2468 9653 2468
9653 2491 9676 2493 9686 2495 9695 2499 9704 2505 9711 2514
9717 2526 9721 2542 14 N F
10135 2542 10156 2542 10156 2282 10123 2282 10123 2468 10066 2468
10066 2491 10089 2493 10100 2495 10109 2499 10117 2505 10125 2514
10130 2526 10135 2542 14 N F
10251 2452 10284 2452 10285 2465 10287 2477 10291 2488 10297 2496
10304 2504 10314 2509 10325 2512 10337 2513 10347 2513 10356 2510
10365 2506 10372 2500 10378 2493 10383 2484 10386 2475 10387 2464

10386 2453 10383 2443 10379 2435 10372 2428 10364 2422 10354 2415
10328 2399 10306 2386 10288 2373 10273 2359 10262 2345 10255 2331
10249 2316 10247 2299 10246 2282 10420 2282 10420 2313 10282 2313
10285 2324 10290 2334 10296 2342 10304 2350 10313 2357 10325 2364
10355 2381 10372 2390 10386 2399 10398 2410 10407 2421 10413 2432
10418 2444 10421 2456 10421 2468 10421 2476 10420 2483 10418 2491
10415 2498 10411 2504 10407 2510 10397 2521 10384 2530 10370 2537
10354 2541 10337 2542 10319 2541 10303 2537 10295 2534 10288 2530
10282 2525 10276 2520 10270 2514 10266 2508 10261 2500 10258 2492
10255 2483 10253 2474 10252 2463 10251 2452 76 N F
10784 2546 10805 2546 10805 2286 10773 2286 10773 2471 10716 2471
10716 2494 10739 2497 10749 2499 10759 2503 10767 2509 10774 2517
10780 2530 10784 2546 14 N F
10931 2462 10899 2462 10901 2481 10906 2498 10909 2506 10913 2513
10917 2519 10922 2525 10928 2530 10934 2534 10940 2538 10948 2541
10963 2545 10981 2546 10998 2545 11013 2541 11027 2536 11038 2528
11048 2518 11052 2512 11055 2506 11058 2499 11060 2491 11061 2484
11061 2475 11061 2466 11059 2457 11057 2449 11053 2442 11048 2435
11041 2429 11033 2424 11023 2420 11034 2415 11044 2410 11052 2403
11058 2396 11063 2388 11066 2379 11068 2369 11069 2359 11068 2344
11064 2330 11058 2316 11054 2310 11049 2304 11044 2298 11037 2293
11030 2289 11021 2285 11012 2282 11002 2280 10991 2279 10979 2278
10969 2279 10959 2280 10950 2282 10942 2284 10934 2288 10927 2292
10921 2296 10916 2301 10911 2307 10907 2313 10903 2320 10900 2327
10896 2343 10895 2360 10928 2360 10928 2351 10930 2342 10932 2333
10937 2324 10944 2317 10953 2312 10966 2308 10981 2307 10994 2307
11006 2310 11015 2315 11022 2321 11028 2329 11032 2338 11034 2348
11035 2359 11033 2372 11032 2378 11029 2383 11027 2387 11023 2391
11015 2397 11004 2402 10992 2405 10978 2406 10962 2406 10962 2433
10977 2433 10991 2435 11002 2437 11011 2440 11018 2445 11023 2452
11026 2462 11027 2473 11026 2482 11024 2491 11021 2498 11016 2505
11010 2510 11002 2514 10992 2516 10981 2517 10971 2517 10962 2515
10953 2511 10946 2506 10940 2499 10936 2489 10933 2477 10931 2462
120 N F
11430 2542 11451 2542 11451 2282 11419 2282 11419 2468 11361 2468
11361 2491 11384 2493 11395 2495 11404 2499 11413 2505 11420 2514
11426 2526 11430 2542 14 N F
11657 2542 11680 2542 11680 2374 11720 2374 11720 2343 11680 2343
11680 2282 11648 2282 11648 2343 11539 2343 11539 2374 11568 2374
11648 2374 11648 2487 11568 2374 11539 2374 11539 2378 11657 2542
18 N F
12081 2547 12102 2547 12102 2287 12069 2287 12069 2472 12012 2472
12012 2495 12035 2498 12045 2500 12055 2504 12063 2510 12070 2518

```
12076 2531 12081 2547 14 N F
12218 2547 12354 2547 12354 2516 12245 2516 12235 2442 12245 2448
12256 2454 12269 2458 12285 2459 12298 2458 12312 2454 12326 2448
12333 2444 12339 2439 12345 2434 12350 2427 12355 2420 12359 2412
12362 2403 12365 2393 12367 2383 12367 2371 12366 2353 12361 2335
12358 2327 12354 2319 12350 2312 12344 2305 12338 2299 12332 2294
12324 2289 12316 2285 12308 2282 12298 2279 12288 2278 12277 2277
12258 2279 12249 2280 12242 2283 12234 2286 12228 2290 12216 2298
12207 2309 12200 2322 12195 2335 12191 2350 12224 2350 12227 2340
12231 2332 12236 2324 12242 2318 12249 2313 12257 2309 12266 2307
12275 2306 12288 2307 12300 2310 12310 2315 12319 2322 12325 2331
12330 2341 12334 2354 12335 2369 12334 2380 12331 2391 12327 2401
12321 2410 12313 2418 12303 2425 12291 2429 12277 2430 12270 2430
12263 2429 12257 2427 12252 2424 12241 2417 12228 2405 12199 2405
12218 2547 79 N F
Gr
S
%%Trailer
%%DocumentFonts:
%%Pages: 1
%%EOF
```

The temporal and spatial variations of simulated cumulative ozone uptake in CK (April)

```
%%Pages: (atend)
%%PageOrder: Ascend
%%Creator: NCAR GKS
%%DocumentFonts: (atend)
%%EndComments
%%BeginProlog

/M3
{
  2 copy lt {pop} {exch pop} ifelse
  2 copy lt {pop} {exch pop} ifelse
} bind def
/Sc
{
  1. exch sub /ylo exch def
  1. exch sub /mag exch def
  1. exch sub /cyn exch def
  ylo mag cyn M3 /b_cmy exch def
  cyn b_cmy sub /c_cmy exch def
  mag b_cmy sub /m_cmy exch def
```

```
  ylo b_cmy sub /y_cmy exch def
  c_cmy m_cmy y_cmy b_cmy setcmykcolor
} bind def
/P
{/n exch def
  newpath
  moveto
  2 1 n {pop lineto} for
  stroke
 } bind def

/F
{/n exch def
  newpath
  moveto
  2 1 n {pop lineto} for
  closepath
  eofill
} bind def

/B
{ /s exch def
  /n exch def
  1 1 n {newpath pop s 0 360 arc stroke} for
} bind def

/Q
{ /s exch def
  /n exch def
  /ms 0 s sub def
  /ts s s add def
  1 1 n {gsave newpath pop translate
  0 0 moveto ms 0 rmoveto ts 0 rlineto
  0 0 moveto 90 rotate ms  0 rmoveto ts 0 rlineto
  stroke grestore} for
} bind def

/Fs
{ /s exch def
  findfont
  s scalefont
  setfont
} bind def
```

```
/V
{ /s exch def
  /n exch def
  /ms 0 s sub def
  /ts s s add def
  1 1 n {gsave newpath pop translate
  0 0 moveto 30 rotate ms 0 rmoveto  ts 0 rlineto
  0 0 moveto 60 rotate ms 0 rmoveto  ts 0 rlineto
  0 0 moveto 60 rotate ms 0 rmoveto  ts 0 rlineto
  stroke grestore} for
} bind def

/X
{ /s exch def
  /n exch def
  /s s 1.25 mul def
  /ms 0 s sub def
  /ts s s add def
  1 1 n {gsave newpath pop translate
  0 0 moveto 45 rotate ms 0 rmoveto  ts 0 rlineto
  0 0 moveto 90 rotate ms 0 rmoveto  ts 0 rlineto
  stroke grestore} for
} bind def

/Ls
{matrix currentmatrix
 currentrgbcolor
 currentlinewidth
 currentlinecap
 currentlinejoin
 currentdash
} bind def

/Lr
{setdash
 setlinejoin
 setlinecap
 setlinewidth
 setrgbcolor
 setmatrix
} bind def
```

```
/M {stroke newpath moveto} bind def
/L {lineto} bind def
/E {eofill} def
/J {setlinejoin} def
/C {setlinecap} def
/W {setlinewidth} def
/S {stroke showpage} def
/A {scale} def
/U {put} def
/D {setdash} def
/I {currentdash} def
/H {currentlinewidth} def
/G {currentrgbcolor} def
/R {setrgbcolor} def
/N {closepath} def
/K {stroke} def
/Z {rotate} def
/As {ashow} def
/Ch {clippath} def
/Ci {colorimage} def
/Co {concat} def
/Cm {currentmatrix} def
/Cp {clip} def
/Ct {currentpoint} def
/Cu {currentfile} def
/Ex {exch} def
/Im {image} def
/In {initgraphics} def
/Ml {setmiterlimit} def
/Mx {matrix} def
/Np {newpath} def
/Ng {neg} def
/Rh {readhexstring} def
/Rm {rmoveto} def
/Sm {setmatrix} def
/Tr {translate} def
/Gs {gsave} def
/Gr {grestore} def

/Ov
{/strn exch def
 /ispc exch def
 /str1 1 string def
```

```
  strn
  {/code exch def
   /chr str1 0 code put str1 def
   0 ispc neg rmoveto
   gsave
   chr stringwidth pop 2 div neg
   0 rmoveto
   chr false charpath stroke
   grestore
  } forall
} bind def

/Oh
{/strn exch def
 /ispc exch def
 /tstr 1 string def
 strn
   {
    /code exch def
    /chr tstr 0 code put tstr def
    chr false charpath
    currentpoint
    /yt exch def /xt exch def
    stroke
    xt ispc add yt moveto
   } forall
 } bind def

/T 256 array def
/O
{ T
  exch
  get
  aload
  pop
  setrgbcolor
} bind def

/Y
{/strn exch def
 /ispc exch def
 /str1 1 string def
  strn
```

```
  {/code exch def
   /chr str1 0 code put str1 def
    0 ispc neg rmoveto
    gsave
      chr stringwidth pop 2 div neg 0 rmoveto
      chr show
    grestore
  } forall
} def

/Bg
{/ury Ex def /urx Ex def /lly Ex def /llx Ex def
 Np Ls Gr Gs 0 O Ch E Lr
 llx lly M urx lly L urx ury L
 llx ury L llx lly L
 Cp Np
} def

/Oc
{/ury exch def
 /urx exch def
 /lly exch def
 /llx exch def
 Np
 llx lly M urx lly L urx ury L  llx ury L llx lly L
 Cp Np
} def

/Cc
{/CellWidth exch def
 /strn CellWidth 1 add string def
 /strn1 CellWidth 1 add 3 mul string def
 systemdict /colorimage known
 { { Cu strn1 Rh pop } false 3 Ci}
 { { Cu strn1 Rh pop
     /ColorStr exch def
     0 1 CellWidth {
       /YIndx exch def
       /GrayVal YIndx 3 mul def
       strn YIndx
         ColorStr GrayVal       get 0.30 mul
         ColorStr GrayVal 1 add get 0.59 mul
         ColorStr GrayVal 2 add get 0.11 mul
```

```
         add add cvi
       put
     } for
     strn
   } Im
 } ifelse
} def

/Ld
{/ury exch def /urx exch def  /lly exch def /llx exch def
 -90 rotate
 lly urx add neg llx lly sub translate
} bind def
/Pt
{/ury exch def /urx exch def  /lly exch def /llx exch def
 90 rotate
 lly urx add lly llx sub translate
} bind def
%%EndProlog

%%BeginSetup
<</PageSize [00612 00792]>> setpagedevice
%%EndSetup

[1. 1. 1.] [0. 0. 0.] [1. 0. 0.] [0. 1. 0.] [0. 0. 1.] [0. 1. 1.] [1. 0. .8]
[1. 1. 0.] [1. .5 0.] [.6 .8 0.] [0. 1. .6] [0. .5 1.] [.5 0. .8] [1. 0. .6]
[.3 .3 .3] [.7 .7 .7] [1. 1. .3] [.7 1. .5] [.5 1. .6] [.2 1. .7] [.3 .8 .8]
[.5 .7 .8] [.7 .5 .8] [1. .3 .9] [.7 .9 .5] [.4 .9 .5] [.2 .9 .7] [.2 .7 .9]
[.2 .5 1.] [.5 .3 1.] [.7 .2 1.] [.9 .1 1.] [.9 1. .2] [.7 1. .3] [.5 1. .3]
[.2 1. .5] [.2 .8 .6] [.2 .7 .7] [.2 .5 .8] [.2 .4 .9] [.4 .3 .9] [.7 .2 .9]
[.8 .2 .8] [.9 .3 .7] [.8 .4 .6] [.8 .6 .5] [.9 .7 .4] [.9 .7 .3] [1. .9 .9]
[.8 1. .1] [.6 1. .2] [.5 1. .2] [.2 .9 .5] [.2 .8 .5] [.2 .7 .7] [.2 .5 .7]
[.2 .4 .9] [.4 .3 .9] [.5 .2 .9] [.8 .2 .7] [1. .2 .7] [1. .3 .6] [1. .4 .5]
[1. .5 .4] [1. .8 .1] [.7 1. .0] [.6 1. .1] [.4 1. .2] [.1 1. .3] [.1 .8 .4]
[.2 .7 .5] [.1 .6 .7] [.1 .5 .7] [.1 .4 .8] [.2 .3 .9] [.2 .2 1.] [.3 .1 1.]
[.5 .1 .9] [.7 .0 .8] [.9 .0 .7] [.9 .7 .1] [.7 .9 .1] [.5 .9 .1] [.3 .9 .2]
[.1 .9 .3] [.2 .7 .4] [.1 .7 .5] [.1 .5 .6] [.1 .4 .7] [.2 .3 .7] [.2 .2 .8]
[.3 .2 .8] [.5 .1 .7] [.7 .1 .7] [.9 .2 .5] [.8 .2 .5] [.8 .7 .0] [.6 .8 .1]
[.4 .9 .1] [.3 .9 .1] [.1 .9 .2] [.2 .8 .2] [.2 .7 .3] [.2 .6 .5] [.1 .5 .5]
[.1 .4 .6] [.2 .3 .7] [.3 .2 .7] [.4 .2 .6] [.7 .2 .5] [.8 .2 .4] [1. .2 .3]
[.7 .7 .0] [.5 .7 .1] [.4 .8 .1] [.2 .9 .1] [.2 .9 .1] [.2 .8 .2] [.1 .7 .2]
[.1 .7 .3] [.1 .6 .4] [.1 .4 .5] [.1 .4 .5] [.2 .3 .5] [.4 .3 .4] [.5 .3 .4]
[.7 .3 .3] [.8 .3 .2] [.7 .6 .0] [.5 .7 .1] [.4 .7 .1] [.2 .7 .2] [.1 .7 .2]
```

[.1 .6 .3] [.1 .5 .4] [.1 .4 .4] [.1 .3 .5] [.1 .2 .7] [.1 .1 .7] [.4 .1 .5]
[.5 .1 .5] [.6 .1 .4] [.7 .1 .4] [.9 .1 .3] [.5 .5 .1] [.4 .6 .1] [.3 .6 .1]
[.2 .6 .2] [.1 .5 .2] [.1 .4 .3] [.1 .4 .4] [.2 .3 .4] [.2 .2 .4] [.2 .2 .5]
[.2 .1 .5] [.3 .1 .5] [.4 .1 .4] [.5 .1 .4] [.7 .1 .3] [.8 .1 .2] [.5 .4 .1]
[.4 .5 .1] [.3 .6 .0] [.2 .6 .1] [.1 .7 .1] [.1 .6 .1] [.1 .5 .2] [.1 .4 .2]
[.1 .4 .2] [.1 .3 .3] [.1 .2 .4] [.1 .2 .5] [.1 .1 .5] [.1 .0 .6] [.3 .0 .5]
[.5 .0 .4] [.7 .3 .0] [.7 .4 .0] [.5 .4 .1] [.3 .4 .2] [.2 .3 .2] [.2 .2 .3]
[.2 .2 .4] [.3 .1 .4] [.5 .1 .3] [.6 .1 .2] [.7 .1 .2] [.8 .0 .2] [1. .0 .2]
[1. .1 .1] [.9 .2 .1] [.8 .2 .1] [.6 .2 .0] [.4 .4 .0] [.2 .4 .1] [.2 .3 .2]
[.2 .2 .2] [.2 .2 .3] [.2 .1 .3] [.3 .1 .3] [.5 .1 .2] [.7 .1 .2] [.8 .0 .2]
[.9 .0 .1] [1. .0 .1] [1. .1 .1] [.8 .2 .0] [.7 .2 .0] [.5 .2 .0] [.4 .3 .0]
[.1 .4 .1] [.1 .4 .1] [.1 .3 .2] [.1 .2 .2] [.1 .2 .3] [.2 .1 .4] [.2 .0 .3]
[.4 .0 .2] [.6 .0 .2] [.7 .0 .1] [.8 .0 .1] [.7 .1 .1] [.7 .1 .1] [.6 .2 .0]
[.4 .2 .0] [.2 .3 .0] [.1 .3 .0] [.1 .2 .1] [.1 .2 .1] [.1 .1 .2] [.1 .1 .3]
[.1 .0 .3] [.1 .0 .2] [.2 .0 .2] [.3 .0 .2] [.3 .0 .1] [.4 .0 .1] [.4 .1 .1]
[.4 .1 .0] [.2 .1 .0] [.0 .0 .0] [.0 .0 .0] [.0 .0 .0] [.0 .0 .0] [.0 .0 .0]
[.0 .0 .0] [.0 .0 .0] [.0 .0 .0] [.0 .0 .0] [.0 .0 .0] [.0 .0 .0] [.0 .0 .0]
[.0 .0 .0] [.0 .0 .0] [.0 .0 .0] [.0 .0 .0] T astore pop
%%Page: 1 1
0.04000 0.04000 A 1 J 1 C 12 W [] 0 D
T   0 [1.000 1.000 1.000] U
T   1 [0.000 0.000 0.000] U
T   2 [0.000 0.000 1.000] U
T   3 [0.000 0.031 1.000] U
T   4 [0.000 0.059 1.000] U
T   5 [0.000 0.090 1.000] U
T   6 [0.000 0.122 1.000] U
T   7 [0.000 0.153 1.000] U
T   8 [0.000 0.180 1.000] U
T   9 [0.000 0.212 1.000] U
T 10 [0.000 0.243 1.000] U
T 11 [0.000 0.275 1.000] U
T 12 [0.000 0.302 1.000] U
T 13 [0.000 0.333 1.000] U
T 14 [0.000 0.365 1.000] U
T 15 [0.000 0.392 1.000] U
T 16 [0.000 0.424 1.000] U
T 17 [0.000 0.455 1.000] U
T 18 [0.000 0.486 1.000] U
T 19 [0.000 0.514 1.000] U
T 20 [0.000 0.545 1.000] U
T 21 [0.000 0.576 1.000] U
T 22 [0.000 0.608 1.000] U

T 23 [0.000 0.635 1.000] U
T 24 [0.000 0.667 1.000] U
T 25 [0.000 0.698 1.000] U
T 26 [0.000 0.725 1.000] U
T 27 [0.000 0.757 1.000] U
T 28 [0.000 0.788 1.000] U
T 29 [0.000 0.820 1.000] U
T 30 [0.000 0.847 1.000] U
T 31 [0.000 0.878 1.000] U
T 32 [0.000 0.910 1.000] U
T 33 [0.000 0.941 1.000] U
T 34 [0.000 0.969 1.000] U
T 35 [0.000 1.000 1.000] U
T 36 [0.000 0.992 0.973] U
T 37 [0.000 0.980 0.941] U
T 38 [0.004 0.973 0.914] U
T 39 [0.004 0.965 0.886] U
T 40 [0.004 0.953 0.859] U
T 41 [0.004 0.945 0.827] U
T 42 [0.008 0.937 0.800] U
T 43 [0.008 0.929 0.773] U
T 44 [0.008 0.918 0.745] U
T 45 [0.008 0.910 0.714] U
T 46 [0.012 0.902 0.686] U
T 47 [0.012 0.890 0.659] U
T 48 [0.012 0.882 0.627] U
T 49 [0.012 0.875 0.600] U
T 50 [0.016 0.863 0.573] U
T 51 [0.016 0.855 0.545] U
T 52 [0.016 0.847 0.514] U
T 53 [0.016 0.839 0.486] U
T 54 [0.020 0.827 0.459] U
T 55 [0.020 0.820 0.431] U
T 56 [0.020 0.812 0.400] U
T 57 [0.020 0.800 0.373] U
T 58 [0.024 0.792 0.345] U
T 59 [0.024 0.784 0.314] U
T 60 [0.024 0.773 0.286] U
T 61 [0.024 0.765 0.259] U
T 62 [0.027 0.757 0.231] U
T 63 [0.027 0.749 0.200] U
T 64 [0.027 0.737 0.173] U
T 65 [0.027 0.729 0.145] U

T 66 [0.031 0.722 0.118] U
T 67 [0.031 0.710 0.086] U
T 68 [0.031 0.702 0.059] U
T 69 [0.059 0.710 0.059] U
T 70 [0.090 0.718 0.055] U
T 71 [0.118 0.729 0.055] U
T 72 [0.145 0.737 0.051] U
T 73 [0.173 0.745 0.051] U
T 74 [0.204 0.753 0.047] U
T 75 [0.231 0.765 0.047] U
T 76 [0.259 0.773 0.043] U
T 77 [0.286 0.780 0.043] U
T 78 [0.318 0.788 0.043] U
T 79 [0.345 0.800 0.039] U
T 80 [0.373 0.808 0.039] U
T 81 [0.400 0.816 0.035] U
T 82 [0.431 0.824 0.035] U
T 83 [0.459 0.835 0.031] U
T 84 [0.486 0.843 0.031] U
T 85 [0.518 0.851 0.031] U
T 86 [0.545 0.859 0.027] U
T 87 [0.573 0.867 0.027] U
T 88 [0.600 0.878 0.024] U
T 89 [0.631 0.886 0.024] U
T 90 [0.659 0.894 0.020] U
T 91 [0.686 0.902 0.020] U
T 92 [0.714 0.914 0.016] U
T 93 [0.745 0.922 0.016] U
T 94 [0.773 0.929 0.016] U
T 95 [0.800 0.937 0.012] U
T 96 [0.827 0.949 0.012] U
T 97 [0.859 0.957 0.008] U
T 98 [0.886 0.965 0.008] U
T 99 [0.914 0.973 0.004] U
T 100 [0.941 0.984 0.004] U
T 101 [0.973 0.992 0.000] U
T 102 [1.000 1.000 0.000] U
T 103 [1.000 0.988 0.000] U
T 104 [1.000 0.980 0.000] U
T 105 [1.000 0.969 0.000] U
T 106 [1.000 0.957 0.000] U
T 107 [1.000 0.945 0.000] U
T 108 [1.000 0.937 0.000] U

T 109 [1.000 0.925 0.000] U
T 110 [1.000 0.914 0.000] U
T 111 [1.000 0.902 0.000] U
T 112 [1.000 0.894 0.000] U
T 113 [1.000 0.882 0.000] U
T 114 [1.000 0.871 0.000] U
T 115 [1.000 0.863 0.000] U
T 116 [1.000 0.851 0.000] U
T 117 [1.000 0.839 0.000] U
T 118 [1.000 0.827 0.000] U
T 119 [1.000 0.820 0.000] U
T 120 [1.000 0.808 0.000] U
T 121 [1.000 0.796 0.000] U
T 122 [1.000 0.784 0.000] U
T 123 [1.000 0.776 0.000] U
T 124 [1.000 0.765 0.000] U
T 125 [1.000 0.753 0.000] U
T 126 [1.000 0.745 0.000] U
T 127 [1.000 0.733 0.000] U
T 128 [1.000 0.722 0.000] U
T 129 [1.000 0.710 0.000] U
T 130 [1.000 0.702 0.000] U
T 131 [1.000 0.690 0.000] U
T 132 [1.000 0.678 0.000] U
T 133 [1.000 0.667 0.000] U
T 134 [1.000 0.659 0.000] U
T 135 [1.000 0.647 0.000] U
T 136 [1.000 0.627 0.000] U
T 137 [1.000 0.608 0.000] U
T 138 [1.000 0.588 0.000] U
T 139 [1.000 0.569 0.000] U
T 140 [1.000 0.549 0.000] U
T 141 [1.000 0.529 0.000] U
T 142 [1.000 0.510 0.000] U
T 143 [1.000 0.490 0.000] U
T 144 [1.000 0.471 0.000] U
T 145 [1.000 0.451 0.000] U
T 146 [1.000 0.431 0.000] U
T 147 [1.000 0.412 0.000] U
T 148 [1.000 0.392 0.000] U
T 149 [1.000 0.373 0.000] U
T 150 [1.000 0.353 0.000] U
T 151 [1.000 0.333 0.000] U

T 152 [1.000 0.314 0.000] U
T 153 [1.000 0.294 0.000] U
T 154 [1.000 0.275 0.000] U
T 155 [1.000 0.255 0.000] U
T 156 [1.000 0.235 0.000] U
T 157 [1.000 0.216 0.000] U
T 158 [1.000 0.196 0.000] U
T 159 [1.000 0.176 0.000] U
T 160 [1.000 0.157 0.000] U
T 161 [1.000 0.137 0.000] U
T 162 [1.000 0.118 0.000] U
T 163 [1.000 0.098 0.000] U
T 164 [1.000 0.078 0.000] U
T 165 [1.000 0.059 0.000] U
T 166 [1.000 0.039 0.000] U
T 167 [1.000 0.020 0.000] U
T 168 [1.000 0.000 0.000] U
T 169 [0.980 0.000 0.016] U
T 170 [0.961 0.000 0.031] U
T 171 [0.941 0.000 0.047] U
T 172 [0.922 0.000 0.063] U
T 173 [0.902 0.000 0.082] U
T 174 [0.878 0.000 0.098] U
T 175 [0.859 0.000 0.114] U
T 176 [0.839 0.000 0.129] U
T 177 [0.820 0.000 0.145] U
T 178 [0.800 0.000 0.161] U
T 179 [0.780 0.000 0.176] U
T 180 [0.761 0.000 0.192] U
T 181 [0.741 0.000 0.212] U
T 182 [0.722 0.000 0.227] U
T 183 [0.702 0.000 0.243] U
T 184 [0.682 0.000 0.259] U
T 185 [0.659 0.000 0.275] U
T 186 [0.639 0.000 0.290] U
T 187 [0.620 0.000 0.306] U
T 188 [0.600 0.000 0.322] U
T 189 [0.580 0.000 0.341] U
T 190 [0.561 0.000 0.357] U
T 191 [0.541 0.000 0.373] U
T 192 [0.522 0.000 0.388] U
T 193 [0.502 0.000 0.404] U
T 194 [0.482 0.000 0.420] U

T 195 [0.463 0.000 0.435] U
T 196 [0.439 0.000 0.451] U
T 197 [0.420 0.000 0.471] U
T 198 [0.400 0.000 0.486] U
T 199 [0.380 0.000 0.502] U
T 200 [0.361 0.000 0.518] U
T 201 [0.341 0.000 0.533] U
-1200 1025 16500 18750 Oc
Gs
1 O
0 O
2300 16170 M 2300 5455 L 2333 5455 L 2366 5455 L 2399 5455 L
2432 5455 L 2466 5455 L 2499 5455 L 2532 5455 L
2565 5455 L 2599 5455 L 2632 5455 L 2665 5455 L
2698 5455 L 2732 5455 L 2765 5455 L 2798 5455 L
2831 5455 L 2864 5455 L 2898 5455 L 2927 5455 L
2926 5459 L 2917 5486 L 2938 5506 L 2967 5505 L
2985 5482 L 2967 5459 L 2967 5455 L 2997 5455 L
3031 5455 L 3064 5455 L 3097 5455 L 3130 5455 L
3164 5455 L 3197 5455 L 3230 5455 L 3263 5455 L
3296 5455 L 3330 5455 L 3363 5455 L 3396 5455 L
3429 5455 L 3463 5455 L 3496 5455 L 3529 5455 L
3562 5455 L 3595 5455 L 3629 5455 L 3662 5455 L
3695 5455 L 3728 5455 L 3762 5455 L 3795 5455 L
3828 5455 L 3861 5455 L 3895 5455 L 3928 5455 L
3961 5455 L 3994 5455 L 4027 5455 L 4061 5455 L
4094 5455 L 4127 5455 L 4160 5455 L 4194 5455 L
4227 5455 L 4260 5455 L 4293 5455 L 4327 5455 L
4360 5455 L 4393 5455 L 4426 5455 L 4459 5455 L
4493 5455 L 4526 5455 L 4559 5455 L 4592 5455 L
4626 5455 L 4659 5455 L 4692 5455 L 4725 5455 L
4759 5455 L 4792 5455 L 4825 5455 L 4858 5455 L
4891 5455 L 4925 5455 L 4958 5455 L 4991 5455 L
5024 5455 L 5058 5455 L 5091 5455 L 5124 5455 L
5157 5455 L 5191 5455 L 5224 5455 L 5257 5455 L
5290 5455 L 5323 5455 L 5357 5455 L 5390 5455 L
5395 5455 L 5407 5466 L 5406 5495 L 5402 5524 L
5404 5553 L 5404 5582 L 5393 5609 L 5369 5625 L
5352 5648 L 5381 5644 L 5387 5616 L 5416 5614 L
5416 5585 L 5411 5557 L 5410 5528 L 5412 5499 L
5430 5476 L 5430 5455 L 5456 5455 L 5490 5455 L
5523 5455 L 5532 5455 L 5539 5469 L 5540 5475 L
5544 5482 L 5551 5487 L 5537 5525 L 5529 5553 L

5547 5530 L 5556 5503 L 5573 5479 L 5592 5457 L
5594 5455 L 5622 5455 L 5644 5455 L 5645 5472 L
5649 5455 L 5656 5455 L 5657 5455 L 5665 5470 L
5675 5497 L 5689 5523 L 5683 5551 L 5659 5567 L
5667 5595 L 5681 5621 L 5681 5650 L 5666 5674 L
5689 5691 L 5674 5715 L 5654 5737 L 5676 5717 L
5703 5726 L 5686 5750 L 5670 5774 L 5650 5795 L
5674 5779 L 5702 5786 L 5688 5811 L 5664 5827 L
5654 5855 L 5627 5866 L 5598 5867 L 5627 5872 L
5655 5876 L 5661 5905 L 5662 5934 L 5645 5957 L
5617 5961 L 5600 5985 L 5627 5975 L 5654 5965 L
5679 5950 L 5677 5921 L 5684 5893 L 5705 5873 L
5723 5896 L 5711 5870 L 5706 5841 L 5712 5830 L
5721 5816 L 5739 5794 L 5745 5765 L 5772 5776 L
5790 5754 L 5797 5725 L 5799 5722 L 5811 5700 L
5823 5674 L 5842 5652 L 5859 5628 L 5853 5600 L
5871 5622 L 5898 5632 L 5926 5625 L 5955 5622 L
5928 5612 L 5902 5598 L 5880 5580 L 5860 5559 L
5880 5538 L 5909 5534 L 5938 5535 L 5967 5536 L
5986 5514 L 6014 5506 L 6043 5507 L 6069 5495 L
6050 5517 L 6058 5545 L 6085 5534 L 6113 5542 L
6119 5571 L 6090 5565 L 6074 5588 L 6051 5570 L
6023 5561 L 6008 5586 L 6036 5590 L 6065 5592 L
6087 5611 L 6061 5624 L 6035 5637 L 6057 5656 L
6085 5663 L 6113 5670 L 6141 5663 L 6159 5640 L
6168 5612 L 6172 5584 L 6200 5590 L 6228 5597 L
6234 5626 L 6207 5636 L 6181 5650 L 6208 5660 L
6209 5689 L 6180 5689 L 6202 5708 L 6216 5734 L
6242 5746 L 6246 5748 L 6256 5751 L 6269 5755 L
6296 5768 L 6324 5762 L 6345 5782 L 6337 5754 L
6314 5736 L 6317 5707 L 6314 5678 L 6325 5651 L
6354 5652 L 6382 5648 L 6403 5628 L 6399 5656 L
6415 5680 L 6431 5705 L 6408 5723 L 6392 5747 L
6417 5732 L 6430 5706 L 6458 5710 L 6472 5735 L
6487 5760 L 6515 5767 L 6544 5767 L 6569 5782 L
6599 5771 L 6577 5763 L 6575 5753 L 6595 5754 L
6614 5755 L 6640 5742 L 6665 5728 L 6637 5727 L
6648 5700 L 6677 5693 L 6705 5699 L 6731 5685 L
6757 5673 L 6754 5702 L 6768 5727 L 6747 5707 L
6730 5731 L 6747 5754 L 6764 5777 L 6785 5797 L
6814 5799 L 6838 5783 L 6866 5775 L 6882 5750 L
6899 5727 L 6921 5747 L 6949 5754 L 6976 5764 L
7003 5775 L 7027 5791 L 7029 5820 L 7046 5796 L

7031 5772 L 7060 5771 L 7082 5790 L 7102 5811 L
7125 5828 L 7149 5844 L 7172 5826 L 7201 5821 L
7229 5828 L 7258 5827 L 7266 5854 L 7287 5874 L
7284 5903 L 7277 5931 L 7291 5957 L 7296 5985 L
7271 5971 L 7268 5999 L 7297 6002 L 7320 5984 L
7341 5964 L 7358 5988 L 7379 5999 L 7361 6015 L
7343 6030 L 7315 6037 L 7292 6055 L 7279 6080 L
7273 6109 L 7245 6113 L 7274 6113 L 7294 6092 L
7317 6074 L 7343 6062 L 7349 6062 L 7372 6062 L
7401 6062 L 7422 6085 L 7434 6099 L 7455 6119 L
7454 6130 L 7455 6156 L 7469 6182 L 7461 6205 L
7487 6216 L 7507 6221 L 7515 6224 L 7527 6217 L
7541 6210 L 7532 6238 L 7559 6227 L 7564 6199 L
7572 6171 L 7590 6186 L 7594 6190 L 7617 6207 L
7641 6223 L 7641 6252 L 7659 6229 L 7651 6201 L
7669 6224 L 7671 6253 L 7676 6281 L 7687 6308 L
7711 6291 L 7724 6307 L 7732 6307 L 7736 6307 L
7759 6291 L 7776 6313 L 7786 6341 L 7785 6369 L
7757 6376 L 7733 6393 L 7762 6390 L 7790 6384 L
7818 6376 L 7840 6357 L 7849 6330 L 7840 6302 L
7838 6273 L 7855 6297 L 7857 6325 L 7860 6354 L
7871 6380 L 7895 6397 L 7891 6426 L 7909 6448 L
7937 6457 L 7946 6430 L 7930 6407 L 7925 6378 L
7937 6404 L 7954 6428 L 7977 6445 L 8001 6460 L
8006 6488 L 8024 6511 L 8013 6537 L 8034 6558 L
8054 6538 L 8077 6556 L 8098 6575 L 8126 6581 L
8120 6609 L 8106 6634 L 8077 6634 L 8074 6663 L
8046 6660 L 8017 6657 L 8000 6661 L 7974 6674 L
7952 6692 L 7941 6704 L 7942 6720 L 7944 6717 L
7954 6704 L 7968 6707 L 7980 6708 L 7997 6709 L
8024 6699 L 8035 6696 L 8051 6691 L 8074 6708 L
8079 6736 L 8054 6749 L 8082 6750 L 8092 6777 L
8109 6754 L 8110 6757 L 8123 6779 L 8125 6808 L
8154 6808 L 8165 6782 L 8157 6754 L 8184 6747 L
8185 6747 L 8198 6773 L 8225 6765 L 8226 6765 L
8253 6770 L 8274 6782 L 8279 6784 L 8273 6812 L
8295 6830 L 8285 6803 L 8310 6790 L 8336 6777 L
8361 6763 L 8360 6734 L 8385 6748 L 8405 6768 L
8394 6794 L 8395 6823 L 8424 6820 L 8437 6845 L
8441 6874 L 8425 6898 L 8403 6880 L 8382 6900 L
8378 6929 L 8355 6945 L 8381 6934 L 8409 6940 L
8402 6968 L 8395 6995 L 8416 6975 L 8417 6947 L
8429 6972 L 8442 6947 L 8437 6919 L 8464 6927 L

8483 6948 L 8511 6944 L 8538 6934 L 8562 6948 L
8534 6955 L 8530 6983 L 8558 6985 L 8587 6984 L
8574 7009 L 8575 7038 L 8562 7012 L 8534 7020 L
8510 7035 L 8501 7062 L 8529 7069 L 8545 7093 L
8562 7116 L 8541 7136 L 8522 7157 L 8548 7168 L
8577 7170 L 8605 7164 L 8588 7140 L 8614 7152 L
8611 7124 L 8601 7097 L 8629 7093 L 8655 7081 L
8655 7081 L 8641 7106 L 8632 7133 L 8648 7157 L
8649 7129 L 8673 7112 L 8701 7114 L 8730 7118 L
8736 7146 L 8753 7151 L 8764 7153 L 8744 7174 L
8727 7197 L 8704 7181 L 8677 7171 L 8687 7198 L
8663 7213 L 8640 7230 L 8619 7249 L 8646 7258 L
8669 7275 L 8694 7289 L 8713 7310 L 8729 7334 L
8741 7360 L 8747 7356 L 8764 7344 L 8754 7317 L
8780 7305 L 8808 7301 L 8813 7273 L 8819 7265 L
8830 7250 L 8827 7221 L 8842 7246 L 8852 7273 L
8879 7264 L 8880 7236 L 8881 7207 L 8889 7214 L
8902 7226 L 8902 7226 L 8908 7254 L 8901 7282 L
8874 7292 L 8868 7320 L 8854 7345 L 8829 7332 L
8821 7360 L 8807 7385 L 8824 7407 L 8850 7418 L
8878 7423 L 8883 7432 L 8892 7448 L 8884 7476 L
8882 7504 L 8891 7531 L 8909 7532 L 8919 7533 L
8928 7560 L 8935 7588 L 8906 7591 L 8881 7603 L
8854 7612 L 8834 7633 L 8850 7656 L 8870 7675 L
8870 7676 L 8887 7699 L 8898 7725 L 8870 7721 L
8893 7737 L 8921 7743 L 8937 7747 L 8949 7750 L
8973 7765 L 8976 7766 L 9001 7771 L 9029 7775 L
9036 7784 L 9046 7797 L 9020 7786 L 8996 7801 L
8979 7824 L 8961 7801 L 8953 7774 L 8927 7762 L
8905 7780 L 8899 7808 L 8886 7833 L 8859 7825 L
8872 7850 L 8895 7867 L 8916 7848 L 8944 7841 L
8967 7824 L 8982 7848 L 8970 7874 L 8966 7902 L
8947 7923 L 8919 7928 L 8891 7928 L 8865 7917 L
8843 7936 L 8841 7964 L 8867 7974 L 8844 7990 L
8849 8017 L 8845 8046 L 8872 8035 L 8894 8017 L
8919 8004 L 8909 8030 L 8901 8058 L 8900 8086 L
8910 8060 L 8927 8037 L 8941 8024 L 8948 8018 L
8953 8046 L 8979 8058 L 8962 8034 L 8990 8027 L
8965 8014 L 8962 7986 L 8968 7984 L 8990 7979 L
9012 7961 L 9017 7989 L 9034 8012 L 9043 8039 L
9071 8036 L 9094 8020 L 9082 7995 L 9057 7982 L
9058 7954 L 9038 7935 L 9009 7933 L 8993 7910 L
8986 7882 L 9014 7882 L 9035 7900 L 9044 7927 L

9072 7930 L 9075 7932 L 9096 7945 L 9100 7947 L
9122 7956 L 9121 7984 L 9142 8004 L 9116 8016 L
9127 8042 L 9102 8056 L 9078 8071 L 9090 8096 L
9118 8091 L 9125 8118 L 9147 8117 L 9153 8116 L
9181 8111 L 9179 8139 L 9152 8146 L 9180 8146 L
9199 8167 L 9204 8195 L 9195 8222 L 9223 8218 L
9238 8222 L 9250 8226 L 9265 8249 L 9251 8274 L
9246 8302 L 9256 8275 L 9280 8261 L 9291 8287 L
9307 8264 L 9313 8274 L 9322 8287 L 9323 8316 L
9326 8321 L 9339 8339 L 9326 8364 L 9332 8391 L
9357 8379 L 9385 8383 L 9384 8411 L 9398 8436 L
9372 8446 L 9359 8471 L 9340 8493 L 9368 8500 L
9387 8520 L 9387 8549 L 9390 8577 L 9418 8580 L
9430 8605 L 9445 8629 L 9463 8651 L 9479 8674 L
9477 8702 L 9452 8716 L 9425 8721 L 9405 8741 L
9377 8746 L 9349 8742 L 9347 8770 L 9369 8752 L
9397 8755 L 9417 8749 L 9424 8748 L 9429 8748 L
9446 8730 L 9474 8731 L 9496 8730 L 9502 8730 L
9529 8727 L 9529 8740 L 9542 8765 L 9540 8793 L
9553 8818 L 9567 8825 L 9578 8831 L 9593 8854 L
9603 8881 L 9581 8898 L 9605 8908 L 9607 8909 L
9616 8936 L 9642 8946 L 9669 8938 L 9652 8916 L
9661 8901 L 9667 8891 L 9676 8865 L 9693 8842 L
9713 8823 L 9734 8842 L 9728 8869 L 9750 8887 L
9762 8912 L 9777 8936 L 9805 8932 L 9806 8931 L
9829 8917 L 9847 8895 L 9859 8920 L 9845 8945 L
9837 8972 L 9823 8996 L 9824 9024 L 9808 9047 L
9796 9073 L 9788 9100 L 9771 9122 L 9744 9129 L
9717 9137 L 9745 9137 L 9773 9137 L 9801 9138 L
9825 9153 L 9837 9166 L 9844 9173 L 9865 9193 L
9857 9220 L 9844 9244 L 9816 9250 L 9801 9274 L
9828 9264 L 9856 9261 L 9874 9282 L 9853 9301 L
9864 9327 L 9839 9339 L 9811 9333 L 9786 9322 L
9807 9340 L 9834 9343 L 9831 9371 L 9804 9377 L
9777 9369 L 9749 9366 L 9750 9394 L 9775 9408 L
9782 9435 L 9787 9462 L 9810 9459 L 9814 9458 L
9832 9436 L 9833 9464 L 9844 9490 L 9836 9463 L
9837 9435 L 9847 9409 L 9874 9416 L 9894 9435 L
9901 9462 L 9899 9490 L 9907 9517 L 9927 9499 L
9918 9472 L 9921 9456 L 9923 9445 L 9925 9417 L
9952 9411 L 9976 9427 L 9980 9432 L 9994 9448 L
9989 9476 L 9964 9488 L 9966 9516 L 9963 9544 L
9989 9554 L 9990 9582 L 9974 9605 L 9977 9633 L

9995 9655 L 9976 9675 L 9949 9668 L 9922 9662 L
9897 9649 L 9896 9621 L 9912 9598 L 9888 9584 L
9881 9611 L 9853 9611 L 9834 9590 L 9808 9582 L
9784 9566 L 9766 9545 L 9775 9572 L 9748 9564 L
9733 9541 L 9724 9568 L 9735 9593 L 9753 9615 L
9780 9609 L 9808 9615 L 9835 9621 L 9857 9639 L
9875 9660 L 9892 9683 L 9915 9698 L 9932 9720 L
9954 9738 L 9968 9761 L 9990 9779 L 10003 9784 L
10010 9787 L 10016 9789 L 10039 9805 L 10046 9832 L
10022 9818 L 9994 9818 L 9966 9817 L 9962 9818 L
9955 9824 L 9944 9834 L 9917 9841 L 9891 9852 L
9864 9857 L 9836 9861 L 9812 9875 L 9798 9899 L
9782 9922 L 9763 9943 L 9746 9965 L 9727 9986 L
9708 10006 L 9685 10022 L 9661 10036 L 9634 10040 L
9606 10039 L 9578 10035 L 9554 10022 L 9531 10005 L
9512 9985 L 9491 9967 L 9467 9953 L 9445 9935 L
9419 9925 L 9391 9925 L 9365 9935 L 9346 9915 L
9371 9902 L 9344 9910 L 9335 9936 L 9341 9964 L
9324 9986 L 9314 10012 L 9326 10037 L 9302 10052 L
9274 10054 L 9246 10051 L 9224 10035 L 9213 10009 L
9197 9987 L 9169 9985 L 9147 10001 L 9128 9981 L
9104 9966 L 9124 9986 L 9141 10008 L 9169 10009 L
9195 10000 L 9194 10028 L 9202 10054 L 9227 10067 L
9254 10073 L 9282 10074 L 9309 10076 L 9337 10076 L
9364 10068 L 9383 10047 L 9411 10050 L 9436 10062 L
9452 10085 L 9468 10107 L 9469 10135 L 9484 10159 L
9504 10178 L 9528 10192 L 9556 10196 L 9571 10219 L
9593 10236 L 9616 10252 L 9643 10256 L 9667 10271 L
9682 10294 L 9702 10313 L 9727 10326 L 9753 10334 L
9780 10341 L 9808 10346 L 9835 10349 L 9863 10352 L
9890 10356 L 9914 10372 L 9920 10399 L 9911 10425 L
9897 10449 L 9884 10474 L 9871 10498 L 9858 10523 L
9844 10547 L 9829 10570 L 9812 10592 L 9789 10608 L
9766 10623 L 9742 10637 L 9714 10637 L 9699 10660 L
9677 10677 L 9651 10689 L 9627 10702 L 9609 10723 L
9595 10747 L 9577 10769 L 9555 10786 L 9531 10799 L
9510 10817 L 9484 10827 L 9458 10836 L 9430 10835 L
9403 10837 L 9378 10849 L 9359 10869 L 9381 10886 L
9368 10911 L 9355 10935 L 9340 10958 L 9312 10961 L
9285 10958 L 9257 10961 L 9231 10970 L 9204 10968 L
9192 10943 L 9165 10937 L 9138 10931 L 9113 10918 L
9087 10910 L 9059 10910 L 9045 10934 L 9072 10928 L
9100 10927 L 9126 10936 L 9147 10954 L 9170 10970 L

9191 10988 L 9215 11002 L 9240 11012 L 9266 11001 L
9293 11001 L 9320 10993 L 9346 11003 L 9361 10980 L
9384 10964 L 9401 10942 L 9420 10922 L 9430 10896 L
9436 10869 L 9462 10861 L 9490 10864 L 9517 10861 L
9532 10884 L 9559 10890 L 9584 10901 L 9610 10910 L
9613 10909 L 9635 10898 L 9662 10892 L 9689 10884 L
9707 10863 L 9732 10852 L 9757 10840 L 9783 10830 L
9810 10826 L 9837 10818 L 9864 10811 L 9889 10821 L
9896 10848 L 9884 10873 L 9870 10897 L 9858 10922 L
9849 10948 L 9829 10968 L 9811 10988 L 9790 11006 L
9765 11019 L 9739 11028 L 9713 11038 L 9686 11038 L
9660 11048 L 9649 11073 L 9629 11092 L 9644 11116 L
9638 11143 L 9638 11171 L 9625 11195 L 9603 11212 L
9578 11222 L 9552 11233 L 9527 11245 L 9502 11255 L
9476 11264 L 9448 11265 L 9429 11285 L 9411 11306 L
9383 11309 L 9399 11332 L 9417 11353 L 9404 11377 L
9378 11387 L 9399 11405 L 9407 11431 L 9406 11459 L
9395 11484 L 9389 11511 L 9379 11537 L 9352 11542 L
9337 11565 L 9332 11592 L 9316 11615 L 9297 11635 L
9286 11660 L 9276 11686 L 9257 11706 L 9251 11732 L
9253 11760 L 9262 11786 L 9240 11802 L 9223 11824 L
9216 11851 L 9220 11878 L 9209 11903 L 9201 11930 L
9175 11939 L 9180 11966 L 9155 11977 L 9155 12004 L
9146 12031 L 9145 12058 L 9136 12084 L 9126 12110 L
9118 12136 L 9110 12162 L 9097 12186 L 9092 12213 L
9078 12237 L 9053 12248 L 9028 12260 L 9002 12266 L
8983 12286 L 8958 12298 L 8932 12307 L 8905 12311 L
8878 12317 L 8865 12293 L 8865 12320 L 8847 12341 L
8820 12347 L 8798 12364 L 8774 12377 L 8751 12391 L
8730 12409 L 8716 12432 L 8712 12459 L 8684 12462 L
8660 12474 L 8635 12461 L 8613 12477 L 8594 12457 L
8602 12484 L 8588 12508 L 8587 12535 L 8596 12561 L
8595 12588 L 8599 12616 L 8614 12639 L 8634 12657 L
8661 12652 L 8673 12677 L 8676 12704 L 8683 12730 L
8692 12757 L 8704 12773 L 8708 12778 L 8733 12791 L
8752 12810 L 8756 12837 L 8770 12861 L 8778 12887 L
8787 12913 L 8781 12939 L 8808 12937 L 8830 12952 L
8840 12927 L 8853 12951 L 8874 12969 L 8890 12947 L
8901 12972 L 8916 12996 L 8927 13020 L 8950 13005 L
8957 13031 L 8955 13059 L 8965 13084 L 8985 13103 L
9010 13113 L 9016 13117 L 9033 13128 L 9026 13102 L
9040 13125 L 9062 13141 L 9074 13166 L 9047 13162 L
9020 13166 L 9036 13189 L 9010 13198 L 8986 13211 L

8978 13237 L 8988 13263 L 9013 13273 L 9039 13264 L
9060 13281 L 9064 13308 L 9082 13288 L 9098 13266 L
9093 13239 L 9083 13213 L 9071 13189 L 9098 13183 L
9124 13192 L 9145 13210 L 9172 13215 L 9199 13219 L
9223 13231 L 9245 13248 L 9240 13275 L 9236 13302 L
9220 13324 L 9226 13351 L 9252 13359 L 9243 13385 L
9258 13408 L 9282 13420 L 9304 13403 L 9328 13390 L
9329 13417 L 9347 13438 L 9346 13465 L 9321 13476 L
9305 13498 L 9279 13492 L 9296 13513 L 9319 13527 L
9345 13520 L 9359 13496 L 9386 13497 L 9386 13525 L
9411 13514 L 9430 13533 L 9453 13548 L 9479 13554 L
9505 13564 L 9530 13574 L 9553 13589 L 9570 13611 L
9566 13637 L 9579 13614 L 9599 13633 L 9626 13636 L
9605 13618 L 9590 13595 L 9617 13602 L 9643 13595 L
9642 13622 L 9668 13632 L 9692 13645 L 9704 13669 L
9729 13680 L 9754 13692 L 9779 13704 L 9768 13729 L
9795 13734 L 9808 13710 L 9818 13685 L 9837 13703 L
9860 13718 L 9859 13745 L 9876 13766 L 9884 13740 L
9867 13719 L 9883 13696 L 9908 13686 L 9881 13682 L
9885 13655 L 9913 13654 L 9930 13651 L 9939 13650 L
9962 13665 L 9969 13671 L 9983 13682 L 9997 13706 L
9998 13706 L 10009 13709 L 10023 13712 L 10012 13687 L
10025 13711 L 10030 13738 L 10029 13765 L 10002 13762 L
9975 13764 L 9995 13782 L 9995 13810 L 10011 13832 L
10034 13846 L 10056 13861 L 10040 13884 L 10033 13910 L
10037 13936 L 10055 13957 L 10066 13968 L 10075 13976 L
10048 13978 L 10024 13965 L 9999 13953 L 9981 13974 L
9955 13966 L 9928 13965 L 9902 13973 L 9878 13987 L
9854 13974 L 9840 13997 L 9849 14023 L 9832 14044 L
9810 14027 L 9787 14012 L 9768 13993 L 9769 13966 L
9754 13989 L 9727 13984 L 9700 13980 L 9684 13958 L
9664 13976 L 9639 13966 L 9612 13961 L 9585 13963 L
9561 13975 L 9542 13995 L 9525 14016 L 9507 14037 L
9532 14048 L 9505 14045 L 9480 14034 L 9453 14031 L
9426 14037 L 9406 14056 L 9402 14083 L 9386 14105 L
9359 14103 L 9342 14125 L 9318 14137 L 9306 14161 L
9279 14158 L 9252 14161 L 9225 14160 L 9204 14142 L
9179 14133 L 9155 14120 L 9128 14115 L 9102 14108 L
9082 14090 L 9057 14081 L 9031 14073 L 9055 14062 L
9051 14035 L 9034 14013 L 9015 13994 L 8996 13974 L
8974 13958 L 8951 13943 L 8925 13936 L 8900 13926 L
8879 13908 L 8866 13884 L 8883 13863 L 8876 13836 L
8856 13817 L 8836 13800 L 8817 13780 L 8790 13773 L

8763 13774 L 8736 13771 L 8711 13782 L 8684 13779 L
8656 13777 L 8629 13778 L 8603 13785 L 8576 13788 L
8555 13806 L 8532 13821 L 8505 13820 L 8502 13847 L
8485 13868 L 8467 13888 L 8473 13915 L 8469 13942 L
8468 13969 L 8470 13996 L 8477 14023 L 8494 14044 L
8511 14065 L 8523 14090 L 8505 14110 L 8480 14121 L
8492 14146 L 8498 14172 L 8491 14198 L 8483 14224 L
8464 14244 L 8459 14271 L 8432 14271 L 8415 14250 L
8424 14276 L 8419 14303 L 8422 14330 L 8408 14353 L
8402 14326 L 8397 14353 L 8373 14340 L 8351 14324 L
8326 14313 L 8302 14301 L 8280 14285 L 8259 14302 L
8232 14306 L 8206 14314 L 8179 14316 L 8152 14313 L
8125 14317 L 8098 14313 L 8075 14327 L 8052 14342 L
8033 14361 L 8010 14376 L 7984 14383 L 7964 14402 L
7945 14422 L 7928 14443 L 7915 14466 L 7903 14491 L
7890 14515 L 7880 14540 L 7870 14565 L 7867 14592 L
7867 14619 L 7872 14646 L 7881 14672 L 7893 14696 L
7906 14720 L 7924 14741 L 7939 14763 L 7964 14774 L
7958 14800 L 7948 14825 L 7967 14845 L 7986 14864 L
8008 14879 L 8034 14888 L 8061 14893 L 8088 14888 L
8111 14875 L 8126 14852 L 8133 14826 L 8151 14806 L
8174 14792 L 8201 14787 L 8223 14803 L 8243 14821 L
8264 14838 L 8284 14856 L 8301 14864 L 8309 14867 L
8326 14867 L 8336 14867 L 8357 14850 L 8371 14850 L
8384 14851 L 8406 14867 L 8406 14867 L 8422 14869 L
8433 14870 L 8458 14880 L 8475 14889 L 8482 14893 L
8504 14909 L 8522 14929 L 8536 14953 L 8553 14974 L
8575 14989 L 8600 15000 L 8583 15021 L 8579 15047 L
8583 15074 L 8575 15100 L 8598 15114 L 8605 15140 L
8613 15166 L 8625 15190 L 8645 15209 L 8667 15225 L
8692 15236 L 8690 15263 L 8710 15281 L 8736 15290 L
8762 15297 L 8788 15305 L 8810 15321 L 8836 15328 L
8851 15350 L 8874 15365 L 8899 15375 L 8925 15383 L
8950 15393 L 8974 15406 L 8998 15418 L 9019 15435 L
9045 15442 L 9071 15449 L 9087 15471 L 9086 15498 L
9099 15521 L 9122 15535 L 9100 15551 L 9122 15567 L
9127 15594 L 9150 15607 L 9164 15630 L 9174 15637 L
9186 15647 L 9204 15667 L 9211 15681 L 9216 15691 L
9228 15705 L 9234 15711 L 9260 15717 L 9262 15720 L
9278 15738 L 9256 15753 L 9267 15777 L 9286 15796 L
9295 15795 L 9311 15793 L 9313 15792 L 9323 15817 L
9342 15836 L 9358 15858 L 9385 15856 L 9407 15840 L
9430 15853 L 9454 15840 L 9481 15841 L 9495 15832 L

9503 15826 L 9523 15807 L 9550 15809 L 9577 15812 L
9598 15828 L 9608 15853 L 9609 15880 L 9629 15898 L
9639 15923 L 9635 15896 L 9622 15872 L 9633 15847 L
9625 15822 L 9639 15798 L 9666 15800 L 9682 15779 L
9705 15764 L 9727 15748 L 9742 15733 L 9754 15732 L
9754 15746 L 9759 15750 L 9769 15746 L 9784 15739 L
9803 15744 L 9801 15751 L 9779 15745 L 9772 15755 L
9766 15762 L 9766 15768 L 9754 15775 L 9732 15791 L
9732 15793 L 9757 15778 L 9777 15766 L 9779 15753 L
9801 15755 L 9806 15746 L 9802 15740 L 9784 15736 L
9760 15748 L 9758 15742 L 9763 15715 L 9754 15698 L
9760 15688 L 9783 15673 L 9803 15655 L 9816 15631 L
9804 15607 L 9786 15586 L 9784 15559 L 9766 15539 L
9752 15516 L 9748 15489 L 9728 15471 L 9715 15447 L
9700 15425 L 9683 15404 L 9667 15382 L 9644 15368 L
9670 15361 L 9651 15342 L 9632 15322 L 9608 15309 L
9581 15310 L 9564 15289 L 9540 15275 L 9518 15261 L
9506 15236 L 9496 15211 L 9517 15195 L 9534 15173 L
9536 15146 L 9534 15125 L 9529 15113 L 9517 15096 L
9494 15087 L 9508 15055 L 9518 15025 L 9541 15011 L
9568 15014 L 9595 15016 L 9619 15028 L 9646 15028 L
9673 15035 L 9700 15033 L 9676 15020 L 9650 15013 L
9627 14998 L 9613 14976 L 9589 14962 L 9563 14955 L
9582 14935 L 9565 14914 L 9587 14898 L 9608 14881 L
9594 14858 L 9568 14849 L 9543 14838 L 9521 14822 L
9495 14819 L 9475 14837 L 9465 14812 L 9451 14789 L
9426 14779 L 9399 14779 L 9372 14772 L 9365 14746 L
9391 14739 L 9369 14722 L 9382 14698 L 9381 14671 L
9402 14654 L 9420 14675 L 9431 14699 L 9458 14699 L
9483 14710 L 9510 14710 L 9536 14719 L 9554 14738 L
9582 14739 L 9609 14741 L 9621 14765 L 9597 14779 L
9571 14785 L 9595 14797 L 9607 14821 L 9629 14838 L
9653 14828 L 9665 14803 L 9672 14799 L 9689 14790 L
9690 14818 L 9679 14842 L 9705 14834 L 9696 14859 L
9716 14841 L 9737 14859 L 9764 14865 L 9756 14891 L
9782 14896 L 9806 14908 L 9782 14920 L 9792 14945 L
9811 14964 L 9834 14978 L 9846 15003 L 9856 15028 L
9879 15042 L 9898 15059 L 9900 15060 L 9915 15059 L
9927 15057 L 9946 15076 L 9950 15103 L 9956 15099 L
9973 15089 L 9991 15110 L 10015 15122 L 10035 15140 L
10042 15146 L 10056 15157 L 10057 15157 L 10071 15163 L
10078 15166 L 10081 15168 L 10088 15170 L 10097 15174 L
10107 15177 L 10131 15189 L 10143 15201 L 10150 15208 L

10170 15216 L 10175 15218 L 10201 15209 L 10226 15219 L
10235 15226 L 10248 15235 L 10223 15246 L 10246 15260 L
10255 15286 L 10257 15285 L 10272 15283 L 10281 15281 L
10284 15281 L 10308 15276 L 10309 15275 L 10321 15268 L
10331 15262 L 10340 15280 L 10343 15286 L 10356 15283 L
10369 15281 L 10383 15304 L 10393 15329 L 10401 15355 L
10403 15328 L 10426 15313 L 10441 15314 L 10453 15314 L
10473 15332 L 10497 15320 L 10524 15318 L 10551 15317 L
10578 15323 L 10600 15338 L 10627 15344 L 10639 15368 L
10644 15341 L 10651 15315 L 10656 15342 L 10661 15369 L
10666 15395 L 10672 15395 L 10693 15397 L 10687 15370 L
10709 15354 L 10733 15342 L 10759 15335 L 10786 15333 L
10796 15307 L 10805 15282 L 10824 15262 L 10813 15237 L
10826 15213 L 10840 15231 L 10843 15234 L 10854 15250 L
10859 15256 L 10856 15283 L 10867 15308 L 10888 15291 L
10914 15283 L 10929 15263 L 10930 15262 L 10954 15275 L
10971 15254 L 10998 15255 L 11015 15243 L 11021 15239 L
11044 15226 L 11070 15234 L 11089 15215 L 11116 15216 L
11139 15230 L 11160 15247 L 11172 15271 L 11177 15283 L
11189 15280 L 11197 15283 L 11202 15289 L 11203 15288 L
11198 15280 L 11189 15277 L 11177 15282 L 11174 15269 L
11163 15241 L 11184 15242 L 11196 15252 L 11225 15269 L
11226 15267 L 11221 15263 L 11197 15251 L 11187 15237 L
11174 15236 L 11150 15223 L 11146 15196 L 11127 15177 L
11128 15150 L 11141 15126 L 11167 15133 L 11153 15109 L
11157 15082 L 11151 15055 L 11144 15029 L 11121 15015 L
11116 14988 L 11125 14962 L 11104 14944 L 11091 14920 L
11082 14895 L 11073 14869 L 11062 14844 L 11070 14818 L
11085 14795 L 11110 14787 L 11103 14813 L 11118 14790 L
11121 14763 L 11147 14772 L 11174 14769 L 11190 14791 L
11214 14779 L 11241 14778 L 11257 14756 L 11230 14752 L
11208 14767 L 11184 14754 L 11157 14751 L 11135 14735 L
11108 14729 L 11085 14715 L 11068 14736 L 11075 14710 L
11071 14683 L 11047 14671 L 11020 14670 L 11031 14645 L
11021 14619 L 11001 14601 L 10991 14576 L 10977 14553 L
10983 14526 L 10984 14499 L 11003 14480 L 11024 14491 L
11034 14487 L 11005 14467 L 11002 14471 L 11004 14478 L
10982 14492 L 10963 14466 L 10943 14449 L 10924 14430 L
10898 14423 L 10924 14416 L 10928 14415 L 10951 14411 L
10960 14414 L 10973 14418 L 10977 14419 L 11002 14429 L
11026 14415 L 11052 14408 L 11079 14411 L 11104 14422 L
11131 14424 L 11156 14414 L 11154 14387 L 11133 14404 L
11106 14399 L 11099 14373 L 11123 14360 L 11098 14350 L

11071 14354 L 11048 14341 L 11063 14318 L 11079 14312 L
11088 14308 L 11114 14300 L 11140 14308 L 11141 14308 L
11160 14322 L 11162 14324 L 11186 14310 L 11181 14283 L
11207 14284 L 11209 14284 L 11217 14310 L 11244 14315 L
11221 14329 L 11248 14331 L 11275 14328 L 11253 14313 L
11268 14290 L 11241 14288 L 11239 14261 L 11212 14254 L
11201 14230 L 11218 14208 L 11229 14233 L 11255 14237 L
11272 14259 L 11286 14268 L 11295 14273 L 11298 14273 L
11322 14271 L 11330 14297 L 11326 14323 L 11323 14351 L
11344 14350 L 11349 14350 L 11350 14350 L 11357 14376 L
11330 14376 L 11304 14386 L 11300 14413 L 11325 14403 L
11352 14404 L 11369 14400 L 11376 14398 L 11379 14397 L
11390 14375 L 11391 14373 L 11400 14366 L 11405 14363 L
11412 14358 L 11413 14357 L 11423 14348 L 11432 14338 L
11449 14316 L 11456 14316 L 11476 14315 L 11479 14342 L
11505 14336 L 11505 14336 L 11509 14309 L 11516 14303 L
11521 14298 L 11529 14290 L 11549 14275 L 11551 14274 L
11557 14300 L 11556 14327 L 11560 14329 L 11582 14336 L
11599 14338 L 11608 14339 L 11629 14357 L 11657 14359 L
11667 14352 L 11679 14344 L 11702 14329 L 11726 14317 L
11753 14322 L 11770 14344 L 11796 14357 L 11778 14334 L
11784 14307 L 11786 14280 L 11798 14256 L 11822 14244 L
11845 14228 L 11867 14214 L 11888 14203 L 11888 14201 L
11865 14211 L 11843 14227 L 11819 14238 L 11795 14251 L
11771 14264 L 11778 14290 L 11765 14314 L 11741 14301 L
11717 14289 L 11722 14262 L 11728 14236 L 11748 14217 L
11774 14210 L 11785 14184 L 11787 14157 L 11763 14145 L
11764 14144 L 11781 14125 L 11803 14108 L 11828 14099 L
11812 14077 L 11837 14065 L 11861 14053 L 11840 14036 L
11813 14032 L 11820 14006 L 11807 13982 L 11823 13960 L
11842 13981 L 11863 13997 L 11868 13970 L 11857 13946 L
11850 13919 L 11877 13913 L 11895 13893 L 11914 13874 L
11941 13868 L 11960 13887 L 11950 13861 L 11928 13845 L
11909 13826 L 11909 13799 L 11895 13822 L 11890 13849 L
11874 13871 L 11847 13876 L 11827 13895 L 11804 13879 L
11780 13892 L 11756 13904 L 11766 13879 L 11741 13891 L
11760 13871 L 11771 13846 L 11750 13863 L 11746 13836 L
11740 13810 L 11739 13837 L 11732 13863 L 11723 13889 L
11697 13880 L 11677 13862 L 11699 13847 L 11717 13827 L
11702 13804 L 11678 13791 L 11693 13769 L 11671 13785 L
11673 13812 L 11666 13838 L 11660 13865 L 11661 13838 L
11636 13826 L 11630 13799 L 11631 13772 L 11604 13766 L
11595 13740 L 11600 13717 L 11601 13714 L 11611 13739 L

11612 13738 L 11621 13734 L 11631 13730 L 11635 13728 L
11634 13700 L 11639 13702 L 11642 13703 L 11652 13706 L
11656 13707 L 11661 13708 L 11679 13688 L 11678 13661 L
11684 13653 L 11689 13647 L 11694 13641 L 11695 13639 L
11697 13630 L 11702 13613 L 11706 13586 L 11720 13566 L
11721 13564 L 11743 13581 L 11732 13606 L 11720 13630 L
11716 13657 L 11691 13668 L 11697 13695 L 11683 13719 L
11702 13738 L 11711 13712 L 11715 13685 L 11733 13706 L
11750 13717 L 11755 13721 L 11761 13694 L 11759 13667 L
11758 13640 L 11782 13627 L 11768 13604 L 11773 13596 L
11779 13585 L 11782 13580 L 11807 13608 L 11806 13593 L
11783 13578 L 11784 13551 L 11788 13548 L 11792 13545 L
11806 13534 L 11793 13510 L 11807 13487 L 11809 13461 L
11810 13459 L 11832 13443 L 11810 13427 L 11826 13421 L
11831 13419 L 11836 13418 L 11847 13408 L 11857 13400 L
11869 13376 L 11889 13357 L 11913 13370 L 11938 13381 L
11960 13397 L 11966 13424 L 11964 13396 L 11944 13379 L
11919 13368 L 11898 13350 L 11873 13338 L 11858 13315 L
11867 13289 L 11877 13291 L 11893 13295 L 11921 13297 L
11947 13303 L 11929 13283 L 11909 13264 L 11925 13241 L
11900 13230 L 11894 13203 L 11867 13202 L 11849 13181 L
11829 13163 L 11810 13143 L 11824 13119 L 11851 13121 L
11878 13126 L 11905 13126 L 11907 13099 L 11880 13101 L
11854 13092 L 11828 13083 L 11816 13059 L 11812 13032 L
11799 13008 L 11825 13003 L 11817 12977 L 11795 12993 L
11789 12966 L 11783 12940 L 11774 12914 L 11800 12907 L
11796 12879 L 11819 12864 L 11845 12857 L 11835 12832 L
11818 12811 L 11799 12831 L 11781 12851 L 11783 12879 L
11770 12854 L 11743 12860 L 11752 12834 L 11764 12822 L
11772 12815 L 11769 12843 L 11789 12824 L 11812 12808 L
11799 12784 L 11774 12774 L 11777 12771 L 11794 12755 L
11811 12777 L 11838 12782 L 11822 12759 L 11833 12734 L
11826 12707 L 11836 12682 L 11860 12695 L 11887 12695 L
11887 12722 L 11878 12748 L 11879 12775 L 11881 12748 L
11905 12735 L 11909 12707 L 11937 12705 L 11912 12694 L
11885 12689 L 11878 12663 L 11851 12667 L 11830 12649 L
11857 12650 L 11884 12647 L 11905 12630 L 11915 12604 L
11888 12602 L 11861 12609 L 11855 12636 L 11828 12634 L
11827 12607 L 11853 12598 L 11873 12580 L 11846 12577 L
11821 12588 L 11806 12612 L 11804 12639 L 11781 12653 L
11772 12627 L 11772 12624 L 11774 12600 L 11787 12575 L
11814 12570 L 11836 12554 L 11848 12549 L 11861 12543 L
11882 12560 L 11910 12559 L 11890 12540 L 11872 12519 L

11879 12493 L 11904 12480 L 11887 12459 L 11910 12444 L
11911 12421 L 11912 12416 L 11934 12433 L 11934 12433 L
11951 12454 L 11955 12481 L 11958 12482 L 11978 12497 L
11981 12510 L 11984 12523 L 12009 12537 L 12009 12564 L
12011 12591 L 12021 12566 L 12024 12539 L 12038 12515 L
12046 12517 L 12064 12522 L 12092 12520 L 12098 12529 L
12108 12542 L 12097 12567 L 12106 12593 L 12109 12621 L
12133 12634 L 12140 12640 L 12153 12653 L 12178 12664 L
12196 12685 L 12222 12693 L 12248 12703 L 12252 12676 L
12234 12655 L 12214 12673 L 12214 12646 L 12215 12619 L
12192 12604 L 12187 12631 L 12176 12606 L 12168 12580 L
12194 12572 L 12209 12595 L 12217 12587 L 12228 12576 L
12245 12554 L 12250 12549 L 12265 12536 L 12277 12561 L
12278 12561 L 12288 12569 L 12299 12577 L 12300 12605 L
12274 12600 L 12277 12627 L 12304 12621 L 12319 12621 L
12331 12621 L 12331 12648 L 12310 12665 L 12287 12681 L
12269 12702 L 12262 12728 L 12285 12743 L 12259 12753 L
12287 12754 L 12313 12761 L 12325 12786 L 12337 12761 L
12346 12748 L 12352 12738 L 12363 12713 L 12354 12688 L
12366 12663 L 12390 12677 L 12389 12704 L 12408 12709 L
12415 12711 L 12442 12705 L 12436 12678 L 12438 12651 L
12447 12654 L 12464 12659 L 12463 12687 L 12454 12713 L
12438 12735 L 12442 12762 L 12437 12789 L 12410 12783 L
12389 12765 L 12365 12778 L 12351 12802 L 12358 12828 L
12375 12807 L 12398 12820 L 12415 12842 L 12442 12848 L
12468 12839 L 12487 12853 L 12490 12855 L 12514 12870 L
12539 12880 L 12546 12906 L 12549 12879 L 12550 12851 L
12551 12851 L 12576 12840 L 12602 12832 L 12629 12832 L
12631 12838 L 12638 12858 L 12646 12854 L 12663 12846 L
12664 12847 L 12688 12856 L 12685 12829 L 12712 12829 L
12719 12803 L 12692 12809 L 12700 12798 L 12708 12787 L
12727 12773 L 12730 12770 L 12741 12795 L 12744 12823 L
12725 12843 L 12726 12871 L 12729 12898 L 12748 12918 L
12730 12939 L 12743 12963 L 12767 12961 L 12771 12961 L
12797 12954 L 12789 12980 L 12783 13006 L 12807 12993 L
12829 12977 L 12833 12977 L 12856 12973 L 12883 12973 L
12895 12976 L 12909 12980 L 12910 12980 L 12911 12980 L
12925 12981 L 12937 12982 L 12952 13005 L 12951 12978 L
12959 12952 L 12980 12969 L 12999 12978 L 12999 13001 L
12998 13007 L 12999 13007 L 12999 14079 L 12999 14085 L
12980 14105 L 12968 14129 L 12961 14156 L 12951 14181 L
12934 14202 L 12915 14222 L 12900 14245 L 12878 14261 L
12864 14284 L 12844 14302 L 12838 14329 L 12819 14348 L

12798 14366 L 12793 14393 L 12772 14411 L 12752 14428 L
12731 14445 L 12712 14465 L 12692 14483 L 12675 14505 L
12656 14524 L 12640 14546 L 12622 14566 L 12606 14588 L
12586 14607 L 12575 14632 L 12563 14657 L 12547 14678 L
12534 14702 L 12519 14725 L 12503 14747 L 12494 14772 L
12476 14793 L 12463 14817 L 12448 14839 L 12438 14865 L
12433 14891 L 12409 14905 L 12384 14913 L 12358 14904 L
12347 14929 L 12328 14948 L 12306 14964 L 12279 14967 L
12256 14981 L 12235 14998 L 12213 15014 L 12196 15036 L
12178 15056 L 12163 15078 L 12147 15100 L 12124 15086 L
12099 15096 L 12072 15102 L 12045 15103 L 12018 15104 L
11995 15119 L 11980 15141 L 11984 15168 L 12007 15183 L
12012 15209 L 11987 15219 L 12013 15226 L 12040 15230 L
12053 15206 L 12045 15232 L 12033 15257 L 12026 15283 L
12029 15310 L 12041 15334 L 12035 15360 L 12009 15368 L
12003 15394 L 12010 15421 L 12022 15445 L 12041 15464 L
12068 15463 L 12081 15487 L 12107 15494 L 12128 15511 L
12149 15529 L 12146 15556 L 12169 15571 L 12182 15594 L
12204 15610 L 12231 15605 L 12258 15601 L 12283 15611 L
12298 15633 L 12324 15624 L 12334 15649 L 12359 15657 L
12377 15678 L 12402 15690 L 12419 15710 L 12445 15720 L
12449 15746 L 12426 15760 L 12434 15786 L 12458 15799 L
12482 15811 L 12506 15823 L 12528 15839 L 12545 15861 L
12561 15882 L 12584 15897 L 12609 15908 L 12621 15932 L
12626 15959 L 12633 15985 L 12638 16012 L 12656 16032 L
12682 16039 L 12698 16061 L 12719 16079 L 12743 16090 L
12767 16102 L 12785 16122 L 12812 16127 L 12836 16139 L
12842 16166 L 12841 16170 L 12833 16170 L 12800 16170 L
12767 16170 L 12734 16170 L 12700 16170 L 12667 16170 L
12634 16170 L 12601 16170 L 12567 16170 L 12534 16170 L
12501 16170 L 12468 16170 L 12435 16170 L 12401 16170 L
12368 16170 L 12335 16170 L 12302 16170 L 12268 16170 L
12235 16170 L 12202 16170 L 12169 16170 L 12135 16170 L
12102 16170 L 12069 16170 L 12036 16170 L 12003 16170 L
11969 16170 L 11936 16170 L 11903 16170 L 11870 16170 L
11836 16170 L 11803 16170 L 11770 16170 L 11737 16170 L
11704 16170 L 11670 16170 L 11637 16170 L 11604 16170 L
11571 16170 L 11537 16170 L 11504 16170 L 11471 16170 L
11438 16170 L 11404 16170 L 11371 16170 L 11338 16170 L
11305 16170 L 11272 16170 L 11238 16170 L 11205 16170 L
11172 16170 L 11139 16170 L 11105 16170 L 11072 16170 L
11039 16170 L 11006 16170 L 10972 16170 L 10939 16170 L
10906 16170 L 10873 16170 L 10840 16170 L 10806 16170 L

10773 16170 L 10740 16170 L 10707 16170 L 10673 16170 L
10640 16170 L 10607 16170 L 10574 16170 L 10540 16170 L
10507 16170 L 10474 16170 L 10441 16170 L 10408 16170 L
10374 16170 L 10341 16170 L 10308 16170 L 10275 16170 L
10241 16170 L 10208 16170 L 10175 16170 L 10142 16170 L
10108 16170 L 10075 16170 L 10042 16170 L 10009 16170 L
9976 16170 L 9942 16170 L 9909 16170 L 9876 16170 L
9843 16170 L 9809 16170 L 9776 16170 L 9743 16170 L
9710 16170 L 9677 16170 L 9643 16170 L 9610 16170 L
9577 16170 L 9544 16170 L 9510 16170 L 9477 16170 L
9444 16170 L 9411 16170 L 9377 16170 L 9344 16170 L
9311 16170 L 9278 16170 L 9245 16170 L 9211 16170 L
9178 16170 L 9145 16170 L 9112 16170 L 9078 16170 L
9045 16170 L 9012 16170 L 8979 16170 L 8945 16170 L
8912 16170 L 8879 16170 L 8846 16170 L 8813 16170 L
8779 16170 L 8746 16170 L 8713 16170 L 8680 16170 L
8646 16170 L 8613 16170 L 8580 16170 L 8547 16170 L
8513 16170 L 8480 16170 L 8447 16170 L 8414 16170 L
8381 16170 L 8347 16170 L 8314 16170 L 8281 16170 L
8248 16170 L 8214 16170 L 8181 16170 L 8148 16170 L
8115 16170 L 8081 16170 L 8048 16170 L 8015 16170 L
7982 16170 L 7949 16170 L 7915 16170 L 7882 16170 L
7849 16170 L 7816 16170 L 7782 16170 L 7749 16170 L
7716 16170 L 7683 16170 L 7650 16170 L 7616 16170 L
7583 16170 L 7550 16170 L 7517 16170 L 7483 16170 L
7450 16170 L 7417 16170 L 7384 16170 L 7350 16170 L
7317 16170 L 7284 16170 L 7251 16170 L 7218 16170 L
7184 16170 L 7151 16170 L 7118 16170 L 7085 16170 L
7051 16170 L 7018 16170 L 6985 16170 L 6952 16170 L
6918 16170 L 6885 16170 L 6852 16170 L 6819 16170 L
6786 16170 L 6752 16170 L 6719 16170 L 6686 16170 L
6653 16170 L 6619 16170 L 6586 16170 L 6553 16170 L
6520 16170 L 6486 16170 L 6453 16170 L 6420 16170 L
6387 16170 L 6354 16170 L 6320 16170 L 6287 16170 L
6254 16170 L 6221 16170 L 6187 16170 L 6154 16170 L
6121 16170 L 6088 16170 L 6054 16170 L 6021 16170 L
5988 16170 L 5955 16170 L 5922 16170 L 5888 16170 L
5855 16170 L 5822 16170 L 5789 16170 L 5755 16170 L
5722 16170 L 5689 16170 L 5656 16170 L 5622 16170 L
5589 16170 L 5556 16170 L 5523 16170 L 5490 16170 L
5456 16170 L 5423 16170 L 5390 16170 L 5357 16170 L
5323 16170 L 5290 16170 L 5257 16170 L 5224 16170 L
5191 16170 L 5157 16170 L 5124 16170 L 5091 16170 L

5058 16170 L 5024 16170 L 4991 16170 L 4958 16170 L
4925 16170 L 4891 16170 L 4858 16170 L 4825 16170 L
4792 16170 L 4759 16170 L 4725 16170 L 4692 16170 L
4659 16170 L 4626 16170 L 4592 16170 L 4559 16170 L
4526 16170 L 4493 16170 L 4459 16170 L 4426 16170 L
4393 16170 L 4360 16170 L 4327 16170 L 4293 16170 L
4260 16170 L 4227 16170 L 4194 16170 L 4160 16170 L
4127 16170 L 4094 16170 L 4061 16170 L 4027 16170 L
3994 16170 L 3961 16170 L 3928 16170 L 3895 16170 L
3861 16170 L 3828 16170 L 3795 16170 L 3762 16170 L
3728 16170 L 3695 16170 L 3662 16170 L 3629 16170 L
3595 16170 L 3562 16170 L 3529 16170 L 3496 16170 L
3463 16170 L 3429 16170 L 3396 16170 L 3363 16170 L
3330 16170 L 3296 16170 L 3263 16170 L 3230 16170 L
3197 16170 L 3164 16170 L 3130 16170 L 3097 16170 L
3064 16170 L 3031 16170 L 2997 16170 L 2964 16170 L
2931 16170 L 2898 16170 L 2864 16170 L 2831 16170 L
2798 16170 L 2765 16170 L 2732 16170 L 2698 16170 L
2665 16170 L 2632 16170 L 2599 16170 L 2565 16170 L
2532 16170 L 2499 16170 L 2466 16170 L 2432 16170 L
2399 16170 L 2366 16170 L 2333 16170 L 2300 16170 L
N E
5542 5477 5541 5470 5543 5466 5545 5461 5547 5457 5548 5455
5556 5455 5575 5455 5575 5458 5573 5462 5571 5466 5569 5471
5567 5475 5563 5478 5559 5480 5556 5483 5552 5486 5549 5482
5545 5480 5542 5477 20 N F
5702 5573 5698 5567 5700 5563 5704 5561 5708 5563 5712 5565
5717 5566 5716 5571 5721 5573 5724 5576 5724 5581 5726 5585
5721 5585 5717 5588 5712 5588 5711 5584 5707 5581 5704 5578
5702 5573 19 N F
5796 5572 5790 5572 5794 5569 5798 5567 5801 5563 5805 5561
5810 5562 5808 5566 5806 5570 5803 5573 5799 5576 5796 5572
12 N F
5817 5461 5816 5455 5822 5455 5855 5455 5865 5455 5871 5458
5879 5459 5885 5461 5890 5456 5890 5455 5910 5455 5903 5461
5904 5468 5908 5474 5902 5477 5909 5480 5915 5478 5917 5485
5916 5492 5916 5499 5921 5504 5924 5511 5930 5507 5936 5511
5937 5519 5933 5524 5927 5520 5921 5516 5914 5515 5908 5511
5903 5506 5897 5502 5893 5496 5886 5497 5879 5495 5872 5491
5866 5488 5862 5494 5855 5495 5847 5494 5843 5488 5838 5483
5832 5479 5825 5477 5827 5471 5823 5465 5817 5461 47 N F
5964 5459 5962 5455 5969 5455 5970 5459 5964 5459 5 N F
5977 5488 5967 5480 5972 5475 5975 5468 5981 5464 5988 5463

5992 5457 5999 5459 6006 5456 6006 5455 6021 5455 6024 5455
6024 5459 6028 5455 6039 5455 6038 5459 6042 5465 6038 5471
6034 5477 6031 5484 6024 5485 6018 5488 6011 5489 6005 5485
5998 5483 5991 5485 5984 5488 5977 5488 28 N F
6055 5463 6054 5459 6056 5458 6058 5457 6058 5455 6059 5455
6060 5456 6062 5456 6063 5458 6064 5460 6064 5462 6064 5465
6063 5467 6061 5466 6059 5465 6057 5465 6055 5463 17 N F
6243 5583 6240 5580 6241 5577 6242 5574 6244 5571 6247 5573
6251 5574 6254 5574 6257 5576 6259 5578 6259 5579 6257 5581
6253 5582 6250 5582 6246 5583 6243 5583 16 N F
6252 5562 6251 5559 6253 5560 6255 5559 6257 5561 6258 5563
6258 5565 6260 5566 6259 5568 6257 5568 6255 5566 6253 5564
6252 5562 13 N F
6256 5651 6252 5647 6253 5644 6257 5644 6259 5641 6260 5638
6263 5640 6262 5643 6260 5646 6257 5647 6256 5651 11 N F
7342 6052 7335 6044 7339 6038 7343 6032 7349 6028 7356 6025
7360 6019 7367 6017 7374 6019 7378 6013 7381 6006 7388 6004
7395 6002 7388 5998 7392 5992 7398 5987 7405 5987 7408 5980
7415 5980 7417 5987 7422 5992 7415 5995 7409 5992 7404 5997
7402 6004 7403 6012 7404 6019 7402 6026 7398 6032 7397 6039
7392 6044 7385 6046 7380 6042 7372 6042 7365 6044 7360 6049
7356 6055 7349 6055 7342 6052 39 N F
7490 6180 7483 6175 7488 6171 7495 6170 7503 6169 7508 6173
7505 6180 7506 6187 7511 6192 7514 6199 7507 6201 7501 6197
7500 6189 7497 6183 7490 6180 15 N F
7498 6166 7491 6165 7493 6158 7499 6154 7506 6153 7513 6151
7520 6149 7522 6156 7523 6163 7527 6169 7533 6172 7527 6180
7520 6179 7513 6179 7510 6173 7506 6167 7498 6166 17 N F
7498 6111 7493 6105 7497 6099 7504 6098 7511 6096 7519 6097
7526 6096 7533 6094 7538 6090 7545 6087 7552 6087 7559 6090
7566 6091 7573 6089 7574 6082 7581 6084 7586 6080 7593 6083
7597 6089 7595 6096 7594 6103 7600 6107 7598 6114 7592 6118
7589 6125 7590 6132 7583 6132 7579 6126 7576 6119 7569 6120
7564 6126 7558 6130 7552 6127 7547 6121 7544 6115 7539 6111
7535 6105 7526 6113 7523 6119 7516 6122 7509 6120 7503 6115
7498 6111 43 N F
7698 6195 7691 6184 7698 6184 7705 6186 7712 6188 7718 6186
7717 6179 7723 6183 7729 6185 7736 6188 7743 6189 7750 6190
7758 6191 7754 6197 7747 6198 7745 6205 7745 6212 7746 6219
7749 6226 7753 6232 7759 6234 7766 6231 7773 6228 7780 6229
7781 6236 7776 6241 7775 6248 7777 6255 7784 6259 7782 6266
7788 6270 7794 6266 7801 6266 7802 6273 7809 6276 7802 6275
7797 6281 7791 6285 7786 6290 7781 6285 7774 6287 7770 6281

7762 6281 7756 6278 7753 6285 7749 6291 7744 6295 7737 6299
7732 6304 7726 6301 7724 6294 7727 6287 7727 6280 7722 6274
7717 6269 7715 6262 7711 6256 7706 6251 7708 6244 7714 6240
7719 6245 7726 6248 7732 6243 7739 6244 7746 6242 7740 6237
7739 6230 7734 6225 7728 6221 7722 6217 7718 6211 7713 6205
7714 6198 7710 6204 7704 6200 7698 6195 76 N F
7944 6713 7942 6708 7943 6705 7945 6702 7948 6700 7950 6699
7951 6699 7953 6702 7950 6704 7948 6707 7946 6710 7944 6713
12 N F
7955 6697 7950 6697 7954 6695 7958 6692 7961 6689 7964 6685
7967 6682 7971 6679 7975 6678 7980 6678 7984 6679 7989 6678
7989 6678 7993 6680 7991 6684 7990 6688 7986 6690 7982 6688
7977 6687 7973 6689 7971 6693 7968 6696 7963 6696 7959 6694
7955 6697 25 N F
7957 6703 7953 6699 7957 6697 7961 6696 7966 6698 7970 6696
7973 6693 7976 6689 7980 6689 7984 6691 7988 6693 7992 6690
7994 6686 7999 6684 8003 6687 8007 6687 8006 6691 8003 6695
7999 6698 7997 6701 7993 6704 7989 6706 7984 6706 7980 6708
7975 6707 7971 6706 7966 6705 7962 6704 7957 6703 29 N F
7984 6676 7979 6675 7983 6673 7987 6671 7991 6669 7996 6669
7997 6673 7993 6675 7989 6677 7984 6676 10 N F
8030 6668 8023 6664 8027 6661 8031 6663 8035 6660 8039 6663
8043 6665 8039 6668 8035 6669 8030 6668 10 N F
8101 6699 8089 6694 8093 6688 8097 6682 8103 6678 8109 6675
8116 6673 8123 6672 8129 6676 8134 6681 8140 6686 8145 6691
8147 6698 8150 6704 8157 6706 8157 6713 8156 6720 8156 6727
8152 6732 8145 6729 8141 6735 8136 6740 8130 6738 8124 6742
8117 6745 8110 6747 8111 6740 8105 6735 8104 6728 8100 6722
8095 6717 8091 6711 8093 6704 8100 6705 8105 6710 8112 6713
8113 6706 8107 6702 8101 6699 39 N F
8169 6668 8165 6666 8167 6664 8171 6664 8174 6663 8176 6666
8179 6667 8182 6666 8185 6668 8185 6671 8188 6673 8191 6672
8193 6675 8194 6679 8195 6682 8193 6685 8190 6687 8187 6689
8184 6690 8181 6688 8178 6688 8175 6687 8174 6683 8174 6680
8173 6677 8170 6675 8169 6672 8169 6668 28 N F
8212 6671 8200 6667 8205 6663 8210 6657 8216 6654 8223 6651
8229 6655 8231 6662 8235 6668 8241 6672 8247 6675 8254 6678
8261 6679 8268 6677 8274 6673 8280 6670 8287 6666 8293 6669
8297 6675 8297 6682 8302 6688 8298 6694 8298 6701 8294 6707
8292 6714 8290 6721 8283 6722 8279 6728 8274 6733 8269 6728
8263 6724 8266 6717 8261 6712 8255 6708 8254 6701 8250 6695
8243 6696 8236 6699 8230 6702 8224 6707 8219 6712 8213 6708
8207 6704 8209 6697 8215 6700 8212 6694 8214 6687 8216 6680

8219 6673 8212 6671 50 N F
8213 6754 8209 6751 8212 6750 8216 6749 8218 6747 8221 6744
8223 6742 8227 6742 8230 6743 8231 6746 8232 6749 8234 6752
8232 6755 8229 6756 8226 6757 8222 6756 8219 6756 8216 6755
8213 6754 19 N F
8275 14738 8272 14733 8276 14734 8279 14737 8283 14740 8285 14743
8288 14747 8290 14751 8293 14754 8294 14758 8295 14762 8293 14766
8291 14763 8289 14759 8286 14756 8283 14753 8282 14749 8281 14745
8279 14741 8275 14738 20 N F
8293 14780 8292 14777 8293 14776 8295 14776 8298 14778 8299 14780
8301 14783 8301 14786 8299 14784 8296 14783 8293 14780 11 N F
8313 14797 8311 14795 8313 14793 8316 14795 8319 14796 8320 14799
8321 14802 8323 14805 8325 14807 8326 14810 8323 14809 8321 14806
8319 14804 8318 14801 8316 14799 8313 14797 16 N F
8362 14788 8356 14786 8361 14787 8365 14788 8369 14790 8372 14793
8375 14796 8378 14799 8379 14803 8377 14807 8373 14810 8371 14813
8369 14809 8370 14805 8374 14804 8375 14799 8371 14797 8368 14794
8365 14791 8362 14788 20 N F
8397 14838 8392 14833 8397 14834 8401 14835 8405 14836 8409 14837
8413 14838 8416 14842 8417 14846 8419 14850 8423 14852 8424 14856
8422 14860 8418 14859 8415 14855 8413 14852 8411 14848 8408 14844
8405 14842 8401 14840 8397 14838 21 N F
8398 14852 8397 14849 8399 14847 8402 14848 8405 14850 8406 14853
8407 14856 8407 14859 8406 14862 8404 14859 8403 14857 8401 14854
8398 14852 13 N F
8431 14850 8426 14849 8430 14847 8434 14849 8438 14850 8442 14851
8446 14853 8450 14856 8453 14859 8456 14862 8458 14866 8461 14869
8464 14872 8467 14875 8470 14878 8473 14881 8475 14886 8471 14884
8467 14882 8463 14881 8460 14878 8456 14877 8455 14873 8452 14870
8448 14868 8445 14865 8441 14862 8437 14860 8437 14856 8434 14853
8431 14850 31 N F
8628 7037 8623 7035 8627 7034 8632 7033 8634 7029 8635 7025
8639 7023 8638 7028 8640 7032 8644 7035 8647 7038 8645 7042
8647 7046 8644 7049 8644 7054 8647 7057 8652 7059 8655 7062
8655 7066 8651 7064 8647 7062 8642 7061 8638 7059 8635 7055
8631 7055 8631 7050 8634 7046 8636 7042 8636 7038 8632 7035
8628 7037 31 N F
8709 12481 8704 12481 8706 12477 8709 12474 8713 12471 8715 12468
8719 12467 8723 12465 8727 12467 8731 12469 8728 12473 8724 12474
8720 12475 8716 12476 8712 12477 8709 12481 16 N F
8717 7287 8712 7276 8717 7270 8721 7265 8721 7258 8724 7251
8731 7254 8736 7258 8743 7258 8744 7266 8741 7272 8742 7279
8741 7286 8743 7293 8746 7300 8748 7306 8747 7313 8750 7320

8750 7327 8750 7334 8747 7340 8740 7337 8737 7331 8733 7325
8727 7321 8727 7314 8725 7307 8721 7301 8720 7294 8717 7287
30 N F
8750 6092 8749 6089 8751 6087 8753 6088 8755 6089 8757 6090
8757 6093 8755 6093 8753 6094 8751 6094 8750 6092 11 N F
8810 7146 8800 7143 8805 7139 8802 7132 8807 7127 8814 7126
8818 7125 8821 7123 8827 7127 8834 7129 8839 7125 8837 7119
8836 7112 8843 7108 8849 7106 8854 7106 8856 7106 8859 7113
8854 7118 8857 7124 8864 7125 8869 7130 8862 7129 8857 7134
8853 7128 8846 7129 8839 7132 8833 7136 8828 7141 8829 7148
8822 7150 8819 7156 8814 7151 8810 7146 34 N F
8809 5985 8802 5982 8805 5978 8807 5974 8808 5970 8812 5968
8812 5972 8816 5975 8819 5978 8820 5983 8818 5987 8813 5987
8809 5985 13 N F
8837 6191 8828 6183 8835 6185 8842 6186 8844 6186 8849 6186
8851 6193 8852 6200 8856 6206 8855 6213 8855 6220 8861 6224
8866 6228 8866 6228 8859 6229 8854 6234 8848 6229 8846 6222
8846 6215 8846 6208 8841 6203 8846 6198 8844 6191 8837 6191
24 N F
8844 6071 8840 6065 8845 6064 8849 6064 8853 6064 8854 6064
8852 6068 8855 6072 8852 6075 8851 6080 8852 6084 8847 6085
8844 6089 8843 6084 8842 6080 8844 6075 8844 6071 17 N F
8848 7641 8842 7629 8849 7629 8854 7628 8856 7628 8863 7626
8870 7625 8876 7621 8882 7618 8889 7618 8896 7620 8895 7628
8894 7634 8891 7641 8888 7648 8882 7651 8875 7654 8870 7658
8864 7654 8857 7652 8852 7646 8848 7641 22 N F
8854 7627 8847 7624 8850 7622 8854 7619 8857 7616 8862 7616
8866 7618 8871 7618 8875 7620 8871 7623 8867 7624 8863 7625
8858 7626 8854 7627 14 N F
8851 6144 8850 6141 8851 6139 8853 6139 8855 6140 8858 6141
8860 6141 8862 6142 8864 6143 8866 6145 8866 6145 8868 6145
8868 6147 8866 6148 8864 6147 8861 6147 8860 6145 8857 6145
8856 6143 8854 6141 8853 6143 8851 6144 22 N F
8853 6017 8850 6015 8852 6014 8854 6014 8855 6016 8853 6017
6 N F
8856 6010 8855 6006 8857 6007 8859 6009 8860 6010 8860 6012
8857 6012 8856 6010 8 N F
8862 7381 8857 7378 8860 7375 8864 7374 8869 7373 8871 7377
8875 7379 8878 7382 8881 7386 8883 7390 8883 7395 8878 7394
8875 7391 8872 7388 8868 7385 8867 7381 8864 7377 8862 7381
18 N F
8859 6071 8857 6069 8859 6068 8860 6068 8861 6068 8862 6070
8864 6071 8866 6071 8869 6071 8869 6073 8868 6075 8866 6076

8864 6075 8861 6075 8861 6073 8859 6071 16 N F
8872 6178 8867 6171 8873 6167 8879 6163 8882 6157 8889 6155
8896 6156 8903 6159 8902 6167 8907 6172 8912 6176 8913 6176
8919 6180 8924 6185 8931 6185 8938 6185 8940 6184 8945 6182
8942 6189 8940 6196 8939 6203 8935 6209 8930 6204 8928 6211
8922 6208 8920 6201 8918 6208 8915 6201 8911 6195 8911 6202
8912 6210 8905 6210 8901 6203 8897 6197 8896 6190 8892 6196
8889 6203 8883 6198 8880 6192 8876 6186 8883 6189 8885 6182
8878 6182 8884 6179 8891 6179 8898 6179 8896 6172 8890 6168
8886 6162 8882 6168 8876 6172 8872 6178 52 N F
8875 6248 8870 6242 8876 6237 8881 6243 8886 6238 8891 6232
8893 6225 8897 6219 8900 6226 8898 6233 8903 6238 8900 6244
8896 6251 8889 6251 8882 6249 8875 6248 16 N F
8897 7964 8893 7958 8896 7954 8898 7950 8898 7946 8903 7946
8905 7950 8908 7948 8913 7946 8917 7947 8921 7948 8926 7949
8927 7953 8931 7956 8934 7959 8939 7960 8943 7961 8946 7965
8945 7969 8941 7971 8937 7970 8933 7967 8933 7963 8930 7959
8926 7961 8927 7965 8923 7965 8919 7964 8914 7965 8910 7966
8906 7965 8901 7965 8897 7964 33 N F
8895 6290 8894 6287 8896 6284 8897 6281 8898 6284 8900 6287
8903 6288 8906 6290 8907 6293 8904 6295 8902 6298 8899 6295
8898 6292 8895 6290 14 N F
8903 7392 8902 7388 8905 7388 8908 7389 8910 7390 8913 7391
8913 7393 8911 7395 8909 7398 8906 7397 8904 7395 8903 7392
12 N F
8908 6007 8906 6005 8908 6004 8910 6003 8912 6003 8913 6006
8912 6008 8910 6008 8908 6007 9 N F
8913 7387 8910 7381 8914 7378 8919 7377 8923 7377 8928 7379
8932 7380 8936 7378 8940 7381 8941 7385 8939 7389 8937 7393
8934 7390 8930 7389 8925 7387 8922 7385 8918 7387 8913 7387
18 N F
8921 7292 8918 7285 8924 7288 8931 7291 8938 7290 8943 7295
8942 7288 8942 7281 8935 7282 8932 7276 8937 7271 8933 7265
8938 7259 8943 7255 8946 7254 8950 7252 8955 7247 8961 7242
8967 7241 8971 7247 8967 7253 8961 7257 8964 7264 8970 7267
8976 7271 8981 7276 8988 7277 8994 7282 9001 7283 9005 7278
9012 7282 9013 7289 9005 7289 8999 7291 8993 7294 8987 7299
8985 7306 8985 7313 8987 7320 8993 7324 8999 7328 9006 7330
9010 7324 9017 7326 9021 7332 9025 7338 9018 7338 9011 7338
9004 7340 8999 7345 8993 7342 8995 7348 9000 7354 8997 7360
8990 7364 8985 7368 8978 7366 8972 7369 8968 7375 8968 7382
8972 7387 8976 7394 8969 7393 8963 7390 8957 7385 8955 7378
8948 7382 8944 7376 8941 7370 8934 7370 8930 7364 8926 7358

8927 7351 8930 7345 8937 7347 8941 7352 8949 7353 8951 7346
8950 7339 8946 7333 8946 7326 8946 7319 8944 7312 8950 7308
8956 7312 8958 7319 8964 7314 8971 7313 8971 7306 8966 7302
8960 7298 8953 7299 8948 7304 8941 7305 8934 7306 8929 7301
8922 7299 8921 7292 98 N F
8928 7204 8926 7202 8926 7198 8929 7196 8927 7192 8927 7189
8928 7190 8929 7192 8933 7193 8935 7195 8938 7197 8935 7197
8933 7200 8931 7203 8934 7205 8933 7209 8930 7207 8928 7204
18 N F
8937 7225 8930 7223 8932 7219 8933 7218 8934 7217 8937 7218
8941 7218 8944 7219 8947 7221 8950 7223 8947 7225 8945 7227
8946 7230 8942 7229 8940 7227 8936 7228 8937 7225 17 N F
8938 6013 8936 6010 8937 6009 8936 6006 8938 6005 8939 6003
8940 6005 8940 6007 8942 6009 8943 6011 8942 6013 8940 6014
8938 6013 13 N F
8952 7939 8948 7937 8949 7934 8951 7931 8954 7931 8957 7929
8960 7930 8963 7932 8965 7934 8969 7934 8972 7934 8974 7936
8976 7939 8974 7941 8970 7942 8968 7944 8964 7943 8961 7942
8958 7941 8955 7941 8952 7939 21 N F
9011 13027 9010 13021 9013 13018 9017 13016 9021 13019 9024 13022
9024 13026 9022 13030 9021 13034 9021 13038 9016 13039 9016 13034
9013 13031 9011 13027 14 N F
9033 7673 9031 7668 9035 7667 9040 7665 9043 7668 9048 7668
9051 7671 9053 7671 9055 7672 9057 7676 9059 7681 9055 7682
9050 7681 9048 7677 9044 7675 9042 7679 9037 7678 9039 7682
9036 7685 9035 7681 9032 7677 9033 7673 22 N F
9046 7575 9042 7573 9044 7570 9046 7568 9050 7569 9052 7571
9055 7573 9053 7576 9050 7576 9046 7575 10 N F
9059 7703 9059 7699 9062 7696 9062 7700 9062 7705 9065 7709
9067 7710 9069 7711 9073 7711 9078 7709 9081 7706 9084 7710
9082 7714 9079 7717 9075 7721 9071 7720 9068 7717 9064 7715
9060 7712 9060 7708 9059 7703 21 N F
9065 7564 9062 7557 9066 7559 9070 7561 9073 7565 9076 7568
9072 7567 9067 7568 9065 7564 9 N F
9088 7876 9084 7869 9089 7868 9093 7869 9095 7873 9099 7872
9104 7873 9104 7878 9104 7882 9105 7887 9100 7887 9099 7883
9095 7885 9091 7884 9087 7881 9088 7876 16 N F
9122 6147 9121 6141 9122 6137 9122 6132 9123 6128 9124 6123
9126 6119 9126 6123 9125 6128 9124 6133 9124 6137 9126 6141
9127 6146 9128 6150 9129 6155 9130 6159 9133 6163 9135 6167
9138 6171 9137 6175 9135 6171 9132 6167 9130 6163 9128 6159
9125 6156 9123 6151 9122 6147 27 N F
9134 7916 9129 7911 9132 7908 9135 7905 9137 7906 9140 7907

9144 7909 9143 7913 9148 7915 9152 7916 9153 7920 9154 7924
9154 7929 9152 7933 9147 7934 9145 7930 9142 7926 9140 7923
9138 7918 9134 7916 20 N F
9140 5927 M 9136 5913 L 9146 5904 L 9161 5907 L 9153 5895 L
9164 5886 L 9176 5877 L 9190 5881 L 9203 5885 L
9217 5890 L 9220 5876 L 9207 5869 L 9193 5867 L
9200 5854 L 9207 5841 L 9211 5827 L 9211 5813 L
9216 5799 L 9221 5786 L 9235 5782 L 9226 5771 L
9232 5757 L 9237 5744 L 9243 5731 L 9249 5718 L
9255 5704 L 9259 5690 L 9259 5676 L 9255 5662 L
9263 5649 L 9276 5643 L 9280 5629 L 9291 5620 L
9302 5610 L 9289 5616 L 9279 5627 L 9272 5639 L
9277 5626 L 9286 5614 L 9296 5603 L 9306 5593 L
9316 5583 L 9328 5575 L 9328 5575 L 9341 5567 L
9347 5580 L 9353 5567 L 9363 5555 L 9373 5546 L
9386 5539 L 9399 5532 L 9412 5525 L 9424 5518 L
9437 5510 L 9447 5500 L 9454 5488 L 9465 5478 L
9472 5465 L 9475 5455 L 9477 5455 L 9510 5455 L
9544 5455 L 9577 5455 L 9608 5455 L 9607 5465 L
9607 5479 L 9609 5494 L 9614 5507 L 9617 5521 L
9622 5535 L 9628 5548 L 9632 5562 L 9635 5576 L
9638 5591 L 9643 5604 L 9646 5619 L 9652 5632 L
9662 5642 L 9665 5657 L 9667 5671 L 9673 5684 L
9685 5693 L 9695 5703 L 9707 5711 L 9720 5718 L
9730 5728 L 9741 5738 L 9751 5749 L 9754 5763 L
9758 5776 L 9753 5790 L 9760 5803 L 9773 5808 L
9784 5818 L 9791 5831 L 9798 5844 L 9802 5858 L
9812 5868 L 9819 5880 L 9824 5894 L 9830 5907 L
9833 5921 L 9838 5935 L 9848 5945 L 9858 5956 L
9859 5970 L 9863 5984 L 9857 5998 L 9859 6012 L
9865 6025 L 9868 6039 L 9872 6053 L 9878 6066 L
9889 6075 L 9891 6089 L 9893 6104 L 9895 6118 L
9899 6132 L 9904 6145 L 9906 6160 L 9911 6173 L
9906 6187 L 9907 6201 L 9910 6215 L 9916 6228 L
9917 6243 L 9917 6257 L 9918 6260 L 9923 6270 L
9924 6285 L 9925 6299 L 9928 6313 L 9934 6327 L
9934 6341 L 9934 6355 L 9940 6369 L 9944 6382 L
9947 6397 L 9949 6411 L 9949 6425 L 9950 6440 L
9951 6454 L 9960 6465 L 9968 6477 L 9960 6489 L
9953 6502 L 9950 6516 L 9955 6530 L 9965 6539 L
9974 6551 L 9973 6565 L 9970 6579 L 9977 6592 L
9989 6601 L 9997 6612 L 10009 6621 L 10014 6634 L
10021 6647 L 10027 6660 L 10026 6674 L 10030 6688 L

10032 6702 L 10034 6716 L 10040 6729 L 10047 6742 L
10058 6751 L 10056 6765 L 10061 6779 L 10073 6787 L
10070 6801 L 10071 6815 L 10068 6829 L 10059 6840 L
10054 6853 L 10050 6867 L 10048 6881 L 10043 6895 L
10039 6909 L 10038 6923 L 10038 6937 L 10039 6951 L
10042 6965 L 10046 6979 L 10054 6991 L 10064 7001 L
10069 7015 L 10080 7024 L 10089 7035 L 10101 7042 L
10104 7044 L 10114 7049 L 10125 7058 L 10117 7070 L
10103 7068 L 10090 7075 L 10089 7090 L 10081 7101 L
10084 7116 L 10073 7125 L 10059 7124 L 10045 7126 L
10031 7122 L 10020 7131 L 10007 7137 L 9994 7131 L
9984 7141 L 9971 7147 L 9960 7156 L 9964 7169 L
9951 7165 L 9940 7174 L 9931 7185 L 9928 7199 L
9916 7207 L 9903 7214 L 9889 7213 L 9875 7209 L
9862 7205 L 9849 7197 L 9840 7186 L 9832 7175 L
9825 7162 L 9817 7150 L 9828 7141 L 9815 7136 L
9806 7126 L 9793 7119 L 9780 7113 L 9766 7111 L
9752 7107 L 9738 7109 L 9724 7103 L 9711 7098 L
9699 7090 L 9687 7083 L 9673 7079 L 9660 7074 L
9649 7065 L 9636 7058 L 9626 7048 L 9618 7033 L
9616 7018 L 9607 7007 L 9599 6995 L 9592 6983 L
9586 6970 L 9582 6956 L 9573 6945 L 9565 6934 L
9571 6921 L 9567 6907 L 9563 6893 L 9554 6882 L
9543 6873 L 9540 6859 L 9529 6849 L 9540 6839 L
9525 6840 L 9511 6838 L 9498 6832 L 9490 6820 L
9496 6807 L 9482 6805 L 9469 6799 L 9464 6785 L
9459 6772 L 9456 6758 L 9452 6744 L 9445 6731 L
9438 6719 L 9427 6709 L 9419 6698 L 9409 6687 L
9404 6674 L 9397 6661 L 9392 6648 L 9386 6634 L
9384 6620 L 9375 6608 L 9371 6595 L 9364 6582 L
9356 6570 L 9368 6562 L 9354 6562 L 9347 6550 L
9340 6537 L 9332 6525 L 9329 6511 L 9325 6497 L
9316 6486 L 9306 6476 L 9296 6465 L 9289 6452 L
9280 6441 L 9274 6429 L 9274 6414 L 9267 6401 L
9262 6388 L 9257 6374 L 9247 6364 L 9256 6353 L
9271 6351 L 9285 6349 L 9299 6352 L 9288 6343 L
9274 6340 L 9259 6339 L 9245 6342 L 9231 6341 L
9222 6329 L 9214 6318 L 9212 6304 L 9210 6289 L
9207 6275 L 9195 6267 L 9189 6254 L 9183 6241 L
9181 6226 L 9180 6212 L 9187 6199 L 9187 6185 L
9193 6172 L 9181 6164 L 9191 6153 L 9188 6139 L
9199 6130 L 9188 6120 L 9192 6106 L 9196 6092 L
9195 6078 L 9192 6064 L 9199 6051 L 9185 6053 L

9183 6039 L 9175 6027 L 9177 6013 L 9166 6004 L
9179 5999 L 9165 5996 L 9164 5982 L 9164 5968 L
9155 5958 L 9169 5954 L 9174 5940 L 9169 5926 L
9158 5917 L 9144 5914 L 9140 5927 L N E
9170 14242 9167 14237 9169 14233 9173 14230 9176 14227 9179 14225
9182 14228 9182 14233 9181 14237 9182 14241 9178 14244 9174 14244
9170 14242 13 N F
9198 15606 9196 15599 9199 15596 9203 15598 9206 15601 9209 15604
9210 15604 9214 15604 9218 15605 9219 15609 9219 15613 9219 15618
9215 15620 9211 15622 9209 15618 9205 15618 9204 15614 9205 15610
9201 15610 9198 15606 20 N F
9209 14252 9205 14249 9207 14246 9210 14243 9212 14239 9216 14236
9220 14236 9223 14233 9226 14237 9225 14241 9224 14245 9221 14249
9217 14248 9214 14251 9209 14252 15 N F
9225 14211 9221 14208 9225 14206 9229 14206 9232 14203 9235 14199
9238 14197 9240 14201 9239 14205 9238 14209 9240 14213 9238 14217
9236 14220 9233 14224 9232 14228 9228 14231 9225 14228 9224 14224
9224 14220 9225 14216 9225 14211 21 N F
9225 14355 9224 14350 9225 14344 9228 14341 9228 14341 9232 14343
9236 14342 9240 14342 9243 14346 9240 14349 9235 14350 9231 14350
9232 14354 9230 14358 9228 14362 9225 14359 9225 14355 17 N F
9227 8137 9225 8132 9227 8128 9230 8124 9234 8122 9238 8119
9242 8117 9246 8117 9250 8119 9254 8121 9259 8122 9263 8123
9259 8125 9255 8127 9254 8131 9252 8135 9250 8139 9246 8140
9243 8143 9238 8144 9234 8143 9230 8140 9227 8137 23 N F
9251 14419 9247 14415 9251 14412 9254 14414 9256 14418 9260 14419
9264 14420 9266 14424 9266 14428 9262 14430 9258 14429 9254 14427
9252 14424 9251 14419 14 N F
9291 14459 9287 14456 9289 14453 9291 14450 9294 14449 9295 14446
9296 14449 9295 14452 9297 14454 9294 14454 9294 14457 9291 14459
12 N F
9291 14471 9289 14466 9291 14467 9292 14467 9295 14466 9298 14468
9301 14467 9301 14470 9299 14472 9298 14475 9295 14475 9294 14472
9291 14471 13 N F
9312 7816 9309 7812 9312 7813 9313 7810 9316 7812 9318 7815
9316 7817 9313 7819 9312 7816 9 N F
9311 14277 9309 14273 9311 14271 9315 14270 9317 14272 9317 14275
9314 14277 9311 14277 8 N F
9318 5482 9316 5477 9320 5475 9324 5477 9327 5481 9330 5484
9334 5487 9335 5491 9332 5494 9328 5495 9324 5493 9320 5490
9321 5486 9318 5482 14 N F
9318 7811 9316 7804 9320 7803 9324 7805 9327 7809 9331 7808
9329 7812 9326 7815 9322 7814 9318 7811 10 N F

9413 15102 9403 15101 9405 15094 9404 15088 9410 15085 9417 15083
9423 15085 9430 15084 9434 15079 9436 15073 9440 15079 9445 15082
9449 15088 9454 15092 9457 15092 9461 15093 9468 15093 9473 15089
9479 15086 9486 15085 9493 15088 9497 15093 9502 15098 9508 15099
9511 15105 9515 15111 9522 15109 9527 15113 9530 15119 9532 15126
9531 15132 9531 15139 9527 15145 9526 15151 9519 15153 9513 15156
9507 15159 9501 15162 9495 15162 9499 15157 9499 15150 9496 15145
9489 15142 9483 15142 9478 15137 9472 15134 9465 15134 9458 15135
9452 15133 9446 15131 9439 15130 9433 15134 9431 15128 9431 15121
9426 15117 9420 15113 9417 15107 9413 15102 58 N F
9421 15022 9416 15017 9422 15014 9429 15012 9433 15017 9435 15024
9440 15028 9445 15033 9449 15039 9450 15045 9456 15049 9462 15049
9469 15049 9471 15042 9467 15038 9462 15032 9458 15027 9455 15021
9452 15015 9459 15013 9465 15011 9472 15010 9477 15006 9484 15007
9490 15010 9490 15017 9494 15022 9497 15029 9497 15035 9494 15042
9488 15046 9482 15049 9489 15048 9496 15048 9502 15050 9500 15059
9497 15065 9491 15067 9485 15069 9478 15071 9472 15070 9466 15066
9462 15071 9457 15075 9450 15074 9446 15068 9443 15062 9443 15055
9439 15050 9432 15049 9425 15049 9423 15042 9425 15036 9419 15035
9422 15029 9421 15022 56 N F
9417 8745 9417 8741 9418 8738 9420 8735 9422 8731 9426 8730
9428 8728 9430 8725 9433 8723 9436 8722 9439 8721 9442 8723
9442 8727 9442 8730 9439 8732 9438 8735 9436 8738 9433 8740
9430 8741 9427 8742 9424 8743 9420 8744 9417 8745 23 N F
9476 8719 9470 8718 9474 8717 9476 8715 9479 8713 9482 8711
9485 8709 9487 8707 9490 8706 9494 8704 9497 8703 9500 8704
9503 8705 9506 8707 9508 8710 9510 8712 9510 8716 9509 8719
9506 8720 9502 8721 9499 8722 9496 8722 9492 8722 9489 8721
9486 8721 9482 8721 9479 8721 9476 8719 28 N F
9546 10862 9538 10853 9542 10848 9548 10843 9553 10839 9558 10834
9562 10828 9566 10823 9571 10818 9578 10821 9584 10818 9590 10815
9595 10810 9600 10806 9606 10802 9611 10797 9616 10792 9621 10787
9626 10783 9632 10780 9638 10776 9645 10774 9649 10768 9655 10765
9662 10764 9668 10762 9673 10757 9680 10755 9686 10752 9692 10749
9699 10747 9705 10745 9711 10740 9717 10737 9723 10734 9728 10729
9735 10728 9742 10726 9747 10722 9753 10718 9756 10717 9760 10716
9767 10715 9774 10716 9780 10715 9787 10713 9794 10711 9800 10708
9806 10706 9813 10702 9819 10699 9825 10697 9832 10695 9838 10692
9845 10691 9852 10689 9859 10688 9860 10688 9866 10688 9872 10691
9878 10694 9881 10700 9885 10706 9891 10710 9892 10717 9893 10723
9894 10730 9892 10737 9886 10740 9880 10745 9874 10747 9867 10748
9860 10748 9853 10749 9847 10752 9840 10753 9834 10757 9828 10760
9823 10765 9817 10768 9811 10771 9805 10775 9798 10777 9792 10779

9785 10781 9779 10783 9772 10785 9765 10787 9758 10789 9753 10792
9746 10795 9742 10801 9739 10807 9734 10812 9728 10816 9723 10821
9717 10823 9710 10826 9704 10828 9698 10832 9691 10834 9685 10836
9678 10838 9672 10842 9668 10847 9665 10853 9662 10860 9658 10865
9655 10871 9650 10876 9645 10881 9639 10885 9633 10888 9626 10890
9619 10891 9613 10893 9606 10893 9599 10892 9592 10891 9585 10889
9579 10887 9572 10884 9567 10879 9562 10875 9557 10870 9551 10866
9546 10862 127 N F
9561 8653 9559 8649 9561 8646 9564 8649 9568 8651 9572 8653
9576 8655 9579 8652 9583 8650 9583 8655 9585 8656 9587 8657
9590 8661 9593 8664 9598 8664 9594 8666 9590 8664 9586 8663
9582 8661 9577 8662 9575 8665 9571 8669 9567 8669 9567 8665
9563 8662 9562 8658 9561 8653 27 N F
9569 8713 9567 8708 9572 8710 9576 8711 9581 8711 9585 8713
9589 8715 9593 8713 9592 8709 9593 8705 9597 8707 9601 8708
9606 8709 9610 8711 9614 8712 9617 8715 9622 8717 9618 8719
9614 8722 9611 8725 9608 8728 9605 8730 9601 8728 9596 8728
9592 8729 9588 8726 9584 8725 9585 8729 9580 8729 9579 8724
9576 8722 9572 8720 9568 8717 9569 8713 34 N F
9585 8438 9580 8435 9583 8431 9587 8429 9590 8426 9591 8430
9590 8434 9594 8432 9598 8431 9602 8429 9607 8429 9610 8426
9610 8430 9607 8434 9603 8435 9600 8439 9597 8442 9592 8444
9589 8447 9585 8447 9588 8444 9592 8442 9587 8442 9585 8438
24 N F
9589 8616 9586 8614 9589 8613 9592 8612 9594 8613 9597 8615
9598 8617 9597 8619 9596 8621 9593 8623 9591 8621 9590 8619
9589 8616 13 N F
9596 8642 9593 8637 9598 8636 9602 8635 9607 8636 9611 8635
9615 8637 9619 8637 9622 8641 9626 8638 9630 8637 9634 8635
9638 8636 9640 8640 9639 8644 9639 8649 9635 8651 9639 8654
9641 8649 9644 8653 9647 8656 9644 8660 9645 8664 9641 8665
9639 8661 9634 8659 9630 8660 9626 8658 9629 8654 9627 8650
9623 8650 9619 8649 9616 8653 9613 8656 9611 8652 9607 8649
9604 8646 9600 8644 9596 8642 39 N F
9618 8816 9608 8814 9611 8807 9611 8800 9614 8794 9618 8788
9623 8783 9622 8776 9616 8773 9622 8769 9621 8762 9627 8764
9634 8764 9641 8767 9641 8768 9647 8770 9654 8772 9653 8779
9660 8777 9665 8772 9668 8779 9662 8783 9664 8790 9670 8786
9676 8789 9678 8783 9685 8784 9681 8790 9687 8793 9688 8800
9681 8800 9675 8804 9671 8810 9672 8817 9670 8823 9667 8830
9670 8837 9674 8842 9681 8843 9679 8849 9673 8854 9667 8857
9661 8861 9654 8860 9648 8858 9643 8852 9642 8845 9644 8839
9638 8835 9633 8829 9628 8825 9622 8821 9618 8816 53 N F

9633 8735 9631 8731 9634 8728 9636 8723 9640 8722 9641 8723
9643 8726 9647 8729 9651 8731 9655 8732 9658 8733 9659 8734
9663 8736 9659 8736 9654 8736 9650 8738 9646 8738 9641 8739
9637 8737 9633 8735 20 N F
9652 8530 9649 8527 9651 8527 9652 8525 9654 8525 9656 8526
9658 8527 9659 8529 9661 8530 9661 8531 9662 8532 9663 8534
9662 8536 9660 8535 9658 8536 9656 8534 9656 8531 9654 8530
9652 8530 19 N F
9657 8524 9655 8522 9657 8520 9659 8522 9660 8523 9661 8525
9661 8528 9659 8528 9657 8526 9657 8524 10 N F
9672 14755 9668 14752 9669 14749 9669 14745 9671 14743 9674 14744
9676 14747 9676 14750 9674 14753 9675 14756 9675 14759 9672 14760
9670 14757 9672 14755 14 N F
9756 10676 9747 10671 9753 10668 9759 10666 9765 10662 9772 10659
9777 10654 9783 10651 9789 10648 9795 10645 9800 10640 9806 10637
9813 10634 9818 10630 9825 10627 9828 10621 9832 10616 9836 10610
9842 10606 9849 10608 9849 10615 9848 10622 9845 10628 9841 10634
9836 10639 9832 10644 9826 10648 9821 10653 9815 10655 9809 10659
9803 10662 9796 10664 9790 10667 9783 10668 9777 10671 9770 10672
9763 10674 9756 10676 38 N F
9792 8796 9791 8793 9791 8789 9794 8790 9797 8792 9798 8795
9800 8798 9803 8798 9806 8800 9807 8803 9806 8806 9802 8805
9800 8802 9798 8800 9795 8798 9792 8796 16 N F
9795 9396 9793 9394 9796 9392 9799 9390 9801 9388 9805 9388
9808 9389 9811 9389 9815 9390 9816 9393 9815 9397 9814 9399
9810 9400 9808 9398 9808 9395 9805 9393 9801 9394 9799 9396
9795 9396 19 N F
9831 9043 9829 9038 9831 9035 9834 9034 9837 9032 9840 9030
9842 9033 9841 9036 9842 9038 9843 9039 9846 9039 9846 9043
9843 9044 9843 9047 9842 9050 9840 9053 9837 9054 9834 9053
9833 9050 9832 9047 9831 9043 21 N F
9842 9027 9838 9024 9839 9021 9842 9019 9845 9018 9848 9020
9850 9022 9849 9025 9845 9026 9842 9027 10 N F
9860 10641 9851 10640 9854 10634 9857 10628 9859 10621 9860 10614
9862 10608 9867 10603 9873 10600 9879 10596 9885 10598 9889 10604
9893 10610 9896 10616 9894 10623 9891 10629 9885 10633 9879 10636
9873 10639 9866 10639 9860 10641 21 N F
9877 14932 9866 14927 9870 14922 9869 14915 9874 14910 9879 14906
9885 14910 9888 14916 9890 14922 9895 14918 9901 14917 9901 14923
9899 14930 9896 14936 9898 14942 9905 14945 9898 14945 9893 14940
9887 14938 9880 14938 9877 14932 21 N F
9913 5703 9905 5701 9908 5697 9910 5691 9913 5686 9919 5684
9922 5679 9928 5680 9927 5686 9928 5691 9930 5697 9929 5703

9924 5705 9918 5705 9913 5703 15 N F
9908 9393 9905 9386 9907 9382 9907 9382 9912 9382 9916 9382
9920 9380 9923 9379 9924 9379 9928 9376 9930 9372 9934 9369
9938 9370 9942 9369 9946 9371 9950 9373 9948 9377 9952 9377
9953 9379 9955 9381 9958 9384 9955 9387 9950 9388 9947 9390
9948 9395 9946 9398 9942 9396 9937 9396 9933 9395 9929 9395
9924 9396 9921 9399 9917 9398 9913 9396 9910 9399 9906 9397
9908 9393 37 N F
9908 9147 9906 9143 9909 9142 9912 9141 9915 9139 9917 9136
9920 9136 9923 9138 9923 9142 9924 9145 9926 9147 9928 9150
9925 9150 9923 9147 9920 9145 9918 9143 9914 9142 9912 9145
9913 9148 9910 9149 9907 9150 9908 9147 22 N F
9911 14976 9907 14974 9911 14974 9914 14972 9916 14971 9916 14971
9918 14968 9921 14969 9925 14969 9922 14972 9920 14973 9916 14973
9915 14976 9911 14976 14 N F
9911 14954 9908 14951 9912 14951 9914 14949 9917 14951 9920 14952
9921 14955 9919 14958 9916 14958 9914 14956 9911 14954 11 N F
9915 9365 9909 9359 9913 9358 9918 9358 9917 9354 9921 9353
9924 9350 9928 9347 9931 9350 9931 9354 9933 9358 9930 9362
9933 9365 9931 9368 9927 9371 9923 9372 9918 9372 9915 9369
9915 9365 19 N F
9913 9882 9909 9875 9914 9870 9916 9863 9922 9859 9929 9860
9934 9865 9940 9869 9945 9873 9951 9877 9953 9883 9951 9890
9949 9897 9948 9903 9944 9909 9937 9912 9934 9918 9930 9924
9923 9924 9916 9922 9915 9916 9912 9909 9910 9903 9910 9896
9912 9889 9913 9882 26 N F
9944 14960 9939 14957 9942 14955 9944 14953 9947 14953 9950 14952
9953 14953 9956 14953 9959 14953 9961 14956 9963 14958 9960 14960
9957 14959 9954 14959 9951 14960 9948 14960 9944 14960 17 N F
9946 14994 9940 14993 9943 14990 9944 14989 9946 14988 9951 14987
9955 14987 9958 14984 9962 14982 9966 14981 9971 14980 9975 14979
9974 14974 9977 14971 9979 14975 9979 14979 9983 14980 9987 14980
9989 14980 9992 14979 9996 14979 10000 14979 10005 14978 10009 14977
10012 14974 10015 14977 10018 14980 10022 14983 10017 14982 10013 14984
10009 14985 10005 14987 10003 14990 9999 14991 9996 14988 9992 14986
9988 14985 9984 14985 9979 14986 9975 14987 9972 14990 9969 14993
9969 14997 9966 14994 9964 14990 9960 14993 9960 14997 9956 14997
9953 14994 9950 14997 9946 14994 51 N F
9943 9192 9940 9190 9942 9187 9944 9190 9947 9192 9950 9194
9952 9196 9952 9199 9952 9203 9954 9205 9958 9204 9956 9207
9956 9210 9953 9213 9950 9213 9947 9211 9946 9208 9947 9204
9947 9201 9943 9201 9945 9198 9945 9194 9943 9192 23 N F
9943 9934 9940 9928 9944 9926 9948 9925 9952 9923 9950 9918

9955 9917 9959 9917 9959 9921 9961 9926 9963 9929 9962 9934
9965 9937 9961 9940 9958 9943 9954 9942 9950 9940 9946 9938
9943 9934 19 N F
9949 9171 9947 9166 9950 9166 9952 9163 9956 9163 9959 9165
9958 9168 9956 9170 9958 9172 9961 9175 9963 9177 9966 9178
9969 9179 9970 9182 9973 9183 9976 9183 9973 9184 9970 9185
9967 9183 9965 9180 9962 9178 9959 9179 9956 9177 9953 9176
9949 9176 9947 9174 9949 9171 27 N F
9951 9397 9949 9390 9953 9390 9957 9388 9959 9384 9962 9380
9967 9380 9970 9377 9970 9372 9970 9368 9968 9364 9965 9360
9970 9362 9970 9362 9974 9362 9978 9362 9979 9362 9983 9360
9982 9356 9980 9351 9985 9350 9988 9348 9990 9350 9992 9351
9996 9352 9994 9356 9994 9361 9995 9365 9999 9367 10003 9369
10000 9373 9996 9373 9998 9376 10002 9379 10005 9382 10004 9387
10002 9391 10002 9395 9999 9399 10002 9402 10001 9407 9998 9410
9994 9412 9990 9414 9988 9418 9985 9422 9980 9422 9977 9420
9976 9415 9972 9413 9968 9410 9965 9408 9961 9405 9957 9403
9953 9401 9951 9397 56 N F
9958 9975 9952 9972 9955 9966 9958 9963 9960 9961 9966 9957
9969 9951 9972 9944 9972 9943 9976 9939 9977 9932 9979 9925
9977 9918 9975 9912 9978 9906 9984 9902 9988 9896 9993 9891
9996 9885 9998 9878 10004 9875 10008 9878 10010 9879 10015 9884
10021 9886 10022 9886 10029 9886 10035 9886 10042 9883 10044 9880
10045 9877 10052 9877 10059 9878 10066 9879 10067 9879 10073 9877
10079 9874 10085 9871 10092 9869 10099 9867 10105 9865 10111 9861
10117 9857 10118 9857 10123 9854 10130 9855 10137 9856 10143 9859
10140 9865 10139 9872 10141 9879 10144 9885 10147 9891 10152 9896
10146 9899 10142 9905 10137 9909 10132 9914 10127 9919 10122 9923
10115 9922 10108 9923 10104 9929 10097 9932 10096 9938 10089 9938
10082 9938 10076 9941 10073 9947 10066 9946 10059 9947 10052 9948
10048 9954 10044 9959 10040 9965 10034 9969 10027 9971 10022 9967
10015 9964 10009 9962 10002 9960 9996 9962 9991 9967 9984 9961
9978 9965 9971 9966 9971 9973 9966 9978 9960 9981 9958 9975
90 N F
9958 10053 9956 10050 9957 10047 9958 10043 9960 10046 9960 10046
9963 10048 9966 10050 9966 10053 9965 10056 9967 10058 9966 10061
9968 10063 9970 10066 9971 10069 9969 10071 9966 10069 9966 10066
9964 10064 9962 10066 9960 10063 9961 10059 9960 10056 9958 10053
24 N F
9958 9834 9956 9826 9959 9823 9962 9820 9967 9819 9971 9820
9975 9821 9980 9822 9984 9824 9988 9826 9989 9830 9988 9835
9986 9838 9981 9839 9979 9843 9978 9847 9974 9849 9972 9853
9968 9852 9967 9848 9962 9846 9959 9843 9958 9839 9958 9834

24 N F
9968 9038 9965 9034 9968 9035 9971 9035 9969 9032 9972 9030
9970 9028 9968 9025 9969 9022 9973 9024 9975 9021 9977 9023
9979 9025 9982 9024 9982 9027 9983 9030 9985 9033 9986 9034
9987 9035 9984 9036 9981 9036 9978 9038 9977 9041 9978 9044
9975 9044 9971 9043 9970 9040 9968 9038 28 N F
9969 8999 9965 8996 9968 8997 9971 8998 9973 9001 9976 8999
9978 9001 9981 9003 9984 9004 9986 9006 9988 9009 9986 9012
9983 9011 9979 9011 9977 9009 9973 9008 9971 9005 9971 9002
9969 8999 19 N F
9979 9766 9978 9762 9979 9758 9979 9753 9982 9750 9986 9750
9991 9750 9995 9750 9997 9754 10000 9758 10001 9762 10005 9765
10006 9769 10009 9773 10010 9777 10007 9781 10003 9782 9999 9780
9993 9777 9989 9774 9986 9772 9982 9769 9979 9766 23 N F
9984 9162 9980 9158 9982 9156 9983 9153 9986 9154 9988 9157
9991 9158 9993 9161 9990 9163 9987 9162 9984 9162 11 N F
9984 14951 9981 14948 9983 14946 9986 14948 9989 14948 9991 14951
9991 14954 9989 14957 9988 14954 9984 14955 9984 14951 11 N F
9991 9986 9987 9982 9990 9983 9994 9982 9995 9979 9998 9977
9995 9975 9993 9972 9997 9972 9999 9974 10002 9976 10004 9978
10007 9980 10009 9983 10011 9985 10009 9988 10009 9991 10007 9994
10004 9993 10001 9991 10001 9988 9999 9991 9995 9992 9993 9990
9994 9987 9991 9986 26 N F
9995 10240 9991 10235 9994 10233 9996 10231 9999 10232 10001 10235
10002 10238 10004 10240 10001 10239 9998 10241 9997 10238 9995 10240
12 N F
10001 10212 9996 10209 9998 10207 10001 10207 10003 10204 10005 10201
10007 10202 10008 10202 10010 10205 10010 10208 10012 10211 10015 10212
10012 10213 10009 10214 10006 10214 10004 10211 10001 10212 17 N F
10003 14965 9997 14961 10000 14958 10004 14958 10007 14961 10011 14960
10016 14962 10020 14962 10024 14962 10028 14961 10028 14957 10026 14953
10024 14949 10024 14945 10027 14948 10031 14951 10034 14954 10038 14954
10038 14958 10042 14959 10045 14960 10046 14961 10050 14963 10046 14965
10044 14969 10040 14971 10042 14975 10039 14972 10036 14968 10032 14970
10028 14970 10024 14969 10020 14968 10016 14966 10011 14966 10007 14966
10003 14965 37 N F
10002 9741 9999 9738 10001 9735 10000 9732 10001 9729 10001 9725
10004 9727 10006 9730 10008 9732 10009 9735 10011 9738 10014 9739
10014 9742 10013 9746 10010 9747 10007 9745 10004 9744 10002 9741
18 N F
10003 9857 10002 9853 10003 9850 10003 9846 10006 9845 10009 9845
10012 9847 10013 9850 10015 9853 10015 9856 10014 9859 10011 9861
10008 9861 10006 9859 10003 9857 15 N F

10018 9415 10017 9408 10021 9409 10025 9409 10027 9413 10030 9417
10033 9414 10038 9414 10041 9417 10044 9420 10040 9422 10035 9421
10031 9423 10032 9427 10036 9429 10033 9432 10036 9436 10031 9436
10028 9439 10026 9435 10027 9431 10025 9427 10028 9425 10024 9423
10022 9419 10017 9419 10018 9415 27 N F
10023 9736 10018 9724 10025 9724 10028 9729 10028 9730 10035 9730
10041 9725 10048 9725 10049 9719 10055 9715 10061 9712 10061 9705
10061 9698 10068 9698 10070 9705 10073 9699 10079 9702 10081 9695
10088 9694 10091 9700 10092 9707 10096 9713 10092 9718 10089 9725
10083 9727 10078 9732 10071 9734 10065 9738 10061 9743 10057 9748
10058 9755 10056 9762 10052 9767 10047 9763 10040 9760 10033 9760
10028 9756 10027 9749 10026 9742 10023 9736 40 N F
10023 10051 10018 10042 10024 10039 10031 10036 10036 10032 10042 10029
10048 10025 10055 10023 10062 10023 10064 10023 10069 10023 10072 10022
10075 10021 10082 10018 10088 10016 10086 10023 10083 10029 10088 10034
10090 10041 10086 10047 10090 10052 10095 10057 10088 10057 10084 10062
10085 10069 10079 10072 10072 10070 10066 10073 10065 10066 10059 10063
10052 10062 10047 10057 10040 10059 10033 10059 10029 10054 10023 10051
36 N F
10021 9880 10020 9877 10021 9874 10020 9871 10022 9868 10025 9869
10028 9871 10031 9872 10029 9875 10027 9878 10025 9880 10021 9880
12 N F
10041 14863 10036 14860 10039 14857 10043 14854 10039 14852 10042 14849
10046 14847 10050 14846 10054 14845 10058 14843 10062 14846 10061 14850
10057 14851 10056 14855 10053 14858 10049 14856 10046 14859 10045 14864
10041 14863 19 N F
10040 9868 10039 9864 10041 9860 10044 9861 10047 9862 10047 9865
10048 9868 10047 9871 10044 9872 10041 9871 10040 9868 11 N F
10054 14965 10051 14964 10054 14963 10057 14963 10059 14965 10057 14967
10054 14965 7 N F
10056 9862 10051 9858 10052 9857 10053 9855 10057 9855 10060 9855
10063 9854 10066 9856 10068 9858 10067 9861 10065 9864 10062 9865
10059 9864 10056 9862 14 N F
10053 9987 10052 9977 10055 9970 10059 9965 10066 9967 10071 9973
10072 9980 10078 9982 10083 9987 10077 9991 10070 9994 10064 9996
10058 9992 10053 9987 14 N F
10059 9871 10054 9867 10057 9866 10060 9868 10062 9867 10063 9867
10067 9868 10070 9866 10072 9863 10074 9861 10077 9862 10078 9866
10077 9869 10074 9871 10071 9873 10069 9876 10067 9877 10064 9876
10062 9873 10059 9871 20 N F
10062 14963 10057 14960 10060 14959 10062 14957 10064 14959 10067 14960
10071 14959 10073 14961 10071 14963 10068 14963 10065 14962 10062 14963
12 N F

10069 10014 10065 10010 10066 10008 10069 10007 10071 10005 10074 10004
10076 10005 10077 10008 10077 10011 10075 10012 10072 10014 10069 10014
12 N F
10072 14881 10067 14880 10069 14878 10072 14876 10075 14878 10078 14878
10081 14876 10083 14879 10080 14881 10078 14883 10075 14881 10072 14881
12 N F
10084 14868 10083 14863 10085 14861 10088 14863 10091 14864 10093 14867
10091 14870 10088 14871 10087 14868 10084 14868 10 N F
10091 9564 10089 9560 10090 9557 10093 9559 10097 9558 10099 9561
10101 9563 10102 9566 10105 9568 10108 9569 10111 9570 10110 9573
10107 9572 10104 9574 10101 9571 10098 9570 10098 9567 10097 9564
10094 9562 10091 9564 20 N F
10096 9724 10094 9721 10097 9720 10100 9718 10103 9717 10104 9717
10106 9715 10109 9713 10112 9711 10114 9709 10115 9712 10119 9713
10122 9712 10125 9712 10127 9710 10130 9707 10130 9711 10132 9714
10129 9715 10125 9715 10122 9714 10119 9716 10116 9717 10113 9718
10110 9720 10107 9720 10106 9724 10104 9726 10101 9726 10099 9728
10095 9728 10096 9724 32 N F
10096 10177 10095 10173 10098 10172 10101 10173 10102 10176 10104 10179
10101 10181 10098 10179 10096 10177 9 N F
10100 6965 10096 6965 10098 6964 10099 6962 10101 6961 10103 6960
10105 6961 10108 6961 10110 6960 10112 6961 10112 6963 10111 6964
10109 6966 10108 6968 10106 6969 10104 6970 10102 6969 10101 6967
10100 6965 19 N F
10102 9763 10097 9758 10099 9757 10101 9757 10104 9753 10106 9749
10109 9746 10112 9743 10116 9741 10120 9738 10124 9739 10128 9738
10132 9736 10135 9739 10140 9739 10144 9739 10145 9743 10140 9742
10140 9747 10136 9748 10132 9745 10128 9746 10123 9746 10119 9748
10116 9751 10112 9754 10115 9757 10117 9761 10113 9763 10110 9766
10105 9766 10102 9763 32 N F
10103 14875 10097 14877 10099 14875 10101 14872 10102 14869 10105 14868
10105 14868 10107 14870 10108 14873 10106 14875 10103 14875 11 N F
10104 9800 10099 9792 10105 9789 10110 9785 10110 9785 10114 9779
10119 9774 10125 9770 10130 9765 10137 9764 10142 9760 10149 9763
10145 9769 10141 9775 10142 9782 10136 9786 10142 9790 10137 9795
10131 9798 10124 9800 10118 9802 10111 9800 10104 9800 23 N F
10103 10245 10100 10242 10101 10241 10103 10241 10105 10242 10106 10243
10109 10245 10108 10248 10105 10249 10106 10246 10103 10245 11 N F
10105 10038 10102 10028 10104 10021 10110 10024 10116 10027 10123 10028
10130 10029 10126 10035 10119 10037 10112 10037 10105 10038 11 N F
10116 10148 10109 10149 10111 10143 10110 10136 10115 10131 10113 10124
10120 10121 10126 10118 10133 10117 10131 10124 10138 10125 10143 10129
10149 10125 10156 10126 10163 10124 10169 10124 10170 10124 10176 10120

10181 10120 10183 10120 10180 10127 10174 10130 10180 10134 10175 10140
10169 10142 10162 10142 10155 10143 10153 10150 10146 10149 10150 10143
10143 10144 10136 10146 10129 10148 10123 10146 10116 10148 35 N F
10122 10025 10112 10020 10118 10015 10122 10020 10129 10022 10136 10020
10142 10022 10149 10024 10153 10030 10156 10031 10160 10032 10165 10036
10163 10030 10165 10023 10171 10019 10177 10022 10181 10028 10178 10035
10175 10041 10169 10043 10162 10042 10155 10042 10148 10040 10141 10040
10134 10039 10133 10032 10128 10027 10122 10025 28 N F
10121 15129 10120 15125 10124 15124 10128 15124 10130 15120 10133 15117
10135 15113 10137 15113 10139 15113 10143 15114 10145 15117 10149 15120
10151 15124 10153 15127 10153 15128 10156 15131 10156 15136 10153 15139
10150 15142 10143 15145 10140 15142 10143 15139 10142 15134 10139 15138
10135 15138 10135 15142 10131 15143 10128 15140 10127 15136 10125 15132
10121 15129 31 N F
10127 10178 10124 10175 10127 10175 10130 10173 10133 10171 10136 10171
10137 10174 10137 10177 10137 10180 10137 10184 10134 10182 10134 10178
10130 10177 10127 10178 14 N F
10131 9815 10128 9810 10131 9808 10136 9806 10140 9806 10145 9807
10148 9810 10150 9810 10153 9811 10152 9815 10156 9818 10154 9822
10150 9821 10145 9820 10141 9822 10137 9821 10133 9820 10131 9815
18 N F
10146 7419 10145 7416 10147 7415 10149 7414 10151 7416 10152 7417
10152 7420 10150 7420 10148 7420 10146 7419 10 N F
10151 9859 10147 9852 10152 9848 10154 9845 10156 9842 10161 9838
10167 9834 10168 9832 10170 9828 10175 9822 10169 9818 10171 9811
10172 9804 10177 9799 10184 9798 10190 9802 10186 9807 10183 9814
10186 9821 10192 9824 10198 9827 10198 9834 10197 9841 10190 9842
10184 9845 10191 9845 10197 9847 10193 9852 10192 9859 10187 9863
10181 9859 10174 9857 10167 9859 10160 9858 10156 9864 10151 9859
36 N F
10151 14985 10148 14984 10149 14981 10152 14980 10153 14981 10155 14982
10157 14985 10153 14988 10151 14985 9 N F
10168 7337 10164 7336 10166 7334 10168 7335 10168 7337 5 N F
10170 15093 10168 15088 10170 15084 10173 15081 10176 15080 10177 15080
10181 15078 10181 15082 10181 15086 10186 15087 10183 15090 10179 15091
10174 15091 10170 15093 14 N F
10171 9884 10168 9881 10169 9877 10173 9874 10177 9874 10178 9878
10176 9883 10177 9887 10179 9890 10184 9890 10188 9891 10186 9895
10182 9897 10180 9901 10182 9904 10181 9909 10178 9905 10174 9904
10170 9903 10171 9899 10170 9895 10168 9888 10171 9884 23 N F
10176 10305 10169 10303 10175 10299 10176 10293 10175 10286 10170 10278
10177 10279 10184 10279 10191 10280 10197 10283 10200 10283 10204 10283
10211 10284 10214 10283 10218 10283 10224 10285 10219 10290 10223 10295

10216 10295 10209 10294 10204 10290 10197 10288 10192 10293 10185 10295
10182 10301 10176 10305 26 N F
10195 10133 10191 10128 10195 10128 10198 10130 10201 10129 10201 10126
10204 10123 10205 10124 10207 10124 10209 10127 10206 10129 10203 10131
10203 10135 10200 10137 10197 10136 10195 10133 16 N F
10205 9862 10204 9858 10206 9855 10207 9852 10211 9852 10211 9856
10209 9859 10209 9862 10205 9862 9 N F
10210 10022 10207 10018 10211 10017 10213 10016 10217 10015 10220 10014
10221 10017 10220 10020 10217 10022 10214 10022 10210 10022 11 N F
10223 14895 10218 14889 10223 14890 10228 14888 10230 14883 10226 14885
10222 14881 10222 14875 10222 14870 10223 14865 10228 14862 10232 14867
10235 14870 10237 14871 10240 14873 10244 14877 10244 14882 10241 14886
10238 14891 10235 14896 10235 14901 10233 14896 10228 14893 10223 14895
24 N F
10231 10264 10230 10257 10234 10256 10238 10254 10243 10254 10240 10258
10244 10260 10247 10263 10244 10266 10241 10269 10237 10271 10234 10267
10231 10264 13 N F
10257 10356 10253 10352 10256 10352 10260 10352 10262 10351 10265 10350
10266 10353 10263 10355 10260 10355 10257 10356 10 N F
10270 10354 10269 10351 10272 10349 10274 10351 10277 10353 10275 10355
10272 10357 10270 10354 8 N F
10285 10371 10281 10369 10284 10368 10286 10366 10290 10366 10290 10366
10293 10366 10295 10369 10298 10369 10299 10372 10295 10373 10292 10371
10290 10373 10287 10375 10284 10377 10284 10373 10285 10371 17 N F
10290 10015 10286 10011 10288 10009 10289 10006 10291 10003 10294 10002
10295 10005 10294 10008 10293 10011 10293 10014 10290 10015 11 N F
10309 10013 10304 10011 10306 10009 10308 10006 10311 10005 10311 10002
10312 9999 10314 10002 10314 10005 10313 10009 10311 10011 10309 10013
12 N F
10321 10015 10317 10011 10319 10008 10321 10005 10324 10003 10324 10007
10322 10010 10323 10013 10321 10015 9 N F
10329 10001 10325 9996 10327 9994 10329 9994 10331 9996 10330 9998
10329 10001 7 N F
10330 10298 10327 10294 10329 10291 10329 10290 10332 10294 10336 10294
10339 10291 10343 10289 10342 10293 10342 10298 10343 10302 10344 10306
10340 10306 10336 10304 10332 10302 10330 10298 16 N F
10340 10341 10334 10339 10337 10336 10340 10338 10340 10338 10343 10340
10340 10341 7 N F
10351 9979 10347 9978 10348 9974 10350 9971 10353 9971 10356 9971
10360 9971 10359 9974 10357 9977 10354 9979 10351 9979 11 N F
10356 10309 10350 10303 10351 10302 10353 10299 10357 10299 10361 10300
10363 10296 10366 10292 10367 10297 10366 10301 10364 10305 10359 10306
10356 10309 13 N F

10430 15272 10429 15268 10433 15268 10435 15270 10438 15270 10441 15272
10445 15271 10448 15272 10446 15274 10444 15277 10441 15278 10438 15277
10436 15275 10433 15274 10430 15272 15 N F
10637 6766 10632 6764 10636 6761 10640 6761 10645 6760 10649 6758
10654 6758 10658 6759 10662 6761 10667 6760 10671 6759 10675 6761
10679 6764 10682 6766 10685 6770 10689 6773 10685 6775 10681 6777
10676 6778 10672 6780 10666 6778 10661 6779 10657 6777 10653 6776
10649 6778 10644 6776 10643 6772 10638 6770 10637 6766 29 N F
10811 15190 10808 15184 10812 15184 10814 15188 10817 15191 10821 15190
10822 15189 10824 15187 10827 15190 10827 15195 10831 15193 10832 15189
10836 15187 10840 15186 10844 15188 10847 15192 10848 15196 10851 15199
10852 15203 10848 15205 10844 15207 10840 15208 10839 15204 10835 15204
10830 15204 10827 15201 10824 15203 10820 15201 10820 15197 10818 15194
10813 15193 10811 15190 32 N F
10812 15136 10811 15131 10814 15131 10813 15128 10816 15125 10819 15126
10822 15126 10824 15129 10824 15133 10821 15135 10820 15138 10820 15141
10817 15139 10815 15137 10812 15136 15 N F
10820 15162 10820 15158 10820 15158 10822 15157 10823 15158 10824 15159
10826 15161 10824 15163 10822 15164 10820 15162 10 N F
10821 15151 10820 15146 10823 15145 10826 15145 10825 15148 10826 15151
10823 15153 10821 15151 8 N F
10848 15183 10846 15179 10848 15175 10846 15170 10850 15167 10853 15170
10857 15171 10857 15176 10859 15179 10856 15182 10858 15186 10854 15188
10850 15186 10848 15183 14 N F
10882 7539 10880 7536 10882 7534 10884 7534 10886 7535 10888 7536
10890 7537 10892 7537 10894 7537 10896 7538 10899 7538 10898 7540
10897 7542 10896 7543 10893 7543 10891 7544 10889 7544 10887 7544
10885 7542 10883 7541 10882 7539 21 N F
10892 14340 10886 14336 10888 14332 10891 14330 10892 14330 10895 14327
10895 14322 10897 14318 10899 14315 10902 14311 10906 14312 10911 14312
10915 14311 10919 14312 10922 14309 10926 14311 10927 14315 10929 14319
10927 14323 10923 14323 10921 14327 10923 14331 10927 14333 10931 14331
10929 14327 10932 14328 10933 14328 10936 14331 10941 14330 10938 14334
10943 14334 10940 14337 10938 14341 10937 14345 10933 14347 10928 14347
10924 14347 10920 14345 10916 14344 10911 14343 10908 14341 10904 14339
10900 14337 10896 14339 10892 14340 45 N F
10908 15175 10903 15169 10908 15169 10911 15173 10914 15175 10918 15175
10922 15177 10925 15180 10927 15184 10931 15186 10934 15189 10935 15194
10939 15194 10938 15198 10942 15199 10946 15198 10948 15200 10949 15201
10952 15204 10951 15209 10946 15208 10942 15207 10945 15211 10941 15212
10944 15215 10940 15216 10942 15220 10939 15222 10938 15226 10941 15230
10945 15229 10945 15233 10941 15235 10938 15238 10933 15238 10929 15239
10926 15236 10922 15234 10926 15231 10925 15227 10921 15225 10924 15222

10925 15218 10921 15215 10917 15213 10916 15209 10911 15210 10908 15207
10905 15203 10904 15199 10908 15201 10912 15202 10916 15203 10920 15202
10918 15198 10913 15197 10915 15193 10919 15192 10920 15187 10918 15183
10914 15184 10909 15184 10909 15179 10908 15175 64 N F
10920 14258 10915 14258 10918 14256 10921 14255 10924 14254 10924 14251
10924 14248 10924 14248 10926 14249 10929 14251 10932 14250 10936 14251
10938 14253 10940 14256 10940 14259 10938 14262 10938 14265 10940 14268
10942 14269 10941 14272 10938 14274 10935 14273 10932 14275 10932 14272
10929 14269 10926 14268 10925 14265 10923 14262 10923 14259 10920 14258
30 N F
10924 7534 10922 7532 10923 7531 10925 7529 10926 7530 10925 7532
10924 7534 7 N F
10934 14642 10928 14640 10931 14637 10935 14636 10939 14637 10937 14641
10938 14645 10942 14642 10946 14642 10949 14645 10952 14642 10956 14640
10959 14638 10963 14636 10967 14634 10972 14634 10972 14638 10975 14635
10979 14635 10982 14638 10979 14642 10977 14646 10975 14649 10971 14651
10971 14656 10967 14656 10963 14656 10959 14658 10955 14660 10951 14661
10948 14664 10947 14660 10944 14657 10942 14653 10938 14652 10933 14651
10933 14647 10934 14642 38 N F
10947 14232 10945 14227 10948 14227 10950 14230 10953 14232 10956 14233
10959 14232 10959 14232 10962 14232 10962 14235 10964 14238 10960 14238
10957 14238 10954 14237 10951 14237 10950 14234 10947 14232 17 N F
10958 7636 10957 7633 10959 7632 10961 7631 10963 7632 10964 7634
10963 7636 10962 7637 10959 7638 10958 7636 10 N F
10969 6638 10966 6636 10968 6636 10971 6637 10973 6638 10975 6639
10973 6640 10970 6640 10969 6638 9 N F
11011 14711 11011 14706 11014 14703 11016 14707 11020 14709 11024 14711
11025 14715 11026 14719 11028 14723 11028 14727 11026 14723 11019 14724
11018 14728 11015 14732 11014 14727 11015 14723 11012 14719 11015 14716
11019 14716 11018 14712 11015 14714 11011 14711 22 N F
11030 6710 11018 6707 11022 6702 11026 6696 11033 6693 11040 6693
11047 6692 11049 6699 11055 6703 11061 6698 11067 6694 11074 6693
11081 6693 11088 6692 11094 6691 11095 6691 11102 6692 11109 6693
11116 6691 11122 6687 11129 6685 11135 6688 11135 6695 11131 6701
11124 6705 11131 6702 11138 6700 11144 6696 11145 6704 11142 6710
11145 6717 11148 6723 11155 6721 11157 6728 11155 6735 11159 6741
11161 6748 11154 6750 11148 6755 11143 6759 11136 6761 11129 6762
11127 6769 11121 6765 11114 6769 11107 6768 11101 6765 11096 6760
11091 6766 11089 6772 11085 6778 11079 6782 11072 6785 11066 6781
11072 6776 11074 6769 11077 6763 11071 6766 11072 6773 11065 6771
11061 6765 11056 6759 11059 6752 11058 6745 11062 6739 11067 6734
11068 6727 11065 6733 11063 6727 11062 6720 11065 6713 11064 6706
11063 6713 11058 6718 11052 6722 11051 6729 11045 6725 11039 6728

11040 6721 11043 6715 11037 6719 11031 6722 11027 6716 11030 6710
84 N F
11059 14266 11058 14263 11061 14261 11064 14262 11067 14261 11070 14261
11071 14264 11072 14265 11073 14267 11074 14270 11077 14268 11078 14271
11078 14274 11079 14277 11079 14281 11076 14280 11073 14280 11069 14280
11066 14279 11069 14277 11072 14276 11071 14273 11071 14269 11068 14270
11065 14270 11062 14272 11062 14269 11059 14266 28 N F
11077 6571 11076 6566 11078 6562 11083 6562 11088 6561 11092 6562
11097 6561 11101 6563 11104 6567 11106 6571 11103 6574 11099 6576
11094 6576 11090 6576 11085 6574 11081 6572 11077 6571 17 N F
11122 14265 11117 14262 11121 14261 11124 14261 11124 14257 11123 14254
11126 14256 11127 14259 11130 14261 11133 14263 11135 14265 11138 14267
11137 14270 11140 14269 11143 14271 11141 14273 11141 14276 11138 14275
11134 14275 11131 14273 11129 14272 11127 14269 11124 14268 11122 14265
24 N F
11157 14243 11152 14241 11156 14241 11159 14239 11161 14237 11163 14235
11166 14236 11163 14238 11165 14241 11163 14243 11160 14244 11157 14243
12 N F
11157 14216 11155 14213 11157 14211 11160 14209 11162 14210 11163 14210
11165 14212 11169 14211 11171 14213 11174 14215 11176 14217 11178 14220
11180 14223 11181 14224 11182 14225 11184 14227 11188 14229 11190 14230
11193 14232 11194 14235 11196 14238 11196 14238 11198 14240 11200 14243
11202 14246 11203 14249 11205 14251 11207 14254 11204 14253 11201 14253
11198 14252 11195 14250 11193 14247 11190 14246 11187 14246 11185 14244
11182 14243 11180 14241 11181 14237 11182 14235 11183 14231 11183 14228
11180 14230 11180 14233 11178 14235 11175 14237 11172 14236 11169 14235
11171 14232 11171 14229 11168 14227 11167 14224 11165 14222 11163 14219
11160 14218 11157 14216 56 N F
11159 6668 11156 6665 11159 6662 11162 6661 11165 6662 11165 6666
11162 6668 11159 6668 8 N F
11166 6676 11164 6670 11168 6671 11170 6674 11172 6676 11173 6680
11170 6681 11168 6679 11166 6676 9 N F
11172 6734 11168 6734 11170 6733 11172 6733 11175 6732 11178 6733
11182 6732 11185 6733 11188 6730 11191 6729 11194 6730 11196 6733
11192 6736 11189 6737 11188 6740 11188 6744 11185 6746 11181 6746
11178 6746 11175 6744 11174 6741 11176 6739 11175 6736 11172 6734
24 N F
11193 6687 11192 6684 11192 6681 11194 6678 11196 6675 11198 6672
11201 6670 11204 6670 11207 6672 11210 6674 11212 6677 11213 6680
11213 6683 11211 6686 11207 6686 11204 6686 11201 6687 11196 6689
11193 6687 19 N F
11229 6801 11225 6794 11231 6789 11237 6791 11237 6791 11243 6796
11250 6797 11255 6792 11259 6786 11264 6781 11263 6773 11260 6767

11253 6765 11249 6759 11254 6754 11261 6752 11268 6750 11273 6745
11280 6741 11287 6740 11293 6743 11298 6748 11299 6755 11306 6754
11312 6750 11319 6753 11322 6759 11323 6766 11323 6773 11323 6780
11324 6787 11323 6795 11319 6801 11323 6807 11319 6813 11319 6820
11323 6826 11328 6831 11333 6837 11333 6844 11338 6849 11342 6855
11345 6861 11343 6868 11347 6874 11352 6879 11357 6884 11358 6891
11357 6898 11358 6905 11351 6905 11344 6906 11343 6899 11342 6892
11338 6886 11334 6880 11331 6873 11336 6869 11333 6862 11332 6855
11330 6848 11323 6850 11316 6848 11313 6841 11306 6839 11306 6832
11303 6825 11305 6818 11299 6814 11292 6814 11285 6813 11279 6809
11272 6809 11265 6807 11263 6814 11259 6820 11252 6823 11252 6816
11250 6809 11246 6803 11241 6807 11233 6806 11229 6801 83 N F
11235 6735 11234 6731 11235 6728 11238 6726 11241 6725 11244 6728
11246 6730 11246 6734 11245 6737 11243 6739 11240 6741 11237 6742
11235 6739 11235 6735 14 N F
11279 12233 11273 12233 11277 12230 11282 12230 11286 12228 11289 12225
11290 12221 11293 12218 11297 12219 11301 12220 11303 12224 11300 12226
11296 12229 11293 12233 11292 12237 11293 12241 11288 12241 11286 12245
11282 12245 11279 12241 11279 12237 11279 12233 22 N F
11291 12571 11288 12567 11289 12562 11292 12559 11293 12563 11295 12567
11300 12567 11301 12571 11303 12575 11304 12580 11302 12584 11298 12586
11298 12581 11296 12577 11294 12573 11291 12571 16 N F
11349 12434 11345 12433 11347 12431 11348 12429 11349 12428 11351 12431
11349 12434 7 N F
11348 12442 11345 12437 11349 12437 11349 12438 11351 12439 11353 12442
11353 12445 11349 12445 11348 12442 9 N F
11353 12417 11348 12420 11350 12417 11352 12414 11354 12411 11355 12408
11358 12410 11361 12409 11364 12411 11363 12414 11360 12415 11357 12417
11355 12414 11353 12417 14 N F
11356 14220 11355 14217 11358 14215 11359 14212 11359 14209 11360 14206
11363 14204 11365 14206 11367 14209 11369 14212 11371 14214 11372 14217
11372 14220 11369 14221 11366 14221 11363 14219 11359 14219 11356 14220
18 N F
11373 12574 11371 12570 11374 12568 11377 12569 11379 12572 11379 12575
11376 12576 11373 12574 8 N F
11383 12581 11379 12576 11382 12576 11384 12579 11386 12581 11383 12581
6 N F
11386 12559 11383 12555 11385 12552 11388 12548 11391 12545 11395 12546
11394 12550 11395 12554 11399 12557 11401 12560 11400 12564 11404 12565
11407 12568 11409 12572 11409 12577 11412 12580 11416 12581 11414 12586
11410 12584 11407 12580 11403 12580 11402 12584 11406 12586 11402 12587
11397 12586 11393 12585 11391 12582 11391 12577 11388 12574 11387 12570
11386 12565 11383 12562 11386 12559 33 N F

11386 14292 11383 14288 11386 14287 11386 14287 11390 14288 11391 14290
11390 14293 11390 14297 11387 14295 11386 14292 10 N F
11400 12617 11396 12617 11399 12615 11402 12614 11402 12614 11400 12617
6 N F
11405 12612 11402 12611 11402 12607 11405 12606 11407 12609 11405 12612
6 N F
11410 12606 11408 12603 11410 12600 11413 12599 11414 12596 11417 12598
11416 12601 11418 12604 11421 12604 11421 12607 11418 12607 11415 12610
11412 12611 11413 12608 11410 12606 15 N F
11415 12560 11414 12557 11417 12556 11421 12557 11423 12560 11425 12562
11426 12565 11423 12566 11419 12565 11418 12562 11415 12560 11 N F
11442 12323 11439 12323 11441 12320 11443 12318 11445 12320 11448 12318
11451 12321 11447 12321 11445 12324 11442 12323 10 N F
11443 14281 11440 14279 11441 14276 11444 14277 11445 14277 11448 14277
11451 14278 11453 14281 11454 14284 11456 14286 11453 14285 11450 14284
11448 14281 11446 14283 11443 14281 15 N F
11502 13908 11501 13904 11502 13901 11505 13899 11505 13902 11506 13905
11506 13909 11509 13907 11512 13908 11511 13911 11508 13912 11505 13913
11502 13911 11502 13908 14 N F
11506 13964 11505 13961 11508 13960 11511 13962 11513 13965 11516 13964
11514 13967 11517 13969 11519 13971 11516 13972 11514 13969 11511 13968
11509 13965 11506 13964 14 N F
11521 13886 11519 13883 11521 13881 11523 13879 11526 13877 11528 13874
11531 13875 11533 13877 11533 13877 11536 13879 11533 13879 11529 13879
11526 13880 11526 13883 11524 13886 11521 13886 16 N F
11530 7007 11528 7005 11531 7003 11534 7002 11537 7001 11539 7000
11540 6999 11541 7003 11539 7006 11536 7007 11533 7009 11530 7007
12 N F
11531 6956 11528 6952 11528 6945 11530 6941 11534 6938 11538 6938
11543 6937 11547 6938 11551 6940 11555 6943 11558 6946 11558 6951
11557 6955 11553 6958 11548 6959 11544 6960 11539 6960 11535 6958
11531 6956 19 N F
11549 14246 11546 14242 11548 14240 11550 14240 11553 14240 11555 14241
11554 14243 11551 14244 11549 14246 9 N F
11553 13923 11551 13919 11552 13920 11554 13920 11557 13919 11554 13918
11553 13915 11556 13913 11559 13914 11561 13917 11563 13919 11564 13922
11563 13925 11560 13927 11557 13926 11554 13926 11553 13923 17 N F
11554 13449 11552 13451 11553 13448 11554 13446 11556 13444 11559 13443
11559 13446 11560 13448 11558 13450 11556 13448 11554 13449 11 N F
11557 13993 11556 13988 11559 13991 11560 13995 11562 13992 11563 13991
11567 13990 11568 13985 11572 13985 11576 13986 11580 13987 11582 13991
11582 13992 11585 13993 11588 13996 11586 14000 11582 14001 11578 14003
11574 14005 11570 14004 11568 14008 11564 14011 11564 14015 11560 14018

11560 14013 11558 14010 11558 14005 11556 14001 11557 13997 11557 13993
30 N F
11559 13969 11558 13965 11560 13962 11560 13961 11562 13959 11565 13959
11567 13959 11568 13959 11570 13961 11569 13964 11570 13967 11567 13967
11565 13969 11562 13971 11559 13969 15 N F
11564 14240 11560 14237 11563 14236 11566 14233 11569 14234 11572 14236
11574 14234 11577 14231 11579 14230 11582 14229 11584 14232 11585 14235
11582 14237 11580 14239 11579 14242 11578 14245 11574 14245 11573 14242
11570 14240 11567 14240 11564 14240 21 N F
11567 13389 11564 13388 11565 13386 11566 13383 11567 13381 11570 13380
11572 13381 11572 13384 11575 13384 11575 13381 11577 13383 11577 13386
11575 13387 11572 13388 11570 13389 11567 13389 16 N F
11573 13691 11570 13689 11571 13686 11573 13688 11575 13689 11578 13690
11581 13689 11583 13690 11583 13692 11584 13693 11586 13695 11585 13697
11585 13700 11582 13701 11583 13698 11581 13696 11580 13694 11577 13694
11574 13693 11573 13691 20 N F
11577 12560 11571 12560 11575 12559 11578 12555 11582 12556 11580 12552
11576 12550 11578 12547 11582 12548 11586 12551 11590 12550 11594 12553
11598 12554 11598 12558 11596 12562 11596 12566 11592 12568 11589 12564
11585 12562 11582 12559 11577 12560 21 N F
11581 14296 11576 14291 11578 14290 11580 14288 11584 14286 11588 14285
11588 14285 11592 14285 11593 14289 11597 14290 11602 14291 11603 14292
11605 14294 11609 14294 11613 14291 11616 14294 11620 14296 11624 14298
11626 14302 11628 14306 11629 14309 11630 14310 11629 14314 11626 14317
11623 14315 11618 14313 11614 14313 11610 14313 11606 14315 11603 14318
11599 14320 11595 14318 11590 14318 11587 14315 11583 14312 11583 14308
11581 14304 11583 14300 11581 14296 39 N F
11588 13442 11583 13442 11585 13440 11587 13438 11589 13437 11589 13440
11588 13442 7 N F
11588 14267 11585 14265 11588 14264 11590 14263 11593 14263 11595 14264
11598 14264 11596 14266 11593 14267 11591 14266 11588 14267 11 N F
11592 13995 11588 13992 11588 13990 11589 13987 11591 13986 11592 13986
11594 13986 11597 13985 11597 13983 11598 13980 11600 13981 11601 13984
11603 13986 11601 13988 11598 13987 11597 13989 11595 13992 11594 13994
11592 13995 19 N F
11596 14229 11591 14228 11592 14227 11593 14225 11595 14223 11597 14220
11600 14219 11603 14217 11606 14217 11609 14215 11607 14218 11605 14220
11604 14223 11605 14227 11605 14230 11602 14230 11599 14229 11596 14229
18 N F
11603 12557 11600 12561 11601 12558 11601 12555 11603 12557 5 N F
11604 13448 11602 13444 11603 13444 11604 13444 11607 13445 11609 13447
11612 13447 11614 13448 11614 13451 11612 13452 11610 13452 11607 13452
11605 13451 11604 13448 14 N F

11603 14276 11602 14274 11602 14270 11604 14269 11605 14270 11606 14271
11605 14274 11603 14276 8 N F
11607 12559 11603 12561 11604 12558 11607 12556 11610 12554 11612 12552
11611 12555 11610 12558 11607 12559 9 N F
11604 12633 11603 12628 11606 12625 11609 12622 11612 12619 11616 12617
11621 12618 11625 12620 11628 12623 11627 12627 11631 12628 11628 12631
11627 12635 11623 12637 11623 12642 11627 12643 11631 12645 11635 12645
11638 12647 11639 12647 11640 12643 11645 12643 11648 12645 11652 12646
11652 12653 11648 12652 11649 12656 11651 12660 11652 12664 11651 12668
11647 12670 11643 12668 11639 12667 11638 12663 11635 12659 11631 12659
11630 12655 11626 12653 11622 12651 11618 12650 11614 12650 11611 12654
11606 12653 11606 12648 11604 12645 11605 12641 11604 12637 11604 12633
48 N F
11616 14262 11607 14262 11610 14259 11614 14257 11617 14254 11621 14252
11625 14251 11625 14251 11629 14249 11633 14246 11633 14246 11633 14246
11636 14243 11636 14239 11635 14235 11638 14232 11641 14229 11643 14232
11647 14235 11649 14239 11653 14241 11655 14245 11655 14249 11654 14253
11651 14256 11647 14258 11643 14258 11638 14259 11635 14262 11631 14264
11628 14267 11628 14271 11628 14276 11629 14280 11632 14283 11629 14287
11625 14286 11622 14284 11617 14282 11614 14280 11615 14275 11615 14271
11615 14267 11616 14262 44 N F
11618 13975 11613 13975 11615 13973 11618 13971 11622 13972 11625 13971
11626 13974 11625 13977 11621 13976 11618 13975 10 N F
11619 12342 11613 12342 11616 12340 11618 12338 11622 12337 11621 12340
11619 12342 7 N F
11619 12615 11617 12611 11618 12606 11618 12602 11619 12599 11619 12598
11622 12594 11625 12597 11627 12593 11630 12590 11634 12592 11637 12588
11641 12587 11642 12587 11645 12588 11649 12591 11649 12595 11652 12598
11655 12601 11656 12605 11660 12605 11660 12604 11661 12609 11663 12613
11656 12614 11652 12617 11649 12620 11644 12620 11642 12623 11641 12627
11638 12631 11636 12627 11635 12623 11631 12620 11634 12617 11632 12613
11631 12617 11627 12618 11623 12617 11619 12615 40 N F
11619 12955 11619 12952 11621 12954 11624 12956 11621 12958 11619 12955
6 N F
11627 12965 11623 12964 11626 12962 11627 12959 11630 12960 11634 12960
11636 12962 11638 12965 11637 12968 11636 12971 11633 12971 11631 12974
11628 12972 11625 12973 11625 12969 11628 12969 11630 12966 11627 12965
18 N F
11630 13972 11626 13970 11628 13968 11631 13968 11635 13967 11638 13966
11639 13963 11642 13962 11644 13965 11641 13966 11639 13969 11638 13972
11635 13973 11633 13975 11630 13972 15 N F
11638 14180 11633 14181 11634 14178 11637 14176 11639 14174 11640 14174
11642 14172 11643 14169 11643 14166 11646 14165 11649 14165 11652 14166

11655 14167 11658 14168 11660 14168 11662 14168 11665 14167 11667 14170
11666 14173 11663 14173 11660 14171 11657 14171 11654 14171 11651 14172
11648 14174 11647 14177 11644 14178 11641 14179 11638 14180 29 N F
11637 12707 11633 12707 11637 12705 11635 12701 11639 12700 11644 12700
11647 12703 11647 12703 11651 12700 11654 12703 11657 12707 11657 12711
11658 12706 11657 12702 11661 12701 11663 12697 11665 12695 11667 12694
11670 12697 11672 12699 11673 12700 11677 12702 11679 12706 11683 12706
11683 12710 11682 12715 11686 12713 11690 12715 11691 12719 11688 12722
11684 12723 11685 12727 11688 12730 11684 12732 11685 12737 11681 12737
11679 12741 11676 12744 11671 12745 11670 12740 11672 12736 11670 12732
11667 12730 11663 12733 11660 12730 11656 12728 11653 12730 11651 12726
11650 12722 11646 12715 11644 12711 11642 12708 11637 12707 53 N F
11635 12364 11635 12360 11638 12360 11641 12358 11640 12361 11643 12364
11645 12366 11646 12364 11646 12360 11648 12362 11648 12365 11647 12369
11643 12369 11640 12368 11637 12366 11635 12364 16 N F
11636 12577 11635 12574 11638 12571 11640 12569 11643 12571 11646 12569
11648 12567 11651 12568 11650 12572 11649 12575 11646 12576 11645 12579
11642 12580 11639 12578 11636 12577 15 N F
11639 12983 11637 12980 11640 12978 11642 12981 11639 12983 5 N F
11640 14024 11637 14019 11641 14017 11644 14015 11649 14014 11651 14014
11653 14013 11657 14013 11662 14014 11665 14016 11667 14020 11663 14022
11661 14018 11656 14019 11652 14021 11648 14021 11644 14023 11640 14024
18 N F
11643 14313 11638 14308 11641 14302 11643 14296 11643 14289 11642 14282
11643 14275 11642 14269 11645 14263 11652 14261 11659 14261 11661 14254
11666 14250 11668 14243 11671 14237 11667 14231 11661 14229 11655 14226
11657 14220 11656 14213 11663 14211 11669 14208 11676 14209 11679 14209
11683 14208 11686 14207 11689 14206 11696 14205 11696 14212 11696 14218
11700 14217 11702 14216 11707 14212 11708 14211 11715 14211 11719 14216
11721 14217 11724 14220 11726 14227 11723 14234 11721 14240 11723 14246
11718 14251 11717 14257 11717 14264 11716 14271 11712 14277 11713 14284
11715 14290 11712 14296 11709 14302 11707 14308 11702 14313 11696 14315
11690 14319 11684 14323 11679 14327 11674 14331 11667 14333 11662 14329
11655 14329 11653 14323 11649 14317 11643 14313 64 N F
11640 12831 11639 12826 11642 12824 11643 12821 11646 12820 11648 12822
11648 12825 11646 12828 11644 12831 11642 12833 11640 12831 11 N F
11644 12555 11642 12553 11643 12553 11646 12553 11646 12553 11649 12554
11650 12550 11648 12547 11649 12544 11652 12545 11652 12548 11652 12551
11652 12554 11654 12557 11654 12557 11651 12557 11648 12556 11644 12555
18 N F
11645 12432 11644 12428 11647 12429 11648 12432 11649 12435 11646 12435
11645 12432 7 N F
11651 13972 11646 13971 11648 13968 11651 13967 11654 13966 11657 13965

11660 13965 11659 13968 11657 13971 11654 13971 11651 13972 11 N F
11649 13144 11647 13141 11650 13140 11651 13142 11652 13145 11649 13144
6 N F
11651 12360 11648 12356 11649 12353 11652 12353 11656 12354 11658 12356
11659 12357 11660 12358 11661 12361 11658 12363 11656 12360 11653 12362
11651 12360 13 N F
11652 12419 11650 12415 11654 12416 11657 12416 11656 12419 11654 12421
11652 12419 7 N F
11658 12816 11652 12813 11656 12813 11661 12813 11661 12809 11664 12812
11667 12815 11669 12811 11671 12810 11673 12810 11677 12809 11681 12806
11683 12810 11686 12813 11685 12817 11687 12820 11689 12824 11685 12826
11681 12827 11681 12832 11681 12836 11685 12838 11686 12842 11684 12846
11686 12849 11686 12854 11690 12855 11694 12856 11697 12859 11701 12861
11697 12863 11695 12860 11690 12860 11686 12859 11682 12859 11681 12854
11678 12851 11676 12848 11674 12844 11671 12840 11667 12838 11663 12837
11659 12836 11657 12832 11656 12828 11653 12824 11657 12822 11661 12820
11658 12816 49 N F
11654 13134 11653 13131 11655 13130 11658 13130 11659 13128 11661 13129
11661 13131 11659 13133 11656 13134 11654 13134 10 N F
11656 12567 11655 12564 11657 12562 11659 12564 11659 12564 11660 12561
11662 12563 11665 12564 11665 12568 11664 12571 11662 12573 11660 12575
11657 12574 11657 12571 11659 12568 11656 12567 16 N F
11657 12400 11655 12396 11658 12396 11659 12396 11661 12398 11660 12401
11657 12400 7 N F
11657 12435 11656 12428 11659 12428 11660 12430 11661 12431 11661 12435
11659 12437 11657 12435 8 N F
11659 12317 11657 12314 11658 12311 11659 12308 11660 12305 11662 12308
11660 12311 11660 12314 11659 12317 9 N F
11659 14135 11658 14131 11661 14129 11664 14128 11665 14125 11665 14122
11668 14120 11670 14118 11673 14119 11674 14122 11677 14123 11679 14121
11679 14118 11682 14119 11683 14119 11685 14118 11687 14115 11688 14112
11690 14115 11692 14118 11693 14121 11692 14124 11691 14127 11688 14128
11685 14130 11683 14133 11680 14133 11677 14134 11676 14131 11673 14129
11670 14130 11668 14132 11666 14135 11664 14137 11660 14137 11659 14135
36 N F
11665 12639 11659 12638 11660 12635 11661 12632 11662 12629 11664 12627
11667 12626 11668 12629 11667 12633 11666 12635 11665 12639 11 N F
11666 12404 11662 12403 11663 12400 11664 12397 11666 12397 11667 12398
11668 12401 11666 12404 8 N F
11666 12396 11662 12394 11664 12391 11668 12391 11671 12391 11669 12394
11666 12396 7 N F
11666 12577 11665 12574 11667 12571 11668 12568 11667 12565 11666 12562
11666 12561 11667 12559 11670 12558 11673 12556 11672 12554 11669 12551

11670 12548 11671 12545 11671 12542 11673 12539 11676 12540 11677 12543
11679 12546 11681 12544 11684 12544 11688 12543 11690 12545 11691 12548
11692 12552 11690 12555 11687 12556 11687 12559 11687 12562 11684 12562
11680 12561 11679 12564 11677 12567 11679 12570 11679 12573 11678 12576
11675 12577 11672 12578 11669 12578 11666 12577 40 N F
11672 12685 11668 12683 11670 12680 11671 12677 11674 12677 11677 12675
11678 12675 11680 12676 11680 12679 11677 12681 11674 12683 11672 12685
12 N F
11670 12381 11669 12378 11670 12375 11674 12375 11675 12373 11677 12370
11680 12369 11680 12373 11682 12375 11682 12379 11680 12376 11678 12379
11676 12381 11673 12382 11670 12381 15 N F
11674 12649 11670 12645 11672 12646 11674 12646 11677 12647 11679 12650
11678 12653 11676 12652 11674 12649 9 N F
11675 12417 11673 12416 11673 12412 11673 12412 11673 12409 11676 12408
11680 12407 11682 12407 11683 12407 11686 12408 11687 12411 11690 12411
11693 12412 11693 12412 11696 12410 11699 12410 11702 12408 11703 12411
11701 12414 11698 12414 11695 12416 11692 12417 11689 12419 11687 12417
11683 12418 11682 12415 11679 12414 11675 12417 28 N F
11677 14174 11674 14170 11676 14168 11677 14171 11679 14173 11679 14176
11677 14174 7 N F
11681 12699 11674 12697 11677 12694 11680 12690 11682 12687 11684 12683
11684 12682 11687 12680 11691 12678 11693 12675 11692 12670 11697 12670
11701 12672 11704 12675 11705 12680 11702 12683 11702 12688 11703 12692
11708 12690 11711 12688 11711 12693 11715 12695 11716 12699 11712 12702
11708 12704 11712 12706 11716 12707 11716 12707 11717 12711 11712 12711
11712 12715 11709 12718 11706 12715 11702 12717 11698 12718 11695 12715
11696 12711 11698 12707 11701 12704 11699 12700 11695 12700 11691 12702
11687 12705 11684 12703 11681 12699 45 N F
11679 12538 11675 12535 11679 12533 11682 12537 11686 12536 11691 12536
11689 12532 11685 12531 11683 12527 11679 12529 11676 12523 11681 12523
11685 12522 11689 12523 11692 12524 11694 12524 11696 12528 11700 12529
11703 12532 11702 12536 11707 12537 11709 12541 11707 12545 11709 12549
11709 12553 11712 12556 11709 12559 11705 12561 11708 12565 11704 12567
11700 12568 11696 12570 11695 12574 11692 12577 11687 12576 11683 12577
11682 12572 11686 12569 11690 12568 11692 12564 11694 12560 11695 12556
11698 12553 11702 12552 11699 12551 11697 12547 11698 12542 11695 12539
11690 12539 11687 12541 11682 12541 11679 12538 52 N F
11682 12405 11677 12404 11678 12401 11680 12399 11682 12401 11685 12401
11685 12404 11682 12405 8 N F
11680 12597 11678 12595 11678 12591 11679 12588 11680 12584 11682 12587
11682 12591 11681 12594 11680 12597 9 N F
11681 13493 11678 13493 11679 13490 11681 13488 11683 13490 11684 13492
11684 13495 11686 13497 11684 13498 11681 13498 11680 13496 11681 13493

12 N F
11684 12675 11680 12674 11680 12673 11681 12671 11683 12668 11687 12669
11685 12672 11684 12675 8 N F
11681 14099 11680 14095 11683 14094 11685 14092 11685 14088 11686 14085
11688 14083 11691 14081 11693 14083 11694 14083 11697 14084 11698 14087
11697 14090 11700 14092 11698 14094 11695 14096 11693 14098 11690 14101
11688 14102 11686 14105 11683 14105 11682 14102 11681 14099 23 N F
11683 12428 11681 12425 11684 12424 11681 12421 11684 12419 11687 12419
11686 12422 11686 12426 11683 12428 9 N F
11688 12654 11681 12652 11685 12651 11688 12648 11689 12646 11690 12644
11692 12640 11693 12640 11697 12640 11701 12638 11700 12634 11700 12629
11704 12629 11704 12629 11707 12627 11711 12630 11713 12633 11718 12634
11719 12630 11722 12627 11721 12623 11725 12621 11728 12621 11730 12621
11734 12621 11736 12625 11735 12629 11735 12634 11733 12637 11731 12641
11733 12644 11729 12646 11727 12650 11723 12652 11720 12649 11715 12649
11712 12652 11710 12656 11705 12657 11702 12654 11698 12656 11693 12657
11692 12653 11688 12654 44 N F
11682 14173 11681 14167 11684 14167 11686 14169 11686 14173 11686 14176
11689 14178 11686 14180 11683 14179 11683 14176 11682 14173 11 N F
11684 12943 11682 12938 11684 12935 11685 12934 11686 12933 11687 12930
11690 12931 11692 12933 11692 12937 11691 12940 11689 12943 11687 12945
11684 12943 13 N F
11686 12495 11682 12495 11683 12493 11684 12492 11686 12490 11686 12486
11688 12484 11689 12485 11690 12485 11692 12488 11693 12491 11696 12492
11698 12495 11698 12498 11695 12496 11694 12499 11697 12501 11694 12502
11692 12505 11689 12504 11688 12501 11687 12498 11686 12495 23 N F
11685 12892 11682 12891 11682 12887 11682 12884 11685 12886 11686 12889
11685 12892 7 N F
11690 12786 11683 12781 11686 12778 11690 12780 11695 12781 11699 12781
11703 12780 11707 12782 11707 12777 11702 12778 11702 12774 11702 12770
11703 12765 11701 12761 11699 12757 11698 12753 11702 12755 11706 12756
11707 12760 11706 12764 11709 12765 11710 12765 11714 12768 11718 12766
11717 12770 11721 12771 11720 12776 11720 12780 11718 12783 11713 12784
11711 12788 11710 12792 11707 12795 11703 12793 11700 12795 11695 12796
11695 12791 11691 12790 11690 12786 39 N F
11684 13483 11683 13481 11684 13478 11687 13478 11687 13478 11687 13480
11688 13483 11689 13485 11686 13485 11684 13483 10 N F
11687 12608 11685 12603 11687 12601 11690 12602 11690 12606 11692 12607
11693 12608 11693 12611 11690 12610 11687 12608 10 N F
11687 12397 11685 12391 11689 12393 11689 12388 11693 12387 11698 12387
11702 12387 11704 12385 11705 12383 11704 12388 11706 12391 11707 12387
11710 12383 11712 12387 11716 12388 11720 12386 11720 12391 11718 12395
11717 12399 11718 12404 11713 12403 11709 12402 11705 12400 11701 12401

11697 12404 11693 12406 11692 12402 11687 12403 11691 12400 11687 12397
30 N F
11687 12640 11685 12635 11689 12634 11691 12637 11691 12640 11689 12643
11687 12640 7 N F
11689 12894 11687 12892 11690 12892 11693 12892 11695 12895 11697 12897
11694 12898 11692 12895 11689 12894 9 N F
11688 14169 11687 14165 11690 14164 11693 14165 11696 14166 11699 14165
11702 14163 11705 14163 11705 14166 11704 14169 11705 14172 11705 14176
11702 14178 11700 14180 11697 14178 11696 14175 11693 14173 11691 14172
11688 14169 19 N F
11695 12667 11687 12665 11691 12664 11693 12663 11696 12663 11699 12660
11703 12662 11707 12662 11711 12659 11713 12656 11716 12652 11720 12654
11724 12655 11726 12659 11725 12664 11723 12668 11726 12670 11724 12674
11720 12674 11719 12679 11716 12681 11714 12677 11716 12673 11712 12673
11708 12671 11704 12669 11700 12668 11695 12667 28 N F
11690 13944 11689 13939 11692 13938 11694 13936 11696 13939 11695 13942
11693 13945 11690 13944 8 N F
11692 12363 11692 12358 11695 12358 11698 12356 11700 12358 11703 12358
11707 12357 11708 12360 11709 12363 11712 12364 11710 12366 11709 12369
11706 12371 11704 12372 11704 12369 11704 12366 11702 12364 11699 12363
11696 12362 11692 12363 20 N F
11698 14154 11693 14153 11696 14150 11700 14149 11702 14145 11701 14140
11706 14140 11705 14144 11707 14148 11709 14151 11707 14155 11702 14154
11698 14154 13 N F
11699 12813 11693 12808 11695 12808 11697 12808 11698 12804 11702 12802
11706 12804 11709 12801 11712 12797 11714 12801 11714 12806 11715 12802
11716 12797 11719 12801 11719 12805 11718 12809 11716 12813 11712 12814
11708 12813 11703 12814 11699 12813 21 N F
11701 12624 11698 12619 11700 12617 11704 12617 11706 12619 11707 12622
11704 12624 11701 12624 8 N F
11703 14032 11701 14026 11704 14022 11705 14018 11706 14014 11706 14010
11710 14012 11711 14013 11713 14015 11715 14012 11715 14007 11719 14009
11721 14013 11724 14016 11725 14020 11727 14024 11731 14025 11731 14030
11730 14034 11729 14038 11725 14039 11720 14039 11716 14039 11712 14039
11711 14035 11707 14033 11703 14032 27 N F
11706 12837 11704 12832 11708 12831 11711 12828 11710 12824 11712 12823
11714 12823 11716 12819 11720 12818 11724 12820 11727 12816 11731 12817
11735 12818 11738 12816 11739 12815 11743 12817 11744 12813 11740 12811
11741 12807 11744 12806 11745 12806 11747 12802 11748 12800 11750 12799
11750 12803 11754 12801 11755 12805 11758 12808 11755 12811 11751 12813
11752 12817 11750 12821 11747 12824 11744 12820 11739 12821 11737 12825
11733 12827 11733 12831 11736 12835 11739 12832 11742 12836 11741 12840
11737 12840 11735 12836 11733 12841 11730 12844 11727 12841 11726 12837

11721 12837 11718 12840 11716 12844 11712 12842 11708 12841 11706 12837
54 N F
11711 13919 11708 13916 11710 13914 11710 13911 11711 13907 11714 13906
11717 13907 11720 13909 11722 13910 11721 13913 11720 13916 11718 13918
11716 13916 11713 13916 11711 13919 15 N F
11711 14135 11709 14130 11712 14128 11715 14127 11716 14130 11716 14134
11714 14136 11711 14135 8 N F
11711 12593 11711 12588 11715 12588 11718 12584 11716 12580 11718 12576
11716 12572 11720 12572 11724 12574 11728 12575 11730 12579 11734 12580
11738 12578 11741 12580 11744 12584 11748 12584 11750 12588 11746 12589
11742 12590 11743 12594 11741 12598 11737 12601 11734 12598 11732 12594
11727 12594 11730 12598 11731 12602 11728 12605 11724 12603 11721 12599
11717 12598 11716 12594 11711 12593 33 N F
11714 12470 11711 12463 11715 12457 11720 12453 11724 12447 11729 12443
11728 12450 11726 12456 11729 12462 11730 12456 11735 12451 11742 12450
11739 12444 11735 12438 11738 12432 11742 12437 11746 12432 11753 12431
11759 12431 11763 12434 11765 12435 11772 12438 11766 12442 11773 12441
11774 12442 11780 12443 11784 12449 11791 12450 11791 12457 11797 12460
11804 12460 11804 12467 11809 12467 11811 12467 11812 12474 11818 12470
11819 12463 11824 12467 11822 12474 11826 12476 11828 12478 11830 12484
11834 12490 11831 12496 11835 12501 11830 12506 11834 12511 11835 12518
11837 12525 11831 12529 11827 12534 11822 12538 11816 12543 11810 12540
11809 12533 11803 12537 11801 12544 11802 12550 11799 12556 11792 12559
11786 12561 11779 12562 11772 12563 11771 12556 11774 12550 11780 12549
11787 12551 11785 12545 11787 12538 11783 12533 11779 12528 11776 12521
11769 12523 11769 12530 11766 12536 11762 12531 11765 12524 11759 12521
11762 12515 11763 12508 11768 12503 11774 12506 11771 12499 11765 12501
11768 12495 11766 12488 11765 12481 11764 12475 11761 12469 11761 12476
11760 12482 11760 12489 11763 12495 11760 12501 11760 12508 11753 12510
11752 12516 11746 12513 11745 12506 11749 12500 11746 12494 11741 12499
11734 12498 11731 12492 11724 12490 11723 12483 11727 12478 11721 12481
11716 12476 11714 12470 110 N F
11713 14150 11712 14145 11713 14141 11714 14141 11717 14139 11721 14139
11723 14143 11725 14143 11727 14143 11730 14147 11734 14148 11739 14147
11743 14148 11747 14150 11748 14154 11750 14158 11750 14162 11747 14165
11745 14169 11741 14171 11737 14172 11733 14172 11729 14173 11725 14175
11721 14177 11717 14175 11715 14171 11716 14167 11715 14163 11713 14159
11715 14154 11713 14150 32 N F
11716 13521 11715 13519 11715 13515 11716 13515 11718 13515 11720 13517
11723 13515 11725 13517 11727 13519 11727 13521 11725 13523 11724 13526
11724 13528 11723 13530 11720 13531 11720 13528 11719 13525 11718 13523
11716 13521 19 N F
11722 13109 11717 13107 11720 13104 11722 13100 11725 13102 11729 13104

11732 13107 11735 13111 11737 13114 11741 13116 11743 13120 11745 13124
11743 13127 11739 13130 11739 13126 11736 13123 11732 13123 11728 13125
11730 13121 11726 13121 11726 13116 11723 13114 11722 13109 23 N F
11730 12615 11729 12610 11732 12609 11735 12609 11738 12610 11740 12613
11742 12610 11744 12613 11745 12616 11743 12618 11745 12620 11747 12623
11744 12624 11740 12623 11738 12621 11736 12618 11736 12615 11733 12616
11730 12615 19 N F
11731 12771 11730 12768 11732 12765 11734 12762 11736 12761 11738 12764
11738 12767 11738 12770 11737 12773 11738 12777 11735 12776 11732 12774
11731 12771 13 N F
11733 13546 11732 13541 11733 13538 11735 13537 11737 13539 11739 13539
11740 13539 11743 13538 11745 13538 11746 13538 11748 13537 11751 13538
11753 13537 11756 13537 11758 13535 11759 13535 11761 13535 11764 13535
11766 13537 11765 13539 11762 13539 11760 13538 11758 13540 11755 13541
11756 13543 11755 13546 11753 13548 11753 13550 11750 13550 11747 13550
11745 13549 11744 13546 11745 13544 11743 13544 11740 13545 11738 13546
11735 13547 11733 13546 38 N F
11746 13257 11742 13255 11745 13254 11748 13254 11746 13257 5 N F
11743 12748 11742 12742 11744 12741 11746 12739 11750 12738 11752 12741
11757 12742 11756 12746 11757 12750 11753 12752 11749 12752 11744 12752
11743 12748 13 N F
11752 14043 11748 14044 11750 14041 11753 14038 11757 14035 11758 14030
11759 14026 11760 14022 11760 14018 11756 14016 11753 14014 11754 14009
11758 14010 11756 14006 11754 14002 11751 13999 11749 13994 11753 13997
11755 14000 11759 13999 11762 13995 11765 13998 11766 14002 11767 14006
11768 14010 11772 14009 11777 14009 11777 14013 11780 14016 11784 14017
11786 14021 11785 14025 11782 14029 11781 14033 11777 14035 11773 14034
11769 14036 11766 14039 11762 14038 11761 14042 11764 14046 11764 14050
11762 14046 11759 14042 11755 14040 11752 14043 46 N F
11751 12791 11748 12792 11750 12788 11752 12785 11752 12781 11752 12778
11750 12775 11753 12774 11756 12775 11760 12775 11762 12777 11761 12780
11761 12783 11759 12786 11757 12788 11755 12791 11751 12791 17 N F
11750 12709 11749 12704 11754 12704 11758 12703 11762 12701 11766 12701
11769 12705 11770 12709 11775 12710 11779 12709 11783 12710 11782 12714
11786 12713 11788 12709 11792 12709 11797 12709 11798 12705 11803 12704
11800 12700 11797 12697 11799 12693 11803 12692 11806 12696 11810 12694
11812 12698 11812 12702 11813 12706 11814 12710 11813 12715 11809 12716
11805 12719 11801 12718 11797 12718 11792 12717 11788 12718 11791 12722
11787 12724 11790 12726 11791 12731 11795 12729 11797 12733 11801 12733
11802 12737 11798 12738 11796 12741 11792 12742 11787 12741 11783 12742
11781 12746 11777 12746 11773 12745 11770 12741 11773 12738 11776 12735
11780 12733 11781 12729 11777 12726 11777 12721 11773 12722 11774 12718
11770 12715 11768 12719 11766 12715 11764 12711 11760 12711 11756 12711

11752 12713 11750 12709 68 N F
11754 13255 11751 13254 11754 13254 11757 13254 11758 13254 11760 13255
11762 13257 11759 13259 11756 13258 11754 13255 10 N F
11758 13238 11756 13234 11758 13232 11760 13229 11763 13231 11763 13234
11761 13237 11758 13238 8 N F
11759 12374 11758 12370 11760 12367 11763 12368 11761 12370 11762 12373
11764 12376 11765 12379 11763 12382 11761 12380 11759 12377 11759 12374
12 N F
11764 12772 11759 12770 11762 12767 11764 12763 11767 12760 11768 12756
11772 12754 11776 12755 11776 12759 11774 12763 11771 12767 11768 12770
11764 12772 13 N F
11762 7073 11761 7064 11763 7061 11766 7057 11771 7055 11775 7054
11779 7055 11783 7058 11787 7059 11791 7057 11796 7057 11800 7058
11804 7060 11806 7064 11805 7068 11804 7073 11801 7076 11800 7081
11796 7083 11791 7084 11787 7086 11783 7088 11779 7090 11774 7090
11771 7087 11771 7083 11771 7078 11771 7074 11766 7072 11762 7073
30 N F
11770 13240 11765 13239 11769 13238 11769 13235 11772 13236 11771 13233
11774 13234 11777 13234 11779 13237 11777 13239 11779 13241 11778 13244
11777 13247 11774 13248 11771 13247 11770 13244 11774 13243 11776 13241
11774 13238 11771 13237 11770 13240 21 N F
11770 13388 11768 13386 11769 13384 11772 13382 11774 13382 11777 13381
11776 13384 11775 13386 11773 13388 11770 13388 10 N F
11776 13263 11771 13262 11774 13261 11774 13261 11777 13261 11781 13260
11779 13263 11776 13263 8 N F
11780 13233 11778 13230 11781 13228 11784 13228 11786 13230 11789 13232
11790 13235 11788 13238 11786 13235 11785 13232 11782 13232 11783 13234
11781 13236 11780 13233 14 N F
11792 13250 11789 13247 11791 13245 11792 13242 11793 13239 11796 13238
11795 13241 11797 13242 11798 13243 11800 13240 11803 13240 11807 13240
11809 13242 11807 13244 11804 13246 11801 13247 11797 13247 11795 13250
11792 13250 19 N F
11797 13204 11793 13201 11796 13200 11797 13196 11799 13194 11801 13196
11801 13200 11800 13203 11797 13204 9 N F
11802 12451 11800 12446 11803 12444 11806 12443 11805 12447 11808 12446
11811 12448 11813 12450 11813 12453 11811 12455 11808 12454 11810 12456
11809 12459 11806 12458 11804 12456 11801 12455 11802 12451 17 N F
11812 11881 11806 11877 11807 11870 11809 11864 11807 11857 11807 11848
11811 11843 11815 11838 11820 11832 11825 11828 11826 11828 11832 11825
11838 11824 11844 11820 11848 11815 11853 11810 11858 11805 11864 11802
11871 11802 11871 11809 11872 11816 11876 11822 11881 11827 11887 11830
11894 11828 11900 11828 11907 11828 11914 11830 11920 11834 11926 11835
11932 11831 11939 11833 11946 11835 11950 11830 11957 11829 11963 11833

11970 11833 11974 11838 11981 11839 11988 11840 11995 11840 12001 11843
12007 11840 12014 11842 12019 11847 12024 11851 12028 11857 12034 11860
12040 11862 12047 11862 12054 11864 12060 11867 12067 11869 12073 11870
12078 11875 12083 11880 12087 11886 12094 11886 12101 11887 12107 11889
12114 11892 12117 11897 12116 11904 12121 11909 12125 11914 12126 11921
12128 11928 12131 11934 12137 11937 12141 11943 12141 11950 12143 11956
12144 11963 12149 11959 12147 11965 12146 11972 12149 11978 12156 11977
12151 11982 12146 11977 12145 11984 12139 11987 12135 11992 12138 11998
12136 12005 12133 12011 12127 12014 12120 12015 12114 12012 12108 12015
12101 12018 12096 12022 12092 12028 12086 12027 12079 12028 12072 12028
12065 12027 12059 12024 12052 12024 12046 12022 12040 12019 12033 12017
12026 12016 12020 12012 12014 12014 12008 12010 12002 12007 11995 12004
11989 12002 11983 11999 11976 11998 11970 11994 11964 11991 11958 11987
11952 11989 11945 11990 11941 11985 11934 11984 11929 11979 11923 11975
11917 11974 11911 11970 11905 11966 11899 11969 11894 11964 11887 11962
11882 11957 11876 11954 11869 11954 11865 11948 11862 11942 11855 11939
11851 11935 11846 11930 11845 11923 11842 11917 11837 11912 11832 11907
11827 11903 11823 11897 11819 11892 11813 11888 11812 11881 143 N F
11809 13260 11807 13259 11809 13257 11811 13258 11812 13260 11809 13260
6 N F
11820 13319 11817 13316 11819 13315 11822 13314 11824 13316 11822 13319
11820 13319 7 N F
11822 13369 11819 13366 11821 13365 11822 13365 11824 13365 11827 13364
11827 13366 11827 13369 11826 13371 11824 13371 11822 13369 11 N F
11826 7122 11819 7120 11823 7114 11827 7109 11830 7102 11835 7096
11839 7091 11842 7084 11835 7081 11835 7074 11837 7067 11834 7061
11828 7058 11825 7051 11832 7048 11837 7043 11840 7037 11836 7031
11825 7031 11829 7025 11834 7020 11840 7015 11847 7013 11848 7021
11850 7014 11855 7009 11862 7012 11869 7014 11876 7016 11882 7019
11889 7021 11896 7022 11903 7023 11910 7024 11917 7023 11924 7025
11931 7027 11937 7022 11933 7028 11928 7033 11924 7039 11918 7044
11911 7044 11904 7046 11898 7048 11894 7055 11887 7057 11880 7056
11874 7058 11867 7060 11863 7066 11865 7073 11860 7078 11853 7081
11849 7087 11848 7094 11844 7100 11839 7104 11834 7110 11829 7115
11826 7122 61 N F
11826 12226 11825 12222 11828 12220 11832 12217 11833 12221 11831 12225
11826 12226 7 N F
11831 13330 11828 13328 11830 13327 11833 13326 11835 13324 11837 13325
11840 13326 11841 13328 11839 13329 11836 13330 11833 13330 11831 13330
12 N F
11840 12220 11834 12216 11838 12215 11841 12211 11843 12207 11847 12207
11845 12211 11847 12215 11851 12216 11852 12221 11848 12222 11844 12219
11840 12220 13 N F

11847 13324 11843 13322 11845 13322 11848 13321 11849 13319 11852 13319
11850 13320 11849 13323 11847 13324 9 N F
11872 13239 11870 13234 11872 13231 11875 13231 11878 13233 11878 13236
11879 13239 11877 13242 11874 13241 11872 13239 10 N F
11906 12354 11904 12351 11907 12350 11908 12353 11910 12356 11914 12356
11914 12359 11916 12362 11914 12364 11911 12366 11910 12363 11908 12360
11907 12357 11906 12354 14 N F
11917 12345 11913 12342 11916 12338 11914 12331 11917 12327 11918 12323
11923 12322 11927 12322 11931 12324 11935 12326 11938 12329 11942 12332
11942 12336 11946 12337 11948 12341 11951 12343 11952 12347 11955 12345
11955 12345 11957 12341 11958 12345 11962 12343 11966 12344 11966 12348
11962 12348 11957 12349 11953 12351 11949 12351 11945 12349 11940 12350
11936 12349 11935 12353 11931 12355 11926 12354 11923 12351 11920 12348
11917 12345 37 N F
11933 12365 11930 12361 11931 12361 11935 12360 11937 12357 11940 12354
11945 12353 11949 12353 11954 12353 11950 12355 11949 12360 11952 12362
11954 12358 11959 12358 11961 12362 11958 12365 11962 12368 11965 12370
11962 12374 11960 12377 11957 12380 11953 12382 11949 12381 11944 12382
11940 12380 11937 12383 11934 12386 11934 12381 11937 12378 11933 12376
11933 12372 11937 12373 11935 12370 11933 12365 34 N F
11949 12392 11949 12386 11952 12386 11955 12388 11958 12386 11960 12389
11963 12389 11962 12392 11961 12395 11958 12396 11955 12396 11952 12394
11949 12392 13 N F
11955 12322 11952 12320 11955 12317 11952 12312 11956 12313 11959 12313
11962 12312 11962 12315 11959 12317 11958 12320 11955 12322 11 N F
11963 12473 11961 12468 11963 12464 11963 12460 11964 12456 11965 12452
11964 12447 11968 12444 11967 12440 11971 12442 11974 12439 11978 12437
11978 12437 11982 12435 11985 12438 11988 12440 11991 12437 11995 12434
11992 12431 11987 12431 11987 12427 11991 12424 11995 12425 11999 12426
12003 12426 12007 12423 12011 12422 12010 12426 12008 12430 12013 12429
12015 12425 12020 12425 12024 12425 12026 12425 12028 12424 12026 12428
12022 12430 12023 12434 12025 12438 12021 12440 12017 12439 12013 12439
12014 12443 12012 12447 12008 12449 12007 12453 12006 12457 12005 12461
12004 12466 12003 12470 12001 12474 11997 12476 11993 12478 11990 12480
11985 12482 11981 12483 11977 12482 11974 12478 11970 12479 11966 12477
11963 12473 61 N F
11974 12344 11969 12341 11972 12337 11974 12333 11975 12329 11978 12332
11981 12328 11983 12324 11987 12323 11989 12326 11989 12331 11991 12334
11990 12339 11994 12341 11993 12345 11990 12343 11989 12347 11987 12351
11982 12352 11983 12356 11987 12358 11991 12359 11993 12363 11990 12365
11986 12367 11982 12368 11978 12371 11974 12368 11974 12364 11970 12364
11971 12359 11975 12360 11977 12356 11977 12352 11974 12349 11974 12344
36 N F

12013 12478 12011 12473 12014 12476 12019 12475 12023 12476 12021 12472
12020 12468 12019 12463 12023 12461 12026 12464 12026 12469 12029 12472
12030 12476 12034 12478 12039 12478 12043 12480 12043 12484 12045 12487
12050 12486 12050 12486 12054 12484 12058 12485 12062 12484 12062 12489
12059 12492 12055 12494 12055 12498 12050 12496 12046 12497 12050 12500
12051 12504 12047 12507 12046 12511 12041 12510 12037 12508 12034 12506
12029 12504 12025 12504 12023 12501 12019 12498 12018 12493 12016 12490
12017 12486 12015 12482 12013 12478 45 N F
12018 12451 12014 12449 12019 12448 12023 12446 12027 12446 12031 12444
12034 12441 12037 12444 12040 12447 12044 12447 12049 12448 12052 12445
12054 12442 12054 12437 12057 12434 12061 12436 12063 12440 12066 12443
12065 12447 12069 12446 12070 12441 12074 12442 12075 12446 12074 12450
12075 12454 12079 12454 12080 12458 12079 12462 12075 12459 12071 12460
12068 12463 12064 12462 12059 12462 12056 12460 12054 12463 12050 12466
12047 12463 12044 12460 12044 12455 12041 12453 12037 12451 12034 12454
12030 12457 12028 12453 12026 12449 12022 12451 12018 12451 47 N F
12023 12380 12022 12377 12023 12374 12022 12368 12025 12366 12027 12364
12029 12366 12029 12370 12031 12372 12032 12375 12030 12378 12029 12381
12026 12382 12023 12380 14 N F
12065 12494 12060 12493 12063 12491 12066 12487 12063 12483 12062 12479
12063 12475 12067 12478 12068 12482 12072 12484 12072 12479 12072 12475
12073 12471 12078 12470 12081 12468 12082 12468 12085 12468 12090 12470
12091 12474 12095 12476 12099 12476 12101 12480 12098 12483 12097 12488
12098 12492 12100 12496 12098 12500 12096 12496 12094 12492 12092 12488
12088 12490 12084 12488 12081 12492 12077 12491 12073 12492 12069 12495
12068 12491 12065 12494 38 N F
12070 12376 12067 12365 12071 12360 12077 12362 12083 12358 12090 12357
12092 12357 12096 12356 12101 12361 12101 12368 12101 12375 12096 12379
12094 12386 12089 12390 12082 12392 12076 12388 12073 12382 12070 12376
18 N F
12110 12268 12107 12262 12109 12258 12113 12258 12117 12259 12120 12263
12118 12267 12114 12269 12110 12268 9 N F
12110 12459 12108 12456 12108 12452 12109 12447 12113 12446 12114 12443
12114 12442 12118 12444 12122 12446 12125 12449 12130 12450 12131 12454
12135 12456 12133 12460 12126 12464 12122 12463 12118 12463 12113 12462
12110 12459 19 N F
12125 12474 12121 12476 12123 12472 12126 12470 12127 12470 12131 12471
12135 12470 12139 12468 12135 12465 12140 12465 12142 12461 12143 12465
12148 12466 12151 12463 12147 12460 12144 12458 12145 12454 12146 12449
12148 12453 12150 12457 12153 12460 12156 12463 12160 12463 12161 12467
12157 12467 12156 12471 12160 12469 12164 12470 12167 12466 12172 12467
12169 12471 12166 12474 12165 12478 12161 12478 12157 12479 12154 12477
12150 12474 12146 12473 12147 12477 12145 12481 12141 12483 12139 12486

12136 12490 12137 12486 12133 12485 12129 12483 12132 12480 12129 12476
12125 12474 49 N F
12136 12530 12129 12528 12133 12525 12136 12522 12136 12521 12137 12518
12137 12513 12140 12510 12143 12507 12145 12511 12144 12515 12148 12514
12147 12509 12149 12505 12150 12501 12152 12505 12154 12509 12155 12513
12154 12517 12152 12521 12150 12525 12148 12529 12144 12531 12140 12532
12136 12530 25 N F
12165 12540 12160 12539 12163 12536 12167 12537 12170 12534 12166 12531
12165 12527 12169 12528 12172 12524 12176 12522 12179 12520 12183 12518
12187 12520 12191 12520 12194 12523 12198 12524 12203 12523 12207 12523
12211 12523 12215 12524 12219 12528 12219 12532 12219 12536 12221 12540
12222 12545 12224 12549 12223 12553 12220 12556 12217 12559 12212 12558
12209 12556 12204 12555 12200 12557 12196 12554 12193 12551 12191 12548
12186 12548 12184 12545 12180 12546 12175 12547 12174 12551 12169 12552
12170 12548 12168 12544 12165 12540 45 N F
12236 12411 12234 12405 12237 12407 12242 12406 12246 12408 12250 12407
12250 12412 12252 12415 12251 12419 12250 12424 12250 12428 12245 12427
12241 12425 12242 12421 12238 12420 12237 12416 12236 12411 17 N F
12274 12325 12269 12321 12271 12317 12272 12313 12276 12311 12277 12306
12279 12303 12283 12300 12287 12300 12291 12299 12294 12303 12290 12302
12286 12304 12284 12308 12281 12311 12279 12315 12277 12319 12279 12323
12278 12328 12274 12325 20 N F
12288 12326 12281 12324 12285 12321 12286 12317 12290 12315 12294 12314
12295 12318 12292 12320 12292 12324 12288 12326 10 N F
12313 12589 12310 12584 12314 12582 12318 12582 12319 12578 12322 12575
12318 12574 12315 12571 12318 12567 12320 12564 12325 12563 12328 12567
12332 12567 12336 12569 12340 12568 12342 12572 12342 12576 12339 12579
12335 12581 12330 12581 12326 12581 12330 12583 12327 12587 12323 12588
12326 12591 12329 12595 12328 12599 12323 12599 12319 12600 12316 12597
12314 12593 12313 12589 32 N F
12323 12552 12321 12547 12326 12547 12330 12548 12334 12549 12337 12545
12332 12545 12328 12546 12329 12542 12333 12540 12338 12541 12342 12539
12342 12535 12340 12531 12344 12528 12347 12532 12350 12534 12354 12536
12358 12537 12362 12540 12363 12544 12361 12548 12360 12552 12355 12553
12353 12557 12349 12559 12346 12563 12345 12558 12341 12557 12337 12554
12334 12557 12329 12558 12326 12560 12321 12559 12325 12556 12323 12552
36 N F
12400 12629 12398 12625 12398 12619 12400 12623 12403 12619 12405 12616
12410 12616 12413 12619 12417 12620 12418 12625 12421 12621 12425 12622
12429 12625 12425 12627 12422 12630 12419 12627 12414 12627 12412 12631
12408 12632 12404 12630 12400 12629 21 N F
12433 12610 12426 12609 12429 12606 12434 12604 12437 12602 12441 12604
12441 12599 12444 12597 12445 12593 12450 12591 12454 12593 12457 12591

12461 12589 12463 12585 12467 12585 12471 12586 12474 12589 12472 12593
12470 12597 12466 12597 12461 12597 12465 12599 12465 12603 12462 12606
12458 12609 12457 12613 12455 12617 12451 12619 12447 12620 12442 12619
12439 12616 12436 12613 12433 12610 33 N F
12458 12794 12457 12787 12458 12780 12461 12774 12465 12769 12470 12764
12472 12758 12477 12753 12480 12747 12477 12740 12475 12734 12477 12733
12482 12732 12484 12725 12489 12721 12496 12720 12503 12721 12509 12724
12509 12726 12511 12731 12507 12737 12505 12743 12511 12747 12516 12751
12523 12752 12524 12746 12524 12739 12525 12732 12526 12731 12528 12726
12530 12719 12536 12718 12539 12718 12543 12718 12545 12720 12549 12722
12556 12723 12560 12717 12566 12720 12571 12721 12573 12721 12569 12726
12566 12732 12570 12737 12574 12743 12572 12750 12571 12757 12570 12763
12569 12770 12572 12776 12572 12783 12566 12787 12560 12785 12555 12789
12542 12787 12536 12784 12533 12778 12527 12775 12522 12770 12517 12774
12515 12781 12509 12783 12502 12783 12503 12790 12498 12795 12495 12801
12496 12808 12501 12812 12504 12818 12505 12825 12503 12831 12500 12837
12494 12841 12487 12842 12481 12839 12476 12835 12472 12829 12472 12822
12476 12817 12470 12815 12463 12813 12463 12806 12463 12799 12458 12794
84 N F
12477 12559 12473 12555 12478 12555 12475 12552 12475 12547 12479 12547
12483 12544 12485 12548 12485 12552 12484 12557 12485 12561 12485 12565
12481 12567 12482 12572 12477 12572 12476 12568 12477 12564 12477 12559
18 N F
12485 8029 12477 8021 12482 8016 12488 8014 12496 8013 12503 8013
12508 8008 12511 8001 12515 7996 12521 7992 12527 7989 12534 7988
12532 7995 12529 8001 12529 8009 12533 8014 12535 8021 12529 8024
12526 8031 12519 8033 12515 8039 12509 8042 12502 8041 12496 8038
12492 8032 12485 8029 26 N F
12525 12806 12523 12802 12524 12798 12526 12794 12528 12790 12532 12789
12536 12788 12541 12788 12545 12789 12549 12790 12550 12794 12554 12796
12558 12794 12562 12792 12566 12793 12570 12791 12574 12792 12578 12789
12582 12789 12581 12793 12577 12796 12573 12797 12570 12800 12569 12804
12571 12808 12570 12813 12566 12815 12562 12817 12558 12819 12560 12823
12556 12824 12552 12821 12551 12817 12550 12813 12550 12808 12548 12804
12545 12807 12545 12811 12545 12816 12546 12820 12549 12824 12550 12828
12551 12832 12551 12836 12547 12835 12542 12833 12537 12829 12535 12825
12534 12821 12532 12817 12529 12814 12528 12810 12525 12806 53 N F
12524 8161 12524 8160 12524 8159 12524 8159 12525 8159 12526 8159
12526 8159 12526 8159 12526 8160 12526 8160 12526 8161 12524 8161
12 N F
12536 8016 12534 8013 12536 8013 12538 8014 12540 8015 12540 8017
12539 8019 12537 8018 12536 8016 9 N F
12543 8019 12542 8015 12545 8016 12546 8018 12545 8020 12543 8019

6 N F
12548 8016 12547 8013 12549 8015 12551 8016 12553 8017 12555 8017
12557 8019 12559 8020 12560 8022 12562 8023 12564 8023 12566 8023
12568 8024 12569 8026 12571 8027 12574 8028 12571 8028 12569 8028
12568 8026 12566 8025 12564 8025 12561 8024 12559 8024 12558 8022
12556 8021 12554 8019 12552 8018 12550 8018 12548 8016 29 N F
12619 12808 12616 12801 12621 12799 12625 12798 12624 12793 12628 12793
12631 12796 12632 12800 12635 12803 12640 12804 12644 12804 12643 12808
12639 12810 12635 12810 12631 12813 12627 12812 12623 12810 12619 12808
18 N F
12639 12792 12633 12789 12637 12788 12638 12784 12642 12784 12645 12781
12649 12781 12653 12779 12654 12783 12651 12787 12656 12787 12660 12788
12663 12791 12660 12794 12656 12796 12653 12799 12650 12802 12646 12804
12642 12802 12639 12799 12636 12796 12639 12792 22 N F
12659 12697 12656 12691 12659 12688 12663 12687 12663 12682 12665 12678
12668 12682 12672 12683 12674 12687 12678 12686 12683 12686 12687 12686
12686 12691 12689 12694 12687 12698 12683 12696 12681 12693 12680 12688
12676 12691 12679 12694 12676 12697 12672 12698 12668 12700 12664 12702
12660 12701 12659 12697 26 N F
12696 8054 12693 8048 12696 8045 12698 8041 12702 8043 12706 8045
12702 8047 12699 8051 12700 8055 12696 8054 10 N F
12721 8183 12715 8170 12722 8173 12729 8174 12735 8177 12733 8184
12727 8187 12721 8183 8 N F
12753 12843 12750 12836 12752 12830 12755 12823 12758 12817 12760 12816
12764 12815 12767 12809 12771 12803 12777 12806 12776 12812 12783 12814
12789 12817 12793 12823 12796 12829 12803 12827 12804 12821 12806 12814
12805 12808 12805 12801 12799 12797 12793 12795 12787 12793 12788 12786
12789 12786 12793 12790 12793 12784 12800 12785 12806 12788 12812 12785
12807 12781 12806 12774 12808 12768 12815 12766 12812 12760 12809 12754
12816 12752 12817 12751 12822 12749 12828 12753 12834 12755 12832 12761
12827 12766 12833 12768 12840 12769 12847 12772 12853 12773 12848 12776
12841 12775 12837 12780 12832 12785 12834 12792 12841 12791 12845 12796
12843 12803 12843 12810 12849 12813 12852 12807 12856 12812 12863 12811
12864 12810 12867 12806 12873 12803 12874 12810 12869 12815 12865 12820
12866 12827 12860 12826 12857 12832 12863 12835 12870 12833 12867 12840
12863 12845 12869 12846 12869 12853 12872 12859 12865 12860 12860 12855
12859 12848 12852 12850 12848 12855 12853 12861 12856 12867 12859 12873
12857 12879 12855 12886 12854 12892 12852 12899 12846 12903 12843 12909
12841 12915 12844 12921 12850 12924 12851 12931 12847 12936 12840 12938
12833 12939 12829 12933 12829 12926 12833 12916 12826 12914 12823 12908
12822 12901 12822 12894 12815 12896 12809 12893 12803 12895 12801 12889
12796 12884 12800 12878 12804 12872 12808 12867 12813 12862 12818 12857
12811 12858 12804 12857 12798 12860 12793 12855 12787 12856 12781 12859

12775 12864 12769 12862 12766 12856 12760 12855 12756 12848 12753 12843
126 N F
12752 12793 12751 12787 12754 12785 12757 12782 12756 12778 12756 12773
12757 12773 12761 12773 12765 12772 12769 12775 12772 12777 12775 12780
12775 12780 12775 12785 12772 12788 12768 12787 12765 12791 12765 12795
12762 12798 12762 12803 12759 12800 12760 12804 12756 12804 12752 12801
12754 12797 12758 12798 12761 12795 12758 12797 12757 12793 12754 12789
12754 12794 12751 12797 12752 12793 33 N F
12757 7950 12754 7945 12756 7941 12760 7939 12760 7943 12761 7948
12757 7950 7 N F
12757 7972 12756 7967 12757 7967 12760 7967 12761 7971 12757 7972
6 N F
12763 12892 12762 12887 12764 12884 12765 12879 12763 12875 12766 12872
12768 12868 12773 12869 12770 12872 12773 12875 12770 12878 12768 12882
12771 12885 12773 12888 12771 12892 12767 12893 12763 12892 17 N F
12772 7966 12769 7962 12773 7959 12775 7955 12780 7957 12783 7959
12781 7963 12779 7967 12775 7969 12772 7966 10 N F
12782 7984 12776 7979 12780 7978 12785 7978 12786 7978 12787 7976
12788 7975 12791 7978 12793 7982 12793 7987 12796 7984 12800 7983
12800 7987 12798 7991 12793 7992 12789 7992 12788 7988 12784 7988
12782 7984 19 N F
12786 7955 12779 7953 12782 7949 12786 7950 12786 7955 5 N F
12787 7949 12783 7944 12786 7940 12787 7945 12787 7949 5 N F
12812 7958 12808 7956 12810 7951 12813 7948 12812 7944 12812 7939
12815 7936 12816 7940 12820 7942 12821 7946 12820 7951 12819 7955
12822 7959 12825 7962 12824 7967 12824 7971 12823 7976 12821 7980
12817 7982 12813 7980 12813 7976 12812 7971 12810 7967 12811 7963
12812 7958 25 N F
12859 7979 12857 7973 12861 7974 12862 7978 12864 7982 12864 7987
12860 7983 12859 7979 8 N F
12885 12955 12882 12951 12886 12950 12889 12947 12889 12943 12892 12939
12888 12937 12892 12935 12896 12934 12897 12930 12896 12925 12899 12922
12901 12923 12904 12923 12905 12927 12903 12931 12903 12936 12904 12940
12906 12944 12907 12948 12906 12953 12905 12957 12900 12956 12898 12960
12895 12962 12892 12959 12888 12958 12885 12955 28 N F
12896 11258 12895 11258 12896 11257 12896 11257 12896 11257 12896 11257
12897 11256 12898 11256 12898 11256 12898 11257 12898 11257 12897 11258
12896 11258 13 N F
12897 11261 12896 11259 12897 11259 12898 11258 12899 11257 12899 11255
12898 11254 12898 11252 12898 11250 12897 11249 12899 11248 12899 11250
12900 11251 12901 11252 12903 11252 12903 11253 12901 11255 12901 11256
12901 11258 12902 11259 12903 11260 12903 11262 12901 11263 12900 11262
12899 11261 12897 11261 26 N F

12904 11267 12903 11266 12903 11265 12904 11263 12905 11264 12906 11265
12906 11265 12906 11266 12906 11268 12906 11268 12905 11268 12904 11267
12904 11267 13 N F
12905 11272 12905 11272 12905 11271 12905 11271 12906 11271 12906 11271
12906 11271 12906 11272 12906 11272 12905 11272 10 N F
12907 11274 12906 11274 12906 11272 12907 11272 12907 11272 12907 11272
12908 11272 12909 11272 12909 11272 12909 11274 12909 11275 12909 11275
12909 11275 12907 11275 12907 11275 12907 11274 16 N F
12909 11279 12909 11279 12909 11278 12910 11277 12910 11277 12910 11277
12910 11276 12911 11276 12912 11276 12912 11277 12912 11277 12911 11278
12911 11279 12911 11279 12909 11279 15 N F
12911 11283 12911 11281 12913 11281 12914 11281 12916 11282 12917 11282
12919 11283 12920 11284 12921 11283 12922 11282 12923 11283 12924 11284
12925 11286 12926 11287 12926 11289 12926 11290 12925 11291 12924 11289
12923 11290 12921 11289 12919 11290 12918 11290 12916 11290 12915 11289
12915 11287 12915 11285 12914 11284 12913 11283 12911 11283 29 N F
12963 7992 12960 7986 12961 7979 12966 7974 12969 7967 12974 7963
12979 7958 12980 7950 12979 7943 12979 7936 12978 7929 12977 7922
12982 7918 12989 7915 12993 7921 12999 7925 12999 7925 12999 8033
12995 8030 12988 8028 12984 8022 12982 8015 12978 8009 12979 8002
12973 7997 12977 7991 12982 7986 12976 7990 12970 7993 12963 7992
30 N F
12974 11621 12970 11609 12977 11607 12984 11607 12988 11602 12995 11601
12999 11606 12999 11607 12999 11637 12993 11637 12998 11632 12991 11634
12994 11627 12988 11623 12987 11616 12994 11619 12999 11623 12995 11617
12995 11610 12988 11612 12983 11616 12980 11622 12976 11628 12974 11621
24 N F
12982 8295 12979 8284 12983 8279 12990 8279 12997 8281 12999 8281
12999 8296 12996 8295 12989 8295 12982 8295 10 N F
12984 11702 12982 11695 12982 11688 12987 11683 12984 11676 12986 11670
12991 11665 12987 11659 12990 11653 12993 11647 12999 11644 12999 11638
12999 11700 12995 11705 12989 11707 12984 11702 16 N F
12983 8119 12983 8112 12985 8105 12988 8099 12992 8093 12996 8086
12999 8084 12999 8119 12996 8116 12991 8121 12986 8126 12983 8119
12 N F
1 O
13000 16171 2298 16171 2298 5454 13000 5454 13000 16171 5 P
Ls Gr Gs Lr
2297 5453 13002 16173 Oc
188 O
2297 6539 2297 6409 2297 6409 2297 7135 2297 7135 2297 7081
2297 6946 2297 6810 2297 6674 2297 6539 10 N F
174 O

2297 7217 2297 7135 2297 7135 2297 7270 2297 7270 2297 7217
6 N F
188 O
2297 7353 2297 7270 2297 7270 2297 7692 2297 7692 2297 7624
2297 7489 2297 7353 8 N F
174 O
2297 7760 2297 7692 2297 7692 2297 8279 2297 8279 2297 8167
2297 8031 2297 7896 2297 7760 9 N F
161 O
2297 8303 2297 8279 2297 8279 2297 8588 2297 8588 2297 8574
2297 8438 2297 8303 8 N F
174 O
2297 8710 2297 8588 2297 8588 2297 9736 2297 9736 2297 9660
2297 9524 2297 9388 2297 9252 2297 9117 2297 8981 2297 8845
2297 8710 13 N F
188 O
2297 9795 2297 9736 2297 9736 2297 9888 2297 9888 2297 9795
6 N F
174 O
2297 9931 2297 9888 2297 9888 2297 9955 2297 9955 2297 9931
6 N F
188 O
2297 10067 2297 9955 2297 9955 2297 11434 2297 11434 2297 11423
2297 11288 2297 11152 2297 11016 2297 10881 2297 10745 2297 10609
2297 10474 2297 10338 2297 10202 2297 10067 16 N F
174 O
2297 11559 2297 11434 2297 11434 2297 11784 2297 11784 2297 11695
2297 11559 7 N F
161 O
2297 11831 2297 11784 2297 11784 2297 11866 2297 11866 2297 11831
6 N F
148 O
2297 11921 2297 11866 2297 11866 2297 11921 2297 11921 5 N F
135 O
2297 11966 2297 11921 2297 11921 2297 11980 2297 11980 2297 11966
6 N F
121 O
2297 12053 2297 11980 2297 11980 2297 12053 2297 12053 5 N F
108 O
2297 12102 2297 12053 2297 12053 2297 12176 2297 12176 2297 12102
6 N F
121 O
2297 12238 2297 12176 2297 12176 2297 12494 2297 12494 2297 12373

2297 12238 7 N F
108 O
2297 12509 2297 12494 2297 12494 2297 12664 2297 12664 2297 12645
2297 12509 7 N F
95 O
2297 12780 2297 12664 2297 12664 2297 12801 2297 12801 2297 12780
6 N F
108 O
2297 12894 2297 12801 2297 12801 2297 12894 2297 12894 5 N F
121 O
2297 12916 2297 12894 2297 12894 2297 12973 2297 12973 2297 12916
6 N F
108 O
2297 13052 2297 12973 2297 12973 2297 13095 2297 13095 2297 13052
6 N F
95 O
2297 13158 2297 13095 2297 13095 2297 13158 2297 13158 5 N F
82 O
2297 13187 2297 13158 2297 13158 2297 13501 2297 13501 2297 13459
2297 13323 2297 13187 8 N F
95 O
2297 13594 2297 13501 2297 13501 2297 13819 2297 13819 2297 13730
2297 13594 7 N F
108 O
2297 13866 2297 13819 2297 13819 2297 13980 2297 13980 2297 13866
6 N F
95 O
2297 14002 2297 13980 2297 13980 2297 14524 2297 14524 2297 14409
2297 14273 2297 14137 2297 14002 9 N F
108 O
2297 14544 2297 14524 2297 14524 2297 15202 2297 15202 2297 15087
2297 14951 2297 14816 2297 14680 2297 14544 10 N F
95 O
2297 15223 2297 15202 2297 15202 2297 15602 2297 15602 2297 15494
2297 15358 2297 15223 8 N F
82 O
2297 15630 2297 15602 2297 15602 2297 15771 2297 15771 2297 15765
2297 15630 7 N F
68 O
2297 15882 2297 15771 2297 15771 2297 15882 2297 15882 5 N F
174 O
2297 6409 2297 5453 2297 5453 2297 5453 2297 6409 2297 6409
6 N F

188 O
2297 7135 2297 6409 2297 6409 2297 7135 2297 7135 5 N F
174 O
2297 7270 2297 7135 2297 7135 2297 7270 2297 7270 5 N F
188 O
2297 7692 2297 7270 2297 7270 2297 7692 2297 7692 5 N F
174 O
2297 8279 2297 7692 2297 7692 2297 8279 2297 8279 5 N F
161 O
2297 8588 2297 8279 2297 8279 2297 8588 2297 8588 5 N F
174 O
2297 9736 2297 8588 2297 8588 2297 9736 2297 9736 5 N F
188 O
2297 9888 2297 9736 2297 9736 2297 9888 2297 9888 5 N F
174 O
2297 9955 2297 9888 2297 9888 2297 9955 2297 9955 5 N F
188 O
2297 11434 2297 9955 2297 9955 2297 11435 2297 11434 5 N F
174 O
2297 11784 2297 11434 2297 11435 2297 11784 2297 11784 5 N F
161 O
2297 11866 2297 11784 2297 11784 2297 11866 2297 11866 5 N F
148 O
2297 11921 2297 11866 2297 11866 2297 11921 2297 11921 5 N F
135 O
2297 11980 2297 11921 2297 11921 2297 11980 2297 11980 5 N F
121 O
2297 12053 2297 11980 2297 11980 2297 12053 2297 12053 5 N F
108 O
2297 12176 2297 12053 2297 12053 2297 12176 2297 12176 5 N F
121 O
2297 12494 2297 12176 2297 12176 2297 12494 2297 12494 5 N F
108 O
2297 12664 2297 12494 2297 12494 2297 12665 2297 12664 5 N F
95 O
2297 12801 2297 12664 2297 12665 2297 12801 2297 12801 5 N F
108 O
2297 12894 2297 12801 2297 12801 2297 12894 2297 12894 5 N F
121 O
2297 12973 2297 12894 2297 12894 2297 12973 2297 12973 5 N F
108 O
2297 13095 2297 12973 2297 12973 2297 13095 2297 13095 5 N F
95 O

2297 13158 2297 13095 2297 13095 2297 13158 2297 13158 5 N F
82 O
2297 13501 2297 13158 2297 13158 2297 13501 2297 13501 5 N F
95 O
2297 13819 2297 13501 2297 13501 2297 13820 2297 13819 5 N F
108 O
2297 13980 2297 13819 2297 13820 2297 13979 2297 13980 5 N F
95 O
2297 14524 2297 13980 2297 13979 2297 14524 2297 14524 5 N F
108 O
2297 15202 2297 14524 2297 14524 2297 15202 2297 15202 5 N F
95 O
2297 15602 2297 15202 2297 15202 2297 15602 2297 15602 5 N F
82 O
2297 15771 2297 15602 2297 15602 2297 15771 2297 15771 5 N F
68 O
2297 15882 2297 15771 2297 15771 2297 15882 2297 15882 5 N F
55 O
2297 16173 2297 15882 2297 15882 2297 16172 2297 16173 2297 16173
6 N F
174 O
2460 5453 M 2297 5453 L 2297 6409 L 2309 6403 L 2391 6267 L
2433 6175 L 2485 6132 L 2568 6013 L 2704 6110 L
2765 6132 L 2839 6165 L 2975 6236 L 3003 6267 L
3045 6403 L 3060 6539 L 2989 6674 L 2975 6702 L
2839 6713 L 2747 6810 L 2839 6901 L 2975 6836 L
3110 6881 L 3216 6946 L 3202 7081 L 3113 7217 L
3110 7223 L 2975 7267 L 2839 7280 L 2704 7290 L
2644 7353 L 2591 7489 L 2568 7529 L 2493 7624 L
2433 7666 L 2297 7692 L 2297 8279 L 2315 8303 L
2349 8438 L 2308 8574 L 2297 8588 L 2297 9736 L
2365 9660 L 2433 9634 L 2568 9648 L 2576 9660 L
2676 9795 L 2704 9816 L 2839 9907 L 2876 9931 L
2975 10007 L 3110 10064 L 3114 10067 L 3246 10176 L
3275 10202 L 3381 10319 L 3403 10338 L 3517 10439 L
3652 10464 L 3690 10474 L 3788 10533 L 3923 10599 L
3923 10396 L 3788 10361 L 3652 10348 L 3631 10338 L
3517 10303 L 3410 10202 L 3381 10164 L 3277 10067 L
3246 10032 L 3110 9949 L 3085 9931 L 2975 9840 L
2916 9795 L 2839 9702 L 2805 9660 L 2750 9524 L
2704 9441 L 2667 9388 L 2568 9308 L 2498 9252 L
2540 9117 L 2568 9085 L 2568 8304 L 2566 8303 L
2568 8298 L 2571 8303 L 2568 8304 L 2568 9085 L

2658 9117 L 2704 9149 L 2839 9247 L 2975 9137 L
3085 9252 L 3110 9272 L 3166 9388 L 3213 9524 L
3177 9660 L 3246 9759 L 3289 9795 L 3381 9830 L
3517 9843 L 3559 9795 L 3517 9735 L 3486 9660 L
3473 9524 L 3517 9429 L 3609 9524 L 3652 9564 L
3693 9660 L 3714 9795 L 3695 9931 L 3733 10067 L
3782 10202 L 3788 10233 L 3923 10288 L 4045 10338 L
3923 10396 L 3923 10599 L 3967 10609 L 3923 10668 L
3847 10745 L 3788 10750 L 3652 10839 L 3600 10881 L
3517 10944 L 3414 11016 L 3381 11063 L 3261 11152 L
3246 11172 L 3169 11288 L 3110 11310 L 2975 11358 L
2839 11417 L 2799 11423 L 2704 11428 L 2568 11436 L
2433 11500 L 2297 11435 L 2297 11784 L 2433 11825 L
2474 11831 L 2557 11966 L 2568 11994 L 2704 11992 L
2704 11829 L 2568 11766 L 2556 11695 L 2568 11673 L
2661 11559 L 2704 11539 L 2839 11541 L 2975 11509 L
3110 11465 L 3246 11452 L 3381 11504 L 3404 11559 L
3381 11585 L 3263 11695 L 3246 11704 L 3110 11738 L
2975 11760 L 2839 11797 L 2704 11829 L 2704 11992 L
2839 11979 L 2975 12008 L 3110 12064 L 3110 11967 L
3107 11966 L 3057 11831 L 3110 11822 L 3246 11813 L
3381 11792 L 3517 11732 L 3553 11695 L 3628 11559 L
3652 11545 L 3788 11501 L 3788 11363 L 3652 11361 L
3562 11288 L 3539 11152 L 3592 11016 L 3652 10962 L
3750 10881 L 3788 10849 L 3923 10855 L 4059 10879 L
4194 10801 L 4194 10489 L 4059 10474 L 4058 10474 L
4059 10465 L 4194 10447 L 4327 10474 L 4194 10489 L
4194 10801 L 4256 10745 L 4330 10655 L 4465 10657 L
4465 10394 L 4348 10338 L 4330 10290 L 4302 10202 L
4194 10181 L 4059 10095 L 4034 10067 L 3962 9931 L
4059 9825 L 4194 9892 L 4330 9898 L 4330 7951 L
4194 7942 L 4059 7906 L 4046 7896 L 4000 7760 L
4013 7624 L 4059 7568 L 4194 7601 L 4233 7624 L
4330 7694 L 4389 7760 L 4450 7896 L 4330 7951 L
4330 9898 L 4465 9910 L 4486 9931 L 4601 10016 L
4645 10067 L 4601 10183 L 4573 10202 L 4586 10338 L
4465 10394 L 4465 10657 L 4601 10742 L 4736 10645 L
4810 10745 L 4742 10881 L 4736 10886 L 4639 11016 L
4601 11049 L 4465 11071 L 4330 11031 L 4194 11084 L
4164 11152 L 4059 11273 L 4018 11288 L 3923 11341 L
3788 11363 L 3788 11501 L 3923 11471 L 4020 11423 L
4059 11415 L 4095 11423 L 4112 11559 L 4059 11593 L
3973 11695 L 3923 11727 L 3788 11717 L 3652 11751 L

3572 11831 L 3517 11856 L 3382 11966 L 3381 11966 L
3376 11966 L 3246 11948 L 3145 11966 L 3110 11967 L
3110 12064 L 3246 12033 L 3381 12009 L 3517 11983 L
3562 11966 L 3652 11892 L 3788 11873 L 3905 11966 L
3923 11976 L 4059 11977 L 4144 11966 L 4194 11948 L
4246 11966 L 4330 12002 L 4421 12102 L 4465 12139 L
4601 12156 L 4726 12102 L 4736 12100 L 4746 12102 L
4838 12238 L 4736 12371 L 4735 12373 L 4601 12440 L
4465 12485 L 4330 12475 L 4194 12404 L 4144 12373 L
4059 12344 L 3923 12348 L 3788 12353 L 3652 12363 L
3618 12373 L 3517 12454 L 3381 12506 L 3369 12509 L
3282 12645 L 3381 12659 L 3517 12687 L 3652 12734 L
3788 12751 L 3866 12780 L 3923 12790 L 4059 12845 L
4194 12916 L 4195 12916 L 4330 12996 L 4465 12963 L
4465 12797 L 4331 12780 L 4330 12779 L 4194 12670 L
4059 12649 L 4042 12645 L 3923 12579 L 3846 12509 L
3923 12487 L 4059 12483 L 4134 12509 L 4194 12593 L
4330 12629 L 4465 12627 L 4545 12645 L 4537 12780 L
4465 12797 L 4465 12963 L 4601 13014 L 4647 13052 L
4736 13152 L 4872 13111 L 4872 12374 L 4811 12373 L
4872 12368 L 4873 12373 L 4872 12374 L 4872 13111 L
5007 13150 L 5007 12237 L 4899 12102 L 5007 12028 L
5129 12102 L 5007 12237 L 5007 13150 L 5057 13187 L
5143 13304 L 5170 13323 L 5278 13399 L 5310 13323 L
5278 13227 L 5249 13187 L 5202 13052 L 5143 12939 L
5123 12916 L 5117 12780 L 5143 12759 L 5166 12780 L
5278 12828 L 5414 12806 L 5549 12868 L 5685 12868 L
5685 12626 L 5549 12605 L 5444 12509 L 5414 12466 L
5331 12373 L 5368 12238 L 5414 12206 L 5506 12102 L
5515 11966 L 5414 11862 L 5331 11831 L 5278 11802 L
5242 11831 L 5143 11866 L 5007 11903 L 4872 11914 L
4736 11932 L 4686 11831 L 4601 11741 L 4465 11760 L
4330 11723 L 4306 11695 L 4330 11665 L 4417 11559 L
4330 11431 L 4322 11423 L 4279 11288 L 4307 11152 L
4330 11142 L 4465 11146 L 4556 11152 L 4601 11201 L
4690 11152 L 4736 11141 L 4872 11058 L 5007 11017 L
5008 11016 L 5061 10881 L 5007 10788 L 4981 10745 L
4945 10609 L 4872 10493 L 4794 10474 L 4736 10407 L
4731 10338 L 4736 10318 L 4830 10202 L 4872 10116 L
4943 10067 L 4872 10002 L 4825 9931 L 4767 9795 L
4806 9660 L 4872 9565 L 5007 9553 L 5044 9524 L
5143 9412 L 5143 7491 L 5137 7489 L 5143 7486 L
5158 7489 L 5143 7491 L 5143 9412 L 5152 9388 L

5230 9252 L 5278 9234 L 5414 9160 L 5414 7245 L
5384 7217 L 5414 7194 L 5470 7217 L 5414 7245 L
5414 9160 L 5549 9157 L 5624 9117 L 5685 9054 L
5685 8863 L 5682 8845 L 5685 8801 L 5685 8676 L
5646 8574 L 5685 8524 L 5685 7788 L 5549 7769 L
5543 7760 L 5529 7624 L 5549 7603 L 5595 7624 L
5685 7703 L 5692 7760 L 5685 7788 L 5685 8524 L
5697 8574 L 5685 8676 L 5685 8801 L 5689 8845 L
5685 8863 L 5685 9054 L 5713 9117 L 5814 9252 L
5727 9388 L 5704 9524 L 5738 9660 L 5820 9713 L
5890 9795 L 5849 9931 L 5820 9994 L 5765 10067 L
5699 10202 L 5777 10338 L 5820 10459 L 5822 10474 L
5834 10609 L 5840 10745 L 5854 10881 L 5821 11016 L
5956 11128 L 5956 9623 L 5885 9524 L 5956 9461 L
5956 9004 L 5936 8981 L 5956 8944 L 5975 8981 L
5956 9004 L 5956 9461 L 6034 9524 L 5956 9623 L
5956 11128 L 6091 11104 L 6091 9457 L 6037 9388 L
6091 9323 L 6142 9388 L 6091 9457 L 6091 11104 L
6227 11104 L 6227 10183 L 6152 10067 L 6227 9956 L
6362 10045 L 6384 10067 L 6362 10100 L 6227 10183 L
6227 11104 L 6281 11152 L 6227 11284 L 6091 11256 L
6059 11288 L 6091 11323 L 6227 11292 L 6312 11423 L
6227 11514 L 6159 11559 L 6227 11631 L 6362 11670 L
6391 11695 L 6362 11700 L 6227 11707 L 6091 11724 L
6073 11695 L 5956 11564 L 5820 11615 L 5765 11695 L
5742 11831 L 5702 11966 L 5717 12102 L 5695 12238 L
5697 12373 L 5733 12509 L 5685 12626 L 5685 12868 L
5820 12812 L 5820 12006 L 5798 11966 L 5820 11928 L
5908 11831 L 5956 11767 L 6002 11831 L 5961 11966 L
5956 11997 L 5820 12006 L 5820 12812 L 5900 12780 L
5942 12645 L 5956 12619 L 5998 12645 L 6044 12780 L
6069 12916 L 6091 12929 L 6189 13052 L 6206 13187 L
6212 13323 L 6171 13459 L 6217 13594 L 6227 13600 L
6344 13730 L 6362 13827 L 6414 13866 L 6498 13987 L
6537 14002 L 6633 14027 L 6769 14045 L 6904 14105 L
7000 14137 L 7040 14155 L 7175 14272 L 7311 14216 L
7446 14224 L 7446 12934 L 7311 12921 L 7278 12916 L
7175 12886 L 7040 12782 L 6904 12896 L 6790 12916 L
6769 12921 L 6633 12930 L 6608 12916 L 6498 12861 L
6362 12790 L 6315 12780 L 6334 12645 L 6254 12509 L
6227 12468 L 6174 12373 L 6227 12282 L 6271 12238 L
6227 12128 L 6219 12102 L 6210 11966 L 6227 11898 L
6362 11897 L 6417 11966 L 6498 12047 L 6554 12102 L

6594 12238 L 6609 12373 L 6609 12509 L 6633 12517 L
6769 12624 L 6801 12645 L 6904 12688 L 7040 12776 L
7057 12645 L 7040 12632 L 6918 12509 L 6904 12499 L
6769 12458 L 6646 12373 L 6654 12238 L 6690 12102 L
6756 11966 L 6672 11831 L 6689 11695 L 6633 11575 L
6624 11559 L 6633 11555 L 6769 11503 L 6847 11559 L
6837 11695 L 6859 11831 L 6904 11901 L 7040 11848 L
7093 11966 L 7128 12102 L 7175 12169 L 7311 12128 L
7402 12238 L 7446 12271 L 7446 12023 L 7340 11966 L
7311 11934 L 7241 11831 L 7252 11695 L 7311 11621 L
7446 11681 L 7462 11695 L 7480 11831 L 7482 11966 L
7446 12023 L 7446 12271 L 7582 12303 L 7717 12308 L
7777 12373 L 7717 12462 L 7690 12509 L 7717 12585 L
7756 12645 L 7717 12780 L 7717 12780 L 7582 12879 L
7491 12916 L 7446 12934 L 7446 14224 L 7582 14198 L
7630 14137 L 7717 14047 L 7823 14002 L 7853 13993 L
7988 13962 L 8124 13938 L 8259 13867 L 8260 13866 L
8352 13730 L 8395 13645 L 8406 13594 L 8395 13508 L
8280 13459 L 8395 13406 L 8419 13323 L 8395 13280 L
8374 13323 L 8259 13456 L 8258 13459 L 8124 13536 L
7988 13486 L 7853 13471 L 7793 13459 L 7717 13431 L
7582 13354 L 7562 13323 L 7582 13279 L 7717 13216 L
7816 13187 L 7853 13168 L 7853 12055 L 7717 12028 L
7669 11966 L 7582 11870 L 7573 11831 L 7582 11782 L
7679 11695 L 7582 11568 L 7548 11559 L 7446 11435 L
7311 11439 L 7175 11436 L 7130 11423 L 7040 11361 L
6904 11319 L 6769 11327 L 6668 11423 L 6633 11432 L
6624 11423 L 6633 11396 L 6738 11288 L 6633 11187 L
6567 11152 L 6498 11117 L 6422 11016 L 6498 10989 L
6498 10892 L 6479 10881 L 6498 10862 L 6512 10881 L
6498 10892 L 6498 10989 L 6627 10881 L 6596 10745 L
6633 10712 L 6719 10745 L 6769 10767 L 6828 10745 L
6873 10609 L 6769 10546 L 6669 10474 L 6633 10428 L
6536 10474 L 6498 10488 L 6362 10484 L 6341 10474 L
6362 10467 L 6465 10338 L 6498 10318 L 6609 10202 L
6633 10171 L 6730 10067 L 6676 9931 L 6633 9873 L
6498 9819 L 6475 9795 L 6485 9660 L 6498 9582 L
6528 9524 L 6498 9486 L 6459 9388 L 6362 9313 L
6319 9252 L 6362 9157 L 6362 7302 L 6330 7217 L
6227 7159 L 6159 7081 L 6227 7050 L 6326 7081 L
6362 7165 L 6420 7217 L 6362 7302 L 6362 9157 L
6456 9252 L 6498 9317 L 6633 9376 L 6750 9252 L
6633 9123 L 6625 9117 L 6498 8986 L 6493 8981 L

6449 8845 L 6498 8768 L 6498 8526 L 6473 8438 L
6498 8407 L 6544 8438 L 6498 8526 L 6498 8768 L
6633 8835 L 6642 8845 L 6769 8917 L 6769 8072 L
6692 8031 L 6644 7896 L 6769 7799 L 6769 7362 L
6687 7353 L 6769 7325 L 6781 7353 L 6769 7362 L
6769 7799 L 6856 7896 L 6797 8031 L 6769 8072 L
6769 8917 L 6848 8981 L 6904 9005 L 6936 8981 L
7040 8892 L 7090 8845 L 7175 8767 L 7175 8507 L
7108 8438 L 7175 8387 L 7233 8438 L 7175 8507 L
7175 8767 L 7311 8778 L 7446 8793 L 7524 8845 L
7582 8886 L 7632 8981 L 7717 9080 L 7853 9054 L
7853 8853 L 7845 8845 L 7853 8835 L 7861 8845 L
7853 8853 L 7853 9054 L 7932 9117 L 7988 9173 L
7988 8724 L 7972 8710 L 7988 8689 L 8005 8710 L
7988 8724 L 7988 9173 L 8033 9252 L 8124 9335 L
8259 9364 L 8262 9388 L 8395 9509 L 8395 9421 L
8358 9388 L 8395 9336 L 8433 9388 L 8395 9421 L
8395 9509 L 8530 9448 L 8530 8488 L 8491 8438 L
8457 8303 L 8530 8258 L 8565 8303 L 8650 8438 L
8530 8488 L 8530 9448 L 8597 9524 L 8584 9660 L
8530 9683 L 8439 9795 L 8395 9901 L 8259 9912 L
8124 9894 L 8084 9931 L 8124 9961 L 8259 9994 L
8350 10067 L 8358 10202 L 8395 10247 L 8470 10202 L
8530 10180 L 8559 10202 L 8530 10286 L 8503 10338 L
8410 10474 L 8395 10553 L 8259 10546 L 8170 10609 L
8132 10745 L 8173 10881 L 8230 11016 L 8256 11152 L
8259 11158 L 8317 11288 L 8275 11423 L 8259 11432 L
8124 11529 L 8099 11559 L 8052 11695 L 8029 11831 L
7988 11947 L 7975 11966 L 7853 12055 L 7853 13168 L
7988 13066 L 8099 13052 L 8124 13049 L 8124 12121 L
8107 12102 L 8124 12084 L 8143 12102 L 8124 12121 L
8124 13049 L 8132 13052 L 8259 13118 L 8395 13131 L
8395 11717 L 8259 11698 L 8257 11695 L 8259 11692 L
8395 11674 L 8417 11695 L 8395 11717 L 8395 13131 L
8530 13089 L 8546 13052 L 8575 12916 L 8570 12780 L
8540 12645 L 8550 12509 L 8666 12399 L 8666 11477 L
8618 11423 L 8666 11359 L 8666 9815 L 8645 9795 L
8666 9777 L 8666 8659 L 8587 8574 L 8666 8445 L
8762 8574 L 8666 8659 L 8666 9777 L 8687 9795 L
8666 9815 L 8666 11359 L 8729 11423 L 8666 11477 L
8666 12399 L 8700 12373 L 8801 12273 L 8937 12251 L
8989 12238 L 9008 12102 L 8962 11966 L 9059 11831 L
9072 11821 L 9130 11695 L 9188 11559 L 9114 11423 L

9072 11329 L 9044 11288 L 8937 11173 L 8801 11172 L
8773 11152 L 8801 11116 L 8817 11016 L 8937 10886 L
8954 10881 L 8937 10877 L 8801 10840 L 8722 10745 L
8801 10662 L 8924 10609 L 8937 10577 L 8950 10474 L
9040 10338 L 9072 10283 L 9072 9470 L 9002 9388 L
9072 9314 L 9161 9388 L 9072 9470 L 9072 10283 L
9168 10202 L 9153 10067 L 9208 10008 L 9227 9931 L
9343 9850 L 9478 9931 L 9479 9931 L 9479 8786 L
9343 8710 L 9343 8710 L 9343 8709 L 9440 8574 L
9479 8550 L 9533 8574 L 9569 8710 L 9479 8786 L
9479 9931 L 9482 9931 L 9577 9795 L 9542 9660 L
9541 9524 L 9498 9388 L 9575 9252 L 9516 9117 L
9614 8992 L 9750 9041 L 9885 8982 L 9885 8630 L
9843 8574 L 9885 8533 L 9928 8574 L 9885 8630 L
9885 8982 L 9896 8981 L 10021 8941 L 10156 8933 L
10156 8789 L 10053 8710 L 10156 8650 L 10256 8710 L
10156 8789 L 10156 8933 L 10252 8845 L 10292 8784 L
10427 8766 L 10478 8710 L 10563 8676 L 10599 8574 L
10698 8504 L 10778 8574 L 10834 8616 L 10969 8623 L
11105 8601 L 11147 8574 L 11240 8504 L 11338 8574 L
11376 8601 L 11409 8574 L 11406 8438 L 11376 8394 L
11240 8314 L 11232 8303 L 11240 8294 L 11347 8167 L
11376 8038 L 11511 8035 L 11647 8134 L 11682 8167 L
11782 8299 L 11786 8303 L 11914 8438 L 11918 8441 L
12007 8574 L 12053 8629 L 12068 8710 L 12085 8845 L
12103 8981 L 12117 9117 L 12129 9252 L 12143 9388 L
12156 9524 L 12168 9660 L 12178 9795 L 12188 9931 L
12189 9941 L 12254 10067 L 12310 10202 L 12324 10238 L
12396 10338 L 12460 10464 L 12465 10474 L 12561 10609 L
12595 10662 L 12664 10745 L 12731 10832 L 12786 10881 L
12866 10950 L 12985 11016 L 13002 11026 L 13002 9526 L
12989 9524 L 12866 9507 L 12731 9464 L 12595 9443 L
12520 9388 L 12460 9343 L 12330 9252 L 12324 9248 L
12244 9117 L 12189 9022 L 12187 8981 L 12179 8845 L
12169 8710 L 12157 8574 L 12151 8438 L 12141 8303 L
12131 8167 L 12126 8031 L 12123 7896 L 12108 7760 L
12077 7624 L 12053 7531 L 11996 7489 L 11918 7472 L
11782 7457 L 11647 7449 L 11511 7436 L 11376 7428 L
11240 7421 L 11105 7433 L 10969 7451 L 10898 7489 L
10834 7554 L 10698 7580 L 10563 7563 L 10427 7605 L
10292 7531 L 10196 7489 L 10156 7484 L 10130 7489 L
10021 7514 L 9885 7547 L 9750 7525 L 9614 7600 L
9520 7624 L 9479 7635 L 9430 7624 L 9343 7606 L

9303 7624 L 9234 7760 L 9274 7896 L 9343 7966 L
9358 8031 L 9343 8043 L 9270 8031 L 9208 7970 L
9167 8031 L 9072 8082 L 8960 8031 L 9053 7896 L
9072 7875 L 9135 7760 L 9072 7690 L 9025 7624 L
9031 7489 L 8937 7406 L 8905 7353 L 8801 7241 L
8788 7217 L 8666 7097 L 8644 7081 L 8530 6967 L
8467 6946 L 8395 6823 L 8387 6810 L 8259 6745 L
8177 6674 L 8124 6639 L 7988 6570 L 7953 6539 L
7853 6452 L 7717 6419 L 7582 6479 L 7525 6539 L
7533 6674 L 7511 6810 L 7458 6946 L 7446 6958 L
7311 6969 L 7275 6946 L 7175 6885 L 7040 6873 L
6954 6810 L 6904 6768 L 6858 6810 L 6883 6946 L
6769 6966 L 6736 6946 L 6633 6894 L 6498 6841 L
6480 6810 L 6416 6674 L 6362 6587 L 6237 6539 L
6227 6536 L 6209 6539 L 6091 6610 L 6066 6674 L
5984 6810 L 5988 6946 L 6027 7081 L 5956 7128 L
5840 7081 L 5820 7057 L 5685 7059 L 5648 6946 L
5685 6811 L 5712 6810 L 5685 6809 L 5549 6692 L
5414 6676 L 5407 6674 L 5340 6539 L 5278 6481 L
5143 6513 L 5007 6477 L 4872 6480 L 4736 6475 L
4646 6403 L 4601 6370 L 4465 6326 L 4334 6403 L
4330 6407 L 4314 6403 L 4194 6383 L 4059 6373 L
3923 6368 L 3830 6403 L 3804 6539 L 3816 6674 L
3788 6772 L 3652 6769 L 3525 6674 L 3517 6645 L
3467 6539 L 3405 6403 L 3381 6282 L 3343 6403 L
3302 6539 L 3246 6564 L 3195 6539 L 3203 6403 L
3246 6345 L 3377 6267 L 3381 6264 L 3502 6132 L
3381 6024 L 3343 5996 L 3359 5860 L 3381 5844 L
3517 5810 L 3652 5851 L 3673 5860 L 3788 5896 L
3923 5911 L 3988 5860 L 4034 5725 L 4059 5715 L
4194 5649 L 4222 5589 L 4330 5507 L 4393 5589 L
4436 5725 L 4465 5837 L 4485 5860 L 4498 5996 L
4601 6028 L 4673 5996 L 4736 5957 L 4872 5981 L
4911 5996 L 5007 6023 L 5143 6069 L 5278 6126 L
5281 6132 L 5388 6267 L 5414 6296 L 5549 6322 L
5608 6267 L 5685 6158 L 5697 6132 L 5820 6019 L
5843 5996 L 5941 5860 L 5956 5849 L 6091 5800 L
6158 5725 L 6204 5589 L 6158 5453 L 6028 5453 L
6025 5453 L 6091 5575 L 6099 5589 L 6091 5600 L
5956 5644 L 5820 5661 L 5770 5725 L 5685 5813 L
5601 5725 L 5549 5613 L 5529 5589 L 5549 5546 L
5652 5453 L 5541 5453 L 5379 5453 L 5217 5453 L
5055 5453 L 4892 5453 L 4730 5453 L 4568 5453 L

4406 5453 L 4244 5453 L 4081 5453 L 3919 5453 L
3757 5453 L 3595 5453 L 3433 5453 L 3271 5453 L
3108 5453 L 2946 5453 L 2784 5453 L 2622 5453 L
2460 5453 L N E
188 O
2309 6403 2297 6409 2297 7135 2318 7217 2297 7270 2297 7692
2433 7666 2493 7624 2568 7529 2591 7489 2644 7353 2704 7290
2839 7280 2975 7267 3110 7223 3113 7217 3202 7081 3216 6946
3110 6881 2975 6836 2839 6901 2747 6810 2839 6713 2975 6702
2989 6674 3060 6539 3045 6403 3003 6267 2975 6236 2839 6165
2765 6132 2704 6110 2568 6013 2485 6132 2433 6175 2391 6267
2309 6403 37 N F
174 O
2297 7135 2297 7270 2318 7217 2297 7135 4 N F
161 O
2297 8279 2297 8588 2308 8574 2349 8438 2315 8303 2297 8279
6 N F
188 O
2365 9660 2297 9736 2297 9888 2337 9931 2297 9955 2297 11435
2433 11500 2568 11436 2704 11428 2799 11423 2839 11417 2975 11358
3110 11310 3169 11288 3246 11172 3261 11152 3381 11063 3414 11016
3517 10944 3600 10881 3652 10839 3788 10750 3847 10745 3923 10668
3967 10609 3923 10599 3788 10533 3690 10474 3652 10464 3517 10439
3403 10338 3381 10319 3275 10202 3246 10176 3114 10067 3110 10064
2975 10007 2876 9931 2839 9907 2704 9816 2676 9795 2576 9660
2568 9648 2433 9634 2365 9660 45 N F
174 O
2297 9888 2297 9955 2337 9931 2297 9888 4 N F
161 O
2297 11784 M 2297 11866 L 2422 11966 L 2433 11992 L 2500 12102 L
2479 12238 L 2568 12328 L 2704 12290 L 2714 12238 L
2728 12102 L 2839 12032 L 2975 12074 L 3030 12102 L
3110 12127 L 3245 12102 L 3246 12102 L 3381 12051 L
3517 12015 L 3651 11966 L 3652 11965 L 3683 11966 L
3788 11970 L 3923 12044 L 4059 12064 L 4146 12102 L
4194 12152 L 4330 12225 L 4344 12238 L 4465 12348 L
4501 12373 L 4465 12386 L 4330 12393 L 4297 12373 L
4194 12286 L 4059 12276 L 3923 12292 L 3788 12306 L
3652 12324 L 3517 12368 L 3511 12373 L 3381 12477 L
3246 12509 L 3245 12509 L 3110 12605 L 3052 12645 L
3064 12780 L 3107 12916 L 3110 12932 L 3236 13052 L
3242 13187 L 3246 13199 L 3381 13248 L 3381 12872 L
3346 12780 L 3381 12763 L 3434 12780 L 3381 12872 L

3381 13248 L 3517 13257 L 3639 13187 L 3652 13181 L
3689 13052 L 3732 12916 L 3788 12881 L 3923 12866 L
4008 12916 L 4050 13052 L 4033 13187 L 4039 13323 L
4059 13376 L 4194 13395 L 4223 13323 L 4313 13187 L
4330 13170 L 4463 13052 L 4465 13051 L 4468 13052 L
4601 13116 L 4669 13187 L 4633 13323 L 4601 13372 L
4556 13459 L 4473 13594 L 4465 13619 L 4389 13730 L
4465 13776 L 4528 13866 L 4601 13950 L 4696 14002 L
4736 14044 L 4872 14049 L 4890 14002 L 4872 13950 L
4822 13866 L 4783 13730 L 4856 13594 L 4819 13459 L
4805 13323 L 4872 13235 L 5007 13243 L 5082 13323 L
5143 13413 L 5193 13459 L 5265 13594 L 5271 13730 L
5278 13819 L 5285 13866 L 5414 13997 L 5419 14002 L
5549 14080 L 5655 14137 L 5685 14156 L 5713 14137 L
5724 14002 L 5685 13976 L 5562 13866 L 5549 13859 L
5414 13818 L 5285 13730 L 5284 13594 L 5346 13459 L
5414 13351 L 5443 13323 L 5414 13294 L 5351 13187 L
5319 13052 L 5278 12922 L 5275 12916 L 5278 12914 L
5414 12861 L 5493 12916 L 5510 13052 L 5549 13186 L
5685 13098 L 5773 13052 L 5820 12981 L 5956 12928 L
6091 13010 L 6125 13052 L 6170 13187 L 6159 13323 L
6091 13415 L 5963 13459 L 6076 13594 L 6091 13603 L
6227 13682 L 6270 13730 L 6326 13866 L 6362 13967 L
6394 14002 L 6362 14091 L 6328 14137 L 6249 14273 L
6228 14409 L 6333 14544 L 6362 14564 L 6486 14680 L
6498 14692 L 6633 14717 L 6769 14709 L 6904 14770 L
6975 14816 L 7040 14877 L 7175 14859 L 7280 14816 L
7311 14797 L 7446 14745 L 7521 14680 L 7582 14636 L
7717 14573 L 7750 14544 L 7836 14409 L 7853 14393 L
7935 14273 L 7988 14228 L 8124 14163 L 8259 14142 L
8273 14137 L 8353 14002 L 8395 13871 L 8395 13866 L
8508 13730 L 8530 13699 L 8666 13613 L 8775 13594 L
8801 13579 L 8937 13494 L 8974 13459 L 8937 13384 L
8914 13323 L 8857 13187 L 8852 13052 L 8801 13017 L
8709 12916 L 8686 12780 L 8666 12750 L 8595 12645 L
8617 12509 L 8666 12463 L 8784 12373 L 8801 12356 L
8937 12315 L 9072 12284 L 9129 12238 L 9105 12102 L
9111 11966 L 9144 11831 L 9208 11759 L 9264 11695 L
9273 11559 L 9343 11460 L 9367 11423 L 9374 11288 L
9479 11167 L 9492 11152 L 9520 11016 L 9479 10995 L
9371 10881 L 9347 10745 L 9343 10686 L 9208 10628 L
9072 10687 L 9010 10609 L 9046 10474 L 9072 10442 L
9208 10395 L 9333 10474 L 9343 10513 L 9346 10474 L

9419 10338 L 9479 10237 L 9485 10202 L 9479 10135 L
9426 10067 L 9479 10051 L 9614 10040 L 9614 9453 L
9569 9388 L 9614 9332 L 9668 9388 L 9614 9453 L
9614 10040 L 9750 10006 L 9750 9718 L 9714 9660 L
9695 9524 L 9750 9407 L 9750 9339 L 9697 9252 L
9750 9201 L 9771 9252 L 9750 9339 L 9750 9407 L
9885 9457 L 9885 9168 L 9842 9117 L 9885 9077 L
9933 9117 L 9885 9168 L 9885 9457 L 9913 9524 L
9885 9564 L 9772 9660 L 9750 9718 L 9750 10006 L
9842 9931 L 9885 9897 L 9950 9931 L 10021 9940 L
10068 9931 L 10085 9795 L 10127 9660 L 10156 9619 L
10244 9524 L 10156 9516 L 10047 9388 L 10156 9282 L
10278 9252 L 10292 9224 L 10313 9117 L 10427 9024 L
10563 9033 L 10656 9117 L 10563 9249 L 10561 9252 L
10498 9388 L 10563 9416 L 10698 9402 L 10698 8897 L
10592 8845 L 10698 8831 L 10745 8845 L 10698 8897 L
10698 9402 L 10834 9419 L 10834 9194 L 10760 9117 L
10834 9058 L 10895 9117 L 10834 9194 L 10834 9419 L
10844 9388 L 10969 9282 L 11105 9286 L 11116 9252 L
11183 9117 L 11105 9018 L 11068 8981 L 10969 8877 L
10937 8845 L 10969 8769 L 11105 8739 L 11240 8750 L
11376 8808 L 11474 8845 L 11511 8858 L 11647 8891 L
11782 8913 L 11884 8981 L 11918 9003 L 12004 9117 L
12053 9207 L 12059 9252 L 12078 9388 L 12097 9524 L
12113 9660 L 12127 9795 L 12139 9931 L 12150 10067 L
12160 10202 L 12172 10338 L 12184 10474 L 12189 10538 L
12215 10609 L 12261 10745 L 12306 10881 L 12324 10931 L
12381 11016 L 12460 11132 L 12476 11152 L 12585 11288 L
12595 11300 L 12714 11423 L 12731 11442 L 12866 11538 L
12917 11559 L 13002 11590 L 13002 11026 L 12985 11016 L
12866 10950 L 12786 10881 L 12731 10832 L 12664 10745 L
12595 10662 L 12561 10609 L 12465 10474 L 12460 10464 L
12396 10338 L 12324 10238 L 12310 10202 L 12254 10067 L
12189 9941 L 12188 9931 L 12178 9795 L 12168 9660 L
12156 9524 L 12143 9388 L 12129 9252 L 12117 9117 L
12103 8981 L 12085 8845 L 12068 8710 L 12053 8629 L
12007 8574 L 11918 8441 L 11914 8438 L 11786 8303 L
11782 8299 L 11682 8167 L 11647 8134 L 11511 8035 L
11376 8038 L 11347 8167 L 11240 8294 L 11232 8303 L
11240 8314 L 11376 8394 L 11406 8438 L 11409 8574 L
11376 8601 L 11338 8574 L 11240 8504 L 11147 8574 L
11105 8601 L 10969 8623 L 10834 8616 L 10778 8574 L
10698 8504 L 10599 8574 L 10563 8676 L 10478 8710 L

10427 8766 L 10292 8784 L 10252 8845 L 10156 8933 L
10021 8941 L 9896 8981 L 9885 8982 L 9750 9041 L
9614 8992 L 9516 9117 L 9575 9252 L 9498 9388 L
9541 9524 L 9542 9660 L 9577 9795 L 9482 9931 L
9479 9931 L 9478 9931 L 9343 9850 L 9227 9931 L
9208 10008 L 9153 10067 L 9168 10202 L 9072 10283 L
9040 10338 L 8950 10474 L 8937 10577 L 8924 10609 L
8801 10662 L 8722 10745 L 8801 10840 L 8937 10877 L
8937 10769 L 8880 10745 L 8937 10719 L 8973 10745 L
8937 10769 L 8937 10877 L 8954 10881 L 8937 10886 L
8817 11016 L 8801 11116 L 8773 11152 L 8801 11172 L
8937 11173 L 9044 11288 L 9072 11329 L 9072 11109 L
8949 11016 L 9072 10922 L 9176 11016 L 9072 11109 L
9072 11329 L 9114 11423 L 9188 11559 L 9130 11695 L
9072 11821 L 9059 11831 L 8962 11966 L 9008 12102 L
8989 12238 L 8937 12251 L 8801 12273 L 8700 12373 L
8666 12399 L 8550 12509 L 8540 12645 L 8570 12780 L
8575 12916 L 8546 13052 L 8530 13089 L 8395 13131 L
8259 13118 L 8132 13052 L 8124 13049 L 8099 13052 L
7988 13066 L 7853 13168 L 7816 13187 L 7717 13216 L
7582 13279 L 7562 13323 L 7582 13354 L 7717 13431 L
7793 13459 L 7853 13471 L 7988 13486 L 8124 13536 L
8258 13459 L 8259 13456 L 8374 13323 L 8395 13280 L
8419 13323 L 8395 13406 L 8280 13459 L 8395 13508 L
8406 13594 L 8395 13645 L 8352 13730 L 8260 13866 L
8259 13867 L 8124 13938 L 7988 13962 L 7853 13993 L
7823 14002 L 7717 14047 L 7630 14137 L 7582 14198 L
7446 14224 L 7311 14216 L 7175 14272 L 7040 14155 L
7000 14137 L 6904 14105 L 6769 14045 L 6633 14027 L
6537 14002 L 6498 13987 L 6414 13866 L 6362 13827 L
6344 13730 L 6227 13600 L 6217 13594 L 6171 13459 L
6212 13323 L 6206 13187 L 6189 13052 L 6091 12929 L
6069 12916 L 6044 12780 L 5998 12645 L 5956 12619 L
5942 12645 L 5900 12780 L 5820 12812 L 5685 12868 L
5549 12868 L 5414 12806 L 5278 12828 L 5166 12780 L
5143 12759 L 5117 12780 L 5123 12916 L 5143 12939 L
5202 13052 L 5249 13187 L 5278 13227 L 5310 13323 L
5278 13399 L 5170 13323 L 5143 13304 L 5057 13187 L
5007 13150 L 4872 13111 L 4736 13152 L 4647 13052 L
4601 13014 L 4465 12963 L 4330 12996 L 4195 12916 L
4194 12916 L 4059 12845 L 3923 12790 L 3866 12780 L
3788 12751 L 3652 12734 L 3517 12687 L 3381 12659 L
3282 12645 L 3369 12509 L 3381 12506 L 3517 12454 L

3618 12373 L 3652 12363 L 3788 12353 L 3923 12348 L
4059 12344 L 4144 12373 L 4194 12404 L 4330 12475 L
4465 12485 L 4601 12440 L 4735 12373 L 4736 12371 L
4838 12238 L 4746 12102 L 4736 12100 L 4726 12102 L
4601 12156 L 4465 12139 L 4421 12102 L 4330 12002 L
4246 11966 L 4194 11948 L 4144 11966 L 4059 11977 L
3923 11976 L 3905 11966 L 3788 11873 L 3652 11892 L
3562 11966 L 3517 11983 L 3381 12009 L 3246 12033 L
3110 12064 L 2975 12008 L 2839 11979 L 2704 11992 L
2568 11994 L 2557 11966 L 2474 11831 L 2433 11825 L
2297 11784 L N E
148 O
2297 11866 M 2297 11921 L 2353 11966 L 2419 12102 L 2388 12238 L
2427 12373 L 2433 12377 L 2568 12428 L 2704 12434 L
2793 12373 L 2779 12238 L 2813 12102 L 2839 12085 L
2888 12102 L 2975 12136 L 3110 12169 L 3246 12126 L
3359 12102 L 3381 12093 L 3517 12048 L 3652 11999 L
3788 12022 L 3912 12102 L 3918 12238 L 3788 12259 L
3652 12286 L 3517 12340 L 3474 12373 L 3381 12448 L
3246 12485 L 3159 12509 L 3110 12543 L 2975 12634 L
2968 12645 L 2975 12700 L 2988 12780 L 3039 12916 L
3079 13052 L 3096 13187 L 3100 13323 L 3110 13360 L
3227 13459 L 3246 13485 L 3332 13594 L 3381 13713 L
3517 13676 L 3628 13594 L 3652 13563 L 3690 13459 L
3788 13351 L 3923 13354 L 3923 13244 L 3798 13187 L
3853 13052 L 3923 12997 L 3947 13052 L 3936 13187 L
3923 13244 L 3923 13354 L 3984 13459 L 3997 13594 L
4059 13699 L 4083 13730 L 4182 13866 L 4194 13879 L
4330 13950 L 4380 14002 L 4399 14137 L 4440 14273 L
4465 14301 L 4601 14384 L 4684 14409 L 4736 14418 L
4872 14467 L 4888 14409 L 4970 14273 L 5007 14193 L
5015 14137 L 5007 14026 L 5003 14002 L 4913 13866 L
4883 13730 L 4963 13594 L 4921 13459 L 4967 13323 L
5007 13313 L 5017 13323 L 5099 13459 L 5143 13529 L
5183 13594 L 5195 13730 L 5244 13866 L 5278 13903 L
5376 14002 L 5414 14042 L 5530 14137 L 5549 14186 L
5685 14267 L 5820 14166 L 5849 14137 L 5820 14059 L
5802 14002 L 5685 13925 L 5619 13866 L 5549 13828 L
5414 13767 L 5360 13730 L 5321 13594 L 5414 13468 L
5549 13463 L 5644 13594 L 5685 13641 L 5793 13730 L
5820 13762 L 5844 13730 L 5907 13594 L 5956 13556 L
5989 13594 L 6091 13650 L 6200 13730 L 6227 13760 L
6285 13866 L 6288 14002 L 6227 14072 L 6190 14137 L

6135 14273 L 6149 14409 L 6227 14536 L 6233 14544 L
6362 14629 L 6417 14680 L 6498 14763 L 6631 14816 L
6633 14816 L 6769 14849 L 6871 14951 L 6904 14993 L
7040 15073 L 7175 15035 L 7311 15039 L 7424 15087 L
7446 15101 L 7495 15087 L 7558 14951 L 7579 14816 L
7582 14813 L 7717 14725 L 7814 14680 L 7853 14647 L
7862 14544 L 7869 14409 L 7988 14285 L 8049 14273 L
8124 14223 L 8259 14195 L 8395 14157 L 8400 14137 L
8407 14002 L 8413 13866 L 8530 13745 L 8618 13730 L
8666 13695 L 8801 13688 L 8937 13695 L 9030 13730 L
9072 13748 L 9208 13735 L 9215 13730 L 9235 13594 L
9208 13511 L 9177 13459 L 9072 13386 L 9021 13323 L
8943 13187 L 8939 13052 L 8937 13047 L 8801 12957 L
8764 12916 L 8743 12780 L 8666 12668 L 8650 12645 L
8666 12575 L 8739 12509 L 8801 12478 L 8937 12390 L
8974 12373 L 9072 12355 L 9208 12262 L 9251 12238 L
9208 12227 L 9154 12102 L 9174 11966 L 9208 11854 L
9220 11831 L 9335 11695 L 9340 11559 L 9343 11555 L
9431 11423 L 9452 11288 L 9479 11257 L 9572 11152 L
9614 11099 L 9750 11057 L 9793 11016 L 9832 10881 L
9753 10745 L 9750 10743 L 9749 10745 L 9614 10850 L
9479 10860 L 9379 10745 L 9395 10609 L 9377 10474 L
9479 10360 L 9495 10338 L 9537 10202 L 9614 10114 L
9750 10127 L 9869 10202 L 9885 10219 L 10017 10338 L
10021 10366 L 10021 10296 L 9979 10202 L 9891 10067 L
10021 9970 L 10147 10067 L 10059 10202 L 10021 10296 L
10021 10366 L 10070 10338 L 10156 10306 L 10219 10202 L
10292 10143 L 10292 9771 L 10199 9660 L 10292 9566 L
10292 9471 L 10156 9452 L 10102 9388 L 10156 9335 L
10292 9305 L 10374 9388 L 10292 9471 L 10292 9566 L
10393 9660 L 10292 9771 L 10292 10143 L 10427 10067 L
10427 10066 L 10427 9331 L 10348 9252 L 10400 9117 L
10427 9095 L 10563 9103 L 10578 9117 L 10563 9138 L
10503 9252 L 10427 9331 L 10427 10066 L 10563 10019 L
10619 9931 L 10698 9849 L 10834 9803 L 10843 9795 L
10969 9677 L 10969 9464 L 10909 9388 L 10969 9337 L
11049 9388 L 10969 9464 L 10969 9677 L 11053 9660 L
11105 9628 L 11158 9524 L 11171 9388 L 11214 9252 L
11240 9199 L 11376 9238 L 11462 9252 L 11511 9262 L
11647 9307 L 11782 9362 L 11818 9388 L 11918 9448 L
11977 9524 L 12053 9631 L 12057 9660 L 12076 9795 L
12091 9931 L 12103 10067 L 12115 10202 L 12126 10338 L
12139 10474 L 12149 10609 L 12160 10745 L 12173 10881 L

12189 10997 L 12198 11016 L 12263 11152 L 12324 11274 L
12335 11288 L 12425 11423 L 12460 11486 L 12513 11559 L
12595 11690 L 12599 11695 L 12701 11831 L 12731 11873 L
12833 11966 L 12866 11989 L 13002 12032 L 13002 11590 L
12917 11559 L 12866 11538 L 12731 11442 L 12714 11423 L
12595 11300 L 12585 11288 L 12476 11152 L 12460 11132 L
12381 11016 L 12324 10931 L 12306 10881 L 12261 10745 L
12215 10609 L 12189 10538 L 12184 10474 L 12172 10338 L
12160 10202 L 12150 10067 L 12139 9931 L 12127 9795 L
12113 9660 L 12097 9524 L 12078 9388 L 12059 9252 L
12053 9207 L 12004 9117 L 11918 9003 L 11884 8981 L
11782 8913 L 11647 8891 L 11511 8858 L 11474 8845 L
11376 8808 L 11240 8750 L 11105 8739 L 10969 8769 L
10937 8845 L 10969 8877 L 11068 8981 L 11105 9018 L
11183 9117 L 11116 9252 L 11105 9286 L 10969 9282 L
10844 9388 L 10834 9419 L 10698 9402 L 10563 9416 L
10498 9388 L 10561 9252 L 10563 9249 L 10656 9117 L
10563 9033 L 10427 9024 L 10313 9117 L 10292 9224 L
10278 9252 L 10156 9282 L 10047 9388 L 10156 9516 L
10244 9524 L 10156 9619 L 10127 9660 L 10085 9795 L
10068 9931 L 10021 9940 L 9950 9931 L 9885 9897 L
9842 9931 L 9750 10006 L 9614 10040 L 9479 10051 L
9426 10067 L 9479 10135 L 9485 10202 L 9479 10237 L
9419 10338 L 9346 10474 L 9343 10513 L 9333 10474 L
9208 10395 L 9072 10442 L 9046 10474 L 9010 10609 L
9072 10687 L 9208 10628 L 9343 10686 L 9347 10745 L
9371 10881 L 9479 10995 L 9520 11016 L 9492 11152 L
9479 11167 L 9374 11288 L 9367 11423 L 9343 11460 L
9273 11559 L 9264 11695 L 9208 11759 L 9144 11831 L
9111 11966 L 9105 12102 L 9129 12238 L 9072 12284 L
8937 12315 L 8801 12356 L 8784 12373 L 8666 12463 L
8617 12509 L 8595 12645 L 8666 12750 L 8686 12780 L
8709 12916 L 8801 13017 L 8852 13052 L 8857 13187 L
8914 13323 L 8937 13384 L 8974 13459 L 8937 13494 L
8801 13579 L 8775 13594 L 8666 13613 L 8530 13699 L
8508 13730 L 8395 13866 L 8395 13871 L 8353 14002 L
8273 14137 L 8259 14142 L 8124 14163 L 7988 14228 L
7935 14273 L 7853 14393 L 7836 14409 L 7750 14544 L
7717 14573 L 7582 14636 L 7521 14680 L 7446 14745 L
7311 14797 L 7280 14816 L 7175 14859 L 7040 14877 L
6975 14816 L 6904 14770 L 6769 14709 L 6633 14717 L
6498 14692 L 6486 14680 L 6362 14564 L 6333 14544 L
6228 14409 L 6249 14273 L 6328 14137 L 6362 14091 L

6394 14002 L 6362 13967 L 6326 13866 L 6270 13730 L
6227 13682 L 6091 13603 L 6076 13594 L 5963 13459 L
6091 13415 L 6091 13342 L 6001 13323 L 5956 13310 L
5860 13187 L 5900 13052 L 5956 13005 L 6031 13052 L
6091 13091 L 6133 13187 L 6105 13323 L 6091 13342 L
6091 13415 L 6159 13323 L 6170 13187 L 6125 13052 L
6091 13010 L 5956 12928 L 5820 12981 L 5773 13052 L
5685 13098 L 5549 13186 L 5510 13052 L 5493 12916 L
5414 12861 L 5278 12914 L 5275 12916 L 5278 12922 L
5319 13052 L 5351 13187 L 5414 13294 L 5414 13089 L
5406 13052 L 5414 12916 L 5414 12916 L 5414 12916 L
5421 13052 L 5414 13089 L 5414 13294 L 5443 13323 L
5414 13351 L 5346 13459 L 5284 13594 L 5285 13730 L
5414 13818 L 5549 13859 L 5562 13866 L 5685 13976 L
5724 14002 L 5713 14137 L 5685 14156 L 5655 14137 L
5549 14080 L 5419 14002 L 5414 13997 L 5285 13866 L
5278 13819 L 5271 13730 L 5265 13594 L 5193 13459 L
5143 13413 L 5082 13323 L 5007 13243 L 4872 13235 L
4805 13323 L 4819 13459 L 4856 13594 L 4783 13730 L
4822 13866 L 4872 13950 L 4890 14002 L 4872 14049 L
4736 14044 L 4696 14002 L 4601 13950 L 4528 13866 L
4465 13776 L 4389 13730 L 4465 13619 L 4465 13304 L
4397 13187 L 4465 13122 L 4581 13187 L 4465 13304 L
4465 13619 L 4473 13594 L 4556 13459 L 4601 13372 L
4633 13323 L 4669 13187 L 4601 13116 L 4468 13052 L
4465 13051 L 4463 13052 L 4330 13170 L 4313 13187 L
4223 13323 L 4194 13395 L 4059 13376 L 4039 13323 L
4033 13187 L 4050 13052 L 4008 12916 L 3923 12866 L
3788 12881 L 3732 12916 L 3689 13052 L 3652 13181 L
3639 13187 L 3517 13257 L 3381 13248 L 3246 13199 L
3242 13187 L 3236 13052 L 3110 12932 L 3107 12916 L
3064 12780 L 3052 12645 L 3110 12605 L 3245 12509 L
3246 12509 L 3381 12477 L 3511 12373 L 3517 12368 L
3652 12324 L 3788 12306 L 3923 12292 L 4059 12276 L
4194 12286 L 4297 12373 L 4330 12393 L 4465 12386 L
4501 12373 L 4465 12348 L 4344 12238 L 4330 12225 L
4194 12152 L 4146 12102 L 4059 12064 L 3923 12044 L
3788 11970 L 3683 11966 L 3652 11965 L 3651 11966 L
3517 12015 L 3381 12051 L 3246 12102 L 3245 12102 L
3110 12127 L 3030 12102 L 2975 12074 L 2839 12032 L
2728 12102 L 2714 12238 L 2704 12290 L 2568 12328 L
2479 12238 L 2500 12102 L 2433 11992 L 2422 11966 L
2297 11866 L N E

135 O
2297 11921 M 2297 11980 L 2373 12102 L 2325 12238 L 2317 12373 L
2433 12441 L 2568 12503 L 2641 12509 L 2704 12516 L
2718 12509 L 2839 12444 L 2892 12373 L 2854 12238 L
2975 12197 L 3110 12210 L 3246 12149 L 3381 12121 L
3455 12102 L 3517 12080 L 3652 12032 L 3788 12074 L
3832 12102 L 3788 12170 L 3697 12238 L 3652 12248 L
3517 12312 L 3438 12373 L 3381 12418 L 3246 12461 L
3110 12490 L 3047 12509 L 2975 12551 L 2912 12645 L
2922 12780 L 2971 12916 L 2975 12923 L 3032 13052 L
3059 13187 L 3059 13323 L 3089 13459 L 3110 13494 L
3196 13594 L 3241 13730 L 3246 13751 L 3319 13866 L
3381 13896 L 3442 13866 L 3517 13815 L 3652 13748 L
3686 13730 L 3788 13634 L 3899 13730 L 3923 13758 L
3980 13866 L 4059 13912 L 4146 14002 L 4194 14072 L
4239 14137 L 4194 14242 L 4059 14237 L 3923 14264 L
3891 14273 L 3846 14409 L 3923 14477 L 4057 14544 L
4059 14546 L 4061 14544 L 4194 14490 L 4330 14488 L
4465 14482 L 4568 14544 L 4601 14557 L 4736 14545 L
4830 14680 L 4872 14713 L 5007 14771 L 5045 14680 L
5027 14544 L 5007 14435 L 4998 14409 L 5007 14395 L
5043 14273 L 5068 14137 L 5050 14002 L 5007 13923 L
4969 13866 L 4945 13730 L 5007 13642 L 5007 13473 L
5002 13459 L 5007 13449 L 5013 13459 L 5007 13473 L
5007 13642 L 5111 13730 L 5143 13750 L 5209 13866 L
5278 13942 L 5338 14002 L 5414 14083 L 5479 14137 L
5536 14273 L 5549 14292 L 5685 14379 L 5758 14409 L
5742 14544 L 5797 14680 L 5820 14721 L 5820 14606 L
5803 14544 L 5820 14508 L 5860 14544 L 5820 14606 L
5820 14721 L 5956 14682 L 5956 14505 L 5851 14409 L
5820 14335 L 5798 14273 L 5820 14256 L 5936 14137 L
5856 14002 L 5820 13868 L 5685 13874 L 5676 13866 L
5549 13798 L 5429 13730 L 5414 13710 L 5358 13594 L
5414 13518 L 5549 13553 L 5579 13594 L 5685 13715 L
5703 13730 L 5820 13865 L 5922 13730 L 5956 13675 L
6091 13698 L 6136 13730 L 6227 13833 L 6245 13866 L
6227 13935 L 6206 14002 L 6122 14137 L 6091 14197 L
6017 14273 L 6056 14409 L 5956 14505 L 5956 14682 L
5957 14680 L 6036 14544 L 6091 14485 L 6116 14544 L
6227 14573 L 6355 14680 L 6362 14693 L 6482 14816 L
6498 14826 L 6633 14853 L 6769 14901 L 6819 14951 L
6904 15059 L 6948 15087 L 7040 15125 L 7175 15126 L
7311 15186 L 7357 15223 L 7410 15358 L 7446 15382 L

7582 15359 L 7583 15358 L 7717 15243 L 7853 15228 L
7872 15223 L 7988 15125 L 8011 15087 L 7988 15058 L
7902 14951 L 7853 14869 L 7775 14816 L 7853 14789 L
7868 14680 L 7879 14544 L 7887 14409 L 7988 14304 L
8124 14277 L 8160 14273 L 8259 14248 L 8395 14228 L
8417 14137 L 8425 14002 L 8431 13866 L 8530 13764 L
8666 13741 L 8801 13740 L 8934 13866 L 8937 13868 L
9072 13899 L 9208 13901 L 9228 13866 L 9343 13782 L
9391 13730 L 9343 13637 L 9328 13594 L 9223 13459 L
9208 13345 L 9173 13323 L 9072 13315 L 8960 13187 L
8979 13052 L 8937 12961 L 8853 12916 L 8801 12789 L
8800 12780 L 8801 12769 L 8884 12645 L 8937 12601 L
9072 12519 L 9102 12509 L 9208 12485 L 9343 12431 L
9454 12373 L 9479 12351 L 9614 12268 L 9647 12238 L
9614 12196 L 9479 12198 L 9343 12123 L 9208 12114 L
9202 12102 L 9208 12076 L 9257 11966 L 9330 11831 L
9343 11814 L 9447 11695 L 9479 11651 L 9586 11559 L
9565 11423 L 9597 11288 L 9614 11265 L 9708 11152 L
9750 11135 L 9874 11016 L 9885 10986 L 9977 10881 L
10021 10811 L 10156 10752 L 10170 10745 L 10292 10665 L
10328 10609 L 10420 10474 L 10427 10468 L 10490 10338 L
10563 10267 L 10580 10202 L 10663 10067 L 10698 10027 L
10732 9931 L 10834 9841 L 10892 9795 L 10969 9722 L
11105 9706 L 11174 9660 L 11229 9524 L 11240 9482 L
11351 9524 L 11376 9532 L 11511 9595 L 11628 9660 L
11647 9672 L 11782 9750 L 11845 9795 L 11918 9855 L
11983 9931 L 12053 10034 L 12056 10067 L 12069 10202 L
12081 10338 L 12095 10474 L 12105 10609 L 12116 10745 L
12127 10881 L 12144 11016 L 12167 11152 L 12189 11261 L
12202 11288 L 12270 11423 L 12324 11556 L 12326 11559 L
12395 11695 L 12450 11831 L 12460 11850 L 12530 11966 L
12595 12073 L 12620 12102 L 12722 12238 L 12731 12247 L
12866 12317 L 13002 12322 L 13002 12032 L 12866 11989 L
12833 11966 L 12731 11873 L 12701 11831 L 12599 11695 L
12595 11690 L 12513 11559 L 12460 11486 L 12425 11423 L
12335 11288 L 12324 11274 L 12263 11152 L 12198 11016 L
12189 10997 L 12173 10881 L 12160 10745 L 12149 10609 L
12139 10474 L 12126 10338 L 12115 10202 L 12103 10067 L
12091 9931 L 12076 9795 L 12057 9660 L 12053 9631 L
11977 9524 L 11918 9448 L 11818 9388 L 11782 9362 L
11647 9307 L 11511 9262 L 11462 9252 L 11376 9238 L
11240 9199 L 11214 9252 L 11171 9388 L 11158 9524 L
11105 9628 L 11053 9660 L 10969 9677 L 10843 9795 L

10834 9803 L 10698 9849 L 10619 9931 L 10563 10019 L
10427 10066 L 10427 10067 L 10292 10143 L 10219 10202 L
10156 10306 L 10070 10338 L 10021 10366 L 10017 10338 L
9885 10219 L 9869 10202 L 9750 10127 L 9614 10114 L
9537 10202 L 9495 10338 L 9479 10360 L 9377 10474 L
9395 10609 L 9379 10745 L 9479 10860 L 9479 10825 L
9410 10745 L 9458 10609 L 9408 10474 L 9479 10394 L
9523 10338 L 9589 10202 L 9614 10173 L 9695 10202 L
9750 10210 L 9877 10338 L 9777 10474 L 9871 10609 L
9750 10621 L 9681 10745 L 9614 10797 L 9479 10825 L
9479 10860 L 9614 10850 L 9749 10745 L 9750 10743 L
9753 10745 L 9832 10881 L 9793 11016 L 9750 11057 L
9614 11099 L 9572 11152 L 9479 11257 L 9452 11288 L
9431 11423 L 9343 11555 L 9340 11559 L 9335 11695 L
9220 11831 L 9208 11854 L 9174 11966 L 9154 12102 L
9208 12227 L 9251 12238 L 9208 12262 L 9072 12355 L
8974 12373 L 8937 12390 L 8801 12478 L 8739 12509 L
8666 12575 L 8650 12645 L 8666 12668 L 8743 12780 L
8764 12916 L 8801 12957 L 8937 13047 L 8939 13052 L
8943 13187 L 9021 13323 L 9072 13386 L 9177 13459 L
9208 13511 L 9235 13594 L 9215 13730 L 9208 13735 L
9072 13748 L 9030 13730 L 8937 13695 L 8801 13688 L
8666 13695 L 8618 13730 L 8530 13745 L 8413 13866 L
8407 14002 L 8400 14137 L 8395 14157 L 8259 14195 L
8124 14223 L 8049 14273 L 7988 14285 L 7869 14409 L
7862 14544 L 7853 14647 L 7814 14680 L 7717 14725 L
7582 14813 L 7579 14816 L 7558 14951 L 7495 15087 L
7446 15101 L 7424 15087 L 7311 15039 L 7175 15035 L
7040 15073 L 6904 14993 L 6871 14951 L 6769 14849 L
6633 14816 L 6631 14816 L 6498 14763 L 6417 14680 L
6362 14629 L 6233 14544 L 6227 14536 L 6149 14409 L
6135 14273 L 6190 14137 L 6227 14072 L 6288 14002 L
6285 13866 L 6227 13760 L 6200 13730 L 6091 13650 L
5989 13594 L 5956 13556 L 5907 13594 L 5844 13730 L
5820 13762 L 5793 13730 L 5685 13641 L 5644 13594 L
5549 13463 L 5414 13468 L 5321 13594 L 5360 13730 L
5414 13767 L 5549 13828 L 5619 13866 L 5685 13925 L
5802 14002 L 5820 14059 L 5849 14137 L 5820 14166 L
5685 14267 L 5549 14186 L 5530 14137 L 5414 14042 L
5376 14002 L 5278 13903 L 5244 13866 L 5195 13730 L
5183 13594 L 5143 13529 L 5099 13459 L 5017 13323 L
5007 13313 L 4967 13323 L 4921 13459 L 4963 13594 L
4883 13730 L 4913 13866 L 5003 14002 L 5007 14026 L

5015 14137 L 5007 14193 L 4970 14273 L 4888 14409 L
4872 14467 L 4736 14418 L 4684 14409 L 4601 14384 L
4465 14301 L 4440 14273 L 4399 14137 L 4380 14002 L
4330 13950 L 4194 13879 L 4182 13866 L 4083 13730 L
4059 13699 L 3997 13594 L 3984 13459 L 3923 13354 L
3788 13351 L 3690 13459 L 3652 13563 L 3628 13594 L
3517 13676 L 3381 13713 L 3332 13594 L 3246 13485 L
3227 13459 L 3110 13360 L 3100 13323 L 3096 13187 L
3079 13052 L 3039 12916 L 2988 12780 L 2975 12700 L
2968 12645 L 2975 12634 L 3110 12543 L 3159 12509 L
3246 12485 L 3381 12448 L 3474 12373 L 3517 12340 L
3652 12286 L 3788 12259 L 3918 12238 L 3912 12102 L
3788 12022 L 3652 11999 L 3517 12048 L 3381 12093 L
3359 12102 L 3246 12126 L 3110 12169 L 2975 12136 L
2888 12102 L 2839 12085 L 2813 12102 L 2779 12238 L
2793 12373 L 2704 12434 L 2568 12428 L 2433 12377 L
2427 12373 L 2388 12238 L 2419 12102 L 2353 11966 L
2297 11921 L N E
121 O
2297 11980 M 2297 12053 L 2328 12102 L 2297 12176 L 2297 12494 L
2433 12506 L 2440 12509 L 2433 12579 L 2426 12645 L
2366 12780 L 2297 12894 L 2297 12973 L 2418 13052 L
2433 13055 L 2568 13140 L 2568 13036 L 2433 12970 L
2372 12916 L 2421 12780 L 2433 12756 L 2568 12698 L
2704 12773 L 2711 12780 L 2704 12794 L 2652 12916 L
2568 13036 L 2568 13140 L 2633 13052 L 2704 12953 L
2729 12916 L 2812 12780 L 2704 12666 L 2638 12645 L
2704 12623 L 2839 12590 L 2856 12645 L 2857 12780 L
2920 12916 L 2975 13030 L 2985 13052 L 3023 13187 L
3017 13323 L 3024 13459 L 3102 13594 L 3110 13603 L
3167 13730 L 3202 13866 L 3246 13944 L 3381 13984 L
3517 13948 L 3652 13952 L 3742 14002 L 3788 14085 L
3832 14002 L 3923 13970 L 3980 14002 L 3941 14137 L
3923 14141 L 3788 14148 L 3652 14262 L 3610 14273 L
3517 14304 L 3412 14409 L 3431 14544 L 3381 14666 L
3272 14544 L 3361 14409 L 3286 14273 L 3273 14137 L
3246 14116 L 3234 14137 L 3110 14213 L 2975 14249 L
2874 14137 L 2874 14002 L 2870 13866 L 2839 13794 L
2815 13866 L 2704 13990 L 2698 14002 L 2678 14137 L
2635 14273 L 2568 14356 L 2466 14409 L 2433 14469 L
2405 14544 L 2433 14659 L 2568 14621 L 2704 14583 L
2807 14680 L 2839 14705 L 2899 14816 L 2975 14874 L
3044 14951 L 3110 15034 L 3165 15087 L 3246 15171 L

3246 15127 L 3207 15087 L 3110 14992 L 3078 14951 L
2975 14836 L 2948 14816 L 2886 14680 L 2839 14584 L
2797 14544 L 2704 14463 L 2631 14409 L 2704 14275 L
2716 14273 L 2839 14258 L 2852 14273 L 2975 14355 L
3110 14352 L 3202 14409 L 3155 14544 L 3187 14680 L
3246 14785 L 3272 14816 L 3344 14951 L 3343 15087 L
3246 15127 L 3246 15171 L 3381 15162 L 3487 15087 L
3517 15020 L 3517 14874 L 3464 14816 L 3517 14756 L
3632 14816 L 3517 14874 L 3517 15020 L 3626 14951 L
3652 14863 L 3656 14816 L 3788 14704 L 3846 14680 L
3923 14643 L 3963 14680 L 4059 14737 L 4194 14726 L
4284 14680 L 4330 14637 L 4465 14636 L 4601 14660 L
4661 14680 L 4736 14726 L 4872 14810 L 4882 14816 L
4998 14951 L 5007 14962 L 5126 15087 L 5143 15100 L
5155 15087 L 5162 14951 L 5143 14816 L 5143 14811 L
5120 14680 L 5082 14544 L 5053 14409 L 5089 14273 L
5121 14137 L 5095 14002 L 5043 13866 L 5143 13813 L
5173 13866 L 5278 13981 L 5299 14002 L 5414 14125 L
5429 14137 L 5494 14273 L 5549 14357 L 5620 14409 L
5682 14544 L 5685 14548 L 5761 14680 L 5820 14784 L
5956 14735 L 6001 14680 L 6091 14582 L 6227 14603 L
6319 14680 L 6362 14755 L 6421 14816 L 6498 14866 L
6633 14891 L 6766 14951 L 6769 14953 L 6882 15087 L
6904 15109 L 7040 15167 L 7175 15198 L 7218 15223 L
7282 15358 L 7311 15407 L 7446 15487 L 7582 15472 L
7699 15358 L 7717 15343 L 7853 15318 L 7988 15307 L
8071 15223 L 8124 15140 L 8184 15087 L 8124 14957 L
8121 14951 L 7988 14942 L 7869 14816 L 7886 14680 L
7896 14544 L 7905 14409 L 7988 14322 L 8124 14297 L
8259 14284 L 8395 14281 L 8403 14273 L 8435 14137 L
8442 14002 L 8449 13866 L 8530 13782 L 8666 13761 L
8801 13757 L 8916 13866 L 8937 13886 L 9072 13991 L
9092 14002 L 9208 14039 L 9343 14008 L 9372 14002 L
9479 13878 L 9493 13866 L 9520 13730 L 9479 13697 L
9401 13594 L 9343 13577 L 9241 13459 L 9225 13323 L
9208 13306 L 9072 13296 L 8976 13187 L 9020 13052 L
9020 12916 L 9072 12821 L 9122 12780 L 9208 12731 L
9343 12652 L 9355 12645 L 9479 12585 L 9614 12522 L
9622 12509 L 9750 12383 L 9760 12373 L 9860 12238 L
9869 12102 L 9885 11987 L 9889 11966 L 9885 11946 L
9750 11864 L 9689 11831 L 9750 11792 L 9885 11737 L
9890 11695 L 9885 11688 L 9822 11559 L 9849 11423 L
9850 11288 L 9885 11186 L 9887 11152 L 10021 11022 L

10026 11016 L 10156 10893 L 10219 10881 L 10292 10828 L
10368 10745 L 10427 10670 L 10443 10609 L 10460 10474 L
10563 10356 L 10583 10338 L 10617 10202 L 10698 10098 L
10735 10067 L 10775 9931 L 10834 9879 L 10940 9795 L
10969 9768 L 11105 9766 L 11240 9740 L 11342 9795 L
11376 9813 L 11511 9894 L 11567 9931 L 11647 10000 L
11716 10067 L 11782 10156 L 11818 10202 L 11918 10326 L
11926 10338 L 12031 10474 L 12053 10510 L 12060 10609 L
12071 10745 L 12081 10881 L 12097 11016 L 12118 11152 L
12141 11288 L 12171 11423 L 12189 11501 L 12214 11559 L
12263 11695 L 12300 11831 L 12324 11929 L 12340 11966 L
12401 12102 L 12451 12238 L 12460 12258 L 12517 12373 L
12587 12509 L 12595 12523 L 12731 12602 L 12866 12606 L
13002 12563 L 13002 12322 L 12866 12317 L 12731 12247 L
12722 12238 L 12620 12102 L 12595 12073 L 12530 11966 L
12460 11850 L 12450 11831 L 12395 11695 L 12326 11559 L
12324 11556 L 12270 11423 L 12202 11288 L 12189 11261 L
12167 11152 L 12144 11016 L 12127 10881 L 12116 10745 L
12105 10609 L 12095 10474 L 12081 10338 L 12069 10202 L
12056 10067 L 12053 10034 L 11983 9931 L 11918 9855 L
11845 9795 L 11782 9750 L 11647 9672 L 11628 9660 L
11511 9595 L 11376 9532 L 11351 9524 L 11240 9482 L
11229 9524 L 11174 9660 L 11105 9706 L 10969 9722 L
10892 9795 L 10834 9841 L 10732 9931 L 10698 10027 L
10663 10067 L 10580 10202 L 10563 10267 L 10490 10338 L
10427 10468 L 10420 10474 L 10328 10609 L 10292 10665 L
10170 10745 L 10156 10752 L 10021 10811 L 9977 10881 L
9885 10986 L 9874 11016 L 9750 11135 L 9708 11152 L
9614 11265 L 9597 11288 L 9565 11423 L 9586 11559 L
9479 11651 L 9447 11695 L 9343 11814 L 9330 11831 L
9257 11966 L 9208 12076 L 9202 12102 L 9208 12114 L
9343 12123 L 9479 12198 L 9479 12083 L 9367 11966 L
9479 11863 L 9578 11966 L 9479 12083 L 9479 12198 L
9614 12196 L 9647 12238 L 9614 12268 L 9479 12351 L
9454 12373 L 9343 12431 L 9208 12485 L 9102 12509 L
9072 12519 L 8937 12601 L 8884 12645 L 8801 12769 L
8800 12780 L 8801 12789 L 8853 12916 L 8937 12961 L
8979 13052 L 8960 13187 L 9072 13315 L 9173 13323 L
9208 13345 L 9223 13459 L 9328 13594 L 9343 13637 L
9391 13730 L 9343 13782 L 9228 13866 L 9208 13901 L
9072 13899 L 8937 13868 L 8934 13866 L 8801 13740 L
8666 13741 L 8530 13764 L 8431 13866 L 8425 14002 L
8417 14137 L 8395 14228 L 8259 14248 L 8160 14273 L

8124 14277 L 7988 14304 L 7887 14409 L 7879 14544 L
7868 14680 L 7853 14789 L 7775 14816 L 7853 14869 L
7902 14951 L 7988 15058 L 8011 15087 L 7988 15125 L
7872 15223 L 7853 15228 L 7717 15243 L 7583 15358 L
7582 15359 L 7446 15382 L 7410 15358 L 7357 15223 L
7311 15186 L 7175 15126 L 7040 15125 L 6948 15087 L
6904 15059 L 6819 14951 L 6769 14901 L 6633 14853 L
6498 14826 L 6482 14816 L 6362 14693 L 6355 14680 L
6227 14573 L 6116 14544 L 6091 14485 L 6036 14544 L
5957 14680 L 5956 14682 L 5820 14721 L 5797 14680 L
5742 14544 L 5758 14409 L 5685 14379 L 5549 14292 L
5536 14273 L 5479 14137 L 5414 14083 L 5338 14002 L
5278 13942 L 5209 13866 L 5143 13750 L 5111 13730 L
5007 13642 L 4945 13730 L 4969 13866 L 5007 13923 L
5007 13737 L 5006 13730 L 5007 13729 L 5009 13730 L
5007 13737 L 5007 13923 L 5050 14002 L 5068 14137 L
5043 14273 L 5007 14395 L 4998 14409 L 5007 14435 L
5027 14544 L 5045 14680 L 5007 14771 L 4872 14713 L
4830 14680 L 4736 14545 L 4601 14557 L 4568 14544 L
4465 14482 L 4330 14488 L 4194 14490 L 4061 14544 L
4059 14546 L 4057 14544 L 3923 14477 L 3846 14409 L
3891 14273 L 3923 14264 L 4059 14237 L 4194 14242 L
4239 14137 L 4194 14072 L 4146 14002 L 4059 13912 L
3980 13866 L 3923 13758 L 3899 13730 L 3788 13634 L
3686 13730 L 3652 13748 L 3517 13815 L 3442 13866 L
3381 13896 L 3319 13866 L 3246 13751 L 3241 13730 L
3196 13594 L 3110 13494 L 3089 13459 L 3059 13323 L
3059 13187 L 3032 13052 L 2975 12923 L 2971 12916 L
2922 12780 L 2912 12645 L 2975 12551 L 3047 12509 L
3110 12490 L 3110 12447 L 2979 12373 L 3110 12259 L
3125 12238 L 3246 12173 L 3381 12144 L 3517 12125 L
3610 12238 L 3517 12283 L 3401 12373 L 3381 12389 L
3246 12438 L 3110 12447 L 3110 12490 L 3246 12461 L
3381 12418 L 3438 12373 L 3517 12312 L 3652 12248 L
3652 12184 L 3547 12102 L 3652 12065 L 3749 12102 L
3652 12184 L 3652 12248 L 3697 12238 L 3788 12170 L
3832 12102 L 3788 12074 L 3652 12032 L 3517 12080 L
3455 12102 L 3381 12121 L 3246 12149 L 3110 12210 L
2975 12197 L 2854 12238 L 2892 12373 L 2839 12444 L
2718 12509 L 2704 12516 L 2641 12509 L 2568 12503 L
2433 12441 L 2317 12373 L 2325 12238 L 2373 12102 L
2297 11980 L N E
108 O

2297 12053 2297 12176 2328 12102 2297 12053 4 N F
2297 12494 2297 12665 2310 12780 2297 12801 2297 12894 2366 12780
2426 12645 2433 12579 2440 12509 2433 12506 2297 12494 11 N F
95 O
2297 12665 2297 12801 2310 12780 2297 12665 4 N F
108 O
2297 12973 M 2297 13095 L 2433 13124 L 2519 13187 L 2568 13260 L
2621 13187 L 2704 13059 L 2707 13052 L 2800 12916 L
2839 12857 L 2868 12916 L 2935 13052 L 2975 13150 L
2986 13187 L 2976 13323 L 2975 13332 L 2960 13459 L
2952 13594 L 2839 13665 L 2799 13730 L 2729 13866 L
2704 13894 L 2647 14002 L 2621 14137 L 2568 14267 L
2562 14273 L 2433 14339 L 2324 14409 L 2297 14524 L
2297 15202 L 2348 15223 L 2433 15353 L 2434 15358 L
2501 15494 L 2568 15507 L 2568 15009 L 2465 14951 L
2433 14925 L 2397 14816 L 2433 14710 L 2568 14812 L
2570 14816 L 2600 14951 L 2568 15009 L 2568 15507 L
2704 15535 L 2704 15384 L 2673 15358 L 2704 15262 L
2839 15352 L 2852 15358 L 2839 15378 L 2704 15384 L
2704 15535 L 2839 15584 L 2875 15630 L 2975 15711 L
3076 15765 L 3110 15783 L 3246 15834 L 3362 15765 L
3268 15630 L 3246 15578 L 3136 15494 L 3110 15486 L
3004 15358 L 2975 15347 L 2839 15269 L 2789 15223 L
2739 15087 L 2732 14951 L 2704 14818 L 2703 14816 L
2704 14809 L 2839 14798 L 2849 14816 L 2975 14911 L
3011 14951 L 3110 15075 L 3122 15087 L 3246 15215 L
3282 15223 L 3381 15278 L 3461 15223 L 3517 15182 L
3652 15135 L 3712 15087 L 3763 14951 L 3788 14885 L
3923 14829 L 4059 14858 L 4169 14816 L 4194 14810 L
4330 14787 L 4465 14788 L 4601 14794 L 4632 14816 L
4736 14912 L 4806 14951 L 4872 14983 L 4961 15087 L
5007 15167 L 5093 15223 L 5143 15241 L 5165 15223 L
5278 15097 L 5281 15087 L 5278 15040 L 5267 14951 L
5194 14816 L 5169 14680 L 5143 14561 L 5137 14544 L
5103 14409 L 5135 14273 L 5143 14242 L 5143 14019 L
5139 14002 L 5143 13952 L 5164 14002 L 5143 14019 L
5143 14242 L 5167 14137 L 5278 14021 L 5386 14137 L
5414 14198 L 5451 14273 L 5517 14409 L 5549 14422 L
5633 14544 L 5685 14609 L 5726 14680 L 5797 14816 L
5820 14893 L 5885 14816 L 5956 14788 L 6045 14680 L
6091 14630 L 6227 14633 L 6283 14680 L 6361 14816 L
6362 14818 L 6498 14906 L 6633 14928 L 6685 14951 L
6769 14998 L 6844 15087 L 6904 15148 L 7040 15209 L

7084 15223 L 7175 15304 L 7208 15358 L 7289 15494 L
7311 15512 L 7446 15554 L 7582 15577 L 7683 15494 L
7717 15461 L 7853 15391 L 7988 15405 L 8124 15392 L
8240 15358 L 8259 15344 L 8357 15223 L 8344 15087 L
8294 14951 L 8259 14942 L 8124 14930 L 7988 14921 L
7889 14816 L 7904 14680 L 7913 14544 L 7923 14409 L
7988 14341 L 8124 14318 L 8259 14306 L 8395 14302 L
8424 14273 L 8452 14137 L 8460 14002 L 8467 13866 L
8530 13801 L 8666 13780 L 8801 13775 L 8898 13866 L
8937 13904 L 9053 14002 L 9072 14020 L 9208 14092 L
9343 14029 L 9460 14002 L 9479 13980 L 9614 13866 L
9614 13862 L 9627 13730 L 9614 13725 L 9479 13606 L
9470 13594 L 9343 13555 L 9259 13459 L 9246 13323 L
9208 13286 L 9072 13278 L 8992 13187 L 9060 13052 L
9072 13010 L 9208 12951 L 9268 12916 L 9343 12864 L
9479 12786 L 9504 12780 L 9614 12717 L 9672 12645 L
9729 12509 L 9750 12489 L 9868 12373 L 9885 12353 L
10021 12246 L 10038 12238 L 10156 12119 L 10168 12102 L
10178 11966 L 10156 11911 L 10117 11831 L 10059 11695 L
10021 11559 L 10021 11559 L 9945 11423 L 9911 11288 L
9920 11152 L 10021 11054 L 10059 11016 L 10156 10924 L
10292 10899 L 10327 10881 L 10427 10775 L 10445 10745 L
10476 10609 L 10494 10474 L 10563 10394 L 10627 10338 L
10654 10202 L 10698 10145 L 10789 10067 L 10818 9931 L
10834 9917 L 10969 9890 L 11054 9931 L 11105 9967 L
11231 10067 L 11240 10076 L 11376 10193 L 11383 10202 L
11485 10338 L 11511 10371 L 11583 10474 L 11647 10548 L
11708 10609 L 11782 10681 L 11838 10745 L 11918 10840 L
11943 10881 L 12037 11016 L 12053 11037 L 12068 11152 L
12088 11288 L 12114 11423 L 12141 11559 L 12169 11695 L
12189 11797 L 12199 11831 L 12216 11966 L 12258 12102 L
12290 12238 L 12324 12351 L 12334 12373 L 12385 12509 L
12422 12645 L 12460 12679 L 12595 12734 L 12731 12751 L
12813 12780 L 12731 12886 L 12595 12892 L 12581 12916 L
12585 13052 L 12595 13112 L 12671 13052 L 12731 12975 L
12742 12916 L 12866 12803 L 13002 12829 L 13002 12563 L
12866 12606 L 12731 12602 L 12595 12523 L 12587 12509 L
12517 12373 L 12460 12258 L 12451 12238 L 12401 12102 L
12340 11966 L 12324 11929 L 12300 11831 L 12263 11695 L
12214 11559 L 12189 11501 L 12171 11423 L 12141 11288 L
12118 11152 L 12097 11016 L 12081 10881 L 12071 10745 L
12060 10609 L 12053 10510 L 12031 10474 L 11926 10338 L
11918 10326 L 11818 10202 L 11782 10156 L 11716 10067 L

11647 10000 L 11567 9931 L 11511 9894 L 11376 9813 L
11342 9795 L 11240 9740 L 11105 9766 L 10969 9768 L
10940 9795 L 10834 9879 L 10775 9931 L 10735 10067 L
10698 10098 L 10617 10202 L 10583 10338 L 10563 10356 L
10460 10474 L 10443 10609 L 10427 10670 L 10368 10745 L
10292 10828 L 10219 10881 L 10156 10893 L 10026 11016 L
10021 11022 L 9887 11152 L 9885 11186 L 9850 11288 L
9849 11423 L 9822 11559 L 9885 11688 L 9890 11695 L
9885 11737 L 9750 11792 L 9689 11831 L 9750 11864 L
9885 11946 L 9889 11966 L 9885 11987 L 9869 12102 L
9860 12238 L 9760 12373 L 9750 12383 L 9622 12509 L
9614 12522 L 9479 12585 L 9355 12645 L 9343 12652 L
9208 12731 L 9122 12780 L 9072 12821 L 9020 12916 L
9020 13052 L 8976 13187 L 9072 13296 L 9208 13306 L
9225 13323 L 9241 13459 L 9343 13577 L 9401 13594 L
9479 13697 L 9520 13730 L 9493 13866 L 9479 13878 L
9372 14002 L 9343 14008 L 9208 14039 L 9092 14002 L
9072 13991 L 8937 13886 L 8916 13866 L 8801 13757 L
8666 13761 L 8530 13782 L 8449 13866 L 8442 14002 L
8435 14137 L 8403 14273 L 8395 14281 L 8259 14284 L
8124 14297 L 7988 14322 L 7905 14409 L 7896 14544 L
7886 14680 L 7869 14816 L 7988 14942 L 8121 14951 L
8124 14957 L 8184 15087 L 8124 15140 L 8071 15223 L
7988 15307 L 7853 15318 L 7717 15343 L 7699 15358 L
7582 15472 L 7446 15487 L 7311 15407 L 7282 15358 L
7218 15223 L 7175 15198 L 7040 15167 L 6904 15109 L
6882 15087 L 6769 14953 L 6766 14951 L 6633 14891 L
6498 14866 L 6421 14816 L 6362 14755 L 6319 14680 L
6227 14603 L 6091 14582 L 6001 14680 L 5956 14735 L
5820 14784 L 5761 14680 L 5685 14548 L 5682 14544 L
5620 14409 L 5549 14357 L 5494 14273 L 5429 14137 L
5414 14125 L 5299 14002 L 5278 13981 L 5173 13866 L
5143 13813 L 5043 13866 L 5095 14002 L 5121 14137 L
5089 14273 L 5053 14409 L 5082 14544 L 5120 14680 L
5143 14811 L 5143 14816 L 5162 14951 L 5155 15087 L
5143 15100 L 5126 15087 L 5007 14962 L 4998 14951 L
4882 14816 L 4872 14810 L 4736 14726 L 4661 14680 L
4601 14660 L 4465 14636 L 4330 14637 L 4284 14680 L
4194 14726 L 4059 14737 L 3963 14680 L 3923 14643 L
3846 14680 L 3788 14704 L 3656 14816 L 3652 14863 L
3626 14951 L 3517 15020 L 3487 15087 L 3381 15162 L
3246 15171 L 3165 15087 L 3110 15034 L 3044 14951 L
2975 14874 L 2899 14816 L 2839 14705 L 2807 14680 L

2704 14583 L 2568 14621 L 2433 14659 L 2405 14544 L
2433 14469 L 2466 14409 L 2568 14356 L 2635 14273 L
2678 14137 L 2698 14002 L 2704 13990 L 2815 13866 L
2839 13794 L 2870 13866 L 2874 14002 L 2874 14137 L
2975 14249 L 2975 14149 L 2964 14137 L 2953 14002 L
2975 13882 L 2993 13866 L 3110 13795 L 3129 13866 L
3191 14002 L 3110 14116 L 3036 14137 L 2975 14149 L
2975 14249 L 3110 14213 L 3234 14137 L 3246 14116 L
3273 14137 L 3286 14273 L 3361 14409 L 3272 14544 L
3381 14666 L 3431 14544 L 3412 14409 L 3517 14304 L
3610 14273 L 3652 14262 L 3788 14148 L 3923 14141 L
3941 14137 L 3980 14002 L 3923 13970 L 3832 14002 L
3788 14085 L 3742 14002 L 3652 13952 L 3517 13948 L
3381 13984 L 3246 13944 L 3202 13866 L 3167 13730 L
3110 13603 L 3102 13594 L 3024 13459 L 3017 13323 L
3023 13187 L 2985 13052 L 2975 13030 L 2920 12916 L
2857 12780 L 2856 12645 L 2839 12590 L 2704 12623 L
2638 12645 L 2704 12666 L 2812 12780 L 2729 12916 L
2704 12953 L 2633 13052 L 2568 13140 L 2433 13055 L
2418 13052 L 2297 12973 L N E
95 O
2297 13095 2297 13158 2413 13187 2433 13212 2487 13323 2568 13406
2635 13323 2704 13218 2713 13187 2776 13052 2839 12960 2886 13052
2943 13187 2933 13323 2904 13459 2839 13576 2826 13594 2745 13730
2704 13800 2657 13866 2595 14002 2568 14118 2556 14137 2433 14244
2315 14137 2357 14002 2386 13866 2391 13730 2424 13594 2297 13501
2297 13820 2311 13866 2297 13979 2297 14524 2324 14409 2433 14339
2562 14273 2568 14267 2621 14137 2647 14002 2704 13894 2729 13866
2799 13730 2839 13665 2952 13594 2960 13459 2975 13332 2976 13323
2986 13187 2975 13150 2935 13052 2868 12916 2839 12857 2800 12916
2707 13052 2704 13059 2621 13187 2568 13260 2519 13187 2433 13124
2297 13095 61 N F
82 O
2297 13158 2297 13501 2424 13594 2391 13730 2386 13866 2357 14002
2315 14137 2433 14244 2433 14084 2418 14002 2433 13949 2478 14002
2433 14084 2433 14244 2556 14137 2568 14118 2568 13924 2503 13866
2488 13730 2523 13594 2568 13537 2665 13459 2704 13408 2741 13323
2768 13187 2839 13058 2898 13187 2890 13323 2847 13459 2839 13472
2750 13594 2704 13692 2678 13730 2591 13866 2568 13924 2568 14118
2595 14002 2657 13866 2704 13800 2745 13730 2826 13594 2839 13576
2904 13459 2933 13323 2943 13187 2886 13052 2839 12960 2776 13052
2713 13187 2704 13218 2635 13323 2568 13406 2487 13323 2433 13212
2413 13187 2297 13158 56 N F

108 O
2311 13866 2297 13820 2297 13979 2311 13866 4 N F
95 O
2297 15202 M 2297 15602 L 2433 15572 L 2568 15587 L 2704 15615 L
2732 15630 L 2839 15705 L 2915 15765 L 2975 15792 L
3110 15875 L 3160 15901 L 3246 15923 L 3381 15980 L
3438 16037 L 3517 16072 L 3517 16033 L 3384 15901 L
3517 15855 L 3574 15901 L 3517 16033 L 3517 16072 L
3652 16143 L 3704 16172 L 3757 16172 L 3919 16172 L
4041 16172 L 4059 16165 L 4194 16136 L 4300 16037 L
4285 15901 L 4194 15825 L 4089 15901 L 4059 15922 L
3923 15959 L 3799 15901 L 3863 15765 L 3788 15672 L
3652 15755 L 3517 15736 L 3391 15630 L 3398 15494 L
3404 15358 L 3517 15266 L 3652 15238 L 3699 15223 L
3788 15166 L 3923 15088 L 3925 15087 L 4059 15041 L
4182 15087 L 4194 15091 L 4272 15087 L 4330 15081 L
4348 15087 L 4465 15110 L 4601 15136 L 4736 15122 L
4872 15155 L 4914 15223 L 5007 15321 L 5103 15358 L
5143 15404 L 5182 15358 L 5278 15287 L 5294 15223 L
5330 15087 L 5325 14951 L 5278 14874 L 5245 14816 L
5207 14680 L 5197 14544 L 5165 14409 L 5177 14273 L
5208 14137 L 5278 14064 L 5346 14137 L 5407 14273 L
5373 14409 L 5414 14442 L 5549 14492 L 5585 14544 L
5685 14670 L 5691 14680 L 5750 14816 L 5757 14951 L
5774 15087 L 5820 15151 L 5881 15223 L 5956 15293 L
5956 15151 L 5916 15087 L 5956 15072 L 5970 15087 L
5956 15151 L 5956 15293 L 6033 15358 L 6091 15408 L
6188 15358 L 6136 15223 L 6091 15128 L 6058 15087 L
5956 14980 L 5911 14951 L 5956 14897 L 5974 14816 L
6089 14680 L 6091 14677 L 6227 14663 L 6247 14680 L
6326 14816 L 6362 14898 L 6498 14946 L 6535 14951 L
6633 14969 L 6769 15044 L 6805 15087 L 6904 15186 L
6983 15223 L 7040 15279 L 7125 15358 L 7175 15422 L
7219 15494 L 7311 15567 L 7446 15617 L 7486 15630 L
7582 15683 L 7667 15630 L 7717 15590 L 7787 15494 L
7853 15452 L 7988 15478 L 8084 15494 L 8124 15505 L
8149 15494 L 8259 15453 L 8395 15405 L 8458 15358 L
8469 15223 L 8436 15087 L 8395 14979 L 8382 14951 L
8259 14920 L 8124 14908 L 7988 14900 L 7909 14816 L
7922 14680 L 7930 14544 L 7941 14409 L 7988 14359 L
8124 14339 L 8259 14327 L 8395 14323 L 8446 14273 L
8470 14137 L 8477 14002 L 8485 13866 L 8530 13819 L
8666 13800 L 8801 13792 L 8880 13866 L 8937 13922 L

9032 14002 L 9072 14041 L 9204 14137 L 9208 14141 L
9212 14137 L 9343 14049 L 9479 14021 L 9499 14002 L
9614 13890 L 9749 13866 L 9695 13730 L 9614 13700 L
9502 13594 L 9479 13570 L 9343 13534 L 9278 13459 L
9266 13323 L 9208 13266 L 9072 13259 L 9009 13187 L
9072 13072 L 9208 13060 L 9217 13052 L 9343 12938 L
9410 12916 L 9479 12862 L 9614 12832 L 9750 12818 L
9758 12780 L 9766 12645 L 9774 12509 L 9885 12399 L
10011 12373 L 10021 12365 L 10156 12351 L 10254 12238 L
10292 12186 L 10337 12102 L 10292 11966 L 10292 11966 L
10224 11831 L 10156 11699 L 10154 11695 L 10054 11559 L
10021 11427 L 10019 11423 L 9943 11288 L 9953 11152 L
10021 11086 L 10092 11016 L 10156 10956 L 10292 10934 L
10393 10881 L 10427 10845 L 10485 10745 L 10509 10609 L
10527 10474 L 10563 10433 L 10671 10338 L 10690 10202 L
10698 10192 L 10834 10172 L 10865 10202 L 10969 10289 L
11023 10338 L 11105 10397 L 11184 10474 L 11240 10525 L
11330 10609 L 11376 10663 L 11448 10745 L 11511 10799 L
11598 10881 L 11647 10927 L 11732 11016 L 11782 11078 L
11848 11152 L 11918 11247 L 11945 11288 L 12053 11403 L
12057 11423 L 12080 11559 L 12104 11695 L 12138 11831 L
12102 11966 L 12163 12102 L 12183 12238 L 12189 12267 L
12221 12373 L 12257 12509 L 12189 12599 L 12164 12645 L
12189 12708 L 12234 12780 L 12324 12838 L 12426 12916 L
12460 12983 L 12492 13052 L 12524 13187 L 12567 13323 L
12496 13459 L 12460 13508 L 12327 13594 L 12460 13645 L
12544 13730 L 12595 13756 L 12595 13605 L 12585 13594 L
12595 13580 L 12606 13594 L 12595 13605 L 12595 13756 L
12715 13730 L 12731 13716 L 12772 13594 L 12788 13459 L
12744 13323 L 12805 13187 L 12866 13111 L 13002 13163 L
13002 12829 L 12866 12803 L 12742 12916 L 12731 12975 L
12671 13052 L 12595 13112 L 12585 13052 L 12581 12916 L
12595 12892 L 12731 12886 L 12813 12780 L 12731 12751 L
12595 12734 L 12460 12679 L 12422 12645 L 12385 12509 L
12334 12373 L 12324 12351 L 12290 12238 L 12258 12102 L
12216 11966 L 12199 11831 L 12189 11797 L 12169 11695 L
12141 11559 L 12114 11423 L 12088 11288 L 12068 11152 L
12053 11037 L 12037 11016 L 11943 10881 L 11918 10840 L
11838 10745 L 11782 10681 L 11708 10609 L 11647 10548 L
11583 10474 L 11511 10371 L 11485 10338 L 11383 10202 L
11376 10193 L 11240 10076 L 11231 10067 L 11105 9967 L
11054 9931 L 10969 9890 L 10834 9917 L 10818 9931 L
10789 10067 L 10698 10145 L 10654 10202 L 10627 10338 L

10563 10394 L 10494 10474 L 10476 10609 L 10445 10745 L
10427 10775 L 10327 10881 L 10292 10899 L 10156 10924 L
10059 11016 L 10021 11054 L 9920 11152 L 9911 11288 L
9945 11423 L 10021 11559 L 10021 11559 L 10059 11695 L
10117 11831 L 10156 11911 L 10178 11966 L 10168 12102 L
10156 12119 L 10038 12238 L 10021 12246 L 9885 12353 L
9868 12373 L 9750 12489 L 9729 12509 L 9672 12645 L
9614 12717 L 9504 12780 L 9479 12786 L 9343 12864 L
9268 12916 L 9208 12951 L 9072 13010 L 9060 13052 L
8992 13187 L 9072 13278 L 9208 13286 L 9246 13323 L
9259 13459 L 9343 13555 L 9470 13594 L 9479 13606 L
9614 13725 L 9627 13730 L 9614 13862 L 9614 13866 L
9479 13980 L 9460 14002 L 9343 14029 L 9208 14092 L
9072 14020 L 9053 14002 L 8937 13904 L 8898 13866 L
8801 13775 L 8666 13780 L 8530 13801 L 8467 13866 L
8460 14002 L 8452 14137 L 8424 14273 L 8395 14302 L
8259 14306 L 8124 14318 L 7988 14341 L 7923 14409 L
7913 14544 L 7904 14680 L 7889 14816 L 7988 14921 L
8124 14930 L 8259 14942 L 8294 14951 L 8344 15087 L
8357 15223 L 8259 15344 L 8240 15358 L 8124 15392 L
7988 15405 L 7853 15391 L 7717 15461 L 7683 15494 L
7582 15577 L 7446 15554 L 7311 15512 L 7289 15494 L
7208 15358 L 7175 15304 L 7084 15223 L 7040 15209 L
6904 15148 L 6844 15087 L 6769 14998 L 6685 14951 L
6633 14928 L 6498 14906 L 6362 14818 L 6361 14816 L
6283 14680 L 6227 14633 L 6091 14630 L 6045 14680 L
5956 14788 L 5885 14816 L 5820 14893 L 5797 14816 L
5726 14680 L 5685 14609 L 5633 14544 L 5549 14422 L
5517 14409 L 5451 14273 L 5414 14198 L 5386 14137 L
5278 14021 L 5167 14137 L 5143 14242 L 5135 14273 L
5103 14409 L 5137 14544 L 5143 14561 L 5169 14680 L
5194 14816 L 5267 14951 L 5278 15040 L 5281 15087 L
5278 15097 L 5165 15223 L 5143 15241 L 5093 15223 L
5007 15167 L 4961 15087 L 4872 14983 L 4806 14951 L
4736 14912 L 4632 14816 L 4601 14794 L 4465 14788 L
4330 14787 L 4194 14810 L 4169 14816 L 4059 14858 L
3923 14829 L 3788 14885 L 3763 14951 L 3712 15087 L
3652 15135 L 3517 15182 L 3461 15223 L 3381 15278 L
3282 15223 L 3246 15215 L 3122 15087 L 3110 15075 L
3011 14951 L 2975 14911 L 2849 14816 L 2839 14798 L
2704 14809 L 2703 14816 L 2704 14818 L 2732 14951 L
2739 15087 L 2789 15223 L 2839 15269 L 2975 15347 L
3004 15358 L 3110 15486 L 3110 15369 L 3102 15358 L

2975 15310 L 2861 15223 L 2839 15195 L 2776 15087 L
2781 14951 L 2839 14870 L 2975 14949 L 2977 14951 L
3088 15087 L 3110 15127 L 3202 15223 L 3155 15358 L
3110 15369 L 3110 15486 L 3136 15494 L 3246 15578 L
3268 15630 L 3362 15765 L 3246 15834 L 3110 15783 L
3076 15765 L 2975 15711 L 2875 15630 L 2839 15584 L
2704 15535 L 2568 15507 L 2501 15494 L 2434 15358 L
2433 15353 L 2348 15223 L 2297 15202 L N E
82 O
2297 15602 2297 15771 2310 15765 2433 15689 2568 15675 2704 15695
2796 15765 2839 15791 2975 15851 3049 15901 3110 15932 3246 15968
3360 16037 3381 16047 3517 16108 3613 16172 3704 16172 3652 16143
3517 16072 3438 16037 3381 15980 3246 15923 3160 15901 3110 15875
2975 15792 2915 15765 2839 15705 2732 15630 2704 15615 2568 15587
2433 15572 2297 15602 32 N F
68 O
2297 15771 2297 15882 2345 15901 2428 16037 2433 16048 2568 16170
2574 16172 2622 16172 2784 16172 2946 16172 2947 16172 2839 16085
2801 16037 2704 15927 2664 15901 2704 15813 2839 15850 2956 15901
2975 15916 3110 15976 3246 16013 3285 16037 3381 16083 3517 16144
3559 16172 3595 16172 3613 16172 3517 16108 3381 16047 3360 16037
3246 15968 3110 15932 3049 15901 2975 15851 2839 15791 2796 15765
2704 15695 2568 15675 2433 15689 2310 15765 2297 15771 41 N F
55 O
2297 15882 2297 16172 2460 16172 2574 16172 2568 16170 2433 16048
2428 16037 2345 15901 2297 15882 9 N F
135 O
2421 12780 2372 12916 2433 12970 2568 13036 2568 12921 2555 12916
2538 12780 2568 12765 2595 12780 2572 12916 2568 12921 2568 13036
2652 12916 2704 12794 2711 12780 2704 12773 2568 12698 2433 12756
2421 12780 19 N F
121 O
2433 14710 2397 14816 2433 14925 2465 14951 2568 15009 2600 14951
2570 14816 2568 14812 2433 14710 9 N F
68 O
2433 14084 2418 14002 2433 13949 2478 14002 2433 14084 5 N F
2503 13866 2488 13730 2523 13594 2568 13537 2665 13459 2704 13408
2741 13323 2768 13187 2839 13058 2898 13187 2890 13323 2847 13459
2839 13472 2839 13346 2847 13323 2852 13187 2839 13159 2824 13187
2825 13323 2839 13346 2839 13472 2750 13594 2704 13692 2678 13730
2591 13866 2568 13924 2568 13735 2571 13730 2568 13725 2567 13730
2568 13735 2568 13924 2503 13866 33 N F
161 O

2568 9308 2498 9252 2540 9117 2568 9085 2658 9117 2704 9149
2839 9247 2975 9137 3085 9252 3110 9272 3166 9388 3213 9524
3177 9660 3246 9759 3289 9795 3381 9830 3517 9843 3559 9795
3517 9735 3486 9660 3473 9524 3517 9429 3609 9524 3652 9564
3693 9660 3714 9795 3695 9931 3733 10067 3782 10202 3788 10233
3923 10288 4045 10338 3923 10396 3788 10361 3652 10348 3631 10338
3517 10303 3517 10225 3652 10212 3661 10202 3652 10188 3560 10067
3517 10035 3418 10067 3493 10202 3517 10225 3517 10303 3410 10202
3381 10164 3277 10067 3246 10032 3110 9949 3085 9931 2975 9840
2916 9795 2839 9702 2805 9660 2750 9524 2704 9441 2667 9388
2568 9308 61 N F
148 O
2568 12765 2538 12780 2555 12916 2568 12921 2572 12916 2595 12780
2568 12765 7 N F
161 O
2568 11766 2556 11695 2568 11673 2661 11559 2704 11539 2839 11541
2975 11509 3110 11465 3246 11452 3381 11504 3404 11559 3381 11585
3263 11695 3246 11704 3110 11738 3110 11578 3246 11564 3251 11559
3246 11556 3110 11552 3094 11559 3110 11578 3110 11738 2975 11760
2839 11797 2704 11829 2704 11710 2839 11703 2879 11695 2839 11682
2704 11677 2688 11695 2704 11710 2704 11829 2568 11766 35 N F
2568 8304 2566 8303 2568 8298 2571 8303 2568 8304 5 N F
55 O
2568 13735 2567 13730 2568 13725 2571 13730 2568 13735 5 N F
135 O
2704 14275 2631 14409 2704 14463 2797 14544 2839 14584 2886 14680
2948 14816 2975 14836 3078 14951 3110 14992 3207 15087 3246 15127
3246 15069 3115 14951 3110 14947 3041 14816 2975 14744 2949 14680
2926 14544 2975 14485 3031 14544 3075 14680 3110 14712 3161 14816
3246 14900 3278 14951 3246 15069 3246 15127 3343 15087 3344 14951
3272 14816 3246 14785 3187 14680 3155 14544 3202 14409 3110 14352
2975 14355 2852 14273 2839 14258 2716 14273 2704 14275 41 N F
55 O
2704 15927 2664 15901 2704 15813 2839 15850 2956 15901 2975 15916
3110 15976 3246 16013 3285 16037 3381 16083 3517 16144 3559 16172
3501 16172 3381 16118 3246 16067 3174 16037 3110 16019 2975 16023
2954 16037 2975 16054 3064 16172 2947 16172 2839 16085 2801 16037
2704 15927 25 N F
121 O
2704 15262 2673 15358 2704 15384 2839 15378 2852 15358 2839 15352
2704 15262 7 N F
148 O
2704 11710 2688 11695 2704 11677 2839 11682 2879 11695 2839 11703

2704 11710 7 N F
82 O
2839 15195 2776 15087 2781 14951 2839 14870 2975 14949 2977 14951
3088 15087 3110 15127 3202 15223 3155 15358 3110 15369 3110 15282
3148 15223 3110 15183 3058 15087 2975 14986 2863 14951 2839 14938
2830 14951 2812 15087 2839 15133 2911 15223 2975 15272 3110 15282
3110 15369 3102 15358 2975 15310 2861 15223 2839 15195 29 N F
68 O
2839 15133 2812 15087 2830 14951 2839 14938 2863 14951 2975 14986
3058 15087 3110 15183 3148 15223 3110 15282 2975 15272 2975 15234
3043 15223 3027 15087 2975 15023 2858 15087 2960 15223 2975 15234
2975 15272 2911 15223 2839 15133 21 N F
55 O
2825 13323 2824 13187 2839 13159 2852 13187 2847 13323 2839 13346
2825 13323 7 N F
2960 15223 2858 15087 2975 15023 3027 15087 3043 15223 2975 15234
2975 15156 2997 15087 2975 15060 2926 15087 2975 15156 2975 15234
2960 15223 13 N F
42 O
2975 15156 2926 15087 2975 15060 2997 15087 2975 15156 5 N F
148 O
2975 14485 2926 14544 2949 14680 2975 14744 3041 14816 3110 14947
3115 14951 3246 15069 3278 14951 3246 14900 3161 14816 3110 14712
3075 14680 3031 14544 2975 14485 15 N F
55 O
2947 16173 2947 16172 3064 16172 3064 16173 2947 16173 5 N F
2947 16173 2947 16173 3064 16173 3064 16173 2975 16173 2947 16173
6 N F
95 O
2964 14137 2953 14002 2975 13882 2993 13866 3110 13795 3129 13866
3191 14002 3110 14116 3036 14137 2975 14149 2964 14137 11 N F
42 O
2975 16054 2954 16037 2975 16023 3110 16019 3174 16037 3246 16067
3381 16118 3501 16172 3433 16172 3423 16172 3381 16153 3246 16131
3173 16172 3108 16172 3064 16172 2975 16054 16 N F
108 O
3110 12447 2979 12373 3110 12259 3125 12238 3246 12173 3381 12144
3517 12125 3610 12238 3517 12283 3401 12373 3381 12389 3246 12438
3246 12414 3353 12373 3381 12355 3517 12255 3552 12238 3517 12195
3381 12168 3246 12197 3169 12238 3110 12326 3056 12373 3110 12404
3246 12414 3246 12438 3110 12447 27 N F
95 O
3110 12404 3056 12373 3110 12326 3169 12238 3246 12197 3381 12168

3517 12195 3552 12238 3517 12255 3381 12355 3353 12373 3246 12414
3246 12390 3290 12373 3381 12315 3494 12238 3381 12192 3246 12220
3214 12238 3151 12373 3246 12390 3246 12414 3110 12404 23 N F
188 O
3110 11822 3057 11831 3107 11966 3110 11967 3145 11966 3246 11948
3376 11966 3381 11966 3382 11966 3517 11856 3572 11831 3652 11751
3788 11717 3923 11727 3973 11695 4059 11593 4112 11559 4095 11423
4059 11415 4020 11423 3923 11471 3788 11501 3652 11545 3628 11559
3553 11695 3517 11732 3381 11792 3246 11813 3110 11822 29 N F
42 O
3064 16173 3064 16172 3108 16172 3173 16172 3173 16173 3108 16173
3064 16173 7 N F
148 O
3110 11578 3094 11559 3110 11552 3246 11556 3251 11559 3246 11564
3110 11578 7 N F
82 O
3246 12390 3151 12373 3214 12238 3246 12220 3381 12192 3494 12238
3381 12315 3381 12275 3436 12238 3381 12215 3276 12238 3381 12275
3381 12315 3290 12373 3246 12390 15 N F
29 O
3271 16173 3173 16173 3173 16172 3271 16172 3423 16172 3423 16173
3271 16173 7 N F
188 O
3203 6403 M 3195 6539 L 3246 6564 L 3302 6539 L 3343 6403 L
3381 6282 L 3405 6403 L 3467 6539 L 3517 6645 L
3517 6272 L 3439 6267 L 3517 6239 L 3521 6267 L
3517 6272 L 3517 6645 L 3525 6674 L 3652 6769 L
3788 6772 L 3816 6674 L 3804 6539 L 3830 6403 L
3923 6368 L 4059 6373 L 4194 6383 L 4314 6403 L
4330 6407 L 4334 6403 L 4465 6326 L 4601 6370 L
4646 6403 L 4736 6475 L 4872 6480 L 5007 6477 L
5143 6513 L 5278 6481 L 5340 6539 L 5407 6674 L
5414 6676 L 5549 6692 L 5685 6809 L 5712 6810 L
5685 6811 L 5648 6946 L 5685 7059 L 5820 7057 L
5840 7081 L 5956 7128 L 6027 7081 L 5988 6946 L
5984 6810 L 6066 6674 L 6091 6610 L 6209 6539 L
6227 6536 L 6237 6539 L 6362 6587 L 6416 6674 L
6480 6810 L 6498 6841 L 6633 6894 L 6736 6946 L
6769 6966 L 6883 6946 L 6858 6810 L 6904 6768 L
6954 6810 L 7040 6873 L 7175 6885 L 7175 6247 L
7040 6179 L 6904 6149 L 6889 6132 L 6904 6107 L
6950 5996 L 7040 5943 L 7175 5972 L 7204 5996 L
7261 6132 L 7175 6247 L 7175 6885 L 7275 6946 L

7311 6969 L 7446 6958 L 7458 6946 L 7511 6810 L
7533 6674 L 7525 6539 L 7582 6479 L 7717 6419 L
7853 6452 L 7953 6539 L 7988 6570 L 8124 6639 L
8177 6674 L 8259 6745 L 8387 6810 L 8395 6823 L
8467 6946 L 8530 6967 L 8644 7081 L 8666 7097 L
8788 7217 L 8801 7241 L 8905 7353 L 8937 7406 L
8937 6152 L 8907 6132 L 8801 6092 L 8666 6039 L
8587 5996 L 8530 5884 L 8517 5860 L 8484 5725 L
8530 5681 L 8666 5619 L 8788 5589 L 8801 5586 L
8937 5579 L 8956 5589 L 9039 5725 L 9072 5771 L
9092 5860 L 9095 5996 L 9072 6070 L 8966 6132 L
8937 6152 L 8937 7406 L 9031 7489 L 9025 7624 L
9072 7690 L 9135 7760 L 9072 7875 L 9053 7896 L
8960 8031 L 9072 8082 L 9167 8031 L 9208 7970 L
9208 5785 L 9161 5725 L 9208 5615 L 9290 5725 L
9208 5785 L 9208 7970 L 9270 8031 L 9343 8043 L
9358 8031 L 9343 7966 L 9274 7896 L 9234 7760 L
9303 7624 L 9343 7606 L 9343 6652 L 9290 6539 L
9343 6446 L 9417 6539 L 9343 6652 L 9343 7606 L
9430 7624 L 9479 7635 L 9479 6993 L 9446 6946 L
9436 6810 L 9479 6767 L 9539 6810 L 9547 6946 L
9479 6993 L 9479 7635 L 9520 7624 L 9614 7600 L
9614 7130 L 9555 7081 L 9614 7000 L 9724 7081 L
9614 7130 L 9614 7600 L 9750 7525 L 9885 7547 L
9885 7153 L 9802 7081 L 9773 6946 L 9750 6935 L
9640 6810 L 9614 6738 L 9568 6674 L 9588 6539 L
9614 6490 L 9620 6403 L 9614 6387 L 9532 6267 L
9488 6132 L 9491 5996 L 9517 5860 L 9614 5729 L
9750 5851 L 9758 5860 L 9836 5996 L 9874 6132 L
9885 6202 L 9917 6267 L 9967 6403 L 9999 6539 L
10021 6632 L 10060 6674 L 10112 6810 L 10083 6946 L
10021 7081 L 10021 7082 L 9885 7153 L 9885 7547 L
10021 7514 L 10130 7489 L 10156 7484 L 10196 7489 L
10292 7531 L 10427 7605 L 10563 7563 L 10698 7580 L
10834 7554 L 10898 7489 L 10969 7451 L 11105 7433 L
11240 7421 L 11376 7428 L 11511 7436 L 11647 7449 L
11782 7457 L 11918 7472 L 11996 7489 L 12053 7531 L
12077 7624 L 12108 7760 L 12123 7896 L 12126 8031 L
12131 8167 L 12141 8303 L 12151 8438 L 12157 8574 L
12169 8710 L 12179 8845 L 12187 8981 L 12189 9022 L
12244 9117 L 12324 9248 L 12330 9252 L 12460 9343 L
12520 9388 L 12595 9443 L 12731 9464 L 12866 9507 L
12989 9524 L 13002 9526 L 13002 5453 L 12839 5453 L

12677 5453 L 12515 5453 L 12353 5453 L 12191 5453 L
12028 5453 L 11866 5453 L 11704 5453 L 11542 5453 L
11380 5453 L 11218 5453 L 11055 5453 L 10893 5453 L
10731 5453 L 10569 5453 L 10407 5453 L 10244 5453 L
10082 5453 L 9920 5453 L 9758 5453 L 9596 5453 L
9434 5453 L 9271 5453 L 9109 5453 L 8947 5453 L
8785 5453 L 8623 5453 L 8460 5453 L 8298 5453 L
8136 5453 L 7974 5453 L 7812 5453 L 7650 5453 L
7487 5453 L 7325 5453 L 7163 5453 L 7001 5453 L
6839 5453 L 6676 5453 L 6514 5453 L 6352 5453 L
6190 5453 L 6158 5453 L 6204 5589 L 6158 5725 L
6091 5800 L 5956 5849 L 5941 5860 L 5843 5996 L
5820 6019 L 5697 6132 L 5685 6158 L 5608 6267 L
5549 6322 L 5414 6296 L 5388 6267 L 5281 6132 L
5278 6126 L 5143 6069 L 5007 6023 L 4911 5996 L
4872 5981 L 4736 5957 L 4673 5996 L 4601 6028 L
4498 5996 L 4485 5860 L 4465 5837 L 4436 5725 L
4393 5589 L 4330 5507 L 4222 5589 L 4194 5649 L
4059 5715 L 4034 5725 L 3988 5860 L 3923 5911 L
3788 5896 L 3673 5860 L 3652 5851 L 3517 5810 L
3381 5844 L 3359 5860 L 3343 5996 L 3381 6024 L
3502 6132 L 3381 6264 L 3377 6267 L 3246 6345 L
3203 6403 L N E
29 O
3173 16172 3246 16131 3381 16153 3423 16172 3271 16172 3173 16172
6 N F
68 O
3381 12275 3276 12238 3381 12215 3436 12238 3381 12275 5 N F
148 O
3381 12872 3346 12780 3381 12763 3434 12780 3381 12872 5 N F
108 O
3517 15855 3384 15901 3517 16033 3574 15901 3517 15855 5 N F
82 O
3517 15736 M 3391 15630 L 3398 15494 L 3404 15358 L 3517 15266 L
3652 15238 L 3699 15223 L 3788 15166 L 3923 15088 L
3925 15087 L 4059 15041 L 4182 15087 L 4194 15091 L
4272 15087 L 4330 15081 L 4348 15087 L 4465 15110 L
4601 15136 L 4736 15122 L 4872 15155 L 4914 15223 L
5007 15321 L 5103 15358 L 5143 15404 L 5182 15358 L
5278 15287 L 5294 15223 L 5330 15087 L 5325 14951 L
5278 14874 L 5245 14816 L 5207 14680 L 5197 14544 L
5165 14409 L 5177 14273 L 5208 14137 L 5278 14064 L
5346 14137 L 5407 14273 L 5373 14409 L 5414 14442 L

5549 14492 L 5585 14544 L 5685 14670 L 5691 14680 L
5750 14816 L 5757 14951 L 5774 15087 L 5820 15151 L
5881 15223 L 5956 15293 L 6033 15358 L 6091 15408 L
6188 15358 L 6136 15223 L 6091 15128 L 6058 15087 L
5956 14980 L 5911 14951 L 5956 14897 L 5974 14816 L
6089 14680 L 6091 14677 L 6227 14663 L 6247 14680 L
6326 14816 L 6362 14898 L 6498 14946 L 6535 14951 L
6633 14969 L 6769 15044 L 6805 15087 L 6904 15186 L
6983 15223 L 7040 15279 L 7125 15358 L 7175 15422 L
7219 15494 L 7311 15567 L 7446 15617 L 7486 15630 L
7582 15683 L 7667 15630 L 7717 15590 L 7787 15494 L
7853 15452 L 7988 15478 L 8084 15494 L 8124 15505 L
8149 15494 L 8259 15453 L 8395 15405 L 8458 15358 L
8469 15223 L 8436 15087 L 8395 14979 L 8382 14951 L
8259 14920 L 8124 14908 L 7988 14900 L 7909 14816 L
7922 14680 L 7930 14544 L 7941 14409 L 7988 14359 L
8124 14339 L 8259 14327 L 8395 14323 L 8446 14273 L
8470 14137 L 8477 14002 L 8485 13866 L 8530 13819 L
8666 13800 L 8801 13792 L 8880 13866 L 8937 13922 L
9032 14002 L 9072 14041 L 9204 14137 L 9208 14141 L
9212 14137 L 9343 14049 L 9479 14021 L 9499 14002 L
9614 13890 L 9749 13866 L 9695 13730 L 9614 13700 L
9502 13594 L 9479 13570 L 9343 13534 L 9278 13459 L
9266 13323 L 9208 13266 L 9072 13259 L 9009 13187 L
9072 13072 L 9208 13060 L 9217 13052 L 9343 12938 L
9410 12916 L 9479 12862 L 9614 12832 L 9750 12818 L
9758 12780 L 9766 12645 L 9774 12509 L 9885 12399 L
10011 12373 L 10021 12365 L 10156 12351 L 10254 12238 L
10292 12186 L 10337 12102 L 10292 11966 L 10292 11966 L
10224 11831 L 10156 11699 L 10154 11695 L 10054 11559 L
10021 11427 L 10019 11423 L 9943 11288 L 9953 11152 L
10021 11086 L 10092 11016 L 10156 10956 L 10292 10934 L
10393 10881 L 10427 10845 L 10485 10745 L 10509 10609 L
10527 10474 L 10563 10433 L 10671 10338 L 10690 10202 L
10698 10192 L 10834 10172 L 10865 10202 L 10969 10289 L
11023 10338 L 11105 10397 L 11184 10474 L 11240 10525 L
11330 10609 L 11376 10663 L 11448 10745 L 11511 10799 L
11598 10881 L 11647 10927 L 11732 11016 L 11782 11078 L
11848 11152 L 11918 11247 L 11945 11288 L 12053 11403 L
12057 11423 L 12080 11559 L 12104 11695 L 12138 11831 L
12102 11966 L 12163 12102 L 12183 12238 L 12189 12267 L
12221 12373 L 12257 12509 L 12189 12599 L 12164 12645 L
12189 12708 L 12234 12780 L 12324 12838 L 12426 12916 L

12460 12983 L 12492 13052 L 12524 13187 L 12567 13323 L
12496 13459 L 12460 13508 L 12327 13594 L 12460 13645 L
12544 13730 L 12595 13756 L 12715 13730 L 12731 13716 L
12772 13594 L 12788 13459 L 12744 13323 L 12805 13187 L
12866 13111 L 13002 13163 L 13002 13459 L 13001 13459 L
13002 13459 L 13002 14028 L 12971 14002 L 12950 13866 L
12900 13730 L 12866 13651 L 12848 13730 L 12731 13830 L
12595 13828 L 12460 13781 L 12324 13785 L 12250 13866 L
12207 14002 L 12189 14008 L 12189 13627 L 12319 13594 L
12200 13459 L 12189 13452 L 12053 13355 L 12047 13459 L
12053 13477 L 12141 13594 L 12189 13627 L 12189 14008 L
12179 14002 L 12053 13898 L 11918 13892 L 11889 13866 L
11918 13760 L 11945 13730 L 11950 13594 L 11938 13459 L
11918 13381 L 11912 13323 L 11918 13282 L 11968 13187 L
12053 13121 L 12152 13187 L 12189 13280 L 12202 13323 L
12324 13407 L 12429 13323 L 12435 13187 L 12398 13052 L
12324 12916 L 12189 12922 L 12185 12916 L 12119 12780 L
12088 12645 L 12156 12509 L 12135 12373 L 12110 12238 L
12100 12102 L 12053 12025 L 12053 11894 L 12081 11831 L
12053 11737 L 11934 11831 L 12053 11894 L 12053 12025 L
11957 11966 L 11918 11862 L 11912 11831 L 11918 11810 L
12004 11695 L 11918 11596 L 11873 11559 L 11782 11468 L
11742 11423 L 11647 11303 L 11634 11288 L 11525 11152 L
11511 11136 L 11388 11016 L 11376 11006 L 11240 10898 L
11219 10881 L 11105 10773 L 11067 10745 L 10969 10661 L
10897 10609 L 10834 10566 L 10717 10474 L 10698 10461 L
10563 10471 L 10561 10474 L 10543 10609 L 10525 10745 L
10465 10881 L 10427 10912 L 10292 10968 L 10156 10987 L
10125 11016 L 10021 11118 L 9986 11152 L 9975 11288 L
10021 11368 L 10063 11423 L 10087 11559 L 10156 11652 L
10216 11695 L 10292 11805 L 10303 11831 L 10359 11966 L
10427 12067 L 10504 12102 L 10563 12199 L 10575 12238 L
10563 12342 L 10546 12373 L 10427 12504 L 10425 12509 L
10292 12526 L 10274 12509 L 10156 12403 L 10021 12405 L
9885 12428 L 9804 12509 L 9796 12645 L 9788 12780 L
9761 12916 L 9750 12928 L 9654 12916 L 9614 12906 L
9572 12916 L 9479 12928 L 9343 12970 L 9252 13052 L
9208 13093 L 9072 13102 L 9025 13187 L 9072 13241 L
9208 13246 L 9287 13323 L 9296 13459 L 9343 13513 L
9479 13543 L 9530 13594 L 9614 13674 L 9750 13723 L
9778 13730 L 9885 13841 L 9894 13866 L 9885 13872 L
9750 13895 L 9614 13915 L 9524 14002 L 9479 14047 L
9343 14069 L 9242 14137 L 9208 14169 L 9175 14137 L

9072 14062 L 9010 14002 L 8937 13940 L 8862 13866 L
8801 13809 L 8666 13819 L 8530 13837 L 8503 13866 L
8495 14002 L 8488 14137 L 8467 14273 L 8395 14344 L
8259 14348 L 8124 14360 L 7988 14378 L 7959 14409 L
7947 14544 L 7940 14680 L 7929 14816 L 7988 14878 L
8124 14886 L 8259 14897 L 8395 14926 L 8419 14951 L
8505 15087 L 8530 15159 L 8540 15223 L 8565 15358 L
8530 15421 L 8414 15494 L 8395 15503 L 8259 15554 L
8124 15621 L 7988 15558 L 7853 15519 L 7763 15630 L
7717 15720 L 7636 15765 L 7582 15789 L 7544 15765 L
7446 15707 L 7330 15630 L 7311 15622 L 7175 15523 L
7040 15550 L 6980 15630 L 6904 15678 L 6771 15765 L
6769 15767 L 6769 15658 L 6904 15632 L 6907 15630 L
6904 15580 L 6769 15568 L 6734 15630 L 6769 15658 L
6769 15767 L 6651 15765 L 6633 15763 L 6585 15630 L
6576 15494 L 6498 15448 L 6397 15358 L 6480 15223 L
6498 15178 L 6533 15223 L 6633 15293 L 6695 15358 L
6769 15435 L 6904 15420 L 7033 15358 L 6904 15233 L
6900 15223 L 6769 15091 L 6763 15087 L 6633 15017 L
6498 15032 L 6362 14976 L 6336 14951 L 6291 14816 L
6227 14706 L 6091 14722 L 6012 14816 L 6004 14951 L
6091 15042 L 6126 15087 L 6188 15223 L 6227 15290 L
6332 15358 L 6227 15432 L 6091 15494 L 5956 15401 L
5936 15358 L 5820 15248 L 5794 15223 L 5698 15087 L
5685 15036 L 5666 14951 L 5685 14893 L 5702 14816 L
5685 14774 L 5649 14680 L 5549 14558 L 5479 14544 L
5414 14524 L 5278 14426 L 5278 14404 L 5355 14273 L
5306 14137 L 5278 14107 L 5250 14137 L 5218 14273 L
5278 14404 L 5278 14426 L 5256 14544 L 5244 14680 L
5278 14779 L 5295 14816 L 5377 14951 L 5380 15087 L
5351 15223 L 5354 15358 L 5414 15438 L 5453 15494 L
5474 15630 L 5414 15749 L 5388 15765 L 5278 15785 L
5143 15790 L 5143 15725 L 5278 15700 L 5374 15630 L
5278 15537 L 5143 15525 L 5007 15515 L 4915 15630 L
5007 15687 L 5143 15725 L 5143 15790 L 5007 15773 L
4872 15771 L 4736 15811 L 4601 15821 L 4465 15843 L
4405 15901 L 4385 16037 L 4330 16161 L 4330 15711 L
4410 15630 L 4465 15598 L 4601 15551 L 4736 15511 L
4766 15494 L 4872 15365 L 4876 15358 L 4872 15354 L
4736 15270 L 4601 15274 L 4465 15298 L 4353 15223 L
4330 15210 L 4296 15223 L 4194 15319 L 4172 15358 L
4141 15494 L 4183 15630 L 4194 15639 L 4330 15711 L
4330 16161 L 4317 16172 L 4244 16172 L 4081 16172 L

4041 16172 L 4059 16165 L 4194 16136 L 4300 16037 L
4285 15901 L 4194 15825 L 4089 15901 L 4059 15922 L
3923 15959 L 3923 15598 L 3997 15494 L 3923 15452 L
3898 15494 L 3923 15598 L 3923 15959 L 3799 15901 L
3863 15765 L 3788 15672 L 3652 15755 L 3517 15736 L
3517 15652 L 3652 15646 L 3671 15630 L 3722 15494 L
3701 15358 L 3652 15334 L 3517 15333 L 3486 15358 L
3454 15494 L 3491 15630 L 3517 15652 L 3517 15736 L
N E
148 O
3493 10202 3418 10067 3517 10035 3560 10067 3652 10188 3661 10202
3652 10212 3517 10225 3493 10202 9 N F
42 O
3423 16172 3423 16173 3433 16173 3501 16173 3501 16172 3433 16172
3423 16172 7 N F
174 O
3517 6272 3439 6267 3517 6239 3521 6267 3517 6272 5 N F
68 O
3491 15630 3454 15494 3486 15358 3517 15333 3652 15334 3701 15358
3722 15494 3671 15630 3652 15646 3517 15652 3517 15514 3543 15494
3517 15471 3510 15494 3517 15514 3517 15652 3491 15630 17 N F
108 O
3517 14874 3464 14816 3517 14756 3632 14816 3517 14874 5 N F
55 O
3501 16172 3501 16173 3560 16173 3559 16172 3501 16172 5 N F
3560 16173 3501 16173 3501 16173 3517 16173 3560 16173 3560 16173
6 N F
3517 15514 3510 15494 3517 15471 3543 15494 3517 15514 5 N F
161 O
3562 11288 3539 11152 3592 11016 3652 10962 3750 10881 3788 10849
3923 10855 4059 10879 4194 10801 4256 10745 4330 10655 4465 10657
4601 10742 4736 10645 4810 10745 4742 10881 4736 10886 4639 11016
4601 11049 4465 11071 4465 11009 4601 10977 4687 10881 4601 10795
4493 10745 4465 10727 4413 10745 4330 10767 4211 10881 4330 10976
4465 11009 4465 11071 4330 11031 4194 11084 4164 11152 4059 11273
4018 11288 3923 11341 3788 11363 3788 11279 3923 11275 4059 11211
4110 11152 4161 11016 4059 10939 3923 10903 3788 10905 3669 11016
3652 11109 3633 11152 3652 11192 3788 11279 3788 11363 3652 11361
3562 11288 55 N F
108 O
3652 12184 3547 12102 3652 12065 3749 12102 3652 12184 3652 12111
3663 12102 3652 12098 3641 12102 3652 12111 3652 12184 11 N F
68 O

3559 16172 3560 16173 3595 16173 3613 16173 3613 16172 3595 16172
3559 16172 7 N F
82 O
3613 16172 3613 16173 3704 16173 3704 16172 3613 16172 5 N F
3704 16173 3613 16173 3613 16173 3652 16173 3704 16173 3704 16173
6 N F
148 O
3652 11192 3633 11152 3652 11109 3669 11016 3788 10905 3923 10903
4059 10939 4161 11016 4110 11152 4059 11211 3923 11275 3923 11245
4057 11152 4059 11134 4083 11016 4059 10998 3923 10931 3788 10940
3707 11016 3700 11152 3788 11228 3923 11245 3923 11275 3788 11279
3652 11192 25 N F
95 O
3652 12111 3641 12102 3652 12098 3663 12102 3652 12111 5 N F
135 O
3788 11228 3700 11152 3707 11016 3788 10940 3923 10931 4059 10998
4083 11016 4059 11134 4057 11152 3923 11245 3923 11215 4014 11152
4024 11016 3923 10959 3788 10976 3745 11016 3760 11152 3788 11176
3923 11215 3923 11245 3788 11228 21 N F
121 O
3760 11152 3745 11016 3788 10976 3923 10959 4024 11016 4014 11152
3923 11215 3923 11185 3971 11152 3974 11016 3923 10988 3788 11011
3782 11016 3788 11047 3832 11152 3923 11185 3923 11215 3788 11176
3760 11152 19 N F
108 O
3788 11047 3782 11016 3788 11011 3923 10988 3974 11016 3971 11152
3923 11185 3923 11155 3928 11152 3924 11016 3923 11016 3921 11016
3915 11152 3923 11155 3923 11185 3832 11152 3788 11047 17 N F
135 O
3923 13244 3798 13187 3853 13052 3923 12997 3947 13052 3936 13187
3923 13244 7 N F
188 O
3923 12487 3846 12509 3923 12579 4042 12645 4059 12649 4194 12670
4330 12779 4331 12780 4465 12797 4537 12780 4545 12645 4465 12627
4330 12629 4194 12593 4134 12509 4059 12483 3923 12487 17 N F
95 O
3923 15452 3898 15494 3923 15598 3997 15494 3923 15452 5 N F
3923 11155 3915 11152 3921 11016 3923 11016 3924 11016 3928 11152
3923 11155 7 N F
161 O
4034 10067 3962 9931 4059 9825 4194 9892 4330 9898 4465 9910
4486 9931 4601 10016 4645 10067 4601 10183 4573 10202 4586 10338
4465 10394 4348 10338 4330 10290 4330 10112 4465 10080 4481 10067

4465 10055 4330 10017 4194 10028 4161 10067 4194 10092 4330 10112
4330 10290 4302 10202 4194 10181 4059 10095 4034 10067 29 N F
4046 7896 4000 7760 4013 7624 4059 7568 4194 7601 4233 7624
4330 7694 4389 7760 4450 7896 4330 7951 4194 7942 4059 7906
4046 7896 13 N F
4059 10474 4058 10474 4059 10465 4194 10447 4327 10474 4194 10489
4059 10474 7 N F
68 O
4183 15630 4141 15494 4172 15358 4194 15319 4296 15223 4330 15210
4353 15223 4465 15298 4601 15274 4736 15270 4872 15354 4876 15358
4872 15365 4766 15494 4736 15511 4601 15551 4465 15598 4410 15630
4330 15711 4330 15568 4465 15506 4500 15494 4465 15470 4374 15358
4330 15325 4296 15358 4251 15494 4330 15568 4330 15711 4194 15639
4183 15630 31 N F
148 O
4194 10092 4161 10067 4194 10028 4330 10017 4465 10055 4481 10067
4465 10080 4330 10112 4194 10092 9 N F
4330 10976 4211 10881 4330 10767 4413 10745 4465 10727 4493 10745
4601 10795 4687 10881 4601 10977 4465 11009 4465 10982 4601 10920
4636 10881 4601 10846 4465 10768 4330 10822 4269 10881 4330 10930
4465 10982 4465 11009 4330 10976 21 N F
55 O
4330 15568 4251 15494 4296 15358 4330 15325 4374 15358 4465 15470
4500 15494 4465 15506 4330 15568 9 N F
135 O
4330 10930 4269 10881 4330 10822 4465 10768 4601 10846 4636 10881
4601 10920 4465 10982 4465 10954 4587 10881 4465 10798 4330 10878
4327 10881 4330 10883 4465 10954 4465 10982 4330 10930 17 N F
188 O
4307 11152 4279 11288 4322 11423 4330 11431 4417 11559 4330 11665
4306 11695 4330 11723 4465 11760 4601 11741 4686 11831 4736 11932
4872 11914 5007 11903 5143 11866 5143 10104 5087 10067 5143 9953
5195 10067 5143 10104 5143 11866 5242 11831 5278 11802 5331 11831
5414 11862 5515 11966 5506 12102 5414 12206 5368 12238 5331 12373
5414 12466 5444 12509 5549 12605 5685 12626 5733 12509 5697 12373
5695 12238 5717 12102 5702 11966 5742 11831 5765 11695 5820 11615
5956 11564 6073 11695 6091 11724 6227 11707 6362 11700 6391 11695
6362 11670 6227 11631 6159 11559 6227 11514 6312 11423 6227 11292
6091 11323 6059 11288 6091 11256 6227 11284 6281 11152 6227 11104
6091 11104 5956 11128 5821 11016 5854 10881 5840 10745 5834 10609
5822 10474 5820 10459 5777 10338 5699 10202 5765 10067 5820 9994
5849 9931 5890 9795 5820 9713 5738 9660 5704 9524 5727 9388
5814 9252 5713 9117 5685 9054 5624 9117 5549 9157 5414 9160

5278 9234 5230 9252 5152 9388 5143 9412 5044 9524 5007 9553
4872 9565 4806 9660 4767 9795 4825 9931 4872 10002 4943 10067
4872 10116 4830 10202 4736 10318 4731 10338 4736 10407 4794 10474
4872 10493 4945 10609 4981 10745 5007 10788 5061 10881 5008 11016
5007 11017 4872 11058 4736 11141 4690 11152 4601 11201 4556 11152
4465 11146 4330 11142 4307 11152 117 N F
68 O
4330 16173 4317 16173 4317 16173 4381 16173 4381 16173 4330 16173
6 N F
4381 16173 4317 16173 4317 16172 4381 16172 4381 16173 5 N F
121 O
4330 10883 4327 10881 4330 10878 4465 10798 4587 10881 4465 10954
4465 10927 4542 10881 4465 10829 4378 10881 4465 10927 4465 10954
4330 10883 13 N F
68 O
4317 16172 M 4330 16161 L 4385 16037 L 4405 15901 L 4465 15843 L
4601 15821 L 4736 15811 L 4872 15771 L 5007 15773 L
5143 15790 L 5278 15785 L 5388 15765 L 5414 15749 L
5474 15630 L 5453 15494 L 5414 15438 L 5354 15358 L
5351 15223 L 5380 15087 L 5377 14951 L 5295 14816 L
5278 14779 L 5244 14680 L 5256 14544 L 5278 14426 L
5414 14524 L 5479 14544 L 5549 14558 L 5649 14680 L
5685 14774 L 5702 14816 L 5685 14893 L 5666 14951 L
5685 15036 L 5698 15087 L 5794 15223 L 5820 15248 L
5936 15358 L 5956 15401 L 6091 15494 L 6227 15432 L
6332 15358 L 6227 15290 L 6188 15223 L 6126 15087 L
6091 15042 L 6004 14951 L 6012 14816 L 6091 14722 L
6227 14706 L 6291 14816 L 6336 14951 L 6362 14976 L
6498 15032 L 6633 15017 L 6763 15087 L 6769 15091 L
6900 15223 L 6904 15233 L 7033 15358 L 6904 15420 L
6769 15435 L 6769 15346 L 6832 15223 L 6769 15160 L
6656 15223 L 6769 15346 L 6769 15435 L 6695 15358 L
6633 15293 L 6633 15183 L 6673 15087 L 6633 15066 L
6563 15087 L 6633 15183 L 6633 15293 L 6533 15223 L
6498 15178 L 6480 15223 L 6397 15358 L 6498 15448 L
6576 15494 L 6585 15630 L 6633 15763 L 6651 15765 L
6769 15767 L 6771 15765 L 6904 15678 L 6980 15630 L
7040 15550 L 7175 15523 L 7311 15622 L 7330 15630 L
7446 15707 L 7544 15765 L 7582 15789 L 7636 15765 L
7717 15720 L 7763 15630 L 7853 15519 L 7988 15558 L
8124 15621 L 8259 15554 L 8395 15503 L 8414 15494 L
8530 15421 L 8565 15358 L 8540 15223 L 8530 15159 L
8505 15087 L 8419 14951 L 8395 14926 L 8259 14897 L

8124 14886 L 7988 14878 L 7929 14816 L 7940 14680 L
7947 14544 L 7959 14409 L 7988 14378 L 8124 14360 L
8259 14348 L 8395 14344 L 8467 14273 L 8488 14137 L
8495 14002 L 8503 13866 L 8530 13837 L 8666 13819 L
8801 13809 L 8862 13866 L 8937 13940 L 9010 14002 L
9072 14062 L 9175 14137 L 9208 14169 L 9242 14137 L
9343 14069 L 9479 14047 L 9524 14002 L 9614 13915 L
9750 13895 L 9885 13872 L 9894 13866 L 9885 13841 L
9778 13730 L 9750 13723 L 9614 13674 L 9530 13594 L
9479 13543 L 9343 13513 L 9296 13459 L 9287 13323 L
9208 13246 L 9072 13241 L 9025 13187 L 9072 13102 L
9208 13093 L 9252 13052 L 9343 12970 L 9479 12928 L
9572 12916 L 9614 12906 L 9654 12916 L 9750 12928 L
9761 12916 L 9788 12780 L 9796 12645 L 9804 12509 L
9885 12428 L 10021 12405 L 10156 12403 L 10274 12509 L
10292 12526 L 10425 12509 L 10427 12504 L 10546 12373 L
10563 12342 L 10575 12238 L 10563 12199 L 10504 12102 L
10427 12067 L 10359 11966 L 10303 11831 L 10292 11805 L
10216 11695 L 10156 11652 L 10087 11559 L 10063 11423 L
10021 11368 L 9975 11288 L 9986 11152 L 10021 11118 L
10125 11016 L 10156 10987 L 10292 10968 L 10427 10912 L
10465 10881 L 10525 10745 L 10543 10609 L 10561 10474 L
10563 10471 L 10698 10461 L 10717 10474 L 10834 10566 L
10897 10609 L 10969 10661 L 11067 10745 L 11105 10773 L
11219 10881 L 11240 10898 L 11376 11006 L 11388 11016 L
11511 11136 L 11525 11152 L 11634 11288 L 11647 11303 L
11742 11423 L 11782 11468 L 11873 11559 L 11918 11596 L
12004 11695 L 11918 11810 L 11912 11831 L 11918 11862 L
11957 11966 L 12053 12025 L 12100 12102 L 12110 12238 L
12135 12373 L 12156 12509 L 12088 12645 L 12119 12780 L
12185 12916 L 12189 12922 L 12324 12916 L 12398 13052 L
12435 13187 L 12429 13323 L 12324 13407 L 12202 13323 L
12189 13280 L 12152 13187 L 12053 13121 L 11968 13187 L
11918 13282 L 11912 13323 L 11918 13381 L 11938 13459 L
11950 13594 L 11945 13730 L 11918 13760 L 11889 13866 L
11918 13892 L 12053 13898 L 12179 14002 L 12189 14008 L
12207 14002 L 12250 13866 L 12324 13785 L 12460 13781 L
12595 13828 L 12731 13830 L 12848 13730 L 12866 13651 L
12900 13730 L 12950 13866 L 12971 14002 L 13002 14028 L
13002 14083 L 12908 14002 L 12866 13913 L 12731 13908 L
12595 13914 L 12460 13948 L 12434 14002 L 12324 14108 L
12263 14137 L 12189 14171 L 12150 14137 L 12053 14061 L
11972 14002 L 11918 13974 L 11800 13866 L 11782 13778 L

11771 13730 L 11782 13707 L 11837 13594 L 11886 13459 L
11876 13323 L 11889 13187 L 11918 13060 L 11929 13052 L
11952 12916 L 11918 12834 L 11914 12780 L 11912 12645 L
11904 12509 L 11918 12483 L 12008 12509 L 12053 12533 L
12064 12509 L 12059 12373 L 12053 12337 L 12015 12238 L
12005 12102 L 11918 12051 L 11854 11966 L 11826 11831 L
11782 11730 L 11678 11695 L 11647 11674 L 11553 11559 L
11511 11512 L 11430 11423 L 11376 11364 L 11295 11288 L
11240 11227 L 11166 11152 L 11105 11102 L 10994 11016 L
10969 10999 L 10834 10896 L 10808 10881 L 10698 10806 L
10563 10769 L 10543 10881 L 10427 10977 L 10292 11003 L
10174 11016 L 10156 11029 L 10021 11150 L 10019 11152 L
10008 11288 L 10021 11311 L 10107 11423 L 10120 11559 L
10156 11608 L 10278 11695 L 10292 11714 L 10345 11831 L
10426 11966 L 10427 11967 L 10563 11982 L 10597 12102 L
10689 12238 L 10698 12255 L 10834 12373 L 10834 12373 L
10918 12509 L 10850 12645 L 10834 12660 L 10698 12678 L
10563 12749 L 10427 12669 L 10402 12645 L 10292 12561 L
10237 12509 L 10156 12436 L 10021 12439 L 9885 12458 L
9834 12509 L 9826 12645 L 9819 12780 L 9799 12916 L
9750 12965 L 9614 12955 L 9479 12970 L 9343 13001 L
9288 13052 L 9208 13125 L 9072 13132 L 9042 13187 L
9072 13222 L 9208 13226 L 9308 13323 L 9315 13459 L
9343 13492 L 9479 13515 L 9557 13594 L 9614 13649 L
9750 13685 L 9885 13717 L 9904 13730 L 9947 13866 L
9885 13907 L 9750 13925 L 9614 13939 L 9550 14002 L
9479 14072 L 9343 14089 L 9272 14137 L 9208 14197 L
9147 14137 L 9072 14083 L 8989 14002 L 8937 13958 L
8843 13866 L 8801 13826 L 8666 13839 L 8530 13856 L
8521 13866 L 8513 14002 L 8505 14137 L 8489 14273 L
8395 14366 L 8259 14370 L 8124 14380 L 7988 14397 L
7977 14409 L 7965 14544 L 7958 14680 L 7949 14816 L
7988 14857 L 8124 14864 L 8259 14874 L 8395 14897 L
8447 14951 L 8530 15063 L 8554 15087 L 8571 15223 L
8643 15358 L 8598 15494 L 8530 15566 L 8395 15598 L
8317 15630 L 8259 15677 L 8191 15765 L 8124 15869 L
8093 15901 L 7988 15952 L 7853 15903 L 7853 15716 L
7952 15630 L 7853 15600 L 7828 15630 L 7853 15716 L
7853 15903 L 7847 15901 L 7717 15860 L 7582 15891 L
7446 15820 L 7360 15765 L 7311 15738 L 7219 15630 L
7175 15599 L 7082 15630 L 7040 15641 L 6904 15725 L
6843 15765 L 6769 15811 L 6633 15816 L 6503 15765 L
6498 15756 L 6449 15630 L 6362 15543 L 6362 15293 L

6416 15223 L 6436 15087 L 6362 15050 L 6257 14951 L
6256 14816 L 6227 14766 L 6091 14767 L 6050 14816 L
6074 14951 L 6091 14970 L 6183 15087 L 6227 15190 L
6257 15223 L 6362 15293 L 6362 15543 L 6227 15520 L
6091 15583 L 5956 15557 L 5914 15494 L 5856 15358 L
5820 15324 L 5714 15223 L 5685 15181 L 5611 15087 L
5599 14951 L 5654 14816 L 5605 14680 L 5549 14611 L
5414 14594 L 5288 14680 L 5345 14816 L 5414 14927 L
5453 14951 L 5455 15087 L 5414 15190 L 5408 15223 L
5414 15247 L 5465 15358 L 5549 15485 L 5557 15494 L
5554 15630 L 5549 15667 L 5532 15765 L 5414 15822 L
5278 15830 L 5143 15826 L 5007 15811 L 4872 15828 L
4736 15884 L 4601 15890 L 4561 15901 L 4465 15991 L
4452 16037 L 4381 16172 L 4317 16172 L N E
108 O
4465 10927 4378 10881 4465 10829 4542 10881 4465 10927 4465 10900
4497 10881 4465 10859 4430 10881 4465 10900 4465 10927 11 N F
55 O
4406 16173 4381 16173 4381 16172 4406 16172 4437 16172 4437 16173
4406 16173 7 N F
135 O
4465 13304 4397 13187 4465 13122 4581 13187 4465 13304 5 N F
55 O
4381 16172 M 4406 16172 L 4437 16172 L 4465 16120 L 4536 16037 L
4601 15973 L 4736 15960 L 4836 15901 L 4872 15885 L
5007 15850 L 5143 15862 L 5278 15874 L 5414 15885 L
5549 15854 L 5623 15765 L 5656 15630 L 5685 15540 L
5791 15494 L 5820 15485 L 5824 15494 L 5887 15630 L
5956 15677 L 6091 15699 L 6160 15630 L 6227 15595 L
6334 15630 L 6362 15668 L 6396 15765 L 6498 15839 L
6633 15867 L 6769 15856 L 6904 15773 L 6920 15765 L
7040 15700 L 7175 15733 L 7202 15765 L 7311 15832 L
7400 15901 L 7446 15944 L 7582 16019 L 7717 16023 L
7759 16037 L 7853 16056 L 7988 16052 L 8006 16037 L
8124 15962 L 8221 15901 L 8259 15849 L 8395 15777 L
8473 15901 L 8513 16037 L 8524 16172 L 8541 16172 L
8666 16067 L 8801 16047 L 8937 16114 L 8937 15780 L
8844 15765 L 8803 15630 L 8937 15524 L 9072 15629 L
9072 15630 L 9076 15765 L 9072 15776 L 8937 15780 L
8937 16114 L 8978 16172 L 9109 16172 L 9271 16172 L
9279 16172 L 9343 16052 L 9465 16037 L 9343 16014 L
9309 15901 L 9208 15792 L 9191 15765 L 9110 15630 L
9081 15494 L 9072 15485 L 8937 15457 L 8820 15358 L

8801 15338 L 8666 15324 L 8602 15223 L 8591 15087 L
8530 15025 L 8475 14951 L 8395 14867 L 8259 14851 L
8124 14842 L 7988 14836 L 7969 14816 L 7977 14680 L
7982 14544 L 7988 14473 L 8055 14409 L 8124 14401 L
8259 14391 L 8395 14387 L 8510 14273 L 8523 14137 L
8530 14002 L 8530 14001 L 8603 13866 L 8666 13858 L
8801 13843 L 8825 13866 L 8937 13976 L 8968 14002 L
9072 14104 L 9118 14137 L 9208 14225 L 9302 14137 L
9343 14110 L 9479 14097 L 9575 14002 L 9614 13964 L
9750 13954 L 9885 13942 L 10001 13866 L 9974 13730 L
9885 13669 L 9750 13647 L 9614 13623 L 9584 13594 L
9479 13488 L 9343 13471 L 9333 13459 L 9328 13323 L
9208 13206 L 9072 13204 L 9058 13187 L 9072 13161 L
9208 13157 L 9323 13052 L 9343 13033 L 9479 13012 L
9614 12999 L 9750 13003 L 9836 12916 L 9850 12780 L
9856 12645 L 9864 12509 L 9885 12488 L 10021 12472 L
10156 12469 L 10200 12509 L 10292 12597 L 10355 12645 L
10427 12714 L 10516 12780 L 10563 12826 L 10610 12780 L
10698 12720 L 10834 12708 L 10900 12645 L 10969 12558 L
11105 12531 L 11180 12509 L 11105 12469 L 11016 12373 L
10969 12343 L 10834 12271 L 10798 12238 L 10698 12176 L
10645 12102 L 10625 11966 L 10563 11903 L 10427 11898 L
10387 11831 L 10354 11695 L 10292 11614 L 10156 11564 L
10153 11559 L 10151 11423 L 10156 11351 L 10221 11288 L
10292 11239 L 10427 11160 L 10563 11182 L 10698 11218 L
10834 11283 L 10842 11288 L 10969 11361 L 11060 11423 L
11105 11464 L 11195 11559 L 11240 11605 L 11327 11695 L
11376 11742 L 11457 11831 L 11511 11890 L 11572 11966 L
11647 12047 L 11733 12102 L 11782 12147 L 11820 12238 L
11909 12373 L 11846 12509 L 11858 12645 L 11862 12780 L
11870 12916 L 11868 13052 L 11846 13187 L 11839 13323 L
11848 13459 L 11782 13566 L 11753 13594 L 11672 13730 L
11741 13866 L 11782 13902 L 11886 14002 L 11918 14059 L
12015 14137 L 11918 14265 L 11909 14273 L 11782 14376 L
11648 14409 L 11664 14544 L 11782 14567 L 11809 14544 L
11918 14423 L 11930 14409 L 11989 14273 L 12053 14257 L
12076 14273 L 12189 14352 L 12252 14273 L 12324 14239 L
12424 14137 L 12460 14102 L 12595 14016 L 12628 14002 L
12731 13973 L 12808 14002 L 12831 14137 L 12866 14186 L
13002 14144 L 13002 14083 L 12908 14002 L 12866 13913 L
12731 13908 L 12595 13914 L 12460 13948 L 12434 14002 L
12324 14108 L 12263 14137 L 12189 14171 L 12150 14137 L
12053 14061 L 11972 14002 L 11918 13974 L 11800 13866 L

11782 13778 L 11771 13730 L 11782 13707 L 11837 13594 L
11886 13459 L 11876 13323 L 11889 13187 L 11918 13060 L
11929 13052 L 11952 12916 L 11918 12834 L 11914 12780 L
11912 12645 L 11904 12509 L 11918 12483 L 12008 12509 L
12053 12533 L 12064 12509 L 12059 12373 L 12053 12337 L
12015 12238 L 12005 12102 L 11918 12051 L 11854 11966 L
11826 11831 L 11782 11730 L 11678 11695 L 11647 11674 L
11553 11559 L 11511 11512 L 11430 11423 L 11376 11364 L
11295 11288 L 11240 11227 L 11166 11152 L 11105 11102 L
10994 11016 L 10969 10999 L 10834 10896 L 10808 10881 L
10698 10806 L 10563 10769 L 10543 10881 L 10427 10977 L
10292 11003 L 10174 11016 L 10156 11029 L 10021 11150 L
10019 11152 L 10008 11288 L 10021 11311 L 10107 11423 L
10120 11559 L 10156 11608 L 10278 11695 L 10292 11714 L
10345 11831 L 10426 11966 L 10427 11967 L 10563 11982 L
10597 12102 L 10689 12238 L 10698 12255 L 10834 12373 L
10834 12373 L 10918 12509 L 10850 12645 L 10834 12660 L
10698 12678 L 10563 12749 L 10427 12669 L 10402 12645 L
10292 12561 L 10237 12509 L 10156 12436 L 10021 12439 L
9885 12458 L 9834 12509 L 9826 12645 L 9819 12780 L
9799 12916 L 9750 12965 L 9614 12955 L 9479 12970 L
9343 13001 L 9288 13052 L 9208 13125 L 9072 13132 L
9042 13187 L 9072 13222 L 9208 13226 L 9308 13323 L
9315 13459 L 9343 13492 L 9479 13515 L 9557 13594 L
9614 13649 L 9750 13685 L 9885 13717 L 9904 13730 L
9947 13866 L 9885 13907 L 9750 13925 L 9614 13939 L
9550 14002 L 9479 14072 L 9343 14089 L 9272 14137 L
9208 14197 L 9147 14137 L 9072 14083 L 8989 14002 L
8937 13958 L 8843 13866 L 8801 13826 L 8666 13839 L
8530 13856 L 8521 13866 L 8513 14002 L 8505 14137 L
8489 14273 L 8395 14366 L 8259 14370 L 8124 14380 L
7988 14397 L 7977 14409 L 7965 14544 L 7958 14680 L
7949 14816 L 7988 14857 L 8124 14864 L 8259 14874 L
8395 14897 L 8447 14951 L 8530 15063 L 8554 15087 L
8571 15223 L 8643 15358 L 8598 15494 L 8530 15566 L
8395 15598 L 8317 15630 L 8259 15677 L 8191 15765 L
8124 15869 L 8093 15901 L 7988 15952 L 7853 15903 L
7847 15901 L 7717 15860 L 7582 15891 L 7446 15820 L
7360 15765 L 7311 15738 L 7219 15630 L 7175 15599 L
7082 15630 L 7040 15641 L 6904 15725 L 6843 15765 L
6769 15811 L 6633 15816 L 6503 15765 L 6498 15756 L
6449 15630 L 6362 15543 L 6227 15520 L 6091 15583 L
5956 15557 L 5914 15494 L 5856 15358 L 5820 15324 L

5714 15223 L 5685 15181 L 5611 15087 L 5599 14951 L
5654 14816 L 5605 14680 L 5549 14611 L 5414 14594 L
5288 14680 L 5345 14816 L 5414 14927 L 5453 14951 L
5455 15087 L 5414 15190 L 5408 15223 L 5414 15247 L
5465 15358 L 5549 15485 L 5549 15295 L 5516 15223 L
5549 15156 L 5549 14926 L 5414 14846 L 5395 14816 L
5383 14680 L 5414 14659 L 5549 14665 L 5562 14680 L
5604 14816 L 5549 14926 L 5549 15156 L 5592 15223 L
5549 15295 L 5549 15485 L 5557 15494 L 5554 15630 L
5549 15667 L 5532 15765 L 5414 15822 L 5278 15830 L
5143 15826 L 5007 15811 L 4872 15828 L 4736 15884 L
4601 15890 L 4561 15901 L 4465 15991 L 4452 16037 L
4381 16172 L N E
95 O
4465 10900 4430 10881 4465 10859 4497 10881 4465 10900 5 N F
42 O
4465 16173 4437 16173 4437 16173 4535 16173 4535 16173 4465 16173
6 N F
4535 16173 4437 16173 4437 16172 4535 16172 4535 16173 5 N F
4437 16172 4465 16120 4536 16037 4601 15973 4736 15960 4836 15901
4872 15885 5007 15850 5143 15862 5278 15874 5414 15885 5549 15854
5623 15765 5656 15630 5685 15540 5791 15494 5820 15485 5824 15494
5887 15630 5956 15677 6091 15699 6160 15630 6227 15595 6334 15630
6362 15668 6396 15765 6498 15839 6633 15867 6769 15856 6904 15773
6920 15765 7040 15700 7175 15733 7202 15765 7311 15832 7400 15901
7446 15944 7582 16019 7717 16023 7759 16037 7853 16056 7988 16052
8006 16037 8124 15962 8221 15901 8259 15849 8395 15777 8473 15901
8513 16037 8524 16172 8460 16172 8403 16172 8395 16143 8362 16037
8259 15963 8124 16034 8119 16037 7988 16143 7853 16165 7717 16133
7582 16113 7446 16045 7441 16037 7311 15914 7273 15901 7175 15848
7055 15765 7040 15759 7029 15765 6904 15831 6769 15900 6760 15901
6633 15939 6498 15937 6362 15958 6227 15971 6091 15911 6084 15901
5956 15781 5924 15765 5820 15707 5738 15765 5685 15810 5617 15901
5549 15929 5414 15949 5278 15925 5163 15901 5143 15899 5007 15888
4964 15901 4872 15957 4737 16037 4736 16037 4601 16085 4535 16172
4437 16172 97 N F
29 O
4535 16172 4568 16172 4730 16172 4822 16172 4866 16037 4872 16033
5007 15943 5143 15955 5278 15988 5414 16013 5549 15980 5685 15934
5731 15901 5820 15820 5951 15901 5956 15904 6064 16037 6091 16159
6102 16172 6190 16172 6352 16172 6514 16172 6676 16172 6679 16172
6662 16037 6769 15983 6878 15901 6904 15888 7040 15833 7128 15901
7175 15933 7311 15978 7374 16037 7446 16138 7518 16172 7650 16172

7812 16172 7974 16172 8045 16172 8124 16106 8259 16058 8333 16172
8403 16172 8395 16143 8362 16037 8259 15963 8124 16034 8119 16037
7988 16143 7853 16165 7717 16133 7582 16113 7446 16045 7441 16037
7311 15914 7273 15901 7175 15848 7055 15765 7040 15759 7029 15765
6904 15831 6769 15900 6760 15901 6633 15939 6498 15937 6362 15958
6227 15971 6091 15911 6084 15901 5956 15781 5924 15765 5820 15707
5738 15765 5685 15810 5617 15901 5549 15929 5414 15949 5278 15925
5163 15901 5143 15899 5007 15888 4964 15901 4872 15957 4737 16037
4736 16037 4601 16085 4535 16172 87 N F
161 O
4872 12374 4811 12373 4872 12368 4873 12373 4872 12374 5 N F
15 O
4822 16172 4866 16037 4872 16033 5007 15943 5143 15955 5278 15988
5414 16013 5549 15980 5685 15934 5731 15901 5820 15820 5951 15901
5956 15904 6064 16037 6091 16159 6102 16172 6028 16172 5909 16172
5956 16096 5976 16037 5956 16013 5820 15926 5685 16005 5549 16031
5530 16037 5485 16172 5379 16172 5217 16172 5156 16172 5234 16037
5143 16012 5007 16006 4962 16037 5007 16153 5080 16172 5055 16172
4892 16172 4822 16172 38 N F
161 O
5007 12237 4899 12102 5007 12028 5129 12102 5007 12237 5 N F
95 O
5007 15515 4915 15630 5007 15687 5143 15725 5278 15700 5374 15630
5278 15537 5143 15525 5007 15515 9 N F
2 O
5007 16153 4962 16037 5007 16006 5143 16012 5234 16037 5156 16172
5080 16172 5007 16153 8 N F
121 O
5007 13473 5002 13459 5007 13449 5013 13459 5007 13473 5 N F
108 O
5007 13737 5006 13730 5007 13729 5009 13730 5007 13737 5 N F
2 O
5080 16173 5080 16172 5156 16172 5156 16173 5080 16173 5 N F
5080 16173 5080 16173 5156 16173 5156 16173 5143 16173 5080 16173
6 N F
174 O
5143 10104 5087 10067 5143 9953 5195 10067 5143 10104 5 N F
188 O
5143 7486 5137 7489 5143 7491 5158 7489 5143 7486 5 N F
95 O
5143 14019 5139 14002 5143 13952 5164 14002 5143 14019 5 N F
68 O
5278 14404 5218 14273 5250 14137 5278 14107 5306 14137 5355 14273

5278 14404 5278 14314 5303 14273 5278 14191 5259 14273 5278 14314
5278 14404 13 N F
55 O
5278 14314 5259 14273 5278 14191 5303 14273 5278 14314 5 N F
121 O
5414 13710 5358 13594 5414 13518 5549 13553 5579 13594 5685 13715
5703 13730 5820 13865 5922 13730 5956 13675 6091 13698 6136 13730
6227 13833 6245 13866 6227 13935 6206 14002 6122 14137 6091 14197
6091 14097 6156 14002 6191 13866 6091 13753 5956 13806 5913 13866
5903 14002 5956 14082 6091 14097 6091 14197 6017 14273 6056 14409
5956 14505 5851 14409 5820 14335 5798 14273 5820 14256 5936 14137
5856 14002 5820 13868 5685 13874 5676 13866 5549 13798 5549 13767
5631 13730 5549 13636 5483 13730 5549 13767 5549 13798 5429 13730
5414 13710 5414 13634 5481 13594 5414 13568 5394 13594 5414 13634
5414 13710 55 N F
42 O
5395 14816 5383 14680 5414 14659 5549 14665 5562 14680 5604 14816
5549 14926 5549 14827 5555 14816 5549 14796 5528 14816 5549 14827
5549 14926 5414 14846 5395 14816 15 N F
188 O
5414 7194 5384 7217 5414 7245 5470 7217 5414 7194 5 N F
108 O
5414 13634 5394 13594 5414 13568 5481 13594 5414 13634 5 N F
135 O
5414 13089 5406 13052 5414 12916 5414 12916 5414 12916 5421 13052
5414 13089 7 N F
108 O
5549 13767 5483 13730 5549 13636 5631 13730 5549 13767 5549 13736
5563 13730 5549 13714 5538 13730 5549 13736 5549 13767 11 N F
42 O
5549 15295 5516 15223 5549 15156 5592 15223 5549 15295 5 N F
29 O
5549 14827 5528 14816 5549 14796 5555 14816 5549 14827 5 N F
161 O
5549 5613 5529 5589 5549 5546 5652 5453 5703 5453 5705 5453
5820 5512 5865 5453 5865 5453 6025 5453 6091 5575 6099 5589
6091 5600 5956 5644 5820 5661 5770 5725 5685 5813 5685 5639
5721 5589 5685 5564 5659 5589 5685 5639 5685 5813 5601 5725
5549 5613 25 N F
188 O
5549 7603 5529 7624 5543 7760 5549 7769 5685 7788 5692 7760
5685 7703 5595 7624 5549 7603 9 N F
2 O

5485 16172 5530 16037 5549 16031 5685 16005 5820 15926 5956 16013
5976 16037 5956 16096 5909 16172 5865 16172 5703 16172 5541 16172
5485 16172 13 N F
95 O
5549 13736 5538 13730 5549 13714 5563 13730 5549 13736 5 N F
188 O
5685 8524 5646 8574 5685 8676 5697 8574 5685 8524 5 N F
161 O
5703 5453 5652 5453 5652 5453 5703 5453 5705 5453 5705 5453
5703 5453 7 N F
148 O
5685 5639 5659 5589 5685 5564 5721 5589 5685 5639 5 N F
188 O
5685 8801 5682 8845 5685 8863 5689 8845 5685 8801 5 N F
174 O
5705 5453 5705 5453 5865 5453 5865 5453 5820 5453 5705 5453
6 N F
5705 5453 5705 5453 5865 5453 5865 5453 5705 5453 5 N F
5865 5453 5705 5453 5820 5512 5865 5453 4 N F
161 O
5820 12006 5798 11966 5820 11928 5908 11831 5956 11767 6002 11831
5961 11966 5956 11997 5820 12006 9 N F
148 O
5820 14508 5803 14544 5820 14606 5860 14544 5820 14508 5 N F
135 O
5956 13310 5860 13187 5900 13052 5956 13005 6031 13052 6091 13091
6133 13187 6105 13323 6091 13342 6091 13211 6097 13187 6091 13174
5956 13140 5938 13187 5956 13210 6091 13211 6091 13342 6001 13323
5956 13310 19 N F
161 O
5865 5453 5865 5453 5865 5453 5865 5453 6025 5453 6025 5453
5865 5453 7 N F
5956 9623 5885 9524 5956 9461 6034 9524 5956 9623 5 N F
108 O
5956 14082 5903 14002 5913 13866 5956 13806 6091 13753 6191 13866
6156 14002 6091 14097 6091 14024 6107 14002 6127 13866 6091 13825
6023 13866 5956 13975 5950 14002 5956 14011 6091 14024 6091 14097
5956 14082 19 N F
15 O
6028 16173 5909 16173 5909 16172 6028 16172 6102 16172 6102 16173
6028 16173 7 N F
108 O
5956 15072 5916 15087 5956 15151 5970 15087 5956 15072 5 N F

161 O
5956 9004 5936 8981 5956 8944 5975 8981 5956 9004 5 N F
121 O
5956 13210 5938 13187 5956 13140 6091 13174 6097 13187 6091 13211
5956 13210 7 N F
95 O
5956 14011 5950 14002 5956 13975 6023 13866 6091 13825 6127 13866
6107 14002 6091 14024 5956 14011 9 N F
174 O
6025 5453 6025 5453 6028 5453 6158 5453 6158 5453 6028 5453
6025 5453 7 N F
161 O
6091 9457 6037 9388 6091 9323 6142 9388 6091 9457 5 N F
55 O
6074 14951 6050 14816 6091 14767 6227 14766 6256 14816 6257 14951
6362 15050 6436 15087 6416 15223 6362 15293 6257 15223 6227 15190
6183 15087 6091 14970 6091 14831 6142 14816 6091 14811 6088 14816
6091 14831 6091 14970 6074 14951 21 N F
42 O
6091 14831 6088 14816 6091 14811 6142 14816 6091 14831 5 N F
161 O
6227 10183 6152 10067 6227 9956 6362 10045 6384 10067 6362 10100
6227 10183 7 N F
188 O
6227 7050 6159 7081 6227 7159 6330 7217 6362 7302 6420 7217
6362 7165 6326 7081 6227 7050 9 N F
161 O
6227 12468 6174 12373 6227 12282 6271 12238 6227 12128 6219 12102
6210 11966 6227 11898 6362 11897 6417 11966 6498 12047 6554 12102
6594 12238 6609 12373 6609 12509 6633 12517 6769 12624 6801 12645
6904 12688 7040 12776 7057 12645 7040 12632 6918 12509 6904 12499
6904 12424 7029 12373 6904 12269 6776 12373 6904 12424 6904 12499
6769 12458 6769 12364 6868 12238 6769 12141 6728 12238 6769 12364
6769 12458 6646 12373 6654 12238 6690 12102 6756 11966 6672 11831
6689 11695 6633 11575 6624 11559 6633 11555 6769 11503 6847 11559
6837 11695 6859 11831 6904 11901 7040 11848 7093 11966 7128 12102
7175 12169 7311 12128 7402 12238 7446 12271 7582 12303 7717 12308
7777 12373 7717 12462 7690 12509 7717 12585 7756 12645 7717 12780
7717 12780 7582 12879 7491 12916 7446 12934 7446 12816 7495 12780
7577 12645 7582 12631 7611 12509 7582 12457 7446 12398 7311 12396
7175 12507 7040 12405 7020 12509 7040 12529 7175 12517 7196 12645
7302 12780 7311 12787 7446 12816 7446 12934 7311 12921 7278 12916
7175 12886 7040 12782 6904 12896 6790 12916 6769 12921 6633 12930

6633 12838 6744 12780 6732 12645 6633 12554 6500 12645 6548 12780
6633 12838 6633 12930 6608 12916 6498 12861 6362 12790 6315 12780
6334 12645 6254 12509 6227 12468 111 N F
188 O
6362 9157 M 6319 9252 L 6362 9313 L 6459 9388 L 6498 9486 L
6528 9524 L 6498 9582 L 6485 9660 L 6475 9795 L
6498 9819 L 6633 9873 L 6676 9931 L 6730 10067 L
6633 10171 L 6609 10202 L 6498 10318 L 6465 10338 L
6362 10467 L 6341 10474 L 6362 10484 L 6498 10488 L
6536 10474 L 6633 10428 L 6669 10474 L 6769 10546 L
6769 9401 L 6713 9388 L 6769 9348 L 6783 9388 L
6769 9401 L 6769 10546 L 6873 10609 L 6828 10745 L
6769 10767 L 6719 10745 L 6633 10712 L 6596 10745 L
6627 10881 L 6498 10989 L 6422 11016 L 6498 11117 L
6567 11152 L 6633 11187 L 6738 11288 L 6633 11396 L
6624 11423 L 6633 11432 L 6668 11423 L 6769 11327 L
6904 11319 L 7040 11361 L 7130 11423 L 7175 11436 L
7311 11439 L 7311 11275 L 7185 11152 L 7175 11121 L
7040 11041 L 7018 11016 L 7040 10996 L 7040 10822 L
6929 10745 L 7040 10658 L 7110 10745 L 7040 10822 L
7040 10996 L 7158 10881 L 7175 10866 L 7175 10715 L
7060 10609 L 7040 10532 L 6984 10474 L 7040 10411 L
7112 10338 L 7175 10273 L 7175 9865 L 7096 9795 L
7040 9722 L 6957 9660 L 7040 9562 L 7175 9573 L
7245 9660 L 7266 9795 L 7175 9865 L 7175 10273 L
7311 10313 L 7311 9939 L 7303 9931 L 7311 9917 L
7318 9931 L 7311 9939 L 7311 10313 L 7342 10338 L
7317 10474 L 7311 10475 L 7292 10609 L 7175 10715 L
7175 10866 L 7232 10881 L 7311 10987 L 7360 11016 L
7423 11152 L 7311 11275 L 7311 11439 L 7446 11435 L
7548 11559 L 7582 11568 L 7582 11541 L 7452 11423 L
7446 11419 L 7361 11288 L 7446 11224 L 7543 11152 L
7582 11035 L 7582 10977 L 7541 10881 L 7574 10745 L
7582 10738 L 7717 10630 L 7853 10671 L 7853 9627 L
7753 9524 L 7853 9409 L 7961 9524 L 7853 9627 L
7853 10671 L 7912 10745 L 7853 10797 L 7732 10881 L
7717 10902 L 7582 10977 L 7582 11035 L 7614 11152 L
7658 11288 L 7668 11423 L 7582 11541 L 7582 11568 L
7679 11695 L 7582 11782 L 7573 11831 L 7582 11870 L
7669 11966 L 7717 12028 L 7853 12055 L 7975 11966 L
7988 11947 L 7988 11411 L 7865 11288 L 7988 11165 L
8113 11288 L 7988 11411 L 7988 11947 L 8029 11831 L
8052 11695 L 8099 11559 L 8124 11529 L 8259 11432 L

8275 11423 L 8317 11288 L 8259 11158 L 8256 11152 L
8230 11016 L 8173 10881 L 8132 10745 L 8170 10609 L
8259 10546 L 8395 10553 L 8410 10474 L 8503 10338 L
8530 10286 L 8559 10202 L 8530 10180 L 8470 10202 L
8395 10247 L 8358 10202 L 8350 10067 L 8259 9994 L
8124 9961 L 8084 9931 L 8124 9894 L 8259 9912 L
8395 9901 L 8439 9795 L 8530 9683 L 8584 9660 L
8597 9524 L 8530 9448 L 8395 9509 L 8262 9388 L
8259 9364 L 8124 9335 L 8033 9252 L 7988 9173 L
7932 9117 L 7853 9054 L 7717 9080 L 7632 8981 L
7582 8886 L 7524 8845 L 7446 8793 L 7311 8778 L
7175 8767 L 7090 8845 L 7040 8892 L 6936 8981 L
6904 9005 L 6848 8981 L 6769 8917 L 6642 8845 L
6633 8835 L 6498 8768 L 6449 8845 L 6493 8981 L
6498 8986 L 6625 9117 L 6633 9123 L 6750 9252 L
6633 9376 L 6498 9317 L 6456 9252 L 6362 9157 L
N E
6498 8407 6473 8438 6498 8526 6544 8438 6498 8407 5 N F
161 O
6498 10892 6479 10881 6498 10862 6512 10881 6498 10892 5 N F
148 O
6548 12780 6500 12645 6633 12554 6732 12645 6744 12780 6633 12838
6633 12755 6692 12645 6633 12591 6555 12645 6633 12755 6633 12838
6548 12780 13 N F
135 O
6633 12755 6555 12645 6633 12591 6692 12645 6633 12755 6633 12678
6651 12645 6633 12628 6609 12645 6633 12678 6633 12755 11 N F
55 O
6633 15183 6563 15087 6633 15066 6673 15087 6633 15183 5 N F
121 O
6633 12678 6609 12645 6633 12628 6651 12645 6633 12678 5 N F
188 O
6769 7799 6644 7896 6692 8031 6769 8072 6797 8031 6856 7896
6769 7799 7 N F
55 O
6769 15346 6656 15223 6769 15160 6832 15223 6769 15346 5 N F
15 O
6679 16172 6662 16037 6769 15983 6878 15901 6904 15888 7040 15833
7128 15901 7175 15933 7311 15978 7374 16037 7446 16138 7518 16172
7487 16172 7394 16172 7311 16045 7296 16037 7175 16000 7040 15918
6904 15996 6840 16037 6904 16160 6912 16172 6839 16172 6679 16172
24 N F
6679 16173 6679 16172 6839 16172 6912 16172 6912 16173 6839 16173

6679 16173 7 N F
188 O
6769 7325 6687 7353 6769 7362 6781 7353 6769 7325 5 N F
174 O
6769 9401 6713 9388 6769 9348 6783 9388 6769 9401 5 N F
148 O
6769 12364 6728 12238 6769 12141 6868 12238 6769 12364 5 N F
95 O
6769 15568 6734 15630 6769 15658 6904 15632 6907 15630 6904 15580
6769 15568 7 N F
148 O
6904 12424 6776 12373 6904 12269 7029 12373 6904 12424 5 N F
2 O
6904 16160 6840 16037 6904 15996 7040 15918 7175 16000 7296 16037
7311 16045 7394 16172 7325 16172 7163 16172 7001 16172 6912 16172
6904 16160 13 N F
174 O
6904 6149 6889 6132 6904 6107 6950 5996 7040 5943 7175 5972
7204 5996 7261 6132 7175 6247 7040 6179 6904 6149 11 N F
7040 10822 6929 10745 7040 10658 7110 10745 7040 10822 5 N F
7040 9722 6957 9660 7040 9562 7175 9573 7245 9660 7266 9795
7175 9865 7096 9795 7040 9722 9 N F
7040 10532 6984 10474 7040 10411 7112 10338 7175 10273 7311 10313
7342 10338 7317 10474 7311 10475 7292 10609 7175 10715 7060 10609
7040 10532 13 N F
7040 11041 7018 11016 7040 10996 7158 10881 7175 10866 7232 10881
7311 10987 7360 11016 7423 11152 7311 11275 7185 11152 7175 11121
7040 11041 13 N F
148 O
7040 12529 7020 12509 7040 12405 7175 12507 7311 12396 7446 12398
7582 12457 7611 12509 7582 12631 7577 12645 7495 12780 7446 12816
7446 12663 7459 12645 7446 12574 7311 12645 7446 12663 7446 12816
7311 12787 7302 12780 7196 12645 7175 12517 7040 12529 23 N F
188 O
7175 8387 7108 8438 7175 8507 7233 8438 7175 8387 5 N F
161 O
7311 11934 7241 11831 7252 11695 7311 11621 7446 11681 7462 11695
7480 11831 7482 11966 7446 12023 7340 11966 7311 11934 11 N F
174 O
7311 9939 7303 9931 7311 9917 7318 9931 7311 9939 5 N F
135 O
7446 12663 7311 12645 7446 12574 7459 12645 7446 12663 5 N F
174 O

7446 11419 7361 11288 7446 11224 7543 11152 7582 11035 7614 11152
7658 11288 7668 11423 7582 11541 7452 11423 7446 11419 11 N F
15 O
7394 16172 7394 16173 7487 16173 7518 16173 7518 16172 7487 16172
7394 16172 7 N F
174 O
7582 10977 7541 10881 7574 10745 7582 10738 7717 10630 7853 10671
7912 10745 7853 10797 7732 10881 7717 10902 7582 10977 11 N F
7853 9627 7753 9524 7853 9409 7961 9524 7853 9627 5 N F
55 O
7853 15716 7828 15630 7853 15600 7952 15630 7853 15716 5 N F
161 O
7853 8853 7845 8845 7853 8835 7861 8845 7853 8853 5 N F
174 O
7988 11411 7865 11288 7988 11165 8113 11288 7988 11411 7988 11310
8011 11288 7988 11265 7966 11288 7988 11310 7988 11411 11 N F
161 O
7988 11310 7966 11288 7988 11265 8011 11288 7988 11310 5 N F
42 O
7988 14836 M 7969 14816 L 7977 14680 L 7982 14544 L 7988 14473 L
8055 14409 L 8124 14401 L 8259 14391 L 8395 14387 L
8510 14273 L 8523 14137 L 8530 14002 L 8530 14001 L
8603 13866 L 8666 13858 L 8801 13843 L 8825 13866 L
8937 13976 L 8968 14002 L 9072 14104 L 9118 14137 L
9208 14225 L 9302 14137 L 9343 14110 L 9479 14097 L
9575 14002 L 9614 13964 L 9750 13954 L 9885 13942 L
10001 13866 L 9974 13730 L 9885 13669 L 9750 13647 L
9614 13623 L 9584 13594 L 9479 13488 L 9343 13471 L
9333 13459 L 9328 13323 L 9208 13206 L 9072 13204 L
9058 13187 L 9072 13161 L 9208 13157 L 9323 13052 L
9343 13033 L 9479 13012 L 9614 12999 L 9750 13003 L
9836 12916 L 9850 12780 L 9856 12645 L 9864 12509 L
9885 12488 L 10021 12472 L 10156 12469 L 10200 12509 L
10292 12597 L 10355 12645 L 10427 12714 L 10516 12780 L
10563 12826 L 10610 12780 L 10698 12720 L 10834 12708 L
10900 12645 L 10969 12558 L 11105 12531 L 11180 12509 L
11105 12469 L 11016 12373 L 10969 12343 L 10834 12271 L
10798 12238 L 10698 12176 L 10645 12102 L 10625 11966 L
10563 11903 L 10427 11898 L 10387 11831 L 10354 11695 L
10292 11614 L 10156 11564 L 10153 11559 L 10151 11423 L
10156 11351 L 10221 11288 L 10292 11239 L 10427 11160 L
10563 11182 L 10698 11218 L 10834 11283 L 10842 11288 L
10969 11361 L 11060 11423 L 11105 11464 L 11195 11559 L

11240 11605 L 11327 11695 L 11376 11742 L 11457 11831 L
11511 11890 L 11572 11966 L 11647 12047 L 11733 12102 L
11782 12147 L 11820 12238 L 11909 12373 L 11846 12509 L
11858 12645 L 11862 12780 L 11870 12916 L 11868 13052 L
11846 13187 L 11839 13323 L 11848 13459 L 11782 13566 L
11753 13594 L 11672 13730 L 11741 13866 L 11782 13902 L
11886 14002 L 11918 14059 L 12015 14137 L 11918 14265 L
11909 14273 L 11782 14376 L 11648 14409 L 11664 14544 L
11782 14567 L 11809 14544 L 11918 14423 L 11930 14409 L
11989 14273 L 12053 14257 L 12076 14273 L 12189 14352 L
12252 14273 L 12324 14239 L 12424 14137 L 12460 14102 L
12595 14016 L 12628 14002 L 12731 13973 L 12808 14002 L
12831 14137 L 12866 14186 L 13002 14144 L 13002 14382 L
12918 14409 L 12866 14437 L 12866 14307 L 12873 14273 L
12866 14271 L 12863 14273 L 12866 14307 L 12866 14437 L
12731 14542 L 12728 14544 L 12595 14670 L 12595 14613 L
12668 14544 L 12731 14489 L 12814 14409 L 12731 14292 L
12661 14409 L 12595 14483 L 12534 14544 L 12595 14613 L
12595 14670 L 12569 14680 L 12460 14751 L 12413 14680 L
12460 14595 L 12483 14544 L 12595 14431 L 12615 14409 L
12685 14273 L 12731 14197 L 12768 14137 L 12731 14067 L
12595 14116 L 12564 14137 L 12460 14197 L 12384 14273 L
12345 14409 L 12324 14456 L 12219 14544 L 12189 14558 L
12060 14544 L 12053 14541 L 12051 14544 L 11918 14617 L
11842 14680 L 11782 14718 L 11647 14774 L 11595 14816 L
11542 14951 L 11511 15067 L 11495 15087 L 11403 15223 L
11376 15318 L 11356 15358 L 11240 15408 L 11116 15358 L
11177 15223 L 11240 15132 L 11280 15087 L 11240 15038 L
11240 14844 L 11363 14816 L 11328 14680 L 11240 14551 L
11205 14680 L 11185 14816 L 11240 14844 L 11240 15038 L
11217 14951 L 11105 14869 L 11048 14816 L 11050 14680 L
11055 14544 L 11078 14409 L 11105 14380 L 11240 14352 L
11376 14351 L 11511 14353 L 11647 14342 L 11758 14273 L
11782 14257 L 11911 14137 L 11838 14002 L 11782 13948 L
11690 13866 L 11647 13794 L 11579 13730 L 11647 13660 L
11691 13594 L 11782 13503 L 11809 13459 L 11803 13323 L
11803 13187 L 11815 13052 L 11816 12916 L 11809 12780 L
11803 12645 L 11788 12509 L 11782 12481 L 11647 12440 L
11563 12373 L 11511 12336 L 11454 12373 L 11458 12509 L
11376 12580 L 11240 12603 L 11240 12299 L 11338 12238 L
11240 12176 L 11143 12102 L 11105 12070 L 10969 11980 L
10943 11966 L 10834 11891 L 10760 11831 L 10698 11793 L
10563 11765 L 10474 11831 L 10563 11834 L 10693 11966 L

10693 12102 L 10698 12109 L 10834 12124 L 10942 12238 L
10969 12253 L 11105 12274 L 11240 12299 L 11240 12603 L
11105 12614 L 10969 12620 L 10950 12645 L 10834 12757 L
10698 12762 L 10672 12780 L 10563 12884 L 10455 12780 L
10427 12759 L 10308 12645 L 10292 12632 L 10164 12509 L
10156 12502 L 10021 12506 L 9994 12509 L 9896 12645 L
9885 12659 L 9880 12780 L 9873 12916 L 9750 13040 L
9614 13044 L 9503 13052 L 9479 13106 L 9359 13187 L
9428 13323 L 9463 13459 L 9479 13460 L 9611 13594 L
9614 13598 L 9750 13610 L 9885 13621 L 10021 13684 L
10063 13730 L 10082 13866 L 10021 13927 L 9885 13977 L
9750 13984 L 9614 13988 L 9601 14002 L 9479 14123 L
9343 14130 L 9332 14137 L 9208 14253 L 9090 14137 L
9072 14125 L 8946 14002 L 8937 13994 L 8807 13866 L
8801 13860 L 8755 13866 L 8692 14002 L 8666 14046 L
8632 14137 L 8549 14273 L 8530 14296 L 8395 14408 L
8375 14409 L 8259 14471 L 8193 14544 L 8140 14680 L
8124 14706 L 8003 14816 L 8124 14820 L 8259 14828 L
8395 14838 L 8504 14951 L 8530 14987 L 8628 15087 L
8633 15223 L 8666 15275 L 8801 15282 L 8870 15358 L
8937 15415 L 9072 15431 L 9132 15494 L 9148 15630 L
9208 15717 L 9255 15765 L 9343 15856 L 9479 15856 L
9614 15859 L 9694 15765 L 9750 15705 L 9822 15630 L
9750 15564 L 9683 15494 L 9692 15358 L 9614 15282 L
9554 15223 L 9545 15087 L 9562 14951 L 9614 14900 L
9750 14881 L 9822 14951 L 9885 15012 L 9959 15087 L
10021 15142 L 10156 15152 L 10225 15223 L 10292 15296 L
10427 15307 L 10563 15329 L 10698 15341 L 10750 15358 L
10834 15468 L 10843 15494 L 10853 15630 L 10834 15660 L
10698 15709 L 10563 15709 L 10427 15635 L 10370 15630 L
10292 15625 L 10254 15630 L 10156 15697 L 10146 15765 L
10156 15793 L 10192 15901 L 10292 16035 L 10293 16037 L
10375 16172 L 10244 16172 L 10235 16172 L 10169 16037 L
10156 16019 L 10080 15901 L 10021 15775 L 10016 15765 L
9916 15630 L 9885 15595 L 9810 15494 L 9767 15358 L
9885 15297 L 10021 15303 L 10146 15358 L 10156 15365 L
10187 15358 L 10162 15223 L 10156 15217 L 10021 15196 L
9899 15087 L 9885 15073 L 9758 14951 L 9750 14943 L
9704 14951 L 9614 15025 L 9602 15087 L 9610 15223 L
9614 15227 L 9747 15358 L 9730 15494 L 9750 15515 L
9876 15630 L 9750 15760 L 9745 15765 L 9662 15901 L
9614 15992 L 9483 16037 L 9614 16117 L 9644 16172 L
9596 16172 L 9434 16172 L 9279 16172 L 9343 16052 L

9465 16037 L 9343 16014 L 9309 15901 L 9208 15792 L
9191 15765 L 9110 15630 L 9081 15494 L 9072 15485 L
8937 15457 L 8820 15358 L 8801 15338 L 8666 15324 L
8602 15223 L 8591 15087 L 8530 15025 L 8475 14951 L
8395 14867 L 8259 14851 L 8124 14842 L 7988 14836 L
N E
161 O
7988 8724 7972 8710 7988 8689 8005 8710 7988 8724 5 N F
29 O
8124 14820 M 8003 14816 L 8124 14706 L 8140 14680 L 8193 14544 L
8259 14471 L 8375 14409 L 8395 14408 L 8530 14296 L
8549 14273 L 8632 14137 L 8666 14046 L 8692 14002 L
8755 13866 L 8801 13860 L 8807 13866 L 8937 13994 L
8946 14002 L 9072 14125 L 9090 14137 L 9208 14253 L
9332 14137 L 9343 14130 L 9479 14123 L 9601 14002 L
9614 13988 L 9750 13984 L 9885 13977 L 10021 13927 L
10082 13866 L 10063 13730 L 10021 13684 L 9885 13621 L
9750 13610 L 9614 13598 L 9611 13594 L 9479 13460 L
9463 13459 L 9428 13323 L 9359 13187 L 9479 13106 L
9503 13052 L 9614 13044 L 9750 13040 L 9873 12916 L
9880 12780 L 9885 12659 L 9896 12645 L 9994 12509 L
10021 12506 L 10156 12502 L 10164 12509 L 10292 12632 L
10308 12645 L 10427 12759 L 10455 12780 L 10563 12884 L
10672 12780 L 10698 12762 L 10834 12757 L 10950 12645 L
10969 12620 L 11105 12614 L 11240 12603 L 11376 12580 L
11458 12509 L 11454 12373 L 11511 12336 L 11563 12373 L
11647 12440 L 11782 12481 L 11788 12509 L 11803 12645 L
11809 12780 L 11816 12916 L 11815 13052 L 11803 13187 L
11803 13323 L 11809 13459 L 11782 13503 L 11691 13594 L
11647 13660 L 11579 13730 L 11647 13794 L 11690 13866 L
11782 13948 L 11838 14002 L 11911 14137 L 11782 14257 L
11758 14273 L 11647 14342 L 11647 14275 L 11649 14273 L
11782 14187 L 11835 14137 L 11790 14002 L 11782 13994 L
11700 14002 L 11647 14136 L 11645 14137 L 11602 14273 L
11647 14275 L 11647 14342 L 11511 14353 L 11376 14351 L
11240 14352 L 11105 14380 L 11078 14409 L 11055 14544 L
11050 14680 L 11048 14816 L 11105 14869 L 11217 14951 L
11240 15038 L 11280 15087 L 11240 15132 L 11177 15223 L
11116 15358 L 11240 15408 L 11356 15358 L 11376 15318 L
11403 15223 L 11495 15087 L 11511 15067 L 11542 14951 L
11595 14816 L 11647 14774 L 11782 14718 L 11842 14680 L
11918 14617 L 12051 14544 L 12053 14541 L 12060 14544 L
12189 14558 L 12219 14544 L 12324 14456 L 12345 14409 L

12384 14273 L 12460 14197 L 12564 14137 L 12595 14116 L
12731 14067 L 12768 14137 L 12731 14197 L 12685 14273 L
12615 14409 L 12595 14431 L 12595 14337 L 12625 14273 L
12595 14220 L 12478 14273 L 12595 14337 L 12595 14431 L
12483 14544 L 12460 14595 L 12460 14463 L 12513 14409 L
12460 14298 L 12429 14409 L 12460 14463 L 12460 14595 L
12413 14680 L 12460 14751 L 12569 14680 L 12595 14670 L
12728 14544 L 12731 14542 L 12866 14437 L 12918 14409 L
13002 14382 L 13002 15415 L 12941 15494 L 12866 15582 L
12780 15630 L 12735 15765 L 12731 15780 L 12690 15901 L
12643 16037 L 12731 16087 L 12784 16172 L 12716 16172 L
12595 16042 L 12592 16037 L 12557 15901 L 12460 15837 L
12324 15791 L 12324 15697 L 12383 15630 L 12324 15575 L
12189 15606 L 12189 15498 L 12258 15494 L 12197 15358 L
12189 15348 L 12053 15268 L 12044 15358 L 12053 15365 L
12186 15494 L 12189 15498 L 12189 15606 L 12125 15494 L
12053 15424 L 11954 15358 L 11965 15223 L 12053 15160 L
12129 15087 L 12189 14999 L 12236 14951 L 12189 14916 L
12125 14951 L 12053 14996 L 11963 15087 L 11918 15157 L
11878 15223 L 11893 15358 L 11918 15424 L 11953 15494 L
12053 15602 L 12131 15630 L 12189 15643 L 12324 15697 L
12324 15791 L 12292 15765 L 12189 15702 L 12053 15665 L
12011 15630 L 11918 15531 L 11885 15494 L 11832 15358 L
11802 15223 L 11860 15087 L 11918 15030 L 11995 14951 L
12053 14874 L 12189 14840 L 12246 14816 L 12324 14746 L
12351 14680 L 12324 14620 L 12189 14657 L 12136 14680 L
12053 14748 L 11918 14757 L 11823 14816 L 11782 14844 L
11670 14951 L 11647 14996 L 11612 15087 L 11579 15223 L
11512 15358 L 11511 15360 L 11443 15494 L 11376 15544 L
11240 15546 L 11105 15528 L 11025 15630 L 11105 15710 L
11132 15765 L 11166 15901 L 11105 15954 L 11015 15901 L
10969 15883 L 10969 15266 L 11004 15223 L 11105 15113 L
11126 15087 L 11115 14951 L 11105 14944 L 10969 14859 L
10834 14854 L 10698 14937 L 10685 14951 L 10563 15063 L
10507 15087 L 10427 15103 L 10380 15087 L 10292 15049 L
10156 15068 L 10021 15004 L 10019 15087 L 10021 15089 L
10156 15087 L 10287 15223 L 10292 15227 L 10427 15227 L
10563 15231 L 10698 15236 L 10834 15250 L 10969 15266 L
10969 15883 L 10834 15851 L 10698 15873 L 10563 15888 L
10427 15852 L 10341 15901 L 10412 16037 L 10427 16061 L
10502 16172 L 10407 16172 L 10375 16172 L 10293 16037 L
10292 16035 L 10192 15901 L 10156 15793 L 10146 15765 L
10156 15697 L 10254 15630 L 10292 15625 L 10370 15630 L

10427 15635 L 10563 15709 L 10698 15709 L 10834 15660 L
10853 15630 L 10843 15494 L 10834 15468 L 10750 15358 L
10698 15341 L 10563 15329 L 10427 15307 L 10292 15296 L
10225 15223 L 10156 15152 L 10021 15142 L 9959 15087 L
9885 15012 L 9822 14951 L 9750 14881 L 9614 14900 L
9562 14951 L 9545 15087 L 9554 15223 L 9614 15282 L
9692 15358 L 9683 15494 L 9750 15564 L 9822 15630 L
9750 15705 L 9694 15765 L 9614 15859 L 9479 15856 L
9479 15800 L 9614 15798 L 9642 15765 L 9750 15649 L
9769 15630 L 9750 15612 L 9636 15494 L 9637 15358 L
9614 15336 L 9498 15223 L 9488 15087 L 9490 14951 L
9614 14831 L 9750 14819 L 9885 14845 L 9906 14816 L
9885 14808 L 9750 14708 L 9734 14680 L 9614 14607 L
9479 14598 L 9346 14680 L 9343 14683 L 9208 14785 L
9130 14816 L 9072 14860 L 8937 14946 L 8929 14951 L
8801 15037 L 8666 15065 L 8665 15087 L 8663 15223 L
8666 15226 L 8801 15227 L 8921 15358 L 8937 15372 L
9072 15378 L 9183 15494 L 9186 15630 L 9208 15661 L
9310 15765 L 9343 15799 L 9479 15800 L 9479 15856 L
9343 15856 L 9255 15765 L 9208 15717 L 9148 15630 L
9132 15494 L 9072 15431 L 8937 15415 L 8870 15358 L
8801 15282 L 8666 15275 L 8633 15223 L 8628 15087 L
8530 14987 L 8504 14951 L 8395 14838 L 8259 14828 L
8124 14820 L N E
15 O
8136 16173 8045 16173 8045 16172 8136 16172 8171 16172 8170 16173
8136 16173 7 N F
161 O
8124 12121 8107 12102 8124 12084 8143 12102 8124 12121 5 N F
15 O
8045 16172 8124 16106 8259 16058 8333 16172 8298 16172 8267 16172
8259 16160 8171 16172 8136 16172 8045 16172 10 N F
2 O
8259 16173 8170 16173 8170 16173 8267 16173 8267 16173 8259 16173
6 N F
8267 16173 8170 16173 8171 16172 8267 16172 8267 16173 5 N F
161 O
8259 11698 8257 11695 8259 11692 8395 11674 8417 11695 8395 11717
8259 11698 7 N F
2 O
8171 16172 8259 16160 8267 16172 8171 16172 4 N F
15 O
8267 16172 8267 16173 8298 16173 8333 16173 8333 16172 8298 16172

8267 16172 7 N F
29 O
8333 16172 8333 16173 8403 16173 8403 16172 8333 16172 5 N F
8403 16173 8333 16173 8333 16173 8395 16173 8403 16173 8403 16173
6 N F
161 O
8395 9421 8358 9388 8395 9336 8433 9388 8395 9421 5 N F
42 O
8403 16172 8403 16173 8460 16173 8524 16173 8524 16172 8460 16172
8403 16172 7 N F
161 O
8491 8438 8457 8303 8530 8258 8565 8303 8650 8438 8530 8488
8491 8438 7 N F
201 O
8530 5681 8484 5725 8517 5860 8530 5884 8587 5996 8666 6039
8801 6092 8907 6132 8937 6152 8966 6132 9072 6070 9095 5996
9092 5860 9072 5771 9039 5725 8956 5589 8937 5579 8801 5586
8788 5589 8666 5619 8530 5681 21 N F
55 O
8524 16172 8524 16173 8541 16173 8541 16172 8524 16172 5 N F
8541 16173 8524 16173 8524 16173 8530 16173 8540 16173 8541 16173
6 N F
161 O
8666 8659 8587 8574 8666 8445 8762 8574 8666 8659 5 N F
8666 11477 8618 11423 8666 11359 8729 11423 8666 11477 5 N F
42 O
8541 16172 8623 16172 8785 16172 8947 16172 8978 16172 8937 16114
8801 16047 8666 16067 8541 16172 9 N F
161 O
8666 9815 8645 9795 8666 9777 8687 9795 8666 9815 5 N F
15 O
8666 15226 8663 15223 8665 15087 8666 15065 8801 15037 8929 14951
8937 14946 9072 14860 9130 14816 9208 14785 9343 14683 9346 14680
9479 14598 9614 14607 9734 14680 9750 14708 9885 14808 9906 14816
9885 14845 9750 14819 9614 14831 9490 14951 9488 15087 9498 15223
9614 15336 9637 15358 9636 15494 9750 15612 9769 15630 9750 15649
9642 15765 9614 15798 9479 15800 9343 15799 9310 15765 9208 15661
9186 15630 9183 15494 9072 15378 8937 15372 8921 15358 8801 15227
8666 15226 43 N F
68 O
8937 15524 8803 15630 8844 15765 8937 15780 9072 15776 9076 15765
9072 15630 9072 15629 8937 15524 9 N F
148 O

8937 10769 8880 10745 8937 10719 8973 10745 8937 10769 5 N F
9072 11109 8949 11016 9072 10922 9176 11016 9072 11109 9072 11057
9118 11016 9072 10975 9018 11016 9072 11057 9072 11109 11 N F
161 O
9072 9470 9002 9388 9072 9314 9161 9388 9072 9470 5 N F
135 O
9072 11057 9018 11016 9072 10975 9118 11016 9072 11057 5 N F
174 O
9208 5785 9161 5725 9208 5615 9290 5725 9208 5785 5 N F
9343 6652 9290 6539 9343 6446 9417 6539 9343 6652 5 N F
161 O
9343 8710 9343 8710 9343 8709 9440 8574 9479 8550 9533 8574
9569 8710 9479 8786 9343 8710 9 N F
108 O
9479 12083 9367 11966 9479 11863 9578 11966 9479 12083 5 N F
121 O
9458 10609 9408 10474 9479 10394 9523 10338 9589 10202 9614 10173
9695 10202 9750 10210 9877 10338 9777 10474 9871 10609 9750 10621
9681 10745 9614 10797 9614 10744 9687 10609 9705 10474 9750 10451
9852 10338 9750 10235 9614 10226 9550 10338 9479 10429 9439 10474
9479 10563 9532 10609 9614 10744 9614 10797 9479 10825 9479 10789
9613 10745 9479 10657 9441 10745 9479 10789 9479 10825 9410 10745
9458 10609 37 N F
174 O
9446 6946 9436 6810 9479 6767 9539 6810 9547 6946 9479 6993
9446 6946 7 N F
108 O
9479 10563 9439 10474 9479 10429 9550 10338 9614 10226 9750 10235
9852 10338 9750 10451 9705 10474 9687 10609 9614 10744 9614 10614
9617 10609 9652 10474 9750 10423 9827 10338 9750 10260 9614 10273
9577 10338 9479 10464 9470 10474 9479 10492 9611 10609 9614 10614
9614 10744 9532 10609 9479 10563 27 N F
9479 10789 9441 10745 9479 10657 9613 10745 9479 10789 9479 10753
9503 10745 9479 10730 9472 10745 9479 10753 9479 10789 11 N F
95 O
9479 10492 9470 10474 9479 10464 9577 10338 9614 10273 9750 10260
9827 10338 9750 10423 9652 10474 9617 10609 9614 10614 9614 10414
9750 10396 9802 10338 9750 10285 9614 10321 9604 10338 9614 10414
9614 10614 9611 10609 9479 10492 21 N F
9479 10753 9472 10745 9479 10730 9503 10745 9479 10753 5 N F
55 O
9614 15992 9483 16037 9614 16117 9644 16172 9758 16172 9885 16172
9885 15927 9870 15901 9869 15765 9885 15748 9900 15765 9900 15901

9885 15927 9885 16172 9920 16172 10082 16172 10235 16172 10169 16037
10156 16019 10080 15901 10021 15775 10016 15765 9916 15630 9885 15595
9810 15494 9767 15358 9885 15297 10021 15303 10146 15358 10156 15365
10187 15358 10162 15223 10156 15217 10021 15196 9899 15087 9885 15073
9758 14951 9750 14943 9704 14951 9614 15025 9602 15087 9610 15223
9614 15227 9747 15358 9730 15494 9750 15515 9876 15630 9750 15760
9745 15765 9662 15901 9614 15992 51 N F
174 O
9532 6267 9488 6132 9491 5996 9517 5860 9614 5729 9750 5851
9758 5860 9836 5996 9874 6132 9885 6202 9917 6267 9967 6403
9999 6539 10021 6632 10060 6674 10112 6810 10083 6946 10021 7081
10021 7082 9885 7153 9885 6882 9979 6810 9979 6674 9951 6539
9891 6403 9885 6391 9844 6267 9824 6132 9764 5996 9750 5973
9655 5860 9614 5822 9586 5860 9523 5996 9513 6132 9606 6267
9614 6280 9659 6403 9635 6539 9634 6674 9750 6808 9765 6810
9885 6882 9885 7153 9802 7081 9773 6946 9750 6935 9640 6810
9614 6738 9568 6674 9588 6539 9614 6490 9620 6403 9614 6387
9532 6267 55 N F
161 O
9606 6267 9513 6132 9523 5996 9586 5860 9614 5822 9655 5860
9750 5973 9764 5996 9824 6132 9844 6267 9885 6391 9891 6403
9951 6539 9979 6674 9979 6810 9885 6882 9765 6810 9750 6808
9750 6779 9885 6773 9927 6674 9903 6539 9885 6501 9830 6403
9791 6267 9774 6132 9750 6081 9709 5996 9614 5892 9555 5996
9539 6132 9614 6236 9686 6267 9698 6403 9659 6539 9659 6674
9750 6779 9750 6808 9634 6674 9635 6539 9659 6403 9614 6280
9606 6267 43 N F
148 O
9614 6236 9539 6132 9555 5996 9614 5892 9709 5996 9750 6081
9774 6132 9791 6267 9830 6403 9885 6501 9903 6539 9927 6674
9885 6773 9750 6779 9750 6750 9876 6674 9861 6539 9770 6403
9750 6320 9737 6403 9682 6539 9684 6674 9750 6750 9750 6779
9659 6674 9659 6539 9698 6403 9686 6267 9614 6236 9614 6201
9722 6132 9659 5996 9614 5947 9586 5996 9564 6132 9614 6201
9614 6236 37 N F
174 O
9614 7130 9555 7081 9614 7000 9724 7081 9614 7130 5 N F
135 O
9614 6201 9564 6132 9586 5996 9614 5947 9659 5996 9722 6132
9614 6201 9614 6166 9668 6132 9614 6010 9589 6132 9614 6166
9614 6201 13 N F
148 O
9614 9453 9569 9388 9614 9332 9668 9388 9614 9453 5 N F

121 O
9614 6166 9589 6132 9614 6010 9668 6132 9614 6166 5 N F
82 O
9614 10414 9604 10338 9614 10321 9750 10285 9802 10338 9750 10396
9750 10369 9778 10338 9750 10310 9663 10338 9750 10369 9750 10396
9614 10414 13 N F
68 O
9750 10369 9663 10338 9750 10310 9778 10338 9750 10369 9750 10341
9753 10338 9750 10335 9740 10338 9750 10341 9750 10369 11 N F
135 O
9684 6674 9682 6539 9737 6403 9750 6320 9770 6403 9861 6539
9876 6674 9750 6750 9750 6721 9828 6674 9823 6539 9750 6438
9705 6539 9709 6674 9750 6721 9750 6750 9684 6674 17 N F
174 O
9750 9407 9695 9524 9714 9660 9750 9718 9772 9660 9885 9564
9913 9524 9885 9457 9750 9407 9 N F
9750 9201 9697 9252 9750 9339 9771 9252 9750 9201 5 N F
121 O
9709 6674 9705 6539 9750 6438 9823 6539 9828 6674 9750 6721
9750 6693 9780 6674 9785 6539 9750 6491 9728 6539 9734 6674
9750 6693 9750 6721 9709 6674 15 N F
108 O
9734 6674 9728 6539 9750 6491 9785 6539 9780 6674 9750 6693
9734 6674 7 N F
55 O
9750 10341 9740 10338 9750 10335 9753 10338 9750 10341 5 N F
148 O
9885 9168 9842 9117 9885 9077 9933 9117 9885 9168 5 N F
161 O
9885 8630 9843 8574 9885 8533 9928 8574 9885 8630 5 N F
68 O
9885 15748 9869 15765 9870 15901 9885 15927 9900 15901 9900 15765
9885 15748 7 N F
135 O
9979 10202 9891 10067 10021 9970 10147 10067 10059 10202 10021 10296
10021 10161 10107 10067 10021 10000 9932 10067 10021 10161 10021 10296
9979 10202 13 N F
121 O
10021 10161 9932 10067 10021 10000 10107 10067 10021 10161 10021 10118
10067 10067 10021 10031 9973 10067 10021 10118 10021 10161 11 N F
108 O
10021 10118 9973 10067 10021 10031 10067 10067 10021 10118 10021 10074
10028 10067 10021 10061 10013 10067 10021 10074 10021 10118 11 N F

95 O
10021 10074 10013 10067 10021 10061 10028 10067 10021 10074 5 N F
15 O
10021 15089 10019 15087 10021 15004 10156 15068 10292 15049 10380 15087
10427 15103 10507 15087 10563 15063 10685 14951 10698 14937 10834 14854
10969 14859 11105 14944 11115 14951 11126 15087 11105 15113 11004 15223
10969 15266 10834 15250 10698 15236 10563 15231 10427 15227 10292 15227
10287 15223 10156 15087 10021 15089 27 N F
161 O
10156 8789 10053 8710 10156 8650 10256 8710 10156 8789 5 N F
135 O
10156 9452 10102 9388 10156 9335 10292 9305 10374 9388 10292 9471
10292 9415 10318 9388 10292 9361 10156 9388 10156 9388 10156 9389
10292 9415 10292 9471 10156 9452 15 N F
121 O
10156 9389 10156 9388 10156 9388 10292 9361 10318 9388 10292 9415
10156 9389 7 N F
135 O
10292 9771 10199 9660 10292 9566 10393 9660 10292 9771 10292 9717
10344 9660 10292 9611 10244 9660 10292 9717 10292 9771 11 N F
42 O
10235 16173 10235 16172 10244 16172 10375 16172 10375 16173 10244 16173
10235 16173 7 N F
121 O
10292 9717 10244 9660 10292 9611 10344 9660 10292 9717 10292 9663
10295 9660 10292 9656 10288 9660 10292 9663 10292 9717 11 N F
108 O
10292 9663 10288 9660 10292 9656 10295 9660 10292 9663 5 N F
15 O
10412 16037 10341 15901 10427 15852 10563 15888 10698 15873 10834 15851
10969 15883 11015 15901 11105 15954 11166 15901 11132 15765 11105 15710
11025 15630 11105 15528 11240 15546 11376 15544 11443 15494 11511 15360
11512 15358 11579 15223 11612 15087 11647 14996 11670 14951 11782 14844
11823 14816 11918 14757 12053 14748 12136 14680 12189 14657 12324 14620
12351 14680 12324 14746 12246 14816 12189 14840 12053 14874 11995 14951
11918 15030 11860 15087 11802 15223 11832 15358 11885 15494 11918 15531
12011 15630 12053 15665 12189 15702 12292 15765 12324 15791 12460 15837
12557 15901 12592 16037 12595 16042 12716 16172 12677 16172 12613 16172
12595 16153 12512 16037 12460 15979 12370 15901 12324 15867 12197 15765
12189 15760 12053 15715 11950 15630 11918 15595 11829 15494 11782 15383
11647 15366 11565 15494 11511 15609 11474 15630 11376 15662 11240 15765
11240 15765 11240 15766 11331 15901 11376 15967 11432 16037 11376 16096
11240 16125 11105 16171 10969 16041 10967 16037 10834 15992 10698 16022

10666 16037 10670 16172 10569 16172 10502 16172 10427 16061 10412 16037
90 N F
135 O
10427 9331 10348 9252 10400 9117 10427 9095 10563 9103 10578 9117
10563 9138 10503 9252 10427 9331 10427 9271 10445 9252 10427 9211
10409 9252 10427 9271 10427 9331 15 N F
29 O
10375 16173 10375 16172 10407 16172 10502 16172 10502 16173 10407 16173
10375 16173 7 N F
121 O
10427 9271 10409 9252 10427 9211 10445 9252 10427 9271 5 N F
29 O
10563 11834 10474 11831 10563 11765 10698 11793 10760 11831 10834 11891
10943 11966 10969 11980 11105 12070 11143 12102 11240 12176 11338 12238
11240 12299 11105 12274 10969 12253 10942 12238 10834 12124 10698 12109
10693 12102 10693 11966 10563 11834 21 N F
15 O
10502 16173 10502 16172 10569 16172 10670 16172 10670 16173 10569 16173
10502 16173 7 N F
174 O
10698 8831 10592 8845 10698 8897 10745 8845 10698 8831 5 N F
2 O
10670 16172 10666 16037 10698 16022 10834 15992 10967 16037 10969 16041
11105 16171 11240 16125 11376 16096 11432 16037 11376 15967 11331 15901
11240 15766 11240 15765 11240 15765 11376 15662 11474 15630 11511 15609
11565 15494 11647 15366 11782 15383 11829 15494 11918 15595 11950 15630
12053 15715 12189 15760 12197 15765 12324 15867 12370 15901 12460 15979
12512 16037 12595 16153 12613 16172 12515 16172 12353 16172 12191 16172
12028 16172 11866 16172 11704 16172 11542 16172 11380 16172 11218 16172
11055 16172 10893 16172 10731 16172 10670 16172 46 N F
148 O
10834 9194 10760 9117 10834 9058 10895 9117 10834 9194 5 N F
135 O
10969 9464 10909 9388 10969 9337 11049 9388 10969 9464 5 N F
55 O
11205 14680 11185 14816 11240 14844 11363 14816 11328 14680 11240 14551
11205 14680 7 N F
15 O
11647 14275 11602 14273 11645 14137 11647 14136 11700 14002 11782 13994
11790 14002 11835 14137 11782 14187 11649 14273 11647 14275 11 N F
42 O
11918 15157 11878 15223 11893 15358 11918 15424 11953 15494 12053 15602
12053 15511 12037 15494 12053 15483 12065 15494 12053 15511 12053 15602

12131 15630 12189 15643 12324 15697 12383 15630 12324 15575 12189 15606
12125 15494 12053 15424 11954 15358 11965 15223 12053 15160 12053 15093
12046 15087 12053 15080 12059 15087 12053 15093 12053 15160 12129 15087
12189 14999 12236 14951 12189 14916 12125 14951 12053 14996 11963 15087
11918 15157 37 N F
68 O
12053 11894 11934 11831 12053 11737 12081 11831 12053 11894 5 N F
55 O
12053 15483 12037 15494 12053 15511 12065 15494 12053 15483 5 N F
15 O
12053 15365 12044 15358 12053 15268 12189 15348 12197 15358 12258 15494
12189 15498 12186 15494 12053 15365 9 N F
55 O
12053 15080 12046 15087 12053 15093 12059 15087 12053 15080 5 N F
95 O
12053 13355 12047 13459 12053 13477 12141 13594 12189 13627 12319 13594
12200 13459 12189 13452 12053 13355 9 N F
15 O
12460 14463 12429 14409 12460 14298 12513 14409 12460 14463 5 N F
12595 14337 12478 14273 12595 14220 12625 14273 12595 14337 5 N F
55 O
12595 14483 12534 14544 12595 14613 12595 14555 12585 14544 12595 14534
12708 14409 12731 14370 12758 14409 12731 14435 12607 14544 12595 14555
12595 14613 12668 14544 12731 14489 12814 14409 12731 14292 12661 14409
12595 14483 19 N F
108 O
12595 13580 12585 13594 12595 13605 12606 13594 12595 13580 5 N F
68 O
12595 14534 12585 14544 12595 14555 12607 14544 12731 14435 12758 14409
12731 14370 12708 14409 12595 14534 9 N F
15 O
12613 16172 12614 16173 12677 16173 12716 16173 12716 16172 12677 16172
12613 16172 7 N F
12731 16087 12643 16037 12690 15901 12731 15780 12735 15765 12780 15630
12866 15582 12941 15494 13002 15415 13002 16172 12839 16172 12784 16172
12731 16087 13 N F
29 O
12716 16172 12716 16173 12785 16173 12784 16172 12716 16172 5 N F
12785 16173 12716 16173 12716 16173 12731 16173 12785 16173 12785 16173
6 N F
12866 14307 12863 14273 12866 14271 12873 14273 12866 14307 5 N F
95 O
13002 13459 13001 13459 13002 13459 13002 13459 4 N F

188 O
13002 5453 13002 5453 13002 9526 13002 9526 13002 5453 13002 5453
6 N F
174 O
13002 11026 13002 9526 13002 9526 13002 11026 13002 11026 5 N F
161 O
13002 11590 13002 11026 13002 11026 13002 11590 13002 11590 5 N F
148 O
13002 12032 13002 11590 13002 11590 13002 12032 13002 12032 5 N F
135 O
13002 12322 13002 12032 13002 12032 13002 12322 13002 12322 5 N F
121 O
13002 12563 13002 12322 13002 12322 13002 12563 13002 12563 5 N F
108 O
13002 12829 13002 12563 13002 12563 13002 12829 13002 12829 5 N F
95 O
13002 13163 13002 12829 13002 12829 13002 13163 13002 13163 5 N F
82 O
13002 13459 13002 13163 13002 13163 13002 13458 13002 13459 5 N F
95 O
13002 13459 13002 13459 13002 13458 13002 13459 13002 13459 5 N F
82 O
13002 14028 13002 13459 13002 13459 13002 14028 13002 14028 5 N F
68 O
13002 14083 13002 14028 13002 14028 13002 14083 13002 14083 5 N F
55 O
13002 14144 13002 14083 13002 14083 13002 14144 13002 14144 5 N F
42 O
13002 14382 13002 14144 13002 14144 13002 14382 13002 14382 5 N F
29 O
13002 15415 13002 14382 13002 14382 13002 15415 13002 15415 5 N F
15 O
13002 16172 13002 15415 13002 15415 13002 16173 13002 16173 13002 16172
6 N F
174 O
13002 9526 13002 11026 13002 11026 13002 11016 13002 10881 13002 10745
13002 10609 13002 10474 13002 10338 13002 10202 13002 10067 13002 9931
13002 9795 13002 9660 13002 9526 13002 9526 16 N F
161 O
13002 11026 13002 11590 13002 11590 13002 11559 13002 11423 13002 11288
13002 11152 13002 11026 13002 11026 9 N F
148 O
13002 11590 13002 12032 13002 12032 13002 11966 13002 11831 13002 11695

13002 11590 13002 11590 8 N F
135 O
13002 12032 13002 12322 13002 12322 13002 12238 13002 12102 13002 12032
13002 12032 7 N F
121 O
13002 12322 13002 12563 13002 12563 13002 12509 13002 12373 13002 12322
13002 12322 7 N F
108 O
13002 12563 13002 12829 13002 12829 13002 12780 13002 12645 13002 12563
13002 12563 7 N F
95 O
13002 12829 13002 13163 13002 13163 13002 13052 13002 12916 13002 12829
13002 12829 7 N F
82 O
13002 13163 13002 13458 13002 13458 13002 13323 13002 13187 13002 13163
13002 13163 7 N F
95 O
13002 13458 13002 13458 13002 13459 13002 13459 13002 13459 13002 13458
6 N F
82 O
13002 13459 13002 14028 13002 14028 13002 14002 13002 13866 13002 13730
13002 13594 13002 13459 13002 13459 9 N F
68 O
13002 14028 13002 14083 13002 14083 13002 14028 13002 14028 5 N F
55 O
13002 14083 13002 14144 13002 14144 13002 14137 13002 14083 13002 14083
6 N F
42 O
13002 14144 13002 14382 13002 14382 13002 14273 13002 14144 13002 14144
6 N F
29 O
13002 14382 13002 15415 13002 15415 13002 15358 13002 15223 13002 15087
13002 14951 13002 14816 13002 14680 13002 14544 13002 14409 13002 14382
13002 14382 13 N F
Ls Gr Gs Lr
-1200 1025 16500 18750 Oc
1 O
10639 15368 10640 15409 10679 15446 10675 15487 10710 15528 10753 15562
10792 15599 10827 15641 10872 15670 10920 15695 10946 15742 10985 15780
11030 15810 11080 15831 11122 15865 11160 15903 11196 15934 11247 15960
18 P
11247 15960 11300 15971 11330 16016 11355 16064 11389 16106 11419 16151
11428 16172 7 P

12840 16172 12841 16165 12836 16139 12812 16127 12785 16122 12767 16102
12743 16090 12718 16078 12698 16061 12682 16039 12656 16031 12637 16011
12633 15985 12626 15958 12621 15932 12608 15908 12584 15897 12561 15882
12544 15860 12528 15839 12506 15823 12482 15811 12458 15799 12434 15786
12425 15760 12449 15746 12444 15720 12419 15710 12402 15689 12377 15677
12359 15657 12333 15649 12323 15624 12298 15633 12283 15610 12257 15601
12230 15604 12204 15610 12182 15594 12168 15570 12146 15555 12149 15528
12128 15511 12107 15494 12081 15487 12067 15463 12040 15464 12022 15444
12010 15421 12003 15394 50 P
12003 15394 12008 15368 12034 15360 12041 15333 12028 15309 12026 15283
12033 15256 12045 15232 12053 15206 12039 15230 12013 15225 11 P
12013 15225 11986 15218 12012 15209 12006 15183 11984 15168 11980 15141
11994 15118 12018 15104 12045 15102 12072 15102 12098 15095 12124 15086
12147 15100 12162 15078 12178 15056 12196 15035 12213 15014 12235 14998
12256 14981 12279 14966 12306 14963 12328 14948 12347 14928 12358 14904
12383 14913 12409 14904 12433 14891 12438 14864 28 P
12438 14864 12448 14839 12463 14816 12476 14793 12494 14772 12503 14747
12519 14725 12534 14702 12546 14678 12563 14656 12575 14632 12586 14607
12605 14588 12622 14566 12640 14546 12656 14524 12675 14505 12691 14483
12712 14465 12731 14445 12752 14428 12772 14410 12793 14393 12798 14366
12818 14348 12838 14328 12844 14302 12864 14284 12878 14261 12900 14245
12915 14222 12934 14202 12951 14181 12960 14155 12967 14129 12980 14105
12999 14085 13001 14061 38 P
13001 13007 12998 13007 13001 12992 3 P
13001 12979 12980 12968 12959 12951 12951 12978 12952 13005 12936 12982
12909 12980 12883 12972 12855 12973 9 P
12855 12973 12828 12977 12806 12993 12783 13006 12789 12980 12797 12953
6:00 PM
12797 12953 12771 12961 12743 12963 12730 12939 12747 12918 12729 12898
12726 12871 12725 12843 12743 12822 12741 12795 12730 12770 12708 12787
12692 12809 12718 12802 12712 12829 12685 12829 12688 12856 12663 12846
12638 12858 12629 12832 12601 12832 12575 12840 12550 12851 12549 12879
12546 12906 12539 12879 12514 12869 12490 12855 12468 12839 12442 12847
12415 12841 12398 12820 12374 12806 12357 12828 12351 12801 12364 12778
12389 12765 12410 12783 12436 12789 12442 12762 12438 12735 12454 12712
12463 12687 12464 12659 12437 12650 12436 12678 12442 12705 12415 12711
12388 12704 12389 12677 50 P
12389 12677 12366 12663 12354 12687 12363 12713 12352 12738 12337 12761
12324 12786 12313 12761 12286 12754 12259 12753 12284 12743 12262 12728
12269 12701 12287 12680 12309 12664 12331 12648 12331 12621 12303 12621
12276 12627 12273 12600 12300 12604 12299 12577 12276 12561 12265 12536
12245 12554 12228 12575 12208 12595 12194 12571 12167 12580 12176 12606
12187 12631 12192 12604 12215 12618 12214 12646 12214 12673 12234 12655

12252 12676 12247 12703 12222 12693 12196 12684 12178 12663 12153 12653
12133 12634 12109 12620 12106 12593 12097 12567 12107 12542 12091 12519
12064 12521 12037 12515 50 P
12037 12515 12024 12538 2 P
12024 12538 12021 12565 12011 12591 12008 12564 12008 12536 11984 12523
11978 12496 11955 12481 11951 12453 11933 12432 11911 12416 11910 12443
11886 12458 11903 12480 11879 12492 11871 12519 11890 12540 11909 12559
11882 12560 11860 12542 11836 12554 11814 12570 11787 12575 11774 12600
11772 12627 11781 12653 11804 12638 11806 12611 11820 12588 11845 12577
11873 12580 11852 12598 11826 12607 11828 12634 11855 12635 11861 12609
11887 12602 11915 12604 11905 12630 11884 12647 11857 12650 11829 12649
11850 12666 11877 12662 11884 12689 11911 12693 11936 12705 11909 12707
11905 12734 11881 12748 50 P
11881 12748 11879 12775 11878 12748 11887 12722 11887 12694 11859 12695
11836 12681 11826 12707 11832 12734 11822 12759 11838 12782 11811 12777
11794 12755 11773 12773 11799 12784 11811 12808 11789 12823 11769 12842
11771 12815 11752 12834 11743 12860 11770 12854 11782 12878 11781 12851
11799 12831 11817 12811 11835 12832 11845 12857 11818 12864 11796 12879
11800 12906 11773 12913 11783 12939 11789 12966 11795 12993 11816 12977
11825 13003 11798 13007 11812 13031 11816 13058 11828 13083 11853 13092
11879 13101 11906 13098 11905 13126 11878 13126 11851 13121 11824 13119
11810 13143 11829 13163 50 P
11829 13163 11849 13181 2 P
11849 13181 11867 13202 11894 13203 11899 13229 11924 13241 11909 13263
11929 13283 11947 13303 11920 13297 11893 13294 11866 13289 11858 13315
11873 13337 11897 13350 11918 13367 11944 13379 11964 13396 11966 13423
11959 13397 11937 13381 11912 13370 11889 13357 11869 13376 11856 13400
11835 13417 11810 13427 11831 13443 11809 13459 11807 13487 11793 13510
11806 13534 11784 13551 11783 13578 11806 13592 11806 13607 11782 13580
11768 13604 11782 13627 11757 13640 11758 13667 11761 13694 11755 13721
11733 13705 11715 13685 11711 13712 11702 13738 11683 13718 11696 13695
11691 13668 11716 13657 50 P
11716 13657 11719 13630 11732 13606 11743 13581 11721 13564 11705 13586
11701 13613 11695 13639 11678 13660 11678 13688 11660 13708 11634 13700
11635 13727 11610 13739 11601 13713 11595 13740 11604 13766 11630 13772
11629 13799 11636 13825 11661 13837 11660 13865 11666 13838 11672 13812
11671 13784 11693 13769 11677 13791 11702 13804 11717 13826 11699 13847
11676 13862 11697 13880 11723 13889 11732 13863 11739 13837 11740 13809
11746 13836 11750 13863 11771 13846 11760 13871 11741 13890 11766 13879
11756 13904 11779 13891 11804 13879 11826 13895 11847 13876 11873 13871
11890 13849 11894 13822 50 P
11894 13822 11909 13799 2 P
11909 13799 11909 13826 11928 13845 11950 13861 11959 13887 11940 13868

11913 13873 11895 13893 11876 13913 11850 13919 11857 13945 11868 13970
11863 13997 11842 13980 11823 13960 11806 13982 11819 14005 11812 14032
11839 14036 11861 14053 11837 14065 11812 14077 11828 14099 11802 14108
11781 14125 11762 14145 11786 14157 11784 14184 11774 14209 11748 14217
11728 14235 11721 14262 11716 14288 11741 14301 11765 14314 11778 14290
11770 14263 11795 14250 11818 14238 11843 14226 11865 14210 11887 14201
11887 14202 11867 14214 11844 14228 11822 14243 11797 14255 11785 14280
11784 14307 11778 14333 50 P
11778 14333 11796 14357 11770 14344 3 P
11770 14344 11753 14322 11726 14317 11702 14329 11679 14344 11656 14359
11629 14357 11608 14339 11581 14335 11555 14327 11557 14300 11550 14273
11528 14289 11509 14309 11505 14335 11479 14342 11476 14314 11449 14316
11432 14338 11413 14357 11391 14373 11379 14397 11352 14404 11325 14403
11300 14413 11304 14386 11329 14375 11356 14376 11350 14349 11323 14350
11326 14323 11330 14296 11322 14271 11294 14273 11271 14259 11255 14237
11228 14233 11217 14208 11201 14229 11212 14254 11239 14261 11241 14288
11268 14290 11253 14312 11275 14328 11248 14331 11221 14329 11244 14314
11217 14309 11208 14284 50 P
11208 14284 11181 14283 11185 14310 11162 14324 11140 14308 11114 14300
11088 14307 11063 14318 11047 14340 11071 14354 11098 14350 11123 14360
11099 14372 11106 14399 11132 14404 11154 14387 11156 14414 11131 14424
11103 14421 11078 14411 11051 14407 11025 14415 11002 14428 10977 14419
10950 14411 10924 14415 10897 14423 10923 14430 10943 14449 10963 14465
10982 14492 11004 14478 11002 14471 11004 14467 11033 14486 11023 14491
11003 14480 10983 14499 10983 14526 10977 14552 10991 14576 11001 14601
11021 14619 11031 14644 11020 14669 11047 14670 11071 14683 11075 14710
11067 14736 11085 14715 50 P
11085 14715 11108 14729 2 P
11108 14729 11134 14735 11157 14750 11184 14754 11207 14767 11230 14752
11257 14756 11241 14778 11214 14778 11190 14791 11174 14769 11147 14771
11121 14763 11118 14790 11103 14813 11110 14786 11084 14795 11070 14818
11062 14844 11073 14869 11082 14895 11091 14920 11104 14944 11125 14962
11116 14988 11121 15014 11144 15029 11151 15055 11157 15081 11153 15108
11167 15133 11140 15126 11127 15150 11126 15177 11146 15196 11150 15223
11174 15236 11187 15237 11197 15251 11220 15263 11226 15267 11225 15269
11195 15252 11184 15242 11163 15240 11173 15268 11177 15282 11188 15277
11198 15279 11203 15287 50 P
11203 15287 11201 15289 11197 15283 11189 15279 11177 15283 11172 15271
11160 15247 11139 15230 11116 15216 11089 15215 11070 15234 11044 15225
11020 15239 10998 15254 10971 15254 10953 15274 10930 15262 10914 15283
10888 15291 10867 15307 10856 15283 10859 15256 10843 15234 10826 15213
10813 15237 10823 15262 10804 15281 10796 15307 10786 15332 10759 15335
10733 15342 10709 15354 10687 15370 10693 15396 10666 15395 10661 15368

10656 15342 10650 15315 10643 15341 10639 15368 40 P
10639 15368 10627 15344 10600 15338 10578 15323 10551 15317 10524 15318
10497 15320 10473 15332 10453 15313 10426 15313 10403 15328 10401 15355
10393 15329 10383 15304 10369 15280 10343 15286 10331 15262 10307 15276
10281 15281 10255 15285 10246 15260 10223 15246 10247 15235 10226 15218
10200 15209 10175 15218 10150 15208 10131 15188 10106 15177 10081 15167
10056 15157 10035 15140 10015 15121 9991 15110 9973 15089 9949 15102
9946 15075 9926 15057 9900 15060 9879 15042 9856 15027 9846 15002
9834 14978 9811 14964 9792 14945 9781 14920 9806 14908 9782 14896
9755 14890 9763 14864 50 P
9763 14864 9737 14859 9716 14841 9696 14859 9704 14833 9679 14842
9690 14817 9688 14790 9665 14803 9653 14828 9628 14838 9607 14821
9595 14797 9571 14785 9597 14779 9620 14765 9608 14741 9581 14738
9554 14738 9535 14718 9510 14710 9483 14710 9458 14698 9431 14699
9419 14675 9402 14653 9381 14670 9382 14697 9369 14722 9391 14738
9364 14745 9372 14771 9398 14779 9425 14779 9451 14789 9464 14812
9474 14837 9494 14818 9521 14822 9543 14838 9568 14848 9594 14857
9607 14881 9587 14898 9564 14914 9581 14935 9563 14954 9589 14961
9612 14975 9627 14998 50 P
9627 14998 9650 15013 2 P
9650 15013 9675 15020 9699 15033 9672 15034 9646 15028 9619 15027
9594 15015 9567 15014 9541 15011 9517 15025 9507 15055 9494 15087
9517 15096 9529 15113 9533 15125 9536 15146 9533 15173 9517 15194
9496 15211 9506 15236 9517 15260 9540 15275 9564 15289 9581 15310
9608 15309 9632 15322 9650 15342 9670 15361 9644 15368 9667 15382
9683 15403 9700 15424 9714 15447 9728 15470 9748 15489 9752 15516
9766 15538 9783 15559 9786 15586 9803 15607 9816 15631 9803 15655
9782 15673 9760 15688 9754 15698 9762 15715 9758 15741 9760 15748
9784 15735 9802 15740 50 P
9802 15740 9806 15746 9801 15754 9778 15753 9777 15766 9756 15778
9731 15793 9732 15791 9754 15775 9766 15768 9766 15762 9772 15754
9779 15745 9801 15751 9802 15743 9784 15738 9768 15746 9759 15750
9754 15746 9754 15732 9742 15733 9727 15748 9705 15764 9682 15779
9665 15800 9638 15798 9625 15821 9633 15847 9622 15872 9634 15896
9639 15923 9629 15898 9608 15880 9608 15853 9598 15827 9576 15811
9549 15809 9523 15806 9503 15826 9481 15841 9454 15840 9430 15853
9406 15840 9384 15855 9357 15858 9342 15836 9323 15817 9313 15792
9286 15796 9267 15776 50 P
9267 15776 9255 15752 2 P
9255 15752 9278 15738 9260 15717 9234 15711 9216 15690 9204 15666
9185 15647 9164 15630 9150 15607 9127 15593 9122 15567 9100 15550
9122 15535 9099 15521 9086 15497 9087 15470 9071 15449 9045 15442
9019 15435 8998 15417 8974 15406 8950 15392 8925 15383 8899 15375

8874 15364 8851 15350 8835 15328 27 P
8835 15328 8810 15320 8788 15304 8762 15297 8736 15290 8710 15280
8690 15263 8691 15236 8666 15225 8645 15209 8625 15190 8612 15166
8605 15140 8597 15114 8575 15100 8583 15074 8578 15047 8583 15020
8600 15000 8575 14989 8553 14973 8536 14952 8522 14929 8503 14909
8482 14893 8458 14880 8433 14870 8406 14867 8384 14851 8357 14849
8336 14866 8309 14867 8284 14856 8264 14837 8242 14821 8222 14803
8201 14787 8174 14791 8151 14806 8132 14826 8126 14852 8111 14874
8087 14888 8060 14892 44 P
8060 14892 8034 14888 8008 14879 7986 14864 7966 14844 7947 14825
7958 14800 7964 14773 7939 14763 7924 14741 7906 14719 7893 14696
7881 14671 7872 14645 7867 14619 7866 14592 7870 14565 17 P
7870 14565 7879 14539 7890 14514 7903 14490 7914 14466 7928 14443
7945 14421 7964 14402 7984 14383 8010 14376 8033 14361 11 P
8033 14361 8052 14342 8074 14327 8098 14313 8125 14316 8152 14313
8179 14316 8206 14314 8232 14306 8259 14302 8280 14285 8301 14301
8326 14313 8351 14324 8373 14340 8397 14352 8402 14326 8408 14352
8422 14329 8418 14302 8424 14275 8415 14250 8432 14271 8459 14271
8464 14244 8482 14224 8490 14198 8498 14172 8492 14146 8480 14121
8505 14110 8523 14089 8510 14065 8494 14043 8476 14023 8470 13996
8468 13969 8469 13942 8472 13915 8467 13888 8485 13868 8502 13846
8505 13819 8532 13820 8555 13806 8576 13787 8603 13785 8629 13778
8656 13777 8683 13779 50 P
8683 13779 8710 13782 8736 13771 8763 13773 8790 13773 8817 13780
8835 13799 8856 13817 8875 13836 8882 13863 8866 13884 8879 13908
8900 13925 8925 13936 8951 13943 8974 13958 8996 13974 9015 13994
9034 14013 9051 14035 9055 14061 9030 14073 9056 14081 9082 14089
9102 14108 9128 14115 9155 14119 9178 14133 9204 14142 9224 14160
9252 14161 9279 14158 9306 14161 9318 14137 9342 14124 9359 14103
9386 14105 9402 14082 9406 14055 9426 14036 9452 14031 9479 14034
9504 14044 9531 14048 9507 14036 9525 14016 9542 13995 9560 13975
9585 13963 9612 13961 50 P
9612 13961 9639 13966 2 P
9639 13966 9664 13976 9684 13957 9700 13979 9727 13984 9754 13988
9769 13966 9767 13993 9787 14012 9810 14027 9831 14043 9849 14023
9840 13997 9854 13974 9878 13987 9901 13972 9928 13965 9955 13966
9981 13974 9999 13953 10023 13964 10047 13977 10075 13976 10055 13957
10037 13936 10032 13909 10040 13883 10056 13861 10034 13846 10010 13832
9995 13809 9995 13782 9974 13764 10002 13761 10029 13765 10029 13738
10025 13711 10012 13687 10023 13712 9996 13706 9983 13682 9961 13665
9939 13650 9913 13654 9885 13655 9881 13682 9908 13685 9883 13696
9867 13718 9883 13740 50 P
9883 13740 9876 13766 9859 13745 9860 13718 9837 13703 9818 13684

9808 13710 9794 13733 9767 13728 9778 13704 9754 13692 9729 13680
9704 13669 9691 13645 9667 13632 9642 13622 9642 13595 9617 13602
9590 13595 9605 13618 9626 13635 9598 13633 9579 13614 9565 13637
9570 13610 9553 13588 9530 13574 9505 13564 9479 13554 9453 13547
9430 13533 9410 13514 9385 13524 9386 13497 9359 13496 9345 13519
9319 13527 9296 13513 9279 13492 9305 13498 9321 13476 9346 13465
9346 13438 9329 13417 9328 13389 9303 13402 9282 13420 9258 13408
9243 13384 9251 13358 50 P
9251 13358 9225 13350 2 P
9225 13350 9220 13323 9235 13301 9240 13274 9244 13248 9223 13231
9198 13218 9171 13215 9144 13210 9124 13192 9098 13183 9071 13188
9082 13213 9093 13238 9098 13265 9082 13288 9064 13308 9060 13281
9038 13264 9013 13273 8987 13263 8978 13237 8986 13211 9009 13197
9035 13189 9020 13166 9047 13162 9074 13165 9062 13141 9040 13125
9026 13101 9033 13128 9010 13113 8985 13103 8965 13084 8954 13058
8957 13031 8949 13005 8927 13020 8915 12996 8901 12972 8890 12947
8873 12969 8853 12951 8840 12927 8830 12952 8808 12936 8781 12939
8787 12913 8778 12887 50 P
8778 12887 8770 12860 8756 12837 8752 12810 8732 12790 8708 12778
8691 12756 8683 12730 8676 12704 8673 12676 8661 12651 8634 12656

12:00 PM

8634 12656 8613 12638 8599 12615 8595 12588 8595 12561 8586 12535
8588 12508 8601 12484 8594 12457 8613 12477 8635 12461 8659 12474
8684 12462 8711 12459 8716 12432 8730 12409 8751 12391 8774 12377
8798 12364 8820 12347 8847 12340 8865 12320 8865 12292 8878 12317
8905 12311 8932 12306 8958 12297 8982 12286 9001 12266 9028 12259
9053 12248 9078 12237 9092 12213 9096 12186 9109 12162 9117 12135
9126 12109 9136 12084 9145 12058 9146 12030 9154 12004 9155 11976
9180 11966 9175 11939 9201 11929 9209 11903 9220 11878 9216 11851
9223 11824 9240 11802 50 P
9240 11802 9262 11785 9252 11759 9250 11732 9257 11705 9276 11686
9285 11660 9297 11635 9316 11614 9331 11592 9337 11565 9351 11541
9378 11537 9389 11511 9395 11484 9405 11458 9407 11431 9398 11405
9378 11386 9404 11377 9417 11353 9398 11332 9383 11309 9411 11306
9429 11285 9448 11265 9476 11264 9501 11255 9527 11245 9552 11233
9577 11222 9603 11211 9625 11195 9638 11170 9638 11143 9643 11116
9628 11092 9648 11073 9660 11048 9686 11038 9713 11038 9739 11028
9765 11019 9789 11006 9810 10988 9829 10968 9848 10948 9857 10922
9870 10897 9884 10873 50 P
9884 10873 9895 10847 2 P
9895 10847 9889 10820 9863 10810 9836 10818 9810 10826 9782 10829
9757 10839 9732 10852 9706 10863 9688 10884 9662 10892 9635 10898
9610 10910 9584 10900 9558 10890 9531 10884 9517 10860 9490 10864

9462 10861 9436 10869 9430 10896 9420 10922 9401 10942 9384 10964
9361 10979 9345 11002 9319 10992 9293 11001 9265 11001 9240 11012
9215 11002 9190 10988 9169 10970 9147 10953 9126 10936 9100 10926
9072 10928 9045 10934 9059 10910 9087 10910 9113 10918 9138 10931
9164 10937 9192 10942 9203 10968 9231 10970 9257 10961 9284 10958
9312 10961 9339 10958 50 P
9339 10958 9355 10935 9368 10910 9380 10886 9359 10869 9377 10849
9403 10837 9430 10835 9458 10836 9484 10826 9510 10817 9531 10799
9555 10785 9577 10768 9595 10747 9609 10723 9627 10702 17 P
9627 10702 9651 10688 9677 10677 9698 10660 9714 10637 9741 10636
9766 10623 9789 10608 9811 10592 9829 10570 9843 10547 9857 10522
9870 10498 9883 10473 9896 10449 9910 10424 9920 10398 9913 10371
9890 10356 9863 10351 9835 10349 9807 10346 9780 10341 9753 10334
9727 10326 9702 10313 9682 10294 9667 10270 9643 10256 9615 10252
30 P
9615 10252 9593 10236 9571 10219 9556 10196 9528 10191 9503 10178
9484 10159 9469 10135 9468 10107 9452 10084 9436 10062 9411 10050
9383 10047 9364 10067 9337 10076 9309 10076 9281 10073 9254 10073
9227 10067 9202 10054 9194 10027 9195 9999 9169 10009 9141 10007
9124 9985 9104 9966 9128 9981 9147 10001 9169 9984 9196 9986
9213 10009 9223 10035 9246 10051 9274 10053 9302 10052 9325 10037
9314 10012 9323 9986 9341 9964 9335 9936 9344 9909 9371 9901
9346 9915 9365 9935 9391 9925 9419 9925 9445 9935 9466 9953
9490 9967 9512 9985 50 P
9512 9985 9531 10005 9553 10022 9578 10034 9606 10039 9633 10039
9661 10036 9685 10021 9708 10005 9727 9986 9746 9965 9763 9942
9782 9922 9798 9899 9812 9875 9836 9861 9863 9856 9891 9852
9917 9841 9944 9834 9955 9824 9962 9818 9966 9817 9994 9817
10022 9818 10046 9832 10039 9805 10016 9789 9990 9779 9968 9761
9954 9737 9931 9720 9914 9698 9892 9682 9874 9660 9856 9638
9834 9621 9807 9614 9780 9609 9753 9615 9735 9593 9724 9567
9732 9541 9747 9564 9774 9572 9766 9545 9784 9566 9807 9582
9834 9590 9853 9610 50 P
9853 9610 9881 9611 2 P
9881 9611 9888 9584 9912 9597 9896 9621 9897 9648 9921 9662
9949 9668 9976 9675 9995 9655 9977 9633 9974 9605 9990 9582
9989 9554 9963 9543 9966 9516 9963 9488 9989 9476 9994 9448
9975 9426 9952 9411 9924 9417 9923 9444 9917 9472 9927 9498
9906 9517 9899 9490 9901 9462 9894 9435 9874 9416 9847 9409
9837 9435 9835 9463 9844 9490 9833 9464 9832 9436 9814 9458
9786 9462 9782 9435 9774 9407 9750 9393 9749 9365 9777 9369
9803 9377 9831 9371 9834 9343 9806 9340 9785 9321 9811 9332
9839 9338 9864 9327 50 P

9864 9327 9853 9301 9874 9282 9855 9261 9827 9264 9801 9274
9816 9250 9843 9244 9856 9219 9864 9192 9844 9173 9825 9153
9801 9138 9773 9137 9745 9136 9717 9137 9743 9129 9771 9122
9787 9100 9795 9073 9808 9047 9823 9024 9822 8996 9836 8971
9845 8945 9859 8920 9847 8895 9828 8916 9805 8931 9777 8936
9762 8912 9749 8887 9728 8869 9734 8842 9713 8823 9693 8842
9675 8864 9667 8891 9652 8915 9669 8938 9642 8945 9616 8936
9606 8909 9581 8898 9603 8881 9593 8854 9578 8830 9553 8817
9540 8792 9541 8764 50 P
9541 8764 9529 8739 2 P
9529 8739 9529 8727 9502 8730 9474 8731 9446 8730 9429 8748
9424 8748 9396 8755 9369 8751 9347 8770 9349 8742 9377 8745
9404 8740 9424 8720 9452 8716 9477 8702 9479 8674 9463 8650
9445 8629 9430 8605 9418 8579 9390 8577 9386 8548 9387 8520
9368 8500 9340 8493 9358 8471 9371 8446 9398 8436 9384 8411
9385 8383 9357 8378 9332 8391 9325 8363 9339 8339 9322 8315
9322 8287 9306 8263 9291 8287 9280 8261 9256 8275 9245 8301
42 P
9245 8301 9251 8274 9265 8249 9250 8226 9222 8218 9195 8222
9204 8195 9199 8167 9180 8146 9151 8146 9179 8139 9181 8110
9153 8116 9125 8118 9118 8090 9090 8096 9078 8070 9102 8056
9127 8042 9116 8015 9142 8004 9121 7984 9122 7956 9096 7945
9072 7930 9043 7927 9035 7900 9014 7881 8985 7882 8993 7910
9009 7933 9037 7934 9058 7953 9056 7982 9082 7995 9094 8020
9070 8036 9042 8039 9033 8012 9017 7989 9012 7961 8990 7979
8962 7985 8965 8013 8989 8027 8962 8034 8978 8057 8952 8046
8948 8018 8927 8037 50 P
8927 8037 8910 8059 8900 8086 8901 8058 8908 8030 8918 8004
8893 8017 8871 8035 8845 8045 8849 8017 8844 7989 8867 7973
8840 7964 8843 7935 8865 7917 8891 7928 8919 7927 8947 7923
8966 7901 8970 7873 8982 7847 8966 7824 8944 7841 8916 7848
8895 7866 8872 7850 8859 7825 8886 7833 8899 7807 8905 7780
8927 7762 8952 7774 8961 7801 8979 7824 8996 7801 9020 7786
9046 7797 9028 7774 9000 7771 8973 7765 8948 7750 8921 7742
8893 7736 8869 7720 8898 7725 8887 7699 8870 7676 8850 7656
8834 7632 8853 7612 50 P
8853 7612 8880 7603 2 P
8880 7603 8906 7591 8934 7588 8927 7560 8919 7533 8891 7531
8882 7504 8884 7475 8892 7448 8878 7423 8850 7418 8824 7407
8807 7384 8821 7359 8828 7332 8854 7344 8868 7319 8874 7291
8901 7282 8907 7254 8902 7226 8881 7207 8880 7236 8879 7264
8852 7272 8841 7246 8827 7221 8830 7250 8813 7273 8808 7301
8779 7304 8754 7317 8764 7344 8740 7359 8729 7333 8713 7310

8693 7289 8669 7275 8646 7257 8619 7249 8640 7230 8663 7213
8687 7198 8677 7171 8704 7180 8726 7197 8744 7174 8763 7153
8736 7146 8730 7118 50 P
8730 7118 8701 7114 8673 7112 8649 7128 8647 7157 8632 7133
8641 7106 8655 7081 8629 7093 8601 7097 8611 7124 8613 7152
8588 7140 8604 7163 8577 7170 8548 7167 8522 7157 8541 7136
8561 7116 8545 7093 8528 7069 8501 7062 8510 7034 8534 7020
8562 7012 8575 7038 8573 7009 8587 6984 8558 6985 8530 6983
8534 6954 8562 6948 8537 6933 8511 6944 8482 6948 8464 6926
8436 6919 8442 6947 8429 6972 8416 6947 8416 6975 8395 6995
8402 6967 8409 6940 8381 6933 8355 6945 8378 6928 8382 6900
8403 6880 8425 6897 50 P
8425 6897 8441 6874 2 P
8441 6874 8437 6845 8424 6820 8395 6822 8394 6794 8405 6768
8384 6747 8359 6734 8360 6762 8336 6777 8310 6789 8284 6803
8295 6830 8273 6812 8278 6784 8253 6770 8225 6765 8198 6773
8185 6747 8157 6754 8165 6781 8154 6808 8125 6808 8123 6779
8109 6754 8092 6777 8082 6750 8053 6749 8079 6736 8074 6708
8051 6691 8023 6698 7997 6708 7968 6707 7953 6704 7941 6720
7940 6703 7951 6692 7974 6674 7999 6661 8017 6656 8045 6659
8074 6663 8077 6634 8106 6634 8120 6609 8126 6581 8098 6575
8077 6556 8054 6538 50 P
8054 6538 8033 6558 8013 6537 8024 6510 8005 6488 8001 6459
7977 6445 7953 6427 7937 6404 7925 6378 7929 6406 7946 6430
7936 6457 7909 6448 7891 6426 7894 6397 7871 6380 7859 6354
7857 6325 7855 6297 7838 6273 7839 6302 7848 6330 7839 6357
7818 6375 7790 6383 7762 6390 7733 6393 7756 6376 7784 6369
7786 6340 7776 6313 7758 6291 7735 6307 7723 6307 7711 6291
7687 6307 7676 6281 7671 6252 7669 6223 7651 6201 7659 6229
7641 6252 7641 6222 7617 6207 45 P
7617 6207 7594 6189 7571 6171 7564 6199 7558 6227 7532 6238
7540 6210 7515 6223 7487 6215 7460 6205 7469 6181 7455 6155
7454 6129 7455 6119 7434 6099 7422 6085 7401 6062 7372 6062
7343 6062 7316 6074 7294 6092 7273 6113 7245 6113 7273 6108
7279 6080 7292 6054 7315 6037 7343 6030 7379 5998 7358 5987
7341 5964 7319 5984 7296 6002 7268 5999 7271 5970 7295 5985
7291 5956 7277 5931 7284 5903 7287 5874 7266 5854 7258 5826
7228 5828 7200 5820 7172 5826 7149 5844 7125 5828 7101 5811
7082 5790 7059 5771 50 P
7059 5771 7030 5771 7046 5796 7029 5819 7027 5791 7003 5774
6976 5764 6949 5754 6921 5746 6899 5727 6882 5750 6866 5774
6838 5783 6814 5799 6785 5797 6764 5777 6747 5753 6729 5730
6747 5707 6768 5727 6754 5701 6757 5672 6730 5685 6705 5698

6676 5693 6648 5700 6636 5727 6665 5728 6640 5742 6614 5755
6575 5753 6576 5763 6599 5771 6568 5782 6543 5766 6515 5767
6487 5760 6472 5735 6458 5710 6430 5706 6417 5732 6392 5747
6408 5723 6431 5705 6415 5680 6399 5656 6403 5627 6382 5648
6353 5652 6324 5651 50 P
6324 5651 6314 5678 2 P
6314 5678 6317 5707 6314 5736 6337 5753 6345 5781 6324 5762
6295 5767 6269 5755 6242 5746 6216 5733 6202 5708 6179 5689
6209 5689 6208 5660 6181 5650 6206 5635 6234 5625 6228 5597
6200 5590 6172 5584 6168 5612 6159 5640 6141 5663 6113 5670
6084 5663 6056 5656 6035 5637 27 P
6035 5637 6061 5624 6086 5611 6065 5592 6036 5590 6008 5586
6023 5561 6051 5569 6074 5588 6090 5564 6118 5570 6112 5542
6084 5534 6057 5545 6049 5516 6069 5495 6042 5507 6013 5506
5985 5513 5967 5535 5937 5535 5908 5534 5880 5538 5859 5559
5880 5579 5902 5598 5928 5612 5955 5622 28 P
5955 5622 5926 5625 5898 5632 5871 5621 5852 5599 5859 5628
5842 5651 5823 5673 5811 5700 5796 5725 5790 5753 5772 5776
5745 5765 5739 5794 5720 5816 5706 5840 5711 5869 5722 5896
5705 5873 5684 5893 5677 5921 5679 5950 5654 5964 5627 5975
5600 5984 5617 5961 5645 5957 5662 5934 5661 5904 5655 5876
5626 5871 5598 5866 5627 5865 5654 5855 5664 5827 5688 5811
5702 5785 5674 5778 5649 5795 5669 5773 5686 5750 5703 5726
5675 5717 5654 5736 5673 5715 5689 5691 5665 5674 5681 5650
5680 5621 5667 5595 50 P
5667 5595 5659 5567 5682 5551 5689 5522 5674 5497 5665 5469
5656 5453 7 P
5649 5453 5644 5472 5643 5453 3 P
5597 5453 5592 5457 5573 5479 5556 5502 5547 5530 5528 5553
5537 5525 5551 5487 5544 5482 5539 5475 5539 5468 5531 5453

12:00 PM

5429 5453 5430 5476 5412 5498 5410 5527 5410 5557 5415 5585
5415 5614 5386 5615 5380 5644 5351 5648 5369 5625 5393 5608
5404 5582 5403 5553 5402 5524 5405 5494 5407 5466 5392 5453
18 P
2966 5453 2967 5459 2985 5482 2967 5505 2938 5506 2916 5486
2926 5459 2928 5453 8 P
13001 8121 12996 8116 12991 8121 12985 8125 12983 8118 12983 8111
12985 8105 12988 8098 12992 8092 12995 8086 13001 8082 13001 8081

12:00 PM

13001 8033 13001 8033 12994 8030 12988 8028 12984 8022 12981 8015
12978 8009 12978 8002 12973 7997 12977 7991 12982 7985 12976 7990
12969 7993 12962 7992 12960 7986 12961 7979 12966 7973 12968 7967

12974 7963 12979 7957 12979 7950 12979 7943 12979 7936 12977 7929
12976 7922 12982 7918 12988 7915 12993 7920 12999 7925 13001 7925
30 P
11825 7031 11829 7025 11833 7019 11839 7015 11846 7013 11847 7020
11850 7013 11855 7009 11862 7012 11869 7014 11876 7015 11882 7019
11889 7021 11896 7021 11903 7023 11910 7024 11917 7023 11924 7025
11931 7026 11936 7021 11932 7027 11927 7033 11923 7039 11918 7044
11911 7044 11904 7046 11897 7048 11894 7054 11887 7057 11880 7055
11873 7058 11866 7060 11863 7066 11865 7073 11859 7078 11853 7080
11849 7086 11847 7093 11844 7100 11838 7104 11833 7110 11829 7115
11826 7121 11819 7120 11823 7114 11827 7108 11830 7102 11835 7096
11839 7091 11842 7084 50 P
11842 7084 11835 7081 11835 7074 11837 7067 11834 7060 11828 7057
11825 7051 11831 7047 11837 7042 11840 7037 11836 7031 11825 7031
12:00 PM
11225 6793 11231 6789 11237 6791 11242 6796 11250 6797 11255 6792
11259 6786 11264 6780 11263 6773 11259 6767 11253 6764 11249 6758
11254 6754 11261 6752 11268 6749 11273 6744 11279 6741 11286 6740
11293 6742 11298 6748 11299 6755 11306 6753 11312 6750 11319 6752
11322 6758 11323 6766 11322 6773 11322 6780 11324 6787 11323 6794
11319 6800 11322 6807 11319 6813 11319 6820 11322 6826 11328 6831
11332 6836 11333 6843 11338 6849 11342 6854 11345 6861 11342 6868
11346 6874 11352 6879 11356 6883 11358 6890 11357 6897 11358 6905
11351 6905 11344 6906 50 P
11344 6906 11343 6899 11341 6892 11338 6886 11334 6880 11331 6873
11336 6868 11333 6862 11331 6855 11329 6848 11322 6850 11316 6847
11313 6841 11306 6839 11306 6831 11302 6825 11305 6818 11299 6813
11292 6814 11285 6812 11279 6808 11272 6809 11265 6807 11262 6814
11259 6820 11252 6822 11252 6815 11250 6809 11246 6802 11240 6807
11233 6806 11228 6801 11225 6793 33 P
11017 6707 11022 6701 11026 6696 11032 6692 11040 6693 11047 6691
11049 6698 11055 6703 11060 6698 11066 6694 11073 6693 11080 6693
11088 6692 11095 6691 11102 6691 11109 6692 11116 6690 11122 6687
11129 6684 11135 6688 11134 6695 11131 6701 11124 6704 11131 6702
11138 6700 11144 6696 11144 6703 11142 6710 11144 6717 11148 6723
11155 6721 11157 6728 11154 6735 11158 6741 11160 6748 11154 6750
11148 6754 11143 6759 11136 6761 11129 6762 11126 6769 11120 6765
11114 6769 11107 6768 11100 6765 11096 6760 11091 6766 11089 6772
11084 6778 11078 6782 50 P
11078 6782 11072 6784 11066 6781 11071 6776 11073 6769 11077 6763
11070 6766 11071 6773 11065 6770 11060 6764 11056 6758 11059 6752
11058 6745 11062 6739 11067 6734 11068 6727 11065 6733 11062 6727
11062 6720 11064 6713 11064 6706 11063 6713 11058 6718 11052 6722

11051 6729 11045 6724 11039 6728 11039 6721 11043 6715 11037 6719
11031 6722 11026 6716 11030 6710 11017 6707 34 P
9618 7032 9615 7018 9606 7007 9598 6995 9592 6982 9585 6969
9582 6955 9572 6945 9564 6933 9571 6920 9567 6907 9563 6893
9553 6882 9543 6873 9539 6859 9529 6848 9539 6839 9525 6839
9511 6837 9498 6831 9490 6820 9496 6807 9482 6804 9469 6799
9464 6785 9459 6771 9456 6757 9451 6744 9445 6731 9438 6719
9427 6709 9419 6697 9409 6687 9403 6674 9397 6661 9391 6647
9386 6634 9384 6620 9375 6608 9371 6595 9363 6582 9356 6570
9368 6562 9354 6562 9346 6550 9339 6537 9332 6525 9329 6511
9324 6497 9315 6486 50 P
9315 6486 9305 6476 9296 6465 9289 6452 9280 6441 9274 6428
9274 6414 9267 6401 9261 6388 9256 6374 9247 6364 9256 6353
9270 6351 9285 6349 9299 6351 9288 6342 9274 6340 9259 6339
9245 6341 9231 6341 9222 6329 9213 6318 9211 6304 9210 6289
9207 6275 9195 6267 9188 6254 9182 6241 9181 6226 9180 6212
9187 6199 9187 6185 9193 6172 9181 6163 9190 6153 9188 6139
9198 6129 9188 6120 9192 6106 9196 6092 9194 6077 9192 6063
9199 6051 9185 6053 9183 6039 9175 6027 9177 6013 9166 6004
9179 5998 9164 5996 50 P
9164 5996 9163 5982 2 P
9163 5982 9164 5967 9155 5958 9169 5954 9173 5940 9169 5926
9158 5917 9144 5914 9140 5927 9136 5913 9146 5903 9160 5906
9153 5895 9163 5885 9175 5877 9189 5881 9203 5885 9217 5890
9220 5876 9207 5869 9193 5866 9200 5853 9207 5841 9211 5827
9211 5812 9216 5799 9221 5785 9235 5782 9226 5771 9231 5757
9237 5744 9243 5730 9249 5717 9255 5704 9258 5690 9258 5676
9255 5661 9263 5649 9276 5643 9280 5629 9291 5620 9302 5610
9289 5615 9279 5627 9271 5639 9277 5625 9286 5614 9296 5603
9306 5593 9316 5582 50 P
9316 5582 9328 5574 9340 5566 9347 5580 9353 5566 9362 5555
9373 5545 9386 5539 9398 5531 9411 5525 9424 5518 9436 5510
9447 5500 9454 5487 9465 5478 9472 5465 9476 5453 17 P
9607 5453 9607 5465 9606 5479 9608 5494 9614 5507 9617 5521
9622 5535 9627 5548 9632 5562 9635 5576 9638 5590 9642 5604
9646 5618 9652 5632 9662 5642 9664 5657 9667 5671 9673 5684
9684 5693 9695 5703 9707 5711 9720 5717 9730 5727 9740 5738
9751 5748 9754 5763 9758 5776 9753 5790 9760 5803 9773 5808
9784 5818 9790 5831 9798 5843 9801 5857 9812 5868 9819 5880
9824 5894 9830 5907 9833 5921 9837 5935 9848 5944 9857 5956
9859 5970 9862 5984 9857 5997 9859 6012 9865 6025 9868 6039
9872 6053 9877 6066 50 P
9877 6066 9889 6075 9891 6089 9893 6103 9894 6118 9899 6132

9903 6145 9906 6160 9911 6173 9906 6186 9907 6201 9910 6215
9916 6228 9917 6242 9917 6257 9922 6270 9923 6285 9925 6299
9928 6313 9934 6326 9934 6341 9934 6355 9940 6368 9944 6382
9947 6397 9949 6411 9949 6425 9949 6439 9951 6454 9960 6465
9968 6477 9960 6489 9953 6502 9950 6516 9955 6529 9965 6539
9974 6551 9973 6565 9970 6579 9977 6592 9988 6601 9997 6612
10008 6621 10014 6634 10021 6646 10027 6659 10025 6674 10030 6688
10032 6702 49 P
10032 6702 10034 6716 10040 6729 10047 6742 10058 6751 10055 6765
10061 6778 10072 6786 10070 6800 10071 6815 10068 6829 10058 6840
10053 6853 10050 6867 10048 6881 10043 6895 10039 6908 10037 6922
10037 6937 10038 6951 10042 6965 10046 6979 10054 6991 10064 7001
10069 7014 10080 7024 10088 7035 10101 7042 10114 7049 10125 7058
10117 7070 10103 7068 10090 7075 10089 7090 10081 7101 10083 7115
10073 7125 10058 7124 10044 7125 10030 7122 10020 7131 10007 7137
9994 7131 9984 7141 9970 7146 9960 7156 9964 7169 9950 7164
9940 7174 9931 7185 50 P
9931 7185 9927 7199 9916 7207 9903 7214 9889 7213 9875 7209
9861 7204 9849 7197 9840 7186 9832 7174 9825 7162 9817 7150
9828 7141 9815 7136 9805 7126 9793 7119 9780 7113 9766 7111
9752 7107 9738 7108 9724 7103 9711 7098 9699 7090 9687 7082
9673 7079 9660 7074 9648 7065 9636 7058 9626 7048 9618 7032
30 P
12970 11609 12977 11607 12983 11607 12988 11602 12995 11600 12999 11606
13001 11607 7 P
13001 11699 13000 11699 12995 11704 12988 11706 12983 11702 12981 11695
12982 11688 12986 11683 12984 11676 12986 11670 12991 11665 12987 11659
12990 11653 12992 11646 12999 11643 12999 11637 12993 11636 12998 11632
12991 11633 12993 11627 12988 11623 12987 11616 12994 11618 12999 11623
12995 11617 12995 11610 12988 11612 12982 11616 12979 11622 12975 11628
12973 11621 12970 11609 32 P
11806 11848 11811 11843 11815 11837 11819 11832 11825 11828 11831 11825
11838 11824 11844 11820 11848 11815 11853 11810 11858 11805 11864 11802
11871 11802 11871 11809 11872 11815 11876 11821 11880 11827 11886 11829
11893 11828 11900 11828 11907 11827 11913 11830 11919 11834 11926 11834
11932 11831 11939 11833 11945 11835 11950 11829 11957 11829 11963 11832
11970 11832 11974 11838 11981 11839 11987 11840 11994 11840 12001 11842
12007 11840 12013 11841 12018 11847 12024 11851 12028 11856 12033 11860
12040 11862 12047 11862 12054 11863 12060 11867 12066 11869 12073 11870
12078 11875 12082 11880 50 P
12082 11880 12087 11886 12093 11886 12100 11887 12107 11889 12113 11891
12117 11897 12116 11904 12121 11908 12125 11914 12126 11921 12127 11928
12131 11934 12136 11937 12141 11942 12141 11949 12142 11956 12143 11963

12148 11959 12147 11965 12146 11972 12149 11978 12155 11977 12151 11982
12146 11977 12145 11984 12138 11986 12134 11992 12138 11998 12136 12005
12132 12011 12126 12014 12119 12014 12113 12012 12107 12015 12101 12017
12096 12022 12092 12028 12085 12026 12079 12027 12072 12028 12065 12027
12059 12023 12052 12023 12046 12021 12039 12019 12033 12016 12026 12015
12020 12012 12013 12014 50 P
12013 12014 12007 12010 2 P
12007 12010 12001 12007 11995 12004 11989 12001 11983 11999 11976 11997
11970 11994 11964 11991 11958 11987 11952 11989 11945 11990 11941 11984
11934 11983 11929 11979 11923 11975 11917 11973 11911 11969 11905 11966
11899 11968 11893 11964 11887 11961 11882 11957 11876 11954 11869 11954
11865 11948 11862 11942 11855 11939 11850 11934 11845 11929 11845 11923
11842 11917 11837 11911 11832 11907 11826 11902 11823 11897 11818 11891
11813 11888 11812 11881 11806 11877 11806 11870 11809 11864 11807 11857
11806 11848 43 P
12979 8284 12983 8279 12990 8279 12997 8281 13001 8281 5 P
13001 8297 12996 8294 12989 8295 12982 8295 12979 8284 5 P
12715 8170 12721 8173 12729 8174 12735 8177 12733 8184 12726 8187
12721 8183 12715 8170 8 P
12477 8021 12481 8016 12488 8014 12495 8013 12502 8012 12508 8008
12510 8001 12515 7995 12521 7992 12527 7989 12534 7988 12532 7995
12529 8001 12529 8008 12533 8014 12535 8020 12529 8024 12526 8030
12519 8032 12515 8038 12509 8042 12502 8041 12496 8037 12491 8032
12485 8029 12477 8021 26 P
12693 8048 12696 8044 12698 8040 12702 8043 12706 8045 12702 8047
12699 8051 12699 8055 12695 8054 12693 8048 10 P
12534 8012 12536 8013 12538 8014 12540 8015 12540 8017 12538 8019
12537 8017 12535 8016 12534 8012 9 P
12542 8015 12544 8016 12546 8018 12545 8020 12543 8019 12542 8015
6:00 PM
12547 8013 12549 8015 12550 8016 12552 8017 12555 8017 12557 8018
12558 8020 12559 8022 12562 8023 12564 8023 12566 8023 12568 8024
12569 8026 12571 8026 12573 8028 12571 8028 12569 8028 12568 8026
12566 8025 12563 8024 12561 8024 12559 8024 12557 8022 12556 8020
12554 8019 12552 8018 12550 8017 12548 8016 12547 8013 29 P
12807 7955 12810 7951 12813 7948 12812 7944 12812 7939 12814 7936
12816 7940 12820 7942 12821 7946 12820 7951 12819 7955 12821 7959
12825 7962 12824 7966 12824 7971 12823 7975 12820 7979 12817 7982
12812 7980 12812 7976 12812 7971 12810 7967 12810 7963 12812 7958
12807 7955 25 P
12776 7979 12780 7978 12785 7978 12788 7975 12791 7978 12792 7982
12792 7987 12796 7984 12800 7982 12800 7987 12797 7991 12793 7991
12788 7992 12788 7987 12784 7987 12781 7984 12776 7979 17 P

12768 7962 12772 7959 12775 7955 12779 7957 12783 7959 12781 7963
12779 7967 12775 7969 12772 7966 12768 7962 10 P
12779 7952 12781 7949 12786 7950 12785 7954 12779 7952 5 P
12783 7944 12785 7940 12787 7944 12786 7949 12783 7944 5 P
12754 7945 12756 7940 12759 7938 12760 7943 12760 7947 12757 7950
12754 7945 7 P
12756 7967 12760 7966 12761 7971 12757 7972 12756 7967 5 P
12857 7972 12861 7973 12861 7978 12864 7982 12864 7986 12860 7983
12859 7979 12857 7972 8 P
12832 12916 12826 12914 12823 12908 12822 12901 12821 12894 12815 12895
12808 12892 12803 12895 12801 12888 12796 12884 12800 12878 12804 12872
12807 12866 12812 12861 12818 12857 12811 12858 12804 12857 12798 12860
12793 12855 12786 12856 12780 12859 12775 12864 12768 12862 12766 12856
12759 12854 12756 12848 12753 12842 12750 12836 12752 12829 12754 12823
12758 12816 12764 12814 12767 12808 12771 12802 12777 12806 12776 12812
12783 12814 12788 12817 12793 12822 12796 12829 12803 12827 12803 12820
12806 12814 12804 12807 12805 12801 12799 12796 12793 12795 12786 12793
12788 12786 12793 12790 50 P
12793 12790 12793 12783 12800 12785 12806 12788 12812 12785 12806 12781
12805 12774 12808 12768 12814 12766 12811 12760 12809 12754 12815 12752
12822 12749 12827 12753 12834 12755 12832 12761 12826 12766 12833 12768
12840 12769 12846 12772 12853 12772 12847 12775 12840 12774 12836 12780
12832 12785 12834 12792 12841 12791 12845 12796 12842 12802 12842 12809
12848 12812 12852 12806 12856 12812 12862 12811 12867 12806 12873 12803
12873 12810 12868 12815 12864 12820 12866 12827 12859 12826 12857 12832
12863 12835 12870 12833 12867 12839 12862 12845 12869 12846 12869 12853
12872 12859 12865 12859 50 P
12865 12859 12860 12855 2 P
12860 12855 12859 12848 12852 12849 12848 12855 12853 12860 12855 12866
12859 12872 12857 12879 12855 12885 12854 12892 12852 12899 12846 12902
12843 12908 12840 12915 12844 12921 12850 12924 12851 12931 12846 12936
12840 12938 12833 12938 12829 12933 12828 12926 12832 12916 23 P
12542 12787 12536 12784 12533 12778 12527 12774 12522 12769 12517 12774
12515 12781 12509 12782 12502 12783 12503 12790 12498 12795 12495 12801
12495 12808 12501 12812 12503 12818 12505 12825 12503 12831 12500 12837
12494 12841 12487 12841 12481 12839 12476 12834 12471 12829 12471 12822
12476 12817 12469 12814 12463 12813 12463 12806 12462 12799 12458 12793
12456 12787 12458 12780 12461 12774 12465 12768 12470 12764 12471 12758
12476 12753 12479 12746 12477 12740 12475 12733 12482 12732 12484 12725
12489 12721 12496 12720 12503 12721 12509 12724 12510 12730 12507 12736
12505 12743 12511 12747 50 P
12511 12747 12516 12750 12523 12752 12524 12745 12523 12739 12525 12732
12528 12725 12529 12719 12536 12717 12543 12718 12549 12722 12556 12722

12560 12717 12566 12720 12573 12721 12569 12726 12565 12732 12570 12737
12573 12743 12572 12749 12570 12756 12569 12763 12569 12770 12572 12776
12571 12782 12566 12787 12559 12785 12555 12789 12542 12787 29 P
12067 12365 12071 12359 12077 12362 12082 12357 12089 12357 12096 12356
12100 12361 12101 12368 12100 12375 12095 12379 12094 12386 12088 12389
12082 12391 12076 12387 12073 12382 12070 12375 12067 12365 17 P
12233 12404 12237 12406 12241 12406 12246 12408 12250 12407 12250 12411
12252 12415 12250 12419 12249 12423 12249 12428 12245 12427 12241 12425
12242 12421 12237 12420 12237 12416 12235 12411 12233 12404 17 P
12160 12539 12162 12536 12167 12537 12169 12534 12166 12531 12165 12527
12169 12528 12172 12524 12176 12522 12179 12519 12183 12517 12187 12520
12191 12519 12194 12523 12198 12523 12202 12523 12207 12523 12211 12523
12215 12524 12218 12528 12219 12532 12219 12536 12221 12540 12222 12544
12223 12548 12222 12552 12220 12556 12216 12559 12212 12558 12208 12556
12204 12555 12200 12556 12196 12554 12193 12551 12191 12548 12186 12548
12183 12544 12179 12546 12175 12547 12173 12551 12169 12551 12170 12547
12168 12543 12165 12540 12160 12539 45 P
12321 12547 12326 12547 12329 12548 12334 12549 12336 12545 12332 12545
12328 12545 12329 12542 12333 12540 12337 12541 12341 12539 12341 12535
12340 12530 12343 12528 12347 12531 12350 12534 12354 12536 12358 12537
12362 12540 12363 12544 12361 12548 12360 12552 12355 12553 12353 12556
12349 12559 12346 12562 12344 12558 12340 12557 12337 12554 12333 12557
12329 12558 12325 12560 12321 12558 12324 12556 12323 12552 12321 12547
36 P
12426 12608 12429 12605 12434 12604 12437 12602 12441 12603 12441 12599
12444 12596 12445 12593 12449 12591 12453 12593 12457 12590 12461 12589
12462 12585 12467 12585 12471 12585 12474 12589 12472 12593 12470 12596
12465 12596 12461 12597 12465 12598 12465 12603 12462 12606 12458 12609
12456 12613 12455 12617 12451 12618 12447 12620 12442 12618 12438 12616
12436 12613 12433 12609 12426 12608 33 P
12473 12555 12477 12555 12475 12551 12475 12547 12479 12547 12483 12544
12485 12548 12484 12552 12484 12556 12485 12561 12484 12565 12481 12567
12482 12571 12477 12571 12476 12568 12477 12563 12476 12559 12473 12555
18 P
11961 12468 11963 12464 11963 12459 11964 12456 11965 12451 11964 12447
11967 12444 11966 12439 11970 12442 11974 12439 11978 12437 11981 12435
11985 12438 11988 12440 11991 12437 11994 12434 11992 12431 11987 12431
11987 12426 11990 12424 11994 12425 11999 12426 12003 12425 12006 12423
12011 12422 12010 12426 12008 12430 12012 12429 12015 12425 12019 12425
12024 12425 12028 12424 12026 12428 12021 12430 12023 12433 12025 12438
12021 12439 12017 12439 12012 12438 12013 12443 12012 12447 12008 12449
12007 12453 12005 12457 12004 12461 12004 12465 12003 12470 12000 12474
11997 12476 11993 12478 50 P

11993 12478 11990 12480 11985 12482 11981 12483 11977 12481 11974 12478
11970 12478 11966 12477 11963 12473 11961 12468 10 P
11711 12463 11715 12457 11720 12452 11723 12446 11729 12443 11728 12450
11725 12456 11729 12462 11730 12456 11735 12451 11742 12450 11738 12444
11735 12438 11737 12432 11742 12437 11745 12431 11752 12431 11759 12431
11765 12435 11771 12437 11766 12442 11773 12441 11779 12443 11784 12449
11790 12450 11790 12456 11797 12459 11803 12459 11804 12466 11811 12467
11812 12474 11817 12469 11819 12463 11824 12467 11822 12474 11828 12477
11830 12484 11834 12489 11831 12495 11835 12501 11830 12505 11834 12511
11835 12518 11836 12524 11831 12528 11827 12534 11822 12538 11816 12542
11810 12540 11809 12533 50 P
11809 12533 11803 12537 11800 12543 11802 12550 11798 12556 11792 12559
11785 12561 11779 12562 11772 12563 11770 12556 11773 12550 11780 12549
11786 12551 11784 12544 11786 12538 11783 12532 11778 12528 11776 12521
11769 12523 11769 12529 11766 12536 11762 12530 11765 12524 11759 12521
11762 12515 11763 12508 11768 12503 11773 12505 11771 12499 11764 12501
11768 12495 11766 12488 11765 12481 11764 12475 11760 12469 11761 12475
11759 12482 11760 12489 11763 12495 11759 12501 11759 12508 11753 12509
11752 12516 11746 12512 11745 12505 11749 12499 11746 12494 11741 12498
11734 12498 11731 12492 50 P
11731 12492 11724 12490 2 P
11724 12490 11723 12483 11727 12478 11721 12481 11715 12476 11714 12470
11711 12463 7 P
11913 12330 11917 12327 11918 12323 11923 12322 11927 12322 11931 12324
11934 12326 11938 12329 11941 12332 11942 12336 11946 12337 11947 12340
11951 12343 11951 12347 11955 12344 11957 12340 11958 12345 11961 12343
11966 12344 11966 12348 11961 12348 11957 12349 11953 12351 11949 12351
11945 12349 11940 12350 11936 12349 11934 12352 11931 12355 11926 12354
11923 12351 11920 12348 11917 12345 11913 12342 11916 12338 11913 12330
36 P
11930 12361 11934 12360 11937 12357 11940 12353 11945 12353 11949 12352
11953 12353 11950 12355 11949 12359 11952 12362 11954 12358 11959 12358
11961 12362 11958 12365 11961 12368 11965 12370 11962 12373 11960 12377
11957 12380 11952 12382 11949 12381 11944 12382 11940 12380 11937 12383
11933 12385 11934 12381 11937 12378 11933 12376 11932 12372 11937 12373
11934 12369 11933 12365 11930 12361 33 P
11969 12340 11972 12337 11974 12333 11974 12329 11978 12331 11981 12328
11983 12324 11986 12323 11989 12326 11989 12330 11991 12334 11990 12338
11994 12340 11993 12345 11989 12343 11988 12347 11986 12351 11982 12352
11983 12356 11987 12357 11991 12358 11993 12362 11990 12365 11986 12367
11981 12368 11978 12370 11974 12368 11974 12364 11970 12363 11971 12359
11975 12360 11977 12356 11977 12352 11974 12349 11973 12344 11969 12340
36 P

12014 12448 12019 12448 12023 12446 12027 12446 12031 12444 12033 12441
12037 12444 12040 12446 12044 12447 12048 12448 12052 12445 12054 12442
12054 12437 12057 12433 12061 12436 12062 12439 12066 12442 12065 12446
12069 12445 12070 12441 12074 12441 12075 12445 12074 12450 12074 12454
12079 12453 12080 12457 12078 12462 12075 12458 12071 12459 12068 12463
12064 12462 12059 12462 12056 12459 12053 12463 12050 12465 12046 12463
12044 12459 12044 12455 12041 12452 12037 12451 12034 12454 12030 12457
12028 12453 12026 12449 12022 12450 12018 12451 12014 12448 47 P
12010 12473 12014 12476 12018 12475 12023 12476 12021 12472 12020 12468
12019 12463 12023 12461 12026 12464 12026 12469 12028 12472 12030 12476
12034 12478 12038 12478 12042 12479 12042 12484 12045 12487 12050 12486
12053 12484 12058 12484 12062 12484 12062 12489 12059 12491 12054 12494
12054 12498 12050 12496 12046 12497 12050 12500 12051 12504 12047 12507
12045 12510 12041 12510 12037 12508 12033 12505 12029 12504 12025 12504
12023 12501 12019 12497 12018 12493 12015 12490 12017 12486 12015 12482
12013 12478 12010 12473 44 P
12059 12493 12063 12490 12065 12486 12063 12483 12062 12479 12063 12475
12066 12477 12068 12482 12072 12483 12072 12479 12072 12475 12073 12470
12077 12470 12081 12468 12085 12468 12089 12470 12091 12474 12094 12475
12099 12475 12100 12479 12098 12483 12097 12488 12098 12492 12099 12496
12098 12500 12096 12496 12094 12492 12092 12488 12088 12489 12084 12488
12081 12491 12077 12491 12072 12492 12069 12495 12067 12490 12065 12494
12059 12493 37 P
12125 12474 12120 12475 12122 12471 12127 12470 12131 12471 12135 12470
12138 12468 12135 12465 12139 12464 12142 12461 12143 12465 12147 12466
12151 12463 12147 12460 12144 12458 12145 12453 12145 12449 12147 12453
12150 12457 12152 12460 12156 12463 12160 12462 12161 12466 12156 12466
12156 12470 12160 12469 12164 12469 12167 12466 12172 12466 12169 12470
12166 12474 12165 12478 12161 12478 12156 12479 12153 12476 12149 12474
12146 12472 12147 12477 12145 12481 12141 12482 12138 12486 12135 12490
12137 12485 12133 12485 12128 12483 12132 12479 12129 12476 12125 12474
48 P
12129 12528 12133 12525 12135 12522 12137 12517 12137 12513 12140 12510
12143 12507 12145 12511 12144 12515 12148 12514 12147 12509 12149 12505
12150 12501 12152 12505 12153 12509 12155 12513 12153 12517 12152 12521
12150 12525 12147 12529 12144 12531 12139 12531 12135 12530 12129 12528
24 P
12896 12925 12899 12922 12904 12922 12905 12927 12903 12931 12903 12935
12904 12940 12905 12944 12907 12948 12906 12952 12905 12957 12900 12956
12898 12960 12894 12962 12892 12958 12887 12957 12884 12954 12882 12951
12886 12950 12889 12947 12888 12943 12892 12939 12888 12937 12892 12935
12896 12934 12897 12930 12896 12925 27 P
12750 12787 12754 12785 12757 12781 12756 12778 12756 12773 12760 12773

12765 12772 12768 12774 12772 12777 12775 12780 12774 12785 12772 12788
12767 12787 12765 12790 12764 12795 12761 12798 12762 12802 12759 12800
12760 12804 12756 12804 12752 12801 12753 12797 12758 12798 12761 12795
12757 12797 12757 12793 12754 12789 12754 12793 12751 12797 12751 12793
12750 12787 31 P
12763 12875 12765 12871 12768 12868 12772 12868 12770 12872 12773 12875
12770 12878 12767 12881 12770 12885 12773 12888 12771 12892 12767 12893
12763 12892 12761 12887 12764 12884 12765 12879 12763 12875 17 P
12616 12800 12620 12799 12624 12798 12624 12793 12628 12793 12631 12796
12632 12800 12635 12803 12639 12803 12644 12803 12643 12808 12639 12809
12635 12810 12631 12812 12626 12811 12623 12809 12618 12808 12616 12800
18 P
12633 12789 12637 12788 12638 12784 12642 12784 12645 12781 12649 12780
12653 12779 12654 12783 12651 12786 12655 12787 12659 12788 12663 12791
12659 12794 12656 12795 12653 12799 12650 12802 12645 12803 12642 12802
12638 12799 12636 12795 12638 12792 12633 12789 22 P
12656 12691 12659 12688 12663 12687 12663 12682 12665 12678 12667 12682
12672 12683 12673 12687 12678 12686 12682 12686 12686 12686 12686 12690
12689 12694 12687 12697 12683 12696 12680 12692 12679 12688 12676 12691
12679 12694 12676 12697 12671 12697 12668 12700 12664 12702 12659 12701
12658 12696 12656 12691 26 P
12537 12829 12535 12825 12534 12821 12532 12817 12529 12813 12528 12809
12524 12806 12523 12802 12524 12798 12525 12794 12528 12790 12532 12789
12536 12787 12541 12787 12545 12788 12549 12790 12550 12794 12554 12795
12558 12794 12562 12792 12566 12792 12570 12790 12574 12791 12577 12788
12582 12789 12581 12793 12577 12795 12572 12797 12569 12800 12569 12804
12570 12808 12569 12813 12566 12815 12562 12816 12558 12819 12559 12823
12555 12823 12552 12821 12551 12816 12550 12812 12550 12808 12548 12804
12545 12807 12544 12811 12545 12815 12546 12820 12548 12823 12550 12827
12551 12832 12550 12836 50 P
12550 12836 12546 12835 12542 12833 12537 12829 4 P
12398 12618 12400 12622 12402 12619 12405 12616 12409 12615 12413 12618
12417 12620 12417 12624 12421 12621 12425 12622 12428 12624 12425 12627
12422 12630 12418 12627 12414 12627 12412 12631 12408 12632 12404 12630
12400 12628 12398 12624 12398 12618 21 P
12310 12584 12314 12582 12318 12582 12319 12578 12322 12575 12318 12574
12315 12571 12317 12567 12320 12563 12324 12563 12327 12567 12331 12567
12336 12569 12340 12568 12342 12572 12342 12576 12339 12579 12334 12581
12330 12581 12326 12581 12329 12583 12327 12587 12323 12588 12326 12591
12329 12595 12327 12598 12323 12599 12319 12600 12316 12596 12314 12593
12313 12589 12310 12584 32 P
12126 12463 12122 12462 12118 12463 12113 12462 12110 12459 12107 12456
12108 12451 12108 12447 12113 12446 12114 12442 12118 12444 12122 12445

12125 12449 12129 12449 12131 12453 12135 12456 12133 12459 12126 12463
18 P
12268 12321 12270 12317 12272 12313 12275 12310 12276 12306 12279 12303
12282 12300 12287 12300 12291 12299 12294 12303 12289 12302 12286 12304
12284 12308 12280 12311 12279 12315 12277 12319 12279 12323 12277 12327
12274 12325 12268 12321 20 P
12281 12324 12284 12321 12286 12317 12290 12315 12294 12313 12295 12318
12292 12320 12292 12324 12288 12325 12281 12324 10 P
12107 12261 12108 12258 12113 12258 12117 12259 12119 12263 12118 12266
12114 12268 12109 12268 12107 12261 9 P
11834 12216 11838 12214 11840 12211 11843 12207 11847 12207 11845 12211
11846 12215 11851 12216 11851 12220 11847 12222 11844 12219 11839 12220
11834 12216 13 P
11824 12221 11828 12219 11832 12217 11833 12221 11830 12225 11826 12226
11824 12221 7 P
11903 12351 11906 12350 11908 12353 11910 12356 11913 12356 11914 12359
11916 12362 11913 12364 11911 12366 11910 12363 11907 12360 11907 12357
11906 12354 11903 12351 14 P
11949 12386 11952 12385 11954 12387 11957 12386 11959 12389 11963 12389
11962 12392 11960 12395 11958 12396 11954 12395 11952 12393 11949 12392
11949 12386 13 P
11952 12312 11956 12312 11959 12312 11962 12311 11961 12314 11959 12317
11958 12320 11955 12322 11952 12319 11954 12317 11952 12312 11 P
12022 12367 12025 12366 12027 12363 12028 12366 12029 12370 12031 12372
12032 12375 12030 12378 12028 12381 12025 12382 12023 12380 12021 12377
12023 12373 12022 12367 14 P
11273 12232 11277 12230 11281 12230 11285 12228 11288 12225 11290 12221
11293 12217 11297 12219 11301 12219 11303 12224 11299 12226 11296 12229
11293 12232 11292 12237 11293 12240 11288 12241 11286 12245 11282 12244
11279 12241 11279 12237 11279 12232 11273 12232 22 P
11439 12323 11441 12320 11442 12317 11445 12319 11448 12318 11450 12320
11447 12321 11445 12324 11442 12323 11439 12323 10 P
11685 12391 11689 12392 11689 12388 11693 12387 11697 12387 11702 12386
11704 12383 11704 12387 11706 12391 11707 12387 11710 12383 11712 12387
11716 12388 11720 12386 11719 12391 11718 12395 11717 12399 11717 12403
11713 12402 11709 12402 11705 12400 11701 12401 11697 12404 11693 12406
11691 12402 11687 12402 11690 12399 11687 12397 11685 12391 29 P
11692 12358 11695 12358 11697 12356 11700 12358 11703 12358 11707 12357
11708 12359 11709 12363 11712 12363 11710 12366 11709 12369 11706 12370
11703 12372 11704 12369 11704 12366 11702 12364 11698 12363 11695 12362
11692 12363 11692 12358 20 P
11648 12356 11649 12353 11652 12353 11655 12353 11658 12356 11660 12358
11661 12361 11658 12362 11655 12360 11653 12362 11650 12360 11648 12356

12:00 PM
11634 12360 11637 12360 11640 12358 11640 12361 11643 12364 11644 12366
11646 12363 11645 12360 11648 12362 11648 12365 11647 12368 11643 12369
11640 12368 11637 12366 11635 12364 11634 12360 16 P
11675 12417 11672 12415 11673 12412 11673 12409 11676 12408 11679 12407
11683 12407 11686 12408 11687 12411 11690 12410 11693 12412 11696 12410
11699 12410 11702 12408 11703 12411 11701 12413 11698 12414 11695 12416
11692 12417 11689 12419 11687 12417 11683 12417 11682 12414 11679 12414
11675 12417 25 P
11679 12376 11678 12378 11676 12381 11673 12382 11670 12380 11669 12377
11670 12375 11674 12375 11675 12372 11677 12370 11680 12369 11680 12372
11682 12375 11682 12378 11679 12376 15 P
11758 12370 11760 12367 11763 12367 11761 12370 11762 12373 11763 12376
11765 12379 11763 12382 11761 12379 11759 12377 11759 12373 11758 12370
12:00 PM
11382 12555 11385 12551 11387 12548 11391 12545 11395 12545 11394 12550
11395 12554 11398 12556 11401 12560 11400 12564 11404 12565 11407 12568
11409 12572 11408 12576 11412 12580 11415 12581 11414 12585 11410 12583
11407 12580 11403 12580 11402 12584 11406 12586 11401 12587 11397 12586
11393 12585 11391 12581 11391 12577 11388 12574 11387 12569 11386 12565
11383 12562 11386 12559 11382 12555 33 P
11408 12602 11410 12600 11413 12599 11414 12596 11416 12598 11416 12601
11418 12604 11421 12603 11421 12607 11418 12607 11415 12609 11412 12611
11413 12608 11410 12605 11408 12602 15 P
11288 12562 11291 12558 11293 12563 11295 12567 11299 12567 11301 12571
11302 12575 11304 12579 11302 12583 11298 12585 11297 12581 11295 12577
11294 12573 11290 12570 11288 12567 11288 12562 16 P
11345 12433 11347 12430 11348 12428 11351 12430 11349 12433 11345 12433
6:00 PM
11345 12437 11348 12437 11351 12439 11353 12441 11352 12444 11349 12444
11348 12442 11345 12437 8 P
11348 12419 11350 12417 11352 12414 11354 12411 11355 12408 11358 12410
11361 12409 11363 12410 11362 12413 11359 12415 11356 12416 11354 12414
11353 12417 11348 12419 14 P
11371 12570 11373 12568 11376 12569 11379 12571 11379 12575 11375 12575
11373 12574 11371 12570 8 P
11379 12576 11382 12576 11383 12579 11386 12581 11383 12581 11379 12576
6:00 PM
11402 12607 11405 12605 11407 12608 11405 12611 11402 12610 11402 12607
6:00 PM
11571 12560 11575 12558 11577 12555 11582 12555 11580 12552 11575 12550
11578 12547 11582 12548 11586 12550 11590 12549 11593 12552 11597 12553
11598 12558 11596 12562 11596 12566 11591 12567 11589 12564 11585 12562

11582 12559 11577 12559 11571 12560 21 P
11655 12614 11651 12616 11649 12620 11644 12620 11642 12623 11640 12627
11638 12630 11635 12627 11634 12623 11631 12620 11634 12617 11632 12613
11631 12617 11627 12618 11623 12616 11618 12615 11617 12610 11617 12606
11617 12602 11618 12597 11621 12594 11625 12597 11627 12593 11630 12590
11634 12591 11637 12588 11641 12587 11645 12588 11649 12591 11648 12595
11652 12597 11655 12601 11656 12605 11660 12604 11661 12608 11663 12613
11655 12614 37 P
11651 12653 11647 12651 11649 12655 11651 12660 11652 12664 11650 12668
11647 12670 11643 12668 11638 12667 11637 12662 11635 12659 11631 12659
11630 12655 11626 12653 11622 12651 11618 12650 11613 12650 11610 12653
11606 12653 11606 12648 11603 12645 11605 12641 11604 12637 11604 12633
11603 12628 11606 12625 11609 12622 11612 12618 11616 12617 11621 12618
11624 12620 11628 12622 11627 12627 11631 12627 11628 12630 11627 12635
11623 12637 11622 12641 11627 12643 11630 12644 11635 12645 11638 12647
11640 12643 11644 12643 11648 12645 11652 12646 11651 12653 47 P
11414 12556 11417 12556 11420 12557 11422 12560 11425 12562 11426 12565
11422 12565 11419 12565 11418 12562 11415 12560 11414 12556 11 P
11396 12616 11399 12614 11402 12614 11400 12617 11396 12616 5 P
11602 12561 11604 12557 11607 12556 11609 12554 11611 12551 11611 12555
11610 12558 11607 12558 11602 12561 9 P
11600 12561 11601 12557 11601 12554 11603 12556 11600 12561 5 P
11635 12573 11637 12571 11640 12569 11643 12571 11645 12569 11648 12567
11650 12568 11650 12571 11649 12574 11646 12576 11644 12579 11642 12580
11639 12578 11636 12577 11635 12573 15 P
11660 12575 11656 12574 11657 12570 11659 12568 11656 12567 11655 12563
11657 12562 11659 12564 11660 12561 11662 12563 11665 12564 11665 12568
11664 12571 11662 12573 11660 12575 15 P
11666 12562 11667 12558 11670 12558 11673 12556 11671 12553 11669 12551
11669 12548 11671 12545 11671 12542 11673 12539 11676 12540 11677 12543
11678 12545 11681 12544 11684 12543 11688 12543 11690 12545 11691 12548
11691 12551 11690 12554 11687 12555 11687 12558 11687 12562 11683 12561
11680 12561 11678 12564 11677 12567 11678 12569 11678 12573 11678 12576
11675 12577 11672 12578 11669 12578 11665 12577 11664 12574 11667 12571
11668 12568 11667 12565 11666 12562 39 P
11676 12523 11681 12522 11685 12522 11689 12522 11693 12524 11696 12528
11700 12529 11703 12532 11702 12536 11707 12537 11709 12541 11707 12545
11709 12549 11709 12553 11712 12556 11709 12559 11705 12561 11708 12564
11703 12567 11699 12568 11695 12569 11695 12574 11691 12576 11687 12576
11683 12576 11682 12572 11685 12569 11689 12568 11691 12564 11694 12560
11694 12556 11698 12553 11702 12552 11698 12550 11696 12547 11697 12542
11695 12539 11690 12538 11687 12541 11682 12541 11679 12537 11675 12535
11679 12533 11682 12536 11686 12535 11690 12536 11689 12531 11685 12530

11683 12527 11679 12528 50 P
11679 12528 11676 12523 2 P
11642 12552 11645 12553 11649 12553 11649 12550 11648 12547 11649 12544
11651 12544 11651 12548 11652 12551 11651 12554 11654 12556 11650 12557
11648 12556 11644 12555 11642 12552 15 P
11710 12588 11715 12588 11717 12584 11716 12580 11717 12576 11716 12572
11720 12571 11723 12574 11728 12575 11730 12579 11734 12580 11737 12577
11741 12580 11744 12584 11748 12584 11750 12588 11746 12589 11742 12590
11743 12594 11741 12598 11737 12601 11734 12597 11731 12594 11727 12594
11730 12597 11731 12602 11728 12604 11723 12603 11721 12599 11717 12597
11716 12593 11711 12592 11710 12588 33 P
11681 12652 11684 12650 11688 12647 11690 12643 11692 12640 11697 12640
11701 12638 11699 12634 11699 12629 11704 12629 11707 12627 11710 12629
11713 12633 11717 12634 11718 12629 11722 12627 11721 12622 11725 12621
11730 12620 11734 12621 11736 12625 11735 12629 11735 12634 11733 12637
11730 12641 11733 12644 11729 12646 11726 12650 11723 12651 11719 12649
11715 12649 11711 12651 11710 12656 11705 12656 11702 12654 11697 12656
11693 12657 11691 12653 11687 12654 11681 12652 40 P
11687 12665 11691 12663 11695 12662 11699 12660 11703 12662 11707 12662
11710 12659 11713 12655 11716 12652 11719 12654 11724 12655 11725 12659
11725 12663 11723 12667 11726 12670 11724 12674 11719 12674 11719 12679
11716 12681 11714 12677 11716 12673 11711 12673 11708 12671 11704 12669
11699 12668 11695 12667 11687 12665 27 P
11674 12697 11677 12693 11679 12690 11682 12686 11683 12682 11687 12680
11691 12678 11693 12674 11692 12670 11697 12670 11701 12672 11703 12675
11705 12680 11702 12683 11701 12687 11703 12692 11707 12690 11711 12688
11711 12692 11715 12695 11715 12699 11711 12701 11708 12704 11712 12706
11716 12707 11716 12711 11712 12710 11712 12715 11709 12718 11706 12715
11702 12717 11698 12718 11695 12715 11696 12711 11698 12707 11701 12703
11699 12700 11695 12700 11690 12701 11687 12704 11683 12702 11681 12699
11674 12697 43 P
11645 12715 11644 12711 11642 12707 11637 12707 11632 12707 11637 12705
11635 12701 11639 12700 11643 12700 11647 12702 11650 12700 11654 12703
11657 12706 11657 12710 11657 12706 11657 12702 11661 12701 11663 12697
11667 12694 11670 12697 11672 12700 11677 12701 11678 12705 11683 12706
11683 12710 11682 12714 11686 12713 11690 12714 11691 12719 11688 12721
11684 12722 11684 12727 11688 12729 11684 12732 11685 12736 11681 12736
11678 12741 11675 12743 11671 12745 11670 12740 11671 12736 11670 12732
11667 12730 11663 12733 11660 12730 11656 12728 11652 12730 11650 12726
11649 12722 11645 12715 50 P
11679 12674 11681 12670 11683 12668 11687 12669 11684 12672 11683 12675
11679 12674 7 P
11668 12683 11669 12680 11671 12677 11674 12677 11676 12674 11679 12675

11679 12679 11677 12681 11674 12682 11671 12684 11668 12683 11 P
11659 12638 11660 12635 11661 12632 11662 12629 11664 12626 11667 12626
11668 12629 11667 12632 11665 12635 11665 12638 11659 12638 11 P
11729 12609 11732 12609 11735 12608 11738 12610 11739 12613 11742 12610
11744 12613 11745 12616 11742 12618 11745 12620 11746 12623 11743 12624
11740 12623 11737 12621 11736 12618 11736 12615 11733 12615 11730 12615
11729 12609 19 P
11698 12618 11700 12616 11703 12616 11705 12619 11707 12622 11704 12624
11701 12623 11698 12618 8 P
11685 12635 11688 12634 11690 12637 11691 12640 11689 12642 11687 12640
11685 12635 7 P
11670 12645 11674 12646 11676 12647 11678 12650 11678 12653 11675 12651
11674 12649 11670 12645 8 P
11685 12603 11687 12600 11689 12602 11690 12605 11692 12607 11693 12610
11690 12610 11687 12608 11685 12603 9 P
11678 12590 11678 12587 11679 12584 11681 12587 11682 12590 11681 12594
11680 12597 11677 12595 11678 12590 9 P
11682 12494 11684 12492 11686 12489 11685 12486 11687 12483 11690 12485
11692 12488 11692 12491 11696 12492 11698 12495 11698 12498 11695 12496
11694 12499 11697 12501 11694 12502 11692 12505 11689 12503 11687 12501
11687 12497 11685 12495 11682 12494 21 P
11799 12445 11802 12444 11805 12443 11804 12446 11808 12446 11811 12448
11813 12450 11813 12453 11811 12455 11808 12453 11810 12456 11809 12459
11806 12458 11803 12456 11800 12454 11802 12451 11799 12445 17 P
11643 12428 11647 12429 11648 12431 11649 12435 11645 12435 11644 12431
11643 12428 7 P
11656 12428 11659 12428 11661 12431 11661 12434 11659 12437 11657 12435
11656 12428 7 P
11655 12396 11658 12396 11661 12398 11660 12400 11657 12399 11655 12396
6:00 PM
11662 12393 11664 12391 11667 12391 11671 12391 11669 12393 11666 12395
11662 12393 7 P
11662 12403 11663 12399 11664 12396 11667 12397 11668 12400 11666 12403
11662 12403 7 P
11650 12415 11654 12416 11657 12416 11656 12419 11654 12421 11651 12418
11650 12415 7 P
11680 12425 11683 12423 11681 12421 11683 12419 11687 12419 11686 12422
11685 12425 11683 12428 11680 12425 9 P
11676 12404 11677 12401 11679 12398 11682 12400 11685 12401 11685 12404
11682 12404 11676 12404 8 P
11613 12342 11616 12339 11618 12338 11621 12337 11621 12340 11618 12342
11613 12342 7 P
11656 12314 11658 12311 11659 12308 11660 12305 11662 12307 11660 12310

11660 12313 11658 12317 11656 12314 9 P
11749 12703 11754 12704 11758 12702 11762 12701 11766 12701 11769 12704
11770 12709 11775 12710 11779 12709 11783 12710 11782 12714 11786 12713
11788 12709 11792 12709 11796 12708 11798 12704 11802 12703 11800 12700
11797 12696 11799 12693 11803 12692 11805 12696 11809 12694 11812 12697
11811 12702 11813 12706 11813 12710 11812 12714 11809 12716 11805 12719
11800 12718 11796 12717 11792 12716 11788 12717 11790 12721 11786 12723
11790 12726 11791 12730 11795 12729 11796 12733 11800 12733 11802 12737
11798 12737 11796 12741 11791 12742 11787 12741 11783 12741 11781 12746
11777 12746 11772 12745 50 P
11772 12745 11770 12741 11772 12737 11776 12735 11779 12733 11781 12728
11777 12726 11777 12721 11773 12722 11773 12717 11770 12715 11768 12719
11765 12715 11764 12711 11759 12711 11755 12710 11752 12713 11749 12709
11749 12703 19 P
11758 12770 11762 12767 11764 12763 11766 12759 11768 12755 11772 12753
11776 12755 11775 12759 11773 12763 11771 12767 11768 12769 11764 12772
11758 12770 13 P
11742 12741 11745 12739 11750 12737 11752 12741 11756 12741 11756 12746
11757 12750 11753 12752 11749 12752 11744 12752 11742 12748 11742 12741

12:00 PM

11652 12813 11656 12813 11661 12813 11661 12808 11664 12812 11667 12815
11669 12811 11672 12809 11677 12808 11681 12806 11683 12810 11686 12813
11684 12816 11687 12820 11689 12824 11685 12825 11681 12827 11681 12832
11681 12836 11685 12838 11686 12842 11683 12846 11686 12849 11685 12853
11689 12855 11694 12856 11697 12859 11701 12861 11697 12863 11695 12859
11690 12859 11686 12859 11682 12859 11681 12854 11678 12851 11675 12848
11674 12844 11671 12840 11667 12838 11663 12837 11658 12835 11656 12832
11655 12828 11653 12824 11657 12822 11661 12820 11658 12816 11652 12813
48 P
11704 12832 11708 12831 11711 12828 11709 12823 11714 12823 11715 12819
11719 12818 11724 12819 11726 12816 11731 12816 11735 12818 11738 12815
11742 12817 11744 12813 11740 12811 11741 12806 11745 12806 11746 12802
11749 12799 11749 12803 11754 12801 11755 12805 11758 12808 11755 12811
11750 12812 11751 12816 11750 12821 11746 12823 11743 12820 11739 12821
11737 12825 11733 12827 11732 12831 11736 12834 11739 12832 11742 12835
11741 12840 11737 12840 11734 12836 11733 12840 11730 12844 11726 12841
11725 12836 11721 12837 11717 12840 11716 12844 11712 12842 11708 12841
11705 12837 11704 12832 50 P
11693 12808 11697 12808 11698 12803 11702 12802 11706 12803 11709 12801
11712 12797 11714 12801 11714 12806 11715 12801 11716 12797 11719 12800
11719 12805 11718 12809 11716 12813 11711 12814 11707 12813 11703 12813
11699 12812 11693 12808 20 P
11683 12780 11686 12778 11690 12780 11695 12781 11699 12781 11703 12779

11707 12781 11707 12777 11702 12778 11701 12774 11702 12769 11703 12765
11701 12761 11699 12757 11698 12753 11702 12754 11706 12756 11707 12760
11705 12764 11710 12765 11714 12768 11717 12766 11717 12770 11721 12771
11720 12775 11720 12780 11717 12783 11713 12784 11711 12788 11710 12792
11707 12795 11703 12793 11699 12795 11695 12795 11695 12791 11691 12789
11689 12785 11683 12780 38 P
11750 12787 11752 12785 11752 12781 11752 12778 11750 12775 11753 12774
11756 12775 11759 12775 11762 12777 11761 12780 11761 12783 11758 12785
11756 12788 11754 12790 11751 12790 11748 12792 11750 12787 17 P
11730 12768 11732 12765 11733 12762 11736 12760 11738 12763 11737 12767
11737 12770 11737 12773 11738 12776 11735 12775 11731 12774 11731 12771
11730 12768 13 P
11717 13106 11719 13103 11722 13099 11725 13102 11729 13104 11732 13107
11735 13111 11737 13114 11741 13116 11743 13119 11744 13123 11742 13127
11739 13130 11739 13125 11736 13123 11731 13123 11727 13125 11730 13121
11725 13120 11726 13116 11723 13113 11722 13109 11717 13106 23 P
11682 12938 11683 12935 11686 12933 11687 12930 11690 12931 11692 12933
11691 12937 11691 12940 11689 12943 11687 12944 11684 12943 11682 12938
12:00 PM
11623 12964 11626 12962 11627 12959 11630 12960 11633 12960 11636 12962
11638 12964 11637 12967 11635 12971 11632 12971 11630 12974 11628 12972
11624 12972 11624 12969 11628 12968 11630 12966 11627 12965 11623 12964
18 P
11621 12958 11619 12955 11618 12952 11621 12954 11624 12955 11621 12958
6:00 PM
11636 12979 11640 12978 11641 12980 11639 12983 11636 12979 5 P
11687 12892 11690 12892 11693 12892 11695 12895 11697 12897 11694 12898
11692 12895 11689 12894 11687 12892 9 P
11682 12884 11685 12885 11686 12888 11685 12892 11682 12891 11682 12887
11682 12884 7 P
11638 12826 11641 12823 11643 12821 11645 12820 11648 12822 11648 12825
11646 12828 11644 12831 11642 12833 11640 12831 11638 12826 11 P
11652 13130 11655 13129 11658 13130 11658 13128 11661 13129 11661 13131
11658 13133 11656 13134 11654 13133 11652 13130 10 P
11647 13140 11650 13139 11651 13142 11651 13144 11649 13144 11647 13140
6:00 PM
11793 13201 11796 13199 11797 13196 11799 13194 11800 13196 11801 13199
11799 13202 11797 13204 11793 13201 9 P
11870 13234 11872 13231 11875 13230 11877 13232 11878 13236 11879 13239
11877 13242 11873 13241 11871 13238 11870 13234 10 P
11789 13247 11790 13244 11792 13242 11792 13238 11796 13237 11795 13241
11798 13242 11800 13239 11803 13239 11806 13240 11809 13242 11806 13244
11804 13245 11800 13247 11797 13247 11795 13249 11791 13250 11789 13247

18 P
11765 13239 11768 13238 11768 13235 11771 13236 11771 13232 11774 13234
11777 13234 11778 13237 11776 13239 11779 13241 11778 13244 11777 13247
11773 13248 11770 13247 11770 13244 11773 13243 11776 13241 11774 13238
11771 13237 11770 13240 11765 13239 21 P
11778 13229 11781 13228 11784 13228 11786 13230 11789 13232 11790 13235
11788 13237 11786 13235 11785 13232 11782 13231 11783 13234 11781 13236
11779 13232 11778 13229 14 P
11756 13234 11758 13231 11760 13229 11763 13230 11763 13234 11761 13236
11758 13238 11756 13234 8 P
11750 13254 11754 13253 11757 13254 11759 13255 11762 13257 11759 13258
11756 13258 11754 13255 11750 13254 9 P
11771 13261 11774 13261 11777 13261 11781 13260 11778 13263 11775 13262
11771 13261 7 P
11741 13255 11744 13254 11748 13254 11746 13257 11741 13255 5 P
11806 13258 11808 13256 11811 13257 11812 13260 11809 13260 11806 13258

6:00 PM

11817 13316 11819 13315 11822 13314 11823 13316 11822 13318 11819 13318
11817 13316 7 P
11843 13322 11845 13322 11848 13321 11849 13318 11852 13318 11850 13320
11849 13323 11846 13324 11843 13322 9 P
11828 13328 11830 13327 11832 13326 11835 13324 11837 13325 11839 13325
11841 13328 11838 13329 11836 13330 11833 13330 11830 13330 11828 13328

12:00 PM

11818 13366 11821 13365 11824 13364 11826 13363 11827 13366 11827 13369
11826 13371 11823 13371 11822 13369 11818 13366 10 P
11768 13386 11769 13383 11771 13382 11774 13382 11777 13381 11776 13384
11775 13386 11772 13388 11770 13387 11768 13386 10 P
11563 13388 11564 13386 11566 13383 11567 13381 11570 13380 11572 13381
11571 13383 11575 13383 11575 13381 11577 13383 11577 13386 11575 13387
11572 13388 11570 13389 11567 13389 11563 13388 16 P
11583 13442 11584 13440 11587 13438 11589 13436 11589 13439 11588 13442
11583 13442 7 P
11601 13444 11604 13444 11607 13445 11609 13446 11611 13447 11614 13448
11614 13450 11612 13452 11609 13452 11607 13452 11604 13450 11603 13448
11601 13444 13 P
11552 13450 11553 13448 11554 13446 11556 13443 11558 13443 11558 13446
11560 13448 11558 13450 11556 13448 11554 13449 11552 13450 11 P
11715 13515 11718 13515 11720 13516 11722 13515 11724 13516 11726 13519
11726 13521 11724 13523 11723 13525 11724 13528 11723 13530 11720 13531
11719 13528 11719 13525 11717 13523 11715 13521 11715 13519 11715 13515
18 P
11731 13541 11732 13538 11735 13537 11737 13539 11740 13539 11742 13538

11745 13538 11748 13537 11750 13538 11753 13537 11756 13536 11758 13535
11761 13535 11763 13535 11765 13536 11765 13539 11762 13539 11759 13538
11757 13540 11755 13540 11756 13543 11755 13546 11753 13547 11752 13550
11750 13550 11747 13550 11745 13548 11744 13546 11745 13543 11742 13543
11740 13545 11737 13546 11735 13546 11732 13546 11731 13541 35 P
11678 13493 11679 13490 11681 13488 11682 13490 11684 13492 11684 13495
11686 13497 11684 13498 11681 13498 11680 13495 11681 13493 11678 13493

12:00 PM

11683 13480 11684 13477 11687 13477 11687 13480 11688 13482 11688 13485
11685 13485 11684 13483 11683 13480 9 P
11569 13688 11571 13686 11573 13687 11575 13689 11577 13690 11580 13689
11583 13690 11583 13693 11586 13694 11584 13697 11584 13700 11582 13701
11582 13698 11581 13695 11579 13694 11576 13694 11574 13693 11572 13691
11569 13688 19 P
11555 13988 11559 13990 11560 13995 11562 13991 11567 13989 11567 13985
11571 13984 11576 13985 11580 13987 11581 13991 11585 13993 11588 13996
11586 14000 11582 14001 11577 14003 11574 14005 11569 14004 11568 14008
11564 14010 11563 14015 11560 14017 11560 14013 11558 14009 11557 14005
11556 14001 11557 13997 11557 13992 11555 13988 28 P
11587 13992 11588 13990 11589 13987 11591 13986 11594 13986 11596 13985
11597 13982 11597 13979 11600 13981 11601 13984 11602 13985 11601 13987
11598 13987 11597 13989 11595 13991 11594 13994 11591 13995 11587 13992
18 P
11701 14026 11703 14022 11704 14018 11705 14014 11706 14009 11710 14012
11713 14015 11715 14011 11715 14007 11718 14009 11721 14012 11723 14016
11725 14020 11726 14024 11731 14025 11731 14029 11729 14034 11729 14038
11724 14039 11720 14039 11716 14039 11711 14038 11711 14034 11707 14032
11703 14032 11701 14026 26 P
11749 13994 11752 13996 11755 14000 11759 13999 11762 13995 11765 13997
11765 14002 11767 14005 11768 14010 11772 14009 11776 14009 11777 14013
11780 14016 11784 14017 11785 14021 11784 14025 11782 14029 11781 14033
11777 14034 11773 14034 11769 14035 11766 14038 11762 14037 11761 14042
11763 14045 11763 14050 11761 14046 11759 14042 11755 14040 11752 14043
11748 14044 11750 14041 11753 14037 11756 14034 11757 14030 11759 14026
11759 14022 11760 14017 11756 14016 11752 14014 11754 14009 11758 14010
11756 14006 11754 14002 11751 13999 11749 13994 46 P
11636 14018 11641 14017 11644 14015 11649 14014 11652 14013 11657 14012
11661 14013 11665 14016 11667 14020 11663 14022 11661 14018 11656 14018
11652 14021 11648 14021 11644 14022 11640 14024 11636 14018 17 P
11646 13971 11648 13968 11650 13967 11654 13965 11657 13965 11660 13965
11659 13968 11657 13970 11654 13971 11651 13972 11646 13971 11 P
11626 13970 11628 13968 11631 13968 11634 13967 11637 13966 11639 13963
11642 13962 11643 13965 11641 13966 11638 13969 11638 13972 11635 13973

11632 13975 11630 13972 11626 13970 15 P
11613 13975 11615 13972 11618 13971 11621 13972 11624 13971 11626 13974
11624 13977 11621 13976 11618 13975 11613 13975 10 P
11557 13964 11560 13962 11561 13959 11564 13958 11568 13959 11570 13961
11568 13964 11570 13967 11567 13967 11565 13969 11562 13971 11559 13969
11557 13964 13 P
11551 13919 11554 13920 11557 13918 11554 13918 11553 13915 11556 13913
11559 13913 11561 13916 11563 13919 11564 13922 11563 13925 11560 13927
11557 13925 11554 13925 11553 13922 11551 13919 16 P
11500 13903 11502 13901 11504 13898 11505 13902 11506 13905 11506 13908
11509 13907 11512 13908 11510 13911 11507 13912 11504 13913 11502 13911
11502 13908 11500 13903 14 P
11529 13879 11526 13879 11526 13883 11523 13885 11520 13886 11519 13883
11520 13880 11523 13878 11526 13876 11527 13873 11530 13875 11533 13877
11536 13879 11533 13878 11529 13879 15 P
11504 13961 11508 13960 11510 13962 11513 13964 11516 13964 11514 13967
11517 13968 11519 13971 11515 13971 11514 13969 11510 13968 11509 13965
11506 13964 11504 13961 14 P
11689 13939 11691 13937 11694 13935 11695 13938 11695 13942 11693 13944
11690 13944 11689 13939 8 P
11707 13916 11709 13913 11710 13910 11710 13907 11714 13906 11716 13907
11719 13908 11722 13910 11721 13913 11720 13916 11717 13918 11716 13916
11712 13916 11711 13919 11707 13916 15 P
11679 14095 11683 14094 11685 14091 11685 14088 11685 14085 11688 14082
11690 14081 11694 14082 11697 14083 11697 14087 11697 14090 11699 14091
11697 14094 11695 14095 11692 14098 11690 14100 11687 14102 11685 14104
11682 14105 11682 14102 11681 14099 11679 14095 22 P
11658 14131 11660 14128 11663 14128 11664 14124 11665 14121 11668 14120
11670 14117 11673 14119 11674 14122 11677 14123 11679 14121 11679 14117
11682 14119 11684 14117 11687 14115 11688 14112 11690 14115 11692 14117
11692 14121 11692 14123 11690 14127 11688 14128 11685 14130 11683 14132
11679 14133 11676 14134 11676 14130 11673 14129 11670 14130 11668 14132
11665 14135 11663 14137 11660 14137 11658 14134 11658 14131 35 P
11711 14145 11713 14141 11716 14139 11721 14139 11723 14143 11727 14143
11730 14146 11734 14148 11738 14147 11743 14148 11746 14150 11748 14154
11750 14157 11750 14162 11747 14165 11745 14169 11741 14170 11737 14172
11732 14172 11728 14173 11724 14175 11721 14177 11717 14175 11715 14171
11716 14167 11715 14162 11713 14159 11714 14154 11713 14150 11711 14145
30 P
11693 14153 11696 14150 11700 14149 11702 14144 11701 14140 11705 14140
11704 14144 11707 14148 11709 14151 11707 14155 11702 14154 11698 14154
11693 14153 13 P
11687 14164 11690 14163 11692 14165 11696 14166 11699 14165 11701 14163

11704 14162 11704 14166 11704 14169 11705 14172 11704 14175 11702 14177
11699 14180 11696 14178 11696 14175 11693 14173 11690 14172 11688 14169
11687 14164 19 P
11709 14130 11711 14128 11715 14127 11716 14130 11716 14134 11714 14136
11711 14134 11709 14130 8 P
11632 14181 11634 14178 11637 14176 11639 14174 11642 14172 11643 14169
11642 14166 11645 14164 11649 14165 11652 14166 11655 14167 11658 14167
11661 14168 11664 14167 11667 14170 11666 14173 11663 14172 11660 14171
11657 14170 11654 14170 11650 14172 11648 14174 11647 14176 11643 14177
11641 14179 11637 14180 11632 14181 27 P
11681 14167 11684 14166 11685 14169 11686 14173 11686 14176 11689 14178
11686 14180 11683 14179 11683 14175 11682 14172 11681 14167 11 P
11674 14170 11676 14168 11677 14170 11679 14173 11679 14175 11677 14174
11674 14170 7 P
11637 14308 11640 14302 11642 14295 11642 14289 11642 14282 11643 14275
11642 14268 11645 14262 11652 14261 11658 14260 11661 14254 11665 14249
11668 14243 11670 14236 11667 14230 11661 14228 11654 14226 11656 14220
11656 14213 11662 14210 11669 14208 11676 14209 11682 14208 11689 14206
11696 14205 11696 14211 11696 14218 11702 14215 11708 14211 11714 14210
11719 14215 11724 14220 11725 14227 11723 14233 11721 14240 11723 14246
11717 14250 11717 14257 11717 14264 11716 14271 11712 14276 11712 14283
11715 14289 11712 14296 11709 14301 11707 14308 11702 14313 11696 14315
11690 14319 11684 14322 50 P
11684 14322 11678 14326 11674 14331 11667 14333 11661 14329 11655 14329
11652 14322 11649 14317 11643 14313 11637 14308 10 P
11607 14262 11610 14259 11614 14256 11617 14254 11621 14252 11625 14250
11629 14248 11632 14246 11636 14243 11636 14239 11634 14235 11637 14232
11641 14229 11643 14232 11647 14235 11649 14238 11652 14241 11655 14245
11655 14249 11654 14253 11650 14256 11647 14258 11642 14258 11638 14259
11635 14261 11631 14263 11628 14267 11628 14271 11628 14275 11629 14280
11631 14283 11629 14287 11625 14286 11621 14283 11617 14282 11614 14279
11614 14275 11615 14271 11615 14266 11615 14262 11607 14262 41 P
11576 14291 11580 14288 11583 14286 11587 14284 11591 14285 11593 14289
11597 14290 11601 14291 11605 14294 11609 14294 11613 14291 11616 14293
11620 14295 11624 14298 11625 14302 11628 14306 11629 14309 11628 14314
11626 14317 11622 14314 11618 14313 11614 14313 11610 14313 11606 14315
11602 14318 11598 14319 11594 14318 11590 14318 11587 14315 11583 14312
11583 14308 11581 14304 11583 14300 11581 14296 11576 14291 35 P
11591 14228 11593 14225 11595 14222 11597 14220 11600 14219 11602 14216
11605 14216 11609 14215 11607 14218 11604 14220 11604 14223 11604 14226
11605 14229 11602 14229 11598 14229 11595 14229 11591 14228 17 P
11560 14237 11563 14235 11566 14233 11568 14234 11571 14235 11574 14233
11576 14231 11579 14229 11582 14229 11583 14232 11584 14235 11582 14236

11580 14239 11578 14242 11577 14245 11574 14245 11573 14241 11570 14240
11567 14240 11563 14240 11560 14237 21 P
11546 14242 11547 14240 11550 14240 11553 14240 11555 14241 11554 14243
11551 14244 11549 14246 11546 14242 9 P
11602 14269 11604 14269 11605 14271 11605 14274 11603 14275 11602 14273
11602 14269 7 P
11585 14265 11587 14263 11590 14263 11593 14263 11595 14264 11598 14264
11596 14266 11593 14266 11590 14266 11588 14267 11585 14265 11 P
10886 14335 10888 14332 10891 14329 10895 14326 10895 14322 10897 14318
10899 14314 10902 14311 10906 14312 10910 14312 10915 14311 10919 14312
10922 14309 10926 14311 10926 14315 10929 14319 10926 14322 10922 14323
10921 14327 10922 14331 10926 14333 10930 14331 10929 14327 10933 14327
10936 14331 10941 14330 10938 14333 10942 14333 10940 14337 10938 14341
10936 14345 10932 14347 10928 14347 10924 14346 10919 14345 10915 14344
10911 14343 10908 14341 10903 14339 10900 14337 10896 14338 10892 14340
10886 14335 43 P
10915 14258 10918 14256 10921 14254 10924 14254 10924 14251 10923 14248
10926 14249 10929 14250 10932 14250 10935 14250 10937 14253 10939 14256
10939 14259 10937 14262 10937 14265 10939 14267 10942 14269 10941 14272
10938 14273 10935 14273 10932 14275 10931 14272 10929 14269 10926 14268
10925 14265 10923 14262 10922 14259 10919 14258 10915 14258 29 P
10945 14226 10948 14227 10950 14230 10952 14232 10956 14233 10958 14232
10962 14232 10962 14235 10963 14238 10960 14238 10957 14238 10953 14236
10951 14237 10950 14234 10947 14232 10945 14226 16 P
11058 14266 11057 14262 11060 14261 11064 14262 11066 14261 11070 14261
11071 14263 11073 14266 11073 14269 11077 14268 11078 14271 11077 14274
11078 14277 11079 14280 11076 14280 11072 14280 11069 14280 11066 14279
11069 14276 11072 14275 11071 14272 11071 14269 11067 14269 11064 14270
11062 14272 11061 14268 11058 14266 27 P
11117 14261 11120 14261 11124 14260 11124 14257 11123 14254 11125 14256
11127 14259 11130 14261 11132 14262 11135 14265 11137 14267 11137 14270
11140 14269 11143 14271 11141 14273 11140 14276 11137 14275 11134 14274
11131 14273 11129 14272 11127 14268 11124 14267 11122 14265 11117 14261
24 P
11154 14212 11157 14210 11159 14208 11163 14209 11165 14212 11168 14211
11171 14213 11174 14215 11176 14217 11177 14220 11179 14222 11182 14225
11184 14227 11187 14228 11190 14230 11192 14232 11194 14235 11195 14238
11198 14240 11200 14242 11201 14246 11203 14248 11205 14251 11207 14254
11204 14253 11200 14253 11198 14252 11195 14249 11193 14247 11190 14245
11187 14246 11185 14243 11182 14242 11179 14240 11180 14237 11182 14234
11183 14231 11183 14228 11180 14230 11180 14233 11178 14235 11175 14237
11172 14236 11169 14235 11171 14232 11171 14229 11168 14227 11166 14224
11165 14221 11163 14219 50 P

11163 14219 11159 14218 11157 14216 11154 14212 4 P
11355 14216 11358 14214 11359 14212 11358 14208 11359 14205 11362 14204
11365 14206 11367 14209 11369 14212 11371 14214 11372 14217 11372 14220
11369 14221 11366 14220 11362 14219 11359 14219 11356 14220 11355 14216
18 P
11439 14279 11441 14276 11444 14277 11447 14277 11450 14278 11453 14280
11454 14283 11456 14286 11453 14285 11450 14283 11448 14281 11446 14282
11443 14281 11439 14279 14 P
11383 14288 11386 14287 11389 14287 11391 14290 11389 14293 11389 14296
11387 14294 11386 14291 11383 14288 9 P
11152 14241 11156 14240 11158 14239 11161 14237 11163 14234 11165 14236
11163 14238 11165 14241 11163 14243 11159 14244 11156 14243 11152 14241

12:00 PM

10928 14640 10931 14637 10935 14636 10939 14637 10936 14641 10938 14644
10942 14642 10946 14642 10949 14645 10951 14642 10955 14639 10959 14637
10963 14635 10967 14634 10971 14633 10971 14638 10974 14635 10978 14634
10981 14638 10979 14641 10977 14645 10975 14649 10971 14651 10971 14655
10967 14655 10963 14655 10959 14658 10955 14659 10951 14661 10948 14664
10946 14659 10943 14656 10942 14652 10937 14651 10933 14651 10933 14646
10934 14642 10928 14640 38 P
11018 14724 11017 14728 11015 14731 11014 14727 11015 14723 11012 14719
11014 14716 11018 14716 11018 14711 11015 14713 11011 14710 11010 14706
11013 14703 11016 14707 11019 14709 11023 14710 11025 14715 11025 14719
11028 14723 11028 14727 11025 14723 11018 14724 22 P
10903 15169 10907 15169 10910 15172 10914 15175 10918 15174 10922 15177
10925 15180 10927 15184 10931 15186 10934 15189 10935 15193 10939 15194
10938 15198 10942 15199 10946 15197 10949 15200 10951 15204 10950 15209
10946 15208 10942 15207 10944 15210 10941 15212 10944 15215 10939 15216
10942 15219 10938 15222 10937 15226 10941 15229 10945 15229 10944 15233
10941 15234 10937 15238 10933 15237 10929 15238 10925 15236 10922 15233
10925 15231 10924 15227 10921 15225 10924 15221 10924 15217 10921 15215
10917 15213 10916 15209 10911 15210 10908 15206 10905 15203 10904 15199
10908 15201 10912 15202 50 P
10912 15202 10916 15203 10920 15201 10917 15198 10913 15197 10915 15192
10919 15191 10919 15187 10918 15183 10914 15183 10909 15184 10909 15179
10908 15175 10903 15169 14 P
10808 15183 10812 15184 10814 15188 10817 15191 10821 15190 10824 15186
10827 15190 10827 15194 10831 15193 10832 15188 10836 15187 10840 15186
10844 15188 10847 15191 10848 15196 10851 15198 10852 15203 10848 15204
10844 15206 10840 15207 10838 15204 10834 15204 10830 15204 10827 15201
10823 15203 10820 15201 10820 15197 10817 15193 10813 15193 10811 15190
10808 15183 31 P
10846 15170 10849 15167 10852 15170 10857 15171 10856 15176 10859 15179

10856 15182 10858 15186 10854 15188 10850 15186 10848 15183 10845 15179
10848 15175 10846 15170 14 P
10810 15131 10814 15131 10813 15127 10816 15125 10819 15126 10822 15126
10823 15129 10824 15132 10821 15134 10820 15137 10820 15140 10817 15139
10815 15137 10811 15135 10810 15131 15 P
10820 15146 10823 15145 10826 15144 10825 15147 10826 15151 10823 15152
10821 15151 10820 15146 8 P
10819 15158 10822 15157 10824 15159 10825 15161 10824 15163 10822 15164
10820 15162 10819 15158 8 P
9403 15100 9404 15094 9404 15087 9410 15084 9416 15082 9423 15085
9429 15084 9433 15079 9436 15073 9439 15078 9445 15082 9449 15088
9454 15092 9460 15092 9467 15093 9473 15089 9479 15086 9486 15085
9492 15087 9496 15093 9501 15098 9508 15099 9511 15105 9515 15111
9521 15108 9527 15113 9530 15119 9531 15125 9531 15132 9531 15139
9527 15145 9526 15151 9519 15153 9513 15155 9507 15159 9501 15162
9494 15162 9498 15157 9499 15150 9495 15145 9489 15141 9483 15141
9478 15137 9472 15134 9465 15134 9458 15134 9452 15133 9445 15131
9438 15130 9433 15134 50 P
9433 15134 9431 15127 9431 15120 9426 15117 9420 15113 9417 15107
9412 15102 9403 15100 8 P
9500 15059 9497 15065 9491 15067 9484 15069 9478 15071 9471 15069
9466 15065 9462 15071 9457 15075 9450 15073 9446 15068 9443 15062
9443 15055 9438 15050 9432 15049 9425 15048 9423 15042 9425 15035
9418 15034 9422 15028 9420 15022 9416 15016 9422 15014 9429 15012
9433 15017 9435 15024 9440 15028 9444 15033 9449 15038 9450 15045
9456 15048 9462 15049 9469 15048 9471 15042 9466 15038 9462 15032
9458 15027 9455 15020 9452 15014 9458 15013 9465 15011 9472 15010
9477 15006 9484 15006 9489 15010 9490 15016 9494 15022 9497 15028
9496 15035 9493 15041 50 P
9493 15041 9488 15046 9482 15048 9489 15047 9496 15047 9501 15050
9500 15059 7 P
9866 14927 9870 14921 9869 14915 9874 14910 9879 14906 9884 14909
9888 14915 9889 14922 9895 14917 9901 14916 9900 14923 9899 14929
9896 14936 9898 14942 9904 14944 9897 14945 9893 14940 9887 14938
9880 14937 9877 14932 9866 14927 21 P
10217 14889 10223 14889 10228 14887 10230 14882 10225 14885 10222 14880
10222 14875 10222 14870 10223 14864 10228 14862 10231 14866 10235 14870
10240 14873 10244 14876 10244 14882 10240 14886 10238 14890 10235 14895
10235 14901 10233 14896 10228 14893 10223 14894 10217 14889 23 P
10036 14860 10039 14856 10042 14854 10038 14851 10042 14849 10045 14846
10050 14846 10054 14844 10058 14843 10061 14845 10061 14849 10056 14850
10055 14855 10052 14858 10049 14856 10045 14859 10045 14863 10041 14863
10036 14860 19 P

10143 15145 10140 15142 10142 15138 10142 15134 10139 15138 10135 15138
10135 15142 10130 15143 10128 15140 10126 15135 10124 15132 10121 15129
10119 15125 10124 15124 10128 15124 10130 15120 10132 15117 10135 15113
10139 15113 10143 15113 10145 15117 10148 15120 10151 15124 10153 15127
10155 15131 10156 15135 10153 15139 10150 15141 10143 15145 29 P
9939 14993 9942 14990 9946 14988 9950 14987 9955 14986 9958 14984
9962 14982 9966 14981 9970 14980 9974 14978 9974 14974 9977 14971
9979 14975 9978 14979 9983 14980 9987 14980 9991 14979 9996 14978
10000 14979 10004 14978 10008 14976 10012 14974 10015 14977 10018 14980
10022 14982 10017 14982 10013 14983 10009 14985 10005 14986 10002 14990
9998 14991 9996 14987 9992 14986 9988 14985 9983 14985 9979 14986
9975 14986 9971 14989 9969 14993 9969 14996 9966 14994 9963 14990
9960 14993 9960 14997 9956 14997 9953 14994 9949 14996 9946 14994
9939 14993 49 P
9996 14961 10000 14958 10004 14958 10007 14961 10011 14960 10015 14961
10020 14961 10024 14962 10028 14961 10027 14957 10026 14953 10024 14949
10024 14945 10027 14948 10030 14950 10034 14953 10038 14953 10038 14958
10042 14959 10046 14961 10050 14963 10046 14965 10044 14969 10040 14970
10042 14974 10038 14972 10036 14968 10032 14969 10028 14969 10023 14969
10020 14967 10016 14966 10011 14966 10007 14966 10003 14965 9996 14961
36 P
10050 14963 10053 14962 10056 14962 10059 14965 10056 14966 10054 14965
10050 14963 7 P
10056 14960 10060 14959 10062 14956 10064 14959 10067 14960 10070 14959
10073 14961 10071 14963 10068 14962 10064 14961 10061 14962 10056 14960
12:00 PM
10167 15088 10170 15084 10172 15081 10177 15080 10181 15077 10181 15081
10181 15086 10185 15086 10183 15089 10178 15091 10174 15091 10170 15093
10167 15088 13 P
10153 14988 10151 14985 10148 14984 10149 14981 10152 14980 10155 14981
10157 14985 10153 14988 8 P
9939 14957 9942 14955 9943 14952 9947 14953 9949 14952 9953 14953
9956 14953 9959 14953 9961 14956 9963 14958 9960 14960 9957 14959
9954 14959 9950 14960 9947 14960 9944 14960 9939 14957 17 P
9981 14948 9983 14946 9985 14947 9988 14948 9990 14950 9990 14954
9989 14956 9988 14954 9984 14954 9984 14951 9981 14948 11 P
10067 14880 10068 14877 10071 14876 10075 14877 10078 14877 10081 14876
10083 14879 10080 14881 10077 14882 10075 14881 10072 14881 10067 14880
12:00 PM
10097 14876 10099 14874 10101 14872 10102 14869 10105 14867 10106 14870
10108 14873 10106 14875 10102 14875 10097 14876 10 P
10083 14863 10085 14861 10088 14862 10090 14864 10092 14867 10090 14870
10088 14871 10087 14868 10083 14867 10083 14863 10 P

10429 15267 10432 15267 10435 15270 10438 15270 10441 15271 10444 15271
10447 15271 10445 15274 10444 15277 10441 15278 10438 15277 10435 15275
10432 15274 10430 15272 10429 15267 15 P
9908 14951 9911 14951 9914 14949 9916 14950 9920 14951 9921 14954
9919 14958 9916 14958 9913 14956 9911 14954 9908 14951 11 P
9907 14974 9910 14973 9913 14972 9916 14970 9918 14968 9921 14968
9924 14969 9922 14971 9919 14973 9916 14973 9914 14976 9911 14975
9907 14974 13 P
9668 14751 9669 14748 9669 14745 9671 14743 9674 14744 9675 14747
9675 14750 9673 14752 9674 14755 9675 14758 9672 14760 9669 14757
9671 14754 9668 14751 14 P
9196 15599 9199 15596 9203 15598 9205 15601 9209 15603 9214 15604
9218 15604 9219 15609 9218 15613 9218 15617 9215 15620 9211 15622
9209 15618 9204 15618 9204 15614 9205 15609 9201 15609 9198 15606
9196 15599 19 P
8356 14785 8360 14787 8364 14788 8368 14790 8371 14793 8375 14796
8378 14799 8379 14803 8377 14807 8373 14809 8370 14813 8368 14809
8370 14804 8374 14803 8375 14799 8371 14797 8368 14794 8365 14790
8361 14788 8356 14785 20 P
8392 14833 8396 14834 8401 14835 8405 14836 8409 14837 8413 14838
8416 14842 8417 14846 8419 14850 8423 14851 8424 14856 8422 14860
8418 14859 8415 14855 8413 14851 8410 14848 8408 14844 8404 14841
8401 14839 8397 14837 8392 14833 21 P
8475 14886 8471 14884 8467 14882 8463 14880 8459 14878 8455 14876
8455 14872 8452 14869 8448 14867 8444 14864 8441 14862 8437 14860
8437 14856 8434 14853 8430 14850 8426 14849 8430 14847 8434 14849
8438 14850 8442 14851 8446 14853 8449 14855 8452 14859 8455 14862
8458 14866 8461 14869 8464 14871 8467 14875 8470 14877 8473 14881
8475 14886 31 P
8397 14849 8399 14847 8402 14848 8404 14850 8405 14853 8407 14856
8407 14859 8405 14862 8404 14859 8402 14856 8400 14854 8398 14851
8397 14849 13 P
8272 14733 8276 14734 8279 14737 8282 14740 8285 14743 8287 14747
8290 14750 8292 14754 8294 14758 8295 14762 8293 14766 8290 14762
8289 14758 8286 14756 8282 14753 8282 14749 8281 14744 8279 14741
8275 14738 8272 14733 20 P
8293 14780 8292 14777 8295 14776 8297 14777 8299 14780 8301 14783
8301 14786 8299 14784 8295 14783 8293 14780 10 P
8310 14794 8313 14793 8315 14795 8319 14796 8320 14799 8321 14802
8322 14804 8324 14807 8326 14810 8323 14808 8321 14806 8319 14804
8318 14801 8315 14799 8313 14797 8310 14794 16 P
9247 14414 9250 14412 9254 14414 9255 14418 9260 14419 9264 14420
9266 14424 9266 14428 9262 14430 9258 14428 9254 14427 9251 14424

9251 14419 9247 14414 14 P
9224 14344 9228 14341 9231 14343 9236 14342 9240 14342 9243 14345
9240 14348 9235 14349 9231 14349 9231 14354 9230 14358 9228 14362
9225 14359 9225 14354 9224 14350 9224 14344 16 P
9167 14236 9169 14233 9172 14229 9176 14227 9179 14225 9181 14228
9181 14233 9181 14237 9181 14241 9177 14243 9173 14244 9170 14242
9167 14236 13 P
9204 14249 9207 14246 9210 14242 9212 14239 9215 14236 9220 14236
9223 14233 9226 14236 9225 14240 9223 14245 9221 14248 9217 14248
9213 14250 9209 14252 9204 14249 15 P
9221 14208 9224 14206 9229 14206 9231 14202 9234 14199 9238 14196
9240 14200 9238 14205 9238 14209 9240 14213 9238 14216 9235 14220
9233 14224 9231 14228 9228 14230 9224 14228 9223 14223 9223 14219
9225 14215 9225 14211 9221 14208 21 P
9309 14273 9311 14271 9314 14269 9317 14272 9317 14275 9314 14276
9310 14277 9309 14273 8 P
9289 14466 9292 14466 9295 14466 9298 14468 9301 14466 9301 14470
9299 14472 9298 14475 9295 14475 9294 14472 9291 14471 9289 14466
12:00 PM
9287 14456 9289 14453 9291 14450 9294 14448 9295 14446 9296 14448
9295 14452 9297 14453 9294 14454 9294 14457 9291 14459 9287 14456
12:00 PM
9010 13021 9013 13017 9017 13016 9020 13019 9024 13021 9024 13026
9021 13030 9021 13034 9020 13038 9016 13038 9015 13034 9013 13030
9010 13027 9010 13021 14 P
8704 12481 8706 12477 8709 12474 8712 12471 8714 12467 8719 12466
8723 12465 8726 12467 8731 12469 8728 12472 8724 12474 8720 12475
8716 12475 8711 12477 8709 12481 8704 12481 16 P
9538 10853 9542 10847 9547 10843 9552 10838 9557 10833 9561 10828
9566 10823 9571 10818 9577 10821 9584 10817 9590 10815 9595 10810
9600 10806 9606 10801 9611 10797 9615 10792 9620 10787 9626 10783
9632 10779 9638 10776 9645 10773 9648 10768 9654 10764 9661 10764
9668 10761 9673 10757 9679 10754 9686 10752 9692 10748 9699 10747
9705 10744 9711 10740 9716 10737 9723 10733 9728 10729 9735 10727
9741 10725 9747 10721 9753 10718 9760 10716 9766 10715 9773 10716
9780 10714 9787 10712 9793 10711 9800 10708 9806 10705 9812 10702
9819 10699 9825 10697 50 P
9825 10697 9832 10694 9838 10692 9845 10691 9852 10689 9859 10688
9866 10687 9872 10691 9877 10694 9880 10700 9884 10706 9890 10710
9892 10717 9893 10723 9894 10730 9892 10737 9886 10740 9880 10745
9874 10747 9867 10747 9860 10747 9853 10749 9847 10751 9840 10753
9834 10757 9828 10760 9823 10765 9816 10767 9810 10771 9805 10774
9798 10776 9792 10779 9785 10781 9778 10783 9772 10785 9765 10787

9758 10788 9752 10792 9746 10795 9741 10800 9739 10806 9733 10811
9728 10816 9723 10820 9717 10823 9710 10825 9704 10828 9698 10831
9691 10833 9685 10836 50 P
9685 10836 9678 10838 2 P
9678 10838 9672 10841 9668 10847 9665 10853 9661 10859 9658 10865
9654 10871 9650 10876 9644 10880 9639 10884 9632 10888 9626 10890
9619 10891 9612 10893 9605 10892 9598 10892 9592 10890 9585 10888
9578 10886 9572 10884 9567 10879 9562 10874 9557 10870 9551 10866
9545 10862 9538 10853 26 P
9747 10671 9753 10668 9759 10665 9765 10662 9772 10659 9776 10654
9782 10651 9789 10648 9795 10644 9800 10640 9806 10636 9813 10634
9818 10629 9825 10627 9828 10621 9832 10615 9836 10610 9842 10606
9848 10608 9849 10615 9848 10622 9845 10628 9840 10633 9836 10639
9832 10644 9826 10648 9821 10653 9814 10655 9809 10659 9802 10662
9796 10664 9789 10667 9783 10668 9776 10671 9769 10672 9763 10674
9756 10676 9747 10671 38 P
9850 10640 9854 10634 9857 10628 9858 10621 9860 10614 9862 10607
9867 10603 9873 10600 9879 10595 9885 10598 9888 10604 9893 10609
9895 10616 9894 10623 9891 10629 9885 10633 9879 10636 9873 10639
9866 10639 9859 10641 9850 10640 21 P
9983 9961 9977 9965 9971 9966 9971 9973 9966 9978 9960 9981
9958 9974 9951 9972 9955 9966 9960 9961 9966 9957 9969 9951
9971 9944 9976 9939 9977 9932 9978 9925 9977 9918 9975 9912
9978 9906 9983 9901 9988 9896 9993 9891 9996 9885 9998 9878
10004 9875 10010 9879 10015 9883 10021 9886 10028 9885 10035 9886
10042 9883 10045 9877 10052 9877 10059 9878 10065 9879 10072 9876
10078 9873 10085 9871 10091 9869 10098 9867 10105 9865 10111 9861
10117 9857 10123 9854 10130 9855 10137 9856 10143 9859 10140 9865
10138 9872 10141 9878 50 P
10141 9878 10144 9885 10146 9891 10152 9895 10145 9899 10142 9905
10136 9908 10132 9914 10127 9919 10122 9923 10115 9922 10108 9923
10104 9929 10097 9931 10096 9938 10089 9938 10082 9938 10076 9940
10072 9947 10065 9946 10059 9947 10052 9948 10048 9954 10043 9959
10040 9965 10034 9969 10027 9971 10022 9967 10015 9964 10008 9962
10002 9959 9995 9961 9990 9966 9983 9961 34 P
10018 10042 10024 10038 10030 10036 10036 10032 10042 10028 10048 10024
10055 10023 10061 10023 10068 10023 10075 10021 10081 10018 10088 10016
10085 10023 10083 10029 10088 10034 10090 10041 10085 10046 10090 10052
10095 10057 10088 10057 10083 10062 10085 10068 10079 10072 10072 10070
10066 10073 10064 10066 10058 10063 10051 10061 10047 10057 10040 10059
10033 10059 10029 10053 10023 10050 10018 10042 34 P
10109 10149 10111 10143 10110 10136 10115 10131 10113 10124 10119 10121
10125 10118 10132 10117 10131 10124 10138 10125 10143 10129 10149 10125

10156 10125 10163 10124 10170 10124 10176 10120 10183 10120 10180 10126
10174 10130 10179 10134 10175 10140 10169 10142 10162 10142 10155 10143
10153 10149 10146 10149 10149 10143 10143 10144 10136 10146 10129 10147
10123 10145 10116 10148 10109 10149 33 P
10170 10278 10177 10279 10184 10279 10190 10280 10197 10283 10204 10283
10211 10283 10218 10283 10224 10285 10218 10289 10223 10295 10216 10295
10209 10294 10204 10290 10197 10288 10192 10293 10185 10295 10182 10301
10176 10304 10169 10303 10175 10299 10176 10292 10175 10285 10170 10278
24 P
10017 9723 10024 9723 10028 9729 10035 9729 10040 9725 10047 9725
10049 9718 10055 9715 10061 9712 10061 9705 10061 9698 10068 9698
10070 9704 10073 9698 10079 9702 10081 9695 10088 9694 10091 9700
10092 9707 10096 9713 10091 9717 10089 9724 10083 9727 10078 9731
10071 9734 10065 9737 10061 9743 10056 9748 10058 9755 10056 9762
10051 9767 10047 9762 10040 9760 10033 9760 10028 9756 10027 9749
10025 9742 10023 9735 10017 9723 39 P
10098 9792 10104 9788 10110 9784 10114 9779 10119 9774 10125 9770
10130 9765 10136 9764 10142 9760 10149 9763 10144 9769 10141 9775
10141 9782 10136 9786 10142 9789 10137 9795 10131 9797 10124 9800
10117 9802 10111 9800 10104 9799 10098 9792 22 P
10146 9852 10152 9848 10156 9842 10161 9838 10167 9834 10170 9827
10175 9822 10169 9817 10170 9811 10172 9804 10177 9799 10184 9797
10190 9802 10185 9807 10183 9814 10186 9820 10192 9824 10198 9827
10198 9834 10196 9841 10190 9841 10183 9845 10190 9845 10197 9846
10192 9852 10192 9859 10186 9862 10181 9859 10173 9857 10167 9859
10160 9858 10156 9863 10151 9859 10146 9852 34 P
9909 9874 9914 9870 9916 9863 9922 9859 9928 9860 9933 9865
9939 9868 9945 9872 9950 9876 9953 9883 9950 9889 9948 9896
9947 9903 9943 9909 9937 9912 9933 9918 9930 9924 9923 9924
9916 9922 9915 9915 9912 9909 9909 9902 9909 9895 9912 9889
9913 9882 9909 9874 26 P
10051 9976 10055 9970 10059 9965 10066 9967 10070 9972 10071 9979
10078 9982 10083 9987 10076 9991 10070 9993 10063 9995 10057 9992
10053 9987 10051 9976 14 P
10112 10019 10117 10015 10122 10020 10129 10021 10136 10020 10142 10021
10149 10024 10153 10030 10160 10032 10165 10036 10163 10030 10165 10023
10170 10019 10176 10022 10181 10028 10178 10034 10175 10040 10168 10043
10161 10042 10155 10041 10148 10040 10141 10039 10134 10039 10132 10032
10128 10027 10122 10025 10112 10019 27 P
10102 10027 10103 10020 10110 10024 10116 10027 10123 10028 10129 10029
10125 10035 10119 10037 10112 10037 10105 10038 10102 10027 11 P
10327 10294 10329 10290 10331 10294 10336 10294 10338 10290 10343 10289
10342 10293 10341 10297 10343 10302 10344 10306 10340 10306 10336 10304

10332 10302 10330 10298 10327 10294 15 P
10350 10303 10352 10299 10357 10299 10361 10299 10363 10295 10366 10292
10366 10296 10365 10301 10364 10305 10359 10306 10356 10309 10350 10303
12:00 PM
10229 10256 10234 10256 10238 10254 10242 10253 10240 10257 10244 10260
10247 10263 10244 10265 10240 10269 10236 10270 10234 10267 10230 10264
10229 10256 13 P
10168 9888 10171 9884 10168 9881 10169 9876 10172 9874 10177 9874
10177 9878 10176 9882 10176 9887 10179 9890 10184 9889 10188 9891
10186 9895 10182 9896 10180 9900 10182 9904 10181 9908 10178 9905
10173 9903 10169 9903 10171 9899 10170 9894 10168 9888 23 P
9956 9826 9959 9822 9962 9819 9967 9819 9971 9820 9975 9821
9980 9822 9984 9823 9988 9826 9988 9830 9988 9834 9985 9838
9981 9839 9978 9842 9977 9847 9974 9849 9972 9853 9968 9852
9966 9847 9962 9846 9959 9843 9958 9839 9958 9834 9956 9826
24 P
9993 9777 9989 9774 9985 9772 9982 9769 9978 9766 9977 9761
9979 9757 9978 9753 9982 9750 9986 9750 9990 9749 9995 9750
9997 9754 10000 9757 10001 9762 10004 9765 10006 9769 10009 9773
10009 9777 10007 9781 10003 9781 9998 9780 9993 9777 23 P
10097 9757 10101 9756 10103 9753 10106 9749 10109 9746 10112 9743
10116 9741 10119 9737 10123 9739 10128 9737 10131 9736 10135 9739
10139 9739 10144 9739 10144 9743 10140 9742 10139 9747 10135 9748
10132 9745 10128 9746 10123 9746 10119 9748 10116 9750 10112 9753
10115 9757 10117 9761 10113 9762 10110 9765 10105 9765 10102 9763
10097 9757 31 P
10128 9809 10131 9807 10136 9806 10140 9806 10144 9807 10148 9809
10152 9811 10152 9815 10156 9818 10154 9822 10150 9821 10145 9820
10141 9821 10137 9821 10132 9820 10131 9815 10128 9809 17 P
9940 9928 9943 9925 9948 9925 9951 9922 9950 9918 9954 9916
9958 9916 9959 9921 9961 9925 9963 9929 9962 9934 9964 9937
9961 9940 9957 9943 9953 9942 9949 9940 9946 9938 9943 9934
9940 9928 19 P
9999 9738 10000 9735 10000 9731 10001 9728 10000 9725 10003 9727
10005 9729 10008 9731 10009 9735 10011 9737 10014 9739 10014 9742
10013 9745 10009 9746 10007 9745 10003 9744 10002 9741 9999 9738
18 P
9987 9982 9990 9983 9993 9982 9995 9979 9997 9977 9995 9975
9993 9972 9996 9972 9999 9974 10002 9975 10004 9978 10007 9980
10008 9983 10011 9985 10009 9987 10008 9991 10007 9994 10003 9993
10000 9991 10000 9988 9998 9991 9995 9992 9993 9990 9994 9987
9990 9986 9987 9982 26 P
9956 10050 9956 10046 9957 10043 9960 10045 9963 10047 9966 10050

9966 10053 9964 10056 9967 10058 9966 10061 9968 10063 9970 10066
9971 10069 9969 10071 9966 10069 9966 10066 9964 10063 9961 10065
9960 10063 9960 10059 9960 10056 9957 10053 9956 10050 23 P
10347 9977 10348 9974 10350 9971 10353 9971 10356 9971 10359 9971
10359 9974 10357 9977 10354 9979 10351 9979 10347 9977 11 P
9949 9390 9953 9390 9957 9387 9959 9384 9962 9380 9966 9380
9969 9377 9970 9372 9970 9367 9968 9364 9965 9360 9969 9361
9974 9361 9978 9361 9982 9360 9981 9356 9980 9351 9984 9350
9988 9348 9991 9351 9996 9352 9994 9356 9994 9360 9995 9365
9999 9366 10003 9369 10000 9372 9995 9373 9998 9376 10002 9379
10005 9382 10003 9386 10002 9390 10002 9395 9999 9398 10002 9402
10001 9406 9997 9410 9994 9412 9990 9414 9987 9418 9984 9421
9980 9422 9976 9419 9976 9415 9972 9413 9968 9410 9964 9407
9961 9405 9957 9403 50 P
9957 9403 9953 9400 9950 9397 9949 9390 4 P
9905 9385 9907 9381 9911 9381 9916 9381 9920 9380 9924 9378
9928 9376 9930 9372 9933 9369 9938 9370 9942 9369 9946 9371
9950 9373 9947 9377 9952 9377 9954 9380 9957 9384 9955 9387
9950 9387 9947 9390 9948 9394 9946 9398 9942 9396 9937 9396
9933 9394 9928 9394 9924 9396 9921 9399 9916 9398 9913 9396
9909 9399 9906 9397 9908 9392 9905 9385 34 P
9908 9359 9913 9358 9917 9358 9916 9354 9921 9353 9923 9350
9927 9347 9931 9350 9931 9354 9933 9358 9930 9361 9933 9365
9930 9368 9927 9371 9922 9372 9918 9372 9915 9369 9915 9364
9908 9359 19 P
10016 9407 10021 9409 10025 9409 10027 9413 10030 9416 10033 9413
10037 9413 10041 9417 10044 9420 10040 9422 10035 9420 10031 9423
10031 9427 10036 9429 10033 9432 10036 9436 10031 9436 10028 9438
10025 9435 10027 9430 10024 9427 10028 9425 10024 9423 10022 9419
10017 9419 10018 9414 10016 9407 27 P
9607 8814 9610 8807 9611 8800 9614 8794 9618 8788 9622 8783
9621 8776 9615 8772 9621 8769 9620 8762 9627 8764 9634 8764
9640 8767 9647 8770 9654 8772 9653 8779 9660 8776 9665 8772
9668 8778 9662 8783 9664 8790 9669 8786 9676 8789 9678 8782
9685 8784 9681 8790 9687 8793 9687 8800 9680 8800 9674 8804
9671 8810 9672 8816 9669 8823 9667 8830 9669 8836 9674 8842
9681 8843 9679 8849 9673 8854 9667 8857 9661 8861 9654 8860
9647 8858 9643 8852 9641 8845 9644 8838 9637 8835 9633 8829
9628 8824 9621 8821 50 P
9621 8821 9618 8816 9607 8814 3 P
9567 8708 9571 8710 9576 8711 9580 8711 9584 8713 9588 8715
9593 8713 9591 8709 9593 8704 9597 8707 9601 8707 9605 8709
9610 8710 9614 8712 9617 8715 9621 8717 9618 8719 9614 8722

9611 8725 9608 8728 9604 8730 9600 8728 9595 8727 9591 8729
9588 8726 9584 8725 9584 8729 9580 8729 9579 8724 9576 8722
9571 8719 9567 8717 9568 8713 9567 8708 34 P
9558 8649 9561 8645 9564 8649 9568 8651 9572 8653 9576 8655
9579 8652 9583 8650 9583 8654 9587 8657 9590 8660 9593 8664
9598 8664 9593 8666 9590 8664 9585 8663 9581 8660 9577 8661
9574 8665 9571 8669 9567 8669 9566 8664 9563 8662 9562 8658
9561 8653 9558 8649 26 P
9593 8637 9598 8636 9602 8634 9606 8636 9611 8634 9614 8637
9619 8637 9621 8640 9626 8638 9630 8637 9634 8634 9638 8636
9639 8640 9639 8644 9639 8649 9635 8651 9639 8653 9640 8649
9644 8652 9646 8656 9644 8659 9645 8664 9640 8665 9638 8661
9634 8659 9630 8660 9626 8658 9629 8654 9627 8650 9622 8650
9618 8649 9616 8652 9613 8656 9611 8651 9607 8649 9604 8645
9600 8644 9596 8641 9593 8637 39 P
9641 8739 9637 8737 9633 8735 9631 8731 9634 8727 9635 8723
9640 8722 9643 8725 9647 8728 9651 8731 9655 8732 9659 8734
9663 8736 9658 8736 9654 8735 9650 8737 9645 8738 9641 8739
18 P
9580 8434 9583 8431 9587 8428 9590 8425 9590 8429 9590 8434
9593 8432 9598 8431 9602 8428 9606 8429 9610 8426 9610 8430
9607 8434 9603 8435 9599 8438 9596 8441 9592 8444 9588 8446
9584 8447 9588 8444 9591 8441 9587 8442 9585 8438 9580 8434
24 P
9904 5701 9908 5697 9910 5691 9913 5686 9919 5684 9922 5679
9927 5680 9926 5685 9928 5691 9929 5697 9929 5703 9924 5704
9918 5705 9913 5703 9904 5701 15 P
9315 5477 9319 5475 9324 5476 9326 5480 9330 5484 9334 5486
9335 5491 9332 5494 9328 5495 9323 5493 9319 5490 9321 5486
9318 5482 9315 5477 14 P
8866 6170 8872 6167 8878 6162 8882 6156 8889 6154 8896 6156
8902 6159 8902 6166 8907 6172 8912 6176 8919 6180 8924 6185
8931 6185 8938 6185 8945 6182 8942 6188 8940 6195 8939 6202
8935 6208 8930 6203 8928 6210 8921 6207 8920 6200 8918 6207
8914 6201 8911 6195 8911 6202 8912 6209 8905 6209 8901 6203
8897 6197 8896 6190 8892 6196 8889 6202 8883 6198 8880 6192
8876 6186 8882 6188 8885 6182 8878 6182 8884 6179 8891 6179
8898 6179 8896 6172 8889 6168 8886 6162 8881 6167 8875 6172
8872 6178 8866 6170 50 P
8870 6241 8875 6237 8880 6242 8886 6238 8891 6232 8893 6225
8897 6219 8899 6226 8898 6232 8902 6238 8899 6244 8896 6251
8889 6251 8882 6249 8875 6248 8870 6241 16 P
8828 6183 8835 6185 8842 6186 8849 6186 8851 6193 8852 6200

8856 6206 8855 6213 8854 6220 8860 6223 8866 6228 8859 6229
8853 6234 8848 6229 8846 6222 8846 6215 8846 6208 8841 6202
8846 6198 8844 6191 8837 6190 8828 6183 22 P
8840 6064 8845 6063 8849 6064 8854 6064 8852 6068 8855 6071
8852 6075 8851 6080 8851 6084 8847 6085 8844 6088 8843 6084
8842 6079 8844 6075 8844 6070 8840 6064 16 P
8801 5982 8805 5978 8807 5974 8807 5970 8811 5967 8812 5972
8816 5975 8819 5978 8820 5983 8818 5987 8813 5987 8809 5985
8801 5982 13 P
8894 6286 8896 6284 8897 6280 8898 6284 8900 6286 8903 6288
8906 6289 8907 6293 8904 6295 8901 6297 8899 6295 8898 6292
8895 6289 8894 6286 14 P
9121 6141 9121 6136 9122 6132 9123 6128 9124 6123 9125 6119
9126 6123 9124 6128 9124 6132 9124 6137 9126 6141 9127 6146
9127 6150 9129 6155 9130 6159 9133 6163 9135 6167 9137 6170
9137 6175 9134 6171 9132 6167 9129 6163 9128 6159 9124 6155
9123 6151 9122 6147 9121 6141 27 P
8918 7285 8924 7288 8931 7291 8938 7290 8943 7295 8942 7288
8942 7280 8935 7282 8932 7275 8937 7271 8933 7264 8938 7259
8943 7255 8950 7252 8955 7247 8960 7242 8967 7240 8971 7247
8967 7253 8961 7257 8963 7264 8969 7267 8976 7271 8981 7276
8988 7277 8993 7282 9000 7283 9005 7278 9012 7281 9012 7288
9005 7289 8999 7290 8992 7294 8987 7299 8985 7306 8985 7313
8987 7319 8993 7324 8999 7328 9006 7330 9010 7324 9016 7326
9021 7332 9025 7338 9018 7337 9010 7337 9003 7339 8999 7345
8992 7342 8995 7348 50 P
8995 7348 9000 7354 8996 7360 8990 7363 8985 7368 8978 7366
8972 7369 8968 7375 8967 7382 8972 7387 8976 7393 8969 7393
8962 7390 8957 7385 8954 7378 8948 7382 8943 7376 8941 7370
8933 7369 8929 7364 8926 7358 8926 7351 8930 7344 8936 7346
8941 7352 8948 7352 8951 7346 8949 7339 8946 7332 8946 7325
8945 7318 8944 7311 8950 7308 8955 7312 8958 7319 8963 7314
8971 7313 8971 7306 8966 7302 8959 7298 8952 7298 8947 7304
8940 7305 8933 7306 8929 7300 8922 7299 8920 7292 8918 7285
48 P
8800 7143 8805 7138 8802 7132 8807 7127 8814 7126 8820 7123
8826 7127 8833 7129 8839 7125 8837 7118 8836 7111 8842 7108
8849 7106 8856 7106 8859 7112 8853 7118 8857 7124 8864 7125
8869 7130 8862 7128 8857 7133 8852 7128 8845 7128 8839 7132
8833 7136 8828 7141 8828 7148 8821 7150 8819 7156 8814 7151
8810 7145 8800 7143 32 P
8200 6667 8205 6662 8209 6657 8216 6654 8223 6651 8229 6655
8231 6661 8235 6667 8241 6672 8247 6675 8254 6677 8261 6678

8268 6677 8274 6673 8280 6669 8286 6665 8293 6669 8297 6675
8297 6682 8302 6687 8298 6693 8297 6701 8294 6707 8292 6714
8289 6720 8282 6722 8279 6728 8274 6733 8269 6728 8263 6724
8265 6717 8260 6711 8254 6708 8254 6701 8249 6695 8242 6696
8235 6698 8229 6702 8224 6707 8219 6711 8213 6708 8207 6703
8208 6696 8215 6700 8212 6693 8214 6687 8216 6679 8219 6672
8212 6671 8200 6667 50 P
8088 6694 8093 6688 8096 6682 8102 6678 8109 6675 8116 6673
8123 6672 8129 6676 8134 6681 8140 6685 8145 6690 8146 6697
8149 6704 8156 6705 8156 6712 8156 6720 8156 6727 8152 6732
8145 6729 8141 6735 8136 6740 8129 6738 8124 6742 8117 6745
8110 6747 8111 6739 8105 6735 8104 6728 8100 6722 8095 6717
8091 6711 8093 6704 8100 6705 8105 6710 8112 6713 8113 6706
8107 6702 8100 6699 8088 6694 39 P
7690 6184 7697 6184 7704 6186 7711 6188 7718 6186 7717 6178
7723 6182 7729 6185 7736 6187 7743 6188 7750 6190 7757 6190
7754 6197 7746 6198 7745 6205 7745 6212 7745 6219 7748 6226
7752 6232 7759 6234 7766 6231 7772 6228 7779 6228 7780 6236
7775 6241 7775 6248 7777 6255 7783 6259 7782 6265 7788 6269
7793 6265 7800 6266 7802 6273 7809 6275 7801 6275 7797 6281
7791 6284 7786 6289 7780 6285 7773 6286 7769 6280 7762 6281
7756 6278 7753 6285 7749 6291 7743 6295 7737 6299 7732 6304
7725 6300 7724 6293 50 P
7724 6293 7726 6287 7726 6279 7722 6274 7717 6268 7715 6262
7710 6256 7705 6251 7708 6243 7714 6240 7718 6245 7725 6247
7731 6243 7738 6243 7745 6242 7740 6237 7738 6230 7733 6225
7728 6221 7722 6216 7717 6210 7713 6205 7714 6198 7710 6203
7703 6200 7698 6195 7690 6184 27 P
7526 6113 7522 6119 7516 6121 7509 6120 7503 6115 7497 6110
7493 6105 7497 6099 7504 6097 7511 6096 7518 6097 7526 6096
7533 6094 7538 6090 7545 6087 7552 6087 7558 6089 7566 6090
7573 6088 7574 6081 7581 6084 7586 6079 7593 6083 7596 6089
7595 6096 7594 6103 7600 6107 7597 6114 7591 6117 7589 6124
7590 6132 7583 6132 7578 6126 7576 6119 7569 6120 7564 6126
7558 6129 7551 6127 7547 6121 7544 6115 7538 6110 7535 6104
7526 6113 43 P
7527 6180 7520 6179 7513 6179 7510 6173 7506 6167 7498 6166
7491 6165 7493 6157 7499 6154 7506 6153 7513 6151 7520 6149
7522 6155 7523 6163 7527 6169 7533 6172 7527 6180 17 P
7483 6175 7488 6170 7495 6169 7502 6168 7508 6173 7505 6180
7506 6187 7511 6192 7514 6199 7507 6200 7501 6196 7500 6189
7496 6183 7490 6180 7483 6175 15 P
7334 6043 7339 6038 7343 6031 7349 6028 7355 6024 7360 6018

7366 6016 7373 6018 7378 6013 7381 6006 7387 6004 7394 6002
7388 5997 7392 5991 7398 5987 7405 5987 7407 5980 7414 5980
7417 5987 7422 5992 7415 5995 7408 5992 7403 5997 7402 6004
7402 6011 7404 6018 7402 6025 7398 6031 7397 6038 7392 6044
7385 6046 7379 6042 7372 6042 7365 6043 7360 6049 7356 6054
7348 6055 7342 6051 7334 6043 39 P
8712 7276 8716 7270 8720 7265 8721 7257 8724 7251 8731 7254
8736 7258 8743 7258 8744 7265 8741 7272 8741 7279 8740 7286
8743 7293 8745 7299 8748 7306 8746 7313 8750 7319 8750 7326
8750 7333 8746 7340 8740 7337 8737 7331 8733 7325 8727 7321
8726 7313 8724 7307 8721 7300 8719 7293 8717 7287 8712 7276
30 P
8841 7629 8848 7628 8855 7628 8862 7626 8869 7625 8875 7621
8881 7618 8889 7617 8895 7620 8895 7627 8893 7634 8890 7641
8888 7647 8882 7650 8875 7653 8869 7658 8864 7654 8857 7652
8852 7646 8847 7640 8841 7629 21 P
8846 7624 8850 7622 8853 7619 8857 7616 8862 7616 8866 7617
8871 7618 8874 7620 8871 7623 8867 7624 8862 7625 8858 7626
8853 7627 8846 7624 14 P
8893 7958 8895 7954 8898 7950 8898 7946 8902 7946 8904 7950
8908 7947 8912 7946 8916 7947 8921 7948 8926 7949 8927 7953
8931 7956 8934 7959 8939 7959 8942 7961 8946 7964 8945 7969
8941 7971 8936 7970 8933 7967 8932 7963 8930 7959 8926 7960
8927 7964 8922 7965 8918 7963 8914 7965 8909 7966 8905 7965
8901 7965 8897 7964 8893 7958 33 P
9225 8131 9227 8128 9230 8124 9234 8122 9238 8119 9242 8117
9246 8117 9250 8119 9254 8121 9258 8122 9263 8122 9259 8124
9255 8127 9254 8131 9252 8135 9250 8139 9245 8139 9242 8143
9238 8143 9234 8143 9230 8139 9227 8137 9225 8131 23 P
10666 6777 10661 6779 10657 6777 10653 6776 10648 6778 10644 6776
10642 6771 10638 6770 10636 6766 10632 6764 10635 6761 10640 6761
10645 6760 10649 6758 10653 6758 10657 6759 10662 6761 10667 6760
10671 6758 10675 6761 10678 6764 10682 6766 10684 6770 10689 6773
10685 6775 10681 6777 10676 6778 10672 6780 10666 6777 29 P
11075 6565 11078 6562 11083 6561 11087 6560 11092 6561 11096 6561
11100 6563 11104 6566 11106 6570 11103 6574 11098 6576 11094 6576
11089 6576 11085 6574 11081 6572 11077 6570 11075 6565 17 P
11528 6945 11530 6941 11534 6938 11538 6938 11543 6937 11547 6938
11551 6940 11555 6943 11557 6946 11558 6951 11556 6955 11553 6958
11548 6959 11543 6960 11539 6960 11535 6958 11531 6955 11528 6952
11528 6945 19 P
11761 7064 11763 7060 11766 7057 11770 7055 11775 7054 11779 7054
11783 7058 11787 7059 11791 7057 11796 7057 11800 7058 11804 7059

11806 7064 11805 7068 11803 7072 11801 7076 11799 7080 11796 7082
11791 7084 11787 7086 11783 7088 11778 7090 11774 7090 11771 7087
11770 7082 11771 7078 11770 7073 11766 7071 11762 7073 11761 7064
30 P
11528 7005 11530 7003 11534 7002 11537 7000 11540 6999 11541 7002
11539 7005 11536 7007 11533 7009 11530 7007 11528 7005 11 P
11195 6689 11193 6687 11192 6684 11192 6680 11194 6677 11196 6675
11198 6672 11201 6670 11204 6670 11207 6672 11210 6674 11212 6676
11213 6679 11213 6683 11211 6686 11207 6686 11204 6686 11200 6687
11195 6689 19 P
11192 6736 11189 6737 11188 6740 11187 6743 11184 6745 11181 6746
11178 6745 11174 6744 11173 6741 11176 6739 11174 6736 11171 6734
11168 6734 11171 6733 11174 6732 11178 6733 11181 6732 11185 6733
11187 6730 11191 6728 11193 6730 11195 6733 11192 6736 23 P
11233 6730 11234 6727 11238 6725 11241 6725 11244 6728 11246 6730
11246 6734 11245 6737 11242 6739 11240 6741 11237 6741 11235 6738
11234 6735 11233 6730 14 P
11156 6664 11158 6662 11161 6661 11165 6662 11164 6665 11162 6668
11158 6668 11156 6664 8 P
11164 6670 11167 6671 11170 6674 11172 6676 11173 6679 11170 6681
11167 6678 11165 6676 11164 6670 9 P
10966 6636 10968 6636 10970 6637 10972 6638 10975 6639 10972 6639
10970 6639 10969 6638 10966 6636 9 P
10880 7536 10881 7534 10884 7534 10886 7534 10888 7535 10889 7537
10892 7536 10894 7537 10896 7537 10898 7537 10898 7540 10896 7541
10895 7543 10893 7543 10891 7544 10889 7543 10887 7543 10885 7542
10883 7541 10881 7539 10880 7536 21 P
10921 7532 10923 7530 10924 7529 10926 7530 10925 7532 10924 7534
10921 7532 7 P
10957 7633 10958 7632 10961 7631 10963 7632 10964 7634 10963 7635
10961 7637 10959 7637 10958 7636 10957 7633 10 P
10145 7416 10147 7415 10149 7414 10150 7416 10152 7417 10152 7419
10150 7420 10148 7420 10145 7419 10145 7416 10 P
10164 7336 10165 7334 10168 7335 10168 7337 10164 7336 5 P
10096 6965 10097 6963 10099 6962 10101 6961 10103 6960 10105 6960
10108 6961 10109 6960 10111 6961 10112 6963 10110 6964 10109 6966
10108 6968 10106 6969 10104 6969 10102 6968 10101 6966 10099 6965
10096 6965 19 P
8935 6010 8937 6008 8936 6006 8938 6004 8939 6003 8940 6005
8940 6007 8942 6009 8942 6011 8942 6013 8940 6014 8938 6013
8935 6010 13 P
8749 6089 8751 6087 8753 6088 8755 6088 8757 6090 8757 6092
8755 6093 8753 6094 8751 6094 8750 6091 8749 6089 11 P

8906 6005 8907 6003 8909 6003 8912 6003 8913 6005 8912 6008
8909 6008 8907 6007 8906 6005 9 P
8857 6069 8859 6067 8861 6067 8862 6069 8864 6071 8866 6071
8868 6071 8869 6073 8868 6075 8866 6075 8864 6075 8861 6075
8860 6073 8859 6071 8857 6069 15 P
8850 6141 8851 6139 8853 6139 8855 6140 8858 6140 8860 6141
8862 6141 8864 6143 8865 6145 8867 6145 8868 6147 8866 6148
8864 6147 8861 6146 8859 6145 8857 6145 8855 6143 8854 6141
8852 6143 8851 6144 8850 6141 21 P
8850 6015 8852 6014 8854 6014 8855 6016 8853 6017 8850 6015

6:00 PM

8854 6006 8857 6007 8859 6008 8860 6010 8859 6012 8857 6012
8856 6010 8854 6006 8 P
8165 6666 8167 6664 8171 6664 8174 6663 8176 6665 8179 6667
8182 6665 8185 6668 8185 6671 8188 6672 8191 6672 8193 6675
8193 6678 8194 6682 8193 6684 8190 6687 8187 6689 8184 6690
8181 6688 8178 6688 8174 6687 8174 6683 8174 6679 8173 6676
8169 6675 8168 6671 8168 6668 8165 6666 28 P
7950 6697 7954 6694 7958 6692 7961 6689 7964 6685 7967 6681
7971 6679 7975 6677 7979 6678 7984 6678 7988 6678 7993 6679
7991 6684 7990 6688 7986 6689 7981 6688 7977 6687 7973 6689
7971 6692 7967 6696 7963 6695 7959 6694 7955 6696 7950 6697
24 P
7953 6699 7957 6696 7961 6696 7965 6698 7970 6696 7973 6693
7975 6689 7980 6689 7984 6691 7988 6692 7992 6690 7994 6685
7998 6684 8003 6687 8007 6687 8006 6691 8003 6694 7999 6697
7997 6701 7993 6704 7988 6705 7984 6706 7979 6708 7975 6707
7971 6706 7966 6705 7961 6703 7957 6702 7953 6699 29 P
8022 6663 8026 6661 8031 6663 8034 6660 8039 6663 8042 6665
8039 6667 8034 6669 8030 6668 8022 6663 10 P
7979 6675 7983 6673 7987 6671 7991 6669 7995 6668 7997 6673
7993 6675 7988 6676 7984 6676 7979 6675 10 P
7942 6708 7943 6704 7945 6701 7947 6700 7951 6698 7952 6702
7950 6704 7947 6707 7946 6709 7944 6712 7942 6708 11 P
8622 7035 8627 7034 8631 7033 8633 7029 8635 7025 8639 7023
8638 7027 8640 7032 8643 7034 8646 7038 8645 7042 8647 7046
8644 7049 8644 7054 8647 7057 8651 7058 8655 7061 8655 7066
8650 7064 8646 7061 8642 7061 8638 7059 8635 7055 8631 7054
8631 7049 8633 7046 8636 7042 8636 7038 8632 7035 8627 7037
8622 7035 31 P
9031 7668 9035 7666 9039 7665 9043 7667 9047 7667 9051 7670
9055 7672 9057 7676 9059 7680 9054 7681 9050 7681 9048 7677
9044 7674 9041 7678 9037 7678 9039 7682 9035 7685 9034 7681

9032 7677 9033 7672 9031 7668 21 P
9075 7721 9070 7720 9067 7717 9063 7714 9060 7712 9060 7707
9059 7703 9058 7699 9062 7695 9062 7700 9062 7705 9064 7708
9068 7710 9073 7711 9077 7709 9081 7706 9083 7709 9082 7714
9079 7716 9075 7721 20 P
9042 7573 9043 7570 9046 7567 9049 7568 9052 7570 9055 7573
9053 7575 9049 7575 9046 7575 9042 7573 10 P
9062 7557 9066 7559 9070 7561 9073 7564 9076 7568 9072 7567
9067 7568 9065 7564 9062 7557 9 P
9316 7804 9320 7802 9324 7805 9326 7808 9331 7807 9329 7811
9326 7815 9322 7814 9318 7811 9316 7804 10 P
9309 7812 9312 7813 9313 7810 9316 7812 9318 7814 9316 7817
9313 7819 9311 7816 9309 7812 9 P
9128 7911 9131 7908 9135 7905 9140 7906 9143 7909 9143 7913
9148 7914 9152 7916 9153 7920 9154 7924 9154 7929 9151 7932
9147 7933 9144 7930 9142 7926 9140 7922 9137 7918 9134 7916
9128 7911 19 P
9084 7869 9088 7868 9093 7868 9095 7873 9099 7872 9103 7873
9104 7877 9104 7882 9104 7886 9100 7887 9099 7883 9095 7885
9090 7883 9087 7881 9087 7876 9084 7869 16 P
8948 7937 8949 7933 8951 7931 8954 7931 8956 7929 8960 7930
8963 7931 8965 7934 8968 7934 8972 7933 8974 7936 8976 7939
8973 7941 8970 7942 8967 7944 8964 7943 8961 7942 8958 7941
8954 7940 8951 7939 8948 7937 21 P
9470 8718 9473 8716 9476 8715 9479 8712 9482 8711 9485 8709
9487 8707 9490 8705 9493 8704 9497 8703 9500 8704 9503 8705
9506 8707 9508 8709 9510 8712 9510 8716 9508 8718 9505 8720
9502 8721 9499 8722 9496 8722 9492 8722 9489 8721 9485 8721
9482 8721 9479 8720 9476 8719 9470 8718 28 P
9422 8731 9425 8730 9427 8727 9429 8724 9432 8723 9436 8722
9439 8721 9442 8723 9442 8726 9442 8730 9439 8732 9437 8735
9436 8738 9433 8740 9430 8741 9426 8742 9424 8743 9420 8744
9417 8744 9417 8741 9418 8738 9419 8735 9422 8731 23 P
9586 8614 9589 8613 9591 8612 9594 8612 9596 8614 9598 8617
9597 8619 9595 8621 9593 8623 9591 8621 9590 8618 9588 8616
9586 8614 13 P
9655 8521 9657 8520 9658 8521 9660 8523 9660 8525 9661 8527
9659 8527 9657 8526 9657 8524 9655 8521 10 P
9648 8527 9651 8527 9651 8525 9653 8524 9655 8525 9657 8527
9659 8528 9660 8530 9662 8532 9663 8534 9662 8535 9660 8535
9657 8535 9656 8533 9655 8531 9654 8530 9652 8530 9648 8527
18 P
9829 9037 9831 9035 9834 9034 9836 9032 9840 9030 9842 9033

9841 9036 9842 9038 9846 9039 9846 9042 9842 9043 9843 9047
9841 9050 9840 9053 9837 9054 9834 9053 9833 9049 9832 9046
9830 9043 9829 9037 20 P
9837 9024 9839 9021 9842 9019 9845 9018 9848 9020 9850 9022
9848 9025 9845 9026 9842 9027 9837 9024 10 P
9964 9034 9968 9034 9971 9035 9969 9032 9971 9030 9970 9027
9967 9025 9969 9022 9973 9023 9974 9021 9977 9023 9979 9025
9982 9023 9982 9027 9983 9030 9985 9033 9987 9035 9984 9036
9981 9036 9977 9037 9977 9041 9977 9044 9974 9043 9971 9043
9970 9040 9967 9037 9964 9034 27 P
9965 8996 9968 8996 9971 8997 9973 9001 9975 8999 9978 9000
9981 9002 9983 9004 9986 9006 9988 9009 9985 9011 9982 9010
9979 9010 9976 9008 9973 9007 9971 9005 9971 9001 9968 8999
9965 8996 19 P
9940 9189 9942 9187 9944 9189 9947 9192 9949 9193 9952 9195
9952 9199 9952 9202 9954 9205 9957 9204 9956 9207 9955 9210
9953 9213 9950 9212 9947 9211 9946 9207 9947 9204 9947 9201
9943 9200 9944 9198 9945 9194 9943 9192 9940 9189 23 P
9946 9165 9950 9165 9952 9163 9955 9163 9958 9164 9958 9168
9955 9170 9958 9172 9960 9175 9962 9177 9966 9178 9969 9178
9969 9182 9973 9182 9976 9183 9973 9184 9969 9184 9967 9182
9964 9180 9962 9178 9958 9178 9956 9176 9953 9176 9949 9176
9947 9174 9949 9171 9946 9165 27 P
9906 9143 9909 9142 9912 9141 9914 9139 9916 9136 9920 9136
9922 9138 9923 9141 9923 9145 9926 9147 9928 9149 9924 9150
9923 9147 9920 9145 9917 9143 9914 9142 9912 9145 9913 9148
9910 9149 9907 9150 9908 9147 9906 9143 22 P
10089 9560 10090 9557 10093 9559 10096 9558 10098 9561 10101 9562
10102 9565 10104 9568 10107 9569 10111 9569 10110 9572 10106 9572
10103 9574 10101 9571 10098 9570 10098 9567 10096 9564 10094 9562
10090 9564 10089 9560 20 P
10094 9721 10097 9720 10100 9718 10103 9717 10106 9715 10109 9713
10111 9711 10114 9709 10115 9712 10118 9713 10122 9712 10125 9712
10127 9709 10130 9707 10130 9710 10131 9714 10128 9715 10125 9714
10122 9714 10119 9716 10116 9716 10112 9717 10110 9720 10106 9720
10106 9723 10104 9726 10101 9726 10098 9728 10095 9727 10096 9724
10094 9721 31 P
10051 9858 10053 9855 10056 9855 10060 9855 10063 9854 10066 9855
10068 9858 10067 9861 10065 9864 10062 9865 10058 9863 10056 9862
10051 9858 13 P
10054 9867 10057 9866 10060 9867 10063 9867 10067 9867 10069 9866
10071 9863 10074 9861 10077 9862 10078 9866 10076 9868 10074 9871
10071 9873 10069 9875 10067 9877 10063 9875 10061 9873 10058 9871

10054 9867 19 P
10039 9863 10040 9860 10044 9861 10047 9861 10047 9865 10048 9868
10047 9871 10043 9872 10041 9871 10040 9867 10039 9863 11 P
10021 9880 10020 9877 10021 9874 10020 9871 10022 9868 10025 9868
10028 9871 10030 9872 10029 9875 10027 9878 10024 9880 10021 9880
12:00 PM
10001 9853 10003 9849 10003 9846 10005 9845 10009 9845 10011 9847
10013 9850 10015 9853 10015 9856 10014 9859 10011 9861 10008 9861
10005 9859 10002 9857 10001 9853 15 P
10280 10369 10284 10368 10286 10366 10290 10365 10293 10366 10294 10368
10298 10368 10298 10372 10295 10372 10292 10370 10290 10372 10287 10375
10284 10376 10283 10373 10285 10370 10280 10369 16 P
10252 10351 10256 10352 10259 10352 10262 10350 10265 10350 10266 10353
10263 10354 10260 10355 10257 10355 10252 10351 10 P
10268 10351 10271 10349 10274 10351 10277 10352 10275 10355 10272 10357
10270 10354 10268 10351 8 P
10334 10338 10337 10336 10339 10337 10343 10339 10339 10341 10334 10338
6:00 PM
9995 10209 9998 10207 10001 10207 10003 10204 10004 10201 10008 10202
10010 10204 10010 10208 10011 10210 10015 10211 10011 10213 10009 10214
10005 10213 10004 10210 10001 10211 9995 10209 16 P
9991 10235 9994 10233 9996 10230 9999 10232 10001 10235 10002 10238
10004 10240 10001 10239 9998 10241 9997 10238 9994 10239 9991 10235
12:00 PM
10100 10241 10103 10241 10106 10243 10109 10245 10108 10248 10104 10249
10106 10245 10102 10245 10100 10241 9 P
10095 10173 10098 10172 10101 10173 10102 10176 10103 10179 10101 10180
10098 10179 10095 10177 10095 10173 9 P
10124 10175 10127 10175 10130 10173 10132 10171 10136 10170 10137 10173
10137 10177 10137 10180 10137 10183 10134 10182 10134 10178 10130 10177
10127 10178 10124 10175 14 P
10191 10128 10194 10128 10197 10129 10200 10129 10201 10125 10204 10123
10207 10124 10209 10127 10205 10129 10203 10131 10203 10134 10200 10137
10197 10136 10195 10132 10191 10128 15 P
10207 10018 10210 10017 10213 10015 10216 10015 10219 10014 10221 10017
10220 10020 10217 10021 10213 10022 10210 10022 10207 10018 11 P
10285 10011 10288 10009 10289 10006 10291 10003 10293 10001 10294 10005
10294 10008 10293 10011 10292 10014 10289 10015 10285 10011 11 P
10304 10011 10305 10008 10307 10006 10311 10005 10311 10002 10312 9999
10314 10001 10313 10005 10313 10008 10311 10011 10309 10013 10304 10011
12:00 PM
10317 10011 10318 10007 10320 10005 10323 10003 10323 10006 10322 10010
10323 10013 10321 10015 10317 10011 9 P

10325 9996 10326 9994 10329 9993 10331 9995 10330 9998 10329 10000
10325 9996 7 P
10204 9858 10206 9855 10207 9852 10210 9852 10211 9855 10209 9859
10209 9862 10205 9862 10204 9858 9 P
10065 10010 10066 10007 10069 10006 10071 10004 10074 10004 10076 10005
10077 10007 10076 10010 10074 10012 10072 10013 10069 10013 10065 10010
12:00 PM
9793 9393 9796 9392 9799 9390 9801 9388 9805 9388 9808 9389
9811 9389 9814 9390 9815 9393 9815 9397 9813 9399 9810 9399
9807 9398 9808 9394 9805 9393 9801 9393 9799 9396 9795 9396
9793 9393 19 P
9980 9158 9982 9155 9982 9152 9985 9154 9988 9156 9990 9158
9993 9160 9990 9162 9987 9161 9983 9161 9980 9158 11 P
9790 8789 9794 8789 9797 8791 9798 8795 9800 8797 9803 8798
9806 8799 9807 8803 9805 8805 9802 8805 9800 8802 9798 8800
9795 8798 9792 8796 9790 8793 9790 8789 16 P
8910 7381 8914 7378 8918 7377 8923 7377 8927 7378 8932 7379
8936 7377 8939 7381 8940 7385 8939 7389 8937 7393 8934 7390
8929 7388 8925 7387 8921 7384 8918 7387 8913 7387 8910 7381
18 P
8857 7378 8860 7375 8864 7374 8869 7373 8871 7377 8875 7379
8878 7382 8880 7386 8883 7390 8883 7395 8878 7394 8875 7391
8871 7388 8868 7385 8866 7381 8864 7377 8862 7381 8857 7378
18 P
8902 7388 8905 7388 8907 7388 8910 7390 8913 7390 8913 7393
8911 7395 8909 7397 8906 7396 8904 7395 8903 7392 8902 7388
12:00 PM
8926 7198 8928 7195 8927 7192 8927 7189 8929 7192 8932 7193
8935 7195 8938 7197 8935 7197 8932 7199 8931 7203 8934 7205
8933 7209 8929 7207 8928 7204 8926 7202 8926 7198 17 P
8930 7223 8932 7219 8934 7217 8937 7218 8940 7218 8943 7219
8947 7220 8949 7222 8947 7224 8945 7227 8945 7230 8942 7229
8939 7227 8936 7228 8936 7225 8930 7223 16 P
8209 6751 8212 6750 8215 6749 8218 6747 8221 6744 8223 6742
8227 6742 8230 6742 8230 6745 8232 6749 8234 6752 8232 6755
8229 6755 8226 6756 8222 6756 8219 6756 8215 6754 8212 6754
8209 6751 19 P
5903 5461 5904 5468 5908 5474 5902 5477 5908 5480 5915 5478
5917 5485 5916 5492 5916 5499 5921 5504 5924 5511 5930 5507
5936 5511 5937 5518 5933 5524 5927 5519 5921 5515 5914 5515
5908 5511 5903 5505 5896 5502 5893 5496 5886 5496 5879 5494
5872 5491 5866 5487 5862 5493 5854 5494 5847 5494 5843 5487
5837 5483 5832 5479 5825 5477 5827 5470 5822 5465 5816 5461

5816 5454 5816 5453 38 P
5839 5453 5846 5454 5847 5453 3 P
5861 5453 5864 5455 5871 5458 5879 5459 5885 5461 5890 5456
5890 5453 7 P
5910 5453 5911 5454 2 P
5911 5454 5903 5461 2 P
5967 5480 5971 5474 5974 5468 5981 5464 5988 5463 5992 5456
5999 5459 6005 5455 6006 5453 9 P
6023 5453 6024 5459 6029 5454 6029 5453 4 P
6040 5453 6038 5458 6042 5465 6038 5470 6034 5477 6031 5483
6024 5485 6017 5488 6010 5489 6004 5484 5997 5483 5990 5485
5984 5488 5977 5488 5967 5480 15 P
5969 5453 5969 5459 5963 5459 5962 5453 5961 5453 5 P
6054 5459 6056 5458 6058 5456 6058 5454 6059 5455 6062 5456
6063 5458 6064 5460 6063 5462 6064 5465 6063 5467 6061 5466
6059 5465 6057 5465 6055 5463 6054 5459 16 P
5540 5469 5543 5465 5545 5461 5547 5457 5549 5453 5 P
5575 5453 5574 5458 5572 5462 5571 5466 5569 5470 5567 5475
5563 5478 5559 5480 5556 5483 5552 5486 5549 5482 5545 5480
5542 5476 5540 5469 14 P
5698 5567 5700 5563 5704 5560 5708 5563 5712 5565 5716 5566
5716 5571 5720 5572 5723 5576 5723 5580 5726 5585 5721 5585
5717 5587 5712 5588 5710 5584 5706 5581 5704 5578 5702 5573
5698 5567 19 P
5790 5572 5794 5569 5798 5566 5800 5562 5805 5561 5809 5561
5808 5566 5806 5570 5802 5573 5799 5575 5795 5572 5790 5572

12:00 PM

6251 5559 6253 5560 6254 5559 6257 5560 6258 5562 6258 5565
6260 5566 6259 5568 6257 5567 6254 5566 6253 5564 6252 5562
6251 5559 13 P
6239 5580 6241 5577 6242 5574 6244 5571 6247 5573 6250 5574
6254 5574 6257 5576 6259 5579 6257 5581 6253 5581 6250 5582
6246 5582 6243 5582 6239 5580 15 P
6252 5647 6253 5644 6257 5644 6259 5641 6260 5638 6263 5640
6262 5643 6259 5646 6256 5647 6256 5651 6252 5647 11 P
12523 8159 12525 8159 12525 8158 12526 8159 12526 8160 12526 8160
12525 8161 12524 8161 12523 8160 12523 8159 10 P
12911 11281 12912 11281 12914 11281 12915 11281 12917 11282 12919 11283
12920 11284 12921 11283 12922 11281 12923 11282 12924 11284 12925 11286
12926 11287 12926 11288 12926 11290 12925 11290 12924 11289 12922 11289
12921 11289 12919 11289 12918 11289 12916 11290 12915 11288 12915 11287
12915 11285 12914 11283 12913 11283 12911 11282 12911 11281 29 P
12896 11256 12897 11256 12898 11256 12898 11256 12897 11258 12895 11258

12895 11257 12896 11256 12896 11256 9 P
12899 11256 12898 11255 12898 11254 12898 11252 12898 11250 12897 11249
12899 11248 12899 11250 12900 11251 12901 11252 12903 11252 12902 11253
12901 11254 12901 11256 12901 11257 12902 11259 12903 11260 12902 11262
12901 11262 12900 11261 12898 11261 12897 11261 12896 11259 12898 11258
12899 11256 25 P
12903 11265 12904 11263 12905 11265 12906 11266 12906 11267 12905 11267
12904 11267 12904 11266 12903 11266 12903 11265 10 P
12905 11271 12906 11271 12906 11272 12905 11272 12905 11271 5 P
12906 11272 12907 11272 12907 11272 12907 11272 12908 11272 12908 11274
12909 11274 12908 11275 12907 11275 12907 11274 12906 11273 12906 11272
12:00 PM
12909 11277 12909 11277 12910 11276 12911 11276 12911 11276 12912 11276
12911 11277 12911 11279 12909 11279 12909 11277 10 P
2297 14405 2329 14399 2320 14346 2297 14349 4 P
2297 14294 2325 14289 2373 14266 2380 14212 2412 14167 2459 14139
2490 14094 2522 14050 2508 13997 2489 13946 2507 13894 2525 13843
2543 13791 2555 13738 2563 13684 2530 13640 2486 13608 2467 13556
2480 13503 2507 13456 2547 13419 2601 13411 2629 13363 2652 13314
2670 13262 2722 13244 2773 13225 2823 13202 2836 13149 2869 13105
2912 13137 2941 13184 2927 13237 2935 13292 2948 13345 3001 13358
3052 13340 3107 13343 3146 13357 3135 13425 3179 13458 3186 13512
3188 13553 3151 13593 3104 13621 3057 13649 46 P
3057 13649 3016 13655 3013 13709 3026 13762 2994 13806 2971 13831
3020 13855 3048 13902 3103 13899 3110 13954 3115 13987 3085 14015
3121 14032 3130 14093 3185 14090 3239 14088 3293 14086 3347 14078
3394 14068 19 P
3394 14068 3397 14013 3416 13962 3466 13939 3513 13912 3560 13885
3610 13863 3651 13826 3684 13784 3718 13740 3770 13723 3819 13701
3861 13666 3905 13634 3957 13618 3972 13565 3946 13517 3950 13463
3938 13409 3930 13369 3863 13335 3862 13280 3850 13227 3859 13173
3879 13122 3873 13067 3866 13013 3812 13014 3757 13016 3702 13017
3648 13018 3593 13014 3540 13026 3490 13049 3450 13011 3464 12958
3483 12907 3452 12862 3397 12864 3342 12866 3288 12873 3233 12869
3186 12897 3153 12941 3120 12985 3065 12992 3011 13000 2944 13010
2907 12969 2904 12914 50 P
2904 12914 2918 12861 2877 12823 2834 12791 2801 12766 2792 12712
2847 12701 2895 12676 2930 12634 2930 12558 2869 12557 2869 12503
2838 12457 2808 12411 2787 12360 2792 12305 2814 12254 2820 12199
2813 12166 2773 12174 2756 12212 2702 12217 2648 12206 2599 12232
2545 12243 2502 12208 2472 12163 2423 12137 2439 12084 2481 12049
2507 12000 2486 11949 2460 11901 2407 11885 2352 11880 2299 11896
2297 11897 37 P

2297 10470 2312 10463 2357 10429 2381 10379 2436 10371 2492 10374
2547 10382 2601 10369 2604 10313 2618 10259 2663 10226 2718 10224
2774 10232 2821 10262 2865 10296 2898 10341 2931 10386 2963 10431
3000 10473 3037 10515 3017 10567 3045 10615 3100 10625 3155 10633
3184 10586 3238 10601 3270 10646 3293 10697 3325 10742 3332 10798
3364 10843 3400 10885 3440 10923 3484 10958 3528 10992 3574 11022
3591 11075 3553 11115 3519 11158 3497 11193 3533 11235 3560 11283
3564 11318 3605 11313 3608 11337 45 P
3608 11337 3611 11358 3556 11364 3501 11371 3448 11356 3396 11336
3357 11321 3315 11358 3278 11398 3253 11448 3219 11492 3164 11499
3140 11548 3125 11579 3081 11546 3043 11517 3009 11560 2980 11607
3000 11626 2967 11670 2917 11693 2862 11701 2808 11693 2753 11695
2698 11687 2646 11705 2599 11734 2561 11775 2534 11779 2536 11827
2481 11824 2420 11828 2410 11788 2354 11791 2297 11812 35 P
3394 14068 3432 14082 3418 14135 3409 14188 3427 14240 3449 14289
3477 14337 3517 14374 3560 14407 3614 14415 3619 14456 11 P
3619 14456 3567 14474 3530 14513 3511 14564 3474 14604 3427 14632
3373 14629 3319 14631 3278 14667 3234 14699 3194 14735 3166 14782
3212 14810 3252 14847 3265 14900 3273 14953 3309 14994 3336 15040
3376 15077 3408 15121 3395 15174 3386 15227 3363 15276 3319 15309
3268 15290 3215 15277 3165 15258 3112 15245 3063 15221 3023 15185
2990 15142 2962 15095 2944 15044 2925 14993 2917 14939 2904 14887
2880 14837 2877 14783 2845 14740 2836 14686 2844 14632 2830 14579
2807 14530 2754 14518 2707 14490 2654 14498 2600 14507 2546 14500
2499 14472 2452 14445 50 P
2452 14445 2398 14443 2344 14447 2297 14459 4 P
3619 14456 3668 14434 3718 14412 3755 14372 3758 14331 3805 14305
3859 14298 3914 14302 3968 14301 4022 14294 4083 14287 4094 14340
4095 14395 4107 14448 4135 14466 4165 14420 4222 14458 4212 14511
4182 14557 4204 14606 4230 14654 4266 14696 4304 14734 4347 14768
4389 14802 4424 14843 4466 14877 4511 14907 4546 14949 4585 14987
4627 15021 4672 15052 4722 15072 4772 15093 4761 15146 4757 15193
4802 15164 4837 15121 4874 15106 4878 15140 4931 15135 4945 15068
4998 15079 5023 15127 5061 15166 5106 15197 5159 15207 5186 15205
5167 15169 5155 15138 50 P
5155 15138 5185 15093 5208 15059 5183 15011 5174 14958 5129 14927
5095 14884 5085 14831 5070 14779 5044 14731 5040 14677 5020 14626
4974 14596 4933 14561 4888 14531 4854 14489 4848 14434 4842 14366
4877 14325 4903 14276 4924 14227 4935 14173 4930 14119 4925 14065
4871 14070 4866 14015 4876 13990 4846 13945 4812 13903 4760 13859
4752 13805 4744 13751 4739 13697 4739 13642 4750 13588 4745 13534
4740 13480 4734 13426 4730 13371 4735 13316 4740 13262 4746 13208
4752 13153 4758 13099 4742 13046 4721 12996 4691 12950 4657 12907

4636 12857 4620 12804 50 P
4620 12804 4594 12756 4584 12702 4578 12647 4557 12597 4580 12562
4615 12529 7 P
4615 12529 4604 12475 4634 12429 4624 12375 4654 12330 4693 12291
4689 12236 4705 12184 4667 12126 4718 12105 4757 12067 4768 12013
4814 11982 4840 11934 4841 11879 4836 11824 4813 11766 17 P
4813 11766 4745 11773 4729 11720 4674 11714 4639 11756 4608 11802
4566 11767 4512 11756 4459 11772 4406 11788 4354 11804 4300 11815
4246 11826 4192 11837 4136 11837 4136 11782 4189 11766 4240 11744
4260 11693 4239 11643 4294 11637 4344 11616 4387 11580 4408 11530
4429 11478 4378 11457 4326 11441 4275 11462 4221 11474 4166 11479
4131 11437 4099 11391 4083 11338 4099 11285 4114 11232 4119 11177
4113 11122 4113 11067 4093 11000 39 P
4093 11000 4037 11001 3981 11001 3943 11041 3909 11084 3874 11127
3835 11167 3784 11189 3742 11225 3700 11261 3662 11301 3638 11326
3608 11337 13 P
4093 11000 4147 10989 4141 10934 4138 10899 4193 10893 4241 10867
4280 10828 4329 10801 4335 10746 4329 10691 4324 10636 4318 10580
4313 10525 4270 10490 4219 10511 4163 10517 4112 10496 4069 10461
4026 10426 3976 10400 3937 10361 3895 10365 3899 10400 3847 10379
3792 10369 3799 10424 3743 10430 3700 10396 3653 10365 3600 10383
3581 10420 3536 10367 3480 10368 3448 10322 3483 10278 3513 10231
3501 10177 3483 10124 3477 10068 3473 10033 3403 10028 3423 9976
3462 9935 3469 9991 3506 10010 3531 9960 3557 9911 3605 9883
3621 9829 3589 9783 50 P
3589 9783 3635 9751 3684 9724 3715 9677 3719 9622 3754 9578
3781 9555 7 P
3781 9555 3787 9611 3819 9657 3840 9709 3867 9758 3903 9801
3939 9844 3991 9822 4024 9812 4040 9865 4090 9892 4145 9902
4201 9902 4254 9886 4297 9850 4347 9824 4401 9835 4453 9857
4458 9913 4431 9961 4452 10013 4504 10035 4555 10057 4625 10051
4621 10009 4677 10004 4732 9999 4788 9994 4824 9952 4871 9921
4922 9901 4978 9891 5033 9886 5087 9871 5127 9833 5168 9794
5204 9751 5248 9717 5302 9702 5357 9715 5412 9711 5467 9718
5510 9755 5555 9788 5632 9783 5629 9741 5601 9743 5581 9690
5577 9621 5630 9601 50 P
5630 9601 5686 9597 5719 9642 5758 9683 5795 9724 5837 9761
5885 9790 5882 9734 5927 9700 5971 9667 5942 9619 5980 9577
5938 9540 5904 9495 5882 9444 5902 9391 5945 9355 5988 9318
6043 9328 6103 9346 20 P
6103 9346 6117 9400 6165 9429 6206 9466 6244 9508 6297 9490
6352 9498 6403 9522 6458 9525 6512 9539 6549 9581 6602 9601
6656 9588 6669 9642 6724 9651 6778 9666 6815 9708 6817 9764

6873 9762 6929 9760 6971 9724 7022 9701 7078 9700 7143 9741
24 P
7143 9741 7127 9794 7118 9849 7119 9905 7104 9959 7059 9991
7044 10045 7045 10101 7046 10143 7008 10183 6979 10213 6981 10269
7022 10308 7062 10345 7106 10380 7076 10427 7034 10463 6986 10491
6933 10509 6883 10532 6841 10568 6819 10603 6804 10642 23 P
6804 10642 6735 10638 6731 10693 6712 10745 6675 10704 6628 10674
6574 10660 6519 10663 6471 10691 6442 10738 6434 10793 6427 10834
6373 10842 6304 10845 6266 10886 6233 10911 6196 10870 6158 10852
6135 10903 6090 10936 6057 10980 6044 11034 6058 11088 6061 11143
6069 11197 6028 11235 5991 11194 5943 11166 5888 11169 5856 11214
5812 11247 5758 11235 5703 11227 5648 11226 5593 11229 5538 11238
5483 11242 5429 11235 5373 11228 5324 11253 5281 11287 5237 11321
5190 11351 5144 11380 5095 11406 5052 11440 5016 11482 4980 11524
4940 11563 4929 11617 50 P
4929 11617 4902 11665 4871 11710 4841 11739 4813 11766 5 P
6804 10642 6817 10697 6840 10747 6880 10785 6924 10819 6977 10833
7060 10839 7067 10895 7069 10950 7070 11005 7071 11061 7067 11116
7069 11171 7076 11226 7082 11274 7054 11272 7053 11230 6998 11232
6963 11233 6923 11195 6878 11227 6844 11271 6790 11279 6742 11306
6744 11361 6746 11417 6708 11457 6658 11480 6610 11508 6613 11563
6615 11611 6656 11610 6666 11570 6721 11578 6796 11583 6804 11638
6822 11690 6818 11745 6841 11795 6888 11824 6941 11839 6948 11894
6949 11948 6940 12003 6942 12058 6982 12095 7037 12099 7084 12072
7132 12045 7147 11992 50 P
7147 11992 7161 11939 7209 11911 7264 11916 7315 11936 7359 11970
7405 12000 7400 12054 7380 12106 7381 12161 7382 12209 7328 12220
7278 12243 7240 12283 7220 12333 7221 12375 16 P
7221 12375 7170 12392 7116 12383 7060 12379 7006 12380 6951 12382
6906 12414 6887 12465 6868 12517 6827 12552 6789 12593 6737 12611
6682 12613 6635 12641 6637 12695 6669 12740 6706 12781 6724 12832
6775 12852 6827 12871 6867 12908 6894 12955 6933 12993 6980 13022
7023 13056 7072 13080 7108 13121 7154 13150 7207 13138 7247 13129
7238 13183 7210 13213 7167 13179 7114 13165 7062 13150 7012 13126
6960 13112 6917 13078 6868 13054 6869 13109 6897 13156 6924 13203
6930 13251 43 P
6930 13251 6887 13218 6859 13216 6836 13265 6782 13272 6738 13239
6695 13206 6654 13242 6599 13244 6545 13247 6498 13275 6451 13303
6399 13321 6350 13344 6300 13368 6246 13376 6206 13382 6165 13388
18 P
6165 13388 6157 13334 6149 13280 6141 13226 6127 13173 6104 13124
6090 13071 6082 13017 6068 12964 6049 12912 6021 12865 5984 12825
5936 12797 5882 12790 5828 12793 5774 12802 5719 12806 5665 12809

5610 12807 5555 12811 5503 12826 5449 12835 5394 12839 5384 12785
5380 12730 5328 12745 5273 12739 5228 12707 5187 12671 5142 12640
5089 12623 5035 12628 4981 12616 4931 12595 4877 12584 4832 12552
4778 12547 4729 12521 4674 12526 4634 12534 4615 12529 41 P
6165 13388 6133 13432 6110 13481 6087 13531 6064 13581 6109 13613
6149 13649 6193 13681 6201 13736 6204 13790 6232 13837 6265 13880
6293 13927 6321 13974 6349 14021 6367 14071 6349 14123 6331 14174
6312 14226 6290 14275 6271 14326 6243 14373 6197 14401 6151 14429
6169 14480 6172 14534 6186 14587 6194 14641 6197 14695 6241 14728
6277 14768 6285 14821 6322 14861 6363 14897 6399 14857 6453 14860
6504 14879 6540 14919 6542 14973 6570 15020 6588 15071 6606 15108
6569 15148 6555 15200 6558 15254 6514 15287 6463 15305 6414 15328
6374 15364 6402 15411 50 P
6402 15411 6434 15455 6487 15468 6539 15481 6588 15504 6620 15548
6566 15550 6513 15558 6520 15612 6524 15680 6483 15681 6486 15735
6440 15776 13 P
6440 15776 6433 15723 6419 15670 6376 15638 6323 15630 6270 15617
6230 15581 6183 15554 6129 15557 6086 15589 6032 15593 5978 15586
5940 15573 5914 15503 5860 15496 5817 15529 5772 15557 5718 15550
5677 15515 5644 15472 5614 15427 5590 15378 5561 15332 5537 15284
5517 15233 5473 15202 5419 15206 5373 15236 5309 15229 5299 15175
5269 15126 5215 15131 5155 15138 33 P
6440 15776 6433 15830 6414 15881 6382 15925 6346 15965 6390 15997
6417 16027 6389 16073 6395 16113 6371 16146 6407 16172 11 P
8148 16172 8163 16144 8184 16095 8200 16043 8252 16058 8304 16048
8358 16053 8413 16053 8460 16027 8514 16028 8545 16003 11 P
8545 16003 8570 16051 8595 16099 8605 16152 8598 16172 5 P
11247 15960 11193 15955 11139 15955 11160 16005 11180 16055 11186 16109
11160 16157 11148 16172 8 P
8545 16003 8508 15964 8474 15921 8427 15895 8465 15858 8414 15841
8384 15766 8426 15732 8468 15698 8520 15683 8551 15639 8599 15614
8653 15615 8693 15615 8705 15562 8712 15509 8729 15457 8755 15410
8786 15365 8835 15328 20 P
8060 14892 8055 14946 8030 14994 7995 15036 8003 15062 8024 15062
8020 15102 7966 15098 7926 15089 7892 15112 7908 15150 7860 15176
7855 15230 7856 15284 7906 15304 7936 15349 7894 15384 7869 15447
7821 15447 7788 15447 20 P
7788 15447 7750 15486 7729 15536 7730 15590 7769 15628 7822 15633
7817 15687 7763 15687 7710 15693 7656 15694 7606 15715 7581 15764
7547 15806 7518 15851 7489 15897 7443 15867 7398 15838 7366 15826
7375 15773 7380 15719 7353 15672 7301 15657 7247 15653 7195 15639
7152 15605 7121 15561 7086 15520 7059 15473 7057 15419 7026 15375
6986 15338 7010 15289 7009 15235 6996 15182 7025 15136 7072 15109

7116 15078 7165 15102 7219 15106 7273 15105 7314 15069 7366 15052
7411 15081 7446 15123 7504 15104 45 P
7504 15104 7555 15124 7594 15162 3 P
7594 15162 7643 15140 7693 15119 7688 15173 7719 15218 7714 15272
7709 15326 7730 15376 8 P
7730 15376 7770 15402 7788 15447 3 P
7504 15104 7514 15051 7558 15020 7568 14967 7573 14913 7593 14862
7567 14815 7531 14773 7505 14725 7494 14672 7495 14618 7507 14565
7534 14518 7583 14494 7637 14490 7691 14497 7744 14510 7796 14524
7832 14543 7870 14565 20 P
6930 13251 6932 13305 6895 13346 6862 13389 6863 13443 6881 13495
6917 13536 6960 13569 7006 13598 7050 13632 7077 13679 7104 13726
7126 13776 7143 13828 7170 13875 7222 13890 7276 13899 7325 13924
7364 13962 7403 14000 7438 14041 7474 14082 7513 14120 7555 14154
7609 14153 7664 14153 7718 14152 7772 14152 7815 14186 7845 14231
7876 14276 7926 14296 7974 14322 8006 14348 8033 14361 35 P
7221 12375 7276 12379 7314 12423 3 P
7314 12423 7320 12478 7332 12531 7381 12556 7434 12571 7488 12576
7538 12554 7577 12515 7615 12475 7630 12423 7651 12372 7676 12323
7725 12349 7746 12365 7788 12330 7822 12307 7875 12323 7926 12344
7960 12386 8009 12412 8064 12406 8099 12364 8114 12311 8114 12270
8169 12270 8220 12292 8236 12344 8235 12385 8274 12424 8308 12448
8356 12422 8382 12471 8403 12522 8423 12573 8438 12625 8493 12621
8548 12627 8585 12666 8626 12667 8634 12656 40 P
7314 12423 7356 12387 7390 12344 7435 12313 7485 12291 7537 12274
7578 12270 7577 12215 7593 12162 7635 12127 7680 12096 7735 12090
7789 12079 7842 12067 7888 12037 7931 11983 7935 11942 7909 11910
7963 11900 8018 11905 8071 11916 8117 11885 8096 11834 8091 11779
8065 11731 8039 11682 8013 11633 8044 11587 8098 11582 8141 11547
8157 11494 8157 11439 8183 11391 8219 11348 8274 11343 8328 11354
8382 11365 8412 11411 8442 11458 8495 11474 8530 11432 8573 11398
8609 11355 8610 11300 8589 11249 8551 11209 8498 11224 8458 11263
8404 11273 8349 11278 50 P
8349 11278 8319 11232 8354 11189 8365 11135 8344 11084 8299 11053
8268 11006 8226 10970 8212 10917 8218 10862 8241 10811 8281 10773
8328 10744 8377 10719 8329 10691 8314 10638 8321 10582 8344 10532
8391 10502 8444 10488 8499 10484 8622 10484 8678 10485 8733 10481
8774 10477 8830 10473 26 P
8830 10473 8881 10495 8936 10491 8973 10449 9009 10407 9056 10377
9108 10357 9162 10370 9209 10340 9216 10285 9261 10317 9314 10335
9338 10385 9391 10403 9446 10405 15 P
9446 10405 9479 10360 9524 10326 9564 10289 9600 10266 9615 10252

6:00 PM

8830 10473 8820 10418 8800 10367 8790 10311 8760 10265 8726 10221
8680 10189 8638 10153 8678 10114 8700 10063 8711 10038 8657 10026
8603 10009 8548 10008 8496 10029 8481 9975 8470 9920 8471 9865
8471 9809 8441 9762 8410 9715 8384 9665 8345 9626 8302 9590
8256 9558 8209 9528 8148 9494 27 P
8148 9494 8108 9533 8065 9569 8010 9580 7954 9585 7899 9591
7844 9603 7795 9629 7779 9683 7758 9735 7705 9751 7678 9784
7629 9758 7612 9705 7585 9656 7534 9635 7484 9609 7428 9605
7393 9649 7416 9700 7463 9730 7499 9773 7503 9815 7457 9846
7402 9853 7358 9818 7314 9783 7265 9758 7218 9728 7176 9733
7143 9741 31 P
8148 9494 8127 9442 8075 9421 8048 9371 8048 9315 8080 9268
8069 9213 8095 9164 8142 9133 8197 9122 8252 9112 8253 9055
8258 8999 8269 8944 8275 8888 8275 8866 16 P
8275 8866 8275 8846 8331 8852 8385 8869 8425 8829 8425 8773
8410 8719 8442 8672 8458 8618 8475 8564 8497 8512 8492 8456
8497 8414 8553 8420 8600 8389 8657 8385 8706 8412 8756 8440
8781 8490 8828 8522 8868 8483 8886 8429 8908 8378 8941 8331
8997 8327 9043 8359 9099 8372 9150 8395 9203 8375 9248 8340
9245 8301 31 P
8275 8866 8219 8866 8219 8810 8180 8770 8133 8738 8084 8711
8030 8695 7978 8673 7938 8634 7894 8598 7854 8638 7819 8682
7765 8666 7711 8650 7684 8600 7648 8557 7603 8522 7547 8522
7547 8466 7588 8457 7587 8401 7597 8345 7608 8289 7581 8240
7549 8193 7501 8162 7447 8147 7391 8137 7347 8101 7310 8058
7304 8002 7292 7946 7312 7893 7338 7843 7291 7812 7249 7773
7227 7721 7226 7678 7268 7677 7251 7623 7214 7580 7169 7544
7124 7509 7096 7460 7064 7413 7040 7361 7023 7306 7010 7251
7003 7194 6966 7151 50 P
6966 7151 6943 7099 6963 7045 6938 7001 6937 6959 5 P
6103 9346 6101 9290 6124 9239 6148 9188 6140 9133 6132 9077
6161 9029 6190 8981 6171 8928 6137 8882 6092 8849 6074 8796
6022 8771 5971 8748 5934 8706 5925 8650 5891 8605 5853 8563
5834 8510 5830 8454 5849 8400 5906 8403 5962 8394 5958 8338
5934 8287 5931 8230 5965 8184 5968 8128 5943 8077 5951 8020
5997 7986 6039 7949 6063 7897 6034 7848 6020 7793 5969 7725
6026 7727 6103 7739 6095 7682 6042 7663 6014 7656 6011 7600
5997 7544 5963 7498 5955 7442 5968 7387 5956 7323 47 P
3781 9555 3775 9499 3753 9448 3746 9392 3757 9337 3721 9294
3776 9282 3742 9213 8 P
3742 9213 3736 9157 3762 9107 3762 9050 3750 8995 3738 8940
3732 8884 3725 8821 3769 8785 3779 8729 3778 8687 3736 8687
3692 8652 3640 8631 3592 8600 3552 8560 3504 8530 3460 8494

3416 8459 3460 8404 3504 8439 3561 8433 3617 8438 3647 8467
3718 8467 3749 8419 3789 8379 3766 8327 3755 8271 3795 8231
3738 8231 3685 8210 3645 8198 3691 8127 3664 8077 3632 8030
3600 7983 3656 7977 3699 7972 3698 7916 3714 7876 3686 7810
42 P
3686 7810 3650 7766 3614 7723 3596 7668 3541 7680 3488 7702
3461 7652 3420 7665 3409 7691 3374 7667 3414 7627 3454 7577
3397 7577 3340 7583 3312 7586 3308 7629 3266 7633 3249 7579
3208 7504 3164 7540 3117 7573 3073 7609 3029 7574 2984 7538
2966 7484 2930 7440 2875 7424 2804 7412 2757 7444 2718 7486
2699 7524 2638 7485 2613 7537 2587 7588 2566 7641 2536 7689
2482 7708 2439 7713 2406 7666 2383 7614 2365 7560 2327 7517
2297 7515 43 P
3686 7810 3743 7810 3800 7804 3792 7733 3849 7727 3857 7798
3904 7830 3944 7870 4001 7870 4006 7813 4051 7777 4106 7766
4150 7802 4190 7842 4237 7874 4277 7914 4333 7926 4377 7890
4429 7912 4482 7934 4532 7908 4580 7877 4612 7830 4602 7774
4580 7721 4581 7665 4636 7649 4686 7645 4681 7588 4626 7570
4611 7515 4600 7460 4590 7403 4546 7366 4507 7325 4467 7284
4431 7240 4395 7195 4407 7139 4421 7114 4471 7141 4502 7190
4542 7231 4595 7210 4632 7166 4644 7110 4656 7054 4640 6999
4626 6950 4684 6946 50 P
4684 6946 4736 6968 4786 6996 4842 6986 4907 6976 5 P
4907 6976 4954 6994 5021 7017 5025 7060 5001 7112 5022 7165
5053 7214 5106 7193 5163 7190 5216 7169 5271 7154 5300 7105
5329 7055 5380 7029 5437 7037 5457 7091 5460 7148 5470 7204
5427 7243 5471 7281 5510 7323 5552 7361 5609 7357 5651 7319
5696 7284 5748 7259 5805 7256 5862 7259 5916 7278 5956 7323
30 P
5956 7323 5985 7273 5999 7218 6056 7221 6112 7230 6169 7238
6224 7252 6280 7261 6315 7215 6353 7173 6325 7141 6356 7111
6308 7079 6253 7062 6210 7025 6165 6989 6138 6938 6098 6897
6058 6857 6104 6823 6150 6789 6196 6755 6250 6774 6307 6777
6359 6803 6411 6778 6444 6834 6501 6838 6558 6836 6612 6856
6666 6876 6719 6896 6765 6863 6786 6809 6841 6791 6853 6757
6909 6773 6899 6829 6889 6886 6901 6935 6937 6959 41 P
4907 6976 4886 6922 4907 6863 4933 6813 4948 6757 4920 6707
4867 6686 4840 6675 4839 6618 4848 6561 4815 6514 4798 6459
4719 6453 4701 6398 4677 6346 4612 6347 4605 6290 4560 6254
4552 6197 4530 6176 4545 6140 4541 6082 4553 6025 4538 5970
4502 5924 4462 5882 4409 5859 4366 5854 4357 5804 4299 5809
4246 5785 4190 5767 4140 5739 4146 5682 4166 5635 4126 5593
4099 5541 4042 5552 3981 5577 3940 5535 3930 5478 3932 5453

42 P
6937 6959 6994 6957 7049 6939 7104 6927 7157 6903 7212 6908
7242 6837 7278 6793 7335 6797 7416 6811 7415 6754 7436 6701
7467 6652 7494 6602 7515 6548 7492 6495 7491 6438 7507 6383
7529 6329 7555 6278 7587 6229 7617 6207 22 P
2297 9444 2313 9434 2364 9410 2418 9397 2474 9389 2529 9397
2582 9417 2627 9451 2650 9502 2683 9547 2740 9545 2794 9532
2850 9541 2894 9575 2931 9617 2959 9666 3009 9691 3063 9673
3072 9617 3097 9567 3147 9592 3197 9617 3250 9600 3306 9593
3315 9538 3325 9483 3323 9426 3333 9371 3380 9339 3419 9298
3412 9242 3432 9190 3442 9163 3479 9207 3500 9244 3499 9187
3492 9131 3483 9090 3538 9078 3590 9056 3645 9066 3672 9115
3684 9171 3714 9200 3742 9213 45 P
5955 5622 5983 5626 6012 5633 6035 5637 4 P
9446 10405 9439 10460 9481 10497 9510 10545 9524 10598 9548 10648
9593 10681 9627 10702 8 P
7594 15162 7599 15188 7602 15216 7587 15238 7565 15253 7544 15271
7542 15298 7528 15321 7528 15346 7554 15343 7581 15343 7602 15342
7627 15355 7652 15362 7679 15362 7705 15369 7730 15376 17 P
3308 4878 3330 4878 3330 4605 3296 4605 3296 4800 3236 4800
3236 4824 3260 4827 3271 4829 3281 4833 3290 4839 3294 4843
3297 4848 3303 4861 3308 4878 15 N F
3523 4878 3545 4878 3545 4605 3511 4605 3511 4800 3450 4800
3450 4824 3475 4827 3486 4829 3496 4833 3504 4839 3508 4843
3512 4848 3518 4861 3523 4878 15 N F
3731 4878 3741 4877 3751 4875 3760 4871 3768 4866 3776 4859
3784 4852 3791 4843 3797 4833 3802 4823 3807 4812 3812 4800
3815 4788 3818 4776 3820 4763 3821 4750 3822 4738 3821 4725
3820 4712 3815 4686 3812 4674 3807 4662 3802 4651 3797 4641
3791 4631 3784 4622 3776 4614 3768 4607 3760 4602 3751 4598
3741 4596 3731 4595 3721 4596 3712 4598 3703 4602 3694 4607
3686 4614 3679 4622 3672 4631 3666 4641 3660 4651 3655 4662
3651 4674 3648 4686 3643 4712 3641 4725 3641 4738 3677 4738
3678 4713 3682 4691 3687 4672 3695 4656 3703 4643 3712 4633
3717 4629 3722 4627 3726 4625 3731 4625 3736 4625 3741 4627
3746 4629 3751 4633 3760 4643 3768 4656 3775 4672 3781 4691
3784 4713 3786 4738 3784 4762 3781 4784 3775 4803 3768 4818
3760 4831 3751 4840 3746 4844 3741 4846 3736 4848 3731 4848
3726 4848 3722 4846 3717 4844 3712 4840 3703 4831 3695 4818
3687 4803 3682 4784 3678 4762 3677 4738 3641 4738 3641 4750
3643 4763 3645 4776 3648 4788 3651 4800 3655 4812 3660 4823
3666 4833 3672 4843 3679 4852 3686 4859 3694 4866 3703 4871
3712 4875 3721 4877 3731 4878 105 N F

3989 4826 3988 4814 3984 4802 3978 4791 3971 4781 3961 4773
3950 4767 3938 4764 3925 4762 3925 4787 3917 4788 3910 4790
3903 4794 3898 4799 3893 4804 3889 4811 3887 4818 3886 4826
3887 4834 3889 4842 3893 4848 3898 4854 3903 4859 3910 4863
3917 4865 3925 4866 3933 4865 3941 4863 3947 4859 3953 4854
3958 4848 3962 4842 3964 4834 3965 4826 3964 4818 3962 4811
3958 4804 3953 4799 3947 4794 3941 4790 3933 4788 3925 4787
3925 4762 3913 4764 3901 4767 3890 4773 3880 4781 3872 4791
3866 4802 3863 4814 3861 4826 3863 4839 3866 4851 3872 4862
3880 4872 3890 4879 3901 4885 3913 4889 3925 4890 3938 4889
3950 4885 3961 4879 3971 4872 3978 4862 3984 4851 3988 4839
3989 4826 67 N F
4042 4886 4237 4886 4237 4854 4079 4854 4079 4764 4231 4764
4231 4731 4079 4731 4079 4637 4243 4637 4243 4605 4042 4605
4042 4886 13 N F
5472 4878 5494 4878 5494 4605 5460 4605 5460 4800 5399 4800
5399 4824 5424 4827 5435 4829 5445 4833 5453 4839 5457 4843
5461 4848 5467 4861 5472 4878 15 N F
5686 4878 5708 4878 5708 4605 5674 4605 5674 4800 5614 4800
5614 4824 5638 4827 5649 4829 5659 4833 5668 4839 5672 4843
5676 4848 5682 4861 5686 4878 15 N F
5925 4878 5949 4878 5949 4701 5991 4701 5991 4669 5949 4669
5949 4605 5915 4605 5915 4669 5801 4669 5801 4701 5831 4701
5915 4701 5915 4820 5831 4701 5801 4701 5801 4705 5925 4878
18 N F
6153 4826 6152 4814 6148 4802 6142 4791 6134 4781 6125 4773
6114 4767 6102 4764 6089 4762 6089 4787 6081 4788 6074 4790
6067 4794 6061 4799 6056 4804 6053 4811 6051 4818 6050 4826
6051 4834 6053 4842 6056 4848 6061 4854 6067 4859 6074 4863
6081 4865 6089 4866 6097 4865 6104 4863 6111 4859 6117 4854
6122 4848 6125 4842 6128 4834 6128 4826 6128 4818 6125 4811
6122 4804 6117 4799 6111 4794 6104 4790 6097 4788 6089 4787
6089 4762 6076 4764 6064 4767 6053 4773 6044 4781 6036 4791
6030 4802 6026 4814 6025 4826 6026 4839 6030 4851 6036 4862
6044 4872 6053 4879 6064 4885 6076 4889 6089 4890 6102 4889
6114 4885 6125 4879 6134 4872 6142 4862 6148 4851 6152 4839
6153 4826 67 N F
6206 4886 6401 4886 6401 4854 6242 4854 6242 4764 6395 4764
6395 4731 6242 4731 6242 4637 6407 4637 6407 4605 6206 4605
6206 4886 13 N F
7620 4878 7642 4878 7642 4605 7608 4605 7608 4800 7548 4800
7548 4824 7572 4827 7583 4829 7593 4833 7602 4839 7606 4843
7610 4848 7616 4861 7620 4878 15 N F

7835 4878 7857 4878 7857 4605 7823 4605 7823 4800 7763 4800
7763 4824 7787 4827 7798 4829 7808 4833 7817 4839 7821 4843
7824 4848 7831 4861 7835 4878 15 N F
8044 4878 8061 4877 8076 4873 8090 4867 8102 4859 8112 4849
8116 4843 8120 4837 8123 4830 8125 4822 8126 4814 8126 4806
8125 4794 8123 4784 8119 4775 8114 4768 8109 4761 8102 4756
8090 4748 8100 4742 8109 4736 8117 4729 8124 4720 8129 4711
8133 4701 8135 4690 8136 4679 8135 4666 8130 4652 8123 4637
8112 4624 8106 4618 8099 4613 8091 4607 8083 4603 8074 4600
8064 4597 8053 4595 8042 4595 8031 4595 8020 4597 8010 4600
8002 4603 7993 4607 7986 4613 7979 4618 7973 4624 7964 4637
7957 4652 7953 4666 7952 4679 7988 4679 7989 4668 7992 4658
7998 4649 8005 4641 8013 4634 8022 4629 8033 4626 8044 4625
8055 4626 8065 4629 8075 4634 8083 4641 8090 4649 8096 4658
8099 4668 8100 4679 8099 4690 8096 4700 8090 4710 8083 4718
8075 4724 8065 4729 8055 4732 8044 4733 8033 4732 8022 4729
8013 4724 8005 4718 7998 4710 7992 4700 7989 4690 7988 4679
7952 4679 7952 4690 7955 4701 7959 4710 7964 4719 7971 4728
7979 4735 7988 4742 7998 4748 7985 4756 7979 4761 7974 4768
7969 4775 7965 4784 7963 4794 7962 4806 7996 4806 7997 4797
8000 4788 8004 4781 8010 4775 8017 4770 8025 4767 8034 4764
8044 4764 8053 4764 8062 4767 8071 4770 8078 4775 8084 4781
8088 4788 8091 4797 8092 4806 8091 4815 8088 4823 8084 4831
8078 4837 8071 4842 8062 4845 8053 4847 8044 4848 8034 4847
8025 4845 8017 4842 8010 4837 8004 4831 8000 4823 7997 4815
7996 4806 7962 4806 7962 4814 7963 4822 7965 4830 7968 4837
7971 4843 7975 4849 7985 4859 7998 4867 8012 4873 8027 4877
8044 4878 145 N F
8302 4826 8300 4814 8297 4802 8291 4791 8283 4781 8274 4773
8263 4767 8251 4764 8238 4762 8238 4787 8230 4788 8223 4790
8216 4794 8210 4799 8205 4804 8202 4811 8199 4818 8199 4826
8199 4834 8202 4842 8205 4848 8210 4854 8216 4859 8223 4863
8230 4865 8238 4866 8246 4865 8253 4863 8260 4859 8266 4854
8271 4848 8274 4842 8276 4834 8277 4826 8276 4818 8274 4811
8271 4804 8266 4799 8260 4794 8253 4790 8246 4788 8238 4787
8238 4762 8225 4764 8213 4767 8202 4773 8193 4781 8185 4791
8179 4802 8175 4814 8174 4826 8175 4839 8179 4851 8185 4862
8193 4872 8202 4879 8213 4885 8225 4889 8238 4890 8251 4889
8263 4885 8274 4879 8283 4872 8291 4862 8297 4851 8300 4839
8302 4826 67 N F
8355 4886 8550 4886 8550 4854 8391 4854 8391 4764 8544 4764
8544 4731 8391 4731 8391 4637 8556 4637 8556 4605 8355 4605
8355 4886 13 N F

9783 4878 9805 4878 9805 4605 9771 4605 9771 4800 9710 4800
9710 4824 9735 4827 9746 4829 9756 4833 9764 4839 9768 4843
9772 4848 9778 4861 9783 4878 15 N F
9905 4784 9939 4784 9940 4797 9943 4810 9947 4821 9953 4830
9961 4838 9971 4843 9982 4847 9995 4848 10006 4847 10015 4844
10024 4840 10032 4834 10039 4826 10043 4818 10047 4807 10048 4796
10047 4784 10044 4774 10039 4766 10032 4759 10024 4752 10013 4744
9985 4727 9962 4714 9943 4700 9928 4686 9917 4671 9908 4656
9903 4640 9900 4623 9899 4605 10082 4605 10082 4637 9937 4637
9941 4649 9946 4660 9952 4668 9960 4676 9970 4684 9982 4691
10014 4709 10032 4718 10047 4728 10059 4739 10068 4751 10075 4763
10080 4775 10083 4788 10084 4800 10083 4808 10082 4816 10080 4824
10077 4832 10073 4838 10069 4845 10063 4851 10058 4856 10045 4866
10030 4872 10013 4877 9995 4878 9977 4877 9968 4875 9959 4872
9951 4869 9944 4865 9937 4861 9931 4855 9925 4849 9920 4842
9916 4834 9912 4826 9909 4816 9907 4806 9906 4795 9905 4784
78 N F
10120 4784 10154 4784 10155 4797 10157 4810 10162 4821 10168 4830
10176 4838 10185 4843 10197 4847 10210 4848 10221 4847 10230 4844
10239 4840 10247 4834 10253 4826 10258 4818 10261 4807 10262 4796
10261 4784 10259 4774 10254 4766 10247 4759 10238 4752 10228 4744
10200 4727 10177 4714 10158 4700 10143 4686 10131 4671 10123 4656
10118 4640 10115 4623 10114 4605 10297 4605 10297 4637 10152 4637
10156 4649 10161 4660 10167 4668 10175 4676 10185 4684 10197 4691
10228 4709 10246 4718 10261 4728 10273 4739 10283 4751 10290 4763
10295 4775 10298 4788 10299 4800 10298 4808 10297 4816 10295 4824
10292 4832 10288 4838 10283 4845 10278 4851 10272 4856 10259 4866
10244 4872 10228 4877 10210 4878 10191 4877 10183 4875 10174 4872
10166 4869 10159 4865 10152 4861 10145 4855 10140 4849 10135 4842
10130 4834 10127 4826 10124 4816 10122 4806 10120 4795 10120 4784
78 N F
10464 4826 10463 4814 10459 4802 10453 4791 10445 4781 10436 4773
10425 4767 10413 4764 10400 4762 10400 4787 10392 4788 10385 4790
10378 4794 10372 4799 10368 4804 10364 4811 10362 4818 10361 4826
10362 4834 10364 4842 10368 4848 10372 4854 10378 4859 10385 4863
10392 4865 10400 4866 10408 4865 10416 4863 10422 4859 10428 4854
10433 4848 10436 4842 10439 4834 10440 4826 10439 4818 10436 4811
10433 4804 10428 4799 10422 4794 10416 4790 10408 4788 10400 4787
10400 4762 10387 4764 10375 4767 10365 4773 10355 4781 10347 4791
10341 4802 10338 4814 10336 4826 10338 4839 10341 4851 10347 4862
10355 4872 10365 4879 10375 4885 10387 4889 10400 4890 10413 4889
10425 4885 10436 4879 10445 4872 10453 4862 10459 4851 10463 4839
10464 4826 67 N F

10517 4886 10712 4886 10712 4854 10553 4854 10553 4764 10706 4764
10706 4731 10553 4731 10553 4637 10718 4637 10718 4605 10517 4605
10517 4886 13 N F
11945 4878 11967 4878 11967 4605 11933 4605 11933 4800 11873 4800
11873 4824 11897 4827 11908 4829 11918 4833 11927 4839 11931 4843
11934 4848 11940 4861 11945 4878 15 N F
12067 4784 12102 4784 12102 4797 12105 4810 12109 4821 12115 4830
12123 4838 12133 4843 12144 4847 12158 4848 12168 4847 12178 4844
12187 4840 12194 4834 12201 4826 12206 4818 12209 4807 12210 4796
12209 4784 12206 4774 12201 4766 12195 4759 12186 4752 12175 4744
12148 4727 12124 4714 12105 4700 12090 4686 12079 4671 12071 4656
12065 4640 12062 4623 12061 4605 12244 4605 12244 4637 12100 4637
12103 4649 12108 4660 12115 4668 12123 4676 12133 4684 12145 4691
12176 4709 12194 4718 12209 4728 12221 4739 12231 4751 12238 4763
12242 4775 12245 4788 12246 4800 12246 4808 12244 4816 12242 4824
12239 4832 12235 4838 12231 4845 12226 4851 12220 4856 12207 4866
12192 4872 12175 4877 12158 4878 12139 4877 12130 4875 12122 4872
12114 4869 12106 4865 12099 4861 12093 4855 12087 4849 12082 4842
12078 4834 12074 4826 12071 4816 12069 4806 12068 4795 12067 4784
78 N F
12455 4808 12421 4808 12418 4817 12415 4825 12410 4832 12405 4838
12398 4842 12391 4845 12384 4847 12377 4848 12369 4848 12362 4846
12356 4844 12350 4841 12344 4837 12339 4833 12335 4827 12330 4821
12323 4806 12318 4789 12315 4768 12314 4746 12323 4754 12331 4761
12339 4767 12347 4770 12355 4773 12363 4775 12381 4776 12394 4774
12408 4770 12421 4764 12434 4754 12440 4748 12445 4742 12450 4734
12454 4727 12457 4718 12459 4708 12461 4698 12461 4687 12427 4685
12426 4696 12422 4707 12417 4718 12410 4727 12402 4734 12392 4740
12382 4744 12371 4746 12359 4744 12349 4740 12340 4734 12331 4727
12324 4718 12319 4707 12315 4696 12314 4685 12315 4674 12319 4663
12324 4653 12331 4644 12340 4636 12349 4630 12359 4626 12371 4625
12382 4626 12392 4630 12402 4636 12410 4644 12417 4653 12422 4663
12426 4674 12427 4685 12461 4687 12460 4671 12455 4655 12449 4639
12444 4632 12439 4625 12433 4619 12426 4613 12419 4608 12411 4603
12402 4600 12392 4597 12382 4595 12371 4595 12358 4595 12347 4598
12337 4601 12327 4605 12319 4611 12311 4618 12305 4626 12299 4634
12294 4644 12289 4655 12286 4666 12283 4678 12279 4704 12278 4731
12279 4748 12280 4764 12282 4779 12285 4793 12288 4806 12293 4818
12298 4829 12304 4839 12311 4848 12318 4856 12326 4862 12335 4868
12344 4872 12354 4876 12365 4878 12377 4878 12392 4877 12407 4873
12420 4866 12431 4857 12440 4847 12447 4835 12452 4822 12455 4808
126 N F
12626 4826 12625 4814 12621 4802 12615 4791 12608 4781 12598 4773

12587 4767 12575 4764 12562 4762 12562 4787 12555 4788 12547 4790
12540 4794 12535 4799 12530 4804 12526 4811 12524 4818 12523 4826
12524 4834 12526 4842 12530 4848 12535 4854 12540 4859 12547 4863
12555 4865 12562 4866 12570 4865 12578 4863 12585 4859 12590 4854
12595 4848 12599 4842 12601 4834 12602 4826 12601 4818 12599 4811
12595 4804 12590 4799 12585 4794 12578 4790 12570 4788 12562 4787
12562 4762 12550 4764 12538 4767 12527 4773 12517 4781 12510 4791
12504 4802 12500 4814 12499 4826 12500 4839 12504 4851 12510 4862
12517 4872 12527 4879 12538 4885 12550 4889 12562 4890 12575 4889
12587 4885 12598 4879 12608 4872 12615 4862 12621 4851 12625 4839
12626 4826 67 N F
12679 4886 12874 4886 12874 4854 12716 4854 12716 4764 12868 4764
12868 4731 12716 4731 12716 4637 12880 4637 12880 4605 12679 4605
12679 4886 13 N F
2316 17019 2338 17019 2338 16745 2304 16745 2304 16940 2244 16940
2244 16964 2268 16967 2279 16970 2289 16973 2298 16980 2301 16984
2305 16989 2311 17002 2316 17019 15 N F
2525 17019 2535 17018 2544 17015 2553 17012 2562 17006 2570 17000
2577 16992 2584 16984 2590 16974 2596 16964 2601 16953 2605 16941
2608 16929 2611 16916 2613 16904 2614 16891 2615 16878 2614 16865
2613 16852 2608 16827 2605 16815 2601 16803 2596 16792 2590 16781
2584 16771 2577 16762 2570 16755 2562 16748 2553 16743 2544 16739
2535 16736 2525 16735 2515 16736 2505 16739 2496 16743 2488 16748
2480 16755 2472 16762 2465 16771 2459 16781 2453 16792 2449 16803
2444 16815 2441 16827 2436 16852 2435 16865 2434 16878 2470 16878
2472 16854 2475 16832 2481 16812 2488 16796 2496 16783 2505 16773
2510 16770 2515 16767 2520 16766 2525 16765 2529 16766 2534 16767
2539 16770 2544 16773 2553 16783 2561 16796 2568 16812 2574 16832
2577 16854 2579 16878 2577 16902 2574 16924 2568 16943 2561 16959
2553 16972 2544 16981 2539 16984 2534 16987 2529 16988 2525 16989
2520 16988 2515 16987 2510 16984 2505 16981 2496 16972 2488 16959
2481 16943 2475 16924 2472 16902 2470 16878 2434 16878 2435 16891
2436 16904 2438 16916 2441 16929 2444 16941 2449 16953 2453 16964
2459 16974 2465 16984 2472 16992 2480 17000 2488 17006 2496 17012
2505 17015 2515 17018 2525 17019 105 N F
2675 17019 2818 17019 2818 16987 2703 16987 2693 16908 2703 16915
2715 16921 2729 16925 2745 16926 2759 16925 2774 16921 2789 16915
2795 16911 2802 16906 2808 16900 2814 16893 2819 16886 2823 16877
2827 16868 2829 16857 2831 16846 2832 16834 2830 16815 2828 16805
2826 16796 2822 16788 2818 16780 2813 16772 2808 16765 2802 16758
2794 16753 2787 16748 2778 16743 2769 16740 2759 16737 2749 16736
2737 16735 2727 16736 2717 16737 2708 16739 2700 16741 2692 16745
2685 16748 2679 16753 2673 16758 2664 16769 2656 16782 2651 16796

2647 16812 2681 16812 2685 16802 2689 16793 2694 16785 2701 16778
2708 16773 2716 16769 2725 16766 2735 16765 2749 16766 2761 16770
2772 16775 2781 16782 2788 16791 2793 16803 2796 16816 2798 16832
2797 16843 2794 16855 2790 16865 2783 16875 2775 16884 2764 16890
2752 16895 2745 16896 2737 16896 2730 16896 2723 16895 2716 16893
2710 16890 2699 16882 2685 16870 2655 16870 2675 17019 83 N F
2997 16967 2996 16954 2992 16942 2986 16931 2978 16922 2969 16914
2958 16908 2946 16904 2933 16903 2933 16928 2925 16928 2918 16931
2911 16934 2905 16939 2901 16945 2897 16952 2895 16959 2894 16967
2895 16975 2897 16982 2901 16989 2905 16995 2911 17000 2918 17003
2925 17006 2933 17006 2941 17006 2949 17003 2955 17000 2961 16995
2966 16989 2970 16982 2972 16975 2973 16967 2972 16959 2970 16952
2966 16945 2961 16939 2955 16934 2949 16931 2941 16928 2933 16928
2933 16903 2920 16904 2908 16908 2898 16914 2888 16922 2880 16931
2874 16942 2871 16954 2869 16967 2871 16980 2874 16992 2880 17003
2888 17012 2898 17020 2908 17026 2920 17030 2933 17031 2946 17030
2958 17026 2969 17020 2978 17012 2986 17003 2992 16992 2996 16980
2997 16967 67 N F
3050 17027 3245 17027 3245 16995 3086 16995 3086 16904 3239 16904
3239 16872 3086 16872 3086 16778 3251 16778 3251 16745 3050 16745
3050 17027 13 N F
4381 17019 4403 17019 4403 16745 4369 16745 4369 16940 4309 16940
4309 16964 4333 16967 4344 16970 4354 16973 4363 16980 4367 16984
4370 16989 4376 17002 4381 17019 15 N F
4596 17019 4618 17019 4618 16745 4584 16745 4584 16940 4523 16940
4523 16964 4548 16967 4559 16970 4569 16973 4577 16980 4581 16984
4585 16989 4591 17002 4596 17019 15 N F
4804 17019 4814 17018 4824 17015 4833 17012 4841 17006 4849 17000
4857 16992 4864 16984 4870 16974 4875 16964 4880 16953 4885 16941
4888 16929 4891 16916 4893 16904 4894 16891 4895 16878 4894 16865
4893 16852 4888 16827 4885 16815 4880 16803 4875 16792 4870 16781
4864 16771 4857 16762 4849 16755 4841 16748 4833 16743 4824 16739
4814 16736 4804 16735 4794 16736 4785 16739 4776 16743 4767 16748
4759 16755 4752 16762 4745 16771 4739 16781 4733 16792 4728 16803
4724 16815 4721 16827 4716 16852 4715 16865 4714 16878 4750 16878
4751 16854 4755 16832 4760 16812 4768 16796 4776 16783 4785 16773
4790 16770 4795 16767 4799 16766 4804 16765 4809 16766 4814 16767
4819 16770 4824 16773 4833 16783 4841 16796 4848 16812 4854 16832
4857 16854 4859 16878 4857 16902 4854 16924 4848 16943 4841 16959
4833 16972 4824 16981 4819 16984 4814 16987 4809 16988 4804 16989
4799 16988 4795 16987 4790 16984 4785 16981 4776 16972 4768 16959
4760 16943 4755 16924 4751 16902 4750 16878 4714 16878 4715 16891
4716 16904 4718 16916 4721 16929 4724 16941 4728 16953 4733 16964

4739 16974 4745 16984 4752 16992 4759 17000 4767 17006 4776 17012
4785 17015 4794 17018 4804 17019 105 N F
5062 16967 5061 16954 5057 16942 5051 16931 5044 16922 5034 16914
5023 16908 5011 16904 4998 16903 4998 16928 4990 16928 4983 16931
4976 16934 4971 16939 4966 16945 4962 16952 4960 16959 4959 16967
4960 16975 4962 16982 4966 16989 4971 16995 4976 17000 4983 17003
4990 17006 4998 17006 5006 17006 5014 17003 5020 17000 5026 16995
5031 16989 5035 16982 5037 16975 5038 16967 5037 16959 5035 16952
5031 16945 5026 16939 5020 16934 5014 16931 5006 16928 4998 16928
4998 16903 4986 16904 4974 16908 4963 16914 4953 16922 4945 16931
4940 16942 4936 16954 4934 16967 4936 16980 4940 16992 4945 17003
4953 17012 4963 17020 4974 17026 4986 17030 4998 17031 5011 17030
5023 17026 5034 17020 5044 17012 5051 17003 5057 16992 5061 16980
5062 16967 67 N F
5115 17027 5310 17027 5310 16995 5152 16995 5152 16904 5304 16904
5304 16872 5152 16872 5152 16778 5316 16778 5316 16745 5115 16745
5115 17027 13 N F
6412 17019 6434 17019 6434 16745 6400 16745 6400 16940 6340 16940
6340 16964 6364 16967 6375 16970 6385 16973 6394 16980 6397 16984
6401 16989 6407 17002 6412 17019 15 N F
6627 17019 6649 17019 6649 16745 6614 16745 6614 16940 6554 16940
6554 16964 6579 16967 6589 16970 6599 16973 6608 16980 6612 16984
6616 16989 6622 17002 6627 17019 15 N F
6771 17019 6914 17019 6914 16987 6799 16987 6789 16908 6799 16915
6811 16921 6825 16925 6841 16926 6855 16925 6870 16921 6884 16915
6891 16911 6898 16906 6904 16900 6910 16893 6915 16886 6919 16877
6923 16868 6925 16857 6927 16846 6928 16834 6926 16815 6924 16805
6922 16796 6918 16788 6914 16780 6909 16772 6904 16765 6897 16758
6890 16753 6883 16748 6874 16743 6865 16740 6855 16737 6845 16736
6833 16735 6823 16736 6813 16737 6804 16739 6796 16741 6788 16745
6781 16748 6775 16753 6769 16758 6760 16769 6752 16782 6747 16796
6743 16812 6777 16812 6781 16802 6785 16793 6790 16785 6797 16778
6804 16773 6812 16769 6821 16766 6831 16765 6845 16766 6857 16770
6868 16775 6877 16782 6884 16791 6889 16803 6892 16816 6893 16832
6893 16843 6890 16855 6886 16865 6879 16875 6871 16884 6860 16890
6848 16895 6841 16896 6833 16896 6825 16896 6819 16895 6812 16893
6806 16890 6795 16882 6781 16870 6751 16870 6771 17019 83 N F
7093 16967 7092 16954 7088 16942 7082 16931 7074 16922 7065 16914
7054 16908 7042 16904 7029 16903 7029 16928 7021 16928 7014 16931
7007 16934 7001 16939 6997 16945 6993 16952 6991 16959 6990 16967
6991 16975 6993 16982 6997 16989 7001 16995 7007 17000 7014 17003
7021 17006 7029 17006 7037 17006 7045 17003 7051 17000 7057 16995
7062 16989 7065 16982 7068 16975 7069 16967 7068 16959 7065 16952

7062 16945 7057 16939 7051 16934 7045 16931 7037 16928 7029 16928
7029 16903 7016 16904 7004 16908 6994 16914 6984 16922 6976 16931
6970 16942 6967 16954 6965 16967 6967 16980 6970 16992 6976 17003
6984 17012 6994 17020 7004 17026 7016 17030 7029 17031 7042 17030
7054 17026 7065 17020 7074 17012 7082 17003 7088 16992 7092 16980
7093 16967 67 N F
7146 17027 7341 17027 7341 16995 7182 16995 7182 16904 7335 16904
7335 16872 7182 16872 7182 16778 7347 16778 7347 16745 7146 16745
7146 17027 13 N F
8433 17019 8455 17019 8455 16745 8421 16745 8421 16940 8361 16940
8361 16964 8385 16967 8396 16970 8406 16973 8415 16980 8419 16984
8422 16989 8428 17002 8433 17019 15 N F
8555 16924 8589 16924 8590 16938 8593 16950 8597 16961 8603 16971
8611 16978 8621 16984 8632 16987 8646 16989 8656 16988 8666 16985
8675 16981 8682 16974 8689 16967 8694 16958 8697 16948 8698 16936
8697 16925 8694 16915 8689 16907 8683 16899 8674 16892 8663 16885
8636 16868 8612 16854 8593 16841 8578 16827 8567 16812 8559 16797
8553 16781 8550 16764 8549 16745 8732 16745 8732 16778 8587 16778
8591 16790 8596 16800 8602 16809 8611 16817 8621 16824 8633 16832
8664 16850 8682 16859 8697 16869 8709 16880 8718 16891 8726 16903
8730 16916 8733 16928 8734 16940 8734 16949 8732 16957 8730 16965
8727 16972 8723 16979 8719 16985 8714 16991 8708 16997 8695 17006
8680 17013 8663 17017 8646 17019 8627 17017 8618 17016 8610 17013
8602 17010 8594 17006 8587 17001 8581 16996 8575 16989 8570 16982
8566 16975 8562 16966 8559 16957 8557 16947 8556 16936 8555 16924
78 N F
8856 17019 8866 17018 8876 17015 8885 17012 8893 17006 8901 17000
8909 16992 8916 16984 8922 16974 8928 16964 8932 16953 8937 16941
8940 16929 8943 16916 8945 16904 8946 16891 8947 16878 8946 16865
8945 16852 8940 16827 8937 16815 8932 16803 8928 16792 8922 16781
8916 16771 8909 16762 8901 16755 8893 16748 8885 16743 8876 16739
8866 16736 8856 16735 8846 16736 8837 16739 8828 16743 8819 16748
8811 16755 8804 16762 8797 16771 8791 16781 8785 16792 8780 16803
8776 16815 8773 16827 8768 16852 8767 16865 8766 16878 8802 16878
8803 16854 8807 16832 8812 16812 8820 16796 8828 16783 8837 16773
8842 16770 8847 16767 8852 16766 8856 16765 8861 16766 8866 16767
8871 16770 8876 16773 8885 16783 8893 16796 8900 16812 8906 16832
8909 16854 8911 16878 8909 16902 8906 16924 8900 16943 8893 16959
8885 16972 8876 16981 8871 16984 8866 16987 8861 16988 8856 16989
8852 16988 8847 16987 8842 16984 8837 16981 8828 16972 8820 16959
8812 16943 8807 16924 8803 16902 8802 16878 8766 16878 8767 16891
8768 16904 8770 16916 8773 16929 8776 16941 8780 16953 8785 16964
8791 16974 8797 16984 8804 16992 8811 17000 8819 17006 8828 17012

8837 17015 8846 17018 8856 17019 105 N F
9114 16967 9113 16954 9109 16942 9103 16931 9096 16922 9086 16914
9075 16908 9063 16904 9050 16903 9050 16928 9042 16928 9035 16931
9028 16934 9023 16939 9018 16945 9014 16952 9012 16959 9011 16967
9012 16975 9014 16982 9018 16989 9023 16995 9028 17000 9035 17003
9042 17006 9050 17006 9058 17006 9066 17003 9072 17000 9078 16995
9083 16989 9087 16982 9089 16975 9090 16967 9089 16959 9087 16952
9083 16945 9078 16939 9072 16934 9066 16931 9058 16928 9050 16928
9050 16903 9038 16904 9026 16908 9015 16914 9005 16922 8997 16931
8992 16942 8988 16954 8986 16967 8988 16980 8992 16992 8997 17003
9005 17012 9015 17020 9026 17026 9038 17030 9050 17031 9063 17030
9075 17026 9086 17020 9096 17012 9103 17003 9109 16992 9113 16980
9114 16967 67 N F
9167 17027 9362 17027 9362 16995 9204 16995 9204 16904 9356 16904
9356 16872 9204 16872 9204 16778 9368 16778 9368 16745 9167 16745
9167 17027 13 N F
10460 17019 10482 17019 10482 16745 10448 16745 10448 16940 10388 16940
10388 16964 10412 16967 10423 16970 10433 16973 10442 16980 10446 16984
10450 16989 10456 17002 10460 17019 15 N F
10583 16924 10617 16924 10618 16938 10620 16950 10625 16961 10631 16971
10639 16978 10648 16984 10660 16987 10673 16989 10683 16988 10693 16985
10702 16981 10710 16974 10716 16967 10721 16958 10724 16948 10725 16936
10724 16925 10721 16915 10717 16907 10710 16899 10701 16892 10691 16885
10663 16868 10640 16854 10621 16841 10606 16827 10594 16812 10586 16797
10581 16781 10578 16764 10577 16745 10759 16745 10759 16778 10615 16778
10618 16790 10623 16800 10630 16809 10638 16817 10648 16824 10660 16832
10691 16850 10709 16859 10724 16869 10736 16880 10746 16891 10753 16903
10758 16916 10761 16928 10761 16940 10761 16949 10760 16957 10757 16965
10754 16972 10751 16979 10746 16985 10741 16991 10735 16997 10722 17006
10707 17013 10691 17017 10673 17019 10654 17017 10645 17016 10637 17013
10629 17010 10622 17006 10615 17001 10608 16996 10603 16989 10598 16982
10593 16975 10590 16966 10587 16957 10585 16947 10583 16936 10583 16924
78 N F
10820 17019 10962 17019 10962 16987 10848 16987 10838 16908 10848 16915
10859 16921 10873 16925 10890 16926 10904 16925 10919 16921 10933 16915
10940 16911 10947 16906 10953 16900 10958 16893 10963 16886 10968 16877
10971 16868 10974 16857 10976 16846 10976 16834 10975 16815 10973 16805
10970 16796 10967 16788 10963 16780 10958 16772 10952 16765 10946 16758
10939 16753 10931 16748 10923 16743 10914 16740 10904 16737 10893 16736
10882 16735 10871 16736 10862 16737 10853 16739 10844 16741 10837 16745
10830 16748 10824 16753 10818 16758 10808 16769 10801 16782 10795 16796
10792 16812 10826 16812 10829 16802 10834 16793 10839 16785 10845 16778
10852 16773 10861 16769 10870 16766 10880 16765 10893 16766 10906 16770

10916 16775 10925 16782 10932 16791 10938 16803 10941 16816 10942 16832
10941 16843 10939 16855 10934 16865 10928 16875 10919 16884 10909 16890
10897 16895 10889 16896 10882 16896 10874 16896 10867 16895 10861 16893
10855 16890 10843 16882 10830 16870 10800 16870 10820 17019 83 N F
11142 16967 11140 16954 11137 16942 11131 16931 11123 16922 11113 16914
11103 16908 11091 16904 11078 16903 11078 16928 11070 16928 11062 16931
11056 16934 11050 16939 11045 16945 11042 16952 11039 16959 11038 16967
11039 16975 11042 16982 11045 16989 11050 16995 11056 17000 11062 17003
11070 17006 11078 17006 11086 17006 11093 17003 11100 17000 11106 16995
11110 16989 11114 16982 11116 16975 11117 16967 11116 16959 11114 16952
11110 16945 11106 16939 11100 16934 11093 16931 11086 16928 11078 16928
11078 16903 11065 16904 11053 16908 11042 16914 11033 16922 11025 16931
11019 16942 11015 16954 11014 16967 11015 16980 11019 16992 11025 17003
11033 17012 11042 17020 11053 17026 11065 17030 11078 17031 11091 17030
11103 17026 11113 17020 11123 17012 11131 17003 11137 16992 11140 16980
11142 16967 67 N F
11195 17027 11390 17027 11390 16995 11231 16995 11231 16904 11383 16904
11383 16872 11231 16872 11231 16778 11396 16778 11396 16745 11195 16745
11195 17027 13 N F
12511 17019 12533 17019 12533 16745 12499 16745 12499 16940 12439 16940
12439 16964 12463 16967 12474 16970 12484 16973 12493 16980 12497 16984
12500 16989 12506 17002 12511 17019 15 N F
12666 16930 12631 16930 12634 16951 12636 16960 12638 16969 12642 16977
12646 16984 12651 16990 12656 16996 12662 17002 12668 17006 12675 17010
12683 17013 12691 17016 12699 17017 12718 17019 12735 17017 12751 17014
12766 17008 12778 17000 12783 16995 12788 16989 12792 16983 12796 16976
12798 16969 12800 16961 12802 16953 12802 16944 12802 16934 12800 16925
12797 16917 12793 16909 12788 16902 12781 16896 12772 16891 12762 16886
12774 16881 12784 16875 12792 16869 12799 16861 12804 16853 12807 16844
12809 16833 12810 16822 12809 16807 12805 16792 12799 16777 12794 16771
12789 16764 12783 16759 12776 16753 12769 16749 12760 16745 12751 16742
12740 16739 12728 16738 12716 16737 12705 16738 12695 16739 12685 16741
12677 16744 12669 16747 12661 16751 12655 16756 12649 16762 12644 16768
12639 16774 12636 16781 12633 16789 12629 16806 12627 16824 12662 16824
12662 16814 12664 16804 12667 16794 12672 16786 12679 16778 12689 16773
12701 16769 12718 16767 12732 16768 12743 16771 12753 16776 12761 16783
12767 16791 12771 16800 12773 16811 12774 16822 12773 16836 12771 16842
12768 16847 12766 16852 12762 16856 12753 16863 12742 16867 12729 16870
12714 16872 12698 16872 12698 16900 12714 16900 12728 16902 12740 16904
12749 16908 12756 16913 12762 16920 12765 16930 12766 16942 12765 16952
12763 16961 12760 16968 12755 16975 12748 16981 12740 16985 12730 16988
12718 16989 12707 16988 12697 16986 12689 16982 12681 16977 12675 16969
12670 16959 12667 16946 12666 16930 123 N F

12934 17019 12944 17018 12954 17015 12963 17012 12971 17006 12979 17000
12987 16992 12994 16984 13000 16974 13006 16964 13011 16953 13015 16941
13018 16929 13021 16916 13023 16904 13024 16891 13025 16878 13024 16865
13023 16852 13018 16827 13015 16815 13011 16803 13006 16792 13000 16781
12994 16771 12987 16762 12979 16755 12971 16748 12963 16743 12954 16739
12944 16736 12934 16735 12925 16736 12915 16739 12906 16743 12898 16748
12890 16755 12882 16762 12875 16771 12869 16781 12863 16792 12858 16803
12854 16815 12851 16827 12846 16852 12845 16865 12844 16878 12880 16878
12882 16854 12885 16832 12891 16812 12898 16796 12906 16783 12915 16773
12920 16770 12925 16767 12930 16766 12934 16765 12939 16766 12944 16767
12949 16770 12954 16773 12963 16783 12971 16796 12978 16812 12984 16832
12987 16854 12989 16878 12987 16902 12984 16924 12978 16943 12971 16959
12963 16972 12954 16981 12949 16984 12944 16987 12939 16988 12934 16989
12930 16988 12925 16987 12920 16984 12915 16981 12906 16972 12898 16959
12891 16943 12885 16924 12882 16902 12880 16878 12844 16878 12845 16891
12846 16904 12848 16916 12851 16929 12854 16941 12858 16953 12863 16964
12869 16974 12875 16984 12882 16992 12890 17000 12898 17006 12906 17012
12915 17015 12925 17018 12934 17019 105 N F
13192 16967 13191 16954 13187 16942 13181 16931 13174 16922 13164 16914
13153 16908 13141 16904 13128 16903 13128 16928 13121 16928 13113 16931
13106 16934 13101 16939 13096 16945 13092 16952 13090 16959 13089 16967
13090 16975 13092 16982 13096 16989 13101 16995 13106 17000 13113 17003
13121 17006 13128 17006 13136 17006 13144 17003 13150 17000 13156 16995
13161 16989 13165 16982 13167 16975 13168 16967 13167 16959 13165 16952
13161 16945 13156 16939 13150 16934 13144 16931 13136 16928 13128 16928
13128 16903 13116 16904 13104 16908 13093 16914 13083 16922 13076 16931
13070 16942 13066 16954 13065 16967 13066 16980 13070 16992 13076 17003
13083 17012 13093 17020 13104 17026 13116 17030 13128 17031 13141 17030
13153 17026 13164 17020 13174 17012 13181 17003 13187 16992 13191 16980
13192 16967 67 N F
13245 17027 13440 17027 13440 16995 13282 16995 13282 16904 13434 16904
13434 16872 13282 16872 13282 16778 13446 16778 13446 16745 13245 16745
13245 17027 13 N F
911 5683 945 5683 946 5696 948 5709 952 5720 959 5729
966 5737 976 5742 988 5746 1001 5747 1011 5746 1021 5743
1030 5739 1038 5733 1044 5725 1049 5717 1052 5706 1053 5695
1052 5683 1049 5673 1045 5665 1038 5658 1029 5651 1018 5643
991 5626 968 5613 949 5599 934 5585 922 5570 914 5555
909 5539 906 5522 905 5504 1087 5504 1087 5536 943 5536
946 5548 951 5559 958 5567 966 5575 976 5583 988 5590
1019 5608 1037 5617 1052 5627 1064 5638 1074 5650 1081 5662
1086 5674 1088 5687 1089 5699 1089 5707 1087 5715 1085 5723
1082 5731 1078 5737 1074 5744 1069 5750 1063 5755 1050 5765

1035 5771 1019 5776 1001 5777 982 5776 973 5774 965 5771
957 5768 949 5764 943 5760 936 5754 931 5748 925 5741
921 5733 917 5725 914 5715 912 5705 911 5694 911 5683
78 N F
1125 5683 1160 5683 1160 5696 1163 5709 1167 5720 1173 5729
1181 5737 1191 5742 1202 5746 1216 5747 1226 5746 1236 5743
1245 5739 1252 5733 1259 5725 1264 5717 1267 5706 1268 5695
1267 5683 1264 5673 1259 5665 1253 5658 1244 5651 1233 5643
1206 5626 1182 5613 1163 5599 1148 5585 1137 5570 1129 5555
1123 5539 1120 5522 1119 5504 1302 5504 1302 5536 1157 5536
1161 5548 1166 5559 1172 5567 1181 5575 1191 5583 1203 5590
1234 5608 1252 5617 1267 5627 1279 5638 1288 5650 1296 5662
1300 5674 1303 5687 1304 5699 1304 5707 1302 5715 1300 5723
1297 5731 1293 5737 1289 5744 1284 5750 1278 5755 1265 5765
1250 5771 1233 5776 1216 5777 1197 5776 1188 5774 1180 5771
1172 5768 1164 5764 1157 5760 1151 5754 1145 5748 1140 5741
1136 5733 1132 5725 1129 5715 1127 5705 1126 5694 1125 5683
78 N F
1470 5725 1468 5713 1465 5701 1459 5690 1451 5680 1441 5672
1430 5666 1419 5663 1406 5661 1406 5686 1398 5687 1390 5689
1384 5693 1378 5698 1373 5703 1369 5710 1367 5717 1366 5725
1367 5733 1369 5741 1373 5747 1378 5753 1384 5758 1390 5762
1398 5764 1406 5765 1414 5764 1421 5762 1428 5758 1434 5753
1438 5747 1442 5741 1444 5733 1445 5725 1444 5717 1442 5710
1438 5703 1434 5698 1428 5693 1421 5689 1414 5687 1406 5686
1406 5661 1393 5663 1381 5666 1370 5672 1361 5680 1353 5690
1347 5701 1343 5713 1342 5725 1343 5738 1347 5750 1353 5761
1361 5771 1370 5778 1381 5784 1393 5788 1406 5789 1419 5788
1430 5784 1441 5778 1451 5771 1459 5761 1465 5750 1468 5738
1470 5725 67 N F
1517 5785 1557 5785 1701 5556 1701 5785 1735 5785 1735 5504
1695 5504 1551 5733 1551 5504 1517 5504 1517 5785 11 N F
911 6859 945 6859 946 6873 948 6886 952 6897 959 6906
966 6913 976 6919 988 6923 1001 6924 1011 6923 1021 6920
1030 6916 1038 6910 1044 6902 1049 6893 1052 6883 1053 6872
1052 6860 1049 6850 1045 6842 1038 6834 1029 6827 1018 6820
991 6803 968 6790 949 6776 934 6762 922 6747 914 6732
909 6716 906 6699 905 6681 1087 6681 1087 6713 943 6713
946 6725 951 6735 958 6744 966 6752 976 6759 988 6767
1019 6785 1037 6794 1052 6804 1064 6815 1074 6827 1081 6839
1086 6851 1088 6863 1089 6876 1089 6884 1087 6892 1085 6900
1082 6907 1078 6914 1074 6921 1069 6927 1063 6932 1050 6941
1035 6948 1019 6952 1001 6954 982 6952 973 6951 965 6948

957 6945 949 6941 943 6936 936 6931 931 6925 925 6918
921 6910 917 6901 914 6892 912 6882 911 6871 911 6859
78 N F
1242 6954 1266 6954 1266 6777 1308 6777 1308 6745 1266 6745
1266 6681 1232 6681 1232 6745 1117 6745 1117 6777 1147 6777
1232 6777 1232 6896 1147 6777 1117 6777 1117 6781 1242 6954
18 N F
1470 6902 1468 6889 1465 6877 1459 6866 1451 6857 1441 6849
1430 6843 1419 6839 1406 6838 1406 6863 1398 6864 1390 6866
1384 6869 1378 6874 1373 6880 1369 6887 1367 6894 1366 6902
1367 6910 1369 6918 1373 6924 1378 6930 1384 6935 1390 6938
1398 6941 1406 6942 1414 6941 1421 6938 1428 6935 1434 6930
1438 6924 1442 6918 1444 6910 1445 6902 1444 6894 1442 6887
1438 6880 1434 6874 1428 6869 1421 6866 1414 6864 1406 6863
1406 6838 1393 6839 1381 6843 1370 6849 1361 6857 1353 6866
1347 6877 1343 6889 1342 6902 1343 6915 1347 6927 1353 6938
1361 6947 1370 6955 1381 6961 1393 6965 1406 6966 1419 6965
1430 6961 1441 6955 1451 6947 1459 6938 1465 6927 1468 6915
1470 6902 67 N F
1517 6962 1557 6962 1701 6733 1701 6962 1735 6962 1735 6681
1695 6681 1551 6910 1551 6681 1517 6681 1517 6962 11 N F
911 8021 945 8021 946 8035 948 8047 952 8058 959 8067
966 8075 976 8081 988 8084 1001 8085 1011 8084 1021 8082
1030 8077 1038 8071 1044 8064 1049 8055 1052 8045 1053 8033
1052 8021 1049 8012 1045 8003 1038 7996 1029 7989 1018 7982
991 7965 968 7951 949 7937 934 7923 922 7909 914 7893
909 7877 906 7860 905 7842 1087 7842 1087 7874 943 7874
946 7887 951 7897 958 7906 966 7913 976 7921 988 7929
1019 7947 1037 7956 1052 7966 1064 7976 1074 7988 1081 8000
1086 8012 1088 8025 1089 8037 1089 8046 1087 8054 1085 8061
1082 8069 1078 8076 1074 8082 1069 8088 1063 8094 1050 8103
1035 8110 1019 8114 1001 8115 982 8114 973 8112 965 8110
957 8106 949 8103 943 8098 936 8092 931 8086 925 8079
921 8071 917 8063 914 8054 912 8044 911 8033 911 8021
78 N F
1298 8045 1264 8045 1262 8054 1258 8062 1253 8069 1248 8075
1241 8079 1235 8083 1227 8085 1220 8085 1212 8085 1206 8084
1199 8081 1193 8078 1188 8074 1182 8070 1178 8064 1174 8058
1167 8043 1162 8026 1159 8006 1157 7983 1166 7992 1174 7999
1182 8004 1190 8008 1198 8010 1206 8012 1224 8013 1237 8012
1251 8008 1265 8001 1277 7991 1283 7986 1288 7979 1293 7972
1297 7964 1300 7955 1303 7946 1304 7935 1304 7925 1270 7923
1269 7934 1265 7945 1260 7955 1253 7964 1245 7972 1235 7978

1225 7981 1214 7983 1203 7981 1192 7978 1183 7972 1174 7964
1167 7955 1162 7945 1159 7934 1157 7923 1159 7911 1162 7900
1167 7890 1174 7881 1183 7873 1192 7867 1203 7864 1214 7862
1225 7864 1235 7867 1245 7873 1253 7881 1260 7890 1265 7900
1269 7911 1270 7923 1304 7925 1303 7908 1299 7892 1292 7877
1287 7869 1282 7862 1276 7856 1270 7850 1262 7845 1254 7841
1245 7837 1235 7834 1225 7833 1214 7832 1201 7833 1190 7835
1180 7838 1170 7843 1162 7848 1154 7855 1148 7863 1142 7872
1137 7881 1133 7892 1129 7903 1126 7915 1123 7941 1121 7969
1122 7985 1123 8001 1125 8016 1128 8030 1132 8043 1136 8055
1141 8066 1147 8076 1154 8085 1161 8093 1169 8100 1178 8105
1188 8110 1198 8113 1208 8115 1220 8115 1236 8114 1250 8110
1263 8103 1274 8095 1283 8084 1290 8072 1295 8059 1298 8045
126 N F
1470 8064 1468 8051 1465 8039 1459 8028 1451 8019 1441 8011
1430 8005 1419 8001 1406 8000 1406 8024 1398 8025 1390 8027
1384 8031 1378 8036 1373 8042 1369 8048 1367 8056 1366 8064
1367 8072 1369 8079 1373 8086 1378 8092 1384 8096 1390 8100
1398 8102 1406 8103 1414 8102 1421 8100 1428 8096 1434 8092
1438 8086 1442 8079 1444 8072 1445 8064 1444 8056 1442 8048
1438 8042 1434 8036 1428 8031 1421 8027 1414 8025 1406 8024
1406 8000 1393 8001 1381 8005 1370 8011 1361 8019 1353 8028
1347 8039 1343 8051 1342 8064 1343 8077 1347 8089 1353 8099
1361 8109 1370 8117 1381 8123 1393 8126 1406 8128 1419 8126
1430 8123 1441 8117 1451 8109 1459 8099 1465 8089 1468 8077
1470 8064 67 N F
1517 8123 1557 8123 1701 7894 1701 8123 1735 8123 1735 7842
1695 7842 1551 8071 1551 7842 1517 7842 1517 8123 11 N F
911 9166 945 9166 946 9180 948 9192 952 9203 959 9213
966 9220 976 9226 988 9229 1001 9231 1011 9230 1021 9227
1030 9223 1038 9217 1044 9209 1049 9200 1052 9190 1053 9178
1052 9167 1049 9157 1045 9149 1038 9141 1029 9134 1018 9127
991 9110 968 9096 949 9083 934 9069 922 9054 914 9039
909 9023 906 9006 905 8987 1087 8987 1087 9020 943 9020
946 9032 951 9042 958 9051 966 9059 976 9066 988 9074
1019 9092 1037 9101 1052 9111 1064 9122 1074 9133 1081 9145
1086 9158 1088 9170 1089 9182 1089 9191 1087 9199 1085 9207
1082 9214 1078 9221 1074 9227 1069 9233 1063 9239 1050 9248
1035 9255 1019 9259 1001 9261 982 9259 973 9258 965 9255
957 9252 949 9248 943 9243 936 9238 931 9231 925 9224
921 9217 917 9208 914 9199 912 9189 911 9178 911 9166
78 N F
1212 9261 1228 9260 1244 9256 1258 9250 1270 9242 1280 9232

1284 9226 1288 9219 1290 9212 1292 9205 1294 9197 1294 9188
1293 9177 1291 9166 1287 9158 1282 9150 1276 9144 1270 9139
1258 9130 1268 9125 1277 9119 1285 9111 1291 9103 1297 9093
1301 9083 1303 9073 1304 9062 1303 9048 1298 9034 1291 9020
1280 9007 1274 9001 1267 8995 1259 8990 1251 8986 1241 8982
1232 8980 1221 8978 1210 8977 1198 8978 1188 8980 1178 8982
1169 8986 1161 8990 1154 8995 1147 9001 1141 9007 1132 9020
1125 9034 1121 9048 1119 9062 1155 9062 1157 9051 1160 9041
1165 9031 1172 9023 1181 9017 1190 9012 1201 9009 1212 9008
1223 9009 1233 9012 1243 9017 1251 9023 1258 9031 1263 9041
1267 9051 1268 9062 1267 9073 1263 9083 1258 9092 1251 9100
1243 9107 1233 9112 1223 9115 1212 9116 1201 9115 1190 9112
1181 9107 1172 9100 1165 9092 1160 9083 1157 9073 1155 9062
1119 9062 1120 9073 1123 9083 1127 9093 1132 9102 1139 9110
1146 9118 1155 9124 1166 9130 1153 9139 1147 9144 1141 9150
1137 9158 1133 9166 1130 9177 1129 9188 1164 9188 1165 9179
1167 9171 1172 9164 1178 9158 1185 9153 1193 9149 1202 9147
1212 9146 1221 9147 1230 9149 1238 9153 1246 9158 1251 9164
1256 9171 1259 9179 1260 9188 1259 9198 1256 9206 1251 9213
1246 9219 1238 9224 1230 9228 1221 9230 1212 9231 1202 9230
1193 9228 1185 9224 1178 9219 1172 9213 1167 9206 1165 9198
1164 9188 1129 9188 1130 9197 1131 9205 1133 9212 1136 9219
1139 9226 1143 9232 1153 9242 1165 9250 1179 9256 1195 9260
1212 9261 145 N F
1470 9209 1468 9196 1465 9184 1459 9173 1451 9164 1441 9156
1430 9150 1419 9146 1406 9145 1406 9170 1398 9170 1390 9173
1384 9176 1378 9181 1373 9187 1369 9194 1367 9201 1366 9209
1367 9217 1369 9224 1373 9231 1378 9237 1384 9242 1390 9245
1398 9248 1406 9248 1414 9248 1421 9245 1428 9242 1434 9237
1438 9231 1442 9224 1444 9217 1445 9209 1444 9201 1442 9194
1438 9187 1434 9181 1428 9176 1421 9173 1414 9170 1406 9170
1406 9145 1393 9146 1381 9150 1370 9156 1361 9164 1353 9173
1347 9184 1343 9196 1342 9209 1343 9222 1347 9234 1353 9245
1361 9254 1370 9262 1381 9268 1393 9272 1406 9273 1419 9272
1430 9268 1441 9262 1451 9254 1459 9245 1465 9234 1468 9222
1470 9209 67 N F
1517 9269 1557 9269 1701 9040 1701 9269 1735 9269 1735 8987
1695 8987 1551 9217 1551 8987 1517 8987 1517 9269 11 N F
943 10308 909 10308 911 10328 913 10337 916 10346 919 10354
923 10361 928 10368 933 10374 939 10379 945 10383 952 10387
960 10390 968 10393 976 10395 995 10396 1012 10395 1028 10391
1043 10385 1055 10377 1060 10372 1065 10366 1069 10360 1073 10354
1076 10346 1078 10339 1079 10330 1079 10322 1079 10311 1077 10302

1074 10294 1070 10286 1065 10280 1058 10274 1049 10268 1039 10263
1051 10258 1061 10253 1069 10246 1076 10239 1081 10230 1085 10221
1087 10210 1087 10199 1086 10184 1082 10169 1076 10155 1072 10148
1066 10142 1060 10136 1054 10131 1046 10126 1037 10122 1028 10119
1017 10117 1006 10115 993 10115 982 10115 972 10116 962 10118
954 10121 946 10124 939 10129 932 10133 926 10139 921 10145
917 10152 913 10159 910 10166 906 10183 905 10201 939 10201
939 10191 941 10181 944 10172 949 10163 956 10156 966 10150
979 10146 995 10145 1009 10145 1021 10148 1030 10153 1038 10160
1044 10168 1048 10177 1050 10188 1051 10199 1050 10213 1048 10219
1046 10225 1043 10229 1039 10233 1030 10240 1019 10244 1006 10247
991 10249 975 10249 975 10277 991 10278 1005 10279 1017 10281
1026 10285 1034 10290 1039 10298 1042 10307 1043 10320 1042 10329
1040 10338 1037 10346 1032 10352 1025 10358 1017 10362 1007 10365
995 10366 984 10365 974 10363 966 10360 958 10354 952 10346
948 10336 944 10323 943 10308 123 N F
1212 10396 1222 10395 1231 10393 1240 10389 1249 10384 1257 10377
1264 10369 1271 10361 1277 10351 1283 10341 1288 10330 1292 10318
1296 10306 1298 10294 1300 10281 1302 10268 1302 10255 1302 10242
1300 10230 1296 10204 1292 10192 1288 10180 1283 10169 1277 10158
1271 10149 1264 10140 1257 10132 1249 10125 1240 10120 1231 10116
1222 10113 1212 10113 1202 10113 1192 10116 1183 10120 1175 10125
1167 10132 1159 10140 1152 10149 1146 10158 1141 10169 1136 10180
1131 10192 1128 10204 1123 10230 1122 10242 1121 10255 1157 10255
1159 10231 1162 10209 1168 10190 1175 10173 1183 10160 1192 10151
1197 10147 1202 10145 1207 10143 1212 10143 1217 10143 1222 10145
1226 10147 1231 10151 1240 10160 1249 10173 1256 10190 1261 10209
1265 10231 1266 10255 1265 10280 1261 10301 1256 10320 1249 10336
1240 10349 1231 10358 1226 10361 1222 10364 1217 10365 1212 10366
1207 10365 1202 10364 1197 10361 1192 10358 1183 10349 1175 10336
1168 10320 1162 10301 1159 10280 1157 10255 1121 10255 1122 10268
1123 10281 1125 10294 1128 10306 1131 10318 1136 10330 1141 10341
1146 10351 1152 10361 1159 10369 1167 10377 1175 10384 1183 10389
1192 10393 1202 10395 1212 10396 105 N F
1470 10344 1468 10331 1465 10319 1459 10308 1451 10299 1441 10291
1430 10285 1419 10281 1406 10280 1406 10305 1398 10306 1390 10308
1384 10312 1378 10316 1373 10322 1369 10329 1367 10336 1366 10344
1367 10352 1369 10360 1373 10366 1378 10372 1384 10377 1390 10380
1398 10383 1406 10384 1414 10383 1421 10380 1428 10377 1434 10372
1438 10366 1442 10360 1444 10352 1445 10344 1444 10336 1442 10329
1438 10322 1434 10316 1428 10312 1421 10308 1414 10306 1406 10305
1406 10280 1393 10281 1381 10285 1370 10291 1361 10299 1353 10308
1347 10319 1343 10331 1342 10344 1343 10357 1347 10369 1353 10380

1361 10389 1370 10397 1381 10403 1393 10407 1406 10408 1419 10407
1430 10403 1441 10397 1451 10389 1459 10380 1465 10369 1468 10357
1470 10344 67 N F
1517 10404 1557 10404 1701 10175 1701 10404 1735 10404 1735 10123
1695 10123 1551 10352 1551 10123 1517 10123 1517 10404 11 N F
943 11433 909 11433 911 11453 913 11462 916 11471 919 11479
923 11486 928 11493 933 11499 939 11504 945 11509 952 11512
960 11516 968 11518 976 11520 995 11521 1012 11520 1028 11516
1043 11510 1055 11502 1060 11497 1065 11491 1069 11485 1073 11479
1076 11472 1078 11464 1079 11456 1079 11447 1079 11437 1077 11427
1074 11419 1070 11412 1065 11405 1058 11399 1049 11393 1039 11388
1051 11384 1061 11378 1069 11371 1076 11364 1081 11355 1085 11346
1087 11336 1087 11324 1086 11309 1082 11294 1076 11280 1072 11273
1066 11267 1060 11261 1054 11256 1046 11251 1037 11247 1028 11244
1017 11242 1006 11240 993 11240 982 11240 972 11241 962 11243
954 11246 946 11250 939 11254 932 11259 926 11264 921 11270
917 11277 913 11284 910 11291 906 11308 905 11326 939 11326
939 11316 941 11306 944 11297 949 11288 956 11281 966 11275
979 11271 995 11270 1009 11270 1021 11273 1030 11278 1038 11285
1044 11293 1048 11303 1050 11313 1051 11324 1050 11338 1048 11344
1046 11350 1043 11355 1039 11359 1030 11365 1019 11370 1006 11372
991 11374 975 11374 975 11403 991 11403 1005 11404 1017 11406
1026 11410 1034 11415 1039 11423 1042 11433 1043 11445 1042 11454
1040 11463 1037 11471 1032 11478 1025 11483 1017 11487 1007 11490
995 11491 984 11490 974 11488 966 11485 958 11479 952 11471
948 11461 944 11448 943 11433 123 N F
1125 11427 1160 11427 1160 11440 1163 11453 1167 11464 1173 11473
1181 11481 1191 11486 1202 11490 1216 11491 1226 11490 1236 11487
1245 11483 1252 11477 1259 11469 1264 11460 1267 11450 1268 11439
1267 11427 1264 11417 1259 11409 1253 11402 1244 11394 1233 11387
1206 11370 1182 11357 1163 11343 1148 11329 1137 11314 1129 11299
1123 11283 1120 11266 1119 11248 1302 11248 1302 11280 1157 11280
1161 11292 1166 11303 1172 11311 1181 11319 1191 11327 1203 11334
1234 11352 1252 11361 1267 11371 1279 11382 1288 11394 1296 11406
1300 11418 1303 11431 1304 11443 1304 11451 1302 11459 1300 11467
1297 11474 1293 11481 1289 11488 1284 11494 1278 11499 1265 11509
1250 11515 1233 11520 1216 11521 1197 11520 1188 11518 1180 11515
1172 11512 1164 11508 1157 11503 1151 11498 1145 11492 1140 11485
1136 11477 1132 11469 1129 11459 1127 11449 1126 11438 1125 11427
78 N F
1470 11469 1468 11457 1465 11445 1459 11434 1451 11424 1441 11416
1430 11410 1419 11407 1406 11405 1406 11430 1398 11431 1390 11433
1384 11437 1378 11442 1373 11447 1369 11454 1367 11461 1366 11469

1367 11477 1369 11485 1373 11491 1378 11497 1384 11502 1390 11506
1398 11508 1406 11509 1414 11508 1421 11506 1428 11502 1434 11497
1438 11491 1442 11485 1444 11477 1445 11469 1444 11461 1442 11454
1438 11447 1434 11442 1428 11437 1421 11433 1414 11431 1406 11430
1406 11405 1393 11407 1381 11410 1370 11416 1361 11424 1353 11434
1347 11445 1343 11457 1342 11469 1343 11482 1347 11494 1353 11505
1361 11515 1370 11522 1381 11528 1393 11532 1406 11533 1419 11532
1430 11528 1441 11522 1451 11515 1459 11505 1465 11494 1468 11482
1470 11469 67 N F
1517 11529 1557 11529 1701 11300 1701 11529 1735 11529 1735 11248
1695 11248 1551 11477 1551 11248 1517 11248 1517 11529 11 N F
943 12551 909 12551 911 12572 913 12581 916 12589 919 12597
923 12605 928 12611 933 12617 939 12622 945 12627 952 12631
960 12634 968 12636 976 12638 995 12640 1012 12638 1028 12635
1043 12629 1055 12620 1060 12615 1065 12610 1069 12604 1073 12597
1076 12590 1078 12582 1079 12574 1079 12565 1079 12555 1077 12546
1074 12538 1070 12530 1065 12523 1058 12517 1049 12512 1039 12507
1051 12502 1061 12496 1069 12490 1076 12482 1081 12474 1085 12464
1087 12454 1087 12443 1086 12427 1082 12412 1076 12398 1072 12392
1066 12385 1060 12379 1054 12374 1046 12370 1037 12366 1028 12362
1017 12360 1006 12359 993 12358 982 12359 972 12360 962 12362
954 12365 946 12368 939 12372 932 12377 926 12382 921 12389
917 12395 913 12402 910 12410 906 12426 905 12445 939 12445
939 12435 941 12425 944 12415 949 12407 956 12399 966 12393
979 12390 995 12388 1009 12389 1021 12392 1030 12397 1038 12403
1044 12412 1048 12421 1050 12431 1051 12443 1050 12457 1048 12463
1046 12468 1043 12473 1039 12477 1030 12483 1019 12488 1006 12491
991 12492 975 12493 975 12521 991 12521 1005 12522 1017 12525
1026 12528 1034 12534 1039 12541 1042 12551 1043 12563 1042 12573
1040 12581 1037 12589 1032 12596 1025 12602 1017 12606 1007 12608
995 12609 984 12609 974 12607 966 12603 958 12598 952 12590
948 12580 944 12567 943 12551 123 N F
1242 12640 1266 12640 1266 12463 1308 12463 1308 12430 1266 12430
1266 12366 1232 12366 1232 12430 1117 12430 1117 12463 1147 12463
1232 12463 1232 12581 1147 12463 1117 12463 1117 12467 1242 12640
18 N F
1470 12588 1468 12575 1465 12563 1459 12552 1451 12543 1441 12535
1430 12529 1419 12525 1406 12524 1406 12548 1398 12549 1390 12551
1384 12555 1378 12560 1373 12566 1369 12572 1367 12580 1366 12588
1367 12596 1369 12603 1373 12610 1378 12616 1384 12620 1390 12624
1398 12626 1406 12627 1414 12626 1421 12624 1428 12620 1434 12616
1438 12610 1442 12603 1444 12596 1445 12588 1444 12580 1442 12572
1438 12566 1434 12560 1428 12555 1421 12551 1414 12549 1406 12548

1406 12524 1393 12525 1381 12529 1370 12535 1361 12543 1353 12552
1347 12563 1343 12575 1342 12588 1343 12601 1347 12613 1353 12623
1361 12633 1370 12641 1381 12647 1393 12650 1406 12652 1419 12650
1430 12647 1441 12641 1451 12633 1459 12623 1465 12613 1468 12601
1470 12588 67 N F
1517 12648 1557 12648 1701 12418 1701 12648 1735 12648 1735 12366
1695 12366 1551 12595 1551 12366 1517 12366 1517 12648 11 N F
943 13664 909 13664 911 13684 913 13694 916 13702 919 13710
923 13717 928 13724 933 13730 939 13735 945 13740 952 13744
960 13747 968 13749 976 13751 995 13752 1012 13751 1028 13747
1043 13741 1055 13733 1060 13728 1065 13723 1069 13717 1073 13710
1076 13703 1078 13695 1079 13687 1079 13678 1079 13668 1077 13659
1074 13650 1070 13643 1065 13636 1058 13630 1049 13624 1039 13620
1051 13615 1061 13609 1069 13602 1076 13595 1081 13587 1085 13577
1087 13567 1087 13555 1086 13540 1082 13525 1076 13511 1072 13504
1066 13498 1060 13492 1054 13487 1046 13482 1037 13478 1028 13475
1017 13473 1006 13471 993 13471 982 13471 972 13473 962 13475
954 13477 946 13481 939 13485 932 13490 926 13495 921 13501
917 13508 913 13515 910 13523 906 13539 905 13557 939 13557
939 13547 941 13538 944 13528 949 13519 956 13512 966 13506
979 13502 995 13501 1009 13502 1021 13504 1030 13509 1038 13516
1044 13524 1048 13534 1050 13544 1051 13555 1050 13570 1048 13576
1046 13581 1043 13586 1039 13590 1030 13596 1019 13601 1006 13604
991 13605 975 13606 975 13634 991 13634 1005 13635 1017 13638
1026 13641 1034 13647 1039 13654 1042 13664 1043 13676 1042 13685
1040 13694 1037 13702 1032 13709 1025 13714 1017 13719 1007 13721
995 13722 984 13722 974 13720 966 13716 958 13710 952 13703
948 13692 944 13680 943 13664 123 N F
1298 13682 1264 13682 1262 13691 1258 13699 1253 13706 1248 13712
1241 13716 1235 13720 1227 13721 1220 13722 1212 13722 1206 13720
1199 13718 1193 13715 1188 13711 1182 13707 1178 13701 1174 13695
1167 13680 1162 13663 1159 13643 1157 13620 1166 13628 1174 13635
1182 13641 1190 13645 1198 13647 1206 13649 1224 13650 1237 13648
1251 13644 1265 13638 1277 13628 1283 13622 1288 13616 1293 13609
1297 13601 1300 13592 1303 13582 1304 13572 1304 13561 1270 13559
1269 13571 1265 13581 1260 13592 1253 13601 1245 13609 1235 13614
1225 13618 1214 13620 1203 13618 1192 13614 1183 13609 1174 13601
1167 13592 1162 13581 1159 13571 1157 13559 1159 13548 1162 13537
1167 13527 1174 13518 1183 13510 1192 13504 1203 13500 1214 13499
1225 13500 1235 13504 1245 13510 1253 13518 1260 13527 1265 13537
1269 13548 1270 13559 1304 13561 1303 13545 1299 13529 1292 13514
1287 13506 1282 13499 1276 13493 1270 13487 1262 13482 1254 13477
1245 13474 1235 13471 1225 13469 1214 13469 1201 13470 1190 13472

1180 13475 1170 13480 1162 13485 1154 13492 1148 13500 1142 13509
1137 13518 1133 13529 1129 13540 1126 13552 1123 13578 1121 13606
1122 13622 1123 13638 1125 13653 1128 13667 1132 13680 1136 13692
1141 13703 1147 13713 1154 13722 1161 13730 1169 13736 1178 13742
1188 13746 1198 13750 1208 13752 1220 13752 1236 13751 1250 13747
1263 13740 1274 13731 1283 13721 1290 13709 1295 13696 1298 13682
126 N F
1470 13701 1468 13688 1465 13676 1459 13665 1451 13655 1441 13647
1430 13642 1419 13638 1406 13637 1406 13661 1398 13662 1390 13664
1384 13668 1378 13673 1373 13678 1369 13685 1367 13693 1366 13701
1367 13708 1369 13716 1373 13723 1378 13728 1384 13733 1390 13737
1398 13739 1406 13740 1414 13739 1421 13737 1428 13733 1434 13728
1438 13723 1442 13716 1444 13708 1445 13701 1444 13693 1442 13685
1438 13678 1434 13673 1428 13668 1421 13664 1414 13662 1406 13661
1406 13637 1393 13638 1381 13642 1370 13647 1361 13655 1353 13665
1347 13676 1343 13688 1342 13701 1343 13713 1347 13725 1353 13736
1361 13746 1370 13754 1381 13759 1393 13763 1406 13765 1419 13763
1430 13759 1441 13754 1451 13746 1459 13736 1465 13725 1468 13713
1470 13701 67 N F
1517 13760 1557 13760 1701 13531 1701 13760 1735 13760 1735 13479
1695 13479 1551 13708 1551 13479 1517 13479 1517 13760 11 N F
943 14770 909 14770 911 14791 913 14800 916 14809 919 14817
923 14824 928 14830 933 14836 939 14842 945 14846 952 14850
960 14853 968 14856 976 14857 995 14859 1012 14858 1028 14854
1043 14848 1055 14840 1060 14835 1065 14829 1069 14823 1073 14816
1076 14809 1078 14801 1079 14793 1079 14784 1079 14774 1077 14765
1074 14757 1070 14749 1065 14742 1058 14736 1049 14731 1039 14726
1051 14721 1061 14715 1069 14709 1076 14701 1081 14693 1085 14684
1087 14673 1087 14662 1086 14647 1082 14632 1076 14617 1072 14611
1066 14604 1060 14599 1054 14593 1046 14589 1037 14585 1028 14582
1017 14579 1006 14578 993 14577 982 14578 972 14579 962 14581
954 14584 946 14587 939 14591 932 14596 926 14602 921 14608
917 14614 913 14621 910 14629 906 14646 905 14664 939 14664
939 14654 941 14644 944 14634 949 14626 956 14618 966 14613
979 14609 995 14608 1009 14608 1021 14611 1030 14616 1038 14623
1044 14631 1048 14640 1050 14651 1051 14662 1050 14676 1048 14682
1046 14687 1043 14692 1039 14696 1030 14703 1019 14707 1006 14710
991 14712 975 14712 975 14740 991 14740 1005 14742 1017 14744
1026 14748 1034 14753 1039 14761 1042 14770 1043 14782 1042 14792
1040 14801 1037 14808 1032 14815 1025 14821 1017 14825 1007 14828
995 14829 984 14828 974 14826 966 14822 958 14817 952 14809
948 14799 944 14786 943 14770 123 N F
1212 14859 1228 14858 1244 14854 1258 14848 1270 14840 1280 14830

1284 14824 1288 14817 1290 14810 1292 14803 1294 14795 1294 14786
1293 14774 1291 14764 1287 14756 1282 14748 1276 14742 1270 14737
1258 14728 1268 14723 1277 14717 1285 14709 1291 14701 1297 14691
1301 14681 1303 14671 1304 14660 1303 14646 1298 14632 1291 14618
1280 14605 1274 14599 1267 14593 1259 14588 1251 14584 1241 14580
1232 14578 1221 14576 1210 14575 1198 14576 1188 14578 1178 14580
1169 14584 1161 14588 1154 14593 1147 14599 1141 14605 1132 14618
1125 14632 1121 14646 1119 14660 1155 14660 1157 14649 1160 14639
1165 14629 1172 14621 1181 14615 1190 14610 1201 14607 1212 14606
1223 14607 1233 14610 1243 14615 1251 14621 1258 14629 1263 14639
1267 14649 1268 14660 1267 14671 1263 14681 1258 14690 1251 14698
1243 14705 1233 14710 1223 14713 1212 14714 1201 14713 1190 14710
1181 14705 1172 14698 1165 14690 1160 14681 1157 14671 1155 14660
1119 14660 1120 14671 1123 14681 1127 14691 1132 14700 1139 14708
1146 14716 1155 14722 1166 14728 1153 14737 1147 14742 1141 14748
1137 14756 1133 14764 1130 14774 1129 14786 1164 14786 1165 14777
1167 14769 1172 14762 1178 14755 1185 14751 1193 14747 1202 14745
1212 14744 1221 14745 1230 14747 1238 14751 1246 14755 1251 14762
1256 14769 1259 14777 1260 14786 1259 14796 1256 14804 1251 14811
1246 14817 1238 14822 1230 14826 1221 14828 1212 14829 1202 14828
1193 14826 1185 14822 1178 14817 1172 14811 1167 14804 1165 14796
1164 14786 1129 14786 1130 14795 1131 14803 1133 14810 1136 14817
1139 14824 1143 14830 1153 14840 1165 14848 1179 14854 1195 14858
1212 14859 145 N F
1470 14807 1468 14794 1465 14782 1459 14771 1451 14762 1441 14754
1430 14748 1419 14744 1406 14743 1406 14768 1398 14768 1390 14771
1384 14774 1378 14779 1373 14785 1369 14792 1367 14799 1366 14807
1367 14815 1369 14822 1373 14829 1378 14835 1384 14840 1390 14843
1398 14846 1406 14846 1414 14846 1421 14843 1428 14840 1434 14835
1438 14829 1442 14822 1444 14815 1445 14807 1444 14799 1442 14792
1438 14785 1434 14779 1428 14774 1421 14771 1414 14768 1406 14768
1406 14743 1393 14744 1381 14748 1370 14754 1361 14762 1353 14771
1347 14782 1343 14794 1342 14807 1343 14820 1347 14832 1353 14843
1361 14852 1370 14860 1381 14866 1393 14870 1406 14871 1419 14870
1430 14866 1441 14860 1451 14852 1459 14843 1465 14832 1468 14820
1470 14807 67 N F
1517 14867 1557 14867 1701 14638 1701 14867 1735 14867 1735 14585
1695 14585 1551 14815 1551 14585 1517 14585 1517 14867 11 N F
1027 15952 1051 15952 1051 15775 1093 15775 1093 15743 1051 15743
1051 15679 1017 15679 1017 15743 903 15743 903 15775 933 15775
1017 15775 1017 15894 933 15775 903 15775 903 15779 1027 15952
18 N F
1212 15952 1222 15951 1231 15949 1240 15945 1249 15940 1257 15933

1264 15925 1271 15917 1277 15907 1283 15897 1288 15886 1292 15874
1296 15862 1298 15850 1300 15837 1302 15824 1302 15811 1302 15798
1300 15785 1296 15760 1292 15748 1288 15736 1283 15725 1277 15714
1271 15705 1264 15696 1257 15688 1249 15681 1240 15676 1231 15672
1222 15669 1212 15669 1202 15669 1192 15672 1183 15676 1175 15681
1167 15688 1159 15696 1152 15705 1146 15714 1141 15725 1136 15736
1131 15748 1128 15760 1123 15785 1122 15798 1121 15811 1157 15811
1159 15787 1162 15765 1168 15746 1175 15729 1183 15716 1192 15707
1197 15703 1202 15701 1207 15699 1212 15699 1217 15699 1222 15701
1226 15703 1231 15707 1240 15716 1249 15729 1256 15746 1261 15765
1265 15787 1266 15811 1265 15836 1261 15857 1256 15876 1249 15892
1240 15905 1231 15914 1226 15917 1222 15920 1217 15921 1212 15922
1207 15921 1202 15920 1197 15917 1192 15914 1183 15905 1175 15892
1168 15876 1162 15857 1159 15836 1157 15811 1121 15811 1122 15824
1123 15837 1125 15850 1128 15862 1131 15874 1136 15886 1141 15897
1146 15907 1152 15917 1159 15925 1167 15933 1175 15940 1183 15945
1192 15949 1202 15951 1212 15952 105 N F
1470 15900 1468 15887 1465 15875 1459 15864 1451 15855 1441 15847
1430 15841 1419 15837 1406 15836 1406 15861 1398 15862 1390 15864
1384 15868 1378 15872 1373 15878 1369 15885 1367 15892 1366 15900
1367 15908 1369 15916 1373 15922 1378 15928 1384 15933 1390 15936
1398 15939 1406 15940 1414 15939 1421 15936 1428 15933 1434 15928
1438 15922 1442 15916 1444 15908 1445 15900 1444 15892 1442 15885
1438 15878 1434 15872 1428 15868 1421 15864 1414 15862 1406 15861
1406 15836 1393 15837 1381 15841 1370 15847 1361 15855 1353 15864
1347 15875 1343 15887 1342 15900 1343 15913 1347 15925 1353 15936
1361 15945 1370 15953 1381 15959 1393 15963 1406 15964 1419 15963
1430 15959 1441 15953 1451 15945 1459 15936 1465 15925 1468 15913
1470 15900 67 N F
1517 15960 1557 15960 1701 15731 1701 15960 1735 15960 1735 15679
1695 15679 1551 15908 1551 15679 1517 15679 1517 15960 11 N F
13570 5585 13604 5585 13605 5599 13607 5611 13611 5622 13618 5632
13625 5639 13635 5645 13647 5648 13660 5650 13670 5649 13680 5646
13689 5642 13697 5636 13703 5628 13708 5619 13711 5609 13712 5597
13711 5586 13708 5576 13704 5568 13697 5560 13688 5553 13677 5546
13650 5529 13627 5515 13608 5502 13593 5488 13581 5473 13573 5458
13567 5442 13564 5425 13564 5406 13746 5406 13746 5439 13602 5439
13605 5451 13610 5461 13617 5470 13625 5478 13635 5485 13647 5493
13678 5511 13696 5520 13711 5530 13723 5541 13733 5552 13740 5564
13745 5577 13747 5589 13748 5601 13748 5610 13746 5618 13744 5626
13741 5633 13737 5640 13733 5646 13728 5652 13722 5658 13709 5667
13694 5674 13678 5678 13660 5680 13641 5678 13632 5677 13624 5674
13616 5671 13608 5667 13602 5662 13595 5657 13589 5650 13584 5643

13580 5636 13576 5627 13573 5618 13571 5608 13570 5597 13570 5585
78 N F
13784 5585 13818 5585 13819 5599 13822 5611 13826 5622 13832 5632
13840 5639 13850 5645 13861 5648 13875 5650 13885 5649 13895 5646
13904 5642 13911 5636 13918 5628 13923 5619 13926 5609 13927 5597
13926 5586 13923 5576 13918 5568 13912 5560 13903 5553 13892 5546
13865 5529 13841 5515 13822 5502 13807 5488 13796 5473 13788 5458
13782 5442 13779 5425 13778 5406 13961 5406 13961 5439 13816 5439
13820 5451 13825 5461 13831 5470 13840 5478 13850 5485 13862 5493
13893 5511 13911 5520 13926 5530 13938 5541 13947 5552 13955 5564
13959 5577 13962 5589 13963 5601 13963 5610 13961 5618 13959 5626
13956 5633 13952 5640 13948 5646 13943 5652 13937 5658 13924 5667
13909 5674 13892 5678 13875 5680 13856 5678 13847 5677 13839 5674
13831 5671 13823 5667 13816 5662 13810 5657 13804 5650 13799 5643
13795 5636 13791 5627 13788 5618 13786 5608 13785 5597 13784 5585
78 N F
14129 5628 14127 5615 14124 5603 14118 5592 14110 5583 14100 5575
14089 5569 14077 5565 14065 5564 14065 5589 14057 5589 14049 5592
14043 5595 14037 5600 14032 5606 14028 5613 14026 5620 14025 5628
14026 5636 14028 5643 14032 5650 14037 5656 14043 5661 14049 5664
14057 5667 14065 5667 14073 5667 14080 5664 14087 5661 14092 5656
14097 5650 14101 5643 14103 5636 14104 5628 14103 5620 14101 5613
14097 5606 14092 5600 14087 5595 14080 5592 14073 5589 14065 5589
14065 5564 14052 5565 14040 5569 14029 5575 14019 5583 14012 5592
14006 5603 14002 5615 14001 5628 14002 5641 14006 5653 14012 5664
14019 5673 14029 5681 14040 5687 14052 5691 14065 5692 14077 5691
14089 5687 14100 5681 14110 5673 14118 5664 14124 5653 14127 5641
14129 5628 67 N F
14176 5688 14216 5688 14360 5459 14360 5688 14394 5688 14394 5406
14354 5406 14210 5636 14210 5406 14176 5406 14176 5688 11 N F
13570 6755 13604 6755 13605 6769 13607 6781 13611 6792 13618 6802
13625 6809 13635 6815 13647 6818 13660 6819 13670 6818 13680 6816
13689 6811 13697 6805 13703 6798 13708 6789 13711 6779 13712 6767
13711 6755 13708 6746 13704 6737 13697 6730 13688 6723 13677 6716
13650 6699 13627 6685 13608 6671 13593 6657 13581 6643 13573 6628
13567 6611 13564 6594 13564 6576 13746 6576 13746 6608 13602 6608
13605 6621 13610 6631 13617 6640 13625 6648 13635 6655 13647 6663
13678 6681 13696 6690 13711 6700 13723 6711 13733 6722 13740 6734
13745 6747 13747 6759 13748 6771 13748 6780 13746 6788 13744 6796
13741 6803 13737 6810 13733 6816 13728 6822 13722 6828 13709 6837
13694 6844 13678 6848 13660 6850 13641 6848 13632 6846 13624 6844
13616 6841 13608 6837 13602 6832 13595 6826 13589 6820 13584 6813
13580 6806 13576 6797 13573 6788 13571 6778 13570 6767 13570 6755

78 N F
13901 6850 13925 6850 13925 6673 13967 6673 13967 6641 13925 6641
13925 6576 13891 6576 13891 6641 13776 6641 13776 6673 13806 6673
13891 6673 13891 6791 13806 6673 13776 6673 13776 6677 13901 6850
18 N F
14129 6798 14127 6785 14124 6773 14118 6762 14110 6753 14100 6745
14089 6739 14077 6735 14065 6734 14065 6758 14057 6759 14049 6762
14043 6765 14037 6770 14032 6776 14028 6782 14026 6790 14025 6798
14026 6806 14028 6813 14032 6820 14037 6826 14043 6830 14049 6834
14057 6836 14065 6837 14073 6836 14080 6834 14087 6830 14092 6826
14097 6820 14101 6813 14103 6806 14104 6798 14103 6790 14101 6782
14097 6776 14092 6770 14087 6765 14080 6762 14073 6759 14065 6758
14065 6734 14052 6735 14040 6739 14029 6745 14019 6753 14012 6762
14006 6773 14002 6785 14001 6798 14002 6811 14006 6823 14012 6834
14019 6843 14029 6851 14040 6857 14052 6861 14065 6862 14077 6861
14089 6857 14100 6851 14110 6843 14118 6834 14124 6823 14127 6811
14129 6798 67 N F
14176 6858 14216 6858 14360 6628 14360 6858 14394 6858 14394 6576
14354 6576 14210 6805 14210 6576 14176 6576 14176 6858 11 N F
13570 7914 13604 7914 13605 7927 13607 7940 13611 7951 13618 7960
13625 7968 13635 7973 13647 7977 13660 7978 13670 7977 13680 7974
13689 7970 13697 7964 13703 7956 13708 7947 13711 7937 13712 7926
13711 7914 13708 7904 13704 7896 13697 7888 13688 7881 13677 7874
13650 7857 13627 7844 13608 7830 13593 7816 13581 7801 13573 7786
13567 7770 13564 7753 13564 7735 13746 7735 13746 7767 13602 7767
13605 7779 13610 7789 13617 7798 13625 7806 13635 7814 13647 7821
13678 7839 13696 7848 13711 7858 13723 7869 13733 7881 13740 7893
13745 7905 13747 7917 13748 7930 13748 7938 13746 7946 13744 7954
13741 7961 13737 7968 13733 7975 13728 7981 13722 7986 13709 7995
13694 8002 13678 8007 13660 8008 13641 8007 13632 8005 13624 8002
13616 7999 13608 7995 13602 7990 13595 7985 13589 7979 13584 7972
13580 7964 13576 7955 13573 7946 13571 7936 13570 7925 13570 7914
78 N F
13957 7938 13923 7938 13921 7947 13917 7955 13912 7962 13907 7968
13900 7972 13893 7975 13886 7977 13879 7978 13871 7977 13864 7976
13858 7974 13852 7971 13846 7967 13841 7962 13837 7957 13833 7951
13826 7936 13821 7919 13817 7898 13816 7875 13825 7884 13833 7891
13841 7896 13849 7900 13857 7903 13865 7905 13883 7906 13896 7904
13910 7900 13924 7893 13936 7884 13942 7878 13947 7872 13952 7864
13956 7856 13959 7848 13962 7838 13963 7828 13963 7817 13929 7815
13928 7826 13924 7837 13919 7847 13912 7857 13904 7864 13894 7870
13884 7874 13873 7875 13862 7874 13851 7870 13842 7864 13833 7857
13826 7847 13821 7837 13818 7826 13816 7815 13818 7804 13821 7793

13826 7783 13833 7774 13842 7766 13851 7760 13862 7756 13873 7755
13884 7756 13894 7760 13904 7766 13912 7774 13919 7783 13924 7793
13928 7804 13929 7815 13963 7817 13962 7801 13958 7785 13951 7769
13946 7762 13941 7755 13935 7749 13929 7743 13921 7738 13913 7733
13904 7730 13894 7727 13884 7725 13873 7725 13860 7725 13849 7727
13839 7731 13829 7735 13821 7741 13813 7748 13807 7756 13801 7764
13796 7774 13792 7785 13788 7796 13785 7808 13782 7834 13780 7861
13781 7878 13782 7894 13784 7908 13787 7922 13791 7935 13795 7948
13800 7959 13806 7969 13813 7978 13820 7985 13828 7992 13837 7998
13846 8002 13857 8005 13867 8007 13879 8008 13895 8007 13909 8002
13922 7996 13933 7987 13942 7977 13949 7965 13954 7952 13957 7938
126 N F
14129 7956 14127 7943 14124 7931 14118 7921 14110 7911 14100 7903
14089 7897 14077 7894 14065 7892 14065 7917 14057 7918 14049 7920
14043 7924 14037 7928 14032 7934 14028 7941 14026 7948 14025 7956
14026 7964 14028 7972 14032 7978 14037 7984 14043 7989 14049 7993
14057 7995 14065 7996 14073 7995 14080 7993 14087 7989 14092 7984
14097 7978 14101 7972 14103 7964 14104 7956 14103 7948 14101 7941
14097 7934 14092 7928 14087 7924 14080 7920 14073 7918 14065 7917
14065 7892 14052 7894 14040 7897 14029 7903 14019 7911 14012 7921
14006 7931 14002 7943 14001 7956 14002 7969 14006 7981 14012 7992
14019 8002 14029 8009 14040 8015 14052 8019 14065 8020 14077 8019
14089 8015 14100 8009 14110 8002 14118 7992 14124 7981 14127 7969
14129 7956 67 N F
14176 8016 14216 8016 14360 7787 14360 8016 14394 8016 14394 7735
14354 7735 14210 7964 14210 7735 14176 7735 14176 8016 11 N F
13570 9056 13604 9056 13605 9069 13607 9082 13611 9093 13618 9102
13625 9110 13635 9115 13647 9119 13660 9120 13670 9119 13680 9116
13689 9112 13697 9106 13703 9098 13708 9090 13711 9079 13712 9068
13711 9056 13708 9046 13704 9038 13697 9031 13688 9024 13677 9016
13650 8999 13627 8986 13608 8972 13593 8958 13581 8943 13573 8928
13567 8912 13564 8895 13564 8877 13746 8877 13746 8909 13602 8909
13605 8921 13610 8932 13617 8940 13625 8948 13635 8956 13647 8963
13678 8981 13696 8990 13711 9000 13723 9011 13733 9023 13740 9035
13745 9047 13747 9060 13748 9072 13748 9080 13746 9088 13744 9096
13741 9104 13737 9110 13733 9117 13728 9123 13722 9128 13709 9138
13694 9144 13678 9149 13660 9150 13641 9149 13632 9147 13624 9145
13616 9141 13608 9137 13602 9133 13595 9127 13589 9121 13584 9114
13580 9106 13576 9098 13573 9088 13571 9078 13570 9067 13570 9056
78 N F
13871 9150 13887 9149 13903 9145 13917 9140 13929 9131 13939 9121
13943 9115 13947 9109 13949 9102 13951 9094 13953 9086 13953 9078
13952 9066 13950 9056 13946 9047 13941 9040 13935 9033 13929 9028

13917 9020 13927 9014 13936 9008 13944 9001 13950 8992 13956 8983
13960 8973 13962 8962 13963 8951 13961 8938 13957 8924 13950 8910
13939 8896 13933 8890 13926 8885 13918 8880 13910 8875 13900 8872
13891 8869 13880 8867 13869 8867 13857 8867 13847 8869 13837 8872
13828 8875 13820 8880 13813 8885 13806 8890 13800 8896 13790 8910
13784 8924 13780 8938 13778 8951 13814 8951 13816 8940 13819 8930
13824 8921 13831 8913 13840 8906 13849 8901 13860 8898 13871 8897
13882 8898 13892 8901 13902 8906 13910 8913 13917 8921 13922 8930
13926 8940 13927 8951 13926 8962 13922 8972 13917 8982 13910 8990
13902 8996 13892 9001 13882 9004 13871 9006 13860 9004 13849 9001
13840 8996 13831 8990 13824 8982 13819 8972 13816 8962 13814 8951
13778 8951 13779 8962 13782 8973 13786 8982 13791 8991 13798 9000
13805 9007 13814 9014 13824 9020 13812 9028 13806 9033 13800 9040
13796 9047 13792 9056 13789 9066 13788 9078 13822 9078 13823 9069
13826 9060 13831 9053 13837 9047 13844 9042 13852 9039 13861 9036
13871 9036 13880 9036 13889 9039 13897 9042 13905 9047 13910 9053
13915 9060 13918 9069 13919 9078 13918 9087 13915 9095 13910 9103
13905 9109 13897 9114 13889 9117 13880 9119 13871 9120 13861 9119
13852 9117 13844 9114 13837 9109 13831 9103 13826 9095 13823 9087
13822 9078 13788 9078 13789 9086 13790 9094 13792 9102 13795 9109
13798 9115 13802 9121 13812 9131 13824 9140 13838 9145 13854 9149
13871 9150 145 N F
14129 9098 14127 9086 14124 9074 14118 9063 14110 9053 14100 9045
14089 9040 14077 9036 14065 9034 14065 9059 14057 9060 14049 9062
14043 9066 14037 9071 14032 9076 14028 9083 14026 9091 14025 9098
14026 9106 14028 9114 14032 9121 14037 9126 14043 9131 14049 9135
14057 9137 14065 9138 14073 9137 14080 9135 14087 9131 14092 9126
14097 9121 14101 9114 14103 9106 14104 9098 14103 9091 14101 9083
14097 9076 14092 9071 14087 9066 14080 9062 14073 9060 14065 9059
14065 9034 14052 9036 14040 9040 14029 9045 14019 9053 14012 9063
14006 9074 14002 9086 14001 9098 14002 9111 14006 9123 14012 9134
14019 9144 14029 9151 14040 9157 14052 9161 14065 9162 14077 9161
14089 9157 14100 9151 14110 9144 14118 9134 14124 9123 14127 9111
14129 9098 67 N F
14176 9158 14216 9158 14360 8929 14360 9158 14394 9158 14394 8877
14354 8877 14210 9106 14210 8877 14176 8877 14176 9158 11 N F
13602 10194 13568 10194 13570 10214 13572 10223 13575 10232 13578 10240
13582 10247 13587 10254 13592 10260 13598 10265 13604 10270 13611 10273
13619 10277 13627 10279 13635 10281 13654 10282 13671 10281 13687 10277
13702 10271 13714 10263 13719 10258 13724 10252 13728 10246 13732 10240
13735 10233 13737 10225 13738 10217 13738 10208 13738 10198 13736 10188
13733 10180 13729 10173 13724 10166 13717 10160 13708 10154 13698 10149
13710 10145 13720 10139 13728 10132 13735 10125 13740 10116 13743 10107

13746 10097 13746 10085 13745 10070 13741 10055 13735 10041 13731 10034
13725 10028 13719 10022 13713 10017 13705 10012 13696 10008 13687 10005
13676 10003 13665 10001 13652 10001 13641 10001 13631 10002 13621 10004
13613 10007 13605 10011 13597 10015 13591 10020 13585 10025 13580 10031
13576 10038 13572 10045 13569 10052 13565 10069 13564 10087 13598 10087
13598 10077 13600 10067 13603 10058 13608 10049 13615 10042 13625 10036
13638 10032 13654 10031 13668 10031 13680 10034 13689 10039 13697 10046
13703 10054 13707 10064 13709 10074 13710 10085 13709 10099 13707 10105
13705 10111 13702 10116 13698 10120 13689 10126 13678 10131 13665 10133
13650 10135 13634 10135 13634 10164 13650 10164 13664 10165 13676 10167
13685 10171 13693 10176 13698 10184 13701 10194 13702 10206 13701 10215
13699 10224 13696 10232 13691 10239 13684 10244 13676 10248 13666 10251
13654 10252 13643 10251 13633 10249 13625 10246 13617 10240 13611 10232
13606 10222 13603 10209 13602 10194 123 N F
13871 10282 13881 10281 13890 10279 13899 10275 13908 10270 13916 10263
13923 10256 13930 10247 13936 10237 13942 10227 13947 10216 13951 10204
13954 10192 13957 10180 13959 10167 13961 10154 13961 10141 13961 10129
13959 10116 13954 10090 13951 10078 13947 10066 13942 10055 13936 10044
13930 10035 13923 10026 13916 10018 13908 10011 13899 10006 13890 10002
13881 10000 13871 9999 13861 10000 13851 10002 13842 10006 13834 10011
13826 10018 13818 10026 13811 10035 13805 10044 13800 10055 13795 10066
13790 10078 13787 10090 13782 10116 13781 10129 13780 10141 13816 10141
13818 10117 13821 10095 13827 10076 13834 10060 13842 10046 13851 10037
13856 10033 13861 10031 13866 10029 13871 10029 13876 10029 13880 10031
13885 10033 13890 10037 13899 10046 13908 10060 13915 10076 13920 10095
13924 10117 13925 10141 13924 10166 13920 10188 13915 10206 13908 10222
13899 10235 13890 10244 13885 10248 13880 10250 13876 10251 13871 10252
13866 10251 13861 10250 13856 10248 13851 10244 13842 10235 13834 10222
13827 10206 13821 10188 13818 10166 13816 10141 13780 10141 13781 10154
13782 10167 13784 10180 13787 10192 13790 10204 13795 10216 13800 10227
13805 10237 13811 10247 13818 10256 13826 10263 13834 10270 13842 10275
13851 10279 13861 10281 13871 10282 105 N F
14129 10230 14127 10218 14124 10206 14118 10195 14110 10185 14100 10177
14089 10171 14077 10168 14065 10166 14065 10191 14057 10192 14049 10194
14043 10198 14037 10202 14032 10208 14028 10215 14026 10222 14025 10230
14026 10238 14028 10246 14032 10252 14037 10258 14043 10263 14049 10267
14057 10269 14065 10270 14073 10269 14080 10267 14087 10263 14092 10258
14097 10252 14101 10246 14103 10238 14104 10230 14103 10222 14101 10215
14097 10208 14092 10202 14087 10198 14080 10194 14073 10192 14065 10191
14065 10166 14052 10168 14040 10171 14029 10177 14019 10185 14012 10195
14006 10206 14002 10218 14001 10230 14002 10243 14006 10255 14012 10266
14019 10276 14029 10283 14040 10289 14052 10293 14065 10294 14077 10293
14089 10289 14100 10283 14110 10276 14118 10266 14124 10255 14127 10243

14129 10230 67 N F
14176 10290 14216 10290 14360 10061 14360 10290 14394 10290 14394 10009
14354 10009 14210 10238 14210 10009 14176 10009 14176 10290 11 N F
13602 11315 13568 11315 13570 11336 13572 11345 13575 11354 13578 11362
13582 11369 13587 11376 13592 11382 13598 11387 13604 11391 13611 11395
13619 11398 13627 11401 13635 11402 13654 11404 13671 11403 13687 11399
13702 11393 13714 11385 13719 11380 13724 11374 13728 11368 13732 11361
13735 11354 13737 11347 13738 11338 13738 11330 13738 11319 13736 11310
13733 11302 13729 11294 13724 11288 13717 11281 13708 11276 13698 11271
13710 11266 13720 11261 13728 11254 13735 11247 13740 11238 13743 11229
13746 11218 13746 11207 13745 11192 13741 11177 13735 11163 13731 11156
13725 11150 13719 11144 13713 11139 13705 11134 13696 11130 13687 11127
13676 11124 13665 11123 13652 11123 13641 11123 13631 11124 13621 11126
13613 11129 13605 11132 13597 11137 13591 11141 13585 11147 13580 11153
13576 11160 13572 11167 13569 11174 13565 11191 13564 11209 13598 11209
13598 11199 13600 11189 13603 11180 13608 11171 13615 11164 13625 11158
13638 11154 13654 11153 13668 11153 13680 11156 13689 11161 13697 11168
13703 11176 13707 11185 13709 11196 13710 11207 13709 11221 13707 11227
13705 11233 13702 11237 13698 11241 13689 11248 13678 11252 13665 11255
13650 11257 13634 11257 13634 11285 13650 11286 13664 11287 13676 11289
13685 11293 13693 11298 13698 11306 13701 11315 13702 11328 13701 11337
13699 11346 13696 11354 13691 11360 13684 11366 13676 11370 13666 11373
13654 11374 13643 11373 13633 11371 13625 11367 13617 11362 13611 11354
13606 11344 13603 11331 13602 11315 123 N F
13784 11309 13818 11309 13819 11323 13822 11336 13826 11347 13832 11356
13840 11363 13850 11369 13861 11373 13875 11374 13885 11373 13895 11370
13904 11366 13911 11360 13918 11352 13923 11343 13926 11333 13927 11321
13926 11310 13923 11300 13918 11292 13912 11284 13903 11277 13892 11270
13865 11253 13841 11240 13822 11226 13807 11212 13796 11197 13788 11182
13782 11166 13779 11149 13778 11131 13961 11131 13961 11163 13816 11163
13820 11175 13825 11185 13831 11194 13840 11202 13850 11209 13862 11217
13893 11235 13911 11244 13926 11254 13938 11265 13947 11277 13955 11289
13959 11301 13962 11313 13963 11326 13963 11334 13961 11342 13959 11350
13956 11357 13952 11364 13948 11371 13943 11377 13937 11382 13924 11391
13909 11398 13892 11402 13875 11404 13856 11402 13847 11401 13839 11398
13831 11395 13823 11391 13816 11386 13810 11381 13804 11375 13799 11368
13795 11360 13791 11351 13788 11342 13786 11332 13785 11321 13784 11309
78 N F
14129 11352 14127 11339 14124 11327 14118 11316 14110 11307 14100 11299
14089 11293 14077 11289 14065 11288 14065 11313 14057 11314 14049 11316
14043 11319 14037 11324 14032 11330 14028 11337 14026 11344 14025 11352
14026 11360 14028 11367 14032 11374 14037 11380 14043 11385 14049 11388
14057 11391 14065 11392 14073 11391 14080 11388 14087 11385 14092 11380

14097 11374 14101 11367 14103 11360 14104 11352 14103 11344 14101 11337
14097 11330 14092 11324 14087 11319 14080 11316 14073 11314 14065 11313
14065 11288 14052 11289 14040 11293 14029 11299 14019 11307 14012 11316
14006 11327 14002 11339 14001 11352 14002 11365 14006 11377 14012 11388
14019 11397 14029 11405 14040 11411 14052 11415 14065 11416 14077 11415
14089 11411 14100 11405 14110 11397 14118 11388 14124 11377 14127 11365
14129 11352 67 N F
14176 11412 14216 11412 14360 11183 14360 11412 14394 11412 14394 11131
14354 11131 14210 11360 14210 11131 14176 11131 14176 11412 11 N F
13602 12430 13568 12430 13570 12451 13572 12460 13575 12469 13578 12477
13582 12484 13587 12490 13592 12496 13598 12502 13604 12506 13611 12510
13619 12513 13627 12515 13635 12517 13654 12519 13671 12517 13687 12514
13702 12508 13714 12500 13719 12495 13724 12489 13728 12483 13732 12476
13735 12469 13737 12461 13738 12453 13738 12444 13738 12434 13736 12425
13733 12417 13729 12409 13724 12402 13717 12396 13708 12391 13698 12386
13710 12381 13720 12375 13728 12369 13735 12361 13740 12353 13743 12344
13746 12333 13746 12322 13745 12307 13741 12292 13735 12277 13731 12271
13725 12264 13719 12259 13713 12253 13705 12249 13696 12245 13687 12242
13676 12239 13665 12238 13652 12237 13641 12238 13631 12239 13621 12241
13613 12244 13605 12247 13597 12251 13591 12256 13585 12262 13580 12268
13576 12274 13572 12281 13569 12289 13565 12306 13564 12324 13598 12324
13598 12314 13600 12304 13603 12294 13608 12286 13615 12278 13625 12273
13638 12269 13654 12267 13668 12268 13680 12271 13689 12276 13697 12282
13703 12291 13707 12300 13709 12311 13710 12322 13709 12336 13707 12342
13705 12347 13702 12352 13698 12356 13689 12363 13678 12367 13665 12370
13650 12372 13634 12372 13634 12400 13650 12400 13664 12402 13676 12404
13685 12408 13693 12413 13698 12420 13701 12430 13702 12442 13701 12452
13699 12461 13696 12468 13691 12475 13684 12481 13676 12485 13666 12488
13654 12489 13643 12488 13633 12486 13625 12482 13617 12477 13611 12469
13606 12459 13603 12446 13602 12430 123 N F
13901 12519 13925 12519 13925 12342 13967 12342 13967 12310 13925 12310
13925 12245 13891 12245 13891 12310 13776 12310 13776 12342 13806 12342
13891 12342 13891 12460 13806 12342 13776 12342 13776 12346 13901 12519
18 N F
14129 12467 14127 12454 14124 12442 14118 12431 14110 12422 14100 12414
14089 12408 14077 12404 14065 12403 14065 12428 14057 12428 14049 12431
14043 12434 14037 12439 14032 12445 14028 12452 14026 12459 14025 12467
14026 12475 14028 12482 14032 12489 14037 12495 14043 12500 14049 12503
14057 12505 14065 12506 14073 12505 14080 12503 14087 12500 14092 12495
14097 12489 14101 12482 14103 12475 14104 12467 14103 12459 14101 12452
14097 12445 14092 12439 14087 12434 14080 12431 14073 12428 14065 12428
14065 12403 14052 12404 14040 12408 14029 12414 14019 12422 14012 12431
14006 12442 14002 12454 14001 12467 14002 12480 14006 12492 14012 12503

14019 12512 14029 12520 14040 12526 14052 12530 14065 12531 14077 12530
14089 12526 14100 12520 14110 12512 14118 12503 14124 12492 14127 12480
14129 12467 67 N F
14176 12527 14216 12527 14360 12298 14360 12527 14394 12527 14394 12245
14354 12245 14210 12474 14210 12245 14176 12245 14176 12527 11 N F
13602 13539 13568 13539 13570 13560 13572 13569 13575 13578 13578 13586
13582 13593 13587 13599 13592 13605 13598 13610 13604 13615 13611 13619
13619 13622 13627 13624 13635 13626 13654 13628 13671 13626 13687 13623
13702 13617 13714 13609 13719 13604 13724 13598 13728 13592 13732 13585
13735 13578 13737 13570 13738 13562 13738 13553 13738 13543 13736 13534
13733 13526 13729 13518 13724 13511 13717 13505 13708 13500 13698 13495
13710 13490 13720 13484 13728 13478 13735 13470 13740 13462 13743 13453
13746 13442 13746 13431 13745 13416 13741 13401 13735 13386 13731 13380
13725 13373 13719 13368 13713 13362 13705 13358 13696 13354 13687 13351
13676 13348 13665 13347 13652 13346 13641 13347 13631 13348 13621 13350
13613 13353 13605 13356 13597 13360 13591 13365 13585 13371 13580 13377
13576 13383 13572 13390 13569 13398 13565 13415 13564 13433 13598 13433
13598 13423 13600 13413 13603 13403 13608 13395 13615 13387 13625 13382
13638 13378 13654 13376 13668 13377 13680 13380 13689 13385 13697 13391
13703 13400 13707 13409 13709 13420 13710 13431 13709 13445 13707 13451
13705 13456 13702 13461 13698 13465 13689 13472 13678 13476 13665 13479
13650 13480 13634 13481 13634 13509 13650 13509 13664 13511 13676 13513
13685 13517 13693 13522 13698 13529 13701 13539 13702 13551 13701 13561
13699 13570 13696 13577 13691 13584 13684 13590 13676 13594 13666 13597
13654 13597 13643 13597 13633 13595 13625 13591 13617 13586 13611 13578
13606 13568 13603 13555 13602 13539 123 N F
13957 13557 13923 13557 13921 13567 13917 13575 13912 13581 13907 13587
13900 13592 13893 13595 13886 13597 13879 13597 13871 13597 13864 13596
13858 13594 13852 13591 13846 13587 13841 13582 13837 13577 13833 13570
13826 13556 13821 13538 13817 13518 13816 13495 13825 13504 13833 13511
13841 13516 13849 13520 13857 13522 13865 13524 13883 13525 13896 13524
13910 13520 13924 13513 13936 13504 13942 13498 13947 13491 13952 13484
13956 13476 13959 13467 13962 13458 13963 13448 13963 13437 13929 13435
13928 13446 13924 13457 13919 13467 13912 13476 13904 13484 13894 13490
13884 13494 13873 13495 13862 13494 13851 13490 13842 13484 13833 13476
13826 13467 13821 13457 13818 13446 13816 13435 13818 13423 13821 13413
13826 13402 13833 13393 13842 13386 13851 13380 13862 13376 13873 13374
13884 13376 13894 13380 13904 13386 13912 13393 13919 13402 13924 13413
13928 13423 13929 13435 13963 13437 13962 13421 13958 13404 13951 13389
13946 13382 13941 13375 13935 13368 13929 13362 13921 13357 13913 13353
13904 13349 13894 13346 13884 13345 13873 13344 13860 13345 13849 13347
13839 13350 13829 13355 13821 13361 13813 13367 13807 13375 13801 13384
13796 13394 13792 13404 13788 13415 13785 13427 13782 13453 13780 13481

13781 13497 13782 13513 13784 13528 13787 13542 13791 13555 13795 13567
13800 13578 13806 13588 13813 13597 13820 13605 13828 13612 13837 13617
13846 13622 13857 13625 13867 13627 13879 13628 13895 13626 13909 13622
13922 13615 13933 13607 13942 13596 13949 13584 13954 13571 13957 13557
126 N F
14129 13576 14127 13563 14124 13551 14118 13540 14110 13531 14100 13523
14089 13517 14077 13513 14065 13512 14065 13536 14057 13537 14049 13540
14043 13543 14037 13548 14032 13554 14028 13561 14026 13568 14025 13576
14026 13584 14028 13591 14032 13598 14037 13604 14043 13609 14049 13612
14057 13614 14065 13615 14073 13614 14080 13612 14087 13609 14092 13604
14097 13598 14101 13591 14103 13584 14104 13576 14103 13568 14101 13561
14097 13554 14092 13548 14087 13543 14080 13540 14073 13537 14065 13536
14065 13512 14052 13513 14040 13517 14029 13523 14019 13531 14012 13540
14006 13551 14002 13563 14001 13576 14002 13589 14006 13601 14012 13612
14019 13621 14029 13629 14040 13635 14052 13639 14065 13640 14077 13639
14089 13635 14100 13629 14110 13621 14118 13612 14124 13601 14127 13589
14129 13576 67 N F
14176 13636 14216 13636 14360 13407 14360 13636 14394 13636 14394 13354
14354 13354 14210 13583 14210 13354 14176 13354 14176 13636 11 N F
13602 14642 13568 14642 13570 14662 13572 14671 13575 14680 13578 14688
13582 14695 13587 14702 13592 14708 13598 14713 13604 14717 13611 14721
13619 14724 13627 14727 13635 14729 13654 14730 13671 14729 13687 14725
13702 14719 13714 14711 13719 14706 13724 14700 13728 14694 13732 14688
13735 14680 13737 14673 13738 14664 13738 14656 13738 14646 13736 14636
13733 14628 13729 14621 13724 14614 13717 14608 13708 14602 13698 14597
13710 14593 13720 14587 13728 14580 13735 14573 13740 14564 13743 14555
13746 14545 13746 14533 13745 14518 13741 14503 13735 14489 13731 14482
13725 14476 13719 14470 13713 14465 13705 14460 13696 14456 13687 14453
13676 14451 13665 14449 13652 14449 13641 14449 13631 14450 13621 14452
13613 14455 13605 14459 13597 14463 13591 14468 13585 14473 13580 14479
13576 14486 13572 14493 13569 14500 13565 14517 13564 14535 13598 14535
13598 14525 13600 14515 13603 14506 13608 14497 13615 14490 13625 14484
13638 14480 13654 14479 13668 14479 13680 14482 13689 14487 13697 14494
13703 14502 13707 14512 13709 14522 13710 14533 13709 14547 13707 14553
13705 14559 13702 14563 13698 14568 13689 14574 13678 14579 13665 14581
13650 14583 13634 14583 13634 14611 13650 14612 13664 14613 13676 14615
13685 14619 13693 14624 13698 14632 13701 14641 13702 14654 13701 14663
13699 14672 13696 14680 13691 14687 13684 14692 13676 14696 13666 14699
13654 14700 13643 14699 13633 14697 13625 14694 13617 14688 13611 14680
13606 14670 13603 14657 13602 14642 123 N F
13871 14730 13887 14729 13903 14725 13917 14719 13929 14711 13939 14701
13943 14695 13947 14689 13949 14682 13951 14674 13953 14666 13953 14658
13952 14646 13950 14636 13946 14627 13941 14620 13935 14613 13929 14608

13917 14599 13927 14594 13936 14588 13944 14580 13950 14572 13956 14563
13960 14553 13962 14542 13963 14531 13961 14518 13957 14503 13950 14489
13939 14476 13933 14470 13926 14464 13918 14459 13910 14455 13900 14452
13891 14449 13880 14447 13869 14447 13857 14447 13847 14449 13837 14452
13828 14455 13820 14459 13813 14464 13806 14470 13800 14476 13790 14489
13784 14503 13780 14518 13778 14531 13814 14531 13816 14520 13819 14510
13824 14501 13831 14493 13840 14486 13849 14481 13860 14478 13871 14477
13882 14478 13892 14481 13902 14486 13910 14493 13917 14501 13922 14510
13926 14520 13927 14531 13926 14542 13922 14552 13917 14562 13910 14570
13902 14576 13892 14581 13882 14584 13871 14585 13860 14584 13849 14581
13840 14576 13831 14570 13824 14562 13819 14552 13816 14542 13814 14531
13778 14531 13779 14542 13782 14553 13786 14562 13791 14571 13798 14580
13805 14587 13814 14594 13824 14599 13812 14608 13806 14613 13800 14620
13796 14627 13792 14636 13789 14646 13788 14658 13822 14658 13823 14648
13826 14640 13831 14633 13837 14627 13844 14622 13852 14618 13861 14616
13871 14616 13880 14616 13889 14618 13897 14622 13905 14627 13910 14633
13915 14640 13918 14648 13919 14658 13918 14667 13915 14675 13910 14683
13905 14689 13897 14693 13889 14697 13880 14699 13871 14700 13861 14699
13852 14697 13844 14693 13837 14689 13831 14683 13826 14675 13823 14667
13822 14658 13788 14658 13789 14666 13790 14674 13792 14682 13795 14689
13798 14695 13802 14701 13812 14711 13824 14719 13838 14725 13854 14729
13871 14730 145 N F
14129 14678 14127 14665 14124 14653 14118 14643 14110 14633 14100 14625
14089 14619 14077 14616 14065 14614 14065 14639 14057 14640 14049 14642
14043 14646 14037 14650 14032 14656 14028 14663 14026 14670 14025 14678
14026 14686 14028 14694 14032 14700 14037 14706 14043 14711 14049 14715
14057 14717 14065 14718 14073 14717 14080 14715 14087 14711 14092 14706
14097 14700 14101 14694 14103 14686 14104 14678 14103 14670 14101 14663
14097 14656 14092 14650 14087 14646 14080 14642 14073 14640 14065 14639
14065 14614 14052 14616 14040 14619 14029 14625 14019 14633 14012 14643
14006 14653 14002 14665 14001 14678 14002 14691 14006 14703 14012 14714
14019 14724 14029 14731 14040 14737 14052 14741 14065 14742 14077 14741
14089 14737 14100 14731 14110 14724 14118 14714 14124 14703 14127 14691
14129 14678 67 N F
14176 14738 14216 14738 14360 14509 14360 14738 14394 14738 14394 14457
14354 14457 14210 14686 14210 14457 14176 14457 14176 14738 11 N F
13688 15828 13712 15828 13712 15651 13754 15651 13754 15619 13712 15619
13712 15555 13678 15555 13678 15619 13564 15619 13564 15651 13594 15651
13678 15651 13678 15770 13594 15651 13564 15651 13564 15655 13688 15828
18 N F
13873 15828 13883 15827 13892 15825 13901 15821 13910 15816 13918 15809
13925 15802 13932 15793 13938 15784 13944 15773 13949 15762 13953 15750
13956 15738 13959 15726 13961 15713 13963 15700 13963 15688 13963 15675

13961 15662 13956 15636 13953 15624 13949 15612 13944 15601 13938 15591
13932 15581 13925 15572 13918 15564 13910 15558 13901 15552 13892 15548
13883 15546 13873 15545 13863 15546 13853 15548 13844 15552 13836 15558
13828 15564 13820 15572 13813 15581 13807 15591 13802 15601 13797 15612
13792 15624 13789 15636 13784 15662 13783 15675 13782 15688 13818 15688
13820 15663 13823 15641 13829 15622 13836 15606 13844 15593 13853 15583
13858 15580 13863 15577 13868 15576 13873 15575 13878 15576 13882 15577
13887 15580 13892 15583 13901 15593 13910 15606 13917 15622 13922 15641
13926 15663 13927 15688 13926 15712 13922 15734 13917 15753 13910 15768
13901 15781 13892 15790 13887 15794 13882 15796 13878 15798 13873 15798
13868 15798 13863 15796 13858 15794 13853 15790 13844 15781 13836 15768
13829 15753 13823 15734 13820 15712 13818 15688 13782 15688 13783 15700
13784 15713 13786 15726 13789 15738 13792 15750 13797 15762 13802 15773
13807 15784 13813 15793 13820 15802 13828 15809 13836 15816 13844 15821
13853 15825 13863 15827 13873 15828 105 N F
14131 15776 14129 15764 14126 15752 14120 15741 14112 15731 14102 15723
14091 15718 14079 15714 14067 15712 14067 15737 14059 15738 14051 15740
14045 15744 14039 15749 14034 15754 14030 15761 14028 15769 14027 15776
14028 15784 14030 15792 14034 15799 14039 15804 14045 15809 14051 15813
14059 15815 14067 15816 14075 15815 14082 15813 14089 15809 14094 15804
14099 15799 14103 15792 14105 15784 14106 15776 14105 15769 14103 15761
14099 15754 14094 15749 14089 15744 14082 15740 14075 15738 14067 15737
14067 15712 14054 15714 14042 15718 14031 15723 14022 15731 14014 15741
14008 15752 14004 15764 14003 15776 14004 15789 14008 15801 14014 15812
14022 15822 14031 15830 14042 15835 14054 15839 14067 15840 14079 15839
14091 15835 14102 15830 14112 15822 14120 15812 14126 15801 14129 15789
14131 15776 67 N F
14178 15836 14218 15836 14362 15607 14362 15836 14396 15836 14396 15555
14356 15555 14212 15784 14212 15555 14178 15555 14178 15836 11 N F
25 W
2646 5172 2646 5453 2 P
3739 5172 3739 5453 2 P
4823 5172 4823 5453 2 P
5903 5172 5903 5453 2 P
6971 5172 6971 5453 2 P
8052 5172 8052 5453 2 P
9133 5172 9133 5453 2 P
10214 5172 10214 5453 2 P
11295 5172 11295 5453 2 P
12376 5172 12376 5453 2 P
2747 16454 2747 16173 2 P
4812 16454 4812 16173 2 P
6843 16454 6843 16173 2 P

8864 16454 8864 16173 2 P
10892 16454 10892 16173 2 P
12942 16454 12942 16173 2 P
2016 5647 2297 5647 2 P
2016 6823 2297 6823 2 P
2016 7980 2297 7980 2 P
2016 9125 2297 9125 2 P
2016 10260 2297 10260 2 P
2016 11387 2297 11387 2 P
2016 12505 2297 12505 2 P
2016 13617 2297 13617 2 P
2016 14723 2297 14723 2 P
2016 15816 2297 15816 2 P
13283 5549 13002 5549 2 P
13283 6719 13002 6719 2 P
13283 7872 13002 7872 2 P
13283 9015 13002 9015 2 P
13283 10147 13002 10147 2 P
13283 11269 13002 11269 2 P
13283 12384 13002 12384 2 P
13283 13492 13002 13492 2 P
13283 14594 13002 14594 2 P
13283 15693 13002 15693 2 P
13002 5453 2297 5453 2297 16173 13002 16173 13002 5453 2297 5453
6:00 PM
12636 17516 12635 17506 12632 17496 12627 17486 12621 17479 12613 17472
12604 17467 12593 17464 12583 17463 12572 17464 12562 17467 12553 17472
12545 17479 12538 17486 12533 17496 12530 17506 12529 17516 12551 17516
12551 17510 12553 17504 12556 17498 12560 17494 12565 17490 12570 17487
12576 17485 12583 17484 12589 17485 12595 17487 12601 17490 12605 17494
12609 17498 12612 17504 12614 17510 12615 17516 12614 17523 12612 17529
12609 17534 12605 17539 12601 17543 12595 17546 12589 17548 12583 17549
12576 17548 12570 17546 12565 17543 12560 17539 12556 17534 12553 17529
12551 17523 12551 17516 12529 17516 12530 17527 12533 17537 12538 17546
12545 17554 12553 17561 12562 17566 12572 17569 12583 17570 12593 17569
12604 17566 12613 17561 12621 17554 12627 17546 12632 17537 12635 17527
12636 17516 67 N F
12997 17447 12993 17462 12989 17476 12984 17489 12979 17501 12973 17512
12966 17521 12958 17530 12949 17537 12940 17544 12930 17549 12920 17554
12908 17558 12896 17561 12884 17563 12870 17564 12856 17564 12838 17563
12820 17561 12804 17556 12788 17550 12774 17543 12761 17534 12748 17523
12737 17511 12728 17498 12719 17484 12712 17469 12705 17452 12700 17435
12697 17417 12694 17398 12693 17378 12694 17356 12696 17335 12700 17315

12706 17297 12713 17281 12721 17265 12730 17251 12740 17239 12752 17228
12764 17218 12778 17210 12792 17204 12807 17198 12823 17195 12839 17193
12856 17192 12875 17192 12892 17195 12907 17198 12921 17203 12934 17209
12946 17216 12956 17224 12965 17233 12973 17243 12980 17254 12986 17266
12991 17278 12995 17291 12998 17304 13000 17318 13002 17332 12956 17332
12951 17307 12944 17286 12940 17277 12936 17269 12931 17262 12925 17256
12919 17250 12912 17245 12904 17241 12896 17238 12887 17236 12878 17234
12856 17233 12842 17233 12828 17235 12815 17239 12804 17244 12793 17250
12783 17257 12775 17265 12767 17275 12760 17285 12755 17296 12750 17308
12746 17321 12743 17334 12741 17348 12740 17363 12739 17378 12740 17395
12741 17411 12744 17426 12748 17440 12753 17453 12759 17464 12765 17475
12773 17485 12781 17494 12790 17502 12800 17508 12810 17514 12821 17518
12832 17521 12844 17523 12856 17524 12874 17522 12891 17518 12906 17512
12920 17503 12931 17493 12936 17486 12941 17479 12944 17472 12947 17464
12949 17456 12951 17447 12997 17447 123 N F
7309 17656 7368 17656 7493 17299 7442 17299 7406 17404 7266 17404
7230 17299 7179 17299 7291 17605 7337 17605 7279 17444 7393 17444
7337 17605 7291 17605 7309 17656 15 N F
7526 17557 7564 17557 7564 17518 7575 17531 7586 17542 7597 17550
7608 17556 7618 17560 7628 17563 7637 17564 7646 17564 7661 17563
7678 17559 7687 17555 7696 17551 7705 17545 7714 17538 7722 17530
7730 17520 7737 17509 7743 17495 7748 17481 7752 17464 7754 17445
7755 17424 7755 17406 7753 17389 7750 17373 7745 17359 7740 17347
7734 17336 7727 17326 7720 17317 7711 17309 7703 17303 7694 17298
7684 17293 7675 17290 7665 17288 7655 17287 7646 17286 7635 17287
7624 17288 7614 17290 7604 17294 7595 17299 7585 17306 7576 17314
7567 17324 7567 17192 7526 17192 7526 17427 7567 17427 7567 17416
7568 17405 7573 17386 7580 17368 7589 17354 7600 17341 7606 17336
7613 17332 7620 17329 7626 17326 7633 17325 7641 17324 7652 17326
7665 17330 7677 17337 7682 17342 7688 17348 7693 17354 7697 17362
7702 17370 7705 17379 7708 17390 7710 17401 7711 17413 7712 17427
7711 17439 7710 17451 7708 17462 7704 17472 7701 17481 7696 17489
7691 17497 7686 17503 7674 17513 7662 17521 7651 17525 7641 17526
7634 17526 7627 17524 7621 17522 7614 17519 7602 17510 7590 17498
7581 17484 7573 17467 7570 17458 7568 17448 7567 17437 7567 17427
7526 17427 7526 17557 104 N F
7806 17557 7842 17557 7842 17508 7857 17529 7872 17544 7884 17554
7896 17561 7906 17563 7915 17564 7922 17562 7929 17559 7929 17521
7908 17519 7891 17514 7884 17511 7877 17508 7871 17503 7866 17499
7861 17493 7857 17488 7852 17474 7848 17459 7847 17442 7847 17299
7806 17299 7806 17557 26 N F
7967 17656 8008 17656 8008 17605 7967 17605 7967 17656 5 N F
7967 17557 8008 17557 8008 17299 7967 17299 7967 17557 5 N F

8079 17656 8120 17656 8120 17299 8079 17299 8079 17656 5 N F
2297 17462 2333 17462 2333 17424 2343 17436 2353 17446 2362 17454
2372 17460 2381 17465 2391 17468 2401 17469 2412 17470 2430 17468
2439 17465 2447 17462 2456 17457 2464 17450 2472 17441 2481 17429
2491 17440 2501 17449 2511 17457 2520 17462 2530 17466 2540 17468
2550 17470 2560 17470 2573 17469 2585 17466 2598 17461 2609 17454
2619 17445 2623 17439 2627 17433 2630 17427 2632 17420 2633 17412
2634 17404 2634 17205 2593 17205 2593 17388 2592 17398 2589 17406
2584 17414 2578 17421 2571 17426 2563 17431 2555 17433 2547 17434
2536 17433 2526 17429 2515 17424 2506 17416 2498 17407 2492 17397
2487 17385 2486 17373 2486 17205 2445 17205 2445 17388 2444 17398
2441 17406 2436 17414 2430 17421 2422 17426 2414 17431 2405 17433
2397 17434 2388 17433 2378 17430 2368 17424 2359 17417 2351 17408
2344 17398 2340 17386 2339 17380 2338 17373 2338 17205 2297 17205
2297 17462 79 N F
2705 17462 2741 17462 2741 17424 2751 17436 2761 17446 2770 17454
2779 17460 2789 17465 2799 17468 2809 17469 2820 17470 2838 17468
2847 17465 2855 17462 2864 17457 2872 17450 2880 17441 2889 17429
2899 17440 2909 17449 2919 17457 2928 17462 2938 17466 2947 17468
2957 17470 2968 17470 2980 17469 2993 17466 3006 17461 3017 17454
3027 17445 3031 17439 3035 17433 3038 17427 3040 17420 3041 17412
3042 17404 3042 17205 3001 17205 3001 17388 3000 17398 2997 17406
2992 17414 2986 17421 2978 17426 2971 17431 2963 17433 2955 17434
2944 17433 2934 17429 2923 17424 2914 17416 2906 17407 2899 17397
2895 17385 2894 17373 2894 17205 2853 17205 2853 17388 2852 17398
2849 17406 2844 17414 2837 17421 2830 17426 2822 17431 2813 17433
2805 17434 2795 17433 2786 17430 2776 17424 2767 17417 2758 17408
2752 17398 2748 17386 2746 17380 2746 17373 2746 17205 2705 17205
2705 17462 79 N F
3210 17470 3222 17469 3233 17467 3244 17464 3255 17459 3265 17453
3275 17446 3284 17438 3292 17428 3300 17418 3307 17408 3313 17396
3318 17384 3322 17371 3325 17358 3326 17344 3327 17330 3326 17315
3325 17302 3322 17288 3318 17276 3313 17264 3307 17252 3300 17242
3292 17232 3284 17223 3275 17215 3265 17208 3255 17203 3244 17198
3233 17195 3222 17192 3210 17192 3198 17192 3186 17195 3175 17198
3165 17203 3154 17208 3145 17215 3136 17223 3127 17232 3120 17242
3113 17252 3107 17264 3102 17276 3098 17288 3095 17302 3093 17315
3093 17330 3136 17330 3137 17310 3141 17291 3147 17275 3156 17260
3161 17253 3166 17247 3172 17242 3179 17238 3186 17235 3193 17232
3201 17231 3210 17230 3218 17231 3226 17232 3234 17235 3241 17238
3247 17242 3253 17247 3259 17253 3264 17260 3272 17275 3279 17291
3282 17310 3284 17330 3282 17349 3279 17368 3272 17385 3264 17401
3259 17407 3253 17413 3247 17419 3241 17423 3234 17427 3226 17429

3218 17431 3210 17432 3201 17431 3193 17429 3186 17427 3179 17423
3172 17419 3166 17413 3161 17407 3156 17401 3147 17385 3141 17368
3137 17349 3136 17330 3093 17330 3093 17344 3095 17358 3098 17371
3102 17384 3107 17396 3113 17408 3120 17418 3127 17428 3136 17438
3145 17446 3154 17453 3165 17459 3175 17464 3186 17467 3198 17469
3210 17470 115 N F
3383 17562 3424 17562 3424 17205 3383 17205 3383 17562 5 N F
3786 17567 3805 17566 3822 17563 3839 17558 3855 17551 3871 17543
3885 17533 3898 17522 3909 17509 3920 17496 3929 17481 3937 17466
3944 17450 3950 17433 3953 17416 3956 17398 3957 17381 3956 17363
3954 17345 3950 17328 3945 17311 3939 17295 3931 17279 3921 17265
3910 17251 3898 17238 3885 17227 3871 17217 3856 17208 3840 17201
3823 17196 3805 17193 3786 17192 3767 17193 3749 17196 3732 17201
3716 17208 3700 17217 3686 17227 3673 17238 3661 17251 3650 17265
3640 17279 3632 17295 3625 17311 3620 17328 3616 17345 3613 17363
3612 17381 3658 17381 3659 17365 3661 17351 3664 17337 3668 17323
3674 17310 3680 17298 3687 17286 3695 17276 3704 17266 3714 17258
3725 17250 3736 17244 3748 17239 3760 17236 3773 17233 3786 17233
3799 17233 3812 17236 3824 17239 3835 17244 3846 17250 3857 17258
3866 17266 3875 17276 3883 17286 3890 17298 3896 17310 3901 17323
3905 17337 3908 17351 3910 17365 3911 17381 3910 17396 3908 17410
3905 17424 3901 17438 3896 17451 3890 17463 3883 17474 3875 17484
3866 17493 3857 17502 3846 17509 3835 17515 3824 17520 3812 17523
3799 17525 3786 17526 3773 17525 3760 17523 3748 17520 3736 17515
3725 17509 3714 17502 3704 17493 3695 17484 3687 17474 3680 17463
3674 17451 3668 17438 3664 17424 3661 17410 3659 17396 3658 17381
3612 17381 3613 17398 3616 17416 3620 17433 3625 17450 3632 17466
3640 17481 3650 17496 3661 17509 3673 17522 3686 17533 3700 17543
3716 17551 3732 17558 3749 17563 3767 17566 3786 17567 131 N F
4038 17439 3995 17439 3996 17453 3997 17465 4000 17477 4004 17488
4008 17498 4013 17507 4019 17516 4026 17523 4033 17530 4041 17536
4050 17540 4060 17544 4070 17548 4081 17550 4092 17551 4104 17552
4116 17551 4127 17550 4137 17548 4147 17545 4156 17542 4165 17538
4173 17533 4181 17527 4188 17521 4194 17514 4199 17506 4203 17498
4207 17489 4209 17479 4211 17468 4211 17457 4211 17444 4209 17433
4205 17422 4200 17413 4193 17404 4185 17396 4174 17389 4160 17383
4176 17377 4188 17370 4199 17361 4207 17352 4214 17341 4218 17329
4221 17316 4222 17302 4220 17282 4218 17273 4215 17263 4212 17254
4207 17245 4202 17237 4195 17229 4188 17221 4179 17215 4169 17209
4158 17204 4146 17200 4133 17197 4118 17195 4102 17194 4088 17195
4075 17196 4063 17199 4052 17202 4042 17207 4033 17212 4024 17218
4017 17225 4011 17233 4005 17241 4000 17250 3996 17260 3993 17270
3991 17281 3990 17292 3990 17304 4033 17304 4034 17291 4036 17279

4039 17267 4046 17256 4050 17251 4055 17246 4061 17242 4067 17239
4075 17236 4084 17234 4093 17233 4104 17233 4114 17233 4122 17233
4130 17235 4137 17237 4144 17240 4149 17243 4155 17247 4159 17252
4167 17262 4172 17274 4175 17287 4176 17302 4175 17311 4174 17320
4172 17327 4169 17334 4165 17340 4161 17345 4155 17350 4149 17354
4143 17357 4135 17359 4119 17363 4100 17365 4079 17365 4079 17401
4099 17401 4117 17403 4132 17406 4144 17411 4149 17414 4154 17417
4157 17422 4160 17427 4163 17433 4164 17439 4166 17455 4165 17467
4162 17478 4158 17488 4151 17496 4143 17504 4132 17509 4119 17512
4104 17513 4091 17513 4078 17510 4067 17505 4058 17498 4054 17494
4050 17489 4044 17476 4040 17459 4038 17439 148 N F
4359 17562 4387 17562 4270 17194 4242 17194 4357 17562 5 N F
4420 17462 4456 17462 4456 17424 4466 17436 4476 17446 4485 17454
4495 17460 4504 17465 4514 17468 4524 17469 4535 17470 4553 17468
4562 17465 4570 17462 4579 17457 4587 17450 4595 17441 4604 17429
4614 17440 4624 17449 4634 17457 4643 17462 4653 17466 4663 17468
4673 17470 4683 17470 4696 17469 4708 17466 4721 17461 4732 17454
4742 17445 4746 17439 4750 17433 4753 17427 4755 17420 4756 17412
4757 17404 4757 17205 4716 17205 4716 17388 4715 17398 4712 17406
4707 17414 4701 17421 4694 17426 4686 17431 4678 17433 4670 17434
4659 17433 4649 17429 4638 17424 4629 17416 4621 17407 4615 17397
4610 17385 4609 17373 4609 17205 4568 17205 4568 17388 4567 17398
4564 17406 4559 17414 4553 17421 4545 17426 4537 17431 4528 17433
4520 17434 4511 17433 4501 17430 4491 17424 4482 17417 4474 17408
4467 17398 4463 17386 4462 17380 4461 17373 4461 17205 4420 17205
4420 17462 79 N F
4815 17432 4859 17432 4860 17449 4863 17465 4869 17479 4876 17491
4881 17496 4886 17500 4892 17504 4899 17507 4906 17510 4913 17512
4921 17513 4930 17513 4943 17512 4956 17509 4967 17503 4977 17495
4985 17486 4991 17475 4995 17462 4996 17447 4995 17432 4992 17420
4985 17409 4977 17400 4966 17391 4952 17382 4917 17360 4888 17343
4875 17334 4864 17325 4854 17317 4845 17308 4837 17298 4830 17289
4824 17279 4820 17270 4816 17260 4813 17249 4809 17228 4808 17205
5040 17205 5040 17245 4856 17245 4861 17261 4867 17274 4875 17285
4886 17295 4898 17305 4914 17314 4953 17337 4976 17349 4995 17361
5010 17375 5023 17390 5032 17405 5038 17421 5041 17437 5042 17452
5042 17463 5040 17473 5037 17483 5033 17492 5029 17501 5023 17509
5016 17517 5009 17524 5001 17530 4992 17536 4983 17540 4973 17544
4963 17547 4952 17550 4941 17551 4930 17552 4918 17551 4906 17550
4895 17548 4884 17544 4874 17540 4865 17535 4856 17529 4848 17522
4841 17514 4834 17506 4829 17496 4824 17485 4820 17473 4818 17460
4816 17446 4815 17432 92 N F
2 O

2458 2789 3107 2789 3107 3744 2458 3744 2458 2789 5 N F
1 O
12 W
2458 2789 3107 2789 3107 3744 2458 3744 2458 2789 5 P
15 O
3107 2789 3756 2789 3756 3744 3107 3744 3107 2789 5 N F
1 O
3107 2789 3756 2789 3756 3744 3107 3744 3107 2789 5 P
29 O
3756 2789 4405 2789 4405 3744 3756 3744 3756 2789 5 N F
1 O
3756 2789 4405 2789 4405 3744 3756 3744 3756 2789 5 P
42 O
4405 2789 5054 2789 5054 3744 4405 3744 4405 2789 5 N F
1 O
4405 2789 5054 2789 5054 3744 4405 3744 4405 2789 5 P
55 O
5054 2789 5703 2789 5703 3744 5054 3744 5054 2789 5 N F
1 O
5054 2789 5703 2789 5703 3744 5054 3744 5054 2789 5 P
68 O
5703 2789 6352 2789 6352 3744 5703 3744 5703 2789 5 N F
1 O
5703 2789 6352 2789 6352 3744 5703 3744 5703 2789 5 P
82 O
6352 2789 7001 2789 7001 3744 6352 3744 6352 2789 5 N F
1 O
6352 2789 7001 2789 7001 3744 6352 3744 6352 2789 5 P
95 O
7001 2789 7650 2789 7650 3744 7001 3744 7001 2789 5 N F
1 O
7001 2789 7650 2789 7650 3744 7001 3744 7001 2789 5 P
108 O
7650 2789 8298 2789 8298 3744 7650 3744 7650 2789 5 N F
1 O
7650 2789 8298 2789 8298 3744 7650 3744 7650 2789 5 P
121 O
8298 2789 8947 2789 8947 3744 8298 3744 8298 2789 5 N F
1 O
8298 2789 8947 2789 8947 3744 8298 3744 8298 2789 5 P
135 O
8947 2789 9596 2789 9596 3744 8947 3744 8947 2789 5 N F
1 O

8947 2789 9596 2789 9596 3744 8947 3744 8947 2789 5 P
148 O
9596 2789 10245 2789 10245 3744 9596 3744 9596 2789 5 N F
1 O
9596 2789 10245 2789 10245 3744 9596 3744 9596 2789 5 P
161 O
10245 2789 10894 2789 10894 3744 10245 3744 10245 2789 5 N F
1 O
10245 2789 10894 2789 10894 3744 10245 3744 10245 2789 5 P
174 O
10894 2789 11543 2789 11543 3744 10894 3744 10894 2789 5 N F
1 O
10894 2789 11543 2789 11543 3744 10894 3744 10894 2789 5 P
188 O
11543 2789 12192 2789 12192 3744 11543 3744 11543 2789 5 N F
1 O
11543 2789 12192 2789 12192 3744 11543 3744 11543 2789 5 P
201 O
12192 2789 12841 2789 12841 3744 12192 3744 12192 2789 5 N F
1 O
12192 2789 12841 2789 12841 3744 12192 3744 12192 2789 5 P
3131 2495 3152 2495 3152 2234 3119 2234 3119 2420 3062 2420
3062 2443 3085 2446 3096 2448 3105 2452 3113 2458 3121 2466
3126 2478 3131 2495 14 N F
3674 2405 3706 2405 3707 2418 3709 2430 3714 2440 3719 2449
3727 2456 3736 2462 3747 2465 3760 2466 3769 2465 3779 2463
3787 2458 3795 2453 3801 2446 3805 2437 3808 2427 3809 2416
3808 2405 3806 2396 3801 2388 3795 2381 3787 2374 3776 2367
3750 2351 3728 2338 3710 2325 3696 2312 3685 2298 3677 2283
3672 2268 3669 2252 3668 2234 3842 2234 3842 2265 3704 2265
3708 2277 3712 2287 3718 2295 3726 2302 3736 2310 3747 2317
3777 2334 3794 2343 3808 2352 3820 2362 3829 2373 3836 2385
3840 2397 3843 2409 3844 2420 3843 2428 3842 2436 3840 2443
3837 2450 3834 2457 3829 2463 3819 2474 3806 2483 3792 2489
3776 2493 3760 2495 3742 2493 3725 2489 3718 2486 3711 2482
3704 2478 3698 2473 3693 2467 3688 2460 3684 2453 3680 2445
3677 2436 3675 2426 3674 2416 3674 2405 76 N F
4354 2414 4322 2414 4324 2434 4328 2451 4332 2459 4335 2465
4340 2472 4345 2477 4350 2482 4356 2487 4363 2490 4370 2493
4386 2497 4404 2499 4420 2497 4436 2494 4449 2488 4461 2480
4471 2470 4475 2465 4478 2458 4481 2451 4483 2444 4484 2436
4484 2428 4484 2418 4482 2409 4480 2401 4476 2394 4471 2388
4464 2382 4456 2377 4446 2372 4457 2368 4467 2362 4475 2356

4481 2349 4486 2341 4489 2332 4491 2322 4492 2311 4491 2297
4487 2282 4481 2269 4477 2262 4472 2256 4466 2251 4460 2246
4452 2242 4444 2238 4435 2235 4425 2233 4414 2231 4402 2231
4392 2231 4382 2232 4373 2234 4365 2237 4357 2240 4350 2244
4344 2249 4338 2254 4334 2260 4329 2266 4323 2280 4319 2296
4318 2313 4350 2313 4351 2304 4352 2294 4355 2285 4360 2277
4367 2270 4376 2264 4388 2261 4404 2259 4417 2260 4428 2263
4438 2267 4445 2274 4450 2282 4454 2291 4457 2301 4457 2311
4456 2325 4454 2330 4452 2335 4449 2340 4446 2344 4438 2350
4427 2354 4415 2357 4400 2358 4385 2359 4385 2386 4400 2386
4413 2387 4425 2389 4434 2393 4441 2398 4446 2405 4449 2414
4450 2426 4449 2435 4447 2443 4444 2451 4439 2457 4433 2463
4425 2467 4415 2469 4404 2470 4394 2469 4384 2467 4376 2464
4369 2459 4363 2451 4359 2442 4356 2429 4354 2414 119 N F
5081 2495 5104 2495 5104 2326 5145 2326 5145 2296 5104 2296
5104 2234 5072 2234 5072 2296 4963 2296 4963 2326 4992 2326
5072 2326 5072 2439 4992 2326 4963 2326 4963 2330 5081 2495
18 N F
5642 2500 5777 2500 5777 2469 5668 2469 5659 2394 5668 2401
5679 2407 5693 2410 5708 2412 5722 2410 5736 2407 5750 2401
5756 2397 5762 2392 5768 2386 5774 2380 5778 2373 5783 2365
5786 2356 5788 2346 5790 2335 5791 2323 5789 2305 5785 2288
5782 2280 5778 2272 5773 2265 5768 2258 5762 2252 5755 2246
5748 2241 5740 2237 5731 2234 5722 2232 5712 2230 5701 2230
5681 2231 5673 2233 5665 2235 5658 2238 5651 2242 5640 2251
5631 2262 5624 2274 5618 2288 5615 2302 5647 2302 5651 2293
5655 2284 5660 2277 5666 2270 5673 2265 5681 2262 5689 2259
5699 2258 5712 2259 5723 2262 5734 2267 5742 2274 5749 2283
5754 2294 5757 2307 5758 2322 5757 2332 5755 2343 5751 2354
5744 2363 5737 2371 5727 2377 5715 2381 5701 2383 5693 2382
5687 2381 5681 2379 5675 2377 5664 2369 5651 2358 5622 2358
5642 2500 79 N F
6433 2433 6400 2433 6398 2441 6395 2449 6390 2456 6385 2461
6379 2465 6372 2468 6365 2470 6358 2471 6351 2470 6345 2469
6339 2467 6333 2464 6328 2461 6323 2456 6318 2451 6314 2445
6308 2431 6303 2414 6300 2395 6299 2373 6307 2382 6315 2388
6322 2393 6330 2397 6338 2399 6346 2401 6362 2402 6375 2401
6388 2397 6401 2390 6413 2381 6419 2376 6424 2370 6428 2363
6432 2355 6435 2347 6437 2338 6438 2328 6439 2318 6406 2316
6405 2327 6402 2337 6397 2347 6390 2355 6382 2363 6373 2368
6363 2372 6353 2373 6342 2372 6332 2368 6323 2363 6315 2355
6308 2347 6303 2337 6300 2327 6299 2316 6300 2305 6303 2295
6308 2285 6315 2276 6323 2269 6332 2263 6342 2260 6353 2258

6363 2260 6373 2263 6382 2269 6390 2276 6397 2285 6402 2295
6405 2305 6406 2316 6439 2318 6437 2302 6433 2287 6427 2272
6423 2265 6418 2259 6412 2253 6406 2247 6399 2242 6391 2238
6383 2234 6373 2232 6363 2230 6353 2230 6341 2230 6330 2232
6320 2235 6311 2240 6303 2245 6296 2252 6290 2259 6284 2268
6279 2277 6275 2287 6272 2297 6269 2309 6266 2333 6265 2360
6265 2376 6266 2391 6268 2405 6271 2418 6274 2430 6279 2442
6284 2452 6289 2462 6296 2471 6303 2478 6310 2485 6319 2490
6328 2494 6337 2497 6347 2499 6358 2500 6373 2498 6387 2494
6399 2488 6410 2480 6419 2470 6426 2458 6430 2446 6433 2433
126 N F
6914 2495 7088 2495 7088 2468 7063 2431 7043 2399 7027 2371
7014 2345 7004 2320 6996 2294 6989 2266 6982 2234 6948 2234
6958 2271 6967 2301 6977 2328 6988 2352 7001 2376 7015 2402
7055 2464 6914 2464 6914 2495 21 N F
7649 2500 7665 2498 7680 2495 7694 2489 7705 2482 7715 2472
7719 2466 7722 2460 7724 2453 7726 2446 7727 2439 7728 2431
7727 2419 7725 2410 7721 2401 7716 2394 7711 2388 7705 2383
7693 2375 7703 2370 7712 2364 7719 2357 7725 2349 7731 2340
7734 2331 7737 2321 7737 2310 7736 2297 7732 2284 7725 2270
7715 2258 7709 2252 7702 2247 7695 2242 7687 2238 7678 2234
7668 2232 7658 2230 7648 2230 7637 2230 7627 2232 7618 2234
7609 2238 7601 2242 7594 2247 7588 2252 7582 2258 7573 2270
7567 2284 7563 2297 7562 2310 7596 2310 7597 2300 7600 2290
7605 2281 7612 2273 7620 2267 7629 2262 7639 2259 7649 2258
7660 2259 7670 2262 7679 2267 7687 2273 7694 2281 7699 2290
7702 2300 7703 2310 7702 2321 7699 2330 7694 2339 7687 2347
7679 2353 7670 2358 7660 2361 7649 2362 7639 2361 7629 2358
7620 2353 7612 2347 7605 2339 7600 2330 7597 2321 7596 2310
7562 2310 7562 2321 7565 2330 7568 2340 7574 2348 7580 2356
7587 2363 7596 2370 7606 2375 7594 2383 7588 2388 7583 2394
7578 2401 7574 2410 7572 2419 7571 2431 7604 2431 7605 2422
7607 2414 7612 2407 7617 2401 7624 2397 7632 2393 7640 2391
7649 2390 7659 2391 7667 2393 7675 2397 7682 2401 7687 2407
7692 2414 7694 2422 7695 2431 7694 2440 7692 2447 7687 2454
7682 2460 7675 2465 7667 2468 7659 2470 7649 2471 7640 2470
7632 2468 7624 2465 7617 2460 7612 2454 7607 2447 7605 2440
7604 2431 7571 2431 7572 2439 7573 2446 7575 2453 7577 2460
7580 2466 7584 2472 7594 2482 7605 2489 7619 2495 7634 2498
7649 2500 145 N F
8297 2500 8307 2499 8316 2498 8325 2496 8333 2493 8340 2489
8347 2484 8354 2478 8360 2471 8366 2463 8370 2454 8375 2443
8378 2432 8381 2419 8383 2405 8385 2390 8385 2373 8385 2353

8383 2334 8381 2317 8377 2302 8372 2289 8367 2277 8361 2267
8355 2258 8348 2251 8340 2245 8332 2240 8324 2236 8316 2233
8307 2231 8290 2230 8277 2231 8264 2234 8253 2239 8243 2246
8234 2256 8226 2268 8220 2282 8215 2299 8248 2299 8251 2289
8255 2281 8259 2274 8264 2268 8270 2264 8276 2261 8284 2259
8292 2258 8299 2259 8306 2260 8312 2263 8318 2266 8323 2270
8328 2275 8336 2287 8343 2302 8347 2318 8350 2336 8351 2356
8342 2347 8333 2340 8325 2335 8318 2332 8311 2329 8304 2328
8292 2327 8282 2328 8272 2330 8264 2332 8256 2335 8248 2339
8241 2344 8235 2349 8230 2355 8225 2361 8221 2367 8215 2382
8212 2397 8211 2413 8244 2413 8245 2403 8248 2393 8252 2384
8259 2375 8266 2367 8275 2361 8286 2357 8297 2356 8309 2357
8320 2361 8329 2367 8336 2375 8343 2384 8347 2393 8350 2403
8351 2413 8350 2424 8347 2434 8343 2443 8336 2452 8329 2460
8320 2466 8309 2470 8297 2471 8286 2470 8275 2466 8266 2460
8259 2452 8252 2443 8248 2434 8245 2424 8244 2413 8211 2413
8212 2422 8212 2430 8214 2438 8217 2446 8220 2454 8224 2461
8228 2468 8234 2474 8240 2479 8246 2484 8253 2489 8261 2493
8270 2496 8278 2498 8288 2499 8297 2500 130 N F
8839 2500 8860 2500 8860 2239 8828 2239 8828 2425 8771 2425
8771 2448 8794 2451 8804 2453 8814 2457 8822 2462 8829 2471
8835 2483 8839 2500 14 N F
9038 2500 9048 2499 9057 2497 9065 2493 9073 2488 9081 2482
9088 2474 9095 2466 9101 2457 9106 2447 9111 2437 9118 2414
9123 2390 9124 2366 9123 2341 9118 2317 9111 2294 9106 2283
9101 2273 9095 2264 9088 2256 9081 2248 9073 2242 9065 2237
9057 2233 9048 2231 9038 2230 9029 2231 9020 2233 9011 2237
9003 2242 8995 2248 8988 2256 8982 2264 8976 2273 8970 2283
8966 2294 8958 2317 8954 2341 8952 2366 8987 2366 8988 2342
8991 2321 8996 2303 9003 2288 9011 2275 9020 2266 9024 2263
9029 2260 9034 2259 9038 2258 9043 2259 9048 2260 9052 2263
9057 2266 9065 2275 9073 2288 9080 2303 9085 2321 9089 2342
9090 2366 9089 2389 9085 2410 9080 2428 9073 2443 9065 2455
9057 2464 9048 2469 9043 2470 9038 2471 9034 2470 9029 2469
9020 2464 9011 2455 9003 2443 8996 2428 8991 2410 8988 2389
8987 2366 8952 2366 8954 2390 8958 2414 8966 2437 8970 2447
8976 2457 8982 2466 8988 2474 8995 2482 9003 2488 9011 2493
9020 2497 9029 2499 9038 2500 93 N F
9518 2495 9539 2495 9539 2234 9506 2234 9506 2420 9449 2420
9449 2443 9472 2446 9483 2448 9492 2452 9501 2458 9508 2466
9514 2478 9518 2495 14 N F
9722 2495 9744 2495 9744 2234 9711 2234 9711 2420 9654 2420
9654 2443 9677 2446 9687 2448 9697 2452 9705 2458 9712 2466

9718 2478 9722 2495 14 N F
10136 2495 10157 2495 10157 2234 10125 2234 10125 2420 10068 2420
10068 2443 10091 2446 10101 2448 10111 2452 10119 2458 10126 2466
10132 2478 10136 2495 14 N F
10253 2405 10285 2405 10286 2418 10289 2430 10293 2440 10299 2449
10306 2456 10315 2462 10326 2465 10339 2466 10349 2465 10358 2463
10366 2458 10374 2453 10380 2446 10385 2437 10388 2427 10389 2416
10388 2405 10385 2396 10380 2388 10374 2381 10366 2374 10356 2367
10329 2351 10307 2338 10289 2325 10275 2312 10264 2298 10256 2283
10251 2268 10248 2252 10247 2234 10421 2234 10421 2265 10283 2265
10287 2277 10292 2287 10298 2295 10305 2302 10315 2310 10327 2317
10356 2334 10373 2343 10388 2352 10399 2362 10408 2373 10415 2385
10420 2397 10422 2409 10423 2420 10423 2428 10421 2436 10419 2443
10416 2450 10413 2457 10409 2463 10398 2474 10386 2483 10371 2489
10356 2493 10339 2495 10321 2493 10305 2489 10297 2486 10290 2482
10283 2478 10277 2473 10272 2467 10267 2460 10263 2453 10259 2445
10257 2436 10255 2426 10253 2416 10253 2405 76 N F
10786 2499 10807 2499 10807 2238 10775 2238 10775 2424 10717 2424
10717 2447 10741 2450 10751 2452 10760 2456 10769 2461 10776 2470
10782 2482 10786 2499 14 N F
10933 2414 10901 2414 10903 2434 10908 2451 10911 2459 10915 2465
10919 2472 10924 2477 10930 2482 10936 2487 10942 2490 10950 2493
10965 2497 10983 2499 11000 2497 11015 2494 11029 2488 11040 2480
11050 2470 11054 2465 11057 2458 11060 2451 11062 2444 11063 2436
11063 2428 11063 2418 11061 2409 11059 2401 11055 2394 11050 2388
11043 2382 11035 2377 11025 2372 11036 2368 11046 2362 11054 2356
11060 2349 11065 2341 11068 2332 11070 2322 11071 2311 11070 2297
11066 2282 11060 2269 11056 2262 11051 2256 11046 2251 11039 2246
11032 2242 11023 2238 11014 2235 11004 2233 10993 2231 10981 2231
10971 2231 10961 2232 10952 2234 10944 2237 10936 2240 10929 2244
10923 2249 10918 2254 10913 2260 10909 2266 10902 2280 10898 2296
10897 2313 10930 2313 10930 2304 10931 2294 10934 2285 10939 2277
10946 2270 10955 2264 10968 2261 10983 2259 10996 2260 11008 2263
11017 2267 11024 2274 11030 2282 11034 2291 11036 2301 11037 2311
11035 2325 11034 2330 11031 2335 11029 2340 11025 2344 11017 2350
11006 2354 10994 2357 10980 2358 10964 2359 10964 2386 10979 2386
10993 2387 11004 2389 11013 2393 11020 2398 11025 2405 11028 2414
11029 2426 11028 2435 11026 2443 11023 2451 11018 2457 11012 2463
11004 2467 10994 2469 10983 2470 10973 2469 10964 2467 10955 2464
10948 2459 10942 2451 10938 2442 10935 2429 10933 2414 119 N F
11432 2495 11453 2495 11453 2234 11421 2234 11421 2420 11363 2420
11363 2443 11387 2446 11397 2448 11406 2452 11415 2458 11422 2466
11428 2478 11432 2495 14 N F

```
11660 2495 11683 2495 11683 2326 11723 2326 11723 2296 11683 2296
11683 2234 11650 2234 11650 2296 11541 2296 11541 2326 11570 2326
11650 2326 11650 2439 11570 2326 11541 2326 11541 2330 11660 2495
18 N F
12083 2500 12104 2500 12104 2239 12072 2239 12072 2425 12014 2425
12014 2448 12037 2451 12048 2453 12057 2457 12066 2462 12073 2471
12079 2483 12083 2500 14 N F
12221 2500 12356 2500 12356 2469 12248 2469 12238 2394 12247 2401
12259 2407 12272 2410 12288 2412 12301 2410 12315 2407 12329 2401
12335 2397 12342 2392 12348 2386 12353 2380 12358 2373 12362 2365
12365 2356 12368 2346 12369 2335 12370 2323 12368 2305 12364 2288
12361 2280 12357 2272 12352 2265 12347 2258 12341 2252 12334 2246
12327 2241 12319 2237 12310 2234 12301 2232 12291 2230 12280 2230
12261 2231 12252 2233 12244 2235 12237 2238 12230 2242 12219 2251
12210 2262 12203 2274 12198 2288 12194 2302 12227 2302 12230 2293
12234 2284 12239 2277 12245 2270 12252 2265 12260 2262 12269 2259
12278 2258 12291 2259 12303 2262 12313 2267 12321 2274 12328 2283
12333 2294 12336 2307 12337 2322 12337 2332 12334 2343 12330 2354
12324 2363 12316 2371 12306 2377 12294 2381 12280 2383 12273 2382
12266 2381 12260 2379 12254 2377 12243 2369 12230 2358 12202 2358
12221 2500 79 N F
Gr
S
%%Trailer
%%DocumentFonts:
%%Pages: 1
%%EOF
```

The temporal and spatial variations of simulated cumulative ozone uptake in CK (May)

```
%%Pages: (atend)
%%PageOrder: Ascend
%%Creator: NCAR GKS
%%DocumentFonts: (atend)
%%EndComments
%%BeginProlog

/M3
{
  2 copy lt {pop} {exch pop} ifelse
  2 copy lt {pop} {exch pop} ifelse
} bind def
/Sc
{
```

```
  1. exch sub /ylo exch def
  1. exch sub /mag exch def
  1. exch sub /cyn exch def
  ylo mag cyn M3 /b_cmy exch def
  cyn b_cmy sub /c_cmy exch def
  mag b_cmy sub /m_cmy exch def
  ylo b_cmy sub /y_cmy exch def
  c_cmy m_cmy y_cmy b_cmy setcmykcolor
} bind def
/P
{/n exch def
  newpath
  moveto
  2 1 n {pop lineto} for
  stroke
 } bind def

/F
{/n exch def
  newpath
  moveto
  2 1 n {pop lineto} for
  closepath
  eofill
} bind def

/B
{ /s exch def
  /n exch def
  1 1 n {newpath pop s 0 360 arc stroke} for
} bind def

/Q
{ /s exch def
  /n exch def
  /ms 0 s sub def
  /ts s s add def
  1 1 n {gsave newpath pop translate
  0 0 moveto ms 0 rmoveto ts 0 rlineto
  0 0 moveto 90 rotate ms  0 rmoveto ts 0 rlineto
  stroke grestore} for
} bind def
```

```
/Fs
{ /s exch def
  findfont
  s scalefont
  setfont
} bind def

/V
{ /s exch def
  /n exch def
  /ms 0 s sub def
  /ts s s add def
  1 1 n {gsave newpath pop translate
  0 0 moveto 30 rotate ms 0 rmoveto  ts 0 rlineto
  0 0 moveto 60 rotate ms 0 rmoveto  ts 0 rlineto
  0 0 moveto 60 rotate ms 0 rmoveto  ts 0 rlineto
  stroke grestore} for
} bind def

/X
{ /s exch def
  /n exch def
  /s s 1.25 mul def
  /ms 0 s sub def
  /ts s s add def
  1 1 n {gsave newpath pop translate
  0 0 moveto 45 rotate ms 0 rmoveto  ts 0 rlineto
  0 0 moveto 90 rotate ms 0 rmoveto  ts 0 rlineto
  stroke grestore} for
} bind def

/Ls
{matrix currentmatrix
 currentrgbcolor
 currentlinewidth
 currentlinecap
 currentlinejoin
 currentdash
} bind def

/Lr
{setdash
 setlinejoin
```

```
 setlinecap
 setlinewidth
 setrgbcolor
 setmatrix
} bind def

/M {stroke newpath moveto} bind def
/L {lineto} bind def
/E {eofill} def
/J {setlinejoin} def
/C {setlinecap} def
/W {setlinewidth} def
/S {stroke showpage} def
/A {scale} def
/U {put} def
/D {setdash} def
/I {currentdash} def
/H {currentlinewidth} def
/G {currentrgbcolor} def
/R {setrgbcolor} def
/N {closepath} def
/K {stroke} def
/Z {rotate} def
/As {ashow} def
/Ch {clippath} def
/Ci {colorimage} def
/Co {concat} def
/Cm {currentmatrix} def
/Cp {clip} def
/Ct {currentpoint} def
/Cu {currentfile} def
/Ex {exch} def
/Im {image} def
/In {initgraphics} def
/Ml {setmiterlimit} def
/Mx {matrix} def
/Np {newpath} def
/Ng {neg} def
/Rh {readhexstring} def
/Rm {rmoveto} def
/Sm {setmatrix} def
/Tr {translate} def
/Gs {gsave} def
```

```
/Gr {grestore} def

/Ov
{/strn exch def
 /ispc exch def
 /str1 1 string def
  strn
  {/code exch def
   /chr str1 0 code put str1 def
   0 ispc neg rmoveto
   gsave
   chr stringwidth pop 2 div neg
   0 rmoveto
   chr false charpath stroke
   grestore
  } forall
} bind def

/Oh
{/strn exch def
 /ispc exch def
 /tstr 1 string def
 strn
   {
    /code exch def
    /chr tstr 0 code put tstr def
    chr false charpath
    currentpoint
    /yt exch def /xt exch def
    stroke
    xt ispc add yt moveto
   } forall
 } bind def

/T 256 array def
/O
{ T
  exch
  get
  aload
  pop
  setrgbcolor
} bind def
```

```
/Y
{/strn exch def
 /ispc exch def
 /str1 1 string def
  strn
  {/code exch def
   /chr str1 0 code put str1 def
    0 ispc neg rmoveto
    gsave
      chr stringwidth pop 2 div neg 0 rmoveto
      chr show
    grestore
  } forall
} def

/Bg
{/ury Ex def /urx Ex def /lly Ex def /llx Ex def
 Np Ls Gr Gs 0 O Ch E Lr
 llx lly M urx lly L urx ury L
 llx ury L llx lly L
 Cp Np
} def

/Oc
{/ury exch def
 /urx exch def
 /lly exch def
 /llx exch def
 Np
 llx lly M urx lly L urx ury L  llx ury L llx lly L
 Cp Np
} def

/Cc
{/CellWidth exch def
 /strn CellWidth 1 add string def
 /strn1 CellWidth 1 add 3 mul string def
 systemdict /colorimage known
 { { Cu strn1 Rh pop } false 3 Ci}
 { { Cu strn1 Rh pop
     /ColorStr exch def
     0 1 CellWidth {
```

```
       /YIndx exch def
       /GrayVal YIndx 3 mul def
       strn YIndx
         ColorStr GrayVal       get 0.30 mul
         ColorStr GrayVal 1 add get 0.59 mul
         ColorStr GrayVal 2 add get 0.11 mul
         add add cvi
       put
     } for
     strn
   } Im
 } ifelse
} def

/Ld
{/ury exch def /urx exch def  /lly exch def /llx exch def
 -90 rotate
 lly urx add neg llx lly sub translate
} bind def
/Pt
{/ury exch def /urx exch def  /lly exch def /llx exch def
 90 rotate
 lly urx add lly llx sub translate
} bind def
%%EndProlog

%%BeginSetup
<</PageSize [00612 00792]>> setpagedevice
%%EndSetup

[1. 1. 1.] [0. 0. 0.] [1. 0. 0.] [0. 1. 0.] [0. 0. 1.] [0. 1. 1.] [1. 0. .8]
[1. 1. 0.] [1. .5 0.] [.6 .8 0.] [0. 1. .6] [0. .5 1.] [.5 0. .8] [1. 0. .6]
[.3 .3 .3] [.7 .7 .7] [1. 1. .3] [.7 1. .5] [.5 1. .6] [.2 1. .7] [.3 .8 .8]
[.5 .7 .8] [.7 .5 .8] [1. .3 .9] [.7 .9 .5] [.4 .9 .5] [.2 .9 .7] [.2 .7 .9]
[.2 .5 1.] [.5 .3 1.] [.7 .2 1.] [.9 .1 1.] [.9 1. .2] [.7 1. .3] [.5 1. .3]
[.2 1. .5] [.2 .8 .6] [.2 .7 .7] [.2 .5 .8] [.2 .4 .9] [.4 .3 .9] [.7 .2 .9]
[.8 .2 .8] [.9 .3 .7] [.8 .4 .6] [.8 .6 .5] [.9 .7 .4] [.9 .7 .3] [1. .9 .9]
[.8 1. .1] [.6 1. .2] [.5 1. .2] [.2 .9 .5] [.2 .8 .5] [.2 .7 .7] [.2 .5 .7]
[.2 .4 .9] [.4 .3 .9] [.5 .2 .9] [.8 .2 .7] [1. .2 .7] [1. .3 .6] [1. .4 .5]
[1. .5 .4] [1. .8 .1] [.7 1. .0] [.6 1. .1] [.4 1. .2] [.1 1. .3] [.1 .8 .4]
[.2 .7 .5] [.1 .6 .7] [.1 .5 .7] [.1 .4 .8] [.2 .3 .9] [.2 .2 1.] [.3 .1 1.]
[.5 .1 .9] [.7 .0 .8] [.9 .0 .7] [.9 .7 .1] [.7 .9 .1] [.5 .9 .1] [.3 .9 .2]
[.1 .9 .3] [.2 .7 .4] [.1 .7 .5] [.1 .5 .6] [.1 .4 .7] [.2 .3 .7] [.2 .2 .8]
```

```
[.3 .2 .8] [.5 .1 .7] [.7 .1 .7] [.9 .2 .5] [.8 .2 .5] [.8 .7 .0] [.6 .8 .1]
[.4 .9 .1] [.3 .9 .1] [.1 .9 .2] [.2 .8 .2] [.2 .7 .3] [.2 .6 .5] [.1 .5 .5]
[.1 .4 .6] [.2 .3 .7] [.3 .2 .7] [.4 .2 .6] [.7 .2 .5] [.8 .2 .4] [1. .2 .3]
[.7 .7 .0] [.5 .7 .1] [.4 .8 .1] [.2 .9 .1] [.2 .9 .1] [.2 .8 .2] [.1 .7 .2]
[.1 .7 .3] [.1 .6 .4] [.1 .4 .5] [.1 .4 .5] [.2 .3 .5] [.4 .3 .4] [.5 .3 .4]
[.7 .3 .3] [.8 .3 .2] [.7 .6 .0] [.5 .7 .1] [.4 .7 .1] [.2 .7 .2] [.1 .7 .2]
[.1 .6 .3] [.1 .5 .4] [.1 .4 .4] [.1 .3 .5] [.1 .2 .7] [.1 .1 .7] [.4 .1 .5]
[.5 .1 .5] [.6 .1 .4] [.7 .1 .4] [.9 .1 .3] [.5 .5 .1] [.4 .6 .1] [.3 .6 .1]
[.2 .6 .2] [.1 .5 .2] [.1 .4 .3] [.1 .4 .4] [.2 .3 .4] [.2 .2 .4] [.2 .2 .5]
[.2 .1 .5] [.3 .1 .5] [.4 .1 .4] [.5 .1 .4] [.7 .1 .3] [.8 .1 .2] [.5 .4 .1]
[.4 .5 .1] [.3 .6 .0] [.2 .6 .1] [.1 .7 .1] [.1 .6 .1] [.1 .5 .2] [.1 .4 .2]
[.1 .4 .2] [.1 .3 .3] [.1 .2 .4] [.1 .2 .5] [.1 .1 .5] [.1 .0 .6] [.3 .0 .5]
[.5 .0 .4] [.7 .3 .0] [.7 .4 .0] [.5 .4 .1] [.3 .4 .2] [.2 .3 .2] [.2 .2 .3]
[.2 .2 .4] [.3 .1 .4] [.5 .1 .3] [.6 .1 .2] [.7 .1 .2] [.8 .0 .2] [1. .0 .2]
[1. .1 .1] [.9 .2 .1] [.8 .2 .1] [.6 .2 .0] [.4 .4 .0] [.2 .4 .1] [.2 .3 .2]
[.2 .2 .2] [.2 .2 .3] [.2 .1 .3] [.3 .1 .3] [.5 .1 .2] [.7 .1 .2] [.8 .0 .2]
[.9 .0 .1] [1. .0 .1] [1. .1 .1] [.8 .2 .0] [.7 .2 .0] [.5 .2 .0] [.4 .3 .0]
[.1 .4 .1] [.1 .4 .1] [.1 .3 .2] [.1 .2 .2] [.1 .2 .3] [.2 .1 .4] [.2 .0 .3]
[.4 .0 .2] [.6 .0 .2] [.7 .0 .1] [.8 .0 .1] [.7 .1 .1] [.7 .1 .1] [.6 .2 .0]
[.4 .2 .0] [.2 .3 .0] [.1 .3 .0] [.1 .2 .1] [.1 .2 .1] [.1 .1 .2] [.1 .1 .3]
[.1 .0 .3] [.1 .0 .2] [.2 .0 .2] [.3 .0 .2] [.3 .0 .1] [.4 .0 .1] [.4 .1 .1]
[.4 .1 .0] [.2 .1 .0] [.0 .0 .0] [.0 .0 .0] [.0 .0 .0] [.0 .0 .0] [.0 .0 .0]
[.0 .0 .0] [.0 .0 .0] [.0 .0 .0] [.0 .0 .0] [.0 .0 .0] [.0 .0 .0] [.0 .0 .0]
[.0 .0 .0] [.0 .0 .0] [.0 .0 .0] [.0 .0 .0]  T astore pop
%%Page: 1 1
0.04000 0.04000 A 1 J 1 C 12 W [] 0 D
T   0 [1.000 1.000 1.000] U
T   1 [0.000 0.000 0.000] U
T   2 [0.000 0.000 1.000] U
T   3 [0.000 0.031 1.000] U
T   4 [0.000 0.059 1.000] U
T   5 [0.000 0.090 1.000] U
T   6 [0.000 0.122 1.000] U
T   7 [0.000 0.153 1.000] U
T   8 [0.000 0.180 1.000] U
T   9 [0.000 0.212 1.000] U
T  10 [0.000 0.243 1.000] U
T  11 [0.000 0.275 1.000] U
T  12 [0.000 0.302 1.000] U
T  13 [0.000 0.333 1.000] U
T  14 [0.000 0.365 1.000] U
T  15 [0.000 0.392 1.000] U
T  16 [0.000 0.424 1.000] U
```

T 17 [0.000 0.455 1.000] U
T 18 [0.000 0.486 1.000] U
T 19 [0.000 0.514 1.000] U
T 20 [0.000 0.545 1.000] U
T 21 [0.000 0.576 1.000] U
T 22 [0.000 0.608 1.000] U
T 23 [0.000 0.635 1.000] U
T 24 [0.000 0.667 1.000] U
T 25 [0.000 0.698 1.000] U
T 26 [0.000 0.725 1.000] U
T 27 [0.000 0.757 1.000] U
T 28 [0.000 0.788 1.000] U
T 29 [0.000 0.820 1.000] U
T 30 [0.000 0.847 1.000] U
T 31 [0.000 0.878 1.000] U
T 32 [0.000 0.910 1.000] U
T 33 [0.000 0.941 1.000] U
T 34 [0.000 0.969 1.000] U
T 35 [0.000 1.000 1.000] U
T 36 [0.000 0.992 0.973] U
T 37 [0.000 0.980 0.941] U
T 38 [0.004 0.973 0.914] U
T 39 [0.004 0.965 0.886] U
T 40 [0.004 0.953 0.859] U
T 41 [0.004 0.945 0.827] U
T 42 [0.008 0.937 0.800] U
T 43 [0.008 0.929 0.773] U
T 44 [0.008 0.918 0.745] U
T 45 [0.008 0.910 0.714] U
T 46 [0.012 0.902 0.686] U
T 47 [0.012 0.890 0.659] U
T 48 [0.012 0.882 0.627] U
T 49 [0.012 0.875 0.600] U
T 50 [0.016 0.863 0.573] U
T 51 [0.016 0.855 0.545] U
T 52 [0.016 0.847 0.514] U
T 53 [0.016 0.839 0.486] U
T 54 [0.020 0.827 0.459] U
T 55 [0.020 0.820 0.431] U
T 56 [0.020 0.812 0.400] U
T 57 [0.020 0.800 0.373] U
T 58 [0.024 0.792 0.345] U
T 59 [0.024 0.784 0.314] U

T 60 [0.024 0.773 0.286] U
T 61 [0.024 0.765 0.259] U
T 62 [0.027 0.757 0.231] U
T 63 [0.027 0.749 0.200] U
T 64 [0.027 0.737 0.173] U
T 65 [0.027 0.729 0.145] U
T 66 [0.031 0.722 0.118] U
T 67 [0.031 0.710 0.086] U
T 68 [0.031 0.702 0.059] U
T 69 [0.059 0.710 0.059] U
T 70 [0.090 0.718 0.055] U
T 71 [0.118 0.729 0.055] U
T 72 [0.145 0.737 0.051] U
T 73 [0.173 0.745 0.051] U
T 74 [0.204 0.753 0.047] U
T 75 [0.231 0.765 0.047] U
T 76 [0.259 0.773 0.043] U
T 77 [0.286 0.780 0.043] U
T 78 [0.318 0.788 0.043] U
T 79 [0.345 0.800 0.039] U
T 80 [0.373 0.808 0.039] U
T 81 [0.400 0.816 0.035] U
T 82 [0.431 0.824 0.035] U
T 83 [0.459 0.835 0.031] U
T 84 [0.486 0.843 0.031] U
T 85 [0.518 0.851 0.031] U
T 86 [0.545 0.859 0.027] U
T 87 [0.573 0.867 0.027] U
T 88 [0.600 0.878 0.024] U
T 89 [0.631 0.886 0.024] U
T 90 [0.659 0.894 0.020] U
T 91 [0.686 0.902 0.020] U
T 92 [0.714 0.914 0.016] U
T 93 [0.745 0.922 0.016] U
T 94 [0.773 0.929 0.016] U
T 95 [0.800 0.937 0.012] U
T 96 [0.827 0.949 0.012] U
T 97 [0.859 0.957 0.008] U
T 98 [0.886 0.965 0.008] U
T 99 [0.914 0.973 0.004] U
T 100 [0.941 0.984 0.004] U
T 101 [0.973 0.992 0.000] U
T 102 [1.000 1.000 0.000] U

T 103 [1.000 0.988 0.000] U
T 104 [1.000 0.980 0.000] U
T 105 [1.000 0.969 0.000] U
T 106 [1.000 0.957 0.000] U
T 107 [1.000 0.945 0.000] U
T 108 [1.000 0.937 0.000] U
T 109 [1.000 0.925 0.000] U
T 110 [1.000 0.914 0.000] U
T 111 [1.000 0.902 0.000] U
T 112 [1.000 0.894 0.000] U
T 113 [1.000 0.882 0.000] U
T 114 [1.000 0.871 0.000] U
T 115 [1.000 0.863 0.000] U
T 116 [1.000 0.851 0.000] U
T 117 [1.000 0.839 0.000] U
T 118 [1.000 0.827 0.000] U
T 119 [1.000 0.820 0.000] U
T 120 [1.000 0.808 0.000] U
T 121 [1.000 0.796 0.000] U
T 122 [1.000 0.784 0.000] U
T 123 [1.000 0.776 0.000] U
T 124 [1.000 0.765 0.000] U
T 125 [1.000 0.753 0.000] U
T 126 [1.000 0.745 0.000] U
T 127 [1.000 0.733 0.000] U
T 128 [1.000 0.722 0.000] U
T 129 [1.000 0.710 0.000] U
T 130 [1.000 0.702 0.000] U
T 131 [1.000 0.690 0.000] U
T 132 [1.000 0.678 0.000] U
T 133 [1.000 0.667 0.000] U
T 134 [1.000 0.659 0.000] U
T 135 [1.000 0.647 0.000] U
T 136 [1.000 0.627 0.000] U
T 137 [1.000 0.608 0.000] U
T 138 [1.000 0.588 0.000] U
T 139 [1.000 0.569 0.000] U
T 140 [1.000 0.549 0.000] U
T 141 [1.000 0.529 0.000] U
T 142 [1.000 0.510 0.000] U
T 143 [1.000 0.490 0.000] U
T 144 [1.000 0.471 0.000] U
T 145 [1.000 0.451 0.000] U

T 146 [1.000 0.431 0.000] U
T 147 [1.000 0.412 0.000] U
T 148 [1.000 0.392 0.000] U
T 149 [1.000 0.373 0.000] U
T 150 [1.000 0.353 0.000] U
T 151 [1.000 0.333 0.000] U
T 152 [1.000 0.314 0.000] U
T 153 [1.000 0.294 0.000] U
T 154 [1.000 0.275 0.000] U
T 155 [1.000 0.255 0.000] U
T 156 [1.000 0.235 0.000] U
T 157 [1.000 0.216 0.000] U
T 158 [1.000 0.196 0.000] U
T 159 [1.000 0.176 0.000] U
T 160 [1.000 0.157 0.000] U
T 161 [1.000 0.137 0.000] U
T 162 [1.000 0.118 0.000] U
T 163 [1.000 0.098 0.000] U
T 164 [1.000 0.078 0.000] U
T 165 [1.000 0.059 0.000] U
T 166 [1.000 0.039 0.000] U
T 167 [1.000 0.020 0.000] U
T 168 [1.000 0.000 0.000] U
T 169 [0.980 0.000 0.016] U
T 170 [0.961 0.000 0.031] U
T 171 [0.941 0.000 0.047] U
T 172 [0.922 0.000 0.063] U
T 173 [0.902 0.000 0.082] U
T 174 [0.878 0.000 0.098] U
T 175 [0.859 0.000 0.114] U
T 176 [0.839 0.000 0.129] U
T 177 [0.820 0.000 0.145] U
T 178 [0.800 0.000 0.161] U
T 179 [0.780 0.000 0.176] U
T 180 [0.761 0.000 0.192] U
T 181 [0.741 0.000 0.212] U
T 182 [0.722 0.000 0.227] U
T 183 [0.702 0.000 0.243] U
T 184 [0.682 0.000 0.259] U
T 185 [0.659 0.000 0.275] U
T 186 [0.639 0.000 0.290] U
T 187 [0.620 0.000 0.306] U
T 188 [0.600 0.000 0.322] U

T 189 [0.580 0.000 0.341] U
T 190 [0.561 0.000 0.357] U
T 191 [0.541 0.000 0.373] U
T 192 [0.522 0.000 0.388] U
T 193 [0.502 0.000 0.404] U
T 194 [0.482 0.000 0.420] U
T 195 [0.463 0.000 0.435] U
T 196 [0.439 0.000 0.451] U
T 197 [0.420 0.000 0.471] U
T 198 [0.400 0.000 0.486] U
T 199 [0.380 0.000 0.502] U
T 200 [0.361 0.000 0.518] U
T 201 [0.341 0.000 0.533] U
-1200 1025 16500 18750 Oc
Gs
1 O
0 O
2300 16170 M 2300 5455 L 2333 5455 L 2366 5455 L 2399 5455 L
2432 5455 L 2466 5455 L 2499 5455 L 2532 5455 L
2565 5455 L 2599 5455 L 2632 5455 L 2665 5455 L
2698 5455 L 2732 5455 L 2765 5455 L 2798 5455 L
2831 5455 L 2864 5455 L 2898 5455 L 2927 5455 L
2926 5459 L 2917 5486 L 2938 5506 L 2967 5505 L
2985 5482 L 2967 5459 L 2967 5455 L 2997 5455 L
3031 5455 L 3064 5455 L 3097 5455 L 3130 5455 L
3164 5455 L 3197 5455 L 3230 5455 L 3263 5455 L
3296 5455 L 3330 5455 L 3363 5455 L 3396 5455 L
3429 5455 L 3463 5455 L 3496 5455 L 3529 5455 L
3562 5455 L 3595 5455 L 3629 5455 L 3662 5455 L
3695 5455 L 3728 5455 L 3762 5455 L 3795 5455 L
3828 5455 L 3861 5455 L 3895 5455 L 3928 5455 L
3961 5455 L 3994 5455 L 4027 5455 L 4061 5455 L
4094 5455 L 4127 5455 L 4160 5455 L 4194 5455 L
4227 5455 L 4260 5455 L 4293 5455 L 4327 5455 L
4360 5455 L 4393 5455 L 4426 5455 L 4459 5455 L
4493 5455 L 4526 5455 L 4559 5455 L 4592 5455 L
4626 5455 L 4659 5455 L 4692 5455 L 4725 5455 L
4759 5455 L 4792 5455 L 4825 5455 L 4858 5455 L
4891 5455 L 4925 5455 L 4958 5455 L 4991 5455 L
5024 5455 L 5058 5455 L 5091 5455 L 5124 5455 L
5157 5455 L 5191 5455 L 5224 5455 L 5257 5455 L
5290 5455 L 5323 5455 L 5357 5455 L 5390 5455 L
5395 5455 L 5407 5466 L 5406 5495 L 5402 5524 L

5404 5553 L 5404 5582 L 5393 5609 L 5369 5625 L
5352 5648 L 5381 5644 L 5387 5616 L 5416 5614 L
5416 5585 L 5411 5557 L 5410 5528 L 5412 5499 L
5430 5476 L 5430 5455 L 5456 5455 L 5490 5455 L
5523 5455 L 5532 5455 L 5539 5469 L 5540 5475 L
5544 5482 L 5551 5487 L 5537 5525 L 5529 5553 L
5547 5530 L 5556 5503 L 5573 5479 L 5592 5457 L
5594 5455 L 5622 5455 L 5644 5455 L 5645 5472 L
5649 5455 L 5656 5455 L 5657 5455 L 5665 5470 L
5675 5497 L 5689 5523 L 5683 5551 L 5659 5567 L
5667 5595 L 5681 5621 L 5681 5650 L 5666 5674 L
5689 5691 L 5674 5715 L 5654 5737 L 5676 5717 L
5703 5726 L 5686 5750 L 5670 5774 L 5650 5795 L
5674 5779 L 5702 5786 L 5688 5811 L 5664 5827 L
5654 5855 L 5627 5866 L 5598 5867 L 5627 5872 L
5655 5876 L 5661 5905 L 5662 5934 L 5645 5957 L
5617 5961 L 5600 5985 L 5627 5975 L 5654 5965 L
5679 5950 L 5677 5921 L 5684 5893 L 5705 5873 L
5723 5896 L 5711 5870 L 5706 5841 L 5712 5830 L
5721 5816 L 5739 5794 L 5745 5765 L 5772 5776 L
5790 5754 L 5797 5725 L 5799 5722 L 5811 5700 L
5823 5674 L 5842 5652 L 5859 5628 L 5853 5600 L
5871 5622 L 5898 5632 L 5926 5625 L 5955 5622 L
5928 5612 L 5902 5598 L 5880 5580 L 5860 5559 L
5880 5538 L 5909 5534 L 5938 5535 L 5967 5536 L
5986 5514 L 6014 5506 L 6043 5507 L 6069 5495 L
6050 5517 L 6058 5545 L 6085 5534 L 6113 5542 L
6119 5571 L 6090 5565 L 6074 5588 L 6051 5570 L
6023 5561 L 6008 5586 L 6036 5590 L 6065 5592 L
6087 5611 L 6061 5624 L 6035 5637 L 6057 5656 L
6085 5663 L 6113 5670 L 6141 5663 L 6159 5640 L
6168 5612 L 6172 5584 L 6200 5590 L 6228 5597 L
6234 5626 L 6207 5636 L 6181 5650 L 6208 5660 L
6209 5689 L 6180 5689 L 6202 5708 L 6216 5734 L
6242 5746 L 6246 5748 L 6256 5751 L 6269 5755 L
6296 5768 L 6324 5762 L 6345 5782 L 6337 5754 L
6314 5736 L 6317 5707 L 6314 5678 L 6325 5651 L
6354 5652 L 6382 5648 L 6403 5628 L 6399 5656 L
6415 5680 L 6431 5705 L 6408 5723 L 6392 5747 L
6417 5732 L 6430 5706 L 6458 5710 L 6472 5735 L
6487 5760 L 6515 5767 L 6544 5767 L 6569 5782 L
6599 5771 L 6577 5763 L 6575 5753 L 6595 5754 L
6614 5755 L 6640 5742 L 6665 5728 L 6637 5727 L

6648 5700 L 6677 5693 L 6705 5699 L 6731 5685 L
6757 5673 L 6754 5702 L 6768 5727 L 6747 5707 L
6730 5731 L 6747 5754 L 6764 5777 L 6785 5797 L
6814 5799 L 6838 5783 L 6866 5775 L 6882 5750 L
6899 5727 L 6921 5747 L 6949 5754 L 6976 5764 L
7003 5775 L 7027 5791 L 7029 5820 L 7046 5796 L
7031 5772 L 7060 5771 L 7082 5790 L 7102 5811 L
7125 5828 L 7149 5844 L 7172 5826 L 7201 5821 L
7229 5828 L 7258 5827 L 7266 5854 L 7287 5874 L
7284 5903 L 7277 5931 L 7291 5957 L 7296 5985 L
7271 5971 L 7268 5999 L 7297 6002 L 7320 5984 L
7341 5964 L 7358 5988 L 7379 5999 L 7361 6015 L
7343 6030 L 7315 6037 L 7292 6055 L 7279 6080 L
7273 6109 L 7245 6113 L 7274 6113 L 7294 6092 L
7317 6074 L 7343 6062 L 7349 6062 L 7372 6062 L
7401 6062 L 7422 6085 L 7434 6099 L 7455 6119 L
7454 6130 L 7455 6156 L 7469 6182 L 7461 6205 L
7487 6216 L 7507 6221 L 7515 6224 L 7527 6217 L
7541 6210 L 7532 6238 L 7559 6227 L 7564 6199 L
7572 6171 L 7590 6186 L 7594 6190 L 7617 6207 L
7641 6223 L 7641 6252 L 7659 6229 L 7651 6201 L
7669 6224 L 7671 6253 L 7676 6281 L 7687 6308 L
7711 6291 L 7724 6307 L 7732 6307 L 7736 6307 L
7759 6291 L 7776 6313 L 7786 6341 L 7785 6369 L
7757 6376 L 7733 6393 L 7762 6390 L 7790 6384 L
7818 6376 L 7840 6357 L 7849 6330 L 7840 6302 L
7838 6273 L 7855 6297 L 7857 6325 L 7860 6354 L
7871 6380 L 7895 6397 L 7891 6426 L 7909 6448 L
7937 6457 L 7946 6430 L 7930 6407 L 7925 6378 L
7937 6404 L 7954 6428 L 7977 6445 L 8001 6460 L
8006 6488 L 8024 6511 L 8013 6537 L 8034 6558 L
8054 6538 L 8077 6556 L 8098 6575 L 8126 6581 L
8120 6609 L 8106 6634 L 8077 6634 L 8074 6663 L
8046 6660 L 8017 6657 L 8000 6661 L 7974 6674 L
7952 6692 L 7941 6704 L 7942 6720 L 7944 6717 L
7954 6704 L 7968 6707 L 7980 6708 L 7997 6709 L
8024 6699 L 8035 6696 L 8051 6691 L 8074 6708 L
8079 6736 L 8054 6749 L 8082 6750 L 8092 6777 L
8109 6754 L 8110 6757 L 8123 6779 L 8125 6808 L
8154 6808 L 8165 6782 L 8157 6754 L 8184 6747 L
8185 6747 L 8198 6773 L 8225 6765 L 8226 6765 L
8253 6770 L 8274 6782 L 8279 6784 L 8273 6812 L
8295 6830 L 8285 6803 L 8310 6790 L 8336 6777 L

8361 6763 L 8360 6734 L 8385 6748 L 8405 6768 L
8394 6794 L 8395 6823 L 8424 6820 L 8437 6845 L
8441 6874 L 8425 6898 L 8403 6880 L 8382 6900 L
8378 6929 L 8355 6945 L 8381 6934 L 8409 6940 L
8402 6968 L 8395 6995 L 8416 6975 L 8417 6947 L
8429 6972 L 8442 6947 L 8437 6919 L 8464 6927 L
8483 6948 L 8511 6944 L 8538 6934 L 8562 6948 L
8534 6955 L 8530 6983 L 8558 6985 L 8587 6984 L
8574 7009 L 8575 7038 L 8562 7012 L 8534 7020 L
8510 7035 L 8501 7062 L 8529 7069 L 8545 7093 L
8562 7116 L 8541 7136 L 8522 7157 L 8548 7168 L
8577 7170 L 8605 7164 L 8588 7140 L 8614 7152 L
8611 7124 L 8601 7097 L 8629 7093 L 8655 7081 L
8655 7081 L 8641 7106 L 8632 7133 L 8648 7157 L
8649 7129 L 8673 7112 L 8701 7114 L 8730 7118 L
8736 7146 L 8753 7151 L 8764 7153 L 8744 7174 L
8727 7197 L 8704 7181 L 8677 7171 L 8687 7198 L
8663 7213 L 8640 7230 L 8619 7249 L 8646 7258 L
8669 7275 L 8694 7289 L 8713 7310 L 8729 7334 L
8741 7360 L 8747 7356 L 8764 7344 L 8754 7317 L
8780 7305 L 8808 7301 L 8813 7273 L 8819 7265 L
8830 7250 L 8827 7221 L 8842 7246 L 8852 7273 L
8879 7264 L 8880 7236 L 8881 7207 L 8889 7214 L
8902 7226 L 8902 7226 L 8908 7254 L 8901 7282 L
8874 7292 L 8868 7320 L 8854 7345 L 8829 7332 L
8821 7360 L 8807 7385 L 8824 7407 L 8850 7418 L
8878 7423 L 8883 7432 L 8892 7448 L 8884 7476 L
8882 7504 L 8891 7531 L 8909 7532 L 8919 7533 L
8928 7560 L 8935 7588 L 8906 7591 L 8881 7603 L
8854 7612 L 8834 7633 L 8850 7656 L 8870 7675 L
8870 7676 L 8887 7699 L 8898 7725 L 8870 7721 L
8893 7737 L 8921 7743 L 8937 7747 L 8949 7750 L
8973 7765 L 8976 7766 L 9001 7771 L 9029 7775 L
9036 7784 L 9046 7797 L 9020 7786 L 8996 7801 L
8979 7824 L 8961 7801 L 8953 7774 L 8927 7762 L
8905 7780 L 8899 7808 L 8886 7833 L 8859 7825 L
8872 7850 L 8895 7867 L 8916 7848 L 8944 7841 L
8967 7824 L 8982 7848 L 8970 7874 L 8966 7902 L
8947 7923 L 8919 7928 L 8891 7928 L 8865 7917 L
8843 7936 L 8841 7964 L 8867 7974 L 8844 7990 L
8849 8017 L 8845 8046 L 8872 8035 L 8894 8017 L
8919 8004 L 8909 8030 L 8901 8058 L 8900 8086 L
8910 8060 L 8927 8037 L 8941 8024 L 8948 8018 L

8953 8046 L 8979 8058 L 8962 8034 L 8990 8027 L
8965 8014 L 8962 7986 L 8968 7984 L 8990 7979 L
9012 7961 L 9017 7989 L 9034 8012 L 9043 8039 L
9071 8036 L 9094 8020 L 9082 7995 L 9057 7982 L
9058 7954 L 9038 7935 L 9009 7933 L 8993 7910 L
8986 7882 L 9014 7882 L 9035 7900 L 9044 7927 L
9072 7930 L 9075 7932 L 9096 7945 L 9100 7947 L
9122 7956 L 9121 7984 L 9142 8004 L 9116 8016 L
9127 8042 L 9102 8056 L 9078 8071 L 9090 8096 L
9118 8091 L 9125 8118 L 9147 8117 L 9153 8116 L
9181 8111 L 9179 8139 L 9152 8146 L 9180 8146 L
9199 8167 L 9204 8195 L 9195 8222 L 9223 8218 L
9238 8222 L 9250 8226 L 9265 8249 L 9251 8274 L
9246 8302 L 9256 8275 L 9280 8261 L 9291 8287 L
9307 8264 L 9313 8274 L 9322 8287 L 9323 8316 L
9326 8321 L 9339 8339 L 9326 8364 L 9332 8391 L
9357 8379 L 9385 8383 L 9384 8411 L 9398 8436 L
9372 8446 L 9359 8471 L 9340 8493 L 9368 8500 L
9387 8520 L 9387 8549 L 9390 8577 L 9418 8580 L
9430 8605 L 9445 8629 L 9463 8651 L 9479 8674 L
9477 8702 L 9452 8716 L 9425 8721 L 9405 8741 L
9377 8746 L 9349 8742 L 9347 8770 L 9369 8752 L
9397 8755 L 9417 8749 L 9424 8748 L 9429 8748 L
9446 8730 L 9474 8731 L 9496 8730 L 9502 8730 L
9529 8727 L 9529 8740 L 9542 8765 L 9540 8793 L
9553 8818 L 9567 8825 L 9578 8831 L 9593 8854 L
9603 8881 L 9581 8898 L 9605 8908 L 9607 8909 L
9616 8936 L 9642 8946 L 9669 8938 L 9652 8916 L
9661 8901 L 9667 8891 L 9676 8865 L 9693 8842 L
9713 8823 L 9734 8842 L 9728 8869 L 9750 8887 L
9762 8912 L 9777 8936 L 9805 8932 L 9806 8931 L
9829 8917 L 9847 8895 L 9859 8920 L 9845 8945 L
9837 8972 L 9823 8996 L 9824 9024 L 9808 9047 L
9796 9073 L 9788 9100 L 9771 9122 L 9744 9129 L
9717 9137 L 9745 9137 L 9773 9137 L 9801 9138 L
9825 9153 L 9837 9166 L 9844 9173 L 9865 9193 L
9857 9220 L 9844 9244 L 9816 9250 L 9801 9274 L
9828 9264 L 9856 9261 L 9874 9282 L 9853 9301 L
9864 9327 L 9839 9339 L 9811 9333 L 9786 9322 L
9807 9340 L 9834 9343 L 9831 9371 L 9804 9377 L
9777 9369 L 9749 9366 L 9750 9394 L 9775 9408 L
9782 9435 L 9787 9462 L 9810 9459 L 9814 9458 L
9832 9436 L 9833 9464 L 9844 9490 L 9836 9463 L

9837 9435 L 9847 9409 L 9874 9416 L 9894 9435 L
9901 9462 L 9899 9490 L 9907 9517 L 9927 9499 L
9918 9472 L 9921 9456 L 9923 9445 L 9925 9417 L
9952 9411 L 9976 9427 L 9980 9432 L 9994 9448 L
9989 9476 L 9964 9488 L 9966 9516 L 9963 9544 L
9989 9554 L 9990 9582 L 9974 9605 L 9977 9633 L
9995 9655 L 9976 9675 L 9949 9668 L 9922 9662 L
9897 9649 L 9896 9621 L 9912 9598 L 9888 9584 L
9881 9611 L 9853 9611 L 9834 9590 L 9808 9582 L
9784 9566 L 9766 9545 L 9775 9572 L 9748 9564 L
9733 9541 L 9724 9568 L 9735 9593 L 9753 9615 L
9780 9609 L 9808 9615 L 9835 9621 L 9857 9639 L
9875 9660 L 9892 9683 L 9915 9698 L 9932 9720 L
9954 9738 L 9968 9761 L 9990 9779 L 10003 9784 L
10010 9787 L 10016 9789 L 10039 9805 L 10046 9832 L
10022 9818 L 9994 9818 L 9966 9817 L 9962 9818 L
9955 9824 L 9944 9834 L 9917 9841 L 9891 9852 L
9864 9857 L 9836 9861 L 9812 9875 L 9798 9899 L
9782 9922 L 9763 9943 L 9746 9965 L 9727 9986 L
9708 10006 L 9685 10022 L 9661 10036 L 9634 10040 L
9606 10039 L 9578 10035 L 9554 10022 L 9531 10005 L
9512 9985 L 9491 9967 L 9467 9953 L 9445 9935 L
9419 9925 L 9391 9925 L 9365 9935 L 9346 9915 L
9371 9902 L 9344 9910 L 9335 9936 L 9341 9964 L
9324 9986 L 9314 10012 L 9326 10037 L 9302 10052 L
9274 10054 L 9246 10051 L 9224 10035 L 9213 10009 L
9197 9987 L 9169 9985 L 9147 10001 L 9128 9981 L
9104 9966 L 9124 9986 L 9141 10008 L 9169 10009 L
9195 10000 L 9194 10028 L 9202 10054 L 9227 10067 L
9254 10073 L 9282 10074 L 9309 10076 L 9337 10076 L
9364 10068 L 9383 10047 L 9411 10050 L 9436 10062 L
9452 10085 L 9468 10107 L 9469 10135 L 9484 10159 L
9504 10178 L 9528 10192 L 9556 10196 L 9571 10219 L
9593 10236 L 9616 10252 L 9643 10256 L 9667 10271 L
9682 10294 L 9702 10313 L 9727 10326 L 9753 10334 L
9780 10341 L 9808 10346 L 9835 10349 L 9863 10352 L
9890 10356 L 9914 10372 L 9920 10399 L 9911 10425 L
9897 10449 L 9884 10474 L 9871 10498 L 9858 10523 L
9844 10547 L 9829 10570 L 9812 10592 L 9789 10608 L
9766 10623 L 9742 10637 L 9714 10637 L 9699 10660 L
9677 10677 L 9651 10689 L 9627 10702 L 9609 10723 L
9595 10747 L 9577 10769 L 9555 10786 L 9531 10799 L
9510 10817 L 9484 10827 L 9458 10836 L 9430 10835 L

9403 10837 L 9378 10849 L 9359 10869 L 9381 10886 L
9368 10911 L 9355 10935 L 9340 10958 L 9312 10961 L
9285 10958 L 9257 10961 L 9231 10970 L 9204 10968 L
9192 10943 L 9165 10937 L 9138 10931 L 9113 10918 L
9087 10910 L 9059 10910 L 9045 10934 L 9072 10928 L
9100 10927 L 9126 10936 L 9147 10954 L 9170 10970 L
9191 10988 L 9215 11002 L 9240 11012 L 9266 11001 L
9293 11001 L 9320 10993 L 9346 11003 L 9361 10980 L
9384 10964 L 9401 10942 L 9420 10922 L 9430 10896 L
9436 10869 L 9462 10861 L 9490 10864 L 9517 10861 L
9532 10884 L 9559 10890 L 9584 10901 L 9610 10910 L
9613 10909 L 9635 10898 L 9662 10892 L 9689 10884 L
9707 10863 L 9732 10852 L 9757 10840 L 9783 10830 L
9810 10826 L 9837 10818 L 9864 10811 L 9889 10821 L
9896 10848 L 9884 10873 L 9870 10897 L 9858 10922 L
9849 10948 L 9829 10968 L 9811 10988 L 9790 11006 L
9765 11019 L 9739 11028 L 9713 11038 L 9686 11038 L
9660 11048 L 9649 11073 L 9629 11092 L 9644 11116 L
9638 11143 L 9638 11171 L 9625 11195 L 9603 11212 L
9578 11222 L 9552 11233 L 9527 11245 L 9502 11255 L
9476 11264 L 9448 11265 L 9429 11285 L 9411 11306 L
9383 11309 L 9399 11332 L 9417 11353 L 9404 11377 L
9378 11387 L 9399 11405 L 9407 11431 L 9406 11459 L
9395 11484 L 9389 11511 L 9379 11537 L 9352 11542 L
9337 11565 L 9332 11592 L 9316 11615 L 9297 11635 L
9286 11660 L 9276 11686 L 9257 11706 L 9251 11732 L
9253 11760 L 9262 11786 L 9240 11802 L 9223 11824 L
9216 11851 L 9220 11878 L 9209 11903 L 9201 11930 L
9175 11939 L 9180 11966 L 9155 11977 L 9155 12004 L
9146 12031 L 9145 12058 L 9136 12084 L 9126 12110 L
9118 12136 L 9110 12162 L 9097 12186 L 9092 12213 L
9078 12237 L 9053 12248 L 9028 12260 L 9002 12266 L
8983 12286 L 8958 12298 L 8932 12307 L 8905 12311 L
8878 12317 L 8865 12293 L 8865 12320 L 8847 12341 L
8820 12347 L 8798 12364 L 8774 12377 L 8751 12391 L
8730 12409 L 8716 12432 L 8712 12459 L 8684 12462 L
8660 12474 L 8635 12461 L 8613 12477 L 8594 12457 L
8602 12484 L 8588 12508 L 8587 12535 L 8596 12561 L
8595 12588 L 8599 12616 L 8614 12639 L 8634 12657 L
8661 12652 L 8673 12677 L 8676 12704 L 8683 12730 L
8692 12757 L 8704 12773 L 8708 12778 L 8733 12791 L
8752 12810 L 8756 12837 L 8770 12861 L 8778 12887 L
8787 12913 L 8781 12939 L 8808 12937 L 8830 12952 L

8840 12927 L 8853 12951 L 8874 12969 L 8890 12947 L
8901 12972 L 8916 12996 L 8927 13020 L 8950 13005 L
8957 13031 L 8955 13059 L 8965 13084 L 8985 13103 L
9010 13113 L 9016 13117 L 9033 13128 L 9026 13102 L
9040 13125 L 9062 13141 L 9074 13166 L 9047 13162 L
9020 13166 L 9036 13189 L 9010 13198 L 8986 13211 L
8978 13237 L 8988 13263 L 9013 13273 L 9039 13264 L
9060 13281 L 9064 13308 L 9082 13288 L 9098 13266 L
9093 13239 L 9083 13213 L 9071 13189 L 9098 13183 L
9124 13192 L 9145 13210 L 9172 13215 L 9199 13219 L
9223 13231 L 9245 13248 L 9240 13275 L 9236 13302 L
9220 13324 L 9226 13351 L 9252 13359 L 9243 13385 L
9258 13408 L 9282 13420 L 9304 13403 L 9328 13390 L
9329 13417 L 9347 13438 L 9346 13465 L 9321 13476 L
9305 13498 L 9279 13492 L 9296 13513 L 9319 13527 L
9345 13520 L 9359 13496 L 9386 13497 L 9386 13525 L
9411 13514 L 9430 13533 L 9453 13548 L 9479 13554 L
9505 13564 L 9530 13574 L 9553 13589 L 9570 13611 L
9566 13637 L 9579 13614 L 9599 13633 L 9626 13636 L
9605 13618 L 9590 13595 L 9617 13602 L 9643 13595 L
9642 13622 L 9668 13632 L 9692 13645 L 9704 13669 L
9729 13680 L 9754 13692 L 9779 13704 L 9768 13729 L
9795 13734 L 9808 13710 L 9818 13685 L 9837 13703 L
9860 13718 L 9859 13745 L 9876 13766 L 9884 13740 L
9867 13719 L 9883 13696 L 9908 13686 L 9881 13682 L
9885 13655 L 9913 13654 L 9930 13651 L 9939 13650 L
9962 13665 L 9969 13671 L 9983 13682 L 9997 13706 L
9998 13706 L 10009 13709 L 10023 13712 L 10012 13687 L
10025 13711 L 10030 13738 L 10029 13765 L 10002 13762 L
9975 13764 L 9995 13782 L 9995 13810 L 10011 13832 L
10034 13846 L 10056 13861 L 10040 13884 L 10033 13910 L
10037 13936 L 10055 13957 L 10066 13968 L 10075 13976 L
10048 13978 L 10024 13965 L 9999 13953 L 9981 13974 L
9955 13966 L 9928 13965 L 9902 13973 L 9878 13987 L
9854 13974 L 9840 13997 L 9849 14023 L 9832 14044 L
9810 14027 L 9787 14012 L 9768 13993 L 9769 13966 L
9754 13989 L 9727 13984 L 9700 13980 L 9684 13958 L
9664 13976 L 9639 13966 L 9612 13961 L 9585 13963 L
9561 13975 L 9542 13995 L 9525 14016 L 9507 14037 L
9532 14048 L 9505 14045 L 9480 14034 L 9453 14031 L
9426 14037 L 9406 14056 L 9402 14083 L 9386 14105 L
9359 14103 L 9342 14125 L 9318 14137 L 9306 14161 L
9279 14158 L 9252 14161 L 9225 14160 L 9204 14142 L

9179 14133 L 9155 14120 L 9128 14115 L 9102 14108 L
9082 14090 L 9057 14081 L 9031 14073 L 9055 14062 L
9051 14035 L 9034 14013 L 9015 13994 L 8996 13974 L
8974 13958 L 8951 13943 L 8925 13936 L 8900 13926 L
8879 13908 L 8866 13884 L 8883 13863 L 8876 13836 L
8856 13817 L 8836 13800 L 8817 13780 L 8790 13773 L
8763 13774 L 8736 13771 L 8711 13782 L 8684 13779 L
8656 13777 L 8629 13778 L 8603 13785 L 8576 13788 L
8555 13806 L 8532 13821 L 8505 13820 L 8502 13847 L
8485 13868 L 8467 13888 L 8473 13915 L 8469 13942 L
8468 13969 L 8470 13996 L 8477 14023 L 8494 14044 L
8511 14065 L 8523 14090 L 8505 14110 L 8480 14121 L
8492 14146 L 8498 14172 L 8491 14198 L 8483 14224 L
8464 14244 L 8459 14271 L 8432 14271 L 8415 14250 L
8424 14276 L 8419 14303 L 8422 14330 L 8408 14353 L
8402 14326 L 8397 14353 L 8373 14340 L 8351 14324 L
8326 14313 L 8302 14301 L 8280 14285 L 8259 14302 L
8232 14306 L 8206 14314 L 8179 14316 L 8152 14313 L
8125 14317 L 8098 14313 L 8075 14327 L 8052 14342 L
8033 14361 L 8010 14376 L 7984 14383 L 7964 14402 L
7945 14422 L 7928 14443 L 7915 14466 L 7903 14491 L
7890 14515 L 7880 14540 L 7870 14565 L 7867 14592 L
7867 14619 L 7872 14646 L 7881 14672 L 7893 14696 L
7906 14720 L 7924 14741 L 7939 14763 L 7964 14774 L
7958 14800 L 7948 14825 L 7967 14845 L 7986 14864 L
8008 14879 L 8034 14888 L 8061 14893 L 8088 14888 L
8111 14875 L 8126 14852 L 8133 14826 L 8151 14806 L
8174 14792 L 8201 14787 L 8223 14803 L 8243 14821 L
8264 14838 L 8284 14856 L 8301 14864 L 8309 14867 L
8326 14867 L 8336 14867 L 8357 14850 L 8371 14850 L
8384 14851 L 8406 14867 L 8406 14867 L 8422 14869 L
8433 14870 L 8458 14880 L 8475 14889 L 8482 14893 L
8504 14909 L 8522 14929 L 8536 14953 L 8553 14974 L
8575 14989 L 8600 15000 L 8583 15021 L 8579 15047 L
8583 15074 L 8575 15100 L 8598 15114 L 8605 15140 L
8613 15166 L 8625 15190 L 8645 15209 L 8667 15225 L
8692 15236 L 8690 15263 L 8710 15281 L 8736 15290 L
8762 15297 L 8788 15305 L 8810 15321 L 8836 15328 L
8851 15350 L 8874 15365 L 8899 15375 L 8925 15383 L
8950 15393 L 8974 15406 L 8998 15418 L 9019 15435 L
9045 15442 L 9071 15449 L 9087 15471 L 9086 15498 L
9099 15521 L 9122 15535 L 9100 15551 L 9122 15567 L
9127 15594 L 9150 15607 L 9164 15630 L 9174 15637 L

9186 15647 L 9204 15667 L 9211 15681 L 9216 15691 L
9228 15705 L 9234 15711 L 9260 15717 L 9262 15720 L
9278 15738 L 9256 15753 L 9267 15777 L 9286 15796 L
9295 15795 L 9311 15793 L 9313 15792 L 9323 15817 L
9342 15836 L 9358 15858 L 9385 15856 L 9407 15840 L
9430 15853 L 9454 15840 L 9481 15841 L 9495 15832 L
9503 15826 L 9523 15807 L 9550 15809 L 9577 15812 L
9598 15828 L 9608 15853 L 9609 15880 L 9629 15898 L
9639 15923 L 9635 15896 L 9622 15872 L 9633 15847 L
9625 15822 L 9639 15798 L 9666 15800 L 9682 15779 L
9705 15764 L 9727 15748 L 9742 15733 L 9754 15732 L
9754 15746 L 9759 15750 L 9769 15746 L 9784 15739 L
9803 15744 L 9801 15751 L 9779 15745 L 9772 15755 L
9766 15762 L 9766 15768 L 9754 15775 L 9732 15791 L
9732 15793 L 9757 15778 L 9777 15766 L 9779 15753 L
9801 15755 L 9806 15746 L 9802 15740 L 9784 15736 L
9760 15748 L 9758 15742 L 9763 15715 L 9754 15698 L
9760 15688 L 9783 15673 L 9803 15655 L 9816 15631 L
9804 15607 L 9786 15586 L 9784 15559 L 9766 15539 L
9752 15516 L 9748 15489 L 9728 15471 L 9715 15447 L
9700 15425 L 9683 15404 L 9667 15382 L 9644 15368 L
9670 15361 L 9651 15342 L 9632 15322 L 9608 15309 L
9581 15310 L 9564 15289 L 9540 15275 L 9518 15261 L
9506 15236 L 9496 15211 L 9517 15195 L 9534 15173 L
9536 15146 L 9534 15125 L 9529 15113 L 9517 15096 L
9494 15087 L 9508 15055 L 9518 15025 L 9541 15011 L
9568 15014 L 9595 15016 L 9619 15028 L 9646 15028 L
9673 15035 L 9700 15033 L 9676 15020 L 9650 15013 L
9627 14998 L 9613 14976 L 9589 14962 L 9563 14955 L
9582 14935 L 9565 14914 L 9587 14898 L 9608 14881 L
9594 14858 L 9568 14849 L 9543 14838 L 9521 14822 L
9495 14819 L 9475 14837 L 9465 14812 L 9451 14789 L
9426 14779 L 9399 14779 L 9372 14772 L 9365 14746 L
9391 14739 L 9369 14722 L 9382 14698 L 9381 14671 L
9402 14654 L 9420 14675 L 9431 14699 L 9458 14699 L
9483 14710 L 9510 14710 L 9536 14719 L 9554 14738 L
9582 14739 L 9609 14741 L 9621 14765 L 9597 14779 L
9571 14785 L 9595 14797 L 9607 14821 L 9629 14838 L
9653 14828 L 9665 14803 L 9672 14799 L 9689 14790 L
9690 14818 L 9679 14842 L 9705 14834 L 9696 14859 L
9716 14841 L 9737 14859 L 9764 14865 L 9756 14891 L
9782 14896 L 9806 14908 L 9782 14920 L 9792 14945 L
9811 14964 L 9834 14978 L 9846 15003 L 9856 15028 L

9879 15042 L 9898 15059 L 9900 15060 L 9915 15059 L
9927 15057 L 9946 15076 L 9950 15103 L 9956 15099 L
9973 15089 L 9991 15110 L 10015 15122 L 10035 15140 L
10042 15146 L 10056 15157 L 10057 15157 L 10071 15163 L
10078 15166 L 10081 15168 L 10088 15170 L 10097 15174 L
10107 15177 L 10131 15189 L 10143 15201 L 10150 15208 L
10170 15216 L 10175 15218 L 10201 15209 L 10226 15219 L
10235 15226 L 10248 15235 L 10223 15246 L 10246 15260 L
10255 15286 L 10257 15285 L 10272 15283 L 10281 15281 L
10284 15281 L 10308 15276 L 10309 15275 L 10321 15268 L
10331 15262 L 10340 15280 L 10343 15286 L 10356 15283 L
10369 15281 L 10383 15304 L 10393 15329 L 10401 15355 L
10403 15328 L 10426 15313 L 10441 15314 L 10453 15314 L
10473 15332 L 10497 15320 L 10524 15318 L 10551 15317 L
10578 15323 L 10600 15338 L 10627 15344 L 10639 15368 L
10644 15341 L 10651 15315 L 10656 15342 L 10661 15369 L
10666 15395 L 10672 15395 L 10693 15397 L 10687 15370 L
10709 15354 L 10733 15342 L 10759 15335 L 10786 15333 L
10796 15307 L 10805 15282 L 10824 15262 L 10813 15237 L
10826 15213 L 10840 15231 L 10843 15234 L 10854 15250 L
10859 15256 L 10856 15283 L 10867 15308 L 10888 15291 L
10914 15283 L 10929 15263 L 10930 15262 L 10954 15275 L
10971 15254 L 10998 15255 L 11015 15243 L 11021 15239 L
11044 15226 L 11070 15234 L 11089 15215 L 11116 15216 L
11139 15230 L 11160 15247 L 11172 15271 L 11177 15283 L
11189 15280 L 11197 15283 L 11202 15289 L 11203 15288 L
11198 15280 L 11189 15277 L 11177 15282 L 11174 15269 L
11163 15241 L 11184 15242 L 11196 15252 L 11225 15269 L
11226 15267 L 11221 15263 L 11197 15251 L 11187 15237 L
11174 15236 L 11150 15223 L 11146 15196 L 11127 15177 L
11128 15150 L 11141 15126 L 11167 15133 L 11153 15109 L
11157 15082 L 11151 15055 L 11144 15029 L 11121 15015 L
11116 14988 L 11125 14962 L 11104 14944 L 11091 14920 L
11082 14895 L 11073 14869 L 11062 14844 L 11070 14818 L
11085 14795 L 11110 14787 L 11103 14813 L 11118 14790 L
11121 14763 L 11147 14772 L 11174 14769 L 11190 14791 L
11214 14779 L 11241 14778 L 11257 14756 L 11230 14752 L
11208 14767 L 11184 14754 L 11157 14751 L 11135 14735 L
11108 14729 L 11085 14715 L 11068 14736 L 11075 14710 L
11071 14683 L 11047 14671 L 11020 14670 L 11031 14645 L
11021 14619 L 11001 14601 L 10991 14576 L 10977 14553 L
10983 14526 L 10984 14499 L 11003 14480 L 11024 14491 L
11034 14487 L 11005 14467 L 11002 14471 L 11004 14478 L

10982 14492 L 10963 14466 L 10943 14449 L 10924 14430 L
10898 14423 L 10924 14416 L 10928 14415 L 10951 14411 L
10960 14414 L 10973 14418 L 10977 14419 L 11002 14429 L
11026 14415 L 11052 14408 L 11079 14411 L 11104 14422 L
11131 14424 L 11156 14414 L 11154 14387 L 11133 14404 L
11106 14399 L 11099 14373 L 11123 14360 L 11098 14350 L
11071 14354 L 11048 14341 L 11063 14318 L 11079 14312 L
11088 14308 L 11114 14300 L 11140 14308 L 11141 14308 L
11160 14322 L 11162 14324 L 11186 14310 L 11181 14283 L
11207 14284 L 11209 14284 L 11217 14310 L 11244 14315 L
11221 14329 L 11248 14331 L 11275 14328 L 11253 14313 L
11268 14290 L 11241 14288 L 11239 14261 L 11212 14254 L
11201 14230 L 11218 14208 L 11229 14233 L 11255 14237 L
11272 14259 L 11286 14268 L 11295 14273 L 11298 14273 L
11322 14271 L 11330 14297 L 11326 14323 L 11323 14351 L
11344 14350 L 11349 14350 L 11350 14350 L 11357 14376 L
11330 14376 L 11304 14386 L 11300 14413 L 11325 14403 L
11352 14404 L 11369 14400 L 11376 14398 L 11379 14397 L
11390 14375 L 11391 14373 L 11400 14366 L 11405 14363 L
11412 14358 L 11413 14357 L 11423 14348 L 11432 14338 L
11449 14316 L 11456 14316 L 11476 14315 L 11479 14342 L
11505 14336 L 11505 14336 L 11509 14309 L 11516 14303 L
11521 14298 L 11529 14290 L 11549 14275 L 11551 14274 L
11557 14300 L 11556 14327 L 11560 14329 L 11582 14336 L
11599 14338 L 11608 14339 L 11629 14357 L 11657 14359 L
11667 14352 L 11679 14344 L 11702 14329 L 11726 14317 L
11753 14322 L 11770 14344 L 11796 14357 L 11778 14334 L
11784 14307 L 11786 14280 L 11798 14256 L 11822 14244 L
11845 14228 L 11867 14214 L 11888 14203 L 11888 14201 L
11865 14211 L 11843 14227 L 11819 14238 L 11795 14251 L
11771 14264 L 11778 14290 L 11765 14314 L 11741 14301 L
11717 14289 L 11722 14262 L 11728 14236 L 11748 14217 L
11774 14210 L 11785 14184 L 11787 14157 L 11763 14145 L
11764 14144 L 11781 14125 L 11803 14108 L 11828 14099 L
11812 14077 L 11837 14065 L 11861 14053 L 11840 14036 L
11813 14032 L 11820 14006 L 11807 13982 L 11823 13960 L
11842 13981 L 11863 13997 L 11868 13970 L 11857 13946 L
11850 13919 L 11877 13913 L 11895 13893 L 11914 13874 L
11941 13868 L 11960 13887 L 11950 13861 L 11928 13845 L
11909 13826 L 11909 13799 L 11895 13822 L 11890 13849 L
11874 13871 L 11847 13876 L 11827 13895 L 11804 13879 L
11780 13892 L 11756 13904 L 11766 13879 L 11741 13891 L
11760 13871 L 11771 13846 L 11750 13863 L 11746 13836 L

11740 13810 L 11739 13837 L 11732 13863 L 11723 13889 L
11697 13880 L 11677 13862 L 11699 13847 L 11717 13827 L
11702 13804 L 11678 13791 L 11693 13769 L 11671 13785 L
11673 13812 L 11666 13838 L 11660 13865 L 11661 13838 L
11636 13826 L 11630 13799 L 11631 13772 L 11604 13766 L
11595 13740 L 11600 13717 L 11601 13714 L 11611 13739 L
11612 13738 L 11621 13734 L 11631 13730 L 11635 13728 L
11634 13700 L 11639 13702 L 11642 13703 L 11652 13706 L
11656 13707 L 11661 13708 L 11679 13688 L 11678 13661 L
11684 13653 L 11689 13647 L 11694 13641 L 11695 13639 L
11697 13630 L 11702 13613 L 11706 13586 L 11720 13566 L
11721 13564 L 11743 13581 L 11732 13606 L 11720 13630 L
11716 13657 L 11691 13668 L 11697 13695 L 11683 13719 L
11702 13738 L 11711 13712 L 11715 13685 L 11733 13706 L
11750 13717 L 11755 13721 L 11761 13694 L 11759 13667 L
11758 13640 L 11782 13627 L 11768 13604 L 11773 13596 L
11779 13585 L 11782 13580 L 11807 13608 L 11806 13593 L
11783 13578 L 11784 13551 L 11788 13548 L 11792 13545 L
11806 13534 L 11793 13510 L 11807 13487 L 11809 13461 L
11810 13459 L 11832 13443 L 11810 13427 L 11826 13421 L
11831 13419 L 11836 13418 L 11847 13408 L 11857 13400 L
11869 13376 L 11889 13357 L 11913 13370 L 11938 13381 L
11960 13397 L 11966 13424 L 11964 13396 L 11944 13379 L
11919 13368 L 11898 13350 L 11873 13338 L 11858 13315 L
11867 13289 L 11877 13291 L 11893 13295 L 11921 13297 L
11947 13303 L 11929 13283 L 11909 13264 L 11925 13241 L
11900 13230 L 11894 13203 L 11867 13202 L 11849 13181 L
11829 13163 L 11810 13143 L 11824 13119 L 11851 13121 L
11878 13126 L 11905 13126 L 11907 13099 L 11880 13101 L
11854 13092 L 11828 13083 L 11816 13059 L 11812 13032 L
11799 13008 L 11825 13003 L 11817 12977 L 11795 12993 L
11789 12966 L 11783 12940 L 11774 12914 L 11800 12907 L
11796 12879 L 11819 12864 L 11845 12857 L 11835 12832 L
11818 12811 L 11799 12831 L 11781 12851 L 11783 12879 L
11770 12854 L 11743 12860 L 11752 12834 L 11764 12822 L
11772 12815 L 11769 12843 L 11789 12824 L 11812 12808 L
11799 12784 L 11774 12774 L 11777 12771 L 11794 12755 L
11811 12777 L 11838 12782 L 11822 12759 L 11833 12734 L
11826 12707 L 11836 12682 L 11860 12695 L 11887 12695 L
11887 12722 L 11878 12748 L 11879 12775 L 11881 12748 L
11905 12735 L 11909 12707 L 11937 12705 L 11912 12694 L
11885 12689 L 11878 12663 L 11851 12667 L 11830 12649 L
11857 12650 L 11884 12647 L 11905 12630 L 11915 12604 L

11888 12602 L 11861 12609 L 11855 12636 L 11828 12634 L
11827 12607 L 11853 12598 L 11873 12580 L 11846 12577 L
11821 12588 L 11806 12612 L 11804 12639 L 11781 12653 L
11772 12627 L 11772 12624 L 11774 12600 L 11787 12575 L
11814 12570 L 11836 12554 L 11848 12549 L 11861 12543 L
11882 12560 L 11910 12559 L 11890 12540 L 11872 12519 L
11879 12493 L 11904 12480 L 11887 12459 L 11910 12444 L
11911 12421 L 11912 12416 L 11934 12433 L 11934 12433 L
11951 12454 L 11955 12481 L 11958 12482 L 11978 12497 L
11981 12510 L 11984 12523 L 12009 12537 L 12009 12564 L
12011 12591 L 12021 12566 L 12024 12539 L 12038 12515 L
12046 12517 L 12064 12522 L 12092 12520 L 12098 12529 L
12108 12542 L 12097 12567 L 12106 12593 L 12109 12621 L
12133 12634 L 12140 12640 L 12153 12653 L 12178 12664 L
12196 12685 L 12222 12693 L 12248 12703 L 12252 12676 L
12234 12655 L 12214 12673 L 12214 12646 L 12215 12619 L
12192 12604 L 12187 12631 L 12176 12606 L 12168 12580 L
12194 12572 L 12209 12595 L 12217 12587 L 12228 12576 L
12245 12554 L 12250 12549 L 12265 12536 L 12277 12561 L
12278 12561 L 12288 12569 L 12299 12577 L 12300 12605 L
12274 12600 L 12277 12627 L 12304 12621 L 12319 12621 L
12331 12621 L 12331 12648 L 12310 12665 L 12287 12681 L
12269 12702 L 12262 12728 L 12285 12743 L 12259 12753 L
12287 12754 L 12313 12761 L 12325 12786 L 12337 12761 L
12346 12748 L 12352 12738 L 12363 12713 L 12354 12688 L
12366 12663 L 12390 12677 L 12389 12704 L 12408 12709 L
12415 12711 L 12442 12705 L 12436 12678 L 12438 12651 L
12447 12654 L 12464 12659 L 12463 12687 L 12454 12713 L
12438 12735 L 12442 12762 L 12437 12789 L 12410 12783 L
12389 12765 L 12365 12778 L 12351 12802 L 12358 12828 L
12375 12807 L 12398 12820 L 12415 12842 L 12442 12848 L
12468 12839 L 12487 12853 L 12490 12855 L 12514 12870 L
12539 12880 L 12546 12906 L 12549 12879 L 12550 12851 L
12551 12851 L 12576 12840 L 12602 12832 L 12629 12832 L
12631 12838 L 12638 12858 L 12646 12854 L 12663 12846 L
12664 12847 L 12688 12856 L 12685 12829 L 12712 12829 L
12719 12803 L 12692 12809 L 12700 12798 L 12708 12787 L
12727 12773 L 12730 12770 L 12741 12795 L 12744 12823 L
12725 12843 L 12726 12871 L 12729 12898 L 12748 12918 L
12730 12939 L 12743 12963 L 12767 12961 L 12771 12961 L
12797 12954 L 12789 12980 L 12783 13006 L 12807 12993 L
12829 12977 L 12833 12977 L 12856 12973 L 12883 12973 L
12895 12976 L 12909 12980 L 12910 12980 L 12911 12980 L

12925 12981 L 12937 12982 L 12952 13005 L 12951 12978 L
12959 12952 L 12980 12969 L 12999 12978 L 12999 13001 L
12998 13007 L 12999 13007 L 12999 14079 L 12999 14085 L
12980 14105 L 12968 14129 L 12961 14156 L 12951 14181 L
12934 14202 L 12915 14222 L 12900 14245 L 12878 14261 L
12864 14284 L 12844 14302 L 12838 14329 L 12819 14348 L
12798 14366 L 12793 14393 L 12772 14411 L 12752 14428 L
12731 14445 L 12712 14465 L 12692 14483 L 12675 14505 L
12656 14524 L 12640 14546 L 12622 14566 L 12606 14588 L
12586 14607 L 12575 14632 L 12563 14657 L 12547 14678 L
12534 14702 L 12519 14725 L 12503 14747 L 12494 14772 L
12476 14793 L 12463 14817 L 12448 14839 L 12438 14865 L
12433 14891 L 12409 14905 L 12384 14913 L 12358 14904 L
12347 14929 L 12328 14948 L 12306 14964 L 12279 14967 L
12256 14981 L 12235 14998 L 12213 15014 L 12196 15036 L
12178 15056 L 12163 15078 L 12147 15100 L 12124 15086 L
12099 15096 L 12072 15102 L 12045 15103 L 12018 15104 L
11995 15119 L 11980 15141 L 11984 15168 L 12007 15183 L
12012 15209 L 11987 15219 L 12013 15226 L 12040 15230 L
12053 15206 L 12045 15232 L 12033 15257 L 12026 15283 L
12029 15310 L 12041 15334 L 12035 15360 L 12009 15368 L
12003 15394 L 12010 15421 L 12022 15445 L 12041 15464 L
12068 15463 L 12081 15487 L 12107 15494 L 12128 15511 L
12149 15529 L 12146 15556 L 12169 15571 L 12182 15594 L
12204 15610 L 12231 15605 L 12258 15601 L 12283 15611 L
12298 15633 L 12324 15624 L 12334 15649 L 12359 15657 L
12377 15678 L 12402 15690 L 12419 15710 L 12445 15720 L
12449 15746 L 12426 15760 L 12434 15786 L 12458 15799 L
12482 15811 L 12506 15823 L 12528 15839 L 12545 15861 L
12561 15882 L 12584 15897 L 12609 15908 L 12621 15932 L
12626 15959 L 12633 15985 L 12638 16012 L 12656 16032 L
12682 16039 L 12698 16061 L 12719 16079 L 12743 16090 L
12767 16102 L 12785 16122 L 12812 16127 L 12836 16139 L
12842 16166 L 12841 16170 L 12833 16170 L 12800 16170 L
12767 16170 L 12734 16170 L 12700 16170 L 12667 16170 L
12634 16170 L 12601 16170 L 12567 16170 L 12534 16170 L
12501 16170 L 12468 16170 L 12435 16170 L 12401 16170 L
12368 16170 L 12335 16170 L 12302 16170 L 12268 16170 L
12235 16170 L 12202 16170 L 12169 16170 L 12135 16170 L
12102 16170 L 12069 16170 L 12036 16170 L 12003 16170 L
11969 16170 L 11936 16170 L 11903 16170 L 11870 16170 L
11836 16170 L 11803 16170 L 11770 16170 L 11737 16170 L
11704 16170 L 11670 16170 L 11637 16170 L 11604 16170 L

11571 16170 L 11537 16170 L 11504 16170 L 11471 16170 L
11438 16170 L 11404 16170 L 11371 16170 L 11338 16170 L
11305 16170 L 11272 16170 L 11238 16170 L 11205 16170 L
11172 16170 L 11139 16170 L 11105 16170 L 11072 16170 L
11039 16170 L 11006 16170 L 10972 16170 L 10939 16170 L
10906 16170 L 10873 16170 L 10840 16170 L 10806 16170 L
10773 16170 L 10740 16170 L 10707 16170 L 10673 16170 L
10640 16170 L 10607 16170 L 10574 16170 L 10540 16170 L
10507 16170 L 10474 16170 L 10441 16170 L 10408 16170 L
10374 16170 L 10341 16170 L 10308 16170 L 10275 16170 L
10241 16170 L 10208 16170 L 10175 16170 L 10142 16170 L
10108 16170 L 10075 16170 L 10042 16170 L 10009 16170 L
9976 16170 L 9942 16170 L 9909 16170 L 9876 16170 L
9843 16170 L 9809 16170 L 9776 16170 L 9743 16170 L
9710 16170 L 9677 16170 L 9643 16170 L 9610 16170 L
9577 16170 L 9544 16170 L 9510 16170 L 9477 16170 L
9444 16170 L 9411 16170 L 9377 16170 L 9344 16170 L
9311 16170 L 9278 16170 L 9245 16170 L 9211 16170 L
9178 16170 L 9145 16170 L 9112 16170 L 9078 16170 L
9045 16170 L 9012 16170 L 8979 16170 L 8945 16170 L
8912 16170 L 8879 16170 L 8846 16170 L 8813 16170 L
8779 16170 L 8746 16170 L 8713 16170 L 8680 16170 L
8646 16170 L 8613 16170 L 8580 16170 L 8547 16170 L
8513 16170 L 8480 16170 L 8447 16170 L 8414 16170 L
8381 16170 L 8347 16170 L 8314 16170 L 8281 16170 L
8248 16170 L 8214 16170 L 8181 16170 L 8148 16170 L
8115 16170 L 8081 16170 L 8048 16170 L 8015 16170 L
7982 16170 L 7949 16170 L 7915 16170 L 7882 16170 L
7849 16170 L 7816 16170 L 7782 16170 L 7749 16170 L
7716 16170 L 7683 16170 L 7650 16170 L 7616 16170 L
7583 16170 L 7550 16170 L 7517 16170 L 7483 16170 L
7450 16170 L 7417 16170 L 7384 16170 L 7350 16170 L
7317 16170 L 7284 16170 L 7251 16170 L 7218 16170 L
7184 16170 L 7151 16170 L 7118 16170 L 7085 16170 L
7051 16170 L 7018 16170 L 6985 16170 L 6952 16170 L
6918 16170 L 6885 16170 L 6852 16170 L 6819 16170 L
6786 16170 L 6752 16170 L 6719 16170 L 6686 16170 L
6653 16170 L 6619 16170 L 6586 16170 L 6553 16170 L
6520 16170 L 6486 16170 L 6453 16170 L 6420 16170 L
6387 16170 L 6354 16170 L 6320 16170 L 6287 16170 L
6254 16170 L 6221 16170 L 6187 16170 L 6154 16170 L
6121 16170 L 6088 16170 L 6054 16170 L 6021 16170 L
5988 16170 L 5955 16170 L 5922 16170 L 5888 16170 L

5855 16170 L 5822 16170 L 5789 16170 L 5755 16170 L
5722 16170 L 5689 16170 L 5656 16170 L 5622 16170 L
5589 16170 L 5556 16170 L 5523 16170 L 5490 16170 L
5456 16170 L 5423 16170 L 5390 16170 L 5357 16170 L
5323 16170 L 5290 16170 L 5257 16170 L 5224 16170 L
5191 16170 L 5157 16170 L 5124 16170 L 5091 16170 L
5058 16170 L 5024 16170 L 4991 16170 L 4958 16170 L
4925 16170 L 4891 16170 L 4858 16170 L 4825 16170 L
4792 16170 L 4759 16170 L 4725 16170 L 4692 16170 L
4659 16170 L 4626 16170 L 4592 16170 L 4559 16170 L
4526 16170 L 4493 16170 L 4459 16170 L 4426 16170 L
4393 16170 L 4360 16170 L 4327 16170 L 4293 16170 L
4260 16170 L 4227 16170 L 4194 16170 L 4160 16170 L
4127 16170 L 4094 16170 L 4061 16170 L 4027 16170 L
3994 16170 L 3961 16170 L 3928 16170 L 3895 16170 L
3861 16170 L 3828 16170 L 3795 16170 L 3762 16170 L
3728 16170 L 3695 16170 L 3662 16170 L 3629 16170 L
3595 16170 L 3562 16170 L 3529 16170 L 3496 16170 L
3463 16170 L 3429 16170 L 3396 16170 L 3363 16170 L
3330 16170 L 3296 16170 L 3263 16170 L 3230 16170 L
3197 16170 L 3164 16170 L 3130 16170 L 3097 16170 L
3064 16170 L 3031 16170 L 2997 16170 L 2964 16170 L
2931 16170 L 2898 16170 L 2864 16170 L 2831 16170 L
2798 16170 L 2765 16170 L 2732 16170 L 2698 16170 L
2665 16170 L 2632 16170 L 2599 16170 L 2565 16170 L
2532 16170 L 2499 16170 L 2466 16170 L 2432 16170 L
2399 16170 L 2366 16170 L 2333 16170 L 2300 16170 L
N E
5542 5477 5541 5470 5543 5466 5545 5461 5547 5457 5548 5455
5556 5455 5575 5455 5575 5458 5573 5462 5571 5466 5569 5471
5567 5475 5563 5478 5559 5480 5556 5483 5552 5486 5549 5482
5545 5480 5542 5477 20 N F
5702 5573 5698 5567 5700 5563 5704 5561 5708 5563 5712 5565
5717 5566 5716 5571 5721 5573 5724 5576 5724 5581 5726 5585
5721 5585 5717 5588 5712 5588 5711 5584 5707 5581 5704 5578
5702 5573 19 N F
5796 5572 5790 5572 5794 5569 5798 5567 5801 5563 5805 5561
5810 5562 5808 5566 5806 5570 5803 5573 5799 5576 5796 5572
12 N F
5817 5461 5816 5455 5822 5455 5855 5455 5865 5455 5871 5458
5879 5459 5885 5461 5890 5456 5890 5455 5910 5455 5903 5461
5904 5468 5908 5474 5902 5477 5909 5480 5915 5478 5917 5485
5916 5492 5916 5499 5921 5504 5924 5511 5930 5507 5936 5511

5937 5519 5933 5524 5927 5520 5921 5516 5914 5515 5908 5511
5903 5506 5897 5502 5893 5496 5886 5497 5879 5495 5872 5491
5866 5488 5862 5494 5855 5495 5847 5494 5843 5488 5838 5483
5832 5479 5825 5477 5827 5471 5823 5465 5817 5461 47 N F
5964 5459 5962 5455 5969 5455 5970 5459 5964 5459 5 N F
5977 5488 5967 5480 5972 5475 5975 5468 5981 5464 5988 5463
5992 5457 5999 5459 6006 5456 6006 5455 6021 5455 6024 5455
6024 5459 6028 5455 6039 5455 6038 5459 6042 5465 6038 5471
6034 5477 6031 5484 6024 5485 6018 5488 6011 5489 6005 5485
5998 5483 5991 5485 5984 5488 5977 5488 28 N F
6055 5463 6054 5459 6056 5458 6058 5457 6058 5455 6059 5455
6060 5456 6062 5456 6063 5458 6064 5460 6064 5462 6064 5465
6063 5467 6061 5466 6059 5465 6057 5465 6055 5463 17 N F
6243 5583 6240 5580 6241 5577 6242 5574 6244 5571 6247 5573
6251 5574 6254 5574 6257 5576 6259 5578 6259 5579 6257 5581
6253 5582 6250 5582 6246 5583 6243 5583 16 N F
6252 5562 6251 5559 6253 5560 6255 5559 6257 5561 6258 5563
6258 5565 6260 5566 6259 5568 6257 5568 6255 5566 6253 5564
6252 5562 13 N F
6256 5651 6252 5647 6253 5644 6257 5644 6259 5641 6260 5638
6263 5640 6262 5643 6260 5646 6257 5647 6256 5651 11 N F
7342 6052 7335 6044 7339 6038 7343 6032 7349 6028 7356 6025
7360 6019 7367 6017 7374 6019 7378 6013 7381 6006 7388 6004
7395 6002 7388 5998 7392 5992 7398 5987 7405 5987 7408 5980
7415 5980 7417 5987 7422 5992 7415 5995 7409 5992 7404 5997
7402 6004 7403 6012 7404 6019 7402 6026 7398 6032 7397 6039
7392 6044 7385 6046 7380 6042 7372 6042 7365 6044 7360 6049
7356 6055 7349 6055 7342 6052 39 N F
7490 6180 7483 6175 7488 6171 7495 6170 7503 6169 7508 6173
7505 6180 7506 6187 7511 6192 7514 6199 7507 6201 7501 6197
7500 6189 7497 6183 7490 6180 15 N F
7498 6166 7491 6165 7493 6158 7499 6154 7506 6153 7513 6151
7520 6149 7522 6156 7523 6163 7527 6169 7533 6172 7527 6180
7520 6179 7513 6179 7510 6173 7506 6167 7498 6166 17 N F
7498 6111 7493 6105 7497 6099 7504 6098 7511 6096 7519 6097
7526 6096 7533 6094 7538 6090 7545 6087 7552 6087 7559 6090
7566 6091 7573 6089 7574 6082 7581 6084 7586 6080 7593 6083
7597 6089 7595 6096 7594 6103 7600 6107 7598 6114 7592 6118
7589 6125 7590 6132 7583 6132 7579 6126 7576 6119 7569 6120
7564 6126 7558 6130 7552 6127 7547 6121 7544 6115 7539 6111
7535 6105 7526 6113 7523 6119 7516 6122 7509 6120 7503 6115
7498 6111 43 N F
7698 6195 7691 6184 7698 6184 7705 6186 7712 6188 7718 6186

7717 6179 7723 6183 7729 6185 7736 6188 7743 6189 7750 6190
7758 6191 7754 6197 7747 6198 7745 6205 7745 6212 7746 6219
7749 6226 7753 6232 7759 6234 7766 6231 7773 6228 7780 6229
7781 6236 7776 6241 7775 6248 7777 6255 7784 6259 7782 6266
7788 6270 7794 6266 7801 6266 7802 6273 7809 6276 7802 6275
7797 6281 7791 6285 7786 6290 7781 6285 7774 6287 7770 6281
7762 6281 7756 6278 7753 6285 7749 6291 7744 6295 7737 6299
7732 6304 7726 6301 7724 6294 7727 6287 7727 6280 7722 6274
7717 6269 7715 6262 7711 6256 7706 6251 7708 6244 7714 6240
7719 6245 7726 6248 7732 6243 7739 6244 7746 6242 7740 6237
7739 6230 7734 6225 7728 6221 7722 6217 7718 6211 7713 6205
7714 6198 7710 6204 7704 6200 7698 6195 76 N F
7944 6713 7942 6708 7943 6705 7945 6702 7948 6700 7950 6699
7951 6699 7953 6702 7950 6704 7948 6707 7946 6710 7944 6713
12 N F
7955 6697 7950 6697 7954 6695 7958 6692 7961 6689 7964 6685
7967 6682 7971 6679 7975 6678 7980 6678 7984 6679 7989 6678
7989 6678 7993 6680 7991 6684 7990 6688 7986 6690 7982 6688
7977 6687 7973 6689 7971 6693 7968 6696 7963 6696 7959 6694
7955 6697 25 N F
7957 6703 7953 6699 7957 6697 7961 6696 7966 6698 7970 6696
7973 6693 7976 6689 7980 6689 7984 6691 7988 6693 7992 6690
7994 6686 7999 6684 8003 6687 8007 6687 8006 6691 8003 6695
7999 6698 7997 6701 7993 6704 7989 6706 7984 6706 7980 6708
7975 6707 7971 6706 7966 6705 7962 6704 7957 6703 29 N F
7984 6676 7979 6675 7983 6673 7987 6671 7991 6669 7996 6669
7997 6673 7993 6675 7989 6677 7984 6676 10 N F
8030 6668 8023 6664 8027 6661 8031 6663 8035 6660 8039 6663
8043 6665 8039 6668 8035 6669 8030 6668 10 N F
8101 6699 8089 6694 8093 6688 8097 6682 8103 6678 8109 6675
8116 6673 8123 6672 8129 6676 8134 6681 8140 6686 8145 6691
8147 6698 8150 6704 8157 6706 8157 6713 8156 6720 8156 6727
8152 6732 8145 6729 8141 6735 8136 6740 8130 6738 8124 6742
8117 6745 8110 6747 8111 6740 8105 6735 8104 6728 8100 6722
8095 6717 8091 6711 8093 6704 8100 6705 8105 6710 8112 6713
8113 6706 8107 6702 8101 6699 39 N F
8169 6668 8165 6666 8167 6664 8171 6664 8174 6663 8176 6666
8179 6667 8182 6666 8185 6668 8185 6671 8188 6673 8191 6672
8193 6675 8194 6679 8195 6682 8193 6685 8190 6687 8187 6689
8184 6690 8181 6688 8178 6688 8175 6687 8174 6683 8174 6680
8173 6677 8170 6675 8169 6672 8169 6668 28 N F
8212 6671 8200 6667 8205 6663 8210 6657 8216 6654 8223 6651
8229 6655 8231 6662 8235 6668 8241 6672 8247 6675 8254 6678

8261 6679 8268 6677 8274 6673 8280 6670 8287 6666 8293 6669
8297 6675 8297 6682 8302 6688 8298 6694 8298 6701 8294 6707
8292 6714 8290 6721 8283 6722 8279 6728 8274 6733 8269 6728
8263 6724 8266 6717 8261 6712 8255 6708 8254 6701 8250 6695
8243 6696 8236 6699 8230 6702 8224 6707 8219 6712 8213 6708
8207 6704 8209 6697 8215 6700 8212 6694 8214 6687 8216 6680
8219 6673 8212 6671 50 N F
8213 6754 8209 6751 8212 6750 8216 6749 8218 6747 8221 6744
8223 6742 8227 6742 8230 6743 8231 6746 8232 6749 8234 6752
8232 6755 8229 6756 8226 6757 8222 6756 8219 6756 8216 6755
8213 6754 19 N F
8275 14738 8272 14733 8276 14734 8279 14737 8283 14740 8285 14743
8288 14747 8290 14751 8293 14754 8294 14758 8295 14762 8293 14766
8291 14763 8289 14759 8286 14756 8283 14753 8282 14749 8281 14745
8279 14741 8275 14738 20 N F
8293 14780 8292 14777 8293 14776 8295 14776 8298 14778 8299 14780
8301 14783 8301 14786 8299 14784 8296 14783 8293 14780 11 N F
8313 14797 8311 14795 8313 14793 8316 14795 8319 14796 8320 14799
8321 14802 8323 14805 8325 14807 8326 14810 8323 14809 8321 14806
8319 14804 8318 14801 8316 14799 8313 14797 16 N F
8362 14788 8356 14786 8361 14787 8365 14788 8369 14790 8372 14793
8375 14796 8378 14799 8379 14803 8377 14807 8373 14810 8371 14813
8369 14809 8370 14805 8374 14804 8375 14799 8371 14797 8368 14794
8365 14791 8362 14788 20 N F
8397 14838 8392 14833 8397 14834 8401 14835 8405 14836 8409 14837
8413 14838 8416 14842 8417 14846 8419 14850 8423 14852 8424 14856
8422 14860 8418 14859 8415 14855 8413 14852 8411 14848 8408 14844
8405 14842 8401 14840 8397 14838 21 N F
8398 14852 8397 14849 8399 14847 8402 14848 8405 14850 8406 14853
8407 14856 8407 14859 8406 14862 8404 14859 8403 14857 8401 14854
8398 14852 13 N F
8431 14850 8426 14849 8430 14847 8434 14849 8438 14850 8442 14851
8446 14853 8450 14856 8453 14859 8456 14862 8458 14866 8461 14869
8464 14872 8467 14875 8470 14878 8473 14881 8475 14886 8471 14884
8467 14882 8463 14881 8460 14878 8456 14877 8455 14873 8452 14870
8448 14868 8445 14865 8441 14862 8437 14860 8437 14856 8434 14853
8431 14850 31 N F
8628 7037 8623 7035 8627 7034 8632 7033 8634 7029 8635 7025
8639 7023 8638 7028 8640 7032 8644 7035 8647 7038 8645 7042
8647 7046 8644 7049 8644 7054 8647 7057 8652 7059 8655 7062
8655 7066 8651 7064 8647 7062 8642 7061 8638 7059 8635 7055
8631 7055 8631 7050 8634 7046 8636 7042 8636 7038 8632 7035
8628 7037 31 N F

8709 12481 8704 12481 8706 12477 8709 12474 8713 12471 8715 12468
8719 12467 8723 12465 8727 12467 8731 12469 8728 12473 8724 12474
8720 12475 8716 12476 8712 12477 8709 12481 16 N F
8717 7287 8712 7276 8717 7270 8721 7265 8721 7258 8724 7251
8731 7254 8736 7258 8743 7258 8744 7266 8741 7272 8742 7279
8741 7286 8743 7293 8746 7300 8748 7306 8747 7313 8750 7320
8750 7327 8750 7334 8747 7340 8740 7337 8737 7331 8733 7325
8727 7321 8727 7314 8725 7307 8721 7301 8720 7294 8717 7287
30 N F
8750 6092 8749 6089 8751 6087 8753 6088 8755 6089 8757 6090
8757 6093 8755 6093 8753 6094 8751 6094 8750 6092 11 N F
8810 7146 8800 7143 8805 7139 8802 7132 8807 7127 8814 7126
8818 7125 8821 7123 8827 7127 8834 7129 8839 7125 8837 7119
8836 7112 8843 7108 8849 7106 8854 7106 8856 7106 8859 7113
8854 7118 8857 7124 8864 7125 8869 7130 8862 7129 8857 7134
8853 7128 8846 7129 8839 7132 8833 7136 8828 7141 8829 7148
8822 7150 8819 7156 8814 7151 8810 7146 34 N F
8809 5985 8802 5982 8805 5978 8807 5974 8808 5970 8812 5968
8812 5972 8816 5975 8819 5978 8820 5983 8818 5987 8813 5987
8809 5985 13 N F
8837 6191 8828 6183 8835 6185 8842 6186 8844 6186 8849 6186
8851 6193 8852 6200 8856 6206 8855 6213 8855 6220 8861 6224
8866 6228 8866 6228 8859 6229 8854 6234 8848 6229 8846 6222
8846 6215 8846 6208 8841 6203 8846 6198 8844 6191 8837 6191
24 N F
8844 6071 8840 6065 8845 6064 8849 6064 8853 6064 8854 6064
8852 6068 8855 6072 8852 6075 8851 6080 8852 6084 8847 6085
8844 6089 8843 6084 8842 6080 8844 6075 8844 6071 17 N F
8848 7641 8842 7629 8849 7629 8854 7628 8856 7628 8863 7626
8870 7625 8876 7621 8882 7618 8889 7618 8896 7620 8895 7628
8894 7634 8891 7641 8888 7648 8882 7651 8875 7654 8870 7658
8864 7654 8857 7652 8852 7646 8848 7641 22 N F
8854 7627 8847 7624 8850 7622 8854 7619 8857 7616 8862 7616
8866 7618 8871 7618 8875 7620 8871 7623 8867 7624 8863 7625
8858 7626 8854 7627 14 N F
8851 6144 8850 6141 8851 6139 8853 6139 8855 6140 8858 6141
8860 6141 8862 6142 8864 6143 8866 6145 8866 6145 8868 6145
8868 6147 8866 6148 8864 6147 8861 6147 8860 6145 8857 6145
8856 6143 8854 6141 8853 6143 8851 6144 22 N F
8853 6017 8850 6015 8852 6014 8854 6014 8855 6016 8853 6017
6 N F
8856 6010 8855 6006 8857 6007 8859 6009 8860 6010 8860 6012
8857 6012 8856 6010 8 N F

8862 7381 8857 7378 8860 7375 8864 7374 8869 7373 8871 7377
8875 7379 8878 7382 8881 7386 8883 7390 8883 7395 8878 7394
8875 7391 8872 7388 8868 7385 8867 7381 8864 7377 8862 7381
18 N F
8859 6071 8857 6069 8859 6068 8860 6068 8861 6068 8862 6070
8864 6071 8866 6071 8869 6071 8869 6073 8868 6075 8866 6076
8864 6075 8861 6075 8861 6073 8859 6071 16 N F
8872 6178 8867 6171 8873 6167 8879 6163 8882 6157 8889 6155
8896 6156 8903 6159 8902 6167 8907 6172 8912 6176 8913 6176
8919 6180 8924 6185 8931 6185 8938 6185 8940 6184 8945 6182
8942 6189 8940 6196 8939 6203 8935 6209 8930 6204 8928 6211
8922 6208 8920 6201 8918 6208 8915 6201 8911 6195 8911 6202
8912 6210 8905 6210 8901 6203 8897 6197 8896 6190 8892 6196
8889 6203 8883 6198 8880 6192 8876 6186 8883 6189 8885 6182
8878 6182 8884 6179 8891 6179 8898 6179 8896 6172 8890 6168
8886 6162 8882 6168 8876 6172 8872 6178 52 N F
8875 6248 8870 6242 8876 6237 8881 6243 8886 6238 8891 6232
8893 6225 8897 6219 8900 6226 8898 6233 8903 6238 8900 6244
8896 6251 8889 6251 8882 6249 8875 6248 16 N F
8897 7964 8893 7958 8896 7954 8898 7950 8898 7946 8903 7946
8905 7950 8908 7948 8913 7946 8917 7947 8921 7948 8926 7949
8927 7953 8931 7956 8934 7959 8939 7960 8943 7961 8946 7965
8945 7969 8941 7971 8937 7970 8933 7967 8933 7963 8930 7959
8926 7961 8927 7965 8923 7965 8919 7964 8914 7965 8910 7966
8906 7965 8901 7965 8897 7964 33 N F
8895 6290 8894 6287 8896 6284 8897 6281 8898 6284 8900 6287
8903 6288 8906 6290 8907 6293 8904 6295 8902 6298 8899 6295
8898 6292 8895 6290 14 N F
8903 7392 8902 7388 8905 7388 8908 7389 8910 7390 8913 7391
8913 7393 8911 7395 8909 7398 8906 7397 8904 7395 8903 7392
12 N F
8908 6007 8906 6005 8908 6004 8910 6003 8912 6003 8913 6006
8912 6008 8910 6008 8908 6007 9 N F
8913 7387 8910 7381 8914 7378 8919 7377 8923 7377 8928 7379
8932 7380 8936 7378 8940 7381 8941 7385 8939 7389 8937 7393
8934 7390 8930 7389 8925 7387 8922 7385 8918 7387 8913 7387
18 N F
8921 7292 8918 7285 8924 7288 8931 7291 8938 7290 8943 7295
8942 7288 8942 7281 8935 7282 8932 7276 8937 7271 8933 7265
8938 7259 8943 7255 8946 7254 8950 7252 8955 7247 8961 7242
8967 7241 8971 7247 8967 7253 8961 7257 8964 7264 8970 7267
8976 7271 8981 7276 8988 7277 8994 7282 9001 7283 9005 7278
9012 7282 9013 7289 9005 7289 8999 7291 8993 7294 8987 7299

8985 7306 8985 7313 8987 7320 8993 7324 8999 7328 9006 7330
9010 7324 9017 7326 9021 7332 9025 7338 9018 7338 9011 7338
9004 7340 8999 7345 8993 7342 8995 7348 9000 7354 8997 7360
8990 7364 8985 7368 8978 7366 8972 7369 8968 7375 8968 7382
8972 7387 8976 7394 8969 7393 8963 7390 8957 7385 8955 7378
8948 7382 8944 7376 8941 7370 8934 7370 8930 7364 8926 7358
8927 7351 8930 7345 8937 7347 8941 7352 8949 7353 8951 7346
8950 7339 8946 7333 8946 7326 8946 7319 8944 7312 8950 7308
8956 7312 8958 7319 8964 7314 8971 7313 8971 7306 8966 7302
8960 7298 8953 7299 8948 7304 8941 7305 8934 7306 8929 7301
8922 7299 8921 7292 98 N F
8928 7204 8926 7202 8926 7198 8929 7196 8927 7192 8927 7189
8928 7190 8929 7192 8933 7193 8935 7195 8938 7197 8935 7197
8933 7200 8931 7203 8934 7205 8933 7209 8930 7207 8928 7204
18 N F
8937 7225 8930 7223 8932 7219 8933 7218 8934 7217 8937 7218
8941 7218 8944 7219 8947 7221 8950 7223 8947 7225 8945 7227
8946 7230 8942 7229 8940 7227 8936 7228 8937 7225 17 N F
8938 6013 8936 6010 8937 6009 8936 6006 8938 6005 8939 6003
8940 6005 8940 6007 8942 6009 8943 6011 8942 6013 8940 6014
8938 6013 13 N F
8952 7939 8948 7937 8949 7934 8951 7931 8954 7931 8957 7929
8960 7930 8963 7932 8965 7934 8969 7934 8972 7934 8974 7936
8976 7939 8974 7941 8970 7942 8968 7944 8964 7943 8961 7942
8958 7941 8955 7941 8952 7939 21 N F
9011 13027 9010 13021 9013 13018 9017 13016 9021 13019 9024 13022
9024 13026 9022 13030 9021 13034 9021 13038 9016 13039 9016 13034
9013 13031 9011 13027 14 N F
9033 7673 9031 7668 9035 7667 9040 7665 9043 7668 9048 7668
9051 7671 9053 7671 9055 7672 9057 7676 9059 7681 9055 7682
9050 7681 9048 7677 9044 7675 9042 7679 9037 7678 9039 7682
9036 7685 9035 7681 9032 7677 9033 7673 22 N F
9046 7575 9042 7573 9044 7570 9046 7568 9050 7569 9052 7571
9055 7573 9053 7576 9050 7576 9046 7575 10 N F
9059 7703 9059 7699 9062 7696 9062 7700 9062 7705 9065 7709
9067 7710 9069 7711 9073 7711 9078 7709 9081 7706 9084 7710
9082 7714 9079 7717 9075 7721 9071 7720 9068 7717 9064 7715
9060 7712 9060 7708 9059 7703 21 N F
9065 7564 9062 7557 9066 7559 9070 7561 9073 7565 9076 7568
9072 7567 9067 7568 9065 7564 9 N F
9088 7876 9084 7869 9089 7868 9093 7869 9095 7873 9099 7872
9104 7873 9104 7878 9104 7882 9105 7887 9100 7887 9099 7883
9095 7885 9091 7884 9087 7881 9088 7876 16 N F

9122 6147 9121 6141 9122 6137 9122 6132 9123 6128 9124 6123
9126 6119 9126 6123 9125 6128 9124 6133 9124 6137 9126 6141
9127 6146 9128 6150 9129 6155 9130 6159 9133 6163 9135 6167
9138 6171 9137 6175 9135 6171 9132 6167 9130 6163 9128 6159
9125 6156 9123 6151 9122 6147 27 N F
9134 7916 9129 7911 9132 7908 9135 7905 9137 7906 9140 7907
9144 7909 9143 7913 9148 7915 9152 7916 9153 7920 9154 7924
9154 7929 9152 7933 9147 7934 9145 7930 9142 7926 9140 7923
9138 7918 9134 7916 20 N F
9140 5927 M 9136 5913 L 9146 5904 L 9161 5907 L 9153 5895 L
9164 5886 L 9176 5877 L 9190 5881 L 9203 5885 L
9217 5890 L 9220 5876 L 9207 5869 L 9193 5867 L
9200 5854 L 9207 5841 L 9211 5827 L 9211 5813 L
9216 5799 L 9221 5786 L 9235 5782 L 9226 5771 L
9232 5757 L 9237 5744 L 9243 5731 L 9249 5718 L
9255 5704 L 9259 5690 L 9259 5676 L 9255 5662 L
9263 5649 L 9276 5643 L 9280 5629 L 9291 5620 L
9302 5610 L 9289 5616 L 9279 5627 L 9272 5639 L
9277 5626 L 9286 5614 L 9296 5603 L 9306 5593 L
9316 5583 L 9328 5575 L 9328 5575 L 9341 5567 L
9347 5580 L 9353 5567 L 9363 5555 L 9373 5546 L
9386 5539 L 9399 5532 L 9412 5525 L 9424 5518 L
9437 5510 L 9447 5500 L 9454 5488 L 9465 5478 L
9472 5465 L 9475 5455 L 9477 5455 L 9510 5455 L
9544 5455 L 9577 5455 L 9608 5455 L 9607 5465 L
9607 5479 L 9609 5494 L 9614 5507 L 9617 5521 L
9622 5535 L 9628 5548 L 9632 5562 L 9635 5576 L
9638 5591 L 9643 5604 L 9646 5619 L 9652 5632 L
9662 5642 L 9665 5657 L 9667 5671 L 9673 5684 L
9685 5693 L 9695 5703 L 9707 5711 L 9720 5718 L
9730 5728 L 9741 5738 L 9751 5749 L 9754 5763 L
9758 5776 L 9753 5790 L 9760 5803 L 9773 5808 L
9784 5818 L 9791 5831 L 9798 5844 L 9802 5858 L
9812 5868 L 9819 5880 L 9824 5894 L 9830 5907 L
9833 5921 L 9838 5935 L 9848 5945 L 9858 5956 L
9859 5970 L 9863 5984 L 9857 5998 L 9859 6012 L
9865 6025 L 9868 6039 L 9872 6053 L 9878 6066 L
9889 6075 L 9891 6089 L 9893 6104 L 9895 6118 L
9899 6132 L 9904 6145 L 9906 6160 L 9911 6173 L
9906 6187 L 9907 6201 L 9910 6215 L 9916 6228 L
9917 6243 L 9917 6257 L 9918 6260 L 9923 6270 L
9924 6285 L 9925 6299 L 9928 6313 L 9934 6327 L
9934 6341 L 9934 6355 L 9940 6369 L 9944 6382 L

9947 6397 L 9949 6411 L 9949 6425 L 9950 6440 L
9951 6454 L 9960 6465 L 9968 6477 L 9960 6489 L
9953 6502 L 9950 6516 L 9955 6530 L 9965 6539 L
9974 6551 L 9973 6565 L 9970 6579 L 9977 6592 L
9989 6601 L 9997 6612 L 10009 6621 L 10014 6634 L
10021 6647 L 10027 6660 L 10026 6674 L 10030 6688 L
10032 6702 L 10034 6716 L 10040 6729 L 10047 6742 L
10058 6751 L 10056 6765 L 10061 6779 L 10073 6787 L
10070 6801 L 10071 6815 L 10068 6829 L 10059 6840 L
10054 6853 L 10050 6867 L 10048 6881 L 10043 6895 L
10039 6909 L 10038 6923 L 10038 6937 L 10039 6951 L
10042 6965 L 10046 6979 L 10054 6991 L 10064 7001 L
10069 7015 L 10080 7024 L 10089 7035 L 10101 7042 L
10104 7044 L 10114 7049 L 10125 7058 L 10117 7070 L
10103 7068 L 10090 7075 L 10089 7090 L 10081 7101 L
10084 7116 L 10073 7125 L 10059 7124 L 10045 7126 L
10031 7122 L 10020 7131 L 10007 7137 L 9994 7131 L
9984 7141 L 9971 7147 L 9960 7156 L 9964 7169 L
9951 7165 L 9940 7174 L 9931 7185 L 9928 7199 L
9916 7207 L 9903 7214 L 9889 7213 L 9875 7209 L
9862 7205 L 9849 7197 L 9840 7186 L 9832 7175 L
9825 7162 L 9817 7150 L 9828 7141 L 9815 7136 L
9806 7126 L 9793 7119 L 9780 7113 L 9766 7111 L
9752 7107 L 9738 7109 L 9724 7103 L 9711 7098 L
9699 7090 L 9687 7083 L 9673 7079 L 9660 7074 L
9649 7065 L 9636 7058 L 9626 7048 L 9618 7033 L
9616 7018 L 9607 7007 L 9599 6995 L 9592 6983 L
9586 6970 L 9582 6956 L 9573 6945 L 9565 6934 L
9571 6921 L 9567 6907 L 9563 6893 L 9554 6882 L
9543 6873 L 9540 6859 L 9529 6849 L 9540 6839 L
9525 6840 L 9511 6838 L 9498 6832 L 9490 6820 L
9496 6807 L 9482 6805 L 9469 6799 L 9464 6785 L
9459 6772 L 9456 6758 L 9452 6744 L 9445 6731 L
9438 6719 L 9427 6709 L 9419 6698 L 9409 6687 L
9404 6674 L 9397 6661 L 9392 6648 L 9386 6634 L
9384 6620 L 9375 6608 L 9371 6595 L 9364 6582 L
9356 6570 L 9368 6562 L 9354 6562 L 9347 6550 L
9340 6537 L 9332 6525 L 9329 6511 L 9325 6497 L
9316 6486 L 9306 6476 L 9296 6465 L 9289 6452 L
9280 6441 L 9274 6429 L 9274 6414 L 9267 6401 L
9262 6388 L 9257 6374 L 9247 6364 L 9256 6353 L
9271 6351 L 9285 6349 L 9299 6352 L 9288 6343 L
9274 6340 L 9259 6339 L 9245 6342 L 9231 6341 L

9222 6329 L 9214 6318 L 9212 6304 L 9210 6289 L
9207 6275 L 9195 6267 L 9189 6254 L 9183 6241 L
9181 6226 L 9180 6212 L 9187 6199 L 9187 6185 L
9193 6172 L 9181 6164 L 9191 6153 L 9188 6139 L
9199 6130 L 9188 6120 L 9192 6106 L 9196 6092 L
9195 6078 L 9192 6064 L 9199 6051 L 9185 6053 L
9183 6039 L 9175 6027 L 9177 6013 L 9166 6004 L
9179 5999 L 9165 5996 L 9164 5982 L 9164 5968 L
9155 5958 L 9169 5954 L 9174 5940 L 9169 5926 L
9158 5917 L 9144 5914 L 9140 5927 L N E
9170 14242 9167 14237 9169 14233 9173 14230 9176 14227 9179 14225
9182 14228 9182 14233 9181 14237 9182 14241 9178 14244 9174 14244
9170 14242 13 N F
9198 15606 9196 15599 9199 15596 9203 15598 9206 15601 9209 15604
9210 15604 9214 15604 9218 15605 9219 15609 9219 15613 9219 15618
9215 15620 9211 15622 9209 15618 9205 15618 9204 15614 9205 15610
9201 15610 9198 15606 20 N F
9209 14252 9205 14249 9207 14246 9210 14243 9212 14239 9216 14236
9220 14236 9223 14233 9226 14237 9225 14241 9224 14245 9221 14249
9217 14248 9214 14251 9209 14252 15 N F
9225 14211 9221 14208 9225 14206 9229 14206 9232 14203 9235 14199
9238 14197 9240 14201 9239 14205 9238 14209 9240 14213 9238 14217
9236 14220 9233 14224 9232 14228 9228 14231 9225 14228 9224 14224
9224 14220 9225 14216 9225 14211 21 N F
9225 14355 9224 14350 9225 14344 9228 14341 9228 14341 9232 14343
9236 14342 9240 14342 9243 14346 9240 14349 9235 14350 9231 14350
9232 14354 9230 14358 9228 14362 9225 14359 9225 14355 17 N F
9227 8137 9225 8132 9227 8128 9230 8124 9234 8122 9238 8119
9242 8117 9246 8117 9250 8119 9254 8121 9259 8122 9263 8123
9259 8125 9255 8127 9254 8131 9252 8135 9250 8139 9246 8140
9243 8143 9238 8144 9234 8143 9230 8140 9227 8137 23 N F
9251 14419 9247 14415 9251 14412 9254 14414 9256 14418 9260 14419
9264 14420 9266 14424 9266 14428 9262 14430 9258 14429 9254 14427
9252 14424 9251 14419 14 N F
9291 14459 9287 14456 9289 14453 9291 14450 9294 14449 9295 14446
9296 14449 9295 14452 9297 14454 9294 14454 9294 14457 9291 14459
12 N F
9291 14471 9289 14466 9291 14467 9292 14467 9295 14466 9298 14468
9301 14467 9301 14470 9299 14472 9298 14475 9295 14475 9294 14472
9291 14471 13 N F
9312 7816 9309 7812 9312 7813 9313 7810 9316 7812 9318 7815
9316 7817 9313 7819 9312 7816 9 N F
9311 14277 9309 14273 9311 14271 9315 14270 9317 14272 9317 14275

9314 14277 9311 14277 8 N F
9318 5482 9316 5477 9320 5475 9324 5477 9327 5481 9330 5484
9334 5487 9335 5491 9332 5494 9328 5495 9324 5493 9320 5490
9321 5486 9318 5482 14 N F
9318 7811 9316 7804 9320 7803 9324 7805 9327 7809 9331 7808
9329 7812 9326 7815 9322 7814 9318 7811 10 N F
9413 15102 9403 15101 9405 15094 9404 15088 9410 15085 9417 15083
9423 15085 9430 15084 9434 15079 9436 15073 9440 15079 9445 15082
9449 15088 9454 15092 9457 15092 9461 15093 9468 15093 9473 15089
9479 15086 9486 15085 9493 15088 9497 15093 9502 15098 9508 15099
9511 15105 9515 15111 9522 15109 9527 15113 9530 15119 9532 15126
9531 15132 9531 15139 9527 15145 9526 15151 9519 15153 9513 15156
9507 15159 9501 15162 9495 15162 9499 15157 9499 15150 9496 15145
9489 15142 9483 15142 9478 15137 9472 15134 9465 15134 9458 15135
9452 15133 9446 15131 9439 15130 9433 15134 9431 15128 9431 15121
9426 15117 9420 15113 9417 15107 9413 15102 58 N F
9421 15022 9416 15017 9422 15014 9429 15012 9433 15017 9435 15024
9440 15028 9445 15033 9449 15039 9450 15045 9456 15049 9462 15049
9469 15049 9471 15042 9467 15038 9462 15032 9458 15027 9455 15021
9452 15015 9459 15013 9465 15011 9472 15010 9477 15006 9484 15007
9490 15010 9490 15017 9494 15022 9497 15029 9497 15035 9494 15042
9488 15046 9482 15049 9489 15048 9496 15048 9502 15050 9500 15059
9497 15065 9491 15067 9485 15069 9478 15071 9472 15070 9466 15066
9462 15071 9457 15075 9450 15074 9446 15068 9443 15062 9443 15055
9439 15050 9432 15049 9425 15049 9423 15042 9425 15036 9419 15035
9422 15029 9421 15022 56 N F
9417 8745 9417 8741 9418 8738 9420 8735 9422 8731 9426 8730
9428 8728 9430 8725 9433 8723 9436 8722 9439 8721 9442 8723
9442 8727 9442 8730 9439 8732 9438 8735 9436 8738 9433 8740
9430 8741 9427 8742 9424 8743 9420 8744 9417 8745 23 N F
9476 8719 9470 8718 9474 8717 9476 8715 9479 8713 9482 8711
9485 8709 9487 8707 9490 8706 9494 8704 9497 8703 9500 8704
9503 8705 9506 8707 9508 8710 9510 8712 9510 8716 9509 8719
9506 8720 9502 8721 9499 8722 9496 8722 9492 8722 9489 8721
9486 8721 9482 8721 9479 8721 9476 8719 28 N F
9546 10862 9538 10853 9542 10848 9548 10843 9553 10839 9558 10834
9562 10828 9566 10823 9571 10818 9578 10821 9584 10818 9590 10815
9595 10810 9600 10806 9606 10802 9611 10797 9616 10792 9621 10787
9626 10783 9632 10780 9638 10776 9645 10774 9649 10768 9655 10765
9662 10764 9668 10762 9673 10757 9680 10755 9686 10752 9692 10749
9699 10747 9705 10745 9711 10740 9717 10737 9723 10734 9728 10729
9735 10728 9742 10726 9747 10722 9753 10718 9756 10717 9760 10716
9767 10715 9774 10716 9780 10715 9787 10713 9794 10711 9800 10708

9806 10706 9813 10702 9819 10699 9825 10697 9832 10695 9838 10692
9845 10691 9852 10689 9859 10688 9860 10688 9866 10688 9872 10691
9878 10694 9881 10700 9885 10706 9891 10710 9892 10717 9893 10723
9894 10730 9892 10737 9886 10740 9880 10745 9874 10747 9867 10748
9860 10748 9853 10749 9847 10752 9840 10753 9834 10757 9828 10760
9823 10765 9817 10768 9811 10771 9805 10775 9798 10777 9792 10779
9785 10781 9779 10783 9772 10785 9765 10787 9758 10789 9753 10792
9746 10795 9742 10801 9739 10807 9734 10812 9728 10816 9723 10821
9717 10823 9710 10826 9704 10828 9698 10832 9691 10834 9685 10836
9678 10838 9672 10842 9668 10847 9665 10853 9662 10860 9658 10865
9655 10871 9650 10876 9645 10881 9639 10885 9633 10888 9626 10890
9619 10891 9613 10893 9606 10893 9599 10892 9592 10891 9585 10889
9579 10887 9572 10884 9567 10879 9562 10875 9557 10870 9551 10866
9546 10862 127 N F
9561 8653 9559 8649 9561 8646 9564 8649 9568 8651 9572 8653
9576 8655 9579 8652 9583 8650 9583 8655 9585 8656 9587 8657
9590 8661 9593 8664 9598 8664 9594 8666 9590 8664 9586 8663
9582 8661 9577 8662 9575 8665 9571 8669 9567 8669 9567 8665
9563 8662 9562 8658 9561 8653 27 N F
9569 8713 9567 8708 9572 8710 9576 8711 9581 8711 9585 8713
9589 8715 9593 8713 9592 8709 9593 8705 9597 8707 9601 8708
9606 8709 9610 8711 9614 8712 9617 8715 9622 8717 9618 8719
9614 8722 9611 8725 9608 8728 9605 8730 9601 8728 9596 8728
9592 8729 9588 8726 9584 8725 9585 8729 9580 8729 9579 8724
9576 8722 9572 8720 9568 8717 9569 8713 34 N F
9585 8438 9580 8435 9583 8431 9587 8429 9590 8426 9591 8430
9590 8434 9594 8432 9598 8431 9602 8429 9607 8429 9610 8426
9610 8430 9607 8434 9603 8435 9600 8439 9597 8442 9592 8444
9589 8447 9585 8447 9588 8444 9592 8442 9587 8442 9585 8438
24 N F
9589 8616 9586 8614 9589 8613 9592 8612 9594 8613 9597 8615
9598 8617 9597 8619 9596 8621 9593 8623 9591 8621 9590 8619
9589 8616 13 N F
9596 8642 9593 8637 9598 8636 9602 8635 9607 8636 9611 8635
9615 8637 9619 8637 9622 8641 9626 8638 9630 8637 9634 8635
9638 8636 9640 8640 9639 8644 9639 8649 9635 8651 9639 8654
9641 8649 9644 8653 9647 8656 9644 8660 9645 8664 9641 8665
9639 8661 9634 8659 9630 8660 9626 8658 9629 8654 9627 8650
9623 8650 9619 8649 9616 8653 9613 8656 9611 8652 9607 8649
9604 8646 9600 8644 9596 8642 39 N F
9618 8816 9608 8814 9611 8807 9611 8800 9614 8794 9618 8788
9623 8783 9622 8776 9616 8773 9622 8769 9621 8762 9627 8764
9634 8764 9641 8767 9641 8768 9647 8770 9654 8772 9653 8779

9660 8777 9665 8772 9668 8779 9662 8783 9664 8790 9670 8786
9676 8789 9678 8783 9685 8784 9681 8790 9687 8793 9688 8800
9681 8800 9675 8804 9671 8810 9672 8817 9670 8823 9667 8830
9670 8837 9674 8842 9681 8843 9679 8849 9673 8854 9667 8857
9661 8861 9654 8860 9648 8858 9643 8852 9642 8845 9644 8839
9638 8835 9633 8829 9628 8825 9622 8821 9618 8816 53 N F
9633 8735 9631 8731 9634 8728 9636 8723 9640 8722 9641 8723
9643 8726 9647 8729 9651 8731 9655 8732 9658 8733 9659 8734
9663 8736 9659 8736 9654 8736 9650 8738 9646 8738 9641 8739
9637 8737 9633 8735 20 N F
9652 8530 9649 8527 9651 8527 9652 8525 9654 8525 9656 8526
9658 8527 9659 8529 9661 8530 9661 8531 9662 8532 9663 8534
9662 8536 9660 8535 9658 8536 9656 8534 9656 8531 9654 8530
9652 8530 19 N F
9657 8524 9655 8522 9657 8520 9659 8522 9660 8523 9661 8525
9661 8528 9659 8528 9657 8526 9657 8524 10 N F
9672 14755 9668 14752 9669 14749 9669 14745 9671 14743 9674 14744
9676 14747 9676 14750 9674 14753 9675 14756 9675 14759 9672 14760
9670 14757 9672 14755 14 N F
9756 10676 9747 10671 9753 10668 9759 10666 9765 10662 9772 10659
9777 10654 9783 10651 9789 10648 9795 10645 9800 10640 9806 10637
9813 10634 9818 10630 9825 10627 9828 10621 9832 10616 9836 10610
9842 10606 9849 10608 9849 10615 9848 10622 9845 10628 9841 10634
9836 10639 9832 10644 9826 10648 9821 10653 9815 10655 9809 10659
9803 10662 9796 10664 9790 10667 9783 10668 9777 10671 9770 10672
9763 10674 9756 10676 38 N F
9792 8796 9791 8793 9791 8789 9794 8790 9797 8792 9798 8795
9800 8798 9803 8798 9806 8800 9807 8803 9806 8806 9802 8805
9800 8802 9798 8800 9795 8798 9792 8796 16 N F
9795 9396 9793 9394 9796 9392 9799 9390 9801 9388 9805 9388
9808 9389 9811 9389 9815 9390 9816 9393 9815 9397 9814 9399
9810 9400 9808 9398 9808 9395 9805 9393 9801 9394 9799 9396
9795 9396 19 N F
9831 9043 9829 9038 9831 9035 9834 9034 9837 9032 9840 9030
9842 9033 9841 9036 9842 9038 9843 9039 9846 9039 9846 9043
9843 9044 9843 9047 9842 9050 9840 9053 9837 9054 9834 9053
9833 9050 9832 9047 9831 9043 21 N F
9842 9027 9838 9024 9839 9021 9842 9019 9845 9018 9848 9020
9850 9022 9849 9025 9845 9026 9842 9027 10 N F
9860 10641 9851 10640 9854 10634 9857 10628 9859 10621 9860 10614
9862 10608 9867 10603 9873 10600 9879 10596 9885 10598 9889 10604
9893 10610 9896 10616 9894 10623 9891 10629 9885 10633 9879 10636
9873 10639 9866 10639 9860 10641 21 N F

9877 14932 9866 14927 9870 14922 9869 14915 9874 14910 9879 14906
9885 14910 9888 14916 9890 14922 9895 14918 9901 14917 9901 14923
9899 14930 9896 14936 9898 14942 9905 14945 9898 14945 9893 14940
9887 14938 9880 14938 9877 14932 21 N F
9913 5703 9905 5701 9908 5697 9910 5691 9913 5686 9919 5684
9922 5679 9928 5680 9927 5686 9928 5691 9930 5697 9929 5703
9924 5705 9918 5705 9913 5703 15 N F
9908 9393 9905 9386 9907 9382 9907 9382 9912 9382 9916 9382
9920 9380 9923 9379 9924 9379 9928 9376 9930 9372 9934 9369
9938 9370 9942 9369 9946 9371 9950 9373 9948 9377 9952 9377
9953 9379 9955 9381 9958 9384 9955 9387 9950 9388 9947 9390
9948 9395 9946 9398 9942 9396 9937 9396 9933 9395 9929 9395
9924 9396 9921 9399 9917 9398 9913 9396 9910 9399 9906 9397
9908 9393 37 N F
9908 9147 9906 9143 9909 9142 9912 9141 9915 9139 9917 9136
9920 9136 9923 9138 9923 9142 9924 9145 9926 9147 9928 9150
9925 9150 9923 9147 9920 9145 9918 9143 9914 9142 9912 9145
9913 9148 9910 9149 9907 9150 9908 9147 22 N F
9911 14976 9907 14974 9911 14974 9914 14972 9916 14971 9916 14971
9918 14968 9921 14969 9925 14969 9922 14972 9920 14973 9916 14973
9915 14976 9911 14976 14 N F
9911 14954 9908 14951 9912 14951 9914 14949 9917 14951 9920 14952
9921 14955 9919 14958 9916 14958 9914 14956 9911 14954 11 N F
9915 9365 9909 9359 9913 9358 9918 9358 9917 9354 9921 9353
9924 9350 9928 9347 9931 9350 9931 9354 9933 9358 9930 9362
9933 9365 9931 9368 9927 9371 9923 9372 9918 9372 9915 9369
9915 9365 19 N F
9913 9882 9909 9875 9914 9870 9916 9863 9922 9859 9929 9860
9934 9865 9940 9869 9945 9873 9951 9877 9953 9883 9951 9890
9949 9897 9948 9903 9944 9909 9937 9912 9934 9918 9930 9924
9923 9924 9916 9922 9915 9916 9912 9909 9910 9903 9910 9896
9912 9889 9913 9882 26 N F
9944 14960 9939 14957 9942 14955 9944 14953 9947 14953 9950 14952
9953 14953 9956 14953 9959 14953 9961 14956 9963 14958 9960 14960
9957 14959 9954 14959 9951 14960 9948 14960 9944 14960 17 N F
9946 14994 9940 14993 9943 14990 9944 14989 9946 14988 9951 14987
9955 14987 9958 14984 9962 14982 9966 14981 9971 14980 9975 14979
9974 14974 9977 14971 9979 14975 9979 14979 9983 14980 9987 14980
9989 14980 9992 14979 9996 14979 10000 14979 10005 14978 10009 14977
10012 14974 10015 14977 10018 14980 10022 14983 10017 14982 10013 14984
10009 14985 10005 14987 10003 14990 9999 14991 9996 14988 9992 14986
9988 14985 9984 14985 9979 14986 9975 14987 9972 14990 9969 14993
9969 14997 9966 14994 9964 14990 9960 14993 9960 14997 9956 14997

9953 14994 9950 14997 9946 14994 51 N F
9943 9192 9940 9190 9942 9187 9944 9190 9947 9192 9950 9194
9952 9196 9952 9199 9952 9203 9954 9205 9958 9204 9956 9207
9956 9210 9953 9213 9950 9213 9947 9211 9946 9208 9947 9204
9947 9201 9943 9201 9945 9198 9945 9194 9943 9192 23 N F
9943 9934 9940 9928 9944 9926 9948 9925 9952 9923 9950 9918
9955 9917 9959 9917 9959 9921 9961 9926 9963 9929 9962 9934
9965 9937 9961 9940 9958 9943 9954 9942 9950 9940 9946 9938
9943 9934 19 N F
9949 9171 9947 9166 9950 9166 9952 9163 9956 9163 9959 9165
9958 9168 9956 9170 9958 9172 9961 9175 9963 9177 9966 9178
9969 9179 9970 9182 9973 9183 9976 9183 9973 9184 9970 9185
9967 9183 9965 9180 9962 9178 9959 9179 9956 9177 9953 9176
9949 9176 9947 9174 9949 9171 27 N F
9951 9397 9949 9390 9953 9390 9957 9388 9959 9384 9962 9380
9967 9380 9970 9377 9970 9372 9970 9368 9968 9364 9965 9360
9970 9362 9970 9362 9974 9362 9978 9362 9979 9362 9983 9360
9982 9356 9980 9351 9985 9350 9988 9348 9990 9350 9992 9351
9996 9352 9994 9356 9994 9361 9995 9365 9999 9367 10003 9369
10000 9373 9996 9373 9998 9376 10002 9379 10005 9382 10004 9387
10002 9391 10002 9395 9999 9399 10002 9402 10001 9407 9998 9410
9994 9412 9990 9414 9988 9418 9985 9422 9980 9422 9977 9420
9976 9415 9972 9413 9968 9410 9965 9408 9961 9405 9957 9403
9953 9401 9951 9397 56 N F
9958 9975 9952 9972 9955 9966 9958 9963 9960 9961 9966 9957
9969 9951 9972 9944 9972 9943 9976 9939 9977 9932 9979 9925
9977 9918 9975 9912 9978 9906 9984 9902 9988 9896 9993 9891
9996 9885 9998 9878 10004 9875 10008 9878 10010 9879 10015 9884
10021 9886 10022 9886 10029 9886 10035 9886 10042 9883 10044 9880
10045 9877 10052 9877 10059 9878 10066 9879 10067 9879 10073 9877
10079 9874 10085 9871 10092 9869 10099 9867 10105 9865 10111 9861
10117 9857 10118 9857 10123 9854 10130 9855 10137 9856 10143 9859
10140 9865 10139 9872 10141 9879 10144 9885 10147 9891 10152 9896
10146 9899 10142 9905 10137 9909 10132 9914 10127 9919 10122 9923
10115 9922 10108 9923 10104 9929 10097 9932 10096 9938 10089 9938
10082 9938 10076 9941 10073 9947 10066 9946 10059 9947 10052 9948
10048 9954 10044 9959 10040 9965 10034 9969 10027 9971 10022 9967
10015 9964 10009 9962 10002 9960 9996 9962 9991 9967 9984 9961
9978 9965 9971 9966 9971 9973 9966 9978 9960 9981 9958 9975
90 N F
9958 10053 9956 10050 9957 10047 9958 10043 9960 10046 9960 10046
9963 10048 9966 10050 9966 10053 9965 10056 9967 10058 9966 10061
9968 10063 9970 10066 9971 10069 9969 10071 9966 10069 9966 10066

9964 10064 9962 10066 9960 10063 9961 10059 9960 10056 9958 10053
24 N F
9958 9834 9956 9826 9959 9823 9962 9820 9967 9819 9971 9820
9975 9821 9980 9822 9984 9824 9988 9826 9989 9830 9988 9835
9986 9838 9981 9839 9979 9843 9978 9847 9974 9849 9972 9853
9968 9852 9967 9848 9962 9846 9959 9843 9958 9839 9958 9834
24 N F
9968 9038 9965 9034 9968 9035 9971 9035 9969 9032 9972 9030
9970 9028 9968 9025 9969 9022 9973 9024 9975 9021 9977 9023
9979 9025 9982 9024 9982 9027 9983 9030 9985 9033 9986 9034
9987 9035 9984 9036 9981 9036 9978 9038 9977 9041 9978 9044
9975 9044 9971 9043 9970 9040 9968 9038 28 N F
9969 8999 9965 8996 9968 8997 9971 8998 9973 9001 9976 8999
9978 9001 9981 9003 9984 9004 9986 9006 9988 9009 9986 9012
9983 9011 9979 9011 9977 9009 9973 9008 9971 9005 9971 9002
9969 8999 19 N F
9979 9766 9978 9762 9979 9758 9979 9753 9982 9750 9986 9750
9991 9750 9995 9750 9997 9754 10000 9758 10001 9762 10005 9765
10006 9769 10009 9773 10010 9777 10007 9781 10003 9782 9999 9780
9993 9777 9989 9774 9986 9772 9982 9769 9979 9766 23 N F
9984 9162 9980 9158 9982 9156 9983 9153 9986 9154 9988 9157
9991 9158 9993 9161 9990 9163 9987 9162 9984 9162 11 N F
9984 14951 9981 14948 9983 14946 9986 14948 9989 14948 9991 14951
9991 14954 9989 14957 9988 14954 9984 14955 9984 14951 11 N F
9991 9986 9987 9982 9990 9983 9994 9982 9995 9979 9998 9977
9995 9975 9993 9972 9997 9972 9999 9974 10002 9976 10004 9978
10007 9980 10009 9983 10011 9985 10009 9988 10009 9991 10007 9994
10004 9993 10001 9991 10001 9988 9999 9991 9995 9992 9993 9990
9994 9987 9991 9986 26 N F
9995 10240 9991 10235 9994 10233 9996 10231 9999 10232 10001 10235
10002 10238 10004 10240 10001 10239 9998 10241 9997 10238 9995 10240
12 N F
10001 10212 9996 10209 9998 10207 10001 10207 10003 10204 10005 10201
10007 10202 10008 10202 10010 10205 10010 10208 10012 10211 10015 10212
10012 10213 10009 10214 10006 10214 10004 10211 10001 10212 17 N F
10003 14965 9997 14961 10000 14958 10004 14958 10007 14961 10011 14960
10016 14962 10020 14962 10024 14962 10028 14961 10028 14957 10026 14953
10024 14949 10024 14945 10027 14948 10031 14951 10034 14954 10038 14954
10038 14958 10042 14959 10045 14960 10046 14961 10050 14963 10046 14965
10044 14969 10040 14971 10042 14975 10039 14972 10036 14968 10032 14970
10028 14970 10024 14969 10020 14968 10016 14966 10011 14966 10007 14966
10003 14965 37 N F
10002 9741 9999 9738 10001 9735 10000 9732 10001 9729 10001 9725

10004 9727 10006 9730 10008 9732 10009 9735 10011 9738 10014 9739
10014 9742 10013 9746 10010 9747 10007 9745 10004 9744 10002 9741
18 N F
10003 9857 10002 9853 10003 9850 10003 9846 10006 9845 10009 9845
10012 9847 10013 9850 10015 9853 10015 9856 10014 9859 10011 9861
10008 9861 10006 9859 10003 9857 15 N F
10018 9415 10017 9408 10021 9409 10025 9409 10027 9413 10030 9417
10033 9414 10038 9414 10041 9417 10044 9420 10040 9422 10035 9421
10031 9423 10032 9427 10036 9429 10033 9432 10036 9436 10031 9436
10028 9439 10026 9435 10027 9431 10025 9427 10028 9425 10024 9423
10022 9419 10017 9419 10018 9415 27 N F
10023 9736 10018 9724 10025 9724 10028 9729 10028 9730 10035 9730
10041 9725 10048 9725 10049 9719 10055 9715 10061 9712 10061 9705
10061 9698 10068 9698 10070 9705 10073 9699 10079 9702 10081 9695
10088 9694 10091 9700 10092 9707 10096 9713 10092 9718 10089 9725
10083 9727 10078 9732 10071 9734 10065 9738 10061 9743 10057 9748
10058 9755 10056 9762 10052 9767 10047 9763 10040 9760 10033 9760
10028 9756 10027 9749 10026 9742 10023 9736 40 N F
10023 10051 10018 10042 10024 10039 10031 10036 10036 10032 10042 10029
10048 10025 10055 10023 10062 10023 10064 10023 10069 10023 10072 10022
10075 10021 10082 10018 10088 10016 10086 10023 10083 10029 10088 10034
10090 10041 10086 10047 10090 10052 10095 10057 10088 10057 10084 10062
10085 10069 10079 10072 10072 10070 10066 10073 10065 10066 10059 10063
10052 10062 10047 10057 10040 10059 10033 10059 10029 10054 10023 10051
36 N F
10021 9880 10020 9877 10021 9874 10020 9871 10022 9868 10025 9869
10028 9871 10031 9872 10029 9875 10027 9878 10025 9880 10021 9880
12 N F
10041 14863 10036 14860 10039 14857 10043 14854 10039 14852 10042 14849
10046 14847 10050 14846 10054 14845 10058 14843 10062 14846 10061 14850
10057 14851 10056 14855 10053 14858 10049 14856 10046 14859 10045 14864
10041 14863 19 N F
10040 9868 10039 9864 10041 9860 10044 9861 10047 9862 10047 9865
10048 9868 10047 9871 10044 9872 10041 9871 10040 9868 11 N F
10054 14965 10051 14964 10054 14963 10057 14963 10059 14965 10057 14967
10054 14965 7 N F
10056 9862 10051 9858 10052 9857 10053 9855 10057 9855 10060 9855
10063 9854 10066 9856 10068 9858 10067 9861 10065 9864 10062 9865
10059 9864 10056 9862 14 N F
10053 9987 10052 9977 10055 9970 10059 9965 10066 9967 10071 9973
10072 9980 10078 9982 10083 9987 10077 9991 10070 9994 10064 9996
10058 9992 10053 9987 14 N F
10059 9871 10054 9867 10057 9866 10060 9868 10062 9867 10063 9867

10067 9868 10070 9866 10072 9863 10074 9861 10077 9862 10078 9866
10077 9869 10074 9871 10071 9873 10069 9876 10067 9877 10064 9876
10062 9873 10059 9871 20 N F
10062 14963 10057 14960 10060 14959 10062 14957 10064 14959 10067 14960
10071 14959 10073 14961 10071 14963 10068 14963 10065 14962 10062 14963
12 N F
10069 10014 10065 10010 10066 10008 10069 10007 10071 10005 10074 10004
10076 10005 10077 10008 10077 10011 10075 10012 10072 10014 10069 10014
12 N F
10072 14881 10067 14880 10069 14878 10072 14876 10075 14878 10078 14878
10081 14876 10083 14879 10080 14881 10078 14883 10075 14881 10072 14881
12 N F
10084 14868 10083 14863 10085 14861 10088 14863 10091 14864 10093 14867
10091 14870 10088 14871 10087 14868 10084 14868 10 N F
10091 9564 10089 9560 10090 9557 10093 9559 10097 9558 10099 9561
10101 9563 10102 9566 10105 9568 10108 9569 10111 9570 10110 9573
10107 9572 10104 9574 10101 9571 10098 9570 10098 9567 10097 9564
10094 9562 10091 9564 20 N F
10096 9724 10094 9721 10097 9720 10100 9718 10103 9717 10104 9717
10106 9715 10109 9713 10112 9711 10114 9709 10115 9712 10119 9713
10122 9712 10125 9712 10127 9710 10130 9707 10130 9711 10132 9714
10129 9715 10125 9715 10122 9714 10119 9716 10116 9717 10113 9718
10110 9720 10107 9720 10106 9724 10104 9726 10101 9726 10099 9728
10095 9728 10096 9724 32 N F
10096 10177 10095 10173 10098 10172 10101 10173 10102 10176 10104 10179
10101 10181 10098 10179 10096 10177 9 N F
10100 6965 10096 6965 10098 6964 10099 6962 10101 6961 10103 6960
10105 6961 10108 6961 10110 6960 10112 6961 10112 6963 10111 6964
10109 6966 10108 6968 10106 6969 10104 6970 10102 6969 10101 6967
10100 6965 19 N F
10102 9763 10097 9758 10099 9757 10101 9757 10104 9753 10106 9749
10109 9746 10112 9743 10116 9741 10120 9738 10124 9739 10128 9738
10132 9736 10135 9739 10140 9739 10144 9739 10145 9743 10140 9742
10140 9747 10136 9748 10132 9745 10128 9746 10123 9746 10119 9748
10116 9751 10112 9754 10115 9757 10117 9761 10113 9763 10110 9766
10105 9766 10102 9763 32 N F
10103 14875 10097 14877 10099 14875 10101 14872 10102 14869 10105 14868
10105 14868 10107 14870 10108 14873 10106 14875 10103 14875 11 N F
10104 9800 10099 9792 10105 9789 10110 9785 10110 9785 10114 9779
10119 9774 10125 9770 10130 9765 10137 9764 10142 9760 10149 9763
10145 9769 10141 9775 10142 9782 10136 9786 10142 9790 10137 9795
10131 9798 10124 9800 10118 9802 10111 9800 10104 9800 23 N F
10103 10245 10100 10242 10101 10241 10103 10241 10105 10242 10106 10243

10109 10245 10108 10248 10105 10249 10106 10246 10103 10245 11 N F
10105 10038 10102 10028 10104 10021 10110 10024 10116 10027 10123 10028
10130 10029 10126 10035 10119 10037 10112 10037 10105 10038 11 N F
10116 10148 10109 10149 10111 10143 10110 10136 10115 10131 10113 10124
10120 10121 10126 10118 10133 10117 10131 10124 10138 10125 10143 10129
10149 10125 10156 10126 10163 10124 10169 10124 10170 10124 10176 10120
10181 10120 10183 10120 10180 10127 10174 10130 10180 10134 10175 10140
10169 10142 10162 10142 10155 10143 10153 10150 10146 10149 10150 10143
10143 10144 10136 10146 10129 10148 10123 10146 10116 10148 35 N F
10122 10025 10112 10020 10118 10015 10122 10020 10129 10022 10136 10020
10142 10022 10149 10024 10153 10030 10156 10031 10160 10032 10165 10036
10163 10030 10165 10023 10171 10019 10177 10022 10181 10028 10178 10035
10175 10041 10169 10043 10162 10042 10155 10042 10148 10040 10141 10040
10134 10039 10133 10032 10128 10027 10122 10025 28 N F
10121 15129 10120 15125 10124 15124 10128 15124 10130 15120 10133 15117
10135 15113 10137 15113 10139 15113 10143 15114 10145 15117 10149 15120
10151 15124 10153 15127 10153 15128 10156 15131 10156 15136 10153 15139
10150 15142 10143 15145 10140 15142 10143 15139 10142 15134 10139 15138
10135 15138 10135 15142 10131 15143 10128 15140 10127 15136 10125 15132
10121 15129 31 N F
10127 10178 10124 10175 10127 10175 10130 10173 10133 10171 10136 10171
10137 10174 10137 10177 10137 10180 10137 10184 10134 10182 10134 10178
10130 10177 10127 10178 14 N F
10131 9815 10128 9810 10131 9808 10136 9806 10140 9806 10145 9807
10148 9810 10150 9810 10153 9811 10152 9815 10156 9818 10154 9822
10150 9821 10145 9820 10141 9822 10137 9821 10133 9820 10131 9815
18 N F
10146 7419 10145 7416 10147 7415 10149 7414 10151 7416 10152 7417
10152 7420 10150 7420 10148 7420 10146 7419 10 N F
10151 9859 10147 9852 10152 9848 10154 9845 10156 9842 10161 9838
10167 9834 10168 9832 10170 9828 10175 9822 10169 9818 10171 9811
10172 9804 10177 9799 10184 9798 10190 9802 10186 9807 10183 9814
10186 9821 10192 9824 10198 9827 10198 9834 10197 9841 10190 9842
10184 9845 10191 9845 10197 9847 10193 9852 10192 9859 10187 9863
10181 9859 10174 9857 10167 9859 10160 9858 10156 9864 10151 9859
36 N F
10151 14985 10148 14984 10149 14981 10152 14980 10153 14981 10155 14982
10157 14985 10153 14988 10151 14985 9 N F
10168 7337 10164 7336 10166 7334 10168 7335 10168 7337 5 N F
10170 15093 10168 15088 10170 15084 10173 15081 10176 15080 10177 15080
10181 15078 10181 15082 10181 15086 10186 15087 10183 15090 10179 15091
10174 15091 10170 15093 14 N F
10171 9884 10168 9881 10169 9877 10173 9874 10177 9874 10178 9878

10176 9883 10177 9887 10179 9890 10184 9890 10188 9891 10186 9895
10182 9897 10180 9901 10182 9904 10181 9909 10178 9905 10174 9904
10170 9903 10171 9899 10170 9895 10168 9888 10171 9884 23 N F
10176 10305 10169 10303 10175 10299 10176 10293 10175 10286 10170 10278
10177 10279 10184 10279 10191 10280 10197 10283 10200 10283 10204 10283
10211 10284 10214 10283 10218 10283 10224 10285 10219 10290 10223 10295
10216 10295 10209 10294 10204 10290 10197 10288 10192 10293 10185 10295
10182 10301 10176 10305 26 N F
10195 10133 10191 10128 10195 10128 10198 10130 10201 10129 10201 10126
10204 10123 10205 10124 10207 10124 10209 10127 10206 10129 10203 10131
10203 10135 10200 10137 10197 10136 10195 10133 16 N F
10205 9862 10204 9858 10206 9855 10207 9852 10211 9852 10211 9856
10209 9859 10209 9862 10205 9862 9 N F
10210 10022 10207 10018 10211 10017 10213 10016 10217 10015 10220 10014
10221 10017 10220 10020 10217 10022 10214 10022 10210 10022 11 N F
10223 14895 10218 14889 10223 14890 10228 14888 10230 14883 10226 14885
10222 14881 10222 14875 10222 14870 10223 14865 10228 14862 10232 14867
10235 14870 10237 14871 10240 14873 10244 14877 10244 14882 10241 14886
10238 14891 10235 14896 10235 14901 10233 14896 10228 14893 10223 14895
24 N F
10231 10264 10230 10257 10234 10256 10238 10254 10243 10254 10240 10258
10244 10260 10247 10263 10244 10266 10241 10269 10237 10271 10234 10267
10231 10264 13 N F
10257 10356 10253 10352 10256 10352 10260 10352 10262 10351 10265 10350
10266 10353 10263 10355 10260 10355 10257 10356 10 N F
10270 10354 10269 10351 10272 10349 10274 10351 10277 10353 10275 10355
10272 10357 10270 10354 8 N F
10285 10371 10281 10369 10284 10368 10286 10366 10290 10366 10290 10366
10293 10366 10295 10369 10298 10369 10299 10372 10295 10373 10292 10371
10290 10373 10287 10375 10284 10377 10284 10373 10285 10371 17 N F
10290 10015 10286 10011 10288 10009 10289 10006 10291 10003 10294 10002
10295 10005 10294 10008 10293 10011 10293 10014 10290 10015 11 N F
10309 10013 10304 10011 10306 10009 10308 10006 10311 10005 10311 10002
10312 9999 10314 10002 10314 10005 10313 10009 10311 10011 10309 10013
12 N F
10321 10015 10317 10011 10319 10008 10321 10005 10324 10003 10324 10007
10322 10010 10323 10013 10321 10015 9 N F
10329 10001 10325 9996 10327 9994 10329 9994 10331 9996 10330 9998
10329 10001 7 N F
10330 10298 10327 10294 10329 10291 10329 10290 10332 10294 10336 10294
10339 10291 10343 10289 10342 10293 10342 10298 10343 10302 10344 10306
10340 10306 10336 10304 10332 10302 10330 10298 16 N F
10340 10341 10334 10339 10337 10336 10340 10338 10340 10338 10343 10340

10340 10341 7 N F
10351 9979 10347 9978 10348 9974 10350 9971 10353 9971 10356 9971
10360 9971 10359 9974 10357 9977 10354 9979 10351 9979 11 N F
10356 10309 10350 10303 10351 10302 10353 10299 10357 10299 10361 10300
10363 10296 10366 10292 10367 10297 10366 10301 10364 10305 10359 10306
10356 10309 13 N F
10430 15272 10429 15268 10433 15268 10435 15270 10438 15270 10441 15272
10445 15271 10448 15272 10446 15274 10444 15277 10441 15278 10438 15277
10436 15275 10433 15274 10430 15272 15 N F
10637 6766 10632 6764 10636 6761 10640 6761 10645 6760 10649 6758
10654 6758 10658 6759 10662 6761 10667 6760 10671 6759 10675 6761
10679 6764 10682 6766 10685 6770 10689 6773 10685 6775 10681 6777
10676 6778 10672 6780 10666 6778 10661 6779 10657 6777 10653 6776
10649 6778 10644 6776 10643 6772 10638 6770 10637 6766 29 N F
10811 15190 10808 15184 10812 15184 10814 15188 10817 15191 10821 15190
10822 15189 10824 15187 10827 15190 10827 15195 10831 15193 10832 15189
10836 15187 10840 15186 10844 15188 10847 15192 10848 15196 10851 15199
10852 15203 10848 15205 10844 15207 10840 15208 10839 15204 10835 15204
10830 15204 10827 15201 10824 15203 10820 15201 10820 15197 10818 15194
10813 15193 10811 15190 32 N F
10812 15136 10811 15131 10814 15131 10813 15128 10816 15125 10819 15126
10822 15126 10824 15129 10824 15133 10821 15135 10820 15138 10820 15141
10817 15139 10815 15137 10812 15136 15 N F
10820 15162 10820 15158 10820 15158 10822 15157 10823 15158 10824 15159
10826 15161 10824 15163 10822 15164 10820 15162 10 N F
10821 15151 10820 15146 10823 15145 10826 15145 10825 15148 10826 15151
10823 15153 10821 15151 8 N F
10848 15183 10846 15179 10848 15175 10846 15170 10850 15167 10853 15170
10857 15171 10857 15176 10859 15179 10856 15182 10858 15186 10854 15188
10850 15186 10848 15183 14 N F
10882 7539 10880 7536 10882 7534 10884 7534 10886 7535 10888 7536
10890 7537 10892 7537 10894 7537 10896 7538 10899 7538 10898 7540
10897 7542 10896 7543 10893 7543 10891 7544 10889 7544 10887 7544
10885 7542 10883 7541 10882 7539 21 N F
10892 14340 10886 14336 10888 14332 10891 14330 10892 14330 10895 14327
10895 14322 10897 14318 10899 14315 10902 14311 10906 14312 10911 14312
10915 14311 10919 14312 10922 14309 10926 14311 10927 14315 10929 14319
10927 14323 10923 14323 10921 14327 10923 14331 10927 14333 10931 14331
10929 14327 10932 14328 10933 14328 10936 14331 10941 14330 10938 14334
10943 14334 10940 14337 10938 14341 10937 14345 10933 14347 10928 14347
10924 14347 10920 14345 10916 14344 10911 14343 10908 14341 10904 14339
10900 14337 10896 14339 10892 14340 45 N F
10908 15175 10903 15169 10908 15169 10911 15173 10914 15175 10918 15175

10922 15177 10925 15180 10927 15184 10931 15186 10934 15189 10935 15194
10939 15194 10938 15198 10942 15199 10946 15198 10948 15200 10949 15201
10952 15204 10951 15209 10946 15208 10942 15207 10945 15211 10941 15212
10944 15215 10940 15216 10942 15220 10939 15222 10938 15226 10941 15230
10945 15229 10945 15233 10941 15235 10938 15238 10933 15238 10929 15239
10926 15236 10922 15234 10926 15231 10925 15227 10921 15225 10924 15222
10925 15218 10921 15215 10917 15213 10916 15209 10911 15210 10908 15207
10905 15203 10904 15199 10908 15201 10912 15202 10916 15203 10920 15202
10918 15198 10913 15197 10915 15193 10919 15192 10920 15187 10918 15183
10914 15184 10909 15184 10909 15179 10908 15175 64 N F
10920 14258 10915 14258 10918 14256 10921 14255 10924 14254 10924 14251
10924 14248 10924 14248 10926 14249 10929 14251 10932 14250 10936 14251
10938 14253 10940 14256 10940 14259 10938 14262 10938 14265 10940 14268
10942 14269 10941 14272 10938 14274 10935 14273 10932 14275 10932 14272
10929 14269 10926 14268 10925 14265 10923 14262 10923 14259 10920 14258
30 N F
10924 7534 10922 7532 10923 7531 10925 7529 10926 7530 10925 7532
10924 7534 7 N F
10934 14642 10928 14640 10931 14637 10935 14636 10939 14637 10937 14641
10938 14645 10942 14642 10946 14642 10949 14645 10952 14642 10956 14640
10959 14638 10963 14636 10967 14634 10972 14634 10972 14638 10975 14635
10979 14635 10982 14638 10979 14642 10977 14646 10975 14649 10971 14651
10971 14656 10967 14656 10963 14656 10959 14658 10955 14660 10951 14661
10948 14664 10947 14660 10944 14657 10942 14653 10938 14652 10933 14651
10933 14647 10934 14642 38 N F
10947 14232 10945 14227 10948 14227 10950 14230 10953 14232 10956 14233
10959 14232 10959 14232 10962 14232 10962 14235 10964 14238 10960 14238
10957 14238 10954 14237 10951 14237 10950 14234 10947 14232 17 N F
10958 7636 10957 7633 10959 7632 10961 7631 10963 7632 10964 7634
10963 7636 10962 7637 10959 7638 10958 7636 10 N F
10969 6638 10966 6636 10968 6636 10971 6637 10973 6638 10975 6639
10973 6640 10970 6640 10969 6638 9 N F
11011 14711 11011 14706 11014 14703 11016 14707 11020 14709 11024 14711
11025 14715 11026 14719 11028 14723 11028 14727 11026 14723 11019 14724
11018 14728 11015 14732 11014 14727 11015 14723 11012 14719 11015 14716
11019 14716 11018 14712 11015 14714 11011 14711 22 N F
11030 6710 11018 6707 11022 6702 11026 6696 11033 6693 11040 6693
11047 6692 11049 6699 11055 6703 11061 6698 11067 6694 11074 6693
11081 6693 11088 6692 11094 6691 11095 6691 11102 6692 11109 6693
11116 6691 11122 6687 11129 6685 11135 6688 11135 6695 11131 6701
11124 6705 11131 6702 11138 6700 11144 6696 11145 6704 11142 6710
11145 6717 11148 6723 11155 6721 11157 6728 11155 6735 11159 6741
11161 6748 11154 6750 11148 6755 11143 6759 11136 6761 11129 6762

11127 6769 11121 6765 11114 6769 11107 6768 11101 6765 11096 6760
11091 6766 11089 6772 11085 6778 11079 6782 11072 6785 11066 6781
11072 6776 11074 6769 11077 6763 11071 6766 11072 6773 11065 6771
11061 6765 11056 6759 11059 6752 11058 6745 11062 6739 11067 6734
11068 6727 11065 6733 11063 6727 11062 6720 11065 6713 11064 6706
11063 6713 11058 6718 11052 6722 11051 6729 11045 6725 11039 6728
11040 6721 11043 6715 11037 6719 11031 6722 11027 6716 11030 6710
84 N F
11059 14266 11058 14263 11061 14261 11064 14262 11067 14261 11070 14261
11071 14264 11072 14265 11073 14267 11074 14270 11077 14268 11078 14271
11078 14274 11079 14277 11079 14281 11076 14280 11073 14280 11069 14280
11066 14279 11069 14277 11072 14276 11071 14273 11071 14269 11068 14270
11065 14270 11062 14272 11062 14269 11059 14266 28 N F
11077 6571 11076 6566 11078 6562 11083 6562 11088 6561 11092 6562
11097 6561 11101 6563 11104 6567 11106 6571 11103 6574 11099 6576
11094 6576 11090 6576 11085 6574 11081 6572 11077 6571 17 N F
11122 14265 11117 14262 11121 14261 11124 14261 11124 14257 11123 14254
11126 14256 11127 14259 11130 14261 11133 14263 11135 14265 11138 14267
11137 14270 11140 14269 11143 14271 11141 14273 11141 14276 11138 14275
11134 14275 11131 14273 11129 14272 11127 14269 11124 14268 11122 14265
24 N F
11157 14243 11152 14241 11156 14241 11159 14239 11161 14237 11163 14235
11166 14236 11163 14238 11165 14241 11163 14243 11160 14244 11157 14243
12 N F
11157 14216 11155 14213 11157 14211 11160 14209 11162 14210 11163 14210
11165 14212 11169 14211 11171 14213 11174 14215 11176 14217 11178 14220
11180 14223 11181 14224 11182 14225 11184 14227 11188 14229 11190 14230
11193 14232 11194 14235 11196 14238 11196 14238 11198 14240 11200 14243
11202 14246 11203 14249 11205 14251 11207 14254 11204 14253 11201 14253
11198 14252 11195 14250 11193 14247 11190 14246 11187 14246 11185 14244
11182 14243 11180 14241 11181 14237 11182 14235 11183 14231 11183 14228
11180 14230 11180 14233 11178 14235 11175 14237 11172 14236 11169 14235
11171 14232 11171 14229 11168 14227 11167 14224 11165 14222 11163 14219
11160 14218 11157 14216 56 N F
11159 6668 11156 6665 11159 6662 11162 6661 11165 6662 11165 6666
11162 6668 11159 6668 8 N F
11166 6676 11164 6670 11168 6671 11170 6674 11172 6676 11173 6680
11170 6681 11168 6679 11166 6676 9 N F
11172 6734 11168 6734 11170 6733 11172 6733 11175 6732 11178 6733
11182 6732 11185 6733 11188 6730 11191 6729 11194 6730 11196 6733
11192 6736 11189 6737 11188 6740 11188 6744 11185 6746 11181 6746
11178 6746 11175 6744 11174 6741 11176 6739 11175 6736 11172 6734
24 N F

11193 6687 11192 6684 11192 6681 11194 6678 11196 6675 11198 6672
11201 6670 11204 6670 11207 6672 11210 6674 11212 6677 11213 6680
11213 6683 11211 6686 11207 6686 11204 6686 11201 6687 11196 6689
11193 6687 19 N F
11229 6801 11225 6794 11231 6789 11237 6791 11237 6791 11243 6796
11250 6797 11255 6792 11259 6786 11264 6781 11263 6773 11260 6767
11253 6765 11249 6759 11254 6754 11261 6752 11268 6750 11273 6745
11280 6741 11287 6740 11293 6743 11298 6748 11299 6755 11306 6754
11312 6750 11319 6753 11322 6759 11323 6766 11323 6773 11323 6780
11324 6787 11323 6795 11319 6801 11323 6807 11319 6813 11319 6820
11323 6826 11328 6831 11333 6837 11333 6844 11338 6849 11342 6855
11345 6861 11343 6868 11347 6874 11352 6879 11357 6884 11358 6891
11357 6898 11358 6905 11351 6905 11344 6906 11343 6899 11342 6892
11338 6886 11334 6880 11331 6873 11336 6869 11333 6862 11332 6855
11330 6848 11323 6850 11316 6848 11313 6841 11306 6839 11306 6832
11303 6825 11305 6818 11299 6814 11292 6814 11285 6813 11279 6809
11272 6809 11265 6807 11263 6814 11259 6820 11252 6823 11252 6816
11250 6809 11246 6803 11241 6807 11233 6806 11229 6801 83 N F
11235 6735 11234 6731 11235 6728 11238 6726 11241 6725 11244 6728
11246 6730 11246 6734 11245 6737 11243 6739 11240 6741 11237 6742
11235 6739 11235 6735 14 N F
11279 12233 11273 12233 11277 12230 11282 12230 11286 12228 11289 12225
11290 12221 11293 12218 11297 12219 11301 12220 11303 12224 11300 12226
11296 12229 11293 12233 11292 12237 11293 12241 11288 12241 11286 12245
11282 12245 11279 12241 11279 12237 11279 12233 22 N F
11291 12571 11288 12567 11289 12562 11292 12559 11293 12563 11295 12567
11300 12567 11301 12571 11303 12575 11304 12580 11302 12584 11298 12586
11298 12581 11296 12577 11294 12573 11291 12571 16 N F
11349 12434 11345 12433 11347 12431 11348 12429 11349 12428 11351 12431
11349 12434 7 N F
11348 12442 11345 12437 11349 12437 11349 12438 11351 12439 11353 12442
11353 12445 11349 12445 11348 12442 9 N F
11353 12417 11348 12420 11350 12417 11352 12414 11354 12411 11355 12408
11358 12410 11361 12409 11364 12411 11363 12414 11360 12415 11357 12417
11355 12414 11353 12417 14 N F
11356 14220 11355 14217 11358 14215 11359 14212 11359 14209 11360 14206
11363 14204 11365 14206 11367 14209 11369 14212 11371 14214 11372 14217
11372 14220 11369 14221 11366 14221 11363 14219 11359 14219 11356 14220
18 N F
11373 12574 11371 12570 11374 12568 11377 12569 11379 12572 11379 12575
11376 12576 11373 12574 8 N F
11383 12581 11379 12576 11382 12576 11384 12579 11386 12581 11383 12581
6 N F

11386 12559 11383 12555 11385 12552 11388 12548 11391 12545 11395 12546
11394 12550 11395 12554 11399 12557 11401 12560 11400 12564 11404 12565
11407 12568 11409 12572 11409 12577 11412 12580 11416 12581 11414 12586
11410 12584 11407 12580 11403 12580 11402 12584 11406 12586 11402 12587
11397 12586 11393 12585 11391 12582 11391 12577 11388 12574 11387 12570
11386 12565 11383 12562 11386 12559 33 N F
11386 14292 11383 14288 11386 14287 11386 14287 11390 14288 11391 14290
11390 14293 11390 14297 11387 14295 11386 14292 10 N F
11400 12617 11396 12617 11399 12615 11402 12614 11402 12614 11400 12617
6 N F
11405 12612 11402 12611 11402 12607 11405 12606 11407 12609 11405 12612
6 N F
11410 12606 11408 12603 11410 12600 11413 12599 11414 12596 11417 12598
11416 12601 11418 12604 11421 12604 11421 12607 11418 12607 11415 12610
11412 12611 11413 12608 11410 12606 15 N F
11415 12560 11414 12557 11417 12556 11421 12557 11423 12560 11425 12562
11426 12565 11423 12566 11419 12565 11418 12562 11415 12560 11 N F
11442 12323 11439 12323 11441 12320 11443 12318 11445 12320 11448 12318
11451 12321 11447 12321 11445 12324 11442 12323 10 N F
11443 14281 11440 14279 11441 14276 11444 14277 11445 14277 11448 14277
11451 14278 11453 14281 11454 14284 11456 14286 11453 14285 11450 14284
11448 14281 11446 14283 11443 14281 15 N F
11502 13908 11501 13904 11502 13901 11505 13899 11505 13902 11506 13905
11506 13909 11509 13907 11512 13908 11511 13911 11508 13912 11505 13913
11502 13911 11502 13908 14 N F
11506 13964 11505 13961 11508 13960 11511 13962 11513 13965 11516 13964
11514 13967 11517 13969 11519 13971 11516 13972 11514 13969 11511 13968
11509 13965 11506 13964 14 N F
11521 13886 11519 13883 11521 13881 11523 13879 11526 13877 11528 13874
11531 13875 11533 13877 11533 13877 11536 13879 11533 13879 11529 13879
11526 13880 11526 13883 11524 13886 11521 13886 16 N F
11530 7007 11528 7005 11531 7003 11534 7002 11537 7001 11539 7000
11540 6999 11541 7003 11539 7006 11536 7007 11533 7009 11530 7007
12 N F
11531 6956 11528 6952 11528 6945 11530 6941 11534 6938 11538 6938
11543 6937 11547 6938 11551 6940 11555 6943 11558 6946 11558 6951
11557 6955 11553 6958 11548 6959 11544 6960 11539 6960 11535 6958
11531 6956 19 N F
11549 14246 11546 14242 11548 14240 11550 14240 11553 14240 11555 14241
11554 14243 11551 14244 11549 14246 9 N F
11553 13923 11551 13919 11552 13920 11554 13920 11557 13919 11554 13918
11553 13915 11556 13913 11559 13914 11561 13917 11563 13919 11564 13922
11563 13925 11560 13927 11557 13926 11554 13926 11553 13923 17 N F

11554 13449 11552 13451 11553 13448 11554 13446 11556 13444 11559 13443
11559 13446 11560 13448 11558 13450 11556 13448 11554 13449 11 N F
11557 13993 11556 13988 11559 13991 11560 13995 11562 13992 11563 13991
11567 13990 11568 13985 11572 13985 11576 13986 11580 13987 11582 13991
11582 13992 11585 13993 11588 13996 11586 14000 11582 14001 11578 14003
11574 14005 11570 14004 11568 14008 11564 14011 11564 14015 11560 14018
11560 14013 11558 14010 11558 14005 11556 14001 11557 13997 11557 13993
30 N F
11559 13969 11558 13965 11560 13962 11560 13961 11562 13959 11565 13959
11567 13959 11568 13959 11570 13961 11569 13964 11570 13967 11567 13967
11565 13969 11562 13971 11559 13969 15 N F
11564 14240 11560 14237 11563 14236 11566 14233 11569 14234 11572 14236
11574 14234 11577 14231 11579 14230 11582 14229 11584 14232 11585 14235
11582 14237 11580 14239 11579 14242 11578 14245 11574 14245 11573 14242
11570 14240 11567 14240 11564 14240 21 N F
11567 13389 11564 13388 11565 13386 11566 13383 11567 13381 11570 13380
11572 13381 11572 13384 11575 13384 11575 13381 11577 13383 11577 13386
11575 13387 11572 13388 11570 13389 11567 13389 16 N F
11573 13691 11570 13689 11571 13686 11573 13688 11575 13689 11578 13690
11581 13689 11583 13690 11583 13692 11584 13693 11586 13695 11585 13697
11585 13700 11582 13701 11583 13698 11581 13696 11580 13694 11577 13694
11574 13693 11573 13691 20 N F
11577 12560 11571 12560 11575 12559 11578 12555 11582 12556 11580 12552
11576 12550 11578 12547 11582 12548 11586 12551 11590 12550 11594 12553
11598 12554 11598 12558 11596 12562 11596 12566 11592 12568 11589 12564
11585 12562 11582 12559 11577 12560 21 N F
11581 14296 11576 14291 11578 14290 11580 14288 11584 14286 11588 14285
11588 14285 11592 14285 11593 14289 11597 14290 11602 14291 11603 14292
11605 14294 11609 14294 11613 14291 11616 14294 11620 14296 11624 14298
11626 14302 11628 14306 11629 14309 11630 14310 11629 14314 11626 14317
11623 14315 11618 14313 11614 14313 11610 14313 11606 14315 11603 14318
11599 14320 11595 14318 11590 14318 11587 14315 11583 14312 11583 14308
11581 14304 11583 14300 11581 14296 39 N F
11588 13442 11583 13442 11585 13440 11587 13438 11589 13437 11589 13440
11588 13442 7 N F
11588 14267 11585 14265 11588 14264 11590 14263 11593 14263 11595 14264
11598 14264 11596 14266 11593 14267 11591 14266 11588 14267 11 N F
11592 13995 11588 13992 11588 13990 11589 13987 11591 13986 11592 13986
11594 13986 11597 13985 11597 13983 11598 13980 11600 13981 11601 13984
11603 13986 11601 13988 11598 13987 11597 13989 11595 13992 11594 13994
11592 13995 19 N F
11596 14229 11591 14228 11592 14227 11593 14225 11595 14223 11597 14220
11600 14219 11603 14217 11606 14217 11609 14215 11607 14218 11605 14220

11604 14223 11605 14227 11605 14230 11602 14230 11599 14229 11596 14229
18 N F
11603 12557 11600 12561 11601 12558 11601 12555 11603 12557 5 N F
11604 13448 11602 13444 11603 13444 11604 13444 11607 13445 11609 13447
11612 13447 11614 13448 11614 13451 11612 13452 11610 13452 11607 13452
11605 13451 11604 13448 14 N F
11603 14276 11602 14274 11602 14270 11604 14269 11605 14270 11606 14271
11605 14274 11603 14276 8 N F
11607 12559 11603 12561 11604 12558 11607 12556 11610 12554 11612 12552
11611 12555 11610 12558 11607 12559 9 N F
11604 12633 11603 12628 11606 12625 11609 12622 11612 12619 11616 12617
11621 12618 11625 12620 11628 12623 11627 12627 11631 12628 11628 12631
11627 12635 11623 12637 11623 12642 11627 12643 11631 12645 11635 12645
11638 12647 11639 12647 11640 12643 11645 12643 11648 12645 11652 12646
11652 12653 11648 12652 11649 12656 11651 12660 11652 12664 11651 12668
11647 12670 11643 12668 11639 12667 11638 12663 11635 12659 11631 12659
11630 12655 11626 12653 11622 12651 11618 12650 11614 12650 11611 12654
11606 12653 11606 12648 11604 12645 11605 12641 11604 12637 11604 12633
48 N F
11616 14262 11607 14262 11610 14259 11614 14257 11617 14254 11621 14252
11625 14251 11625 14251 11629 14249 11633 14246 11633 14246 11633 14246
11636 14243 11636 14239 11635 14235 11638 14232 11641 14229 11643 14232
11647 14235 11649 14239 11653 14241 11655 14245 11655 14249 11654 14253
11651 14256 11647 14258 11643 14258 11638 14259 11635 14262 11631 14264
11628 14267 11628 14271 11628 14276 11629 14280 11632 14283 11629 14287
11625 14286 11622 14284 11617 14282 11614 14280 11615 14275 11615 14271
11615 14267 11616 14262 44 N F
11618 13975 11613 13975 11615 13973 11618 13971 11622 13972 11625 13971
11626 13974 11625 13977 11621 13976 11618 13975 10 N F
11619 12342 11613 12342 11616 12340 11618 12338 11622 12337 11621 12340
11619 12342 7 N F
11619 12615 11617 12611 11618 12606 11618 12602 11619 12599 11619 12598
11622 12594 11625 12597 11627 12593 11630 12590 11634 12592 11637 12588
11641 12587 11642 12587 11645 12588 11649 12591 11649 12595 11652 12598
11655 12601 11656 12605 11660 12605 11660 12604 11661 12609 11663 12613
11656 12614 11652 12617 11649 12620 11644 12620 11642 12623 11641 12627
11638 12631 11636 12627 11635 12623 11631 12620 11634 12617 11632 12613
11631 12617 11627 12618 11623 12617 11619 12615 40 N F
11619 12955 11619 12952 11621 12954 11624 12956 11621 12958 11619 12955
6 N F
11627 12965 11623 12964 11626 12962 11627 12959 11630 12960 11634 12960
11636 12962 11638 12965 11637 12968 11636 12971 11633 12971 11631 12974
11628 12972 11625 12973 11625 12969 11628 12969 11630 12966 11627 12965

18 N F
11630 13972 11626 13970 11628 13968 11631 13968 11635 13967 11638 13966
11639 13963 11642 13962 11644 13965 11641 13966 11639 13969 11638 13972
11635 13973 11633 13975 11630 13972 15 N F
11638 14180 11633 14181 11634 14178 11637 14176 11639 14174 11640 14174
11642 14172 11643 14169 11643 14166 11646 14165 11649 14165 11652 14166
11655 14167 11658 14168 11660 14168 11662 14168 11665 14167 11667 14170
11666 14173 11663 14173 11660 14171 11657 14171 11654 14171 11651 14172
11648 14174 11647 14177 11644 14178 11641 14179 11638 14180 29 N F
11637 12707 11633 12707 11637 12705 11635 12701 11639 12700 11644 12700
11647 12703 11647 12703 11651 12700 11654 12703 11657 12707 11657 12711
11658 12706 11657 12702 11661 12701 11663 12697 11665 12695 11667 12694
11670 12697 11672 12699 11673 12700 11677 12702 11679 12706 11683 12706
11683 12710 11682 12715 11686 12713 11690 12715 11691 12719 11688 12722
11684 12723 11685 12727 11688 12730 11684 12732 11685 12737 11681 12737
11679 12741 11676 12744 11671 12745 11670 12740 11672 12736 11670 12732
11667 12730 11663 12733 11660 12730 11656 12728 11653 12730 11651 12726
11650 12722 11646 12715 11644 12711 11642 12708 11637 12707 53 N F
11635 12364 11635 12360 11638 12360 11641 12358 11640 12361 11643 12364
11645 12366 11646 12364 11646 12360 11648 12362 11648 12365 11647 12369
11643 12369 11640 12368 11637 12366 11635 12364 16 N F
11636 12577 11635 12574 11638 12571 11640 12569 11643 12571 11646 12569
11648 12567 11651 12568 11650 12572 11649 12575 11646 12576 11645 12579
11642 12580 11639 12578 11636 12577 15 N F
11639 12983 11637 12980 11640 12978 11642 12981 11639 12983 5 N F
11640 14024 11637 14019 11641 14017 11644 14015 11649 14014 11651 14014
11653 14013 11657 14013 11662 14014 11665 14016 11667 14020 11663 14022
11661 14018 11656 14019 11652 14021 11648 14021 11644 14023 11640 14024
18 N F
11643 14313 11638 14308 11641 14302 11643 14296 11643 14289 11642 14282
11643 14275 11642 14269 11645 14263 11652 14261 11659 14261 11661 14254
11666 14250 11668 14243 11671 14237 11667 14231 11661 14229 11655 14226
11657 14220 11656 14213 11663 14211 11669 14208 11676 14209 11679 14209
11683 14208 11686 14207 11689 14206 11696 14205 11696 14212 11696 14218
11700 14217 11702 14216 11707 14212 11708 14211 11715 14211 11719 14216
11721 14217 11724 14220 11726 14227 11723 14234 11721 14240 11723 14246
11718 14251 11717 14257 11717 14264 11716 14271 11712 14277 11713 14284
11715 14290 11712 14296 11709 14302 11707 14308 11702 14313 11696 14315
11690 14319 11684 14323 11679 14327 11674 14331 11667 14333 11662 14329
11655 14329 11653 14323 11649 14317 11643 14313 64 N F
11640 12831 11639 12826 11642 12824 11643 12821 11646 12820 11648 12822
11648 12825 11646 12828 11644 12831 11642 12833 11640 12831 11 N F
11644 12555 11642 12553 11643 12553 11646 12553 11646 12553 11649 12554

11650 12550 11648 12547 11649 12544 11652 12545 11652 12548 11652 12551
11652 12554 11654 12557 11654 12557 11651 12557 11648 12556 11644 12555
18 N F
11645 12432 11644 12428 11647 12429 11648 12432 11649 12435 11646 12435
11645 12432 7 N F
11651 13972 11646 13971 11648 13968 11651 13967 11654 13966 11657 13965
11660 13965 11659 13968 11657 13971 11654 13971 11651 13972 11 N F
11649 13144 11647 13141 11650 13140 11651 13142 11652 13145 11649 13144
6 N F
11651 12360 11648 12356 11649 12353 11652 12353 11656 12354 11658 12356
11659 12357 11660 12358 11661 12361 11658 12363 11656 12360 11653 12362
11651 12360 13 N F
11652 12419 11650 12415 11654 12416 11657 12416 11656 12419 11654 12421
11652 12419 7 N F
11658 12816 11652 12813 11656 12813 11661 12813 11661 12809 11664 12812
11667 12815 11669 12811 11671 12810 11673 12810 11677 12809 11681 12806
11683 12810 11686 12813 11685 12817 11687 12820 11689 12824 11685 12826
11681 12827 11681 12832 11681 12836 11685 12838 11686 12842 11684 12846
11686 12849 11686 12854 11690 12855 11694 12856 11697 12859 11701 12861
11697 12863 11695 12860 11690 12860 11686 12859 11682 12859 11681 12854
11678 12851 11676 12848 11674 12844 11671 12840 11667 12838 11663 12837
11659 12836 11657 12832 11656 12828 11653 12824 11657 12822 11661 12820
11658 12816 49 N F
11654 13134 11653 13131 11655 13130 11658 13130 11659 13128 11661 13129
11661 13131 11659 13133 11656 13134 11654 13134 10 N F
11656 12567 11655 12564 11657 12562 11659 12564 11659 12564 11660 12561
11662 12563 11665 12564 11665 12568 11664 12571 11662 12573 11660 12575
11657 12574 11657 12571 11659 12568 11656 12567 16 N F
11657 12400 11655 12396 11658 12396 11659 12396 11661 12398 11660 12401
11657 12400 7 N F
11657 12435 11656 12428 11659 12428 11660 12430 11661 12431 11661 12435
11659 12437 11657 12435 8 N F
11659 12317 11657 12314 11658 12311 11659 12308 11660 12305 11662 12308
11660 12311 11660 12314 11659 12317 9 N F
11659 14135 11658 14131 11661 14129 11664 14128 11665 14125 11665 14122
11668 14120 11670 14118 11673 14119 11674 14122 11677 14123 11679 14121
11679 14118 11682 14119 11683 14119 11685 14118 11687 14115 11688 14112
11690 14115 11692 14118 11693 14121 11692 14124 11691 14127 11688 14128
11685 14130 11683 14133 11680 14133 11677 14134 11676 14131 11673 14129
11670 14130 11668 14132 11666 14135 11664 14137 11660 14137 11659 14135
36 N F
11665 12639 11659 12638 11660 12635 11661 12632 11662 12629 11664 12627
11667 12626 11668 12629 11667 12633 11666 12635 11665 12639 11 N F

11666 12404 11662 12403 11663 12400 11664 12397 11666 12397 11667 12398
11668 12401 11666 12404 8 N F
11666 12396 11662 12394 11664 12391 11668 12391 11671 12391 11669 12394
11666 12396 7 N F
11666 12577 11665 12574 11667 12571 11668 12568 11667 12565 11666 12562
11666 12561 11667 12559 11670 12558 11673 12556 11672 12554 11669 12551
11670 12548 11671 12545 11671 12542 11673 12539 11676 12540 11677 12543
11679 12546 11681 12544 11684 12544 11688 12543 11690 12545 11691 12548
11692 12552 11690 12555 11687 12556 11687 12559 11687 12562 11684 12562
11680 12561 11679 12564 11677 12567 11679 12570 11679 12573 11678 12576
11675 12577 11672 12578 11669 12578 11666 12577 40 N F
11672 12685 11668 12683 11670 12680 11671 12677 11674 12677 11677 12675
11678 12675 11680 12676 11680 12679 11677 12681 11674 12683 11672 12685
12 N F
11670 12381 11669 12378 11670 12375 11674 12375 11675 12373 11677 12370
11680 12369 11680 12373 11682 12375 11682 12379 11680 12376 11678 12379
11676 12381 11673 12382 11670 12381 15 N F
11674 12649 11670 12645 11672 12646 11674 12646 11677 12647 11679 12650
11678 12653 11676 12652 11674 12649 9 N F
11675 12417 11673 12416 11673 12412 11673 12412 11673 12409 11676 12408
11680 12407 11682 12407 11683 12407 11686 12408 11687 12411 11690 12411
11693 12412 11693 12412 11696 12410 11699 12410 11702 12408 11703 12411
11701 12414 11698 12414 11695 12416 11692 12417 11689 12419 11687 12417
11683 12418 11682 12415 11679 12414 11675 12417 28 N F
11677 14174 11674 14170 11676 14168 11677 14171 11679 14173 11679 14176
11677 14174 7 N F
11681 12699 11674 12697 11677 12694 11680 12690 11682 12687 11684 12683
11684 12682 11687 12680 11691 12678 11693 12675 11692 12670 11697 12670
11701 12672 11704 12675 11705 12680 11702 12683 11702 12688 11703 12692
11708 12690 11711 12688 11711 12693 11715 12695 11716 12699 11712 12702
11708 12704 11712 12706 11716 12707 11716 12707 11717 12711 11712 12711
11712 12715 11709 12718 11706 12715 11702 12717 11698 12718 11695 12715
11696 12711 11698 12707 11701 12704 11699 12700 11695 12700 11691 12702
11687 12705 11684 12703 11681 12699 45 N F
11679 12538 11675 12535 11679 12533 11682 12537 11686 12536 11691 12536
11689 12532 11685 12531 11683 12527 11679 12529 11676 12523 11681 12523
11685 12522 11689 12523 11692 12524 11694 12524 11696 12528 11700 12529
11703 12532 11702 12536 11707 12537 11709 12541 11707 12545 11709 12549
11709 12553 11712 12556 11709 12559 11705 12561 11708 12565 11704 12567
11700 12568 11696 12570 11695 12574 11692 12577 11687 12576 11683 12577
11682 12572 11686 12569 11690 12568 11692 12564 11694 12560 11695 12556
11698 12553 11702 12552 11699 12551 11697 12547 11698 12542 11695 12539
11690 12539 11687 12541 11682 12541 11679 12538 52 N F

11682 12405 11677 12404 11678 12401 11680 12399 11682 12401 11685 12401
11685 12404 11682 12405 8 N F
11680 12597 11678 12595 11678 12591 11679 12588 11680 12584 11682 12587
11682 12591 11681 12594 11680 12597 9 N F
11681 13493 11678 13493 11679 13490 11681 13488 11683 13490 11684 13492
11684 13495 11686 13497 11684 13498 11681 13498 11680 13496 11681 13493
12 N F
11684 12675 11680 12674 11680 12673 11681 12671 11683 12668 11687 12669
11685 12672 11684 12675 8 N F
11681 14099 11680 14095 11683 14094 11685 14092 11685 14088 11686 14085
11688 14083 11691 14081 11693 14083 11694 14083 11697 14084 11698 14087
11697 14090 11700 14092 11698 14094 11695 14096 11693 14098 11690 14101
11688 14102 11686 14105 11683 14105 11682 14102 11681 14099 23 N F
11683 12428 11681 12425 11684 12424 11681 12421 11684 12419 11687 12419
11686 12422 11686 12426 11683 12428 9 N F
11688 12654 11681 12652 11685 12651 11688 12648 11689 12646 11690 12644
11692 12640 11693 12640 11697 12640 11701 12638 11700 12634 11700 12629
11704 12629 11704 12629 11707 12627 11711 12630 11713 12633 11718 12634
11719 12630 11722 12627 11721 12623 11725 12621 11728 12621 11730 12621
11734 12621 11736 12625 11735 12629 11735 12634 11733 12637 11731 12641
11733 12644 11729 12646 11727 12650 11723 12652 11720 12649 11715 12649
11712 12652 11710 12656 11705 12657 11702 12654 11698 12656 11693 12657
11692 12653 11688 12654 44 N F
11682 14173 11681 14167 11684 14167 11686 14169 11686 14173 11686 14176
11689 14178 11686 14180 11683 14179 11683 14176 11682 14173 11 N F
11684 12943 11682 12938 11684 12935 11685 12934 11686 12933 11687 12930
11690 12931 11692 12933 11692 12937 11691 12940 11689 12943 11687 12945
11684 12943 13 N F
11686 12495 11682 12495 11683 12493 11684 12492 11686 12490 11686 12486
11688 12484 11689 12485 11690 12485 11692 12488 11693 12491 11696 12492
11698 12495 11698 12498 11695 12496 11694 12499 11697 12501 11694 12502
11692 12505 11689 12504 11688 12501 11687 12498 11686 12495 23 N F
11685 12892 11682 12891 11682 12887 11682 12884 11685 12886 11686 12889
11685 12892 7 N F
11690 12786 11683 12781 11686 12778 11690 12780 11695 12781 11699 12781
11703 12780 11707 12782 11707 12777 11702 12778 11702 12774 11702 12770
11703 12765 11701 12761 11699 12757 11698 12753 11702 12755 11706 12756
11707 12760 11706 12764 11709 12765 11710 12765 11714 12768 11718 12766
11717 12770 11721 12771 11720 12776 11720 12780 11718 12783 11713 12784
11711 12788 11710 12792 11707 12795 11703 12793 11700 12795 11695 12796
11695 12791 11691 12790 11690 12786 39 N F
11684 13483 11683 13481 11684 13478 11687 13478 11687 13478 11687 13480
11688 13483 11689 13485 11686 13485 11684 13483 10 N F

11687 12608 11685 12603 11687 12601 11690 12602 11690 12606 11692 12607
11693 12608 11693 12611 11690 12610 11687 12608 10 N F
11687 12397 11685 12391 11689 12393 11689 12388 11693 12387 11698 12387
11702 12387 11704 12385 11705 12383 11704 12388 11706 12391 11707 12387
11710 12383 11712 12387 11716 12388 11720 12386 11720 12391 11718 12395
11717 12399 11718 12404 11713 12403 11709 12402 11705 12400 11701 12401
11697 12404 11693 12406 11692 12402 11687 12403 11691 12400 11687 12397
30 N F
11687 12640 11685 12635 11689 12634 11691 12637 11691 12640 11689 12643
11687 12640 7 N F
11689 12894 11687 12892 11690 12892 11693 12892 11695 12895 11697 12897
11694 12898 11692 12895 11689 12894 9 N F
11688 14169 11687 14165 11690 14164 11693 14165 11696 14166 11699 14165
11702 14163 11705 14163 11705 14166 11704 14169 11705 14172 11705 14176
11702 14178 11700 14180 11697 14178 11696 14175 11693 14173 11691 14172
11688 14169 19 N F
11695 12667 11687 12665 11691 12664 11693 12663 11696 12663 11699 12660
11703 12662 11707 12662 11711 12659 11713 12656 11716 12652 11720 12654
11724 12655 11726 12659 11725 12664 11723 12668 11726 12670 11724 12674
11720 12674 11719 12679 11716 12681 11714 12677 11716 12673 11712 12673
11708 12671 11704 12669 11700 12668 11695 12667 28 N F
11690 13944 11689 13939 11692 13938 11694 13936 11696 13939 11695 13942
11693 13945 11690 13944 8 N F
11692 12363 11692 12358 11695 12358 11698 12356 11700 12358 11703 12358
11707 12357 11708 12360 11709 12363 11712 12364 11710 12366 11709 12369
11706 12371 11704 12372 11704 12369 11704 12366 11702 12364 11699 12363
11696 12362 11692 12363 20 N F
11698 14154 11693 14153 11696 14150 11700 14149 11702 14145 11701 14140
11706 14140 11705 14144 11707 14148 11709 14151 11707 14155 11702 14154
11698 14154 13 N F
11699 12813 11693 12808 11695 12808 11697 12808 11698 12804 11702 12802
11706 12804 11709 12801 11712 12797 11714 12801 11714 12806 11715 12802
11716 12797 11719 12801 11719 12805 11718 12809 11716 12813 11712 12814
11708 12813 11703 12814 11699 12813 21 N F
11701 12624 11698 12619 11700 12617 11704 12617 11706 12619 11707 12622
11704 12624 11701 12624 8 N F
11703 14032 11701 14026 11704 14022 11705 14018 11706 14014 11706 14010
11710 14012 11711 14013 11713 14015 11715 14012 11715 14007 11719 14009
11721 14013 11724 14016 11725 14020 11727 14024 11731 14025 11731 14030
11730 14034 11729 14038 11725 14039 11720 14039 11716 14039 11712 14039
11711 14035 11707 14033 11703 14032 27 N F
11706 12837 11704 12832 11708 12831 11711 12828 11710 12824 11712 12823
11714 12823 11716 12819 11720 12818 11724 12820 11727 12816 11731 12817

11735 12818 11738 12816 11739 12815 11743 12817 11744 12813 11740 12811
11741 12807 11744 12806 11745 12806 11747 12802 11748 12800 11750 12799
11750 12803 11754 12801 11755 12805 11758 12808 11755 12811 11751 12813
11752 12817 11750 12821 11747 12824 11744 12820 11739 12821 11737 12825
11733 12827 11733 12831 11736 12835 11739 12832 11742 12836 11741 12840
11737 12840 11735 12836 11733 12841 11730 12844 11727 12841 11726 12837
11721 12837 11718 12840 11716 12844 11712 12842 11708 12841 11706 12837
54 N F
11711 13919 11708 13916 11710 13914 11710 13911 11711 13907 11714 13906
11717 13907 11720 13909 11722 13910 11721 13913 11720 13916 11718 13918
11716 13916 11713 13916 11711 13919 15 N F
11711 14135 11709 14130 11712 14128 11715 14127 11716 14130 11716 14134
11714 14136 11711 14135 8 N F
11711 12593 11711 12588 11715 12588 11718 12584 11716 12580 11718 12576
11716 12572 11720 12572 11724 12574 11728 12575 11730 12579 11734 12580
11738 12578 11741 12580 11744 12584 11748 12584 11750 12588 11746 12589
11742 12590 11743 12594 11741 12598 11737 12601 11734 12598 11732 12594
11727 12594 11730 12598 11731 12602 11728 12605 11724 12603 11721 12599
11717 12598 11716 12594 11711 12593 33 N F
11714 12470 11711 12463 11715 12457 11720 12453 11724 12447 11729 12443
11728 12450 11726 12456 11729 12462 11730 12456 11735 12451 11742 12450
11739 12444 11735 12438 11738 12432 11742 12437 11746 12432 11753 12431
11759 12431 11763 12434 11765 12435 11772 12438 11766 12442 11773 12441
11774 12442 11780 12443 11784 12449 11791 12450 11791 12457 11797 12460
11804 12460 11804 12467 11809 12467 11811 12467 11812 12474 11818 12470
11819 12463 11824 12467 11822 12474 11826 12476 11828 12478 11830 12484
11834 12490 11831 12496 11835 12501 11830 12506 11834 12511 11835 12518
11837 12525 11831 12529 11827 12534 11822 12538 11816 12543 11810 12540
11809 12533 11803 12537 11801 12544 11802 12550 11799 12556 11792 12559
11786 12561 11779 12562 11772 12563 11771 12556 11774 12550 11780 12549
11787 12551 11785 12545 11787 12538 11783 12533 11779 12528 11776 12521
11769 12523 11769 12530 11766 12536 11762 12531 11765 12524 11759 12521
11762 12515 11763 12508 11768 12503 11774 12506 11771 12499 11765 12501
11768 12495 11766 12488 11765 12481 11764 12475 11761 12469 11761 12476
11760 12482 11760 12489 11763 12495 11760 12501 11760 12508 11753 12510
11752 12516 11746 12513 11745 12506 11749 12500 11746 12494 11741 12499
11734 12498 11731 12492 11724 12490 11723 12483 11727 12478 11721 12481
11716 12476 11714 12470 110 N F
11713 14150 11712 14145 11713 14141 11714 14141 11717 14139 11721 14139
11723 14143 11725 14143 11727 14143 11730 14147 11734 14148 11739 14147
11743 14148 11747 14150 11748 14154 11750 14158 11750 14162 11747 14165
11745 14169 11741 14171 11737 14172 11733 14172 11729 14173 11725 14175
11721 14177 11717 14175 11715 14171 11716 14167 11715 14163 11713 14159

11715 14154 11713 14150 32 N F
11716 13521 11715 13519 11715 13515 11716 13515 11718 13515 11720 13517
11723 13515 11725 13517 11727 13519 11727 13521 11725 13523 11724 13526
11724 13528 11723 13530 11720 13531 11720 13528 11719 13525 11718 13523
11716 13521 19 N F
11722 13109 11717 13107 11720 13104 11722 13100 11725 13102 11729 13104
11732 13107 11735 13111 11737 13114 11741 13116 11743 13120 11745 13124
11743 13127 11739 13130 11739 13126 11736 13123 11732 13123 11728 13125
11730 13121 11726 13121 11726 13116 11723 13114 11722 13109 23 N F
11730 12615 11729 12610 11732 12609 11735 12609 11738 12610 11740 12613
11742 12610 11744 12613 11745 12616 11743 12618 11745 12620 11747 12623
11744 12624 11740 12623 11738 12621 11736 12618 11736 12615 11733 12616
11730 12615 19 N F
11731 12771 11730 12768 11732 12765 11734 12762 11736 12761 11738 12764
11738 12767 11738 12770 11737 12773 11738 12777 11735 12776 11732 12774
11731 12771 13 N F
11733 13546 11732 13541 11733 13538 11735 13537 11737 13539 11739 13539
11740 13539 11743 13538 11745 13538 11746 13538 11748 13537 11751 13538
11753 13537 11756 13537 11758 13535 11759 13535 11761 13535 11764 13535
11766 13537 11765 13539 11762 13539 11760 13538 11758 13540 11755 13541
11756 13543 11755 13546 11753 13548 11753 13550 11750 13550 11747 13550
11745 13549 11744 13546 11745 13544 11743 13544 11740 13545 11738 13546
11735 13547 11733 13546 38 N F
11746 13257 11742 13255 11745 13254 11748 13254 11746 13257 5 N F
11743 12748 11742 12742 11744 12741 11746 12739 11750 12738 11752 12741
11757 12742 11756 12746 11757 12750 11753 12752 11749 12752 11744 12752
11743 12748 13 N F
11752 14043 11748 14044 11750 14041 11753 14038 11757 14035 11758 14030
11759 14026 11760 14022 11760 14018 11756 14016 11753 14014 11754 14009
11758 14010 11756 14006 11754 14002 11751 13999 11749 13994 11753 13997
11755 14000 11759 13999 11762 13995 11765 13998 11766 14002 11767 14006
11768 14010 11772 14009 11777 14009 11777 14013 11780 14016 11784 14017
11786 14021 11785 14025 11782 14029 11781 14033 11777 14035 11773 14034
11769 14036 11766 14039 11762 14038 11761 14042 11764 14046 11764 14050
11762 14046 11759 14042 11755 14040 11752 14043 46 N F
11751 12791 11748 12792 11750 12788 11752 12785 11752 12781 11752 12778
11750 12775 11753 12774 11756 12775 11760 12775 11762 12777 11761 12780
11761 12783 11759 12786 11757 12788 11755 12791 11751 12791 17 N F
11750 12709 11749 12704 11754 12704 11758 12703 11762 12701 11766 12701
11769 12705 11770 12709 11775 12710 11779 12709 11783 12710 11782 12714
11786 12713 11788 12709 11792 12709 11797 12709 11798 12705 11803 12704
11800 12700 11797 12697 11799 12693 11803 12692 11806 12696 11810 12694
11812 12698 11812 12702 11813 12706 11814 12710 11813 12715 11809 12716

11805 12719 11801 12718 11797 12718 11792 12717 11788 12718 11791 12722
11787 12724 11790 12726 11791 12731 11795 12729 11797 12733 11801 12733
11802 12737 11798 12738 11796 12741 11792 12742 11787 12741 11783 12742
11781 12746 11777 12746 11773 12745 11770 12741 11773 12738 11776 12735
11780 12733 11781 12729 11777 12726 11777 12721 11773 12722 11774 12718
11770 12715 11768 12719 11766 12715 11764 12711 11760 12711 11756 12711
11752 12713 11750 12709 68 N F
11754 13255 11751 13254 11754 13254 11757 13254 11758 13254 11760 13255
11762 13257 11759 13259 11756 13258 11754 13255 10 N F
11758 13238 11756 13234 11758 13232 11760 13229 11763 13231 11763 13234
11761 13237 11758 13238 8 N F
11759 12374 11758 12370 11760 12367 11763 12368 11761 12370 11762 12373
11764 12376 11765 12379 11763 12382 11761 12380 11759 12377 11759 12374
12 N F
11764 12772 11759 12770 11762 12767 11764 12763 11767 12760 11768 12756
11772 12754 11776 12755 11776 12759 11774 12763 11771 12767 11768 12770
11764 12772 13 N F
11762 7073 11761 7064 11763 7061 11766 7057 11771 7055 11775 7054
11779 7055 11783 7058 11787 7059 11791 7057 11796 7057 11800 7058
11804 7060 11806 7064 11805 7068 11804 7073 11801 7076 11800 7081
11796 7083 11791 7084 11787 7086 11783 7088 11779 7090 11774 7090
11771 7087 11771 7083 11771 7078 11771 7074 11766 7072 11762 7073
30 N F
11770 13240 11765 13239 11769 13238 11769 13235 11772 13236 11771 13233
11774 13234 11777 13234 11779 13237 11777 13239 11779 13241 11778 13244
11777 13247 11774 13248 11771 13247 11770 13244 11774 13243 11776 13241
11774 13238 11771 13237 11770 13240 21 N F
11770 13388 11768 13386 11769 13384 11772 13382 11774 13382 11777 13381
11776 13384 11775 13386 11773 13388 11770 13388 10 N F
11776 13263 11771 13262 11774 13261 11774 13261 11777 13261 11781 13260
11779 13263 11776 13263 8 N F
11780 13233 11778 13230 11781 13228 11784 13228 11786 13230 11789 13232
11790 13235 11788 13238 11786 13235 11785 13232 11782 13232 11783 13234
11781 13236 11780 13233 14 N F
11792 13250 11789 13247 11791 13245 11792 13242 11793 13239 11796 13238
11795 13241 11797 13242 11798 13243 11800 13240 11803 13240 11807 13240
11809 13242 11807 13244 11804 13246 11801 13247 11797 13247 11795 13250
11792 13250 19 N F
11797 13204 11793 13201 11796 13200 11797 13196 11799 13194 11801 13196
11801 13200 11800 13203 11797 13204 9 N F
11802 12451 11800 12446 11803 12444 11806 12443 11805 12447 11808 12446
11811 12448 11813 12450 11813 12453 11811 12455 11808 12454 11810 12456
11809 12459 11806 12458 11804 12456 11801 12455 11802 12451 17 N F

11812 11881 11806 11877 11807 11870 11809 11864 11807 11857 11807 11848
11811 11843 11815 11838 11820 11832 11825 11828 11826 11828 11832 11825
11838 11824 11844 11820 11848 11815 11853 11810 11858 11805 11864 11802
11871 11802 11871 11809 11872 11816 11876 11822 11881 11827 11887 11830
11894 11828 11900 11828 11907 11828 11914 11830 11920 11834 11926 11835
11932 11831 11939 11833 11946 11835 11950 11830 11957 11829 11963 11833
11970 11833 11974 11838 11981 11839 11988 11840 11995 11840 12001 11843
12007 11840 12014 11842 12019 11847 12024 11851 12028 11857 12034 11860
12040 11862 12047 11862 12054 11864 12060 11867 12067 11869 12073 11870
12078 11875 12083 11880 12087 11886 12094 11886 12101 11887 12107 11889
12114 11892 12117 11897 12116 11904 12121 11909 12125 11914 12126 11921
12128 11928 12131 11934 12137 11937 12141 11943 12141 11950 12143 11956
12144 11963 12149 11959 12147 11965 12146 11972 12149 11978 12156 11977
12151 11982 12146 11977 12145 11984 12139 11987 12135 11992 12138 11998
12136 12005 12133 12011 12127 12014 12120 12015 12114 12012 12108 12015
12101 12018 12096 12022 12092 12028 12086 12027 12079 12028 12072 12028
12065 12027 12059 12024 12052 12024 12046 12022 12040 12019 12033 12017
12026 12016 12020 12012 12014 12014 12008 12010 12002 12007 11995 12004
11989 12002 11983 11999 11976 11998 11970 11994 11964 11991 11958 11987
11952 11989 11945 11990 11941 11985 11934 11984 11929 11979 11923 11975
11917 11974 11911 11970 11905 11966 11899 11969 11894 11964 11887 11962
11882 11957 11876 11954 11869 11954 11865 11948 11862 11942 11855 11939
11851 11935 11846 11930 11845 11923 11842 11917 11837 11912 11832 11907
11827 11903 11823 11897 11819 11892 11813 11888 11812 11881 143 N F
11809 13260 11807 13259 11809 13257 11811 13258 11812 13260 11809 13260
6 N F
11820 13319 11817 13316 11819 13315 11822 13314 11824 13316 11822 13319
11820 13319 7 N F
11822 13369 11819 13366 11821 13365 11822 13365 11824 13365 11827 13364
11827 13366 11827 13369 11826 13371 11824 13371 11822 13369 11 N F
11826 7122 11819 7120 11823 7114 11827 7109 11830 7102 11835 7096
11839 7091 11842 7084 11835 7081 11835 7074 11837 7067 11834 7061
11828 7058 11825 7051 11832 7048 11837 7043 11840 7037 11836 7031
11825 7031 11829 7025 11834 7020 11840 7015 11847 7013 11848 7021
11850 7014 11855 7009 11862 7012 11869 7014 11876 7016 11882 7019
11889 7021 11896 7022 11903 7023 11910 7024 11917 7023 11924 7025
11931 7027 11937 7022 11933 7028 11928 7033 11924 7039 11918 7044
11911 7044 11904 7046 11898 7048 11894 7055 11887 7057 11880 7056
11874 7058 11867 7060 11863 7066 11865 7073 11860 7078 11853 7081
11849 7087 11848 7094 11844 7100 11839 7104 11834 7110 11829 7115
11826 7122 61 N F
11826 12226 11825 12222 11828 12220 11832 12217 11833 12221 11831 12225
11826 12226 7 N F

11831 13330 11828 13328 11830 13327 11833 13326 11835 13324 11837 13325
11840 13326 11841 13328 11839 13329 11836 13330 11833 13330 11831 13330
12 N F
11840 12220 11834 12216 11838 12215 11841 12211 11843 12207 11847 12207
11845 12211 11847 12215 11851 12216 11852 12221 11848 12222 11844 12219
11840 12220 13 N F
11847 13324 11843 13322 11845 13322 11848 13321 11849 13319 11852 13319
11850 13320 11849 13323 11847 13324 9 N F
11872 13239 11870 13234 11872 13231 11875 13231 11878 13233 11878 13236
11879 13239 11877 13242 11874 13241 11872 13239 10 N F
11906 12354 11904 12351 11907 12350 11908 12353 11910 12356 11914 12356
11914 12359 11916 12362 11914 12364 11911 12366 11910 12363 11908 12360
11907 12357 11906 12354 14 N F
11917 12345 11913 12342 11916 12338 11914 12331 11917 12327 11918 12323
11923 12322 11927 12322 11931 12324 11935 12326 11938 12329 11942 12332
11942 12336 11946 12337 11948 12341 11951 12343 11952 12347 11955 12345
11955 12345 11957 12341 11958 12345 11962 12343 11966 12344 11966 12348
11962 12348 11957 12349 11953 12351 11949 12351 11945 12349 11940 12350
11936 12349 11935 12353 11931 12355 11926 12354 11923 12351 11920 12348
11917 12345 37 N F
11933 12365 11930 12361 11931 12361 11935 12360 11937 12357 11940 12354
11945 12353 11949 12353 11954 12353 11950 12355 11949 12360 11952 12362
11954 12358 11959 12358 11961 12362 11958 12365 11962 12368 11965 12370
11962 12374 11960 12377 11957 12380 11953 12382 11949 12381 11944 12382
11940 12380 11937 12383 11934 12386 11934 12381 11937 12378 11933 12376
11933 12372 11937 12373 11935 12370 11933 12365 34 N F
11949 12392 11949 12386 11952 12386 11955 12388 11958 12386 11960 12389
11963 12389 11962 12392 11961 12395 11958 12396 11955 12396 11952 12394
11949 12392 13 N F
11955 12322 11952 12320 11955 12317 11952 12312 11956 12313 11959 12313
11962 12312 11962 12315 11959 12317 11958 12320 11955 12322 11 N F
11963 12473 11961 12468 11963 12464 11963 12460 11964 12456 11965 12452
11964 12447 11968 12444 11967 12440 11971 12442 11974 12439 11978 12437
11978 12437 11982 12435 11985 12438 11988 12440 11991 12437 11995 12434
11992 12431 11987 12431 11987 12427 11991 12424 11995 12425 11999 12426
12003 12426 12007 12423 12011 12422 12010 12426 12008 12430 12013 12429
12015 12425 12020 12425 12024 12425 12026 12425 12028 12424 12026 12428
12022 12430 12023 12434 12025 12438 12021 12440 12017 12439 12013 12439
12014 12443 12012 12447 12008 12449 12007 12453 12006 12457 12005 12461
12004 12466 12003 12470 12001 12474 11997 12476 11993 12478 11990 12480
11985 12482 11981 12483 11977 12482 11974 12478 11970 12479 11966 12477
11963 12473 61 N F
11974 12344 11969 12341 11972 12337 11974 12333 11975 12329 11978 12332

11981 12328 11983 12324 11987 12323 11989 12326 11989 12331 11991 12334
11990 12339 11994 12341 11993 12345 11990 12343 11989 12347 11987 12351
11982 12352 11983 12356 11987 12358 11991 12359 11993 12363 11990 12365
11986 12367 11982 12368 11978 12371 11974 12368 11974 12364 11970 12364
11971 12359 11975 12360 11977 12356 11977 12352 11974 12349 11974 12344
36 N F
12013 12478 12011 12473 12014 12476 12019 12475 12023 12476 12021 12472
12020 12468 12019 12463 12023 12461 12026 12464 12026 12469 12029 12472
12030 12476 12034 12478 12039 12478 12043 12480 12043 12484 12045 12487
12050 12486 12050 12486 12054 12484 12058 12485 12062 12484 12062 12489
12059 12492 12055 12494 12055 12498 12050 12496 12046 12497 12050 12500
12051 12504 12047 12507 12046 12511 12041 12510 12037 12508 12034 12506
12029 12504 12025 12504 12023 12501 12019 12498 12018 12493 12016 12490
12017 12486 12015 12482 12013 12478 45 N F
12018 12451 12014 12449 12019 12448 12023 12446 12027 12446 12031 12444
12034 12441 12037 12444 12040 12447 12044 12447 12049 12448 12052 12445
12054 12442 12054 12437 12057 12434 12061 12436 12063 12440 12066 12443
12065 12447 12069 12446 12070 12441 12074 12442 12075 12446 12074 12450
12075 12454 12079 12454 12080 12458 12079 12462 12075 12459 12071 12460
12068 12463 12064 12462 12059 12462 12056 12460 12054 12463 12050 12466
12047 12463 12044 12460 12044 12455 12041 12453 12037 12451 12034 12454
12030 12457 12028 12453 12026 12449 12022 12451 12018 12451 47 N F
12023 12380 12022 12377 12023 12374 12022 12368 12025 12366 12027 12364
12029 12366 12029 12370 12031 12372 12032 12375 12030 12378 12029 12381
12026 12382 12023 12380 14 N F
12065 12494 12060 12493 12063 12491 12066 12487 12063 12483 12062 12479
12063 12475 12067 12478 12068 12482 12072 12484 12072 12479 12072 12475
12073 12471 12078 12470 12081 12468 12082 12468 12085 12468 12090 12470
12091 12474 12095 12476 12099 12476 12101 12480 12098 12483 12097 12488
12098 12492 12100 12496 12098 12500 12096 12496 12094 12492 12092 12488
12088 12490 12084 12488 12081 12492 12077 12491 12073 12492 12069 12495
12068 12491 12065 12494 38 N F
12070 12376 12067 12365 12071 12360 12077 12362 12083 12358 12090 12357
12092 12357 12096 12356 12101 12361 12101 12368 12101 12375 12096 12379
12094 12386 12089 12390 12082 12392 12076 12388 12073 12382 12070 12376
18 N F
12110 12268 12107 12262 12109 12258 12113 12258 12117 12259 12120 12263
12118 12267 12114 12269 12110 12268 9 N F
12110 12459 12108 12456 12108 12452 12109 12447 12113 12446 12114 12443
12114 12442 12118 12444 12122 12446 12125 12449 12130 12450 12131 12454
12135 12456 12133 12460 12126 12464 12122 12463 12118 12463 12113 12462
12110 12459 19 N F
12125 12474 12121 12476 12123 12472 12126 12470 12127 12470 12131 12471

12135 12470 12139 12468 12135 12465 12140 12465 12142 12461 12143 12465
12148 12466 12151 12463 12147 12460 12144 12458 12145 12454 12146 12449
12148 12453 12150 12457 12153 12460 12156 12463 12160 12463 12161 12467
12157 12467 12156 12471 12160 12469 12164 12470 12167 12466 12172 12467
12169 12471 12166 12474 12165 12478 12161 12478 12157 12479 12154 12477
12150 12474 12146 12473 12147 12477 12145 12481 12141 12483 12139 12486
12136 12490 12137 12486 12133 12485 12129 12483 12132 12480 12129 12476
12125 12474 49 N F
12136 12530 12129 12528 12133 12525 12136 12522 12136 12521 12137 12518
12137 12513 12140 12510 12143 12507 12145 12511 12144 12515 12148 12514
12147 12509 12149 12505 12150 12501 12152 12505 12154 12509 12155 12513
12154 12517 12152 12521 12150 12525 12148 12529 12144 12531 12140 12532
12136 12530 25 N F
12165 12540 12160 12539 12163 12536 12167 12537 12170 12534 12166 12531
12165 12527 12169 12528 12172 12524 12176 12522 12179 12520 12183 12518
12187 12520 12191 12520 12194 12523 12198 12524 12203 12523 12207 12523
12211 12523 12215 12524 12219 12528 12219 12532 12219 12536 12221 12540
12222 12545 12224 12549 12223 12553 12220 12556 12217 12559 12212 12558
12209 12556 12204 12555 12200 12557 12196 12554 12193 12551 12191 12548
12186 12548 12184 12545 12180 12546 12175 12547 12174 12551 12169 12552
12170 12548 12168 12544 12165 12540 45 N F
12236 12411 12234 12405 12237 12407 12242 12406 12246 12408 12250 12407
12250 12412 12252 12415 12251 12419 12250 12424 12250 12428 12245 12427
12241 12425 12242 12421 12238 12420 12237 12416 12236 12411 17 N F
12274 12325 12269 12321 12271 12317 12272 12313 12276 12311 12277 12306
12279 12303 12283 12300 12287 12300 12291 12299 12294 12303 12290 12302
12286 12304 12284 12308 12281 12311 12279 12315 12277 12319 12279 12323
12278 12328 12274 12325 20 N F
12288 12326 12281 12324 12285 12321 12286 12317 12290 12315 12294 12314
12295 12318 12292 12320 12292 12324 12288 12326 10 N F
12313 12589 12310 12584 12314 12582 12318 12582 12319 12578 12322 12575
12318 12574 12315 12571 12318 12567 12320 12564 12325 12563 12328 12567
12332 12567 12336 12569 12340 12568 12342 12572 12342 12576 12339 12579
12335 12581 12330 12581 12326 12581 12330 12583 12327 12587 12323 12588
12326 12591 12329 12595 12328 12599 12323 12599 12319 12600 12316 12597
12314 12593 12313 12589 32 N F
12323 12552 12321 12547 12326 12547 12330 12548 12334 12549 12337 12545
12332 12545 12328 12546 12329 12542 12333 12540 12338 12541 12342 12539
12342 12535 12340 12531 12344 12528 12347 12532 12350 12534 12354 12536
12358 12537 12362 12540 12363 12544 12361 12548 12360 12552 12355 12553
12353 12557 12349 12559 12346 12563 12345 12558 12341 12557 12337 12554
12334 12557 12329 12558 12326 12560 12321 12559 12325 12556 12323 12552
36 N F

12400 12629 12398 12625 12398 12619 12400 12623 12403 12619 12405 12616
12410 12616 12413 12619 12417 12620 12418 12625 12421 12621 12425 12622
12429 12625 12425 12627 12422 12630 12419 12627 12414 12627 12412 12631
12408 12632 12404 12630 12400 12629 21 N F
12433 12610 12426 12609 12429 12606 12434 12604 12437 12602 12441 12604
12441 12599 12444 12597 12445 12593 12450 12591 12454 12593 12457 12591
12461 12589 12463 12585 12467 12585 12471 12586 12474 12589 12472 12593
12470 12597 12466 12597 12461 12597 12465 12599 12465 12603 12462 12606
12458 12609 12457 12613 12455 12617 12451 12619 12447 12620 12442 12619
12439 12616 12436 12613 12433 12610 33 N F
12458 12794 12457 12787 12458 12780 12461 12774 12465 12769 12470 12764
12472 12758 12477 12753 12480 12747 12477 12740 12475 12734 12477 12733
12482 12732 12484 12725 12489 12721 12496 12720 12503 12721 12509 12724
12509 12726 12511 12731 12507 12737 12505 12743 12511 12747 12516 12751
12523 12752 12524 12746 12524 12739 12525 12732 12526 12731 12528 12726
12530 12719 12536 12718 12539 12718 12543 12718 12545 12720 12549 12722
12556 12723 12560 12717 12566 12720 12571 12721 12573 12721 12569 12726
12566 12732 12570 12737 12574 12743 12572 12750 12571 12757 12570 12763
12569 12770 12572 12776 12572 12783 12566 12787 12560 12785 12555 12789
12542 12787 12536 12784 12533 12778 12527 12775 12522 12770 12517 12774
12515 12781 12509 12783 12502 12783 12503 12790 12498 12795 12495 12801
12496 12808 12501 12812 12504 12818 12505 12825 12503 12831 12500 12837
12494 12841 12487 12842 12481 12839 12476 12835 12472 12829 12472 12822
12476 12817 12470 12815 12463 12813 12463 12806 12463 12799 12458 12794
84 N F
12477 12559 12473 12555 12478 12555 12475 12552 12475 12547 12479 12547
12483 12544 12485 12548 12485 12552 12484 12557 12485 12561 12485 12565
12481 12567 12482 12572 12477 12572 12476 12568 12477 12564 12477 12559
18 N F
12485 8029 12477 8021 12482 8016 12488 8014 12496 8013 12503 8013
12508 8008 12511 8001 12515 7996 12521 7992 12527 7989 12534 7988
12532 7995 12529 8001 12529 8009 12533 8014 12535 8021 12529 8024
12526 8031 12519 8033 12515 8039 12509 8042 12502 8041 12496 8038
12492 8032 12485 8029 26 N F
12525 12806 12523 12802 12524 12798 12526 12794 12528 12790 12532 12789
12536 12788 12541 12788 12545 12789 12549 12790 12550 12794 12554 12796
12558 12794 12562 12792 12566 12793 12570 12791 12574 12792 12578 12789
12582 12789 12581 12793 12577 12796 12573 12797 12570 12800 12569 12804
12571 12808 12570 12813 12566 12815 12562 12817 12558 12819 12560 12823
12556 12824 12552 12821 12551 12817 12550 12813 12550 12808 12548 12804
12545 12807 12545 12811 12545 12816 12546 12820 12549 12824 12550 12828
12551 12832 12551 12836 12547 12835 12542 12833 12537 12829 12535 12825
12534 12821 12532 12817 12529 12814 12528 12810 12525 12806 53 N F

12524 8161 12524 8160 12524 8159 12524 8159 12525 8159 12526 8159
12526 8159 12526 8159 12526 8160 12526 8160 12526 8161 12524 8161
12 N F
12536 8016 12534 8013 12536 8013 12538 8014 12540 8015 12540 8017
12539 8019 12537 8018 12536 8016 9 N F
12543 8019 12542 8015 12545 8016 12546 8018 12545 8020 12543 8019
6 N F
12548 8016 12547 8013 12549 8015 12551 8016 12553 8017 12555 8017
12557 8019 12559 8020 12560 8022 12562 8023 12564 8023 12566 8023
12568 8024 12569 8026 12571 8027 12574 8028 12571 8028 12569 8028
12568 8026 12566 8025 12564 8025 12561 8024 12559 8024 12558 8022
12556 8021 12554 8019 12552 8018 12550 8018 12548 8016 29 N F
12619 12808 12616 12801 12621 12799 12625 12798 12624 12793 12628 12793
12631 12796 12632 12800 12635 12803 12640 12804 12644 12804 12643 12808
12639 12810 12635 12810 12631 12813 12627 12812 12623 12810 12619 12808
18 N F
12639 12792 12633 12789 12637 12788 12638 12784 12642 12784 12645 12781
12649 12781 12653 12779 12654 12783 12651 12787 12656 12787 12660 12788
12663 12791 12660 12794 12656 12796 12653 12799 12650 12802 12646 12804
12642 12802 12639 12799 12636 12796 12639 12792 22 N F
12659 12697 12656 12691 12659 12688 12663 12687 12663 12682 12665 12678
12668 12682 12672 12683 12674 12687 12678 12686 12683 12686 12687 12686
12686 12691 12689 12694 12687 12698 12683 12696 12681 12693 12680 12688
12676 12691 12679 12694 12676 12697 12672 12698 12668 12700 12664 12702
12660 12701 12659 12697 26 N F
12696 8054 12693 8048 12696 8045 12698 8041 12702 8043 12706 8045
12702 8047 12699 8051 12700 8055 12696 8054 10 N F
12721 8183 12715 8170 12722 8173 12729 8174 12735 8177 12733 8184
12727 8187 12721 8183 8 N F
12753 12843 12750 12836 12752 12830 12755 12823 12758 12817 12760 12816
12764 12815 12767 12809 12771 12803 12777 12806 12776 12812 12783 12814
12789 12817 12793 12823 12796 12829 12803 12827 12804 12821 12806 12814
12805 12808 12805 12801 12799 12797 12793 12795 12787 12793 12788 12786
12789 12786 12793 12790 12793 12784 12800 12785 12806 12788 12812 12785
12807 12781 12806 12774 12808 12768 12815 12766 12812 12760 12809 12754
12816 12752 12817 12751 12822 12749 12828 12753 12834 12755 12832 12761
12827 12766 12833 12768 12840 12769 12847 12772 12853 12773 12848 12776
12841 12775 12837 12780 12832 12785 12834 12792 12841 12791 12845 12796
12843 12803 12843 12810 12849 12813 12852 12807 12856 12812 12863 12811
12864 12810 12867 12806 12873 12803 12874 12810 12869 12815 12865 12820
12866 12827 12860 12826 12857 12832 12863 12835 12870 12833 12867 12840
12863 12845 12869 12846 12869 12853 12872 12859 12865 12860 12860 12855
12859 12848 12852 12850 12848 12855 12853 12861 12856 12867 12859 12873

12857 12879 12855 12886 12854 12892 12852 12899 12846 12903 12843 12909
12841 12915 12844 12921 12850 12924 12851 12931 12847 12936 12840 12938
12833 12939 12829 12933 12829 12926 12833 12916 12826 12914 12823 12908
12822 12901 12822 12894 12815 12896 12809 12893 12803 12895 12801 12889
12796 12884 12800 12878 12804 12872 12808 12867 12813 12862 12818 12857
12811 12858 12804 12857 12798 12860 12793 12855 12787 12856 12781 12859
12775 12864 12769 12862 12766 12856 12760 12855 12756 12848 12753 12843
126 N F
12752 12793 12751 12787 12754 12785 12757 12782 12756 12778 12756 12773
12757 12773 12761 12773 12765 12772 12769 12775 12772 12777 12775 12780
12775 12780 12775 12785 12772 12788 12768 12787 12765 12791 12765 12795
12762 12798 12762 12803 12759 12800 12760 12804 12756 12804 12752 12801
12754 12797 12758 12798 12761 12795 12758 12797 12757 12793 12754 12789
12754 12794 12751 12797 12752 12793 33 N F
12757 7950 12754 7945 12756 7941 12760 7939 12760 7943 12761 7948
12757 7950 7 N F
12757 7972 12756 7967 12757 7967 12760 7967 12761 7971 12757 7972
6 N F
12763 12892 12762 12887 12764 12884 12765 12879 12763 12875 12766 12872
12768 12868 12773 12869 12770 12872 12773 12875 12770 12878 12768 12882
12771 12885 12773 12888 12771 12892 12767 12893 12763 12892 17 N F
12772 7966 12769 7962 12773 7959 12775 7955 12780 7957 12783 7959
12781 7963 12779 7967 12775 7969 12772 7966 10 N F
12782 7984 12776 7979 12780 7978 12785 7978 12786 7978 12787 7976
12788 7975 12791 7978 12793 7982 12793 7987 12796 7984 12800 7983
12800 7987 12798 7991 12793 7992 12789 7992 12788 7988 12784 7988
12782 7984 19 N F
12786 7955 12779 7953 12782 7949 12786 7950 12786 7955 5 N F
12787 7949 12783 7944 12786 7940 12787 7945 12787 7949 5 N F
12812 7958 12808 7956 12810 7951 12813 7948 12812 7944 12812 7939
12815 7936 12816 7940 12820 7942 12821 7946 12820 7951 12819 7955
12822 7959 12825 7962 12824 7967 12824 7971 12823 7976 12821 7980
12817 7982 12813 7980 12813 7976 12812 7971 12810 7967 12811 7963
12812 7958 25 N F
12859 7979 12857 7973 12861 7974 12862 7978 12864 7982 12864 7987
12860 7983 12859 7979 8 N F
12885 12955 12882 12951 12886 12950 12889 12947 12889 12943 12892 12939
12888 12937 12892 12935 12896 12934 12897 12930 12896 12925 12899 12922
12901 12923 12904 12923 12905 12927 12903 12931 12903 12936 12904 12940
12906 12944 12907 12948 12906 12953 12905 12957 12900 12956 12898 12960
12895 12962 12892 12959 12888 12958 12885 12955 28 N F
12896 11258 12895 11258 12896 11257 12896 11257 12896 11257 12896 11257
12897 11256 12898 11256 12898 11256 12898 11257 12898 11257 12897 11258

12896 11258 13 N F
12897 11261 12896 11259 12897 11259 12898 11258 12899 11257 12899 11255
12898 11254 12898 11252 12898 11250 12897 11249 12899 11248 12899 11250
12900 11251 12901 11252 12903 11252 12903 11253 12901 11255 12901 11256
12901 11258 12902 11259 12903 11260 12903 11262 12901 11263 12900 11262
12899 11261 12897 11261 26 N F
12904 11267 12903 11266 12903 11265 12904 11263 12905 11264 12906 11265
12906 11265 12906 11266 12906 11268 12906 11268 12905 11268 12904 11267
12904 11267 13 N F
12905 11272 12905 11272 12905 11271 12905 11271 12906 11271 12906 11271
12906 11271 12906 11272 12906 11272 12905 11272 10 N F
12907 11274 12906 11274 12906 11272 12907 11272 12907 11272 12907 11272
12908 11272 12909 11272 12909 11272 12909 11274 12909 11275 12909 11275
12909 11275 12907 11275 12907 11275 12907 11274 16 N F
12909 11279 12909 11279 12909 11278 12910 11277 12910 11277 12910 11277
12910 11276 12911 11276 12912 11276 12912 11277 12912 11277 12911 11278
12911 11279 12911 11279 12909 11279 15 N F
12911 11283 12911 11281 12913 11281 12914 11281 12916 11282 12917 11282
12919 11283 12920 11284 12921 11283 12922 11282 12923 11283 12924 11284
12925 11286 12926 11287 12926 11289 12926 11290 12925 11291 12924 11289
12923 11290 12921 11289 12919 11290 12918 11290 12916 11290 12915 11289
12915 11287 12915 11285 12914 11284 12913 11283 12911 11283 29 N F
12963 7992 12960 7986 12961 7979 12966 7974 12969 7967 12974 7963
12979 7958 12980 7950 12979 7943 12979 7936 12978 7929 12977 7922
12982 7918 12989 7915 12993 7921 12999 7925 12999 7925 12999 8033
12995 8030 12988 8028 12984 8022 12982 8015 12978 8009 12979 8002
12973 7997 12977 7991 12982 7986 12976 7990 12970 7993 12963 7992
30 N F
12974 11621 12970 11609 12977 11607 12984 11607 12988 11602 12995 11601
12999 11606 12999 11607 12999 11637 12993 11637 12998 11632 12991 11634
12994 11627 12988 11623 12987 11616 12994 11619 12999 11623 12995 11617
12995 11610 12988 11612 12983 11616 12980 11622 12976 11628 12974 11621
24 N F
12982 8295 12979 8284 12983 8279 12990 8279 12997 8281 12999 8281
12999 8296 12996 8295 12989 8295 12982 8295 10 N F
12984 11702 12982 11695 12982 11688 12987 11683 12984 11676 12986 11670
12991 11665 12987 11659 12990 11653 12993 11647 12999 11644 12999 11638
12999 11700 12995 11705 12989 11707 12984 11702 16 N F
12983 8119 12983 8112 12985 8105 12988 8099 12992 8093 12996 8086
12999 8084 12999 8119 12996 8116 12991 8121 12986 8126 12983 8119
12 N F
1 O
13000 16171 2298 16171 2298 5454 13000 5454 13000 16171 5 P

Ls Gr Gs Lr
2297 5453 13002 16173 Oc
147 O
2297 6810 2297 6785 2297 6785 2297 11860 2297 11860 2297 11831
2297 11695 2297 11559 2297 11423 2297 11288 2297 11152 2297 11016
2297 10881 2297 10745 2297 10609 2297 10474 2297 10338 2297 10202
2297 10067 2297 9931 2297 9795 2297 9660 2297 9524 2297 9388
2297 9252 2297 9117 2297 8981 2297 8845 2297 8710 2297 8574
2297 8438 2297 8303 2297 8167 2297 8031 2297 7896 2297 7760
2297 7624 2297 7489 2297 7353 2297 7217 2297 7081 2297 6946
2297 6810 43 N F
129 O
2297 11966 2297 11860 2297 11860 2297 13110 2297 13110 2297 13052
2297 12916 2297 12780 2297 12645 2297 12509 2297 12373 2297 12238
2297 12102 2297 11966 14 N F
111 O
2297 13187 2297 13110 2297 13110 2297 13543 2297 13543 2297 13459
2297 13323 2297 13187 8 N F
129 O
2297 13594 2297 13543 2297 13543 2297 15617 2297 15617 2297 15494
2297 15358 2297 15223 2297 15087 2297 14951 2297 14816 2297 14680
2297 14544 2297 14409 2297 14273 2297 14137 2297 14002 2297 13866
2297 13730 2297 13594 20 N F
111 O
2297 15630 2297 15617 2297 15617 2297 16016 2297 16016 2297 15901
2297 15765 2297 15630 8 N F
92 O
2297 16037 2297 16016 2297 16016 2297 16085 2297 16085 2297 16037
6 N F
129 O
2297 6785 2297 5453 2297 5453 2297 5453 2297 6785 2297 6785
6 N F
147 O
2297 11860 2297 6785 2297 6785 2297 11860 2297 11860 5 N F
129 O
2297 13110 2297 11860 2297 11860 2297 13110 2297 13110 5 N F
111 O
2297 13543 2297 13110 2297 13110 2297 13543 2297 13543 5 N F
129 O
2297 15617 2297 13543 2297 13543 2297 15616 2297 15617 5 N F
111 O
2297 16016 2297 15617 2297 15616 2297 16016 2297 16016 5 N F
92 O

2297 16085 2297 16016 2297 16016 2297 16085 2297 16085 5 N F
111 O
2297 16173 2297 16085 2297 16085 2297 16172 2297 16173 2297 16173
6 N F
129 O
2489 5453 2297 5453 2297 6785 2433 6739 2497 6674 2568 6569
2704 6605 2839 6610 2903 6539 2975 6412 3026 6539 3110 6647
3246 6671 3256 6674 3381 6750 3424 6810 3441 6946 3428 7081
3517 7195 3652 7100 3764 7081 3788 7077 3913 6946 3899 6810
3822 6674 3788 6619 3754 6539 3788 6460 3923 6510 3972 6539
4058 6674 4059 6676 4156 6810 4166 6946 4059 7067 4049 7081
4059 7098 4194 7107 4255 7081 4276 6946 4330 6936 4351 6946
4465 7017 4601 7026 4722 6946 4736 6928 4785 6810 4872 6688
5007 6716 5050 6674 5103 6539 5143 6448 5182 6403 5278 6363
5359 6403 5356 6539 5325 6674 5414 6729 5482 6674 5549 6602
5629 6539 5667 6403 5685 6386 5779 6267 5820 6225 5956 6159
6091 6266 6094 6267 6227 6304 6338 6403 6362 6444 6395 6403
6385 6267 6362 6152 6359 6132 6345 5996 6362 5962 6432 5860
6498 5825 6540 5860 6633 5948 6769 5970 6793 5996 6824 6132
6904 6216 7040 6251 7074 6267 7175 6328 7311 6312 7399 6267
7446 6227 7488 6132 7446 6074 7391 5996 7311 5918 7242 5860
7192 5725 7175 5628 7040 5602 6904 5627 6769 5605 6642 5589
6769 5551 6904 5518 7040 5559 7175 5562 7311 5563 7446 5554
7582 5531 7717 5472 7811 5453 7650 5453 7458 5453 7267 5453
7076 5453 6885 5453 6694 5453 6503 5453 6312 5453 6120 5453
6074 5453 6091 5485 6139 5589 6091 5655 5956 5693 5820 5720
5817 5725 5691 5860 5685 5865 5675 5860 5554 5725 5549 5714
5447 5589 5488 5453 5356 5453 5165 5453 4973 5453 4782 5453
4591 5453 4400 5453 4209 5453 4018 5453 3827 5453 3635 5453
3444 5453 3253 5453 3062 5453 2871 5453 2680 5453 2489 5453
150 N F
147 O
2433 6739 M 2297 6785 L 2297 11860 L 2429 11966 L 2433 11976 L
2507 12102 L 2497 12238 L 2568 12312 L 2704 12272 L
2712 12238 L 2727 12102 L 2839 12047 L 2975 12087 L
3006 12102 L 3110 12140 L 3246 12107 L 3275 12102 L
3381 12072 L 3381 7225 L 3375 7217 L 3381 7195 L
3427 7217 L 3381 7225 L 3381 12072 L 3517 12036 L
3652 11977 L 3788 11987 L 3923 12075 L 3923 11262 L
3788 11252 L 3681 11152 L 3704 11016 L 3788 10941 L
3923 10931 L 4059 10996 L 4089 11016 L 4080 11152 L
4059 11180 L 3923 11262 L 3923 12075 L 4059 12092 L
4081 12102 L 4194 12212 L 4194 7795 L 4131 7760 L

4194 7721 L 4236 7760 L 4194 7795 L 4194 12212 L
4234 12238 L 4194 12255 L 4059 12254 L 3923 12268 L
3788 12285 L 3652 12308 L 3517 12360 L 3498 12373 L
3381 12468 L 3246 12504 L 3224 12509 L 3110 12589 L
3036 12645 L 3057 12780 L 3095 12916 L 3110 12994 L
3152 13052 L 3166 13187 L 3183 13323 L 3246 13388 L
3355 13459 L 3381 13498 L 3517 13569 L 3609 13459 L
3652 13417 L 3728 13323 L 3721 13187 L 3747 13052 L
3788 12946 L 3842 12916 L 3923 12907 L 3941 12916 L
4005 13052 L 3987 13187 L 3960 13323 L 4023 13459 L
4056 13594 L 4059 13600 L 4152 13730 L 4194 13788 L
4194 13649 L 4074 13594 L 4194 13558 L 4280 13594 L
4194 13649 L 4194 13788 L 4330 13863 L 4330 13507 L
4316 13459 L 4318 13323 L 4330 13307 L 4373 13187 L
4465 13103 L 4465 10997 L 4330 10932 L 4268 10881 L
4330 10822 L 4465 10760 L 4601 10843 L 4643 10881 L
4601 10936 L 4465 10997 L 4465 13103 L 4601 13163 L
4622 13187 L 4601 13254 L 4513 13323 L 4484 13459 L
4465 13488 L 4330 13507 L 4330 13863 L 4333 13866 L
4464 14002 L 4465 14008 L 4503 14137 L 4553 14273 L
4601 14308 L 4736 14326 L 4872 14393 L 4953 14273 L
5007 14182 L 5014 14137 L 5011 14002 L 5007 13994 L
4913 13866 L 4879 13730 L 4983 13594 L 4923 13459 L
4971 13323 L 5007 13314 L 5016 13323 L 5097 13459 L
5143 13520 L 5178 13594 L 5193 13730 L 5249 13866 L
5278 13899 L 5391 14002 L 5414 14026 L 5549 14117 L
5599 14137 L 5685 14190 L 5793 14137 L 5779 14002 L
5685 13940 L 5604 13866 L 5549 13837 L 5414 13776 L
5355 13730 L 5333 13594 L 5414 13467 L 5549 13492 L
5549 11885 L 5496 11831 L 5414 11782 L 5278 11698 L
5149 11831 L 5143 11832 L 5007 11837 L 4973 11831 L
4872 11735 L 4855 11695 L 4824 11559 L 4736 11495 L
4699 11423 L 4736 11390 L 4803 11288 L 4872 11206 L
4944 11152 L 5007 11114 L 5048 11152 L 5039 11288 L
5007 11388 L 4872 11378 L 4788 11423 L 4872 11527 L
5007 11445 L 5143 11448 L 5143 10872 L 5057 10745 L
5007 10699 L 4982 10609 L 4947 10474 L 5007 10448 L
5030 10474 L 5143 10595 L 5167 10609 L 5176 10745 L
5143 10872 L 5143 11448 L 5278 11536 L 5313 11559 L
5414 11609 L 5414 6969 L 5383 6946 L 5414 6886 L
5471 6946 L 5414 6969 L 5414 11609 L 5536 11695 L
5549 11726 L 5589 11695 L 5685 11653 L 5691 11695 L
5685 11768 L 5634 11831 L 5549 11885 L 5549 13492 L

5639 13594 L 5685 13636 L 5685 13336 L 5669 13323 L
5685 13311 L 5694 13323 L 5685 13336 L 5685 13636 L
5807 13730 L 5820 13745 L 5831 13730 L 5862 13594 L
5956 13526 L 6018 13594 L 6091 13639 L 6091 13369 L
5956 13323 L 5956 13323 L 5875 13187 L 5946 13052 L
5956 13044 L 5956 11438 L 5820 11427 L 5801 11423 L
5685 11349 L 5670 11288 L 5615 11152 L 5631 11016 L
5593 10881 L 5549 10867 L 5541 10745 L 5549 10734 L
5624 10609 L 5685 10525 L 5820 10579 L 5840 10609 L
5839 10745 L 5820 10808 L 5693 10881 L 5739 11016 L
5820 11105 L 5956 11138 L 5956 7767 L 5950 7760 L
5916 7624 L 5956 7579 L 5987 7624 L 5963 7760 L
5956 7767 L 5956 11138 L 6091 11146 L 6120 11152 L
6091 11160 L 5973 11288 L 6000 11423 L 5956 11438 L
5956 13044 L 5988 13052 L 6091 13078 L 6134 13187 L
6125 13323 L 6091 13369 L 6091 13639 L 6227 13723 L
6234 13730 L 6303 13866 L 6362 13994 L 6373 14002 L
6362 14036 L 6293 14137 L 6290 14273 L 6227 14343 L
6215 14409 L 6227 14431 L 6292 14544 L 6362 14590 L
6457 14680 L 6498 14729 L 6633 14794 L 6664 14816 L
6769 14841 L 6769 14680 L 6769 14680 L 6633 14565 L
6603 14544 L 6498 14503 L 6362 14441 L 6277 14409 L
6362 14329 L 6400 14273 L 6498 14224 L 6633 14191 L
6741 14273 L 6769 14343 L 6769 10524 L 6715 10474 L
6672 10338 L 6769 10303 L 6904 10292 L 6968 10338 L
6904 10415 L 6866 10474 L 6769 10524 L 6769 14343 L
6795 14409 L 6797 14544 L 6770 14680 L 6769 14680 L
6769 14841 L 6880 14951 L 6904 14987 L 7005 14951 L
7040 14829 L 7175 14855 L 7311 14846 L 7400 14816 L
7446 14801 L 7582 14680 L 7582 7368 L 7569 7353 L
7582 7332 L 7597 7353 L 7582 7368 L 7582 14680 L
7586 14680 L 7717 14664 L 7853 14635 L 7853 6837 L
7789 6810 L 7717 6743 L 7684 6674 L 7717 6599 L
7766 6539 L 7853 6445 L 7948 6539 L 7988 6569 L
8109 6674 L 7988 6770 L 7920 6810 L 7853 6837 L
7853 14635 L 7861 14544 L 7871 14409 L 7988 14289 L
8051 14273 L 8124 14227 L 8259 14210 L 8395 14189 L
8403 14137 L 8410 14002 L 8412 13866 L 8530 13736 L
8552 13730 L 8666 13633 L 8801 13599 L 8822 13594 L
8937 13485 L 8967 13459 L 8937 13444 L 8884 13323 L
8849 13187 L 8849 13052 L 8801 13013 L 8712 12916 L
8672 12780 L 8666 12771 L 8585 12645 L 8595 12509 L
8666 12445 L 8666 8644 L 8602 8574 L 8530 8443 L

8527 8438 L 8530 8397 L 8530 7165 L 8486 7081 L
8530 7040 L 8575 7081 L 8530 7165 L 8530 8397 L
8666 8429 L 8674 8438 L 8759 8574 L 8666 8644 L
8666 12445 L 8763 12373 L 8801 12333 L 8937 12291 L
9055 12238 L 9072 12200 L 9089 12102 L 9103 11966 L
9127 11831 L 9208 11735 L 9241 11695 L 9257 11559 L
9343 11433 L 9350 11423 L 9366 11288 L 9343 11175 L
9319 11152 L 9208 11058 L 9072 11104 L 8937 11057 L
8893 11016 L 8937 10960 L 9072 10887 L 9095 10881 L
9072 10853 L 8937 10846 L 8801 10796 L 8760 10745 L
8801 10696 L 8801 7598 L 8666 7596 L 8585 7489 L
8640 7353 L 8565 7217 L 8666 7153 L 8729 7217 L
8703 7353 L 8801 7422 L 8841 7489 L 8801 7598 L
8801 10696 L 8937 10629 L 8951 10609 L 8982 10474 L
9072 10359 L 9072 9940 L 9070 9931 L 9072 9929 L
9090 9931 L 9072 9940 L 9072 10359 L 9150 10338 L
9208 10260 L 9343 10210 L 9348 10202 L 9343 10198 L
9208 10141 L 9145 10067 L 9208 9979 L 9221 9931 L
9343 9867 L 9448 9931 L 9479 9951 L 9575 9931 L
9614 9893 L 9614 9514 L 9546 9388 L 9614 9288 L
9719 9388 L 9614 9514 L 9614 9893 L 9709 9795 L
9750 9759 L 9808 9660 L 9885 9583 L 9913 9524 L
9885 9455 L 9805 9388 L 9783 9252 L 9750 9210 L
9614 9186 L 9546 9117 L 9552 8981 L 9497 8845 L
9479 8837 L 9343 8752 L 9314 8710 L 9335 8574 L
9343 8558 L 9343 6796 L 9276 6674 L 9235 6539 L
9232 6403 L 9246 6267 L 9343 6205 L 9369 6267 L
9439 6403 L 9479 6491 L 9502 6539 L 9479 6652 L
9472 6674 L 9343 6796 L 9343 8558 L 9479 8506 L
9580 8574 L 9614 8629 L 9700 8710 L 9750 8740 L
9750 7242 L 9725 7217 L 9614 7158 L 9512 7081 L
9479 7054 L 9393 6946 L 9353 6810 L 9479 6679 L
9557 6810 L 9614 6893 L 9741 6946 L 9750 6955 L
9786 6946 L 9750 6931 L 9713 6810 L 9633 6674 L
9633 6539 L 9655 6403 L 9614 6313 L 9571 6267 L
9511 6132 L 9519 5996 L 9562 5860 L 9614 5788 L
9723 5860 L 9750 5902 L 9818 5996 L 9874 6132 L
9885 6230 L 9906 6267 L 9948 6403 L 9989 6539 L
10021 6643 L 10071 6674 L 10102 6810 L 10064 6946 L
10031 7081 L 10021 7097 L 9912 7217 L 9885 7241 L
9750 7242 L 9750 8740 L 9883 8845 L 9885 8848 L
9885 8583 L 9883 8574 L 9885 8572 L 9887 8574 L
9885 8583 L 9885 8848 L 10021 8850 L 10156 8889 L

10156 8772 L 10078 8710 L 10156 8688 L 10194 8710 L
10156 8772 L 10156 8889 L 10243 8845 L 10292 8818 L
10407 8845 L 10427 8849 L 10563 8877 L 10698 8944 L
10790 8981 L 10834 8987 L 10969 9070 L 11007 9117 L
11105 9131 L 11115 9117 L 11105 9104 L 10979 8981 L
10969 8971 L 10852 8845 L 10834 8813 L 10698 8772 L
10631 8710 L 10688 8574 L 10698 8566 L 10707 8574 L
10834 8672 L 10969 8681 L 11105 8679 L 11240 8631 L
11376 8698 L 11511 8653 L 11647 8689 L 11678 8710 L
11782 8755 L 11918 8817 L 11918 8284 L 11782 8169 L
11780 8167 L 11647 8125 L 11511 8054 L 11473 8031 L
11376 7900 L 11372 7896 L 11240 7809 L 11221 7760 L
11234 7624 L 11240 7567 L 11376 7585 L 11511 7569 L
11647 7549 L 11781 7624 L 11782 7625 L 11789 7624 L
11918 7607 L 12045 7624 L 12053 7626 L 12089 7760 L
12102 7896 L 12055 8031 L 12053 8037 L 11963 8167 L
11918 8284 L 11918 8817 L 11930 8845 L 12053 8912 L
12069 8981 L 12091 9117 L 12115 9252 L 12137 9388 L
12141 9524 L 12154 9660 L 12169 9795 L 12189 9911 L
12204 9931 L 12305 10067 L 12324 10098 L 12324 6951 L
12319 6946 L 12324 6925 L 12331 6946 L 12324 6951 L
12324 10098 L 12417 10202 L 12460 10256 L 12556 10338 L
12595 10398 L 12634 10474 L 12731 10584 L 12758 10609 L
12866 10686 L 12948 10745 L 13002 10788 L 13002 8465 L
12989 8438 L 12980 8303 L 12923 8167 L 12927 8031 L
12890 7896 L 12884 7760 L 12866 7711 L 12790 7624 L
12800 7489 L 12731 7399 L 12677 7353 L 12694 7217 L
12601 7081 L 12595 7051 L 12553 6946 L 12460 6889 L
12350 6810 L 12406 6674 L 12324 6639 L 12189 6572 L
12069 6539 L 12100 6403 L 12053 6387 L 11953 6403 L
11918 6409 L 11885 6403 L 11860 6267 L 11782 6187 L
11647 6203 L 11511 6154 L 11448 6132 L 11435 5996 L
11376 5964 L 11337 5996 L 11240 6070 L 11105 6057 L
10969 6028 L 10848 5996 L 10834 5991 L 10815 5996 L
10779 6132 L 10698 6150 L 10582 6132 L 10563 6121 L
10427 6070 L 10292 6029 L 10156 6078 L 10021 6047 L
10000 5996 L 9930 5860 L 9920 5725 L 9921 5589 L
9885 5531 L 9840 5453 L 9752 5453 L 9561 5453 L
9370 5453 L 9263 5453 L 9343 5486 L 9405 5589 L
9417 5725 L 9344 5860 L 9343 5862 L 9208 5943 L
9164 5860 L 9091 5725 L 9105 5589 L 9165 5453 L
8987 5453 L 8796 5453 L 8605 5453 L 8414 5453 L
8223 5453 L 8032 5453 L 7841 5453 L 7811 5453 L

7717 5472 L 7582 5531 L 7446 5554 L 7311 5563 L
7175 5562 L 7040 5559 L 6904 5518 L 6769 5551 L
6642 5589 L 6769 5605 L 6904 5627 L 7040 5602 L
7175 5628 L 7192 5725 L 7242 5860 L 7311 5918 L
7391 5996 L 7446 6074 L 7488 6132 L 7446 6227 L
7399 6267 L 7311 6312 L 7175 6328 L 7074 6267 L
7040 6251 L 6904 6216 L 6824 6132 L 6793 5996 L
6769 5970 L 6633 5948 L 6540 5860 L 6498 5825 L
6432 5860 L 6362 5962 L 6345 5996 L 6359 6132 L
6362 6152 L 6385 6267 L 6395 6403 L 6362 6444 L
6338 6403 L 6227 6304 L 6094 6267 L 6091 6266 L
5956 6159 L 5820 6225 L 5779 6267 L 5685 6386 L
5667 6403 L 5629 6539 L 5549 6602 L 5482 6674 L
5414 6729 L 5325 6674 L 5356 6539 L 5359 6403 L
5278 6363 L 5182 6403 L 5143 6448 L 5103 6539 L
5050 6674 L 5007 6716 L 4872 6688 L 4785 6810 L
4736 6928 L 4722 6946 L 4601 7026 L 4465 7017 L
4351 6946 L 4330 6936 L 4276 6946 L 4255 7081 L
4194 7107 L 4059 7098 L 4049 7081 L 4059 7067 L
4166 6946 L 4156 6810 L 4059 6676 L 4058 6674 L
3972 6539 L 3923 6510 L 3788 6460 L 3754 6539 L
3788 6619 L 3822 6674 L 3899 6810 L 3913 6946 L
3788 7077 L 3764 7081 L 3652 7100 L 3517 7195 L
3428 7081 L 3441 6946 L 3424 6810 L 3381 6750 L
3256 6674 L 3246 6671 L 3110 6647 L 3026 6539 L
2975 6412 L 2903 6539 L 2839 6610 L 2704 6605 L
2568 6569 L 2497 6674 L 2433 6739 L N E
129 O
2297 11860 M 2297 13110 L 2433 13151 L 2473 13187 L 2524 13323 L
2568 13389 L 2610 13323 L 2678 13187 L 2704 13145 L
2734 13052 L 2838 12916 L 2839 12914 L 2841 12916 L
2919 13052 L 2960 13187 L 2946 13323 L 2911 13459 L
2839 13570 L 2820 13594 L 2741 13730 L 2704 13789 L
2653 13866 L 2590 14002 L 2568 14073 L 2529 14137 L
2433 14225 L 2298 14137 L 2345 14002 L 2383 13866 L
2384 13730 L 2402 13594 L 2297 13543 L 2297 15616 L
2433 15596 L 2568 15619 L 2632 15630 L 2704 15638 L
2839 15739 L 2873 15765 L 2975 15802 L 3101 15901 L
3110 15905 L 3110 15331 L 2975 15304 L 2877 15223 L
2839 15170 L 2787 15087 L 2808 14951 L 2839 14910 L
2922 14951 L 2975 14960 L 3089 15087 L 3110 15146 L
3184 15223 L 3110 15331 L 3110 15905 L 3246 15932 L
3246 14970 L 3227 14951 L 3246 14943 L 3252 14951 L

3246 14970 L 3246 15932 L 3381 16010 L 3407 16037 L
3517 16074 L 3517 15672 L 3460 15630 L 3436 15494 L
3463 15358 L 3517 15312 L 3652 15313 L 3747 15358 L
3759 15494 L 3693 15630 L 3652 15666 L 3517 15672 L
3517 16074 L 3649 16172 L 3827 16172 L 4018 16172 L
4209 16172 L 4273 16172 L 4330 16134 L 4330 15758 L
4194 15710 L 4103 15630 L 4109 15494 L 4112 15358 L
4059 15266 L 4009 15223 L 4059 15169 L 4194 15159 L
4330 15125 L 4465 15191 L 4601 15177 L 4736 15152 L
4851 15223 L 4872 15239 L 4966 15358 L 4872 15472 L
4851 15494 L 4736 15568 L 4601 15595 L 4475 15630 L
4465 15636 L 4330 15758 L 4330 16134 L 4380 16037 L
4401 15901 L 4465 15831 L 4601 15791 L 4736 15786 L
4866 15765 L 4872 15761 L 4886 15765 L 5007 15768 L
5143 15775 L 5278 15772 L 5339 15765 L 5414 15723 L
5468 15630 L 5439 15494 L 5414 15454 L 5335 15358 L
5329 15223 L 5354 15087 L 5350 14951 L 5279 14816 L
5278 14815 L 5219 14680 L 5227 14544 L 5218 14409 L
5183 14273 L 5206 14137 L 5278 14055 L 5357 14137 L
5414 14268 L 5417 14273 L 5414 14287 L 5352 14409 L
5414 14455 L 5549 14495 L 5587 14544 L 5685 14660 L
5698 14680 L 5757 14816 L 5739 14951 L 5758 15087 L
5820 15184 L 5852 15223 L 5956 15326 L 5994 15358 L
6091 15440 L 6227 15375 L 6251 15358 L 6227 15344 L
6151 15223 L 6098 15087 L 6091 15079 L 5962 14951 L
5982 14816 L 6091 14702 L 6227 14683 L 6323 14816 L
6362 14944 L 6378 14951 L 6498 15000 L 6633 14993 L
6769 15072 L 6782 15087 L 6903 15223 L 6904 15228 L
7017 15358 L 6904 15431 L 6769 15463 L 6669 15358 L
6633 15322 L 6498 15298 L 6481 15358 L 6498 15373 L
6633 15439 L 6694 15494 L 6656 15630 L 6769 15751 L
6904 15670 L 6993 15630 L 7040 15574 L 7175 15543 L
7278 15630 L 7311 15672 L 7446 15762 L 7450 15765 L
7582 15837 L 7717 15831 L 7853 15869 L 7882 15901 L
7988 15938 L 8124 15904 L 8129 15901 L 8259 15774 L
8281 15765 L 8395 15676 L 8530 15710 L 8573 15765 L
8596 15901 L 8666 15968 L 8801 15919 L 8832 15901 L
8937 15827 L 9045 15765 L 9024 15630 L 8937 15535 L
8845 15494 L 8801 15484 L 8758 15494 L 8739 15630 L
8666 15707 L 8576 15630 L 8640 15494 L 8623 15358 L
8535 15223 L 8530 15127 L 8511 15087 L 8406 14951 L
8395 14935 L 8259 14916 L 8124 14900 L 7988 14899 L
7900 14816 L 7927 14680 L 7944 14544 L 7979 14409 L

7988 14399 L 8124 14373 L 8259 14358 L 8395 14344 L
8460 14273 L 8491 14137 L 8504 14002 L 8522 13866 L
8530 13857 L 8666 13798 L 8801 13764 L 8902 13866 L
8937 13900 L 9044 14002 L 9072 14025 L 9199 14137 L
9208 14143 L 9219 14137 L 9343 14035 L 9479 14012 L
9491 14002 L 9614 13886 L 9750 13876 L 9868 13866 L
9750 13798 L 9703 13730 L 9614 13710 L 9486 13594 L
9479 13587 L 9343 13557 L 9249 13459 L 9241 13323 L
9208 13291 L 9072 13296 L 8969 13187 L 9007 13052 L
8998 12916 L 9052 12780 L 9072 12743 L 9152 12645 L
9208 12591 L 9275 12509 L 9343 12450 L 9444 12373 L
9479 12323 L 9548 12238 L 9479 12196 L 9390 12102 L
9365 11966 L 9479 11853 L 9529 11831 L 9614 11760 L
9614 10818 L 9479 10774 L 9456 10745 L 9420 10609 L
9369 10474 L 9479 10360 L 9536 10338 L 9591 10202 L
9614 10174 L 9695 10202 L 9750 10209 L 9881 10338 L
9868 10474 L 9877 10609 L 9750 10682 L 9718 10745 L
9614 10818 L 9614 11760 L 9667 11695 L 9730 11559 L
9750 11466 L 9756 11423 L 9783 11288 L 9829 11152 L
9885 11067 L 9919 11016 L 9996 10881 L 10021 10834 L
10021 10115 L 9976 10067 L 10021 10038 L 10067 10067 L
10021 10115 L 10021 10834 L 10156 10764 L 10191 10745 L
10292 10673 L 10292 9678 L 10277 9660 L 10292 9644 L
10309 9660 L 10292 9678 L 10292 10673 L 10334 10609 L
10427 10475 L 10427 10474 L 10520 10338 L 10563 10297 L
10592 10202 L 10696 10067 L 10698 10064 L 10769 9931 L
10834 9884 L 10965 9795 L 10969 9792 L 11007 9795 L
11105 9847 L 11199 9931 L 11240 9969 L 11376 10051 L
11400 10067 L 11511 10163 L 11567 10202 L 11647 10238 L
11761 10338 L 11782 10355 L 11918 10435 L 11969 10474 L
12053 10550 L 12062 10609 L 12082 10745 L 12101 10881 L
12128 11016 L 12167 11152 L 12189 11252 L 12208 11288 L
12284 11423 L 12324 11516 L 12350 11559 L 12415 11695 L
12460 11809 L 12469 11831 L 12521 11966 L 12581 12102 L
12595 12141 L 12656 12238 L 12731 12348 L 12804 12373 L
12866 12389 L 12907 12373 L 13002 12349 L 13002 10788 L
12948 10745 L 12866 10686 L 12758 10609 L 12731 10584 L
12634 10474 L 12595 10398 L 12556 10338 L 12460 10256 L
12417 10202 L 12324 10098 L 12305 10067 L 12204 9931 L
12189 9911 L 12169 9795 L 12154 9660 L 12141 9524 L
12137 9388 L 12115 9252 L 12091 9117 L 12069 8981 L
12053 8912 L 11930 8845 L 11918 8817 L 11782 8755 L
11678 8710 L 11647 8689 L 11511 8653 L 11376 8698 L

11240 8631 L 11105 8679 L 10969 8681 L 10834 8672 L
10707 8574 L 10698 8566 L 10688 8574 L 10631 8710 L
10698 8772 L 10834 8813 L 10852 8845 L 10969 8971 L
10979 8981 L 11105 9104 L 11115 9117 L 11105 9131 L
11007 9117 L 10969 9070 L 10834 8987 L 10790 8981 L
10698 8944 L 10563 8877 L 10427 8849 L 10407 8845 L
10292 8818 L 10243 8845 L 10156 8889 L 10021 8850 L
9885 8848 L 9883 8845 L 9750 8740 L 9700 8710 L
9614 8629 L 9580 8574 L 9479 8506 L 9343 8558 L
9335 8574 L 9314 8710 L 9343 8752 L 9479 8837 L
9497 8845 L 9552 8981 L 9546 9117 L 9614 9186 L
9750 9210 L 9783 9252 L 9805 9388 L 9885 9455 L
9913 9524 L 9885 9583 L 9808 9660 L 9750 9759 L
9709 9795 L 9614 9893 L 9575 9931 L 9479 9951 L
9448 9931 L 9343 9867 L 9221 9931 L 9208 9979 L
9145 10067 L 9208 10141 L 9343 10198 L 9348 10202 L
9343 10210 L 9208 10260 L 9150 10338 L 9072 10359 L
8982 10474 L 8951 10609 L 8937 10629 L 8801 10696 L
8760 10745 L 8801 10796 L 8937 10846 L 9072 10853 L
9095 10881 L 9072 10887 L 8937 10960 L 8893 11016 L
8937 11057 L 9072 11104 L 9208 11058 L 9319 11152 L
9343 11175 L 9366 11288 L 9350 11423 L 9343 11433 L
9257 11559 L 9241 11695 L 9208 11735 L 9127 11831 L
9103 11966 L 9089 12102 L 9072 12200 L 9055 12238 L
8937 12291 L 8801 12333 L 8763 12373 L 8666 12445 L
8595 12509 L 8585 12645 L 8666 12771 L 8672 12780 L
8712 12916 L 8801 13013 L 8849 13052 L 8849 13187 L
8884 13323 L 8937 13444 L 8967 13459 L 8937 13485 L
8822 13594 L 8801 13599 L 8666 13633 L 8552 13730 L
8530 13736 L 8412 13866 L 8410 14002 L 8403 14137 L
8395 14189 L 8259 14210 L 8124 14227 L 8051 14273 L
7988 14289 L 7871 14409 L 7861 14544 L 7853 14635 L
7717 14664 L 7586 14680 L 7582 14680 L 7446 14801 L
7400 14816 L 7311 14846 L 7175 14855 L 7040 14829 L
7005 14951 L 6904 14987 L 6880 14951 L 6769 14841 L
6664 14816 L 6633 14794 L 6498 14729 L 6457 14680 L
6362 14590 L 6292 14544 L 6227 14431 L 6215 14409 L
6227 14343 L 6290 14273 L 6293 14137 L 6362 14036 L
6373 14002 L 6362 13994 L 6303 13866 L 6234 13730 L
6227 13723 L 6091 13639 L 6018 13594 L 5956 13526 L
5862 13594 L 5831 13730 L 5820 13745 L 5807 13730 L
5685 13636 L 5639 13594 L 5549 13492 L 5414 13467 L
5333 13594 L 5355 13730 L 5414 13776 L 5549 13837 L

5604 13866 L 5685 13940 L 5779 14002 L 5793 14137 L
5685 14190 L 5599 14137 L 5549 14117 L 5414 14026 L
5391 14002 L 5278 13899 L 5249 13866 L 5193 13730 L
5178 13594 L 5143 13520 L 5097 13459 L 5016 13323 L
5007 13314 L 4971 13323 L 4923 13459 L 4983 13594 L
4879 13730 L 4913 13866 L 5007 13994 L 5011 14002 L
5014 14137 L 5007 14182 L 4953 14273 L 4872 14393 L
4736 14326 L 4601 14308 L 4553 14273 L 4503 14137 L
4465 14008 L 4464 14002 L 4333 13866 L 4330 13863 L
4194 13788 L 4152 13730 L 4059 13600 L 4056 13594 L
4023 13459 L 3960 13323 L 3987 13187 L 4005 13052 L
3941 12916 L 3923 12907 L 3842 12916 L 3788 12946 L
3747 13052 L 3721 13187 L 3728 13323 L 3652 13417 L
3609 13459 L 3517 13569 L 3381 13498 L 3355 13459 L
3246 13388 L 3183 13323 L 3166 13187 L 3152 13052 L
3110 12994 L 3095 12916 L 3057 12780 L 3036 12645 L
3110 12589 L 3224 12509 L 3246 12504 L 3246 12416 L
3110 12391 L 3080 12373 L 3110 12347 L 3171 12238 L
3246 12192 L 3381 12153 L 3517 12219 L 3532 12238 L
3517 12246 L 3381 12351 L 3334 12373 L 3246 12416 L
3246 12504 L 3381 12468 L 3498 12373 L 3517 12360 L
3652 12308 L 3788 12285 L 3923 12268 L 4059 12254 L
4194 12255 L 4234 12238 L 4194 12212 L 4081 12102 L
4059 12092 L 3923 12075 L 3788 11987 L 3652 11977 L
3517 12036 L 3381 12072 L 3275 12102 L 3246 12107 L
3110 12140 L 3006 12102 L 2975 12087 L 2839 12047 L
2727 12102 L 2712 12238 L 2704 12272 L 2568 12312 L
2497 12238 L 2507 12102 L 2433 11976 L 2429 11966 L
2297 11860 L N E
111 O
2297 13110 2297 13543 2402 13594 2384 13730 2383 13866 2345 14002
2298 14137 2433 14225 2529 14137 2568 14073 2590 14002 2653 13866
2704 13789 2741 13730 2820 13594 2839 13570 2839 13294 2819 13187
2839 13151 2856 13187 2839 13294 2839 13570 2911 13459 2946 13323
2960 13187 2919 13052 2841 12916 2839 12914 2838 12916 2734 13052
2704 13145 2678 13187 2610 13323 2568 13389 2524 13323 2473 13187
2433 13151 2297 13110 38 N F
2297 15616 2297 16016 2313 16037 2297 16085 2297 16172 2489 16172
2680 16172 2871 16172 3045 16172 2975 16051 2961 16037 2975 16027
3110 16011 3179 16037 3246 16054 3381 16086 3515 16172 3635 16172
3649 16172 3517 16074 3407 16037 3381 16010 3246 15932 3110 15905
3101 15901 2975 15802 2873 15765 2839 15739 2704 15638 2632 15630
2568 15619 2433 15596 2297 15616 33 N F

92 O
2297 16016 2297 16085 2313 16037 2297 16016 4 N F
111 O
2839 15170 2787 15087 2808 14951 2839 14910 2922 14951 2975 14960
3089 15087 3110 15146 3184 15223 3110 15331 2975 15304 2975 15166
3015 15087 2975 15042 2913 15087 2975 15166 2975 15304 2877 15223
2839 15170 19 N F
92 O
2839 13294 2819 13187 2839 13151 2856 13187 2839 13294 5 N F
2975 15166 2913 15087 2975 15042 3015 15087 2975 15166 5 N F
2975 16051 2961 16037 2975 16027 3110 16011 3179 16037 3246 16054
3381 16086 3515 16172 3444 16172 3431 16172 3381 16140 3246 16125
3179 16172 3062 16172 3045 16172 2975 16051 16 N F
3045 16173 3045 16172 3062 16172 3179 16172 3179 16173 3062 16173
3045 16173 7 N F
111 O
3110 12391 3080 12373 3110 12347 3171 12238 3246 12192 3381 12153
3517 12219 3532 12238 3517 12246 3381 12351 3381 12266 3417 12238
3381 12217 3325 12238 3381 12266 3381 12351 3334 12373 3246 12416
3110 12391 19 N F
74 O
3253 16173 3179 16173 3179 16172 3253 16172 3431 16172 3431 16173
3253 16173 7 N F
147 O
3246 14943 3227 14951 3246 14970 3252 14951 3246 14943 5 N F
74 O
3179 16172 3246 16125 3381 16140 3431 16172 3253 16172 3179 16172
6 N F
92 O
3381 12266 3325 12238 3381 12217 3417 12238 3381 12266 5 N F
129 O
3381 7225 3375 7217 3381 7195 3427 7217 3381 7225 5 N F
92 O
3431 16172 3431 16173 3444 16173 3515 16173 3515 16172 3444 16172
3431 16172 7 N F
111 O
3460 15630 3436 15494 3463 15358 3517 15312 3652 15313 3747 15358
3759 15494 3693 15630 3652 15666 3517 15672 3460 15630 11 N F
3515 16172 3515 16173 3635 16173 3649 16173 3649 16172 3635 16172
3515 16172 7 N F
129 O
3788 11252 3681 11152 3704 11016 3788 10941 3923 10931 4059 10996
4089 11016 4080 11152 4059 11180 3923 11262 3788 11252 11 N F

111 O
4059 15266 4009 15223 4059 15169 4194 15159 4330 15125 4465 15191
4601 15177 4736 15152 4851 15223 4872 15239 4966 15358 4872 15472
4851 15494 4736 15568 4601 15595 4475 15630 4465 15636 4330 15758
4194 15710 4103 15630 4109 15494 4112 15358 4059 15266 23 N F
129 O
4194 13649 4074 13594 4194 13558 4280 13594 4194 13649 5 N F
4194 7795 4131 7760 4194 7721 4236 7760 4194 7795 5 N F
4330 10932 4268 10881 4330 10822 4465 10760 4601 10843 4643 10881
4601 10936 4465 10997 4465 10887 4475 10881 4465 10874 4455 10881
4465 10887 4465 10997 4330 10932 15 N F
111 O
4400 16173 4273 16173 4273 16172 4400 16172 4454 16172 4454 16173
4400 16173 7 N F
129 O
4330 13507 4316 13459 4318 13323 4330 13307 4373 13187 4465 13103
4601 13163 4622 13187 4601 13254 4513 13323 4484 13459 4465 13488
4330 13507 13 N F
111 O
4273 16172 M 4330 16134 L 4380 16037 L 4401 15901 L 4465 15831 L
4601 15791 L 4736 15786 L 4866 15765 L 4872 15761 L
4886 15765 L 5007 15768 L 5143 15775 L 5278 15772 L
5339 15765 L 5414 15723 L 5468 15630 L 5439 15494 L
5414 15454 L 5335 15358 L 5329 15223 L 5354 15087 L
5350 14951 L 5279 14816 L 5278 14815 L 5278 14278 L
5282 14273 L 5278 14262 L 5276 14273 L 5278 14278 L
5278 14815 L 5219 14680 L 5227 14544 L 5218 14409 L
5183 14273 L 5206 14137 L 5278 14055 L 5357 14137 L
5414 14268 L 5417 14273 L 5414 14287 L 5352 14409 L
5414 14455 L 5549 14495 L 5587 14544 L 5685 14660 L
5698 14680 L 5757 14816 L 5739 14951 L 5758 15087 L
5820 15184 L 5852 15223 L 5956 15326 L 5994 15358 L
6091 15440 L 6227 15375 L 6251 15358 L 6227 15344 L
6151 15223 L 6098 15087 L 6091 15079 L 6091 14843 L
6227 14839 L 6231 14816 L 6227 14810 L 6091 14808 L
6084 14816 L 6091 14843 L 6091 15079 L 5962 14951 L
5982 14816 L 6091 14702 L 6227 14683 L 6323 14816 L
6362 14944 L 6378 14951 L 6498 15000 L 6633 14993 L
6769 15072 L 6782 15087 L 6903 15223 L 6904 15228 L
7017 15358 L 6904 15431 L 6769 15463 L 6669 15358 L
6633 15322 L 6498 15298 L 6481 15358 L 6498 15373 L
6633 15439 L 6694 15494 L 6656 15630 L 6769 15751 L
6904 15670 L 6993 15630 L 7040 15574 L 7175 15543 L

7278 15630 L 7311 15672 L 7446 15762 L 7450 15765 L
7582 15837 L 7717 15831 L 7853 15869 L 7882 15901 L
7988 15938 L 8124 15904 L 8129 15901 L 8259 15774 L
8281 15765 L 8395 15676 L 8530 15710 L 8573 15765 L
8596 15901 L 8666 15968 L 8801 15919 L 8832 15901 L
8937 15827 L 9045 15765 L 9024 15630 L 8937 15535 L
8845 15494 L 8801 15484 L 8758 15494 L 8739 15630 L
8666 15707 L 8576 15630 L 8640 15494 L 8623 15358 L
8535 15223 L 8530 15127 L 8511 15087 L 8406 14951 L
8395 14935 L 8259 14916 L 8124 14900 L 7988 14899 L
7900 14816 L 7927 14680 L 7944 14544 L 7979 14409 L
7988 14399 L 8124 14373 L 8259 14358 L 8395 14344 L
8460 14273 L 8491 14137 L 8504 14002 L 8522 13866 L
8530 13857 L 8666 13798 L 8801 13764 L 8902 13866 L
8937 13900 L 9044 14002 L 9072 14025 L 9199 14137 L
9208 14143 L 9219 14137 L 9343 14035 L 9479 14012 L
9491 14002 L 9614 13886 L 9750 13876 L 9868 13866 L
9750 13798 L 9703 13730 L 9614 13710 L 9486 13594 L
9479 13587 L 9343 13557 L 9249 13459 L 9241 13323 L
9208 13291 L 9072 13296 L 8969 13187 L 9007 13052 L
8998 12916 L 9052 12780 L 9072 12743 L 9152 12645 L
9208 12591 L 9275 12509 L 9343 12450 L 9444 12373 L
9479 12323 L 9548 12238 L 9479 12196 L 9390 12102 L
9365 11966 L 9479 11853 L 9529 11831 L 9614 11760 L
9667 11695 L 9730 11559 L 9750 11466 L 9756 11423 L
9783 11288 L 9829 11152 L 9885 11067 L 9919 11016 L
9996 10881 L 10021 10834 L 10156 10764 L 10191 10745 L
10292 10673 L 10334 10609 L 10427 10475 L 10427 10474 L
10520 10338 L 10563 10297 L 10592 10202 L 10696 10067 L
10698 10064 L 10769 9931 L 10834 9884 L 10965 9795 L
10969 9792 L 11007 9795 L 11105 9847 L 11199 9931 L
11240 9969 L 11376 10051 L 11400 10067 L 11511 10163 L
11567 10202 L 11647 10238 L 11761 10338 L 11782 10355 L
11918 10435 L 11969 10474 L 12053 10550 L 12062 10609 L
12082 10745 L 12101 10881 L 12128 11016 L 12167 11152 L
12189 11252 L 12208 11288 L 12284 11423 L 12324 11516 L
12350 11559 L 12415 11695 L 12460 11809 L 12469 11831 L
12521 11966 L 12581 12102 L 12595 12141 L 12656 12238 L
12731 12348 L 12804 12373 L 12866 12389 L 12907 12373 L
13002 12349 L 13002 14040 L 12909 14002 L 12866 13951 L
12731 13946 L 12596 14002 L 12595 14002 L 12595 13786 L
12727 13730 L 12731 13722 L 12755 13594 L 12749 13459 L
12731 13412 L 12595 13383 L 12595 13256 L 12672 13187 L

12595 13086 L 12575 13187 L 12595 13256 L 12595 13383 L
12509 13459 L 12460 13488 L 12324 13492 L 12295 13459 L
12189 13398 L 12097 13323 L 12074 13187 L 12053 13168 L
12027 13187 L 11989 13323 L 11989 13459 L 12043 13594 L
12053 13614 L 12189 13708 L 12324 13665 L 12460 13709 L
12476 13730 L 12595 13786 L 12595 14002 L 12460 14092 L
12406 14137 L 12324 14207 L 12189 14236 L 12107 14137 L
12053 14092 L 11918 14004 L 11917 14002 L 11782 13874 L
11771 13866 L 11647 13731 L 11646 13730 L 11647 13730 L
11769 13594 L 11782 13580 L 11893 13459 L 11890 13323 L
11893 13187 L 11899 13052 L 11906 12916 L 11904 12780 L
11904 12645 L 11902 12509 L 11918 12475 L 11976 12509 L
12053 12582 L 12153 12509 L 12122 12373 L 12083 12238 L
12067 12102 L 12053 12091 L 12053 11866 L 12096 11831 L
12053 11705 L 12008 11831 L 12053 11866 L 12053 12091 L
11918 12046 L 11866 11966 L 11869 11831 L 11918 11748 L
12045 11695 L 11949 11559 L 11918 11524 L 11830 11423 L
11782 11374 L 11701 11288 L 11647 11242 L 11524 11152 L
11511 11141 L 11376 11035 L 11351 11016 L 11240 10930 L
11174 10881 L 11105 10834 L 10969 10757 L 10950 10745 L
10834 10676 L 10735 10609 L 10698 10582 L 10563 10572 L
10553 10609 L 10526 10745 L 10460 10881 L 10427 10910 L
10292 10965 L 10156 10995 L 10134 11016 L 10021 11136 L
10006 11152 L 9989 11288 L 10021 11350 L 10058 11423 L
10068 11559 L 10156 11677 L 10177 11695 L 10222 11831 L
10259 11966 L 10262 12102 L 10176 12238 L 10156 12261 L
10021 12354 L 10003 12373 L 9885 12401 L 9782 12509 L
9772 12645 L 9764 12780 L 9750 12870 L 9614 12884 L
9543 12916 L 9479 12927 L 9343 12966 L 9249 13052 L
9208 13089 L 9072 13086 L 9013 13187 L 9072 13250 L
9208 13238 L 9296 13323 L 9302 13459 L 9343 13501 L
9479 13534 L 9535 13594 L 9614 13666 L 9750 13695 L
9885 13712 L 9931 13730 L 10009 13866 L 9885 13905 L
9750 13918 L 9614 13926 L 9534 14002 L 9479 14049 L
9343 14081 L 9274 14137 L 9208 14174 L 9157 14137 L
9072 14062 L 8998 14002 L 8937 13944 L 8857 13866 L
8801 13810 L 8689 13866 L 8666 13942 L 8636 14002 L
8596 14137 L 8543 14273 L 8530 14302 L 8445 14409 L
8395 14472 L 8286 14544 L 8259 14569 L 8177 14680 L
8124 14732 L 7988 14735 L 7967 14816 L 7988 14835 L
8124 14836 L 8259 14867 L 8395 14891 L 8437 14951 L
8530 15056 L 8555 15087 L 8561 15223 L 8666 15335 L
8801 15344 L 8814 15358 L 8937 15477 L 9072 15483 L

9083 15494 L 9087 15630 L 9208 15759 L 9214 15765 L
9343 15895 L 9479 15895 L 9614 15895 L 9741 15765 L
9750 15754 L 9872 15630 L 9750 15507 L 9739 15494 L
9739 15358 L 9614 15231 L 9607 15223 L 9605 15087 L
9611 14951 L 9614 14948 L 9750 14945 L 9756 14951 L
9885 15082 L 9890 15087 L 10021 15214 L 10156 15223 L
10156 15223 L 10259 15358 L 10292 15392 L 10427 15377 L
10563 15485 L 10698 15438 L 10834 15485 L 10839 15494 L
10858 15630 L 10834 15672 L 10698 15697 L 10577 15630 L
10563 15545 L 10427 15586 L 10292 15577 L 10168 15630 L
10180 15765 L 10225 15901 L 10292 15990 L 10321 16037 L
10410 16172 L 10326 16172 L 10134 16172 L 9943 16172 L
9752 16172 L 9561 16172 L 9370 16172 L 9179 16172 L
8987 16172 L 8796 16172 L 8605 16172 L 8420 16172 L
8395 16117 L 8356 16037 L 8259 15973 L 8141 16037 L
8124 16041 L 7988 16111 L 7853 16110 L 7717 16075 L
7582 16054 L 7545 16037 L 7446 15968 L 7363 15901 L
7311 15862 L 7175 15783 L 7144 15765 L 7040 15711 L
6950 15765 L 6904 15781 L 6769 15847 L 6633 15856 L
6498 15827 L 6406 15765 L 6362 15644 L 6362 15232 L
6370 15223 L 6362 15192 L 6350 15223 L 6362 15232 L
6362 15644 L 6353 15630 L 6227 15579 L 6149 15630 L
6091 15693 L 5956 15674 L 5879 15630 L 5830 15494 L
5820 15470 L 5791 15494 L 5686 15630 L 5685 15630 L
5641 15765 L 5549 15859 L 5549 15284 L 5617 15223 L
5549 15122 L 5549 14963 L 5550 14951 L 5652 14816 L
5607 14680 L 5549 14618 L 5414 14627 L 5353 14680 L
5379 14816 L 5414 14863 L 5548 14951 L 5549 14963 L
5549 15122 L 5511 15223 L 5549 15284 L 5549 15859 L
5414 15875 L 5278 15863 L 5143 15844 L 5007 15837 L
4881 15901 L 4872 15907 L 4736 15978 L 4601 16007 L
4573 16037 L 4465 16157 L 4454 16172 L 4400 16172 L
4273 16172 L N E
92 O
4591 16173 4454 16173 4454 16172 4591 16172 4625 16172 4625 16173
4591 16173 7 N F
111 O
4465 10887 4455 10881 4465 10874 4475 10881 4465 10887 5 N F
92 O
4454 16172 4465 16157 4573 16037 4601 16007 4736 15978 4872 15907
4881 15901 5007 15837 5143 15844 5278 15863 5414 15875 5549 15859
5641 15765 5685 15630 5686 15630 5791 15494 5820 15470 5830 15494
5879 15630 5956 15674 6091 15693 6149 15630 6227 15579 6353 15630

6362 15644 6406 15765 6498 15827 6633 15856 6769 15847 6904 15781
6950 15765 7040 15711 7144 15765 7175 15783 7311 15862 7363 15901
7446 15968 7545 16037 7582 16054 7717 16075 7853 16110 7988 16111
8124 16041 8141 16037 8259 15973 8356 16037 8395 16117 8420 16172
8414 16172 8355 16172 8259 16062 8124 16098 8026 16172 7841 16172
7676 16172 7582 16144 7446 16067 7426 16037 7311 15929 7199 15901
7175 15886 7040 15786 6904 15834 6799 15901 6769 15922 6633 15954
6498 15973 6362 16024 6227 16014 6091 15936 6067 15901 5956 15803
5833 15765 5820 15758 5812 15765 5685 15869 5653 15901 5549 15926
5414 15941 5278 15928 5143 15907 5007 15904 4872 15994 4810 16037
4736 16125 4625 16172 4591 16172 4454 16172 88 N F
74 O
4782 16173 4625 16173 4625 16172 4782 16172 4889 16172 4889 16173
4782 16173 7 N F
165 O
4736 11390 4699 11423 4736 11495 4824 11559 4855 11695 4872 11735
4973 11831 5007 11837 5143 11832 5149 11831 5278 11698 5414 11782
5496 11831 5549 11885 5634 11831 5685 11768 5691 11695 5685 11653
5589 11695 5549 11726 5536 11695 5414 11609 5313 11559 5278 11536
5143 11448 5007 11445 4872 11527 4788 11423 4872 11378 5007 11388
5039 11288 5048 11152 5007 11114 4944 11152 4872 11206 4803 11288
4736 11390 37 N F
74 O
4625 16172 4736 16125 4810 16037 4872 15994 5007 15904 5143 15907
5278 15928 5414 15941 5549 15926 5653 15901 5685 15869 5812 15765
5820 15758 5833 15765 5956 15803 6067 15901 6091 15936 6227 16014
6362 16024 6498 15973 6633 15954 6769 15922 6799 15901 6904 15834
7040 15786 7175 15886 7199 15901 7311 15929 7426 16037 7446 16067
7582 16144 7676 16172 7650 16172 7514 16172 7446 16129 7383 16037
7311 15969 7175 15931 7124 15901 7040 15834 6904 15887 6883 15901
6769 15981 6649 16037 6633 16121 6609 16172 6503 16172 6312 16172
6187 16172 6091 16084 6079 16037 5969 15901 5956 15890 5820 15819
5732 15901 5685 15928 5549 15961 5414 15993 5278 15974 5143 15939
5007 15940 4893 16037 4889 16172 4782 16172 4625 16172 65 N F
56 O
4973 16173 4889 16173 4889 16172 4974 16172 4994 16172 4994 16173
4973 16173 7 N F
4889 16172 4893 16037 5007 15940 5143 15939 5278 15974 5414 15993
5549 15961 5685 15928 5732 15901 5820 15819 5956 15890 5969 15901
6079 16037 6091 16084 6187 16172 6120 16172 6036 16172 6030 16037
5956 15950 5867 15901 5820 15876 5793 15901 5685 15963 5549 15995
5428 16037 5420 16172 5356 16172 5242 16172 5278 16119 5378 16037
5278 16019 5143 15971 5007 15976 4935 16037 4994 16172 4974 16172

4889 16172 37 N F
38 O
4994 16172 4935 16037 5007 15976 5143 15971 5278 16019 5378 16037
5278 16119 5242 16172 5165 16172 5143 16172 5143 16142 5229 16037
5143 16003 5007 16012 4978 16037 5007 16097 5143 16142 5143 16172
4994 16172 19 N F
165 O
5007 10448 4947 10474 4982 10609 5007 10699 5057 10745 5143 10872
5176 10745 5167 10609 5143 10595 5030 10474 5007 10448 11 N F
20 O
5007 16097 4978 16037 5007 16012 5143 16003 5229 16037 5143 16142
5143 16044 5148 16037 5143 16035 5117 16037 5143 16044 5143 16142
5007 16097 13 N F
2 O
5143 16044 5117 16037 5143 16035 5148 16037 5143 16044 5 N F
56 O
5356 16173 5242 16173 5242 16172 5356 16172 5420 16172 5420 16173
5356 16173 7 N F
92 O
5278 14278 5276 14273 5278 14262 5282 14273 5278 14278 5 N F
5379 14816 5353 14680 5414 14627 5549 14618 5607 14680 5652 14816
5550 14951 5549 14963 5549 14862 5584 14816 5549 14733 5490 14816
5549 14862 5549 14963 5548 14951 5414 14863 5379 14816 17 N F
129 O
5414 6969 5383 6946 5414 6886 5471 6946 5414 6969 5 N F
38 O
5480 16173 5420 16173 5420 16173 5480 16173 5480 16173 5 N F
5480 16173 5420 16173 5420 16172 5480 16172 5480 16173 5 N F
5420 16172 5428 16037 5549 15995 5685 15963 5793 15901 5820 15876
5867 15901 5956 15950 6030 16037 6036 16172 5934 16172 5956 16126
5982 16037 5956 16007 5820 15935 5685 15997 5549 16030 5528 16037
5480 16172 5420 16172 20 N F
111 O
5549 5714 5447 5589 5488 5453 5547 5453 5738 5453 5796 5453
5820 5465 5829 5453 5929 5453 6074 5453 6091 5485 6139 5589
6091 5655 5956 5693 5820 5720 5817 5725 5691 5860 5685 5865
5685 5635 5717 5589 5685 5568 5661 5589 5685 5635 5685 5865
5675 5860 5554 5725 5549 5714 27 N F
20 O
5540 16173 5480 16173 5480 16173 5540 16173 5540 16173 5 N F
5540 16173 5480 16173 5480 16172 5540 16172 5540 16173 5 N F
74 O
5549 14862 5490 14816 5549 14733 5584 14816 5549 14862 5 N F

92 O
5549 15284 5511 15223 5549 15122 5617 15223 5549 15284 5 N F
20 O
5480 16172 5528 16037 5549 16030 5685 15997 5820 15935 5956 16007
5982 16037 5956 16126 5934 16172 5929 16172 5854 16172 5900 16037
5820 15996 5685 16032 5665 16037 5549 16150 5540 16172 5480 16172
18 N F
165 O
5549 10734 5541 10745 5549 10867 5593 10881 5631 11016 5615 11152
5670 11288 5685 11349 5801 11423 5820 11427 5956 11438 6000 11423
5973 11288 6091 11160 6120 11152 6091 11146 5956 11138 5820 11105
5739 11016 5693 10881 5820 10808 5839 10745 5840 10609 5820 10579
5685 10525 5624 10609 5549 10734 27 N F
2 O
5540 16172 5547 16172 5738 16172 5854 16172 5900 16037 5820 15996
5685 16032 5665 16037 5549 16150 5540 16172 10 N F
92 O
5685 5635 5661 5589 5685 5568 5717 5589 5685 5635 5 N F
129 O
5685 13336 5669 13323 5685 13311 5694 13323 5685 13336 5 N F
5796 5453 5796 5453 5829 5453 5829 5453 5820 5453 5796 5453
6 N F
5796 5453 5796 5453 5829 5453 5829 5453 5796 5453 5 N F
5829 5453 5796 5453 5820 5465 5829 5453 4 N F
111 O
5929 5453 5829 5453 5829 5453 5929 5453 6074 5453 6074 5453
5929 5453 7 N F
20 O
5929 16173 5854 16173 5854 16172 5929 16172 5934 16172 5934 16173
5929 16173 7 N F
129 O
5956 13323 5875 13187 5946 13052 5956 13044 5988 13052 6091 13078
6134 13187 6125 13323 6091 13369 5956 13323 5956 13323 11 N F
5950 7760 5916 7624 5956 7579 5987 7624 5963 7760 5956 7767
5950 7760 7 N F
38 O
5956 16173 5934 16173 5934 16173 6036 16173 6036 16173 5956 16173
6 N F
6036 16173 5934 16173 5934 16172 6036 16172 6036 16173 5 N F
56 O
6036 16172 6036 16173 6120 16173 6187 16173 6187 16172 6120 16172
6036 16172 7 N F
92 O

6091 14843 6084 14816 6091 14808 6227 14810 6231 14816 6227 14839
6091 14843 7 N F
129 O
6362 14441 6277 14409 6362 14329 6400 14273 6498 14224 6633 14191
6741 14273 6769 14343 6795 14409 6797 14544 6770 14680 6769 14680
6769 14680 6633 14565 6603 14544 6498 14503 6362 14441 17 N F
92 O
6362 15232 6350 15223 6362 15192 6370 15223 6362 15232 5 N F
56 O
6694 16173 6609 16173 6609 16172 6694 16172 6797 16172 6797 16173
6694 16173 7 N F
6609 16172 6633 16121 6649 16037 6769 15981 6883 15901 6904 15887
7040 15834 7124 15901 7175 15931 7311 15969 7383 16037 7446 16129
7514 16172 7458 16172 7435 16172 7340 16037 7311 16009 7175 15966
7065 15901 7040 15881 6991 15901 6904 15948 6773 16037 6797 16172
6694 16172 6609 16172 26 N F
165 O
6769 10303 6672 10338 6715 10474 6769 10524 6866 10474 6904 10415
6968 10338 6904 10292 6769 10303 9 N F
38 O
6797 16172 6773 16037 6904 15948 6991 15901 7040 15881 7065 15901
7175 15966 7311 16009 7340 16037 7435 16172 7397 16172 7311 16053
7279 16037 7175 16001 7040 15946 6904 16012 6867 16037 6904 16124
6930 16172 6885 16172 6797 16172 21 N F
6797 16173 6797 16172 6885 16172 6930 16172 6930 16173 6885 16173
6797 16173 7 N F
20 O
6904 16124 6867 16037 6904 16012 7040 15946 7175 16001 7279 16037
7311 16053 7397 16172 7360 16172 7311 16105 7179 16037 7175 16036
7040 16023 7010 16037 7040 16130 7048 16172 6930 16172 6904 16124
18 N F
6930 16173 6930 16172 7048 16172 7048 16173 6930 16173 5 N F
6930 16173 6930 16173 7048 16173 7048 16173 7040 16173 6930 16173
6 N F
2 O
7040 16130 7010 16037 7040 16023 7175 16036 7179 16037 7311 16105
7360 16172 7267 16172 7076 16172 7048 16172 7040 16130 11 N F
20 O
7360 16172 7360 16173 7397 16173 7397 16172 7360 16172 5 N F
7397 16173 7360 16173 7360 16173 7397 16173 7397 16173 5 N F
38 O
7397 16172 7397 16173 7435 16173 7435 16172 7397 16172 5 N F
7435 16173 7397 16173 7397 16173 7435 16173 7435 16173 5 N F

56 O
7435 16172 7435 16173 7458 16173 7514 16173 7514 16172 7458 16172
7435 16172 7 N F
74 O
7514 16172 7514 16173 7650 16173 7676 16173 7676 16172 7650 16172
7514 16172 7 N F
129 O
7582 7368 7569 7353 7582 7332 7597 7353 7582 7368 5 N F
92 O
7676 16172 7676 16173 7841 16173 8026 16173 8026 16172 7841 16172
7676 16172 7 N F
129 O
7717 6743 7684 6674 7717 6599 7766 6539 7853 6445 7948 6539
7988 6569 8109 6674 7988 6770 7920 6810 7853 6837 7789 6810
7717 6743 13 N F
92 O
7988 14835 M 7967 14816 L 7988 14735 L 8124 14732 L 8177 14680 L
8259 14569 L 8286 14544 L 8395 14472 L 8445 14409 L
8530 14302 L 8543 14273 L 8596 14137 L 8636 14002 L
8666 13942 L 8689 13866 L 8801 13810 L 8857 13866 L
8937 13944 L 8998 14002 L 9072 14062 L 9157 14137 L
9208 14174 L 9274 14137 L 9343 14081 L 9479 14049 L
9534 14002 L 9614 13926 L 9750 13918 L 9885 13905 L
10009 13866 L 9931 13730 L 9885 13712 L 9750 13695 L
9614 13666 L 9535 13594 L 9479 13534 L 9343 13501 L
9302 13459 L 9296 13323 L 9208 13238 L 9072 13250 L
9072 13204 L 9198 13187 L 9072 13161 L 9057 13187 L
9072 13204 L 9072 13250 L 9013 13187 L 9072 13086 L
9208 13089 L 9249 13052 L 9343 12966 L 9479 12927 L
9543 12916 L 9614 12884 L 9750 12870 L 9764 12780 L
9772 12645 L 9782 12509 L 9885 12401 L 10003 12373 L
10021 12354 L 10156 12261 L 10176 12238 L 10262 12102 L
10259 11966 L 10222 11831 L 10177 11695 L 10156 11677 L
10068 11559 L 10058 11423 L 10021 11350 L 9989 11288 L
10006 11152 L 10021 11136 L 10134 11016 L 10156 10995 L
10292 10965 L 10427 10910 L 10460 10881 L 10526 10745 L
10553 10609 L 10563 10572 L 10698 10582 L 10735 10609 L
10834 10676 L 10950 10745 L 10969 10757 L 11105 10834 L
11174 10881 L 11240 10930 L 11351 11016 L 11376 11035 L
11511 11141 L 11524 11152 L 11647 11242 L 11701 11288 L
11782 11374 L 11830 11423 L 11918 11524 L 11949 11559 L
12045 11695 L 11918 11748 L 11869 11831 L 11866 11966 L
11918 12046 L 12053 12091 L 12067 12102 L 12083 12238 L

12122 12373 L 12153 12509 L 12053 12582 L 11976 12509 L
11918 12475 L 11902 12509 L 11904 12645 L 11904 12780 L
11906 12916 L 11899 13052 L 11893 13187 L 11890 13323 L
11893 13459 L 11782 13580 L 11769 13594 L 11647 13730 L
11646 13730 L 11647 13731 L 11771 13866 L 11782 13874 L
11917 14002 L 11918 14004 L 12053 14092 L 12107 14137 L
12189 14236 L 12324 14207 L 12406 14137 L 12460 14092 L
12595 14002 L 12596 14002 L 12731 13946 L 12866 13951 L
12909 14002 L 13002 14040 L 13002 14114 L 12959 14137 L
12866 14184 L 12740 14273 L 12787 14409 L 12731 14459 L
12647 14544 L 12595 14594 L 12595 14550 L 12601 14544 L
12731 14411 L 12733 14409 L 12731 14401 L 12728 14409 L
12595 14537 L 12588 14544 L 12595 14550 L 12595 14594 L
12475 14680 L 12460 14691 L 12447 14680 L 12460 14657 L
12529 14544 L 12595 14480 L 12669 14409 L 12726 14273 L
12731 14264 L 12822 14137 L 12731 14037 L 12595 14119 L
12572 14137 L 12460 14191 L 12373 14273 L 12335 14409 L
12324 14437 L 12211 14544 L 12189 14559 L 12053 14582 L
11976 14680 L 11918 14701 L 11782 14815 L 11781 14816 L
11659 14951 L 11647 14991 L 11610 15087 L 11581 15223 L
11535 15358 L 11511 15458 L 11495 15494 L 11376 15572 L
11240 15569 L 11240 15465 L 11376 15432 L 11402 15358 L
11440 15223 L 11511 15108 L 11515 15087 L 11529 14951 L
11537 14816 L 11647 14704 L 11718 14680 L 11782 14651 L
11838 14544 L 11782 14411 L 11762 14409 L 11647 14408 L
11511 14409 L 11376 14404 L 11240 14402 L 11152 14409 L
11105 14508 L 11104 14544 L 11102 14680 L 11101 14816 L
11105 14820 L 11240 14951 L 11240 14957 L 11299 15087 L
11240 15128 L 11159 15223 L 11105 15292 L 11051 15358 L
11105 15397 L 11240 15465 L 11240 15569 L 11105 15580 L
11054 15630 L 11105 15685 L 11140 15765 L 11215 15901 L
11105 15958 L 10969 15904 L 10959 15901 L 10834 15860 L
10698 15887 L 10613 15901 L 10563 15912 L 10465 16037 L
10551 16172 L 10517 16172 L 10427 16172 L 10427 15908 L
10453 15901 L 10427 15898 L 10424 15901 L 10427 15908 L
10427 16172 L 10410 16172 L 10321 16037 L 10292 15990 L
10225 15901 L 10180 15765 L 10168 15630 L 10292 15577 L
10427 15586 L 10563 15545 L 10577 15630 L 10698 15697 L
10834 15672 L 10858 15630 L 10839 15494 L 10834 15485 L
10698 15438 L 10563 15485 L 10427 15377 L 10292 15392 L
10259 15358 L 10156 15223 L 10156 15223 L 10021 15214 L
9890 15087 L 9885 15082 L 9756 14951 L 9750 14945 L
9614 14948 L 9611 14951 L 9605 15087 L 9607 15223 L

9614 15231 L 9739 15358 L 9739 15494 L 9750 15507 L
9872 15630 L 9750 15754 L 9741 15765 L 9614 15895 L
9479 15895 L 9479 15878 L 9614 15877 L 9723 15765 L
9750 15733 L 9851 15630 L 9750 15528 L 9722 15494 L
9720 15358 L 9614 15250 L 9589 15223 L 9586 15087 L
9590 14951 L 9614 14928 L 9750 14923 L 9779 14951 L
9885 15058 L 9911 15087 L 10021 15194 L 10156 15203 L
10175 15223 L 10292 15340 L 10427 15341 L 10563 15344 L
10698 15344 L 10834 15345 L 10969 15344 L 11088 15223 L
11105 15206 L 11222 15087 L 11218 14951 L 11105 14841 L
11079 14816 L 11079 14680 L 11080 14544 L 11083 14409 L
11105 14385 L 11240 14377 L 11376 14379 L 11511 14384 L
11647 14385 L 11782 14325 L 11866 14273 L 11918 14244 L
12008 14137 L 11918 14059 L 11896 14002 L 11782 13894 L
11745 13866 L 11647 13759 L 11618 13730 L 11647 13697 L
11739 13594 L 11782 13548 L 11863 13459 L 11859 13323 L
11862 13187 L 11867 13052 L 11873 12916 L 11868 12780 L
11861 12645 L 11846 12509 L 11882 12373 L 11794 12238 L
11782 12195 L 11731 12102 L 11647 12019 L 11601 11966 L
11527 11831 L 11511 11797 L 11442 11695 L 11376 11627 L
11311 11559 L 11240 11503 L 11110 11423 L 11105 11421 L
10969 11360 L 10834 11297 L 10801 11288 L 10698 11257 L
10563 11243 L 10427 11280 L 10418 11288 L 10292 11392 L
10266 11423 L 10204 11559 L 10292 11621 L 10330 11695 L
10351 11831 L 10427 11952 L 10563 11966 L 10563 11966 L
10589 12102 L 10665 12238 L 10698 12313 L 10755 12373 L
10698 12453 L 10634 12509 L 10698 12560 L 10794 12645 L
10698 12653 L 10563 12679 L 10427 12648 L 10423 12645 L
10292 12542 L 10251 12509 L 10156 12426 L 10021 12446 L
9885 12488 L 9865 12509 L 9849 12645 L 9836 12780 L
9818 12916 L 9750 12989 L 9614 13003 L 9479 13030 L
9402 13052 L 9343 13113 L 9225 13187 L 9343 13267 L
9373 13323 L 9392 13459 L 9479 13481 L 9584 13594 L
9614 13621 L 9750 13648 L 9885 13667 L 10021 13719 L
10029 13730 L 10053 13866 L 10021 13908 L 9885 13945 L
9750 13961 L 9614 13967 L 9577 14002 L 9479 14086 L
9343 14126 L 9330 14137 L 9208 14206 L 9115 14137 L
9072 14100 L 8952 14002 L 8937 13987 L 8812 13866 L
8801 13855 L 8779 13866 L 8767 14002 L 8720 14137 L
8666 14270 L 8665 14273 L 8598 14409 L 8542 14544 L
8530 14563 L 8435 14680 L 8395 14716 L 8259 14806 L
8249 14816 L 8259 14818 L 8395 14847 L 8467 14951 L
8530 15022 L 8582 15087 L 8587 15223 L 8666 15307 L

8801 15321 L 8833 15358 L 8937 15459 L 9072 15466 L
9101 15494 L 9104 15630 L 9208 15741 L 9231 15765 L
9343 15878 L 9479 15878 L 9479 15895 L 9343 15895 L
9214 15765 L 9208 15759 L 9087 15630 L 9083 15494 L
9072 15483 L 8937 15477 L 8814 15358 L 8801 15344 L
8666 15335 L 8561 15223 L 8555 15087 L 8530 15056 L
8437 14951 L 8395 14891 L 8259 14867 L 8124 14836 L
7988 14835 L N E
74 O
8032 16173 8026 16173 8026 16172 8032 16172 8101 16172 8101 16173
8032 16173 7 N F
8026 16172 8032 16172 8101 16172 8124 16155 8259 16116 8309 16172
8355 16172 8259 16062 8124 16098 8026 16172 10 N F
56 O
8223 16173 8101 16173 8101 16172 8223 16172 8248 16172 8248 16173
8223 16173 7 N F
8101 16172 8124 16155 8259 16116 8309 16172 8262 16172 8259 16169
8248 16172 8223 16172 8101 16172 9 N F
38 O
8259 16173 8248 16173 8248 16173 8262 16173 8262 16173 8259 16173
6 N F
8262 16173 8248 16173 8248 16172 8262 16172 8262 16173 5 N F
74 O
8259 14818 M 8249 14816 L 8259 14806 L 8395 14716 L 8435 14680 L
8530 14563 L 8542 14544 L 8598 14409 L 8665 14273 L
8666 14270 L 8720 14137 L 8767 14002 L 8779 13866 L
8801 13855 L 8812 13866 L 8937 13987 L 8952 14002 L
9072 14100 L 9115 14137 L 9208 14206 L 9330 14137 L
9343 14126 L 9479 14086 L 9577 14002 L 9614 13967 L
9750 13961 L 9885 13945 L 10021 13908 L 10053 13866 L
10029 13730 L 10021 13719 L 9885 13667 L 9750 13648 L
9614 13621 L 9584 13594 L 9479 13481 L 9392 13459 L
9373 13323 L 9343 13267 L 9225 13187 L 9343 13113 L
9402 13052 L 9479 13030 L 9614 13003 L 9750 12989 L
9818 12916 L 9836 12780 L 9849 12645 L 9865 12509 L
9885 12488 L 10021 12446 L 10156 12426 L 10251 12509 L
10292 12542 L 10423 12645 L 10427 12648 L 10563 12679 L
10698 12653 L 10794 12645 L 10698 12560 L 10634 12509 L
10698 12453 L 10755 12373 L 10698 12313 L 10665 12238 L
10589 12102 L 10563 11966 L 10563 11966 L 10427 11952 L
10351 11831 L 10330 11695 L 10292 11621 L 10204 11559 L
10266 11423 L 10292 11392 L 10418 11288 L 10427 11280 L
10563 11243 L 10698 11257 L 10801 11288 L 10834 11297 L

10969 11360 L 11105 11421 L 11110 11423 L 11240 11503 L
11311 11559 L 11376 11627 L 11442 11695 L 11511 11797 L
11527 11831 L 11601 11966 L 11647 12019 L 11731 12102 L
11782 12195 L 11794 12238 L 11882 12373 L 11846 12509 L
11861 12645 L 11868 12780 L 11873 12916 L 11867 13052 L
11862 13187 L 11859 13323 L 11863 13459 L 11782 13548 L
11739 13594 L 11647 13697 L 11618 13730 L 11647 13759 L
11745 13866 L 11782 13894 L 11896 14002 L 11918 14059 L
12008 14137 L 11918 14244 L 11866 14273 L 11782 14325 L
11647 14385 L 11511 14384 L 11376 14379 L 11240 14377 L
11105 14385 L 11083 14409 L 11080 14544 L 11079 14680 L
11079 14816 L 11105 14841 L 11218 14951 L 11222 15087 L
11105 15206 L 11088 15223 L 10969 15344 L 10834 15345 L
10698 15344 L 10563 15344 L 10427 15341 L 10292 15340 L
10175 15223 L 10156 15203 L 10021 15194 L 9911 15087 L
9885 15058 L 9779 14951 L 9750 14923 L 9614 14928 L
9590 14951 L 9586 15087 L 9589 15223 L 9614 15250 L
9720 15358 L 9722 15494 L 9750 15528 L 9851 15630 L
9750 15733 L 9723 15765 L 9614 15877 L 9479 15878 L
9479 15862 L 9614 15860 L 9706 15765 L 9750 15712 L
9830 15630 L 9750 15549 L 9704 15494 L 9702 15358 L
9614 15269 L 9572 15223 L 9566 15087 L 9570 14951 L
9614 14907 L 9750 14901 L 9802 14951 L 9885 15035 L
9932 15087 L 10021 15174 L 10156 15183 L 10194 15223 L
10292 15321 L 10427 15323 L 10563 15326 L 10698 15325 L
10834 15325 L 10969 15322 L 11067 15223 L 11105 15185 L
11202 15087 L 11197 14951 L 11105 14862 L 11058 14816 L
11057 14680 L 11057 14544 L 11059 14409 L 11105 14359 L
11240 14352 L 11376 14355 L 11511 14359 L 11647 14363 L
11770 14273 L 11782 14266 L 11918 14168 L 11944 14137 L
11918 14115 L 11875 14002 L 11782 13914 L 11719 13866 L
11647 13787 L 11590 13730 L 11647 13664 L 11710 13594 L
11782 13516 L 11834 13459 L 11829 13323 L 11831 13187 L
11835 13052 L 11840 12916 L 11833 12780 L 11819 12645 L
11790 12509 L 11782 12480 L 11647 12469 L 11533 12373 L
11511 12353 L 11382 12238 L 11376 12231 L 11240 12124 L
11213 12102 L 11105 11999 L 11062 11966 L 10969 11853 L
10932 11831 L 10834 11790 L 10698 11758 L 10563 11770 L
10484 11831 L 10563 11840 L 10675 11966 L 10672 12102 L
10698 12138 L 10834 12194 L 10866 12238 L 10969 12307 L
11068 12373 L 11105 12415 L 11240 12463 L 11346 12509 L
11240 12526 L 11105 12551 L 10969 12569 L 10905 12645 L
10834 12709 L 10698 12714 L 10610 12780 L 10563 12822 L

10513 12780 L 10427 12714 L 10345 12645 L 10292 12603 L
10176 12509 L 10156 12492 L 10077 12509 L 10034 12645 L
10021 12683 L 9987 12780 L 9931 12916 L 9885 12992 L
9846 13052 L 9750 13186 L 9749 13187 L 9705 13323 L
9700 13459 L 9716 13594 L 9750 13601 L 9885 13623 L
10021 13662 L 10075 13730 L 10088 13866 L 10021 13954 L
9885 13986 L 9759 14002 L 9750 14005 L 9614 14021 L
9479 14123 L 9434 14137 L 9343 14191 L 9208 14237 L
9072 14137 L 9072 14137 L 8937 14089 L 8870 14137 L
8801 14232 L 8782 14273 L 8710 14409 L 8666 14520 L
8656 14544 L 8572 14680 L 8530 14739 L 8444 14816 L
8498 14951 L 8530 14988 L 8608 15087 L 8614 15223 L
8666 15279 L 8801 15299 L 8851 15358 L 8937 15441 L
9072 15449 L 9118 15494 L 9120 15630 L 9208 15724 L
9248 15765 L 9343 15861 L 9479 15862 L 9479 15878 L
9343 15878 L 9231 15765 L 9208 15741 L 9104 15630 L
9101 15494 L 9072 15466 L 8937 15459 L 8833 15358 L
8801 15321 L 8666 15307 L 8587 15223 L 8582 15087 L
8530 15022 L 8467 14951 L 8395 14847 L 8259 14818 L
N E
38 O
8248 16172 8259 16169 8262 16172 8248 16172 4 N F
56 O
8262 16172 8262 16173 8309 16173 8309 16172 8262 16172 5 N F
8309 16173 8262 16173 8262 16173 8309 16173 8309 16173 5 N F
74 O
8309 16172 8309 16173 8355 16173 8355 16172 8309 16172 5 N F
8355 16173 8309 16173 8309 16173 8355 16173 8355 16173 5 N F
92 O
8355 16172 8355 16173 8414 16173 8420 16173 8420 16172 8414 16172
8355 16172 7 N F
56 O
8498 14951 M 8444 14816 L 8530 14739 L 8572 14680 L 8656 14544 L
8666 14520 L 8710 14409 L 8782 14273 L 8801 14232 L
8870 14137 L 8937 14089 L 9072 14137 L 9072 14137 L
9208 14237 L 9343 14191 L 9434 14137 L 9479 14123 L
9614 14021 L 9750 14005 L 9759 14002 L 9885 13986 L
10021 13954 L 10088 13866 L 10075 13730 L 10021 13662 L
9885 13623 L 9750 13601 L 9716 13594 L 9700 13459 L
9705 13323 L 9749 13187 L 9750 13186 L 9846 13052 L
9885 12992 L 9931 12916 L 9987 12780 L 10021 12683 L
10034 12645 L 10077 12509 L 10156 12492 L 10176 12509 L
10292 12603 L 10345 12645 L 10427 12714 L 10513 12780 L

10563 12822 L 10610 12780 L 10698 12714 L 10834 12709 L
10905 12645 L 10969 12569 L 11105 12551 L 11240 12526 L
11346 12509 L 11240 12463 L 11105 12415 L 11068 12373 L
10969 12307 L 10866 12238 L 10834 12194 L 10698 12138 L
10672 12102 L 10675 11966 L 10563 11840 L 10484 11831 L
10563 11770 L 10698 11758 L 10834 11790 L 10932 11831 L
10969 11853 L 11062 11966 L 11105 11999 L 11213 12102 L
11240 12124 L 11376 12231 L 11382 12238 L 11511 12353 L
11533 12373 L 11647 12469 L 11782 12480 L 11790 12509 L
11819 12645 L 11833 12780 L 11840 12916 L 11835 13052 L
11831 13187 L 11829 13323 L 11834 13459 L 11782 13516 L
11710 13594 L 11647 13664 L 11590 13730 L 11647 13787 L
11719 13866 L 11782 13914 L 11875 14002 L 11918 14115 L
11944 14137 L 11918 14168 L 11782 14266 L 11770 14273 L
11647 14363 L 11511 14359 L 11376 14355 L 11240 14352 L
11105 14359 L 11059 14409 L 11057 14544 L 11057 14680 L
11058 14816 L 11105 14862 L 11197 14951 L 11202 15087 L
11105 15185 L 11067 15223 L 10969 15322 L 10834 15325 L
10698 15325 L 10563 15326 L 10427 15323 L 10292 15321 L
10194 15223 L 10156 15183 L 10021 15174 L 9932 15087 L
9885 15035 L 9802 14951 L 9750 14901 L 9614 14907 L
9570 14951 L 9566 15087 L 9572 15223 L 9614 15269 L
9702 15358 L 9704 15494 L 9750 15549 L 9830 15630 L
9750 15712 L 9706 15765 L 9614 15860 L 9479 15862 L
9479 15846 L 9614 15842 L 9689 15765 L 9750 15691 L
9809 15630 L 9750 15570 L 9687 15494 L 9683 15358 L
9614 15288 L 9554 15223 L 9547 15087 L 9549 14951 L
9614 14887 L 9750 14879 L 9825 14951 L 9885 15012 L
9952 15087 L 10021 15154 L 10156 15163 L 10213 15223 L
10292 15302 L 10427 15304 L 10563 15307 L 10698 15306 L
10834 15304 L 10969 15301 L 11046 15223 L 11105 15165 L
11181 15087 L 11175 14951 L 11105 14883 L 11037 14816 L
11034 14680 L 11033 14544 L 11034 14409 L 11105 14332 L
11240 14327 L 11376 14330 L 11511 14335 L 11647 14340 L
11739 14273 L 11782 14247 L 11905 14137 L 11854 14002 L
11782 13934 L 11693 13866 L 11647 13816 L 11562 13730 L
11647 13631 L 11680 13594 L 11782 13483 L 11805 13459 L
11799 13323 L 11800 13187 L 11804 13052 L 11806 12916 L
11797 12780 L 11782 12675 L 11647 12778 L 11511 12721 L
11376 12705 L 11240 12693 L 11105 12721 L 10969 12726 L
10834 12776 L 10698 12775 L 10691 12780 L 10563 12894 L
10428 12780 L 10427 12780 L 10416 12780 L 10292 12889 L
10281 12916 L 10265 13052 L 10292 13099 L 10361 13187 L

10292 13269 L 10246 13323 L 10156 13427 L 10123 13459 L
10021 13521 L 9974 13594 L 10021 13605 L 10121 13730 L
10123 13866 L 10021 13999 L 10011 14002 L 9885 14066 L
9750 14116 L 9668 14137 L 9614 14158 L 9479 14213 L
9343 14261 L 9208 14268 L 9072 14247 L 8937 14252 L
8921 14273 L 8828 14409 L 8801 14456 L 8764 14544 L
8684 14680 L 8666 14712 L 8600 14816 L 8530 14937 L
8528 14951 L 8530 14954 L 8635 15087 L 8640 15223 L
8666 15250 L 8801 15277 L 8870 15358 L 8937 15423 L
9072 15432 L 9135 15494 L 9136 15630 L 9208 15706 L
9264 15765 L 9343 15844 L 9479 15846 L 9479 15862 L
9343 15861 L 9248 15765 L 9208 15724 L 9120 15630 L
9118 15494 L 9072 15449 L 8937 15441 L 8851 15358 L
8801 15299 L 8666 15279 L 8614 15223 L 8608 15087 L
8530 14988 L 8498 14951 L N E
129 O
8530 7165 8486 7081 8530 7040 8575 7081 8530 7165 5 N F
8530 8443 8527 8438 8530 8397 8666 8429 8674 8438 8759 8574
8666 8644 8602 8574 8530 8443 9 N F
38 O
8530 14954 M 8528 14951 L 8530 14937 L 8600 14816 L 8666 14712 L
8684 14680 L 8764 14544 L 8801 14456 L 8828 14409 L
8921 14273 L 8937 14252 L 9072 14247 L 9208 14268 L
9343 14261 L 9479 14213 L 9614 14158 L 9668 14137 L
9750 14116 L 9885 14066 L 10011 14002 L 10021 13999 L
10123 13866 L 10121 13730 L 10021 13605 L 9974 13594 L
10021 13521 L 10123 13459 L 10156 13427 L 10246 13323 L
10292 13269 L 10361 13187 L 10292 13099 L 10265 13052 L
10281 12916 L 10292 12889 L 10416 12780 L 10427 12780 L
10428 12780 L 10563 12894 L 10691 12780 L 10698 12775 L
10834 12776 L 10969 12726 L 11105 12721 L 11240 12693 L
11376 12705 L 11511 12721 L 11647 12778 L 11782 12675 L
11797 12780 L 11806 12916 L 11804 13052 L 11800 13187 L
11799 13323 L 11805 13459 L 11782 13483 L 11680 13594 L
11647 13631 L 11562 13730 L 11647 13816 L 11693 13866 L
11782 13934 L 11854 14002 L 11905 14137 L 11782 14247 L
11739 14273 L 11647 14340 L 11511 14335 L 11376 14330 L
11240 14327 L 11105 14332 L 11034 14409 L 11033 14544 L
11034 14680 L 11037 14816 L 11105 14883 L 11175 14951 L
11181 15087 L 11105 15165 L 11046 15223 L 10969 15301 L
10834 15304 L 10698 15306 L 10563 15307 L 10427 15304 L
10292 15302 L 10213 15223 L 10156 15163 L 10021 15154 L
9952 15087 L 9885 15012 L 9825 14951 L 9750 14879 L

9614 14887 L 9549 14951 L 9547 15087 L 9554 15223 L
9614 15288 L 9683 15358 L 9687 15494 L 9750 15570 L
9809 15630 L 9750 15691 L 9689 15765 L 9614 15842 L
9479 15846 L 9479 15830 L 9614 15824 L 9671 15765 L
9750 15669 L 9788 15630 L 9750 15591 L 9670 15494 L
9664 15358 L 9614 15307 L 9537 15223 L 9528 15087 L
9528 14951 L 9614 14866 L 9750 14857 L 9848 14951 L
9885 14988 L 9973 15087 L 10021 15133 L 10156 15143 L
10232 15223 L 10292 15283 L 10427 15286 L 10563 15289 L
10698 15287 L 10834 15284 L 10969 15280 L 11025 15223 L
11105 15144 L 11161 15087 L 11153 14951 L 11105 14904 L
11016 14816 L 11011 14680 L 11010 14544 L 11010 14409 L
11105 14305 L 11240 14302 L 11376 14305 L 11511 14310 L
11647 14317 L 11708 14273 L 11782 14228 L 11884 14137 L
11833 14002 L 11782 13953 L 11667 13866 L 11647 13844 L
11534 13730 L 11647 13598 L 11650 13594 L 11647 13562 L
11530 13459 L 11511 13424 L 11377 13323 L 11376 13322 L
11372 13323 L 11240 13370 L 11105 13399 L 10969 13434 L
10834 13429 L 10768 13459 L 10698 13492 L 10563 13557 L
10498 13594 L 10427 13632 L 10292 13724 L 10280 13730 L
10174 13866 L 10156 13888 L 10121 14002 L 10021 14116 L
9973 14137 L 9885 14206 L 9775 14273 L 9750 14289 L
9614 14325 L 9479 14348 L 9343 14373 L 9208 14393 L
9125 14409 L 9072 14423 L 8937 14462 L 8888 14544 L
8806 14680 L 8801 14687 L 8732 14816 L 8673 14951 L
8666 14989 L 8662 15087 L 8666 15199 L 8669 15223 L
8801 15254 L 8889 15358 L 8937 15404 L 9072 15415 L
9153 15494 L 9152 15630 L 9208 15689 L 9281 15765 L
9343 15828 L 9479 15830 L 9479 15846 L 9343 15844 L
9264 15765 L 9208 15706 L 9136 15630 L 9135 15494 L
9072 15432 L 8937 15423 L 8870 15358 L 8801 15277 L
8666 15250 L 8640 15223 L 8635 15087 L 8530 14954 L
N E
129 O
8640 7353 8565 7217 8666 7153 8729 7217 8703 7353 8801 7422
8841 7489 8801 7598 8666 7596 8585 7489 8640 7353 11 N F
20 O
8666 15199 8662 15087 8666 14989 8673 14951 8732 14816 8801 14687
8806 14680 8888 14544 8937 14462 9072 14423 9125 14409 9208 14393
9343 14373 9479 14348 9614 14325 9750 14289 9775 14273 9885 14206
9973 14137 10021 14116 10121 14002 10156 13888 10174 13866 10280 13730
10292 13724 10427 13632 10498 13594 10563 13557 10698 13492 10768 13459
10834 13429 10969 13434 11105 13399 11240 13370 11372 13323 11376 13322

11377 13323 11511 13424 11530 13459 11647 13562 11650 13594 11647 13598
11534 13730 11647 13844 11667 13866 11782 13953 11833 14002 11884 14137
11782 14228 11708 14273 11647 14317 11511 14310 11376 14305 11240 14302
11105 14305 11010 14409 11010 14544 11011 14680 11016 14816 11105 14904
11153 14951 11161 15087 11105 15144 11025 15223 10969 15280 10834 15284
10698 15287 10563 15289 10427 15286 10292 15283 10232 15223 10156 15143
10021 15133 9973 15087 9885 14988 9848 14951 9750 14857 9614 14866
9528 14951 9528 15087 9537 15223 9614 15307 9664 15358 9670 15494
9750 15591 9788 15630 9750 15669 9671 15765 9614 15824 9479 15830
9479 15813 9614 15806 9654 15765 9750 15648 9767 15630 9750 15612
9653 15494 9645 15358 9614 15327 9519 15223 9508 15087 9507 14951
9614 14846 9750 14835 9872 14951 9885 14965 9994 15087 10021 15113
10156 15123 10252 15223 10292 15263 10427 15268 10563 15270 10698 15269
10834 15263 10969 15259 11005 15223 11105 15123 11140 15087 11132 14951
11105 14925 10994 14816 10988 14680 10987 14544 10985 14409 11105 14278
11240 14276 11376 14281 11511 14286 11647 14295 11676 14273 11782 14209
11862 14137 11812 14002 11782 13973 11651 14002 11647 14012 11616 14002
11533 13866 11511 13838 11428 13866 11376 13909 11321 14002 11376 14120
11382 14137 11376 14141 11240 14208 11105 14158 11083 14137 11023 14002
10969 13977 10834 13933 10698 13891 10563 13900 10427 13977 10383 14002
10292 14059 10214 14137 10156 14178 10069 14273 10021 14331 9941 14409
9885 14457 9750 14529 9614 14514 9479 14512 9343 14535 9319 14544
9208 14598 9072 14663 9036 14680 8937 14736 8888 14816 8806 14951
8801 14977 8786 15087 8763 15223 8801 15232 8908 15358 8937 15386
9072 15398 9170 15494 9169 15630 9208 15672 9298 15765 9343 15811
9479 15813 9479 15830 9343 15828 9281 15765 9208 15689 9152 15630
9153 15494 9072 15415 8937 15404 8889 15358 8801 15254 8669 15223
8666 15199 199 N F
2 O
8801 15232 8763 15223 8786 15087 8801 14977 8806 14951 8888 14816
8937 14736 9036 14680 9072 14663 9208 14598 9319 14544 9343 14535
9479 14512 9614 14514 9750 14529 9885 14457 9941 14409 10021 14331
10069 14273 10156 14178 10214 14137 10292 14059 10383 14002 10427 13977
10563 13900 10698 13891 10834 13933 10969 13977 11023 14002 11083 14137
11105 14158 11240 14208 11376 14141 11382 14137 11376 14120 11321 14002
11376 13909 11428 13866 11511 13838 11533 13866 11616 14002 11647 14012
11651 14002 11782 13973 11812 14002 11862 14137 11782 14209 11676 14273
11647 14295 11511 14286 11376 14281 11240 14276 11105 14278 10985 14409
10987 14544 10988 14680 10994 14816 11105 14925 11132 14951 11140 15087
11105 15123 11005 15223 10969 15259 10834 15263 10698 15269 10563 15270
10427 15268 10292 15263 10252 15223 10156 15123 10021 15113 9994 15087
9885 14965 9872 14951 9750 14835 9614 14846 9507 14951 9508 15087
9519 15223 9614 15327 9645 15358 9653 15494 9750 15612 9767 15630

9750 15648 9654 15765 9614 15806 9479 15813 9343 15811 9298 15765
9208 15672 9169 15630 9170 15494 9072 15398 8937 15386 8908 15358
8801 15232 97 N F
74 O
9072 13204 9057 13187 9072 13161 9198 13187 9072 13204 5 N F
129 O
9072 9940 9070 9931 9072 9929 9090 9931 9072 9940 5 N F
9164 5860 9091 5725 9105 5589 9165 5453 9179 5453 9263 5453
9343 5486 9405 5589 9417 5725 9344 5860 9343 5862 9208 5943
9208 5757 9251 5725 9208 5669 9183 5725 9208 5757 9208 5943
9164 5860 19 N F
9179 5453 9165 5453 9165 5453 9179 5453 9263 5453 9263 5453
9179 5453 7 N F
111 O
9208 5757 9183 5725 9208 5669 9251 5725 9208 5757 5 N F
129 O
9235 6539 9232 6403 9246 6267 9343 6205 9369 6267 9439 6403
9479 6491 9502 6539 9479 6652 9472 6674 9343 6796 9276 6674
9235 6539 13 N F
9393 6946 9353 6810 9479 6679 9557 6810 9614 6893 9741 6946
9750 6955 9786 6946 9750 6931 9750 6743 9881 6674 9856 6539
9757 6403 9750 6366 9745 6403 9690 6539 9694 6674 9750 6743
9750 6931 9713 6810 9633 6674 9633 6539 9655 6403 9614 6313
9614 6196 9715 6132 9647 5996 9614 5957 9594 5996 9572 6132
9614 6196 9614 6313 9571 6267 9511 6132 9519 5996 9562 5860
9614 5788 9723 5860 9750 5902 9818 5996 9874 6132 9885 6230
9906 6267 9948 6403 9989 6539 10021 6643 10071 6674 10102 6810
10064 6946 10031 7081 10021 7097 9912 7217 9885 7241 9750 7242
9725 7217 9614 7158 9512 7081 9479 7054 9393 6946 59 N F
111 O
9420 10609 9369 10474 9479 10360 9536 10338 9591 10202 9614 10174
9695 10202 9750 10209 9881 10338 9868 10474 9877 10609 9750 10682
9750 10409 9813 10338 9750 10276 9651 10338 9750 10409 9750 10682
9718 10745 9614 10818 9614 10662 9643 10609 9649 10474 9614 10411
9479 10406 9413 10474 9479 10571 9579 10609 9614 10662 9614 10818
9479 10774 9456 10745 9420 10609 33 N F
92 O
9479 10571 9413 10474 9479 10406 9614 10411 9649 10474 9643 10609
9614 10662 9579 10609 9479 10571 9479 10505 9534 10474 9479 10451
9457 10474 9479 10505 9479 10571 15 N F
74 O
9479 10505 9457 10474 9479 10451 9534 10474 9479 10505 5 N F
129 O

9614 9514 9546 9388 9614 9288 9719 9388 9614 9514 5 N F
111 O
9614 6196 9572 6132 9594 5996 9614 5957 9647 5996 9715 6132
9614 6196 7 N F
92 O
9750 10409 9651 10338 9750 10276 9813 10338 9750 10409 5 N F
111 O
9694 6674 9690 6539 9745 6403 9750 6366 9757 6403 9856 6539
9881 6674 9750 6743 9750 6581 9754 6539 9750 6533 9747 6539
9750 6581 9750 6743 9694 6674 15 N F
92 O
9750 6581 9747 6539 9750 6533 9754 6539 9750 6581 5 N F
129 O
9885 5531 9840 5453 9943 5453 10134 5453 10325 5453 10517 5453
10708 5453 10899 5453 11090 5453 11281 5453 11472 5453 11664 5453
11855 5453 12046 5453 12237 5453 12428 5453 12619 5453 12810 5453
13002 5453 13002 8465 12989 8438 12980 8303 12923 8167 12927 8031
12890 7896 12884 7760 12866 7711 12790 7624 12800 7489 12731 7399
12677 7353 12694 7217 12601 7081 12595 7051 12553 6946 12460 6889
12350 6810 12406 6674 12324 6639 12189 6572 12069 6539 12100 6403
12053 6387 11953 6403 11918 6409 11885 6403 11860 6267 11782 6187
11647 6203 11511 6154 11448 6132 11435 5996 11376 5964 11337 5996
11240 6070 11105 6057 10969 6028 10848 5996 10834 5991 10815 5996
10779 6132 10698 6150 10582 6132 10563 6121 10427 6070 10292 6029
10156 6078 10021 6047 10000 5996 9930 5860 9920 5725 9921 5589
9885 5531 73 N F
9885 8583 9883 8574 9885 8572 9887 8574 9885 8583 5 N F
111 O
10021 10115 9976 10067 10021 10038 10067 10067 10021 10115 5 N F
129 O
10156 8772 10078 8710 10156 8688 10194 8710 10156 8772 5 N F
111 O
10292 9678 10277 9660 10292 9644 10309 9660 10292 9678 5 N F
74 O
10427 15908 10424 15901 10427 15898 10453 15901 10427 15908 5 N F
10551 16172 10465 16037 10563 15912 10613 15901 10698 15887 10834 15860
10959 15901 10969 15904 11105 15958 11215 15901 11140 15765 11105 15685
11054 15630 11105 15580 11240 15569 11376 15572 11495 15494 11511 15458
11535 15358 11581 15223 11610 15087 11647 14991 11659 14951 11781 14816
11782 14815 11918 14701 11976 14680 12053 14582 12189 14559 12211 14544
12324 14437 12335 14409 12373 14273 12460 14191 12572 14137 12595 14119
12731 14037 12822 14137 12731 14264 12731 14177 12759 14137 12731 14106
12683 14137 12731 14177 12731 14264 12726 14273 12669 14409 12595 14480

12529 14544 12460 14657 12460 14562 12471 14544 12595 14423 12610 14409
12677 14273 12595 14183 12460 14261 12447 14273 12390 14409 12399 14544
12460 14562 12460 14657 12447 14680 12460 14691 12475 14680 12595 14594
12647 14544 12731 14459 12787 14409 12740 14273 12866 14184 12959 14137
13002 14114 13002 14241 12866 14251 12836 14273 12841 14409 12731 14507
12694 14544 12595 14638 12537 14680 12460 14735 12335 14816 12324 14825
12245 14951 12189 15010 12121 15087 12053 15156 11986 15223 11974 15358
12053 15435 12108 15494 12189 15580 12324 15579 12379 15630 12372 15765
12460 15878 12480 15901 12595 16021 12607 16037 12731 16166 12736 16172
12716 16172 12595 16060 12579 16037 12460 15922 12426 15901 12324 15829
12324 15787 12340 15765 12353 15630 12324 15603 12189 15604 12085 15494
12053 15460 11948 15358 11952 15223 12053 15122 12087 15087 12189 14972
12209 14951 12189 14918 12108 14951 12053 14988 11918 15087 11917 15087
11839 15223 11853 15358 11894 15494 11918 15532 12013 15630 12053 15649
12189 15691 12284 15765 12324 15787 12324 15829 12209 15765 12189 15750
12053 15670 11970 15630 11918 15577 11866 15494 11798 15358 11782 15291
11782 15170 11824 15087 11918 15020 12002 14951 12053 14872 12189 14857
12298 14816 12324 14801 12394 14680 12324 14565 12189 14651 12146 14680
12053 14762 11918 14792 11889 14816 11782 14902 11737 14951 11725 15087
11782 15170 11782 15291 11647 15266 11607 15358 11542 15494 11511 15614
11479 15630 11376 15646 11240 15713 11225 15765 11240 15787 11304 15901
11376 16001 11402 16037 11376 16061 11240 16079 11105 16113 11016 16037
10969 16005 10834 15954 10698 15994 10627 16037 10671 16172 10551 16172
186 N F
10551 16173 10551 16172 10671 16172 10671 16173 10551 16173 5 N F
10551 16173 10551 16173 10671 16173 10671 16173 10563 16173 10551 16173
6 N F
56 O
10671 16172 10627 16037 10698 15994 10834 15954 10969 16005 11016 16037
11105 16113 11240 16079 11376 16061 11402 16037 11376 16001 11304 15901
11240 15787 11225 15765 11240 15713 11376 15646 11479 15630 11511 15614
11542 15494 11607 15358 11647 15266 11782 15291 11798 15358 11866 15494
11918 15577 11970 15630 12053 15670 12189 15750 12209 15765 12324 15829
12426 15901 12460 15922 12579 16037 12595 16060 12716 16172 12660 16172
12595 16112 12541 16037 12460 15958 12368 15901 12324 15870 12189 15782
12165 15765 12053 15691 11926 15630 11918 15621 11838 15494 11782 15394
11647 15383 11576 15494 11537 15630 11511 15671 11376 15690 11284 15765
11373 15901 11376 15905 11473 16037 11376 16126 11240 16150 11190 16172
11090 16172 10899 16172 10834 16172 10834 16090 10843 16037 10834 16033
10823 16037 10834 16090 10834 16172 10708 16172 10671 16172 71 N F
38 O
10834 16090 10823 16037 10834 16033 10843 16037 10834 16090 5 N F
111 O

11105 15292 11051 15358 11105 15397 11240 15465 11376 15432 11402 15358
11440 15223 11511 15108 11515 15087 11529 14951 11537 14816 11647 14704
11718 14680 11782 14651 11838 14544 11782 14411 11762 14409 11647 14408
11511 14409 11376 14404 11240 14402 11152 14409 11105 14508 11104 14544
11102 14680 11101 14816 11105 14820 11240 14951 11240 14957 11299 15087
11240 15128 11159 15223 11105 15292 33 N F
129 O
11240 7809 11221 7760 11234 7624 11240 7567 11376 7585 11511 7569
11647 7549 11781 7624 11782 7625 11789 7624 11918 7607 12045 7624
12053 7626 12089 7760 12102 7896 12055 8031 12053 8037 11963 8167
11918 8284 11782 8169 11780 8167 11647 8125 11511 8054 11473 8031
11376 7900 11372 7896 11240 7809 27 N F
38 O
11190 16172 11240 16150 11376 16126 11473 16037 11376 15905 11373 15901
11284 15765 11376 15690 11511 15671 11537 15630 11576 15494 11647 15383
11782 15394 11838 15494 11918 15621 11926 15630 12053 15691 12165 15765
12189 15782 12324 15870 12368 15901 12460 15958 12541 16037 12595 16112
12660 16172 12619 16172 12604 16172 12595 16164 12504 16037 12460 15994
12324 15909 12317 15901 12189 15805 12134 15765 12053 15712 11918 15644
11899 15630 11810 15494 11782 15444 11647 15438 11611 15494 11567 15630
11511 15717 11376 15734 11337 15765 11376 15824 11433 15901 11511 16001
11525 16037 11517 16172 11472 16172 11281 16172 11190 16172 53 N F
20 O
11376 15824 11337 15765 11376 15734 11511 15717 11567 15630 11611 15494
11647 15438 11782 15444 11810 15494 11899 15630 11918 15644 12053 15712
12134 15765 12189 15805 12317 15901 12324 15909 12460 15994 12504 16037
12595 16164 12604 16172 12575 16172 12467 16037 12460 16030 12324 15940
12286 15901 12189 15828 12102 15765 12053 15733 11918 15661 11875 15630
11783 15494 11782 15493 11647 15493 11646 15494 11596 15630 11511 15764
11495 15765 11494 15901 11511 15923 11555 16037 11563 16172 11517 16172
11525 16037 11511 16001 11433 15901 11376 15824 46 N F
2 O
11511 15923 11494 15901 11495 15765 11511 15764 11596 15630 11646 15494
11647 15493 11782 15493 11783 15494 11875 15630 11918 15661 12053 15733
12102 15765 12189 15828 12286 15901 12324 15940 12460 16030 12467 16037
12575 16172 12428 16172 12237 16172 12046 16172 11855 16172 11664 16172
11563 16172 11555 16037 11511 15923 27 N F
20 O
11563 16173 11517 16173 11517 16173 11563 16173 11563 16173 5 N F
11563 16173 11517 16173 11517 16172 11563 16172 11563 16173 5 N F
56 O
11782 15170 11725 15087 11737 14951 11782 14902 11889 14816 11918 14792
12053 14762 12146 14680 12189 14651 12324 14565 12394 14680 12324 14801

12324 14710 12341 14680 12324 14651 12280 14680 12324 14710 12324 14801
12298 14816 12189 14857 12053 14872 12002 14951 11918 15020 11918 14953
11919 14951 11918 14948 11912 14951 11918 14953 11918 15020 11824 15087
11782 15170 31 N F
92 O
11917 15087 11839 15223 11853 15358 11894 15494 11918 15532 12013 15630
12053 15649 12189 15691 12284 15765 12324 15787 12324 15656 12189 15632
12150 15630 12053 15604 11958 15494 11918 15429 11909 15358 11918 15231
11922 15358 12053 15485 12061 15494 12189 15629 12324 15627 12327 15630
12324 15656 12324 15787 12340 15765 12353 15630 12324 15603 12189 15604
12085 15494 12053 15460 11948 15358 11952 15223 12053 15122 12053 15088
12050 15087 12053 15085 12054 15087 12053 15088 12053 15122 12087 15087
12189 14972 12209 14951 12189 14918 12108 14951 12053 14988 11918 15087
11917 15087 49 N F
111 O
11918 15231 11909 15358 11918 15429 11958 15494 12053 15604 12150 15630
12189 15632 12324 15656 12327 15630 12324 15627 12189 15629 12061 15494
12053 15485 11922 15358 11918 15231 15 N F
38 O
11918 14953 11912 14951 11918 14948 11919 14951 11918 14953 5 N F
56 O
12053 15435 11974 15358 11986 15223 12053 15156 12121 15087 12189 15010
12245 14951 12324 14825 12335 14816 12460 14735 12537 14680 12595 14638
12694 14544 12731 14507 12841 14409 12836 14273 12866 14251 13002 14241
13002 14375 12958 14409 12866 14520 12818 14544 12731 14591 12612 14680
12595 14696 12460 14780 12404 14816 12324 14882 12281 14951 12189 15048
12154 15087 12053 15190 12020 15223 11999 15358 12053 15410 12131 15494
12189 15555 12324 15555 12406 15630 12403 15765 12460 15838 12515 15901
12595 15984 12633 16037 12731 16138 12758 16172 12736 16172 12731 16166
12607 16037 12595 16021 12480 15901 12460 15878 12372 15765 12379 15630
12324 15579 12189 15580 12108 15494 12053 15435 58 N F
129 O
12027 13187 11989 13323 11989 13459 12043 13594 12053 13614 12189 13708
12324 13665 12460 13709 12476 13730 12595 13786 12727 13730 12731 13722
12755 13594 12749 13459 12731 13412 12595 13383 12509 13459 12460 13488
12324 13492 12295 13459 12189 13398 12097 13323 12074 13187 12053 13168
12027 13187 25 N F
38 O
12053 15410 11999 15358 12020 15223 12053 15190 12154 15087 12189 15048
12281 14951 12324 14882 12404 14816 12460 14780 12595 14696 12612 14680
12731 14591 12818 14544 12866 14520 12958 14409 13002 14375 13002 14729
12902 14816 12866 14842 12731 14904 12595 14937 12568 14951 12460 15002
12382 14951 12324 14940 12317 14951 12189 15086 12188 15087 12064 15223

12053 15228 12025 15358 12053 15385 12154 15494 12189 15530 12324 15530
12432 15630 12434 15765 12460 15798 12551 15901 12595 15947 12659 16037
12731 16111 12780 16172 12758 16172 12731 16138 12633 16037 12595 15984
12515 15901 12460 15838 12403 15765 12406 15630 12324 15555 12189 15555
12131 15494 12053 15410 56 N F
92 O
12053 11866 12008 11831 12053 11705 12096 11831 12053 11866 5 N F
20 O
12053 15385 12025 15358 12053 15228 12064 15223 12188 15087 12189 15086
12317 14951 12324 14940 12382 14951 12460 15002 12568 14951 12595 14937
12731 14904 12866 14842 12902 14816 13002 14729 13002 15064 12959 15087
12866 15144 12743 15223 12731 15233 12615 15358 12595 15384 12460 15410
12324 15396 12232 15358 12189 15338 12053 15349 12051 15358 12053 15360
12178 15494 12189 15506 12324 15506 12458 15630 12460 15663 12494 15765
12586 15901 12595 15911 12685 16037 12731 16084 12801 16172 12780 16172
12731 16111 12659 16037 12595 15947 12551 15901 12460 15798 12434 15765
12432 15630 12324 15530 12189 15530 12154 15494 12053 15385 53 N F
111 O
12053 15085 12050 15087 12053 15088 12054 15087 12053 15085 5 N F
2 O
12053 15360 12051 15358 12053 15349 12189 15338 12232 15358 12324 15396
12460 15410 12595 15384 12615 15358 12731 15233 12743 15223 12866 15144
12959 15087 13002 15064 13002 16172 12810 16172 12801 16172 12731 16084
12685 16037 12595 15911 12586 15901 12494 15765 12460 15663 12458 15630
12324 15506 12189 15506 12178 15494 12053 15360 28 N F
38 O
12324 14710 12280 14680 12324 14651 12341 14680 12324 14710 5 N F
129 O
12324 6951 12319 6946 12324 6925 12331 6946 12324 6951 5 N F
56 O
12399 14544 12390 14409 12447 14273 12460 14261 12595 14183 12677 14273
12610 14409 12595 14423 12595 14337 12628 14273 12595 14236 12534 14273
12595 14337 12595 14423 12471 14544 12460 14562 12460 14443 12496 14409
12460 14374 12444 14409 12460 14443 12460 14562 12399 14544 23 N F
38 O
12460 14443 12444 14409 12460 14374 12496 14409 12460 14443 5 N F
12595 14337 12534 14273 12595 14236 12628 14273 12595 14337 5 N F
20 O
12575 16172 12575 16173 12604 16173 12604 16172 12575 16172 5 N F
12604 16173 12575 16173 12575 16173 12595 16173 12604 16173 12604 16173
6 N F
129 O
12595 13086 12575 13187 12595 13256 12672 13187 12595 13086 5 N F

111 O
12595 14537 12588 14544 12595 14550 12601 14544 12731 14411 12733 14409
12731 14401 12728 14409 12595 14537 9 N F
38 O
12604 16172 12604 16173 12619 16173 12660 16173 12660 16172 12619 16172
12604 16172 7 N F
56 O
12660 16172 12660 16173 12717 16173 12716 16172 12660 16172 5 N F
12717 16173 12660 16173 12660 16173 12717 16173 12717 16173 5 N F
12731 14177 12683 14137 12731 14106 12759 14137 12731 14177 5 N F
74 O
12716 16172 12717 16173 12736 16173 12736 16172 12716 16172 5 N F
12736 16173 12717 16173 12717 16173 12731 16173 12736 16173 12736 16173
6 N F
56 O
12736 16173 12736 16172 12758 16172 12758 16173 12736 16173 5 N F
12736 16173 12736 16173 12758 16173 12758 16173 12736 16173 5 N F
38 O
12758 16173 12758 16172 12780 16172 12780 16173 12758 16173 5 N F
12758 16173 12758 16173 12780 16173 12780 16173 12758 16173 5 N F
20 O
12780 16173 12780 16172 12801 16172 12801 16173 12780 16173 5 N F
12780 16173 12780 16173 12801 16173 12802 16173 12780 16173 5 N F
129 O
13002 5453 13002 5453 13002 8465 13002 8465 13002 5453 13002 5453
6 N F
147 O
13002 10788 13002 8465 13002 8465 13002 10788 13002 10788 5 N F
129 O
13002 12349 13002 10788 13002 10788 13002 12349 13002 12349 5 N F
111 O
13002 14040 13002 12349 13002 12349 13002 14040 13002 14040 5 N F
92 O
13002 14114 13002 14040 13002 14040 13002 14114 13002 14114 5 N F
74 O
13002 14241 13002 14114 13002 14114 13002 14241 13002 14241 5 N F
56 O
13002 14375 13002 14241 13002 14241 13002 14375 13002 14375 5 N F
38 O
13002 14729 13002 14375 13002 14375 13002 14729 13002 14729 5 N F
20 O
13002 15064 13002 14729 13002 14729 13002 15064 13002 15064 5 N F
2 O

13002 16172 13002 15064 13002 15064 13002 16173 13002 16173 13002 16172
6 N F
147 O
13002 8465 13002 8465 13002 10788 13002 10788 13002 10745 13002 10609
13002 10474 13002 10338 13002 10202 13002 10067 13002 9931 13002 9795
13002 9660 13002 9524 13002 9388 13002 9252 13002 9117 13002 8981
13002 8845 13002 8710 13002 8574 13002 8465 22 N F
129 O
13002 10788 13002 12349 13002 12349 13002 12238 13002 12102 13002 11966
13002 11831 13002 11695 13002 11559 13002 11423 13002 11288 13002 11152
13002 11016 13002 10881 13002 10788 13002 10788 16 N F
111 O
13002 12349 13002 14040 13002 14040 13002 14002 13002 13866 13002 13730
13002 13594 13002 13459 13002 13323 13002 13187 13002 13052 13002 12916
13002 12780 13002 12645 13002 12509 13002 12373 13002 12349 13002 12349
18 N F
92 O
13002 14040 13002 14114 13002 14114 13002 14040 13002 14040 5 N F
74 O
13002 14114 13002 14241 13002 14241 13002 14137 13002 14114 13002 14114
6 N F
56 O
13002 14241 13002 14375 13002 14375 13002 14273 13002 14241 13002 14241
6 N F
38 O
13002 14375 13002 14729 13002 14729 13002 14680 13002 14544 13002 14409
13002 14375 13002 14375 8 N F
20 O
13002 14729 13002 15064 13002 15064 13002 14951 13002 14816 13002 14729
13002 14729 7 N F
Ls Gr Gs Lr
-1200 1025 16500 18750 Oc
1 O
10639 15368 10640 15409 10679 15446 10675 15487 10710 15528 10753 15562
10792 15599 10827 15641 10872 15670 10920 15695 10946 15742 10985 15780
11030 15810 11080 15831 11122 15865 11160 15903 11196 15934 11247 15960
18 P
11247 15960 11300 15971 11330 16016 11355 16064 11389 16106 11419 16151
11428 16172 7 P
12840 16172 12841 16165 12836 16139 12812 16127 12785 16122 12767 16102
12743 16090 12718 16078 12698 16061 12682 16039 12656 16031 12637 16011
12633 15985 12626 15958 12621 15932 12608 15908 12584 15897 12561 15882
12544 15860 12528 15839 12506 15823 12482 15811 12458 15799 12434 15786

12425 15760 12449 15746 12444 15720 12419 15710 12402 15689 12377 15677
12359 15657 12333 15649 12323 15624 12298 15633 12283 15610 12257 15601
12230 15604 12204 15610 12182 15594 12168 15570 12146 15555 12149 15528
12128 15511 12107 15494 12081 15487 12067 15463 12040 15464 12022 15444
12010 15421 12003 15394 50 P
12003 15394 12008 15368 12034 15360 12041 15333 12028 15309 12026 15283
12033 15256 12045 15232 12053 15206 12039 15230 12013 15225 11 P
12013 15225 11986 15218 12012 15209 12006 15183 11984 15168 11980 15141
11994 15118 12018 15104 12045 15102 12072 15102 12098 15095 12124 15086
12147 15100 12162 15078 12178 15056 12196 15035 12213 15014 12235 14998
12256 14981 12279 14966 12306 14963 12328 14948 12347 14928 12358 14904
12383 14913 12409 14904 12433 14891 12438 14864 28 P
12438 14864 12448 14839 12463 14816 12476 14793 12494 14772 12503 14747
12519 14725 12534 14702 12546 14678 12563 14656 12575 14632 12586 14607
12605 14588 12622 14566 12640 14546 12656 14524 12675 14505 12691 14483
12712 14465 12731 14445 12752 14428 12772 14410 12793 14393 12798 14366
12818 14348 12838 14328 12844 14302 12864 14284 12878 14261 12900 14245
12915 14222 12934 14202 12951 14181 12960 14155 12967 14129 12980 14105
12999 14085 13001 14061 38 P
13001 13007 12998 13007 13001 12992 3 P
13001 12979 12980 12968 12959 12951 12951 12978 12952 13005 12936 12982
12909 12980 12883 12972 12855 12973 9 P
12855 12973 12828 12977 12806 12993 12783 13006 12789 12980 12797 12953
6:00 PM
12797 12953 12771 12961 12743 12963 12730 12939 12747 12918 12729 12898
12726 12871 12725 12843 12743 12822 12741 12795 12730 12770 12708 12787
12692 12809 12718 12802 12712 12829 12685 12829 12688 12856 12663 12846
12638 12858 12629 12832 12601 12832 12575 12840 12550 12851 12549 12879
12546 12906 12539 12879 12514 12869 12490 12855 12468 12839 12442 12847
12415 12841 12398 12820 12374 12806 12357 12828 12351 12801 12364 12778
12389 12765 12410 12783 12436 12789 12442 12762 12438 12735 12454 12712
12463 12687 12464 12659 12437 12650 12436 12678 12442 12705 12415 12711
12388 12704 12389 12677 50 P
12389 12677 12366 12663 12354 12687 12363 12713 12352 12738 12337 12761
12324 12786 12313 12761 12286 12754 12259 12753 12284 12743 12262 12728
12269 12701 12287 12680 12309 12664 12331 12648 12331 12621 12303 12621
12276 12627 12273 12600 12300 12604 12299 12577 12276 12561 12265 12536
12245 12554 12228 12575 12208 12595 12194 12571 12167 12580 12176 12606
12187 12631 12192 12604 12215 12618 12214 12646 12214 12673 12234 12655
12252 12676 12247 12703 12222 12693 12196 12684 12178 12663 12153 12653
12133 12634 12109 12620 12106 12593 12097 12567 12107 12542 12091 12519
12064 12521 12037 12515 50 P
12037 12515 12024 12538 2 P

12024 12538 12021 12565 12011 12591 12008 12564 12008 12536 11984 12523
11978 12496 11955 12481 11951 12453 11933 12432 11911 12416 11910 12443
11886 12458 11903 12480 11879 12492 11871 12519 11890 12540 11909 12559
11882 12560 11860 12542 11836 12554 11814 12570 11787 12575 11774 12600
11772 12627 11781 12653 11804 12638 11806 12611 11820 12588 11845 12577
11873 12580 11852 12598 11826 12607 11828 12634 11855 12635 11861 12609
11887 12602 11915 12604 11905 12630 11884 12647 11857 12650 11829 12649
11850 12666 11877 12662 11884 12689 11911 12693 11936 12705 11909 12707
11905 12734 11881 12748 50 P
11881 12748 11879 12775 11878 12748 11887 12722 11887 12694 11859 12695
11836 12681 11826 12707 11832 12734 11822 12759 11838 12782 11811 12777
11794 12755 11773 12773 11799 12784 11811 12808 11789 12823 11769 12842
11771 12815 11752 12834 11743 12860 11770 12854 11782 12878 11781 12851
11799 12831 11817 12811 11835 12832 11845 12857 11818 12864 11796 12879
11800 12906 11773 12913 11783 12939 11789 12966 11795 12993 11816 12977
11825 13003 11798 13007 11812 13031 11816 13058 11828 13083 11853 13092
11879 13101 11906 13098 11905 13126 11878 13126 11851 13121 11824 13119
11810 13143 11829 13163 50 P
11829 13163 11849 13181 2 P
11849 13181 11867 13202 11894 13203 11899 13229 11924 13241 11909 13263
11929 13283 11947 13303 11920 13297 11893 13294 11866 13289 11858 13315
11873 13337 11897 13350 11918 13367 11944 13379 11964 13396 11966 13423
11959 13397 11937 13381 11912 13370 11889 13357 11869 13376 11856 13400
11835 13417 11810 13427 11831 13443 11809 13459 11807 13487 11793 13510
11806 13534 11784 13551 11783 13578 11806 13592 11806 13607 11782 13580
11768 13604 11782 13627 11757 13640 11758 13667 11761 13694 11755 13721
11733 13705 11715 13685 11711 13712 11702 13738 11683 13718 11696 13695
11691 13668 11716 13657 50 P
11716 13657 11719 13630 11732 13606 11743 13581 11721 13564 11705 13586
11701 13613 11695 13639 11678 13660 11678 13688 11660 13708 11634 13700
11635 13727 11610 13739 11601 13713 11595 13740 11604 13766 11630 13772
11629 13799 11636 13825 11661 13837 11660 13865 11666 13838 11672 13812
11671 13784 11693 13769 11677 13791 11702 13804 11717 13826 11699 13847
11676 13862 11697 13880 11723 13889 11732 13863 11739 13837 11740 13809
11746 13836 11750 13863 11771 13846 11760 13871 11741 13890 11766 13879
11756 13904 11779 13891 11804 13879 11826 13895 11847 13876 11873 13871
11890 13849 11894 13822 50 P
11894 13822 11909 13799 2 P
11909 13799 11909 13826 11928 13845 11950 13861 11959 13887 11940 13868
11913 13873 11895 13893 11876 13913 11850 13919 11857 13945 11868 13970
11863 13997 11842 13980 11823 13960 11806 13982 11819 14005 11812 14032
11839 14036 11861 14053 11837 14065 11812 14077 11828 14099 11802 14108
11781 14125 11762 14145 11786 14157 11784 14184 11774 14209 11748 14217

11728 14235 11721 14262 11716 14288 11741 14301 11765 14314 11778 14290
11770 14263 11795 14250 11818 14238 11843 14226 11865 14210 11887 14201
11887 14202 11867 14214 11844 14228 11822 14243 11797 14255 11785 14280
11784 14307 11778 14333 50 P
11778 14333 11796 14357 11770 14344 3 P
11770 14344 11753 14322 11726 14317 11702 14329 11679 14344 11656 14359
11629 14357 11608 14339 11581 14335 11555 14327 11557 14300 11550 14273
11528 14289 11509 14309 11505 14335 11479 14342 11476 14314 11449 14316
11432 14338 11413 14357 11391 14373 11379 14397 11352 14404 11325 14403
11300 14413 11304 14386 11329 14375 11356 14376 11350 14349 11323 14350
11326 14323 11330 14296 11322 14271 11294 14273 11271 14259 11255 14237
11228 14233 11217 14208 11201 14229 11212 14254 11239 14261 11241 14288
11268 14290 11253 14312 11275 14328 11248 14331 11221 14329 11244 14314
11217 14309 11208 14284 50 P
11208 14284 11181 14283 11185 14310 11162 14324 11140 14308 11114 14300
11088 14307 11063 14318 11047 14340 11071 14354 11098 14350 11123 14360
11099 14372 11106 14399 11132 14404 11154 14387 11156 14414 11131 14424
11103 14421 11078 14411 11051 14407 11025 14415 11002 14428 10977 14419
10950 14411 10924 14415 10897 14423 10923 14430 10943 14449 10963 14465
10982 14492 11004 14478 11002 14471 11004 14467 11033 14486 11023 14491
11003 14480 10983 14499 10983 14526 10977 14552 10991 14576 11001 14601
11021 14619 11031 14644 11020 14669 11047 14670 11071 14683 11075 14710
11067 14736 11085 14715 50 P
11085 14715 11108 14729 2 P
11108 14729 11134 14735 11157 14750 11184 14754 11207 14767 11230 14752
11257 14756 11241 14778 11214 14778 11190 14791 11174 14769 11147 14771
11121 14763 11118 14790 11103 14813 11110 14786 11084 14795 11070 14818
11062 14844 11073 14869 11082 14895 11091 14920 11104 14944 11125 14962
11116 14988 11121 15014 11144 15029 11151 15055 11157 15081 11153 15108
11167 15133 11140 15126 11127 15150 11126 15177 11146 15196 11150 15223
11174 15236 11187 15237 11197 15251 11220 15263 11226 15267 11225 15269
11195 15252 11184 15242 11163 15240 11173 15268 11177 15282 11188 15277
11198 15279 11203 15287 50 P
11203 15287 11201 15289 11197 15283 11189 15279 11177 15283 11172 15271
11160 15247 11139 15230 11116 15216 11089 15215 11070 15234 11044 15225
11020 15239 10998 15254 10971 15254 10953 15274 10930 15262 10914 15283
10888 15291 10867 15307 10856 15283 10859 15256 10843 15234 10826 15213
10813 15237 10823 15262 10804 15281 10796 15307 10786 15332 10759 15335
10733 15342 10709 15354 10687 15370 10693 15396 10666 15395 10661 15368
10656 15342 10650 15315 10643 15341 10639 15368 40 P
10639 15368 10627 15344 10600 15338 10578 15323 10551 15317 10524 15318
10497 15320 10473 15332 10453 15313 10426 15313 10403 15328 10401 15355
10393 15329 10383 15304 10369 15280 10343 15286 10331 15262 10307 15276

10281 15281 10255 15285 10246 15260 10223 15246 10247 15235 10226 15218
10200 15209 10175 15218 10150 15208 10131 15188 10106 15177 10081 15167
10056 15157 10035 15140 10015 15121 9991 15110 9973 15089 9949 15102
9946 15075 9926 15057 9900 15060 9879 15042 9856 15027 9846 15002
9834 14978 9811 14964 9792 14945 9781 14920 9806 14908 9782 14896
9755 14890 9763 14864 50 P
9763 14864 9737 14859 9716 14841 9696 14859 9704 14833 9679 14842
9690 14817 9688 14790 9665 14803 9653 14828 9628 14838 9607 14821
9595 14797 9571 14785 9597 14779 9620 14765 9608 14741 9581 14738
9554 14738 9535 14718 9510 14710 9483 14710 9458 14698 9431 14699
9419 14675 9402 14653 9381 14670 9382 14697 9369 14722 9391 14738
9364 14745 9372 14771 9398 14779 9425 14779 9451 14789 9464 14812
9474 14837 9494 14818 9521 14822 9543 14838 9568 14848 9594 14857
9607 14881 9587 14898 9564 14914 9581 14935 9563 14954 9589 14961
9612 14975 9627 14998 50 P
9627 14998 9650 15013 2 P
9650 15013 9675 15020 9699 15033 9672 15034 9646 15028 9619 15027
9594 15015 9567 15014 9541 15011 9517 15025 9507 15055 9494 15087
9517 15096 9529 15113 9533 15125 9536 15146 9533 15173 9517 15194
9496 15211 9506 15236 9517 15260 9540 15275 9564 15289 9581 15310
9608 15309 9632 15322 9650 15342 9670 15361 9644 15368 9667 15382
9683 15403 9700 15424 9714 15447 9728 15470 9748 15489 9752 15516
9766 15538 9783 15559 9786 15586 9803 15607 9816 15631 9803 15655
9782 15673 9760 15688 9754 15698 9762 15715 9758 15741 9760 15748
9784 15735 9802 15740 50 P
9802 15740 9806 15746 9801 15754 9778 15753 9777 15766 9756 15778
9731 15793 9732 15791 9754 15775 9766 15768 9766 15762 9772 15754
9779 15745 9801 15751 9802 15743 9784 15738 9768 15746 9759 15750
9754 15746 9754 15732 9742 15733 9727 15748 9705 15764 9682 15779
9665 15800 9638 15798 9625 15821 9633 15847 9622 15872 9634 15896
9639 15923 9629 15898 9608 15880 9608 15853 9598 15827 9576 15811
9549 15809 9523 15806 9503 15826 9481 15841 9454 15840 9430 15853
9406 15840 9384 15855 9357 15858 9342 15836 9323 15817 9313 15792
9286 15796 9267 15776 50 P
9267 15776 9255 15752 2 P
9255 15752 9278 15738 9260 15717 9234 15711 9216 15690 9204 15666
9185 15647 9164 15630 9150 15607 9127 15593 9122 15567 9100 15550
9122 15535 9099 15521 9086 15497 9087 15470 9071 15449 9045 15442
9019 15435 8998 15417 8974 15406 8950 15392 8925 15383 8899 15375
8874 15364 8851 15350 8835 15328 27 P
8835 15328 8810 15320 8788 15304 8762 15297 8736 15290 8710 15280
8690 15263 8691 15236 8666 15225 8645 15209 8625 15190 8612 15166
8605 15140 8597 15114 8575 15100 8583 15074 8578 15047 8583 15020

8600 15000 8575 14989 8553 14973 8536 14952 8522 14929 8503 14909
8482 14893 8458 14880 8433 14870 8406 14867 8384 14851 8357 14849
8336 14866 8309 14867 8284 14856 8264 14837 8242 14821 8222 14803
8201 14787 8174 14791 8151 14806 8132 14826 8126 14852 8111 14874
8087 14888 8060 14892 44 P
8060 14892 8034 14888 8008 14879 7986 14864 7966 14844 7947 14825
7958 14800 7964 14773 7939 14763 7924 14741 7906 14719 7893 14696
7881 14671 7872 14645 7867 14619 7866 14592 7870 14565 17 P
7870 14565 7879 14539 7890 14514 7903 14490 7914 14466 7928 14443
7945 14421 7964 14402 7984 14383 8010 14376 8033 14361 11 P
8033 14361 8052 14342 8074 14327 8098 14313 8125 14316 8152 14313
8179 14316 8206 14314 8232 14306 8259 14302 8280 14285 8301 14301
8326 14313 8351 14324 8373 14340 8397 14352 8402 14326 8408 14352
8422 14329 8418 14302 8424 14275 8415 14250 8432 14271 8459 14271
8464 14244 8482 14224 8490 14198 8498 14172 8492 14146 8480 14121
8505 14110 8523 14089 8510 14065 8494 14043 8476 14023 8470 13996
8468 13969 8469 13942 8472 13915 8467 13888 8485 13868 8502 13846
8505 13819 8532 13820 8555 13806 8576 13787 8603 13785 8629 13778
8656 13777 8683 13779 50 P
8683 13779 8710 13782 8736 13771 8763 13773 8790 13773 8817 13780
8835 13799 8856 13817 8875 13836 8882 13863 8866 13884 8879 13908
8900 13925 8925 13936 8951 13943 8974 13958 8996 13974 9015 13994
9034 14013 9051 14035 9055 14061 9030 14073 9056 14081 9082 14089
9102 14108 9128 14115 9155 14119 9178 14133 9204 14142 9224 14160
9252 14161 9279 14158 9306 14161 9318 14137 9342 14124 9359 14103
9386 14105 9402 14082 9406 14055 9426 14036 9452 14031 9479 14034
9504 14044 9531 14048 9507 14036 9525 14016 9542 13995 9560 13975
9585 13963 9612 13961 50 P
9612 13961 9639 13966 2 P
9639 13966 9664 13976 9684 13957 9700 13979 9727 13984 9754 13988
9769 13966 9767 13993 9787 14012 9810 14027 9831 14043 9849 14023
9840 13997 9854 13974 9878 13987 9901 13972 9928 13965 9955 13966
9981 13974 9999 13953 10023 13964 10047 13977 10075 13976 10055 13957
10037 13936 10032 13909 10040 13883 10056 13861 10034 13846 10010 13832
9995 13809 9995 13782 9974 13764 10002 13761 10029 13765 10029 13738
10025 13711 10012 13687 10023 13712 9996 13706 9983 13682 9961 13665
9939 13650 9913 13654 9885 13655 9881 13682 9908 13685 9883 13696
9867 13718 9883 13740 50 P
9883 13740 9876 13766 9859 13745 9860 13718 9837 13703 9818 13684
9808 13710 9794 13733 9767 13728 9778 13704 9754 13692 9729 13680
9704 13669 9691 13645 9667 13632 9642 13622 9642 13595 9617 13602
9590 13595 9605 13618 9626 13635 9598 13633 9579 13614 9565 13637
9570 13610 9553 13588 9530 13574 9505 13564 9479 13554 9453 13547

9430 13533 9410 13514 9385 13524 9386 13497 9359 13496 9345 13519
9319 13527 9296 13513 9279 13492 9305 13498 9321 13476 9346 13465
9346 13438 9329 13417 9328 13389 9303 13402 9282 13420 9258 13408
9243 13384 9251 13358 50 P
9251 13358 9225 13350 2 P
9225 13350 9220 13323 9235 13301 9240 13274 9244 13248 9223 13231
9198 13218 9171 13215 9144 13210 9124 13192 9098 13183 9071 13188
9082 13213 9093 13238 9098 13265 9082 13288 9064 13308 9060 13281
9038 13264 9013 13273 8987 13263 8978 13237 8986 13211 9009 13197
9035 13189 9020 13166 9047 13162 9074 13165 9062 13141 9040 13125
9026 13101 9033 13128 9010 13113 8985 13103 8965 13084 8954 13058
8957 13031 8949 13005 8927 13020 8915 12996 8901 12972 8890 12947
8873 12969 8853 12951 8840 12927 8830 12952 8808 12936 8781 12939
8787 12913 8778 12887 50 P
8778 12887 8770 12860 8756 12837 8752 12810 8732 12790 8708 12778
8691 12756 8683 12730 8676 12704 8673 12676 8661 12651 8634 12656

12:00 PM

8634 12656 8613 12638 8599 12615 8595 12588 8595 12561 8586 12535
8588 12508 8601 12484 8594 12457 8613 12477 8635 12461 8659 12474
8684 12462 8711 12459 8716 12432 8730 12409 8751 12391 8774 12377
8798 12364 8820 12347 8847 12340 8865 12320 8865 12292 8878 12317
8905 12311 8932 12306 8958 12297 8982 12286 9001 12266 9028 12259
9053 12248 9078 12237 9092 12213 9096 12186 9109 12162 9117 12135
9126 12109 9136 12084 9145 12058 9146 12030 9154 12004 9155 11976
9180 11966 9175 11939 9201 11929 9209 11903 9220 11878 9216 11851
9223 11824 9240 11802 50 P
9240 11802 9262 11785 9252 11759 9250 11732 9257 11705 9276 11686
9285 11660 9297 11635 9316 11614 9331 11592 9337 11565 9351 11541
9378 11537 9389 11511 9395 11484 9405 11458 9407 11431 9398 11405
9378 11386 9404 11377 9417 11353 9398 11332 9383 11309 9411 11306
9429 11285 9448 11265 9476 11264 9501 11255 9527 11245 9552 11233
9577 11222 9603 11211 9625 11195 9638 11170 9638 11143 9643 11116
9628 11092 9648 11073 9660 11048 9686 11038 9713 11038 9739 11028
9765 11019 9789 11006 9810 10988 9829 10968 9848 10948 9857 10922
9870 10897 9884 10873 50 P
9884 10873 9895 10847 2 P
9895 10847 9889 10820 9863 10810 9836 10818 9810 10826 9782 10829
9757 10839 9732 10852 9706 10863 9688 10884 9662 10892 9635 10898
9610 10910 9584 10900 9558 10890 9531 10884 9517 10860 9490 10864
9462 10861 9436 10869 9430 10896 9420 10922 9401 10942 9384 10964
9361 10979 9345 11002 9319 10992 9293 11001 9265 11001 9240 11012
9215 11002 9190 10988 9169 10970 9147 10953 9126 10936 9100 10926
9072 10928 9045 10934 9059 10910 9087 10910 9113 10918 9138 10931

9164 10937 9192 10942 9203 10968 9231 10970 9257 10961 9284 10958
9312 10961 9339 10958 50 P
9339 10958 9355 10935 9368 10910 9380 10886 9359 10869 9377 10849
9403 10837 9430 10835 9458 10836 9484 10826 9510 10817 9531 10799
9555 10785 9577 10768 9595 10747 9609 10723 9627 10702 17 P
9627 10702 9651 10688 9677 10677 9698 10660 9714 10637 9741 10636
9766 10623 9789 10608 9811 10592 9829 10570 9843 10547 9857 10522
9870 10498 9883 10473 9896 10449 9910 10424 9920 10398 9913 10371
9890 10356 9863 10351 9835 10349 9807 10346 9780 10341 9753 10334
9727 10326 9702 10313 9682 10294 9667 10270 9643 10256 9615 10252
30 P
9615 10252 9593 10236 9571 10219 9556 10196 9528 10191 9503 10178
9484 10159 9469 10135 9468 10107 9452 10084 9436 10062 9411 10050
9383 10047 9364 10067 9337 10076 9309 10076 9281 10073 9254 10073
9227 10067 9202 10054 9194 10027 9195 9999 9169 10009 9141 10007
9124 9985 9104 9966 9128 9981 9147 10001 9169 9984 9196 9986
9213 10009 9223 10035 9246 10051 9274 10053 9302 10052 9325 10037
9314 10012 9323 9986 9341 9964 9335 9936 9344 9909 9371 9901
9346 9915 9365 9935 9391 9925 9419 9925 9445 9935 9466 9953
9490 9967 9512 9985 50 P
9512 9985 9531 10005 9553 10022 9578 10034 9606 10039 9633 10039
9661 10036 9685 10021 9708 10005 9727 9986 9746 9965 9763 9942
9782 9922 9798 9899 9812 9875 9836 9861 9863 9856 9891 9852
9917 9841 9944 9834 9955 9824 9962 9818 9966 9817 9994 9817
10022 9818 10046 9832 10039 9805 10016 9789 9990 9779 9968 9761
9954 9737 9931 9720 9914 9698 9892 9682 9874 9660 9856 9638
9834 9621 9807 9614 9780 9609 9753 9615 9735 9593 9724 9567
9732 9541 9747 9564 9774 9572 9766 9545 9784 9566 9807 9582
9834 9590 9853 9610 50 P
9853 9610 9881 9611 2 P
9881 9611 9888 9584 9912 9597 9896 9621 9897 9648 9921 9662
9949 9668 9976 9675 9995 9655 9977 9633 9974 9605 9990 9582
9989 9554 9963 9543 9966 9516 9963 9488 9989 9476 9994 9448
9975 9426 9952 9411 9924 9417 9923 9444 9917 9472 9927 9498
9906 9517 9899 9490 9901 9462 9894 9435 9874 9416 9847 9409
9837 9435 9835 9463 9844 9490 9833 9464 9832 9436 9814 9458
9786 9462 9782 9435 9774 9407 9750 9393 9749 9365 9777 9369
9803 9377 9831 9371 9834 9343 9806 9340 9785 9321 9811 9332
9839 9338 9864 9327 50 P
9864 9327 9853 9301 9874 9282 9855 9261 9827 9264 9801 9274
9816 9250 9843 9244 9856 9219 9864 9192 9844 9173 9825 9153
9801 9138 9773 9137 9745 9136 9717 9137 9743 9129 9771 9122
9787 9100 9795 9073 9808 9047 9823 9024 9822 8996 9836 8971

9845 8945 9859 8920 9847 8895 9828 8916 9805 8931 9777 8936
9762 8912 9749 8887 9728 8869 9734 8842 9713 8823 9693 8842
9675 8864 9667 8891 9652 8915 9669 8938 9642 8945 9616 8936
9606 8909 9581 8898 9603 8881 9593 8854 9578 8830 9553 8817
9540 8792 9541 8764 50 P
9541 8764 9529 8739 2 P
9529 8739 9529 8727 9502 8730 9474 8731 9446 8730 9429 8748
9424 8748 9396 8755 9369 8751 9347 8770 9349 8742 9377 8745
9404 8740 9424 8720 9452 8716 9477 8702 9479 8674 9463 8650
9445 8629 9430 8605 9418 8579 9390 8577 9386 8548 9387 8520
9368 8500 9340 8493 9358 8471 9371 8446 9398 8436 9384 8411
9385 8383 9357 8378 9332 8391 9325 8363 9339 8339 9322 8315
9322 8287 9306 8263 9291 8287 9280 8261 9256 8275 9245 8301
42 P
9245 8301 9251 8274 9265 8249 9250 8226 9222 8218 9195 8222
9204 8195 9199 8167 9180 8146 9151 8146 9179 8139 9181 8110
9153 8116 9125 8118 9118 8090 9090 8096 9078 8070 9102 8056
9127 8042 9116 8015 9142 8004 9121 7984 9122 7956 9096 7945
9072 7930 9043 7927 9035 7900 9014 7881 8985 7882 8993 7910
9009 7933 9037 7934 9058 7953 9056 7982 9082 7995 9094 8020
9070 8036 9042 8039 9033 8012 9017 7989 9012 7961 8990 7979
8962 7985 8965 8013 8989 8027 8962 8034 8978 8057 8952 8046
8948 8018 8927 8037 50 P
8927 8037 8910 8059 8900 8086 8901 8058 8908 8030 8918 8004
8893 8017 8871 8035 8845 8045 8849 8017 8844 7989 8867 7973
8840 7964 8843 7935 8865 7917 8891 7928 8919 7927 8947 7923
8966 7901 8970 7873 8982 7847 8966 7824 8944 7841 8916 7848
8895 7866 8872 7850 8859 7825 8886 7833 8899 7807 8905 7780
8927 7762 8952 7774 8961 7801 8979 7824 8996 7801 9020 7786
9046 7797 9028 7774 9000 7771 8973 7765 8948 7750 8921 7742
8893 7736 8869 7720 8898 7725 8887 7699 8870 7676 8850 7656
8834 7632 8853 7612 50 P
8853 7612 8880 7603 2 P
8880 7603 8906 7591 8934 7588 8927 7560 8919 7533 8891 7531
8882 7504 8884 7475 8892 7448 8878 7423 8850 7418 8824 7407
8807 7384 8821 7359 8828 7332 8854 7344 8868 7319 8874 7291
8901 7282 8907 7254 8902 7226 8881 7207 8880 7236 8879 7264
8852 7272 8841 7246 8827 7221 8830 7250 8813 7273 8808 7301
8779 7304 8754 7317 8764 7344 8740 7359 8729 7333 8713 7310
8693 7289 8669 7275 8646 7257 8619 7249 8640 7230 8663 7213
8687 7198 8677 7171 8704 7180 8726 7197 8744 7174 8763 7153
8736 7146 8730 7118 50 P
8730 7118 8701 7114 8673 7112 8649 7128 8647 7157 8632 7133

8641 7106 8655 7081 8629 7093 8601 7097 8611 7124 8613 7152
8588 7140 8604 7163 8577 7170 8548 7167 8522 7157 8541 7136
8561 7116 8545 7093 8528 7069 8501 7062 8510 7034 8534 7020
8562 7012 8575 7038 8573 7009 8587 6984 8558 6985 8530 6983
8534 6954 8562 6948 8537 6933 8511 6944 8482 6948 8464 6926
8436 6919 8442 6947 8429 6972 8416 6947 8416 6975 8395 6995
8402 6967 8409 6940 8381 6933 8355 6945 8378 6928 8382 6900
8403 6880 8425 6897 50 P
8425 6897 8441 6874 2 P
8441 6874 8437 6845 8424 6820 8395 6822 8394 6794 8405 6768
8384 6747 8359 6734 8360 6762 8336 6777 8310 6789 8284 6803
8295 6830 8273 6812 8278 6784 8253 6770 8225 6765 8198 6773
8185 6747 8157 6754 8165 6781 8154 6808 8125 6808 8123 6779
8109 6754 8092 6777 8082 6750 8053 6749 8079 6736 8074 6708
8051 6691 8023 6698 7997 6708 7968 6707 7953 6704 7941 6720
7940 6703 7951 6692 7974 6674 7999 6661 8017 6656 8045 6659
8074 6663 8077 6634 8106 6634 8120 6609 8126 6581 8098 6575
8077 6556 8054 6538 50 P
8054 6538 8033 6558 8013 6537 8024 6510 8005 6488 8001 6459
7977 6445 7953 6427 7937 6404 7925 6378 7929 6406 7946 6430
7936 6457 7909 6448 7891 6426 7894 6397 7871 6380 7859 6354
7857 6325 7855 6297 7838 6273 7839 6302 7848 6330 7839 6357
7818 6375 7790 6383 7762 6390 7733 6393 7756 6376 7784 6369
7786 6340 7776 6313 7758 6291 7735 6307 7723 6307 7711 6291
7687 6307 7676 6281 7671 6252 7669 6223 7651 6201 7659 6229
7641 6252 7641 6222 7617 6207 45 P
7617 6207 7594 6189 7571 6171 7564 6199 7558 6227 7532 6238
7540 6210 7515 6223 7487 6215 7460 6205 7469 6181 7455 6155
7454 6129 7455 6119 7434 6099 7422 6085 7401 6062 7372 6062
7343 6062 7316 6074 7294 6092 7273 6113 7245 6113 7273 6108
7279 6080 7292 6054 7315 6037 7343 6030 7379 5998 7358 5987
7341 5964 7319 5984 7296 6002 7268 5999 7271 5970 7295 5985
7291 5956 7277 5931 7284 5903 7287 5874 7266 5854 7258 5826
7228 5828 7200 5820 7172 5826 7149 5844 7125 5828 7101 5811
7082 5790 7059 5771 50 P
7059 5771 7030 5771 7046 5796 7029 5819 7027 5791 7003 5774
6976 5764 6949 5754 6921 5746 6899 5727 6882 5750 6866 5774
6838 5783 6814 5799 6785 5797 6764 5777 6747 5753 6729 5730
6747 5707 6768 5727 6754 5701 6757 5672 6730 5685 6705 5698
6676 5693 6648 5700 6636 5727 6665 5728 6640 5742 6614 5755
6575 5753 6576 5763 6599 5771 6568 5782 6543 5766 6515 5767
6487 5760 6472 5735 6458 5710 6430 5706 6417 5732 6392 5747
6408 5723 6431 5705 6415 5680 6399 5656 6403 5627 6382 5648

6353 5652 6324 5651 50 P
6324 5651 6314 5678 2 P
6314 5678 6317 5707 6314 5736 6337 5753 6345 5781 6324 5762
6295 5767 6269 5755 6242 5746 6216 5733 6202 5708 6179 5689
6209 5689 6208 5660 6181 5650 6206 5635 6234 5625 6228 5597
6200 5590 6172 5584 6168 5612 6159 5640 6141 5663 6113 5670
6084 5663 6056 5656 6035 5637 27 P
6035 5637 6061 5624 6086 5611 6065 5592 6036 5590 6008 5586
6023 5561 6051 5569 6074 5588 6090 5564 6118 5570 6112 5542
6084 5534 6057 5545 6049 5516 6069 5495 6042 5507 6013 5506
5985 5513 5967 5535 5937 5535 5908 5534 5880 5538 5859 5559
5880 5579 5902 5598 5928 5612 5955 5622 28 P
5955 5622 5926 5625 5898 5632 5871 5621 5852 5599 5859 5628
5842 5651 5823 5673 5811 5700 5796 5725 5790 5753 5772 5776
5745 5765 5739 5794 5720 5816 5706 5840 5711 5869 5722 5896
5705 5873 5684 5893 5677 5921 5679 5950 5654 5964 5627 5975
5600 5984 5617 5961 5645 5957 5662 5934 5661 5904 5655 5876
5626 5871 5598 5866 5627 5865 5654 5855 5664 5827 5688 5811
5702 5785 5674 5778 5649 5795 5669 5773 5686 5750 5703 5726
5675 5717 5654 5736 5673 5715 5689 5691 5665 5674 5681 5650
5680 5621 5667 5595 50 P
5667 5595 5659 5567 5682 5551 5689 5522 5674 5497 5665 5469
5656 5453 7 P
5649 5453 5644 5472 5643 5453 3 P
5597 5453 5592 5457 5573 5479 5556 5502 5547 5530 5528 5553
5537 5525 5551 5487 5544 5482 5539 5475 5539 5468 5531 5453

12:00 PM

5429 5453 5430 5476 5412 5498 5410 5527 5410 5557 5415 5585
5415 5614 5386 5615 5380 5644 5351 5648 5369 5625 5393 5608
5404 5582 5403 5553 5402 5524 5405 5494 5407 5466 5392 5453
18 P
2966 5453 2967 5459 2985 5482 2967 5505 2938 5506 2916 5486
2926 5459 2928 5453 8 P
13001 8121 12996 8116 12991 8121 12985 8125 12983 8118 12983 8111
12985 8105 12988 8098 12992 8092 12995 8086 13001 8082 13001 8081

12:00 PM

13001 8033 13001 8033 12994 8030 12988 8028 12984 8022 12981 8015
12978 8009 12978 8002 12973 7997 12977 7991 12982 7985 12976 7990
12969 7993 12962 7992 12960 7986 12961 7979 12966 7973 12968 7967
12974 7963 12979 7957 12979 7950 12979 7943 12979 7936 12977 7929
12976 7922 12982 7918 12988 7915 12993 7920 12999 7925 13001 7925
30 P
11825 7031 11829 7025 11833 7019 11839 7015 11846 7013 11847 7020

11850 7013 11855 7009 11862 7012 11869 7014 11876 7015 11882 7019
11889 7021 11896 7021 11903 7023 11910 7024 11917 7023 11924 7025
11931 7026 11936 7021 11932 7027 11927 7033 11923 7039 11918 7044
11911 7044 11904 7046 11897 7048 11894 7054 11887 7057 11880 7055
11873 7058 11866 7060 11863 7066 11865 7073 11859 7078 11853 7080
11849 7086 11847 7093 11844 7100 11838 7104 11833 7110 11829 7115
11826 7121 11819 7120 11823 7114 11827 7108 11830 7102 11835 7096
11839 7091 11842 7084 50 P
11842 7084 11835 7081 11835 7074 11837 7067 11834 7060 11828 7057
11825 7051 11831 7047 11837 7042 11840 7037 11836 7031 11825 7031

12:00 PM

11225 6793 11231 6789 11237 6791 11242 6796 11250 6797 11255 6792
11259 6786 11264 6780 11263 6773 11259 6767 11253 6764 11249 6758
11254 6754 11261 6752 11268 6749 11273 6744 11279 6741 11286 6740
11293 6742 11298 6748 11299 6755 11306 6753 11312 6750 11319 6752
11322 6758 11323 6766 11322 6773 11322 6780 11324 6787 11323 6794
11319 6800 11322 6807 11319 6813 11319 6820 11322 6826 11328 6831
11332 6836 11333 6843 11338 6849 11342 6854 11345 6861 11342 6868
11346 6874 11352 6879 11356 6883 11358 6890 11357 6897 11358 6905
11351 6905 11344 6906 50 P
11344 6906 11343 6899 11341 6892 11338 6886 11334 6880 11331 6873
11336 6868 11333 6862 11331 6855 11329 6848 11322 6850 11316 6847
11313 6841 11306 6839 11306 6831 11302 6825 11305 6818 11299 6813
11292 6814 11285 6812 11279 6808 11272 6809 11265 6807 11262 6814
11259 6820 11252 6822 11252 6815 11250 6809 11246 6802 11240 6807
11233 6806 11228 6801 11225 6793 33 P
11017 6707 11022 6701 11026 6696 11032 6692 11040 6693 11047 6691
11049 6698 11055 6703 11060 6698 11066 6694 11073 6693 11080 6693
11088 6692 11095 6691 11102 6691 11109 6692 11116 6690 11122 6687
11129 6684 11135 6688 11134 6695 11131 6701 11124 6704 11131 6702
11138 6700 11144 6696 11144 6703 11142 6710 11144 6717 11148 6723
11155 6721 11157 6728 11154 6735 11158 6741 11160 6748 11154 6750
11148 6754 11143 6759 11136 6761 11129 6762 11126 6769 11120 6765
11114 6769 11107 6768 11100 6765 11096 6760 11091 6766 11089 6772
11084 6778 11078 6782 50 P
11078 6782 11072 6784 11066 6781 11071 6776 11073 6769 11077 6763
11070 6766 11071 6773 11065 6770 11060 6764 11056 6758 11059 6752
11058 6745 11062 6739 11067 6734 11068 6727 11065 6733 11062 6727
11062 6720 11064 6713 11064 6706 11063 6713 11058 6718 11052 6722
11051 6729 11045 6724 11039 6728 11039 6721 11043 6715 11037 6719
11031 6722 11026 6716 11030 6710 11017 6707 34 P
9618 7032 9615 7018 9606 7007 9598 6995 9592 6982 9585 6969
9582 6955 9572 6945 9564 6933 9571 6920 9567 6907 9563 6893

9553 6882 9543 6873 9539 6859 9529 6848 9539 6839 9525 6839
9511 6837 9498 6831 9490 6820 9496 6807 9482 6804 9469 6799
9464 6785 9459 6771 9456 6757 9451 6744 9445 6731 9438 6719
9427 6709 9419 6697 9409 6687 9403 6674 9397 6661 9391 6647
9386 6634 9384 6620 9375 6608 9371 6595 9363 6582 9356 6570
9368 6562 9354 6562 9346 6550 9339 6537 9332 6525 9329 6511
9324 6497 9315 6486 50 P
9315 6486 9305 6476 9296 6465 9289 6452 9280 6441 9274 6428
9274 6414 9267 6401 9261 6388 9256 6374 9247 6364 9256 6353
9270 6351 9285 6349 9299 6351 9288 6342 9274 6340 9259 6339
9245 6341 9231 6341 9222 6329 9213 6318 9211 6304 9210 6289
9207 6275 9195 6267 9188 6254 9182 6241 9181 6226 9180 6212
9187 6199 9187 6185 9193 6172 9181 6163 9190 6153 9188 6139
9198 6129 9188 6120 9192 6106 9196 6092 9194 6077 9192 6063
9199 6051 9185 6053 9183 6039 9175 6027 9177 6013 9166 6004
9179 5998 9164 5996 50 P
9164 5996 9163 5982 2 P
9163 5982 9164 5967 9155 5958 9169 5954 9173 5940 9169 5926
9158 5917 9144 5914 9140 5927 9136 5913 9146 5903 9160 5906
9153 5895 9163 5885 9175 5877 9189 5881 9203 5885 9217 5890
9220 5876 9207 5869 9193 5866 9200 5853 9207 5841 9211 5827
9211 5812 9216 5799 9221 5785 9235 5782 9226 5771 9231 5757
9237 5744 9243 5730 9249 5717 9255 5704 9258 5690 9258 5676
9255 5661 9263 5649 9276 5643 9280 5629 9291 5620 9302 5610
9289 5615 9279 5627 9271 5639 9277 5625 9286 5614 9296 5603
9306 5593 9316 5582 50 P
9316 5582 9328 5574 9340 5566 9347 5580 9353 5566 9362 5555
9373 5545 9386 5539 9398 5531 9411 5525 9424 5518 9436 5510
9447 5500 9454 5487 9465 5478 9472 5465 9476 5453 17 P
9607 5453 9607 5465 9606 5479 9608 5494 9614 5507 9617 5521
9622 5535 9627 5548 9632 5562 9635 5576 9638 5590 9642 5604
9646 5618 9652 5632 9662 5642 9664 5657 9667 5671 9673 5684
9684 5693 9695 5703 9707 5711 9720 5717 9730 5727 9740 5738
9751 5748 9754 5763 9758 5776 9753 5790 9760 5803 9773 5808
9784 5818 9790 5831 9798 5843 9801 5857 9812 5868 9819 5880
9824 5894 9830 5907 9833 5921 9837 5935 9848 5944 9857 5956
9859 5970 9862 5984 9857 5997 9859 6012 9865 6025 9868 6039
9872 6053 9877 6066 50 P
9877 6066 9889 6075 9891 6089 9893 6103 9894 6118 9899 6132
9903 6145 9906 6160 9911 6173 9906 6186 9907 6201 9910 6215
9916 6228 9917 6242 9917 6257 9922 6270 9923 6285 9925 6299
9928 6313 9934 6326 9934 6341 9934 6355 9940 6368 9944 6382
9947 6397 9949 6411 9949 6425 9949 6439 9951 6454 9960 6465

9968 6477 9960 6489 9953 6502 9950 6516 9955 6529 9965 6539
9974 6551 9973 6565 9970 6579 9977 6592 9988 6601 9997 6612
10008 6621 10014 6634 10021 6646 10027 6659 10025 6674 10030 6688
10032 6702 49 P
10032 6702 10034 6716 10040 6729 10047 6742 10058 6751 10055 6765
10061 6778 10072 6786 10070 6800 10071 6815 10068 6829 10058 6840
10053 6853 10050 6867 10048 6881 10043 6895 10039 6908 10037 6922
10037 6937 10038 6951 10042 6965 10046 6979 10054 6991 10064 7001
10069 7014 10080 7024 10088 7035 10101 7042 10114 7049 10125 7058
10117 7070 10103 7068 10090 7075 10089 7090 10081 7101 10083 7115
10073 7125 10058 7124 10044 7125 10030 7122 10020 7131 10007 7137
9994 7131 9984 7141 9970 7146 9960 7156 9964 7169 9950 7164
9940 7174 9931 7185 50 P
9931 7185 9927 7199 9916 7207 9903 7214 9889 7213 9875 7209
9861 7204 9849 7197 9840 7186 9832 7174 9825 7162 9817 7150
9828 7141 9815 7136 9805 7126 9793 7119 9780 7113 9766 7111
9752 7107 9738 7108 9724 7103 9711 7098 9699 7090 9687 7082
9673 7079 9660 7074 9648 7065 9636 7058 9626 7048 9618 7032
30 P
12970 11609 12977 11607 12983 11607 12988 11602 12995 11600 12999 11606
13001 11607 7 P
13001 11699 13000 11699 12995 11704 12988 11706 12983 11702 12981 11695
12982 11688 12986 11683 12984 11676 12986 11670 12991 11665 12987 11659
12990 11653 12992 11646 12999 11643 12999 11637 12993 11636 12998 11632
12991 11633 12993 11627 12988 11623 12987 11616 12994 11618 12999 11623
12995 11617 12995 11610 12988 11612 12982 11616 12979 11622 12975 11628
12973 11621 12970 11609 32 P
11806 11848 11811 11843 11815 11837 11819 11832 11825 11828 11831 11825
11838 11824 11844 11820 11848 11815 11853 11810 11858 11805 11864 11802
11871 11802 11871 11809 11872 11815 11876 11821 11880 11827 11886 11829
11893 11828 11900 11828 11907 11827 11913 11830 11919 11834 11926 11834
11932 11831 11939 11833 11945 11835 11950 11829 11957 11829 11963 11832
11970 11832 11974 11838 11981 11839 11987 11840 11994 11840 12001 11842
12007 11840 12013 11841 12018 11847 12024 11851 12028 11856 12033 11860
12040 11862 12047 11862 12054 11863 12060 11867 12066 11869 12073 11870
12078 11875 12082 11880 50 P
12082 11880 12087 11886 12093 11886 12100 11887 12107 11889 12113 11891
12117 11897 12116 11904 12121 11908 12125 11914 12126 11921 12127 11928
12131 11934 12136 11937 12141 11942 12141 11949 12142 11956 12143 11963
12148 11959 12147 11965 12146 11972 12149 11978 12155 11977 12151 11982
12146 11977 12145 11984 12138 11986 12134 11992 12138 11998 12136 12005
12132 12011 12126 12014 12119 12014 12113 12012 12107 12015 12101 12017
12096 12022 12092 12028 12085 12026 12079 12027 12072 12028 12065 12027

12059 12023 12052 12023 12046 12021 12039 12019 12033 12016 12026 12015
12020 12012 12013 12014 50 P
12013 12014 12007 12010 2 P
12007 12010 12001 12007 11995 12004 11989 12001 11983 11999 11976 11997
11970 11994 11964 11991 11958 11987 11952 11989 11945 11990 11941 11984
11934 11983 11929 11979 11923 11975 11917 11973 11911 11969 11905 11966
11899 11968 11893 11964 11887 11961 11882 11957 11876 11954 11869 11954
11865 11948 11862 11942 11855 11939 11850 11934 11845 11929 11845 11923
11842 11917 11837 11911 11832 11907 11826 11902 11823 11897 11818 11891
11813 11888 11812 11881 11806 11877 11806 11870 11809 11864 11807 11857
11806 11848 43 P
12979 8284 12983 8279 12990 8279 12997 8281 13001 8281 5 P
13001 8297 12996 8294 12989 8295 12982 8295 12979 8284 5 P
12715 8170 12721 8173 12729 8174 12735 8177 12733 8184 12726 8187
12721 8183 12715 8170 8 P
12477 8021 12481 8016 12488 8014 12495 8013 12502 8012 12508 8008
12510 8001 12515 7995 12521 7992 12527 7989 12534 7988 12532 7995
12529 8001 12529 8008 12533 8014 12535 8020 12529 8024 12526 8030
12519 8032 12515 8038 12509 8042 12502 8041 12496 8037 12491 8032
12485 8029 12477 8021 26 P
12693 8048 12696 8044 12698 8040 12702 8043 12706 8045 12702 8047
12699 8051 12699 8055 12695 8054 12693 8048 10 P
12534 8012 12536 8013 12538 8014 12540 8015 12540 8017 12538 8019
12537 8017 12535 8016 12534 8012 9 P
12542 8015 12544 8016 12546 8018 12545 8020 12543 8019 12542 8015
6:00 PM
12547 8013 12549 8015 12550 8016 12552 8017 12555 8017 12557 8018
12558 8020 12559 8022 12562 8023 12564 8023 12566 8023 12568 8024
12569 8026 12571 8026 12573 8028 12571 8028 12569 8028 12568 8026
12566 8025 12563 8024 12561 8024 12559 8024 12557 8022 12556 8020
12554 8019 12552 8018 12550 8017 12548 8016 12547 8013 29 P
12807 7955 12810 7951 12813 7948 12812 7944 12812 7939 12814 7936
12816 7940 12820 7942 12821 7946 12820 7951 12819 7955 12821 7959
12825 7962 12824 7966 12824 7971 12823 7975 12820 7979 12817 7982
12812 7980 12812 7976 12812 7971 12810 7967 12810 7963 12812 7958
12807 7955 25 P
12776 7979 12780 7978 12785 7978 12788 7975 12791 7978 12792 7982
12792 7987 12796 7984 12800 7982 12800 7987 12797 7991 12793 7991
12788 7992 12788 7987 12784 7987 12781 7984 12776 7979 17 P
12768 7962 12772 7959 12775 7955 12779 7957 12783 7959 12781 7963
12779 7967 12775 7969 12772 7966 12768 7962 10 P
12779 7952 12781 7949 12786 7950 12785 7954 12779 7952 5 P
12783 7944 12785 7940 12787 7944 12786 7949 12783 7944 5 P

12754 7945 12756 7940 12759 7938 12760 7943 12760 7947 12757 7950
12754 7945 7 P
12756 7967 12760 7966 12761 7971 12757 7972 12756 7967 5 P
12857 7972 12861 7973 12861 7978 12864 7982 12864 7986 12860 7983
12859 7979 12857 7972 8 P
12832 12916 12826 12914 12823 12908 12822 12901 12821 12894 12815 12895
12808 12892 12803 12895 12801 12888 12796 12884 12800 12878 12804 12872
12807 12866 12812 12861 12818 12857 12811 12858 12804 12857 12798 12860
12793 12855 12786 12856 12780 12859 12775 12864 12768 12862 12766 12856
12759 12854 12756 12848 12753 12842 12750 12836 12752 12829 12754 12823
12758 12816 12764 12814 12767 12808 12771 12802 12777 12806 12776 12812
12783 12814 12788 12817 12793 12822 12796 12829 12803 12827 12803 12820
12806 12814 12804 12807 12805 12801 12799 12796 12793 12795 12786 12793
12788 12786 12793 12790 50 P
12793 12790 12793 12783 12800 12785 12806 12788 12812 12785 12806 12781
12805 12774 12808 12768 12814 12766 12811 12760 12809 12754 12815 12752
12822 12749 12827 12753 12834 12755 12832 12761 12826 12766 12833 12768
12840 12769 12846 12772 12853 12772 12847 12775 12840 12774 12836 12780
12832 12785 12834 12792 12841 12791 12845 12796 12842 12802 12842 12809
12848 12812 12852 12806 12856 12812 12862 12811 12867 12806 12873 12803
12873 12810 12868 12815 12864 12820 12866 12827 12859 12826 12857 12832
12863 12835 12870 12833 12867 12839 12862 12845 12869 12846 12869 12853
12872 12859 12865 12859 50 P
12865 12859 12860 12855 2 P
12860 12855 12859 12848 12852 12849 12848 12855 12853 12860 12855 12866
12859 12872 12857 12879 12855 12885 12854 12892 12852 12899 12846 12902
12843 12908 12840 12915 12844 12921 12850 12924 12851 12931 12846 12936
12840 12938 12833 12938 12829 12933 12828 12926 12832 12916 23 P
12542 12787 12536 12784 12533 12778 12527 12774 12522 12769 12517 12774
12515 12781 12509 12782 12502 12783 12503 12790 12498 12795 12495 12801
12495 12808 12501 12812 12503 12818 12505 12825 12503 12831 12500 12837
12494 12841 12487 12841 12481 12839 12476 12834 12471 12829 12471 12822
12476 12817 12469 12814 12463 12813 12463 12806 12462 12799 12458 12793
12456 12787 12458 12780 12461 12774 12465 12768 12470 12764 12471 12758
12476 12753 12479 12746 12477 12740 12475 12733 12482 12732 12484 12725
12489 12721 12496 12720 12503 12721 12509 12724 12510 12730 12507 12736
12505 12743 12511 12747 50 P
12511 12747 12516 12750 12523 12752 12524 12745 12523 12739 12525 12732
12528 12725 12529 12719 12536 12717 12543 12718 12549 12722 12556 12722
12560 12717 12566 12720 12573 12721 12569 12726 12565 12732 12570 12737
12573 12743 12572 12749 12570 12756 12569 12763 12569 12770 12572 12776
12571 12782 12566 12787 12559 12785 12555 12789 12542 12787 29 P
12067 12365 12071 12359 12077 12362 12082 12357 12089 12357 12096 12356

12100 12361 12101 12368 12100 12375 12095 12379 12094 12386 12088 12389
12082 12391 12076 12387 12073 12382 12070 12375 12067 12365 17 P
12233 12404 12237 12406 12241 12406 12246 12408 12250 12407 12250 12411
12252 12415 12250 12419 12249 12423 12249 12428 12245 12427 12241 12425
12242 12421 12237 12420 12237 12416 12235 12411 12233 12404 17 P
12160 12539 12162 12536 12167 12537 12169 12534 12166 12531 12165 12527
12169 12528 12172 12524 12176 12522 12179 12519 12183 12517 12187 12520
12191 12519 12194 12523 12198 12523 12202 12523 12207 12523 12211 12523
12215 12524 12218 12528 12219 12532 12219 12536 12221 12540 12222 12544
12223 12548 12222 12552 12220 12556 12216 12559 12212 12558 12208 12556
12204 12555 12200 12556 12196 12554 12193 12551 12191 12548 12186 12548
12183 12544 12179 12546 12175 12547 12173 12551 12169 12551 12170 12547
12168 12543 12165 12540 12160 12539 45 P
12321 12547 12326 12547 12329 12548 12334 12549 12336 12545 12332 12545
12328 12545 12329 12542 12333 12540 12337 12541 12341 12539 12341 12535
12340 12530 12343 12528 12347 12531 12350 12534 12354 12536 12358 12537
12362 12540 12363 12544 12361 12548 12360 12552 12355 12553 12353 12556
12349 12559 12346 12562 12344 12558 12340 12557 12337 12554 12333 12557
12329 12558 12325 12560 12321 12558 12324 12556 12323 12552 12321 12547
36 P
12426 12608 12429 12605 12434 12604 12437 12602 12441 12603 12441 12599
12444 12596 12445 12593 12449 12591 12453 12593 12457 12590 12461 12589
12462 12585 12467 12585 12471 12585 12474 12589 12472 12593 12470 12596
12465 12596 12461 12597 12465 12598 12465 12603 12462 12606 12458 12609
12456 12613 12455 12617 12451 12618 12447 12620 12442 12618 12438 12616
12436 12613 12433 12609 12426 12608 33 P
12473 12555 12477 12555 12475 12551 12475 12547 12479 12547 12483 12544
12485 12548 12484 12552 12484 12556 12485 12561 12484 12565 12481 12567
12482 12571 12477 12571 12476 12568 12477 12563 12476 12559 12473 12555
18 P
11961 12468 11963 12464 11963 12459 11964 12456 11965 12451 11964 12447
11967 12444 11966 12439 11970 12442 11974 12439 11978 12437 11981 12435
11985 12438 11988 12440 11991 12437 11994 12434 11992 12431 11987 12431
11987 12426 11990 12424 11994 12425 11999 12426 12003 12425 12006 12423
12011 12422 12010 12426 12008 12430 12012 12429 12015 12425 12019 12425
12024 12425 12028 12424 12026 12428 12021 12430 12023 12433 12025 12438
12021 12439 12017 12439 12012 12438 12013 12443 12012 12447 12008 12449
12007 12453 12005 12457 12004 12461 12004 12465 12003 12470 12000 12474
11997 12476 11993 12478 50 P
11993 12478 11990 12480 11985 12482 11981 12483 11977 12481 11974 12478
11970 12478 11966 12477 11963 12473 11961 12468 10 P
11711 12463 11715 12457 11720 12452 11723 12446 11729 12443 11728 12450
11725 12456 11729 12462 11730 12456 11735 12451 11742 12450 11738 12444

11735 12438 11737 12432 11742 12437 11745 12431 11752 12431 11759 12431
11765 12435 11771 12437 11766 12442 11773 12441 11779 12443 11784 12449
11790 12450 11790 12456 11797 12459 11803 12459 11804 12466 11811 12467
11812 12474 11817 12469 11819 12463 11824 12467 11822 12474 11828 12477
11830 12484 11834 12489 11831 12495 11835 12501 11830 12505 11834 12511
11835 12518 11836 12524 11831 12528 11827 12534 11822 12538 11816 12542
11810 12540 11809 12533 50 P
11809 12533 11803 12537 11800 12543 11802 12550 11798 12556 11792 12559
11785 12561 11779 12562 11772 12563 11770 12556 11773 12550 11780 12549
11786 12551 11784 12544 11786 12538 11783 12532 11778 12528 11776 12521
11769 12523 11769 12529 11766 12536 11762 12530 11765 12524 11759 12521
11762 12515 11763 12508 11768 12503 11773 12505 11771 12499 11764 12501
11768 12495 11766 12488 11765 12481 11764 12475 11760 12469 11761 12475
11759 12482 11760 12489 11763 12495 11759 12501 11759 12508 11753 12509
11752 12516 11746 12512 11745 12505 11749 12499 11746 12494 11741 12498
11734 12498 11731 12492 50 P
11731 12492 11724 12490 2 P
11724 12490 11723 12483 11727 12478 11721 12481 11715 12476 11714 12470
11711 12463 7 P
11913 12330 11917 12327 11918 12323 11923 12322 11927 12322 11931 12324
11934 12326 11938 12329 11941 12332 11942 12336 11946 12337 11947 12340
11951 12343 11951 12347 11955 12344 11957 12340 11958 12345 11961 12343
11966 12344 11966 12348 11961 12348 11957 12349 11953 12351 11949 12351
11945 12349 11940 12350 11936 12349 11934 12352 11931 12355 11926 12354
11923 12351 11920 12348 11917 12345 11913 12342 11916 12338 11913 12330
36 P
11930 12361 11934 12360 11937 12357 11940 12353 11945 12353 11949 12352
11953 12353 11950 12355 11949 12359 11952 12362 11954 12358 11959 12358
11961 12362 11958 12365 11961 12368 11965 12370 11962 12373 11960 12377
11957 12380 11952 12382 11949 12381 11944 12382 11940 12380 11937 12383
11933 12385 11934 12381 11937 12378 11933 12376 11932 12372 11937 12373
11934 12369 11933 12365 11930 12361 33 P
11969 12340 11972 12337 11974 12333 11974 12329 11978 12331 11981 12328
11983 12324 11986 12323 11989 12326 11989 12330 11991 12334 11990 12338
11994 12340 11993 12345 11989 12343 11988 12347 11986 12351 11982 12352
11983 12356 11987 12357 11991 12358 11993 12362 11990 12365 11986 12367
11981 12368 11978 12370 11974 12368 11974 12364 11970 12363 11971 12359
11975 12360 11977 12356 11977 12352 11974 12349 11973 12344 11969 12340
36 P
12014 12448 12019 12448 12023 12446 12027 12446 12031 12444 12033 12441
12037 12444 12040 12446 12044 12447 12048 12448 12052 12445 12054 12442
12054 12437 12057 12433 12061 12436 12062 12439 12066 12442 12065 12446
12069 12445 12070 12441 12074 12441 12075 12445 12074 12450 12074 12454

12079 12453 12080 12457 12078 12462 12075 12458 12071 12459 12068 12463
12064 12462 12059 12462 12056 12459 12053 12463 12050 12465 12046 12463
12044 12459 12044 12455 12041 12452 12037 12451 12034 12454 12030 12457
12028 12453 12026 12449 12022 12450 12018 12451 12014 12448 47 P
12010 12473 12014 12476 12018 12475 12023 12476 12021 12472 12020 12468
12019 12463 12023 12461 12026 12464 12026 12469 12028 12472 12030 12476
12034 12478 12038 12478 12042 12479 12042 12484 12045 12487 12050 12486
12053 12484 12058 12484 12062 12484 12062 12489 12059 12491 12054 12494
12054 12498 12050 12496 12046 12497 12050 12500 12051 12504 12047 12507
12045 12510 12041 12510 12037 12508 12033 12505 12029 12504 12025 12504
12023 12501 12019 12497 12018 12493 12015 12490 12017 12486 12015 12482
12013 12478 12010 12473 44 P
12059 12493 12063 12490 12065 12486 12063 12483 12062 12479 12063 12475
12066 12477 12068 12482 12072 12483 12072 12479 12072 12475 12073 12470
12077 12470 12081 12468 12085 12468 12089 12470 12091 12474 12094 12475
12099 12475 12100 12479 12098 12483 12097 12488 12098 12492 12099 12496
12098 12500 12096 12496 12094 12492 12092 12488 12088 12489 12084 12488
12081 12491 12077 12491 12072 12492 12069 12495 12067 12490 12065 12494
12059 12493 37 P
12125 12474 12120 12475 12122 12471 12127 12470 12131 12471 12135 12470
12138 12468 12135 12465 12139 12464 12142 12461 12143 12465 12147 12466
12151 12463 12147 12460 12144 12458 12145 12453 12145 12449 12147 12453
12150 12457 12152 12460 12156 12463 12160 12462 12161 12466 12156 12466
12156 12470 12160 12469 12164 12469 12167 12466 12172 12466 12169 12470
12166 12474 12165 12478 12161 12478 12156 12479 12153 12476 12149 12474
12146 12472 12147 12477 12145 12481 12141 12482 12138 12486 12135 12490
12137 12485 12133 12485 12128 12483 12132 12479 12129 12476 12125 12474
48 P
12129 12528 12133 12525 12135 12522 12137 12517 12137 12513 12140 12510
12143 12507 12145 12511 12144 12515 12148 12514 12147 12509 12149 12505
12150 12501 12152 12505 12153 12509 12155 12513 12153 12517 12152 12521
12150 12525 12147 12529 12144 12531 12139 12531 12135 12530 12129 12528
24 P
12896 12925 12899 12922 12904 12922 12905 12927 12903 12931 12903 12935
12904 12940 12905 12944 12907 12948 12906 12952 12905 12957 12900 12956
12898 12960 12894 12962 12892 12958 12887 12957 12884 12954 12882 12951
12886 12950 12889 12947 12888 12943 12892 12939 12888 12937 12892 12935
12896 12934 12897 12930 12896 12925 27 P
12750 12787 12754 12785 12757 12781 12756 12778 12756 12773 12760 12773
12765 12772 12768 12774 12772 12777 12775 12780 12774 12785 12772 12788
12767 12787 12765 12790 12764 12795 12761 12798 12762 12802 12759 12800
12760 12804 12756 12804 12752 12801 12753 12797 12758 12798 12761 12795
12757 12797 12757 12793 12754 12789 12754 12793 12751 12797 12751 12793

12750 12787 31 P
12763 12875 12765 12871 12768 12868 12772 12868 12770 12872 12773 12875
12770 12878 12767 12881 12770 12885 12773 12888 12771 12892 12767 12893
12763 12892 12761 12887 12764 12884 12765 12879 12763 12875 17 P
12616 12800 12620 12799 12624 12798 12624 12793 12628 12793 12631 12796
12632 12800 12635 12803 12639 12803 12644 12803 12643 12808 12639 12809
12635 12810 12631 12812 12626 12811 12623 12809 12618 12808 12616 12800
18 P
12633 12789 12637 12788 12638 12784 12642 12784 12645 12781 12649 12780
12653 12779 12654 12783 12651 12786 12655 12787 12659 12788 12663 12791
12659 12794 12656 12795 12653 12799 12650 12802 12645 12803 12642 12802
12638 12799 12636 12795 12638 12792 12633 12789 22 P
12656 12691 12659 12688 12663 12687 12663 12682 12665 12678 12667 12682
12672 12683 12673 12687 12678 12686 12682 12686 12686 12686 12686 12690
12689 12694 12687 12697 12683 12696 12680 12692 12679 12688 12676 12691
12679 12694 12676 12697 12671 12697 12668 12700 12664 12702 12659 12701
12658 12696 12656 12691 26 P
12537 12829 12535 12825 12534 12821 12532 12817 12529 12813 12528 12809
12524 12806 12523 12802 12524 12798 12525 12794 12528 12790 12532 12789
12536 12787 12541 12787 12545 12788 12549 12790 12550 12794 12554 12795
12558 12794 12562 12792 12566 12792 12570 12790 12574 12791 12577 12788
12582 12789 12581 12793 12577 12795 12572 12797 12569 12800 12569 12804
12570 12808 12569 12813 12566 12815 12562 12816 12558 12819 12559 12823
12555 12823 12552 12821 12551 12816 12550 12812 12550 12808 12548 12804
12545 12807 12544 12811 12545 12815 12546 12820 12548 12823 12550 12827
12551 12832 12550 12836 50 P
12550 12836 12546 12835 12542 12833 12537 12829 4 P
12398 12618 12400 12622 12402 12619 12405 12616 12409 12615 12413 12618
12417 12620 12417 12624 12421 12621 12425 12622 12428 12624 12425 12627
12422 12630 12418 12627 12414 12627 12412 12631 12408 12632 12404 12630
12400 12628 12398 12624 12398 12618 21 P
12310 12584 12314 12582 12318 12582 12319 12578 12322 12575 12318 12574
12315 12571 12317 12567 12320 12563 12324 12563 12327 12567 12331 12567
12336 12569 12340 12568 12342 12572 12342 12576 12339 12579 12334 12581
12330 12581 12326 12581 12329 12583 12327 12587 12323 12588 12326 12591
12329 12595 12327 12598 12323 12599 12319 12600 12316 12596 12314 12593
12313 12589 12310 12584 32 P
12126 12463 12122 12462 12118 12463 12113 12462 12110 12459 12107 12456
12108 12451 12108 12447 12113 12446 12114 12442 12118 12444 12122 12445
12125 12449 12129 12449 12131 12453 12135 12456 12133 12459 12126 12463
18 P
12268 12321 12270 12317 12272 12313 12275 12310 12276 12306 12279 12303
12282 12300 12287 12300 12291 12299 12294 12303 12289 12302 12286 12304

12284 12308 12280 12311 12279 12315 12277 12319 12279 12323 12277 12327
12274 12325 12268 12321 20 P
12281 12324 12284 12321 12286 12317 12290 12315 12294 12313 12295 12318
12292 12320 12292 12324 12288 12325 12281 12324 10 P
12107 12261 12108 12258 12113 12258 12117 12259 12119 12263 12118 12266
12114 12268 12109 12268 12107 12261 9 P
11834 12216 11838 12214 11840 12211 11843 12207 11847 12207 11845 12211
11846 12215 11851 12216 11851 12220 11847 12222 11844 12219 11839 12220
11834 12216 13 P
11824 12221 11828 12219 11832 12217 11833 12221 11830 12225 11826 12226
11824 12221 7 P
11903 12351 11906 12350 11908 12353 11910 12356 11913 12356 11914 12359
11916 12362 11913 12364 11911 12366 11910 12363 11907 12360 11907 12357
11906 12354 11903 12351 14 P
11949 12386 11952 12385 11954 12387 11957 12386 11959 12389 11963 12389
11962 12392 11960 12395 11958 12396 11954 12395 11952 12393 11949 12392
11949 12386 13 P
11952 12312 11956 12312 11959 12312 11962 12311 11961 12314 11959 12317
11958 12320 11955 12322 11952 12319 11954 12317 11952 12312 11 P
12022 12367 12025 12366 12027 12363 12028 12366 12029 12370 12031 12372
12032 12375 12030 12378 12028 12381 12025 12382 12023 12380 12021 12377
12023 12373 12022 12367 14 P
11273 12232 11277 12230 11281 12230 11285 12228 11288 12225 11290 12221
11293 12217 11297 12219 11301 12219 11303 12224 11299 12226 11296 12229
11293 12232 11292 12237 11293 12240 11288 12241 11286 12245 11282 12244
11279 12241 11279 12237 11279 12232 11273 12232 22 P
11439 12323 11441 12320 11442 12317 11445 12319 11448 12318 11450 12320
11447 12321 11445 12324 11442 12323 11439 12323 10 P
11685 12391 11689 12392 11689 12388 11693 12387 11697 12387 11702 12386
11704 12383 11704 12387 11706 12391 11707 12387 11710 12383 11712 12387
11716 12388 11720 12386 11719 12391 11718 12395 11717 12399 11717 12403
11713 12402 11709 12402 11705 12400 11701 12401 11697 12404 11693 12406
11691 12402 11687 12402 11690 12399 11687 12397 11685 12391 29 P
11692 12358 11695 12358 11697 12356 11700 12358 11703 12358 11707 12357
11708 12359 11709 12363 11712 12363 11710 12366 11709 12369 11706 12370
11703 12372 11704 12369 11704 12366 11702 12364 11698 12363 11695 12362
11692 12363 11692 12358 20 P
11648 12356 11649 12353 11652 12353 11655 12353 11658 12356 11660 12358
11661 12361 11658 12362 11655 12360 11653 12362 11650 12360 11648 12356

12:00 PM

11634 12360 11637 12360 11640 12358 11640 12361 11643 12364 11644 12366
11646 12363 11645 12360 11648 12362 11648 12365 11647 12368 11643 12369
11640 12368 11637 12366 11635 12364 11634 12360 16 P

11675 12417 11672 12415 11673 12412 11673 12409 11676 12408 11679 12407
11683 12407 11686 12408 11687 12411 11690 12410 11693 12412 11696 12410
11699 12410 11702 12408 11703 12411 11701 12413 11698 12414 11695 12416
11692 12417 11689 12419 11687 12417 11683 12417 11682 12414 11679 12414
11675 12417 25 P
11679 12376 11678 12378 11676 12381 11673 12382 11670 12380 11669 12377
11670 12375 11674 12375 11675 12372 11677 12370 11680 12369 11680 12372
11682 12375 11682 12378 11679 12376 15 P
11758 12370 11760 12367 11763 12367 11761 12370 11762 12373 11763 12376
11765 12379 11763 12382 11761 12379 11759 12377 11759 12373 11758 12370
12:00 PM
11382 12555 11385 12551 11387 12548 11391 12545 11395 12545 11394 12550
11395 12554 11398 12556 11401 12560 11400 12564 11404 12565 11407 12568
11409 12572 11408 12576 11412 12580 11415 12581 11414 12585 11410 12583
11407 12580 11403 12580 11402 12584 11406 12586 11401 12587 11397 12586
11393 12585 11391 12581 11391 12577 11388 12574 11387 12569 11386 12565
11383 12562 11386 12559 11382 12555 33 P
11408 12602 11410 12600 11413 12599 11414 12596 11416 12598 11416 12601
11418 12604 11421 12603 11421 12607 11418 12607 11415 12609 11412 12611
11413 12608 11410 12605 11408 12602 15 P
11288 12562 11291 12558 11293 12563 11295 12567 11299 12567 11301 12571
11302 12575 11304 12579 11302 12583 11298 12585 11297 12581 11295 12577
11294 12573 11290 12570 11288 12567 11288 12562 16 P
11345 12433 11347 12430 11348 12428 11351 12430 11349 12433 11345 12433
6:00 PM
11345 12437 11348 12437 11351 12439 11353 12441 11352 12444 11349 12444
11348 12442 11345 12437 8 P
11348 12419 11350 12417 11352 12414 11354 12411 11355 12408 11358 12410
11361 12409 11363 12410 11362 12413 11359 12415 11356 12416 11354 12414
11353 12417 11348 12419 14 P
11371 12570 11373 12568 11376 12569 11379 12571 11379 12575 11375 12575
11373 12574 11371 12570 8 P
11379 12576 11382 12576 11383 12579 11386 12581 11383 12581 11379 12576
6:00 PM
11402 12607 11405 12605 11407 12608 11405 12611 11402 12610 11402 12607
6:00 PM
11571 12560 11575 12558 11577 12555 11582 12555 11580 12552 11575 12550
11578 12547 11582 12548 11586 12550 11590 12549 11593 12552 11597 12553
11598 12558 11596 12562 11596 12566 11591 12567 11589 12564 11585 12562
11582 12559 11577 12559 11571 12560 21 P
11655 12614 11651 12616 11649 12620 11644 12620 11642 12623 11640 12627
11638 12630 11635 12627 11634 12623 11631 12620 11634 12617 11632 12613
11631 12617 11627 12618 11623 12616 11618 12615 11617 12610 11617 12606

11617 12602 11618 12597 11621 12594 11625 12597 11627 12593 11630 12590
11634 12591 11637 12588 11641 12587 11645 12588 11649 12591 11648 12595
11652 12597 11655 12601 11656 12605 11660 12604 11661 12608 11663 12613
11655 12614 37 P
11651 12653 11647 12651 11649 12655 11651 12660 11652 12664 11650 12668
11647 12670 11643 12668 11638 12667 11637 12662 11635 12659 11631 12659
11630 12655 11626 12653 11622 12651 11618 12650 11613 12650 11610 12653
11606 12653 11606 12648 11603 12645 11605 12641 11604 12637 11604 12633
11603 12628 11606 12625 11609 12622 11612 12618 11616 12617 11621 12618
11624 12620 11628 12622 11627 12627 11631 12627 11628 12630 11627 12635
11623 12637 11622 12641 11627 12643 11630 12644 11635 12645 11638 12647
11640 12643 11644 12643 11648 12645 11652 12646 11651 12653 47 P
11414 12556 11417 12556 11420 12557 11422 12560 11425 12562 11426 12565
11422 12565 11419 12565 11418 12562 11415 12560 11414 12556 11 P
11396 12616 11399 12614 11402 12614 11400 12617 11396 12616 5 P
11602 12561 11604 12557 11607 12556 11609 12554 11611 12551 11611 12555
11610 12558 11607 12558 11602 12561 9 P
11600 12561 11601 12557 11601 12554 11603 12556 11600 12561 5 P
11635 12573 11637 12571 11640 12569 11643 12571 11645 12569 11648 12567
11650 12568 11650 12571 11649 12574 11646 12576 11644 12579 11642 12580
11639 12578 11636 12577 11635 12573 15 P
11660 12575 11656 12574 11657 12570 11659 12568 11656 12567 11655 12563
11657 12562 11659 12564 11660 12561 11662 12563 11665 12564 11665 12568
11664 12571 11662 12573 11660 12575 15 P
11666 12562 11667 12558 11670 12558 11673 12556 11671 12553 11669 12551
11669 12548 11671 12545 11671 12542 11673 12539 11676 12540 11677 12543
11678 12545 11681 12544 11684 12543 11688 12543 11690 12545 11691 12548
11691 12551 11690 12554 11687 12555 11687 12558 11687 12562 11683 12561
11680 12561 11678 12564 11677 12567 11678 12569 11678 12573 11678 12576
11675 12577 11672 12578 11669 12578 11665 12577 11664 12574 11667 12571
11668 12568 11667 12565 11666 12562 39 P
11676 12523 11681 12522 11685 12522 11689 12522 11693 12524 11696 12528
11700 12529 11703 12532 11702 12536 11707 12537 11709 12541 11707 12545
11709 12549 11709 12553 11712 12556 11709 12559 11705 12561 11708 12564
11703 12567 11699 12568 11695 12569 11695 12574 11691 12576 11687 12576
11683 12576 11682 12572 11685 12569 11689 12568 11691 12564 11694 12560
11694 12556 11698 12553 11702 12552 11698 12550 11696 12547 11697 12542
11695 12539 11690 12538 11687 12541 11682 12541 11679 12537 11675 12535
11679 12533 11682 12536 11686 12535 11690 12536 11689 12531 11685 12530
11683 12527 11679 12528 50 P
11679 12528 11676 12523 2 P
11642 12552 11645 12553 11649 12553 11649 12550 11648 12547 11649 12544
11651 12544 11651 12548 11652 12551 11651 12554 11654 12556 11650 12557

11648 12556 11644 12555 11642 12552 15 P
11710 12588 11715 12588 11717 12584 11716 12580 11717 12576 11716 12572
11720 12571 11723 12574 11728 12575 11730 12579 11734 12580 11737 12577
11741 12580 11744 12584 11748 12584 11750 12588 11746 12589 11742 12590
11743 12594 11741 12598 11737 12601 11734 12597 11731 12594 11727 12594
11730 12597 11731 12602 11728 12604 11723 12603 11721 12599 11717 12597
11716 12593 11711 12592 11710 12588 33 P
11681 12652 11684 12650 11688 12647 11690 12643 11692 12640 11697 12640
11701 12638 11699 12634 11699 12629 11704 12629 11707 12627 11710 12629
11713 12633 11717 12634 11718 12629 11722 12627 11721 12622 11725 12621
11730 12620 11734 12621 11736 12625 11735 12629 11735 12634 11733 12637
11730 12641 11733 12644 11729 12646 11726 12650 11723 12651 11719 12649
11715 12649 11711 12651 11710 12656 11705 12656 11702 12654 11697 12656
11693 12657 11691 12653 11687 12654 11681 12652 40 P
11687 12665 11691 12663 11695 12662 11699 12660 11703 12662 11707 12662
11710 12659 11713 12655 11716 12652 11719 12654 11724 12655 11725 12659
11725 12663 11723 12667 11726 12670 11724 12674 11719 12674 11719 12679
11716 12681 11714 12677 11716 12673 11711 12673 11708 12671 11704 12669
11699 12668 11695 12667 11687 12665 27 P
11674 12697 11677 12693 11679 12690 11682 12686 11683 12682 11687 12680
11691 12678 11693 12674 11692 12670 11697 12670 11701 12672 11703 12675
11705 12680 11702 12683 11701 12687 11703 12692 11707 12690 11711 12688
11711 12692 11715 12695 11715 12699 11711 12701 11708 12704 11712 12706
11716 12707 11716 12711 11712 12710 11712 12715 11709 12718 11706 12715
11702 12717 11698 12718 11695 12715 11696 12711 11698 12707 11701 12703
11699 12700 11695 12700 11690 12701 11687 12704 11683 12702 11681 12699
11674 12697 43 P
11645 12715 11644 12711 11642 12707 11637 12707 11632 12707 11637 12705
11635 12701 11639 12700 11643 12700 11647 12702 11650 12700 11654 12703
11657 12706 11657 12710 11657 12706 11657 12702 11661 12701 11663 12697
11667 12694 11670 12697 11672 12700 11677 12701 11678 12705 11683 12706
11683 12710 11682 12714 11686 12713 11690 12714 11691 12719 11688 12721
11684 12722 11684 12727 11688 12729 11684 12732 11685 12736 11681 12736
11678 12741 11675 12743 11671 12745 11670 12740 11671 12736 11670 12732
11667 12730 11663 12733 11660 12730 11656 12728 11652 12730 11650 12726
11649 12722 11645 12715 50 P
11679 12674 11681 12670 11683 12668 11687 12669 11684 12672 11683 12675
11679 12674 7 P
11668 12683 11669 12680 11671 12677 11674 12677 11676 12674 11679 12675
11679 12679 11677 12681 11674 12682 11671 12684 11668 12683 11 P
11659 12638 11660 12635 11661 12632 11662 12629 11664 12626 11667 12626
11668 12629 11667 12632 11665 12635 11665 12638 11659 12638 11 P
11729 12609 11732 12609 11735 12608 11738 12610 11739 12613 11742 12610

11744 12613 11745 12616 11742 12618 11745 12620 11746 12623 11743 12624
11740 12623 11737 12621 11736 12618 11736 12615 11733 12615 11730 12615
11729 12609 19 P
11698 12618 11700 12616 11703 12616 11705 12619 11707 12622 11704 12624
11701 12623 11698 12618 8 P
11685 12635 11688 12634 11690 12637 11691 12640 11689 12642 11687 12640
11685 12635 7 P
11670 12645 11674 12646 11676 12647 11678 12650 11678 12653 11675 12651
11674 12649 11670 12645 8 P
11685 12603 11687 12600 11689 12602 11690 12605 11692 12607 11693 12610
11690 12610 11687 12608 11685 12603 9 P
11678 12590 11678 12587 11679 12584 11681 12587 11682 12590 11681 12594
11680 12597 11677 12595 11678 12590 9 P
11682 12494 11684 12492 11686 12489 11685 12486 11687 12483 11690 12485
11692 12488 11692 12491 11696 12492 11698 12495 11698 12498 11695 12496
11694 12499 11697 12501 11694 12502 11692 12505 11689 12503 11687 12501
11687 12497 11685 12495 11682 12494 21 P
11799 12445 11802 12444 11805 12443 11804 12446 11808 12446 11811 12448
11813 12450 11813 12453 11811 12455 11808 12453 11810 12456 11809 12459
11806 12458 11803 12456 11800 12454 11802 12451 11799 12445 17 P
11643 12428 11647 12429 11648 12431 11649 12435 11645 12435 11644 12431
11643 12428 7 P
11656 12428 11659 12428 11661 12431 11661 12434 11659 12437 11657 12435
11656 12428 7 P
11655 12396 11658 12396 11661 12398 11660 12400 11657 12399 11655 12396
6:00 PM
11662 12393 11664 12391 11667 12391 11671 12391 11669 12393 11666 12395
11662 12393 7 P
11662 12403 11663 12399 11664 12396 11667 12397 11668 12400 11666 12403
11662 12403 7 P
11650 12415 11654 12416 11657 12416 11656 12419 11654 12421 11651 12418
11650 12415 7 P
11680 12425 11683 12423 11681 12421 11683 12419 11687 12419 11686 12422
11685 12425 11683 12428 11680 12425 9 P
11676 12404 11677 12401 11679 12398 11682 12400 11685 12401 11685 12404
11682 12404 11676 12404 8 P
11613 12342 11616 12339 11618 12338 11621 12337 11621 12340 11618 12342
11613 12342 7 P
11656 12314 11658 12311 11659 12308 11660 12305 11662 12307 11660 12310
11660 12313 11658 12317 11656 12314 9 P
11749 12703 11754 12704 11758 12702 11762 12701 11766 12701 11769 12704
11770 12709 11775 12710 11779 12709 11783 12710 11782 12714 11786 12713
11788 12709 11792 12709 11796 12708 11798 12704 11802 12703 11800 12700

11797 12696 11799 12693 11803 12692 11805 12696 11809 12694 11812 12697
11811 12702 11813 12706 11813 12710 11812 12714 11809 12716 11805 12719
11800 12718 11796 12717 11792 12716 11788 12717 11790 12721 11786 12723
11790 12726 11791 12730 11795 12729 11796 12733 11800 12733 11802 12737
11798 12737 11796 12741 11791 12742 11787 12741 11783 12741 11781 12746
11777 12746 11772 12745 50 P
11772 12745 11770 12741 11772 12737 11776 12735 11779 12733 11781 12728
11777 12726 11777 12721 11773 12722 11773 12717 11770 12715 11768 12719
11765 12715 11764 12711 11759 12711 11755 12710 11752 12713 11749 12709
11749 12703 19 P
11758 12770 11762 12767 11764 12763 11766 12759 11768 12755 11772 12753
11776 12755 11775 12759 11773 12763 11771 12767 11768 12769 11764 12772
11758 12770 13 P
11742 12741 11745 12739 11750 12737 11752 12741 11756 12741 11756 12746
11757 12750 11753 12752 11749 12752 11744 12752 11742 12748 11742 12741
12:00 PM
11652 12813 11656 12813 11661 12813 11661 12808 11664 12812 11667 12815
11669 12811 11672 12809 11677 12808 11681 12806 11683 12810 11686 12813
11684 12816 11687 12820 11689 12824 11685 12825 11681 12827 11681 12832
11681 12836 11685 12838 11686 12842 11683 12846 11686 12849 11685 12853
11689 12855 11694 12856 11697 12859 11701 12861 11697 12863 11695 12859
11690 12859 11686 12859 11682 12859 11681 12854 11678 12851 11675 12848
11674 12844 11671 12840 11667 12838 11663 12837 11658 12835 11656 12832
11655 12828 11653 12824 11657 12822 11661 12820 11658 12816 11652 12813
48 P
11704 12832 11708 12831 11711 12828 11709 12823 11714 12823 11715 12819
11719 12818 11724 12819 11726 12816 11731 12816 11735 12818 11738 12815
11742 12817 11744 12813 11740 12811 11741 12806 11745 12806 11746 12802
11749 12799 11749 12803 11754 12801 11755 12805 11758 12808 11755 12811
11750 12812 11751 12816 11750 12821 11746 12823 11743 12820 11739 12821
11737 12825 11733 12827 11732 12831 11736 12834 11739 12832 11742 12835
11741 12840 11737 12840 11734 12836 11733 12840 11730 12844 11726 12841
11725 12836 11721 12837 11717 12840 11716 12844 11712 12842 11708 12841
11705 12837 11704 12832 50 P
11693 12808 11697 12808 11698 12803 11702 12802 11706 12803 11709 12801
11712 12797 11714 12801 11714 12806 11715 12801 11716 12797 11719 12800
11719 12805 11718 12809 11716 12813 11711 12814 11707 12813 11703 12813
11699 12812 11693 12808 20 P
11683 12780 11686 12778 11690 12780 11695 12781 11699 12781 11703 12779
11707 12781 11707 12777 11702 12778 11701 12774 11702 12769 11703 12765
11701 12761 11699 12757 11698 12753 11702 12754 11706 12756 11707 12760
11705 12764 11710 12765 11714 12768 11717 12766 11717 12770 11721 12771
11720 12775 11720 12780 11717 12783 11713 12784 11711 12788 11710 12792

11707 12795 11703 12793 11699 12795 11695 12795 11695 12791 11691 12789
11689 12785 11683 12780 38 P
11750 12787 11752 12785 11752 12781 11752 12778 11750 12775 11753 12774
11756 12775 11759 12775 11762 12777 11761 12780 11761 12783 11758 12785
11756 12788 11754 12790 11751 12790 11748 12792 11750 12787 17 P
11730 12768 11732 12765 11733 12762 11736 12760 11738 12763 11737 12767
11737 12770 11737 12773 11738 12776 11735 12775 11731 12774 11731 12771
11730 12768 13 P
11717 13106 11719 13103 11722 13099 11725 13102 11729 13104 11732 13107
11735 13111 11737 13114 11741 13116 11743 13119 11744 13123 11742 13127
11739 13130 11739 13125 11736 13123 11731 13123 11727 13125 11730 13121
11725 13120 11726 13116 11723 13113 11722 13109 11717 13106 23 P
11682 12938 11683 12935 11686 12933 11687 12930 11690 12931 11692 12933
11691 12937 11691 12940 11689 12943 11687 12944 11684 12943 11682 12938
12:00 PM
11623 12964 11626 12962 11627 12959 11630 12960 11633 12960 11636 12962
11638 12964 11637 12967 11635 12971 11632 12971 11630 12974 11628 12972
11624 12972 11624 12969 11628 12968 11630 12966 11627 12965 11623 12964
18 P
11621 12958 11619 12955 11618 12952 11621 12954 11624 12955 11621 12958
6:00 PM
11636 12979 11640 12978 11641 12980 11639 12983 11636 12979 5 P
11687 12892 11690 12892 11693 12892 11695 12895 11697 12897 11694 12898
11692 12895 11689 12894 11687 12892 9 P
11682 12884 11685 12885 11686 12888 11685 12892 11682 12891 11682 12887
11682 12884 7 P
11638 12826 11641 12823 11643 12821 11645 12820 11648 12822 11648 12825
11646 12828 11644 12831 11642 12833 11640 12831 11638 12826 11 P
11652 13130 11655 13129 11658 13130 11658 13128 11661 13129 11661 13131
11658 13133 11656 13134 11654 13133 11652 13130 10 P
11647 13140 11650 13139 11651 13142 11651 13144 11649 13144 11647 13140
6:00 PM
11793 13201 11796 13199 11797 13196 11799 13194 11800 13196 11801 13199
11799 13202 11797 13204 11793 13201 9 P
11870 13234 11872 13231 11875 13230 11877 13232 11878 13236 11879 13239
11877 13242 11873 13241 11871 13238 11870 13234 10 P
11789 13247 11790 13244 11792 13242 11792 13238 11796 13237 11795 13241
11798 13242 11800 13239 11803 13239 11806 13240 11809 13242 11806 13244
11804 13245 11800 13247 11797 13247 11795 13249 11791 13250 11789 13247
18 P
11765 13239 11768 13238 11768 13235 11771 13236 11771 13232 11774 13234
11777 13234 11778 13237 11776 13239 11779 13241 11778 13244 11777 13247
11773 13248 11770 13247 11770 13244 11773 13243 11776 13241 11774 13238

11771 13237 11770 13240 11765 13239 21 P
11778 13229 11781 13228 11784 13228 11786 13230 11789 13232 11790 13235
11788 13237 11786 13235 11785 13232 11782 13231 11783 13234 11781 13236
11779 13232 11778 13229 14 P
11756 13234 11758 13231 11760 13229 11763 13230 11763 13234 11761 13236
11758 13238 11756 13234 8 P
11750 13254 11754 13253 11757 13254 11759 13255 11762 13257 11759 13258
11756 13258 11754 13255 11750 13254 9 P
11771 13261 11774 13261 11777 13261 11781 13260 11778 13263 11775 13262
11771 13261 7 P
11741 13255 11744 13254 11748 13254 11746 13257 11741 13255 5 P
11806 13258 11808 13256 11811 13257 11812 13260 11809 13260 11806 13258
6:00 PM
11817 13316 11819 13315 11822 13314 11823 13316 11822 13318 11819 13318
11817 13316 7 P
11843 13322 11845 13322 11848 13321 11849 13318 11852 13318 11850 13320
11849 13323 11846 13324 11843 13322 9 P
11828 13328 11830 13327 11832 13326 11835 13324 11837 13325 11839 13325
11841 13328 11838 13329 11836 13330 11833 13330 11830 13330 11828 13328
12:00 PM
11818 13366 11821 13365 11824 13364 11826 13363 11827 13366 11827 13369
11826 13371 11823 13371 11822 13369 11818 13366 10 P
11768 13386 11769 13383 11771 13382 11774 13382 11777 13381 11776 13384
11775 13386 11772 13388 11770 13387 11768 13386 10 P
11563 13388 11564 13386 11566 13383 11567 13381 11570 13380 11572 13381
11571 13383 11575 13383 11575 13381 11577 13383 11577 13386 11575 13387
11572 13388 11570 13389 11567 13389 11563 13388 16 P
11583 13442 11584 13440 11587 13438 11589 13436 11589 13439 11588 13442
11583 13442 7 P
11601 13444 11604 13444 11607 13445 11609 13446 11611 13447 11614 13448
11614 13450 11612 13452 11609 13452 11607 13452 11604 13450 11603 13448
11601 13444 13 P
11552 13450 11553 13448 11554 13446 11556 13443 11558 13443 11558 13446
11560 13448 11558 13450 11556 13448 11554 13449 11552 13450 11 P
11715 13515 11718 13515 11720 13516 11722 13515 11724 13516 11726 13519
11726 13521 11724 13523 11723 13525 11724 13528 11723 13530 11720 13531
11719 13528 11719 13525 11717 13523 11715 13521 11715 13519 11715 13515
18 P
11731 13541 11732 13538 11735 13537 11737 13539 11740 13539 11742 13538
11745 13538 11748 13537 11750 13538 11753 13537 11756 13536 11758 13535
11761 13535 11763 13535 11765 13536 11765 13539 11762 13539 11759 13538
11757 13540 11755 13540 11756 13543 11755 13546 11753 13547 11752 13550
11750 13550 11747 13550 11745 13548 11744 13546 11745 13543 11742 13543

11740 13545 11737 13546 11735 13546 11732 13546 11731 13541 35 P
11678 13493 11679 13490 11681 13488 11682 13490 11684 13492 11684 13495
11686 13497 11684 13498 11681 13498 11680 13495 11681 13493 11678 13493

12:00 PM

11683 13480 11684 13477 11687 13477 11687 13480 11688 13482 11688 13485
11685 13485 11684 13483 11683 13480 9 P
11569 13688 11571 13686 11573 13687 11575 13689 11577 13690 11580 13689
11583 13690 11583 13693 11586 13694 11584 13697 11584 13700 11582 13701
11582 13698 11581 13695 11579 13694 11576 13694 11574 13693 11572 13691
11569 13688 19 P
11555 13988 11559 13990 11560 13995 11562 13991 11567 13989 11567 13985
11571 13984 11576 13985 11580 13987 11581 13991 11585 13993 11588 13996
11586 14000 11582 14001 11577 14003 11574 14005 11569 14004 11568 14008
11564 14010 11563 14015 11560 14017 11560 14013 11558 14009 11557 14005
11556 14001 11557 13997 11557 13992 11555 13988 28 P
11587 13992 11588 13990 11589 13987 11591 13986 11594 13986 11596 13985
11597 13982 11597 13979 11600 13981 11601 13984 11602 13985 11601 13987
11598 13987 11597 13989 11595 13991 11594 13994 11591 13995 11587 13992
18 P
11701 14026 11703 14022 11704 14018 11705 14014 11706 14009 11710 14012
11713 14015 11715 14011 11715 14007 11718 14009 11721 14012 11723 14016
11725 14020 11726 14024 11731 14025 11731 14029 11729 14034 11729 14038
11724 14039 11720 14039 11716 14039 11711 14038 11711 14034 11707 14032
11703 14032 11701 14026 26 P
11749 13994 11752 13996 11755 14000 11759 13999 11762 13995 11765 13997
11765 14002 11767 14005 11768 14010 11772 14009 11776 14009 11777 14013
11780 14016 11784 14017 11785 14021 11784 14025 11782 14029 11781 14033
11777 14034 11773 14034 11769 14035 11766 14038 11762 14037 11761 14042
11763 14045 11763 14050 11761 14046 11759 14042 11755 14040 11752 14043
11748 14044 11750 14041 11753 14037 11756 14034 11757 14030 11759 14026
11759 14022 11760 14017 11756 14016 11752 14014 11754 14009 11758 14010
11756 14006 11754 14002 11751 13999 11749 13994 46 P
11636 14018 11641 14017 11644 14015 11649 14014 11652 14013 11657 14012
11661 14013 11665 14016 11667 14020 11663 14022 11661 14018 11656 14018
11652 14021 11648 14021 11644 14022 11640 14024 11636 14018 17 P
11646 13971 11648 13968 11650 13967 11654 13965 11657 13965 11660 13965
11659 13968 11657 13970 11654 13971 11651 13972 11646 13971 11 P
11626 13970 11628 13968 11631 13968 11634 13967 11637 13966 11639 13963
11642 13962 11643 13965 11641 13966 11638 13969 11638 13972 11635 13973
11632 13975 11630 13972 11626 13970 15 P
11613 13975 11615 13972 11618 13971 11621 13972 11624 13971 11626 13974
11624 13977 11621 13976 11618 13975 11613 13975 10 P
11557 13964 11560 13962 11561 13959 11564 13958 11568 13959 11570 13961

11568 13964 11570 13967 11567 13967 11565 13969 11562 13971 11559 13969
11557 13964 13 P
11551 13919 11554 13920 11557 13918 11554 13918 11553 13915 11556 13913
11559 13913 11561 13916 11563 13919 11564 13922 11563 13925 11560 13927
11557 13925 11554 13925 11553 13922 11551 13919 16 P
11500 13903 11502 13901 11504 13898 11505 13902 11506 13905 11506 13908
11509 13907 11512 13908 11510 13911 11507 13912 11504 13913 11502 13911
11502 13908 11500 13903 14 P
11529 13879 11526 13879 11526 13883 11523 13885 11520 13886 11519 13883
11520 13880 11523 13878 11526 13876 11527 13873 11530 13875 11533 13877
11536 13879 11533 13878 11529 13879 15 P
11504 13961 11508 13960 11510 13962 11513 13964 11516 13964 11514 13967
11517 13968 11519 13971 11515 13971 11514 13969 11510 13968 11509 13965
11506 13964 11504 13961 14 P
11689 13939 11691 13937 11694 13935 11695 13938 11695 13942 11693 13944
11690 13944 11689 13939 8 P
11707 13916 11709 13913 11710 13910 11710 13907 11714 13906 11716 13907
11719 13908 11722 13910 11721 13913 11720 13916 11717 13918 11716 13916
11712 13916 11711 13919 11707 13916 15 P
11679 14095 11683 14094 11685 14091 11685 14088 11685 14085 11688 14082
11690 14081 11694 14082 11697 14083 11697 14087 11697 14090 11699 14091
11697 14094 11695 14095 11692 14098 11690 14100 11687 14102 11685 14104
11682 14105 11682 14102 11681 14099 11679 14095 22 P
11658 14131 11660 14128 11663 14128 11664 14124 11665 14121 11668 14120
11670 14117 11673 14119 11674 14122 11677 14123 11679 14121 11679 14117
11682 14119 11684 14117 11687 14115 11688 14112 11690 14115 11692 14117
11692 14121 11692 14123 11690 14127 11688 14128 11685 14130 11683 14132
11679 14133 11676 14134 11676 14130 11673 14129 11670 14130 11668 14132
11665 14135 11663 14137 11660 14137 11658 14134 11658 14131 35 P
11711 14145 11713 14141 11716 14139 11721 14139 11723 14143 11727 14143
11730 14146 11734 14148 11738 14147 11743 14148 11746 14150 11748 14154
11750 14157 11750 14162 11747 14165 11745 14169 11741 14170 11737 14172
11732 14172 11728 14173 11724 14175 11721 14177 11717 14175 11715 14171
11716 14167 11715 14162 11713 14159 11714 14154 11713 14150 11711 14145
30 P
11693 14153 11696 14150 11700 14149 11702 14144 11701 14140 11705 14140
11704 14144 11707 14148 11709 14151 11707 14155 11702 14154 11698 14154
11693 14153 13 P
11687 14164 11690 14163 11692 14165 11696 14166 11699 14165 11701 14163
11704 14162 11704 14166 11704 14169 11705 14172 11704 14175 11702 14177
11699 14180 11696 14178 11696 14175 11693 14173 11690 14172 11688 14169
11687 14164 19 P
11709 14130 11711 14128 11715 14127 11716 14130 11716 14134 11714 14136

11711 14134 11709 14130 8 P
11632 14181 11634 14178 11637 14176 11639 14174 11642 14172 11643 14169
11642 14166 11645 14164 11649 14165 11652 14166 11655 14167 11658 14167
11661 14168 11664 14167 11667 14170 11666 14173 11663 14172 11660 14171
11657 14170 11654 14170 11650 14172 11648 14174 11647 14176 11643 14177
11641 14179 11637 14180 11632 14181 27 P
11681 14167 11684 14166 11685 14169 11686 14173 11686 14176 11689 14178
11686 14180 11683 14179 11683 14175 11682 14172 11681 14167 11 P
11674 14170 11676 14168 11677 14170 11679 14173 11679 14175 11677 14174
11674 14170 7 P
11637 14308 11640 14302 11642 14295 11642 14289 11642 14282 11643 14275
11642 14268 11645 14262 11652 14261 11658 14260 11661 14254 11665 14249
11668 14243 11670 14236 11667 14230 11661 14228 11654 14226 11656 14220
11656 14213 11662 14210 11669 14208 11676 14209 11682 14208 11689 14206
11696 14205 11696 14211 11696 14218 11702 14215 11708 14211 11714 14210
11719 14215 11724 14220 11725 14227 11723 14233 11721 14240 11723 14246
11717 14250 11717 14257 11717 14264 11716 14271 11712 14276 11712 14283
11715 14289 11712 14296 11709 14301 11707 14308 11702 14313 11696 14315
11690 14319 11684 14322 50 P
11684 14322 11678 14326 11674 14331 11667 14333 11661 14329 11655 14329
11652 14322 11649 14317 11643 14313 11637 14308 10 P
11607 14262 11610 14259 11614 14256 11617 14254 11621 14252 11625 14250
11629 14248 11632 14246 11636 14243 11636 14239 11634 14235 11637 14232
11641 14229 11643 14232 11647 14235 11649 14238 11652 14241 11655 14245
11655 14249 11654 14253 11650 14256 11647 14258 11642 14258 11638 14259
11635 14261 11631 14263 11628 14267 11628 14271 11628 14275 11629 14280
11631 14283 11629 14287 11625 14286 11621 14283 11617 14282 11614 14279
11614 14275 11615 14271 11615 14266 11615 14262 11607 14262 41 P
11576 14291 11580 14288 11583 14286 11587 14284 11591 14285 11593 14289
11597 14290 11601 14291 11605 14294 11609 14294 11613 14291 11616 14293
11620 14295 11624 14298 11625 14302 11628 14306 11629 14309 11628 14314
11626 14317 11622 14314 11618 14313 11614 14313 11610 14313 11606 14315
11602 14318 11598 14319 11594 14318 11590 14318 11587 14315 11583 14312
11583 14308 11581 14304 11583 14300 11581 14296 11576 14291 35 P
11591 14228 11593 14225 11595 14222 11597 14220 11600 14219 11602 14216
11605 14216 11609 14215 11607 14218 11604 14220 11604 14223 11604 14226
11605 14229 11602 14229 11598 14229 11595 14229 11591 14228 17 P
11560 14237 11563 14235 11566 14233 11568 14234 11571 14235 11574 14233
11576 14231 11579 14229 11582 14229 11583 14232 11584 14235 11582 14236
11580 14239 11578 14242 11577 14245 11574 14245 11573 14241 11570 14240
11567 14240 11563 14240 11560 14237 21 P
11546 14242 11547 14240 11550 14240 11553 14240 11555 14241 11554 14243
11551 14244 11549 14246 11546 14242 9 P

11602 14269 11604 14269 11605 14271 11605 14274 11603 14275 11602 14273
11602 14269 7 P
11585 14265 11587 14263 11590 14263 11593 14263 11595 14264 11598 14264
11596 14266 11593 14266 11590 14266 11588 14267 11585 14265 11 P
10886 14335 10888 14332 10891 14329 10895 14326 10895 14322 10897 14318
10899 14314 10902 14311 10906 14312 10910 14312 10915 14311 10919 14312
10922 14309 10926 14311 10926 14315 10929 14319 10926 14322 10922 14323
10921 14327 10922 14331 10926 14333 10930 14331 10929 14327 10933 14327
10936 14331 10941 14330 10938 14333 10942 14333 10940 14337 10938 14341
10936 14345 10932 14347 10928 14347 10924 14346 10919 14345 10915 14344
10911 14343 10908 14341 10903 14339 10900 14337 10896 14338 10892 14340
10886 14335 43 P
10915 14258 10918 14256 10921 14254 10924 14254 10924 14251 10923 14248
10926 14249 10929 14250 10932 14250 10935 14250 10937 14253 10939 14256
10939 14259 10937 14262 10937 14265 10939 14267 10942 14269 10941 14272
10938 14273 10935 14273 10932 14275 10931 14272 10929 14269 10926 14268
10925 14265 10923 14262 10922 14259 10919 14258 10915 14258 29 P
10945 14226 10948 14227 10950 14230 10952 14232 10956 14233 10958 14232
10962 14232 10962 14235 10963 14238 10960 14238 10957 14238 10953 14236
10951 14237 10950 14234 10947 14232 10945 14226 16 P
11058 14266 11057 14262 11060 14261 11064 14262 11066 14261 11070 14261
11071 14263 11073 14266 11073 14269 11077 14268 11078 14271 11077 14274
11078 14277 11079 14280 11076 14280 11072 14280 11069 14280 11066 14279
11069 14276 11072 14275 11071 14272 11071 14269 11067 14269 11064 14270
11062 14272 11061 14268 11058 14266 27 P
11117 14261 11120 14261 11124 14260 11124 14257 11123 14254 11125 14256
11127 14259 11130 14261 11132 14262 11135 14265 11137 14267 11137 14270
11140 14269 11143 14271 11141 14273 11140 14276 11137 14275 11134 14274
11131 14273 11129 14272 11127 14268 11124 14267 11122 14265 11117 14261
24 P
11154 14212 11157 14210 11159 14208 11163 14209 11165 14212 11168 14211
11171 14213 11174 14215 11176 14217 11177 14220 11179 14222 11182 14225
11184 14227 11187 14228 11190 14230 11192 14232 11194 14235 11195 14238
11198 14240 11200 14242 11201 14246 11203 14248 11205 14251 11207 14254
11204 14253 11200 14253 11198 14252 11195 14249 11193 14247 11190 14245
11187 14246 11185 14243 11182 14242 11179 14240 11180 14237 11182 14234
11183 14231 11183 14228 11180 14230 11180 14233 11178 14235 11175 14237
11172 14236 11169 14235 11171 14232 11171 14229 11168 14227 11166 14224
11165 14221 11163 14219 50 P
11163 14219 11159 14218 11157 14216 11154 14212 4 P
11355 14216 11358 14214 11359 14212 11358 14208 11359 14205 11362 14204
11365 14206 11367 14209 11369 14212 11371 14214 11372 14217 11372 14220
11369 14221 11366 14220 11362 14219 11359 14219 11356 14220 11355 14216

18 P
11439 14279 11441 14276 11444 14277 11447 14277 11450 14278 11453 14280
11454 14283 11456 14286 11453 14285 11450 14283 11448 14281 11446 14282
11443 14281 11439 14279 14 P
11383 14288 11386 14287 11389 14287 11391 14290 11389 14293 11389 14296
11387 14294 11386 14291 11383 14288 9 P
11152 14241 11156 14240 11158 14239 11161 14237 11163 14234 11165 14236
11163 14238 11165 14241 11163 14243 11159 14244 11156 14243 11152 14241
12:00 PM
10928 14640 10931 14637 10935 14636 10939 14637 10936 14641 10938 14644
10942 14642 10946 14642 10949 14645 10951 14642 10955 14639 10959 14637
10963 14635 10967 14634 10971 14633 10971 14638 10974 14635 10978 14634
10981 14638 10979 14641 10977 14645 10975 14649 10971 14651 10971 14655
10967 14655 10963 14655 10959 14658 10955 14659 10951 14661 10948 14664
10946 14659 10943 14656 10942 14652 10937 14651 10933 14651 10933 14646
10934 14642 10928 14640 38 P
11018 14724 11017 14728 11015 14731 11014 14727 11015 14723 11012 14719
11014 14716 11018 14716 11018 14711 11015 14713 11011 14710 11010 14706
11013 14703 11016 14707 11019 14709 11023 14710 11025 14715 11025 14719
11028 14723 11028 14727 11025 14723 11018 14724 22 P
10903 15169 10907 15169 10910 15172 10914 15175 10918 15174 10922 15177
10925 15180 10927 15184 10931 15186 10934 15189 10935 15193 10939 15194
10938 15198 10942 15199 10946 15197 10949 15200 10951 15204 10950 15209
10946 15208 10942 15207 10944 15210 10941 15212 10944 15215 10939 15216
10942 15219 10938 15222 10937 15226 10941 15229 10945 15229 10944 15233
10941 15234 10937 15238 10933 15237 10929 15238 10925 15236 10922 15233
10925 15231 10924 15227 10921 15225 10924 15221 10924 15217 10921 15215
10917 15213 10916 15209 10911 15210 10908 15206 10905 15203 10904 15199
10908 15201 10912 15202 50 P
10912 15202 10916 15203 10920 15201 10917 15198 10913 15197 10915 15192
10919 15191 10919 15187 10918 15183 10914 15183 10909 15184 10909 15179
10908 15175 10903 15169 14 P
10808 15183 10812 15184 10814 15188 10817 15191 10821 15190 10824 15186
10827 15190 10827 15194 10831 15193 10832 15188 10836 15187 10840 15186
10844 15188 10847 15191 10848 15196 10851 15198 10852 15203 10848 15204
10844 15206 10840 15207 10838 15204 10834 15204 10830 15204 10827 15201
10823 15203 10820 15201 10820 15197 10817 15193 10813 15193 10811 15190
10808 15183 31 P
10846 15170 10849 15167 10852 15170 10857 15171 10856 15176 10859 15179
10856 15182 10858 15186 10854 15188 10850 15186 10848 15183 10845 15179
10848 15175 10846 15170 14 P
10810 15131 10814 15131 10813 15127 10816 15125 10819 15126 10822 15126
10823 15129 10824 15132 10821 15134 10820 15137 10820 15140 10817 15139

10815 15137 10811 15135 10810 15131 15 P
10820 15146 10823 15145 10826 15144 10825 15147 10826 15151 10823 15152
10821 15151 10820 15146 8 P
10819 15158 10822 15157 10824 15159 10825 15161 10824 15163 10822 15164
10820 15162 10819 15158 8 P
9403 15100 9404 15094 9404 15087 9410 15084 9416 15082 9423 15085
9429 15084 9433 15079 9436 15073 9439 15078 9445 15082 9449 15088
9454 15092 9460 15092 9467 15093 9473 15089 9479 15086 9486 15085
9492 15087 9496 15093 9501 15098 9508 15099 9511 15105 9515 15111
9521 15108 9527 15113 9530 15119 9531 15125 9531 15132 9531 15139
9527 15145 9526 15151 9519 15153 9513 15155 9507 15159 9501 15162
9494 15162 9498 15157 9499 15150 9495 15145 9489 15141 9483 15141
9478 15137 9472 15134 9465 15134 9458 15134 9452 15133 9445 15131
9438 15130 9433 15134 50 P
9433 15134 9431 15127 9431 15120 9426 15117 9420 15113 9417 15107
9412 15102 9403 15100 8 P
9500 15059 9497 15065 9491 15067 9484 15069 9478 15071 9471 15069
9466 15065 9462 15071 9457 15075 9450 15073 9446 15068 9443 15062
9443 15055 9438 15050 9432 15049 9425 15048 9423 15042 9425 15035
9418 15034 9422 15028 9420 15022 9416 15016 9422 15014 9429 15012
9433 15017 9435 15024 9440 15028 9444 15033 9449 15038 9450 15045
9456 15048 9462 15049 9469 15048 9471 15042 9466 15038 9462 15032
9458 15027 9455 15020 9452 15014 9458 15013 9465 15011 9472 15010
9477 15006 9484 15006 9489 15010 9490 15016 9494 15022 9497 15028
9496 15035 9493 15041 50 P
9493 15041 9488 15046 9482 15048 9489 15047 9496 15047 9501 15050
9500 15059 7 P
9866 14927 9870 14921 9869 14915 9874 14910 9879 14906 9884 14909
9888 14915 9889 14922 9895 14917 9901 14916 9900 14923 9899 14929
9896 14936 9898 14942 9904 14944 9897 14945 9893 14940 9887 14938
9880 14937 9877 14932 9866 14927 21 P
10217 14889 10223 14889 10228 14887 10230 14882 10225 14885 10222 14880
10222 14875 10222 14870 10223 14864 10228 14862 10231 14866 10235 14870
10240 14873 10244 14876 10244 14882 10240 14886 10238 14890 10235 14895
10235 14901 10233 14896 10228 14893 10223 14894 10217 14889 23 P
10036 14860 10039 14856 10042 14854 10038 14851 10042 14849 10045 14846
10050 14846 10054 14844 10058 14843 10061 14845 10061 14849 10056 14850
10055 14855 10052 14858 10049 14856 10045 14859 10045 14863 10041 14863
10036 14860 19 P
10143 15145 10140 15142 10142 15138 10142 15134 10139 15138 10135 15138
10135 15142 10130 15143 10128 15140 10126 15135 10124 15132 10121 15129
10119 15125 10124 15124 10128 15124 10130 15120 10132 15117 10135 15113
10139 15113 10143 15113 10145 15117 10148 15120 10151 15124 10153 15127

10155 15131 10156 15135 10153 15139 10150 15141 10143 15145 29 P
9939 14993 9942 14990 9946 14988 9950 14987 9955 14986 9958 14984
9962 14982 9966 14981 9970 14980 9974 14978 9974 14974 9977 14971
9979 14975 9978 14979 9983 14980 9987 14980 9991 14979 9996 14978
10000 14979 10004 14978 10008 14976 10012 14974 10015 14977 10018 14980
10022 14982 10017 14982 10013 14983 10009 14985 10005 14986 10002 14990
9998 14991 9996 14987 9992 14986 9988 14985 9983 14985 9979 14986
9975 14986 9971 14989 9969 14993 9969 14996 9966 14994 9963 14990
9960 14993 9960 14997 9956 14997 9953 14994 9949 14996 9946 14994
9939 14993 49 P
9996 14961 10000 14958 10004 14958 10007 14961 10011 14960 10015 14961
10020 14961 10024 14962 10028 14961 10027 14957 10026 14953 10024 14949
10024 14945 10027 14948 10030 14950 10034 14953 10038 14953 10038 14958
10042 14959 10046 14961 10050 14963 10046 14965 10044 14969 10040 14970
10042 14974 10038 14972 10036 14968 10032 14969 10028 14969 10023 14969
10020 14967 10016 14966 10011 14966 10007 14966 10003 14965 9996 14961
36 P
10050 14963 10053 14962 10056 14962 10059 14965 10056 14966 10054 14965
10050 14963 7 P
10056 14960 10060 14959 10062 14956 10064 14959 10067 14960 10070 14959
10073 14961 10071 14963 10068 14962 10064 14961 10061 14962 10056 14960
12:00 PM
10167 15088 10170 15084 10172 15081 10177 15080 10181 15077 10181 15081
10181 15086 10185 15086 10183 15089 10178 15091 10174 15091 10170 15093
10167 15088 13 P
10153 14988 10151 14985 10148 14984 10149 14981 10152 14980 10155 14981
10157 14985 10153 14988 8 P
9939 14957 9942 14955 9943 14952 9947 14953 9949 14952 9953 14953
9956 14953 9959 14953 9961 14956 9963 14958 9960 14960 9957 14959
9954 14959 9950 14960 9947 14960 9944 14960 9939 14957 17 P
9981 14948 9983 14946 9985 14947 9988 14948 9990 14950 9990 14954
9989 14956 9988 14954 9984 14954 9984 14951 9981 14948 11 P
10067 14880 10068 14877 10071 14876 10075 14877 10078 14877 10081 14876
10083 14879 10080 14881 10077 14882 10075 14881 10072 14881 10067 14880
12:00 PM
10097 14876 10099 14874 10101 14872 10102 14869 10105 14867 10106 14870
10108 14873 10106 14875 10102 14875 10097 14876 10 P
10083 14863 10085 14861 10088 14862 10090 14864 10092 14867 10090 14870
10088 14871 10087 14868 10083 14867 10083 14863 10 P
10429 15267 10432 15267 10435 15270 10438 15270 10441 15271 10444 15271
10447 15271 10445 15274 10444 15277 10441 15278 10438 15277 10435 15275
10432 15274 10430 15272 10429 15267 15 P
9908 14951 9911 14951 9914 14949 9916 14950 9920 14951 9921 14954

9919 14958 9916 14958 9913 14956 9911 14954 9908 14951 11 P
9907 14974 9910 14973 9913 14972 9916 14970 9918 14968 9921 14968
9924 14969 9922 14971 9919 14973 9916 14973 9914 14976 9911 14975
9907 14974 13 P
9668 14751 9669 14748 9669 14745 9671 14743 9674 14744 9675 14747
9675 14750 9673 14752 9674 14755 9675 14758 9672 14760 9669 14757
9671 14754 9668 14751 14 P
9196 15599 9199 15596 9203 15598 9205 15601 9209 15603 9214 15604
9218 15604 9219 15609 9218 15613 9218 15617 9215 15620 9211 15622
9209 15618 9204 15618 9204 15614 9205 15609 9201 15609 9198 15606
9196 15599 19 P
8356 14785 8360 14787 8364 14788 8368 14790 8371 14793 8375 14796
8378 14799 8379 14803 8377 14807 8373 14809 8370 14813 8368 14809
8370 14804 8374 14803 8375 14799 8371 14797 8368 14794 8365 14790
8361 14788 8356 14785 20 P
8392 14833 8396 14834 8401 14835 8405 14836 8409 14837 8413 14838
8416 14842 8417 14846 8419 14850 8423 14851 8424 14856 8422 14860
8418 14859 8415 14855 8413 14851 8410 14848 8408 14844 8404 14841
8401 14839 8397 14837 8392 14833 21 P
8475 14886 8471 14884 8467 14882 8463 14880 8459 14878 8455 14876
8455 14872 8452 14869 8448 14867 8444 14864 8441 14862 8437 14860
8437 14856 8434 14853 8430 14850 8426 14849 8430 14847 8434 14849
8438 14850 8442 14851 8446 14853 8449 14855 8452 14859 8455 14862
8458 14866 8461 14869 8464 14871 8467 14875 8470 14877 8473 14881
8475 14886 31 P
8397 14849 8399 14847 8402 14848 8404 14850 8405 14853 8407 14856
8407 14859 8405 14862 8404 14859 8402 14856 8400 14854 8398 14851
8397 14849 13 P
8272 14733 8276 14734 8279 14737 8282 14740 8285 14743 8287 14747
8290 14750 8292 14754 8294 14758 8295 14762 8293 14766 8290 14762
8289 14758 8286 14756 8282 14753 8282 14749 8281 14744 8279 14741
8275 14738 8272 14733 20 P
8293 14780 8292 14777 8295 14776 8297 14777 8299 14780 8301 14783
8301 14786 8299 14784 8295 14783 8293 14780 10 P
8310 14794 8313 14793 8315 14795 8319 14796 8320 14799 8321 14802
8322 14804 8324 14807 8326 14810 8323 14808 8321 14806 8319 14804
8318 14801 8315 14799 8313 14797 8310 14794 16 P
9247 14414 9250 14412 9254 14414 9255 14418 9260 14419 9264 14420
9266 14424 9266 14428 9262 14430 9258 14428 9254 14427 9251 14424
9251 14419 9247 14414 14 P
9224 14344 9228 14341 9231 14343 9236 14342 9240 14342 9243 14345
9240 14348 9235 14349 9231 14349 9231 14354 9230 14358 9228 14362
9225 14359 9225 14354 9224 14350 9224 14344 16 P

9167 14236 9169 14233 9172 14229 9176 14227 9179 14225 9181 14228
9181 14233 9181 14237 9181 14241 9177 14243 9173 14244 9170 14242
9167 14236 13 P
9204 14249 9207 14246 9210 14242 9212 14239 9215 14236 9220 14236
9223 14233 9226 14236 9225 14240 9223 14245 9221 14248 9217 14248
9213 14250 9209 14252 9204 14249 15 P
9221 14208 9224 14206 9229 14206 9231 14202 9234 14199 9238 14196
9240 14200 9238 14205 9238 14209 9240 14213 9238 14216 9235 14220
9233 14224 9231 14228 9228 14230 9224 14228 9223 14223 9223 14219
9225 14215 9225 14211 9221 14208 21 P
9309 14273 9311 14271 9314 14269 9317 14272 9317 14275 9314 14276
9310 14277 9309 14273 8 P
9289 14466 9292 14466 9295 14466 9298 14468 9301 14466 9301 14470
9299 14472 9298 14475 9295 14475 9294 14472 9291 14471 9289 14466

12:00 PM

9287 14456 9289 14453 9291 14450 9294 14448 9295 14446 9296 14448
9295 14452 9297 14453 9294 14454 9294 14457 9291 14459 9287 14456

12:00 PM

9010 13021 9013 13017 9017 13016 9020 13019 9024 13021 9024 13026
9021 13030 9021 13034 9020 13038 9016 13038 9015 13034 9013 13030
9010 13027 9010 13021 14 P
8704 12481 8706 12477 8709 12474 8712 12471 8714 12467 8719 12466
8723 12465 8726 12467 8731 12469 8728 12472 8724 12474 8720 12475
8716 12475 8711 12477 8709 12481 8704 12481 16 P
9538 10853 9542 10847 9547 10843 9552 10838 9557 10833 9561 10828
9566 10823 9571 10818 9577 10821 9584 10817 9590 10815 9595 10810
9600 10806 9606 10801 9611 10797 9615 10792 9620 10787 9626 10783
9632 10779 9638 10776 9645 10773 9648 10768 9654 10764 9661 10764
9668 10761 9673 10757 9679 10754 9686 10752 9692 10748 9699 10747
9705 10744 9711 10740 9716 10737 9723 10733 9728 10729 9735 10727
9741 10725 9747 10721 9753 10718 9760 10716 9766 10715 9773 10716
9780 10714 9787 10712 9793 10711 9800 10708 9806 10705 9812 10702
9819 10699 9825 10697 50 P
9825 10697 9832 10694 9838 10692 9845 10691 9852 10689 9859 10688
9866 10687 9872 10691 9877 10694 9880 10700 9884 10706 9890 10710
9892 10717 9893 10723 9894 10730 9892 10737 9886 10740 9880 10745
9874 10747 9867 10747 9860 10747 9853 10749 9847 10751 9840 10753
9834 10757 9828 10760 9823 10765 9816 10767 9810 10771 9805 10774
9798 10776 9792 10779 9785 10781 9778 10783 9772 10785 9765 10787
9758 10788 9752 10792 9746 10795 9741 10800 9739 10806 9733 10811
9728 10816 9723 10820 9717 10823 9710 10825 9704 10828 9698 10831
9691 10833 9685 10836 50 P
9685 10836 9678 10838 2 P

9678 10838 9672 10841 9668 10847 9665 10853 9661 10859 9658 10865
9654 10871 9650 10876 9644 10880 9639 10884 9632 10888 9626 10890
9619 10891 9612 10893 9605 10892 9598 10892 9592 10890 9585 10888
9578 10886 9572 10884 9567 10879 9562 10874 9557 10870 9551 10866
9545 10862 9538 10853 26 P
9747 10671 9753 10668 9759 10665 9765 10662 9772 10659 9776 10654
9782 10651 9789 10648 9795 10644 9800 10640 9806 10636 9813 10634
9818 10629 9825 10627 9828 10621 9832 10615 9836 10610 9842 10606
9848 10608 9849 10615 9848 10622 9845 10628 9840 10633 9836 10639
9832 10644 9826 10648 9821 10653 9814 10655 9809 10659 9802 10662
9796 10664 9789 10667 9783 10668 9776 10671 9769 10672 9763 10674
9756 10676 9747 10671 38 P
9850 10640 9854 10634 9857 10628 9858 10621 9860 10614 9862 10607
9867 10603 9873 10600 9879 10595 9885 10598 9888 10604 9893 10609
9895 10616 9894 10623 9891 10629 9885 10633 9879 10636 9873 10639
9866 10639 9859 10641 9850 10640 21 P
9983 9961 9977 9965 9971 9966 9971 9973 9966 9978 9960 9981
9958 9974 9951 9972 9955 9966 9960 9961 9966 9957 9969 9951
9971 9944 9976 9939 9977 9932 9978 9925 9977 9918 9975 9912
9978 9906 9983 9901 9988 9896 9993 9891 9996 9885 9998 9878
10004 9875 10010 9879 10015 9883 10021 9886 10028 9885 10035 9886
10042 9883 10045 9877 10052 9877 10059 9878 10065 9879 10072 9876
10078 9873 10085 9871 10091 9869 10098 9867 10105 9865 10111 9861
10117 9857 10123 9854 10130 9855 10137 9856 10143 9859 10140 9865
10138 9872 10141 9878 50 P
10141 9878 10144 9885 10146 9891 10152 9895 10145 9899 10142 9905
10136 9908 10132 9914 10127 9919 10122 9923 10115 9922 10108 9923
10104 9929 10097 9931 10096 9938 10089 9938 10082 9938 10076 9940
10072 9947 10065 9946 10059 9947 10052 9948 10048 9954 10043 9959
10040 9965 10034 9969 10027 9971 10022 9967 10015 9964 10008 9962
10002 9959 9995 9961 9990 9966 9983 9961 34 P
10018 10042 10024 10038 10030 10036 10036 10032 10042 10028 10048 10024
10055 10023 10061 10023 10068 10023 10075 10021 10081 10018 10088 10016
10085 10023 10083 10029 10088 10034 10090 10041 10085 10046 10090 10052
10095 10057 10088 10057 10083 10062 10085 10068 10079 10072 10072 10070
10066 10073 10064 10066 10058 10063 10051 10061 10047 10057 10040 10059
10033 10059 10029 10053 10023 10050 10018 10042 34 P
10109 10149 10111 10143 10110 10136 10115 10131 10113 10124 10119 10121
10125 10118 10132 10117 10131 10124 10138 10125 10143 10129 10149 10125
10156 10125 10163 10124 10170 10124 10176 10120 10183 10120 10180 10126
10174 10130 10179 10134 10175 10140 10169 10142 10162 10142 10155 10143
10153 10149 10146 10149 10149 10143 10143 10144 10136 10146 10129 10147
10123 10145 10116 10148 10109 10149 33 P

10170 10278 10177 10279 10184 10279 10190 10280 10197 10283 10204 10283
10211 10283 10218 10283 10224 10285 10218 10289 10223 10295 10216 10295
10209 10294 10204 10290 10197 10288 10192 10293 10185 10295 10182 10301
10176 10304 10169 10303 10175 10299 10176 10292 10175 10285 10170 10278
24 P
10017 9723 10024 9723 10028 9729 10035 9729 10040 9725 10047 9725
10049 9718 10055 9715 10061 9712 10061 9705 10061 9698 10068 9698
10070 9704 10073 9698 10079 9702 10081 9695 10088 9694 10091 9700
10092 9707 10096 9713 10091 9717 10089 9724 10083 9727 10078 9731
10071 9734 10065 9737 10061 9743 10056 9748 10058 9755 10056 9762
10051 9767 10047 9762 10040 9760 10033 9760 10028 9756 10027 9749
10025 9742 10023 9735 10017 9723 39 P
10098 9792 10104 9788 10110 9784 10114 9779 10119 9774 10125 9770
10130 9765 10136 9764 10142 9760 10149 9763 10144 9769 10141 9775
10141 9782 10136 9786 10142 9789 10137 9795 10131 9797 10124 9800
10117 9802 10111 9800 10104 9799 10098 9792 22 P
10146 9852 10152 9848 10156 9842 10161 9838 10167 9834 10170 9827
10175 9822 10169 9817 10170 9811 10172 9804 10177 9799 10184 9797
10190 9802 10185 9807 10183 9814 10186 9820 10192 9824 10198 9827
10198 9834 10196 9841 10190 9841 10183 9845 10190 9845 10197 9846
10192 9852 10192 9859 10186 9862 10181 9859 10173 9857 10167 9859
10160 9858 10156 9863 10151 9859 10146 9852 34 P
9909 9874 9914 9870 9916 9863 9922 9859 9928 9860 9933 9865
9939 9868 9945 9872 9950 9876 9953 9883 9950 9889 9948 9896
9947 9903 9943 9909 9937 9912 9933 9918 9930 9924 9923 9924
9916 9922 9915 9915 9912 9909 9909 9902 9909 9895 9912 9889
9913 9882 9909 9874 26 P
10051 9976 10055 9970 10059 9965 10066 9967 10070 9972 10071 9979
10078 9982 10083 9987 10076 9991 10070 9993 10063 9995 10057 9992
10053 9987 10051 9976 14 P
10112 10019 10117 10015 10122 10020 10129 10021 10136 10020 10142 10021
10149 10024 10153 10030 10160 10032 10165 10036 10163 10030 10165 10023
10170 10019 10176 10022 10181 10028 10178 10034 10175 10040 10168 10043
10161 10042 10155 10041 10148 10040 10141 10039 10134 10039 10132 10032
10128 10027 10122 10025 10112 10019 27 P
10102 10027 10103 10020 10110 10024 10116 10027 10123 10028 10129 10029
10125 10035 10119 10037 10112 10037 10105 10038 10102 10027 11 P
10327 10294 10329 10290 10331 10294 10336 10294 10338 10290 10343 10289
10342 10293 10341 10297 10343 10302 10344 10306 10340 10306 10336 10304
10332 10302 10330 10298 10327 10294 15 P
10350 10303 10352 10299 10357 10299 10361 10299 10363 10295 10366 10292
10366 10296 10365 10301 10364 10305 10359 10306 10356 10309 10350 10303

10229 10256 10234 10256 10238 10254 10242 10253 10240 10257 10244 10260
10247 10263 10244 10265 10240 10269 10236 10270 10234 10267 10230 10264
10229 10256 13 P
10168 9888 10171 9884 10168 9881 10169 9876 10172 9874 10177 9874
10177 9878 10176 9882 10176 9887 10179 9890 10184 9889 10188 9891
10186 9895 10182 9896 10180 9900 10182 9904 10181 9908 10178 9905
10173 9903 10169 9903 10171 9899 10170 9894 10168 9888 23 P
9956 9826 9959 9822 9962 9819 9967 9819 9971 9820 9975 9821
9980 9822 9984 9823 9988 9826 9988 9830 9988 9834 9985 9838
9981 9839 9978 9842 9977 9847 9974 9849 9972 9853 9968 9852
9966 9847 9962 9846 9959 9843 9958 9839 9958 9834 9956 9826
24 P
9993 9777 9989 9774 9985 9772 9982 9769 9978 9766 9977 9761
9979 9757 9978 9753 9982 9750 9986 9750 9990 9749 9995 9750
9997 9754 10000 9757 10001 9762 10004 9765 10006 9769 10009 9773
10009 9777 10007 9781 10003 9781 9998 9780 9993 9777 23 P
10097 9757 10101 9756 10103 9753 10106 9749 10109 9746 10112 9743
10116 9741 10119 9737 10123 9739 10128 9737 10131 9736 10135 9739
10139 9739 10144 9739 10144 9743 10140 9742 10139 9747 10135 9748
10132 9745 10128 9746 10123 9746 10119 9748 10116 9750 10112 9753
10115 9757 10117 9761 10113 9762 10110 9765 10105 9765 10102 9763
10097 9757 31 P
10128 9809 10131 9807 10136 9806 10140 9806 10144 9807 10148 9809
10152 9811 10152 9815 10156 9818 10154 9822 10150 9821 10145 9820
10141 9821 10137 9821 10132 9820 10131 9815 10128 9809 17 P
9940 9928 9943 9925 9948 9925 9951 9922 9950 9918 9954 9916
9958 9916 9959 9921 9961 9925 9963 9929 9962 9934 9964 9937
9961 9940 9957 9943 9953 9942 9949 9940 9946 9938 9943 9934
9940 9928 19 P
9999 9738 10000 9735 10000 9731 10001 9728 10000 9725 10003 9727
10005 9729 10008 9731 10009 9735 10011 9737 10014 9739 10014 9742
10013 9745 10009 9746 10007 9745 10003 9744 10002 9741 9999 9738
18 P
9987 9982 9990 9983 9993 9982 9995 9979 9997 9977 9995 9975
9993 9972 9996 9972 9999 9974 10002 9975 10004 9978 10007 9980
10008 9983 10011 9985 10009 9987 10008 9991 10007 9994 10003 9993
10000 9991 10000 9988 9998 9991 9995 9992 9993 9990 9994 9987
9990 9986 9987 9982 26 P
9956 10050 9956 10046 9957 10043 9960 10045 9963 10047 9966 10050
9966 10053 9964 10056 9967 10058 9966 10061 9968 10063 9970 10066
9971 10069 9969 10071 9966 10069 9966 10066 9964 10063 9961 10065
9960 10063 9960 10059 9960 10056 9957 10053 9956 10050 23 P
10347 9977 10348 9974 10350 9971 10353 9971 10356 9971 10359 9971

10359 9974 10357 9977 10354 9979 10351 9979 10347 9977 11 P
9949 9390 9953 9390 9957 9387 9959 9384 9962 9380 9966 9380
9969 9377 9970 9372 9970 9367 9968 9364 9965 9360 9969 9361
9974 9361 9978 9361 9982 9360 9981 9356 9980 9351 9984 9350
9988 9348 9991 9351 9996 9352 9994 9356 9994 9360 9995 9365
9999 9366 10003 9369 10000 9372 9995 9373 9998 9376 10002 9379
10005 9382 10003 9386 10002 9390 10002 9395 9999 9398 10002 9402
10001 9406 9997 9410 9994 9412 9990 9414 9987 9418 9984 9421
9980 9422 9976 9419 9976 9415 9972 9413 9968 9410 9964 9407
9961 9405 9957 9403 50 P
9957 9403 9953 9400 9950 9397 9949 9390 4 P
9905 9385 9907 9381 9911 9381 9916 9381 9920 9380 9924 9378
9928 9376 9930 9372 9933 9369 9938 9370 9942 9369 9946 9371
9950 9373 9947 9377 9952 9377 9954 9380 9957 9384 9955 9387
9950 9387 9947 9390 9948 9394 9946 9398 9942 9396 9937 9396
9933 9394 9928 9394 9924 9396 9921 9399 9916 9398 9913 9396
9909 9399 9906 9397 9908 9392 9905 9385 34 P
9908 9359 9913 9358 9917 9358 9916 9354 9921 9353 9923 9350
9927 9347 9931 9350 9931 9354 9933 9358 9930 9361 9933 9365
9930 9368 9927 9371 9922 9372 9918 9372 9915 9369 9915 9364
9908 9359 19 P
10016 9407 10021 9409 10025 9409 10027 9413 10030 9416 10033 9413
10037 9413 10041 9417 10044 9420 10040 9422 10035 9420 10031 9423
10031 9427 10036 9429 10033 9432 10036 9436 10031 9436 10028 9438
10025 9435 10027 9430 10024 9427 10028 9425 10024 9423 10022 9419
10017 9419 10018 9414 10016 9407 27 P
9607 8814 9610 8807 9611 8800 9614 8794 9618 8788 9622 8783
9621 8776 9615 8772 9621 8769 9620 8762 9627 8764 9634 8764
9640 8767 9647 8770 9654 8772 9653 8779 9660 8776 9665 8772
9668 8778 9662 8783 9664 8790 9669 8786 9676 8789 9678 8782
9685 8784 9681 8790 9687 8793 9687 8800 9680 8800 9674 8804
9671 8810 9672 8816 9669 8823 9667 8830 9669 8836 9674 8842
9681 8843 9679 8849 9673 8854 9667 8857 9661 8861 9654 8860
9647 8858 9643 8852 9641 8845 9644 8838 9637 8835 9633 8829
9628 8824 9621 8821 50 P
9621 8821 9618 8816 9607 8814 3 P
9567 8708 9571 8710 9576 8711 9580 8711 9584 8713 9588 8715
9593 8713 9591 8709 9593 8704 9597 8707 9601 8707 9605 8709
9610 8710 9614 8712 9617 8715 9621 8717 9618 8719 9614 8722
9611 8725 9608 8728 9604 8730 9600 8728 9595 8727 9591 8729
9588 8726 9584 8725 9584 8729 9580 8729 9579 8724 9576 8722
9571 8719 9567 8717 9568 8713 9567 8708 34 P
9558 8649 9561 8645 9564 8649 9568 8651 9572 8653 9576 8655

9579 8652 9583 8650 9583 8654 9587 8657 9590 8660 9593 8664
9598 8664 9593 8666 9590 8664 9585 8663 9581 8660 9577 8661
9574 8665 9571 8669 9567 8669 9566 8664 9563 8662 9562 8658
9561 8653 9558 8649 26 P
9593 8637 9598 8636 9602 8634 9606 8636 9611 8634 9614 8637
9619 8637 9621 8640 9626 8638 9630 8637 9634 8634 9638 8636
9639 8640 9639 8644 9639 8649 9635 8651 9639 8653 9640 8649
9644 8652 9646 8656 9644 8659 9645 8664 9640 8665 9638 8661
9634 8659 9630 8660 9626 8658 9629 8654 9627 8650 9622 8650
9618 8649 9616 8652 9613 8656 9611 8651 9607 8649 9604 8645
9600 8644 9596 8641 9593 8637 39 P
9641 8739 9637 8737 9633 8735 9631 8731 9634 8727 9635 8723
9640 8722 9643 8725 9647 8728 9651 8731 9655 8732 9659 8734
9663 8736 9658 8736 9654 8735 9650 8737 9645 8738 9641 8739
18 P
9580 8434 9583 8431 9587 8428 9590 8425 9590 8429 9590 8434
9593 8432 9598 8431 9602 8428 9606 8429 9610 8426 9610 8430
9607 8434 9603 8435 9599 8438 9596 8441 9592 8444 9588 8446
9584 8447 9588 8444 9591 8441 9587 8442 9585 8438 9580 8434
24 P
9904 5701 9908 5697 9910 5691 9913 5686 9919 5684 9922 5679
9927 5680 9926 5685 9928 5691 9929 5697 9929 5703 9924 5704
9918 5705 9913 5703 9904 5701 15 P
9315 5477 9319 5475 9324 5476 9326 5480 9330 5484 9334 5486
9335 5491 9332 5494 9328 5495 9323 5493 9319 5490 9321 5486
9318 5482 9315 5477 14 P
8866 6170 8872 6167 8878 6162 8882 6156 8889 6154 8896 6156
8902 6159 8902 6166 8907 6172 8912 6176 8919 6180 8924 6185
8931 6185 8938 6185 8945 6182 8942 6188 8940 6195 8939 6202
8935 6208 8930 6203 8928 6210 8921 6207 8920 6200 8918 6207
8914 6201 8911 6195 8911 6202 8912 6209 8905 6209 8901 6203
8897 6197 8896 6190 8892 6196 8889 6202 8883 6198 8880 6192
8876 6186 8882 6188 8885 6182 8878 6182 8884 6179 8891 6179
8898 6179 8896 6172 8889 6168 8886 6162 8881 6167 8875 6172
8872 6178 8866 6170 50 P
8870 6241 8875 6237 8880 6242 8886 6238 8891 6232 8893 6225
8897 6219 8899 6226 8898 6232 8902 6238 8899 6244 8896 6251
8889 6251 8882 6249 8875 6248 8870 6241 16 P
8828 6183 8835 6185 8842 6186 8849 6186 8851 6193 8852 6200
8856 6206 8855 6213 8854 6220 8860 6223 8866 6228 8859 6229
8853 6234 8848 6229 8846 6222 8846 6215 8846 6208 8841 6202
8846 6198 8844 6191 8837 6190 8828 6183 22 P
8840 6064 8845 6063 8849 6064 8854 6064 8852 6068 8855 6071

8852 6075 8851 6080 8851 6084 8847 6085 8844 6088 8843 6084
8842 6079 8844 6075 8844 6070 8840 6064 16 P
8801 5982 8805 5978 8807 5974 8807 5970 8811 5967 8812 5972
8816 5975 8819 5978 8820 5983 8818 5987 8813 5987 8809 5985
8801 5982 13 P
8894 6286 8896 6284 8897 6280 8898 6284 8900 6286 8903 6288
8906 6289 8907 6293 8904 6295 8901 6297 8899 6295 8898 6292
8895 6289 8894 6286 14 P
9121 6141 9121 6136 9122 6132 9123 6128 9124 6123 9125 6119
9126 6123 9124 6128 9124 6132 9124 6137 9126 6141 9127 6146
9127 6150 9129 6155 9130 6159 9133 6163 9135 6167 9137 6170
9137 6175 9134 6171 9132 6167 9129 6163 9128 6159 9124 6155
9123 6151 9122 6147 9121 6141 27 P
8918 7285 8924 7288 8931 7291 8938 7290 8943 7295 8942 7288
8942 7280 8935 7282 8932 7275 8937 7271 8933 7264 8938 7259
8943 7255 8950 7252 8955 7247 8960 7242 8967 7240 8971 7247
8967 7253 8961 7257 8963 7264 8969 7267 8976 7271 8981 7276
8988 7277 8993 7282 9000 7283 9005 7278 9012 7281 9012 7288
9005 7289 8999 7290 8992 7294 8987 7299 8985 7306 8985 7313
8987 7319 8993 7324 8999 7328 9006 7330 9010 7324 9016 7326
9021 7332 9025 7338 9018 7337 9010 7337 9003 7339 8999 7345
8992 7342 8995 7348 50 P
8995 7348 9000 7354 8996 7360 8990 7363 8985 7368 8978 7366
8972 7369 8968 7375 8967 7382 8972 7387 8976 7393 8969 7393
8962 7390 8957 7385 8954 7378 8948 7382 8943 7376 8941 7370
8933 7369 8929 7364 8926 7358 8926 7351 8930 7344 8936 7346
8941 7352 8948 7352 8951 7346 8949 7339 8946 7332 8946 7325
8945 7318 8944 7311 8950 7308 8955 7312 8958 7319 8963 7314
8971 7313 8971 7306 8966 7302 8959 7298 8952 7298 8947 7304
8940 7305 8933 7306 8929 7300 8922 7299 8920 7292 8918 7285
48 P
8800 7143 8805 7138 8802 7132 8807 7127 8814 7126 8820 7123
8826 7127 8833 7129 8839 7125 8837 7118 8836 7111 8842 7108
8849 7106 8856 7106 8859 7112 8853 7118 8857 7124 8864 7125
8869 7130 8862 7128 8857 7133 8852 7128 8845 7128 8839 7132
8833 7136 8828 7141 8828 7148 8821 7150 8819 7156 8814 7151
8810 7145 8800 7143 32 P
8200 6667 8205 6662 8209 6657 8216 6654 8223 6651 8229 6655
8231 6661 8235 6667 8241 6672 8247 6675 8254 6677 8261 6678
8268 6677 8274 6673 8280 6669 8286 6665 8293 6669 8297 6675
8297 6682 8302 6687 8298 6693 8297 6701 8294 6707 8292 6714
8289 6720 8282 6722 8279 6728 8274 6733 8269 6728 8263 6724
8265 6717 8260 6711 8254 6708 8254 6701 8249 6695 8242 6696

8235 6698 8229 6702 8224 6707 8219 6711 8213 6708 8207 6703
8208 6696 8215 6700 8212 6693 8214 6687 8216 6679 8219 6672
8212 6671 8200 6667 50 P
8088 6694 8093 6688 8096 6682 8102 6678 8109 6675 8116 6673
8123 6672 8129 6676 8134 6681 8140 6685 8145 6690 8146 6697
8149 6704 8156 6705 8156 6712 8156 6720 8156 6727 8152 6732
8145 6729 8141 6735 8136 6740 8129 6738 8124 6742 8117 6745
8110 6747 8111 6739 8105 6735 8104 6728 8100 6722 8095 6717
8091 6711 8093 6704 8100 6705 8105 6710 8112 6713 8113 6706
8107 6702 8100 6699 8088 6694 39 P
7690 6184 7697 6184 7704 6186 7711 6188 7718 6186 7717 6178
7723 6182 7729 6185 7736 6187 7743 6188 7750 6190 7757 6190
7754 6197 7746 6198 7745 6205 7745 6212 7745 6219 7748 6226
7752 6232 7759 6234 7766 6231 7772 6228 7779 6228 7780 6236
7775 6241 7775 6248 7777 6255 7783 6259 7782 6265 7788 6269
7793 6265 7800 6266 7802 6273 7809 6275 7801 6275 7797 6281
7791 6284 7786 6289 7780 6285 7773 6286 7769 6280 7762 6281
7756 6278 7753 6285 7749 6291 7743 6295 7737 6299 7732 6304
7725 6300 7724 6293 50 P
7724 6293 7726 6287 7726 6279 7722 6274 7717 6268 7715 6262
7710 6256 7705 6251 7708 6243 7714 6240 7718 6245 7725 6247
7731 6243 7738 6243 7745 6242 7740 6237 7738 6230 7733 6225
7728 6221 7722 6216 7717 6210 7713 6205 7714 6198 7710 6203
7703 6200 7698 6195 7690 6184 27 P
7526 6113 7522 6119 7516 6121 7509 6120 7503 6115 7497 6110
7493 6105 7497 6099 7504 6097 7511 6096 7518 6097 7526 6096
7533 6094 7538 6090 7545 6087 7552 6087 7558 6089 7566 6090
7573 6088 7574 6081 7581 6084 7586 6079 7593 6083 7596 6089
7595 6096 7594 6103 7600 6107 7597 6114 7591 6117 7589 6124
7590 6132 7583 6132 7578 6126 7576 6119 7569 6120 7564 6126
7558 6129 7551 6127 7547 6121 7544 6115 7538 6110 7535 6104
7526 6113 43 P
7527 6180 7520 6179 7513 6179 7510 6173 7506 6167 7498 6166
7491 6165 7493 6157 7499 6154 7506 6153 7513 6151 7520 6149
7522 6155 7523 6163 7527 6169 7533 6172 7527 6180 17 P
7483 6175 7488 6170 7495 6169 7502 6168 7508 6173 7505 6180
7506 6187 7511 6192 7514 6199 7507 6200 7501 6196 7500 6189
7496 6183 7490 6180 7483 6175 15 P
7334 6043 7339 6038 7343 6031 7349 6028 7355 6024 7360 6018
7366 6016 7373 6018 7378 6013 7381 6006 7387 6004 7394 6002
7388 5997 7392 5991 7398 5987 7405 5987 7407 5980 7414 5980
7417 5987 7422 5992 7415 5995 7408 5992 7403 5997 7402 6004
7402 6011 7404 6018 7402 6025 7398 6031 7397 6038 7392 6044

7385 6046 7379 6042 7372 6042 7365 6043 7360 6049 7356 6054
7348 6055 7342 6051 7334 6043 39 P
8712 7276 8716 7270 8720 7265 8721 7257 8724 7251 8731 7254
8736 7258 8743 7258 8744 7265 8741 7272 8741 7279 8740 7286
8743 7293 8745 7299 8748 7306 8746 7313 8750 7319 8750 7326
8750 7333 8746 7340 8740 7337 8737 7331 8733 7325 8727 7321
8726 7313 8724 7307 8721 7300 8719 7293 8717 7287 8712 7276
30 P
8841 7629 8848 7628 8855 7628 8862 7626 8869 7625 8875 7621
8881 7618 8889 7617 8895 7620 8895 7627 8893 7634 8890 7641
8888 7647 8882 7650 8875 7653 8869 7658 8864 7654 8857 7652
8852 7646 8847 7640 8841 7629 21 P
8846 7624 8850 7622 8853 7619 8857 7616 8862 7616 8866 7617
8871 7618 8874 7620 8871 7623 8867 7624 8862 7625 8858 7626
8853 7627 8846 7624 14 P
8893 7958 8895 7954 8898 7950 8898 7946 8902 7946 8904 7950
8908 7947 8912 7946 8916 7947 8921 7948 8926 7949 8927 7953
8931 7956 8934 7959 8939 7959 8942 7961 8946 7964 8945 7969
8941 7971 8936 7970 8933 7967 8932 7963 8930 7959 8926 7960
8927 7964 8922 7965 8918 7963 8914 7965 8909 7966 8905 7965
8901 7965 8897 7964 8893 7958 33 P
9225 8131 9227 8128 9230 8124 9234 8122 9238 8119 9242 8117
9246 8117 9250 8119 9254 8121 9258 8122 9263 8122 9259 8124
9255 8127 9254 8131 9252 8135 9250 8139 9245 8139 9242 8143
9238 8143 9234 8143 9230 8139 9227 8137 9225 8131 23 P
10666 6777 10661 6779 10657 6777 10653 6776 10648 6778 10644 6776
10642 6771 10638 6770 10636 6766 10632 6764 10635 6761 10640 6761
10645 6760 10649 6758 10653 6758 10657 6759 10662 6761 10667 6760
10671 6758 10675 6761 10678 6764 10682 6766 10684 6770 10689 6773
10685 6775 10681 6777 10676 6778 10672 6780 10666 6777 29 P
11075 6565 11078 6562 11083 6561 11087 6560 11092 6561 11096 6561
11100 6563 11104 6566 11106 6570 11103 6574 11098 6576 11094 6576
11089 6576 11085 6574 11081 6572 11077 6570 11075 6565 17 P
11528 6945 11530 6941 11534 6938 11538 6938 11543 6937 11547 6938
11551 6940 11555 6943 11557 6946 11558 6951 11556 6955 11553 6958
11548 6959 11543 6960 11539 6960 11535 6958 11531 6955 11528 6952
11528 6945 19 P
11761 7064 11763 7060 11766 7057 11770 7055 11775 7054 11779 7054
11783 7058 11787 7059 11791 7057 11796 7057 11800 7058 11804 7059
11806 7064 11805 7068 11803 7072 11801 7076 11799 7080 11796 7082
11791 7084 11787 7086 11783 7088 11778 7090 11774 7090 11771 7087
11770 7082 11771 7078 11770 7073 11766 7071 11762 7073 11761 7064
30 P

11528 7005 11530 7003 11534 7002 11537 7000 11540 6999 11541 7002
11539 7005 11536 7007 11533 7009 11530 7007 11528 7005 11 P
11195 6689 11193 6687 11192 6684 11192 6680 11194 6677 11196 6675
11198 6672 11201 6670 11204 6670 11207 6672 11210 6674 11212 6676
11213 6679 11213 6683 11211 6686 11207 6686 11204 6686 11200 6687
11195 6689 19 P
11192 6736 11189 6737 11188 6740 11187 6743 11184 6745 11181 6746
11178 6745 11174 6744 11173 6741 11176 6739 11174 6736 11171 6734
11168 6734 11171 6733 11174 6732 11178 6733 11181 6732 11185 6733
11187 6730 11191 6728 11193 6730 11195 6733 11192 6736 23 P
11233 6730 11234 6727 11238 6725 11241 6725 11244 6728 11246 6730
11246 6734 11245 6737 11242 6739 11240 6741 11237 6741 11235 6738
11234 6735 11233 6730 14 P
11156 6664 11158 6662 11161 6661 11165 6662 11164 6665 11162 6668
11158 6668 11156 6664 8 P
11164 6670 11167 6671 11170 6674 11172 6676 11173 6679 11170 6681
11167 6678 11165 6676 11164 6670 9 P
10966 6636 10968 6636 10970 6637 10972 6638 10975 6639 10972 6639
10970 6639 10969 6638 10966 6636 9 P
10880 7536 10881 7534 10884 7534 10886 7534 10888 7535 10889 7537
10892 7536 10894 7537 10896 7537 10898 7537 10898 7540 10896 7541
10895 7543 10893 7543 10891 7544 10889 7543 10887 7543 10885 7542
10883 7541 10881 7539 10880 7536 21 P
10921 7532 10923 7530 10924 7529 10926 7530 10925 7532 10924 7534
10921 7532 7 P
10957 7633 10958 7632 10961 7631 10963 7632 10964 7634 10963 7635
10961 7637 10959 7637 10958 7636 10957 7633 10 P
10145 7416 10147 7415 10149 7414 10150 7416 10152 7417 10152 7419
10150 7420 10148 7420 10145 7419 10145 7416 10 P
10164 7336 10165 7334 10168 7335 10168 7337 10164 7336 5 P
10096 6965 10097 6963 10099 6962 10101 6961 10103 6960 10105 6960
10108 6961 10109 6960 10111 6961 10112 6963 10110 6964 10109 6966
10108 6968 10106 6969 10104 6969 10102 6968 10101 6966 10099 6965
10096 6965 19 P
8935 6010 8937 6008 8936 6006 8938 6004 8939 6003 8940 6005
8940 6007 8942 6009 8942 6011 8942 6013 8940 6014 8938 6013
8935 6010 13 P
8749 6089 8751 6087 8753 6088 8755 6088 8757 6090 8757 6092
8755 6093 8753 6094 8751 6094 8750 6091 8749 6089 11 P
8906 6005 8907 6003 8909 6003 8912 6003 8913 6005 8912 6008
8909 6008 8907 6007 8906 6005 9 P
8857 6069 8859 6067 8861 6067 8862 6069 8864 6071 8866 6071
8868 6071 8869 6073 8868 6075 8866 6075 8864 6075 8861 6075

8860 6073 8859 6071 8857 6069 15 P
8850 6141 8851 6139 8853 6139 8855 6140 8858 6140 8860 6141
8862 6141 8864 6143 8865 6145 8867 6145 8868 6147 8866 6148
8864 6147 8861 6146 8859 6145 8857 6145 8855 6143 8854 6141
8852 6143 8851 6144 8850 6141 21 P
8850 6015 8852 6014 8854 6014 8855 6016 8853 6017 8850 6015

6:00 PM

8854 6006 8857 6007 8859 6008 8860 6010 8859 6012 8857 6012
8856 6010 8854 6006 8 P
8165 6666 8167 6664 8171 6664 8174 6663 8176 6665 8179 6667
8182 6665 8185 6668 8185 6671 8188 6672 8191 6672 8193 6675
8193 6678 8194 6682 8193 6684 8190 6687 8187 6689 8184 6690
8181 6688 8178 6688 8174 6687 8174 6683 8174 6679 8173 6676
8169 6675 8168 6671 8168 6668 8165 6666 28 P
7950 6697 7954 6694 7958 6692 7961 6689 7964 6685 7967 6681
7971 6679 7975 6677 7979 6678 7984 6678 7988 6678 7993 6679
7991 6684 7990 6688 7986 6689 7981 6688 7977 6687 7973 6689
7971 6692 7967 6696 7963 6695 7959 6694 7955 6696 7950 6697
24 P
7953 6699 7957 6696 7961 6696 7965 6698 7970 6696 7973 6693
7975 6689 7980 6689 7984 6691 7988 6692 7992 6690 7994 6685
7998 6684 8003 6687 8007 6687 8006 6691 8003 6694 7999 6697
7997 6701 7993 6704 7988 6705 7984 6706 7979 6708 7975 6707
7971 6706 7966 6705 7961 6703 7957 6702 7953 6699 29 P
8022 6663 8026 6661 8031 6663 8034 6660 8039 6663 8042 6665
8039 6667 8034 6669 8030 6668 8022 6663 10 P
7979 6675 7983 6673 7987 6671 7991 6669 7995 6668 7997 6673
7993 6675 7988 6676 7984 6676 7979 6675 10 P
7942 6708 7943 6704 7945 6701 7947 6700 7951 6698 7952 6702
7950 6704 7947 6707 7946 6709 7944 6712 7942 6708 11 P
8622 7035 8627 7034 8631 7033 8633 7029 8635 7025 8639 7023
8638 7027 8640 7032 8643 7034 8646 7038 8645 7042 8647 7046
8644 7049 8644 7054 8647 7057 8651 7058 8655 7061 8655 7066
8650 7064 8646 7061 8642 7061 8638 7059 8635 7055 8631 7054
8631 7049 8633 7046 8636 7042 8636 7038 8632 7035 8627 7037
8622 7035 31 P
9031 7668 9035 7666 9039 7665 9043 7667 9047 7667 9051 7670
9055 7672 9057 7676 9059 7680 9054 7681 9050 7681 9048 7677
9044 7674 9041 7678 9037 7678 9039 7682 9035 7685 9034 7681
9032 7677 9033 7672 9031 7668 21 P
9075 7721 9070 7720 9067 7717 9063 7714 9060 7712 9060 7707
9059 7703 9058 7699 9062 7695 9062 7700 9062 7705 9064 7708
9068 7710 9073 7711 9077 7709 9081 7706 9083 7709 9082 7714

9079 7716 9075 7721 20 P
9042 7573 9043 7570 9046 7567 9049 7568 9052 7570 9055 7573
9053 7575 9049 7575 9046 7575 9042 7573 10 P
9062 7557 9066 7559 9070 7561 9073 7564 9076 7568 9072 7567
9067 7568 9065 7564 9062 7557 9 P
9316 7804 9320 7802 9324 7805 9326 7808 9331 7807 9329 7811
9326 7815 9322 7814 9318 7811 9316 7804 10 P
9309 7812 9312 7813 9313 7810 9316 7812 9318 7814 9316 7817
9313 7819 9311 7816 9309 7812 9 P
9128 7911 9131 7908 9135 7905 9140 7906 9143 7909 9143 7913
9148 7914 9152 7916 9153 7920 9154 7924 9154 7929 9151 7932
9147 7933 9144 7930 9142 7926 9140 7922 9137 7918 9134 7916
9128 7911 19 P
9084 7869 9088 7868 9093 7868 9095 7873 9099 7872 9103 7873
9104 7877 9104 7882 9104 7886 9100 7887 9099 7883 9095 7885
9090 7883 9087 7881 9087 7876 9084 7869 16 P
8948 7937 8949 7933 8951 7931 8954 7931 8956 7929 8960 7930
8963 7931 8965 7934 8968 7934 8972 7933 8974 7936 8976 7939
8973 7941 8970 7942 8967 7944 8964 7943 8961 7942 8958 7941
8954 7940 8951 7939 8948 7937 21 P
9470 8718 9473 8716 9476 8715 9479 8712 9482 8711 9485 8709
9487 8707 9490 8705 9493 8704 9497 8703 9500 8704 9503 8705
9506 8707 9508 8709 9510 8712 9510 8716 9508 8718 9505 8720
9502 8721 9499 8722 9496 8722 9492 8722 9489 8721 9485 8721
9482 8721 9479 8720 9476 8719 9470 8718 28 P
9422 8731 9425 8730 9427 8727 9429 8724 9432 8723 9436 8722
9439 8721 9442 8723 9442 8726 9442 8730 9439 8732 9437 8735
9436 8738 9433 8740 9430 8741 9426 8742 9424 8743 9420 8744
9417 8744 9417 8741 9418 8738 9419 8735 9422 8731 23 P
9586 8614 9589 8613 9591 8612 9594 8612 9596 8614 9598 8617
9597 8619 9595 8621 9593 8623 9591 8621 9590 8618 9588 8616
9586 8614 13 P
9655 8521 9657 8520 9658 8521 9660 8523 9660 8525 9661 8527
9659 8527 9657 8526 9657 8524 9655 8521 10 P
9648 8527 9651 8527 9651 8525 9653 8524 9655 8525 9657 8527
9659 8528 9660 8530 9662 8532 9663 8534 9662 8535 9660 8535
9657 8535 9656 8533 9655 8531 9654 8530 9652 8530 9648 8527
18 P
9829 9037 9831 9035 9834 9034 9836 9032 9840 9030 9842 9033
9841 9036 9842 9038 9846 9039 9846 9042 9842 9043 9843 9047
9841 9050 9840 9053 9837 9054 9834 9053 9833 9049 9832 9046
9830 9043 9829 9037 20 P
9837 9024 9839 9021 9842 9019 9845 9018 9848 9020 9850 9022

9848 9025 9845 9026 9842 9027 9837 9024 10 P
9964 9034 9968 9034 9971 9035 9969 9032 9971 9030 9970 9027
9967 9025 9969 9022 9973 9023 9974 9021 9977 9023 9979 9025
9982 9023 9982 9027 9983 9030 9985 9033 9987 9035 9984 9036
9981 9036 9977 9037 9977 9041 9977 9044 9974 9043 9971 9043
9970 9040 9967 9037 9964 9034 27 P
9965 8996 9968 8996 9971 8997 9973 9001 9975 8999 9978 9000
9981 9002 9983 9004 9986 9006 9988 9009 9985 9011 9982 9010
9979 9010 9976 9008 9973 9007 9971 9005 9971 9001 9968 8999
9965 8996 19 P
9940 9189 9942 9187 9944 9189 9947 9192 9949 9193 9952 9195
9952 9199 9952 9202 9954 9205 9957 9204 9956 9207 9955 9210
9953 9213 9950 9212 9947 9211 9946 9207 9947 9204 9947 9201
9943 9200 9944 9198 9945 9194 9943 9192 9940 9189 23 P
9946 9165 9950 9165 9952 9163 9955 9163 9958 9164 9958 9168
9955 9170 9958 9172 9960 9175 9962 9177 9966 9178 9969 9178
9969 9182 9973 9182 9976 9183 9973 9184 9969 9184 9967 9182
9964 9180 9962 9178 9958 9178 9956 9176 9953 9176 9949 9176
9947 9174 9949 9171 9946 9165 27 P
9906 9143 9909 9142 9912 9141 9914 9139 9916 9136 9920 9136
9922 9138 9923 9141 9923 9145 9926 9147 9928 9149 9924 9150
9923 9147 9920 9145 9917 9143 9914 9142 9912 9145 9913 9148
9910 9149 9907 9150 9908 9147 9906 9143 22 P
10089 9560 10090 9557 10093 9559 10096 9558 10098 9561 10101 9562
10102 9565 10104 9568 10107 9569 10111 9569 10110 9572 10106 9572
10103 9574 10101 9571 10098 9570 10098 9567 10096 9564 10094 9562
10090 9564 10089 9560 20 P
10094 9721 10097 9720 10100 9718 10103 9717 10106 9715 10109 9713
10111 9711 10114 9709 10115 9712 10118 9713 10122 9712 10125 9712
10127 9709 10130 9707 10130 9710 10131 9714 10128 9715 10125 9714
10122 9714 10119 9716 10116 9716 10112 9717 10110 9720 10106 9720
10106 9723 10104 9726 10101 9726 10098 9728 10095 9727 10096 9724
10094 9721 31 P
10051 9858 10053 9855 10056 9855 10060 9855 10063 9854 10066 9855
10068 9858 10067 9861 10065 9864 10062 9865 10058 9863 10056 9862
10051 9858 13 P
10054 9867 10057 9866 10060 9867 10063 9867 10067 9867 10069 9866
10071 9863 10074 9861 10077 9862 10078 9866 10076 9868 10074 9871
10071 9873 10069 9875 10067 9877 10063 9875 10061 9873 10058 9871
10054 9867 19 P
10039 9863 10040 9860 10044 9861 10047 9861 10047 9865 10048 9868
10047 9871 10043 9872 10041 9871 10040 9867 10039 9863 11 P
10021 9880 10020 9877 10021 9874 10020 9871 10022 9868 10025 9868

10028 9871 10030 9872 10029 9875 10027 9878 10024 9880 10021 9880
12:00 PM
10001 9853 10003 9849 10003 9846 10005 9845 10009 9845 10011 9847
10013 9850 10015 9853 10015 9856 10014 9859 10011 9861 10008 9861
10005 9859 10002 9857 10001 9853 15 P
10280 10369 10284 10368 10286 10366 10290 10365 10293 10366 10294 10368
10298 10368 10298 10372 10295 10372 10292 10370 10290 10372 10287 10375
10284 10376 10283 10373 10285 10370 10280 10369 16 P
10252 10351 10256 10352 10259 10352 10262 10350 10265 10350 10266 10353
10263 10354 10260 10355 10257 10355 10252 10351 10 P
10268 10351 10271 10349 10274 10351 10277 10352 10275 10355 10272 10357
10270 10354 10268 10351 8 P
10334 10338 10337 10336 10339 10337 10343 10339 10339 10341 10334 10338
6:00 PM
9995 10209 9998 10207 10001 10207 10003 10204 10004 10201 10008 10202
10010 10204 10010 10208 10011 10210 10015 10211 10011 10213 10009 10214
10005 10213 10004 10210 10001 10211 9995 10209 16 P
9991 10235 9994 10233 9996 10230 9999 10232 10001 10235 10002 10238
10004 10240 10001 10239 9998 10241 9997 10238 9994 10239 9991 10235
12:00 PM
10100 10241 10103 10241 10106 10243 10109 10245 10108 10248 10104 10249
10106 10245 10102 10245 10100 10241 9 P
10095 10173 10098 10172 10101 10173 10102 10176 10103 10179 10101 10180
10098 10179 10095 10177 10095 10173 9 P
10124 10175 10127 10175 10130 10173 10132 10171 10136 10170 10137 10173
10137 10177 10137 10180 10137 10183 10134 10182 10134 10178 10130 10177
10127 10178 10124 10175 14 P
10191 10128 10194 10128 10197 10129 10200 10129 10201 10125 10204 10123
10207 10124 10209 10127 10205 10129 10203 10131 10203 10134 10200 10137
10197 10136 10195 10132 10191 10128 15 P
10207 10018 10210 10017 10213 10015 10216 10015 10219 10014 10221 10017
10220 10020 10217 10021 10213 10022 10210 10022 10207 10018 11 P
10285 10011 10288 10009 10289 10006 10291 10003 10293 10001 10294 10005
10294 10008 10293 10011 10292 10014 10289 10015 10285 10011 11 P
10304 10011 10305 10008 10307 10006 10311 10005 10311 10002 10312 9999
10314 10001 10313 10005 10313 10008 10311 10011 10309 10013 10304 10011
12:00 PM
10317 10011 10318 10007 10320 10005 10323 10003 10323 10006 10322 10010
10323 10013 10321 10015 10317 10011 9 P
10325 9996 10326 9994 10329 9993 10331 9995 10330 9998 10329 10000
10325 9996 7 P
10204 9858 10206 9855 10207 9852 10210 9852 10211 9855 10209 9859
10209 9862 10205 9862 10204 9858 9 P

10065 10010 10066 10007 10069 10006 10071 10004 10074 10004 10076 10005
10077 10007 10076 10010 10074 10012 10072 10013 10069 10013 10065 10010
12:00 PM
9793 9393 9796 9392 9799 9390 9801 9388 9805 9388 9808 9389
9811 9389 9814 9390 9815 9393 9815 9397 9813 9399 9810 9399
9807 9398 9808 9394 9805 9393 9801 9393 9799 9396 9795 9396
9793 9393 19 P
9980 9158 9982 9155 9982 9152 9985 9154 9988 9156 9990 9158
9993 9160 9990 9162 9987 9161 9983 9161 9980 9158 11 P
9790 8789 9794 8789 9797 8791 9798 8795 9800 8797 9803 8798
9806 8799 9807 8803 9805 8805 9802 8805 9800 8802 9798 8800
9795 8798 9792 8796 9790 8793 9790 8789 16 P
8910 7381 8914 7378 8918 7377 8923 7377 8927 7378 8932 7379
8936 7377 8939 7381 8940 7385 8939 7389 8937 7393 8934 7390
8929 7388 8925 7387 8921 7384 8918 7387 8913 7387 8910 7381
18 P
8857 7378 8860 7375 8864 7374 8869 7373 8871 7377 8875 7379
8878 7382 8880 7386 8883 7390 8883 7395 8878 7394 8875 7391
8871 7388 8868 7385 8866 7381 8864 7377 8862 7381 8857 7378
18 P
8902 7388 8905 7388 8907 7388 8910 7390 8913 7390 8913 7393
8911 7395 8909 7397 8906 7396 8904 7395 8903 7392 8902 7388
12:00 PM
8926 7198 8928 7195 8927 7192 8927 7189 8929 7192 8932 7193
8935 7195 8938 7197 8935 7197 8932 7199 8931 7203 8934 7205
8933 7209 8929 7207 8928 7204 8926 7202 8926 7198 17 P
8930 7223 8932 7219 8934 7217 8937 7218 8940 7218 8943 7219
8947 7220 8949 7222 8947 7224 8945 7227 8945 7230 8942 7229
8939 7227 8936 7228 8936 7225 8930 7223 16 P
8209 6751 8212 6750 8215 6749 8218 6747 8221 6744 8223 6742
8227 6742 8230 6742 8230 6745 8232 6749 8234 6752 8232 6755
8229 6755 8226 6756 8222 6756 8219 6756 8215 6754 8212 6754
8209 6751 19 P
5903 5461 5904 5468 5908 5474 5902 5477 5908 5480 5915 5478
5917 5485 5916 5492 5916 5499 5921 5504 5924 5511 5930 5507
5936 5511 5937 5518 5933 5524 5927 5519 5921 5515 5914 5515
5908 5511 5903 5505 5896 5502 5893 5496 5886 5496 5879 5494
5872 5491 5866 5487 5862 5493 5854 5494 5847 5494 5843 5487
5837 5483 5832 5479 5825 5477 5827 5470 5822 5465 5816 5461
5816 5454 5816 5453 38 P
5839 5453 5846 5454 5847 5453 3 P
5861 5453 5864 5455 5871 5458 5879 5459 5885 5461 5890 5456
5890 5453 7 P

5910 5453 5911 5454 2 P
5911 5454 5903 5461 2 P
5967 5480 5971 5474 5974 5468 5981 5464 5988 5463 5992 5456
5999 5459 6005 5455 6006 5453 9 P
6023 5453 6024 5459 6029 5454 6029 5453 4 P
6040 5453 6038 5458 6042 5465 6038 5470 6034 5477 6031 5483
6024 5485 6017 5488 6010 5489 6004 5484 5997 5483 5990 5485
5984 5488 5977 5488 5967 5480 15 P
5969 5453 5969 5459 5963 5459 5962 5453 5961 5453 5 P
6054 5459 6056 5458 6058 5456 6058 5454 6059 5455 6062 5456
6063 5458 6064 5460 6063 5462 6064 5465 6063 5467 6061 5466
6059 5465 6057 5465 6055 5463 6054 5459 16 P
5540 5469 5543 5465 5545 5461 5547 5457 5549 5453 5 P
5575 5453 5574 5458 5572 5462 5571 5466 5569 5470 5567 5475
5563 5478 5559 5480 5556 5483 5552 5486 5549 5482 5545 5480
5542 5476 5540 5469 14 P
5698 5567 5700 5563 5704 5560 5708 5563 5712 5565 5716 5566
5716 5571 5720 5572 5723 5576 5723 5580 5726 5585 5721 5585
5717 5587 5712 5588 5710 5584 5706 5581 5704 5578 5702 5573
5698 5567 19 P
5790 5572 5794 5569 5798 5566 5800 5562 5805 5561 5809 5561
5808 5566 5806 5570 5802 5573 5799 5575 5795 5572 5790 5572
12:00 PM
6251 5559 6253 5560 6254 5559 6257 5560 6258 5562 6258 5565
6260 5566 6259 5568 6257 5567 6254 5566 6253 5564 6252 5562
6251 5559 13 P
6239 5580 6241 5577 6242 5574 6244 5571 6247 5573 6250 5574
6254 5574 6257 5576 6259 5579 6257 5581 6253 5581 6250 5582
6246 5582 6243 5582 6239 5580 15 P
6252 5647 6253 5644 6257 5644 6259 5641 6260 5638 6263 5640
6262 5643 6259 5646 6256 5647 6256 5651 6252 5647 11 P
12523 8159 12525 8159 12525 8158 12526 8159 12526 8160 12526 8160
12525 8161 12524 8161 12523 8160 12523 8159 10 P
12911 11281 12912 11281 12914 11281 12915 11281 12917 11282 12919 11283
12920 11284 12921 11283 12922 11281 12923 11282 12924 11284 12925 11286
12926 11287 12926 11288 12926 11290 12925 11290 12924 11289 12922 11289
12921 11289 12919 11289 12918 11289 12916 11290 12915 11288 12915 11287
12915 11285 12914 11283 12913 11283 12911 11282 12911 11281 29 P
12896 11256 12897 11256 12898 11256 12898 11256 12897 11258 12895 11258
12895 11257 12896 11256 12896 11256 9 P
12899 11256 12898 11255 12898 11254 12898 11252 12898 11250 12897 11249
12899 11248 12899 11250 12900 11251 12901 11252 12903 11252 12902 11253
12901 11254 12901 11256 12901 11257 12902 11259 12903 11260 12902 11262

12901 11262 12900 11261 12898 11261 12897 11261 12896 11259 12898 11258
12899 11256 25 P
12903 11265 12904 11263 12905 11265 12906 11266 12906 11267 12905 11267
12904 11267 12904 11266 12903 11266 12903 11265 10 P
12905 11271 12906 11271 12906 11272 12905 11272 12905 11271 5 P
12906 11272 12907 11272 12907 11272 12907 11272 12908 11272 12908 11274
12909 11274 12908 11275 12907 11275 12907 11274 12906 11273 12906 11272
12:00 PM
12909 11277 12909 11277 12910 11276 12911 11276 12911 11276 12912 11276
12911 11277 12911 11279 12909 11279 12909 11277 10 P
2297 14405 2329 14399 2320 14346 2297 14349 4 P
2297 14294 2325 14289 2373 14266 2380 14212 2412 14167 2459 14139
2490 14094 2522 14050 2508 13997 2489 13946 2507 13894 2525 13843
2543 13791 2555 13738 2563 13684 2530 13640 2486 13608 2467 13556
2480 13503 2507 13456 2547 13419 2601 13411 2629 13363 2652 13314
2670 13262 2722 13244 2773 13225 2823 13202 2836 13149 2869 13105
2912 13137 2941 13184 2927 13237 2935 13292 2948 13345 3001 13358
3052 13340 3107 13343 3146 13357 3135 13425 3179 13458 3186 13512
3188 13553 3151 13593 3104 13621 3057 13649 46 P
3057 13649 3016 13655 3013 13709 3026 13762 2994 13806 2971 13831
3020 13855 3048 13902 3103 13899 3110 13954 3115 13987 3085 14015
3121 14032 3130 14093 3185 14090 3239 14088 3293 14086 3347 14078
3394 14068 19 P
3394 14068 3397 14013 3416 13962 3466 13939 3513 13912 3560 13885
3610 13863 3651 13826 3684 13784 3718 13740 3770 13723 3819 13701
3861 13666 3905 13634 3957 13618 3972 13565 3946 13517 3950 13463
3938 13409 3930 13369 3863 13335 3862 13280 3850 13227 3859 13173
3879 13122 3873 13067 3866 13013 3812 13014 3757 13016 3702 13017
3648 13018 3593 13014 3540 13026 3490 13049 3450 13011 3464 12958
3483 12907 3452 12862 3397 12864 3342 12866 3288 12873 3233 12869
3186 12897 3153 12941 3120 12985 3065 12992 3011 13000 2944 13010
2907 12969 2904 12914 50 P
2904 12914 2918 12861 2877 12823 2834 12791 2801 12766 2792 12712
2847 12701 2895 12676 2930 12634 2930 12558 2869 12557 2869 12503
2838 12457 2808 12411 2787 12360 2792 12305 2814 12254 2820 12199
2813 12166 2773 12174 2756 12212 2702 12217 2648 12206 2599 12232
2545 12243 2502 12208 2472 12163 2423 12137 2439 12084 2481 12049
2507 12000 2486 11949 2460 11901 2407 11885 2352 11880 2299 11896
2297 11897 37 P
2297 10470 2312 10463 2357 10429 2381 10379 2436 10371 2492 10374
2547 10382 2601 10369 2604 10313 2618 10259 2663 10226 2718 10224
2774 10232 2821 10262 2865 10296 2898 10341 2931 10386 2963 10431
3000 10473 3037 10515 3017 10567 3045 10615 3100 10625 3155 10633

3184 10586 3238 10601 3270 10646 3293 10697 3325 10742 3332 10798
3364 10843 3400 10885 3440 10923 3484 10958 3528 10992 3574 11022
3591 11075 3553 11115 3519 11158 3497 11193 3533 11235 3560 11283
3564 11318 3605 11313 3608 11337 45 P
3608 11337 3611 11358 3556 11364 3501 11371 3448 11356 3396 11336
3357 11321 3315 11358 3278 11398 3253 11448 3219 11492 3164 11499
3140 11548 3125 11579 3081 11546 3043 11517 3009 11560 2980 11607
3000 11626 2967 11670 2917 11693 2862 11701 2808 11693 2753 11695
2698 11687 2646 11705 2599 11734 2561 11775 2534 11779 2536 11827
2481 11824 2420 11828 2410 11788 2354 11791 2297 11812 35 P
3394 14068 3432 14082 3418 14135 3409 14188 3427 14240 3449 14289
3477 14337 3517 14374 3560 14407 3614 14415 3619 14456 11 P
3619 14456 3567 14474 3530 14513 3511 14564 3474 14604 3427 14632
3373 14629 3319 14631 3278 14667 3234 14699 3194 14735 3166 14782
3212 14810 3252 14847 3265 14900 3273 14953 3309 14994 3336 15040
3376 15077 3408 15121 3395 15174 3386 15227 3363 15276 3319 15309
3268 15290 3215 15277 3165 15258 3112 15245 3063 15221 3023 15185
2990 15142 2962 15095 2944 15044 2925 14993 2917 14939 2904 14887
2880 14837 2877 14783 2845 14740 2836 14686 2844 14632 2830 14579
2807 14530 2754 14518 2707 14490 2654 14498 2600 14507 2546 14500
2499 14472 2452 14445 50 P
2452 14445 2398 14443 2344 14447 2297 14459 4 P
3619 14456 3668 14434 3718 14412 3755 14372 3758 14331 3805 14305
3859 14298 3914 14302 3968 14301 4022 14294 4083 14287 4094 14340
4095 14395 4107 14448 4135 14466 4165 14420 4222 14458 4212 14511
4182 14557 4204 14606 4230 14654 4266 14696 4304 14734 4347 14768
4389 14802 4424 14843 4466 14877 4511 14907 4546 14949 4585 14987
4627 15021 4672 15052 4722 15072 4772 15093 4761 15146 4757 15193
4802 15164 4837 15121 4874 15106 4878 15140 4931 15135 4945 15068
4998 15079 5023 15127 5061 15166 5106 15197 5159 15207 5186 15205
5167 15169 5155 15138 50 P
5155 15138 5185 15093 5208 15059 5183 15011 5174 14958 5129 14927
5095 14884 5085 14831 5070 14779 5044 14731 5040 14677 5020 14626
4974 14596 4933 14561 4888 14531 4854 14489 4848 14434 4842 14366
4877 14325 4903 14276 4924 14227 4935 14173 4930 14119 4925 14065
4871 14070 4866 14015 4876 13990 4846 13945 4812 13903 4760 13859
4752 13805 4744 13751 4739 13697 4739 13642 4750 13588 4745 13534
4740 13480 4734 13426 4730 13371 4735 13316 4740 13262 4746 13208
4752 13153 4758 13099 4742 13046 4721 12996 4691 12950 4657 12907
4636 12857 4620 12804 50 P
4620 12804 4594 12756 4584 12702 4578 12647 4557 12597 4580 12562
4615 12529 7 P
4615 12529 4604 12475 4634 12429 4624 12375 4654 12330 4693 12291

4689 12236 4705 12184 4667 12126 4718 12105 4757 12067 4768 12013
4814 11982 4840 11934 4841 11879 4836 11824 4813 11766 17 P
4813 11766 4745 11773 4729 11720 4674 11714 4639 11756 4608 11802
4566 11767 4512 11756 4459 11772 4406 11788 4354 11804 4300 11815
4246 11826 4192 11837 4136 11837 4136 11782 4189 11766 4240 11744
4260 11693 4239 11643 4294 11637 4344 11616 4387 11580 4408 11530
4429 11478 4378 11457 4326 11441 4275 11462 4221 11474 4166 11479
4131 11437 4099 11391 4083 11338 4099 11285 4114 11232 4119 11177
4113 11122 4113 11067 4093 11000 39 P
4093 11000 4037 11001 3981 11001 3943 11041 3909 11084 3874 11127
3835 11167 3784 11189 3742 11225 3700 11261 3662 11301 3638 11326
3608 11337 13 P
4093 11000 4147 10989 4141 10934 4138 10899 4193 10893 4241 10867
4280 10828 4329 10801 4335 10746 4329 10691 4324 10636 4318 10580
4313 10525 4270 10490 4219 10511 4163 10517 4112 10496 4069 10461
4026 10426 3976 10400 3937 10361 3895 10365 3899 10400 3847 10379
3792 10369 3799 10424 3743 10430 3700 10396 3653 10365 3600 10383
3581 10420 3536 10367 3480 10368 3448 10322 3483 10278 3513 10231
3501 10177 3483 10124 3477 10068 3473 10033 3403 10028 3423 9976
3462 9935 3469 9991 3506 10010 3531 9960 3557 9911 3605 9883
3621 9829 3589 9783 50 P
3589 9783 3635 9751 3684 9724 3715 9677 3719 9622 3754 9578
3781 9555 7 P
3781 9555 3787 9611 3819 9657 3840 9709 3867 9758 3903 9801
3939 9844 3991 9822 4024 9812 4040 9865 4090 9892 4145 9902
4201 9902 4254 9886 4297 9850 4347 9824 4401 9835 4453 9857
4458 9913 4431 9961 4452 10013 4504 10035 4555 10057 4625 10051
4621 10009 4677 10004 4732 9999 4788 9994 4824 9952 4871 9921
4922 9901 4978 9891 5033 9886 5087 9871 5127 9833 5168 9794
5204 9751 5248 9717 5302 9702 5357 9715 5412 9711 5467 9718
5510 9755 5555 9788 5632 9783 5629 9741 5601 9743 5581 9690
5577 9621 5630 9601 50 P
5630 9601 5686 9597 5719 9642 5758 9683 5795 9724 5837 9761
5885 9790 5882 9734 5927 9700 5971 9667 5942 9619 5980 9577
5938 9540 5904 9495 5882 9444 5902 9391 5945 9355 5988 9318
6043 9328 6103 9346 20 P
6103 9346 6117 9400 6165 9429 6206 9466 6244 9508 6297 9490
6352 9498 6403 9522 6458 9525 6512 9539 6549 9581 6602 9601
6656 9588 6669 9642 6724 9651 6778 9666 6815 9708 6817 9764
6873 9762 6929 9760 6971 9724 7022 9701 7078 9700 7143 9741
24 P
7143 9741 7127 9794 7118 9849 7119 9905 7104 9959 7059 9991
7044 10045 7045 10101 7046 10143 7008 10183 6979 10213 6981 10269

7022 10308 7062 10345 7106 10380 7076 10427 7034 10463 6986 10491
6933 10509 6883 10532 6841 10568 6819 10603 6804 10642 23 P
6804 10642 6735 10638 6731 10693 6712 10745 6675 10704 6628 10674
6574 10660 6519 10663 6471 10691 6442 10738 6434 10793 6427 10834
6373 10842 6304 10845 6266 10886 6233 10911 6196 10870 6158 10852
6135 10903 6090 10936 6057 10980 6044 11034 6058 11088 6061 11143
6069 11197 6028 11235 5991 11194 5943 11166 5888 11169 5856 11214
5812 11247 5758 11235 5703 11227 5648 11226 5593 11229 5538 11238
5483 11242 5429 11235 5373 11228 5324 11253 5281 11287 5237 11321
5190 11351 5144 11380 5095 11406 5052 11440 5016 11482 4980 11524
4940 11563 4929 11617 50 P
4929 11617 4902 11665 4871 11710 4841 11739 4813 11766 5 P
6804 10642 6817 10697 6840 10747 6880 10785 6924 10819 6977 10833
7060 10839 7067 10895 7069 10950 7070 11005 7071 11061 7067 11116
7069 11171 7076 11226 7082 11274 7054 11272 7053 11230 6998 11232
6963 11233 6923 11195 6878 11227 6844 11271 6790 11279 6742 11306
6744 11361 6746 11417 6708 11457 6658 11480 6610 11508 6613 11563
6615 11611 6656 11610 6666 11570 6721 11578 6796 11583 6804 11638
6822 11690 6818 11745 6841 11795 6888 11824 6941 11839 6948 11894
6949 11948 6940 12003 6942 12058 6982 12095 7037 12099 7084 12072
7132 12045 7147 11992 50 P
7147 11992 7161 11939 7209 11911 7264 11916 7315 11936 7359 11970
7405 12000 7400 12054 7380 12106 7381 12161 7382 12209 7328 12220
7278 12243 7240 12283 7220 12333 7221 12375 16 P
7221 12375 7170 12392 7116 12383 7060 12379 7006 12380 6951 12382
6906 12414 6887 12465 6868 12517 6827 12552 6789 12593 6737 12611
6682 12613 6635 12641 6637 12695 6669 12740 6706 12781 6724 12832
6775 12852 6827 12871 6867 12908 6894 12955 6933 12993 6980 13022
7023 13056 7072 13080 7108 13121 7154 13150 7207 13138 7247 13129
7238 13183 7210 13213 7167 13179 7114 13165 7062 13150 7012 13126
6960 13112 6917 13078 6868 13054 6869 13109 6897 13156 6924 13203
6930 13251 43 P
6930 13251 6887 13218 6859 13216 6836 13265 6782 13272 6738 13239
6695 13206 6654 13242 6599 13244 6545 13247 6498 13275 6451 13303
6399 13321 6350 13344 6300 13368 6246 13376 6206 13382 6165 13388
18 P
6165 13388 6157 13334 6149 13280 6141 13226 6127 13173 6104 13124
6090 13071 6082 13017 6068 12964 6049 12912 6021 12865 5984 12825
5936 12797 5882 12790 5828 12793 5774 12802 5719 12806 5665 12809
5610 12807 5555 12811 5503 12826 5449 12835 5394 12839 5384 12785
5380 12730 5328 12745 5273 12739 5228 12707 5187 12671 5142 12640
5089 12623 5035 12628 4981 12616 4931 12595 4877 12584 4832 12552
4778 12547 4729 12521 4674 12526 4634 12534 4615 12529 41 P

6165 13388 6133 13432 6110 13481 6087 13531 6064 13581 6109 13613
6149 13649 6193 13681 6201 13736 6204 13790 6232 13837 6265 13880
6293 13927 6321 13974 6349 14021 6367 14071 6349 14123 6331 14174
6312 14226 6290 14275 6271 14326 6243 14373 6197 14401 6151 14429
6169 14480 6172 14534 6186 14587 6194 14641 6197 14695 6241 14728
6277 14768 6285 14821 6322 14861 6363 14897 6399 14857 6453 14860
6504 14879 6540 14919 6542 14973 6570 15020 6588 15071 6606 15108
6569 15148 6555 15200 6558 15254 6514 15287 6463 15305 6414 15328
6374 15364 6402 15411 50 P
6402 15411 6434 15455 6487 15468 6539 15481 6588 15504 6620 15548
6566 15550 6513 15558 6520 15612 6524 15680 6483 15681 6486 15735
6440 15776 13 P
6440 15776 6433 15723 6419 15670 6376 15638 6323 15630 6270 15617
6230 15581 6183 15554 6129 15557 6086 15589 6032 15593 5978 15586
5940 15573 5914 15503 5860 15496 5817 15529 5772 15557 5718 15550
5677 15515 5644 15472 5614 15427 5590 15378 5561 15332 5537 15284
5517 15233 5473 15202 5419 15206 5373 15236 5309 15229 5299 15175
5269 15126 5215 15131 5155 15138 33 P
6440 15776 6433 15830 6414 15881 6382 15925 6346 15965 6390 15997
6417 16027 6389 16073 6395 16113 6371 16146 6407 16172 11 P
8148 16172 8163 16144 8184 16095 8200 16043 8252 16058 8304 16048
8358 16053 8413 16053 8460 16027 8514 16028 8545 16003 11 P
8545 16003 8570 16051 8595 16099 8605 16152 8598 16172 5 P
11247 15960 11193 15955 11139 15955 11160 16005 11180 16055 11186 16109
11160 16157 11148 16172 8 P
8545 16003 8508 15964 8474 15921 8427 15895 8465 15858 8414 15841
8384 15766 8426 15732 8468 15698 8520 15683 8551 15639 8599 15614
8653 15615 8693 15615 8705 15562 8712 15509 8729 15457 8755 15410
8786 15365 8835 15328 20 P
8060 14892 8055 14946 8030 14994 7995 15036 8003 15062 8024 15062
8020 15102 7966 15098 7926 15089 7892 15112 7908 15150 7860 15176
7855 15230 7856 15284 7906 15304 7936 15349 7894 15384 7869 15447
7821 15447 7788 15447 20 P
7788 15447 7750 15486 7729 15536 7730 15590 7769 15628 7822 15633
7817 15687 7763 15687 7710 15693 7656 15694 7606 15715 7581 15764
7547 15806 7518 15851 7489 15897 7443 15867 7398 15838 7366 15826
7375 15773 7380 15719 7353 15672 7301 15657 7247 15653 7195 15639
7152 15605 7121 15561 7086 15520 7059 15473 7057 15419 7026 15375
6986 15338 7010 15289 7009 15235 6996 15182 7025 15136 7072 15109
7116 15078 7165 15102 7219 15106 7273 15105 7314 15069 7366 15052
7411 15081 7446 15123 7504 15104 45 P
7504 15104 7555 15124 7594 15162 3 P
7594 15162 7643 15140 7693 15119 7688 15173 7719 15218 7714 15272

7709 15326 7730 15376 8 P
7730 15376 7770 15402 7788 15447 3 P
7504 15104 7514 15051 7558 15020 7568 14967 7573 14913 7593 14862
7567 14815 7531 14773 7505 14725 7494 14672 7495 14618 7507 14565
7534 14518 7583 14494 7637 14490 7691 14497 7744 14510 7796 14524
7832 14543 7870 14565 20 P
6930 13251 6932 13305 6895 13346 6862 13389 6863 13443 6881 13495
6917 13536 6960 13569 7006 13598 7050 13632 7077 13679 7104 13726
7126 13776 7143 13828 7170 13875 7222 13890 7276 13899 7325 13924
7364 13962 7403 14000 7438 14041 7474 14082 7513 14120 7555 14154
7609 14153 7664 14153 7718 14152 7772 14152 7815 14186 7845 14231
7876 14276 7926 14296 7974 14322 8006 14348 8033 14361 35 P
7221 12375 7276 12379 7314 12423 3 P
7314 12423 7320 12478 7332 12531 7381 12556 7434 12571 7488 12576
7538 12554 7577 12515 7615 12475 7630 12423 7651 12372 7676 12323
7725 12349 7746 12365 7788 12330 7822 12307 7875 12323 7926 12344
7960 12386 8009 12412 8064 12406 8099 12364 8114 12311 8114 12270
8169 12270 8220 12292 8236 12344 8235 12385 8274 12424 8308 12448
8356 12422 8382 12471 8403 12522 8423 12573 8438 12625 8493 12621
8548 12627 8585 12666 8626 12667 8634 12656 40 P
7314 12423 7356 12387 7390 12344 7435 12313 7485 12291 7537 12274
7578 12270 7577 12215 7593 12162 7635 12127 7680 12096 7735 12090
7789 12079 7842 12067 7888 12037 7931 11983 7935 11942 7909 11910
7963 11900 8018 11905 8071 11916 8117 11885 8096 11834 8091 11779
8065 11731 8039 11682 8013 11633 8044 11587 8098 11582 8141 11547
8157 11494 8157 11439 8183 11391 8219 11348 8274 11343 8328 11354
8382 11365 8412 11411 8442 11458 8495 11474 8530 11432 8573 11398
8609 11355 8610 11300 8589 11249 8551 11209 8498 11224 8458 11263
8404 11273 8349 11278 50 P
8349 11278 8319 11232 8354 11189 8365 11135 8344 11084 8299 11053
8268 11006 8226 10970 8212 10917 8218 10862 8241 10811 8281 10773
8328 10744 8377 10719 8329 10691 8314 10638 8321 10582 8344 10532
8391 10502 8444 10488 8499 10484 8622 10484 8678 10485 8733 10481
8774 10477 8830 10473 26 P
8830 10473 8881 10495 8936 10491 8973 10449 9009 10407 9056 10377
9108 10357 9162 10370 9209 10340 9216 10285 9261 10317 9314 10335
9338 10385 9391 10403 9446 10405 15 P
9446 10405 9479 10360 9524 10326 9564 10289 9600 10266 9615 10252

6:00 PM

8830 10473 8820 10418 8800 10367 8790 10311 8760 10265 8726 10221
8680 10189 8638 10153 8678 10114 8700 10063 8711 10038 8657 10026
8603 10009 8548 10008 8496 10029 8481 9975 8470 9920 8471 9865
8471 9809 8441 9762 8410 9715 8384 9665 8345 9626 8302 9590

8256 9558 8209 9528 8148 9494 27 P
8148 9494 8108 9533 8065 9569 8010 9580 7954 9585 7899 9591
7844 9603 7795 9629 7779 9683 7758 9735 7705 9751 7678 9784
7629 9758 7612 9705 7585 9656 7534 9635 7484 9609 7428 9605
7393 9649 7416 9700 7463 9730 7499 9773 7503 9815 7457 9846
7402 9853 7358 9818 7314 9783 7265 9758 7218 9728 7176 9733
7143 9741 31 P
8148 9494 8127 9442 8075 9421 8048 9371 8048 9315 8080 9268
8069 9213 8095 9164 8142 9133 8197 9122 8252 9112 8253 9055
8258 8999 8269 8944 8275 8888 8275 8866 16 P
8275 8866 8275 8846 8331 8852 8385 8869 8425 8829 8425 8773
8410 8719 8442 8672 8458 8618 8475 8564 8497 8512 8492 8456
8497 8414 8553 8420 8600 8389 8657 8385 8706 8412 8756 8440
8781 8490 8828 8522 8868 8483 8886 8429 8908 8378 8941 8331
8997 8327 9043 8359 9099 8372 9150 8395 9203 8375 9248 8340
9245 8301 31 P
8275 8866 8219 8866 8219 8810 8180 8770 8133 8738 8084 8711
8030 8695 7978 8673 7938 8634 7894 8598 7854 8638 7819 8682
7765 8666 7711 8650 7684 8600 7648 8557 7603 8522 7547 8522
7547 8466 7588 8457 7587 8401 7597 8345 7608 8289 7581 8240
7549 8193 7501 8162 7447 8147 7391 8137 7347 8101 7310 8058
7304 8002 7292 7946 7312 7893 7338 7843 7291 7812 7249 7773
7227 7721 7226 7678 7268 7677 7251 7623 7214 7580 7169 7544
7124 7509 7096 7460 7064 7413 7040 7361 7023 7306 7010 7251
7003 7194 6966 7151 50 P
6966 7151 6943 7099 6963 7045 6938 7001 6937 6959 5 P
6103 9346 6101 9290 6124 9239 6148 9188 6140 9133 6132 9077
6161 9029 6190 8981 6171 8928 6137 8882 6092 8849 6074 8796
6022 8771 5971 8748 5934 8706 5925 8650 5891 8605 5853 8563
5834 8510 5830 8454 5849 8400 5906 8403 5962 8394 5958 8338
5934 8287 5931 8230 5965 8184 5968 8128 5943 8077 5951 8020
5997 7986 6039 7949 6063 7897 6034 7848 6020 7793 5969 7725
6026 7727 6103 7739 6095 7682 6042 7663 6014 7656 6011 7600
5997 7544 5963 7498 5955 7442 5968 7387 5956 7323 47 P
3781 9555 3775 9499 3753 9448 3746 9392 3757 9337 3721 9294
3776 9282 3742 9213 8 P
3742 9213 3736 9157 3762 9107 3762 9050 3750 8995 3738 8940
3732 8884 3725 8821 3769 8785 3779 8729 3778 8687 3736 8687
3692 8652 3640 8631 3592 8600 3552 8560 3504 8530 3460 8494
3416 8459 3460 8404 3504 8439 3561 8433 3617 8438 3647 8467
3718 8467 3749 8419 3789 8379 3766 8327 3755 8271 3795 8231
3738 8231 3685 8210 3645 8198 3691 8127 3664 8077 3632 8030
3600 7983 3656 7977 3699 7972 3698 7916 3714 7876 3686 7810

42 P
3686 7810 3650 7766 3614 7723 3596 7668 3541 7680 3488 7702
3461 7652 3420 7665 3409 7691 3374 7667 3414 7627 3454 7577
3397 7577 3340 7583 3312 7586 3308 7629 3266 7633 3249 7579
3208 7504 3164 7540 3117 7573 3073 7609 3029 7574 2984 7538
2966 7484 2930 7440 2875 7424 2804 7412 2757 7444 2718 7486
2699 7524 2638 7485 2613 7537 2587 7588 2566 7641 2536 7689
2482 7708 2439 7713 2406 7666 2383 7614 2365 7560 2327 7517
2297 7515 43 P
3686 7810 3743 7810 3800 7804 3792 7733 3849 7727 3857 7798
3904 7830 3944 7870 4001 7870 4006 7813 4051 7777 4106 7766
4150 7802 4190 7842 4237 7874 4277 7914 4333 7926 4377 7890
4429 7912 4482 7934 4532 7908 4580 7877 4612 7830 4602 7774
4580 7721 4581 7665 4636 7649 4686 7645 4681 7588 4626 7570
4611 7515 4600 7460 4590 7403 4546 7366 4507 7325 4467 7284
4431 7240 4395 7195 4407 7139 4421 7114 4471 7141 4502 7190
4542 7231 4595 7210 4632 7166 4644 7110 4656 7054 4640 6999
4626 6950 4684 6946 50 P
4684 6946 4736 6968 4786 6996 4842 6986 4907 6976 5 P
4907 6976 4954 6994 5021 7017 5025 7060 5001 7112 5022 7165
5053 7214 5106 7193 5163 7190 5216 7169 5271 7154 5300 7105
5329 7055 5380 7029 5437 7037 5457 7091 5460 7148 5470 7204
5427 7243 5471 7281 5510 7323 5552 7361 5609 7357 5651 7319
5696 7284 5748 7259 5805 7256 5862 7259 5916 7278 5956 7323
30 P
5956 7323 5985 7273 5999 7218 6056 7221 6112 7230 6169 7238
6224 7252 6280 7261 6315 7215 6353 7173 6325 7141 6356 7111
6308 7079 6253 7062 6210 7025 6165 6989 6138 6938 6098 6897
6058 6857 6104 6823 6150 6789 6196 6755 6250 6774 6307 6777
6359 6803 6411 6778 6444 6834 6501 6838 6558 6836 6612 6856
6666 6876 6719 6896 6765 6863 6786 6809 6841 6791 6853 6757
6909 6773 6899 6829 6889 6886 6901 6935 6937 6959 41 P
4907 6976 4886 6922 4907 6863 4933 6813 4948 6757 4920 6707
4867 6686 4840 6675 4839 6618 4848 6561 4815 6514 4798 6459
4719 6453 4701 6398 4677 6346 4612 6347 4605 6290 4560 6254
4552 6197 4530 6176 4545 6140 4541 6082 4553 6025 4538 5970
4502 5924 4462 5882 4409 5859 4366 5854 4357 5804 4299 5809
4246 5785 4190 5767 4140 5739 4146 5682 4166 5635 4126 5593
4099 5541 4042 5552 3981 5577 3940 5535 3930 5478 3932 5453
42 P
6937 6959 6994 6957 7049 6939 7104 6927 7157 6903 7212 6908
7242 6837 7278 6793 7335 6797 7416 6811 7415 6754 7436 6701
7467 6652 7494 6602 7515 6548 7492 6495 7491 6438 7507 6383

7529 6329 7555 6278 7587 6229 7617 6207 22 P
2297 9444 2313 9434 2364 9410 2418 9397 2474 9389 2529 9397
2582 9417 2627 9451 2650 9502 2683 9547 2740 9545 2794 9532
2850 9541 2894 9575 2931 9617 2959 9666 3009 9691 3063 9673
3072 9617 3097 9567 3147 9592 3197 9617 3250 9600 3306 9593
3315 9538 3325 9483 3323 9426 3333 9371 3380 9339 3419 9298
3412 9242 3432 9190 3442 9163 3479 9207 3500 9244 3499 9187
3492 9131 3483 9090 3538 9078 3590 9056 3645 9066 3672 9115
3684 9171 3714 9200 3742 9213 45 P
5955 5622 5983 5626 6012 5633 6035 5637 4 P
9446 10405 9439 10460 9481 10497 9510 10545 9524 10598 9548 10648
9593 10681 9627 10702 8 P
7594 15162 7599 15188 7602 15216 7587 15238 7565 15253 7544 15271
7542 15298 7528 15321 7528 15346 7554 15343 7581 15343 7602 15342
7627 15355 7652 15362 7679 15362 7705 15369 7730 15376 17 P
3308 4878 3330 4878 3330 4605 3296 4605 3296 4800 3236 4800
3236 4824 3260 4827 3271 4829 3281 4833 3290 4839 3294 4843
3297 4848 3303 4861 3308 4878 15 N F
3523 4878 3545 4878 3545 4605 3511 4605 3511 4800 3450 4800
3450 4824 3475 4827 3486 4829 3496 4833 3504 4839 3508 4843
3512 4848 3518 4861 3523 4878 15 N F
3731 4878 3741 4877 3751 4875 3760 4871 3768 4866 3776 4859
3784 4852 3791 4843 3797 4833 3802 4823 3807 4812 3812 4800
3815 4788 3818 4776 3820 4763 3821 4750 3822 4738 3821 4725
3820 4712 3815 4686 3812 4674 3807 4662 3802 4651 3797 4641
3791 4631 3784 4622 3776 4614 3768 4607 3760 4602 3751 4598
3741 4596 3731 4595 3721 4596 3712 4598 3703 4602 3694 4607
3686 4614 3679 4622 3672 4631 3666 4641 3660 4651 3655 4662
3651 4674 3648 4686 3643 4712 3641 4725 3641 4738 3677 4738
3678 4713 3682 4691 3687 4672 3695 4656 3703 4643 3712 4633
3717 4629 3722 4627 3726 4625 3731 4625 3736 4625 3741 4627
3746 4629 3751 4633 3760 4643 3768 4656 3775 4672 3781 4691
3784 4713 3786 4738 3784 4762 3781 4784 3775 4803 3768 4818
3760 4831 3751 4840 3746 4844 3741 4846 3736 4848 3731 4848
3726 4848 3722 4846 3717 4844 3712 4840 3703 4831 3695 4818
3687 4803 3682 4784 3678 4762 3677 4738 3641 4738 3641 4750
3643 4763 3645 4776 3648 4788 3651 4800 3655 4812 3660 4823
3666 4833 3672 4843 3679 4852 3686 4859 3694 4866 3703 4871
3712 4875 3721 4877 3731 4878 105 N F
3989 4826 3988 4814 3984 4802 3978 4791 3971 4781 3961 4773
3950 4767 3938 4764 3925 4762 3925 4787 3917 4788 3910 4790
3903 4794 3898 4799 3893 4804 3889 4811 3887 4818 3886 4826
3887 4834 3889 4842 3893 4848 3898 4854 3903 4859 3910 4863

3917 4865 3925 4866 3933 4865 3941 4863 3947 4859 3953 4854
3958 4848 3962 4842 3964 4834 3965 4826 3964 4818 3962 4811
3958 4804 3953 4799 3947 4794 3941 4790 3933 4788 3925 4787
3925 4762 3913 4764 3901 4767 3890 4773 3880 4781 3872 4791
3866 4802 3863 4814 3861 4826 3863 4839 3866 4851 3872 4862
3880 4872 3890 4879 3901 4885 3913 4889 3925 4890 3938 4889
3950 4885 3961 4879 3971 4872 3978 4862 3984 4851 3988 4839
3989 4826 67 N F
4042 4886 4237 4886 4237 4854 4079 4854 4079 4764 4231 4764
4231 4731 4079 4731 4079 4637 4243 4637 4243 4605 4042 4605
4042 4886 13 N F
5472 4878 5494 4878 5494 4605 5460 4605 5460 4800 5399 4800
5399 4824 5424 4827 5435 4829 5445 4833 5453 4839 5457 4843
5461 4848 5467 4861 5472 4878 15 N F
5686 4878 5708 4878 5708 4605 5674 4605 5674 4800 5614 4800
5614 4824 5638 4827 5649 4829 5659 4833 5668 4839 5672 4843
5676 4848 5682 4861 5686 4878 15 N F
5925 4878 5949 4878 5949 4701 5991 4701 5991 4669 5949 4669
5949 4605 5915 4605 5915 4669 5801 4669 5801 4701 5831 4701
5915 4701 5915 4820 5831 4701 5801 4701 5801 4705 5925 4878
18 N F
6153 4826 6152 4814 6148 4802 6142 4791 6134 4781 6125 4773
6114 4767 6102 4764 6089 4762 6089 4787 6081 4788 6074 4790
6067 4794 6061 4799 6056 4804 6053 4811 6051 4818 6050 4826
6051 4834 6053 4842 6056 4848 6061 4854 6067 4859 6074 4863
6081 4865 6089 4866 6097 4865 6104 4863 6111 4859 6117 4854
6122 4848 6125 4842 6128 4834 6128 4826 6128 4818 6125 4811
6122 4804 6117 4799 6111 4794 6104 4790 6097 4788 6089 4787
6089 4762 6076 4764 6064 4767 6053 4773 6044 4781 6036 4791
6030 4802 6026 4814 6025 4826 6026 4839 6030 4851 6036 4862
6044 4872 6053 4879 6064 4885 6076 4889 6089 4890 6102 4889
6114 4885 6125 4879 6134 4872 6142 4862 6148 4851 6152 4839
6153 4826 67 N F
6206 4886 6401 4886 6401 4854 6242 4854 6242 4764 6395 4764
6395 4731 6242 4731 6242 4637 6407 4637 6407 4605 6206 4605
6206 4886 13 N F
7620 4878 7642 4878 7642 4605 7608 4605 7608 4800 7548 4800
7548 4824 7572 4827 7583 4829 7593 4833 7602 4839 7606 4843
7610 4848 7616 4861 7620 4878 15 N F
7835 4878 7857 4878 7857 4605 7823 4605 7823 4800 7763 4800
7763 4824 7787 4827 7798 4829 7808 4833 7817 4839 7821 4843
7824 4848 7831 4861 7835 4878 15 N F
8044 4878 8061 4877 8076 4873 8090 4867 8102 4859 8112 4849

8116 4843 8120 4837 8123 4830 8125 4822 8126 4814 8126 4806
8125 4794 8123 4784 8119 4775 8114 4768 8109 4761 8102 4756
8090 4748 8100 4742 8109 4736 8117 4729 8124 4720 8129 4711
8133 4701 8135 4690 8136 4679 8135 4666 8130 4652 8123 4637
8112 4624 8106 4618 8099 4613 8091 4607 8083 4603 8074 4600
8064 4597 8053 4595 8042 4595 8031 4595 8020 4597 8010 4600
8002 4603 7993 4607 7986 4613 7979 4618 7973 4624 7964 4637
7957 4652 7953 4666 7952 4679 7988 4679 7989 4668 7992 4658
7998 4649 8005 4641 8013 4634 8022 4629 8033 4626 8044 4625
8055 4626 8065 4629 8075 4634 8083 4641 8090 4649 8096 4658
8099 4668 8100 4679 8099 4690 8096 4700 8090 4710 8083 4718
8075 4724 8065 4729 8055 4732 8044 4733 8033 4732 8022 4729
8013 4724 8005 4718 7998 4710 7992 4700 7989 4690 7988 4679
7952 4679 7952 4690 7955 4701 7959 4710 7964 4719 7971 4728
7979 4735 7988 4742 7998 4748 7985 4756 7979 4761 7974 4768
7969 4775 7965 4784 7963 4794 7962 4806 7996 4806 7997 4797
8000 4788 8004 4781 8010 4775 8017 4770 8025 4767 8034 4764
8044 4764 8053 4764 8062 4767 8071 4770 8078 4775 8084 4781
8088 4788 8091 4797 8092 4806 8091 4815 8088 4823 8084 4831
8078 4837 8071 4842 8062 4845 8053 4847 8044 4848 8034 4847
8025 4845 8017 4842 8010 4837 8004 4831 8000 4823 7997 4815
7996 4806 7962 4806 7962 4814 7963 4822 7965 4830 7968 4837
7971 4843 7975 4849 7985 4859 7998 4867 8012 4873 8027 4877
8044 4878 145 N F
8302 4826 8300 4814 8297 4802 8291 4791 8283 4781 8274 4773
8263 4767 8251 4764 8238 4762 8238 4787 8230 4788 8223 4790
8216 4794 8210 4799 8205 4804 8202 4811 8199 4818 8199 4826
8199 4834 8202 4842 8205 4848 8210 4854 8216 4859 8223 4863
8230 4865 8238 4866 8246 4865 8253 4863 8260 4859 8266 4854
8271 4848 8274 4842 8276 4834 8277 4826 8276 4818 8274 4811
8271 4804 8266 4799 8260 4794 8253 4790 8246 4788 8238 4787
8238 4762 8225 4764 8213 4767 8202 4773 8193 4781 8185 4791
8179 4802 8175 4814 8174 4826 8175 4839 8179 4851 8185 4862
8193 4872 8202 4879 8213 4885 8225 4889 8238 4890 8251 4889
8263 4885 8274 4879 8283 4872 8291 4862 8297 4851 8300 4839
8302 4826 67 N F
8355 4886 8550 4886 8550 4854 8391 4854 8391 4764 8544 4764
8544 4731 8391 4731 8391 4637 8556 4637 8556 4605 8355 4605
8355 4886 13 N F
9783 4878 9805 4878 9805 4605 9771 4605 9771 4800 9710 4800
9710 4824 9735 4827 9746 4829 9756 4833 9764 4839 9768 4843
9772 4848 9778 4861 9783 4878 15 N F
9905 4784 9939 4784 9940 4797 9943 4810 9947 4821 9953 4830

9961 4838 9971 4843 9982 4847 9995 4848 10006 4847 10015 4844
10024 4840 10032 4834 10039 4826 10043 4818 10047 4807 10048 4796
10047 4784 10044 4774 10039 4766 10032 4759 10024 4752 10013 4744
9985 4727 9962 4714 9943 4700 9928 4686 9917 4671 9908 4656
9903 4640 9900 4623 9899 4605 10082 4605 10082 4637 9937 4637
9941 4649 9946 4660 9952 4668 9960 4676 9970 4684 9982 4691
10014 4709 10032 4718 10047 4728 10059 4739 10068 4751 10075 4763
10080 4775 10083 4788 10084 4800 10083 4808 10082 4816 10080 4824
10077 4832 10073 4838 10069 4845 10063 4851 10058 4856 10045 4866
10030 4872 10013 4877 9995 4878 9977 4877 9968 4875 9959 4872
9951 4869 9944 4865 9937 4861 9931 4855 9925 4849 9920 4842
9916 4834 9912 4826 9909 4816 9907 4806 9906 4795 9905 4784
78 N F
10120 4784 10154 4784 10155 4797 10157 4810 10162 4821 10168 4830
10176 4838 10185 4843 10197 4847 10210 4848 10221 4847 10230 4844
10239 4840 10247 4834 10253 4826 10258 4818 10261 4807 10262 4796
10261 4784 10259 4774 10254 4766 10247 4759 10238 4752 10228 4744
10200 4727 10177 4714 10158 4700 10143 4686 10131 4671 10123 4656
10118 4640 10115 4623 10114 4605 10297 4605 10297 4637 10152 4637
10156 4649 10161 4660 10167 4668 10175 4676 10185 4684 10197 4691
10228 4709 10246 4718 10261 4728 10273 4739 10283 4751 10290 4763
10295 4775 10298 4788 10299 4800 10298 4808 10297 4816 10295 4824
10292 4832 10288 4838 10283 4845 10278 4851 10272 4856 10259 4866
10244 4872 10228 4877 10210 4878 10191 4877 10183 4875 10174 4872
10166 4869 10159 4865 10152 4861 10145 4855 10140 4849 10135 4842
10130 4834 10127 4826 10124 4816 10122 4806 10120 4795 10120 4784
78 N F
10464 4826 10463 4814 10459 4802 10453 4791 10445 4781 10436 4773
10425 4767 10413 4764 10400 4762 10400 4787 10392 4788 10385 4790
10378 4794 10372 4799 10368 4804 10364 4811 10362 4818 10361 4826
10362 4834 10364 4842 10368 4848 10372 4854 10378 4859 10385 4863
10392 4865 10400 4866 10408 4865 10416 4863 10422 4859 10428 4854
10433 4848 10436 4842 10439 4834 10440 4826 10439 4818 10436 4811
10433 4804 10428 4799 10422 4794 10416 4790 10408 4788 10400 4787
10400 4762 10387 4764 10375 4767 10365 4773 10355 4781 10347 4791
10341 4802 10338 4814 10336 4826 10338 4839 10341 4851 10347 4862
10355 4872 10365 4879 10375 4885 10387 4889 10400 4890 10413 4889
10425 4885 10436 4879 10445 4872 10453 4862 10459 4851 10463 4839
10464 4826 67 N F
10517 4886 10712 4886 10712 4854 10553 4854 10553 4764 10706 4764
10706 4731 10553 4731 10553 4637 10718 4637 10718 4605 10517 4605
10517 4886 13 N F
11945 4878 11967 4878 11967 4605 11933 4605 11933 4800 11873 4800

11873 4824 11897 4827 11908 4829 11918 4833 11927 4839 11931 4843
11934 4848 11940 4861 11945 4878 15 N F
12067 4784 12102 4784 12102 4797 12105 4810 12109 4821 12115 4830
12123 4838 12133 4843 12144 4847 12158 4848 12168 4847 12178 4844
12187 4840 12194 4834 12201 4826 12206 4818 12209 4807 12210 4796
12209 4784 12206 4774 12201 4766 12195 4759 12186 4752 12175 4744
12148 4727 12124 4714 12105 4700 12090 4686 12079 4671 12071 4656
12065 4640 12062 4623 12061 4605 12244 4605 12244 4637 12100 4637
12103 4649 12108 4660 12115 4668 12123 4676 12133 4684 12145 4691
12176 4709 12194 4718 12209 4728 12221 4739 12231 4751 12238 4763
12242 4775 12245 4788 12246 4800 12246 4808 12244 4816 12242 4824
12239 4832 12235 4838 12231 4845 12226 4851 12220 4856 12207 4866
12192 4872 12175 4877 12158 4878 12139 4877 12130 4875 12122 4872
12114 4869 12106 4865 12099 4861 12093 4855 12087 4849 12082 4842
12078 4834 12074 4826 12071 4816 12069 4806 12068 4795 12067 4784
78 N F
12455 4808 12421 4808 12418 4817 12415 4825 12410 4832 12405 4838
12398 4842 12391 4845 12384 4847 12377 4848 12369 4848 12362 4846
12356 4844 12350 4841 12344 4837 12339 4833 12335 4827 12330 4821
12323 4806 12318 4789 12315 4768 12314 4746 12323 4754 12331 4761
12339 4767 12347 4770 12355 4773 12363 4775 12381 4776 12394 4774
12408 4770 12421 4764 12434 4754 12440 4748 12445 4742 12450 4734
12454 4727 12457 4718 12459 4708 12461 4698 12461 4687 12427 4685
12426 4696 12422 4707 12417 4718 12410 4727 12402 4734 12392 4740
12382 4744 12371 4746 12359 4744 12349 4740 12340 4734 12331 4727
12324 4718 12319 4707 12315 4696 12314 4685 12315 4674 12319 4663
12324 4653 12331 4644 12340 4636 12349 4630 12359 4626 12371 4625
12382 4626 12392 4630 12402 4636 12410 4644 12417 4653 12422 4663
12426 4674 12427 4685 12461 4687 12460 4671 12455 4655 12449 4639
12444 4632 12439 4625 12433 4619 12426 4613 12419 4608 12411 4603
12402 4600 12392 4597 12382 4595 12371 4595 12358 4595 12347 4598
12337 4601 12327 4605 12319 4611 12311 4618 12305 4626 12299 4634
12294 4644 12289 4655 12286 4666 12283 4678 12279 4704 12278 4731
12279 4748 12280 4764 12282 4779 12285 4793 12288 4806 12293 4818
12298 4829 12304 4839 12311 4848 12318 4856 12326 4862 12335 4868
12344 4872 12354 4876 12365 4878 12377 4878 12392 4877 12407 4873
12420 4866 12431 4857 12440 4847 12447 4835 12452 4822 12455 4808
126 N F
12626 4826 12625 4814 12621 4802 12615 4791 12608 4781 12598 4773
12587 4767 12575 4764 12562 4762 12562 4787 12555 4788 12547 4790
12540 4794 12535 4799 12530 4804 12526 4811 12524 4818 12523 4826
12524 4834 12526 4842 12530 4848 12535 4854 12540 4859 12547 4863
12555 4865 12562 4866 12570 4865 12578 4863 12585 4859 12590 4854

12595 4848 12599 4842 12601 4834 12602 4826 12601 4818 12599 4811
12595 4804 12590 4799 12585 4794 12578 4790 12570 4788 12562 4787
12562 4762 12550 4764 12538 4767 12527 4773 12517 4781 12510 4791
12504 4802 12500 4814 12499 4826 12500 4839 12504 4851 12510 4862
12517 4872 12527 4879 12538 4885 12550 4889 12562 4890 12575 4889
12587 4885 12598 4879 12608 4872 12615 4862 12621 4851 12625 4839
12626 4826 67 N F
12679 4886 12874 4886 12874 4854 12716 4854 12716 4764 12868 4764
12868 4731 12716 4731 12716 4637 12880 4637 12880 4605 12679 4605
12679 4886 13 N F
2316 17019 2338 17019 2338 16745 2304 16745 2304 16940 2244 16940
2244 16964 2268 16967 2279 16970 2289 16973 2298 16980 2301 16984
2305 16989 2311 17002 2316 17019 15 N F
2525 17019 2535 17018 2544 17015 2553 17012 2562 17006 2570 17000
2577 16992 2584 16984 2590 16974 2596 16964 2601 16953 2605 16941
2608 16929 2611 16916 2613 16904 2614 16891 2615 16878 2614 16865
2613 16852 2608 16827 2605 16815 2601 16803 2596 16792 2590 16781
2584 16771 2577 16762 2570 16755 2562 16748 2553 16743 2544 16739
2535 16736 2525 16735 2515 16736 2505 16739 2496 16743 2488 16748
2480 16755 2472 16762 2465 16771 2459 16781 2453 16792 2449 16803
2444 16815 2441 16827 2436 16852 2435 16865 2434 16878 2470 16878
2472 16854 2475 16832 2481 16812 2488 16796 2496 16783 2505 16773
2510 16770 2515 16767 2520 16766 2525 16765 2529 16766 2534 16767
2539 16770 2544 16773 2553 16783 2561 16796 2568 16812 2574 16832
2577 16854 2579 16878 2577 16902 2574 16924 2568 16943 2561 16959
2553 16972 2544 16981 2539 16984 2534 16987 2529 16988 2525 16989
2520 16988 2515 16987 2510 16984 2505 16981 2496 16972 2488 16959
2481 16943 2475 16924 2472 16902 2470 16878 2434 16878 2435 16891
2436 16904 2438 16916 2441 16929 2444 16941 2449 16953 2453 16964
2459 16974 2465 16984 2472 16992 2480 17000 2488 17006 2496 17012
2505 17015 2515 17018 2525 17019 105 N F
2675 17019 2818 17019 2818 16987 2703 16987 2693 16908 2703 16915
2715 16921 2729 16925 2745 16926 2759 16925 2774 16921 2789 16915
2795 16911 2802 16906 2808 16900 2814 16893 2819 16886 2823 16877
2827 16868 2829 16857 2831 16846 2832 16834 2830 16815 2828 16805
2826 16796 2822 16788 2818 16780 2813 16772 2808 16765 2802 16758
2794 16753 2787 16748 2778 16743 2769 16740 2759 16737 2749 16736
2737 16735 2727 16736 2717 16737 2708 16739 2700 16741 2692 16745
2685 16748 2679 16753 2673 16758 2664 16769 2656 16782 2651 16796
2647 16812 2681 16812 2685 16802 2689 16793 2694 16785 2701 16778
2708 16773 2716 16769 2725 16766 2735 16765 2749 16766 2761 16770
2772 16775 2781 16782 2788 16791 2793 16803 2796 16816 2798 16832
2797 16843 2794 16855 2790 16865 2783 16875 2775 16884 2764 16890

2752 16895 2745 16896 2737 16896 2730 16896 2723 16895 2716 16893
2710 16890 2699 16882 2685 16870 2655 16870 2675 17019 83 N F
2997 16967 2996 16954 2992 16942 2986 16931 2978 16922 2969 16914
2958 16908 2946 16904 2933 16903 2933 16928 2925 16928 2918 16931
2911 16934 2905 16939 2901 16945 2897 16952 2895 16959 2894 16967
2895 16975 2897 16982 2901 16989 2905 16995 2911 17000 2918 17003
2925 17006 2933 17006 2941 17006 2949 17003 2955 17000 2961 16995
2966 16989 2970 16982 2972 16975 2973 16967 2972 16959 2970 16952
2966 16945 2961 16939 2955 16934 2949 16931 2941 16928 2933 16928
2933 16903 2920 16904 2908 16908 2898 16914 2888 16922 2880 16931
2874 16942 2871 16954 2869 16967 2871 16980 2874 16992 2880 17003
2888 17012 2898 17020 2908 17026 2920 17030 2933 17031 2946 17030
2958 17026 2969 17020 2978 17012 2986 17003 2992 16992 2996 16980
2997 16967 67 N F
3050 17027 3245 17027 3245 16995 3086 16995 3086 16904 3239 16904
3239 16872 3086 16872 3086 16778 3251 16778 3251 16745 3050 16745
3050 17027 13 N F
4381 17019 4403 17019 4403 16745 4369 16745 4369 16940 4309 16940
4309 16964 4333 16967 4344 16970 4354 16973 4363 16980 4367 16984
4370 16989 4376 17002 4381 17019 15 N F
4596 17019 4618 17019 4618 16745 4584 16745 4584 16940 4523 16940
4523 16964 4548 16967 4559 16970 4569 16973 4577 16980 4581 16984
4585 16989 4591 17002 4596 17019 15 N F
4804 17019 4814 17018 4824 17015 4833 17012 4841 17006 4849 17000
4857 16992 4864 16984 4870 16974 4875 16964 4880 16953 4885 16941
4888 16929 4891 16916 4893 16904 4894 16891 4895 16878 4894 16865
4893 16852 4888 16827 4885 16815 4880 16803 4875 16792 4870 16781
4864 16771 4857 16762 4849 16755 4841 16748 4833 16743 4824 16739
4814 16736 4804 16735 4794 16736 4785 16739 4776 16743 4767 16748
4759 16755 4752 16762 4745 16771 4739 16781 4733 16792 4728 16803
4724 16815 4721 16827 4716 16852 4715 16865 4714 16878 4750 16878
4751 16854 4755 16832 4760 16812 4768 16796 4776 16783 4785 16773
4790 16770 4795 16767 4799 16766 4804 16765 4809 16766 4814 16767
4819 16770 4824 16773 4833 16783 4841 16796 4848 16812 4854 16832
4857 16854 4859 16878 4857 16902 4854 16924 4848 16943 4841 16959
4833 16972 4824 16981 4819 16984 4814 16987 4809 16988 4804 16989
4799 16988 4795 16987 4790 16984 4785 16981 4776 16972 4768 16959
4760 16943 4755 16924 4751 16902 4750 16878 4714 16878 4715 16891
4716 16904 4718 16916 4721 16929 4724 16941 4728 16953 4733 16964
4739 16974 4745 16984 4752 16992 4759 17000 4767 17006 4776 17012
4785 17015 4794 17018 4804 17019 105 N F
5062 16967 5061 16954 5057 16942 5051 16931 5044 16922 5034 16914
5023 16908 5011 16904 4998 16903 4998 16928 4990 16928 4983 16931

4976 16934 4971 16939 4966 16945 4962 16952 4960 16959 4959 16967
4960 16975 4962 16982 4966 16989 4971 16995 4976 17000 4983 17003
4990 17006 4998 17006 5006 17006 5014 17003 5020 17000 5026 16995
5031 16989 5035 16982 5037 16975 5038 16967 5037 16959 5035 16952
5031 16945 5026 16939 5020 16934 5014 16931 5006 16928 4998 16928
4998 16903 4986 16904 4974 16908 4963 16914 4953 16922 4945 16931
4940 16942 4936 16954 4934 16967 4936 16980 4940 16992 4945 17003
4953 17012 4963 17020 4974 17026 4986 17030 4998 17031 5011 17030
5023 17026 5034 17020 5044 17012 5051 17003 5057 16992 5061 16980
5062 16967 67 N F
5115 17027 5310 17027 5310 16995 5152 16995 5152 16904 5304 16904
5304 16872 5152 16872 5152 16778 5316 16778 5316 16745 5115 16745
5115 17027 13 N F
6412 17019 6434 17019 6434 16745 6400 16745 6400 16940 6340 16940
6340 16964 6364 16967 6375 16970 6385 16973 6394 16980 6397 16984
6401 16989 6407 17002 6412 17019 15 N F
6627 17019 6649 17019 6649 16745 6614 16745 6614 16940 6554 16940
6554 16964 6579 16967 6589 16970 6599 16973 6608 16980 6612 16984
6616 16989 6622 17002 6627 17019 15 N F
6771 17019 6914 17019 6914 16987 6799 16987 6789 16908 6799 16915
6811 16921 6825 16925 6841 16926 6855 16925 6870 16921 6884 16915
6891 16911 6898 16906 6904 16900 6910 16893 6915 16886 6919 16877
6923 16868 6925 16857 6927 16846 6928 16834 6926 16815 6924 16805
6922 16796 6918 16788 6914 16780 6909 16772 6904 16765 6897 16758
6890 16753 6883 16748 6874 16743 6865 16740 6855 16737 6845 16736
6833 16735 6823 16736 6813 16737 6804 16739 6796 16741 6788 16745
6781 16748 6775 16753 6769 16758 6760 16769 6752 16782 6747 16796
6743 16812 6777 16812 6781 16802 6785 16793 6790 16785 6797 16778
6804 16773 6812 16769 6821 16766 6831 16765 6845 16766 6857 16770
6868 16775 6877 16782 6884 16791 6889 16803 6892 16816 6893 16832
6893 16843 6890 16855 6886 16865 6879 16875 6871 16884 6860 16890
6848 16895 6841 16896 6833 16896 6825 16896 6819 16895 6812 16893
6806 16890 6795 16882 6781 16870 6751 16870 6771 17019 83 N F
7093 16967 7092 16954 7088 16942 7082 16931 7074 16922 7065 16914
7054 16908 7042 16904 7029 16903 7029 16928 7021 16928 7014 16931
7007 16934 7001 16939 6997 16945 6993 16952 6991 16959 6990 16967
6991 16975 6993 16982 6997 16989 7001 16995 7007 17000 7014 17003
7021 17006 7029 17006 7037 17006 7045 17003 7051 17000 7057 16995
7062 16989 7065 16982 7068 16975 7069 16967 7068 16959 7065 16952
7062 16945 7057 16939 7051 16934 7045 16931 7037 16928 7029 16928
7029 16903 7016 16904 7004 16908 6994 16914 6984 16922 6976 16931
6970 16942 6967 16954 6965 16967 6967 16980 6970 16992 6976 17003
6984 17012 6994 17020 7004 17026 7016 17030 7029 17031 7042 17030

7054 17026 7065 17020 7074 17012 7082 17003 7088 16992 7092 16980
7093 16967 67 N F
7146 17027 7341 17027 7341 16995 7182 16995 7182 16904 7335 16904
7335 16872 7182 16872 7182 16778 7347 16778 7347 16745 7146 16745
7146 17027 13 N F
8433 17019 8455 17019 8455 16745 8421 16745 8421 16940 8361 16940
8361 16964 8385 16967 8396 16970 8406 16973 8415 16980 8419 16984
8422 16989 8428 17002 8433 17019 15 N F
8555 16924 8589 16924 8590 16938 8593 16950 8597 16961 8603 16971
8611 16978 8621 16984 8632 16987 8646 16989 8656 16988 8666 16985
8675 16981 8682 16974 8689 16967 8694 16958 8697 16948 8698 16936
8697 16925 8694 16915 8689 16907 8683 16899 8674 16892 8663 16885
8636 16868 8612 16854 8593 16841 8578 16827 8567 16812 8559 16797
8553 16781 8550 16764 8549 16745 8732 16745 8732 16778 8587 16778
8591 16790 8596 16800 8602 16809 8611 16817 8621 16824 8633 16832
8664 16850 8682 16859 8697 16869 8709 16880 8718 16891 8726 16903
8730 16916 8733 16928 8734 16940 8734 16949 8732 16957 8730 16965
8727 16972 8723 16979 8719 16985 8714 16991 8708 16997 8695 17006
8680 17013 8663 17017 8646 17019 8627 17017 8618 17016 8610 17013
8602 17010 8594 17006 8587 17001 8581 16996 8575 16989 8570 16982
8566 16975 8562 16966 8559 16957 8557 16947 8556 16936 8555 16924
78 N F
8856 17019 8866 17018 8876 17015 8885 17012 8893 17006 8901 17000
8909 16992 8916 16984 8922 16974 8928 16964 8932 16953 8937 16941
8940 16929 8943 16916 8945 16904 8946 16891 8947 16878 8946 16865
8945 16852 8940 16827 8937 16815 8932 16803 8928 16792 8922 16781
8916 16771 8909 16762 8901 16755 8893 16748 8885 16743 8876 16739
8866 16736 8856 16735 8846 16736 8837 16739 8828 16743 8819 16748
8811 16755 8804 16762 8797 16771 8791 16781 8785 16792 8780 16803
8776 16815 8773 16827 8768 16852 8767 16865 8766 16878 8802 16878
8803 16854 8807 16832 8812 16812 8820 16796 8828 16783 8837 16773
8842 16770 8847 16767 8852 16766 8856 16765 8861 16766 8866 16767
8871 16770 8876 16773 8885 16783 8893 16796 8900 16812 8906 16832
8909 16854 8911 16878 8909 16902 8906 16924 8900 16943 8893 16959
8885 16972 8876 16981 8871 16984 8866 16987 8861 16988 8856 16989
8852 16988 8847 16987 8842 16984 8837 16981 8828 16972 8820 16959
8812 16943 8807 16924 8803 16902 8802 16878 8766 16878 8767 16891
8768 16904 8770 16916 8773 16929 8776 16941 8780 16953 8785 16964
8791 16974 8797 16984 8804 16992 8811 17000 8819 17006 8828 17012
8837 17015 8846 17018 8856 17019 105 N F
9114 16967 9113 16954 9109 16942 9103 16931 9096 16922 9086 16914
9075 16908 9063 16904 9050 16903 9050 16928 9042 16928 9035 16931
9028 16934 9023 16939 9018 16945 9014 16952 9012 16959 9011 16967

9012 16975 9014 16982 9018 16989 9023 16995 9028 17000 9035 17003
9042 17006 9050 17006 9058 17006 9066 17003 9072 17000 9078 16995
9083 16989 9087 16982 9089 16975 9090 16967 9089 16959 9087 16952
9083 16945 9078 16939 9072 16934 9066 16931 9058 16928 9050 16928
9050 16903 9038 16904 9026 16908 9015 16914 9005 16922 8997 16931
8992 16942 8988 16954 8986 16967 8988 16980 8992 16992 8997 17003
9005 17012 9015 17020 9026 17026 9038 17030 9050 17031 9063 17030
9075 17026 9086 17020 9096 17012 9103 17003 9109 16992 9113 16980
9114 16967 67 N F
9167 17027 9362 17027 9362 16995 9204 16995 9204 16904 9356 16904
9356 16872 9204 16872 9204 16778 9368 16778 9368 16745 9167 16745
9167 17027 13 N F
10460 17019 10482 17019 10482 16745 10448 16745 10448 16940 10388 16940
10388 16964 10412 16967 10423 16970 10433 16973 10442 16980 10446 16984
10450 16989 10456 17002 10460 17019 15 N F
10583 16924 10617 16924 10618 16938 10620 16950 10625 16961 10631 16971
10639 16978 10648 16984 10660 16987 10673 16989 10683 16988 10693 16985
10702 16981 10710 16974 10716 16967 10721 16958 10724 16948 10725 16936
10724 16925 10721 16915 10717 16907 10710 16899 10701 16892 10691 16885
10663 16868 10640 16854 10621 16841 10606 16827 10594 16812 10586 16797
10581 16781 10578 16764 10577 16745 10759 16745 10759 16778 10615 16778
10618 16790 10623 16800 10630 16809 10638 16817 10648 16824 10660 16832
10691 16850 10709 16859 10724 16869 10736 16880 10746 16891 10753 16903
10758 16916 10761 16928 10761 16940 10761 16949 10760 16957 10757 16965
10754 16972 10751 16979 10746 16985 10741 16991 10735 16997 10722 17006
10707 17013 10691 17017 10673 17019 10654 17017 10645 17016 10637 17013
10629 17010 10622 17006 10615 17001 10608 16996 10603 16989 10598 16982
10593 16975 10590 16966 10587 16957 10585 16947 10583 16936 10583 16924
78 N F
10820 17019 10962 17019 10962 16987 10848 16987 10838 16908 10848 16915
10859 16921 10873 16925 10890 16926 10904 16925 10919 16921 10933 16915
10940 16911 10947 16906 10953 16900 10958 16893 10963 16886 10968 16877
10971 16868 10974 16857 10976 16846 10976 16834 10975 16815 10973 16805
10970 16796 10967 16788 10963 16780 10958 16772 10952 16765 10946 16758
10939 16753 10931 16748 10923 16743 10914 16740 10904 16737 10893 16736
10882 16735 10871 16736 10862 16737 10853 16739 10844 16741 10837 16745
10830 16748 10824 16753 10818 16758 10808 16769 10801 16782 10795 16796
10792 16812 10826 16812 10829 16802 10834 16793 10839 16785 10845 16778
10852 16773 10861 16769 10870 16766 10880 16765 10893 16766 10906 16770
10916 16775 10925 16782 10932 16791 10938 16803 10941 16816 10942 16832
10941 16843 10939 16855 10934 16865 10928 16875 10919 16884 10909 16890
10897 16895 10889 16896 10882 16896 10874 16896 10867 16895 10861 16893
10855 16890 10843 16882 10830 16870 10800 16870 10820 17019 83 N F

11142 16967 11140 16954 11137 16942 11131 16931 11123 16922 11113 16914
11103 16908 11091 16904 11078 16903 11078 16928 11070 16928 11062 16931
11056 16934 11050 16939 11045 16945 11042 16952 11039 16959 11038 16967
11039 16975 11042 16982 11045 16989 11050 16995 11056 17000 11062 17003
11070 17006 11078 17006 11086 17006 11093 17003 11100 17000 11106 16995
11110 16989 11114 16982 11116 16975 11117 16967 11116 16959 11114 16952
11110 16945 11106 16939 11100 16934 11093 16931 11086 16928 11078 16928
11078 16903 11065 16904 11053 16908 11042 16914 11033 16922 11025 16931
11019 16942 11015 16954 11014 16967 11015 16980 11019 16992 11025 17003
11033 17012 11042 17020 11053 17026 11065 17030 11078 17031 11091 17030
11103 17026 11113 17020 11123 17012 11131 17003 11137 16992 11140 16980
11142 16967 67 N F
11195 17027 11390 17027 11390 16995 11231 16995 11231 16904 11383 16904
11383 16872 11231 16872 11231 16778 11396 16778 11396 16745 11195 16745
11195 17027 13 N F
12511 17019 12533 17019 12533 16745 12499 16745 12499 16940 12439 16940
12439 16964 12463 16967 12474 16970 12484 16973 12493 16980 12497 16984
12500 16989 12506 17002 12511 17019 15 N F
12666 16930 12631 16930 12634 16951 12636 16960 12638 16969 12642 16977
12646 16984 12651 16990 12656 16996 12662 17002 12668 17006 12675 17010
12683 17013 12691 17016 12699 17017 12718 17019 12735 17017 12751 17014
12766 17008 12778 17000 12783 16995 12788 16989 12792 16983 12796 16976
12798 16969 12800 16961 12802 16953 12802 16944 12802 16934 12800 16925
12797 16917 12793 16909 12788 16902 12781 16896 12772 16891 12762 16886
12774 16881 12784 16875 12792 16869 12799 16861 12804 16853 12807 16844
12809 16833 12810 16822 12809 16807 12805 16792 12799 16777 12794 16771
12789 16764 12783 16759 12776 16753 12769 16749 12760 16745 12751 16742
12740 16739 12728 16738 12716 16737 12705 16738 12695 16739 12685 16741
12677 16744 12669 16747 12661 16751 12655 16756 12649 16762 12644 16768
12639 16774 12636 16781 12633 16789 12629 16806 12627 16824 12662 16824
12662 16814 12664 16804 12667 16794 12672 16786 12679 16778 12689 16773
12701 16769 12718 16767 12732 16768 12743 16771 12753 16776 12761 16783
12767 16791 12771 16800 12773 16811 12774 16822 12773 16836 12771 16842
12768 16847 12766 16852 12762 16856 12753 16863 12742 16867 12729 16870
12714 16872 12698 16872 12698 16900 12714 16900 12728 16902 12740 16904
12749 16908 12756 16913 12762 16920 12765 16930 12766 16942 12765 16952
12763 16961 12760 16968 12755 16975 12748 16981 12740 16985 12730 16988
12718 16989 12707 16988 12697 16986 12689 16982 12681 16977 12675 16969
12670 16959 12667 16946 12666 16930 123 N F
12934 17019 12944 17018 12954 17015 12963 17012 12971 17006 12979 17000
12987 16992 12994 16984 13000 16974 13006 16964 13011 16953 13015 16941
13018 16929 13021 16916 13023 16904 13024 16891 13025 16878 13024 16865
13023 16852 13018 16827 13015 16815 13011 16803 13006 16792 13000 16781

12994 16771 12987 16762 12979 16755 12971 16748 12963 16743 12954 16739
12944 16736 12934 16735 12925 16736 12915 16739 12906 16743 12898 16748
12890 16755 12882 16762 12875 16771 12869 16781 12863 16792 12858 16803
12854 16815 12851 16827 12846 16852 12845 16865 12844 16878 12880 16878
12882 16854 12885 16832 12891 16812 12898 16796 12906 16783 12915 16773
12920 16770 12925 16767 12930 16766 12934 16765 12939 16766 12944 16767
12949 16770 12954 16773 12963 16783 12971 16796 12978 16812 12984 16832
12987 16854 12989 16878 12987 16902 12984 16924 12978 16943 12971 16959
12963 16972 12954 16981 12949 16984 12944 16987 12939 16988 12934 16989
12930 16988 12925 16987 12920 16984 12915 16981 12906 16972 12898 16959
12891 16943 12885 16924 12882 16902 12880 16878 12844 16878 12845 16891
12846 16904 12848 16916 12851 16929 12854 16941 12858 16953 12863 16964
12869 16974 12875 16984 12882 16992 12890 17000 12898 17006 12906 17012
12915 17015 12925 17018 12934 17019 105 N F
13192 16967 13191 16954 13187 16942 13181 16931 13174 16922 13164 16914
13153 16908 13141 16904 13128 16903 13128 16928 13121 16928 13113 16931
13106 16934 13101 16939 13096 16945 13092 16952 13090 16959 13089 16967
13090 16975 13092 16982 13096 16989 13101 16995 13106 17000 13113 17003
13121 17006 13128 17006 13136 17006 13144 17003 13150 17000 13156 16995
13161 16989 13165 16982 13167 16975 13168 16967 13167 16959 13165 16952
13161 16945 13156 16939 13150 16934 13144 16931 13136 16928 13128 16928
13128 16903 13116 16904 13104 16908 13093 16914 13083 16922 13076 16931
13070 16942 13066 16954 13065 16967 13066 16980 13070 16992 13076 17003
13083 17012 13093 17020 13104 17026 13116 17030 13128 17031 13141 17030
13153 17026 13164 17020 13174 17012 13181 17003 13187 16992 13191 16980
13192 16967 67 N F
13245 17027 13440 17027 13440 16995 13282 16995 13282 16904 13434 16904
13434 16872 13282 16872 13282 16778 13446 16778 13446 16745 13245 16745
13245 17027 13 N F
911 5683 945 5683 946 5696 948 5709 952 5720 959 5729
966 5737 976 5742 988 5746 1001 5747 1011 5746 1021 5743
1030 5739 1038 5733 1044 5725 1049 5717 1052 5706 1053 5695
1052 5683 1049 5673 1045 5665 1038 5658 1029 5651 1018 5643
991 5626 968 5613 949 5599 934 5585 922 5570 914 5555
909 5539 906 5522 905 5504 1087 5504 1087 5536 943 5536
946 5548 951 5559 958 5567 966 5575 976 5583 988 5590
1019 5608 1037 5617 1052 5627 1064 5638 1074 5650 1081 5662
1086 5674 1088 5687 1089 5699 1089 5707 1087 5715 1085 5723
1082 5731 1078 5737 1074 5744 1069 5750 1063 5755 1050 5765
1035 5771 1019 5776 1001 5777 982 5776 973 5774 965 5771
957 5768 949 5764 943 5760 936 5754 931 5748 925 5741
921 5733 917 5725 914 5715 912 5705 911 5694 911 5683
78 N F

1125 5683 1160 5683 1160 5696 1163 5709 1167 5720 1173 5729
1181 5737 1191 5742 1202 5746 1216 5747 1226 5746 1236 5743
1245 5739 1252 5733 1259 5725 1264 5717 1267 5706 1268 5695
1267 5683 1264 5673 1259 5665 1253 5658 1244 5651 1233 5643
1206 5626 1182 5613 1163 5599 1148 5585 1137 5570 1129 5555
1123 5539 1120 5522 1119 5504 1302 5504 1302 5536 1157 5536
1161 5548 1166 5559 1172 5567 1181 5575 1191 5583 1203 5590
1234 5608 1252 5617 1267 5627 1279 5638 1288 5650 1296 5662
1300 5674 1303 5687 1304 5699 1304 5707 1302 5715 1300 5723
1297 5731 1293 5737 1289 5744 1284 5750 1278 5755 1265 5765
1250 5771 1233 5776 1216 5777 1197 5776 1188 5774 1180 5771
1172 5768 1164 5764 1157 5760 1151 5754 1145 5748 1140 5741
1136 5733 1132 5725 1129 5715 1127 5705 1126 5694 1125 5683
78 N F
1470 5725 1468 5713 1465 5701 1459 5690 1451 5680 1441 5672
1430 5666 1419 5663 1406 5661 1406 5686 1398 5687 1390 5689
1384 5693 1378 5698 1373 5703 1369 5710 1367 5717 1366 5725
1367 5733 1369 5741 1373 5747 1378 5753 1384 5758 1390 5762
1398 5764 1406 5765 1414 5764 1421 5762 1428 5758 1434 5753
1438 5747 1442 5741 1444 5733 1445 5725 1444 5717 1442 5710
1438 5703 1434 5698 1428 5693 1421 5689 1414 5687 1406 5686
1406 5661 1393 5663 1381 5666 1370 5672 1361 5680 1353 5690
1347 5701 1343 5713 1342 5725 1343 5738 1347 5750 1353 5761
1361 5771 1370 5778 1381 5784 1393 5788 1406 5789 1419 5788
1430 5784 1441 5778 1451 5771 1459 5761 1465 5750 1468 5738
1470 5725 67 N F
1517 5785 1557 5785 1701 5556 1701 5785 1735 5785 1735 5504
1695 5504 1551 5733 1551 5504 1517 5504 1517 5785 11 N F
911 6859 945 6859 946 6873 948 6886 952 6897 959 6906
966 6913 976 6919 988 6923 1001 6924 1011 6923 1021 6920
1030 6916 1038 6910 1044 6902 1049 6893 1052 6883 1053 6872
1052 6860 1049 6850 1045 6842 1038 6834 1029 6827 1018 6820
991 6803 968 6790 949 6776 934 6762 922 6747 914 6732
909 6716 906 6699 905 6681 1087 6681 1087 6713 943 6713
946 6725 951 6735 958 6744 966 6752 976 6759 988 6767
1019 6785 1037 6794 1052 6804 1064 6815 1074 6827 1081 6839
1086 6851 1088 6863 1089 6876 1089 6884 1087 6892 1085 6900
1082 6907 1078 6914 1074 6921 1069 6927 1063 6932 1050 6941
1035 6948 1019 6952 1001 6954 982 6952 973 6951 965 6948
957 6945 949 6941 943 6936 936 6931 931 6925 925 6918
921 6910 917 6901 914 6892 912 6882 911 6871 911 6859
78 N F
1242 6954 1266 6954 1266 6777 1308 6777 1308 6745 1266 6745

1266 6681 1232 6681 1232 6745 1117 6745 1117 6777 1147 6777
1232 6777 1232 6896 1147 6777 1117 6777 1117 6781 1242 6954
18 N F
1470 6902 1468 6889 1465 6877 1459 6866 1451 6857 1441 6849
1430 6843 1419 6839 1406 6838 1406 6863 1398 6864 1390 6866
1384 6869 1378 6874 1373 6880 1369 6887 1367 6894 1366 6902
1367 6910 1369 6918 1373 6924 1378 6930 1384 6935 1390 6938
1398 6941 1406 6942 1414 6941 1421 6938 1428 6935 1434 6930
1438 6924 1442 6918 1444 6910 1445 6902 1444 6894 1442 6887
1438 6880 1434 6874 1428 6869 1421 6866 1414 6864 1406 6863
1406 6838 1393 6839 1381 6843 1370 6849 1361 6857 1353 6866
1347 6877 1343 6889 1342 6902 1343 6915 1347 6927 1353 6938
1361 6947 1370 6955 1381 6961 1393 6965 1406 6966 1419 6965
1430 6961 1441 6955 1451 6947 1459 6938 1465 6927 1468 6915
1470 6902 67 N F
1517 6962 1557 6962 1701 6733 1701 6962 1735 6962 1735 6681
1695 6681 1551 6910 1551 6681 1517 6681 1517 6962 11 N F
911 8021 945 8021 946 8035 948 8047 952 8058 959 8067
966 8075 976 8081 988 8084 1001 8085 1011 8084 1021 8082
1030 8077 1038 8071 1044 8064 1049 8055 1052 8045 1053 8033
1052 8021 1049 8012 1045 8003 1038 7996 1029 7989 1018 7982
991 7965 968 7951 949 7937 934 7923 922 7909 914 7893
909 7877 906 7860 905 7842 1087 7842 1087 7874 943 7874
946 7887 951 7897 958 7906 966 7913 976 7921 988 7929
1019 7947 1037 7956 1052 7966 1064 7976 1074 7988 1081 8000
1086 8012 1088 8025 1089 8037 1089 8046 1087 8054 1085 8061
1082 8069 1078 8076 1074 8082 1069 8088 1063 8094 1050 8103
1035 8110 1019 8114 1001 8115 982 8114 973 8112 965 8110
957 8106 949 8103 943 8098 936 8092 931 8086 925 8079
921 8071 917 8063 914 8054 912 8044 911 8033 911 8021
78 N F
1298 8045 1264 8045 1262 8054 1258 8062 1253 8069 1248 8075
1241 8079 1235 8083 1227 8085 1220 8085 1212 8085 1206 8084
1199 8081 1193 8078 1188 8074 1182 8070 1178 8064 1174 8058
1167 8043 1162 8026 1159 8006 1157 7983 1166 7992 1174 7999
1182 8004 1190 8008 1198 8010 1206 8012 1224 8013 1237 8012
1251 8008 1265 8001 1277 7991 1283 7986 1288 7979 1293 7972
1297 7964 1300 7955 1303 7946 1304 7935 1304 7925 1270 7923
1269 7934 1265 7945 1260 7955 1253 7964 1245 7972 1235 7978
1225 7981 1214 7983 1203 7981 1192 7978 1183 7972 1174 7964
1167 7955 1162 7945 1159 7934 1157 7923 1159 7911 1162 7900
1167 7890 1174 7881 1183 7873 1192 7867 1203 7864 1214 7862
1225 7864 1235 7867 1245 7873 1253 7881 1260 7890 1265 7900

1269 7911 1270 7923 1304 7925 1303 7908 1299 7892 1292 7877
1287 7869 1282 7862 1276 7856 1270 7850 1262 7845 1254 7841
1245 7837 1235 7834 1225 7833 1214 7832 1201 7833 1190 7835
1180 7838 1170 7843 1162 7848 1154 7855 1148 7863 1142 7872
1137 7881 1133 7892 1129 7903 1126 7915 1123 7941 1121 7969
1122 7985 1123 8001 1125 8016 1128 8030 1132 8043 1136 8055
1141 8066 1147 8076 1154 8085 1161 8093 1169 8100 1178 8105
1188 8110 1198 8113 1208 8115 1220 8115 1236 8114 1250 8110
1263 8103 1274 8095 1283 8084 1290 8072 1295 8059 1298 8045
126 N F
1470 8064 1468 8051 1465 8039 1459 8028 1451 8019 1441 8011
1430 8005 1419 8001 1406 8000 1406 8024 1398 8025 1390 8027
1384 8031 1378 8036 1373 8042 1369 8048 1367 8056 1366 8064
1367 8072 1369 8079 1373 8086 1378 8092 1384 8096 1390 8100
1398 8102 1406 8103 1414 8102 1421 8100 1428 8096 1434 8092
1438 8086 1442 8079 1444 8072 1445 8064 1444 8056 1442 8048
1438 8042 1434 8036 1428 8031 1421 8027 1414 8025 1406 8024
1406 8000 1393 8001 1381 8005 1370 8011 1361 8019 1353 8028
1347 8039 1343 8051 1342 8064 1343 8077 1347 8089 1353 8099
1361 8109 1370 8117 1381 8123 1393 8126 1406 8128 1419 8126
1430 8123 1441 8117 1451 8109 1459 8099 1465 8089 1468 8077
1470 8064 67 N F
1517 8123 1557 8123 1701 7894 1701 8123 1735 8123 1735 7842
1695 7842 1551 8071 1551 7842 1517 7842 1517 8123 11 N F
911 9166 945 9166 946 9180 948 9192 952 9203 959 9213
966 9220 976 9226 988 9229 1001 9231 1011 9230 1021 9227
1030 9223 1038 9217 1044 9209 1049 9200 1052 9190 1053 9178
1052 9167 1049 9157 1045 9149 1038 9141 1029 9134 1018 9127
991 9110 968 9096 949 9083 934 9069 922 9054 914 9039
909 9023 906 9006 905 8987 1087 8987 1087 9020 943 9020
946 9032 951 9042 958 9051 966 9059 976 9066 988 9074
1019 9092 1037 9101 1052 9111 1064 9122 1074 9133 1081 9145
1086 9158 1088 9170 1089 9182 1089 9191 1087 9199 1085 9207
1082 9214 1078 9221 1074 9227 1069 9233 1063 9239 1050 9248
1035 9255 1019 9259 1001 9261 982 9259 973 9258 965 9255
957 9252 949 9248 943 9243 936 9238 931 9231 925 9224
921 9217 917 9208 914 9199 912 9189 911 9178 911 9166
78 N F
1212 9261 1228 9260 1244 9256 1258 9250 1270 9242 1280 9232
1284 9226 1288 9219 1290 9212 1292 9205 1294 9197 1294 9188
1293 9177 1291 9166 1287 9158 1282 9150 1276 9144 1270 9139
1258 9130 1268 9125 1277 9119 1285 9111 1291 9103 1297 9093
1301 9083 1303 9073 1304 9062 1303 9048 1298 9034 1291 9020

1280 9007 1274 9001 1267 8995 1259 8990 1251 8986 1241 8982
1232 8980 1221 8978 1210 8977 1198 8978 1188 8980 1178 8982
1169 8986 1161 8990 1154 8995 1147 9001 1141 9007 1132 9020
1125 9034 1121 9048 1119 9062 1155 9062 1157 9051 1160 9041
1165 9031 1172 9023 1181 9017 1190 9012 1201 9009 1212 9008
1223 9009 1233 9012 1243 9017 1251 9023 1258 9031 1263 9041
1267 9051 1268 9062 1267 9073 1263 9083 1258 9092 1251 9100
1243 9107 1233 9112 1223 9115 1212 9116 1201 9115 1190 9112
1181 9107 1172 9100 1165 9092 1160 9083 1157 9073 1155 9062
1119 9062 1120 9073 1123 9083 1127 9093 1132 9102 1139 9110
1146 9118 1155 9124 1166 9130 1153 9139 1147 9144 1141 9150
1137 9158 1133 9166 1130 9177 1129 9188 1164 9188 1165 9179
1167 9171 1172 9164 1178 9158 1185 9153 1193 9149 1202 9147
1212 9146 1221 9147 1230 9149 1238 9153 1246 9158 1251 9164
1256 9171 1259 9179 1260 9188 1259 9198 1256 9206 1251 9213
1246 9219 1238 9224 1230 9228 1221 9230 1212 9231 1202 9230
1193 9228 1185 9224 1178 9219 1172 9213 1167 9206 1165 9198
1164 9188 1129 9188 1130 9197 1131 9205 1133 9212 1136 9219
1139 9226 1143 9232 1153 9242 1165 9250 1179 9256 1195 9260
1212 9261 145 N F
1470 9209 1468 9196 1465 9184 1459 9173 1451 9164 1441 9156
1430 9150 1419 9146 1406 9145 1406 9170 1398 9170 1390 9173
1384 9176 1378 9181 1373 9187 1369 9194 1367 9201 1366 9209
1367 9217 1369 9224 1373 9231 1378 9237 1384 9242 1390 9245
1398 9248 1406 9248 1414 9248 1421 9245 1428 9242 1434 9237
1438 9231 1442 9224 1444 9217 1445 9209 1444 9201 1442 9194
1438 9187 1434 9181 1428 9176 1421 9173 1414 9170 1406 9170
1406 9145 1393 9146 1381 9150 1370 9156 1361 9164 1353 9173
1347 9184 1343 9196 1342 9209 1343 9222 1347 9234 1353 9245
1361 9254 1370 9262 1381 9268 1393 9272 1406 9273 1419 9272
1430 9268 1441 9262 1451 9254 1459 9245 1465 9234 1468 9222
1470 9209 67 N F
1517 9269 1557 9269 1701 9040 1701 9269 1735 9269 1735 8987
1695 8987 1551 9217 1551 8987 1517 8987 1517 9269 11 N F
943 10308 909 10308 911 10328 913 10337 916 10346 919 10354
923 10361 928 10368 933 10374 939 10379 945 10383 952 10387
960 10390 968 10393 976 10395 995 10396 1012 10395 1028 10391
1043 10385 1055 10377 1060 10372 1065 10366 1069 10360 1073 10354
1076 10346 1078 10339 1079 10330 1079 10322 1079 10311 1077 10302
1074 10294 1070 10286 1065 10280 1058 10274 1049 10268 1039 10263
1051 10258 1061 10253 1069 10246 1076 10239 1081 10230 1085 10221
1087 10210 1087 10199 1086 10184 1082 10169 1076 10155 1072 10148
1066 10142 1060 10136 1054 10131 1046 10126 1037 10122 1028 10119

1017 10117 1006 10115 993 10115 982 10115 972 10116 962 10118
954 10121 946 10124 939 10129 932 10133 926 10139 921 10145
917 10152 913 10159 910 10166 906 10183 905 10201 939 10201
939 10191 941 10181 944 10172 949 10163 956 10156 966 10150
979 10146 995 10145 1009 10145 1021 10148 1030 10153 1038 10160
1044 10168 1048 10177 1050 10188 1051 10199 1050 10213 1048 10219
1046 10225 1043 10229 1039 10233 1030 10240 1019 10244 1006 10247
991 10249 975 10249 975 10277 991 10278 1005 10279 1017 10281
1026 10285 1034 10290 1039 10298 1042 10307 1043 10320 1042 10329
1040 10338 1037 10346 1032 10352 1025 10358 1017 10362 1007 10365
995 10366 984 10365 974 10363 966 10360 958 10354 952 10346
948 10336 944 10323 943 10308 123 N F
1212 10396 1222 10395 1231 10393 1240 10389 1249 10384 1257 10377
1264 10369 1271 10361 1277 10351 1283 10341 1288 10330 1292 10318
1296 10306 1298 10294 1300 10281 1302 10268 1302 10255 1302 10242
1300 10230 1296 10204 1292 10192 1288 10180 1283 10169 1277 10158
1271 10149 1264 10140 1257 10132 1249 10125 1240 10120 1231 10116
1222 10113 1212 10113 1202 10113 1192 10116 1183 10120 1175 10125
1167 10132 1159 10140 1152 10149 1146 10158 1141 10169 1136 10180
1131 10192 1128 10204 1123 10230 1122 10242 1121 10255 1157 10255
1159 10231 1162 10209 1168 10190 1175 10173 1183 10160 1192 10151
1197 10147 1202 10145 1207 10143 1212 10143 1217 10143 1222 10145
1226 10147 1231 10151 1240 10160 1249 10173 1256 10190 1261 10209
1265 10231 1266 10255 1265 10280 1261 10301 1256 10320 1249 10336
1240 10349 1231 10358 1226 10361 1222 10364 1217 10365 1212 10366
1207 10365 1202 10364 1197 10361 1192 10358 1183 10349 1175 10336
1168 10320 1162 10301 1159 10280 1157 10255 1121 10255 1122 10268
1123 10281 1125 10294 1128 10306 1131 10318 1136 10330 1141 10341
1146 10351 1152 10361 1159 10369 1167 10377 1175 10384 1183 10389
1192 10393 1202 10395 1212 10396 105 N F
1470 10344 1468 10331 1465 10319 1459 10308 1451 10299 1441 10291
1430 10285 1419 10281 1406 10280 1406 10305 1398 10306 1390 10308
1384 10312 1378 10316 1373 10322 1369 10329 1367 10336 1366 10344
1367 10352 1369 10360 1373 10366 1378 10372 1384 10377 1390 10380
1398 10383 1406 10384 1414 10383 1421 10380 1428 10377 1434 10372
1438 10366 1442 10360 1444 10352 1445 10344 1444 10336 1442 10329
1438 10322 1434 10316 1428 10312 1421 10308 1414 10306 1406 10305
1406 10280 1393 10281 1381 10285 1370 10291 1361 10299 1353 10308
1347 10319 1343 10331 1342 10344 1343 10357 1347 10369 1353 10380
1361 10389 1370 10397 1381 10403 1393 10407 1406 10408 1419 10407
1430 10403 1441 10397 1451 10389 1459 10380 1465 10369 1468 10357
1470 10344 67 N F
1517 10404 1557 10404 1701 10175 1701 10404 1735 10404 1735 10123

1695 10123 1551 10352 1551 10123 1517 10123 1517 10404 11 N F
943 11433 909 11433 911 11453 913 11462 916 11471 919 11479
923 11486 928 11493 933 11499 939 11504 945 11509 952 11512
960 11516 968 11518 976 11520 995 11521 1012 11520 1028 11516
1043 11510 1055 11502 1060 11497 1065 11491 1069 11485 1073 11479
1076 11472 1078 11464 1079 11456 1079 11447 1079 11437 1077 11427
1074 11419 1070 11412 1065 11405 1058 11399 1049 11393 1039 11388
1051 11384 1061 11378 1069 11371 1076 11364 1081 11355 1085 11346
1087 11336 1087 11324 1086 11309 1082 11294 1076 11280 1072 11273
1066 11267 1060 11261 1054 11256 1046 11251 1037 11247 1028 11244
1017 11242 1006 11240 993 11240 982 11240 972 11241 962 11243
954 11246 946 11250 939 11254 932 11259 926 11264 921 11270
917 11277 913 11284 910 11291 906 11308 905 11326 939 11326
939 11316 941 11306 944 11297 949 11288 956 11281 966 11275
979 11271 995 11270 1009 11270 1021 11273 1030 11278 1038 11285
1044 11293 1048 11303 1050 11313 1051 11324 1050 11338 1048 11344
1046 11350 1043 11355 1039 11359 1030 11365 1019 11370 1006 11372
991 11374 975 11374 975 11403 991 11403 1005 11404 1017 11406
1026 11410 1034 11415 1039 11423 1042 11433 1043 11445 1042 11454
1040 11463 1037 11471 1032 11478 1025 11483 1017 11487 1007 11490
995 11491 984 11490 974 11488 966 11485 958 11479 952 11471
948 11461 944 11448 943 11433 123 N F
1125 11427 1160 11427 1160 11440 1163 11453 1167 11464 1173 11473
1181 11481 1191 11486 1202 11490 1216 11491 1226 11490 1236 11487
1245 11483 1252 11477 1259 11469 1264 11460 1267 11450 1268 11439
1267 11427 1264 11417 1259 11409 1253 11402 1244 11394 1233 11387
1206 11370 1182 11357 1163 11343 1148 11329 1137 11314 1129 11299
1123 11283 1120 11266 1119 11248 1302 11248 1302 11280 1157 11280
1161 11292 1166 11303 1172 11311 1181 11319 1191 11327 1203 11334
1234 11352 1252 11361 1267 11371 1279 11382 1288 11394 1296 11406
1300 11418 1303 11431 1304 11443 1304 11451 1302 11459 1300 11467
1297 11474 1293 11481 1289 11488 1284 11494 1278 11499 1265 11509
1250 11515 1233 11520 1216 11521 1197 11520 1188 11518 1180 11515
1172 11512 1164 11508 1157 11503 1151 11498 1145 11492 1140 11485
1136 11477 1132 11469 1129 11459 1127 11449 1126 11438 1125 11427
78 N F
1470 11469 1468 11457 1465 11445 1459 11434 1451 11424 1441 11416
1430 11410 1419 11407 1406 11405 1406 11430 1398 11431 1390 11433
1384 11437 1378 11442 1373 11447 1369 11454 1367 11461 1366 11469
1367 11477 1369 11485 1373 11491 1378 11497 1384 11502 1390 11506
1398 11508 1406 11509 1414 11508 1421 11506 1428 11502 1434 11497
1438 11491 1442 11485 1444 11477 1445 11469 1444 11461 1442 11454
1438 11447 1434 11442 1428 11437 1421 11433 1414 11431 1406 11430

1406 11405 1393 11407 1381 11410 1370 11416 1361 11424 1353 11434
1347 11445 1343 11457 1342 11469 1343 11482 1347 11494 1353 11505
1361 11515 1370 11522 1381 11528 1393 11532 1406 11533 1419 11532
1430 11528 1441 11522 1451 11515 1459 11505 1465 11494 1468 11482
1470 11469 67 N F
1517 11529 1557 11529 1701 11300 1701 11529 1735 11529 1735 11248
1695 11248 1551 11477 1551 11248 1517 11248 1517 11529 11 N F
943 12551 909 12551 911 12572 913 12581 916 12589 919 12597
923 12605 928 12611 933 12617 939 12622 945 12627 952 12631
960 12634 968 12636 976 12638 995 12640 1012 12638 1028 12635
1043 12629 1055 12620 1060 12615 1065 12610 1069 12604 1073 12597
1076 12590 1078 12582 1079 12574 1079 12565 1079 12555 1077 12546
1074 12538 1070 12530 1065 12523 1058 12517 1049 12512 1039 12507
1051 12502 1061 12496 1069 12490 1076 12482 1081 12474 1085 12464
1087 12454 1087 12443 1086 12427 1082 12412 1076 12398 1072 12392
1066 12385 1060 12379 1054 12374 1046 12370 1037 12366 1028 12362
1017 12360 1006 12359 993 12358 982 12359 972 12360 962 12362
954 12365 946 12368 939 12372 932 12377 926 12382 921 12389
917 12395 913 12402 910 12410 906 12426 905 12445 939 12445
939 12435 941 12425 944 12415 949 12407 956 12399 966 12393
979 12390 995 12388 1009 12389 1021 12392 1030 12397 1038 12403
1044 12412 1048 12421 1050 12431 1051 12443 1050 12457 1048 12463
1046 12468 1043 12473 1039 12477 1030 12483 1019 12488 1006 12491
991 12492 975 12493 975 12521 991 12521 1005 12522 1017 12525
1026 12528 1034 12534 1039 12541 1042 12551 1043 12563 1042 12573
1040 12581 1037 12589 1032 12596 1025 12602 1017 12606 1007 12608
995 12609 984 12609 974 12607 966 12603 958 12598 952 12590
948 12580 944 12567 943 12551 123 N F
1242 12640 1266 12640 1266 12463 1308 12463 1308 12430 1266 12430
1266 12366 1232 12366 1232 12430 1117 12430 1117 12463 1147 12463
1232 12463 1232 12581 1147 12463 1117 12463 1117 12467 1242 12640
18 N F
1470 12588 1468 12575 1465 12563 1459 12552 1451 12543 1441 12535
1430 12529 1419 12525 1406 12524 1406 12548 1398 12549 1390 12551
1384 12555 1378 12560 1373 12566 1369 12572 1367 12580 1366 12588
1367 12596 1369 12603 1373 12610 1378 12616 1384 12620 1390 12624
1398 12626 1406 12627 1414 12626 1421 12624 1428 12620 1434 12616
1438 12610 1442 12603 1444 12596 1445 12588 1444 12580 1442 12572
1438 12566 1434 12560 1428 12555 1421 12551 1414 12549 1406 12548
1406 12524 1393 12525 1381 12529 1370 12535 1361 12543 1353 12552
1347 12563 1343 12575 1342 12588 1343 12601 1347 12613 1353 12623
1361 12633 1370 12641 1381 12647 1393 12650 1406 12652 1419 12650
1430 12647 1441 12641 1451 12633 1459 12623 1465 12613 1468 12601

1470 12588 67 N F
1517 12648 1557 12648 1701 12418 1701 12648 1735 12648 1735 12366
1695 12366 1551 12595 1551 12366 1517 12366 1517 12648 11 N F
943 13664 909 13664 911 13684 913 13694 916 13702 919 13710
923 13717 928 13724 933 13730 939 13735 945 13740 952 13744
960 13747 968 13749 976 13751 995 13752 1012 13751 1028 13747
1043 13741 1055 13733 1060 13728 1065 13723 1069 13717 1073 13710
1076 13703 1078 13695 1079 13687 1079 13678 1079 13668 1077 13659
1074 13650 1070 13643 1065 13636 1058 13630 1049 13624 1039 13620
1051 13615 1061 13609 1069 13602 1076 13595 1081 13587 1085 13577
1087 13567 1087 13555 1086 13540 1082 13525 1076 13511 1072 13504
1066 13498 1060 13492 1054 13487 1046 13482 1037 13478 1028 13475
1017 13473 1006 13471 993 13471 982 13471 972 13473 962 13475
954 13477 946 13481 939 13485 932 13490 926 13495 921 13501
917 13508 913 13515 910 13523 906 13539 905 13557 939 13557
939 13547 941 13538 944 13528 949 13519 956 13512 966 13506
979 13502 995 13501 1009 13502 1021 13504 1030 13509 1038 13516
1044 13524 1048 13534 1050 13544 1051 13555 1050 13570 1048 13576
1046 13581 1043 13586 1039 13590 1030 13596 1019 13601 1006 13604
991 13605 975 13606 975 13634 991 13634 1005 13635 1017 13638
1026 13641 1034 13647 1039 13654 1042 13664 1043 13676 1042 13685
1040 13694 1037 13702 1032 13709 1025 13714 1017 13719 1007 13721
995 13722 984 13722 974 13720 966 13716 958 13710 952 13703
948 13692 944 13680 943 13664 123 N F
1298 13682 1264 13682 1262 13691 1258 13699 1253 13706 1248 13712
1241 13716 1235 13720 1227 13721 1220 13722 1212 13722 1206 13720
1199 13718 1193 13715 1188 13711 1182 13707 1178 13701 1174 13695
1167 13680 1162 13663 1159 13643 1157 13620 1166 13628 1174 13635
1182 13641 1190 13645 1198 13647 1206 13649 1224 13650 1237 13648
1251 13644 1265 13638 1277 13628 1283 13622 1288 13616 1293 13609
1297 13601 1300 13592 1303 13582 1304 13572 1304 13561 1270 13559
1269 13571 1265 13581 1260 13592 1253 13601 1245 13609 1235 13614
1225 13618 1214 13620 1203 13618 1192 13614 1183 13609 1174 13601
1167 13592 1162 13581 1159 13571 1157 13559 1159 13548 1162 13537
1167 13527 1174 13518 1183 13510 1192 13504 1203 13500 1214 13499
1225 13500 1235 13504 1245 13510 1253 13518 1260 13527 1265 13537
1269 13548 1270 13559 1304 13561 1303 13545 1299 13529 1292 13514
1287 13506 1282 13499 1276 13493 1270 13487 1262 13482 1254 13477
1245 13474 1235 13471 1225 13469 1214 13469 1201 13470 1190 13472
1180 13475 1170 13480 1162 13485 1154 13492 1148 13500 1142 13509
1137 13518 1133 13529 1129 13540 1126 13552 1123 13578 1121 13606
1122 13622 1123 13638 1125 13653 1128 13667 1132 13680 1136 13692
1141 13703 1147 13713 1154 13722 1161 13730 1169 13736 1178 13742

1188 13746 1198 13750 1208 13752 1220 13752 1236 13751 1250 13747
1263 13740 1274 13731 1283 13721 1290 13709 1295 13696 1298 13682
126 N F
1470 13701 1468 13688 1465 13676 1459 13665 1451 13655 1441 13647
1430 13642 1419 13638 1406 13637 1406 13661 1398 13662 1390 13664
1384 13668 1378 13673 1373 13678 1369 13685 1367 13693 1366 13701
1367 13708 1369 13716 1373 13723 1378 13728 1384 13733 1390 13737
1398 13739 1406 13740 1414 13739 1421 13737 1428 13733 1434 13728
1438 13723 1442 13716 1444 13708 1445 13701 1444 13693 1442 13685
1438 13678 1434 13673 1428 13668 1421 13664 1414 13662 1406 13661
1406 13637 1393 13638 1381 13642 1370 13647 1361 13655 1353 13665
1347 13676 1343 13688 1342 13701 1343 13713 1347 13725 1353 13736
1361 13746 1370 13754 1381 13759 1393 13763 1406 13765 1419 13763
1430 13759 1441 13754 1451 13746 1459 13736 1465 13725 1468 13713
1470 13701 67 N F
1517 13760 1557 13760 1701 13531 1701 13760 1735 13760 1735 13479
1695 13479 1551 13708 1551 13479 1517 13479 1517 13760 11 N F
943 14770 909 14770 911 14791 913 14800 916 14809 919 14817
923 14824 928 14830 933 14836 939 14842 945 14846 952 14850
960 14853 968 14856 976 14857 995 14859 1012 14858 1028 14854
1043 14848 1055 14840 1060 14835 1065 14829 1069 14823 1073 14816
1076 14809 1078 14801 1079 14793 1079 14784 1079 14774 1077 14765
1074 14757 1070 14749 1065 14742 1058 14736 1049 14731 1039 14726
1051 14721 1061 14715 1069 14709 1076 14701 1081 14693 1085 14684
1087 14673 1087 14662 1086 14647 1082 14632 1076 14617 1072 14611
1066 14604 1060 14599 1054 14593 1046 14589 1037 14585 1028 14582
1017 14579 1006 14578 993 14577 982 14578 972 14579 962 14581
954 14584 946 14587 939 14591 932 14596 926 14602 921 14608
917 14614 913 14621 910 14629 906 14646 905 14664 939 14664
939 14654 941 14644 944 14634 949 14626 956 14618 966 14613
979 14609 995 14608 1009 14608 1021 14611 1030 14616 1038 14623
1044 14631 1048 14640 1050 14651 1051 14662 1050 14676 1048 14682
1046 14687 1043 14692 1039 14696 1030 14703 1019 14707 1006 14710
991 14712 975 14712 975 14740 991 14740 1005 14742 1017 14744
1026 14748 1034 14753 1039 14761 1042 14770 1043 14782 1042 14792
1040 14801 1037 14808 1032 14815 1025 14821 1017 14825 1007 14828
995 14829 984 14828 974 14826 966 14822 958 14817 952 14809
948 14799 944 14786 943 14770 123 N F
1212 14859 1228 14858 1244 14854 1258 14848 1270 14840 1280 14830
1284 14824 1288 14817 1290 14810 1292 14803 1294 14795 1294 14786
1293 14774 1291 14764 1287 14756 1282 14748 1276 14742 1270 14737
1258 14728 1268 14723 1277 14717 1285 14709 1291 14701 1297 14691
1301 14681 1303 14671 1304 14660 1303 14646 1298 14632 1291 14618

1280 14605 1274 14599 1267 14593 1259 14588 1251 14584 1241 14580
1232 14578 1221 14576 1210 14575 1198 14576 1188 14578 1178 14580
1169 14584 1161 14588 1154 14593 1147 14599 1141 14605 1132 14618
1125 14632 1121 14646 1119 14660 1155 14660 1157 14649 1160 14639
1165 14629 1172 14621 1181 14615 1190 14610 1201 14607 1212 14606
1223 14607 1233 14610 1243 14615 1251 14621 1258 14629 1263 14639
1267 14649 1268 14660 1267 14671 1263 14681 1258 14690 1251 14698
1243 14705 1233 14710 1223 14713 1212 14714 1201 14713 1190 14710
1181 14705 1172 14698 1165 14690 1160 14681 1157 14671 1155 14660
1119 14660 1120 14671 1123 14681 1127 14691 1132 14700 1139 14708
1146 14716 1155 14722 1166 14728 1153 14737 1147 14742 1141 14748
1137 14756 1133 14764 1130 14774 1129 14786 1164 14786 1165 14777
1167 14769 1172 14762 1178 14755 1185 14751 1193 14747 1202 14745
1212 14744 1221 14745 1230 14747 1238 14751 1246 14755 1251 14762
1256 14769 1259 14777 1260 14786 1259 14796 1256 14804 1251 14811
1246 14817 1238 14822 1230 14826 1221 14828 1212 14829 1202 14828
1193 14826 1185 14822 1178 14817 1172 14811 1167 14804 1165 14796
1164 14786 1129 14786 1130 14795 1131 14803 1133 14810 1136 14817
1139 14824 1143 14830 1153 14840 1165 14848 1179 14854 1195 14858
1212 14859 145 N F
1470 14807 1468 14794 1465 14782 1459 14771 1451 14762 1441 14754
1430 14748 1419 14744 1406 14743 1406 14768 1398 14768 1390 14771
1384 14774 1378 14779 1373 14785 1369 14792 1367 14799 1366 14807
1367 14815 1369 14822 1373 14829 1378 14835 1384 14840 1390 14843
1398 14846 1406 14846 1414 14846 1421 14843 1428 14840 1434 14835
1438 14829 1442 14822 1444 14815 1445 14807 1444 14799 1442 14792
1438 14785 1434 14779 1428 14774 1421 14771 1414 14768 1406 14768
1406 14743 1393 14744 1381 14748 1370 14754 1361 14762 1353 14771
1347 14782 1343 14794 1342 14807 1343 14820 1347 14832 1353 14843
1361 14852 1370 14860 1381 14866 1393 14870 1406 14871 1419 14870
1430 14866 1441 14860 1451 14852 1459 14843 1465 14832 1468 14820
1470 14807 67 N F
1517 14867 1557 14867 1701 14638 1701 14867 1735 14867 1735 14585
1695 14585 1551 14815 1551 14585 1517 14585 1517 14867 11 N F
1027 15952 1051 15952 1051 15775 1093 15775 1093 15743 1051 15743
1051 15679 1017 15679 1017 15743 903 15743 903 15775 933 15775
1017 15775 1017 15894 933 15775 903 15775 903 15779 1027 15952
18 N F
1212 15952 1222 15951 1231 15949 1240 15945 1249 15940 1257 15933
1264 15925 1271 15917 1277 15907 1283 15897 1288 15886 1292 15874
1296 15862 1298 15850 1300 15837 1302 15824 1302 15811 1302 15798
1300 15785 1296 15760 1292 15748 1288 15736 1283 15725 1277 15714
1271 15705 1264 15696 1257 15688 1249 15681 1240 15676 1231 15672

1222 15669 1212 15669 1202 15669 1192 15672 1183 15676 1175 15681
1167 15688 1159 15696 1152 15705 1146 15714 1141 15725 1136 15736
1131 15748 1128 15760 1123 15785 1122 15798 1121 15811 1157 15811
1159 15787 1162 15765 1168 15746 1175 15729 1183 15716 1192 15707
1197 15703 1202 15701 1207 15699 1212 15699 1217 15699 1222 15701
1226 15703 1231 15707 1240 15716 1249 15729 1256 15746 1261 15765
1265 15787 1266 15811 1265 15836 1261 15857 1256 15876 1249 15892
1240 15905 1231 15914 1226 15917 1222 15920 1217 15921 1212 15922
1207 15921 1202 15920 1197 15917 1192 15914 1183 15905 1175 15892
1168 15876 1162 15857 1159 15836 1157 15811 1121 15811 1122 15824
1123 15837 1125 15850 1128 15862 1131 15874 1136 15886 1141 15897
1146 15907 1152 15917 1159 15925 1167 15933 1175 15940 1183 15945
1192 15949 1202 15951 1212 15952 105 N F
1470 15900 1468 15887 1465 15875 1459 15864 1451 15855 1441 15847
1430 15841 1419 15837 1406 15836 1406 15861 1398 15862 1390 15864
1384 15868 1378 15872 1373 15878 1369 15885 1367 15892 1366 15900
1367 15908 1369 15916 1373 15922 1378 15928 1384 15933 1390 15936
1398 15939 1406 15940 1414 15939 1421 15936 1428 15933 1434 15928
1438 15922 1442 15916 1444 15908 1445 15900 1444 15892 1442 15885
1438 15878 1434 15872 1428 15868 1421 15864 1414 15862 1406 15861
1406 15836 1393 15837 1381 15841 1370 15847 1361 15855 1353 15864
1347 15875 1343 15887 1342 15900 1343 15913 1347 15925 1353 15936
1361 15945 1370 15953 1381 15959 1393 15963 1406 15964 1419 15963
1430 15959 1441 15953 1451 15945 1459 15936 1465 15925 1468 15913
1470 15900 67 N F
1517 15960 1557 15960 1701 15731 1701 15960 1735 15960 1735 15679
1695 15679 1551 15908 1551 15679 1517 15679 1517 15960 11 N F
13570 5585 13604 5585 13605 5599 13607 5611 13611 5622 13618 5632
13625 5639 13635 5645 13647 5648 13660 5650 13670 5649 13680 5646
13689 5642 13697 5636 13703 5628 13708 5619 13711 5609 13712 5597
13711 5586 13708 5576 13704 5568 13697 5560 13688 5553 13677 5546
13650 5529 13627 5515 13608 5502 13593 5488 13581 5473 13573 5458
13567 5442 13564 5425 13564 5406 13746 5406 13746 5439 13602 5439
13605 5451 13610 5461 13617 5470 13625 5478 13635 5485 13647 5493
13678 5511 13696 5520 13711 5530 13723 5541 13733 5552 13740 5564
13745 5577 13747 5589 13748 5601 13748 5610 13746 5618 13744 5626
13741 5633 13737 5640 13733 5646 13728 5652 13722 5658 13709 5667
13694 5674 13678 5678 13660 5680 13641 5678 13632 5677 13624 5674
13616 5671 13608 5667 13602 5662 13595 5657 13589 5650 13584 5643
13580 5636 13576 5627 13573 5618 13571 5608 13570 5597 13570 5585
78 N F
13784 5585 13818 5585 13819 5599 13822 5611 13826 5622 13832 5632
13840 5639 13850 5645 13861 5648 13875 5650 13885 5649 13895 5646

13904 5642 13911 5636 13918 5628 13923 5619 13926 5609 13927 5597
13926 5586 13923 5576 13918 5568 13912 5560 13903 5553 13892 5546
13865 5529 13841 5515 13822 5502 13807 5488 13796 5473 13788 5458
13782 5442 13779 5425 13778 5406 13961 5406 13961 5439 13816 5439
13820 5451 13825 5461 13831 5470 13840 5478 13850 5485 13862 5493
13893 5511 13911 5520 13926 5530 13938 5541 13947 5552 13955 5564
13959 5577 13962 5589 13963 5601 13963 5610 13961 5618 13959 5626
13956 5633 13952 5640 13948 5646 13943 5652 13937 5658 13924 5667
13909 5674 13892 5678 13875 5680 13856 5678 13847 5677 13839 5674
13831 5671 13823 5667 13816 5662 13810 5657 13804 5650 13799 5643
13795 5636 13791 5627 13788 5618 13786 5608 13785 5597 13784 5585
78 N F
14129 5628 14127 5615 14124 5603 14118 5592 14110 5583 14100 5575
14089 5569 14077 5565 14065 5564 14065 5589 14057 5589 14049 5592
14043 5595 14037 5600 14032 5606 14028 5613 14026 5620 14025 5628
14026 5636 14028 5643 14032 5650 14037 5656 14043 5661 14049 5664
14057 5667 14065 5667 14073 5667 14080 5664 14087 5661 14092 5656
14097 5650 14101 5643 14103 5636 14104 5628 14103 5620 14101 5613
14097 5606 14092 5600 14087 5595 14080 5592 14073 5589 14065 5589
14065 5564 14052 5565 14040 5569 14029 5575 14019 5583 14012 5592
14006 5603 14002 5615 14001 5628 14002 5641 14006 5653 14012 5664
14019 5673 14029 5681 14040 5687 14052 5691 14065 5692 14077 5691
14089 5687 14100 5681 14110 5673 14118 5664 14124 5653 14127 5641
14129 5628 67 N F
14176 5688 14216 5688 14360 5459 14360 5688 14394 5688 14394 5406
14354 5406 14210 5636 14210 5406 14176 5406 14176 5688 11 N F
13570 6755 13604 6755 13605 6769 13607 6781 13611 6792 13618 6802
13625 6809 13635 6815 13647 6818 13660 6819 13670 6818 13680 6816
13689 6811 13697 6805 13703 6798 13708 6789 13711 6779 13712 6767
13711 6755 13708 6746 13704 6737 13697 6730 13688 6723 13677 6716
13650 6699 13627 6685 13608 6671 13593 6657 13581 6643 13573 6628
13567 6611 13564 6594 13564 6576 13746 6576 13746 6608 13602 6608
13605 6621 13610 6631 13617 6640 13625 6648 13635 6655 13647 6663
13678 6681 13696 6690 13711 6700 13723 6711 13733 6722 13740 6734
13745 6747 13747 6759 13748 6771 13748 6780 13746 6788 13744 6796
13741 6803 13737 6810 13733 6816 13728 6822 13722 6828 13709 6837
13694 6844 13678 6848 13660 6850 13641 6848 13632 6846 13624 6844
13616 6841 13608 6837 13602 6832 13595 6826 13589 6820 13584 6813
13580 6806 13576 6797 13573 6788 13571 6778 13570 6767 13570 6755
78 N F
13901 6850 13925 6850 13925 6673 13967 6673 13967 6641 13925 6641
13925 6576 13891 6576 13891 6641 13776 6641 13776 6673 13806 6673
13891 6673 13891 6791 13806 6673 13776 6673 13776 6677 13901 6850

18 N F
14129 6798 14127 6785 14124 6773 14118 6762 14110 6753 14100 6745
14089 6739 14077 6735 14065 6734 14065 6758 14057 6759 14049 6762
14043 6765 14037 6770 14032 6776 14028 6782 14026 6790 14025 6798
14026 6806 14028 6813 14032 6820 14037 6826 14043 6830 14049 6834
14057 6836 14065 6837 14073 6836 14080 6834 14087 6830 14092 6826
14097 6820 14101 6813 14103 6806 14104 6798 14103 6790 14101 6782
14097 6776 14092 6770 14087 6765 14080 6762 14073 6759 14065 6758
14065 6734 14052 6735 14040 6739 14029 6745 14019 6753 14012 6762
14006 6773 14002 6785 14001 6798 14002 6811 14006 6823 14012 6834
14019 6843 14029 6851 14040 6857 14052 6861 14065 6862 14077 6861
14089 6857 14100 6851 14110 6843 14118 6834 14124 6823 14127 6811
14129 6798 67 N F
14176 6858 14216 6858 14360 6628 14360 6858 14394 6858 14394 6576
14354 6576 14210 6805 14210 6576 14176 6576 14176 6858 11 N F
13570 7914 13604 7914 13605 7927 13607 7940 13611 7951 13618 7960
13625 7968 13635 7973 13647 7977 13660 7978 13670 7977 13680 7974
13689 7970 13697 7964 13703 7956 13708 7947 13711 7937 13712 7926
13711 7914 13708 7904 13704 7896 13697 7888 13688 7881 13677 7874
13650 7857 13627 7844 13608 7830 13593 7816 13581 7801 13573 7786
13567 7770 13564 7753 13564 7735 13746 7735 13746 7767 13602 7767
13605 7779 13610 7789 13617 7798 13625 7806 13635 7814 13647 7821
13678 7839 13696 7848 13711 7858 13723 7869 13733 7881 13740 7893
13745 7905 13747 7917 13748 7930 13748 7938 13746 7946 13744 7954
13741 7961 13737 7968 13733 7975 13728 7981 13722 7986 13709 7995
13694 8002 13678 8007 13660 8008 13641 8007 13632 8005 13624 8002
13616 7999 13608 7995 13602 7990 13595 7985 13589 7979 13584 7972
13580 7964 13576 7955 13573 7946 13571 7936 13570 7925 13570 7914
78 N F
13957 7938 13923 7938 13921 7947 13917 7955 13912 7962 13907 7968
13900 7972 13893 7975 13886 7977 13879 7978 13871 7977 13864 7976
13858 7974 13852 7971 13846 7967 13841 7962 13837 7957 13833 7951
13826 7936 13821 7919 13817 7898 13816 7875 13825 7884 13833 7891
13841 7896 13849 7900 13857 7903 13865 7905 13883 7906 13896 7904
13910 7900 13924 7893 13936 7884 13942 7878 13947 7872 13952 7864
13956 7856 13959 7848 13962 7838 13963 7828 13963 7817 13929 7815
13928 7826 13924 7837 13919 7847 13912 7857 13904 7864 13894 7870
13884 7874 13873 7875 13862 7874 13851 7870 13842 7864 13833 7857
13826 7847 13821 7837 13818 7826 13816 7815 13818 7804 13821 7793
13826 7783 13833 7774 13842 7766 13851 7760 13862 7756 13873 7755
13884 7756 13894 7760 13904 7766 13912 7774 13919 7783 13924 7793
13928 7804 13929 7815 13963 7817 13962 7801 13958 7785 13951 7769
13946 7762 13941 7755 13935 7749 13929 7743 13921 7738 13913 7733

13904 7730 13894 7727 13884 7725 13873 7725 13860 7725 13849 7727
13839 7731 13829 7735 13821 7741 13813 7748 13807 7756 13801 7764
13796 7774 13792 7785 13788 7796 13785 7808 13782 7834 13780 7861
13781 7878 13782 7894 13784 7908 13787 7922 13791 7935 13795 7948
13800 7959 13806 7969 13813 7978 13820 7985 13828 7992 13837 7998
13846 8002 13857 8005 13867 8007 13879 8008 13895 8007 13909 8002
13922 7996 13933 7987 13942 7977 13949 7965 13954 7952 13957 7938
126 N F
14129 7956 14127 7943 14124 7931 14118 7921 14110 7911 14100 7903
14089 7897 14077 7894 14065 7892 14065 7917 14057 7918 14049 7920
14043 7924 14037 7928 14032 7934 14028 7941 14026 7948 14025 7956
14026 7964 14028 7972 14032 7978 14037 7984 14043 7989 14049 7993
14057 7995 14065 7996 14073 7995 14080 7993 14087 7989 14092 7984
14097 7978 14101 7972 14103 7964 14104 7956 14103 7948 14101 7941
14097 7934 14092 7928 14087 7924 14080 7920 14073 7918 14065 7917
14065 7892 14052 7894 14040 7897 14029 7903 14019 7911 14012 7921
14006 7931 14002 7943 14001 7956 14002 7969 14006 7981 14012 7992
14019 8002 14029 8009 14040 8015 14052 8019 14065 8020 14077 8019
14089 8015 14100 8009 14110 8002 14118 7992 14124 7981 14127 7969
14129 7956 67 N F
14176 8016 14216 8016 14360 7787 14360 8016 14394 8016 14394 7735
14354 7735 14210 7964 14210 7735 14176 7735 14176 8016 11 N F
13570 9056 13604 9056 13605 9069 13607 9082 13611 9093 13618 9102
13625 9110 13635 9115 13647 9119 13660 9120 13670 9119 13680 9116
13689 9112 13697 9106 13703 9098 13708 9090 13711 9079 13712 9068
13711 9056 13708 9046 13704 9038 13697 9031 13688 9024 13677 9016
13650 8999 13627 8986 13608 8972 13593 8958 13581 8943 13573 8928
13567 8912 13564 8895 13564 8877 13746 8877 13746 8909 13602 8909
13605 8921 13610 8932 13617 8940 13625 8948 13635 8956 13647 8963
13678 8981 13696 8990 13711 9000 13723 9011 13733 9023 13740 9035
13745 9047 13747 9060 13748 9072 13748 9080 13746 9088 13744 9096
13741 9104 13737 9110 13733 9117 13728 9123 13722 9128 13709 9138
13694 9144 13678 9149 13660 9150 13641 9149 13632 9147 13624 9145
13616 9141 13608 9137 13602 9133 13595 9127 13589 9121 13584 9114
13580 9106 13576 9098 13573 9088 13571 9078 13570 9067 13570 9056
78 N F
13871 9150 13887 9149 13903 9145 13917 9140 13929 9131 13939 9121
13943 9115 13947 9109 13949 9102 13951 9094 13953 9086 13953 9078
13952 9066 13950 9056 13946 9047 13941 9040 13935 9033 13929 9028
13917 9020 13927 9014 13936 9008 13944 9001 13950 8992 13956 8983
13960 8973 13962 8962 13963 8951 13961 8938 13957 8924 13950 8910
13939 8896 13933 8890 13926 8885 13918 8880 13910 8875 13900 8872
13891 8869 13880 8867 13869 8867 13857 8867 13847 8869 13837 8872

13828 8875 13820 8880 13813 8885 13806 8890 13800 8896 13790 8910
13784 8924 13780 8938 13778 8951 13814 8951 13816 8940 13819 8930
13824 8921 13831 8913 13840 8906 13849 8901 13860 8898 13871 8897
13882 8898 13892 8901 13902 8906 13910 8913 13917 8921 13922 8930
13926 8940 13927 8951 13926 8962 13922 8972 13917 8982 13910 8990
13902 8996 13892 9001 13882 9004 13871 9006 13860 9004 13849 9001
13840 8996 13831 8990 13824 8982 13819 8972 13816 8962 13814 8951
13778 8951 13779 8962 13782 8973 13786 8982 13791 8991 13798 9000
13805 9007 13814 9014 13824 9020 13812 9028 13806 9033 13800 9040
13796 9047 13792 9056 13789 9066 13788 9078 13822 9078 13823 9069
13826 9060 13831 9053 13837 9047 13844 9042 13852 9039 13861 9036
13871 9036 13880 9036 13889 9039 13897 9042 13905 9047 13910 9053
13915 9060 13918 9069 13919 9078 13918 9087 13915 9095 13910 9103
13905 9109 13897 9114 13889 9117 13880 9119 13871 9120 13861 9119
13852 9117 13844 9114 13837 9109 13831 9103 13826 9095 13823 9087
13822 9078 13788 9078 13789 9086 13790 9094 13792 9102 13795 9109
13798 9115 13802 9121 13812 9131 13824 9140 13838 9145 13854 9149
13871 9150 145 N F
14129 9098 14127 9086 14124 9074 14118 9063 14110 9053 14100 9045
14089 9040 14077 9036 14065 9034 14065 9059 14057 9060 14049 9062
14043 9066 14037 9071 14032 9076 14028 9083 14026 9091 14025 9098
14026 9106 14028 9114 14032 9121 14037 9126 14043 9131 14049 9135
14057 9137 14065 9138 14073 9137 14080 9135 14087 9131 14092 9126
14097 9121 14101 9114 14103 9106 14104 9098 14103 9091 14101 9083
14097 9076 14092 9071 14087 9066 14080 9062 14073 9060 14065 9059
14065 9034 14052 9036 14040 9040 14029 9045 14019 9053 14012 9063
14006 9074 14002 9086 14001 9098 14002 9111 14006 9123 14012 9134
14019 9144 14029 9151 14040 9157 14052 9161 14065 9162 14077 9161
14089 9157 14100 9151 14110 9144 14118 9134 14124 9123 14127 9111
14129 9098 67 N F
14176 9158 14216 9158 14360 8929 14360 9158 14394 9158 14394 8877
14354 8877 14210 9106 14210 8877 14176 8877 14176 9158 11 N F
13602 10194 13568 10194 13570 10214 13572 10223 13575 10232 13578 10240
13582 10247 13587 10254 13592 10260 13598 10265 13604 10270 13611 10273
13619 10277 13627 10279 13635 10281 13654 10282 13671 10281 13687 10277
13702 10271 13714 10263 13719 10258 13724 10252 13728 10246 13732 10240
13735 10233 13737 10225 13738 10217 13738 10208 13738 10198 13736 10188
13733 10180 13729 10173 13724 10166 13717 10160 13708 10154 13698 10149
13710 10145 13720 10139 13728 10132 13735 10125 13740 10116 13743 10107
13746 10097 13746 10085 13745 10070 13741 10055 13735 10041 13731 10034
13725 10028 13719 10022 13713 10017 13705 10012 13696 10008 13687 10005
13676 10003 13665 10001 13652 10001 13641 10001 13631 10002 13621 10004
13613 10007 13605 10011 13597 10015 13591 10020 13585 10025 13580 10031

13576 10038 13572 10045 13569 10052 13565 10069 13564 10087 13598 10087
13598 10077 13600 10067 13603 10058 13608 10049 13615 10042 13625 10036
13638 10032 13654 10031 13668 10031 13680 10034 13689 10039 13697 10046
13703 10054 13707 10064 13709 10074 13710 10085 13709 10099 13707 10105
13705 10111 13702 10116 13698 10120 13689 10126 13678 10131 13665 10133
13650 10135 13634 10135 13634 10164 13650 10164 13664 10165 13676 10167
13685 10171 13693 10176 13698 10184 13701 10194 13702 10206 13701 10215
13699 10224 13696 10232 13691 10239 13684 10244 13676 10248 13666 10251
13654 10252 13643 10251 13633 10249 13625 10246 13617 10240 13611 10232
13606 10222 13603 10209 13602 10194 123 N F
13871 10282 13881 10281 13890 10279 13899 10275 13908 10270 13916 10263
13923 10256 13930 10247 13936 10237 13942 10227 13947 10216 13951 10204
13954 10192 13957 10180 13959 10167 13961 10154 13961 10141 13961 10129
13959 10116 13954 10090 13951 10078 13947 10066 13942 10055 13936 10044
13930 10035 13923 10026 13916 10018 13908 10011 13899 10006 13890 10002
13881 10000 13871 9999 13861 10000 13851 10002 13842 10006 13834 10011
13826 10018 13818 10026 13811 10035 13805 10044 13800 10055 13795 10066
13790 10078 13787 10090 13782 10116 13781 10129 13780 10141 13816 10141
13818 10117 13821 10095 13827 10076 13834 10060 13842 10046 13851 10037
13856 10033 13861 10031 13866 10029 13871 10029 13876 10029 13880 10031
13885 10033 13890 10037 13899 10046 13908 10060 13915 10076 13920 10095
13924 10117 13925 10141 13924 10166 13920 10188 13915 10206 13908 10222
13899 10235 13890 10244 13885 10248 13880 10250 13876 10251 13871 10252
13866 10251 13861 10250 13856 10248 13851 10244 13842 10235 13834 10222
13827 10206 13821 10188 13818 10166 13816 10141 13780 10141 13781 10154
13782 10167 13784 10180 13787 10192 13790 10204 13795 10216 13800 10227
13805 10237 13811 10247 13818 10256 13826 10263 13834 10270 13842 10275
13851 10279 13861 10281 13871 10282 105 N F
14129 10230 14127 10218 14124 10206 14118 10195 14110 10185 14100 10177
14089 10171 14077 10168 14065 10166 14065 10191 14057 10192 14049 10194
14043 10198 14037 10202 14032 10208 14028 10215 14026 10222 14025 10230
14026 10238 14028 10246 14032 10252 14037 10258 14043 10263 14049 10267
14057 10269 14065 10270 14073 10269 14080 10267 14087 10263 14092 10258
14097 10252 14101 10246 14103 10238 14104 10230 14103 10222 14101 10215
14097 10208 14092 10202 14087 10198 14080 10194 14073 10192 14065 10191
14065 10166 14052 10168 14040 10171 14029 10177 14019 10185 14012 10195
14006 10206 14002 10218 14001 10230 14002 10243 14006 10255 14012 10266
14019 10276 14029 10283 14040 10289 14052 10293 14065 10294 14077 10293
14089 10289 14100 10283 14110 10276 14118 10266 14124 10255 14127 10243
14129 10230 67 N F
14176 10290 14216 10290 14360 10061 14360 10290 14394 10290 14394 10009
14354 10009 14210 10238 14210 10009 14176 10009 14176 10290 11 N F
13602 11315 13568 11315 13570 11336 13572 11345 13575 11354 13578 11362

13582 11369 13587 11376 13592 11382 13598 11387 13604 11391 13611 11395
13619 11398 13627 11401 13635 11402 13654 11404 13671 11403 13687 11399
13702 11393 13714 11385 13719 11380 13724 11374 13728 11368 13732 11361
13735 11354 13737 11347 13738 11338 13738 11330 13738 11319 13736 11310
13733 11302 13729 11294 13724 11288 13717 11281 13708 11276 13698 11271
13710 11266 13720 11261 13728 11254 13735 11247 13740 11238 13743 11229
13746 11218 13746 11207 13745 11192 13741 11177 13735 11163 13731 11156
13725 11150 13719 11144 13713 11139 13705 11134 13696 11130 13687 11127
13676 11124 13665 11123 13652 11123 13641 11123 13631 11124 13621 11126
13613 11129 13605 11132 13597 11137 13591 11141 13585 11147 13580 11153
13576 11160 13572 11167 13569 11174 13565 11191 13564 11209 13598 11209
13598 11199 13600 11189 13603 11180 13608 11171 13615 11164 13625 11158
13638 11154 13654 11153 13668 11153 13680 11156 13689 11161 13697 11168
13703 11176 13707 11185 13709 11196 13710 11207 13709 11221 13707 11227
13705 11233 13702 11237 13698 11241 13689 11248 13678 11252 13665 11255
13650 11257 13634 11257 13634 11285 13650 11286 13664 11287 13676 11289
13685 11293 13693 11298 13698 11306 13701 11315 13702 11328 13701 11337
13699 11346 13696 11354 13691 11360 13684 11366 13676 11370 13666 11373
13654 11374 13643 11373 13633 11371 13625 11367 13617 11362 13611 11354
13606 11344 13603 11331 13602 11315 123 N F
13784 11309 13818 11309 13819 11323 13822 11336 13826 11347 13832 11356
13840 11363 13850 11369 13861 11373 13875 11374 13885 11373 13895 11370
13904 11366 13911 11360 13918 11352 13923 11343 13926 11333 13927 11321
13926 11310 13923 11300 13918 11292 13912 11284 13903 11277 13892 11270
13865 11253 13841 11240 13822 11226 13807 11212 13796 11197 13788 11182
13782 11166 13779 11149 13778 11131 13961 11131 13961 11163 13816 11163
13820 11175 13825 11185 13831 11194 13840 11202 13850 11209 13862 11217
13893 11235 13911 11244 13926 11254 13938 11265 13947 11277 13955 11289
13959 11301 13962 11313 13963 11326 13963 11334 13961 11342 13959 11350
13956 11357 13952 11364 13948 11371 13943 11377 13937 11382 13924 11391
13909 11398 13892 11402 13875 11404 13856 11402 13847 11401 13839 11398
13831 11395 13823 11391 13816 11386 13810 11381 13804 11375 13799 11368
13795 11360 13791 11351 13788 11342 13786 11332 13785 11321 13784 11309
78 N F
14129 11352 14127 11339 14124 11327 14118 11316 14110 11307 14100 11299
14089 11293 14077 11289 14065 11288 14065 11313 14057 11314 14049 11316
14043 11319 14037 11324 14032 11330 14028 11337 14026 11344 14025 11352
14026 11360 14028 11367 14032 11374 14037 11380 14043 11385 14049 11388
14057 11391 14065 11392 14073 11391 14080 11388 14087 11385 14092 11380
14097 11374 14101 11367 14103 11360 14104 11352 14103 11344 14101 11337
14097 11330 14092 11324 14087 11319 14080 11316 14073 11314 14065 11313
14065 11288 14052 11289 14040 11293 14029 11299 14019 11307 14012 11316
14006 11327 14002 11339 14001 11352 14002 11365 14006 11377 14012 11388

14019 11397 14029 11405 14040 11411 14052 11415 14065 11416 14077 11415
14089 11411 14100 11405 14110 11397 14118 11388 14124 11377 14127 11365
14129 11352 67 N F
14176 11412 14216 11412 14360 11183 14360 11412 14394 11412 14394 11131
14354 11131 14210 11360 14210 11131 14176 11131 14176 11412 11 N F
13602 12430 13568 12430 13570 12451 13572 12460 13575 12469 13578 12477
13582 12484 13587 12490 13592 12496 13598 12502 13604 12506 13611 12510
13619 12513 13627 12515 13635 12517 13654 12519 13671 12517 13687 12514
13702 12508 13714 12500 13719 12495 13724 12489 13728 12483 13732 12476
13735 12469 13737 12461 13738 12453 13738 12444 13738 12434 13736 12425
13733 12417 13729 12409 13724 12402 13717 12396 13708 12391 13698 12386
13710 12381 13720 12375 13728 12369 13735 12361 13740 12353 13743 12344
13746 12333 13746 12322 13745 12307 13741 12292 13735 12277 13731 12271
13725 12264 13719 12259 13713 12253 13705 12249 13696 12245 13687 12242
13676 12239 13665 12238 13652 12237 13641 12238 13631 12239 13621 12241
13613 12244 13605 12247 13597 12251 13591 12256 13585 12262 13580 12268
13576 12274 13572 12281 13569 12289 13565 12306 13564 12324 13598 12324
13598 12314 13600 12304 13603 12294 13608 12286 13615 12278 13625 12273
13638 12269 13654 12267 13668 12268 13680 12271 13689 12276 13697 12282
13703 12291 13707 12300 13709 12311 13710 12322 13709 12336 13707 12342
13705 12347 13702 12352 13698 12356 13689 12363 13678 12367 13665 12370
13650 12372 13634 12372 13634 12400 13650 12400 13664 12402 13676 12404
13685 12408 13693 12413 13698 12420 13701 12430 13702 12442 13701 12452
13699 12461 13696 12468 13691 12475 13684 12481 13676 12485 13666 12488
13654 12489 13643 12488 13633 12486 13625 12482 13617 12477 13611 12469
13606 12459 13603 12446 13602 12430 123 N F
13901 12519 13925 12519 13925 12342 13967 12342 13967 12310 13925 12310
13925 12245 13891 12245 13891 12310 13776 12310 13776 12342 13806 12342
13891 12342 13891 12460 13806 12342 13776 12342 13776 12346 13901 12519
18 N F
14129 12467 14127 12454 14124 12442 14118 12431 14110 12422 14100 12414
14089 12408 14077 12404 14065 12403 14065 12428 14057 12428 14049 12431
14043 12434 14037 12439 14032 12445 14028 12452 14026 12459 14025 12467
14026 12475 14028 12482 14032 12489 14037 12495 14043 12500 14049 12503
14057 12505 14065 12506 14073 12505 14080 12503 14087 12500 14092 12495
14097 12489 14101 12482 14103 12475 14104 12467 14103 12459 14101 12452
14097 12445 14092 12439 14087 12434 14080 12431 14073 12428 14065 12428
14065 12403 14052 12404 14040 12408 14029 12414 14019 12422 14012 12431
14006 12442 14002 12454 14001 12467 14002 12480 14006 12492 14012 12503
14019 12512 14029 12520 14040 12526 14052 12530 14065 12531 14077 12530
14089 12526 14100 12520 14110 12512 14118 12503 14124 12492 14127 12480
14129 12467 67 N F
14176 12527 14216 12527 14360 12298 14360 12527 14394 12527 14394 12245

14354 12245 14210 12474 14210 12245 14176 12245 14176 12527 11 N F
13602 13539 13568 13539 13570 13560 13572 13569 13575 13578 13578 13586
13582 13593 13587 13599 13592 13605 13598 13610 13604 13615 13611 13619
13619 13622 13627 13624 13635 13626 13654 13628 13671 13626 13687 13623
13702 13617 13714 13609 13719 13604 13724 13598 13728 13592 13732 13585
13735 13578 13737 13570 13738 13562 13738 13553 13738 13543 13736 13534
13733 13526 13729 13518 13724 13511 13717 13505 13708 13500 13698 13495
13710 13490 13720 13484 13728 13478 13735 13470 13740 13462 13743 13453
13746 13442 13746 13431 13745 13416 13741 13401 13735 13386 13731 13380
13725 13373 13719 13368 13713 13362 13705 13358 13696 13354 13687 13351
13676 13348 13665 13347 13652 13346 13641 13347 13631 13348 13621 13350
13613 13353 13605 13356 13597 13360 13591 13365 13585 13371 13580 13377
13576 13383 13572 13390 13569 13398 13565 13415 13564 13433 13598 13433
13598 13423 13600 13413 13603 13403 13608 13395 13615 13387 13625 13382
13638 13378 13654 13376 13668 13377 13680 13380 13689 13385 13697 13391
13703 13400 13707 13409 13709 13420 13710 13431 13709 13445 13707 13451
13705 13456 13702 13461 13698 13465 13689 13472 13678 13476 13665 13479
13650 13480 13634 13481 13634 13509 13650 13509 13664 13511 13676 13513
13685 13517 13693 13522 13698 13529 13701 13539 13702 13551 13701 13561
13699 13570 13696 13577 13691 13584 13684 13590 13676 13594 13666 13597
13654 13597 13643 13597 13633 13595 13625 13591 13617 13586 13611 13578
13606 13568 13603 13555 13602 13539 123 N F
13957 13557 13923 13557 13921 13567 13917 13575 13912 13581 13907 13587
13900 13592 13893 13595 13886 13597 13879 13597 13871 13597 13864 13596
13858 13594 13852 13591 13846 13587 13841 13582 13837 13577 13833 13570
13826 13556 13821 13538 13817 13518 13816 13495 13825 13504 13833 13511
13841 13516 13849 13520 13857 13522 13865 13524 13883 13525 13896 13524
13910 13520 13924 13513 13936 13504 13942 13498 13947 13491 13952 13484
13956 13476 13959 13467 13962 13458 13963 13448 13963 13437 13929 13435
13928 13446 13924 13457 13919 13467 13912 13476 13904 13484 13894 13490
13884 13494 13873 13495 13862 13494 13851 13490 13842 13484 13833 13476
13826 13467 13821 13457 13818 13446 13816 13435 13818 13423 13821 13413
13826 13402 13833 13393 13842 13386 13851 13380 13862 13376 13873 13374
13884 13376 13894 13380 13904 13386 13912 13393 13919 13402 13924 13413
13928 13423 13929 13435 13963 13437 13962 13421 13958 13404 13951 13389
13946 13382 13941 13375 13935 13368 13929 13362 13921 13357 13913 13353
13904 13349 13894 13346 13884 13345 13873 13344 13860 13345 13849 13347
13839 13350 13829 13355 13821 13361 13813 13367 13807 13375 13801 13384
13796 13394 13792 13404 13788 13415 13785 13427 13782 13453 13780 13481
13781 13497 13782 13513 13784 13528 13787 13542 13791 13555 13795 13567
13800 13578 13806 13588 13813 13597 13820 13605 13828 13612 13837 13617
13846 13622 13857 13625 13867 13627 13879 13628 13895 13626 13909 13622
13922 13615 13933 13607 13942 13596 13949 13584 13954 13571 13957 13557

126 N F
14129 13576 14127 13563 14124 13551 14118 13540 14110 13531 14100 13523
14089 13517 14077 13513 14065 13512 14065 13536 14057 13537 14049 13540
14043 13543 14037 13548 14032 13554 14028 13561 14026 13568 14025 13576
14026 13584 14028 13591 14032 13598 14037 13604 14043 13609 14049 13612
14057 13614 14065 13615 14073 13614 14080 13612 14087 13609 14092 13604
14097 13598 14101 13591 14103 13584 14104 13576 14103 13568 14101 13561
14097 13554 14092 13548 14087 13543 14080 13540 14073 13537 14065 13536
14065 13512 14052 13513 14040 13517 14029 13523 14019 13531 14012 13540
14006 13551 14002 13563 14001 13576 14002 13589 14006 13601 14012 13612
14019 13621 14029 13629 14040 13635 14052 13639 14065 13640 14077 13639
14089 13635 14100 13629 14110 13621 14118 13612 14124 13601 14127 13589
14129 13576 67 N F
14176 13636 14216 13636 14360 13407 14360 13636 14394 13636 14394 13354
14354 13354 14210 13583 14210 13354 14176 13354 14176 13636 11 N F
13602 14642 13568 14642 13570 14662 13572 14671 13575 14680 13578 14688
13582 14695 13587 14702 13592 14708 13598 14713 13604 14717 13611 14721
13619 14724 13627 14727 13635 14729 13654 14730 13671 14729 13687 14725
13702 14719 13714 14711 13719 14706 13724 14700 13728 14694 13732 14688
13735 14680 13737 14673 13738 14664 13738 14656 13738 14646 13736 14636
13733 14628 13729 14621 13724 14614 13717 14608 13708 14602 13698 14597
13710 14593 13720 14587 13728 14580 13735 14573 13740 14564 13743 14555
13746 14545 13746 14533 13745 14518 13741 14503 13735 14489 13731 14482
13725 14476 13719 14470 13713 14465 13705 14460 13696 14456 13687 14453
13676 14451 13665 14449 13652 14449 13641 14449 13631 14450 13621 14452
13613 14455 13605 14459 13597 14463 13591 14468 13585 14473 13580 14479
13576 14486 13572 14493 13569 14500 13565 14517 13564 14535 13598 14535
13598 14525 13600 14515 13603 14506 13608 14497 13615 14490 13625 14484
13638 14480 13654 14479 13668 14479 13680 14482 13689 14487 13697 14494
13703 14502 13707 14512 13709 14522 13710 14533 13709 14547 13707 14553
13705 14559 13702 14563 13698 14568 13689 14574 13678 14579 13665 14581
13650 14583 13634 14583 13634 14611 13650 14612 13664 14613 13676 14615
13685 14619 13693 14624 13698 14632 13701 14641 13702 14654 13701 14663
13699 14672 13696 14680 13691 14687 13684 14692 13676 14696 13666 14699
13654 14700 13643 14699 13633 14697 13625 14694 13617 14688 13611 14680
13606 14670 13603 14657 13602 14642 123 N F
13871 14730 13887 14729 13903 14725 13917 14719 13929 14711 13939 14701
13943 14695 13947 14689 13949 14682 13951 14674 13953 14666 13953 14658
13952 14646 13950 14636 13946 14627 13941 14620 13935 14613 13929 14608
13917 14599 13927 14594 13936 14588 13944 14580 13950 14572 13956 14563
13960 14553 13962 14542 13963 14531 13961 14518 13957 14503 13950 14489
13939 14476 13933 14470 13926 14464 13918 14459 13910 14455 13900 14452
13891 14449 13880 14447 13869 14447 13857 14447 13847 14449 13837 14452

13828 14455 13820 14459 13813 14464 13806 14470 13800 14476 13790 14489
13784 14503 13780 14518 13778 14531 13814 14531 13816 14520 13819 14510
13824 14501 13831 14493 13840 14486 13849 14481 13860 14478 13871 14477
13882 14478 13892 14481 13902 14486 13910 14493 13917 14501 13922 14510
13926 14520 13927 14531 13926 14542 13922 14552 13917 14562 13910 14570
13902 14576 13892 14581 13882 14584 13871 14585 13860 14584 13849 14581
13840 14576 13831 14570 13824 14562 13819 14552 13816 14542 13814 14531
13778 14531 13779 14542 13782 14553 13786 14562 13791 14571 13798 14580
13805 14587 13814 14594 13824 14599 13812 14608 13806 14613 13800 14620
13796 14627 13792 14636 13789 14646 13788 14658 13822 14658 13823 14648
13826 14640 13831 14633 13837 14627 13844 14622 13852 14618 13861 14616
13871 14616 13880 14616 13889 14618 13897 14622 13905 14627 13910 14633
13915 14640 13918 14648 13919 14658 13918 14667 13915 14675 13910 14683
13905 14689 13897 14693 13889 14697 13880 14699 13871 14700 13861 14699
13852 14697 13844 14693 13837 14689 13831 14683 13826 14675 13823 14667
13822 14658 13788 14658 13789 14666 13790 14674 13792 14682 13795 14689
13798 14695 13802 14701 13812 14711 13824 14719 13838 14725 13854 14729
13871 14730 145 N F
14129 14678 14127 14665 14124 14653 14118 14643 14110 14633 14100 14625
14089 14619 14077 14616 14065 14614 14065 14639 14057 14640 14049 14642
14043 14646 14037 14650 14032 14656 14028 14663 14026 14670 14025 14678
14026 14686 14028 14694 14032 14700 14037 14706 14043 14711 14049 14715
14057 14717 14065 14718 14073 14717 14080 14715 14087 14711 14092 14706
14097 14700 14101 14694 14103 14686 14104 14678 14103 14670 14101 14663
14097 14656 14092 14650 14087 14646 14080 14642 14073 14640 14065 14639
14065 14614 14052 14616 14040 14619 14029 14625 14019 14633 14012 14643
14006 14653 14002 14665 14001 14678 14002 14691 14006 14703 14012 14714
14019 14724 14029 14731 14040 14737 14052 14741 14065 14742 14077 14741
14089 14737 14100 14731 14110 14724 14118 14714 14124 14703 14127 14691
14129 14678 67 N F
14176 14738 14216 14738 14360 14509 14360 14738 14394 14738 14394 14457
14354 14457 14210 14686 14210 14457 14176 14457 14176 14738 11 N F
13688 15828 13712 15828 13712 15651 13754 15651 13754 15619 13712 15619
13712 15555 13678 15555 13678 15619 13564 15619 13564 15651 13594 15651
13678 15651 13678 15770 13594 15651 13564 15651 13564 15655 13688 15828
18 N F
13873 15828 13883 15827 13892 15825 13901 15821 13910 15816 13918 15809
13925 15802 13932 15793 13938 15784 13944 15773 13949 15762 13953 15750
13956 15738 13959 15726 13961 15713 13963 15700 13963 15688 13963 15675
13961 15662 13956 15636 13953 15624 13949 15612 13944 15601 13938 15591
13932 15581 13925 15572 13918 15564 13910 15558 13901 15552 13892 15548
13883 15546 13873 15545 13863 15546 13853 15548 13844 15552 13836 15558
13828 15564 13820 15572 13813 15581 13807 15591 13802 15601 13797 15612

13792 15624 13789 15636 13784 15662 13783 15675 13782 15688 13818 15688
13820 15663 13823 15641 13829 15622 13836 15606 13844 15593 13853 15583
13858 15580 13863 15577 13868 15576 13873 15575 13878 15576 13882 15577
13887 15580 13892 15583 13901 15593 13910 15606 13917 15622 13922 15641
13926 15663 13927 15688 13926 15712 13922 15734 13917 15753 13910 15768
13901 15781 13892 15790 13887 15794 13882 15796 13878 15798 13873 15798
13868 15798 13863 15796 13858 15794 13853 15790 13844 15781 13836 15768
13829 15753 13823 15734 13820 15712 13818 15688 13782 15688 13783 15700
13784 15713 13786 15726 13789 15738 13792 15750 13797 15762 13802 15773
13807 15784 13813 15793 13820 15802 13828 15809 13836 15816 13844 15821
13853 15825 13863 15827 13873 15828 105 N F
14131 15776 14129 15764 14126 15752 14120 15741 14112 15731 14102 15723
14091 15718 14079 15714 14067 15712 14067 15737 14059 15738 14051 15740
14045 15744 14039 15749 14034 15754 14030 15761 14028 15769 14027 15776
14028 15784 14030 15792 14034 15799 14039 15804 14045 15809 14051 15813
14059 15815 14067 15816 14075 15815 14082 15813 14089 15809 14094 15804
14099 15799 14103 15792 14105 15784 14106 15776 14105 15769 14103 15761
14099 15754 14094 15749 14089 15744 14082 15740 14075 15738 14067 15737
14067 15712 14054 15714 14042 15718 14031 15723 14022 15731 14014 15741
14008 15752 14004 15764 14003 15776 14004 15789 14008 15801 14014 15812
14022 15822 14031 15830 14042 15835 14054 15839 14067 15840 14079 15839
14091 15835 14102 15830 14112 15822 14120 15812 14126 15801 14129 15789
14131 15776 67 N F
14178 15836 14218 15836 14362 15607 14362 15836 14396 15836 14396 15555
14356 15555 14212 15784 14212 15555 14178 15555 14178 15836 11 N F
25 W
2646 5172 2646 5453 2 P
3739 5172 3739 5453 2 P
4823 5172 4823 5453 2 P
5903 5172 5903 5453 2 P
6971 5172 6971 5453 2 P
8052 5172 8052 5453 2 P
9133 5172 9133 5453 2 P
10214 5172 10214 5453 2 P
11295 5172 11295 5453 2 P
12376 5172 12376 5453 2 P
2747 16454 2747 16173 2 P
4812 16454 4812 16173 2 P
6843 16454 6843 16173 2 P
8864 16454 8864 16173 2 P
10892 16454 10892 16173 2 P
12942 16454 12942 16173 2 P
2016 5647 2297 5647 2 P

2016 6823 2297 6823 2 P
2016 7980 2297 7980 2 P
2016 9125 2297 9125 2 P
2016 10260 2297 10260 2 P
2016 11387 2297 11387 2 P
2016 12505 2297 12505 2 P
2016 13617 2297 13617 2 P
2016 14723 2297 14723 2 P
2016 15816 2297 15816 2 P
13283 5549 13002 5549 2 P
13283 6719 13002 6719 2 P
13283 7872 13002 7872 2 P
13283 9015 13002 9015 2 P
13283 10147 13002 10147 2 P
13283 11269 13002 11269 2 P
13283 12384 13002 12384 2 P
13283 13492 13002 13492 2 P
13283 14594 13002 14594 2 P
13283 15693 13002 15693 2 P
13002 5453 2297 5453 2297 16173 13002 16173 13002 5453 2297 5453
6:00 PM
12636 17516 12635 17506 12632 17496 12627 17486 12621 17479 12613 17472
12604 17467 12593 17464 12583 17463 12572 17464 12562 17467 12553 17472
12545 17479 12538 17486 12533 17496 12530 17506 12529 17516 12551 17516
12551 17510 12553 17504 12556 17498 12560 17494 12565 17490 12570 17487
12576 17485 12583 17484 12589 17485 12595 17487 12601 17490 12605 17494
12609 17498 12612 17504 12614 17510 12615 17516 12614 17523 12612 17529
12609 17534 12605 17539 12601 17543 12595 17546 12589 17548 12583 17549
12576 17548 12570 17546 12565 17543 12560 17539 12556 17534 12553 17529
12551 17523 12551 17516 12529 17516 12530 17527 12533 17537 12538 17546
12545 17554 12553 17561 12562 17566 12572 17569 12583 17570 12593 17569
12604 17566 12613 17561 12621 17554 12627 17546 12632 17537 12635 17527
12636 17516 67 N F
12997 17447 12993 17462 12989 17476 12984 17489 12979 17501 12973 17512
12966 17521 12958 17530 12949 17537 12940 17544 12930 17549 12920 17554
12908 17558 12896 17561 12884 17563 12870 17564 12856 17564 12838 17563
12820 17561 12804 17556 12788 17550 12774 17543 12761 17534 12748 17523
12737 17511 12728 17498 12719 17484 12712 17469 12705 17452 12700 17435
12697 17417 12694 17398 12693 17378 12694 17356 12696 17335 12700 17315
12706 17297 12713 17281 12721 17265 12730 17251 12740 17239 12752 17228
12764 17218 12778 17210 12792 17204 12807 17198 12823 17195 12839 17193
12856 17192 12875 17192 12892 17195 12907 17198 12921 17203 12934 17209
12946 17216 12956 17224 12965 17233 12973 17243 12980 17254 12986 17266

12991 17278 12995 17291 12998 17304 13000 17318 13002 17332 12956 17332
12951 17307 12944 17286 12940 17277 12936 17269 12931 17262 12925 17256
12919 17250 12912 17245 12904 17241 12896 17238 12887 17236 12878 17234
12856 17233 12842 17233 12828 17235 12815 17239 12804 17244 12793 17250
12783 17257 12775 17265 12767 17275 12760 17285 12755 17296 12750 17308
12746 17321 12743 17334 12741 17348 12740 17363 12739 17378 12740 17395
12741 17411 12744 17426 12748 17440 12753 17453 12759 17464 12765 17475
12773 17485 12781 17494 12790 17502 12800 17508 12810 17514 12821 17518
12832 17521 12844 17523 12856 17524 12874 17522 12891 17518 12906 17512
12920 17503 12931 17493 12936 17486 12941 17479 12944 17472 12947 17464
12949 17456 12951 17447 12997 17447 123 N F
7210 17656 7274 17656 7378 17345 7483 17656 7546 17656 7546 17299
7503 17299 7503 17598 7401 17299 7353 17299 7253 17598 7253 17299
7210 17299 7210 17656 14 N F
7613 17480 7614 17496 7618 17511 7621 17518 7625 17525 7630 17531
7636 17538 7642 17543 7650 17549 7658 17553 7667 17557 7678 17560
7689 17562 7701 17564 7714 17564 7729 17564 7745 17562 7761 17558
7776 17552 7783 17548 7790 17543 7796 17538 7801 17532 7805 17525
7809 17518 7811 17510 7811 17501 7811 17337 7812 17333 7815 17329
7818 17325 7823 17323 7828 17321 7833 17320 7838 17321 7842 17322
7842 17291 7832 17289 7816 17289 7805 17289 7797 17290 7790 17292
7785 17295 7781 17299 7777 17305 7771 17322 7749 17306 7739 17300
7730 17296 7721 17293 7711 17290 7700 17289 7686 17289 7675 17289
7665 17290 7655 17292 7647 17295 7639 17298 7633 17302 7627 17306
7622 17311 7617 17316 7614 17322 7609 17334 7606 17347 7605 17360
7643 17360 7644 17353 7647 17346 7651 17339 7657 17333 7665 17329
7674 17325 7685 17323 7697 17322 7714 17324 7729 17329 7741 17335
7752 17342 7760 17350 7766 17359 7769 17367 7771 17376 7771 17427
7751 17419 7736 17416 7689 17409 7675 17405 7665 17400 7657 17394
7651 17388 7647 17382 7645 17375 7643 17368 7643 17360 7605 17360
7606 17379 7609 17395 7614 17407 7622 17417 7631 17425 7643 17431
7656 17435 7671 17439 7737 17450 7753 17454 7764 17459 7767 17461
7769 17464 7770 17467 7771 17470 7771 17493 7770 17497 7768 17502
7764 17508 7758 17513 7750 17518 7740 17522 7729 17525 7714 17526
7699 17525 7687 17522 7676 17517 7668 17511 7661 17504 7657 17496
7654 17488 7653 17480 7613 17480 129 N F
7862 17557 7908 17557 7972 17358 8043 17557 8089 17557 7969 17233
7961 17219 7953 17209 7944 17202 7936 17197 7928 17194 7920 17192
7903 17192 7894 17192 7880 17197 7880 17233 7896 17230 7903 17229
7911 17230 7918 17233 7924 17237 7929 17244 7934 17253 7949 17296
7862 17557 25 N F
2297 17462 2333 17462 2333 17424 2343 17436 2353 17446 2362 17454
2372 17460 2381 17465 2391 17468 2401 17469 2412 17470 2430 17468

2439 17465 2447 17462 2456 17457 2464 17450 2472 17441 2481 17429
2491 17440 2501 17449 2511 17457 2520 17462 2530 17466 2540 17468
2550 17470 2560 17470 2573 17469 2585 17466 2598 17461 2609 17454
2619 17445 2623 17439 2627 17433 2630 17427 2632 17420 2633 17412
2634 17404 2634 17205 2593 17205 2593 17388 2592 17398 2589 17406
2584 17414 2578 17421 2571 17426 2563 17431 2555 17433 2547 17434
2536 17433 2526 17429 2515 17424 2506 17416 2498 17407 2492 17397
2487 17385 2486 17373 2486 17205 2445 17205 2445 17388 2444 17398
2441 17406 2436 17414 2430 17421 2422 17426 2414 17431 2405 17433
2397 17434 2388 17433 2378 17430 2368 17424 2359 17417 2351 17408
2344 17398 2340 17386 2339 17380 2338 17373 2338 17205 2297 17205
2297 17462 79 N F
2705 17462 2741 17462 2741 17424 2751 17436 2761 17446 2770 17454
2779 17460 2789 17465 2799 17468 2809 17469 2820 17470 2838 17468
2847 17465 2855 17462 2864 17457 2872 17450 2880 17441 2889 17429
2899 17440 2909 17449 2919 17457 2928 17462 2938 17466 2947 17468
2957 17470 2968 17470 2980 17469 2993 17466 3006 17461 3017 17454
3027 17445 3031 17439 3035 17433 3038 17427 3040 17420 3041 17412
3042 17404 3042 17205 3001 17205 3001 17388 3000 17398 2997 17406
2992 17414 2986 17421 2978 17426 2971 17431 2963 17433 2955 17434
2944 17433 2934 17429 2923 17424 2914 17416 2906 17407 2899 17397
2895 17385 2894 17373 2894 17205 2853 17205 2853 17388 2852 17398
2849 17406 2844 17414 2837 17421 2830 17426 2822 17431 2813 17433
2805 17434 2795 17433 2786 17430 2776 17424 2767 17417 2758 17408
2752 17398 2748 17386 2746 17380 2746 17373 2746 17205 2705 17205
2705 17462 79 N F
3210 17470 3222 17469 3233 17467 3244 17464 3255 17459 3265 17453
3275 17446 3284 17438 3292 17428 3300 17418 3307 17408 3313 17396
3318 17384 3322 17371 3325 17358 3326 17344 3327 17330 3326 17315
3325 17302 3322 17288 3318 17276 3313 17264 3307 17252 3300 17242
3292 17232 3284 17223 3275 17215 3265 17208 3255 17203 3244 17198
3233 17195 3222 17192 3210 17192 3198 17192 3186 17195 3175 17198
3165 17203 3154 17208 3145 17215 3136 17223 3127 17232 3120 17242
3113 17252 3107 17264 3102 17276 3098 17288 3095 17302 3093 17315
3093 17330 3136 17330 3137 17310 3141 17291 3147 17275 3156 17260
3161 17253 3166 17247 3172 17242 3179 17238 3186 17235 3193 17232
3201 17231 3210 17230 3218 17231 3226 17232 3234 17235 3241 17238
3247 17242 3253 17247 3259 17253 3264 17260 3272 17275 3279 17291
3282 17310 3284 17330 3282 17349 3279 17368 3272 17385 3264 17401
3259 17407 3253 17413 3247 17419 3241 17423 3234 17427 3226 17429
3218 17431 3210 17432 3201 17431 3193 17429 3186 17427 3179 17423
3172 17419 3166 17413 3161 17407 3156 17401 3147 17385 3141 17368
3137 17349 3136 17330 3093 17330 3093 17344 3095 17358 3098 17371

3102 17384 3107 17396 3113 17408 3120 17418 3127 17428 3136 17438
3145 17446 3154 17453 3165 17459 3175 17464 3186 17467 3198 17469
3210 17470 115 N F
3383 17562 3424 17562 3424 17205 3383 17205 3383 17562 5 N F
3786 17567 3805 17566 3822 17563 3839 17558 3855 17551 3871 17543
3885 17533 3898 17522 3909 17509 3920 17496 3929 17481 3937 17466
3944 17450 3950 17433 3953 17416 3956 17398 3957 17381 3956 17363
3954 17345 3950 17328 3945 17311 3939 17295 3931 17279 3921 17265
3910 17251 3898 17238 3885 17227 3871 17217 3856 17208 3840 17201
3823 17196 3805 17193 3786 17192 3767 17193 3749 17196 3732 17201
3716 17208 3700 17217 3686 17227 3673 17238 3661 17251 3650 17265
3640 17279 3632 17295 3625 17311 3620 17328 3616 17345 3613 17363
3612 17381 3658 17381 3659 17365 3661 17351 3664 17337 3668 17323
3674 17310 3680 17298 3687 17286 3695 17276 3704 17266 3714 17258
3725 17250 3736 17244 3748 17239 3760 17236 3773 17233 3786 17233
3799 17233 3812 17236 3824 17239 3835 17244 3846 17250 3857 17258
3866 17266 3875 17276 3883 17286 3890 17298 3896 17310 3901 17323
3905 17337 3908 17351 3910 17365 3911 17381 3910 17396 3908 17410
3905 17424 3901 17438 3896 17451 3890 17463 3883 17474 3875 17484
3866 17493 3857 17502 3846 17509 3835 17515 3824 17520 3812 17523
3799 17525 3786 17526 3773 17525 3760 17523 3748 17520 3736 17515
3725 17509 3714 17502 3704 17493 3695 17484 3687 17474 3680 17463
3674 17451 3668 17438 3664 17424 3661 17410 3659 17396 3658 17381
3612 17381 3613 17398 3616 17416 3620 17433 3625 17450 3632 17466
3640 17481 3650 17496 3661 17509 3673 17522 3686 17533 3700 17543
3716 17551 3732 17558 3749 17563 3767 17566 3786 17567 131 N F
4038 17439 3995 17439 3996 17453 3997 17465 4000 17477 4004 17488
4008 17498 4013 17507 4019 17516 4026 17523 4033 17530 4041 17536
4050 17540 4060 17544 4070 17548 4081 17550 4092 17551 4104 17552
4116 17551 4127 17550 4137 17548 4147 17545 4156 17542 4165 17538
4173 17533 4181 17527 4188 17521 4194 17514 4199 17506 4203 17498
4207 17489 4209 17479 4211 17468 4211 17457 4211 17444 4209 17433
4205 17422 4200 17413 4193 17404 4185 17396 4174 17389 4160 17383
4176 17377 4188 17370 4199 17361 4207 17352 4214 17341 4218 17329
4221 17316 4222 17302 4220 17282 4218 17273 4215 17263 4212 17254
4207 17245 4202 17237 4195 17229 4188 17221 4179 17215 4169 17209
4158 17204 4146 17200 4133 17197 4118 17195 4102 17194 4088 17195
4075 17196 4063 17199 4052 17202 4042 17207 4033 17212 4024 17218
4017 17225 4011 17233 4005 17241 4000 17250 3996 17260 3993 17270
3991 17281 3990 17292 3990 17304 4033 17304 4034 17291 4036 17279
4039 17267 4046 17256 4050 17251 4055 17246 4061 17242 4067 17239
4075 17236 4084 17234 4093 17233 4104 17233 4114 17233 4122 17233
4130 17235 4137 17237 4144 17240 4149 17243 4155 17247 4159 17252

4167 17262 4172 17274 4175 17287 4176 17302 4175 17311 4174 17320
4172 17327 4169 17334 4165 17340 4161 17345 4155 17350 4149 17354
4143 17357 4135 17359 4119 17363 4100 17365 4079 17365 4079 17401
4099 17401 4117 17403 4132 17406 4144 17411 4149 17414 4154 17417
4157 17422 4160 17427 4163 17433 4164 17439 4166 17455 4165 17467
4162 17478 4158 17488 4151 17496 4143 17504 4132 17509 4119 17512
4104 17513 4091 17513 4078 17510 4067 17505 4058 17498 4054 17494
4050 17489 4044 17476 4040 17459 4038 17439 148 N F
4359 17562 4387 17562 4270 17194 4242 17194 4357 17562 5 N F
4420 17462 4456 17462 4456 17424 4466 17436 4476 17446 4485 17454
4495 17460 4504 17465 4514 17468 4524 17469 4535 17470 4553 17468
4562 17465 4570 17462 4579 17457 4587 17450 4595 17441 4604 17429
4614 17440 4624 17449 4634 17457 4643 17462 4653 17466 4663 17468
4673 17470 4683 17470 4696 17469 4708 17466 4721 17461 4732 17454
4742 17445 4746 17439 4750 17433 4753 17427 4755 17420 4756 17412
4757 17404 4757 17205 4716 17205 4716 17388 4715 17398 4712 17406
4707 17414 4701 17421 4694 17426 4686 17431 4678 17433 4670 17434
4659 17433 4649 17429 4638 17424 4629 17416 4621 17407 4615 17397
4610 17385 4609 17373 4609 17205 4568 17205 4568 17388 4567 17398
4564 17406 4559 17414 4553 17421 4545 17426 4537 17431 4528 17433
4520 17434 4511 17433 4501 17430 4491 17424 4482 17417 4474 17408
4467 17398 4463 17386 4462 17380 4461 17373 4461 17205 4420 17205
4420 17462 79 N F
4815 17432 4859 17432 4860 17449 4863 17465 4869 17479 4876 17491
4881 17496 4886 17500 4892 17504 4899 17507 4906 17510 4913 17512
4921 17513 4930 17513 4943 17512 4956 17509 4967 17503 4977 17495
4985 17486 4991 17475 4995 17462 4996 17447 4995 17432 4992 17420
4985 17409 4977 17400 4966 17391 4952 17382 4917 17360 4888 17343
4875 17334 4864 17325 4854 17317 4845 17308 4837 17298 4830 17289
4824 17279 4820 17270 4816 17260 4813 17249 4809 17228 4808 17205
5040 17205 5040 17245 4856 17245 4861 17261 4867 17274 4875 17285
4886 17295 4898 17305 4914 17314 4953 17337 4976 17349 4995 17361
5010 17375 5023 17390 5032 17405 5038 17421 5041 17437 5042 17452
5042 17463 5040 17473 5037 17483 5033 17492 5029 17501 5023 17509
5016 17517 5009 17524 5001 17530 4992 17536 4983 17540 4973 17544
4963 17547 4952 17550 4941 17551 4930 17552 4918 17551 4906 17550
4895 17548 4884 17544 4874 17540 4865 17535 4856 17529 4848 17522
4841 17514 4834 17506 4829 17496 4824 17485 4820 17473 4818 17460
4816 17446 4815 17432 92 N F
2 O
2458 2789 3323 2789 3323 3744 2458 3744 2458 2789 5 N F
1 O
12 W

2458 2789 3323 2789 3323 3744 2458 3744 2458 2789 5 P
20 O
3323 2789 4188 2789 4188 3744 3323 3744 3323 2789 5 N F
1 O
3323 2789 4188 2789 4188 3744 3323 3744 3323 2789 5 P
38 O
4188 2789 5054 2789 5054 3744 4188 3744 4188 2789 5 N F
1 O
4188 2789 5054 2789 5054 3744 4188 3744 4188 2789 5 P
56 O
5054 2789 5919 2789 5919 3744 5054 3744 5054 2789 5 N F
1 O
5054 2789 5919 2789 5919 3744 5054 3744 5054 2789 5 P
74 O
5919 2789 6784 2789 6784 3744 5919 3744 5919 2789 5 N F
1 O
5919 2789 6784 2789 6784 3744 5919 3744 5919 2789 5 P
92 O
6784 2789 7650 2789 7650 3744 6784 3744 6784 2789 5 N F
1 O
6784 2789 7650 2789 7650 3744 6784 3744 6784 2789 5 P
111 O
7650 2789 8515 2789 8515 3744 7650 3744 7650 2789 5 N F
1 O
7650 2789 8515 2789 8515 3744 7650 3744 7650 2789 5 P
129 O
8515 2789 9380 2789 9380 3744 8515 3744 8515 2789 5 N F
1 O
8515 2789 9380 2789 9380 3744 8515 3744 8515 2789 5 P
147 O
9380 2789 10245 2789 10245 3744 9380 3744 9380 2789 5 N F
1 O
9380 2789 10245 2789 10245 3744 9380 3744 9380 2789 5 P
165 O
10245 2789 11111 2789 11111 3744 10245 3744 10245 2789 5 N F
1 O
10245 2789 11111 2789 11111 3744 10245 3744 10245 2789 5 P
183 O
11111 2789 11976 2789 11976 3744 11111 3744 11111 2789 5 N F
1 O
11111 2789 11976 2789 11976 3744 11111 3744 11111 2789 5 P
201 O
11976 2789 12841 2789 12841 3744 11976 3744 11976 2789 5 N F

1 O
11976 2789 12841 2789 12841 3744 11976 3744 11976 2789 5 P
3323 2500 3339 2498 3354 2495 3367 2489 3379 2482 3388 2472
3392 2466 3396 2460 3398 2453 3400 2446 3401 2439 3402 2431
3401 2419 3398 2410 3395 2401 3390 2394 3385 2388 3379 2383
3367 2375 3377 2370 3385 2364 3393 2357 3399 2349 3404 2340
3408 2331 3410 2321 3411 2310 3410 2297 3405 2284 3398 2270
3388 2258 3382 2252 3376 2247 3368 2242 3360 2238 3352 2234
3342 2232 3332 2230 3321 2230 3311 2230 3301 2232 3291 2234
3283 2238 3275 2242 3268 2247 3262 2252 3256 2258 3247 2270
3240 2284 3237 2297 3235 2310 3270 2310 3271 2300 3274 2290
3279 2281 3286 2273 3294 2267 3303 2262 3313 2259 3323 2258
3334 2259 3344 2262 3353 2267 3361 2273 3367 2281 3372 2290
3376 2300 3377 2310 3376 2321 3372 2330 3367 2339 3361 2347
3353 2353 3344 2358 3334 2361 3323 2362 3313 2361 3303 2358
3294 2353 3286 2347 3279 2339 3274 2330 3271 2321 3270 2310
3235 2310 3236 2321 3238 2330 3242 2340 3247 2348 3254 2356
3261 2363 3270 2370 3279 2375 3267 2383 3262 2388 3256 2394
3252 2401 3248 2410 3246 2419 3245 2431 3277 2431 3278 2422
3281 2414 3285 2407 3291 2401 3298 2397 3306 2393 3314 2391
3323 2390 3332 2391 3341 2393 3349 2397 3355 2401 3361 2407
3365 2414 3368 2422 3369 2431 3368 2440 3365 2447 3361 2454
3355 2460 3349 2465 3341 2468 3332 2470 3323 2471 3314 2470
3306 2468 3298 2465 3291 2460 3285 2454 3281 2447 3278 2440
3277 2431 3245 2431 3245 2439 3246 2446 3248 2453 3251 2460
3254 2466 3258 2472 3268 2482 3279 2489 3292 2495 3307 2498
3323 2500 145 N F
4188 2500 4197 2499 4206 2498 4215 2496 4223 2493 4230 2489
4238 2484 4244 2478 4250 2471 4256 2463 4260 2454 4265 2443
4268 2432 4271 2419 4273 2405 4275 2390 4275 2373 4275 2353
4273 2334 4271 2317 4267 2302 4263 2289 4257 2277 4251 2267
4245 2258 4238 2251 4230 2245 4222 2240 4214 2236 4206 2233
4197 2231 4180 2230 4167 2231 4154 2234 4143 2239 4133 2246
4124 2256 4116 2268 4110 2282 4105 2299 4138 2299 4141 2289
4145 2281 4149 2274 4154 2268 4160 2264 4166 2261 4174 2259
4182 2258 4189 2259 4196 2260 4202 2263 4208 2266 4213 2270
4218 2275 4226 2287 4233 2302 4237 2318 4240 2336 4241 2356
4232 2347 4223 2340 4215 2335 4208 2332 4201 2329 4194 2328
4182 2327 4172 2328 4162 2330 4154 2332 4146 2335 4138 2339
4132 2344 4125 2349 4120 2355 4115 2361 4111 2367 4105 2382
4102 2397 4102 2413 4134 2413 4135 2403 4138 2393 4142 2384
4149 2375 4156 2367 4165 2361 4176 2357 4188 2356 4199 2357
4210 2361 4219 2367 4226 2375 4233 2384 4237 2393 4240 2403

4241 2413 4240 2424 4237 2434 4233 2443 4226 2452 4219 2460
4210 2466 4199 2470 4188 2471 4176 2470 4165 2466 4156 2460
4149 2452 4142 2443 4138 2434 4135 2424 4134 2413 4102 2413
4102 2422 4102 2430 4104 2438 4107 2446 4110 2454 4114 2461
4118 2468 4124 2474 4130 2479 4136 2484 4143 2489 4151 2493
4160 2496 4168 2498 4178 2499 4188 2500 130 N F
4946 2500 4967 2500 4967 2239 4934 2239 4934 2425 4877 2425
4877 2448 4900 2451 4910 2453 4920 2457 4928 2462 4936 2471
4941 2483 4946 2500 14 N F
5145 2500 5154 2499 5163 2497 5172 2493 5180 2488 5187 2482
5194 2474 5201 2466 5207 2457 5212 2447 5217 2437 5224 2414
5229 2390 5231 2366 5229 2341 5224 2317 5217 2294 5212 2283
5207 2273 5201 2264 5194 2256 5187 2248 5180 2242 5172 2237
5163 2233 5154 2231 5145 2230 5135 2231 5126 2233 5117 2237
5109 2242 5102 2248 5095 2256 5088 2264 5082 2273 5077 2283
5072 2294 5065 2317 5060 2341 5059 2366 5093 2366 5094 2342
5097 2321 5103 2303 5109 2288 5117 2275 5126 2266 5131 2263
5135 2260 5140 2259 5145 2258 5149 2259 5154 2260 5159 2263
5163 2266 5172 2275 5180 2288 5186 2303 5192 2321 5195 2342
5196 2366 5195 2389 5192 2410 5186 2428 5180 2443 5172 2455
5163 2464 5154 2469 5149 2470 5145 2471 5140 2470 5135 2469
5126 2464 5117 2455 5109 2443 5103 2428 5097 2410 5094 2389
5093 2366 5059 2366 5060 2390 5065 2414 5072 2437 5077 2447
5082 2457 5088 2466 5095 2474 5102 2482 5109 2488 5117 2493
5126 2497 5135 2499 5145 2500 93 N F
5841 2495 5862 2495 5862 2234 5829 2234 5829 2420 5772 2420
5772 2443 5795 2446 5805 2448 5815 2452 5823 2458 5830 2466
5836 2478 5841 2495 14 N F
6045 2495 6066 2495 6066 2234 6034 2234 6034 2420 5976 2420
5976 2443 5999 2446 6010 2448 6019 2452 6028 2458 6035 2466
6041 2478 6045 2495 14 N F
6675 2495 6696 2495 6696 2234 6664 2234 6664 2420 6606 2420
6606 2443 6630 2446 6640 2448 6649 2452 6658 2458 6665 2466
6671 2478 6675 2495 14 N F
6792 2405 6824 2405 6825 2418 6828 2430 6832 2440 6838 2449
6845 2456 6854 2462 6865 2465 6878 2466 6888 2465 6897 2463
6905 2458 6913 2453 6919 2446 6924 2437 6927 2427 6928 2416
6927 2405 6924 2396 6919 2388 6913 2381 6905 2374 6895 2367
6868 2351 6846 2338 6828 2325 6814 2312 6803 2298 6795 2283
6790 2268 6787 2252 6786 2234 6960 2234 6960 2265 6822 2265
6826 2277 6831 2287 6837 2295 6844 2302 6854 2310 6865 2317
6895 2334 6912 2343 6927 2352 6938 2362 6947 2373 6954 2385
6959 2397 6961 2409 6962 2420 6962 2428 6960 2436 6958 2443

6955 2450 6952 2457 6947 2463 6937 2474 6925 2483 6910 2489
6895 2493 6878 2495 6860 2493 6844 2489 6836 2486 6829 2482
6822 2478 6816 2473 6811 2467 6806 2460 6802 2453 6798 2445
6796 2436 6794 2426 6792 2416 6792 2405 76 N F
7542 2499 7563 2499 7563 2238 7530 2238 7530 2424 7473 2424
7473 2447 7496 2450 7506 2452 7516 2456 7524 2461 7531 2470
7537 2482 7542 2499 14 N F
7689 2414 7656 2414 7658 2434 7663 2451 7666 2459 7670 2465
7674 2472 7679 2477 7685 2482 7691 2487 7698 2490 7705 2493
7721 2497 7738 2499 7755 2497 7770 2494 7784 2488 7796 2480
7805 2470 7809 2465 7813 2458 7815 2451 7817 2444 7818 2436
7819 2428 7818 2418 7817 2409 7814 2401 7810 2394 7805 2388
7799 2382 7790 2377 7780 2372 7792 2368 7801 2362 7809 2356
7816 2349 7820 2341 7824 2332 7826 2322 7826 2311 7825 2297
7822 2282 7816 2269 7811 2262 7806 2256 7801 2251 7794 2246
7787 2242 7779 2238 7770 2235 7760 2233 7749 2231 7736 2231
7726 2231 7716 2232 7707 2234 7699 2237 7692 2240 7685 2244
7678 2249 7673 2254 7668 2260 7664 2266 7657 2280 7654 2296
7652 2313 7685 2313 7685 2304 7687 2294 7690 2285 7694 2277
7701 2270 7711 2264 7723 2261 7738 2259 7752 2260 7763 2263
7772 2267 7779 2274 7785 2282 7789 2291 7791 2301 7792 2311
7791 2325 7789 2330 7787 2335 7784 2340 7781 2344 7772 2350
7762 2354 7749 2357 7735 2358 7719 2359 7719 2386 7735 2386
7748 2387 7759 2389 7768 2393 7775 2398 7780 2405 7783 2414
7784 2426 7784 2435 7782 2443 7778 2451 7774 2457 7767 2463
7759 2467 7750 2469 7738 2470 7728 2469 7719 2467 7711 2464
7703 2459 7698 2451 7693 2442 7690 2429 7689 2414 119 N F
8404 2495 8425 2495 8425 2234 8392 2234 8392 2420 8335 2420
8335 2443 8358 2446 8369 2448 8378 2452 8386 2458 8394 2466
8400 2478 8404 2495 14 N F
8631 2495 8654 2495 8654 2326 8694 2326 8694 2296 8654 2296
8654 2234 8622 2234 8622 2296 8513 2296 8513 2326 8542 2326
8622 2326 8622 2439 8542 2326 8513 2326 8513 2330 8631 2495
18 N F
9271 2500 9292 2500 9292 2239 9260 2239 9260 2425 9202 2425
9202 2448 9225 2451 9236 2453 9245 2457 9254 2462 9261 2471
9267 2483 9271 2500 14 N F
9409 2500 9544 2500 9544 2469 9435 2469 9426 2394 9435 2401
9446 2407 9460 2410 9476 2412 9489 2410 9503 2407 9517 2401
9523 2397 9530 2392 9535 2386 9541 2380 9546 2373 9550 2365
9553 2356 9556 2346 9557 2335 9558 2323 9556 2305 9552 2288
9549 2280 9545 2272 9540 2265 9535 2258 9529 2252 9522 2246
9515 2241 9507 2237 9498 2234 9489 2232 9479 2230 9468 2230

9449 2231 9440 2233 9432 2235 9425 2238 9418 2242 9407 2251
9398 2262 9391 2274 9386 2288 9382 2302 9414 2302 9418 2293
9422 2284 9427 2277 9433 2270 9440 2265 9448 2262 9456 2259
9466 2258 9479 2259 9491 2262 9501 2267 9509 2274 9516 2283
9521 2294 9524 2307 9525 2322 9524 2332 9522 2343 9518 2354
9512 2363 9504 2371 9494 2377 9482 2381 9468 2383 9461 2382
9454 2381 9448 2379 9442 2377 9431 2369 9418 2358 9390 2358
9409 2500 79 N F
10136 2500 10157 2500 10157 2239 10125 2239 10125 2425 10068 2425
10068 2448 10091 2451 10101 2453 10111 2457 10119 2462 10126 2471
10132 2483 10136 2500 14 N F
10417 2433 10385 2433 10383 2441 10379 2449 10375 2456 10370 2461
10363 2465 10357 2468 10350 2470 10343 2471 10336 2470 10329 2469
10323 2467 10317 2464 10312 2461 10307 2456 10303 2451 10299 2445
10292 2431 10287 2414 10284 2395 10283 2373 10291 2382 10299 2388
10307 2393 10314 2397 10322 2399 10330 2401 10347 2402 10359 2401
10373 2397 10386 2390 10398 2381 10403 2376 10408 2370 10413 2363
10416 2355 10419 2347 10422 2338 10423 2328 10423 2318 10391 2316
10389 2327 10386 2337 10381 2347 10375 2355 10367 2363 10357 2368
10348 2372 10337 2373 10326 2372 10317 2368 10307 2363 10300 2355
10293 2347 10288 2337 10285 2327 10283 2316 10285 2305 10288 2295
10293 2285 10300 2276 10307 2269 10317 2263 10326 2260 10337 2258
10348 2260 10357 2263 10367 2269 10375 2276 10381 2285 10386 2295
10389 2305 10391 2316 10423 2318 10422 2302 10418 2287 10411 2272
10407 2265 10402 2259 10397 2253 10390 2247 10383 2242 10375 2238
10367 2234 10358 2232 10348 2230 10337 2230 10325 2230 10315 2232
10305 2235 10296 2240 10288 2245 10281 2252 10274 2259 10269 2268
10264 2277 10260 2287 10256 2297 10254 2309 10250 2333 10249 2360
10249 2376 10251 2391 10253 2405 10255 2418 10259 2430 10263 2442
10268 2452 10274 2462 10280 2471 10287 2478 10295 2485 10303 2490
10312 2494 10322 2497 10332 2499 10343 2500 10358 2498 10372 2494
10384 2488 10394 2480 10403 2470 10410 2458 10415 2446 10417 2433
126 N F
11001 2495 11022 2495 11022 2234 10989 2234 10989 2420 10932 2420
10932 2443 10955 2446 10965 2448 10975 2452 10983 2458 10990 2466
10996 2478 11001 2495 14 N F
11115 2495 11289 2495 11289 2468 11265 2431 11245 2399 11229 2371
11216 2345 11206 2320 11198 2294 11191 2266 11184 2234 11150 2234
11159 2271 11169 2301 11179 2328 11190 2352 11202 2376 11217 2402
11257 2464 11115 2464 11115 2495 21 N F
11867 2500 11888 2500 11888 2239 11855 2239 11855 2425 11798 2425
11798 2448 11821 2451 11832 2453 11841 2457 11849 2462 11857 2471
11862 2483 11867 2500 14 N F

```
12066 2500 12082 2498 12096 2495 12110 2489 12121 2482 12131 2472
12135 2466 12138 2460 12141 2453 12142 2446 12144 2439 12144 2431
12143 2419 12141 2410 12137 2401 12133 2394 12127 2388 12121 2383
12110 2375 12119 2370 12128 2364 12135 2357 12142 2349 12147 2340
12150 2331 12153 2321 12154 2310 12152 2297 12148 2284 12141 2270
12131 2258 12125 2252 12118 2247 12111 2242 12103 2238 12094 2234
12085 2232 12074 2230 12064 2230 12053 2230 12043 2232 12034 2234
12025 2238 12018 2242 12010 2247 12004 2252 11998 2258 11989 2270
11983 2284 11979 2297 11978 2310 12012 2310 12013 2300 12016 2290
12021 2281 12028 2273 12036 2267 12045 2262 12055 2259 12066 2258
12076 2259 12086 2262 12095 2267 12103 2273 12110 2281 12115 2290
12118 2300 12119 2310 12118 2321 12115 2330 12110 2339 12103 2347
12095 2353 12086 2358 12076 2361 12066 2362 12055 2361 12045 2358
12036 2353 12028 2347 12021 2339 12016 2330 12013 2321 12012 2310
11978 2310 11978 2321 11981 2330 11985 2340 11990 2348 11996 2356
12003 2363 12012 2370 12022 2375 12010 2383 12004 2388 11999 2394
11994 2401 11990 2410 11988 2419 11987 2431 12020 2431 12021 2422
12023 2414 12028 2407 12033 2401 12040 2397 12048 2393 12057 2391
12066 2390 12075 2391 12083 2393 12091 2397 12098 2401 12104 2407
12108 2414 12111 2422 12111 2431 12111 2440 12108 2447 12104 2454
12098 2460 12091 2465 12083 2468 12075 2470 12066 2471 12057 2470
12048 2468 12040 2465 12033 2460 12028 2454 12023 2447 12021 2440
12020 2431 11987 2431 11988 2439 11989 2446 11991 2453 11993 2460
11997 2466 12000 2472 12010 2482 12022 2489 12035 2495 12050 2498
12066 2500 145 N F
Gr
S
%%Trailer
%%DocumentFonts:
%%Pages: 1
%%EOF
```

The temporal and spatial variations of simulated cumulative ozone uptake in T1 (March)

```
%%Pages: (atend)
%%PageOrder: Ascend
%%Creator: NCAR GKS
%%DocumentFonts: (atend)
%%EndComments
%%BeginProlog

/M3
{
  2 copy lt {pop} {exch pop} ifelse
```

```
  2 copy lt {pop} {exch pop} ifelse
} bind def
/Sc
{
  1. exch sub /ylo exch def
  1. exch sub /mag exch def
  1. exch sub /cyn exch def
  ylo mag cyn M3 /b_cmy exch def
  cyn b_cmy sub /c_cmy exch def
  mag b_cmy sub /m_cmy exch def
  ylo b_cmy sub /y_cmy exch def
  c_cmy m_cmy y_cmy b_cmy setcmykcolor
} bind def
/P
{/n exch def
  newpath
  moveto
  2 1 n {pop lineto} for
  stroke
 } bind def

/F
{/n exch def
  newpath
  moveto
  2 1 n {pop lineto} for
  closepath
  eofill
} bind def

/B
{ /s exch def
  /n exch def
  1 1 n {newpath pop s 0 360 arc stroke} for
} bind def

/Q
{ /s exch def
  /n exch def
  /ms 0 s sub def
  /ts s s add def
  1 1 n {gsave newpath pop translate
  0 0 moveto ms 0 rmoveto ts 0 rlineto
```

```
  0 0 moveto 90 rotate ms  0 rmoveto ts 0 rlineto
  stroke grestore} for
} bind def

/Fs
{ /s exch def
  findfont
  s scalefont
  setfont
} bind def

/V
{ /s exch def
  /n exch def
  /ms 0 s sub def
  /ts s s add def
  1 1 n {gsave newpath pop translate
  0 0 moveto 30 rotate ms 0 rmoveto  ts 0 rlineto
  0 0 moveto 60 rotate ms 0 rmoveto  ts 0 rlineto
  0 0 moveto 60 rotate ms 0 rmoveto  ts 0 rlineto
  stroke grestore} for
} bind def

/X
{ /s exch def
  /n exch def
  /s s 1.25 mul def
  /ms 0 s sub def
  /ts s s add def
  1 1 n {gsave newpath pop translate
  0 0 moveto 45 rotate ms 0 rmoveto  ts 0 rlineto
  0 0 moveto 90 rotate ms 0 rmoveto  ts 0 rlineto
  stroke grestore} for
} bind def

/Ls
{matrix currentmatrix
 currentrgbcolor
 currentlinewidth
 currentlinecap
 currentlinejoin
 currentdash
} bind def
```

```
/Lr
{setdash
 setlinejoin
 setlinecap
 setlinewidth
 setrgbcolor
 setmatrix
} bind def

/M {stroke newpath moveto} bind def
/L {lineto} bind def
/E {eofill} def
/J {setlinejoin} def
/C {setlinecap} def
/W {setlinewidth} def
/S {stroke showpage} def
/A {scale} def
/U {put} def
/D {setdash} def
/I {currentdash} def
/H {currentlinewidth} def
/G {currentrgbcolor} def
/R {setrgbcolor} def
/N {closepath} def
/K {stroke} def
/Z {rotate} def
/As {ashow} def
/Ch {clippath} def
/Ci {colorimage} def
/Co {concat} def
/Cm {currentmatrix} def
/Cp {clip} def
/Ct {currentpoint} def
/Cu {currentfile} def
/Ex {exch} def
/Im {image} def
/In {initgraphics} def
/Ml {setmiterlimit} def
/Mx {matrix} def
/Np {newpath} def
/Ng {neg} def
/Rh {readhexstring} def
```

```
/Rm {rmoveto} def
/Sm {setmatrix} def
/Tr {translate} def
/Gs {gsave} def
/Gr {grestore} def

/Ov
{/strn exch def
 /ispc exch def
 /str1 1 string def
  strn
  {/code exch def
   /chr str1 0 code put str1 def
   0 ispc neg rmoveto
   gsave
   chr stringwidth pop 2 div neg
   0 rmoveto
   chr false charpath stroke
   grestore
  } forall
} bind def

/Oh
{/strn exch def
 /ispc exch def
 /tstr 1 string def
 strn
   {
    /code exch def
    /chr tstr 0 code put tstr def
    chr false charpath
    currentpoint
    /yt exch def /xt exch def
    stroke
    xt ispc add yt moveto
   } forall
 } bind def

/T 256 array def
/O
{ T
  exch
  get
```

```
  aload
  pop
  setrgbcolor
} bind def

/Y
{/strn exch def
 /ispc exch def
 /str1 1 string def
  strn
  {/code exch def
   /chr str1 0 code put str1 def
    0 ispc neg rmoveto
    gsave
      chr stringwidth pop 2 div neg 0 rmoveto
      chr show
    grestore
  } forall
} def

/Bg
{/ury Ex def /urx Ex def /lly Ex def /llx Ex def
 Np Ls Gr Gs 0 O Ch E Lr
 llx lly M urx lly L urx ury L
 llx ury L llx lly L
 Cp Np
} def

/Oc
{/ury exch def
 /urx exch def
 /lly exch def
 /llx exch def
 Np
 llx lly M urx lly L urx ury L  llx ury L llx lly L
 Cp Np
} def

/Cc
{/CellWidth exch def
 /strn CellWidth 1 add string def
 /strn1 CellWidth 1 add 3 mul string def
 systemdict /colorimage known
```

```
 { { Cu strn1 Rh pop } false 3 Ci}
 { { Cu strn1 Rh pop
     /ColorStr exch def
     0 1 CellWidth {
       /YIndx exch def
       /GrayVal YIndx 3 mul def
       strn YIndx
         ColorStr GrayVal       get 0.30 mul
         ColorStr GrayVal 1 add get 0.59 mul
         ColorStr GrayVal 2 add get 0.11 mul
         add add cvi
       put
     } for
     strn
   } Im
 } ifelse
} def

/Ld
{/ury exch def /urx exch def  /lly exch def /llx exch def
 -90 rotate
 lly urx add neg llx lly sub translate
} bind def
/Pt
{/ury exch def /urx exch def  /lly exch def /llx exch def
 90 rotate
 lly urx add lly llx sub translate
} bind def
%%EndProlog

%%BeginSetup
<</PageSize [00612 00792]>> setpagedevice
%%EndSetup

[1. 1. 1.] [0. 0. 0.] [1. 0. 0.] [0. 1. 0.] [0. 0. 1.] [0. 1. 1.] [1. 0. .8]
[1. 1. 0.] [1. .5 0.] [.6 .8 0.] [0. 1. .6] [0. .5 1.] [.5 0. .8] [1. 0. .6]
[.3 .3 .3] [.7 .7 .7] [1. 1. .3] [.7 1. .5] [.5 1. .6] [.2 1. .7] [.3 .8 .8]
[.5 .7 .8] [.7 .5 .8] [1. .3 .9] [.7 .9 .5] [.4 .9 .5] [.2 .9 .7] [.2 .7 .9]
[.2 .5 1.] [.5 .3 1.] [.7 .2 1.] [.9 .1 1.] [.9 1. .2] [.7 1. .3] [.5 1. .3]
[.2 1. .5] [.2 .8 .6] [.2 .7 .7] [.2 .5 .8] [.2 .4 .9] [.4 .3 .9] [.7 .2 .9]
[.8 .2 .8] [.9 .3 .7] [.8 .4 .6] [.8 .6 .5] [.9 .7 .4] [.9 .7 .3] [1. .9 .9]
[.8 1. .1] [.6 1. .2] [.5 1. .2] [.2 .9 .5] [.2 .8 .5] [.2 .7 .7] [.2 .5 .7]
[.2 .4 .9] [.4 .3 .9] [.5 .2 .9] [.8 .2 .7] [1. .2 .7] [1. .3 .6] [1. .4 .5]
```

[1. .5 .4] [1. .8 .1] [.7 1. .0] [.6 1. .1] [.4 1. .2] [.1 1. .3] [.1 .8 .4]
[.2 .7 .5] [.1 .6 .7] [.1 .5 .7] [.1 .4 .8] [.2 .3 .9] [.2 .2 1.] [.3 .1 1.]
[.5 .1 .9] [.7 .0 .8] [.9 .0 .7] [.9 .7 .1] [.7 .9 .1] [.5 .9 .1] [.3 .9 .2]
[.1 .9 .3] [.2 .7 .4] [.1 .7 .5] [.1 .5 .6] [.1 .4 .7] [.2 .3 .7] [.2 .2 .8]
[.3 .2 .8] [.5 .1 .7] [.7 .1 .7] [.9 .2 .5] [.8 .2 .5] [.8 .7 .0] [.6 .8 .1]
[.4 .9 .1] [.3 .9 .1] [.1 .9 .2] [.2 .8 .2] [.2 .7 .3] [.2 .6 .5] [.1 .5 .5]
[.1 .4 .6] [.2 .3 .7] [.3 .2 .7] [.4 .2 .6] [.7 .2 .5] [.8 .2 .4] [1. .2 .3]
[.7 .7 .0] [.5 .7 .1] [.4 .8 .1] [.2 .9 .1] [.2 .9 .1] [.2 .8 .2] [.1 .7 .2]
[.1 .7 .3] [.1 .6 .4] [.1 .4 .5] [.1 .4 .5] [.2 .3 .5] [.4 .3 .4] [.5 .3 .4]
[.7 .3 .3] [.8 .3 .2] [.7 .6 .0] [.5 .7 .1] [.4 .7 .1] [.2 .7 .2] [.1 .7 .2]
[.1 .6 .3] [.1 .5 .4] [.1 .4 .4] [.1 .3 .5] [.1 .2 .7] [.1 .1 .7] [.4 .1 .5]
[.5 .1 .5] [.6 .1 .4] [.7 .1 .4] [.9 .1 .3] [.5 .5 .1] [.4 .6 .1] [.3 .6 .1]
[.2 .6 .2] [.1 .5 .2] [.1 .4 .3] [.1 .4 .4] [.2 .3 .4] [.2 .2 .4] [.2 .2 .5]
[.2 .1 .5] [.3 .1 .5] [.4 .1 .4] [.5 .1 .4] [.7 .1 .3] [.8 .1 .2] [.5 .4 .1]
[.4 .5 .1] [.3 .6 .0] [.2 .6 .1] [.1 .7 .1] [.1 .6 .1] [.1 .5 .2] [.1 .4 .2]
[.1 .4 .2] [.1 .3 .3] [.1 .2 .4] [.1 .2 .5] [.1 .1 .5] [.1 .0 .6] [.3 .0 .5]
[.5 .0 .4] [.7 .3 .0] [.7 .4 .0] [.5 .4 .1] [.3 .4 .2] [.2 .3 .2] [.2 .2 .3]
[.2 .2 .4] [.3 .1 .4] [.5 .1 .3] [.6 .1 .2] [.7 .1 .2] [.8 .0 .2] [1. .0 .2]
[1. .1 .1] [.9 .2 .1] [.8 .2 .1] [.6 .2 .0] [.4 .4 .0] [.2 .4 .1] [.2 .3 .2]
[.2 .2 .2] [.2 .2 .3] [.2 .1 .3] [.3 .1 .3] [.5 .1 .2] [.7 .1 .2] [.8 .0 .2]
[.9 .0 .1] [1. .0 .1] [1. .1 .1] [.8 .2 .0] [.7 .2 .0] [.5 .2 .0] [.4 .3 .0]
[.1 .4 .1] [.1 .4 .1] [.1 .3 .2] [.1 .2 .2] [.1 .2 .3] [.2 .1 .4] [.2 .0 .3]
[.4 .0 .2] [.6 .0 .2] [.7 .0 .1] [.8 .0 .1] [.7 .1 .1] [.7 .1 .1] [.6 .2 .0]
[.4 .2 .0] [.2 .3 .0] [.1 .3 .0] [.1 .2 .1] [.1 .2 .1] [.1 .1 .2] [.1 .1 .3]
[.1 .0 .3] [.1 .0 .2] [.2 .0 .2] [.3 .0 .2] [.3 .0 .1] [.4 .0 .1] [.4 .1 .1]
[.4 .1 .0] [.2 .1 .0] [.0 .0 .0] [.0 .0 .0] [.0 .0 .0] [.0 .0 .0] [.0 .0 .0]
[.0 .0 .0] [.0 .0 .0] [.0 .0 .0] [.0 .0 .0] [.0 .0 .0] [.0 .0 .0] [.0 .0 .0]
[.0 .0 .0] [.0 .0 .0] [.0 .0 .0] [.0 .0 .0] T astore pop
%%Page: 1 1
0.04000 0.04000 A 1 J 1 C 12 W [] 0 D
T   0 [1.000 1.000 1.000] U
T   1 [0.000 0.000 0.000] U
T   2 [0.000 0.000 1.000] U
T   3 [0.000 0.031 1.000] U
T   4 [0.000 0.059 1.000] U
T   5 [0.000 0.090 1.000] U
T   6 [0.000 0.122 1.000] U
T   7 [0.000 0.153 1.000] U
T   8 [0.000 0.180 1.000] U
T   9 [0.000 0.212 1.000] U
T 10 [0.000 0.243 1.000] U
T 11 [0.000 0.275 1.000] U
T 12 [0.000 0.302 1.000] U

T 13 [0.000 0.333 1.000] U
T 14 [0.000 0.365 1.000] U
T 15 [0.000 0.392 1.000] U
T 16 [0.000 0.424 1.000] U
T 17 [0.000 0.455 1.000] U
T 18 [0.000 0.486 1.000] U
T 19 [0.000 0.514 1.000] U
T 20 [0.000 0.545 1.000] U
T 21 [0.000 0.576 1.000] U
T 22 [0.000 0.608 1.000] U
T 23 [0.000 0.635 1.000] U
T 24 [0.000 0.667 1.000] U
T 25 [0.000 0.698 1.000] U
T 26 [0.000 0.725 1.000] U
T 27 [0.000 0.757 1.000] U
T 28 [0.000 0.788 1.000] U
T 29 [0.000 0.820 1.000] U
T 30 [0.000 0.847 1.000] U
T 31 [0.000 0.878 1.000] U
T 32 [0.000 0.910 1.000] U
T 33 [0.000 0.941 1.000] U
T 34 [0.000 0.969 1.000] U
T 35 [0.000 1.000 1.000] U
T 36 [0.000 0.992 0.973] U
T 37 [0.000 0.980 0.941] U
T 38 [0.004 0.973 0.914] U
T 39 [0.004 0.965 0.886] U
T 40 [0.004 0.953 0.859] U
T 41 [0.004 0.945 0.827] U
T 42 [0.008 0.937 0.800] U
T 43 [0.008 0.929 0.773] U
T 44 [0.008 0.918 0.745] U
T 45 [0.008 0.910 0.714] U
T 46 [0.012 0.902 0.686] U
T 47 [0.012 0.890 0.659] U
T 48 [0.012 0.882 0.627] U
T 49 [0.012 0.875 0.600] U
T 50 [0.016 0.863 0.573] U
T 51 [0.016 0.855 0.545] U
T 52 [0.016 0.847 0.514] U
T 53 [0.016 0.839 0.486] U
T 54 [0.020 0.827 0.459] U
T 55 [0.020 0.820 0.431] U

T 56 [0.020 0.812 0.400] U
T 57 [0.020 0.800 0.373] U
T 58 [0.024 0.792 0.345] U
T 59 [0.024 0.784 0.314] U
T 60 [0.024 0.773 0.286] U
T 61 [0.024 0.765 0.259] U
T 62 [0.027 0.757 0.231] U
T 63 [0.027 0.749 0.200] U
T 64 [0.027 0.737 0.173] U
T 65 [0.027 0.729 0.145] U
T 66 [0.031 0.722 0.118] U
T 67 [0.031 0.710 0.086] U
T 68 [0.031 0.702 0.059] U
T 69 [0.059 0.710 0.059] U
T 70 [0.090 0.718 0.055] U
T 71 [0.118 0.729 0.055] U
T 72 [0.145 0.737 0.051] U
T 73 [0.173 0.745 0.051] U
T 74 [0.204 0.753 0.047] U
T 75 [0.231 0.765 0.047] U
T 76 [0.259 0.773 0.043] U
T 77 [0.286 0.780 0.043] U
T 78 [0.318 0.788 0.043] U
T 79 [0.345 0.800 0.039] U
T 80 [0.373 0.808 0.039] U
T 81 [0.400 0.816 0.035] U
T 82 [0.431 0.824 0.035] U
T 83 [0.459 0.835 0.031] U
T 84 [0.486 0.843 0.031] U
T 85 [0.518 0.851 0.031] U
T 86 [0.545 0.859 0.027] U
T 87 [0.573 0.867 0.027] U
T 88 [0.600 0.878 0.024] U
T 89 [0.631 0.886 0.024] U
T 90 [0.659 0.894 0.020] U
T 91 [0.686 0.902 0.020] U
T 92 [0.714 0.914 0.016] U
T 93 [0.745 0.922 0.016] U
T 94 [0.773 0.929 0.016] U
T 95 [0.800 0.937 0.012] U
T 96 [0.827 0.949 0.012] U
T 97 [0.859 0.957 0.008] U
T 98 [0.886 0.965 0.008] U

T 99 [0.914 0.973 0.004] U
T 100 [0.941 0.984 0.004] U
T 101 [0.973 0.992 0.000] U
T 102 [1.000 1.000 0.000] U
T 103 [1.000 0.988 0.000] U
T 104 [1.000 0.980 0.000] U
T 105 [1.000 0.969 0.000] U
T 106 [1.000 0.957 0.000] U
T 107 [1.000 0.945 0.000] U
T 108 [1.000 0.937 0.000] U
T 109 [1.000 0.925 0.000] U
T 110 [1.000 0.914 0.000] U
T 111 [1.000 0.902 0.000] U
T 112 [1.000 0.894 0.000] U
T 113 [1.000 0.882 0.000] U
T 114 [1.000 0.871 0.000] U
T 115 [1.000 0.863 0.000] U
T 116 [1.000 0.851 0.000] U
T 117 [1.000 0.839 0.000] U
T 118 [1.000 0.827 0.000] U
T 119 [1.000 0.820 0.000] U
T 120 [1.000 0.808 0.000] U
T 121 [1.000 0.796 0.000] U
T 122 [1.000 0.784 0.000] U
T 123 [1.000 0.776 0.000] U
T 124 [1.000 0.765 0.000] U
T 125 [1.000 0.753 0.000] U
T 126 [1.000 0.745 0.000] U
T 127 [1.000 0.733 0.000] U
T 128 [1.000 0.722 0.000] U
T 129 [1.000 0.710 0.000] U
T 130 [1.000 0.702 0.000] U
T 131 [1.000 0.690 0.000] U
T 132 [1.000 0.678 0.000] U
T 133 [1.000 0.667 0.000] U
T 134 [1.000 0.659 0.000] U
T 135 [1.000 0.647 0.000] U
T 136 [1.000 0.627 0.000] U
T 137 [1.000 0.608 0.000] U
T 138 [1.000 0.588 0.000] U
T 139 [1.000 0.569 0.000] U
T 140 [1.000 0.549 0.000] U
T 141 [1.000 0.529 0.000] U

T 142 [1.000 0.510 0.000] U
T 143 [1.000 0.490 0.000] U
T 144 [1.000 0.471 0.000] U
T 145 [1.000 0.451 0.000] U
T 146 [1.000 0.431 0.000] U
T 147 [1.000 0.412 0.000] U
T 148 [1.000 0.392 0.000] U
T 149 [1.000 0.373 0.000] U
T 150 [1.000 0.353 0.000] U
T 151 [1.000 0.333 0.000] U
T 152 [1.000 0.314 0.000] U
T 153 [1.000 0.294 0.000] U
T 154 [1.000 0.275 0.000] U
T 155 [1.000 0.255 0.000] U
T 156 [1.000 0.235 0.000] U
T 157 [1.000 0.216 0.000] U
T 158 [1.000 0.196 0.000] U
T 159 [1.000 0.176 0.000] U
T 160 [1.000 0.157 0.000] U
T 161 [1.000 0.137 0.000] U
T 162 [1.000 0.118 0.000] U
T 163 [1.000 0.098 0.000] U
T 164 [1.000 0.078 0.000] U
T 165 [1.000 0.059 0.000] U
T 166 [1.000 0.039 0.000] U
T 167 [1.000 0.020 0.000] U
T 168 [1.000 0.000 0.000] U
T 169 [0.980 0.000 0.016] U
T 170 [0.961 0.000 0.031] U
T 171 [0.941 0.000 0.047] U
T 172 [0.922 0.000 0.063] U
T 173 [0.902 0.000 0.082] U
T 174 [0.878 0.000 0.098] U
T 175 [0.859 0.000 0.114] U
T 176 [0.839 0.000 0.129] U
T 177 [0.820 0.000 0.145] U
T 178 [0.800 0.000 0.161] U
T 179 [0.780 0.000 0.176] U
T 180 [0.761 0.000 0.192] U
T 181 [0.741 0.000 0.212] U
T 182 [0.722 0.000 0.227] U
T 183 [0.702 0.000 0.243] U
T 184 [0.682 0.000 0.259] U

T 185 [0.659 0.000 0.275] U
T 186 [0.639 0.000 0.290] U
T 187 [0.620 0.000 0.306] U
T 188 [0.600 0.000 0.322] U
T 189 [0.580 0.000 0.341] U
T 190 [0.561 0.000 0.357] U
T 191 [0.541 0.000 0.373] U
T 192 [0.522 0.000 0.388] U
T 193 [0.502 0.000 0.404] U
T 194 [0.482 0.000 0.420] U
T 195 [0.463 0.000 0.435] U
T 196 [0.439 0.000 0.451] U
T 197 [0.420 0.000 0.471] U
T 198 [0.400 0.000 0.486] U
T 199 [0.380 0.000 0.502] U
T 200 [0.361 0.000 0.518] U
T 201 [0.341 0.000 0.533] U
-1150 1075 16450 18700 Oc
Gs
1 O
0 O
2303 16210 M 2303 5501 L 2336 5501 L 2369 5501 L 2402 5501 L
2435 5501 L 2468 5501 L 2501 5501 L 2534 5501 L
2568 5501 L 2601 5501 L 2634 5501 L 2667 5501 L
2700 5501 L 2733 5501 L 2766 5501 L 2799 5501 L
2832 5501 L 2865 5501 L 2899 5501 L 2930 5501 L
2929 5504 L 2919 5532 L 2941 5551 L 2970 5550 L
2988 5528 L 2970 5505 L 2970 5501 L 2998 5501 L
3031 5501 L 3064 5501 L 3097 5501 L 3130 5501 L
3163 5501 L 3197 5501 L 3230 5501 L 3263 5501 L
3296 5501 L 3329 5501 L 3362 5501 L 3395 5501 L
3428 5501 L 3461 5501 L 3495 5501 L 3528 5501 L
3561 5501 L 3594 5501 L 3627 5501 L 3660 5501 L
3693 5501 L 3726 5501 L 3759 5501 L 3792 5501 L
3826 5501 L 3859 5501 L 3892 5501 L 3925 5501 L
3958 5501 L 3991 5501 L 4024 5501 L 4057 5501 L
4090 5501 L 4124 5501 L 4157 5501 L 4190 5501 L
4223 5501 L 4256 5501 L 4289 5501 L 4322 5501 L
4355 5501 L 4388 5501 L 4422 5501 L 4455 5501 L
4488 5501 L 4521 5501 L 4554 5501 L 4587 5501 L
4620 5501 L 4653 5501 L 4686 5501 L 4719 5501 L
4753 5501 L 4786 5501 L 4819 5501 L 4852 5501 L
4885 5501 L 4918 5501 L 4951 5501 L 4984 5501 L

5017 5501 L 5051 5501 L 5084 5501 L 5117 5501 L
5150 5501 L 5183 5501 L 5216 5501 L 5249 5501 L
5282 5501 L 5315 5501 L 5349 5501 L 5382 5501 L
5396 5501 L 5409 5511 L 5407 5540 L 5404 5569 L
5405 5598 L 5405 5628 L 5395 5654 L 5371 5671 L
5353 5694 L 5382 5690 L 5388 5661 L 5417 5660 L
5417 5631 L 5412 5602 L 5411 5573 L 5413 5544 L
5432 5522 L 5431 5501 L 5448 5501 L 5481 5501 L
5514 5501 L 5533 5501 L 5540 5514 L 5541 5521 L
5545 5528 L 5552 5533 L 5538 5570 L 5530 5598 L
5548 5576 L 5557 5548 L 5574 5525 L 5593 5503 L
5595 5501 L 5613 5501 L 5645 5501 L 5646 5518 L
5650 5501 L 5658 5501 L 5666 5516 L 5676 5543 L
5690 5568 L 5684 5597 L 5660 5613 L 5668 5641 L
5682 5666 L 5683 5695 L 5667 5720 L 5691 5736 L
5675 5761 L 5655 5782 L 5677 5762 L 5704 5772 L
5687 5795 L 5671 5819 L 5651 5840 L 5675 5824 L
5703 5831 L 5689 5857 L 5665 5873 L 5655 5900 L
5628 5911 L 5599 5912 L 5628 5917 L 5656 5922 L
5662 5950 L 5664 5979 L 5646 6002 L 5618 6006 L
5601 6030 L 5629 6021 L 5655 6010 L 5681 5996 L
5678 5967 L 5685 5938 L 5706 5919 L 5724 5942 L
5712 5915 L 5707 5886 L 5714 5875 L 5722 5862 L
5740 5839 L 5746 5811 L 5773 5821 L 5791 5799 L
5798 5771 L 5800 5767 L 5812 5746 L 5824 5719 L
5843 5697 L 5860 5674 L 5854 5645 L 5872 5667 L
5899 5678 L 5927 5671 L 5956 5668 L 5929 5658 L
5903 5644 L 5881 5625 L 5861 5605 L 5881 5584 L
5910 5580 L 5939 5580 L 5968 5581 L 5986 5559 L
6014 5551 L 6044 5553 L 6070 5541 L 6051 5562 L
6059 5590 L 6086 5580 L 6114 5588 L 6119 5616 L
6091 5610 L 6074 5634 L 6052 5615 L 6024 5607 L
6009 5631 L 6037 5636 L 6066 5637 L 6088 5657 L
6062 5670 L 6036 5683 L 6058 5702 L 6086 5709 L
6114 5715 L 6142 5708 L 6160 5686 L 6169 5658 L
6173 5629 L 6201 5636 L 6229 5643 L 6235 5671 L
6208 5681 L 6182 5695 L 6209 5706 L 6210 5735 L
6181 5735 L 6203 5754 L 6216 5779 L 6243 5792 L
6247 5793 L 6257 5796 L 6270 5801 L 6296 5813 L
6325 5807 L 6346 5827 L 6338 5799 L 6315 5781 L
6318 5753 L 6315 5724 L 6325 5697 L 6354 5697 L
6383 5694 L 6403 5673 L 6400 5702 L 6416 5726 L
6431 5750 L 6409 5768 L 6393 5793 L 6418 5778 L

6430 5751 L 6459 5755 L 6473 5781 L 6487 5806 L
6516 5813 L 6545 5812 L 6569 5828 L 6599 5816 L
6578 5808 L 6576 5799 L 6595 5800 L 6615 5801 L
6641 5787 L 6666 5774 L 6637 5772 L 6649 5746 L
6677 5739 L 6706 5744 L 6731 5731 L 6758 5718 L
6754 5747 L 6768 5772 L 6747 5753 L 6730 5776 L
6748 5799 L 6765 5823 L 6786 5842 L 6815 5844 L
6839 5828 L 6867 5820 L 6882 5796 L 6900 5773 L
6921 5792 L 6949 5800 L 6977 5809 L 7003 5820 L
7027 5836 L 7030 5865 L 7046 5842 L 7031 5817 L
7060 5817 L 7082 5835 L 7102 5856 L 7125 5874 L
7150 5889 L 7173 5872 L 7201 5866 L 7229 5874 L
7258 5872 L 7267 5900 L 7288 5920 L 7284 5948 L
7278 5977 L 7291 6002 L 7296 6031 L 7271 6016 L
7268 6045 L 7297 6047 L 7320 6029 L 7341 6010 L
7358 6033 L 7379 6044 L 7361 6060 L 7343 6076 L
7315 6082 L 7292 6100 L 7279 6126 L 7274 6154 L
7245 6159 L 7274 6158 L 7294 6137 L 7317 6120 L
7343 6108 L 7349 6108 L 7372 6108 L 7401 6107 L
7423 6131 L 7434 6144 L 7455 6164 L 7454 6175 L
7455 6201 L 7469 6227 L 7461 6250 L 7487 6261 L
7507 6266 L 7515 6269 L 7527 6263 L 7541 6255 L
7532 6283 L 7559 6273 L 7564 6244 L 7572 6216 L
7590 6232 L 7594 6235 L 7617 6252 L 7641 6268 L
7641 6297 L 7659 6274 L 7651 6247 L 7669 6269 L
7671 6298 L 7676 6326 L 7687 6353 L 7711 6337 L
7723 6353 L 7732 6352 L 7736 6352 L 7759 6336 L
7776 6359 L 7786 6386 L 7785 6415 L 7757 6421 L
7733 6438 L 7762 6435 L 7790 6429 L 7818 6421 L
7840 6402 L 7848 6375 L 7840 6347 L 7838 6319 L
7855 6342 L 7857 6370 L 7859 6399 L 7871 6426 L
7894 6442 L 7891 6471 L 7909 6493 L 7936 6502 L
7946 6475 L 7929 6452 L 7925 6423 L 7937 6449 L
7954 6473 L 7977 6490 L 8001 6505 L 8005 6533 L
8024 6556 L 8013 6582 L 8033 6603 L 8054 6583 L
8077 6601 L 8098 6620 L 8126 6626 L 8120 6654 L
8106 6679 L 8077 6679 L 8074 6708 L 8045 6705 L
8017 6702 L 7999 6706 L 7974 6719 L 7952 6737 L
7940 6749 L 7942 6764 L 7944 6761 L 7953 6749 L
7968 6752 L 7980 6753 L 7996 6754 L 8023 6744 L
8034 6741 L 8051 6736 L 8074 6753 L 8079 6781 L
8053 6794 L 8082 6795 L 8092 6822 L 8109 6799 L
8110 6802 L 8123 6824 L 8125 6853 L 8154 6853 L

8164 6827 L 8157 6799 L 8184 6792 L 8185 6792 L
8197 6818 L 8225 6810 L 8225 6810 L 8253 6815 L
8273 6827 L 8278 6829 L 8272 6857 L 8295 6875 L
8285 6848 L 8310 6835 L 8336 6822 L 8360 6807 L
8359 6779 L 8384 6792 L 8405 6813 L 8393 6839 L
8395 6868 L 8423 6865 L 8437 6890 L 8440 6919 L
8425 6943 L 8402 6925 L 8382 6945 L 8378 6974 L
8354 6990 L 8380 6978 L 8408 6984 L 8401 7012 L
8395 7040 L 8415 7020 L 8416 6992 L 8429 7017 L
8441 6992 L 8436 6963 L 8464 6971 L 8482 6993 L
8510 6989 L 8537 6978 L 8561 6993 L 8534 7000 L
8529 7028 L 8558 7030 L 8586 7028 L 8573 7054 L
8574 7082 L 8561 7057 L 8534 7065 L 8509 7080 L
8501 7107 L 8528 7114 L 8545 7138 L 8561 7161 L
8541 7181 L 8521 7202 L 8548 7212 L 8576 7215 L
8604 7208 L 8587 7185 L 8613 7197 L 8611 7168 L
8601 7142 L 8629 7138 L 8654 7126 L 8655 7126 L
8641 7151 L 8632 7178 L 8647 7202 L 8649 7173 L
8672 7157 L 8701 7159 L 8729 7163 L 8735 7191 L
8753 7196 L 8763 7198 L 8743 7219 L 8726 7242 L
8703 7225 L 8676 7216 L 8686 7242 L 8662 7258 L
8639 7275 L 8618 7294 L 8646 7302 L 8668 7319 L
8693 7334 L 8713 7354 L 8729 7378 L 8740 7404 L
8746 7400 L 8764 7389 L 8754 7362 L 8779 7349 L
8807 7346 L 8812 7318 L 8818 7309 L 8829 7295 L
8827 7266 L 8841 7291 L 8851 7317 L 8879 7309 L
8879 7280 L 8880 7252 L 8889 7259 L 8901 7270 L
8902 7271 L 8907 7299 L 8900 7327 L 8873 7336 L
8867 7364 L 8853 7389 L 8828 7377 L 8820 7404 L
8806 7429 L 8823 7452 L 8850 7463 L 8878 7468 L
8882 7476 L 8891 7493 L 8883 7520 L 8881 7549 L
8890 7576 L 8908 7577 L 8918 7578 L 8927 7605 L
8934 7632 L 8906 7635 L 8880 7648 L 8853 7656 L
8833 7677 L 8849 7701 L 8869 7720 L 8870 7720 L
8886 7743 L 8897 7769 L 8869 7765 L 8893 7781 L
8920 7787 L 8937 7791 L 8948 7795 L 8972 7810 L
8975 7810 L 9000 7815 L 9028 7819 L 9035 7828 L
9045 7842 L 9019 7831 L 8995 7846 L 8978 7868 L
8961 7846 L 8952 7819 L 8926 7806 L 8904 7824 L
8898 7852 L 8885 7877 L 8858 7869 L 8872 7894 L
8894 7911 L 8916 7892 L 8943 7886 L 8966 7868 L
8981 7892 L 8970 7918 L 8966 7946 L 8946 7967 L
8919 7972 L 8890 7972 L 8864 7962 L 8842 7980 L

8840 8008 L 8867 8018 L 8843 8034 L 8848 8062 L
8845 8090 L 8871 8079 L 8893 8061 L 8918 8048 L
8908 8075 L 8901 8102 L 8899 8130 L 8909 8104 L
8926 8081 L 8941 8068 L 8947 8062 L 8952 8090 L
8978 8102 L 8962 8079 L 8989 8071 L 8964 8058 L
8962 8030 L 8967 8029 L 8989 8023 L 9011 8005 L
9017 8033 L 9033 8056 L 9042 8083 L 9070 8080 L
9094 8064 L 9081 8039 L 9056 8026 L 9058 7998 L
9037 7979 L 9008 7978 L 8993 7954 L 8985 7927 L
9013 7926 L 9034 7945 L 9043 7972 L 9071 7974 L
9074 7976 L 9095 7989 L 9099 7991 L 9121 8000 L
9120 8029 L 9141 8048 L 9115 8060 L 9126 8086 L
9102 8100 L 9077 8115 L 9090 8140 L 9117 8135 L
9124 8162 L 9146 8161 L 9153 8160 L 9180 8155 L
9178 8183 L 9151 8190 L 9179 8190 L 9198 8211 L
9203 8239 L 9194 8266 L 9222 8262 L 9237 8266 L
9249 8270 L 9264 8293 L 9250 8318 L 9245 8346 L
9255 8319 L 9279 8305 L 9290 8331 L 9306 8308 L
9312 8318 L 9321 8332 L 9322 8360 L 9325 8365 L
9338 8383 L 9325 8408 L 9331 8435 L 9356 8423 L
9384 8427 L 9383 8455 L 9397 8480 L 9371 8490 L
9358 8515 L 9339 8537 L 9367 8544 L 9386 8564 L
9386 8593 L 9389 8620 L 9417 8624 L 9429 8649 L
9444 8673 L 9462 8694 L 9478 8718 L 9476 8746 L
9451 8760 L 9423 8765 L 9404 8785 L 9376 8789 L
9348 8786 L 9346 8814 L 9368 8796 L 9396 8798 L
9416 8793 L 9423 8791 L 9428 8792 L 9445 8774 L
9473 8775 L 9495 8774 L 9501 8773 L 9528 8771 L
9527 8784 L 9541 8808 L 9539 8837 L 9552 8861 L
9566 8868 L 9577 8874 L 9592 8898 L 9602 8924 L
9580 8942 L 9603 8952 L 9606 8953 L 9615 8979 L
9641 8990 L 9668 8982 L 9651 8959 L 9660 8945 L
9666 8935 L 9674 8908 L 9692 8886 L 9712 8867 L
9733 8885 L 9727 8913 L 9748 8931 L 9761 8956 L
9776 8980 L 9804 8975 L 9804 8975 L 9827 8960 L
9845 8939 L 9857 8964 L 9844 8989 L 9835 9015 L
9821 9040 L 9823 9068 L 9807 9091 L 9794 9116 L
9786 9143 L 9770 9166 L 9743 9173 L 9716 9181 L
9744 9181 L 9772 9181 L 9800 9182 L 9824 9197 L
9836 9209 L 9843 9217 L 9864 9236 L 9855 9263 L
9842 9288 L 9815 9294 L 9800 9318 L 9826 9308 L
9854 9305 L 9873 9326 L 9852 9345 L 9863 9371 L
9837 9382 L 9810 9376 L 9784 9365 L 9805 9384 L

9833 9387 L 9830 9415 L 9803 9420 L 9776 9413 L
9748 9409 L 9749 9437 L 9773 9451 L 9781 9478 L
9785 9506 L 9809 9502 L 9813 9501 L 9831 9480 L
9832 9508 L 9843 9533 L 9834 9507 L 9836 9479 L
9845 9452 L 9872 9460 L 9893 9479 L 9900 9506 L
9898 9534 L 9905 9561 L 9926 9542 L 9917 9515 L
9920 9499 L 9922 9488 L 9923 9460 L 9951 9455 L
9974 9470 L 9979 9476 L 9992 9491 L 9987 9519 L
9962 9531 L 9965 9559 L 9962 9587 L 9988 9597 L
9989 9625 L 9973 9649 L 9976 9676 L 9993 9698 L
9974 9719 L 9947 9711 L 9920 9705 L 9896 9692 L
9895 9664 L 9911 9641 L 9887 9627 L 9880 9654 L
9852 9654 L 9833 9633 L 9806 9625 L 9783 9610 L
9765 9589 L 9773 9615 L 9746 9608 L 9731 9584 L
9723 9611 L 9734 9637 L 9752 9658 L 9779 9653 L
9806 9658 L 9834 9665 L 9855 9682 L 9873 9704 L
9890 9726 L 9913 9742 L 9931 9764 L 9953 9781 L
9967 9805 L 9989 9822 L 10002 9827 L 10008 9830 L
10015 9833 L 10038 9848 L 10045 9875 L 10021 9861 L
9993 9861 L 9965 9860 L 9961 9861 L 9954 9867 L
9943 9877 L 9916 9885 L 9890 9895 L 9863 9900 L
9835 9904 L 9811 9919 L 9797 9942 L 9781 9965 L
9762 9986 L 9745 10008 L 9726 10029 L 9707 10049 L
9684 10065 L 9660 10079 L 9633 10083 L 9605 10082 L
9577 10078 L 9553 10065 L 9530 10048 L 9511 10028 L
9489 10011 L 9466 9996 L 9444 9979 L 9418 9968 L
9390 9968 L 9364 9978 L 9345 9958 L 9370 9945 L
9343 9953 L 9334 9980 L 9340 10007 L 9323 10029 L
9313 10055 L 9325 10081 L 9301 10095 L 9273 10097 L
9245 10094 L 9223 10078 L 9212 10052 L 9196 10030 L
9168 10028 L 9146 10044 L 9127 10024 L 9104 10009 L
9123 10029 L 9140 10051 L 9168 10052 L 9194 10043 L
9193 10071 L 9201 10097 L 9226 10110 L 9253 10116 L
9281 10117 L 9308 10119 L 9336 10119 L 9363 10111 L
9382 10090 L 9410 10093 L 9435 10105 L 9451 10128 L
9467 10150 L 9468 10178 L 9483 10202 L 9503 10221 L
9527 10235 L 9554 10239 L 9570 10262 L 9592 10279 L
9615 10295 L 9642 10299 L 9666 10314 L 9681 10337 L
9701 10356 L 9726 10369 L 9752 10377 L 9779 10384 L
9806 10389 L 9834 10392 L 9862 10395 L 9889 10399 L
9912 10414 L 9919 10442 L 9909 10468 L 9895 10492 L
9882 10516 L 9869 10541 L 9856 10565 L 9843 10590 L
9828 10613 L 9810 10635 L 9788 10651 L 9765 10666 L

9740 10680 L 9713 10680 L 9697 10703 L 9675 10720 L
9650 10731 L 9626 10745 L 9608 10766 L 9594 10790 L
9576 10811 L 9554 10828 L 9530 10842 L 9509 10859 L
9483 10869 L 9457 10879 L 9429 10878 L 9402 10879 L
9377 10891 L 9358 10912 L 9380 10929 L 9367 10953 L
9354 10978 L 9339 11001 L 9311 11004 L 9284 11001 L
9256 11004 L 9230 11013 L 9203 11010 L 9191 10985 L
9164 10980 L 9137 10973 L 9113 10961 L 9086 10953 L
9058 10953 L 9044 10977 L 9071 10971 L 9099 10969 L
9125 10979 L 9146 10996 L 9169 11013 L 9190 11031 L
9214 11044 L 9239 11055 L 9265 11044 L 9292 11043 L
9319 11035 L 9345 11045 L 9360 11022 L 9383 11007 L
9400 10985 L 9419 10964 L 9429 10939 L 9435 10912 L
9461 10904 L 9489 10907 L 9516 10903 L 9531 10927 L
9558 10933 L 9583 10943 L 9609 10953 L 9611 10951 L
9634 10941 L 9661 10935 L 9688 10926 L 9705 10905 L
9731 10894 L 9756 10882 L 9782 10872 L 9809 10869 L
9835 10860 L 9862 10853 L 9888 10863 L 9894 10890 L
9883 10915 L 9869 10939 L 9857 10964 L 9847 10990 L
9828 11010 L 9810 11031 L 9788 11049 L 9764 11062 L
9738 11071 L 9712 11081 L 9685 11080 L 9659 11090 L
9648 11116 L 9628 11135 L 9642 11158 L 9637 11185 L
9637 11213 L 9624 11238 L 9602 11254 L 9576 11264 L
9551 11275 L 9526 11287 L 9501 11297 L 9475 11307 L
9447 11308 L 9428 11327 L 9410 11348 L 9382 11352 L
9398 11375 L 9416 11395 L 9403 11420 L 9377 11429 L
9398 11447 L 9406 11473 L 9405 11501 L 9394 11526 L
9388 11553 L 9378 11579 L 9351 11584 L 9336 11607 L
9331 11634 L 9315 11657 L 9296 11677 L 9285 11702 L
9275 11728 L 9256 11748 L 9250 11774 L 9252 11802 L
9261 11828 L 9239 11844 L 9222 11866 L 9215 11893 L
9219 11920 L 9208 11945 L 9200 11972 L 9174 11981 L
9180 12008 L 9154 12019 L 9154 12046 L 9145 12072 L
9144 12100 L 9135 12126 L 9125 12152 L 9117 12178 L
9109 12204 L 9096 12228 L 9091 12255 L 9078 12279 L
9053 12290 L 9028 12301 L 9001 12308 L 8982 12328 L
8957 12339 L 8931 12348 L 8904 12353 L 8878 12359 L
8865 12334 L 8864 12362 L 8846 12383 L 8820 12389 L
8798 12405 L 8774 12418 L 8750 12433 L 8729 12450 L
8715 12474 L 8711 12501 L 8684 12503 L 8659 12515 L
8635 12503 L 8613 12519 L 8594 12499 L 8601 12526 L
8587 12549 L 8586 12577 L 8595 12603 L 8595 12630 L
8599 12657 L 8613 12680 L 8634 12698 L 8661 12693 L

8672 12718 L 8675 12746 L 8683 12772 L 8691 12798 L
8704 12815 L 8708 12820 L 8732 12832 L 8751 12852 L
8755 12879 L 8769 12902 L 8778 12928 L 8786 12954 L
8780 12981 L 8807 12978 L 8830 12994 L 8839 12968 L
8852 12993 L 8873 13010 L 8889 12989 L 8901 13014 L
8915 13037 L 8926 13062 L 8949 13046 L 8957 13073 L
8954 13100 L 8965 13125 L 8984 13144 L 9009 13155 L
9016 13158 L 9033 13169 L 9025 13143 L 9039 13166 L
9061 13183 L 9073 13207 L 9046 13204 L 9019 13208 L
9035 13230 L 9009 13239 L 8985 13252 L 8977 13278 L
8987 13304 L 9012 13314 L 9038 13305 L 9059 13322 L
9063 13349 L 9082 13329 L 9097 13307 L 9092 13280 L
9082 13255 L 9071 13230 L 9097 13225 L 9123 13233 L
9144 13251 L 9171 13256 L 9198 13260 L 9222 13272 L
9244 13289 L 9239 13316 L 9235 13343 L 9219 13365 L
9225 13392 L 9251 13400 L 9242 13426 L 9257 13449 L
9282 13461 L 9303 13444 L 9327 13431 L 9328 13458 L
9346 13479 L 9345 13506 L 9320 13517 L 9304 13540 L
9278 13533 L 9295 13554 L 9318 13568 L 9344 13561 L
9358 13537 L 9385 13538 L 9385 13566 L 9410 13555 L
9429 13574 L 9452 13589 L 9478 13595 L 9504 13605 L
9529 13615 L 9552 13630 L 9569 13651 L 9565 13678 L
9578 13655 L 9598 13674 L 9625 13677 L 9604 13659 L
9589 13636 L 9615 13643 L 9642 13636 L 9641 13663 L
9667 13673 L 9690 13686 L 9703 13710 L 9728 13721 L
9753 13733 L 9777 13745 L 9766 13770 L 9793 13775 L
9807 13751 L 9816 13725 L 9836 13744 L 9859 13759 L
9858 13786 L 9875 13807 L 9883 13781 L 9866 13759 L
9882 13737 L 9907 13727 L 9880 13723 L 9884 13696 L
9911 13695 L 9929 13692 L 9938 13690 L 9960 13706 L
9968 13712 L 9982 13723 L 9995 13747 L 9997 13747 L
10007 13750 L 10022 13753 L 10011 13728 L 10024 13752 L
10028 13779 L 10027 13806 L 10000 13802 L 9973 13805 L
9993 13823 L 9994 13850 L 10009 13873 L 10033 13887 L
10055 13902 L 10039 13924 L 10031 13950 L 10036 13977 L
10054 13998 L 10065 14009 L 10073 14017 L 10046 14019 L
10022 14005 L 9998 13994 L 9980 14015 L 9954 14007 L
9927 14006 L 9900 14014 L 9877 14027 L 9853 14015 L
9839 14038 L 9848 14064 L 9830 14084 L 9809 14067 L
9786 14053 L 9766 14034 L 9768 14007 L 9753 14029 L
9726 14025 L 9699 14020 L 9683 13999 L 9663 14017 L
9638 14007 L 9611 14002 L 9584 14004 L 9560 14016 L
9541 14036 L 9524 14057 L 9506 14077 L 9531 14089 L

9504 14085 L 9479 14075 L 9452 14072 L 9425 14077 L
9405 14096 L 9401 14123 L 9385 14146 L 9358 14144 L
9341 14165 L 9317 14178 L 9305 14202 L 9278 14199 L
9251 14202 L 9224 14201 L 9203 14183 L 9178 14174 L
9154 14160 L 9127 14156 L 9101 14149 L 9082 14130 L
9056 14122 L 9030 14114 L 9054 14102 L 9050 14075 L
9033 14054 L 9014 14035 L 8995 14015 L 8973 13999 L
8951 13984 L 8925 13977 L 8899 13966 L 8878 13949 L
8866 13925 L 8882 13904 L 8875 13877 L 8856 13858 L
8835 13841 L 8816 13821 L 8790 13814 L 8762 13814 L
8735 13812 L 8710 13823 L 8683 13820 L 8656 13818 L
8629 13819 L 8602 13826 L 8575 13829 L 8555 13847 L
8532 13861 L 8505 13860 L 8502 13887 L 8485 13908 L
8467 13929 L 8472 13956 L 8469 13983 L 8467 14010 L
8470 14037 L 8476 14064 L 8494 14085 L 8510 14106 L
8522 14130 L 8505 14151 L 8480 14162 L 8492 14186 L
8498 14213 L 8490 14239 L 8482 14265 L 8463 14285 L
8459 14311 L 8432 14312 L 8414 14291 L 8424 14316 L
8418 14343 L 8422 14370 L 8407 14393 L 8402 14367 L
8396 14393 L 8372 14381 L 8351 14365 L 8326 14354 L
8301 14342 L 8279 14326 L 8258 14343 L 8232 14347 L
8206 14355 L 8179 14357 L 8152 14354 L 8125 14358 L
8098 14354 L 8074 14367 L 8052 14383 L 8033 14402 L
8010 14417 L 7984 14424 L 7964 14443 L 7945 14462 L
7928 14483 L 7915 14507 L 7903 14531 L 7890 14555 L
7880 14580 L 7870 14606 L 7867 14633 L 7867 14660 L
7872 14686 L 7881 14712 L 7893 14736 L 7906 14760 L
7924 14781 L 7939 14803 L 7964 14814 L 7958 14840 L
7948 14866 L 7967 14885 L 7986 14904 L 8008 14919 L
8034 14928 L 8061 14933 L 8087 14928 L 8111 14915 L
8126 14892 L 8132 14866 L 8151 14846 L 8174 14832 L
8201 14827 L 8222 14843 L 8242 14862 L 8264 14878 L
8284 14896 L 8301 14904 L 8309 14908 L 8326 14907 L
8336 14907 L 8357 14890 L 8370 14891 L 8384 14891 L
8405 14907 L 8406 14907 L 8422 14909 L 8433 14910 L
8458 14920 L 8475 14929 L 8482 14933 L 8503 14950 L
8521 14970 L 8536 14993 L 8553 15014 L 8575 15029 L
8600 15040 L 8582 15061 L 8578 15088 L 8582 15114 L
8574 15140 L 8597 15154 L 8605 15180 L 8612 15206 L
8625 15230 L 8644 15249 L 8666 15266 L 8691 15276 L
8689 15303 L 8710 15321 L 8735 15330 L 8761 15337 L
8787 15345 L 8809 15361 L 8835 15368 L 8851 15390 L
8874 15405 L 8899 15415 L 8924 15423 L 8950 15433 L

8973 15446 L 8998 15458 L 9018 15475 L 9044 15482 L
9070 15489 L 9086 15511 L 9085 15538 L 9098 15561 L
9122 15575 L 9099 15591 L 9121 15607 L 9126 15633 L
9150 15647 L 9163 15670 L 9173 15677 L 9185 15686 L
9203 15706 L 9210 15721 L 9215 15731 L 9227 15745 L
9233 15751 L 9259 15757 L 9261 15760 L 9277 15777 L
9255 15792 L 9266 15817 L 9285 15836 L 9294 15835 L
9310 15832 L 9312 15832 L 9322 15857 L 9341 15876 L
9357 15898 L 9384 15896 L 9406 15880 L 9429 15893 L
9453 15880 L 9480 15881 L 9493 15872 L 9502 15865 L
9522 15846 L 9548 15849 L 9575 15851 L 9597 15867 L
9607 15893 L 9608 15920 L 9628 15938 L 9638 15963 L
9634 15936 L 9621 15912 L 9632 15887 L 9624 15861 L
9637 15838 L 9664 15840 L 9681 15818 L 9704 15804 L
9726 15788 L 9741 15773 L 9753 15772 L 9753 15785 L
9758 15790 L 9767 15786 L 9783 15778 L 9802 15784 L
9800 15790 L 9777 15784 L 9771 15795 L 9765 15802 L
9765 15808 L 9753 15815 L 9730 15831 L 9730 15833 L
9755 15818 L 9776 15806 L 9777 15793 L 9800 15794 L
9805 15786 L 9801 15780 L 9783 15775 L 9759 15788 L
9757 15781 L 9762 15755 L 9753 15738 L 9759 15728 L
9782 15712 L 9802 15695 L 9815 15671 L 9803 15647 L
9785 15626 L 9783 15599 L 9765 15579 L 9750 15556 L
9747 15529 L 9727 15511 L 9713 15487 L 9699 15465 L
9682 15444 L 9666 15422 L 9643 15408 L 9669 15401 L
9649 15382 L 9631 15362 L 9607 15349 L 9580 15350 L
9563 15329 L 9539 15315 L 9517 15301 L 9505 15276 L
9494 15251 L 9516 15235 L 9533 15213 L 9535 15186 L
9533 15165 L 9528 15153 L 9516 15136 L 9493 15127 L
9506 15095 L 9516 15065 L 9540 15051 L 9567 15054 L
9594 15056 L 9618 15068 L 9645 15068 L 9671 15075 L
9698 15073 L 9675 15061 L 9649 15053 L 9626 15039 L
9611 15016 L 9588 15002 L 9562 14995 L 9580 14975 L
9563 14954 L 9586 14939 L 9607 14921 L 9593 14898 L
9567 14889 L 9542 14878 L 9520 14862 L 9493 14859 L
9474 14878 L 9464 14852 L 9450 14829 L 9425 14819 L
9397 14819 L 9371 14812 L 9364 14786 L 9390 14779 L
9368 14762 L 9381 14738 L 9380 14711 L 9401 14694 L
9419 14715 L 9430 14740 L 9457 14739 L 9482 14751 L
9509 14750 L 9534 14759 L 9553 14778 L 9580 14779 L
9607 14781 L 9620 14806 L 9596 14820 L 9570 14825 L
9594 14837 L 9606 14862 L 9627 14879 L 9652 14868 L
9664 14843 L 9671 14840 L 9688 14831 L 9689 14858 L

9678 14882 L 9703 14874 L 9695 14900 L 9715 14882 L
9736 14899 L 9762 14905 L 9754 14931 L 9781 14936 L
9805 14948 L 9781 14960 L 9791 14985 L 9810 15004 L
9833 15018 L 9844 15043 L 9855 15068 L 9878 15082 L
9897 15099 L 9898 15100 L 9913 15099 L 9925 15098 L
9945 15116 L 9948 15143 L 9955 15139 L 9972 15129 L
9990 15150 L 10014 15162 L 10033 15181 L 10041 15186 L
10055 15197 L 10055 15197 L 10069 15203 L 10076 15206 L
10080 15208 L 10086 15210 L 10095 15214 L 10105 15217 L
10130 15229 L 10142 15241 L 10149 15248 L 10169 15256 L
10174 15258 L 10199 15249 L 10225 15259 L 10234 15266 L
10246 15275 L 10221 15286 L 10245 15300 L 10253 15326 L
10255 15325 L 10271 15323 L 10280 15321 L 10283 15321 L
10306 15316 L 10307 15315 L 10320 15308 L 10330 15302 L
10338 15320 L 10341 15326 L 10355 15323 L 10368 15321 L
10381 15344 L 10392 15369 L 10400 15395 L 10401 15368 L
10424 15353 L 10440 15354 L 10451 15354 L 10471 15372 L
10496 15360 L 10523 15358 L 10550 15357 L 10576 15363 L
10598 15378 L 10625 15384 L 10637 15408 L 10642 15381 L
10649 15355 L 10654 15382 L 10659 15408 L 10665 15435 L
10670 15435 L 10692 15437 L 10686 15410 L 10707 15394 L
10731 15382 L 10758 15375 L 10784 15373 L 10794 15347 L
10803 15322 L 10822 15302 L 10811 15277 L 10824 15253 L
10838 15271 L 10841 15275 L 10853 15290 L 10857 15296 L
10854 15323 L 10865 15348 L 10886 15331 L 10912 15323 L
10927 15303 L 10928 15302 L 10952 15315 L 10969 15294 L
10996 15295 L 11013 15283 L 11019 15279 L 11042 15266 L
11068 15274 L 11087 15255 L 11114 15256 L 11137 15270 L
11158 15287 L 11170 15311 L 11175 15323 L 11187 15320 L
11195 15323 L 11200 15329 L 11201 15328 L 11196 15320 L
11187 15317 L 11175 15322 L 11171 15309 L 11161 15281 L
11182 15282 L 11194 15292 L 11223 15309 L 11224 15307 L
11219 15303 L 11195 15291 L 11185 15277 L 11172 15276 L
11148 15263 L 11144 15236 L 11125 15217 L 11126 15190 L
11139 15166 L 11165 15173 L 11151 15149 L 11155 15122 L
11149 15095 L 11142 15069 L 11119 15055 L 11114 15028 L
11123 15002 L 11102 14984 L 11089 14961 L 11080 14935 L
11071 14910 L 11060 14885 L 11068 14859 L 11083 14836 L
11108 14827 L 11101 14853 L 11116 14831 L 11119 14804 L
11145 14812 L 11172 14809 L 11188 14831 L 11212 14819 L
11239 14819 L 11255 14796 L 11228 14792 L 11206 14807 L
11182 14795 L 11155 14791 L 11133 14775 L 11106 14769 L
11083 14755 L 11066 14777 L 11073 14750 L 11069 14724 L

11045 14711 L 11018 14710 L 11029 14685 L 11019 14660 L
10999 14642 L 10989 14616 L 10975 14593 L 10981 14567 L
10982 14539 L 11001 14521 L 11022 14532 L 11032 14527 L
11003 14508 L 11000 14511 L 11002 14519 L 10980 14532 L
10961 14506 L 10941 14490 L 10922 14471 L 10896 14463 L
10922 14456 L 10926 14455 L 10949 14452 L 10958 14455 L
10971 14458 L 10975 14459 L 11000 14469 L 11024 14456 L
11050 14448 L 11077 14452 L 11102 14462 L 11129 14465 L
11154 14455 L 11152 14428 L 11131 14444 L 11104 14440 L
11097 14413 L 11121 14401 L 11096 14391 L 11069 14395 L
11046 14381 L 11061 14359 L 11077 14352 L 11086 14348 L
11112 14341 L 11138 14349 L 11139 14349 L 11158 14363 L
11160 14365 L 11184 14351 L 11179 14324 L 11205 14325 L
11207 14325 L 11215 14350 L 11242 14355 L 11219 14370 L
11246 14371 L 11273 14369 L 11251 14353 L 11266 14331 L
11239 14329 L 11237 14302 L 11210 14295 L 11199 14270 L
11215 14249 L 11227 14273 L 11253 14278 L 11270 14299 L
11284 14308 L 11293 14314 L 11296 14313 L 11320 14311 L
11328 14337 L 11324 14364 L 11321 14391 L 11341 14391 L
11347 14390 L 11348 14390 L 11355 14417 L 11327 14416 L
11302 14427 L 11298 14454 L 11323 14443 L 11350 14444 L
11367 14440 L 11374 14438 L 11377 14438 L 11388 14416 L
11389 14413 L 11398 14407 L 11403 14403 L 11410 14398 L
11411 14398 L 11420 14388 L 11430 14378 L 11447 14357 L
11454 14357 L 11474 14355 L 11476 14383 L 11502 14376 L
11503 14376 L 11507 14350 L 11513 14343 L 11518 14339 L
11526 14330 L 11547 14315 L 11548 14314 L 11555 14340 L
11553 14368 L 11558 14369 L 11579 14376 L 11596 14379 L
11606 14380 L 11627 14397 L 11654 14400 L 11665 14392 L
11677 14384 L 11700 14370 L 11724 14358 L 11751 14363 L
11768 14384 L 11794 14398 L 11776 14374 L 11781 14348 L
11783 14321 L 11795 14296 L 11820 14285 L 11842 14269 L
11865 14255 L 11885 14243 L 11885 14242 L 11863 14251 L
11841 14267 L 11816 14279 L 11792 14291 L 11768 14304 L
11776 14331 L 11763 14355 L 11739 14341 L 11714 14329 L
11719 14303 L 11726 14276 L 11746 14258 L 11772 14250 L
11782 14225 L 11785 14198 L 11760 14186 L 11761 14185 L
11779 14166 L 11800 14149 L 11826 14140 L 11810 14118 L
11834 14106 L 11859 14094 L 11837 14077 L 11810 14073 L
11817 14046 L 11804 14023 L 11821 14001 L 11839 14021 L
11861 14038 L 11866 14011 L 11854 13986 L 11848 13960 L
11874 13954 L 11893 13934 L 11911 13914 L 11938 13909 L
11957 13928 L 11948 13902 L 11926 13886 L 11906 13867 L

11907 13840 L 11892 13863 L 11887 13890 L 11871 13912 L
11845 13917 L 11824 13935 L 11802 13920 L 11777 13932 L
11753 13945 L 11764 13920 L 11739 13931 L 11758 13912 L
11769 13887 L 11748 13904 L 11744 13877 L 11738 13850 L
11737 13878 L 11730 13904 L 11721 13930 L 11695 13921 L
11674 13903 L 11697 13888 L 11715 13867 L 11699 13845 L
11675 13832 L 11691 13810 L 11669 13826 L 11670 13853 L
11664 13879 L 11658 13906 L 11658 13879 L 11634 13866 L
11627 13840 L 11628 13813 L 11602 13807 L 11592 13781 L
11598 13758 L 11598 13755 L 11608 13780 L 11610 13779 L
11619 13775 L 11628 13771 L 11633 13769 L 11632 13741 L
11637 13743 L 11640 13744 L 11650 13747 L 11654 13748 L
11658 13749 L 11676 13729 L 11676 13702 L 11681 13694 L
11686 13688 L 11692 13682 L 11693 13680 L 11695 13670 L
11699 13654 L 11703 13627 L 11718 13607 L 11719 13605 L
11740 13622 L 11730 13647 L 11718 13671 L 11714 13698 L
11689 13709 L 11694 13736 L 11680 13760 L 11700 13779 L
11708 13753 L 11713 13726 L 11731 13746 L 11747 13758 L
11753 13762 L 11759 13735 L 11756 13708 L 11755 13681 L
11779 13668 L 11766 13645 L 11770 13637 L 11776 13626 L
11779 13621 L 11805 13649 L 11804 13634 L 11781 13619 L
11782 13592 L 11785 13589 L 11789 13586 L 11804 13575 L
11791 13551 L 11805 13528 L 11807 13503 L 11807 13501 L
11829 13484 L 11807 13468 L 11824 13462 L 11828 13460 L
11833 13459 L 11844 13449 L 11854 13441 L 11867 13417 L
11886 13398 L 11910 13411 L 11935 13422 L 11957 13438 L
11963 13465 L 11962 13437 L 11941 13420 L 11916 13409 L
11895 13391 L 11871 13379 L 11856 13356 L 11864 13330 L
11875 13332 L 11891 13336 L 11918 13338 L 11945 13344 L
11926 13324 L 11907 13305 L 11922 13282 L 11897 13271 L
11892 13244 L 11865 13243 L 11847 13222 L 11826 13204 L
11808 13184 L 11821 13161 L 11849 13162 L 11876 13168 L
11903 13167 L 11904 13140 L 11877 13142 L 11851 13133 L
11825 13125 L 11814 13100 L 11809 13073 L 11796 13049 L
11823 13044 L 11814 13018 L 11792 13034 L 11786 13008 L
11781 12981 L 11771 12955 L 11798 12948 L 11794 12921 L
11816 12905 L 11843 12899 L 11833 12873 L 11815 12852 L
11797 12873 L 11778 12893 L 11780 12920 L 11768 12896 L
11741 12902 L 11750 12876 L 11762 12864 L 11769 12857 L
11767 12884 L 11787 12865 L 11809 12850 L 11797 12826 L
11771 12815 L 11774 12812 L 11792 12797 L 11808 12819 L
11835 12824 L 11820 12801 L 11830 12776 L 11824 12749 L
11833 12723 L 11857 12737 L 11885 12736 L 11885 12764 L

11876 12789 L 11877 12817 L 11879 12789 L 11903 12776 L
11907 12749 L 11934 12747 L 11909 12735 L 11882 12731 L
11875 12704 L 11848 12708 L 11827 12690 L 11854 12692 L
11882 12689 L 11903 12672 L 11913 12646 L 11885 12644 L
11859 12650 L 11853 12677 L 11825 12676 L 11824 12648 L
11850 12640 L 11871 12621 L 11843 12619 L 11818 12630 L
11804 12653 L 11802 12681 L 11778 12695 L 11770 12669 L
11770 12666 L 11772 12641 L 11785 12617 L 11812 12612 L
11833 12595 L 11845 12590 L 11859 12584 L 11880 12602 L
11907 12601 L 11887 12581 L 11869 12561 L 11877 12534 L
11901 12522 L 11884 12500 L 11907 12485 L 11909 12463 L
11909 12458 L 11931 12474 L 11931 12474 L 11949 12495 L
11953 12522 L 11955 12524 L 11975 12538 L 11979 12552 L
11982 12565 L 12006 12578 L 12006 12606 L 12008 12633 L
12019 12608 L 12021 12580 L 12035 12556 L 12043 12558 L
12062 12563 L 12089 12562 L 12096 12571 L 12105 12584 L
12094 12609 L 12103 12635 L 12107 12662 L 12130 12676 L
12137 12682 L 12151 12694 L 12176 12705 L 12194 12726 L
12220 12735 L 12245 12745 L 12249 12718 L 12231 12697 L
12211 12715 L 12212 12688 L 12213 12660 L 12189 12646 L
12185 12673 L 12174 12648 L 12165 12622 L 12191 12613 L
12206 12636 L 12214 12629 L 12226 12617 L 12242 12595 L
12247 12591 L 12263 12577 L 12274 12602 L 12275 12603 L
12285 12611 L 12296 12619 L 12298 12646 L 12271 12641 L
12274 12669 L 12301 12663 L 12316 12663 L 12328 12662 L
12329 12690 L 12307 12706 L 12284 12722 L 12267 12743 L
12259 12769 L 12282 12785 L 12257 12794 L 12284 12795 L
12310 12803 L 12322 12827 L 12335 12803 L 12343 12789 L
12349 12780 L 12361 12755 L 12351 12729 L 12363 12705 L
12387 12718 L 12386 12746 L 12405 12751 L 12412 12752 L
12439 12747 L 12434 12720 L 12435 12692 L 12444 12695 L
12461 12701 L 12460 12728 L 12451 12754 L 12436 12777 L
12439 12804 L 12434 12831 L 12407 12824 L 12386 12807 L
12362 12819 L 12348 12843 L 12355 12870 L 12372 12848 L
12396 12861 L 12412 12883 L 12439 12889 L 12465 12881 L
12485 12895 L 12488 12897 L 12511 12911 L 12536 12921 L
12544 12947 L 12546 12920 L 12548 12893 L 12548 12893 L
12573 12882 L 12599 12873 L 12626 12874 L 12628 12880 L
12635 12900 L 12643 12896 L 12660 12888 L 12661 12888 L
12685 12898 L 12682 12870 L 12709 12871 L 12716 12844 L
12689 12850 L 12697 12840 L 12705 12828 L 12724 12814 L
12727 12812 L 12739 12837 L 12741 12864 L 12722 12885 L
12723 12912 L 12726 12939 L 12745 12959 L 12727 12980 L

12740 13004 L 12764 13003 L 12768 13003 L 12794 12995 L
12786 13021 L 12780 13048 L 12804 13035 L 12826 13019 L
12830 13018 L 12853 13015 L 12880 13014 L 12892 13017 L
12906 13021 L 12906 13022 L 12908 13022 L 12922 13023 L
12934 13024 L 12949 13047 L 12948 13019 L 12956 12993 L
12977 13010 L 12996 13020 L 12996 13042 L 12995 13049 L
12996 13048 L 12996 14120 L 12996 14126 L 12977 14146 L
12964 14170 L 12958 14196 L 12948 14222 L 12931 14243 L
12912 14262 L 12897 14285 L 12875 14302 L 12861 14325 L
12841 14343 L 12835 14369 L 12815 14389 L 12795 14407 L
12790 14433 L 12769 14451 L 12749 14469 L 12728 14486 L
12709 14506 L 12689 14524 L 12672 14546 L 12653 14565 L
12637 14587 L 12619 14607 L 12603 14629 L 12584 14648 L
12572 14672 L 12560 14697 L 12544 14718 L 12531 14743 L
12516 14765 L 12500 14787 L 12491 14813 L 12473 14833 L
12460 14857 L 12446 14880 L 12435 14905 L 12430 14931 L
12407 14945 L 12381 14954 L 12355 14944 L 12344 14969 L
12325 14989 L 12303 15004 L 12276 15007 L 12253 15021 L
12232 15039 L 12210 15054 L 12194 15076 L 12176 15096 L
12160 15118 L 12144 15140 L 12121 15126 L 12096 15136 L
12070 15142 L 12042 15143 L 12015 15145 L 11992 15159 L
11978 15181 L 11981 15208 L 12004 15223 L 12010 15249 L
11984 15259 L 12010 15266 L 12037 15270 L 12051 15246 L
12042 15272 L 12031 15297 L 12023 15323 L 12026 15350 L
12039 15374 L 12032 15400 L 12006 15408 L 12000 15434 L
12007 15461 L 12020 15484 L 12038 15504 L 12065 15503 L
12079 15527 L 12105 15534 L 12126 15551 L 12146 15568 L
12144 15595 L 12166 15611 L 12180 15634 L 12202 15650 L
12228 15644 L 12255 15641 L 12280 15651 L 12296 15673 L
12321 15664 L 12331 15689 L 12357 15697 L 12375 15717 L
12399 15729 L 12417 15750 L 12442 15759 L 12446 15786 L
12423 15800 L 12431 15826 L 12455 15839 L 12479 15851 L
12504 15863 L 12525 15879 L 12542 15900 L 12558 15922 L
12581 15936 L 12606 15948 L 12618 15972 L 12623 15998 L
12630 16025 L 12635 16051 L 12653 16071 L 12679 16079 L
12695 16101 L 12716 16118 L 12740 16130 L 12764 16142 L
12782 16162 L 12809 16167 L 12833 16179 L 12839 16205 L
12838 16210 L 12831 16210 L 12798 16210 L 12765 16210 L
12731 16210 L 12698 16210 L 12665 16210 L 12632 16210 L
12599 16210 L 12566 16210 L 12533 16210 L 12500 16210 L
12467 16210 L 12434 16210 L 12400 16210 L 12367 16210 L
12334 16210 L 12301 16210 L 12268 16210 L 12235 16210 L
12202 16210 L 12169 16210 L 12136 16210 L 12102 16210 L

12069 16210 L 12036 16210 L 12003 16210 L 11970 16210 L
11937 16210 L 11904 16210 L 11871 16210 L 11838 16210 L
11804 16210 L 11771 16210 L 11738 16210 L 11705 16210 L
11672 16210 L 11639 16210 L 11606 16210 L 11573 16210 L
11540 16210 L 11507 16210 L 11473 16210 L 11440 16210 L
11407 16210 L 11374 16210 L 11341 16210 L 11308 16210 L
11275 16210 L 11242 16210 L 11209 16210 L 11175 16210 L
11142 16210 L 11109 16210 L 11076 16210 L 11043 16210 L
11010 16210 L 10977 16210 L 10944 16210 L 10911 16210 L
10877 16210 L 10844 16210 L 10811 16210 L 10778 16210 L
10745 16210 L 10712 16210 L 10679 16210 L 10646 16210 L
10613 16210 L 10580 16210 L 10546 16210 L 10513 16210 L
10480 16210 L 10447 16210 L 10414 16210 L 10381 16210 L
10348 16210 L 10315 16210 L 10282 16210 L 10248 16210 L
10215 16210 L 10182 16210 L 10149 16210 L 10116 16210 L
10083 16210 L 10050 16210 L 10017 16210 L 9984 16210 L
9950 16210 L 9917 16210 L 9884 16210 L 9851 16210 L
9818 16210 L 9785 16210 L 9752 16210 L 9719 16210 L
9686 16210 L 9653 16210 L 9619 16210 L 9586 16210 L
9553 16210 L 9520 16210 L 9487 16210 L 9454 16210 L
9421 16210 L 9388 16210 L 9355 16210 L 9321 16210 L
9288 16210 L 9255 16210 L 9222 16210 L 9189 16210 L
9156 16210 L 9123 16210 L 9090 16210 L 9057 16210 L
9023 16210 L 8990 16210 L 8957 16210 L 8924 16210 L
8891 16210 L 8858 16210 L 8825 16210 L 8792 16210 L
8759 16210 L 8725 16210 L 8692 16210 L 8659 16210 L
8626 16210 L 8593 16210 L 8560 16210 L 8527 16210 L
8494 16210 L 8461 16210 L 8428 16210 L 8394 16210 L
8361 16210 L 8328 16210 L 8295 16210 L 8262 16210 L
8229 16210 L 8196 16210 L 8163 16210 L 8130 16210 L
8096 16210 L 8063 16210 L 8030 16210 L 7997 16210 L
7964 16210 L 7931 16210 L 7898 16210 L 7865 16210 L
7832 16210 L 7798 16210 L 7765 16210 L 7732 16210 L
7699 16210 L 7666 16210 L 7633 16210 L 7600 16210 L
7567 16210 L 7534 16210 L 7501 16210 L 7467 16210 L
7434 16210 L 7401 16210 L 7368 16210 L 7335 16210 L
7302 16210 L 7269 16210 L 7236 16210 L 7203 16210 L
7169 16210 L 7136 16210 L 7103 16210 L 7070 16210 L
7037 16210 L 7004 16210 L 6971 16210 L 6938 16210 L
6905 16210 L 6871 16210 L 6838 16210 L 6805 16210 L
6772 16210 L 6739 16210 L 6706 16210 L 6673 16210 L
6640 16210 L 6607 16210 L 6574 16210 L 6540 16210 L
6507 16210 L 6474 16210 L 6441 16210 L 6408 16210 L

6375 16210 L 6342 16210 L 6309 16210 L 6276 16210 L
6242 16210 L 6209 16210 L 6176 16210 L 6143 16210 L
6110 16210 L 6077 16210 L 6044 16210 L 6011 16210 L
5978 16210 L 5944 16210 L 5911 16210 L 5878 16210 L
5845 16210 L 5812 16210 L 5779 16210 L 5746 16210 L
5713 16210 L 5680 16210 L 5646 16210 L 5613 16210 L
5580 16210 L 5547 16210 L 5514 16210 L 5481 16210 L
5448 16210 L 5415 16210 L 5382 16210 L 5349 16210 L
5315 16210 L 5282 16210 L 5249 16210 L 5216 16210 L
5183 16210 L 5150 16210 L 5117 16210 L 5084 16210 L
5051 16210 L 5017 16210 L 4984 16210 L 4951 16210 L
4918 16210 L 4885 16210 L 4852 16210 L 4819 16210 L
4786 16210 L 4753 16210 L 4719 16210 L 4686 16210 L
4653 16210 L 4620 16210 L 4587 16210 L 4554 16210 L
4521 16210 L 4488 16210 L 4455 16210 L 4422 16210 L
4388 16210 L 4355 16210 L 4322 16210 L 4289 16210 L
4256 16210 L 4223 16210 L 4190 16210 L 4157 16210 L
4124 16210 L 4090 16210 L 4057 16210 L 4024 16210 L
3991 16210 L 3958 16210 L 3925 16210 L 3892 16210 L
3859 16210 L 3826 16210 L 3792 16210 L 3759 16210 L
3726 16210 L 3693 16210 L 3660 16210 L 3627 16210 L
3594 16210 L 3561 16210 L 3528 16210 L 3495 16210 L
3461 16210 L 3428 16210 L 3395 16210 L 3362 16210 L
3329 16210 L 3296 16210 L 3263 16210 L 3230 16210 L
3197 16210 L 3163 16210 L 3130 16210 L 3097 16210 L
3064 16210 L 3031 16210 L 2998 16210 L 2965 16210 L
2932 16210 L 2899 16210 L 2865 16210 L 2832 16210 L
2799 16210 L 2766 16210 L 2733 16210 L 2700 16210 L
2667 16210 L 2634 16210 L 2601 16210 L 2568 16210 L
2534 16210 L 2501 16210 L 2468 16210 L 2435 16210 L
2402 16210 L 2369 16210 L 2336 16210 L 2303 16210 L
N E
5544 5522 5542 5515 5544 5511 5546 5507 5549 5503 5549 5501
5576 5501 5576 5504 5574 5508 5572 5512 5570 5516 5568 5520
5564 5523 5561 5526 5557 5529 5553 5532 5551 5528 5546 5526
5544 5522 19 N F
5703 5619 5699 5613 5701 5609 5705 5606 5709 5609 5713 5610
5718 5612 5718 5616 5722 5618 5725 5622 5725 5626 5727 5631
5722 5631 5718 5633 5714 5634 5712 5629 5708 5627 5705 5623
5703 5619 19 N F
5797 5618 5791 5617 5795 5615 5799 5612 5802 5609 5806 5607
5811 5607 5809 5612 5807 5616 5804 5619 5800 5621 5797 5618
12 N F

5818 5507 5817 5501 5845 5501 5866 5501 5872 5504 5880 5504
5886 5507 5891 5502 5891 5501 5911 5501 5904 5506 5905 5514
5909 5520 5903 5523 5910 5526 5916 5523 5918 5530 5917 5537
5917 5545 5922 5550 5925 5557 5931 5553 5937 5557 5938 5564
5934 5570 5928 5565 5922 5562 5915 5561 5909 5556 5904 5551
5898 5548 5894 5542 5887 5542 5880 5540 5873 5537 5867 5533
5863 5539 5856 5540 5848 5540 5844 5533 5839 5529 5833 5525
5826 5523 5828 5516 5824 5511 5818 5507 46 N F
5965 5504 5963 5501 5970 5501 5971 5505 5965 5504 5 N F
5978 5533 5968 5526 5973 5520 5976 5514 5982 5510 5989 5509
5993 5503 6000 5504 6007 5501 6007 5501 6011 5501 6025 5501
6025 5505 6029 5501 6040 5501 6039 5504 6043 5510 6039 5517
6035 5523 6032 5529 6025 5530 6019 5534 6011 5535 6006 5530
5999 5529 5992 5531 5985 5534 5978 5533 28 N F
6056 5509 6055 5505 6057 5503 6059 5502 6059 5501 6060 5501
6061 5501 6063 5502 6064 5504 6065 5506 6065 5508 6065 5510
6064 5512 6062 5512 6060 5510 6058 5510 6056 5509 17 N F
6244 5628 6241 5626 6242 5623 6243 5620 6245 5617 6248 5619
6251 5620 6255 5620 6258 5622 6260 5624 6260 5624 6257 5627
6254 5627 6251 5628 6247 5628 6244 5628 16 N F
6253 5608 6252 5605 6254 5606 6256 5605 6257 5606 6259 5608
6259 5610 6261 5612 6260 5614 6258 5613 6256 5612 6254 5610
6253 5608 13 N F
6257 5696 6253 5693 6254 5690 6258 5689 6260 5687 6261 5683
6264 5685 6263 5689 6260 5691 6257 5693 6257 5696 11 N F
7343 6097 7335 6089 7339 6083 7343 6077 7349 6074 7356 6070
7360 6064 7367 6062 7374 6064 7378 6058 7381 6052 7388 6049
7395 6047 7389 6043 7393 6037 7398 6033 7405 6032 7408 6025
7415 6025 7417 6032 7422 6037 7415 6040 7409 6037 7404 6043
7402 6050 7403 6057 7404 6064 7402 6071 7398 6077 7397 6084
7392 6089 7385 6092 7380 6087 7372 6087 7365 6089 7361 6094
7356 6100 7349 6100 7343 6097 39 N F
7490 6225 7483 6221 7488 6216 7496 6215 7503 6214 7508 6219
7506 6225 7506 6232 7511 6238 7514 6244 7507 6246 7501 6242
7500 6235 7497 6228 7490 6225 15 N F
7499 6211 7491 6210 7493 6203 7499 6199 7506 6198 7513 6196
7520 6194 7522 6201 7523 6208 7527 6214 7533 6217 7527 6226
7520 6224 7513 6225 7510 6218 7506 6212 7499 6211 17 N F
7498 6156 7493 6150 7497 6144 7504 6143 7511 6141 7519 6142
7526 6142 7533 6140 7538 6135 7545 6132 7552 6132 7559 6135
7566 6136 7573 6134 7575 6127 7581 6130 7586 6125 7593 6128
7597 6134 7595 6142 7594 6149 7600 6152 7598 6159 7592 6163
7589 6170 7590 6177 7583 6177 7579 6171 7576 6164 7569 6166

7565 6171 7559 6175 7552 6173 7548 6167 7545 6160 7539 6156
7535 6150 7526 6158 7523 6164 7516 6167 7509 6165 7503 6161
7498 6156 43 N F
7698 6241 7691 6229 7698 6229 7705 6231 7712 6233 7718 6231
7717 6224 7723 6228 7729 6231 7736 6233 7743 6234 7750 6236
7758 6236 7754 6242 7747 6243 7745 6250 7745 6257 7746 6264
7748 6271 7753 6277 7759 6279 7766 6277 7772 6274 7780 6274
7781 6281 7775 6286 7775 6293 7777 6300 7784 6304 7782 6311
7788 6315 7794 6311 7801 6312 7802 6319 7809 6321 7801 6321
7797 6326 7791 6330 7786 6335 7780 6330 7773 6332 7770 6326
7762 6327 7756 6323 7753 6330 7749 6336 7744 6341 7737 6344
7732 6349 7725 6346 7724 6339 7727 6332 7727 6325 7722 6319
7717 6314 7715 6307 7711 6301 7706 6296 7708 6289 7714 6285
7719 6291 7726 6293 7732 6289 7739 6289 7746 6287 7740 6283
7739 6276 7734 6270 7728 6266 7722 6262 7718 6256 7713 6250
7714 6243 7710 6249 7704 6245 7698 6241 76 N F
7944 6758 7942 6753 7943 6750 7945 6746 7948 6745 7950 6744
7951 6744 7952 6747 7950 6749 7947 6752 7946 6755 7944 6758
12 N F
7955 6742 7950 6742 7954 6740 7958 6737 7961 6734 7964 6730
7967 6727 7971 6724 7975 6723 7979 6723 7984 6724 7988 6723
7989 6723 7993 6725 7991 6729 7990 6733 7986 6735 7982 6733
7977 6732 7973 6734 7971 6738 7968 6741 7963 6741 7959 6739
7955 6742 25 N F
7957 6748 7953 6744 7957 6742 7961 6741 7965 6743 7970 6741
7973 6738 7975 6734 7980 6734 7984 6736 7988 6738 7992 6735
7994 6731 7998 6729 8002 6732 8007 6732 8006 6736 8003 6740
7999 6743 7996 6746 7993 6749 7988 6750 7984 6751 7980 6753
7975 6752 7970 6751 7966 6750 7962 6749 7957 6748 29 N F
7984 6721 7979 6720 7983 6718 7987 6716 7991 6714 7996 6714
7997 6718 7993 6720 7988 6722 7984 6721 10 N F
8030 6713 8022 6709 8026 6706 8031 6708 8035 6705 8039 6708
8043 6710 8039 6713 8034 6714 8030 6713 10 N F
8100 6744 8089 6739 8093 6733 8096 6727 8103 6723 8109 6720
8116 6718 8123 6717 8129 6721 8134 6726 8140 6730 8145 6736
8146 6743 8149 6749 8156 6751 8156 6758 8156 6765 8156 6772
8152 6777 8145 6774 8141 6780 8136 6785 8129 6783 8124 6787
8117 6790 8110 6792 8110 6784 8105 6780 8104 6773 8100 6767
8095 6762 8091 6756 8093 6749 8100 6750 8105 6755 8112 6758
8113 6751 8107 6747 8100 6744 39 N F
8168 6713 8164 6711 8167 6709 8171 6709 8174 6708 8176 6711
8179 6712 8182 6711 8184 6713 8185 6716 8187 6718 8191 6717
8193 6720 8193 6723 8194 6727 8193 6730 8190 6732 8187 6734

8184 6735 8181 6733 8177 6733 8174 6732 8174 6728 8173 6725
8173 6722 8169 6720 8168 6717 8168 6713 28 N F
8211 6716 8200 6712 8205 6708 8209 6702 8216 6699 8223 6696
8229 6700 8231 6707 8235 6712 8241 6717 8247 6720 8254 6723
8261 6724 8268 6722 8274 6718 8280 6715 8286 6711 8292 6714
8297 6720 8297 6727 8302 6732 8298 6738 8297 6746 8294 6752
8292 6759 8289 6766 8282 6767 8278 6773 8273 6778 8269 6773
8263 6769 8265 6762 8260 6757 8254 6753 8253 6746 8249 6740
8242 6741 8235 6744 8229 6747 8224 6752 8219 6757 8212 6753
8207 6749 8208 6742 8215 6745 8212 6738 8214 6732 8216 6725
8218 6718 8211 6716 50 N F
8212 6799 8209 6796 8212 6795 8215 6794 8218 6792 8221 6789
8223 6787 8227 6787 8230 6787 8231 6791 8231 6794 8234 6797
8232 6800 8229 6801 8225 6802 8222 6801 8219 6801 8215 6800
8212 6799 19 N F
8275 14779 8272 14774 8276 14774 8279 14777 8282 14780 8285 14784
8287 14787 8290 14791 8292 14794 8294 14798 8295 14803 8293 14807
8291 14803 8289 14799 8285 14797 8282 14794 8282 14789 8281 14785
8278 14781 8275 14779 20 N F
8292 14820 8291 14817 8293 14817 8294 14816 8297 14818 8299 14821
8301 14823 8301 14827 8298 14825 8295 14823 8292 14820 11 N F
8313 14837 8310 14835 8313 14833 8315 14835 8318 14837 8320 14839
8320 14842 8322 14845 8325 14847 8326 14850 8323 14849 8321 14847
8318 14844 8318 14841 8315 14840 8313 14837 16 N F
8361 14829 8356 14826 8360 14827 8364 14829 8368 14830 8371 14834
8375 14836 8378 14839 8379 14844 8376 14847 8373 14850 8370 14853
8368 14849 8370 14845 8374 14844 8374 14839 8371 14837 8368 14834
8365 14831 8361 14829 20 N F
8396 14878 8392 14874 8396 14874 8400 14876 8405 14877 8409 14878
8413 14879 8415 14882 8417 14886 8419 14890 8423 14892 8424 14896
8422 14900 8417 14899 8415 14895 8412 14892 8410 14888 8408 14885
8404 14882 8400 14880 8396 14878 21 N F
8398 14892 8396 14889 8399 14887 8402 14888 8404 14890 8405 14893
8406 14896 8407 14900 8405 14903 8404 14900 8402 14897 8400 14894
8398 14892 13 N F
8430 14891 8426 14890 8430 14887 8434 14889 8438 14890 8442 14892
8446 14893 8449 14896 8452 14899 8455 14902 8458 14906 8460 14909
8464 14912 8467 14915 8470 14918 8473 14921 8475 14926 8471 14925
8467 14922 8463 14921 8459 14919 8455 14917 8455 14913 8452 14910
8448 14908 8444 14905 8441 14902 8437 14901 8437 14896 8434 14893
8430 14891 31 N F
8627 7082 8622 7080 8626 7079 8631 7078 8633 7074 8634 7069
8638 7068 8637 7072 8639 7076 8643 7079 8646 7083 8644 7087

8647 7091 8644 7094 8644 7099 8647 7102 8651 7103 8655 7106
8654 7111 8650 7108 8646 7107 8641 7106 8638 7104 8635 7100
8630 7099 8631 7095 8633 7091 8636 7087 8635 7082 8631 7080
8627 7082 31 N F
8709 12522 8704 12523 8705 12519 8708 12515 8712 12513 8714 12509
8718 12508 8722 12507 8726 12509 8730 12511 8728 12514 8724 12515
8719 12516 8715 12517 8711 12519 8709 12522 16 N F
8716 7332 8712 7321 8716 7315 8720 7309 8720 7302 8724 7296
8730 7299 8736 7303 8743 7303 8743 7310 8741 7317 8741 7324
8740 7331 8742 7338 8745 7344 8748 7351 8746 7358 8749 7364
8750 7371 8749 7378 8746 7385 8740 7382 8736 7376 8733 7370
8727 7366 8726 7358 8724 7352 8720 7345 8719 7338 8716 7332
30 N F
8750 6137 8748 6134 8750 6133 8752 6133 8755 6134 8756 6136
8757 6138 8755 6138 8753 6140 8751 6139 8750 6137 11 N F
8809 7190 8799 7188 8805 7183 8802 7177 8807 7172 8814 7171
8817 7169 8820 7168 8826 7172 8833 7174 8839 7170 8836 7163
8836 7156 8842 7153 8849 7151 8853 7151 8856 7151 8858 7157
8853 7162 8856 7169 8864 7169 8868 7175 8861 7173 8856 7178
8852 7173 8845 7173 8839 7177 8832 7180 8828 7186 8828 7193
8821 7194 8818 7201 8814 7196 8809 7190 34 N F
8808 6031 8801 6027 8804 6024 8806 6020 8807 6015 8811 6013
8812 6017 8815 6020 8818 6024 8819 6028 8817 6032 8813 6032
8808 6031 13 N F
8836 6236 8827 6228 8835 6230 8841 6232 8843 6231 8849 6231
8851 6238 8851 6245 8856 6251 8855 6258 8854 6265 8860 6269
8865 6273 8866 6274 8858 6275 8853 6279 8848 6274 8845 6268
8846 6260 8845 6253 8840 6248 8846 6243 8844 6237 8836 6236
24 N F
8843 6116 8840 6110 8844 6109 8849 6110 8853 6110 8853 6109
8851 6113 8854 6117 8851 6121 8851 6125 8851 6130 8846 6130
8843 6134 8843 6129 8842 6125 8843 6121 8843 6116 17 N F
8847 7685 8841 7674 8848 7673 8853 7673 8855 7672 8862 7670
8869 7670 8875 7666 8881 7662 8888 7662 8895 7665 8895 7672
8893 7679 8890 7685 8887 7692 8881 7695 8875 7698 8869 7702
8863 7698 8856 7696 8851 7691 8847 7685 22 N F
8853 7671 8846 7669 8850 7666 8853 7663 8857 7661 8861 7661
8866 7662 8870 7663 8874 7665 8871 7668 8866 7669 8862 7670
8857 7670 8853 7671 14 N F
8850 6190 8849 6186 8850 6184 8853 6184 8855 6185 8857 6186
8859 6186 8861 6187 8863 6188 8865 6190 8865 6190 8867 6190
8867 6192 8865 6193 8863 6192 8861 6192 8859 6190 8857 6190
8855 6188 8853 6187 8852 6188 8850 6190 22 N F

8853 6063 8850 6060 8851 6059 8854 6059 8854 6061 8853 6063
6 N F
8855 6055 8854 6052 8856 6052 8858 6054 8860 6056 8859 6058
8857 6057 8855 6055 8 N F
8861 7426 8856 7423 8859 7420 8864 7418 8868 7418 8870 7422
8874 7423 8877 7427 8880 7431 8882 7435 8882 7439 8878 7439
8875 7436 8871 7433 8868 7430 8866 7426 8864 7422 8861 7426
18 N F
8858 6117 8856 6114 8858 6113 8859 6113 8860 6113 8861 6115
8863 6116 8866 6116 8868 6116 8868 6119 8868 6121 8865 6121
8863 6121 8861 6120 8860 6118 8858 6117 16 N F
8871 6223 8866 6216 8872 6212 8878 6208 8882 6202 8889 6200
8896 6202 8902 6205 8901 6212 8906 6217 8911 6221 8912 6222
8918 6225 8923 6230 8930 6230 8938 6230 8939 6229 8944 6227
8941 6234 8940 6241 8938 6248 8934 6254 8929 6249 8927 6256
8921 6253 8919 6246 8918 6253 8914 6247 8910 6240 8910 6248
8911 6255 8904 6255 8900 6249 8896 6243 8895 6235 8891 6241
8888 6248 8882 6244 8879 6237 8875 6231 8882 6234 8884 6227
8877 6228 8883 6224 8890 6224 8898 6224 8895 6217 8889 6214
8885 6207 8881 6213 8875 6217 8871 6223 52 N F
8875 6293 8869 6287 8875 6282 8880 6288 8885 6283 8890 6277
8892 6270 8896 6264 8899 6271 8897 6278 8902 6283 8899 6289
8896 6296 8889 6297 8882 6294 8875 6293 16 N F
8896 8008 8893 8003 8895 7999 8897 7995 8897 7990 8902 7990
8904 7994 8908 7992 8912 7990 8916 7991 8921 7993 8925 7993
8927 7997 8930 8001 8933 8003 8938 8004 8942 8006 8945 8009
8945 8013 8941 8015 8936 8015 8933 8012 8932 8007 8929 8003
8925 8005 8927 8009 8922 8009 8918 8008 8914 8009 8909 8010
8905 8009 8900 8009 8896 8008 33 N F
8894 6335 8893 6332 8896 6329 8896 6326 8897 6329 8899 6332
8902 6334 8905 6335 8906 6338 8904 6340 8901 6343 8899 6340
8897 6337 8894 6335 14 N F
8902 7437 8901 7433 8904 7433 8907 7433 8910 7434 8912 7435
8912 7438 8910 7440 8908 7442 8906 7441 8903 7440 8902 7437
12 N F
8907 6052 8905 6050 8907 6049 8909 6048 8911 6049 8912 6051
8911 6053 8909 6053 8907 6052 9 N F
8913 7431 8910 7425 8913 7423 8918 7422 8922 7422 8927 7423
8931 7424 8935 7422 8939 7425 8940 7430 8938 7434 8937 7438
8933 7435 8929 7433 8925 7432 8921 7429 8917 7432 8913 7431
18 N F
8920 7337 8917 7329 8924 7332 8930 7336 8937 7335 8943 7339
8941 7332 8941 7325 8934 7327 8931 7320 8936 7315 8933 7309

8938 7304 8943 7299 8945 7299 8949 7297 8954 7292 8960 7287
8967 7285 8970 7292 8967 7298 8961 7302 8963 7308 8969 7312
8975 7315 8981 7320 8988 7322 8993 7327 9000 7328 9005 7323
9011 7326 9012 7333 9005 7333 8998 7335 8992 7339 8986 7344
8984 7351 8984 7358 8986 7364 8992 7369 8998 7372 9005 7374
9009 7369 9016 7371 9020 7377 9024 7382 9017 7382 9010 7382
9003 7384 8998 7389 8992 7386 8995 7393 8999 7399 8996 7405
8990 7408 8984 7413 8977 7411 8971 7413 8967 7419 8967 7427
8972 7432 8975 7438 8968 7438 8962 7434 8957 7429 8954 7423
8947 7426 8943 7421 8940 7414 8933 7414 8929 7408 8925 7403
8926 7396 8929 7389 8936 7391 8941 7397 8948 7397 8950 7391
8949 7384 8945 7377 8946 7370 8945 7363 8944 7356 8950 7352
8955 7357 8958 7364 8963 7359 8970 7358 8971 7351 8965 7347
8959 7343 8952 7343 8947 7348 8940 7350 8933 7351 8928 7346
8921 7344 8920 7337 98 N F
8928 7249 8925 7246 8925 7242 8928 7240 8926 7237 8927 7234
8927 7235 8929 7236 8932 7238 8934 7240 8938 7242 8934 7242
8932 7244 8931 7248 8933 7250 8932 7253 8929 7252 8928 7249
18 N F
8936 7269 8930 7267 8931 7264 8932 7263 8933 7262 8936 7263
8940 7263 8943 7264 8946 7266 8949 7267 8946 7269 8944 7272
8945 7275 8942 7274 8939 7272 8936 7273 8936 7269 17 N F
8937 6058 8935 6056 8936 6054 8935 6052 8937 6050 8938 6048
8939 6050 8940 6053 8941 6054 8942 6056 8942 6059 8939 6059
8937 6058 13 N F
8951 7984 8947 7981 8948 7978 8950 7975 8954 7975 8956 7973
8959 7975 8962 7976 8964 7979 8968 7978 8971 7978 8974 7980
8975 7983 8973 7985 8970 7986 8967 7988 8964 7987 8960 7986
8957 7985 8954 7985 8951 7984 21 N F
9010 13068 9010 13062 9012 13059 9016 13058 9020 13061 9023 13063
9024 13068 9021 13071 9020 13075 9020 13080 9016 13080 9015 13076
9013 13072 9010 13068 14 N F
9032 7717 9030 7712 9035 7711 9039 7709 9042 7712 9047 7712
9050 7715 9052 7716 9055 7717 9057 7721 9058 7725 9054 7726
9049 7726 9047 7722 9043 7719 9041 7723 9036 7722 9038 7727
9035 7730 9034 7725 9031 7722 9032 7717 22 N F
9045 7620 9042 7617 9043 7614 9046 7612 9049 7613 9051 7615
9054 7617 9052 7620 9049 7620 9045 7620 10 N F
9059 7748 9058 7743 9061 7740 9061 7745 9061 7749 9064 7753
9066 7754 9068 7755 9072 7755 9077 7754 9080 7751 9083 7754
9082 7759 9078 7761 9074 7765 9070 7765 9067 7761 9063 7759
9059 7757 9059 7752 9059 7748 21 N F
9064 7608 9061 7602 9065 7604 9069 7605 9072 7609 9075 7612

9071 7612 9066 7613 9064 7608 9 N F
9087 7921 9084 7914 9088 7913 9092 7913 9094 7917 9098 7916
9103 7917 9103 7922 9104 7926 9104 7931 9099 7931 9098 7927
9094 7930 9090 7928 9086 7925 9087 7921 16 N F
9121 6192 9120 6187 9121 6182 9121 6178 9122 6173 9123 6169
9125 6164 9125 6169 9124 6173 9123 6178 9123 6182 9125 6187
9126 6191 9127 6196 9128 6200 9129 6205 9132 6208 9135 6212
9137 6216 9136 6221 9134 6217 9131 6213 9129 6209 9127 6205
9124 6201 9123 6197 9121 6192 27 N F
9133 7960 9128 7956 9131 7953 9135 7950 9136 7950 9139 7951
9143 7953 9143 7958 9147 7959 9151 7960 9152 7964 9154 7969
9153 7973 9151 7977 9146 7978 9144 7974 9141 7971 9139 7967
9137 7963 9133 7960 20 N F
9139 5973 M 9135 5959 L 9146 5949 L 9160 5952 L 9152 5940 L
9163 5931 L 9175 5923 L 9189 5927 L 9203 5931 L
9216 5935 L 9220 5921 L 9207 5915 L 9192 5912 L
9199 5899 L 9207 5887 L 9211 5873 L 9211 5858 L
9215 5845 L 9220 5831 L 9235 5828 L 9226 5816 L
9231 5803 L 9236 5789 L 9242 5776 L 9248 5763 L
9254 5750 L 9258 5736 L 9258 5721 L 9254 5707 L
9262 5695 L 9275 5689 L 9279 5675 L 9290 5665 L
9301 5656 L 9288 5661 L 9279 5672 L 9271 5685 L
9276 5671 L 9285 5660 L 9295 5649 L 9305 5639 L
9315 5628 L 9327 5621 L 9327 5620 L 9340 5612 L
9346 5625 L 9352 5612 L 9362 5601 L 9372 5591 L
9385 5585 L 9398 5577 L 9411 5571 L 9423 5564 L
9435 5556 L 9446 5546 L 9453 5533 L 9464 5524 L
9471 5511 L 9474 5501 L 9487 5501 L 9520 5501 L
9553 5501 L 9586 5501 L 9607 5501 L 9606 5510 L
9605 5525 L 9607 5539 L 9613 5553 L 9615 5567 L
9621 5581 L 9627 5594 L 9631 5608 L 9634 5622 L
9637 5636 L 9642 5650 L 9645 5664 L 9651 5677 L
9661 5688 L 9663 5702 L 9666 5716 L 9672 5730 L
9683 5738 L 9694 5748 L 9706 5756 L 9719 5763 L
9729 5773 L 9739 5784 L 9749 5794 L 9753 5808 L
9757 5822 L 9752 5836 L 9759 5849 L 9772 5854 L
9783 5863 L 9789 5876 L 9796 5889 L 9800 5903 L
9811 5913 L 9818 5926 L 9823 5939 L 9829 5952 L
9831 5967 L 9836 5980 L 9847 5990 L 9856 6001 L
9858 6016 L 9861 6030 L 9856 6043 L 9858 6057 L
9864 6070 L 9867 6085 L 9871 6098 L 9876 6112 L
9888 6120 L 9890 6135 L 9891 6149 L 9893 6163 L
9898 6177 L 9902 6191 L 9905 6205 L 9910 6218 L

9904 6232 L 9906 6246 L 9909 6260 L 9915 6274 L
9916 6288 L 9916 6302 L 9917 6305 L 9922 6316 L
9923 6330 L 9924 6344 L 9927 6359 L 9932 6372 L
9933 6386 L 9933 6401 L 9938 6414 L 9943 6428 L
9946 6442 L 9948 6456 L 9948 6470 L 9948 6485 L
9950 6499 L 9959 6510 L 9967 6522 L 9959 6534 L
9952 6547 L 9949 6561 L 9954 6575 L 9964 6585 L
9973 6596 L 9971 6610 L 9969 6624 L 9976 6637 L
9987 6646 L 9996 6657 L 10007 6666 L 10012 6679 L
10020 6691 L 10025 6705 L 10025 6719 L 10029 6733 L
10031 6747 L 10033 6761 L 10039 6774 L 10046 6787 L
10057 6796 L 10054 6810 L 10059 6824 L 10071 6831 L
10069 6845 L 10069 6860 L 10067 6874 L 10057 6885 L
10052 6898 L 10049 6912 L 10047 6926 L 10042 6940 L
10038 6953 L 10036 6968 L 10036 6982 L 10037 6996 L
10040 7010 L 10045 7024 L 10053 7036 L 10063 7046 L
10068 7059 L 10079 7069 L 10087 7080 L 10100 7087 L
10103 7089 L 10112 7094 L 10124 7103 L 10116 7114 L
10101 7113 L 10089 7120 L 10087 7134 L 10079 7146 L
10082 7160 L 10072 7170 L 10057 7169 L 10043 7170 L
10029 7167 L 10018 7176 L 10005 7182 L 9992 7175 L
9983 7186 L 9970 7192 L 9958 7200 L 9963 7214 L
9949 7209 L 9939 7219 L 9930 7230 L 9926 7244 L
9915 7252 L 9902 7259 L 9888 7258 L 9874 7254 L
9860 7249 L 9848 7242 L 9839 7231 L 9831 7219 L
9823 7207 L 9816 7195 L 9827 7186 L 9814 7181 L
9804 7171 L 9792 7164 L 9779 7158 L 9765 7155 L
9751 7152 L 9737 7153 L 9723 7148 L 9710 7142 L
9698 7135 L 9686 7127 L 9672 7124 L 9659 7119 L
9648 7110 L 9635 7103 L 9625 7093 L 9617 7077 L
9614 7063 L 9606 7052 L 9597 7040 L 9591 7027 L
9585 7015 L 9581 7001 L 9572 6990 L 9563 6978 L
9570 6965 L 9566 6952 L 9562 6938 L 9552 6927 L
9542 6918 L 9539 6904 L 9528 6893 L 9539 6883 L
9524 6884 L 9510 6882 L 9497 6877 L 9489 6865 L
9495 6852 L 9481 6849 L 9468 6844 L 9463 6830 L
9458 6817 L 9455 6803 L 9451 6789 L 9444 6776 L
9437 6764 L 9426 6754 L 9418 6743 L 9408 6732 L
9402 6719 L 9396 6706 L 9391 6692 L 9385 6679 L
9383 6665 L 9374 6653 L 9370 6640 L 9363 6627 L
9355 6615 L 9367 6607 L 9353 6607 L 9346 6595 L
9339 6582 L 9331 6570 L 9328 6556 L 9324 6542 L
9315 6531 L 9305 6521 L 9295 6510 L 9288 6497 L

9279 6486 L 9273 6474 L 9273 6459 L 9266 6446 L
9261 6433 L 9256 6420 L 9246 6409 L 9255 6398 L
9270 6396 L 9284 6394 L 9298 6397 L 9287 6388 L
9273 6385 L 9258 6385 L 9244 6387 L 9230 6386 L
9222 6374 L 9213 6363 L 9211 6349 L 9209 6335 L
9206 6320 L 9194 6312 L 9188 6299 L 9182 6286 L
9180 6272 L 9179 6257 L 9186 6245 L 9186 6230 L
9192 6217 L 9180 6209 L 9190 6198 L 9187 6184 L
9198 6175 L 9187 6165 L 9191 6151 L 9195 6138 L
9194 6123 L 9192 6109 L 9198 6096 L 9184 6099 L
9183 6084 L 9175 6072 L 9176 6058 L 9165 6049 L
9178 6044 L 9164 6042 L 9163 6027 L 9164 6013 L
9154 6003 L 9168 5999 L 9173 5985 L 9168 5972 L
9157 5963 L 9143 5959 L 9139 5973 L N E
9169 14283 9167 14277 9168 14273 9172 14270 9175 14268 9179 14265
9181 14269 9181 14273 9180 14278 9181 14282 9177 14284 9173 14285
9169 14283 13 N F
9197 15646 9195 15639 9198 15636 9202 15638 9205 15641 9208 15643
9209 15644 9213 15644 9217 15645 9219 15649 9218 15653 9218 15657
9214 15660 9210 15662 9208 15658 9204 15658 9203 15654 9204 15650
9200 15650 9197 15646 20 N F
9208 14292 9204 14290 9206 14287 9209 14283 9211 14279 9215 14277
9219 14277 9222 14274 9225 14277 9224 14281 9223 14285 9220 14289
9216 14289 9213 14291 9208 14292 15 N F
9225 14252 9220 14249 9224 14246 9228 14247 9231 14243 9234 14240
9237 14237 9239 14241 9238 14245 9237 14250 9239 14254 9237 14257
9235 14261 9233 14265 9231 14269 9227 14271 9224 14269 9223 14265
9223 14260 9224 14256 9225 14252 21 N F
9224 14395 9223 14391 9224 14384 9227 14382 9227 14382 9231 14384
9235 14383 9240 14383 9242 14386 9239 14389 9235 14390 9230 14390
9231 14394 9230 14399 9227 14402 9225 14399 9224 14395 17 N F
9226 8181 9224 8176 9226 8172 9230 8169 9233 8166 9237 8164
9241 8161 9245 8161 9249 8163 9253 8166 9258 8167 9262 8167
9258 8169 9254 8171 9253 8175 9251 8179 9249 8183 9245 8184
9242 8187 9237 8188 9233 8187 9229 8184 9226 8181 23 N F
9250 14460 9246 14455 9250 14452 9253 14455 9255 14459 9259 14460
9263 14460 9265 14464 9265 14469 9261 14470 9257 14469 9253 14468
9251 14464 9250 14460 14 N F
9290 14499 9286 14496 9288 14494 9290 14491 9293 14489 9295 14486
9295 14489 9294 14492 9296 14494 9293 14495 9293 14498 9290 14499
12 N F
9290 14511 9288 14507 9290 14507 9291 14507 9294 14507 9297 14509
9300 14507 9301 14510 9298 14512 9297 14516 9294 14516 9293 14513

9290 14511 13 N F
9311 7860 9308 7856 9311 7857 9312 7854 9315 7856 9317 7859
9315 7862 9312 7863 9311 7860 9 N F
9310 14318 9308 14314 9311 14312 9314 14310 9316 14312 9316 14316
9313 14317 9310 14318 8 N F
9317 5528 9315 5523 9319 5521 9323 5522 9326 5526 9329 5530
9333 5532 9334 5536 9331 5540 9327 5541 9323 5539 9319 5536
9320 5531 9317 5528 14 N F
9317 7856 9315 7849 9319 7847 9323 7850 9326 7853 9330 7852
9328 7856 9325 7859 9321 7858 9317 7856 10 N F
9412 15142 9402 15141 9404 15134 9403 15128 9409 15125 9415 15123
9422 15125 9428 15125 9433 15119 9435 15113 9438 15119 9444 15123
9448 15128 9453 15132 9456 15133 9460 15133 9467 15133 9472 15129
9478 15126 9485 15126 9491 15128 9495 15133 9500 15138 9507 15139
9510 15145 9514 15151 9520 15149 9526 15153 9529 15159 9531 15166
9530 15172 9530 15179 9526 15185 9525 15191 9518 15193 9512 15196
9506 15199 9500 15202 9493 15203 9498 15197 9498 15191 9494 15185
9488 15182 9482 15182 9477 15177 9471 15174 9464 15174 9457 15175
9451 15173 9444 15171 9438 15171 9432 15174 9430 15168 9430 15161
9425 15157 9419 15153 9416 15147 9412 15142 58 N F
9420 15062 9415 15057 9421 15054 9428 15052 9432 15057 9434 15064
9439 15068 9444 15073 9448 15079 9449 15085 9455 15089 9461 15089
9468 15089 9470 15082 9465 15078 9461 15072 9457 15067 9454 15061
9451 15055 9458 15053 9464 15051 9471 15050 9476 15046 9483 15047
9489 15050 9489 15057 9493 15063 9496 15069 9496 15076 9493 15082
9487 15086 9481 15089 9488 15088 9495 15088 9501 15090 9499 15099
9496 15105 9490 15107 9484 15110 9477 15112 9471 15110 9465 15106
9461 15111 9456 15115 9449 15114 9445 15108 9442 15102 9442 15096
9438 15090 9431 15089 9424 15089 9422 15082 9424 15076 9418 15075
9421 15069 9420 15062 56 N F
9416 8788 9416 8785 9417 8782 9419 8779 9421 8775 9424 8774
9427 8771 9428 8769 9431 8767 9435 8766 9438 8765 9441 8767
9441 8771 9441 8774 9438 8776 9437 8779 9435 8782 9432 8784
9429 8785 9426 8786 9423 8787 9419 8788 9416 8788 23 N F
9475 8763 9469 8762 9472 8760 9475 8758 9478 8757 9481 8755
9484 8753 9486 8751 9489 8749 9493 8748 9496 8747 9499 8748
9502 8749 9505 8751 9507 8753 9509 8756 9509 8759 9507 8762
9505 8764 9501 8765 9498 8765 9495 8766 9491 8766 9488 8765
9485 8765 9481 8765 9478 8765 9475 8763 28 N F
9544 10905 9537 10896 9541 10890 9546 10886 9552 10881 9556 10876
9561 10871 9565 10865 9570 10861 9576 10864 9582 10860 9589 10858
9594 10853 9599 10848 9605 10844 9610 10840 9615 10835 9619 10830
9625 10826 9631 10822 9637 10819 9644 10816 9647 10811 9653 10807

9660 10807 9667 10804 9672 10800 9678 10797 9685 10795 9691 10791
9698 10790 9704 10787 9710 10783 9716 10780 9722 10776 9727 10772
9734 10770 9740 10768 9746 10764 9752 10761 9755 10760 9759 10759
9765 10758 9772 10759 9779 10757 9786 10755 9792 10753 9799 10751
9805 10748 9811 10745 9818 10742 9824 10739 9831 10737 9837 10735
9844 10734 9851 10732 9857 10731 9858 10731 9864 10730 9871 10733
9876 10737 9879 10743 9883 10749 9889 10752 9891 10759 9892 10766
9893 10773 9890 10779 9885 10783 9879 10787 9873 10790 9866 10790
9859 10790 9852 10792 9846 10794 9839 10796 9833 10800 9827 10803
9822 10807 9816 10810 9810 10814 9803 10817 9797 10819 9790 10821
9784 10824 9777 10826 9771 10828 9764 10830 9757 10831 9751 10835
9745 10838 9741 10843 9738 10849 9733 10854 9727 10859 9722 10863
9716 10866 9709 10868 9703 10871 9697 10874 9690 10876 9684 10878
9677 10881 9671 10884 9667 10890 9664 10896 9661 10902 9657 10908
9653 10914 9649 10919 9643 10923 9638 10927 9632 10931 9625 10933
9618 10934 9611 10935 9605 10935 9598 10935 9591 10933 9584 10931
9578 10929 9571 10926 9566 10922 9561 10917 9556 10913 9550 10909
9544 10905 127 N F
9560 8697 9557 8693 9560 8689 9563 8693 9567 8695 9571 8697
9575 8699 9578 8696 9582 8694 9582 8699 9583 8699 9586 8701
9589 8705 9592 8708 9592 8708 9597 8708 9593 8710 9589 8708
9584 8707 9581 8704 9576 8706 9573 8709 9570 8713 9566 8713
9566 8708 9562 8706 9561 8702 9560 8697 28 N F
9567 8757 9566 8752 9570 8754 9575 8755 9579 8755 9583 8757
9588 8758 9592 8757 9590 8753 9592 8748 9596 8751 9600 8751
9605 8752 9609 8754 9613 8756 9616 8759 9620 8761 9617 8763
9613 8765 9610 8769 9607 8772 9603 8774 9599 8772 9595 8772
9591 8773 9587 8770 9583 8769 9584 8773 9579 8773 9578 8768
9575 8766 9570 8764 9567 8761 9567 8757 34 N F
9584 8482 9579 8479 9582 8475 9586 8473 9589 8470 9589 8474
9589 8478 9593 8476 9597 8475 9601 8473 9605 8473 9609 8470
9609 8474 9606 8478 9602 8479 9599 8483 9595 8486 9591 8487
9588 8491 9583 8491 9587 8488 9590 8485 9586 8486 9584 8482
24 N F
9588 8660 9585 8658 9588 8657 9591 8656 9593 8657 9595 8658
9597 8660 9596 8663 9595 8665 9592 8667 9590 8665 9589 8663
9588 8660 13 N F
9595 8686 9592 8681 9597 8680 9601 8679 9605 8680 9610 8679
9614 8681 9618 8681 9621 8684 9625 8682 9629 8681 9633 8678
9637 8680 9638 8684 9638 8688 9638 8693 9634 8695 9638 8697
9639 8693 9643 8696 9646 8700 9643 8703 9644 8708 9640 8709
9637 8705 9633 8703 9629 8704 9625 8702 9628 8698 9626 8694
9622 8694 9617 8693 9615 8696 9612 8700 9610 8696 9606 8693

9603 8690 9599 8688 9595 8686 39 N F
9616 8860 9607 8858 9609 8851 9610 8844 9613 8838 9617 8832
9621 8827 9621 8820 9614 8816 9620 8813 9619 8806 9626 8808
9633 8808 9639 8811 9640 8811 9646 8814 9653 8816 9652 8823
9659 8820 9664 8816 9667 8823 9661 8827 9663 8833 9669 8830
9675 8833 9677 8826 9684 8828 9680 8834 9686 8837 9687 8844
9680 8844 9674 8848 9670 8853 9671 8860 9669 8867 9666 8874
9669 8880 9673 8886 9680 8887 9678 8893 9672 8897 9666 8901
9660 8904 9653 8904 9646 8902 9642 8896 9641 8889 9642 8882
9636 8879 9632 8873 9627 8869 9621 8865 9616 8860 53 N F
9632 8778 9630 8775 9633 8771 9635 8767 9639 8766 9640 8767
9642 8769 9646 8772 9650 8775 9654 8776 9656 8777 9658 8778
9662 8780 9657 8780 9653 8779 9649 8781 9644 8782 9640 8783
9636 8781 9632 8778 20 N F
9651 8574 9647 8571 9650 8571 9650 8569 9653 8568 9655 8569
9656 8571 9658 8572 9659 8574 9660 8575 9661 8576 9662 8578
9661 8579 9659 8579 9656 8579 9655 8578 9655 8575 9653 8574
9651 8574 19 N F
9655 8568 9654 8565 9656 8564 9658 8565 9659 8567 9660 8569
9660 8572 9658 8572 9656 8570 9655 8568 10 N F
9670 14795 9667 14792 9668 14789 9668 14786 9670 14783 9673 14784
9675 14787 9674 14790 9673 14793 9673 14796 9674 14799 9671 14800
9669 14798 9670 14795 14 N F
9755 10719 9746 10714 9752 10711 9758 10708 9764 10705 9770 10702
9775 10697 9782 10694 9788 10691 9794 10687 9799 10683 9805 10679
9812 10677 9817 10673 9823 10670 9827 10664 9831 10658 9835 10653
9841 10649 9847 10651 9848 10658 9847 10665 9844 10671 9839 10676
9835 10682 9831 10687 9825 10691 9820 10696 9813 10698 9808 10702
9802 10705 9795 10707 9789 10710 9782 10711 9775 10714 9769 10715
9762 10717 9755 10719 38 N F
9791 8840 9790 8837 9790 8833 9793 8833 9796 8835 9797 8839
9799 8841 9802 8842 9805 8843 9806 8847 9804 8850 9801 8849
9799 8846 9797 8844 9794 8842 9791 8840 16 N F
9794 9440 9792 9437 9795 9436 9798 9434 9800 9431 9804 9432
9807 9432 9810 9433 9813 9434 9814 9437 9814 9440 9812 9443
9809 9443 9806 9442 9807 9438 9804 9437 9800 9437 9798 9439
9794 9440 19 N F
9830 9087 9828 9081 9830 9079 9833 9078 9836 9075 9839 9074
9841 9076 9839 9079 9841 9081 9842 9082 9845 9083 9845 9086
9842 9087 9842 9091 9840 9094 9839 9097 9836 9097 9833 9097
9831 9093 9831 9090 9830 9087 21 N F
9841 9071 9837 9068 9838 9065 9841 9063 9844 9062 9847 9063
9849 9066 9847 9069 9844 9069 9841 9071 10 N F

9858 10684 9850 10683 9853 10677 9856 10671 9857 10664 9859 10657
9861 10650 9866 10646 9872 10643 9878 10638 9884 10641 9887 10647
9891 10653 9894 10659 9893 10666 9890 10672 9884 10676 9878 10679
9872 10682 9865 10682 9858 10684 21 N F
9876 14972 9865 14967 9869 14962 9868 14955 9873 14951 9878 14946
9884 14950 9887 14956 9888 14962 9894 14958 9900 14957 9899 14963
9897 14970 9895 14976 9897 14983 9903 14985 9897 14985 9892 14980
9885 14979 9879 14978 9876 14972 21 N F
9912 5749 9903 5747 9907 5742 9909 5737 9912 5732 9917 5730
9921 5725 9926 5725 9925 5731 9927 5737 9928 5742 9928 5748
9923 5750 9917 5751 9912 5749 15 N F
9907 9436 9904 9429 9906 9425 9906 9425 9910 9425 9915 9425
9919 9424 9921 9423 9923 9422 9927 9420 9929 9416 9932 9413
9937 9414 9941 9412 9945 9415 9949 9416 9946 9420 9951 9421
9952 9422 9953 9424 9956 9427 9953 9431 9949 9431 9945 9434
9947 9438 9945 9441 9940 9440 9936 9439 9932 9438 9927 9438
9923 9440 9920 9443 9916 9442 9912 9439 9909 9443 9905 9440
9907 9436 37 N F
9907 9191 9904 9187 9908 9186 9911 9185 9913 9182 9915 9180
9919 9180 9921 9182 9922 9185 9922 9188 9925 9190 9927 9193
9923 9194 9922 9191 9919 9189 9916 9187 9913 9186 9911 9188
9912 9192 9909 9193 9906 9194 9907 9191 22 N F
9910 15016 9906 15014 9909 15014 9912 15013 9915 15011 9915 15011
9917 15008 9920 15009 9923 15009 9921 15012 9918 15013 9915 15014
9913 15016 9910 15016 14 N F
9910 14994 9907 14991 9910 14991 9913 14989 9915 14991 9919 14992
9919 14995 9918 14998 9915 14998 9912 14997 9910 14994 11 N F
9914 9408 9907 9403 9912 9402 9916 9402 9915 9397 9920 9397
9922 9393 9926 9391 9930 9393 9930 9398 9931 9402 9929 9405
9932 9408 9929 9412 9926 9414 9921 9416 9917 9415 9914 9412
9914 9408 19 N F
9912 9925 9908 9918 9913 9913 9915 9906 9920 9902 9927 9903
9932 9908 9938 9912 9944 9916 9949 9920 9952 9927 9950 9933
9947 9940 9946 9947 9942 9952 9936 9955 9932 9961 9929 9967
9922 9967 9915 9965 9914 9959 9911 9952 9908 9946 9909 9939
9911 9932 9912 9925 26 N F
9943 15000 9938 14998 9941 14996 9942 14993 9945 14994 9948 14993
9951 14994 9955 14993 9958 14994 9960 14996 9962 14999 9959 15000
9956 14999 9953 15000 9949 15000 9946 15000 9943 15000 17 N F
9945 15034 9938 15033 9941 15030 9943 15029 9945 15028 9949 15027
9954 15027 9957 15024 9961 15022 9965 15021 9969 15021 9973 15019
9973 15014 9976 15011 9978 15015 9978 15019 9982 15020 9986 15020
9988 15020 9990 15019 9995 15019 9999 15019 10003 15018 10007 15017

10011 15014 10014 15017 10017 15020 10020 15023 10016 15023 10012 15024
10008 15025 10004 15027 10001 15030 9997 15032 9995 15028 9991 15027
9986 15025 9982 15025 9978 15026 9974 15027 9971 15030 9968 15033
9968 15037 9965 15034 9963 15031 9959 15033 9959 15037 9955 15037
9952 15034 9948 15037 9945 15034 51 N F
9942 9235 9938 9233 9941 9231 9943 9233 9945 9235 9948 9237
9951 9239 9951 9243 9951 9246 9953 9249 9956 9248 9955 9251
9954 9254 9952 9256 9949 9256 9946 9254 9945 9251 9946 9248
9945 9244 9942 9244 9943 9241 9944 9238 9942 9235 23 N F
9942 9978 9939 9972 9942 9969 9947 9968 9950 9966 9949 9961
9953 9960 9958 9960 9958 9964 9959 9969 9962 9973 9961 9977
9963 9981 9960 9983 9956 9986 9952 9985 9948 9983 9944 9981
9942 9978 19 N F
9948 9215 9945 9209 9949 9209 9951 9207 9954 9207 9957 9208
9957 9211 9954 9214 9957 9216 9959 9218 9961 9221 9965 9222
9968 9222 9968 9225 9972 9226 9975 9227 9972 9228 9968 9228
9966 9226 9963 9224 9961 9222 9957 9222 9955 9220 9951 9220
9948 9220 9946 9217 9948 9215 27 N F
9950 9440 9947 9433 9952 9433 9956 9431 9958 9427 9961 9424
9965 9423 9968 9420 9969 9416 9969 9411 9967 9407 9964 9404
9968 9405 9968 9405 9973 9405 9977 9405 9977 9405 9981 9403
9980 9399 9979 9395 9983 9394 9987 9392 9989 9394 9990 9395
9995 9396 9992 9400 9993 9404 9994 9409 9998 9410 10001 9413
9999 9416 9994 9416 9997 9420 10001 9422 10004 9426 10002 9430
10001 9434 10001 9438 9998 9442 10001 9446 10000 9450 9996 9453
9993 9456 9989 9458 9986 9461 9983 9465 9979 9466 9975 9463
9974 9459 9971 9456 9967 9454 9963 9451 9960 9449 9956 9447
9952 9444 9950 9440 56 N F
9957 10018 9950 10015 9954 10009 9956 10006 9959 10004 9964 10000
9967 9994 9970 9987 9971 9986 9975 9982 9976 9975 9977 9968
9976 9962 9973 9955 9977 9949 9982 9945 9987 9940 9991 9934
9995 9928 9997 9921 10003 9918 10007 9921 10009 9922 10014 9927
10020 9929 10020 9929 10027 9929 10034 9930 10040 9926 10042 9923
10044 9920 10051 9921 10058 9921 10065 9922 10065 9922 10071 9920
10077 9917 10084 9914 10090 9912 10097 9910 10104 9908 10110 9904
10115 9900 10116 9900 10122 9897 10129 9898 10135 9899 10142 9902
10139 9908 10137 9915 10140 9922 10143 9928 10145 9935 10151 9939
10144 9942 10141 9948 10135 9952 10130 9957 10126 9962 10120 9966
10113 9965 10106 9966 10102 9972 10096 9975 10095 9981 10088 9981
10081 9981 10074 9984 10071 9990 10065 9989 10058 9990 10051 9991
10047 9997 10042 10002 10038 10008 10033 10013 10026 10014 10020 10010
10014 10007 10007 10005 10001 10003 9994 10005 9989 10010 9982 10004
9976 10008 9970 10009 9970 10016 9965 10021 9959 10024 9957 10018

90 N F
9956 10096 9955 10093 9955 10090 9956 10086 9959 10089 9959 10089
9962 10091 9964 10093 9965 10096 9963 10099 9966 10101 9964 10104
9967 10106 9969 10109 9970 10112 9968 10114 9965 10113 9965 10109
9963 10107 9960 10109 9959 10106 9959 10102 9958 10099 9956 10096
24 N F
9957 9877 9955 9869 9958 9866 9961 9863 9965 9862 9970 9863
9974 9865 9978 9865 9983 9867 9987 9869 9987 9873 9987 9878
9984 9881 9980 9883 9977 9886 9976 9890 9972 9892 9971 9896
9967 9895 9965 9891 9961 9889 9958 9886 9957 9882 9957 9877
24 N F
9966 9081 9963 9078 9967 9078 9970 9079 9968 9076 9971 9074
9969 9071 9966 9069 9968 9066 9971 9067 9973 9064 9976 9067
9978 9069 9981 9067 9981 9071 9982 9074 9984 9077 9985 9078
9986 9079 9983 9080 9979 9080 9976 9081 9976 9085 9977 9088
9973 9087 9970 9087 9969 9083 9966 9081 28 N F
9967 9043 9964 9040 9967 9040 9970 9041 9972 9044 9974 9042
9977 9044 9979 9046 9982 9048 9985 9050 9987 9053 9985 9055
9981 9055 9978 9054 9975 9052 9972 9051 9970 9049 9970 9045
9967 9043 19 N F
9978 9809 9976 9805 9978 9801 9977 9796 9980 9794 9985 9793
9989 9793 9994 9794 9996 9797 9998 9801 10000 9805 10003 9808
10005 9812 10008 9816 10008 9820 10006 9824 10002 9825 9997 9823
9992 9820 9988 9818 9984 9815 9981 9812 9978 9809 23 N F
9982 9205 9978 9202 9981 9199 9981 9196 9984 9198 9986 9200
9989 9201 9991 9204 9989 9206 9986 9205 9982 9205 11 N F
9983 14991 9979 14988 9982 14986 9984 14988 9987 14989 9989 14991
9989 14994 9988 14997 9986 14994 9983 14995 9983 14991 11 N F
9989 10029 9986 10025 9989 10026 9992 10025 9994 10022 9996 10020
9994 10018 9992 10015 9995 10015 9998 10017 10001 10019 10003 10021
10005 10023 10007 10026 10010 10028 10007 10031 10007 10034 10005 10037
10002 10036 9999 10034 9999 10031 9997 10034 9994 10035 9991 10033
9993 10030 9989 10029 26 N F
9993 10282 9990 10278 9993 10276 9995 10274 9998 10275 9999 10278
10001 10281 10003 10283 10000 10282 9997 10284 9996 10281 9993 10282
12 N F
9999 10254 9994 10252 9997 10250 10000 10250 10002 10247 10003 10244
10005 10245 10006 10245 10009 10248 10009 10251 10011 10254 10014 10255
10011 10256 10007 10257 10004 10257 10003 10254 9999 10254 17 N F
10002 15006 9995 15001 9998 14998 10003 14998 10006 15001 10010 15001
10014 15002 10019 15002 10023 15003 10027 15002 10026 14997 10025 14993
10023 14989 10023 14985 10026 14988 10029 14991 10032 14994 10037 14994
10037 14998 10041 14999 10044 15000 10045 15001 10048 15003 10045 15006

10043 15009 10039 15011 10041 15015 10037 15012 10035 15008 10031 15010
10027 15010 10022 15009 10018 15008 10014 15006 10010 15006 10006 15007
10002 15006 37 N F
10001 9784 9998 9782 9999 9779 9999 9775 10000 9772 9999 9769
10002 9770 10004 9773 10007 9775 10008 9778 10010 9781 10013 9782
10013 9786 10012 9789 10008 9790 10005 9789 10002 9787 10001 9784
18 N F
10001 9900 10000 9896 10002 9893 10002 9890 10005 9888 10008 9888
10010 9890 10012 9893 10014 9896 10014 9899 10013 9902 10010 9904
10007 9904 10004 9902 10001 9900 15 N F
10017 9458 10015 9451 10019 9452 10024 9452 10026 9457 10029 9460
10032 9457 10036 9457 10040 9460 10043 9464 10038 9465 10034 9464
10030 9466 10030 9471 10035 9472 10032 9476 10035 9479 10030 9480
10026 9482 10024 9478 10026 9474 10023 9471 10027 9468 10023 9466
10021 9463 10016 9462 10017 9458 27 N F
10021 9779 10016 9767 10023 9767 10026 9772 10027 9773 10034 9773
10039 9768 10046 9769 10047 9762 10054 9759 10060 9755 10060 9748
10060 9741 10067 9741 10069 9748 10072 9742 10078 9745 10080 9738
10086 9737 10090 9743 10091 9750 10095 9756 10090 9761 10088 9768
10082 9770 10077 9775 10070 9777 10064 9781 10060 9786 10055 9792
10057 9799 10055 9805 10050 9811 10045 9806 10039 9804 10032 9804
10026 9799 10026 9792 10024 9785 10021 9779 40 N F
10022 10094 10017 10085 10023 10082 10029 10079 10035 10075 10041 10072
10046 10068 10053 10066 10060 10066 10062 10066 10067 10066 10071 10065
10074 10064 10080 10061 10087 10059 10084 10066 10082 10072 10087 10077
10088 10084 10084 10090 10089 10095 10094 10100 10087 10100 10082 10105
10084 10112 10078 10115 10071 10113 10065 10116 10063 10109 10057 10106
10050 10105 10045 10100 10039 10102 10032 10102 10028 10097 10022 10094
36 N F
10020 9923 10018 9920 10020 9917 10019 9914 10020 9911 10024 9912
10026 9914 10029 9915 10028 9918 10026 9921 10023 9923 10020 9923
12 N F
10039 14903 10035 14900 10038 14897 10041 14894 10037 14892 10041 14890
10044 14887 10048 14886 10053 14885 10057 14883 10060 14886 10059 14890
10055 14891 10054 14895 10051 14898 10047 14896 10044 14900 10044 14904
10039 14903 19 N F
10039 9911 10038 9907 10039 9904 10042 9904 10046 9905 10046 9908
10047 9911 10046 9914 10042 9915 10039 9914 10039 9911 11 N F
10052 15005 10049 15004 10052 15003 10055 15003 10058 15005 10055 15007
10052 15005 7 N F
10055 9905 10050 9901 10050 9901 10052 9899 10055 9898 10059 9898
10062 9897 10065 9899 10067 9901 10065 9904 10064 9907 10061 9908
10057 9907 10055 9905 14 N F

10052 10030 10050 10020 10053 10013 10058 10008 10065 10010 10069 10016
10070 10023 10077 10025 10081 10030 10075 10034 10069 10037 10062 10039
10056 10035 10052 10030 14 N F
10057 9914 10053 9910 10056 9909 10059 9911 10061 9910 10062 9910
10065 9911 10068 9909 10071 9907 10073 9904 10076 9906 10077 9909
10075 9912 10072 9914 10070 9916 10068 9919 10065 9920 10062 9919
10060 9916 10057 9914 20 N F
10060 15003 10055 15000 10058 14999 10061 14997 10063 14999 10066 15000
10069 15000 10072 15001 10069 15003 10066 15003 10063 15002 10060 15003
12 N F
10068 10057 10064 10053 10065 10051 10068 10050 10070 10048 10072 10047
10075 10048 10076 10051 10075 10054 10073 10055 10071 10057 10068 10057
12 N F
10071 14922 10065 14921 10067 14918 10070 14917 10073 14918 10076 14918
10079 14917 10081 14919 10079 14921 10076 14923 10074 14921 10071 14922
12 N F
10082 14908 10081 14904 10084 14901 10087 14903 10089 14905 10091 14907
10089 14910 10086 14911 10085 14908 10082 14908 10 N F
10089 9607 10088 9604 10089 9601 10092 9602 10095 9601 10097 9604
10100 9606 10100 9609 10103 9611 10106 9613 10109 9613 10109 9616
10105 9616 10102 9617 10100 9615 10097 9614 10097 9610 10095 9608
10092 9606 10089 9607 20 N F
10095 9768 10093 9764 10096 9763 10099 9761 10102 9760 10102 9760
10105 9759 10107 9756 10110 9754 10113 9752 10114 9755 10117 9756
10120 9755 10124 9755 10126 9753 10128 9751 10129 9754 10130 9757
10127 9758 10124 9758 10120 9757 10118 9759 10114 9760 10111 9761
10109 9763 10105 9764 10105 9767 10102 9769 10099 9769 10097 9771
10094 9771 10095 9768 32 N F
10094 10220 10093 10216 10096 10215 10100 10216 10101 10219 10102 10222
10099 10224 10097 10222 10094 10220 9 N F
10098 7010 10094 7010 10096 7009 10098 7007 10100 7006 10102 7005
10104 7005 10106 7006 10108 7005 10110 7006 10111 7008 10109 7009
10108 7011 10107 7013 10105 7014 10103 7015 10101 7014 10100 7011
10098 7010 19 N F
10101 14915 10095 14917 10098 14915 10100 14912 10101 14909 10103 14908
10104 14908 10105 14911 10107 14913 10105 14916 10101 14915 11 N F
10100 9806 10095 9801 10097 9800 10100 9800 10102 9796 10105 9792
10107 9789 10111 9786 10115 9784 10118 9781 10122 9783 10126 9781
10130 9779 10134 9782 10138 9783 10143 9782 10143 9786 10139 9786
10138 9790 10134 9791 10131 9788 10126 9790 10122 9790 10118 9791
10114 9794 10111 9797 10114 9800 10116 9804 10112 9806 10108 9809
10104 9809 10100 9806 32 N F
10103 9843 10097 9836 10103 9832 10108 9828 10109 9828 10112 9822

10118 9817 10123 9814 10128 9809 10135 9807 10141 9803 10147 9806
10143 9812 10140 9818 10140 9825 10134 9829 10140 9833 10136 9839
10129 9841 10123 9843 10116 9845 10110 9843 10103 9843 23 N F
10101 10288 10099 10284 10099 10284 10102 10284 10104 10285 10105 10286
10108 10288 10106 10291 10103 10292 10105 10289 10101 10288 11 N F
10104 10081 10101 10071 10102 10064 10108 10067 10114 10070 10121 10072
10128 10072 10124 10078 10118 10080 10111 10080 10104 10081 11 N F
10115 10191 10107 10192 10110 10186 10108 10179 10113 10174 10112 10167
10118 10164 10124 10161 10131 10160 10130 10167 10136 10168 10142 10172
10148 10168 10155 10169 10161 10167 10167 10167 10168 10167 10174 10163
10179 10163 10181 10163 10179 10170 10173 10173 10178 10177 10174 10183
10167 10185 10160 10185 10154 10186 10152 10193 10145 10192 10148 10186
10141 10187 10135 10189 10128 10191 10121 10189 10115 10191 35 N F
10120 10068 10111 10063 10116 10058 10121 10064 10128 10065 10134 10063
10141 10065 10147 10067 10152 10073 10155 10074 10159 10075 10164 10079
10161 10073 10164 10066 10169 10062 10175 10065 10179 10071 10177 10078
10174 10084 10167 10086 10160 10085 10153 10085 10146 10083 10139 10083
10133 10082 10132 10075 10127 10070 10120 10068 28 N F
10120 15169 10118 15165 10123 15164 10127 15164 10129 15160 10131 15157
10133 15153 10135 15153 10138 15154 10142 15154 10144 15157 10147 15161
10150 15164 10152 15168 10152 15168 10154 15171 10155 15176 10152 15179
10148 15182 10142 15185 10139 15182 10141 15179 10140 15174 10137 15178
10133 15178 10133 15182 10129 15183 10127 15180 10125 15176 10123 15172
10120 15169 31 N F
10126 10221 10123 10218 10126 10218 10129 10216 10131 10214 10135 10214
10136 10217 10135 10220 10136 10223 10135 10227 10133 10224 10132 10221
10129 10220 10126 10221 14 N F
10130 9859 10126 9853 10130 9851 10134 9849 10139 9849 10143 9850
10147 9853 10148 9853 10151 9854 10151 9859 10155 9861 10153 9865
10148 9864 10144 9863 10140 9865 10135 9864 10131 9863 10130 9859
18 N F
10144 7464 10144 7461 10146 7460 10148 7459 10149 7460 10151 7462
10151 7464 10148 7465 10146 7465 10144 7464 10 N F
10150 9902 10145 9896 10151 9891 10153 9888 10154 9885 10160 9881
10166 9877 10166 9876 10168 9871 10173 9866 10168 9861 10169 9854
10171 9848 10176 9842 10183 9841 10188 9845 10184 9851 10182 9857
10185 9864 10190 9867 10197 9870 10197 9877 10195 9884 10188 9885
10182 9888 10189 9888 10196 9890 10191 9895 10191 9902 10185 9906
10179 9902 10172 9901 10166 9903 10159 9901 10155 9907 10150 9902
36 N F
10149 15026 10147 15024 10148 15021 10151 15020 10152 15021 10154 15022
10155 15025 10152 15028 10149 15026 9 N F
10166 7382 10163 7381 10164 7379 10167 7379 10166 7382 5 N F

10169 15133 10166 15128 10169 15125 10171 15121 10174 15120 10176 15120
10179 15118 10180 15122 10180 15126 10184 15127 10182 15130 10177 15131
10173 15131 10169 15133 14 N F
10169 9928 10166 9924 10167 9920 10171 9917 10176 9917 10176 9921
10175 9926 10175 9930 10178 9933 10182 9933 10187 9934 10185 9938
10181 9940 10179 9944 10181 9948 10179 9952 10177 9948 10172 9947
10168 9947 10169 9942 10169 9938 10167 9931 10169 9928 23 N F
10174 10347 10168 10346 10174 10342 10175 10335 10174 10329 10169 10321
10176 10322 10182 10322 10189 10323 10196 10326 10199 10326 10203 10326
10209 10327 10212 10326 10216 10326 10223 10328 10217 10333 10222 10338
10215 10338 10208 10337 10202 10333 10196 10331 10190 10336 10184 10338
10180 10344 10174 10347 26 N F
10194 10176 10190 10171 10193 10171 10196 10173 10199 10172 10200 10169
10202 10166 10204 10167 10206 10167 10207 10170 10204 10172 10202 10174
10201 10178 10199 10180 10196 10179 10194 10176 16 N F
10204 9905 10203 9901 10204 9898 10206 9895 10209 9896 10209 9899
10208 9902 10207 9905 10204 9905 9 N F
10209 10065 10206 10061 10209 10060 10212 10059 10215 10058 10218 10057
10220 10060 10219 10063 10215 10065 10212 10065 10209 10065 11 N F
10222 14935 10216 14930 10222 14930 10227 14928 10229 14923 10224 14925
10221 14921 10221 14916 10220 14910 10222 14905 10227 14902 10230 14907
10234 14911 10235 14911 10239 14913 10242 14917 10243 14922 10239 14927
10236 14931 10234 14936 10234 14941 10232 14936 10227 14934 10222 14935
24 N F
10229 10307 10228 10300 10233 10299 10237 10297 10241 10297 10239 10300
10242 10303 10246 10306 10242 10308 10239 10312 10235 10314 10233 10310
10229 10307 13 N F
10255 10398 10251 10395 10255 10395 10258 10395 10261 10393 10264 10393
10265 10396 10262 10397 10258 10398 10255 10398 10 N F
10269 10397 10267 10394 10270 10392 10273 10394 10276 10395 10274 10398
10271 10400 10269 10397 8 N F
10284 10414 10279 10412 10282 10411 10285 10409 10288 10409 10288 10409
10292 10409 10293 10412 10297 10412 10297 10415 10294 10416 10291 10414
10288 10416 10286 10418 10283 10420 10282 10416 10284 10414 17 N F
10288 10058 10284 10054 10287 10052 10288 10049 10290 10046 10292 10045
10293 10048 10293 10051 10292 10054 10291 10058 10288 10058 11 N F
10307 10056 10302 10054 10304 10052 10306 10049 10309 10048 10310 10045
10311 10042 10313 10045 10312 10048 10312 10052 10309 10054 10307 10056
12 N F
10320 10058 10316 10054 10317 10051 10319 10048 10322 10047 10322 10050
10320 10053 10322 10056 10320 10058 9 N F
10327 10044 10324 10039 10325 10037 10328 10037 10330 10039 10328 10041
10327 10044 7 N F

10328 10341 10325 10337 10327 10334 10328 10333 10330 10337 10335 10337
10337 10334 10341 10332 10341 10336 10340 10341 10342 10345 10343 10349
10338 10349 10335 10347 10331 10345 10328 10341 16 N F
10338 10384 10333 10381 10335 10379 10338 10381 10338 10381 10341 10382
10338 10384 7 N F
10349 10023 10346 10021 10347 10017 10348 10015 10352 10014 10355 10014
10358 10014 10358 10017 10356 10020 10353 10022 10349 10023 11 N F
10355 10352 10349 10346 10349 10345 10351 10342 10355 10342 10360 10343
10362 10338 10365 10335 10365 10340 10364 10344 10362 10348 10358 10349
10355 10352 13 N F
10429 15312 10428 15308 10431 15308 10434 15310 10437 15310 10440 15312
10443 15311 10446 15312 10444 15314 10443 15317 10440 15318 10437 15317
10434 15315 10431 15314 10429 15312 15 N F
10635 6811 10631 6809 10634 6806 10639 6806 10643 6805 10647 6803
10652 6803 10656 6804 10661 6806 10665 6805 10669 6804 10674 6806
10677 6809 10681 6811 10683 6815 10687 6818 10683 6820 10679 6822
10675 6823 10670 6825 10664 6822 10660 6824 10656 6822 10651 6821
10647 6823 10643 6821 10641 6817 10637 6815 10635 6811 29 N F
10809 15230 10806 15224 10810 15225 10812 15228 10815 15231 10819 15230
10820 15229 10823 15227 10825 15230 10825 15235 10830 15233 10830 15229
10834 15227 10838 15227 10842 15228 10845 15232 10846 15236 10849 15239
10850 15243 10846 15245 10842 15247 10838 15248 10837 15244 10833 15244
10828 15244 10826 15241 10822 15243 10818 15241 10819 15237 10816 15234
10811 15233 10809 15230 32 N F
10810 15176 10809 15171 10812 15171 10811 15168 10814 15166 10817 15166
10820 15167 10822 15169 10822 15173 10820 15175 10818 15178 10818 15181
10815 15179 10813 15177 10810 15176 15 N F
10819 15202 10818 15198 10818 15198 10820 15197 10822 15198 10822 15199
10824 15201 10822 15203 10820 15204 10819 15202 10 N F
10819 15191 10818 15186 10821 15185 10824 15185 10823 15188 10824 15191
10822 15193 10819 15191 8 N F
10846 15223 10844 15219 10846 15215 10845 15210 10848 15207 10851 15210
10855 15211 10855 15216 10857 15219 10854 15223 10856 15226 10853 15228
10849 15226 10846 15223 14 N F
10880 7584 10878 7580 10880 7578 10882 7578 10884 7579 10886 7580
10888 7582 10890 7581 10892 7581 10895 7582 10897 7582 10897 7584
10895 7586 10894 7588 10891 7588 10889 7588 10887 7588 10885 7588
10883 7587 10881 7585 10880 7584 21 N F
10890 14381 10885 14376 10886 14372 10889 14370 10890 14370 10893 14367
10893 14363 10895 14359 10897 14355 10900 14352 10904 14353 10909 14352
10913 14351 10917 14353 10921 14350 10925 14351 10925 14356 10927 14359
10925 14363 10921 14364 10919 14368 10921 14372 10925 14374 10929 14372
10927 14368 10930 14368 10931 14368 10935 14371 10939 14371 10936 14374

10941 14374 10938 14378 10936 14382 10935 14386 10931 14387 10926 14387
10922 14387 10918 14385 10914 14385 10910 14384 10906 14382 10902 14380
10898 14377 10894 14379 10890 14381 45 N F
10906 15215 10901 15209 10906 15210 10909 15213 10912 15215 10917 15215
10920 15217 10923 15220 10925 15224 10929 15226 10932 15230 10933 15234
10937 15234 10936 15238 10940 15239 10944 15238 10946 15240 10947 15241
10950 15244 10949 15249 10944 15248 10940 15247 10943 15251 10939 15252
10942 15256 10938 15256 10940 15260 10937 15262 10936 15266 10939 15270
10943 15269 10943 15273 10939 15275 10936 15278 10931 15278 10927 15279
10924 15276 10920 15274 10924 15271 10923 15267 10919 15265 10922 15262
10923 15258 10919 15255 10915 15253 10914 15249 10909 15250 10906 15247
10904 15244 10902 15239 10906 15241 10910 15242 10914 15244 10918 15242
10916 15238 10912 15237 10913 15233 10917 15232 10918 15227 10916 15223
10912 15224 10908 15224 10907 15219 10906 15215 64 N F
10918 14298 10913 14298 10916 14297 10919 14295 10922 14295 10923 14292
10922 14289 10922 14289 10925 14290 10927 14292 10931 14291 10934 14292
10936 14294 10938 14297 10938 14300 10936 14303 10936 14306 10938 14308
10941 14310 10939 14313 10936 14314 10933 14313 10930 14315 10930 14312
10928 14310 10925 14309 10923 14306 10921 14303 10921 14300 10918 14298
30 N F
10922 7579 10920 7577 10921 7575 10923 7574 10925 7575 10923 7577
10922 7579 7 N F
10932 14683 10926 14681 10929 14678 10933 14677 10937 14678 10935 14681
10936 14685 10940 14682 10944 14683 10947 14686 10950 14682 10954 14680
10958 14678 10961 14676 10965 14674 10970 14674 10970 14678 10973 14675
10977 14675 10980 14678 10977 14682 10975 14686 10973 14689 10969 14692
10970 14696 10965 14696 10961 14696 10958 14699 10953 14700 10949 14701
10946 14704 10945 14700 10942 14697 10940 14693 10936 14692 10931 14692
10932 14687 10932 14683 38 N F
10946 14273 10943 14267 10946 14268 10948 14271 10951 14272 10954 14274
10957 14273 10958 14273 10960 14272 10960 14276 10962 14279 10958 14279
10955 14278 10952 14277 10949 14278 10948 14275 10946 14273 17 N F
10956 7681 10955 7678 10957 7676 10959 7676 10961 7676 10962 7678
10961 7680 10960 7682 10958 7682 10956 7681 10 N F
10967 6683 10964 6681 10967 6681 10969 6682 10971 6683 10973 6684
10971 6685 10968 6685 10967 6683 9 N F
11009 14751 11009 14747 11012 14743 11014 14747 11018 14749 11022 14751
11023 14755 11024 14760 11026 14763 11026 14768 11024 14764 11017 14764
11016 14769 11013 14772 11012 14768 11013 14763 11010 14760 11013 14756
11017 14756 11017 14752 11013 14754 11009 14751 22 N F
11028 6755 11016 6752 11020 6746 11024 6741 11031 6738 11038 6738
11045 6737 11047 6743 11053 6748 11059 6743 11065 6739 11072 6738
11079 6738 11086 6737 11092 6736 11093 6736 11100 6737 11107 6738

11114 6735 11120 6732 11127 6730 11133 6733 11133 6740 11129 6746
11122 6749 11129 6747 11136 6745 11142 6741 11143 6748 11140 6755
11143 6762 11146 6768 11153 6766 11155 6773 11153 6780 11157 6786
11159 6793 11152 6795 11146 6800 11141 6804 11134 6806 11127 6807
11125 6814 11119 6810 11112 6814 11105 6813 11099 6810 11094 6805
11089 6810 11087 6817 11083 6823 11077 6827 11070 6830 11064 6826
11070 6821 11072 6814 11075 6808 11069 6811 11070 6818 11063 6816
11059 6810 11054 6804 11057 6797 11056 6790 11060 6784 11065 6779
11066 6772 11063 6778 11061 6772 11060 6764 11063 6758 11062 6751
11061 6758 11056 6763 11050 6767 11049 6774 11043 6770 11037 6773
11038 6766 11041 6760 11035 6764 11029 6767 11025 6761 11028 6755
84 N F
11057 14306 11056 14303 11059 14302 11062 14303 11065 14301 11068 14302
11069 14305 11070 14306 11071 14307 11072 14310 11075 14309 11076 14312
11076 14315 11077 14318 11077 14321 11074 14321 11071 14321 11067 14321
11064 14320 11067 14318 11070 14316 11069 14313 11069 14310 11066 14310
11063 14311 11060 14312 11060 14309 11057 14306 28 N F
11075 6616 11074 6611 11076 6607 11081 6607 11086 6606 11090 6607
11095 6606 11099 6608 11102 6612 11104 6616 11101 6619 11097 6621
11092 6621 11088 6621 11083 6619 11079 6617 11075 6616 17 N F
11120 14306 11115 14302 11119 14302 11122 14301 11122 14298 11121 14295
11124 14297 11125 14300 11128 14302 11131 14303 11133 14305 11136 14307
11135 14311 11138 14310 11141 14311 11139 14314 11139 14317 11136 14316
11132 14315 11129 14314 11126 14312 11125 14309 11122 14308 11120 14306
24 N F
11154 14284 11150 14282 11154 14281 11156 14280 11159 14278 11161 14275
11164 14277 11161 14279 11163 14281 11161 14284 11158 14285 11154 14284
12 N F
11155 14257 11153 14253 11155 14251 11158 14249 11160 14250 11161 14250
11163 14252 11167 14252 11169 14254 11172 14256 11174 14258 11176 14261
11178 14263 11179 14265 11180 14266 11182 14268 11185 14269 11188 14271
11191 14273 11192 14276 11194 14279 11194 14279 11196 14281 11198 14283
11199 14286 11201 14289 11203 14292 11205 14295 11202 14293 11199 14294
11196 14292 11193 14290 11191 14288 11188 14286 11185 14287 11183 14284
11180 14283 11178 14281 11179 14278 11180 14275 11181 14272 11181 14269
11178 14271 11178 14274 11176 14276 11173 14278 11170 14277 11167 14276
11169 14273 11169 14270 11166 14268 11165 14265 11163 14262 11161 14260
11158 14259 11155 14257 56 N F
11157 6713 11154 6710 11157 6707 11160 6706 11163 6707 11163 6711
11160 6713 11157 6713 8 N F
11163 6721 11162 6715 11166 6716 11168 6719 11170 6721 11171 6725
11168 6726 11166 6724 11163 6721 9 N F
11170 6779 11166 6779 11168 6778 11170 6778 11173 6777 11176 6777

11180 6777 11183 6778 11186 6775 11189 6774 11192 6775 11194 6778
11190 6781 11187 6782 11186 6785 11185 6789 11182 6790 11179 6791
11176 6791 11173 6789 11171 6786 11174 6784 11173 6781 11170 6779
24 N F
11191 6732 11190 6729 11190 6726 11192 6723 11194 6720 11196 6717
11199 6715 11202 6715 11205 6717 11208 6719 11210 6722 11211 6725
11211 6728 11209 6731 11205 6731 11202 6731 11199 6732 11194 6734
11191 6732 19 N F
11227 6846 11223 6838 11229 6834 11234 6836 11235 6836 11241 6841
11248 6842 11253 6837 11257 6831 11262 6826 11261 6818 11258 6812
11251 6810 11247 6804 11252 6799 11259 6797 11266 6794 11271 6790
11277 6786 11284 6785 11291 6788 11296 6793 11297 6800 11304 6798
11310 6795 11317 6797 11320 6804 11321 6811 11321 6818 11320 6825
11322 6832 11321 6839 11317 6846 11321 6852 11317 6858 11317 6865
11321 6871 11326 6876 11330 6882 11331 6889 11336 6894 11340 6900
11343 6906 11341 6913 11344 6919 11350 6923 11355 6929 11356 6936
11355 6943 11356 6950 11348 6950 11341 6951 11341 6944 11340 6937
11336 6930 11332 6925 11329 6918 11334 6914 11331 6907 11329 6900
11328 6893 11321 6895 11314 6892 11311 6886 11304 6884 11304 6877
11301 6870 11303 6863 11297 6858 11290 6859 11283 6857 11277 6853
11270 6854 11263 6852 11261 6859 11257 6865 11250 6868 11250 6860
11248 6854 11244 6847 11238 6852 11231 6851 11227 6846 83 N F
11233 6780 11232 6776 11233 6772 11236 6770 11239 6770 11242 6773
11244 6775 11244 6779 11243 6782 11241 6784 11238 6786 11234 6787
11233 6784 11233 6780 14 N F
11277 12274 11271 12274 11275 12272 11279 12272 11283 12270 11287 12267
11288 12263 11291 12259 11295 12261 11299 12261 11301 12266 11298 12268
11294 12271 11291 12274 11290 12278 11291 12283 11286 12283 11284 12287
11280 12286 11277 12283 11277 12279 11277 12274 22 N F
11288 12612 11286 12608 11287 12604 11289 12601 11291 12605 11293 12608
11298 12609 11299 12613 11301 12617 11302 12621 11300 12625 11296 12627
11295 12623 11294 12619 11292 12615 11288 12612 16 N F
11347 12475 11343 12475 11345 12472 11346 12470 11347 12469 11348 12472
11347 12475 7 N F
11346 12483 11343 12479 11347 12479 11347 12479 11349 12481 11351 12483
11350 12486 11347 12486 11346 12483 9 N F
11351 12459 11346 12461 11348 12459 11350 12456 11352 12453 11353 12450
11356 12452 11359 12451 11361 12452 11361 12456 11358 12457 11355 12458
11353 12456 11351 12459 14 N F
11354 14261 11353 14257 11356 14255 11357 14252 11356 14249 11357 14246
11360 14245 11363 14247 11365 14250 11367 14252 11368 14255 11370 14258
11370 14261 11367 14262 11364 14261 11360 14260 11357 14260 11354 14261
18 N F

11371 12616 11369 12612 11371 12610 11374 12611 11377 12613 11377 12616
11374 12617 11371 12616 8 N F
11381 12622 11377 12618 11380 12618 11382 12621 11384 12623 11381 12622
6 N F
11384 12601 11380 12597 11383 12593 11386 12590 11389 12587 11393 12587
11392 12591 11393 12596 11396 12598 11399 12602 11398 12606 11402 12607
11405 12610 11407 12614 11406 12618 11409 12622 11414 12623 11412 12627
11408 12625 11405 12622 11401 12621 11400 12626 11404 12628 11399 12629
11395 12628 11391 12627 11388 12623 11389 12619 11386 12615 11385 12611
11384 12607 11381 12604 11384 12601 33 N F
11384 14332 11381 14329 11384 14328 11384 14328 11387 14328 11389 14331
11388 14334 11388 14337 11385 14335 11384 14332 10 N F
11398 12659 11394 12658 11396 12656 11399 12656 11400 12656 11398 12659
6 N F
11403 12653 11400 12652 11400 12649 11403 12647 11405 12650 11403 12653
6 N F
11408 12647 11406 12644 11408 12642 11411 12641 11412 12637 11415 12640
11414 12643 11415 12646 11419 12645 11419 12649 11416 12649 11413 12651
11410 12653 11411 12649 11408 12647 15 N F
11413 12602 11412 12598 11415 12598 11418 12599 11420 12601 11423 12604
11424 12607 11420 12607 11417 12607 11416 12604 11413 12602 11 N F
11440 12364 11436 12364 11439 12362 11441 12359 11443 12361 11446 12360
11448 12362 11445 12363 11443 12365 11440 12364 10 N F
11441 14322 11437 14319 11439 14317 11442 14318 11443 14318 11445 14318
11449 14319 11451 14321 11452 14324 11454 14327 11451 14325 11448 14324
11446 14321 11443 14323 11441 14322 15 N F
11500 13949 11498 13945 11500 13942 11503 13940 11503 13943 11504 13946
11503 13949 11507 13948 11510 13949 11508 13952 11505 13953 11502 13954
11500 13952 11500 13949 14 N F
11504 14005 11502 14002 11506 14001 11508 14003 11510 14005 11514 14005
11512 14008 11515 14009 11517 14012 11513 14013 11512 14010 11508 14009
11507 14006 11504 14005 14 N F
11518 13927 11517 13924 11518 13921 11521 13919 11524 13917 11525 13915
11528 13916 11531 13918 11531 13918 11534 13920 11530 13920 11527 13920
11524 13921 11524 13924 11522 13926 11518 13927 16 N F
11528 7052 11525 7050 11529 7048 11532 7047 11535 7045 11537 7045
11538 7044 11539 7048 11537 7050 11534 7052 11531 7054 11528 7052
12 N F
11529 7000 11526 6997 11526 6990 11528 6986 11532 6983 11536 6983
11541 6982 11545 6982 11549 6985 11552 6988 11556 6991 11556 6996
11554 7000 11551 7003 11546 7004 11542 7005 11537 7005 11533 7003
11529 7000 19 N F
11547 14286 11544 14283 11545 14281 11548 14281 11551 14280 11553 14282

11552 14284 11549 14285 11547 14286 9 N F
11551 13963 11549 13960 11550 13960 11552 13961 11555 13959 11552 13959
11551 13956 11554 13954 11557 13955 11559 13957 11561 13960 11562 13963
11561 13966 11558 13968 11555 13967 11552 13967 11551 13963 17 N F
11552 13490 11550 13492 11551 13489 11552 13487 11554 13485 11556 13484
11557 13487 11558 13489 11556 13491 11554 13489 11552 13490 11 N F
11555 14033 11553 14029 11557 14031 11558 14036 11560 14033 11560 14032
11564 14030 11565 14026 11570 14025 11574 14026 11578 14028 11579 14032
11580 14032 11583 14034 11586 14037 11584 14040 11579 14042 11575 14044
11572 14046 11567 14045 11566 14049 11562 14051 11561 14056 11558 14058
11558 14054 11556 14050 11555 14046 11554 14042 11555 14038 11555 14033
30 N F
11557 14010 11555 14005 11558 14003 11558 14002 11559 14000 11562 14000
11565 14000 11566 14000 11568 14002 11566 14005 11568 14008 11565 14008
11563 14010 11560 14012 11557 14010 15 N F
11562 14281 11558 14278 11561 14276 11563 14274 11566 14275 11569 14276
11572 14274 11574 14272 11577 14270 11580 14270 11582 14272 11583 14276
11580 14277 11578 14280 11576 14283 11575 14286 11572 14285 11571 14282
11568 14281 11565 14280 11562 14281 21 N F
11565 13430 11561 13429 11563 13427 11564 13424 11565 13422 11568 13421
11570 13422 11570 13425 11572 13425 11573 13422 11575 13424 11575 13427
11573 13428 11570 13429 11568 13430 11565 13430 16 N F
11570 13732 11567 13729 11569 13727 11571 13729 11573 13730 11576 13730
11578 13730 11581 13731 11581 13732 11581 13734 11583 13736 11582 13738
11583 13741 11580 13742 11580 13739 11579 13737 11577 13735 11574 13735
11572 13734 11570 13732 20 N F
11575 12601 11569 12602 11573 12600 11575 12597 11580 12597 11577 12594
11573 12592 11576 12588 11580 12590 11584 12592 11588 12591 11591 12594
11596 12595 11596 12599 11594 12603 11594 12608 11589 12609 11587 12606
11583 12604 11579 12601 11575 12601 21 N F
11578 14337 11574 14331 11575 14330 11578 14329 11581 14327 11585 14325
11586 14325 11590 14326 11591 14330 11595 14331 11599 14332 11601 14333
11603 14334 11607 14335 11610 14332 11614 14334 11618 14336 11622 14339
11624 14342 11626 14346 11627 14350 11627 14350 11626 14355 11624 14358
11620 14355 11616 14354 11612 14354 11608 14354 11604 14356 11600 14358
11596 14360 11592 14359 11588 14358 11584 14356 11581 14353 11581 14349
11579 14345 11581 14341 11578 14337 39 N F
11585 13483 11581 13483 11582 13481 11585 13479 11587 13478 11587 13481
11585 13483 7 N F
11586 14308 11583 14305 11585 14304 11588 14304 11591 14304 11593 14305
11596 14305 11594 14307 11591 14307 11588 14307 11586 14308 11 N F
11589 14036 11585 14033 11586 14031 11587 14028 11589 14027 11589 14027
11592 14027 11594 14026 11595 14023 11595 14021 11598 14022 11599 14025

11600 14027 11598 14028 11596 14028 11595 14030 11593 14032 11591 14034
11589 14036 19 N F
11593 14270 11589 14268 11589 14267 11591 14266 11592 14263 11595 14261
11598 14259 11600 14258 11604 14257 11607 14256 11605 14259 11602 14261
11602 14264 11602 14267 11603 14270 11600 14270 11596 14269 11593 14270
18 N F
11601 12598 11598 12602 11599 12599 11599 12596 11601 12598 5 N F
11601 13489 11599 13485 11600 13485 11602 13485 11605 13486 11607 13488
11609 13488 11612 13489 11612 13492 11610 13494 11607 13493 11605 13493
11602 13492 11601 13489 14 N F
11601 14316 11599 14314 11599 14310 11602 14310 11603 14311 11604 14312
11603 14315 11601 14316 8 N F
11605 12600 11600 12602 11602 12599 11605 12598 11607 12596 11610 12593
11609 12596 11608 12600 11605 12600 9 N F
11602 12674 11601 12670 11604 12667 11607 12663 11610 12660 11614 12659
11618 12660 11622 12662 11626 12664 11624 12668 11629 12669 11626 12672
11625 12677 11621 12679 11620 12683 11624 12685 11628 12686 11633 12687
11636 12688 11637 12689 11638 12685 11642 12685 11646 12687 11650 12688
11649 12694 11645 12693 11647 12697 11649 12701 11650 12706 11648 12710
11645 12712 11640 12710 11636 12708 11636 12704 11633 12700 11629 12701
11628 12697 11624 12694 11620 12693 11616 12692 11611 12692 11608 12695
11604 12694 11604 12690 11601 12687 11603 12683 11602 12678 11602 12674
48 N F
11613 14303 11605 14302 11608 14300 11612 14298 11615 14294 11619 14292
11622 14292 11623 14291 11627 14289 11630 14287 11630 14287 11630 14287
11634 14284 11634 14280 11632 14276 11635 14273 11638 14270 11641 14273
11644 14276 11647 14279 11651 14282 11653 14285 11653 14290 11652 14294
11649 14297 11645 14298 11640 14299 11636 14299 11633 14302 11629 14304
11626 14307 11626 14312 11626 14316 11627 14320 11629 14324 11627 14327
11623 14327 11619 14324 11615 14323 11612 14320 11612 14316 11613 14312
11612 14307 11613 14303 44 N F
11616 14016 11611 14016 11613 14013 11616 14012 11619 14013 11622 14012
11624 14015 11622 14018 11619 14017 11616 14016 10 N F
11616 12384 11611 12383 11614 12382 11616 12380 11619 12379 11619 12382
11616 12384 7 N F
11617 12656 11615 12652 11615 12648 11616 12644 11616 12641 11617 12639
11619 12636 11623 12639 11625 12635 11628 12632 11632 12633 11635 12630
11639 12628 11639 12629 11643 12630 11646 12633 11646 12637 11650 12639
11653 12642 11654 12647 11657 12646 11658 12646 11659 12650 11661 12654
11653 12656 11650 12658 11646 12661 11642 12661 11640 12665 11638 12669
11636 12672 11633 12669 11632 12664 11629 12662 11632 12659 11630 12655
11629 12659 11625 12660 11621 12658 11617 12656 40 N F
11617 12996 11616 12993 11619 12995 11622 12997 11619 12999 11617 12996

6 N F
11625 13007 11621 13006 11624 13004 11625 13001 11628 13001 11631 13001
11634 13004 11636 13006 11635 13009 11633 13012 11630 13013 11628 13015
11626 13014 11622 13014 11622 13011 11626 13010 11628 13008 11625 13007
18 N F
11628 14013 11624 14011 11626 14009 11629 14009 11632 14008 11635 14007
11637 14004 11640 14003 11641 14006 11638 14007 11636 14010 11636 14013
11633 14014 11630 14016 11628 14013 15 N F
11635 14221 11630 14222 11632 14219 11635 14217 11637 14215 11638 14214
11640 14213 11641 14210 11640 14206 11643 14205 11647 14206 11650 14207
11653 14208 11656 14208 11658 14208 11659 14209 11662 14208 11664 14211
11664 14214 11661 14213 11658 14212 11655 14211 11651 14211 11648 14212
11646 14215 11644 14217 11641 14219 11638 14220 11635 14221 29 N F
11635 12749 11631 12749 11635 12747 11633 12743 11637 12742 11641 12742
11645 12744 11645 12744 11649 12742 11652 12745 11655 12748 11655 12752
11655 12748 11655 12744 11659 12742 11661 12738 11663 12737 11664 12736
11667 12739 11669 12740 11671 12742 11675 12743 11677 12747 11681 12747
11680 12752 11680 12756 11684 12754 11688 12756 11689 12760 11686 12763
11681 12764 11682 12768 11686 12771 11682 12774 11683 12778 11678 12778
11677 12782 11673 12785 11669 12786 11668 12782 11669 12778 11668 12774
11664 12772 11661 12775 11658 12772 11654 12770 11650 12772 11648 12768
11647 12763 11643 12757 11642 12753 11639 12749 11635 12749 53 N F
11633 12406 11632 12402 11636 12402 11638 12400 11638 12403 11641 12405
11643 12408 11644 12405 11643 12402 11646 12404 11645 12407 11644 12410
11641 12411 11638 12410 11635 12408 11633 12406 16 N F
11634 12619 11633 12615 11635 12613 11638 12611 11641 12612 11644 12611
11645 12608 11648 12610 11648 12613 11647 12616 11644 12618 11643 12621
11639 12622 11637 12620 11634 12619 15 N F
11637 13024 11634 13021 11637 13019 11639 13022 11637 13024 5 N F
11638 14065 11634 14060 11638 14058 11642 14056 11646 14055 11649 14054
11651 14054 11655 14054 11659 14054 11663 14057 11664 14061 11661 14063
11658 14059 11654 14060 11650 14062 11646 14062 11642 14063 11638 14065
18 N F
11641 14354 11635 14349 11638 14343 11640 14336 11640 14330 11640 14323
11641 14316 11640 14309 11643 14303 11650 14302 11656 14301 11658 14295
11663 14290 11666 14284 11668 14277 11665 14272 11659 14269 11652 14267
11654 14261 11654 14254 11660 14252 11667 14249 11673 14250 11677 14249
11680 14249 11684 14248 11687 14247 11693 14245 11693 14252 11694 14259
11697 14257 11700 14256 11704 14253 11705 14252 11712 14251 11717 14256
11719 14258 11722 14261 11723 14268 11721 14274 11719 14280 11721 14287
11715 14291 11715 14298 11715 14305 11713 14311 11710 14317 11710 14324
11713 14330 11710 14337 11706 14342 11705 14349 11700 14354 11694 14356
11688 14359 11682 14363 11677 14367 11672 14372 11665 14373 11659 14370

11652 14370 11651 14363 11647 14358 11641 14354 64 N F
11637 12872 11636 12867 11639 12865 11641 12862 11644 12861 11646 12864
11646 12867 11644 12870 11642 12872 11640 12875 11637 12872 11 N F
11642 12597 11640 12594 11641 12594 11643 12595 11643 12595 11647 12595
11647 12592 11646 12589 11646 12585 11650 12586 11650 12590 11650 12593
11649 12596 11652 12598 11652 12598 11648 12598 11645 12597 11642 12597
18 N F
11642 12473 11641 12470 11645 12470 11646 12473 11647 12476 11643 12477
11642 12473 7 N F
11649 14013 11644 14012 11646 14009 11648 14007 11652 14007 11655 14006
11658 14006 11657 14009 11655 14011 11652 14012 11649 14013 11 N F
11647 13185 11645 13182 11648 13181 11649 13183 11650 13186 11647 13185
6 N F
11648 12402 11646 12398 11647 12395 11650 12395 11653 12395 11656 12398
11656 12398 11658 12400 11659 12403 11656 12404 11653 12402 11651 12404
11648 12402 13 N F
11650 12460 11648 12457 11651 12458 11655 12458 11654 12461 11652 12463
11650 12460 7 N F
11656 12857 11650 12855 11654 12854 11658 12855 11659 12850 11662 12854
11665 12857 11667 12853 11669 12852 11671 12851 11675 12850 11679 12848
11680 12852 11684 12854 11682 12858 11685 12862 11687 12866 11683 12867
11679 12869 11679 12873 11679 12877 11683 12879 11684 12884 11681 12887
11684 12891 11683 12895 11687 12897 11692 12898 11695 12901 11699 12902
11695 12905 11692 12901 11688 12901 11684 12900 11679 12900 11679 12896
11676 12893 11673 12889 11671 12885 11669 12882 11665 12880 11661 12879
11656 12877 11654 12874 11653 12869 11651 12866 11655 12863 11658 12861
11656 12857 49 N F
11651 13175 11651 13172 11653 13171 11656 13171 11656 13169 11659 13170
11658 13173 11656 13175 11654 13176 11651 13175 10 N F
11654 12608 11653 12605 11655 12603 11657 12606 11657 12606 11657 12603
11660 12605 11663 12606 11663 12609 11662 12612 11660 12615 11657 12617
11654 12616 11654 12612 11657 12610 11654 12608 16 N F
11655 12442 11653 12438 11656 12437 11656 12437 11659 12440 11658 12443
11655 12442 7 N F
11655 12476 11654 12470 11657 12470 11658 12472 11659 12473 11659 12476
11657 12479 11655 12476 8 N F
11656 12359 11654 12356 11656 12353 11657 12350 11658 12347 11660 12350
11658 12352 11657 12356 11656 12359 9 N F
11656 14175 11656 14172 11658 14169 11661 14169 11663 14166 11663 14162
11665 14160 11668 14158 11671 14160 11672 14163 11675 14164 11677 14162
11677 14158 11679 14160 11680 14160 11682 14158 11685 14156 11686 14153
11687 14156 11690 14158 11690 14161 11690 14165 11688 14168 11685 14169
11683 14171 11681 14173 11677 14174 11674 14175 11674 14171 11671 14170

11668 14171 11666 14173 11664 14176 11661 14178 11658 14178 11656 14175
36 N F
11663 12680 11657 12680 11658 12677 11658 12674 11660 12671 11662 12668
11665 12668 11666 12671 11665 12674 11663 12677 11663 12680 11 N F
11664 12445 11660 12444 11661 12441 11662 12438 11664 12439 11665 12439
11666 12442 11664 12445 8 N F
11664 12437 11660 12435 11662 12433 11665 12433 11669 12433 11667 12436
11664 12437 7 N F
11663 12619 11662 12615 11664 12613 11666 12610 11665 12607 11664 12604
11664 12603 11665 12600 11668 12600 11671 12598 11669 12595 11667 12593
11667 12590 11669 12587 11669 12584 11671 12581 11674 12581 11675 12585
11676 12588 11679 12585 11682 12585 11685 12585 11688 12587 11689 12590
11689 12593 11688 12596 11685 12597 11684 12600 11684 12604 11681 12603
11678 12603 11676 12606 11674 12608 11676 12611 11677 12614 11676 12618
11673 12619 11670 12620 11666 12620 11663 12619 40 N F
11669 12726 11666 12725 11667 12722 11669 12719 11672 12719 11674 12716
11676 12717 11677 12717 11677 12721 11675 12722 11672 12724 11669 12726
12 N F
11668 12422 11667 12419 11668 12416 11671 12417 11673 12414 11675 12411
11678 12411 11678 12414 11680 12417 11680 12420 11677 12417 11676 12420
11674 12423 11671 12424 11668 12422 15 N F
11671 12690 11668 12687 11670 12687 11671 12688 11674 12689 11676 12692
11676 12695 11673 12693 11671 12690 9 N F
11673 12459 11670 12457 11671 12454 11671 12451 11674 12449 11677 12449
11679 12449 11681 12449 11684 12449 11685 12453 11688 12452 11691 12454
11691 12454 11694 12452 11697 12451 11700 12450 11701 12453 11699 12455
11696 12456 11693 12458 11690 12459 11687 12461 11684 12459 11681 12459
11680 12456 11677 12456 11673 12459 27 N F
11675 14215 11672 14211 11674 14209 11675 14212 11677 14214 11677 14216
11675 14215 7 N F
11679 12740 11672 12739 11675 12735 11677 12731 11680 12728 11681 12724
11681 12724 11685 12721 11689 12720 11691 12716 11690 12712 11694 12712
11698 12714 11701 12717 11703 12721 11700 12725 11699 12729 11701 12733
11705 12732 11709 12730 11709 12734 11713 12736 11713 12741 11710 12743
11706 12746 11710 12748 11713 12748 11714 12748 11714 12753 11710 12752
11710 12757 11707 12760 11704 12757 11700 12759 11696 12760 11692 12757
11694 12753 11696 12749 11699 12745 11697 12741 11692 12742 11688 12743
11685 12746 11681 12744 11679 12740 45 N F
11677 12579 11673 12577 11677 12575 11680 12578 11684 12577 11688 12578
11687 12574 11683 12572 11681 12568 11677 12570 11674 12565 11678 12564
11683 12564 11687 12564 11690 12565 11691 12566 11694 12569 11698 12571
11701 12574 11700 12578 11704 12579 11706 12583 11704 12587 11707 12590
11707 12595 11710 12598 11707 12601 11703 12603 11705 12606 11701 12608

11697 12610 11693 12611 11693 12616 11689 12618 11685 12618 11680 12618
11680 12614 11683 12611 11687 12609 11690 12606 11692 12602 11692 12597
11696 12595 11700 12594 11696 12592 11694 12588 11695 12584 11692 12581
11688 12580 11684 12583 11680 12582 11677 12579 52 N F
11679 12446 11674 12446 11675 12443 11677 12440 11680 12442 11683 12443
11683 12446 11679 12446 8 N F
11678 12639 11675 12636 11676 12632 11677 12629 11678 12626 11679 12629
11679 12632 11679 12635 11678 12639 9 N F
11678 13534 11676 13534 11677 13531 11679 13529 11680 13531 11682 13533
11682 13536 11684 13538 11681 13539 11679 13539 11678 13537 11678 13534
12 N F
11681 12717 11677 12715 11678 12714 11679 12712 11681 12710 11684 12711
11682 12714 11681 12717 8 N F
11679 14140 11678 14136 11681 14135 11683 14132 11683 14129 11683 14126
11685 14123 11688 14122 11691 14123 11691 14123 11695 14124 11695 14127
11695 14131 11698 14133 11695 14135 11692 14136 11690 14139 11688 14141
11685 14143 11684 14145 11680 14146 11680 14143 11679 14140 23 N F
11681 12470 11678 12467 11681 12465 11679 12463 11681 12461 11684 12461
11684 12464 11683 12467 11681 12470 9 N F
11685 12696 11678 12694 11682 12692 11686 12689 11687 12688 11688 12685
11690 12682 11691 12682 11694 12682 11698 12680 11697 12675 11697 12671
11702 12671 11702 12671 11705 12668 11708 12671 11711 12675 11715 12675
11716 12671 11720 12669 11719 12664 11723 12663 11725 12662 11727 12662
11732 12662 11733 12667 11733 12671 11733 12675 11731 12679 11728 12682
11731 12686 11727 12688 11725 12691 11721 12693 11717 12690 11713 12691
11709 12693 11707 12697 11703 12698 11699 12696 11695 12697 11691 12699
11690 12695 11685 12696 44 N F
11680 14213 11679 14208 11682 14207 11684 14210 11684 14213 11684 14217
11686 14219 11684 14221 11681 14220 11681 14216 11680 14213 11 N F
11682 12984 11679 12979 11681 12977 11683 12975 11684 12975 11684 12971
11688 12972 11690 12975 11689 12978 11689 12981 11687 12984 11684 12986
11682 12984 13 N F
11683 12536 11679 12536 11681 12535 11682 12534 11684 12531 11683 12528
11685 12525 11687 12526 11688 12527 11690 12529 11691 12533 11693 12534
11696 12536 11696 12540 11693 12538 11692 12541 11695 12542 11692 12544
11690 12547 11687 12545 11685 12543 11685 12539 11683 12536 23 N F
11683 12933 11680 12932 11680 12929 11680 12925 11683 12927 11684 12930
11683 12933 7 N F
11687 12827 11680 12822 11684 12820 11688 12822 11692 12823 11697 12822
11701 12821 11705 12823 11704 12819 11700 12820 11699 12815 11700 12811
11700 12807 11699 12803 11697 12799 11696 12795 11700 12796 11704 12797
11705 12802 11704 12806 11707 12807 11708 12807 11711 12809 11715 12807
11715 12812 11719 12813 11718 12817 11718 12822 11715 12825 11711 12826

11709 12829 11708 12834 11705 12837 11701 12834 11697 12837 11693 12837
11693 12833 11689 12831 11687 12827 39 N F
11681 13524 11681 13522 11682 13519 11684 13519 11685 13519 11685 13521
11686 13524 11686 13526 11684 13526 11681 13524 10 N F
11685 12650 11683 12645 11685 12642 11687 12644 11688 12647 11689 12648
11690 12649 11691 12652 11687 12652 11685 12650 10 N F
11685 12438 11683 12433 11687 12435 11687 12430 11691 12429 11695 12429
11700 12428 11701 12427 11702 12425 11702 12429 11704 12433 11705 12429
11708 12425 11710 12429 11714 12430 11718 12428 11717 12432 11716 12437
11715 12441 11715 12445 11711 12444 11707 12444 11703 12442 11698 12443
11695 12446 11691 12448 11689 12444 11685 12444 11688 12441 11685 12438
30 N F
11685 12681 11683 12677 11686 12676 11688 12679 11689 12682 11687 12684
11685 12681 7 N F
11687 12936 11684 12934 11688 12934 11691 12934 11693 12936 11695 12939
11692 12940 11690 12937 11687 12936 9 N F
11686 14210 11685 14205 11688 14204 11690 14206 11694 14207 11697 14206
11699 14204 11702 14203 11702 14207 11702 14210 11703 14213 11702 14216
11700 14219 11697 14220 11694 14219 11694 14216 11691 14214 11688 14212
11686 14210 19 N F
11693 12709 11685 12707 11689 12705 11691 12705 11693 12704 11697 12702
11701 12703 11705 12704 11708 12701 11711 12697 11713 12694 11717 12696
11722 12697 11723 12701 11723 12705 11721 12709 11724 12712 11722 12716
11718 12716 11717 12720 11713 12722 11711 12718 11714 12715 11709 12715
11705 12713 11702 12711 11697 12710 11693 12709 28 N F
11688 13985 11686 13980 11689 13978 11692 13976 11693 13979 11693 13982
11691 13985 11688 13985 8 N F
11690 12404 11690 12400 11693 12400 11695 12398 11698 12400 11701 12400
11704 12398 11706 12401 11707 12404 11710 12405 11708 12408 11707 12411
11704 12412 11701 12414 11702 12411 11702 12408 11700 12405 11696 12405
11693 12404 11690 12404 20 N F
11696 14195 11691 14194 11694 14191 11698 14189 11700 14185 11699 14181
11703 14180 11702 14185 11705 14188 11707 14192 11704 14195 11700 14195
11696 14195 13 N F
11697 12854 11691 12850 11693 12850 11695 12849 11696 12845 11700 12844
11704 12845 11707 12842 11710 12839 11711 12843 11711 12847 11713 12843
11714 12839 11717 12842 11717 12846 11716 12851 11714 12855 11709 12855
11705 12854 11701 12855 11697 12854 21 N F
11699 12665 11696 12661 11698 12658 11701 12658 11703 12661 11704 12664
11702 12666 11699 12665 8 N F
11701 14073 11699 14067 11701 14063 11702 14059 11703 14055 11704 14050
11708 14053 11709 14054 11711 14056 11713 14052 11712 14048 11716 14050
11719 14054 11721 14057 11723 14061 11725 14065 11729 14066 11729 14070

11727 14074 11727 14079 11722 14080 11718 14080 11714 14080 11709 14080
11709 14075 11705 14073 11701 14073 27 N F
11704 12879 11702 12873 11706 12873 11709 12869 11707 12865 11709 12865
11712 12864 11713 12861 11718 12860 11722 12861 11725 12858 11729 12858
11733 12860 11736 12857 11736 12857 11740 12859 11742 12855 11738 12852
11738 12848 11742 12848 11743 12847 11744 12843 11746 12842 11747 12840
11747 12845 11751 12843 11753 12846 11756 12850 11752 12852 11748 12854
11749 12858 11748 12862 11744 12865 11741 12862 11737 12862 11735 12866
11731 12868 11730 12873 11733 12876 11737 12874 11740 12877 11739 12882
11735 12882 11732 12878 11731 12882 11728 12885 11725 12883 11723 12878
11719 12879 11715 12882 11714 12886 11710 12883 11706 12883 11704 12879
54 N F
11709 13960 11705 13957 11707 13955 11708 13951 11708 13948 11711 13947
11714 13948 11717 13949 11720 13951 11719 13954 11718 13957 11715 13959
11713 13957 11710 13957 11709 13960 15 N F
11709 14175 11707 14171 11709 14169 11712 14168 11714 14171 11714 14174
11712 14177 11709 14175 8 N F
11709 12634 11708 12630 11713 12629 11715 12626 11714 12622 11715 12618
11714 12614 11718 12613 11721 12616 11726 12617 11728 12621 11732 12622
11735 12619 11739 12622 11742 12625 11746 12626 11748 12630 11744 12631
11739 12632 11740 12636 11738 12640 11735 12642 11732 12639 11729 12636
11725 12636 11728 12639 11729 12644 11725 12646 11721 12644 11719 12641
11715 12639 11713 12635 11709 12634 33 N F
11711 12512 11709 12505 11713 12499 11718 12494 11721 12488 11727 12485
11726 12491 11724 12498 11726 12504 11728 12497 11733 12493 11740 12492
11736 12486 11733 12480 11735 12474 11739 12479 11743 12473 11750 12473
11757 12473 11761 12475 11763 12477 11769 12479 11764 12484 11771 12483
11772 12483 11778 12485 11781 12490 11788 12491 11788 12498 11794 12501
11801 12501 11802 12508 11807 12509 11809 12509 11810 12515 11815 12511
11817 12505 11822 12509 11820 12516 11824 12518 11826 12519 11828 12526
11832 12531 11829 12537 11833 12543 11828 12547 11832 12553 11833 12560
11834 12566 11829 12570 11825 12576 11819 12580 11814 12584 11808 12581
11806 12575 11801 12579 11798 12585 11800 12592 11796 12598 11790 12601
11783 12603 11777 12604 11770 12604 11768 12598 11771 12591 11778 12591
11785 12593 11782 12586 11784 12580 11781 12574 11776 12569 11773 12563
11767 12564 11766 12571 11764 12578 11760 12572 11763 12566 11757 12563
11760 12557 11761 12550 11765 12544 11772 12547 11769 12541 11762 12542
11766 12536 11764 12530 11763 12523 11762 12516 11758 12510 11759 12517
11757 12524 11758 12531 11761 12537 11757 12543 11757 12550 11751 12551
11750 12558 11744 12554 11743 12547 11746 12541 11744 12535 11739 12540
11732 12540 11728 12534 11722 12532 11720 12525 11725 12520 11719 12522
11713 12518 11711 12512 110 N F
11711 14191 11709 14186 11711 14182 11712 14181 11714 14179 11719 14180

11721 14184 11725 14184 11728 14187 11732 14189 11736 14188 11740 14189
11744 14191 11745 14195 11748 14198 11748 14203 11745 14206 11743 14210
11739 14212 11735 14213 11730 14212 11726 14214 11722 14216 11719 14218
11715 14216 11713 14212 11713 14208 11713 14203 11711 14199 11712 14195
11711 14191 31 N F
11713 13562 11713 13560 11713 13556 11714 13556 11716 13556 11718 13558
11720 13556 11722 13558 11724 13560 11724 13562 11722 13564 11721 13567
11722 13569 11720 13571 11718 13572 11717 13569 11717 13566 11715 13564
11713 13562 19 N F
11720 13151 11714 13148 11717 13145 11719 13141 11723 13144 11727 13145
11730 13149 11732 13152 11735 13156 11739 13157 11741 13161 11742 13165
11740 13169 11737 13171 11737 13167 11734 13164 11729 13165 11725 13166
11728 13163 11723 13162 11724 13158 11720 13155 11720 13151 23 N F
11728 12657 11726 12651 11730 12651 11733 12650 11736 12652 11737 12654
11739 12652 11742 12654 11743 12657 11740 12660 11743 12662 11744 12665
11741 12666 11738 12665 11735 12663 11734 12660 11734 12657 11731 12657
11728 12657 19 N F
11729 12813 11728 12809 11730 12807 11731 12804 11734 12802 11736 12805
11735 12808 11735 12812 11735 12815 11736 12818 11733 12817 11729 12816
11729 12813 13 N F
11730 13587 11729 13582 11730 13579 11733 13578 11735 13580 11737 13580
11738 13580 11740 13579 11743 13579 11744 13579 11745 13578 11748 13579
11751 13578 11754 13578 11756 13576 11757 13576 11758 13576 11761 13576
11764 13578 11763 13580 11760 13580 11757 13579 11755 13581 11753 13582
11754 13584 11753 13587 11751 13589 11750 13591 11747 13591 11745 13591
11743 13590 11742 13587 11743 13585 11740 13585 11738 13586 11735 13587
11733 13588 11730 13587 38 N F
11744 13298 11739 13296 11742 13295 11746 13295 11744 13298 5 N F
11740 12790 11740 12783 11741 12782 11743 12781 11747 12779 11750 12783
11754 12783 11754 12787 11755 12792 11751 12793 11746 12793 11742 12793
11740 12790 13 N F
11750 14084 11745 14085 11748 14081 11751 14078 11754 14075 11755 14071
11757 14067 11757 14063 11758 14058 11754 14057 11750 14054 11752 14050
11756 14051 11754 14047 11752 14043 11749 14040 11747 14035 11750 14037
11753 14041 11757 14040 11759 14036 11763 14038 11763 14043 11765 14047
11766 14051 11770 14050 11774 14050 11774 14054 11778 14057 11782 14058
11783 14062 11782 14066 11780 14069 11779 14074 11775 14075 11771 14075
11767 14076 11764 14079 11759 14078 11759 14083 11761 14086 11761 14091
11759 14087 11757 14083 11753 14081 11750 14084 46 N F
11749 12832 11746 12833 11748 12829 11750 12826 11750 12823 11750 12819
11748 12817 11751 12816 11754 12817 11757 12816 11760 12818 11759 12822
11759 12825 11756 12827 11754 12830 11752 12832 11749 12832 17 N F
11747 12751 11747 12745 11751 12745 11755 12744 11760 12743 11764 12743

11766 12746 11768 12750 11772 12752 11776 12751 11781 12752 11779 12756
11784 12755 11786 12751 11790 12751 11794 12750 11796 12746 11800 12745
11798 12741 11795 12738 11797 12734 11801 12734 11803 12737 11807 12736
11810 12739 11809 12743 11811 12748 11811 12752 11810 12756 11806 12758
11803 12760 11799 12760 11794 12759 11790 12758 11786 12759 11788 12763
11784 12765 11788 12768 11789 12772 11793 12770 11794 12775 11798 12775
11800 12779 11796 12779 11793 12783 11789 12784 11785 12783 11781 12783
11779 12787 11774 12787 11770 12786 11767 12783 11770 12779 11774 12777
11778 12774 11778 12770 11775 12767 11775 12763 11771 12764 11771 12759
11768 12757 11765 12760 11764 12757 11762 12753 11757 12753 11753 12752
11750 12755 11747 12751 68 N F
11752 13297 11748 13296 11751 13295 11755 13295 11756 13295 11757 13296
11760 13298 11757 13300 11754 13299 11752 13297 10 N F
11756 13279 11754 13275 11756 13273 11758 13270 11761 13272 11760 13275
11758 13278 11756 13279 8 N F
11757 12415 11755 12411 11758 12409 11761 12409 11759 12412 11760 12415
11761 12418 11762 12421 11761 12424 11758 12421 11757 12418 11757 12415
12 N F
11762 12814 11756 12811 11759 12808 11762 12805 11764 12801 11766 12797
11770 12795 11774 12797 11773 12801 11771 12805 11769 12808 11765 12811
11762 12814 13 N F
11760 7118 11759 7109 11761 7105 11764 7102 11768 7100 11773 7099
11777 7100 11780 7103 11785 7104 11789 7102 11793 7102 11798 7102
11802 7104 11804 7109 11803 7113 11801 7117 11799 7121 11797 7125
11793 7128 11789 7129 11785 7131 11781 7133 11776 7134 11772 7135
11768 7132 11768 7127 11769 7123 11768 7118 11764 7116 11760 7118
30 N F
11767 13281 11763 13281 11766 13280 11766 13276 11769 13277 11769 13274
11772 13276 11775 13275 11776 13278 11774 13281 11777 13282 11776 13285
11774 13288 11771 13289 11768 13288 11768 13285 11771 13284 11773 13282
11772 13279 11769 13278 11767 13281 21 N F
11768 13429 11765 13427 11767 13425 11769 13423 11772 13423 11775 13423
11774 13425 11772 13427 11770 13429 11768 13429 10 N F
11773 13304 11769 13303 11771 13302 11772 13302 11775 13302 11778 13302
11776 13304 11773 13304 8 N F
11777 13274 11776 13271 11778 13269 11782 13269 11784 13272 11787 13273
11787 13276 11785 13279 11784 13276 11783 13273 11779 13273 11781 13276
11778 13277 11777 13274 14 N F
11789 13291 11786 13289 11788 13286 11790 13283 11790 13280 11793 13279
11793 13282 11795 13283 11796 13284 11798 13281 11801 13281 11804 13282
11807 13284 11804 13286 11801 13287 11798 13288 11795 13288 11793 13291
11789 13291 19 N F
11795 13246 11791 13242 11794 13241 11795 13238 11797 13235 11798 13238

11799 13241 11797 13244 11795 13246 9 N F
11799 12493 11797 12487 11800 12486 11803 12485 11802 12488 11805 12488
11808 12489 11811 12492 11811 12495 11808 12497 11806 12495 11808 12498
11807 12501 11804 12500 11801 12498 11798 12496 11799 12493 17 N F
11810 11923 11804 11919 11804 11912 11806 11906 11805 11899 11805 11890
11809 11885 11813 11880 11817 11874 11823 11870 11824 11870 11829 11867
11836 11866 11842 11862 11846 11857 11851 11852 11856 11847 11862 11844
11868 11844 11869 11851 11870 11858 11874 11864 11878 11869 11884 11872
11891 11870 11898 11870 11905 11869 11911 11872 11917 11876 11924 11877
11930 11873 11936 11875 11943 11877 11948 11872 11955 11871 11961 11875
11967 11875 11972 11880 11978 11881 11985 11882 11992 11882 11999 11885
12005 11882 12011 11884 12016 11889 12021 11893 12025 11899 12031 11902
12038 11904 12045 11904 12051 11906 12058 11909 12064 11911 12071 11912
12075 11917 12080 11922 12084 11928 12091 11928 12098 11929 12105 11931
12111 11933 12115 11939 12114 11946 12119 11950 12122 11956 12124 11963
12125 11970 12128 11976 12134 11979 12139 11985 12138 11991 12140 11998
12141 12005 12146 12001 12144 12007 12143 12014 12146 12020 12153 12019
12148 12024 12143 12019 12142 12026 12136 12028 12132 12034 12135 12040
12134 12047 12130 12053 12124 12056 12117 12057 12111 12054 12105 12057
12099 12060 12094 12064 12090 12070 12083 12068 12076 12070 12069 12070
12063 12069 12057 12066 12050 12066 12043 12063 12037 12061 12031 12058
12024 12058 12018 12054 12011 12056 12005 12052 11999 12049 11993 12046
11987 12044 11980 12041 11974 12039 11968 12036 11961 12033 11956 12029
11949 12031 11942 12032 11939 12027 11932 12026 11927 12021 11921 12017
11914 12016 11909 12012 11903 12008 11896 12010 11891 12006 11885 12004
11879 11999 11873 11996 11866 11996 11863 11990 11859 11984 11853 11981
11848 11977 11843 11972 11843 11965 11839 11959 11835 11953 11830 11949
11824 11945 11821 11939 11816 11934 11810 11930 11810 11923 143 N F
11807 13301 11804 13300 11806 13298 11809 13299 11810 13301 11807 13301
6 N F
11817 13360 11814 13357 11817 13356 11820 13356 11821 13358 11820 13360
11817 13360 7 N F
11819 13410 11816 13407 11819 13406 11820 13406 11822 13406 11824 13405
11825 13407 11825 13410 11824 13413 11821 13412 11819 13410 11 N F
11824 7166 11817 7165 11820 7159 11825 7153 11828 7147 11832 7141
11837 7135 11839 7129 11833 7126 11832 7119 11834 7112 11832 7105
11825 7102 11823 7096 11829 7092 11834 7087 11838 7081 11834 7076
11823 7076 11826 7069 11831 7064 11837 7060 11844 7058 11845 7065
11847 7058 11853 7054 11860 7057 11866 7059 11873 7060 11880 7064
11887 7066 11894 7067 11901 7068 11908 7068 11915 7068 11922 7070
11929 7071 11934 7066 11930 7072 11925 7078 11921 7083 11916 7088
11909 7089 11902 7091 11895 7093 11892 7099 11885 7102 11878 7101
11871 7103 11864 7105 11861 7111 11862 7118 11857 7123 11850 7125

11847 7131 11845 7138 11842 7145 11836 7149 11831 7154 11827 7160
11824 7166 61 N F
11824 12268 11822 12263 11826 12261 11830 12259 11831 12263 11828 12267
11824 12268 7 N F
11828 13372 11825 13369 11828 13369 11830 13367 11832 13365 11835 13366
11837 13367 11839 13369 11836 13370 11834 13371 11831 13371 11828 13372
12 N F
11837 12262 11832 12258 11836 12256 11839 12253 11840 12249 11845 12249
11843 12253 11844 12257 11848 12258 11849 12262 11845 12264 11842 12261
11837 12262 13 N F
11844 13365 11840 13363 11843 13363 11846 13362 11847 13360 11850 13360
11847 13362 11847 13364 11844 13365 9 N F
11869 13280 11867 13275 11869 13272 11873 13272 11875 13274 11876 13277
11877 13280 11875 13283 11871 13283 11869 13280 10 N F
11903 12396 11901 12393 11904 12392 11906 12395 11908 12398 11911 12397
11912 12401 11914 12403 11911 12406 11909 12408 11907 12405 11905 12402
11905 12399 11903 12396 14 N F
11914 12387 11911 12384 11913 12380 11911 12372 11914 12369 11916 12365
11920 12364 11925 12364 11928 12366 11932 12368 11936 12371 11939 12374
11940 12378 11944 12378 11945 12383 11949 12384 11949 12389 11953 12386
11953 12386 11955 12382 11955 12387 11959 12385 11964 12386 11963 12390
11959 12390 11955 12390 11951 12393 11946 12393 11942 12391 11938 12392
11934 12390 11932 12395 11928 12397 11924 12396 11920 12393 11917 12390
11914 12387 37 N F
11931 12407 11928 12403 11928 12403 11932 12402 11935 12398 11938 12395
11942 12395 11947 12394 11951 12395 11947 12397 11946 12401 11950 12404
11952 12400 11956 12400 11959 12404 11956 12407 11959 12410 11963 12412
11960 12415 11957 12419 11954 12422 11950 12424 11946 12423 11942 12424
11938 12422 11935 12425 11931 12427 11932 12423 11935 12420 11931 12418
11930 12414 11934 12415 11932 12411 11931 12407 34 N F
11947 12434 11946 12428 11950 12427 11952 12430 11955 12428 11957 12431
11960 12431 11960 12434 11958 12437 11955 12438 11952 12437 11949 12435
11947 12434 13 N F
11953 12363 11950 12361 11952 12359 11950 12354 11953 12355 11956 12354
11959 12353 11959 12357 11957 12359 11955 12362 11953 12363 11 N F
11961 12515 11958 12510 11960 12506 11960 12502 11961 12497 11963 12493
11962 12489 11965 12486 11964 12482 11968 12483 11972 12481 11975 12479
11976 12479 11979 12477 11982 12480 11986 12482 11989 12479 11992 12476
11989 12473 11985 12473 11985 12468 11988 12465 11992 12467 11996 12468
12001 12467 12004 12465 12008 12463 12008 12468 12006 12472 12010 12470
12013 12467 12017 12467 12021 12467 12023 12466 12026 12466 12023 12470
12019 12471 12021 12475 12023 12479 12019 12481 12014 12481 12010 12481
12011 12485 12010 12489 12005 12490 12005 12495 12003 12499 12002 12503

12002 12507 12001 12512 11998 12515 11995 12517 11991 12520 11987 12522
11983 12524 11979 12525 11975 12523 11972 12520 11967 12520 11963 12519
11961 12515 61 N F
11971 12386 11967 12383 11969 12379 11972 12375 11972 12371 11976 12373
11979 12370 11980 12366 11984 12364 11987 12368 11987 12372 11989 12376
11988 12380 11991 12383 11991 12387 11987 12385 11986 12389 11984 12393
11980 12394 11980 12398 11984 12399 11989 12400 11991 12404 11987 12407
11984 12409 11979 12410 11975 12412 11972 12410 11972 12405 11967 12405
11968 12401 11973 12402 11975 12398 11975 12394 11972 12390 11971 12386
36 N F
12010 12520 12008 12515 12012 12517 12016 12517 12020 12518 12019 12514
12017 12509 12017 12505 12020 12503 12023 12506 12024 12510 12026 12514
12028 12518 12032 12520 12036 12520 12040 12521 12040 12526 12043 12529
12047 12528 12048 12528 12051 12526 12055 12526 12060 12526 12059 12530
12056 12533 12052 12535 12052 12540 12048 12538 12044 12539 12047 12542
12048 12546 12045 12548 12043 12552 12039 12552 12035 12550 12031 12547
12027 12546 12023 12546 12020 12542 12017 12539 12016 12535 12013 12532
12015 12528 12012 12524 12010 12520 45 N F
12015 12493 12012 12490 12016 12490 12020 12488 12025 12488 12028 12486
12031 12482 12034 12486 12037 12489 12042 12489 12046 12490 12050 12487
12052 12483 12052 12479 12054 12476 12059 12477 12060 12481 12064 12484
12063 12488 12067 12487 12067 12483 12072 12483 12073 12487 12071 12492
12072 12496 12076 12495 12078 12499 12076 12503 12073 12501 12068 12501
12066 12505 12061 12504 12057 12504 12054 12501 12051 12505 12048 12507
12044 12505 12041 12501 12042 12497 12038 12494 12034 12493 12031 12496
12028 12499 12026 12495 12024 12491 12020 12492 12015 12493 47 N F
12020 12422 12019 12419 12020 12415 12020 12409 12022 12408 12025 12405
12026 12408 12026 12411 12029 12414 12029 12417 12028 12420 12026 12423
12023 12423 12020 12422 14 N F
12063 12536 12057 12535 12061 12532 12063 12529 12060 12525 12060 12521
12061 12516 12064 12519 12066 12524 12070 12525 12069 12521 12069 12517
12071 12512 12075 12512 12079 12509 12079 12509 12083 12510 12087 12512
12088 12516 12092 12517 12097 12517 12098 12521 12096 12525 12095 12529
12095 12534 12097 12538 12096 12542 12094 12538 12091 12534 12090 12530
12086 12531 12082 12530 12079 12533 12074 12533 12070 12534 12067 12536
12065 12532 12063 12536 38 N F
12068 12417 12064 12407 12068 12401 12075 12403 12080 12399 12087 12399
12090 12398 12094 12398 12098 12403 12099 12410 12098 12417 12093 12421
12092 12428 12086 12431 12079 12433 12074 12429 12070 12424 12068 12417
18 N F
12107 12310 12104 12304 12106 12300 12111 12299 12115 12301 12117 12304
12115 12308 12111 12310 12107 12310 9 N F
12108 12501 12105 12497 12106 12493 12106 12489 12110 12488 12111 12485

12112 12484 12116 12486 12120 12487 12123 12490 12127 12491 12128 12495
12132 12497 12130 12501 12124 12505 12119 12504 12115 12505 12111 12504
12108 12501 19 N F
12122 12516 12118 12517 12120 12513 12124 12512 12124 12512 12128 12513
12132 12512 12136 12509 12133 12507 12137 12506 12140 12503 12141 12507
12145 12508 12148 12505 12145 12502 12141 12499 12142 12495 12143 12491
12145 12495 12147 12498 12150 12502 12153 12505 12158 12504 12159 12508
12154 12508 12153 12512 12157 12511 12162 12511 12165 12508 12169 12508
12167 12512 12163 12515 12163 12520 12158 12520 12154 12521 12151 12518
12147 12516 12143 12514 12144 12518 12142 12522 12138 12524 12136 12528
12133 12531 12135 12527 12130 12527 12126 12525 12129 12521 12126 12518
12122 12516 49 N F
12133 12571 12127 12569 12130 12567 12133 12563 12133 12563 12135 12559
12135 12555 12137 12551 12141 12548 12142 12553 12141 12557 12145 12555
12145 12551 12146 12547 12148 12543 12150 12547 12151 12551 12152 12555
12151 12559 12149 12563 12148 12567 12145 12571 12141 12573 12137 12573
12133 12571 25 N F
12163 12582 12157 12581 12160 12578 12165 12579 12167 12575 12164 12573
12163 12569 12167 12569 12169 12566 12173 12564 12177 12561 12181 12559
12184 12562 12189 12561 12191 12565 12196 12565 12200 12565 12204 12565
12209 12565 12213 12566 12216 12569 12217 12574 12216 12578 12218 12582
12219 12586 12221 12590 12220 12594 12217 12598 12214 12601 12210 12600
12206 12597 12202 12597 12197 12598 12194 12596 12190 12593 12188 12589
12184 12590 12181 12586 12177 12588 12173 12589 12171 12593 12167 12593
12167 12589 12165 12585 12163 12582 45 N F
12233 12453 12231 12446 12235 12448 12239 12448 12243 12449 12248 12449
12248 12453 12250 12457 12248 12461 12247 12465 12247 12470 12243 12469
12239 12467 12239 12463 12235 12462 12234 12457 12233 12453 17 N F
12271 12366 12266 12363 12268 12359 12269 12355 12273 12352 12274 12348
12277 12344 12280 12342 12284 12342 12289 12341 12291 12344 12287 12344
12283 12346 12281 12350 12278 12353 12276 12357 12274 12361 12276 12365
12275 12369 12271 12366 20 N F
12285 12367 12279 12366 12282 12363 12284 12359 12288 12357 12292 12355
12293 12360 12289 12362 12290 12366 12285 12367 10 N F
12310 12630 12307 12626 12311 12624 12315 12624 12316 12619 12320 12617
12315 12616 12313 12612 12315 12609 12318 12605 12322 12605 12325 12608
12329 12609 12333 12611 12337 12610 12339 12614 12339 12618 12336 12621
12332 12623 12328 12623 12323 12623 12327 12625 12324 12628 12320 12630
12324 12633 12326 12636 12325 12640 12321 12641 12316 12642 12314 12638
12311 12635 12310 12630 32 N F
12320 12594 12318 12588 12323 12589 12327 12590 12331 12591 12334 12587
12329 12587 12325 12587 12327 12583 12331 12581 12335 12583 12339 12581
12339 12576 12337 12572 12341 12570 12344 12573 12348 12575 12351 12578

12356 12579 12359 12582 12360 12586 12359 12590 12357 12594 12353 12595
12350 12598 12346 12601 12343 12604 12342 12600 12338 12599 12334 12596
12331 12599 12327 12600 12323 12602 12319 12600 12322 12598 12320 12594
36 N F
12397 12670 12395 12666 12396 12660 12397 12664 12400 12661 12403 12657
12407 12657 12410 12660 12414 12662 12415 12666 12418 12663 12422 12664
12426 12666 12423 12669 12419 12672 12416 12669 12411 12669 12409 12673
12405 12674 12402 12671 12397 12670 21 N F
12430 12651 12424 12650 12427 12647 12431 12646 12435 12643 12438 12645
12438 12641 12441 12638 12443 12634 12447 12633 12451 12634 12454 12632
12459 12631 12460 12627 12464 12627 12469 12627 12471 12631 12470 12635
12467 12638 12463 12638 12458 12639 12462 12640 12462 12645 12459 12648
12456 12651 12454 12655 12452 12659 12448 12660 12444 12661 12440 12660
12436 12658 12433 12655 12430 12651 33 N F
12456 12835 12454 12829 12455 12822 12458 12816 12462 12810 12468 12806
12469 12799 12474 12794 12477 12788 12474 12782 12472 12775 12475 12775
12479 12773 12481 12767 12487 12763 12493 12762 12500 12762 12506 12766
12506 12767 12508 12772 12504 12778 12502 12785 12508 12788 12513 12792
12520 12794 12521 12787 12521 12780 12522 12773 12523 12772 12525 12767
12527 12760 12533 12759 12536 12760 12540 12760 12542 12761 12546 12763
12553 12764 12557 12759 12564 12761 12569 12762 12570 12762 12566 12768
12563 12774 12567 12779 12571 12785 12569 12791 12568 12798 12567 12805
12566 12812 12570 12817 12569 12824 12563 12828 12557 12826 12552 12831
12540 12828 12533 12826 12530 12819 12524 12816 12520 12811 12515 12816
12513 12822 12506 12824 12499 12825 12500 12832 12495 12836 12492 12842
12493 12849 12498 12853 12501 12860 12502 12866 12501 12873 12497 12879
12491 12882 12485 12883 12478 12881 12473 12876 12469 12871 12469 12864
12473 12859 12467 12856 12460 12854 12460 12847 12460 12840 12456 12835
84 N F
12474 12601 12470 12597 12475 12597 12472 12593 12472 12589 12477 12589
12480 12586 12482 12590 12482 12594 12481 12598 12482 12603 12482 12607
12478 12609 12479 12613 12475 12613 12473 12609 12474 12605 12474 12601
18 N F
12482 8073 12474 8065 12479 8060 12486 8058 12493 8058 12500 8057
12505 8052 12508 8046 12512 8040 12518 8036 12524 8033 12532 8032
12529 8039 12527 8046 12526 8053 12530 8058 12533 8065 12526 8069
12523 8075 12516 8077 12512 8083 12506 8087 12499 8085 12493 8082
12489 8076 12482 8073 26 N F
12522 12848 12520 12844 12521 12840 12523 12836 12525 12832 12529 12830
12533 12829 12538 12829 12542 12830 12546 12832 12547 12836 12551 12837
12555 12835 12559 12833 12563 12834 12567 12832 12572 12833 12575 12830
12579 12830 12578 12835 12574 12837 12570 12839 12567 12842 12566 12846
12568 12850 12567 12854 12563 12857 12559 12858 12555 12861 12557 12865

12553 12865 12550 12862 12548 12858 12547 12854 12547 12850 12545 12846
12542 12848 12542 12853 12543 12857 12543 12861 12546 12865 12547 12869
12549 12873 12548 12878 12544 12876 12540 12875 12534 12871 12532 12867
12531 12862 12529 12859 12527 12855 12525 12851 12522 12848 53 N F
12522 8205 12521 8204 12521 8203 12521 8203 12522 8203 12523 8203
12523 8203 12523 8203 12523 8204 12524 8205 12523 8205 12522 8205
12 N F
12533 8060 12531 8057 12533 8058 12535 8058 12537 8059 12537 8061
12536 8063 12534 8062 12533 8060 9 N F
12540 8063 12540 8060 12542 8060 12543 8062 12542 8064 12540 8063
6 N F
12546 8060 12545 8057 12546 8059 12548 8060 12550 8061 12552 8061
12554 8063 12556 8064 12557 8066 12559 8067 12561 8068 12563 8068
12565 8069 12566 8071 12569 8071 12571 8072 12569 8073 12566 8072
12565 8070 12563 8069 12561 8069 12559 8069 12556 8068 12555 8066
12553 8065 12552 8063 12549 8063 12547 8062 12546 8060 29 N F
12616 12850 12614 12842 12618 12841 12622 12839 12621 12835 12625 12834
12628 12837 12629 12842 12632 12845 12637 12845 12641 12845 12640 12850
12636 12851 12632 12852 12628 12854 12624 12853 12620 12851 12616 12850
18 N F
12636 12834 12630 12830 12634 12830 12635 12826 12639 12826 12642 12823
12647 12822 12651 12820 12651 12825 12648 12828 12653 12829 12657 12829
12660 12833 12657 12836 12653 12837 12650 12841 12647 12843 12643 12845
12639 12844 12636 12841 12633 12837 12636 12834 22 N F
12656 12738 12653 12733 12656 12730 12660 12728 12660 12724 12662 12720
12665 12723 12669 12725 12671 12728 12675 12728 12680 12727 12684 12728
12683 12732 12686 12735 12684 12739 12680 12738 12678 12734 12677 12730
12673 12733 12676 12736 12673 12739 12669 12739 12665 12742 12661 12743
12657 12742 12656 12738 26 N F
12693 8098 12690 8092 12693 8089 12695 8085 12699 8087 12703 8090
12699 8091 12696 8095 12697 8100 12693 8098 10 N F
12718 8227 12712 8215 12719 8217 12726 8218 12732 8221 12730 8228
12724 8231 12718 8227 8 N F
12750 12884 12748 12878 12749 12871 12752 12865 12755 12858 12757 12858
12761 12856 12764 12850 12768 12844 12774 12847 12773 12854 12780 12856
12786 12859 12790 12864 12793 12870 12800 12869 12801 12862 12803 12856
12802 12849 12802 12842 12796 12838 12790 12837 12784 12834 12785 12827
12786 12828 12790 12832 12790 12825 12797 12826 12803 12829 12809 12826
12804 12822 12803 12816 12805 12809 12812 12808 12809 12802 12806 12795
12813 12793 12814 12793 12819 12791 12825 12795 12831 12796 12829 12803
12824 12807 12830 12810 12837 12811 12843 12813 12850 12814 12844 12817
12838 12816 12833 12821 12829 12827 12831 12833 12838 12833 12842 12838
12840 12844 12840 12851 12846 12854 12849 12848 12853 12854 12860 12853

12861 12852 12864 12847 12870 12845 12871 12852 12866 12856 12861 12862
12863 12868 12857 12867 12854 12873 12860 12877 12867 12875 12864 12881
12860 12886 12866 12888 12866 12894 12869 12901 12862 12901 12857 12896
12856 12890 12849 12891 12845 12897 12850 12902 12853 12908 12856 12914
12854 12920 12852 12927 12851 12934 12849 12940 12843 12944 12840 12950
12838 12957 12841 12963 12847 12965 12848 12972 12844 12977 12837 12980
12830 12980 12826 12974 12826 12968 12830 12958 12823 12956 12820 12949
12819 12943 12819 12936 12812 12937 12806 12934 12800 12937 12798 12930
12793 12925 12797 12919 12801 12914 12805 12908 12810 12903 12815 12899
12808 12900 12801 12899 12795 12902 12790 12896 12784 12898 12778 12901
12772 12905 12766 12904 12763 12897 12757 12896 12753 12890 12750 12884
126 N F
12749 12834 12748 12829 12751 12826 12754 12823 12753 12819 12754 12815
12754 12815 12758 12814 12762 12814 12766 12816 12769 12819 12772 12822
12772 12822 12772 12826 12769 12829 12765 12828 12762 12832 12762 12836
12759 12840 12759 12844 12756 12842 12757 12846 12753 12846 12749 12843
12751 12839 12755 12840 12758 12837 12755 12839 12754 12834 12751 12831
12751 12835 12748 12838 12749 12834 33 N F
12754 7994 12751 7989 12753 7985 12757 7983 12757 7988 12758 7992
12754 7994 7 N F
12754 8016 12753 8011 12754 8011 12757 8011 12758 8015 12754 8016
6 N F
12760 12933 12759 12929 12761 12925 12762 12921 12760 12917 12763 12913
12765 12909 12770 12910 12767 12914 12770 12916 12767 12919 12765 12923
12768 12926 12770 12930 12768 12934 12764 12935 12760 12933 17 N F
12769 8010 12766 8006 12770 8003 12772 7999 12777 8001 12780 8003
12778 8007 12776 8011 12772 8013 12769 8010 10 N F
12779 8028 12773 8023 12777 8023 12782 8022 12783 8022 12784 8021
12785 8019 12788 8022 12790 8027 12790 8031 12793 8028 12797 8027
12797 8031 12795 8035 12790 8036 12786 8036 12785 8032 12781 8032
12779 8028 19 N F
12783 7999 12776 7997 12779 7993 12783 7995 12783 7999 5 N F
12784 7993 12780 7988 12783 7985 12784 7989 12784 7993 5 N F
12809 8003 12805 8000 12807 7996 12810 7993 12809 7988 12809 7984
12812 7980 12813 7984 12817 7986 12818 7991 12817 7995 12816 8000
12819 8003 12822 8006 12821 8011 12821 8015 12820 8020 12818 8024
12814 8026 12810 8025 12810 8020 12809 8016 12807 8012 12808 8007
12809 8003 25 N F
12856 8023 12854 8017 12858 8018 12859 8022 12861 8026 12861 8031
12857 8028 12856 8023 8 N F
12882 12996 12879 12992 12883 12991 12886 12988 12886 12984 12889 12981
12885 12978 12889 12976 12893 12975 12893 12971 12893 12966 12896 12964
12898 12964 12901 12964 12902 12969 12900 12973 12900 12977 12901 12981

12903 12986 12904 12990 12903 12994 12902 12998 12897 12997 12895 13001
12892 13004 12889 13000 12885 12999 12882 12996 28 N F
12893 11300 12892 11300 12893 11299 12893 11299 12893 11299 12893 11299
12894 11298 12895 11298 12895 11299 12895 11299 12895 11300 12894 11301
12893 11300 13 N F
12894 11303 12893 11302 12894 11301 12895 11301 12896 11299 12896 11298
12895 11296 12895 11294 12895 11293 12894 11291 12896 11291 12896 11292
12897 11294 12898 11294 12900 11294 12900 11296 12898 11297 12898 11299
12898 11300 12899 11301 12900 11303 12900 11304 12898 11305 12897 11304
12896 11303 12894 11303 26 N F
12901 11309 12900 11308 12900 11307 12901 11306 12902 11306 12903 11307
12903 11308 12903 11309 12903 11310 12903 11310 12902 11310 12901 11309
12901 11309 13 N F
12902 11315 12902 11314 12902 11313 12902 11313 12903 11313 12903 11313
12903 11314 12903 11315 12903 11315 12902 11315 10 N F
12904 11317 12903 11316 12903 11314 12903 11314 12904 11314 12904 11315
12905 11314 12906 11314 12906 11315 12906 11316 12906 11317 12906 11317
12906 11318 12904 11317 12904 11317 12904 11317 16 N F
12906 11321 12906 11321 12906 11320 12907 11320 12907 11319 12907 11319
12907 11318 12908 11318 12908 11318 12909 11319 12909 11320 12908 11320
12908 11321 12908 11322 12906 11321 15 N F
12908 11325 12908 11323 12910 11323 12911 11324 12913 11324 12914 11324
12916 11325 12917 11326 12918 11325 12919 11324 12920 11325 12921 11327
12922 11328 12923 11329 12923 11331 12923 11333 12922 11333 12921 11332
12919 11332 12918 11332 12916 11332 12915 11332 12913 11332 12912 11331
12912 11329 12912 11328 12911 11326 12910 11326 12908 11325 29 N F
12960 8037 12957 8030 12958 8023 12963 8018 12966 8011 12971 8007
12976 8002 12977 7995 12976 7988 12976 7980 12974 7974 12974 7967
12979 7962 12986 7960 12990 7965 12996 7969 12996 7969 12996 8077
12992 8075 12985 8072 12981 8066 12978 8060 12975 8053 12975 8046
12970 8041 12974 8035 12979 8030 12973 8034 12967 8037 12960 8037
30 N F
12971 11664 12967 11651 12974 11649 12981 11649 12985 11644 12992 11643
12996 11649 12996 11649 12996 11679 12990 11679 12995 11674 12988 11676
12991 11669 12985 11665 12984 11658 12991 11661 12996 11665 12992 11659
12992 11652 12985 11654 12980 11658 12976 11664 12973 11670 12971 11664
24 N F
12979 8339 12976 8328 12980 8323 12987 8323 12994 8325 12996 8325
12996 8340 12993 8339 12986 8339 12979 8339 10 N F
12981 11744 12978 11737 12979 11731 12984 11725 12981 11719 12983 11712
12988 11707 12984 11701 12987 11695 12989 11689 12996 11686 12996 11680
12996 11742 12992 11747 12985 11749 12981 11744 16 N F
12980 8163 12980 8156 12982 8149 12985 8143 12989 8137 12993 8131

12996 8128 12996 8164 12993 8161 12988 8165 12982 8170 12980 8163
12 N F
1 O
12997 16211 2301 16211 2301 5500 12997 5500 12997 16211 5 P
Ls Gr Gs Lr
2301 5499 12998 16212 Oc
68 O
2301 5770 2301 5659 2301 5659 2301 5967 2301 5967 2301 5906
2301 5770 7 N F
55 O
2301 6041 2301 5967 2301 5967 2301 6460 2301 6460 2301 6448
2301 6313 2301 6177 2301 6041 9 N F
68 O
2301 6584 2301 6460 2301 6460 2301 6883 2301 6883 2301 6855
2301 6719 2301 6584 8 N F
55 O
2301 6991 2301 6883 2301 6883 2301 7471 2301 7471 2301 7397
2301 7262 2301 7126 2301 6991 9 N F
68 O
2301 7533 2301 7471 2301 7471 2301 7655 2301 7655 2301 7533
6 N F
55 O
2301 7669 2301 7655 2301 7655 2301 7871 2301 7871 2301 7804
2301 7669 7 N F
42 O
2301 7940 2301 7871 2301 7871 2301 8196 2301 8196 2301 8075
2301 7940 7 N F
29 O
2301 8211 2301 8196 2301 8196 2301 8680 2301 8680 2301 8618
2301 8482 2301 8347 2301 8211 9 N F
42 O
2301 8754 2301 8680 2301 8680 2301 9379 2301 9379 2301 9296
2301 9160 2301 9025 2301 8889 2301 8754 10 N F
29 O
2301 9432 2301 9379 2301 9379 2301 9452 2301 9452 2301 9432
6 N F
42 O
2301 9567 2301 9452 2301 9452 2301 9638 2301 9638 2301 9567
6 N F
55 O
2301 9703 2301 9638 2301 9638 2301 10162 2301 10162 2301 10110
2301 9974 2301 9838 2301 9703 9 N F
68 O

2301 10245 2301 10162 2301 10162 2301 10611 2301 10611 2301 10516
2301 10381 2301 10245 8 N F
82 O
2301 10652 2301 10611 2301 10611 2301 10802 2301 10802 2301 10788
2301 10652 7 N F
68 O
2301 10923 2301 10802 2301 10802 2301 11354 2301 11354 2301 11330
2301 11194 2301 11059 2301 10923 9 N F
55 O
2301 11466 2301 11354 2301 11354 2301 11737 2301 11737 2301 11737
2301 11601 2301 11466 8 N F
42 O
2301 11838 2301 11737 2301 11737 2301 11838 2301 11838 5 N F
29 O
2301 11873 2301 11838 2301 11838 2301 11947 2301 11947 2301 11873
6 N F
15 O
2301 12008 2301 11947 2301 11947 2301 13192 2301 13192 2301 13093
2301 12957 2301 12822 2301 12686 2301 12551 2301 12415 2301 12279
2301 12144 2301 12008 14 N F
2 O
2301 13229 2301 13192 2301 13192 2301 13906 2301 13906 2301 13771
2301 13635 2301 13500 2301 13364 2301 13229 10 N F
15 O
2301 13907 2301 13906 2301 13906 2301 13908 2301 13908 2301 13907
6 N F
55 O
2301 5659 2301 5499 2301 5499 2301 5499 2301 5659 2301 5659
6 N F
68 O
2301 5967 2301 5659 2301 5659 2301 5967 2301 5967 5 N F
55 O
2301 6460 2301 5967 2301 5967 2301 6460 2301 6460 5 N F
68 O
2301 6883 2301 6460 2301 6460 2301 6883 2301 6883 5 N F
55 O
2301 7471 2301 6883 2301 6883 2301 7472 2301 7471 5 N F
68 O
2301 7655 2301 7471 2301 7472 2301 7655 2301 7655 5 N F
55 O
2301 7871 2301 7655 2301 7655 2301 7871 2301 7871 5 N F
42 O
2301 8196 2301 7871 2301 7871 2301 8196 2301 8196 5 N F

29 O
2301 8680 2301 8196 2301 8196 2301 8680 2301 8680 5 N F
42 O
2301 9379 2301 8680 2301 8680 2301 9379 2301 9379 5 N F
29 O
2301 9452 2301 9379 2301 9379 2301 9452 2301 9452 5 N F
42 O
2301 9638 2301 9452 2301 9452 2301 9638 2301 9638 5 N F
55 O
2301 10162 2301 9638 2301 9638 2301 10162 2301 10162 5 N F
68 O
2301 10611 2301 10162 2301 10162 2301 10611 2301 10611 5 N F
82 O
2301 10802 2301 10611 2301 10611 2301 10802 2301 10802 5 N F
68 O
2301 11354 2301 10802 2301 10802 2301 11354 2301 11354 5 N F
55 O
2301 11737 2301 11354 2301 11354 2301 11737 2301 11737 5 N F
42 O
2301 11838 2301 11737 2301 11737 2301 11838 2301 11838 5 N F
29 O
2301 11947 2301 11838 2301 11838 2301 11947 2301 11947 5 N F
15 O
2301 13192 2301 11947 2301 11947 2301 13192 2301 13192 5 N F
2 O
2301 13906 2301 13192 2301 13192 2301 13906 2301 13906 5 N F
15 O
2301 13908 2301 13906 2301 13906 2301 13907 2301 13908 5 N F
2 O
2301 16212 2301 13908 2301 13907 2301 16212 2301 16212 2301 16212
6 N F
55 O
2479 5499 2301 5499 2301 5659 2380 5634 2436 5627 2450 5634
2571 5739 2616 5770 2707 5905 2823 5770 2790 5634 2728 5499
2657 5499 2479 5499 14 N F
68 O
2380 5634 M 2301 5659 L 2301 5967 L 2436 5991 L 2559 6041 L
2571 6044 L 2629 6177 L 2599 6313 L 2707 6408 L
2839 6313 L 2842 6309 L 2842 6115 L 2762 6041 L
2710 5906 L 2842 5815 L 2882 5906 L 2933 6041 L
2842 6115 L 2842 6309 L 2911 6177 L 2978 6093 L
3075 6177 L 3113 6190 L 3248 6260 L 3384 6211 L
3519 6295 L 3532 6313 L 3519 6351 L 3492 6448 L

3519 6484 L 3655 6529 L 3745 6448 L 3790 6356 L
3926 6389 L 4061 6408 L 4196 6440 L 4239 6448 L
4332 6479 L 4380 6448 L 4467 6407 L 4603 6413 L
4691 6448 L 4738 6495 L 4873 6549 L 5009 6542 L
5117 6584 L 5144 6597 L 5280 6638 L 5309 6719 L
5415 6783 L 5551 6780 L 5638 6855 L 5622 6991 L
5672 7126 L 5686 7149 L 5821 7142 L 5957 7200 L
6028 7126 L 6055 6991 L 6092 6948 L 6207 6855 L
6228 6839 L 6294 6855 L 6363 6918 L 6394 6991 L
6363 7043 L 6228 7071 L 6166 7126 L 6228 7181 L
6341 7262 L 6363 7286 L 6494 7397 L 6498 7400 L
6634 7432 L 6769 7460 L 6905 7458 L 6905 7334 L
6858 7262 L 6905 7231 L 6970 7262 L 6905 7334 L
6905 7458 L 7025 7533 L 7040 7539 L 7049 7533 L
7136 7397 L 7176 7316 L 7311 7393 L 7378 7397 L
7446 7401 L 7448 7397 L 7446 7386 L 7410 7262 L
7446 7231 L 7582 7194 L 7633 7262 L 7717 7386 L
7738 7397 L 7853 7423 L 7929 7397 L 7988 7354 L
8123 7349 L 8259 7381 L 8361 7397 L 8394 7402 L
8530 7410 L 8587 7397 L 8665 7351 L 8707 7397 L
8801 7451 L 8925 7533 L 8929 7669 L 8936 7685 L
9071 7749 L 9135 7804 L 9121 7940 L 9108 8075 L
9207 8093 L 9342 8091 L 9356 8075 L 9342 8041 L
9222 7940 L 9228 7804 L 9271 7669 L 9342 7633 L
9478 7650 L 9613 7636 L 9748 7541 L 9771 7533 L
9884 7517 L 10019 7501 L 10155 7496 L 10290 7505 L
10426 7524 L 10449 7533 L 10561 7580 L 10696 7591 L
10832 7563 L 10862 7533 L 10967 7477 L 11103 7463 L
11238 7451 L 11373 7458 L 11509 7469 L 11644 7481 L
11780 7496 L 11915 7515 L 11999 7533 L 12051 7580 L
12071 7669 L 12092 7804 L 12108 7940 L 12117 8075 L
12126 8211 L 12136 8347 L 12139 8482 L 12146 8618 L
12152 8754 L 12158 8889 L 12166 9025 L 12175 9160 L
12186 9269 L 12202 9296 L 12257 9432 L 12294 9567 L
12321 9639 L 12359 9703 L 12436 9838 L 12457 9872 L
12540 9974 L 12592 10034 L 12666 10110 L 12728 10181 L
12787 10245 L 12863 10321 L 12953 10381 L 12998 10425 L
12998 9511 L 12876 9432 L 12863 9416 L 12739 9296 L
12728 9286 L 12592 9252 L 12457 9178 L 12434 9160 L
12321 9030 L 12320 9025 L 12254 8889 L 12197 8754 L
12186 8728 L 12182 8618 L 12176 8482 L 12175 8347 L
12168 8211 L 12161 8075 L 12154 7940 L 12144 7804 L
12134 7669 L 12113 7533 L 12051 7470 L 11915 7451 L

11780 7441 L 11644 7429 L 11509 7421 L 11373 7410 L
11238 7405 L 11103 7408 L 10967 7417 L 10832 7456 L
10696 7481 L 10561 7480 L 10426 7444 L 10290 7425 L
10155 7411 L 10019 7410 L 9884 7420 L 9748 7430 L
9613 7454 L 9478 7460 L 9342 7462 L 9207 7459 L
9071 7433 L 8966 7397 L 8936 7380 L 8801 7281 L
8782 7262 L 8665 7130 L 8660 7126 L 8534 6991 L
8530 6987 L 8518 6991 L 8394 7070 L 8301 7126 L
8259 7262 L 8259 7262 L 8259 7262 L 8239 7126 L
8123 7012 L 7988 7022 L 7853 7062 L 7746 6991 L
7717 6964 L 7623 6855 L 7582 6800 L 7560 6855 L
7508 6991 L 7446 7044 L 7311 7068 L 7176 7006 L
7123 6991 L 7040 6961 L 6947 6855 L 6905 6804 L
6769 6837 L 6746 6855 L 6634 6892 L 6538 6855 L
6498 6817 L 6455 6719 L 6402 6584 L 6363 6556 L
6228 6530 L 6092 6506 L 5975 6584 L 5957 6621 L
5821 6626 L 5769 6584 L 5792 6448 L 5741 6313 L
5697 6177 L 5806 6041 L 5821 6026 L 5864 5906 L
5957 5790 L 5980 5770 L 6092 5717 L 6133 5634 L
6092 5509 L 6088 5499 L 6045 5499 L 6002 5499 L
6000 5634 L 5957 5654 L 5821 5669 L 5747 5770 L
5686 5848 L 5582 5770 L 5551 5745 L 5454 5770 L
5415 5818 L 5280 5867 L 5211 5770 L 5144 5691 L
5009 5739 L 4965 5634 L 5009 5551 L 5128 5499 L
4975 5499 L 4797 5499 L 4618 5499 L 4440 5499 L
4333 5499 L 4386 5634 L 4377 5770 L 4332 5844 L
4196 5804 L 4126 5770 L 4196 5662 L 4201 5634 L
4327 5499 L 4262 5499 L 4084 5499 L 3905 5499 L
3727 5499 L 3549 5499 L 3370 5499 L 3192 5499 L
3014 5499 L 2836 5499 L 2728 5499 L 2790 5634 L
2823 5770 L 2707 5905 L 2616 5770 L 2571 5739 L
2450 5634 L 2436 5627 L 2380 5634 L N E
55 O
2301 5967 M 2301 6460 L 2341 6448 L 2436 6390 L 2554 6448 L
2571 6540 L 2580 6584 L 2571 6593 L 2520 6719 L
2478 6855 L 2436 6879 L 2301 6883 L 2301 7472 L
2367 7533 L 2301 7655 L 2301 7871 L 2436 7814 L
2456 7804 L 2571 7730 L 2603 7669 L 2657 7533 L
2707 7427 L 2747 7397 L 2842 7359 L 2948 7397 L
2978 7415 L 3028 7397 L 3113 7286 L 3116 7262 L
3243 7126 L 3248 7116 L 3248 6901 L 3194 6855 L
3248 6789 L 3364 6855 L 3248 6901 L 3248 7116 L
3268 7126 L 3384 7156 L 3510 7262 L 3519 7268 L

3570 7262 L 3655 7220 L 3723 7126 L 3790 7092 L
3926 7090 L 4061 7101 L 4061 6784 L 4010 6719 L
4061 6657 L 4115 6719 L 4061 6784 L 4061 7101 L
4196 7113 L 4332 7092 L 4426 6991 L 4467 6947 L
4601 6991 L 4603 7006 L 4613 7126 L 4603 7152 L
4570 7262 L 4603 7283 L 4700 7397 L 4738 7466 L
4815 7533 L 4873 7569 L 5009 7610 L 5009 7324 L
4906 7262 L 4873 7186 L 4835 7126 L 4873 7032 L
5009 7074 L 5056 7126 L 5144 7248 L 5157 7262 L
5144 7278 L 5009 7324 L 5009 7610 L 5144 7640 L
5230 7669 L 5280 7705 L 5415 7770 L 5415 7312 L
5356 7262 L 5280 7147 L 5262 7126 L 5144 7024 L
5114 6991 L 5144 6960 L 5219 6991 L 5280 7077 L
5297 7126 L 5415 7209 L 5415 7013 L 5401 6991 L
5415 6973 L 5449 6991 L 5415 7013 L 5415 7209 L
5498 7262 L 5415 7312 L 5415 7770 L 5437 7804 L
5525 7940 L 5551 7960 L 5686 7947 L 5690 7940 L
5736 7804 L 5753 7669 L 5744 7533 L 5699 7397 L
5821 7333 L 5902 7397 L 5957 7455 L 6092 7449 L
6228 7520 L 6248 7533 L 6363 7600 L 6498 7628 L
6559 7669 L 6587 7804 L 6592 7940 L 6621 8075 L
6634 8085 L 6765 8211 L 6769 8218 L 6905 8345 L
6907 8347 L 6978 8482 L 7040 8544 L 7110 8618 L
7113 8754 L 7096 8889 L 7076 9025 L 7040 9073 L
6905 9144 L 6769 9100 L 6649 9025 L 6634 9015 L
6533 9025 L 6498 9027 L 6401 9160 L 6363 9202 L
6341 9296 L 6363 9331 L 6495 9432 L 6498 9456 L
6587 9567 L 6634 9622 L 6661 9567 L 6672 9432 L
6634 9405 L 6605 9296 L 6634 9229 L 6769 9295 L
6905 9233 L 6934 9296 L 7040 9370 L 7075 9432 L
7176 9551 L 7197 9567 L 7311 9686 L 7390 9567 L
7446 9522 L 7446 8083 L 7427 8075 L 7446 8069 L
7518 8075 L 7446 8083 L 7446 9522 L 7513 9567 L
7451 9703 L 7446 9706 L 7311 9722 L 7258 9838 L
7176 9895 L 7097 9838 L 7040 9756 L 6905 9741 L
6798 9838 L 6832 9974 L 6843 10110 L 6769 10157 L
6634 10226 L 6611 10245 L 6501 10381 L 6634 10426 L
6713 10516 L 6769 10566 L 6878 10652 L 6877 10788 L
6769 10845 L 6635 10788 L 6634 10787 L 6633 10788 L
6634 10792 L 6657 10923 L 6634 10978 L 6498 11017 L
6443 11059 L 6498 11157 L 6543 11194 L 6634 11275 L
6674 11330 L 6634 11370 L 6591 11466 L 6634 11507 L
6762 11466 L 6769 11459 L 6905 11451 L 6933 11466 L

7040 11520 L 7176 11536 L 7311 11537 L 7401 11601 L
7446 11634 L 7446 11514 L 7311 11484 L 7176 11469 L
7162 11466 L 7040 11378 L 6905 11335 L 6884 11330 L
6801 11194 L 6905 11079 L 7040 11173 L 7084 11194 L
7176 11263 L 7176 11063 L 7157 11059 L 7176 11034 L
7176 10754 L 7078 10652 L 7176 10540 L 7286 10652 L
7176 10754 L 7176 11034 L 7181 11059 L 7176 11063 L
7176 11263 L 7311 11221 L 7322 11194 L 7446 11108 L
7564 11194 L 7453 11330 L 7547 11466 L 7446 11514 L
7446 11634 L 7578 11737 L 7541 11873 L 7572 12008 L
7582 12067 L 7614 12144 L 7657 12279 L 7717 12394 L
7733 12415 L 7782 12551 L 7843 12686 L 7853 12751 L
7853 12102 L 7717 12068 L 7687 12008 L 7582 11874 L
7581 11873 L 7582 11871 L 7582 8750 L 7470 8618 L
7582 8509 L 7582 7696 L 7558 7669 L 7582 7639 L
7683 7669 L 7582 7696 L 7582 8509 L 7692 8618 L
7582 8750 L 7582 11871 L 7717 11787 L 7853 11742 L
7863 11737 L 7935 11601 L 7877 11466 L 7853 11385 L
7717 11331 L 7717 11330 L 7655 11194 L 7717 11166 L
7788 11194 L 7853 11313 L 7853 9660 L 7750 9567 L
7853 9446 L 7853 9005 L 7722 8889 L 7717 8883 L
7584 8754 L 7717 8639 L 7717 8241 L 7653 8211 L
7717 8182 L 7831 8211 L 7717 8241 L 7717 8639 L
7853 8743 L 7988 8633 L 7988 8016 L 7853 7955 L
7838 7940 L 7801 7804 L 7853 7746 L 7887 7804 L
7988 7902 L 8029 7940 L 7988 8016 L 7988 8633 L
8095 8754 L 7988 8882 L 7983 8889 L 7853 9005 L
7853 9446 L 7943 9567 L 7853 9660 L 7853 11313 L
7853 11330 L 7988 11464 L 7988 11424 L 7893 11330 L
7988 11234 L 8082 11330 L 7988 11424 L 7988 11464 L
8121 11330 L 8123 11220 L 8259 11314 L 8274 11330 L
8259 11375 L 8148 11466 L 8123 11477 L 8020 11601 L
8026 11737 L 8003 11873 L 7988 11913 L 7944 12008 L
7853 12102 L 7853 12751 L 7988 12759 L 8123 12736 L
8259 12749 L 8394 12726 L 8477 12686 L 8530 12615 L
8646 12551 L 8665 12542 L 8791 12551 L 8801 12551 L
8807 12551 L 8936 12541 L 8987 12551 L 9071 12572 L
9207 12613 L 9342 12590 L 9478 12582 L 9524 12551 L
9613 12489 L 9740 12415 L 9748 12411 L 9831 12279 L
9748 12171 L 9613 12153 L 9478 12224 L 9342 12196 L
9207 12213 L 9071 12201 L 8963 12144 L 8936 12122 L
8824 12008 L 8801 11879 L 8794 11873 L 8801 11865 L
8841 11737 L 8936 11650 L 9032 11601 L 8967 11466 L

8936 11428 L 8801 11385 L 8759 11330 L 8665 11210 L
8653 11194 L 8628 11059 L 8665 11032 L 8665 9944 L
8561 9838 L 8665 9735 L 8785 9838 L 8665 9944 L
8665 11032 L 8801 10969 L 8839 10923 L 8858 10788 L
8936 10730 L 8936 9846 L 8932 9838 L 8936 9835 L
8942 9838 L 8936 9846 L 8936 10730 L 9071 10668 L
9071 10027 L 9022 9974 L 9071 9931 L 9139 9974 L
9071 10027 L 9071 10668 L 9090 10652 L 9176 10516 L
9207 10453 L 9262 10381 L 9300 10245 L 9342 10236 L
9478 10146 L 9613 10126 L 9748 10131 L 9796 10110 L
9884 10005 L 9926 9974 L 9884 9929 L 9748 9964 L
9710 9974 L 9613 10012 L 9563 9974 L 9478 9896 L
9422 9838 L 9342 9781 L 9228 9703 L 9207 9664 L
9071 9627 L 9037 9567 L 8944 9432 L 9071 9296 L
9071 9296 L 9086 9160 L 9071 9150 L 9020 9025 L
8936 8957 L 8804 8889 L 8801 8885 L 8797 8889 L
8801 8893 L 8871 9025 L 8936 9117 L 9058 9160 L
8936 9183 L 8801 9162 L 8665 9200 L 8530 9257 L
8445 9296 L 8496 9432 L 8394 9524 L 8281 9432 L
8320 9296 L 8259 9247 L 8202 9160 L 8259 9091 L
8394 9039 L 8406 9025 L 8430 8889 L 8440 8754 L
8394 8700 L 8340 8618 L 8259 8484 L 8257 8482 L
8259 8481 L 8278 8347 L 8393 8211 L 8373 8075 L
8394 8063 L 8530 7987 L 8530 7701 L 8461 7669 L
8394 7623 L 8259 7601 L 8123 7631 L 7988 7668 L
7894 7533 L 7988 7504 L 8123 7493 L 8259 7511 L
8394 7506 L 8530 7525 L 8589 7533 L 8665 7635 L
8679 7669 L 8665 7680 L 8530 7701 L 8530 7987 L
8602 7940 L 8665 7881 L 8696 7940 L 8682 8075 L
8665 8094 L 8530 8177 L 8397 8211 L 8530 8223 L
8637 8347 L 8665 8404 L 8801 8452 L 8863 8482 L
8912 8618 L 8936 8645 L 8973 8618 L 8997 8482 L
9071 8438 L 9193 8347 L 9207 8334 L 9342 8316 L
9375 8347 L 9478 8432 L 9541 8482 L 9613 8504 L
9695 8482 L 9613 8380 L 9589 8347 L 9478 8282 L
9410 8211 L 9478 8125 L 9478 7973 L 9469 7940 L
9478 7928 L 9493 7940 L 9478 7973 L 9478 8125 L
9613 8127 L 9743 8211 L 9748 8216 L 9754 8211 L
9748 8208 L 9649 8075 L 9748 7970 L 9884 8009 L
10006 8075 L 10019 8102 L 10091 8211 L 10155 8249 L
10155 7939 L 10019 7895 L 9884 7895 L 9793 7804 L
9884 7723 L 9980 7669 L 10019 7639 L 10076 7669 L
10155 7698 L 10290 7724 L 10414 7804 L 10290 7904 L

10155 7939 L 10155 8249 L 10196 8211 L 10290 8111 L
10426 8192 L 10561 8160 L 10601 8075 L 10561 7997 L
10478 7940 L 10437 7804 L 10556 7669 L 10561 7667 L
10567 7669 L 10696 7741 L 10832 7680 L 10967 7701 L
10975 7669 L 10997 7533 L 11103 7518 L 11238 7497 L
11373 7506 L 11509 7517 L 11644 7533 L 11645 7533 L
11780 7597 L 11887 7669 L 11915 7692 L 12007 7804 L
12051 7868 L 12061 7940 L 12073 8075 L 12084 8211 L
12096 8347 L 12102 8482 L 12110 8618 L 12117 8754 L
12124 8889 L 12132 9025 L 12141 9160 L 12152 9296 L
12161 9432 L 12168 9567 L 12177 9703 L 12185 9838 L
12186 9850 L 12238 9974 L 12291 10110 L 12321 10202 L
12343 10245 L 12408 10381 L 12457 10492 L 12473 10516 L
12547 10652 L 12592 10730 L 12632 10788 L 12728 10902 L
12754 10923 L 12863 11025 L 12900 11059 L 12998 11153 L
12998 10425 L 12953 10381 L 12863 10321 L 12787 10245 L
12728 10181 L 12666 10110 L 12592 10034 L 12540 9974 L
12457 9872 L 12436 9838 L 12359 9703 L 12321 9639 L
12294 9567 L 12257 9432 L 12202 9296 L 12186 9269 L
12175 9160 L 12166 9025 L 12158 8889 L 12152 8754 L
12146 8618 L 12139 8482 L 12136 8347 L 12126 8211 L
12117 8075 L 12108 7940 L 12092 7804 L 12071 7669 L
12051 7580 L 11999 7533 L 11915 7515 L 11780 7496 L
11644 7481 L 11509 7469 L 11373 7458 L 11238 7451 L
11103 7463 L 10967 7477 L 10862 7533 L 10832 7563 L
10696 7591 L 10561 7580 L 10449 7533 L 10426 7524 L
10290 7505 L 10155 7496 L 10019 7501 L 9884 7517 L
9771 7533 L 9748 7541 L 9613 7636 L 9478 7650 L
9342 7633 L 9271 7669 L 9228 7804 L 9222 7940 L
9342 8041 L 9356 8075 L 9342 8091 L 9207 8093 L
9108 8075 L 9121 7940 L 9135 7804 L 9071 7749 L
8936 7685 L 8929 7669 L 8925 7533 L 8801 7451 L
8707 7397 L 8665 7351 L 8587 7397 L 8530 7410 L
8394 7402 L 8361 7397 L 8259 7381 L 8123 7349 L
7988 7354 L 7929 7397 L 7853 7423 L 7738 7397 L
7717 7386 L 7633 7262 L 7582 7194 L 7446 7231 L
7410 7262 L 7446 7386 L 7448 7397 L 7446 7401 L
7378 7397 L 7311 7393 L 7176 7316 L 7136 7397 L
7049 7533 L 7040 7539 L 7025 7533 L 6905 7458 L
6769 7460 L 6634 7432 L 6498 7400 L 6494 7397 L
6363 7286 L 6341 7262 L 6228 7181 L 6166 7126 L
6228 7071 L 6363 7043 L 6394 6991 L 6363 6918 L
6294 6855 L 6228 6839 L 6207 6855 L 6092 6948 L

6055 6991 L 6028 7126 L 5957 7200 L 5821 7142 L
5686 7149 L 5672 7126 L 5622 6991 L 5638 6855 L
5551 6780 L 5415 6783 L 5309 6719 L 5280 6638 L
5144 6597 L 5117 6584 L 5009 6542 L 4873 6549 L
4738 6495 L 4691 6448 L 4603 6413 L 4467 6407 L
4380 6448 L 4332 6479 L 4239 6448 L 4196 6440 L
4061 6408 L 3926 6389 L 3790 6356 L 3745 6448 L
3655 6529 L 3519 6484 L 3492 6448 L 3519 6351 L
3532 6313 L 3519 6295 L 3384 6211 L 3248 6260 L
3113 6190 L 3075 6177 L 2978 6093 L 2911 6177 L
2842 6309 L 2839 6313 L 2707 6408 L 2599 6313 L
2629 6177 L 2571 6044 L 2559 6041 L 2436 5991 L
2301 5967 L N E
68 O
2341 6448 2301 6460 2301 6883 2436 6879 2478 6855 2520 6719
2571 6593 2580 6584 2571 6540 2554 6448 2436 6390 2341 6448
12 N F
2367 7533 2301 7472 2301 7655 2367 7533 4 N F
42 O
2301 7871 M 2301 8196 L 2436 8166 L 2571 8079 L 2585 8075 L
2707 8050 L 2730 8075 L 2787 8211 L 2842 8278 L
2904 8347 L 2968 8482 L 2978 8505 L 2995 8482 L
3113 8451 L 3219 8347 L 3113 8286 L 3030 8211 L
2978 8081 L 2968 8075 L 2978 8071 L 3113 7942 L
3119 7940 L 3248 7907 L 3248 7584 L 3234 7533 L
3246 7397 L 3248 7392 L 3270 7397 L 3384 7454 L
3435 7533 L 3384 7569 L 3248 7584 L 3248 7907 L
3384 7829 L 3432 7804 L 3519 7746 L 3655 7778 L
3679 7804 L 3732 7940 L 3762 8075 L 3790 8125 L
3836 8211 L 3926 8296 L 4061 8309 L 4174 8211 L
4196 8192 L 4247 8211 L 4332 8238 L 4467 8333 L
4467 8067 L 4332 8046 L 4196 8030 L 4061 7973 L
4002 7940 L 3988 7804 L 3934 7669 L 3926 7650 L
3890 7533 L 3926 7461 L 4061 7509 L 4117 7533 L
4196 7567 L 4196 7409 L 4170 7397 L 4061 7284 L
4026 7262 L 4061 7204 L 4099 7262 L 4196 7355 L
4217 7397 L 4196 7409 L 4196 7567 L 4332 7627 L
4378 7669 L 4467 7797 L 4476 7804 L 4562 7940 L
4467 8067 L 4467 8333 L 4491 8347 L 4603 8419 L
4738 8437 L 4814 8482 L 4768 8618 L 4738 8644 L
4679 8754 L 4603 8862 L 4467 8845 L 4403 8754 L
4332 8670 L 4274 8618 L 4196 8538 L 4103 8482 L
4061 8406 L 3976 8482 L 4033 8618 L 4040 8754 L

4046 8889 L 4058 9025 L 4061 9032 L 4123 9160 L
4196 9215 L 4321 9296 L 4332 9306 L 4425 9432 L
4467 9518 L 4514 9567 L 4467 9651 L 4332 9622 L
4196 9663 L 4182 9703 L 4196 9778 L 4332 9790 L
4467 9767 L 4489 9838 L 4603 9930 L 4603 9201 L
4495 9160 L 4595 9025 L 4603 9007 L 4738 8921 L
4850 9025 L 4738 9092 L 4653 9160 L 4603 9201 L
4603 9930 L 4633 9974 L 4689 10110 L 4668 10245 L
4628 10381 L 4603 10448 L 4467 10509 L 4453 10516 L
4332 10642 L 4196 10587 L 4061 10568 L 3926 10522 L
3914 10516 L 3790 10462 L 3655 10434 L 3519 10390 L
3509 10381 L 3384 10271 L 3360 10245 L 3248 10141 L
3206 10110 L 3113 10040 L 3001 9974 L 2978 9954 L
2842 9867 L 2805 9838 L 2719 9703 L 2707 9681 L
2635 9567 L 2571 9474 L 2436 9452 L 2301 9452 L
2301 9638 L 2436 9621 L 2571 9666 L 2597 9703 L
2669 9838 L 2707 9881 L 2842 9964 L 2857 9974 L
2978 10055 L 3102 10110 L 3113 10116 L 3248 10213 L
3282 10245 L 3384 10353 L 3416 10381 L 3519 10472 L
3655 10501 L 3700 10516 L 3790 10564 L 3926 10628 L
4021 10652 L 4061 10734 L 4071 10788 L 4061 10794 L
3993 10788 L 3926 10759 L 3897 10788 L 3790 10804 L
3655 10905 L 3630 10923 L 3519 11023 L 3460 11059 L
3384 11158 L 3329 11194 L 3285 11330 L 3248 11343 L
3113 11394 L 2978 11439 L 2903 11466 L 2842 11482 L
2707 11492 L 2571 11516 L 2476 11601 L 2439 11737 L
2436 11740 L 2301 11737 L 2301 11838 L 2436 11871 L
2444 11873 L 2556 12008 L 2571 12037 L 2707 12053 L
2707 11868 L 2571 11797 L 2552 11737 L 2571 11674 L
2611 11601 L 2707 11566 L 2842 11567 L 2978 11538 L
3113 11502 L 3248 11475 L 3341 11466 L 3384 11459 L
3407 11466 L 3440 11601 L 3384 11665 L 3279 11737 L
3248 11748 L 3113 11772 L 2978 11791 L 2842 11830 L
2707 11868 L 2707 12053 L 2842 12035 L 2978 12071 L
3113 12113 L 3248 12094 L 3384 12081 L 3519 12039 L
3564 12008 L 3655 11943 L 3790 11895 L 3926 11959 L
4061 11914 L 4196 11912 L 4332 11984 L 4390 12008 L
4465 12144 L 4467 12146 L 4603 12184 L 4738 12167 L
4873 12182 L 4899 12144 L 5009 12086 L 5144 12080 L
5177 12144 L 5144 12182 L 5058 12279 L 5009 12338 L
4873 12396 L 4738 12370 L 4657 12415 L 4603 12449 L
4467 12516 L 4332 12503 L 4196 12427 L 4177 12415 L
4061 12357 L 3926 12358 L 3790 12368 L 3655 12387 L

3605 12415 L 3519 12474 L 3384 12539 L 3360 12551 L
3248 12677 L 3231 12686 L 3248 12696 L 3370 12686 L
3384 12681 L 3388 12686 L 3519 12712 L 3655 12754 L
3790 12769 L 3926 12804 L 3971 12822 L 4061 12862 L
4196 12908 L 4314 12957 L 4332 12967 L 4467 12987 L
4467 12884 L 4332 12861 L 4255 12822 L 4196 12784 L
4061 12753 L 3926 12712 L 3857 12686 L 3790 12646 L
3655 12614 L 3548 12551 L 3655 12496 L 3790 12462 L
3926 12438 L 4061 12442 L 4196 12518 L 4250 12551 L
4332 12609 L 4467 12618 L 4603 12612 L 4738 12557 L
4873 12661 L 4890 12686 L 4873 12713 L 4738 12740 L
4686 12822 L 4603 12871 L 4467 12884 L 4467 12987 L
4603 13018 L 4720 13093 L 4738 13111 L 4873 13134 L
5009 13177 L 5116 13229 L 5144 13246 L 5168 13229 L
5167 13093 L 5144 13042 L 5089 12957 L 5133 12822 L
5144 12807 L 5280 12795 L 5415 12812 L 5458 12822 L
5551 12845 L 5551 12712 L 5415 12697 L 5387 12686 L
5280 12581 L 5239 12551 L 5165 12415 L 5275 12279 L
5280 12273 L 5415 12184 L 5436 12144 L 5468 12008 L
5415 11925 L 5280 11891 L 5144 11943 L 5009 11973 L
4873 11990 L 4781 12008 L 4738 12017 L 4727 12008 L
4610 11873 L 4603 11867 L 4467 11861 L 4406 11873 L
4332 11879 L 4322 11873 L 4231 11737 L 4196 11689 L
4061 11710 L 4035 11737 L 3926 11815 L 3790 11791 L
3661 11873 L 3655 11875 L 3519 11951 L 3454 12008 L
3384 12034 L 3248 12040 L 3113 12053 L 2985 12008 L
2978 11989 L 2934 11873 L 2978 11855 L 3113 11828 L
3248 11810 L 3384 11778 L 3458 11737 L 3519 11660 L
3556 11601 L 3655 11534 L 3655 11424 L 3543 11330 L
3537 11194 L 3585 11059 L 3655 10995 L 3728 10923 L
3790 10874 L 3926 10871 L 4061 10894 L 4196 10820 L
4226 10788 L 4332 10656 L 4467 10689 L 4603 10725 L
4732 10652 L 4738 10648 L 4747 10652 L 4847 10788 L
4761 10923 L 4738 10937 L 4641 11059 L 4603 11086 L
4467 11098 L 4332 11073 L 4196 11127 L 4159 11194 L
4061 11329 L 4058 11330 L 3926 11385 L 3790 11409 L
3655 11424 L 3655 11534 L 3790 11498 L 3887 11466 L
3926 11456 L 4061 11408 L 4192 11330 L 4196 11320 L
4239 11194 L 4332 11137 L 4467 11151 L 4603 11153 L
4735 11059 L 4738 11055 L 4873 11001 L 5009 10956 L
5029 10923 L 5009 10864 L 4982 10788 L 4945 10652 L
4873 10546 L 4803 10516 L 4738 10451 L 4721 10381 L
4738 10341 L 4816 10245 L 4873 10175 L 5009 10174 L

5144 10191 L 5258 10245 L 5280 10290 L 5415 10275 L
5418 10245 L 5415 10241 L 5362 10110 L 5347 9974 L
5415 9911 L 5494 9838 L 5439 9703 L 5458 9567 L
5546 9432 L 5551 9414 L 5629 9296 L 5686 9262 L
5686 8705 L 5626 8618 L 5686 8506 L 5707 8618 L
5686 8705 L 5686 9262 L 5700 9296 L 5695 9432 L
5686 9507 L 5634 9567 L 5686 9640 L 5696 9703 L
5821 9832 L 5828 9838 L 5838 9974 L 5821 10009 L
5758 10110 L 5688 10245 L 5769 10381 L 5778 10516 L
5745 10652 L 5792 10788 L 5821 10882 L 5829 10923 L
5821 10951 L 5801 11059 L 5821 11121 L 5957 11187 L
5957 11127 L 5873 11059 L 5930 10923 L 5896 10788 L
5939 10652 L 5884 10516 L 5901 10381 L 5957 10249 L
6034 10245 L 6092 10225 L 6097 10110 L 6092 10085 L
5965 9974 L 6059 9838 L 5957 9819 L 5834 9703 L
5821 9686 L 5755 9567 L 5789 9432 L 5821 9390 L
5957 9304 L 5957 9284 L 5898 9160 L 5842 9025 L
5871 8889 L 5826 8754 L 5930 8618 L 5892 8482 L
5924 8347 L 5906 8211 L 5937 8075 L 5932 7940 L
5944 7804 L 5957 7786 L 5996 7804 L 6016 7940 L
6015 8075 L 6045 8211 L 6056 8347 L 6021 8482 L
6092 8577 L 6092 7753 L 6041 7669 L 6092 7636 L
6144 7669 L 6092 7753 L 6092 8577 L 6111 8618 L
6144 8754 L 6108 8889 L 6092 8920 L 6061 9025 L
6007 9160 L 5957 9284 L 5957 9304 L 5961 9296 L
6092 9205 L 6228 9251 L 6228 9042 L 6209 9025 L
6228 8963 L 6308 9025 L 6228 9042 L 6228 9251 L
6245 9296 L 6283 9432 L 6363 9492 L 6407 9567 L
6418 9703 L 6363 9785 L 6265 9838 L 6243 9974 L
6354 10110 L 6351 10245 L 6228 10361 L 6217 10381 L
6126 10516 L 6092 10589 L 6023 10652 L 6092 10661 L
6129 10652 L 6228 10593 L 6363 10630 L 6498 10570 L
6634 10532 L 6769 10642 L 6783 10652 L 6769 10688 L
6634 10690 L 6498 10779 L 6494 10788 L 6498 10803 L
6544 10923 L 6498 10951 L 6363 11027 L 6340 11059 L
6228 11091 L 6092 11096 L 5957 11127 L 5957 11187 L
6092 11155 L 6228 11156 L 6281 11194 L 6363 11322 L
6409 11330 L 6369 11466 L 6391 11601 L 6498 11688 L
6498 11571 L 6445 11466 L 6498 11406 L 6537 11466 L
6498 11571 L 6498 11688 L 6540 11737 L 6535 11873 L
6634 12008 L 6634 12008 L 6634 12009 L 6633 12008 L
6498 11929 L 6463 11873 L 6363 11788 L 6228 11776 L
6092 11804 L 6049 11737 L 5957 11612 L 5821 11674 L

5780 11737 L 5759 11873 L 5758 12008 L 5731 12144 L
5701 12279 L 5722 12415 L 5743 12551 L 5710 12686 L
5686 12711 L 5551 12712 L 5551 12845 L 5686 12830 L
5697 12822 L 5821 12687 L 5822 12686 L 5842 12551 L
5828 12415 L 5828 12279 L 5821 12232 L 5815 12144 L
5821 12127 L 5867 12008 L 5832 11873 L 5880 11737 L
5957 11697 L 5986 11737 L 6070 11873 L 6074 12008 L
6092 12063 L 6111 12008 L 6129 11873 L 6228 11831 L
6363 11866 L 6371 11873 L 6464 12008 L 6498 12043 L
6602 12144 L 6634 12226 L 6665 12144 L 6720 12008 L
6634 11874 L 6633 11873 L 6632 11737 L 6527 11601 L
6634 11560 L 6769 11521 L 6890 11601 L 6896 11737 L
6905 11767 L 6990 11737 L 7040 11718 L 7151 11737 L
7176 11740 L 7177 11737 L 7190 11601 L 7311 11590 L
7326 11601 L 7446 11687 L 7510 11737 L 7500 11873 L
7511 12008 L 7446 12126 L 7405 12144 L 7446 12199 L
7491 12279 L 7582 12324 L 7651 12415 L 7678 12551 L
7717 12614 L 7768 12686 L 7751 12822 L 7717 12860 L
7582 12887 L 7446 12938 L 7311 12940 L 7176 12910 L
7040 12862 L 6905 12951 L 6888 12957 L 6769 12989 L
6634 13004 L 6523 12957 L 6498 12944 L 6363 12906 L
6228 12913 L 6157 12957 L 6168 13093 L 6192 13229 L
6201 13364 L 6178 13500 L 6228 13621 L 6236 13635 L
6332 13771 L 6363 13841 L 6424 13907 L 6498 14034 L
6511 14042 L 6634 14156 L 6769 14112 L 6905 14128 L
7040 14110 L 7176 14131 L 7311 14046 L 7311 13486 L
7176 13477 L 7040 13412 L 6941 13364 L 6905 13353 L
6769 13325 L 6634 13351 L 6577 13364 L 6498 13397 L
6400 13364 L 6363 13354 L 6307 13229 L 6281 13093 L
6363 13027 L 6498 13035 L 6634 13068 L 6769 13074 L
6905 13063 L 7040 13039 L 7176 13056 L 7311 13063 L
7446 13051 L 7582 13055 L 7639 13093 L 7582 13138 L
7446 13220 L 7433 13229 L 7347 13364 L 7311 13486 L
7311 14046 L 7326 14042 L 7446 14018 L 7582 13972 L
7717 13929 L 7853 13920 L 7893 13907 L 7988 13881 L
8123 13835 L 8189 13771 L 8210 13635 L 8123 13626 L
7988 13567 L 7853 13513 L 7828 13500 L 7717 13424 L
7672 13364 L 7717 13320 L 7853 13251 L 7871 13229 L
7916 13093 L 7988 13053 L 8123 13042 L 8259 13069 L
8355 13093 L 8379 13229 L 8391 13364 L 8394 13380 L
8530 13389 L 8665 13416 L 8754 13364 L 8801 13285 L
8841 13229 L 8936 13198 L 9071 13106 L 9207 13098 L
9213 13093 L 9342 12977 L 9392 12957 L 9478 12889 L

9613 12869 L 9748 12863 L 9757 12822 L 9756 12686 L
9753 12551 L 9884 12425 L 9933 12415 L 10019 12362 L
10155 12368 L 10209 12279 L 10267 12144 L 10221 12008 L
10155 11881 L 10149 11873 L 10063 11737 L 10019 11648 L
9951 11601 L 9900 11466 L 9884 11394 L 9851 11330 L
9849 11194 L 9884 11165 L 10019 11062 L 10019 10199 L
9930 10110 L 10019 10022 L 10104 10110 L 10019 10199 L
10019 11062 L 10023 11059 L 10155 10936 L 10226 10923 L
10290 10880 L 10290 9799 L 10199 9703 L 10290 9600 L
10290 9537 L 10155 9531 L 10059 9432 L 10155 9332 L
10155 8805 L 10051 8754 L 10155 8687 L 10239 8754 L
10155 8805 L 10155 9332 L 10251 9296 L 10265 9160 L
10290 9135 L 10426 9030 L 10561 9041 L 10679 9160 L
10633 9296 L 10561 9345 L 10426 9423 L 10415 9432 L
10290 9537 L 10290 9600 L 10391 9703 L 10290 9799 L
10290 10880 L 10362 10788 L 10426 10690 L 10433 10652 L
10441 10516 L 10500 10381 L 10561 10296 L 10573 10245 L
10614 10110 L 10696 9987 L 10701 9974 L 10832 9839 L
10832 9838 L 10877 9703 L 10832 9659 L 10719 9567 L
10832 9480 L 10832 9271 L 10716 9160 L 10832 9042 L
10943 9160 L 10832 9271 L 10832 9480 L 10843 9432 L
10967 9312 L 11012 9296 L 11103 9184 L 11109 9160 L
11103 9153 L 10967 9069 L 10910 9025 L 10832 8981 L
10754 8889 L 10696 8866 L 10561 8815 L 10488 8754 L
10561 8674 L 10574 8618 L 10696 8516 L 10819 8618 L
10832 8628 L 10967 8639 L 11024 8618 L 11103 8539 L
11220 8482 L 11138 8347 L 11238 8236 L 11260 8211 L
11238 8173 L 11103 8140 L 11044 8075 L 11103 8024 L
11157 7940 L 11161 7804 L 11185 7669 L 11238 7584 L
11373 7611 L 11509 7661 L 11525 7669 L 11644 7738 L
11751 7804 L 11780 7826 L 11897 7940 L 11915 7965 L
11961 8075 L 12016 8211 L 12051 8284 L 12057 8347 L
12065 8482 L 12074 8618 L 12082 8754 L 12090 8889 L
12098 9025 L 12106 9160 L 12116 9296 L 12123 9432 L
12130 9567 L 12136 9703 L 12142 9838 L 12152 9974 L
12161 10110 L 12174 10245 L 12186 10363 L 12192 10381 L
12225 10516 L 12256 10652 L 12294 10788 L 12321 10877 L
12343 10923 L 12417 11059 L 12457 11135 L 12499 11194 L
12592 11323 L 12599 11330 L 12715 11466 L 12728 11480 L
12863 11579 L 12918 11601 L 12998 11636 L 12998 11153 L
12900 11059 L 12863 11025 L 12754 10923 L 12728 10902 L
12632 10788 L 12592 10730 L 12547 10652 L 12473 10516 L
12457 10492 L 12408 10381 L 12343 10245 L 12321 10202 L

12291 10110 L 12238 9974 L 12186 9850 L 12185 9838 L
12177 9703 L 12168 9567 L 12161 9432 L 12152 9296 L
12141 9160 L 12132 9025 L 12124 8889 L 12117 8754 L
12110 8618 L 12102 8482 L 12096 8347 L 12084 8211 L
12073 8075 L 12061 7940 L 12051 7868 L 12007 7804 L
11915 7692 L 11887 7669 L 11780 7597 L 11645 7533 L
11644 7533 L 11509 7517 L 11373 7506 L 11238 7497 L
11103 7518 L 10997 7533 L 10975 7669 L 10967 7701 L
10832 7680 L 10696 7741 L 10567 7669 L 10561 7667 L
10556 7669 L 10437 7804 L 10478 7940 L 10561 7997 L
10601 8075 L 10561 8160 L 10426 8192 L 10290 8111 L
10196 8211 L 10155 8249 L 10091 8211 L 10019 8102 L
10006 8075 L 9884 8009 L 9748 7970 L 9649 8075 L
9748 8208 L 9754 8211 L 9748 8216 L 9743 8211 L
9613 8127 L 9478 8125 L 9410 8211 L 9478 8282 L
9589 8347 L 9613 8380 L 9695 8482 L 9613 8504 L
9541 8482 L 9478 8432 L 9375 8347 L 9342 8316 L
9207 8334 L 9193 8347 L 9071 8438 L 8997 8482 L
8973 8618 L 8936 8645 L 8912 8618 L 8863 8482 L
8801 8452 L 8665 8404 L 8637 8347 L 8530 8223 L
8397 8211 L 8530 8177 L 8665 8094 L 8682 8075 L
8696 7940 L 8665 7881 L 8602 7940 L 8530 7987 L
8394 8063 L 8373 8075 L 8393 8211 L 8278 8347 L
8259 8481 L 8257 8482 L 8259 8484 L 8340 8618 L
8394 8700 L 8440 8754 L 8430 8889 L 8406 9025 L
8394 9039 L 8259 9091 L 8202 9160 L 8259 9247 L
8320 9296 L 8281 9432 L 8394 9524 L 8394 9481 L
8334 9432 L 8394 9351 L 8448 9432 L 8394 9481 L
8394 9524 L 8496 9432 L 8445 9296 L 8530 9257 L
8530 8565 L 8458 8482 L 8394 8396 L 8352 8347 L
8394 8297 L 8530 8295 L 8575 8347 L 8631 8482 L
8530 8565 L 8530 9257 L 8665 9200 L 8665 8694 L
8586 8618 L 8665 8520 L 8745 8618 L 8665 8694 L
8665 9200 L 8801 9162 L 8936 9183 L 9058 9160 L
8936 9117 L 8871 9025 L 8801 8893 L 8797 8889 L
8801 8885 L 8804 8889 L 8936 8957 L 9020 9025 L
9071 9150 L 9086 9160 L 9071 9296 L 9071 9296 L
8944 9432 L 9037 9567 L 9071 9627 L 9071 9521 L
8991 9432 L 9071 9345 L 9194 9432 L 9071 9521 L
9071 9627 L 9207 9664 L 9228 9703 L 9342 9781 L
9342 8764 L 9334 8754 L 9342 8744 L 9350 8754 L
9342 8764 L 9342 9781 L 9422 9838 L 9478 9896 L
9563 9974 L 9613 10012 L 9613 9556 L 9516 9432 L

9600 9296 L 9613 9271 L 9626 9296 L 9714 9432 L
9613 9556 L 9613 10012 L 9710 9974 L 9748 9964 L
9884 9929 L 9884 9252 L 9784 9160 L 9884 9075 L
9884 8892 L 9883 8889 L 9884 8888 L 9884 8682 L
9824 8618 L 9884 8505 L 9948 8618 L 9884 8682 L
9884 8888 L 9887 8889 L 9884 8892 L 9884 9075 L
9970 9160 L 9884 9252 L 9884 9929 L 9926 9974 L
9884 10005 L 9796 10110 L 9748 10131 L 9613 10126 L
9478 10146 L 9342 10236 L 9300 10245 L 9262 10381 L
9207 10453 L 9176 10516 L 9090 10652 L 9071 10668 L
8936 10730 L 8858 10788 L 8839 10923 L 8801 10969 L
8665 11032 L 8628 11059 L 8653 11194 L 8665 11210 L
8759 11330 L 8801 11385 L 8936 11428 L 8967 11466 L
9032 11601 L 8936 11650 L 8841 11737 L 8801 11865 L
8794 11873 L 8801 11879 L 8824 12008 L 8936 12122 L
8963 12144 L 9071 12201 L 9071 11161 L 8979 11059 L
9071 10959 L 9175 11059 L 9071 11161 L 9071 12201 L
9207 12213 L 9342 12196 L 9478 12224 L 9478 12060 L
9390 12008 L 9478 11940 L 9478 10908 L 9365 10788 L
9427 10652 L 9466 10516 L 9478 10501 L 9503 10381 L
9613 10248 L 9748 10268 L 9856 10381 L 9782 10516 L
9780 10652 L 9748 10704 L 9638 10788 L 9613 10809 L
9478 10908 L 9478 11940 L 9524 12008 L 9478 12060 L
9478 12224 L 9613 12153 L 9748 12171 L 9831 12279 L
9748 12411 L 9740 12415 L 9613 12489 L 9524 12551 L
9478 12582 L 9342 12590 L 9207 12613 L 9071 12572 L
8987 12551 L 8936 12541 L 8807 12551 L 8801 12551 L
8791 12551 L 8665 12542 L 8646 12551 L 8530 12615 L
8477 12686 L 8394 12726 L 8259 12749 L 8123 12736 L
7988 12759 L 7853 12751 L 7843 12686 L 7782 12551 L
7733 12415 L 7717 12394 L 7657 12279 L 7614 12144 L
7582 12067 L 7572 12008 L 7541 11873 L 7578 11737 L
7446 11634 L 7401 11601 L 7311 11537 L 7176 11536 L
7040 11520 L 6933 11466 L 6905 11451 L 6769 11459 L
6762 11466 L 6634 11507 L 6591 11466 L 6634 11370 L
6674 11330 L 6634 11275 L 6543 11194 L 6498 11157 L
6443 11059 L 6498 11017 L 6634 10978 L 6657 10923 L
6634 10792 L 6633 10788 L 6634 10787 L 6635 10788 L
6769 10845 L 6877 10788 L 6878 10652 L 6769 10566 L
6713 10516 L 6634 10426 L 6501 10381 L 6611 10245 L
6634 10226 L 6769 10157 L 6843 10110 L 6832 9974 L
6798 9838 L 6905 9741 L 7040 9756 L 7097 9838 L
7176 9895 L 7258 9838 L 7311 9722 L 7446 9706 L

7451 9703 L 7513 9567 L 7446 9522 L 7390 9567 L
7311 9686 L 7197 9567 L 7176 9551 L 7075 9432 L
7040 9370 L 6934 9296 L 6905 9233 L 6769 9295 L
6634 9229 L 6605 9296 L 6634 9405 L 6672 9432 L
6661 9567 L 6634 9622 L 6587 9567 L 6498 9456 L
6495 9432 L 6363 9331 L 6341 9296 L 6363 9202 L
6401 9160 L 6498 9027 L 6498 8514 L 6481 8482 L
6498 8464 L 6519 8482 L 6498 8514 L 6498 9027 L
6533 9025 L 6634 9015 L 6649 9025 L 6769 9100 L
6905 9144 L 7040 9073 L 7076 9025 L 7096 8889 L
7113 8754 L 7110 8618 L 7040 8544 L 6978 8482 L
6907 8347 L 6905 8345 L 6769 8218 L 6765 8211 L
6634 8085 L 6621 8075 L 6592 7940 L 6587 7804 L
6559 7669 L 6498 7628 L 6363 7600 L 6248 7533 L
6228 7520 L 6092 7449 L 5957 7455 L 5902 7397 L
5821 7333 L 5699 7397 L 5744 7533 L 5753 7669 L
5736 7804 L 5690 7940 L 5686 7947 L 5551 7960 L
5525 7940 L 5437 7804 L 5415 7770 L 5280 7705 L
5230 7669 L 5144 7640 L 5009 7610 L 4873 7569 L
4815 7533 L 4738 7466 L 4700 7397 L 4603 7283 L
4570 7262 L 4603 7152 L 4613 7126 L 4603 7006 L
4601 6991 L 4467 6947 L 4426 6991 L 4332 7092 L
4196 7113 L 4061 7101 L 3926 7090 L 3790 7092 L
3723 7126 L 3655 7220 L 3570 7262 L 3519 7268 L
3510 7262 L 3384 7156 L 3268 7126 L 3248 7116 L
3243 7126 L 3116 7262 L 3113 7286 L 3028 7397 L
2978 7415 L 2948 7397 L 2842 7359 L 2747 7397 L
2707 7427 L 2657 7533 L 2603 7669 L 2571 7730 L
2456 7804 L 2436 7814 L 2301 7871 L N E
29 O
2301 8196 2301 8680 2388 8618 2436 8554 2436 8386 2404 8347
2436 8289 2460 8347 2436 8386 2436 8554 2571 8505 2671 8618
2707 8666 2842 8662 2978 8700 3035 8754 2978 8832 2842 8849
2707 8868 2571 8887 2566 8889 2436 8958 2390 9025 2397 9160
2352 9296 2301 9379 2301 9452 2436 9452 2571 9474 2635 9567
2707 9681 2719 9703 2805 9838 2842 9867 2978 9954 3001 9974
3113 10040 3113 9939 2978 9843 2970 9838 2842 9705 2841 9703
2827 9567 2842 9508 2978 9445 3002 9432 3113 9361 3142 9432
3201 9567 3154 9703 3242 9838 3113 9939 3113 10040 3206 10110
3248 10141 3360 10245 3384 10271 3509 10381 3519 10390 3655 10434
3655 10347 3519 10300 3456 10245 3384 10166 3321 10110 3248 10027
3143 9974 3248 9844 3384 9897 3519 9971 3519 9680 3483 9567
3519 9473 3617 9567 3519 9680 3519 9971 3534 9974 3655 9989

3655 9922 3626 9838 3543 9703 3655 9607 3684 9703 3677 9838
3655 9922 3655 9989 3708 10110 3763 10245 3655 10347 3655 10434
3790 10462 3914 10516 3926 10522 4061 10568 4196 10587 4332 10642
4453 10516 4467 10509 4467 10204 4332 10186 4196 10166 4096 10110
4061 10048 4008 9974 4061 9907 4196 9957 4332 9973 4340 9974
4467 9998 4547 10110 4467 10204 4467 10509 4603 10448 4628 10381
4668 10245 4689 10110 4633 9974 4603 9930 4489 9838 4467 9767
4332 9790 4196 9778 4182 9703 4196 9663 4332 9622 4467 9651
4514 9567 4467 9518 4425 9432 4332 9306 4321 9296 4196 9215
4123 9160 4061 9032 4058 9025 4046 8889 4040 8754 4033 8618
3976 8482 4061 8406 4103 8482 4196 8538 4274 8618 4332 8670
4403 8754 4467 8845 4603 8862 4679 8754 4738 8644 4768 8618
4814 8482 4738 8437 4603 8419 4491 8347 4467 8333 4332 8238
4247 8211 4196 8192 4174 8211 4061 8309 3926 8296 3836 8211
3790 8125 3762 8075 3732 7940 3679 7804 3655 7778 3519 7746
3432 7804 3384 7829 3248 7907 3119 7940 3113 7942 2978 8071
2968 8075 2978 8081 3030 8211 3113 8286 3219 8347 3113 8451
2995 8482 2978 8505 2968 8482 2904 8347 2842 8278 2787 8211
2730 8075 2707 8050 2585 8075 2571 8079 2436 8166 2301 8196
192 N F
42 O
2388 8618 2301 8680 2301 9379 2352 9296 2397 9160 2390 9025
2436 8958 2566 8889 2571 8887 2707 8868 2842 8849 2978 8832
3035 8754 2978 8700 2842 8662 2707 8666 2671 8618 2571 8505
2436 8554 2388 8618 20 N F
55 O
2436 9621 2301 9638 2301 10162 2436 10212 2456 10245 2571 10373
2576 10381 2673 10516 2707 10545 2842 10648 2855 10652 2978 10690
3113 10693 3113 10276 3027 10245 3113 10238 3125 10245 3113 10276
3113 10693 3243 10788 3248 10811 3269 10923 3248 10959 3204 11059
3113 11167 3101 11194 2978 11323 2945 11330 2842 11347 2707 11378
2571 11375 2436 11398 2301 11354 2301 11737 2436 11740 2439 11737
2476 11601 2571 11516 2707 11492 2842 11482 2903 11466 2978 11439
3113 11394 3248 11343 3285 11330 3329 11194 3384 11158 3460 11059
3519 11023 3519 10890 3416 10788 3468 10652 3519 10620 3655 10603
3755 10652 3678 10788 3655 10804 3519 10890 3519 11023 3630 10923
3655 10905 3790 10804 3897 10788 3926 10759 3993 10788 4061 10794
4071 10788 4061 10734 4021 10652 3926 10628 3790 10564 3700 10516
3655 10501 3519 10472 3416 10381 3384 10353 3282 10245 3248 10213
3113 10116 3102 10110 2978 10055 2857 9974 2842 9964 2707 9881
2669 9838 2597 9703 2571 9666 2436 9621 88 N F
68 O
2436 10212 2301 10162 2301 10611 2349 10652 2334 10788 2301 10802

2301 11354 2436 11398 2571 11375 2707 11378 2842 11347 2945 11330
2978 11323 3101 11194 3113 11167 3204 11059 3248 10959 3269 10923
3248 10811 3243 10788 3113 10693 2978 10690 2855 10652 2842 10648
2707 10545 2673 10516 2576 10381 2571 10373 2456 10245 2436 10212
30 N F
82 O
2349 10652 2301 10611 2301 10802 2334 10788 2349 10652 5 N F
29 O
2301 11838 M 2301 11947 L 2395 12008 L 2436 12079 L 2469 12144 L
2445 12279 L 2571 12410 L 2655 12415 L 2707 12419 L
2711 12415 L 2767 12279 L 2767 12144 L 2842 12112 L
2978 12138 L 2999 12144 L 3113 12205 L 3248 12155 L
3278 12144 L 3384 12128 L 3519 12103 L 3655 12014 L
3728 12008 L 3790 12005 L 3796 12008 L 3926 12115 L
4061 12131 L 4089 12144 L 4061 12265 L 4048 12279 L
3926 12286 L 3790 12296 L 3655 12315 L 3519 12377 L
3477 12415 L 3384 12467 L 3248 12522 L 3179 12551 L
3113 12590 L 2999 12686 L 3017 12822 L 3092 12957 L
3113 13050 L 3146 13093 L 3188 13229 L 3248 13328 L
3384 13356 L 3384 12903 L 3349 12822 L 3384 12808 L
3466 12822 L 3384 12903 L 3384 13356 L 3519 13340 L
3655 13235 L 3657 13229 L 3685 13093 L 3718 12957 L
3790 12920 L 3926 12913 L 4033 12957 L 4060 13093 L
4018 13229 L 4012 13364 L 4060 13500 L 4061 13505 L
4071 13500 L 4196 13450 L 4210 13364 L 4283 13229 L
4332 13169 L 4458 13093 L 4467 13091 L 4472 13093 L
4603 13178 L 4654 13229 L 4629 13364 L 4603 13386 L
4543 13500 L 4467 13609 L 4429 13635 L 4332 13737 L
4251 13771 L 4332 13811 L 4464 13907 L 4467 13909 L
4583 14042 L 4603 14076 L 4738 14103 L 4873 14049 L
4876 14042 L 4873 14034 L 4832 13907 L 4801 13771 L
4815 13635 L 4826 13500 L 4783 13364 L 4873 13285 L
5009 13321 L 5066 13364 L 5144 13469 L 5280 13499 L
5403 13364 L 5391 13229 L 5291 13093 L 5284 12957 L
5415 12934 L 5493 12957 L 5551 13069 L 5686 12968 L
5689 12957 L 5821 12886 L 5938 12957 L 5957 12969 L
5957 12933 L 5896 12822 L 5928 12686 L 5957 12603 L
5957 12055 L 5951 12008 L 5917 11873 L 5957 11808 L
5993 11873 L 5964 12008 L 5957 12055 L 5957 12603 L
6061 12686 L 6030 12822 L 5957 12933 L 5957 12969 L
6075 13093 L 6092 13115 L 6122 13229 L 6113 13364 L
6092 13412 L 5971 13500 L 6092 13622 L 6099 13635 L
6228 13763 L 6234 13771 L 6289 13907 L 6358 14042 L

6291 14178 L 6247 14314 L 6262 14449 L 6324 14585 L
6363 14617 L 6474 14720 L 6498 14746 L 6634 14788 L
6769 14851 L 6780 14856 L 6905 14992 L 6905 14992 L
7040 15039 L 7176 15012 L 7213 14992 L 7311 14872 L
7348 14856 L 7446 14834 L 7582 14744 L 7618 14720 L
7717 14660 L 7826 14585 L 7853 14565 L 7872 14449 L
7962 14314 L 7988 14290 L 8123 14259 L 8259 14218 L
8366 14178 L 8394 14123 L 8402 14042 L 8412 13907 L
8530 13783 L 8620 13771 L 8665 13757 L 8791 13771 L
8801 13772 L 8808 13771 L 8936 13735 L 9071 13710 L
9207 13718 L 9333 13635 L 9231 13500 L 9234 13364 L
9207 13338 L 9071 13329 L 8994 13229 L 9071 13161 L
9207 13157 L 9277 13093 L 9342 13035 L 9478 12993 L
9613 12977 L 9748 13005 L 9796 12957 L 9815 12822 L
9816 12686 L 9817 12551 L 9884 12486 L 10019 12466 L
10155 12472 L 10240 12551 L 10290 12600 L 10406 12686 L
10426 12706 L 10561 12780 L 10696 12720 L 10832 12704 L
10850 12686 L 10882 12551 L 10832 12491 L 10768 12415 L
10696 12364 L 10634 12279 L 10562 12144 L 10561 12141 L
10426 12073 L 10375 12008 L 10306 11873 L 10290 11848 L
10208 11737 L 10155 11698 L 10076 11601 L 10019 11469 L
10017 11466 L 9942 11330 L 9944 11194 L 10019 11124 L
10087 11059 L 10155 10996 L 10290 10978 L 10379 10923 L
10426 10869 L 10473 10788 L 10491 10652 L 10496 10516 L
10561 10428 L 10607 10381 L 10628 10245 L 10696 10137 L
10723 10110 L 10759 9974 L 10832 9899 L 10894 9838 L
10967 9752 L 10967 9505 L 10907 9432 L 10967 9374 L
11077 9432 L 10967 9505 L 10967 9752 L 11088 9703 L
11103 9686 L 11145 9567 L 11123 9432 L 11140 9296 L
11171 9160 L 11103 9089 L 11029 9025 L 10967 8967 L
10876 8889 L 10832 8836 L 10696 8797 L 10635 8754 L
10655 8618 L 10696 8584 L 10738 8618 L 10832 8696 L
10967 8702 L 11103 8664 L 11157 8618 L 11238 8555 L
11329 8618 L 11373 8654 L 11413 8618 L 11387 8482 L
11373 8466 L 11238 8380 L 11214 8347 L 11238 8320 L
11333 8211 L 11295 8075 L 11293 7940 L 11373 7867 L
11509 7931 L 11521 7940 L 11644 8059 L 11660 8075 L
11774 8211 L 11780 8218 L 11867 8347 L 11915 8461 L
11921 8482 L 11972 8618 L 12027 8754 L 12051 8809 L
12055 8889 L 12063 9025 L 12071 9160 L 12079 9296 L
12085 9432 L 12091 9567 L 12095 9703 L 12099 9838 L
12105 9974 L 12110 10110 L 12118 10245 L 12126 10381 L
12132 10516 L 12140 10652 L 12148 10788 L 12159 10923 L

12174 11059 L 12186 11163 L 12195 11194 L 12240 11330 L
12282 11466 L 12321 11598 L 12323 11601 L 12383 11737 L
12404 11873 L 12449 12008 L 12457 12038 L 12504 12144 L
12591 12279 L 12592 12281 L 12728 12378 L 12838 12415 L
12863 12424 L 12998 12435 L 12998 11636 L 12918 11601 L
12863 11579 L 12728 11480 L 12715 11466 L 12599 11330 L
12592 11323 L 12499 11194 L 12457 11135 L 12417 11059 L
12343 10923 L 12321 10877 L 12294 10788 L 12256 10652 L
12225 10516 L 12192 10381 L 12186 10363 L 12174 10245 L
12161 10110 L 12152 9974 L 12142 9838 L 12136 9703 L
12130 9567 L 12123 9432 L 12116 9296 L 12106 9160 L
12098 9025 L 12090 8889 L 12082 8754 L 12074 8618 L
12065 8482 L 12057 8347 L 12051 8284 L 12016 8211 L
11961 8075 L 11915 7965 L 11897 7940 L 11780 7826 L
11751 7804 L 11644 7738 L 11525 7669 L 11509 7661 L
11373 7611 L 11238 7584 L 11185 7669 L 11161 7804 L
11157 7940 L 11103 8024 L 11044 8075 L 11103 8140 L
11238 8173 L 11260 8211 L 11238 8236 L 11138 8347 L
11220 8482 L 11103 8539 L 11024 8618 L 10967 8639 L
10832 8628 L 10819 8618 L 10696 8516 L 10574 8618 L
10561 8674 L 10488 8754 L 10561 8815 L 10696 8866 L
10754 8889 L 10832 8981 L 10910 9025 L 10967 9069 L
11103 9153 L 11109 9160 L 11103 9184 L 11012 9296 L
10967 9312 L 10843 9432 L 10832 9480 L 10719 9567 L
10832 9659 L 10877 9703 L 10832 9838 L 10832 9839 L
10701 9974 L 10696 9987 L 10614 10110 L 10573 10245 L
10561 10296 L 10500 10381 L 10441 10516 L 10433 10652 L
10426 10690 L 10362 10788 L 10290 10880 L 10226 10923 L
10155 10936 L 10023 11059 L 10019 11062 L 9884 11165 L
9849 11194 L 9851 11330 L 9884 11394 L 9900 11466 L
9951 11601 L 10019 11648 L 10063 11737 L 10149 11873 L
10155 11881 L 10221 12008 L 10267 12144 L 10209 12279 L
10155 12368 L 10019 12362 L 9933 12415 L 9884 12425 L
9753 12551 L 9756 12686 L 9757 12822 L 9748 12863 L
9613 12869 L 9478 12889 L 9392 12957 L 9342 12977 L
9213 13093 L 9207 13098 L 9071 13106 L 8936 13198 L
8841 13229 L 8801 13285 L 8754 13364 L 8665 13416 L
8530 13389 L 8394 13380 L 8391 13364 L 8379 13229 L
8355 13093 L 8259 13069 L 8123 13042 L 7988 13053 L
7916 13093 L 7871 13229 L 7853 13251 L 7717 13320 L
7672 13364 L 7717 13424 L 7828 13500 L 7853 13513 L
7988 13567 L 8123 13626 L 8210 13635 L 8189 13771 L
8123 13835 L 7988 13881 L 7893 13907 L 7853 13920 L

7717 13929 L 7582 13972 L 7446 14018 L 7326 14042 L
7311 14046 L 7176 14131 L 7040 14110 L 6905 14128 L
6769 14112 L 6634 14156 L 6511 14042 L 6498 14034 L
6424 13907 L 6363 13841 L 6332 13771 L 6236 13635 L
6228 13621 L 6178 13500 L 6201 13364 L 6192 13229 L
6168 13093 L 6157 12957 L 6228 12913 L 6363 12906 L
6498 12944 L 6523 12957 L 6634 13004 L 6634 12931 L
6498 12865 L 6381 12822 L 6363 12774 L 6325 12686 L
6228 12558 L 6221 12551 L 6166 12415 L 6228 12325 L
6286 12279 L 6229 12144 L 6228 12124 L 6223 12008 L
6228 11989 L 6363 11969 L 6390 12008 L 6498 12117 L
6526 12144 L 6561 12279 L 6563 12415 L 6578 12551 L
6634 12610 L 6727 12686 L 6769 12745 L 6905 12808 L
6927 12822 L 6905 12840 L 6769 12880 L 6634 12931 L
6634 13004 L 6769 12989 L 6888 12957 L 6905 12951 L
7040 12862 L 7040 12737 L 6905 12724 L 6849 12686 L
6769 12622 L 6681 12551 L 6769 12509 L 6897 12551 L
6905 12562 L 7040 12677 L 7053 12686 L 7040 12737 L
7040 12862 L 7176 12910 L 7311 12940 L 7446 12938 L
7446 12849 L 7311 12834 L 7289 12822 L 7176 12699 L
7168 12686 L 7040 12596 L 6991 12551 L 6905 12483 L
6769 12450 L 6694 12415 L 6692 12279 L 6740 12144 L
6769 12078 L 6769 11932 L 6735 11873 L 6753 11737 L
6683 11601 L 6769 11578 L 6804 11601 L 6784 11737 L
6812 11873 L 6769 11932 L 6769 12078 L 6886 12144 L
6905 12161 L 6986 12279 L 7040 12330 L 7040 12068 L
6905 12019 L 6903 12008 L 6905 12007 L 7040 11941 L
7058 12008 L 7040 12068 L 7040 12330 L 7138 12415 L
7176 12456 L 7209 12415 L 7247 12279 L 7311 12232 L
7352 12279 L 7446 12364 L 7570 12415 L 7582 12428 L
7612 12551 L 7615 12686 L 7582 12707 L 7494 12822 L
7446 12849 L 7446 12938 L 7582 12887 L 7717 12860 L
7751 12822 L 7768 12686 L 7717 12614 L 7678 12551 L
7651 12415 L 7582 12324 L 7491 12279 L 7446 12199 L
7405 12144 L 7446 12126 L 7446 12014 L 7431 12008 L
7311 11905 L 7288 11873 L 7298 11737 L 7311 11720 L
7395 11737 L 7446 11761 L 7459 11873 L 7449 12008 L
7446 12014 L 7446 12126 L 7511 12008 L 7500 11873 L
7510 11737 L 7446 11687 L 7326 11601 L 7311 11590 L
7190 11601 L 7177 11737 L 7176 11740 L 7151 11737 L
7040 11718 L 6990 11737 L 6905 11767 L 6896 11737 L
6890 11601 L 6769 11521 L 6634 11560 L 6527 11601 L
6632 11737 L 6633 11873 L 6634 11874 L 6720 12008 L

6665 12144 L 6634 12226 L 6602 12144 L 6498 12043 L
6464 12008 L 6371 11873 L 6363 11866 L 6228 11831 L
6129 11873 L 6111 12008 L 6092 12063 L 6074 12008 L
6070 11873 L 5986 11737 L 5957 11697 L 5880 11737 L
5832 11873 L 5867 12008 L 5821 12127 L 5815 12144 L
5821 12232 L 5828 12279 L 5828 12415 L 5842 12551 L
5822 12686 L 5821 12687 L 5697 12822 L 5686 12830 L
5551 12845 L 5458 12822 L 5415 12812 L 5280 12795 L
5144 12807 L 5133 12822 L 5089 12957 L 5144 13042 L
5167 13093 L 5168 13229 L 5144 13246 L 5116 13229 L
5009 13177 L 4873 13134 L 4738 13111 L 4720 13093 L
4603 13018 L 4467 12987 L 4332 12967 L 4314 12957 L
4196 12908 L 4061 12862 L 3971 12822 L 3926 12804 L
3790 12769 L 3655 12754 L 3519 12712 L 3388 12686 L
3384 12681 L 3370 12686 L 3248 12696 L 3231 12686 L
3248 12677 L 3360 12551 L 3384 12539 L 3519 12474 L
3605 12415 L 3655 12387 L 3790 12368 L 3926 12358 L
4061 12357 L 4177 12415 L 4196 12427 L 4332 12503 L
4332 12303 L 4267 12279 L 4332 12271 L 4340 12279 L
4332 12303 L 4332 12503 L 4467 12516 L 4603 12449 L
4657 12415 L 4738 12370 L 4873 12396 L 5009 12338 L
5058 12279 L 5144 12182 L 5177 12144 L 5144 12080 L
5009 12086 L 4899 12144 L 4873 12182 L 4738 12167 L
4603 12184 L 4467 12146 L 4465 12144 L 4390 12008 L
4332 11984 L 4196 11912 L 4061 11914 L 3926 11959 L
3790 11895 L 3655 11943 L 3564 12008 L 3519 12039 L
3384 12081 L 3248 12094 L 3113 12113 L 2978 12071 L
2842 12035 L 2707 12053 L 2571 12037 L 2556 12008 L
2444 11873 L 2436 11871 L 2301 11838 L N E
15 O
2301 11947 M 2301 13192 L 2436 13212 L 2462 13229 L 2571 13363 L
2696 13229 L 2707 13214 L 2787 13093 L 2842 13009 L
2877 13093 L 2954 13229 L 2952 13364 L 2931 13500 L
2854 13635 L 2842 13646 L 2781 13771 L 2711 13907 L
2707 13914 L 2663 14042 L 2639 14178 L 2597 14314 L
2571 14347 L 2436 14436 L 2418 14449 L 2369 14585 L
2374 14720 L 2305 14856 L 2303 14992 L 2436 15073 L
2571 15111 L 2674 14992 L 2608 14856 L 2571 14756 L
2537 14720 L 2571 14702 L 2707 14650 L 2787 14720 L
2842 14773 L 2873 14856 L 2978 14943 L 2978 14238 L
2927 14178 L 2947 14042 L 2978 13971 L 3113 13960 L
3184 14042 L 3115 14178 L 3113 14180 L 2978 14238 L
2978 14943 L 3017 14992 L 3113 15118 L 3122 15127 L

3248 15260 L 3286 15263 L 3384 15290 L 3412 15263 L
3519 15174 L 3655 15147 L 3677 15127 L 3749 14992 L
3790 14894 L 3867 14856 L 3926 14834 L 3976 14856 L
4061 14883 L 4135 14856 L 4196 14843 L 4332 14814 L
4467 14824 L 4603 14828 L 4640 14856 L 4738 14963 L
4806 14992 L 4873 15019 L 4955 15127 L 4980 15263 L
5009 15292 L 5144 15327 L 5223 15263 L 5280 15179 L
5286 15127 L 5281 14992 L 5280 14988 L 5191 14856 L
5163 14720 L 5151 14585 L 5144 14550 L 5117 14449 L
5142 14314 L 5144 14304 L 5175 14178 L 5280 14103 L
5369 14178 L 5412 14314 L 5404 14449 L 5415 14457 L
5551 14499 L 5605 14585 L 5686 14699 L 5686 14228 L
5560 14178 L 5551 14174 L 5421 14042 L 5415 14036 L
5319 13907 L 5415 13835 L 5551 13896 L 5571 13907 L
5686 13978 L 5754 14042 L 5748 14178 L 5686 14228 L
5686 14699 L 5698 14720 L 5782 14856 L 5806 14992 L
5817 15127 L 5821 15135 L 5929 15263 L 5957 15288 L
6092 15375 L 6102 15263 L 6092 15232 L 6008 15127 L
5957 15072 L 5841 14992 L 5957 14887 L 5962 14856 L
6066 14720 L 6092 14689 L 6228 14678 L 6280 14720 L
6356 14856 L 6363 14879 L 6498 14952 L 6634 14973 L
6677 14992 L 6769 15054 L 6827 15127 L 6905 15203 L
7008 15263 L 7040 15299 L 7141 15398 L 7176 15441 L
7225 15534 L 7311 15616 L 7421 15670 L 7446 15686 L
7582 15752 L 7666 15670 L 7717 15609 L 7775 15534 L
7853 15491 L 7976 15534 L 7988 15539 L 8123 15584 L
8259 15548 L 8309 15534 L 8394 15482 L 8510 15398 L
8475 15263 L 8443 15127 L 8395 14992 L 8394 14991 L
8259 14944 L 8123 14932 L 7988 14921 L 7925 14856 L
7941 14720 L 7950 14585 L 7958 14449 L 7988 14411 L
8123 14404 L 8259 14394 L 8394 14380 L 8462 14314 L
8494 14178 L 8499 14042 L 8505 13907 L 8530 13881 L
8665 13872 L 8801 13872 L 8852 13907 L 8936 13991 L
8997 14042 L 9071 14116 L 9140 14178 L 9207 14244 L
9277 14178 L 9342 14134 L 9478 14134 L 9571 14042 L
9613 13992 L 9748 13973 L 9884 13945 L 9923 13907 L
9906 13771 L 9884 13747 L 9748 13723 L 9613 13690 L
9573 13635 L 9478 13538 L 9342 13526 L 9321 13500 L
9325 13364 L 9207 13247 L 9071 13247 L 9057 13229 L
9071 13216 L 9207 13216 L 9341 13093 L 9342 13092 L
9478 13080 L 9613 13074 L 9748 13077 L 9868 12957 L
9873 12822 L 9876 12686 L 9880 12551 L 9884 12547 L
10019 12536 L 10155 12536 L 10171 12551 L 10290 12670 L

10312 12686 L 10426 12798 L 10457 12822 L 10561 12925 L
10665 12822 L 10696 12801 L 10832 12797 L 10943 12686 L
10967 12653 L 11103 12635 L 11238 12602 L 11327 12551 L
11238 12500 L 11103 12457 L 11057 12415 L 10967 12332 L
10832 12281 L 10829 12279 L 10696 12178 L 10672 12144 L
10653 12008 L 10561 11918 L 10426 11911 L 10402 11873 L
10356 11737 L 10290 11669 L 10155 11617 L 10142 11601 L
10128 11466 L 10019 11349 L 10008 11330 L 10010 11194 L
10019 11186 L 10152 11059 L 10155 11056 L 10290 11045 L
10426 11021 L 10524 10923 L 10546 10788 L 10549 10652 L
10551 10516 L 10561 10502 L 10678 10381 L 10683 10245 L
10696 10223 L 10808 10110 L 10818 9974 L 10832 9960 L
10955 9838 L 10967 9824 L 11103 9805 L 11204 9703 L
11217 9567 L 11214 9432 L 11219 9296 L 11232 9160 L
11103 9025 L 11103 9025 L 10967 8899 L 10956 8889 L
10967 8834 L 11060 8754 L 11103 8746 L 11238 8693 L
11373 8745 L 11509 8646 L 11631 8754 L 11644 8771 L
11714 8889 L 11780 8968 L 11821 9025 L 11915 9149 L
11920 9160 L 11959 9296 L 12011 9432 L 12051 9520 L
12052 9567 L 12054 9703 L 12056 9838 L 12058 9974 L
12059 10110 L 12061 10245 L 12063 10381 L 12065 10516 L
12066 10652 L 12067 10788 L 12069 10923 L 12071 11059 L
12074 11194 L 12077 11330 L 12079 11466 L 12084 11601 L
12091 11737 L 12094 11873 L 12099 12008 L 12097 12144 L
12110 12279 L 12130 12415 L 12165 12551 L 12134 12686 L
12160 12822 L 12186 12921 L 12227 12957 L 12321 13050 L
12337 13093 L 12347 13229 L 12321 13355 L 12186 13234 L
12051 13262 L 11915 13362 L 11913 13364 L 11913 13500 L
11893 13635 L 11808 13771 L 11780 13810 L 11747 13907 L
11780 13932 L 11910 14042 L 11915 14048 L 12051 14087 L
12186 14154 L 12321 14093 L 12379 14042 L 12455 13907 L
12457 13906 L 12461 13907 L 12592 13922 L 12728 13923 L
12863 13916 L 12905 14042 L 12998 14145 L 12998 12435 L
12863 12424 L 12838 12415 L 12728 12378 L 12592 12281 L
12591 12279 L 12504 12144 L 12457 12038 L 12449 12008 L
12404 11873 L 12383 11737 L 12323 11601 L 12321 11598 L
12282 11466 L 12240 11330 L 12195 11194 L 12186 11163 L
12174 11059 L 12159 10923 L 12148 10788 L 12140 10652 L
12132 10516 L 12126 10381 L 12118 10245 L 12110 10110 L
12105 9974 L 12099 9838 L 12095 9703 L 12091 9567 L
12085 9432 L 12079 9296 L 12071 9160 L 12063 9025 L
12055 8889 L 12051 8809 L 12027 8754 L 11972 8618 L
11921 8482 L 11915 8461 L 11867 8347 L 11780 8218 L

11774 8211 L 11660 8075 L 11644 8059 L 11521 7940 L
11509 7931 L 11373 7867 L 11293 7940 L 11295 8075 L
11333 8211 L 11238 8320 L 11214 8347 L 11238 8380 L
11373 8466 L 11387 8482 L 11413 8618 L 11373 8654 L
11329 8618 L 11238 8555 L 11157 8618 L 11103 8664 L
10967 8702 L 10832 8696 L 10738 8618 L 10696 8584 L
10655 8618 L 10635 8754 L 10696 8797 L 10832 8836 L
10876 8889 L 10967 8967 L 11029 9025 L 11103 9089 L
11171 9160 L 11140 9296 L 11123 9432 L 11145 9567 L
11103 9686 L 11088 9703 L 10967 9752 L 10894 9838 L
10832 9899 L 10759 9974 L 10723 10110 L 10696 10137 L
10628 10245 L 10607 10381 L 10561 10428 L 10496 10516 L
10491 10652 L 10473 10788 L 10426 10869 L 10379 10923 L
10290 10978 L 10155 10996 L 10087 11059 L 10019 11124 L
9944 11194 L 9942 11330 L 10017 11466 L 10019 11469 L
10076 11601 L 10155 11698 L 10208 11737 L 10290 11848 L
10306 11873 L 10375 12008 L 10426 12073 L 10561 12141 L
10562 12144 L 10634 12279 L 10696 12364 L 10768 12415 L
10832 12491 L 10882 12551 L 10850 12686 L 10832 12704 L
10696 12720 L 10561 12780 L 10426 12706 L 10406 12686 L
10290 12600 L 10240 12551 L 10155 12472 L 10019 12466 L
9884 12486 L 9817 12551 L 9816 12686 L 9815 12822 L
9796 12957 L 9748 13005 L 9613 12977 L 9478 12993 L
9342 13035 L 9277 13093 L 9207 13157 L 9071 13161 L
8994 13229 L 9071 13329 L 9207 13338 L 9234 13364 L
9231 13500 L 9333 13635 L 9207 13718 L 9071 13710 L
8936 13735 L 8808 13771 L 8801 13772 L 8791 13771 L
8665 13757 L 8620 13771 L 8530 13783 L 8412 13907 L
8402 14042 L 8394 14123 L 8366 14178 L 8259 14218 L
8123 14259 L 7988 14290 L 7962 14314 L 7872 14449 L
7853 14565 L 7826 14585 L 7717 14660 L 7618 14720 L
7582 14744 L 7446 14834 L 7348 14856 L 7311 14872 L
7213 14992 L 7176 15012 L 7040 15039 L 6905 14992 L
6905 14992 L 6780 14856 L 6769 14851 L 6634 14788 L
6498 14746 L 6474 14720 L 6363 14617 L 6324 14585 L
6262 14449 L 6247 14314 L 6291 14178 L 6358 14042 L
6289 13907 L 6234 13771 L 6228 13763 L 6099 13635 L
6092 13622 L 5971 13500 L 6092 13412 L 6113 13364 L
6122 13229 L 6092 13115 L 6075 13093 L 5957 12969 L
5938 12957 L 5821 12886 L 5689 12957 L 5686 12968 L
5551 13069 L 5493 12957 L 5415 12934 L 5284 12957 L
5291 13093 L 5391 13229 L 5403 13364 L 5280 13499 L
5144 13469 L 5066 13364 L 5009 13321 L 4873 13285 L

4783 13364 L 4826 13500 L 4815 13635 L 4801 13771 L
4832 13907 L 4873 14034 L 4876 14042 L 4873 14049 L
4738 14103 L 4603 14076 L 4583 14042 L 4467 13909 L
4464 13907 L 4332 13811 L 4251 13771 L 4332 13737 L
4429 13635 L 4467 13609 L 4543 13500 L 4603 13386 L
4629 13364 L 4654 13229 L 4603 13178 L 4472 13093 L
4467 13091 L 4458 13093 L 4332 13169 L 4283 13229 L
4210 13364 L 4196 13450 L 4071 13500 L 4061 13505 L
4060 13500 L 4012 13364 L 4018 13229 L 4060 13093 L
4033 12957 L 3926 12913 L 3790 12920 L 3718 12957 L
3685 13093 L 3657 13229 L 3655 13235 L 3519 13340 L
3384 13356 L 3248 13328 L 3188 13229 L 3146 13093 L
3113 13050 L 3092 12957 L 3017 12822 L 2999 12686 L
3113 12590 L 3179 12551 L 3248 12522 L 3248 12415 L
3247 12415 L 3236 12279 L 3248 12274 L 3384 12249 L
3457 12279 L 3384 12334 L 3249 12415 L 3248 12415 L
3248 12522 L 3384 12467 L 3477 12415 L 3519 12377 L
3655 12315 L 3790 12296 L 3926 12286 L 4048 12279 L
4061 12265 L 4089 12144 L 4061 12131 L 3926 12115 L
3796 12008 L 3790 12005 L 3728 12008 L 3655 12014 L
3519 12103 L 3384 12128 L 3278 12144 L 3248 12155 L
3113 12205 L 2999 12144 L 2978 12138 L 2842 12112 L
2767 12144 L 2767 12279 L 2711 12415 L 2707 12419 L
2655 12415 L 2571 12410 L 2445 12279 L 2469 12144 L
2436 12079 L 2395 12008 L 2301 11947 L N E
2 O
2301 13192 M 2301 13906 L 2301 13907 L 2301 13907 L 2301 16212 L
2479 16212 L 2657 16212 L 2707 16212 L 2707 15559 L
2628 15534 L 2571 15428 L 2559 15398 L 2571 15373 L
2687 15263 L 2707 15164 L 2711 15263 L 2842 15374 L
2915 15398 L 2842 15492 L 2800 15534 L 2707 15559 L
2707 16212 L 2836 16212 L 3014 16212 L 3192 16212 L
3370 16212 L 3549 16212 L 3681 16212 L 3655 16195 L
3519 16099 L 3454 16076 L 3384 15960 L 3308 15941 L
3248 15913 L 3113 15811 L 3106 15805 L 2978 15747 L
2908 15670 L 2978 15567 L 3113 15656 L 3132 15670 L
3248 15747 L 3323 15805 L 3384 15902 L 3519 15884 L
3604 15941 L 3655 15989 L 3790 16045 L 3926 16062 L
4061 16056 L 4164 16076 L 4061 16117 L 3963 16212 L
4084 16212 L 4262 16212 L 4440 16212 L 4618 16212 L
4797 16212 L 4975 16212 L 5153 16212 L 5332 16212 L
5510 16212 L 5688 16212 L 5867 16212 L 6045 16212 L
6223 16212 L 6401 16212 L 6580 16212 L 6758 16212 L

6936 16212 L 7115 16212 L 7293 16212 L 7471 16212 L
7650 16212 L 7828 16212 L 8006 16212 L 8184 16212 L
8363 16212 L 8541 16212 L 8719 16212 L 8898 16212 L
9076 16212 L 9254 16212 L 9432 16212 L 9611 16212 L
9789 16212 L 9967 16212 L 10146 16212 L 10324 16212 L
10502 16212 L 10681 16212 L 10859 16212 L 11037 16212 L
11215 16212 L 11394 16212 L 11572 16212 L 11750 16212 L
11929 16212 L 12107 16212 L 12285 16212 L 12463 16212 L
12642 16212 L 12728 16212 L 12728 14480 L 12713 14449 L
12728 14420 L 12763 14449 L 12728 14480 L 12728 16212 L
12820 16212 L 12998 16212 L 12998 14145 L 12905 14042 L
12863 13916 L 12728 13923 L 12592 13922 L 12461 13907 L
12457 13906 L 12455 13907 L 12379 14042 L 12321 14093 L
12186 14154 L 12051 14087 L 11915 14048 L 11910 14042 L
11780 13932 L 11747 13907 L 11780 13810 L 11808 13771 L
11893 13635 L 11913 13500 L 11913 13364 L 11915 13362 L
12051 13262 L 12186 13234 L 12321 13355 L 12347 13229 L
12337 13093 L 12321 13050 L 12227 12957 L 12186 12921 L
12160 12822 L 12134 12686 L 12165 12551 L 12130 12415 L
12110 12279 L 12097 12144 L 12099 12008 L 12094 11873 L
12091 11737 L 12084 11601 L 12079 11466 L 12077 11330 L
12074 11194 L 12071 11059 L 12069 10923 L 12067 10788 L
12066 10652 L 12065 10516 L 12063 10381 L 12061 10245 L
12059 10110 L 12058 9974 L 12056 9838 L 12054 9703 L
12052 9567 L 12051 9520 L 12011 9432 L 11959 9296 L
11920 9160 L 11915 9149 L 11821 9025 L 11780 8968 L
11714 8889 L 11644 8771 L 11631 8754 L 11509 8646 L
11373 8745 L 11238 8693 L 11103 8746 L 11060 8754 L
10967 8834 L 10956 8889 L 10967 8899 L 11103 9025 L
11103 9025 L 11232 9160 L 11219 9296 L 11214 9432 L
11217 9567 L 11204 9703 L 11103 9805 L 10967 9824 L
10955 9838 L 10832 9960 L 10818 9974 L 10808 10110 L
10696 10223 L 10683 10245 L 10678 10381 L 10561 10502 L
10551 10516 L 10549 10652 L 10546 10788 L 10524 10923 L
10426 11021 L 10290 11045 L 10155 11056 L 10152 11059 L
10019 11186 L 10010 11194 L 10008 11330 L 10019 11349 L
10128 11466 L 10142 11601 L 10155 11617 L 10290 11669 L
10356 11737 L 10402 11873 L 10426 11911 L 10561 11918 L
10653 12008 L 10672 12144 L 10696 12178 L 10829 12279 L
10832 12281 L 10967 12332 L 11057 12415 L 11103 12457 L
11238 12500 L 11327 12551 L 11238 12602 L 11103 12635 L
10967 12653 L 10943 12686 L 10832 12797 L 10696 12801 L
10665 12822 L 10561 12925 L 10457 12822 L 10426 12798 L

10312 12686 L 10290 12670 L 10171 12551 L 10155 12536 L
10019 12536 L 9884 12547 L 9880 12551 L 9876 12686 L
9873 12822 L 9868 12957 L 9748 13077 L 9613 13074 L
9478 13080 L 9342 13092 L 9341 13093 L 9207 13216 L
9071 13216 L 9057 13229 L 9071 13247 L 9207 13247 L
9325 13364 L 9321 13500 L 9342 13526 L 9478 13538 L
9573 13635 L 9613 13690 L 9748 13723 L 9884 13747 L
9906 13771 L 9923 13907 L 9884 13945 L 9748 13973 L
9613 13992 L 9571 14042 L 9478 14134 L 9342 14134 L
9277 14178 L 9207 14244 L 9140 14178 L 9071 14116 L
8997 14042 L 8936 13991 L 8852 13907 L 8801 13872 L
8665 13872 L 8530 13881 L 8505 13907 L 8499 14042 L
8494 14178 L 8462 14314 L 8394 14380 L 8259 14394 L
8123 14404 L 7988 14411 L 7958 14449 L 7950 14585 L
7941 14720 L 7925 14856 L 7988 14921 L 8123 14932 L
8259 14944 L 8394 14991 L 8395 14992 L 8443 15127 L
8475 15263 L 8510 15398 L 8394 15482 L 8309 15534 L
8259 15548 L 8123 15584 L 7988 15539 L 7976 15534 L
7853 15491 L 7775 15534 L 7717 15609 L 7666 15670 L
7582 15752 L 7446 15686 L 7421 15670 L 7311 15616 L
7225 15534 L 7176 15441 L 7141 15398 L 7040 15299 L
7008 15263 L 6905 15203 L 6827 15127 L 6769 15054 L
6677 14992 L 6634 14973 L 6498 14952 L 6363 14879 L
6356 14856 L 6280 14720 L 6228 14678 L 6092 14689 L
6066 14720 L 5962 14856 L 5957 14887 L 5841 14992 L
5957 15072 L 6008 15127 L 6092 15232 L 6102 15263 L
6092 15375 L 5957 15288 L 5929 15263 L 5821 15135 L
5817 15127 L 5806 14992 L 5782 14856 L 5698 14720 L
5686 14699 L 5605 14585 L 5551 14499 L 5415 14457 L
5404 14449 L 5412 14314 L 5369 14178 L 5280 14103 L
5175 14178 L 5144 14304 L 5142 14314 L 5117 14449 L
5144 14550 L 5151 14585 L 5163 14720 L 5191 14856 L
5280 14988 L 5281 14992 L 5286 15127 L 5280 15179 L
5223 15263 L 5144 15327 L 5009 15292 L 4980 15263 L
4955 15127 L 4873 15019 L 4806 14992 L 4738 14963 L
4640 14856 L 4603 14828 L 4467 14824 L 4332 14814 L
4196 14843 L 4135 14856 L 4061 14883 L 3976 14856 L
3926 14834 L 3867 14856 L 3790 14894 L 3749 14992 L
3677 15127 L 3655 15147 L 3519 15174 L 3412 15263 L
3384 15290 L 3286 15263 L 3248 15260 L 3122 15127 L
3113 15118 L 3017 14992 L 2978 14943 L 2873 14856 L
2842 14773 L 2787 14720 L 2707 14650 L 2571 14702 L
2537 14720 L 2571 14756 L 2608 14856 L 2674 14992 L

2571 15111 L 2436 15073 L 2303 14992 L 2305 14856 L
2374 14720 L 2369 14585 L 2418 14449 L 2436 14436 L
2571 14347 L 2597 14314 L 2639 14178 L 2663 14042 L
2707 13914 L 2711 13907 L 2781 13771 L 2842 13646 L
2854 13635 L 2931 13500 L 2952 13364 L 2954 13229 L
2877 13093 L 2842 13009 L 2787 13093 L 2707 13214 L
2696 13229 L 2571 13363 L 2462 13229 L 2436 13212 L
2301 13192 L N E
15 O
2301 13907 2301 13906 2301 13907 2301 13907 4 N F
42 O
2436 8289 2404 8347 2436 8386 2460 8347 2436 8289 5 N F
29 O
2571 11797 2552 11737 2571 11674 2611 11601 2707 11566 2842 11567
2978 11538 3113 11502 3248 11475 3341 11466 3384 11459 3407 11466
3440 11601 3384 11665 3279 11737 3248 11748 3248 11613 3268 11601
3248 11592 3113 11600 3109 11601 3113 11605 3248 11613 3248 11748
3113 11772 2978 11791 2842 11830 2707 11868 2571 11797 29 N F
15 O
2571 15373 2559 15398 2571 15428 2628 15534 2707 15559 2800 15534
2842 15492 2915 15398 2842 15374 2711 15263 2707 15164 2687 15263
2571 15373 13 N F
55 O
2762 6041 2710 5906 2842 5815 2882 5906 2933 6041 2842 6115
2762 6041 7 N F
15 O
2841 9703 2827 9567 2842 9508 2978 9445 3002 9432 3113 9361
3142 9432 3201 9567 3154 9703 3242 9838 3113 9939 2978 9843
2970 9838 2842 9705 2841 9703 15 N F
2978 15567 2908 15670 2978 15747 3106 15805 3113 15811 3248 15913
3308 15941 3384 15960 3454 16076 3519 16099 3655 16195 3681 16212
3727 16212 3905 16212 3963 16212 4061 16117 4164 16076 4061 16056
3926 16062 3790 16045 3655 15989 3604 15941 3519 15884 3384 15902
3323 15805 3248 15747 3132 15670 3113 15656 2978 15567 29 N F
2 O
2978 14238 2927 14178 2947 14042 2978 13971 3113 13960 3184 14042
3115 14178 3113 14180 2978 14238 9 N F
55 O
2978 11855 M 2934 11873 L 2978 11989 L 2985 12008 L 3113 12053 L
3248 12040 L 3384 12034 L 3454 12008 L 3519 11951 L
3655 11875 L 3661 11873 L 3790 11791 L 3926 11815 L
3926 11627 L 3790 11620 L 3764 11601 L 3790 11594 L
3926 11592 L 3948 11601 L 3926 11627 L 3926 11815 L

4035 11737 L 4061 11710 L 4196 11689 L 4231 11737 L
4322 11873 L 4332 11879 L 4406 11873 L 4467 11861 L
4603 11867 L 4610 11873 L 4727 12008 L 4738 12017 L
4781 12008 L 4873 11990 L 5009 11973 L 5009 11907 L
4907 11873 L 4873 11832 L 4817 11737 L 4738 11672 L
4603 11716 L 4545 11601 L 4584 11466 L 4467 11383 L
4387 11330 L 4467 11249 L 4567 11330 L 4603 11410 L
4714 11330 L 4738 11322 L 4847 11194 L 4873 11182 L
5009 11107 L 5074 11059 L 5144 10941 L 5280 10969 L
5280 10549 L 5179 10516 L 5280 10425 L 5350 10516 L
5280 10549 L 5280 10969 L 5368 11059 L 5415 11089 L
5442 11059 L 5504 10923 L 5483 10788 L 5551 10729 L
5612 10788 L 5686 10857 L 5726 10923 L 5728 11059 L
5745 11194 L 5821 11267 L 5910 11330 L 5957 11441 L
6078 11466 L 5957 11475 L 5821 11547 L 5792 11601 L
5716 11737 L 5688 11873 L 5686 11881 L 5551 11876 L
5549 11873 L 5415 11773 L 5280 11769 L 5144 11850 L
5114 11873 L 5009 11907 L 5009 11973 L 5144 11943 L
5280 11891 L 5415 11925 L 5468 12008 L 5436 12144 L
5415 12184 L 5280 12273 L 5275 12279 L 5165 12415 L
5239 12551 L 5280 12581 L 5387 12686 L 5415 12697 L
5551 12712 L 5686 12711 L 5710 12686 L 5743 12551 L
5722 12415 L 5701 12279 L 5731 12144 L 5758 12008 L
5759 11873 L 5780 11737 L 5821 11674 L 5957 11612 L
6049 11737 L 6092 11804 L 6092 11373 L 6039 11330 L
6092 11275 L 6219 11330 L 6092 11373 L 6092 11804 L
6228 11776 L 6228 11469 L 6122 11466 L 6228 11462 L
6231 11466 L 6228 11469 L 6228 11776 L 6363 11788 L
6463 11873 L 6498 11929 L 6633 12008 L 6634 12009 L
6634 12008 L 6634 12008 L 6535 11873 L 6540 11737 L
6498 11688 L 6391 11601 L 6369 11466 L 6409 11330 L
6363 11322 L 6281 11194 L 6228 11156 L 6092 11155 L
5957 11187 L 5821 11121 L 5801 11059 L 5821 10951 L
5829 10923 L 5821 10882 L 5792 10788 L 5745 10652 L
5778 10516 L 5769 10381 L 5688 10245 L 5758 10110 L
5821 10009 L 5838 9974 L 5828 9838 L 5821 9832 L
5696 9703 L 5686 9640 L 5634 9567 L 5686 9507 L
5695 9432 L 5700 9296 L 5686 9262 L 5629 9296 L
5551 9414 L 5546 9432 L 5458 9567 L 5439 9703 L
5494 9838 L 5415 9911 L 5347 9974 L 5362 10110 L
5415 10241 L 5418 10245 L 5415 10275 L 5280 10290 L
5258 10245 L 5144 10191 L 5009 10174 L 4873 10175 L
4816 10245 L 4738 10341 L 4721 10381 L 4738 10451 L

4803 10516 L 4873 10546 L 4945 10652 L 4982 10788 L
5009 10864 L 5029 10923 L 5009 10956 L 4873 11001 L
4738 11055 L 4735 11059 L 4603 11153 L 4467 11151 L
4332 11137 L 4239 11194 L 4196 11320 L 4192 11330 L
4061 11408 L 3926 11456 L 3887 11466 L 3790 11498 L
3655 11534 L 3556 11601 L 3519 11660 L 3458 11737 L
3384 11778 L 3248 11810 L 3113 11828 L 2978 11855 L
N E
68 O
3113 10238 3027 10245 3113 10276 3125 10245 3113 10238 5 N F
15 O
3113 11605 3109 11601 3113 11600 3248 11592 3268 11601 3248 11613
3113 11605 7 N F
3248 10027 3143 9974 3248 9844 3384 9897 3519 9971 3534 9974
3655 9989 3708 10110 3763 10245 3655 10347 3519 10300 3456 10245
3384 10166 3321 10110 3248 10027 15 N F
42 O
3248 6901 3194 6855 3248 6789 3364 6855 3248 6901 5 N F
29 O
3248 7584 3234 7533 3246 7397 3248 7392 3270 7397 3384 7454
3435 7533 3384 7569 3248 7584 9 N F
2 O
3247 12415 3236 12279 3248 12274 3384 12249 3457 12279 3384 12334
3249 12415 3248 12415 3247 12415 9 N F
15 O
3384 12903 3349 12822 3384 12808 3466 12822 3384 12903 5 N F
68 O
3468 10652 3416 10788 3519 10890 3655 10804 3678 10788 3755 10652
3655 10603 3519 10620 3468 10652 9 N F
15 O
3519 9680 3483 9567 3519 9473 3617 9567 3519 9680 5 N F
29 O
3543 11330 3537 11194 3585 11059 3655 10995 3728 10923 3790 10874
3926 10871 4061 10894 4196 10820 4226 10788 4332 10656 4467 10689
4603 10725 4732 10652 4738 10648 4747 10652 4847 10788 4761 10923
4738 10937 4641 11059 4603 11086 4467 11098 4467 11034 4603 10996
4662 10923 4603 10841 4501 10788 4467 10775 4393 10788 4332 10807
4226 10923 4332 10990 4467 11034 4467 11098 4332 11073 4196 11127
4159 11194 4061 11329 4058 11330 3926 11385 3790 11409 3790 11314
3926 11306 4061 11241 4095 11194 4139 11059 4061 11000 3926 10960
3790 10958 3686 11059 3655 11191 3654 11194 3655 11197 3790 11314
3790 11409 3655 11424 3543 11330 57 N F
15 O

3626 9838 3543 9703 3655 9607 3684 9703 3677 9838 3655 9922
3626 9838 7 N F
55 O
3655 12496 3548 12551 3655 12614 3790 12646 3857 12686 3926 12712
3926 12558 3911 12551 3926 12546 4061 12548 4065 12551 4061 12557
3926 12558 3926 12712 4061 12753 4196 12784 4255 12822 4332 12861
4467 12884 4603 12871 4686 12822 4738 12740 4873 12713 4890 12686
4873 12661 4738 12557 4603 12612 4467 12618 4332 12609 4250 12551
4196 12518 4061 12442 3926 12438 3790 12462 3655 12496 35 N F
15 O
3655 11197 3654 11194 3655 11191 3686 11059 3790 10958 3926 10960
4061 11000 4139 11059 4095 11194 4061 11241 3926 11306 3926 11198
3935 11194 3944 11059 3926 11052 3873 11059 3911 11194 3926 11198
3926 11306 3790 11314 3655 11197 21 N F
68 O
3790 11594 3764 11601 3790 11620 3926 11627 3948 11601 3926 11592
3790 11594 7 N F
2 O
3911 11194 3873 11059 3926 11052 3944 11059 3935 11194 3926 11198
3911 11194 7 N F
29 O
3926 7650 3890 7533 3926 7461 4061 7509 4117 7533 4196 7567
4332 7627 4378 7669 4467 7797 4476 7804 4562 7940 4467 8067
4332 8046 4196 8030 4196 7807 4200 7804 4196 7801 4193 7804
4196 7807 4196 8030 4061 7973 4002 7940 3988 7804 3934 7669
3926 7650 25 N F
68 O
3926 12546 3911 12551 3926 12558 4061 12557 4065 12551 4061 12548
3926 12546 7 N F
15 O
4061 10048 4008 9974 4061 9907 4196 9957 4332 9973 4340 9974
4467 9998 4547 10110 4467 10204 4332 10186 4196 10166 4096 10110
4061 10048 13 N F
42 O
4061 6784 4010 6719 4061 6657 4115 6719 4061 6784 5 N F
55 O
4061 7204 4026 7262 4061 7284 4170 7397 4196 7409 4217 7397
4196 7355 4099 7262 4061 7204 9 N F
82 O
4196 5662 4126 5770 4196 5804 4332 5844 4377 5770 4386 5634
4333 5499 4327 5499 4201 5634 4196 5662 10 N F
15 O
4196 7807 4193 7804 4196 7801 4200 7804 4196 7807 5 N F

4332 10990 4226 10923 4332 10807 4393 10788 4467 10775 4501 10788
4603 10841 4662 10923 4603 10996 4467 11034 4467 10933 4498 10923
4467 10910 4430 10923 4467 10933 4467 11034 4332 10990 17 N F
4332 12303 4267 12279 4332 12271 4340 12279 4332 12303 5 N F
82 O
4333 5499 4327 5499 4327 5499 4333 5499 4333 5499 5 N F
4327 5499 4327 5499 4333 5499 4333 5499 4332 5499 4327 5499
6 N F
68 O
4467 11249 4387 11330 4467 11383 4584 11466 4545 11601 4603 11716
4738 11672 4817 11737 4873 11832 4907 11873 5009 11907 5114 11873
5144 11850 5144 11664 5009 11629 5001 11601 5009 11497 5012 11466
5009 11465 4922 11330 5009 11214 5144 11209 5239 11330 5280 11461
5283 11466 5291 11601 5280 11605 5144 11664 5144 11850 5280 11769
5415 11773 5549 11873 5551 11876 5686 11881 5688 11873 5716 11737
5792 11601 5821 11547 5957 11475 6078 11466 5957 11441 5910 11330
5821 11267 5745 11194 5728 11059 5726 10923 5686 10857 5612 10788
5551 10729 5483 10788 5504 10923 5442 11059 5415 11089 5368 11059
5280 10969 5144 10941 5074 11059 5009 11107 4873 11182 4847 11194
4738 11322 4714 11330 4603 11410 4567 11330 4467 11249 65 N F
2 O
4467 10933 4430 10923 4467 10910 4498 10923 4467 10933 5 N F
29 O
4603 9201 4495 9160 4595 9025 4603 9007 4738 8921 4850 9025
4738 9092 4653 9160 4603 9201 9 N F
42 O
4873 7186 4835 7126 4873 7032 5009 7074 5056 7126 5144 7248
5157 7262 5144 7278 5009 7324 4906 7262 4873 7186 11 N F
82 O
5009 11214 4922 11330 5009 11465 5012 11466 5009 11497 5001 11601
5009 11629 5144 11664 5280 11605 5291 11601 5283 11466 5280 11461
5239 11330 5144 11209 5009 11214 15 N F
55 O
5009 5739 4965 5634 5009 5551 5128 5499 5153 5499 5294 5499
5415 5634 5496 5499 5510 5499 5688 5499 5773 5499 5821 5570
5858 5499 5867 5499 6002 5499 6000 5634 5957 5654 5821 5669
5747 5770 5686 5848 5686 5643 5692 5634 5686 5627 5675 5634
5686 5643 5686 5848 5582 5770 5551 5745 5454 5770 5415 5818
5280 5867 5211 5770 5144 5691 5009 5739 34 N F
68 O
5144 6960 5114 6991 5144 7024 5262 7126 5280 7147 5356 7262
5415 7312 5498 7262 5415 7209 5297 7126 5280 7077 5219 6991
5144 6960 13 N F

55 O
5153 5499 5128 5499 5128 5499 5153 5499 5294 5499 5294 5499
5153 5499 7 N F
42 O
5280 10549 5179 10516 5280 10425 5350 10516 5280 10549 5 N F
68 O
5294 5499 5294 5499 5332 5499 5496 5499 5496 5499 5332 5499
5294 5499 7 N F
5332 5499 5294 5499 5415 5634 5496 5499 5332 5499 5 N F
29 O
5415 13835 5319 13907 5415 14036 5421 14042 5551 14174 5560 14178
5686 14228 5748 14178 5754 14042 5686 13978 5571 13907 5551 13896
5415 13835 13 N F
42 O
5415 7013 5401 6991 5415 6973 5449 6991 5415 7013 5 N F
55 O
5686 8506 5626 8618 5686 8705 5707 8618 5686 8506 5 N F
42 O
5686 5643 5675 5634 5686 5627 5692 5634 5686 5643 5 N F
82 O
5806 6041 M 5697 6177 L 5741 6313 L 5792 6448 L 5769 6584 L
5821 6626 L 5957 6621 L 5957 6557 L 5832 6448 L
5957 6403 L 6017 6448 L 5957 6557 L 5957 6621 L
5975 6584 L 6092 6506 L 6228 6530 L 6363 6556 L
6402 6584 L 6455 6719 L 6498 6817 L 6538 6855 L
6634 6892 L 6746 6855 L 6769 6837 L 6905 6804 L
6947 6855 L 7040 6961 L 7123 6991 L 7176 7006 L
7311 7068 L 7311 6838 L 7176 6731 L 7167 6719 L
7120 6584 L 7040 6472 L 7026 6448 L 7040 6400 L
7059 6448 L 7176 6537 L 7244 6584 L 7311 6624 L
7446 6644 L 7482 6719 L 7446 6815 L 7311 6838 L
7311 7068 L 7446 7044 L 7508 6991 L 7560 6855 L
7582 6800 L 7623 6855 L 7717 6964 L 7717 6727 L
7706 6719 L 7717 6710 L 7728 6719 L 7717 6727 L
7717 6964 L 7746 6991 L 7853 7062 L 7988 7022 L
8123 7012 L 8239 7126 L 8259 7262 L 8259 7262 L
8259 7262 L 8301 7126 L 8394 7070 L 8518 6991 L
8530 6987 L 8534 6991 L 8660 7126 L 8665 7130 L
8782 7262 L 8801 7281 L 8936 7380 L 8966 7397 L
9071 7433 L 9207 7459 L 9342 7462 L 9478 7460 L
9613 7454 L 9748 7430 L 9884 7420 L 10019 7410 L
10019 7186 L 9903 7126 L 9884 7116 L 9823 6991 L
9748 6951 L 9661 6855 L 9613 6744 L 9598 6719 L

9613 6634 L 9617 6584 L 9623 6448 L 9613 6431 L
9546 6313 L 9502 6177 L 9504 6041 L 9521 5906 L
9592 5770 L 9613 5750 L 9632 5770 L 9748 5867 L
9783 5906 L 9832 6041 L 9881 6177 L 9884 6186 L
9929 6313 L 9958 6448 L 9994 6584 L 10019 6646 L
10079 6719 L 10103 6855 L 10081 6991 L 10063 7126 L
10019 7186 L 10019 7410 L 10155 7411 L 10290 7425 L
10426 7444 L 10561 7480 L 10696 7481 L 10832 7456 L
10967 7417 L 11103 7408 L 11238 7405 L 11373 7410 L
11509 7421 L 11644 7429 L 11780 7441 L 11915 7451 L
12051 7470 L 12113 7533 L 12134 7669 L 12144 7804 L
12154 7940 L 12161 8075 L 12168 8211 L 12175 8347 L
12176 8482 L 12182 8618 L 12186 8728 L 12197 8754 L
12254 8889 L 12320 9025 L 12321 9030 L 12321 7778 L
12276 7669 L 12225 7533 L 12321 7434 L 12375 7533 L
12443 7669 L 12321 7778 L 12321 9030 L 12434 9160 L
12457 9178 L 12592 9252 L 12592 7818 L 12577 7804 L
12592 7798 L 12599 7804 L 12592 7818 L 12592 9252 L
12728 9286 L 12739 9296 L 12863 9416 L 12876 9432 L
12998 9511 L 12998 5499 L 12820 5499 L 12776 5499 L
12863 5613 L 12871 5634 L 12907 5770 L 12876 5906 L
12890 6041 L 12881 6177 L 12894 6313 L 12868 6448 L
12863 6504 L 12810 6584 L 12740 6719 L 12741 6855 L
12728 6879 L 12688 6991 L 12592 7083 L 12457 7109 L
12377 7126 L 12321 7194 L 12261 7126 L 12186 7118 L
12156 7126 L 12090 7262 L 12051 7357 L 11915 7305 L
11780 7311 L 11644 7295 L 11509 7268 L 11474 7262 L
11373 7243 L 11238 7207 L 11103 7197 L 10967 7220 L
10832 7227 L 10743 7262 L 10696 7288 L 10561 7285 L
10426 7269 L 10408 7262 L 10290 7168 L 10268 7126 L
10194 6991 L 10170 6855 L 10155 6822 L 10141 6719 L
10064 6584 L 10019 6567 L 9987 6448 L 9974 6313 L
9966 6177 L 9884 6101 L 9862 6041 L 9831 5906 L
9748 5814 L 9696 5770 L 9613 5685 L 9524 5770 L
9487 5906 L 9478 6026 L 9476 6041 L 9476 6177 L
9478 6179 L 9518 6313 L 9571 6448 L 9544 6584 L
9532 6719 L 9613 6851 L 9615 6855 L 9680 6991 L
9613 7021 L 9571 6991 L 9603 6855 L 9478 6803 L
9413 6855 L 9385 6991 L 9393 7126 L 9342 7237 L
9207 7220 L 9071 7164 L 9007 7126 L 8936 7084 L
8801 6997 L 8794 6991 L 8665 6908 L 8605 6855 L
8530 6810 L 8394 6731 L 8345 6719 L 8259 6697 L
8151 6584 L 8123 6555 L 7988 6477 L 7863 6448 L

7853 6447 L 7717 6432 L 7618 6448 L 7582 6452 L
7446 6482 L 7389 6448 L 7311 6360 L 7292 6313 L
7311 6271 L 7327 6177 L 7311 6151 L 7220 6041 L
7176 6006 L 7040 5929 L 6905 5912 L 6775 6041 L
6769 6046 L 6753 6041 L 6634 5963 L 6498 6022 L
6470 6041 L 6363 6111 L 6285 6041 L 6228 5958 L
6193 5906 L 6188 5770 L 6194 5634 L 6175 5499 L
6088 5499 L 6092 5509 L 6133 5634 L 6092 5717 L
5980 5770 L 5957 5790 L 5864 5906 L 5821 6026 L
5806 6041 L N E
29 O
5821 9686 5755 9567 5789 9432 5821 9390 5957 9304 5961 9296
6092 9205 6228 9251 6245 9296 6283 9432 6363 9492 6407 9567
6418 9703 6363 9785 6265 9838 6243 9974 6354 10110 6351 10245
6228 10361 6228 10196 6292 10110 6228 10039 6161 10110 6228 10196
6228 10361 6217 10381 6126 10516 6092 10589 6023 10652 6092 10661
6129 10652 6228 10593 6363 10630 6498 10570 6634 10532 6769 10642
6783 10652 6769 10688 6634 10690 6498 10779 6494 10788 6498 10803
6544 10923 6498 10951 6363 11027 6363 10848 6408 10788 6363 10737
6292 10788 6363 10848 6363 11027 6340 11059 6228 11091 6092 11096
6092 10797 6101 10788 6092 10784 6080 10788 6092 10797 6092 11096
5957 11127 5957 11068 5996 11059 5957 11036 5946 11059 5957 11068
5957 11127 5873 11059 5930 10923 5896 10788 5939 10652 5884 10516
5901 10381 5957 10249 6034 10245 6092 10225 6097 10110 6092 10085
5965 9974 6059 9838 5957 9819 5957 9726 5993 9703 6092 9589
6115 9567 6147 9432 6092 9320 6029 9432 5957 9492 5841 9567
5933 9703 5957 9726 5957 9819 5834 9703 5821 9686 95 N F
68 O
5773 5499 5773 5499 5858 5499 5858 5499 5821 5499 5773 5499
6 N F
5773 5499 5773 5499 5858 5499 5858 5499 5773 5499 5 N F
5858 5499 5773 5499 5821 5570 5858 5499 4 N F
29 O
5871 8889 5826 8754 5930 8618 5892 8482 5924 8347 5906 8211
5937 8075 5932 7940 5944 7804 5957 7786 5996 7804 6016 7940
6015 8075 6045 8211 6056 8347 6021 8482 6092 8577 6111 8618
6144 8754 6108 8889 6092 8920 6061 9025 6007 9160 5957 9284
5957 9069 5984 9025 5957 8941 5957 8833 5992 8754 5957 8721
5926 8754 5957 8833 5957 8941 5927 9025 5957 9069 5957 9284
5898 9160 5842 9025 5871 8889 39 N F
68 O
5957 6557 5832 6448 5957 6403 6017 6448 5957 6557 5 N F
15 O

5933 9703 5841 9567 5957 9492 6029 9432 6092 9320 6147 9432
6115 9567 6092 9589 5993 9703 5957 9726 5933 9703 11 N F
55 O
5867 5499 5858 5499 5858 5499 5867 5499 6002 5499 6002 5499
5867 5499 7 N F
15 O
5957 12933 5896 12822 5928 12686 5957 12603 6061 12686 6030 12822
5957 12933 7 N F
5951 12008 5917 11873 5957 11808 5993 11873 5964 12008 5957 12055
5951 12008 7 N F
5957 8833 5926 8754 5957 8721 5992 8754 5957 8833 5 N F
5957 9069 5927 9025 5957 8941 5984 9025 5957 9069 5 N F
5957 11068 5946 11059 5957 11036 5996 11059 5957 11068 5 N F
68 O
6045 5499 6002 5499 6002 5499 6045 5499 6088 5499 6088 5499
6045 5499 7 N F
42 O
6092 11373 6039 11330 6092 11275 6219 11330 6092 11373 5 N F
29 O
6092 7753 6041 7669 6092 7636 6144 7669 6092 7753 5 N F
15 O
6092 10797 6080 10788 6092 10784 6101 10788 6092 10797 5 N F
82 O
6088 5499 6088 5499 6175 5499 6175 5499 6092 5499 6088 5499
6 N F
6088 5499 6088 5499 6175 5499 6175 5499 6088 5499 5 N F
68 O
6228 11462 6122 11466 6228 11469 6231 11466 6228 11462 5 N F
15 O
6228 10196 6161 10110 6228 10039 6292 10110 6228 10196 6228 10111
6229 10110 6228 10108 6226 10110 6228 10111 6228 10196 11 N F
6221 12551 6166 12415 6228 12325 6286 12279 6229 12144 6228 12124
6223 12008 6228 11989 6363 11969 6390 12008 6498 12117 6526 12144
6561 12279 6563 12415 6578 12551 6634 12610 6727 12686 6769 12745
6905 12808 6927 12822 6905 12840 6769 12880 6634 12931 6634 12836
6663 12822 6634 12765 6601 12822 6634 12836 6634 12931 6498 12865
6381 12822 6363 12774 6325 12686 6228 12558 6221 12551 35 N F
95 O
6223 5499 M 6175 5499 L 6194 5634 L 6188 5770 L 6193 5906 L
6228 5958 L 6285 6041 L 6363 6111 L 6470 6041 L
6498 6022 L 6634 5963 L 6753 6041 L 6769 6046 L
6775 6041 L 6905 5912 L 7040 5929 L 7176 6006 L
7220 6041 L 7311 6151 L 7327 6177 L 7311 6271 L

7292 6313 L 7311 6360 L 7389 6448 L 7446 6482 L
7582 6452 L 7618 6448 L 7717 6432 L 7853 6447 L
7863 6448 L 7988 6477 L 8123 6555 L 8151 6584 L
8259 6697 L 8345 6719 L 8394 6731 L 8530 6810 L
8605 6855 L 8665 6908 L 8794 6991 L 8801 6997 L
8936 7084 L 9007 7126 L 9071 7164 L 9207 7220 L
9342 7237 L 9342 6696 L 9299 6584 L 9342 6491 L
9407 6584 L 9342 6696 L 9342 7237 L 9393 7126 L
9385 6991 L 9413 6855 L 9478 6803 L 9603 6855 L
9571 6991 L 9613 7021 L 9680 6991 L 9615 6855 L
9613 6851 L 9532 6719 L 9544 6584 L 9571 6448 L
9518 6313 L 9478 6179 L 9476 6177 L 9476 6041 L
9478 6026 L 9487 5906 L 9524 5770 L 9613 5685 L
9696 5770 L 9748 5814 L 9831 5906 L 9862 6041 L
9884 6101 L 9966 6177 L 9974 6313 L 9987 6448 L
10019 6567 L 10019 6462 L 10016 6448 L 10019 6314 L
10039 6448 L 10019 6462 L 10019 6567 L 10064 6584 L
10141 6719 L 10155 6822 L 10155 5866 L 10133 5770 L
10155 5680 L 10204 5770 L 10155 5866 L 10155 6822 L
10170 6855 L 10194 6991 L 10268 7126 L 10290 7168 L
10408 7262 L 10426 7269 L 10561 7285 L 10696 7288 L
10696 6900 L 10561 6881 L 10491 6855 L 10426 6811 L
10374 6719 L 10360 6584 L 10307 6448 L 10290 6413 L
10269 6313 L 10290 6259 L 10351 6313 L 10426 6359 L
10477 6448 L 10561 6537 L 10650 6584 L 10696 6622 L
10824 6719 L 10832 6799 L 10930 6719 L 10967 6708 L
10973 6719 L 10967 6725 L 10842 6855 L 10832 6860 L
10696 6900 L 10696 7288 L 10743 7262 L 10832 7227 L
10967 7220 L 11103 7197 L 11238 7207 L 11373 7243 L
11373 6674 L 11238 6672 L 11155 6584 L 11103 6535 L
10989 6448 L 10967 6371 L 10944 6313 L 10922 6177 L
10832 6111 L 10696 6060 L 10675 6041 L 10561 5934 L
10484 5906 L 10500 5770 L 10561 5736 L 10696 5702 L
10832 5674 L 10967 5703 L 11037 5634 L 11103 5624 L
11238 5585 L 11360 5634 L 11373 5645 L 11509 5760 L
11515 5770 L 11509 5816 L 11479 5906 L 11463 6041 L
11373 6145 L 11332 6177 L 11373 6204 L 11433 6313 L
11437 6448 L 11479 6584 L 11373 6674 L 11373 7243 L
11474 7262 L 11509 7268 L 11644 7295 L 11780 7311 L
11915 7305 L 12051 7357 L 12090 7262 L 12156 7126 L
12186 7118 L 12261 7126 L 12321 7194 L 12377 7126 L
12457 7109 L 12592 7083 L 12688 6991 L 12728 6879 L
12741 6855 L 12740 6719 L 12810 6584 L 12863 6504 L

12868 6448 L 12894 6313 L 12881 6177 L 12890 6041 L
12876 5906 L 12907 5770 L 12871 5634 L 12863 5613 L
12776 5499 L 12642 5499 L 12463 5499 L 12285 5499 L
12107 5499 L 11929 5499 L 11750 5499 L 11572 5499 L
11394 5499 L 11215 5499 L 11037 5499 L 10859 5499 L
10681 5499 L 10502 5499 L 10324 5499 L 10146 5499 L
9967 5499 L 9852 5499 L 9884 5601 L 9906 5634 L
9899 5770 L 9900 5906 L 9884 5940 L 9880 5906 L
9857 5770 L 9748 5727 L 9661 5634 L 9613 5610 L
9486 5634 L 9478 5640 L 9467 5634 L 9342 5619 L
9207 5577 L 9171 5634 L 9145 5770 L 9207 5862 L
9207 5807 L 9182 5770 L 9207 5687 L 9275 5770 L
9207 5807 L 9207 5862 L 9342 5809 L 9418 5906 L
9421 6041 L 9401 6177 L 9478 6274 L 9489 6313 L
9507 6448 L 9478 6548 L 9421 6448 L 9342 6338 L
9266 6448 L 9242 6584 L 9246 6719 L 9207 6831 L
9071 6843 L 8936 6810 L 8801 6779 L 8692 6719 L
8665 6700 L 8530 6613 L 8486 6584 L 8394 6520 L
8286 6448 L 8259 6428 L 8123 6328 L 8098 6313 L
7988 6271 L 7853 6264 L 7717 6247 L 7582 6240 L
7463 6177 L 7446 6162 L 7364 6041 L 7311 5992 L
7208 5906 L 7176 5864 L 7040 5800 L 6905 5781 L
6832 5770 L 6769 5738 L 6634 5678 L 6498 5661 L
6460 5634 L 6498 5557 L 6634 5563 L 6769 5558 L
6905 5531 L 6980 5499 L 6936 5499 L 6758 5499 L
6580 5499 L 6401 5499 L 6223 5499 L N E
55 O
6228 8963 6209 9025 6228 9042 6308 9025 6228 8963 5 N F
2 O
6228 10111 6226 10110 6228 10108 6229 10110 6228 10111 5 N F
55 O
6363 13027 6281 13093 6307 13229 6363 13354 6400 13364 6498 13397
6577 13364 6634 13351 6769 13325 6905 13353 6941 13364 7040 13412
7176 13477 7311 13486 7347 13364 7433 13229 7446 13220 7582 13138
7639 13093 7582 13055 7446 13051 7311 13063 7176 13056 7040 13039
6905 13063 6769 13074 6634 13068 6498 13035 6363 13027 29 N F
15 O
6363 10848 6292 10788 6363 10737 6408 10788 6363 10848 5 N F
29 O
6498 11571 6445 11466 6498 11406 6537 11466 6498 11571 5 N F
108 O
6498 5557 6460 5634 6498 5661 6634 5678 6769 5738 6832 5770
6905 5781 7040 5800 7176 5864 7208 5906 7311 5992 7364 6041

7446 6162 7463 6177 7582 6240 7717 6247 7853 6264 7988 6271
8098 6313 8123 6328 8259 6428 8286 6448 8394 6520 8486 6584
8530 6613 8665 6700 8692 6719 8801 6779 8936 6810 9071 6843
9207 6831 9246 6719 9242 6584 9266 6448 9342 6338 9421 6448
9478 6548 9507 6448 9489 6313 9478 6274 9401 6177 9421 6041
9418 5906 9342 5809 9207 5862 9145 5770 9171 5634 9207 5577
9342 5619 9467 5634 9478 5640 9486 5634 9613 5610 9661 5634
9748 5727 9857 5770 9880 5906 9884 5940 9900 5906 9899 5770
9906 5634 9884 5601 9852 5499 9789 5499 9615 5499 9613 5500
9478 5538 9342 5528 9260 5499 9254 5499 9166 5499 9111 5634
9108 5770 9189 5906 9207 5957 9342 5927 9367 6041 9342 6134
9208 6177 9207 6177 9150 6313 9124 6448 9071 6553 8936 6533
8801 6489 8742 6448 8665 6381 8585 6313 8530 6264 8408 6177
8394 6170 8259 6109 8123 6054 8064 6041 7988 5975 7921 6041
7853 6054 7717 6087 7582 6094 7519 6041 7446 5921 7426 5906
7446 5895 7582 5860 7717 5859 7853 5817 7988 5791 8000 5770
8123 5727 8259 5641 8269 5634 8394 5578 8530 5524 8665 5502
8682 5499 8541 5499 8363 5499 8184 5499 8006 5499 7828 5499
7650 5499 7471 5499 7293 5499 7115 5499 6980 5499 6905 5531
6769 5558 6634 5563 6498 5557 129 N F
55 O
6498 8464 6481 8482 6498 8514 6519 8482 6498 8464 5 N F
2 O
6634 12836 6601 12822 6634 12765 6663 12822 6634 12836 5 N F
42 O
6769 12509 6681 12551 6769 12622 6849 12686 6905 12724 7040 12737
7053 12686 7040 12677 6905 12562 6897 12551 6769 12509 11 N F
15 O
6753 11737 6683 11601 6769 11578 6804 11601 6784 11737 6812 11873
6769 11932 6735 11873 6753 11737 9 N F
6694 12415 6692 12279 6740 12144 6769 12078 6886 12144 6905 12161
6986 12279 7040 12330 7138 12415 7176 12456 7209 12415 7247 12279
7311 12232 7352 12279 7446 12364 7570 12415 7582 12428 7612 12551
7615 12686 7582 12707 7494 12822 7446 12849 7311 12834 7289 12822
7176 12699 7168 12686 7040 12596 6991 12551 6905 12483 6769 12450
6694 12415 31 N F
68 O
6905 11079 6801 11194 6884 11330 6905 11335 7040 11378 7162 11466
7176 11469 7311 11484 7446 11514 7547 11466 7453 11330 7564 11194
7446 11108 7322 11194 7311 11221 7176 11263 7084 11194 7040 11173
6905 11079 19 N F
55 O
6905 7334 6858 7262 6905 7231 6970 7262 6905 7334 5 N F

15 O
6905 12019 6903 12008 6905 12007 7040 11941 7058 12008 7040 12068
6905 12019 7 N F
95 O
7040 6400 7026 6448 7040 6472 7120 6584 7167 6719 7176 6731
7311 6838 7446 6815 7482 6719 7446 6644 7311 6624 7244 6584
7176 6537 7059 6448 7040 6400 15 N F
42 O
7176 10754 7078 10652 7176 10540 7286 10652 7176 10754 7176 10713
7241 10652 7176 10585 7117 10652 7176 10713 7176 10754 11 N F
29 O
7176 10713 7117 10652 7176 10585 7241 10652 7176 10713 7176 10672
7197 10652 7176 10630 7156 10652 7176 10672 7176 10713 11 N F
15 O
7176 10672 7156 10652 7176 10630 7197 10652 7176 10672 5 N F
42 O
7176 11063 7157 11059 7176 11034 7181 11059 7176 11063 5 N F
15 O
7311 11905 7288 11873 7298 11737 7311 11720 7395 11737 7446 11761
7459 11873 7449 12008 7446 12014 7431 12008 7311 11905 11 N F
121 O
7446 5895 7426 5906 7446 5921 7519 6041 7582 6094 7717 6087
7853 6054 7921 6041 7988 5975 8064 6041 8123 6054 8259 6109
8394 6170 8408 6177 8530 6264 8585 6313 8665 6381 8742 6448
8801 6489 8936 6533 8936 6173 8801 6120 8680 6041 8665 6025
8582 5906 8580 5770 8665 5716 8801 5678 8936 5666 9063 5770
9071 5776 9108 5906 9118 6041 9071 6151 8936 6173 8936 6533
9071 6553 9124 6448 9150 6313 9207 6177 9208 6177 9342 6134
9367 6041 9342 5927 9207 5957 9189 5906 9108 5770 9111 5634
9166 5499 9076 5499 8898 5499 8719 5499 8682 5499 8665 5502
8530 5524 8394 5578 8269 5634 8259 5641 8123 5727 8000 5770
7988 5791 7853 5817 7717 5859 7582 5860 7446 5895 65 N F
42 O
7446 8083 7427 8075 7446 8069 7518 8075 7446 8083 5 N F
7582 8750 7470 8618 7582 8509 7692 8618 7582 8750 7582 8663
7619 8618 7582 8581 7544 8618 7582 8663 7582 8750 11 N F
29 O
7582 8663 7544 8618 7582 8581 7619 8618 7582 8663 5 N F
42 O
7582 7696 7558 7669 7582 7639 7683 7669 7582 7696 5 N F
68 O
7582 11871 7581 11873 7582 11874 7687 12008 7717 12068 7853 12102
7944 12008 7988 11913 8003 11873 8026 11737 8020 11601 8123 11477

8148 11466 8259 11375 8274 11330 8259 11314 8123 11220 8121 11330
7988 11464 7853 11330 7853 11313 7788 11194 7717 11166 7655 11194
7717 11330 7717 11331 7853 11385 7877 11466 7935 11601 7863 11737
7853 11742 7717 11787 7582 11871 33 N F
42 O
7717 8883 7584 8754 7717 8639 7853 8743 7988 8633 8095 8754
7988 8882 7988 8803 8030 8754 7988 8707 7934 8754 7988 8803
7988 8882 7983 8889 7853 9005 7853 8950 7921 8889 7853 8817
7785 8889 7853 8950 7853 9005 7722 8889 7717 8883 7717 8815
7783 8754 7717 8699 7654 8754 7717 8815 7717 8883 29 N F
7717 8241 7653 8211 7717 8182 7831 8211 7717 8241 5 N F
29 O
7717 8815 7654 8754 7717 8699 7783 8754 7717 8815 5 N F
68 O
7717 6727 7706 6719 7717 6710 7728 6719 7717 6727 5 N F
42 O
7853 9660 7750 9567 7853 9446 7943 9567 7853 9660 7853 9613
7898 9567 7853 9507 7802 9567 7853 9613 7853 9660 11 N F
29 O
7853 8950 7785 8889 7853 8817 7921 8889 7853 8950 7853 8894
7859 8889 7853 8883 7847 8889 7853 8894 7853 8950 11 N F
42 O
7838 7940 7801 7804 7853 7746 7887 7804 7988 7902 8029 7940
7988 8016 7853 7955 7838 7940 9 N F
29 O
7853 9613 7802 9567 7853 9507 7898 9567 7853 9613 5 N F
15 O
7853 8894 7847 8889 7853 8883 7859 8889 7853 8894 5 N F
42 O
7988 11424 7893 11330 7988 11234 8082 11330 7988 11424 7988 11384
8042 11330 7988 11275 7933 11330 7988 11384 7988 11424 11 N F
7988 7668 7894 7533 7988 7504 8123 7493 8259 7511 8394 7506
8530 7525 8589 7533 8665 7635 8679 7669 8665 7680 8530 7701
8461 7669 8394 7623 8259 7601 8123 7631 7988 7668 17 N F
29 O
7988 11384 7933 11330 7988 11275 8042 11330 7988 11384 7988 11344
8002 11330 7988 11315 7973 11330 7988 11344 7988 11384 11 N F
7988 8803 7934 8754 7988 8707 8030 8754 7988 8803 5 N F
15 O
7988 11344 7973 11330 7988 11315 8002 11330 7988 11344 5 N F
29 O
8394 9481 8334 9432 8394 9351 8448 9432 8394 9481 8394 9437
8400 9432 8394 9423 8388 9432 8394 9437 8394 9481 11 N F

8394 8396 8352 8347 8394 8297 8530 8295 8575 8347 8631 8482
8530 8565 8530 8491 8540 8482 8530 8442 8522 8482 8530 8491
8530 8565 8458 8482 8394 8396 15 N F
15 O
8394 9437 8388 9432 8394 9423 8400 9432 8394 9437 5 N F
8530 8491 8522 8482 8530 8442 8540 8482 8530 8491 5 N F
42 O
8665 9944 8561 9838 8665 9735 8785 9838 8665 9944 8665 9894
8728 9838 8665 9784 8610 9838 8665 9894 8665 9944 11 N F
135 O
8665 5716 8580 5770 8582 5906 8665 6025 8680 6041 8801 6120
8936 6173 9071 6151 9118 6041 9108 5906 9071 5776 9063 5770
8936 5666 8801 5678 8665 5716 15 N F
29 O
8665 8694 8586 8618 8665 8520 8745 8618 8665 8694 5 N F
8665 9894 8610 9838 8665 9784 8728 9838 8665 9894 8665 9844
8671 9838 8665 9833 8660 9838 8665 9844 8665 9894 11 N F
15 O
8665 9844 8660 9838 8665 9833 8671 9838 8665 9844 5 N F
42 O
8936 9846 8932 9838 8936 9835 8942 9838 8936 9846 5 N F
29 O
9071 11161 8979 11059 9071 10959 9175 11059 9071 11161 9071 11110
9123 11059 9071 11009 9025 11059 9071 11110 9071 11161 11 N F
9071 9521 8991 9432 9071 9345 9194 9432 9071 9521 9071 9470
9124 9432 9071 9395 9037 9432 9071 9470 9071 9521 11 N F
42 O
9071 10027 9022 9974 9071 9931 9139 9974 9071 10027 5 N F
15 O
9071 11110 9025 11059 9071 11009 9123 11059 9071 11110 5 N F
9071 9470 9037 9432 9071 9395 9124 9432 9071 9470 5 N F
108 O
9254 5499 9166 5499 9166 5499 9254 5499 9260 5499 9260 5499
9254 5499 7 N F
82 O
9207 5807 9182 5770 9207 5687 9275 5770 9207 5807 5 N F
121 O
9432 5499 9260 5499 9342 5528 9478 5538 9613 5500 9615 5499
9611 5499 9432 5499 8 N F
82 O
9342 6696 9299 6584 9342 6491 9407 6584 9342 6696 5 N F
29 O
9342 8764 9334 8754 9342 8744 9350 8754 9342 8764 5 N F

9478 10908 9365 10788 9427 10652 9466 10516 9478 10501 9503 10381
9613 10248 9748 10268 9856 10381 9782 10516 9780 10652 9748 10704
9638 10788 9613 10809 9613 10516 9748 10468 9809 10381 9748 10317
9613 10305 9551 10381 9613 10516 9613 10809 9478 10908 9478 10853
9553 10788 9478 10702 9417 10788 9478 10853 9478 10908 29 N F
9478 12060 9390 12008 9478 11940 9524 12008 9478 12060 5 N F
15 O
9478 10853 9417 10788 9478 10702 9553 10788 9478 10853 9478 10797
9489 10788 9478 10775 9468 10788 9478 10797 9478 10853 11 N F
2 O
9478 10797 9468 10788 9478 10775 9489 10788 9478 10797 5 N F
42 O
9478 7973 9469 7940 9478 7928 9493 7940 9478 7973 5 N F
68 O
9546 6313 9502 6177 9504 6041 9521 5906 9592 5770 9613 5750
9632 5770 9748 5867 9783 5906 9832 6041 9881 6177 9884 6186
9929 6313 9958 6448 9994 6584 10019 6646 10079 6719 10103 6855
10081 6991 10063 7126 10019 7186 9903 7126 9884 7116 9884 6969
10019 6942 10048 6855 10019 6724 10019 6719 9965 6584 9929 6448
9884 6313 9884 6312 9846 6177 9802 6041 9748 5924 9729 5906
9613 5809 9555 5906 9530 6041 9527 6177 9575 6313 9613 6381
9654 6448 9646 6584 9638 6719 9707 6855 9748 6900 9884 6969
9884 7116 9823 6991 9748 6951 9661 6855 9613 6744 9598 6719
9613 6634 9617 6584 9623 6448 9613 6431 9546 6313 59 N F
29 O
9613 9556 9516 9432 9600 9296 9613 9271 9626 9296 9714 9432
9613 9556 9613 9489 9659 9432 9613 9368 9568 9432 9613 9489
9613 9556 13 N F
55 O
9575 6313 9527 6177 9530 6041 9555 5906 9613 5809 9729 5906
9748 5924 9802 6041 9846 6177 9884 6312 9884 6313 9929 6448
9965 6584 10019 6719 10019 6724 10048 6855 10019 6942 9884 6969
9884 6910 9973 6855 9974 6719 9935 6584 9900 6448 9884 6400
9838 6313 9811 6177 9771 6041 9748 5991 9662 5906 9613 5865
9589 5906 9557 6041 9552 6177 9603 6313 9613 6330 9684 6448
9674 6584 9670 6719 9748 6849 9763 6855 9884 6910 9884 6969
9748 6900 9707 6855 9638 6719 9646 6584 9654 6448 9613 6381
9575 6313 49 N F
15 O
9613 10516 9551 10381 9613 10305 9748 10317 9809 10381 9748 10468
9613 10516 9613 10414 9748 10401 9763 10381 9748 10366 9613 10362
9598 10381 9613 10414 9613 10516 15 N F
42 O

9603 6313 9552 6177 9557 6041 9589 5906 9613 5865 9662 5906
9748 5991 9771 6041 9811 6177 9838 6313 9884 6400 9900 6448
9935 6584 9974 6719 9973 6855 9884 6910 9884 6846 9929 6719
9906 6584 9884 6500 9844 6448 9792 6313 9776 6177 9748 6073
9724 6041 9613 5933 9584 6041 9577 6177 9613 6270 9669 6313
9715 6448 9703 6584 9702 6719 9748 6796 9884 6846 9884 6910
9763 6855 9748 6849 9670 6719 9674 6584 9684 6448 9613 6330
9603 6313 43 N F
15 O
9613 9489 9568 9432 9613 9368 9659 9432 9613 9489 5 N F
29 O
9613 6270 9577 6177 9584 6041 9613 5933 9724 6041 9748 6073
9776 6177 9792 6313 9844 6448 9884 6500 9906 6584 9929 6719
9884 6846 9748 6796 9748 6744 9884 6722 9885 6719 9884 6711
9841 6584 9758 6448 9748 6364 9745 6448 9732 6584 9734 6719
9748 6744 9748 6796 9702 6719 9703 6584 9715 6448 9669 6313
9613 6270 9613 6205 9703 6177 9621 6041 9613 6034 9611 6041
9602 6177 9613 6205 9613 6270 39 N F
2 O
9613 10414 9598 10381 9613 10362 9748 10366 9763 10381 9748 10401
9613 10414 7 N F
15 O
9613 6205 9602 6177 9611 6041 9613 6034 9621 6041 9703 6177
9613 6205 7 N F
108 O
9789 5499 9615 5499 9615 5499 9789 5499 9852 5499 9852 5499
9789 5499 7 N F
15 O
9734 6719 9732 6584 9745 6448 9748 6364 9758 6448 9841 6584
9884 6711 9885 6719 9884 6722 9748 6744 9734 6719 11 N F
29 O
9884 9252 9784 9160 9884 9075 9970 9160 9884 9252 9884 9177
9899 9160 9884 9145 9866 9160 9884 9177 9884 9252 11 N F
42 O
9884 7895 9793 7804 9884 7723 9980 7669 10019 7639 10076 7669
10155 7698 10290 7724 10414 7804 10290 7904 10155 7939 10019 7895
9884 7895 13 N F
29 O
9884 8682 9824 8618 9884 8505 9948 8618 9884 8682 5 N F
15 O
9884 9177 9866 9160 9884 9145 9899 9160 9884 9177 5 N F
55 O
9884 8888 9883 8889 9884 8892 9887 8889 9884 8888 5 N F

29 O
10019 10199 9930 10110 10019 10022 10104 10110 10019 10199 10019 10143
10051 10110 10019 10077 9986 10110 10019 10143 10019 10199 11 N F
15 O
10019 10143 9986 10110 10019 10077 10051 10110 10019 10143 5 N F
108 O
10019 6314 10016 6448 10019 6462 10039 6448 10019 6314 5 N F
29 O
10155 8805 10051 8754 10155 8687 10239 8754 10155 8805 5 N F
10155 9531 10059 9432 10155 9332 10251 9296 10265 9160 10290 9135
10426 9030 10561 9041 10679 9160 10633 9296 10561 9345 10426 9423
10426 9365 10505 9296 10561 9224 10612 9160 10561 9109 10426 9099
10357 9160 10342 9296 10426 9365 10426 9423 10415 9432 10290 9537
10290 9479 10346 9432 10290 9361 10155 9393 10117 9432 10155 9470
10290 9479 10290 9537 10155 9531 33 N F
15 O
10155 9470 10117 9432 10155 9393 10290 9361 10346 9432 10290 9479
10155 9470 7 N F
108 O
10155 5680 10133 5770 10155 5866 10204 5770 10155 5680 5 N F
29 O
10290 9799 10199 9703 10290 9600 10391 9703 10290 9799 10290 9739
10328 9703 10290 9664 10256 9703 10290 9739 10290 9799 11 N F
15 O
10290 9739 10256 9703 10290 9664 10328 9703 10290 9739 5 N F
108 O
10290 6259 10269 6313 10290 6413 10307 6448 10360 6584 10374 6719
10426 6811 10491 6855 10561 6881 10696 6900 10832 6860 10842 6855
10967 6725 10973 6719 10967 6708 10930 6719 10832 6799 10824 6719
10696 6622 10650 6584 10561 6537 10477 6448 10426 6359 10351 6313
10290 6259 25 N F
15 O
10426 9365 10342 9296 10357 9160 10426 9099 10561 9109 10612 9160
10561 9224 10505 9296 10426 9365 10426 9307 10439 9296 10426 9207
10412 9296 10426 9307 10426 9365 15 N F
2 O
10426 9307 10412 9296 10426 9207 10439 9296 10426 9307 5 N F
108 O
10500 5770 10484 5906 10561 5934 10675 6041 10696 6060 10832 6111
10922 6177 10944 6313 10967 6371 10989 6448 11103 6535 11103 5806
11013 5770 11103 5678 11136 5770 11103 5806 11103 6535 11155 6584
11238 6672 11373 6674 11479 6584 11437 6448 11433 6313 11373 6204
11332 6177 11373 6145 11463 6041 11479 5906 11509 5816 11515 5770

11509 5760 11373 5645 11360 5634 11238 5585 11103 5624 11037 5634
10967 5703 10832 5674 10696 5702 10561 5736 10500 5770 41 N F
29 O
10832 9271 10716 9160 10832 9042 10943 9160 10832 9271 10832 9198
10869 9160 10832 9120 10793 9160 10832 9198 10832 9271 11 N F
15 O
10832 9198 10793 9160 10832 9120 10869 9160 10832 9198 5 N F
10967 9505 10907 9432 10967 9374 11077 9432 10967 9505 5 N F
95 O
11103 5806 11013 5770 11103 5678 11136 5770 11103 5806 5 N F
12321 7434 12225 7533 12276 7669 12321 7778 12443 7669 12375 7533
12321 7434 7 N F
12592 7798 12577 7804 12592 7818 12599 7804 12592 7798 5 N F
15 O
12728 14420 12713 14449 12728 14480 12763 14449 12728 14420 5 N F
82 O
12998 5499 12998 5499 12998 9511 12998 9511 12998 5499 12998 5499
6 N F
68 O
12998 10425 12998 9511 12998 9511 12998 10426 12998 10425 5 N F
55 O
12998 11153 12998 10425 12998 10426 12998 11153 12998 11153 5 N F
42 O
12998 11636 12998 11153 12998 11153 12998 11636 12998 11636 5 N F
29 O
12998 12435 12998 11636 12998 11636 12998 12435 12998 12435 5 N F
15 O
12998 14145 12998 12435 12998 12435 12998 14145 12998 14145 5 N F
2 O
12998 16212 12998 14145 12998 14145 12998 16212 12998 16212 12998 16212
6 N F
68 O
12998 9511 12998 10426 12998 10426 12998 10381 12998 10245 12998 10110
12998 9974 12998 9838 12998 9703 12998 9567 12998 9511 12998 9511
12 N F
55 O
12998 10426 12998 11153 12998 11153 12998 11059 12998 10923 12998 10788
12998 10652 12998 10516 12998 10426 12998 10426 10 N F
42 O
12998 11153 12998 11636 12998 11636 12998 11601 12998 11466 12998 11330
12998 11194 12998 11153 12998 11153 9 N F
29 O
12998 11636 12998 12435 12998 12435 12998 12415 12998 12279 12998 12144

12998 12008 12998 11873 12998 11737 12998 11636 12998 11636 11 N F
15 O
12998 12435 12998 14145 12998 14145 12998 14042 12998 13907 12998 13771
12998 13635 12998 13500 12998 13364 12998 13229 12998 13093 12998 12957
12998 12822 12998 12686 12998 12551 12998 12435 12998 12435 17 N F
Ls Gr Gs Lr
-1150 1075 16450 18700 Oc
1 O
10637 15408 10638 15449 10677 15486 10674 15526 10709 15568 10751 15601
10790 15639 10825 15680 10870 15710 10918 15735 10944 15783 10983 15820
11028 15850 11078 15870 11120 15905 11158 15942 11194 15973 11245 16000
18 P
11245 16000 11297 16011 11327 16055 11353 16103 11387 16145 11417 16190
11426 16212 7 P
12837 16212 12838 16205 12833 16178 12809 16167 12782 16162 12764 16142
12740 16129 12715 16118 12694 16100 12678 16079 12653 16071 12634 16051
12630 16024 12623 15998 12618 15971 12605 15948 12581 15936 12558 15921
12542 15900 12525 15879 12503 15863 12479 15851 12455 15838 12431 15826
12423 15800 12446 15786 12441 15759 12416 15750 12398 15729 12374 15717
12357 15697 12331 15689 12321 15664 12295 15673 12280 15650 12255 15641
12228 15644 12201 15650 12179 15634 12166 15610 12143 15595 12146 15568
12125 15551 12105 15533 12078 15526 12065 15503 12038 15504 12020 15484
12007 15460 12000 15434 50 P
12000 15434 12006 15408 12032 15400 12038 15374 12026 15350 12023 15323
12031 15296 12042 15272 12050 15246 12037 15269 12010 15266 11 P
12010 15266 11984 15258 12010 15249 12004 15223 11981 15208 11977 15181
11992 15159 12015 15145 12042 15142 12069 15142 12096 15135 12121 15126
12144 15140 12159 15118 12176 15096 12193 15075 12210 15054 12232 15039
12253 15021 12276 15006 12303 15004 12325 14988 12344 14969 12355 14944
12381 14953 12407 14944 12430 14931 12435 14905 28 P
12435 14905 12445 14879 12460 14857 12473 14833 12491 14812 12500 14787
12516 14765 12531 14742 12544 14718 12560 14697 12571 14672 12583 14648
12603 14628 12619 14606 12637 14587 12653 14565 12672 14545 12689 14523
12708 14505 12728 14486 12749 14468 12769 14451 12790 14433 12795 14407
12815 14388 12835 14369 12841 14343 12861 14324 12875 14301 12896 14285
12911 14262 12931 14243 12947 14222 12957 14196 12964 14169 12976 14145
12995 14126 12998 14101 38 P
12998 13048 12994 13048 12998 13033 3 P
12998 13020 12976 13010 12955 12993 12948 13019 12949 13046 12933 13024
12906 13022 12880 13013 12852 13015 9 P
12852 13015 12826 13018 12803 13034 12779 13047 12786 13021 12794 12995
6:00 PM
12794 12995 12768 13002 12740 13004 12727 12980 12744 12959 12726 12939

12723 12912 12722 12884 12740 12864 12738 12837 12727 12812 12705 12828
12689 12850 12715 12844 12709 12870 12682 12870 12685 12897 12660 12888
12635 12899 12626 12874 12598 12873 12573 12882 12547 12892 12546 12920
12544 12947 12536 12920 12511 12911 12487 12897 12465 12881 12439 12889
12412 12883 12395 12861 12372 12848 12354 12869 12348 12843 12362 12819
12386 12806 12407 12824 12433 12830 12439 12804 12435 12776 12451 12754
12460 12728 12461 12700 12435 12692 12433 12720 12439 12746 12412 12752
12386 12746 12387 12718 50 P
12387 12718 12363 12704 12351 12729 12360 12755 12349 12779 12334 12803
12322 12827 12310 12803 12284 12795 12256 12794 12282 12784 12259 12769
12266 12743 12284 12722 12307 12706 12329 12690 12328 12662 12301 12662
12274 12668 12271 12641 12298 12646 12295 12619 12274 12602 12263 12577
12242 12595 12225 12617 12206 12636 12191 12613 12165 12621 12173 12648
12185 12672 12189 12646 12212 12660 12212 12688 12211 12715 12231 12696
12249 12718 12245 12745 12219 12734 12193 12726 12176 12705 12150 12694
12130 12676 12106 12662 12103 12635 12094 12609 12105 12584 12089 12561
12061 12563 12035 12556 50 P
12035 12556 12021 12580 2 P
12021 12580 12019 12607 12008 12633 12006 12605 12006 12578 11982 12565
11975 12538 11953 12522 11948 12495 11931 12474 11909 12458 11907 12485
11884 12500 11901 12522 11876 12534 11869 12561 11887 12581 11907 12600
11880 12601 11858 12584 11833 12595 11811 12612 11785 12617 11772 12641
11770 12668 11778 12695 11801 12680 11804 12653 11818 12629 11843 12619
11871 12621 11850 12639 11824 12648 11825 12676 11852 12677 11858 12650
11885 12643 11912 12646 11903 12671 11881 12689 11854 12692 11827 12690
11848 12708 11875 12704 11882 12731 11909 12735 11934 12747 11906 12749
11903 12776 11879 12789 50 P
11879 12789 11876 12817 11875 12789 11884 12763 11884 12736 11857 12736
11833 12723 11823 12749 11830 12775 11819 12800 11835 12823 11808 12819
11792 12797 11771 12815 11796 12825 11809 12849 11786 12864 11766 12884
11769 12856 11749 12875 11741 12901 11767 12895 11780 12920 11778 12892
11796 12873 11815 12852 11832 12873 11843 12898 11816 12905 11794 12920
11797 12948 11771 12955 11780 12981 11786 13008 11792 13034 11814 13018
11823 13044 11796 13049 11809 13073 11813 13100 11825 13124 11851 13133
11877 13142 11904 13140 11903 13167 11875 13167 11848 13162 11821 13160
11807 13184 11826 13204 50 P
11826 13204 11846 13222 2 P
11846 13222 11865 13243 11892 13244 11897 13271 11922 13282 11906 13304
11926 13324 11945 13344 11918 13338 11890 13336 11864 13330 11855 13356
11871 13379 11895 13391 11916 13408 11941 13420 11962 13437 11963 13464
11957 13438 11935 13422 11910 13411 11886 13398 11866 13417 11854 13441
11833 13458 11807 13467 11829 13484 11807 13500 11804 13528 11790 13551
11803 13575 11782 13592 11780 13619 11803 13633 11804 13649 11779 13621

11765 13644 11779 13668 11755 13680 11756 13708 11758 13735 11753 13762
11730 13746 11713 13726 11708 13753 11700 13778 11680 13760 11694 13736
11689 13709 11714 13698 50 P
11714 13698 11717 13671 11729 13647 11740 13621 11719 13605 11703 13627
11699 13654 11692 13680 11676 13701 11676 13729 11658 13749 11632 13741
11633 13768 11608 13780 11598 13754 11592 13781 11601 13806 11628 13812
11627 13840 11634 13866 11658 13878 11658 13905 11664 13879 11670 13853
11669 13825 11691 13810 11675 13832 11699 13845 11715 13867 11696 13888
11674 13903 11695 13921 11721 13930 11729 13904 11737 13877 11738 13850
11744 13877 11748 13904 11768 13886 11758 13912 11739 13931 11764 13920
11753 13945 11777 13932 11801 13920 11824 13935 11844 13917 11871 13911
11887 13890 11892 13863 50 P
11892 13863 11906 13840 2 P
11906 13840 11906 13867 11926 13886 11947 13902 11957 13927 11938 13909
11911 13914 11893 13934 11874 13954 11847 13960 11854 13986 11866 14011
11861 14038 11839 14021 11821 14001 11804 14022 11817 14046 11810 14073
11837 14077 11858 14094 11834 14105 11810 14118 11825 14140 11800 14149
11779 14166 11760 14186 11784 14197 11782 14225 11772 14250 11745 14258
11725 14276 11719 14302 11714 14329 11738 14341 11762 14354 11775 14330
11768 14304 11792 14291 11816 14279 11840 14267 11862 14251 11885 14241
11885 14243 11865 14254 11842 14268 11819 14285 11795 14296 11783 14320
11781 14347 11775 14374 50 P
11775 14374 11793 14397 11767 14384 3 P
11767 14384 11751 14363 11724 14358 11699 14369 11677 14384 11654 14400
11627 14397 11606 14380 11579 14376 11553 14367 11554 14340 11548 14314
11526 14330 11507 14349 11503 14376 11476 14382 11474 14355 11447 14357
11430 14378 11411 14397 11389 14413 11376 14437 11350 14444 11323 14443
11298 14453 11302 14427 11327 14416 11354 14416 11348 14390 11320 14391
11324 14364 11328 14337 11319 14311 11293 14314 11269 14299 11253 14278
11226 14273 11215 14248 11199 14270 11210 14295 11236 14301 11239 14329
11266 14331 11251 14353 11273 14368 11246 14371 11218 14369 11242 14355
11215 14350 11206 14324 50 P
11206 14324 11179 14324 11184 14351 11160 14365 11138 14349 11112 14340
11086 14348 11061 14359 11045 14381 11069 14394 11096 14390 11121 14401
11097 14413 11103 14439 11130 14444 11152 14428 11153 14454 11128 14464
11101 14462 11077 14451 11049 14448 11023 14456 11000 14469 10975 14459
10948 14452 10921 14456 10896 14463 10921 14471 10941 14489 10961 14506
10979 14532 11001 14518 11000 14511 11003 14507 11032 14527 11021 14531
11001 14521 10982 14539 10981 14566 10975 14593 10989 14616 10999 14641
11019 14659 11029 14685 11018 14709 11045 14711 11069 14723 11073 14750
11065 14776 11083 14755 50 P
11083 14755 11106 14769 2 P
11106 14769 11132 14775 11155 14791 11181 14794 11206 14807 11228 14792

11255 14796 11239 14818 11212 14819 11188 14831 11172 14809 11145 14812
11119 14803 11116 14830 11101 14853 11108 14827 11083 14835 11068 14858
11060 14884 11071 14909 11079 14935 11089 14960 11102 14984 11122 15002
11114 15028 11119 15055 11142 15069 11149 15095 11155 15121 11151 15148
11165 15173 11138 15166 11126 15190 11124 15217 11144 15235 11148 15262
11172 15276 11185 15277 11194 15291 11218 15303 11224 15307 11223 15309
11194 15292 11182 15281 11161 15281 11171 15308 11174 15322 11186 15317
11196 15319 11201 15327 50 P
11201 15327 11200 15329 11194 15323 11187 15319 11174 15323 11170 15311
11158 15287 11137 15270 11114 15255 11087 15255 11067 15274 11042 15266
11018 15279 10996 15295 10969 15294 10951 15315 10928 15302 10912 15323
10885 15331 10864 15348 10854 15323 10857 15296 10841 15274 10824 15253
10811 15277 10821 15302 10803 15321 10794 15347 10784 15372 10757 15375
10731 15381 10707 15394 10685 15410 10692 15437 10665 15435 10659 15408
10654 15381 10649 15355 10642 15381 10637 15408 40 P
10637 15408 10625 15383 10598 15378 10576 15363 10549 15357 10522 15358
10495 15360 10471 15372 10451 15353 10424 15353 10401 15368 10399 15395
10391 15369 10381 15344 10368 15320 10341 15326 10329 15302 10306 15316
10280 15321 10253 15325 10245 15300 10221 15286 10246 15275 10225 15259
10199 15249 10174 15258 10148 15248 10129 15229 10105 15217 10080 15208
10054 15197 10033 15180 10014 15161 9989 15150 9972 15129 9948 15142
9945 15116 9925 15097 9898 15100 9878 15082 9855 15068 9844 15042
9833 15018 9810 15004 9791 14984 9781 14960 9805 14948 9781 14936
9754 14931 9762 14905 50 P
9762 14905 9735 14899 9715 14882 9695 14899 9703 14873 9677 14882
9689 14857 9688 14830 9663 14843 9652 14868 9627 14878 9606 14861
9594 14837 9569 14825 9596 14819 9619 14806 9607 14781 9580 14779
9553 14778 9534 14758 9508 14750 9481 14750 9457 14738 9430 14739
9419 14715 9401 14694 9380 14711 9380 14738 9368 14762 9390 14779
9364 14786 9371 14812 9397 14819 9424 14819 9450 14829 9464 14852
9473 14877 9493 14859 9520 14862 9542 14878 9567 14889 9593 14898
9607 14921 9586 14938 9563 14954 9580 14975 9562 14995 9588 15002
9611 15016 9626 15038 50 P
9626 15038 9648 15053 2 P
9648 15053 9674 15060 9698 15073 9671 15075 9645 15068 9618 15068
9594 15056 9566 15054 9539 15051 9516 15064 9506 15095 9493 15127
9516 15136 9528 15153 9532 15164 9535 15186 9532 15213 9516 15234
9494 15251 9505 15276 9516 15301 9539 15315 9562 15329 9580 15350
9607 15349 9631 15362 9649 15382 9669 15400 9643 15408 9666 15422
9682 15443 9698 15465 9713 15487 9727 15510 9747 15529 9750 15556
9764 15578 9782 15599 9785 15626 9803 15646 9814 15671 9801 15694
9781 15712 9759 15728 9753 15737 9761 15755 9756 15781 9759 15787
9783 15775 9800 15779 50 P

9800 15779 9805 15785 9799 15794 9777 15793 9776 15806 9755 15817
9730 15833 9730 15831 9753 15814 9764 15808 9764 15802 9771 15794
9777 15784 9800 15790 9801 15784 9783 15778 9767 15786 9758 15790
9753 15785 9753 15772 9741 15772 9725 15787 9704 15804 9681 15818
9664 15840 9637 15838 9624 15861 9632 15887 9621 15912 9633 15936
9638 15962 9627 15937 9608 15919 9607 15892 9597 15867 9575 15851
9548 15849 9521 15846 9502 15865 9480 15881 9453 15880 9429 15892
9405 15880 9383 15895 9357 15898 9341 15876 9322 15857 9312 15831
9285 15836 9266 15816 50 P
9266 15816 9254 15792 2 P
9254 15792 9277 15777 9259 15757 9233 15751 9214 15730 9203 15706
9184 15686 9163 15670 9149 15646 9126 15633 9121 15607 9099 15591
9121 15575 9098 15561 9085 15537 9086 15510 9070 15488 9044 15482
9018 15475 8997 15458 8973 15446 8950 15432 8924 15423 8898 15415
8873 15404 8851 15390 8835 15368 27 P
8835 15368 8809 15361 8787 15344 8761 15337 8735 15330 8710 15320
8689 15303 8691 15276 8665 15265 8644 15249 8625 15230 8612 15206
8604 15180 8597 15154 8574 15140 8582 15114 8578 15088 8582 15061
8599 15040 8574 15029 8552 15014 8535 14992 8521 14969 8503 14949
8481 14933 8458 14920 8432 14910 8406 14907 8383 14891 8357 14890
8335 14906 8308 14907 8284 14896 8264 14878 8242 14861 8222 14843
8200 14827 8173 14832 8150 14846 8132 14866 8126 14892 8111 14915
8087 14928 8060 14933 44 P
8060 14933 8034 14928 8008 14919 7985 14904 7966 14885 7947 14865
7957 14840 7963 14814 7939 14803 7923 14780 7906 14760 7893 14736
7881 14712 7872 14686 7867 14659 7866 14632 7869 14605 17 P
7869 14605 7879 14580 7890 14555 7902 14531 7915 14507 7927 14483
7945 14462 7964 14443 7983 14424 8010 14417 8032 14402 11 P
8032 14402 8052 14382 8074 14367 8098 14354 8125 14357 8151 14354
8178 14357 8205 14354 8231 14346 8258 14343 8279 14325 8301 14342
8325 14353 8350 14364 8372 14380 8396 14393 8402 14366 8407 14393
8421 14370 8418 14343 8423 14316 8414 14290 8431 14311 8458 14311
8463 14285 8482 14265 8490 14239 8498 14212 8491 14186 8480 14162
8504 14151 8522 14130 8510 14106 8493 14084 8476 14063 8469 14037
8467 14010 8469 13983 8472 13955 8467 13929 8484 13908 8502 13887
8504 13860 8532 13861 8554 13847 8575 13828 8602 13825 8628 13819
8656 13818 8683 13819 50 P
8683 13819 8710 13822 8735 13812 8762 13814 8790 13814 8816 13821
8835 13840 8855 13858 8875 13877 8882 13903 8865 13925 8878 13949
8899 13966 8924 13976 8951 13984 8973 13999 8995 14015 9014 14034
9033 14054 9050 14075 9054 14102 9030 14113 9055 14122 9081 14130
9101 14149 9127 14155 9154 14160 9177 14174 9203 14182 9224 14201
9251 14201 9278 14198 9305 14202 9317 14177 9341 14165 9358 14144

9385 14145 9400 14123 9405 14096 9424 14077 9451 14072 9478 14075
9503 14085 9530 14089 9506 14077 9524 14056 9541 14035 9559 14016
9584 14003 9611 14002 50 P
9611 14002 9638 14007 2 P
9638 14007 9663 14017 9683 13998 9699 14020 9726 14025 9753 14029
9768 14006 9766 14034 9786 14053 9808 14067 9830 14084 9848 14063
9839 14038 9853 14014 9877 14027 9900 14013 9926 14006 9954 14007
9980 14014 9998 13994 10022 14005 10046 14018 10073 14017 10054 13997
10036 13977 10031 13950 10039 13924 10054 13902 10032 13886 10009 13872
9993 13850 9993 13823 9973 13805 10000 13802 10027 13806 10028 13779
10024 13752 10010 13728 10021 13753 9995 13747 9981 13723 9960 13706
9938 13690 9911 13695 9884 13696 9879 13723 9907 13726 9882 13737
9866 13759 9882 13781 50 P
9882 13781 9875 13807 9857 13786 9859 13758 9836 13744 9816 13725
9807 13751 9793 13775 9766 13770 9777 13744 9753 13733 9728 13721
9703 13710 9690 13686 9666 13673 9641 13663 9641 13636 9615 13643
9589 13636 9604 13659 9624 13676 9597 13674 9578 13655 9564 13678
9568 13651 9552 13629 9529 13615 9503 13605 9478 13595 9452 13588
9429 13574 9409 13555 9384 13565 9385 13538 9358 13537 9344 13561
9318 13568 9294 13554 9278 13533 9304 13539 9320 13516 9345 13506
9345 13479 9328 13458 9327 13430 9303 13444 9281 13460 9257 13449
9242 13426 9250 13400 50 P
9250 13400 9225 13392 2 P
9225 13392 9219 13365 9234 13343 9239 13316 9243 13289 9222 13272
9197 13260 9170 13256 9144 13251 9123 13233 9097 13224 9070 13230
9082 13254 9092 13280 9097 13307 9081 13329 9063 13349 9059 13322
9038 13305 9012 13314 8987 13304 8977 13278 8985 13252 9009 13238
9034 13230 9019 13207 9046 13203 9073 13207 9061 13182 9039 13166
9025 13143 9032 13169 9009 13154 8984 13144 8964 13125 8954 13100
8957 13073 8949 13046 8926 13061 8915 13037 8900 13013 8889 12988
8873 13010 8852 12993 8839 12968 8829 12994 8807 12977 8780 12981
8786 12954 8778 12928 50 P
8778 12928 8769 12902 8755 12878 8751 12852 8732 12832 8707 12819
8691 12798 8682 12771 8675 12745 8672 12718 8661 12693 8634 12698

12:00 PM

8634 12698 8613 12680 8598 12657 8595 12630 8595 12603 8586 12576
8587 12549 8600 12525 8593 12499 8612 12519 8634 12502 8659 12515
8684 12503 8711 12501 8715 12473 8729 12450 8750 12433 8773 12418
8798 12405 8820 12388 8846 12382 8864 12362 8865 12334 8877 12358
8904 12352 8931 12348 8957 12339 8982 12327 9001 12307 9027 12301
9052 12290 9077 12279 9091 12255 9096 12228 9109 12203 9117 12178
9125 12151 9135 12125 9144 12100 9145 12072 9153 12046 9154 12018
9180 12008 9174 11981 9200 11972 9208 11945 9219 11920 9215 11893

9222 11866 9239 11844 50 P
9239 11844 9261 11827 9251 11802 9249 11774 9256 11747 9275 11728
9285 11702 9296 11677 9315 11656 9330 11634 9336 11607 9350 11583
9378 11578 9387 11553 9393 11526 9405 11500 9406 11473 9398 11447
9377 11428 9402 11419 9416 11395 9398 11375 9382 11351 9409 11348
9428 11327 9446 11307 9474 11306 9500 11297 9526 11287 9551 11275
9576 11264 9602 11254 9624 11237 9637 11213 9636 11185 9642 11158
9627 11135 9647 11115 9659 11090 9684 11080 9712 11080 9738 11071
9764 11061 9788 11048 9810 11030 9828 11010 9847 10990 9856 10964
9869 10939 9883 10915 50 P
9883 10915 9894 10890 2 P
9894 10890 9888 10863 9862 10853 9835 10860 9809 10868 9781 10872
9755 10882 9731 10894 9705 10905 9687 10926 9661 10935 9634 10940
9609 10952 9583 10943 9557 10932 9530 10926 9516 10903 9488 10907
9461 10903 9435 10911 9429 10938 9419 10964 9400 10985 9383 11006
9360 11022 9344 11045 9319 11035 9292 11043 9264 11043 9239 11054
9213 11044 9189 11030 9168 11013 9146 10996 9125 10979 9098 10969
9071 10971 9044 10976 9058 10953 9086 10953 9112 10960 9137 10973
9164 10980 9191 10985 9203 11010 9230 11013 9256 11003 9284 11001
9311 11003 9338 11001 50 P
9338 11001 9354 10977 9366 10953 9380 10929 9358 10911 9377 10891
9401 10879 9429 10878 9457 10879 9482 10869 9509 10859 9530 10841
9554 10828 9576 10811 9594 10790 9608 10766 9626 10745 17 P
9626 10745 9650 10731 9675 10720 9697 10703 9712 10680 9740 10679
9764 10666 9788 10651 9810 10634 9828 10613 9842 10589 9856 10565
9869 10541 9882 10516 9895 10491 9909 10468 9919 10441 9912 10414
9889 10399 9862 10394 9834 10391 9806 10389 9779 10384 9752 10377
9725 10369 9701 10356 9681 10336 9666 10313 9642 10299 9615 10294
30 P
9615 10294 9591 10279 9569 10262 9554 10239 9526 10234 9502 10221
9482 10202 9467 10178 9467 10150 9451 10127 9435 10105 9409 10093
9382 10090 9363 10110 9336 10119 9308 10119 9280 10116 9253 10116
9225 10110 9200 10097 9193 10070 9194 10043 9168 10052 9140 10051
9123 10028 9103 10009 9127 10024 9146 10044 9168 10027 9196 10029
9212 10052 9222 10078 9245 10094 9273 10097 9300 10094 9325 10080
9313 10055 9322 10029 9340 10007 9334 9979 9343 9953 9370 9945
9345 9958 9364 9978 9390 9968 9417 9968 9444 9978 9465 9996
9489 10010 9511 10028 50 P
9511 10028 9530 10048 9552 10065 9577 10078 9604 10082 9632 10083
9660 10079 9684 10064 9706 10049 9726 10029 9745 10008 9762 9986
9781 9965 9796 9942 9811 9918 9835 9904 9862 9900 9890 9895
9915 9884 9943 9877 9954 9867 9960 9861 9965 9860 9993 9860
10021 9861 10045 9875 10037 9848 10014 9832 9988 9822 9967 9804

9952 9781 9930 9764 9913 9742 9890 9725 9873 9703 9855 9682
9833 9665 9806 9658 9779 9652 9752 9658 9734 9636 9723 9610
9731 9584 9746 9608 9773 9615 9764 9588 9783 9609 9806 9625
9833 9633 9851 9654 50 P
9851 9654 9879 9654 2 P
9879 9654 9886 9627 9911 9640 9895 9664 9895 9691 9920 9705
9947 9711 9974 9718 9993 9698 9976 9676 9973 9648 9988 9625
9987 9597 9962 9587 9964 9559 9962 9531 9987 9519 9992 9491
9974 9470 9950 9455 9923 9460 9922 9488 9916 9515 9926 9541
9905 9560 9898 9533 9900 9505 9893 9478 9872 9460 9845 9452
9836 9478 9834 9506 9842 9533 9831 9508 9830 9480 9813 9501
9785 9505 9781 9478 9773 9451 9749 9437 9748 9409 9776 9412
9803 9420 9830 9414 9833 9386 9805 9384 9784 9365 9810 9376
9837 9382 9863 9370 50 P
9863 9370 9852 9345 9872 9326 9854 9304 9826 9307 9800 9317
9815 9293 9842 9288 9855 9263 9863 9236 9843 9217 9824 9197
9800 9182 9771 9180 9743 9180 9716 9180 9742 9173 9770 9165
9786 9143 9794 9116 9806 9091 9822 9068 9821 9040 9835 9015
9844 8988 9857 8964 9845 8938 9827 8960 9804 8975 9776 8979
9761 8956 9748 8931 9726 8913 9733 8885 9712 8866 9691 8886
9674 8908 9666 8935 9651 8959 9668 8981 9641 8990 9615 8979
9605 8953 9580 8942 9602 8924 9592 8898 9577 8874 9552 8861
9538 8836 9540 8808 50 P
9540 8808 9527 8784 2 P
9527 8784 9528 8771 9501 8773 9473 8774 9445 8774 9428 8792
9423 8791 9395 8798 9367 8795 9346 8814 9348 8786 9376 8789
9403 8785 9423 8764 9451 8759 9475 8746 9478 8718 9462 8694
9444 8673 9429 8649 9417 8623 9389 8620 9385 8592 9386 8564
9366 8544 9339 8537 9357 8515 9370 8490 9397 8480 9383 8455
9384 8427 9356 8422 9330 8435 9325 8408 9338 8382 9321 8360
9321 8331 9306 8307 9290 8331 9279 8305 9255 8319 9244 8345
42 P
9244 8345 9250 8318 9264 8293 9249 8269 9221 8262 9193 8266
9203 8239 9198 8211 9179 8190 9151 8190 9178 8183 9180 8155
9152 8160 9124 8162 9117 8135 9089 8140 9077 8114 9101 8100
9126 8086 9115 8060 9141 8048 9120 8028 9121 8000 9095 7989
9071 7974 9043 7971 9034 7944 9013 7926 8985 7927 8992 7954
9008 7977 9037 7978 9057 7998 9056 8026 9081 8039 9094 8064
9070 8080 9041 8083 9032 8056 9016 8033 9011 8005 8989 8023
8961 8029 8964 8058 8989 8071 8961 8078 8978 8101 8952 8090
8947 8062 8926 8081 50 P
8926 8081 8909 8104 8899 8130 8900 8101 8908 8075 8917 8048
8893 8061 8871 8079 8844 8090 8848 8061 8843 8034 8866 8018

8840 8008 8842 7979 8864 7961 8890 7972 8918 7972 8946 7966
8965 7946 8969 7918 8981 7892 8966 7868 8943 7885 8915 7892
8894 7911 8871 7894 8858 7869 8885 7877 8898 7852 8904 7824
8926 7806 8952 7819 8960 7845 8978 7868 8995 7845 9019 7830
9045 7841 9027 7819 9000 7815 8972 7809 8947 7794 8920 7787
8892 7781 8869 7765 8897 7769 8886 7743 8869 7720 8849 7700
8833 7677 8852 7656 50 P
8852 7656 8880 7648 2 P
8880 7648 8906 7635 8933 7632 8927 7604 8918 7578 8890 7575
8881 7549 8883 7520 8891 7493 8878 7467 8849 7462 8823 7452
8806 7429 8820 7404 8828 7376 8853 7389 8867 7364 8873 7336
8900 7326 8907 7299 8901 7270 8880 7252 8879 7280 8878 7309
8851 7317 8841 7290 8826 7266 8829 7294 8812 7317 8807 7345
8779 7349 8754 7361 8763 7388 8740 7404 8728 7378 8713 7354
8693 7334 8668 7319 8646 7302 8618 7294 8639 7275 8662 7258
8686 7242 8676 7216 8703 7225 8726 7241 8743 7218 8763 7198
8735 7191 8729 7163 50 P
8729 7163 8700 7159 8672 7157 8648 7173 8647 7202 8631 7177
8641 7150 8655 7126 8628 7138 8600 7141 8611 7168 8613 7197
8587 7185 8604 7208 8576 7215 8547 7212 8521 7202 8540 7181
8561 7161 8544 7137 8528 7114 8500 7106 8509 7079 8533 7064
8561 7057 8574 7082 8573 7054 8586 7028 8558 7030 8529 7028
8533 6999 8561 6993 8537 6978 8510 6989 8482 6993 8464 6971
8436 6963 8441 6991 8429 7017 8416 6991 8415 7020 8395 7040
8401 7012 8408 6984 8380 6978 8354 6990 8378 6974 8382 6945
8402 6925 8425 6942 50 P
8425 6942 8440 6918 2 P
8440 6918 8437 6890 8423 6864 8395 6868 8393 6839 8404 6813
8384 6792 8359 6778 8360 6807 8335 6822 8309 6834 8284 6848
8294 6875 8272 6857 8278 6829 8253 6815 8224 6810 8197 6818
8185 6792 8157 6799 8164 6826 8154 6853 8125 6853 8122 6824
8108 6799 8091 6822 8082 6795 8053 6794 8079 6781 8074 6752
8050 6736 8023 6743 7996 6753 7968 6752 7953 6749 7941 6764
7940 6748 7951 6737 7974 6719 7999 6706 8017 6701 8045 6705
8074 6707 8077 6679 8105 6679 8119 6654 8126 6626 8098 6620
8076 6601 8054 6583 50 P
8054 6583 8033 6603 8013 6582 8024 6556 8005 6533 8001 6504
7976 6490 7953 6473 7937 6449 7925 6423 7929 6451 7946 6475
7936 6502 7909 6493 7890 6471 7894 6442 7871 6425 7859 6399
7857 6370 7855 6342 7838 6318 7839 6347 7848 6375 7839 6402
7817 6421 7790 6429 7762 6435 7733 6438 7757 6421 7785 6414
7786 6386 7776 6358 7758 6336 7735 6352 7723 6352 7711 6336
7687 6352 7676 6326 7671 6297 7669 6269 7651 6246 7659 6274

7641 6296 7641 6268 7616 6252 45 P
7616 6252 7594 6234 7572 6216 7564 6244 7558 6272 7532 6283
7540 6255 7515 6268 7487 6261 7461 6250 7469 6226 7455 6201
7454 6175 7455 6164 7434 6144 7423 6130 7401 6107 7372 6108
7343 6108 7317 6119 7294 6137 7274 6158 7245 6159 7273 6154
7279 6125 7292 6099 7315 6082 7343 6075 7379 6044 7358 6033
7341 6009 7319 6029 7297 6047 7268 6045 7271 6016 7296 6030
7291 6002 7278 5976 7284 5948 7287 5919 7266 5899 7258 5872
7229 5874 7201 5866 7172 5871 7149 5889 7125 5874 7102 5856
7082 5835 7060 5817 50 P
7060 5817 7031 5817 7046 5841 7029 5865 7027 5836 7003 5820
6976 5809 6949 5800 6921 5792 6899 5772 6882 5796 6866 5820
6839 5828 6815 5844 6786 5842 6765 5822 6747 5799 6730 5776
6747 5753 6768 5772 6754 5747 6757 5718 6731 5730 6706 5744
6677 5739 6649 5746 6637 5772 6666 5773 6641 5787 6615 5800
6576 5799 6577 5808 6599 5816 6569 5827 6544 5812 6515 5812
6487 5806 6472 5780 6459 5755 6430 5751 6417 5777 6392 5792
6408 5768 6431 5750 6415 5726 6399 5701 6403 5673 6383 5694
6354 5697 6325 5697 50 P
6325 5697 6314 5723 2 P
6314 5723 6317 5752 6315 5781 6338 5799 6346 5827 6325 5807
6296 5813 6270 5800 6242 5791 6216 5779 6202 5754 6180 5735
6209 5734 6209 5705 6182 5695 6208 5681 6234 5671 6229 5642
6201 5635 6173 5629 6169 5658 6160 5685 6142 5708 6114 5715
6086 5708 6057 5701 6036 5682 27 P
6036 5682 6061 5669 6088 5657 6066 5637 6037 5636 6008 5631
6024 5607 6052 5615 6074 5634 6091 5610 6119 5616 6114 5587
6086 5580 6058 5590 6050 5562 6070 5541 6043 5552 6014 5551
5986 5559 5967 5581 5938 5580 5909 5580 5880 5584 5861 5604
5881 5625 5903 5644 5928 5657 5956 5668 28 P
5956 5668 5927 5670 5899 5678 5872 5667 5853 5645 5860 5673
5843 5697 5824 5719 5812 5746 5797 5770 5791 5799 5773 5821
5746 5811 5740 5839 5721 5861 5706 5886 5712 5914 5724 5941
5706 5918 5684 5938 5678 5967 5680 5995 5655 6010 5629 6020
5601 6030 5617 6006 5646 6002 5663 5979 5662 5950 5656 5921
5627 5917 5599 5912 5628 5911 5655 5900 5665 5872 5689 5856
5703 5831 5675 5824 5651 5840 5671 5819 5687 5795 5704 5771
5676 5762 5655 5782 5675 5761 5690 5736 5667 5720 5682 5695
5682 5666 5668 5641 50 P
5668 5641 5660 5613 5684 5597 5690 5568 5676 5543 5666 5515
5657 5499 7 P
5650 5499 5645 5518 5645 5499 3 P
5597 5499 5593 5502 5574 5524 5557 5548 5548 5576 5530 5598

5538 5570 5552 5533 5545 5527 5540 5520 5540 5514 5532 5499

12:00 PM

5430 5499 5431 5521 5413 5544 5411 5573 5412 5602 5416 5630
5416 5659 5387 5661 5381 5690 5353 5694 5370 5670 5394 5654
5405 5627 5405 5598 5404 5569 5407 5540 5408 5511 5393 5499
18 P
2969 5499 2970 5505 2988 5527 2970 5550 2941 5551 2919 5531
2928 5504 2931 5499 8 P
12998 8165 12993 8160 12988 8165 12982 8169 12980 8163 12980 8156
12982 8149 12985 8142 12989 8136 12992 8131 12998 8126 12998 8125

12:00 PM

12998 8078 12998 8077 12991 8075 12985 8072 12981 8066 12978 8060
12975 8053 12975 8046 12970 8041 12974 8035 12979 8030 12973 8034
12967 8037 12959 8036 12957 8030 12958 8023 12962 8018 12965 8011
12971 8007 12976 8001 12976 7994 12976 7987 12976 7980 12974 7973
12973 7966 12979 7962 12986 7959 12990 7965 12996 7969 12998 7969
30 P
11823 7075 11826 7069 11831 7064 11837 7060 11844 7058 11845 7065
11847 7058 11853 7054 11859 7056 11866 7059 11873 7060 11880 7063
11886 7066 11894 7066 11901 7068 11908 7068 11915 7068 11922 7070
11929 7071 11934 7066 11930 7072 11925 7077 11921 7083 11916 7088
11909 7089 11902 7090 11895 7093 11891 7099 11884 7102 11877 7100
11871 7103 11864 7104 11861 7111 11862 7118 11857 7123 11850 7125
11846 7131 11845 7138 11841 7145 11836 7149 11831 7154 11826 7160
11824 7166 11817 7164 11820 7159 11825 7153 11828 7147 11832 7141
11836 7135 11839 7128 50 P
11839 7128 11832 7126 11832 7119 11834 7112 11831 7105 11825 7102
11823 7095 11829 7092 11834 7087 11838 7081 11833 7075 11823 7075

12:00 PM

11223 6838 11228 6834 11235 6836 11240 6841 11247 6842 11253 6837
11257 6831 11261 6825 11261 6818 11257 6812 11251 6809 11247 6804
11252 6798 11259 6797 11266 6794 11271 6790 11277 6786 11284 6785
11291 6787 11296 6793 11297 6800 11304 6798 11310 6794 11317 6797
11320 6804 11321 6811 11320 6818 11320 6825 11322 6832 11320 6839
11317 6845 11320 6851 11317 6858 11317 6865 11320 6871 11326 6876
11330 6882 11331 6889 11336 6894 11340 6899 11343 6906 11340 6912
11344 6918 11349 6923 11354 6928 11355 6935 11355 6942 11355 6950
11348 6949 11341 6951 50 P
11341 6951 11340 6943 11339 6936 11336 6930 11331 6925 11329 6918
11334 6913 11331 6907 11329 6900 11327 6893 11320 6895 11314 6892
11310 6886 11304 6884 11303 6876 11301 6870 11302 6863 11297 6858
11290 6858 11283 6857 11277 6853 11270 6854 11263 6852 11260 6859
11257 6865 11250 6868 11250 6860 11247 6854 11244 6847 11238 6852

11231 6851 11226 6846 11223 6838 33 P
11015 6752 11020 6746 11024 6741 11030 6737 11038 6738 11045 6736
11047 6743 11053 6748 11058 6743 11064 6739 11071 6738 11079 6738
11086 6737 11093 6736 11100 6736 11107 6737 11114 6735 11120 6731
11127 6729 11133 6733 11132 6740 11128 6746 11122 6749 11129 6747
11136 6745 11142 6741 11142 6748 11140 6755 11142 6762 11146 6768
11153 6766 11155 6773 11152 6779 11156 6786 11158 6792 11152 6795
11146 6799 11141 6804 11134 6806 11127 6807 11124 6814 11118 6810
11112 6814 11105 6813 11098 6810 11093 6805 11089 6810 11087 6817
11083 6822 11077 6827 50 P
11077 6827 11070 6829 11064 6825 11070 6821 11071 6814 11075 6808
11069 6811 11069 6818 11063 6815 11058 6809 11054 6804 11057 6797
11056 6790 11060 6784 11065 6779 11066 6772 11063 6778 11061 6771
11059 6764 11063 6758 11062 6751 11061 6758 11056 6763 11050 6767
11049 6774 11043 6769 11037 6773 11037 6766 11041 6759 11035 6764
11029 6767 11025 6761 11028 6755 11015 6752 34 P
9617 7077 9614 7063 9605 7052 9597 7040 9591 7027 9584 7014
9581 7000 9572 6990 9563 6978 9569 6965 9566 6951 9561 6937
9552 6927 9542 6917 9538 6903 9528 6893 9538 6883 9524 6884
9510 6882 9497 6877 9488 6865 9495 6852 9481 6849 9467 6843
9463 6830 9458 6816 9455 6802 9450 6788 9444 6776 9436 6764
9426 6754 9417 6742 9408 6731 9402 6719 9396 6705 9391 6692
9385 6679 9383 6665 9374 6653 9370 6639 9363 6627 9355 6615
9367 6607 9353 6607 9345 6594 9338 6582 9331 6570 9328 6556
9323 6542 9314 6531 50 P
9314 6531 9304 6521 9294 6510 9288 6497 9279 6486 9272 6473
9272 6459 9266 6446 9261 6433 9256 6420 9246 6409 9255 6397
9269 6396 9284 6394 9298 6396 9287 6387 9272 6385 9258 6384
9244 6387 9229 6386 9221 6374 9212 6363 9211 6348 9209 6335
9206 6320 9194 6312 9188 6299 9182 6286 9180 6272 9179 6257
9185 6244 9186 6230 9191 6217 9180 6209 9190 6198 9186 6184
9198 6175 9187 6165 9191 6151 9195 6137 9193 6123 9191 6109
9198 6096 9184 6098 9182 6084 9174 6072 9176 6058 9164 6049
9178 6044 9164 6041 50 P
9164 6041 9162 6027 2 P
9162 6027 9163 6013 9154 6003 9168 5999 9173 5985 9168 5971
9157 5962 9143 5959 9139 5973 9135 5959 9145 5949 9160 5952
9152 5940 9163 5931 9175 5922 9189 5927 9202 5931 9216 5935
9219 5921 9206 5914 9192 5912 9198 5899 9206 5886 9210 5872
9210 5858 9215 5844 9220 5831 9234 5827 9225 5816 9231 5803
9236 5789 9242 5776 9248 5763 9254 5749 9258 5735 9257 5721
9254 5707 9262 5694 9275 5688 9279 5675 9290 5665 9301 5656
9287 5661 9278 5672 9270 5684 9276 5671 9285 5659 9294 5649

9305 5638 9315 5628 50 P
9315 5628 9327 5620 9339 5612 9346 5625 9352 5612 9361 5601
9372 5591 9385 5584 9397 5577 9410 5571 9423 5564 9435 5556
9445 5545 9453 5533 9464 5523 9471 5511 9475 5499 17 P
9607 5499 9605 5510 9605 5525 9607 5539 9613 5552 9615 5567
9620 5580 9626 5594 9631 5607 9634 5622 9637 5636 9641 5650
9645 5664 9651 5677 9661 5687 9663 5702 9666 5716 9672 5729
9683 5738 9694 5748 9706 5756 9719 5763 9729 5773 9739 5783
9749 5794 9753 5808 9757 5821 9752 5835 9758 5848 9772 5854
9783 5863 9789 5876 9796 5889 9800 5903 9810 5913 9818 5925
9823 5939 9828 5952 9831 5967 9836 5980 9847 5990 9856 6001
9858 6016 9861 6030 9856 6043 9857 6057 9864 6070 9866 6084
9871 6098 9876 6111 50 P
9876 6111 9888 6120 9890 6134 9891 6149 9893 6163 9897 6177
9902 6190 9905 6205 9909 6218 9904 6232 9905 6246 9909 6260
9914 6273 9916 6288 9916 6302 9921 6315 9922 6330 9923 6344
9926 6358 9932 6372 9933 6386 9933 6400 9938 6414 9943 6428
9945 6442 9948 6456 9948 6470 9948 6485 9950 6499 9958 6510
9966 6522 9959 6534 9951 6546 9949 6560 9954 6574 9964 6584
9972 6596 9971 6610 9969 6624 9976 6637 9987 6645 9995 6657
10007 6665 10012 6679 10020 6691 10025 6705 10024 6719 10029 6733
10031 6747 49 P
10031 6747 10032 6761 10038 6774 10045 6786 10056 6796 10054 6810
10059 6823 10071 6831 10068 6845 10069 6859 10066 6873 10057 6884
10052 6898 10049 6912 10046 6926 10042 6940 10037 6953 10036 6967
10036 6982 10037 6996 10040 7010 10045 7024 10053 7035 10063 7046
10067 7059 10078 7068 10087 7080 10100 7087 10112 7093 10123 7102
10115 7114 10101 7113 10089 7120 10087 7134 10079 7146 10082 7160
10072 7169 10057 7169 10043 7170 10029 7167 10018 7176 10005 7182
9992 7175 9983 7185 9969 7191 9958 7200 9963 7213 9949 7209
9938 7218 9930 7230 50 P
9930 7230 9926 7244 9914 7252 9902 7259 9887 7258 9873 7253
9860 7249 9848 7242 9839 7231 9830 7219 9823 7207 9816 7195
9827 7185 9814 7181 9804 7170 9791 7164 9778 7157 9764 7155
9750 7152 9737 7153 9723 7148 9710 7142 9698 7135 9685 7127
9672 7124 9659 7118 9647 7110 9634 7103 9624 7093 9617 7077
30 P
12967 11650 12974 11649 12981 11649 12985 11643 12991 11642 12995 11648
12998 11649 7 P
12998 11741 12997 11742 12992 11747 12985 11748 12980 11744 12978 11737
12979 11730 12983 11725 12981 11718 12982 11712 12988 11707 12984 11701
12987 11695 12989 11689 12995 11685 12996 11678 12989 11678 12994 11674
12988 11675 12990 11669 12985 11665 12984 11658 12990 11660 12996 11664

12992 11659 12991 11652 12985 11654 12979 11658 12976 11664 12972 11670
12970 11663 12967 11650 32 P
11804 11890 11809 11885 11813 11879 11817 11874 11823 11870 11829 11867
11836 11866 11841 11862 11845 11856 11850 11852 11855 11847 11861 11844
11868 11844 11868 11851 11869 11858 11873 11863 11878 11868 11884 11872
11891 11869 11898 11870 11904 11869 11911 11872 11917 11875 11924 11876
11930 11873 11936 11875 11943 11876 11947 11872 11954 11871 11960 11874
11967 11874 11971 11880 11978 11881 11985 11882 11992 11882 11998 11884
12004 11881 12011 11883 12016 11888 12021 11893 12025 11898 12031 11902
12038 11904 12045 11904 12051 11905 12057 11908 12064 11911 12070 11912
12075 11917 12080 11922 50 P
12080 11922 12084 11927 12091 11927 12098 11929 12104 11931 12111 11933
12114 11939 12113 11946 12119 11950 12122 11956 12123 11963 12125 11969
12128 11976 12134 11979 12139 11984 12138 11991 12140 11998 12141 12005
12146 12001 12144 12007 12143 12014 12146 12020 12153 12019 12148 12024
12143 12019 12142 12026 12136 12028 12132 12034 12135 12040 12133 12047
12130 12053 12123 12055 12117 12056 12111 12053 12105 12057 12098 12059
12093 12064 12090 12069 12083 12068 12076 12069 12069 12069 12062 12069
12056 12065 12049 12065 12043 12063 12036 12060 12030 12058 12024 12058
12018 12054 12011 12055 50 P
12011 12055 12005 12052 2 P
12005 12052 11999 12049 11992 12046 11986 12043 11980 12040 11973 12039
11967 12036 11961 12033 11955 12029 11949 12031 11942 12032 11938 12026
11932 12025 11926 12021 11920 12017 11914 12015 11908 12011 11903 12008
11896 12010 11891 12006 11884 12003 11879 11999 11873 11996 11866 11995
11862 11990 11859 11983 11853 11981 11848 11976 11843 11972 11843 11965
11839 11959 11835 11953 11829 11949 11824 11945 11821 11939 11816 11933
11810 11930 11809 11923 11803 11919 11804 11912 11806 11905 11804 11899
11804 11890 43 P
12975 8328 12980 8323 12987 8323 12994 8325 12998 8325 5 P
12998 8341 12993 8339 12986 8339 12979 8339 12975 8328 5 P
12712 8214 12719 8217 12726 8218 12732 8221 12730 8228 12723 8231
12718 8227 12712 8214 8 P
12474 8065 12479 8060 12486 8058 12492 8057 12499 8057 12505 8052
12508 8046 12512 8040 12518 8036 12524 8033 12531 8032 12529 8039
12526 8046 12526 8053 12530 8058 12532 8065 12526 8068 12523 8075
12516 8077 12512 8083 12506 8086 12499 8085 12493 8082 12488 8076
12482 8073 12474 8065 26 P
12690 8092 12693 8089 12695 8085 12699 8087 12703 8090 12699 8091
12696 8095 12697 8099 12692 8098 12690 8092 10 P
12531 8056 12533 8057 12535 8058 12537 8059 12537 8061 12536 8063
12534 8062 12532 8060 12531 8056 9 P
12539 8060 12541 8060 12543 8062 12542 8064 12540 8063 12539 8060

6:00 PM

12544 8057 12546 8059 12548 8060 12550 8061 12552 8061 12554 8063
12555 8064 12556 8066 12559 8067 12561 8067 12563 8068 12565 8068
12566 8070 12568 8071 12570 8071 12568 8072 12566 8072 12564 8070
12563 8069 12561 8069 12559 8068 12556 8068 12555 8066 12553 8064
12552 8063 12549 8062 12547 8062 12545 8060 12544 8057 29 P
12805 7999 12806 7996 12810 7992 12809 7988 12809 7983 12812 7979
12813 7984 12817 7986 12818 7990 12817 7995 12816 7999 12818 8003
12822 8006 12821 8011 12821 8015 12820 8020 12817 8023 12814 8026
12809 8024 12809 8020 12809 8015 12807 8011 12807 8007 12808 8003
12805 7999 25 P
12773 8023 12777 8022 12782 8022 12785 8019 12787 8022 12790 8026
12789 8031 12793 8028 12797 8027 12797 8031 12794 8035 12790 8036
12785 8036 12785 8032 12780 8032 12778 8028 12773 8023 17 P
12766 8006 12770 8003 12772 7999 12776 8001 12780 8003 12778 8007
12776 8011 12772 8013 12769 8010 12766 8006 10 P
12776 7997 12778 7993 12783 7994 12783 7999 12776 7997 5 P
12780 7988 12783 7984 12784 7989 12783 7993 12780 7988 5 P
12751 7989 12752 7985 12757 7983 12757 7987 12757 7992 12754 7994
12751 7989 7 P
12752 8011 12757 8011 12758 8015 12754 8016 12752 8011 5 P
12853 8016 12858 8018 12858 8022 12860 8026 12860 8030 12857 8027
12856 8023 12853 8016 8 P
12829 12958 12823 12955 12820 12949 12819 12942 12819 12935 12812 12937
12806 12934 12799 12937 12798 12930 12793 12925 12797 12919 12801 12913
12805 12908 12809 12903 12814 12898 12808 12899 12801 12898 12795 12901
12790 12896 12784 12897 12777 12901 12772 12905 12765 12903 12763 12897
12756 12896 12753 12890 12750 12884 12747 12877 12749 12871 12751 12864
12755 12858 12761 12856 12764 12850 12768 12844 12774 12847 12773 12854
12779 12855 12786 12859 12790 12864 12793 12870 12799 12869 12800 12862
12803 12855 12801 12849 12802 12842 12796 12838 12790 12837 12783 12834
12785 12827 12790 12832 50 P
12790 12832 12790 12825 12797 12826 12803 12829 12809 12826 12803 12822
12802 12816 12805 12809 12812 12807 12808 12802 12806 12795 12813 12793
12819 12790 12824 12795 12831 12796 12829 12803 12823 12807 12830 12810
12837 12811 12843 12813 12850 12814 12844 12817 12837 12816 12833 12821
12829 12826 12831 12833 12838 12832 12842 12838 12839 12844 12839 12851
12845 12854 12849 12848 12853 12853 12859 12852 12864 12847 12870 12845
12870 12852 12865 12856 12861 12862 12863 12868 12856 12867 12854 12873
12860 12876 12866 12875 12864 12881 12859 12886 12866 12887 12866 12894
12869 12901 12862 12901 50 P
12862 12901 12857 12896 2 P
12857 12896 12856 12889 12849 12891 12845 12897 12850 12902 12853 12908

12856 12914 12853 12920 12852 12927 12851 12933 12849 12940 12843 12944
12840 12950 12837 12956 12841 12962 12846 12965 12848 12972 12843 12977
12837 12979 12830 12980 12826 12974 12826 12967 12829 12958 23 P
12539 12828 12533 12825 12530 12819 12524 12816 12519 12811 12515 12816
12512 12822 12506 12824 12499 12825 12500 12831 12495 12836 12491 12842
12492 12849 12498 12853 12501 12859 12502 12866 12501 12873 12497 12878
12491 12882 12484 12883 12478 12880 12473 12876 12468 12870 12469 12863
12473 12858 12467 12856 12460 12854 12460 12847 12460 12840 12455 12835
12453 12828 12455 12821 12458 12816 12462 12810 12467 12805 12469 12799
12474 12794 12476 12788 12474 12782 12472 12775 12479 12773 12481 12767
12487 12762 12493 12761 12500 12762 12506 12766 12508 12772 12504 12778
12502 12784 12508 12788 50 P
12508 12788 12513 12792 12520 12793 12521 12786 12520 12780 12522 12773
12525 12767 12526 12760 12533 12759 12540 12760 12546 12763 12553 12764
12557 12759 12563 12761 12570 12762 12566 12768 12563 12774 12567 12778
12571 12784 12569 12791 12568 12798 12567 12805 12566 12811 12569 12817
12569 12824 12563 12828 12556 12826 12552 12831 12539 12828 29 P
12064 12407 12068 12401 12074 12403 12080 12399 12086 12399 12093 12398
12098 12402 12099 12409 12098 12416 12093 12421 12091 12427 12086 12431
12079 12433 12074 12429 12070 12423 12068 12417 12064 12407 17 P
12230 12446 12235 12448 12239 12448 12243 12449 12248 12449 12248 12453
12250 12457 12248 12461 12246 12465 12246 12470 12242 12469 12238 12467
12239 12463 12235 12462 12234 12457 12233 12453 12230 12446 17 P
12157 12581 12160 12578 12164 12578 12167 12575 12163 12572 12162 12568
12166 12569 12169 12565 12173 12564 12177 12561 12180 12559 12184 12562
12188 12561 12191 12565 12195 12565 12200 12564 12204 12564 12208 12564
12213 12566 12215 12569 12216 12573 12216 12578 12218 12582 12219 12586
12221 12590 12220 12594 12217 12598 12214 12600 12209 12599 12206 12597
12201 12597 12197 12598 12193 12596 12190 12593 12187 12589 12183 12590
12181 12586 12177 12587 12172 12589 12171 12593 12166 12593 12167 12589
12165 12585 12162 12582 12157 12581 45 P
12318 12588 12323 12589 12327 12590 12331 12590 12334 12586 12329 12586
12325 12587 12327 12583 12330 12581 12335 12583 12338 12580 12338 12576
12337 12572 12340 12570 12344 12573 12347 12575 12351 12577 12356 12578
12359 12582 12360 12585 12358 12590 12357 12594 12352 12594 12350 12598
12346 12600 12343 12604 12342 12600 12337 12598 12334 12596 12330 12598
12326 12599 12323 12601 12318 12600 12322 12598 12320 12593 12318 12588
36 P
12423 12650 12426 12647 12431 12646 12434 12643 12438 12645 12438 12641
12441 12638 12443 12634 12446 12632 12451 12634 12454 12632 12458 12631
12460 12627 12464 12627 12468 12627 12471 12631 12469 12634 12467 12638
12462 12638 12458 12639 12462 12640 12462 12644 12459 12648 12455 12650
12454 12654 12452 12658 12448 12660 12444 12661 12439 12660 12436 12657

12433 12654 12430 12651 12423 12650 33 P
12470 12597 12474 12597 12472 12593 12472 12589 12476 12589 12480 12586
12482 12590 12481 12594 12481 12598 12482 12603 12482 12607 12478 12608
12479 12613 12474 12613 12473 12609 12474 12605 12474 12600 12470 12597
18 P
11958 12510 11960 12506 11960 12501 11961 12497 11962 12493 11962 12488
11965 12485 11964 12482 11968 12483 11971 12480 11975 12479 11979 12477
11982 12479 11985 12482 11989 12478 11992 12476 11989 12472 11984 12472
11984 12468 11988 12465 11992 12467 11996 12468 12000 12467 12004 12464
12008 12463 12007 12468 12006 12471 12010 12470 12012 12466 12017 12466
12021 12466 12025 12466 12023 12470 12019 12471 12020 12475 12023 12479
12019 12481 12014 12480 12010 12480 12011 12485 12009 12488 12005 12490
12004 12494 12003 12498 12002 12502 12002 12507 12000 12512 11998 12515
11994 12517 11990 12520 50 P
11990 12520 11987 12522 11983 12523 11978 12525 11975 12523 11971 12520
11967 12520 11963 12518 11961 12515 11958 12510 10 P
11709 12504 11713 12499 11717 12494 11721 12488 11727 12484 11726 12491
11723 12498 11726 12504 11728 12497 11732 12493 11739 12491 11736 12485
11732 12480 11735 12473 11739 12479 11743 12473 11750 12472 11757 12473
11763 12477 11769 12479 11764 12484 11771 12483 11777 12484 11781 12490
11788 12491 11788 12498 11794 12501 11801 12501 11802 12508 11808 12508
11809 12515 11815 12511 11816 12504 11822 12509 11819 12515 11825 12519
11828 12526 11832 12531 11828 12537 11832 12542 11828 12547 11832 12553
11832 12560 11834 12566 11828 12570 11825 12576 11819 12579 11814 12584
11808 12581 11806 12575 50 P
11806 12575 11801 12579 11798 12585 11800 12592 11796 12598 11790 12601
11783 12603 11777 12603 11770 12604 11768 12598 11771 12591 11778 12591
11784 12593 11782 12586 11784 12580 11780 12574 11776 12569 11773 12563
11766 12564 11766 12571 11764 12577 11760 12572 11763 12565 11756 12563
11759 12556 11761 12550 11765 12544 11771 12547 11768 12541 11762 12542
11765 12536 11764 12529 11763 12523 11761 12516 11758 12510 11758 12517
11757 12523 11758 12530 11761 12537 11757 12542 11757 12549 11750 12551
11749 12558 11744 12554 11743 12547 11746 12541 11743 12535 11738 12540
11732 12540 11728 12534 50 P
11728 12534 11722 12532 2 P
11722 12532 11720 12525 11725 12520 11718 12522 11713 12518 11711 12511
11709 12504 7 P
11911 12372 11914 12369 11916 12365 11920 12364 11924 12364 11928 12366
11932 12367 11935 12370 11939 12373 11939 12378 11944 12378 11945 12383
11948 12384 11949 12388 11952 12386 11955 12382 11955 12386 11959 12385
11963 12386 11963 12390 11959 12390 11954 12390 11951 12392 11946 12392
11942 12391 11938 12391 11933 12390 11932 12394 11928 12397 11924 12395
11920 12393 11917 12390 11914 12386 11911 12384 11913 12380 11911 12372

36 P
11927 12402 11932 12401 11934 12398 11938 12395 11942 12394 11946 12394
11951 12394 11947 12397 11946 12401 11949 12404 11952 12400 11956 12400
11958 12404 11955 12407 11959 12409 11962 12412 11959 12415 11957 12419
11954 12422 11950 12423 11946 12422 11941 12423 11938 12422 11935 12425
11931 12427 11932 12423 11934 12420 11931 12417 11930 12413 11934 12415
11932 12411 11931 12407 11927 12402 33 P
11967 12382 11969 12379 11971 12375 11972 12371 11976 12373 11978 12370
11980 12365 11984 12364 11987 12367 11987 12372 11988 12376 11988 12380
11991 12383 11991 12387 11987 12385 11986 12389 11984 12393 11980 12393
11980 12398 11984 12399 11989 12400 11990 12404 11987 12407 11983 12409
11979 12410 11975 12412 11971 12409 11971 12405 11967 12405 11968 12401
11973 12401 11975 12398 11975 12393 11971 12390 11971 12386 11967 12382
36 P
12012 12490 12016 12490 12020 12488 12024 12488 12028 12485 12031 12482
12034 12485 12037 12488 12041 12489 12046 12489 12049 12487 12052 12483
12052 12479 12054 12476 12058 12477 12060 12481 12063 12484 12062 12488
12067 12487 12067 12483 12071 12483 12072 12487 12071 12491 12072 12495
12076 12495 12077 12499 12076 12503 12072 12500 12068 12501 12065 12504
12061 12504 12056 12504 12053 12501 12051 12505 12047 12507 12044 12505
12041 12501 12042 12497 12038 12494 12034 12493 12031 12496 12027 12498
12025 12494 12024 12491 12019 12492 12015 12493 12012 12490 47 P
12008 12515 12011 12517 12016 12516 12020 12518 12018 12513 12017 12509
12017 12505 12020 12502 12023 12506 12024 12510 12026 12514 12027 12518
12032 12520 12036 12520 12040 12521 12040 12525 12043 12528 12047 12528
12050 12526 12055 12526 12060 12526 12059 12530 12056 12533 12052 12535
12052 12540 12048 12538 12043 12539 12047 12541 12048 12546 12045 12548
12043 12552 12039 12551 12034 12549 12031 12547 12027 12546 12023 12546
12020 12542 12017 12539 12016 12535 12013 12532 12014 12527 12012 12523
12010 12520 12008 12515 44 P
12057 12534 12061 12532 12063 12528 12060 12525 12060 12520 12061 12516
12064 12519 12065 12523 12069 12525 12069 12521 12069 12516 12070 12512
12075 12512 12078 12509 12083 12510 12086 12512 12088 12516 12092 12517
12096 12517 12098 12521 12095 12525 12094 12529 12095 12533 12097 12537
12095 12541 12093 12537 12091 12534 12090 12529 12085 12531 12082 12529
12078 12533 12074 12533 12070 12534 12066 12536 12064 12532 12062 12536
12057 12534 37 P
12122 12516 12118 12517 12120 12513 12124 12511 12128 12513 12132 12512
12136 12509 12133 12506 12137 12506 12140 12502 12141 12507 12145 12508
12148 12505 12144 12501 12141 12499 12142 12495 12143 12491 12145 12494
12147 12498 12150 12502 12153 12505 12157 12504 12158 12508 12154 12508
12153 12512 12157 12511 12162 12511 12164 12508 12169 12508 12166 12512
12163 12515 12163 12519 12158 12519 12154 12521 12151 12518 12147 12516

12143 12514 12144 12518 12142 12522 12138 12524 12136 12528 12133 12531
12134 12527 12130 12526 12126 12525 12129 12521 12126 12518 12122 12516
48 P
12127 12569 12130 12566 12133 12563 12134 12559 12135 12555 12137 12551
12140 12548 12142 12553 12141 12557 12145 12555 12144 12551 12146 12547
12148 12542 12149 12546 12151 12550 12152 12555 12151 12559 12149 12563
12147 12567 12145 12570 12141 12573 12137 12573 12133 12571 12127 12569
24 P
12893 12966 12896 12963 12901 12964 12902 12968 12900 12973 12900 12977
12901 12981 12902 12986 12903 12989 12903 12994 12901 12998 12897 12997
12895 13001 12891 13003 12889 12999 12885 12998 12881 12996 12879 12992
12883 12991 12886 12988 12886 12983 12888 12980 12885 12978 12888 12976
12893 12975 12893 12970 12893 12966 27 P
12747 12828 12751 12826 12754 12823 12752 12819 12753 12814 12758 12814
12762 12813 12765 12816 12769 12819 12772 12821 12771 12826 12769 12829
12764 12828 12762 12832 12762 12836 12758 12840 12759 12844 12756 12841
12757 12846 12752 12846 12749 12842 12751 12838 12755 12839 12758 12837
12754 12838 12754 12834 12751 12831 12751 12835 12748 12838 12749 12834
12747 12828 31 P
12760 12916 12763 12913 12765 12909 12769 12910 12766 12913 12770 12916
12767 12919 12765 12923 12768 12926 12770 12930 12768 12933 12764 12934
12759 12933 12759 12928 12761 12925 12762 12920 12760 12916 17 P
12613 12842 12618 12840 12621 12839 12621 12834 12625 12834 12628 12837
12629 12841 12632 12845 12636 12845 12641 12845 12640 12849 12636 12851
12632 12851 12628 12854 12624 12853 12620 12851 12615 12849 12613 12842
18 P
12630 12830 12634 12830 12635 12825 12639 12826 12642 12823 12646 12822
12650 12820 12651 12825 12648 12828 12653 12828 12657 12829 12660 12832
12657 12835 12653 12837 12650 12840 12647 12843 12642 12845 12639 12844
12635 12840 12633 12837 12635 12833 12630 12830 22 P
12653 12733 12656 12729 12660 12728 12660 12724 12662 12720 12664 12723
12669 12724 12671 12728 12675 12727 12679 12727 12684 12728 12683 12732
12686 12735 12684 12739 12680 12738 12678 12734 12677 12729 12673 12732
12676 12735 12673 12739 12669 12739 12665 12742 12661 12743 12657 12742
12655 12738 12653 12733 26 P
12534 12870 12532 12867 12531 12862 12529 12859 12526 12855 12525 12850
12522 12848 12520 12844 12521 12839 12523 12835 12525 12831 12529 12830
12533 12829 12538 12829 12542 12830 12546 12831 12547 12835 12551 12837
12555 12835 12559 12833 12563 12834 12567 12832 12571 12833 12575 12830
12579 12830 12577 12834 12574 12837 12570 12839 12567 12841 12566 12846
12567 12849 12567 12854 12563 12856 12559 12858 12555 12861 12557 12864
12553 12865 12549 12862 12548 12858 12547 12854 12547 12849 12545 12845
12542 12848 12542 12853 12542 12857 12543 12861 12546 12865 12547 12869

12548 12873 12548 12877 50 P
12548 12877 12544 12876 12539 12875 12534 12870 4 P
12395 12660 12397 12664 12400 12661 12402 12657 12407 12657 12410 12660
12414 12662 12415 12666 12418 12663 12422 12663 12425 12666 12422 12669
12419 12671 12416 12668 12411 12669 12409 12672 12405 12674 12401 12671
12397 12670 12395 12666 12395 12660 21 P
12307 12626 12311 12624 12315 12624 12316 12619 12320 12617 12315 12615
12312 12612 12315 12608 12317 12605 12322 12605 12324 12608 12329 12608
12333 12611 12337 12610 12339 12613 12339 12618 12336 12620 12332 12622
12327 12622 12323 12622 12327 12625 12324 12628 12320 12629 12323 12633
12326 12636 12325 12640 12320 12641 12316 12641 12313 12638 12311 12634
12310 12630 12307 12626 32 P
12123 12505 12119 12504 12115 12505 12111 12504 12107 12501 12105 12497
12105 12493 12106 12488 12110 12487 12112 12484 12115 12485 12120 12487
12122 12490 12127 12491 12128 12495 12132 12497 12130 12501 12123 12505
18 P
12266 12363 12268 12358 12269 12355 12273 12352 12274 12348 12277 12344
12280 12342 12284 12342 12288 12341 12291 12344 12287 12344 12282 12345
12281 12350 12278 12352 12276 12357 12274 12360 12275 12365 12274 12369
12271 12366 12266 12363 20 P
12278 12366 12282 12363 12284 12359 12287 12357 12291 12355 12292 12359
12288 12362 12289 12366 12285 12367 12278 12366 10 P
12104 12303 12106 12299 12110 12299 12114 12301 12117 12304 12115 12308
12111 12310 12107 12310 12104 12303 9 P
11831 12258 11836 12256 11838 12252 11840 12249 11845 12249 11843 12252
11844 12257 11848 12258 11849 12262 11845 12264 11841 12261 11837 12262
11831 12258 13 P
11822 12263 11826 12261 11829 12259 11831 12263 11828 12266 11824 12267
11822 12263 7 P
11901 12393 11904 12392 11905 12395 11908 12397 11911 12397 11911 12400
11913 12403 11911 12405 11909 12407 11907 12405 11905 12402 11904 12399
11903 12395 11901 12393 14 P
11946 12428 11949 12427 11952 12429 11955 12428 11957 12430 11960 12430
11960 12434 11958 12437 11955 12438 11952 12437 11949 12435 11946 12434
11946 12428 13 P
11949 12353 11953 12354 11956 12354 11959 12353 11959 12356 11956 12358
11955 12362 11952 12363 11949 12361 11952 12359 11949 12353 11 P
12019 12409 12022 12407 12024 12405 12026 12408 12026 12411 12028 12413
12029 12416 12027 12420 12026 12422 12023 12423 12020 12421 12019 12419
12020 12415 12019 12409 14 P
11271 12274 11275 12272 11279 12271 11283 12270 11287 12267 11288 12263
11290 12259 11295 12260 11299 12261 11301 12265 11297 12268 11294 12271
11291 12274 11289 12278 11290 12282 11286 12282 11284 12287 11280 12286

11276 12283 11277 12279 11276 12274 11271 12274 22 P
11436 12364 11439 12362 11440 12359 11443 12361 11446 12360 11448 12362
11445 12363 11442 12365 11439 12364 11436 12364 10 P
11683 12433 11687 12434 11686 12430 11691 12429 11695 12429 11699 12428
11702 12424 11701 12429 11704 12433 11705 12428 11707 12425 11709 12429
11714 12430 11717 12428 11717 12432 11716 12436 11715 12441 11715 12445
11710 12444 11706 12443 11702 12442 11698 12443 11695 12445 11691 12448
11689 12444 11685 12444 11688 12441 11685 12438 11683 12433 29 P
11689 12400 11693 12400 11695 12398 11698 12400 11701 12399 11704 12398
11706 12401 11707 12404 11710 12405 11708 12407 11707 12411 11703 12412
11701 12414 11701 12411 11702 12407 11699 12405 11696 12405 11693 12404
11690 12404 11689 12400 20 P
11645 12398 11647 12395 11650 12394 11653 12395 11655 12398 11658 12400
11658 12403 11655 12404 11653 12401 11651 12404 11648 12402 11645 12398

12:00 PM

11632 12401 11635 12401 11638 12400 11638 12403 11640 12405 11642 12408
11644 12405 11643 12402 11646 12404 11645 12407 11644 12410 11641 12411
11637 12409 11635 12408 11633 12406 11632 12401 16 P
11673 12458 11670 12457 11670 12454 11671 12450 11674 12449 11677 12449
11680 12449 11684 12449 11685 12452 11688 12452 11691 12454 11693 12451
11696 12451 11699 12449 11701 12452 11699 12455 11695 12456 11693 12457
11689 12458 11687 12461 11684 12458 11681 12459 11680 12456 11677 12456
11673 12458 25 P
11677 12417 11676 12420 11673 12423 11671 12423 11667 12422 11666 12419
11667 12416 11671 12416 11673 12414 11674 12411 11678 12411 11678 12414
11680 12417 11679 12420 11677 12417 15 P
11755 12411 11758 12409 11761 12409 11759 12412 11760 12415 11761 12418
11762 12421 11760 12423 11758 12421 11757 12418 11756 12415 11755 12411

12:00 PM

11380 12597 11383 12593 11385 12590 11389 12587 11393 12587 11392 12591
11392 12596 11396 12598 11398 12601 11397 12606 11402 12607 11405 12610
11406 12614 11406 12618 11409 12621 11413 12623 11412 12627 11408 12625
11405 12621 11401 12621 11399 12625 11403 12627 11399 12628 11395 12627
11391 12627 11388 12623 11389 12619 11385 12615 11385 12611 11384 12607
11381 12604 11384 12600 11380 12597 33 P
11405 12644 11408 12641 11411 12640 11412 12637 11414 12639 11414 12643
11415 12646 11418 12645 11419 12648 11416 12649 11413 12651 11410 12652
11411 12649 11408 12647 11405 12644 15 P
11286 12604 11289 12600 11291 12604 11293 12608 11297 12608 11299 12612
11301 12617 11302 12621 11300 12625 11296 12627 11295 12622 11293 12619
11292 12614 11288 12612 11286 12608 11286 12604 16 P
11343 12475 11345 12472 11346 12469 11348 12472 11347 12475 11343 12475

6:00 PM

11343 12478 11346 12478 11349 12480 11351 12483 11350 12486 11347 12486
11346 12483 11343 12478 8 P
11346 12461 11348 12458 11349 12456 11351 12453 11353 12450 11355 12451
11359 12450 11361 12452 11360 12455 11357 12456 11354 12458 11352 12456
11351 12458 11346 12461 14 P
11368 12612 11371 12610 11374 12611 11376 12613 11376 12616 11373 12617
11370 12615 11368 12612 8 P
11377 12618 11380 12618 11381 12621 11384 12622 11381 12622 11377 12618
6:00 PM
11400 12648 11403 12647 11404 12650 11403 12653 11399 12652 11400 12648
6:00 PM
11569 12601 11573 12600 11575 12597 11579 12597 11577 12593 11573 12592
11576 12588 11580 12590 11584 12592 11588 12591 11591 12594 11595 12595
11595 12599 11593 12603 11593 12607 11589 12609 11586 12605 11583 12604
11579 12600 11575 12601 11569 12601 21 P
11653 12656 11649 12658 11646 12661 11642 12661 11640 12664 11638 12669
11635 12672 11633 12669 11632 12664 11629 12662 11631 12658 11630 12654
11629 12658 11625 12660 11620 12658 11616 12656 11615 12652 11615 12648
11615 12643 11616 12639 11619 12636 11622 12639 11625 12634 11628 12632
11631 12633 11635 12629 11638 12628 11643 12629 11646 12632 11646 12636
11650 12639 11652 12642 11654 12646 11658 12646 11659 12650 11660 12654
11653 12656 37 P
11649 12694 11645 12693 11647 12697 11649 12701 11650 12705 11648 12710
11644 12711 11640 12710 11636 12708 11635 12704 11633 12700 11628 12700
11628 12696 11623 12694 11620 12692 11615 12692 11611 12691 11608 12695
11604 12694 11604 12690 11601 12686 11603 12682 11601 12678 11601 12674
11600 12670 11604 12667 11607 12663 11609 12660 11614 12658 11618 12660
11622 12661 11626 12664 11624 12668 11628 12669 11626 12672 11625 12676
11621 12678 11620 12683 11624 12684 11628 12686 11633 12686 11636 12689
11637 12684 11642 12685 11645 12686 11650 12688 11649 12694 47 P
11412 12598 11415 12598 11418 12598 11420 12601 11423 12603 11423 12606
11420 12607 11417 12607 11416 12604 11413 12602 11412 12598 11 P
11394 12658 11396 12656 11399 12656 11398 12658 11394 12658 5 P
11600 12602 11601 12599 11605 12598 11607 12596 11609 12593 11608 12596
11607 12599 11604 12600 11600 12602 9 P
11598 12602 11599 12599 11598 12596 11601 12598 11598 12602 5 P
11633 12615 11635 12613 11637 12611 11641 12612 11643 12611 11645 12608
11648 12610 11648 12613 11647 12616 11643 12618 11642 12621 11639 12622
11637 12619 11634 12618 11633 12615 15 P
11657 12617 11654 12615 11654 12612 11657 12610 11654 12608 11652 12605
11655 12603 11657 12606 11657 12603 11659 12605 11663 12606 11663 12609
11662 12612 11660 12615 11657 12617 15 P
11663 12603 11664 12600 11667 12599 11671 12598 11669 12595 11667 12593

11667 12590 11669 12586 11669 12583 11671 12580 11674 12581 11674 12584
11676 12587 11679 12585 11682 12585 11685 12584 11687 12586 11688 12590
11689 12593 11687 12596 11685 12597 11684 12600 11684 12604 11681 12603
11678 12603 11676 12605 11674 12608 11676 12611 11676 12614 11676 12618
11672 12619 11670 12620 11666 12619 11663 12618 11662 12615 11664 12612
11665 12610 11664 12606 11663 12603 39 P
11674 12565 11678 12564 11683 12564 11687 12564 11691 12565 11693 12569
11698 12570 11701 12573 11700 12578 11704 12579 11706 12583 11704 12586
11706 12590 11707 12594 11710 12598 11706 12600 11702 12603 11705 12606
11701 12608 11697 12610 11693 12611 11692 12615 11689 12618 11685 12618
11680 12618 11680 12614 11683 12611 11687 12609 11689 12605 11692 12601
11692 12597 11695 12595 11700 12594 11696 12592 11694 12588 11695 12584
11692 12580 11688 12580 11684 12583 11680 12582 11677 12579 11673 12577
11677 12575 11679 12578 11684 12577 11688 12577 11687 12573 11683 12572
11680 12568 11677 12570 50 P
11677 12570 11674 12565 2 P
11640 12594 11643 12594 11647 12595 11647 12592 11646 12589 11646 12585
11649 12586 11649 12589 11649 12592 11649 12596 11651 12598 11648 12598
11645 12597 11642 12597 11640 12594 15 P
11708 12629 11713 12629 11715 12626 11714 12621 11715 12618 11714 12613
11718 12613 11721 12616 11725 12617 11727 12620 11731 12621 11735 12619
11738 12622 11741 12625 11746 12626 11748 12629 11743 12631 11739 12632
11740 12635 11738 12639 11735 12642 11731 12639 11729 12635 11725 12635
11728 12639 11728 12643 11725 12646 11721 12644 11718 12641 11714 12639
11713 12635 11709 12634 11708 12629 33 P
11678 12693 11682 12692 11686 12689 11688 12685 11690 12681 11694 12682
11698 12679 11697 12675 11697 12671 11701 12671 11705 12668 11708 12671
11710 12675 11715 12675 11716 12671 11720 12668 11719 12664 11723 12662
11727 12662 11731 12662 11733 12667 11732 12671 11733 12675 11731 12679
11728 12682 11730 12686 11727 12688 11724 12691 11720 12693 11717 12690
11713 12691 11709 12693 11707 12697 11703 12698 11699 12696 11695 12697
11691 12699 11689 12695 11685 12695 11678 12693 40 P
11685 12706 11689 12705 11693 12704 11696 12702 11701 12703 11705 12704
11708 12700 11710 12697 11713 12693 11717 12695 11721 12697 11723 12700
11722 12705 11720 12709 11724 12712 11722 12715 11717 12715 11717 12720
11713 12722 11711 12718 11713 12714 11709 12714 11705 12712 11701 12711
11697 12710 11693 12708 11685 12706 27 P
11672 12738 11674 12735 11677 12731 11680 12728 11681 12724 11685 12721
11688 12720 11691 12716 11690 12711 11694 12711 11698 12713 11701 12717
11702 12721 11700 12725 11699 12729 11701 12733 11705 12732 11709 12729
11709 12734 11713 12736 11713 12741 11709 12743 11706 12746 11709 12747
11714 12748 11714 12753 11709 12752 11710 12756 11707 12760 11703 12756
11700 12759 11695 12759 11692 12756 11694 12753 11696 12749 11699 12745

11696 12741 11692 12741 11688 12743 11685 12746 11681 12744 11679 12740
11672 12738 43 P
11643 12757 11641 12753 11639 12749 11635 12748 11630 12748 11634 12747
11633 12743 11637 12742 11641 12741 11644 12744 11648 12742 11652 12744
11655 12748 11655 12752 11655 12748 11655 12743 11659 12742 11660 12738
11664 12735 11667 12738 11670 12741 11674 12743 11676 12747 11681 12747
11680 12752 11679 12756 11684 12754 11687 12756 11688 12760 11685 12763
11681 12764 11682 12768 11686 12771 11682 12774 11683 12778 11678 12778
11676 12782 11673 12785 11669 12786 11667 12782 11669 12777 11668 12774
11664 12771 11660 12775 11657 12771 11654 12769 11650 12771 11648 12768
11647 12763 11643 12757 50 P
11677 12715 11679 12712 11681 12710 11684 12711 11682 12713 11681 12717
11677 12715 7 P
11665 12724 11667 12721 11669 12719 11672 12719 11674 12716 11677 12717
11677 12720 11674 12722 11671 12724 11669 12726 11665 12724 11 P
11657 12680 11658 12677 11658 12674 11659 12670 11662 12668 11665 12668
11666 12671 11665 12674 11663 12677 11663 12680 11657 12680 11 P
11726 12651 11729 12650 11732 12650 11736 12651 11737 12654 11739 12651
11742 12654 11743 12657 11740 12659 11742 12662 11744 12664 11741 12665
11738 12664 11735 12662 11734 12660 11734 12656 11730 12657 11727 12656
11726 12651 19 P
11695 12660 11698 12658 11701 12658 11703 12661 11704 12664 11702 12665
11699 12665 11695 12660 8 P
11683 12676 11686 12676 11688 12678 11688 12682 11686 12684 11685 12681
11683 12676 7 P
11668 12686 11671 12688 11674 12689 11676 12691 11676 12695 11673 12693
11671 12690 11668 12686 8 P
11683 12644 11684 12642 11687 12643 11687 12647 11690 12649 11691 12652
11687 12651 11685 12649 11683 12644 9 P
11676 12632 11677 12629 11677 12626 11679 12628 11679 12632 11678 12635
11678 12638 11675 12636 11676 12632 9 P
11679 12536 11681 12534 11684 12531 11683 12528 11685 12525 11687 12527
11690 12529 11690 12533 11693 12534 11695 12536 11695 12540 11693 12537
11692 12541 11694 12542 11692 12544 11690 12547 11687 12545 11685 12542
11684 12539 11683 12536 11679 12536 21 P
11797 12487 11800 12485 11803 12485 11802 12488 11805 12487 11808 12489
11810 12491 11810 12494 11808 12497 11805 12495 11808 12498 11807 12501
11803 12500 11801 12498 11798 12496 11799 12493 11797 12487 17 P
11641 12470 11644 12470 11646 12473 11647 12476 11643 12476 11642 12473
11641 12470 7 P
11654 12470 11657 12470 11658 12473 11659 12476 11657 12478 11654 12476
11654 12470 7 P
11652 12437 11656 12437 11658 12439 11658 12442 11655 12441 11652 12437

6:00 PM
11659 12435 11662 12433 11665 12432 11668 12433 11666 12435 11664 12437
11659 12435 7 P
11659 12444 11660 12441 11662 12438 11664 12439 11665 12442 11664 12445
11659 12444 7 P
11648 12457 11651 12457 11654 12457 11654 12461 11651 12463 11649 12460
11648 12457 7 P
11678 12466 11681 12465 11679 12463 11681 12461 11684 12461 11684 12464
11683 12467 11681 12470 11678 12466 9 P
11674 12446 11675 12443 11677 12440 11679 12442 11683 12442 11683 12446
11679 12446 11674 12446 8 P
11611 12383 11613 12381 11616 12379 11619 12378 11619 12381 11616 12384
11611 12383 7 P
11654 12356 11656 12353 11657 12350 11658 12346 11659 12349 11658 12352
11657 12355 11656 12358 11654 12356 9 P
11746 12745 11751 12745 11755 12744 11759 12742 11764 12742 11766 12746
11768 12750 11772 12751 11776 12750 11780 12752 11779 12756 11783 12754
11785 12750 11790 12750 11794 12750 11796 12746 11800 12745 11797 12741
11795 12738 11796 12734 11801 12733 11803 12737 11807 12735 11810 12739
11809 12743 11811 12747 11811 12752 11810 12756 11806 12757 11803 12760
11799 12759 11794 12759 11790 12758 11786 12759 11788 12763 11784 12765
11788 12768 11789 12772 11793 12770 11794 12775 11798 12775 11800 12778
11795 12779 11793 12783 11789 12784 11785 12783 11780 12783 11779 12787
11774 12787 11770 12786 50 P
11770 12786 11767 12782 11770 12779 11774 12776 11777 12774 11778 12770
11775 12767 11774 12763 11771 12763 11771 12759 11767 12756 11765 12760
11763 12756 11761 12752 11757 12752 11753 12752 11749 12754 11747 12750
11746 12745 19 P
11756 12811 11759 12808 11762 12805 11764 12801 11766 12797 11770 12795
11774 12796 11773 12800 11771 12805 11768 12808 11765 12811 11761 12813
11756 12811 13 P
11739 12783 11743 12781 11747 12779 11750 12782 11754 12783 11753 12787
11754 12791 11750 12793 11746 12793 11742 12793 11740 12789 11739 12783
12:00 PM
11649 12854 11654 12854 11658 12854 11659 12850 11662 12854 11665 12856
11666 12853 11670 12851 11674 12850 11678 12848 11680 12852 11684 12854
11682 12858 11685 12861 11687 12866 11683 12867 11678 12868 11679 12873
11679 12877 11683 12879 11684 12883 11681 12887 11684 12890 11683 12895
11687 12897 11691 12897 11695 12901 11699 12902 11695 12904 11692 12901
11688 12901 11684 12900 11679 12900 11679 12896 11676 12892 11673 12889
11671 12885 11669 12882 11664 12880 11660 12878 11656 12877 11654 12873
11653 12869 11651 12866 11655 12863 11658 12861 11656 12857 11649 12854
48 P

11701 12873 11706 12873 11708 12869 11707 12865 11712 12864 11713 12860
11717 12860 11722 12861 11724 12857 11729 12857 11732 12860 11736 12856
11740 12859 11742 12855 11738 12852 11738 12848 11742 12847 11744 12843
11747 12840 11747 12844 11751 12842 11753 12846 11756 12849 11752 12852
11748 12854 11749 12858 11748 12862 11744 12865 11741 12862 11737 12862
11734 12866 11731 12868 11730 12873 11733 12876 11737 12874 11739 12877
11739 12881 11734 12881 11732 12877 11730 12882 11728 12885 11724 12882
11723 12878 11718 12878 11715 12881 11714 12885 11710 12883 11706 12882
11703 12878 11701 12873 50 P
11691 12849 11695 12849 11695 12845 11700 12844 11703 12845 11707 12842
11709 12839 11711 12842 11711 12847 11712 12843 11714 12839 11717 12842
11717 12846 11715 12850 11714 12854 11709 12855 11705 12854 11701 12855
11696 12854 11691 12849 20 P
11680 12822 11684 12819 11688 12821 11692 12823 11696 12822 11700 12821
11705 12823 11704 12818 11700 12819 11699 12815 11699 12811 11700 12806
11699 12802 11696 12798 11695 12794 11700 12796 11704 12797 11705 12802
11703 12805 11708 12806 11711 12809 11715 12807 11714 12811 11718 12813
11718 12817 11718 12821 11715 12825 11711 12826 11708 12829 11708 12833
11705 12837 11701 12834 11697 12837 11693 12837 11693 12833 11688 12831
11687 12827 11680 12822 38 P
11748 12829 11749 12826 11750 12823 11750 12819 11748 12817 11751 12816
11754 12816 11757 12816 11759 12818 11759 12821 11758 12825 11756 12827
11754 12830 11752 12832 11749 12832 11745 12833 11748 12829 17 P
11728 12809 11730 12807 11731 12804 11734 12802 11736 12805 11735 12808
11735 12811 11735 12814 11736 12818 11732 12817 11729 12816 11729 12812
11728 12809 13 P
11714 13148 11717 13145 11719 13141 11723 13144 11727 13145 11730 13148
11732 13152 11735 13155 11739 13157 11741 13161 11742 13165 11740 13168
11736 13171 11737 13167 11733 13164 11729 13164 11725 13166 11728 13162
11723 13162 11723 13158 11720 13155 11720 13151 11714 13148 23 P
11679 12979 11681 12976 11684 12974 11684 12971 11687 12972 11689 12974
11689 12977 11688 12981 11687 12984 11684 12986 11681 12984 11679 12979
12:00 PM
11621 13005 11623 13003 11625 13001 11628 13001 11631 13001 11633 13003
11636 13006 11635 13009 11633 13012 11630 13012 11628 13015 11626 13013
11622 13014 11622 13010 11626 13010 11628 13008 11625 13006 11621 13005
18 P
11619 12999 11617 12996 11616 12993 11619 12995 11621 12997 11619 12999
6:00 PM
11634 13021 11637 13019 11639 13022 11636 13024 11634 13021 5 P
11684 12933 11687 12933 11691 12933 11692 12936 11694 12939 11691 12939
11689 12937 11687 12935 11684 12933 9 P
11680 12925 11683 12927 11684 12930 11683 12933 11680 12932 11680 12928

11680 12925 7 P
11636 12867 11639 12865 11640 12862 11643 12861 11645 12863 11645 12867
11644 12869 11642 12872 11640 12874 11637 12872 11636 12867 11 P
11650 13172 11653 13171 11656 13171 11656 13168 11659 13169 11658 13172
11656 13174 11654 13175 11651 13174 11650 13172 10 P
11645 13181 11648 13181 11649 13183 11649 13186 11647 13185 11645 13181
6:00 PM
11790 13242 11794 13240 11795 13237 11796 13235 11798 13237 11799 13240
11797 13244 11794 13245 11790 13242 9 P
11867 13274 11869 13272 11872 13272 11875 13274 11876 13277 11876 13280
11874 13282 11871 13282 11869 13280 11867 13274 10 P
11786 13288 11788 13286 11789 13283 11790 13280 11793 13279 11793 13282
11795 13283 11797 13281 11801 13280 11804 13281 11807 13283 11804 13285
11801 13287 11798 13288 11795 13288 11793 13290 11789 13291 11786 13288
18 P
11763 13280 11766 13279 11766 13276 11769 13277 11768 13274 11772 13275
11775 13275 11776 13278 11774 13280 11777 13282 11776 13285 11774 13288
11771 13289 11768 13288 11768 13285 11771 13284 11773 13282 11772 13279
11768 13278 11767 13281 11763 13280 21 P
11775 13271 11778 13269 11781 13269 11783 13271 11786 13273 11787 13276
11785 13279 11783 13276 11782 13273 11779 13273 11781 13275 11778 13277
11777 13274 11775 13271 14 P
11753 13275 11756 13273 11758 13270 11760 13272 11760 13275 11758 13278
11756 13279 11753 13275 8 P
11748 13295 11751 13294 11754 13295 11757 13296 11760 13298 11757 13300
11753 13299 11751 13296 11748 13295 9 P
11768 13302 11772 13302 11775 13301 11778 13301 11776 13304 11773 13303
11768 13302 7 P
11739 13296 11742 13295 11745 13295 11743 13297 11739 13296 5 P
11804 13300 11806 13297 11808 13299 11809 13301 11807 13301 11804 13300
6:00 PM
11814 13357 11817 13356 11819 13355 11821 13357 11819 13360 11817 13359
11814 13357 7 P
11840 13363 11843 13363 11845 13362 11847 13359 11850 13360 11847 13361
11846 13364 11844 13365 11840 13363 9 P
11825 13369 11828 13368 11830 13367 11832 13365 11835 13366 11837 13367
11838 13369 11836 13370 11833 13371 11831 13371 11828 13371 11825 13369
12:00 PM
11816 13407 11818 13406 11821 13406 11824 13405 11825 13407 11824 13410
11824 13412 11821 13412 11819 13410 11816 13407 10 P
11765 13427 11767 13424 11769 13423 11772 13423 11774 13422 11773 13425
11772 13427 11770 13429 11767 13429 11765 13427 10 P
11561 13429 11562 13427 11563 13424 11565 13422 11568 13421 11570 13422

11569 13424 11572 13425 11573 13422 11575 13424 11575 13427 11572 13428
11570 13429 11567 13430 11564 13430 11561 13429 16 P
11580 13483 11582 13481 11584 13479 11586 13478 11586 13480 11585 13483
11580 13483 7 P
11599 13485 11602 13485 11604 13486 11607 13487 11609 13488 11612 13488
11612 13491 11609 13493 11607 13492 11605 13493 11602 13492 11601 13489
11599 13485 13 P
11550 13492 11550 13489 11551 13486 11554 13485 11556 13484 11556 13486
11557 13489 11555 13491 11554 13488 11551 13490 11550 13492 11 P
11713 13556 11715 13556 11717 13557 11720 13556 11722 13558 11724 13559
11724 13562 11722 13564 11721 13566 11722 13569 11720 13571 11717 13572
11717 13569 11716 13566 11715 13564 11713 13562 11713 13559 11713 13556
18 P
11729 13581 11730 13579 11732 13578 11735 13580 11737 13580 11740 13578
11743 13579 11745 13578 11748 13578 11751 13578 11753 13578 11756 13576
11758 13576 11761 13576 11763 13578 11763 13580 11760 13579 11757 13579
11755 13580 11752 13581 11753 13584 11752 13587 11751 13588 11750 13591
11747 13591 11744 13591 11742 13589 11741 13587 11743 13584 11740 13584
11738 13586 11735 13587 11732 13587 11730 13587 11729 13581 35 P
11676 13534 11677 13531 11678 13529 11680 13531 11682 13533 11682 13536
11684 13538 11681 13539 11679 13539 11678 13536 11678 13534 11676 13534

12:00 PM

11681 13521 11681 13519 11684 13519 11685 13521 11686 13523 11686 13526
11683 13526 11681 13524 11681 13521 9 P
11567 13729 11569 13727 11571 13728 11572 13730 11575 13730 11578 13730
11580 13731 11581 13734 11583 13735 11582 13738 11582 13741 11580 13742
11580 13739 11579 13736 11577 13735 11574 13735 11571 13734 11570 13732
11567 13729 19 P
11553 14028 11557 14031 11558 14035 11560 14032 11564 14030 11565 14026
11569 14025 11573 14026 11578 14027 11579 14032 11583 14034 11586 14037
11583 14040 11579 14041 11575 14044 11571 14046 11567 14045 11565 14049
11562 14051 11561 14055 11557 14058 11558 14054 11556 14050 11555 14046
11554 14041 11555 14038 11555 14033 11553 14028 28 P
11585 14033 11586 14030 11586 14027 11589 14027 11591 14027 11594 14026
11595 14023 11595 14020 11597 14022 11598 14024 11600 14026 11598 14028
11595 14027 11594 14030 11593 14032 11591 14034 11589 14036 11585 14033
18 P
11699 14067 11701 14063 11702 14059 11703 14054 11704 14050 11707 14053
11710 14056 11713 14052 11712 14048 11716 14049 11718 14053 11721 14057
11723 14061 11724 14065 11729 14066 11728 14070 11727 14074 11726 14078
11722 14080 11718 14080 11714 14080 11709 14080 11708 14075 11705 14073
11700 14073 11699 14067 26 P
11747 14034 11750 14037 11753 14041 11757 14039 11759 14035 11763 14038

11763 14042 11765 14046 11765 14051 11770 14049 11774 14049 11774 14054
11778 14056 11782 14058 11783 14061 11782 14066 11779 14069 11779 14074
11775 14075 11771 14075 11766 14076 11763 14079 11759 14078 11759 14082
11761 14086 11761 14090 11759 14087 11757 14083 11753 14081 11750 14084
11745 14085 11748 14081 11751 14078 11754 14075 11755 14071 11757 14067
11757 14062 11758 14058 11753 14056 11750 14054 11751 14050 11756 14051
11753 14047 11752 14042 11749 14039 11747 14034 46 P
11634 14059 11638 14058 11642 14055 11646 14055 11650 14054 11655 14053
11659 14054 11662 14057 11664 14061 11660 14063 11658 14059 11654 14059
11650 14061 11645 14062 11641 14063 11637 14065 11634 14059 17 P
11644 14012 11645 14009 11648 14007 11651 14006 11655 14005 11658 14005
11657 14009 11655 14011 11651 14012 11648 14013 11644 14012 11 P
11623 14011 11626 14008 11629 14009 11632 14008 11635 14006 11637 14004
11640 14003 11641 14006 11638 14007 11636 14009 11636 14012 11633 14014
11630 14016 11628 14013 11623 14011 15 P
11611 14016 11613 14013 11616 14012 11619 14012 11622 14011 11623 14014
11622 14017 11619 14017 11616 14016 11611 14016 10 P
11555 14005 11557 14003 11559 14000 11562 13999 11565 13999 11568 14002
11566 14005 11568 14007 11564 14007 11563 14010 11560 14011 11557 14010
11555 14005 13 P
11549 13960 11552 13961 11555 13959 11551 13959 11551 13955 11554 13954
11557 13954 11559 13957 11561 13960 11562 13963 11561 13966 11558 13968
11555 13967 11551 13966 11550 13963 11549 13960 16 P
11498 13944 11500 13941 11503 13939 11503 13942 11503 13946 11503 13949
11506 13948 11510 13949 11508 13952 11505 13953 11502 13954 11499 13952
11500 13949 11498 13944 14 P
11527 13920 11524 13920 11524 13924 11521 13926 11518 13927 11516 13924
11518 13921 11521 13919 11523 13917 11525 13914 11528 13916 11531 13918
11533 13919 11530 13919 11527 13920 15 P
11502 14002 11505 14001 11508 14003 11510 14005 11513 14005 11512 14007
11514 14009 11516 14012 11513 14012 11511 14010 11508 14009 11507 14005
11504 14005 11502 14002 14 P
11686 13980 11689 13978 11692 13976 11693 13979 11692 13982 11691 13985
11687 13985 11686 13980 8 P
11705 13957 11707 13954 11708 13951 11708 13948 11711 13947 11714 13948
11717 13949 11720 13950 11718 13954 11717 13957 11715 13959 11713 13956
11710 13957 11708 13960 11705 13957 15 P
11677 14136 11680 14135 11683 14132 11683 14129 11683 14126 11685 14123
11688 14122 11691 14123 11694 14124 11695 14127 11694 14131 11697 14132
11695 14134 11692 14136 11690 14139 11688 14141 11685 14143 11683 14145
11680 14146 11679 14143 11678 14139 11677 14136 22 P
11656 14172 11658 14169 11661 14168 11662 14165 11663 14162 11665 14160
11667 14158 11670 14160 11672 14163 11674 14164 11677 14161 11677 14158

11679 14160 11682 14158 11684 14156 11686 14153 11687 14156 11689 14158
11690 14161 11689 14165 11688 14167 11685 14168 11683 14170 11680 14173
11677 14174 11674 14174 11673 14171 11671 14169 11667 14170 11665 14173
11663 14175 11661 14177 11658 14177 11656 14175 11656 14172 35 P
11709 14186 11710 14181 11714 14179 11718 14180 11720 14184 11725 14184
11728 14187 11731 14189 11736 14188 11740 14189 11744 14190 11745 14195
11748 14198 11748 14202 11745 14205 11743 14209 11738 14211 11735 14212
11730 14212 11726 14214 11722 14215 11718 14218 11715 14216 11713 14212
11713 14208 11713 14203 11711 14199 11712 14195 11710 14191 11709 14186
30 P
11691 14194 11694 14191 11698 14189 11699 14185 11699 14181 11703 14180
11702 14184 11705 14188 11707 14192 11704 14195 11700 14195 11695 14195
11691 14194 13 P
11685 14205 11687 14204 11690 14206 11693 14207 11696 14205 11699 14203
11702 14203 11702 14207 11701 14210 11702 14213 11702 14216 11700 14218
11697 14220 11694 14219 11693 14216 11691 14214 11688 14212 11686 14210
11685 14205 19 P
11707 14170 11709 14168 11712 14168 11714 14171 11714 14174 11712 14176
11708 14175 11707 14170 8 P
11630 14222 11632 14219 11634 14217 11637 14215 11639 14212 11640 14209
11640 14206 11643 14205 11647 14205 11650 14207 11652 14208 11656 14208
11659 14208 11662 14208 11664 14210 11664 14214 11660 14213 11657 14212
11654 14211 11651 14211 11648 14212 11645 14214 11644 14217 11641 14218
11638 14219 11635 14221 11630 14222 27 P
11678 14208 11681 14207 11684 14210 11684 14213 11684 14216 11686 14218
11684 14221 11680 14219 11680 14216 11680 14213 11678 14208 11 P
11672 14211 11673 14209 11675 14211 11677 14214 11677 14216 11674 14215
11672 14211 7 P
11635 14349 11638 14343 11640 14336 11640 14329 11640 14323 11641 14316
11640 14309 11643 14303 11649 14302 11656 14301 11658 14294 11663 14290
11666 14283 11668 14277 11665 14272 11658 14269 11652 14267 11654 14260
11654 14253 11660 14251 11666 14248 11673 14250 11680 14248 11686 14247
11693 14245 11693 14252 11694 14259 11700 14256 11705 14252 11712 14251
11717 14256 11722 14261 11723 14267 11721 14274 11718 14280 11720 14287
11715 14291 11714 14298 11715 14304 11713 14311 11710 14317 11710 14324
11713 14330 11710 14336 11706 14342 11705 14349 11700 14353 11693 14356
11687 14359 11682 14363 50 P
11682 14363 11676 14367 11671 14372 11665 14373 11659 14369 11652 14369
11650 14363 11647 14357 11641 14353 11635 14349 10 P
11605 14302 11608 14299 11612 14297 11615 14294 11619 14292 11623 14291
11627 14289 11630 14287 11634 14283 11634 14280 11632 14275 11635 14273
11638 14269 11641 14273 11644 14275 11647 14279 11650 14281 11653 14285
11653 14289 11652 14294 11648 14296 11644 14298 11640 14298 11636 14299

11633 14302 11629 14304 11626 14307 11626 14311 11626 14316 11627 14320
11629 14324 11627 14327 11622 14326 11619 14324 11615 14323 11612 14320
11612 14316 11612 14311 11612 14307 11613 14303 11605 14302 41 P
11573 14331 11577 14329 11581 14326 11585 14325 11589 14325 11591 14330
11595 14331 11599 14331 11602 14334 11607 14335 11610 14332 11614 14334
11618 14336 11621 14338 11623 14342 11626 14346 11627 14350 11626 14354
11623 14358 11620 14355 11616 14354 11612 14353 11607 14353 11604 14356
11600 14358 11596 14360 11592 14359 11587 14358 11584 14356 11581 14353
11580 14349 11579 14344 11580 14340 11578 14337 11573 14331 35 P
11589 14268 11591 14266 11592 14263 11594 14261 11597 14259 11600 14257
11604 14257 11606 14256 11605 14259 11602 14260 11601 14264 11602 14267
11602 14270 11599 14270 11596 14269 11593 14270 11589 14268 17 P
11558 14278 11561 14276 11563 14274 11566 14275 11569 14276 11572 14274
11574 14272 11577 14270 11580 14269 11582 14272 11582 14275 11579 14277
11577 14280 11576 14282 11575 14286 11572 14285 11570 14282 11568 14280
11564 14280 11561 14281 11558 14278 21 P
11543 14283 11545 14280 11548 14281 11550 14280 11553 14281 11551 14284
11549 14285 11547 14286 11543 14283 9 P
11599 14310 11602 14309 11604 14312 11602 14315 11601 14316 11599 14314
11599 14310 7 P
11583 14305 11585 14304 11587 14303 11590 14304 11593 14304 11595 14305
11593 14307 11591 14307 11588 14307 11585 14307 11583 14305 11 P
10884 14376 10886 14372 10889 14370 10892 14367 10893 14363 10895 14359
10897 14355 10900 14352 10904 14352 10909 14352 10913 14351 10917 14352
10920 14350 10924 14351 10925 14356 10927 14359 10925 14363 10920 14364
10919 14368 10920 14372 10925 14373 10928 14372 10927 14367 10931 14368
10934 14371 10939 14371 10936 14374 10940 14374 10938 14378 10936 14381
10934 14385 10931 14387 10926 14387 10922 14387 10918 14385 10913 14385
10909 14383 10905 14381 10902 14380 10898 14377 10894 14379 10890 14381
10884 14376 43 P
10913 14298 10915 14296 10919 14295 10922 14295 10922 14292 10921 14288
10924 14290 10927 14292 10930 14290 10933 14292 10935 14294 10938 14296
10938 14300 10935 14302 10935 14305 10938 14308 10940 14310 10939 14312
10936 14314 10933 14313 10930 14315 10929 14312 10927 14309 10924 14308
10923 14305 10921 14303 10920 14300 10918 14298 10913 14298 29 P
10943 14267 10946 14268 10948 14271 10951 14272 10954 14274 10957 14273
10960 14272 10960 14275 10961 14278 10958 14279 10955 14278 10952 14277
10949 14278 10948 14274 10945 14273 10943 14267 16 P
11056 14306 11056 14303 11058 14302 11062 14302 11064 14301 11068 14301
11069 14304 11071 14307 11072 14310 11074 14309 11076 14311 11076 14315
11077 14318 11077 14321 11073 14320 11070 14321 11067 14321 11064 14319
11066 14317 11070 14316 11069 14313 11069 14310 11066 14310 11063 14311
11059 14312 11059 14309 11056 14306 27 P

11115 14302 11119 14302 11122 14301 11122 14297 11121 14295 11123 14296
11125 14299 11128 14302 11130 14303 11133 14305 11135 14307 11135 14310
11138 14309 11141 14311 11139 14314 11138 14317 11135 14315 11132 14315
11129 14314 11126 14312 11125 14309 11122 14308 11120 14305 11115 14302
24 P
11152 14253 11155 14251 11157 14249 11160 14250 11163 14252 11166 14252
11169 14253 11172 14255 11174 14258 11175 14261 11178 14263 11180 14265
11182 14268 11185 14269 11188 14271 11191 14273 11192 14275 11194 14279
11196 14281 11198 14283 11199 14286 11201 14289 11203 14292 11204 14294
11201 14293 11199 14294 11195 14292 11193 14290 11191 14288 11188 14286
11185 14287 11183 14284 11180 14283 11177 14281 11178 14278 11180 14275
11180 14272 11180 14268 11178 14271 11178 14274 11175 14276 11173 14278
11170 14276 11167 14275 11169 14273 11168 14269 11166 14268 11164 14265
11163 14262 11160 14260 50 P
11160 14260 11158 14258 11155 14257 11152 14253 4 P
11353 14257 11355 14255 11357 14252 11356 14249 11357 14246 11360 14245
11362 14247 11365 14250 11367 14252 11368 14255 11370 14258 11370 14261
11367 14261 11363 14261 11360 14260 11357 14260 11354 14260 11353 14257
18 P
11437 14319 11439 14316 11442 14317 11445 14318 11448 14318 11450 14321
11452 14324 11454 14326 11450 14325 11448 14324 11446 14321 11443 14323
11441 14321 11437 14319 14 P
11381 14329 11384 14328 11387 14328 11389 14331 11388 14334 11388 14337
11385 14335 11384 14332 11381 14329 9 P
11150 14281 11153 14281 11156 14280 11159 14278 11160 14275 11163 14276
11161 14279 11163 14281 11160 14283 11157 14285 11154 14283 11150 14281

12:00 PM

10926 14680 10929 14678 10933 14677 10937 14677 10934 14681 10936 14685
10940 14682 10944 14683 10947 14685 10949 14682 10954 14680 10957 14678
10961 14676 10965 14674 10969 14673 10969 14678 10972 14675 10977 14674
10979 14678 10977 14681 10975 14686 10972 14689 10969 14691 10969 14695
10965 14695 10961 14695 10957 14698 10953 14700 10949 14701 10946 14704
10944 14700 10941 14697 10940 14693 10935 14692 10931 14691 10931 14687
10932 14683 10926 14680 38 P
11016 14764 11015 14768 11013 14772 11012 14768 11012 14763 11010 14759
11012 14756 11016 14756 11016 14751 11012 14754 11009 14751 11008 14747
11011 14743 11014 14747 11018 14749 11021 14751 11023 14755 11023 14759
11026 14763 11026 14768 11023 14763 11016 14764 22 P
10901 15209 10905 15209 10909 15212 10912 15215 10916 15215 10920 15217
10923 15220 10925 15224 10929 15226 10932 15229 10933 15233 10937 15234
10936 15238 10940 15239 10944 15237 10947 15240 10949 15244 10948 15248
10944 15248 10940 15247 10942 15251 10939 15252 10942 15255 10938 15256
10940 15260 10936 15262 10935 15266 10939 15269 10943 15268 10942 15273

10939 15275 10935 15277 10931 15277 10927 15279 10924 15275 10920 15274
10924 15271 10922 15267 10919 15265 10921 15262 10922 15258 10919 15255
10915 15253 10913 15249 10909 15249 10906 15247 10903 15243 10902 15239
10906 15241 10910 15242 50 P
10910 15242 10914 15243 10918 15241 10915 15238 10911 15237 10913 15232
10917 15232 10918 15227 10916 15223 10912 15223 10907 15224 10907 15219
10906 15215 10901 15209 14 P
10806 15224 10810 15224 10812 15228 10815 15231 10819 15230 10823 15226
10825 15230 10825 15234 10829 15233 10830 15229 10834 15227 10838 15226
10842 15228 10845 15231 10846 15235 10849 15239 10850 15243 10846 15245
10842 15246 10838 15247 10837 15244 10832 15244 10828 15244 10825 15241
10821 15243 10818 15241 10818 15237 10816 15233 10811 15233 10809 15230
10806 15224 31 P
10845 15210 10847 15207 10851 15210 10855 15211 10855 15216 10857 15219
10854 15222 10856 15226 10852 15228 10848 15226 10846 15223 10844 15219
10846 15215 10845 15210 14 P
10809 15171 10812 15170 10811 15167 10814 15165 10817 15166 10820 15166
10821 15169 10822 15173 10819 15175 10818 15177 10818 15181 10815 15179
10813 15177 10810 15175 10809 15171 15 P
10818 15186 10821 15184 10824 15184 10823 15187 10824 15190 10821 15192
10819 15190 10818 15186 8 P
10817 15198 10820 15197 10822 15199 10824 15201 10822 15203 10820 15204
10818 15202 10817 15198 8 P
9401 15141 9403 15134 9403 15127 9409 15125 9415 15123 9422 15125
9428 15124 9432 15119 9435 15113 9438 15118 9444 15122 9448 15128
9453 15132 9460 15132 9466 15133 9472 15129 9478 15126 9485 15125
9491 15127 9495 15133 9500 15138 9507 15139 9510 15145 9514 15151
9520 15149 9526 15153 9529 15159 9530 15166 9529 15172 9530 15179
9526 15184 9524 15191 9518 15193 9512 15196 9506 15199 9500 15202
9493 15202 9497 15197 9498 15190 9494 15184 9488 15182 9481 15182
9477 15176 9471 15174 9464 15174 9457 15174 9451 15173 9444 15170
9437 15170 9431 15174 50 P
9431 15174 9429 15167 9430 15161 9424 15157 9419 15153 9416 15147
9412 15142 9401 15141 8 P
9499 15099 9496 15105 9489 15107 9484 15109 9477 15111 9470 15110
9465 15105 9461 15111 9456 15115 9449 15113 9445 15108 9442 15102
9442 15095 9437 15090 9431 15089 9424 15089 9422 15082 9424 15076
9417 15075 9421 15069 9420 15062 9415 15057 9421 15054 9428 15052
9432 15057 9434 15063 9439 15068 9443 15073 9448 15078 9449 15085
9455 15089 9461 15089 9468 15089 9470 15082 9465 15077 9461 15072
9457 15067 9454 15061 9451 15055 9457 15053 9464 15051 9471 15050
9475 15046 9482 15047 9488 15050 9489 15057 9493 15062 9495 15069
9495 15075 9492 15082 50 P

9492 15082 9487 15085 9481 15089 9488 15088 9494 15088 9501 15090
9499 15099 7 P
9865 14967 9869 14961 9868 14955 9873 14950 9878 14946 9884 14949
9886 14956 9888 14962 9893 14958 9900 14956 9899 14963 9897 14970
9894 14976 9897 14982 9903 14984 9897 14985 9892 14980 9885 14978
9878 14978 9876 14971 9865 14967 21 P
10216 14929 10222 14929 10226 14928 10229 14922 10224 14925 10220 14921
10220 14915 10220 14910 10222 14905 10226 14902 10230 14906 10233 14911
10238 14913 10242 14917 10242 14922 10239 14926 10236 14931 10233 14936
10233 14941 10231 14936 10226 14933 10221 14934 10216 14929 23 P
10035 14900 10037 14897 10041 14894 10037 14892 10041 14889 10044 14886
10048 14886 10052 14885 10057 14883 10060 14885 10059 14890 10055 14891
10054 14895 10051 14898 10047 14896 10044 14899 10043 14904 10039 14903
10035 14900 19 P
10141 15185 10138 15182 10141 15178 10140 15174 10137 15177 10133 15177
10133 15182 10129 15183 10126 15180 10125 15176 10123 15172 10119 15169
10118 15165 10122 15164 10126 15164 10129 15160 10131 15156 10133 15153
10137 15153 10141 15153 10144 15157 10147 15160 10150 15164 10151 15168
10154 15171 10154 15175 10152 15178 10148 15181 10141 15185 29 P
9938 15033 9941 15030 9945 15028 9949 15027 9954 15026 9957 15024
9960 15022 9965 15021 9969 15020 9973 15018 9973 15014 9976 15011
9978 15015 9977 15019 9981 15020 9986 15020 9990 15019 9994 15018
9999 15019 10003 15018 10007 15017 10010 15014 10014 15017 10016 15020
10020 15022 10016 15022 10012 15024 10007 15025 10003 15026 10001 15030
9997 15031 9994 15028 9991 15026 9986 15025 9982 15025 9978 15026
9973 15026 9970 15029 9967 15033 9967 15037 9964 15034 9962 15031
9959 15033 9959 15037 9955 15037 9951 15034 9948 15037 9945 15034
9938 15033 49 P
9995 15001 9998 14998 10002 14998 10006 15001 10010 15000 10014 15002
10018 15002 10022 15003 10027 15002 10026 14997 10024 14993 10023 14989
10023 14985 10025 14988 10029 14991 10032 14993 10036 14993 10036 14998
10041 14999 10044 15001 10048 15003 10045 15005 10042 15009 10038 15011
10041 15014 10037 15012 10035 15008 10031 15010 10027 15010 10022 15009
10018 15007 10014 15006 10010 15006 10006 15006 10001 15005 9995 15001
36 P
10049 15004 10052 15003 10055 15003 10058 15005 10055 15006 10052 15005
10049 15004 7 P
10055 15000 10058 14999 10060 14997 10063 14999 10066 15000 10069 14999
10072 15001 10069 15003 10066 15003 10063 15002 10060 15003 10055 15000

12:00 PM

10166 15128 10168 15124 10171 15121 10175 15120 10179 15118 10180 15122
10180 15126 10184 15126 10181 15130 10177 15131 10173 15131 10169 15133
10166 15128 13 P

10151 15028 10149 15025 10146 15024 10147 15021 10151 15020 10153 15021
10155 15025 10151 15028 8 P
9938 14997 9941 14995 9942 14992 9945 14993 9948 14992 9951 14993
9955 14993 9958 14993 9960 14996 9962 14998 9959 14999 9956 14999
9952 14999 9949 15000 9946 15000 9943 15000 9938 14997 17 P
9979 14988 9981 14985 9984 14988 9987 14988 9989 14991 9989 14994
9987 14997 9986 14994 9983 14995 9983 14991 9979 14988 11 P
10065 14920 10067 14918 10070 14917 10073 14918 10076 14918 10079 14917
10081 14919 10079 14921 10076 14922 10073 14921 10070 14921 10065 14920
12:00 PM
10095 14917 10097 14914 10100 14912 10101 14909 10103 14907 10105 14911
10107 14913 10104 14915 10101 14915 10095 14917 10 P
10081 14903 10083 14901 10086 14903 10089 14905 10091 14907 10089 14910
10086 14911 10085 14908 10082 14907 10081 14903 10 P
10428 15308 10431 15308 10433 15310 10436 15310 10440 15311 10443 15311
10446 15311 10444 15313 10442 15317 10440 15318 10436 15317 10434 15315
10431 15314 10428 15312 10428 15308 15 P
9907 14991 9910 14991 9913 14989 9915 14991 9919 14991 9919 14995
9918 14998 9915 14998 9912 14996 9910 14994 9907 14991 11 P
9906 15014 9909 15013 9912 15012 9915 15011 9916 15008 9920 15009
9923 15009 9921 15011 9918 15013 9915 15013 9913 15016 9910 15016
9906 15014 13 P
9667 14792 9668 14789 9668 14785 9670 14783 9673 14784 9674 14787
9674 14790 9672 14793 9673 14796 9674 14799 9670 14800 9668 14798
9670 14794 9667 14792 14 P
9195 15639 9198 15636 9202 15638 9205 15641 9209 15643 9213 15644
9217 15644 9218 15649 9218 15653 9218 15657 9214 15659 9210 15662
9208 15658 9204 15658 9203 15653 9204 15649 9200 15649 9197 15646
9195 15639 19 P
8356 14826 8360 14827 8364 14828 8368 14830 8371 14833 8374 14836
8378 14839 8379 14843 8376 14847 8373 14850 8370 14853 8368 14849
8370 14845 8374 14843 8374 14839 8371 14837 8367 14834 8365 14830
8361 14828 8356 14826 20 P
8392 14873 8396 14874 8400 14876 8404 14876 8409 14877 8412 14878
8415 14882 8416 14886 8418 14890 8423 14892 8424 14896 8422 14900
8417 14899 8415 14895 8412 14892 8410 14888 8408 14884 8404 14882
8400 14879 8396 14878 8392 14873 21 P
8474 14926 8470 14925 8467 14922 8462 14920 8459 14918 8455 14917
8454 14913 8451 14910 8447 14907 8444 14905 8440 14902 8437 14900
8436 14896 8433 14892 8430 14890 8426 14889 8430 14887 8433 14889
8438 14890 8441 14891 8446 14893 8449 14896 8452 14899 8455 14902
8457 14906 8460 14909 8464 14912 8466 14915 8469 14918 8473 14921
8474 14926 31 P

8396 14889 8399 14887 8402 14888 8404 14890 8405 14893 8406 14896
8407 14899 8405 14903 8403 14899 8402 14897 8400 14894 8398 14892
8396 14889 13 P
8271 14773 8276 14774 8279 14777 8282 14780 8285 14783 8287 14787
8289 14791 8292 14794 8294 14798 8294 14802 8293 14806 8290 14802
8289 14799 8285 14796 8282 14793 8281 14789 8280 14785 8278 14781
8275 14778 8271 14773 20 P
8292 14820 8291 14817 8294 14816 8297 14818 8299 14820 8300 14823
8301 14826 8298 14825 8295 14823 8292 14820 10 P
8310 14835 8313 14833 8315 14835 8318 14836 8320 14839 8320 14842
8322 14845 8324 14847 8325 14850 8323 14849 8321 14846 8318 14844
8317 14841 8315 14839 8313 14837 8310 14835 16 P
9246 14455 9249 14452 9253 14454 9255 14459 9259 14459 9263 14460
9265 14464 9265 14468 9261 14470 9257 14469 9253 14468 9250 14464
9250 14460 9246 14455 14 P
9224 14384 9227 14381 9231 14383 9235 14382 9239 14382 9242 14386
9239 14389 9234 14390 9230 14390 9231 14394 9229 14399 9227 14402
9224 14399 9224 14395 9223 14390 9224 14384 16 P
9166 14277 9168 14273 9171 14270 9175 14268 9178 14265 9181 14269
9181 14273 9180 14278 9181 14282 9177 14284 9173 14285 9169 14282
9166 14277 13 P
9203 14290 9206 14287 9209 14283 9211 14279 9214 14276 9219 14276
9222 14274 9225 14277 9224 14281 9222 14285 9220 14289 9216 14289
9212 14291 9208 14292 9203 14290 15 P
9220 14248 9224 14246 9228 14246 9231 14243 9233 14240 9237 14237
9239 14241 9238 14245 9237 14250 9239 14253 9237 14257 9234 14261
9232 14265 9231 14268 9227 14271 9224 14268 9222 14264 9222 14260
9224 14256 9224 14252 9220 14248 21 P
9308 14314 9310 14311 9313 14310 9316 14312 9315 14315 9313 14317
9309 14317 9308 14314 8 P
9287 14507 9291 14507 9294 14507 9297 14509 9299 14507 9300 14510
9298 14512 9297 14515 9294 14516 9293 14513 9290 14511 9287 14507
12:00 PM
9286 14496 9288 14494 9290 14491 9292 14489 9294 14486 9295 14489
9293 14492 9296 14494 9293 14494 9293 14498 9290 14499 9286 14496
12:00 PM
9009 13062 9012 13059 9016 13058 9019 13060 9023 13063 9023 13067
9021 13070 9020 13075 9019 13079 9015 13080 9015 13075 9012 13072
9010 13068 9009 13062 14 P
8704 12522 8705 12518 8708 12515 8712 12513 8714 12509 8718 12508
8722 12506 8726 12509 8730 12510 8727 12514 8723 12515 8719 12516
8715 12517 8711 12519 8708 12522 8704 12522 16 P
9537 10895 9541 10890 9546 10886 9551 10881 9556 10876 9561 10871

9565 10865 9570 10860 9576 10864 9582 10860 9589 10858 9594 10853
9599 10848 9604 10844 9610 10839 9615 10834 9619 10829 9625 10825
9631 10822 9637 10819 9644 10816 9647 10810 9653 10807 9660 10807
9667 10804 9672 10800 9678 10797 9685 10795 9691 10791 9697 10790
9704 10787 9709 10783 9716 10780 9721 10776 9727 10772 9734 10770
9740 10768 9746 10764 9752 10761 9759 10758 9765 10758 9772 10759
9779 10757 9785 10755 9792 10753 9799 10751 9805 10748 9811 10745
9817 10741 9824 10739 50 P
9824 10739 9830 10737 9837 10734 9844 10733 9850 10732 9857 10731
9864 10730 9870 10733 9876 10737 9879 10743 9883 10748 9889 10752
9890 10759 9892 10766 9893 10773 9890 10779 9884 10783 9879 10787
9872 10789 9865 10790 9858 10790 9852 10791 9846 10794 9839 10795
[truncated: 563,964 more chars]
