# Supplementary material for: The Effect of Elevated Ozone Concentrations with Varying Shading on Dry Matter Loss in a Winter Wheat-Producing Region in China
Source: PLoS One. 2016 Jan 13;11(1):e0145446. doi: 10.1371/journal.pone.0145446 (PMC4711948; doi:10.1371/journal.pone.0145446)
Supplement: S2 File — (PDF) [file pone.0145446.s012.pdf]

S2 File. Regional loss of dry matter for winter wheat, simulated under elevated ozone concentrations
and reduced solar radiation (from March to May of 2006).

Regional loss of dry matter in T1 (March)

```
%%Pages: (atend)
%%PageOrder: Ascend
%%Creator: NCAR GKS
%%DocumentFonts: (atend)
%%EndComments
%%BeginProlog

/M3
{
  2 copy lt {pop} {exch pop} ifelse
  2 copy lt {pop} {exch pop} ifelse
} bind def
/Sc
{
  1. exch sub /ylo exch def
  1. exch sub /mag exch def
  1. exch sub /cyn exch def
  ylo mag cyn M3 /b_cmy exch def
  cyn b_cmy sub /c_cmy exch def
  mag b_cmy sub /m_cmy exch def
  ylo b_cmy sub /y_cmy exch def
  c_cmy m_cmy y_cmy b_cmy setcmykcolor
} bind def
/P
{/n exch def
  newpath
  moveto
  2 1 n {pop lineto} for
  stroke
 } bind def

/F
{/n exch def
  newpath
  moveto
  2 1 n {pop lineto} for
  closepath
  eofill
} bind def
```

```
/B
{ /s exch def
  /n exch def
  1 1 n {newpath pop s 0 360 arc stroke} for
} bind def

/Q
{ /s exch def
  /n exch def
  /ms 0 s sub def
  /ts s s add def
  1 1 n {gsave newpath pop translate
  0 0 moveto ms 0 rmoveto ts 0 rlineto
  0 0 moveto 90 rotate ms  0 rmoveto ts 0 rlineto
  stroke grestore} for
} bind def

/Fs
{ /s exch def
  findfont
  s scalefont
  setfont
} bind def

/V
{ /s exch def
  /n exch def
  /ms 0 s sub def
  /ts s s add def
  1 1 n {gsave newpath pop translate
  0 0 moveto 30 rotate ms 0 rmoveto  ts 0 rlineto
  0 0 moveto 60 rotate ms 0 rmoveto  ts 0 rlineto
  0 0 moveto 60 rotate ms 0 rmoveto  ts 0 rlineto
  stroke grestore} for
} bind def

/X
{ /s exch def
  /n exch def
  /s s 1.25 mul def
  /ms 0 s sub def
  /ts s s add def
```

```
  1 1 n {gsave newpath pop translate
  0 0 moveto 45 rotate ms 0 rmoveto  ts 0 rlineto
  0 0 moveto 90 rotate ms 0 rmoveto  ts 0 rlineto
  stroke grestore} for
} bind def

/Ls
{matrix currentmatrix
 currentrgbcolor
 currentlinewidth
 currentlinecap
 currentlinejoin
 currentdash
} bind def

/Lr
{setdash
 setlinejoin
 setlinecap
 setlinewidth
 setrgbcolor
 setmatrix
} bind def

/M {stroke newpath moveto} bind def
/L {lineto} bind def
/E {eofill} def
/J {setlinejoin} def
/C {setlinecap} def
/W {setlinewidth} def
/S {stroke showpage} def
/A {scale} def
/U {put} def
/D {setdash} def
/I {currentdash} def
/H {currentlinewidth} def
/G {currentrgbcolor} def
/R {setrgbcolor} def
/N {closepath} def
/K {stroke} def
/Z {rotate} def
/As {ashow} def
/Ch {clippath} def
```

```
/Ci {colorimage} def
/Co {concat} def
/Cm {currentmatrix} def
/Cp {clip} def
/Ct {currentpoint} def
/Cu {currentfile} def
/Ex {exch} def
/Im {image} def
/In {initgraphics} def
/Ml {setmiterlimit} def
/Mx {matrix} def
/Np {newpath} def
/Ng {neg} def
/Rh {readhexstring} def
/Rm {rmoveto} def
/Sm {setmatrix} def
/Tr {translate} def
/Gs {gsave} def
/Gr {grestore} def

/Ov
{/strn exch def
 /ispc exch def
 /str1 1 string def
  strn
  {/code exch def
   /chr str1 0 code put str1 def
   0 ispc neg rmoveto
   gsave
   chr stringwidth pop 2 div neg
   0 rmoveto
   chr false charpath stroke
   grestore
  } forall
} bind def

/Oh
{/strn exch def
 /ispc exch def
 /tstr 1 string def
 strn
   {
    /code exch def
```

```
    /chr tstr 0 code put tstr def
    chr false charpath
    currentpoint
    /yt exch def /xt exch def
    stroke
    xt ispc add yt moveto
   } forall
 } bind def

/T 256 array def
/O
{ T
  exch
  get
  aload
  pop
  setrgbcolor
} bind def

/Y
{/strn exch def
 /ispc exch def
 /str1 1 string def
  strn
  {/code exch def
   /chr str1 0 code put str1 def
    0 ispc neg rmoveto
    gsave
      chr stringwidth pop 2 div neg 0 rmoveto
      chr show
    grestore
  } forall
} def

/Bg
{/ury Ex def /urx Ex def /lly Ex def /llx Ex def
 Np Ls Gr Gs 0 O Ch E Lr
 llx lly M urx lly L urx ury L
 llx ury L llx lly L
 Cp Np
} def

/Oc
```

```
{/ury exch def
 /urx exch def
 /lly exch def
 /llx exch def
 Np
 llx lly M urx lly L urx ury L  llx ury L llx lly L
 Cp Np
} def

/Cc
{/CellWidth exch def
 /strn CellWidth 1 add string def
 /strn1 CellWidth 1 add 3 mul string def
 systemdict /colorimage known
 { { Cu strn1 Rh pop } false 3 Ci}
 { { Cu strn1 Rh pop
     /ColorStr exch def
     0 1 CellWidth {
       /YIndx exch def
       /GrayVal YIndx 3 mul def
       strn YIndx
         ColorStr GrayVal       get 0.30 mul
         ColorStr GrayVal 1 add get 0.59 mul
         ColorStr GrayVal 2 add get 0.11 mul
         add add cvi
       put
     } for
     strn
   } Im
 } ifelse
} def

/Ld
{/ury exch def /urx exch def  /lly exch def /llx exch def
 -90 rotate
 lly urx add neg llx lly sub translate
} bind def
/Pt
{/ury exch def /urx exch def  /lly exch def /llx exch def
 90 rotate
 lly urx add lly llx sub translate
} bind def
%%EndProlog
```

```
%%BeginSetup
<</PageSize [00612 00792]>> setpagedevice
%%EndSetup

[1. 1. 1.] [0. 0. 0.] [1. 0. 0.] [0. 1. 0.] [0. 0. 1.] [0. 1. 1.] [1. 0. .8]
[1. 1. 0.] [1. .5 0.] [.6 .8 0.] [0. 1. .6] [0. .5 1.] [.5 0. .8] [1. 0. .6]
[.3 .3 .3] [.7 .7 .7] [1. 1. .3] [.7 1. .5] [.5 1. .6] [.2 1. .7] [.3 .8 .8]
[.5 .7 .8] [.7 .5 .8] [1. .3 .9] [.7 .9 .5] [.4 .9 .5] [.2 .9 .7] [.2 .7 .9]
[.2 .5 1.] [.5 .3 1.] [.7 .2 1.] [.9 .1 1.] [.9 1. .2] [.7 1. .3] [.5 1. .3]
[.2 1. .5] [.2 .8 .6] [.2 .7 .7] [.2 .5 .8] [.2 .4 .9] [.4 .3 .9] [.7 .2 .9]
[.8 .2 .8] [.9 .3 .7] [.8 .4 .6] [.8 .6 .5] [.9 .7 .4] [.9 .7 .3] [1. .9 .9]
[.8 1. .1] [.6 1. .2] [.5 1. .2] [.2 .9 .5] [.2 .8 .5] [.2 .7 .7] [.2 .5 .7]
[.2 .4 .9] [.4 .3 .9] [.5 .2 .9] [.8 .2 .7] [1. .2 .7] [1. .3 .6] [1. .4 .5]
[1. .5 .4] [1. .8 .1] [.7 1. .0] [.6 1. .1] [.4 1. .2] [.1 1. .3] [.1 .8 .4]
[.2 .7 .5] [.1 .6 .7] [.1 .5 .7] [.1 .4 .8] [.2 .3 .9] [.2 .2 1.] [.3 .1 1.]
[.5 .1 .9] [.7 .0 .8] [.9 .0 .7] [.9 .7 .1] [.7 .9 .1] [.5 .9 .1] [.3 .9 .2]
[.1 .9 .3] [.2 .7 .4] [.1 .7 .5] [.1 .5 .6] [.1 .4 .7] [.2 .3 .7] [.2 .2 .8]
[.3 .2 .8] [.5 .1 .7] [.7 .1 .7] [.9 .2 .5] [.8 .2 .5] [.8 .7 .0] [.6 .8 .1]
[.4 .9 .1] [.3 .9 .1] [.1 .9 .2] [.2 .8 .2] [.2 .7 .3] [.2 .6 .5] [.1 .5 .5]
[.1 .4 .6] [.2 .3 .7] [.3 .2 .7] [.4 .2 .6] [.7 .2 .5] [.8 .2 .4] [1. .2 .3]
[.7 .7 .0] [.5 .7 .1] [.4 .8 .1] [.2 .9 .1] [.2 .9 .1] [.2 .8 .2] [.1 .7 .2]
[.1 .7 .3] [.1 .6 .4] [.1 .4 .5] [.1 .4 .5] [.2 .3 .5] [.4 .3 .4] [.5 .3 .4]
[.7 .3 .3] [.8 .3 .2] [.7 .6 .0] [.5 .7 .1] [.4 .7 .1] [.2 .7 .2] [.1 .7 .2]
[.1 .6 .3] [.1 .5 .4] [.1 .4 .4] [.1 .3 .5] [.1 .2 .7] [.1 .1 .7] [.4 .1 .5]
[.5 .1 .5] [.6 .1 .4] [.7 .1 .4] [.9 .1 .3] [.5 .5 .1] [.4 .6 .1] [.3 .6 .1]
[.2 .6 .2] [.1 .5 .2] [.1 .4 .3] [.1 .4 .4] [.2 .3 .4] [.2 .2 .4] [.2 .2 .5]
[.2 .1 .5] [.3 .1 .5] [.4 .1 .4] [.5 .1 .4] [.7 .1 .3] [.8 .1 .2] [.5 .4 .1]
[.4 .5 .1] [.3 .6 .0] [.2 .6 .1] [.1 .7 .1] [.1 .6 .1] [.1 .5 .2] [.1 .4 .2]
[.1 .4 .2] [.1 .3 .3] [.1 .2 .4] [.1 .2 .5] [.1 .1 .5] [.1 .0 .6] [.3 .0 .5]
[.5 .0 .4] [.7 .3 .0] [.7 .4 .0] [.5 .4 .1] [.3 .4 .2] [.2 .3 .2] [.2 .2 .3]
[.2 .2 .4] [.3 .1 .4] [.5 .1 .3] [.6 .1 .2] [.7 .1 .2] [.8 .0 .2] [1. .0 .2]
[1. .1 .1] [.9 .2 .1] [.8 .2 .1] [.6 .2 .0] [.4 .4 .0] [.2 .4 .1] [.2 .3 .2]
[.2 .2 .2] [.2 .2 .3] [.2 .1 .3] [.3 .1 .3] [.5 .1 .2] [.7 .1 .2] [.8 .0 .2]
[.9 .0 .1] [1. .0 .1] [1. .1 .1] [.8 .2 .0] [.7 .2 .0] [.5 .2 .0] [.4 .3 .0]
[.1 .4 .1] [.1 .4 .1] [.1 .3 .2] [.1 .2 .2] [.1 .2 .3] [.2 .1 .4] [.2 .0 .3]
[.4 .0 .2] [.6 .0 .2] [.7 .0 .1] [.8 .0 .1] [.7 .1 .1] [.7 .1 .1] [.6 .2 .0]
[.4 .2 .0] [.2 .3 .0] [.1 .3 .0] [.1 .2 .1] [.1 .2 .1] [.1 .1 .2] [.1 .1 .3]
[.1 .0 .3] [.1 .0 .2] [.2 .0 .2] [.3 .0 .2] [.3 .0 .1] [.4 .0 .1] [.4 .1 .1]
[.4 .1 .0] [.2 .1 .0] [.0 .0 .0] [.0 .0 .0] [.0 .0 .0] [.0 .0 .0] [.0 .0 .0]
[.0 .0 .0] [.0 .0 .0] [.0 .0 .0] [.0 .0 .0] [.0 .0 .0] [.0 .0 .0] [.0 .0 .0]
[.0 .0 .0] [.0 .0 .0] [.0 .0 .0] [.0 .0 .0]  T astore pop
%%Page: 1 1
```

0.04000 0.04000 A 1 J 1 C 12 W [] 0 D
T 0 [1.000 1.000 1.000] U
T 1 [0.000 0.000 0.000] U
T 2 [0.000 0.000 1.000] U
T 3 [0.000 0.031 1.000] U
T 4 [0.000 0.059 1.000] U
T 5 [0.000 0.090 1.000] U
T 6 [0.000 0.122 1.000] U
T 7 [0.000 0.153 1.000] U
T 8 [0.000 0.180 1.000] U
T 9 [0.000 0.212 1.000] U
T 10 [0.000 0.243 1.000] U
T 11 [0.000 0.275 1.000] U
T 12 [0.000 0.302 1.000] U
T 13 [0.000 0.333 1.000] U
T 14 [0.000 0.365 1.000] U
T 15 [0.000 0.392 1.000] U
T 16 [0.000 0.424 1.000] U
T 17 [0.000 0.455 1.000] U
T 18 [0.000 0.486 1.000] U
T 19 [0.000 0.514 1.000] U
T 20 [0.000 0.545 1.000] U
T 21 [0.000 0.576 1.000] U
T 22 [0.000 0.608 1.000] U
T 23 [0.000 0.635 1.000] U
T 24 [0.000 0.667 1.000] U
T 25 [0.000 0.698 1.000] U
T 26 [0.000 0.725 1.000] U
T 27 [0.000 0.757 1.000] U
T 28 [0.000 0.788 1.000] U
T 29 [0.000 0.820 1.000] U
T 30 [0.000 0.847 1.000] U
T 31 [0.000 0.878 1.000] U
T 32 [0.000 0.910 1.000] U
T 33 [0.000 0.941 1.000] U
T 34 [0.000 0.969 1.000] U
T 35 [0.000 1.000 1.000] U
T 36 [0.000 0.992 0.973] U
T 37 [0.000 0.980 0.941] U
T 38 [0.004 0.973 0.914] U
T 39 [0.004 0.965 0.886] U
T 40 [0.004 0.953 0.859] U
T 41 [0.004 0.945 0.827] U

T 42 [0.008 0.937 0.800] U
T 43 [0.008 0.929 0.773] U
T 44 [0.008 0.918 0.745] U
T 45 [0.008 0.910 0.714] U
T 46 [0.012 0.902 0.686] U
T 47 [0.012 0.890 0.659] U
T 48 [0.012 0.882 0.627] U
T 49 [0.012 0.875 0.600] U
T 50 [0.016 0.863 0.573] U
T 51 [0.016 0.855 0.545] U
T 52 [0.016 0.847 0.514] U
T 53 [0.016 0.839 0.486] U
T 54 [0.020 0.827 0.459] U
T 55 [0.020 0.820 0.431] U
T 56 [0.020 0.812 0.400] U
T 57 [0.020 0.800 0.373] U
T 58 [0.024 0.792 0.345] U
T 59 [0.024 0.784 0.314] U
T 60 [0.024 0.773 0.286] U
T 61 [0.024 0.765 0.259] U
T 62 [0.027 0.757 0.231] U
T 63 [0.027 0.749 0.200] U
T 64 [0.027 0.737 0.173] U
T 65 [0.027 0.729 0.145] U
T 66 [0.031 0.722 0.118] U
T 67 [0.031 0.710 0.086] U
T 68 [0.031 0.702 0.059] U
T 69 [0.059 0.710 0.059] U
T 70 [0.090 0.718 0.055] U
T 71 [0.118 0.729 0.055] U
T 72 [0.145 0.737 0.051] U
T 73 [0.173 0.745 0.051] U
T 74 [0.204 0.753 0.047] U
T 75 [0.231 0.765 0.047] U
T 76 [0.259 0.773 0.043] U
T 77 [0.286 0.780 0.043] U
T 78 [0.318 0.788 0.043] U
T 79 [0.345 0.800 0.039] U
T 80 [0.373 0.808 0.039] U
T 81 [0.400 0.816 0.035] U
T 82 [0.431 0.824 0.035] U
T 83 [0.459 0.835 0.031] U
T 84 [0.486 0.843 0.031] U

T 85 [0.518 0.851 0.031] U
T 86 [0.545 0.859 0.027] U
T 87 [0.573 0.867 0.027] U
T 88 [0.600 0.878 0.024] U
T 89 [0.631 0.886 0.024] U
T 90 [0.659 0.894 0.020] U
T 91 [0.686 0.902 0.020] U
T 92 [0.714 0.914 0.016] U
T 93 [0.745 0.922 0.016] U
T 94 [0.773 0.929 0.016] U
T 95 [0.800 0.937 0.012] U
T 96 [0.827 0.949 0.012] U
T 97 [0.859 0.957 0.008] U
T 98 [0.886 0.965 0.008] U
T 99 [0.914 0.973 0.004] U
T 100 [0.941 0.984 0.004] U
T 101 [0.973 0.992 0.000] U
T 102 [1.000 1.000 0.000] U
T 103 [1.000 0.988 0.000] U
T 104 [1.000 0.980 0.000] U
T 105 [1.000 0.969 0.000] U
T 106 [1.000 0.957 0.000] U
T 107 [1.000 0.945 0.000] U
T 108 [1.000 0.937 0.000] U
T 109 [1.000 0.925 0.000] U
T 110 [1.000 0.914 0.000] U
T 111 [1.000 0.902 0.000] U
T 112 [1.000 0.894 0.000] U
T 113 [1.000 0.882 0.000] U
T 114 [1.000 0.871 0.000] U
T 115 [1.000 0.863 0.000] U
T 116 [1.000 0.851 0.000] U
T 117 [1.000 0.839 0.000] U
T 118 [1.000 0.827 0.000] U
T 119 [1.000 0.820 0.000] U
T 120 [1.000 0.808 0.000] U
T 121 [1.000 0.796 0.000] U
T 122 [1.000 0.784 0.000] U
T 123 [1.000 0.776 0.000] U
T 124 [1.000 0.765 0.000] U
T 125 [1.000 0.753 0.000] U
T 126 [1.000 0.745 0.000] U
T 127 [1.000 0.733 0.000] U

T 128 [1.000 0.722 0.000] U
T 129 [1.000 0.710 0.000] U
T 130 [1.000 0.702 0.000] U
T 131 [1.000 0.690 0.000] U
T 132 [1.000 0.678 0.000] U
T 133 [1.000 0.667 0.000] U
T 134 [1.000 0.659 0.000] U
T 135 [1.000 0.647 0.000] U
T 136 [1.000 0.627 0.000] U
T 137 [1.000 0.608 0.000] U
T 138 [1.000 0.588 0.000] U
T 139 [1.000 0.569 0.000] U
T 140 [1.000 0.549 0.000] U
T 141 [1.000 0.529 0.000] U
T 142 [1.000 0.510 0.000] U
T 143 [1.000 0.490 0.000] U
T 144 [1.000 0.471 0.000] U
T 145 [1.000 0.451 0.000] U
T 146 [1.000 0.431 0.000] U
T 147 [1.000 0.412 0.000] U
T 148 [1.000 0.392 0.000] U
T 149 [1.000 0.373 0.000] U
T 150 [1.000 0.353 0.000] U
T 151 [1.000 0.333 0.000] U
T 152 [1.000 0.314 0.000] U
T 153 [1.000 0.294 0.000] U
T 154 [1.000 0.275 0.000] U
T 155 [1.000 0.255 0.000] U
T 156 [1.000 0.235 0.000] U
T 157 [1.000 0.216 0.000] U
T 158 [1.000 0.196 0.000] U
T 159 [1.000 0.176 0.000] U
T 160 [1.000 0.157 0.000] U
T 161 [1.000 0.137 0.000] U
T 162 [1.000 0.118 0.000] U
T 163 [1.000 0.098 0.000] U
T 164 [1.000 0.078 0.000] U
T 165 [1.000 0.059 0.000] U
T 166 [1.000 0.039 0.000] U
T 167 [1.000 0.020 0.000] U
T 168 [1.000 0.000 0.000] U
T 169 [0.980 0.000 0.016] U
T 170 [0.961 0.000 0.031] U

T 171 [0.941 0.000 0.047] U
T 172 [0.922 0.000 0.063] U
T 173 [0.902 0.000 0.082] U
T 174 [0.878 0.000 0.098] U
T 175 [0.859 0.000 0.114] U
T 176 [0.839 0.000 0.129] U
T 177 [0.820 0.000 0.145] U
T 178 [0.800 0.000 0.161] U
T 179 [0.780 0.000 0.176] U
T 180 [0.761 0.000 0.192] U
T 181 [0.741 0.000 0.212] U
T 182 [0.722 0.000 0.227] U
T 183 [0.702 0.000 0.243] U
T 184 [0.682 0.000 0.259] U
T 185 [0.659 0.000 0.275] U
T 186 [0.639 0.000 0.290] U
T 187 [0.620 0.000 0.306] U
T 188 [0.600 0.000 0.322] U
T 189 [0.580 0.000 0.341] U
T 190 [0.561 0.000 0.357] U
T 191 [0.541 0.000 0.373] U
T 192 [0.522 0.000 0.388] U
T 193 [0.502 0.000 0.404] U
T 194 [0.482 0.000 0.420] U
T 195 [0.463 0.000 0.435] U
T 196 [0.439 0.000 0.451] U
T 197 [0.420 0.000 0.471] U
T 198 [0.400 0.000 0.486] U
T 199 [0.380 0.000 0.502] U
T 200 [0.361 0.000 0.518] U
T 201 [0.341 0.000 0.533] U
-1150 1075 16450 18700 Oc
Gs
1 O
0 O
2300 16217 M 2300 5503 L 2333 5503 L 2367 5503 L 2400 5503 L
2433 5503 L 2466 5503 L 2499 5503 L 2533 5503 L
2566 5503 L 2599 5503 L 2632 5503 L 2666 5503 L
2699 5503 L 2732 5503 L 2765 5503 L 2798 5503 L
2832 5503 L 2865 5503 L 2898 5503 L 2928 5503 L
2927 5506 L 2917 5534 L 2939 5553 L 2968 5552 L
2986 5530 L 2968 5507 L 2967 5503 L 2998 5503 L
3031 5503 L 3064 5503 L 3098 5503 L 3131 5503 L

3164 5503 L 3197 5503 L 3230 5503 L 3264 5503 L
3297 5503 L 3330 5503 L 3363 5503 L 3397 5503 L
3430 5503 L 3463 5503 L 3496 5503 L 3529 5503 L
3563 5503 L 3596 5503 L 3629 5503 L 3662 5503 L
3696 5503 L 3729 5503 L 3762 5503 L 3795 5503 L
3828 5503 L 3862 5503 L 3895 5503 L 3928 5503 L
3961 5503 L 3995 5503 L 4028 5503 L 4061 5503 L
4094 5503 L 4128 5503 L 4161 5503 L 4194 5503 L
4227 5503 L 4260 5503 L 4294 5503 L 4327 5503 L
4360 5503 L 4393 5503 L 4427 5503 L 4460 5503 L
4493 5503 L 4526 5503 L 4559 5503 L 4593 5503 L
4626 5503 L 4659 5503 L 4692 5503 L 4726 5503 L
4759 5503 L 4792 5503 L 4825 5503 L 4859 5503 L
4892 5503 L 4925 5503 L 4958 5503 L 4991 5503 L
5025 5503 L 5058 5503 L 5091 5503 L 5124 5503 L
5158 5503 L 5191 5503 L 5224 5503 L 5257 5503 L
5290 5503 L 5324 5503 L 5357 5503 L 5390 5503 L
5395 5503 L 5408 5513 L 5406 5542 L 5403 5571 L
5404 5600 L 5404 5630 L 5394 5657 L 5369 5673 L
5352 5696 L 5381 5692 L 5387 5663 L 5416 5662 L
5416 5633 L 5411 5604 L 5410 5575 L 5412 5546 L
5430 5524 L 5430 5503 L 5457 5503 L 5490 5503 L
5523 5503 L 5532 5503 L 5539 5516 L 5540 5523 L
5544 5530 L 5551 5535 L 5537 5572 L 5529 5600 L
5547 5578 L 5556 5550 L 5573 5527 L 5592 5505 L
5594 5503 L 5623 5503 L 5644 5503 L 5645 5520 L
5649 5503 L 5656 5503 L 5658 5503 L 5665 5518 L
5675 5545 L 5689 5570 L 5683 5599 L 5659 5615 L
5668 5643 L 5681 5668 L 5682 5698 L 5666 5722 L
5690 5738 L 5674 5763 L 5654 5784 L 5676 5765 L
5703 5774 L 5686 5797 L 5670 5821 L 5650 5842 L
5674 5826 L 5702 5833 L 5688 5859 L 5664 5875 L
5654 5902 L 5627 5913 L 5598 5914 L 5627 5919 L
5655 5924 L 5661 5952 L 5663 5981 L 5645 6005 L
5617 6009 L 5600 6032 L 5628 6023 L 5654 6012 L
5680 5998 L 5678 5969 L 5684 5941 L 5705 5921 L
5723 5944 L 5711 5917 L 5706 5889 L 5713 5877 L
5721 5864 L 5740 5842 L 5745 5813 L 5772 5824 L
5790 5801 L 5797 5773 L 5799 5769 L 5811 5748 L
5823 5721 L 5842 5699 L 5859 5676 L 5853 5647 L
5872 5669 L 5898 5680 L 5926 5673 L 5955 5670 L
5928 5660 L 5902 5646 L 5880 5627 L 5860 5607 L
5880 5586 L 5909 5582 L 5938 5582 L 5967 5584 L

5986 5561 L 6014 5553 L 6043 5555 L 6069 5543 L
6050 5564 L 6058 5592 L 6085 5582 L 6113 5590 L
6119 5618 L 6090 5612 L 6074 5636 L 6051 5617 L
6024 5609 L 6008 5633 L 6037 5638 L 6066 5639 L
6087 5659 L 6061 5672 L 6035 5685 L 6057 5704 L
6085 5711 L 6113 5717 L 6141 5711 L 6159 5688 L
6169 5660 L 6172 5631 L 6200 5638 L 6229 5645 L
6234 5673 L 6207 5683 L 6182 5697 L 6209 5708 L
6209 5737 L 6180 5737 L 6202 5756 L 6216 5782 L
6242 5794 L 6247 5795 L 6256 5799 L 6270 5803 L
6296 5815 L 6324 5810 L 6345 5829 L 6338 5801 L
6315 5784 L 6317 5755 L 6314 5726 L 6325 5699 L
6354 5700 L 6382 5696 L 6403 5675 L 6399 5704 L
6415 5728 L 6431 5752 L 6408 5771 L 6392 5795 L
6417 5780 L 6430 5754 L 6458 5757 L 6472 5783 L
6487 5808 L 6515 5815 L 6544 5814 L 6569 5830 L
6599 5819 L 6577 5810 L 6575 5801 L 6595 5802 L
6614 5803 L 6640 5790 L 6666 5776 L 6637 5775 L
6649 5748 L 6677 5741 L 6705 5746 L 6731 5733 L
6757 5720 L 6754 5749 L 6768 5774 L 6747 5755 L
6730 5778 L 6747 5801 L 6764 5825 L 6785 5845 L
6814 5847 L 6838 5830 L 6866 5823 L 6882 5798 L
6899 5775 L 6921 5794 L 6949 5802 L 6976 5811 L
7003 5823 L 7027 5839 L 7029 5867 L 7046 5844 L
7031 5819 L 7060 5819 L 7082 5837 L 7102 5858 L
7125 5876 L 7149 5891 L 7173 5874 L 7201 5868 L
7229 5876 L 7258 5874 L 7266 5902 L 7287 5922 L
7284 5951 L 7277 5979 L 7291 6004 L 7296 6033 L
7271 6018 L 7268 6047 L 7297 6049 L 7320 6032 L
7341 6012 L 7358 6035 L 7379 6046 L 7361 6062 L
7343 6078 L 7315 6085 L 7292 6102 L 7279 6128 L
7273 6156 L 7245 6161 L 7274 6160 L 7294 6140 L
7317 6122 L 7343 6110 L 7349 6110 L 7372 6110 L
7401 6110 L 7422 6133 L 7434 6147 L 7455 6167 L
7454 6177 L 7455 6203 L 7469 6229 L 7461 6252 L
7487 6263 L 7507 6269 L 7515 6271 L 7527 6265 L
7541 6258 L 7532 6285 L 7559 6275 L 7564 6246 L
7572 6219 L 7590 6234 L 7594 6237 L 7617 6255 L
7641 6270 L 7641 6299 L 7659 6277 L 7651 6249 L
7669 6271 L 7671 6300 L 7676 6329 L 7687 6355 L
7711 6339 L 7724 6355 L 7732 6355 L 7736 6354 L
7759 6338 L 7776 6361 L 7786 6388 L 7785 6417 L
7757 6424 L 7733 6441 L 7762 6438 L 7790 6431 L

7818 6423 L 7840 6405 L 7849 6377 L 7840 6350 L
7838 6321 L 7855 6344 L 7857 6373 L 7860 6402 L
7871 6428 L 7894 6445 L 7891 6473 L 7909 6496 L
7937 6504 L 7946 6477 L 7930 6454 L 7925 6426 L
7937 6452 L 7954 6475 L 7977 6493 L 8001 6507 L
8006 6536 L 8024 6558 L 8013 6585 L 8034 6605 L
8054 6586 L 8077 6604 L 8098 6623 L 8126 6629 L
8120 6657 L 8106 6682 L 8077 6682 L 8074 6710 L
8046 6708 L 8017 6704 L 8000 6709 L 7974 6722 L
7952 6740 L 7940 6751 L 7942 6767 L 7944 6764 L
7954 6752 L 7968 6755 L 7980 6755 L 7997 6756 L
8023 6746 L 8035 6743 L 8051 6739 L 8074 6755 L
8079 6784 L 8054 6797 L 8082 6798 L 8092 6825 L
8109 6802 L 8110 6804 L 8123 6827 L 8125 6855 L
8154 6856 L 8165 6829 L 8157 6801 L 8184 6795 L
8185 6795 L 8198 6821 L 8225 6813 L 8226 6813 L
8253 6818 L 8274 6829 L 8278 6832 L 8273 6860 L
8295 6878 L 8285 6851 L 8310 6837 L 8336 6825 L
8361 6810 L 8359 6781 L 8385 6795 L 8405 6815 L
8394 6842 L 8395 6870 L 8424 6867 L 8437 6893 L
8441 6921 L 8425 6945 L 8403 6928 L 8382 6948 L
8378 6976 L 8355 6993 L 8381 6981 L 8409 6987 L
8402 7015 L 8395 7043 L 8416 7023 L 8416 6994 L
8429 7020 L 8442 6994 L 8437 6966 L 8464 6974 L
8483 6996 L 8511 6992 L 8537 6981 L 8562 6996 L
8534 7002 L 8530 7031 L 8558 7033 L 8587 7031 L
8573 7056 L 8575 7085 L 8562 7060 L 8534 7067 L
8510 7082 L 8501 7110 L 8529 7117 L 8545 7140 L
8561 7164 L 8541 7184 L 8522 7205 L 8548 7215 L
8577 7218 L 8604 7211 L 8588 7188 L 8614 7200 L
8611 7171 L 8601 7145 L 8629 7141 L 8654 7129 L
8655 7129 L 8641 7154 L 8632 7181 L 8648 7205 L
8649 7176 L 8673 7160 L 8701 7162 L 8730 7166 L
8736 7194 L 8753 7198 L 8763 7201 L 8744 7221 L
8727 7245 L 8704 7228 L 8677 7219 L 8687 7245 L
8663 7261 L 8640 7278 L 8619 7297 L 8646 7305 L
8669 7322 L 8693 7337 L 8713 7357 L 8729 7381 L
8740 7407 L 8747 7403 L 8764 7391 L 8754 7365 L
8780 7352 L 8808 7349 L 8813 7320 L 8819 7312 L
8830 7297 L 8827 7269 L 8842 7294 L 8852 7320 L
8879 7312 L 8880 7283 L 8881 7255 L 8889 7262 L
8902 7273 L 8902 7274 L 8907 7302 L 8901 7330 L
8874 7339 L 8868 7367 L 8854 7392 L 8828 7380 L

8821 7407 L 8807 7432 L 8824 7455 L 8850 7466 L
8878 7471 L 8883 7479 L 8892 7496 L 8884 7523 L
8882 7552 L 8891 7579 L 8909 7580 L 8919 7581 L
8927 7608 L 8935 7635 L 8906 7638 L 8880 7651 L
8853 7659 L 8834 7680 L 8850 7704 L 8869 7723 L
8870 7723 L 8887 7746 L 8898 7773 L 8870 7768 L
8893 7784 L 8921 7790 L 8937 7795 L 8948 7798 L
8973 7813 L 8976 7813 L 9000 7818 L 9028 7822 L
9036 7831 L 9046 7845 L 9020 7834 L 8996 7849 L
8979 7871 L 8961 7849 L 8953 7822 L 8927 7809 L
8905 7827 L 8899 7855 L 8886 7880 L 8859 7872 L
8872 7897 L 8895 7914 L 8916 7895 L 8944 7889 L
8966 7872 L 8982 7895 L 8970 7921 L 8966 7949 L
8947 7970 L 8919 7975 L 8891 7976 L 8865 7965 L
8843 7983 L 8841 8011 L 8867 8021 L 8844 8037 L
8849 8065 L 8845 8093 L 8872 8083 L 8893 8065 L
8918 8051 L 8909 8078 L 8901 8105 L 8900 8134 L
8910 8107 L 8927 8084 L 8941 8072 L 8948 8066 L
8953 8094 L 8978 8105 L 8962 8082 L 8990 8075 L
8965 8061 L 8962 8033 L 8967 8032 L 8990 8026 L
9012 8008 L 9017 8036 L 9033 8060 L 9043 8086 L
9071 8083 L 9094 8068 L 9082 8042 L 9057 8030 L
9058 8001 L 9037 7982 L 9009 7981 L 8993 7957 L
8985 7930 L 9014 7929 L 9035 7948 L 9044 7975 L
9072 7977 L 9075 7979 L 9096 7993 L 9100 7994 L
9122 8004 L 9121 8032 L 9142 8051 L 9116 8063 L
9127 8089 L 9102 8104 L 9078 8118 L 9090 8143 L
9118 8138 L 9125 8166 L 9147 8164 L 9153 8164 L
9181 8158 L 9179 8186 L 9152 8194 L 9180 8194 L
9199 8215 L 9204 8243 L 9195 8269 L 9223 8266 L
9238 8270 L 9250 8273 L 9265 8297 L 9251 8321 L
9246 8349 L 9256 8323 L 9280 8308 L 9291 8335 L
9307 8311 L 9313 8321 L 9322 8335 L 9323 8363 L
9326 8368 L 9339 8386 L 9326 8411 L 9332 8439 L
9357 8426 L 9385 8430 L 9384 8459 L 9398 8483 L
9371 8494 L 9358 8519 L 9340 8540 L 9368 8548 L
9387 8568 L 9387 8596 L 9390 8624 L 9418 8627 L
9430 8653 L 9445 8677 L 9463 8698 L 9479 8721 L
9477 8750 L 9452 8763 L 9424 8768 L 9405 8788 L
9377 8793 L 9349 8789 L 9347 8817 L 9368 8799 L
9397 8802 L 9417 8797 L 9424 8795 L 9429 8796 L
9446 8778 L 9474 8778 L 9496 8777 L 9502 8777 L
9529 8775 L 9528 8787 L 9542 8812 L 9540 8840 L

9553 8865 L 9567 8872 L 9578 8878 L 9593 8902 L
9603 8928 L 9581 8945 L 9604 8956 L 9607 8957 L
9616 8983 L 9642 8993 L 9669 8985 L 9652 8963 L
9661 8948 L 9667 8939 L 9675 8912 L 9693 8890 L
9713 8870 L 9734 8889 L 9728 8916 L 9749 8935 L
9762 8960 L 9777 8983 L 9805 8979 L 9805 8979 L
9829 8964 L 9846 8942 L 9859 8968 L 9845 8992 L
9836 9019 L 9823 9044 L 9824 9072 L 9808 9095 L
9795 9120 L 9788 9147 L 9771 9170 L 9744 9177 L
9717 9184 L 9745 9184 L 9773 9185 L 9801 9186 L
9825 9200 L 9837 9213 L 9844 9221 L 9865 9240 L
9856 9267 L 9843 9292 L 9816 9297 L 9801 9321 L
9827 9312 L 9855 9308 L 9874 9330 L 9853 9349 L
9864 9375 L 9838 9386 L 9811 9380 L 9785 9369 L
9806 9388 L 9834 9391 L 9831 9418 L 9804 9424 L
9777 9417 L 9749 9413 L 9750 9441 L 9774 9455 L
9782 9482 L 9786 9510 L 9810 9506 L 9814 9505 L
9832 9484 L 9833 9512 L 9844 9537 L 9836 9511 L
9837 9483 L 9847 9456 L 9873 9464 L 9894 9482 L
9901 9510 L 9899 9538 L 9906 9565 L 9927 9546 L
9918 9519 L 9921 9503 L 9923 9492 L 9924 9464 L
9952 9459 L 9975 9474 L 9980 9479 L 9993 9495 L
9989 9523 L 9963 9535 L 9966 9563 L 9963 9591 L
9989 9601 L 9990 9629 L 9974 9652 L 9977 9680 L
9995 9702 L 9976 9723 L 9949 9715 L 9921 9709 L
9897 9696 L 9896 9668 L 9912 9645 L 9888 9631 L
9881 9658 L 9853 9658 L 9834 9637 L 9807 9629 L
9784 9614 L 9766 9593 L 9774 9619 L 9747 9612 L
9732 9588 L 9724 9615 L 9735 9641 L 9753 9662 L
9780 9656 L 9807 9662 L 9835 9669 L 9856 9686 L
9874 9708 L 9891 9730 L 9914 9746 L 9932 9768 L
9954 9785 L 9968 9809 L 9990 9826 L 10003 9831 L
10010 9834 L 10016 9837 L 10039 9852 L 10046 9879 L
10022 9865 L 9994 9865 L 9966 9864 L 9962 9865 L
9955 9871 L 9944 9882 L 9917 9889 L 9891 9899 L
9864 9904 L 9836 9909 L 9812 9923 L 9798 9947 L
9782 9969 L 9763 9990 L 9746 10012 L 9727 10033 L
9708 10053 L 9685 10069 L 9661 10084 L 9634 10087 L
9606 10086 L 9578 10082 L 9553 10069 L 9531 10053 L
9512 10032 L 9490 10015 L 9467 10000 L 9445 9983 L
9419 9973 L 9391 9972 L 9365 9983 L 9346 9962 L
9371 9949 L 9344 9957 L 9335 9984 L 9341 10011 L
9323 10033 L 9314 10059 L 9326 10085 L 9302 10099 L

9274 10101 L 9246 10099 L 9223 10082 L 9213 10057 L
9197 10034 L 9169 10032 L 9147 10049 L 9128 10028 L
9104 10013 L 9124 10033 L 9141 10055 L 9169 10056 L
9195 10047 L 9194 10075 L 9202 10102 L 9226 10114 L
9254 10121 L 9281 10121 L 9309 10123 L 9337 10123 L
9364 10115 L 9383 10095 L 9411 10097 L 9436 10110 L
9452 10132 L 9468 10155 L 9469 10183 L 9484 10206 L
9503 10226 L 9528 10239 L 9555 10243 L 9571 10267 L
9593 10283 L 9616 10299 L 9643 10303 L 9667 10318 L
9682 10341 L 9702 10360 L 9727 10373 L 9753 10381 L
9780 10388 L 9807 10393 L 9835 10396 L 9863 10399 L
9890 10403 L 9913 10419 L 9920 10446 L 9910 10472 L
9896 10496 L 9884 10521 L 9870 10545 L 9857 10570 L
9844 10594 L 9829 10618 L 9811 10639 L 9789 10656 L
9766 10670 L 9741 10684 L 9714 10684 L 9698 10708 L
9676 10725 L 9651 10736 L 9627 10749 L 9609 10770 L
9595 10794 L 9577 10816 L 9555 10833 L 9531 10846 L
9510 10864 L 9484 10874 L 9458 10884 L 9430 10882 L
9403 10884 L 9378 10896 L 9359 10916 L 9381 10933 L
9367 10958 L 9355 10982 L 9340 11006 L 9312 11008 L
9285 11006 L 9257 11008 L 9231 11018 L 9203 11015 L
9192 10990 L 9165 10984 L 9138 10978 L 9113 10965 L
9087 10957 L 9059 10957 L 9045 10981 L 9072 10976 L
9100 10974 L 9126 10984 L 9147 11001 L 9169 11017 L
9190 11035 L 9215 11049 L 9240 11059 L 9265 11048 L
9293 11048 L 9320 11040 L 9345 11050 L 9361 11027 L
9384 11011 L 9401 10989 L 9420 10969 L 9430 10943 L
9436 10916 L 9462 10908 L 9490 10911 L 9517 10908 L
9531 10931 L 9558 10937 L 9584 10948 L 9610 10957 L
9612 10956 L 9635 10945 L 9662 10939 L 9689 10931 L
9706 10910 L 9732 10899 L 9757 10887 L 9783 10877 L
9810 10873 L 9837 10865 L 9863 10858 L 9889 10868 L
9895 10895 L 9884 10920 L 9870 10944 L 9858 10969 L
9848 10995 L 9829 11015 L 9811 11035 L 9790 11053 L
9765 11066 L 9739 11075 L 9713 11085 L 9686 11085 L
9660 11095 L 9648 11120 L 9629 11139 L 9643 11163 L
9637 11190 L 9638 11218 L 9625 11242 L 9603 11259 L
9577 11269 L 9552 11280 L 9527 11292 L 9502 11302 L
9475 11311 L 9448 11312 L 9429 11332 L 9411 11353 L
9383 11357 L 9399 11380 L 9417 11400 L 9404 11424 L
9378 11434 L 9399 11452 L 9407 11478 L 9405 11506 L
9395 11531 L 9389 11558 L 9379 11584 L 9352 11589 L
9337 11612 L 9331 11639 L 9316 11662 L 9297 11682 L

9286 11707 L 9276 11733 L 9257 11753 L 9250 11779 L
9252 11807 L 9262 11833 L 9240 11849 L 9223 11871 L
9216 11898 L 9220 11925 L 9209 11951 L 9201 11977 L
9175 11986 L 9180 12013 L 9155 12024 L 9154 12051 L
9146 12078 L 9145 12105 L 9136 12131 L 9126 12157 L
9118 12183 L 9109 12209 L 9096 12233 L 9092 12260 L
9078 12284 L 9053 12295 L 9028 12307 L 9001 12313 L
8983 12333 L 8958 12345 L 8932 12354 L 8905 12358 L
8878 12364 L 8865 12340 L 8865 12367 L 8847 12388 L
8820 12394 L 8798 12411 L 8774 12424 L 8751 12438 L
8730 12456 L 8716 12479 L 8712 12506 L 8684 12509 L
8660 12521 L 8635 12508 L 8613 12524 L 8594 12504 L
8601 12531 L 8588 12555 L 8587 12582 L 8595 12608 L
8595 12635 L 8599 12663 L 8614 12686 L 8634 12704 L
8661 12699 L 8673 12724 L 8676 12751 L 8683 12777 L
8692 12803 L 8704 12820 L 8708 12825 L 8733 12838 L
8752 12857 L 8755 12884 L 8770 12908 L 8778 12934 L
8787 12960 L 8781 12986 L 8808 12983 L 8830 12999 L
8840 12974 L 8853 12998 L 8873 13016 L 8890 12994 L
8901 13019 L 8916 13042 L 8927 13067 L 8950 13052 L
8957 13078 L 8955 13106 L 8965 13131 L 8985 13150 L
9010 13160 L 9016 13164 L 9033 13175 L 9026 13148 L
9040 13172 L 9062 13188 L 9074 13213 L 9047 13209 L
9020 13213 L 9035 13236 L 9010 13244 L 8986 13258 L
8978 13284 L 8987 13310 L 9013 13320 L 9038 13311 L
9060 13328 L 9064 13355 L 9082 13335 L 9098 13312 L
9093 13286 L 9083 13260 L 9071 13235 L 9098 13230 L
9124 13239 L 9145 13257 L 9171 13262 L 9198 13266 L
9223 13278 L 9244 13295 L 9240 13322 L 9236 13349 L
9220 13371 L 9225 13397 L 9251 13406 L 9243 13432 L
9258 13455 L 9282 13467 L 9304 13450 L 9328 13437 L
9329 13464 L 9347 13485 L 9346 13512 L 9321 13523 L
9305 13545 L 9279 13539 L 9296 13560 L 9319 13574 L
9345 13566 L 9359 13543 L 9386 13544 L 9385 13571 L
9411 13561 L 9430 13580 L 9453 13595 L 9479 13601 L
9505 13611 L 9530 13621 L 9553 13636 L 9570 13657 L
9566 13684 L 9579 13661 L 9599 13680 L 9626 13682 L
9605 13665 L 9590 13642 L 9616 13649 L 9643 13642 L
9642 13669 L 9668 13679 L 9691 13692 L 9704 13716 L
9729 13727 L 9754 13739 L 9778 13751 L 9768 13776 L
9794 13781 L 9808 13757 L 9817 13731 L 9837 13750 L
9860 13765 L 9859 13792 L 9876 13813 L 9884 13787 L
9867 13765 L 9883 13743 L 9908 13733 L 9881 13729 L

9885 13702 L 9912 13701 L 9930 13698 L 9939 13696 L
9961 13712 L 9969 13718 L 9983 13729 L 9996 13753 L
9998 13753 L 10009 13756 L 10023 13759 L 10012 13734 L
10025 13758 L 10029 13785 L 10028 13812 L 10002 13808 L
9974 13811 L 9995 13829 L 9995 13856 L 10010 13879 L
10034 13893 L 10056 13908 L 10040 13930 L 10032 13956 L
10037 13983 L 10055 14004 L 10066 14015 L 10074 14023 L
10047 14025 L 10023 14011 L 9999 14000 L 9981 14021 L
9955 14013 L 9928 14012 L 9902 14020 L 9878 14033 L
9854 14021 L 9840 14044 L 9849 14070 L 9831 14091 L
9810 14074 L 9787 14059 L 9767 14040 L 9769 14013 L
9754 14035 L 9727 14031 L 9700 14026 L 9684 14005 L
9664 14023 L 9639 14013 L 9612 14008 L 9585 14010 L
9561 14022 L 9542 14042 L 9525 14063 L 9507 14083 L
9532 14095 L 9505 14092 L 9479 14081 L 9452 14078 L
9426 14084 L 9406 14103 L 9402 14129 L 9386 14152 L
9359 14150 L 9342 14171 L 9318 14184 L 9306 14208 L
9279 14205 L 9252 14208 L 9225 14207 L 9204 14189 L
9179 14180 L 9155 14166 L 9128 14162 L 9102 14155 L
9082 14136 L 9056 14128 L 9030 14120 L 9055 14108 L
9051 14082 L 9034 14060 L 9015 14041 L 8996 14021 L
8974 14005 L 8951 13990 L 8925 13983 L 8900 13973 L
8879 13955 L 8866 13931 L 8883 13910 L 8876 13883 L
8856 13864 L 8835 13847 L 8816 13827 L 8790 13820 L
8763 13820 L 8736 13818 L 8711 13828 L 8683 13826 L
8656 13824 L 8629 13825 L 8603 13832 L 8576 13835 L
8555 13853 L 8532 13867 L 8505 13866 L 8502 13893 L
8485 13914 L 8467 13935 L 8472 13962 L 8469 13989 L
8468 14016 L 8470 14043 L 8477 14070 L 8494 14091 L
8511 14112 L 8523 14137 L 8505 14157 L 8480 14168 L
8492 14193 L 8498 14219 L 8491 14245 L 8483 14271 L
8464 14291 L 8459 14318 L 8432 14318 L 8415 14297 L
8424 14323 L 8419 14349 L 8422 14376 L 8408 14399 L
8402 14373 L 8397 14399 L 8373 14387 L 8351 14371 L
8326 14360 L 8302 14348 L 8280 14332 L 8259 14349 L
8232 14353 L 8206 14361 L 8179 14363 L 8152 14360 L
8125 14364 L 8098 14360 L 8074 14374 L 8052 14389 L
8033 14408 L 8010 14423 L 7984 14430 L 7964 14449 L
7945 14468 L 7928 14489 L 7915 14513 L 7903 14537 L
7890 14561 L 7880 14587 L 7870 14612 L 7867 14639 L
7867 14666 L 7872 14693 L 7881 14718 L 7893 14743 L
7906 14767 L 7924 14787 L 7939 14810 L 7964 14820 L
7958 14847 L 7948 14872 L 7967 14892 L 7986 14911 L

8008 14926 L 8034 14935 L 8061 14939 L 8087 14935 L
8111 14921 L 8126 14899 L 8133 14873 L 8151 14853 L
8174 14839 L 8201 14834 L 8223 14850 L 8243 14868 L
8264 14884 L 8284 14903 L 8301 14911 L 8309 14914 L
8326 14914 L 8336 14913 L 8357 14896 L 8370 14897 L
8384 14898 L 8406 14914 L 8406 14914 L 8422 14916 L
8433 14917 L 8458 14927 L 8475 14936 L 8482 14940 L
8504 14956 L 8522 14976 L 8536 14999 L 8553 15020 L
8575 15036 L 8600 15047 L 8583 15067 L 8579 15094 L
8583 15121 L 8574 15147 L 8598 15161 L 8605 15187 L
8613 15213 L 8625 15237 L 8645 15256 L 8666 15272 L
8691 15283 L 8690 15310 L 8710 15327 L 8736 15337 L
8762 15344 L 8788 15351 L 8810 15368 L 8836 15375 L
8851 15397 L 8874 15411 L 8899 15422 L 8925 15430 L
8950 15439 L 8974 15453 L 8998 15464 L 9019 15482 L
9045 15489 L 9071 15496 L 9087 15517 L 9086 15544 L
9099 15568 L 9122 15582 L 9100 15597 L 9122 15614 L
9127 15640 L 9150 15653 L 9164 15677 L 9173 15684 L
9186 15693 L 9204 15713 L 9211 15728 L 9216 15738 L
9228 15752 L 9234 15758 L 9260 15764 L 9262 15767 L
9278 15784 L 9255 15799 L 9267 15824 L 9286 15843 L
9295 15842 L 9311 15839 L 9313 15839 L 9323 15864 L
9342 15883 L 9358 15905 L 9384 15903 L 9406 15887 L
9430 15900 L 9454 15887 L 9481 15888 L 9494 15879 L
9503 15872 L 9522 15853 L 9549 15856 L 9576 15858 L
9598 15874 L 9608 15900 L 9609 15927 L 9629 15945 L
9639 15970 L 9635 15943 L 9622 15919 L 9633 15894 L
9625 15868 L 9638 15845 L 9665 15847 L 9682 15825 L
9705 15811 L 9727 15795 L 9742 15780 L 9754 15779 L
9754 15792 L 9759 15797 L 9769 15793 L 9784 15785 L
9803 15791 L 9801 15797 L 9778 15791 L 9772 15801 L
9766 15809 L 9766 15815 L 9754 15822 L 9731 15838 L
9731 15840 L 9756 15825 L 9777 15813 L 9778 15800 L
9801 15801 L 9806 15793 L 9802 15787 L 9784 15782 L
9760 15795 L 9758 15788 L 9763 15762 L 9754 15745 L
9760 15735 L 9783 15719 L 9803 15701 L 9816 15678 L
9804 15653 L 9786 15633 L 9784 15606 L 9766 15585 L
9751 15563 L 9748 15536 L 9728 15517 L 9714 15494 L
9700 15471 L 9683 15450 L 9667 15429 L 9644 15414 L
9670 15407 L 9650 15389 L 9632 15369 L 9608 15356 L
9581 15356 L 9563 15336 L 9540 15322 L 9517 15307 L
9506 15283 L 9495 15258 L 9517 15241 L 9533 15220 L
9536 15193 L 9533 15171 L 9529 15160 L 9517 15143 L

9494 15134 L 9507 15102 L 9517 15071 L 9541 15058 L
9568 15061 L 9595 15063 L 9619 15074 L 9646 15075 L
9672 15081 L 9699 15080 L 9676 15067 L 9650 15060 L
9627 15045 L 9612 15022 L 9589 15008 L 9563 15001 L
9581 14982 L 9564 14960 L 9587 14945 L 9608 14928 L
9594 14904 L 9568 14895 L 9543 14885 L 9521 14869 L
9494 14865 L 9475 14884 L 9465 14859 L 9451 14836 L
9425 14826 L 9398 14826 L 9372 14818 L 9365 14792 L
9391 14785 L 9369 14769 L 9381 14745 L 9381 14717 L
9402 14700 L 9419 14721 L 9431 14746 L 9458 14745 L
9483 14757 L 9510 14757 L 9535 14765 L 9554 14785 L
9581 14786 L 9608 14788 L 9620 14812 L 9597 14826 L
9571 14832 L 9595 14844 L 9607 14868 L 9628 14885 L
9653 14874 L 9665 14850 L 9672 14846 L 9689 14837 L
9690 14864 L 9679 14889 L 9704 14880 L 9696 14906 L
9716 14888 L 9737 14906 L 9763 14912 L 9755 14937 L
9782 14943 L 9806 14955 L 9782 14966 L 9792 14991 L
9811 15011 L 9834 15025 L 9846 15050 L 9856 15075 L
9879 15089 L 9898 15105 L 9899 15107 L 9914 15105 L
9926 15104 L 9946 15123 L 9949 15149 L 9956 15146 L
9973 15136 L 9991 15157 L 10015 15168 L 10035 15187 L
10042 15193 L 10056 15204 L 10056 15204 L 10071 15210 L
10078 15213 L 10081 15214 L 10088 15217 L 10097 15220 L
10106 15224 L 10131 15235 L 10143 15248 L 10150 15255 L
10170 15263 L 10175 15265 L 10201 15256 L 10226 15265 L
10235 15272 L 10247 15282 L 10223 15293 L 10246 15307 L
10254 15332 L 10256 15332 L 10272 15330 L 10281 15328 L
10284 15327 L 10308 15323 L 10308 15322 L 10321 15314 L
10331 15308 L 10340 15326 L 10343 15333 L 10356 15330 L
10369 15327 L 10383 15351 L 10393 15376 L 10401 15402 L
10403 15375 L 10426 15360 L 10441 15360 L 10453 15360 L
10473 15379 L 10497 15367 L 10524 15365 L 10551 15364 L
10578 15370 L 10600 15385 L 10626 15391 L 10639 15415 L
10644 15388 L 10650 15362 L 10656 15388 L 10660 15415 L
10666 15442 L 10672 15442 L 10693 15443 L 10687 15417 L
10709 15401 L 10733 15388 L 10759 15382 L 10786 15379 L
10796 15354 L 10804 15328 L 10823 15309 L 10813 15284 L
10826 15260 L 10840 15278 L 10843 15281 L 10854 15297 L
10859 15303 L 10855 15330 L 10867 15355 L 10887 15337 L
10914 15330 L 10929 15310 L 10930 15308 L 10953 15321 L
10971 15301 L 10998 15302 L 11015 15290 L 11020 15286 L
11044 15272 L 11069 15281 L 11089 15262 L 11116 15262 L
11139 15277 L 11160 15294 L 11172 15318 L 11176 15330 L

11189 15326 L 11196 15330 L 11202 15336 L 11203 15334 L
11198 15326 L 11188 15324 L 11176 15329 L 11173 15315 L
11163 15287 L 11184 15288 L 11196 15299 L 11225 15316 L
11226 15314 L 11220 15310 L 11196 15298 L 11187 15284 L
11174 15283 L 11150 15269 L 11146 15242 L 11126 15224 L
11127 15197 L 11140 15173 L 11167 15180 L 11153 15155 L
11156 15128 L 11151 15102 L 11144 15076 L 11121 15061 L
11116 15035 L 11124 15009 L 11104 14991 L 11091 14967 L
11082 14942 L 11073 14916 L 11062 14891 L 11070 14865 L
11084 14842 L 11110 14833 L 11103 14860 L 11118 14837 L
11121 14810 L 11147 14819 L 11174 14816 L 11190 14838 L
11214 14825 L 11241 14825 L 11257 14803 L 11230 14799 L
11207 14814 L 11183 14801 L 11156 14797 L 11134 14782 L
11108 14776 L 11084 14762 L 11067 14783 L 11075 14757 L
11071 14730 L 11047 14717 L 11020 14716 L 11031 14691 L
11021 14666 L 11001 14648 L 10991 14623 L 10977 14599 L
10983 14573 L 10983 14546 L 11003 14527 L 11023 14538 L
11033 14533 L 11004 14514 L 11002 14517 L 11003 14525 L
10981 14539 L 10963 14512 L 10943 14496 L 10923 14477 L
10897 14470 L 10923 14462 L 10928 14462 L 10950 14458 L
10960 14461 L 10972 14464 L 10976 14466 L 11002 14476 L
11025 14462 L 11051 14454 L 11078 14458 L 11103 14469 L
11130 14471 L 11156 14461 L 11154 14434 L 11132 14451 L
11106 14446 L 11099 14419 L 11123 14407 L 11098 14397 L
11071 14401 L 11047 14388 L 11063 14365 L 11079 14358 L
11088 14355 L 11114 14347 L 11140 14355 L 11140 14355 L
11159 14369 L 11162 14371 L 11185 14357 L 11181 14330 L
11206 14331 L 11208 14331 L 11217 14357 L 11244 14361 L
11221 14376 L 11248 14378 L 11275 14375 L 11253 14359 L
11268 14337 L 11241 14335 L 11238 14308 L 11212 14301 L
11201 14276 L 11217 14255 L 11228 14280 L 11255 14284 L
11271 14306 L 11286 14315 L 11294 14320 L 11298 14320 L
11322 14318 L 11330 14343 L 11326 14370 L 11323 14397 L
11343 14397 L 11349 14397 L 11350 14397 L 11356 14423 L
11329 14423 L 11304 14433 L 11300 14460 L 11325 14449 L
11352 14451 L 11369 14446 L 11375 14445 L 11378 14444 L
11389 14422 L 11391 14420 L 11400 14413 L 11405 14410 L
11412 14404 L 11413 14404 L 11422 14394 L 11432 14384 L
11449 14363 L 11456 14363 L 11476 14362 L 11478 14389 L
11504 14383 L 11505 14383 L 11509 14356 L 11515 14350 L
11520 14345 L 11528 14337 L 11549 14321 L 11550 14320 L
11557 14347 L 11555 14374 L 11560 14375 L 11581 14382 L
11598 14385 L 11608 14386 L 11629 14403 L 11656 14406 L

11667 14398 L 11679 14391 L 11702 14376 L 11726 14364 L
11753 14369 L 11770 14390 L 11796 14404 L 11778 14381 L
11783 14354 L 11785 14327 L 11797 14302 L 11822 14291 L
11844 14275 L 11867 14261 L 11887 14249 L 11887 14248 L
11865 14258 L 11843 14273 L 11818 14285 L 11794 14298 L
11770 14311 L 11778 14337 L 11765 14361 L 11741 14348 L
11716 14336 L 11721 14309 L 11728 14282 L 11748 14264 L
11774 14256 L 11784 14231 L 11787 14204 L 11762 14192 L
11763 14191 L 11781 14172 L 11802 14155 L 11828 14146 L
11812 14124 L 11836 14112 L 11861 14100 L 11839 14083 L
11812 14079 L 11819 14053 L 11806 14029 L 11823 14007 L
11841 14027 L 11863 14044 L 11868 14017 L 11857 13992 L
11850 13966 L 11876 13960 L 11895 13940 L 11913 13920 L
11940 13915 L 11959 13934 L 11950 13908 L 11928 13892 L
11908 13873 L 11909 13846 L 11894 13869 L 11889 13896 L
11873 13918 L 11847 13923 L 11826 13941 L 11804 13926 L
11779 13938 L 11755 13951 L 11766 13926 L 11741 13938 L
11760 13918 L 11771 13893 L 11750 13910 L 11746 13883 L
11740 13856 L 11739 13884 L 11732 13910 L 11723 13936 L
11697 13927 L 11676 13909 L 11699 13894 L 11717 13873 L
11701 13851 L 11677 13838 L 11693 13816 L 11671 13831 L
11672 13859 L 11666 13885 L 11660 13912 L 11660 13885 L
11636 13872 L 11629 13846 L 11630 13819 L 11604 13813 L
11594 13787 L 11600 13764 L 11600 13761 L 11610 13786 L
11612 13785 L 11621 13781 L 11630 13777 L 11635 13775 L
11634 13747 L 11639 13749 L 11642 13750 L 11651 13753 L
11656 13754 L 11660 13755 L 11678 13735 L 11678 13708 L
11683 13700 L 11688 13694 L 11694 13688 L 11695 13686 L
11697 13676 L 11701 13660 L 11705 13633 L 11720 13613 L
11721 13611 L 11742 13628 L 11732 13653 L 11720 13677 L
11716 13704 L 11691 13715 L 11696 13742 L 11682 13766 L
11702 13785 L 11710 13759 L 11715 13732 L 11733 13752 L
11749 13764 L 11755 13768 L 11761 13741 L 11758 13714 L
11757 13687 L 11781 13674 L 11768 13651 L 11772 13643 L
11778 13632 L 11781 13627 L 11807 13655 L 11806 13639 L
11783 13625 L 11784 13598 L 11787 13595 L 11791 13592 L
11806 13581 L 11793 13557 L 11807 13534 L 11809 13508 L
11809 13506 L 11831 13490 L 11809 13474 L 11826 13468 L
11830 13466 L 11835 13464 L 11846 13455 L 11856 13447 L
11869 13423 L 11888 13404 L 11912 13417 L 11937 13428 L
11959 13444 L 11966 13471 L 11964 13443 L 11943 13425 L
11918 13414 L 11897 13397 L 11873 13385 L 11858 13362 L
11866 13336 L 11877 13338 L 11893 13342 L 11920 13344 L

11947 13350 L 11928 13330 L 11909 13311 L 11924 13288 L
11899 13277 L 11894 13250 L 11867 13249 L 11849 13228 L
11828 13210 L 11810 13190 L 11823 13166 L 11851 13168 L
11878 13173 L 11905 13173 L 11906 13146 L 11879 13148 L
11853 13139 L 11827 13130 L 11816 13106 L 11811 13079 L
11798 13055 L 11825 13050 L 11816 13024 L 11794 13040 L
11788 13013 L 11783 12987 L 11773 12961 L 11800 12953 L
11796 12926 L 11818 12911 L 11845 12904 L 11835 12879 L
11817 12858 L 11799 12878 L 11780 12898 L 11782 12925 L
11770 12901 L 11743 12907 L 11752 12881 L 11764 12869 L
11771 12862 L 11769 12889 L 11789 12870 L 11811 12855 L
11799 12831 L 11773 12821 L 11776 12818 L 11794 12802 L
11810 12824 L 11837 12829 L 11822 12806 L 11832 12781 L
11826 12754 L 11835 12729 L 11859 12742 L 11887 12742 L
11887 12769 L 11878 12795 L 11879 12822 L 11881 12795 L
11905 12782 L 11909 12754 L 11936 12752 L 11911 12740 L
11884 12736 L 11877 12710 L 11850 12713 L 11829 12696 L
11856 12697 L 11884 12694 L 11905 12677 L 11915 12651 L
11887 12649 L 11861 12656 L 11855 12683 L 11827 12681 L
11826 12654 L 11852 12645 L 11873 12627 L 11845 12624 L
11820 12635 L 11806 12659 L 11804 12686 L 11780 12700 L
11772 12674 L 11772 12671 L 11774 12647 L 11787 12622 L
11814 12617 L 11835 12601 L 11847 12596 L 11861 12590 L
11882 12607 L 11909 12606 L 11890 12587 L 11871 12566 L
11879 12540 L 11903 12527 L 11886 12506 L 11909 12491 L
11911 12468 L 11911 12463 L 11933 12480 L 11933 12480 L
11951 12501 L 11955 12528 L 11957 12529 L 11977 12544 L
11981 12557 L 11984 12570 L 12008 12583 L 12008 12611 L
12011 12638 L 12021 12613 L 12023 12586 L 12037 12562 L
12045 12564 L 12064 12568 L 12091 12567 L 12098 12576 L
12107 12589 L 12096 12614 L 12105 12640 L 12109 12668 L
12133 12681 L 12139 12687 L 12153 12700 L 12178 12711 L
12196 12731 L 12222 12740 L 12247 12750 L 12251 12723 L
12234 12702 L 12213 12720 L 12214 12693 L 12215 12666 L
12191 12651 L 12187 12678 L 12176 12653 L 12167 12627 L
12193 12619 L 12208 12642 L 12216 12634 L 12228 12623 L
12244 12601 L 12249 12596 L 12265 12583 L 12276 12608 L
12277 12608 L 12288 12616 L 12298 12624 L 12300 12652 L
12273 12647 L 12276 12674 L 12303 12668 L 12319 12668 L
12331 12668 L 12331 12695 L 12309 12712 L 12287 12727 L
12269 12749 L 12261 12775 L 12284 12790 L 12259 12800 L
12286 12801 L 12313 12808 L 12324 12833 L 12337 12808 L
12346 12795 L 12352 12785 L 12363 12760 L 12353 12735 L

12366 12710 L 12389 12724 L 12388 12751 L 12407 12756 L
12415 12758 L 12441 12752 L 12436 12725 L 12437 12698 L
12446 12701 L 12464 12706 L 12463 12734 L 12453 12760 L
12438 12782 L 12442 12809 L 12436 12836 L 12409 12830 L
12389 12812 L 12364 12825 L 12351 12849 L 12357 12875 L
12374 12854 L 12398 12867 L 12415 12889 L 12441 12895 L
12468 12886 L 12487 12900 L 12490 12902 L 12513 12917 L
12539 12926 L 12546 12953 L 12549 12926 L 12550 12898 L
12550 12898 L 12575 12887 L 12601 12879 L 12628 12879 L
12631 12885 L 12638 12905 L 12645 12901 L 12662 12893 L
12664 12894 L 12688 12903 L 12684 12876 L 12712 12876 L
12718 12850 L 12692 12856 L 12699 12845 L 12708 12834 L
12726 12820 L 12730 12817 L 12741 12842 L 12743 12870 L
12725 12890 L 12726 12918 L 12728 12945 L 12747 12965 L
12730 12986 L 12743 13010 L 12766 13008 L 12770 13008 L
12797 13001 L 12789 13027 L 12782 13053 L 12806 13040 L
12828 13024 L 12833 13024 L 12855 13020 L 12883 13020 L
12894 13023 L 12908 13027 L 12909 13027 L 12911 13027 L
12924 13028 L 12936 13029 L 12951 13052 L 12951 13025 L
12958 12999 L 12979 13016 L 12999 13025 L 12999 13048 L
12997 13054 L 12999 13054 L 12999 14126 L 12998 14132 L
12980 14152 L 12967 14176 L 12960 14202 L 12950 14228 L
12933 14249 L 12914 14269 L 12899 14291 L 12878 14308 L
12864 14331 L 12843 14349 L 12837 14376 L 12818 14395 L
12798 14413 L 12793 14440 L 12772 14457 L 12751 14475 L
12730 14492 L 12711 14512 L 12691 14530 L 12675 14552 L
12656 14571 L 12640 14593 L 12621 14613 L 12605 14635 L
12586 14654 L 12574 14679 L 12563 14703 L 12546 14725 L
12534 14749 L 12519 14772 L 12503 14794 L 12494 14819 L
12476 14839 L 12463 14863 L 12448 14886 L 12438 14911 L
12432 14938 L 12409 14952 L 12383 14960 L 12358 14951 L
12347 14976 L 12328 14995 L 12305 15011 L 12278 15013 L
12256 15028 L 12235 15045 L 12213 15061 L 12196 15082 L
12178 15103 L 12162 15125 L 12146 15147 L 12123 15133 L
12098 15142 L 12072 15149 L 12045 15149 L 12018 15151 L
11994 15165 L 11980 15188 L 11983 15215 L 12006 15230 L
12012 15256 L 11986 15265 L 12012 15273 L 12039 15276 L
12053 15253 L 12044 15279 L 12033 15303 L 12026 15329 L
12028 15356 L 12041 15381 L 12034 15407 L 12008 15415 L
12002 15441 L 12009 15467 L 12022 15491 L 12040 15511 L
12067 15510 L 12081 15533 L 12107 15541 L 12128 15558 L
12149 15575 L 12146 15602 L 12168 15618 L 12182 15641 L
12204 15657 L 12230 15651 L 12257 15648 L 12282 15658 L

12298 15680 L 12323 15671 L 12333 15696 L 12359 15704 L
12377 15724 L 12401 15736 L 12419 15757 L 12444 15766 L
12448 15793 L 12425 15807 L 12434 15833 L 12457 15846 L
12482 15858 L 12506 15870 L 12528 15886 L 12544 15907 L
12561 15929 L 12584 15943 L 12608 15955 L 12621 15979 L
12626 16005 L 12633 16032 L 12637 16058 L 12655 16078 L
12681 16086 L 12697 16108 L 12718 16125 L 12743 16137 L
12767 16149 L 12785 16169 L 12811 16174 L 12836 16186 L
12841 16213 L 12840 16217 L 12833 16217 L 12800 16217 L
12766 16217 L 12733 16217 L 12700 16217 L 12667 16217 L
12633 16217 L 12600 16217 L 12567 16217 L 12534 16217 L
12501 16217 L 12467 16217 L 12434 16217 L 12401 16217 L
12368 16217 L 12334 16217 L 12301 16217 L 12268 16217 L
12235 16217 L 12201 16217 L 12168 16217 L 12135 16217 L
12102 16217 L 12069 16217 L 12035 16217 L 12002 16217 L
11969 16217 L 11936 16217 L 11902 16217 L 11869 16217 L
11836 16217 L 11803 16217 L 11770 16217 L 11736 16217 L
11703 16217 L 11670 16217 L 11637 16217 L 11603 16217 L
11570 16217 L 11537 16217 L 11504 16217 L 11471 16217 L
11437 16217 L 11404 16217 L 11371 16217 L 11338 16217 L
11304 16217 L 11271 16217 L 11238 16217 L 11205 16217 L
11171 16217 L 11138 16217 L 11105 16217 L 11072 16217 L
11039 16217 L 11005 16217 L 10972 16217 L 10939 16217 L
10906 16217 L 10872 16217 L 10839 16217 L 10806 16217 L
10773 16217 L 10740 16217 L 10706 16217 L 10673 16217 L
10640 16217 L 10607 16217 L 10573 16217 L 10540 16217 L
10507 16217 L 10474 16217 L 10440 16217 L 10407 16217 L
10374 16217 L 10341 16217 L 10308 16217 L 10274 16217 L
10241 16217 L 10208 16217 L 10175 16217 L 10141 16217 L
10108 16217 L 10075 16217 L 10042 16217 L 10009 16217 L
9975 16217 L 9942 16217 L 9909 16217 L 9876 16217 L
9842 16217 L 9809 16217 L 9776 16217 L 9743 16217 L
9710 16217 L 9676 16217 L 9643 16217 L 9610 16217 L
9577 16217 L 9543 16217 L 9510 16217 L 9477 16217 L
9444 16217 L 9410 16217 L 9377 16217 L 9344 16217 L
9311 16217 L 9278 16217 L 9244 16217 L 9211 16217 L
9178 16217 L 9145 16217 L 9111 16217 L 9078 16217 L
9045 16217 L 9012 16217 L 8979 16217 L 8945 16217 L
8912 16217 L 8879 16217 L 8846 16217 L 8812 16217 L
8779 16217 L 8746 16217 L 8713 16217 L 8680 16217 L
8646 16217 L 8613 16217 L 8580 16217 L 8547 16217 L
8513 16217 L 8480 16217 L 8447 16217 L 8414 16217 L
8380 16217 L 8347 16217 L 8314 16217 L 8281 16217 L

8248 16217 L 8214 16217 L 8181 16217 L 8148 16217 L
8115 16217 L 8081 16217 L 8048 16217 L 8015 16217 L
7982 16217 L 7949 16217 L 7915 16217 L 7882 16217 L
7849 16217 L 7816 16217 L 7782 16217 L 7749 16217 L
7716 16217 L 7683 16217 L 7650 16217 L 7616 16217 L
7583 16217 L 7550 16217 L 7517 16217 L 7483 16217 L
7450 16217 L 7417 16217 L 7384 16217 L 7350 16217 L
7317 16217 L 7284 16217 L 7251 16217 L 7218 16217 L
7184 16217 L 7151 16217 L 7118 16217 L 7085 16217 L
7051 16217 L 7018 16217 L 6985 16217 L 6952 16217 L
6919 16217 L 6885 16217 L 6852 16217 L 6819 16217 L
6786 16217 L 6752 16217 L 6719 16217 L 6686 16217 L
6653 16217 L 6619 16217 L 6586 16217 L 6553 16217 L
6520 16217 L 6487 16217 L 6453 16217 L 6420 16217 L
6387 16217 L 6354 16217 L 6320 16217 L 6287 16217 L
6254 16217 L 6221 16217 L 6188 16217 L 6154 16217 L
6121 16217 L 6088 16217 L 6055 16217 L 6021 16217 L
5988 16217 L 5955 16217 L 5922 16217 L 5889 16217 L
5855 16217 L 5822 16217 L 5789 16217 L 5756 16217 L
5722 16217 L 5689 16217 L 5656 16217 L 5623 16217 L
5589 16217 L 5556 16217 L 5523 16217 L 5490 16217 L
5457 16217 L 5423 16217 L 5390 16217 L 5357 16217 L
5324 16217 L 5290 16217 L 5257 16217 L 5224 16217 L
5191 16217 L 5158 16217 L 5124 16217 L 5091 16217 L
5058 16217 L 5025 16217 L 4991 16217 L 4958 16217 L
4925 16217 L 4892 16217 L 4859 16217 L 4825 16217 L
4792 16217 L 4759 16217 L 4726 16217 L 4692 16217 L
4659 16217 L 4626 16217 L 4593 16217 L 4559 16217 L
4526 16217 L 4493 16217 L 4460 16217 L 4427 16217 L
4393 16217 L 4360 16217 L 4327 16217 L 4294 16217 L
4260 16217 L 4227 16217 L 4194 16217 L 4161 16217 L
4128 16217 L 4094 16217 L 4061 16217 L 4028 16217 L
3995 16217 L 3961 16217 L 3928 16217 L 3895 16217 L
3862 16217 L 3828 16217 L 3795 16217 L 3762 16217 L
3729 16217 L 3696 16217 L 3662 16217 L 3629 16217 L
3596 16217 L 3563 16217 L 3529 16217 L 3496 16217 L
3463 16217 L 3430 16217 L 3397 16217 L 3363 16217 L
3330 16217 L 3297 16217 L 3264 16217 L 3230 16217 L
3197 16217 L 3164 16217 L 3131 16217 L 3098 16217 L
3064 16217 L 3031 16217 L 2998 16217 L 2965 16217 L
2931 16217 L 2898 16217 L 2865 16217 L 2832 16217 L
2798 16217 L 2765 16217 L 2732 16217 L 2699 16217 L
2666 16217 L 2632 16217 L 2599 16217 L 2566 16217 L

2533 16217 L 2499 16217 L 2466 16217 L 2433 16217 L
2400 16217 L 2367 16217 L 2333 16217 L 2300 16217 L
N E
5543 5524 5541 5517 5543 5513 5545 5509 5547 5505 5548 5503
5556 5503 5575 5503 5575 5505 5573 5510 5571 5514 5569 5518
5567 5522 5563 5525 5560 5528 5556 5531 5552 5534 5550 5530
5545 5528 5543 5524 20 N F
5702 5621 5699 5615 5700 5611 5704 5608 5708 5611 5712 5612
5717 5614 5717 5618 5721 5620 5724 5624 5724 5628 5726 5633
5721 5633 5717 5635 5713 5636 5711 5631 5707 5629 5704 5625
5702 5621 19 N F
5796 5620 5790 5619 5794 5617 5798 5614 5801 5611 5805 5609
5810 5609 5808 5614 5806 5618 5803 5621 5799 5623 5796 5620
12 N F
5817 5509 5816 5503 5822 5503 5855 5503 5865 5503 5872 5506
5879 5506 5885 5509 5890 5504 5890 5503 5910 5503 5904 5508
5904 5516 5908 5522 5902 5525 5909 5528 5916 5525 5918 5532
5916 5539 5916 5547 5921 5552 5924 5559 5930 5555 5936 5559
5937 5566 5933 5572 5927 5567 5921 5563 5914 5563 5908 5558
5903 5553 5897 5550 5893 5544 5886 5544 5879 5542 5872 5539
5866 5535 5862 5541 5855 5542 5848 5542 5844 5535 5838 5531
5832 5527 5825 5525 5828 5518 5823 5513 5817 5509 47 N F
5964 5506 5963 5503 5970 5503 5970 5507 5964 5506 5 N F
5977 5535 5967 5528 5972 5522 5975 5516 5981 5512 5988 5511
5992 5505 5999 5506 6006 5503 6006 5503 6021 5503 6024 5503
6024 5507 6028 5503 6039 5503 6038 5506 6042 5512 6038 5519
6034 5525 6031 5531 6024 5532 6018 5536 6011 5537 6005 5532
5998 5531 5991 5533 5984 5536 5977 5535 28 N F
6055 5511 6054 5507 6056 5505 6058 5504 6058 5503 6060 5503
6060 5503 6062 5504 6064 5506 6065 5508 6064 5510 6064 5512
6063 5514 6061 5514 6059 5512 6057 5512 6055 5511 17 N F
6243 5630 6240 5628 6241 5625 6242 5622 6245 5619 6248 5621
6251 5622 6254 5622 6257 5624 6259 5626 6259 5626 6257 5629
6253 5629 6250 5630 6247 5630 6243 5630 16 N F
6252 5610 6251 5607 6253 5608 6255 5607 6257 5608 6258 5610
6259 5612 6260 5614 6259 5616 6257 5615 6255 5614 6254 5612
6252 5610 13 N F
6256 5699 6252 5695 6253 5692 6257 5691 6259 5689 6260 5685
6263 5688 6262 5691 6260 5693 6257 5695 6256 5699 11 N F
7342 6099 7335 6091 7339 6086 7343 6079 7349 6076 7356 6072
7360 6066 7367 6064 7374 6066 7378 6060 7381 6054 7388 6051
7395 6049 7388 6045 7392 6039 7398 6035 7405 6034 7408 6027
7415 6027 7417 6034 7422 6040 7415 6042 7409 6040 7404 6045

7402 6052 7403 6059 7404 6066 7402 6073 7398 6079 7397 6086
7392 6092 7385 6094 7380 6090 7372 6090 7365 6091 7361 6097
7356 6102 7349 6103 7342 6099 39 N F
7490 6228 7483 6223 7488 6218 7496 6217 7503 6216 7508 6221
7505 6228 7506 6235 7511 6240 7514 6247 7507 6248 7501 6244
7500 6237 7497 6231 7490 6228 15 N F
7498 6214 7491 6212 7493 6205 7499 6201 7506 6200 7513 6199
7520 6197 7522 6203 7523 6211 7527 6217 7533 6220 7527 6228
7520 6226 7513 6227 7510 6220 7506 6215 7498 6214 17 N F
7498 6158 7493 6153 7497 6147 7504 6145 7511 6144 7519 6145
7526 6144 7533 6142 7538 6138 7545 6135 7552 6135 7559 6137
7566 6138 7573 6136 7574 6129 7581 6132 7586 6127 7593 6131
7597 6137 7595 6144 7594 6151 7600 6155 7598 6162 7592 6165
7589 6172 7590 6179 7583 6179 7579 6173 7576 6167 7569 6168
7564 6173 7558 6177 7552 6175 7548 6169 7544 6163 7539 6158
7535 6152 7526 6161 7523 6167 7516 6169 7509 6167 7503 6163
7498 6158 43 N F
7698 6243 7691 6232 7698 6232 7705 6233 7712 6236 7718 6233
7717 6226 7723 6230 7729 6233 7736 6235 7743 6236 7750 6238
7758 6238 7754 6245 7747 6245 7745 6252 7745 6260 7746 6267
7749 6273 7753 6279 7759 6282 7766 6279 7773 6276 7780 6276
7781 6283 7775 6289 7775 6296 7777 6303 7784 6306 7782 6313
7788 6317 7794 6313 7801 6314 7802 6321 7809 6323 7802 6323
7797 6329 7791 6332 7786 6337 7781 6333 7774 6334 7770 6328
7762 6329 7756 6326 7753 6333 7749 6339 7744 6343 7737 6346
7732 6351 7725 6348 7724 6341 7727 6334 7727 6327 7722 6322
7717 6316 7715 6309 7711 6304 7706 6298 7708 6291 7714 6288
7719 6293 7726 6295 7732 6291 7739 6291 7746 6290 7740 6285
7739 6278 7734 6273 7728 6268 7722 6264 7718 6258 7713 6253
7714 6246 7710 6251 7704 6248 7698 6243 76 N F
7944 6760 7942 6755 7943 6752 7945 6749 7948 6747 7950 6747
7951 6746 7953 6749 7950 6752 7948 6754 7946 6757 7944 6760
12 N F
7955 6744 7950 6745 7954 6742 7958 6740 7961 6736 7964 6733
7967 6729 7971 6727 7975 6725 7980 6726 7984 6726 7989 6726
7989 6726 7993 6727 7991 6731 7990 6736 7986 6737 7982 6735
7977 6734 7973 6737 7971 6740 7968 6744 7963 6743 7959 6741
7955 6744 25 N F
7957 6750 7953 6747 7957 6744 7961 6743 7966 6745 7970 6744
7973 6741 7975 6737 7980 6736 7984 6739 7988 6740 7992 6737
7994 6733 7999 6732 8002 6734 8007 6734 8006 6739 8003 6742
7999 6745 7997 6749 7993 6752 7989 6753 7984 6754 7980 6755
7975 6755 7971 6754 7966 6753 7962 6751 7957 6750 29 N F

7984 6724 7979 6723 7983 6721 7987 6719 7991 6716 7996 6716
7997 6721 7993 6723 7989 6724 7984 6724 10 N F
8030 6716 8023 6711 8027 6709 8031 6710 8035 6708 8039 6710
8043 6713 8039 6715 8035 6717 8030 6716 10 N F
8100 6747 8089 6741 8093 6735 8097 6729 8103 6726 8109 6723
8116 6721 8123 6719 8129 6724 8134 6729 8140 6733 8145 6738
8147 6745 8150 6752 8157 6753 8157 6760 8156 6767 8156 6775
8152 6780 8145 6777 8141 6782 8136 6788 8130 6786 8124 6790
8117 6793 8110 6794 8111 6787 8105 6783 8104 6776 8100 6769
8095 6764 8091 6758 8093 6751 8100 6753 8105 6758 8112 6761
8113 6754 8107 6750 8100 6747 39 N F
8169 6716 8165 6714 8167 6712 8171 6712 8174 6710 8176 6713
8179 6715 8182 6713 8185 6715 8185 6718 8188 6720 8191 6720
8193 6723 8194 6726 8195 6729 8193 6732 8190 6734 8187 6736
8184 6737 8181 6735 8178 6735 8175 6734 8174 6731 8174 6727
8173 6724 8170 6723 8169 6719 8169 6716 28 N F
8212 6719 8200 6715 8205 6710 8210 6704 8216 6701 8223 6699
8229 6702 8231 6709 8235 6715 8241 6719 8247 6723 8254 6725
8261 6726 8268 6725 8274 6721 8280 6717 8286 6713 8293 6717
8297 6723 8297 6730 8302 6735 8298 6741 8298 6748 8294 6754
8292 6761 8290 6768 8283 6770 8279 6776 8274 6781 8269 6775
8263 6771 8266 6765 8261 6759 8255 6755 8254 6748 8250 6742
8243 6744 8236 6746 8230 6750 8224 6755 8219 6759 8213 6756
8207 6751 8209 6744 8215 6748 8212 6741 8214 6734 8216 6727
8219 6720 8212 6719 50 N F
8212 6801 8209 6799 8212 6798 8215 6796 8218 6794 8221 6792
8223 6790 8227 6789 8230 6790 8231 6793 8232 6797 8234 6799
8232 6803 8229 6803 8226 6804 8222 6804 8219 6804 8216 6802
8212 6801 19 N F
8275 14785 8272 14780 8276 14781 8279 14784 8283 14787 8285 14790
8287 14794 8290 14797 8293 14801 8294 14805 8295 14809 8293 14813
8291 14809 8289 14805 8286 14803 8282 14800 8282 14796 8281 14791
8279 14788 8275 14785 20 N F
8293 14827 8292 14824 8293 14823 8295 14823 8298 14824 8299 14827
8301 14830 8301 14833 8299 14831 8296 14830 8293 14827 11 N F
8313 14844 8311 14841 8313 14839 8316 14841 8318 14843 8320 14846
8321 14849 8323 14852 8325 14854 8326 14857 8323 14855 8321 14853
8319 14851 8318 14848 8316 14846 8313 14844 16 N F
8362 14835 8356 14833 8361 14834 8365 14835 8369 14837 8372 14840
8375 14843 8378 14846 8379 14850 8377 14854 8373 14856 8370 14860
8369 14856 8370 14852 8374 14850 8375 14846 8371 14844 8368 14841
8365 14837 8362 14835 20 N F
8397 14885 8392 14880 8397 14881 8401 14882 8405 14883 8409 14884

8413 14885 8416 14889 8417 14893 8419 14897 8423 14899 8424 14903
8422 14906 8418 14905 8415 14902 8413 14898 8410 14895 8408 14891
8405 14888 8401 14886 8397 14885 21 N F
8398 14898 8397 14896 8399 14894 8402 14895 8405 14897 8406 14900
8407 14903 8407 14906 8406 14909 8404 14906 8402 14904 8401 14901
8398 14898 13 N F
8431 14897 8426 14896 8430 14894 8434 14896 8438 14897 8442 14898
8446 14900 8450 14903 8452 14906 8456 14909 8458 14913 8461 14916
8464 14919 8467 14922 8470 14925 8473 14928 8475 14933 8471 14931
8467 14929 8463 14927 8459 14925 8455 14924 8455 14919 8452 14916
8448 14914 8445 14912 8441 14909 8437 14907 8437 14903 8434 14899
8431 14897 31 N F
8628 7084 8623 7083 8627 7081 8631 7081 8634 7077 8635 7072
8639 7071 8638 7075 8640 7079 8643 7082 8646 7086 8645 7090
8647 7094 8644 7097 8644 7101 8647 7105 8652 7106 8655 7109
8654 7114 8651 7111 8646 7109 8642 7109 8638 7106 8635 7103
8631 7102 8631 7097 8633 7093 8636 7090 8636 7085 8632 7083
8628 7084 31 N F
8709 12528 8704 12528 8706 12524 8709 12521 8712 12518 8715 12515
8719 12514 8723 12512 8727 12514 8731 12516 8728 12519 8724 12521
8720 12522 8716 12522 8711 12524 8709 12528 16 N F
8717 7335 8712 7324 8716 7318 8721 7312 8721 7305 8724 7299
8731 7302 8736 7306 8743 7306 8744 7313 8741 7320 8742 7327
8741 7334 8743 7341 8746 7347 8748 7354 8747 7361 8750 7367
8750 7374 8750 7381 8747 7388 8740 7385 8737 7379 8733 7372
8727 7368 8727 7361 8724 7355 8721 7348 8719 7341 8717 7335
30 N F
8750 6139 8749 6136 8751 6135 8753 6136 8755 6136 8757 6138
8757 6140 8755 6141 8753 6142 8751 6141 8750 6139 11 N F
8810 7193 8800 7191 8805 7186 8802 7180 8807 7174 8814 7174
8818 7172 8821 7171 8827 7175 8833 7177 8839 7173 8837 7166
8836 7159 8842 7156 8849 7153 8853 7153 8856 7153 8859 7160
8854 7165 8857 7171 8864 7172 8869 7178 8862 7176 8857 7181
8853 7175 8845 7176 8839 7180 8833 7183 8828 7189 8828 7196
8822 7197 8819 7204 8814 7199 8810 7193 34 N F
8809 6033 8802 6029 8805 6026 8807 6022 8808 6017 8812 6015
8812 6020 8816 6022 8819 6026 8820 6030 8818 6035 8813 6034
8809 6033 13 N F
8837 6238 8828 6231 8835 6232 8842 6234 8844 6234 8849 6233
8851 6240 8852 6247 8856 6253 8855 6260 8854 6268 8860 6272
8866 6276 8866 6276 8859 6277 8853 6282 8848 6277 8846 6270
8846 6263 8846 6255 8841 6250 8846 6246 8844 6239 8837 6238
24 N F

8844 6118 8840 6113 8845 6111 8849 6112 8853 6112 8854 6112
8852 6116 8855 6119 8852 6123 8851 6127 8851 6132 8847 6133
8844 6136 8843 6132 8842 6127 8844 6123 8844 6118 17 N F
8848 7688 8842 7677 8849 7676 8854 7676 8856 7675 8863 7673
8870 7673 8875 7669 8882 7665 8889 7665 8895 7668 8895 7675
8894 7682 8890 7688 8888 7695 8882 7698 8875 7701 8869 7705
8863 7701 8857 7699 8852 7694 8848 7688 22 N F
8854 7674 8846 7672 8850 7669 8853 7666 8857 7664 8862 7664
8866 7665 8871 7666 8875 7668 8871 7671 8867 7672 8862 7673
8858 7673 8854 7674 14 N F
8851 6192 8850 6188 8851 6186 8853 6187 8855 6187 8858 6188
8860 6188 8862 6189 8864 6191 8865 6192 8866 6192 8868 6192
8868 6195 8866 6195 8864 6195 8861 6194 8860 6193 8857 6192
8856 6191 8854 6189 8853 6191 8851 6192 22 N F
8853 6065 8850 6063 8852 6061 8854 6061 8855 6064 8853 6065
6 N F
8856 6058 8855 6054 8857 6055 8859 6056 8860 6058 8859 6060
8857 6060 8856 6058 8 N F
8862 7429 8857 7426 8860 7422 8864 7421 8869 7421 8871 7425
8875 7426 8878 7430 8880 7434 8883 7437 8883 7442 8878 7442
8875 7438 8872 7436 8868 7433 8867 7429 8864 7425 8862 7429
18 N F
8859 6119 8857 6117 8859 6115 8859 6115 8861 6115 8862 6117
8864 6119 8866 6119 8869 6119 8869 6121 8868 6123 8866 6123
8864 6123 8861 6122 8861 6120 8859 6119 16 N F
8872 6226 8867 6219 8873 6215 8878 6210 8882 6204 8889 6202
8896 6204 8903 6207 8902 6214 8907 6220 8912 6224 8913 6224
8919 6228 8924 6233 8931 6233 8938 6232 8940 6231 8945 6229
8942 6236 8940 6243 8939 6250 8935 6256 8930 6251 8928 6258
8921 6255 8920 6248 8918 6255 8915 6249 8911 6243 8910 6250
8912 6257 8905 6257 8901 6251 8897 6245 8896 6238 8892 6244
8889 6250 8883 6246 8880 6240 8876 6234 8883 6236 8885 6229
8878 6230 8884 6226 8891 6227 8898 6227 8896 6220 8890 6216
8886 6210 8881 6215 8875 6219 8872 6226 52 N F
8875 6296 8870 6289 8876 6285 8881 6290 8886 6285 8891 6280
8893 6273 8897 6267 8900 6273 8898 6280 8903 6285 8900 6292
8896 6298 8889 6299 8882 6297 8875 6296 16 N F
8897 8011 8893 8006 8895 8002 8898 7998 8898 7993 8903 7993
8904 7998 8908 7995 8912 7994 8917 7995 8921 7996 8926 7996
8927 8001 8930 8004 8934 8006 8939 8007 8943 8009 8946 8012
8945 8017 8941 8018 8937 8018 8933 8015 8932 8010 8930 8007
8926 8008 8927 8012 8923 8012 8918 8011 8914 8013 8910 8013
8905 8012 8901 8012 8897 8011 33 N F

8895 6337 8894 6334 8896 6332 8897 6328 8898 6331 8900 6334
8903 6336 8906 6337 8907 6341 8904 6343 8902 6345 8899 6343
8898 6340 8895 6337 14 N F
8903 7440 8902 7436 8905 7436 8908 7436 8910 7437 8913 7438
8913 7441 8910 7442 8909 7445 8906 7444 8904 7443 8903 7440
12 N F
8908 6054 8906 6053 8908 6051 8910 6050 8912 6051 8913 6053
8912 6055 8910 6055 8908 6054 9 N F
8913 7434 8910 7428 8914 7426 8918 7425 8923 7425 8928 7426
8932 7427 8936 7425 8939 7428 8941 7433 8939 7437 8937 7441
8934 7438 8930 7436 8925 7434 8921 7432 8918 7435 8913 7434
18 N F
8921 7339 8918 7332 8924 7335 8931 7338 8938 7338 8943 7342
8942 7335 8942 7328 8935 7330 8932 7323 8937 7318 8933 7312
8938 7307 8943 7302 8945 7301 8950 7300 8955 7295 8960 7290
8967 7288 8971 7294 8967 7301 8961 7304 8964 7311 8970 7315
8976 7318 8981 7323 8988 7325 8993 7329 9000 7330 9005 7325
9011 7329 9012 7336 9005 7336 8998 7338 8992 7342 8987 7347
8985 7353 8985 7360 8987 7367 8993 7372 8999 7375 9006 7377
9010 7372 9017 7374 9021 7379 9025 7385 9018 7385 9011 7385
9004 7387 8999 7392 8992 7389 8995 7396 9000 7401 8996 7408
8990 7411 8985 7416 8978 7413 8972 7416 8968 7422 8968 7429
8972 7435 8976 7441 8969 7441 8963 7437 8957 7432 8954 7426
8948 7429 8943 7424 8941 7417 8934 7417 8930 7411 8926 7406
8926 7398 8930 7392 8937 7394 8941 7400 8948 7400 8951 7394
8950 7387 8946 7380 8946 7373 8945 7366 8944 7359 8950 7355
8955 7360 8958 7366 8964 7362 8971 7361 8971 7354 8966 7350
8960 7346 8952 7346 8948 7351 8941 7352 8934 7354 8929 7348
8922 7346 8921 7339 98 N F
8928 7252 8926 7249 8926 7245 8929 7243 8927 7240 8927 7237
8928 7237 8929 7239 8932 7241 8935 7243 8938 7244 8935 7245
8933 7247 8931 7250 8934 7253 8933 7256 8930 7255 8928 7252
18 N F
8936 7272 8930 7270 8932 7267 8933 7266 8934 7264 8937 7266
8940 7265 8944 7267 8947 7268 8950 7270 8947 7272 8944 7274
8945 7278 8942 7277 8940 7275 8936 7276 8936 7272 17 N F
8938 6061 8936 6058 8937 6056 8936 6054 8937 6052 8939 6051
8940 6053 8940 6055 8942 6056 8943 6059 8942 6061 8940 6061
8938 6061 13 N F
8951 7987 8948 7984 8949 7981 8951 7978 8954 7978 8957 7976
8960 7978 8963 7979 8965 7982 8968 7982 8972 7981 8974 7983
8976 7986 8973 7989 8970 7990 8967 7992 8964 7990 8961 7989
8958 7988 8955 7988 8951 7987 21 N F

9011 13074 9010 13068 9013 13064 9017 13063 9020 13066 9024 13069
9024 13073 9022 13077 9021 13081 9021 13085 9016 13086 9015 13081
9013 13078 9011 13074 14 N F
9033 7720 9031 7716 9035 7714 9039 7712 9043 7715 9047 7715
9051 7718 9053 7719 9055 7720 9057 7724 9059 7728 9054 7729
9050 7729 9048 7725 9044 7722 9041 7726 9037 7725 9039 7730
9036 7733 9035 7728 9032 7725 9033 7720 22 N F
9046 7623 9042 7620 9044 7617 9046 7615 9049 7616 9052 7618
9055 7620 9053 7623 9049 7623 9046 7623 10 N F
9059 7751 9058 7746 9062 7743 9062 7748 9062 7752 9064 7756
9067 7757 9069 7758 9073 7759 9077 7757 9081 7754 9083 7757
9082 7762 9079 7764 9075 7768 9071 7768 9067 7764 9064 7762
9060 7760 9060 7755 9059 7751 21 N F
9065 7611 9062 7605 9066 7607 9070 7608 9073 7612 9076 7615
9071 7615 9067 7616 9065 7611 9 N F
9087 7924 9084 7917 9089 7916 9093 7916 9095 7920 9099 7919
9103 7920 9104 7925 9104 7929 9104 7934 9100 7934 9098 7930
9095 7933 9091 7931 9087 7928 9087 7924 16 N F
9122 6194 9121 6189 9121 6184 9122 6180 9123 6175 9124 6171
9125 6166 9126 6171 9125 6175 9124 6180 9124 6185 9126 6189
9127 6193 9127 6198 9129 6202 9130 6207 9132 6211 9135 6214
9138 6218 9137 6223 9134 6219 9132 6215 9130 6211 9128 6207
9125 6203 9123 6199 9122 6194 27 N F
9134 7964 9128 7959 9132 7956 9135 7953 9137 7953 9140 7954
9144 7956 9143 7961 9148 7962 9152 7963 9152 7968 9154 7972
9154 7976 9152 7980 9147 7981 9145 7977 9142 7974 9140 7970
9138 7966 9134 7964 20 N F
9140 5975 M 9136 5961 L 9146 5951 L 9160 5955 L 9152 5942 L
9164 5933 L 9175 5925 L 9189 5929 L 9203 5933 L
9217 5938 L 9220 5923 L 9207 5917 L 9193 5914 L
9200 5901 L 9207 5889 L 9211 5875 L 9211 5860 L
9216 5847 L 9221 5833 L 9235 5830 L 9226 5818 L
9232 5805 L 9237 5792 L 9243 5778 L 9249 5765 L
9255 5752 L 9259 5738 L 9258 5723 L 9255 5709 L
9263 5697 L 9276 5691 L 9280 5677 L 9291 5667 L
9302 5658 L 9289 5663 L 9279 5674 L 9271 5687 L
9277 5673 L 9286 5662 L 9296 5651 L 9306 5641 L
9316 5630 L 9328 5623 L 9328 5622 L 9340 5614 L
9347 5627 L 9353 5614 L 9362 5603 L 9373 5593 L
9386 5587 L 9398 5579 L 9412 5573 L 9424 5566 L
9436 5558 L 9447 5548 L 9454 5535 L 9465 5526 L
9472 5513 L 9475 5503 L 9477 5503 L 9510 5503 L
9543 5503 L 9577 5503 L 9607 5503 L 9607 5512 L

9606 5527 L 9608 5541 L 9614 5555 L 9616 5569 L
9622 5583 L 9628 5596 L 9632 5610 L 9635 5624 L
9638 5638 L 9643 5652 L 9646 5666 L 9652 5679 L
9662 5690 L 9664 5704 L 9667 5718 L 9673 5732 L
9684 5740 L 9695 5750 L 9707 5758 L 9720 5765 L
9730 5775 L 9740 5786 L 9750 5796 L 9754 5810 L
9758 5824 L 9753 5838 L 9760 5851 L 9773 5856 L
9784 5865 L 9790 5878 L 9797 5891 L 9801 5905 L
9812 5915 L 9819 5928 L 9824 5941 L 9830 5955 L
9832 5969 L 9838 5982 L 9848 5992 L 9857 6003 L
9859 6018 L 9863 6032 L 9857 6045 L 9859 6060 L
9865 6073 L 9868 6087 L 9872 6101 L 9878 6114 L
9889 6123 L 9891 6137 L 9892 6151 L 9894 6166 L
9899 6179 L 9903 6193 L 9906 6207 L 9911 6221 L
9906 6234 L 9907 6249 L 9910 6263 L 9916 6276 L
9917 6290 L 9917 6305 L 9918 6307 L 9923 6318 L
9924 6332 L 9925 6347 L 9928 6361 L 9934 6374 L
9934 6388 L 9934 6403 L 9939 6416 L 9944 6430 L
9947 6444 L 9949 6458 L 9949 6473 L 9949 6487 L
9951 6502 L 9960 6513 L 9968 6525 L 9960 6537 L
9953 6549 L 9950 6563 L 9955 6577 L 9965 6587 L
9974 6598 L 9972 6613 L 9970 6627 L 9977 6640 L
9988 6648 L 9997 6660 L 10008 6668 L 10014 6682 L
10021 6694 L 10026 6707 L 10026 6722 L 10030 6735 L
10032 6749 L 10034 6764 L 10040 6777 L 10047 6789 L
10058 6799 L 10055 6813 L 10060 6826 L 10073 6834 L
10070 6848 L 10071 6862 L 10068 6877 L 10058 6887 L
10053 6901 L 10050 6915 L 10048 6929 L 10043 6942 L
10039 6956 L 10037 6970 L 10038 6985 L 10039 6999 L
10041 7013 L 10046 7027 L 10054 7038 L 10064 7049 L
10069 7062 L 10080 7071 L 10088 7083 L 10101 7090 L
10104 7091 L 10113 7097 L 10125 7105 L 10117 7117 L
10103 7116 L 10090 7123 L 10089 7137 L 10081 7149 L
10083 7163 L 10073 7173 L 10059 7172 L 10044 7173 L
10030 7170 L 10020 7179 L 10006 7185 L 9994 7178 L
9984 7189 L 9971 7194 L 9959 7203 L 9964 7217 L
9950 7212 L 9940 7221 L 9931 7233 L 9927 7247 L
9916 7255 L 9903 7262 L 9889 7260 L 9875 7257 L
9862 7252 L 9849 7245 L 9840 7234 L 9832 7222 L
9824 7210 L 9817 7198 L 9828 7189 L 9815 7184 L
9805 7173 L 9793 7167 L 9780 7161 L 9766 7158 L
9752 7155 L 9738 7156 L 9724 7151 L 9711 7145 L
9699 7138 L 9687 7130 L 9673 7127 L 9660 7121 L

9649 7112 L 9636 7106 L 9626 7096 L 9618 7080 L
9615 7066 L 9607 7055 L 9598 7043 L 9592 7030 L
9586 7017 L 9582 7003 L 9573 6993 L 9564 6981 L
9571 6968 L 9567 6954 L 9562 6941 L 9553 6930 L
9543 6920 L 9540 6906 L 9529 6896 L 9540 6886 L
9525 6887 L 9511 6885 L 9498 6879 L 9490 6868 L
9496 6855 L 9482 6852 L 9469 6846 L 9464 6833 L
9459 6819 L 9455 6805 L 9452 6792 L 9445 6779 L
9438 6767 L 9427 6757 L 9419 6745 L 9409 6734 L
9403 6721 L 9397 6708 L 9392 6695 L 9386 6682 L
9384 6668 L 9375 6656 L 9371 6642 L 9363 6630 L
9356 6618 L 9368 6610 L 9354 6610 L 9347 6597 L
9340 6585 L 9332 6573 L 9329 6559 L 9324 6545 L
9315 6534 L 9305 6523 L 9296 6513 L 9289 6500 L
9280 6489 L 9274 6476 L 9274 6462 L 9267 6449 L
9261 6436 L 9257 6422 L 9247 6411 L 9256 6400 L
9270 6398 L 9285 6397 L 9299 6399 L 9288 6390 L
9274 6388 L 9259 6387 L 9245 6389 L 9231 6389 L
9222 6377 L 9213 6366 L 9212 6351 L 9210 6337 L
9207 6323 L 9195 6314 L 9188 6302 L 9183 6288 L
9181 6274 L 9180 6260 L 9187 6247 L 9187 6232 L
9192 6219 L 9181 6211 L 9190 6201 L 9188 6187 L
9199 6177 L 9188 6168 L 9192 6154 L 9196 6140 L
9194 6126 L 9192 6111 L 9199 6099 L 9185 6101 L
9183 6087 L 9175 6075 L 9177 6060 L 9166 6052 L
9179 6046 L 9165 6044 L 9163 6030 L 9164 6015 L
9155 6006 L 9169 6001 L 9173 5988 L 9169 5974 L
9158 5965 L 9144 5961 L 9140 5975 L N E
9170 14289 9167 14284 9169 14280 9172 14277 9176 14274 9179 14272
9181 14275 9181 14280 9181 14284 9181 14288 9178 14291 9173 14291
9170 14289 13 N F
9198 15653 9196 15646 9199 15643 9203 15645 9206 15648 9209 15650
9209 15650 9214 15651 9218 15652 9219 15656 9219 15660 9219 15664
9215 15667 9211 15669 9209 15665 9205 15665 9204 15661 9205 15657
9201 15656 9198 15653 20 N F
9209 14298 9204 14296 9207 14293 9210 14290 9212 14286 9215 14283
9220 14283 9223 14280 9226 14283 9225 14288 9223 14292 9221 14295
9217 14295 9213 14298 9209 14298 15 N F
9225 14258 9221 14255 9225 14253 9229 14253 9232 14250 9234 14246
9238 14244 9240 14247 9239 14252 9238 14256 9240 14260 9238 14264
9235 14267 9233 14271 9232 14275 9228 14278 9225 14275 9223 14271
9224 14266 9225 14262 9225 14258 21 N F
9225 14401 9224 14397 9224 14391 9228 14388 9228 14388 9232 14390

9236 14389 9240 14389 9243 14392 9240 14395 9235 14396 9231 14396
9231 14401 9230 14405 9228 14409 9225 14406 9225 14401 17 N F
9227 8184 9225 8179 9227 8175 9230 8172 9234 8169 9238 8167
9242 8164 9246 8165 9250 8167 9254 8169 9259 8170 9263 8170
9259 8172 9255 8174 9253 8178 9252 8183 9250 8187 9246 8187
9242 8191 9238 8191 9233 8190 9230 8187 9227 8184 23 N F
9251 14466 9247 14461 9250 14459 9254 14461 9256 14465 9260 14466
9264 14467 9266 14471 9266 14475 9262 14477 9258 14476 9254 14474
9251 14471 9251 14466 14 N F
9291 14506 9287 14503 9289 14500 9291 14497 9293 14495 9295 14493
9296 14496 9295 14499 9297 14501 9294 14501 9294 14504 9291 14506
12 N F
9291 14518 9289 14513 9291 14513 9292 14514 9295 14513 9298 14515
9301 14513 9301 14517 9299 14519 9298 14522 9295 14522 9294 14519
9291 14518 13 N F
9311 7864 9309 7859 9312 7861 9313 7857 9316 7859 9318 7862
9316 7865 9313 7867 9311 7864 9 N F
9311 14324 9309 14320 9311 14318 9314 14317 9317 14319 9317 14322
9314 14323 9311 14324 8 N F
9318 5530 9315 5525 9320 5523 9324 5524 9326 5528 9330 5532
9333 5534 9335 5538 9332 5542 9328 5543 9323 5541 9320 5538
9320 5533 9318 5530 14 N F
9318 7859 9316 7852 9320 7850 9324 7853 9326 7856 9331 7855
9329 7859 9326 7862 9322 7861 9318 7859 10 N F
9412 15149 9403 15148 9405 15141 9404 15134 9410 15131 9416 15129
9423 15132 9429 15131 9433 15126 9436 15119 9439 15125 9445 15129
9449 15135 9454 15139 9457 15139 9461 15139 9468 15140 9473 15136
9479 15133 9486 15132 9492 15134 9496 15140 9501 15144 9508 15146
9511 15152 9515 15157 9521 15156 9527 15160 9530 15166 9532 15172
9531 15179 9531 15186 9527 15192 9526 15198 9519 15200 9513 15203
9507 15206 9501 15209 9494 15209 9499 15204 9499 15197 9495 15192
9489 15189 9482 15189 9478 15183 9472 15181 9465 15181 9458 15181
9452 15180 9445 15177 9439 15177 9433 15181 9431 15174 9431 15168
9426 15164 9420 15160 9417 15154 9412 15149 58 N F
9420 15069 9416 15064 9422 15061 9429 15059 9433 15064 9435 15070
9440 15075 9445 15080 9449 15085 9450 15092 9456 15096 9462 15096
9469 15095 9471 15089 9466 15084 9462 15079 9458 15074 9455 15067
9452 15062 9458 15060 9465 15058 9472 15057 9477 15053 9484 15053
9489 15057 9490 15064 9494 15069 9497 15075 9496 15082 9493 15088
9488 15093 9482 15095 9489 15094 9495 15094 9502 15097 9500 15106
9497 15112 9491 15114 9484 15116 9478 15118 9471 15116 9466 15112
9462 15118 9457 15122 9450 15120 9446 15115 9443 15109 9443 15102
9439 15097 9432 15096 9425 15095 9423 15089 9425 15082 9418 15081

9422 15075 9420 15069 56 N F
9417 8792 9417 8788 9418 8785 9419 8782 9422 8779 9425 8778
9427 8775 9429 8772 9432 8771 9436 8769 9439 8769 9441 8771
9441 8774 9442 8777 9439 8779 9437 8782 9436 8785 9433 8787
9430 8789 9427 8790 9424 8791 9420 8791 9417 8792 23 N F
9476 8767 9470 8765 9473 8764 9476 8762 9479 8760 9482 8758
9485 8757 9487 8754 9490 8753 9493 8752 9496 8750 9500 8751
9503 8753 9506 8754 9508 8757 9510 8760 9510 8763 9508 8766
9505 8768 9502 8769 9499 8769 9496 8770 9492 8769 9489 8769
9486 8769 9482 8769 9479 8768 9476 8767 28 N F
9545 10909 9538 10900 9542 10895 9547 10890 9552 10886 9557 10881
9562 10875 9566 10870 9571 10865 9577 10868 9583 10865 9590 10862
9595 10858 9600 10853 9606 10849 9611 10844 9616 10839 9620 10834
9626 10830 9632 10827 9638 10824 9645 10821 9648 10815 9654 10812
9661 10811 9668 10809 9673 10804 9679 10802 9686 10799 9692 10796
9699 10794 9705 10792 9711 10787 9717 10784 9723 10781 9728 10776
9735 10775 9741 10773 9747 10769 9753 10765 9756 10764 9760 10763
9766 10762 9773 10763 9780 10762 9787 10760 9793 10758 9800 10756
9806 10753 9812 10749 9819 10746 9825 10744 9832 10742 9838 10739
9845 10738 9852 10736 9858 10735 9859 10735 9865 10735 9872 10738
9878 10741 9880 10748 9885 10753 9890 10757 9892 10764 9893 10771
9894 10777 9891 10784 9886 10787 9880 10792 9874 10794 9867 10795
9860 10795 9853 10796 9847 10799 9840 10800 9834 10804 9828 10807
9823 10812 9817 10815 9811 10818 9805 10822 9798 10824 9791 10826
9785 10829 9778 10831 9772 10832 9765 10834 9758 10836 9752 10839
9746 10842 9742 10848 9739 10854 9734 10859 9728 10863 9723 10868
9717 10870 9710 10873 9704 10876 9698 10879 9691 10881 9685 10883
9678 10885 9672 10889 9668 10894 9665 10900 9662 10907 9658 10912
9654 10918 9650 10924 9644 10928 9639 10932 9632 10935 9626 10937
9619 10938 9612 10940 9605 10940 9599 10939 9592 10938 9585 10936
9579 10934 9572 10931 9567 10927 9562 10922 9557 10917 9551 10913
9545 10909 127 N F
9561 8701 9558 8697 9561 8693 9564 8696 9568 8698 9572 8701
9576 8703 9579 8700 9583 8698 9583 8702 9584 8703 9587 8705
9590 8708 9593 8711 9593 8711 9598 8711 9594 8714 9590 8711
9585 8710 9581 8708 9577 8709 9574 8713 9571 8716 9567 8716
9567 8712 9563 8710 9562 8705 9561 8701 28 N F
9568 8760 9567 8756 9571 8757 9576 8758 9580 8759 9584 8761
9589 8762 9593 8760 9591 8756 9593 8752 9597 8755 9601 8755
9606 8756 9610 8758 9614 8760 9617 8763 9621 8764 9618 8767
9614 8769 9611 8773 9608 8776 9604 8778 9600 8775 9596 8775
9591 8776 9588 8774 9584 8772 9584 8777 9580 8776 9579 8772
9575 8769 9571 8767 9568 8765 9568 8760 34 N F

9585 8486 9580 8482 9583 8479 9587 8476 9590 8473 9590 8477
9590 8482 9594 8480 9598 8478 9602 8476 9606 8476 9610 8473
9610 8478 9607 8481 9603 8483 9599 8486 9596 8489 9592 8491
9589 8494 9584 8495 9588 8492 9591 8489 9587 8490 9585 8486
24 N F
9588 8664 9586 8661 9589 8661 9591 8659 9594 8660 9596 8662
9598 8664 9597 8667 9595 8669 9593 8670 9591 8669 9590 8666
9588 8664 13 N F
9596 8689 9593 8684 9598 8684 9602 8682 9606 8683 9611 8682
9614 8685 9619 8684 9622 8688 9626 8686 9630 8684 9634 8682
9638 8683 9639 8687 9639 8692 9639 8696 9635 8699 9639 8701
9640 8697 9643 8700 9647 8703 9644 8707 9645 8711 9641 8712
9638 8708 9634 8706 9630 8708 9626 8705 9629 8702 9627 8698
9623 8698 9618 8696 9616 8700 9613 8703 9611 8699 9607 8697
9604 8693 9600 8691 9596 8689 39 N F
9617 8863 9608 8861 9610 8855 9611 8848 9614 8841 9618 8836
9622 8830 9622 8823 9615 8820 9621 8816 9620 8809 9627 8811
9634 8812 9640 8815 9641 8815 9647 8817 9654 8819 9653 8826
9660 8824 9665 8820 9668 8826 9662 8830 9664 8837 9670 8833
9676 8836 9678 8830 9685 8832 9681 8837 9687 8840 9688 8848
9681 8848 9675 8851 9671 8857 9672 8864 9670 8871 9667 8877
9670 8884 9674 8889 9681 8890 9679 8897 9673 8901 9667 8904
9661 8908 9654 8907 9647 8905 9643 8900 9642 8893 9643 8886
9637 8882 9633 8877 9628 8872 9622 8869 9617 8863 53 N F
9633 8782 9631 8778 9634 8775 9636 8771 9640 8770 9641 8770
9643 8773 9647 8776 9651 8778 9655 8779 9657 8781 9659 8781
9663 8783 9658 8784 9654 8783 9650 8785 9645 8785 9641 8787
9637 8784 9633 8782 20 N F
9652 8577 9648 8575 9651 8574 9651 8572 9654 8572 9656 8573
9657 8574 9659 8576 9660 8578 9661 8579 9662 8579 9663 8581
9662 8583 9660 8582 9657 8583 9656 8581 9656 8579 9654 8578
9652 8577 19 N F
9656 8571 9655 8569 9657 8567 9658 8569 9660 8571 9661 8573
9661 8575 9659 8575 9657 8573 9656 8571 10 N F
9671 14801 9668 14798 9669 14795 9669 14792 9671 14790 9674 14791
9676 14794 9675 14797 9674 14799 9674 14802 9675 14806 9672 14806
9670 14804 9671 14801 14 N F
9756 10723 9747 10718 9753 10715 9759 10713 9765 10709 9771 10706
9776 10701 9783 10698 9789 10695 9795 10692 9800 10687 9806 10684
9813 10681 9818 10677 9824 10674 9828 10668 9832 10663 9836 10657
9842 10653 9848 10656 9849 10663 9848 10669 9845 10676 9840 10681
9836 10686 9832 10691 9826 10695 9821 10700 9814 10702 9809 10707
9803 10709 9796 10712 9790 10714 9783 10716 9776 10718 9770 10720

9763 10721 9756 10723 38 N F
9792 8843 9791 8841 9791 8837 9794 8837 9797 8839 9798 8842
9800 8845 9803 8845 9806 8847 9807 8850 9805 8853 9802 8853
9800 8850 9798 8847 9795 8846 9792 8843 16 N F
9795 9444 9793 9441 9796 9439 9799 9438 9801 9435 9805 9436
9808 9436 9811 9437 9814 9438 9815 9441 9815 9444 9813 9447
9810 9447 9807 9445 9808 9442 9805 9441 9801 9441 9799 9443
9795 9444 19 N F
9831 9091 9829 9085 9831 9083 9834 9081 9837 9079 9840 9078
9842 9080 9841 9083 9842 9085 9843 9086 9846 9086 9846 9090
9843 9091 9843 9094 9841 9097 9840 9100 9837 9101 9834 9100
9832 9097 9832 9094 9831 9091 21 N F
9842 9074 9838 9072 9839 9069 9842 9066 9845 9065 9848 9067
9850 9069 9848 9072 9845 9073 9842 9074 10 N F
9859 10689 9851 10687 9854 10681 9857 10675 9858 10668 9860 10662
9862 10655 9867 10650 9873 10647 9879 10643 9885 10645 9889 10651
9893 10657 9895 10663 9894 10670 9891 10677 9885 10680 9879 10683
9873 10686 9866 10687 9859 10689 21 N F
9877 14978 9866 14974 9870 14968 9869 14962 9874 14957 9879 14953
9885 14956 9888 14962 9889 14969 9895 14965 9901 14963 9900 14970
9898 14976 9896 14983 9898 14989 9904 14991 9898 14992 9893 14987
9886 14985 9880 14985 9877 14978 21 N F
9913 5751 9904 5749 9908 5744 9910 5739 9913 5734 9918 5732
9922 5727 9927 5728 9926 5733 9928 5739 9929 5744 9929 5750
9924 5752 9918 5753 9913 5751 15 N F
9908 9440 9905 9433 9907 9429 9907 9429 9911 9429 9916 9429
9920 9428 9923 9427 9924 9426 9928 9424 9930 9420 9933 9417
9938 9417 9942 9416 9946 9419 9950 9420 9947 9424 9952 9425
9953 9426 9954 9428 9957 9431 9955 9435 9950 9435 9947 9438
9948 9442 9946 9445 9942 9444 9937 9443 9933 9442 9928 9442
9924 9443 9921 9447 9917 9445 9913 9443 9910 9446 9906 9444
9908 9440 37 N F
9908 9195 9905 9190 9909 9189 9912 9189 9914 9186 9916 9184
9920 9184 9923 9185 9923 9189 9923 9192 9926 9194 9928 9197
9924 9198 9923 9195 9920 9193 9917 9190 9914 9190 9912 9192
9913 9195 9910 9196 9907 9198 9908 9195 22 N F
9911 15022 9907 15021 9910 15020 9913 15019 9916 15017 9916 15017
9918 15015 9921 15016 9924 15016 9922 15018 9919 15020 9916 15020
9914 15023 9911 15022 14 N F
9911 15001 9908 14998 9911 14998 9914 14996 9917 14998 9920 14998
9920 15001 9919 15004 9916 15005 9913 15003 9911 15001 11 N F
9915 9412 9908 9406 9913 9405 9917 9406 9916 9401 9921 9401
9924 9397 9927 9395 9931 9397 9931 9401 9933 9405 9930 9409

9933 9412 9930 9415 9927 9418 9923 9420 9918 9419 9915 9416
9915 9412 19 N F
9913 9929 9909 9922 9914 9917 9916 9911 9922 9906 9928 9907
9933 9912 9939 9916 9945 9920 9950 9924 9953 9931 9951 9937
9948 9944 9947 9951 9943 9956 9937 9959 9933 9965 9930 9971
9923 9971 9916 9970 9915 9963 9912 9956 9909 9950 9910 9943
9912 9936 9913 9929 26 N F
9944 15007 9939 15004 9942 15002 9943 14999 9946 15000 9950 14999
9953 15000 9956 15000 9959 15000 9961 15003 9963 15005 9960 15006
9957 15006 9954 15006 9951 15007 9947 15007 9944 15007 17 N F
9946 15041 9939 15040 9942 15037 9944 15036 9946 15035 9950 15034
9955 15033 9958 15031 9962 15029 9966 15028 9971 15027 9974 15025
9974 15021 9977 15018 9979 15022 9979 15026 9983 15027 9987 15027
9989 15027 9991 15026 9996 15025 10000 15026 10004 15025 10008 15023
10012 15021 10015 15024 10018 15027 10022 15029 10017 15029 10013 15030
10009 15032 10005 15033 10002 15037 9998 15038 9996 15034 9992 15033
9988 15032 9983 15032 9979 15033 9975 15033 9972 15036 9969 15039
9969 15044 9966 15041 9964 15037 9960 15040 9960 15044 9956 15044
9953 15041 9950 15044 9946 15041 51 N F
9943 9239 9939 9237 9942 9234 9944 9237 9947 9239 9949 9241
9952 9243 9952 9247 9952 9250 9954 9252 9957 9252 9956 9254
9955 9258 9953 9260 9950 9260 9947 9258 9946 9255 9947 9252
9946 9248 9943 9248 9944 9245 9945 9242 9943 9239 23 N F
9943 9982 9940 9976 9943 9973 9948 9973 9951 9970 9950 9966
9954 9964 9959 9964 9959 9969 9961 9973 9963 9977 9962 9981
9964 9985 9961 9987 9958 9990 9953 9989 9950 9987 9946 9985
9943 9982 19 N F
9949 9218 9946 9213 9950 9213 9952 9211 9955 9210 9958 9212
9958 9215 9955 9218 9958 9220 9960 9222 9963 9225 9966 9226
9969 9226 9969 9229 9973 9230 9976 9230 9973 9232 9970 9232
9967 9230 9964 9228 9962 9226 9958 9226 9956 9224 9952 9223
9949 9224 9947 9221 9949 9218 27 N F
9951 9444 9949 9437 9953 9437 9957 9435 9959 9431 9962 9428
9966 9427 9969 9424 9970 9420 9970 9415 9968 9411 9965 9408
9969 9409 9970 9409 9974 9409 9978 9409 9978 9409 9982 9407
9981 9403 9980 9399 9984 9398 9988 9395 9990 9397 9991 9399
9996 9400 9994 9403 9994 9408 9995 9412 9999 9414 10003 9416
10000 9420 9995 9420 9998 9424 10002 9426 10005 9429 10004 9434
10002 9438 10002 9442 9999 9446 10002 9450 10001 9454 9997 9457
9994 9459 9990 9462 9987 9465 9984 9469 9980 9469 9977 9467
9976 9463 9972 9460 9968 9458 9964 9455 9961 9453 9957 9451
9953 9448 9951 9444 56 N F
9958 10022 9951 10019 9955 10013 9958 10011 9960 10008 9966 10004

9969 9998 9971 9992 9972 9991 9976 9986 9977 9979 9978 9973
9977 9966 9975 9959 9978 9953 9984 9949 9988 9944 9992 9938
9996 9932 9998 9925 10004 9922 10008 9925 10010 9926 10015 9931
10021 9933 10021 9933 10028 9933 10035 9934 10042 9931 10044 9927
10045 9924 10052 9925 10059 9926 10066 9926 10066 9926 10072 9924
10079 9921 10085 9918 10092 9916 10098 9915 10105 9912 10111 9908
10117 9904 10117 9904 10123 9901 10130 9902 10137 9904 10143 9906
10140 9912 10139 9919 10141 9926 10144 9932 10146 9939 10152 9943
10145 9946 10142 9952 10136 9956 10132 9961 10127 9967 10121 9970
10114 9969 10108 9970 10104 9976 10097 9979 10096 9986 10089 9985
10082 9985 10076 9988 10073 9994 10066 9994 10059 9994 10052 9996
10048 10001 10043 10006 10039 10012 10034 10017 10027 10018 10021 10014
10015 10011 10008 10010 10002 10007 9995 10009 9990 10014 9984 10008
9978 10012 9971 10013 9971 10020 9966 10025 9960 10029 9958 10022
90 N F
9957 10101 9956 10097 9957 10094 9958 10091 9960 10093 9960 10093
9963 10095 9965 10097 9966 10100 9964 10103 9967 10106 9966 10109
9968 10110 9970 10113 9971 10116 9969 10119 9966 10117 9966 10113
9964 10111 9961 10113 9960 10110 9960 10107 9959 10103 9957 10101
24 N F
9958 9881 9956 9873 9959 9870 9962 9867 9967 9866 9971 9867
9975 9869 9980 9869 9984 9871 9988 9873 9988 9877 9988 9882
9985 9885 9981 9887 9978 9890 9977 9894 9974 9896 9972 9901
9968 9899 9966 9895 9962 9893 9959 9890 9958 9886 9958 9881
24 N F
9967 9085 9965 9081 9968 9082 9971 9083 9969 9080 9972 9078
9970 9075 9967 9072 9969 9070 9972 9071 9974 9068 9977 9070
9979 9073 9982 9071 9982 9074 9983 9077 9985 9080 9986 9081
9987 9083 9984 9083 9980 9083 9978 9085 9977 9088 9978 9092
9974 9091 9971 9090 9970 9087 9967 9085 28 N F
9968 9047 9965 9043 9968 9044 9971 9045 9973 9048 9975 9046
9978 9048 9981 9050 9983 9052 9986 9054 9988 9056 9986 9059
9983 9058 9979 9058 9976 9056 9973 9055 9971 9052 9971 9049
9968 9047 19 N F
9979 9813 9978 9809 9979 9805 9978 9800 9982 9798 9986 9797
9990 9797 9995 9798 9997 9801 10000 9805 10001 9809 10004 9812
10006 9816 10009 9820 10010 9824 10007 9828 10003 9829 9999 9827
9993 9824 9989 9822 9985 9819 9982 9816 9979 9813 23 N F
9983 9209 9979 9206 9982 9203 9982 9200 9985 9202 9987 9204
9991 9205 9992 9208 9990 9210 9987 9209 9983 9209 11 N F
9984 14998 9980 14995 9983 14992 9985 14994 9988 14995 9990 14998
9991 15001 9989 15004 9987 15001 9984 15001 9984 14998 11 N F
9990 10033 9987 10029 9990 10031 9993 10029 9995 10026 9997 10024

9995 10022 9993 10019 9996 10019 9999 10021 10002 10023 10004 10025
10007 10028 10008 10030 10011 10033 10009 10035 10008 10038 10006 10041
10003 10040 10000 10039 10000 10035 9998 10038 9995 10039 9992 10037
9994 10034 9990 10033 26 N F
9994 10287 9991 10283 9994 10280 9996 10278 9999 10279 10000 10282
10002 10285 10004 10287 10001 10287 9998 10288 9997 10285 9994 10287
12 N F
10001 10259 9995 10256 9998 10254 10001 10255 10003 10252 10004 10249
10006 10249 10008 10249 10010 10252 10010 10255 10012 10258 10015 10259
10012 10260 10009 10261 10005 10261 10004 10258 10001 10259 17 N F
10003 15012 9996 15008 9999 15005 10004 15005 10007 15008 10011 15007
10015 15008 10020 15009 10024 15009 10028 15008 10027 15004 10026 15000
10024 14996 10024 14992 10027 14995 10030 14997 10034 15000 10038 15000
10038 15005 10042 15006 10045 15007 10046 15008 10050 15010 10046 15012
10044 15016 10040 15018 10042 15021 10039 15019 10036 15015 10032 15017
10028 15016 10024 15016 10020 15014 10016 15013 10011 15013 10007 15013
10003 15012 37 N F
10002 9788 9999 9786 10000 9783 10000 9779 10001 9776 10000 9773
10003 9774 10006 9777 10008 9779 10009 9782 10011 9785 10014 9786
10014 9790 10013 9793 10010 9794 10006 9793 10003 9791 10002 9788
18 N F
10002 9904 10001 9900 10003 9897 10003 9894 10006 9892 10009 9892
10011 9894 10013 9897 10015 9900 10015 9903 10014 9907 10011 9908
10008 9908 10005 9906 10002 9904 15 N F
10018 9462 10016 9455 10021 9456 10025 9456 10027 9460 10030 9464
10033 9461 10038 9461 10041 9464 10044 9468 10040 9469 10035 9468
10031 9470 10032 9475 10036 9476 10033 9479 10036 9483 10031 9483
10027 9486 10025 9482 10027 9478 10024 9474 10028 9472 10024 9470
10022 9466 10017 9466 10018 9462 27 N F
10023 9783 10017 9771 10024 9771 10027 9776 10028 9777 10035 9777
10040 9772 10047 9773 10049 9766 10055 9763 10061 9759 10061 9752
10061 9745 10068 9745 10070 9752 10073 9746 10079 9749 10081 9742
10088 9741 10091 9747 10092 9754 10096 9760 10091 9765 10089 9772
10083 9774 10078 9779 10071 9781 10065 9785 10061 9790 10056 9796
10058 9803 10056 9809 10052 9815 10047 9810 10040 9808 10033 9808
10028 9803 10027 9796 10025 9789 10023 9783 40 N F
10023 10098 10018 10090 10024 10086 10030 10083 10036 10080 10042 10076
10048 10072 10054 10071 10061 10070 10064 10070 10068 10070 10072 10069
10075 10068 10081 10066 10088 10064 10085 10070 10083 10077 10088 10082
10090 10088 10085 10094 10090 10099 10095 10104 10088 10104 10083 10109
10085 10116 10079 10119 10072 10117 10066 10120 10065 10114 10058 10110
10052 10109 10047 10104 10040 10107 10033 10106 10029 10101 10023 10098
36 N F

10021 9928 10019 9925 10021 9922 10020 9918 10021 9915 10025 9916
10027 9918 10030 9919 10029 9923 10027 9925 10025 9927 10021 9928
12 N F
10040 14910 10036 14907 10039 14904 10042 14901 10039 14899 10042 14896
10045 14893 10050 14892 10054 14891 10058 14890 10061 14892 10061 14897
10056 14898 10055 14902 10052 14905 10048 14903 10046 14906 10045 14910
10040 14910 19 N F
10040 9915 10039 9911 10040 9908 10044 9908 10047 9909 10047 9912
10048 9915 10047 9918 10044 9919 10040 9918 10040 9915 11 N F
10054 15012 10050 15011 10053 15010 10057 15009 10059 15011 10056 15013
10054 15012 7 N F
10056 9909 10051 9905 10052 9905 10053 9903 10056 9902 10060 9902
10063 9902 10066 9903 10068 9906 10067 9908 10065 9911 10062 9912
10059 9911 10056 9909 14 N F
10053 10034 10051 10024 10054 10017 10059 10012 10066 10015 10070 10020
10071 10027 10078 10029 10083 10035 10076 10038 10070 10041 10064 10043
10058 10040 10053 10034 14 N F
10059 9918 10054 9914 10057 9913 10060 9915 10062 9914 10063 9914
10066 9915 10069 9913 10072 9911 10074 9908 10077 9910 10078 9913
10076 9916 10074 9918 10071 9920 10069 9923 10066 9925 10063 9923
10061 9920 10059 9918 20 N F
10061 15010 10056 15007 10059 15005 10062 15003 10064 15006 10067 15007
10070 15006 10073 15008 10071 15010 10067 15010 10064 15008 10061 15010
12 N F
10069 10061 10065 10057 10066 10055 10069 10054 10071 10052 10074 10052
10076 10052 10077 10055 10076 10058 10074 10060 10072 10061 10069 10061
12 N F
10072 14928 10067 14927 10068 14925 10071 14923 10074 14924 10078 14925
10081 14923 10083 14926 10080 14928 10078 14929 10075 14928 10072 14928
12 N F
10083 14914 10083 14910 10085 14908 10088 14909 10090 14911 10092 14914
10091 14917 10088 14918 10087 14915 10083 14914 10 N F
10090 9611 10089 9608 10090 9605 10093 9606 10096 9605 10098 9608
10101 9610 10102 9613 10104 9615 10107 9617 10111 9617 10110 9620
10106 9619 10103 9621 10101 9619 10098 9618 10098 9614 10096 9611
10093 9610 10090 9611 20 N F
10096 9772 10094 9769 10097 9767 10100 9765 10103 9764 10103 9764
10106 9763 10109 9761 10111 9758 10114 9756 10115 9759 10118 9760
10122 9759 10125 9759 10127 9757 10130 9755 10130 9758 10131 9761
10128 9762 10125 9762 10122 9761 10119 9763 10116 9764 10112 9765
10110 9767 10107 9768 10106 9771 10104 9773 10100 9773 10098 9776
10095 9775 10096 9772 32 N F
10095 10224 10094 10220 10098 10220 10101 10220 10102 10223 10103 10226

10101 10228 10098 10226 10095 10224 9 N F
10099 7012 10096 7013 10097 7011 10099 7010 10101 7009 10103 7008
10105 7008 10107 7008 10109 7008 10112 7008 10112 7011 10110 7012
10109 7014 10108 7016 10106 7017 10104 7017 10102 7016 10101 7014
10099 7012 19 N F
10102 14922 10097 14924 10099 14921 10101 14919 10102 14916 10104 14915
10105 14914 10106 14917 10108 14920 10106 14922 10102 14922 11 N F
10101 9810 10097 9805 10098 9805 10101 9804 10103 9800 10106 9796
10109 9793 10112 9790 10116 9788 10119 9785 10123 9787 10127 9785
10132 9783 10135 9786 10139 9787 10144 9786 10144 9790 10140 9790
10140 9794 10135 9795 10132 9792 10128 9794 10123 9794 10119 9795
10115 9798 10112 9801 10115 9804 10117 9808 10113 9810 10110 9813
10105 9813 10101 9810 32 N F
10104 9847 10098 9840 10104 9836 10110 9832 10110 9832 10113 9826
10119 9821 10125 9818 10129 9813 10136 9811 10142 9808 10149 9810
10145 9816 10141 9822 10141 9829 10136 9833 10141 9837 10137 9843
10131 9845 10124 9847 10117 9849 10111 9847 10104 9847 23 N F
10102 10292 10100 10289 10101 10289 10103 10288 10105 10289 10106 10290
10109 10292 10107 10295 10104 10296 10106 10293 10102 10292 11 N F
10105 10085 10102 10075 10103 10068 10110 10071 10116 10074 10122 10076
10129 10077 10125 10082 10119 10084 10112 10084 10105 10085 11 N F
10116 10195 10108 10197 10111 10190 10110 10183 10114 10178 10113 10171
10119 10169 10126 10165 10133 10165 10131 10171 10138 10172 10143 10176
10149 10173 10156 10173 10163 10171 10168 10171 10170 10171 10175 10168
10181 10167 10182 10167 10180 10174 10174 10177 10179 10182 10175 10187
10169 10190 10162 10189 10155 10190 10153 10197 10146 10196 10149 10190
10142 10191 10136 10193 10129 10195 10123 10193 10116 10195 35 N F
10122 10072 10112 10067 10117 10062 10122 10068 10129 10069 10135 10067
10142 10069 10149 10072 10153 10077 10156 10078 10160 10079 10165 10084
10163 10077 10165 10071 10170 10066 10176 10070 10180 10075 10178 10082
10175 10088 10168 10090 10161 10089 10154 10089 10148 10087 10141 10087
10134 10087 10133 10080 10128 10075 10122 10072 28 N F
10121 15176 10120 15172 10124 15171 10128 15171 10130 15167 10132 15163
10134 15160 10137 15160 10139 15160 10143 15160 10145 15164 10148 15167
10151 15171 10153 15174 10153 15174 10155 15178 10156 15182 10153 15185
10150 15188 10143 15192 10140 15189 10142 15185 10141 15181 10139 15184
10134 15184 10135 15189 10130 15190 10128 15187 10126 15183 10124 15179
10121 15176 31 N F
10127 10225 10124 10223 10127 10223 10130 10220 10133 10219 10136 10218
10137 10221 10137 10224 10137 10228 10137 10231 10134 10229 10133 10225
10130 10224 10127 10225 14 N F
10131 9863 10127 9857 10131 9855 10136 9854 10140 9853 10144 9855
10148 9857 10150 9857 10152 9858 10152 9863 10156 9865 10154 9869

10149 9868 10145 9867 10141 9869 10137 9869 10132 9867 10131 9863
18 N F
10145 7467 10145 7464 10147 7463 10149 7461 10150 7463 10152 7465
10152 7467 10150 7468 10147 7468 10145 7467 10 N F
10151 9907 10147 9900 10152 9895 10154 9892 10156 9889 10161 9885
10167 9881 10168 9880 10170 9875 10175 9870 10169 9865 10170 9858
10172 9852 10177 9847 10184 9845 10190 9849 10185 9855 10183 9861
10186 9868 10192 9872 10198 9875 10198 9882 10196 9888 10189 9889
10183 9893 10190 9892 10197 9894 10193 9899 10192 9906 10186 9910
10180 9906 10174 9905 10167 9907 10160 9905 10156 9911 10151 9907
36 N F
10151 15032 10148 15031 10149 15028 10152 15027 10153 15027 10155 15029
10156 15031 10153 15035 10151 15032 9 N F
10168 7385 10164 7383 10166 7382 10168 7382 10168 7385 5 N F
10170 15140 10167 15135 10170 15131 10173 15128 10176 15127 10177 15127
10180 15124 10181 15129 10181 15133 10185 15133 10183 15137 10178 15137
10174 15138 10170 15140 14 N F
10171 9932 10168 9928 10169 9924 10172 9922 10177 9921 10177 9926
10176 9930 10176 9934 10179 9938 10184 9937 10188 9938 10186 9942
10182 9944 10180 9948 10182 9952 10181 9956 10178 9952 10174 9951
10169 9951 10171 9946 10170 9942 10168 9935 10171 9932 23 N F
10176 10352 10169 10350 10175 10347 10176 10340 10175 10333 10170 10326
10177 10326 10184 10326 10190 10328 10197 10330 10200 10330 10204 10330
10211 10331 10213 10330 10218 10330 10224 10333 10219 10337 10223 10342
10216 10342 10209 10341 10204 10337 10197 10336 10192 10340 10185 10342
10182 10349 10176 10352 26 N F
10195 10180 10191 10176 10194 10176 10197 10177 10201 10176 10201 10173
10204 10171 10205 10171 10207 10172 10208 10174 10206 10176 10203 10179
10203 10182 10200 10184 10197 10183 10195 10180 16 N F
10205 9909 10204 9905 10206 9902 10207 9899 10210 9900 10211 9903
10209 9906 10209 9909 10205 9909 9 N F
10210 10069 10207 10066 10210 10065 10213 10063 10216 10062 10220 10061
10221 10064 10220 10068 10217 10069 10213 10069 10210 10069 11 N F
10223 14941 10217 14936 10223 14936 10228 14934 10230 14929 10225 14932
10222 14927 10222 14922 10221 14917 10223 14911 10228 14909 10231 14913
10235 14917 10236 14918 10240 14920 10244 14924 10244 14929 10241 14933
10238 14938 10235 14942 10235 14948 10233 14943 10228 14940 10223 14941
24 N F
10230 10312 10229 10304 10234 10303 10238 10302 10242 10301 10240 10305
10243 10307 10247 10310 10244 10313 10240 10316 10236 10318 10234 10314
10230 10312 13 N F
10256 10403 10253 10399 10256 10399 10259 10400 10262 10398 10265 10397
10266 10400 10263 10402 10260 10402 10256 10403 10 N F

10270 10401 10268 10398 10271 10397 10274 10399 10277 10400 10275 10402
10272 10404 10270 10401 8 N F
10285 10418 10280 10416 10284 10415 10286 10413 10289 10413 10289 10413
10293 10413 10295 10416 10298 10416 10298 10419 10295 10420 10292 10418
10290 10420 10287 10422 10284 10424 10283 10421 10285 10418 17 N F
10289 10062 10285 10058 10288 10057 10289 10053 10291 10050 10294 10049
10295 10052 10294 10055 10293 10058 10293 10062 10289 10062 11 N F
10308 10061 10304 10059 10305 10056 10307 10053 10311 10053 10311 10049
10312 10046 10314 10049 10314 10052 10313 10056 10310 10058 10308 10061
12 N F
10321 10063 10317 10058 10318 10055 10320 10052 10323 10051 10323 10054
10322 10057 10323 10060 10321 10063 9 N F
10328 10048 10325 10044 10326 10041 10329 10041 10331 10043 10330 10045
10328 10048 7 N F
10330 10345 10327 10341 10328 10338 10329 10337 10331 10341 10336 10342
10338 10338 10342 10336 10342 10341 10342 10345 10343 10349 10344 10353
10340 10353 10336 10351 10332 10349 10330 10345 16 N F
10340 10389 10334 10386 10336 10383 10340 10385 10340 10385 10342 10387
10340 10389 7 N F
10351 10027 10347 10025 10348 10022 10350 10019 10353 10018 10356 10018
10359 10018 10359 10022 10357 10024 10354 10026 10351 10027 11 N F
10356 10357 10350 10350 10351 10349 10352 10347 10357 10346 10361 10347
10363 10343 10366 10339 10366 10344 10365 10348 10363 10352 10359 10353
10356 10357 13 N F
10430 15319 10429 15314 10432 15314 10435 15316 10438 15317 10441 15318
10444 15318 10448 15318 10445 15321 10444 15324 10441 15325 10438 15324
10435 15322 10432 15321 10430 15319 15 N F
10636 6813 10632 6812 10635 6809 10640 6809 10644 6807 10649 6806
10653 6806 10658 6807 10662 6808 10666 6808 10671 6806 10675 6808
10678 6811 10682 6814 10685 6818 10689 6820 10685 6823 10680 6825
10676 6826 10672 6827 10665 6825 10661 6827 10657 6824 10653 6824
10648 6825 10644 6824 10642 6819 10638 6818 10636 6813 29 N F
10811 15236 10808 15230 10812 15231 10814 15235 10817 15238 10821 15236
10822 15236 10824 15234 10827 15237 10827 15241 10831 15240 10832 15235
10836 15234 10840 15233 10844 15235 10847 15238 10848 15242 10851 15245
10852 15250 10848 15251 10844 15253 10840 15255 10839 15250 10834 15250
10830 15251 10827 15248 10823 15250 10820 15248 10820 15244 10817 15240
10813 15240 10811 15236 32 N F
10812 15182 10810 15178 10813 15177 10813 15174 10816 15172 10819 15173
10822 15173 10823 15176 10824 15179 10821 15181 10820 15184 10820 15188
10817 15186 10815 15184 10812 15182 15 N F
10820 15209 10819 15205 10820 15205 10822 15204 10823 15205 10824 15206
10826 15208 10824 15210 10822 15211 10820 15209 10 N F

10820 15197 10820 15193 10823 15191 10826 15191 10825 15194 10826 15197
10823 15199 10820 15197 8 N F
10848 15229 10845 15226 10848 15222 10846 15217 10849 15214 10852 15217
10857 15218 10856 15222 10859 15226 10856 15229 10858 15233 10854 15235
10850 15233 10848 15229 14 N F
10881 7587 10880 7583 10881 7581 10884 7581 10886 7582 10888 7583
10889 7585 10892 7584 10894 7584 10896 7585 10898 7585 10898 7587
10896 7589 10895 7591 10893 7591 10891 7591 10889 7591 10886 7591
10885 7590 10883 7588 10881 7587 21 N F
10892 14387 10886 14383 10888 14379 10891 14377 10891 14376 10894 14373
10895 14369 10897 14365 10899 14361 10902 14358 10906 14359 10910 14359
10915 14358 10919 14359 10922 14356 10926 14358 10927 14362 10929 14366
10926 14369 10922 14370 10920 14374 10922 14378 10926 14380 10930 14378
10929 14374 10932 14374 10933 14375 10936 14378 10940 14377 10938 14381
10942 14380 10940 14384 10938 14388 10936 14392 10932 14394 10928 14394
10924 14393 10920 14392 10915 14391 10911 14390 10907 14388 10903 14386
10900 14384 10896 14385 10892 14387 45 N F
10908 15222 10903 15216 10907 15216 10910 15219 10914 15222 10918 15222
10922 15224 10925 15227 10927 15231 10931 15233 10933 15236 10935 15240
10939 15241 10938 15245 10942 15246 10946 15244 10948 15246 10949 15247
10951 15251 10950 15255 10946 15255 10942 15254 10944 15257 10941 15259
10944 15262 10939 15263 10942 15266 10938 15269 10937 15273 10940 15276
10945 15275 10944 15280 10941 15282 10937 15285 10933 15284 10929 15285
10925 15282 10922 15281 10925 15278 10924 15274 10921 15272 10924 15269
10924 15264 10921 15262 10917 15260 10915 15256 10911 15256 10908 15254
10905 15250 10904 15246 10908 15248 10912 15249 10916 15250 10920 15248
10917 15245 10913 15243 10915 15239 10919 15238 10919 15234 10918 15230
10913 15230 10909 15230 10909 15226 10908 15222 64 N F
10919 14305 10915 14305 10918 14303 10921 14301 10924 14301 10924 14298
10923 14295 10924 14295 10926 14296 10929 14298 10932 14297 10935 14298
10937 14300 10939 14303 10939 14306 10937 14309 10937 14312 10939 14315
10942 14316 10940 14319 10938 14320 10934 14320 10932 14322 10931 14318
10929 14316 10926 14315 10925 14312 10923 14309 10922 14306 10919 14305
30 N F
10924 7582 10921 7580 10923 7578 10924 7577 10926 7578 10925 7580
10924 7582 7 N F
10933 14689 10928 14687 10931 14684 10935 14683 10939 14684 10936 14687
10938 14691 10941 14689 10946 14689 10949 14692 10951 14688 10955 14687
10959 14685 10963 14682 10967 14681 10971 14680 10971 14685 10974 14681
10979 14681 10981 14685 10979 14688 10977 14692 10974 14696 10971 14698
10971 14702 10967 14702 10962 14702 10959 14705 10955 14707 10951 14708
10948 14711 10946 14707 10943 14703 10941 14700 10937 14698 10933 14698
10933 14694 10933 14689 38 N F

10947 14279 10945 14273 10948 14274 10950 14277 10953 14279 10955 14280
10958 14279 10959 14279 10962 14278 10962 14282 10963 14285 10960 14285
10957 14284 10954 14283 10950 14284 10949 14281 10947 14279 17 N F
10958 7684 10957 7681 10958 7679 10961 7679 10963 7679 10964 7681
10963 7683 10961 7685 10959 7685 10958 7684 10 N F
10968 6686 10966 6684 10968 6683 10970 6684 10972 6686 10974 6687
10972 6687 10970 6687 10968 6686 9 N F
11011 14757 11010 14753 11013 14750 11016 14753 11019 14756 11023 14758
11025 14762 11025 14766 11027 14770 11027 14774 11025 14770 11018 14771
11017 14775 11015 14778 11014 14774 11014 14770 11012 14766 11014 14762
11019 14763 11018 14758 11014 14760 11011 14757 22 N F
11030 6758 11017 6755 11022 6749 11026 6743 11033 6740 11040 6741
11047 6739 11049 6746 11055 6750 11060 6746 11066 6742 11073 6741
11081 6741 11088 6740 11094 6739 11095 6738 11102 6739 11109 6740
11116 6738 11122 6734 11129 6732 11135 6736 11135 6743 11130 6749
11124 6752 11131 6750 11138 6748 11144 6744 11144 6751 11142 6758
11144 6765 11148 6771 11155 6769 11157 6776 11154 6782 11158 6788
11160 6795 11154 6798 11148 6802 11143 6807 11136 6809 11129 6810
11126 6816 11120 6813 11114 6816 11107 6816 11100 6813 11095 6807
11091 6813 11089 6820 11084 6826 11078 6830 11072 6832 11066 6828
11071 6824 11073 6817 11077 6811 11070 6813 11071 6820 11064 6818
11060 6812 11056 6806 11059 6800 11058 6793 11062 6787 11067 6782
11068 6774 11065 6781 11062 6774 11061 6767 11064 6761 11063 6754
11063 6761 11058 6766 11052 6770 11050 6777 11045 6772 11039 6776
11040 6769 11043 6762 11037 6767 11031 6770 11026 6764 11030 6758
84 N F
11058 14312 11057 14309 11060 14308 11063 14309 11066 14308 11070 14308
11071 14311 11072 14312 11073 14313 11074 14317 11076 14315 11078 14318
11077 14321 11078 14324 11079 14327 11076 14327 11072 14327 11069 14327
11066 14326 11069 14324 11072 14323 11071 14319 11071 14316 11068 14316
11064 14317 11061 14319 11061 14315 11058 14312 28 N F
11077 6618 11075 6613 11078 6610 11083 6609 11087 6608 11092 6609
11096 6609 11100 6611 11104 6614 11106 6618 11103 6622 11098 6623
11094 6624 11089 6624 11085 6622 11081 6620 11077 6618 17 N F
11122 14312 11117 14308 11120 14308 11124 14307 11124 14304 11123 14301
11125 14303 11127 14306 11129 14308 11132 14310 11135 14311 11137 14314
11137 14317 11140 14316 11143 14317 11141 14320 11140 14323 11137 14322
11134 14321 11131 14320 11128 14319 11127 14315 11124 14314 11122 14312
24 N F
11156 14290 11152 14288 11155 14287 11158 14286 11161 14284 11162 14281
11165 14283 11163 14285 11165 14288 11162 14290 11159 14291 11156 14290
12 N F
11157 14263 11154 14259 11157 14257 11159 14255 11162 14256 11162 14257

11165 14258 11168 14258 11171 14260 11174 14262 11176 14264 11177 14267
11179 14270 11181 14271 11182 14272 11184 14274 11187 14275 11190 14277
11192 14279 11194 14282 11195 14285 11196 14285 11198 14287 11200 14290
11201 14293 11203 14296 11205 14298 11206 14301 11203 14300 11200 14300
11197 14299 11195 14297 11193 14294 11190 14292 11187 14293 11185 14291
11182 14289 11179 14287 11180 14284 11182 14281 11182 14278 11182 14275
11180 14277 11180 14280 11177 14282 11175 14284 11172 14283 11169 14282
11171 14279 11170 14276 11168 14274 11166 14271 11165 14269 11162 14266
11159 14265 11157 14263 56 N F
11158 6715 11156 6712 11158 6710 11161 6708 11164 6710 11164 6713
11162 6715 11158 6715 8 N F
11165 6723 11164 6718 11167 6719 11170 6721 11172 6724 11173 6727
11170 6729 11167 6726 11165 6723 9 N F
11171 6782 11168 6781 11170 6781 11171 6781 11175 6780 11178 6780
11181 6780 11185 6780 11187 6778 11190 6776 11193 6778 11196 6781
11192 6784 11189 6785 11188 6788 11187 6791 11184 6793 11181 6794
11177 6793 11174 6792 11173 6789 11176 6787 11174 6784 11171 6782
24 N F
11193 6735 11192 6732 11192 6728 11194 6725 11196 6722 11198 6720
11201 6717 11204 6718 11207 6720 11209 6722 11212 6724 11213 6727
11212 6731 11211 6734 11207 6734 11204 6734 11200 6735 11196 6737
11193 6735 19 N F
11228 6848 11225 6841 11230 6836 11236 6839 11237 6839 11242 6844
11250 6845 11255 6840 11259 6834 11263 6828 11263 6821 11259 6815
11253 6812 11249 6806 11254 6801 11261 6800 11268 6797 11273 6792
11279 6789 11286 6787 11293 6790 11298 6796 11299 6803 11306 6801
11312 6798 11319 6800 11322 6806 11323 6813 11322 6821 11322 6828
11323 6835 11323 6842 11319 6848 11322 6854 11319 6861 11319 6868
11323 6874 11328 6879 11332 6884 11333 6891 11338 6897 11342 6902
11345 6909 11342 6915 11346 6921 11351 6926 11356 6931 11358 6938
11357 6945 11357 6953 11350 6952 11343 6954 11343 6947 11341 6939
11338 6933 11333 6928 11331 6921 11336 6916 11333 6910 11331 6903
11329 6896 11322 6898 11316 6895 11312 6889 11306 6886 11305 6879
11302 6873 11304 6866 11299 6861 11292 6861 11285 6860 11279 6856
11272 6857 11265 6855 11262 6862 11259 6868 11252 6870 11252 6863
11249 6856 11246 6850 11240 6855 11233 6854 11228 6848 83 N F
11234 6783 11233 6778 11235 6775 11237 6773 11241 6773 11243 6775
11246 6778 11246 6781 11245 6784 11243 6787 11240 6789 11236 6789
11235 6786 11234 6783 14 N F
11279 12280 11273 12280 11277 12277 11281 12277 11285 12275 11288 12272
11290 12268 11293 12265 11297 12266 11301 12267 11303 12271 11299 12273
11296 12276 11293 12280 11291 12284 11292 12288 11288 12288 11286 12292
11282 12292 11279 12288 11279 12284 11279 12280 22 N F

11290 12618 11288 12614 11288 12609 11291 12606 11293 12610 11295 12614
11299 12614 11301 12618 11302 12622 11304 12626 11302 12631 11298 12633
11297 12628 11295 12624 11294 12620 11290 12618 16 N F
11349 12481 11345 12480 11347 12478 11348 12476 11349 12475 11350 12478
11349 12481 7 N F
11348 12489 11345 12484 11348 12484 11349 12485 11351 12486 11353 12488
11352 12492 11349 12492 11348 12489 9 N F
11353 12464 11348 12467 11350 12464 11352 12461 11353 12458 11355 12455
11358 12457 11360 12456 11363 12458 11362 12461 11359 12462 11356 12464
11354 12461 11353 12464 14 N F
11356 14267 11355 14263 11357 14262 11359 14259 11358 14255 11359 14252
11362 14251 11365 14253 11366 14256 11369 14258 11370 14261 11372 14264
11372 14267 11369 14268 11365 14267 11362 14266 11359 14266 11356 14267
18 N F
11372 12621 11371 12617 11373 12615 11376 12616 11378 12619 11379 12622
11375 12622 11372 12621 8 N F
11383 12628 11379 12623 11382 12623 11383 12626 11386 12628 11383 12628
6 N F
11386 12606 11382 12602 11385 12599 11387 12595 11391 12592 11395 12593
11394 12597 11394 12601 11398 12603 11401 12607 11399 12611 11404 12612
11407 12615 11409 12619 11408 12624 11411 12627 11415 12628 11414 12632
11410 12631 11407 12627 11403 12627 11402 12631 11405 12633 11401 12634
11397 12633 11393 12632 11390 12628 11391 12624 11388 12621 11387 12617
11386 12612 11383 12609 11386 12606 33 N F
11386 14338 11383 14335 11386 14334 11386 14334 11389 14334 11391 14337
11390 14340 11389 14344 11387 14342 11386 14338 10 N F
11400 12664 11396 12664 11398 12662 11401 12661 11402 12661 11400 12664
6 N F
11405 12659 11402 12658 11402 12654 11405 12653 11406 12656 11405 12659
6 N F
11410 12653 11408 12650 11410 12647 11413 12646 11414 12643 11416 12645
11416 12648 11417 12651 11420 12651 11421 12654 11417 12654 11415 12657
11412 12658 11413 12655 11410 12653 15 N F
11415 12607 11414 12604 11417 12603 11420 12604 11422 12607 11425 12609
11425 12612 11422 12613 11419 12612 11417 12609 11415 12607 11 N F
11441 12370 11438 12370 11441 12367 11442 12365 11445 12367 11448 12365
11450 12368 11447 12368 11445 12371 11441 12370 10 N F
11443 14328 11439 14325 11441 14323 11444 14324 11445 14324 11447 14324
11450 14325 11453 14327 11454 14330 11456 14333 11453 14332 11450 14330
11448 14328 11445 14329 11443 14328 15 N F
11502 13955 11500 13951 11502 13948 11504 13946 11505 13949 11505 13952
11505 13955 11508 13954 11512 13955 11510 13958 11507 13959 11504 13960
11502 13958 11502 13955 14 N F

11506 14011 11504 14008 11507 14007 11510 14009 11512 14011 11516 14011
11514 14014 11517 14015 11518 14018 11515 14019 11513 14016 11510 14015
11509 14012 11506 14011 14 N F
11520 13933 11518 13930 11520 13927 11523 13925 11525 13923 11527 13921
11530 13922 11533 13924 11533 13924 11535 13926 11532 13926 11529 13926
11525 13927 11526 13930 11523 13932 11520 13933 16 N F
11530 7055 11527 7052 11530 7051 11534 7050 11537 7048 11539 7047
11540 7047 11541 7050 11539 7053 11536 7055 11533 7056 11530 7055
12 N F
11531 7003 11528 7000 11528 6993 11530 6989 11533 6986 11538 6985
11542 6985 11547 6985 11551 6987 11554 6991 11557 6994 11558 6999
11556 7003 11552 7005 11548 7007 11544 7007 11539 7008 11535 7006
11531 7003 19 N F
11549 14293 11546 14289 11547 14287 11550 14287 11553 14287 11555 14288
11554 14290 11551 14291 11549 14293 9 N F
11553 13969 11551 13966 11552 13966 11554 13967 11557 13965 11554 13965
11553 13962 11556 13960 11559 13961 11561 13963 11563 13966 11564 13969
11562 13972 11560 13974 11557 13973 11554 13973 11553 13969 17 N F
11554 13496 11552 13498 11553 13495 11554 13493 11556 13491 11558 13490
11558 13492 11559 13495 11558 13497 11556 13495 11554 13496 11 N F
11557 14039 11555 14035 11559 14038 11560 14042 11562 14039 11562 14038
11566 14036 11567 14032 11571 14031 11576 14032 11580 14034 11581 14038
11582 14038 11585 14040 11588 14043 11585 14047 11581 14048 11577 14050
11574 14052 11569 14051 11568 14055 11564 14058 11563 14062 11560 14065
11560 14060 11558 14056 11557 14052 11556 14048 11557 14044 11557 14039
30 N F
11559 14016 11557 14012 11559 14009 11560 14008 11561 14006 11564 14006
11567 14006 11568 14006 11570 14008 11568 14011 11570 14014 11567 14014
11565 14016 11562 14018 11559 14016 15 N F
11563 14287 11560 14284 11563 14282 11565 14280 11568 14281 11571 14282
11574 14280 11576 14278 11579 14276 11582 14276 11583 14279 11584 14282
11582 14283 11580 14286 11578 14289 11577 14292 11574 14291 11573 14289
11570 14287 11567 14287 11563 14287 21 N F
11567 13436 11563 13435 11565 13433 11566 13430 11567 13428 11570 13427
11572 13428 11572 13431 11574 13431 11575 13428 11577 13430 11577 13433
11575 13434 11572 13435 11569 13436 11567 13436 16 N F
11572 13738 11569 13735 11571 13733 11573 13734 11575 13736 11577 13736
11580 13736 11583 13737 11583 13738 11583 13740 11585 13742 11584 13744
11584 13747 11582 13748 11582 13745 11581 13743 11579 13741 11576 13741
11574 13740 11572 13738 20 N F
11577 12606 11571 12607 11575 12606 11577 12602 11582 12603 11579 12599
11575 12597 11578 12594 11582 12595 11586 12598 11590 12597 11593 12600
11597 12600 11598 12605 11596 12609 11595 12613 11591 12615 11589 12611

11585 12609 11581 12606 11577 12606 21 N F
11580 14343 11576 14337 11577 14337 11580 14335 11583 14333 11587 14331
11588 14331 11591 14332 11593 14336 11597 14337 11601 14338 11603 14339
11605 14341 11609 14341 11612 14338 11616 14340 11620 14343 11624 14345
11626 14349 11628 14353 11629 14356 11629 14357 11628 14361 11626 14364
11622 14362 11618 14360 11614 14360 11609 14360 11606 14362 11602 14365
11598 14366 11594 14365 11590 14365 11586 14362 11583 14359 11583 14355
11581 14351 11582 14347 11580 14343 39 N F
11587 13489 11583 13489 11584 13487 11586 13485 11589 13484 11589 13486
11587 13489 7 N F
11588 14314 11585 14311 11587 14311 11590 14310 11592 14310 11595 14311
11598 14311 11596 14313 11593 14313 11590 14313 11588 14314 11 N F
11591 14042 11587 14039 11588 14037 11588 14034 11591 14033 11591 14033
11594 14033 11596 14032 11597 14029 11597 14027 11600 14028 11601 14031
11602 14033 11600 14034 11598 14034 11597 14036 11595 14038 11593 14040
11591 14042 19 N F
11595 14276 11591 14275 11591 14274 11593 14272 11594 14269 11597 14267
11600 14265 11602 14264 11606 14264 11608 14262 11607 14265 11604 14267
11604 14270 11604 14273 11605 14277 11602 14276 11598 14276 11595 14276
18 N F
11603 12604 11600 12608 11601 12605 11601 12602 11603 12604 5 N F
11603 13495 11601 13491 11602 13491 11604 13491 11606 13492 11609 13493
11611 13494 11614 13495 11614 13497 11612 13499 11609 13499 11607 13499
11604 13498 11603 13495 14 N F
11603 14323 11601 14320 11601 14316 11604 14316 11605 14317 11606 14318
11605 14321 11603 14323 8 N F
11606 12606 11602 12608 11604 12605 11607 12603 11609 12601 11612 12599
11611 12602 11610 12605 11606 12606 9 N F
11604 12680 11603 12675 11606 12672 11609 12669 11612 12666 11616 12664
11620 12665 11624 12667 11628 12669 11626 12674 11631 12675 11628 12678
11627 12682 11623 12684 11622 12689 11626 12690 11630 12692 11635 12692
11638 12694 11639 12694 11640 12690 11644 12690 11648 12692 11652 12693
11651 12700 11647 12699 11649 12703 11651 12707 11652 12711 11650 12715
11646 12717 11642 12715 11638 12714 11637 12710 11635 12706 11631 12706
11630 12702 11626 12700 11622 12698 11618 12697 11613 12697 11610 12700
11606 12700 11606 12695 11603 12692 11605 12688 11604 12684 11604 12680
48 N F
11615 14309 11607 14309 11610 14306 11614 14304 11617 14301 11621 14299
11624 14298 11625 14298 11629 14295 11632 14293 11632 14293 11632 14293
11636 14290 11636 14286 11634 14282 11637 14279 11640 14276 11643 14279
11646 14282 11649 14285 11653 14288 11655 14292 11655 14296 11654 14300
11650 14303 11646 14304 11642 14305 11638 14306 11635 14308 11631 14311
11628 14314 11628 14318 11628 14322 11629 14327 11631 14330 11629 14334

11625 14333 11621 14330 11617 14329 11614 14326 11614 14322 11615 14318
11614 14313 11615 14309 44 N F
11618 14022 11613 14022 11615 14019 11618 14018 11621 14019 11624 14018
11626 14021 11624 14024 11621 14023 11618 14022 10 N F
11618 12389 11613 12389 11615 12387 11618 12385 11621 12384 11621 12387
11618 12389 7 N F
11618 12662 11617 12658 11617 12653 11617 12649 11618 12646 11619 12645
11621 12641 11625 12644 11627 12640 11630 12637 11634 12639 11637 12635
11641 12634 11641 12634 11645 12635 11648 12638 11648 12642 11652 12644
11655 12648 11656 12652 11659 12651 11660 12651 11661 12656 11663 12660
11655 12661 11651 12664 11648 12667 11644 12667 11642 12670 11640 12674
11638 12678 11635 12674 11634 12670 11631 12667 11634 12664 11632 12660
11631 12664 11627 12665 11622 12664 11618 12662 40 N F
11619 13002 11618 12999 11621 13001 11624 13003 11621 13005 11619 13002
6 N F
11627 13012 11623 13011 11626 13009 11627 13006 11630 13007 11633 13007
11636 13009 11638 13012 11637 13015 11635 13018 11632 13018 11630 13021
11628 13019 11624 13020 11624 13016 11628 13016 11630 13013 11627 13012
18 N F
11630 14019 11626 14017 11628 14015 11631 14015 11634 14014 11637 14013
11639 14010 11642 14009 11643 14012 11640 14013 11638 14016 11638 14019
11635 14020 11632 14022 11630 14019 15 N F
11637 14227 11632 14228 11634 14225 11636 14223 11639 14221 11640 14221
11641 14219 11643 14216 11642 14212 11645 14212 11649 14212 11652 14213
11655 14214 11658 14214 11660 14215 11661 14215 11664 14214 11666 14217
11666 14220 11663 14219 11660 14218 11657 14217 11653 14217 11650 14218
11648 14221 11646 14224 11643 14225 11640 14226 11637 14227 29 N F
11637 12754 11633 12754 11636 12752 11635 12748 11639 12747 11643 12747
11646 12749 11647 12750 11650 12747 11654 12750 11657 12753 11657 12758
11657 12753 11657 12749 11661 12748 11663 12744 11665 12742 11666 12741
11669 12744 11671 12746 11672 12747 11677 12749 11678 12752 11683 12753
11682 12757 11682 12762 11686 12760 11690 12761 11691 12766 11688 12769
11683 12770 11684 12774 11688 12777 11684 12779 11685 12784 11680 12784
11679 12788 11675 12791 11671 12791 11670 12787 11671 12783 11670 12779
11666 12777 11663 12780 11660 12777 11656 12775 11652 12777 11650 12773
11649 12769 11645 12762 11643 12758 11641 12755 11637 12754 53 N F
11635 12411 11634 12407 11637 12407 11640 12405 11640 12408 11642 12411
11644 12413 11646 12411 11645 12407 11648 12409 11647 12412 11646 12415
11643 12416 11640 12415 11637 12413 11635 12411 16 N F
11636 12624 11635 12621 11637 12618 11640 12616 11643 12618 11645 12616
11647 12614 11650 12615 11650 12619 11648 12622 11646 12623 11644 12626
11641 12627 11639 12625 11636 12624 15 N F
11639 13029 11636 13027 11639 13025 11641 13027 11639 13029 5 N F

11640 14071 11636 14066 11640 14064 11644 14062 11648 14061 11651 14061
11653 14060 11657 14060 11661 14060 11665 14063 11666 14067 11662 14069
11660 14065 11656 14066 11652 14068 11648 14068 11644 14069 11640 14071
18 N F
11643 14360 11637 14355 11640 14349 11642 14343 11642 14336 11642 14329
11643 14322 11642 14316 11645 14310 11652 14308 11658 14307 11660 14301
11665 14296 11668 14290 11670 14284 11667 14278 11661 14275 11654 14273
11656 14267 11656 14260 11662 14258 11669 14255 11675 14256 11679 14255
11682 14255 11686 14254 11689 14253 11695 14252 11695 14258 11696 14265
11699 14264 11702 14262 11706 14259 11707 14258 11714 14258 11719 14263
11721 14264 11724 14267 11725 14274 11723 14280 11721 14287 11723 14293
11717 14298 11717 14304 11717 14311 11715 14318 11712 14324 11712 14330
11715 14337 11712 14343 11708 14348 11707 14355 11702 14360 11695 14362
11690 14366 11684 14369 11679 14373 11674 14378 11667 14380 11661 14376
11654 14376 11653 14369 11649 14364 11643 14360 64 N F
11639 12878 11638 12873 11641 12871 11643 12868 11646 12867 11648 12869
11648 12872 11646 12875 11644 12878 11642 12880 11639 12878 11 N F
11644 12602 11642 12600 11643 12600 11645 12600 11645 12600 11648 12600
11649 12597 11648 12594 11648 12591 11652 12592 11652 12595 11652 12598
11651 12601 11653 12604 11654 12604 11650 12604 11647 12603 11644 12602
18 N F
11644 12479 11643 12475 11647 12476 11648 12479 11649 12482 11645 12482
11644 12479 7 N F
11651 14019 11646 14018 11648 14015 11650 14014 11654 14013 11657 14012
11660 14012 11659 14015 11657 14017 11654 14018 11651 14019 11 N F
11649 13191 11647 13187 11650 13186 11651 13189 11651 13192 11649 13191
6 N F
11650 12407 11647 12403 11649 12400 11652 12400 11655 12401 11658 12403
11658 12404 11660 12405 11661 12408 11658 12410 11655 12407 11653 12409
11650 12407 13 N F
11652 12466 11650 12462 11653 12463 11656 12463 11656 12466 11653 12468
11652 12466 7 N F
11658 12863 11652 12860 11656 12860 11660 12860 11661 12856 11664 12859
11667 12862 11668 12858 11671 12857 11673 12857 11677 12856 11681 12853
11682 12857 11686 12860 11684 12864 11687 12867 11689 12871 11685 12873
11681 12874 11681 12879 11681 12883 11685 12885 11686 12889 11683 12893
11686 12896 11685 12901 11689 12902 11694 12903 11697 12906 11701 12908
11697 12910 11694 12907 11690 12907 11686 12906 11681 12906 11681 12901
11678 12898 11675 12895 11673 12891 11671 12887 11667 12885 11663 12884
11658 12883 11656 12879 11655 12875 11653 12871 11657 12869 11660 12867
11658 12863 49 N F
11653 13180 11653 13177 11655 13176 11658 13177 11658 13175 11661 13176
11660 13178 11658 13180 11656 13181 11653 13180 10 N F

11656 12614 11654 12611 11657 12609 11659 12611 11659 12611 11659 12608
11662 12610 11665 12611 11665 12615 11664 12618 11662 12620 11659 12622
11656 12621 11656 12618 11659 12615 11656 12614 16 N F
11657 12447 11655 12443 11658 12443 11658 12443 11661 12445 11660 12448
11657 12447 7 N F
11656 12482 11655 12475 11659 12475 11660 12477 11661 12478 11661 12482
11659 12484 11656 12482 8 N F
11658 12364 11656 12361 11658 12358 11659 12355 11660 12352 11662 12355
11660 12358 11659 12361 11658 12364 9 N F
11658 14181 11658 14178 11660 14176 11663 14175 11664 14172 11665 14169
11667 14166 11670 14164 11673 14166 11674 14169 11677 14170 11679 14168
11679 14164 11681 14166 11682 14166 11684 14165 11686 14162 11688 14159
11689 14162 11692 14164 11692 14168 11692 14171 11690 14174 11687 14175
11685 14177 11683 14179 11679 14180 11676 14181 11676 14177 11673 14176
11670 14177 11667 14179 11666 14182 11663 14184 11660 14184 11658 14181
36 N F
11665 12686 11659 12685 11660 12682 11660 12679 11662 12676 11664 12673
11667 12673 11668 12676 11667 12679 11665 12682 11665 12686 11 N F
11666 12451 11661 12450 11662 12447 11664 12444 11666 12444 11667 12444
11667 12448 11666 12451 8 N F
11666 12443 11662 12441 11664 12438 11667 12438 11670 12438 11669 12441
11666 12443 7 N F
11665 12624 11664 12621 11666 12618 11668 12615 11667 12612 11666 12609
11666 12608 11667 12606 11670 12605 11673 12603 11671 12601 11669 12598
11669 12595 11671 12592 11671 12589 11673 12586 11676 12587 11677 12590
11678 12593 11681 12591 11684 12591 11687 12590 11690 12592 11690 12595
11691 12599 11690 12602 11687 12603 11686 12606 11686 12609 11683 12609
11680 12608 11678 12611 11676 12614 11678 12617 11678 12620 11678 12623
11675 12624 11672 12625 11668 12625 11665 12624 40 N F
11671 12732 11668 12730 11669 12727 11671 12724 11674 12724 11676 12722
11678 12722 11679 12723 11679 12726 11677 12728 11674 12729 11671 12732
12 N F
11670 12428 11669 12425 11670 12422 11673 12422 11675 12420 11676 12417
11680 12416 11680 12420 11682 12422 11682 12426 11679 12423 11678 12426
11676 12428 11673 12429 11670 12428 15 N F
11673 12696 11670 12692 11672 12693 11673 12693 11676 12694 11678 12697
11678 12700 11675 12699 11673 12696 9 N F
11675 12464 11672 12463 11673 12459 11673 12456 11676 12455 11679 12454
11681 12454 11683 12454 11686 12455 11687 12458 11690 12458 11693 12459
11693 12459 11696 12457 11699 12457 11701 12455 11703 12458 11701 12460
11698 12461 11695 12463 11692 12464 11689 12466 11686 12464 11683 12465
11682 12462 11679 12461 11675 12464 27 N F
11677 14221 11674 14217 11676 14215 11677 14218 11679 14220 11679 14223

11677 14221 7 N F
11681 12746 11674 12744 11677 12740 11679 12737 11682 12734 11683 12730
11683 12729 11687 12727 11691 12725 11693 12721 11692 12717 11696 12717
11700 12719 11703 12722 11705 12727 11702 12730 11701 12735 11703 12739
11707 12737 11711 12735 11711 12740 11715 12742 11715 12746 11711 12749
11708 12751 11712 12753 11715 12754 11716 12754 11716 12758 11712 12758
11712 12762 11709 12765 11706 12762 11702 12764 11698 12765 11694 12762
11696 12758 11698 12754 11701 12751 11699 12747 11694 12747 11690 12749
11687 12752 11683 12750 11681 12746 45 N F
11679 12585 11675 12582 11679 12580 11682 12584 11686 12583 11690 12583
11689 12579 11685 12577 11683 12574 11679 12575 11676 12570 11680 12570
11685 12569 11689 12570 11692 12571 11693 12571 11696 12575 11700 12576
11703 12579 11702 12583 11706 12584 11708 12588 11706 12592 11708 12596
11709 12600 11712 12603 11709 12606 11705 12608 11707 12612 11703 12614
11699 12615 11695 12617 11695 12621 11691 12624 11687 12623 11682 12624
11682 12619 11685 12616 11689 12615 11691 12611 11694 12607 11694 12603
11698 12600 11702 12599 11698 12598 11696 12594 11697 12589 11694 12586
11690 12586 11686 12588 11682 12588 11679 12585 52 N F
11681 12451 11676 12451 11677 12448 11679 12446 11682 12448 11685 12448
11685 12451 11681 12451 8 N F
11680 12644 11677 12642 11678 12638 11679 12634 11680 12631 11681 12634
11681 12638 11680 12641 11680 12644 9 N F
11680 13540 11678 13540 11679 13537 11681 13535 11682 13537 11684 13539
11684 13542 11686 13544 11683 13545 11681 13545 11680 13542 11680 13540
12 N F
11683 12722 11679 12721 11680 12720 11681 12718 11683 12715 11686 12716
11684 12719 11683 12722 8 N F
11681 14146 11680 14142 11683 14141 11685 14139 11685 14135 11685 14132
11687 14130 11690 14128 11693 14129 11693 14130 11697 14130 11697 14134
11697 14137 11699 14139 11697 14141 11694 14142 11692 14145 11690 14147
11687 14149 11685 14152 11682 14152 11682 14149 11681 14146 23 N F
11683 12475 11680 12472 11683 12471 11681 12468 11683 12466 11686 12466
11686 12469 11685 12473 11683 12475 9 N F
11687 12701 11680 12699 11684 12697 11688 12695 11688 12693 11690 12691
11692 12687 11693 12687 11696 12687 11700 12685 11699 12681 11699 12676
11704 12676 11704 12676 11707 12674 11710 12677 11713 12680 11717 12681
11718 12677 11722 12674 11721 12670 11725 12668 11727 12668 11729 12668
11734 12668 11735 12672 11735 12676 11735 12681 11733 12684 11730 12688
11733 12691 11729 12693 11727 12697 11723 12699 11719 12696 11715 12696
11711 12699 11709 12703 11705 12704 11701 12701 11697 12703 11693 12704
11691 12700 11687 12701 44 N F
11682 14219 11681 14214 11684 14213 11686 14216 11686 14219 11686 14223
11688 14225 11686 14227 11683 14226 11683 14222 11682 14219 11 N F

11684 12990 11681 12985 11683 12982 11685 12981 11686 12980 11686 12977
11690 12978 11692 12980 11691 12984 11691 12987 11689 12990 11686 12991
11684 12990 13 N F
11685 12542 11681 12541 11683 12540 11684 12539 11686 12537 11685 12533
11687 12530 11689 12532 11690 12532 11692 12535 11692 12538 11695 12539
11698 12542 11698 12545 11695 12543 11694 12546 11697 12548 11694 12549
11692 12552 11689 12551 11687 12548 11686 12545 11685 12542 23 N F
11685 12939 11682 12938 11682 12934 11682 12931 11685 12933 11686 12936
11685 12939 7 N F
11689 12832 11682 12828 11686 12825 11690 12827 11694 12828 11699 12828
11703 12826 11707 12828 11706 12824 11702 12825 11701 12821 11702 12816
11702 12812 11701 12808 11699 12804 11698 12800 11702 12802 11706 12803
11707 12807 11705 12811 11709 12812 11710 12812 11713 12815 11717 12813
11716 12817 11721 12818 11720 12823 11720 12827 11717 12830 11713 12831
11711 12835 11710 12839 11707 12842 11703 12840 11699 12842 11695 12843
11695 12838 11691 12836 11689 12832 39 N F
11683 13530 11683 13527 11684 13525 11686 13524 11686 13524 11687 13527
11688 13530 11688 13532 11685 13532 11683 13530 10 N F
11687 12655 11685 12650 11686 12648 11689 12649 11690 12652 11691 12654
11692 12655 11693 12658 11689 12657 11687 12655 10 N F
11687 12444 11685 12438 11689 12440 11689 12435 11693 12434 11697 12434
11702 12434 11703 12432 11704 12430 11704 12435 11706 12438 11707 12434
11710 12430 11712 12434 11716 12435 11720 12433 11719 12438 11718 12442
11717 12446 11717 12451 11713 12450 11709 12449 11704 12447 11700 12448
11697 12451 11693 12453 11691 12449 11687 12450 11690 12447 11687 12444
30 N F
11687 12687 11685 12682 11688 12681 11690 12684 11691 12687 11688 12690
11687 12687 7 N F
11689 12941 11686 12939 11690 12939 11693 12939 11695 12942 11697 12944
11694 12945 11692 12942 11689 12941 9 N F
11688 14216 11687 14212 11690 14210 11692 14212 11696 14213 11699 14212
11701 14210 11704 14210 11704 14213 11704 14216 11705 14219 11704 14222
11702 14225 11699 14227 11696 14225 11696 14222 11693 14220 11690 14219
11688 14216 19 N F
11695 12714 11687 12712 11691 12711 11693 12710 11695 12710 11699 12707
11703 12709 11707 12709 11710 12706 11713 12703 11715 12699 11719 12701
11723 12702 11725 12706 11725 12711 11723 12715 11726 12717 11724 12721
11719 12721 11719 12726 11715 12728 11713 12724 11716 12720 11711 12720
11707 12718 11704 12716 11699 12715 11695 12714 28 N F
11690 13991 11688 13986 11691 13984 11694 13983 11695 13985 11695 13989
11693 13991 11690 13991 8 N F
11692 12410 11692 12405 11695 12405 11697 12403 11700 12405 11703 12405
11706 12404 11708 12407 11709 12410 11712 12410 11710 12413 11709 12416

11706 12418 11703 12419 11703 12416 11704 12413 11702 12411 11698 12410
11695 12409 11692 12410 20 N F
11698 14201 11693 14200 11696 14197 11700 14196 11701 14192 11701 14187
11705 14186 11704 14191 11707 14195 11709 14198 11706 14202 11702 14201
11698 14201 13 N F
11699 12859 11693 12855 11695 12855 11697 12855 11698 12851 11702 12849
11706 12851 11709 12848 11711 12844 11713 12848 11713 12853 11714 12849
11716 12844 11719 12848 11719 12852 11718 12856 11716 12860 11711 12861
11707 12860 11703 12861 11699 12859 21 N F
11701 12671 11698 12666 11700 12664 11703 12664 11705 12666 11706 12669
11704 12671 11701 12671 8 N F
11703 14079 11701 14073 11703 14069 11704 14065 11705 14061 11706 14057
11710 14059 11711 14060 11713 14062 11715 14058 11714 14054 11718 14056
11721 14060 11723 14063 11725 14067 11727 14071 11731 14072 11730 14076
11729 14081 11728 14085 11724 14086 11720 14086 11716 14086 11711 14086
11711 14081 11707 14079 11703 14079 27 N F
11705 12884 11703 12879 11708 12878 11711 12875 11709 12871 11711 12870
11714 12870 11715 12866 11719 12865 11724 12866 11727 12863 11731 12864
11735 12865 11738 12863 11738 12862 11742 12864 11744 12860 11740 12858
11740 12854 11744 12853 11745 12853 11746 12849 11748 12847 11749 12846
11749 12850 11753 12848 11755 12852 11758 12855 11754 12858 11750 12860
11751 12864 11750 12868 11746 12871 11743 12867 11739 12868 11737 12872
11733 12874 11732 12878 11735 12881 11739 12879 11742 12883 11741 12887
11737 12887 11734 12883 11733 12888 11730 12891 11727 12888 11725 12884
11721 12884 11717 12887 11716 12891 11712 12889 11708 12888 11705 12884
54 N F
11711 13966 11707 13963 11709 13961 11710 13957 11710 13954 11713 13953
11716 13954 11719 13955 11722 13957 11721 13960 11720 13963 11717 13965
11715 13963 11712 13963 11711 13966 15 N F
11710 14182 11709 14177 11711 14175 11714 14174 11716 14177 11716 14181
11713 14183 11710 14182 8 N F
11711 12639 11710 12635 11715 12635 11717 12631 11716 12627 11717 12623
11716 12619 11720 12619 11723 12621 11728 12622 11729 12626 11734 12627
11737 12625 11741 12627 11744 12631 11748 12631 11750 12635 11746 12636
11741 12637 11742 12641 11740 12645 11737 12648 11734 12645 11731 12641
11727 12641 11730 12645 11731 12649 11727 12652 11723 12650 11721 12646
11717 12645 11715 12641 11711 12639 33 N F
11713 12517 11711 12510 11715 12504 11720 12500 11723 12494 11729 12490
11728 12497 11726 12503 11728 12509 11730 12503 11735 12498 11742 12497
11738 12491 11735 12485 11737 12479 11741 12484 11745 12479 11752 12478
11759 12478 11763 12481 11765 12482 11771 12485 11766 12489 11773 12488
11774 12489 11779 12490 11783 12496 11790 12497 11790 12504 11796 12507
11803 12507 11804 12514 11809 12514 11811 12514 11812 12521 11817 12517

11819 12510 11824 12514 11822 12521 11826 12523 11828 12524 11830 12531
11834 12537 11831 12543 11835 12548 11830 12553 11834 12558 11835 12565
11836 12572 11831 12576 11827 12581 11821 12585 11816 12590 11810 12587
11808 12580 11803 12584 11800 12591 11802 12597 11798 12603 11792 12606
11785 12608 11779 12609 11772 12610 11770 12603 11773 12597 11780 12596
11787 12598 11784 12592 11786 12585 11783 12580 11778 12575 11775 12568
11769 12570 11768 12577 11766 12583 11762 12577 11765 12571 11759 12568
11762 12562 11763 12555 11767 12550 11774 12552 11771 12546 11764 12548
11768 12542 11766 12535 11765 12528 11764 12522 11760 12516 11761 12523
11759 12529 11760 12536 11763 12542 11759 12548 11759 12555 11753 12557
11752 12563 11746 12560 11745 12553 11748 12547 11746 12541 11741 12546
11734 12545 11730 12539 11724 12537 11722 12530 11727 12525 11721 12528
11715 12523 11713 12517 110 N F
11713 14197 11711 14192 11713 14188 11713 14187 11716 14185 11721 14186
11723 14190 11724 14190 11727 14190 11730 14193 11734 14195 11738 14194
11742 14195 11746 14197 11747 14201 11750 14205 11750 14209 11747 14212
11745 14216 11741 14218 11737 14219 11732 14219 11728 14220 11724 14222
11721 14224 11717 14222 11715 14218 11715 14214 11715 14209 11713 14205
11714 14201 11713 14197 32 N F
11715 13568 11715 13566 11715 13562 11716 13562 11717 13562 11720 13564
11722 13562 11724 13564 11726 13566 11726 13568 11724 13570 11723 13572
11724 13575 11722 13577 11720 13578 11719 13575 11719 13572 11717 13570
11715 13568 19 N F
11722 13156 11716 13154 11719 13150 11721 13147 11725 13149 11729 13151
11732 13154 11734 13158 11737 13161 11741 13163 11743 13167 11744 13171
11742 13174 11739 13177 11739 13173 11735 13170 11731 13170 11727 13172
11730 13168 11725 13168 11726 13163 11722 13161 11722 13156 23 N F
11730 12662 11728 12657 11732 12656 11735 12656 11738 12657 11739 12660
11741 12657 11744 12660 11745 12663 11742 12665 11744 12667 11746 12670
11743 12671 11740 12670 11737 12668 11736 12665 11736 12662 11733 12663
11730 12662 19 N F
11731 12818 11730 12815 11732 12812 11733 12809 11736 12808 11738 12810
11737 12814 11737 12817 11737 12820 11738 12823 11735 12822 11731 12821
11731 12818 13 N F
11732 13593 11731 13588 11732 13585 11735 13584 11737 13586 11739 13586
11740 13586 11742 13585 11745 13585 11746 13585 11747 13584 11750 13585
11753 13584 11756 13584 11758 13582 11759 13582 11760 13582 11763 13582
11765 13584 11765 13586 11762 13586 11759 13585 11757 13587 11755 13588
11756 13590 11755 13593 11753 13595 11752 13597 11749 13597 11747 13597
11745 13595 11744 13593 11745 13591 11742 13590 11740 13592 11737 13593
11735 13593 11732 13593 38 N F
11746 13304 11741 13302 11744 13301 11748 13301 11746 13304 5 N F
11742 12795 11742 12789 11743 12788 11745 12786 11749 12785 11752 12788

11756 12788 11756 12793 11757 12797 11753 12799 11748 12799 11744 12799
11742 12795 13 N F
11752 14090 11747 14091 11750 14088 11753 14085 11756 14081 11757 14077
11759 14073 11759 14069 11760 14064 11756 14063 11752 14060 11754 14056
11758 14057 11756 14053 11754 14049 11751 14046 11749 14041 11752 14044
11755 14047 11759 14046 11761 14042 11765 14044 11765 14049 11767 14053
11768 14057 11772 14056 11776 14056 11776 14060 11780 14063 11784 14064
11785 14068 11784 14072 11782 14076 11781 14080 11777 14081 11773 14081
11769 14082 11766 14086 11761 14084 11761 14089 11763 14092 11763 14097
11761 14093 11759 14089 11755 14087 11752 14090 46 N F
11751 12838 11748 12839 11750 12834 11752 12832 11752 12828 11752 12825
11750 12822 11753 12821 11756 12822 11759 12822 11762 12824 11761 12827
11761 12830 11758 12833 11756 12835 11754 12838 11751 12838 17 N F
11749 12756 11749 12751 11753 12751 11757 12750 11762 12748 11766 12748
11768 12752 11770 12756 11774 12757 11778 12756 11783 12757 11781 12761
11786 12760 11788 12756 11792 12756 11796 12756 11798 12752 11802 12750
11800 12747 11797 12744 11799 12740 11803 12739 11805 12743 11809 12741
11812 12745 11811 12749 11813 12753 11813 12757 11812 12762 11808 12763
11805 12766 11801 12765 11796 12765 11792 12764 11788 12765 11790 12769
11786 12771 11790 12773 11791 12777 11795 12776 11796 12780 11800 12780
11802 12784 11798 12785 11795 12788 11791 12789 11787 12788 11783 12789
11781 12793 11776 12793 11772 12792 11769 12788 11772 12785 11776 12782
11779 12780 11780 12776 11777 12773 11777 12768 11773 12769 11773 12765
11770 12762 11767 12766 11765 12762 11764 12758 11759 12758 11755 12758
11751 12760 11749 12756 68 N F
11754 13302 11750 13301 11753 13300 11757 13301 11758 13301 11759 13302
11762 13304 11759 13306 11756 13305 11754 13302 10 N F
11758 13285 11756 13281 11758 13279 11760 13276 11763 13278 11762 13281
11760 13284 11758 13285 8 N F
11759 12421 11757 12417 11760 12414 11763 12415 11761 12417 11762 12420
11763 12423 11764 12426 11763 12429 11760 12427 11759 12424 11759 12421
12 N F
11764 12819 11758 12817 11761 12814 11764 12810 11766 12807 11768 12803
11772 12801 11776 12802 11775 12806 11773 12810 11771 12814 11767 12817
11764 12819 13 N F
11762 7120 11761 7112 11763 7108 11766 7105 11770 7103 11775 7101
11779 7102 11782 7105 11787 7107 11791 7105 11795 7104 11800 7105
11804 7107 11806 7111 11805 7116 11803 7120 11801 7124 11799 7128
11795 7131 11791 7132 11787 7133 11783 7136 11778 7137 11774 7138
11770 7135 11770 7130 11771 7126 11770 7121 11766 7119 11762 7120
30 N F
11769 13287 11765 13286 11768 13285 11768 13282 11771 13283 11771 13280
11774 13281 11777 13281 11778 13284 11776 13286 11779 13288 11778 13291

11776 13294 11773 13295 11770 13294 11770 13291 11773 13290 11775 13288
11774 13285 11771 13284 11769 13287 21 N F
11770 13435 11767 13433 11769 13430 11771 13429 11774 13429 11777 13428
11776 13431 11774 13433 11772 13435 11770 13435 10 N F
11775 13310 11771 13308 11773 13308 11774 13308 11777 13307 11780 13307
11778 13310 11775 13310 8 N F
11779 13280 11778 13277 11780 13275 11784 13275 11786 13277 11789 13279
11789 13282 11787 13285 11786 13282 11785 13279 11781 13278 11783 13281
11780 13283 11779 13280 14 N F
11791 13297 11788 13294 11790 13292 11792 13289 11792 13285 11795 13285
11795 13288 11797 13289 11798 13289 11800 13287 11803 13286 11806 13287
11809 13289 11806 13291 11803 13293 11800 13294 11797 13294 11795 13296
11791 13297 19 N F
11797 13251 11793 13248 11796 13246 11797 13243 11799 13241 11800 13243
11801 13247 11799 13250 11797 13251 9 N F
11801 12498 11799 12493 11802 12491 11805 12490 11804 12494 11807 12493
11810 12495 11813 12497 11813 12500 11810 12502 11808 12501 11810 12503
11809 12506 11806 12505 11803 12503 11800 12502 11801 12498 17 N F
11812 11928 11806 11924 11806 11918 11808 11911 11807 11904 11807 11895
11811 11890 11815 11885 11819 11879 11825 11875 11826 11875 11831 11872
11838 11871 11844 11867 11848 11862 11853 11857 11858 11852 11864 11849
11870 11849 11871 11856 11872 11863 11876 11869 11880 11874 11886 11877
11893 11875 11900 11875 11907 11875 11913 11877 11919 11881 11926 11882
11932 11878 11939 11880 11945 11882 11950 11877 11957 11876 11963 11880
11970 11880 11974 11885 11980 11886 11987 11887 11994 11887 12001 11890
12007 11887 12013 11889 12018 11894 12023 11898 12027 11904 12033 11907
12040 11909 12047 11909 12054 11911 12060 11914 12066 11916 12073 11917
12077 11923 12082 11927 12086 11933 12093 11933 12100 11934 12107 11936
12113 11939 12117 11944 12116 11951 12121 11956 12124 11961 12126 11968
12127 11975 12130 11981 12136 11985 12141 11990 12141 11997 12142 12003
12143 12010 12148 12006 12147 12012 12145 12019 12149 12025 12155 12024
12151 12029 12146 12024 12145 12031 12138 12034 12134 12039 12138 12045
12136 12052 12132 12058 12126 12061 12119 12062 12113 12059 12107 12062
12101 12065 12096 12069 12092 12075 12085 12074 12078 12075 12072 12075
12065 12074 12059 12071 12052 12071 12045 12069 12039 12066 12033 12064
12026 12063 12020 12059 12013 12061 12007 12057 12001 12054 11995 12051
11989 12049 11982 12046 11976 12045 11970 12041 11963 12038 11958 12034
11951 12036 11945 12038 11941 12032 11934 12031 11929 12026 11923 12022
11916 12021 11911 12017 11905 12013 11898 12016 11893 12011 11887 12009
11881 12005 11875 12001 11868 12001 11865 11995 11861 11989 11855 11987
11850 11982 11845 11977 11845 11970 11841 11964 11837 11959 11832 11954
11826 11950 11823 11944 11818 11939 11812 11935 11812 11928 143 N F
11809 13307 11806 13306 11808 13304 11811 13305 11812 13307 11809 13307

6 N F
11819 13366 11816 13363 11819 13362 11822 13361 11823 13363 11822 13366
11819 13366 7 N F
11821 13416 11818 13413 11821 13412 11822 13412 11824 13411 11826 13410
11827 13413 11827 13416 11826 13418 11823 13418 11821 13416 11 N F
11826 7169 11819 7168 11823 7162 11827 7156 11830 7150 11834 7144
11839 7138 11841 7132 11835 7129 11834 7122 11836 7115 11834 7108
11827 7105 11825 7098 11831 7095 11836 7090 11840 7084 11836 7078
11825 7078 11828 7072 11833 7067 11839 7063 11846 7061 11847 7068
11849 7061 11855 7057 11862 7059 11868 7062 11875 7063 11882 7066
11889 7069 11896 7069 11903 7071 11910 7071 11917 7071 11924 7073
11931 7074 11936 7069 11932 7075 11927 7080 11923 7086 11918 7091
11911 7091 11904 7093 11897 7096 11894 7102 11887 7105 11880 7103
11873 7106 11866 7107 11863 7114 11865 7121 11859 7125 11853 7128
11849 7134 11847 7141 11844 7147 11838 7152 11833 7157 11829 7162
11826 7169 61 N F
11826 12273 11824 12269 11828 12267 11832 12264 11833 12268 11830 12272
11826 12273 7 N F
11830 13377 11827 13375 11830 13374 11832 13373 11834 13371 11837 13372
11839 13373 11841 13375 11838 13376 11836 13377 11833 13377 11830 13377
12 N F
11839 12267 11834 12263 11838 12262 11841 12258 11842 12254 11847 12254
11845 12258 11846 12262 11850 12263 11851 12268 11847 12269 11844 12266
11839 12267 13 N F
11846 13371 11842 13369 11845 13369 11848 13368 11849 13366 11852 13366
11849 13367 11849 13370 11846 13371 9 N F
11871 13286 11869 13280 11871 13278 11875 13278 11877 13280 11878 13283
11879 13286 11877 13289 11873 13288 11871 13286 10 N F
11905 12401 11903 12398 11906 12397 11908 12400 11910 12403 11913 12403
11914 12406 11916 12409 11913 12411 11911 12413 11909 12410 11908 12407
11907 12404 11905 12401 14 N F
11916 12392 11913 12389 11915 12385 11913 12378 11916 12374 11918 12370
11922 12369 11927 12369 11930 12371 11934 12373 11938 12376 11941 12379
11942 12383 11946 12384 11947 12388 11951 12390 11951 12394 11955 12392
11955 12392 11957 12388 11958 12392 11961 12390 11966 12391 11966 12395
11961 12395 11957 12396 11953 12398 11949 12398 11944 12396 11940 12397
11936 12396 11934 12400 11930 12402 11926 12401 11923 12398 11919 12395
11916 12392 37 N F
11933 12412 11930 12408 11930 12408 11934 12407 11937 12404 11940 12401
11944 12400 11949 12400 11953 12400 11949 12402 11948 12407 11952 12409
11954 12405 11958 12405 11961 12409 11958 12412 11961 12415 11965 12417
11962 12421 11959 12424 11956 12427 11952 12429 11948 12428 11944 12429
11940 12427 11937 12430 11933 12433 11934 12428 11937 12425 11933 12423

11932 12419 11936 12420 11934 12417 11933 12412 34 N F
11949 12439 11949 12433 11952 12433 11954 12435 11957 12433 11959 12436
11962 12436 11962 12439 11960 12442 11957 12443 11954 12443 11952 12441
11949 12439 13 N F
11955 12368 11952 12367 11954 12364 11952 12359 11955 12360 11958 12360
11962 12359 11961 12362 11959 12364 11958 12367 11955 12368 11 N F
11963 12520 11961 12515 11962 12511 11962 12507 11963 12503 11965 12498
11964 12494 11967 12491 11966 12487 11970 12489 11974 12486 11977 12484
11978 12484 11981 12482 11984 12485 11988 12487 11991 12484 11994 12481
11991 12478 11987 12478 11987 12474 11990 12471 11994 12472 11998 12473
12003 12473 12006 12470 12010 12469 12010 12473 12008 12477 12012 12476
12015 12472 12019 12472 12023 12472 12025 12472 12028 12471 12025 12475
12021 12477 12023 12481 12025 12485 12021 12487 12017 12486 12012 12486
12013 12490 12012 12494 12008 12496 12007 12500 12005 12504 12004 12508
12004 12513 12003 12517 12000 12521 11997 12523 11993 12525 11989 12527
11985 12529 11981 12530 11977 12528 11974 12525 11969 12526 11965 12524
11963 12520 61 N F
11973 12391 11969 12388 11972 12384 11974 12380 11974 12376 11978 12379
11981 12375 11982 12371 11986 12370 11989 12373 11989 12378 11991 12381
11990 12386 11994 12388 11993 12392 11989 12390 11988 12394 11986 12398
11982 12399 11982 12403 11987 12405 11991 12406 11993 12409 11989 12412
11986 12414 11981 12415 11977 12418 11974 12415 11974 12411 11969 12411
11970 12406 11975 12407 11977 12403 11977 12399 11974 12396 11973 12391
36 N F
12012 12525 12010 12520 12014 12523 12018 12522 12022 12523 12021 12519
12020 12515 12019 12510 12023 12508 12025 12511 12026 12516 12028 12519
12030 12523 12034 12525 12038 12525 12042 12527 12042 12531 12045 12534
12049 12533 12050 12533 12053 12531 12057 12532 12062 12531 12062 12536
12058 12539 12054 12541 12054 12545 12050 12543 12046 12544 12049 12547
12051 12551 12047 12554 12045 12558 12041 12557 12037 12555 12033 12553
12029 12551 12025 12551 12022 12548 12019 12545 12018 12540 12015 12537
12017 12533 12015 12529 12012 12525 45 N F
12017 12498 12014 12495 12018 12495 12022 12493 12027 12493 12030 12491
12033 12488 12036 12491 12039 12494 12044 12494 12048 12495 12052 12492
12054 12489 12054 12484 12057 12481 12061 12483 12063 12487 12066 12490
12065 12494 12069 12493 12070 12488 12074 12489 12075 12493 12074 12497
12074 12501 12079 12501 12080 12505 12078 12509 12075 12506 12070 12507
12068 12510 12063 12509 12059 12509 12056 12507 12053 12510 12050 12513
12046 12510 12044 12507 12044 12502 12041 12500 12036 12498 12033 12501
12030 12504 12028 12500 12026 12496 12022 12497 12017 12498 47 N F
12022 12427 12021 12424 12022 12421 12022 12415 12024 12413 12027 12411
12028 12413 12029 12417 12031 12419 12031 12422 12030 12425 12029 12428
12025 12429 12022 12427 14 N F

12065 12541 12059 12540 12063 12538 12065 12534 12063 12530 12062 12526
12063 12522 12066 12525 12068 12529 12072 12531 12071 12526 12072 12522
12073 12518 12077 12517 12081 12515 12082 12515 12085 12515 12089 12517
12090 12521 12094 12523 12099 12523 12100 12527 12098 12530 12097 12535
12097 12539 12099 12543 12098 12547 12096 12543 12093 12539 12092 12535
12088 12537 12084 12535 12081 12539 12076 12538 12072 12539 12069 12542
12067 12538 12065 12541 38 N F
12070 12422 12067 12412 12070 12406 12077 12409 12082 12405 12089 12404
12092 12404 12096 12403 12100 12408 12101 12415 12100 12422 12095 12426
12094 12433 12088 12437 12082 12439 12076 12435 12072 12429 12070 12422
18 N F
12109 12315 12107 12309 12108 12305 12113 12305 12117 12306 12119 12310
12117 12314 12114 12316 12109 12315 9 N F
12110 12506 12107 12503 12108 12498 12108 12494 12113 12493 12114 12490
12114 12489 12118 12491 12122 12493 12125 12496 12129 12497 12131 12501
12134 12503 12133 12507 12126 12510 12122 12510 12117 12510 12113 12509
12110 12506 19 N F
12125 12521 12120 12523 12122 12519 12126 12517 12126 12517 12130 12518
12135 12517 12138 12515 12135 12512 12139 12512 12142 12508 12143 12512
12147 12513 12150 12510 12147 12507 12143 12505 12144 12501 12145 12496
12147 12500 12150 12504 12152 12507 12156 12510 12160 12509 12161 12514
12157 12513 12156 12518 12160 12516 12164 12517 12167 12513 12171 12514
12169 12517 12166 12521 12165 12525 12161 12525 12156 12526 12153 12524
12149 12521 12145 12520 12146 12524 12145 12528 12141 12530 12138 12533
12135 12537 12137 12533 12132 12532 12128 12530 12131 12527 12129 12523
12125 12521 49 N F
12135 12577 12129 12575 12132 12572 12135 12569 12135 12568 12137 12565
12137 12560 12139 12557 12143 12554 12144 12558 12143 12562 12148 12561
12147 12556 12149 12552 12150 12548 12152 12552 12153 12556 12155 12560
12153 12564 12151 12568 12150 12572 12147 12576 12144 12578 12139 12579
12135 12577 25 N F
12165 12587 12159 12586 12162 12583 12167 12584 12169 12581 12166 12578
12165 12574 12169 12575 12171 12571 12175 12569 12179 12567 12183 12565
12186 12567 12191 12567 12193 12570 12198 12571 12202 12570 12206 12570
12211 12570 12215 12571 12218 12575 12219 12579 12219 12583 12220 12587
12222 12592 12223 12596 12222 12600 12219 12603 12216 12606 12212 12605
12208 12603 12204 12602 12200 12603 12196 12601 12193 12598 12190 12595
12186 12595 12183 12592 12179 12593 12175 12594 12173 12598 12169 12599
12170 12594 12167 12591 12165 12587 45 N F
12235 12458 12233 12452 12237 12454 12241 12453 12245 12455 12250 12454
12250 12459 12252 12462 12250 12466 12249 12471 12249 12475 12245 12474
12241 12472 12241 12468 12237 12467 12237 12463 12235 12458 17 N F
12274 12372 12268 12368 12270 12364 12272 12360 12275 12358 12276 12353

12279 12350 12282 12347 12287 12347 12291 12346 12293 12350 12289 12349
12285 12351 12284 12355 12280 12358 12278 12362 12277 12366 12278 12370
12277 12374 12274 12372 20 N F
12288 12373 12281 12371 12284 12368 12286 12364 12290 12362 12294 12361
12295 12365 12291 12367 12292 12371 12288 12373 10 N F
12313 12636 12309 12631 12313 12629 12318 12629 12318 12625 12322 12622
12318 12621 12315 12618 12317 12614 12320 12611 12324 12610 12327 12614
12331 12614 12335 12616 12340 12615 12342 12619 12341 12623 12338 12626
12334 12628 12330 12628 12325 12628 12329 12630 12327 12634 12323 12635
12326 12638 12328 12642 12327 12646 12323 12646 12319 12647 12316 12644
12313 12640 12313 12636 32 N F
12323 12599 12321 12594 12325 12594 12329 12595 12334 12596 12336 12592
12332 12592 12327 12593 12329 12589 12333 12587 12337 12588 12341 12586
12341 12582 12340 12578 12343 12575 12346 12579 12350 12581 12354 12583
12358 12584 12361 12587 12362 12591 12361 12595 12359 12599 12355 12600
12352 12604 12348 12606 12346 12610 12344 12605 12340 12604 12337 12601
12333 12604 12329 12605 12325 12607 12321 12606 12324 12603 12323 12599
36 N F
12400 12676 12397 12672 12398 12666 12400 12670 12402 12666 12405 12663
12409 12663 12412 12666 12416 12667 12417 12672 12420 12668 12425 12669
12428 12672 12425 12674 12421 12677 12418 12674 12414 12674 12412 12678
12407 12679 12404 12677 12400 12676 21 N F
12432 12657 12426 12656 12429 12653 12433 12651 12437 12649 12441 12651
12440 12646 12444 12644 12445 12640 12449 12638 12453 12640 12457 12638
12461 12636 12462 12632 12467 12632 12471 12633 12474 12636 12472 12640
12470 12644 12465 12644 12461 12644 12465 12646 12465 12650 12461 12653
12458 12656 12456 12660 12455 12664 12450 12666 12446 12667 12442 12666
12438 12663 12435 12660 12432 12657 33 N F
12458 12841 12456 12834 12458 12827 12461 12821 12465 12816 12470 12811
12471 12804 12476 12800 12479 12794 12477 12787 12475 12781 12477 12780
12481 12779 12484 12772 12489 12768 12496 12767 12502 12768 12508 12771
12509 12772 12510 12778 12507 12784 12504 12790 12510 12794 12516 12798
12523 12799 12524 12792 12523 12786 12525 12779 12525 12778 12527 12772
12529 12766 12536 12765 12538 12765 12543 12765 12544 12766 12548 12769
12555 12770 12560 12764 12566 12767 12571 12767 12573 12768 12569 12773
12565 12779 12570 12784 12573 12790 12572 12797 12570 12804 12569 12810
12568 12817 12572 12823 12571 12830 12566 12834 12559 12832 12554 12836
12542 12834 12536 12831 12533 12825 12527 12822 12522 12817 12517 12821
12515 12828 12508 12830 12502 12830 12502 12837 12498 12842 12494 12848
12495 12855 12501 12859 12503 12865 12505 12872 12503 12878 12500 12884
12494 12888 12487 12889 12481 12886 12475 12882 12471 12876 12471 12869
12476 12864 12469 12862 12463 12860 12463 12853 12462 12846 12458 12841
84 N F

12476 12606 12473 12602 12477 12602 12475 12599 12475 12594 12479 12594
12482 12591 12485 12595 12484 12599 12484 12604 12485 12608 12484 12612
12480 12614 12481 12619 12477 12619 12475 12615 12477 12611 12476 12606
18 N F
12485 8076 12477 8069 12481 8063 12488 8061 12495 8061 12502 8060
12507 8055 12510 8049 12514 8043 12520 8039 12527 8036 12534 8035
12531 8042 12529 8049 12528 8056 12532 8062 12535 8068 12529 8072
12525 8078 12519 8080 12515 8086 12509 8090 12502 8089 12495 8085
12491 8080 12485 8076 26 N F
12524 12853 12523 12849 12524 12845 12525 12841 12527 12837 12532 12836
12536 12835 12540 12835 12544 12836 12549 12837 12549 12841 12554 12843
12557 12841 12561 12839 12566 12840 12570 12838 12574 12838 12577 12836
12582 12836 12580 12840 12577 12843 12572 12844 12569 12847 12568 12851
12570 12855 12569 12860 12566 12862 12561 12864 12558 12866 12559 12870
12555 12871 12552 12868 12550 12864 12549 12859 12550 12855 12548 12851
12545 12854 12544 12858 12545 12863 12546 12867 12548 12871 12550 12875
12551 12879 12550 12883 12546 12882 12542 12880 12537 12876 12535 12872
12534 12868 12532 12864 12529 12861 12527 12856 12524 12853 53 N F
12524 8208 12523 8207 12523 8207 12523 8206 12525 8206 12525 8206
12525 8206 12526 8206 12526 8207 12526 8208 12525 8208 12524 8208
12 N F
12535 8063 12533 8060 12535 8061 12538 8062 12540 8062 12540 8065
12538 8067 12537 8065 12535 8063 9 N F
12542 8067 12542 8063 12544 8064 12546 8065 12544 8067 12542 8067
6 N F
12548 8064 12547 8061 12548 8062 12550 8064 12552 8064 12555 8065
12556 8066 12558 8068 12559 8070 12561 8070 12563 8071 12566 8071
12568 8072 12569 8074 12571 8074 12573 8075 12571 8076 12569 8075
12567 8074 12565 8072 12563 8072 12561 8072 12559 8071 12557 8070
12556 8068 12554 8066 12552 8066 12550 8065 12548 8064 29 N F
12618 12855 12616 12848 12620 12846 12624 12845 12623 12840 12628 12840
12631 12843 12632 12847 12635 12850 12639 12851 12643 12851 12643 12855
12639 12857 12634 12857 12631 12859 12626 12858 12622 12857 12618 12855
18 N F
12638 12839 12633 12836 12637 12835 12637 12831 12642 12831 12645 12828
12649 12828 12653 12826 12654 12830 12651 12833 12655 12834 12659 12835
12663 12838 12659 12841 12655 12843 12653 12846 12649 12849 12645 12851
12641 12849 12638 12846 12635 12843 12638 12839 22 N F
12658 12744 12656 12738 12659 12735 12663 12734 12662 12729 12664 12725
12667 12729 12671 12730 12673 12734 12678 12733 12682 12733 12686 12733
12686 12738 12689 12741 12687 12745 12683 12743 12680 12739 12679 12735
12676 12738 12679 12741 12676 12744 12671 12745 12668 12747 12664 12749
12659 12748 12658 12744 26 N F

12695 8101 12693 8096 12696 8092 12698 8088 12702 8090 12706 8093
12701 8094 12699 8098 12699 8103 12695 8101 10 N F
12721 8230 12715 8218 12721 8221 12728 8221 12735 8225 12733 8231
12726 8234 12721 8230 8 N F
12752 12890 12750 12883 12752 12876 12754 12870 12757 12864 12759 12863
12764 12862 12767 12856 12771 12850 12777 12853 12776 12859 12782 12861
12788 12864 12793 12870 12795 12876 12802 12874 12803 12868 12806 12861
12804 12855 12805 12848 12799 12844 12792 12842 12786 12840 12788 12833
12788 12833 12793 12837 12793 12831 12800 12832 12806 12835 12812 12832
12806 12828 12805 12821 12808 12815 12814 12813 12811 12807 12809 12801
12815 12799 12816 12798 12822 12796 12827 12800 12834 12802 12831 12808
12826 12813 12833 12815 12840 12816 12846 12819 12853 12819 12847 12823
12840 12821 12836 12827 12832 12832 12834 12839 12840 12838 12845 12843
12842 12850 12842 12857 12848 12859 12852 12853 12856 12859 12862 12858
12863 12857 12866 12853 12873 12850 12873 12857 12868 12862 12864 12867
12866 12874 12859 12873 12857 12879 12863 12882 12869 12880 12866 12886
12862 12892 12869 12893 12869 12900 12872 12906 12865 12907 12860 12902
12858 12895 12852 12897 12848 12902 12852 12908 12855 12914 12859 12920
12856 12926 12854 12933 12854 12939 12851 12946 12846 12950 12843 12956
12840 12962 12843 12968 12849 12971 12850 12978 12846 12983 12840 12985
12833 12986 12829 12980 12828 12973 12832 12963 12826 12961 12823 12955
12822 12948 12821 12941 12814 12943 12808 12939 12802 12942 12800 12936
12796 12931 12800 12925 12803 12919 12807 12914 12812 12909 12817 12904
12810 12905 12804 12904 12797 12907 12793 12902 12786 12903 12780 12906
12775 12911 12768 12909 12766 12903 12759 12902 12756 12895 12752 12890
126 N F
12751 12840 12750 12834 12754 12832 12757 12829 12755 12825 12756 12820
12756 12820 12760 12820 12765 12819 12768 12822 12772 12824 12775 12827
12775 12827 12774 12832 12771 12835 12767 12834 12765 12838 12764 12842
12761 12845 12762 12850 12758 12847 12759 12851 12755 12851 12752 12848
12753 12844 12758 12845 12761 12842 12757 12844 12756 12840 12754 12836
12754 12841 12751 12844 12751 12840 33 N F
12756 7997 12754 7992 12755 7988 12759 7986 12759 7991 12760 7995
12756 7997 7 N F
12756 8020 12755 8015 12756 8014 12759 8014 12761 8019 12756 8020
6 N F
12762 12939 12761 12934 12764 12931 12764 12926 12763 12922 12765 12919
12768 12915 12772 12916 12769 12919 12773 12922 12769 12925 12767 12929
12770 12932 12773 12935 12771 12939 12766 12940 12762 12939 17 N F
12771 8013 12768 8009 12772 8006 12775 8003 12779 8004 12783 8007
12781 8011 12779 8015 12775 8016 12771 8013 10 N F
12781 8031 12775 8026 12780 8026 12784 8026 12785 8025 12786 8024
12788 8022 12790 8026 12792 8030 12792 8034 12795 8031 12800 8030

12800 8034 12797 8038 12793 8039 12788 8039 12788 8035 12783 8035
12781 8031 19 N F
12785 8002 12779 8000 12781 7996 12786 7998 12785 8002 5 N F
12786 7996 12783 7991 12785 7988 12787 7992 12786 7996 5 N F
12811 8006 12807 8003 12809 7999 12812 7996 12812 7991 12812 7987
12814 7983 12816 7987 12820 7989 12821 7994 12820 7998 12819 8003
12821 8007 12824 8010 12823 8014 12824 8019 12823 8023 12820 8027
12816 8029 12812 8028 12812 8023 12811 8019 12809 8015 12810 8010
12811 8006 25 N F
12859 8026 12856 8020 12861 8021 12861 8026 12863 8029 12863 8034
12860 8031 12859 8026 8 N F
12884 13002 12881 12998 12886 12997 12889 12994 12888 12989 12891 12986
12888 12984 12891 12982 12896 12981 12896 12976 12896 12972 12899 12969
12901 12969 12903 12970 12904 12974 12903 12978 12903 12983 12904 12987
12905 12991 12906 12995 12906 13000 12904 13004 12900 13003 12898 13007
12894 13009 12892 13006 12887 13004 12884 13002 28 N F
12895 11305 12895 11305 12895 11304 12895 11304 12896 11304 12896 11304
12897 11303 12897 11303 12898 11303 12897 11304 12897 11304 12896 11305
12895 11305 13 N F
12896 11308 12896 11307 12896 11306 12897 11305 12898 11304 12898 11302
12898 11301 12898 11299 12897 11298 12897 11296 12898 11295 12899 11297
12899 11299 12901 11299 12903 11299 12902 11301 12901 11302 12900 11303
12901 11305 12902 11306 12903 11308 12902 11309 12901 11310 12900 11309
12898 11308 12896 11308 26 N F
12903 11314 12903 11313 12903 11312 12904 11311 12904 11311 12905 11312
12905 11312 12906 11313 12906 11315 12905 11315 12905 11315 12904 11314
12903 11314 13 N F
12904 11319 12904 11319 12904 11318 12905 11318 12905 11318 12905 11318
12905 11318 12905 11319 12905 11320 12904 11319 10 N F
12906 11322 12906 11321 12906 11319 12906 11319 12906 11319 12907 11319
12907 11319 12908 11319 12908 11319 12908 11321 12909 11322 12909 11322
12908 11322 12907 11322 12906 11322 12906 11322 16 N F
12909 11326 12909 11326 12909 11325 12909 11325 12909 11324 12910 11324
12910 11323 12910 11323 12911 11323 12911 11324 12911 11324 12911 11325
12911 11326 12911 11326 12909 11326 15 N F
12911 11330 12911 11328 12912 11328 12914 11329 12915 11329 12917 11329
12918 11330 12919 11331 12921 11330 12922 11329 12923 11330 12923 11331
12924 11333 12925 11334 12926 11336 12926 11337 12924 11338 12924 11336
12922 11337 12920 11336 12919 11337 12917 11337 12915 11337 12915 11336
12915 11334 12915 11332 12914 11331 12912 11330 12911 11330 29 N F
12962 8040 12959 8033 12961 8026 12965 8021 12968 8014 12974 8010
12979 8005 12979 7998 12979 7991 12979 7984 12977 7977 12976 7970
12982 7965 12988 7963 12993 7968 12999 7972 12999 7972 12999 8080

12994 8078 12988 8075 12984 8069 12981 8063 12978 8056 12978 8049
12973 8044 12977 8039 12981 8033 12976 8038 12969 8040 12962 8040
30 N F
12973 11668 12970 11656 12977 11654 12983 11654 12988 11649 12994 11648
12998 11653 12999 11654 12999 11684 12992 11684 12997 11679 12991 11681
12993 11674 12988 11670 12987 11663 12993 11666 12999 11670 12995 11664
12994 11657 12988 11659 12982 11663 12979 11669 12975 11675 12973 11668
24 N F
12981 8342 12978 8332 12983 8326 12990 8326 12996 8328 12999 8328
12999 8343 12996 8342 12988 8343 12981 8342 10 N F
12983 11749 12981 11742 12982 11736 12986 11730 12984 11724 12985 11717
12990 11712 12987 11706 12990 11700 12992 11694 12998 11691 12999 11685
12999 11747 12995 11752 12988 11754 12983 11749 16 N F
12983 8166 12983 8159 12985 8152 12988 8146 12992 8140 12995 8134
12999 8131 12999 8167 12996 8164 12991 8169 12985 8173 12983 8166
12 N F
1 O
13000 16218 2299 16218 2299 5502 13000 5502 13000 16218 5 P
Ls Gr Gs Lr
2298 5501 13001 16219 Oc
111 O
2298 5772 2298 5638 2298 5638 2298 6263 2298 6263 2298 6179
2298 6044 2298 5908 2298 5772 9 N F
129 O
2298 6315 2298 6263 2298 6263 2298 6345 2298 6345 2298 6315
6 N F
111 O
2298 6451 2298 6345 2298 6345 2298 6972 2298 6972 2298 6858
2298 6722 2298 6586 2298 6451 9 N F
129 O
2298 6993 2298 6972 2298 6972 2298 7347 2298 7347 2298 7265
2298 7129 2298 6993 8 N F
111 O
2298 7400 2298 7347 2298 7347 2298 7744 2298 7744 2298 7672
2298 7536 2298 7400 8 N F
129 O
2298 7807 2298 7744 2298 7744 2298 8006 2298 8006 2298 7943
2298 7807 7 N F
147 O
2298 8079 2298 8006 2298 8006 2298 9587 2298 9587 2298 9571
2298 9435 2298 9300 2298 9164 2298 9028 2298 8893 2298 8757
2298 8621 2298 8486 2298 8350 2298 8214 2298 8079 17 N F
129 O

2298 9707 2298 9587 2298 9587 2298 10166 2298 10166 2298 10114
2298 9978 2298 9842 2298 9707 9 N F
111 O
2298 10249 2298 10166 2298 10166 2298 11391 2298 11391 2298 11335
2298 11199 2298 11064 2298 10928 2298 10792 2298 10656 2298 10521
2298 10385 2298 10249 14 N F
129 O
2298 11471 2298 11391 2298 11391 2298 11772 2298 11772 2298 11742
2298 11606 2298 11471 8 N F
147 O
2298 11878 2298 11772 2298 11772 2298 11890 2298 11890 2298 11878
6 N F
165 O
2298 12013 2298 11890 2298 11890 2298 12072 2298 12072 2298 12013
6 N F
183 O
2298 12149 2298 12072 2298 12072 2298 12273 2298 12273 2298 12149
6 N F
165 O
2298 12285 2298 12273 2298 12273 2298 12519 2298 12519 2298 12420
2298 12285 7 N F
129 O
2298 5638 2298 5501 2298 5501 2298 5501 2298 5638 2298 5638
6 N F
111 O
2298 6263 2298 5638 2298 5638 2298 6263 2298 6263 5 N F
129 O
2298 6345 2298 6263 2298 6263 2298 6345 2298 6345 5 N F
111 O
2298 6972 2298 6345 2298 6345 2298 6972 2298 6972 5 N F
129 O
2298 7347 2298 6972 2298 6972 2298 7347 2298 7347 5 N F
111 O
2298 7744 2298 7347 2298 7347 2298 7744 2298 7744 5 N F
129 O
2298 8006 2298 7744 2298 7744 2298 8006 2298 8006 5 N F
147 O
2298 9587 2298 8006 2298 8006 2298 9587 2298 9587 5 N F
129 O
2298 10166 2298 9587 2298 9587 2298 10166 2298 10166 5 N F
111 O
2298 11391 2298 10166 2298 10166 2298 11391 2298 11391 5 N F
129 O

2298 11772 2298 11391 2298 11391 2298 11772 2298 11772 5 N F
147 O
2298 11890 2298 11772 2298 11772 2298 11890 2298 11890 5 N F
165 O
2298 12072 2298 11890 2298 11890 2298 12073 2298 12072 5 N F
183 O
2298 12273 2298 12072 2298 12073 2298 12273 2298 12273 5 N F
165 O
2298 12519 2298 12273 2298 12273 2298 12519 2298 12519 5 N F
183 O
2298 16219 2298 12519 2298 12519 2298 16219 2298 16219 2298 16219
6 N F
129 O
2489 5501 2298 5501 2298 5638 2304 5637 2433 5622 2459 5637
2569 5744 2616 5772 2704 5871 2761 5772 2734 5637 2704 5543
2695 5501 2680 5501 2489 5501 15 N F
111 O
2298 5638 M 2298 6263 L 2359 6179 L 2433 6099 L 2549 6179 L
2433 6309 L 2423 6315 L 2298 6345 L 2298 6972 L
2433 6993 L 2434 6993 L 2569 7109 L 2613 6993 L
2617 6858 L 2660 6722 L 2704 6675 L 2840 6653 L
2921 6586 L 2918 6451 L 2975 6339 L 3111 6335 L
3190 6451 L 3246 6555 L 3382 6571 L 3395 6586 L
3510 6722 L 3517 6735 L 3608 6858 L 3653 6894 L
3731 6858 L 3788 6821 L 3844 6722 L 3851 6586 L
3901 6451 L 3924 6443 L 3945 6451 L 4059 6515 L
4175 6586 L 4195 6609 L 4195 5973 L 4059 5962 L
3999 5908 L 4020 5772 L 4059 5712 L 4195 5642 L
4198 5637 L 4330 5508 L 4390 5637 L 4370 5772 L
4330 5851 L 4218 5908 L 4195 5973 L 4195 6609 L
4330 6648 L 4403 6586 L 4466 6537 L 4601 6573 L
4617 6586 L 4670 6722 L 4601 6846 L 4593 6858 L
4601 6861 L 4737 6870 L 4756 6858 L 4827 6722 L
4872 6681 L 5008 6592 L 5143 6658 L 5220 6722 L
5231 6858 L 5143 6916 L 5073 6993 L 5143 7084 L
5194 7129 L 5279 7246 L 5349 7129 L 5297 6993 L
5414 6865 L 5468 6858 L 5550 6844 L 5570 6858 L
5593 6993 L 5622 7129 L 5685 7238 L 5820 7213 L
5906 7265 L 5956 7303 L 6000 7265 L 6091 7134 L
6214 7265 L 6218 7400 L 6227 7405 L 6227 7017 L
6114 6993 L 6227 6913 L 6279 6993 L 6227 7017 L
6227 7405 L 6362 7428 L 6498 7464 L 6633 7477 L
6769 7515 L 6844 7536 L 6904 7612 L 7040 7638 L

7155 7536 L 7175 7511 L 7311 7478 L 7446 7467 L
7497 7400 L 7582 7287 L 7671 7400 L 7717 7449 L
7853 7462 L 7988 7424 L 8124 7416 L 8259 7441 L
8395 7445 L 8530 7454 L 8666 7445 L 8801 7526 L
8816 7536 L 8870 7672 L 8937 7792 L 8967 7807 L
8937 7844 L 8801 7919 L 8794 7943 L 8801 8031 L
8858 8079 L 8937 8102 L 9072 8188 L 9208 8151 L
9343 8139 L 9397 8079 L 9358 7943 L 9343 7905 L
9306 7807 L 9343 7750 L 9477 7807 L 9479 7808 L
9481 7807 L 9614 7778 L 9746 7672 L 9749 7670 L
9885 7656 L 10020 7574 L 10156 7608 L 10291 7637 L
10427 7666 L 10562 7632 L 10698 7654 L 10833 7616 L
10915 7536 L 10969 7505 L 11104 7486 L 11240 7466 L
11375 7476 L 11511 7486 L 11646 7498 L 11782 7513 L
11917 7535 L 11923 7536 L 12053 7663 L 12055 7672 L
12077 7807 L 12090 7943 L 12099 8079 L 12110 8214 L
12121 8350 L 12127 8486 L 12135 8621 L 12141 8757 L
12148 8893 L 12155 9028 L 12164 9164 L 12177 9300 L
12185 9435 L 12188 9483 L 12221 9571 L 12275 9707 L
12317 9842 L 12324 9858 L 12425 9978 L 12459 10019 L
12573 10114 L 12595 10138 L 12715 10249 L 12730 10263 L
12866 10377 L 12879 10385 L 13001 10501 L 13001 9479 L
12896 9435 L 13001 9310 L 13001 9289 L 12983 9300 L
12866 9388 L 12807 9300 L 12730 9253 L 12595 9271 L
12459 9236 L 12324 9177 L 12316 9164 L 12254 9028 L
12188 8914 L 12187 8893 L 12181 8757 L 12176 8621 L
12169 8486 L 12165 8350 L 12157 8214 L 12149 8079 L
12143 7943 L 12137 7807 L 12127 7672 L 12107 7536 L
12053 7480 L 11917 7460 L 11782 7449 L 11646 7437 L
11511 7430 L 11375 7421 L 11240 7413 L 11104 7422 L
10969 7435 L 10833 7495 L 10698 7527 L 10562 7533 L
10427 7489 L 10291 7466 L 10156 7449 L 10020 7452 L
9885 7465 L 9749 7483 L 9614 7531 L 9479 7524 L
9343 7528 L 9208 7519 L 9072 7497 L 8937 7452 L
8859 7400 L 8801 7354 L 8720 7265 L 8666 7196 L
8574 7129 L 8530 7070 L 8482 7129 L 8395 7257 L
8390 7265 L 8259 7319 L 8124 7267 L 8116 7265 L
8101 7129 L 7988 7079 L 7881 7129 L 7853 7238 L
7793 7129 L 7717 7088 L 7582 7001 L 7446 7101 L
7366 7129 L 7311 7169 L 7253 7129 L 7175 7091 L
7040 7041 L 6911 6993 L 6904 6978 L 6769 6948 L
6633 6920 L 6498 6864 L 6493 6858 L 6427 6722 L
6362 6621 L 6303 6586 L 6227 6557 L 6091 6530 L

5978 6451 L 5956 6435 L 5925 6451 L 5956 6492 L
6008 6586 L 5956 6696 L 5820 6693 L 5685 6624 L
5629 6586 L 5572 6451 L 5590 6315 L 5596 6179 L
5622 6044 L 5685 5942 L 5685 5688 L 5566 5637 L
5685 5594 L 5718 5637 L 5685 5688 L 5685 5942 L
5721 5908 L 5811 5772 L 5820 5756 L 5956 5708 L
6091 5642 L 6094 5637 L 6091 5631 L 6021 5501 L
5929 5501 L 5738 5501 L 5547 5501 L 5356 5501 L
5165 5501 L 4974 5501 L 4783 5501 L 4592 5501 L
4400 5501 L 4209 5501 L 4018 5501 L 3827 5501 L
3636 5501 L 3445 5501 L 3254 5501 L 3063 5501 L
2871 5501 L 2695 5501 L 2704 5543 L 2734 5637 L
2761 5772 L 2704 5871 L 2616 5772 L 2569 5744 L
2459 5637 L 2433 5622 L 2304 5637 L 2298 5638 L
N E
129 O
2359 6179 2298 6263 2298 6345 2423 6315 2433 6309 2549 6179
2433 6099 2359 6179 8 N F
2433 6993 M 2298 6972 L 2298 7347 L 2338 7400 L 2433 7523 L
2446 7536 L 2436 7672 L 2433 7673 L 2298 7744 L
2298 8006 L 2433 7959 L 2460 7943 L 2569 7892 L
2650 7807 L 2674 7672 L 2704 7575 L 2745 7536 L
2840 7442 L 2975 7484 L 3111 7459 L 3140 7400 L
3164 7265 L 3246 7192 L 3382 7248 L 3407 7265 L
3517 7326 L 3653 7377 L 3732 7265 L 3788 7219 L
3858 7265 L 3924 7294 L 4059 7398 L 4061 7400 L
4195 7465 L 4195 7007 L 4171 6993 L 4195 6972 L
4245 6993 L 4195 7007 L 4195 7465 L 4293 7400 L
4249 7265 L 4330 7208 L 4422 7129 L 4466 7088 L
4500 7129 L 4466 7204 L 4418 7265 L 4466 7288 L
4601 7400 L 4601 7400 L 4674 7536 L 4737 7651 L
4781 7672 L 4737 7694 L 4642 7807 L 4713 7943 L
4737 7969 L 4872 8050 L 4872 7404 L 4869 7400 L
4872 7397 L 4888 7400 L 4872 7404 L 4872 8050 L
5008 8033 L 5008 7877 L 4953 7807 L 5008 7743 L
5118 7807 L 5008 7877 L 5008 8033 L 5143 8009 L
5143 7545 L 5113 7536 L 5143 7508 L 5279 7535 L
5281 7536 L 5279 7537 L 5143 7545 L 5143 8009 L
5200 8079 L 5279 8188 L 5319 8214 L 5413 8350 L
5414 8351 L 5414 7360 L 5292 7265 L 5414 7182 L
5550 7258 L 5577 7265 L 5550 7279 L 5414 7360 L
5414 8351 L 5450 8486 L 5471 8621 L 5529 8757 L
5550 8777 L 5550 7707 L 5533 7672 L 5550 7638 L

5578 7672 L 5550 7707 L 5550 8777 L 5572 8893 L
5550 8961 L 5423 9028 L 5414 9034 L 5390 9028 L
5308 8893 L 5299 8757 L 5321 8621 L 5279 8580 L
5143 8568 L 5078 8621 L 5098 8757 L 5143 8807 L
5244 8893 L 5143 9023 L 5116 9028 L 5008 9035 L
4872 9084 L 4737 9142 L 4708 9164 L 4601 9281 L
4561 9300 L 4534 9435 L 4601 9486 L 4627 9571 L
4632 9707 L 4614 9842 L 4694 9978 L 4721 10114 L
4705 10249 L 4655 10385 L 4630 10521 L 4737 10590 L
4872 10655 L 4873 10656 L 4908 10792 L 4872 10901 L
4859 10928 L 4737 11017 L 4704 11064 L 4601 11137 L
4466 11138 L 4330 11121 L 4202 11199 L 4195 11226 L
4159 11335 L 4059 11392 L 3924 11445 L 3828 11471 L
3788 11484 L 3653 11520 L 3517 11598 L 3514 11606 L
3427 11742 L 3382 11768 L 3246 11804 L 3111 11824 L
2975 11849 L 2903 11878 L 2927 12013 L 2975 12027 L
3111 12070 L 3246 12054 L 3382 12044 L 3458 12013 L
3517 11959 L 3653 11885 L 3673 11878 L 3788 11814 L
3924 11840 L 3924 11652 L 3788 11627 L 3759 11606 L
3788 11598 L 3924 11589 L 3959 11606 L 3924 11652 L
3924 11840 L 4059 11748 L 4195 11766 L 4291 11878 L
4330 11903 L 4466 11914 L 4466 11401 L 4363 11335 L
4466 11224 L 4592 11335 L 4466 11401 L 4466 11914 L
4601 11930 L 4678 12013 L 4737 12059 L 4872 12021 L
4887 12013 L 5008 11990 L 5143 11962 L 5279 11915 L
5414 11965 L 5442 12013 L 5417 12149 L 5414 12156 L
5279 12238 L 5241 12285 L 5143 12376 L 5096 12420 L
5143 12542 L 5157 12556 L 5279 12612 L 5360 12692 L
5414 12709 L 5550 12733 L 5685 12741 L 5685 11925 L
5550 11916 L 5536 11878 L 5414 11779 L 5279 11768 L
5143 11848 L 5104 11878 L 5008 11908 L 4924 11878 L
4872 11820 L 4823 11742 L 4737 11672 L 4601 11716 L
4528 11606 L 4567 11471 L 4601 11358 L 4622 11335 L
4737 11284 L 4804 11199 L 4872 11159 L 5008 11097 L
5055 11064 L 5117 10928 L 5143 10814 L 5143 10720 L
5008 10671 L 5002 10656 L 5008 10627 L 5008 10415 L
4966 10385 L 5008 10355 L 5025 10385 L 5008 10415 L
5008 10627 L 5143 10647 L 5174 10656 L 5143 10720 L
5143 10814 L 5279 10836 L 5402 10792 L 5414 10786 L
5550 10675 L 5646 10792 L 5685 10832 L 5740 10928 L
5738 11064 L 5767 11199 L 5820 11246 L 5947 11335 L
5956 11363 L 6091 11453 L 6091 11348 L 6076 11335 L
6091 11318 L 6128 11335 L 6091 11348 L 6091 11453 L

6227 11436 L 6264 11471 L 6227 11506 L 6091 11538 L
5956 11508 L 5820 11601 L 5818 11606 L 5730 11742 L
5699 11878 L 5685 11925 L 5685 12741 L 5742 12692 L
5773 12556 L 5746 12420 L 5726 12285 L 5753 12149 L
5804 12013 L 5782 11878 L 5803 11742 L 5820 11715 L
5956 11645 L 6025 11742 L 6091 11842 L 6227 11804 L
6227 11743 L 6182 11742 L 6227 11738 L 6307 11742 L
6227 11743 L 6227 11804 L 6362 11825 L 6362 11277 L
6314 11199 L 6227 11138 L 6091 11144 L 5956 11174 L
5820 11072 L 5817 11064 L 5820 11046 L 5850 10928 L
5823 10792 L 5820 10783 L 5780 10656 L 5820 10544 L
5826 10521 L 5844 10385 L 5840 10249 L 5867 10114 L
5904 9978 L 5920 9842 L 5820 9743 L 5788 9707 L
5729 9571 L 5750 9435 L 5820 9347 L 5888 9300 L
5820 9181 L 5807 9164 L 5763 9028 L 5810 8893 L
5734 8757 L 5763 8621 L 5740 8486 L 5734 8350 L
5768 8214 L 5775 8079 L 5820 7992 L 5835 7943 L
5820 7889 L 5806 7807 L 5811 7672 L 5820 7563 L
5956 7570 L 6060 7536 L 6091 7531 L 6100 7536 L
6227 7637 L 6321 7672 L 6274 7807 L 6243 7943 L
6306 8079 L 6362 8115 L 6498 8196 L 6526 8214 L
6528 8350 L 6498 8354 L 6380 8486 L 6368 8621 L
6362 8628 L 6295 8757 L 6227 8797 L 6164 8893 L
6106 9028 L 6227 9138 L 6256 9164 L 6286 9300 L
6362 9431 L 6368 9435 L 6445 9571 L 6473 9707 L
6439 9842 L 6498 9916 L 6633 9940 L 6664 9978 L
6634 10114 L 6633 10114 L 6498 10193 L 6481 10249 L
6440 10385 L 6498 10444 L 6633 10457 L 6690 10521 L
6769 10592 L 6769 7998 L 6734 7943 L 6769 7874 L
6834 7943 L 6769 7998 L 6769 10592 L 6855 10656 L
6833 10792 L 6769 10831 L 6661 10792 L 6633 10782 L
6620 10792 L 6633 10928 L 6498 11009 L 6419 11064 L
6473 11199 L 6362 11277 L 6362 11825 L 6425 11878 L
6498 11993 L 6576 11878 L 6580 11742 L 6498 11646 L
6445 11606 L 6398 11471 L 6498 11357 L 6572 11471 L
6633 11531 L 6769 11490 L 6904 11507 L 7040 11562 L
7175 11565 L 7311 11560 L 7377 11606 L 7446 11656 L
7552 11742 L 7525 11878 L 7553 12013 L 7566 12149 L
7582 12185 L 7620 12285 L 7710 12420 L 7717 12464 L
7760 12556 L 7839 12692 L 7853 12773 L 7853 12134 L
7717 12088 L 7673 12013 L 7582 11911 L 7572 11878 L
7582 11845 L 7700 11742 L 7646 11606 L 7582 11524 L
7446 11551 L 7311 11498 L 7175 11488 L 7115 11471 L

7040 11417 L 6904 11367 L 6778 11335 L 6786 11199 L
6904 11068 L 7040 11165 L 7040 10809 L 7035 10792 L
7019 10656 L 7040 10636 L 7044 10656 L 7084 10792 L
7040 10809 L 7040 11165 L 7123 11199 L 7175 11228 L
7175 10747 L 7089 10656 L 7175 10559 L 7175 8555 L
7106 8486 L 7175 8408 L 7175 7974 L 7140 7943 L
7111 7807 L 7175 7703 L 7311 7784 L 7373 7807 L
7311 7832 L 7194 7943 L 7175 7974 L 7175 8408 L
7297 8486 L 7175 8555 L 7175 10559 L 7270 10656 L
7175 10747 L 7175 11228 L 7225 11199 L 7311 11127 L
7446 11072 L 7582 11109 L 7582 10466 L 7446 10407 L
7429 10385 L 7446 10361 L 7446 8238 L 7435 8214 L
7446 8206 L 7456 8214 L 7446 8238 L 7446 10361 L
7551 10249 L 7582 10215 L 7599 10249 L 7647 10385 L
7582 10466 L 7582 11109 L 7717 11081 L 7815 11064 L
7827 10928 L 7778 10792 L 7717 10737 L 7634 10656 L
7672 10521 L 7717 10488 L 7853 10457 L 7988 10418 L
7988 9848 L 7972 9842 L 7853 9808 L 7756 9707 L
7853 9701 L 7853 9648 L 7768 9571 L 7853 9468 L
7853 8982 L 7751 8893 L 7717 8860 L 7618 8757 L
7582 8712 L 7509 8621 L 7582 8547 L 7657 8621 L
7717 8671 L 7822 8757 L 7853 8788 L 7888 8757 L
7988 8662 L 8073 8757 L 7988 8855 L 7955 8893 L
7853 8982 L 7853 9468 L 7929 9571 L 7853 9648 L
7853 9701 L 7982 9571 L 7988 9523 L 8067 9435 L
8124 9400 L 8180 9435 L 8224 9571 L 8259 9617 L
8286 9571 L 8395 9564 L 8395 9512 L 8305 9435 L
8395 9318 L 8476 9435 L 8395 9512 L 8395 9564 L
8530 9447 L 8596 9571 L 8530 9632 L 8404 9707 L
8395 9722 L 8259 9786 L 8124 9789 L 8003 9842 L
7988 9848 L 7988 10418 L 8055 10385 L 8002 10249 L
8124 10199 L 8178 10249 L 8259 10302 L 8395 10280 L
8530 10311 L 8666 10320 L 8666 9931 L 8578 9842 L
8666 9756 L 8767 9842 L 8666 9931 L 8666 10320 L
8709 10385 L 8666 10469 L 8538 10521 L 8530 10524 L
8458 10656 L 8395 10706 L 8259 10753 L 8190 10792 L
8259 10913 L 8262 10928 L 8320 11064 L 8395 11182 L
8414 11199 L 8530 11291 L 8575 11335 L 8636 11471 L
8666 11547 L 8679 11606 L 8666 11643 L 8609 11742 L
8530 11800 L 8395 11844 L 8259 11828 L 8128 11878 L
8124 11881 L 8005 12013 L 7988 12030 L 7853 12134 L
7853 12773 L 7988 12787 L 8124 12766 L 8259 12766 L
8395 12753 L 8530 12728 L 8649 12692 L 8666 12685 L

8801 12687 L 8841 12692 L 8902 12827 L 8937 12850 L
8981 12827 L 9072 12771 L 9208 12814 L 9208 12164 L
9113 12149 L 9072 12134 L 8937 12018 L 8932 12013 L
8937 11999 L 9072 11898 L 9072 11190 L 8957 11064 L
9072 10939 L 9203 11064 L 9072 11190 L 9072 11898 L
9208 12010 L 9316 11878 L 9343 11866 L 9479 11818 L
9550 11878 L 9587 12013 L 9480 12149 L 9479 12150 L
9472 12149 L 9343 12113 L 9275 12149 L 9208 12164 L
9208 12814 L 9247 12827 L 9343 12849 L 9418 12827 L
9479 12805 L 9614 12757 L 9651 12692 L 9686 12556 L
9749 12504 L 9749 11878 L 9749 11878 L 9749 11877 L
9750 11878 L 9749 11878 L 9749 12504 L 9847 12420 L
9885 12379 L 10020 12301 L 10064 12285 L 10156 12152 L
10157 12149 L 10156 12115 L 10148 12013 L 10057 11878 L
10020 11786 L 9951 11742 L 9885 11710 L 9779 11606 L
9749 11529 L 9626 11471 L 9614 11465 L 9479 11355 L
9343 11378 L 9238 11335 L 9274 11199 L 9265 11064 L
9244 10928 L 9208 10851 L 9177 10792 L 9208 10744 L
9248 10656 L 9339 10521 L 9343 10513 L 9405 10385 L
9443 10249 L 9479 10229 L 9614 10168 L 9749 10191 L
9818 10249 L 9885 10343 L 9932 10385 L 9885 10499 L
9882 10521 L 9885 10528 L 10020 10597 L 10020 10244 L
9890 10114 L 10020 9986 L 10143 10114 L 10020 10244 L
10020 10597 L 10091 10656 L 10156 10745 L 10291 10689 L
10307 10656 L 10291 10595 L 10252 10521 L 10291 10431 L
10301 10385 L 10291 10339 L 10275 10249 L 10291 10215 L
10378 10114 L 10342 9978 L 10291 9910 L 10156 9843 L
10156 9842 L 10156 9840 L 10157 9707 L 10156 9694 L
10020 9639 L 9885 9615 L 9837 9707 L 9801 9842 L
9749 9879 L 9614 9888 L 9562 9842 L 9479 9783 L
9388 9707 L 9378 9571 L 9343 9494 L 9208 9549 L
9072 9563 L 8958 9435 L 9072 9305 L 9099 9300 L
9072 9178 L 9021 9164 L 9006 9028 L 8937 8965 L
8846 8893 L 8801 8829 L 8666 8878 L 8648 8893 L
8666 8895 L 8795 9028 L 8666 9133 L 8630 9164 L
8530 9215 L 8395 9246 L 8283 9164 L 8395 9099 L
8451 9028 L 8507 8893 L 8515 8757 L 8409 8621 L
8398 8486 L 8395 8481 L 8286 8350 L 8395 8228 L
8530 8237 L 8530 8143 L 8455 8079 L 8530 8030 L
8614 8079 L 8530 8143 L 8530 8237 L 8628 8350 L
8666 8422 L 8801 8477 L 8817 8486 L 8905 8621 L
8937 8654 L 8996 8621 L 9068 8486 L 9072 8483 L
9208 8405 L 9317 8486 L 9343 8504 L 9479 8507 L

9614 8538 L 9749 8535 L 9780 8486 L 9749 8415 L
9695 8350 L 9614 8235 L 9577 8214 L 9614 8207 L
9719 8079 L 9749 8050 L 9816 8079 L 9885 8188 L
9905 8214 L 9946 8350 L 10020 8360 L 10156 8374 L
10156 7856 L 10020 7808 L 9885 7821 L 9872 7807 L
9885 7796 L 10020 7806 L 10156 7772 L 10245 7807 L
10156 7856 L 10156 8374 L 10177 8350 L 10291 8267 L
10391 8350 L 10427 8393 L 10562 8432 L 10677 8350 L
10698 8259 L 10698 8208 L 10657 8079 L 10638 7943 L
10698 7890 L 10813 7807 L 10833 7795 L 10847 7807 L
10833 7842 L 10774 7943 L 10818 8079 L 10698 8208 L
10698 8259 L 10715 8350 L 10727 8486 L 10833 8537 L
10886 8486 L 10893 8350 L 10867 8214 L 10900 8079 L
10969 8061 L 11104 7968 L 11121 7943 L 11119 7807 L
11124 7672 L 11159 7536 L 11240 7519 L 11375 7531 L
11436 7536 L 11511 7564 L 11646 7626 L 11740 7672 L
11782 7697 L 11906 7807 L 11917 7818 L 11994 7943 L
12043 8079 L 12053 8103 L 12064 8214 L 12077 8350 L
12085 8486 L 12094 8621 L 12101 8757 L 12109 8893 L
12117 9028 L 12125 9164 L 12136 9300 L 12143 9435 L
12150 9571 L 12158 9707 L 12165 9842 L 12177 9978 L
12188 10100 L 12194 10114 L 12251 10249 L 12294 10385 L
12324 10498 L 12333 10521 L 12384 10656 L 12441 10792 L
12459 10841 L 12508 10928 L 12595 11047 L 12610 11064 L
12730 11190 L 12740 11199 L 12857 11335 L 12866 11345 L
13001 11422 L 13001 10501 L 12879 10385 L 12866 10377 L
12730 10263 L 12715 10249 L 12595 10138 L 12573 10114 L
12459 10019 L 12425 9978 L 12324 9858 L 12317 9842 L
12275 9707 L 12221 9571 L 12188 9483 L 12185 9435 L
12177 9300 L 12164 9164 L 12155 9028 L 12148 8893 L
12141 8757 L 12135 8621 L 12127 8486 L 12121 8350 L
12110 8214 L 12099 8079 L 12090 7943 L 12077 7807 L
12055 7672 L 12053 7663 L 11923 7536 L 11917 7535 L
11782 7513 L 11646 7498 L 11511 7486 L 11375 7476 L
11240 7466 L 11104 7486 L 10969 7505 L 10915 7536 L
10833 7616 L 10698 7654 L 10562 7632 L 10427 7666 L
10291 7637 L 10156 7608 L 10020 7574 L 9885 7656 L
9749 7670 L 9746 7672 L 9614 7778 L 9481 7807 L
9479 7808 L 9477 7807 L 9343 7750 L 9306 7807 L
9343 7905 L 9358 7943 L 9397 8079 L 9343 8139 L
9208 8151 L 9072 8188 L 8937 8102 L 8858 8079 L
8801 8031 L 8794 7943 L 8801 7919 L 8937 7844 L
8967 7807 L 8937 7792 L 8870 7672 L 8816 7536 L

8801 7526 L 8666 7445 L 8530 7454 L 8395 7445 L
8259 7441 L 8124 7416 L 7988 7424 L 7853 7462 L
7717 7449 L 7671 7400 L 7582 7287 L 7497 7400 L
7446 7467 L 7311 7478 L 7175 7511 L 7155 7536 L
7040 7638 L 6904 7612 L 6844 7536 L 6769 7515 L
6633 7477 L 6498 7464 L 6362 7428 L 6227 7405 L
6218 7400 L 6214 7265 L 6091 7134 L 6000 7265 L
5956 7303 L 5906 7265 L 5820 7213 L 5685 7238 L
5622 7129 L 5593 6993 L 5570 6858 L 5550 6844 L
5468 6858 L 5414 6865 L 5297 6993 L 5349 7129 L
5279 7246 L 5194 7129 L 5143 7084 L 5073 6993 L
5143 6916 L 5231 6858 L 5220 6722 L 5143 6658 L
5008 6592 L 4872 6681 L 4827 6722 L 4756 6858 L
4737 6870 L 4601 6861 L 4593 6858 L 4601 6846 L
4670 6722 L 4617 6586 L 4601 6573 L 4466 6537 L
4403 6586 L 4330 6648 L 4195 6609 L 4175 6586 L
4059 6515 L 3945 6451 L 3924 6443 L 3901 6451 L
3851 6586 L 3844 6722 L 3788 6821 L 3731 6858 L
3653 6894 L 3608 6858 L 3517 6735 L 3510 6722 L
3395 6586 L 3382 6571 L 3246 6555 L 3190 6451 L
3111 6335 L 2975 6339 L 2918 6451 L 2921 6586 L
2840 6653 L 2704 6675 L 2660 6722 L 2617 6858 L
2613 6993 L 2569 7109 L 2434 6993 L 2433 6993 L
N E
111 O
2298 7347 2298 7744 2433 7673 2436 7672 2446 7536 2433 7523
2338 7400 2298 7347 8 N F
147 O
2433 7959 M 2298 8006 L 2298 9587 L 2343 9571 L 2433 9542 L
2542 9571 L 2569 9581 L 2652 9707 L 2704 9783 L
2747 9842 L 2840 9906 L 2942 9978 L 2975 10002 L
3111 10076 L 3162 10114 L 3246 10176 L 3321 10249 L
3382 10316 L 3460 10385 L 3517 10435 L 3653 10470 L
3788 10518 L 3796 10521 L 3924 10575 L 3924 10423 L
3788 10408 L 3653 10396 L 3621 10385 L 3517 10334 L
3421 10249 L 3382 10204 L 3287 10114 L 3246 10069 L
3111 9990 L 3091 9978 L 2975 9876 L 2923 9842 L
2840 9758 L 2802 9707 L 2748 9571 L 2704 9480 L
2685 9435 L 2631 9300 L 2704 9263 L 2840 9194 L
2858 9164 L 2975 9073 L 3111 9106 L 3111 8097 L
3106 8079 L 3111 8073 L 3127 8079 L 3111 8097 L
3111 9106 L 3187 9164 L 3205 9300 L 3246 9395 L
3382 9425 L 3473 9300 L 3446 9164 L 3382 9099 L

3339 9028 L 3346 8893 L 3382 8790 L 3517 8813 L
3517 8393 L 3423 8350 L 3517 8268 L 3606 8350 L
3517 8393 L 3517 8813 L 3653 8865 L 3788 8844 L
3788 7624 L 3740 7536 L 3788 7519 L 3794 7536 L
3788 7624 L 3788 8844 L 3803 8893 L 3825 9028 L
3805 9164 L 3788 9186 L 3687 9300 L 3686 9435 L
3737 9571 L 3784 9707 L 3788 9726 L 3924 9836 L
4059 9783 L 4059 8137 L 4010 8079 L 4059 8054 L
4113 8079 L 4059 8137 L 4059 9783 L 4088 9842 L
4195 9913 L 4195 9069 L 4171 9028 L 4195 8974 L
4307 9028 L 4195 9069 L 4195 9913 L 4330 9933 L
4330 8955 L 4316 8893 L 4195 8825 L 4150 8757 L
4183 8621 L 4195 8617 L 4195 7900 L 4059 7824 L
4052 7807 L 4036 7672 L 4059 7642 L 4177 7672 L
4195 7678 L 4305 7807 L 4195 7900 L 4195 8617 L
4199 8621 L 4330 8753 L 4334 8757 L 4343 8893 L
4330 8955 L 4330 9933 L 4466 9957 L 4466 8643 L
4447 8621 L 4330 8517 L 4299 8486 L 4330 8447 L
4380 8486 L 4466 8589 L 4483 8621 L 4466 8643 L
4466 9957 L 4488 9978 L 4588 10114 L 4498 10249 L
4466 10317 L 4412 10249 L 4330 10230 L 4195 10197 L
4059 10143 L 4032 10114 L 3926 9978 L 3924 9890 L
3906 9978 L 3788 10033 L 3771 10114 L 3788 10146 L
3849 10249 L 3924 10330 L 4012 10385 L 3924 10423 L
3924 10575 L 4059 10614 L 4185 10656 L 4144 10792 L
4059 10846 L 3924 10828 L 3788 10843 L 3672 10928 L
3653 10946 L 3522 11064 L 3517 11076 L 3409 11199 L
3395 11335 L 3382 11345 L 3246 11388 L 3111 11433 L
3002 11471 L 2975 11478 L 2840 11512 L 2704 11520 L
2569 11551 L 2509 11606 L 2466 11742 L 2433 11778 L
2298 11772 L 2298 11890 L 2433 11952 L 2483 12013 L
2550 12149 L 2569 12234 L 2616 12149 L 2704 12120 L
2704 11814 L 2620 11742 L 2704 11607 L 2840 11619 L
2874 11606 L 2975 11590 L 3111 11557 L 3246 11537 L
3382 11588 L 3388 11606 L 3382 11615 L 3246 11701 L
3176 11742 L 3111 11755 L 2975 11772 L 2840 11796 L
2704 11814 L 2704 12120 L 2840 12077 L 2975 12107 L
3111 12144 L 3246 12120 L 3382 12103 L 3517 12062 L
3588 12013 L 3653 11969 L 3788 11941 L 3905 12013 L
3924 12027 L 4059 12036 L 4151 12013 L 4195 11998 L
4247 12013 L 4330 12077 L 4361 12149 L 4466 12261 L
4601 12260 L 4737 12239 L 4814 12285 L 4737 12320 L
4601 12346 L 4560 12420 L 4466 12469 L 4330 12463 L

4256 12420 L 4195 12370 L 4059 12335 L 3924 12339 L
3788 12347 L 3653 12367 L 3559 12420 L 3517 12447 L
3382 12514 L 3297 12556 L 3246 12613 L 3111 12678 L
3096 12692 L 3111 12814 L 3246 12773 L 3382 12740 L
3517 12761 L 3653 12806 L 3788 12815 L 3846 12827 L
3924 12843 L 4059 12903 L 4195 12962 L 4197 12963 L
4330 13047 L 4466 13034 L 4466 12919 L 4330 12901 L
4199 12827 L 4195 12824 L 4059 12783 L 3924 12737 L
3809 12692 L 3788 12680 L 3653 12654 L 3517 12586 L
3489 12556 L 3517 12543 L 3653 12471 L 3788 12443 L
3924 12422 L 4059 12421 L 4195 12495 L 4292 12556 L
4330 12582 L 4466 12597 L 4601 12577 L 4648 12556 L
4737 12505 L 4872 12539 L 4929 12556 L 4997 12692 L
4915 12827 L 4872 12890 L 4737 12867 L 4601 12915 L
4466 12919 L 4466 13034 L 4601 13068 L 4650 13099 L
4737 13189 L 4872 13190 L 4980 13234 L 5008 13246 L
5143 13328 L 5255 13370 L 5279 13379 L 5287 13370 L
5291 13234 L 5279 13219 L 5215 13099 L 5161 12963 L
5279 12850 L 5414 12845 L 5550 12880 L 5685 12871 L
5752 12827 L 5820 12773 L 5876 12692 L 5912 12556 L
5887 12420 L 5894 12285 L 5890 12149 L 5907 12013 L
5874 11878 L 5956 11743 L 6031 11878 L 6023 12013 L
6051 12149 L 6059 12285 L 6091 12382 L 6126 12285 L
6123 12149 L 6166 12013 L 6195 11878 L 6227 11865 L
6274 11878 L 6362 11924 L 6423 12013 L 6498 12087 L
6563 12149 L 6617 12285 L 6633 12368 L 6633 12103 L
6532 12013 L 6633 11956 L 6670 12013 L 6633 12103 L
6633 12368 L 6647 12285 L 6703 12149 L 6765 12013 L
6690 11878 L 6703 11742 L 6633 11647 L 6602 11606 L
6498 11498 L 6483 11471 L 6498 11454 L 6508 11471 L
6633 11594 L 6769 11555 L 6850 11606 L 6830 11742 L
6870 11878 L 6904 11925 L 6970 11878 L 7040 11830 L
7127 11878 L 7118 12013 L 7147 12149 L 7175 12193 L
7311 12153 L 7311 12130 L 7276 12013 L 7202 11878 L
7242 11742 L 7311 11653 L 7446 11720 L 7474 11742 L
7477 11878 L 7484 12013 L 7446 12087 L 7311 12130 L
7311 12153 L 7419 12285 L 7446 12310 L 7582 12376 L
7618 12420 L 7651 12556 L 7717 12664 L 7739 12692 L
7717 12810 L 7686 12827 L 7582 12847 L 7446 12916 L
7311 12918 L 7175 12857 L 7095 12827 L 7094 12692 L
7040 12652 L 6939 12556 L 6904 12527 L 6769 12487 L
6633 12484 L 6623 12556 L 6633 12566 L 6769 12668 L
6801 12692 L 6904 12759 L 7027 12827 L 6904 12923 L

6769 12959 L 6754 12963 L 6633 12981 L 6586 12963 L
6498 12922 L 6362 12881 L 6227 12881 L 6109 12963 L
6147 13099 L 6174 13234 L 6181 13370 L 6140 13506 L
6201 13641 L 6227 13666 L 6307 13777 L 6362 13911 L
6364 13913 L 6461 14048 L 6498 14153 L 6527 14184 L
6602 14320 L 6633 14340 L 6769 14326 L 6777 14320 L
6904 14268 L 7040 14245 L 7175 14309 L 7175 13442 L
7040 13397 L 6965 13370 L 6904 13355 L 6769 13329 L
6633 13352 L 6544 13370 L 6498 13389 L 6451 13370 L
6362 13347 L 6301 13234 L 6274 13099 L 6362 13023 L
6498 13030 L 6633 13063 L 6769 13070 L 6904 13063 L
7040 13037 L 7175 13062 L 7311 13068 L 7446 13051 L
7582 13059 L 7636 13099 L 7582 13140 L 7446 13217 L
7416 13234 L 7314 13370 L 7311 13380 L 7175 13442 L
7175 14309 L 7311 14221 L 7446 14220 L 7504 14184 L
7582 14114 L 7695 14048 L 7717 14040 L 7853 14024 L
7988 14000 L 8124 14017 L 8124 13509 L 8109 13506 L
7988 13463 L 7860 13370 L 7979 13234 L 7988 13211 L
8124 13191 L 8145 13234 L 8254 13370 L 8130 13506 L
8124 13509 L 8124 14017 L 8259 13973 L 8290 13913 L
8395 13833 L 8446 13777 L 8530 13684 L 8666 13656 L
8732 13641 L 8801 13610 L 8937 13566 L 9041 13506 L
9065 13370 L 8964 13234 L 9072 13139 L 9208 13137 L
9249 13099 L 9343 13015 L 9479 12966 L 9501 12963 L
9614 12946 L 9653 12963 L 9749 12989 L 9776 12963 L
9798 12827 L 9799 12692 L 9800 12556 L 9885 12475 L
10020 12456 L 10156 12465 L 10256 12556 L 10291 12591 L
10427 12672 L 10558 12556 L 10562 12549 L 10660 12420 L
10575 12285 L 10562 12261 L 10468 12149 L 10427 12127 L
10335 12013 L 10291 11920 L 10268 11878 L 10175 11742 L
10156 11727 L 10061 11606 L 10020 11518 L 9983 11471 L
9923 11335 L 9918 11199 L 10020 11102 L 10061 11064 L
10156 10976 L 10291 10965 L 10354 10928 L 10427 10850 L
10460 10792 L 10479 10656 L 10478 10521 L 10562 10408 L
10584 10385 L 10610 10249 L 10698 10116 L 10700 10114 L
10741 9978 L 10833 9884 L 10833 9233 L 10759 9164 L
10833 9092 L 10899 9164 L 10833 9233 L 10833 9884 L
10875 9842 L 10969 9727 L 11024 9707 L 11104 9615 L
11118 9571 L 11104 9498 L 10969 9542 L 10881 9435 L
10969 9353 L 11104 9345 L 11111 9300 L 11153 9164 L
11104 9112 L 11005 9028 L 10969 8996 L 10843 8893 L
10833 8882 L 10698 8830 L 10592 8757 L 10621 8621 L
10698 8560 L 10772 8621 L 10833 8673 L 10969 8682 L

11104 8636 L 11122 8621 L 11240 8532 L 11331 8486 L
11240 8423 L 11186 8350 L 11240 8296 L 11314 8214 L
11240 8090 L 11209 8079 L 11221 7943 L 11230 7807 L
11240 7763 L 11375 7751 L 11475 7807 L 11511 7829 L
11646 7929 L 11666 7943 L 11782 8049 L 11803 8079 L
11894 8214 L 11917 8250 L 11955 8350 L 11997 8486 L
12053 8618 L 12053 8621 L 12062 8757 L 12070 8893 L
12078 9028 L 12086 9164 L 12095 9300 L 12101 9435 L
12106 9571 L 12111 9707 L 12116 9842 L 12123 9978 L
12130 10114 L 12139 10249 L 12148 10385 L 12156 10521 L
12163 10656 L 12173 10792 L 12184 10928 L 12188 10962 L
12219 11064 L 12257 11199 L 12299 11335 L 12324 11411 L
12355 11471 L 12422 11606 L 12459 11704 L 12477 11742 L
12508 11878 L 12561 12013 L 12595 12066 L 12686 12149 L
12730 12186 L 12866 12261 L 12993 12285 L 13001 12286 L
13001 11422 L 12866 11345 L 12857 11335 L 12740 11199 L
12730 11190 L 12610 11064 L 12595 11047 L 12508 10928 L
12459 10841 L 12441 10792 L 12384 10656 L 12333 10521 L
12324 10498 L 12294 10385 L 12251 10249 L 12194 10114 L
12188 10100 L 12177 9978 L 12165 9842 L 12158 9707 L
12150 9571 L 12143 9435 L 12136 9300 L 12125 9164 L
12117 9028 L 12109 8893 L 12101 8757 L 12094 8621 L
12085 8486 L 12077 8350 L 12064 8214 L 12053 8103 L
12043 8079 L 11994 7943 L 11917 7818 L 11906 7807 L
11782 7697 L 11740 7672 L 11646 7626 L 11511 7564 L
11436 7536 L 11375 7531 L 11240 7519 L 11159 7536 L
11124 7672 L 11119 7807 L 11121 7943 L 11104 7968 L
10969 8061 L 10900 8079 L 10867 8214 L 10893 8350 L
10886 8486 L 10833 8537 L 10727 8486 L 10715 8350 L
10698 8259 L 10677 8350 L 10562 8432 L 10427 8393 L
10391 8350 L 10291 8267 L 10177 8350 L 10156 8374 L
10020 8360 L 9946 8350 L 9905 8214 L 9885 8188 L
9816 8079 L 9749 8050 L 9719 8079 L 9614 8207 L
9577 8214 L 9614 8235 L 9695 8350 L 9749 8415 L
9749 8302 L 9631 8214 L 9749 8134 L 9865 8214 L
9749 8302 L 9749 8415 L 9780 8486 L 9749 8535 L
9614 8538 L 9479 8507 L 9343 8504 L 9317 8486 L
9208 8405 L 9072 8483 L 9068 8486 L 8996 8621 L
8937 8654 L 8905 8621 L 8817 8486 L 8801 8477 L
8666 8422 L 8628 8350 L 8530 8237 L 8395 8228 L
8286 8350 L 8395 8481 L 8398 8486 L 8409 8621 L
8515 8757 L 8507 8893 L 8451 9028 L 8395 9099 L
8283 9164 L 8395 9246 L 8530 9215 L 8630 9164 L

8666 9133 L 8795 9028 L 8666 8895 L 8648 8893 L
8666 8878 L 8666 8675 L 8611 8621 L 8530 8546 L
8476 8486 L 8395 8374 L 8375 8350 L 8395 8328 L
8530 8321 L 8555 8350 L 8606 8486 L 8666 8551 L
8725 8621 L 8666 8675 L 8666 8878 L 8801 8829 L
8846 8893 L 8937 8965 L 9006 9028 L 9021 9164 L
9072 9178 L 9099 9300 L 9072 9305 L 8958 9435 L
9072 9563 L 9072 9501 L 9013 9435 L 9072 9368 L
9175 9435 L 9072 9501 L 9072 9563 L 9208 9549 L
9343 9494 L 9378 9571 L 9388 9707 L 9479 9783 L
9562 9842 L 9614 9888 L 9614 9545 L 9534 9435 L
9614 9303 L 9700 9435 L 9614 9545 L 9614 9888 L
9749 9879 L 9801 9842 L 9837 9707 L 9885 9615 L
9885 9230 L 9810 9164 L 9885 9105 L 9885 8961 L
9848 8893 L 9885 8850 L 9995 8893 L 9885 8961 L
9885 9105 L 9943 9164 L 9885 9230 L 9885 9615 L
10020 9639 L 10156 9694 L 10157 9707 L 10156 9840 L
10156 9842 L 10156 9843 L 10291 9910 L 10291 9772 L
10228 9707 L 10291 9634 L 10291 9516 L 10156 9509 L
10083 9435 L 10156 9360 L 10156 8765 L 10139 8757 L
10156 8747 L 10168 8757 L 10156 8765 L 10156 9360 L
10291 9305 L 10296 9300 L 10310 9164 L 10427 9064 L
10562 9079 L 10645 9164 L 10562 9277 L 10545 9300 L
10427 9401 L 10387 9435 L 10291 9516 L 10291 9634 L
10360 9707 L 10291 9772 L 10291 9910 L 10342 9978 L
10378 10114 L 10291 10215 L 10275 10249 L 10291 10339 L
10301 10385 L 10291 10431 L 10252 10521 L 10291 10595 L
10307 10656 L 10291 10689 L 10156 10745 L 10091 10656 L
10020 10597 L 9885 10528 L 9882 10521 L 9885 10499 L
9932 10385 L 9885 10343 L 9818 10249 L 9749 10191 L
9614 10168 L 9479 10229 L 9443 10249 L 9405 10385 L
9343 10513 L 9339 10521 L 9248 10656 L 9208 10744 L
9177 10792 L 9208 10851 L 9244 10928 L 9265 11064 L
9274 11199 L 9238 11335 L 9343 11378 L 9479 11355 L
9479 10879 L 9396 10792 L 9479 10671 L 9518 10656 L
9536 10521 L 9530 10385 L 9614 10283 L 9749 10296 L
9833 10385 L 9749 10517 L 9740 10521 L 9720 10656 L
9614 10731 L 9588 10792 L 9479 10879 L 9479 11355 L
9614 11465 L 9626 11471 L 9749 11529 L 9779 11606 L
9885 11710 L 9951 11742 L 10020 11786 L 10057 11878 L
10148 12013 L 10156 12115 L 10157 12149 L 10156 12152 L
10064 12285 L 10020 12301 L 9885 12379 L 9847 12420 L
9749 12504 L 9686 12556 L 9651 12692 L 9614 12757 L

9479 12805 L 9418 12827 L 9343 12849 L 9247 12827 L
9208 12814 L 9072 12771 L 8981 12827 L 8937 12850 L
8902 12827 L 8841 12692 L 8801 12687 L 8666 12685 L
8649 12692 L 8530 12728 L 8395 12753 L 8259 12766 L
8124 12766 L 7988 12787 L 7853 12773 L 7839 12692 L
7760 12556 L 7717 12464 L 7710 12420 L 7620 12285 L
7582 12185 L 7566 12149 L 7553 12013 L 7525 11878 L
7552 11742 L 7446 11656 L 7377 11606 L 7311 11560 L
7175 11565 L 7040 11562 L 6904 11507 L 6769 11490 L
6633 11531 L 6572 11471 L 6498 11357 L 6398 11471 L
6445 11606 L 6498 11646 L 6580 11742 L 6576 11878 L
6498 11993 L 6425 11878 L 6362 11825 L 6227 11804 L
6091 11842 L 6025 11742 L 5956 11645 L 5820 11715 L
5803 11742 L 5782 11878 L 5804 12013 L 5753 12149 L
5726 12285 L 5746 12420 L 5773 12556 L 5742 12692 L
5685 12741 L 5550 12733 L 5414 12709 L 5360 12692 L
5279 12612 L 5157 12556 L 5143 12542 L 5096 12420 L
5143 12376 L 5241 12285 L 5279 12238 L 5414 12156 L
5417 12149 L 5442 12013 L 5414 11965 L 5279 11915 L
5143 11962 L 5008 11990 L 4887 12013 L 4872 12021 L
4737 12059 L 4678 12013 L 4601 11930 L 4466 11914 L
4330 11903 L 4291 11878 L 4195 11766 L 4059 11748 L
3924 11840 L 3788 11814 L 3673 11878 L 3653 11885 L
3517 11959 L 3458 12013 L 3382 12044 L 3246 12054 L
3111 12070 L 2975 12027 L 2927 12013 L 2903 11878 L
2975 11849 L 3111 11824 L 3246 11804 L 3382 11768 L
3427 11742 L 3514 11606 L 3517 11598 L 3653 11520 L
3788 11484 L 3788 11376 L 3653 11365 L 3621 11335 L
3595 11199 L 3637 11064 L 3653 11050 L 3784 10928 L
3788 10925 L 3830 10928 L 3924 10936 L 4059 10971 L
4166 10928 L 4195 10913 L 4307 10792 L 4330 10766 L
4466 10761 L 4544 10792 L 4601 10821 L 4601 10683 L
4466 10659 L 4458 10656 L 4466 10649 L 4601 10569 L
4647 10656 L 4601 10683 L 4601 10821 L 4690 10928 L
4601 11050 L 4550 11064 L 4466 11073 L 4417 11064 L
4330 11033 L 4214 11064 L 4195 11070 L 4125 11199 L
4059 11292 L 3979 11335 L 3924 11360 L 3788 11376 L
3788 11484 L 3828 11471 L 3924 11445 L 4059 11392 L
4159 11335 L 4195 11226 L 4202 11199 L 4330 11121 L
4466 11138 L 4601 11137 L 4704 11064 L 4737 11017 L
4859 10928 L 4872 10901 L 4908 10792 L 4873 10656 L
4872 10655 L 4737 10590 L 4630 10521 L 4655 10385 L
4705 10249 L 4721 10114 L 4694 9978 L 4614 9842 L

4632 9707 L 4627 9571 L 4601 9486 L 4534 9435 L
4561 9300 L 4601 9281 L 4601 7675 L 4597 7672 L
4601 7670 L 4609 7672 L 4601 7675 L 4601 9281 L
4708 9164 L 4737 9142 L 4872 9084 L 5008 9035 L
5116 9028 L 5143 9023 L 5244 8893 L 5143 8807 L
5098 8757 L 5078 8621 L 5143 8568 L 5279 8580 L
5321 8621 L 5299 8757 L 5308 8893 L 5390 9028 L
5414 9034 L 5423 9028 L 5550 8961 L 5572 8893 L
5550 8777 L 5529 8757 L 5471 8621 L 5450 8486 L
5414 8351 L 5413 8350 L 5319 8214 L 5279 8188 L
5200 8079 L 5143 8009 L 5008 8033 L 4872 8050 L
4737 7969 L 4713 7943 L 4642 7807 L 4737 7694 L
4781 7672 L 4737 7651 L 4674 7536 L 4601 7400 L
4601 7400 L 4466 7288 L 4418 7265 L 4466 7204 L
4500 7129 L 4466 7088 L 4422 7129 L 4330 7208 L
4249 7265 L 4293 7400 L 4195 7465 L 4061 7400 L
4059 7398 L 3924 7294 L 3858 7265 L 3788 7219 L
3732 7265 L 3653 7377 L 3517 7326 L 3407 7265 L
3382 7248 L 3246 7192 L 3164 7265 L 3140 7400 L
3111 7459 L 2975 7484 L 2840 7442 L 2745 7536 L
2704 7575 L 2674 7672 L 2650 7807 L 2569 7892 L
2460 7943 L 2433 7959 L N E
129 O
2298 9587 2298 10166 2433 10182 2549 10249 2569 10261 2642 10385
2704 10463 2775 10521 2840 10566 2975 10612 3111 10551 3174 10656
3246 10737 3333 10656 3246 10592 3136 10521 3111 10495 2975 10410
2926 10385 2840 10284 2737 10249 2704 10184 2569 10184 2549 10114
2569 10090 2704 10107 2840 10091 2975 10113 2977 10114 3111 10187
3212 10249 3246 10309 3301 10385 3382 10488 3446 10521 3517 10547
3653 10559 3788 10631 3845 10656 3788 10707 3725 10792 3653 10839
3521 10928 3517 10931 3487 10928 3382 10841 3326 10928 3246 11011
3215 11064 3111 11199 3111 11199 2997 11335 2975 11343 2840 11371
2704 11399 2569 11397 2433 11424 2298 11391 2298 11772 2433 11778
2466 11742 2509 11606 2569 11551 2704 11520 2840 11512 2975 11478
3002 11471 3111 11433 3246 11388 3382 11345 3395 11335 3409 11199
3517 11076 3522 11064 3653 10946 3672 10928 3788 10843 3924 10828
4059 10846 4144 10792 4185 10656 4059 10614 3924 10575 3796 10521
3788 10518 3653 10470 3517 10435 3460 10385 3382 10316 3321 10249
3246 10176 3162 10114 3111 10076 2975 10002 2942 9978 2840 9906
2747 9842 2704 9783 2652 9707 2569 9581 2542 9571 2433 9542
2343 9571 2298 9587 104 N F
111 O
2298 10166 2298 11391 2433 11424 2569 11397 2704 11399 2840 11371

2975 11343 2997 11335 3111 11199 3111 11199 3215 11064 3246 11011
3326 10928 3382 10841 3487 10928 3517 10931 3521 10928 3653 10839
3725 10792 3788 10707 3845 10656 3788 10631 3653 10559 3517 10547
3446 10521 3382 10488 3301 10385 3246 10309 3212 10249 3111 10187
2977 10114 2975 10113 2840 10091 2704 10107 2569 10090 2549 10114
2569 10184 2704 10184 2737 10249 2840 10284 2926 10385 2975 10410
3111 10495 3136 10521 3246 10592 3333 10656 3246 10737 3174 10656
3111 10551 2975 10612 2840 10566 2775 10521 2704 10463 2642 10385
2569 10261 2549 10249 2433 10182 2298 10166 58 N F
165 O
2433 11952 M 2298 11890 L 2298 12073 L 2343 12149 L 2298 12273 L
2298 12519 L 2433 12529 L 2474 12556 L 2537 12692 L
2433 12740 L 2389 12827 L 2302 12963 L 2433 13071 L
2482 13099 L 2569 13159 L 2619 13099 L 2704 12986 L
2720 12963 L 2789 12827 L 2704 12694 L 2690 12692 L
2704 12690 L 2840 12658 L 2850 12692 L 2868 12827 L
2940 12963 L 2975 13031 L 2998 13099 L 3029 13234 L
3024 13370 L 3032 13506 L 3111 13622 L 3129 13641 L
3192 13777 L 3246 13883 L 3271 13913 L 3382 13966 L
3517 13913 L 3522 13913 L 3653 13890 L 3696 13913 L
3788 13961 L 3924 13962 L 4059 14022 L 4093 14048 L
4195 14152 L 4223 14184 L 4195 14298 L 4059 14272 L
3945 14320 L 3924 14351 L 3899 14455 L 3924 14480 L
4059 14471 L 4195 14477 L 4330 14478 L 4466 14496 L
4466 13262 L 4451 13234 L 4466 13223 L 4483 13234 L
4466 13262 L 4466 14496 L 4601 14582 L 4737 14572 L
4770 14591 L 4872 14720 L 4919 14727 L 5008 14738 L
5014 14727 L 5012 14591 L 5008 14569 L 4982 14455 L
5008 14414 L 5046 14320 L 5089 14184 L 5075 14048 L
5008 13922 L 5004 13913 L 4993 13777 L 5008 13725 L
5041 13777 L 5143 13875 L 5160 13913 L 5279 14037 L
5288 14048 L 5414 14152 L 5448 14184 L 5520 14320 L
5550 14355 L 5550 14054 L 5543 14048 L 5550 14041 L
5565 14048 L 5550 14054 L 5550 14355 L 5685 14433 L
5685 13800 L 5550 13799 L 5498 13777 L 5414 13672 L
5383 13641 L 5414 13616 L 5518 13506 L 5550 13417 L
5579 13506 L 5610 13641 L 5685 13762 L 5708 13777 L
5685 13800 L 5685 14433 L 5808 14320 L 5820 14311 L
5929 14184 L 5898 14048 L 5849 13913 L 5956 13791 L
5956 13290 L 5896 13234 L 5956 13107 L 6051 13234 L
5956 13290 L 5956 13791 L 5972 13777 L 6091 13746 L
6126 13777 L 6227 13902 L 6231 13913 L 6227 13956 L
6191 14048 L 6142 14184 L 6098 14320 L 6131 14455 L

6181 14591 L 6227 14604 L 6362 14709 L 6380 14727 L
6498 14847 L 6537 14862 L 6633 14901 L 6769 14964 L
6801 14998 L 6904 15124 L 6917 15134 L 7040 15216 L
7175 15261 L 7192 15269 L 7258 15405 L 7311 15511 L
7368 15541 L 7446 15572 L 7556 15541 L 7582 15529 L
7694 15405 L 7717 15387 L 7853 15379 L 7988 15392 L
8099 15269 L 8124 15218 L 8208 15134 L 8124 15032 L
8108 14998 L 7988 14971 L 7884 14862 L 7908 14727 L
7921 14591 L 7932 14455 L 7988 14377 L 8124 14361 L
8259 14339 L 8353 14320 L 8395 14311 L 8464 14184 L
8473 14048 L 8478 13913 L 8530 13858 L 8666 13845 L
8801 13849 L 8907 13913 L 8937 13943 L 9072 14046 L
9075 14048 L 9208 14146 L 9343 14101 L 9479 14107 L
9539 14048 L 9614 13965 L 9749 13937 L 9837 13913 L
9749 13793 L 9742 13777 L 9614 13729 L 9546 13641 L
9479 13572 L 9343 13554 L 9305 13506 L 9307 13370 L
9208 13272 L 9072 13272 L 9042 13234 L 9072 13208 L
9208 13209 L 9326 13099 L 9343 13084 L 9479 13071 L
9614 13065 L 9749 13074 L 9860 12963 L 9867 12827 L
9868 12692 L 9872 12556 L 9885 12543 L 10020 12535 L
10156 12536 L 10178 12556 L 10291 12669 L 10324 12692 L
10427 12794 L 10472 12827 L 10562 12917 L 10562 12728 L
10458 12692 L 10562 12564 L 10698 12613 L 10803 12692 L
10698 12708 L 10562 12728 L 10562 12917 L 10653 12827 L
10698 12799 L 10833 12792 L 10934 12692 L 10969 12644 L
11104 12618 L 11240 12574 L 11271 12556 L 11240 12538 L
11104 12487 L 11028 12420 L 10969 12366 L 10833 12308 L
10801 12285 L 10698 12205 L 10657 12149 L 10626 12013 L
10562 11951 L 10427 11944 L 10389 11878 L 10298 11742 L
10291 11736 L 10156 11636 L 10133 11606 L 10110 11471 L
10020 11376 L 9997 11335 L 9997 11199 L 10020 11177 L
10138 11064 L 10156 11047 L 10291 11041 L 10427 11006 L
10506 10928 L 10538 10792 L 10542 10656 L 10543 10521 L
10562 10495 L 10667 10385 L 10675 10249 L 10698 10215 L
10796 10114 L 10811 9978 L 10833 9955 L 10947 9842 L
10969 9816 L 11104 9792 L 11189 9707 L 11207 9571 L
11200 9435 L 11210 9300 L 11229 9164 L 11104 9031 L
11101 9028 L 10969 8909 L 10948 8893 L 10833 8760 L
10815 8757 L 10833 8755 L 10969 8757 L 11104 8738 L
11238 8621 L 11240 8620 L 11241 8621 L 11375 8737 L
11375 8624 L 11373 8621 L 11375 8612 L 11377 8621 L
11375 8624 L 11375 8737 L 11493 8621 L 11502 8486 L
11511 8423 L 11593 8486 L 11646 8525 L 11701 8621 L

11782 8756 L 11782 8757 L 11859 8893 L 11917 8959 L
11946 9028 L 11999 9164 L 12053 9287 L 12053 9300 L
12059 9435 L 12062 9571 L 12064 9707 L 12066 9842 L
12068 9978 L 12070 10114 L 12073 10249 L 12075 10385 L
12078 10521 L 12080 10656 L 12082 10792 L 12084 10928 L
12088 11064 L 12092 11199 L 12096 11335 L 12100 11471 L
12106 11606 L 12116 11742 L 12119 11878 L 12126 12013 L
12127 12149 L 12149 12285 L 12177 12420 L 12188 12456 L
12221 12556 L 12188 12608 L 12154 12692 L 12188 12808 L
12196 12827 L 12324 12944 L 12352 12963 L 12411 13099 L
12439 13234 L 12425 13370 L 12324 13472 L 12188 13438 L
12053 13392 L 12021 13506 L 12019 13641 L 12037 13777 L
12053 13871 L 12188 13803 L 12206 13777 L 12324 13725 L
12459 13762 L 12480 13777 L 12595 13833 L 12730 13818 L
12785 13777 L 12854 13641 L 12866 13619 L 12881 13641 L
12941 13777 L 12983 13913 L 12982 14048 L 13001 14070 L
13001 12286 L 12993 12285 L 12866 12261 L 12730 12186 L
12686 12149 L 12595 12066 L 12561 12013 L 12508 11878 L
12477 11742 L 12459 11704 L 12422 11606 L 12355 11471 L
12324 11411 L 12299 11335 L 12257 11199 L 12219 11064 L
12188 10962 L 12184 10928 L 12173 10792 L 12163 10656 L
12156 10521 L 12148 10385 L 12139 10249 L 12130 10114 L
12123 9978 L 12116 9842 L 12111 9707 L 12106 9571 L
12101 9435 L 12095 9300 L 12086 9164 L 12078 9028 L
12070 8893 L 12062 8757 L 12053 8621 L 12053 8618 L
11997 8486 L 11955 8350 L 11917 8250 L 11894 8214 L
11803 8079 L 11782 8049 L 11666 7943 L 11646 7929 L
11511 7829 L 11475 7807 L 11375 7751 L 11240 7763 L
11230 7807 L 11221 7943 L 11209 8079 L 11240 8090 L
11314 8214 L 11240 8296 L 11186 8350 L 11240 8423 L
11331 8486 L 11240 8532 L 11122 8621 L 11104 8636 L
10969 8682 L 10833 8673 L 10772 8621 L 10698 8560 L
10621 8621 L 10592 8757 L 10698 8830 L 10833 8882 L
10843 8893 L 10969 8996 L 11005 9028 L 11104 9112 L
11153 9164 L 11111 9300 L 11104 9345 L 10969 9353 L
10881 9435 L 10969 9542 L 10969 9443 L 10963 9435 L
10969 9430 L 10979 9435 L 10969 9443 L 10969 9542 L
11104 9498 L 11118 9571 L 11104 9615 L 11024 9707 L
10969 9727 L 10875 9842 L 10833 9884 L 10741 9978 L
10700 10114 L 10698 10116 L 10610 10249 L 10584 10385 L
10562 10408 L 10478 10521 L 10479 10656 L 10460 10792 L
10427 10850 L 10354 10928 L 10291 10965 L 10156 10976 L
10061 11064 L 10020 11102 L 9918 11199 L 9923 11335 L

9983 11471 L 10020 11518 L 10061 11606 L 10156 11727 L
10175 11742 L 10268 11878 L 10291 11920 L 10335 12013 L
10427 12127 L 10468 12149 L 10562 12261 L 10575 12285 L
10660 12420 L 10562 12549 L 10558 12556 L 10427 12672 L
10291 12591 L 10256 12556 L 10156 12465 L 10020 12456 L
9885 12475 L 9800 12556 L 9799 12692 L 9798 12827 L
9776 12963 L 9749 12989 L 9653 12963 L 9614 12946 L
9501 12963 L 9479 12966 L 9343 13015 L 9249 13099 L
9208 13137 L 9072 13139 L 8964 13234 L 9065 13370 L
9041 13506 L 8937 13566 L 8801 13610 L 8732 13641 L
8666 13656 L 8530 13684 L 8446 13777 L 8395 13833 L
8290 13913 L 8259 13973 L 8124 14017 L 7988 14000 L
7853 14024 L 7717 14040 L 7695 14048 L 7582 14114 L
7504 14184 L 7446 14220 L 7311 14221 L 7175 14309 L
7040 14245 L 6904 14268 L 6777 14320 L 6769 14326 L
6633 14340 L 6602 14320 L 6527 14184 L 6498 14153 L
6461 14048 L 6364 13913 L 6362 13911 L 6307 13777 L
6227 13666 L 6201 13641 L 6140 13506 L 6181 13370 L
6174 13234 L 6147 13099 L 6109 12963 L 6227 12881 L
6362 12881 L 6498 12922 L 6586 12963 L 6633 12981 L
6633 12867 L 6524 12827 L 6498 12771 L 6471 12692 L
6362 12558 L 6361 12556 L 6337 12420 L 6362 12403 L
6386 12420 L 6498 12511 L 6510 12556 L 6633 12686 L
6640 12692 L 6725 12827 L 6633 12867 L 6633 12981 L
6754 12963 L 6769 12959 L 6904 12923 L 7027 12827 L
6904 12759 L 6801 12692 L 6769 12668 L 6633 12566 L
6623 12556 L 6633 12484 L 6769 12487 L 6904 12527 L
6904 12435 L 6805 12420 L 6769 12382 L 6753 12285 L
6769 12233 L 6826 12285 L 6904 12360 L 6975 12420 L
6904 12435 L 6904 12527 L 6939 12556 L 7040 12652 L
7094 12692 L 7095 12827 L 7175 12857 L 7311 12918 L
7446 12916 L 7446 12755 L 7311 12732 L 7278 12692 L
7289 12556 L 7311 12523 L 7446 12493 L 7517 12556 L
7492 12692 L 7446 12755 L 7446 12916 L 7582 12847 L
7686 12827 L 7717 12810 L 7739 12692 L 7717 12664 L
7651 12556 L 7618 12420 L 7582 12376 L 7446 12310 L
7419 12285 L 7311 12153 L 7175 12193 L 7147 12149 L
7118 12013 L 7127 11878 L 7040 11830 L 6970 11878 L
6904 11925 L 6870 11878 L 6830 11742 L 6850 11606 L
6769 11555 L 6633 11594 L 6508 11471 L 6498 11454 L
6483 11471 L 6498 11498 L 6602 11606 L 6633 11647 L
6703 11742 L 6690 11878 L 6765 12013 L 6703 12149 L
6647 12285 L 6633 12368 L 6617 12285 L 6563 12149 L

6498 12087 L 6423 12013 L 6362 11924 L 6274 11878 L
6227 11865 L 6195 11878 L 6166 12013 L 6123 12149 L
6126 12285 L 6091 12382 L 6059 12285 L 6051 12149 L
6023 12013 L 6031 11878 L 5956 11743 L 5874 11878 L
5907 12013 L 5890 12149 L 5894 12285 L 5887 12420 L
5912 12556 L 5876 12692 L 5820 12773 L 5752 12827 L
5685 12871 L 5550 12880 L 5414 12845 L 5279 12850 L
5161 12963 L 5215 13099 L 5279 13219 L 5291 13234 L
5287 13370 L 5279 13379 L 5255 13370 L 5143 13328 L
5008 13246 L 4980 13234 L 4872 13190 L 4737 13189 L
4650 13099 L 4601 13068 L 4466 13034 L 4330 13047 L
4197 12963 L 4195 12962 L 4059 12903 L 3924 12843 L
3846 12827 L 3788 12815 L 3653 12806 L 3517 12761 L
3382 12740 L 3246 12773 L 3111 12814 L 3096 12692 L
3111 12678 L 3111 12476 L 2987 12420 L 3111 12312 L
3132 12285 L 3246 12243 L 3382 12202 L 3496 12149 L
3517 12146 L 3653 12109 L 3788 12140 L 3803 12149 L
3788 12163 L 3653 12276 L 3644 12285 L 3517 12314 L
3395 12420 L 3382 12427 L 3246 12458 L 3111 12476 L
3111 12678 L 3246 12613 L 3297 12556 L 3382 12514 L
3517 12447 L 3559 12420 L 3653 12367 L 3788 12347 L
3924 12339 L 4059 12335 L 4195 12370 L 4256 12420 L
4330 12463 L 4466 12469 L 4560 12420 L 4601 12346 L
4737 12320 L 4814 12285 L 4737 12239 L 4601 12260 L
4466 12261 L 4361 12149 L 4330 12077 L 4247 12013 L
4195 11998 L 4151 12013 L 4059 12036 L 3924 12027 L
3905 12013 L 3788 11941 L 3653 11969 L 3588 12013 L
3517 12062 L 3382 12103 L 3246 12120 L 3111 12144 L
2975 12107 L 2840 12077 L 2704 12120 L 2616 12149 L
2569 12234 L 2550 12149 L 2483 12013 L 2433 11952 L
N E
183 O
2343 12149 2298 12073 2298 12273 2343 12149 4 N F
2433 12529 M 2298 12519 L 2298 16219 L 2489 16219 L 2680 16219 L
2704 16219 L 2704 14458 L 2701 14455 L 2704 14451 L
2809 14455 L 2704 14458 L 2704 16219 L 2871 16219 L
3063 16219 L 3246 16219 L 3246 15154 L 3226 15134 L
3111 15029 L 3087 14998 L 2975 14869 L 2967 14862 L
2902 14727 L 2866 14591 L 2975 14476 L 3092 14591 L
3111 14635 L 3144 14727 L 3226 14862 L 3246 14885 L
3321 14998 L 3278 15134 L 3246 15154 L 3246 16219 L
3254 16219 L 3445 16219 L 3636 16219 L 3827 16219 L
4018 16219 L 4209 16219 L 4400 16219 L 4592 16219 L

4783 16219 L 4974 16219 L 5165 16219 L 5356 16219 L
5547 16219 L 5738 16219 L 5820 16219 L 5820 14747 L
5805 14727 L 5733 14591 L 5751 14455 L 5820 14364 L
5845 14455 L 5950 14591 L 5875 14727 L 5820 14747 L
5820 16219 L 5929 16219 L 6121 16219 L 6312 16219 L
6503 16219 L 6694 16219 L 6885 16219 L 7076 16219 L
7267 16219 L 7458 16219 L 7650 16219 L 7841 16219 L
8032 16219 L 8223 16219 L 8414 16219 L 8605 16219 L
8796 16219 L 8987 16219 L 9178 16219 L 9370 16219 L
9561 16219 L 9752 16219 L 9943 16219 L 10134 16219 L
10325 16219 L 10516 16219 L 10707 16219 L 10899 16219 L
11090 16219 L 11281 16219 L 11472 16219 L 11663 16219 L
11854 16219 L 11917 16219 L 11917 13956 L 11853 13913 L
11917 13829 L 12048 13913 L 11917 13956 L 11917 16219 L
12045 16219 L 12236 16219 L 12428 16219 L 12619 16219 L
12810 16219 L 13001 16219 L 13001 14070 L 12982 14048 L
12983 13913 L 12941 13777 L 12881 13641 L 12866 13619 L
12854 13641 L 12785 13777 L 12730 13818 L 12595 13833 L
12480 13777 L 12459 13762 L 12324 13725 L 12206 13777 L
12188 13803 L 12053 13871 L 12037 13777 L 12019 13641 L
12021 13506 L 12053 13392 L 12188 13438 L 12324 13472 L
12425 13370 L 12439 13234 L 12411 13099 L 12352 12963 L
12324 12944 L 12196 12827 L 12188 12808 L 12154 12692 L
12188 12608 L 12221 12556 L 12188 12456 L 12177 12420 L
12149 12285 L 12127 12149 L 12126 12013 L 12119 11878 L
12116 11742 L 12106 11606 L 12100 11471 L 12096 11335 L
12092 11199 L 12088 11064 L 12084 10928 L 12082 10792 L
12080 10656 L 12078 10521 L 12075 10385 L 12073 10249 L
12070 10114 L 12068 9978 L 12066 9842 L 12064 9707 L
12062 9571 L 12059 9435 L 12053 9300 L 12053 9287 L
11999 9164 L 11946 9028 L 11917 8959 L 11859 8893 L
11782 8757 L 11782 8756 L 11701 8621 L 11646 8525 L
11593 8486 L 11511 8423 L 11502 8486 L 11493 8621 L
11375 8737 L 11241 8621 L 11240 8620 L 11238 8621 L
11104 8738 L 10969 8757 L 10833 8755 L 10815 8757 L
10833 8760 L 10948 8893 L 10969 8909 L 11101 9028 L
11104 9031 L 11229 9164 L 11210 9300 L 11200 9435 L
11207 9571 L 11189 9707 L 11104 9792 L 10969 9816 L
10947 9842 L 10833 9955 L 10811 9978 L 10796 10114 L
10698 10215 L 10675 10249 L 10667 10385 L 10562 10495 L
10543 10521 L 10542 10656 L 10538 10792 L 10506 10928 L
10427 11006 L 10291 11041 L 10156 11047 L 10138 11064 L
10020 11177 L 9997 11199 L 9997 11335 L 10020 11376 L

10110 11471 L 10133 11606 L 10156 11636 L 10291 11736 L
10298 11742 L 10389 11878 L 10427 11944 L 10562 11951 L
10626 12013 L 10657 12149 L 10698 12205 L 10801 12285 L
10833 12308 L 10969 12366 L 11028 12420 L 11104 12487 L
11240 12538 L 11271 12556 L 11240 12574 L 11104 12618 L
10969 12644 L 10934 12692 L 10833 12792 L 10698 12799 L
10653 12827 L 10562 12917 L 10472 12827 L 10427 12794 L
10324 12692 L 10291 12669 L 10178 12556 L 10156 12536 L
10020 12535 L 9885 12543 L 9872 12556 L 9868 12692 L
9867 12827 L 9860 12963 L 9749 13074 L 9614 13065 L
9479 13071 L 9343 13084 L 9326 13099 L 9208 13209 L
9072 13208 L 9042 13234 L 9072 13272 L 9208 13272 L
9307 13370 L 9305 13506 L 9343 13554 L 9479 13572 L
9546 13641 L 9614 13729 L 9742 13777 L 9749 13793 L
9837 13913 L 9749 13937 L 9614 13965 L 9539 14048 L
9479 14107 L 9343 14101 L 9208 14146 L 9075 14048 L
9072 14046 L 8937 13943 L 8907 13913 L 8801 13849 L
8666 13845 L 8530 13858 L 8478 13913 L 8473 14048 L
8464 14184 L 8395 14311 L 8353 14320 L 8259 14339 L
8124 14361 L 7988 14377 L 7932 14455 L 7921 14591 L
7908 14727 L 7884 14862 L 7988 14971 L 8108 14998 L
8124 15032 L 8208 15134 L 8124 15218 L 8099 15269 L
7988 15392 L 7853 15379 L 7717 15387 L 7694 15405 L
7582 15529 L 7556 15541 L 7446 15572 L 7368 15541 L
7311 15511 L 7258 15405 L 7192 15269 L 7175 15261 L
7040 15216 L 6917 15134 L 6904 15124 L 6801 14998 L
6769 14964 L 6633 14901 L 6537 14862 L 6498 14847 L
6380 14727 L 6362 14709 L 6227 14604 L 6181 14591 L
6131 14455 L 6098 14320 L 6142 14184 L 6191 14048 L
6227 13956 L 6231 13913 L 6227 13902 L 6126 13777 L
6091 13746 L 5972 13777 L 5956 13791 L 5849 13913 L
5898 14048 L 5929 14184 L 5820 14311 L 5808 14320 L
5685 14433 L 5550 14355 L 5520 14320 L 5448 14184 L
5414 14152 L 5288 14048 L 5279 14037 L 5160 13913 L
5143 13875 L 5041 13777 L 5008 13725 L 4993 13777 L
5004 13913 L 5008 13922 L 5075 14048 L 5089 14184 L
5046 14320 L 5008 14414 L 4982 14455 L 5008 14569 L
5012 14591 L 5014 14727 L 5008 14738 L 4919 14727 L
4872 14720 L 4770 14591 L 4737 14572 L 4601 14582 L
4466 14496 L 4330 14478 L 4195 14477 L 4059 14471 L
3924 14480 L 3899 14455 L 3924 14351 L 3945 14320 L
4059 14272 L 4195 14298 L 4223 14184 L 4195 14152 L
4093 14048 L 4059 14022 L 3924 13962 L 3788 13961 L

3696 13913 L 3653 13890 L 3522 13913 L 3517 13913 L
3382 13966 L 3271 13913 L 3246 13883 L 3192 13777 L
3129 13641 L 3111 13622 L 3032 13506 L 3024 13370 L
3029 13234 L 2998 13099 L 2975 13031 L 2940 12963 L
2868 12827 L 2850 12692 L 2840 12658 L 2704 12690 L
2690 12692 L 2704 12694 L 2789 12827 L 2720 12963 L
2704 12986 L 2619 13099 L 2569 13159 L 2482 13099 L
2433 13071 L 2302 12963 L 2389 12827 L 2433 12740 L
2537 12692 L 2474 12556 L 2433 12529 L N E
165 O
2704 11607 2620 11742 2704 11814 2840 11796 2975 11772 3111 11755
3176 11742 3246 11701 3382 11615 3388 11606 3382 11588 3246 11537
3111 11557 2975 11590 2874 11606 2840 11619 2704 11607 17 N F
2704 9263 2631 9300 2685 9435 2704 9480 2748 9571 2802 9707
2840 9758 2923 9842 2975 9876 3091 9978 3111 9990 3246 10069
3287 10114 3382 10204 3382 9836 3261 9707 3382 9618 3435 9707
3382 9836 3382 10204 3421 10249 3517 10334 3517 9846 3449 9842
3517 9838 3520 9842 3517 9846 3517 10334 3621 10385 3653 10396
3788 10408 3924 10423 4012 10385 3924 10330 3849 10249 3788 10146
3771 10114 3788 10033 3906 9978 3924 9890 3926 9978 4032 10114
4059 10143 4195 10197 4330 10230 4412 10249 4466 10317 4498 10249
4588 10114 4488 9978 4466 9957 4330 9933 4195 9913 4088 9842
4059 9783 3924 9836 3788 9726 3784 9707 3737 9571 3686 9435
3687 9300 3788 9186 3805 9164 3825 9028 3803 8893 3788 8844
3653 8865 3517 8813 3382 8790 3346 8893 3339 9028 3382 9099
3446 9164 3473 9300 3382 9425 3246 9395 3205 9300 3187 9164
3111 9106 2975 9073 2858 9164 2840 9194 2704 9263 83 N F
2704 14458 2701 14455 2704 14451 2809 14455 2704 14458 5 N F
2902 14727 2866 14591 2975 14476 3092 14591 3111 14635 3144 14727
3226 14862 3246 14885 3321 14998 3278 15134 3246 15154 3226 15134
3111 15029 3087 14998 2975 14869 2967 14862 2902 14727 17 N F
183 O
3111 12312 2987 12420 3111 12476 3246 12458 3382 12427 3395 12420
3517 12314 3644 12285 3653 12276 3788 12163 3803 12149 3788 12140
3653 12109 3517 12146 3496 12149 3382 12202 3246 12243 3132 12285
3111 12312 19 N F
165 O
3111 8073 3106 8079 3111 8097 3127 8079 3111 8073 5 N F
147 O
3382 9836 3261 9707 3382 9618 3435 9707 3382 9836 5 N F
165 O
3517 8268 3423 8350 3517 8393 3606 8350 3517 8268 5 N F
147 O

3517 9846 3449 9842 3517 9838 3520 9842 3517 9846 5 N F
129 O
3517 12586 3489 12556 3517 12543 3653 12471 3788 12443 3924 12422
4059 12421 4195 12495 4292 12556 4330 12582 4466 12597 4601 12577
4648 12556 4737 12505 4872 12539 4929 12556 4997 12692 4915 12827
4872 12890 4737 12867 4601 12915 4466 12919 4330 12901 4199 12827
4195 12824 4059 12783 4059 12603 4091 12556 4059 12540 3924 12539
3866 12556 3924 12584 4059 12603 4059 12783 3924 12737 3809 12692
3788 12680 3653 12654 3517 12586 39 N F
165 O
3637 11064 3595 11199 3621 11335 3653 11365 3788 11376 3924 11360
3924 11234 3788 11201 3787 11199 3778 11064 3788 11054 3924 11042
3984 11064 4015 11199 3924 11234 3924 11360 3979 11335 4059 11292
4125 11199 4195 11070 4214 11064 4330 11033 4417 11064 4466 11073
4466 10955 4361 10928 4466 10896 4544 10928 4466 10955 4466 11073
4550 11064 4601 11050 4690 10928 4601 10821 4544 10792 4466 10761
4330 10766 4307 10792 4195 10913 4166 10928 4059 10971 3924 10936
3830 10928 3788 10925 3784 10928 3653 11050 3637 11064 47 N F
129 O
3788 7624 3740 7536 3788 7519 3794 7536 3788 7624 5 N F
111 O
3788 11627 3759 11606 3788 11598 3924 11589 3959 11606 3924 11652
3788 11627 7 N F
183 O
3788 11054 3778 11064 3787 11199 3788 11201 3924 11234 4015 11199
3984 11064 3924 11042 3788 11054 9 N F
111 O
3924 12584 3866 12556 3924 12539 4059 12540 4091 12556 4059 12603
3924 12584 7 N F
92 O
4059 5962 3999 5908 4020 5772 4059 5712 4195 5642 4198 5637
4330 5508 4390 5637 4370 5772 4330 5851 4218 5908 4195 5973
4059 5962 13 N F
129 O
4059 8137 4010 8079 4059 8054 4113 8079 4059 8137 5 N F
165 O
4059 7642 4036 7672 4052 7807 4059 7824 4195 7900 4305 7807
4195 7678 4177 7672 4059 7642 9 N F
129 O
4195 8825 4150 8757 4183 8621 4195 8617 4199 8621 4330 8753
4334 8757 4343 8893 4330 8955 4316 8893 4195 8825 11 N F
4195 9069 4171 9028 4195 8974 4307 9028 4195 9069 5 N F
111 O

4195 7007 4171 6993 4195 6972 4245 6993 4195 7007 5 N F
165 O
4330 8447 4299 8486 4330 8517 4447 8621 4466 8643 4483 8621
4466 8589 4380 8486 4330 8447 9 N F
183 O
4466 10896 4361 10928 4466 10955 4544 10928 4466 10896 5 N F
111 O
4466 11401 4363 11335 4466 11224 4592 11335 4466 11401 5 N F
183 O
4466 13223 4451 13234 4466 13262 4483 13234 4466 13223 5 N F
129 O
4466 10659 4458 10656 4466 10649 4601 10569 4647 10656 4601 10683
4466 10659 7 N F
111 O
4601 11716 4528 11606 4567 11471 4601 11358 4622 11335 4737 11284
4804 11199 4872 11159 5008 11097 5055 11064 5117 10928 5143 10814
5279 10836 5402 10792 5414 10786 5550 10675 5646 10792 5685 10832
5740 10928 5738 11064 5767 11199 5820 11246 5947 11335 5956 11363
6091 11453 6227 11436 6264 11471 6227 11506 6091 11538 5956 11508
5820 11601 5818 11606 5730 11742 5699 11878 5685 11925 5685 11622
5691 11606 5705 11471 5685 11427 5605 11471 5633 11606 5685 11622
5685 11925 5550 11916 5536 11878 5414 11779 5279 11768 5143 11848
5143 11608 5148 11606 5179 11471 5215 11335 5150 11199 5143 11193
5008 11198 5006 11199 4919 11335 5008 11445 5094 11471 5138 11606
5143 11608 5143 11848 5104 11878 5008 11908 4924 11878 4872 11820
4823 11742 4737 11672 4601 11716 69 N F
129 O
4601 7675 4597 7672 4601 7670 4609 7672 4601 7675 5 N F
111 O
4872 7404 4869 7400 4872 7397 4888 7400 4872 7404 5 N F
92 O
5008 11445 4919 11335 5006 11199 5008 11198 5143 11193 5150 11199
5215 11335 5179 11471 5148 11606 5143 11608 5138 11606 5094 11471
5008 11445 13 N F
147 O
5008 7743 4953 7807 5008 7877 5118 7807 5008 7743 5 N F
111 O
5008 10415 4966 10385 5008 10355 5025 10385 5008 10415 5 N F
5008 10671 5002 10656 5008 10627 5143 10647 5174 10656 5143 10720
5008 10671 7 N F
5143 7545 5113 7536 5143 7508 5279 7535 5281 7536 5279 7537
5143 7545 7 N F
5414 7360 5292 7265 5414 7182 5550 7258 5577 7265 5550 7279

5414 7360 7 N F
183 O
5414 13616 5383 13641 5414 13672 5498 13777 5550 13799 5685 13800
5708 13777 5685 13762 5610 13641 5579 13506 5550 13417 5518 13506
5414 13616 13 N F
111 O
5550 7707 5533 7672 5550 7638 5578 7672 5550 7707 5 N F
147 O
5550 14054 5543 14048 5550 14041 5565 14048 5550 14054 5 N F
129 O
5685 5594 5566 5637 5685 5688 5718 5637 5685 5594 5 N F
92 O
5629 6586 M 5572 6451 L 5590 6315 L 5596 6179 L 5622 6044 L
5685 5942 L 5721 5908 L 5811 5772 L 5820 5756 L
5956 5708 L 6091 5642 L 6094 5637 L 6091 5631 L
6021 5501 L 6121 5501 L 6127 5501 L 6161 5637 L
6130 5772 L 6091 5887 L 6065 5908 L 6091 5926 L
6207 6044 L 6227 6058 L 6341 6179 L 6362 6195 L
6498 6212 L 6633 6214 L 6660 6179 L 6769 6078 L
6809 6044 L 6904 5949 L 7040 5956 L 7175 6030 L
7192 6044 L 7239 6179 L 7175 6283 L 7040 6275 L
6973 6315 L 6946 6451 L 7012 6586 L 7040 6599 L
7113 6722 L 7175 6808 L 7226 6858 L 7311 6921 L
7446 6915 L 7481 6858 L 7534 6722 L 7468 6586 L
7582 6524 L 7717 6513 L 7853 6567 L 7895 6586 L
7988 6680 L 8124 6614 L 8197 6722 L 8259 6791 L
8395 6780 L 8530 6838 L 8560 6858 L 8666 6958 L
8706 6993 L 8801 7105 L 8836 7129 L 8937 7192 L
9043 7265 L 9072 7280 L 9208 7323 L 9343 7336 L
9479 7351 L 9614 7314 L 9705 7265 L 9614 7236 L
9497 7129 L 9479 7097 L 9439 6993 L 9450 6858 L
9479 6835 L 9558 6722 L 9577 6586 L 9599 6451 L
9530 6315 L 9489 6179 L 9488 6044 L 9498 5908 L
9538 5772 L 9614 5688 L 9706 5772 L 9749 5806 L
9837 5908 L 9865 6044 L 9885 6099 L 9971 6179 L
9984 6315 L 9992 6451 L 10020 6563 L 10095 6586 L
10145 6722 L 10156 6813 L 10174 6858 L 10173 6993 L
10210 7129 L 10283 7265 L 10291 7272 L 10427 7363 L
10535 7400 L 10562 7402 L 10637 7400 L 10698 7381 L
10753 7265 L 10833 7215 L 10969 7200 L 11104 7168 L
11240 7176 L 11375 7204 L 11511 7200 L 11646 7235 L
11782 7253 L 11865 7129 L 11917 7083 L 12053 7068 L
12188 7024 L 12324 7003 L 12341 6993 L 12396 6858 L

12459 6771 L 12595 6832 L 12667 6722 L 12683 6586 L
12730 6542 L 12852 6451 L 12866 6411 L 12876 6315 L
12866 6216 L 12856 6179 L 12836 6044 L 12820 5908 L
12859 5772 L 12777 5637 L 12745 5501 L 12810 5501 L
13001 5501 L 13001 9289 L 12983 9300 L 12866 9388 L
12807 9300 L 12730 9253 L 12595 9271 L 12459 9236 L
12324 9177 L 12324 8143 L 12459 8126 L 12595 8080 L
12595 8079 L 12595 8078 L 12459 8055 L 12395 7943 L
12367 7807 L 12377 7672 L 12324 7551 L 12273 7672 L
12283 7807 L 12274 7943 L 12300 8079 L 12324 8143 L
12324 9177 L 12316 9164 L 12254 9028 L 12188 8914 L
12187 8893 L 12181 8757 L 12176 8621 L 12169 8486 L
12165 8350 L 12157 8214 L 12149 8079 L 12143 7943 L
12137 7807 L 12127 7672 L 12107 7536 L 12053 7480 L
11917 7460 L 11782 7449 L 11646 7437 L 11511 7430 L
11375 7421 L 11240 7413 L 11104 7422 L 10969 7435 L
10833 7495 L 10698 7527 L 10562 7533 L 10427 7489 L
10291 7466 L 10156 7449 L 10020 7452 L 10020 7112 L
10055 6993 L 10096 6858 L 10079 6722 L 10020 6651 L
9992 6586 L 9958 6451 L 9929 6315 L 9885 6200 L
9879 6179 L 9830 6044 L 9779 5908 L 9749 5874 L
9621 5772 L 9614 5765 L 9608 5772 L 9535 5908 L
9517 6044 L 9516 6179 L 9562 6315 L 9614 6407 L
9640 6451 L 9632 6586 L 9623 6722 L 9683 6858 L
9749 6932 L 9869 6993 L 9885 7022 L 10020 7112 L
10020 7452 L 9885 7465 L 9749 7483 L 9749 7230 L
9785 7129 L 9759 6993 L 9749 6988 L 9633 6858 L
9614 6812 L 9528 6858 L 9521 6993 L 9614 7065 L
9711 7129 L 9749 7230 L 9749 7483 L 9614 7531 L
9479 7524 L 9343 7528 L 9208 7519 L 9072 7497 L
8937 7452 L 8859 7400 L 8801 7354 L 8720 7265 L
8666 7196 L 8574 7129 L 8530 7070 L 8482 7129 L
8395 7257 L 8390 7265 L 8259 7319 L 8124 7267 L
8116 7265 L 8101 7129 L 7988 7079 L 7988 6938 L
8118 6858 L 7988 6735 L 7853 6794 L 7776 6858 L
7853 6936 L 7988 6938 L 7988 7079 L 7881 7129 L
7853 7238 L 7793 7129 L 7717 7088 L 7582 7001 L
7446 7101 L 7366 7129 L 7311 7169 L 7253 7129 L
7175 7091 L 7040 7041 L 6911 6993 L 6904 6978 L
6769 6948 L 6633 6920 L 6498 6864 L 6493 6858 L
6427 6722 L 6362 6621 L 6303 6586 L 6227 6557 L
6091 6530 L 5978 6451 L 5956 6435 L 5925 6451 L
5956 6492 L 6008 6586 L 5956 6696 L 5820 6693 L

5685 6624 L 5629 6586 L N E
5633 11606 5605 11471 5685 11427 5705 11471 5691 11606 5685 11622
5633 11606 7 N F
147 O
5750 9435 5729 9571 5788 9707 5820 9743 5920 9842 5904 9978
5867 10114 5840 10249 5844 10385 5826 10521 5820 10544 5780 10656
5820 10783 5823 10792 5850 10928 5820 11046 5817 11064 5820 11072
5956 11174 5956 11103 5906 11064 5956 10944 6005 10928 5956 10900
5927 10792 5956 10713 5956 10609 5920 10521 5956 10390 5956 9759
5903 9707 5820 9594 5808 9571 5820 9523 5956 9462 5956 9113
5894 9028 5956 8893 5883 8757 5956 8690 6028 8757 5956 8893
6008 9028 5956 9113 5956 9462 5987 9435 6053 9300 6091 9272
6144 9300 6181 9435 6167 9571 6091 9643 6042 9707 5956 9759
5956 10390 5965 10385 6091 10345 6134 10385 6091 10453 6042 10521
5956 10609 5956 10713 6091 10702 6227 10688 6227 10230 6132 10114
6227 10016 6316 10114 6227 10230 6227 10688 6362 10671 6391 10656
6498 10605 6633 10623 6685 10656 6633 10669 6498 10748 6475 10792
6498 10892 6507 10928 6498 10933 6383 10928 6362 10926 6227 10850
6132 10928 6227 11042 6242 11064 6227 11068 6091 11078 5956 11103
5956 11174 6091 11144 6227 11138 6314 11199 6362 11277 6473 11199
6419 11064 6498 11009 6633 10928 6620 10792 6633 10782 6661 10792
6769 10831 6833 10792 6855 10656 6769 10592 6690 10521 6633 10457
6498 10444 6440 10385 6481 10249 6498 10193 6633 10114 6634 10114
6664 9978 6633 9940 6498 9916 6439 9842 6473 9707 6445 9571
6368 9435 6362 9431 6286 9300 6256 9164 6227 9138 6106 9028
6164 8893 6227 8797 6295 8757 6362 8628 6368 8621 6380 8486
6498 8354 6528 8350 6526 8214 6498 8196 6362 8115 6306 8079
6243 7943 6274 7807 6321 7672 6227 7637 6100 7536 6091 7531
6060 7536 5956 7570 5820 7563 5811 7672 5806 7807 5820 7889
5835 7943 5820 7992 5775 8079 5768 8214 5734 8350 5740 8486
5763 8621 5734 8757 5810 8893 5763 9028 5807 9164 5820 9181
5888 9300 5820 9347 5750 9435 165 N F
165 O
5805 14727 5733 14591 5751 14455 5820 14364 5845 14455 5950 14591
5875 14727 5820 14747 5805 14727 9 N F
5820 9523 5808 9571 5820 9594 5903 9707 5956 9759 6042 9707
6091 9643 6167 9571 6181 9435 6144 9300 6091 9272 6053 9300
5987 9435 5956 9462 5820 9523 15 N F
5956 8690 5883 8757 5956 8893 5894 9028 5956 9113 6008 9028
5956 8893 6028 8757 5956 8690 9 N F
183 O
5956 13107 5896 13234 5956 13290 6051 13234 5956 13107 5 N F
165 O

5956 10944 5906 11064 5956 11103 6091 11078 6227 11068 6242 11064
6227 11042 6132 10928 6227 10850 6362 10926 6383 10928 6498 10933
6507 10928 6498 10892 6475 10792 6498 10748 6633 10669 6685 10656
6633 10623 6498 10605 6391 10656 6362 10671 6227 10688 6091 10702
5956 10713 5927 10792 5956 10900 6005 10928 5956 10944 29 N F
5956 10390 5920 10521 5956 10609 6042 10521 6091 10453 6134 10385
6091 10345 5965 10385 5956 10390 9 N F
92 O
6021 5501 6021 5501 6120 5501 6127 5501 6127 5501 6121 5501
6021 5501 7 N F
74 O
6091 5926 M 6065 5908 L 6091 5887 L 6130 5772 L 6161 5637 L
6127 5501 L 6312 5501 L 6313 5501 L 6254 5637 L
6362 5686 L 6498 5733 L 6633 5735 L 6716 5772 L
6769 5787 L 6904 5796 L 7040 5815 L 7175 5871 L
7205 5908 L 7311 6001 L 7357 6044 L 7430 6179 L
7446 6208 L 7582 6262 L 7717 6259 L 7853 6264 L
7988 6274 L 8074 6315 L 8124 6345 L 8259 6449 L
8262 6451 L 8395 6539 L 8462 6586 L 8530 6627 L
8666 6719 L 8669 6722 L 8801 6819 L 8895 6858 L
8937 6876 L 9072 6907 L 9208 6901 L 9267 6858 L
9266 6722 L 9245 6586 L 9281 6451 L 9343 6388 L
9390 6451 L 9463 6586 L 9465 6722 L 9479 6728 L
9483 6722 L 9493 6586 L 9529 6451 L 9499 6315 L
9479 6246 L 9430 6179 L 9441 6044 L 9442 5908 L
9359 5772 L 9479 5665 L 9493 5637 L 9614 5592 L
9727 5637 L 9749 5653 L 9784 5637 L 9792 5501 L
9943 5501 L 10134 5501 L 10325 5501 L 10516 5501 L
10707 5501 L 10899 5501 L 11090 5501 L 11281 5501 L
11472 5501 L 11663 5501 L 11854 5501 L 12045 5501 L
12236 5501 L 12428 5501 L 12619 5501 L 12745 5501 L
12777 5637 L 12859 5772 L 12820 5908 L 12836 6044 L
12856 6179 L 12866 6216 L 12876 6315 L 12866 6411 L
12852 6451 L 12730 6542 L 12683 6586 L 12667 6722 L
12595 6832 L 12459 6771 L 12459 5914 L 12472 5908 L
12533 5772 L 12459 5733 L 12436 5772 L 12448 5908 L
12459 5914 L 12459 6771 L 12396 6858 L 12341 6993 L
12324 7003 L 12188 7024 L 12053 7068 L 11917 7083 L
11865 7129 L 11782 7253 L 11646 7235 L 11511 7200 L
11375 7204 L 11240 7176 L 11104 7168 L 10969 7200 L
10833 7215 L 10753 7265 L 10698 7381 L 10637 7400 L
10562 7402 L 10535 7400 L 10427 7363 L 10291 7272 L
10283 7265 L 10210 7129 L 10173 6993 L 10174 6858 L

10156 6813 L 10145 6722 L 10095 6586 L 10020 6563 L
9992 6451 L 9984 6315 L 9971 6179 L 9885 6099 L
9865 6044 L 9837 5908 L 9749 5806 L 9706 5772 L
9614 5688 L 9538 5772 L 9498 5908 L 9488 6044 L
9489 6179 L 9530 6315 L 9599 6451 L 9577 6586 L
9558 6722 L 9479 6835 L 9450 6858 L 9439 6993 L
9479 7097 L 9497 7129 L 9614 7236 L 9705 7265 L
9614 7314 L 9479 7351 L 9343 7336 L 9343 6648 L
9376 6586 L 9343 6535 L 9316 6586 L 9343 6648 L
9343 7336 L 9208 7323 L 9072 7280 L 9043 7265 L
8937 7192 L 8836 7129 L 8801 7105 L 8706 6993 L
8666 6958 L 8560 6858 L 8530 6838 L 8395 6780 L
8259 6791 L 8197 6722 L 8124 6614 L 7988 6680 L
7895 6586 L 7853 6567 L 7717 6513 L 7582 6524 L
7468 6586 L 7534 6722 L 7481 6858 L 7446 6915 L
7311 6921 L 7226 6858 L 7175 6808 L 7113 6722 L
7040 6599 L 7012 6586 L 6946 6451 L 6973 6315 L
7040 6275 L 7175 6283 L 7239 6179 L 7192 6044 L
7175 6030 L 7040 5956 L 6904 5949 L 6809 6044 L
6769 6078 L 6660 6179 L 6633 6214 L 6498 6212 L
6362 6195 L 6341 6179 L 6227 6058 L 6207 6044 L
6091 5926 L N E
147 O
6091 11318 6076 11335 6091 11348 6128 11335 6091 11318 5 N F
129 O
6227 6913 6114 6993 6227 7017 6279 6993 6227 6913 5 N F
74 O
6127 5501 6127 5501 6312 5501 6313 5501 6313 5501 6312 5501
6127 5501 7 N F
165 O
6227 10016 6132 10114 6227 10230 6227 10136 6209 10114 6227 10096
6243 10114 6227 10136 6227 10230 6316 10114 6227 10016 11 N F
111 O
6227 11743 6182 11742 6227 11738 6307 11742 6227 11743 5 N F
183 O
6227 10096 6209 10114 6227 10136 6243 10114 6227 10096 5 N F
56 O
6362 5686 6254 5637 6313 5501 6503 5501 6531 5501 6633 5529
6769 5535 6904 5506 6923 5501 7076 5501 7267 5501 7431 5501
7446 5503 7582 5540 7717 5552 7853 5552 7988 5545 8124 5537
8259 5510 8287 5501 8414 5501 8605 5501 8796 5501 8868 5501
8801 5532 8666 5561 8530 5593 8406 5637 8395 5643 8259 5723
8171 5772 8124 5851 8069 5908 8124 5964 8178 6044 8259 6085

8395 6170 8406 6179 8530 6302 8546 6315 8666 6416 8720 6451
8801 6499 8937 6529 9072 6534 9109 6451 9149 6315 9208 6223
9343 6205 9353 6179 9385 6044 9361 5908 9343 5883 9208 5902
9208 5837 9340 5772 9208 5641 9162 5772 9208 5837 9208 5902
9116 5772 9130 5637 9175 5501 9178 5501 9266 5501 9343 5521
9479 5520 9522 5501 9561 5501 9752 5501 9792 5501 9784 5637
9749 5653 9727 5637 9614 5592 9493 5637 9479 5665 9359 5772
9442 5908 9441 6044 9430 6179 9479 6246 9499 6315 9529 6451
9493 6586 9483 6722 9479 6728 9465 6722 9463 6586 9390 6451
9343 6388 9281 6451 9245 6586 9266 6722 9267 6858 9208 6901
9072 6907 8937 6876 8895 6858 8801 6819 8669 6722 8666 6719
8530 6627 8462 6586 8395 6539 8262 6451 8259 6449 8124 6345
8074 6315 7988 6274 7853 6264 7717 6259 7582 6262 7582 6045
7590 6044 7582 6038 7580 6044 7582 6045 7582 6262 7446 6208
7430 6179 7357 6044 7311 6001 7205 5908 7175 5871 7040 5815
6904 5796 6769 5787 6716 5772 6633 5735 6498 5733 6362 5686
132 N F
129 O
6301 13234 6274 13099 6362 13023 6498 13030 6633 13063 6769 13070
6904 13063 7040 13037 7175 13062 7311 13068 7446 13051 7582 13059
7636 13099 7582 13140 7446 13217 7416 13234 7314 13370 7311 13380
7175 13442 7040 13397 6965 13370 6904 13355 6769 13329 6633 13352
6544 13370 6498 13389 6451 13370 6362 13347 6301 13234 29 N F
56 O
6503 5501 6313 5501 6313 5501 6503 5501 6531 5501 6531 5501
6503 5501 7 N F
183 O
6362 12403 6337 12420 6361 12556 6362 12558 6471 12692 6498 12771
6524 12827 6633 12867 6725 12827 6640 12692 6633 12686 6510 12556
6498 12511 6386 12420 6362 12403 15 N F
74 O
6694 5501 6531 5501 6633 5529 6769 5535 6904 5506 6923 5501
6885 5501 6694 5501 8 N F
129 O
6633 12103 6532 12013 6633 11956 6670 12013 6633 12103 5 N F
111 O
6769 7998 6734 7943 6769 7874 6834 7943 6769 7998 5 N F
183 O
6769 12233 6753 12285 6769 12382 6805 12420 6904 12435 6975 12420
6904 12360 6826 12285 6769 12233 9 N F
111 O
6904 11367 6778 11335 6786 11199 6904 11068 7040 11165 7123 11199
7175 11228 7225 11199 7311 11127 7446 11072 7582 11109 7717 11081

7815 11064 7827 10928 7778 10792 7717 10737 7634 10656 7672 10521
7717 10488 7853 10457 7988 10418 8055 10385 8002 10249 8124 10199
8178 10249 8259 10302 8395 10280 8530 10311 8666 10320 8709 10385
8666 10469 8538 10521 8530 10524 8458 10656 8395 10706 8259 10753
8190 10792 8259 10913 8262 10928 8320 11064 8395 11182 8414 11199
8530 11291 8575 11335 8636 11471 8666 11547 8679 11606 8666 11643
8609 11742 8530 11800 8395 11844 8259 11828 8128 11878 8124 11881
8005 12013 7988 12030 7988 11455 8106 11335 7988 11211 7865 11335
7988 11455 7988 12030 7853 12134 7717 12088 7673 12013 7582 11911
7572 11878 7582 11845 7700 11742 7646 11606 7582 11524 7582 11390
7606 11335 7582 11305 7557 11335 7582 11390 7582 11524 7446 11551
7311 11498 7175 11488 7115 11471 7040 11417 6904 11367 83 N F
7035 10792 7019 10656 7040 10636 7044 10656 7084 10792 7040 10809
7035 10792 7 N F
147 O
7175 10559 7089 10656 7175 10747 7175 10700 7134 10656 7175 10610
7221 10656 7175 10700 7175 10747 7270 10656 7175 10559 11 N F
111 O
7175 8555 7106 8486 7175 8408 7297 8486 7175 8555 5 N F
7140 7943 7111 7807 7175 7703 7311 7784 7373 7807 7311 7832
7194 7943 7175 7974 7140 7943 9 N F
165 O
7175 10610 7134 10656 7175 10700 7221 10656 7175 10610 5 N F
7242 11742 7202 11878 7276 12013 7311 12130 7446 12087 7484 12013
7477 11878 7474 11742 7446 11720 7311 11653 7242 11742 11 N F
183 O
7289 12556 7278 12692 7311 12732 7446 12755 7492 12692 7517 12556
7446 12493 7311 12523 7289 12556 9 N F
111 O
7446 10407 7429 10385 7446 10361 7551 10249 7582 10215 7599 10249
7647 10385 7582 10466 7446 10407 9 N F
74 O
7458 5501 7431 5501 7446 5503 7582 5540 7717 5552 7853 5552
7988 5545 8124 5537 8259 5510 8287 5501 8223 5501 8032 5501
7841 5501 7650 5501 7458 5501 15 N F
111 O
7446 8238 7435 8214 7446 8206 7456 8214 7446 8238 5 N F
147 O
7582 8547 7509 8621 7582 8712 7618 8757 7717 8860 7751 8893
7853 8982 7853 8915 7827 8893 7717 8784 7691 8757 7717 8734
7745 8757 7853 8866 7978 8757 7988 8747 7997 8757 7988 8767
7879 8893 7853 8915 7853 8982 7955 8893 7988 8855 8073 8757
7988 8662 7888 8757 7853 8788 7822 8757 7717 8671 7657 8621

7582 8547 31 N F
129 O
7582 11305 7557 11335 7582 11390 7606 11335 7582 11305 5 N F
38 O
7582 6045 7580 6044 7582 6038 7590 6044 7582 6045 5 N F
165 O
7717 8734 7691 8757 7717 8784 7827 8893 7853 8915 7879 8893
7988 8767 7997 8757 7988 8747 7978 8757 7853 8866 7745 8757
7717 8734 13 N F
111 O
7853 9808 7756 9707 7853 9701 7982 9571 7988 9523 8067 9435
8124 9400 8180 9435 8224 9571 8259 9617 8286 9571 8395 9564
8530 9447 8596 9571 8530 9632 8404 9707 8395 9722 8259 9786
8124 9789 8003 9842 7988 9848 7972 9842 7853 9808 23 N F
147 O
7853 9468 7768 9571 7853 9648 7853 9595 7827 9571 7853 9539
7876 9571 7853 9595 7853 9648 7929 9571 7853 9468 11 N F
74 O
7853 6936 7776 6858 7853 6794 7988 6735 8118 6858 7988 6938
7853 6936 7 N F
165 O
7853 9539 7827 9571 7853 9595 7876 9571 7853 9539 5 N F
7979 13234 7860 13370 7988 13463 8109 13506 8124 13509 8130 13506
8254 13370 8145 13234 8124 13191 7988 13211 7979 13234 11 N F
129 O
7988 11211 7865 11335 7988 11455 7988 11411 7910 11335 7988 11256
8063 11335 7988 11411 7988 11455 8106 11335 7988 11211 11 N F
147 O
7988 11256 7910 11335 7988 11411 7988 11367 7955 11335 7988 11302
8020 11335 7988 11367 7988 11411 8063 11335 7988 11256 11 N F
165 O
7988 11302 7955 11335 7988 11367 8020 11335 7988 11302 5 N F
38 O
8124 5964 8069 5908 8124 5851 8171 5772 8259 5723 8395 5643
8406 5637 8530 5593 8666 5561 8801 5532 8868 5501 8987 5501
9175 5501 9130 5637 9116 5772 9208 5902 9343 5883 9361 5908
9385 6044 9353 6179 9343 6205 9208 6223 9149 6315 9109 6451
9072 6534 8937 6529 8937 6172 9072 6160 9144 6044 9123 5908
9072 5782 9050 5772 8937 5695 8801 5721 8675 5772 8666 5813
8653 5908 8666 5950 8701 6044 8801 6116 8937 6172 8937 6529
8801 6499 8720 6451 8666 6416 8546 6315 8530 6302 8406 6179
8395 6170 8259 6085 8178 6044 8124 5964 52 N F
147 O

8395 9318 8305 9435 8395 9512 8395 9460 8365 9435 8395 9397
8421 9435 8395 9460 8395 9512 8476 9435 8395 9318 11 N F
165 O
8395 9397 8365 9435 8395 9460 8421 9435 8395 9397 5 N F
8395 8328 8375 8350 8395 8374 8476 8486 8530 8546 8611 8621
8666 8675 8725 8621 8666 8551 8606 8486 8555 8350 8530 8321
8395 8328 13 N F
147 O
8530 8030 8455 8079 8530 8143 8614 8079 8530 8030 5 N F
8666 9756 8578 9842 8666 9931 8666 9873 8636 9842 8666 9813
8700 9842 8666 9873 8666 9931 8767 9842 8666 9756 11 N F
165 O
8666 9813 8636 9842 8666 9873 8700 9842 8666 9813 5 N F
20 O
8666 5950 8653 5908 8666 5813 8675 5772 8801 5721 8937 5695
9050 5772 9072 5782 9123 5908 9144 6044 9072 6160 8937 6172
8801 6116 8701 6044 8666 5950 15 N F
38 O
8987 5501 8868 5501 8869 5501 8987 5501 9175 5501 9175 5501
8987 5501 7 N F
147 O
8937 11999 8932 12013 8937 12018 9072 12134 9113 12149 9208 12164
9275 12149 9343 12113 9472 12149 9479 12150 9480 12149 9587 12013
9550 11878 9479 11818 9343 11866 9316 11878 9208 12010 9072 11898
8937 11999 19 N F
9072 10939 8957 11064 9072 11190 9072 11132 9010 11064 9072 10996
9143 11064 9072 11132 9072 11190 9203 11064 9072 10939 11 N F
165 O
9072 10996 9010 11064 9072 11132 9072 11074 9062 11064 9072 11053
9083 11064 9072 11074 9072 11132 9143 11064 9072 10996 11 N F
9072 9368 9013 9435 9072 9501 9072 9439 9068 9435 9072 9431
9078 9435 9072 9439 9072 9501 9175 9435 9072 9368 11 N F
183 O
9072 11053 9062 11064 9072 11074 9083 11064 9072 11053 5 N F
9072 9431 9068 9435 9072 9439 9078 9435 9072 9431 5 N F
74 O
9208 5641 9162 5772 9208 5837 9340 5772 9208 5641 5 N F
56 O
9178 5501 9175 5501 9175 5501 9179 5501 9266 5501 9266 5501
9178 5501 7 N F
38 O
9266 5501 9266 5501 9370 5501 9522 5501 9522 5501 9370 5501
9266 5501 7 N F

9343 5521 9266 5501 9370 5501 9522 5501 9479 5520 9343 5521
6 N F
92 O
9343 6535 9316 6586 9343 6648 9376 6586 9343 6535 5 N F
165 O
9479 10671 9396 10792 9479 10879 9479 10815 9457 10792 9479 10760
9507 10792 9479 10815 9479 10879 9588 10792 9614 10731 9614 10447
9588 10385 9614 10354 9749 10357 9776 10385 9749 10427 9614 10447
9614 10731 9720 10656 9740 10521 9749 10517 9833 10385 9749 10296
9614 10283 9530 10385 9536 10521 9518 10656 9479 10671 29 N F
183 O
9479 10760 9457 10792 9479 10815 9507 10792 9479 10760 5 N F
111 O
9517 6044 9516 6179 9562 6315 9614 6407 9640 6451 9632 6586
9623 6722 9683 6858 9749 6932 9869 6993 9885 7022 9885 6941
9749 6876 9733 6858 9659 6722 9664 6586 9673 6451 9614 6352
9593 6315 9544 6179 9546 6044 9572 5908 9614 5832 9709 5908
9749 5945 9795 6044 9838 6179 9875 6315 9885 6333 9924 6451
9958 6586 10015 6722 10020 6778 10030 6858 10020 6882 9885 6941
9885 7022 10020 7112 10055 6993 10096 6858 10079 6722 10020 6651
9992 6586 9958 6451 9929 6315 9885 6200 9879 6179 9830 6044
9779 5908 9749 5874 9621 5772 9614 5765 9608 5772 9535 5908
9517 6044 55 N F
74 O
9614 7065 9521 6993 9528 6858 9614 6812 9633 6858 9749 6988
9759 6993 9785 7129 9749 7230 9711 7129 9614 7065 11 N F
165 O
9614 9303 9534 9435 9614 9545 9614 9457 9598 9435 9614 9410
9631 9435 9614 9457 9614 9545 9700 9435 9614 9303 11 N F
129 O
9546 6044 9544 6179 9593 6315 9614 6352 9673 6451 9664 6586
9659 6722 9733 6858 9749 6876 9885 6941 9885 6880 9838 6858
9749 6816 9694 6722 9696 6586 9706 6451 9642 6315 9614 6291
9571 6179 9575 6044 9608 5908 9614 5898 9627 5908 9749 6019
9761 6044 9797 6179 9819 6315 9885 6435 9890 6451 9924 6586
9959 6722 9925 6858 9885 6880 9885 6941 10020 6882 10030 6858
10020 6778 10015 6722 9958 6586 9924 6451 9885 6333 9875 6315
9838 6179 9795 6044 9749 5945 9709 5908 9614 5832 9572 5908
9546 6044 49 N F
147 O
9575 6044 9571 6179 9614 6291 9642 6315 9706 6451 9696 6586
9694 6722 9749 6816 9838 6858 9885 6880 9885 6769 9749 6755
9730 6722 9728 6586 9739 6451 9728 6315 9614 6219 9599 6179

9604 6044 9614 6005 9660 6044 9749 6151 9757 6179 9764 6315
9785 6451 9885 6569 9889 6586 9904 6722 9885 6769 9885 6880
9925 6858 9959 6722 9924 6586 9890 6451 9885 6435 9819 6315
9797 6179 9761 6044 9749 6019 9627 5908 9614 5898 9608 5908
9575 6044 43 N F
183 O
9614 10354 9588 10385 9614 10447 9749 10427 9776 10385 9749 10357
9614 10354 7 N F
9614 9410 9598 9435 9614 9457 9631 9435 9614 9410 5 N F
165 O
9604 6044 9599 6179 9614 6219 9728 6315 9739 6451 9728 6586
9730 6722 9749 6755 9885 6769 9904 6722 9889 6586 9885 6569
9785 6451 9764 6315 9757 6179 9749 6151 9660 6044 9614 6005
9604 6044 19 N F
129 O
9749 8302 9631 8214 9749 8134 9865 8214 9749 8302 5 N F
147 O
9749 11877 9749 11878 9749 11878 9750 11878 9749 11877 5 N F
165 O
9885 9105 9810 9164 9885 9230 9943 9164 9885 9105 5 N F
129 O
9885 8961 9848 8893 9885 8850 9995 8893 9885 8961 5 N F
147 O
9885 7796 9872 7807 9885 7821 10020 7808 10156 7856 10245 7807
10156 7772 10020 7806 9885 7796 9 N F
10020 9986 9890 10114 10020 10244 10020 10175 9959 10114 10020 10054
10078 10114 10020 10175 10020 10244 10143 10114 10020 9986 11 N F
165 O
10020 10054 9959 10114 10020 10175 10078 10114 10020 10054 5 N F
10156 9360 10083 9435 10156 9509 10291 9516 10291 9443 10175 9435
10291 9424 10393 9300 10420 9164 10427 9158 10536 9164 10457 9300
10427 9326 10300 9435 10291 9443 10291 9516 10387 9435 10427 9401
10545 9300 10562 9277 10645 9164 10562 9079 10427 9064 10310 9164
10296 9300 10291 9305 10156 9360 27 N F
10156 8747 10139 8757 10156 8765 10168 8757 10156 8747 5 N F
183 O
10291 9424 10175 9435 10291 9443 10300 9435 10427 9326 10457 9300
10536 9164 10427 9158 10420 9164 10393 9300 10291 9424 11 N F
165 O
10291 9634 10228 9707 10291 9772 10360 9707 10291 9634 5 N F
147 O
10562 12728 10458 12692 10562 12564 10698 12613 10803 12692 10698 12708
10562 12728 7 N F

10698 7890 10638 7943 10657 8079 10698 8208 10818 8079 10774 7943
10833 7842 10847 7807 10833 7795 10813 7807 10698 7890 11 N F
165 O
10833 9092 10759 9164 10833 9233 10899 9164 10833 9092 5 N F
183 O
10969 9430 10963 9435 10969 9443 10979 9435 10969 9430 5 N F
147 O
11375 8624 11373 8621 11375 8612 11377 8621 11375 8624 5 N F
165 O
11917 13956 11853 13913 11917 13829 12048 13913 11917 13956 5 N F
74 O
12283 7807 12273 7672 12324 7551 12377 7672 12367 7807 12395 7943
12459 8055 12595 8078 12595 8079 12595 8080 12459 8126 12324 8143
12300 8079 12274 7943 12283 7807 15 N F
92 O
12459 5733 12436 5772 12448 5908 12459 5914 12472 5908 12533 5772
12459 5733 7 N F
13001 9479 12896 9435 13001 9310 13001 9479 4 N F
13001 5501 13001 5501 13001 9289 13001 9289 13001 5501 13001 5501
6 N F
111 O
13001 9310 13001 9289 13001 9289 13001 9310 13001 9310 5 N F
92 O
13001 9479 13001 9310 13001 9310 13001 9479 13001 9479 5 N F
111 O
13001 10501 13001 9479 13001 9479 13001 10501 13001 10501 5 N F
129 O
13001 11422 13001 10501 13001 10501 13001 11422 13001 11422 5 N F
147 O
13001 12286 13001 11422 13001 11422 13001 12286 13001 12286 5 N F
165 O
13001 14070 13001 12286 13001 12286 13001 14070 13001 14070 5 N F
183 O
13001 16219 13001 14070 13001 14070 13001 16219 13001 16219 13001 16219
6 N F
111 O
13001 9289 13001 9289 13001 9310 13001 9310 13001 9300 13001 9289
6 N F
92 O
13001 9310 13001 9479 13001 9479 13001 9435 13001 9310 13001 9310
6 N F
111 O
13001 9479 13001 9479 13001 10501 13001 10501 13001 10385 13001 10249

13001 10114 13001 9978 13001 9842 13001 9707 13001 9571 13001 9479
12 N F
129 O
13001 10501 13001 10501 13001 11422 13001 11422 13001 11335 13001 11199
13001 11064 13001 10928 13001 10792 13001 10656 13001 10521 13001 10501
12 N F
147 O
13001 11422 13001 11422 13001 12286 13001 12286 13001 12285 13001 12149
13001 12013 13001 11878 13001 11742 13001 11606 13001 11471 13001 11422
12 N F
165 O
13001 12286 13001 12286 13001 14070 13001 14070 13001 14048 13001 13913
13001 13777 13001 13641 13001 13506 13001 13370 13001 13234 13001 13099
13001 12963 13001 12827 13001 12692 13001 12556 13001 12420 13001 12286
18 N F
Ls Gr Gs Lr
-1150 1075 16450 18700 Oc
1 O
10638 15415 10639 15455 10678 15493 10675 15533 10710 15574 10753 15608
10791 15646 10826 15687 10872 15717 10920 15742 10946 15789 10984 15827
11030 15857 11080 15877 11122 15912 11160 15949 11196 15980 11246 16007
18 P
11246 16007 11300 16018 11330 16063 11355 16110 11389 16152 11419 16197
11427 16219 7 P
12839 16219 12841 16212 12835 16186 12811 16174 12785 16169 12766 16149
12742 16136 12718 16125 12697 16107 12681 16086 12655 16078 12637 16058
12633 16031 12625 16005 12620 15978 12608 15955 12583 15943 12561 15928
12544 15907 12527 15885 12505 15870 12481 15858 12457 15845 12433 15833
12425 15807 12448 15793 12444 15766 12418 15757 12401 15736 12376 15724
12359 15704 12333 15696 12323 15671 12298 15680 12282 15657 12257 15648
12230 15651 12204 15657 12181 15641 12168 15617 12146 15602 12148 15575
12127 15558 12107 15540 12081 15533 12067 15510 12040 15511 12022 15491
12009 15467 12002 15441 50 P
12002 15441 12008 15415 12034 15407 12040 15380 12028 15356 12025 15329
12033 15303 12044 15279 12053 15253 12039 15276 12012 15272 11 P
12012 15272 11986 15265 12012 15256 12006 15230 11983 15215 11980 15188
11994 15165 12017 15151 12045 15149 12071 15148 12098 15142 12123 15132
12146 15147 12162 15125 12178 15103 12195 15082 12212 15061 12234 15045
12255 15027 12278 15013 12305 15010 12328 14995 12346 14975 12357 14950
12383 14960 12409 14951 12432 14938 12437 14911 28 P
12437 14911 12447 14886 12462 14863 12475 14839 12493 14819 12502 14793
12518 14771 12533 14749 12546 14725 12562 14703 12574 14678 12586 14654
12605 14635 12621 14613 12640 14593 12655 14571 12675 14552 12691 14530

12711 14511 12730 14492 12751 14475 12772 14457 12792 14439 12798 14413
12817 14395 12837 14375 12843 14349 12863 14331 12878 14308 12899 14291
12914 14268 12933 14249 12950 14227 12960 14202 12967 14176 12979 14152
12998 14132 13001 14107 38 P
13001 13054 12997 13054 13001 13039 3 P
13001 13026 12979 13016 12958 12998 12950 13025 12951 13052 12936 13029
12909 13027 12882 13019 12855 13020 9 P
12855 13020 12828 13024 12806 13040 12782 13053 12788 13026 12797 13000
6:00 PM
12797 13000 12770 13008 12743 13010 12729 12986 12747 12965 12728 12945
12726 12917 12725 12890 12743 12869 12741 12842 12729 12817 12707 12833
12691 12856 12718 12849 12712 12876 12684 12876 12687 12903 12662 12893
12637 12905 12628 12879 12601 12878 12575 12887 12549 12898 12548 12925
12546 12953 12539 12926 12513 12917 12490 12902 12467 12886 12441 12894
12415 12888 12398 12867 12374 12853 12357 12875 12350 12848 12364 12825
12388 12812 12409 12830 12436 12836 12441 12809 12438 12782 12453 12759
12462 12733 12463 12706 12437 12698 12436 12725 12441 12752 12415 12757
12388 12751 12389 12724 50 P
12389 12724 12365 12710 12353 12734 12363 12760 12351 12785 12337 12808
12324 12833 12312 12808 12286 12800 12258 12799 12284 12790 12261 12775
12269 12748 12286 12727 12309 12712 12331 12695 12330 12668 12303 12668
12276 12674 12273 12647 12300 12651 12298 12624 12276 12607 12265 12583
12244 12600 12228 12622 12208 12642 12193 12618 12167 12627 12176 12653
12187 12678 12191 12651 12215 12665 12214 12693 12213 12720 12234 12702
12251 12723 12247 12750 12222 12740 12195 12731 12178 12711 12152 12699
12132 12681 12108 12667 12105 12640 12096 12614 12107 12589 12091 12566
12063 12568 12037 12562 50 P
12037 12562 12023 12585 2 P
12023 12585 12021 12613 12010 12638 12008 12611 12008 12583 11984 12570
11977 12543 11955 12528 11951 12500 11933 12479 11911 12463 11909 12490
11886 12506 11903 12527 11879 12540 11871 12566 11889 12586 11909 12606
11881 12607 11860 12590 11835 12600 11814 12617 11786 12622 11774 12647
11771 12674 11780 12700 11803 12686 11806 12658 11820 12635 11845 12624
11872 12627 11852 12645 11826 12654 11827 12681 11854 12683 11860 12656
11887 12649 11915 12651 11905 12677 11883 12694 11856 12697 11829 12696
11850 12713 11877 12710 11884 12736 11911 12740 11936 12752 11909 12754
11905 12781 11881 12795 50 P
11881 12795 11879 12822 11877 12795 11887 12769 11886 12741 11859 12742
11835 12728 11825 12754 11832 12781 11822 12806 11837 12829 11810 12824
11794 12802 11773 12820 11799 12831 11811 12855 11788 12870 11768 12889
11771 12862 11751 12881 11743 12907 11770 12901 11782 12925 11780 12898
11799 12878 11817 12857 11835 12878 11845 12904 11818 12910 11796 12926
11800 12953 11773 12960 11782 12986 11788 13013 11794 13040 11816 13023

11825 13050 11798 13054 11811 13079 11815 13105 11827 13130 11853 13139
11879 13148 11906 13145 11904 13173 11877 13173 11851 13168 11823 13166
11809 13190 11828 13209 50 P
11828 13209 11848 13228 2 P
11848 13228 11867 13249 11894 13250 11899 13277 11924 13288 11909 13310
11928 13330 11947 13350 11920 13344 11893 13341 11866 13336 11858 13361
11873 13385 11897 13396 11918 13414 11943 13425 11963 13443 11965 13470
11959 13444 11937 13428 11912 13417 11888 13403 11868 13422 11856 13447
11835 13464 11809 13473 11831 13490 11809 13506 11807 13533 11793 13557
11806 13581 11783 13598 11782 13625 11806 13639 11806 13655 11781 13627
11767 13650 11781 13674 11757 13686 11758 13714 11760 13741 11755 13768
11732 13752 11715 13732 11710 13758 11701 13784 11682 13765 11696 13742
11691 13715 11716 13704 50 P
11716 13704 11720 13677 11731 13652 11742 13627 11721 13611 11705 13633
11701 13659 11694 13686 11677 13707 11678 13735 11660 13755 11634 13747
11635 13774 11610 13786 11600 13760 11594 13787 11604 13812 11630 13818
11629 13846 11636 13872 11660 13884 11659 13912 11665 13885 11672 13859
11670 13831 11693 13815 11677 13838 11701 13850 11716 13873 11699 13893
11676 13909 11696 13927 11722 13935 11731 13910 11738 13883 11739 13856
11745 13883 11749 13910 11771 13892 11760 13918 11741 13937 11766 13926
11755 13951 11779 13938 11803 13926 11826 13941 11846 13923 11873 13918
11889 13896 11894 13869 50 P
11894 13869 11909 13846 2 P
11909 13846 11908 13873 11927 13892 11949 13908 11959 13934 11940 13914
11913 13920 11895 13940 11876 13960 11850 13966 11856 13992 11868 14017
11863 14044 11841 14027 11823 14007 11806 14028 11819 14052 11812 14078
11839 14083 11860 14099 11836 14112 11812 14124 11828 14146 11802 14155
11781 14172 11762 14191 11786 14204 11784 14231 11774 14256 11748 14264
11728 14282 11721 14309 11716 14335 11741 14347 11764 14360 11778 14337
11770 14310 11794 14297 11818 14285 11843 14273 11865 14257 11887 14248
11887 14249 11867 14260 11844 14275 11822 14290 11797 14302 11785 14326
11783 14354 11777 14380 50 P
11777 14380 11795 14404 11770 14390 3 P
11770 14390 11752 14369 11725 14364 11701 14376 11678 14390 11656 14406
11629 14403 11608 14386 11581 14382 11555 14374 11556 14346 11550 14320
11528 14336 11509 14356 11504 14382 11478 14388 11476 14361 11448 14363
11432 14384 11412 14403 11390 14420 11378 14444 11352 14450 11325 14449
11300 14459 11304 14432 11329 14422 11356 14423 11349 14396 11323 14397
11325 14370 11330 14343 11322 14317 11294 14319 11271 14305 11255 14283
11228 14279 11217 14254 11200 14276 11212 14301 11238 14308 11240 14335
11268 14337 11252 14359 11274 14375 11247 14377 11221 14375 11243 14361
11217 14357 11208 14331 50 P
11208 14331 11181 14330 11185 14357 11161 14371 11139 14355 11114 14347

11087 14354 11063 14365 11047 14387 11071 14401 11098 14397 11123 14407
11099 14419 11105 14445 11132 14450 11153 14434 11156 14461 11130 14471
11103 14468 11078 14458 11051 14454 11025 14462 11001 14475 10976 14465
10950 14458 10923 14462 10897 14469 10923 14477 10942 14496 10962 14512
10981 14538 11003 14525 11001 14517 11004 14514 11033 14533 11023 14538
11003 14527 10983 14545 10983 14573 10976 14599 10991 14622 11000 14648
11021 14666 11030 14691 11020 14716 11047 14717 11071 14730 11074 14757
11067 14783 11084 14762 50 P
11084 14762 11108 14775 2 P
11108 14775 11134 14782 11156 14797 11183 14801 11207 14814 11230 14799
11257 14802 11240 14825 11214 14825 11189 14837 11173 14815 11146 14819
11121 14809 11118 14837 11102 14860 11109 14833 11084 14842 11070 14865
11062 14891 11072 14916 11081 14941 11091 14967 11104 14991 11124 15009
11116 15034 11121 15061 11144 15075 11151 15102 11156 15128 11152 15155
11166 15180 11140 15173 11127 15197 11126 15224 11146 15242 11150 15269
11173 15282 11186 15284 11196 15297 11220 15310 11226 15313 11224 15316
11195 15299 11184 15288 11163 15287 11173 15315 11176 15329 11188 15324
11197 15326 11203 15334 50 P
11203 15334 11201 15336 11196 15330 11188 15326 11176 15330 11172 15318
11159 15294 11138 15276 11116 15262 11088 15261 11069 15281 11043 15272
11020 15286 10998 15301 10971 15301 10953 15321 10929 15308 10913 15330
10887 15337 10866 15354 10855 15330 10859 15303 10842 15281 10825 15260
10812 15284 10823 15309 10804 15328 10796 15354 10786 15379 10759 15382
10732 15388 10709 15401 10687 15417 10693 15443 10666 15442 10660 15415
10656 15388 10650 15362 10643 15388 10638 15415 40 P
10638 15415 10626 15390 10600 15385 10577 15369 10551 15364 10524 15365
10497 15367 10472 15379 10452 15360 10425 15360 10403 15374 10400 15402
10393 15375 10383 15351 10369 15327 10342 15332 10331 15308 10307 15322
10281 15328 10254 15332 10246 15306 10222 15293 10247 15282 10226 15265
10201 15255 10175 15265 10150 15254 10131 15235 10106 15224 10081 15214
10056 15203 10034 15187 10015 15168 9991 15156 9973 15136 9949 15149
9946 15122 9926 15104 9899 15106 9879 15089 9856 15074 9845 15049
9834 15025 9811 15011 9792 14991 9781 14966 9806 14955 9782 14942
9755 14937 9763 14911 50 P
9763 14911 9737 14905 9716 14888 9696 14906 9704 14880 9678 14889
9690 14864 9688 14837 9665 14850 9653 14874 9628 14885 9607 14868
9595 14843 9571 14832 9597 14826 9620 14812 9608 14787 9581 14785
9554 14785 9535 14765 9509 14756 9482 14757 9458 14745 9431 14745
9419 14721 9402 14700 9381 14717 9381 14744 9369 14769 9391 14785
9364 14792 9372 14818 9398 14826 9425 14826 9451 14835 9464 14859
9474 14884 9494 14865 9521 14869 9543 14885 9568 14895 9594 14904
9607 14927 9587 14945 9564 14960 9581 14981 9562 15001 9589 15008
9612 15022 9627 15045 50 P

9627 15045 9649 15060 2 P
9649 15060 9675 15067 9699 15080 9672 15081 9646 15075 9619 15074
9595 15062 9567 15061 9540 15057 9517 15071 9507 15102 9494 15134
9517 15143 9529 15160 9533 15171 9536 15192 9533 15219 9517 15241
9495 15258 9506 15282 9517 15307 9540 15322 9563 15336 9581 15356
9608 15355 9632 15368 9650 15388 9670 15407 9644 15414 9667 15428
9683 15450 9699 15471 9714 15494 9728 15517 9748 15536 9751 15562
9766 15585 9783 15606 9786 15632 9804 15653 9815 15678 9803 15701
9782 15719 9760 15734 9754 15744 9762 15762 9757 15788 9760 15794
9784 15782 9801 15786 50 P
9801 15786 9806 15792 9800 15801 9778 15800 9777 15813 9756 15824
9731 15840 9731 15837 9754 15821 9766 15815 9766 15808 9772 15801
9778 15791 9801 15797 9803 15791 9784 15785 9768 15793 9759 15797
9754 15792 9754 15779 9742 15779 9726 15794 9705 15811 9682 15825
9665 15847 9638 15845 9624 15868 9633 15894 9622 15919 9634 15943
9639 15969 9629 15944 9609 15926 9608 15899 9598 15874 9576 15858
9549 15856 9522 15853 9503 15872 9481 15888 9454 15887 9430 15899
9406 15886 9384 15902 9357 15905 9342 15883 9322 15864 9313 15838
9286 15843 9267 15823 50 P
9267 15823 9255 15799 2 P
9255 15799 9278 15784 9260 15764 9233 15758 9215 15737 9204 15713
9185 15693 9164 15677 9150 15653 9126 15640 9121 15614 9100 15597
9122 15582 9099 15568 9086 15544 9087 15517 9071 15495 9045 15489
9018 15482 8998 15464 8974 15453 8950 15439 8924 15430 8899 15422
8874 15411 8851 15396 8835 15374 27 P
8835 15374 8809 15367 8787 15351 8762 15344 8735 15337 8710 15327
8690 15309 8691 15282 8666 15272 8645 15255 8625 15237 8612 15213
8605 15187 8597 15161 8574 15147 8583 15121 8578 15094 8582 15067
8600 15046 8575 15036 8553 15020 8535 14999 8522 14976 8503 14956
8482 14940 8458 14927 8433 14917 8406 14914 8384 14898 8357 14896
8336 14913 8309 14914 8284 14903 8264 14884 8242 14868 8222 14849
8200 14833 8174 14839 8151 14853 8132 14872 8126 14899 8111 14921
8087 14934 8061 14939 44 P
8061 14939 8034 14934 8008 14926 7985 14911 7967 14891 7948 14872
7958 14847 7964 14820 7939 14809 7923 14787 7906 14766 7893 14742
7881 14718 7872 14692 7867 14666 7867 14638 7869 14612 17 P
7869 14612 7879 14586 7890 14561 7902 14537 7915 14513 7928 14489
7945 14468 7964 14449 7984 14430 8010 14423 8033 14408 11 P
8033 14408 8052 14389 8074 14373 8098 14360 8125 14364 8151 14360
8179 14363 8206 14361 8231 14353 8258 14349 8279 14331 8301 14347
8325 14360 8351 14371 8372 14387 8396 14399 8402 14373 8407 14399
8422 14376 8418 14349 8424 14322 8414 14297 8432 14318 8459 14317
8464 14290 8482 14271 8490 14245 8498 14219 8492 14193 8480 14168

8505 14157 8523 14136 8510 14112 8494 14090 8476 14069 8470 14043
8468 14016 8469 13989 8472 13962 8467 13935 8484 13914 8502 13893
8505 13866 8532 13867 8555 13853 8575 13834 8603 13832 8629 13825
8656 13824 8683 13826 50 P
8683 13826 8710 13828 8735 13818 8763 13820 8790 13820 8816 13827
8835 13846 8856 13864 8875 13883 8882 13909 8866 13931 8879 13955
8900 13972 8925 13982 8951 13990 8974 14005 8996 14021 9015 14041
9034 14060 9051 14081 9055 14108 9030 14120 9056 14128 9082 14136
9102 14155 9128 14162 9155 14166 9178 14180 9204 14189 9225 14207
9251 14208 9279 14205 9306 14208 9318 14184 9342 14171 9359 14150
9386 14152 9401 14129 9406 14102 9426 14083 9452 14078 9479 14081
9504 14091 9531 14095 9507 14083 9525 14063 9542 14041 9560 14021
9584 14010 9612 14008 50 P
9612 14008 9639 14013 2 P
9639 14013 9664 14023 9683 14004 9700 14026 9727 14031 9754 14035
9769 14013 9767 14040 9787 14059 9810 14073 9831 14090 9849 14070
9840 14044 9854 14020 9878 14033 9901 14019 9927 14012 9955 14013
9981 14020 9999 14000 10023 14011 10047 14024 10074 14023 10055 14004
10037 13983 10032 13956 10040 13930 10056 13908 10034 13893 10010 13878
9994 13856 9994 13829 9974 13811 10001 13808 10028 13812 10029 13785
10025 13758 10012 13734 10023 13759 9996 13753 9983 13729 9961 13712
9939 13696 9912 13701 9885 13702 9880 13729 9908 13732 9883 13743
9867 13765 9883 13787 50 P
9883 13787 9876 13813 9858 13792 9860 13764 9837 13750 9817 13731
9808 13757 9794 13781 9767 13776 9778 13750 9753 13739 9729 13727
9704 13716 9691 13692 9667 13679 9642 13669 9643 13642 9616 13649
9590 13642 9604 13665 9625 13682 9598 13680 9579 13661 9565 13684
9569 13657 9553 13635 9530 13621 9504 13611 9479 13601 9452 13594
9429 13580 9410 13561 9385 13571 9386 13544 9358 13543 9345 13566
9319 13574 9296 13560 9279 13538 9305 13545 9320 13522 9345 13512
9347 13485 9329 13464 9327 13436 9303 13449 9282 13466 9257 13455
9243 13431 9251 13406 50 P
9251 13406 9225 13397 2 P
9225 13397 9219 13371 9235 13348 9240 13321 9244 13294 9222 13278
9198 13265 9171 13261 9144 13257 9124 13238 9098 13230 9071 13235
9082 13260 9092 13286 9098 13312 9082 13335 9063 13355 9060 13328
9038 13311 9012 13320 8987 13309 8978 13284 8986 13258 9009 13244
9035 13236 9020 13213 9047 13209 9074 13212 9061 13188 9040 13172
9025 13148 9033 13174 9010 13160 8985 13150 8965 13131 8954 13105
8957 13078 8950 13052 8927 13067 8915 13042 8901 13019 8890 12994
8873 13016 8852 12998 8840 12974 8830 12999 8808 12983 8780 12986
8787 12960 8778 12933 50 P
8778 12933 8770 12908 8755 12884 8751 12857 8732 12838 8708 12825

8691 12803 8683 12777 8676 12751 8672 12724 8661 12699 8634 12704
12:00 PM
8634 12704 8613 12685 8599 12662 8595 12635 8595 12608 8587 12582
8588 12554 8601 12530 8594 12504 8613 12524 8635 12508 8660 12520
8684 12508 8711 12506 8715 12479 8730 12456 8750 12438 8774 12423
8798 12411 8820 12394 8846 12387 8865 12367 8865 12339 8878 12364
8904 12358 8932 12353 8958 12344 8982 12333 9001 12313 9028 12306
9053 12295 9078 12284 9092 12260 9096 12233 9109 12209 9117 12182
9126 12157 9136 12131 9145 12105 9146 12078 9154 12051 9155 12024
9180 12013 9175 11986 9200 11976 9209 11950 9220 11925 9215 11898
9222 11871 9239 11849 50 P
9239 11849 9261 11833 9252 11806 9250 11779 9257 11752 9276 11733
9285 11707 9297 11682 9316 11662 9331 11639 9336 11612 9351 11589
9378 11584 9388 11558 9394 11531 9405 11505 9407 11478 9398 11451
9378 11433 9403 11424 9417 11400 9398 11379 9383 11356 9410 11353
9428 11332 9448 11312 9475 11311 9501 11302 9527 11292 9552 11280
9577 11269 9603 11258 9625 11242 9638 11217 9637 11189 9643 11163
9629 11139 9648 11120 9660 11095 9685 11085 9713 11085 9739 11075
9765 11066 9789 11053 9811 11035 9829 11015 9848 10995 9857 10968
9870 10944 9884 10920 50 P
9884 10920 9895 10895 2 P
9895 10895 9889 10867 9863 10858 9836 10865 9810 10873 9782 10876
9756 10887 9732 10898 9706 10909 9688 10931 9662 10939 9635 10945
9610 10957 9584 10948 9558 10937 9531 10931 9517 10908 9489 10911
9462 10908 9435 10916 9430 10943 9420 10969 9401 10989 9384 11011
9361 11027 9345 11050 9319 11039 9293 11047 9265 11048 9240 11059
9214 11049 9190 11035 9169 11017 9147 11001 9125 10983 9099 10974
9072 10975 9045 10981 9059 10957 9087 10957 9113 10965 9138 10978
9164 10984 9192 10989 9203 11015 9231 11017 9257 11008 9284 11005
9312 11008 9340 11005 50 P
9340 11005 9354 10982 9367 10957 9380 10933 9358 10916 9377 10896
9402 10884 9430 10882 9458 10883 9484 10874 9509 10864 9531 10846
9555 10832 9577 10815 9595 10794 9609 10770 9626 10749 17 P
9626 10749 9651 10736 9676 10724 9698 10708 9713 10684 9741 10684
9766 10670 9789 10655 9811 10639 9829 10617 9843 10594 9857 10570
9870 10545 9883 10520 9896 10496 9910 10472 9920 10446 9913 10419
9890 10403 9863 10399 9835 10396 9807 10393 9780 10388 9753 10381
9726 10373 9702 10360 9682 10341 9667 10318 9643 10303 9616 10299
30 P
9616 10299 9593 10283 9571 10266 9555 10243 9528 10239 9503 10226
9484 10206 9468 10182 9468 10155 9452 10131 9435 10109 9410 10097
9383 10094 9363 10115 9337 10123 9309 10123 9281 10121 9253 10120
9226 10114 9202 10101 9194 10074 9195 10046 9168 10056 9140 10055

9124 10032 9104 10013 9127 10028 9146 10049 9169 10031 9196 10034
9212 10056 9223 10082 9246 10098 9273 10101 9301 10099 9325 10085
9314 10059 9323 10032 9340 10011 9335 9984 9344 9957 9370 9949
9346 9962 9365 9982 9391 9972 9419 9972 9444 9982 9466 10000
9490 10014 9511 10032 50 P
9511 10032 9531 10052 9553 10069 9578 10082 9605 10086 9633 10087
9661 10084 9684 10069 9707 10052 9727 10032 9746 10012 9762 9989
9782 9969 9797 9946 9812 9922 9836 9908 9863 9903 9891 9899
9916 9888 9944 9881 9955 9871 9962 9865 9966 9864 9994 9865
10022 9865 10046 9879 10039 9852 10015 9836 9989 9826 9968 9808
9954 9785 9931 9767 9914 9745 9891 9730 9874 9707 9856 9686
9834 9668 9807 9661 9780 9656 9753 9662 9735 9640 9724 9615
9732 9588 9747 9611 9774 9619 9766 9593 9784 9613 9807 9629
9834 9637 9853 9658 50 P
9853 9658 9880 9658 2 P
9880 9658 9887 9631 9912 9645 9896 9668 9897 9696 9921 9709
9948 9715 9976 9722 9994 9702 9977 9680 9974 9652 9989 9629
9988 9601 9963 9591 9965 9563 9963 9535 9988 9523 9993 9495
9975 9474 9951 9459 9924 9463 9923 9491 9918 9519 9927 9546
9906 9565 9899 9537 9901 9509 9894 9482 9873 9463 9846 9456
9837 9482 9835 9510 9843 9537 9832 9511 9832 9483 9814 9505
9786 9510 9782 9482 9774 9455 9750 9441 9749 9413 9777 9416
9804 9424 9831 9418 9834 9390 9806 9388 9785 9369 9811 9380
9838 9386 9864 9374 50 P
9864 9374 9853 9348 9873 9329 9855 9308 9827 9311 9801 9321
9815 9297 9843 9291 9856 9267 9864 9240 9844 9220 9825 9200
9801 9185 9772 9184 9745 9184 9717 9184 9743 9176 9771 9169
9787 9147 9795 9120 9807 9094 9824 9071 9822 9043 9836 9019
9845 8992 9858 8967 9846 8942 9828 8964 9805 8979 9777 8983
9762 8959 9749 8934 9727 8916 9734 8889 9713 8870 9692 8889
9675 8912 9667 8938 9652 8963 9669 8985 9642 8993 9616 8983
9607 8956 9581 8945 9603 8928 9593 8901 9578 8878 9553 8865
9539 8840 9542 8812 50 P
9542 8812 9528 8787 2 P
9528 8787 9529 8774 9502 8777 9474 8778 9445 8777 9429 8795
9423 8795 9397 8802 9368 8799 9347 8817 9349 8789 9377 8793
9404 8788 9424 8768 9452 8763 9477 8749 9479 8721 9463 8697
9444 8676 9430 8652 9418 8627 9390 8624 9386 8596 9387 8567
9367 8547 9340 8540 9358 8518 9371 8494 9398 8483 9384 8459
9385 8430 9357 8426 9332 8439 9326 8411 9338 8386 9322 8363
9321 8334 9306 8311 9291 8334 9280 8308 9255 8323 9245 8349
42 P
9245 8349 9250 8321 9265 8297 9250 8273 9222 8265 9195 8269

9204 8242 9199 8214 9180 8193 9152 8193 9179 8186 9181 8158
9153 8163 9125 8165 9118 8138 9090 8143 9077 8118 9102 8103
9126 8089 9116 8063 9141 8051 9121 8032 9122 8003 9096 7992
9072 7977 9044 7975 9035 7948 9014 7929 8985 7929 8993 7957
9009 7980 9037 7982 9058 8001 9057 8029 9082 8042 9094 8068
9070 8083 9043 8086 9033 8060 9017 8036 9011 8008 8989 8026
8962 8033 8965 8061 8989 8075 8962 8082 8978 8105 8952 8093
8948 8065 8927 8084 50 P
8927 8084 8910 8107 8900 8133 8901 8105 8908 8078 8918 8051
8893 8064 8871 8082 8845 8093 8849 8064 8843 8037 8867 8021
8841 8011 8843 7983 8864 7964 8891 7976 8919 7975 8947 7970
8966 7949 8970 7921 8982 7895 8966 7871 8944 7888 8916 7895
8895 7914 8872 7897 8858 7872 8886 7880 8899 7855 8904 7827
8927 7809 8952 7822 8961 7849 8979 7871 8996 7848 9019 7833
9046 7844 9028 7822 9000 7818 8972 7813 8948 7798 8921 7790
8893 7784 8870 7768 8898 7772 8887 7746 8870 7723 8850 7703
8834 7680 8853 7659 50 P
8853 7659 8880 7650 2 P
8880 7650 8906 7638 8934 7635 8927 7608 8919 7580 8891 7579
8882 7551 8884 7523 8892 7495 8878 7471 8850 7466 8824 7454
8807 7432 8821 7407 8828 7379 8854 7392 8867 7367 8874 7339
8900 7329 8907 7302 8902 7274 8881 7254 8879 7283 8879 7311
8852 7320 8842 7293 8827 7269 8829 7297 8813 7320 8808 7348
8779 7352 8754 7365 8764 7391 8740 7407 8729 7381 8713 7357
8693 7337 8669 7322 8646 7305 8619 7297 8640 7277 8663 7261
8686 7245 8676 7218 8703 7228 8727 7245 8743 7221 8763 7201
8735 7194 8729 7166 50 P
8729 7166 8701 7161 8672 7160 8649 7176 8647 7204 8632 7180
8641 7153 8655 7128 8629 7140 8600 7144 8611 7171 8613 7199
8588 7188 8604 7211 8576 7217 8548 7215 8522 7204 8541 7183
8561 7163 8545 7140 8529 7117 8501 7109 8510 7082 8534 7067
8561 7060 8575 7085 8573 7056 8587 7031 8558 7033 8530 7031
8534 7002 8562 6996 8537 6981 8511 6991 8482 6996 8464 6974
8436 6966 8441 6994 8429 7020 8416 6994 8416 7022 8395 7042
8402 7015 8409 6987 8381 6981 8354 6992 8378 6976 8382 6948
8402 6928 8425 6945 50 P
8425 6945 8440 6921 2 P
8440 6921 8437 6892 8424 6867 8395 6870 8393 6841 8405 6815
8385 6795 8359 6781 8360 6810 8336 6825 8310 6837 8285 6851
8295 6877 8272 6859 8278 6832 8253 6818 8225 6812 8197 6820
8185 6794 8157 6801 8164 6829 8154 6856 8125 6855 8123 6827
8108 6801 8091 6825 8082 6798 8053 6796 8079 6784 8074 6755
8051 6738 8023 6746 7996 6756 7968 6755 7953 6751 7942 6767

7940 6751 7952 6740 7974 6722 7999 6709 8017 6704 8045 6707
8074 6710 8077 6681 8105 6681 8120 6657 8126 6629 8098 6622
8077 6603 8054 6585 50 P
8054 6585 8033 6605 8013 6585 8024 6558 8005 6536 8001 6507
7976 6493 7953 6475 7937 6452 7925 6425 7929 6454 7946 6477
7936 6504 7909 6495 7891 6473 7894 6445 7871 6428 7859 6401
7857 6373 7855 6344 7838 6321 7839 6350 7849 6377 7839 6404
7817 6423 7790 6431 7762 6437 7733 6440 7757 6423 7785 6417
7786 6388 7776 6361 7758 6338 7735 6354 7723 6355 7711 6339
7687 6355 7676 6329 7671 6300 7669 6271 7651 6248 7659 6276
7641 6299 7641 6270 7616 6254 45 P
7616 6254 7594 6237 7571 6218 7564 6246 7558 6275 7532 6285
7540 6258 7515 6271 7487 6263 7461 6252 7469 6229 7455 6203
7454 6177 7455 6166 7434 6146 7422 6133 7401 6109 7372 6110
7343 6110 7317 6122 7294 6139 7273 6160 7245 6161 7273 6156
7279 6127 7292 6102 7315 6084 7343 6077 7379 6046 7358 6035
7340 6012 7319 6031 7297 6049 7268 6047 7271 6018 7296 6032
7291 6004 7277 5978 7284 5950 7287 5921 7266 5902 7258 5874
7229 5876 7201 5868 7172 5874 7149 5891 7124 5876 7101 5858
7081 5837 7059 5818 50 P
7059 5818 7030 5819 7046 5843 7029 5867 7027 5838 7003 5822
6976 5811 6949 5802 6921 5794 6899 5775 6882 5798 6866 5822
6838 5830 6814 5846 6785 5844 6764 5825 6747 5801 6730 5778
6746 5754 6768 5774 6753 5749 6757 5720 6731 5733 6705 5746
6676 5741 6648 5748 6636 5774 6665 5776 6640 5789 6614 5803
6575 5801 6577 5810 6599 5818 6569 5829 6544 5814 6515 5814
6487 5808 6472 5783 6458 5757 6429 5753 6417 5779 6392 5794
6408 5770 6430 5752 6415 5728 6399 5704 6403 5675 6382 5696
6353 5699 6324 5698 50 P
6324 5698 6314 5726 2 P
6314 5726 6317 5754 6314 5783 6338 5801 6345 5829 6324 5810
6295 5815 6269 5803 6242 5793 6216 5781 6202 5756 6180 5737
6209 5736 6208 5707 6181 5697 6207 5683 6234 5673 6229 5644
6200 5637 6172 5631 6168 5660 6159 5687 6141 5710 6113 5717
6085 5711 6057 5704 6035 5684 27 P
6035 5684 6061 5671 6087 5659 6065 5639 6036 5638 6008 5633
6023 5609 6051 5617 6073 5636 6090 5612 6118 5618 6113 5590
6085 5582 6058 5592 6050 5564 6069 5543 6043 5555 6014 5553
5985 5561 5967 5583 5938 5582 5908 5581 5880 5586 5860 5606
5880 5627 5902 5645 5928 5659 5955 5669 28 P
5955 5669 5926 5672 5898 5680 5871 5669 5853 5647 5859 5676
5842 5699 5823 5721 5811 5748 5797 5772 5790 5801 5771 5824
5745 5813 5739 5841 5720 5863 5706 5888 5711 5917 5723 5943

5705 5920 5684 5940 5677 5969 5680 5997 5654 6012 5627 6023
5600 6032 5617 6009 5645 6004 5662 5981 5661 5952 5655 5924
5626 5919 5598 5914 5627 5913 5654 5902 5664 5875 5688 5858
5702 5833 5674 5826 5650 5842 5670 5821 5686 5797 5703 5773
5675 5764 5654 5784 5674 5762 5689 5738 5666 5722 5681 5697
5681 5668 5667 5642 50 P
5667 5642 5659 5615 5683 5598 5689 5570 5675 5544 5665 5517
5656 5501 7 P
5649 5501 5645 5520 5644 5501 3 P
5596 5501 5592 5505 5573 5527 5556 5550 5547 5578 5529 5600
5537 5572 5551 5535 5544 5529 5540 5522 5539 5516 5531 5501

12:00 PM

5429 5501 5430 5523 5412 5546 5410 5575 5410 5604 5415 5633
5415 5662 5386 5663 5380 5691 5352 5696 5369 5672 5393 5656
5404 5629 5404 5600 5402 5571 5406 5542 5407 5513 5392 5501
18 P
2967 5501 2968 5507 2986 5529 2968 5552 2939 5553 2917 5534
2926 5506 2928 5501 8 P
13001 8169 12995 8163 12990 8169 12985 8172 12982 8166 12982 8159
12985 8152 12988 8146 12991 8140 12995 8134 13001 8129 13001 8128

12:00 PM

13001 8081 13000 8081 12994 8078 12987 8075 12983 8069 12981 8063
12978 8056 12978 8049 12973 8044 12976 8038 12981 8033 12976 8037
12969 8040 12962 8040 12959 8033 12960 8026 12965 8021 12968 8014
12973 8010 12978 8005 12979 7998 12979 7991 12978 7983 12976 7977
12976 7969 12981 7965 12988 7963 12993 7968 12998 7972 13001 7972
30 P
11825 7078 11828 7072 11833 7067 11839 7063 11846 7061 11847 7068
11849 7061 11855 7056 11861 7059 11868 7062 11875 7063 11882 7066
11888 7068 11895 7069 11902 7070 11910 7071 11917 7070 11924 7072
11931 7074 11936 7069 11932 7075 11927 7080 11923 7086 11918 7091
11910 7091 11904 7093 11897 7096 11894 7102 11887 7104 11880 7103
11873 7105 11866 7107 11863 7113 11864 7120 11859 7125 11852 7128
11848 7134 11847 7141 11844 7147 11838 7152 11833 7157 11829 7162
11825 7169 11818 7168 11822 7161 11826 7156 11830 7149 11834 7143
11838 7138 11841 7131 50 P
11841 7131 11835 7128 11834 7121 11836 7114 11833 7108 11827 7105
11824 7098 11831 7095 11836 7090 11840 7084 11836 7078 11825 7078

12:00 PM

11224 6841 11230 6836 11237 6839 11242 6843 11249 6844 11254 6840
11259 6834 11263 6828 11262 6821 11259 6814 11252 6812 11249 6806
11254 6801 11261 6800 11267 6797 11273 6792 11279 6788 11286 6787
11293 6790 11297 6795 11298 6802 11305 6801 11311 6797 11318 6800

11322 6806 11323 6813 11322 6820 11322 6828 11323 6835 11323 6842
11319 6848 11322 6854 11318 6861 11318 6868 11322 6873 11327 6879
11332 6884 11332 6891 11337 6896 11342 6902 11345 6908 11342 6915
11346 6921 11351 6926 11356 6931 11358 6938 11357 6945 11357 6953
11350 6952 11343 6954 50 P
11343 6954 11343 6946 11341 6939 11338 6933 11333 6927 11331 6921
11336 6916 11332 6910 11331 6903 11329 6896 11322 6897 11316 6895
11312 6889 11305 6886 11305 6879 11302 6872 11304 6865 11299 6861
11291 6861 11284 6860 11279 6856 11272 6857 11265 6855 11262 6861
11259 6868 11252 6870 11251 6863 11249 6856 11245 6850 11240 6855
11233 6854 11228 6848 11224 6841 33 P
11017 6755 11021 6749 11026 6743 11032 6740 11040 6740 11047 6739
11049 6746 11055 6750 11060 6745 11066 6742 11073 6741 11080 6740
11087 6740 11094 6738 11101 6739 11109 6740 11115 6738 11122 6734
11128 6732 11135 6735 11134 6743 11130 6749 11124 6752 11130 6749
11137 6747 11144 6744 11144 6751 11142 6758 11144 6764 11148 6770
11155 6768 11156 6775 11154 6782 11158 6788 11160 6795 11153 6798
11148 6802 11142 6806 11135 6808 11128 6809 11126 6816 11120 6813
11114 6816 11107 6815 11100 6813 11095 6807 11091 6813 11088 6820
11084 6825 11078 6829 50 P
11078 6829 11071 6832 11065 6828 11071 6823 11073 6816 11077 6811
11070 6813 11071 6820 11064 6818 11060 6812 11056 6806 11059 6800
11058 6793 11062 6787 11066 6781 11067 6774 11064 6780 11062 6774
11061 6767 11064 6761 11063 6753 11062 6761 11057 6765 11051 6770
11050 6777 11045 6772 11038 6776 11039 6769 11042 6762 11037 6766
11030 6770 11026 6764 11029 6757 11017 6755 34 P
9618 7079 9615 7066 9607 7054 9598 7042 9592 7030 9585 7017
9582 7003 9572 6992 9564 6981 9571 6968 9567 6954 9562 6940
9553 6929 9542 6920 9539 6906 9529 6896 9539 6886 9525 6887
9511 6885 9497 6879 9489 6868 9496 6855 9481 6852 9468 6846
9464 6833 9459 6819 9455 6805 9451 6791 9444 6779 9437 6766
9427 6756 9419 6745 9409 6734 9403 6721 9397 6708 9391 6695
9386 6681 9384 6667 9375 6656 9371 6642 9363 6630 9356 6617
9368 6609 9354 6609 9347 6597 9339 6585 9332 6572 9329 6558
9324 6545 9315 6534 50 P
9315 6534 9305 6523 9296 6513 9289 6500 9280 6489 9273 6476
9273 6461 9267 6449 9261 6435 9256 6422 9247 6411 9256 6400
9270 6398 9284 6396 9299 6399 9287 6390 9273 6387 9259 6387
9245 6389 9231 6388 9222 6376 9213 6365 9212 6351 9210 6337
9206 6323 9195 6314 9188 6301 9182 6288 9181 6274 9180 6259
9186 6247 9186 6232 9192 6219 9181 6211 9190 6201 9187 6186
9198 6177 9188 6167 9192 6153 9196 6140 9194 6125 9192 6111
9199 6098 9185 6101 9183 6087 9175 6074 9177 6060 9166 6051

9179 6046 9164 6044 50 P
9164 6044 9163 6030 2 P
9163 6030 9164 6015 9155 6005 9169 6001 9173 5988 9169 5974
9157 5964 9144 5961 9139 5975 9135 5961 9146 5951 9160 5954
9152 5942 9163 5933 9175 5925 9189 5929 9203 5933 9217 5938
9220 5923 9207 5917 9193 5914 9199 5901 9207 5889 9211 5875
9211 5860 9216 5847 9221 5833 9235 5829 9226 5818 9231 5805
9237 5791 9242 5778 9249 5765 9255 5751 9258 5737 9258 5723
9255 5709 9262 5697 9276 5690 9279 5676 9291 5667 9302 5658
9289 5663 9279 5674 9271 5686 9277 5673 9286 5662 9296 5651
9306 5641 9316 5630 50 P
9316 5630 9328 5622 9340 5614 9347 5627 9352 5614 9362 5603
9373 5593 9386 5586 9398 5579 9411 5573 9424 5566 9436 5558
9446 5548 9454 5535 9465 5526 9472 5513 9475 5501 17 P
9608 5501 9607 5512 9606 5527 9608 5541 9614 5555 9616 5569
9622 5583 9627 5595 9632 5609 9634 5624 9638 5638 9643 5652
9646 5666 9652 5679 9662 5690 9664 5704 9667 5718 9673 5732
9684 5740 9695 5750 9707 5758 9720 5765 9730 5775 9740 5785
9750 5796 9754 5810 9758 5824 9753 5838 9759 5850 9772 5856
9784 5865 9790 5878 9797 5891 9801 5905 9811 5915 9819 5927
9824 5941 9829 5954 9832 5969 9837 5982 9848 5992 9857 6003
9859 6018 9862 6032 9857 6045 9858 6059 9865 6073 9868 6087
9872 6101 9877 6114 50 P
9877 6114 9889 6122 9891 6137 9892 6151 9894 6165 9898 6179
9903 6193 9906 6207 9911 6220 9905 6234 9906 6248 9910 6262
9915 6276 9917 6290 9917 6304 9922 6318 9923 6332 9924 6346
9927 6361 9933 6374 9934 6388 9934 6403 9939 6416 9944 6430
9947 6444 9949 6458 9949 6473 9949 6487 9951 6501 9959 6513
9967 6524 9960 6537 9952 6549 9950 6563 9955 6577 9965 6587
9973 6598 9972 6613 9970 6627 9977 6639 9988 6648 9996 6659
10008 6668 10013 6681 10021 6694 10026 6707 10025 6721 10030 6735
10032 6749 49 P
10032 6749 10034 6763 10039 6777 10046 6789 10057 6799 10055 6813
10060 6826 10072 6834 10070 6848 10071 6862 10068 6876 10058 6887
10053 6900 10050 6914 10048 6928 10043 6942 10039 6956 10037 6970
10037 6984 10038 6999 10041 7013 10046 7026 10054 7038 10064 7048
10068 7062 10080 7071 10088 7083 10101 7090 10113 7096 10124 7105
10116 7117 10102 7116 10090 7123 10088 7137 10080 7149 10083 7163
10073 7172 10058 7171 10044 7173 10030 7169 10019 7178 10006 7184
9993 7178 9984 7188 9970 7194 9959 7203 9964 7217 9950 7212
9940 7221 9931 7232 50 P
9931 7232 9927 7246 9915 7255 9903 7261 9889 7260 9875 7256
9861 7252 9849 7245 9840 7234 9832 7222 9824 7210 9817 7197

9828 7188 9815 7184 9805 7173 9792 7167 9779 7160 9766 7158
9752 7154 9737 7156 9724 7150 9711 7145 9698 7138 9687 7130
9673 7126 9659 7121 9648 7112 9636 7106 9625 7096 9618 7079
30 P
12969 11655 12976 11654 12983 11654 12987 11648 12994 11647 12998 11653
13001 11654 7 P
13001 11746 12999 11747 12994 11752 12988 11754 12983 11749 12981 11742
12982 11735 12986 11730 12983 11723 12985 11717 12990 11712 12986 11706
12989 11700 12992 11693 12998 11690 12999 11683 12992 11683 12997 11678
12990 11681 12993 11674 12987 11670 12987 11663 12993 11666 12998 11669
12994 11664 12994 11657 12988 11659 12982 11663 12979 11669 12975 11675
12973 11668 12969 11655 32 P
11806 11895 11811 11890 11815 11884 11819 11879 11825 11875 11831 11872
11838 11871 11844 11867 11847 11861 11852 11857 11858 11852 11864 11848
11870 11849 11871 11855 11872 11862 11875 11868 11880 11873 11886 11876
11893 11875 11900 11875 11906 11874 11913 11877 11919 11880 11926 11881
11932 11878 11938 11880 11945 11882 11949 11876 11956 11876 11962 11879
11969 11880 11974 11885 11980 11886 11987 11887 11994 11887 12000 11889
12006 11887 12013 11888 12018 11894 12023 11898 12027 11903 12033 11907
12040 11909 12047 11909 12053 11910 12060 11913 12066 11916 12072 11917
12077 11922 12082 11927 50 P
12082 11927 12086 11933 12093 11933 12100 11934 12106 11936 12113 11938
12117 11944 12115 11951 12121 11955 12124 11961 12126 11968 12127 11975
12130 11981 12136 11984 12141 11989 12140 11996 12142 12003 12143 12010
12148 12005 12147 12012 12145 12019 12148 12025 12155 12024 12150 12029
12145 12024 12144 12031 12138 12033 12134 12039 12137 12045 12135 12052
12132 12058 12126 12061 12119 12061 12113 12058 12107 12062 12100 12065
12096 12069 12092 12075 12085 12073 12078 12074 12071 12074 12064 12074
12058 12071 12052 12071 12045 12068 12039 12066 12032 12063 12026 12062
12019 12059 12013 12061 50 P
12013 12061 12007 12057 2 P
12007 12057 12001 12053 11995 12051 11988 12049 11982 12045 11975 12044
11969 12041 11963 12038 11958 12034 11951 12036 11944 12037 11940 12032
11933 12030 11929 12026 11923 12022 11916 12021 11910 12017 11904 12013
11898 12015 11893 12011 11887 12009 11881 12004 11875 12001 11868 12001
11865 11995 11861 11989 11855 11986 11850 11981 11845 11976 11845 11970
11841 11964 11837 11958 11831 11954 11826 11950 11823 11944 11818 11939
11812 11935 11811 11928 11806 11924 11806 11917 11808 11911 11807 11904
11806 11895 43 P
12978 8331 12982 8326 12989 8326 12996 8328 13001 8328 5 P
13001 8344 12995 8342 12988 8342 12981 8342 12978 8331 5 P
12715 8218 12721 8220 12728 8221 12735 8224 12733 8231 12726 8234
12720 8230 12715 8218 8 P

12476 8068 12481 8063 12488 8061 12495 8061 12502 8060 12507 8055
12510 8049 12514 8043 12520 8039 12526 8036 12534 8035 12531 8042
12528 8049 12528 8056 12532 8061 12535 8068 12528 8071 12525 8078
12518 8080 12515 8086 12509 8090 12501 8089 12495 8085 12491 8079
12484 8076 12476 8068 26 P
12692 8096 12696 8092 12698 8088 12701 8090 12705 8093 12701 8094
12699 8098 12699 8103 12695 8101 12692 8096 10 P
12533 8060 12535 8061 12537 8061 12539 8062 12539 8064 12538 8067
12537 8065 12535 8063 12533 8060 9 P
12542 8063 12544 8063 12545 8065 12544 8067 12542 8067 12542 8063
6:00 PM
12547 8060 12548 8062 12550 8063 12552 8064 12554 8064 12556 8066
12558 8067 12559 8069 12561 8070 12563 8070 12566 8071 12568 8071
12569 8074 12571 8074 12573 8075 12571 8076 12569 8075 12567 8074
12565 8072 12563 8072 12561 8071 12559 8071 12557 8069 12555 8068
12554 8066 12552 8065 12549 8065 12548 8063 12547 8060 29 P
12807 8003 12809 7999 12812 7996 12812 7991 12812 7986 12814 7983
12815 7987 12819 7989 12820 7993 12820 7998 12819 8003 12821 8006
12824 8009 12823 8014 12823 8018 12822 8023 12820 8027 12816 8029
12812 8027 12812 8023 12811 8019 12809 8014 12810 8010 12811 8006
12807 8003 25 P
12775 8026 12780 8026 12784 8025 12787 8022 12790 8026 12792 8029
12792 8034 12795 8031 12799 8029 12799 8034 12797 8038 12792 8039
12788 8039 12787 8035 12783 8035 12781 8031 12775 8026 17 P
12768 8008 12772 8006 12774 8003 12779 8004 12783 8006 12780 8011
12778 8014 12774 8016 12771 8013 12768 8008 10 P
12778 8000 12781 7996 12785 7998 12785 8002 12778 8000 5 P
12782 7991 12785 7987 12786 7992 12786 7996 12782 7991 5 P
12754 7992 12755 7988 12759 7986 12759 7991 12760 7995 12756 7997
12754 7992 7 P
12755 8014 12759 8014 12760 8018 12756 8019 12755 8014 5 P
12856 8020 12860 8021 12861 8025 12863 8029 12863 8034 12859 8030
12858 8026 12856 8020 8 P
12832 12963 12826 12961 12822 12955 12822 12948 12821 12941 12814 12942
12808 12939 12802 12942 12800 12935 12795 12931 12799 12925 12803 12919
12807 12913 12812 12909 12817 12904 12810 12905 12803 12904 12797 12907
12793 12902 12786 12903 12780 12906 12774 12911 12768 12909 12765 12903
12759 12901 12756 12895 12752 12889 12750 12883 12751 12876 12754 12870
12757 12863 12763 12861 12766 12855 12770 12849 12777 12852 12776 12859
12782 12861 12788 12864 12792 12869 12795 12876 12802 12874 12803 12867
12806 12861 12804 12854 12804 12848 12799 12844 12792 12842 12786 12839
12787 12833 12793 12837 50 P
12793 12837 12793 12830 12799 12831 12805 12834 12812 12832 12806 12828

12805 12821 12807 12814 12814 12813 12810 12807 12809 12800 12815 12798
12821 12796 12827 12800 12834 12802 12831 12808 12826 12813 12832 12815
12839 12816 12846 12818 12852 12819 12846 12823 12840 12821 12836 12826
12831 12832 12834 12838 12840 12838 12845 12843 12842 12849 12842 12856
12848 12859 12851 12853 12856 12859 12862 12858 12866 12853 12873 12850
12873 12857 12868 12862 12864 12867 12865 12874 12859 12873 12856 12878
12863 12882 12869 12880 12866 12886 12862 12892 12868 12893 12868 12899
12871 12906 12865 12906 50 P
12865 12906 12859 12902 2 P
12859 12902 12858 12895 12851 12896 12848 12902 12852 12908 12855 12913
12859 12919 12856 12926 12854 12932 12853 12939 12851 12946 12845 12949
12842 12955 12840 12962 12843 12968 12849 12970 12850 12977 12846 12983
12839 12985 12832 12986 12829 12980 12828 12973 12832 12963 23 P
12542 12834 12535 12831 12532 12825 12526 12821 12522 12817 12517 12821
12515 12827 12508 12830 12501 12830 12502 12837 12497 12842 12494 12848
12495 12854 12501 12859 12503 12865 12504 12871 12503 12878 12499 12884
12494 12888 12487 12889 12480 12886 12475 12881 12471 12876 12471 12869
12475 12864 12469 12861 12462 12860 12462 12853 12462 12846 12458 12840
12456 12834 12457 12827 12460 12821 12465 12816 12469 12811 12471 12804
12476 12799 12479 12793 12476 12787 12474 12781 12481 12778 12483 12772
12489 12768 12495 12767 12502 12768 12508 12771 12510 12778 12506 12783
12504 12790 12510 12793 50 P
12510 12793 12516 12798 12522 12799 12524 12792 12523 12785 12525 12778
12527 12772 12529 12766 12535 12764 12542 12765 12548 12769 12555 12769
12559 12764 12566 12767 12573 12768 12568 12773 12565 12779 12569 12784
12573 12790 12571 12797 12570 12803 12569 12810 12568 12817 12571 12823
12571 12830 12566 12833 12559 12831 12554 12836 12542 12834 29 P
12066 12412 12070 12406 12076 12408 12082 12405 12089 12404 12096 12402
12100 12408 12101 12415 12100 12422 12095 12426 12093 12433 12088 12436
12081 12438 12076 12435 12072 12429 12070 12422 12066 12412 17 P
12233 12451 12237 12454 12241 12453 12245 12454 12250 12454 12250 12458
12252 12462 12250 12466 12249 12470 12249 12475 12244 12474 12241 12472
12241 12468 12237 12467 12236 12462 12235 12458 12233 12451 17 P
12159 12586 12162 12583 12166 12584 12169 12580 12166 12578 12164 12573
12169 12575 12171 12571 12175 12569 12179 12566 12183 12564 12186 12567
12191 12566 12193 12570 12198 12570 12202 12570 12206 12570 12211 12570
12215 12571 12218 12575 12219 12579 12219 12583 12220 12587 12221 12591
12223 12596 12222 12599 12219 12603 12216 12606 12212 12605 12208 12603
12204 12602 12199 12603 12195 12601 12192 12598 12190 12594 12185 12595
12183 12591 12179 12593 12175 12594 12173 12598 12169 12599 12169 12594
12167 12591 12164 12587 12159 12586 45 P
12321 12593 12325 12594 12329 12595 12334 12596 12336 12592 12331 12592
12327 12592 12329 12589 12332 12586 12337 12588 12341 12586 12341 12582

12339 12577 12343 12575 12346 12578 12350 12580 12353 12583 12358 12584
12361 12587 12362 12591 12361 12595 12359 12599 12354 12600 12352 12604
12348 12606 12345 12609 12344 12605 12340 12604 12336 12601 12333 12604
12329 12605 12325 12607 12321 12606 12324 12603 12322 12599 12321 12593
36 P
12426 12655 12429 12653 12433 12651 12437 12649 12440 12650 12440 12646
12444 12643 12445 12639 12449 12637 12453 12640 12457 12637 12461 12636
12462 12632 12466 12632 12470 12632 12474 12636 12472 12640 12469 12643
12465 12643 12460 12644 12465 12646 12465 12650 12461 12653 12458 12656
12456 12660 12454 12664 12450 12665 12446 12667 12441 12665 12438 12663
12435 12660 12432 12656 12426 12655 33 P
12473 12602 12477 12602 12474 12598 12474 12594 12479 12594 12482 12591
12484 12595 12484 12599 12483 12604 12484 12608 12484 12612 12480 12614
12481 12618 12477 12619 12475 12614 12476 12610 12476 12606 12473 12602
18 P
11960 12515 11962 12511 11962 12507 11963 12502 11965 12498 11964 12494
11967 12491 11966 12486 11970 12488 11974 12486 11977 12484 11981 12482
11984 12485 11988 12487 11991 12484 11994 12481 11991 12478 11987 12478
11987 12473 11990 12471 11994 12472 11998 12473 12003 12472 12006 12470
12010 12469 12010 12473 12008 12477 12012 12476 12014 12472 12019 12472
12023 12472 12027 12471 12025 12475 12021 12477 12023 12480 12025 12485
12020 12486 12016 12486 12012 12486 12013 12490 12011 12494 12007 12495
12006 12500 12005 12504 12004 12508 12004 12512 12003 12517 12000 12520
11996 12522 11992 12525 50 P
11992 12525 11989 12527 11985 12529 11981 12530 11976 12528 11974 12525
11969 12526 11965 12523 11963 12520 11960 12515 10 P
11710 12509 11715 12504 11720 12499 11723 12493 11729 12490 11728 12497
11725 12503 11728 12509 11729 12502 11735 12498 11742 12497 11738 12491
11734 12485 11737 12479 11741 12484 11745 12478 11752 12478 11759 12478
11765 12482 11771 12484 11766 12489 11773 12488 11779 12490 11783 12495
11790 12497 11790 12503 11796 12506 11803 12507 11803 12513 11810 12514
11811 12521 11817 12516 11818 12509 11823 12514 11822 12521 11828 12524
11830 12530 11833 12536 11830 12542 11835 12548 11830 12553 11834 12558
11835 12565 11836 12571 11830 12575 11826 12581 11821 12585 11816 12589
11809 12586 11808 12580 50 P
11808 12580 11802 12584 11800 12590 11801 12597 11798 12603 11792 12606
11785 12608 11778 12608 11772 12610 11770 12603 11773 12597 11780 12596
11786 12598 11784 12592 11786 12585 11782 12579 11778 12575 11775 12568
11768 12570 11768 12576 11766 12583 11761 12577 11765 12571 11758 12568
11761 12562 11763 12555 11767 12550 11773 12552 11771 12546 11764 12547
11767 12541 11766 12535 11765 12528 11763 12521 11760 12515 11760 12522
11759 12529 11760 12536 11763 12542 11759 12548 11759 12555 11752 12556
11751 12563 11746 12560 11745 12553 11748 12547 11745 12540 11741 12546

11734 12545 11730 12539 50 P
11730 12539 11724 12537 2 P
11724 12537 11722 12530 11727 12525 11720 12527 11715 12523 11713 12516
11710 12509 7 P
11913 12377 11916 12374 11918 12370 11922 12369 11926 12369 11930 12371
11934 12373 11938 12376 11941 12379 11941 12383 11946 12384 11947 12388
11951 12390 11951 12394 11954 12391 11956 12387 11958 12392 11961 12390
11966 12391 11965 12395 11961 12395 11956 12395 11953 12398 11948 12398
11944 12396 11940 12397 11935 12395 11934 12400 11930 12401 11926 12401
11922 12398 11919 12395 11916 12392 11913 12389 11915 12385 11913 12377
36 P
11930 12408 11934 12407 11937 12404 11940 12400 11944 12400 11948 12399
11953 12400 11949 12402 11948 12407 11952 12409 11954 12405 11958 12405
11960 12409 11958 12412 11961 12415 11965 12417 11961 12420 11959 12424
11956 12427 11952 12429 11948 12428 11944 12429 11940 12427 11937 12430
11933 12433 11933 12428 11937 12424 11932 12423 11932 12419 11936 12420
11934 12416 11933 12412 11930 12408 33 P
11969 12387 11971 12384 11974 12380 11974 12376 11978 12378 11981 12375
11982 12371 11986 12369 11989 12373 11989 12377 11990 12381 11989 12385
11994 12387 11993 12392 11989 12390 11988 12394 11986 12398 11982 12399
11982 12403 11987 12405 11991 12405 11992 12409 11989 12412 11985 12414
11981 12415 11977 12417 11974 12415 11974 12411 11969 12411 11970 12406
11975 12407 11976 12403 11977 12399 11974 12395 11973 12391 11969 12387
36 P
12014 12495 12018 12495 12022 12493 12026 12493 12030 12491 12033 12487
12036 12491 12039 12494 12043 12494 12048 12494 12052 12492 12054 12488
12053 12484 12056 12480 12060 12483 12062 12486 12065 12489 12064 12493
12069 12492 12069 12488 12074 12488 12075 12492 12073 12497 12074 12501
12078 12500 12079 12505 12078 12508 12075 12506 12070 12506 12068 12509
12063 12509 12058 12509 12055 12506 12053 12510 12049 12513 12046 12510
12043 12506 12044 12502 12040 12499 12036 12498 12033 12501 12029 12504
12027 12500 12026 12496 12021 12497 12017 12498 12014 12495 47 P
12010 12520 12013 12522 12018 12522 12022 12522 12020 12519 12019 12514
12019 12510 12023 12508 12025 12511 12026 12515 12028 12519 12029 12523
12034 12525 12038 12525 12042 12526 12042 12530 12045 12534 12049 12533
12053 12531 12057 12532 12062 12531 12061 12535 12058 12539 12054 12541
12054 12545 12050 12543 12046 12544 12049 12547 12050 12551 12047 12554
12045 12557 12040 12557 12036 12555 12033 12553 12029 12551 12024 12551
12022 12547 12019 12544 12018 12540 12015 12537 12017 12533 12014 12529
12012 12525 12010 12520 44 P
12059 12540 12063 12537 12065 12534 12062 12530 12062 12526 12063 12521
12066 12525 12068 12529 12071 12530 12071 12526 12071 12522 12072 12518
12077 12517 12081 12514 12085 12515 12089 12517 12090 12521 12094 12522

12098 12522 12100 12527 12097 12530 12097 12534 12097 12539 12099 12543
12097 12547 12096 12543 12093 12539 12092 12535 12088 12536 12084 12535
12081 12539 12076 12538 12072 12539 12068 12542 12067 12537 12064 12541
12059 12540 37 P
12124 12521 12120 12522 12122 12519 12126 12516 12130 12518 12134 12517
12138 12514 12135 12512 12139 12511 12142 12508 12143 12512 12147 12513
12150 12510 12147 12507 12143 12505 12144 12500 12145 12496 12147 12500
12149 12504 12152 12507 12155 12510 12159 12509 12161 12513 12156 12513
12155 12518 12159 12516 12164 12516 12166 12513 12171 12514 12169 12517
12165 12520 12165 12525 12161 12525 12156 12526 12153 12523 12149 12521
12145 12519 12146 12523 12144 12528 12140 12529 12138 12533 12135 12536
12136 12532 12132 12532 12128 12530 12131 12527 12128 12523 12124 12521
48 P
12129 12575 12132 12572 12135 12569 12136 12564 12137 12560 12139 12556
12143 12554 12144 12558 12143 12562 12147 12561 12147 12556 12148 12552
12150 12548 12151 12551 12153 12556 12154 12560 12153 12564 12151 12568
12149 12572 12147 12576 12143 12578 12139 12578 12135 12577 12129 12575
24 P
12895 12972 12899 12969 12903 12969 12904 12974 12903 12978 12902 12982
12903 12987 12905 12991 12906 12995 12906 12999 12904 13003 12900 13003
12897 13006 12894 13009 12891 13005 12887 13004 12884 13001 12881 12998
12885 12997 12888 12994 12888 12989 12891 12986 12887 12984 12891 12982
12895 12981 12896 12976 12895 12972 27 P
12750 12834 12754 12831 12756 12828 12755 12824 12756 12820 12760 12820
12764 12819 12768 12821 12771 12824 12774 12827 12774 12831 12771 12834
12767 12834 12764 12837 12764 12842 12761 12845 12762 12849 12758 12847
12759 12851 12755 12851 12752 12848 12753 12844 12757 12845 12761 12842
12757 12844 12756 12839 12754 12836 12754 12840 12751 12844 12751 12839
12750 12834 31 P
12762 12922 12765 12918 12768 12915 12772 12916 12769 12919 12772 12921
12769 12925 12767 12928 12770 12932 12773 12935 12770 12939 12766 12940
12762 12938 12761 12934 12764 12931 12764 12926 12762 12922 17 P
12615 12847 12620 12846 12624 12845 12623 12840 12627 12839 12631 12842
12632 12847 12634 12850 12639 12850 12643 12850 12642 12855 12638 12856
12634 12857 12631 12859 12626 12858 12622 12856 12618 12855 12615 12847
18 P
12632 12835 12636 12835 12637 12831 12641 12831 12645 12828 12649 12827
12653 12826 12654 12830 12650 12833 12655 12834 12659 12835 12662 12838
12659 12841 12655 12842 12653 12846 12649 12849 12645 12850 12641 12849
12638 12846 12635 12842 12638 12839 12632 12835 22 P
12655 12738 12658 12735 12663 12733 12662 12729 12664 12725 12667 12728
12671 12730 12673 12734 12677 12733 12682 12733 12686 12733 12685 12738
12689 12741 12686 12745 12683 12743 12680 12739 12679 12735 12676 12738

12678 12741 12675 12744 12671 12745 12668 12747 12663 12749 12659 12748
12658 12743 12655 12738 26 P
12537 12876 12534 12872 12533 12868 12531 12864 12528 12860 12527 12856
12524 12853 12522 12849 12524 12845 12525 12841 12527 12837 12531 12835
12535 12834 12540 12834 12544 12835 12548 12837 12549 12841 12553 12842
12557 12840 12561 12838 12566 12839 12569 12838 12574 12838 12577 12835
12581 12835 12580 12840 12576 12842 12572 12844 12569 12847 12568 12851
12570 12855 12569 12860 12565 12862 12561 12863 12557 12866 12559 12870
12555 12871 12552 12868 12550 12863 12549 12859 12549 12855 12547 12851
12544 12854 12544 12858 12545 12862 12545 12867 12548 12870 12549 12874
12551 12878 12550 12883 50 P
12550 12883 12546 12882 12542 12880 12537 12876 4 P
12397 12665 12400 12669 12402 12666 12405 12663 12409 12662 12412 12665
12416 12667 12417 12671 12420 12668 12424 12669 12428 12671 12425 12674
12421 12677 12418 12674 12414 12674 12411 12678 12407 12679 12404 12677
12400 12675 12397 12671 12397 12665 21 P
12309 12631 12313 12629 12317 12629 12318 12625 12322 12622 12317 12621
12315 12618 12317 12614 12320 12611 12324 12610 12327 12613 12331 12614
12335 12616 12339 12615 12342 12619 12341 12623 12338 12626 12334 12628
12330 12628 12325 12628 12329 12630 12327 12634 12322 12635 12325 12638
12328 12642 12327 12646 12323 12646 12318 12647 12316 12643 12313 12640
12312 12635 12309 12631 32 P
12126 12510 12121 12509 12117 12510 12113 12509 12110 12506 12107 12502
12107 12498 12108 12494 12112 12493 12114 12489 12118 12491 12122 12492
12125 12495 12129 12497 12130 12500 12134 12502 12132 12506 12126 12510
18 P
12268 12367 12270 12364 12271 12359 12275 12357 12276 12353 12279 12349
12282 12346 12286 12347 12291 12346 12293 12349 12289 12349 12285 12351
12284 12355 12280 12358 12278 12362 12277 12366 12278 12370 12277 12374
12273 12371 12268 12367 20 P
12281 12371 12284 12368 12286 12364 12289 12362 12294 12360 12295 12365
12291 12367 12292 12371 12287 12372 12281 12371 10 P
12106 12309 12108 12305 12112 12305 12117 12306 12119 12309 12117 12313
12113 12315 12109 12315 12106 12309 9 P
11833 12263 11838 12262 11840 12258 11842 12254 11846 12254 11844 12258
11846 12262 11850 12263 11851 12267 11847 12269 11843 12266 11839 12267
11833 12263 13 P
11824 12269 11828 12266 11831 12264 11833 12268 11830 12272 11825 12273
11824 12269 7 P
11903 12398 11906 12397 11908 12400 11910 12402 11913 12402 11913 12406
11915 12408 11913 12411 11911 12413 11909 12410 11907 12407 11906 12404
11905 12401 11903 12398 14 P
11948 12433 11952 12433 11954 12435 11957 12433 11959 12436 11962 12436

11962 12439 11960 12442 11957 12443 11954 12442 11951 12440 11948 12439
11948 12433 13 P
11952 12359 11955 12359 11958 12359 11961 12358 11961 12362 11959 12364
11958 12367 11954 12368 11952 12366 11954 12364 11952 12359 11 P
12021 12414 12024 12413 12026 12411 12028 12413 12028 12416 12031 12419
12031 12422 12029 12424 12028 12428 12025 12428 12022 12427 12021 12423
12022 12420 12021 12414 14 P
11273 12279 11276 12277 11281 12277 11285 12275 11288 12272 11290 12268
11292 12264 11296 12266 11301 12266 11302 12271 11299 12273 11296 12276
11293 12279 11291 12284 11292 12288 11288 12288 11286 12292 11281 12292
11279 12288 11279 12284 11279 12279 11273 12279 22 P
11438 12370 11440 12367 11442 12364 11445 12366 11448 12365 11450 12367
11447 12368 11445 12371 11441 12370 11438 12370 10 P
11685 12438 11689 12440 11688 12435 11693 12434 11697 12434 11701 12434
11704 12430 11703 12434 11706 12438 11707 12434 11709 12430 11712 12434
11716 12435 11720 12433 11719 12437 11717 12442 11716 12446 11717 12450
11713 12449 11708 12449 11704 12447 11700 12448 11696 12451 11693 12453
11691 12449 11687 12449 11690 12447 11687 12443 11685 12438 29 P
11692 12405 11695 12405 11697 12403 11700 12405 11703 12405 11706 12404
11707 12406 11708 12409 11712 12410 11710 12413 11709 12416 11706 12417
11703 12419 11703 12416 11703 12413 11701 12411 11698 12410 11695 12409
11692 12409 11692 12405 20 P
11647 12403 11649 12400 11652 12400 11655 12400 11657 12403 11660 12405
11660 12408 11657 12409 11655 12407 11652 12409 11650 12407 11647 12403
12:00 PM
11634 12407 11637 12407 11640 12405 11640 12408 11642 12411 11644 12413
11646 12411 11645 12407 11648 12409 11647 12412 11646 12415 11643 12416
11640 12415 11637 12413 11634 12411 11634 12407 16 P
11675 12464 11672 12462 11672 12459 11673 12456 11676 12455 11679 12454
11682 12454 11686 12455 11687 12457 11689 12457 11693 12459 11695 12457
11699 12456 11701 12455 11702 12458 11700 12460 11697 12461 11694 12463
11691 12464 11688 12466 11686 12464 11683 12464 11681 12461 11678 12461
11675 12464 25 P
11679 12422 11677 12426 11676 12428 11672 12429 11670 12428 11669 12424
11670 12421 11673 12422 11674 12419 11676 12416 11679 12416 11680 12419
11682 12422 11681 12425 11679 12422 15 P
11757 12416 11759 12414 11763 12414 11761 12417 11762 12420 11763 12423
11764 12426 11763 12429 11760 12427 11759 12423 11758 12420 11757 12416
12:00 PM
11382 12602 11384 12598 11387 12595 11390 12592 11395 12592 11394 12597
11394 12601 11398 12603 11401 12607 11399 12611 11404 12612 11406 12615
11408 12619 11408 12624 11411 12627 11415 12628 11413 12632 11410 12631
11407 12627 11403 12627 11402 12631 11405 12633 11401 12634 11397 12633

11392 12632 11390 12628 11390 12624 11388 12621 11387 12617 11386 12612
11383 12609 11385 12606 11382 12602 33 P
11408 12649 11410 12647 11412 12646 11413 12643 11416 12645 11416 12648
11417 12651 11420 12650 11420 12654 11417 12654 11414 12656 11412 12658
11412 12655 11410 12653 11408 12649 15 P
11288 12609 11291 12606 11293 12610 11295 12613 11299 12614 11301 12618
11302 12622 11303 12626 11302 12631 11298 12632 11297 12628 11295 12624
11294 12620 11290 12618 11288 12614 11288 12609 16 P
11345 12480 11347 12477 11348 12475 11350 12477 11349 12480 11345 12480
6:00 PM
11345 12484 11348 12484 11351 12486 11353 12488 11352 12491 11349 12492
11347 12488 11345 12484 8 P
11348 12466 11349 12464 11352 12461 11353 12458 11354 12455 11357 12457
11360 12456 11363 12457 11362 12461 11359 12462 11356 12463 11354 12461
11353 12464 11348 12466 14 P
11370 12617 11373 12615 11376 12616 11378 12618 11378 12621 11375 12622
11372 12621 11370 12617 8 P
11378 12623 11382 12623 11383 12626 11386 12628 11383 12627 11378 12623
6:00 PM
11402 12654 11405 12653 11406 12655 11405 12658 11402 12657 11402 12654
6:00 PM
11571 12607 11575 12605 11577 12602 11582 12603 11579 12599 11575 12597
11578 12593 11582 12595 11585 12597 11590 12596 11593 12599 11597 12600
11597 12605 11595 12608 11595 12613 11591 12614 11589 12611 11584 12609
11581 12606 11577 12606 11571 12607 21 P
11655 12661 11651 12663 11648 12667 11644 12666 11641 12670 11640 12674
11637 12678 11635 12674 11634 12670 11630 12667 11634 12664 11631 12660
11631 12664 11627 12665 11622 12663 11618 12662 11616 12657 11617 12653
11617 12649 11619 12644 11621 12641 11625 12644 11626 12640 11629 12637
11634 12639 11636 12635 11641 12634 11645 12635 11648 12637 11648 12642
11651 12644 11655 12647 11656 12651 11660 12651 11660 12655 11663 12660
11655 12661 37 P
11651 12699 11647 12698 11649 12703 11650 12706 11652 12711 11650 12715
11646 12717 11642 12715 11638 12714 11637 12710 11635 12706 11630 12706
11629 12702 11626 12699 11622 12698 11618 12697 11613 12697 11610 12700
11606 12699 11606 12695 11603 12692 11605 12688 11604 12684 11604 12679
11602 12675 11606 12672 11608 12669 11612 12665 11616 12664 11620 12665
11624 12667 11628 12669 11626 12674 11630 12674 11628 12678 11627 12682
11622 12684 11622 12689 11626 12690 11630 12691 11635 12692 11638 12694
11640 12690 11644 12690 11648 12692 11652 12693 11651 12699 47 P
11413 12604 11417 12603 11420 12604 11422 12606 11424 12608 11425 12612
11422 12612 11419 12612 11417 12609 11414 12607 11413 12604 11 P
11396 12663 11398 12661 11401 12661 11400 12664 11396 12663 5 P

11602 12607 11604 12605 11606 12603 11609 12601 11611 12598 11610 12601
11609 12605 11606 12605 11602 12607 9 P
11599 12607 11601 12605 11600 12601 11602 12604 11599 12607 5 P
11635 12620 11637 12618 11640 12616 11642 12618 11645 12616 11647 12613
11650 12615 11650 12618 11648 12621 11645 12623 11644 12626 11641 12627
11639 12625 11636 12624 11635 12620 15 P
11659 12622 11656 12621 11656 12618 11658 12615 11656 12613 11654 12611
11657 12608 11659 12611 11659 12608 11662 12610 11665 12611 11665 12614
11664 12618 11662 12620 11659 12622 15 P
11665 12608 11666 12606 11670 12605 11672 12603 11671 12600 11669 12598
11669 12595 11671 12592 11671 12589 11672 12586 11676 12586 11676 12590
11678 12593 11680 12591 11684 12591 11687 12590 11689 12592 11690 12595
11691 12598 11689 12601 11686 12603 11686 12606 11686 12609 11683 12608
11680 12608 11678 12611 11676 12614 11678 12617 11678 12620 11678 12623
11674 12624 11671 12625 11668 12625 11665 12624 11664 12621 11666 12618
11667 12615 11666 12612 11665 12608 39 P
11676 12570 11680 12569 11684 12569 11689 12569 11693 12571 11695 12575
11699 12576 11703 12579 11702 12583 11706 12584 11708 12588 11706 12592
11708 12596 11708 12600 11712 12603 11708 12606 11705 12608 11707 12611
11703 12613 11699 12615 11695 12617 11694 12621 11691 12624 11686 12623
11682 12624 11681 12619 11685 12616 11689 12614 11691 12611 11693 12607
11694 12603 11698 12600 11702 12599 11698 12597 11696 12593 11697 12589
11694 12586 11689 12585 11686 12588 11681 12587 11678 12584 11675 12582
11679 12580 11681 12583 11686 12583 11690 12583 11688 12579 11685 12577
11683 12573 11679 12575 50 P
11679 12575 11676 12570 2 P
11642 12599 11645 12600 11648 12600 11649 12597 11648 12594 11648 12591
11651 12591 11651 12594 11651 12598 11651 12601 11654 12604 11650 12604
11647 12603 11644 12602 11642 12599 15 P
11710 12635 11714 12634 11717 12631 11716 12627 11717 12623 11715 12619
11720 12618 11723 12621 11728 12622 11729 12626 11734 12627 11737 12625
11741 12627 11743 12631 11748 12631 11750 12635 11745 12636 11741 12637
11742 12641 11740 12644 11737 12648 11734 12644 11731 12641 11727 12641
11730 12644 11730 12649 11727 12651 11723 12650 11721 12646 11716 12644
11715 12640 11711 12639 11710 12635 33 P
11680 12699 11684 12697 11687 12695 11689 12691 11692 12687 11696 12687
11700 12685 11699 12681 11699 12676 11703 12676 11707 12674 11710 12676
11713 12680 11717 12681 11718 12676 11721 12674 11721 12669 11725 12668
11729 12667 11734 12668 11735 12672 11735 12676 11735 12681 11732 12684
11730 12688 11732 12691 11729 12693 11727 12697 11722 12698 11719 12696
11715 12696 11711 12698 11709 12703 11705 12704 11701 12701 11697 12703
11693 12704 11691 12700 11687 12700 11680 12699 40 P
11687 12712 11691 12710 11695 12710 11699 12707 11702 12708 11707 12709

11710 12706 11712 12703 11715 12699 11719 12701 11723 12702 11725 12706
11724 12711 11722 12714 11725 12717 11723 12721 11719 12721 11719 12725
11715 12727 11713 12724 11715 12720 11711 12720 11707 12718 11703 12716
11699 12715 11695 12714 11687 12712 27 P
11674 12743 11676 12740 11679 12736 11681 12733 11683 12729 11686 12726
11691 12725 11693 12721 11692 12717 11696 12717 11700 12719 11703 12722
11704 12726 11702 12730 11701 12734 11703 12738 11707 12737 11711 12735
11710 12739 11714 12742 11715 12746 11711 12748 11708 12751 11712 12753
11716 12754 11716 12758 11712 12757 11712 12762 11709 12765 11706 12762
11702 12764 11698 12765 11694 12762 11695 12758 11698 12754 11700 12750
11699 12747 11694 12747 11690 12748 11687 12752 11683 12749 11681 12746
11674 12743 43 P
11645 12762 11643 12758 11641 12754 11637 12754 11632 12754 11636 12752
11634 12748 11638 12747 11643 12747 11647 12749 11650 12747 11654 12750
11656 12753 11657 12757 11657 12753 11657 12749 11661 12748 11663 12743
11666 12741 11669 12743 11672 12747 11677 12748 11678 12752 11683 12753
11682 12757 11681 12761 11685 12760 11689 12761 11691 12766 11687 12768
11683 12769 11684 12774 11687 12776 11684 12779 11684 12783 11680 12784
11678 12788 11675 12791 11671 12791 11670 12787 11671 12783 11670 12779
11666 12777 11663 12780 11659 12777 11656 12775 11652 12777 11650 12773
11649 12769 11645 12762 50 P
11679 12720 11681 12718 11683 12715 11686 12716 11684 12719 11683 12722
11679 12720 7 P
11667 12730 11669 12727 11671 12724 11674 12724 11676 12721 11679 12722
11679 12726 11676 12728 11673 12729 11671 12731 11667 12730 11 P
11658 12685 11659 12682 11660 12679 11662 12676 11663 12673 11667 12673
11667 12676 11666 12679 11665 12682 11664 12685 11658 12685 11 P
11728 12656 11731 12656 11735 12655 11738 12657 11739 12660 11741 12657
11743 12660 11744 12663 11742 12665 11744 12667 11746 12670 11743 12671
11740 12670 11737 12668 11736 12665 11736 12662 11732 12662 11729 12662
11728 12656 19 P
11698 12665 11700 12663 11703 12663 11705 12666 11706 12669 11703 12671
11700 12670 11698 12665 8 P
11685 12682 11688 12681 11690 12684 11691 12687 11688 12690 11687 12686
11685 12682 7 P
11670 12692 11673 12693 11676 12694 11678 12697 11678 12700 11675 12698
11673 12696 11670 12692 8 P
11685 12650 11686 12647 11689 12649 11689 12652 11692 12654 11692 12657
11689 12657 11686 12655 11685 12650 9 P
11678 12637 11678 12634 11679 12631 11681 12634 11681 12637 11680 12641
11679 12644 11677 12641 11678 12637 9 P
11681 12541 11684 12539 11685 12536 11685 12533 11687 12530 11689 12532
11692 12535 11692 12538 11695 12539 11698 12542 11698 12545 11695 12543

11694 12546 11696 12548 11694 12549 11692 12552 11689 12550 11687 12548
11686 12544 11685 12541 11681 12541 21 P
11799 12493 11802 12491 11805 12490 11804 12493 11807 12493 11810 12494
11812 12497 11812 12500 11810 12502 11807 12500 11809 12503 11809 12506
11806 12505 11803 12503 11800 12501 11801 12498 11799 12493 17 P
11643 12475 11646 12476 11648 12478 11648 12482 11645 12482 11644 12478
11643 12475 7 P
11655 12475 11658 12475 11660 12478 11660 12481 11658 12484 11656 12482
11655 12475 7 P
11655 12443 11658 12442 11660 12444 11659 12448 11656 12447 11655 12443
6:00 PM
11662 12440 11664 12438 11667 12437 11670 12438 11669 12441 11665 12442
11662 12440 7 P
11661 12449 11662 12447 11663 12443 11666 12444 11667 12448 11666 12450
11661 12449 7 P
11650 12462 11653 12463 11656 12463 11656 12466 11653 12468 11651 12465
11650 12462 7 P
11680 12472 11683 12470 11680 12468 11683 12466 11686 12466 11686 12469
11685 12472 11683 12475 11680 12472 9 P
11676 12451 11677 12448 11679 12445 11681 12448 11685 12448 11685 12451
11681 12451 11676 12451 8 P
11613 12388 11615 12387 11618 12385 11621 12384 11620 12387 11618 12389
11613 12388 7 P
11656 12361 11657 12358 11658 12355 11659 12352 11662 12355 11660 12357
11659 12360 11658 12364 11656 12361 9 P
11749 12750 11753 12750 11757 12749 11761 12748 11766 12748 11768 12752
11770 12755 11774 12757 11778 12756 11782 12757 11781 12761 11785 12760
11787 12756 11792 12756 11796 12755 11797 12752 11802 12750 11800 12747
11796 12743 11799 12739 11803 12739 11805 12743 11809 12741 11811 12745
11811 12749 11812 12753 11813 12757 11812 12761 11808 12763 11804 12766
11800 12765 11796 12764 11792 12763 11787 12764 11790 12768 11786 12770
11790 12773 11790 12777 11795 12776 11796 12780 11800 12780 11802 12784
11797 12784 11795 12788 11791 12789 11787 12788 11782 12788 11780 12792
11776 12792 11772 12791 50 P
11772 12791 11769 12788 11772 12785 11775 12782 11779 12779 11780 12775
11777 12773 11777 12768 11772 12769 11773 12764 11770 12762 11767 12766
11765 12762 11764 12758 11759 12758 11755 12757 11751 12760 11749 12756
11749 12750 19 P
11758 12817 11761 12813 11764 12810 11766 12806 11767 12802 11772 12800
11776 12802 11775 12806 11773 12810 11771 12813 11767 12817 11764 12819
11758 12817 13 P
11742 12788 11745 12786 11749 12784 11751 12788 11756 12788 11756 12792
11757 12797 11752 12799 11748 12798 11744 12799 11742 12795 11742 12788

11651 12860 11656 12860 11660 12860 11661 12855 11663 12859 11666 12862
11668 12858 11672 12856 11677 12855 11680 12853 11682 12857 11686 12860
11684 12863 11687 12867 11688 12871 11685 12872 11680 12874 11681 12878
11680 12883 11685 12885 11686 12889 11683 12892 11686 12896 11685 12901
11689 12902 11693 12903 11696 12906 11701 12908 11697 12910 11694 12906
11690 12906 11686 12905 11681 12905 11681 12901 11678 12898 11675 12895
11673 12890 11670 12887 11666 12885 11662 12884 11658 12882 11656 12879
11655 12875 11652 12871 11656 12869 11660 12867 11658 12863 11651 12860
48 P
11703 12878 11708 12878 11710 12875 11709 12870 11713 12870 11715 12866
11719 12865 11723 12866 11727 12863 11731 12863 11735 12865 11738 12862
11742 12864 11743 12860 11740 12857 11740 12853 11744 12853 11746 12849
11749 12845 11749 12850 11753 12848 11754 12852 11758 12855 11754 12857
11750 12859 11751 12863 11749 12868 11746 12870 11743 12867 11739 12868
11736 12871 11732 12874 11732 12878 11735 12881 11739 12879 11742 12882
11741 12887 11736 12887 11734 12883 11732 12887 11730 12891 11726 12888
11725 12884 11721 12884 11717 12887 11715 12891 11712 12889 11707 12888
11705 12884 11703 12878 50 P
11692 12855 11697 12855 11698 12850 11701 12849 11706 12850 11709 12848
11711 12844 11713 12848 11713 12853 11714 12848 11716 12844 11718 12847
11718 12852 11717 12856 11715 12860 11711 12861 11707 12860 11702 12860
11699 12859 11692 12855 20 P
11682 12827 11686 12825 11689 12827 11694 12828 11698 12828 11702 12826
11706 12828 11706 12824 11702 12825 11701 12820 11701 12816 11702 12812
11701 12808 11699 12804 11698 12800 11701 12802 11706 12803 11707 12807
11705 12811 11709 12812 11713 12814 11717 12813 11716 12817 11721 12818
11720 12823 11720 12827 11717 12830 11713 12831 11710 12835 11710 12839
11706 12842 11702 12840 11699 12842 11694 12842 11694 12838 11691 12836
11689 12832 11682 12827 38 P
11750 12834 11751 12831 11751 12828 11752 12825 11750 12822 11752 12821
11756 12822 11759 12821 11761 12824 11760 12827 11760 12830 11758 12832
11756 12835 11754 12838 11751 12837 11748 12838 11750 12834 17 P
11730 12814 11732 12812 11733 12809 11736 12807 11737 12810 11737 12813
11737 12817 11737 12820 11737 12823 11734 12822 11731 12821 11731 12818
11730 12814 13 P
11716 13153 11719 13150 11721 13146 11724 13149 11729 13151 11731 13154
11734 13158 11737 13161 11741 13162 11742 13166 11744 13171 11742 13174
11738 13176 11739 13172 11735 13169 11731 13170 11727 13172 11729 13168
11725 13167 11725 13163 11722 13160 11722 13156 11716 13153 23 P
11681 12984 11683 12982 11686 12980 11686 12977 11689 12977 11692 12980
11691 12983 11691 12987 11689 12989 11686 12991 11683 12990 11681 12984

11623 13011 11626 13009 11627 13006 11630 13007 11633 13006 11635 13009
11637 13011 11636 13015 11635 13017 11632 13018 11630 13021 11627 13019
11624 13019 11624 13016 11627 13015 11630 13013 11627 13012 11623 13011
18 P
11621 13004 11619 13002 11618 12998 11621 13001 11623 13003 11621 13004
6:00 PM
11636 13026 11639 13025 11641 13027 11638 13029 11636 13026 5 P
11686 12939 11689 12939 11693 12939 11694 12942 11696 12944 11693 12945
11692 12942 11688 12941 11686 12939 9 P
11682 12931 11685 12932 11685 12935 11685 12939 11681 12937 11682 12934
11682 12931 7 P
11638 12873 11641 12870 11642 12868 11645 12867 11648 12869 11648 12872
11646 12875 11644 12877 11641 12880 11639 12878 11638 12873 11 P
11652 13177 11655 13176 11657 13177 11658 13174 11660 13175 11660 13178
11658 13180 11656 13181 11653 13180 11652 13177 10 P
11647 13187 11649 13186 11651 13188 11651 13191 11649 13191 11647 13187
6:00 PM
11793 13247 11796 13246 11797 13243 11799 13240 11800 13243 11801 13246
11799 13249 11796 13251 11793 13247 9 P
11869 13280 11871 13278 11874 13278 11877 13279 11877 13282 11879 13286
11876 13288 11873 13288 11871 13286 11869 13280 10 P
11788 13294 11790 13292 11792 13288 11792 13285 11795 13284 11795 13287
11797 13289 11800 13287 11803 13286 11806 13287 11809 13289 11806 13291
11803 13293 11800 13294 11797 13294 11794 13296 11791 13296 11788 13294
18 P
11765 13286 11768 13285 11768 13282 11771 13282 11771 13279 11773 13281
11777 13281 11778 13283 11776 13286 11779 13288 11778 13291 11776 13294
11773 13295 11770 13294 11770 13290 11773 13290 11775 13287 11773 13285
11770 13284 11769 13287 11765 13286 21 P
11778 13277 11780 13274 11783 13274 11786 13277 11788 13279 11789 13282
11787 13285 11785 13282 11785 13279 11781 13278 11783 13281 11780 13283
11779 13280 11778 13277 14 P
11756 13281 11758 13279 11760 13276 11763 13277 11762 13281 11760 13283
11757 13285 11756 13281 8 P
11750 13301 11753 13300 11756 13300 11759 13302 11761 13304 11759 13306
11756 13304 11753 13302 11750 13301 9 P
11770 13308 11773 13308 11777 13307 11780 13307 11778 13310 11775 13309
11770 13308 7 P
11741 13301 11744 13301 11748 13301 11745 13303 11741 13301 5 P
11806 13306 11808 13303 11810 13304 11811 13307 11809 13307 11806 13306
6:00 PM
11816 13363 11818 13361 11821 13361 11823 13363 11822 13365 11819 13365
11816 13363 7 P

11842 13368 11845 13368 11847 13368 11848 13365 11851 13366 11849 13367
11848 13370 11846 13371 11842 13368 9 P
11827 13375 11830 13374 11832 13373 11834 13371 11837 13372 11839 13373
11840 13375 11838 13375 11836 13377 11833 13377 11830 13377 11827 13375

12:00 PM

11818 13413 11821 13412 11823 13411 11826 13410 11826 13413 11826 13415
11825 13418 11823 13418 11821 13416 11818 13413 10 P
11767 13432 11768 13430 11771 13429 11774 13429 11776 13428 11775 13431
11774 13433 11772 13435 11770 13434 11767 13432 10 P
11563 13435 11564 13432 11565 13430 11567 13428 11569 13427 11572 13428
11571 13430 11574 13430 11575 13428 11577 13430 11577 13432 11575 13434
11572 13435 11569 13436 11566 13436 11563 13435 16 P
11583 13489 11584 13487 11586 13485 11589 13484 11589 13486 11587 13488
11583 13489 7 P
11601 13491 11604 13491 11606 13492 11608 13493 11611 13494 11614 13494
11614 13497 11612 13499 11609 13498 11606 13499 11604 13498 11603 13495
11601 13491 13 P
11551 13498 11553 13495 11554 13492 11555 13491 11558 13490 11558 13492
11559 13495 11557 13497 11556 13494 11554 13496 11551 13498 11 P
11715 13562 11717 13562 11720 13563 11722 13562 11724 13563 11726 13565
11726 13568 11724 13570 11723 13572 11724 13575 11722 13577 11720 13577
11719 13574 11718 13572 11717 13570 11715 13568 11714 13565 11715 13562
18 P
11731 13587 11732 13585 11735 13584 11737 13586 11739 13585 11742 13584
11745 13585 11747 13584 11750 13584 11752 13584 11755 13584 11758 13582
11760 13581 11763 13582 11765 13584 11764 13586 11761 13585 11759 13585
11757 13586 11754 13587 11756 13590 11754 13592 11753 13594 11752 13597
11749 13597 11746 13597 11744 13595 11743 13593 11745 13590 11742 13590
11739 13592 11737 13593 11735 13593 11732 13592 11731 13587 35 P
11677 13540 11679 13537 11680 13535 11682 13537 11684 13539 11684 13542
11686 13544 11683 13545 11680 13545 11680 13542 11680 13540 11677 13540

12:00 PM

11683 13527 11684 13524 11686 13524 11686 13527 11687 13529 11688 13532
11685 13532 11683 13530 11683 13527 9 P
11569 13735 11570 13733 11573 13734 11575 13736 11577 13736 11580 13736
11582 13737 11583 13740 11585 13741 11584 13744 11584 13747 11582 13748
11582 13745 11581 13742 11579 13741 11576 13741 11573 13740 11572 13738
11569 13735 19 P
11555 14035 11558 14037 11560 14041 11562 14038 11566 14036 11567 14032
11571 14031 11576 14032 11579 14033 11581 14038 11585 14040 11588 14043
11585 14046 11581 14048 11577 14049 11573 14052 11569 14051 11567 14055
11564 14058 11563 14062 11560 14064 11560 14060 11557 14056 11557 14052
11556 14048 11557 14044 11557 14039 11555 14035 28 P

11587 14039 11587 14037 11588 14034 11591 14033 11593 14033 11596 14032
11597 14029 11597 14026 11599 14028 11600 14031 11602 14032 11600 14034
11598 14033 11597 14036 11595 14038 11593 14040 11591 14042 11587 14039
18 P
11701 14073 11703 14069 11704 14065 11705 14061 11706 14056 11709 14059
11713 14062 11715 14058 11714 14054 11718 14056 11720 14060 11723 14063
11724 14067 11727 14071 11730 14072 11730 14076 11729 14080 11728 14084
11724 14086 11720 14085 11715 14086 11711 14085 11710 14081 11707 14079
11702 14078 11701 14073 26 P
11749 14040 11752 14043 11754 14047 11759 14046 11761 14042 11765 14044
11765 14048 11766 14053 11767 14057 11772 14055 11776 14055 11776 14060
11779 14063 11783 14063 11785 14068 11784 14072 11781 14075 11781 14080
11777 14081 11772 14081 11768 14082 11765 14085 11761 14084 11760 14088
11763 14092 11763 14096 11761 14092 11759 14089 11755 14087 11751 14090
11747 14091 11750 14087 11753 14084 11756 14081 11757 14077 11758 14073
11759 14068 11759 14064 11756 14063 11752 14060 11753 14056 11758 14056
11756 14053 11754 14049 11751 14046 11749 14040 46 P
11636 14066 11640 14064 11644 14061 11648 14061 11652 14060 11657 14060
11661 14060 11664 14063 11666 14067 11662 14069 11660 14065 11656 14066
11652 14067 11648 14068 11643 14069 11640 14071 11636 14066 17 P
11646 14018 11648 14015 11650 14013 11654 14012 11656 14012 11660 14012
11659 14014 11657 14017 11654 14018 11650 14019 11646 14018 11 P
11626 14017 11628 14014 11631 14015 11634 14014 11637 14013 11638 14010
11642 14009 11643 14012 11640 14013 11638 14016 11638 14019 11635 14020
11632 14021 11630 14019 11626 14017 15 P
11612 14021 11615 14019 11618 14018 11621 14018 11624 14018 11626 14020
11624 14024 11621 14023 11618 14022 11612 14021 10 P
11557 14011 11559 14009 11561 14006 11564 14005 11568 14005 11570 14008
11568 14011 11570 14014 11566 14013 11564 14016 11562 14017 11558 14016
11557 14011 13 P
11551 13966 11554 13967 11557 13965 11554 13964 11553 13961 11556 13960
11558 13961 11561 13963 11562 13966 11563 13969 11562 13972 11560 13974
11557 13973 11554 13973 11553 13969 11551 13966 16 P
11500 13950 11501 13948 11504 13946 11505 13949 11505 13952 11505 13955
11508 13954 11511 13955 11510 13958 11507 13959 11504 13960 11501 13958
11501 13955 11500 13950 14 P
11528 13926 11525 13926 11525 13930 11523 13932 11520 13933 11518 13930
11520 13927 11522 13925 11525 13923 11527 13920 11530 13921 11533 13924
11535 13926 11532 13925 11528 13926 15 P
11504 14007 11507 14007 11510 14009 11512 14011 11515 14011 11514 14013
11517 14015 11518 14018 11515 14018 11513 14016 11510 14014 11508 14012
11506 14011 11504 14007 14 P
11688 13985 11691 13984 11694 13982 11695 13985 11694 13988 11693 13991

11689 13991 11688 13985 8 P
11707 13963 11709 13960 11709 13957 11710 13954 11713 13953 11716 13954
11719 13955 11722 13957 11720 13960 11720 13963 11717 13965 11715 13962
11712 13963 11710 13966 11707 13963 15 P
11679 14142 11683 14141 11684 14138 11685 14135 11685 14132 11687 14129
11690 14128 11693 14129 11696 14130 11697 14133 11696 14137 11699 14138
11697 14141 11694 14142 11692 14145 11690 14147 11687 14148 11685 14151
11682 14152 11681 14148 11680 14146 11679 14142 22 P
11658 14178 11660 14175 11663 14174 11664 14172 11665 14168 11667 14166
11670 14164 11672 14166 11673 14169 11677 14170 11679 14167 11678 14164
11681 14166 11684 14165 11686 14162 11687 14159 11689 14162 11692 14164
11692 14167 11692 14170 11690 14173 11687 14175 11685 14177 11683 14179
11679 14180 11676 14180 11676 14177 11672 14176 11669 14176 11667 14179
11665 14182 11663 14184 11659 14184 11658 14181 11658 14178 35 P
11711 14191 11713 14188 11716 14185 11720 14186 11722 14190 11727 14190
11730 14193 11734 14195 11738 14194 11742 14195 11746 14196 11747 14201
11750 14204 11750 14209 11746 14212 11744 14216 11741 14217 11736 14219
11732 14218 11728 14219 11724 14222 11721 14224 11717 14222 11715 14218
11715 14214 11714 14209 11713 14205 11714 14201 11713 14197 11711 14191
30 P
11692 14200 11696 14197 11700 14195 11701 14191 11701 14187 11705 14186
11704 14190 11706 14194 11709 14198 11706 14201 11702 14201 11698 14201
11692 14200 13 P
11686 14211 11689 14210 11692 14212 11695 14212 11699 14211 11701 14210
11704 14209 11704 14212 11703 14216 11705 14219 11704 14222 11702 14224
11699 14226 11696 14225 11695 14222 11693 14220 11690 14218 11688 14216
11686 14211 19 P
11708 14177 11711 14175 11714 14174 11715 14177 11715 14180 11713 14182
11710 14181 11708 14177 8 P
11632 14227 11634 14225 11636 14223 11639 14221 11641 14218 11642 14216
11642 14212 11645 14211 11648 14212 11651 14212 11655 14214 11658 14214
11661 14215 11664 14214 11666 14217 11666 14220 11663 14219 11659 14218
11656 14217 11653 14217 11650 14218 11648 14221 11646 14223 11643 14224
11640 14225 11637 14226 11632 14227 27 P
11680 14214 11684 14213 11685 14216 11686 14219 11686 14223 11688 14225
11685 14226 11683 14225 11683 14222 11681 14219 11680 14214 11 P
11673 14217 11676 14215 11677 14217 11679 14219 11678 14222 11676 14221
11673 14217 7 P
11637 14355 11640 14349 11642 14343 11642 14336 11642 14329 11643 14322
11642 14315 11645 14309 11651 14308 11658 14307 11660 14301 11665 14296
11667 14290 11670 14283 11667 14278 11660 14275 11654 14273 11656 14267
11656 14260 11662 14258 11669 14255 11675 14255 11682 14254 11688 14253
11695 14251 11695 14258 11695 14265 11702 14262 11707 14258 11714 14258

11718 14262 11723 14267 11725 14274 11723 14280 11720 14286 11722 14293
11717 14297 11716 14304 11717 14311 11715 14317 11712 14323 11712 14330
11715 14336 11712 14343 11708 14348 11706 14355 11702 14360 11695 14362
11689 14365 11684 14369 50 P
11684 14369 11678 14373 11673 14378 11667 14379 11661 14376 11654 14375
11652 14369 11648 14364 11643 14360 11637 14355 10 P
11607 14308 11610 14305 11614 14303 11617 14301 11621 14298 11625 14297
11629 14295 11632 14293 11635 14290 11636 14286 11634 14282 11637 14279
11640 14275 11643 14279 11646 14282 11649 14285 11652 14288 11655 14292
11655 14296 11654 14300 11650 14302 11646 14304 11642 14304 11637 14305
11634 14308 11630 14310 11628 14314 11627 14318 11628 14322 11629 14326
11631 14330 11629 14333 11625 14333 11621 14330 11617 14329 11613 14326
11614 14322 11614 14317 11614 14313 11615 14309 11607 14308 41 P
11576 14337 11579 14335 11583 14332 11587 14331 11591 14332 11592 14336
11597 14337 11601 14337 11604 14340 11608 14340 11612 14338 11616 14340
11620 14343 11623 14345 11626 14349 11627 14352 11629 14356 11628 14360
11626 14364 11622 14361 11618 14360 11614 14360 11609 14360 11605 14362
11602 14365 11598 14366 11594 14365 11590 14364 11586 14361 11583 14359
11582 14354 11580 14351 11582 14346 11580 14343 11576 14337 35 P
11590 14274 11592 14272 11594 14269 11597 14267 11599 14265 11602 14264
11605 14264 11608 14262 11607 14265 11604 14267 11604 14270 11604 14273
11605 14276 11601 14276 11598 14275 11595 14276 11590 14274 17 P
11560 14283 11563 14282 11565 14280 11568 14281 11571 14282 11573 14280
11576 14278 11578 14276 11582 14275 11583 14279 11584 14281 11582 14283
11579 14286 11578 14289 11577 14292 11574 14291 11572 14288 11570 14287
11566 14287 11563 14287 11560 14283 21 P
11546 14289 11547 14287 11550 14287 11553 14286 11555 14288 11554 14290
11551 14290 11549 14292 11546 14289 9 P
11601 14316 11604 14316 11605 14318 11605 14321 11602 14322 11601 14320
11601 14316 7 P
11584 14311 11587 14310 11590 14310 11592 14310 11595 14311 11598 14311
11595 14312 11593 14313 11590 14313 11587 14314 11584 14311 11 P
10886 14382 10887 14378 10891 14376 10894 14373 10895 14369 10897 14365
10899 14361 10902 14358 10906 14359 10910 14358 10914 14357 10919 14359
10922 14356 10926 14357 10926 14361 10928 14365 10926 14369 10922 14370
10920 14374 10922 14378 10926 14380 10930 14378 10928 14374 10933 14374
10936 14377 10940 14376 10938 14380 10942 14380 10940 14383 10938 14388
10936 14392 10932 14393 10928 14393 10924 14393 10919 14392 10915 14391
10911 14390 10907 14388 10903 14386 10899 14383 10896 14385 10892 14387
10886 14382 43 P
10914 14304 10917 14302 10920 14301 10924 14301 10924 14297 10923 14295
10926 14296 10928 14297 10932 14297 10935 14297 10937 14300 10939 14303
10939 14306 10937 14309 10937 14312 10939 14314 10942 14316 10940 14318

10938 14320 10934 14319 10932 14322 10931 14318 10929 14316 10926 14315
10925 14311 10922 14309 10922 14305 10919 14304 10914 14304 29 P
10944 14273 10948 14274 10949 14276 10953 14279 10955 14280 10958 14279
10961 14278 10962 14281 10963 14285 10960 14285 10956 14284 10954 14283
10950 14284 10949 14281 10947 14279 10944 14273 16 P
11058 14312 11057 14309 11060 14308 11063 14309 11066 14307 11070 14308
11071 14310 11072 14313 11073 14316 11076 14315 11078 14318 11077 14321
11078 14324 11079 14327 11076 14326 11072 14326 11069 14327 11066 14325
11068 14324 11071 14322 11071 14319 11071 14316 11067 14316 11064 14317
11061 14318 11061 14315 11058 14312 27 P
11117 14308 11120 14308 11123 14307 11124 14304 11122 14301 11125 14303
11127 14305 11129 14308 11132 14309 11135 14311 11137 14314 11136 14317
11139 14316 11143 14317 11141 14319 11140 14323 11137 14322 11134 14321
11131 14320 11128 14318 11127 14315 11124 14314 11122 14312 11117 14308
24 P
11154 14259 11157 14257 11159 14255 11162 14257 11165 14258 11168 14258
11171 14260 11173 14261 11175 14264 11177 14267 11179 14269 11181 14272
11184 14274 11187 14275 11190 14276 11192 14279 11194 14282 11195 14285
11197 14287 11200 14289 11201 14293 11202 14295 11204 14298 11206 14301
11203 14300 11200 14300 11197 14298 11195 14296 11193 14294 11190 14292
11187 14293 11185 14290 11181 14289 11179 14287 11180 14284 11181 14281
11182 14278 11182 14275 11179 14277 11180 14280 11177 14282 11174 14284
11172 14283 11168 14281 11171 14279 11170 14276 11167 14274 11166 14271
11164 14268 11162 14266 50 P
11162 14266 11159 14265 11156 14262 11154 14259 4 P
11354 14263 11357 14261 11359 14258 11358 14255 11359 14252 11362 14251
11365 14253 11366 14255 11368 14258 11370 14261 11372 14264 11372 14267
11368 14267 11365 14267 11362 14266 11359 14266 11356 14267 11354 14263
18 P
11439 14325 11441 14323 11444 14324 11447 14324 11450 14325 11453 14327
11454 14330 11455 14333 11453 14331 11449 14330 11448 14328 11445 14329
11442 14328 11439 14325 14 P
11383 14335 11385 14334 11389 14334 11391 14337 11389 14340 11389 14343
11387 14342 11386 14338 11383 14335 9 P
11152 14288 11155 14287 11158 14286 11160 14284 11162 14281 11165 14283
11163 14285 11164 14287 11162 14290 11159 14290 11156 14289 11152 14288

12:00 PM

10927 14687 10931 14684 10934 14683 10939 14684 10936 14687 10938 14691
10941 14688 10946 14689 10949 14692 10951 14688 10955 14686 10959 14684
10963 14682 10967 14680 10971 14680 10971 14684 10974 14681 10978 14681
10981 14685 10978 14688 10977 14692 10974 14695 10970 14698 10971 14702
10967 14702 10962 14702 10959 14705 10955 14706 10950 14707 10948 14711
10946 14706 10943 14703 10941 14699 10937 14698 10933 14698 10933 14693

10933 14689 10927 14687 38 P
11018 14771 11017 14775 11014 14778 11014 14774 11014 14770 11012 14766
11014 14762 11018 14762 11018 14758 11014 14760 11011 14757 11010 14753
11013 14750 11015 14753 11019 14755 11023 14757 11025 14762 11025 14766
11027 14770 11027 14774 11025 14770 11018 14771 22 P
10903 15216 10907 15216 10910 15219 10913 15222 10918 15222 10921 15224
10925 15227 10927 15231 10931 15232 10933 15236 10934 15240 10939 15241
10938 15245 10942 15246 10946 15244 10949 15247 10951 15251 10950 15255
10946 15255 10942 15253 10944 15257 10940 15259 10943 15262 10939 15263
10942 15266 10938 15268 10937 15273 10940 15276 10944 15275 10944 15279
10940 15281 10937 15284 10933 15284 10928 15285 10925 15282 10921 15280
10925 15278 10924 15274 10920 15272 10923 15268 10924 15264 10920 15261
10917 15260 10915 15256 10911 15256 10907 15253 10905 15250 10904 15246
10907 15248 10912 15249 50 P
10912 15249 10915 15250 10919 15248 10917 15245 10913 15243 10914 15239
10919 15238 10919 15234 10918 15230 10913 15230 10909 15230 10909 15226
10907 15222 10903 15216 14 P
10808 15230 10812 15231 10813 15235 10817 15238 10820 15236 10824 15233
10826 15237 10827 15241 10831 15239 10831 15235 10835 15233 10840 15233
10844 15234 10846 15238 10847 15242 10851 15245 10852 15249 10847 15251
10844 15253 10840 15254 10838 15250 10834 15250 10830 15251 10827 15247
10823 15249 10819 15247 10820 15243 10817 15240 10813 15240 10810 15236
10808 15230 31 P
10846 15217 10849 15213 10852 15217 10856 15218 10856 15222 10859 15226
10855 15229 10857 15233 10854 15234 10850 15233 10848 15229 10845 15225
10848 15222 10846 15217 14 P
10810 15177 10813 15177 10813 15174 10815 15172 10818 15173 10821 15173
10823 15176 10824 15179 10821 15181 10819 15184 10819 15187 10817 15185
10815 15183 10811 15182 10810 15177 15 P
10819 15192 10823 15191 10826 15191 10825 15194 10825 15197 10823 15199
10820 15197 10819 15192 8 P
10819 15205 10821 15204 10824 15205 10825 15208 10824 15210 10821 15211
10820 15209 10819 15205 8 P
9402 15147 9404 15141 9403 15134 9409 15131 9416 15129 9422 15132
9429 15131 9433 15126 9436 15119 9439 15125 9445 15129 9449 15134
9454 15139 9460 15139 9467 15139 9473 15135 9479 15132 9486 15132
9492 15134 9496 15140 9501 15144 9508 15146 9511 15152 9515 15157
9521 15155 9526 15159 9529 15166 9531 15172 9530 15178 9530 15185
9527 15191 9525 15198 9519 15199 9513 15202 9507 15206 9501 15209
9494 15209 9499 15204 9499 15197 9495 15191 9489 15188 9482 15188
9478 15183 9472 15181 9465 15181 9458 15181 9451 15180 9445 15177
9438 15177 9432 15181 50 P
9432 15181 9430 15174 9431 15167 9426 15163 9420 15160 9417 15154

9412 15149 9402 15147 8 P
9500 15106 9497 15112 9490 15113 9484 15116 9478 15118 9471 15116
9466 15112 9462 15118 9456 15121 9450 15120 9446 15114 9442 15109
9442 15102 9438 15097 9431 15096 9425 15095 9423 15089 9425 15082
9418 15081 9421 15075 9420 15069 9416 15063 9422 15060 9429 15059
9433 15064 9435 15070 9440 15075 9444 15080 9449 15085 9450 15092
9455 15095 9462 15096 9469 15095 9471 15089 9466 15084 9462 15078
9458 15073 9455 15067 9452 15061 9458 15060 9465 15058 9471 15057
9477 15053 9484 15053 9489 15056 9490 15063 9494 15069 9496 15075
9496 15082 9493 15088 50 P
9493 15088 9488 15092 9482 15095 9488 15094 9495 15094 9501 15097
9500 15106 7 P
9866 14974 9870 14968 9869 14961 9874 14957 9879 14952 9885 14956
9887 14962 9889 14969 9894 14964 9901 14963 9900 14970 9898 14976
9895 14983 9898 14989 9904 14991 9898 14991 9893 14986 9886 14985
9879 14984 9877 14978 9866 14974 21 P
10217 14936 10223 14936 10227 14934 10230 14929 10225 14932 10222 14927
10222 14922 10221 14917 10223 14911 10228 14908 10231 14913 10235 14917
10240 14919 10244 14924 10244 14929 10240 14933 10238 14938 10235 14942
10234 14948 10233 14942 10228 14940 10223 14941 10217 14936 23 P
10036 14906 10039 14903 10042 14900 10038 14898 10042 14896 10045 14893
10049 14892 10053 14891 10058 14890 10061 14892 10060 14896 10056 14897
10055 14901 10052 14905 10048 14903 10045 14906 10044 14910 10040 14910
10036 14906 19 P
10143 15191 10139 15189 10142 15185 10141 15181 10138 15184 10134 15184
10135 15189 10130 15190 10128 15187 10126 15182 10124 15178 10121 15176
10119 15171 10124 15170 10128 15170 10130 15167 10132 15163 10134 15159
10138 15160 10143 15160 10145 15164 10148 15167 10151 15170 10153 15174
10155 15178 10155 15182 10153 15185 10150 15188 10143 15191 29 P
9939 15040 9942 15036 9946 15034 9950 15034 9955 15033 9958 15031
9962 15028 9966 15027 9970 15027 9974 15025 9974 15021 9977 15018
9979 15021 9978 15026 9983 15026 9987 15027 9991 15026 9995 15025
10000 15025 10004 15025 10008 15023 10012 15020 10015 15024 10017 15027
10021 15029 10017 15029 10013 15030 10009 15031 10005 15033 10002 15036
9998 15038 9995 15034 9992 15033 9987 15032 9983 15032 9979 15033
9974 15033 9971 15036 9969 15039 9969 15043 9966 15041 9963 15037
9960 15039 9960 15043 9956 15043 9952 15041 9949 15043 9946 15041
9939 15040 49 P
9996 15007 9999 15004 10003 15005 10007 15007 10011 15007 10015 15008
10020 15009 10024 15009 10028 15008 10027 15004 10025 14999 10024 14996
10024 14991 10027 14995 10030 14997 10034 15000 10038 15000 10038 15004
10042 15005 10046 15007 10049 15010 10046 15012 10044 15016 10039 15017
10042 15021 10038 15018 10036 15014 10032 15016 10028 15016 10023 15016

10020 15014 10015 15012 10011 15013 10007 15013 10002 15012 9996 15007
36 P
10050 15010 10053 15010 10056 15009 10059 15011 10056 15013 10053 15012
10050 15010 7 P
10056 15006 10059 15005 10061 15003 10064 15005 10067 15006 10070 15006
10073 15007 10071 15010 10067 15009 10064 15008 10061 15010 10056 15006
12:00 PM
10167 15134 10169 15131 10172 15127 10176 15126 10180 15124 10181 15128
10181 15133 10185 15133 10182 15137 10178 15137 10174 15138 10170 15139
10167 15134 13 P
10152 15034 10151 15032 10147 15031 10148 15027 10152 15027 10154 15028
10156 15031 10152 15034 8 P
9939 15004 9942 15002 9943 14999 9946 15000 9949 14999 9952 15000
9956 14999 9959 15000 9961 15003 9963 15005 9960 15006 9957 15005
9954 15006 9950 15006 9947 15006 9944 15006 9939 15004 17 P
9980 14995 9983 14992 9985 14994 9988 14995 9990 14997 9990 15000
9988 15003 9987 15000 9984 15001 9984 14998 9980 14995 11 P
10066 14927 10068 14925 10071 14923 10074 14924 10078 14925 10080 14923
10082 14925 10080 14928 10077 14929 10075 14927 10072 14928 10066 14927
12:00 PM
10096 14924 10099 14921 10101 14919 10102 14915 10104 14914 10106 14917
10108 14920 10106 14922 10102 14922 10096 14924 10 P
10082 14910 10085 14907 10088 14909 10090 14911 10092 14914 10090 14917
10087 14918 10086 14914 10083 14914 10082 14910 10 P
10429 15314 10432 15314 10435 15316 10438 15317 10441 15318 10444 15318
10447 15318 10445 15320 10444 15323 10441 15325 10438 15324 10435 15322
10432 15320 10429 15319 10429 15314 15 P
9908 14998 9911 14998 9914 14996 9916 14997 9920 14998 9920 15001
9919 15004 9916 15005 9913 15003 9911 15000 9908 14998 11 P
9907 15021 9910 15020 9913 15019 9916 15017 9918 15014 9921 15016
9924 15016 9922 15018 9919 15019 9916 15020 9914 15022 9911 15022
9907 15021 13 P
9668 14798 9669 14795 9669 14792 9670 14789 9674 14791 9675 14793
9675 14797 9673 14799 9674 14802 9675 14805 9672 14806 9669 14804
9671 14801 9668 14798 14 P
9196 15646 9199 15643 9203 15644 9205 15648 9209 15650 9213 15651
9218 15651 9219 15656 9219 15660 9218 15664 9214 15666 9211 15668
9209 15665 9204 15665 9204 15660 9205 15656 9200 15656 9198 15653
9196 15646 19 P
8356 14833 8360 14833 8365 14835 8368 14836 8371 14840 8374 14843
8378 14846 8379 14850 8377 14854 8373 14856 8370 14860 8368 14855
8370 14851 8374 14850 8374 14846 8371 14844 8368 14841 8365 14837
8361 14835 8356 14833 20 P

8392 14880 8396 14880 8401 14882 8404 14883 8409 14884 8413 14885
8415 14889 8417 14893 8419 14897 8423 14898 8424 14903 8422 14906
8417 14905 8415 14901 8412 14898 8410 14894 8408 14891 8404 14888
8401 14886 8396 14884 8392 14880 21 P
8475 14933 8470 14931 8467 14929 8463 14927 8459 14925 8455 14924
8455 14919 8452 14916 8448 14914 8444 14911 8441 14908 8437 14907
8437 14903 8434 14899 8430 14897 8426 14896 8430 14893 8433 14896
8438 14897 8442 14898 8446 14899 8450 14902 8452 14906 8455 14908
8458 14912 8460 14915 8464 14919 8467 14921 8470 14925 8473 14928
8475 14933 31 P
8396 14895 8399 14893 8402 14894 8404 14897 8406 14899 8407 14903
8407 14906 8406 14909 8404 14906 8402 14903 8400 14901 8398 14898
8396 14895 13 P
8272 14780 8276 14780 8279 14783 8283 14786 8285 14790 8287 14793
8289 14797 8292 14800 8294 14805 8295 14809 8293 14813 8291 14809
8289 14805 8285 14802 8282 14800 8282 14796 8281 14791 8278 14787
8275 14785 8272 14780 20 P
8293 14827 8292 14823 8294 14822 8297 14824 8299 14827 8301 14829
8301 14833 8298 14831 8295 14829 8293 14827 10 P
8310 14841 8313 14839 8315 14841 8318 14843 8320 14846 8321 14849
8322 14851 8324 14854 8326 14857 8323 14855 8321 14853 8318 14851
8318 14848 8315 14846 8313 14843 8310 14841 16 P
9247 14461 9250 14458 9254 14461 9255 14465 9260 14466 9264 14466
9266 14470 9266 14475 9262 14477 9257 14475 9254 14474 9251 14470
9250 14466 9247 14461 14 P
9224 14390 9227 14388 9232 14389 9236 14389 9240 14388 9242 14392
9239 14395 9235 14396 9231 14396 9231 14401 9230 14404 9228 14408
9225 14406 9225 14401 9224 14397 9224 14390 16 P
9167 14283 9169 14280 9172 14276 9176 14274 9179 14271 9181 14275
9181 14279 9181 14283 9181 14288 9177 14290 9173 14291 9169 14289
9167 14283 13 P
9204 14296 9207 14293 9210 14289 9212 14286 9215 14283 9219 14282
9223 14280 9226 14283 9225 14287 9223 14292 9221 14295 9217 14295
9213 14297 9209 14298 9204 14296 15 P
9221 14255 9225 14252 9229 14253 9232 14249 9234 14246 9238 14243
9240 14247 9239 14251 9238 14255 9240 14259 9238 14264 9235 14267
9233 14271 9231 14275 9228 14278 9225 14275 9223 14271 9224 14266
9225 14262 9225 14258 9221 14255 21 P
9309 14320 9311 14317 9314 14316 9316 14318 9316 14322 9314 14323
9311 14324 9309 14320 8 P
9289 14513 9292 14513 9295 14513 9298 14515 9300 14513 9301 14516
9299 14518 9298 14522 9294 14522 9293 14519 9291 14517 9289 14513

9287 14502 9289 14500 9291 14497 9293 14495 9295 14493 9296 14495
9294 14499 9297 14500 9294 14501 9293 14504 9291 14506 9287 14502

12:00 PM

9010 13068 9012 13064 9017 13063 9020 13066 9024 13068 9024 13073
9021 13076 9021 13081 9021 13085 9016 13085 9015 13081 9013 13077
9010 13074 9010 13068 14 P
8704 12528 8706 12523 8708 12520 8712 12518 8714 12514 8719 12514
8723 12512 8726 12514 8730 12515 8728 12519 8724 12521 8720 12521
8715 12522 8711 12524 8709 12528 8704 12528 16 P
9538 10900 9542 10895 9547 10890 9552 10886 9557 10880 9561 10875
9566 10869 9571 10865 9577 10868 9583 10865 9589 10862 9595 10857
9600 10853 9605 10848 9611 10844 9615 10839 9620 10834 9626 10830
9632 10826 9638 10823 9644 10821 9648 10815 9654 10811 9661 10811
9668 10809 9673 10804 9679 10802 9685 10799 9691 10796 9698 10794
9705 10791 9710 10787 9717 10784 9723 10781 9728 10776 9734 10775
9741 10773 9747 10768 9753 10765 9759 10763 9766 10762 9773 10763
9780 10761 9786 10759 9793 10758 9800 10755 9806 10753 9812 10749
9818 10746 9825 10744 50 P
9825 10744 9832 10741 9838 10739 9844 10738 9851 10736 9858 10735
9865 10734 9871 10738 9877 10741 9880 10747 9884 10753 9890 10756
9891 10763 9893 10770 9894 10777 9891 10783 9885 10787 9880 10791
9873 10794 9866 10795 9860 10794 9853 10796 9847 10798 9840 10800
9834 10804 9828 10807 9822 10812 9817 10815 9811 10818 9804 10822
9798 10823 9791 10826 9785 10829 9778 10830 9771 10832 9765 10834
9758 10836 9752 10839 9746 10842 9741 10847 9738 10854 9733 10858
9728 10863 9723 10868 9717 10870 9710 10872 9704 10875 9698 10879
9691 10881 9684 10883 50 P
9684 10883 9678 10885 2 P
9678 10885 9672 10889 9668 10894 9665 10900 9661 10907 9658 10912
9654 10918 9649 10923 9644 10928 9638 10931 9632 10935 9626 10937
9619 10938 9612 10940 9605 10939 9598 10939 9591 10937 9585 10936
9578 10933 9572 10931 9567 10926 9562 10922 9557 10917 9551 10913
9545 10909 9538 10900 26 P
9746 10718 9753 10715 9759 10712 9765 10709 9771 10706 9776 10701
9782 10698 9789 10695 9795 10691 9800 10687 9806 10683 9812 10681
9818 10677 9824 10674 9827 10668 9832 10662 9836 10657 9842 10653
9848 10655 9848 10662 9848 10669 9844 10675 9840 10681 9836 10686
9832 10691 9826 10695 9821 10700 9814 10702 9808 10706 9803 10709
9796 10711 9789 10714 9783 10715 9776 10718 9769 10719 9763 10721
9756 10723 9746 10718 38 P
9850 10687 9854 10681 9856 10675 9858 10668 9860 10661 9862 10655
9867 10650 9873 10647 9878 10642 9885 10645 9889 10651 9892 10657
9895 10663 9894 10670 9891 10676 9885 10680 9879 10683 9872 10686

9865 10686 9859 10688 9850 10687 21 P
9983 10008 9977 10012 9971 10013 9970 10020 9966 10025 9960 10028
9957 10022 9951 10019 9955 10013 9960 10008 9965 10004 9969 9998
9971 9991 9976 9986 9977 9979 9978 9972 9977 9965 9974 9959
9978 9953 9983 9949 9988 9943 9992 9938 9996 9932 9998 9925
10004 9922 10009 9926 10015 9931 10021 9933 10028 9932 10035 9934
10041 9930 10045 9924 10052 9924 10058 9925 10065 9926 10072 9924
10078 9921 10085 9918 10092 9916 10098 9914 10104 9912 10110 9908
10116 9904 10123 9901 10130 9902 10136 9903 10143 9906 10139 9912
10138 9919 10141 9925 50 P
10141 9925 10144 9932 10146 9938 10152 9943 10145 9946 10142 9952
10136 9956 10131 9961 10127 9966 10121 9970 10114 9969 10107 9970
10103 9976 10097 9979 10095 9985 10088 9985 10081 9985 10075 9988
10072 9994 10065 9993 10058 9994 10052 9995 10048 10001 10043 10006
10039 10012 10034 10016 10027 10017 10021 10014 10015 10010 10008 10009
10002 10007 9995 10009 9990 10014 9983 10008 34 P
10017 10090 10023 10086 10030 10083 10036 10079 10042 10076 10047 10071
10054 10070 10061 10070 10068 10070 10075 10068 10081 10065 10088 10063
10085 10070 10083 10077 10088 10081 10089 10088 10085 10094 10090 10099
10095 10104 10088 10104 10083 10109 10085 10116 10079 10119 10072 10116
10066 10120 10064 10113 10058 10110 10051 10108 10046 10104 10040 10106
10033 10106 10029 10101 10022 10098 10017 10090 34 P
10108 10197 10111 10190 10109 10183 10114 10178 10113 10171 10119 10168
10125 10165 10132 10164 10130 10171 10137 10172 10143 10176 10148 10172
10155 10173 10162 10171 10169 10171 10175 10168 10182 10167 10180 10174
10174 10177 10179 10181 10175 10187 10168 10189 10161 10189 10154 10190
10153 10197 10146 10196 10149 10190 10142 10191 10136 10193 10129 10195
10122 10193 10116 10195 10108 10197 33 P
10169 10325 10176 10326 10183 10326 10190 10327 10197 10330 10203 10330
10210 10330 10217 10330 10224 10333 10218 10336 10223 10342 10216 10342
10209 10341 10203 10336 10196 10335 10191 10340 10185 10342 10181 10348
10175 10351 10168 10350 10175 10346 10176 10340 10175 10333 10169 10325
24 P
10017 9771 10024 9771 10028 9776 10035 9777 10040 9772 10047 9772
10048 9766 10054 9762 10061 9759 10060 9752 10060 9745 10067 9745
10070 9752 10073 9745 10079 9749 10081 9742 10087 9740 10090 9747
10092 9754 10096 9760 10091 9765 10089 9772 10082 9774 10078 9779
10071 9781 10065 9785 10060 9790 10056 9795 10058 9802 10056 9809
10051 9814 10046 9809 10039 9808 10032 9807 10027 9803 10027 9796
10025 9789 10022 9783 10017 9771 39 P
10098 9839 10104 9836 10110 9832 10113 9825 10118 9821 10124 9817
10129 9813 10136 9811 10142 9807 10148 9810 10144 9816 10141 9822
10141 9829 10136 9833 10141 9837 10137 9842 10130 9845 10124 9846

10117 9849 10110 9847 10103 9846 10098 9839 22 P
10146 9899 10152 9895 10155 9889 10161 9885 10167 9881 10169 9874
10174 9870 10169 9865 10170 9858 10172 9851 10177 9846 10183 9845
10189 9849 10185 9854 10183 9861 10186 9867 10191 9871 10198 9874
10198 9881 10196 9888 10189 9889 10183 9892 10190 9892 10197 9894
10193 9899 10192 9906 10186 9909 10180 9906 10173 9904 10167 9906
10160 9905 10156 9911 10151 9906 10146 9899 34 P
9909 9922 9914 9917 9916 9910 9921 9906 9928 9907 9933 9912
9939 9916 9945 9920 9950 9924 9953 9930 9950 9937 9948 9944
9947 9950 9943 9956 9937 9959 9933 9965 9930 9971 9923 9971
9916 9970 9914 9963 9912 9956 9909 9950 9909 9943 9912 9936
9913 9929 9909 9922 26 P
10051 10023 10054 10017 10059 10012 10066 10014 10070 10020 10071 10027
10078 10029 10082 10034 10076 10037 10070 10041 10063 10043 10057 10039
10053 10034 10051 10023 14 P
10112 10067 10117 10062 10122 10067 10129 10069 10135 10067 10142 10069
10148 10071 10153 10077 10159 10079 10165 10083 10162 10077 10165 10070
10170 10066 10176 10069 10180 10075 10177 10081 10175 10088 10168 10090
10161 10089 10154 10088 10147 10087 10140 10087 10133 10086 10132 10079
10128 10074 10121 10072 10112 10067 27 P
10101 10074 10103 10067 10109 10071 10115 10074 10122 10076 10129 10076
10125 10082 10118 10084 10111 10084 10104 10085 10101 10074 11 P
10326 10341 10328 10337 10331 10341 10335 10341 10338 10337 10342 10336
10342 10340 10341 10344 10343 10349 10344 10353 10340 10353 10336 10351
10332 10349 10329 10345 10326 10341 15 P
10350 10350 10352 10346 10356 10346 10361 10347 10363 10342 10365 10339
10366 10343 10365 10348 10363 10352 10359 10353 10356 10356 10350 10350
12:00 PM
10229 10304 10233 10303 10238 10301 10242 10301 10240 10305 10243 10307
10247 10310 10244 10313 10240 10315 10236 10318 10234 10314 10230 10311
10229 10304 13 P
10168 9935 10170 9931 10167 9928 10168 9924 10172 9921 10176 9921
10177 9925 10176 9929 10176 9934 10179 9937 10183 9937 10188 9938
10186 9942 10182 9944 10180 9948 10182 9951 10180 9956 10177 9952
10173 9951 10169 9950 10170 9946 10170 9942 10168 9935 23 P
9956 9873 9959 9870 9962 9866 9966 9866 9971 9867 9975 9868
9979 9869 9984 9871 9987 9873 9988 9877 9988 9881 9985 9885
9981 9886 9978 9890 9977 9894 9973 9896 9972 9900 9968 9899
9966 9895 9962 9893 9958 9890 9958 9886 9958 9881 9956 9873
24 P
9993 9824 9989 9822 9985 9819 9981 9816 9978 9813 9977 9809
9979 9804 9978 9800 9981 9797 9986 9797 9990 9797 9994 9797
9997 9801 9999 9805 10001 9809 10004 9812 10006 9816 10008 9819

10009 9824 10007 9828 10002 9829 9999 9827 9993 9824 23 P
10096 9805 10101 9803 10103 9800 10106 9796 10108 9793 10112 9790
10116 9788 10119 9785 10123 9786 10127 9785 10131 9783 10135 9786
10139 9786 10144 9786 10144 9790 10140 9789 10139 9794 10135 9795
10132 9792 10128 9793 10123 9793 10119 9795 10115 9797 10112 9801
10115 9804 10117 9808 10112 9810 10109 9813 10105 9813 10101 9810
10096 9805 31 P
10127 9857 10131 9854 10135 9853 10140 9853 10144 9854 10148 9857
10152 9858 10152 9863 10155 9865 10153 9869 10149 9868 10145 9867
10141 9868 10136 9868 10132 9867 10131 9863 10127 9857 17 P
9940 9975 9943 9973 9948 9972 9951 9970 9950 9965 9954 9964
9958 9964 9959 9968 9960 9972 9963 9977 9962 9981 9964 9985
9960 9987 9957 9990 9953 9989 9949 9987 9945 9985 9942 9981
9940 9975 19 P
9999 9785 10000 9782 10000 9779 10001 9776 10000 9772 10003 9774
10005 9776 10008 9779 10009 9782 10010 9785 10014 9786 10014 9789
10013 9793 10009 9794 10006 9793 10003 9791 10002 9788 9999 9785
18 P
9987 10029 9990 10030 9993 10029 9995 10026 9997 10024 9994 10022
9993 10019 9996 10019 9999 10021 10002 10023 10004 10025 10006 10027
10008 10030 10010 10032 10008 10035 10008 10038 10006 10041 10003 10040
10000 10038 10000 10035 9998 10038 9995 10038 9992 10037 9993 10034
9990 10033 9987 10029 26 P
9955 10097 9956 10094 9957 10091 9960 10093 9963 10094 9965 10097
9966 10100 9964 10103 9966 10105 9965 10108 9968 10110 9970 10113
9971 10116 9969 10119 9966 10116 9966 10113 9964 10110 9961 10113
9960 10109 9960 10106 9959 10103 9957 10100 9955 10097 23 P
10347 10024 10348 10021 10349 10019 10353 10017 10356 10018 10359 10018
10358 10021 10357 10024 10354 10026 10350 10027 10347 10024 11 P
9948 9437 9953 9437 9957 9435 9959 9431 9962 9427 9966 9427
9969 9424 9970 9419 9970 9415 9967 9411 9965 9407 9969 9409
9973 9409 9978 9409 9982 9407 9981 9403 9980 9398 9984 9397
9988 9395 9991 9398 9995 9399 9993 9403 9994 9407 9994 9412
9999 9413 10002 9416 10000 9420 9995 9420 9998 9423 10002 9426
10005 9429 10003 9433 10001 9438 10001 9442 9999 9446 10001 9449
10001 9454 9997 9456 9993 9459 9989 9461 9987 9465 9984 9468
9980 9469 9976 9467 9976 9462 9972 9460 9968 9457 9964 9455
9960 9452 9956 9451 50 P
9956 9451 9952 9448 9950 9444 9948 9437 4 P
9905 9433 9907 9428 9911 9428 9915 9429 9920 9427 9924 9426
9928 9424 9930 9419 9933 9417 9937 9417 9942 9416 9945 9418
9950 9420 9947 9424 9951 9424 9954 9428 9957 9431 9954 9434
9950 9434 9947 9438 9948 9441 9945 9445 9941 9444 9937 9443

9933 9441 9928 9442 9924 9443 9921 9446 9916 9445 9913 9443
9909 9446 9906 9444 9907 9440 9905 9433 34 P
9908 9406 9913 9405 9917 9405 9916 9401 9921 9400 9923 9397
9927 9395 9931 9397 9930 9401 9933 9405 9929 9409 9933 9412
9930 9415 9927 9418 9922 9419 9918 9419 9914 9416 9915 9412
9908 9406 19 P
10016 9455 10020 9456 10025 9456 10027 9460 10029 9463 10033 9461
10037 9461 10041 9464 10044 9467 10039 9469 10035 9468 10031 9470
10031 9474 10036 9476 10033 9479 10035 9483 10031 9483 10027 9485
10025 9482 10027 9477 10024 9474 10028 9472 10024 9470 10021 9466
10017 9466 10017 9462 10016 9455 27 P
9607 8861 9610 8855 9610 8848 9614 8841 9617 8835 9622 8830
9622 8823 9615 8820 9621 8816 9620 8809 9627 8811 9634 8811
9640 8814 9647 8817 9653 8819 9653 8826 9659 8824 9665 8820
9668 8826 9662 8830 9663 8837 9669 8833 9676 8836 9678 8830
9685 8831 9681 8837 9687 8840 9687 8848 9680 8848 9674 8851
9670 8857 9672 8864 9669 8871 9667 8877 9669 8884 9674 8889
9681 8890 9678 8896 9673 8901 9667 8904 9661 8908 9654 8907
9647 8905 9643 8899 9641 8893 9643 8886 9637 8882 9633 8877
9627 8872 9622 8869 50 P
9622 8869 9617 8863 9607 8861 3 P
9567 8756 9571 8757 9575 8758 9580 8758 9584 8760 9588 8761
9593 8760 9591 8756 9593 8752 9596 8754 9601 8754 9605 8756
9609 8758 9614 8759 9617 8763 9621 8764 9617 8766 9614 8768
9611 8772 9608 8775 9604 8777 9600 8775 9596 8775 9591 8776
9588 8773 9583 8772 9584 8777 9580 8776 9579 8771 9575 8769
9571 8767 9567 8765 9568 8760 9567 8756 34 P
9558 8696 9561 8693 9564 8696 9567 8698 9572 8700 9575 8702
9579 8700 9583 8697 9583 8702 9587 8704 9589 8708 9593 8711
9597 8711 9594 8714 9589 8711 9585 8710 9581 8708 9577 8709
9574 8713 9571 8716 9566 8716 9566 8711 9562 8709 9562 8705
9561 8701 9558 8696 26 P
9593 8684 9597 8683 9602 8682 9606 8683 9610 8682 9614 8685
9619 8684 9622 8688 9625 8686 9630 8684 9633 8682 9638 8683
9639 8687 9639 8692 9638 8696 9634 8699 9639 8701 9640 8696
9643 8700 9646 8703 9644 8707 9645 8711 9640 8712 9638 8708
9634 8706 9630 8707 9626 8705 9629 8702 9627 8697 9623 8697
9618 8696 9616 8700 9612 8703 9610 8699 9607 8696 9604 8693
9600 8691 9596 8689 9593 8684 39 P
9640 8786 9637 8784 9633 8781 9631 8778 9634 8775 9636 8771
9640 8770 9643 8773 9646 8775 9651 8778 9655 8779 9659 8781
9662 8783 9658 8784 9654 8782 9649 8785 9645 8785 9640 8786
18 P

9580 8482 9583 8479 9587 8476 9590 8473 9590 8477 9589 8481
9593 8480 9597 8478 9602 8476 9606 8476 9610 8473 9609 8477
9607 8481 9602 8482 9599 8486 9596 8489 9592 8490 9588 8494
9584 8494 9588 8491 9591 8489 9587 8489 9585 8486 9580 8482
24 P
9904 5748 9908 5744 9910 5739 9913 5734 9918 5732 9921 5727
9927 5727 9926 5733 9928 5739 9929 5744 9929 5750 9923 5752
9918 5753 9913 5750 9904 5748 15 P
9315 5524 9319 5522 9323 5524 9326 5528 9329 5531 9333 5534
9335 5538 9332 5542 9327 5543 9323 5541 9319 5538 9320 5533
9318 5529 9315 5524 14 P
8866 6218 8872 6214 8878 6210 8882 6204 8889 6202 8896 6203
8902 6206 8902 6214 8907 6219 8912 6224 8918 6227 8923 6232
8931 6232 8938 6232 8945 6229 8942 6236 8940 6243 8938 6250
8935 6256 8930 6251 8928 6258 8921 6255 8920 6248 8918 6255
8914 6249 8910 6243 8910 6250 8911 6257 8904 6257 8901 6251
8896 6245 8896 6237 8892 6244 8888 6250 8882 6246 8880 6239
8875 6233 8882 6236 8885 6229 8878 6230 8884 6226 8891 6226
8898 6226 8895 6219 8889 6216 8886 6209 8881 6215 8875 6219
8872 6225 8866 6218 50 P
8870 6289 8875 6284 8880 6290 8886 6285 8891 6279 8893 6273
8896 6266 8899 6273 8898 6280 8902 6285 8899 6291 8896 6298
8889 6299 8882 6296 8875 6295 8870 6289 16 P
8828 6231 8835 6232 8842 6234 8849 6233 8851 6240 8852 6247
8856 6253 8855 6260 8854 6267 8860 6271 8866 6276 8859 6277
8853 6281 8848 6276 8845 6269 8846 6262 8845 6255 8841 6250
8846 6245 8844 6239 8837 6238 8828 6231 22 P
8840 6112 8844 6111 8849 6112 8853 6111 8851 6116 8855 6119
8852 6123 8851 6127 8851 6132 8846 6132 8844 6136 8843 6131
8842 6127 8844 6123 8843 6118 8840 6112 16 P
8801 6029 8805 6026 8807 6022 8807 6017 8811 6015 8812 6019
8816 6022 8819 6026 8820 6030 8817 6034 8813 6034 8809 6032
8801 6029 13 P
8894 6334 8896 6331 8896 6328 8898 6331 8900 6334 8902 6336
8906 6337 8907 6340 8904 6343 8901 6345 8899 6343 8898 6339
8895 6337 8894 6334 14 P
9120 6189 9121 6184 9121 6180 9123 6175 9124 6170 9125 6166
9126 6171 9124 6175 9124 6180 9124 6184 9125 6189 9127 6193
9127 6198 9128 6202 9130 6206 9132 6210 9135 6214 9138 6218
9137 6223 9134 6219 9132 6215 9130 6211 9127 6206 9124 6203
9123 6198 9121 6194 9120 6189 27 P
8917 7332 8924 7335 8930 7338 8937 7338 8943 7342 8942 7335
8942 7328 8935 7329 8931 7323 8937 7318 8933 7312 8938 7306

8943 7302 8950 7299 8955 7294 8960 7290 8967 7288 8971 7294
8967 7301 8961 7304 8964 7311 8969 7315 8976 7318 8981 7323
8988 7324 8993 7329 9000 7330 9005 7325 9011 7329 9012 7336
9005 7336 8998 7338 8992 7341 8987 7346 8985 7353 8985 7360
8987 7367 8993 7372 8998 7375 9005 7377 9010 7372 9016 7373
9021 7379 9025 7385 9017 7385 9010 7384 9003 7387 8998 7392
8992 7389 8995 7396 50 P
8995 7396 9000 7401 8996 7408 8990 7411 8985 7416 8978 7413
8972 7416 8967 7422 8967 7429 8972 7434 8975 7440 8968 7440
8963 7437 8957 7432 8954 7426 8948 7429 8943 7424 8940 7417
8933 7417 8930 7411 8925 7405 8926 7398 8930 7392 8937 7394
8941 7400 8948 7400 8951 7393 8949 7386 8946 7380 8946 7373
8945 7366 8944 7359 8950 7355 8955 7360 8958 7366 8964 7361
8971 7361 8971 7353 8965 7350 8959 7346 8952 7346 8947 7351
8940 7352 8933 7353 8929 7348 8922 7346 8921 7339 8917 7332
48 P
8799 7191 8805 7186 8802 7179 8807 7174 8814 7174 8821 7170
8826 7175 8833 7177 8839 7173 8836 7166 8836 7159 8842 7155
8849 7153 8856 7153 8858 7160 8853 7165 8857 7171 8864 7172
8869 7177 8862 7176 8857 7181 8852 7175 8845 7176 8839 7180
8833 7183 8828 7188 8828 7196 8821 7197 8819 7204 8814 7198
8809 7193 8799 7191 32 P
8200 6714 8205 6710 8209 6704 8216 6701 8222 6698 8229 6702
8231 6709 8235 6715 8241 6719 8247 6723 8254 6725 8261 6726
8268 6724 8274 6721 8280 6717 8286 6713 8293 6716 8297 6722
8297 6729 8302 6735 8298 6741 8298 6748 8294 6754 8292 6761
8289 6768 8283 6770 8279 6776 8273 6780 8269 6775 8263 6771
8265 6764 8260 6759 8255 6755 8253 6748 8249 6742 8242 6744
8235 6746 8229 6750 8224 6754 8219 6759 8212 6756 8207 6751
8208 6744 8215 6748 8212 6741 8214 6734 8216 6727 8218 6720
8211 6719 8200 6714 50 P
8089 6741 8092 6735 8097 6729 8103 6726 8109 6722 8116 6721
8123 6719 8129 6723 8134 6728 8140 6733 8144 6738 8147 6745
8149 6751 8156 6753 8156 6760 8156 6767 8156 6775 8151 6780
8145 6777 8141 6782 8136 6788 8129 6785 8124 6790 8117 6792
8110 6794 8111 6787 8105 6783 8104 6776 8100 6769 8095 6764
8091 6758 8093 6751 8100 6752 8105 6757 8112 6761 8113 6753
8107 6750 8100 6747 8089 6741 39 P
7690 6231 7698 6231 7705 6233 7712 6235 7718 6233 7716 6226
7722 6230 7729 6233 7736 6235 7743 6236 7750 6238 7757 6238
7754 6244 7746 6245 7745 6252 7745 6259 7745 6266 7748 6273
7752 6279 7759 6282 7766 6279 7772 6276 7779 6276 7780 6283
7775 6288 7775 6295 7777 6302 7784 6306 7781 6313 7787 6317

7793 6313 7801 6314 7802 6321 7808 6323 7801 6323 7797 6329
7790 6332 7786 6337 7780 6332 7773 6334 7770 6328 7762 6329
7756 6325 7753 6332 7749 6338 7743 6343 7737 6346 7732 6351
7726 6348 7724 6341 50 P
7724 6341 7727 6334 7727 6327 7722 6322 7717 6316 7714 6309
7710 6303 7705 6298 7707 6291 7714 6287 7719 6293 7726 6295
7731 6290 7738 6291 7745 6289 7740 6285 7738 6277 7734 6273
7728 6268 7722 6264 7717 6258 7713 6253 7714 6245 7710 6251
7703 6247 7698 6243 7690 6231 27 P
7526 6160 7522 6166 7515 6169 7509 6167 7503 6163 7498 6158
7493 6153 7497 6146 7504 6145 7511 6144 7518 6144 7525 6144
7532 6142 7538 6138 7544 6134 7552 6134 7558 6137 7566 6138
7572 6136 7574 6129 7581 6132 7586 6127 7592 6131 7596 6137
7595 6144 7593 6151 7599 6154 7598 6161 7591 6165 7589 6172
7590 6179 7583 6179 7578 6173 7576 6166 7569 6168 7564 6173
7558 6177 7551 6175 7547 6169 7544 6162 7539 6158 7534 6152
7526 6160 43 P
7527 6227 7520 6226 7513 6227 7510 6220 7505 6215 7498 6213
7491 6212 7492 6205 7499 6201 7506 6200 7513 6198 7520 6196
7522 6203 7523 6210 7526 6217 7533 6219 7527 6227 17 P
7483 6223 7488 6218 7495 6217 7503 6216 7508 6220 7505 6227
7506 6234 7511 6240 7513 6246 7506 6248 7500 6244 7500 6237
7497 6230 7490 6227 7483 6223 15 P
7334 6091 7339 6085 7343 6079 7349 6075 7355 6072 7360 6066
7366 6064 7373 6066 7378 6060 7381 6054 7388 6051 7395 6049
7388 6045 7392 6039 7398 6035 7405 6034 7407 6027 7415 6027
7417 6034 7421 6039 7415 6042 7409 6039 7404 6045 7402 6052
7403 6059 7404 6066 7402 6073 7398 6079 7397 6086 7392 6091
7385 6094 7379 6089 7372 6089 7365 6091 7360 6096 7356 6102
7348 6102 7342 6099 7334 6091 39 P
8712 7324 8716 7318 8720 7312 8721 7305 8724 7298 8730 7302
8736 7305 8743 7305 8743 7313 8741 7319 8741 7326 8740 7333
8742 7340 8746 7347 8748 7353 8747 7360 8750 7367 8750 7374
8749 7381 8747 7388 8740 7384 8736 7379 8733 7372 8727 7368
8726 7361 8724 7354 8721 7348 8719 7341 8717 7334 8712 7324
30 P
8842 7677 8849 7676 8856 7675 8862 7673 8870 7673 8875 7668
8881 7665 8888 7665 8895 7667 8895 7675 8893 7681 8890 7688
8888 7695 8881 7698 8875 7701 8869 7705 8863 7701 8857 7699
8852 7693 8848 7688 8842 7677 21 P
8846 7671 8850 7669 8853 7666 8857 7663 8862 7664 8866 7665
8870 7665 8874 7667 8871 7670 8866 7672 8862 7672 8858 7673
8853 7674 8846 7671 14 P

8893 8005 8895 8001 8898 7998 8898 7993 8902 7993 8904 7997
8908 7995 8912 7993 8917 7994 8921 7996 8925 7996 8927 8000
8930 8004 8934 8006 8938 8007 8943 8008 8945 8012 8945 8016
8941 8018 8936 8018 8933 8014 8932 8010 8930 8006 8925 8008
8927 8012 8923 8012 8918 8011 8914 8012 8909 8013 8905 8012
8901 8012 8896 8011 8893 8005 33 P
9225 8179 9227 8175 9230 8171 9234 8169 9238 8167 9241 8164
9246 8164 9250 8167 9254 8169 9258 8170 9263 8170 9259 8172
9255 8174 9253 8178 9252 8182 9250 8186 9245 8187 9242 8190
9238 8191 9233 8190 9230 8187 9226 8184 9225 8179 23 P
10665 6825 10661 6826 10657 6824 10652 6823 10648 6825 10644 6823
10642 6819 10638 6818 10636 6813 10632 6812 10635 6808 10640 6808
10644 6807 10649 6806 10653 6806 10658 6806 10661 6808 10666 6807
10671 6806 10675 6808 10678 6811 10682 6814 10685 6818 10688 6820
10685 6822 10680 6825 10676 6826 10672 6827 10665 6825 29 P
11075 6613 11078 6609 11083 6609 11087 6608 11092 6609 11096 6608
11100 6610 11103 6614 11105 6618 11102 6622 11098 6623 11093 6623
11089 6623 11085 6622 11081 6619 11076 6618 11075 6613 17 P
11528 6993 11529 6989 11533 6986 11537 6985 11542 6984 11547 6985
11551 6987 11554 6990 11557 6993 11557 6998 11556 7003 11552 7005
11548 7006 11543 7007 11539 7007 11534 7005 11531 7003 11528 6999
11528 6993 19 P
11760 7112 11763 7108 11766 7104 11770 7103 11774 7101 11779 7102
11782 7105 11787 7106 11790 7105 11795 7104 11800 7105 11804 7107
11806 7111 11805 7116 11803 7120 11801 7124 11799 7128 11795 7130
11790 7131 11786 7133 11782 7135 11778 7137 11773 7138 11770 7134
11770 7130 11770 7125 11770 7121 11766 7119 11761 7120 11760 7112
30 P
11527 7052 11530 7050 11533 7049 11536 7048 11540 7047 11541 7050
11539 7053 11536 7055 11533 7056 11530 7055 11527 7052 11 P
11195 6736 11193 6735 11192 6731 11192 6728 11194 6725 11196 6722
11197 6719 11200 6717 11204 6717 11207 6719 11209 6722 11212 6724
11213 6727 11212 6730 11210 6734 11207 6734 11203 6734 11200 6735
11195 6736 19 P
11192 6783 11189 6785 11187 6787 11187 6791 11184 6793 11180 6793
11177 6793 11174 6792 11173 6788 11175 6786 11174 6783 11171 6781
11167 6781 11171 6780 11174 6780 11178 6780 11181 6779 11185 6780
11187 6778 11190 6776 11193 6778 11195 6780 11192 6783 23 P
11233 6778 11234 6775 11237 6773 11240 6772 11243 6775 11245 6777
11246 6781 11245 6784 11242 6787 11239 6788 11236 6789 11235 6786
11234 6783 11233 6778 14 P
11156 6712 11158 6709 11161 6708 11164 6709 11164 6713 11161 6715
11158 6715 11156 6712 8 P

11164 6717 11167 6719 11169 6721 11172 6723 11173 6727 11170 6728
11167 6726 11165 6723 11164 6717 9 P
10965 6684 10968 6683 10970 6684 10972 6685 10974 6686 10972 6687
10970 6687 10968 6686 10965 6684 9 P
10880 7583 10881 7581 10883 7581 10885 7582 10888 7583 10889 7585
10891 7584 10893 7584 10896 7585 10898 7585 10898 7587 10896 7589
10895 7590 10893 7590 10891 7591 10889 7591 10886 7591 10884 7589
10883 7588 10881 7586 10880 7583 21 P
10921 7580 10922 7578 10924 7576 10926 7578 10925 7579 10924 7581
10921 7580 7 P
10956 7680 10958 7679 10960 7678 10962 7679 10963 7681 10962 7683
10961 7685 10959 7685 10957 7684 10956 7680 10 P
10145 7464 10147 7462 10148 7461 10150 7463 10152 7465 10152 7467
10150 7467 10147 7467 10145 7466 10145 7464 10 P
10164 7383 10165 7381 10167 7382 10167 7384 10164 7383 5 P
10095 7013 10097 7011 10099 7010 10101 7008 10103 7007 10105 7008
10107 7008 10109 7007 10111 7008 10112 7011 10110 7012 10109 7013
10108 7015 10106 7017 10103 7017 10101 7016 10101 7014 10099 7012
10095 7013 19 P
8935 6058 8937 6056 8936 6054 8937 6052 8939 6051 8939 6053
8940 6055 8942 6056 8943 6058 8942 6061 8940 6061 8938 6060
8935 6058 13 P
8749 6136 8750 6135 8752 6135 8755 6136 8756 6138 8757 6140
8755 6140 8753 6141 8751 6141 8750 6139 8749 6136 11 P
8906 6052 8907 6051 8909 6050 8911 6051 8913 6053 8911 6055
8909 6055 8907 6054 8906 6052 9 P
8857 6117 8859 6115 8861 6115 8862 6117 8864 6118 8866 6118
8868 6118 8869 6120 8868 6123 8865 6123 8863 6123 8861 6122
8860 6120 8858 6119 8857 6117 15 P
8849 6188 8851 6186 8853 6186 8855 6187 8857 6188 8859 6188
8862 6189 8863 6190 8865 6192 8867 6192 8867 6194 8865 6195
8863 6194 8861 6194 8859 6193 8857 6192 8856 6190 8853 6189
8852 6190 8850 6191 8849 6188 21 P
8850 6062 8852 6061 8854 6061 8855 6063 8853 6064 8850 6062

6:00 PM

8855 6054 8857 6054 8858 6056 8860 6058 8859 6060 8857 6059
8856 6058 8855 6054 8 P
8164 6713 8167 6712 8171 6711 8173 6710 8176 6713 8179 6715
8182 6713 8184 6715 8185 6718 8187 6720 8191 6720 8192 6722
8193 6726 8194 6729 8193 6732 8190 6734 8187 6736 8184 6737
8181 6735 8177 6735 8175 6734 8174 6730 8173 6727 8172 6724
8170 6722 8168 6719 8169 6715 8164 6713 28 P
7950 6744 7954 6742 7958 6740 7961 6736 7964 6733 7967 6729

7970 6727 7975 6725 7980 6726 7984 6726 7989 6726 7993 6727
7990 6731 7990 6736 7985 6737 7982 6735 7977 6734 7973 6736
7970 6740 7968 6743 7963 6743 7959 6741 7955 6744 7950 6744
24 P
7953 6747 7956 6744 7961 6743 7965 6745 7969 6744 7973 6740
7975 6736 7980 6736 7984 6738 7988 6740 7991 6737 7994 6733
7998 6731 8002 6734 8007 6734 8005 6738 8003 6742 7999 6745
7996 6749 7992 6751 7988 6753 7984 6753 7980 6755 7975 6755
7970 6754 7966 6752 7962 6751 7957 6750 7953 6747 29 P
8023 6711 8026 6709 8031 6710 8034 6708 8039 6710 8042 6712
8039 6715 8034 6716 8030 6715 8023 6711 10 P
7979 6722 7983 6721 7987 6718 7991 6716 7996 6716 7997 6720
7992 6722 7988 6724 7984 6724 7979 6722 10 P
7942 6755 7943 6752 7945 6749 7947 6747 7951 6746 7952 6749
7950 6751 7947 6754 7946 6757 7944 6760 7942 6755 11 P
8623 7082 8627 7081 8631 7080 8633 7076 8634 7072 8639 7070
8638 7075 8640 7079 8643 7082 8646 7085 8645 7090 8647 7093
8644 7097 8644 7101 8647 7104 8652 7106 8655 7109 8654 7113
8650 7111 8646 7109 8642 7109 8638 7106 8635 7103 8631 7102
8631 7097 8633 7093 8636 7090 8635 7085 8632 7083 8627 7084
8623 7082 31 P
9031 7715 9035 7714 9039 7712 9043 7715 9047 7715 9051 7717
9055 7720 9057 7723 9059 7728 9054 7729 9050 7729 9047 7724
9044 7722 9041 7725 9037 7725 9039 7729 9036 7732 9034 7728
9032 7724 9032 7720 9031 7715 21 P
9075 7768 9070 7767 9067 7764 9063 7762 9059 7759 9060 7755
9059 7750 9058 7746 9061 7743 9062 7748 9062 7752 9064 7756
9068 7758 9073 7758 9077 7757 9081 7753 9083 7757 9082 7762
9079 7764 9075 7768 20 P
9042 7620 9044 7617 9046 7615 9049 7616 9052 7618 9054 7620
9053 7623 9049 7623 9046 7622 9042 7620 10 P
9061 7604 9066 7607 9070 7608 9073 7611 9076 7615 9071 7614
9067 7615 9065 7611 9061 7604 9 P
9315 7851 9320 7850 9323 7852 9326 7856 9330 7855 9329 7859
9326 7862 9321 7861 9318 7858 9315 7851 10 P
9309 7859 9312 7861 9313 7857 9315 7859 9318 7862 9316 7865
9313 7866 9311 7863 9309 7859 9 P
9128 7958 9132 7956 9135 7952 9139 7954 9144 7956 9143 7961
9147 7962 9152 7963 9152 7968 9154 7971 9154 7976 9151 7980
9147 7981 9145 7977 9142 7973 9139 7970 9138 7965 9133 7963
9128 7958 19 P
9084 7916 9088 7915 9093 7916 9095 7920 9099 7919 9103 7920
9104 7925 9104 7929 9104 7934 9099 7934 9098 7930 9095 7933

9090 7931 9087 7928 9087 7923 9084 7916 16 P
8947 7984 8949 7981 8951 7978 8954 7978 8957 7976 8960 7977
8963 7979 8965 7982 8968 7982 8972 7980 8974 7983 8976 7986
8973 7988 8970 7990 8967 7991 8964 7990 8961 7989 8958 7988
8954 7988 8951 7986 8947 7984 21 P
9470 8765 9473 8764 9476 8761 9479 8760 9482 8758 9485 8756
9487 8754 9490 8752 9493 8751 9496 8750 9500 8751 9503 8752
9506 8754 9508 8757 9510 8759 9510 8763 9508 8766 9505 8767
9502 8768 9499 8768 9495 8770 9492 8769 9489 8768 9485 8768
9482 8768 9479 8768 9475 8766 9470 8765 28 P
9422 8778 9425 8777 9427 8774 9429 8772 9432 8770 9435 8769
9438 8768 9441 8771 9441 8774 9442 8777 9439 8779 9437 8782
9436 8785 9433 8787 9430 8788 9427 8789 9423 8791 9420 8791
9416 8792 9416 8788 9417 8785 9419 8782 9422 8778 23 P
9586 8661 9589 8660 9591 8659 9594 8660 9596 8661 9598 8664
9597 8666 9595 8668 9593 8670 9590 8668 9589 8666 9588 8664
9586 8661 13 P
9655 8569 9656 8567 9658 8569 9660 8571 9660 8573 9661 8575
9659 8575 9657 8573 9656 8571 9655 8569 10 P
9648 8574 9650 8574 9651 8572 9653 8572 9655 8573 9657 8574
9659 8576 9660 8578 9661 8579 9663 8581 9661 8582 9659 8582
9657 8583 9656 8581 9655 8579 9654 8578 9651 8577 9648 8574
18 P
9829 9085 9830 9082 9834 9081 9836 9079 9839 9077 9842 9080
9840 9083 9842 9085 9846 9086 9846 9090 9842 9091 9843 9094
9841 9097 9840 9100 9836 9101 9833 9100 9832 9097 9832 9093
9830 9090 9829 9085 20 P
9837 9071 9839 9069 9841 9066 9844 9065 9848 9066 9850 9069
9848 9072 9845 9073 9842 9074 9837 9071 10 P
9964 9081 9967 9082 9971 9082 9969 9079 9971 9077 9970 9075
9967 9072 9969 9070 9972 9071 9974 9068 9977 9070 9979 9072
9982 9071 9981 9074 9983 9077 9985 9080 9987 9083 9984 9083
9980 9083 9977 9085 9977 9088 9977 9092 9974 9091 9971 9090
9970 9087 9967 9085 9964 9081 27 P
9964 9043 9968 9044 9971 9045 9972 9048 9975 9045 9978 9048
9980 9050 9983 9051 9986 9054 9988 9056 9986 9058 9982 9058
9979 9058 9976 9056 9973 9055 9971 9052 9971 9049 9968 9047
9964 9043 19 P
9939 9236 9942 9234 9944 9236 9947 9239 9949 9241 9951 9243
9951 9246 9951 9250 9954 9252 9957 9251 9956 9254 9955 9257
9953 9260 9949 9260 9947 9258 9946 9255 9947 9251 9946 9248
9943 9248 9944 9244 9944 9241 9942 9239 9939 9236 23 P
9946 9213 9949 9213 9952 9210 9955 9210 9958 9212 9958 9215

9955 9217 9958 9220 9960 9222 9962 9225 9965 9226 9969 9226
9969 9229 9972 9229 9976 9230 9973 9232 9969 9232 9966 9230
9964 9227 9962 9225 9958 9226 9956 9224 9952 9223 9949 9224
9947 9221 9948 9218 9946 9213 27 P
9905 9190 9908 9189 9912 9188 9914 9186 9916 9183 9920 9183
9922 9185 9923 9189 9923 9192 9926 9194 9928 9197 9924 9197
9922 9194 9920 9192 9917 9190 9914 9190 9912 9192 9913 9195
9909 9196 9906 9197 9908 9194 9905 9190 22 P
10088 9607 10090 9604 10093 9606 10096 9605 10098 9608 10101 9610
10101 9613 10104 9615 10107 9616 10110 9617 10109 9620 10106 9619
10103 9621 10101 9618 10097 9617 10098 9614 10096 9611 10093 9609
10090 9611 10088 9607 20 P
10094 9768 10097 9767 10100 9765 10103 9764 10106 9762 10108 9760
10111 9758 10114 9756 10115 9759 10118 9760 10121 9759 10125 9759
10127 9757 10129 9754 10130 9758 10131 9761 10128 9762 10125 9761
10122 9761 10118 9763 10115 9764 10112 9765 10110 9767 10107 9767
10106 9771 10103 9773 10100 9773 10098 9775 10095 9775 10095 9771
10094 9768 31 P
10051 9905 10053 9902 10056 9902 10059 9902 10063 9901 10066 9903
10068 9905 10066 9908 10065 9911 10061 9912 10058 9911 10056 9909
10051 9905 13 P
10053 9914 10057 9913 10060 9915 10063 9914 10066 9915 10069 9913
10072 9910 10074 9908 10077 9909 10078 9913 10076 9916 10073 9917
10071 9920 10069 9923 10066 9924 10063 9923 10061 9920 10058 9918
10053 9914 19 P
10039 9910 10040 9907 10043 9908 10046 9908 10047 9912 10048 9915
10046 9918 10043 9919 10040 9918 10039 9915 10039 9910 11 P
10021 9927 10019 9924 10021 9921 10020 9918 10021 9915 10024 9915
10027 9917 10030 9919 10029 9922 10027 9925 10024 9927 10021 9927
12:00 PM
10001 9900 10002 9897 10002 9893 10006 9892 10009 9892 10011 9894
10013 9897 10015 9900 10015 9903 10014 9906 10011 9908 10008 9908
10005 9906 10002 9904 10001 9900 15 P
10280 10416 10283 10415 10286 10413 10289 10413 10292 10413 10295 10415
10298 10415 10298 10419 10295 10420 10292 10418 10289 10420 10287 10422
10284 10424 10283 10420 10285 10418 10280 10416 16 P
10252 10399 10256 10399 10259 10399 10262 10397 10265 10397 10266 10400
10263 10401 10260 10401 10256 10403 10252 10399 10 P
10268 10398 10271 10396 10274 10398 10277 10399 10275 10402 10272 10404
10270 10401 10268 10398 8 P
10334 10385 10336 10383 10339 10385 10342 10386 10339 10389 10334 10385
6:00 PM
9995 10256 9998 10254 10001 10254 10002 10251 10004 10248 10007 10249

10009 10251 10009 10255 10012 10257 10015 10259 10012 10260 10008 10261
10005 10261 10003 10257 10000 10258 9995 10256 16 P
9991 10283 9993 10280 9995 10278 9999 10279 10000 10282 10001 10285
10004 10287 10001 10286 9998 10288 9997 10285 9994 10286 9991 10283
12:00 PM
10100 10289 10103 10288 10106 10290 10108 10292 10107 10295 10104 10296
10106 10293 10102 10292 10100 10289 9 P
10094 10220 10097 10219 10101 10220 10102 10223 10103 10226 10100 10228
10097 10226 10095 10224 10094 10220 9 P
10124 10222 10127 10222 10130 10220 10132 10219 10136 10218 10137 10221
10136 10224 10137 10227 10136 10230 10133 10228 10133 10225 10130 10224
10127 10225 10124 10222 14 P
10191 10175 10194 10175 10197 10177 10200 10176 10201 10172 10203 10170
10206 10171 10208 10174 10205 10176 10203 10178 10202 10181 10200 10184
10197 10183 10195 10180 10191 10175 15 P
10207 10065 10210 10064 10213 10063 10216 10062 10219 10061 10221 10064
10219 10067 10217 10069 10213 10069 10210 10069 10207 10065 11 P
10285 10058 10288 10056 10289 10053 10290 10050 10294 10049 10295 10052
10294 10055 10293 10058 10292 10062 10289 10062 10285 10058 11 P
10303 10058 10305 10056 10307 10053 10310 10052 10311 10049 10312 10046
10313 10049 10313 10052 10313 10056 10310 10058 10308 10060 10303 10058
12:00 PM
10317 10058 10318 10055 10320 10052 10323 10050 10323 10053 10321 10057
10323 10060 10321 10063 10317 10058 9 P
10325 10043 10326 10041 10329 10041 10331 10043 10329 10045 10328 10048
10325 10043 7 P
10204 9905 10205 9902 10207 9899 10210 9900 10210 9903 10209 9906
10208 9909 10205 9909 10204 9905 9 P
10065 10057 10066 10055 10068 10053 10071 10051 10073 10051 10076 10052
10077 10055 10076 10057 10074 10059 10072 10060 10069 10060 10065 10057
12:00 PM
9793 9441 9796 9439 9799 9438 9801 9435 9804 9435 9807 9436
9811 9437 9814 9438 9815 9440 9814 9444 9813 9446 9810 9447
9807 9445 9807 9442 9804 9440 9801 9441 9798 9443 9795 9443
9793 9441 19 P
9979 9205 9981 9203 9982 9200 9985 9201 9987 9204 9991 9205
9992 9207 9990 9210 9987 9208 9983 9208 9979 9205 11 P
9790 8836 9794 8837 9797 8838 9797 8842 9799 8845 9803 8845
9805 8846 9806 8850 9805 8853 9801 8852 9800 8850 9797 8847
9795 8845 9792 8843 9790 8840 9790 8836 16 P
8910 7428 8914 7425 8918 7425 8923 7425 8927 7426 8932 7427
8936 7425 8939 7428 8940 7432 8938 7437 8937 7440 8933 7438
8929 7436 8925 7434 8921 7432 8917 7434 8913 7434 8910 7428

18 P
8857 7425 8859 7422 8864 7421 8869 7421 8871 7425 8875 7426
8878 7430 8880 7433 8882 7437 8882 7442 8878 7441 8875 7438
8871 7436 8868 7432 8866 7428 8864 7424 8862 7429 8857 7425
18 P
8902 7436 8904 7436 8907 7436 8910 7437 8913 7438 8913 7440
8910 7442 8909 7445 8906 7444 8903 7443 8903 7440 8902 7436
12:00 PM
8926 7245 8928 7242 8927 7240 8927 7236 8929 7239 8932 7240
8935 7242 8938 7244 8935 7245 8932 7247 8931 7250 8933 7253
8932 7256 8929 7254 8928 7252 8926 7249 8926 7245 17 P
8930 7270 8931 7267 8933 7264 8937 7266 8940 7265 8943 7267
8946 7268 8949 7270 8947 7272 8944 7274 8945 7277 8942 7276
8939 7275 8936 7275 8936 7272 8930 7270 16 P
8209 6799 8212 6797 8215 6796 8218 6794 8221 6792 8223 6790
8227 6789 8230 6790 8230 6793 8231 6797 8234 6799 8232 6802
8229 6803 8226 6804 8222 6804 8219 6804 8215 6802 8212 6801
8209 6799 19 P
5904 5508 5904 5515 5908 5521 5901 5524 5908 5527 5915 5525
5918 5532 5916 5539 5916 5547 5921 5552 5924 5558 5930 5554
5936 5559 5937 5566 5933 5572 5927 5567 5921 5563 5914 5563
5908 5558 5903 5553 5897 5550 5893 5543 5885 5544 5878 5542
5872 5539 5866 5535 5862 5541 5855 5542 5847 5541 5843 5535
5838 5530 5832 5527 5825 5525 5827 5518 5822 5513 5817 5508
5816 5501 5816 5501 38 P
5839 5501 5846 5502 5847 5501 3 P
5861 5501 5864 5502 5871 5505 5878 5506 5885 5509 5890 5503
5891 5501 7 P
5911 5501 5912 5501 2 P
5912 5501 5904 5508 2 P
5967 5528 5972 5522 5974 5515 5981 5512 5988 5510 5992 5505
5999 5506 6006 5503 6006 5501 9 P
6023 5501 6024 5506 6029 5502 6030 5501 4 P
6039 5501 6038 5506 6042 5512 6038 5519 6034 5524 6031 5531
6024 5532 6017 5536 6010 5536 6005 5532 5998 5531 5991 5533
5984 5536 5977 5535 5967 5528 15 P
5969 5501 5970 5507 5964 5506 5962 5501 5962 5501 5 P
6054 5507 6056 5505 6058 5504 6058 5502 6060 5503 6062 5503
6063 5506 6064 5508 6064 5510 6064 5512 6063 5514 6061 5514
6059 5512 6057 5512 6055 5510 6054 5507 16 P
5540 5517 5543 5513 5545 5509 5547 5505 5549 5501 5 P
5575 5501 5574 5505 5573 5509 5571 5514 5569 5518 5567 5522
5563 5525 5559 5528 5556 5530 5552 5534 5549 5530 5545 5528

5542 5524 5540 5517 14 P
5698 5615 5700 5611 5704 5608 5708 5611 5712 5612 5717 5614
5717 5618 5720 5620 5724 5623 5724 5628 5726 5632 5721 5633
5717 5635 5712 5635 5710 5631 5706 5629 5704 5625 5702 5621
5698 5615 19 P
5790 5619 5794 5616 5798 5614 5800 5611 5805 5609 5810 5609
5808 5613 5806 5618 5803 5621 5798 5623 5796 5620 5790 5619
12:00 PM
6251 5606 6253 5608 6255 5607 6256 5608 6258 5610 6258 5612
6260 5614 6259 5616 6256 5615 6255 5614 6253 5612 6252 5610
6251 5606 13 P
6240 5628 6241 5625 6242 5621 6244 5619 6247 5620 6251 5622
6254 5622 6257 5623 6259 5626 6256 5628 6253 5629 6249 5629
6246 5630 6243 5630 6240 5628 15 P
6252 5694 6253 5692 6256 5691 6259 5689 6260 5685 6263 5687
6262 5691 6260 5693 6256 5695 6256 5698 6252 5694 11 P
12523 8206 12524 8206 12525 8206 12525 8206 12525 8207 12526 8207
12525 8208 12524 8208 12523 8207 12523 8206 10 P
12910 11328 12912 11328 12914 11328 12915 11329 12917 11329 12918 11330
12919 11331 12921 11330 12921 11328 12923 11330 12923 11331 12924 11333
12925 11334 12925 11335 12925 11337 12924 11337 12923 11336 12922 11336
12920 11336 12918 11336 12917 11337 12915 11337 12915 11335 12915 11334
12915 11332 12914 11331 12912 11330 12910 11329 12910 11328 29 P
12896 11304 12896 11303 12897 11303 12897 11304 12896 11305 12895 11305
12895 11304 12895 11304 12896 11304 9 P
12898 11304 12898 11302 12897 11300 12897 11299 12897 11297 12896 11295
12898 11295 12899 11297 12899 11298 12901 11299 12902 11299 12902 11300
12901 11301 12900 11303 12900 11305 12901 11306 12902 11307 12902 11309
12901 11309 12899 11308 12898 11308 12896 11308 12896 11306 12897 11305
12898 11304 25 P
12902 11312 12903 11311 12905 11312 12906 11313 12906 11314 12904 11314
12903 11314 12903 11313 12902 11313 12902 11312 10 P
12904 11318 12905 11318 12905 11319 12904 11319 12904 11318 5 P
12906 11319 12906 11319 12907 11319 12907 11319 12908 11319 12908 11321
12908 11321 12908 11322 12907 11322 12906 11321 12906 11321 12906 11319
12:00 PM
12909 11325 12909 11325 12910 11323 12910 11323 12911 11323 12911 11323
12911 11325 12910 11326 12909 11326 12909 11325 10 P
2298 14452 2330 14446 2321 14393 2298 14396 4 P
2298 14341 2325 14336 2374 14312 2381 14258 2413 14214 2459 14185
2491 14141 2523 14097 2509 14044 2490 13993 2507 13941 2526 13890
2543 13838 2556 13785 2563 13730 2530 13687 2486 13655 2468 13604
2480 13550 2508 13503 2548 13466 2602 13458 2630 13410 2653 13361

2671 13309 2722 13290 2774 13272 2823 13249 2837 13195 2869 13152
2913 13184 2941 13231 2927 13285 2935 13338 2948 13392 3001 13405
3053 13387 3108 13390 3146 13404 3135 13472 3179 13505 3187 13559
3188 13600 3151 13640 3104 13668 3057 13696 46 P
3057 13696 3017 13701 3014 13756 3027 13809 2994 13853 2971 13878
3021 13902 3048 13948 3103 13946 3111 14000 3115 14034 3085 14061
3122 14079 3130 14140 3185 14137 3239 14135 3294 14133 3348 14125
3394 14115 19 P
3394 14115 3397 14060 3417 14009 3466 13986 3513 13959 3561 13932
3610 13909 3651 13874 3685 13831 3718 13788 3770 13770 3820 13748
3861 13712 3906 13681 3958 13664 3973 13612 3946 13564 3950 13509
3939 13456 3930 13416 3863 13382 3862 13327 3850 13274 3860 13220
3880 13169 3873 13115 3867 13060 3812 13061 3757 13062 3702 13064
3648 13065 3593 13061 3540 13073 3490 13096 3451 13058 3464 13005
3484 12954 3452 12909 3397 12911 3343 12913 3288 12920 3234 12917
3186 12944 3153 12988 3120 13032 3066 13039 3012 13047 2944 13057
2907 13016 2905 12961 50 P
2905 12961 2918 12908 2878 12871 2834 12838 2801 12813 2793 12759
2847 12748 2895 12722 2931 12681 2931 12605 2869 12605 2869 12549
2839 12504 2809 12458 2788 12407 2793 12352 2815 12301 2820 12246
2813 12213 2773 12221 2757 12259 2702 12264 2648 12253 2600 12279
2546 12290 2503 12256 2472 12210 2424 12184 2439 12131 2482 12096
2507 12047 2486 11996 2460 11948 2407 11932 2352 11927 2299 11944
2298 11944 37 P
2298 10517 2313 10510 2357 10477 2382 10426 2436 10418 2492 10421
2547 10429 2601 10416 2605 10360 2619 10306 2663 10273 2719 10271
2774 10279 2822 10309 2866 10343 2899 10388 2931 10433 2964 10478
3000 10520 3037 10562 3018 10614 3046 10662 3100 10672 3155 10681
3185 10633 3238 10648 3271 10694 3293 10744 3325 10790 3332 10845
3364 10890 3401 10932 3441 10971 3484 11005 3528 11039 3575 11069
3592 11122 3554 11162 3519 11205 3497 11240 3533 11283 3560 11330
3564 11365 3606 11360 3608 11384 45 P
3608 11384 3611 11405 3556 11411 3501 11418 3448 11403 3396 11383
3358 11369 3316 11405 3278 11445 3253 11494 3220 11539 3165 11546
3141 11596 3125 11626 3082 11593 3043 11563 3010 11607 2981 11655
3001 11674 2968 11718 2917 11741 2862 11748 2808 11740 2753 11742
2699 11734 2646 11753 2599 11781 2562 11822 2535 11826 2537 11874
2482 11872 2420 11875 2410 11835 2355 11838 2298 11859 35 P
3394 14115 3432 14129 3418 14181 3410 14235 3427 14287 3450 14336
3477 14383 3517 14421 3560 14453 3614 14463 3619 14503 11 P
3619 14503 3568 14521 3531 14560 3512 14611 3475 14651 3427 14678
3373 14675 3319 14677 3279 14714 3235 14745 3194 14782 3166 14828
3213 14857 3253 14893 3266 14946 3273 15000 3309 15040 3337 15087

3377 15124 3409 15168 3395 15220 3387 15274 3363 15323 3319 15355
3268 15337 3216 15323 3165 15305 3113 15291 3063 15268 3023 15232
2991 15189 2963 15142 2944 15091 2926 15040 2918 14986 2904 14933
2881 14884 2878 14830 2845 14786 2837 14733 2845 14679 2831 14626
2808 14577 2755 14564 2708 14537 2654 14545 2600 14553 2547 14546
2500 14519 2453 14492 50 P
2453 14492 2398 14490 2344 14494 2298 14506 4 P
3619 14503 3669 14481 3719 14458 3756 14419 3759 14378 3806 14351
3860 14345 3914 14349 3968 14347 4023 14341 4083 14334 4095 14387
4096 14442 4107 14495 4136 14513 4165 14467 4222 14505 4213 14558
4183 14603 4205 14654 4231 14701 4266 14743 4305 14780 4347 14814
4389 14849 4424 14890 4466 14924 4512 14954 4546 14996 4585 15034
4627 15068 4672 15098 4722 15119 4772 15140 4762 15193 4757 15240
4802 15210 4837 15168 4874 15153 4878 15187 4931 15181 4945 15115
4998 15126 5023 15174 5061 15212 5106 15243 5159 15254 5186 15252
5168 15216 5155 15184 50 P
5155 15184 5185 15140 5209 15106 5184 15058 5174 15005 5129 14974
5095 14932 5085 14878 5070 14826 5045 14778 5040 14723 5019 14673
4975 14643 4933 14608 4888 14577 4854 14535 4849 14481 4842 14413
4877 14372 4903 14324 4924 14273 4935 14220 4930 14166 4925 14111
4871 14117 4866 14062 4877 14037 4847 13991 4813 13949 4760 13906
4753 13852 4744 13798 4739 13743 4740 13689 4750 13635 4745 13581
4740 13527 4735 13472 4730 13418 4735 13364 4741 13309 4747 13254
4752 13200 4758 13146 4742 13093 4721 13043 4691 12997 4657 12954
4636 12904 4620 12851 50 P
4620 12851 4595 12803 4584 12749 4578 12695 4558 12643 4581 12610
4614 12576 7 P
4614 12576 4604 12522 4635 12476 4624 12422 4655 12377 4694 12338
4689 12283 4705 12230 4667 12173 4718 12152 4757 12114 4768 12060
4814 12030 4841 11981 4841 11926 4836 11871 4813 11813 17 P
4813 11813 4745 11820 4729 11767 4675 11761 4639 11803 4609 11849
4566 11814 4512 11803 4459 11819 4407 11835 4354 11851 4300 11862
4246 11873 4192 11884 4137 11884 4136 11830 4189 11813 4240 11791
4261 11740 4239 11690 4294 11684 4345 11663 4388 11627 4409 11577
4430 11526 4379 11504 4326 11489 4275 11510 4221 11521 4166 11526
4131 11484 4100 11438 4083 11385 4099 11332 4114 11279 4120 11224
4114 11169 4113 11114 4093 11047 39 P
4093 11047 4038 11047 3982 11048 3943 11088 3909 11131 3874 11174
3836 11214 3785 11236 3743 11272 3700 11308 3662 11348 3638 11372
3608 11384 13 P
4093 11047 4147 11036 4141 10981 4138 10946 4193 10940 4242 10914
4281 10875 4330 10848 4335 10794 4330 10738 4324 10683 4319 10627
4313 10572 4270 10537 4219 10558 4164 10564 4112 10543 4069 10507

4026 10472 3976 10447 3937 10408 3895 10412 3899 10447 3847 10426
3793 10416 3799 10471 3744 10477 3700 10443 3654 10413 3600 10429
3582 10467 3536 10414 3481 10415 3449 10369 3483 10326 3513 10278
3501 10224 3484 10171 3477 10115 3473 10081 3404 10076 3424 10023
3462 9983 3469 10038 3506 10057 3532 10008 3557 9958 3606 9931
3621 9877 3590 9831 50 P
3590 9831 3635 9799 3684 9772 3715 9725 3720 9669 3755 9625
3781 9603 7 P
3781 9603 3787 9658 3819 9704 3841 9756 3868 9806 3904 9848
3939 9891 3991 9870 4024 9859 4041 9913 4090 9939 4145 9949
4201 9949 4255 9933 4298 9897 4347 9871 4402 9882 4453 9904
4458 9960 4432 10009 4453 10060 4504 10082 4556 10104 4625 10098
4621 10056 4677 10051 4733 10046 4788 10042 4824 9999 4871 9968
4923 9948 4978 9938 5033 9934 5087 9918 5127 9880 5168 9841
5204 9799 5248 9765 5302 9750 5357 9762 5413 9758 5468 9766
5510 9802 5556 9835 5632 9830 5629 9788 5602 9790 5582 9738
5578 9668 5630 9648 50 P
5630 9648 5686 9645 5719 9689 5757 9730 5796 9772 5837 9809
5885 9837 5882 9781 5927 9747 5971 9714 5942 9666 5980 9624
5938 9587 5905 9542 5882 9491 5902 9439 5945 9402 5987 9366
6043 9376 6103 9393 20 P
6103 9393 6117 9447 6165 9476 6207 9514 6244 9555 6297 9537
6352 9545 6403 9569 6458 9573 6513 9587 6550 9629 6602 9648
6657 9635 6670 9690 6724 9698 6779 9713 6815 9755 6817 9811
6873 9809 6928 9808 6971 9771 7022 9748 7078 9747 7143 9788
24 P
7143 9788 7128 9842 7118 9897 7119 9952 7105 10006 7059 10038
7044 10092 7046 10148 7047 10190 7008 10230 6979 10261 6981 10317
7022 10355 7062 10393 7106 10427 7076 10474 7034 10511 6986 10538
6933 10556 6883 10578 6841 10615 6819 10651 6804 10689 23 P
6804 10689 6735 10685 6731 10740 6712 10793 6675 10751 6628 10722
6574 10708 6519 10710 6471 10738 6442 10786 6434 10840 6428 10881
6373 10889 6304 10893 6267 10933 6233 10958 6196 10917 6158 10900
6135 10950 6090 10982 6058 11027 6044 11081 6058 11135 6061 11190
6070 11245 6029 11282 5991 11241 5943 11213 5889 11216 5856 11261
5812 11294 5758 11281 5703 11274 5648 11272 5593 11276 5538 11285
5484 11289 5429 11282 5374 11275 5325 11300 5281 11334 5238 11368
5191 11398 5144 11427 5095 11453 5052 11487 5016 11529 4980 11571
4941 11610 4929 11663 50 P
4929 11663 4902 11712 4871 11758 4842 11787 4813 11813 5 P
6804 10689 6817 10744 6840 10794 6880 10832 6924 10866 6977 10881
7061 10886 7067 10942 7069 10997 7070 11052 7071 11108 7068 11163
7069 11218 7076 11273 7082 11321 7054 11319 7053 11278 6998 11279

6963 11280 6923 11242 6878 11274 6845 11318 6790 11326 6742 11353
6744 11408 6746 11464 6708 11504 6658 11527 6610 11555 6613 11610
6615 11659 6656 11657 6666 11617 6721 11625 6796 11630 6804 11685
6822 11737 6818 11792 6841 11842 6888 11872 6941 11886 6948 11940
6949 11995 6940 12050 6942 12105 6982 12143 7037 12146 7084 12119
7132 12092 7147 12039 50 P
7147 12039 7162 11986 7209 11959 7264 11963 7315 11983 7359 12017
7405 12047 7401 12102 7380 12153 7381 12208 7382 12256 7329 12267
7278 12289 7240 12329 7221 12380 7222 12422 16 P
7222 12422 7170 12439 7115 12429 7061 12426 7006 12427 6951 12429
6906 12461 6887 12512 6868 12563 6826 12600 6789 12640 6737 12657
6682 12660 6635 12688 6637 12742 6670 12786 6706 12827 6724 12880
6775 12898 6826 12918 6866 12955 6893 13002 6934 13040 6980 13069
7023 13102 7072 13126 7108 13168 7154 13197 7208 13185 7247 13176
7238 13230 7210 13260 7167 13226 7114 13211 7062 13197 7013 13173
6960 13159 6917 13125 6868 13101 6869 13155 6897 13203 6924 13250
6930 13298 43 P
6930 13298 6887 13265 6860 13263 6835 13312 6782 13319 6738 13286
6695 13253 6654 13289 6599 13292 6545 13294 6498 13322 6451 13350
6399 13368 6350 13391 6301 13414 6247 13422 6206 13429 6166 13435
18 P
6166 13435 6158 13381 6149 13327 6141 13273 6128 13220 6104 13171
6090 13117 6082 13064 6068 13010 6050 12959 6021 12912 5984 12872
5937 12844 5883 12837 5828 12840 5774 12849 5719 12853 5665 12856
5610 12854 5556 12858 5502 12873 5449 12882 5394 12886 5385 12832
5380 12777 5328 12792 5274 12785 5228 12754 5187 12719 5142 12687
5090 12670 5035 12675 4981 12663 4931 12642 4878 12631 4832 12600
4778 12594 4729 12568 4675 12573 4635 12580 4614 12576 41 P
6166 13435 6133 13479 6110 13528 6087 13578 6065 13627 6109 13659
6149 13696 6193 13728 6201 13782 6204 13837 6232 13883 6265 13927
6293 13974 6321 14020 6349 14067 6368 14118 6349 14170 6331 14221
6313 14272 6290 14322 6272 14373 6244 14420 6197 14447 6151 14476
6169 14527 6172 14581 6186 14634 6194 14688 6197 14742 6241 14774
6277 14814 6285 14868 6322 14908 6362 14944 6399 14904 6453 14907
6504 14925 6540 14965 6543 15020 6570 15067 6588 15118 6606 15155
6569 15195 6556 15247 6558 15301 6514 15333 6463 15352 6414 15375
6374 15411 6402 15458 50 P
6402 15458 6434 15501 6487 15515 6540 15528 6588 15551 6620 15595
6566 15597 6513 15605 6521 15658 6523 15726 6483 15728 6486 15782
6441 15823 13 P
6441 15823 6433 15770 6420 15717 6376 15685 6323 15677 6270 15664
6230 15628 6183 15600 6129 15603 6086 15636 6032 15639 5978 15632
5940 15619 5914 15549 5860 15542 5818 15575 5771 15604 5718 15597

5677 15561 5644 15519 5615 15473 5590 15425 5561 15379 5537 15331
5517 15280 5473 15249 5419 15253 5373 15282 5310 15275 5300 15222
5269 15173 5215 15178 5155 15184 33 P
6441 15823 6433 15877 6414 15928 6382 15971 6346 16011 6390 16043
6417 16074 6389 16120 6395 16160 6371 16192 6407 16219 11 P
8148 16219 8163 16191 8184 16141 8200 16090 8252 16105 8305 16095
8358 16100 8412 16100 8460 16075 8514 16075 8546 16049 11 P
8546 16049 8570 16097 8595 16145 8605 16198 8598 16219 5 P
11246 16007 11193 16001 11138 16002 11159 16052 11180 16101 11186 16155
11160 16203 11148 16219 8 P
8546 16049 8508 16010 8474 15968 8427 15942 8466 15904 8414 15888
8384 15813 8426 15778 8468 15745 8520 15730 8551 15685 8599 15660
8653 15661 8693 15662 8705 15609 8712 15555 8728 15504 8755 15457
8786 15412 8835 15374 20 P
8061 14939 8055 14993 8030 15041 7995 15083 8003 15109 8024 15109
8019 15149 7966 15144 7926 15137 7892 15159 7908 15197 7860 15223
7855 15276 7855 15331 7905 15351 7936 15396 7894 15431 7868 15493
7821 15494 7787 15494 20 P
7787 15494 7750 15532 7729 15583 7730 15637 7768 15675 7822 15680
7817 15734 7763 15734 7709 15740 7656 15741 7606 15762 7581 15810
7547 15852 7518 15898 7489 15943 7443 15914 7398 15885 7366 15872
7376 15819 7380 15765 7353 15718 7301 15703 7247 15700 7195 15685
7152 15652 7121 15608 7086 15567 7059 15520 7057 15466 7026 15422
6986 15384 7011 15336 7008 15282 6997 15229 7025 15183 7072 15156
7116 15124 7165 15149 7218 15153 7273 15151 7314 15116 7366 15099
7411 15128 7446 15169 7504 15151 45 P
7504 15151 7555 15171 7593 15209 3 P
7593 15209 7643 15187 7693 15166 7688 15220 7719 15265 7714 15319
7709 15373 7730 15423 8 P
7730 15423 7770 15449 7787 15494 3 P
7504 15151 7514 15097 7558 15067 7568 15013 7573 14959 7593 14908
7567 14861 7532 14820 7505 14772 7494 14719 7495 14665 7507 14612
7534 14565 7583 14541 7637 14537 7691 14544 7744 14557 7796 14571
7832 14589 7869 14612 20 P
6930 13298 6932 13352 6895 13392 6861 13436 6863 13490 6881 13542
6917 13583 6960 13616 7006 13645 7049 13678 7077 13726 7104 13774
7126 13823 7143 13875 7170 13922 7222 13936 7276 13946 7324 13971
7364 14009 7403 14046 7438 14088 7474 14129 7513 14167 7555 14201
7609 14200 7664 14200 7718 14199 7773 14198 7815 14233 7845 14278
7876 14323 7926 14343 7974 14369 8006 14395 8033 14408 35 P
7222 12422 7276 12426 7314 12470 3 P
7314 12470 7321 12525 7332 12578 7381 12603 7434 12618 7489 12622
7539 12601 7577 12562 7615 12522 7630 12470 7651 12419 7676 12370

7725 12395 7746 12413 7788 12377 7822 12354 7875 12370 7926 12391
7961 12433 8009 12459 8063 12454 8098 12411 8114 12358 8114 12317
8169 12317 8220 12338 8236 12391 8235 12433 8274 12471 8308 12494
8357 12469 8382 12518 8402 12569 8423 12619 8438 12672 8493 12668
8547 12674 8585 12713 8626 12713 8634 12704 40 P
7314 12470 7356 12435 7390 12392 7435 12360 7485 12338 7538 12322
7578 12317 7577 12262 7593 12209 7635 12174 7680 12143 7735 12137
7788 12125 7842 12115 7888 12083 7931 12030 7935 11989 7909 11958
7962 11946 8017 11952 8071 11962 8117 11932 8096 11881 8091 11826
8065 11777 8039 11729 8013 11681 8043 11634 8098 11629 8141 11594
8157 11541 8157 11486 8183 11437 8219 11395 8273 11390 8328 11401
8381 11412 8412 11458 8442 11505 8495 11521 8530 11479 8573 11444
8609 11403 8610 11347 8589 11295 8551 11256 8497 11271 8458 11310
8404 11320 8349 11325 50 P
8349 11325 8318 11279 8354 11236 8365 11183 8344 11131 8299 11100
8268 11054 8226 11017 8212 10964 8218 10909 8241 10859 8281 10820
8328 10790 8377 10766 8329 10738 8314 10685 8321 10630 8344 10579
8390 10549 8444 10535 8500 10531 8622 10532 8678 10532 8733 10528
8775 10525 8830 10520 26 P
8830 10520 8881 10542 8936 10538 8972 10496 9009 10454 9055 10424
9108 10404 9162 10417 9209 10387 9216 10332 9261 10364 9314 10382
9338 10432 9391 10450 9446 10452 15 P
9446 10452 9479 10407 9523 10374 9564 10335 9600 10314 9616 10299

6:00 PM

8830 10520 8820 10466 8800 10413 8790 10359 8760 10312 8726 10268
8680 10236 8638 10200 8677 10162 8700 10110 8711 10085 8656 10073
8603 10056 8547 10056 8496 10076 8480 10023 8470 9968 8470 9912
8471 9856 8440 9809 8410 9762 8384 9713 8345 9673 8302 9637
8256 9606 8209 9575 8148 9541 27 P
8148 9541 8108 9581 8065 9616 8010 9627 7954 9633 7899 9639
7844 9650 7795 9676 7779 9730 7758 9782 7705 9799 7679 9831
7629 9806 7612 9752 7585 9703 7534 9682 7484 9657 7428 9652
7394 9696 7416 9747 7463 9778 7499 9820 7503 9862 7457 9894
7402 9900 7358 9865 7315 9831 7265 9805 7218 9775 7176 9780
7143 9788 31 P
8148 9541 8127 9490 8075 9468 8048 9419 8048 9362 8079 9316
8069 9261 8095 9211 8142 9180 8197 9169 8252 9158 8252 9102
8258 9047 8270 8991 8275 8935 8276 8913 16 P
8276 8913 8276 8893 8331 8899 8385 8916 8425 8877 8425 8820
8410 8766 8441 8720 8458 8666 8475 8611 8497 8560 8493 8503
8497 8461 8553 8467 8600 8437 8657 8432 8706 8459 8756 8487
8781 8537 8828 8569 8868 8530 8886 8476 8908 8425 8940 8378
8997 8374 9043 8406 9098 8419 9150 8442 9203 8422 9247 8388

9245 8349 31 P
8276 8913 8219 8913 8219 8857 8179 8817 8133 8785 8083 8759
8030 8743 7977 8721 7938 8681 7894 8645 7854 8686 7818 8729
7765 8713 7710 8697 7684 8648 7648 8604 7604 8569 7547 8570
7547 8514 7588 8505 7587 8448 7598 8392 7608 8337 7580 8287
7549 8241 7502 8210 7447 8194 7391 8184 7347 8149 7310 8106
7304 8049 7292 7994 7312 7941 7338 7890 7290 7859 7250 7820
7227 7768 7226 7725 7268 7724 7251 7671 7214 7627 7169 7592
7124 7557 7097 7507 7064 7460 7041 7408 7022 7354 7010 7298
7003 7241 6966 7198 50 P
6966 7198 6942 7146 6963 7092 6938 7049 6937 7006 5 P
6103 9393 6101 9337 6124 9286 6149 9235 6140 9180 6132 9124
6161 9076 6190 9028 6171 8974 6138 8929 6093 8896 6073 8843
6023 8819 5972 8795 5934 8753 5925 8697 5891 8652 5853 8610
5834 8558 5831 8501 5849 8448 5906 8450 5962 8441 5959 8385
5935 8334 5932 8277 5965 8232 5968 8175 5943 8124 5951 8068
5997 8034 6039 7996 6063 7944 6034 7895 6020 7841 5969 7772
6026 7775 6103 7786 6095 7730 6042 7711 6014 7704 6011 7647
5997 7592 5963 7546 5955 7489 5969 7434 5956 7370 47 P
3781 9603 3775 9547 3753 9495 3747 9439 3757 9384 3721 9341
3776 9329 3743 9260 8 P
3743 9260 3736 9204 3762 9154 3762 9098 3750 9043 3738 8987
3733 8931 3726 8869 3769 8832 3779 8777 3779 8734 3737 8735
3693 8699 3640 8678 3593 8647 3553 8608 3505 8577 3461 8542
3417 8507 3460 8451 3505 8487 3561 8480 3617 8485 3648 8515
3719 8514 3749 8467 3789 8426 3767 8374 3756 8318 3795 8278
3738 8278 3686 8257 3645 8245 3692 8174 3664 8124 3633 8077
3600 8030 3657 8024 3699 8020 3699 7963 3715 7923 3687 7858
42 P
3687 7858 3650 7814 3614 7770 3597 7716 3541 7728 3489 7750
3461 7699 3420 7712 3410 7738 3374 7715 3414 7674 3455 7624
3397 7624 3341 7631 3312 7634 3309 7676 3266 7681 3249 7627
3208 7551 3164 7588 3118 7620 3073 7657 3029 7621 2984 7586
2967 7531 2930 7488 2875 7472 2805 7459 2758 7492 2718 7533
2699 7572 2639 7533 2613 7584 2587 7635 2567 7688 2536 7737
2482 7755 2440 7760 2407 7714 2383 7661 2365 7608 2328 7565
2298 7563 43 P
3687 7858 3744 7857 3800 7851 3793 7780 3850 7774 3857 7845
3904 7877 3945 7917 4002 7917 4007 7860 4051 7824 4107 7813
4150 7849 4191 7890 4238 7921 4278 7962 4334 7973 4378 7937
4430 7959 4482 7982 4532 7956 4580 7925 4612 7878 4602 7822
4581 7769 4582 7712 4636 7696 4686 7692 4681 7635 4627 7618
4611 7563 4601 7507 4590 7451 4547 7414 4507 7373 4467 7332

4431 7287 4396 7242 4407 7187 4421 7161 4471 7189 4502 7237
4542 7278 4596 7257 4632 7213 4645 7157 4656 7101 4641 7046
4627 6998 4684 6993 50 P
4684 6993 4736 7016 4786 7044 4843 7034 4907 7024 5 P
4907 7024 4954 7042 5022 7064 5025 7107 5002 7160 5022 7213
5053 7261 5106 7241 5163 7237 5217 7217 5272 7202 5300 7152
5329 7102 5380 7077 5437 7084 5457 7138 5460 7195 5470 7252
5428 7291 5471 7329 5510 7370 5552 7408 5609 7405 5651 7366
5697 7332 5748 7307 5805 7304 5862 7306 5916 7325 5956 7370
30 P
5956 7370 5985 7321 5999 7266 6056 7268 6113 7277 6169 7286
6224 7299 6281 7308 6315 7262 6354 7220 6325 7189 6356 7159
6308 7126 6254 7109 6210 7072 6166 7036 6139 6985 6099 6945
6058 6904 6104 6870 6150 6836 6196 6802 6251 6822 6307 6825
6359 6850 6411 6826 6444 6882 6501 6885 6558 6883 6612 6904
6666 6924 6719 6944 6766 6911 6786 6857 6840 6839 6853 6805
6909 6820 6899 6877 6889 6933 6900 6982 6937 7006 41 P
4907 7024 4886 6970 4907 6911 4933 6860 4949 6805 4921 6754
4867 6734 4840 6723 4839 6665 4849 6609 4816 6562 4798 6507
4719 6501 4701 6446 4678 6393 4613 6395 4606 6338 4560 6302
4553 6245 4531 6224 4546 6187 4541 6130 4554 6073 4538 6017
4503 5971 4462 5929 4409 5906 4366 5902 4357 5851 4299 5856
4246 5833 4191 5815 4140 5787 4147 5729 4167 5683 4126 5641
4099 5589 4042 5600 3981 5625 3940 5583 3930 5526 3932 5501
42 P
6937 7006 6994 7005 7049 6986 7105 6974 7157 6951 7211 6956
7242 6885 7278 6840 7335 6844 7416 6859 7415 6802 7436 6748
7467 6700 7493 6649 7515 6595 7492 6543 7491 6485 7507 6430
7529 6376 7555 6325 7587 6277 7616 6254 22 P
2298 9491 2313 9481 2364 9457 2419 9444 2475 9436 2530 9445
2583 9464 2628 9498 2651 9549 2684 9594 2740 9592 2795 9579
2850 9588 2895 9622 2932 9665 2960 9713 3010 9738 3063 9720
3072 9665 3097 9615 3147 9639 3198 9665 3251 9647 3306 9640
3316 9585 3325 9530 3324 9474 3334 9418 3380 9386 3419 9346
3412 9290 3433 9237 3443 9211 3480 9254 3500 9291 3499 9234
3492 9178 3483 9137 3539 9125 3590 9103 3645 9113 3672 9163
3684 9218 3714 9247 3743 9260 45 P
5955 5669 5984 5673 6012 5681 6035 5684 4 P
9446 10452 9439 10507 9481 10544 9510 10591 9524 10645 9548 10695
9593 10728 9626 10749 8 P
7593 15209 7599 15235 7602 15262 7587 15285 7565 15300 7544 15318
7542 15345 7527 15367 7528 15393 7554 15389 7582 15389 7603 15389
7626 15401 7652 15409 7679 15409 7706 15416 7730 15423 17 P

3308 4926 3330 4926 3330 4653 3296 4653 3296 4847 3236 4847
3236 4872 3260 4874 3271 4877 3281 4881 3290 4887 3294 4891
3298 4896 3304 4909 3308 4926 15 N F
3523 4926 3545 4926 3545 4653 3511 4653 3511 4847 3451 4847
3451 4872 3475 4874 3486 4877 3496 4881 3505 4887 3509 4891
3512 4896 3518 4909 3523 4926 15 N F
3732 4926 3742 4925 3751 4923 3760 4919 3769 4913 3777 4907
3784 4899 3791 4891 3797 4881 3803 4871 3808 4860 3812 4848
3816 4836 3818 4824 3820 4811 3822 4798 3822 4785 3822 4772
3820 4759 3818 4747 3816 4734 3812 4722 3808 4710 3803 4699
3797 4688 3791 4678 3784 4670 3777 4662 3769 4655 3760 4650
3751 4646 3742 4643 3732 4643 3722 4643 3712 4646 3703 4650
3695 4655 3687 4662 3679 4670 3673 4678 3666 4688 3661 4699
3656 4710 3652 4722 3648 4734 3645 4747 3643 4759 3642 4772
3641 4785 3678 4785 3679 4761 3682 4739 3688 4720 3695 4703
3703 4690 3712 4681 3717 4677 3722 4675 3727 4673 3732 4673
3737 4673 3742 4675 3746 4677 3751 4681 3760 4690 3769 4703
3776 4720 3781 4739 3785 4761 3786 4785 3785 4810 3781 4831
3776 4850 3769 4866 3760 4879 3751 4888 3746 4891 3742 4894
3737 4895 3732 4896 3727 4895 3722 4894 3717 4891 3712 4888
3703 4879 3695 4866 3688 4850 3682 4831 3679 4810 3678 4785
3641 4785 3642 4798 3643 4811 3645 4824 3648 4836 3652 4848
3656 4860 3661 4871 3666 4881 3673 4891 3679 4899 3687 4907
3695 4913 3703 4919 3712 4923 3722 4925 3732 4926 107 N F
3990 4874 3988 4861 3985 4849 3979 4838 3971 4829 3961 4821
3951 4815 3939 4811 3926 4810 3926 4835 3918 4836 3910 4838
3904 4841 3898 4846 3893 4852 3889 4859 3887 4866 3886 4874
3887 4882 3889 4889 3893 4896 3898 4902 3904 4907 3910 4910
3918 4913 3926 4914 3934 4913 3941 4910 3948 4907 3954 4902
3958 4896 3962 4889 3964 4882 3965 4874 3964 4866 3962 4859
3958 4852 3954 4846 3948 4841 3941 4838 3934 4836 3926 4835
3926 4810 3913 4811 3901 4815 3890 4821 3881 4829 3873 4838
3867 4849 3863 4861 3862 4874 3863 4887 3867 4899 3873 4910
3881 4919 3890 4927 3901 4933 3913 4937 3926 4938 3939 4937
3951 4933 3961 4927 3971 4919 3979 4910 3985 4899 3988 4887
3990 4874 67 N F
4043 4934 4237 4934 4237 4902 4079 4902 4079 4811 4231 4811
4231 4779 4079 4779 4079 4685 4243 4685 4243 4653 4043 4653
4043 4934 13 N F
5472 4926 5494 4926 5494 4653 5460 4653 5460 4847 5400 4847
5400 4872 5424 4874 5435 4877 5445 4881 5454 4887 5458 4891
5461 4896 5467 4909 5472 4926 15 N F
5687 4926 5709 4926 5709 4653 5675 4653 5675 4847 5614 4847

5614 4872 5639 4874 5650 4877 5660 4881 5668 4887 5672 4891
5676 4896 5682 4909 5687 4926 15 N F
5925 4926 5949 4926 5949 4749 5992 4749 5992 4717 5949 4717
5949 4653 5915 4653 5915 4717 5801 4717 5801 4749 5831 4749
5915 4749 5915 4868 5831 4749 5801 4749 5801 4753 5925 4926
18 N F
6153 4874 6152 4861 6148 4849 6142 4838 6134 4829 6125 4821
6114 4815 6102 4811 6089 4810 6089 4835 6081 4836 6074 4838
6067 4841 6061 4846 6057 4852 6053 4859 6051 4866 6050 4874
6051 4882 6053 4889 6057 4896 6061 4902 6067 4907 6074 4910
6081 4913 6089 4914 6097 4913 6105 4910 6111 4907 6117 4902
6122 4896 6126 4889 6128 4882 6129 4874 6128 4866 6126 4859
6122 4852 6117 4846 6111 4841 6105 4838 6097 4836 6089 4835
6089 4810 6076 4811 6064 4815 6054 4821 6044 4829 6036 4838
6030 4849 6027 4861 6025 4874 6027 4887 6030 4899 6036 4910
6044 4919 6054 4927 6064 4933 6076 4937 6089 4938 6102 4937
6114 4933 6125 4927 6134 4919 6142 4910 6148 4899 6152 4887
6153 4874 67 N F
6206 4934 6401 4934 6401 4902 6242 4902 6242 4811 6395 4811
6395 4779 6242 4779 6242 4685 6407 4685 6407 4653 6206 4653
6206 4934 13 N F
7620 4926 7642 4926 7642 4653 7608 4653 7608 4847 7548 4847
7548 4872 7572 4874 7583 4877 7593 4881 7602 4887 7606 4891
7610 4896 7616 4909 7620 4926 15 N F
7835 4926 7857 4926 7857 4653 7823 4653 7823 4847 7763 4847
7763 4872 7787 4874 7798 4877 7808 4881 7817 4887 7821 4891
7824 4896 7831 4909 7835 4926 15 N F
8044 4926 8061 4925 8076 4921 8090 4915 8102 4907 8108 4902
8112 4897 8116 4891 8120 4884 8122 4877 8124 4870 8126 4862
8126 4854 8125 4842 8123 4831 8119 4823 8114 4815 8109 4809
8102 4804 8090 4795 8100 4790 8109 4784 8117 4776 8124 4768
8129 4759 8133 4749 8135 4738 8136 4727 8135 4713 8130 4699
8123 4685 8112 4672 8106 4666 8099 4660 8091 4655 8083 4651
8074 4647 8064 4645 8053 4643 8042 4643 8031 4643 8020 4645
8010 4647 8001 4651 7993 4655 7986 4660 7979 4666 7973 4672
7964 4685 7957 4699 7953 4713 7952 4727 7988 4727 7989 4716
7992 4706 7998 4696 8004 4688 8013 4682 8022 4677 8033 4674
8044 4673 8055 4674 8065 4677 8075 4682 8083 4688 8090 4696
8096 4706 8099 4716 8100 4727 8099 4738 8096 4748 8090 4757
8083 4765 8075 4772 8065 4777 8055 4780 8044 4781 8033 4780
8022 4777 8013 4772 8004 4765 7998 4757 7992 4748 7989 4738
7988 4727 7952 4727 7952 4738 7955 4748 7959 4758 7964 4767
7971 4775 7979 4783 7988 4790 7998 4795 7985 4804 7979 4809

7974 4815 7969 4823 7965 4831 7962 4842 7962 4854 7996 4854
7997 4844 8000 4836 8004 4829 8010 4823 8017 4818 8025 4814
8034 4812 8044 4811 8053 4812 8062 4814 8071 4818 8078 4823
8084 4829 8088 4836 8091 4844 8092 4854 8091 4863 8088 4871
8084 4878 8078 4884 8071 4889 8062 4893 8053 4895 8044 4896
8034 4895 8025 4893 8017 4889 8010 4884 8004 4878 8000 4871
7997 4863 7996 4854 7962 4854 7962 4862 7963 4870 7965 4877
7968 4884 7971 4891 7975 4897 7980 4902 7985 4907 7998 4915
8012 4921 8027 4925 8044 4926 147 N F
8302 4874 8300 4861 8297 4849 8291 4838 8283 4829 8273 4821
8263 4815 8251 4811 8238 4810 8238 4835 8230 4836 8222 4838
8216 4841 8210 4846 8205 4852 8202 4859 8199 4866 8198 4874
8199 4882 8202 4889 8205 4896 8210 4902 8216 4907 8222 4910
8230 4913 8238 4914 8246 4913 8253 4910 8260 4907 8266 4902
8270 4896 8274 4889 8276 4882 8277 4874 8276 4866 8274 4859
8270 4852 8266 4846 8260 4841 8253 4838 8246 4836 8238 4835
8238 4810 8225 4811 8213 4815 8202 4821 8193 4829 8185 4838
8179 4849 8175 4861 8174 4874 8175 4887 8179 4899 8185 4910
8193 4919 8202 4927 8213 4933 8225 4937 8238 4938 8251 4937
8263 4933 8273 4927 8283 4919 8291 4910 8297 4899 8300 4887
8302 4874 67 N F
8355 4934 8549 4934 8549 4902 8391 4902 8391 4811 8543 4811
8543 4779 8391 4779 8391 4685 8556 4685 8556 4653 8355 4653
8355 4934 13 N F
9783 4926 9805 4926 9805 4653 9770 4653 9770 4847 9710 4847
9710 4872 9735 4874 9745 4877 9755 4881 9764 4887 9768 4891
9772 4896 9778 4909 9783 4926 15 N F
9905 4831 9939 4831 9940 4845 9942 4858 9947 4869 9953 4878
9961 4885 9970 4891 9982 4895 9995 4896 10006 4895 10015 4892
10024 4888 10032 4882 10038 4874 10043 4865 10046 4855 10047 4843
10046 4832 10044 4822 10039 4814 10032 4806 10023 4799 10013 4792
9985 4775 9962 4762 9943 4748 9928 4734 9916 4719 9908 4704
9903 4688 9900 4671 9899 4653 10082 4653 10082 4685 9937 4685
9941 4697 9946 4707 9952 4716 9960 4724 9970 4731 9982 4739
10013 4757 10031 4766 10046 4776 10058 4787 10068 4799 10075 4811
10080 4823 10083 4835 10084 4847 10083 4856 10082 4864 10080 4872
10077 4879 10073 4886 10068 4893 10063 4899 10057 4904 10044 4913
10029 4920 10013 4924 9995 4926 9976 4924 9968 4923 9959 4920
9951 4917 9944 4913 9937 4908 9931 4903 9925 4897 9920 4890
9915 4882 9912 4873 9909 4864 9907 4854 9905 4843 9905 4831
78 N F
10120 4831 10154 4831 10155 4845 10157 4858 10161 4869 10168 4878
10175 4885 10185 4891 10197 4895 10210 4896 10220 4895 10230 4892

10239 4888 10247 4882 10253 4874 10258 4865 10261 4855 10262 4843
10261 4832 10258 4822 10254 4814 10247 4806 10238 4799 10227 4792
10200 4775 10177 4762 10158 4748 10143 4734 10131 4719 10123 4704
10118 4688 10115 4671 10114 4653 10296 4653 10296 4685 10152 4685
10155 4697 10160 4707 10167 4716 10175 4724 10185 4731 10197 4739
10228 4757 10246 4766 10261 4776 10273 4787 10283 4799 10290 4811
10295 4823 10297 4835 10298 4847 10298 4856 10296 4864 10294 4872
10291 4879 10287 4886 10283 4893 10278 4899 10272 4904 10259 4913
10244 4920 10228 4924 10210 4926 10191 4924 10182 4923 10174 4920
10166 4917 10158 4913 10152 4908 10145 4903 10140 4897 10134 4890
10130 4882 10126 4873 10124 4864 10121 4854 10120 4843 10120 4831
78 N F
10464 4874 10463 4861 10459 4849 10453 4838 10445 4829 10436 4821
10425 4815 10413 4811 10400 4810 10400 4835 10392 4836 10385 4838
10378 4841 10372 4846 10367 4852 10364 4859 10361 4866 10361 4874
10361 4882 10364 4889 10367 4896 10372 4902 10378 4907 10385 4910
10392 4913 10400 4914 10408 4913 10415 4910 10422 4907 10428 4902
10433 4896 10436 4889 10438 4882 10439 4874 10438 4866 10436 4859
10433 4852 10428 4846 10422 4841 10415 4838 10408 4836 10400 4835
10400 4810 10387 4811 10375 4815 10364 4821 10355 4829 10347 4838
10341 4849 10337 4861 10336 4874 10337 4887 10341 4899 10347 4910
10355 4919 10364 4927 10375 4933 10387 4937 10400 4938 10413 4937
10425 4933 10436 4927 10445 4919 10453 4910 10459 4899 10463 4887
10464 4874 67 N F
10517 4934 10712 4934 10712 4902 10553 4902 10553 4811 10706 4811
10706 4779 10553 4779 10553 4685 10718 4685 10718 4653 10517 4653
10517 4934 13 N F
11945 4926 11967 4926 11967 4653 11933 4653 11933 4847 11872 4847
11872 4872 11897 4874 11908 4877 11917 4881 11926 4887 11930 4891
11934 4896 11940 4909 11945 4926 15 N F
12067 4831 12101 4831 12102 4845 12105 4858 12109 4869 12115 4878
12123 4885 12132 4891 12144 4895 12157 4896 12168 4895 12177 4892
12186 4888 12194 4882 12200 4874 12205 4865 12208 4855 12209 4843
12209 4832 12206 4822 12201 4814 12194 4806 12185 4799 12175 4792
12147 4775 12124 4762 12105 4748 12090 4734 12079 4719 12070 4704
12065 4688 12062 4671 12061 4653 12244 4653 12244 4685 12099 4685
12103 4697 12108 4707 12114 4716 12122 4724 12132 4731 12144 4739
12175 4757 12193 4766 12208 4776 12221 4787 12230 4799 12237 4811
12242 4823 12245 4835 12246 4847 12245 4856 12244 4864 12242 4872
12239 4879 12235 4886 12230 4893 12225 4899 12220 4904 12206 4913
12191 4920 12175 4924 12157 4926 12138 4924 12130 4923 12121 4920
12113 4917 12106 4913 12099 4908 12093 4903 12087 4897 12082 4890
12077 4882 12074 4873 12071 4864 12069 4854 12067 4843 12067 4831

78 N F
12454 4856 12420 4856 12418 4865 12414 4873 12410 4880 12404 4885
12398 4890 12391 4893 12384 4895 12376 4896 12369 4895 12362 4894
12355 4892 12349 4889 12344 4885 12339 4880 12334 4875 12330 4869
12323 4854 12318 4836 12315 4816 12314 4793 12322 4802 12330 4809
12338 4814 12346 4818 12354 4821 12363 4822 12380 4823 12393 4822
12407 4818 12421 4811 12434 4802 12440 4796 12445 4789 12449 4782
12453 4774 12457 4765 12459 4756 12460 4746 12460 4735 12426 4733
12425 4744 12422 4755 12416 4765 12409 4774 12401 4782 12392 4788
12381 4792 12370 4793 12359 4792 12349 4788 12339 4782 12331 4774
12324 4765 12318 4755 12315 4744 12314 4733 12315 4722 12318 4711
12324 4701 12331 4691 12339 4684 12349 4678 12359 4674 12370 4673
12381 4674 12392 4678 12401 4684 12409 4691 12416 4701 12422 4711
12425 4722 12426 4733 12460 4735 12459 4719 12455 4703 12448 4687
12444 4680 12439 4673 12433 4666 12426 4661 12419 4655 12410 4651
12401 4647 12392 4645 12381 4643 12370 4643 12358 4643 12346 4645
12336 4649 12327 4653 12318 4659 12311 4666 12304 4673 12298 4682
12293 4692 12289 4702 12285 4714 12283 4726 12279 4751 12278 4779
12278 4796 12279 4811 12281 4826 12284 4840 12288 4853 12292 4865
12298 4876 12304 4886 12310 4895 12318 4903 12326 4910 12334 4916
12344 4920 12354 4923 12365 4925 12376 4926 12392 4924 12406 4920
12419 4914 12430 4905 12439 4895 12447 4883 12452 4870 12454 4856
126 N F
12626 4874 12625 4861 12621 4849 12615 4838 12607 4829 12598 4821
12587 4815 12575 4811 12562 4810 12562 4835 12554 4836 12547 4838
12540 4841 12534 4846 12529 4852 12526 4859 12523 4866 12523 4874
12523 4882 12526 4889 12529 4896 12534 4902 12540 4907 12547 4910
12554 4913 12562 4914 12570 4913 12577 4910 12584 4907 12590 4902
12595 4896 12598 4889 12601 4882 12601 4874 12601 4866 12598 4859
12595 4852 12590 4846 12584 4841 12577 4838 12570 4836 12562 4835
12562 4810 12549 4811 12537 4815 12526 4821 12517 4829 12509 4838
12503 4849 12499 4861 12498 4874 12499 4887 12503 4899 12509 4910
12517 4919 12526 4927 12537 4933 12549 4937 12562 4938 12575 4937
12587 4933 12598 4927 12607 4919 12615 4910 12621 4899 12625 4887
12626 4874 67 N F
12679 4934 12874 4934 12874 4902 12715 4902 12715 4811 12868 4811
12868 4779 12715 4779 12715 4685 12880 4685 12880 4653 12679 4653
12679 4934 13 N F
2316 17065 2338 17065 2338 16792 2304 16792 2304 16987 2244 16987
2244 17011 2268 17014 2279 17016 2289 17020 2298 17026 2302 17030
2306 17035 2312 17048 2316 17065 15 N F
2525 17065 2535 17064 2545 17062 2554 17058 2562 17053 2570 17046
2577 17039 2584 17030 2591 17021 2596 17010 2601 16999 2605 16987

2609 16975 2612 16963 2614 16950 2615 16937 2615 16925 2615 16912
2614 16899 2612 16886 2609 16873 2605 16861 2601 16849 2596 16838
2591 16828 2584 16818 2577 16809 2570 16801 2562 16795 2554 16789
2545 16785 2535 16783 2525 16782 2515 16783 2506 16785 2497 16789
2488 16795 2480 16801 2473 16809 2466 16818 2460 16828 2454 16838
2449 16849 2445 16861 2441 16873 2438 16886 2436 16899 2435 16912
2435 16925 2471 16925 2472 16900 2476 16878 2481 16859 2488 16843
2496 16830 2506 16820 2510 16817 2515 16814 2520 16813 2525 16812
2530 16813 2535 16814 2540 16817 2545 16820 2554 16830 2562 16843
2569 16859 2574 16878 2578 16900 2579 16925 2578 16949 2574 16971
2569 16990 2562 17005 2554 17018 2545 17027 2540 17031 2535 17033
2530 17035 2525 17035 2520 17035 2515 17033 2510 17031 2506 17027
2496 17018 2488 17005 2481 16990 2476 16971 2472 16949 2471 16925
2435 16925 2435 16937 2436 16950 2438 16963 2441 16975 2445 16987
2449 16999 2454 17010 2460 17021 2466 17030 2473 17039 2480 17046
2488 17053 2497 17058 2506 17062 2515 17064 2525 17065 107 N F
2676 17065 2818 17065 2818 17033 2704 17033 2694 16955 2703 16962
2715 16968 2729 16971 2746 16973 2760 16972 2775 16968 2789 16962
2796 16957 2803 16952 2809 16946 2814 16940 2819 16932 2824 16924
2827 16914 2830 16904 2832 16893 2832 16880 2831 16861 2829 16852
2826 16843 2823 16834 2819 16826 2814 16819 2808 16812 2802 16805
2795 16799 2787 16794 2779 16790 2770 16787 2760 16784 2749 16782
2738 16782 2727 16782 2718 16783 2709 16785 2700 16788 2693 16791
2686 16795 2679 16799 2674 16804 2664 16816 2657 16829 2651 16843
2647 16858 2682 16858 2685 16848 2690 16839 2695 16831 2701 16825
2708 16819 2717 16815 2726 16813 2736 16812 2749 16813 2762 16816
2772 16821 2781 16829 2788 16838 2794 16849 2797 16863 2798 16878
2797 16890 2795 16901 2790 16912 2784 16922 2775 16930 2765 16937
2752 16941 2745 16942 2738 16943 2730 16942 2723 16941 2717 16939
2711 16936 2699 16928 2686 16917 2656 16917 2676 17065 83 N F
2998 17014 2996 17001 2993 16989 2987 16978 2979 16968 2969 16960
2959 16955 2947 16951 2934 16949 2934 16974 2926 16975 2918 16977
2912 16981 2906 16986 2901 16991 2898 16998 2895 17006 2894 17014
2895 17021 2898 17029 2901 17036 2906 17041 2912 17046 2918 17050
2926 17052 2934 17053 2942 17052 2949 17050 2956 17046 2962 17041
2966 17036 2970 17029 2972 17021 2973 17014 2972 17006 2970 16998
2966 16991 2962 16986 2956 16981 2949 16977 2942 16975 2934 16974
2934 16949 2921 16951 2909 16955 2898 16960 2889 16968 2881 16978
2875 16989 2871 17001 2870 17014 2871 17026 2875 17038 2881 17049
2889 17059 2898 17067 2909 17072 2921 17076 2934 17078 2947 17076
2959 17072 2969 17067 2979 17059 2987 17049 2993 17038 2996 17026
2998 17014 67 N F
3051 17073 3245 17073 3245 17041 3087 17041 3087 16951 3239 16951

3239 16919 3087 16919 3087 16824 3251 16824 3251 16792 3051 16792
3051 17073 13 N F
4381 17065 4403 17065 4403 16792 4369 16792 4369 16987 4309 16987
4309 17011 4333 17014 4344 17016 4354 17020 4363 17026 4367 17030
4371 17035 4377 17048 4381 17065 15 N F
4596 17065 4618 17065 4618 16792 4584 16792 4584 16987 4524 16987
4524 17011 4548 17014 4559 17016 4569 17020 4578 17026 4582 17030
4585 17035 4591 17048 4596 17065 15 N F
4805 17065 4815 17064 4824 17062 4833 17058 4842 17053 4850 17046
4857 17039 4864 17030 4870 17021 4876 17010 4881 16999 4885 16987
4889 16975 4891 16963 4893 16950 4895 16937 4895 16925 4895 16912
4893 16899 4891 16886 4889 16873 4885 16861 4881 16849 4876 16838
4870 16828 4864 16818 4857 16809 4850 16801 4842 16795 4833 16789
4824 16785 4815 16783 4805 16782 4795 16783 4785 16785 4776 16789
4768 16795 4760 16801 4752 16809 4745 16818 4739 16828 4734 16838
4729 16849 4724 16861 4721 16873 4718 16886 4716 16899 4715 16912
4714 16925 4751 16925 4752 16900 4755 16878 4761 16859 4768 16843
4776 16830 4785 16820 4790 16817 4795 16814 4800 16813 4805 16812
4810 16813 4815 16814 4819 16817 4824 16820 4833 16830 4842 16843
4849 16859 4854 16878 4858 16900 4859 16925 4858 16949 4854 16971
4849 16990 4842 17005 4833 17018 4824 17027 4819 17031 4815 17033
4810 17035 4805 17035 4800 17035 4795 17033 4790 17031 4785 17027
4776 17018 4768 17005 4761 16990 4755 16971 4752 16949 4751 16925
4714 16925 4715 16937 4716 16950 4718 16963 4721 16975 4724 16987
4729 16999 4734 17010 4739 17021 4745 17030 4752 17039 4760 17046
4768 17053 4776 17058 4785 17062 4795 17064 4805 17065 107 N F
5063 17014 5061 17001 5058 16989 5052 16978 5044 16968 5034 16960
5023 16955 5011 16951 4999 16949 4999 16974 4991 16975 4983 16977
4977 16981 4971 16986 4966 16991 4962 16998 4960 17006 4959 17014
4960 17021 4962 17029 4966 17036 4971 17041 4977 17046 4983 17050
4991 17052 4999 17053 5007 17052 5014 17050 5021 17046 5026 17041
5031 17036 5035 17029 5037 17021 5038 17014 5037 17006 5035 16998
5031 16991 5026 16986 5021 16981 5014 16977 5007 16975 4999 16974
4999 16949 4986 16951 4974 16955 4963 16960 4954 16968 4946 16978
4940 16989 4936 17001 4935 17014 4936 17026 4940 17038 4946 17049
4954 17059 4963 17067 4974 17072 4986 17076 4999 17078 5011 17076
5023 17072 5034 17067 5044 17059 5052 17049 5058 17038 5061 17026
5063 17014 67 N F
5116 17073 5310 17073 5310 17041 5152 17041 5152 16951 5304 16951
5304 16919 5152 16919 5152 16824 5316 16824 5316 16792 5116 16792
5116 17073 13 N F
6412 17065 6434 17065 6434 16792 6400 16792 6400 16987 6340 16987
6340 17011 6364 17014 6375 17016 6385 17020 6394 17026 6398 17030

6401 17035 6407 17048 6412 17065 15 N F
6627 17065 6649 17065 6649 16792 6615 16792 6615 16987 6554 16987
6554 17011 6579 17014 6590 17016 6600 17020 6608 17026 6612 17030
6616 17035 6622 17048 6627 17065 15 N F
6771 17065 6914 17065 6914 17033 6799 17033 6789 16955 6799 16962
6811 16968 6825 16971 6841 16973 6855 16972 6870 16968 6885 16962
6891 16957 6898 16952 6904 16946 6910 16940 6915 16932 6919 16924
6923 16914 6925 16904 6927 16893 6928 16880 6926 16861 6924 16852
6922 16843 6918 16834 6914 16826 6909 16819 6904 16812 6898 16805
6891 16799 6883 16794 6874 16790 6865 16787 6855 16784 6845 16782
6833 16782 6823 16782 6813 16783 6804 16785 6796 16788 6788 16791
6781 16795 6775 16799 6769 16804 6760 16816 6752 16829 6747 16843
6743 16858 6777 16858 6781 16848 6785 16839 6790 16831 6797 16825
6804 16819 6812 16815 6821 16813 6831 16812 6845 16813 6857 16816
6868 16821 6877 16829 6884 16838 6889 16849 6892 16863 6894 16878
6893 16890 6890 16901 6886 16912 6879 16922 6871 16930 6861 16937
6848 16941 6841 16942 6833 16943 6826 16942 6819 16941 6812 16939
6806 16936 6795 16928 6781 16917 6751 16917 6771 17065 83 N F
7093 17014 7092 17001 7088 16989 7082 16978 7074 16968 7065 16960
7054 16955 7042 16951 7029 16949 7029 16974 7021 16975 7014 16977
7007 16981 7001 16986 6997 16991 6993 16998 6991 17006 6990 17014
6991 17021 6993 17029 6997 17036 7001 17041 7007 17046 7014 17050
7021 17052 7029 17053 7037 17052 7045 17050 7051 17046 7057 17041
7062 17036 7066 17029 7068 17021 7069 17014 7068 17006 7066 16998
7062 16991 7057 16986 7051 16981 7045 16977 7037 16975 7029 16974
7029 16949 7016 16951 7005 16955 6994 16960 6984 16968 6976 16978
6970 16989 6967 17001 6965 17014 6967 17026 6970 17038 6976 17049
6984 17059 6994 17067 7005 17072 7016 17076 7029 17078 7042 17076
7054 17072 7065 17067 7074 17059 7082 17049 7088 17038 7092 17026
7093 17014 67 N F
7146 17073 7341 17073 7341 17041 7182 17041 7182 16951 7335 16951
7335 16919 7182 16919 7182 16824 7347 16824 7347 16792 7146 16792
7146 17073 13 N F
8433 17065 8455 17065 8455 16792 8421 16792 8421 16987 8361 16987
8361 17011 8385 17014 8396 17016 8406 17020 8415 17026 8419 17030
8422 17035 8428 17048 8433 17065 15 N F
8555 16971 8589 16971 8590 16985 8593 16997 8597 17008 8603 17017
8611 17025 8621 17030 8632 17034 8646 17035 8656 17034 8666 17031
8674 17027 8682 17021 8689 17014 8694 17005 8697 16994 8698 16983
8697 16971 8694 16961 8689 16953 8682 16946 8674 16939 8663 16931
8636 16915 8612 16901 8593 16887 8578 16873 8567 16859 8559 16843
8553 16827 8550 16810 8549 16792 8732 16792 8732 16824 8587 16824
8591 16836 8596 16847 8602 16855 8610 16863 8620 16871 8633 16878

8664 16896 8682 16905 8697 16915 8709 16926 8718 16938 8725 16950
8730 16962 8733 16975 8734 16987 8733 16995 8732 17004 8730 17011
8727 17019 8723 17026 8719 17032 8714 17038 8708 17043 8695 17053
8680 17060 8663 17064 8646 17065 8627 17064 8618 17062 8609 17060
8602 17056 8594 17052 8587 17048 8581 17042 8575 17036 8570 17029
8566 17021 8562 17013 8559 17003 8557 16993 8556 16982 8555 16971
78 N F
8856 17065 8866 17064 8876 17062 8885 17058 8893 17053 8901 17046
8909 17039 8916 17030 8922 17021 8927 17010 8932 16999 8937 16987
8940 16975 8943 16963 8945 16950 8946 16937 8947 16925 8946 16912
8945 16899 8943 16886 8940 16873 8937 16861 8932 16849 8927 16838
8922 16828 8916 16818 8909 16809 8901 16801 8893 16795 8885 16789
8876 16785 8866 16783 8856 16782 8846 16783 8837 16785 8828 16789
8819 16795 8811 16801 8804 16809 8797 16818 8791 16828 8785 16838
8780 16849 8776 16861 8772 16873 8770 16886 8768 16899 8766 16912
8766 16925 8802 16925 8803 16900 8807 16878 8812 16859 8819 16843
8828 16830 8837 16820 8842 16817 8846 16814 8851 16813 8856 16812
8861 16813 8866 16814 8871 16817 8876 16820 8885 16830 8893 16843
8900 16859 8906 16878 8909 16900 8910 16925 8909 16949 8906 16971
8900 16990 8893 17005 8885 17018 8876 17027 8871 17031 8866 17033
8861 17035 8856 17035 8851 17035 8846 17033 8842 17031 8837 17027
8828 17018 8819 17005 8812 16990 8807 16971 8803 16949 8802 16925
8766 16925 8766 16937 8768 16950 8770 16963 8772 16975 8776 16987
8780 16999 8785 17010 8791 17021 8797 17030 8804 17039 8811 17046
8819 17053 8828 17058 8837 17062 8846 17064 8856 17065 107 N F
9114 17014 9113 17001 9109 16989 9103 16978 9095 16968 9086 16960
9075 16955 9063 16951 9050 16949 9050 16974 9042 16975 9035 16977
9028 16981 9022 16986 9018 16991 9014 16998 9012 17006 9011 17014
9012 17021 9014 17029 9018 17036 9022 17041 9028 17046 9035 17050
9042 17052 9050 17053 9058 17052 9066 17050 9072 17046 9078 17041
9083 17036 9087 17029 9089 17021 9090 17014 9089 17006 9087 16998
9083 16991 9078 16986 9072 16981 9066 16977 9058 16975 9050 16974
9050 16949 9037 16951 9025 16955 9015 16960 9005 16968 8997 16978
8991 16989 8988 17001 8986 17014 8988 17026 8991 17038 8997 17049
9005 17059 9015 17067 9025 17072 9037 17076 9050 17078 9063 17076
9075 17072 9086 17067 9095 17059 9103 17049 9109 17038 9113 17026
9114 17014 67 N F
9167 17073 9362 17073 9362 17041 9203 17041 9203 16951 9356 16951
9356 16919 9203 16919 9203 16824 9368 16824 9368 16792 9167 16792
9167 17073 13 N F
10460 17065 10482 17065 10482 16792 10448 16792 10448 16987 10388 16987
10388 17011 10412 17014 10423 17016 10433 17020 10442 17026 10446 17030
10449 17035 10455 17048 10460 17065 15 N F

10582 16971 10617 16971 10617 16985 10620 16997 10624 17008 10630 17017
10638 17025 10648 17030 10659 17034 10673 17035 10683 17034 10693 17031
10702 17027 10709 17021 10716 17014 10721 17005 10724 16994 10725 16983
10724 16971 10721 16961 10716 16953 10710 16946 10701 16939 10690 16931
10663 16915 10639 16901 10620 16887 10605 16873 10594 16859 10586 16843
10580 16827 10577 16810 10576 16792 10759 16792 10759 16824 10615 16824
10618 16836 10623 16847 10630 16855 10638 16863 10648 16871 10660 16878
10691 16896 10709 16905 10724 16915 10736 16926 10746 16938 10753 16950
10757 16962 10760 16975 10761 16987 10761 16995 10759 17004 10757 17011
10754 17019 10750 17026 10746 17032 10741 17038 10735 17043 10722 17053
10707 17060 10690 17064 10673 17065 10654 17064 10645 17062 10637 17060
10629 17056 10621 17052 10614 17048 10608 17042 10602 17036 10597 17029
10593 17021 10589 17013 10586 17003 10584 16993 10583 16982 10582 16971
78 N F
10819 17065 10962 17065 10962 17033 10847 17033 10837 16955 10847 16962
10859 16968 10873 16971 10890 16973 10904 16972 10918 16968 10933 16962
10940 16957 10946 16952 10952 16946 10958 16940 10963 16932 10967 16924
10971 16914 10974 16904 10975 16893 10976 16880 10974 16861 10972 16852
10970 16843 10966 16834 10962 16826 10958 16819 10952 16812 10946 16805
10939 16799 10931 16794 10922 16790 10913 16787 10903 16784 10893 16782
10882 16782 10871 16782 10861 16783 10852 16785 10844 16788 10836 16791
10829 16795 10823 16799 10818 16804 10808 16816 10801 16829 10795 16843
10791 16858 10825 16858 10829 16848 10833 16839 10839 16831 10845 16825
10852 16819 10860 16815 10869 16813 10879 16812 10893 16813 10905 16816
10916 16821 10925 16829 10932 16838 10937 16849 10941 16863 10942 16878
10941 16890 10938 16901 10934 16912 10927 16922 10919 16930 10909 16937
10896 16941 10889 16942 10882 16943 10874 16942 10867 16941 10861 16939
10855 16936 10843 16928 10829 16917 10799 16917 10819 17065 83 N F
11141 17014 11140 17001 11136 16989 11130 16978 11123 16968 11113 16960
11102 16955 11090 16951 11077 16949 11077 16974 11070 16975 11062 16977
11055 16981 11050 16986 11045 16991 11041 16998 11039 17006 11038 17014
11039 17021 11041 17029 11045 17036 11050 17041 11055 17046 11062 17050
11070 17052 11077 17053 11085 17052 11093 17050 11099 17046 11105 17041
11110 17036 11114 17029 11116 17021 11117 17014 11116 17006 11114 16998
11110 16991 11105 16986 11099 16981 11093 16977 11085 16975 11077 16974
11077 16949 11065 16951 11053 16955 11042 16960 11032 16968 11025 16978
11019 16989 11015 17001 11014 17014 11015 17026 11019 17038 11025 17049
11032 17059 11042 17067 11053 17072 11065 17076 11077 17078 11090 17076
11102 17072 11113 17067 11123 17059 11130 17049 11136 17038 11140 17026
11141 17014 67 N F
11194 17073 11389 17073 11389 17041 11231 17041 11231 16951 11383 16951
11383 16919 11231 16919 11231 16824 11395 16824 11395 16792 11194 16792
11194 17073 13 N F

12511 17065 12533 17065 12533 16792 12498 16792 12498 16987 12438 16987
12438 17011 12463 17014 12473 17016 12483 17020 12492 17026 12496 17030
12500 17035 12506 17048 12511 17065 15 N F
12665 16977 12631 16977 12633 16997 12635 17007 12638 17015 12641 17023
12645 17030 12650 17037 12655 17043 12661 17048 12668 17053 12674 17056
12682 17060 12690 17062 12699 17064 12717 17065 12735 17064 12751 17060
12765 17054 12777 17046 12783 17041 12788 17036 12792 17030 12795 17023
12798 17016 12800 17008 12801 17000 12801 16991 12801 16981 12799 16972
12797 16963 12793 16956 12787 16949 12780 16943 12772 16937 12761 16933
12773 16928 12783 16922 12792 16915 12798 16908 12803 16900 12807 16890
12809 16880 12810 16868 12808 16853 12805 16838 12798 16824 12794 16817
12789 16811 12783 16805 12776 16800 12768 16795 12760 16791 12750 16788
12739 16786 12728 16784 12715 16784 12704 16784 12694 16786 12685 16788
12676 16790 12668 16794 12661 16798 12654 16803 12648 16808 12643 16814
12639 16821 12635 16828 12632 16836 12628 16852 12627 16870 12661 16870
12661 16860 12663 16851 12666 16841 12671 16832 12678 16825 12688 16819
12701 16815 12717 16814 12731 16815 12743 16817 12753 16822 12760 16829
12766 16837 12770 16847 12773 16857 12773 16868 12772 16883 12770 16889
12768 16894 12765 16899 12761 16903 12753 16909 12742 16914 12729 16917
12714 16918 12697 16919 12697 16947 12713 16947 12727 16948 12739 16951
12749 16954 12756 16960 12761 16967 12764 16977 12765 16989 12765 16998
12763 17007 12759 17015 12754 17022 12747 17027 12739 17032 12729 17034
12717 17035 12706 17035 12697 17033 12688 17029 12681 17023 12674 17016
12670 17005 12667 16993 12665 16977 123 N F
12934 17065 12944 17064 12953 17062 12962 17058 12971 17053 12979 17046
12986 17039 12993 17030 12999 17021 13005 17010 13010 16999 13014 16987
13018 16975 13021 16963 13023 16950 13024 16937 13024 16925 13024 16912
13023 16899 13021 16886 13018 16873 13014 16861 13010 16849 13005 16838
12999 16828 12993 16818 12986 16809 12979 16801 12971 16795 12962 16789
12953 16785 12944 16783 12934 16782 12924 16783 12915 16785 12905 16789
12897 16795 12889 16801 12882 16809 12875 16818 12868 16828 12863 16838
12858 16849 12854 16861 12850 16873 12847 16886 12845 16899 12844 16912
12844 16925 12880 16925 12881 16900 12885 16878 12890 16859 12897 16843
12905 16830 12914 16820 12919 16817 12924 16814 12929 16813 12934 16812
12939 16813 12944 16814 12949 16817 12953 16820 12963 16830 12971 16843
12978 16859 12983 16878 12987 16900 12988 16925 12987 16949 12983 16971
12978 16990 12971 17005 12963 17018 12953 17027 12949 17031 12944 17033
12939 17035 12934 17035 12929 17035 12924 17033 12919 17031 12914 17027
12905 17018 12897 17005 12890 16990 12885 16971 12881 16949 12880 16925
12844 16925 12844 16937 12845 16950 12847 16963 12850 16975 12854 16987
12858 16999 12863 17010 12868 17021 12875 17030 12882 17039 12889 17046
12897 17053 12905 17058 12915 17062 12924 17064 12934 17065 107 N F
13192 17014 13191 17001 13187 16989 13181 16978 13173 16968 13164 16960

13153 16955 13141 16951 13128 16949 13128 16974 13120 16975 13113 16977
13106 16981 13100 16986 13095 16991 13092 16998 13089 17006 13089 17014
13089 17021 13092 17029 13095 17036 13100 17041 13106 17046 13113 17050
13120 17052 13128 17053 13136 17052 13143 17050 13150 17046 13156 17041
13161 17036 13164 17029 13166 17021 13167 17014 13166 17006 13164 16998
13161 16991 13156 16986 13150 16981 13143 16977 13136 16975 13128 16974
13128 16949 13115 16951 13103 16955 13092 16960 13083 16968 13075 16978
13069 16989 13065 17001 13064 17014 13065 17026 13069 17038 13075 17049
13083 17059 13092 17067 13103 17072 13115 17076 13128 17078 13141 17076
13153 17072 13164 17067 13173 17059 13181 17049 13187 17038 13191 17026
13192 17014 67 N F
13245 17073 13440 17073 13440 17041 13281 17041 13281 16951 13434 16951
13434 16919 13281 16919 13281 16824 13446 16824 13446 16792 13245 16792
13245 17073 13 N F
911 5730 945 5730 946 5744 949 5756 953 5767 959 5777
967 5784 977 5790 988 5793 1002 5795 1012 5794 1022 5791
1030 5787 1038 5781 1045 5773 1050 5764 1053 5754 1054 5742
1053 5731 1050 5721 1045 5713 1038 5705 1030 5698 1019 5691
992 5674 968 5660 949 5647 934 5633 923 5618 915 5603
909 5587 906 5570 905 5551 1088 5551 1088 5584 943 5584
947 5596 952 5606 958 5615 967 5623 977 5630 989 5638
1020 5656 1038 5665 1053 5675 1065 5686 1074 5697 1081 5709
1086 5722 1089 5734 1090 5746 1089 5755 1088 5763 1086 5771
1083 5778 1079 5785 1075 5792 1070 5797 1064 5803 1051 5812
1036 5819 1019 5823 1002 5825 983 5823 974 5822 965 5819
958 5816 950 5812 943 5807 937 5802 931 5795 926 5788
922 5781 918 5772 915 5763 913 5753 912 5742 911 5730
78 N F
1126 5730 1160 5730 1161 5744 1164 5756 1168 5767 1174 5777
1182 5784 1191 5790 1203 5793 1216 5795 1227 5794 1236 5791
1245 5787 1253 5781 1259 5773 1264 5764 1267 5754 1269 5742
1268 5731 1265 5721 1260 5713 1253 5705 1245 5698 1234 5691
1206 5674 1183 5660 1164 5647 1149 5633 1138 5618 1129 5603
1124 5587 1121 5570 1120 5551 1303 5551 1303 5584 1158 5584
1162 5596 1167 5606 1173 5615 1181 5623 1191 5630 1203 5638
1234 5656 1252 5665 1267 5675 1280 5686 1289 5697 1296 5709
1301 5722 1304 5734 1305 5746 1304 5755 1303 5763 1301 5771
1298 5778 1294 5785 1289 5792 1284 5797 1279 5803 1265 5812
1250 5819 1234 5823 1216 5825 1197 5823 1189 5822 1180 5819
1172 5816 1165 5812 1158 5807 1152 5802 1146 5795 1141 5788
1136 5781 1133 5772 1130 5763 1128 5753 1126 5742 1126 5730
78 N F
1470 5773 1469 5760 1465 5748 1459 5737 1451 5728 1442 5720

1431 5714 1419 5710 1406 5709 1406 5734 1398 5734 1391 5737
1384 5740 1378 5745 1374 5751 1370 5758 1368 5765 1367 5773
1368 5781 1370 5788 1374 5795 1378 5801 1384 5806 1391 5809
1398 5812 1406 5812 1414 5812 1422 5809 1428 5806 1434 5801
1439 5795 1443 5788 1445 5781 1446 5773 1445 5765 1443 5758
1439 5751 1434 5745 1428 5740 1422 5737 1414 5734 1406 5734
1406 5709 1393 5710 1381 5714 1371 5720 1361 5728 1353 5737
1347 5748 1344 5760 1342 5773 1344 5786 1347 5798 1353 5809
1361 5818 1371 5826 1381 5832 1393 5836 1406 5837 1419 5836
1431 5832 1442 5826 1451 5818 1459 5809 1465 5798 1469 5786
1470 5773 67 N F
1517 5833 1557 5833 1702 5604 1702 5833 1736 5833 1736 5551
1696 5551 1551 5781 1551 5551 1517 5551 1517 5833 11 N F
911 6907 945 6907 946 6921 949 6933 953 6944 959 6953
967 6961 977 6967 988 6970 1002 6971 1012 6970 1022 6968
1030 6963 1038 6957 1045 6950 1050 6941 1053 6930 1054 6919
1053 6907 1050 6898 1045 6889 1038 6882 1030 6875 1019 6868
992 6851 968 6837 949 6823 934 6809 923 6795 915 6779
909 6763 906 6746 905 6728 1088 6728 1088 6760 943 6760
947 6773 952 6783 958 6792 967 6799 977 6807 989 6814
1020 6833 1038 6841 1053 6852 1065 6862 1074 6874 1081 6886
1086 6898 1089 6911 1090 6923 1089 6932 1088 6940 1086 6947
1083 6955 1079 6962 1075 6968 1070 6974 1064 6980 1051 6989
1036 6996 1019 7000 1002 7001 983 7000 974 6998 965 6996
958 6992 950 6988 943 6984 937 6978 931 6972 926 6965
922 6957 918 6949 915 6940 913 6929 912 6919 911 6907
78 N F
1242 7001 1267 7001 1267 6825 1309 6825 1309 6792 1267 6792
1267 6728 1232 6728 1232 6792 1118 6792 1118 6825 1148 6825
1232 6825 1232 6943 1148 6825 1118 6825 1118 6829 1242 7001
18 N F
1470 6950 1469 6937 1465 6925 1459 6914 1451 6904 1442 6897
1431 6891 1419 6887 1406 6886 1406 6910 1398 6911 1391 6913
1384 6917 1378 6922 1374 6928 1370 6934 1368 6942 1367 6950
1368 6958 1370 6965 1374 6972 1378 6978 1384 6982 1391 6986
1398 6988 1406 6989 1414 6988 1422 6986 1428 6982 1434 6978
1439 6972 1443 6965 1445 6958 1446 6950 1445 6942 1443 6934
1439 6928 1434 6922 1428 6917 1422 6913 1414 6911 1406 6910
1406 6886 1393 6887 1381 6891 1371 6897 1361 6904 1353 6914
1347 6925 1344 6937 1342 6950 1344 6962 1347 6974 1353 6985
1361 6995 1371 7003 1381 7009 1393 7012 1406 7014 1419 7012
1431 7009 1442 7003 1451 6995 1459 6985 1465 6974 1469 6962
1470 6950 67 N F

1517 7009 1557 7009 1702 6780 1702 7009 1736 7009 1736 6728
1696 6728 1551 6957 1551 6728 1517 6728 1517 7009 11 N F
911 8068 945 8068 946 8082 949 8095 953 8105 959 8115
967 8122 977 8128 988 8131 1002 8133 1012 8132 1022 8129
1030 8125 1038 8119 1045 8111 1050 8102 1053 8092 1054 8080
1053 8069 1050 8059 1045 8051 1038 8043 1030 8036 1019 8029
992 8012 968 7999 949 7985 934 7971 923 7956 915 7941
909 7925 906 7908 905 7890 1088 7890 1088 7922 943 7922
947 7934 952 7944 958 7953 967 7961 977 7968 989 7976
1020 7994 1038 8003 1053 8013 1065 8024 1074 8035 1081 8048
1086 8060 1089 8072 1090 8084 1089 8093 1088 8101 1086 8109
1083 8116 1079 8123 1075 8130 1070 8136 1064 8141 1051 8150
1036 8157 1019 8161 1002 8163 983 8161 974 8160 965 8157
958 8154 950 8150 943 8145 937 8140 931 8133 926 8127
922 8119 918 8110 915 8101 913 8091 912 8080 911 8068
78 N F
1299 8092 1265 8092 1262 8102 1259 8110 1254 8117 1248 8122
1242 8127 1235 8130 1228 8132 1220 8133 1213 8132 1206 8131
1200 8129 1194 8126 1188 8122 1183 8117 1178 8112 1174 8106
1167 8091 1162 8073 1159 8053 1158 8030 1166 8039 1174 8046
1182 8051 1191 8055 1199 8058 1207 8059 1224 8060 1238 8059
1252 8055 1265 8048 1278 8039 1284 8033 1289 8026 1294 8019
1298 8011 1301 8002 1303 7993 1304 7983 1305 7972 1271 7970
1269 7981 1266 7992 1261 8002 1254 8011 1245 8019 1236 8025
1225 8029 1214 8030 1203 8029 1193 8025 1183 8019 1175 8011
1168 8002 1163 7992 1159 7981 1158 7970 1159 7959 1163 7948
1168 7938 1175 7928 1183 7921 1193 7915 1203 7911 1214 7910
1225 7911 1236 7915 1245 7921 1254 7928 1261 7938 1266 7948
1269 7959 1271 7970 1305 7972 1303 7956 1299 7940 1292 7924
1288 7917 1283 7910 1277 7903 1270 7898 1263 7892 1255 7888
1246 7884 1236 7882 1226 7880 1214 7879 1202 7880 1191 7882
1180 7886 1171 7890 1163 7896 1155 7903 1148 7910 1143 7919
1138 7929 1133 7939 1130 7951 1127 7963 1123 7988 1122 8016
1122 8033 1124 8048 1126 8063 1129 8077 1132 8090 1137 8102
1142 8113 1148 8123 1155 8132 1162 8140 1170 8147 1179 8153
1188 8157 1198 8160 1209 8162 1220 8163 1236 8161 1251 8157
1263 8151 1275 8142 1284 8132 1291 8120 1296 8106 1299 8092
126 N F
1470 8111 1469 8098 1465 8086 1459 8075 1451 8066 1442 8058
1431 8052 1419 8048 1406 8047 1406 8072 1398 8072 1391 8075
1384 8078 1378 8083 1374 8089 1370 8096 1368 8103 1367 8111
1368 8119 1370 8126 1374 8133 1378 8139 1384 8144 1391 8147
1398 8150 1406 8150 1414 8150 1422 8147 1428 8144 1434 8139

1439 8133 1443 8126 1445 8119 1446 8111 1445 8103 1443 8096
1439 8089 1434 8083 1428 8078 1422 8075 1414 8072 1406 8072
1406 8047 1393 8048 1381 8052 1371 8058 1361 8066 1353 8075
1347 8086 1344 8098 1342 8111 1344 8124 1347 8136 1353 8147
1361 8156 1371 8164 1381 8170 1393 8174 1406 8175 1419 8174
1431 8170 1442 8164 1451 8156 1459 8147 1465 8136 1469 8124
1470 8111 67 N F
1517 8171 1557 8171 1702 7942 1702 8171 1736 8171 1736 7890
1696 7890 1551 8119 1551 7890 1517 7890 1517 8171 11 N F
911 9214 945 9214 946 9227 949 9240 953 9251 959 9260
967 9268 977 9273 988 9277 1002 9278 1012 9277 1022 9274
1030 9270 1038 9264 1045 9256 1050 9247 1053 9237 1054 9226
1053 9214 1050 9204 1045 9196 1038 9188 1030 9181 1019 9174
992 9157 968 9144 949 9130 934 9116 923 9101 915 9086
909 9070 906 9053 905 9035 1088 9035 1088 9067 943 9067
947 9079 952 9089 958 9098 967 9106 977 9113 989 9121
1020 9139 1038 9148 1053 9158 1065 9169 1074 9181 1081 9193
1086 9205 1089 9217 1090 9230 1089 9238 1088 9246 1086 9254
1083 9261 1079 9268 1075 9275 1070 9281 1064 9286 1051 9295
1036 9302 1019 9307 1002 9308 983 9307 974 9305 965 9302
958 9299 950 9295 943 9290 937 9285 931 9279 926 9272
922 9264 918 9255 915 9246 913 9236 912 9225 911 9214
78 N F
1212 9308 1229 9307 1245 9303 1259 9297 1271 9289 1276 9284
1281 9279 1285 9273 1288 9266 1291 9260 1293 9252 1294 9244
1295 9236 1294 9224 1291 9214 1287 9205 1283 9197 1277 9191
1271 9186 1258 9177 1269 9172 1278 9166 1285 9158 1292 9150
1297 9141 1301 9131 1304 9120 1305 9109 1303 9096 1299 9081
1291 9067 1281 9054 1275 9048 1268 9042 1260 9037 1251 9033
1242 9030 1232 9027 1222 9025 1210 9025 1199 9025 1189 9027
1179 9030 1170 9033 1162 9037 1154 9042 1148 9048 1142 9054
1132 9067 1125 9081 1121 9096 1120 9109 1156 9109 1157 9098
1161 9088 1166 9079 1173 9071 1181 9064 1191 9059 1201 9056
1212 9055 1223 9056 1234 9059 1243 9064 1252 9071 1259 9079
1264 9088 1267 9098 1269 9109 1267 9120 1264 9130 1259 9139
1252 9148 1243 9154 1234 9159 1223 9162 1212 9163 1201 9162
1191 9159 1181 9154 1173 9148 1166 9139 1161 9130 1157 9120
1156 9109 1120 9109 1121 9120 1123 9130 1127 9140 1133 9149
1139 9158 1147 9165 1156 9172 1166 9177 1154 9186 1148 9191
1142 9197 1137 9205 1133 9214 1131 9224 1130 9236 1164 9236
1165 9226 1168 9218 1173 9211 1178 9205 1186 9200 1194 9196
1203 9194 1212 9193 1222 9194 1231 9196 1239 9200 1246 9205
1252 9211 1257 9218 1260 9226 1261 9236 1260 9245 1257 9253

1252 9261 1246 9267 1239 9271 1231 9275 1222 9277 1212 9278
1203 9277 1194 9275 1186 9271 1178 9267 1173 9261 1168 9253
1165 9245 1164 9236 1130 9236 1130 9244 1132 9252 1134 9260
1136 9266 1140 9273 1144 9279 1149 9284 1154 9289 1166 9297
1180 9303 1196 9307 1212 9308 147 N F
1470 9256 1469 9243 1465 9231 1459 9221 1451 9211 1442 9203
1431 9197 1419 9194 1406 9192 1406 9217 1398 9218 1391 9220
1384 9224 1378 9228 1374 9234 1370 9241 1368 9248 1367 9256
1368 9264 1370 9272 1374 9278 1378 9284 1384 9289 1391 9293
1398 9295 1406 9296 1414 9295 1422 9293 1428 9289 1434 9284
1439 9278 1443 9272 1445 9264 1446 9256 1445 9248 1443 9241
1439 9234 1434 9228 1428 9224 1422 9220 1414 9218 1406 9217
1406 9192 1393 9194 1381 9197 1371 9203 1361 9211 1353 9221
1347 9231 1344 9243 1342 9256 1344 9269 1347 9281 1353 9292
1361 9301 1371 9309 1381 9315 1393 9319 1406 9320 1419 9319
1431 9315 1442 9309 1451 9301 1459 9292 1465 9281 1469 9269
1470 9256 67 N F
1517 9316 1557 9316 1702 9087 1702 9316 1736 9316 1736 9035
1696 9035 1551 9264 1551 9035 1517 9035 1517 9316 11 N F
943 10355 909 10355 911 10375 914 10384 916 10393 920 10401
924 10408 928 10415 934 10421 939 10426 946 10430 953 10434
960 10437 968 10440 977 10442 996 10443 1013 10442 1029 10438
1043 10432 1056 10424 1061 10419 1066 10413 1070 10407 1073 10401
1076 10394 1078 10386 1079 10378 1080 10369 1079 10359 1078 10349
1075 10341 1071 10334 1066 10327 1059 10321 1050 10315 1040 10310
1052 10306 1062 10300 1070 10293 1077 10286 1082 10277 1085 10268
1087 10258 1088 10246 1087 10231 1083 10216 1077 10202 1072 10195
1067 10189 1061 10183 1054 10178 1047 10173 1038 10169 1028 10166
1018 10164 1006 10162 994 10162 983 10162 973 10163 963 10165
954 10168 946 10172 939 10176 933 10181 927 10186 922 10192
917 10199 914 10206 911 10213 907 10230 905 10248 939 10248
940 10238 941 10228 944 10219 949 10210 957 10203 967 10197
979 10193 996 10192 1010 10192 1021 10195 1031 10200 1039 10207
1045 10215 1049 10225 1051 10235 1052 10246 1050 10260 1049 10266
1046 10272 1043 10277 1040 10281 1031 10287 1020 10292 1007 10294
992 10296 976 10296 976 10325 992 10325 1006 10326 1017 10328
1027 10332 1034 10337 1040 10345 1043 10355 1044 10367 1043 10376
1041 10385 1038 10393 1032 10400 1026 10405 1018 10409 1007 10412
996 10413 985 10412 975 10410 966 10407 959 10401 953 10393
948 10383 945 10370 943 10355 123 N F
1212 10443 1222 10442 1232 10440 1241 10436 1249 10431 1257 10424
1265 10417 1272 10408 1278 10398 1283 10388 1288 10377 1293 10365
1296 10353 1299 10341 1301 10328 1302 10315 1303 10302 1302 10290

1301 10277 1299 10264 1296 10251 1293 10239 1288 10227 1283 10216
1278 10205 1272 10196 1265 10187 1257 10179 1249 10172 1241 10167
1232 10163 1222 10161 1212 10160 1202 10161 1193 10163 1184 10167
1175 10172 1167 10179 1160 10187 1153 10196 1147 10205 1141 10216
1136 10227 1132 10239 1129 10251 1126 10264 1124 10277 1122 10290
1122 10302 1158 10302 1159 10278 1163 10256 1168 10237 1175 10221
1184 10207 1193 10198 1198 10194 1203 10192 1207 10190 1212 10190
1217 10190 1222 10192 1227 10194 1232 10198 1241 10207 1249 10221
1256 10237 1262 10256 1265 10278 1267 10302 1265 10327 1262 10349
1256 10367 1249 10383 1241 10396 1232 10405 1227 10409 1222 10411
1217 10412 1212 10413 1207 10412 1203 10411 1198 10409 1193 10405
1184 10396 1175 10383 1168 10367 1163 10349 1159 10327 1158 10302
1122 10302 1122 10315 1124 10328 1126 10341 1129 10353 1132 10365
1136 10377 1141 10388 1147 10398 1153 10408 1160 10417 1167 10424
1175 10431 1184 10436 1193 10440 1202 10442 1212 10443 107 N F
1470 10391 1469 10379 1465 10367 1459 10356 1451 10346 1442 10338
1431 10332 1419 10329 1406 10327 1406 10352 1398 10353 1391 10355
1384 10359 1378 10363 1374 10369 1370 10376 1368 10383 1367 10391
1368 10399 1370 10407 1374 10413 1378 10419 1384 10424 1391 10428
1398 10430 1406 10431 1414 10430 1422 10428 1428 10424 1434 10419
1439 10413 1443 10407 1445 10399 1446 10391 1445 10383 1443 10376
1439 10369 1434 10363 1428 10359 1422 10355 1414 10353 1406 10352
1406 10327 1393 10329 1381 10332 1371 10338 1361 10346 1353 10356
1347 10367 1344 10379 1342 10391 1344 10404 1347 10416 1353 10427
1361 10437 1371 10444 1381 10450 1393 10454 1406 10455 1419 10454
1431 10450 1442 10444 1451 10437 1459 10427 1465 10416 1469 10404
1470 10391 67 N F
1517 10451 1557 10451 1702 10222 1702 10451 1736 10451 1736 10170
1696 10170 1551 10399 1551 10170 1517 10170 1517 10451 11 N F
943 11480 909 11480 911 11500 914 11510 916 11518 920 11526
924 11533 928 11540 934 11546 939 11551 946 11556 953 11559
960 11563 968 11565 977 11567 996 11568 1013 11567 1029 11563
1043 11557 1056 11549 1061 11544 1066 11539 1070 11532 1073 11526
1076 11519 1078 11511 1079 11503 1080 11494 1079 11484 1078 11475
1075 11466 1071 11459 1066 11452 1059 11446 1050 11440 1040 11436
1052 11431 1062 11425 1070 11418 1077 11411 1082 11403 1085 11393
1087 11383 1088 11371 1087 11356 1083 11341 1077 11327 1072 11320
1067 11314 1061 11308 1054 11303 1047 11298 1038 11294 1028 11291
1018 11289 1006 11287 994 11287 983 11287 973 11288 963 11290
954 11293 946 11297 939 11301 933 11306 927 11311 922 11317
917 11324 914 11331 911 11339 907 11355 905 11373 939 11373
940 11363 941 11353 944 11344 949 11335 957 11328 967 11322
979 11318 996 11317 1010 11317 1021 11320 1031 11325 1039 11332

1045 11340 1049 11350 1051 11360 1052 11371 1050 11386 1049 11392
1046 11397 1043 11402 1040 11406 1031 11412 1020 11417 1007 11420
992 11421 976 11421 976 11450 992 11450 1006 11451 1017 11453
1027 11457 1034 11463 1040 11470 1043 11480 1044 11492 1043 11501
1041 11510 1038 11518 1032 11525 1026 11530 1018 11534 1007 11537
996 11538 985 11537 975 11535 966 11532 959 11526 953 11519
948 11508 945 11496 943 11480 123 N F
1126 11474 1160 11474 1161 11487 1164 11500 1168 11511 1174 11520
1182 11528 1191 11533 1203 11537 1216 11538 1227 11537 1236 11534
1245 11530 1253 11524 1259 11516 1264 11508 1267 11497 1269 11486
1268 11474 1265 11464 1260 11456 1253 11449 1245 11442 1234 11434
1206 11417 1183 11404 1164 11390 1149 11376 1138 11361 1129 11346
1124 11330 1121 11313 1120 11295 1303 11295 1303 11327 1158 11327
1162 11339 1167 11350 1173 11358 1181 11366 1191 11374 1203 11381
1234 11399 1252 11408 1267 11418 1280 11429 1289 11441 1296 11453
1301 11465 1304 11478 1305 11490 1304 11498 1303 11506 1301 11514
1298 11522 1294 11528 1289 11535 1284 11541 1279 11546 1265 11556
1250 11562 1234 11567 1216 11568 1197 11567 1189 11565 1180 11562
1172 11559 1165 11555 1158 11551 1152 11545 1146 11539 1141 11532
1136 11524 1133 11516 1130 11506 1128 11496 1126 11485 1126 11474
78 N F
1470 11516 1469 11504 1465 11492 1459 11481 1451 11471 1442 11463
1431 11457 1419 11454 1406 11452 1406 11477 1398 11478 1391 11480
1384 11484 1378 11489 1374 11494 1370 11501 1368 11508 1367 11516
1368 11524 1370 11532 1374 11538 1378 11544 1384 11549 1391 11553
1398 11555 1406 11556 1414 11555 1422 11553 1428 11549 1434 11544
1439 11538 1443 11532 1445 11524 1446 11516 1445 11508 1443 11501
1439 11494 1434 11489 1428 11484 1422 11480 1414 11478 1406 11477
1406 11452 1393 11454 1381 11457 1371 11463 1361 11471 1353 11481
1347 11492 1344 11504 1342 11516 1344 11529 1347 11541 1353 11552
1361 11562 1371 11569 1381 11575 1393 11579 1406 11580 1419 11579
1431 11575 1442 11569 1451 11562 1459 11552 1465 11541 1469 11529
1470 11516 67 N F
1517 11576 1557 11576 1702 11347 1702 11576 1736 11576 1736 11295
1696 11295 1551 11524 1551 11295 1517 11295 1517 11576 11 N F
943 12598 909 12598 911 12619 914 12628 916 12636 920 12644
924 12652 928 12658 934 12664 939 12669 946 12674 953 12678
960 12681 968 12683 977 12685 996 12686 1013 12685 1029 12682
1043 12676 1056 12667 1061 12662 1066 12657 1070 12651 1073 12644
1076 12637 1078 12629 1079 12621 1080 12612 1079 12602 1078 12593
1075 12584 1071 12577 1066 12570 1059 12564 1050 12559 1040 12554
1052 12549 1062 12543 1070 12537 1077 12529 1082 12521 1085 12511
1087 12501 1088 12490 1087 12474 1083 12459 1077 12445 1072 12438

1067 12432 1061 12426 1054 12421 1047 12417 1038 12413 1028 12409
1018 12407 1006 12406 994 12405 983 12406 973 12407 963 12409
954 12412 946 12415 939 12419 933 12424 927 12429 922 12436
917 12442 914 12449 911 12457 907 12473 905 12492 939 12492
940 12482 941 12472 944 12462 949 12454 957 12446 967 12440
979 12437 996 12435 1010 12436 1021 12439 1031 12444 1039 12450
1045 12459 1049 12468 1051 12478 1052 12490 1050 12504 1049 12510
1046 12515 1043 12520 1040 12524 1031 12530 1020 12535 1007 12538
992 12539 976 12540 976 12568 992 12568 1006 12569 1017 12572
1027 12575 1034 12581 1040 12588 1043 12598 1044 12610 1043 12620
1041 12628 1038 12636 1032 12643 1026 12649 1018 12653 1007 12655
996 12656 985 12656 975 12654 966 12650 959 12645 953 12637
948 12627 945 12614 943 12598 123 N F
1242 12686 1267 12686 1267 12510 1309 12510 1309 12477 1267 12477
1267 12413 1232 12413 1232 12477 1118 12477 1118 12510 1148 12510
1232 12510 1232 12628 1148 12510 1118 12510 1118 12514 1242 12686
18 N F
1470 12635 1469 12622 1465 12610 1459 12599 1451 12590 1442 12582
1431 12576 1419 12572 1406 12571 1406 12595 1398 12596 1391 12598
1384 12602 1378 12607 1374 12613 1370 12619 1368 12627 1367 12635
1368 12643 1370 12650 1374 12657 1378 12663 1384 12667 1391 12671
1398 12673 1406 12674 1414 12673 1422 12671 1428 12667 1434 12663
1439 12657 1443 12650 1445 12643 1446 12635 1445 12627 1443 12619
1439 12613 1434 12607 1428 12602 1422 12598 1414 12596 1406 12595
1406 12571 1393 12572 1381 12576 1371 12582 1361 12590 1353 12599
1347 12610 1344 12622 1342 12635 1344 12648 1347 12660 1353 12670
1361 12680 1371 12688 1381 12694 1393 12697 1406 12699 1419 12697
1431 12694 1442 12688 1451 12680 1459 12670 1465 12660 1469 12648
1470 12635 67 N F
1517 12694 1557 12694 1702 12465 1702 12694 1736 12694 1736 12413
1696 12413 1551 12642 1551 12413 1517 12413 1517 12694 11 N F
943 13711 909 13711 911 13731 914 13741 916 13749 920 13757
924 13764 928 13771 934 13777 939 13782 946 13787 953 13790
960 13794 968 13796 977 13798 996 13799 1013 13798 1029 13794
1043 13788 1056 13780 1061 13775 1066 13769 1070 13763 1073 13757
1076 13750 1078 13742 1079 13734 1080 13725 1079 13715 1078 13705
1075 13697 1071 13690 1066 13683 1059 13677 1050 13671 1040 13666
1052 13662 1062 13656 1070 13649 1077 13642 1082 13633 1085 13624
1087 13614 1088 13602 1087 13587 1083 13572 1077 13558 1072 13551
1067 13545 1061 13539 1054 13534 1047 13529 1038 13525 1028 13522
1018 13520 1006 13518 994 13518 983 13518 973 13519 963 13521
954 13524 946 13528 939 13532 933 13537 927 13542 922 13548
917 13555 914 13562 911 13569 907 13586 905 13604 939 13604

940 13594 941 13584 944 13575 949 13566 957 13559 967 13553
979 13549 996 13548 1010 13548 1021 13551 1031 13556 1039 13563
1045 13571 1049 13581 1051 13591 1052 13602 1050 13616 1049 13622
1046 13628 1043 13633 1040 13637 1031 13643 1020 13648 1007 13651
992 13652 976 13652 976 13681 992 13681 1006 13682 1017 13684
1027 13688 1034 13694 1040 13701 1043 13711 1044 13723 1043 13732
1041 13741 1038 13749 1032 13756 1026 13761 1018 13765 1007 13768
996 13769 985 13768 975 13766 966 13763 959 13757 953 13749
948 13739 945 13726 943 13711 123 N F
1299 13729 1265 13729 1262 13738 1259 13746 1254 13753 1248 13759
1242 13763 1235 13766 1228 13768 1220 13769 1213 13769 1206 13767
1200 13765 1194 13762 1188 13758 1183 13754 1178 13748 1174 13742
1167 13727 1162 13710 1159 13689 1158 13666 1166 13675 1174 13682
1182 13688 1191 13691 1199 13694 1207 13696 1224 13697 1238 13695
1252 13691 1265 13685 1278 13675 1284 13669 1289 13663 1294 13655
1298 13647 1301 13639 1303 13629 1304 13619 1305 13608 1271 13606
1269 13617 1266 13628 1261 13639 1254 13648 1245 13655 1236 13661
1225 13665 1214 13666 1203 13665 1193 13661 1183 13655 1175 13648
1168 13639 1163 13628 1159 13617 1158 13606 1159 13595 1163 13584
1168 13574 1175 13565 1183 13557 1193 13551 1203 13547 1214 13546
1225 13547 1236 13551 1245 13557 1254 13565 1261 13574 1266 13584
1269 13595 1271 13606 1305 13608 1303 13592 1299 13576 1292 13560
1288 13553 1283 13546 1277 13540 1270 13534 1263 13529 1255 13524
1246 13521 1236 13518 1226 13516 1214 13516 1202 13516 1191 13519
1180 13522 1171 13526 1163 13532 1155 13539 1148 13547 1143 13555
1138 13565 1133 13576 1130 13587 1127 13599 1123 13625 1122 13652
1122 13669 1124 13685 1126 13700 1129 13714 1132 13727 1137 13739
1142 13750 1148 13760 1155 13769 1162 13777 1170 13783 1179 13789
1188 13793 1198 13797 1209 13798 1220 13799 1236 13798 1251 13794
1263 13787 1275 13778 1284 13768 1291 13756 1296 13743 1299 13729
126 N F
1470 13747 1469 13735 1465 13723 1459 13712 1451 13702 1442 13694
1431 13688 1419 13685 1406 13683 1406 13708 1398 13709 1391 13711
1384 13715 1378 13720 1374 13725 1370 13732 1368 13739 1367 13747
1368 13755 1370 13763 1374 13769 1378 13775 1384 13780 1391 13784
1398 13786 1406 13787 1414 13786 1422 13784 1428 13780 1434 13775
1439 13769 1443 13763 1445 13755 1446 13747 1445 13739 1443 13732
1439 13725 1434 13720 1428 13715 1422 13711 1414 13709 1406 13708
1406 13683 1393 13685 1381 13688 1371 13694 1361 13702 1353 13712
1347 13723 1344 13735 1342 13747 1344 13760 1347 13772 1353 13783
1361 13793 1371 13800 1381 13806 1393 13810 1406 13811 1419 13810
1431 13806 1442 13800 1451 13793 1459 13783 1465 13772 1469 13760
1470 13747 67 N F

1517 13807 1557 13807 1702 13578 1702 13807 1736 13807 1736 13526
1696 13526 1551 13755 1551 13526 1517 13526 1517 13807 11 N F
943 14817 909 14817 911 14838 914 14847 916 14855 920 14863
924 14871 928 14877 934 14883 939 14888 946 14893 953 14897
960 14900 968 14902 977 14904 996 14905 1013 14904 1029 14901
1043 14895 1056 14886 1061 14881 1066 14876 1070 14870 1073 14863
1076 14856 1078 14848 1079 14840 1080 14831 1079 14821 1078 14812
1075 14804 1071 14796 1066 14789 1059 14783 1050 14778 1040 14773
1052 14768 1062 14762 1070 14756 1077 14748 1082 14740 1085 14730
1087 14720 1088 14709 1087 14693 1083 14678 1077 14664 1072 14658
1067 14651 1061 14645 1054 14640 1047 14636 1038 14632 1028 14628
1018 14626 1006 14625 994 14624 983 14625 973 14626 963 14628
954 14631 946 14634 939 14638 933 14643 927 14649 922 14655
917 14661 914 14668 911 14676 907 14692 905 14711 939 14711
940 14701 941 14691 944 14681 949 14673 957 14665 967 14659
979 14656 996 14654 1010 14655 1021 14658 1031 14663 1039 14669
1045 14678 1049 14687 1051 14697 1052 14709 1050 14723 1049 14729
1046 14734 1043 14739 1040 14743 1031 14749 1020 14754 1007 14757
992 14758 976 14759 976 14787 992 14787 1006 14788 1017 14791
1027 14794 1034 14800 1040 14807 1043 14817 1044 14829 1043 14839
1041 14847 1038 14855 1032 14862 1026 14868 1018 14872 1007 14874
996 14875 985 14875 975 14873 966 14869 959 14864 953 14856
948 14846 945 14833 943 14817 123 N F
1212 14905 1229 14904 1245 14901 1259 14895 1271 14887 1276 14882
1281 14876 1285 14870 1288 14864 1291 14857 1293 14850 1294 14842
1295 14833 1294 14821 1291 14811 1287 14802 1283 14795 1277 14789
1271 14783 1258 14775 1269 14770 1278 14763 1285 14756 1292 14747
1297 14738 1301 14728 1304 14718 1305 14707 1303 14693 1299 14679
1291 14665 1281 14652 1275 14645 1268 14640 1260 14635 1251 14631
1242 14627 1232 14624 1222 14623 1210 14622 1199 14623 1189 14624
1179 14627 1170 14631 1162 14635 1154 14640 1148 14645 1142 14652
1132 14665 1125 14679 1121 14693 1120 14707 1156 14707 1157 14696
1161 14685 1166 14676 1173 14668 1181 14661 1191 14657 1201 14653
1212 14652 1223 14653 1234 14657 1243 14661 1252 14668 1259 14676
1264 14685 1267 14696 1269 14707 1267 14718 1264 14728 1259 14737
1252 14745 1243 14752 1234 14757 1223 14760 1212 14761 1201 14760
1191 14757 1181 14752 1173 14745 1166 14737 1161 14728 1157 14718
1156 14707 1120 14707 1121 14718 1123 14728 1127 14738 1133 14747
1139 14755 1147 14762 1156 14769 1166 14775 1154 14783 1148 14789
1142 14795 1137 14802 1133 14811 1131 14821 1130 14833 1164 14833
1165 14824 1168 14816 1173 14808 1178 14802 1186 14797 1194 14794
1203 14792 1212 14791 1222 14792 1231 14794 1239 14797 1246 14802
1252 14808 1257 14816 1260 14824 1261 14833 1260 14842 1257 14851

1252 14858 1246 14864 1239 14869 1231 14872 1222 14875 1212 14875
1203 14875 1194 14872 1186 14869 1178 14864 1173 14858 1168 14851
1165 14842 1164 14833 1130 14833 1130 14842 1132 14850 1134 14857
1136 14864 1140 14870 1144 14876 1149 14882 1154 14887 1166 14895
1180 14901 1196 14904 1212 14905 147 N F
1470 14854 1469 14841 1465 14829 1459 14818 1451 14809 1442 14801
1431 14795 1419 14791 1406 14790 1406 14814 1398 14815 1391 14817
1384 14821 1378 14826 1374 14832 1370 14838 1368 14846 1367 14854
1368 14862 1370 14869 1374 14876 1378 14882 1384 14886 1391 14890
1398 14892 1406 14893 1414 14892 1422 14890 1428 14886 1434 14882
1439 14876 1443 14869 1445 14862 1446 14854 1445 14846 1443 14838
1439 14832 1434 14826 1428 14821 1422 14817 1414 14815 1406 14814
1406 14790 1393 14791 1381 14795 1371 14801 1361 14809 1353 14818
1347 14829 1344 14841 1342 14854 1344 14867 1347 14879 1353 14889
1361 14899 1371 14907 1381 14913 1393 14916 1406 14918 1419 14916
1431 14913 1442 14907 1451 14899 1459 14889 1465 14879 1469 14867
1470 14854 67 N F
1517 14914 1557 14914 1702 14684 1702 14914 1736 14914 1736 14632
1696 14632 1551 14861 1551 14632 1517 14632 1517 14914 11 N F
1028 15999 1052 15999 1052 15822 1094 15822 1094 15790 1052 15790
1052 15725 1018 15725 1018 15790 903 15790 903 15822 933 15822
1018 15822 1018 15940 933 15822 903 15822 903 15826 1028 15999
18 N F
1212 15999 1222 15998 1232 15995 1241 15991 1249 15986 1257 15980
1265 15972 1272 15963 1278 15954 1283 15943 1288 15932 1293 15921
1296 15909 1299 15896 1301 15884 1302 15871 1303 15858 1302 15845
1301 15832 1299 15819 1296 15807 1293 15795 1288 15783 1283 15771
1278 15761 1272 15751 1265 15742 1257 15735 1249 15728 1241 15723
1232 15719 1222 15716 1212 15715 1202 15716 1193 15719 1184 15723
1175 15728 1167 15735 1160 15742 1153 15751 1147 15761 1141 15771
1136 15783 1132 15795 1129 15807 1126 15819 1124 15832 1122 15845
1122 15858 1158 15858 1159 15833 1163 15811 1168 15792 1175 15776
1184 15763 1193 15753 1198 15750 1203 15747 1207 15746 1212 15745
1217 15746 1222 15747 1227 15750 1232 15753 1241 15763 1249 15776
1256 15792 1262 15811 1265 15833 1267 15858 1265 15882 1262 15904
1256 15923 1249 15939 1241 15951 1232 15961 1227 15964 1222 15966
1217 15968 1212 15968 1207 15968 1203 15966 1198 15964 1193 15961
1184 15951 1175 15939 1168 15923 1163 15904 1159 15882 1158 15858
1122 15858 1122 15871 1124 15884 1126 15896 1129 15909 1132 15921
1136 15932 1141 15943 1147 15954 1153 15963 1160 15972 1167 15980
1175 15986 1184 15991 1193 15995 1202 15998 1212 15999 107 N F
1470 15947 1469 15934 1465 15922 1459 15911 1451 15902 1442 15894
1431 15888 1419 15884 1406 15883 1406 15907 1398 15908 1391 15911

1384 15914 1378 15919 1374 15925 1370 15931 1368 15939 1367 15947
1368 15955 1370 15962 1374 15969 1378 15975 1384 15980 1391 15983
1398 15985 1406 15986 1414 15985 1422 15983 1428 15980 1434 15975
1439 15969 1443 15962 1445 15955 1446 15947 1445 15939 1443 15931
1439 15925 1434 15919 1428 15914 1422 15911 1414 15908 1406 15907
1406 15883 1393 15884 1381 15888 1371 15894 1361 15902 1353 15911
1347 15922 1344 15934 1342 15947 1344 15960 1347 15972 1353 15983
1361 15992 1371 16000 1381 16006 1393 16010 1406 16011 1419 16010
1431 16006 1442 16000 1451 15992 1459 15983 1465 15972 1469 15960
1470 15947 67 N F
1517 16007 1557 16007 1702 15778 1702 16007 1736 16007 1736 15725
1696 15725 1551 15954 1551 15725 1517 15725 1517 16007 11 N F
13569 5633 13603 5633 13604 5647 13607 5659 13611 5670 13617 5679
13625 5687 13634 5693 13646 5696 13659 5697 13670 5696 13679 5694
13688 5689 13696 5683 13702 5676 13707 5667 13710 5656 13711 5645
13711 5633 13708 5624 13703 5615 13696 5608 13687 5601 13677 5593
13649 5577 13626 5563 13607 5549 13592 5535 13581 5521 13572 5505
13567 5489 13564 5472 13563 5454 13746 5454 13746 5486 13601 5486
13605 5499 13610 5509 13616 5518 13624 5525 13634 5533 13646 5540
13677 5559 13695 5567 13710 5577 13723 5588 13732 5600 13739 5612
13744 5624 13747 5637 13748 5649 13747 5657 13746 5666 13744 5673
13741 5681 13737 5688 13732 5694 13727 5700 13722 5705 13708 5715
13693 5722 13677 5726 13659 5727 13640 5726 13632 5724 13623 5722
13615 5718 13608 5714 13601 5710 13595 5704 13589 5698 13584 5691
13579 5683 13576 5675 13573 5665 13571 5655 13569 5645 13569 5633
78 N F
13784 5633 13818 5633 13819 5647 13821 5659 13826 5670 13832 5679
13840 5687 13849 5693 13861 5696 13874 5697 13884 5696 13894 5694
13903 5689 13911 5683 13917 5676 13922 5667 13925 5656 13926 5645
13925 5633 13922 5624 13918 5615 13911 5608 13902 5601 13892 5593
13864 5577 13841 5563 13822 5549 13807 5535 13795 5521 13787 5505
13782 5489 13779 5472 13778 5454 13960 5454 13960 5486 13816 5486
13819 5499 13824 5509 13831 5518 13839 5525 13849 5533 13861 5540
13892 5559 13910 5567 13925 5577 13937 5588 13947 5600 13954 5612
13959 5624 13961 5637 13962 5649 13962 5657 13961 5666 13958 5673
13955 5681 13952 5688 13947 5694 13942 5700 13936 5705 13923 5715
13908 5722 13892 5726 13874 5727 13855 5726 13846 5724 13838 5722
13830 5718 13823 5714 13816 5710 13809 5704 13804 5698 13799 5691
13794 5683 13791 5675 13788 5665 13785 5655 13784 5645 13784 5633
78 N F
14128 5676 14127 5663 14123 5651 14117 5640 14109 5630 14100 5623
14089 5617 14077 5613 14064 5612 14064 5636 14056 5637 14049 5639
14042 5643 14036 5648 14031 5654 14028 5660 14025 5668 14025 5676

14025 5684 14028 5691 14031 5698 14036 5703 14042 5708 14049 5712
14056 5714 14064 5715 14072 5714 14079 5712 14086 5708 14092 5703
14097 5698 14100 5691 14103 5684 14103 5676 14103 5668 14100 5660
14097 5654 14092 5648 14086 5643 14079 5639 14072 5637 14064 5636
14064 5612 14051 5613 14039 5617 14028 5623 14019 5630 14011 5640
14005 5651 14001 5663 14000 5676 14001 5688 14005 5700 14011 5711
14019 5721 14028 5729 14039 5735 14051 5738 14064 5740 14077 5738
14089 5735 14100 5729 14109 5721 14117 5711 14123 5700 14127 5688
14128 5676 67 N F
14175 5735 14215 5735 14360 5506 14360 5735 14394 5735 14394 5454
14354 5454 14209 5683 14209 5454 14175 5454 14175 5735 11 N F
13569 6803 13603 6803 13604 6816 13607 6829 13611 6840 13617 6849
13625 6857 13634 6862 13646 6866 13659 6867 13670 6866 13679 6863
13688 6859 13696 6853 13702 6845 13707 6836 13710 6826 13711 6815
13711 6803 13708 6793 13703 6785 13696 6777 13687 6770 13677 6763
13649 6746 13626 6733 13607 6719 13592 6705 13581 6690 13572 6675
13567 6659 13564 6642 13563 6624 13746 6624 13746 6656 13601 6656
13605 6668 13610 6679 13616 6687 13624 6695 13634 6703 13646 6710
13677 6728 13695 6737 13710 6747 13723 6758 13732 6770 13739 6782
13744 6794 13747 6806 13748 6819 13747 6827 13746 6835 13744 6843
13741 6850 13737 6857 13732 6864 13727 6870 13722 6875 13708 6884
13693 6891 13677 6896 13659 6897 13640 6896 13632 6894 13623 6891
13615 6888 13608 6884 13601 6879 13595 6874 13589 6868 13584 6861
13579 6853 13576 6844 13573 6835 13571 6825 13569 6814 13569 6803
78 N F
13900 6897 13924 6897 13924 6720 13966 6720 13966 6688 13924 6688
13924 6624 13890 6624 13890 6688 13776 6688 13776 6720 13806 6720
13890 6720 13890 6839 13806 6720 13776 6720 13776 6724 13900 6897
18 N F
14128 6845 14127 6832 14123 6820 14117 6810 14109 6800 14100 6792
14089 6786 14077 6783 14064 6781 14064 6806 14056 6807 14049 6809
14042 6813 14036 6817 14031 6823 14028 6830 14025 6837 14025 6845
14025 6853 14028 6861 14031 6867 14036 6873 14042 6878 14049 6882
14056 6884 14064 6885 14072 6884 14079 6882 14086 6878 14092 6873
14097 6867 14100 6861 14103 6853 14103 6845 14103 6837 14100 6830
14097 6823 14092 6817 14086 6813 14079 6809 14072 6807 14064 6806
14064 6781 14051 6783 14039 6786 14028 6792 14019 6800 14011 6810
14005 6820 14001 6832 14000 6845 14001 6858 14005 6870 14011 6881
14019 6891 14028 6898 14039 6904 14051 6908 14064 6909 14077 6908
14089 6904 14100 6898 14109 6891 14117 6881 14123 6870 14127 6858
14128 6845 67 N F
14175 6905 14215 6905 14360 6676 14360 6905 14394 6905 14394 6624
14354 6624 14209 6853 14209 6624 14175 6624 14175 6905 11 N F

13569 7961 13603 7961 13604 7975 13607 7987 13611 7998 13617 8007
13625 8015 13634 8021 13646 8024 13659 8025 13670 8024 13679 8022
13688 8017 13696 8011 13702 8004 13707 7995 13710 7985 13711 7973
13711 7961 13708 7952 13703 7943 13696 7936 13687 7929 13677 7922
13649 7905 13626 7891 13607 7877 13592 7863 13581 7849 13572 7833
13567 7817 13564 7800 13563 7782 13746 7782 13746 7814 13601 7814
13605 7827 13610 7837 13616 7846 13624 7853 13634 7861 13646 7869
13677 7887 13695 7896 13710 7906 13723 7916 13732 7928 13739 7940
13744 7952 13747 7965 13748 7977 13747 7986 13746 7994 13744 8001
13741 8009 13737 8016 13732 8022 13727 8028 13722 8034 13708 8043
13693 8050 13677 8054 13659 8055 13640 8054 13632 8052 13623 8050
13615 8046 13608 8043 13601 8038 13595 8032 13589 8026 13584 8019
13579 8011 13576 8003 13573 7994 13571 7984 13569 7973 13569 7961
78 N F
13956 7985 13922 7985 13920 7994 13916 8002 13912 8009 13906 8015
13900 8019 13893 8023 13886 8025 13878 8025 13871 8025 13864 8024
13857 8021 13851 8018 13846 8014 13841 8010 13836 8004 13832 7998
13825 7983 13820 7966 13817 7946 13816 7923 13824 7932 13832 7939
13840 7944 13848 7948 13856 7950 13865 7952 13882 7953 13895 7952
13909 7948 13923 7941 13936 7931 13942 7926 13947 7919 13951 7912
13955 7904 13959 7895 13961 7886 13962 7875 13962 7865 13928 7863
13927 7874 13924 7885 13918 7895 13911 7904 13903 7912 13894 7918
13883 7921 13872 7923 13861 7921 13851 7918 13841 7912 13833 7904
13826 7895 13820 7885 13817 7874 13816 7863 13817 7851 13820 7840
13826 7830 13833 7821 13841 7813 13851 7807 13861 7804 13872 7802
13883 7804 13894 7807 13903 7813 13911 7821 13918 7830 13924 7840
13927 7851 13928 7863 13962 7865 13961 7848 13957 7832 13950 7817
13946 7809 13941 7802 13935 7796 13928 7790 13921 7785 13912 7781
13903 7777 13894 7774 13883 7773 13872 7772 13860 7773 13848 7775
13838 7778 13829 7783 13820 7788 13813 7795 13806 7803 13800 7812
13795 7821 13791 7832 13787 7843 13785 7855 13781 7881 13780 7909
13780 7925 13781 7941 13783 7956 13786 7970 13790 7983 13794 7995
13800 8006 13806 8016 13812 8025 13820 8033 13828 8040 13836 8045
13846 8050 13856 8053 13867 8055 13878 8055 13894 8054 13908 8050
13921 8043 13932 8035 13941 8024 13949 8012 13954 7999 13956 7985
126 N F
14128 8004 14127 7991 14123 7979 14117 7968 14109 7959 14100 7951
14089 7945 14077 7941 14064 7940 14064 7964 14056 7965 14049 7967
14042 7971 14036 7976 14031 7982 14028 7988 14025 7996 14025 8004
14025 8012 14028 8019 14031 8026 14036 8032 14042 8036 14049 8040
14056 8042 14064 8043 14072 8042 14079 8040 14086 8036 14092 8032
14097 8026 14100 8019 14103 8012 14103 8004 14103 7996 14100 7988
14097 7982 14092 7976 14086 7971 14079 7967 14072 7965 14064 7964

14064 7940 14051 7941 14039 7945 14028 7951 14019 7959 14011 7968
14005 7979 14001 7991 14000 8004 14001 8017 14005 8029 14011 8039
14019 8049 14028 8057 14039 8063 14051 8066 14064 8068 14077 8066
14089 8063 14100 8057 14109 8049 14117 8039 14123 8029 14127 8017
14128 8004 67 N F
14175 8063 14215 8063 14360 7834 14360 8063 14394 8063 14394 7782
14354 7782 14209 8011 14209 7782 14175 7782 14175 8063 11 N F
13569 9103 13603 9103 13604 9117 13607 9129 13611 9140 13617 9150
13625 9157 13634 9163 13646 9166 13659 9167 13670 9166 13679 9164
13688 9159 13696 9153 13702 9146 13707 9137 13710 9127 13711 9115
13711 9103 13708 9094 13703 9085 13696 9078 13687 9071 13677 9064
13649 9047 13626 9033 13607 9019 13592 9005 13581 8991 13572 8976
13567 8959 13564 8942 13563 8924 13746 8924 13746 8956 13601 8956
13605 8969 13610 8979 13616 8988 13624 8996 13634 9003 13646 9011
13677 9029 13695 9038 13710 9048 13723 9059 13732 9070 13739 9082
13744 9095 13747 9107 13748 9119 13747 9128 13746 9136 13744 9144
13741 9151 13737 9158 13732 9164 13727 9170 13722 9176 13708 9185
13693 9192 13677 9196 13659 9197 13640 9196 13632 9194 13623 9192
13615 9189 13608 9185 13601 9180 13595 9174 13589 9168 13584 9161
13579 9153 13576 9145 13573 9136 13571 9126 13569 9115 13569 9103
78 N F
13870 9197 13887 9196 13902 9193 13916 9187 13928 9179 13934 9174
13938 9168 13943 9162 13946 9156 13949 9149 13951 9142 13952 9134
13952 9125 13951 9113 13949 9103 13945 9094 13940 9087 13935 9081
13929 9075 13916 9067 13926 9062 13935 9055 13943 9048 13950 9039
13955 9030 13959 9020 13962 9010 13962 8999 13961 8985 13956 8971
13949 8957 13939 8944 13932 8937 13925 8932 13917 8927 13909 8923
13900 8919 13890 8916 13879 8915 13868 8914 13857 8915 13846 8916
13837 8919 13828 8923 13820 8927 13812 8932 13805 8937 13800 8944
13790 8957 13783 8971 13779 8985 13778 8999 13814 8999 13815 8988
13818 8977 13824 8968 13831 8960 13839 8953 13849 8949 13859 8945
13870 8944 13881 8945 13892 8949 13901 8953 13909 8960 13916 8968
13922 8977 13925 8988 13926 8999 13925 9010 13922 9020 13916 9029
13909 9037 13901 9044 13892 9049 13881 9052 13870 9053 13859 9052
13849 9049 13839 9044 13831 9037 13824 9029 13818 9020 13815 9010
13814 8999 13778 8999 13779 9010 13781 9020 13785 9030 13790 9039
13797 9047 13805 9054 13814 9061 13824 9067 13811 9075 13805 9081
13800 9087 13795 9094 13791 9103 13789 9113 13788 9125 13822 9125
13823 9116 13826 9108 13830 9100 13836 9094 13843 9089 13851 9086
13860 9084 13870 9083 13880 9084 13889 9086 13897 9089 13904 9094
13910 9100 13914 9108 13917 9116 13918 9125 13917 9134 13914 9143
13910 9150 13904 9156 13897 9161 13889 9164 13880 9167 13870 9167
13860 9167 13851 9164 13843 9161 13836 9156 13830 9150 13826 9143

13823 9134 13822 9125 13788 9125 13788 9134 13789 9142 13791 9149
13794 9156 13798 9162 13802 9168 13806 9174 13812 9179 13824 9187
13838 9193 13853 9196 13870 9197 147 N F
14128 9146 14127 9133 14123 9121 14117 9110 14109 9101 14100 9093
14089 9087 14077 9083 14064 9082 14064 9106 14056 9107 14049 9109
14042 9113 14036 9118 14031 9124 14028 9130 14025 9138 14025 9146
14025 9154 14028 9161 14031 9168 14036 9174 14042 9178 14049 9182
14056 9184 14064 9185 14072 9184 14079 9182 14086 9178 14092 9174
14097 9168 14100 9161 14103 9154 14103 9146 14103 9138 14100 9130
14097 9124 14092 9118 14086 9113 14079 9109 14072 9107 14064 9106
14064 9082 14051 9083 14039 9087 14028 9093 14019 9101 14011 9110
14005 9121 14001 9133 14000 9146 14001 9159 14005 9171 14011 9181
14019 9191 14028 9199 14039 9205 14051 9208 14064 9210 14077 9208
14089 9205 14100 9199 14109 9191 14117 9181 14123 9171 14127 9159
14128 9146 67 N F
14175 9206 14215 9206 14360 8976 14360 9206 14394 9206 14394 8924
14354 8924 14209 9153 14209 8924 14175 8924 14175 9206 11 N F
13601 10241 13567 10241 13569 10261 13571 10271 13574 10279 13577 10287
13581 10294 13586 10301 13591 10307 13597 10312 13604 10317 13611 10321
13618 10324 13626 10326 13635 10328 13653 10329 13671 10328 13687 10324
13701 10318 13713 10310 13719 10305 13724 10300 13728 10294 13731 10287
13734 10280 13736 10272 13737 10264 13738 10255 13737 10245 13736 10236
13733 10227 13729 10220 13723 10213 13716 10207 13708 10201 13697 10197
13709 10192 13719 10186 13728 10179 13734 10172 13739 10164 13743 10154
13745 10144 13746 10132 13744 10117 13741 10102 13734 10088 13730 10081
13725 10075 13719 10069 13712 10064 13704 10059 13696 10055 13686 10052
13675 10050 13664 10048 13651 10048 13640 10048 13630 10050 13621 10052
13612 10054 13604 10058 13597 10062 13590 10067 13585 10072 13579 10078
13575 10085 13571 10092 13568 10100 13564 10116 13563 10134 13597 10134
13598 10124 13599 10115 13602 10105 13607 10096 13614 10089 13624 10083
13637 10079 13653 10078 13667 10079 13679 10081 13689 10086 13696 10093
13702 10101 13706 10111 13709 10121 13709 10132 13708 10147 13706 10153
13704 10158 13701 10163 13698 10167 13689 10173 13678 10178 13665 10181
13650 10182 13633 10183 13633 10211 13649 10211 13663 10212 13675 10215
13685 10218 13692 10224 13697 10231 13700 10241 13701 10253 13701 10262
13699 10271 13695 10279 13690 10286 13684 10291 13675 10296 13665 10298
13653 10299 13642 10299 13633 10297 13624 10293 13617 10287 13611 10280
13606 10269 13603 10257 13601 10241 123 N F
13870 10329 13880 10328 13889 10326 13898 10322 13907 10317 13915 10310
13922 10303 13929 10294 13935 10285 13941 10274 13946 10263 13950 10252
13954 10239 13957 10227 13959 10214 13960 10201 13960 10189 13960 10176
13959 10163 13957 10150 13954 10138 13950 10125 13946 10113 13941 10102
13935 10092 13929 10082 13922 10073 13915 10065 13907 10059 13898 10053

13889 10049 13880 10047 13870 10046 13860 10047 13851 10049 13842 10053
13833 10059 13825 10065 13818 10073 13811 10082 13805 10092 13799 10102
13794 10113 13790 10125 13786 10138 13783 10150 13781 10163 13780 10176
13780 10189 13816 10189 13817 10164 13821 10142 13826 10123 13833 10107
13841 10094 13851 10084 13855 10081 13860 10078 13865 10077 13870 10076
13875 10077 13880 10078 13885 10081 13889 10084 13899 10094 13907 10107
13914 10123 13919 10142 13923 10164 13924 10189 13923 10213 13919 10235
13914 10254 13907 10270 13899 10282 13889 10291 13885 10295 13880 10297
13875 10299 13870 10299 13865 10299 13860 10297 13855 10295 13851 10291
13841 10282 13833 10270 13826 10254 13821 10235 13817 10213 13816 10189
13780 10189 13780 10201 13781 10214 13783 10227 13786 10239 13790 10252
13794 10263 13799 10274 13805 10285 13811 10294 13818 10303 13825 10310
13833 10317 13842 10322 13851 10326 13860 10328 13870 10329 107 N F
14128 10278 14127 10265 14123 10253 14117 10242 14109 10232 14100 10225
14089 10219 14077 10215 14064 10214 14064 10238 14056 10239 14049 10241
14042 10245 14036 10250 14031 10256 14028 10262 14025 10270 14025 10278
14025 10286 14028 10293 14031 10300 14036 10305 14042 10310 14049 10314
14056 10316 14064 10317 14072 10316 14079 10314 14086 10310 14092 10305
14097 10300 14100 10293 14103 10286 14103 10278 14103 10270 14100 10262
14097 10256 14092 10250 14086 10245 14079 10241 14072 10239 14064 10238
14064 10214 14051 10215 14039 10219 14028 10225 14019 10232 14011 10242
14005 10253 14001 10265 14000 10278 14001 10290 14005 10302 14011 10313
14019 10323 14028 10331 14039 10337 14051 10340 14064 10342 14077 10340
14089 10337 14100 10331 14109 10323 14117 10313 14123 10302 14127 10290
14128 10278 67 N F
14175 10337 14215 10337 14360 10108 14360 10337 14394 10337 14394 10056
14354 10056 14209 10285 14209 10056 14175 10056 14175 10337 11 N F
13601 11363 13567 11363 13569 11383 13571 11392 13574 11401 13577 11409
13581 11416 13586 11423 13591 11429 13597 11434 13604 11438 13611 11442
13618 11445 13626 11448 13635 11450 13653 11451 13671 11450 13687 11446
13701 11440 13713 11432 13719 11427 13724 11421 13728 11415 13731 11409
13734 11401 13736 11394 13737 11385 13738 11377 13737 11367 13736 11357
13733 11349 13729 11341 13723 11335 13716 11329 13708 11323 13697 11318
13709 11313 13719 11308 13728 11301 13734 11294 13739 11285 13743 11276
13745 11266 13746 11254 13744 11239 13741 11224 13734 11210 13730 11203
13725 11197 13719 11191 13712 11186 13704 11181 13696 11177 13686 11174
13675 11172 13664 11170 13651 11170 13640 11170 13630 11171 13621 11173
13612 11176 13604 11180 13597 11184 13590 11189 13585 11194 13579 11200
13575 11207 13571 11214 13568 11221 13564 11238 13563 11256 13597 11256
13598 11246 13599 11236 13602 11227 13607 11218 13614 11211 13624 11205
13637 11201 13653 11200 13667 11200 13679 11203 13689 11208 13696 11215
13702 11223 13706 11233 13709 11243 13709 11254 13708 11268 13706 11274
13704 11280 13701 11284 13698 11288 13689 11295 13678 11299 13665 11302

13650 11304 13633 11304 13633 11332 13649 11333 13663 11334 13675 11336
13685 11340 13692 11345 13697 11353 13700 11362 13701 11375 13701 11384
13699 11393 13695 11401 13690 11407 13684 11413 13675 11417 13665 11420
13653 11421 13642 11420 13633 11418 13624 11415 13617 11409 13611 11401
13606 11391 13603 11378 13601 11363 123 N F
13784 11356 13818 11356 13819 11370 13821 11383 13826 11394 13832 11403
13840 11411 13849 11416 13861 11420 13874 11421 13884 11420 13894 11417
13903 11413 13911 11407 13917 11399 13922 11390 13925 11380 13926 11369
13925 11357 13922 11347 13918 11339 13911 11331 13902 11324 13892 11317
13864 11300 13841 11287 13822 11273 13807 11259 13795 11244 13787 11229
13782 11213 13779 11196 13778 11178 13960 11178 13960 11210 13816 11210
13819 11222 13824 11232 13831 11241 13839 11249 13849 11256 13861 11264
13892 11282 13910 11291 13925 11301 13937 11312 13947 11324 13954 11336
13959 11348 13961 11360 13962 11373 13962 11381 13961 11389 13958 11397
13955 11404 13952 11411 13947 11418 13942 11424 13936 11429 13923 11438
13908 11445 13892 11449 13874 11451 13855 11450 13846 11448 13838 11445
13830 11442 13823 11438 13816 11433 13809 11428 13804 11422 13799 11415
13794 11407 13791 11398 13788 11389 13785 11379 13784 11368 13784 11356
78 N F
14128 11399 14127 11386 14123 11374 14117 11363 14109 11354 14100 11346
14089 11340 14077 11337 14064 11335 14064 11360 14056 11361 14049 11363
14042 11367 14036 11371 14031 11377 14028 11384 14025 11391 14025 11399
14025 11407 14028 11415 14031 11421 14036 11427 14042 11432 14049 11436
14056 11438 14064 11439 14072 11438 14079 11436 14086 11432 14092 11427
14097 11421 14100 11415 14103 11407 14103 11399 14103 11391 14100 11384
14097 11377 14092 11371 14086 11367 14079 11363 14072 11361 14064 11360
14064 11335 14051 11337 14039 11340 14028 11346 14019 11354 14011 11363
14005 11374 14001 11386 14000 11399 14001 11412 14005 11424 14011 11435
14019 11444 14028 11452 14039 11458 14051 11462 14064 11463 14077 11462
14089 11458 14100 11452 14109 11444 14117 11435 14123 11424 14127 11412
14128 11399 67 N F
14175 11459 14215 11459 14360 11230 14360 11459 14394 11459 14394 11178
14354 11178 14209 11407 14209 11178 14175 11178 14175 11459 11 N F
13601 12477 13567 12477 13569 12498 13571 12507 13574 12516 13577 12524
13581 12531 13586 12537 13591 12543 13597 12548 13604 12553 13611 12557
13618 12560 13626 12562 13635 12564 13653 12566 13671 12564 13687 12561
13701 12555 13713 12547 13719 12542 13724 12536 13728 12530 13731 12523
13734 12516 13736 12508 13737 12500 13738 12491 13737 12481 13736 12472
13733 12464 13729 12456 13723 12449 13716 12443 13708 12438 13697 12433
13709 12428 13719 12422 13728 12416 13734 12408 13739 12400 13743 12391
13745 12380 13746 12369 13744 12354 13741 12339 13734 12324 13730 12318
13725 12311 13719 12306 13712 12300 13704 12296 13696 12292 13686 12289
13675 12286 13664 12285 13651 12284 13640 12285 13630 12286 13621 12288

13612 12291 13604 12294 13597 12298 13590 12303 13585 12309 13579 12315
13575 12321 13571 12328 13568 12336 13564 12353 13563 12371 13597 12371
13598 12361 13599 12351 13602 12341 13607 12333 13614 12325 13624 12320
13637 12316 13653 12314 13667 12315 13679 12318 13689 12323 13696 12329
13702 12338 13706 12347 13709 12358 13709 12369 13708 12383 13706 12389
13704 12394 13701 12399 13698 12403 13689 12410 13678 12414 13665 12417
13650 12418 13633 12419 13633 12447 13649 12447 13663 12449 13675 12451
13685 12455 13692 12460 13697 12467 13700 12477 13701 12489 13701 12499
13699 12507 13695 12515 13690 12522 13684 12528 13675 12532 13665 12535
13653 12535 13642 12535 13633 12533 13624 12529 13617 12524 13611 12516
13606 12506 13603 12493 13601 12477 123 N F
13900 12566 13924 12566 13924 12389 13966 12389 13966 12357 13924 12357
13924 12292 13890 12292 13890 12357 13776 12357 13776 12389 13806 12389
13890 12389 13890 12507 13806 12389 13776 12389 13776 12393 13900 12566
18 N F
14128 12514 14127 12501 14123 12489 14117 12478 14109 12469 14100 12461
14089 12455 14077 12451 14064 12450 14064 12474 14056 12475 14049 12478
14042 12481 14036 12486 14031 12492 14028 12499 14025 12506 14025 12514
14025 12522 14028 12529 14031 12536 14036 12542 14042 12547 14049 12550
14056 12552 14064 12553 14072 12552 14079 12550 14086 12547 14092 12542
14097 12536 14100 12529 14103 12522 14103 12514 14103 12506 14100 12499
14097 12492 14092 12486 14086 12481 14079 12478 14072 12475 14064 12474
14064 12450 14051 12451 14039 12455 14028 12461 14019 12469 14011 12478
14005 12489 14001 12501 14000 12514 14001 12527 14005 12539 14011 12550
14019 12559 14028 12567 14039 12573 14051 12577 14064 12578 14077 12577
14089 12573 14100 12567 14109 12559 14117 12550 14123 12539 14127 12527
14128 12514 67 N F
14175 12574 14215 12574 14360 12345 14360 12574 14394 12574 14394 12292
14354 12292 14209 12521 14209 12292 14175 12292 14175 12574 11 N F
13601 13586 13567 13586 13569 13607 13571 13616 13574 13624 13577 13632
13581 13640 13586 13646 13591 13652 13597 13657 13604 13662 13611 13666
13618 13669 13626 13671 13635 13673 13653 13675 13671 13673 13687 13670
13701 13664 13713 13655 13719 13650 13724 13645 13728 13639 13731 13632
13734 13625 13736 13617 13737 13609 13738 13600 13737 13590 13736 13581
13733 13573 13729 13565 13723 13558 13716 13552 13708 13547 13697 13542
13709 13537 13719 13531 13728 13525 13734 13517 13739 13509 13743 13499
13745 13489 13746 13478 13744 13462 13741 13447 13734 13433 13730 13427
13725 13420 13719 13414 13712 13409 13704 13405 13696 13401 13686 13397
13675 13395 13664 13394 13651 13393 13640 13394 13630 13395 13621 13397
13612 13400 13604 13403 13597 13407 13590 13412 13585 13418 13579 13424
13575 13430 13571 13437 13568 13445 13564 13461 13563 13480 13597 13480
13598 13470 13599 13460 13602 13450 13607 13442 13614 13434 13624 13428
13637 13425 13653 13423 13667 13424 13679 13427 13689 13432 13696 13438

13702 13447 13706 13456 13709 13466 13709 13478 13708 13492 13706 13498
13704 13503 13701 13508 13698 13512 13689 13518 13678 13523 13665 13526
13650 13527 13633 13528 13633 13556 13649 13556 13663 13557 13675 13560
13685 13563 13692 13569 13697 13576 13700 13586 13701 13598 13701 13608
13699 13616 13695 13624 13690 13631 13684 13637 13675 13641 13665 13643
13653 13644 13642 13644 13633 13642 13624 13638 13617 13633 13611 13625
13606 13615 13603 13602 13601 13586 123 N F
13956 13604 13922 13604 13920 13613 13916 13621 13912 13628 13906 13634
13900 13639 13893 13642 13886 13644 13878 13644 13871 13644 13864 13643
13857 13640 13851 13637 13846 13634 13841 13629 13836 13623 13832 13617
13825 13603 13820 13585 13817 13565 13816 13542 13824 13551 13832 13558
13840 13563 13848 13567 13856 13569 13865 13571 13882 13572 13895 13571
13909 13567 13923 13560 13936 13550 13942 13545 13947 13538 13951 13531
13955 13523 13959 13514 13961 13505 13962 13495 13962 13484 13928 13482
13927 13493 13924 13504 13918 13514 13911 13523 13903 13531 13894 13537
13883 13541 13872 13542 13861 13541 13851 13537 13841 13531 13833 13523
13826 13514 13820 13504 13817 13493 13816 13482 13817 13470 13820 13459
13826 13449 13833 13440 13841 13432 13851 13426 13861 13423 13872 13421
13883 13423 13894 13426 13903 13432 13911 13440 13918 13449 13924 13459
13927 13470 13928 13482 13962 13484 13961 13467 13957 13451 13950 13436
13946 13428 13941 13422 13935 13415 13928 13409 13921 13404 13912 13400
13903 13396 13894 13393 13883 13392 13872 13391 13860 13392 13848 13394
13838 13397 13829 13402 13820 13407 13813 13414 13806 13422 13800 13431
13795 13440 13791 13451 13787 13462 13785 13474 13781 13500 13780 13528
13780 13544 13781 13560 13783 13575 13786 13589 13790 13602 13794 13614
13800 13625 13806 13635 13812 13644 13820 13652 13828 13659 13836 13664
13846 13669 13856 13672 13867 13674 13878 13675 13894 13673 13908 13669
13921 13662 13932 13654 13941 13643 13949 13631 13954 13618 13956 13604
126 N F
14128 13623 14127 13610 14123 13598 14117 13587 14109 13578 14100 13570
14089 13564 14077 13560 14064 13559 14064 13583 14056 13584 14049 13586
14042 13590 14036 13595 14031 13601 14028 13607 14025 13615 14025 13623
14025 13631 14028 13638 14031 13645 14036 13651 14042 13655 14049 13659
14056 13661 14064 13662 14072 13661 14079 13659 14086 13655 14092 13651
14097 13645 14100 13638 14103 13631 14103 13623 14103 13615 14100 13607
14097 13601 14092 13595 14086 13590 14079 13586 14072 13584 14064 13583
14064 13559 14051 13560 14039 13564 14028 13570 14019 13578 14011 13587
14005 13598 14001 13610 14000 13623 14001 13636 14005 13648 14011 13658
14019 13668 14028 13676 14039 13682 14051 13685 14064 13687 14077 13685
14089 13682 14100 13676 14109 13668 14117 13658 14123 13648 14127 13636
14128 13623 67 N F
14175 13683 14215 13683 14360 13453 14360 13683 14394 13683 14394 13401
14354 13401 14209 13630 14209 13401 14175 13401 14175 13683 11 N F

13601 14688 13567 14688 13569 14709 13571 14718 13574 14727 13577 14735
13581 14742 13586 14749 13591 14754 13597 14760 13604 14764 13611 14768
13618 14771 13626 14774 13635 14775 13653 14777 13671 14776 13687 14772
13701 14766 13713 14758 13719 14753 13724 14747 13728 14741 13731 14734
13734 14727 13736 14720 13737 14711 13738 14702 13737 14692 13736 14683
13733 14675 13729 14667 13723 14661 13716 14654 13708 14649 13697 14644
13709 14639 13719 14634 13728 14627 13734 14620 13739 14611 13743 14602
13745 14591 13746 14580 13744 14565 13741 14550 13734 14536 13730 14529
13725 14523 13719 14517 13712 14512 13704 14507 13696 14503 13686 14500
13675 14497 13664 14496 13651 14495 13640 14496 13630 14497 13621 14499
13612 14502 13604 14505 13597 14510 13590 14514 13585 14520 13579 14526
13575 14532 13571 14540 13568 14547 13564 14564 13563 14582 13597 14582
13598 14572 13599 14562 13602 14553 13607 14544 13614 14537 13624 14531
13637 14527 13653 14526 13667 14526 13679 14529 13689 14534 13696 14541
13702 14549 13706 14558 13709 14569 13709 14580 13708 14594 13706 14600
13704 14606 13701 14610 13698 14614 13689 14621 13678 14625 13665 14628
13650 14630 13633 14630 13633 14658 13649 14659 13663 14660 13675 14662
13685 14666 13692 14671 13697 14679 13700 14688 13701 14700 13701 14710
13699 14719 13695 14727 13690 14733 13684 14739 13675 14743 13665 14746
13653 14747 13642 14746 13633 14744 13624 14740 13617 14735 13611 14727
13606 14717 13603 14704 13601 14688 123 N F
13870 14777 13887 14776 13902 14772 13916 14766 13928 14758 13934 14753
13938 14748 13943 14742 13946 14735 13949 14728 13951 14721 13952 14713
13952 14704 13951 14693 13949 14682 13945 14674 13940 14666 13935 14660
13929 14655 13916 14646 13926 14641 13935 14635 13943 14627 13950 14619
13955 14610 13959 14600 13962 14589 13962 14578 13961 14564 13956 14550
13949 14536 13939 14523 13932 14517 13925 14511 13917 14506 13909 14502
13900 14498 13890 14496 13879 14494 13868 14493 13857 14494 13846 14496
13837 14498 13828 14502 13820 14506 13812 14511 13805 14517 13800 14523
13790 14536 13783 14550 13779 14564 13778 14578 13814 14578 13815 14567
13818 14557 13824 14547 13831 14539 13839 14533 13849 14528 13859 14525
13870 14524 13881 14525 13892 14528 13901 14533 13909 14539 13916 14547
13922 14557 13925 14567 13926 14578 13925 14589 13922 14599 13916 14608
13909 14616 13901 14623 13892 14628 13881 14631 13870 14632 13859 14631
13849 14628 13839 14623 13831 14616 13824 14608 13818 14599 13815 14589
13814 14578 13778 14578 13779 14589 13781 14599 13785 14609 13790 14618
13797 14626 13805 14634 13814 14640 13824 14646 13811 14655 13805 14660
13800 14666 13795 14674 13791 14682 13789 14693 13788 14704 13822 14704
13823 14695 13826 14687 13830 14680 13836 14674 13843 14669 13851 14665
13860 14663 13870 14662 13880 14663 13889 14665 13897 14669 13904 14674
13910 14680 13914 14687 13917 14695 13918 14704 13917 14714 13914 14722
13910 14729 13904 14735 13897 14740 13889 14744 13880 14746 13870 14747
13860 14746 13851 14744 13843 14740 13836 14735 13830 14729 13826 14722

13823 14714 13822 14704 13788 14704 13788 14713 13789 14721 13791 14728
13794 14735 13798 14742 13802 14748 13806 14753 13812 14758 13824 14766
13838 14772 13853 14776 13870 14777 147 N F
14128 14725 14127 14712 14123 14700 14117 14689 14109 14680 14100 14672
14089 14666 14077 14662 14064 14661 14064 14686 14056 14686 14049 14689
14042 14692 14036 14697 14031 14703 14028 14710 14025 14717 14025 14725
14025 14733 14028 14740 14031 14747 14036 14753 14042 14758 14049 14761
14056 14764 14064 14764 14072 14764 14079 14761 14086 14758 14092 14753
14097 14747 14100 14740 14103 14733 14103 14725 14103 14717 14100 14710
14097 14703 14092 14697 14086 14692 14079 14689 14072 14686 14064 14686
14064 14661 14051 14662 14039 14666 14028 14672 14019 14680 14011 14689
14005 14700 14001 14712 14000 14725 14001 14738 14005 14750 14011 14761
14019 14770 14028 14778 14039 14784 14051 14788 14064 14789 14077 14788
14089 14784 14100 14778 14109 14770 14117 14761 14123 14750 14127 14738
14128 14725 67 N F
14175 14785 14215 14785 14360 14556 14360 14785 14394 14785 14394 14504
14354 14504 14209 14733 14209 14504 14175 14504 14175 14785 11 N F
13687 15875 13711 15875 13711 15698 13754 15698 13754 15666 13711 15666
13711 15602 13677 15602 13677 15666 13563 15666 13563 15698 13593 15698
13677 15698 13677 15817 13593 15698 13563 15698 13563 15702 13687 15875
18 N F
13872 15875 13882 15874 13891 15872 13900 15868 13909 15863 13917 15856
13924 15848 13931 15840 13938 15830 13943 15820 13948 15809 13952 15797
13956 15785 13959 15773 13961 15760 13962 15747 13962 15734 13962 15721
13961 15708 13959 15696 13956 15683 13952 15671 13948 15659 13943 15648
13938 15637 13931 15627 13924 15619 13917 15611 13909 15604 13900 15599
13891 15595 13882 15592 13872 15592 13862 15592 13853 15595 13844 15599
13835 15604 13827 15611 13820 15619 13813 15627 13807 15637 13801 15648
13796 15659 13792 15671 13788 15683 13785 15696 13783 15708 13782 15721
13782 15734 13818 15734 13819 15710 13823 15688 13828 15669 13835 15652
13843 15639 13853 15630 13857 15626 13862 15624 13867 15622 13872 15622
13877 15622 13882 15624 13887 15626 13892 15630 13901 15639 13909 15652
13916 15669 13921 15688 13925 15710 13926 15734 13925 15759 13921 15780
13916 15799 13909 15815 13901 15828 13892 15837 13887 15840 13882 15843
13877 15844 13872 15845 13867 15844 13862 15843 13857 15840 13853 15837
13843 15828 13835 15815 13828 15799 13823 15780 13819 15759 13818 15734
13782 15734 13782 15747 13783 15760 13785 15773 13788 15785 13792 15797
13796 15809 13801 15820 13807 15830 13813 15840 13820 15848 13827 15856
13835 15863 13844 15868 13853 15872 13862 15874 13872 15875 107 N F
14130 15823 14129 15810 14125 15798 14119 15787 14111 15778 14102 15770
14091 15764 14079 15760 14066 15759 14066 15784 14058 15785 14051 15787
14044 15790 14038 15795 14033 15801 14030 15808 14027 15815 14027 15823
14027 15831 14030 15838 14033 15845 14038 15851 14044 15856 14051 15859

14058 15862 14066 15863 14074 15862 14081 15859 14088 15856 14094 15851
14099 15845 14102 15838 14105 15831 14105 15823 14105 15815 14102 15808
14099 15801 14094 15795 14088 15790 14081 15787 14074 15785 14066 15784
14066 15759 14053 15760 14041 15764 14030 15770 14021 15778 14013 15787
14007 15798 14003 15810 14002 15823 14003 15836 14007 15848 14013 15859
14021 15868 14030 15876 14041 15882 14053 15886 14066 15887 14079 15886
14091 15882 14102 15876 14111 15868 14119 15859 14125 15848 14129 15836
14130 15823 67 N F
14177 15883 14217 15883 14362 15654 14362 15883 14396 15883 14396 15602
14356 15602 14211 15831 14211 15602 14177 15602 14177 15883 11 N F
25 W
2647 5219 2647 5501 2 P
3740 5219 3740 5501 2 P
4824 5219 4824 5501 2 P
5903 5219 5903 5501 2 P
6971 5219 6971 5501 2 P
8052 5219 8052 5501 2 P
9133 5219 9133 5501 2 P
10214 5219 10214 5501 2 P
11295 5219 11295 5501 2 P
12376 5219 12376 5501 2 P
2748 16501 2748 16219 2 P
4813 16501 4813 16219 2 P
6843 16501 6843 16219 2 P
8864 16501 8864 16219 2 P
10891 16501 10891 16219 2 P
12942 16501 12942 16219 2 P
2017 5694 2298 5694 2 P
2017 6871 2298 6871 2 P
2017 8027 2298 8027 2 P
2017 9172 2298 9172 2 P
2017 10308 2298 10308 2 P
2017 11434 2298 11434 2 P
2017 12552 2298 12552 2 P
2017 13664 2298 13664 2 P
2017 14770 2298 14770 2 P
2017 15863 2298 15863 2 P
13282 5597 13001 5597 2 P
13282 6767 13001 6767 2 P
13282 7920 13001 7920 2 P
13282 9062 13001 9062 2 P
13282 10194 13001 10194 2 P
13282 11316 13001 11316 2 P

13282 12431 13001 12431 2 P
13282 13539 13001 13539 2 P
13282 14641 13001 14641 2 P
13282 15739 13001 15739 2 P
13001 5501 2298 5501 2298 16219 13001 16219 13001 5501 2298 5501

6:00 PM

7006 17608 7070 17608 7174 17297 7279 17608 7342 17608 7342 17251
7299 17251 7299 17550 7197 17251 7149 17251 7049 17550 7049 17251
7006 17251 7006 17608 14 N F
7409 17432 7410 17448 7414 17463 7417 17470 7421 17477 7426 17483
7432 17490 7438 17495 7446 17501 7454 17505 7464 17509 7474 17512
7485 17515 7497 17516 7511 17516 7525 17516 7541 17514 7557 17510
7572 17504 7580 17500 7586 17495 7592 17490 7597 17484 7602 17477
7605 17470 7607 17462 7607 17453 7607 17289 7608 17285 7611 17281
7614 17278 7619 17275 7624 17273 7629 17272 7634 17273 7638 17274
7638 17243 7628 17241 7613 17241 7601 17241 7593 17242 7586 17244
7581 17247 7577 17251 7573 17257 7567 17274 7545 17258 7536 17252
7527 17248 7517 17245 7507 17242 7496 17241 7483 17241 7471 17241
7461 17242 7451 17244 7443 17247 7435 17250 7429 17254 7423 17258
7418 17263 7414 17268 7410 17274 7405 17286 7402 17299 7401 17312
7439 17312 7440 17305 7443 17298 7447 17291 7454 17286 7461 17281
7470 17277 7481 17275 7493 17274 7510 17276 7525 17281 7537 17287
7548 17294 7556 17302 7562 17311 7565 17320 7567 17328 7567 17379
7547 17372 7532 17368 7485 17361 7471 17357 7461 17352 7453 17346
7447 17340 7443 17334 7441 17327 7440 17320 7439 17312 7401 17312
7402 17331 7405 17347 7411 17359 7418 17369 7427 17377 7439 17383
7452 17387 7467 17391 7534 17402 7549 17406 7560 17411 7563 17414
7565 17416 7566 17419 7567 17422 7567 17445 7566 17449 7564 17454
7560 17460 7554 17465 7546 17470 7537 17474 7525 17477 7511 17478
7496 17477 7483 17474 7472 17469 7464 17463 7457 17456 7453 17448
7450 17440 7449 17432 7409 17432 129 N F
7684 17509 7720 17509 7720 17460 7735 17481 7749 17496 7762 17507
7773 17513 7784 17515 7792 17516 7800 17514 7806 17511 7806 17473
7786 17471 7769 17466 7761 17463 7755 17460 7749 17455 7743 17451
7739 17445 7735 17440 7732 17433 7729 17426 7726 17411 7725 17394
7725 17251 7684 17251 7684 17509 27 N F
8043 17422 8002 17422 8001 17433 7997 17443 7992 17452 7985 17461
7977 17468 7967 17473 7955 17477 7941 17478 7933 17478 7925 17476
7918 17474 7911 17470 7904 17466 7899 17461 7893 17455 7888 17449
7884 17442 7880 17434 7875 17416 7871 17396 7870 17374 7871 17352
7875 17333 7881 17316 7885 17309 7890 17302 7895 17296 7900 17291
7906 17287 7913 17283 7920 17280 7927 17278 7935 17277 7944 17277
7952 17277 7959 17278 7966 17280 7972 17283 7982 17290 7991 17299

7997 17309 8002 17320 8004 17330 8005 17340 8046 17340 8045 17330
8044 17321 8042 17311 8039 17302 8035 17293 8031 17285 8025 17277
8019 17269 8012 17263 8004 17256 7995 17251 7986 17247 7975 17243
7964 17240 7952 17239 7939 17238 7924 17239 7911 17242 7898 17245
7887 17251 7877 17257 7867 17265 7859 17273 7852 17283 7846 17293
7841 17305 7836 17316 7833 17329 7830 17341 7828 17355 7827 17381
7828 17400 7831 17416 7835 17432 7840 17445 7846 17458 7853 17469
7861 17479 7870 17487 7879 17494 7888 17501 7897 17506 7907 17510
7916 17513 7925 17515 7941 17516 7959 17515 7977 17511 7985 17508
7993 17505 8000 17500 8008 17495 8014 17489 8020 17482 8026 17474
8031 17466 8035 17456 8039 17446 8041 17434 8043 17422 107 N F
8092 17608 8132 17608 8132 17473 8132 17473 8145 17487 8157 17497
8168 17505 8179 17510 8189 17513 8198 17515 8214 17516 8229 17515
8244 17511 8257 17505 8269 17497 8279 17487 8286 17477 8291 17465
8293 17459 8293 17453 8293 17251 8252 17251 8252 17437 8251 17443
8249 17450 8245 17457 8239 17464 8231 17469 8222 17474 8211 17477
8199 17478 8192 17478 8185 17476 8178 17474 8172 17472 8161 17464
8151 17454 8143 17443 8137 17430 8134 17416 8132 17402 8132 17251
8092 17251 8092 17608 44 N F
2298 17596 2446 17596 2455 17595 2465 17593 2476 17591 2487 17587
2499 17581 2510 17575 2521 17567 2531 17557 2542 17546 2551 17533
2559 17518 2567 17502 2572 17484 2577 17463 2580 17441 2581 17417
2580 17393 2577 17370 2573 17350 2568 17332 2561 17316 2553 17301
2544 17288 2534 17277 2524 17267 2513 17259 2502 17252 2490 17247
2479 17243 2468 17240 2456 17239 2446 17238 2298 17238 2298 17555
2344 17555 2344 17279 2438 17279 2449 17280 2460 17282 2470 17285
2479 17289 2488 17294 2496 17301 2503 17308 2509 17316 2515 17326
2520 17336 2525 17348 2528 17360 2531 17373 2533 17387 2535 17402
2535 17417 2534 17434 2533 17449 2530 17464 2527 17477 2523 17490
2518 17501 2512 17511 2505 17520 2498 17528 2491 17535 2482 17541
2474 17546 2465 17550 2455 17553 2445 17554 2436 17555 2344 17555
2298 17555 2298 17596 74 N F
2645 17596 2708 17596 2813 17284 2917 17596 2981 17596 2981 17238
2938 17238 2938 17537 2836 17238 2787 17238 2688 17537 2688 17238
2645 17238 2645 17596 14 N F
2 O
2458 2837 3324 2837 3324 3792 2458 3792 2458 2837 5 N F
1 O
12 W
2458 2837 3324 2837 3324 3792 2458 3792 2458 2837 5 P
20 O
3324 2837 4189 2837 4189 3792 3324 3792 3324 2837 5 N F
1 O

3324 2837 4189 2837 4189 3792 3324 3792 3324 2837 5 P
38 O
4189 2837 5054 2837 5054 3792 4189 3792 4189 2837 5 N F
1 O
4189 2837 5054 2837 5054 3792 4189 3792 4189 2837 5 P
56 O
5054 2837 5919 2837 5919 3792 5054 3792 5054 2837 5 N F
1 O
5054 2837 5919 2837 5919 3792 5054 3792 5054 2837 5 P
74 O
5919 2837 6784 2837 6784 3792 5919 3792 5919 2837 5 N F
1 O
5919 2837 6784 2837 6784 3792 5919 3792 5919 2837 5 P
92 O
6784 2837 7650 2837 7650 3792 6784 3792 6784 2837 5 N F
1 O
6784 2837 7650 2837 7650 3792 6784 3792 6784 2837 5 P
111 O
7650 2837 8515 2837 8515 3792 7650 3792 7650 2837 5 N F
1 O
7650 2837 8515 2837 8515 3792 7650 3792 7650 2837 5 P
129 O
8515 2837 9380 2837 9380 3792 8515 3792 8515 2837 5 N F
1 O
8515 2837 9380 2837 9380 3792 8515 3792 8515 2837 5 P
147 O
9380 2837 10245 2837 10245 3792 9380 3792 9380 2837 5 N F
1 O
9380 2837 10245 2837 10245 3792 9380 3792 9380 2837 5 P
165 O
10245 2837 11110 2837 11110 3792 10245 3792 10245 2837 5 N F
1 O
10245 2837 11110 2837 11110 3792 10245 3792 10245 2837 5 P
183 O
11110 2837 11975 2837 11975 3792 11110 3792 11110 2837 5 N F
1 O
11110 2837 11975 2837 11975 3792 11110 3792 11110 2837 5 P
201 O
11975 2837 12841 2837 12841 3792 11975 3792 11975 2837 5 N F
1 O
11975 2837 12841 2837 12841 3792 11975 3792 11975 2837 5 P
3170 2547 3179 2547 3188 2544 3197 2541 3205 2536 3213 2530
3220 2522 3226 2514 3232 2505 3237 2495 3242 2485 3250 2462

3254 2438 3256 2414 3254 2389 3250 2365 3242 2342 3237 2331
3232 2321 3226 2312 3220 2303 3213 2296 3205 2290 3197 2285
3188 2281 3179 2278 3170 2278 3160 2278 3151 2281 3143 2285
3135 2290 3127 2296 3120 2303 3113 2312 3107 2321 3102 2331
3097 2342 3090 2365 3085 2389 3084 2414 3118 2414 3119 2390
3123 2369 3128 2351 3135 2336 3143 2323 3151 2314 3156 2311
3160 2308 3165 2307 3170 2306 3174 2307 3179 2308 3184 2311
3188 2314 3197 2323 3205 2336 3212 2351 3217 2369 3220 2390
3221 2414 3220 2437 3217 2457 3212 2475 3205 2491 3197 2503
3188 2511 3184 2515 3179 2517 3174 2518 3170 2519 3165 2518
3160 2517 3156 2515 3151 2511 3143 2503 3135 2491 3128 2475
3123 2457 3119 2437 3118 2414 3084 2414 3085 2438 3090 2462
3097 2485 3102 2495 3107 2505 3113 2514 3120 2522 3127 2530
3135 2536 3143 2541 3151 2544 3160 2547 3170 2547 95 N F
3305 2325 3344 2325 3344 2287 3305 2287 3305 2325 5 N F
3476 2547 3492 2546 3506 2543 3520 2537 3531 2530 3541 2520
3545 2514 3548 2508 3550 2501 3552 2494 3554 2487 3554 2479
3553 2467 3551 2458 3547 2449 3542 2442 3537 2436 3531 2431
3520 2423 3529 2418 3538 2412 3545 2405 3552 2397 3557 2388
3560 2379 3563 2369 3564 2358 3562 2345 3558 2332 3551 2318
3541 2306 3535 2300 3528 2294 3521 2290 3513 2286 3504 2282
3495 2280 3484 2278 3474 2278 3463 2278 3453 2280 3444 2282
3435 2286 3427 2290 3420 2294 3414 2300 3408 2306 3399 2318
3393 2332 3389 2345 3388 2358 3422 2358 3423 2348 3426 2338
3431 2329 3438 2321 3446 2315 3455 2310 3465 2307 3476 2306
3486 2307 3496 2310 3505 2315 3513 2321 3520 2329 3525 2338
3528 2348 3529 2358 3528 2368 3525 2378 3520 2387 3513 2395
3505 2401 3496 2406 3486 2409 3476 2410 3465 2409 3455 2406
3446 2401 3438 2395 3431 2387 3426 2378 3423 2368 3422 2358
3388 2358 3388 2368 3391 2378 3395 2388 3400 2396 3406 2404
3413 2411 3422 2418 3432 2423 3420 2431 3414 2436 3409 2442
3404 2449 3400 2458 3398 2467 3397 2479 3430 2479 3431 2470
3433 2462 3438 2455 3443 2449 3450 2445 3458 2441 3466 2439
3476 2438 3485 2439 3493 2441 3501 2445 3508 2449 3514 2455
3518 2462 3521 2470 3521 2479 3521 2487 3518 2495 3514 2502
3508 2508 3501 2513 3493 2516 3485 2518 3476 2519 3466 2518
3458 2516 3450 2513 3443 2508 3438 2502 3433 2495 3431 2487
3430 2479 3397 2479 3398 2487 3399 2494 3401 2501 3403 2508
3407 2514 3410 2520 3420 2530 3431 2537 3445 2543 3460 2546
3476 2547 145 N F
3933 2547 3942 2547 3951 2544 3960 2541 3968 2536 3976 2530
3983 2522 3989 2514 3995 2505 4000 2495 4005 2485 4013 2462
4017 2438 4019 2414 4017 2389 4013 2365 4005 2342 4000 2331

3995 2321 3989 2312 3983 2303 3976 2296 3968 2290 3960 2285
3951 2281 3942 2278 3933 2278 3923 2278 3914 2281 3906 2285
3898 2290 3890 2296 3883 2303 3876 2312 3870 2321 3865 2331
3860 2342 3853 2365 3848 2389 3847 2414 3881 2414 3882 2390
3886 2369 3891 2351 3898 2336 3905 2323 3914 2314 3919 2311
3923 2308 3928 2307 3933 2306 3937 2307 3942 2308 3947 2311
3951 2314 3960 2323 3968 2336 3975 2351 3980 2369 3983 2390
3984 2414 3983 2437 3980 2457 3975 2475 3968 2491 3960 2503
3951 2511 3947 2515 3942 2517 3937 2518 3933 2519 3928 2518
3923 2517 3919 2515 3914 2511 3905 2503 3898 2491 3891 2475
3886 2457 3882 2437 3881 2414 3847 2414 3848 2438 3853 2462
3860 2485 3865 2495 3870 2505 3876 2514 3883 2522 3890 2530
3898 2536 3906 2541 3914 2544 3923 2547 3933 2547 95 N F
4068 2325 4107 2325 4107 2287 4068 2287 4068 2325 5 N F
4239 2547 4255 2546 4269 2543 4283 2537 4294 2530 4304 2520
4308 2514 4311 2508 4313 2501 4315 2494 4316 2487 4317 2479
4316 2467 4314 2458 4310 2449 4305 2442 4300 2436 4294 2431
4282 2423 4292 2418 4301 2412 4308 2405 4314 2397 4320 2388
4323 2379 4326 2369 4326 2358 4325 2345 4321 2332 4314 2318
4304 2306 4298 2300 4291 2294 4284 2290 4276 2286 4267 2282
4257 2280 4247 2278 4237 2278 4226 2278 4216 2280 4207 2282
4198 2286 4190 2290 4183 2294 4177 2300 4171 2306 4162 2318
4156 2332 4152 2345 4151 2358 4185 2358 4186 2348 4189 2338
4194 2329 4201 2321 4209 2315 4218 2310 4228 2307 4239 2306
4249 2307 4259 2310 4268 2315 4276 2321 4283 2329 4288 2338
4291 2348 4292 2358 4291 2368 4288 2378 4283 2387 4276 2395
4268 2401 4259 2406 4249 2409 4239 2410 4228 2409 4218 2406
4209 2401 4201 2395 4194 2387 4189 2378 4186 2368 4185 2358
4151 2358 4151 2368 4154 2378 4157 2388 4163 2396 4169 2404
4176 2411 4185 2418 4195 2423 4183 2431 4177 2436 4172 2442
4167 2449 4163 2458 4161 2467 4160 2479 4193 2479 4194 2470
4196 2462 4201 2455 4206 2449 4213 2445 4221 2441 4229 2439
4239 2438 4248 2439 4256 2441 4264 2445 4271 2449 4276 2455
4281 2462 4283 2470 4284 2479 4283 2487 4281 2495 4276 2502
4271 2508 4264 2513 4256 2516 4248 2518 4239 2519 4229 2518
4221 2516 4213 2513 4206 2508 4201 2502 4196 2495 4194 2487
4193 2479 4160 2479 4161 2487 4162 2494 4164 2501 4166 2508
4169 2514 4173 2520 4183 2530 4194 2537 4208 2543 4223 2546
4239 2547 145 N F
4361 2458 4393 2458 4394 2471 4397 2482 4401 2493 4406 2502
4414 2509 4423 2514 4434 2518 4447 2519 4457 2518 4466 2515
4474 2511 4482 2505 4488 2498 4493 2490 4496 2480 4497 2469
4496 2458 4493 2449 4488 2441 4482 2434 4474 2427 4463 2420

4437 2404 4415 2391 4397 2378 4383 2364 4372 2351 4364 2336
4359 2321 4356 2305 4355 2287 4529 2287 4529 2318 4391 2318
4395 2330 4400 2339 4406 2348 4413 2355 4423 2362 4434 2369
4464 2387 4481 2395 4495 2405 4507 2415 4516 2426 4523 2438
4527 2449 4530 2461 4531 2473 4531 2481 4529 2489 4527 2496
4524 2503 4521 2510 4516 2516 4506 2527 4494 2535 4479 2542
4464 2546 4447 2547 4429 2546 4412 2542 4405 2539 4398 2535
4391 2531 4385 2525 4380 2520 4375 2513 4371 2506 4367 2497
4365 2489 4362 2479 4361 2469 4361 2458 76 N F
4796 2547 4805 2547 4814 2544 4823 2541 4831 2536 4839 2530
4846 2522 4852 2514 4858 2505 4864 2495 4868 2485 4876 2462
4880 2438 4882 2414 4880 2389 4876 2365 4868 2342 4864 2331
4858 2321 4852 2312 4846 2303 4839 2296 4831 2290 4823 2285
4814 2281 4805 2278 4796 2278 4787 2278 4777 2281 4769 2285
4761 2290 4753 2296 4746 2303 4740 2312 4734 2321 4728 2331
4724 2342 4716 2365 4712 2389 4710 2414 4744 2414 4746 2390
4749 2369 4754 2351 4761 2336 4769 2323 4777 2314 4782 2311
4787 2308 4791 2307 4796 2306 4801 2307 4805 2308 4810 2311
4815 2314 4823 2323 4831 2336 4838 2351 4843 2369 4846 2390
4848 2414 4846 2437 4843 2457 4838 2475 4831 2491 4823 2503
4815 2511 4810 2515 4805 2517 4801 2518 4796 2519 4791 2518
4787 2517 4782 2515 4777 2511 4769 2503 4761 2491 4754 2475
4749 2457 4746 2437 4744 2414 4710 2414 4712 2438 4716 2462
4724 2485 4728 2495 4734 2505 4740 2514 4746 2522 4753 2530
4761 2536 4769 2541 4777 2544 4787 2547 4796 2547 95 N F
4932 2325 4970 2325 4970 2287 4932 2287 4932 2325 5 N F
5102 2547 5118 2546 5133 2543 5146 2537 5157 2530 5167 2520
5171 2514 5174 2508 5177 2501 5179 2494 5180 2487 5180 2479
5179 2467 5177 2458 5173 2449 5169 2442 5163 2436 5158 2431
5146 2423 5155 2418 5164 2412 5171 2405 5178 2397 5183 2388
5187 2379 5189 2369 5190 2358 5188 2345 5184 2332 5177 2318
5167 2306 5161 2300 5154 2294 5147 2290 5139 2286 5130 2282
5121 2280 5111 2278 5100 2278 5089 2278 5079 2280 5070 2282
5061 2286 5054 2290 5047 2294 5040 2300 5035 2306 5025 2318
5019 2332 5015 2345 5014 2358 5048 2358 5049 2348 5053 2338
5058 2329 5064 2321 5072 2315 5081 2310 5091 2307 5102 2306
5112 2307 5122 2310 5131 2315 5139 2321 5146 2329 5151 2338
5154 2348 5155 2358 5154 2368 5151 2378 5146 2387 5139 2395
5131 2401 5122 2406 5112 2409 5102 2410 5091 2409 5081 2406
5072 2401 5064 2395 5058 2387 5053 2378 5049 2368 5048 2358
5014 2358 5015 2368 5017 2378 5021 2388 5026 2396 5032 2404
5040 2411 5048 2418 5058 2423 5046 2431 5040 2436 5035 2442
5030 2449 5027 2458 5024 2467 5023 2479 5056 2479 5057 2470

5060 2462 5064 2455 5070 2449 5076 2445 5084 2441 5093 2439
5102 2438 5111 2439 5119 2441 5127 2445 5134 2449 5140 2455
5144 2462 5147 2470 5148 2479 5147 2487 5144 2495 5140 2502
5134 2508 5127 2513 5119 2516 5111 2518 5102 2519 5093 2518
5084 2516 5076 2513 5070 2508 5064 2502 5060 2495 5057 2487
5056 2479 5023 2479 5024 2487 5025 2494 5027 2501 5029 2508
5033 2514 5037 2520 5046 2530 5058 2537 5071 2543 5086 2546
5102 2547 145 N F
5335 2547 5358 2547 5358 2379 5398 2379 5398 2348 5358 2348
5358 2287 5325 2287 5325 2348 5216 2348 5216 2379 5245 2379
5325 2379 5325 2492 5245 2379 5216 2379 5216 2383 5335 2547
18 N F
5663 2547 5673 2547 5682 2544 5690 2541 5698 2536 5706 2530
5713 2522 5719 2514 5725 2505 5731 2495 5735 2485 5743 2462
5747 2438 5749 2414 5747 2389 5743 2365 5735 2342 5731 2331
5725 2321 5719 2312 5713 2303 5706 2296 5698 2290 5690 2285
5682 2281 5673 2278 5663 2278 5654 2278 5645 2281 5636 2285
5628 2290 5620 2296 5613 2303 5607 2312 5601 2321 5595 2331
5591 2342 5583 2365 5579 2389 5577 2414 5611 2414 5613 2390
5616 2369 5621 2351 5628 2336 5636 2323 5645 2314 5649 2311
5654 2308 5658 2307 5663 2306 5668 2307 5672 2308 5677 2311
5682 2314 5690 2323 5698 2336 5705 2351 5710 2369 5713 2390
5715 2414 5713 2437 5710 2457 5705 2475 5698 2491 5690 2503
5682 2511 5677 2515 5672 2517 5668 2518 5663 2519 5658 2518
5654 2517 5649 2515 5645 2511 5636 2503 5628 2491 5621 2475
5616 2457 5613 2437 5611 2414 5577 2414 5579 2438 5583 2462
5591 2485 5595 2495 5601 2505 5607 2514 5613 2522 5620 2530
5628 2536 5636 2541 5645 2544 5654 2547 5663 2547 95 N F
5799 2325 5837 2325 5837 2287 5799 2287 5799 2325 5 N F
5969 2547 5985 2546 6000 2543 6013 2537 6025 2530 6034 2520
6038 2514 6041 2508 6044 2501 6046 2494 6047 2487 6047 2479
6046 2467 6044 2458 6040 2449 6036 2442 6030 2436 6025 2431
6013 2423 6022 2418 6031 2412 6038 2405 6045 2397 6050 2388
6054 2379 6056 2369 6057 2358 6055 2345 6051 2332 6044 2318
6034 2306 6028 2300 6021 2294 6014 2290 6006 2286 5997 2282
5988 2280 5978 2278 5967 2278 5956 2278 5946 2280 5937 2282
5929 2286 5921 2290 5914 2294 5907 2300 5902 2306 5893 2318
5886 2332 5882 2345 5881 2358 5915 2358 5916 2348 5920 2338
5925 2329 5931 2321 5939 2315 5948 2310 5958 2307 5969 2306
5979 2307 5989 2310 5998 2315 6006 2321 6013 2329 6018 2338
6021 2348 6022 2358 6021 2368 6018 2378 6013 2387 6006 2395
5998 2401 5989 2406 5979 2409 5969 2410 5958 2409 5948 2406
5939 2401 5931 2395 5925 2387 5920 2378 5916 2368 5915 2358

5881 2358 5882 2368 5884 2378 5888 2388 5893 2396 5899 2404
5907 2411 5915 2418 5925 2423 5913 2431 5907 2436 5902 2442
5897 2449 5894 2458 5891 2467 5890 2479 5923 2479 5924 2470
5927 2462 5931 2455 5937 2449 5943 2445 5951 2441 5960 2439
5969 2438 5978 2439 5987 2441 5994 2445 6001 2449 6007 2455
6011 2462 6014 2470 6015 2479 6014 2487 6011 2495 6007 2502
6001 2508 5994 2513 5987 2516 5978 2518 5969 2519 5960 2518
5951 2516 5943 2513 5937 2508 5931 2502 5927 2495 5924 2487
5923 2479 5890 2479 5891 2487 5892 2494 5894 2501 5897 2508
5900 2514 5904 2520 5913 2530 5925 2537 5938 2543 5953 2546
5969 2547 145 N F
6256 2481 6223 2481 6221 2489 6218 2497 6213 2504 6208 2509
6202 2513 6195 2516 6188 2518 6181 2519 6174 2518 6167 2517
6161 2515 6156 2512 6150 2508 6145 2504 6141 2499 6137 2493
6130 2479 6126 2462 6123 2443 6122 2421 6130 2430 6137 2436
6145 2441 6153 2445 6160 2447 6168 2449 6185 2450 6198 2449
6211 2445 6224 2438 6236 2429 6241 2424 6246 2418 6251 2411
6255 2403 6258 2395 6260 2386 6261 2376 6261 2366 6229 2364
6228 2374 6224 2385 6219 2395 6213 2403 6205 2411 6196 2416
6186 2420 6175 2421 6165 2420 6155 2416 6146 2411 6138 2403
6131 2395 6126 2385 6123 2374 6122 2364 6123 2353 6126 2343
6131 2333 6138 2324 6146 2317 6155 2311 6165 2308 6175 2306
6186 2308 6196 2311 6205 2317 6213 2324 6219 2333 6224 2343
6228 2353 6229 2364 6261 2366 6260 2350 6256 2335 6250 2320
6245 2313 6240 2307 6235 2300 6229 2295 6221 2290 6214 2286
6205 2282 6196 2280 6186 2278 6175 2278 6164 2278 6153 2280
6143 2283 6134 2288 6126 2293 6119 2300 6113 2307 6107 2315
6102 2325 6098 2335 6095 2345 6092 2357 6088 2381 6087 2408
6088 2424 6089 2439 6091 2453 6094 2466 6097 2478 6101 2490
6106 2500 6112 2510 6118 2518 6125 2526 6133 2532 6141 2538
6150 2542 6160 2545 6170 2547 6181 2547 6196 2546 6210 2542
6222 2536 6233 2528 6241 2518 6248 2506 6253 2494 6256 2481
126 N F
6528 2547 6538 2547 6547 2544 6555 2541 6563 2536 6571 2530
6578 2522 6585 2514 6591 2505 6596 2495 6601 2485 6608 2462
6613 2438 6614 2414 6613 2389 6608 2365 6601 2342 6596 2331
6591 2321 6585 2312 6578 2303 6571 2296 6563 2290 6555 2285
6547 2281 6538 2278 6528 2278 6519 2278 6510 2281 6501 2285
6493 2290 6485 2296 6478 2303 6472 2312 6466 2321 6461 2331
6456 2342 6448 2365 6444 2389 6442 2414 6477 2414 6478 2390
6481 2369 6486 2351 6493 2336 6501 2323 6510 2314 6514 2311
6519 2308 6524 2307 6528 2306 6533 2307 6538 2308 6542 2311
6547 2314 6555 2323 6563 2336 6570 2351 6575 2369 6579 2390

6580 2414 6579 2437 6575 2457 6570 2475 6563 2491 6555 2503
6547 2511 6542 2515 6538 2517 6533 2518 6528 2519 6524 2518
6519 2517 6514 2515 6510 2511 6501 2503 6493 2491 6486 2475
6481 2457 6478 2437 6477 2414 6442 2414 6444 2438 6448 2462
6456 2485 6461 2495 6466 2505 6472 2514 6478 2522 6485 2530
6493 2536 6501 2541 6510 2544 6519 2547 6528 2547 95 N F
6664 2325 6702 2325 6702 2287 6664 2287 6664 2325 5 N F
6834 2547 6850 2546 6865 2543 6878 2537 6890 2530 6899 2520
6903 2514 6906 2508 6909 2501 6911 2494 6912 2487 6912 2479
6912 2467 6909 2458 6906 2449 6901 2442 6896 2436 6890 2431
6878 2423 6888 2418 6896 2412 6904 2405 6910 2397 6915 2388
6919 2379 6921 2369 6922 2358 6921 2345 6916 2332 6909 2318
6899 2306 6893 2300 6887 2294 6879 2290 6871 2286 6862 2282
6853 2280 6843 2278 6832 2278 6821 2278 6811 2280 6802 2282
6794 2286 6786 2290 6779 2294 6773 2300 6767 2306 6758 2318
6751 2332 6747 2345 6746 2358 6781 2358 6782 2348 6785 2338
6790 2329 6797 2321 6804 2315 6814 2310 6823 2307 6834 2306
6845 2307 6854 2310 6864 2315 6872 2321 6878 2329 6883 2338
6886 2348 6888 2358 6886 2368 6883 2378 6878 2387 6872 2395
6864 2401 6854 2406 6845 2409 6834 2410 6823 2409 6814 2406
6804 2401 6797 2395 6790 2387 6785 2378 6782 2368 6781 2358
6746 2358 6747 2368 6749 2378 6753 2388 6758 2396 6764 2404
6772 2411 6780 2418 6790 2423 6778 2431 6772 2436 6767 2442
6763 2449 6759 2458 6757 2467 6756 2479 6788 2479 6789 2470
6792 2462 6796 2455 6802 2449 6809 2445 6816 2441 6825 2439
6834 2438 6843 2439 6852 2441 6859 2445 6866 2449 6872 2455
6876 2462 6879 2470 6880 2479 6879 2487 6876 2495 6872 2502
6866 2508 6859 2513 6852 2516 6843 2518 6834 2519 6825 2518
6816 2516 6809 2513 6802 2508 6796 2502 6792 2495 6789 2487
6788 2479 6756 2479 6756 2487 6757 2494 6759 2501 6762 2508
6765 2514 6769 2520 6778 2530 6790 2537 6803 2543 6818 2546
6834 2547 145 N F
7039 2547 7055 2546 7069 2543 7083 2537 7094 2530 7104 2520
7108 2514 7111 2508 7113 2501 7115 2494 7116 2487 7117 2479
7116 2467 7114 2458 7110 2449 7105 2442 7100 2436 7094 2431
7082 2423 7092 2418 7101 2412 7108 2405 7115 2397 7120 2388
7123 2379 7126 2369 7126 2358 7125 2345 7121 2332 7114 2318
7104 2306 7098 2300 7091 2294 7084 2290 7076 2286 7067 2282
7057 2280 7047 2278 7037 2278 7026 2278 7016 2280 7007 2282
6998 2286 6990 2290 6983 2294 6977 2300 6971 2306 6962 2318
6956 2332 6952 2345 6951 2358 6985 2358 6986 2348 6989 2338
6994 2329 7001 2321 7009 2315 7018 2310 7028 2307 7039 2306
7049 2307 7059 2310 7068 2315 7076 2321 7083 2329 7088 2338

7091 2348 7092 2358 7091 2368 7088 2378 7083 2387 7076 2395
7068 2401 7059 2406 7049 2409 7039 2410 7028 2409 7018 2406
7009 2401 7001 2395 6994 2387 6989 2378 6986 2368 6985 2358
6951 2358 6951 2368 6954 2378 6957 2388 6963 2396 6969 2404
6976 2411 6985 2418 6995 2423 6983 2431 6977 2436 6972 2442
6967 2449 6963 2458 6961 2467 6960 2479 6993 2479 6994 2470
6996 2462 7001 2455 7006 2449 7013 2445 7021 2441 7029 2439
7039 2438 7048 2439 7056 2441 7064 2445 7071 2449 7076 2455
7081 2462 7083 2470 7084 2479 7083 2487 7081 2495 7076 2502
7071 2508 7064 2513 7056 2516 7048 2518 7039 2519 7029 2518
7021 2516 7013 2513 7006 2508 7001 2502 6996 2495 6994 2487
6993 2479 6960 2479 6961 2487 6962 2494 6964 2501 6966 2508
6969 2514 6973 2520 6983 2530 6994 2537 7008 2543 7023 2546
7039 2547 145 N F
7496 2547 7505 2547 7514 2544 7523 2541 7531 2536 7538 2530
7546 2522 7552 2514 7558 2505 7563 2495 7568 2485 7575 2462
7580 2438 7582 2414 7580 2389 7575 2365 7568 2342 7563 2331
7558 2321 7552 2312 7546 2303 7538 2296 7531 2290 7523 2285
7514 2281 7505 2278 7496 2278 7486 2278 7477 2281 7469 2285
7460 2290 7453 2296 7446 2303 7439 2312 7433 2321 7428 2331
7423 2342 7416 2365 7411 2389 7410 2414 7444 2414 7445 2390
7449 2369 7454 2351 7461 2336 7468 2323 7477 2314 7482 2311
7486 2308 7491 2307 7496 2306 7500 2307 7505 2308 7510 2311
7514 2314 7523 2323 7531 2336 7537 2351 7543 2369 7546 2390
7547 2414 7546 2437 7543 2457 7537 2475 7531 2491 7523 2503
7514 2511 7510 2515 7505 2517 7500 2518 7496 2519 7491 2518
7486 2517 7482 2515 7477 2511 7468 2503 7461 2491 7454 2475
7449 2457 7445 2437 7444 2414 7410 2414 7411 2438 7416 2462
7423 2485 7428 2495 7433 2505 7439 2514 7446 2522 7453 2530
7460 2536 7469 2541 7477 2544 7486 2547 7496 2547 95 N F
7631 2325 7670 2325 7670 2287 7631 2287 7631 2325 5 N F
7801 2547 7811 2547 7820 2546 7829 2544 7837 2541 7844 2537
7851 2532 7858 2526 7864 2519 7869 2511 7874 2502 7879 2491
7882 2480 7885 2467 7887 2453 7889 2438 7889 2421 7889 2401
7887 2382 7884 2365 7881 2350 7876 2337 7871 2325 7865 2315
7859 2306 7852 2299 7844 2293 7836 2288 7828 2284 7820 2281
7811 2279 7794 2278 7781 2279 7768 2282 7757 2287 7746 2294
7737 2304 7730 2316 7724 2330 7719 2347 7752 2347 7755 2337
7759 2329 7763 2322 7768 2316 7774 2312 7780 2309 7788 2307
7796 2306 7803 2307 7810 2308 7816 2311 7822 2314 7827 2318
7832 2323 7840 2335 7847 2350 7851 2366 7854 2384 7855 2404
7846 2395 7837 2388 7829 2383 7822 2380 7815 2377 7808 2376
7796 2375 7786 2376 7776 2378 7768 2380 7760 2383 7752 2387

7745 2392 7739 2397 7734 2403 7729 2409 7725 2415 7719 2430
7716 2445 7715 2461 7748 2461 7749 2451 7752 2441 7756 2431
7763 2423 7770 2415 7779 2409 7790 2405 7801 2404 7813 2405
7824 2409 7833 2415 7840 2423 7847 2431 7851 2441 7854 2451
7855 2461 7854 2472 7851 2482 7847 2491 7840 2500 7833 2508
7824 2514 7813 2517 7801 2519 7790 2517 7779 2514 7770 2508
7763 2500 7756 2491 7752 2482 7749 2472 7748 2461 7715 2461
7716 2470 7716 2478 7718 2486 7721 2494 7724 2502 7728 2509
7732 2515 7738 2522 7744 2527 7750 2532 7757 2537 7765 2540
7773 2543 7782 2546 7792 2547 7801 2547 130 N F
8259 2547 8268 2547 8277 2544 8286 2541 8294 2536 8301 2530
8308 2522 8315 2514 8321 2505 8326 2495 8331 2485 8338 2462
8343 2438 8345 2414 8343 2389 8338 2365 8331 2342 8326 2331
8321 2321 8315 2312 8308 2303 8301 2296 8294 2290 8286 2285
8277 2281 8268 2278 8259 2278 8249 2278 8240 2281 8231 2285
8223 2290 8216 2296 8209 2303 8202 2312 8196 2321 8191 2331
8186 2342 8179 2365 8174 2389 8173 2414 8207 2414 8208 2390
8212 2369 8217 2351 8223 2336 8231 2323 8240 2314 8245 2311
8249 2308 8254 2307 8259 2306 8263 2307 8268 2308 8273 2311
8277 2314 8286 2323 8294 2336 8300 2351 8306 2369 8309 2390
8310 2414 8309 2437 8306 2457 8300 2475 8294 2491 8286 2503
8277 2511 8273 2515 8268 2517 8263 2518 8259 2519 8254 2518
8249 2517 8245 2515 8240 2511 8231 2503 8223 2491 8217 2475
8212 2457 8208 2437 8207 2414 8173 2414 8174 2438 8179 2462
8186 2485 8191 2495 8196 2505 8202 2514 8209 2522 8216 2530
8223 2536 8231 2541 8240 2544 8249 2547 8259 2547 95 N F
8394 2325 8432 2325 8432 2287 8394 2287 8394 2325 5 N F
8564 2547 8574 2547 8583 2546 8591 2544 8600 2541 8607 2537
8614 2532 8621 2526 8627 2519 8632 2511 8637 2502 8641 2491
8645 2480 8648 2467 8650 2453 8652 2438 8652 2421 8652 2401
8650 2382 8647 2365 8644 2350 8639 2337 8634 2325 8628 2315
8622 2306 8615 2299 8607 2293 8599 2288 8591 2284 8583 2281
8574 2279 8557 2278 8543 2279 8531 2282 8520 2287 8509 2294
8500 2304 8493 2316 8487 2330 8482 2347 8515 2347 8518 2337
8522 2329 8526 2322 8531 2316 8537 2312 8543 2309 8550 2307
8559 2306 8566 2307 8573 2308 8579 2311 8585 2314 8590 2318
8595 2323 8603 2335 8610 2350 8614 2366 8617 2384 8618 2404
8609 2395 8600 2388 8592 2383 8585 2380 8578 2377 8571 2376
8559 2375 8549 2376 8539 2378 8530 2380 8522 2383 8515 2387
8508 2392 8502 2397 8497 2403 8492 2409 8488 2415 8482 2430
8479 2445 8478 2461 8511 2461 8512 2451 8515 2441 8519 2431
8525 2423 8533 2415 8542 2409 8553 2405 8564 2404 8576 2405
8586 2409 8596 2415 8603 2423 8610 2431 8614 2441 8617 2451

8618 2461 8617 2472 8614 2482 8610 2491 8603 2500 8596 2508
8586 2514 8576 2517 8564 2519 8553 2517 8542 2514 8533 2508
8525 2500 8519 2491 8515 2482 8512 2472 8511 2461 8478 2461
8478 2470 8479 2478 8481 2486 8483 2494 8487 2502 8491 2509
8495 2515 8501 2522 8507 2527 8513 2532 8520 2537 8528 2540
8536 2543 8545 2546 8555 2547 8564 2547 130 N F
8687 2458 8719 2458 8720 2471 8722 2482 8727 2493 8732 2502
8740 2509 8749 2514 8760 2518 8773 2519 8783 2518 8792 2515
8800 2511 8808 2505 8814 2498 8818 2490 8821 2480 8822 2469
8821 2458 8819 2449 8814 2441 8808 2434 8800 2427 8789 2420
8763 2404 8741 2391 8723 2378 8709 2364 8698 2351 8690 2336
8685 2321 8682 2305 8681 2287 8855 2287 8855 2318 8717 2318
8721 2330 8725 2339 8732 2348 8739 2355 8749 2362 8760 2369
8790 2387 8807 2395 8821 2405 8833 2415 8842 2426 8849 2438
8853 2449 8856 2461 8857 2473 8856 2481 8855 2489 8853 2496
8850 2503 8847 2510 8842 2516 8832 2527 8819 2535 8805 2542
8789 2546 8773 2547 8755 2546 8738 2542 8731 2539 8724 2535
8717 2531 8711 2525 8706 2520 8701 2513 8697 2506 8693 2497
8690 2489 8688 2479 8687 2469 8687 2458 76 N F
9122 2547 9131 2547 9140 2544 9149 2541 9157 2536 9165 2530
9172 2522 9178 2514 9184 2505 9190 2495 9194 2485 9202 2462
9206 2438 9208 2414 9206 2389 9202 2365 9194 2342 9190 2331
9184 2321 9178 2312 9172 2303 9165 2296 9157 2290 9149 2285
9140 2281 9131 2278 9122 2278 9112 2278 9103 2281 9095 2285
9087 2290 9079 2296 9072 2303 9065 2312 9059 2321 9054 2331
9049 2342 9042 2365 9037 2389 9036 2414 9070 2414 9071 2390
9075 2369 9080 2351 9087 2336 9095 2323 9103 2314 9108 2311
9112 2308 9117 2307 9122 2306 9126 2307 9131 2308 9136 2311
9140 2314 9149 2323 9157 2336 9164 2351 9169 2369 9172 2390
9173 2414 9172 2437 9169 2457 9164 2475 9157 2491 9149 2503
9140 2511 9136 2515 9131 2517 9126 2518 9122 2519 9117 2518
9112 2517 9108 2515 9103 2511 9095 2503 9087 2491 9080 2475
9075 2457 9071 2437 9070 2414 9036 2414 9037 2438 9042 2462
9049 2485 9054 2495 9059 2505 9065 2514 9072 2522 9079 2530
9087 2536 9095 2541 9103 2544 9112 2547 9122 2547 95 N F
9258 2325 9296 2325 9296 2287 9258 2287 9258 2325 5 N F
9428 2547 9437 2547 9446 2546 9455 2544 9463 2541 9470 2537
9478 2532 9484 2526 9490 2519 9496 2511 9500 2502 9505 2491
9508 2480 9511 2467 9513 2453 9515 2438 9516 2421 9515 2401
9513 2382 9511 2365 9507 2350 9503 2337 9497 2325 9491 2315
9485 2306 9478 2299 9470 2293 9462 2288 9454 2284 9446 2281
9437 2279 9420 2278 9407 2279 9394 2282 9383 2287 9373 2294
9364 2304 9356 2316 9350 2330 9345 2347 9378 2347 9381 2337

9385 2329 9389 2322 9394 2316 9400 2312 9406 2309 9414 2307
9422 2306 9429 2307 9436 2308 9442 2311 9448 2314 9453 2318
9458 2323 9467 2335 9473 2350 9478 2366 9480 2384 9481 2404
9472 2395 9464 2388 9456 2383 9448 2380 9441 2377 9434 2376
9422 2375 9412 2376 9402 2378 9394 2380 9386 2383 9378 2387
9372 2392 9366 2397 9360 2403 9356 2409 9352 2415 9346 2430
9342 2445 9342 2461 9374 2461 9375 2451 9378 2441 9382 2431
9389 2423 9396 2415 9406 2409 9416 2405 9428 2404 9439 2405
9450 2409 9459 2415 9467 2423 9473 2431 9477 2441 9480 2451
9481 2461 9480 2472 9477 2482 9473 2491 9467 2500 9459 2508
9450 2514 9439 2517 9428 2519 9416 2517 9406 2514 9396 2508
9389 2500 9382 2491 9378 2482 9375 2472 9374 2461 9342 2461
9342 2470 9343 2478 9344 2486 9347 2494 9350 2502 9354 2509
9359 2515 9364 2522 9370 2527 9376 2532 9384 2537 9391 2540
9400 2543 9408 2546 9418 2547 9428 2547 130 N F
9661 2547 9684 2547 9684 2379 9724 2379 9724 2348 9684 2348
9684 2287 9651 2287 9651 2348 9542 2348 9542 2379 9571 2379
9651 2379 9651 2492 9571 2379 9542 2379 9542 2383 9661 2547
18 N F
9989 2547 9998 2547 10007 2544 10016 2541 10024 2536 10032 2530
10039 2522 10045 2514 10051 2505 10057 2495 10061 2485 10069 2462
10073 2438 10075 2414 10073 2389 10069 2365 10061 2342 10057 2331
10051 2321 10045 2312 10039 2303 10032 2296 10024 2290 10016 2285
10007 2281 9998 2278 9989 2278 9979 2278 9970 2281 9962 2285
9954 2290 9946 2296 9939 2303 9932 2312 9927 2321 9921 2331
9916 2342 9909 2365 9904 2389 9903 2414 9937 2414 9938 2390
9942 2369 9947 2351 9954 2336 9962 2323 9970 2314 9975 2311
9980 2308 9984 2307 9989 2306 9994 2307 9998 2308 10003 2311
10007 2314 10016 2323 10024 2336 10031 2351 10036 2369 10039 2390
10041 2414 10039 2437 10036 2457 10031 2475 10024 2491 10016 2503
10007 2511 10003 2515 9998 2517 9994 2518 9989 2519 9984 2518
9980 2517 9975 2515 9970 2511 9962 2503 9954 2491 9947 2475
9942 2457 9938 2437 9937 2414 9903 2414 9904 2438 9909 2462
9916 2485 9921 2495 9927 2505 9932 2514 9939 2522 9946 2530
9954 2536 9962 2541 9970 2544 9979 2547 9989 2547 95 N F
10125 2325 10163 2325 10163 2287 10125 2287 10125 2325 5 N F
10295 2547 10304 2547 10313 2546 10322 2544 10330 2541 10338 2537
10345 2532 10351 2526 10357 2519 10363 2511 10368 2502 10372 2491
10375 2480 10378 2467 10380 2453 10382 2438 10383 2421 10382 2401
10380 2382 10378 2365 10374 2350 10370 2337 10364 2325 10359 2315
10352 2306 10345 2299 10337 2293 10330 2288 10321 2284 10313 2281
10304 2279 10287 2278 10274 2279 10261 2282 10250 2287 10240 2294
10231 2304 10223 2316 10217 2330 10213 2347 10245 2347 10248 2337

10252 2329 10256 2322 10261 2316 10267 2312 10273 2309 10281 2307
10289 2306 10296 2307 10303 2308 10309 2311 10315 2314 10320 2318
10325 2323 10334 2335 10340 2350 10345 2366 10347 2384 10348 2404
10339 2395 10331 2388 10323 2383 10315 2380 10308 2377 10301 2376
10289 2375 10279 2376 10270 2378 10261 2380 10253 2383 10245 2387
10239 2392 10233 2397 10227 2403 10223 2409 10219 2415 10213 2430
10209 2445 10209 2461 10241 2461 10242 2451 10245 2441 10250 2431
10256 2423 10263 2415 10273 2409 10283 2405 10295 2404 10306 2405
10317 2409 10326 2415 10334 2423 10340 2431 10344 2441 10347 2451
10348 2461 10347 2472 10344 2482 10340 2491 10334 2500 10326 2508
10317 2514 10306 2517 10295 2519 10283 2517 10273 2514 10263 2508
10256 2500 10250 2491 10245 2482 10242 2472 10241 2461 10209 2461
10209 2470 10210 2478 10211 2486 10214 2494 10217 2502 10221 2509
10226 2515 10231 2522 10237 2527 10243 2532 10251 2537 10258 2540
10267 2543 10276 2546 10285 2547 10295 2547 130 N F
10581 2481 10549 2481 10547 2489 10543 2497 10539 2504 10534 2509
10528 2513 10521 2516 10514 2518 10507 2519 10500 2518 10493 2517
10487 2515 10481 2512 10476 2508 10471 2504 10467 2499 10463 2493
10456 2479 10451 2462 10449 2443 10448 2421 10455 2430 10463 2436
10471 2441 10478 2445 10486 2447 10494 2449 10511 2450 10523 2449
10537 2445 10550 2438 10562 2429 10567 2424 10572 2418 10577 2411
10581 2403 10584 2395 10586 2386 10587 2376 10587 2366 10555 2364
10554 2374 10550 2385 10545 2395 10539 2403 10531 2411 10522 2416
10512 2420 10501 2421 10491 2420 10481 2416 10472 2411 10464 2403
10457 2395 10452 2385 10449 2374 10448 2364 10449 2353 10452 2343
10457 2333 10464 2324 10472 2317 10481 2311 10491 2308 10501 2306
10512 2308 10522 2311 10531 2317 10539 2324 10545 2333 10550 2343
10554 2353 10555 2364 10587 2366 10586 2350 10582 2335 10576 2320
10571 2313 10566 2307 10561 2300 10554 2295 10547 2290 10540 2286
10531 2282 10522 2280 10512 2278 10501 2278 10489 2278 10479 2280
10469 2283 10460 2288 10452 2293 10445 2300 10438 2307 10433 2315
10428 2325 10424 2335 10421 2345 10418 2357 10414 2381 10413 2408
10414 2424 10415 2439 10417 2453 10420 2466 10423 2478 10427 2490
10432 2500 10438 2510 10444 2518 10451 2526 10459 2532 10467 2538
10476 2542 10486 2545 10496 2547 10507 2547 10522 2546 10536 2542
10548 2536 10558 2528 10567 2518 10574 2506 10579 2494 10581 2481
126 N F
10854 2547 10864 2547 10873 2544 10881 2541 10889 2536 10897 2530
10904 2522 10910 2514 10916 2505 10922 2495 10926 2485 10934 2462
10938 2438 10940 2414 10938 2389 10934 2365 10926 2342 10922 2331
10916 2321 10910 2312 10904 2303 10897 2296 10889 2290 10881 2285
10873 2281 10864 2278 10854 2278 10845 2278 10836 2281 10827 2285
10819 2290 10811 2296 10804 2303 10798 2312 10792 2321 10786 2331

10782 2342 10774 2365 10770 2389 10768 2414 10802 2414 10804 2390
10807 2369 10812 2351 10819 2336 10827 2323 10836 2314 10840 2311
10845 2308 10849 2307 10854 2306 10859 2307 10863 2308 10868 2311
10873 2314 10881 2323 10889 2336 10896 2351 10901 2369 10904 2390
10906 2414 10904 2437 10901 2457 10896 2475 10889 2491 10881 2503
10873 2511 10868 2515 10863 2517 10859 2518 10854 2519 10849 2518
10845 2517 10840 2515 10836 2511 10827 2503 10819 2491 10812 2475
10807 2457 10804 2437 10802 2414 10768 2414 10770 2438 10774 2462
10782 2485 10786 2495 10792 2505 10798 2514 10804 2522 10811 2530
10819 2536 10827 2541 10836 2544 10845 2547 10854 2547 95 N F
10990 2325 11028 2325 11028 2287 10990 2287 10990 2325 5 N F
11160 2547 11169 2547 11178 2546 11187 2544 11195 2541 11203 2537
11210 2532 11216 2526 11222 2519 11228 2511 11233 2502 11237 2491
11241 2480 11243 2467 11246 2453 11247 2438 11248 2421 11247 2401
11246 2382 11243 2365 11239 2350 11235 2337 11230 2325 11224 2315
11217 2306 11210 2299 11203 2293 11195 2288 11187 2284 11178 2281
11170 2279 11152 2278 11139 2279 11127 2282 11115 2287 11105 2294
11096 2304 11088 2316 11082 2330 11078 2347 11110 2347 11113 2337
11117 2329 11121 2322 11126 2316 11132 2312 11139 2309 11146 2307
11154 2306 11161 2307 11168 2308 11175 2311 11180 2314 11186 2318
11191 2323 11199 2335 11205 2350 11210 2366 11212 2384 11213 2404
11204 2395 11196 2388 11188 2383 11180 2380 11173 2377 11166 2376
11154 2375 11144 2376 11135 2378 11126 2380 11118 2383 11111 2387
11104 2392 11098 2397 11092 2403 11088 2409 11084 2415 11078 2430
11074 2445 11074 2461 11106 2461 11107 2451 11110 2441 11115 2431
11121 2423 11129 2415 11138 2409 11148 2405 11160 2404 11171 2405
11182 2409 11191 2415 11199 2423 11205 2431 11210 2441 11212 2451
11213 2461 11212 2472 11210 2482 11205 2491 11199 2500 11191 2508
11182 2514 11171 2517 11160 2519 11148 2517 11138 2514 11129 2508
11121 2500 11115 2491 11110 2482 11107 2472 11106 2461 11074 2461
11074 2470 11075 2478 11077 2486 11079 2494 11082 2502 11086 2509
11091 2515 11096 2522 11102 2527 11109 2532 11116 2537 11124 2540
11132 2543 11141 2546 11150 2547 11160 2547 130 N F
11364 2547 11380 2546 11395 2543 11408 2537 11420 2530 11430 2520
11433 2514 11437 2508 11439 2501 11441 2494 11442 2487 11443 2479
11442 2467 11440 2458 11436 2449 11431 2442 11426 2436 11420 2431
11408 2423 11418 2418 11426 2412 11434 2405 11440 2397 11445 2388
11449 2379 11452 2369 11452 2358 11451 2345 11447 2332 11439 2318
11430 2306 11424 2300 11417 2294 11410 2290 11401 2286 11393 2282
11383 2280 11373 2278 11362 2278 11352 2278 11342 2280 11333 2282
11324 2286 11316 2290 11309 2294 11303 2300 11297 2306 11288 2318
11282 2332 11278 2345 11276 2358 11311 2358 11312 2348 11315 2338
11320 2329 11327 2321 11335 2315 11344 2310 11354 2307 11364 2306

11375 2307 11385 2310 11394 2315 11402 2321 11409 2329 11414 2338
11417 2348 11418 2358 11417 2368 11414 2378 11409 2387 11402 2395
11394 2401 11385 2406 11375 2409 11364 2410 11354 2409 11344 2406
11335 2401 11327 2395 11320 2387 11315 2378 11312 2368 11311 2358
11276 2358 11277 2368 11280 2378 11283 2388 11288 2396 11295 2404
11302 2411 11311 2418 11320 2423 11309 2431 11303 2436 11297 2442
11293 2449 11289 2458 11287 2467 11286 2479 11319 2479 11319 2470
11322 2462 11326 2455 11332 2449 11339 2445 11347 2441 11355 2439
11364 2438 11373 2439 11382 2441 11390 2445 11397 2449 11402 2455
11407 2462 11409 2470 11410 2479 11409 2487 11407 2495 11402 2502
11397 2508 11390 2513 11382 2516 11373 2518 11364 2519 11355 2518
11347 2516 11339 2513 11332 2508 11326 2502 11322 2495 11319 2487
11319 2479 11286 2479 11286 2487 11288 2494 11289 2501 11292 2508
11295 2514 11299 2520 11309 2530 11320 2537 11334 2543 11348 2546
11364 2547 145 N F
11999 2543 12020 2543 12020 2282 11988 2282 11988 2468 11930 2468
11930 2491 11954 2494 11964 2496 11973 2500 11982 2505 11989 2514
11995 2526 11999 2543 14 N F
Gr
S
%%Trailer
%%DocumentFonts:
%%Pages: 1
%%EOF

Regional loss of dry matter in T1 (April)
%%Pages: (atend)
%%PageOrder: Ascend
%%Creator: NCAR GKS
%%DocumentFonts: (atend)
%%EndComments
%%BeginProlog

/M3
{
  2 copy lt {pop} {exch pop} ifelse
  2 copy lt {pop} {exch pop} ifelse
} bind def
/Sc
{
  1. exch sub /ylo exch def
  1. exch sub /mag exch def
  1. exch sub /cyn exch def

```
  ylo mag cyn M3 /b_cmy exch def
  cyn b_cmy sub /c_cmy exch def
  mag b_cmy sub /m_cmy exch def
  ylo b_cmy sub /y_cmy exch def
  c_cmy m_cmy y_cmy b_cmy setcmykcolor
} bind def
/P
{/n exch def
  newpath
  moveto
  2 1 n {pop lineto} for
  stroke
 } bind def

/F
{/n exch def
  newpath
  moveto
  2 1 n {pop lineto} for
  closepath
  eofill
} bind def

/B
{ /s exch def
  /n exch def
  1 1 n {newpath pop s 0 360 arc stroke} for
} bind def

/Q
{ /s exch def
  /n exch def
  /ms 0 s sub def
  /ts s s add def
  1 1 n {gsave newpath pop translate
  0 0 moveto ms 0 rmoveto ts 0 rlineto
  0 0 moveto 90 rotate ms  0 rmoveto ts 0 rlineto
  stroke grestore} for
} bind def

/Fs
{ /s exch def
  findfont
```

```
  s scalefont
  setfont
} bind def

/V
{ /s exch def
  /n exch def
  /ms 0 s sub def
  /ts s s add def
  1 1 n {gsave newpath pop translate
  0 0 moveto 30 rotate ms 0 rmoveto  ts 0 rlineto
  0 0 moveto 60 rotate ms 0 rmoveto  ts 0 rlineto
  0 0 moveto 60 rotate ms 0 rmoveto  ts 0 rlineto
  stroke grestore} for
} bind def

/X
{ /s exch def
  /n exch def
  /s s 1.25 mul def
  /ms 0 s sub def
  /ts s s add def
  1 1 n {gsave newpath pop translate
  0 0 moveto 45 rotate ms 0 rmoveto  ts 0 rlineto
  0 0 moveto 90 rotate ms 0 rmoveto  ts 0 rlineto
  stroke grestore} for
} bind def

/Ls
{matrix currentmatrix
 currentrgbcolor
 currentlinewidth
 currentlinecap
 currentlinejoin
 currentdash
} bind def

/Lr
{setdash
 setlinejoin
 setlinecap
 setlinewidth
 setrgbcolor
```

```
 setmatrix
} bind def

/M {stroke newpath moveto} bind def
/L {lineto} bind def
/E {eofill} def
/J {setlinejoin} def
/C {setlinecap} def
/W {setlinewidth} def
/S {stroke showpage} def
/A {scale} def
/U {put} def
/D {setdash} def
/I {currentdash} def
/H {currentlinewidth} def
/G {currentrgbcolor} def
/R {setrgbcolor} def
/N {closepath} def
/K {stroke} def
/Z {rotate} def
/As {ashow} def
/Ch {clippath} def
/Ci {colorimage} def
/Co {concat} def
/Cm {currentmatrix} def
/Cp {clip} def
/Ct {currentpoint} def
/Cu {currentfile} def
/Ex {exch} def
/Im {image} def
/In {initgraphics} def
/Ml {setmiterlimit} def
/Mx {matrix} def
/Np {newpath} def
/Ng {neg} def
/Rh {readhexstring} def
/Rm {rmoveto} def
/Sm {setmatrix} def
/Tr {translate} def
/Gs {gsave} def
/Gr {grestore} def

/Ov
```

```
{/strn exch def
 /ispc exch def
 /str1 1 string def
  strn
  {/code exch def
   /chr str1 0 code put str1 def
   0 ispc neg rmoveto
   gsave
   chr stringwidth pop 2 div neg
   0 rmoveto
   chr false charpath stroke
   grestore
  } forall
} bind def

/Oh
{/strn exch def
 /ispc exch def
 /tstr 1 string def
 strn
   {
    /code exch def
    /chr tstr 0 code put tstr def
    chr false charpath
    currentpoint
    /yt exch def /xt exch def
    stroke
    xt ispc add yt moveto
   } forall
 } bind def

/T 256 array def
/O
{ T
  exch
  get
  aload
  pop
  setrgbcolor
} bind def

/Y
{/strn exch def
```

```
 /ispc exch def
 /str1 1 string def
  strn
  {/code exch def
   /chr str1 0 code put str1 def
    0 ispc neg rmoveto
    gsave
      chr stringwidth pop 2 div neg 0 rmoveto
      chr show
    grestore
  } forall
} def

/Bg
{/ury Ex def /urx Ex def /lly Ex def /llx Ex def
 Np Ls Gr Gs 0 O Ch E Lr
 llx lly M urx lly L urx ury L
 llx ury L llx lly L
 Cp Np
} def

/Oc
{/ury exch def
 /urx exch def
 /lly exch def
 /llx exch def
 Np
 llx lly M urx lly L urx ury L  llx ury L llx lly L
 Cp Np
} def

/Cc
{/CellWidth exch def
 /strn CellWidth 1 add string def
 /strn1 CellWidth 1 add 3 mul string def
 systemdict /colorimage known
 { { Cu strn1 Rh pop } false 3 Ci}
 { { Cu strn1 Rh pop
    /ColorStr exch def
    0 1 CellWidth {
      /YIndx exch def
      /GrayVal YIndx 3 mul def
      strn YIndx
```

```
        ColorStr GrayVal       get 0.30 mul
        ColorStr GrayVal 1 add get 0.59 mul
        ColorStr GrayVal 2 add get 0.11 mul
        add add cvi
      put
    } for
    strn
  } Im
 } ifelse
} def

/Ld
{/ury exch def /urx exch def  /lly exch def /llx exch def
 -90 rotate
 lly urx add neg llx lly sub translate
} bind def
/Pt
{/ury exch def /urx exch def  /lly exch def /llx exch def
 90 rotate
 lly urx add lly llx sub translate
} bind def
%%EndProlog

%%BeginSetup
<</PageSize [00612 00792]>> setpagedevice
%%EndSetup

[1. 1. 1.] [0. 0. 0.] [1. 0. 0.] [0. 1. 0.] [0. 0. 1.] [0. 1. 1.] [1. 0. .8]
[1. 1. 0.] [1. .5 0.] [.6 .8 0.] [0. 1. .6] [0. .5 1.] [.5 0. .8] [1. 0. .6]
[.3 .3 .3] [.7 .7 .7] [1. 1. .3] [.7 1. .5] [.5 1. .6] [.2 1. .7] [.3 .8 .8]
[.5 .7 .8] [.7 .5 .8] [1. .3 .9] [.7 .9 .5] [.4 .9 .5] [.2 .9 .7] [.2 .7 .9]
[.2 .5 1.] [.5 .3 1.] [.7 .2 1.] [.9 .1 1.] [.9 1. .2] [.7 1. .3] [.5 1. .3]
[.2 1. .5] [.2 .8 .6] [.2 .7 .7] [.2 .5 .8] [.2 .4 .9] [.4 .3 .9] [.7 .2 .9]
[.8 .2 .8] [.9 .3 .7] [.8 .4 .6] [.8 .6 .5] [.9 .7 .4] [.9 .7 .3] [1. .9 .9]
[.8 1. .1] [.6 1. .2] [.5 1. .2] [.2 .9 .5] [.2 .8 .5] [.2 .7 .7] [.2 .5 .7]
[.2 .4 .9] [.4 .3 .9] [.5 .2 .9] [.8 .2 .7] [1. .2 .7] [1. .3 .6] [1. .4 .5]
[1. .5 .4] [1. .8 .1] [.7 1. .0] [.6 1. .1] [.4 1. .2] [.1 1. .3] [.1 .8 .4]
[.2 .7 .5] [.1 .6 .7] [.1 .5 .7] [.1 .4 .8] [.2 .3 .9] [.2 .2 1.] [.3 .1 1.]
[.5 .1 .9] [.7 .0 .8] [.9 .0 .7] [.9 .7 .1] [.7 .9 .1] [.5 .9 .1] [.3 .9 .2]
[.1 .9 .3] [.2 .7 .4] [.1 .7 .5] [.1 .5 .6] [.1 .4 .7] [.2 .3 .7] [.2 .2 .8]
[.3 .2 .8] [.5 .1 .7] [.7 .1 .7] [.9 .2 .5] [.8 .2 .5] [.8 .7 .0] [.6 .8 .1]
[.4 .9 .1] [.3 .9 .1] [.1 .9 .2] [.2 .8 .2] [.2 .7 .3] [.2 .6 .5] [.1 .5 .5]
[.1 .4 .6] [.2 .3 .7] [.3 .2 .7] [.4 .2 .6] [.7 .2 .5] [.8 .2 .4] [1. .2 .3]
```

```
[.7 .7 .0] [.5 .7 .1] [.4 .8 .1] [.2 .9 .1] [.2 .9 .1] [.2 .8 .2] [.1 .7 .2]
[.1 .7 .3] [.1 .6 .4] [.1 .4 .5] [.1 .4 .5] [.2 .3 .5] [.4 .3 .4] [.5 .3 .4]
[.7 .3 .3] [.8 .3 .2] [.7 .6 .0] [.5 .7 .1] [.4 .7 .1] [.2 .7 .2] [.1 .7 .2]
[.1 .6 .3] [.1 .5 .4] [.1 .4 .4] [.1 .3 .5] [.1 .2 .7] [.1 .1 .7] [.4 .1 .5]
[.5 .1 .5] [.6 .1 .4] [.7 .1 .4] [.9 .1 .3] [.5 .5 .1] [.4 .6 .1] [.3 .6 .1]
[.2 .6 .2] [.1 .5 .2] [.1 .4 .3] [.1 .4 .4] [.2 .3 .4] [.2 .2 .4] [.2 .2 .5]
[.2 .1 .5] [.3 .1 .5] [.4 .1 .4] [.5 .1 .4] [.7 .1 .3] [.8 .1 .2] [.5 .4 .1]
[.4 .5 .1] [.3 .6 .0] [.2 .6 .1] [.1 .7 .1] [.1 .6 .1] [.1 .5 .2] [.1 .4 .2]
[.1 .4 .2] [.1 .3 .3] [.1 .2 .4] [.1 .2 .5] [.1 .1 .5] [.1 .0 .6] [.3 .0 .5]
[.5 .0 .4] [.7 .3 .0] [.7 .4 .0] [.5 .4 .1] [.3 .4 .2] [.2 .3 .2] [.2 .2 .3]
[.2 .2 .4] [.3 .1 .4] [.5 .1 .3] [.6 .1 .2] [.7 .1 .2] [.8 .0 .2] [1. .0 .2]
[1. .1 .1] [.9 .2 .1] [.8 .2 .1] [.6 .2 .0] [.4 .4 .0] [.2 .4 .1] [.2 .3 .2]
[.2 .2 .2] [.2 .2 .3] [.2 .1 .3] [.3 .1 .3] [.5 .1 .2] [.7 .1 .2] [.8 .0 .2]
[.9 .0 .1] [1. .0 .1] [1. .1 .1] [.8 .2 .0] [.7 .2 .0] [.5 .2 .0] [.4 .3 .0]
[.1 .4 .1] [.1 .4 .1] [.1 .3 .2] [.1 .2 .2] [.1 .2 .3] [.2 .1 .4] [.2 .0 .3]
[.4 .0 .2] [.6 .0 .2] [.7 .0 .1] [.8 .0 .1] [.7 .1 .1] [.7 .1 .1] [.6 .2 .0]
[.4 .2 .0] [.2 .3 .0] [.1 .3 .0] [.1 .2 .1] [.1 .2 .1] [.1 .1 .2] [.1 .1 .3]
[.1 .0 .3] [.1 .0 .2] [.2 .0 .2] [.3 .0 .2] [.3 .0 .1] [.4 .0 .1] [.4 .1 .1]
[.4 .1 .0] [.2 .1 .0] [.0 .0 .0] [.0 .0 .0] [.0 .0 .0] [.0 .0 .0] [.0 .0 .0]
[.0 .0 .0] [.0 .0 .0] [.0 .0 .0] [.0 .0 .0] [.0 .0 .0] [.0 .0 .0] [.0 .0 .0]
[.0 .0 .0] [.0 .0 .0] [.0 .0 .0] [.0 .0 .0]  T astore pop
%%Page: 1 1
0.04000 0.04000 A 1 J 1 C 12 W [] 0 D
T   0 [1.000 1.000 1.000] U
T   1 [0.000 0.000 0.000] U
T   2 [0.000 0.000 1.000] U
T   3 [0.000 0.031 1.000] U
T   4 [0.000 0.059 1.000] U
T   5 [0.000 0.090 1.000] U
T   6 [0.000 0.122 1.000] U
T   7 [0.000 0.153 1.000] U
T   8 [0.000 0.180 1.000] U
T   9 [0.000 0.212 1.000] U
T  10 [0.000 0.243 1.000] U
T  11 [0.000 0.275 1.000] U
T  12 [0.000 0.302 1.000] U
T  13 [0.000 0.333 1.000] U
T  14 [0.000 0.365 1.000] U
T  15 [0.000 0.392 1.000] U
T  16 [0.000 0.424 1.000] U
T  17 [0.000 0.455 1.000] U
T  18 [0.000 0.486 1.000] U
T  19 [0.000 0.514 1.000] U
```

T 20 [0.000 0.545 1.000] U
T 21 [0.000 0.576 1.000] U
T 22 [0.000 0.608 1.000] U
T 23 [0.000 0.635 1.000] U
T 24 [0.000 0.667 1.000] U
T 25 [0.000 0.698 1.000] U
T 26 [0.000 0.725 1.000] U
T 27 [0.000 0.757 1.000] U
T 28 [0.000 0.788 1.000] U
T 29 [0.000 0.820 1.000] U
T 30 [0.000 0.847 1.000] U
T 31 [0.000 0.878 1.000] U
T 32 [0.000 0.910 1.000] U
T 33 [0.000 0.941 1.000] U
T 34 [0.000 0.969 1.000] U
T 35 [0.000 1.000 1.000] U
T 36 [0.000 0.992 0.973] U
T 37 [0.000 0.980 0.941] U
T 38 [0.004 0.973 0.914] U
T 39 [0.004 0.965 0.886] U
T 40 [0.004 0.953 0.859] U
T 41 [0.004 0.945 0.827] U
T 42 [0.008 0.937 0.800] U
T 43 [0.008 0.929 0.773] U
T 44 [0.008 0.918 0.745] U
T 45 [0.008 0.910 0.714] U
T 46 [0.012 0.902 0.686] U
T 47 [0.012 0.890 0.659] U
T 48 [0.012 0.882 0.627] U
T 49 [0.012 0.875 0.600] U
T 50 [0.016 0.863 0.573] U
T 51 [0.016 0.855 0.545] U
T 52 [0.016 0.847 0.514] U
T 53 [0.016 0.839 0.486] U
T 54 [0.020 0.827 0.459] U
T 55 [0.020 0.820 0.431] U
T 56 [0.020 0.812 0.400] U
T 57 [0.020 0.800 0.373] U
T 58 [0.024 0.792 0.345] U
T 59 [0.024 0.784 0.314] U
T 60 [0.024 0.773 0.286] U
T 61 [0.024 0.765 0.259] U
T 62 [0.027 0.757 0.231] U

T 63 [0.027 0.749 0.200] U
T 64 [0.027 0.737 0.173] U
T 65 [0.027 0.729 0.145] U
T 66 [0.031 0.722 0.118] U
T 67 [0.031 0.710 0.086] U
T 68 [0.031 0.702 0.059] U
T 69 [0.059 0.710 0.059] U
T 70 [0.090 0.718 0.055] U
T 71 [0.118 0.729 0.055] U
T 72 [0.145 0.737 0.051] U
T 73 [0.173 0.745 0.051] U
T 74 [0.204 0.753 0.047] U
T 75 [0.231 0.765 0.047] U
T 76 [0.259 0.773 0.043] U
T 77 [0.286 0.780 0.043] U
T 78 [0.318 0.788 0.043] U
T 79 [0.345 0.800 0.039] U
T 80 [0.373 0.808 0.039] U
T 81 [0.400 0.816 0.035] U
T 82 [0.431 0.824 0.035] U
T 83 [0.459 0.835 0.031] U
T 84 [0.486 0.843 0.031] U
T 85 [0.518 0.851 0.031] U
T 86 [0.545 0.859 0.027] U
T 87 [0.573 0.867 0.027] U
T 88 [0.600 0.878 0.024] U
T 89 [0.631 0.886 0.024] U
T 90 [0.659 0.894 0.020] U
T 91 [0.686 0.902 0.020] U
T 92 [0.714 0.914 0.016] U
T 93 [0.745 0.922 0.016] U
T 94 [0.773 0.929 0.016] U
T 95 [0.800 0.937 0.012] U
T 96 [0.827 0.949 0.012] U
T 97 [0.859 0.957 0.008] U
T 98 [0.886 0.965 0.008] U
T 99 [0.914 0.973 0.004] U
T 100 [0.941 0.984 0.004] U
T 101 [0.973 0.992 0.000] U
T 102 [1.000 1.000 0.000] U
T 103 [1.000 0.988 0.000] U
T 104 [1.000 0.980 0.000] U
T 105 [1.000 0.969 0.000] U

T 106 [1.000 0.957 0.000] U
T 107 [1.000 0.945 0.000] U
T 108 [1.000 0.937 0.000] U
T 109 [1.000 0.925 0.000] U
T 110 [1.000 0.914 0.000] U
T 111 [1.000 0.902 0.000] U
T 112 [1.000 0.894 0.000] U
T 113 [1.000 0.882 0.000] U
T 114 [1.000 0.871 0.000] U
T 115 [1.000 0.863 0.000] U
T 116 [1.000 0.851 0.000] U
T 117 [1.000 0.839 0.000] U
T 118 [1.000 0.827 0.000] U
T 119 [1.000 0.820 0.000] U
T 120 [1.000 0.808 0.000] U
T 121 [1.000 0.796 0.000] U
T 122 [1.000 0.784 0.000] U
T 123 [1.000 0.776 0.000] U
T 124 [1.000 0.765 0.000] U
T 125 [1.000 0.753 0.000] U
T 126 [1.000 0.745 0.000] U
T 127 [1.000 0.733 0.000] U
T 128 [1.000 0.722 0.000] U
T 129 [1.000 0.710 0.000] U
T 130 [1.000 0.702 0.000] U
T 131 [1.000 0.690 0.000] U
T 132 [1.000 0.678 0.000] U
T 133 [1.000 0.667 0.000] U
T 134 [1.000 0.659 0.000] U
T 135 [1.000 0.647 0.000] U
T 136 [1.000 0.627 0.000] U
T 137 [1.000 0.608 0.000] U
T 138 [1.000 0.588 0.000] U
T 139 [1.000 0.569 0.000] U
T 140 [1.000 0.549 0.000] U
T 141 [1.000 0.529 0.000] U
T 142 [1.000 0.510 0.000] U
T 143 [1.000 0.490 0.000] U
T 144 [1.000 0.471 0.000] U
T 145 [1.000 0.451 0.000] U
T 146 [1.000 0.431 0.000] U
T 147 [1.000 0.412 0.000] U
T 148 [1.000 0.392 0.000] U

T 149 [1.000 0.373 0.000] U
T 150 [1.000 0.353 0.000] U
T 151 [1.000 0.333 0.000] U
T 152 [1.000 0.314 0.000] U
T 153 [1.000 0.294 0.000] U
T 154 [1.000 0.275 0.000] U
T 155 [1.000 0.255 0.000] U
T 156 [1.000 0.235 0.000] U
T 157 [1.000 0.216 0.000] U
T 158 [1.000 0.196 0.000] U
T 159 [1.000 0.176 0.000] U
T 160 [1.000 0.157 0.000] U
T 161 [1.000 0.137 0.000] U
T 162 [1.000 0.118 0.000] U
T 163 [1.000 0.098 0.000] U
T 164 [1.000 0.078 0.000] U
T 165 [1.000 0.059 0.000] U
T 166 [1.000 0.039 0.000] U
T 167 [1.000 0.020 0.000] U
T 168 [1.000 0.000 0.000] U
T 169 [0.980 0.000 0.016] U
T 170 [0.961 0.000 0.031] U
T 171 [0.941 0.000 0.047] U
T 172 [0.922 0.000 0.063] U
T 173 [0.902 0.000 0.082] U
T 174 [0.878 0.000 0.098] U
T 175 [0.859 0.000 0.114] U
T 176 [0.839 0.000 0.129] U
T 177 [0.820 0.000 0.145] U
T 178 [0.800 0.000 0.161] U
T 179 [0.780 0.000 0.176] U
T 180 [0.761 0.000 0.192] U
T 181 [0.741 0.000 0.212] U
T 182 [0.722 0.000 0.227] U
T 183 [0.702 0.000 0.243] U
T 184 [0.682 0.000 0.259] U
T 185 [0.659 0.000 0.275] U
T 186 [0.639 0.000 0.290] U
T 187 [0.620 0.000 0.306] U
T 188 [0.600 0.000 0.322] U
T 189 [0.580 0.000 0.341] U
T 190 [0.561 0.000 0.357] U
T 191 [0.541 0.000 0.373] U

T 192 [0.522 0.000 0.388] U
T 193 [0.502 0.000 0.404] U
T 194 [0.482 0.000 0.420] U
T 195 [0.463 0.000 0.435] U
T 196 [0.439 0.000 0.451] U
T 197 [0.420 0.000 0.471] U
T 198 [0.400 0.000 0.486] U
T 199 [0.380 0.000 0.502] U
T 200 [0.361 0.000 0.518] U
T 201 [0.341 0.000 0.533] U
-1200 1025 16500 18750 Oc
Gs
1 O
0 O
2300 16170 M 2300 5455 L 2333 5455 L 2366 5455 L 2399 5455 L
2432 5455 L 2466 5455 L 2499 5455 L 2532 5455 L
2565 5455 L 2599 5455 L 2632 5455 L 2665 5455 L
2698 5455 L 2732 5455 L 2765 5455 L 2798 5455 L
2831 5455 L 2864 5455 L 2898 5455 L 2927 5455 L
2926 5459 L 2917 5486 L 2938 5506 L 2967 5505 L
2985 5482 L 2967 5459 L 2967 5455 L 2997 5455 L
3031 5455 L 3064 5455 L 3097 5455 L 3130 5455 L
3164 5455 L 3197 5455 L 3230 5455 L 3263 5455 L
3296 5455 L 3330 5455 L 3363 5455 L 3396 5455 L
3429 5455 L 3463 5455 L 3496 5455 L 3529 5455 L
3562 5455 L 3595 5455 L 3629 5455 L 3662 5455 L
3695 5455 L 3728 5455 L 3762 5455 L 3795 5455 L
3828 5455 L 3861 5455 L 3895 5455 L 3928 5455 L
3961 5455 L 3994 5455 L 4027 5455 L 4061 5455 L
4094 5455 L 4127 5455 L 4160 5455 L 4194 5455 L
4227 5455 L 4260 5455 L 4293 5455 L 4327 5455 L
4360 5455 L 4393 5455 L 4426 5455 L 4459 5455 L
4493 5455 L 4526 5455 L 4559 5455 L 4592 5455 L
4626 5455 L 4659 5455 L 4692 5455 L 4725 5455 L
4759 5455 L 4792 5455 L 4825 5455 L 4858 5455 L
4891 5455 L 4925 5455 L 4958 5455 L 4991 5455 L
5024 5455 L 5058 5455 L 5091 5455 L 5124 5455 L
5157 5455 L 5191 5455 L 5224 5455 L 5257 5455 L
5290 5455 L 5323 5455 L 5357 5455 L 5390 5455 L
5395 5455 L 5407 5466 L 5406 5495 L 5402 5524 L
5404 5553 L 5404 5582 L 5393 5609 L 5369 5625 L
5352 5648 L 5381 5644 L 5387 5616 L 5416 5614 L
5416 5585 L 5411 5557 L 5410 5528 L 5412 5499 L

5430 5476 L 5430 5455 L 5456 5455 L 5490 5455 L
5523 5455 L 5532 5455 L 5539 5469 L 5540 5475 L
5544 5482 L 5551 5487 L 5537 5525 L 5529 5553 L
5547 5530 L 5556 5503 L 5573 5479 L 5592 5457 L
5594 5455 L 5622 5455 L 5644 5455 L 5645 5472 L
5649 5455 L 5656 5455 L 5657 5455 L 5665 5470 L
5675 5497 L 5689 5523 L 5683 5551 L 5659 5567 L
5667 5595 L 5681 5621 L 5681 5650 L 5666 5674 L
5689 5691 L 5674 5715 L 5654 5737 L 5676 5717 L
5703 5726 L 5686 5750 L 5670 5774 L 5650 5795 L
5674 5779 L 5702 5786 L 5688 5811 L 5664 5827 L
5654 5855 L 5627 5866 L 5598 5867 L 5627 5872 L
5655 5876 L 5661 5905 L 5662 5934 L 5645 5957 L
5617 5961 L 5600 5985 L 5627 5975 L 5654 5965 L
5679 5950 L 5677 5921 L 5684 5893 L 5705 5873 L
5723 5896 L 5711 5870 L 5706 5841 L 5712 5830 L
5721 5816 L 5739 5794 L 5745 5765 L 5772 5776 L
5790 5754 L 5797 5725 L 5799 5722 L 5811 5700 L
5823 5674 L 5842 5652 L 5859 5628 L 5853 5600 L
5871 5622 L 5898 5632 L 5926 5625 L 5955 5622 L
5928 5612 L 5902 5598 L 5880 5580 L 5860 5559 L
5880 5538 L 5909 5534 L 5938 5535 L 5967 5536 L
5986 5514 L 6014 5506 L 6043 5507 L 6069 5495 L
6050 5517 L 6058 5545 L 6085 5534 L 6113 5542 L
6119 5571 L 6090 5565 L 6074 5588 L 6051 5570 L
6023 5561 L 6008 5586 L 6036 5590 L 6065 5592 L
6087 5611 L 6061 5624 L 6035 5637 L 6057 5656 L
6085 5663 L 6113 5670 L 6141 5663 L 6159 5640 L
6168 5612 L 6172 5584 L 6200 5590 L 6228 5597 L
6234 5626 L 6207 5636 L 6181 5650 L 6208 5660 L
6209 5689 L 6180 5689 L 6202 5708 L 6216 5734 L
6242 5746 L 6246 5748 L 6256 5751 L 6269 5755 L
6296 5768 L 6324 5762 L 6345 5782 L 6337 5754 L
6314 5736 L 6317 5707 L 6314 5678 L 6325 5651 L
6354 5652 L 6382 5648 L 6403 5628 L 6399 5656 L
6415 5680 L 6431 5705 L 6408 5723 L 6392 5747 L
6417 5732 L 6430 5706 L 6458 5710 L 6472 5735 L
6487 5760 L 6515 5767 L 6544 5767 L 6569 5782 L
6599 5771 L 6577 5763 L 6575 5753 L 6595 5754 L
6614 5755 L 6640 5742 L 6665 5728 L 6637 5727 L
6648 5700 L 6677 5693 L 6705 5699 L 6731 5685 L
6757 5673 L 6754 5702 L 6768 5727 L 6747 5707 L
6730 5731 L 6747 5754 L 6764 5777 L 6785 5797 L

6814 5799 L 6838 5783 L 6866 5775 L 6882 5750 L
6899 5727 L 6921 5747 L 6949 5754 L 6976 5764 L
7003 5775 L 7027 5791 L 7029 5820 L 7046 5796 L
7031 5772 L 7060 5771 L 7082 5790 L 7102 5811 L
7125 5828 L 7149 5844 L 7172 5826 L 7201 5821 L
7229 5828 L 7258 5827 L 7266 5854 L 7287 5874 L
7284 5903 L 7277 5931 L 7291 5957 L 7296 5985 L
7271 5971 L 7268 5999 L 7297 6002 L 7320 5984 L
7341 5964 L 7358 5988 L 7379 5999 L 7361 6015 L
7343 6030 L 7315 6037 L 7292 6055 L 7279 6080 L
7273 6109 L 7245 6113 L 7274 6113 L 7294 6092 L
7317 6074 L 7343 6062 L 7349 6062 L 7372 6062 L
7401 6062 L 7422 6085 L 7434 6099 L 7455 6119 L
7454 6130 L 7455 6156 L 7469 6182 L 7461 6205 L
7487 6216 L 7507 6221 L 7515 6224 L 7527 6217 L
7541 6210 L 7532 6238 L 7559 6227 L 7564 6199 L
7572 6171 L 7590 6186 L 7594 6190 L 7617 6207 L
7641 6223 L 7641 6252 L 7659 6229 L 7651 6201 L
7669 6224 L 7671 6253 L 7676 6281 L 7687 6308 L
7711 6291 L 7724 6307 L 7732 6307 L 7736 6307 L
7759 6291 L 7776 6313 L 7786 6341 L 7785 6369 L
7757 6376 L 7733 6393 L 7762 6390 L 7790 6384 L
7818 6376 L 7840 6357 L 7849 6330 L 7840 6302 L
7838 6273 L 7855 6297 L 7857 6325 L 7860 6354 L
7871 6380 L 7895 6397 L 7891 6426 L 7909 6448 L
7937 6457 L 7946 6430 L 7930 6407 L 7925 6378 L
7937 6404 L 7954 6428 L 7977 6445 L 8001 6460 L
8006 6488 L 8024 6511 L 8013 6537 L 8034 6558 L
8054 6538 L 8077 6556 L 8098 6575 L 8126 6581 L
8120 6609 L 8106 6634 L 8077 6634 L 8074 6663 L
8046 6660 L 8017 6657 L 8000 6661 L 7974 6674 L
7952 6692 L 7941 6704 L 7942 6720 L 7944 6717 L
7954 6704 L 7968 6707 L 7980 6708 L 7997 6709 L
8024 6699 L 8035 6696 L 8051 6691 L 8074 6708 L
8079 6736 L 8054 6749 L 8082 6750 L 8092 6777 L
8109 6754 L 8110 6757 L 8123 6779 L 8125 6808 L
8154 6808 L 8165 6782 L 8157 6754 L 8184 6747 L
8185 6747 L 8198 6773 L 8225 6765 L 8226 6765 L
8253 6770 L 8274 6782 L 8279 6784 L 8273 6812 L
8295 6830 L 8285 6803 L 8310 6790 L 8336 6777 L
8361 6763 L 8360 6734 L 8385 6748 L 8405 6768 L
8394 6794 L 8395 6823 L 8424 6820 L 8437 6845 L
8441 6874 L 8425 6898 L 8403 6880 L 8382 6900 L

8378 6929 L 8355 6945 L 8381 6934 L 8409 6940 L
8402 6968 L 8395 6995 L 8416 6975 L 8417 6947 L
8429 6972 L 8442 6947 L 8437 6919 L 8464 6927 L
8483 6948 L 8511 6944 L 8538 6934 L 8562 6948 L
8534 6955 L 8530 6983 L 8558 6985 L 8587 6984 L
8574 7009 L 8575 7038 L 8562 7012 L 8534 7020 L
8510 7035 L 8501 7062 L 8529 7069 L 8545 7093 L
8562 7116 L 8541 7136 L 8522 7157 L 8548 7168 L
8577 7170 L 8605 7164 L 8588 7140 L 8614 7152 L
8611 7124 L 8601 7097 L 8629 7093 L 8655 7081 L
8655 7081 L 8641 7106 L 8632 7133 L 8648 7157 L
8649 7129 L 8673 7112 L 8701 7114 L 8730 7118 L
8736 7146 L 8753 7151 L 8764 7153 L 8744 7174 L
8727 7197 L 8704 7181 L 8677 7171 L 8687 7198 L
8663 7213 L 8640 7230 L 8619 7249 L 8646 7258 L
8669 7275 L 8694 7289 L 8713 7310 L 8729 7334 L
8741 7360 L 8747 7356 L 8764 7344 L 8754 7317 L
8780 7305 L 8808 7301 L 8813 7273 L 8819 7265 L
8830 7250 L 8827 7221 L 8842 7246 L 8852 7273 L
8879 7264 L 8880 7236 L 8881 7207 L 8889 7214 L
8902 7226 L 8902 7226 L 8908 7254 L 8901 7282 L
8874 7292 L 8868 7320 L 8854 7345 L 8829 7332 L
8821 7360 L 8807 7385 L 8824 7407 L 8850 7418 L
8878 7423 L 8883 7432 L 8892 7448 L 8884 7476 L
8882 7504 L 8891 7531 L 8909 7532 L 8919 7533 L
8928 7560 L 8935 7588 L 8906 7591 L 8881 7603 L
8854 7612 L 8834 7633 L 8850 7656 L 8870 7675 L
8870 7676 L 8887 7699 L 8898 7725 L 8870 7721 L
8893 7737 L 8921 7743 L 8937 7747 L 8949 7750 L
8973 7765 L 8976 7766 L 9001 7771 L 9029 7775 L
9036 7784 L 9046 7797 L 9020 7786 L 8996 7801 L
8979 7824 L 8961 7801 L 8953 7774 L 8927 7762 L
8905 7780 L 8899 7808 L 8886 7833 L 8859 7825 L
8872 7850 L 8895 7867 L 8916 7848 L 8944 7841 L
8967 7824 L 8982 7848 L 8970 7874 L 8966 7902 L
8947 7923 L 8919 7928 L 8891 7928 L 8865 7917 L
8843 7936 L 8841 7964 L 8867 7974 L 8844 7990 L
8849 8017 L 8845 8046 L 8872 8035 L 8894 8017 L
8919 8004 L 8909 8030 L 8901 8058 L 8900 8086 L
8910 8060 L 8927 8037 L 8941 8024 L 8948 8018 L
8953 8046 L 8979 8058 L 8962 8034 L 8990 8027 L
8965 8014 L 8962 7986 L 8968 7984 L 8990 7979 L
9012 7961 L 9017 7989 L 9034 8012 L 9043 8039 L

9071 8036 L 9094 8020 L 9082 7995 L 9057 7982 L
9058 7954 L 9038 7935 L 9009 7933 L 8993 7910 L
8986 7882 L 9014 7882 L 9035 7900 L 9044 7927 L
9072 7930 L 9075 7932 L 9096 7945 L 9100 7947 L
9122 7956 L 9121 7984 L 9142 8004 L 9116 8016 L
9127 8042 L 9102 8056 L 9078 8071 L 9090 8096 L
9118 8091 L 9125 8118 L 9147 8117 L 9153 8116 L
9181 8111 L 9179 8139 L 9152 8146 L 9180 8146 L
9199 8167 L 9204 8195 L 9195 8222 L 9223 8218 L
9238 8222 L 9250 8226 L 9265 8249 L 9251 8274 L
9246 8302 L 9256 8275 L 9280 8261 L 9291 8287 L
9307 8264 L 9313 8274 L 9322 8287 L 9323 8316 L
9326 8321 L 9339 8339 L 9326 8364 L 9332 8391 L
9357 8379 L 9385 8383 L 9384 8411 L 9398 8436 L
9372 8446 L 9359 8471 L 9340 8493 L 9368 8500 L
9387 8520 L 9387 8549 L 9390 8577 L 9418 8580 L
9430 8605 L 9445 8629 L 9463 8651 L 9479 8674 L
9477 8702 L 9452 8716 L 9425 8721 L 9405 8741 L
9377 8746 L 9349 8742 L 9347 8770 L 9369 8752 L
9397 8755 L 9417 8749 L 9424 8748 L 9429 8748 L
9446 8730 L 9474 8731 L 9496 8730 L 9502 8730 L
9529 8727 L 9529 8740 L 9542 8765 L 9540 8793 L
9553 8818 L 9567 8825 L 9578 8831 L 9593 8854 L
9603 8881 L 9581 8898 L 9605 8908 L 9607 8909 L
9616 8936 L 9642 8946 L 9669 8938 L 9652 8916 L
9661 8901 L 9667 8891 L 9676 8865 L 9693 8842 L
9713 8823 L 9734 8842 L 9728 8869 L 9750 8887 L
9762 8912 L 9777 8936 L 9805 8932 L 9806 8931 L
9829 8917 L 9847 8895 L 9859 8920 L 9845 8945 L
9837 8972 L 9823 8996 L 9824 9024 L 9808 9047 L
9796 9073 L 9788 9100 L 9771 9122 L 9744 9129 L
9717 9137 L 9745 9137 L 9773 9137 L 9801 9138 L
9825 9153 L 9837 9166 L 9844 9173 L 9865 9193 L
9857 9220 L 9844 9244 L 9816 9250 L 9801 9274 L
9828 9264 L 9856 9261 L 9874 9282 L 9853 9301 L
9864 9327 L 9839 9339 L 9811 9333 L 9786 9322 L
9807 9340 L 9834 9343 L 9831 9371 L 9804 9377 L
9777 9369 L 9749 9366 L 9750 9394 L 9775 9408 L
9782 9435 L 9787 9462 L 9810 9459 L 9814 9458 L
9832 9436 L 9833 9464 L 9844 9490 L 9836 9463 L
9837 9435 L 9847 9409 L 9874 9416 L 9894 9435 L
9901 9462 L 9899 9490 L 9907 9517 L 9927 9499 L
9918 9472 L 9921 9456 L 9923 9445 L 9925 9417 L

9952 9411 L 9976 9427 L 9980 9432 L 9994 9448 L
9989 9476 L 9964 9488 L 9966 9516 L 9963 9544 L
9989 9554 L 9990 9582 L 9974 9605 L 9977 9633 L
9995 9655 L 9976 9675 L 9949 9668 L 9922 9662 L
9897 9649 L 9896 9621 L 9912 9598 L 9888 9584 L
9881 9611 L 9853 9611 L 9834 9590 L 9808 9582 L
9784 9566 L 9766 9545 L 9775 9572 L 9748 9564 L
9733 9541 L 9724 9568 L 9735 9593 L 9753 9615 L
9780 9609 L 9808 9615 L 9835 9621 L 9857 9639 L
9875 9660 L 9892 9683 L 9915 9698 L 9932 9720 L
9954 9738 L 9968 9761 L 9990 9779 L 10003 9784 L
10010 9787 L 10016 9789 L 10039 9805 L 10046 9832 L
10022 9818 L 9994 9818 L 9966 9817 L 9962 9818 L
9955 9824 L 9944 9834 L 9917 9841 L 9891 9852 L
9864 9857 L 9836 9861 L 9812 9875 L 9798 9899 L
9782 9922 L 9763 9943 L 9746 9965 L 9727 9986 L
9708 10006 L 9685 10022 L 9661 10036 L 9634 10040 L
9606 10039 L 9578 10035 L 9554 10022 L 9531 10005 L
9512 9985 L 9491 9967 L 9467 9953 L 9445 9935 L
9419 9925 L 9391 9925 L 9365 9935 L 9346 9915 L
9371 9902 L 9344 9910 L 9335 9936 L 9341 9964 L
9324 9986 L 9314 10012 L 9326 10037 L 9302 10052 L
9274 10054 L 9246 10051 L 9224 10035 L 9213 10009 L
9197 9987 L 9169 9985 L 9147 10001 L 9128 9981 L
9104 9966 L 9124 9986 L 9141 10008 L 9169 10009 L
9195 10000 L 9194 10028 L 9202 10054 L 9227 10067 L
9254 10073 L 9282 10074 L 9309 10076 L 9337 10076 L
9364 10068 L 9383 10047 L 9411 10050 L 9436 10062 L
9452 10085 L 9468 10107 L 9469 10135 L 9484 10159 L
9504 10178 L 9528 10192 L 9556 10196 L 9571 10219 L
9593 10236 L 9616 10252 L 9643 10256 L 9667 10271 L
9682 10294 L 9702 10313 L 9727 10326 L 9753 10334 L
9780 10341 L 9808 10346 L 9835 10349 L 9863 10352 L
9890 10356 L 9914 10372 L 9920 10399 L 9911 10425 L
9897 10449 L 9884 10474 L 9871 10498 L 9858 10523 L
9844 10547 L 9829 10570 L 9812 10592 L 9789 10608 L
9766 10623 L 9742 10637 L 9714 10637 L 9699 10660 L
9677 10677 L 9651 10689 L 9627 10702 L 9609 10723 L
9595 10747 L 9577 10769 L 9555 10786 L 9531 10799 L
9510 10817 L 9484 10827 L 9458 10836 L 9430 10835 L
9403 10837 L 9378 10849 L 9359 10869 L 9381 10886 L
9368 10911 L 9355 10935 L 9340 10958 L 9312 10961 L
9285 10958 L 9257 10961 L 9231 10970 L 9204 10968 L

9192 10943 L 9165 10937 L 9138 10931 L 9113 10918 L
9087 10910 L 9059 10910 L 9045 10934 L 9072 10928 L
9100 10927 L 9126 10936 L 9147 10954 L 9170 10970 L
9191 10988 L 9215 11002 L 9240 11012 L 9266 11001 L
9293 11001 L 9320 10993 L 9346 11003 L 9361 10980 L
9384 10964 L 9401 10942 L 9420 10922 L 9430 10896 L
9436 10869 L 9462 10861 L 9490 10864 L 9517 10861 L
9532 10884 L 9559 10890 L 9584 10901 L 9610 10910 L
9613 10909 L 9635 10898 L 9662 10892 L 9689 10884 L
9707 10863 L 9732 10852 L 9757 10840 L 9783 10830 L
9810 10826 L 9837 10818 L 9864 10811 L 9889 10821 L
9896 10848 L 9884 10873 L 9870 10897 L 9858 10922 L
9849 10948 L 9829 10968 L 9811 10988 L 9790 11006 L
9765 11019 L 9739 11028 L 9713 11038 L 9686 11038 L
9660 11048 L 9649 11073 L 9629 11092 L 9644 11116 L
9638 11143 L 9638 11171 L 9625 11195 L 9603 11212 L
9578 11222 L 9552 11233 L 9527 11245 L 9502 11255 L
9476 11264 L 9448 11265 L 9429 11285 L 9411 11306 L
9383 11309 L 9399 11332 L 9417 11353 L 9404 11377 L
9378 11387 L 9399 11405 L 9407 11431 L 9406 11459 L
9395 11484 L 9389 11511 L 9379 11537 L 9352 11542 L
9337 11565 L 9332 11592 L 9316 11615 L 9297 11635 L
9286 11660 L 9276 11686 L 9257 11706 L 9251 11732 L
9253 11760 L 9262 11786 L 9240 11802 L 9223 11824 L
9216 11851 L 9220 11878 L 9209 11903 L 9201 11930 L
9175 11939 L 9180 11966 L 9155 11977 L 9155 12004 L
9146 12031 L 9145 12058 L 9136 12084 L 9126 12110 L
9118 12136 L 9110 12162 L 9097 12186 L 9092 12213 L
9078 12237 L 9053 12248 L 9028 12260 L 9002 12266 L
8983 12286 L 8958 12298 L 8932 12307 L 8905 12311 L
8878 12317 L 8865 12293 L 8865 12320 L 8847 12341 L
8820 12347 L 8798 12364 L 8774 12377 L 8751 12391 L
8730 12409 L 8716 12432 L 8712 12459 L 8684 12462 L
8660 12474 L 8635 12461 L 8613 12477 L 8594 12457 L
8602 12484 L 8588 12508 L 8587 12535 L 8596 12561 L
8595 12588 L 8599 12616 L 8614 12639 L 8634 12657 L
8661 12652 L 8673 12677 L 8676 12704 L 8683 12730 L
8692 12757 L 8704 12773 L 8708 12778 L 8733 12791 L
8752 12810 L 8756 12837 L 8770 12861 L 8778 12887 L
8787 12913 L 8781 12939 L 8808 12937 L 8830 12952 L
8840 12927 L 8853 12951 L 8874 12969 L 8890 12947 L
8901 12972 L 8916 12996 L 8927 13020 L 8950 13005 L
8957 13031 L 8955 13059 L 8965 13084 L 8985 13103 L

9010 13113 L 9016 13117 L 9033 13128 L 9026 13102 L
9040 13125 L 9062 13141 L 9074 13166 L 9047 13162 L
9020 13166 L 9036 13189 L 9010 13198 L 8986 13211 L
8978 13237 L 8988 13263 L 9013 13273 L 9039 13264 L
9060 13281 L 9064 13308 L 9082 13288 L 9098 13266 L
9093 13239 L 9083 13213 L 9071 13189 L 9098 13183 L
9124 13192 L 9145 13210 L 9172 13215 L 9199 13219 L
9223 13231 L 9245 13248 L 9240 13275 L 9236 13302 L
9220 13324 L 9226 13351 L 9252 13359 L 9243 13385 L
9258 13408 L 9282 13420 L 9304 13403 L 9328 13390 L
9329 13417 L 9347 13438 L 9346 13465 L 9321 13476 L
9305 13498 L 9279 13492 L 9296 13513 L 9319 13527 L
9345 13520 L 9359 13496 L 9386 13497 L 9386 13525 L
9411 13514 L 9430 13533 L 9453 13548 L 9479 13554 L
9505 13564 L 9530 13574 L 9553 13589 L 9570 13611 L
9566 13637 L 9579 13614 L 9599 13633 L 9626 13636 L
9605 13618 L 9590 13595 L 9617 13602 L 9643 13595 L
9642 13622 L 9668 13632 L 9692 13645 L 9704 13669 L
9729 13680 L 9754 13692 L 9779 13704 L 9768 13729 L
9795 13734 L 9808 13710 L 9818 13685 L 9837 13703 L
9860 13718 L 9859 13745 L 9876 13766 L 9884 13740 L
9867 13719 L 9883 13696 L 9908 13686 L 9881 13682 L
9885 13655 L 9913 13654 L 9930 13651 L 9939 13650 L
9962 13665 L 9969 13671 L 9983 13682 L 9997 13706 L
9998 13706 L 10009 13709 L 10023 13712 L 10012 13687 L
10025 13711 L 10030 13738 L 10029 13765 L 10002 13762 L
9975 13764 L 9995 13782 L 9995 13810 L 10011 13832 L
10034 13846 L 10056 13861 L 10040 13884 L 10033 13910 L
10037 13936 L 10055 13957 L 10066 13968 L 10075 13976 L
10048 13978 L 10024 13965 L 9999 13953 L 9981 13974 L
9955 13966 L 9928 13965 L 9902 13973 L 9878 13987 L
9854 13974 L 9840 13997 L 9849 14023 L 9832 14044 L
9810 14027 L 9787 14012 L 9768 13993 L 9769 13966 L
9754 13989 L 9727 13984 L 9700 13980 L 9684 13958 L
9664 13976 L 9639 13966 L 9612 13961 L 9585 13963 L
9561 13975 L 9542 13995 L 9525 14016 L 9507 14037 L
9532 14048 L 9505 14045 L 9480 14034 L 9453 14031 L
9426 14037 L 9406 14056 L 9402 14083 L 9386 14105 L
9359 14103 L 9342 14125 L 9318 14137 L 9306 14161 L
9279 14158 L 9252 14161 L 9225 14160 L 9204 14142 L
9179 14133 L 9155 14120 L 9128 14115 L 9102 14108 L
9082 14090 L 9057 14081 L 9031 14073 L 9055 14062 L
9051 14035 L 9034 14013 L 9015 13994 L 8996 13974 L

8974 13958 L 8951 13943 L 8925 13936 L 8900 13926 L
8879 13908 L 8866 13884 L 8883 13863 L 8876 13836 L
8856 13817 L 8836 13800 L 8817 13780 L 8790 13773 L
8763 13774 L 8736 13771 L 8711 13782 L 8684 13779 L
8656 13777 L 8629 13778 L 8603 13785 L 8576 13788 L
8555 13806 L 8532 13821 L 8505 13820 L 8502 13847 L
8485 13868 L 8467 13888 L 8473 13915 L 8469 13942 L
8468 13969 L 8470 13996 L 8477 14023 L 8494 14044 L
8511 14065 L 8523 14090 L 8505 14110 L 8480 14121 L
8492 14146 L 8498 14172 L 8491 14198 L 8483 14224 L
8464 14244 L 8459 14271 L 8432 14271 L 8415 14250 L
8424 14276 L 8419 14303 L 8422 14330 L 8408 14353 L
8402 14326 L 8397 14353 L 8373 14340 L 8351 14324 L
8326 14313 L 8302 14301 L 8280 14285 L 8259 14302 L
8232 14306 L 8206 14314 L 8179 14316 L 8152 14313 L
8125 14317 L 8098 14313 L 8075 14327 L 8052 14342 L
8033 14361 L 8010 14376 L 7984 14383 L 7964 14402 L
7945 14422 L 7928 14443 L 7915 14466 L 7903 14491 L
7890 14515 L 7880 14540 L 7870 14565 L 7867 14592 L
7867 14619 L 7872 14646 L 7881 14672 L 7893 14696 L
7906 14720 L 7924 14741 L 7939 14763 L 7964 14774 L
7958 14800 L 7948 14825 L 7967 14845 L 7986 14864 L
8008 14879 L 8034 14888 L 8061 14893 L 8088 14888 L
8111 14875 L 8126 14852 L 8133 14826 L 8151 14806 L
8174 14792 L 8201 14787 L 8223 14803 L 8243 14821 L
8264 14838 L 8284 14856 L 8301 14864 L 8309 14867 L
8326 14867 L 8336 14867 L 8357 14850 L 8371 14850 L
8384 14851 L 8406 14867 L 8406 14867 L 8422 14869 L
8433 14870 L 8458 14880 L 8475 14889 L 8482 14893 L
8504 14909 L 8522 14929 L 8536 14953 L 8553 14974 L
8575 14989 L 8600 15000 L 8583 15021 L 8579 15047 L
8583 15074 L 8575 15100 L 8598 15114 L 8605 15140 L
8613 15166 L 8625 15190 L 8645 15209 L 8667 15225 L
8692 15236 L 8690 15263 L 8710 15281 L 8736 15290 L
8762 15297 L 8788 15305 L 8810 15321 L 8836 15328 L
8851 15350 L 8874 15365 L 8899 15375 L 8925 15383 L
8950 15393 L 8974 15406 L 8998 15418 L 9019 15435 L
9045 15442 L 9071 15449 L 9087 15471 L 9086 15498 L
9099 15521 L 9122 15535 L 9100 15551 L 9122 15567 L
9127 15594 L 9150 15607 L 9164 15630 L 9174 15637 L
9186 15647 L 9204 15667 L 9211 15681 L 9216 15691 L
9228 15705 L 9234 15711 L 9260 15717 L 9262 15720 L
9278 15738 L 9256 15753 L 9267 15777 L 9286 15796 L

9295 15795 L 9311 15793 L 9313 15792 L 9323 15817 L
9342 15836 L 9358 15858 L 9385 15856 L 9407 15840 L
9430 15853 L 9454 15840 L 9481 15841 L 9495 15832 L
9503 15826 L 9523 15807 L 9550 15809 L 9577 15812 L
9598 15828 L 9608 15853 L 9609 15880 L 9629 15898 L
9639 15923 L 9635 15896 L 9622 15872 L 9633 15847 L
9625 15822 L 9639 15798 L 9666 15800 L 9682 15779 L
9705 15764 L 9727 15748 L 9742 15733 L 9754 15732 L
9754 15746 L 9759 15750 L 9769 15746 L 9784 15739 L
9803 15744 L 9801 15751 L 9779 15745 L 9772 15755 L
9766 15762 L 9766 15768 L 9754 15775 L 9732 15791 L
9732 15793 L 9757 15778 L 9777 15766 L 9779 15753 L
9801 15755 L 9806 15746 L 9802 15740 L 9784 15736 L
9760 15748 L 9758 15742 L 9763 15715 L 9754 15698 L
9760 15688 L 9783 15673 L 9803 15655 L 9816 15631 L
9804 15607 L 9786 15586 L 9784 15559 L 9766 15539 L
9752 15516 L 9748 15489 L 9728 15471 L 9715 15447 L
9700 15425 L 9683 15404 L 9667 15382 L 9644 15368 L
9670 15361 L 9651 15342 L 9632 15322 L 9608 15309 L
9581 15310 L 9564 15289 L 9540 15275 L 9518 15261 L
9506 15236 L 9496 15211 L 9517 15195 L 9534 15173 L
9536 15146 L 9534 15125 L 9529 15113 L 9517 15096 L
9494 15087 L 9508 15055 L 9518 15025 L 9541 15011 L
9568 15014 L 9595 15016 L 9619 15028 L 9646 15028 L
9673 15035 L 9700 15033 L 9676 15020 L 9650 15013 L
9627 14998 L 9613 14976 L 9589 14962 L 9563 14955 L
9582 14935 L 9565 14914 L 9587 14898 L 9608 14881 L
9594 14858 L 9568 14849 L 9543 14838 L 9521 14822 L
9495 14819 L 9475 14837 L 9465 14812 L 9451 14789 L
9426 14779 L 9399 14779 L 9372 14772 L 9365 14746 L
9391 14739 L 9369 14722 L 9382 14698 L 9381 14671 L
9402 14654 L 9420 14675 L 9431 14699 L 9458 14699 L
9483 14710 L 9510 14710 L 9536 14719 L 9554 14738 L
9582 14739 L 9609 14741 L 9621 14765 L 9597 14779 L
9571 14785 L 9595 14797 L 9607 14821 L 9629 14838 L
9653 14828 L 9665 14803 L 9672 14799 L 9689 14790 L
9690 14818 L 9679 14842 L 9705 14834 L 9696 14859 L
9716 14841 L 9737 14859 L 9764 14865 L 9756 14891 L
9782 14896 L 9806 14908 L 9782 14920 L 9792 14945 L
9811 14964 L 9834 14978 L 9846 15003 L 9856 15028 L
9879 15042 L 9898 15059 L 9900 15060 L 9915 15059 L
9927 15057 L 9946 15076 L 9950 15103 L 9956 15099 L
9973 15089 L 9991 15110 L 10015 15122 L 10035 15140 L

10042 15146 L 10056 15157 L 10057 15157 L 10071 15163 L
10078 15166 L 10081 15168 L 10088 15170 L 10097 15174 L
10107 15177 L 10131 15189 L 10143 15201 L 10150 15208 L
10170 15216 L 10175 15218 L 10201 15209 L 10226 15219 L
10235 15226 L 10248 15235 L 10223 15246 L 10246 15260 L
10255 15286 L 10257 15285 L 10272 15283 L 10281 15281 L
10284 15281 L 10308 15276 L 10309 15275 L 10321 15268 L
10331 15262 L 10340 15280 L 10343 15286 L 10356 15283 L
10369 15281 L 10383 15304 L 10393 15329 L 10401 15355 L
10403 15328 L 10426 15313 L 10441 15314 L 10453 15314 L
10473 15332 L 10497 15320 L 10524 15318 L 10551 15317 L
10578 15323 L 10600 15338 L 10627 15344 L 10639 15368 L
10644 15341 L 10651 15315 L 10656 15342 L 10661 15369 L
10666 15395 L 10672 15395 L 10693 15397 L 10687 15370 L
10709 15354 L 10733 15342 L 10759 15335 L 10786 15333 L
10796 15307 L 10805 15282 L 10824 15262 L 10813 15237 L
10826 15213 L 10840 15231 L 10843 15234 L 10854 15250 L
10859 15256 L 10856 15283 L 10867 15308 L 10888 15291 L
10914 15283 L 10929 15263 L 10930 15262 L 10954 15275 L
10971 15254 L 10998 15255 L 11015 15243 L 11021 15239 L
11044 15226 L 11070 15234 L 11089 15215 L 11116 15216 L
11139 15230 L 11160 15247 L 11172 15271 L 11177 15283 L
11189 15280 L 11197 15283 L 11202 15289 L 11203 15288 L
11198 15280 L 11189 15277 L 11177 15282 L 11174 15269 L
11163 15241 L 11184 15242 L 11196 15252 L 11225 15269 L
11226 15267 L 11221 15263 L 11197 15251 L 11187 15237 L
11174 15236 L 11150 15223 L 11146 15196 L 11127 15177 L
11128 15150 L 11141 15126 L 11167 15133 L 11153 15109 L
11157 15082 L 11151 15055 L 11144 15029 L 11121 15015 L
11116 14988 L 11125 14962 L 11104 14944 L 11091 14920 L
11082 14895 L 11073 14869 L 11062 14844 L 11070 14818 L
11085 14795 L 11110 14787 L 11103 14813 L 11118 14790 L
11121 14763 L 11147 14772 L 11174 14769 L 11190 14791 L
11214 14779 L 11241 14778 L 11257 14756 L 11230 14752 L
11208 14767 L 11184 14754 L 11157 14751 L 11135 14735 L
11108 14729 L 11085 14715 L 11068 14736 L 11075 14710 L
11071 14683 L 11047 14671 L 11020 14670 L 11031 14645 L
11021 14619 L 11001 14601 L 10991 14576 L 10977 14553 L
10983 14526 L 10984 14499 L 11003 14480 L 11024 14491 L
11034 14487 L 11005 14467 L 11002 14471 L 11004 14478 L
10982 14492 L 10963 14466 L 10943 14449 L 10924 14430 L
10898 14423 L 10924 14416 L 10928 14415 L 10951 14411 L
10960 14414 L 10973 14418 L 10977 14419 L 11002 14429 L

11026 14415 L 11052 14408 L 11079 14411 L 11104 14422 L
11131 14424 L 11156 14414 L 11154 14387 L 11133 14404 L
11106 14399 L 11099 14373 L 11123 14360 L 11098 14350 L
11071 14354 L 11048 14341 L 11063 14318 L 11079 14312 L
11088 14308 L 11114 14300 L 11140 14308 L 11141 14308 L
11160 14322 L 11162 14324 L 11186 14310 L 11181 14283 L
11207 14284 L 11209 14284 L 11217 14310 L 11244 14315 L
11221 14329 L 11248 14331 L 11275 14328 L 11253 14313 L
11268 14290 L 11241 14288 L 11239 14261 L 11212 14254 L
11201 14230 L 11218 14208 L 11229 14233 L 11255 14237 L
11272 14259 L 11286 14268 L 11295 14273 L 11298 14273 L
11322 14271 L 11330 14297 L 11326 14323 L 11323 14351 L
11344 14350 L 11349 14350 L 11350 14350 L 11357 14376 L
11330 14376 L 11304 14386 L 11300 14413 L 11325 14403 L
11352 14404 L 11369 14400 L 11376 14398 L 11379 14397 L
11390 14375 L 11391 14373 L 11400 14366 L 11405 14363 L
11412 14358 L 11413 14357 L 11423 14348 L 11432 14338 L
11449 14316 L 11456 14316 L 11476 14315 L 11479 14342 L
11505 14336 L 11505 14336 L 11509 14309 L 11516 14303 L
11521 14298 L 11529 14290 L 11549 14275 L 11551 14274 L
11557 14300 L 11556 14327 L 11560 14329 L 11582 14336 L
11599 14338 L 11608 14339 L 11629 14357 L 11657 14359 L
11667 14352 L 11679 14344 L 11702 14329 L 11726 14317 L
11753 14322 L 11770 14344 L 11796 14357 L 11778 14334 L
11784 14307 L 11786 14280 L 11798 14256 L 11822 14244 L
11845 14228 L 11867 14214 L 11888 14203 L 11888 14201 L
11865 14211 L 11843 14227 L 11819 14238 L 11795 14251 L
11771 14264 L 11778 14290 L 11765 14314 L 11741 14301 L
11717 14289 L 11722 14262 L 11728 14236 L 11748 14217 L
11774 14210 L 11785 14184 L 11787 14157 L 11763 14145 L
11764 14144 L 11781 14125 L 11803 14108 L 11828 14099 L
11812 14077 L 11837 14065 L 11861 14053 L 11840 14036 L
11813 14032 L 11820 14006 L 11807 13982 L 11823 13960 L
11842 13981 L 11863 13997 L 11868 13970 L 11857 13946 L
11850 13919 L 11877 13913 L 11895 13893 L 11914 13874 L
11941 13868 L 11960 13887 L 11950 13861 L 11928 13845 L
11909 13826 L 11909 13799 L 11895 13822 L 11890 13849 L
11874 13871 L 11847 13876 L 11827 13895 L 11804 13879 L
11780 13892 L 11756 13904 L 11766 13879 L 11741 13891 L
11760 13871 L 11771 13846 L 11750 13863 L 11746 13836 L
11740 13810 L 11739 13837 L 11732 13863 L 11723 13889 L
11697 13880 L 11677 13862 L 11699 13847 L 11717 13827 L
11702 13804 L 11678 13791 L 11693 13769 L 11671 13785 L

11673 13812 L 11666 13838 L 11660 13865 L 11661 13838 L
11636 13826 L 11630 13799 L 11631 13772 L 11604 13766 L
11595 13740 L 11600 13717 L 11601 13714 L 11611 13739 L
11612 13738 L 11621 13734 L 11631 13730 L 11635 13728 L
11634 13700 L 11639 13702 L 11642 13703 L 11652 13706 L
11656 13707 L 11661 13708 L 11679 13688 L 11678 13661 L
11684 13653 L 11689 13647 L 11694 13641 L 11695 13639 L
11697 13630 L 11702 13613 L 11706 13586 L 11720 13566 L
11721 13564 L 11743 13581 L 11732 13606 L 11720 13630 L
11716 13657 L 11691 13668 L 11697 13695 L 11683 13719 L
11702 13738 L 11711 13712 L 11715 13685 L 11733 13706 L
11750 13717 L 11755 13721 L 11761 13694 L 11759 13667 L
11758 13640 L 11782 13627 L 11768 13604 L 11773 13596 L
11779 13585 L 11782 13580 L 11807 13608 L 11806 13593 L
11783 13578 L 11784 13551 L 11788 13548 L 11792 13545 L
11806 13534 L 11793 13510 L 11807 13487 L 11809 13461 L
11810 13459 L 11832 13443 L 11810 13427 L 11826 13421 L
11831 13419 L 11836 13418 L 11847 13408 L 11857 13400 L
11869 13376 L 11889 13357 L 11913 13370 L 11938 13381 L
11960 13397 L 11966 13424 L 11964 13396 L 11944 13379 L
11919 13368 L 11898 13350 L 11873 13338 L 11858 13315 L
11867 13289 L 11877 13291 L 11893 13295 L 11921 13297 L
11947 13303 L 11929 13283 L 11909 13264 L 11925 13241 L
11900 13230 L 11894 13203 L 11867 13202 L 11849 13181 L
11829 13163 L 11810 13143 L 11824 13119 L 11851 13121 L
11878 13126 L 11905 13126 L 11907 13099 L 11880 13101 L
11854 13092 L 11828 13083 L 11816 13059 L 11812 13032 L
11799 13008 L 11825 13003 L 11817 12977 L 11795 12993 L
11789 12966 L 11783 12940 L 11774 12914 L 11800 12907 L
11796 12879 L 11819 12864 L 11845 12857 L 11835 12832 L
11818 12811 L 11799 12831 L 11781 12851 L 11783 12879 L
11770 12854 L 11743 12860 L 11752 12834 L 11764 12822 L
11772 12815 L 11769 12843 L 11789 12824 L 11812 12808 L
11799 12784 L 11774 12774 L 11777 12771 L 11794 12755 L
11811 12777 L 11838 12782 L 11822 12759 L 11833 12734 L
11826 12707 L 11836 12682 L 11860 12695 L 11887 12695 L
11887 12722 L 11878 12748 L 11879 12775 L 11881 12748 L
11905 12735 L 11909 12707 L 11937 12705 L 11912 12694 L
11885 12689 L 11878 12663 L 11851 12667 L 11830 12649 L
11857 12650 L 11884 12647 L 11905 12630 L 11915 12604 L
11888 12602 L 11861 12609 L 11855 12636 L 11828 12634 L
11827 12607 L 11853 12598 L 11873 12580 L 11846 12577 L
11821 12588 L 11806 12612 L 11804 12639 L 11781 12653 L

11772 12627 L 11772 12624 L 11774 12600 L 11787 12575 L
11814 12570 L 11836 12554 L 11848 12549 L 11861 12543 L
11882 12560 L 11910 12559 L 11890 12540 L 11872 12519 L
11879 12493 L 11904 12480 L 11887 12459 L 11910 12444 L
11911 12421 L 11912 12416 L 11934 12433 L 11934 12433 L
11951 12454 L 11955 12481 L 11958 12482 L 11978 12497 L
11981 12510 L 11984 12523 L 12009 12537 L 12009 12564 L
12011 12591 L 12021 12566 L 12024 12539 L 12038 12515 L
12046 12517 L 12064 12522 L 12092 12520 L 12098 12529 L
12108 12542 L 12097 12567 L 12106 12593 L 12109 12621 L
12133 12634 L 12140 12640 L 12153 12653 L 12178 12664 L
12196 12685 L 12222 12693 L 12248 12703 L 12252 12676 L
12234 12655 L 12214 12673 L 12214 12646 L 12215 12619 L
12192 12604 L 12187 12631 L 12176 12606 L 12168 12580 L
12194 12572 L 12209 12595 L 12217 12587 L 12228 12576 L
12245 12554 L 12250 12549 L 12265 12536 L 12277 12561 L
12278 12561 L 12288 12569 L 12299 12577 L 12300 12605 L
12274 12600 L 12277 12627 L 12304 12621 L 12319 12621 L
12331 12621 L 12331 12648 L 12310 12665 L 12287 12681 L
12269 12702 L 12262 12728 L 12285 12743 L 12259 12753 L
12287 12754 L 12313 12761 L 12325 12786 L 12337 12761 L
12346 12748 L 12352 12738 L 12363 12713 L 12354 12688 L
12366 12663 L 12390 12677 L 12389 12704 L 12408 12709 L
12415 12711 L 12442 12705 L 12436 12678 L 12438 12651 L
12447 12654 L 12464 12659 L 12463 12687 L 12454 12713 L
12438 12735 L 12442 12762 L 12437 12789 L 12410 12783 L
12389 12765 L 12365 12778 L 12351 12802 L 12358 12828 L
12375 12807 L 12398 12820 L 12415 12842 L 12442 12848 L
12468 12839 L 12487 12853 L 12490 12855 L 12514 12870 L
12539 12880 L 12546 12906 L 12549 12879 L 12550 12851 L
12551 12851 L 12576 12840 L 12602 12832 L 12629 12832 L
12631 12838 L 12638 12858 L 12646 12854 L 12663 12846 L
12664 12847 L 12688 12856 L 12685 12829 L 12712 12829 L
12719 12803 L 12692 12809 L 12700 12798 L 12708 12787 L
12727 12773 L 12730 12770 L 12741 12795 L 12744 12823 L
12725 12843 L 12726 12871 L 12729 12898 L 12748 12918 L
12730 12939 L 12743 12963 L 12767 12961 L 12771 12961 L
12797 12954 L 12789 12980 L 12783 13006 L 12807 12993 L
12829 12977 L 12833 12977 L 12856 12973 L 12883 12973 L
12895 12976 L 12909 12980 L 12910 12980 L 12911 12980 L
12925 12981 L 12937 12982 L 12952 13005 L 12951 12978 L
12959 12952 L 12980 12969 L 12999 12978 L 12999 13001 L
12998 13007 L 12999 13007 L 12999 14079 L 12999 14085 L

12980 14105 L 12968 14129 L 12961 14156 L 12951 14181 L
12934 14202 L 12915 14222 L 12900 14245 L 12878 14261 L
12864 14284 L 12844 14302 L 12838 14329 L 12819 14348 L
12798 14366 L 12793 14393 L 12772 14411 L 12752 14428 L
12731 14445 L 12712 14465 L 12692 14483 L 12675 14505 L
12656 14524 L 12640 14546 L 12622 14566 L 12606 14588 L
12586 14607 L 12575 14632 L 12563 14657 L 12547 14678 L
12534 14702 L 12519 14725 L 12503 14747 L 12494 14772 L
12476 14793 L 12463 14817 L 12448 14839 L 12438 14865 L
12433 14891 L 12409 14905 L 12384 14913 L 12358 14904 L
12347 14929 L 12328 14948 L 12306 14964 L 12279 14967 L
12256 14981 L 12235 14998 L 12213 15014 L 12196 15036 L
12178 15056 L 12163 15078 L 12147 15100 L 12124 15086 L
12099 15096 L 12072 15102 L 12045 15103 L 12018 15104 L
11995 15119 L 11980 15141 L 11984 15168 L 12007 15183 L
12012 15209 L 11987 15219 L 12013 15226 L 12040 15230 L
12053 15206 L 12045 15232 L 12033 15257 L 12026 15283 L
12029 15310 L 12041 15334 L 12035 15360 L 12009 15368 L
12003 15394 L 12010 15421 L 12022 15445 L 12041 15464 L
12068 15463 L 12081 15487 L 12107 15494 L 12128 15511 L
12149 15529 L 12146 15556 L 12169 15571 L 12182 15594 L
12204 15610 L 12231 15605 L 12258 15601 L 12283 15611 L
12298 15633 L 12324 15624 L 12334 15649 L 12359 15657 L
12377 15678 L 12402 15690 L 12419 15710 L 12445 15720 L
12449 15746 L 12426 15760 L 12434 15786 L 12458 15799 L
12482 15811 L 12506 15823 L 12528 15839 L 12545 15861 L
12561 15882 L 12584 15897 L 12609 15908 L 12621 15932 L
12626 15959 L 12633 15985 L 12638 16012 L 12656 16032 L
12682 16039 L 12698 16061 L 12719 16079 L 12743 16090 L
12767 16102 L 12785 16122 L 12812 16127 L 12836 16139 L
12842 16166 L 12841 16170 L 12833 16170 L 12800 16170 L
12767 16170 L 12734 16170 L 12700 16170 L 12667 16170 L
12634 16170 L 12601 16170 L 12567 16170 L 12534 16170 L
12501 16170 L 12468 16170 L 12435 16170 L 12401 16170 L
12368 16170 L 12335 16170 L 12302 16170 L 12268 16170 L
12235 16170 L 12202 16170 L 12169 16170 L 12135 16170 L
12102 16170 L 12069 16170 L 12036 16170 L 12003 16170 L
11969 16170 L 11936 16170 L 11903 16170 L 11870 16170 L
11836 16170 L 11803 16170 L 11770 16170 L 11737 16170 L
11704 16170 L 11670 16170 L 11637 16170 L 11604 16170 L
11571 16170 L 11537 16170 L 11504 16170 L 11471 16170 L
11438 16170 L 11404 16170 L 11371 16170 L 11338 16170 L
11305 16170 L 11272 16170 L 11238 16170 L 11205 16170 L

11172 16170 L 11139 16170 L 11105 16170 L 11072 16170 L
11039 16170 L 11006 16170 L 10972 16170 L 10939 16170 L
10906 16170 L 10873 16170 L 10840 16170 L 10806 16170 L
10773 16170 L 10740 16170 L 10707 16170 L 10673 16170 L
10640 16170 L 10607 16170 L 10574 16170 L 10540 16170 L
10507 16170 L 10474 16170 L 10441 16170 L 10408 16170 L
10374 16170 L 10341 16170 L 10308 16170 L 10275 16170 L
10241 16170 L 10208 16170 L 10175 16170 L 10142 16170 L
10108 16170 L 10075 16170 L 10042 16170 L 10009 16170 L
9976 16170 L 9942 16170 L 9909 16170 L 9876 16170 L
9843 16170 L 9809 16170 L 9776 16170 L 9743 16170 L
9710 16170 L 9677 16170 L 9643 16170 L 9610 16170 L
9577 16170 L 9544 16170 L 9510 16170 L 9477 16170 L
9444 16170 L 9411 16170 L 9377 16170 L 9344 16170 L
9311 16170 L 9278 16170 L 9245 16170 L 9211 16170 L
9178 16170 L 9145 16170 L 9112 16170 L 9078 16170 L
9045 16170 L 9012 16170 L 8979 16170 L 8945 16170 L
8912 16170 L 8879 16170 L 8846 16170 L 8813 16170 L
8779 16170 L 8746 16170 L 8713 16170 L 8680 16170 L
8646 16170 L 8613 16170 L 8580 16170 L 8547 16170 L
8513 16170 L 8480 16170 L 8447 16170 L 8414 16170 L
8381 16170 L 8347 16170 L 8314 16170 L 8281 16170 L
8248 16170 L 8214 16170 L 8181 16170 L 8148 16170 L
8115 16170 L 8081 16170 L 8048 16170 L 8015 16170 L
7982 16170 L 7949 16170 L 7915 16170 L 7882 16170 L
7849 16170 L 7816 16170 L 7782 16170 L 7749 16170 L
7716 16170 L 7683 16170 L 7650 16170 L 7616 16170 L
7583 16170 L 7550 16170 L 7517 16170 L 7483 16170 L
7450 16170 L 7417 16170 L 7384 16170 L 7350 16170 L
7317 16170 L 7284 16170 L 7251 16170 L 7218 16170 L
7184 16170 L 7151 16170 L 7118 16170 L 7085 16170 L
7051 16170 L 7018 16170 L 6985 16170 L 6952 16170 L
6918 16170 L 6885 16170 L 6852 16170 L 6819 16170 L
6786 16170 L 6752 16170 L 6719 16170 L 6686 16170 L
6653 16170 L 6619 16170 L 6586 16170 L 6553 16170 L
6520 16170 L 6486 16170 L 6453 16170 L 6420 16170 L
6387 16170 L 6354 16170 L 6320 16170 L 6287 16170 L
6254 16170 L 6221 16170 L 6187 16170 L 6154 16170 L
6121 16170 L 6088 16170 L 6054 16170 L 6021 16170 L
5988 16170 L 5955 16170 L 5922 16170 L 5888 16170 L
5855 16170 L 5822 16170 L 5789 16170 L 5755 16170 L
5722 16170 L 5689 16170 L 5656 16170 L 5622 16170 L
5589 16170 L 5556 16170 L 5523 16170 L 5490 16170 L

5456 16170 L 5423 16170 L 5390 16170 L 5357 16170 L
5323 16170 L 5290 16170 L 5257 16170 L 5224 16170 L
5191 16170 L 5157 16170 L 5124 16170 L 5091 16170 L
5058 16170 L 5024 16170 L 4991 16170 L 4958 16170 L
4925 16170 L 4891 16170 L 4858 16170 L 4825 16170 L
4792 16170 L 4759 16170 L 4725 16170 L 4692 16170 L
4659 16170 L 4626 16170 L 4592 16170 L 4559 16170 L
4526 16170 L 4493 16170 L 4459 16170 L 4426 16170 L
4393 16170 L 4360 16170 L 4327 16170 L 4293 16170 L
4260 16170 L 4227 16170 L 4194 16170 L 4160 16170 L
4127 16170 L 4094 16170 L 4061 16170 L 4027 16170 L
3994 16170 L 3961 16170 L 3928 16170 L 3895 16170 L
3861 16170 L 3828 16170 L 3795 16170 L 3762 16170 L
3728 16170 L 3695 16170 L 3662 16170 L 3629 16170 L
3595 16170 L 3562 16170 L 3529 16170 L 3496 16170 L
3463 16170 L 3429 16170 L 3396 16170 L 3363 16170 L
3330 16170 L 3296 16170 L 3263 16170 L 3230 16170 L
3197 16170 L 3164 16170 L 3130 16170 L 3097 16170 L
3064 16170 L 3031 16170 L 2997 16170 L 2964 16170 L
2931 16170 L 2898 16170 L 2864 16170 L 2831 16170 L
2798 16170 L 2765 16170 L 2732 16170 L 2698 16170 L
2665 16170 L 2632 16170 L 2599 16170 L 2565 16170 L
2532 16170 L 2499 16170 L 2466 16170 L 2432 16170 L
2399 16170 L 2366 16170 L 2333 16170 L 2300 16170 L
N E
5542 5477 5541 5470 5543 5466 5545 5461 5547 5457 5548 5455
5556 5455 5575 5455 5575 5458 5573 5462 5571 5466 5569 5471
5567 5475 5563 5478 5559 5480 5556 5483 5552 5486 5549 5482
5545 5480 5542 5477 20 N F
5702 5573 5698 5567 5700 5563 5704 5561 5708 5563 5712 5565
5717 5566 5716 5571 5721 5573 5724 5576 5724 5581 5726 5585
5721 5585 5717 5588 5712 5588 5711 5584 5707 5581 5704 5578
5702 5573 19 N F
5796 5572 5790 5572 5794 5569 5798 5567 5801 5563 5805 5561
5810 5562 5808 5566 5806 5570 5803 5573 5799 5576 5796 5572
12 N F
5817 5461 5816 5455 5822 5455 5855 5455 5865 5455 5871 5458
5879 5459 5885 5461 5890 5456 5890 5455 5910 5455 5903 5461
5904 5468 5908 5474 5902 5477 5909 5480 5915 5478 5917 5485
5916 5492 5916 5499 5921 5504 5924 5511 5930 5507 5936 5511
5937 5519 5933 5524 5927 5520 5921 5516 5914 5515 5908 5511
5903 5506 5897 5502 5893 5496 5886 5497 5879 5495 5872 5491
5866 5488 5862 5494 5855 5495 5847 5494 5843 5488 5838 5483

5832 5479 5825 5477 5827 5471 5823 5465 5817 5461 47 N F
5964 5459 5962 5455 5969 5455 5970 5459 5964 5459 5 N F
5977 5488 5967 5480 5972 5475 5975 5468 5981 5464 5988 5463
5992 5457 5999 5459 6006 5456 6006 5455 6021 5455 6024 5455
6024 5459 6028 5455 6039 5455 6038 5459 6042 5465 6038 5471
6034 5477 6031 5484 6024 5485 6018 5488 6011 5489 6005 5485
5998 5483 5991 5485 5984 5488 5977 5488 28 N F
6055 5463 6054 5459 6056 5458 6058 5457 6058 5455 6059 5455
6060 5456 6062 5456 6063 5458 6064 5460 6064 5462 6064 5465
6063 5467 6061 5466 6059 5465 6057 5465 6055 5463 17 N F
6243 5583 6240 5580 6241 5577 6242 5574 6244 5571 6247 5573
6251 5574 6254 5574 6257 5576 6259 5578 6259 5579 6257 5581
6253 5582 6250 5582 6246 5583 6243 5583 16 N F
6252 5562 6251 5559 6253 5560 6255 5559 6257 5561 6258 5563
6258 5565 6260 5566 6259 5568 6257 5568 6255 5566 6253 5564
6252 5562 13 N F
6256 5651 6252 5647 6253 5644 6257 5644 6259 5641 6260 5638
6263 5640 6262 5643 6260 5646 6257 5647 6256 5651 11 N F
7342 6052 7335 6044 7339 6038 7343 6032 7349 6028 7356 6025
7360 6019 7367 6017 7374 6019 7378 6013 7381 6006 7388 6004
7395 6002 7388 5998 7392 5992 7398 5987 7405 5987 7408 5980
7415 5980 7417 5987 7422 5992 7415 5995 7409 5992 7404 5997
7402 6004 7403 6012 7404 6019 7402 6026 7398 6032 7397 6039
7392 6044 7385 6046 7380 6042 7372 6042 7365 6044 7360 6049
7356 6055 7349 6055 7342 6052 39 N F
7490 6180 7483 6175 7488 6171 7495 6170 7503 6169 7508 6173
7505 6180 7506 6187 7511 6192 7514 6199 7507 6201 7501 6197
7500 6189 7497 6183 7490 6180 15 N F
7498 6166 7491 6165 7493 6158 7499 6154 7506 6153 7513 6151
7520 6149 7522 6156 7523 6163 7527 6169 7533 6172 7527 6180
7520 6179 7513 6179 7510 6173 7506 6167 7498 6166 17 N F
7498 6111 7493 6105 7497 6099 7504 6098 7511 6096 7519 6097
7526 6096 7533 6094 7538 6090 7545 6087 7552 6087 7559 6090
7566 6091 7573 6089 7574 6082 7581 6084 7586 6080 7593 6083
7597 6089 7595 6096 7594 6103 7600 6107 7598 6114 7592 6118
7589 6125 7590 6132 7583 6132 7579 6126 7576 6119 7569 6120
7564 6126 7558 6130 7552 6127 7547 6121 7544 6115 7539 6111
7535 6105 7526 6113 7523 6119 7516 6122 7509 6120 7503 6115
7498 6111 43 N F
7698 6195 7691 6184 7698 6184 7705 6186 7712 6188 7718 6186
7717 6179 7723 6183 7729 6185 7736 6188 7743 6189 7750 6190
7758 6191 7754 6197 7747 6198 7745 6205 7745 6212 7746 6219
7749 6226 7753 6232 7759 6234 7766 6231 7773 6228 7780 6229

7781 6236 7776 6241 7775 6248 7777 6255 7784 6259 7782 6266
7788 6270 7794 6266 7801 6266 7802 6273 7809 6276 7802 6275
7797 6281 7791 6285 7786 6290 7781 6285 7774 6287 7770 6281
7762 6281 7756 6278 7753 6285 7749 6291 7744 6295 7737 6299
7732 6304 7726 6301 7724 6294 7727 6287 7727 6280 7722 6274
7717 6269 7715 6262 7711 6256 7706 6251 7708 6244 7714 6240
7719 6245 7726 6248 7732 6243 7739 6244 7746 6242 7740 6237
7739 6230 7734 6225 7728 6221 7722 6217 7718 6211 7713 6205
7714 6198 7710 6204 7704 6200 7698 6195 76 N F
7944 6713 7942 6708 7943 6705 7945 6702 7948 6700 7950 6699
7951 6699 7953 6702 7950 6704 7948 6707 7946 6710 7944 6713
12 N F
7955 6697 7950 6697 7954 6695 7958 6692 7961 6689 7964 6685
7967 6682 7971 6679 7975 6678 7980 6678 7984 6679 7989 6678
7989 6678 7993 6680 7991 6684 7990 6688 7986 6690 7982 6688
7977 6687 7973 6689 7971 6693 7968 6696 7963 6696 7959 6694
7955 6697 25 N F
7957 6703 7953 6699 7957 6697 7961 6696 7966 6698 7970 6696
7973 6693 7976 6689 7980 6689 7984 6691 7988 6693 7992 6690
7994 6686 7999 6684 8003 6687 8007 6687 8006 6691 8003 6695
7999 6698 7997 6701 7993 6704 7989 6706 7984 6706 7980 6708
7975 6707 7971 6706 7966 6705 7962 6704 7957 6703 29 N F
7984 6676 7979 6675 7983 6673 7987 6671 7991 6669 7996 6669
7997 6673 7993 6675 7989 6677 7984 6676 10 N F
8030 6668 8023 6664 8027 6661 8031 6663 8035 6660 8039 6663
8043 6665 8039 6668 8035 6669 8030 6668 10 N F
8101 6699 8089 6694 8093 6688 8097 6682 8103 6678 8109 6675
8116 6673 8123 6672 8129 6676 8134 6681 8140 6686 8145 6691
8147 6698 8150 6704 8157 6706 8157 6713 8156 6720 8156 6727
8152 6732 8145 6729 8141 6735 8136 6740 8130 6738 8124 6742
8117 6745 8110 6747 8111 6740 8105 6735 8104 6728 8100 6722
8095 6717 8091 6711 8093 6704 8100 6705 8105 6710 8112 6713
8113 6706 8107 6702 8101 6699 39 N F
8169 6668 8165 6666 8167 6664 8171 6664 8174 6663 8176 6666
8179 6667 8182 6666 8185 6668 8185 6671 8188 6673 8191 6672
8193 6675 8194 6679 8195 6682 8193 6685 8190 6687 8187 6689
8184 6690 8181 6688 8178 6688 8175 6687 8174 6683 8174 6680
8173 6677 8170 6675 8169 6672 8169 6668 28 N F
8212 6671 8200 6667 8205 6663 8210 6657 8216 6654 8223 6651
8229 6655 8231 6662 8235 6668 8241 6672 8247 6675 8254 6678
8261 6679 8268 6677 8274 6673 8280 6670 8287 6666 8293 6669
8297 6675 8297 6682 8302 6688 8298 6694 8298 6701 8294 6707
8292 6714 8290 6721 8283 6722 8279 6728 8274 6733 8269 6728

8263 6724 8266 6717 8261 6712 8255 6708 8254 6701 8250 6695
8243 6696 8236 6699 8230 6702 8224 6707 8219 6712 8213 6708
8207 6704 8209 6697 8215 6700 8212 6694 8214 6687 8216 6680
8219 6673 8212 6671 50 N F
8213 6754 8209 6751 8212 6750 8216 6749 8218 6747 8221 6744
8223 6742 8227 6742 8230 6743 8231 6746 8232 6749 8234 6752
8232 6755 8229 6756 8226 6757 8222 6756 8219 6756 8216 6755
8213 6754 19 N F
8275 14738 8272 14733 8276 14734 8279 14737 8283 14740 8285 14743
8288 14747 8290 14751 8293 14754 8294 14758 8295 14762 8293 14766
8291 14763 8289 14759 8286 14756 8283 14753 8282 14749 8281 14745
8279 14741 8275 14738 20 N F
8293 14780 8292 14777 8293 14776 8295 14776 8298 14778 8299 14780
8301 14783 8301 14786 8299 14784 8296 14783 8293 14780 11 N F
8313 14797 8311 14795 8313 14793 8316 14795 8319 14796 8320 14799
8321 14802 8323 14805 8325 14807 8326 14810 8323 14809 8321 14806
8319 14804 8318 14801 8316 14799 8313 14797 16 N F
8362 14788 8356 14786 8361 14787 8365 14788 8369 14790 8372 14793
8375 14796 8378 14799 8379 14803 8377 14807 8373 14810 8371 14813
8369 14809 8370 14805 8374 14804 8375 14799 8371 14797 8368 14794
8365 14791 8362 14788 20 N F
8397 14838 8392 14833 8397 14834 8401 14835 8405 14836 8409 14837
8413 14838 8416 14842 8417 14846 8419 14850 8423 14852 8424 14856
8422 14860 8418 14859 8415 14855 8413 14852 8411 14848 8408 14844
8405 14842 8401 14840 8397 14838 21 N F
8398 14852 8397 14849 8399 14847 8402 14848 8405 14850 8406 14853
8407 14856 8407 14859 8406 14862 8404 14859 8403 14857 8401 14854
8398 14852 13 N F
8431 14850 8426 14849 8430 14847 8434 14849 8438 14850 8442 14851
8446 14853 8450 14856 8453 14859 8456 14862 8458 14866 8461 14869
8464 14872 8467 14875 8470 14878 8473 14881 8475 14886 8471 14884
8467 14882 8463 14881 8460 14878 8456 14877 8455 14873 8452 14870
8448 14868 8445 14865 8441 14862 8437 14860 8437 14856 8434 14853
8431 14850 31 N F
8628 7037 8623 7035 8627 7034 8632 7033 8634 7029 8635 7025
8639 7023 8638 7028 8640 7032 8644 7035 8647 7038 8645 7042
8647 7046 8644 7049 8644 7054 8647 7057 8652 7059 8655 7062
8655 7066 8651 7064 8647 7062 8642 7061 8638 7059 8635 7055
8631 7055 8631 7050 8634 7046 8636 7042 8636 7038 8632 7035
8628 7037 31 N F
8709 12481 8704 12481 8706 12477 8709 12474 8713 12471 8715 12468
8719 12467 8723 12465 8727 12467 8731 12469 8728 12473 8724 12474
8720 12475 8716 12476 8712 12477 8709 12481 16 N F

8717 7287 8712 7276 8717 7270 8721 7265 8721 7258 8724 7251
8731 7254 8736 7258 8743 7258 8744 7266 8741 7272 8742 7279
8741 7286 8743 7293 8746 7300 8748 7306 8747 7313 8750 7320
8750 7327 8750 7334 8747 7340 8740 7337 8737 7331 8733 7325
8727 7321 8727 7314 8725 7307 8721 7301 8720 7294 8717 7287
30 N F
8750 6092 8749 6089 8751 6087 8753 6088 8755 6089 8757 6090
8757 6093 8755 6093 8753 6094 8751 6094 8750 6092 11 N F
8810 7146 8800 7143 8805 7139 8802 7132 8807 7127 8814 7126
8818 7125 8821 7123 8827 7127 8834 7129 8839 7125 8837 7119
8836 7112 8843 7108 8849 7106 8854 7106 8856 7106 8859 7113
8854 7118 8857 7124 8864 7125 8869 7130 8862 7129 8857 7134
8853 7128 8846 7129 8839 7132 8833 7136 8828 7141 8829 7148
8822 7150 8819 7156 8814 7151 8810 7146 34 N F
8809 5985 8802 5982 8805 5978 8807 5974 8808 5970 8812 5968
8812 5972 8816 5975 8819 5978 8820 5983 8818 5987 8813 5987
8809 5985 13 N F
8837 6191 8828 6183 8835 6185 8842 6186 8844 6186 8849 6186
8851 6193 8852 6200 8856 6206 8855 6213 8855 6220 8861 6224
8866 6228 8866 6228 8859 6229 8854 6234 8848 6229 8846 6222
8846 6215 8846 6208 8841 6203 8846 6198 8844 6191 8837 6191
24 N F
8844 6071 8840 6065 8845 6064 8849 6064 8853 6064 8854 6064
8852 6068 8855 6072 8852 6075 8851 6080 8852 6084 8847 6085
8844 6089 8843 6084 8842 6080 8844 6075 8844 6071 17 N F
8848 7641 8842 7629 8849 7629 8854 7628 8856 7628 8863 7626
8870 7625 8876 7621 8882 7618 8889 7618 8896 7620 8895 7628
8894 7634 8891 7641 8888 7648 8882 7651 8875 7654 8870 7658
8864 7654 8857 7652 8852 7646 8848 7641 22 N F
8854 7627 8847 7624 8850 7622 8854 7619 8857 7616 8862 7616
8866 7618 8871 7618 8875 7620 8871 7623 8867 7624 8863 7625
8858 7626 8854 7627 14 N F
8851 6144 8850 6141 8851 6139 8853 6139 8855 6140 8858 6141
8860 6141 8862 6142 8864 6143 8866 6145 8866 6145 8868 6145
8868 6147 8866 6148 8864 6147 8861 6147 8860 6145 8857 6145
8856 6143 8854 6141 8853 6143 8851 6144 22 N F
8853 6017 8850 6015 8852 6014 8854 6014 8855 6016 8853 6017
6 N F
8856 6010 8855 6006 8857 6007 8859 6009 8860 6010 8860 6012
8857 6012 8856 6010 8 N F
8862 7381 8857 7378 8860 7375 8864 7374 8869 7373 8871 7377
8875 7379 8878 7382 8881 7386 8883 7390 8883 7395 8878 7394
8875 7391 8872 7388 8868 7385 8867 7381 8864 7377 8862 7381

18 N F
8859 6071 8857 6069 8859 6068 8860 6068 8861 6068 8862 6070
8864 6071 8866 6071 8869 6071 8869 6073 8868 6075 8866 6076
8864 6075 8861 6075 8861 6073 8859 6071 16 N F
8872 6178 8867 6171 8873 6167 8879 6163 8882 6157 8889 6155
8896 6156 8903 6159 8902 6167 8907 6172 8912 6176 8913 6176
8919 6180 8924 6185 8931 6185 8938 6185 8940 6184 8945 6182
8942 6189 8940 6196 8939 6203 8935 6209 8930 6204 8928 6211
8922 6208 8920 6201 8918 6208 8915 6201 8911 6195 8911 6202
8912 6210 8905 6210 8901 6203 8897 6197 8896 6190 8892 6196
8889 6203 8883 6198 8880 6192 8876 6186 8883 6189 8885 6182
8878 6182 8884 6179 8891 6179 8898 6179 8896 6172 8890 6168
8886 6162 8882 6168 8876 6172 8872 6178 52 N F
8875 6248 8870 6242 8876 6237 8881 6243 8886 6238 8891 6232
8893 6225 8897 6219 8900 6226 8898 6233 8903 6238 8900 6244
8896 6251 8889 6251 8882 6249 8875 6248 16 N F
8897 7964 8893 7958 8896 7954 8898 7950 8898 7946 8903 7946
8905 7950 8908 7948 8913 7946 8917 7947 8921 7948 8926 7949
8927 7953 8931 7956 8934 7959 8939 7960 8943 7961 8946 7965
8945 7969 8941 7971 8937 7970 8933 7967 8933 7963 8930 7959
8926 7961 8927 7965 8923 7965 8919 7964 8914 7965 8910 7966
8906 7965 8901 7965 8897 7964 33 N F
8895 6290 8894 6287 8896 6284 8897 6281 8898 6284 8900 6287
8903 6288 8906 6290 8907 6293 8904 6295 8902 6298 8899 6295
8898 6292 8895 6290 14 N F
8903 7392 8902 7388 8905 7388 8908 7389 8910 7390 8913 7391
8913 7393 8911 7395 8909 7398 8906 7397 8904 7395 8903 7392
12 N F
8908 6007 8906 6005 8908 6004 8910 6003 8912 6003 8913 6006
8912 6008 8910 6008 8908 6007 9 N F
8913 7387 8910 7381 8914 7378 8919 7377 8923 7377 8928 7379
8932 7380 8936 7378 8940 7381 8941 7385 8939 7389 8937 7393
8934 7390 8930 7389 8925 7387 8922 7385 8918 7387 8913 7387
18 N F
8921 7292 8918 7285 8924 7288 8931 7291 8938 7290 8943 7295
8942 7288 8942 7281 8935 7282 8932 7276 8937 7271 8933 7265
8938 7259 8943 7255 8946 7254 8950 7252 8955 7247 8961 7242
8967 7241 8971 7247 8967 7253 8961 7257 8964 7264 8970 7267
8976 7271 8981 7276 8988 7277 8994 7282 9001 7283 9005 7278
9012 7282 9013 7289 9005 7289 8999 7291 8993 7294 8987 7299
8985 7306 8985 7313 8987 7320 8993 7324 8999 7328 9006 7330
9010 7324 9017 7326 9021 7332 9025 7338 9018 7338 9011 7338
9004 7340 8999 7345 8993 7342 8995 7348 9000 7354 8997 7360

8990 7364 8985 7368 8978 7366 8972 7369 8968 7375 8968 7382
8972 7387 8976 7394 8969 7393 8963 7390 8957 7385 8955 7378
8948 7382 8944 7376 8941 7370 8934 7370 8930 7364 8926 7358
8927 7351 8930 7345 8937 7347 8941 7352 8949 7353 8951 7346
8950 7339 8946 7333 8946 7326 8946 7319 8944 7312 8950 7308
8956 7312 8958 7319 8964 7314 8971 7313 8971 7306 8966 7302
8960 7298 8953 7299 8948 7304 8941 7305 8934 7306 8929 7301
8922 7299 8921 7292 98 N F
8928 7204 8926 7202 8926 7198 8929 7196 8927 7192 8927 7189
8928 7190 8929 7192 8933 7193 8935 7195 8938 7197 8935 7197
8933 7200 8931 7203 8934 7205 8933 7209 8930 7207 8928 7204
18 N F
8937 7225 8930 7223 8932 7219 8933 7218 8934 7217 8937 7218
8941 7218 8944 7219 8947 7221 8950 7223 8947 7225 8945 7227
8946 7230 8942 7229 8940 7227 8936 7228 8937 7225 17 N F
8938 6013 8936 6010 8937 6009 8936 6006 8938 6005 8939 6003
8940 6005 8940 6007 8942 6009 8943 6011 8942 6013 8940 6014
8938 6013 13 N F
8952 7939 8948 7937 8949 7934 8951 7931 8954 7931 8957 7929
8960 7930 8963 7932 8965 7934 8969 7934 8972 7934 8974 7936
8976 7939 8974 7941 8970 7942 8968 7944 8964 7943 8961 7942
8958 7941 8955 7941 8952 7939 21 N F
9011 13027 9010 13021 9013 13018 9017 13016 9021 13019 9024 13022
9024 13026 9022 13030 9021 13034 9021 13038 9016 13039 9016 13034
9013 13031 9011 13027 14 N F
9033 7673 9031 7668 9035 7667 9040 7665 9043 7668 9048 7668
9051 7671 9053 7671 9055 7672 9057 7676 9059 7681 9055 7682
9050 7681 9048 7677 9044 7675 9042 7679 9037 7678 9039 7682
9036 7685 9035 7681 9032 7677 9033 7673 22 N F
9046 7575 9042 7573 9044 7570 9046 7568 9050 7569 9052 7571
9055 7573 9053 7576 9050 7576 9046 7575 10 N F
9059 7703 9059 7699 9062 7696 9062 7700 9062 7705 9065 7709
9067 7710 9069 7711 9073 7711 9078 7709 9081 7706 9084 7710
9082 7714 9079 7717 9075 7721 9071 7720 9068 7717 9064 7715
9060 7712 9060 7708 9059 7703 21 N F
9065 7564 9062 7557 9066 7559 9070 7561 9073 7565 9076 7568
9072 7567 9067 7568 9065 7564 9 N F
9088 7876 9084 7869 9089 7868 9093 7869 9095 7873 9099 7872
9104 7873 9104 7878 9104 7882 9105 7887 9100 7887 9099 7883
9095 7885 9091 7884 9087 7881 9088 7876 16 N F
9122 6147 9121 6141 9122 6137 9122 6132 9123 6128 9124 6123
9126 6119 9126 6123 9125 6128 9124 6133 9124 6137 9126 6141
9127 6146 9128 6150 9129 6155 9130 6159 9133 6163 9135 6167

9138 6171 9137 6175 9135 6171 9132 6167 9130 6163 9128 6159
9125 6156 9123 6151 9122 6147 27 N F
9134 7916 9129 7911 9132 7908 9135 7905 9137 7906 9140 7907
9144 7909 9143 7913 9148 7915 9152 7916 9153 7920 9154 7924
9154 7929 9152 7933 9147 7934 9145 7930 9142 7926 9140 7923
9138 7918 9134 7916 20 N F
9140 5927 M 9136 5913 L 9146 5904 L 9161 5907 L 9153 5895 L
9164 5886 L 9176 5877 L 9190 5881 L 9203 5885 L
9217 5890 L 9220 5876 L 9207 5869 L 9193 5867 L
9200 5854 L 9207 5841 L 9211 5827 L 9211 5813 L
9216 5799 L 9221 5786 L 9235 5782 L 9226 5771 L
9232 5757 L 9237 5744 L 9243 5731 L 9249 5718 L
9255 5704 L 9259 5690 L 9259 5676 L 9255 5662 L
9263 5649 L 9276 5643 L 9280 5629 L 9291 5620 L
9302 5610 L 9289 5616 L 9279 5627 L 9272 5639 L
9277 5626 L 9286 5614 L 9296 5603 L 9306 5593 L
9316 5583 L 9328 5575 L 9328 5575 L 9341 5567 L
9347 5580 L 9353 5567 L 9363 5555 L 9373 5546 L
9386 5539 L 9399 5532 L 9412 5525 L 9424 5518 L
9437 5510 L 9447 5500 L 9454 5488 L 9465 5478 L
9472 5465 L 9475 5455 L 9477 5455 L 9510 5455 L
9544 5455 L 9577 5455 L 9608 5455 L 9607 5465 L
9607 5479 L 9609 5494 L 9614 5507 L 9617 5521 L
9622 5535 L 9628 5548 L 9632 5562 L 9635 5576 L
9638 5591 L 9643 5604 L 9646 5619 L 9652 5632 L
9662 5642 L 9665 5657 L 9667 5671 L 9673 5684 L
9685 5693 L 9695 5703 L 9707 5711 L 9720 5718 L
9730 5728 L 9741 5738 L 9751 5749 L 9754 5763 L
9758 5776 L 9753 5790 L 9760 5803 L 9773 5808 L
9784 5818 L 9791 5831 L 9798 5844 L 9802 5858 L
9812 5868 L 9819 5880 L 9824 5894 L 9830 5907 L
9833 5921 L 9838 5935 L 9848 5945 L 9858 5956 L
9859 5970 L 9863 5984 L 9857 5998 L 9859 6012 L
9865 6025 L 9868 6039 L 9872 6053 L 9878 6066 L
9889 6075 L 9891 6089 L 9893 6104 L 9895 6118 L
9899 6132 L 9904 6145 L 9906 6160 L 9911 6173 L
9906 6187 L 9907 6201 L 9910 6215 L 9916 6228 L
9917 6243 L 9917 6257 L 9918 6260 L 9923 6270 L
9924 6285 L 9925 6299 L 9928 6313 L 9934 6327 L
9934 6341 L 9934 6355 L 9940 6369 L 9944 6382 L
9947 6397 L 9949 6411 L 9949 6425 L 9950 6440 L
9951 6454 L 9960 6465 L 9968 6477 L 9960 6489 L
9953 6502 L 9950 6516 L 9955 6530 L 9965 6539 L

9974 6551 L 9973 6565 L 9970 6579 L 9977 6592 L
9989 6601 L 9997 6612 L 10009 6621 L 10014 6634 L
10021 6647 L 10027 6660 L 10026 6674 L 10030 6688 L
10032 6702 L 10034 6716 L 10040 6729 L 10047 6742 L
10058 6751 L 10056 6765 L 10061 6779 L 10073 6787 L
10070 6801 L 10071 6815 L 10068 6829 L 10059 6840 L
10054 6853 L 10050 6867 L 10048 6881 L 10043 6895 L
10039 6909 L 10038 6923 L 10038 6937 L 10039 6951 L
10042 6965 L 10046 6979 L 10054 6991 L 10064 7001 L
10069 7015 L 10080 7024 L 10089 7035 L 10101 7042 L
10104 7044 L 10114 7049 L 10125 7058 L 10117 7070 L
10103 7068 L 10090 7075 L 10089 7090 L 10081 7101 L
10084 7116 L 10073 7125 L 10059 7124 L 10045 7126 L
10031 7122 L 10020 7131 L 10007 7137 L 9994 7131 L
9984 7141 L 9971 7147 L 9960 7156 L 9964 7169 L
9951 7165 L 9940 7174 L 9931 7185 L 9928 7199 L
9916 7207 L 9903 7214 L 9889 7213 L 9875 7209 L
9862 7205 L 9849 7197 L 9840 7186 L 9832 7175 L
9825 7162 L 9817 7150 L 9828 7141 L 9815 7136 L
9806 7126 L 9793 7119 L 9780 7113 L 9766 7111 L
9752 7107 L 9738 7109 L 9724 7103 L 9711 7098 L
9699 7090 L 9687 7083 L 9673 7079 L 9660 7074 L
9649 7065 L 9636 7058 L 9626 7048 L 9618 7033 L
9616 7018 L 9607 7007 L 9599 6995 L 9592 6983 L
9586 6970 L 9582 6956 L 9573 6945 L 9565 6934 L
9571 6921 L 9567 6907 L 9563 6893 L 9554 6882 L
9543 6873 L 9540 6859 L 9529 6849 L 9540 6839 L
9525 6840 L 9511 6838 L 9498 6832 L 9490 6820 L
9496 6807 L 9482 6805 L 9469 6799 L 9464 6785 L
9459 6772 L 9456 6758 L 9452 6744 L 9445 6731 L
9438 6719 L 9427 6709 L 9419 6698 L 9409 6687 L
9404 6674 L 9397 6661 L 9392 6648 L 9386 6634 L
9384 6620 L 9375 6608 L 9371 6595 L 9364 6582 L
9356 6570 L 9368 6562 L 9354 6562 L 9347 6550 L
9340 6537 L 9332 6525 L 9329 6511 L 9325 6497 L
9316 6486 L 9306 6476 L 9296 6465 L 9289 6452 L
9280 6441 L 9274 6429 L 9274 6414 L 9267 6401 L
9262 6388 L 9257 6374 L 9247 6364 L 9256 6353 L
9271 6351 L 9285 6349 L 9299 6352 L 9288 6343 L
9274 6340 L 9259 6339 L 9245 6342 L 9231 6341 L
9222 6329 L 9214 6318 L 9212 6304 L 9210 6289 L
9207 6275 L 9195 6267 L 9189 6254 L 9183 6241 L
9181 6226 L 9180 6212 L 9187 6199 L 9187 6185 L

9193 6172 L 9181 6164 L 9191 6153 L 9188 6139 L
9199 6130 L 9188 6120 L 9192 6106 L 9196 6092 L
9195 6078 L 9192 6064 L 9199 6051 L 9185 6053 L
9183 6039 L 9175 6027 L 9177 6013 L 9166 6004 L
9179 5999 L 9165 5996 L 9164 5982 L 9164 5968 L
9155 5958 L 9169 5954 L 9174 5940 L 9169 5926 L
9158 5917 L 9144 5914 L 9140 5927 L N E
9170 14242 9167 14237 9169 14233 9173 14230 9176 14227 9179 14225
9182 14228 9182 14233 9181 14237 9182 14241 9178 14244 9174 14244
9170 14242 13 N F
9198 15606 9196 15599 9199 15596 9203 15598 9206 15601 9209 15604
9210 15604 9214 15604 9218 15605 9219 15609 9219 15613 9219 15618
9215 15620 9211 15622 9209 15618 9205 15618 9204 15614 9205 15610
9201 15610 9198 15606 20 N F
9209 14252 9205 14249 9207 14246 9210 14243 9212 14239 9216 14236
9220 14236 9223 14233 9226 14237 9225 14241 9224 14245 9221 14249
9217 14248 9214 14251 9209 14252 15 N F
9225 14211 9221 14208 9225 14206 9229 14206 9232 14203 9235 14199
9238 14197 9240 14201 9239 14205 9238 14209 9240 14213 9238 14217
9236 14220 9233 14224 9232 14228 9228 14231 9225 14228 9224 14224
9224 14220 9225 14216 9225 14211 21 N F
9225 14355 9224 14350 9225 14344 9228 14341 9228 14341 9232 14343
9236 14342 9240 14342 9243 14346 9240 14349 9235 14350 9231 14350
9232 14354 9230 14358 9228 14362 9225 14359 9225 14355 17 N F
9227 8137 9225 8132 9227 8128 9230 8124 9234 8122 9238 8119
9242 8117 9246 8117 9250 8119 9254 8121 9259 8122 9263 8123
9259 8125 9255 8127 9254 8131 9252 8135 9250 8139 9246 8140
9243 8143 9238 8144 9234 8143 9230 8140 9227 8137 23 N F
9251 14419 9247 14415 9251 14412 9254 14414 9256 14418 9260 14419
9264 14420 9266 14424 9266 14428 9262 14430 9258 14429 9254 14427
9252 14424 9251 14419 14 N F
9291 14459 9287 14456 9289 14453 9291 14450 9294 14449 9295 14446
9296 14449 9295 14452 9297 14454 9294 14454 9294 14457 9291 14459
12 N F
9291 14471 9289 14466 9291 14467 9292 14467 9295 14466 9298 14468
9301 14467 9301 14470 9299 14472 9298 14475 9295 14475 9294 14472
9291 14471 13 N F
9312 7816 9309 7812 9312 7813 9313 7810 9316 7812 9318 7815
9316 7817 9313 7819 9312 7816 9 N F
9311 14277 9309 14273 9311 14271 9315 14270 9317 14272 9317 14275
9314 14277 9311 14277 8 N F
9318 5482 9316 5477 9320 5475 9324 5477 9327 5481 9330 5484
9334 5487 9335 5491 9332 5494 9328 5495 9324 5493 9320 5490

9321 5486 9318 5482 14 N F
9318 7811 9316 7804 9320 7803 9324 7805 9327 7809 9331 7808
9329 7812 9326 7815 9322 7814 9318 7811 10 N F
9413 15102 9403 15101 9405 15094 9404 15088 9410 15085 9417 15083
9423 15085 9430 15084 9434 15079 9436 15073 9440 15079 9445 15082
9449 15088 9454 15092 9457 15092 9461 15093 9468 15093 9473 15089
9479 15086 9486 15085 9493 15088 9497 15093 9502 15098 9508 15099
9511 15105 9515 15111 9522 15109 9527 15113 9530 15119 9532 15126
9531 15132 9531 15139 9527 15145 9526 15151 9519 15153 9513 15156
9507 15159 9501 15162 9495 15162 9499 15157 9499 15150 9496 15145
9489 15142 9483 15142 9478 15137 9472 15134 9465 15134 9458 15135
9452 15133 9446 15131 9439 15130 9433 15134 9431 15128 9431 15121
9426 15117 9420 15113 9417 15107 9413 15102 58 N F
9421 15022 9416 15017 9422 15014 9429 15012 9433 15017 9435 15024
9440 15028 9445 15033 9449 15039 9450 15045 9456 15049 9462 15049
9469 15049 9471 15042 9467 15038 9462 15032 9458 15027 9455 15021
9452 15015 9459 15013 9465 15011 9472 15010 9477 15006 9484 15007
9490 15010 9490 15017 9494 15022 9497 15029 9497 15035 9494 15042
9488 15046 9482 15049 9489 15048 9496 15048 9502 15050 9500 15059
9497 15065 9491 15067 9485 15069 9478 15071 9472 15070 9466 15066
9462 15071 9457 15075 9450 15074 9446 15068 9443 15062 9443 15055
9439 15050 9432 15049 9425 15049 9423 15042 9425 15036 9419 15035
9422 15029 9421 15022 56 N F
9417 8745 9417 8741 9418 8738 9420 8735 9422 8731 9426 8730
9428 8728 9430 8725 9433 8723 9436 8722 9439 8721 9442 8723
9442 8727 9442 8730 9439 8732 9438 8735 9436 8738 9433 8740
9430 8741 9427 8742 9424 8743 9420 8744 9417 8745 23 N F
9476 8719 9470 8718 9474 8717 9476 8715 9479 8713 9482 8711
9485 8709 9487 8707 9490 8706 9494 8704 9497 8703 9500 8704
9503 8705 9506 8707 9508 8710 9510 8712 9510 8716 9509 8719
9506 8720 9502 8721 9499 8722 9496 8722 9492 8722 9489 8721
9486 8721 9482 8721 9479 8721 9476 8719 28 N F
9546 10862 9538 10853 9542 10848 9548 10843 9553 10839 9558 10834
9562 10828 9566 10823 9571 10818 9578 10821 9584 10818 9590 10815
9595 10810 9600 10806 9606 10802 9611 10797 9616 10792 9621 10787
9626 10783 9632 10780 9638 10776 9645 10774 9649 10768 9655 10765
9662 10764 9668 10762 9673 10757 9680 10755 9686 10752 9692 10749
9699 10747 9705 10745 9711 10740 9717 10737 9723 10734 9728 10729
9735 10728 9742 10726 9747 10722 9753 10718 9756 10717 9760 10716
9767 10715 9774 10716 9780 10715 9787 10713 9794 10711 9800 10708
9806 10706 9813 10702 9819 10699 9825 10697 9832 10695 9838 10692
9845 10691 9852 10689 9859 10688 9860 10688 9866 10688 9872 10691
9878 10694 9881 10700 9885 10706 9891 10710 9892 10717 9893 10723

9894 10730 9892 10737 9886 10740 9880 10745 9874 10747 9867 10748
9860 10748 9853 10749 9847 10752 9840 10753 9834 10757 9828 10760
9823 10765 9817 10768 9811 10771 9805 10775 9798 10777 9792 10779
9785 10781 9779 10783 9772 10785 9765 10787 9758 10789 9753 10792
9746 10795 9742 10801 9739 10807 9734 10812 9728 10816 9723 10821
9717 10823 9710 10826 9704 10828 9698 10832 9691 10834 9685 10836
9678 10838 9672 10842 9668 10847 9665 10853 9662 10860 9658 10865
9655 10871 9650 10876 9645 10881 9639 10885 9633 10888 9626 10890
9619 10891 9613 10893 9606 10893 9599 10892 9592 10891 9585 10889
9579 10887 9572 10884 9567 10879 9562 10875 9557 10870 9551 10866
9546 10862 127 N F
9561 8653 9559 8649 9561 8646 9564 8649 9568 8651 9572 8653
9576 8655 9579 8652 9583 8650 9583 8655 9585 8656 9587 8657
9590 8661 9593 8664 9598 8664 9594 8666 9590 8664 9586 8663
9582 8661 9577 8662 9575 8665 9571 8669 9567 8669 9567 8665
9563 8662 9562 8658 9561 8653 27 N F
9569 8713 9567 8708 9572 8710 9576 8711 9581 8711 9585 8713
9589 8715 9593 8713 9592 8709 9593 8705 9597 8707 9601 8708
9606 8709 9610 8711 9614 8712 9617 8715 9622 8717 9618 8719
9614 8722 9611 8725 9608 8728 9605 8730 9601 8728 9596 8728
9592 8729 9588 8726 9584 8725 9585 8729 9580 8729 9579 8724
9576 8722 9572 8720 9568 8717 9569 8713 34 N F
9585 8438 9580 8435 9583 8431 9587 8429 9590 8426 9591 8430
9590 8434 9594 8432 9598 8431 9602 8429 9607 8429 9610 8426
9610 8430 9607 8434 9603 8435 9600 8439 9597 8442 9592 8444
9589 8447 9585 8447 9588 8444 9592 8442 9587 8442 9585 8438
24 N F
9589 8616 9586 8614 9589 8613 9592 8612 9594 8613 9597 8615
9598 8617 9597 8619 9596 8621 9593 8623 9591 8621 9590 8619
9589 8616 13 N F
9596 8642 9593 8637 9598 8636 9602 8635 9607 8636 9611 8635
9615 8637 9619 8637 9622 8641 9626 8638 9630 8637 9634 8635
9638 8636 9640 8640 9639 8644 9639 8649 9635 8651 9639 8654
9641 8649 9644 8653 9647 8656 9644 8660 9645 8664 9641 8665
9639 8661 9634 8659 9630 8660 9626 8658 9629 8654 9627 8650
9623 8650 9619 8649 9616 8653 9613 8656 9611 8652 9607 8649
9604 8646 9600 8644 9596 8642 39 N F
9618 8816 9608 8814 9611 8807 9611 8800 9614 8794 9618 8788
9623 8783 9622 8776 9616 8773 9622 8769 9621 8762 9627 8764
9634 8764 9641 8767 9641 8768 9647 8770 9654 8772 9653 8779
9660 8777 9665 8772 9668 8779 9662 8783 9664 8790 9670 8786
9676 8789 9678 8783 9685 8784 9681 8790 9687 8793 9688 8800
9681 8800 9675 8804 9671 8810 9672 8817 9670 8823 9667 8830

9670 8837 9674 8842 9681 8843 9679 8849 9673 8854 9667 8857
9661 8861 9654 8860 9648 8858 9643 8852 9642 8845 9644 8839
9638 8835 9633 8829 9628 8825 9622 8821 9618 8816 53 N F
9633 8735 9631 8731 9634 8728 9636 8723 9640 8722 9641 8723
9643 8726 9647 8729 9651 8731 9655 8732 9658 8733 9659 8734
9663 8736 9659 8736 9654 8736 9650 8738 9646 8738 9641 8739
9637 8737 9633 8735 20 N F
9652 8530 9649 8527 9651 8527 9652 8525 9654 8525 9656 8526
9658 8527 9659 8529 9661 8530 9661 8531 9662 8532 9663 8534
9662 8536 9660 8535 9658 8536 9656 8534 9656 8531 9654 8530
9652 8530 19 N F
9657 8524 9655 8522 9657 8520 9659 8522 9660 8523 9661 8525
9661 8528 9659 8528 9657 8526 9657 8524 10 N F
9672 14755 9668 14752 9669 14749 9669 14745 9671 14743 9674 14744
9676 14747 9676 14750 9674 14753 9675 14756 9675 14759 9672 14760
9670 14757 9672 14755 14 N F
9756 10676 9747 10671 9753 10668 9759 10666 9765 10662 9772 10659
9777 10654 9783 10651 9789 10648 9795 10645 9800 10640 9806 10637
9813 10634 9818 10630 9825 10627 9828 10621 9832 10616 9836 10610
9842 10606 9849 10608 9849 10615 9848 10622 9845 10628 9841 10634
9836 10639 9832 10644 9826 10648 9821 10653 9815 10655 9809 10659
9803 10662 9796 10664 9790 10667 9783 10668 9777 10671 9770 10672
9763 10674 9756 10676 38 N F
9792 8796 9791 8793 9791 8789 9794 8790 9797 8792 9798 8795
9800 8798 9803 8798 9806 8800 9807 8803 9806 8806 9802 8805
9800 8802 9798 8800 9795 8798 9792 8796 16 N F
9795 9396 9793 9394 9796 9392 9799 9390 9801 9388 9805 9388
9808 9389 9811 9389 9815 9390 9816 9393 9815 9397 9814 9399
9810 9400 9808 9398 9808 9395 9805 9393 9801 9394 9799 9396
9795 9396 19 N F
9831 9043 9829 9038 9831 9035 9834 9034 9837 9032 9840 9030
9842 9033 9841 9036 9842 9038 9843 9039 9846 9039 9846 9043
9843 9044 9843 9047 9842 9050 9840 9053 9837 9054 9834 9053
9833 9050 9832 9047 9831 9043 21 N F
9842 9027 9838 9024 9839 9021 9842 9019 9845 9018 9848 9020
9850 9022 9849 9025 9845 9026 9842 9027 10 N F
9860 10641 9851 10640 9854 10634 9857 10628 9859 10621 9860 10614
9862 10608 9867 10603 9873 10600 9879 10596 9885 10598 9889 10604
9893 10610 9896 10616 9894 10623 9891 10629 9885 10633 9879 10636
9873 10639 9866 10639 9860 10641 21 N F
9877 14932 9866 14927 9870 14922 9869 14915 9874 14910 9879 14906
9885 14910 9888 14916 9890 14922 9895 14918 9901 14917 9901 14923
9899 14930 9896 14936 9898 14942 9905 14945 9898 14945 9893 14940

9887 14938 9880 14938 9877 14932 21 N F
9913 5703 9905 5701 9908 5697 9910 5691 9913 5686 9919 5684
9922 5679 9928 5680 9927 5686 9928 5691 9930 5697 9929 5703
9924 5705 9918 5705 9913 5703 15 N F
9908 9393 9905 9386 9907 9382 9907 9382 9912 9382 9916 9382
9920 9380 9923 9379 9924 9379 9928 9376 9930 9372 9934 9369
9938 9370 9942 9369 9946 9371 9950 9373 9948 9377 9952 9377
9953 9379 9955 9381 9958 9384 9955 9387 9950 9388 9947 9390
9948 9395 9946 9398 9942 9396 9937 9396 9933 9395 9929 9395
9924 9396 9921 9399 9917 9398 9913 9396 9910 9399 9906 9397
9908 9393 37 N F
9908 9147 9906 9143 9909 9142 9912 9141 9915 9139 9917 9136
9920 9136 9923 9138 9923 9142 9924 9145 9926 9147 9928 9150
9925 9150 9923 9147 9920 9145 9918 9143 9914 9142 9912 9145
9913 9148 9910 9149 9907 9150 9908 9147 22 N F
9911 14976 9907 14974 9911 14974 9914 14972 9916 14971 9916 14971
9918 14968 9921 14969 9925 14969 9922 14972 9920 14973 9916 14973
9915 14976 9911 14976 14 N F
9911 14954 9908 14951 9912 14951 9914 14949 9917 14951 9920 14952
9921 14955 9919 14958 9916 14958 9914 14956 9911 14954 11 N F
9915 9365 9909 9359 9913 9358 9918 9358 9917 9354 9921 9353
9924 9350 9928 9347 9931 9350 9931 9354 9933 9358 9930 9362
9933 9365 9931 9368 9927 9371 9923 9372 9918 9372 9915 9369
9915 9365 19 N F
9913 9882 9909 9875 9914 9870 9916 9863 9922 9859 9929 9860
9934 9865 9940 9869 9945 9873 9951 9877 9953 9883 9951 9890
9949 9897 9948 9903 9944 9909 9937 9912 9934 9918 9930 9924
9923 9924 9916 9922 9915 9916 9912 9909 9910 9903 9910 9896
9912 9889 9913 9882 26 N F
9944 14960 9939 14957 9942 14955 9944 14953 9947 14953 9950 14952
9953 14953 9956 14953 9959 14953 9961 14956 9963 14958 9960 14960
9957 14959 9954 14959 9951 14960 9948 14960 9944 14960 17 N F
9946 14994 9940 14993 9943 14990 9944 14989 9946 14988 9951 14987
9955 14987 9958 14984 9962 14982 9966 14981 9971 14980 9975 14979
9974 14974 9977 14971 9979 14975 9979 14979 9983 14980 9987 14980
9989 14980 9992 14979 9996 14979 10000 14979 10005 14978 10009 14977
10012 14974 10015 14977 10018 14980 10022 14983 10017 14982 10013 14984
10009 14985 10005 14987 10003 14990 9999 14991 9996 14988 9992 14986
9988 14985 9984 14985 9979 14986 9975 14987 9972 14990 9969 14993
9969 14997 9966 14994 9964 14990 9960 14993 9960 14997 9956 14997
9953 14994 9950 14997 9946 14994 51 N F
9943 9192 9940 9190 9942 9187 9944 9190 9947 9192 9950 9194
9952 9196 9952 9199 9952 9203 9954 9205 9958 9204 9956 9207

9956 9210 9953 9213 9950 9213 9947 9211 9946 9208 9947 9204
9947 9201 9943 9201 9945 9198 9945 9194 9943 9192 23 N F
9943 9934 9940 9928 9944 9926 9948 9925 9952 9923 9950 9918
9955 9917 9959 9917 9959 9921 9961 9926 9963 9929 9962 9934
9965 9937 9961 9940 9958 9943 9954 9942 9950 9940 9946 9938
9943 9934 19 N F
9949 9171 9947 9166 9950 9166 9952 9163 9956 9163 9959 9165
9958 9168 9956 9170 9958 9172 9961 9175 9963 9177 9966 9178
9969 9179 9970 9182 9973 9183 9976 9183 9973 9184 9970 9185
9967 9183 9965 9180 9962 9178 9959 9179 9956 9177 9953 9176
9949 9176 9947 9174 9949 9171 27 N F
9951 9397 9949 9390 9953 9390 9957 9388 9959 9384 9962 9380
9967 9380 9970 9377 9970 9372 9970 9368 9968 9364 9965 9360
9970 9362 9970 9362 9974 9362 9978 9362 9979 9362 9983 9360
9982 9356 9980 9351 9985 9350 9988 9348 9990 9350 9992 9351
9996 9352 9994 9356 9994 9361 9995 9365 9999 9367 10003 9369
10000 9373 9996 9373 9998 9376 10002 9379 10005 9382 10004 9387
10002 9391 10002 9395 9999 9399 10002 9402 10001 9407 9998 9410
9994 9412 9990 9414 9988 9418 9985 9422 9980 9422 9977 9420
9976 9415 9972 9413 9968 9410 9965 9408 9961 9405 9957 9403
9953 9401 9951 9397 56 N F
9958 9975 9952 9972 9955 9966 9958 9963 9960 9961 9966 9957
9969 9951 9972 9944 9972 9943 9976 9939 9977 9932 9979 9925
9977 9918 9975 9912 9978 9906 9984 9902 9988 9896 9993 9891
9996 9885 9998 9878 10004 9875 10008 9878 10010 9879 10015 9884
10021 9886 10022 9886 10029 9886 10035 9886 10042 9883 10044 9880
10045 9877 10052 9877 10059 9878 10066 9879 10067 9879 10073 9877
10079 9874 10085 9871 10092 9869 10099 9867 10105 9865 10111 9861
10117 9857 10118 9857 10123 9854 10130 9855 10137 9856 10143 9859
10140 9865 10139 9872 10141 9879 10144 9885 10147 9891 10152 9896
10146 9899 10142 9905 10137 9909 10132 9914 10127 9919 10122 9923
10115 9922 10108 9923 10104 9929 10097 9932 10096 9938 10089 9938
10082 9938 10076 9941 10073 9947 10066 9946 10059 9947 10052 9948
10048 9954 10044 9959 10040 9965 10034 9969 10027 9971 10022 9967
10015 9964 10009 9962 10002 9960 9996 9962 9991 9967 9984 9961
9978 9965 9971 9966 9971 9973 9966 9978 9960 9981 9958 9975
90 N F
9958 10053 9956 10050 9957 10047 9958 10043 9960 10046 9960 10046
9963 10048 9966 10050 9966 10053 9965 10056 9967 10058 9966 10061
9968 10063 9970 10066 9971 10069 9969 10071 9966 10069 9966 10066
9964 10064 9962 10066 9960 10063 9961 10059 9960 10056 9958 10053
24 N F
9958 9834 9956 9826 9959 9823 9962 9820 9967 9819 9971 9820

9975 9821 9980 9822 9984 9824 9988 9826 9989 9830 9988 9835
9986 9838 9981 9839 9979 9843 9978 9847 9974 9849 9972 9853
9968 9852 9967 9848 9962 9846 9959 9843 9958 9839 9958 9834
24 N F
9968 9038 9965 9034 9968 9035 9971 9035 9969 9032 9972 9030
9970 9028 9968 9025 9969 9022 9973 9024 9975 9021 9977 9023
9979 9025 9982 9024 9982 9027 9983 9030 9985 9033 9986 9034
9987 9035 9984 9036 9981 9036 9978 9038 9977 9041 9978 9044
9975 9044 9971 9043 9970 9040 9968 9038 28 N F
9969 8999 9965 8996 9968 8997 9971 8998 9973 9001 9976 8999
9978 9001 9981 9003 9984 9004 9986 9006 9988 9009 9986 9012
9983 9011 9979 9011 9977 9009 9973 9008 9971 9005 9971 9002
9969 8999 19 N F
9979 9766 9978 9762 9979 9758 9979 9753 9982 9750 9986 9750
9991 9750 9995 9750 9997 9754 10000 9758 10001 9762 10005 9765
10006 9769 10009 9773 10010 9777 10007 9781 10003 9782 9999 9780
9993 9777 9989 9774 9986 9772 9982 9769 9979 9766 23 N F
9984 9162 9980 9158 9982 9156 9983 9153 9986 9154 9988 9157
9991 9158 9993 9161 9990 9163 9987 9162 9984 9162 11 N F
9984 14951 9981 14948 9983 14946 9986 14948 9989 14948 9991 14951
9991 14954 9989 14957 9988 14954 9984 14955 9984 14951 11 N F
9991 9986 9987 9982 9990 9983 9994 9982 9995 9979 9998 9977
9995 9975 9993 9972 9997 9972 9999 9974 10002 9976 10004 9978
10007 9980 10009 9983 10011 9985 10009 9988 10009 9991 10007 9994
10004 9993 10001 9991 10001 9988 9999 9991 9995 9992 9993 9990
9994 9987 9991 9986 26 N F
9995 10240 9991 10235 9994 10233 9996 10231 9999 10232 10001 10235
10002 10238 10004 10240 10001 10239 9998 10241 9997 10238 9995 10240
12 N F
10001 10212 9996 10209 9998 10207 10001 10207 10003 10204 10005 10201
10007 10202 10008 10202 10010 10205 10010 10208 10012 10211 10015 10212
10012 10213 10009 10214 10006 10214 10004 10211 10001 10212 17 N F
10003 14965 9997 14961 10000 14958 10004 14958 10007 14961 10011 14960
10016 14962 10020 14962 10024 14962 10028 14961 10028 14957 10026 14953
10024 14949 10024 14945 10027 14948 10031 14951 10034 14954 10038 14954
10038 14958 10042 14959 10045 14960 10046 14961 10050 14963 10046 14965
10044 14969 10040 14971 10042 14975 10039 14972 10036 14968 10032 14970
10028 14970 10024 14969 10020 14968 10016 14966 10011 14966 10007 14966
10003 14965 37 N F
10002 9741 9999 9738 10001 9735 10000 9732 10001 9729 10001 9725
10004 9727 10006 9730 10008 9732 10009 9735 10011 9738 10014 9739
10014 9742 10013 9746 10010 9747 10007 9745 10004 9744 10002 9741
18 N F

10003 9857 10002 9853 10003 9850 10003 9846 10006 9845 10009 9845
10012 9847 10013 9850 10015 9853 10015 9856 10014 9859 10011 9861
10008 9861 10006 9859 10003 9857 15 N F
10018 9415 10017 9408 10021 9409 10025 9409 10027 9413 10030 9417
10033 9414 10038 9414 10041 9417 10044 9420 10040 9422 10035 9421
10031 9423 10032 9427 10036 9429 10033 9432 10036 9436 10031 9436
10028 9439 10026 9435 10027 9431 10025 9427 10028 9425 10024 9423
10022 9419 10017 9419 10018 9415 27 N F
10023 9736 10018 9724 10025 9724 10028 9729 10028 9730 10035 9730
10041 9725 10048 9725 10049 9719 10055 9715 10061 9712 10061 9705
10061 9698 10068 9698 10070 9705 10073 9699 10079 9702 10081 9695
10088 9694 10091 9700 10092 9707 10096 9713 10092 9718 10089 9725
10083 9727 10078 9732 10071 9734 10065 9738 10061 9743 10057 9748
10058 9755 10056 9762 10052 9767 10047 9763 10040 9760 10033 9760
10028 9756 10027 9749 10026 9742 10023 9736 40 N F
10023 10051 10018 10042 10024 10039 10031 10036 10036 10032 10042 10029
10048 10025 10055 10023 10062 10023 10064 10023 10069 10023 10072 10022
10075 10021 10082 10018 10088 10016 10086 10023 10083 10029 10088 10034
10090 10041 10086 10047 10090 10052 10095 10057 10088 10057 10084 10062
10085 10069 10079 10072 10072 10070 10066 10073 10065 10066 10059 10063
10052 10062 10047 10057 10040 10059 10033 10059 10029 10054 10023 10051
36 N F
10021 9880 10020 9877 10021 9874 10020 9871 10022 9868 10025 9869
10028 9871 10031 9872 10029 9875 10027 9878 10025 9880 10021 9880
12 N F
10041 14863 10036 14860 10039 14857 10043 14854 10039 14852 10042 14849
10046 14847 10050 14846 10054 14845 10058 14843 10062 14846 10061 14850
10057 14851 10056 14855 10053 14858 10049 14856 10046 14859 10045 14864
10041 14863 19 N F
10040 9868 10039 9864 10041 9860 10044 9861 10047 9862 10047 9865
10048 9868 10047 9871 10044 9872 10041 9871 10040 9868 11 N F
10054 14965 10051 14964 10054 14963 10057 14963 10059 14965 10057 14967
10054 14965 7 N F
10056 9862 10051 9858 10052 9857 10053 9855 10057 9855 10060 9855
10063 9854 10066 9856 10068 9858 10067 9861 10065 9864 10062 9865
10059 9864 10056 9862 14 N F
10053 9987 10052 9977 10055 9970 10059 9965 10066 9967 10071 9973
10072 9980 10078 9982 10083 9987 10077 9991 10070 9994 10064 9996
10058 9992 10053 9987 14 N F
10059 9871 10054 9867 10057 9866 10060 9868 10062 9867 10063 9867
10067 9868 10070 9866 10072 9863 10074 9861 10077 9862 10078 9866
10077 9869 10074 9871 10071 9873 10069 9876 10067 9877 10064 9876
10062 9873 10059 9871 20 N F

10062 14963 10057 14960 10060 14959 10062 14957 10064 14959 10067 14960
10071 14959 10073 14961 10071 14963 10068 14963 10065 14962 10062 14963
12 N F
10069 10014 10065 10010 10066 10008 10069 10007 10071 10005 10074 10004
10076 10005 10077 10008 10077 10011 10075 10012 10072 10014 10069 10014
12 N F
10072 14881 10067 14880 10069 14878 10072 14876 10075 14878 10078 14878
10081 14876 10083 14879 10080 14881 10078 14883 10075 14881 10072 14881
12 N F
10084 14868 10083 14863 10085 14861 10088 14863 10091 14864 10093 14867
10091 14870 10088 14871 10087 14868 10084 14868 10 N F
10091 9564 10089 9560 10090 9557 10093 9559 10097 9558 10099 9561
10101 9563 10102 9566 10105 9568 10108 9569 10111 9570 10110 9573
10107 9572 10104 9574 10101 9571 10098 9570 10098 9567 10097 9564
10094 9562 10091 9564 20 N F
10096 9724 10094 9721 10097 9720 10100 9718 10103 9717 10104 9717
10106 9715 10109 9713 10112 9711 10114 9709 10115 9712 10119 9713
10122 9712 10125 9712 10127 9710 10130 9707 10130 9711 10132 9714
10129 9715 10125 9715 10122 9714 10119 9716 10116 9717 10113 9718
10110 9720 10107 9720 10106 9724 10104 9726 10101 9726 10099 9728
10095 9728 10096 9724 32 N F
10096 10177 10095 10173 10098 10172 10101 10173 10102 10176 10104 10179
10101 10181 10098 10179 10096 10177 9 N F
10100 6965 10096 6965 10098 6964 10099 6962 10101 6961 10103 6960
10105 6961 10108 6961 10110 6960 10112 6961 10112 6963 10111 6964
10109 6966 10108 6968 10106 6969 10104 6970 10102 6969 10101 6967
10100 6965 19 N F
10102 9763 10097 9758 10099 9757 10101 9757 10104 9753 10106 9749
10109 9746 10112 9743 10116 9741 10120 9738 10124 9739 10128 9738
10132 9736 10135 9739 10140 9739 10144 9739 10145 9743 10140 9742
10140 9747 10136 9748 10132 9745 10128 9746 10123 9746 10119 9748
10116 9751 10112 9754 10115 9757 10117 9761 10113 9763 10110 9766
10105 9766 10102 9763 32 N F
10103 14875 10097 14877 10099 14875 10101 14872 10102 14869 10105 14868
10105 14868 10107 14870 10108 14873 10106 14875 10103 14875 11 N F
10104 9800 10099 9792 10105 9789 10110 9785 10110 9785 10114 9779
10119 9774 10125 9770 10130 9765 10137 9764 10142 9760 10149 9763
10145 9769 10141 9775 10142 9782 10136 9786 10142 9790 10137 9795
10131 9798 10124 9800 10118 9802 10111 9800 10104 9800 23 N F
10103 10245 10100 10242 10101 10241 10103 10241 10105 10242 10106 10243
10109 10245 10108 10248 10105 10249 10106 10246 10103 10245 11 N F
10105 10038 10102 10028 10104 10021 10110 10024 10116 10027 10123 10028
10130 10029 10126 10035 10119 10037 10112 10037 10105 10038 11 N F

10116 10148 10109 10149 10111 10143 10110 10136 10115 10131 10113 10124
10120 10121 10126 10118 10133 10117 10131 10124 10138 10125 10143 10129
10149 10125 10156 10126 10163 10124 10169 10124 10170 10124 10176 10120
10181 10120 10183 10120 10180 10127 10174 10130 10180 10134 10175 10140
10169 10142 10162 10142 10155 10143 10153 10150 10146 10149 10150 10143
10143 10144 10136 10146 10129 10148 10123 10146 10116 10148 35 N F
10122 10025 10112 10020 10118 10015 10122 10020 10129 10022 10136 10020
10142 10022 10149 10024 10153 10030 10156 10031 10160 10032 10165 10036
10163 10030 10165 10023 10171 10019 10177 10022 10181 10028 10178 10035
10175 10041 10169 10043 10162 10042 10155 10042 10148 10040 10141 10040
10134 10039 10133 10032 10128 10027 10122 10025 28 N F
10121 15129 10120 15125 10124 15124 10128 15124 10130 15120 10133 15117
10135 15113 10137 15113 10139 15113 10143 15114 10145 15117 10149 15120
10151 15124 10153 15127 10153 15128 10156 15131 10156 15136 10153 15139
10150 15142 10143 15145 10140 15142 10143 15139 10142 15134 10139 15138
10135 15138 10135 15142 10131 15143 10128 15140 10127 15136 10125 15132
10121 15129 31 N F
10127 10178 10124 10175 10127 10175 10130 10173 10133 10171 10136 10171
10137 10174 10137 10177 10137 10180 10137 10184 10134 10182 10134 10178
10130 10177 10127 10178 14 N F
10131 9815 10128 9810 10131 9808 10136 9806 10140 9806 10145 9807
10148 9810 10150 9810 10153 9811 10152 9815 10156 9818 10154 9822
10150 9821 10145 9820 10141 9822 10137 9821 10133 9820 10131 9815
18 N F
10146 7419 10145 7416 10147 7415 10149 7414 10151 7416 10152 7417
10152 7420 10150 7420 10148 7420 10146 7419 10 N F
10151 9859 10147 9852 10152 9848 10154 9845 10156 9842 10161 9838
10167 9834 10168 9832 10170 9828 10175 9822 10169 9818 10171 9811
10172 9804 10177 9799 10184 9798 10190 9802 10186 9807 10183 9814
10186 9821 10192 9824 10198 9827 10198 9834 10197 9841 10190 9842
10184 9845 10191 9845 10197 9847 10193 9852 10192 9859 10187 9863
10181 9859 10174 9857 10167 9859 10160 9858 10156 9864 10151 9859
36 N F
10151 14985 10148 14984 10149 14981 10152 14980 10153 14981 10155 14982
10157 14985 10153 14988 10151 14985 9 N F
10168 7337 10164 7336 10166 7334 10168 7335 10168 7337 5 N F
10170 15093 10168 15088 10170 15084 10173 15081 10176 15080 10177 15080
10181 15078 10181 15082 10181 15086 10186 15087 10183 15090 10179 15091
10174 15091 10170 15093 14 N F
10171 9884 10168 9881 10169 9877 10173 9874 10177 9874 10178 9878
10176 9883 10177 9887 10179 9890 10184 9890 10188 9891 10186 9895
10182 9897 10180 9901 10182 9904 10181 9909 10178 9905 10174 9904
10170 9903 10171 9899 10170 9895 10168 9888 10171 9884 23 N F

10176 10305 10169 10303 10175 10299 10176 10293 10175 10286 10170 10278
10177 10279 10184 10279 10191 10280 10197 10283 10200 10283 10204 10283
10211 10284 10214 10283 10218 10283 10224 10285 10219 10290 10223 10295
10216 10295 10209 10294 10204 10290 10197 10288 10192 10293 10185 10295
10182 10301 10176 10305 26 N F
10195 10133 10191 10128 10195 10128 10198 10130 10201 10129 10201 10126
10204 10123 10205 10124 10207 10124 10209 10127 10206 10129 10203 10131
10203 10135 10200 10137 10197 10136 10195 10133 16 N F
10205 9862 10204 9858 10206 9855 10207 9852 10211 9852 10211 9856
10209 9859 10209 9862 10205 9862 9 N F
10210 10022 10207 10018 10211 10017 10213 10016 10217 10015 10220 10014
10221 10017 10220 10020 10217 10022 10214 10022 10210 10022 11 N F
10223 14895 10218 14889 10223 14890 10228 14888 10230 14883 10226 14885
10222 14881 10222 14875 10222 14870 10223 14865 10228 14862 10232 14867
10235 14870 10237 14871 10240 14873 10244 14877 10244 14882 10241 14886
10238 14891 10235 14896 10235 14901 10233 14896 10228 14893 10223 14895
24 N F
10231 10264 10230 10257 10234 10256 10238 10254 10243 10254 10240 10258
10244 10260 10247 10263 10244 10266 10241 10269 10237 10271 10234 10267
10231 10264 13 N F
10257 10356 10253 10352 10256 10352 10260 10352 10262 10351 10265 10350
10266 10353 10263 10355 10260 10355 10257 10356 10 N F
10270 10354 10269 10351 10272 10349 10274 10351 10277 10353 10275 10355
10272 10357 10270 10354 8 N F
10285 10371 10281 10369 10284 10368 10286 10366 10290 10366 10290 10366
10293 10366 10295 10369 10298 10369 10299 10372 10295 10373 10292 10371
10290 10373 10287 10375 10284 10377 10284 10373 10285 10371 17 N F
10290 10015 10286 10011 10288 10009 10289 10006 10291 10003 10294 10002
10295 10005 10294 10008 10293 10011 10293 10014 10290 10015 11 N F
10309 10013 10304 10011 10306 10009 10308 10006 10311 10005 10311 10002
10312 9999 10314 10002 10314 10005 10313 10009 10311 10011 10309 10013
12 N F
10321 10015 10317 10011 10319 10008 10321 10005 10324 10003 10324 10007
10322 10010 10323 10013 10321 10015 9 N F
10329 10001 10325 9996 10327 9994 10329 9994 10331 9996 10330 9998
10329 10001 7 N F
10330 10298 10327 10294 10329 10291 10329 10290 10332 10294 10336 10294
10339 10291 10343 10289 10342 10293 10342 10298 10343 10302 10344 10306
10340 10306 10336 10304 10332 10302 10330 10298 16 N F
10340 10341 10334 10339 10337 10336 10340 10338 10340 10338 10343 10340
10340 10341 7 N F
10351 9979 10347 9978 10348 9974 10350 9971 10353 9971 10356 9971
10360 9971 10359 9974 10357 9977 10354 9979 10351 9979 11 N F

10356 10309 10350 10303 10351 10302 10353 10299 10357 10299 10361 10300
10363 10296 10366 10292 10367 10297 10366 10301 10364 10305 10359 10306
10356 10309 13 N F
10430 15272 10429 15268 10433 15268 10435 15270 10438 15270 10441 15272
10445 15271 10448 15272 10446 15274 10444 15277 10441 15278 10438 15277
10436 15275 10433 15274 10430 15272 15 N F
10637 6766 10632 6764 10636 6761 10640 6761 10645 6760 10649 6758
10654 6758 10658 6759 10662 6761 10667 6760 10671 6759 10675 6761
10679 6764 10682 6766 10685 6770 10689 6773 10685 6775 10681 6777
10676 6778 10672 6780 10666 6778 10661 6779 10657 6777 10653 6776
10649 6778 10644 6776 10643 6772 10638 6770 10637 6766 29 N F
10811 15190 10808 15184 10812 15184 10814 15188 10817 15191 10821 15190
10822 15189 10824 15187 10827 15190 10827 15195 10831 15193 10832 15189
10836 15187 10840 15186 10844 15188 10847 15192 10848 15196 10851 15199
10852 15203 10848 15205 10844 15207 10840 15208 10839 15204 10835 15204
10830 15204 10827 15201 10824 15203 10820 15201 10820 15197 10818 15194
10813 15193 10811 15190 32 N F
10812 15136 10811 15131 10814 15131 10813 15128 10816 15125 10819 15126
10822 15126 10824 15129 10824 15133 10821 15135 10820 15138 10820 15141
10817 15139 10815 15137 10812 15136 15 N F
10820 15162 10820 15158 10820 15158 10822 15157 10823 15158 10824 15159
10826 15161 10824 15163 10822 15164 10820 15162 10 N F
10821 15151 10820 15146 10823 15145 10826 15145 10825 15148 10826 15151
10823 15153 10821 15151 8 N F
10848 15183 10846 15179 10848 15175 10846 15170 10850 15167 10853 15170
10857 15171 10857 15176 10859 15179 10856 15182 10858 15186 10854 15188
10850 15186 10848 15183 14 N F
10882 7539 10880 7536 10882 7534 10884 7534 10886 7535 10888 7536
10890 7537 10892 7537 10894 7537 10896 7538 10899 7538 10898 7540
10897 7542 10896 7543 10893 7543 10891 7544 10889 7544 10887 7544
10885 7542 10883 7541 10882 7539 21 N F
10892 14340 10886 14336 10888 14332 10891 14330 10892 14330 10895 14327
10895 14322 10897 14318 10899 14315 10902 14311 10906 14312 10911 14312
10915 14311 10919 14312 10922 14309 10926 14311 10927 14315 10929 14319
10927 14323 10923 14323 10921 14327 10923 14331 10927 14333 10931 14331
10929 14327 10932 14328 10933 14328 10936 14331 10941 14330 10938 14334
10943 14334 10940 14337 10938 14341 10937 14345 10933 14347 10928 14347
10924 14347 10920 14345 10916 14344 10911 14343 10908 14341 10904 14339
10900 14337 10896 14339 10892 14340 45 N F
10908 15175 10903 15169 10908 15169 10911 15173 10914 15175 10918 15175
10922 15177 10925 15180 10927 15184 10931 15186 10934 15189 10935 15194
10939 15194 10938 15198 10942 15199 10946 15198 10948 15200 10949 15201
10952 15204 10951 15209 10946 15208 10942 15207 10945 15211 10941 15212

10944 15215 10940 15216 10942 15220 10939 15222 10938 15226 10941 15230
10945 15229 10945 15233 10941 15235 10938 15238 10933 15238 10929 15239
10926 15236 10922 15234 10926 15231 10925 15227 10921 15225 10924 15222
10925 15218 10921 15215 10917 15213 10916 15209 10911 15210 10908 15207
10905 15203 10904 15199 10908 15201 10912 15202 10916 15203 10920 15202
10918 15198 10913 15197 10915 15193 10919 15192 10920 15187 10918 15183
10914 15184 10909 15184 10909 15179 10908 15175 64 N F
10920 14258 10915 14258 10918 14256 10921 14255 10924 14254 10924 14251
10924 14248 10924 14248 10926 14249 10929 14251 10932 14250 10936 14251
10938 14253 10940 14256 10940 14259 10938 14262 10938 14265 10940 14268
10942 14269 10941 14272 10938 14274 10935 14273 10932 14275 10932 14272
10929 14269 10926 14268 10925 14265 10923 14262 10923 14259 10920 14258
30 N F
10924 7534 10922 7532 10923 7531 10925 7529 10926 7530 10925 7532
10924 7534 7 N F
10934 14642 10928 14640 10931 14637 10935 14636 10939 14637 10937 14641
10938 14645 10942 14642 10946 14642 10949 14645 10952 14642 10956 14640
10959 14638 10963 14636 10967 14634 10972 14634 10972 14638 10975 14635
10979 14635 10982 14638 10979 14642 10977 14646 10975 14649 10971 14651
10971 14656 10967 14656 10963 14656 10959 14658 10955 14660 10951 14661
10948 14664 10947 14660 10944 14657 10942 14653 10938 14652 10933 14651
10933 14647 10934 14642 38 N F
10947 14232 10945 14227 10948 14227 10950 14230 10953 14232 10956 14233
10959 14232 10959 14232 10962 14232 10962 14235 10964 14238 10960 14238
10957 14238 10954 14237 10951 14237 10950 14234 10947 14232 17 N F
10958 7636 10957 7633 10959 7632 10961 7631 10963 7632 10964 7634
10963 7636 10962 7637 10959 7638 10958 7636 10 N F
10969 6638 10966 6636 10968 6636 10971 6637 10973 6638 10975 6639
10973 6640 10970 6640 10969 6638 9 N F
11011 14711 11011 14706 11014 14703 11016 14707 11020 14709 11024 14711
11025 14715 11026 14719 11028 14723 11028 14727 11026 14723 11019 14724
11018 14728 11015 14732 11014 14727 11015 14723 11012 14719 11015 14716
11019 14716 11018 14712 11015 14714 11011 14711 22 N F
11030 6710 11018 6707 11022 6702 11026 6696 11033 6693 11040 6693
11047 6692 11049 6699 11055 6703 11061 6698 11067 6694 11074 6693
11081 6693 11088 6692 11094 6691 11095 6691 11102 6692 11109 6693
11116 6691 11122 6687 11129 6685 11135 6688 11135 6695 11131 6701
11124 6705 11131 6702 11138 6700 11144 6696 11145 6704 11142 6710
11145 6717 11148 6723 11155 6721 11157 6728 11155 6735 11159 6741
11161 6748 11154 6750 11148 6755 11143 6759 11136 6761 11129 6762
11127 6769 11121 6765 11114 6769 11107 6768 11101 6765 11096 6760
11091 6766 11089 6772 11085 6778 11079 6782 11072 6785 11066 6781
11072 6776 11074 6769 11077 6763 11071 6766 11072 6773 11065 6771

11061 6765 11056 6759 11059 6752 11058 6745 11062 6739 11067 6734
11068 6727 11065 6733 11063 6727 11062 6720 11065 6713 11064 6706
11063 6713 11058 6718 11052 6722 11051 6729 11045 6725 11039 6728
11040 6721 11043 6715 11037 6719 11031 6722 11027 6716 11030 6710
84 N F
11059 14266 11058 14263 11061 14261 11064 14262 11067 14261 11070 14261
11071 14264 11072 14265 11073 14267 11074 14270 11077 14268 11078 14271
11078 14274 11079 14277 11079 14281 11076 14280 11073 14280 11069 14280
11066 14279 11069 14277 11072 14276 11071 14273 11071 14269 11068 14270
11065 14270 11062 14272 11062 14269 11059 14266 28 N F
11077 6571 11076 6566 11078 6562 11083 6562 11088 6561 11092 6562
11097 6561 11101 6563 11104 6567 11106 6571 11103 6574 11099 6576
11094 6576 11090 6576 11085 6574 11081 6572 11077 6571 17 N F
11122 14265 11117 14262 11121 14261 11124 14261 11124 14257 11123 14254
11126 14256 11127 14259 11130 14261 11133 14263 11135 14265 11138 14267
11137 14270 11140 14269 11143 14271 11141 14273 11141 14276 11138 14275
11134 14275 11131 14273 11129 14272 11127 14269 11124 14268 11122 14265
24 N F
11157 14243 11152 14241 11156 14241 11159 14239 11161 14237 11163 14235
11166 14236 11163 14238 11165 14241 11163 14243 11160 14244 11157 14243
12 N F
11157 14216 11155 14213 11157 14211 11160 14209 11162 14210 11163 14210
11165 14212 11169 14211 11171 14213 11174 14215 11176 14217 11178 14220
11180 14223 11181 14224 11182 14225 11184 14227 11188 14229 11190 14230
11193 14232 11194 14235 11196 14238 11196 14238 11198 14240 11200 14243
11202 14246 11203 14249 11205 14251 11207 14254 11204 14253 11201 14253
11198 14252 11195 14250 11193 14247 11190 14246 11187 14246 11185 14244
11182 14243 11180 14241 11181 14237 11182 14235 11183 14231 11183 14228
11180 14230 11180 14233 11178 14235 11175 14237 11172 14236 11169 14235
11171 14232 11171 14229 11168 14227 11167 14224 11165 14222 11163 14219
11160 14218 11157 14216 56 N F
11159 6668 11156 6665 11159 6662 11162 6661 11165 6662 11165 6666
11162 6668 11159 6668 8 N F
11166 6676 11164 6670 11168 6671 11170 6674 11172 6676 11173 6680
11170 6681 11168 6679 11166 6676 9 N F
11172 6734 11168 6734 11170 6733 11172 6733 11175 6732 11178 6733
11182 6732 11185 6733 11188 6730 11191 6729 11194 6730 11196 6733
11192 6736 11189 6737 11188 6740 11188 6744 11185 6746 11181 6746
11178 6746 11175 6744 11174 6741 11176 6739 11175 6736 11172 6734
24 N F
11193 6687 11192 6684 11192 6681 11194 6678 11196 6675 11198 6672
11201 6670 11204 6670 11207 6672 11210 6674 11212 6677 11213 6680
11213 6683 11211 6686 11207 6686 11204 6686 11201 6687 11196 6689

11193 6687 19 N F
11229 6801 11225 6794 11231 6789 11237 6791 11237 6791 11243 6796
11250 6797 11255 6792 11259 6786 11264 6781 11263 6773 11260 6767
11253 6765 11249 6759 11254 6754 11261 6752 11268 6750 11273 6745
11280 6741 11287 6740 11293 6743 11298 6748 11299 6755 11306 6754
11312 6750 11319 6753 11322 6759 11323 6766 11323 6773 11323 6780
11324 6787 11323 6795 11319 6801 11323 6807 11319 6813 11319 6820
11323 6826 11328 6831 11333 6837 11333 6844 11338 6849 11342 6855
11345 6861 11343 6868 11347 6874 11352 6879 11357 6884 11358 6891
11357 6898 11358 6905 11351 6905 11344 6906 11343 6899 11342 6892
11338 6886 11334 6880 11331 6873 11336 6869 11333 6862 11332 6855
11330 6848 11323 6850 11316 6848 11313 6841 11306 6839 11306 6832
11303 6825 11305 6818 11299 6814 11292 6814 11285 6813 11279 6809
11272 6809 11265 6807 11263 6814 11259 6820 11252 6823 11252 6816
11250 6809 11246 6803 11241 6807 11233 6806 11229 6801 83 N F
11235 6735 11234 6731 11235 6728 11238 6726 11241 6725 11244 6728
11246 6730 11246 6734 11245 6737 11243 6739 11240 6741 11237 6742
11235 6739 11235 6735 14 N F
11279 12233 11273 12233 11277 12230 11282 12230 11286 12228 11289 12225
11290 12221 11293 12218 11297 12219 11301 12220 11303 12224 11300 12226
11296 12229 11293 12233 11292 12237 11293 12241 11288 12241 11286 12245
11282 12245 11279 12241 11279 12237 11279 12233 22 N F
11291 12571 11288 12567 11289 12562 11292 12559 11293 12563 11295 12567
11300 12567 11301 12571 11303 12575 11304 12580 11302 12584 11298 12586
11298 12581 11296 12577 11294 12573 11291 12571 16 N F
11349 12434 11345 12433 11347 12431 11348 12429 11349 12428 11351 12431
11349 12434 7 N F
11348 12442 11345 12437 11349 12437 11349 12438 11351 12439 11353 12442
11353 12445 11349 12445 11348 12442 9 N F
11353 12417 11348 12420 11350 12417 11352 12414 11354 12411 11355 12408
11358 12410 11361 12409 11364 12411 11363 12414 11360 12415 11357 12417
11355 12414 11353 12417 14 N F
11356 14220 11355 14217 11358 14215 11359 14212 11359 14209 11360 14206
11363 14204 11365 14206 11367 14209 11369 14212 11371 14214 11372 14217
11372 14220 11369 14221 11366 14221 11363 14219 11359 14219 11356 14220
18 N F
11373 12574 11371 12570 11374 12568 11377 12569 11379 12572 11379 12575
11376 12576 11373 12574 8 N F
11383 12581 11379 12576 11382 12576 11384 12579 11386 12581 11383 12581
6 N F
11386 12559 11383 12555 11385 12552 11388 12548 11391 12545 11395 12546
11394 12550 11395 12554 11399 12557 11401 12560 11400 12564 11404 12565
11407 12568 11409 12572 11409 12577 11412 12580 11416 12581 11414 12586

11410 12584 11407 12580 11403 12580 11402 12584 11406 12586 11402 12587
11397 12586 11393 12585 11391 12582 11391 12577 11388 12574 11387 12570
11386 12565 11383 12562 11386 12559 33 N F
11386 14292 11383 14288 11386 14287 11386 14287 11390 14288 11391 14290
11390 14293 11390 14297 11387 14295 11386 14292 10 N F
11400 12617 11396 12617 11399 12615 11402 12614 11402 12614 11400 12617
6 N F
11405 12612 11402 12611 11402 12607 11405 12606 11407 12609 11405 12612
6 N F
11410 12606 11408 12603 11410 12600 11413 12599 11414 12596 11417 12598
11416 12601 11418 12604 11421 12604 11421 12607 11418 12607 11415 12610
11412 12611 11413 12608 11410 12606 15 N F
11415 12560 11414 12557 11417 12556 11421 12557 11423 12560 11425 12562
11426 12565 11423 12566 11419 12565 11418 12562 11415 12560 11 N F
11442 12323 11439 12323 11441 12320 11443 12318 11445 12320 11448 12318
11451 12321 11447 12321 11445 12324 11442 12323 10 N F
11443 14281 11440 14279 11441 14276 11444 14277 11445 14277 11448 14277
11451 14278 11453 14281 11454 14284 11456 14286 11453 14285 11450 14284
11448 14281 11446 14283 11443 14281 15 N F
11502 13908 11501 13904 11502 13901 11505 13899 11505 13902 11506 13905
11506 13909 11509 13907 11512 13908 11511 13911 11508 13912 11505 13913
11502 13911 11502 13908 14 N F
11506 13964 11505 13961 11508 13960 11511 13962 11513 13965 11516 13964
11514 13967 11517 13969 11519 13971 11516 13972 11514 13969 11511 13968
11509 13965 11506 13964 14 N F
11521 13886 11519 13883 11521 13881 11523 13879 11526 13877 11528 13874
11531 13875 11533 13877 11533 13877 11536 13879 11533 13879 11529 13879
11526 13880 11526 13883 11524 13886 11521 13886 16 N F
11530 7007 11528 7005 11531 7003 11534 7002 11537 7001 11539 7000
11540 6999 11541 7003 11539 7006 11536 7007 11533 7009 11530 7007
12 N F
11531 6956 11528 6952 11528 6945 11530 6941 11534 6938 11538 6938
11543 6937 11547 6938 11551 6940 11555 6943 11558 6946 11558 6951
11557 6955 11553 6958 11548 6959 11544 6960 11539 6960 11535 6958
11531 6956 19 N F
11549 14246 11546 14242 11548 14240 11550 14240 11553 14240 11555 14241
11554 14243 11551 14244 11549 14246 9 N F
11553 13923 11551 13919 11552 13920 11554 13920 11557 13919 11554 13918
11553 13915 11556 13913 11559 13914 11561 13917 11563 13919 11564 13922
11563 13925 11560 13927 11557 13926 11554 13926 11553 13923 17 N F
11554 13449 11552 13451 11553 13448 11554 13446 11556 13444 11559 13443
11559 13446 11560 13448 11558 13450 11556 13448 11554 13449 11 N F
11557 13993 11556 13988 11559 13991 11560 13995 11562 13992 11563 13991

11567 13990 11568 13985 11572 13985 11576 13986 11580 13987 11582 13991
11582 13992 11585 13993 11588 13996 11586 14000 11582 14001 11578 14003
11574 14005 11570 14004 11568 14008 11564 14011 11564 14015 11560 14018
11560 14013 11558 14010 11558 14005 11556 14001 11557 13997 11557 13993
30 N F
11559 13969 11558 13965 11560 13962 11560 13961 11562 13959 11565 13959
11567 13959 11568 13959 11570 13961 11569 13964 11570 13967 11567 13967
11565 13969 11562 13971 11559 13969 15 N F
11564 14240 11560 14237 11563 14236 11566 14233 11569 14234 11572 14236
11574 14234 11577 14231 11579 14230 11582 14229 11584 14232 11585 14235
11582 14237 11580 14239 11579 14242 11578 14245 11574 14245 11573 14242
11570 14240 11567 14240 11564 14240 21 N F
11567 13389 11564 13388 11565 13386 11566 13383 11567 13381 11570 13380
11572 13381 11572 13384 11575 13384 11575 13381 11577 13383 11577 13386
11575 13387 11572 13388 11570 13389 11567 13389 16 N F
11573 13691 11570 13689 11571 13686 11573 13688 11575 13689 11578 13690
11581 13689 11583 13690 11583 13692 11584 13693 11586 13695 11585 13697
11585 13700 11582 13701 11583 13698 11581 13696 11580 13694 11577 13694
11574 13693 11573 13691 20 N F
11577 12560 11571 12560 11575 12559 11578 12555 11582 12556 11580 12552
11576 12550 11578 12547 11582 12548 11586 12551 11590 12550 11594 12553
11598 12554 11598 12558 11596 12562 11596 12566 11592 12568 11589 12564
11585 12562 11582 12559 11577 12560 21 N F
11581 14296 11576 14291 11578 14290 11580 14288 11584 14286 11588 14285
11588 14285 11592 14285 11593 14289 11597 14290 11602 14291 11603 14292
11605 14294 11609 14294 11613 14291 11616 14294 11620 14296 11624 14298
11626 14302 11628 14306 11629 14309 11630 14310 11629 14314 11626 14317
11623 14315 11618 14313 11614 14313 11610 14313 11606 14315 11603 14318
11599 14320 11595 14318 11590 14318 11587 14315 11583 14312 11583 14308
11581 14304 11583 14300 11581 14296 39 N F
11588 13442 11583 13442 11585 13440 11587 13438 11589 13437 11589 13440
11588 13442 7 N F
11588 14267 11585 14265 11588 14264 11590 14263 11593 14263 11595 14264
11598 14264 11596 14266 11593 14267 11591 14266 11588 14267 11 N F
11592 13995 11588 13992 11588 13990 11589 13987 11591 13986 11592 13986
11594 13986 11597 13985 11597 13983 11598 13980 11600 13981 11601 13984
11603 13986 11601 13988 11598 13987 11597 13989 11595 13992 11594 13994
11592 13995 19 N F
11596 14229 11591 14228 11592 14227 11593 14225 11595 14223 11597 14220
11600 14219 11603 14217 11606 14217 11609 14215 11607 14218 11605 14220
11604 14223 11605 14227 11605 14230 11602 14230 11599 14229 11596 14229
18 N F
11603 12557 11600 12561 11601 12558 11601 12555 11603 12557 5 N F

11604 13448 11602 13444 11603 13444 11604 13444 11607 13445 11609 13447
11612 13447 11614 13448 11614 13451 11612 13452 11610 13452 11607 13452
11605 13451 11604 13448 14 N F
11603 14276 11602 14274 11602 14270 11604 14269 11605 14270 11606 14271
11605 14274 11603 14276 8 N F
11607 12559 11603 12561 11604 12558 11607 12556 11610 12554 11612 12552
11611 12555 11610 12558 11607 12559 9 N F
11604 12633 11603 12628 11606 12625 11609 12622 11612 12619 11616 12617
11621 12618 11625 12620 11628 12623 11627 12627 11631 12628 11628 12631
11627 12635 11623 12637 11623 12642 11627 12643 11631 12645 11635 12645
11638 12647 11639 12647 11640 12643 11645 12643 11648 12645 11652 12646
11652 12653 11648 12652 11649 12656 11651 12660 11652 12664 11651 12668
11647 12670 11643 12668 11639 12667 11638 12663 11635 12659 11631 12659
11630 12655 11626 12653 11622 12651 11618 12650 11614 12650 11611 12654
11606 12653 11606 12648 11604 12645 11605 12641 11604 12637 11604 12633
48 N F
11616 14262 11607 14262 11610 14259 11614 14257 11617 14254 11621 14252
11625 14251 11625 14251 11629 14249 11633 14246 11633 14246 11633 14246
11636 14243 11636 14239 11635 14235 11638 14232 11641 14229 11643 14232
11647 14235 11649 14239 11653 14241 11655 14245 11655 14249 11654 14253
11651 14256 11647 14258 11643 14258 11638 14259 11635 14262 11631 14264
11628 14267 11628 14271 11628 14276 11629 14280 11632 14283 11629 14287
11625 14286 11622 14284 11617 14282 11614 14280 11615 14275 11615 14271
11615 14267 11616 14262 44 N F
11618 13975 11613 13975 11615 13973 11618 13971 11622 13972 11625 13971
11626 13974 11625 13977 11621 13976 11618 13975 10 N F
11619 12342 11613 12342 11616 12340 11618 12338 11622 12337 11621 12340
11619 12342 7 N F
11619 12615 11617 12611 11618 12606 11618 12602 11619 12599 11619 12598
11622 12594 11625 12597 11627 12593 11630 12590 11634 12592 11637 12588
11641 12587 11642 12587 11645 12588 11649 12591 11649 12595 11652 12598
11655 12601 11656 12605 11660 12605 11660 12604 11661 12609 11663 12613
11656 12614 11652 12617 11649 12620 11644 12620 11642 12623 11641 12627
11638 12631 11636 12627 11635 12623 11631 12620 11634 12617 11632 12613
11631 12617 11627 12618 11623 12617 11619 12615 40 N F
11619 12955 11619 12952 11621 12954 11624 12956 11621 12958 11619 12955
6 N F
11627 12965 11623 12964 11626 12962 11627 12959 11630 12960 11634 12960
11636 12962 11638 12965 11637 12968 11636 12971 11633 12971 11631 12974
11628 12972 11625 12973 11625 12969 11628 12969 11630 12966 11627 12965
18 N F
11630 13972 11626 13970 11628 13968 11631 13968 11635 13967 11638 13966
11639 13963 11642 13962 11644 13965 11641 13966 11639 13969 11638 13972

11635 13973 11633 13975 11630 13972 15 N F
11638 14180 11633 14181 11634 14178 11637 14176 11639 14174 11640 14174
11642 14172 11643 14169 11643 14166 11646 14165 11649 14165 11652 14166
11655 14167 11658 14168 11660 14168 11662 14168 11665 14167 11667 14170
11666 14173 11663 14173 11660 14171 11657 14171 11654 14171 11651 14172
11648 14174 11647 14177 11644 14178 11641 14179 11638 14180 29 N F
11637 12707 11633 12707 11637 12705 11635 12701 11639 12700 11644 12700
11647 12703 11647 12703 11651 12700 11654 12703 11657 12707 11657 12711
11658 12706 11657 12702 11661 12701 11663 12697 11665 12695 11667 12694
11670 12697 11672 12699 11673 12700 11677 12702 11679 12706 11683 12706
11683 12710 11682 12715 11686 12713 11690 12715 11691 12719 11688 12722
11684 12723 11685 12727 11688 12730 11684 12732 11685 12737 11681 12737
11679 12741 11676 12744 11671 12745 11670 12740 11672 12736 11670 12732
11667 12730 11663 12733 11660 12730 11656 12728 11653 12730 11651 12726
11650 12722 11646 12715 11644 12711 11642 12708 11637 12707 53 N F
11635 12364 11635 12360 11638 12360 11641 12358 11640 12361 11643 12364
11645 12366 11646 12364 11646 12360 11648 12362 11648 12365 11647 12369
11643 12369 11640 12368 11637 12366 11635 12364 16 N F
11636 12577 11635 12574 11638 12571 11640 12569 11643 12571 11646 12569
11648 12567 11651 12568 11650 12572 11649 12575 11646 12576 11645 12579
11642 12580 11639 12578 11636 12577 15 N F
11639 12983 11637 12980 11640 12978 11642 12981 11639 12983 5 N F
11640 14024 11637 14019 11641 14017 11644 14015 11649 14014 11651 14014
11653 14013 11657 14013 11662 14014 11665 14016 11667 14020 11663 14022
11661 14018 11656 14019 11652 14021 11648 14021 11644 14023 11640 14024
18 N F
11643 14313 11638 14308 11641 14302 11643 14296 11643 14289 11642 14282
11643 14275 11642 14269 11645 14263 11652 14261 11659 14261 11661 14254
11666 14250 11668 14243 11671 14237 11667 14231 11661 14229 11655 14226
11657 14220 11656 14213 11663 14211 11669 14208 11676 14209 11679 14209
11683 14208 11686 14207 11689 14206 11696 14205 11696 14212 11696 14218
11700 14217 11702 14216 11707 14212 11708 14211 11715 14211 11719 14216
11721 14217 11724 14220 11726 14227 11723 14234 11721 14240 11723 14246
11718 14251 11717 14257 11717 14264 11716 14271 11712 14277 11713 14284
11715 14290 11712 14296 11709 14302 11707 14308 11702 14313 11696 14315
11690 14319 11684 14323 11679 14327 11674 14331 11667 14333 11662 14329
11655 14329 11653 14323 11649 14317 11643 14313 64 N F
11640 12831 11639 12826 11642 12824 11643 12821 11646 12820 11648 12822
11648 12825 11646 12828 11644 12831 11642 12833 11640 12831 11 N F
11644 12555 11642 12553 11643 12553 11646 12553 11646 12553 11649 12554
11650 12550 11648 12547 11649 12544 11652 12545 11652 12548 11652 12551
11652 12554 11654 12557 11654 12557 11651 12557 11648 12556 11644 12555
18 N F

11645 12432 11644 12428 11647 12429 11648 12432 11649 12435 11646 12435
11645 12432 7 N F
11651 13972 11646 13971 11648 13968 11651 13967 11654 13966 11657 13965
11660 13965 11659 13968 11657 13971 11654 13971 11651 13972 11 N F
11649 13144 11647 13141 11650 13140 11651 13142 11652 13145 11649 13144
6 N F
11651 12360 11648 12356 11649 12353 11652 12353 11656 12354 11658 12356
11659 12357 11660 12358 11661 12361 11658 12363 11656 12360 11653 12362
11651 12360 13 N F
11652 12419 11650 12415 11654 12416 11657 12416 11656 12419 11654 12421
11652 12419 7 N F
11658 12816 11652 12813 11656 12813 11661 12813 11661 12809 11664 12812
11667 12815 11669 12811 11671 12810 11673 12810 11677 12809 11681 12806
11683 12810 11686 12813 11685 12817 11687 12820 11689 12824 11685 12826
11681 12827 11681 12832 11681 12836 11685 12838 11686 12842 11684 12846
11686 12849 11686 12854 11690 12855 11694 12856 11697 12859 11701 12861
11697 12863 11695 12860 11690 12860 11686 12859 11682 12859 11681 12854
11678 12851 11676 12848 11674 12844 11671 12840 11667 12838 11663 12837
11659 12836 11657 12832 11656 12828 11653 12824 11657 12822 11661 12820
11658 12816 49 N F
11654 13134 11653 13131 11655 13130 11658 13130 11659 13128 11661 13129
11661 13131 11659 13133 11656 13134 11654 13134 10 N F
11656 12567 11655 12564 11657 12562 11659 12564 11659 12564 11660 12561
11662 12563 11665 12564 11665 12568 11664 12571 11662 12573 11660 12575
11657 12574 11657 12571 11659 12568 11656 12567 16 N F
11657 12400 11655 12396 11658 12396 11659 12396 11661 12398 11660 12401
11657 12400 7 N F
11657 12435 11656 12428 11659 12428 11660 12430 11661 12431 11661 12435
11659 12437 11657 12435 8 N F
11659 12317 11657 12314 11658 12311 11659 12308 11660 12305 11662 12308
11660 12311 11660 12314 11659 12317 9 N F
11659 14135 11658 14131 11661 14129 11664 14128 11665 14125 11665 14122
11668 14120 11670 14118 11673 14119 11674 14122 11677 14123 11679 14121
11679 14118 11682 14119 11683 14119 11685 14118 11687 14115 11688 14112
11690 14115 11692 14118 11693 14121 11692 14124 11691 14127 11688 14128
11685 14130 11683 14133 11680 14133 11677 14134 11676 14131 11673 14129
11670 14130 11668 14132 11666 14135 11664 14137 11660 14137 11659 14135
36 N F
11665 12639 11659 12638 11660 12635 11661 12632 11662 12629 11664 12627
11667 12626 11668 12629 11667 12633 11666 12635 11665 12639 11 N F
11666 12404 11662 12403 11663 12400 11664 12397 11666 12397 11667 12398
11668 12401 11666 12404 8 N F
11666 12396 11662 12394 11664 12391 11668 12391 11671 12391 11669 12394

11666 12396 7 N F
11666 12577 11665 12574 11667 12571 11668 12568 11667 12565 11666 12562
11666 12561 11667 12559 11670 12558 11673 12556 11672 12554 11669 12551
11670 12548 11671 12545 11671 12542 11673 12539 11676 12540 11677 12543
11679 12546 11681 12544 11684 12544 11688 12543 11690 12545 11691 12548
11692 12552 11690 12555 11687 12556 11687 12559 11687 12562 11684 12562
11680 12561 11679 12564 11677 12567 11679 12570 11679 12573 11678 12576
11675 12577 11672 12578 11669 12578 11666 12577 40 N F
11672 12685 11668 12683 11670 12680 11671 12677 11674 12677 11677 12675
11678 12675 11680 12676 11680 12679 11677 12681 11674 12683 11672 12685
12 N F
11670 12381 11669 12378 11670 12375 11674 12375 11675 12373 11677 12370
11680 12369 11680 12373 11682 12375 11682 12379 11680 12376 11678 12379
11676 12381 11673 12382 11670 12381 15 N F
11674 12649 11670 12645 11672 12646 11674 12646 11677 12647 11679 12650
11678 12653 11676 12652 11674 12649 9 N F
11675 12417 11673 12416 11673 12412 11673 12412 11673 12409 11676 12408
11680 12407 11682 12407 11683 12407 11686 12408 11687 12411 11690 12411
11693 12412 11693 12412 11696 12410 11699 12410 11702 12408 11703 12411
11701 12414 11698 12414 11695 12416 11692 12417 11689 12419 11687 12417
11683 12418 11682 12415 11679 12414 11675 12417 28 N F
11677 14174 11674 14170 11676 14168 11677 14171 11679 14173 11679 14176
11677 14174 7 N F
11681 12699 11674 12697 11677 12694 11680 12690 11682 12687 11684 12683
11684 12682 11687 12680 11691 12678 11693 12675 11692 12670 11697 12670
11701 12672 11704 12675 11705 12680 11702 12683 11702 12688 11703 12692
11708 12690 11711 12688 11711 12693 11715 12695 11716 12699 11712 12702
11708 12704 11712 12706 11716 12707 11716 12707 11717 12711 11712 12711
11712 12715 11709 12718 11706 12715 11702 12717 11698 12718 11695 12715
11696 12711 11698 12707 11701 12704 11699 12700 11695 12700 11691 12702
11687 12705 11684 12703 11681 12699 45 N F
11679 12538 11675 12535 11679 12533 11682 12537 11686 12536 11691 12536
11689 12532 11685 12531 11683 12527 11679 12529 11676 12523 11681 12523
11685 12522 11689 12523 11692 12524 11694 12524 11696 12528 11700 12529
11703 12532 11702 12536 11707 12537 11709 12541 11707 12545 11709 12549
11709 12553 11712 12556 11709 12559 11705 12561 11708 12565 11704 12567
11700 12568 11696 12570 11695 12574 11692 12577 11687 12576 11683 12577
11682 12572 11686 12569 11690 12568 11692 12564 11694 12560 11695 12556
11698 12553 11702 12552 11699 12551 11697 12547 11698 12542 11695 12539
11690 12539 11687 12541 11682 12541 11679 12538 52 N F
11682 12405 11677 12404 11678 12401 11680 12399 11682 12401 11685 12401
11685 12404 11682 12405 8 N F
11680 12597 11678 12595 11678 12591 11679 12588 11680 12584 11682 12587

11682 12591 11681 12594 11680 12597 9 N F
11681 13493 11678 13493 11679 13490 11681 13488 11683 13490 11684 13492
11684 13495 11686 13497 11684 13498 11681 13498 11680 13496 11681 13493
12 N F
11684 12675 11680 12674 11680 12673 11681 12671 11683 12668 11687 12669
11685 12672 11684 12675 8 N F
11681 14099 11680 14095 11683 14094 11685 14092 11685 14088 11686 14085
11688 14083 11691 14081 11693 14083 11694 14083 11697 14084 11698 14087
11697 14090 11700 14092 11698 14094 11695 14096 11693 14098 11690 14101
11688 14102 11686 14105 11683 14105 11682 14102 11681 14099 23 N F
11683 12428 11681 12425 11684 12424 11681 12421 11684 12419 11687 12419
11686 12422 11686 12426 11683 12428 9 N F
11688 12654 11681 12652 11685 12651 11688 12648 11689 12646 11690 12644
11692 12640 11693 12640 11697 12640 11701 12638 11700 12634 11700 12629
11704 12629 11704 12629 11707 12627 11711 12630 11713 12633 11718 12634
11719 12630 11722 12627 11721 12623 11725 12621 11728 12621 11730 12621
11734 12621 11736 12625 11735 12629 11735 12634 11733 12637 11731 12641
11733 12644 11729 12646 11727 12650 11723 12652 11720 12649 11715 12649
11712 12652 11710 12656 11705 12657 11702 12654 11698 12656 11693 12657
11692 12653 11688 12654 44 N F
11682 14173 11681 14167 11684 14167 11686 14169 11686 14173 11686 14176
11689 14178 11686 14180 11683 14179 11683 14176 11682 14173 11 N F
11684 12943 11682 12938 11684 12935 11685 12934 11686 12933 11687 12930
11690 12931 11692 12933 11692 12937 11691 12940 11689 12943 11687 12945
11684 12943 13 N F
11686 12495 11682 12495 11683 12493 11684 12492 11686 12490 11686 12486
11688 12484 11689 12485 11690 12485 11692 12488 11693 12491 11696 12492
11698 12495 11698 12498 11695 12496 11694 12499 11697 12501 11694 12502
11692 12505 11689 12504 11688 12501 11687 12498 11686 12495 23 N F
11685 12892 11682 12891 11682 12887 11682 12884 11685 12886 11686 12889
11685 12892 7 N F
11690 12786 11683 12781 11686 12778 11690 12780 11695 12781 11699 12781
11703 12780 11707 12782 11707 12777 11702 12778 11702 12774 11702 12770
11703 12765 11701 12761 11699 12757 11698 12753 11702 12755 11706 12756
11707 12760 11706 12764 11709 12765 11710 12765 11714 12768 11718 12766
11717 12770 11721 12771 11720 12776 11720 12780 11718 12783 11713 12784
11711 12788 11710 12792 11707 12795 11703 12793 11700 12795 11695 12796
11695 12791 11691 12790 11690 12786 39 N F
11684 13483 11683 13481 11684 13478 11687 13478 11687 13478 11687 13480
11688 13483 11689 13485 11686 13485 11684 13483 10 N F
11687 12608 11685 12603 11687 12601 11690 12602 11690 12606 11692 12607
11693 12608 11693 12611 11690 12610 11687 12608 10 N F
11687 12397 11685 12391 11689 12393 11689 12388 11693 12387 11698 12387

11702 12387 11704 12385 11705 12383 11704 12388 11706 12391 11707 12387
11710 12383 11712 12387 11716 12388 11720 12386 11720 12391 11718 12395
11717 12399 11718 12404 11713 12403 11709 12402 11705 12400 11701 12401
11697 12404 11693 12406 11692 12402 11687 12403 11691 12400 11687 12397
30 N F
11687 12640 11685 12635 11689 12634 11691 12637 11691 12640 11689 12643
11687 12640 7 N F
11689 12894 11687 12892 11690 12892 11693 12892 11695 12895 11697 12897
11694 12898 11692 12895 11689 12894 9 N F
11688 14169 11687 14165 11690 14164 11693 14165 11696 14166 11699 14165
11702 14163 11705 14163 11705 14166 11704 14169 11705 14172 11705 14176
11702 14178 11700 14180 11697 14178 11696 14175 11693 14173 11691 14172
11688 14169 19 N F
11695 12667 11687 12665 11691 12664 11693 12663 11696 12663 11699 12660
11703 12662 11707 12662 11711 12659 11713 12656 11716 12652 11720 12654
11724 12655 11726 12659 11725 12664 11723 12668 11726 12670 11724 12674
11720 12674 11719 12679 11716 12681 11714 12677 11716 12673 11712 12673
11708 12671 11704 12669 11700 12668 11695 12667 28 N F
11690 13944 11689 13939 11692 13938 11694 13936 11696 13939 11695 13942
11693 13945 11690 13944 8 N F
11692 12363 11692 12358 11695 12358 11698 12356 11700 12358 11703 12358
11707 12357 11708 12360 11709 12363 11712 12364 11710 12366 11709 12369
11706 12371 11704 12372 11704 12369 11704 12366 11702 12364 11699 12363
11696 12362 11692 12363 20 N F
11698 14154 11693 14153 11696 14150 11700 14149 11702 14145 11701 14140
11706 14140 11705 14144 11707 14148 11709 14151 11707 14155 11702 14154
11698 14154 13 N F
11699 12813 11693 12808 11695 12808 11697 12808 11698 12804 11702 12802
11706 12804 11709 12801 11712 12797 11714 12801 11714 12806 11715 12802
11716 12797 11719 12801 11719 12805 11718 12809 11716 12813 11712 12814
11708 12813 11703 12814 11699 12813 21 N F
11701 12624 11698 12619 11700 12617 11704 12617 11706 12619 11707 12622
11704 12624 11701 12624 8 N F
11703 14032 11701 14026 11704 14022 11705 14018 11706 14014 11706 14010
11710 14012 11711 14013 11713 14015 11715 14012 11715 14007 11719 14009
11721 14013 11724 14016 11725 14020 11727 14024 11731 14025 11731 14030
11730 14034 11729 14038 11725 14039 11720 14039 11716 14039 11712 14039
11711 14035 11707 14033 11703 14032 27 N F
11706 12837 11704 12832 11708 12831 11711 12828 11710 12824 11712 12823
11714 12823 11716 12819 11720 12818 11724 12820 11727 12816 11731 12817
11735 12818 11738 12816 11739 12815 11743 12817 11744 12813 11740 12811
11741 12807 11744 12806 11745 12806 11747 12802 11748 12800 11750 12799
11750 12803 11754 12801 11755 12805 11758 12808 11755 12811 11751 12813

11752 12817 11750 12821 11747 12824 11744 12820 11739 12821 11737 12825
11733 12827 11733 12831 11736 12835 11739 12832 11742 12836 11741 12840
11737 12840 11735 12836 11733 12841 11730 12844 11727 12841 11726 12837
11721 12837 11718 12840 11716 12844 11712 12842 11708 12841 11706 12837
54 N F
11711 13919 11708 13916 11710 13914 11710 13911 11711 13907 11714 13906
11717 13907 11720 13909 11722 13910 11721 13913 11720 13916 11718 13918
11716 13916 11713 13916 11711 13919 15 N F
11711 14135 11709 14130 11712 14128 11715 14127 11716 14130 11716 14134
11714 14136 11711 14135 8 N F
11711 12593 11711 12588 11715 12588 11718 12584 11716 12580 11718 12576
11716 12572 11720 12572 11724 12574 11728 12575 11730 12579 11734 12580
11738 12578 11741 12580 11744 12584 11748 12584 11750 12588 11746 12589
11742 12590 11743 12594 11741 12598 11737 12601 11734 12598 11732 12594
11727 12594 11730 12598 11731 12602 11728 12605 11724 12603 11721 12599
11717 12598 11716 12594 11711 12593 33 N F
11714 12470 11711 12463 11715 12457 11720 12453 11724 12447 11729 12443
11728 12450 11726 12456 11729 12462 11730 12456 11735 12451 11742 12450
11739 12444 11735 12438 11738 12432 11742 12437 11746 12432 11753 12431
11759 12431 11763 12434 11765 12435 11772 12438 11766 12442 11773 12441
11774 12442 11780 12443 11784 12449 11791 12450 11791 12457 11797 12460
11804 12460 11804 12467 11809 12467 11811 12467 11812 12474 11818 12470
11819 12463 11824 12467 11822 12474 11826 12476 11828 12478 11830 12484
11834 12490 11831 12496 11835 12501 11830 12506 11834 12511 11835 12518
11837 12525 11831 12529 11827 12534 11822 12538 11816 12543 11810 12540
11809 12533 11803 12537 11801 12544 11802 12550 11799 12556 11792 12559
11786 12561 11779 12562 11772 12563 11771 12556 11774 12550 11780 12549
11787 12551 11785 12545 11787 12538 11783 12533 11779 12528 11776 12521
11769 12523 11769 12530 11766 12536 11762 12531 11765 12524 11759 12521
11762 12515 11763 12508 11768 12503 11774 12506 11771 12499 11765 12501
11768 12495 11766 12488 11765 12481 11764 12475 11761 12469 11761 12476
11760 12482 11760 12489 11763 12495 11760 12501 11760 12508 11753 12510
11752 12516 11746 12513 11745 12506 11749 12500 11746 12494 11741 12499
11734 12498 11731 12492 11724 12490 11723 12483 11727 12478 11721 12481
11716 12476 11714 12470 110 N F
11713 14150 11712 14145 11713 14141 11714 14141 11717 14139 11721 14139
11723 14143 11725 14143 11727 14143 11730 14147 11734 14148 11739 14147
11743 14148 11747 14150 11748 14154 11750 14158 11750 14162 11747 14165
11745 14169 11741 14171 11737 14172 11733 14172 11729 14173 11725 14175
11721 14177 11717 14175 11715 14171 11716 14167 11715 14163 11713 14159
11715 14154 11713 14150 32 N F
11716 13521 11715 13519 11715 13515 11716 13515 11718 13515 11720 13517
11723 13515 11725 13517 11727 13519 11727 13521 11725 13523 11724 13526

11724 13528 11723 13530 11720 13531 11720 13528 11719 13525 11718 13523
11716 13521 19 N F
11722 13109 11717 13107 11720 13104 11722 13100 11725 13102 11729 13104
11732 13107 11735 13111 11737 13114 11741 13116 11743 13120 11745 13124
11743 13127 11739 13130 11739 13126 11736 13123 11732 13123 11728 13125
11730 13121 11726 13121 11726 13116 11723 13114 11722 13109 23 N F
11730 12615 11729 12610 11732 12609 11735 12609 11738 12610 11740 12613
11742 12610 11744 12613 11745 12616 11743 12618 11745 12620 11747 12623
11744 12624 11740 12623 11738 12621 11736 12618 11736 12615 11733 12616
11730 12615 19 N F
11731 12771 11730 12768 11732 12765 11734 12762 11736 12761 11738 12764
11738 12767 11738 12770 11737 12773 11738 12777 11735 12776 11732 12774
11731 12771 13 N F
11733 13546 11732 13541 11733 13538 11735 13537 11737 13539 11739 13539
11740 13539 11743 13538 11745 13538 11746 13538 11748 13537 11751 13538
11753 13537 11756 13537 11758 13535 11759 13535 11761 13535 11764 13535
11766 13537 11765 13539 11762 13539 11760 13538 11758 13540 11755 13541
11756 13543 11755 13546 11753 13548 11753 13550 11750 13550 11747 13550
11745 13549 11744 13546 11745 13544 11743 13544 11740 13545 11738 13546
11735 13547 11733 13546 38 N F
11746 13257 11742 13255 11745 13254 11748 13254 11746 13257 5 N F
11743 12748 11742 12742 11744 12741 11746 12739 11750 12738 11752 12741
11757 12742 11756 12746 11757 12750 11753 12752 11749 12752 11744 12752
11743 12748 13 N F
11752 14043 11748 14044 11750 14041 11753 14038 11757 14035 11758 14030
11759 14026 11760 14022 11760 14018 11756 14016 11753 14014 11754 14009
11758 14010 11756 14006 11754 14002 11751 13999 11749 13994 11753 13997
11755 14000 11759 13999 11762 13995 11765 13998 11766 14002 11767 14006
11768 14010 11772 14009 11777 14009 11777 14013 11780 14016 11784 14017
11786 14021 11785 14025 11782 14029 11781 14033 11777 14035 11773 14034
11769 14036 11766 14039 11762 14038 11761 14042 11764 14046 11764 14050
11762 14046 11759 14042 11755 14040 11752 14043 46 N F
11751 12791 11748 12792 11750 12788 11752 12785 11752 12781 11752 12778
11750 12775 11753 12774 11756 12775 11760 12775 11762 12777 11761 12780
11761 12783 11759 12786 11757 12788 11755 12791 11751 12791 17 N F
11750 12709 11749 12704 11754 12704 11758 12703 11762 12701 11766 12701
11769 12705 11770 12709 11775 12710 11779 12709 11783 12710 11782 12714
11786 12713 11788 12709 11792 12709 11797 12709 11798 12705 11803 12704
11800 12700 11797 12697 11799 12693 11803 12692 11806 12696 11810 12694
11812 12698 11812 12702 11813 12706 11814 12710 11813 12715 11809 12716
11805 12719 11801 12718 11797 12718 11792 12717 11788 12718 11791 12722
11787 12724 11790 12726 11791 12731 11795 12729 11797 12733 11801 12733
11802 12737 11798 12738 11796 12741 11792 12742 11787 12741 11783 12742

11781 12746 11777 12746 11773 12745 11770 12741 11773 12738 11776 12735
11780 12733 11781 12729 11777 12726 11777 12721 11773 12722 11774 12718
11770 12715 11768 12719 11766 12715 11764 12711 11760 12711 11756 12711
11752 12713 11750 12709 68 N F
11754 13255 11751 13254 11754 13254 11757 13254 11758 13254 11760 13255
11762 13257 11759 13259 11756 13258 11754 13255 10 N F
11758 13238 11756 13234 11758 13232 11760 13229 11763 13231 11763 13234
11761 13237 11758 13238 8 N F
11759 12374 11758 12370 11760 12367 11763 12368 11761 12370 11762 12373
11764 12376 11765 12379 11763 12382 11761 12380 11759 12377 11759 12374
12 N F
11764 12772 11759 12770 11762 12767 11764 12763 11767 12760 11768 12756
11772 12754 11776 12755 11776 12759 11774 12763 11771 12767 11768 12770
11764 12772 13 N F
11762 7073 11761 7064 11763 7061 11766 7057 11771 7055 11775 7054
11779 7055 11783 7058 11787 7059 11791 7057 11796 7057 11800 7058
11804 7060 11806 7064 11805 7068 11804 7073 11801 7076 11800 7081
11796 7083 11791 7084 11787 7086 11783 7088 11779 7090 11774 7090
11771 7087 11771 7083 11771 7078 11771 7074 11766 7072 11762 7073
30 N F
11770 13240 11765 13239 11769 13238 11769 13235 11772 13236 11771 13233
11774 13234 11777 13234 11779 13237 11777 13239 11779 13241 11778 13244
11777 13247 11774 13248 11771 13247 11770 13244 11774 13243 11776 13241
11774 13238 11771 13237 11770 13240 21 N F
11770 13388 11768 13386 11769 13384 11772 13382 11774 13382 11777 13381
11776 13384 11775 13386 11773 13388 11770 13388 10 N F
11776 13263 11771 13262 11774 13261 11774 13261 11777 13261 11781 13260
11779 13263 11776 13263 8 N F
11780 13233 11778 13230 11781 13228 11784 13228 11786 13230 11789 13232
11790 13235 11788 13238 11786 13235 11785 13232 11782 13232 11783 13234
11781 13236 11780 13233 14 N F
11792 13250 11789 13247 11791 13245 11792 13242 11793 13239 11796 13238
11795 13241 11797 13242 11798 13243 11800 13240 11803 13240 11807 13240
11809 13242 11807 13244 11804 13246 11801 13247 11797 13247 11795 13250
11792 13250 19 N F
11797 13204 11793 13201 11796 13200 11797 13196 11799 13194 11801 13196
11801 13200 11800 13203 11797 13204 9 N F
11802 12451 11800 12446 11803 12444 11806 12443 11805 12447 11808 12446
11811 12448 11813 12450 11813 12453 11811 12455 11808 12454 11810 12456
11809 12459 11806 12458 11804 12456 11801 12455 11802 12451 17 N F
11812 11881 11806 11877 11807 11870 11809 11864 11807 11857 11807 11848
11811 11843 11815 11838 11820 11832 11825 11828 11826 11828 11832 11825
11838 11824 11844 11820 11848 11815 11853 11810 11858 11805 11864 11802

11871 11802 11871 11809 11872 11816 11876 11822 11881 11827 11887 11830
11894 11828 11900 11828 11907 11828 11914 11830 11920 11834 11926 11835
11932 11831 11939 11833 11946 11835 11950 11830 11957 11829 11963 11833
11970 11833 11974 11838 11981 11839 11988 11840 11995 11840 12001 11843
12007 11840 12014 11842 12019 11847 12024 11851 12028 11857 12034 11860
12040 11862 12047 11862 12054 11864 12060 11867 12067 11869 12073 11870
12078 11875 12083 11880 12087 11886 12094 11886 12101 11887 12107 11889
12114 11892 12117 11897 12116 11904 12121 11909 12125 11914 12126 11921
12128 11928 12131 11934 12137 11937 12141 11943 12141 11950 12143 11956
12144 11963 12149 11959 12147 11965 12146 11972 12149 11978 12156 11977
12151 11982 12146 11977 12145 11984 12139 11987 12135 11992 12138 11998
12136 12005 12133 12011 12127 12014 12120 12015 12114 12012 12108 12015
12101 12018 12096 12022 12092 12028 12086 12027 12079 12028 12072 12028
12065 12027 12059 12024 12052 12024 12046 12022 12040 12019 12033 12017
12026 12016 12020 12012 12014 12014 12008 12010 12002 12007 11995 12004
11989 12002 11983 11999 11976 11998 11970 11994 11964 11991 11958 11987
11952 11989 11945 11990 11941 11985 11934 11984 11929 11979 11923 11975
11917 11974 11911 11970 11905 11966 11899 11969 11894 11964 11887 11962
11882 11957 11876 11954 11869 11954 11865 11948 11862 11942 11855 11939
11851 11935 11846 11930 11845 11923 11842 11917 11837 11912 11832 11907
11827 11903 11823 11897 11819 11892 11813 11888 11812 11881 143 N F
11809 13260 11807 13259 11809 13257 11811 13258 11812 13260 11809 13260
6 N F
11820 13319 11817 13316 11819 13315 11822 13314 11824 13316 11822 13319
11820 13319 7 N F
11822 13369 11819 13366 11821 13365 11822 13365 11824 13365 11827 13364
11827 13366 11827 13369 11826 13371 11824 13371 11822 13369 11 N F
11826 7122 11819 7120 11823 7114 11827 7109 11830 7102 11835 7096
11839 7091 11842 7084 11835 7081 11835 7074 11837 7067 11834 7061
11828 7058 11825 7051 11832 7048 11837 7043 11840 7037 11836 7031
11825 7031 11829 7025 11834 7020 11840 7015 11847 7013 11848 7021
11850 7014 11855 7009 11862 7012 11869 7014 11876 7016 11882 7019
11889 7021 11896 7022 11903 7023 11910 7024 11917 7023 11924 7025
11931 7027 11937 7022 11933 7028 11928 7033 11924 7039 11918 7044
11911 7044 11904 7046 11898 7048 11894 7055 11887 7057 11880 7056
11874 7058 11867 7060 11863 7066 11865 7073 11860 7078 11853 7081
11849 7087 11848 7094 11844 7100 11839 7104 11834 7110 11829 7115
11826 7122 61 N F
11826 12226 11825 12222 11828 12220 11832 12217 11833 12221 11831 12225
11826 12226 7 N F
11831 13330 11828 13328 11830 13327 11833 13326 11835 13324 11837 13325
11840 13326 11841 13328 11839 13329 11836 13330 11833 13330 11831 13330
12 N F

11840 12220 11834 12216 11838 12215 11841 12211 11843 12207 11847 12207
11845 12211 11847 12215 11851 12216 11852 12221 11848 12222 11844 12219
11840 12220 13 N F
11847 13324 11843 13322 11845 13322 11848 13321 11849 13319 11852 13319
11850 13320 11849 13323 11847 13324 9 N F
11872 13239 11870 13234 11872 13231 11875 13231 11878 13233 11878 13236
11879 13239 11877 13242 11874 13241 11872 13239 10 N F
11906 12354 11904 12351 11907 12350 11908 12353 11910 12356 11914 12356
11914 12359 11916 12362 11914 12364 11911 12366 11910 12363 11908 12360
11907 12357 11906 12354 14 N F
11917 12345 11913 12342 11916 12338 11914 12331 11917 12327 11918 12323
11923 12322 11927 12322 11931 12324 11935 12326 11938 12329 11942 12332
11942 12336 11946 12337 11948 12341 11951 12343 11952 12347 11955 12345
11955 12345 11957 12341 11958 12345 11962 12343 11966 12344 11966 12348
11962 12348 11957 12349 11953 12351 11949 12351 11945 12349 11940 12350
11936 12349 11935 12353 11931 12355 11926 12354 11923 12351 11920 12348
11917 12345 37 N F
11933 12365 11930 12361 11931 12361 11935 12360 11937 12357 11940 12354
11945 12353 11949 12353 11954 12353 11950 12355 11949 12360 11952 12362
11954 12358 11959 12358 11961 12362 11958 12365 11962 12368 11965 12370
11962 12374 11960 12377 11957 12380 11953 12382 11949 12381 11944 12382
11940 12380 11937 12383 11934 12386 11934 12381 11937 12378 11933 12376
11933 12372 11937 12373 11935 12370 11933 12365 34 N F
11949 12392 11949 12386 11952 12386 11955 12388 11958 12386 11960 12389
11963 12389 11962 12392 11961 12395 11958 12396 11955 12396 11952 12394
11949 12392 13 N F
11955 12322 11952 12320 11955 12317 11952 12312 11956 12313 11959 12313
11962 12312 11962 12315 11959 12317 11958 12320 11955 12322 11 N F
11963 12473 11961 12468 11963 12464 11963 12460 11964 12456 11965 12452
11964 12447 11968 12444 11967 12440 11971 12442 11974 12439 11978 12437
11978 12437 11982 12435 11985 12438 11988 12440 11991 12437 11995 12434
11992 12431 11987 12431 11987 12427 11991 12424 11995 12425 11999 12426
12003 12426 12007 12423 12011 12422 12010 12426 12008 12430 12013 12429
12015 12425 12020 12425 12024 12425 12026 12425 12028 12424 12026 12428
12022 12430 12023 12434 12025 12438 12021 12440 12017 12439 12013 12439
12014 12443 12012 12447 12008 12449 12007 12453 12006 12457 12005 12461
12004 12466 12003 12470 12001 12474 11997 12476 11993 12478 11990 12480
11985 12482 11981 12483 11977 12482 11974 12478 11970 12479 11966 12477
11963 12473 61 N F
11974 12344 11969 12341 11972 12337 11974 12333 11975 12329 11978 12332
11981 12328 11983 12324 11987 12323 11989 12326 11989 12331 11991 12334
11990 12339 11994 12341 11993 12345 11990 12343 11989 12347 11987 12351
11982 12352 11983 12356 11987 12358 11991 12359 11993 12363 11990 12365

11986 12367 11982 12368 11978 12371 11974 12368 11974 12364 11970 12364
11971 12359 11975 12360 11977 12356 11977 12352 11974 12349 11974 12344
36 N F
12013 12478 12011 12473 12014 12476 12019 12475 12023 12476 12021 12472
12020 12468 12019 12463 12023 12461 12026 12464 12026 12469 12029 12472
12030 12476 12034 12478 12039 12478 12043 12480 12043 12484 12045 12487
12050 12486 12050 12486 12054 12484 12058 12485 12062 12484 12062 12489
12059 12492 12055 12494 12055 12498 12050 12496 12046 12497 12050 12500
12051 12504 12047 12507 12046 12511 12041 12510 12037 12508 12034 12506
12029 12504 12025 12504 12023 12501 12019 12498 12018 12493 12016 12490
12017 12486 12015 12482 12013 12478 45 N F
12018 12451 12014 12449 12019 12448 12023 12446 12027 12446 12031 12444
12034 12441 12037 12444 12040 12447 12044 12447 12049 12448 12052 12445
12054 12442 12054 12437 12057 12434 12061 12436 12063 12440 12066 12443
12065 12447 12069 12446 12070 12441 12074 12442 12075 12446 12074 12450
12075 12454 12079 12454 12080 12458 12079 12462 12075 12459 12071 12460
12068 12463 12064 12462 12059 12462 12056 12460 12054 12463 12050 12466
12047 12463 12044 12460 12044 12455 12041 12453 12037 12451 12034 12454
12030 12457 12028 12453 12026 12449 12022 12451 12018 12451 47 N F
12023 12380 12022 12377 12023 12374 12022 12368 12025 12366 12027 12364
12029 12366 12029 12370 12031 12372 12032 12375 12030 12378 12029 12381
12026 12382 12023 12380 14 N F
12065 12494 12060 12493 12063 12491 12066 12487 12063 12483 12062 12479
12063 12475 12067 12478 12068 12482 12072 12484 12072 12479 12072 12475
12073 12471 12078 12470 12081 12468 12082 12468 12085 12468 12090 12470
12091 12474 12095 12476 12099 12476 12101 12480 12098 12483 12097 12488
12098 12492 12100 12496 12098 12500 12096 12496 12094 12492 12092 12488
12088 12490 12084 12488 12081 12492 12077 12491 12073 12492 12069 12495
12068 12491 12065 12494 38 N F
12070 12376 12067 12365 12071 12360 12077 12362 12083 12358 12090 12357
12092 12357 12096 12356 12101 12361 12101 12368 12101 12375 12096 12379
12094 12386 12089 12390 12082 12392 12076 12388 12073 12382 12070 12376
18 N F
12110 12268 12107 12262 12109 12258 12113 12258 12117 12259 12120 12263
12118 12267 12114 12269 12110 12268 9 N F
12110 12459 12108 12456 12108 12452 12109 12447 12113 12446 12114 12443
12114 12442 12118 12444 12122 12446 12125 12449 12130 12450 12131 12454
12135 12456 12133 12460 12126 12464 12122 12463 12118 12463 12113 12462
12110 12459 19 N F
12125 12474 12121 12476 12123 12472 12126 12470 12127 12470 12131 12471
12135 12470 12139 12468 12135 12465 12140 12465 12142 12461 12143 12465
12148 12466 12151 12463 12147 12460 12144 12458 12145 12454 12146 12449
12148 12453 12150 12457 12153 12460 12156 12463 12160 12463 12161 12467

12157 12467 12156 12471 12160 12469 12164 12470 12167 12466 12172 12467
12169 12471 12166 12474 12165 12478 12161 12478 12157 12479 12154 12477
12150 12474 12146 12473 12147 12477 12145 12481 12141 12483 12139 12486
12136 12490 12137 12486 12133 12485 12129 12483 12132 12480 12129 12476
12125 12474 49 N F
12136 12530 12129 12528 12133 12525 12136 12522 12136 12521 12137 12518
12137 12513 12140 12510 12143 12507 12145 12511 12144 12515 12148 12514
12147 12509 12149 12505 12150 12501 12152 12505 12154 12509 12155 12513
12154 12517 12152 12521 12150 12525 12148 12529 12144 12531 12140 12532
12136 12530 25 N F
12165 12540 12160 12539 12163 12536 12167 12537 12170 12534 12166 12531
12165 12527 12169 12528 12172 12524 12176 12522 12179 12520 12183 12518
12187 12520 12191 12520 12194 12523 12198 12524 12203 12523 12207 12523
12211 12523 12215 12524 12219 12528 12219 12532 12219 12536 12221 12540
12222 12545 12224 12549 12223 12553 12220 12556 12217 12559 12212 12558
12209 12556 12204 12555 12200 12557 12196 12554 12193 12551 12191 12548
12186 12548 12184 12545 12180 12546 12175 12547 12174 12551 12169 12552
12170 12548 12168 12544 12165 12540 45 N F
12236 12411 12234 12405 12237 12407 12242 12406 12246 12408 12250 12407
12250 12412 12252 12415 12251 12419 12250 12424 12250 12428 12245 12427
12241 12425 12242 12421 12238 12420 12237 12416 12236 12411 17 N F
12274 12325 12269 12321 12271 12317 12272 12313 12276 12311 12277 12306
12279 12303 12283 12300 12287 12300 12291 12299 12294 12303 12290 12302
12286 12304 12284 12308 12281 12311 12279 12315 12277 12319 12279 12323
12278 12328 12274 12325 20 N F
12288 12326 12281 12324 12285 12321 12286 12317 12290 12315 12294 12314
12295 12318 12292 12320 12292 12324 12288 12326 10 N F
12313 12589 12310 12584 12314 12582 12318 12582 12319 12578 12322 12575
12318 12574 12315 12571 12318 12567 12320 12564 12325 12563 12328 12567
12332 12567 12336 12569 12340 12568 12342 12572 12342 12576 12339 12579
12335 12581 12330 12581 12326 12581 12330 12583 12327 12587 12323 12588
12326 12591 12329 12595 12328 12599 12323 12599 12319 12600 12316 12597
12314 12593 12313 12589 32 N F
12323 12552 12321 12547 12326 12547 12330 12548 12334 12549 12337 12545
12332 12545 12328 12546 12329 12542 12333 12540 12338 12541 12342 12539
12342 12535 12340 12531 12344 12528 12347 12532 12350 12534 12354 12536
12358 12537 12362 12540 12363 12544 12361 12548 12360 12552 12355 12553
12353 12557 12349 12559 12346 12563 12345 12558 12341 12557 12337 12554
12334 12557 12329 12558 12326 12560 12321 12559 12325 12556 12323 12552
36 N F
12400 12629 12398 12625 12398 12619 12400 12623 12403 12619 12405 12616
12410 12616 12413 12619 12417 12620 12418 12625 12421 12621 12425 12622
12429 12625 12425 12627 12422 12630 12419 12627 12414 12627 12412 12631

12408 12632 12404 12630 12400 12629 21 N F
12433 12610 12426 12609 12429 12606 12434 12604 12437 12602 12441 12604
12441 12599 12444 12597 12445 12593 12450 12591 12454 12593 12457 12591
12461 12589 12463 12585 12467 12585 12471 12586 12474 12589 12472 12593
12470 12597 12466 12597 12461 12597 12465 12599 12465 12603 12462 12606
12458 12609 12457 12613 12455 12617 12451 12619 12447 12620 12442 12619
12439 12616 12436 12613 12433 12610 33 N F
12458 12794 12457 12787 12458 12780 12461 12774 12465 12769 12470 12764
12472 12758 12477 12753 12480 12747 12477 12740 12475 12734 12477 12733
12482 12732 12484 12725 12489 12721 12496 12720 12503 12721 12509 12724
12509 12726 12511 12731 12507 12737 12505 12743 12511 12747 12516 12751
12523 12752 12524 12746 12524 12739 12525 12732 12526 12731 12528 12726
12530 12719 12536 12718 12539 12718 12543 12718 12545 12720 12549 12722
12556 12723 12560 12717 12566 12720 12571 12721 12573 12721 12569 12726
12566 12732 12570 12737 12574 12743 12572 12750 12571 12757 12570 12763
12569 12770 12572 12776 12572 12783 12566 12787 12560 12785 12555 12789
12542 12787 12536 12784 12533 12778 12527 12775 12522 12770 12517 12774
12515 12781 12509 12783 12502 12783 12503 12790 12498 12795 12495 12801
12496 12808 12501 12812 12504 12818 12505 12825 12503 12831 12500 12837
12494 12841 12487 12842 12481 12839 12476 12835 12472 12829 12472 12822
12476 12817 12470 12815 12463 12813 12463 12806 12463 12799 12458 12794
84 N F
12477 12559 12473 12555 12478 12555 12475 12552 12475 12547 12479 12547
12483 12544 12485 12548 12485 12552 12484 12557 12485 12561 12485 12565
12481 12567 12482 12572 12477 12572 12476 12568 12477 12564 12477 12559
18 N F
12485 8029 12477 8021 12482 8016 12488 8014 12496 8013 12503 8013
12508 8008 12511 8001 12515 7996 12521 7992 12527 7989 12534 7988
12532 7995 12529 8001 12529 8009 12533 8014 12535 8021 12529 8024
12526 8031 12519 8033 12515 8039 12509 8042 12502 8041 12496 8038
12492 8032 12485 8029 26 N F
12525 12806 12523 12802 12524 12798 12526 12794 12528 12790 12532 12789
12536 12788 12541 12788 12545 12789 12549 12790 12550 12794 12554 12796
12558 12794 12562 12792 12566 12793 12570 12791 12574 12792 12578 12789
12582 12789 12581 12793 12577 12796 12573 12797 12570 12800 12569 12804
12571 12808 12570 12813 12566 12815 12562 12817 12558 12819 12560 12823
12556 12824 12552 12821 12551 12817 12550 12813 12550 12808 12548 12804
12545 12807 12545 12811 12545 12816 12546 12820 12549 12824 12550 12828
12551 12832 12551 12836 12547 12835 12542 12833 12537 12829 12535 12825
12534 12821 12532 12817 12529 12814 12528 12810 12525 12806 53 N F
12524 8161 12524 8160 12524 8159 12524 8159 12525 8159 12526 8159
12526 8159 12526 8159 12526 8160 12526 8160 12526 8161 12524 8161
12 N F

12536 8016 12534 8013 12536 8013 12538 8014 12540 8015 12540 8017
12539 8019 12537 8018 12536 8016 9 N F
12543 8019 12542 8015 12545 8016 12546 8018 12545 8020 12543 8019
6 N F
12548 8016 12547 8013 12549 8015 12551 8016 12553 8017 12555 8017
12557 8019 12559 8020 12560 8022 12562 8023 12564 8023 12566 8023
12568 8024 12569 8026 12571 8027 12574 8028 12571 8028 12569 8028
12568 8026 12566 8025 12564 8025 12561 8024 12559 8024 12558 8022
12556 8021 12554 8019 12552 8018 12550 8018 12548 8016 29 N F
12619 12808 12616 12801 12621 12799 12625 12798 12624 12793 12628 12793
12631 12796 12632 12800 12635 12803 12640 12804 12644 12804 12643 12808
12639 12810 12635 12810 12631 12813 12627 12812 12623 12810 12619 12808
18 N F
12639 12792 12633 12789 12637 12788 12638 12784 12642 12784 12645 12781
12649 12781 12653 12779 12654 12783 12651 12787 12656 12787 12660 12788
12663 12791 12660 12794 12656 12796 12653 12799 12650 12802 12646 12804
12642 12802 12639 12799 12636 12796 12639 12792 22 N F
12659 12697 12656 12691 12659 12688 12663 12687 12663 12682 12665 12678
12668 12682 12672 12683 12674 12687 12678 12686 12683 12686 12687 12686
12686 12691 12689 12694 12687 12698 12683 12696 12681 12693 12680 12688
12676 12691 12679 12694 12676 12697 12672 12698 12668 12700 12664 12702
12660 12701 12659 12697 26 N F
12696 8054 12693 8048 12696 8045 12698 8041 12702 8043 12706 8045
12702 8047 12699 8051 12700 8055 12696 8054 10 N F
12721 8183 12715 8170 12722 8173 12729 8174 12735 8177 12733 8184
12727 8187 12721 8183 8 N F
12753 12843 12750 12836 12752 12830 12755 12823 12758 12817 12760 12816
12764 12815 12767 12809 12771 12803 12777 12806 12776 12812 12783 12814
12789 12817 12793 12823 12796 12829 12803 12827 12804 12821 12806 12814
12805 12808 12805 12801 12799 12797 12793 12795 12787 12793 12788 12786
12789 12786 12793 12790 12793 12784 12800 12785 12806 12788 12812 12785
12807 12781 12806 12774 12808 12768 12815 12766 12812 12760 12809 12754
12816 12752 12817 12751 12822 12749 12828 12753 12834 12755 12832 12761
12827 12766 12833 12768 12840 12769 12847 12772 12853 12773 12848 12776
12841 12775 12837 12780 12832 12785 12834 12792 12841 12791 12845 12796
12843 12803 12843 12810 12849 12813 12852 12807 12856 12812 12863 12811
12864 12810 12867 12806 12873 12803 12874 12810 12869 12815 12865 12820
12866 12827 12860 12826 12857 12832 12863 12835 12870 12833 12867 12840
12863 12845 12869 12846 12869 12853 12872 12859 12865 12860 12860 12855
12859 12848 12852 12850 12848 12855 12853 12861 12856 12867 12859 12873
12857 12879 12855 12886 12854 12892 12852 12899 12846 12903 12843 12909
12841 12915 12844 12921 12850 12924 12851 12931 12847 12936 12840 12938
12833 12939 12829 12933 12829 12926 12833 12916 12826 12914 12823 12908

12822 12901 12822 12894 12815 12896 12809 12893 12803 12895 12801 12889
12796 12884 12800 12878 12804 12872 12808 12867 12813 12862 12818 12857
12811 12858 12804 12857 12798 12860 12793 12855 12787 12856 12781 12859
12775 12864 12769 12862 12766 12856 12760 12855 12756 12848 12753 12843
126 N F
12752 12793 12751 12787 12754 12785 12757 12782 12756 12778 12756 12773
12757 12773 12761 12773 12765 12772 12769 12775 12772 12777 12775 12780
12775 12780 12775 12785 12772 12788 12768 12787 12765 12791 12765 12795
12762 12798 12762 12803 12759 12800 12760 12804 12756 12804 12752 12801
12754 12797 12758 12798 12761 12795 12758 12797 12757 12793 12754 12789
12754 12794 12751 12797 12752 12793 33 N F
12757 7950 12754 7945 12756 7941 12760 7939 12760 7943 12761 7948
12757 7950 7 N F
12757 7972 12756 7967 12757 7967 12760 7967 12761 7971 12757 7972
6 N F
12763 12892 12762 12887 12764 12884 12765 12879 12763 12875 12766 12872
12768 12868 12773 12869 12770 12872 12773 12875 12770 12878 12768 12882
12771 12885 12773 12888 12771 12892 12767 12893 12763 12892 17 N F
12772 7966 12769 7962 12773 7959 12775 7955 12780 7957 12783 7959
12781 7963 12779 7967 12775 7969 12772 7966 10 N F
12782 7984 12776 7979 12780 7978 12785 7978 12786 7978 12787 7976
12788 7975 12791 7978 12793 7982 12793 7987 12796 7984 12800 7983
12800 7987 12798 7991 12793 7992 12789 7992 12788 7988 12784 7988
12782 7984 19 N F
12786 7955 12779 7953 12782 7949 12786 7950 12786 7955 5 N F
12787 7949 12783 7944 12786 7940 12787 7945 12787 7949 5 N F
12812 7958 12808 7956 12810 7951 12813 7948 12812 7944 12812 7939
12815 7936 12816 7940 12820 7942 12821 7946 12820 7951 12819 7955
12822 7959 12825 7962 12824 7967 12824 7971 12823 7976 12821 7980
12817 7982 12813 7980 12813 7976 12812 7971 12810 7967 12811 7963
12812 7958 25 N F
12859 7979 12857 7973 12861 7974 12862 7978 12864 7982 12864 7987
12860 7983 12859 7979 8 N F
12885 12955 12882 12951 12886 12950 12889 12947 12889 12943 12892 12939
12888 12937 12892 12935 12896 12934 12897 12930 12896 12925 12899 12922
12901 12923 12904 12923 12905 12927 12903 12931 12903 12936 12904 12940
12906 12944 12907 12948 12906 12953 12905 12957 12900 12956 12898 12960
12895 12962 12892 12959 12888 12958 12885 12955 28 N F
12896 11258 12895 11258 12896 11257 12896 11257 12896 11257 12896 11257
12897 11256 12898 11256 12898 11256 12898 11257 12898 11257 12897 11258
12896 11258 13 N F
12897 11261 12896 11259 12897 11259 12898 11258 12899 11257 12899 11255
12898 11254 12898 11252 12898 11250 12897 11249 12899 11248 12899 11250

12900 11251 12901 11252 12903 11252 12903 11253 12901 11255 12901 11256
12901 11258 12902 11259 12903 11260 12903 11262 12901 11263 12900 11262
12899 11261 12897 11261 26 N F
12904 11267 12903 11266 12903 11265 12904 11263 12905 11264 12906 11265
12906 11265 12906 11266 12906 11268 12906 11268 12905 11268 12904 11267
12904 11267 13 N F
12905 11272 12905 11272 12905 11271 12905 11271 12906 11271 12906 11271
12906 11271 12906 11272 12906 11272 12905 11272 10 N F
12907 11274 12906 11274 12906 11272 12907 11272 12907 11272 12907 11272
12908 11272 12909 11272 12909 11272 12909 11274 12909 11275 12909 11275
12909 11275 12907 11275 12907 11275 12907 11274 16 N F
12909 11279 12909 11279 12909 11278 12910 11277 12910 11277 12910 11277
12910 11276 12911 11276 12912 11276 12912 11277 12912 11277 12911 11278
12911 11279 12911 11279 12909 11279 15 N F
12911 11283 12911 11281 12913 11281 12914 11281 12916 11282 12917 11282
12919 11283 12920 11284 12921 11283 12922 11282 12923 11283 12924 11284
12925 11286 12926 11287 12926 11289 12926 11290 12925 11291 12924 11289
12923 11290 12921 11289 12919 11290 12918 11290 12916 11290 12915 11289
12915 11287 12915 11285 12914 11284 12913 11283 12911 11283 29 N F
12963 7992 12960 7986 12961 7979 12966 7974 12969 7967 12974 7963
12979 7958 12980 7950 12979 7943 12979 7936 12978 7929 12977 7922
12982 7918 12989 7915 12993 7921 12999 7925 12999 7925 12999 8033
12995 8030 12988 8028 12984 8022 12982 8015 12978 8009 12979 8002
12973 7997 12977 7991 12982 7986 12976 7990 12970 7993 12963 7992
30 N F
12974 11621 12970 11609 12977 11607 12984 11607 12988 11602 12995 11601
12999 11606 12999 11607 12999 11637 12993 11637 12998 11632 12991 11634
12994 11627 12988 11623 12987 11616 12994 11619 12999 11623 12995 11617
12995 11610 12988 11612 12983 11616 12980 11622 12976 11628 12974 11621
24 N F
12982 8295 12979 8284 12983 8279 12990 8279 12997 8281 12999 8281
12999 8296 12996 8295 12989 8295 12982 8295 10 N F
12984 11702 12982 11695 12982 11688 12987 11683 12984 11676 12986 11670
12991 11665 12987 11659 12990 11653 12993 11647 12999 11644 12999 11638
12999 11700 12995 11705 12989 11707 12984 11702 16 N F
12983 8119 12983 8112 12985 8105 12988 8099 12992 8093 12996 8086
12999 8084 12999 8119 12996 8116 12991 8121 12986 8126 12983 8119
12 N F
1 O
13000 16171 2298 16171 2298 5454 13000 5454 13000 16171 5 P
Ls Gr Gs Lr
2297 5453 13002 16173 Oc
56 O

2297 5589 2297 5554 2297 5554 2297 7777 2297 7777 2297 7760
2297 7624 2297 7489 2297 7353 2297 7217 2297 7081 2297 6946
2297 6810 2297 6674 2297 6539 2297 6403 2297 6267 2297 6132
2297 5996 2297 5860 2297 5725 2297 5589 22 N F
74 O
2297 7896 2297 7777 2297 7777 2297 8034 2297 8034 2297 8031
2297 7896 7 N F
92 O
2297 8167 2297 8034 2297 8034 2297 8993 2297 8993 2297 8981
2297 8845 2297 8710 2297 8574 2297 8438 2297 8303 2297 8167
12 N F
74 O
2297 9117 2297 8993 2297 8993 2297 9213 2297 9213 2297 9117
6 N F
92 O
2297 9252 2297 9213 2297 9213 2297 9486 2297 9486 2297 9388
2297 9252 7 N F
74 O
2297 9524 2297 9486 2297 9486 2297 9673 2297 9673 2297 9660
2297 9524 7 N F
56 O
2297 9795 2297 9673 2297 9673 2297 9884 2297 9884 2297 9795
6 N F
74 O
2297 9931 2297 9884 2297 9884 2297 9973 2297 9973 2297 9931
6 N F
56 O
2297 10067 2297 9973 2297 9973 2297 10592 2297 10592 2297 10474
2297 10338 2297 10202 2297 10067 9 N F
38 O
2297 10609 2297 10592 2297 10592 2297 10734 2297 10734 2297 10609
6 N F
56 O
2297 10745 2297 10734 2297 10734 2297 11425 2297 11425 2297 11423
2297 11288 2297 11152 2297 11016 2297 10881 2297 10745 11 N F
74 O
2297 11559 2297 11425 2297 11425 2297 11726 2297 11726 2297 11695
2297 11559 7 N F
92 O
2297 11806 2297 11726 2297 11726 2297 11806 2297 11806 5 N F
111 O
2297 11831 2297 11806 2297 11806 2297 11889 2297 11889 2297 11831
6 N F

129 O
2297 11966 2297 11889 2297 11889 2297 11981 2297 11981 2297 11966
6 N F
147 O
2297 12102 2297 11981 2297 11981 2297 12330 2297 12330 2297 12238
2297 12102 7 N F
129 O
2297 12373 2297 12330 2297 12330 2297 12395 2297 12395 2297 12373
6 N F
147 O
2297 12509 2297 12395 2297 12395 2297 13160 2297 13160 2297 13052
2297 12916 2297 12780 2297 12645 2297 12509 10 N F
165 O
2297 13187 2297 13160 2297 13160 2297 13617 2297 13617 2297 13594
2297 13459 2297 13323 2297 13187 9 N F
147 O
2297 13730 2297 13617 2297 13617 2297 14053 2297 14053 2297 14002
2297 13866 2297 13730 8 N F
165 O
2297 14137 2297 14053 2297 14053 2297 14378 2297 14378 2297 14273
2297 14137 7 N F
147 O
2297 14409 2297 14378 2297 14378 2297 15268 2297 15268 2297 15223
2297 15087 2297 14951 2297 14816 2297 14680 2297 14544 2297 14409
12 N F
165 O
2297 15358 2297 15268 2297 15268 2297 15451 2297 15451 2297 15358
6 N F
147 O
2297 15494 2297 15451 2297 15451 2297 15528 2297 15528 2297 15494
6 N F
165 O
2297 15630 2297 15528 2297 15528 2297 15974 2297 15974 2297 15901
2297 15765 2297 15630 8 N F
183 O
2297 16037 2297 15974 2297 15974 2297 16110 2297 16110 2297 16037
6 N F
74 O
2297 5554 2297 5453 2297 5453 2297 5453 2297 5554 2297 5554
6 N F
56 O
2297 7777 2297 5554 2297 5554 2297 7777 2297 7777 5 N F
74 O

2297 8034 2297 7777 2297 7777 2297 8034 2297 8034 5 N F
92 O
2297 8993 2297 8034 2297 8034 2297 8993 2297 8993 5 N F
74 O
2297 9213 2297 8993 2297 8993 2297 9213 2297 9213 5 N F
92 O
2297 9486 2297 9213 2297 9213 2297 9486 2297 9486 5 N F
74 O
2297 9673 2297 9486 2297 9486 2297 9673 2297 9673 5 N F
56 O
2297 9884 2297 9673 2297 9673 2297 9884 2297 9884 5 N F
74 O
2297 9973 2297 9884 2297 9884 2297 9973 2297 9973 5 N F
56 O
2297 10592 2297 9973 2297 9973 2297 10592 2297 10592 5 N F
38 O
2297 10734 2297 10592 2297 10592 2297 10733 2297 10734 5 N F
56 O
2297 11425 2297 10734 2297 10733 2297 11425 2297 11425 5 N F
74 O
2297 11726 2297 11425 2297 11425 2297 11726 2297 11726 5 N F
92 O
2297 11806 2297 11726 2297 11726 2297 11806 2297 11806 5 N F
111 O
2297 11889 2297 11806 2297 11806 2297 11889 2297 11889 5 N F
129 O
2297 11981 2297 11889 2297 11889 2297 11981 2297 11981 5 N F
147 O
2297 12330 2297 11981 2297 11981 2297 12330 2297 12330 5 N F
129 O
2297 12395 2297 12330 2297 12330 2297 12395 2297 12395 5 N F
147 O
2297 13160 2297 12395 2297 12395 2297 13160 2297 13160 5 N F
165 O
2297 13617 2297 13160 2297 13160 2297 13618 2297 13617 5 N F
147 O
2297 14053 2297 13617 2297 13618 2297 14053 2297 14053 5 N F
165 O
2297 14378 2297 14053 2297 14053 2297 14378 2297 14378 5 N F
147 O
2297 15268 2297 14378 2297 14378 2297 15269 2297 15268 5 N F
165 O
2297 15451 2297 15268 2297 15269 2297 15451 2297 15451 5 N F

147 O
2297 15528 2297 15451 2297 15451 2297 15528 2297 15528 5 N F
165 O
2297 15974 2297 15528 2297 15528 2297 15974 2297 15974 5 N F
183 O
2297 16110 2297 15974 2297 15974 2297 16110 2297 16110 5 N F
165 O
2297 16173 2297 16110 2297 16110 2297 16172 2297 16173 2297 16173
6 N F
74 O
2470 5453 2297 5453 2297 5554 2433 5561 2504 5589 2568 5633
2704 5725 2704 5725 2704 5725 2704 5724 2655 5589 2697 5453
2643 5453 2470 5453 14 N F
56 O
2297 5554 M 2297 7777 L 2356 7760 L 2433 7727 L 2568 7634 L
2573 7624 L 2629 7489 L 2694 7353 L 2704 7339 L
2839 7295 L 2839 6854 L 2794 6810 L 2839 6753 L
2934 6810 L 2839 6854 L 2839 7295 L 2975 7327 L
3095 7217 L 3110 7206 L 3195 7081 L 3243 6946 L
3110 6844 L 3020 6810 L 3070 6674 L 3099 6539 L
3103 6403 L 3110 6393 L 3115 6403 L 3122 6539 L
3246 6614 L 3381 6618 L 3417 6674 L 3499 6810 L
3517 6924 L 3652 6941 L 3788 6876 L 3923 6864 L
3953 6810 L 3923 6776 L 3863 6674 L 3862 6539 L
3923 6448 L 4059 6474 L 4177 6539 L 4194 6554 L
4330 6623 L 4429 6539 L 4465 6510 L 4559 6539 L
4601 6650 L 4607 6674 L 4601 6697 L 4582 6810 L
4601 6816 L 4736 6830 L 4771 6810 L 4787 6674 L
4872 6569 L 4942 6539 L 5007 6528 L 5035 6539 L
5143 6595 L 5235 6674 L 5276 6810 L 5278 6821 L
5280 6810 L 5414 6743 L 5549 6747 L 5615 6810 L
5605 6946 L 5625 7081 L 5685 7183 L 5820 7134 L
5947 7217 L 5956 7227 L 5956 6324 L 5839 6267 L
5898 6132 L 5956 6030 L 6046 6132 L 6039 6267 L
5956 6324 L 5956 7227 L 5970 7217 L 6091 7099 L
6194 7217 L 6148 7353 L 6227 7392 L 6227 6979 L
6148 6946 L 6186 6810 L 6227 6763 L 6227 6434 L
6091 6425 L 6060 6403 L 6091 6349 L 6227 6325 L
6288 6403 L 6227 6434 L 6227 6763 L 6362 6802 L
6366 6810 L 6362 6851 L 6334 6946 L 6227 6979 L
6227 7392 L 6362 7415 L 6498 7419 L 6633 7442 L
6769 7473 L 6832 7489 L 6889 7624 L 6904 7638 L
6904 7231 L 6898 7217 L 6904 7211 L 6917 7217 L

6904 7231 L 6904 7638 L 7040 7627 L 7052 7624 L
7142 7489 L 7123 7353 L 7175 7263 L 7311 7336 L
7419 7217 L 7446 7185 L 7582 7129 L 7626 7217 L
7711 7353 L 7717 7361 L 7853 7391 L 7985 7353 L
7988 7350 L 7988 7135 L 7951 7081 L 7988 7053 L
8044 7081 L 7988 7135 L 7988 7350 L 8124 7298 L
8259 7316 L 8395 7293 L 8530 7275 L 8666 7266 L
8734 7353 L 8801 7397 L 8901 7489 L 8893 7624 L
8907 7760 L 8808 7896 L 8844 8031 L 8937 8148 L
8965 8167 L 9072 8202 L 9113 8167 L 9208 8106 L
9343 8097 L 9425 8031 L 9386 7896 L 9343 7818 L
9306 7760 L 9343 7710 L 9477 7760 L 9479 7761 L
9482 7760 L 9614 7732 L 9750 7636 L 9885 7626 L
9889 7624 L 10021 7542 L 10156 7568 L 10292 7571 L
10427 7616 L 10563 7576 L 10698 7591 L 10834 7571 L
10941 7489 L 10969 7475 L 11105 7456 L 11240 7438 L
11376 7445 L 11511 7455 L 11647 7467 L 11782 7482 L
11827 7489 L 11918 7530 L 12031 7624 L 12053 7646 L
12079 7760 L 12098 7896 L 12109 8031 L 12117 8167 L
12127 8303 L 12134 8438 L 12141 8574 L 12151 8710 L
12159 8845 L 12169 8981 L 12177 9117 L 12189 9245 L
12194 9252 L 12293 9388 L 12324 9502 L 12334 9524 L
12424 9660 L 12460 9724 L 12529 9795 L 12595 9864 L
12670 9931 L 12731 9983 L 12807 10067 L 12866 10136 L
12970 10202 L 13002 10223 L 13002 8595 L 12985 8710 L
12944 8845 L 12866 8919 L 12731 8884 L 12640 8845 L
12595 8818 L 12460 8771 L 12324 8728 L 12308 8710 L
12192 8574 L 12189 8566 L 12185 8438 L 12181 8303 L
12176 8167 L 12173 8031 L 12168 7896 L 12155 7760 L
12143 7624 L 12121 7489 L 12053 7418 L 11918 7401 L
11782 7394 L 11647 7385 L 11511 7379 L 11376 7372 L
11240 7369 L 11105 7372 L 10969 7383 L 10834 7421 L
10698 7437 L 10563 7432 L 10427 7403 L 10292 7387 L
10156 7371 L 10021 7382 L 9885 7381 L 9750 7390 L
9614 7415 L 9479 7416 L 9343 7406 L 9208 7387 L
9085 7353 L 9072 7348 L 8937 7281 L 8869 7217 L
8801 7169 L 8703 7081 L 8666 7045 L 8569 6946 L
8530 6906 L 8420 6810 L 8395 6793 L 8259 6741 L
8167 6674 L 8124 6641 L 7988 6544 L 7983 6539 L
7853 6443 L 7717 6405 L 7582 6441 L 7484 6539 L
7535 6674 L 7499 6810 L 7446 6908 L 7311 6906 L
7175 6830 L 7040 6811 L 7039 6810 L 7040 6790 L
7044 6674 L 7040 6668 L 6904 6564 L 6769 6547 L

6758 6539 L 6638 6403 L 6769 6277 L 6904 6314 L
6933 6267 L 7040 6217 L 7128 6267 L 7175 6367 L
7253 6267 L 7309 6132 L 7221 5996 L 7175 5961 L
7040 5909 L 6904 5923 L 6857 5996 L 6769 6095 L
6737 6132 L 6737 6267 L 6633 6388 L 6498 6390 L
6406 6267 L 6362 6242 L 6237 6132 L 6227 6120 L
6091 6017 L 6062 5996 L 6035 5860 L 6091 5835 L
6173 5725 L 6195 5589 L 6141 5453 L 6096 5453 L
6044 5453 L 6091 5530 L 6123 5589 L 6091 5646 L
5956 5703 L 5855 5725 L 5820 5749 L 5728 5860 L
5685 5894 L 5550 5996 L 5549 5998 L 5414 6053 L
5374 5996 L 5322 5860 L 5278 5829 L 5216 5725 L
5143 5629 L 5070 5589 L 5012 5453 L 4887 5453 L
4715 5453 L 4542 5453 L 4369 5453 L 4197 5453 L
4024 5453 L 3851 5453 L 3679 5453 L 3506 5453 L
3333 5453 L 3161 5453 L 2988 5453 L 2815 5453 L
2697 5453 L 2655 5589 L 2704 5724 L 2704 5725 L
2704 5725 L 2704 5725 L 2568 5633 L 2504 5589 L
2433 5561 L 2297 5554 L N E
74 O
2356 7760 M 2297 7777 L 2297 8034 L 2305 8031 L 2433 8003 L
2568 8008 L 2704 7942 L 2801 8031 L 2801 8167 L
2839 8223 L 2913 8303 L 2953 8438 L 2975 8491 L
3110 8445 L 3117 8438 L 3230 8303 L 3110 8238 L
3028 8167 L 2975 8043 L 2927 8031 L 2975 8022 L
3110 7917 L 3195 7896 L 3246 7879 L 3246 7573 L
3214 7489 L 3246 7373 L 3381 7396 L 3449 7489 L
3381 7533 L 3246 7573 L 3246 7879 L 3381 7793 L
3438 7760 L 3517 7690 L 3652 7716 L 3703 7760 L
3775 7896 L 3776 8031 L 3788 8051 L 3849 8167 L
3923 8249 L 4059 8229 L 4092 8167 L 4166 8031 L
4059 7984 L 3932 7896 L 3941 7760 L 3923 7688 L
3888 7624 L 3872 7489 L 3923 7392 L 4059 7423 L
4194 7481 L 4194 6986 L 4084 6946 L 4194 6895 L
4276 6946 L 4194 6986 L 4194 7481 L 4224 7489 L
4330 7517 L 4368 7489 L 4336 7353 L 4330 7329 L
4300 7217 L 4330 7183 L 4419 7081 L 4465 7004 L
4517 7081 L 4465 7157 L 4390 7217 L 4465 7248 L
4601 7327 L 4620 7353 L 4684 7489 L 4736 7591 L
4811 7624 L 4736 7654 L 4601 7640 L 4571 7760 L
4579 7896 L 4601 7992 L 4623 8031 L 4605 8167 L
4736 8298 L 4796 8303 L 4870 8438 L 4809 8574 L
4736 8649 L 4700 8710 L 4736 8792 L 4872 8738 L

4967 8845 L 4946 8981 L 4872 8998 L 4736 9104 L
4722 9117 L 4601 9216 L 4484 9252 L 4465 9338 L
4463 9388 L 4465 9394 L 4601 9502 L 4605 9524 L
4601 9545 L 4537 9660 L 4568 9795 L 4601 9822 L
4682 9931 L 4733 10067 L 4724 10202 L 4679 10338 L
4658 10474 L 4736 10520 L 4872 10573 L 4895 10609 L
4908 10745 L 4872 10862 L 4865 10881 L 4736 10973 L
4705 11016 L 4601 11102 L 4465 11101 L 4330 11085 L
4206 11152 L 4194 11186 L 4156 11288 L 4059 11346 L
3923 11398 L 3813 11423 L 3788 11433 L 3652 11475 L
3521 11559 L 3517 11566 L 3411 11695 L 3381 11710 L
3246 11761 L 3110 11780 L 2975 11807 L 2914 11831 L
2954 11966 L 2975 11972 L 3110 12017 L 3246 12005 L
3246 11892 L 3135 11831 L 3246 11821 L 3381 11819 L
3466 11831 L 3381 11889 L 3246 11892 L 3246 12005 L
3381 11998 L 3487 11966 L 3517 11938 L 3652 11848 L
3705 11831 L 3788 11779 L 3923 11804 L 3923 11700 L
3893 11695 L 3788 11629 L 3717 11559 L 3788 11538 L
3923 11525 L 3998 11559 L 3931 11695 L 3923 11700 L
3923 11804 L 4059 11713 L 4194 11732 L 4269 11831 L
4330 11872 L 4465 11886 L 4601 11895 L 4640 11966 L
4736 12027 L 4872 12018 L 5007 11967 L 5009 11966 L
5143 11930 L 5278 11903 L 5414 11962 L 5417 11966 L
5414 11976 L 5278 12058 L 5245 12102 L 5196 12238 L
5143 12290 L 5063 12373 L 5084 12509 L 5143 12590 L
5204 12645 L 5278 12686 L 5414 12724 L 5549 12754 L
5685 12755 L 5685 12577 L 5549 12568 L 5472 12509 L
5414 12425 L 5375 12373 L 5412 12238 L 5414 12236 L
5510 12102 L 5518 11966 L 5416 11831 L 5414 11829 L
5278 11780 L 5202 11831 L 5143 11853 L 5007 11891 L
4872 11872 L 4736 11862 L 4718 11831 L 4736 11762 L
4755 11695 L 4736 11678 L 4679 11695 L 4601 11718 L
4496 11695 L 4494 11559 L 4514 11423 L 4465 11376 L
4345 11288 L 4330 11235 L 4322 11152 L 4330 11148 L
4465 11150 L 4486 11152 L 4601 11268 L 4736 11192 L
4764 11152 L 4872 11091 L 5007 11034 L 5028 11016 L
5088 10881 L 5054 10745 L 5007 10686 L 4989 10609 L
5000 10474 L 4893 10338 L 5007 10264 L 5007 7770 L
4994 7760 L 5007 7740 L 5033 7760 L 5007 7770 L
5007 10264 L 5089 10338 L 5022 10474 L 5143 10528 L
5278 10564 L 5278 7542 L 5143 7524 L 5007 7491 L
5001 7489 L 4872 7375 L 4856 7353 L 4872 7340 L
5007 7349 L 5007 7231 L 4980 7217 L 4996 7081 L

5007 7079 L 5010 7081 L 5045 7217 L 5007 7231 L
5007 7349 L 5047 7353 L 5143 7391 L 5143 7023 L
5073 6946 L 5143 6873 L 5261 6946 L 5143 7023 L
5143 7391 L 5278 7449 L 5278 7145 L 5233 7081 L
5278 6984 L 5327 7081 L 5278 7145 L 5278 7449 L
5368 7489 L 5278 7542 L 5278 10564 L 5402 10609 L
5414 10613 L 5414 7319 L 5308 7217 L 5414 7122 L
5549 7178 L 5632 7217 L 5549 7282 L 5414 7319 L
5414 10613 L 5420 10609 L 5526 10474 L 5549 10460 L
5549 7680 L 5526 7624 L 5549 7566 L 5600 7624 L
5549 7680 L 5549 10460 L 5685 10363 L 5685 10098 L
5619 10067 L 5685 10018 L 5703 10067 L 5685 10098 L
5685 10363 L 5713 10474 L 5695 10609 L 5735 10745 L
5787 10881 L 5771 11016 L 5820 11141 L 5956 11152 L
6091 11130 L 6227 11137 L 6247 11152 L 6227 11211 L
6091 11203 L 6000 11288 L 6091 11363 L 6227 11339 L
6302 11423 L 6227 11509 L 6131 11559 L 6227 11631 L
6362 11638 L 6362 11168 L 6352 11152 L 6227 11058 L
6091 11064 L 5956 11093 L 5858 11016 L 5914 10881 L
5878 10745 L 5903 10609 L 5857 10474 L 5869 10338 L
5895 10202 L 5930 10067 L 5931 9931 L 5944 9795 L
5827 9660 L 5820 9651 L 5744 9524 L 5765 9388 L
5820 9315 L 5902 9252 L 5867 9117 L 5820 9021 L
5802 8981 L 5820 8941 L 5845 8845 L 5820 8766 L
5795 8710 L 5803 8574 L 5801 8438 L 5820 8368 L
5860 8303 L 5868 8167 L 5865 8031 L 5865 7896 L
5820 7803 L 5807 7760 L 5790 7624 L 5820 7496 L
5820 7454 L 5802 7353 L 5820 7343 L 5830 7353 L
5820 7454 L 5820 7496 L 5956 7501 L 6091 7519 L
6219 7624 L 6183 7760 L 6172 7896 L 6179 8031 L
6091 8159 L 6088 8167 L 6091 8176 L 6194 8303 L
6191 8438 L 6146 8574 L 6153 8710 L 6137 8845 L
6091 8933 L 6081 8981 L 6064 9117 L 6091 9130 L
6227 9123 L 6271 9252 L 6352 9388 L 6362 9398 L
6362 8362 L 6303 8303 L 6292 8167 L 6362 8149 L
6391 8167 L 6392 8303 L 6362 8362 L 6362 9398 L
6433 9524 L 6428 9660 L 6362 9769 L 6313 9795 L
6329 9931 L 6362 9957 L 6477 10067 L 6498 10122 L
6524 10202 L 6498 10229 L 6362 10326 L 6341 10338 L
6227 10412 L 6167 10474 L 6227 10528 L 6362 10572 L
6498 10526 L 6633 10495 L 6633 10341 L 6623 10338 L
6633 10328 L 6686 10338 L 6633 10341 L 6633 10495 L
6769 10588 L 6799 10609 L 6769 10667 L 6633 10648 L

6513 10745 L 6582 10881 L 6498 10941 L 6362 10997 L
6352 11016 L 6362 11058 L 6382 11152 L 6362 11168 L
6362 11638 L 6444 11695 L 6362 11709 L 6227 11709 L
6091 11719 L 6076 11695 L 6035 11559 L 5956 11530 L
5913 11559 L 5820 11596 L 5755 11695 L 5730 11831 L
5697 11966 L 5705 12102 L 5685 12187 L 5647 12238 L
5672 12373 L 5685 12398 L 5707 12509 L 5685 12577 L
5685 12755 L 5799 12645 L 5820 12541 L 5827 12509 L
5820 12480 L 5800 12373 L 5806 12238 L 5802 12102 L
5779 11966 L 5820 11831 L 5820 11826 L 5856 11695 L
5956 11641 L 5999 11695 L 6083 11831 L 6087 11966 L
6091 11982 L 6095 11966 L 6106 11831 L 6227 11774 L
6362 11809 L 6389 11831 L 6482 11966 L 6498 11981 L
6623 12102 L 6633 12129 L 6642 12102 L 6680 11966 L
6633 11895 L 6588 11831 L 6592 11695 L 6498 11572 L
6483 11559 L 6419 11423 L 6498 11339 L 6498 11067 L
6468 11016 L 6498 11000 L 6591 11016 L 6498 11067 L
6498 11339 L 6557 11423 L 6633 11497 L 6633 11434 L
6622 11423 L 6633 11395 L 6670 11423 L 6633 11434 L
6633 11497 L 6769 11456 L 6904 11522 L 7040 11548 L
7175 11533 L 7311 11523 L 7358 11559 L 7446 11619 L
7540 11695 L 7524 11831 L 7546 11966 L 7549 12102 L
7582 12156 L 7610 12238 L 7717 12295 L 7788 12373 L
7761 12509 L 7830 12645 L 7845 12780 L 7853 12801 L
7853 12110 L 7846 12102 L 7717 12054 L 7661 11966 L
7582 11869 L 7572 11831 L 7582 11790 L 7687 11695 L
7582 11571 L 7513 11559 L 7446 11539 L 7311 11452 L
7175 11446 L 7095 11423 L 7040 11382 L 6904 11331 L
6769 11325 L 6740 11288 L 6633 11159 L 6624 11152 L
6633 11124 L 6693 11016 L 6708 10881 L 6769 10839 L
6904 10812 L 6904 10511 L 6769 10499 L 6738 10474 L
6769 10352 L 6769 7983 L 6701 7896 L 6769 7808 L
6850 7896 L 6769 7983 L 6769 10352 L 6791 10338 L
6904 10270 L 6984 10338 L 6919 10474 L 6904 10511 L
6904 10812 L 7040 10821 L 7040 10659 L 6972 10609 L
7040 10577 L 7048 10609 L 7040 10659 L 7040 10821 L
7091 10881 L 7040 10940 L 6973 11016 L 7040 11074 L
7175 11147 L 7175 10797 L 7114 10745 L 7175 10733 L
7175 10684 L 7099 10609 L 7175 10514 L 7257 10609 L
7175 10684 L 7175 10733 L 7311 10671 L 7355 10745 L
7311 10785 L 7175 10797 L 7175 11147 L 7182 11152 L
7311 11233 L 7371 11152 L 7436 11016 L 7446 10997 L
7559 11016 L 7582 11022 L 7586 11016 L 7717 10971 L

7808 10881 L 7853 10806 L 7900 10745 L 7853 10658 L
7717 10615 L 7689 10609 L 7661 10474 L 7582 10417 L
7446 10355 L 7436 10338 L 7311 10229 L 7266 10202 L
7175 10141 L 7050 10067 L 7092 9931 L 7175 9920 L
7175 8478 L 7138 8438 L 7175 8395 L 7218 8438 L
7175 8478 L 7175 9920 L 7205 9931 L 7311 10022 L
7390 9931 L 7368 9795 L 7446 9736 L 7446 7384 L
7353 7353 L 7446 7277 L 7465 7353 L 7446 7384 L
7446 9736 L 7582 9679 L 7582 9280 L 7554 9252 L
7582 9244 L 7607 9252 L 7582 9280 L 7582 9679 L
7596 9660 L 7661 9524 L 7717 9474 L 7727 9524 L
7853 9643 L 7853 9584 L 7789 9524 L 7853 9445 L
7853 9303 L 7767 9252 L 7853 9181 L 7853 8939 L
7754 8845 L 7717 8807 L 7617 8710 L 7582 8666 L
7499 8574 L 7582 8489 L 7677 8574 L 7717 8606 L
7836 8710 L 7853 8725 L 7870 8710 L 7988 8593 L
8090 8710 L 7988 8815 L 7960 8845 L 7853 8939 L
7853 9181 L 7938 9252 L 7853 9303 L 7853 9445 L
7916 9524 L 7853 9584 L 7853 9643 L 7977 9524 L
7988 9395 L 7998 9388 L 8124 9364 L 8171 9388 L
8199 9524 L 8259 9555 L 8296 9524 L 8395 9517 L
8511 9524 L 8414 9660 L 8395 9793 L 8394 9795 L
8259 9898 L 8124 9848 L 8000 9931 L 7988 9943 L
7853 9977 L 7723 9931 L 7717 9929 L 7716 9931 L
7698 10067 L 7644 10202 L 7717 10308 L 7781 10338 L
7853 10385 L 7988 10408 L 8062 10338 L 7988 10244 L
7952 10202 L 7988 10176 L 8095 10067 L 8124 10038 L
8181 10067 L 8258 10202 L 8259 10203 L 8395 10265 L
8530 10220 L 8607 10338 L 8530 10354 L 8420 10474 L
8395 10573 L 8259 10566 L 8124 10602 L 8118 10609 L
8124 10653 L 8126 10745 L 8149 10881 L 8216 11016 L
8245 11152 L 8259 11174 L 8318 11288 L 8280 11423 L
8259 11440 L 8133 11559 L 8124 11593 L 8099 11695 L
8079 11831 L 8026 11966 L 7988 12018 L 7863 12102 L
7853 12110 L 7853 12801 L 7988 12796 L 8124 12796 L
8124 12112 L 8117 12102 L 8124 12094 L 8134 12102 L
8124 12112 L 8124 12796 L 8259 12796 L 8319 12780 L
8395 12756 L 8471 12645 L 8508 12509 L 8530 12490 L
8666 12383 L 8678 12373 L 8801 12257 L 8937 12239 L
8939 12238 L 8944 12102 L 8937 12091 L 8827 11966 L
8801 11880 L 8721 11831 L 8799 11695 L 8801 11682 L
8864 11559 L 8831 11423 L 8801 11411 L 8792 11423 L
8666 11500 L 8605 11423 L 8666 11308 L 8693 11288 L

8666 11258 L 8615 11152 L 8574 11016 L 8666 10952 L
8666 9887 L 8572 9795 L 8666 9707 L 8767 9795 L
8666 9887 L 8666 10952 L 8801 10894 L 8813 10881 L
8801 10876 L 8705 10745 L 8801 10651 L 8878 10609 L
8937 10504 L 8946 10474 L 9013 10338 L 9072 10208 L
9077 10202 L 9072 10176 L 9043 10067 L 8999 9931 L
9072 9867 L 9208 9852 L 9343 9810 L 9398 9795 L
9343 9746 L 9274 9660 L 9208 9586 L 9072 9569 L
9027 9524 L 8948 9388 L 9072 9255 L 9084 9252 L
9197 9117 L 9072 9014 L 8947 9117 L 8937 9121 L
8801 9132 L 8666 9136 L 8530 9200 L 8398 9252 L
8487 9388 L 8395 9469 L 8301 9388 L 8391 9252 L
8259 9141 L 8242 9117 L 8259 9095 L 8395 9009 L
8417 8981 L 8415 8845 L 8523 8710 L 8505 8574 L
8423 8438 L 8395 8391 L 8315 8303 L 8395 8220 L
8530 8195 L 8530 8096 L 8452 8031 L 8530 7978 L
8624 8031 L 8530 8096 L 8530 8195 L 8616 8303 L
8666 8374 L 8779 8438 L 8801 8483 L 8852 8574 L
8937 8668 L 9072 8685 L 9208 8626 L 9229 8574 L
9208 8546 L 9119 8438 L 9208 8389 L 9317 8438 L
9343 8458 L 9479 8467 L 9614 8509 L 9750 8534 L
9833 8438 L 9885 8380 L 10016 8438 L 10021 8448 L
10156 8523 L 10292 8506 L 10427 8441 L 10563 8446 L
10563 8042 L 10541 8031 L 10563 8019 L 10569 8031 L
10563 8042 L 10563 8446 L 10698 8445 L 10834 8517 L
10930 8438 L 10969 8306 L 11088 8438 L 11105 8442 L
11117 8438 L 11128 8303 L 11240 8188 L 11259 8167 L
11240 8133 L 11105 8104 L 10981 8167 L 10969 8288 L
10961 8167 L 10969 8157 L 11039 8031 L 11105 7985 L
11163 7896 L 11165 7760 L 11192 7624 L 11240 7558 L
11376 7574 L 11511 7612 L 11551 7624 L 11647 7661 L
11782 7725 L 11833 7760 L 11918 7836 L 11960 7896 L
12019 8031 L 12053 8125 L 12057 8167 L 12072 8303 L
12083 8438 L 12093 8574 L 12106 8710 L 12117 8845 L
12127 8981 L 12137 9117 L 12148 9252 L 12161 9388 L
12169 9524 L 12180 9660 L 12187 9795 L 12189 9806 L
12257 9931 L 12316 10067 L 12324 10091 L 12385 10202 L
12460 10337 L 12461 10338 L 12531 10474 L 12595 10582 L
12614 10609 L 12712 10745 L 12731 10767 L 12851 10881 L
12866 10898 L 13000 11016 L 13002 11017 L 13002 10223 L
12970 10202 L 12866 10136 L 12807 10067 L 12731 9983 L
12670 9931 L 12595 9864 L 12529 9795 L 12460 9724 L
12424 9660 L 12334 9524 L 12324 9502 L 12293 9388 L

12194 9252 L 12189 9245 L 12177 9117 L 12169 8981 L
12159 8845 L 12151 8710 L 12141 8574 L 12134 8438 L
12127 8303 L 12117 8167 L 12109 8031 L 12098 7896 L
12079 7760 L 12053 7646 L 12031 7624 L 11918 7530 L
11827 7489 L 11782 7482 L 11647 7467 L 11511 7455 L
11376 7445 L 11240 7438 L 11105 7456 L 10969 7475 L
10941 7489 L 10834 7571 L 10698 7591 L 10563 7576 L
10427 7616 L 10292 7571 L 10156 7568 L 10021 7542 L
9889 7624 L 9885 7626 L 9750 7636 L 9614 7732 L
9482 7760 L 9479 7761 L 9477 7760 L 9343 7710 L
9306 7760 L 9343 7818 L 9386 7896 L 9425 8031 L
9343 8097 L 9208 8106 L 9113 8167 L 9072 8202 L
8965 8167 L 8937 8148 L 8844 8031 L 8808 7896 L
8907 7760 L 8893 7624 L 8901 7489 L 8801 7397 L
8734 7353 L 8666 7266 L 8530 7275 L 8395 7293 L
8259 7316 L 8124 7298 L 7988 7350 L 7985 7353 L
7853 7391 L 7717 7361 L 7711 7353 L 7626 7217 L
7582 7129 L 7446 7185 L 7419 7217 L 7311 7336 L
7175 7263 L 7123 7353 L 7142 7489 L 7052 7624 L
7040 7627 L 6904 7638 L 6889 7624 L 6832 7489 L
6769 7473 L 6633 7442 L 6498 7419 L 6362 7415 L
6227 7392 L 6148 7353 L 6194 7217 L 6091 7099 L
5970 7217 L 5956 7227 L 5947 7217 L 5820 7134 L
5685 7183 L 5625 7081 L 5605 6946 L 5615 6810 L
5549 6747 L 5414 6743 L 5280 6810 L 5278 6821 L
5276 6810 L 5235 6674 L 5143 6595 L 5035 6539 L
5007 6528 L 4942 6539 L 4872 6569 L 4787 6674 L
4771 6810 L 4736 6830 L 4601 6816 L 4582 6810 L
4601 6697 L 4607 6674 L 4601 6650 L 4559 6539 L
4465 6510 L 4429 6539 L 4330 6623 L 4194 6554 L
4177 6539 L 4059 6474 L 3923 6448 L 3862 6539 L
3863 6674 L 3923 6776 L 3953 6810 L 3923 6864 L
3788 6876 L 3652 6941 L 3517 6924 L 3499 6810 L
3417 6674 L 3381 6618 L 3246 6614 L 3122 6539 L
3115 6403 L 3110 6393 L 3103 6403 L 3099 6539 L
3070 6674 L 3020 6810 L 3110 6844 L 3243 6946 L
3195 7081 L 3110 7206 L 3095 7217 L 2975 7327 L
2839 7295 L 2704 7339 L 2694 7353 L 2629 7489 L
2573 7624 L 2568 7634 L 2433 7727 L 2356 7760 L
N E
92 O
2305 8031 M 2297 8034 L 2297 8993 L 2341 9117 L 2297 9213 L
2297 9486 L 2433 9443 L 2568 9457 L 2615 9524 L

2704 9657 L 2705 9660 L 2791 9795 L 2839 9834 L
2975 9923 L 2985 9931 L 3110 10015 L 3186 10067 L
3246 10114 L 3342 10202 L 3381 10244 L 3479 10338 L
3517 10373 L 3652 10415 L 3788 10453 L 3834 10474 L
3923 10519 L 4059 10564 L 4194 10607 L 4194 10493 L
4059 10477 L 4050 10474 L 3923 10394 L 3788 10361 L
3652 10349 L 3623 10338 L 3517 10293 L 3424 10202 L
3381 10152 L 3286 10067 L 3246 10027 L 3110 9947 L
3086 9931 L 2975 9839 L 2913 9795 L 2839 9695 L
2815 9660 L 2781 9524 L 2815 9388 L 2839 9369 L
2933 9252 L 2975 9218 L 3061 9252 L 3110 9267 L
3110 8886 L 2975 8863 L 2880 8845 L 2839 8836 L
2704 8830 L 2568 8820 L 2446 8845 L 2433 8848 L
2422 8845 L 2348 8710 L 2433 8592 L 2447 8574 L
2568 8506 L 2649 8574 L 2704 8622 L 2839 8594 L
2975 8616 L 3056 8710 L 3110 8792 L 3210 8845 L
3110 8886 L 3110 9267 L 3173 9388 L 3243 9524 L
3204 9660 L 3246 9718 L 3341 9795 L 3381 9812 L
3469 9795 L 3448 9660 L 3430 9524 L 3480 9388 L
3517 9341 L 3630 9388 L 3652 9400 L 3705 9524 L
3761 9660 L 3788 9787 L 3794 9795 L 3836 9931 L
3788 9964 L 3759 10067 L 3788 10159 L 3806 10202 L
3923 10283 L 4055 10338 L 4059 10369 L 4194 10441 L
4330 10434 L 4352 10338 L 4330 10265 L 4313 10202 L
4194 10171 L 4059 10101 L 4028 10067 L 3942 9931 L
4002 9795 L 4059 9767 L 4075 9795 L 4194 9872 L
4330 9875 L 4330 9680 L 4275 9660 L 4330 9637 L
4330 9230 L 4194 9170 L 4124 9117 L 4059 9005 L
4047 8981 L 4040 8845 L 4059 8767 L 4071 8710 L
4059 8659 L 4031 8574 L 3949 8438 L 4059 8341 L
4111 8438 L 4194 8502 L 4258 8574 L 4330 8640 L
4330 7902 L 4293 7896 L 4194 7877 L 4059 7815 L
4037 7760 L 4039 7624 L 4059 7597 L 4194 7612 L
4217 7624 L 4330 7724 L 4358 7760 L 4352 7896 L
4330 7902 L 4330 8640 L 4387 8710 L 4465 8827 L
4465 8259 L 4372 8167 L 4465 8107 L 4465 7659 L
4439 7624 L 4465 7588 L 4588 7624 L 4465 7659 L
4465 8107 L 4598 8167 L 4465 8259 L 4465 8827 L
4601 8844 L 4601 8334 L 4545 8303 L 4601 8176 L
4727 8303 L 4601 8334 L 4601 8844 L 4607 8845 L
4601 8848 L 4496 8981 L 4465 9024 L 4419 9117 L
4330 9230 L 4330 9637 L 4381 9660 L 4330 9680 L
4330 9875 L 4465 9883 L 4517 9931 L 4601 10003 L

4649 10067 L 4610 10202 L 4602 10338 L 4601 10341 L
4465 10400 L 4338 10474 L 4330 10483 L 4194 10493 L
4194 10607 L 4199 10609 L 4194 10619 L 4145 10745 L
4059 10790 L 3923 10782 L 3788 10792 L 3678 10881 L
3652 10904 L 3522 11016 L 3517 11030 L 3408 11152 L
3383 11288 L 3381 11289 L 3246 11333 L 3110 11375 L
2989 11423 L 2975 11428 L 2839 11462 L 2704 11470 L
2568 11506 L 2510 11559 L 2470 11695 L 2433 11733 L
2297 11726 L 2297 11806 L 2425 11831 L 2433 11834 L
2543 11966 L 2568 12018 L 2704 12007 L 2704 11829 L
2568 11701 L 2567 11695 L 2568 11692 L 2642 11559 L
2704 11534 L 2839 11533 L 2975 11506 L 3110 11467 L
3246 11442 L 3381 11442 L 3421 11559 L 3381 11602 L
3259 11695 L 3246 11701 L 3110 11728 L 2975 11751 L
2839 11791 L 2704 11829 L 2704 12007 L 2839 11990 L
2975 12028 L 3110 12073 L 3246 12052 L 3381 12036 L
3517 12000 L 3572 11966 L 3652 11905 L 3788 11873 L
3923 11948 L 4059 11938 L 4194 11912 L 4321 11966 L
4330 11973 L 4420 12102 L 4465 12148 L 4601 12177 L
4736 12154 L 4839 12238 L 4736 12313 L 4672 12373 L
4601 12409 L 4465 12462 L 4330 12449 L 4194 12383 L
4177 12373 L 4059 12316 L 3923 12316 L 3788 12323 L
3652 12339 L 3587 12373 L 3517 12423 L 3381 12489 L
3336 12509 L 3246 12610 L 3169 12645 L 3246 12696 L
3381 12671 L 3517 12705 L 3652 12756 L 3788 12768 L
3830 12780 L 3923 12800 L 4059 12861 L 4193 12916 L
4194 12919 L 4330 12994 L 4465 12980 L 4601 13024 L
4640 13052 L 4736 13152 L 4736 12967 L 4601 12927 L
4532 12916 L 4465 12903 L 4330 12880 L 4194 12813 L
4066 12780 L 4059 12778 L 3923 12713 L 3788 12665 L
3652 12650 L 3636 12645 L 3517 12566 L 3476 12509 L
3517 12492 L 3652 12413 L 3788 12373 L 3791 12373 L
3923 12370 L 3990 12373 L 4059 12379 L 4194 12455 L
4302 12509 L 4330 12529 L 4465 12548 L 4583 12509 L
4601 12502 L 4736 12454 L 4872 12482 L 4929 12509 L
5007 12628 L 5007 12240 L 4995 12238 L 4926 12102 L
5007 12054 L 5106 12102 L 5010 12238 L 5007 12240 L
5007 12628 L 5036 12645 L 5007 12672 L 4955 12780 L
4921 12916 L 4872 12949 L 4736 12967 L 4736 13152 L
4872 13128 L 5007 13176 L 5025 13187 L 5143 13307 L
5269 13187 L 5212 13052 L 5143 12924 L 5136 12916 L
5143 12843 L 5278 12831 L 5414 12817 L 5549 12866 L
5685 12861 L 5820 12796 L 5852 12780 L 5914 12645 L

5956 12551 L 5956 12389 L 5952 12373 L 5956 12357 L
5956 12090 L 5924 11966 L 5904 11831 L 5956 11752 L
6004 11831 L 5973 11966 L 5956 12090 L 5956 12357 L
5962 12373 L 5956 12389 L 5956 12551 L 6091 12630 L
6209 12509 L 6149 12373 L 6227 12260 L 6254 12238 L
6227 12170 L 6208 12102 L 6205 11966 L 6227 11883 L
6362 11912 L 6404 11966 L 6498 12056 L 6545 12102 L
6587 12238 L 6602 12373 L 6602 12509 L 6633 12530 L
6769 12636 L 6769 12530 L 6735 12509 L 6769 12497 L
6805 12509 L 6769 12530 L 6769 12636 L 6782 12645 L
6904 12718 L 7011 12780 L 6904 12866 L 6769 12908 L
6732 12916 L 6633 12929 L 6607 12916 L 6498 12859 L
6362 12806 L 6227 12787 L 6091 12800 L 6071 12916 L
6091 12931 L 6165 13052 L 6185 13187 L 6194 13323 L
6149 13459 L 6207 13594 L 6227 13609 L 6327 13730 L
6362 13846 L 6381 13866 L 6481 14002 L 6498 14072 L
6578 14137 L 6633 14189 L 6769 14205 L 6904 14224 L
6904 13758 L 6876 13730 L 6831 13594 L 6769 13504 L
6698 13459 L 6633 13439 L 6498 13425 L 6362 13444 L
6330 13323 L 6257 13187 L 6227 13064 L 6226 13052 L
6227 13041 L 6328 12916 L 6362 12911 L 6380 12916 L
6498 12957 L 6633 13005 L 6769 13011 L 6904 12998 L
7040 12965 L 7175 12991 L 7311 13005 L 7446 12999 L
7446 12886 L 7311 12881 L 7175 12801 L 7136 12780 L
7086 12645 L 7040 12608 L 6940 12509 L 6904 12480 L
6769 12437 L 6655 12373 L 6666 12238 L 6711 12102 L
6769 11973 L 6783 11966 L 6769 11959 L 6691 11831 L
6708 11695 L 6633 11559 L 6769 11517 L 6829 11559 L
6822 11695 L 6853 11831 L 6904 11905 L 7020 11831 L
7040 11817 L 7063 11831 L 7098 11966 L 7109 12102 L
7175 12199 L 7311 12135 L 7401 12238 L 7446 12275 L
7446 12016 L 7335 11966 L 7311 11943 L 7237 11831 L
7259 11695 L 7311 11631 L 7446 11681 L 7463 11695 L
7476 11831 L 7475 11966 L 7446 12016 L 7446 12275 L
7582 12324 L 7671 12373 L 7666 12509 L 7717 12599 L
7748 12645 L 7717 12757 L 7688 12780 L 7582 12823 L
7446 12886 L 7446 12999 L 7582 12996 L 7672 13052 L
7582 13109 L 7462 13187 L 7446 13270 L 7431 13323 L
7446 13370 L 7464 13459 L 7484 13594 L 7446 13626 L
7311 13677 L 7225 13730 L 7175 13754 L 7041 13730 L
7040 13730 L 7036 13730 L 6904 13758 L 6904 14224 L
7040 14264 L 7051 14273 L 7175 14345 L 7311 14314 L
7446 14302 L 7582 14298 L 7621 14273 L 7706 14137 L

7717 14127 L 7853 14041 L 7952 14002 L 7988 13990 L
8124 13965 L 8124 13504 L 7994 13459 L 7988 13456 L
7853 13347 L 7717 13332 L 7708 13323 L 7717 13315 L
7853 13304 L 7913 13187 L 7988 13092 L 8117 13052 L
8124 13051 L 8127 13052 L 8259 13159 L 8339 13187 L
8352 13323 L 8259 13442 L 8243 13459 L 8124 13504 L
8124 13965 L 8259 13887 L 8268 13866 L 8395 13734 L
8398 13730 L 8430 13594 L 8415 13459 L 8481 13323 L
8530 13258 L 8666 13247 L 8681 13187 L 8791 13052 L
8694 12916 L 8675 12780 L 8666 12767 L 8613 12645 L
8636 12509 L 8666 12482 L 8801 12373 L 8801 12373 L
8937 12342 L 9072 12321 L 9072 11132 L 8948 11016 L
9072 10905 L 9196 11016 L 9072 11132 L 9072 12321 L
9193 12238 L 9103 12102 L 9076 11966 L 9137 11831 L
9208 11759 L 9274 11695 L 9271 11559 L 9341 11423 L
9303 11288 L 9320 11152 L 9295 11016 L 9304 10881 L
9298 10745 L 9208 10632 L 9072 10654 L 9048 10609 L
9072 10537 L 9121 10474 L 9208 10431 L 9307 10474 L
9343 10524 L 9351 10474 L 9429 10338 L 9479 10246 L
9489 10202 L 9614 10078 L 9614 9524 L 9515 9388 L
9614 9255 L 9719 9388 L 9614 9524 L 9614 10078 L
9688 10067 L 9750 10041 L 9874 9931 L 9885 9921 L
9885 9232 L 9776 9117 L 9885 9023 L 9993 9117 L
9885 9232 L 9885 9921 L 9915 9931 L 10021 9938 L
10068 9931 L 10078 9795 L 10119 9660 L 10117 9524 L
10032 9388 L 10156 9262 L 10156 8730 L 10125 8710 L
10156 8693 L 10183 8710 L 10156 8730 L 10156 9262 L
10182 9252 L 10191 9117 L 10292 9029 L 10394 8981 L
10427 8956 L 10480 8981 L 10563 8989 L 10698 9087 L
10834 8999 L 10955 9117 L 10969 9201 L 11105 9128 L
11110 9117 L 11105 9111 L 10969 8981 L 10969 8981 L
10834 8876 L 10819 8845 L 10698 8794 L 10582 8710 L
10626 8574 L 10698 8522 L 10761 8574 L 10834 8631 L
10969 8636 L 11105 8605 L 11146 8574 L 11240 8507 L
11342 8574 L 11376 8600 L 11404 8574 L 11388 8438 L
11376 8420 L 11240 8326 L 11223 8303 L 11240 8286 L
11342 8167 L 11335 8031 L 11369 7896 L 11376 7889 L
11390 7896 L 11511 7952 L 11615 8031 L 11647 8056 L
11749 8167 L 11782 8206 L 11868 8303 L 11918 8364 L
11949 8438 L 12014 8574 L 12053 8635 L 12062 8710 L
12074 8845 L 12086 8981 L 12096 9117 L 12107 9252 L
12119 9388 L 12128 9524 L 12137 9660 L 12144 9795 L
12157 9931 L 12169 10067 L 12181 10202 L 12189 10271 L

12215 10338 L 12258 10474 L 12299 10609 L 12324 10690 L
12350 10745 L 12411 10881 L 12460 10970 L 12494 11016 L
12592 11152 L 12595 11157 L 12708 11288 L 12731 11314 L
12859 11423 L 12866 11428 L 13002 11493 L 13002 11017 L
13000 11016 L 12866 10898 L 12851 10881 L 12731 10767 L
12712 10745 L 12614 10609 L 12595 10582 L 12531 10474 L
12461 10338 L 12460 10337 L 12385 10202 L 12324 10091 L
12316 10067 L 12257 9931 L 12189 9806 L 12187 9795 L
12180 9660 L 12169 9524 L 12161 9388 L 12148 9252 L
12137 9117 L 12127 8981 L 12117 8845 L 12106 8710 L
12093 8574 L 12083 8438 L 12072 8303 L 12057 8167 L
12053 8125 L 12019 8031 L 11960 7896 L 11918 7836 L
11833 7760 L 11782 7725 L 11647 7661 L 11551 7624 L
11511 7612 L 11376 7574 L 11240 7558 L 11192 7624 L
11165 7760 L 11163 7896 L 11105 7985 L 11039 8031 L
10969 8157 L 10961 8167 L 10969 8288 L 10981 8167 L
11105 8104 L 11240 8133 L 11259 8167 L 11240 8188 L
11128 8303 L 11117 8438 L 11105 8442 L 11088 8438 L
10969 8306 L 10930 8438 L 10834 8517 L 10698 8445 L
10563 8446 L 10427 8441 L 10292 8506 L 10156 8523 L
10021 8448 L 10016 8438 L 9885 8380 L 9833 8438 L
9750 8534 L 9614 8509 L 9479 8467 L 9343 8458 L
9317 8438 L 9208 8389 L 9119 8438 L 9208 8546 L
9229 8574 L 9208 8626 L 9072 8685 L 8937 8668 L
8852 8574 L 8801 8483 L 8779 8438 L 8666 8374 L
8616 8303 L 8530 8195 L 8395 8220 L 8315 8303 L
8395 8391 L 8423 8438 L 8505 8574 L 8523 8710 L
8415 8845 L 8417 8981 L 8395 9009 L 8259 9095 L
8242 9117 L 8259 9141 L 8391 9252 L 8301 9388 L
8395 9469 L 8395 9422 L 8356 9388 L 8395 9332 L
8433 9388 L 8395 9422 L 8395 9469 L 8487 9388 L
8398 9252 L 8530 9200 L 8666 9136 L 8666 8636 L
8605 8574 L 8530 8481 L 8498 8438 L 8506 8303 L
8530 8297 L 8535 8303 L 8598 8438 L 8666 8488 L
8733 8574 L 8666 8636 L 8666 9136 L 8801 9132 L
8937 9121 L 8937 9110 L 8801 8986 L 8801 8981 L
8715 8845 L 8801 8725 L 8918 8845 L 8937 8851 L
9050 8981 L 8937 9110 L 8937 9121 L 8947 9117 L
9072 9014 L 9197 9117 L 9084 9252 L 9072 9255 L
8948 9388 L 9027 9524 L 9072 9569 L 9072 9469 L
9002 9388 L 9072 9313 L 9171 9388 L 9072 9469 L
9072 9569 L 9208 9586 L 9274 9660 L 9343 9746 L
9398 9795 L 9343 9810 L 9208 9852 L 9072 9867 L

8999 9931 L 9043 10067 L 9072 10176 L 9077 10202 L
9072 10208 L 9013 10338 L 8946 10474 L 8937 10504 L
8878 10609 L 8801 10651 L 8705 10745 L 8801 10876 L
8813 10881 L 8801 10894 L 8666 10952 L 8574 11016 L
8615 11152 L 8666 11258 L 8693 11288 L 8666 11308 L
8605 11423 L 8666 11500 L 8792 11423 L 8801 11411 L
8831 11423 L 8864 11559 L 8801 11682 L 8799 11695 L
8721 11831 L 8801 11880 L 8827 11966 L 8937 12091 L
8944 12102 L 8939 12238 L 8937 12239 L 8801 12257 L
8678 12373 L 8666 12383 L 8530 12490 L 8508 12509 L
8471 12645 L 8395 12756 L 8319 12780 L 8259 12796 L
8124 12796 L 7988 12796 L 7853 12801 L 7845 12780 L
7830 12645 L 7761 12509 L 7788 12373 L 7717 12295 L
7610 12238 L 7582 12156 L 7549 12102 L 7546 11966 L
7524 11831 L 7540 11695 L 7446 11619 L 7358 11559 L
7311 11523 L 7175 11533 L 7040 11548 L 6904 11522 L
6769 11456 L 6633 11497 L 6557 11423 L 6498 11339 L
6419 11423 L 6483 11559 L 6498 11572 L 6592 11695 L
6588 11831 L 6633 11895 L 6680 11966 L 6642 12102 L
6633 12129 L 6623 12102 L 6498 11981 L 6482 11966 L
6389 11831 L 6362 11809 L 6227 11774 L 6106 11831 L
6095 11966 L 6091 11982 L 6087 11966 L 6083 11831 L
5999 11695 L 5956 11641 L 5856 11695 L 5820 11826 L
5820 11831 L 5779 11966 L 5802 12102 L 5806 12238 L
5800 12373 L 5820 12480 L 5827 12509 L 5820 12541 L
5799 12645 L 5685 12755 L 5549 12754 L 5414 12724 L
5278 12686 L 5204 12645 L 5143 12590 L 5084 12509 L
5063 12373 L 5143 12290 L 5196 12238 L 5245 12102 L
5278 12058 L 5414 11976 L 5417 11966 L 5414 11962 L
5278 11903 L 5143 11930 L 5009 11966 L 5007 11967 L
4872 12018 L 4736 12027 L 4640 11966 L 4601 11895 L
4465 11886 L 4330 11872 L 4269 11831 L 4194 11732 L
4059 11713 L 3923 11804 L 3788 11779 L 3705 11831 L
3652 11848 L 3517 11938 L 3487 11966 L 3381 11998 L
3246 12005 L 3110 12017 L 2975 11972 L 2954 11966 L
2914 11831 L 2975 11807 L 3110 11780 L 3246 11761 L
3381 11710 L 3411 11695 L 3517 11566 L 3521 11559 L
3652 11475 L 3652 11361 L 3571 11288 L 3551 11152 L
3601 11016 L 3652 10972 L 3754 10881 L 3788 10853 L
3923 10855 L 4059 10873 L 4194 10808 L 4259 10745 L
4330 10652 L 4465 10665 L 4601 10723 L 4601 10618 L
4543 10609 L 4601 10578 L 4615 10609 L 4601 10618 L
4601 10723 L 4736 10648 L 4800 10745 L 4736 10856 L

4727 10881 L 4626 11016 L 4601 11037 L 4465 11052 L
4330 11022 L 4194 11071 L 4147 11152 L 4059 11269 L
4019 11288 L 3923 11332 L 3788 11355 L 3652 11361 L
3652 11475 L 3788 11433 L 3813 11423 L 3923 11398 L
4059 11346 L 4156 11288 L 4194 11186 L 4206 11152 L
4330 11085 L 4465 11101 L 4601 11102 L 4705 11016 L
4736 10973 L 4865 10881 L 4872 10862 L 4908 10745 L
4895 10609 L 4872 10573 L 4736 10520 L 4658 10474 L
4679 10338 L 4724 10202 L 4733 10067 L 4682 9931 L
4601 9822 L 4568 9795 L 4537 9660 L 4601 9545 L
4605 9524 L 4601 9502 L 4465 9394 L 4463 9388 L
4465 9338 L 4484 9252 L 4601 9216 L 4722 9117 L
4736 9104 L 4872 8998 L 4946 8981 L 4967 8845 L
4872 8738 L 4736 8792 L 4700 8710 L 4736 8649 L
4809 8574 L 4870 8438 L 4796 8303 L 4736 8298 L
4605 8167 L 4623 8031 L 4601 7992 L 4579 7896 L
4571 7760 L 4601 7640 L 4736 7654 L 4811 7624 L
4736 7591 L 4684 7489 L 4620 7353 L 4601 7327 L
4465 7248 L 4390 7217 L 4465 7157 L 4517 7081 L
4465 7004 L 4419 7081 L 4330 7183 L 4300 7217 L
4330 7329 L 4336 7353 L 4368 7489 L 4330 7517 L
4224 7489 L 4194 7481 L 4059 7423 L 3923 7392 L
3872 7489 L 3888 7624 L 3923 7688 L 3941 7760 L
3932 7896 L 4059 7984 L 4166 8031 L 4092 8167 L
4059 8229 L 3923 8249 L 3849 8167 L 3788 8051 L
3776 8031 L 3775 7896 L 3703 7760 L 3652 7716 L
3517 7690 L 3438 7760 L 3381 7793 L 3246 7879 L
3195 7896 L 3110 7917 L 2975 8022 L 2927 8031 L
2975 8043 L 3028 8167 L 3110 8238 L 3230 8303 L
3117 8438 L 3110 8445 L 2975 8491 L 2953 8438 L
2913 8303 L 2839 8223 L 2801 8167 L 2801 8031 L
2704 7942 L 2568 8008 L 2433 8003 L 2305 8031 L
N E
74 O
2297 8993 2297 9213 2341 9117 2297 8993 4 N F
2297 9486 2297 9673 2313 9660 2433 9632 2533 9660 2568 9682
2631 9795 2704 9863 2829 9931 2839 9939 2975 10036 3046 10067
3110 10106 3246 10194 3255 10202 3381 10336 3383 10338 3517 10463
3584 10474 3652 10492 3788 10565 3883 10609 3788 10693 3748 10745
3652 10808 3558 10881 3517 10913 3381 10928 3304 11016 3246 11056
3147 11152 3110 11214 3046 11288 2975 11314 2839 11350 2704 11379
2568 11386 2476 11423 2433 11454 2297 11425 2297 11726 2433 11733
2470 11695 2510 11559 2568 11506 2704 11470 2839 11462 2975 11428

2989 11423 3110 11375 3246 11333 3381 11289 3383 11288 3408 11152
3517 11030 3522 11016 3652 10904 3678 10881 3788 10792 3923 10782
4059 10790 4145 10745 4194 10619 4199 10609 4194 10607 4059 10564
3923 10519 3834 10474 3788 10453 3652 10415 3517 10373 3479 10338
3381 10244 3342 10202 3246 10114 3186 10067 3110 10015 2985 9931
2975 9923 2839 9834 2791 9795 2705 9660 2704 9657 2615 9524
2568 9457 2433 9443 2297 9486 87 N F
56 O
2297 9673 2297 9884 2380 9931 2297 9973 2297 10592 2328 10609
2297 10733 2297 11425 2433 11454 2433 11044 2400 11016 2385 10881
2433 10771 2568 10823 2594 10881 2568 10962 2534 11016 2433 11044
2433 11454 2476 11423 2568 11386 2704 11379 2839 11350 2975 11314
3046 11288 3110 11214 3147 11152 3246 11056 3304 11016 3381 10928
3517 10913 3558 10881 3652 10808 3748 10745 3788 10693 3883 10609
3788 10565 3652 10492 3584 10474 3517 10463 3383 10338 3381 10336
3255 10202 3246 10194 3110 10106 3046 10067 2975 10036 2839 9939
2829 9931 2704 9863 2631 9795 2568 9682 2533 9660 2433 9632
2313 9660 2297 9673 56 N F
74 O
2380 9931 2297 9884 2297 9973 2380 9931 4 N F
38 O
2297 10592 2297 10733 2328 10609 2297 10592 4 N F
111 O
2425 11831 M 2297 11806 L 2297 11889 L 2409 11966 L 2433 12010 L
2483 12102 L 2451 12238 L 2568 12349 L 2704 12334 L
2740 12238 L 2741 12102 L 2839 12053 L 2975 12084 L
3029 12102 L 3110 12140 L 3236 12102 L 3246 12100 L
3381 12074 L 3517 12045 L 3645 11966 L 3652 11961 L
3788 11956 L 3804 11966 L 3923 12050 L 4059 12068 L
4131 12102 L 4194 12182 L 4330 12213 L 4354 12238 L
4362 12373 L 4330 12374 L 4328 12373 L 4194 12261 L
4059 12255 L 3923 12263 L 3788 12272 L 3652 12290 L
3517 12356 L 3496 12373 L 3381 12443 L 3246 12500 L
3221 12509 L 3110 12573 L 3018 12645 L 3029 12780 L
3098 12916 L 3110 12971 L 3186 13052 L 3226 13187 L
3246 13227 L 3381 13269 L 3381 12850 L 3352 12780 L
3381 12767 L 3442 12780 L 3381 12850 L 3381 13269 L
3517 13269 L 3649 13187 L 3652 13186 L 3691 13052 L
3735 12916 L 3788 12888 L 3923 12875 L 4009 12916 L
4051 13052 L 4023 13187 L 4033 13323 L 4059 13384 L
4194 13396 L 4217 13323 L 4303 13187 L 4330 13159 L
4465 13052 L 4601 13133 L 4653 13187 L 4631 13323 L
4601 13365 L 4552 13459 L 4467 13594 L 4465 13601 L

4391 13730 L 4465 13773 L 4536 13866 L 4601 13942 L
4726 14002 L 4736 14011 L 4872 14030 L 4883 14002 L
4872 13968 L 4824 13866 L 4784 13730 L 4846 13594 L
4830 13459 L 4806 13323 L 4872 13242 L 5007 13268 L
5062 13323 L 5143 13423 L 5193 13459 L 5278 13567 L
5278 13370 L 5177 13323 L 5278 13208 L 5306 13323 L
5278 13370 L 5278 13567 L 5351 13459 L 5414 13340 L
5426 13323 L 5414 13305 L 5370 13187 L 5316 13052 L
5283 12916 L 5414 12883 L 5480 12916 L 5518 13052 L
5549 13137 L 5685 13088 L 5736 13052 L 5798 12916 L
5820 12906 L 5873 12916 L 5956 12935 L 6091 13032 L
6103 13052 L 6138 13187 L 6131 13323 L 6091 13393 L
5958 13459 L 6088 13594 L 6091 13596 L 6227 13697 L
6254 13730 L 6302 13866 L 6362 13975 L 6384 14002 L
6362 14062 L 6298 14137 L 6240 14273 L 6243 14409 L
6321 14544 L 6362 14578 L 6471 14680 L 6498 14709 L
6633 14744 L 6769 14776 L 6878 14816 L 6904 14837 L
7032 14951 L 7040 14953 L 7062 14951 L 7175 14931 L
7311 14848 L 7364 14816 L 7446 14782 L 7579 14680 L
7582 14678 L 7717 14600 L 7777 14544 L 7853 14451 L
7858 14409 L 7946 14273 L 7988 14236 L 8124 14180 L
8259 14147 L 8285 14137 L 8360 14002 L 8387 13866 L
8395 13858 L 8508 13730 L 8530 13699 L 8666 13636 L
8801 13615 L 8873 13594 L 8937 13566 L 9068 13459 L
8998 13323 L 8942 13187 L 8977 13052 L 8974 12916 L
9052 12780 L 9072 12753 L 9208 12719 L 9343 12670 L
9398 12645 L 9479 12622 L 9614 12559 L 9632 12509 L
9750 12390 L 9767 12373 L 9868 12238 L 9827 12102 L
9750 11997 L 9614 11995 L 9529 12102 L 9479 12125 L
9410 12102 L 9343 12065 L 9255 11966 L 9335 11831 L
9343 11823 L 9458 11695 L 9479 11668 L 9479 10847 L
9389 10745 L 9445 10609 L 9436 10474 L 9479 10421 L
9516 10338 L 9595 10202 L 9614 10184 L 9673 10202 L
9750 10214 L 9871 10338 L 9791 10474 L 9827 10609 L
9750 10643 L 9667 10745 L 9614 10786 L 9479 10847 L
9479 11668 L 9577 11695 L 9614 11716 L 9637 11695 L
9625 11559 L 9614 11529 L 9558 11423 L 9590 11288 L
9614 11257 L 9701 11152 L 9750 11135 L 9879 11016 L
9885 11000 L 9973 10881 L 10021 10806 L 10021 10168 L
9924 10067 L 10021 9989 L 10112 10067 L 10021 10168 L
10021 10806 L 10156 10769 L 10223 10745 L 10292 10706 L
10292 9747 L 10215 9660 L 10292 9576 L 10292 9472 L
10156 9457 L 10095 9388 L 10156 9326 L 10292 9282 L

10318 9252 L 10345 9117 L 10427 9051 L 10563 9060 L
10625 9117 L 10563 9206 L 10532 9252 L 10427 9350 L
10386 9388 L 10292 9472 L 10292 9576 L 10379 9660 L
10292 9747 L 10292 10706 L 10345 10609 L 10415 10474 L
10427 10461 L 10465 10338 L 10563 10217 L 10566 10202 L
10610 10067 L 10698 9951 L 10705 9931 L 10834 9804 L
10834 9163 L 10788 9117 L 10834 9079 L 10873 9117 L
10834 9163 L 10834 9804 L 10844 9795 L 10969 9662 L
10975 9660 L 11105 9552 L 11118 9524 L 11105 9444 L
10969 9499 L 10883 9388 L 10969 9312 L 11105 9347 L
11130 9252 L 11185 9117 L 11105 9024 L 11060 8981 L
10969 8890 L 10922 8845 L 10834 8721 L 10765 8710 L
10834 8703 L 10969 8707 L 11105 8689 L 11240 8626 L
11376 8694 L 11506 8574 L 11511 8540 L 11589 8574 L
11647 8600 L 11772 8710 L 11782 8717 L 11912 8845 L
11918 8850 L 11996 8981 L 12053 9081 L 12056 9117 L
12066 9252 L 12078 9388 L 12087 9524 L 12094 9660 L
12101 9795 L 12111 9931 L 12120 10067 L 12129 10202 L
12140 10338 L 12152 10474 L 12164 10609 L 12176 10745 L
12189 10863 L 12194 10881 L 12237 11016 L 12283 11152 L
12324 11244 L 12352 11288 L 12429 11423 L 12460 11488 L
12505 11559 L 12591 11695 L 12595 11702 L 12693 11831 L
12731 11879 L 12832 11966 L 12866 11990 L 13002 12037 L
13002 11493 L 12866 11428 L 12859 11423 L 12731 11314 L
12708 11288 L 12595 11157 L 12592 11152 L 12494 11016 L
12460 10970 L 12411 10881 L 12350 10745 L 12324 10690 L
12299 10609 L 12258 10474 L 12215 10338 L 12189 10271 L
12181 10202 L 12169 10067 L 12157 9931 L 12144 9795 L
12137 9660 L 12128 9524 L 12119 9388 L 12107 9252 L
12096 9117 L 12086 8981 L 12074 8845 L 12062 8710 L
12053 8635 L 12014 8574 L 11949 8438 L 11918 8364 L
11868 8303 L 11782 8206 L 11749 8167 L 11647 8056 L
11615 8031 L 11511 7952 L 11390 7896 L 11376 7889 L
11369 7896 L 11335 8031 L 11342 8167 L 11240 8286 L
11223 8303 L 11240 8326 L 11376 8420 L 11388 8438 L
11404 8574 L 11376 8600 L 11342 8574 L 11240 8507 L
11146 8574 L 11105 8605 L 10969 8636 L 10834 8631 L
10761 8574 L 10698 8522 L 10626 8574 L 10582 8710 L
10698 8794 L 10819 8845 L 10834 8876 L 10969 8981 L
10969 8981 L 11105 9111 L 11110 9117 L 11105 9128 L
10969 9201 L 10955 9117 L 10834 8999 L 10698 9087 L
10563 8989 L 10480 8981 L 10427 8956 L 10394 8981 L
10292 9029 L 10191 9117 L 10182 9252 L 10156 9262 L

10032 9388 L 10117 9524 L 10119 9660 L 10078 9795 L
10068 9931 L 10021 9938 L 9915 9931 L 9885 9921 L
9874 9931 L 9750 10041 L 9688 10067 L 9614 10078 L
9489 10202 L 9479 10246 L 9429 10338 L 9351 10474 L
9343 10524 L 9307 10474 L 9208 10431 L 9121 10474 L
9072 10537 L 9048 10609 L 9072 10654 L 9208 10632 L
9298 10745 L 9304 10881 L 9295 11016 L 9320 11152 L
9303 11288 L 9341 11423 L 9271 11559 L 9274 11695 L
9208 11759 L 9137 11831 L 9076 11966 L 9103 12102 L
9193 12238 L 9072 12321 L 8937 12342 L 8801 12373 L
8801 12373 L 8666 12482 L 8636 12509 L 8613 12645 L
8666 12767 L 8675 12780 L 8694 12916 L 8791 13052 L
8681 13187 L 8666 13247 L 8530 13258 L 8481 13323 L
8415 13459 L 8430 13594 L 8398 13730 L 8395 13734 L
8268 13866 L 8259 13887 L 8124 13965 L 7988 13990 L
7952 14002 L 7853 14041 L 7717 14127 L 7706 14137 L
7621 14273 L 7582 14298 L 7446 14302 L 7311 14314 L
7175 14345 L 7051 14273 L 7040 14264 L 6904 14224 L
6769 14205 L 6633 14189 L 6578 14137 L 6498 14072 L
6481 14002 L 6381 13866 L 6362 13846 L 6327 13730 L
6227 13609 L 6207 13594 L 6149 13459 L 6194 13323 L
6185 13187 L 6165 13052 L 6091 12931 L 6071 12916 L
6091 12800 L 6227 12787 L 6227 12757 L 6123 12645 L
6227 12546 L 6278 12645 L 6227 12757 L 6227 12787 L
6362 12806 L 6498 12859 L 6607 12916 L 6633 12929 L
6633 12846 L 6509 12780 L 6498 12753 L 6458 12645 L
6406 12509 L 6362 12411 L 6348 12373 L 6362 12362 L
6380 12373 L 6498 12448 L 6519 12509 L 6633 12584 L
6708 12645 L 6765 12780 L 6633 12846 L 6633 12929 L
6732 12916 L 6769 12908 L 6904 12866 L 7011 12780 L
6904 12718 L 6782 12645 L 6769 12636 L 6633 12530 L
6602 12509 L 6602 12373 L 6587 12238 L 6545 12102 L
6498 12056 L 6404 11966 L 6362 11912 L 6227 11883 L
6205 11966 L 6208 12102 L 6227 12170 L 6254 12238 L
6227 12260 L 6149 12373 L 6209 12509 L 6091 12630 L
5956 12551 L 5914 12645 L 5852 12780 L 5820 12796 L
5685 12861 L 5549 12866 L 5414 12817 L 5278 12831 L
5143 12843 L 5136 12916 L 5143 12924 L 5212 13052 L
5269 13187 L 5143 13307 L 5025 13187 L 5007 13176 L
4872 13128 L 4736 13152 L 4640 13052 L 4601 13024 L
4465 12980 L 4330 12994 L 4194 12919 L 4193 12916 L
4059 12861 L 3923 12800 L 3830 12780 L 3788 12768 L
3652 12756 L 3517 12705 L 3381 12671 L 3246 12696 L

3169 12645 L 3246 12610 L 3336 12509 L 3381 12489 L
3517 12423 L 3587 12373 L 3652 12339 L 3788 12323 L
3923 12316 L 4059 12316 L 4177 12373 L 4194 12383 L
4330 12449 L 4465 12462 L 4601 12409 L 4672 12373 L
4736 12313 L 4839 12238 L 4736 12154 L 4601 12177 L
4465 12148 L 4420 12102 L 4330 11973 L 4321 11966 L
4194 11912 L 4059 11938 L 3923 11948 L 3788 11873 L
3652 11905 L 3572 11966 L 3517 12000 L 3381 12036 L
3246 12052 L 3110 12073 L 2975 12028 L 2839 11990 L
2704 12007 L 2568 12018 L 2543 11966 L 2433 11834 L
2425 11831 L N E
129 O
2409 11966 M 2297 11889 L 2297 11981 L 2368 12102 L 2313 12238 L
2297 12330 L 2297 12395 L 2433 12459 L 2529 12509 L
2568 12583 L 2704 12562 L 2794 12509 L 2839 12474 L
2903 12373 L 2891 12238 L 2975 12203 L 3110 12218 L
3246 12162 L 3381 12121 L 3447 12102 L 3517 12090 L
3652 12043 L 3788 12079 L 3827 12102 L 3788 12152 L
3670 12238 L 3652 12240 L 3517 12291 L 3420 12373 L
3381 12397 L 3246 12442 L 3110 12474 L 2992 12509 L
2975 12517 L 2885 12645 L 2896 12780 L 2964 12916 L
2975 12939 L 3016 13052 L 3048 13187 L 3054 13323 L
3095 13459 L 3110 13482 L 3201 13594 L 3246 13711 L
3257 13730 L 3354 13866 L 3381 13878 L 3408 13866 L
3517 13800 L 3652 13730 L 3652 13730 L 3788 13628 L
3788 13523 L 3778 13459 L 3788 13449 L 3870 13459 L
3788 13523 L 3788 13628 L 3921 13730 L 3923 13733 L
4006 13866 L 4059 13895 L 4168 14002 L 4194 14032 L
4270 14137 L 4314 14273 L 4194 14378 L 4115 14273 L
4059 14254 L 3970 14273 L 3923 14300 L 3878 14409 L
3923 14461 L 4059 14430 L 4194 14423 L 4330 14450 L
4465 14432 L 4599 14544 L 4601 14545 L 4604 14544 L
4736 14515 L 4776 14544 L 4846 14680 L 4872 14701 L
5007 14749 L 5037 14680 L 5022 14544 L 5007 14471 L
4989 14409 L 5007 14380 L 5043 14273 L 5072 14137 L
5048 14002 L 5007 13929 L 4973 13866 L 4951 13730 L
5007 13626 L 5007 13507 L 4997 13459 L 5007 13440 L
5019 13459 L 5007 13507 L 5007 13626 L 5121 13730 L
5143 13746 L 5194 13866 L 5278 13956 L 5278 13764 L
5274 13730 L 5278 13704 L 5284 13730 L 5278 13764 L
5278 13956 L 5322 14002 L 5414 14094 L 5464 14137 L
5534 14273 L 5549 14296 L 5685 14394 L 5685 14188 L
5606 14137 L 5549 14109 L 5418 14002 L 5414 13997 L

5291 13866 L 5414 13793 L 5549 13851 L 5573 13866 L
5685 13949 L 5751 14002 L 5753 14137 L 5685 14188 L
5685 14394 L 5721 14409 L 5727 14544 L 5790 14680 L
5820 14731 L 5956 14685 L 5960 14680 L 6054 14544 L
5956 14489 L 5873 14409 L 5855 14273 L 5943 14137 L
5870 14002 L 5820 13872 L 5781 13866 L 5685 13840 L
5549 13780 L 5441 13730 L 5414 13690 L 5369 13594 L
5414 13534 L 5549 13510 L 5597 13594 L 5685 13700 L
5724 13730 L 5820 13861 L 5928 13730 L 5956 13693 L
5956 13203 L 5943 13187 L 5956 13143 L 6049 13187 L
5956 13203 L 5956 13693 L 6091 13699 L 6136 13730 L
6227 13842 L 6236 13866 L 6227 13933 L 6205 14002 L
6122 14137 L 6091 14199 L 6001 14273 L 6063 14409 L
6091 14477 L 6115 14544 L 6227 14569 L 6358 14680 L
6362 14688 L 6475 14816 L 6498 14831 L 6633 14865 L
6769 14915 L 6806 14951 L 6904 15071 L 6932 15087 L
7040 15141 L 7175 15148 L 7295 15223 L 7311 15274 L
7350 15358 L 7446 15417 L 7582 15377 L 7599 15358 L
7717 15253 L 7853 15230 L 7873 15223 L 7988 15126 L
8011 15087 L 7988 15054 L 7900 14951 L 7853 14878 L
7793 14816 L 7853 14786 L 7872 14680 L 7890 14544 L
7904 14409 L 7988 14312 L 8124 14277 L 8150 14273 L
8259 14249 L 8395 14230 L 8426 14137 L 8434 14002 L
8440 13866 L 8530 13771 L 8666 13745 L 8801 13751 L
8933 13866 L 8937 13869 L 9072 13924 L 9206 14002 L
9208 14002 L 9212 14002 L 9327 13866 L 9343 13856 L
9479 13753 L 9487 13730 L 9479 13720 L 9415 13594 L
9343 13574 L 9242 13459 L 9231 13323 L 9208 13300 L
9072 13290 L 8987 13187 L 9072 13077 L 9208 13066 L
9223 13052 L 9343 12944 L 9414 12916 L 9479 12865 L
9614 12844 L 9750 12865 L 9767 12780 L 9768 12645 L
9767 12509 L 9885 12392 L 9984 12373 L 10021 12351 L
10114 12373 L 10156 12377 L 10177 12373 L 10281 12238 L
10292 12222 L 10359 12102 L 10295 11966 L 10292 11957 L
10213 11831 L 10156 11730 L 10135 11695 L 10040 11559 L
10021 11499 L 9969 11423 L 9915 11288 L 9920 11152 L
10021 11056 L 10061 11016 L 10156 10925 L 10292 10904 L
10332 10881 L 10427 10779 L 10448 10745 L 10475 10609 L
10487 10474 L 10563 10377 L 10603 10338 L 10629 10202 L
10698 10102 L 10734 10067 L 10771 9931 L 10834 9870 L
10915 9795 L 10969 9738 L 11105 9705 L 11158 9660 L
11202 9524 L 11207 9388 L 11229 9252 L 11240 9211 L
11376 9228 L 11511 9236 L 11647 9234 L 11719 9252 L

11782 9275 L 11903 9388 L 11918 9404 L 11971 9524 L
12031 9660 L 12053 9700 L 12058 9795 L 12065 9931 L
12071 10067 L 12077 10202 L 12083 10338 L 12092 10474 L
12099 10609 L 12106 10745 L 12113 10881 L 12125 11016 L
12140 11152 L 12160 11288 L 12184 11423 L 12189 11450 L
12222 11559 L 12265 11695 L 12291 11831 L 12318 11966 L
12324 11992 L 12367 12102 L 12416 12238 L 12460 12344 L
12476 12373 L 12549 12509 L 12595 12604 L 12660 12645 L
12731 12658 L 12866 12661 L 12921 12645 L 13002 12611 L
13002 12037 L 12866 11990 L 12832 11966 L 12731 11879 L
12693 11831 L 12595 11702 L 12591 11695 L 12505 11559 L
12460 11488 L 12429 11423 L 12352 11288 L 12324 11244 L
12283 11152 L 12237 11016 L 12194 10881 L 12189 10863 L
12176 10745 L 12164 10609 L 12152 10474 L 12140 10338 L
12129 10202 L 12120 10067 L 12111 9931 L 12101 9795 L
12094 9660 L 12087 9524 L 12078 9388 L 12066 9252 L
12056 9117 L 12053 9081 L 11996 8981 L 11918 8850 L
11912 8845 L 11782 8717 L 11772 8710 L 11647 8600 L
11589 8574 L 11511 8540 L 11506 8574 L 11376 8694 L
11240 8626 L 11105 8689 L 10969 8707 L 10834 8703 L
10765 8710 L 10834 8721 L 10922 8845 L 10969 8890 L
11060 8981 L 11105 9024 L 11185 9117 L 11130 9252 L
11105 9347 L 10969 9312 L 10883 9388 L 10969 9499 L
10969 9404 L 10957 9388 L 10969 9377 L 10990 9388 L
10969 9404 L 10969 9499 L 11105 9444 L 11118 9524 L
11105 9552 L 10975 9660 L 10969 9662 L 10844 9795 L
10834 9804 L 10705 9931 L 10698 9951 L 10610 10067 L
10566 10202 L 10563 10217 L 10465 10338 L 10427 10461 L
10415 10474 L 10345 10609 L 10292 10706 L 10223 10745 L
10156 10769 L 10021 10806 L 9973 10881 L 9885 11000 L
9879 11016 L 9750 11135 L 9701 11152 L 9614 11257 L
9590 11288 L 9558 11423 L 9614 11529 L 9625 11559 L
9637 11695 L 9614 11716 L 9577 11695 L 9479 11668 L
9458 11695 L 9343 11823 L 9335 11831 L 9255 11966 L
9343 12065 L 9410 12102 L 9479 12125 L 9529 12102 L
9614 11995 L 9750 11997 L 9827 12102 L 9868 12238 L
9767 12373 L 9750 12390 L 9632 12509 L 9614 12559 L
9479 12622 L 9398 12645 L 9343 12670 L 9208 12719 L
9072 12753 L 9052 12780 L 8974 12916 L 8977 13052 L
8942 13187 L 8998 13323 L 9068 13459 L 8937 13566 L
8873 13594 L 8801 13615 L 8666 13636 L 8530 13699 L
8508 13730 L 8395 13858 L 8387 13866 L 8360 14002 L
8285 14137 L 8259 14147 L 8124 14180 L 7988 14236 L

7946 14273 L 7858 14409 L 7853 14451 L 7777 14544 L
7717 14600 L 7582 14678 L 7579 14680 L 7446 14782 L
7364 14816 L 7311 14848 L 7175 14931 L 7062 14951 L
7040 14953 L 7032 14951 L 6904 14837 L 6878 14816 L
6769 14776 L 6633 14744 L 6498 14709 L 6471 14680 L
6362 14578 L 6321 14544 L 6243 14409 L 6240 14273 L
6298 14137 L 6362 14062 L 6384 14002 L 6362 13975 L
6302 13866 L 6254 13730 L 6227 13697 L 6091 13596 L
6088 13594 L 5958 13459 L 6091 13393 L 6131 13323 L
6138 13187 L 6103 13052 L 6091 13032 L 5956 12935 L
5873 12916 L 5820 12906 L 5798 12916 L 5736 13052 L
5685 13088 L 5549 13137 L 5518 13052 L 5480 12916 L
5414 12883 L 5283 12916 L 5316 13052 L 5370 13187 L
5414 13305 L 5426 13323 L 5414 13340 L 5351 13459 L
5278 13567 L 5193 13459 L 5143 13423 L 5062 13323 L
5007 13268 L 4872 13242 L 4806 13323 L 4830 13459 L
4846 13594 L 4784 13730 L 4824 13866 L 4872 13968 L
4883 14002 L 4872 14030 L 4736 14011 L 4726 14002 L
4601 13942 L 4536 13866 L 4465 13773 L 4391 13730 L
4465 13601 L 4467 13594 L 4552 13459 L 4601 13365 L
4631 13323 L 4653 13187 L 4601 13133 L 4465 13052 L
4330 13159 L 4303 13187 L 4217 13323 L 4194 13396 L
4059 13384 L 4033 13323 L 4023 13187 L 4051 13052 L
4009 12916 L 3923 12875 L 3788 12888 L 3735 12916 L
3691 13052 L 3652 13186 L 3649 13187 L 3517 13269 L
3381 13269 L 3246 13227 L 3226 13187 L 3186 13052 L
3110 12971 L 3098 12916 L 3029 12780 L 3018 12645 L
3110 12573 L 3221 12509 L 3246 12500 L 3381 12443 L
3496 12373 L 3517 12356 L 3652 12290 L 3788 12272 L
3923 12263 L 4059 12255 L 4194 12261 L 4328 12373 L
4330 12374 L 4362 12373 L 4354 12238 L 4330 12213 L
4194 12182 L 4131 12102 L 4059 12068 L 3923 12050 L
3804 11966 L 3788 11956 L 3652 11961 L 3645 11966 L
3517 12045 L 3381 12074 L 3246 12100 L 3236 12102 L
3110 12140 L 3029 12102 L 2975 12084 L 2839 12053 L
2741 12102 L 2740 12238 L 2704 12334 L 2568 12349 L
2451 12238 L 2483 12102 L 2433 12010 L 2409 11966 L
N E
147 O
2368 12102 2297 11981 2297 12330 2313 12238 2368 12102 5 N F
2433 12459 M 2297 12395 L 2297 13160 L 2433 13187 L 2434 13187 L
2516 13323 L 2568 13372 L 2568 13057 L 2560 13052 L
2433 12991 L 2346 12916 L 2405 12780 L 2433 12718 L

2568 12665 L 2704 12725 L 2752 12780 L 2704 12856 L
2678 12916 L 2572 13052 L 2568 13057 L 2568 13372 L
2610 13323 L 2704 13199 L 2708 13187 L 2779 13052 L
2839 12967 L 2882 13052 L 2944 13187 L 2938 13323 L
2912 13459 L 2839 13591 L 2837 13594 L 2760 13730 L
2704 13847 L 2692 13866 L 2628 14002 L 2599 14137 L
2568 14215 L 2503 14273 L 2433 14310 L 2297 14378 L
2297 15269 L 2375 15358 L 2297 15451 L 2297 15528 L
2433 15524 L 2568 15557 L 2704 15597 L 2783 15630 L
2839 15672 L 2955 15765 L 2975 15772 L 3110 15869 L
3110 15425 L 3065 15358 L 2975 15327 L 2839 15232 L
2828 15223 L 2754 15087 L 2754 14951 L 2839 14836 L
2975 14940 L 2984 14951 L 3098 15087 L 3110 15109 L
3217 15223 L 3246 15348 L 3246 15127 L 3208 15087 L
3110 14991 L 3077 14951 L 2975 14832 L 2956 14816 L
2896 14680 L 2849 14544 L 2839 14490 L 2704 14410 L
2702 14409 L 2704 14405 L 2839 14374 L 2975 14403 L
3000 14409 L 3110 14505 L 3110 14037 L 3042 14002 L
3110 13962 L 3133 14002 L 3110 14037 L 3110 14505 L
3120 14544 L 3160 14680 L 3233 14816 L 3246 14829 L
3334 14951 L 3324 15087 L 3246 15127 L 3246 15348 L
3315 15358 L 3246 15396 L 3110 15425 L 3110 15869 L
3164 15901 L 3246 15918 L 3381 15978 L 3431 16037 L
3517 16076 L 3652 16165 L 3666 16172 L 3679 16172 L
3851 16172 L 4024 16172 L 4105 16172 L 4194 16145 L
4299 16037 L 4278 15901 L 4194 15834 L 4109 15901 L
4059 15935 L 3923 15968 L 3788 15910 L 3772 15901 L
3652 15766 L 3650 15765 L 3517 15752 L 3381 15680 L
3328 15630 L 3381 15523 L 3387 15494 L 3385 15358 L
3517 15244 L 3641 15223 L 3652 15220 L 3788 15122 L
3848 15087 L 3901 14951 L 3923 14937 L 3983 14951 L
4059 14978 L 4194 15030 L 4330 15005 L 4443 15087 L
4465 15097 L 4601 15140 L 4736 15097 L 4872 15106 L
4928 15223 L 5007 15307 L 5114 15358 L 5143 15387 L
5179 15358 L 5278 15282 L 5292 15223 L 5324 15087 L
5319 14951 L 5278 14880 L 5240 14816 L 5201 14680 L
5190 14544 L 5160 14409 L 5172 14273 L 5207 14137 L
5278 14071 L 5345 14137 L 5401 14273 L 5367 14409 L
5414 14445 L 5549 14497 L 5581 14544 L 5685 14675 L
5688 14680 L 5746 14816 L 5748 14951 L 5772 15087 L
5820 15157 L 5876 15223 L 5956 15298 L 6028 15358 L
6091 15411 L 6201 15358 L 6138 15223 L 6091 15119 L
6065 15087 L 5956 14974 L 5920 14951 L 5956 14913 L

5977 14816 L 6091 14682 L 6101 14680 L 6227 14660 L
6250 14680 L 6324 14816 L 6362 14909 L 6498 14950 L
6508 14951 L 6633 14975 L 6769 15048 L 6801 15087 L
6904 15190 L 6973 15223 L 7040 15289 L 7123 15358 L
7175 15420 L 7216 15494 L 7311 15578 L 7442 15630 L
7446 15633 L 7582 15707 L 7685 15630 L 7717 15600 L
7790 15494 L 7853 15452 L 7988 15478 L 8084 15494 L
8124 15504 L 8145 15494 L 8259 15448 L 8395 15389 L
8441 15358 L 8458 15223 L 8421 15087 L 8395 15019 L
8364 14951 L 8259 14919 L 8124 14906 L 7988 14896 L
7913 14816 L 7929 14680 L 7940 14544 L 7951 14409 L
7988 14366 L 8124 14343 L 8259 14329 L 8395 14326 L
8449 14273 L 8478 14137 L 8484 14002 L 8489 13866 L
8530 13823 L 8666 13805 L 8801 13805 L 8871 13866 L
8937 13931 L 9022 14002 L 9072 14052 L 9184 14137 L
9208 14161 L 9233 14137 L 9343 14063 L 9479 14049 L
9527 14002 L 9614 13912 L 9750 13890 L 9845 13866 L
9750 13736 L 9747 13730 L 9614 13679 L 9535 13594 L
9479 13538 L 9343 13513 L 9296 13459 L 9291 13323 L
9208 13242 L 9072 13237 L 9031 13187 L 9072 13135 L
9208 13129 L 9291 13052 L 9343 13005 L 9479 12969 L
9614 12956 L 9750 12976 L 9810 12916 L 9827 12780 L
9828 12645 L 9832 12509 L 9885 12456 L 10021 12437 L
10156 12442 L 10229 12509 L 10292 12571 L 10388 12645 L
10427 12683 L 10556 12780 L 10563 12787 L 10569 12780 L
10698 12694 L 10834 12677 L 10867 12645 L 10969 12515 L
10993 12509 L 10969 12483 L 10891 12373 L 10834 12346 L
10715 12238 L 10698 12227 L 10604 12102 L 10563 11978 L
10447 11966 L 10427 11965 L 10342 11831 L 10292 11734 L
10263 11695 L 10156 11623 L 10105 11559 L 10079 11423 L
10021 11356 L 9979 11288 L 9986 11152 L 10021 11119 L
10126 11016 L 10156 10987 L 10292 10973 L 10427 10910 L
10460 10881 L 10525 10745 L 10537 10609 L 10548 10474 L
10563 10455 L 10681 10338 L 10691 10202 L 10698 10192 L
10826 10067 L 10834 9989 L 10969 9967 L 11105 10017 L
11210 10067 L 11240 10092 L 11338 10202 L 11376 10306 L
11385 10338 L 11440 10474 L 11511 10569 L 11543 10609 L
11647 10726 L 11665 10745 L 11767 10881 L 11782 10899 L
11864 11016 L 11918 11094 L 11955 11152 L 12037 11288 L
12053 11308 L 12061 11423 L 12072 11559 L 12083 11695 L
12107 11831 L 12089 11966 L 12127 12102 L 12143 12238 L
12167 12373 L 12189 12495 L 12192 12509 L 12189 12516 L
12144 12645 L 12187 12780 L 12189 12786 L 12324 12882 L

12368 12916 L 12431 13052 L 12460 13156 L 12467 13187 L
12460 13245 L 12446 13323 L 12324 13431 L 12189 13358 L
12135 13323 L 12053 13224 L 11985 13323 L 11987 13459 L
12002 13594 L 12029 13730 L 12053 13783 L 12189 13776 L
12234 13730 L 12324 13698 L 12425 13730 L 12460 13739 L
12595 13800 L 12731 13783 L 12793 13730 L 12833 13594 L
12860 13459 L 12866 13431 L 12875 13459 L 12941 13594 L
12992 13730 L 13002 13772 L 13002 12611 L 12921 12645 L
12866 12661 L 12731 12658 L 12660 12645 L 12595 12604 L
12549 12509 L 12476 12373 L 12460 12344 L 12416 12238 L
12367 12102 L 12324 11992 L 12318 11966 L 12291 11831 L
12265 11695 L 12222 11559 L 12189 11450 L 12184 11423 L
12160 11288 L 12140 11152 L 12125 11016 L 12113 10881 L
12106 10745 L 12099 10609 L 12092 10474 L 12083 10338 L
12077 10202 L 12071 10067 L 12065 9931 L 12058 9795 L
12053 9700 L 12031 9660 L 11971 9524 L 11918 9404 L
11903 9388 L 11782 9275 L 11719 9252 L 11647 9234 L
11511 9236 L 11376 9228 L 11240 9211 L 11229 9252 L
11207 9388 L 11202 9524 L 11158 9660 L 11105 9705 L
10969 9738 L 10915 9795 L 10834 9870 L 10771 9931 L
10734 10067 L 10698 10102 L 10629 10202 L 10603 10338 L
10563 10377 L 10487 10474 L 10475 10609 L 10448 10745 L
10427 10779 L 10332 10881 L 10292 10904 L 10156 10925 L
10061 11016 L 10021 11056 L 9920 11152 L 9915 11288 L
9969 11423 L 10021 11499 L 10040 11559 L 10135 11695 L
10156 11730 L 10213 11831 L 10292 11957 L 10295 11966 L
10359 12102 L 10292 12222 L 10281 12238 L 10177 12373 L
10156 12377 L 10114 12373 L 10021 12351 L 9984 12373 L
9885 12392 L 9767 12509 L 9768 12645 L 9767 12780 L
9750 12865 L 9614 12844 L 9479 12865 L 9414 12916 L
9343 12944 L 9223 13052 L 9208 13066 L 9072 13077 L
8987 13187 L 9072 13290 L 9208 13300 L 9231 13323 L
9242 13459 L 9343 13574 L 9415 13594 L 9479 13720 L
9487 13730 L 9479 13753 L 9343 13856 L 9327 13866 L
9212 14002 L 9208 14002 L 9206 14002 L 9072 13924 L
8937 13869 L 8933 13866 L 8801 13751 L 8666 13745 L
8530 13771 L 8440 13866 L 8434 14002 L 8426 14137 L
8395 14230 L 8259 14249 L 8150 14273 L 8124 14277 L
7988 14312 L 7904 14409 L 7890 14544 L 7872 14680 L
7853 14786 L 7793 14816 L 7853 14878 L 7900 14951 L
7988 15054 L 8011 15087 L 7988 15126 L 7873 15223 L
7853 15230 L 7717 15253 L 7599 15358 L 7582 15377 L
7446 15417 L 7350 15358 L 7311 15274 L 7295 15223 L

7175 15148 L 7040 15141 L 6932 15087 L 6904 15071 L
6806 14951 L 6769 14915 L 6633 14865 L 6498 14831 L
6475 14816 L 6362 14688 L 6358 14680 L 6227 14569 L
6115 14544 L 6091 14477 L 6063 14409 L 6001 14273 L
6091 14199 L 6122 14137 L 6205 14002 L 6227 13933 L
6236 13866 L 6227 13842 L 6136 13730 L 6091 13699 L
5956 13693 L 5928 13730 L 5820 13861 L 5724 13730 L
5685 13700 L 5597 13594 L 5549 13510 L 5414 13534 L
5369 13594 L 5414 13690 L 5441 13730 L 5549 13780 L
5685 13840 L 5781 13866 L 5820 13872 L 5870 14002 L
5943 14137 L 5855 14273 L 5873 14409 L 5956 14489 L
6054 14544 L 5960 14680 L 5956 14685 L 5820 14731 L
5790 14680 L 5727 14544 L 5721 14409 L 5685 14394 L
5549 14296 L 5534 14273 L 5464 14137 L 5414 14094 L
5322 14002 L 5278 13956 L 5194 13866 L 5143 13746 L
5121 13730 L 5007 13626 L 4951 13730 L 4973 13866 L
5007 13929 L 5048 14002 L 5072 14137 L 5043 14273 L
5007 14380 L 4989 14409 L 5007 14471 L 5022 14544 L
5037 14680 L 5007 14749 L 4872 14701 L 4846 14680 L
4776 14544 L 4736 14515 L 4604 14544 L 4601 14545 L
4599 14544 L 4465 14432 L 4330 14450 L 4194 14423 L
4059 14430 L 3923 14461 L 3878 14409 L 3923 14300 L
3970 14273 L 4059 14254 L 4115 14273 L 4194 14378 L
4314 14273 L 4270 14137 L 4194 14032 L 4168 14002 L
4059 13895 L 4006 13866 L 3923 13733 L 3921 13730 L
3788 13628 L 3652 13730 L 3652 13730 L 3517 13800 L
3408 13866 L 3381 13878 L 3354 13866 L 3257 13730 L
3246 13711 L 3201 13594 L 3110 13482 L 3095 13459 L
3054 13323 L 3048 13187 L 3016 13052 L 2975 12939 L
2964 12916 L 2896 12780 L 2885 12645 L 2975 12517 L
2992 12509 L 3110 12474 L 3246 12442 L 3246 12384 L
3169 12373 L 3217 12238 L 3246 12225 L 3381 12195 L
3484 12238 L 3381 12310 L 3283 12373 L 3246 12384 L
3246 12442 L 3381 12397 L 3420 12373 L 3517 12291 L
3652 12240 L 3670 12238 L 3788 12152 L 3827 12102 L
3788 12079 L 3652 12043 L 3517 12090 L 3447 12102 L
3381 12121 L 3246 12162 L 3110 12218 L 2975 12203 L
2891 12238 L 2903 12373 L 2839 12474 L 2794 12509 L
2704 12562 L 2568 12583 L 2529 12509 L 2433 12459 L
N E
165 O
2433 13187 2297 13160 2297 13618 2317 13730 2345 13866 2317 14002
2297 14053 2297 14378 2433 14310 2503 14273 2568 14215 2599 14137

2628 14002 2692 13866 2704 13847 2760 13730 2837 13594 2839 13591
2912 13459 2938 13323 2944 13187 2882 13052 2839 12967 2779 13052
2708 13187 2704 13199 2610 13323 2568 13372 2516 13323 2434 13187
2433 13187 31 N F
147 O
2297 13618 2297 14053 2317 14002 2345 13866 2317 13730 2297 13618
6 N F
165 O
2375 15358 2297 15269 2297 15451 2375 15358 4 N F
2433 15524 2297 15528 2297 15974 2344 16037 2297 16110 2297 16172
2470 16172 2643 16172 2815 16172 2988 16172 3037 16172 2975 16096
2897 16037 2839 15953 2793 15901 2839 15890 2864 15901 2975 15993
3110 16010 3192 16037 3246 16065 3381 16119 3500 16172 3506 16172
3666 16172 3652 16165 3517 16076 3431 16037 3381 15978 3246 15918
3164 15901 3110 15869 2975 15772 2955 15765 2839 15672 2783 15630
2704 15597 2568 15557 2433 15524 39 N F
183 O
2344 16037 2297 15974 2297 16110 2344 16037 4 N F
129 O
2433 12991 2346 12916 2405 12780 2433 12718 2568 12665 2704 12725
2752 12780 2704 12856 2678 12916 2572 13052 2568 13057 2560 13052
2433 12991 13 N F
74 O
2422 8845 2348 8710 2433 8592 2447 8574 2568 8506 2649 8574
2704 8622 2839 8594 2975 8616 3056 8710 3110 8792 3210 8845
3110 8886 2975 8863 2880 8845 2839 8836 2704 8830 2568 8820
2446 8845 2433 8848 2422 8845 21 N F
38 O
2400 11016 2385 10881 2433 10771 2568 10823 2594 10881 2568 10962
2534 11016 2433 11044 2400 11016 9 N F
111 O
2568 11692 2567 11695 2568 11701 2704 11829 2704 11713 2686 11695
2704 11668 2839 11666 2975 11691 3058 11559 3110 11544 3246 11538
3283 11559 3246 11586 3110 11608 2978 11695 2975 11695 2839 11709
2704 11713 2704 11829 2839 11791 2975 11751 3110 11728 3246 11701
3259 11695 3381 11602 3421 11559 3381 11442 3246 11442 3110 11467
2975 11506 2839 11533 2704 11534 2642 11559 2568 11692 35 N F
129 O
2704 11668 2686 11695 2704 11713 2839 11709 2975 11695 2978 11695
3110 11608 3246 11586 3283 11559 3246 11538 3110 11544 3058 11559
2975 11691 2839 11666 2704 11668 15 N F
2704 14410 2702 14409 2704 14405 2839 14374 2975 14403 3000 14409
3110 14505 3120 14544 3160 14680 3233 14816 3246 14829 3334 14951

3324 15087 3246 15127 3208 15087 3110 14991 3077 14951 2975 14832
2956 14816 2896 14680 2849 14544 2839 14490 2704 14410 23 N F
165 O
2754 14951 2754 15087 2828 15223 2839 15232 2975 15327 2975 15204
2892 15087 2975 15050 3006 15087 2975 15204 2975 15327 3065 15358
3110 15425 3246 15396 3315 15358 3246 15348 3217 15223 3110 15109
3098 15087 2984 14951 2975 14940 2839 14836 2754 14951 23 N F
111 O
2815 9388 2781 9524 2815 9660 2839 9695 2913 9795 2975 9839
3086 9931 3110 9947 3246 10027 3286 10067 3381 10152 3424 10202
3517 10293 3623 10338 3652 10349 3652 10230 3517 10218 3501 10202
3401 10067 3517 10017 3598 10067 3652 10140 3678 10202 3652 10230
3652 10349 3788 10361 3923 10394 4050 10474 4059 10477 4194 10493
4330 10483 4338 10474 4465 10400 4601 10341 4602 10338 4610 10202
4649 10067 4601 10003 4517 9931 4465 9883 4330 9875 4194 9872
4075 9795 4059 9767 4002 9795 3942 9931 4028 10067 4059 10101
4194 10171 4313 10202 4330 10265 4330 10115 4194 10095 4149 10067
4194 10002 4330 9998 4465 10028 4513 10067 4465 10106 4330 10115
4330 10265 4352 10338 4330 10434 4194 10441 4059 10369 4055 10338
3923 10283 3806 10202 3788 10159 3759 10067 3788 9964 3836 9931
3794 9795 3788 9787 3761 9660 3705 9524 3652 9400 3630 9388
3517 9341 3480 9388 3430 9524 3448 9660 3469 9795 3381 9812
3341 9795 3246 9718 3204 9660 3243 9524 3173 9388 3110 9267
3061 9252 2975 9218 2933 9252 2839 9369 2815 9388 95 N F
183 O
2839 15890 2793 15901 2839 15953 2897 16037 2975 16096 3037 16172
3161 16172 3333 16172 3500 16172 3381 16119 3246 16065 3192 16037
3110 16010 2975 15993 2864 15901 2839 15890 16 N F
74 O
2839 6753 2794 6810 2839 6854 2934 6810 2839 6753 5 N F
183 O
2975 15050 2892 15087 2975 15204 3006 15087 2975 15050 5 N F
165 O
3110 13962 3042 14002 3110 14037 3133 14002 3110 13962 5 N F
56 O
3246 11892 3135 11831 3246 11821 3381 11819 3466 11831 3381 11889
3246 11892 7 N F
165 O
3217 12238 3169 12373 3246 12384 3283 12373 3381 12310 3484 12238
3381 12195 3246 12225 3217 12238 9 N F
92 O
3246 7373 3214 7489 3246 7573 3381 7533 3449 7489 3381 7396
3246 7373 7 N F

165 O
3381 15523 M 3328 15630 L 3381 15680 L 3517 15752 L 3650 15765 L
3652 15766 L 3772 15901 L 3788 15910 L 3788 15853 L
3656 15765 L 3788 15699 L 3843 15765 L 3788 15853 L
3788 15910 L 3923 15968 L 3923 15601 L 3891 15494 L
3923 15438 L 4024 15494 L 3923 15601 L 3923 15968 L
4059 15935 L 4109 15901 L 4194 15834 L 4278 15901 L
4299 16037 L 4194 16145 L 4105 16172 L 4197 16172 L
4369 16172 L 4425 16172 L 4465 16094 L 4515 16037 L
4601 15955 L 4736 15945 L 4811 15901 L 4872 15874 L
5007 15845 L 5143 15860 L 5143 15711 L 5007 15672 L
4951 15630 L 5007 15551 L 5143 15549 L 5278 15564 L
5352 15630 L 5278 15683 L 5143 15711 L 5143 15860 L
5278 15868 L 5414 15878 L 5549 15836 L 5549 15340 L
5497 15223 L 5549 15121 L 5549 14937 L 5414 14861 L
5386 14816 L 5366 14680 L 5414 14649 L 5549 14659 L
5566 14680 L 5605 14816 L 5549 14937 L 5549 15121 L
5607 15223 L 5549 15340 L 5549 15836 L 5604 15765 L
5636 15630 L 5682 15494 L 5685 15463 L 5820 15468 L
5830 15494 L 5902 15630 L 5956 15669 L 6091 15687 L
6147 15630 L 6227 15588 L 6351 15630 L 6362 15647 L
6400 15765 L 6498 15836 L 6633 15864 L 6769 15854 L
6769 15672 L 6713 15630 L 6769 15525 L 6850 15494 L
6904 15488 L 6927 15494 L 6915 15630 L 6904 15637 L
6769 15672 L 6769 15854 L 6904 15774 L 6920 15765 L
7040 15703 L 7175 15761 L 7178 15765 L 7311 15839 L
7389 15901 L 7446 15956 L 7577 16037 L 7582 16040 L
7717 16043 L 7853 16066 L 7988 16057 L 8013 16037 L
8124 15965 L 8234 15901 L 8259 15867 L 8395 15817 L
8434 15901 L 8474 16037 L 8489 16172 L 8513 16172 L
8591 16172 L 8666 16104 L 8801 16073 L 8931 16172 L
9031 16172 L 9203 16172 L 9288 16172 L 9343 16078 L
9477 16037 L 9419 15901 L 9343 15900 L 9218 15765 L
9208 15755 L 9132 15630 L 9104 15494 L 9072 15459 L
8937 15425 L 8854 15358 L 8801 15295 L 8666 15286 L
8630 15223 L 8622 15087 L 8530 14994 L 8500 14951 L
8395 14842 L 8259 14832 L 8124 14827 L 7988 14825 L
7980 14816 L 7986 14680 L 7988 14591 L 8026 14544 L
8124 14439 L 8149 14409 L 8259 14403 L 8395 14400 L
8523 14273 L 8530 14137 L 8530 14133 L 8591 14002 L
8665 13866 L 8666 13866 L 8801 13859 L 8809 13866 L
8937 13993 L 8947 14002 L 9072 14125 L 9088 14137 L
9208 14256 L 9333 14137 L 9343 14131 L 9479 14126 L

9604 14002 L 9614 13992 L 9750 13988 L 9885 13982 L
10021 13911 L 10067 13866 L 10065 13730 L 10021 13677 L
9885 13610 L 9750 13601 L 9614 13595 L 9614 13594 L
9542 13459 L 9571 13323 L 9614 13294 L 9750 13215 L
9774 13187 L 9750 13166 L 9614 13059 L 9612 13052 L
9614 13052 L 9750 13049 L 9883 12916 L 9885 12830 L
9920 12780 L 9980 12645 L 10021 12520 L 10033 12509 L
10156 12507 L 10158 12509 L 10292 12641 L 10296 12645 L
10427 12774 L 10436 12780 L 10563 12905 L 10691 12780 L
10698 12775 L 10834 12772 L 10964 12645 L 10969 12639 L
11105 12636 L 11240 12631 L 11376 12617 L 11491 12509 L
11511 12385 L 11647 12485 L 11782 12496 L 11785 12509 L
11801 12645 L 11809 12780 L 11818 12916 L 11813 13052 L
11791 13187 L 11792 13323 L 11800 13459 L 11782 13489 L
11675 13594 L 11647 13643 L 11564 13730 L 11647 13806 L
11673 13866 L 11782 13959 L 11827 14002 L 11904 14137 L
11782 14258 L 11754 14273 L 11647 14342 L 11511 14360 L
11376 14350 L 11240 14345 L 11105 14367 L 11067 14409 L
11040 14544 L 11038 14680 L 11036 14816 L 11105 14876 L
11225 14951 L 11240 14987 L 11376 15064 L 11466 14951 L
11511 14841 L 11514 14816 L 11647 14702 L 11740 14680 L
11782 14671 L 11918 14558 L 11918 13886 L 11896 13866 L
11918 13813 L 12006 13866 L 11918 13886 L 11918 14558 L
11943 14544 L 12053 14424 L 12189 14489 L 12293 14409 L
12324 14377 L 12360 14273 L 12460 14171 L 12520 14137 L
12595 14083 L 12731 14028 L 12785 14137 L 12866 14269 L
12882 14273 L 13002 14369 L 13002 14002 L 13001 14002 L
13002 14000 L 13002 13772 L 12992 13730 L 12941 13594 L
12875 13459 L 12866 13431 L 12860 13459 L 12833 13594 L
12793 13730 L 12731 13783 L 12595 13800 L 12460 13739 L
12425 13730 L 12324 13698 L 12234 13730 L 12189 13776 L
12053 13783 L 12029 13730 L 12002 13594 L 11987 13459 L
11985 13323 L 12053 13224 L 12135 13323 L 12189 13358 L
12324 13431 L 12446 13323 L 12460 13245 L 12467 13187 L
12460 13156 L 12431 13052 L 12368 12916 L 12324 12882 L
12189 12786 L 12187 12780 L 12144 12645 L 12189 12516 L
12192 12509 L 12189 12495 L 12167 12373 L 12143 12238 L
12127 12102 L 12089 11966 L 12107 11831 L 12083 11695 L
12072 11559 L 12061 11423 L 12053 11308 L 12037 11288 L
11955 11152 L 11918 11094 L 11864 11016 L 11782 10899 L
11767 10881 L 11665 10745 L 11647 10726 L 11543 10609 L
11511 10569 L 11440 10474 L 11385 10338 L 11376 10306 L
11338 10202 L 11240 10092 L 11210 10067 L 11105 10017 L

10969 9967 L 10834 9989 L 10826 10067 L 10698 10192 L
10691 10202 L 10681 10338 L 10563 10455 L 10548 10474 L
10537 10609 L 10525 10745 L 10460 10881 L 10427 10910 L
10292 10973 L 10156 10987 L 10126 11016 L 10021 11119 L
9986 11152 L 9979 11288 L 10021 11356 L 10079 11423 L
10105 11559 L 10156 11623 L 10263 11695 L 10292 11734 L
10342 11831 L 10427 11965 L 10447 11966 L 10563 11978 L
10604 12102 L 10698 12227 L 10715 12238 L 10834 12346 L
10891 12373 L 10969 12483 L 10969 12241 L 10963 12238 L
10834 12105 L 10746 12102 L 10698 12029 L 10697 11966 L
10563 11834 L 10427 11832 L 10426 11831 L 10424 11695 L
10427 11642 L 10563 11664 L 10624 11695 L 10698 11755 L
10755 11831 L 10764 11966 L 10834 12011 L 10969 12092 L
10980 12102 L 11105 12232 L 11114 12238 L 11105 12238 L
10969 12241 L 10969 12483 L 10993 12509 L 10969 12515 L
10867 12645 L 10834 12677 L 10698 12694 L 10569 12780 L
10563 12787 L 10556 12780 L 10427 12683 L 10388 12645 L
10292 12571 L 10229 12509 L 10156 12442 L 10021 12437 L
9885 12456 L 9832 12509 L 9828 12645 L 9827 12780 L
9810 12916 L 9750 12976 L 9614 12956 L 9479 12969 L
9343 13005 L 9291 13052 L 9208 13129 L 9072 13135 L
9031 13187 L 9072 13237 L 9208 13242 L 9291 13323 L
9296 13459 L 9343 13513 L 9479 13538 L 9535 13594 L
9614 13679 L 9747 13730 L 9750 13736 L 9845 13866 L
9750 13890 L 9614 13912 L 9527 14002 L 9479 14049 L
9343 14063 L 9233 14137 L 9208 14161 L 9184 14137 L
9072 14052 L 9022 14002 L 8937 13931 L 8871 13866 L
8801 13805 L 8666 13805 L 8530 13823 L 8489 13866 L
8484 14002 L 8478 14137 L 8449 14273 L 8395 14326 L
8259 14329 L 8124 14343 L 7988 14366 L 7951 14409 L
7940 14544 L 7929 14680 L 7913 14816 L 7988 14896 L
8124 14906 L 8259 14919 L 8364 14951 L 8395 15019 L
8421 15087 L 8458 15223 L 8441 15358 L 8395 15389 L
8259 15448 L 8145 15494 L 8124 15504 L 8084 15494 L
7988 15478 L 7853 15452 L 7790 15494 L 7717 15600 L
7685 15630 L 7582 15707 L 7446 15633 L 7442 15630 L
7311 15578 L 7216 15494 L 7175 15420 L 7123 15358 L
7040 15289 L 6973 15223 L 6904 15190 L 6801 15087 L
6769 15048 L 6633 14975 L 6508 14951 L 6498 14950 L
6362 14909 L 6324 14816 L 6250 14680 L 6227 14660 L
6101 14680 L 6091 14682 L 5977 14816 L 5956 14913 L
5920 14951 L 5956 14974 L 6065 15087 L 6091 15119 L
6091 14853 L 6082 14816 L 6091 14805 L 6175 14816 L

6091 14853 L 6091 15119 L 6138 15223 L 6201 15358 L
6091 15411 L 6028 15358 L 5956 15298 L 5876 15223 L
5820 15157 L 5772 15087 L 5748 14951 L 5746 14816 L
5688 14680 L 5685 14675 L 5581 14544 L 5549 14497 L
5414 14445 L 5367 14409 L 5401 14273 L 5345 14137 L
5278 14071 L 5207 14137 L 5172 14273 L 5160 14409 L
5190 14544 L 5201 14680 L 5240 14816 L 5278 14880 L
5319 14951 L 5324 15087 L 5292 15223 L 5278 15282 L
5179 15358 L 5143 15387 L 5114 15358 L 5007 15307 L
4928 15223 L 4872 15106 L 4736 15097 L 4601 15140 L
4465 15097 L 4443 15087 L 4330 15005 L 4194 15030 L
4059 14978 L 3983 14951 L 3923 14937 L 3901 14951 L
3848 15087 L 3788 15122 L 3652 15220 L 3641 15223 L
3517 15244 L 3385 15358 L 3387 15494 L 3381 15523 L
N E
129 O
3381 12767 3352 12780 3381 12850 3442 12780 3381 12767 5 N F
3517 10017 3401 10067 3501 10202 3517 10218 3652 10230 3678 10202
3652 10140 3598 10067 3517 10017 9 N F
74 O
3517 12566 3476 12509 3517 12492 3652 12413 3788 12373 3791 12373
3923 12370 3990 12373 4059 12379 4194 12455 4302 12509 4330 12529
4465 12548 4583 12509 4601 12502 4736 12454 4872 12482 4929 12509
5007 12628 5036 12645 5007 12672 4955 12780 4921 12916 4872 12949
4736 12967 4601 12927 4532 12916 4465 12903 4465 12790 4512 12780
4534 12645 4465 12629 4330 12628 4194 12589 4136 12509 4059 12476
3923 12472 3789 12509 3923 12587 4046 12645 4059 12648 4194 12672
4330 12742 4391 12780 4465 12790 4465 12903 4330 12880 4194 12813
4066 12780 4059 12778 3923 12713 3788 12665 3652 12650 3636 12645
3517 12566 55 N F
165 O
3500 16173 3500 16172 3506 16172 3666 16172 3666 16173 3506 16173
3500 16173 7 N F
111 O
3601 11016 3551 11152 3571 11288 3652 11361 3788 11355 3788 11278
3652 11179 3643 11152 3652 11121 3678 11016 3788 10918 3923 10916
4059 10951 4150 11016 4095 11152 4059 11200 3923 11267 3788 11278
3788 11355 3923 11332 4019 11288 4059 11269 4147 11152 4194 11071
4330 11022 4465 11052 4465 11000 4330 10956 4215 10881 4330 10769
4396 10745 4465 10729 4498 10745 4601 10805 4664 10881 4601 10961
4465 11000 4465 11052 4601 11037 4626 11016 4727 10881 4736 10856
4800 10745 4736 10648 4601 10723 4465 10665 4330 10652 4259 10745
4194 10808 4059 10873 3923 10855 3788 10853 3754 10881 3652 10972

3601 11016 55 N F
129 O
3652 11121 3643 11152 3652 11179 3788 11278 3923 11267 3923 11202
3788 11171 3766 11152 3753 11016 3788 10985 3923 10971 4037 11016
4020 11152 3923 11202 3923 11267 4059 11200 4095 11152 4150 11016
4059 10951 3923 10916 3788 10918 3678 11016 3652 11121 23 N F
147 O
3788 15853 3656 15765 3788 15699 3843 15765 3788 15853 5 N F
56 O
3788 11629 3717 11559 3788 11538 3923 11525 3998 11559 3931 11695
3923 11700 3893 11695 3788 11629 9 N F
147 O
3788 10985 3753 11016 3766 11152 3788 11171 3923 11202 4020 11152
4037 11016 3923 10971 3788 10985 9 N F
3788 13449 3778 13459 3788 13523 3870 13459 3788 13449 5 N F
56 O
3923 12587 3789 12509 3923 12472 4059 12476 4136 12509 4194 12589
4330 12628 4465 12629 4534 12645 4512 12780 4465 12790 4391 12780
4330 12742 4194 12672 4059 12648 4046 12645 3923 12587 17 N F
147 O
3923 15601 3891 15494 3923 15438 4024 15494 3923 15601 5 N F
74 O
4031 8574 3949 8438 4059 8341 4111 8438 4194 8502 4258 8574
4330 8640 4387 8710 4465 8827 4601 8844 4607 8845 4601 8848
4496 8981 4465 9024 4419 9117 4330 9230 4194 9170 4124 9117
4059 9005 4047 8981 4040 8845 4059 8767 4071 8710 4059 8659
4031 8574 25 N F
111 O
4039 7624 4037 7760 4059 7815 4194 7877 4293 7896 4330 7902
4352 7896 4358 7760 4330 7724 4217 7624 4194 7612 4059 7597
4039 7624 13 N F
56 O
4194 6986 4084 6946 4194 6895 4276 6946 4194 6986 5 N F
129 O
4194 10002 4149 10067 4194 10095 4330 10115 4465 10106 4513 10067
4465 10028 4330 9998 4194 10002 9 N F
4330 10769 4215 10881 4330 10956 4465 11000 4465 10938 4330 10889
4317 10881 4330 10869 4465 10806 4599 10881 4465 10938 4465 11000
4601 10961 4664 10881 4601 10805 4498 10745 4465 10729 4396 10745
4330 10769 19 N F
74 O
4330 9680 4275 9660 4330 9637 4381 9660 4330 9680 5 N F
147 O

4330 10869 4317 10881 4330 10889 4465 10938 4599 10881 4465 10806
4330 10869 7 N F
56 O
4330 11235 4322 11152 4330 11148 4465 11150 4486 11152 4601 11268
4736 11192 4764 11152 4872 11091 5007 11034 5028 11016 5088 10881
5054 10745 5007 10686 4989 10609 5000 10474 4893 10338 5007 10264
5089 10338 5022 10474 5143 10528 5278 10564 5402 10609 5414 10613
5420 10609 5526 10474 5549 10460 5685 10363 5713 10474 5695 10609
5735 10745 5787 10881 5771 11016 5820 11141 5956 11152 6091 11130
6227 11137 6247 11152 6227 11211 6091 11203 6000 11288 6091 11363
6227 11339 6302 11423 6227 11509 6131 11559 6227 11631 6362 11638
6444 11695 6362 11709 6227 11709 6091 11719 6076 11695 6035 11559
5956 11530 5913 11559 5820 11596 5755 11695 5730 11831 5697 11966
5705 12102 5685 12187 5647 12238 5672 12373 5685 12398 5707 12509
5685 12577 5549 12568 5472 12509 5414 12425 5375 12373 5412 12238
5414 12236 5510 12102 5518 11966 5416 11831 5414 11829 5278 11780
5202 11831 5143 11853 5007 11891 5007 11714 5044 11695 5143 11649
5278 11582 5367 11559 5414 11440 5484 11559 5549 11589 5685 11613
5701 11559 5717 11423 5685 11366 5549 11417 5414 11417 5278 11376
5240 11288 5143 11162 5007 11158 4909 11288 5005 11423 4981 11559
4992 11695 5007 11714 5007 11891 4872 11872 4736 11862 4718 11831
4736 11762 4755 11695 4736 11678 4679 11695 4601 11718 4496 11695
4494 11559 4514 11423 4465 11376 4345 11288 4330 11235 119 N F
74 O
4465 8259 4372 8167 4465 8107 4598 8167 4465 8259 5 N F
183 O
4465 16094 4425 16172 4542 16172 4715 16172 4887 16172 5060 16172
5232 16172 5405 16172 5578 16172 5750 16172 5923 16172 6096 16172
6268 16172 6441 16172 6614 16172 6786 16172 6959 16172 7132 16172
7304 16172 7477 16172 7650 16172 7822 16172 7995 16172 8167 16172
8340 16172 8489 16172 8474 16037 8434 15901 8395 15817 8259 15867
8234 15901 8124 15965 8013 16037 7988 16057 7853 16066 7717 16043
7582 16040 7577 16037 7446 15956 7389 15901 7311 15839 7178 15765
7175 15761 7040 15703 6920 15765 6904 15774 6769 15854 6633 15864
6498 15836 6400 15765 6362 15647 6351 15630 6227 15588 6147 15630
6091 15687 5956 15669 5902 15630 5830 15494 5820 15468 5685 15463
5682 15494 5636 15630 5604 15765 5549 15836 5414 15878 5278 15868
5143 15860 5007 15845 4872 15874 4811 15901 4736 15945 4601 15955
4515 16037 4465 16094 74 N F
74 O
4465 7659 4439 7624 4465 7588 4588 7624 4465 7659 5 N F
4601 10618 4543 10609 4601 10578 4615 10609 4601 10618 5 N F
4601 8334 4545 8303 4601 8176 4727 8303 4601 8334 5 N F

56 O
4872 7375 4856 7353 4872 7340 5007 7349 5047 7353 5143 7391
5278 7449 5368 7489 5278 7542 5143 7524 5007 7491 5001 7489
4872 7375 13 N F
38 O
5005 11423 4909 11288 5007 11158 5143 11162 5240 11288 5278 11376
5414 11417 5549 11417 5685 11366 5717 11423 5701 11559 5685 11613
5549 11589 5484 11559 5414 11440 5367 11559 5278 11582 5143 11649
5044 11695 5007 11714 4992 11695 4981 11559 5005 11423 23 N F
111 O
5007 12054 4926 12102 4995 12238 5007 12240 5010 12238 5106 12102
5007 12054 7 N F
147 O
5007 15672 4951 15630 5007 15551 5143 15549 5278 15564 5352 15630
5278 15683 5143 15711 5007 15672 9 N F
92 O
4996 7081 4980 7217 5007 7231 5045 7217 5010 7081 5007 7079
4996 7081 7 N F
5007 7740 4994 7760 5007 7770 5033 7760 5007 7740 5 N F
147 O
5007 13440 4997 13459 5007 13507 5019 13459 5007 13440 5 N F
74 O
5060 5453 5012 5453 5070 5589 5143 5629 5216 5725 5278 5829
5322 5860 5374 5996 5414 6053 5549 5998 5550 5996 5685 5894
5685 5724 5565 5589 5685 5500 5786 5589 5685 5724 5685 5894
5728 5860 5820 5749 5855 5725 5956 5703 6091 5646 6123 5589
6091 5530 6044 5453 5962 5453 5956 5529 5946 5453 5923 5453
5750 5453 5578 5453 5405 5453 5232 5453 5060 5453 35 N F
56 O
5143 7023 5073 6946 5143 6873 5261 6946 5143 7023 5 N F
92 O
5278 13370 5177 13323 5278 13208 5306 13323 5278 13370 5 N F
56 O
5278 7145 5233 7081 5278 6984 5327 7081 5278 7145 5 N F
111 O
5278 13764 5274 13730 5278 13704 5284 13730 5278 13764 5 N F
5414 13997 5291 13866 5414 13793 5549 13851 5573 13866 5685 13949
5751 14002 5753 14137 5685 14188 5606 14137 5549 14109 5418 14002
5414 13997 13 N F
56 O
5414 7319 5308 7217 5414 7122 5549 7178 5632 7217 5549 7282
5414 7319 7 N F
183 O

5414 14649 5366 14680 5386 14816 5414 14861 5549 14937 5605 14816
5566 14680 5549 14659 5414 14649 9 N F
5549 15121 5497 15223 5549 15340 5607 15223 5549 15121 5 N F
56 O
5549 7680 5526 7624 5549 7566 5600 7624 5549 7680 5 N F
92 O
5685 5500 5565 5589 5685 5724 5786 5589 5685 5500 5 N F
56 O
5685 10098 5619 10067 5685 10018 5703 10067 5685 10098 5 N F
92 O
5765 9388 5744 9524 5820 9651 5827 9660 5944 9795 5931 9931
5930 10067 5895 10202 5869 10338 5857 10474 5903 10609 5878 10745
5914 10881 5858 11016 5956 11093 5956 11034 5933 11016 5956 10973
5956 10496 5942 10474 5956 10398 5956 9697 5922 9660 5820 9532
5815 9524 5820 9504 5956 9405 5956 9057 5896 8981 5956 8847
5956 8843 5908 8710 5956 8661 6002 8710 5956 8843 5956 8847
6008 8981 5956 9057 5956 9405 5976 9388 6082 9252 6091 9247
6101 9252 6184 9388 6135 9524 6091 9562 6005 9660 5956 9697
5956 10398 6072 10338 6091 10334 6094 10338 6091 10341 5977 10474
5956 10496 5956 10973 6025 11016 5956 11034 5956 11093 6091 11064
6091 10764 6058 10745 6091 10738 6091 10609 6091 10609 6091 10609
6091 10609 6091 10609 6091 10738 6105 10745 6091 10764 6091 11064
6227 11058 6352 11152 6362 11168 6382 11152 6362 11058 6352 11016
6362 10997 6498 10941 6498 10882 6495 10881 6362 10809 6284 10745
6362 10684 6420 10745 6498 10879 6499 10881 6498 10882 6498 10941
6582 10881 6513 10745 6633 10648 6769 10667 6799 10609 6769 10588
6633 10495 6498 10526 6362 10572 6227 10528 6167 10474 6227 10412
6227 10182 6148 10067 6227 9968 6336 10067 6227 10182 6227 10412
6341 10338 6362 10326 6498 10229 6524 10202 6498 10122 6477 10067
6362 9957 6329 9931 6313 9795 6362 9769 6428 9660 6433 9524
6362 9398 6352 9388 6271 9252 6227 9123 6091 9130 6064 9117
6081 8981 6091 8933 6137 8845 6153 8710 6146 8574 6191 8438
6194 8303 6091 8176 6088 8167 6091 8159 6179 8031 6172 7896
6183 7760 6219 7624 6091 7519 5956 7501 5820 7496 5790 7624
5807 7760 5820 7803 5865 7896 5865 8031 5868 8167 5860 8303
5820 8368 5801 8438 5803 8574 5795 8710 5820 8766 5845 8845
5820 8941 5802 8981 5820 9021 5867 9117 5902 9252 5820 9315
5765 9388 163 N F
5820 7343 5802 7353 5820 7454 5830 7353 5820 7343 5 N F
111 O
5820 9504 5815 9524 5820 9532 5922 9660 5956 9697 5956 9560
5929 9524 5956 9501 6090 9388 6091 9386 6092 9388 6091 9390
5988 9524 5956 9560 5956 9697 6005 9660 6091 9562 6135 9524

6184 9388 6101 9252 6091 9247 6082 9252 5976 9388 5956 9405
5820 9504 25 N F
38 O
5956 6324 5839 6267 5898 6132 5956 6030 6046 6132 6039 6267
5956 6324 7 N F
111 O
5956 8847 5896 8981 5956 9057 6008 8981 5956 8847 5 N F
5956 11752 5904 11831 5924 11966 5956 12090 5973 11966 6004 11831
5956 11752 7 N F
5956 8661 5908 8710 5956 8843 6002 8710 5956 8661 5 N F
129 O
5956 9501 5929 9524 5956 9560 5988 9524 6091 9390 6092 9388
6091 9386 6090 9388 5956 9501 9 N F
111 O
5956 10973 5933 11016 5956 11034 6025 11016 5956 10973 5 N F
5956 10398 5942 10474 5956 10496 5977 10474 6091 10341 6094 10338
6091 10334 6072 10338 5956 10398 9 N F
147 O
5956 13143 5943 13187 5956 13203 6049 13187 5956 13143 5 N F
92 O
5945 5453 5945 5453 5962 5453 5962 5453 5956 5453 5945 5453
6 N F
5945 5453 5946 5453 5962 5453 5962 5453 5945 5453 5 N F
5962 5453 5946 5453 5956 5529 5962 5453 4 N F
111 O
5956 12357 5952 12373 5956 12389 5962 12373 5956 12357 5 N F
74 O
6044 5453 5962 5453 5962 5453 6044 5453 6044 5453 5 N F
5962 5453 5962 5453 6044 5453 6044 5453 5962 5453 5 N F
38 O
6062 5996 M 6035 5860 L 6091 5835 L 6173 5725 L 6195 5589 L
6141 5453 L 6268 5453 L 6441 5453 L 6614 5453 L
6786 5453 L 6959 5453 L 7024 5453 L 6904 5499 L
6769 5553 L 6696 5589 L 6769 5601 L 6904 5628 L
7040 5665 L 7135 5725 L 7175 5738 L 7279 5860 L
7311 5886 L 7427 5996 L 7446 6027 L 7558 6132 L
7582 6145 L 7717 6154 L 7853 6162 L 7988 6186 L
8124 6254 L 8145 6267 L 8259 6340 L 8331 6403 L
8395 6457 L 8503 6539 L 8530 6558 L 8666 6655 L
8693 6674 L 8801 6730 L 8937 6773 L 9038 6810 L
9072 6820 L 9208 6832 L 9227 6810 L 9240 6674 L
9228 6539 L 9237 6403 L 9335 6267 L 9343 6264 L
9396 6132 L 9418 5996 L 9427 5860 L 9343 5779 L

9208 5839 L 9208 5746 L 9240 5725 L 9208 5688 L
9192 5725 L 9208 5746 L 9208 5839 L 9126 5725 L
9163 5589 L 9208 5525 L 9343 5564 L 9479 5585 L
9614 5559 L 9664 5589 L 9750 5708 L 9794 5725 L
9875 5860 L 9884 5996 L 9885 6000 L 9913 5996 L
9933 5860 L 9929 5725 L 9936 5589 L 9900 5453 L
10067 5453 L 10239 5453 L 10412 5453 L 10584 5453 L
10757 5453 L 10930 5453 L 11102 5453 L 11275 5453 L
11448 5453 L 11620 5453 L 11793 5453 L 11966 5453 L
12138 5453 L 12311 5453 L 12484 5453 L 12656 5453 L
12829 5453 L 13002 5453 L 13002 6628 L 12991 6674 L
12996 6810 L 13002 6843 L 13002 7374 L 12975 7489 L
12991 7624 L 13002 7655 L 13002 8595 L 12985 8710 L
12944 8845 L 12866 8919 L 12731 8884 L 12640 8845 L
12595 8818 L 12460 8771 L 12324 8728 L 12308 8710 L
12192 8574 L 12189 8566 L 12185 8438 L 12181 8303 L
12176 8167 L 12173 8031 L 12168 7896 L 12155 7760 L
12143 7624 L 12121 7489 L 12053 7418 L 11918 7401 L
11782 7394 L 11647 7385 L 11511 7379 L 11376 7372 L
11240 7369 L 11240 6673 L 11376 6583 L 11418 6539 L
11394 6403 L 11390 6267 L 11376 6246 L 11376 6007 L
11386 5996 L 11376 5880 L 11349 5996 L 11376 6007 L
11376 6246 L 11240 6256 L 11126 6132 L 11142 5996 L
11240 5911 L 11367 5860 L 11376 5855 L 11443 5725 L
11376 5680 L 11249 5589 L 11240 5585 L 11229 5589 L
11213 5725 L 11105 5823 L 10969 5783 L 10926 5725 L
10834 5653 L 10698 5668 L 10563 5668 L 10427 5688 L
10311 5725 L 10292 5739 L 10215 5860 L 10292 5929 L
10427 5865 L 10563 5895 L 10698 5995 L 10701 5996 L
10834 6037 L 10969 6022 L 11065 6132 L 10969 6228 L
10955 6267 L 10969 6339 L 10981 6403 L 11105 6487 L
11160 6539 L 11240 6673 L 11240 7369 L 11105 7372 L
10969 7383 L 10834 7421 L 10698 7437 L 10698 6880 L
10834 6810 L 10836 6810 L 10969 6735 L 11023 6674 L
10969 6582 L 10834 6593 L 10834 6464 L 10933 6403 L
10834 6373 L 10802 6403 L 10834 6464 L 10834 6593 L
10723 6539 L 10698 6528 L 10563 6464 L 10514 6403 L
10452 6267 L 10427 6228 L 10332 6132 L 10292 6059 L
10156 6108 L 10156 5828 L 10286 5725 L 10193 5589 L
10156 5570 L 10136 5589 L 10115 5725 L 10156 5828 L
10156 6108 L 10134 6132 L 10156 6148 L 10209 6267 L
10258 6403 L 10292 6493 L 10317 6539 L 10351 6674 L
10420 6810 L 10427 6821 L 10563 6864 L 10698 6880 L

10698 7437 L 10563 7432 L 10427 7403 L 10292 7387 L
10156 7371 L 10021 7382 L 9885 7381 L 9885 7216 L
10021 7158 L 10085 7081 L 10105 6946 L 10119 6810 L
10082 6674 L 10021 6606 L 10021 6438 L 10076 6403 L
10101 6267 L 10021 6175 L 10014 6267 L 10015 6403 L
10021 6438 L 10021 6606 L 10001 6539 L 9977 6403 L
9944 6267 L 9885 6132 L 9885 6132 L 9847 5996 L
9800 5860 L 9750 5805 L 9659 5725 L 9614 5676 L
9567 5725 L 9508 5860 L 9493 5996 L 9492 6132 L
9528 6267 L 9614 6397 L 9617 6403 L 9614 6457 L
9593 6539 L 9565 6674 L 9479 6796 L 9479 6454 L
9497 6403 L 9488 6267 L 9479 6223 L 9348 6267 L
9450 6403 L 9479 6454 L 9479 6796 L 9463 6810 L
9477 6946 L 9479 6948 L 9569 7081 L 9614 7124 L
9735 7081 L 9614 7032 L 9481 6946 L 9505 6810 L
9614 6747 L 9639 6810 L 9750 6936 L 9770 6946 L
9760 7081 L 9885 7216 L 9885 7381 L 9750 7390 L
9614 7415 L 9479 7416 L 9343 7406 L 9343 6610 L
9389 6539 L 9343 6477 L 9311 6539 L 9343 6610 L
9343 7406 L 9208 7387 L 9085 7353 L 9072 7348 L
8937 7281 L 8869 7217 L 8801 7169 L 8703 7081 L
8666 7045 L 8569 6946 L 8530 6906 L 8420 6810 L
8395 6793 L 8259 6741 L 8167 6674 L 8124 6641 L
7988 6544 L 7983 6539 L 7853 6443 L 7717 6405 L
7582 6441 L 7484 6539 L 7535 6674 L 7499 6810 L
7446 6908 L 7311 6906 L 7175 6830 L 7040 6811 L
7039 6810 L 7040 6790 L 7044 6674 L 7040 6668 L
6904 6564 L 6769 6547 L 6758 6539 L 6638 6403 L
6769 6277 L 6904 6314 L 6933 6267 L 7040 6217 L
7128 6267 L 7175 6367 L 7253 6267 L 7309 6132 L
7221 5996 L 7175 5961 L 7040 5909 L 6904 5923 L
6857 5996 L 6769 6095 L 6737 6132 L 6737 6267 L
6633 6388 L 6498 6390 L 6406 6267 L 6362 6242 L
6237 6132 L 6227 6120 L 6091 6017 L 6062 5996 L
N E
56 O
6044 5453 6044 5453 6096 5453 6141 5453 6141 5453 6096 5453
6044 5453 7 N F
111 O
6091 10738 6058 10745 6091 10764 6105 10745 6091 10738 5 N F
38 O
6091 6425 6060 6403 6091 6349 6227 6325 6288 6403 6227 6434
6091 6425 7 N F

183 O
6091 14805 6082 14816 6091 14853 6175 14816 6091 14805 5 N F
74 O
6091 10609 6091 10609 6091 10609 6091 10609 6091 10609 5 N F
92 O
6227 12757 6123 12645 6227 12546 6278 12645 6227 12757 5 N F
111 O
6227 9968 6148 10067 6227 10182 6227 10092 6209 10067 6227 10045
6251 10067 6227 10092 6227 10182 6336 10067 6227 9968 11 N F
74 O
6186 6810 6148 6946 6227 6979 6334 6946 6362 6851 6366 6810
6362 6802 6227 6763 6186 6810 9 N F
129 O
6227 10045 6209 10067 6227 10092 6251 10067 6227 10045 5 N F
74 O
6227 13064 6226 13052 6227 13041 6328 12916 6362 12911 6380 12916
6498 12957 6633 13005 6769 13011 6904 12998 7040 12965 7175 12991
7311 13005 7446 12999 7582 12996 7672 13052 7582 13109 7462 13187
7446 13270 7431 13323 7446 13370 7464 13459 7484 13594 7446 13626
7311 13677 7225 13730 7175 13754 7041 13730 7040 13730 7036 13730
6904 13758 6876 13730 6831 13594 6769 13504 6698 13459 6633 13439
6498 13425 6362 13444 6330 13323 6257 13187 6227 13064 41 N F
111 O
6362 10684 6284 10745 6362 10809 6495 10881 6498 10882 6499 10881
6498 10879 6420 10745 6362 10684 9 N F
92 O
6362 8149 6292 8167 6303 8303 6362 8362 6392 8303 6391 8167
6362 8149 7 N F
129 O
6362 12362 6348 12373 6362 12411 6406 12509 6458 12645 6498 12753
6509 12780 6633 12846 6633 12688 6616 12645 6633 12639 6640 12645
6633 12688 6633 12846 6765 12780 6708 12645 6633 12584 6519 12509
6498 12448 6380 12373 6362 12362 21 N F
56 O
6498 11067 6468 11016 6498 11000 6591 11016 6498 11067 5 N F
147 O
6633 12639 6616 12645 6633 12688 6640 12645 6633 12639 5 N F
56 O
6633 11434 6622 11423 6633 11395 6670 11423 6633 11434 5 N F
6633 10341 6623 10338 6633 10328 6686 10338 6633 10341 5 N F
6633 11159 6624 11152 6633 11124 6693 11016 6708 10881 6769 10839
6904 10812 7040 10821 7091 10881 7040 10940 6973 11016 7040 11074
7175 11147 7182 11152 7311 11233 7371 11152 7436 11016 7446 10997

7559 11016 7582 11022 7586 11016 7717 10971 7808 10881 7853 10806
7900 10745 7853 10658 7717 10615 7689 10609 7661 10474 7582 10417
7446 10355 7436 10338 7311 10229 7266 10202 7175 10141 7050 10067
7092 9931 7175 9920 7205 9931 7311 10022 7390 9931 7368 9795
7446 9736 7582 9679 7596 9660 7661 9524 7717 9474 7727 9524
7853 9643 7977 9524 7988 9395 7998 9388 8124 9364 8171 9388
8199 9524 8259 9555 8296 9524 8395 9517 8511 9524 8414 9660
8395 9793 8394 9795 8259 9898 8124 9848 8000 9931 7988 9943
7853 9977 7723 9931 7717 9929 7716 9931 7698 10067 7644 10202
7717 10308 7781 10338 7853 10385 7988 10408 8062 10338 7988 10244
7952 10202 7988 10176 8095 10067 8124 10038 8181 10067 8258 10202
8259 10203 8395 10265 8530 10220 8607 10338 8530 10354 8420 10474
8395 10573 8259 10566 8124 10602 8118 10609 8124 10653 8126 10745
8149 10881 8216 11016 8245 11152 8259 11174 8318 11288 8280 11423
8259 11440 8133 11559 8124 11593 8099 11695 8079 11831 8026 11966
7988 12018 7988 11413 8116 11288 7988 11160 7861 11288 7988 11413
7988 12018 7863 12102 7853 12110 7846 12102 7717 12054 7661 11966
7582 11869 7572 11831 7582 11790 7687 11695 7582 11571 7582 11352
7603 11288 7582 11234 7535 11288 7582 11352 7582 11571 7513 11559
7446 11539 7311 11452 7175 11446 7095 11423 7040 11382 6904 11331
6769 11325 6740 11288 6633 11159 141 N F
111 O
6769 11517 6633 11559 6708 11695 6691 11831 6769 11959 6783 11966
6769 11973 6711 12102 6666 12238 6655 12373 6769 12437 6904 12480
6940 12509 7040 12608 7086 12645 7136 12780 7175 12801 7311 12881
7446 12886 7446 12800 7327 12780 7311 12775 7196 12645 7175 12570
7040 12528 7021 12509 6904 12413 6769 12378 6761 12373 6733 12238
6769 12134 6876 12238 6904 12263 7040 12371 7042 12373 7175 12491
7299 12373 7311 12345 7434 12373 7446 12375 7582 12464 7599 12509
7582 12608 7568 12645 7479 12780 7446 12800 7446 12886 7582 12823
7688 12780 7717 12757 7748 12645 7717 12599 7666 12509 7671 12373
7582 12324 7446 12275 7401 12238 7311 12135 7175 12199 7109 12102
7098 11966 7063 11831 7040 11817 7020 11831 6904 11905 6853 11831
6822 11695 6829 11559 6769 11517 69 N F
20 O
6769 5601 6696 5589 6769 5553 6904 5499 7024 5453 7132 5453
7304 5453 7477 5453 7650 5453 7822 5453 7995 5453 8167 5453
8340 5453 8513 5453 8685 5453 8858 5453 9031 5453 9203 5453
9376 5453 9549 5453 9721 5453 9894 5453 9900 5453 9936 5589
9929 5725 9933 5860 9913 5996 9885 6000 9884 5996 9875 5860
9794 5725 9750 5708 9664 5589 9614 5559 9479 5585 9343 5564
9208 5525 9163 5589 9126 5725 9208 5839 9343 5779 9427 5860
9418 5996 9396 6132 9343 6264 9335 6267 9237 6403 9228 6539

9240 6674 9227 6810 9208 6832 9072 6820 9038 6810 8937 6773
8937 5897 8985 5860 8937 5822 8801 5837 8787 5860 8801 5873
8937 5897 8937 6773 8801 6730 8693 6674 8666 6655 8530 6558
8503 6539 8395 6457 8331 6403 8259 6340 8145 6267 8124 6254
7988 6186 7853 6162 7717 6154 7582 6145 7558 6132 7446 6027
7427 5996 7311 5886 7279 5860 7175 5738 7135 5725 7040 5665
6904 5628 6769 5601 86 N F
56 O
6769 7983 6701 7896 6769 7808 6850 7896 6769 7983 5 N F
147 O
6769 15672 6713 15630 6769 15525 6850 15494 6904 15488 6927 15494
6915 15630 6904 15637 6769 15672 9 N F
129 O
6769 12134 6733 12238 6761 12373 6769 12378 6904 12413 7021 12509
7040 12528 7175 12570 7196 12645 7311 12775 7327 12780 7446 12800
7479 12780 7568 12645 7582 12608 7599 12509 7582 12464 7446 12375
7434 12373 7311 12345 7299 12373 7175 12491 7042 12373 7040 12371
6904 12263 6876 12238 6769 12134 27 N F
74 O
6769 12530 6735 12509 6769 12497 6805 12509 6769 12530 5 N F
56 O
6769 10499 6738 10474 6769 10352 6791 10338 6904 10270 6984 10338
6919 10474 6904 10511 6769 10499 9 N F
74 O
6904 7211 6898 7217 6904 7231 6917 7217 6904 7211 5 N F
56 O
7040 10659 6972 10609 7040 10577 7048 10609 7040 10659 5 N F
92 O
7175 10514 7099 10609 7175 10684 7175 10634 7150 10609 7175 10578
7202 10609 7175 10634 7175 10684 7257 10609 7175 10514 11 N F
56 O
7175 10797 7114 10745 7175 10733 7311 10671 7355 10745 7311 10785
7175 10797 7 N F
7175 8478 7138 8438 7175 8395 7218 8438 7175 8478 5 N F
111 O
7175 10578 7150 10609 7175 10634 7202 10609 7175 10578 5 N F
7259 11695 7237 11831 7311 11943 7335 11966 7446 12016 7475 11966
7476 11831 7463 11695 7446 11681 7311 11631 7259 11695 11 N F
56 O
7446 7384 7353 7353 7446 7277 7465 7353 7446 7384 5 N F
92 O
7582 8489 7499 8574 7582 8666 7617 8710 7717 8807 7754 8845
7853 8939 7853 8868 7828 8845 7717 8728 7699 8710 7717 8691

7739 8710 7853 8816 7968 8710 7988 8690 8006 8710 7988 8728
7879 8845 7853 8868 7853 8939 7960 8845 7988 8815 8090 8710
7988 8593 7870 8710 7853 8725 7836 8710 7717 8606 7677 8574
7582 8489 31 N F
74 O
7582 11234 7535 11288 7582 11352 7603 11288 7582 11234 5 N F
56 O
7582 9280 7554 9252 7582 9244 7607 9252 7582 9280 5 N F
111 O
7717 8691 7699 8710 7717 8728 7828 8845 7853 8868 7879 8845
7988 8728 8006 8710 7988 8690 7968 8710 7853 8816 7739 8710
7717 8691 13 N F
7717 13315 7708 13323 7717 13332 7853 13347 7988 13456 7994 13459
8124 13504 8243 13459 8259 13442 8352 13323 8339 13187 8259 13159
8127 13052 8124 13051 8117 13052 7988 13092 7913 13187 7853 13304
7717 13315 19 N F
56 O
7853 9303 7767 9252 7853 9181 7938 9252 7853 9303 5 N F
92 O
7853 9445 7789 9524 7853 9584 7853 9525 7851 9524 7853 9522
7854 9524 7853 9525 7853 9584 7916 9524 7853 9445 11 N F
111 O
7853 9522 7851 9524 7853 9525 7854 9524 7853 9522 5 N F
74 O
7988 11160 7861 11288 7988 11413 7988 11365 7910 11288 7988 11209
8067 11288 7988 11365 7988 11413 8116 11288 7988 11160 11 N F
92 O
7988 11209 7910 11288 7988 11365 7988 11318 7958 11288 7988 11257
8019 11288 7988 11318 7988 11365 8067 11288 7988 11209 11 N F
74 O
7988 7053 7951 7081 7988 7135 8044 7081 7988 7053 5 N F
111 O
7988 11257 7958 11288 7988 11318 8019 11288 7988 11257 5 N F
183 O
7986 14680 M 7980 14816 L 7988 14825 L 8124 14827 L 8259 14832 L
8395 14842 L 8500 14951 L 8530 14994 L 8622 15087 L
8630 15223 L 8666 15286 L 8801 15295 L 8854 15358 L
8937 15425 L 9072 15459 L 9104 15494 L 9132 15630 L
9208 15755 L 9218 15765 L 9343 15900 L 9419 15901 L
9477 16037 L 9343 16078 L 9288 16172 L 9376 16172 L
9549 16172 L 9667 16172 L 9614 16094 L 9480 16037 L
9614 15968 L 9639 15901 L 9724 15765 L 9750 15735 L
9750 15555 L 9694 15494 L 9705 15358 L 9614 15268 L

9566 15223 L 9556 15087 L 9586 14951 L 9614 14924 L
9750 14899 L 9806 14951 L 9885 15026 L 9944 15087 L
10021 15153 L 10156 15183 L 10193 15223 L 10292 15334 L
10404 15358 L 10339 15494 L 10292 15501 L 10268 15494 L
10156 15432 L 10021 15365 L 9885 15462 L 9880 15494 L
9750 15555 L 9750 15735 L 9836 15630 L 9885 15501 L
10004 15630 L 10021 15657 L 10077 15765 L 10134 15901 L
10156 15943 L 10212 16037 L 10282 16172 L 10412 16172 L
10584 16172 L 10757 16172 L 10930 16172 L 11102 16172 L
11275 16172 L 11448 16172 L 11620 16172 L 11793 16172 L
11966 16172 L 12053 16172 L 12053 15515 L 12032 15494 L
12053 15472 L 12053 15126 L 12020 15087 L 12053 15052 L
12097 15087 L 12053 15126 L 12053 15472 L 12076 15494 L
12053 15515 L 12053 16172 L 12138 16172 L 12311 16172 L
12460 16172 L 12460 14712 L 12448 14680 L 12460 14657 L
12515 14680 L 12460 14712 L 12460 16172 L 12484 16172 L
12595 16172 L 12595 14663 L 12505 14544 L 12595 14449 L
12720 14544 L 12595 14663 L 12595 16172 L 12656 16172 L
12731 16172 L 12731 14535 L 12631 14409 L 12702 14273 L
12731 14222 L 12857 14273 L 12866 14358 L 12887 14409 L
12866 14423 L 12731 14535 L 12731 16172 L 12829 16172 L
13002 16172 L 13002 14369 L 12882 14273 L 12866 14269 L
12785 14137 L 12731 14028 L 12595 14083 L 12520 14137 L
12460 14171 L 12360 14273 L 12324 14377 L 12293 14409 L
12189 14489 L 12053 14424 L 11943 14544 L 11918 14558 L
11782 14671 L 11740 14680 L 11647 14702 L 11514 14816 L
11511 14841 L 11466 14951 L 11376 15064 L 11240 14987 L
11225 14951 L 11105 14876 L 11036 14816 L 11038 14680 L
11040 14544 L 11067 14409 L 11105 14367 L 11240 14345 L
11376 14350 L 11511 14360 L 11647 14342 L 11754 14273 L
11782 14258 L 11904 14137 L 11827 14002 L 11782 13959 L
11673 13866 L 11647 13806 L 11564 13730 L 11647 13643 L
11675 13594 L 11782 13489 L 11800 13459 L 11792 13323 L
11791 13187 L 11813 13052 L 11818 12916 L 11809 12780 L
11801 12645 L 11785 12509 L 11782 12496 L 11647 12485 L
11511 12385 L 11491 12509 L 11376 12617 L 11240 12631 L
11105 12636 L 10969 12639 L 10964 12645 L 10834 12772 L
10698 12775 L 10691 12780 L 10563 12905 L 10436 12780 L
10427 12774 L 10296 12645 L 10292 12641 L 10158 12509 L
10156 12507 L 10033 12509 L 10021 12520 L 9980 12645 L
9920 12780 L 9885 12830 L 9883 12916 L 9750 13049 L
9614 13052 L 9612 13052 L 9614 13059 L 9750 13166 L
9774 13187 L 9750 13215 L 9614 13294 L 9571 13323 L

9542 13459 L 9614 13594 L 9614 13595 L 9750 13601 L
9885 13610 L 10021 13677 L 10065 13730 L 10067 13866 L
10021 13911 L 9885 13982 L 9750 13988 L 9614 13992 L
9604 14002 L 9479 14126 L 9343 14131 L 9333 14137 L
9208 14256 L 9088 14137 L 9072 14125 L 8947 14002 L
8937 13993 L 8809 13866 L 8801 13859 L 8666 13866 L
8665 13866 L 8591 14002 L 8530 14133 L 8530 14137 L
8523 14273 L 8395 14400 L 8259 14403 L 8149 14409 L
8124 14439 L 8026 14544 L 7988 14591 L 7986 14680 L
N E
92 O
8124 12094 8117 12102 8124 12112 8134 12102 8124 12094 5 N F
111 O
8395 9332 8356 9388 8395 9422 8433 9388 8395 9332 5 N F
92 O
8530 7978 8452 8031 8530 8096 8624 8031 8530 7978 5 N F
165 O
8489 16173 8489 16172 8513 16172 8591 16172 8591 16173 8513 16173
8489 16173 7 N F
111 O
8506 8303 8498 8438 8530 8481 8605 8574 8666 8636 8733 8574
8666 8488 8598 8438 8535 8303 8530 8297 8506 8303 11 N F
92 O
8666 9707 8572 9795 8666 9887 8666 9824 8637 9795 8666 9768
8697 9795 8666 9824 8666 9887 8767 9795 8666 9707 11 N F
183 O
8666 16104 8591 16172 8685 16172 8858 16172 8931 16172 8801 16073
8666 16104 7 N F
111 O
8666 9768 8637 9795 8666 9824 8697 9795 8666 9768 5 N F
74 O
8801 8981 8715 8845 8801 8725 8918 8845 8937 8851 9050 8981
8937 9110 8801 8986 8801 8981 9 N F
2 O
8801 5873 8787 5860 8801 5837 8937 5822 8985 5860 8937 5897
8801 5873 7 N F
111 O
9072 10905 8948 11016 9072 11132 9072 11077 9007 11016 9072 10957
9138 11016 9072 11077 9072 11132 9196 11016 9072 10905 11 N F
9072 9313 9002 9388 9072 9469 9072 9406 9056 9388 9072 9371
9095 9388 9072 9406 9072 9469 9171 9388 9072 9313 11 N F
129 O
9072 10957 9007 11016 9072 11077 9072 11023 9065 11016 9072 11010

9079 11016 9072 11023 9072 11077 9138 11016 9072 10957 11 N F
9072 9371 9056 9388 9072 9406 9095 9388 9072 9371 5 N F
147 O
9072 11010 9065 11016 9072 11023 9079 11016 9072 11010 5 N F
56 O
9208 5688 9192 5725 9208 5746 9240 5725 9208 5688 5 N F
9343 6477 9311 6539 9343 6610 9389 6539 9343 6477 5 N F
20 O
9450 6403 9348 6267 9479 6223 9488 6267 9497 6403 9479 6454
9450 6403 7 N F
129 O
9445 10609 9389 10745 9479 10847 9479 10791 9438 10745 9479 10673
9614 10609 9565 10474 9563 10338 9614 10261 9750 10265 9821 10338
9750 10427 9651 10474 9614 10609 9614 10610 9550 10745 9479 10791
9479 10847 9614 10786 9667 10745 9750 10643 9827 10609 9791 10474
9871 10338 9750 10214 9673 10202 9614 10184 9595 10202 9516 10338
9479 10421 9436 10474 9445 10609 33 N F
147 O
9479 10673 9438 10745 9479 10791 9550 10745 9614 10610 9614 10609
9651 10474 9750 10427 9750 10366 9614 10353 9610 10338 9614 10332
9750 10315 9772 10338 9750 10366 9750 10427 9821 10338 9750 10265
9614 10261 9563 10338 9565 10474 9614 10609 9479 10673 23 N F
56 O
9479 6796 9463 6810 9477 6946 9479 6948 9569 7081 9614 7124
9735 7081 9614 7032 9481 6946 9505 6810 9614 6747 9639 6810
9750 6936 9770 6946 9760 7081 9885 7216 9885 6930 9750 6863
9703 6810 9631 6674 9638 6539 9649 6403 9614 6337 9568 6267
9518 6132 9521 5996 9546 5860 9614 5755 9728 5860 9750 5884
9810 5996 9849 6132 9878 6267 9885 6281 9938 6403 9968 6539
10004 6674 10021 6742 10048 6810 10021 6892 9885 6930 9885 7216
10021 7158 10085 7081 10105 6946 10119 6810 10082 6674 10021 6606
10001 6539 9977 6403 9944 6267 9885 6132 9885 6132 9847 5996
9800 5860 9750 5805 9659 5725 9614 5676 9567 5725 9508 5860
9493 5996 9492 6132 9528 6267 9614 6397 9617 6403 9614 6457
9593 6539 9565 6674 9479 6796 69 N F
165 O
9614 16094 9480 16037 9614 15968 9639 15901 9724 15765 9750 15735
9836 15630 9885 15501 10004 15630 10021 15657 10077 15765 10134 15901
10156 15943 10212 16037 10282 16172 10239 16172 10067 16172 9894 16172
9721 16172 9667 16172 9614 16094 21 N F
111 O
9614 9255 9515 9388 9614 9524 9614 9445 9572 9388 9614 9332
9658 9388 9614 9445 9614 9524 9719 9388 9614 9255 11 N F

74 O
9521 5996 9518 6132 9568 6267 9614 6337 9649 6403 9638 6539
9631 6674 9703 6810 9750 6863 9885 6930 9885 6862 9786 6810
9750 6798 9662 6674 9666 6539 9681 6403 9614 6277 9608 6267
9544 6132 9548 5996 9584 5860 9614 5813 9665 5860 9750 5954
9772 5996 9814 6132 9834 6267 9885 6372 9899 6403 9935 6539
9960 6674 9960 6810 9885 6862 9885 6930 10021 6892 10048 6810
10021 6742 10004 6674 9968 6539 9938 6403 9885 6281 9878 6267
9849 6132 9810 5996 9750 5884 9728 5860 9614 5755 9546 5860
9521 5996 49 N F
92 O
9548 5996 9544 6132 9608 6267 9614 6277 9681 6403 9666 6539
9662 6674 9750 6798 9786 6810 9885 6862 9885 6777 9750 6755
9692 6674 9694 6539 9713 6403 9680 6267 9614 6223 9569 6132
9575 5996 9614 5876 9720 5996 9750 6041 9778 6132 9789 6267
9836 6403 9885 6479 9902 6539 9917 6674 9885 6777 9885 6862
9960 6810 9960 6674 9935 6539 9899 6403 9885 6372 9834 6267
9814 6132 9772 5996 9750 5954 9665 5860 9614 5813 9584 5860
9548 5996 43 N F
165 O
9566 15223 9556 15087 9586 14951 9614 14924 9750 14899 9806 14951
9885 15026 9944 15087 10021 15153 10156 15183 10193 15223 10292 15334
10404 15358 10339 15494 10292 15501 10268 15494 10156 15432 10021 15365
9885 15462 9880 15494 9750 15555 9694 15494 9705 15358 9614 15268
9566 15223 25 N F
111 O
9575 5996 9569 6132 9614 6223 9614 6171 9595 6132 9602 5996
9614 5959 9647 5996 9721 6132 9614 6171 9614 6223 9680 6267
9713 6403 9694 6539 9692 6674 9750 6755 9750 6713 9723 6674
9721 6539 9745 6403 9750 6321 9762 6403 9841 6539 9854 6674
9750 6713 9750 6755 9885 6777 9917 6674 9902 6539 9885 6479
9836 6403 9789 6267 9778 6132 9750 6041 9720 5996 9614 5876
9575 5996 37 N F
129 O
9614 9332 9572 9388 9614 9445 9658 9388 9614 9332 5 N F
9602 5996 9595 6132 9614 6171 9721 6132 9647 5996 9614 5959
9602 5996 7 N F
165 O
9614 10332 9610 10338 9614 10353 9750 10366 9772 10338 9750 10315
9614 10332 7 N F
129 O
9745 6403 9721 6539 9723 6674 9750 6713 9750 6563 9749 6539
9750 6535 9752 6539 9750 6563 9750 6713 9854 6674 9841 6539

9762 6403 9750 6321 9745 6403 15 N F
147 O
9750 6535 9749 6539 9750 6563 9752 6539 9750 6535 5 N F
111 O
9885 9023 9776 9117 9885 9232 9885 9133 9870 9117 9885 9104
9900 9117 9885 9133 9885 9232 9993 9117 9885 9023 11 N F
129 O
9885 9104 9870 9117 9885 9133 9900 9117 9885 9104 5 N F
10021 9989 9924 10067 10021 10168 10021 10100 9989 10067 10021 10041
10051 10067 10021 10100 10021 10168 10112 10067 10021 9989 11 N F
147 O
10021 10041 9989 10067 10021 10100 10051 10067 10021 10041 5 N F
20 O
10015 6403 10014 6267 10021 6175 10101 6267 10076 6403 10021 6438
10015 6403 7 N F
129 O
10156 9326 10095 9388 10156 9457 10292 9472 10292 9404 10172 9388
10292 9368 10397 9252 10427 9149 10456 9252 10427 9280 10309 9388
10292 9404 10292 9472 10386 9388 10427 9350 10532 9252 10563 9206
10625 9117 10563 9060 10427 9051 10345 9117 10318 9252 10292 9282
10156 9326 25 N F
20 O
10156 5828 10115 5725 10136 5589 10156 5570 10193 5589 10286 5725
10156 5828 7 N F
111 O
10156 8693 10125 8710 10156 8730 10183 8710 10156 8693 5 N F
20 O
10156 6148 10134 6132 10156 6108 10292 6059 10332 6132 10427 6228
10452 6267 10514 6403 10563 6464 10698 6528 10723 6539 10834 6593
10969 6582 11023 6674 10969 6735 10836 6810 10834 6810 10698 6880
10563 6864 10427 6821 10420 6810 10351 6674 10317 6539 10292 6493
10258 6403 10209 6267 10156 6148 27 N F
147 O
10292 9368 10172 9388 10292 9404 10309 9388 10427 9280 10456 9252
10427 9149 10397 9252 10292 9368 9 N F
20 O
10292 5929 10215 5860 10292 5739 10311 5725 10427 5688 10563 5668
10698 5668 10834 5653 10926 5725 10969 5783 11105 5823 11213 5725
11229 5589 11240 5585 11249 5589 11376 5680 11443 5725 11376 5855
11367 5860 11240 5911 11142 5996 11126 6132 11240 6256 11376 6246
11390 6267 11394 6403 11418 6539 11376 6583 11240 6673 11160 6539
11105 6487 10981 6403 10969 6339 10955 6267 10969 6228 11065 6132
10969 6022 10834 6037 10701 5996 10698 5995 10563 5895 10427 5865

10292 5929 43 N F
129 O
10292 9576 10215 9660 10292 9747 10292 9676 10277 9660 10292 9644
10308 9660 10292 9676 10292 9747 10379 9660 10292 9576 11 N F
147 O
10292 9644 10277 9660 10292 9676 10308 9660 10292 9644 5 N F
183 O
10427 11642 10424 11695 10426 11831 10427 11832 10563 11834 10697 11966
10698 12029 10746 12102 10834 12105 10963 12238 10969 12241 11105 12238
11114 12238 11105 12232 10980 12102 10969 12092 10834 12011 10764 11966
10755 11831 10698 11755 10624 11695 10563 11664 10427 11642 23 N F
56 O
10563 8042 10541 8031 10563 8019 10569 8031 10563 8042 5 N F
129 O
10834 9079 10788 9117 10834 9163 10873 9117 10834 9079 5 N F
20 O
10834 6464 10802 6403 10834 6373 10933 6403 10834 6464 5 N F
147 O
10969 9377 10957 9388 10969 9404 10990 9388 10969 9377 5 N F
20 O
11376 6007 11349 5996 11376 5880 11386 5996 11376 6007 5 N F
147 O
11918 13886 11896 13866 11918 13813 12006 13866 11918 13886 5 N F
165 O
12053 15126 12020 15087 12053 15052 12097 15087 12053 15126 5 N F
12053 15515 12032 15494 12053 15472 12076 15494 12053 15515 5 N F
12460 14712 12448 14680 12460 14657 12515 14680 12460 14712 5 N F
12595 14663 12505 14544 12595 14449 12720 14544 12595 14663 5 N F
12731 14535 12631 14409 12702 14273 12731 14222 12857 14273 12866 14358
12887 14409 12866 14423 12731 14535 9 N F
56 O
13002 7374 12975 7489 12991 7624 13002 7655 13002 7374 5 N F
13002 6628 12991 6674 12996 6810 13002 6843 13002 6628 5 N F
147 O
13002 14002 13001 14002 13002 14000 13002 14002 4 N F
38 O
13002 5453 13002 5453 13002 6628 13002 6628 13002 5453 13002 5453
6 N F
56 O
13002 6843 13002 6628 13002 6628 13002 6843 13002 6843 5 N F
38 O
13002 7374 13002 6843 13002 6843 13002 7374 13002 7374 5 N F
56 O

13002 7655 13002 7374 13002 7374 13002 7655 13002 7655 5 N F
38 O
13002 8595 13002 7655 13002 7655 13002 8595 13002 8595 5 N F
56 O
13002 10223 13002 8595 13002 8595 13002 10223 13002 10223 5 N F
74 O
13002 11017 13002 10223 13002 10223 13002 11017 13002 11017 5 N F
92 O
13002 11493 13002 11017 13002 11017 13002 11493 13002 11493 5 N F
111 O
13002 12037 13002 11493 13002 11493 13002 12037 13002 12037 5 N F
129 O
13002 12611 13002 12037 13002 12037 13002 12611 13002 12611 5 N F
147 O
13002 13772 13002 12611 13002 12611 13002 13773 13002 13772 5 N F
165 O
13002 14000 13002 13772 13002 13773 13002 13998 13002 14000 5 N F
147 O
13002 14002 13002 14000 13002 13998 13002 14002 13002 14002 5 N F
165 O
13002 14369 13002 14002 13002 14002 13002 14369 13002 14369 5 N F
183 O
13002 16172 13002 14369 13002 14369 13002 16173 13002 16173 13002 16172
6 N F
56 O
13002 6628 13002 6628 13002 6843 13002 6843 13002 6810 13002 6674
13002 6628 7 N F
38 O
13002 6843 13002 7374 13002 7374 13002 7353 13002 7217 13002 7081
13002 6946 13002 6843 13002 6843 9 N F
56 O
13002 7374 13002 7374 13002 7655 13002 7655 13002 7624 13002 7489
13002 7374 7 N F
38 O
13002 7655 13002 8595 13002 8594 13002 8574 13002 8438 13002 8303
13002 8167 13002 8031 13002 7896 13002 7760 13002 7655 13002 7655
12 N F
56 O
13002 8594 13002 8595 13002 10223 13002 10223 13002 10202 13002 10067
13002 9931 13002 9795 13002 9660 13002 9524 13002 9388 13002 9252
13002 9117 13002 8981 13002 8845 13002 8710 13002 8594 17 N F
74 O
13002 10223 13002 10223 13002 11017 13002 11017 13002 11016 13002 10881

13002 10745 13002 10609 13002 10474 13002 10338 13002 10223 11 N F
92 O
13002 11017 13002 11017 13002 11493 13002 11493 13002 11423 13002 11288
13002 11152 13002 11017 8 N F
111 O
13002 11493 13002 11493 13002 12037 13002 12037 13002 11966 13002 11831
13002 11695 13002 11559 13002 11493 9 N F
129 O
13002 12037 13002 12037 13002 12611 13002 12611 13002 12509 13002 12373
13002 12238 13002 12102 13002 12037 9 N F
147 O
13002 12611 13002 12611 13002 13773 13002 13773 13002 13730 13002 13594
13002 13459 13002 13323 13002 13187 13002 13052 13002 12916 13002 12780
13002 12645 13002 12611 14 N F
165 O
13002 13773 13002 13773 13002 13998 13002 13997 13002 13866 13002 13773
6 N F
147 O
13002 13998 13002 14002 13002 14002 13002 14002 13002 13997 13002 13998
6 N F
165 O
13002 14002 13002 14002 13002 14369 13002 14369 13002 14273 13002 14137
13002 14002 7 N F
Ls Gr Gs Lr
-1200 1025 16500 18750 Oc
1 O
10639 15368 10640 15409 10679 15446 10675 15487 10710 15528 10753 15562
10792 15599 10827 15641 10872 15670 10920 15695 10946 15742 10985 15780
11030 15810 11080 15831 11122 15865 11160 15903 11196 15934 11247 15960
18 P
11247 15960 11300 15971 11330 16016 11355 16064 11389 16106 11419 16151
11428 16172 7 P
12840 16172 12841 16165 12836 16139 12812 16127 12785 16122 12767 16102
12743 16090 12718 16078 12698 16061 12682 16039 12656 16031 12637 16011
12633 15985 12626 15958 12621 15932 12608 15908 12584 15897 12561 15882
12544 15860 12528 15839 12506 15823 12482 15811 12458 15799 12434 15786
12425 15760 12449 15746 12444 15720 12419 15710 12402 15689 12377 15677
12359 15657 12333 15649 12323 15624 12298 15633 12283 15610 12257 15601
12230 15604 12204 15610 12182 15594 12168 15570 12146 15555 12149 15528
12128 15511 12107 15494 12081 15487 12067 15463 12040 15464 12022 15444
12010 15421 12003 15394 50 P
12003 15394 12008 15368 12034 15360 12041 15333 12028 15309 12026 15283
12033 15256 12045 15232 12053 15206 12039 15230 12013 15225 11 P

12013 15225 11986 15218 12012 15209 12006 15183 11984 15168 11980 15141
11994 15118 12018 15104 12045 15102 12072 15102 12098 15095 12124 15086
12147 15100 12162 15078 12178 15056 12196 15035 12213 15014 12235 14998
12256 14981 12279 14966 12306 14963 12328 14948 12347 14928 12358 14904
12383 14913 12409 14904 12433 14891 12438 14864 28 P
12438 14864 12448 14839 12463 14816 12476 14793 12494 14772 12503 14747
12519 14725 12534 14702 12546 14678 12563 14656 12575 14632 12586 14607
12605 14588 12622 14566 12640 14546 12656 14524 12675 14505 12691 14483
12712 14465 12731 14445 12752 14428 12772 14410 12793 14393 12798 14366
12818 14348 12838 14328 12844 14302 12864 14284 12878 14261 12900 14245
12915 14222 12934 14202 12951 14181 12960 14155 12967 14129 12980 14105
12999 14085 13001 14061 38 P
13001 13007 12998 13007 13001 12992 3 P
13001 12979 12980 12968 12959 12951 12951 12978 12952 13005 12936 12982
12909 12980 12883 12972 12855 12973 9 P
12855 12973 12828 12977 12806 12993 12783 13006 12789 12980 12797 12953
6:00 PM
12797 12953 12771 12961 12743 12963 12730 12939 12747 12918 12729 12898
12726 12871 12725 12843 12743 12822 12741 12795 12730 12770 12708 12787
12692 12809 12718 12802 12712 12829 12685 12829 12688 12856 12663 12846
12638 12858 12629 12832 12601 12832 12575 12840 12550 12851 12549 12879
12546 12906 12539 12879 12514 12869 12490 12855 12468 12839 12442 12847
12415 12841 12398 12820 12374 12806 12357 12828 12351 12801 12364 12778
12389 12765 12410 12783 12436 12789 12442 12762 12438 12735 12454 12712
12463 12687 12464 12659 12437 12650 12436 12678 12442 12705 12415 12711
12388 12704 12389 12677 50 P
12389 12677 12366 12663 12354 12687 12363 12713 12352 12738 12337 12761
12324 12786 12313 12761 12286 12754 12259 12753 12284 12743 12262 12728
12269 12701 12287 12680 12309 12664 12331 12648 12331 12621 12303 12621
12276 12627 12273 12600 12300 12604 12299 12577 12276 12561 12265 12536
12245 12554 12228 12575 12208 12595 12194 12571 12167 12580 12176 12606
12187 12631 12192 12604 12215 12618 12214 12646 12214 12673 12234 12655
12252 12676 12247 12703 12222 12693 12196 12684 12178 12663 12153 12653
12133 12634 12109 12620 12106 12593 12097 12567 12107 12542 12091 12519
12064 12521 12037 12515 50 P
12037 12515 12024 12538 2 P
12024 12538 12021 12565 12011 12591 12008 12564 12008 12536 11984 12523
11978 12496 11955 12481 11951 12453 11933 12432 11911 12416 11910 12443
11886 12458 11903 12480 11879 12492 11871 12519 11890 12540 11909 12559
11882 12560 11860 12542 11836 12554 11814 12570 11787 12575 11774 12600
11772 12627 11781 12653 11804 12638 11806 12611 11820 12588 11845 12577
11873 12580 11852 12598 11826 12607 11828 12634 11855 12635 11861 12609
11887 12602 11915 12604 11905 12630 11884 12647 11857 12650 11829 12649

11850 12666 11877 12662 11884 12689 11911 12693 11936 12705 11909 12707
11905 12734 11881 12748 50 P
11881 12748 11879 12775 11878 12748 11887 12722 11887 12694 11859 12695
11836 12681 11826 12707 11832 12734 11822 12759 11838 12782 11811 12777
11794 12755 11773 12773 11799 12784 11811 12808 11789 12823 11769 12842
11771 12815 11752 12834 11743 12860 11770 12854 11782 12878 11781 12851
11799 12831 11817 12811 11835 12832 11845 12857 11818 12864 11796 12879
11800 12906 11773 12913 11783 12939 11789 12966 11795 12993 11816 12977
11825 13003 11798 13007 11812 13031 11816 13058 11828 13083 11853 13092
11879 13101 11906 13098 11905 13126 11878 13126 11851 13121 11824 13119
11810 13143 11829 13163 50 P
11829 13163 11849 13181 2 P
11849 13181 11867 13202 11894 13203 11899 13229 11924 13241 11909 13263
11929 13283 11947 13303 11920 13297 11893 13294 11866 13289 11858 13315
11873 13337 11897 13350 11918 13367 11944 13379 11964 13396 11966 13423
11959 13397 11937 13381 11912 13370 11889 13357 11869 13376 11856 13400
11835 13417 11810 13427 11831 13443 11809 13459 11807 13487 11793 13510
11806 13534 11784 13551 11783 13578 11806 13592 11806 13607 11782 13580
11768 13604 11782 13627 11757 13640 11758 13667 11761 13694 11755 13721
11733 13705 11715 13685 11711 13712 11702 13738 11683 13718 11696 13695
11691 13668 11716 13657 50 P
11716 13657 11719 13630 11732 13606 11743 13581 11721 13564 11705 13586
11701 13613 11695 13639 11678 13660 11678 13688 11660 13708 11634 13700
11635 13727 11610 13739 11601 13713 11595 13740 11604 13766 11630 13772
11629 13799 11636 13825 11661 13837 11660 13865 11666 13838 11672 13812
11671 13784 11693 13769 11677 13791 11702 13804 11717 13826 11699 13847
11676 13862 11697 13880 11723 13889 11732 13863 11739 13837 11740 13809
11746 13836 11750 13863 11771 13846 11760 13871 11741 13890 11766 13879
11756 13904 11779 13891 11804 13879 11826 13895 11847 13876 11873 13871
11890 13849 11894 13822 50 P
11894 13822 11909 13799 2 P
11909 13799 11909 13826 11928 13845 11950 13861 11959 13887 11940 13868
11913 13873 11895 13893 11876 13913 11850 13919 11857 13945 11868 13970
11863 13997 11842 13980 11823 13960 11806 13982 11819 14005 11812 14032
11839 14036 11861 14053 11837 14065 11812 14077 11828 14099 11802 14108
11781 14125 11762 14145 11786 14157 11784 14184 11774 14209 11748 14217
11728 14235 11721 14262 11716 14288 11741 14301 11765 14314 11778 14290
11770 14263 11795 14250 11818 14238 11843 14226 11865 14210 11887 14201
11887 14202 11867 14214 11844 14228 11822 14243 11797 14255 11785 14280
11784 14307 11778 14333 50 P
11778 14333 11796 14357 11770 14344 3 P
11770 14344 11753 14322 11726 14317 11702 14329 11679 14344 11656 14359
11629 14357 11608 14339 11581 14335 11555 14327 11557 14300 11550 14273

11528 14289 11509 14309 11505 14335 11479 14342 11476 14314 11449 14316
11432 14338 11413 14357 11391 14373 11379 14397 11352 14404 11325 14403
11300 14413 11304 14386 11329 14375 11356 14376 11350 14349 11323 14350
11326 14323 11330 14296 11322 14271 11294 14273 11271 14259 11255 14237
11228 14233 11217 14208 11201 14229 11212 14254 11239 14261 11241 14288
11268 14290 11253 14312 11275 14328 11248 14331 11221 14329 11244 14314
11217 14309 11208 14284 50 P
11208 14284 11181 14283 11185 14310 11162 14324 11140 14308 11114 14300
11088 14307 11063 14318 11047 14340 11071 14354 11098 14350 11123 14360
11099 14372 11106 14399 11132 14404 11154 14387 11156 14414 11131 14424
11103 14421 11078 14411 11051 14407 11025 14415 11002 14428 10977 14419
10950 14411 10924 14415 10897 14423 10923 14430 10943 14449 10963 14465
10982 14492 11004 14478 11002 14471 11004 14467 11033 14486 11023 14491
11003 14480 10983 14499 10983 14526 10977 14552 10991 14576 11001 14601
11021 14619 11031 14644 11020 14669 11047 14670 11071 14683 11075 14710
11067 14736 11085 14715 50 P
11085 14715 11108 14729 2 P
11108 14729 11134 14735 11157 14750 11184 14754 11207 14767 11230 14752
11257 14756 11241 14778 11214 14778 11190 14791 11174 14769 11147 14771
11121 14763 11118 14790 11103 14813 11110 14786 11084 14795 11070 14818
11062 14844 11073 14869 11082 14895 11091 14920 11104 14944 11125 14962
11116 14988 11121 15014 11144 15029 11151 15055 11157 15081 11153 15108
11167 15133 11140 15126 11127 15150 11126 15177 11146 15196 11150 15223
11174 15236 11187 15237 11197 15251 11220 15263 11226 15267 11225 15269
11195 15252 11184 15242 11163 15240 11173 15268 11177 15282 11188 15277
11198 15279 11203 15287 50 P
11203 15287 11201 15289 11197 15283 11189 15279 11177 15283 11172 15271
11160 15247 11139 15230 11116 15216 11089 15215 11070 15234 11044 15225
11020 15239 10998 15254 10971 15254 10953 15274 10930 15262 10914 15283
10888 15291 10867 15307 10856 15283 10859 15256 10843 15234 10826 15213
10813 15237 10823 15262 10804 15281 10796 15307 10786 15332 10759 15335
10733 15342 10709 15354 10687 15370 10693 15396 10666 15395 10661 15368
10656 15342 10650 15315 10643 15341 10639 15368 40 P
10639 15368 10627 15344 10600 15338 10578 15323 10551 15317 10524 15318
10497 15320 10473 15332 10453 15313 10426 15313 10403 15328 10401 15355
10393 15329 10383 15304 10369 15280 10343 15286 10331 15262 10307 15276
10281 15281 10255 15285 10246 15260 10223 15246 10247 15235 10226 15218
10200 15209 10175 15218 10150 15208 10131 15188 10106 15177 10081 15167
10056 15157 10035 15140 10015 15121 9991 15110 9973 15089 9949 15102
9946 15075 9926 15057 9900 15060 9879 15042 9856 15027 9846 15002
9834 14978 9811 14964 9792 14945 9781 14920 9806 14908 9782 14896
9755 14890 9763 14864 50 P
9763 14864 9737 14859 9716 14841 9696 14859 9704 14833 9679 14842

9690 14817 9688 14790 9665 14803 9653 14828 9628 14838 9607 14821
9595 14797 9571 14785 9597 14779 9620 14765 9608 14741 9581 14738
9554 14738 9535 14718 9510 14710 9483 14710 9458 14698 9431 14699
9419 14675 9402 14653 9381 14670 9382 14697 9369 14722 9391 14738
9364 14745 9372 14771 9398 14779 9425 14779 9451 14789 9464 14812
9474 14837 9494 14818 9521 14822 9543 14838 9568 14848 9594 14857
9607 14881 9587 14898 9564 14914 9581 14935 9563 14954 9589 14961
9612 14975 9627 14998 50 P
9627 14998 9650 15013 2 P
9650 15013 9675 15020 9699 15033 9672 15034 9646 15028 9619 15027
9594 15015 9567 15014 9541 15011 9517 15025 9507 15055 9494 15087
9517 15096 9529 15113 9533 15125 9536 15146 9533 15173 9517 15194
9496 15211 9506 15236 9517 15260 9540 15275 9564 15289 9581 15310
9608 15309 9632 15322 9650 15342 9670 15361 9644 15368 9667 15382
9683 15403 9700 15424 9714 15447 9728 15470 9748 15489 9752 15516
9766 15538 9783 15559 9786 15586 9803 15607 9816 15631 9803 15655
9782 15673 9760 15688 9754 15698 9762 15715 9758 15741 9760 15748
9784 15735 9802 15740 50 P
9802 15740 9806 15746 9801 15754 9778 15753 9777 15766 9756 15778
9731 15793 9732 15791 9754 15775 9766 15768 9766 15762 9772 15754
9779 15745 9801 15751 9802 15743 9784 15738 9768 15746 9759 15750
9754 15746 9754 15732 9742 15733 9727 15748 9705 15764 9682 15779
9665 15800 9638 15798 9625 15821 9633 15847 9622 15872 9634 15896
9639 15923 9629 15898 9608 15880 9608 15853 9598 15827 9576 15811
9549 15809 9523 15806 9503 15826 9481 15841 9454 15840 9430 15853
9406 15840 9384 15855 9357 15858 9342 15836 9323 15817 9313 15792
9286 15796 9267 15776 50 P
9267 15776 9255 15752 2 P
9255 15752 9278 15738 9260 15717 9234 15711 9216 15690 9204 15666
9185 15647 9164 15630 9150 15607 9127 15593 9122 15567 9100 15550
9122 15535 9099 15521 9086 15497 9087 15470 9071 15449 9045 15442
9019 15435 8998 15417 8974 15406 8950 15392 8925 15383 8899 15375
8874 15364 8851 15350 8835 15328 27 P
8835 15328 8810 15320 8788 15304 8762 15297 8736 15290 8710 15280
8690 15263 8691 15236 8666 15225 8645 15209 8625 15190 8612 15166
8605 15140 8597 15114 8575 15100 8583 15074 8578 15047 8583 15020
8600 15000 8575 14989 8553 14973 8536 14952 8522 14929 8503 14909
8482 14893 8458 14880 8433 14870 8406 14867 8384 14851 8357 14849
8336 14866 8309 14867 8284 14856 8264 14837 8242 14821 8222 14803
8201 14787 8174 14791 8151 14806 8132 14826 8126 14852 8111 14874
8087 14888 8060 14892 44 P
8060 14892 8034 14888 8008 14879 7986 14864 7966 14844 7947 14825
7958 14800 7964 14773 7939 14763 7924 14741 7906 14719 7893 14696

7881 14671 7872 14645 7867 14619 7866 14592 7870 14565 17 P
7870 14565 7879 14539 7890 14514 7903 14490 7914 14466 7928 14443
7945 14421 7964 14402 7984 14383 8010 14376 8033 14361 11 P
8033 14361 8052 14342 8074 14327 8098 14313 8125 14316 8152 14313
8179 14316 8206 14314 8232 14306 8259 14302 8280 14285 8301 14301
8326 14313 8351 14324 8373 14340 8397 14352 8402 14326 8408 14352
8422 14329 8418 14302 8424 14275 8415 14250 8432 14271 8459 14271
8464 14244 8482 14224 8490 14198 8498 14172 8492 14146 8480 14121
8505 14110 8523 14089 8510 14065 8494 14043 8476 14023 8470 13996
8468 13969 8469 13942 8472 13915 8467 13888 8485 13868 8502 13846
8505 13819 8532 13820 8555 13806 8576 13787 8603 13785 8629 13778
8656 13777 8683 13779 50 P
8683 13779 8710 13782 8736 13771 8763 13773 8790 13773 8817 13780
8835 13799 8856 13817 8875 13836 8882 13863 8866 13884 8879 13908
8900 13925 8925 13936 8951 13943 8974 13958 8996 13974 9015 13994
9034 14013 9051 14035 9055 14061 9030 14073 9056 14081 9082 14089
9102 14108 9128 14115 9155 14119 9178 14133 9204 14142 9224 14160
9252 14161 9279 14158 9306 14161 9318 14137 9342 14124 9359 14103
9386 14105 9402 14082 9406 14055 9426 14036 9452 14031 9479 14034
9504 14044 9531 14048 9507 14036 9525 14016 9542 13995 9560 13975
9585 13963 9612 13961 50 P
9612 13961 9639 13966 2 P
9639 13966 9664 13976 9684 13957 9700 13979 9727 13984 9754 13988
9769 13966 9767 13993 9787 14012 9810 14027 9831 14043 9849 14023
9840 13997 9854 13974 9878 13987 9901 13972 9928 13965 9955 13966
9981 13974 9999 13953 10023 13964 10047 13977 10075 13976 10055 13957
10037 13936 10032 13909 10040 13883 10056 13861 10034 13846 10010 13832
9995 13809 9995 13782 9974 13764 10002 13761 10029 13765 10029 13738
10025 13711 10012 13687 10023 13712 9996 13706 9983 13682 9961 13665
9939 13650 9913 13654 9885 13655 9881 13682 9908 13685 9883 13696
9867 13718 9883 13740 50 P
9883 13740 9876 13766 9859 13745 9860 13718 9837 13703 9818 13684
9808 13710 9794 13733 9767 13728 9778 13704 9754 13692 9729 13680
9704 13669 9691 13645 9667 13632 9642 13622 9642 13595 9617 13602
9590 13595 9605 13618 9626 13635 9598 13633 9579 13614 9565 13637
9570 13610 9553 13588 9530 13574 9505 13564 9479 13554 9453 13547
9430 13533 9410 13514 9385 13524 9386 13497 9359 13496 9345 13519
9319 13527 9296 13513 9279 13492 9305 13498 9321 13476 9346 13465
9346 13438 9329 13417 9328 13389 9303 13402 9282 13420 9258 13408
9243 13384 9251 13358 50 P
9251 13358 9225 13350 2 P
9225 13350 9220 13323 9235 13301 9240 13274 9244 13248 9223 13231
9198 13218 9171 13215 9144 13210 9124 13192 9098 13183 9071 13188

9082 13213 9093 13238 9098 13265 9082 13288 9064 13308 9060 13281
9038 13264 9013 13273 8987 13263 8978 13237 8986 13211 9009 13197
9035 13189 9020 13166 9047 13162 9074 13165 9062 13141 9040 13125
9026 13101 9033 13128 9010 13113 8985 13103 8965 13084 8954 13058
8957 13031 8949 13005 8927 13020 8915 12996 8901 12972 8890 12947
8873 12969 8853 12951 8840 12927 8830 12952 8808 12936 8781 12939
8787 12913 8778 12887 50 P
8778 12887 8770 12860 8756 12837 8752 12810 8732 12790 8708 12778
8691 12756 8683 12730 8676 12704 8673 12676 8661 12651 8634 12656
12:00 PM
8634 12656 8613 12638 8599 12615 8595 12588 8595 12561 8586 12535
8588 12508 8601 12484 8594 12457 8613 12477 8635 12461 8659 12474
8684 12462 8711 12459 8716 12432 8730 12409 8751 12391 8774 12377
8798 12364 8820 12347 8847 12340 8865 12320 8865 12292 8878 12317
8905 12311 8932 12306 8958 12297 8982 12286 9001 12266 9028 12259
9053 12248 9078 12237 9092 12213 9096 12186 9109 12162 9117 12135
9126 12109 9136 12084 9145 12058 9146 12030 9154 12004 9155 11976
9180 11966 9175 11939 9201 11929 9209 11903 9220 11878 9216 11851
9223 11824 9240 11802 50 P
9240 11802 9262 11785 9252 11759 9250 11732 9257 11705 9276 11686
9285 11660 9297 11635 9316 11614 9331 11592 9337 11565 9351 11541
9378 11537 9389 11511 9395 11484 9405 11458 9407 11431 9398 11405
9378 11386 9404 11377 9417 11353 9398 11332 9383 11309 9411 11306
9429 11285 9448 11265 9476 11264 9501 11255 9527 11245 9552 11233
9577 11222 9603 11211 9625 11195 9638 11170 9638 11143 9643 11116
9628 11092 9648 11073 9660 11048 9686 11038 9713 11038 9739 11028
9765 11019 9789 11006 9810 10988 9829 10968 9848 10948 9857 10922
9870 10897 9884 10873 50 P
9884 10873 9895 10847 2 P
9895 10847 9889 10820 9863 10810 9836 10818 9810 10826 9782 10829
9757 10839 9732 10852 9706 10863 9688 10884 9662 10892 9635 10898
9610 10910 9584 10900 9558 10890 9531 10884 9517 10860 9490 10864
9462 10861 9436 10869 9430 10896 9420 10922 9401 10942 9384 10964
9361 10979 9345 11002 9319 10992 9293 11001 9265 11001 9240 11012
9215 11002 9190 10988 9169 10970 9147 10953 9126 10936 9100 10926
9072 10928 9045 10934 9059 10910 9087 10910 9113 10918 9138 10931
9164 10937 9192 10942 9203 10968 9231 10970 9257 10961 9284 10958
9312 10961 9339 10958 50 P
9339 10958 9355 10935 9368 10910 9380 10886 9359 10869 9377 10849
9403 10837 9430 10835 9458 10836 9484 10826 9510 10817 9531 10799
9555 10785 9577 10768 9595 10747 9609 10723 9627 10702 17 P
9627 10702 9651 10688 9677 10677 9698 10660 9714 10637 9741 10636
9766 10623 9789 10608 9811 10592 9829 10570 9843 10547 9857 10522

9870 10498 9883 10473 9896 10449 9910 10424 9920 10398 9913 10371
9890 10356 9863 10351 9835 10349 9807 10346 9780 10341 9753 10334
9727 10326 9702 10313 9682 10294 9667 10270 9643 10256 9615 10252
30 P
9615 10252 9593 10236 9571 10219 9556 10196 9528 10191 9503 10178
9484 10159 9469 10135 9468 10107 9452 10084 9436 10062 9411 10050
9383 10047 9364 10067 9337 10076 9309 10076 9281 10073 9254 10073
9227 10067 9202 10054 9194 10027 9195 9999 9169 10009 9141 10007
9124 9985 9104 9966 9128 9981 9147 10001 9169 9984 9196 9986
9213 10009 9223 10035 9246 10051 9274 10053 9302 10052 9325 10037
9314 10012 9323 9986 9341 9964 9335 9936 9344 9909 9371 9901
9346 9915 9365 9935 9391 9925 9419 9925 9445 9935 9466 9953
9490 9967 9512 9985 50 P
9512 9985 9531 10005 9553 10022 9578 10034 9606 10039 9633 10039
9661 10036 9685 10021 9708 10005 9727 9986 9746 9965 9763 9942
9782 9922 9798 9899 9812 9875 9836 9861 9863 9856 9891 9852
9917 9841 9944 9834 9955 9824 9962 9818 9966 9817 9994 9817
10022 9818 10046 9832 10039 9805 10016 9789 9990 9779 9968 9761
9954 9737 9931 9720 9914 9698 9892 9682 9874 9660 9856 9638
9834 9621 9807 9614 9780 9609 9753 9615 9735 9593 9724 9567
9732 9541 9747 9564 9774 9572 9766 9545 9784 9566 9807 9582
9834 9590 9853 9610 50 P
9853 9610 9881 9611 2 P
9881 9611 9888 9584 9912 9597 9896 9621 9897 9648 9921 9662
9949 9668 9976 9675 9995 9655 9977 9633 9974 9605 9990 9582
9989 9554 9963 9543 9966 9516 9963 9488 9989 9476 9994 9448
9975 9426 9952 9411 9924 9417 9923 9444 9917 9472 9927 9498
9906 9517 9899 9490 9901 9462 9894 9435 9874 9416 9847 9409
9837 9435 9835 9463 9844 9490 9833 9464 9832 9436 9814 9458
9786 9462 9782 9435 9774 9407 9750 9393 9749 9365 9777 9369
9803 9377 9831 9371 9834 9343 9806 9340 9785 9321 9811 9332
9839 9338 9864 9327 50 P
9864 9327 9853 9301 9874 9282 9855 9261 9827 9264 9801 9274
9816 9250 9843 9244 9856 9219 9864 9192 9844 9173 9825 9153
9801 9138 9773 9137 9745 9136 9717 9137 9743 9129 9771 9122
9787 9100 9795 9073 9808 9047 9823 9024 9822 8996 9836 8971
9845 8945 9859 8920 9847 8895 9828 8916 9805 8931 9777 8936
9762 8912 9749 8887 9728 8869 9734 8842 9713 8823 9693 8842
9675 8864 9667 8891 9652 8915 9669 8938 9642 8945 9616 8936
9606 8909 9581 8898 9603 8881 9593 8854 9578 8830 9553 8817
9540 8792 9541 8764 50 P
9541 8764 9529 8739 2 P
9529 8739 9529 8727 9502 8730 9474 8731 9446 8730 9429 8748

9424 8748 9396 8755 9369 8751 9347 8770 9349 8742 9377 8745
9404 8740 9424 8720 9452 8716 9477 8702 9479 8674 9463 8650
9445 8629 9430 8605 9418 8579 9390 8577 9386 8548 9387 8520
9368 8500 9340 8493 9358 8471 9371 8446 9398 8436 9384 8411
9385 8383 9357 8378 9332 8391 9325 8363 9339 8339 9322 8315
9322 8287 9306 8263 9291 8287 9280 8261 9256 8275 9245 8301
42 P
9245 8301 9251 8274 9265 8249 9250 8226 9222 8218 9195 8222
9204 8195 9199 8167 9180 8146 9151 8146 9179 8139 9181 8110
9153 8116 9125 8118 9118 8090 9090 8096 9078 8070 9102 8056
9127 8042 9116 8015 9142 8004 9121 7984 9122 7956 9096 7945
9072 7930 9043 7927 9035 7900 9014 7881 8985 7882 8993 7910
9009 7933 9037 7934 9058 7953 9056 7982 9082 7995 9094 8020
9070 8036 9042 8039 9033 8012 9017 7989 9012 7961 8990 7979
8962 7985 8965 8013 8989 8027 8962 8034 8978 8057 8952 8046
8948 8018 8927 8037 50 P
8927 8037 8910 8059 8900 8086 8901 8058 8908 8030 8918 8004
8893 8017 8871 8035 8845 8045 8849 8017 8844 7989 8867 7973
8840 7964 8843 7935 8865 7917 8891 7928 8919 7927 8947 7923
8966 7901 8970 7873 8982 7847 8966 7824 8944 7841 8916 7848
8895 7866 8872 7850 8859 7825 8886 7833 8899 7807 8905 7780
8927 7762 8952 7774 8961 7801 8979 7824 8996 7801 9020 7786
9046 7797 9028 7774 9000 7771 8973 7765 8948 7750 8921 7742
8893 7736 8869 7720 8898 7725 8887 7699 8870 7676 8850 7656
8834 7632 8853 7612 50 P
8853 7612 8880 7603 2 P
8880 7603 8906 7591 8934 7588 8927 7560 8919 7533 8891 7531
8882 7504 8884 7475 8892 7448 8878 7423 8850 7418 8824 7407
8807 7384 8821 7359 8828 7332 8854 7344 8868 7319 8874 7291
8901 7282 8907 7254 8902 7226 8881 7207 8880 7236 8879 7264
8852 7272 8841 7246 8827 7221 8830 7250 8813 7273 8808 7301
8779 7304 8754 7317 8764 7344 8740 7359 8729 7333 8713 7310
8693 7289 8669 7275 8646 7257 8619 7249 8640 7230 8663 7213
8687 7198 8677 7171 8704 7180 8726 7197 8744 7174 8763 7153
8736 7146 8730 7118 50 P
8730 7118 8701 7114 8673 7112 8649 7128 8647 7157 8632 7133
8641 7106 8655 7081 8629 7093 8601 7097 8611 7124 8613 7152
8588 7140 8604 7163 8577 7170 8548 7167 8522 7157 8541 7136
8561 7116 8545 7093 8528 7069 8501 7062 8510 7034 8534 7020
8562 7012 8575 7038 8573 7009 8587 6984 8558 6985 8530 6983
8534 6954 8562 6948 8537 6933 8511 6944 8482 6948 8464 6926
8436 6919 8442 6947 8429 6972 8416 6947 8416 6975 8395 6995
8402 6967 8409 6940 8381 6933 8355 6945 8378 6928 8382 6900

8403 6880 8425 6897 50 P
8425 6897 8441 6874 2 P
8441 6874 8437 6845 8424 6820 8395 6822 8394 6794 8405 6768
8384 6747 8359 6734 8360 6762 8336 6777 8310 6789 8284 6803
8295 6830 8273 6812 8278 6784 8253 6770 8225 6765 8198 6773
8185 6747 8157 6754 8165 6781 8154 6808 8125 6808 8123 6779
8109 6754 8092 6777 8082 6750 8053 6749 8079 6736 8074 6708
8051 6691 8023 6698 7997 6708 7968 6707 7953 6704 7941 6720
7940 6703 7951 6692 7974 6674 7999 6661 8017 6656 8045 6659
8074 6663 8077 6634 8106 6634 8120 6609 8126 6581 8098 6575
8077 6556 8054 6538 50 P
8054 6538 8033 6558 8013 6537 8024 6510 8005 6488 8001 6459
7977 6445 7953 6427 7937 6404 7925 6378 7929 6406 7946 6430
7936 6457 7909 6448 7891 6426 7894 6397 7871 6380 7859 6354
7857 6325 7855 6297 7838 6273 7839 6302 7848 6330 7839 6357
7818 6375 7790 6383 7762 6390 7733 6393 7756 6376 7784 6369
7786 6340 7776 6313 7758 6291 7735 6307 7723 6307 7711 6291
7687 6307 7676 6281 7671 6252 7669 6223 7651 6201 7659 6229
7641 6252 7641 6222 7617 6207 45 P
7617 6207 7594 6189 7571 6171 7564 6199 7558 6227 7532 6238
7540 6210 7515 6223 7487 6215 7460 6205 7469 6181 7455 6155
7454 6129 7455 6119 7434 6099 7422 6085 7401 6062 7372 6062
7343 6062 7316 6074 7294 6092 7273 6113 7245 6113 7273 6108
7279 6080 7292 6054 7315 6037 7343 6030 7379 5998 7358 5987
7341 5964 7319 5984 7296 6002 7268 5999 7271 5970 7295 5985
7291 5956 7277 5931 7284 5903 7287 5874 7266 5854 7258 5826
7228 5828 7200 5820 7172 5826 7149 5844 7125 5828 7101 5811
7082 5790 7059 5771 50 P
7059 5771 7030 5771 7046 5796 7029 5819 7027 5791 7003 5774
6976 5764 6949 5754 6921 5746 6899 5727 6882 5750 6866 5774
6838 5783 6814 5799 6785 5797 6764 5777 6747 5753 6729 5730
6747 5707 6768 5727 6754 5701 6757 5672 6730 5685 6705 5698
6676 5693 6648 5700 6636 5727 6665 5728 6640 5742 6614 5755
6575 5753 6576 5763 6599 5771 6568 5782 6543 5766 6515 5767
6487 5760 6472 5735 6458 5710 6430 5706 6417 5732 6392 5747
6408 5723 6431 5705 6415 5680 6399 5656 6403 5627 6382 5648
6353 5652 6324 5651 50 P
6324 5651 6314 5678 2 P
6314 5678 6317 5707 6314 5736 6337 5753 6345 5781 6324 5762
6295 5767 6269 5755 6242 5746 6216 5733 6202 5708 6179 5689
6209 5689 6208 5660 6181 5650 6206 5635 6234 5625 6228 5597
6200 5590 6172 5584 6168 5612 6159 5640 6141 5663 6113 5670
6084 5663 6056 5656 6035 5637 27 P

6035 5637 6061 5624 6086 5611 6065 5592 6036 5590 6008 5586
6023 5561 6051 5569 6074 5588 6090 5564 6118 5570 6112 5542
6084 5534 6057 5545 6049 5516 6069 5495 6042 5507 6013 5506
5985 5513 5967 5535 5937 5535 5908 5534 5880 5538 5859 5559
5880 5579 5902 5598 5928 5612 5955 5622 28 P
5955 5622 5926 5625 5898 5632 5871 5621 5852 5599 5859 5628
5842 5651 5823 5673 5811 5700 5796 5725 5790 5753 5772 5776
5745 5765 5739 5794 5720 5816 5706 5840 5711 5869 5722 5896
5705 5873 5684 5893 5677 5921 5679 5950 5654 5964 5627 5975
5600 5984 5617 5961 5645 5957 5662 5934 5661 5904 5655 5876
5626 5871 5598 5866 5627 5865 5654 5855 5664 5827 5688 5811
5702 5785 5674 5778 5649 5795 5669 5773 5686 5750 5703 5726
5675 5717 5654 5736 5673 5715 5689 5691 5665 5674 5681 5650
5680 5621 5667 5595 50 P
5667 5595 5659 5567 5682 5551 5689 5522 5674 5497 5665 5469
5656 5453 7 P
5649 5453 5644 5472 5643 5453 3 P
5597 5453 5592 5457 5573 5479 5556 5502 5547 5530 5528 5553
5537 5525 5551 5487 5544 5482 5539 5475 5539 5468 5531 5453
12:00 PM
5429 5453 5430 5476 5412 5498 5410 5527 5410 5557 5415 5585
5415 5614 5386 5615 5380 5644 5351 5648 5369 5625 5393 5608
5404 5582 5403 5553 5402 5524 5405 5494 5407 5466 5392 5453
18 P
2966 5453 2967 5459 2985 5482 2967 5505 2938 5506 2916 5486
2926 5459 2928 5453 8 P
13001 8121 12996 8116 12991 8121 12985 8125 12983 8118 12983 8111
12985 8105 12988 8098 12992 8092 12995 8086 13001 8082 13001 8081
12:00 PM
13001 8033 13001 8033 12994 8030 12988 8028 12984 8022 12981 8015
12978 8009 12978 8002 12973 7997 12977 7991 12982 7985 12976 7990
12969 7993 12962 7992 12960 7986 12961 7979 12966 7973 12968 7967
12974 7963 12979 7957 12979 7950 12979 7943 12979 7936 12977 7929
12976 7922 12982 7918 12988 7915 12993 7920 12999 7925 13001 7925
30 P
11825 7031 11829 7025 11833 7019 11839 7015 11846 7013 11847 7020
11850 7013 11855 7009 11862 7012 11869 7014 11876 7015 11882 7019
11889 7021 11896 7021 11903 7023 11910 7024 11917 7023 11924 7025
11931 7026 11936 7021 11932 7027 11927 7033 11923 7039 11918 7044
11911 7044 11904 7046 11897 7048 11894 7054 11887 7057 11880 7055
11873 7058 11866 7060 11863 7066 11865 7073 11859 7078 11853 7080
11849 7086 11847 7093 11844 7100 11838 7104 11833 7110 11829 7115
11826 7121 11819 7120 11823 7114 11827 7108 11830 7102 11835 7096

11839 7091 11842 7084 50 P
11842 7084 11835 7081 11835 7074 11837 7067 11834 7060 11828 7057
11825 7051 11831 7047 11837 7042 11840 7037 11836 7031 11825 7031

12:00 PM

11225 6793 11231 6789 11237 6791 11242 6796 11250 6797 11255 6792
11259 6786 11264 6780 11263 6773 11259 6767 11253 6764 11249 6758
11254 6754 11261 6752 11268 6749 11273 6744 11279 6741 11286 6740
11293 6742 11298 6748 11299 6755 11306 6753 11312 6750 11319 6752
11322 6758 11323 6766 11322 6773 11322 6780 11324 6787 11323 6794
11319 6800 11322 6807 11319 6813 11319 6820 11322 6826 11328 6831
11332 6836 11333 6843 11338 6849 11342 6854 11345 6861 11342 6868
11346 6874 11352 6879 11356 6883 11358 6890 11357 6897 11358 6905
11351 6905 11344 6906 50 P
11344 6906 11343 6899 11341 6892 11338 6886 11334 6880 11331 6873
11336 6868 11333 6862 11331 6855 11329 6848 11322 6850 11316 6847
11313 6841 11306 6839 11306 6831 11302 6825 11305 6818 11299 6813
11292 6814 11285 6812 11279 6808 11272 6809 11265 6807 11262 6814
11259 6820 11252 6822 11252 6815 11250 6809 11246 6802 11240 6807
11233 6806 11228 6801 11225 6793 33 P
11017 6707 11022 6701 11026 6696 11032 6692 11040 6693 11047 6691
11049 6698 11055 6703 11060 6698 11066 6694 11073 6693 11080 6693
11088 6692 11095 6691 11102 6691 11109 6692 11116 6690 11122 6687
11129 6684 11135 6688 11134 6695 11131 6701 11124 6704 11131 6702
11138 6700 11144 6696 11144 6703 11142 6710 11144 6717 11148 6723
11155 6721 11157 6728 11154 6735 11158 6741 11160 6748 11154 6750
11148 6754 11143 6759 11136 6761 11129 6762 11126 6769 11120 6765
11114 6769 11107 6768 11100 6765 11096 6760 11091 6766 11089 6772
11084 6778 11078 6782 50 P
11078 6782 11072 6784 11066 6781 11071 6776 11073 6769 11077 6763
11070 6766 11071 6773 11065 6770 11060 6764 11056 6758 11059 6752
11058 6745 11062 6739 11067 6734 11068 6727 11065 6733 11062 6727
11062 6720 11064 6713 11064 6706 11063 6713 11058 6718 11052 6722
11051 6729 11045 6724 11039 6728 11039 6721 11043 6715 11037 6719
11031 6722 11026 6716 11030 6710 11017 6707 34 P
9618 7032 9615 7018 9606 7007 9598 6995 9592 6982 9585 6969
9582 6955 9572 6945 9564 6933 9571 6920 9567 6907 9563 6893
9553 6882 9543 6873 9539 6859 9529 6848 9539 6839 9525 6839
9511 6837 9498 6831 9490 6820 9496 6807 9482 6804 9469 6799
9464 6785 9459 6771 9456 6757 9451 6744 9445 6731 9438 6719
9427 6709 9419 6697 9409 6687 9403 6674 9397 6661 9391 6647
9386 6634 9384 6620 9375 6608 9371 6595 9363 6582 9356 6570
9368 6562 9354 6562 9346 6550 9339 6537 9332 6525 9329 6511
9324 6497 9315 6486 50 P

9315 6486 9305 6476 9296 6465 9289 6452 9280 6441 9274 6428
9274 6414 9267 6401 9261 6388 9256 6374 9247 6364 9256 6353
9270 6351 9285 6349 9299 6351 9288 6342 9274 6340 9259 6339
9245 6341 9231 6341 9222 6329 9213 6318 9211 6304 9210 6289
9207 6275 9195 6267 9188 6254 9182 6241 9181 6226 9180 6212
9187 6199 9187 6185 9193 6172 9181 6163 9190 6153 9188 6139
9198 6129 9188 6120 9192 6106 9196 6092 9194 6077 9192 6063
9199 6051 9185 6053 9183 6039 9175 6027 9177 6013 9166 6004
9179 5998 9164 5996 50 P
9164 5996 9163 5982 2 P
9163 5982 9164 5967 9155 5958 9169 5954 9173 5940 9169 5926
9158 5917 9144 5914 9140 5927 9136 5913 9146 5903 9160 5906
9153 5895 9163 5885 9175 5877 9189 5881 9203 5885 9217 5890
9220 5876 9207 5869 9193 5866 9200 5853 9207 5841 9211 5827
9211 5812 9216 5799 9221 5785 9235 5782 9226 5771 9231 5757
9237 5744 9243 5730 9249 5717 9255 5704 9258 5690 9258 5676
9255 5661 9263 5649 9276 5643 9280 5629 9291 5620 9302 5610
9289 5615 9279 5627 9271 5639 9277 5625 9286 5614 9296 5603
9306 5593 9316 5582 50 P
9316 5582 9328 5574 9340 5566 9347 5580 9353 5566 9362 5555
9373 5545 9386 5539 9398 5531 9411 5525 9424 5518 9436 5510
9447 5500 9454 5487 9465 5478 9472 5465 9476 5453 17 P
9607 5453 9607 5465 9606 5479 9608 5494 9614 5507 9617 5521
9622 5535 9627 5548 9632 5562 9635 5576 9638 5590 9642 5604
9646 5618 9652 5632 9662 5642 9664 5657 9667 5671 9673 5684
9684 5693 9695 5703 9707 5711 9720 5717 9730 5727 9740 5738
9751 5748 9754 5763 9758 5776 9753 5790 9760 5803 9773 5808
9784 5818 9790 5831 9798 5843 9801 5857 9812 5868 9819 5880
9824 5894 9830 5907 9833 5921 9837 5935 9848 5944 9857 5956
9859 5970 9862 5984 9857 5997 9859 6012 9865 6025 9868 6039
9872 6053 9877 6066 50 P
9877 6066 9889 6075 9891 6089 9893 6103 9894 6118 9899 6132
9903 6145 9906 6160 9911 6173 9906 6186 9907 6201 9910 6215
9916 6228 9917 6242 9917 6257 9922 6270 9923 6285 9925 6299
9928 6313 9934 6326 9934 6341 9934 6355 9940 6368 9944 6382
9947 6397 9949 6411 9949 6425 9949 6439 9951 6454 9960 6465
9968 6477 9960 6489 9953 6502 9950 6516 9955 6529 9965 6539
9974 6551 9973 6565 9970 6579 9977 6592 9988 6601 9997 6612
10008 6621 10014 6634 10021 6646 10027 6659 10025 6674 10030 6688
10032 6702 49 P
10032 6702 10034 6716 10040 6729 10047 6742 10058 6751 10055 6765
10061 6778 10072 6786 10070 6800 10071 6815 10068 6829 10058 6840
10053 6853 10050 6867 10048 6881 10043 6895 10039 6908 10037 6922

10037 6937 10038 6951 10042 6965 10046 6979 10054 6991 10064 7001
10069 7014 10080 7024 10088 7035 10101 7042 10114 7049 10125 7058
10117 7070 10103 7068 10090 7075 10089 7090 10081 7101 10083 7115
10073 7125 10058 7124 10044 7125 10030 7122 10020 7131 10007 7137
9994 7131 9984 7141 9970 7146 9960 7156 9964 7169 9950 7164
9940 7174 9931 7185 50 P
9931 7185 9927 7199 9916 7207 9903 7214 9889 7213 9875 7209
9861 7204 9849 7197 9840 7186 9832 7174 9825 7162 9817 7150
9828 7141 9815 7136 9805 7126 9793 7119 9780 7113 9766 7111
9752 7107 9738 7108 9724 7103 9711 7098 9699 7090 9687 7082
9673 7079 9660 7074 9648 7065 9636 7058 9626 7048 9618 7032
30 P
12970 11609 12977 11607 12983 11607 12988 11602 12995 11600 12999 11606
13001 11607 7 P
13001 11699 13000 11699 12995 11704 12988 11706 12983 11702 12981 11695
12982 11688 12986 11683 12984 11676 12986 11670 12991 11665 12987 11659
12990 11653 12992 11646 12999 11643 12999 11637 12993 11636 12998 11632
12991 11633 12993 11627 12988 11623 12987 11616 12994 11618 12999 11623
12995 11617 12995 11610 12988 11612 12982 11616 12979 11622 12975 11628
12973 11621 12970 11609 32 P
11806 11848 11811 11843 11815 11837 11819 11832 11825 11828 11831 11825
11838 11824 11844 11820 11848 11815 11853 11810 11858 11805 11864 11802
11871 11802 11871 11809 11872 11815 11876 11821 11880 11827 11886 11829
11893 11828 11900 11828 11907 11827 11913 11830 11919 11834 11926 11834
11932 11831 11939 11833 11945 11835 11950 11829 11957 11829 11963 11832
11970 11832 11974 11838 11981 11839 11987 11840 11994 11840 12001 11842
12007 11840 12013 11841 12018 11847 12024 11851 12028 11856 12033 11860
12040 11862 12047 11862 12054 11863 12060 11867 12066 11869 12073 11870
12078 11875 12082 11880 50 P
12082 11880 12087 11886 12093 11886 12100 11887 12107 11889 12113 11891
12117 11897 12116 11904 12121 11908 12125 11914 12126 11921 12127 11928
12131 11934 12136 11937 12141 11942 12141 11949 12142 11956 12143 11963
12148 11959 12147 11965 12146 11972 12149 11978 12155 11977 12151 11982
12146 11977 12145 11984 12138 11986 12134 11992 12138 11998 12136 12005
12132 12011 12126 12014 12119 12014 12113 12012 12107 12015 12101 12017
12096 12022 12092 12028 12085 12026 12079 12027 12072 12028 12065 12027
12059 12023 12052 12023 12046 12021 12039 12019 12033 12016 12026 12015
12020 12012 12013 12014 50 P
12013 12014 12007 12010 2 P
12007 12010 12001 12007 11995 12004 11989 12001 11983 11999 11976 11997
11970 11994 11964 11991 11958 11987 11952 11989 11945 11990 11941 11984
11934 11983 11929 11979 11923 11975 11917 11973 11911 11969 11905 11966
11899 11968 11893 11964 11887 11961 11882 11957 11876 11954 11869 11954

11865 11948 11862 11942 11855 11939 11850 11934 11845 11929 11845 11923
11842 11917 11837 11911 11832 11907 11826 11902 11823 11897 11818 11891
11813 11888 11812 11881 11806 11877 11806 11870 11809 11864 11807 11857
11806 11848 43 P
12979 8284 12983 8279 12990 8279 12997 8281 13001 8281 5 P
13001 8297 12996 8294 12989 8295 12982 8295 12979 8284 5 P
12715 8170 12721 8173 12729 8174 12735 8177 12733 8184 12726 8187
12721 8183 12715 8170 8 P
12477 8021 12481 8016 12488 8014 12495 8013 12502 8012 12508 8008
12510 8001 12515 7995 12521 7992 12527 7989 12534 7988 12532 7995
12529 8001 12529 8008 12533 8014 12535 8020 12529 8024 12526 8030
12519 8032 12515 8038 12509 8042 12502 8041 12496 8037 12491 8032
12485 8029 12477 8021 26 P
12693 8048 12696 8044 12698 8040 12702 8043 12706 8045 12702 8047
12699 8051 12699 8055 12695 8054 12693 8048 10 P
12534 8012 12536 8013 12538 8014 12540 8015 12540 8017 12538 8019
12537 8017 12535 8016 12534 8012 9 P
12542 8015 12544 8016 12546 8018 12545 8020 12543 8019 12542 8015

6:00 PM

12547 8013 12549 8015 12550 8016 12552 8017 12555 8017 12557 8018
12558 8020 12559 8022 12562 8023 12564 8023 12566 8023 12568 8024
12569 8026 12571 8026 12573 8028 12571 8028 12569 8028 12568 8026
12566 8025 12563 8024 12561 8024 12559 8024 12557 8022 12556 8020
12554 8019 12552 8018 12550 8017 12548 8016 12547 8013 29 P
12807 7955 12810 7951 12813 7948 12812 7944 12812 7939 12814 7936
12816 7940 12820 7942 12821 7946 12820 7951 12819 7955 12821 7959
12825 7962 12824 7966 12824 7971 12823 7975 12820 7979 12817 7982
12812 7980 12812 7976 12812 7971 12810 7967 12810 7963 12812 7958
12807 7955 25 P
12776 7979 12780 7978 12785 7978 12788 7975 12791 7978 12792 7982
12792 7987 12796 7984 12800 7982 12800 7987 12797 7991 12793 7991
12788 7992 12788 7987 12784 7987 12781 7984 12776 7979 17 P
12768 7962 12772 7959 12775 7955 12779 7957 12783 7959 12781 7963
12779 7967 12775 7969 12772 7966 12768 7962 10 P
12779 7952 12781 7949 12786 7950 12785 7954 12779 7952 5 P
12783 7944 12785 7940 12787 7944 12786 7949 12783 7944 5 P
12754 7945 12756 7940 12759 7938 12760 7943 12760 7947 12757 7950
12754 7945 7 P
12756 7967 12760 7966 12761 7971 12757 7972 12756 7967 5 P
12857 7972 12861 7973 12861 7978 12864 7982 12864 7986 12860 7983
12859 7979 12857 7972 8 P
12832 12916 12826 12914 12823 12908 12822 12901 12821 12894 12815 12895
12808 12892 12803 12895 12801 12888 12796 12884 12800 12878 12804 12872

12807 12866 12812 12861 12818 12857 12811 12858 12804 12857 12798 12860
12793 12855 12786 12856 12780 12859 12775 12864 12768 12862 12766 12856
12759 12854 12756 12848 12753 12842 12750 12836 12752 12829 12754 12823
12758 12816 12764 12814 12767 12808 12771 12802 12777 12806 12776 12812
12783 12814 12788 12817 12793 12822 12796 12829 12803 12827 12803 12820
12806 12814 12804 12807 12805 12801 12799 12796 12793 12795 12786 12793
12788 12786 12793 12790 50 P
12793 12790 12793 12783 12800 12785 12806 12788 12812 12785 12806 12781
12805 12774 12808 12768 12814 12766 12811 12760 12809 12754 12815 12752
12822 12749 12827 12753 12834 12755 12832 12761 12826 12766 12833 12768
12840 12769 12846 12772 12853 12772 12847 12775 12840 12774 12836 12780
12832 12785 12834 12792 12841 12791 12845 12796 12842 12802 12842 12809
12848 12812 12852 12806 12856 12812 12862 12811 12867 12806 12873 12803
12873 12810 12868 12815 12864 12820 12866 12827 12859 12826 12857 12832
12863 12835 12870 12833 12867 12839 12862 12845 12869 12846 12869 12853
12872 12859 12865 12859 50 P
12865 12859 12860 12855 2 P
12860 12855 12859 12848 12852 12849 12848 12855 12853 12860 12855 12866
12859 12872 12857 12879 12855 12885 12854 12892 12852 12899 12846 12902
12843 12908 12840 12915 12844 12921 12850 12924 12851 12931 12846 12936
12840 12938 12833 12938 12829 12933 12828 12926 12832 12916 23 P
12542 12787 12536 12784 12533 12778 12527 12774 12522 12769 12517 12774
12515 12781 12509 12782 12502 12783 12503 12790 12498 12795 12495 12801
12495 12808 12501 12812 12503 12818 12505 12825 12503 12831 12500 12837
12494 12841 12487 12841 12481 12839 12476 12834 12471 12829 12471 12822
12476 12817 12469 12814 12463 12813 12463 12806 12462 12799 12458 12793
12456 12787 12458 12780 12461 12774 12465 12768 12470 12764 12471 12758
12476 12753 12479 12746 12477 12740 12475 12733 12482 12732 12484 12725
12489 12721 12496 12720 12503 12721 12509 12724 12510 12730 12507 12736
12505 12743 12511 12747 50 P
12511 12747 12516 12750 12523 12752 12524 12745 12523 12739 12525 12732
12528 12725 12529 12719 12536 12717 12543 12718 12549 12722 12556 12722
12560 12717 12566 12720 12573 12721 12569 12726 12565 12732 12570 12737
12573 12743 12572 12749 12570 12756 12569 12763 12569 12770 12572 12776
12571 12782 12566 12787 12559 12785 12555 12789 12542 12787 29 P
12067 12365 12071 12359 12077 12362 12082 12357 12089 12357 12096 12356
12100 12361 12101 12368 12100 12375 12095 12379 12094 12386 12088 12389
12082 12391 12076 12387 12073 12382 12070 12375 12067 12365 17 P
12233 12404 12237 12406 12241 12406 12246 12408 12250 12407 12250 12411
12252 12415 12250 12419 12249 12423 12249 12428 12245 12427 12241 12425
12242 12421 12237 12420 12237 12416 12235 12411 12233 12404 17 P
12160 12539 12162 12536 12167 12537 12169 12534 12166 12531 12165 12527
12169 12528 12172 12524 12176 12522 12179 12519 12183 12517 12187 12520

12191 12519 12194 12523 12198 12523 12202 12523 12207 12523 12211 12523
12215 12524 12218 12528 12219 12532 12219 12536 12221 12540 12222 12544
12223 12548 12222 12552 12220 12556 12216 12559 12212 12558 12208 12556
12204 12555 12200 12556 12196 12554 12193 12551 12191 12548 12186 12548
12183 12544 12179 12546 12175 12547 12173 12551 12169 12551 12170 12547
12168 12543 12165 12540 12160 12539 45 P
12321 12547 12326 12547 12329 12548 12334 12549 12336 12545 12332 12545
12328 12545 12329 12542 12333 12540 12337 12541 12341 12539 12341 12535
12340 12530 12343 12528 12347 12531 12350 12534 12354 12536 12358 12537
12362 12540 12363 12544 12361 12548 12360 12552 12355 12553 12353 12556
12349 12559 12346 12562 12344 12558 12340 12557 12337 12554 12333 12557
12329 12558 12325 12560 12321 12558 12324 12556 12323 12552 12321 12547
36 P
12426 12608 12429 12605 12434 12604 12437 12602 12441 12603 12441 12599
12444 12596 12445 12593 12449 12591 12453 12593 12457 12590 12461 12589
12462 12585 12467 12585 12471 12585 12474 12589 12472 12593 12470 12596
12465 12596 12461 12597 12465 12598 12465 12603 12462 12606 12458 12609
12456 12613 12455 12617 12451 12618 12447 12620 12442 12618 12438 12616
12436 12613 12433 12609 12426 12608 33 P
12473 12555 12477 12555 12475 12551 12475 12547 12479 12547 12483 12544
12485 12548 12484 12552 12484 12556 12485 12561 12484 12565 12481 12567
12482 12571 12477 12571 12476 12568 12477 12563 12476 12559 12473 12555
18 P
11961 12468 11963 12464 11963 12459 11964 12456 11965 12451 11964 12447
11967 12444 11966 12439 11970 12442 11974 12439 11978 12437 11981 12435
11985 12438 11988 12440 11991 12437 11994 12434 11992 12431 11987 12431
11987 12426 11990 12424 11994 12425 11999 12426 12003 12425 12006 12423
12011 12422 12010 12426 12008 12430 12012 12429 12015 12425 12019 12425
12024 12425 12028 12424 12026 12428 12021 12430 12023 12433 12025 12438
12021 12439 12017 12439 12012 12438 12013 12443 12012 12447 12008 12449
12007 12453 12005 12457 12004 12461 12004 12465 12003 12470 12000 12474
11997 12476 11993 12478 50 P
11993 12478 11990 12480 11985 12482 11981 12483 11977 12481 11974 12478
11970 12478 11966 12477 11963 12473 11961 12468 10 P
11711 12463 11715 12457 11720 12452 11723 12446 11729 12443 11728 12450
11725 12456 11729 12462 11730 12456 11735 12451 11742 12450 11738 12444
11735 12438 11737 12432 11742 12437 11745 12431 11752 12431 11759 12431
11765 12435 11771 12437 11766 12442 11773 12441 11779 12443 11784 12449
11790 12450 11790 12456 11797 12459 11803 12459 11804 12466 11811 12467
11812 12474 11817 12469 11819 12463 11824 12467 11822 12474 11828 12477
11830 12484 11834 12489 11831 12495 11835 12501 11830 12505 11834 12511
11835 12518 11836 12524 11831 12528 11827 12534 11822 12538 11816 12542
11810 12540 11809 12533 50 P

11809 12533 11803 12537 11800 12543 11802 12550 11798 12556 11792 12559
11785 12561 11779 12562 11772 12563 11770 12556 11773 12550 11780 12549
11786 12551 11784 12544 11786 12538 11783 12532 11778 12528 11776 12521
11769 12523 11769 12529 11766 12536 11762 12530 11765 12524 11759 12521
11762 12515 11763 12508 11768 12503 11773 12505 11771 12499 11764 12501
11768 12495 11766 12488 11765 12481 11764 12475 11760 12469 11761 12475
11759 12482 11760 12489 11763 12495 11759 12501 11759 12508 11753 12509
11752 12516 11746 12512 11745 12505 11749 12499 11746 12494 11741 12498
11734 12498 11731 12492 50 P
11731 12492 11724 12490 2 P
11724 12490 11723 12483 11727 12478 11721 12481 11715 12476 11714 12470
11711 12463 7 P
11913 12330 11917 12327 11918 12323 11923 12322 11927 12322 11931 12324
11934 12326 11938 12329 11941 12332 11942 12336 11946 12337 11947 12340
11951 12343 11951 12347 11955 12344 11957 12340 11958 12345 11961 12343
11966 12344 11966 12348 11961 12348 11957 12349 11953 12351 11949 12351
11945 12349 11940 12350 11936 12349 11934 12352 11931 12355 11926 12354
11923 12351 11920 12348 11917 12345 11913 12342 11916 12338 11913 12330
36 P
11930 12361 11934 12360 11937 12357 11940 12353 11945 12353 11949 12352
11953 12353 11950 12355 11949 12359 11952 12362 11954 12358 11959 12358
11961 12362 11958 12365 11961 12368 11965 12370 11962 12373 11960 12377
11957 12380 11952 12382 11949 12381 11944 12382 11940 12380 11937 12383
11933 12385 11934 12381 11937 12378 11933 12376 11932 12372 11937 12373
11934 12369 11933 12365 11930 12361 33 P
11969 12340 11972 12337 11974 12333 11974 12329 11978 12331 11981 12328
11983 12324 11986 12323 11989 12326 11989 12330 11991 12334 11990 12338
11994 12340 11993 12345 11989 12343 11988 12347 11986 12351 11982 12352
11983 12356 11987 12357 11991 12358 11993 12362 11990 12365 11986 12367
11981 12368 11978 12370 11974 12368 11974 12364 11970 12363 11971 12359
11975 12360 11977 12356 11977 12352 11974 12349 11973 12344 11969 12340
36 P
12014 12448 12019 12448 12023 12446 12027 12446 12031 12444 12033 12441
12037 12444 12040 12446 12044 12447 12048 12448 12052 12445 12054 12442
12054 12437 12057 12433 12061 12436 12062 12439 12066 12442 12065 12446
12069 12445 12070 12441 12074 12441 12075 12445 12074 12450 12074 12454
12079 12453 12080 12457 12078 12462 12075 12458 12071 12459 12068 12463
12064 12462 12059 12462 12056 12459 12053 12463 12050 12465 12046 12463
12044 12459 12044 12455 12041 12452 12037 12451 12034 12454 12030 12457
12028 12453 12026 12449 12022 12450 12018 12451 12014 12448 47 P
12010 12473 12014 12476 12018 12475 12023 12476 12021 12472 12020 12468
12019 12463 12023 12461 12026 12464 12026 12469 12028 12472 12030 12476
12034 12478 12038 12478 12042 12479 12042 12484 12045 12487 12050 12486

12053 12484 12058 12484 12062 12484 12062 12489 12059 12491 12054 12494
12054 12498 12050 12496 12046 12497 12050 12500 12051 12504 12047 12507
12045 12510 12041 12510 12037 12508 12033 12505 12029 12504 12025 12504
12023 12501 12019 12497 12018 12493 12015 12490 12017 12486 12015 12482
12013 12478 12010 12473 44 P
12059 12493 12063 12490 12065 12486 12063 12483 12062 12479 12063 12475
12066 12477 12068 12482 12072 12483 12072 12479 12072 12475 12073 12470
12077 12470 12081 12468 12085 12468 12089 12470 12091 12474 12094 12475
12099 12475 12100 12479 12098 12483 12097 12488 12098 12492 12099 12496
12098 12500 12096 12496 12094 12492 12092 12488 12088 12489 12084 12488
12081 12491 12077 12491 12072 12492 12069 12495 12067 12490 12065 12494
12059 12493 37 P
12125 12474 12120 12475 12122 12471 12127 12470 12131 12471 12135 12470
12138 12468 12135 12465 12139 12464 12142 12461 12143 12465 12147 12466
12151 12463 12147 12460 12144 12458 12145 12453 12145 12449 12147 12453
12150 12457 12152 12460 12156 12463 12160 12462 12161 12466 12156 12466
12156 12470 12160 12469 12164 12469 12167 12466 12172 12466 12169 12470
12166 12474 12165 12478 12161 12478 12156 12479 12153 12476 12149 12474
12146 12472 12147 12477 12145 12481 12141 12482 12138 12486 12135 12490
12137 12485 12133 12485 12128 12483 12132 12479 12129 12476 12125 12474
48 P
12129 12528 12133 12525 12135 12522 12137 12517 12137 12513 12140 12510
12143 12507 12145 12511 12144 12515 12148 12514 12147 12509 12149 12505
12150 12501 12152 12505 12153 12509 12155 12513 12153 12517 12152 12521
12150 12525 12147 12529 12144 12531 12139 12531 12135 12530 12129 12528
24 P
12896 12925 12899 12922 12904 12922 12905 12927 12903 12931 12903 12935
12904 12940 12905 12944 12907 12948 12906 12952 12905 12957 12900 12956
12898 12960 12894 12962 12892 12958 12887 12957 12884 12954 12882 12951
12886 12950 12889 12947 12888 12943 12892 12939 12888 12937 12892 12935
12896 12934 12897 12930 12896 12925 27 P
12750 12787 12754 12785 12757 12781 12756 12778 12756 12773 12760 12773
12765 12772 12768 12774 12772 12777 12775 12780 12774 12785 12772 12788
12767 12787 12765 12790 12764 12795 12761 12798 12762 12802 12759 12800
12760 12804 12756 12804 12752 12801 12753 12797 12758 12798 12761 12795
12757 12797 12757 12793 12754 12789 12754 12793 12751 12797 12751 12793
12750 12787 31 P
12763 12875 12765 12871 12768 12868 12772 12868 12770 12872 12773 12875
12770 12878 12767 12881 12770 12885 12773 12888 12771 12892 12767 12893
12763 12892 12761 12887 12764 12884 12765 12879 12763 12875 17 P
12616 12800 12620 12799 12624 12798 12624 12793 12628 12793 12631 12796
12632 12800 12635 12803 12639 12803 12644 12803 12643 12808 12639 12809
12635 12810 12631 12812 12626 12811 12623 12809 12618 12808 12616 12800

18 P
12633 12789 12637 12788 12638 12784 12642 12784 12645 12781 12649 12780
12653 12779 12654 12783 12651 12786 12655 12787 12659 12788 12663 12791
12659 12794 12656 12795 12653 12799 12650 12802 12645 12803 12642 12802
12638 12799 12636 12795 12638 12792 12633 12789 22 P
12656 12691 12659 12688 12663 12687 12663 12682 12665 12678 12667 12682
12672 12683 12673 12687 12678 12686 12682 12686 12686 12686 12686 12690
12689 12694 12687 12697 12683 12696 12680 12692 12679 12688 12676 12691
12679 12694 12676 12697 12671 12697 12668 12700 12664 12702 12659 12701
12658 12696 12656 12691 26 P
12537 12829 12535 12825 12534 12821 12532 12817 12529 12813 12528 12809
12524 12806 12523 12802 12524 12798 12525 12794 12528 12790 12532 12789
12536 12787 12541 12787 12545 12788 12549 12790 12550 12794 12554 12795
12558 12794 12562 12792 12566 12792 12570 12790 12574 12791 12577 12788
12582 12789 12581 12793 12577 12795 12572 12797 12569 12800 12569 12804
12570 12808 12569 12813 12566 12815 12562 12816 12558 12819 12559 12823
12555 12823 12552 12821 12551 12816 12550 12812 12550 12808 12548 12804
12545 12807 12544 12811 12545 12815 12546 12820 12548 12823 12550 12827
12551 12832 12550 12836 50 P
12550 12836 12546 12835 12542 12833 12537 12829 4 P
12398 12618 12400 12622 12402 12619 12405 12616 12409 12615 12413 12618
12417 12620 12417 12624 12421 12621 12425 12622 12428 12624 12425 12627
12422 12630 12418 12627 12414 12627 12412 12631 12408 12632 12404 12630
12400 12628 12398 12624 12398 12618 21 P
12310 12584 12314 12582 12318 12582 12319 12578 12322 12575 12318 12574
12315 12571 12317 12567 12320 12563 12324 12563 12327 12567 12331 12567
12336 12569 12340 12568 12342 12572 12342 12576 12339 12579 12334 12581
12330 12581 12326 12581 12329 12583 12327 12587 12323 12588 12326 12591
12329 12595 12327 12598 12323 12599 12319 12600 12316 12596 12314 12593
12313 12589 12310 12584 32 P
12126 12463 12122 12462 12118 12463 12113 12462 12110 12459 12107 12456
12108 12451 12108 12447 12113 12446 12114 12442 12118 12444 12122 12445
12125 12449 12129 12449 12131 12453 12135 12456 12133 12459 12126 12463
18 P
12268 12321 12270 12317 12272 12313 12275 12310 12276 12306 12279 12303
12282 12300 12287 12300 12291 12299 12294 12303 12289 12302 12286 12304
12284 12308 12280 12311 12279 12315 12277 12319 12279 12323 12277 12327
12274 12325 12268 12321 20 P
12281 12324 12284 12321 12286 12317 12290 12315 12294 12313 12295 12318
12292 12320 12292 12324 12288 12325 12281 12324 10 P
12107 12261 12108 12258 12113 12258 12117 12259 12119 12263 12118 12266
12114 12268 12109 12268 12107 12261 9 P
11834 12216 11838 12214 11840 12211 11843 12207 11847 12207 11845 12211

11846 12215 11851 12216 11851 12220 11847 12222 11844 12219 11839 12220
11834 12216 13 P
11824 12221 11828 12219 11832 12217 11833 12221 11830 12225 11826 12226
11824 12221 7 P
11903 12351 11906 12350 11908 12353 11910 12356 11913 12356 11914 12359
11916 12362 11913 12364 11911 12366 11910 12363 11907 12360 11907 12357
11906 12354 11903 12351 14 P
11949 12386 11952 12385 11954 12387 11957 12386 11959 12389 11963 12389
11962 12392 11960 12395 11958 12396 11954 12395 11952 12393 11949 12392
11949 12386 13 P
11952 12312 11956 12312 11959 12312 11962 12311 11961 12314 11959 12317
11958 12320 11955 12322 11952 12319 11954 12317 11952 12312 11 P
12022 12367 12025 12366 12027 12363 12028 12366 12029 12370 12031 12372
12032 12375 12030 12378 12028 12381 12025 12382 12023 12380 12021 12377
12023 12373 12022 12367 14 P
11273 12232 11277 12230 11281 12230 11285 12228 11288 12225 11290 12221
11293 12217 11297 12219 11301 12219 11303 12224 11299 12226 11296 12229
11293 12232 11292 12237 11293 12240 11288 12241 11286 12245 11282 12244
11279 12241 11279 12237 11279 12232 11273 12232 22 P
11439 12323 11441 12320 11442 12317 11445 12319 11448 12318 11450 12320
11447 12321 11445 12324 11442 12323 11439 12323 10 P
11685 12391 11689 12392 11689 12388 11693 12387 11697 12387 11702 12386
11704 12383 11704 12387 11706 12391 11707 12387 11710 12383 11712 12387
11716 12388 11720 12386 11719 12391 11718 12395 11717 12399 11717 12403
11713 12402 11709 12402 11705 12400 11701 12401 11697 12404 11693 12406
11691 12402 11687 12402 11690 12399 11687 12397 11685 12391 29 P
11692 12358 11695 12358 11697 12356 11700 12358 11703 12358 11707 12357
11708 12359 11709 12363 11712 12363 11710 12366 11709 12369 11706 12370
11703 12372 11704 12369 11704 12366 11702 12364 11698 12363 11695 12362
11692 12363 11692 12358 20 P
11648 12356 11649 12353 11652 12353 11655 12353 11658 12356 11660 12358
11661 12361 11658 12362 11655 12360 11653 12362 11650 12360 11648 12356
12:00 PM
11634 12360 11637 12360 11640 12358 11640 12361 11643 12364 11644 12366
11646 12363 11645 12360 11648 12362 11648 12365 11647 12368 11643 12369
11640 12368 11637 12366 11635 12364 11634 12360 16 P
11675 12417 11672 12415 11673 12412 11673 12409 11676 12408 11679 12407
11683 12407 11686 12408 11687 12411 11690 12410 11693 12412 11696 12410
11699 12410 11702 12408 11703 12411 11701 12413 11698 12414 11695 12416
11692 12417 11689 12419 11687 12417 11683 12417 11682 12414 11679 12414
11675 12417 25 P
11679 12376 11678 12378 11676 12381 11673 12382 11670 12380 11669 12377
11670 12375 11674 12375 11675 12372 11677 12370 11680 12369 11680 12372

11682 12375 11682 12378 11679 12376 15 P
11758 12370 11760 12367 11763 12367 11761 12370 11762 12373 11763 12376
11765 12379 11763 12382 11761 12379 11759 12377 11759 12373 11758 12370
12:00 PM
11382 12555 11385 12551 11387 12548 11391 12545 11395 12545 11394 12550
11395 12554 11398 12556 11401 12560 11400 12564 11404 12565 11407 12568
11409 12572 11408 12576 11412 12580 11415 12581 11414 12585 11410 12583
11407 12580 11403 12580 11402 12584 11406 12586 11401 12587 11397 12586
11393 12585 11391 12581 11391 12577 11388 12574 11387 12569 11386 12565
11383 12562 11386 12559 11382 12555 33 P
11408 12602 11410 12600 11413 12599 11414 12596 11416 12598 11416 12601
11418 12604 11421 12603 11421 12607 11418 12607 11415 12609 11412 12611
11413 12608 11410 12605 11408 12602 15 P
11288 12562 11291 12558 11293 12563 11295 12567 11299 12567 11301 12571
11302 12575 11304 12579 11302 12583 11298 12585 11297 12581 11295 12577
11294 12573 11290 12570 11288 12567 11288 12562 16 P
11345 12433 11347 12430 11348 12428 11351 12430 11349 12433 11345 12433
6:00 PM
11345 12437 11348 12437 11351 12439 11353 12441 11352 12444 11349 12444
11348 12442 11345 12437 8 P
11348 12419 11350 12417 11352 12414 11354 12411 11355 12408 11358 12410
11361 12409 11363 12410 11362 12413 11359 12415 11356 12416 11354 12414
11353 12417 11348 12419 14 P
11371 12570 11373 12568 11376 12569 11379 12571 11379 12575 11375 12575
11373 12574 11371 12570 8 P
11379 12576 11382 12576 11383 12579 11386 12581 11383 12581 11379 12576
6:00 PM
11402 12607 11405 12605 11407 12608 11405 12611 11402 12610 11402 12607
6:00 PM
11571 12560 11575 12558 11577 12555 11582 12555 11580 12552 11575 12550
11578 12547 11582 12548 11586 12550 11590 12549 11593 12552 11597 12553
11598 12558 11596 12562 11596 12566 11591 12567 11589 12564 11585 12562
11582 12559 11577 12559 11571 12560 21 P
11655 12614 11651 12616 11649 12620 11644 12620 11642 12623 11640 12627
11638 12630 11635 12627 11634 12623 11631 12620 11634 12617 11632 12613
11631 12617 11627 12618 11623 12616 11618 12615 11617 12610 11617 12606
11617 12602 11618 12597 11621 12594 11625 12597 11627 12593 11630 12590
11634 12591 11637 12588 11641 12587 11645 12588 11649 12591 11648 12595
11652 12597 11655 12601 11656 12605 11660 12604 11661 12608 11663 12613
11655 12614 37 P
11651 12653 11647 12651 11649 12655 11651 12660 11652 12664 11650 12668
11647 12670 11643 12668 11638 12667 11637 12662 11635 12659 11631 12659
11630 12655 11626 12653 11622 12651 11618 12650 11613 12650 11610 12653

11606 12653 11606 12648 11603 12645 11605 12641 11604 12637 11604 12633
11603 12628 11606 12625 11609 12622 11612 12618 11616 12617 11621 12618
11624 12620 11628 12622 11627 12627 11631 12627 11628 12630 11627 12635
11623 12637 11622 12641 11627 12643 11630 12644 11635 12645 11638 12647
11640 12643 11644 12643 11648 12645 11652 12646 11651 12653 47 P
11414 12556 11417 12556 11420 12557 11422 12560 11425 12562 11426 12565
11422 12565 11419 12565 11418 12562 11415 12560 11414 12556 11 P
11396 12616 11399 12614 11402 12614 11400 12617 11396 12616 5 P
11602 12561 11604 12557 11607 12556 11609 12554 11611 12551 11611 12555
11610 12558 11607 12558 11602 12561 9 P
11600 12561 11601 12557 11601 12554 11603 12556 11600 12561 5 P
11635 12573 11637 12571 11640 12569 11643 12571 11645 12569 11648 12567
11650 12568 11650 12571 11649 12574 11646 12576 11644 12579 11642 12580
11639 12578 11636 12577 11635 12573 15 P
11660 12575 11656 12574 11657 12570 11659 12568 11656 12567 11655 12563
11657 12562 11659 12564 11660 12561 11662 12563 11665 12564 11665 12568
11664 12571 11662 12573 11660 12575 15 P
11666 12562 11667 12558 11670 12558 11673 12556 11671 12553 11669 12551
11669 12548 11671 12545 11671 12542 11673 12539 11676 12540 11677 12543
11678 12545 11681 12544 11684 12543 11688 12543 11690 12545 11691 12548
11691 12551 11690 12554 11687 12555 11687 12558 11687 12562 11683 12561
11680 12561 11678 12564 11677 12567 11678 12569 11678 12573 11678 12576
11675 12577 11672 12578 11669 12578 11665 12577 11664 12574 11667 12571
11668 12568 11667 12565 11666 12562 39 P
11676 12523 11681 12522 11685 12522 11689 12522 11693 12524 11696 12528
11700 12529 11703 12532 11702 12536 11707 12537 11709 12541 11707 12545
11709 12549 11709 12553 11712 12556 11709 12559 11705 12561 11708 12564
11703 12567 11699 12568 11695 12569 11695 12574 11691 12576 11687 12576
11683 12576 11682 12572 11685 12569 11689 12568 11691 12564 11694 12560
11694 12556 11698 12553 11702 12552 11698 12550 11696 12547 11697 12542
11695 12539 11690 12538 11687 12541 11682 12541 11679 12537 11675 12535
11679 12533 11682 12536 11686 12535 11690 12536 11689 12531 11685 12530
11683 12527 11679 12528 50 P
11679 12528 11676 12523 2 P
11642 12552 11645 12553 11649 12553 11649 12550 11648 12547 11649 12544
11651 12544 11651 12548 11652 12551 11651 12554 11654 12556 11650 12557
11648 12556 11644 12555 11642 12552 15 P
11710 12588 11715 12588 11717 12584 11716 12580 11717 12576 11716 12572
11720 12571 11723 12574 11728 12575 11730 12579 11734 12580 11737 12577
11741 12580 11744 12584 11748 12584 11750 12588 11746 12589 11742 12590
11743 12594 11741 12598 11737 12601 11734 12597 11731 12594 11727 12594
11730 12597 11731 12602 11728 12604 11723 12603 11721 12599 11717 12597
11716 12593 11711 12592 11710 12588 33 P

11681 12652 11684 12650 11688 12647 11690 12643 11692 12640 11697 12640
11701 12638 11699 12634 11699 12629 11704 12629 11707 12627 11710 12629
11713 12633 11717 12634 11718 12629 11722 12627 11721 12622 11725 12621
11730 12620 11734 12621 11736 12625 11735 12629 11735 12634 11733 12637
11730 12641 11733 12644 11729 12646 11726 12650 11723 12651 11719 12649
11715 12649 11711 12651 11710 12656 11705 12656 11702 12654 11697 12656
11693 12657 11691 12653 11687 12654 11681 12652 40 P
11687 12665 11691 12663 11695 12662 11699 12660 11703 12662 11707 12662
11710 12659 11713 12655 11716 12652 11719 12654 11724 12655 11725 12659
11725 12663 11723 12667 11726 12670 11724 12674 11719 12674 11719 12679
11716 12681 11714 12677 11716 12673 11711 12673 11708 12671 11704 12669
11699 12668 11695 12667 11687 12665 27 P
11674 12697 11677 12693 11679 12690 11682 12686 11683 12682 11687 12680
11691 12678 11693 12674 11692 12670 11697 12670 11701 12672 11703 12675
11705 12680 11702 12683 11701 12687 11703 12692 11707 12690 11711 12688
11711 12692 11715 12695 11715 12699 11711 12701 11708 12704 11712 12706
11716 12707 11716 12711 11712 12710 11712 12715 11709 12718 11706 12715
11702 12717 11698 12718 11695 12715 11696 12711 11698 12707 11701 12703
11699 12700 11695 12700 11690 12701 11687 12704 11683 12702 11681 12699
11674 12697 43 P
11645 12715 11644 12711 11642 12707 11637 12707 11632 12707 11637 12705
11635 12701 11639 12700 11643 12700 11647 12702 11650 12700 11654 12703
11657 12706 11657 12710 11657 12706 11657 12702 11661 12701 11663 12697
11667 12694 11670 12697 11672 12700 11677 12701 11678 12705 11683 12706
11683 12710 11682 12714 11686 12713 11690 12714 11691 12719 11688 12721
11684 12722 11684 12727 11688 12729 11684 12732 11685 12736 11681 12736
11678 12741 11675 12743 11671 12745 11670 12740 11671 12736 11670 12732
11667 12730 11663 12733 11660 12730 11656 12728 11652 12730 11650 12726
11649 12722 11645 12715 50 P
11679 12674 11681 12670 11683 12668 11687 12669 11684 12672 11683 12675
11679 12674 7 P
11668 12683 11669 12680 11671 12677 11674 12677 11676 12674 11679 12675
11679 12679 11677 12681 11674 12682 11671 12684 11668 12683 11 P
11659 12638 11660 12635 11661 12632 11662 12629 11664 12626 11667 12626
11668 12629 11667 12632 11665 12635 11665 12638 11659 12638 11 P
11729 12609 11732 12609 11735 12608 11738 12610 11739 12613 11742 12610
11744 12613 11745 12616 11742 12618 11745 12620 11746 12623 11743 12624
11740 12623 11737 12621 11736 12618 11736 12615 11733 12615 11730 12615
11729 12609 19 P
11698 12618 11700 12616 11703 12616 11705 12619 11707 12622 11704 12624
11701 12623 11698 12618 8 P
11685 12635 11688 12634 11690 12637 11691 12640 11689 12642 11687 12640
11685 12635 7 P

11670 12645 11674 12646 11676 12647 11678 12650 11678 12653 11675 12651
11674 12649 11670 12645 8 P
11685 12603 11687 12600 11689 12602 11690 12605 11692 12607 11693 12610
11690 12610 11687 12608 11685 12603 9 P
11678 12590 11678 12587 11679 12584 11681 12587 11682 12590 11681 12594
11680 12597 11677 12595 11678 12590 9 P
11682 12494 11684 12492 11686 12489 11685 12486 11687 12483 11690 12485
11692 12488 11692 12491 11696 12492 11698 12495 11698 12498 11695 12496
11694 12499 11697 12501 11694 12502 11692 12505 11689 12503 11687 12501
11687 12497 11685 12495 11682 12494 21 P
11799 12445 11802 12444 11805 12443 11804 12446 11808 12446 11811 12448
11813 12450 11813 12453 11811 12455 11808 12453 11810 12456 11809 12459
11806 12458 11803 12456 11800 12454 11802 12451 11799 12445 17 P
11643 12428 11647 12429 11648 12431 11649 12435 11645 12435 11644 12431
11643 12428 7 P
11656 12428 11659 12428 11661 12431 11661 12434 11659 12437 11657 12435
11656 12428 7 P
11655 12396 11658 12396 11661 12398 11660 12400 11657 12399 11655 12396
6:00 PM
11662 12393 11664 12391 11667 12391 11671 12391 11669 12393 11666 12395
11662 12393 7 P
11662 12403 11663 12399 11664 12396 11667 12397 11668 12400 11666 12403
11662 12403 7 P
11650 12415 11654 12416 11657 12416 11656 12419 11654 12421 11651 12418
11650 12415 7 P
11680 12425 11683 12423 11681 12421 11683 12419 11687 12419 11686 12422
11685 12425 11683 12428 11680 12425 9 P
11676 12404 11677 12401 11679 12398 11682 12400 11685 12401 11685 12404
11682 12404 11676 12404 8 P
11613 12342 11616 12339 11618 12338 11621 12337 11621 12340 11618 12342
11613 12342 7 P
11656 12314 11658 12311 11659 12308 11660 12305 11662 12307 11660 12310
11660 12313 11658 12317 11656 12314 9 P
11749 12703 11754 12704 11758 12702 11762 12701 11766 12701 11769 12704
11770 12709 11775 12710 11779 12709 11783 12710 11782 12714 11786 12713
11788 12709 11792 12709 11796 12708 11798 12704 11802 12703 11800 12700
11797 12696 11799 12693 11803 12692 11805 12696 11809 12694 11812 12697
11811 12702 11813 12706 11813 12710 11812 12714 11809 12716 11805 12719
11800 12718 11796 12717 11792 12716 11788 12717 11790 12721 11786 12723
11790 12726 11791 12730 11795 12729 11796 12733 11800 12733 11802 12737
11798 12737 11796 12741 11791 12742 11787 12741 11783 12741 11781 12746
11777 12746 11772 12745 50 P
11772 12745 11770 12741 11772 12737 11776 12735 11779 12733 11781 12728

11777 12726 11777 12721 11773 12722 11773 12717 11770 12715 11768 12719
11765 12715 11764 12711 11759 12711 11755 12710 11752 12713 11749 12709
11749 12703 19 P
11758 12770 11762 12767 11764 12763 11766 12759 11768 12755 11772 12753
11776 12755 11775 12759 11773 12763 11771 12767 11768 12769 11764 12772
11758 12770 13 P
11742 12741 11745 12739 11750 12737 11752 12741 11756 12741 11756 12746
11757 12750 11753 12752 11749 12752 11744 12752 11742 12748 11742 12741
12:00 PM
11652 12813 11656 12813 11661 12813 11661 12808 11664 12812 11667 12815
11669 12811 11672 12809 11677 12808 11681 12806 11683 12810 11686 12813
11684 12816 11687 12820 11689 12824 11685 12825 11681 12827 11681 12832
11681 12836 11685 12838 11686 12842 11683 12846 11686 12849 11685 12853
11689 12855 11694 12856 11697 12859 11701 12861 11697 12863 11695 12859
11690 12859 11686 12859 11682 12859 11681 12854 11678 12851 11675 12848
11674 12844 11671 12840 11667 12838 11663 12837 11658 12835 11656 12832
11655 12828 11653 12824 11657 12822 11661 12820 11658 12816 11652 12813
48 P
11704 12832 11708 12831 11711 12828 11709 12823 11714 12823 11715 12819
11719 12818 11724 12819 11726 12816 11731 12816 11735 12818 11738 12815
11742 12817 11744 12813 11740 12811 11741 12806 11745 12806 11746 12802
11749 12799 11749 12803 11754 12801 11755 12805 11758 12808 11755 12811
11750 12812 11751 12816 11750 12821 11746 12823 11743 12820 11739 12821
11737 12825 11733 12827 11732 12831 11736 12834 11739 12832 11742 12835
11741 12840 11737 12840 11734 12836 11733 12840 11730 12844 11726 12841
11725 12836 11721 12837 11717 12840 11716 12844 11712 12842 11708 12841
11705 12837 11704 12832 50 P
11693 12808 11697 12808 11698 12803 11702 12802 11706 12803 11709 12801
11712 12797 11714 12801 11714 12806 11715 12801 11716 12797 11719 12800
11719 12805 11718 12809 11716 12813 11711 12814 11707 12813 11703 12813
11699 12812 11693 12808 20 P
11683 12780 11686 12778 11690 12780 11695 12781 11699 12781 11703 12779
11707 12781 11707 12777 11702 12778 11701 12774 11702 12769 11703 12765
11701 12761 11699 12757 11698 12753 11702 12754 11706 12756 11707 12760
11705 12764 11710 12765 11714 12768 11717 12766 11717 12770 11721 12771
11720 12775 11720 12780 11717 12783 11713 12784 11711 12788 11710 12792
11707 12795 11703 12793 11699 12795 11695 12795 11695 12791 11691 12789
11689 12785 11683 12780 38 P
11750 12787 11752 12785 11752 12781 11752 12778 11750 12775 11753 12774
11756 12775 11759 12775 11762 12777 11761 12780 11761 12783 11758 12785
11756 12788 11754 12790 11751 12790 11748 12792 11750 12787 17 P
11730 12768 11732 12765 11733 12762 11736 12760 11738 12763 11737 12767
11737 12770 11737 12773 11738 12776 11735 12775 11731 12774 11731 12771

11730 12768 13 P
11717 13106 11719 13103 11722 13099 11725 13102 11729 13104 11732 13107
11735 13111 11737 13114 11741 13116 11743 13119 11744 13123 11742 13127
11739 13130 11739 13125 11736 13123 11731 13123 11727 13125 11730 13121
11725 13120 11726 13116 11723 13113 11722 13109 11717 13106 23 P
11682 12938 11683 12935 11686 12933 11687 12930 11690 12931 11692 12933
11691 12937 11691 12940 11689 12943 11687 12944 11684 12943 11682 12938
12:00 PM
11623 12964 11626 12962 11627 12959 11630 12960 11633 12960 11636 12962
11638 12964 11637 12967 11635 12971 11632 12971 11630 12974 11628 12972
11624 12972 11624 12969 11628 12968 11630 12966 11627 12965 11623 12964
18 P
11621 12958 11619 12955 11618 12952 11621 12954 11624 12955 11621 12958
6:00 PM
11636 12979 11640 12978 11641 12980 11639 12983 11636 12979 5 P
11687 12892 11690 12892 11693 12892 11695 12895 11697 12897 11694 12898
11692 12895 11689 12894 11687 12892 9 P
11682 12884 11685 12885 11686 12888 11685 12892 11682 12891 11682 12887
11682 12884 7 P
11638 12826 11641 12823 11643 12821 11645 12820 11648 12822 11648 12825
11646 12828 11644 12831 11642 12833 11640 12831 11638 12826 11 P
11652 13130 11655 13129 11658 13130 11658 13128 11661 13129 11661 13131
11658 13133 11656 13134 11654 13133 11652 13130 10 P
11647 13140 11650 13139 11651 13142 11651 13144 11649 13144 11647 13140
6:00 PM
11793 13201 11796 13199 11797 13196 11799 13194 11800 13196 11801 13199
11799 13202 11797 13204 11793 13201 9 P
11870 13234 11872 13231 11875 13230 11877 13232 11878 13236 11879 13239
11877 13242 11873 13241 11871 13238 11870 13234 10 P
11789 13247 11790 13244 11792 13242 11792 13238 11796 13237 11795 13241
11798 13242 11800 13239 11803 13239 11806 13240 11809 13242 11806 13244
11804 13245 11800 13247 11797 13247 11795 13249 11791 13250 11789 13247
18 P
11765 13239 11768 13238 11768 13235 11771 13236 11771 13232 11774 13234
11777 13234 11778 13237 11776 13239 11779 13241 11778 13244 11777 13247
11773 13248 11770 13247 11770 13244 11773 13243 11776 13241 11774 13238
11771 13237 11770 13240 11765 13239 21 P
11778 13229 11781 13228 11784 13228 11786 13230 11789 13232 11790 13235
11788 13237 11786 13235 11785 13232 11782 13231 11783 13234 11781 13236
11779 13232 11778 13229 14 P
11756 13234 11758 13231 11760 13229 11763 13230 11763 13234 11761 13236
11758 13238 11756 13234 8 P
11750 13254 11754 13253 11757 13254 11759 13255 11762 13257 11759 13258

11756 13258 11754 13255 11750 13254 9 P
11771 13261 11774 13261 11777 13261 11781 13260 11778 13263 11775 13262
11771 13261 7 P
11741 13255 11744 13254 11748 13254 11746 13257 11741 13255 5 P
11806 13258 11808 13256 11811 13257 11812 13260 11809 13260 11806 13258

6:00 PM

11817 13316 11819 13315 11822 13314 11823 13316 11822 13318 11819 13318
11817 13316 7 P
11843 13322 11845 13322 11848 13321 11849 13318 11852 13318 11850 13320
11849 13323 11846 13324 11843 13322 9 P
11828 13328 11830 13327 11832 13326 11835 13324 11837 13325 11839 13325
11841 13328 11838 13329 11836 13330 11833 13330 11830 13330 11828 13328

12:00 PM

11818 13366 11821 13365 11824 13364 11826 13363 11827 13366 11827 13369
11826 13371 11823 13371 11822 13369 11818 13366 10 P
11768 13386 11769 13383 11771 13382 11774 13382 11777 13381 11776 13384
11775 13386 11772 13388 11770 13387 11768 13386 10 P
11563 13388 11564 13386 11566 13383 11567 13381 11570 13380 11572 13381
11571 13383 11575 13383 11575 13381 11577 13383 11577 13386 11575 13387
11572 13388 11570 13389 11567 13389 11563 13388 16 P
11583 13442 11584 13440 11587 13438 11589 13436 11589 13439 11588 13442
11583 13442 7 P
11601 13444 11604 13444 11607 13445 11609 13446 11611 13447 11614 13448
11614 13450 11612 13452 11609 13452 11607 13452 11604 13450 11603 13448
11601 13444 13 P
11552 13450 11553 13448 11554 13446 11556 13443 11558 13443 11558 13446
11560 13448 11558 13450 11556 13448 11554 13449 11552 13450 11 P
11715 13515 11718 13515 11720 13516 11722 13515 11724 13516 11726 13519
11726 13521 11724 13523 11723 13525 11724 13528 11723 13530 11720 13531
11719 13528 11719 13525 11717 13523 11715 13521 11715 13519 11715 13515
18 P
11731 13541 11732 13538 11735 13537 11737 13539 11740 13539 11742 13538
11745 13538 11748 13537 11750 13538 11753 13537 11756 13536 11758 13535
11761 13535 11763 13535 11765 13536 11765 13539 11762 13539 11759 13538
11757 13540 11755 13540 11756 13543 11755 13546 11753 13547 11752 13550
11750 13550 11747 13550 11745 13548 11744 13546 11745 13543 11742 13543
11740 13545 11737 13546 11735 13546 11732 13546 11731 13541 35 P
11678 13493 11679 13490 11681 13488 11682 13490 11684 13492 11684 13495
11686 13497 11684 13498 11681 13498 11680 13495 11681 13493 11678 13493

12:00 PM

11683 13480 11684 13477 11687 13477 11687 13480 11688 13482 11688 13485
11685 13485 11684 13483 11683 13480 9 P
11569 13688 11571 13686 11573 13687 11575 13689 11577 13690 11580 13689

11583 13690 11583 13693 11586 13694 11584 13697 11584 13700 11582 13701 11582 13698 11581 13695 11579 13694 11576 13694 11574 13693 11572 13691 11569 13688 19 P
11555 13988 11559 13990 11560 13995 11562 13991 11567 13989 11567 13985 11571 13984 11576 13985 11580 13987 11581 13991 11585 13993 11588 13996 11586 14000 11582 14001 11577 14003 11574 14005 11569 14004 11568 14008 11564 14010 11563 14015 11560 14017 11560 14013 11558 14009 11557 14005 11556 14001 11557 13997 11557 13992 11555 13988 28 P
11587 13992 11588 13990 11589 13987 11591 13986 11594 13986 11596 13985 11597 13982 11597 13979 11600 13981 11601 13984 11602 13985 11601 13987 11598 13987 11597 13989 11595 13991 11594 13994 11591 13995 11587 13992 18 P
11701 14026 11703 14022 11704 14018 11705 14014 11706 14009 11710 14012 11713 14015 11715 14011 11715 14007 11718 14009 11721 14012 11723 14016 11725 14020 11726 14024 11731 14025 11731 14029 11729 14034 11729 14038 11724 14039 11720 14039 11716 14039 11711 14038 11711 14034 11707 14032 11703 14032 11701 14026 26 P
11749 13994 11752 13996 11755 14000 11759 13999 11762 13995 11765 13997 11765 14002 11767 14005 11768 14010 11772 14009 11776 14009 11777 14013 11780 14016 11784 14017 11785 14021 11784 14025 11782 14029 11781 14033 11777 14034 11773 14034 11769 14035 11766 14038 11762 14037 11761 14042 11763 14045 11763 14050 11761 14046 11759 14042 11755 14040 11752 14043 11748 14044 11750 14041 11753 14037 11756 14034 11757 14030 11759 14026 11759 14022 11760 14017 11756 14016 11752 14014 11754 14009 11758 14010 11756 14006 11754 14002 11751 13999 11749 13994 46 P
11636 14018 11641 14017 11644 14015 11649 14014 11652 14013 11657 14012 11661 14013 11665 14016 11667 14020 11663 14022 11661 14018 11656 14018 11652 14021 11648 14021 11644 14022 11640 14024 11636 14018 17 P
11646 13971 11648 13968 11650 13967 11654 13965 11657 13965 11660 13965 11659 13968 11657 13970 11654 13971 11651 13972 11646 13971 11 P
11626 13970 11628 13968 11631 13968 11634 13967 11637 13966 11639 13963 11642 13962 11643 13965 11641 13966 11638 13969 11638 13972 11635 13973 11632 13975 11630 13972 11626 13970 15 P
11613 13975 11615 13972 11618 13971 11621 13972 11624 13971 11626 13974 11624 13977 11621 13976 11618 13975 11613 13975 10 P
11557 13964 11560 13962 11561 13959 11564 13958 11568 13959 11570 13961 11568 13964 11570 13967 11567 13967 11565 13969 11562 13971 11559 13969 11557 13964 13 P
11551 13919 11554 13920 11557 13918 11554 13918 11553 13915 11556 13913 11559 13913 11561 13916 11563 13919 11564 13922 11563 13925 11560 13927 11557 13925 11554 13925 11553 13922 11551 13919 16 P
11500 13903 11502 13901 11504 13898 11505 13902 11506 13905 11506 13908 11509 13907 11512 13908 11510 13911 11507 13912 11504 13913 11502 13911

11502 13908 11500 13903 14 P
11529 13879 11526 13879 11526 13883 11523 13885 11520 13886 11519 13883
11520 13880 11523 13878 11526 13876 11527 13873 11530 13875 11533 13877
11536 13879 11533 13878 11529 13879 15 P
11504 13961 11508 13960 11510 13962 11513 13964 11516 13964 11514 13967
11517 13968 11519 13971 11515 13971 11514 13969 11510 13968 11509 13965
11506 13964 11504 13961 14 P
11689 13939 11691 13937 11694 13935 11695 13938 11695 13942 11693 13944
11690 13944 11689 13939 8 P
11707 13916 11709 13913 11710 13910 11710 13907 11714 13906 11716 13907
11719 13908 11722 13910 11721 13913 11720 13916 11717 13918 11716 13916
11712 13916 11711 13919 11707 13916 15 P
11679 14095 11683 14094 11685 14091 11685 14088 11685 14085 11688 14082
11690 14081 11694 14082 11697 14083 11697 14087 11697 14090 11699 14091
11697 14094 11695 14095 11692 14098 11690 14100 11687 14102 11685 14104
11682 14105 11682 14102 11681 14099 11679 14095 22 P
11658 14131 11660 14128 11663 14128 11664 14124 11665 14121 11668 14120
11670 14117 11673 14119 11674 14122 11677 14123 11679 14121 11679 14117
11682 14119 11684 14117 11687 14115 11688 14112 11690 14115 11692 14117
11692 14121 11692 14123 11690 14127 11688 14128 11685 14130 11683 14132
11679 14133 11676 14134 11676 14130 11673 14129 11670 14130 11668 14132
11665 14135 11663 14137 11660 14137 11658 14134 11658 14131 35 P
11711 14145 11713 14141 11716 14139 11721 14139 11723 14143 11727 14143
11730 14146 11734 14148 11738 14147 11743 14148 11746 14150 11748 14154
11750 14157 11750 14162 11747 14165 11745 14169 11741 14170 11737 14172
11732 14172 11728 14173 11724 14175 11721 14177 11717 14175 11715 14171
11716 14167 11715 14162 11713 14159 11714 14154 11713 14150 11711 14145
30 P
11693 14153 11696 14150 11700 14149 11702 14144 11701 14140 11705 14140
11704 14144 11707 14148 11709 14151 11707 14155 11702 14154 11698 14154
11693 14153 13 P
11687 14164 11690 14163 11692 14165 11696 14166 11699 14165 11701 14163
11704 14162 11704 14166 11704 14169 11705 14172 11704 14175 11702 14177
11699 14180 11696 14178 11696 14175 11693 14173 11690 14172 11688 14169
11687 14164 19 P
11709 14130 11711 14128 11715 14127 11716 14130 11716 14134 11714 14136
11711 14134 11709 14130 8 P
11632 14181 11634 14178 11637 14176 11639 14174 11642 14172 11643 14169
11642 14166 11645 14164 11649 14165 11652 14166 11655 14167 11658 14167
11661 14168 11664 14167 11667 14170 11666 14173 11663 14172 11660 14171
11657 14170 11654 14170 11650 14172 11648 14174 11647 14176 11643 14177
11641 14179 11637 14180 11632 14181 27 P
11681 14167 11684 14166 11685 14169 11686 14173 11686 14176 11689 14178

11686 14180 11683 14179 11683 14175 11682 14172 11681 14167 11 P
11674 14170 11676 14168 11677 14170 11679 14173 11679 14175 11677 14174
11674 14170 7 P
11637 14308 11640 14302 11642 14295 11642 14289 11642 14282 11643 14275
11642 14268 11645 14262 11652 14261 11658 14260 11661 14254 11665 14249
11668 14243 11670 14236 11667 14230 11661 14228 11654 14226 11656 14220
11656 14213 11662 14210 11669 14208 11676 14209 11682 14208 11689 14206
11696 14205 11696 14211 11696 14218 11702 14215 11708 14211 11714 14210
11719 14215 11724 14220 11725 14227 11723 14233 11721 14240 11723 14246
11717 14250 11717 14257 11717 14264 11716 14271 11712 14276 11712 14283
11715 14289 11712 14296 11709 14301 11707 14308 11702 14313 11696 14315
11690 14319 11684 14322 50 P
11684 14322 11678 14326 11674 14331 11667 14333 11661 14329 11655 14329
11652 14322 11649 14317 11643 14313 11637 14308 10 P
11607 14262 11610 14259 11614 14256 11617 14254 11621 14252 11625 14250
11629 14248 11632 14246 11636 14243 11636 14239 11634 14235 11637 14232
11641 14229 11643 14232 11647 14235 11649 14238 11652 14241 11655 14245
11655 14249 11654 14253 11650 14256 11647 14258 11642 14258 11638 14259
11635 14261 11631 14263 11628 14267 11628 14271 11628 14275 11629 14280
11631 14283 11629 14287 11625 14286 11621 14283 11617 14282 11614 14279
11614 14275 11615 14271 11615 14266 11615 14262 11607 14262 41 P
11576 14291 11580 14288 11583 14286 11587 14284 11591 14285 11593 14289
11597 14290 11601 14291 11605 14294 11609 14294 11613 14291 11616 14293
11620 14295 11624 14298 11625 14302 11628 14306 11629 14309 11628 14314
11626 14317 11622 14314 11618 14313 11614 14313 11610 14313 11606 14315
11602 14318 11598 14319 11594 14318 11590 14318 11587 14315 11583 14312
11583 14308 11581 14304 11583 14300 11581 14296 11576 14291 35 P
11591 14228 11593 14225 11595 14222 11597 14220 11600 14219 11602 14216
11605 14216 11609 14215 11607 14218 11604 14220 11604 14223 11604 14226
11605 14229 11602 14229 11598 14229 11595 14229 11591 14228 17 P
11560 14237 11563 14235 11566 14233 11568 14234 11571 14235 11574 14233
11576 14231 11579 14229 11582 14229 11583 14232 11584 14235 11582 14236
11580 14239 11578 14242 11577 14245 11574 14245 11573 14241 11570 14240
11567 14240 11563 14240 11560 14237 21 P
11546 14242 11547 14240 11550 14240 11553 14240 11555 14241 11554 14243
11551 14244 11549 14246 11546 14242 9 P
11602 14269 11604 14269 11605 14271 11605 14274 11603 14275 11602 14273
11602 14269 7 P
11585 14265 11587 14263 11590 14263 11593 14263 11595 14264 11598 14264
11596 14266 11593 14266 11590 14266 11588 14267 11585 14265 11 P
10886 14335 10888 14332 10891 14329 10895 14326 10895 14322 10897 14318
10899 14314 10902 14311 10906 14312 10910 14312 10915 14311 10919 14312
10922 14309 10926 14311 10926 14315 10929 14319 10926 14322 10922 14323

10921 14327 10922 14331 10926 14333 10930 14331 10929 14327 10933 14327
10936 14331 10941 14330 10938 14333 10942 14333 10940 14337 10938 14341
10936 14345 10932 14347 10928 14347 10924 14346 10919 14345 10915 14344
10911 14343 10908 14341 10903 14339 10900 14337 10896 14338 10892 14340
10886 14335 43 P
10915 14258 10918 14256 10921 14254 10924 14254 10924 14251 10923 14248
10926 14249 10929 14250 10932 14250 10935 14250 10937 14253 10939 14256
10939 14259 10937 14262 10937 14265 10939 14267 10942 14269 10941 14272
10938 14273 10935 14273 10932 14275 10931 14272 10929 14269 10926 14268
10925 14265 10923 14262 10922 14259 10919 14258 10915 14258 29 P
10945 14226 10948 14227 10950 14230 10952 14232 10956 14233 10958 14232
10962 14232 10962 14235 10963 14238 10960 14238 10957 14238 10953 14236
10951 14237 10950 14234 10947 14232 10945 14226 16 P
11058 14266 11057 14262 11060 14261 11064 14262 11066 14261 11070 14261
11071 14263 11073 14266 11073 14269 11077 14268 11078 14271 11077 14274
11078 14277 11079 14280 11076 14280 11072 14280 11069 14280 11066 14279
11069 14276 11072 14275 11071 14272 11071 14269 11067 14269 11064 14270
11062 14272 11061 14268 11058 14266 27 P
11117 14261 11120 14261 11124 14260 11124 14257 11123 14254 11125 14256
11127 14259 11130 14261 11132 14262 11135 14265 11137 14267 11137 14270
11140 14269 11143 14271 11141 14273 11140 14276 11137 14275 11134 14274
11131 14273 11129 14272 11127 14268 11124 14267 11122 14265 11117 14261
24 P
11154 14212 11157 14210 11159 14208 11163 14209 11165 14212 11168 14211
11171 14213 11174 14215 11176 14217 11177 14220 11179 14222 11182 14225
11184 14227 11187 14228 11190 14230 11192 14232 11194 14235 11195 14238
11198 14240 11200 14242 11201 14246 11203 14248 11205 14251 11207 14254
11204 14253 11200 14253 11198 14252 11195 14249 11193 14247 11190 14245
11187 14246 11185 14243 11182 14242 11179 14240 11180 14237 11182 14234
11183 14231 11183 14228 11180 14230 11180 14233 11178 14235 11175 14237
11172 14236 11169 14235 11171 14232 11171 14229 11168 14227 11166 14224
11165 14221 11163 14219 50 P
11163 14219 11159 14218 11157 14216 11154 14212 4 P
11355 14216 11358 14214 11359 14212 11358 14208 11359 14205 11362 14204
11365 14206 11367 14209 11369 14212 11371 14214 11372 14217 11372 14220
11369 14221 11366 14220 11362 14219 11359 14219 11356 14220 11355 14216
18 P
11439 14279 11441 14276 11444 14277 11447 14277 11450 14278 11453 14280
11454 14283 11456 14286 11453 14285 11450 14283 11448 14281 11446 14282
11443 14281 11439 14279 14 P
11383 14288 11386 14287 11389 14287 11391 14290 11389 14293 11389 14296
11387 14294 11386 14291 11383 14288 9 P
11152 14241 11156 14240 11158 14239 11161 14237 11163 14234 11165 14236

11163 14238 11165 14241 11163 14243 11159 14244 11156 14243 11152 14241

12:00 PM

10928 14640 10931 14637 10935 14636 10939 14637 10936 14641 10938 14644
10942 14642 10946 14642 10949 14645 10951 14642 10955 14639 10959 14637
10963 14635 10967 14634 10971 14633 10971 14638 10974 14635 10978 14634
10981 14638 10979 14641 10977 14645 10975 14649 10971 14651 10971 14655
10967 14655 10963 14655 10959 14658 10955 14659 10951 14661 10948 14664
10946 14659 10943 14656 10942 14652 10937 14651 10933 14651 10933 14646
10934 14642 10928 14640 38 P
11018 14724 11017 14728 11015 14731 11014 14727 11015 14723 11012 14719
11014 14716 11018 14716 11018 14711 11015 14713 11011 14710 11010 14706
11013 14703 11016 14707 11019 14709 11023 14710 11025 14715 11025 14719
11028 14723 11028 14727 11025 14723 11018 14724 22 P
10903 15169 10907 15169 10910 15172 10914 15175 10918 15174 10922 15177
10925 15180 10927 15184 10931 15186 10934 15189 10935 15193 10939 15194
10938 15198 10942 15199 10946 15197 10949 15200 10951 15204 10950 15209
10946 15208 10942 15207 10944 15210 10941 15212 10944 15215 10939 15216
10942 15219 10938 15222 10937 15226 10941 15229 10945 15229 10944 15233
10941 15234 10937 15238 10933 15237 10929 15238 10925 15236 10922 15233
10925 15231 10924 15227 10921 15225 10924 15221 10924 15217 10921 15215
10917 15213 10916 15209 10911 15210 10908 15206 10905 15203 10904 15199
10908 15201 10912 15202 50 P
10912 15202 10916 15203 10920 15201 10917 15198 10913 15197 10915 15192
10919 15191 10919 15187 10918 15183 10914 15183 10909 15184 10909 15179
10908 15175 10903 15169 14 P
10808 15183 10812 15184 10814 15188 10817 15191 10821 15190 10824 15186
10827 15190 10827 15194 10831 15193 10832 15188 10836 15187 10840 15186
10844 15188 10847 15191 10848 15196 10851 15198 10852 15203 10848 15204
10844 15206 10840 15207 10838 15204 10834 15204 10830 15204 10827 15201
10823 15203 10820 15201 10820 15197 10817 15193 10813 15193 10811 15190
10808 15183 31 P
10846 15170 10849 15167 10852 15170 10857 15171 10856 15176 10859 15179
10856 15182 10858 15186 10854 15188 10850 15186 10848 15183 10845 15179
10848 15175 10846 15170 14 P
10810 15131 10814 15131 10813 15127 10816 15125 10819 15126 10822 15126
10823 15129 10824 15132 10821 15134 10820 15137 10820 15140 10817 15139
10815 15137 10811 15135 10810 15131 15 P
10820 15146 10823 15145 10826 15144 10825 15147 10826 15151 10823 15152
10821 15151 10820 15146 8 P
10819 15158 10822 15157 10824 15159 10825 15161 10824 15163 10822 15164
10820 15162 10819 15158 8 P
9403 15100 9404 15094 9404 15087 9410 15084 9416 15082 9423 15085
9429 15084 9433 15079 9436 15073 9439 15078 9445 15082 9449 15088

9454 15092 9460 15092 9467 15093 9473 15089 9479 15086 9486 15085
9492 15087 9496 15093 9501 15098 9508 15099 9511 15105 9515 15111
9521 15108 9527 15113 9530 15119 9531 15125 9531 15132 9531 15139
9527 15145 9526 15151 9519 15153 9513 15155 9507 15159 9501 15162
9494 15162 9498 15157 9499 15150 9495 15145 9489 15141 9483 15141
9478 15137 9472 15134 9465 15134 9458 15134 9452 15133 9445 15131
9438 15130 9433 15134 50 P
9433 15134 9431 15127 9431 15120 9426 15117 9420 15113 9417 15107
9412 15102 9403 15100 8 P
9500 15059 9497 15065 9491 15067 9484 15069 9478 15071 9471 15069
9466 15065 9462 15071 9457 15075 9450 15073 9446 15068 9443 15062
9443 15055 9438 15050 9432 15049 9425 15048 9423 15042 9425 15035
9418 15034 9422 15028 9420 15022 9416 15016 9422 15014 9429 15012
9433 15017 9435 15024 9440 15028 9444 15033 9449 15038 9450 15045
9456 15048 9462 15049 9469 15048 9471 15042 9466 15038 9462 15032
9458 15027 9455 15020 9452 15014 9458 15013 9465 15011 9472 15010
9477 15006 9484 15006 9489 15010 9490 15016 9494 15022 9497 15028
9496 15035 9493 15041 50 P
9493 15041 9488 15046 9482 15048 9489 15047 9496 15047 9501 15050
9500 15059 7 P
9866 14927 9870 14921 9869 14915 9874 14910 9879 14906 9884 14909
9888 14915 9889 14922 9895 14917 9901 14916 9900 14923 9899 14929
9896 14936 9898 14942 9904 14944 9897 14945 9893 14940 9887 14938
9880 14937 9877 14932 9866 14927 21 P
10217 14889 10223 14889 10228 14887 10230 14882 10225 14885 10222 14880
10222 14875 10222 14870 10223 14864 10228 14862 10231 14866 10235 14870
10240 14873 10244 14876 10244 14882 10240 14886 10238 14890 10235 14895
10235 14901 10233 14896 10228 14893 10223 14894 10217 14889 23 P
10036 14860 10039 14856 10042 14854 10038 14851 10042 14849 10045 14846
10050 14846 10054 14844 10058 14843 10061 14845 10061 14849 10056 14850
10055 14855 10052 14858 10049 14856 10045 14859 10045 14863 10041 14863
10036 14860 19 P
10143 15145 10140 15142 10142 15138 10142 15134 10139 15138 10135 15138
10135 15142 10130 15143 10128 15140 10126 15135 10124 15132 10121 15129
10119 15125 10124 15124 10128 15124 10130 15120 10132 15117 10135 15113
10139 15113 10143 15113 10145 15117 10148 15120 10151 15124 10153 15127
10155 15131 10156 15135 10153 15139 10150 15141 10143 15145 29 P
9939 14993 9942 14990 9946 14988 9950 14987 9955 14986 9958 14984
9962 14982 9966 14981 9970 14980 9974 14978 9974 14974 9977 14971
9979 14975 9978 14979 9983 14980 9987 14980 9991 14979 9996 14978
10000 14979 10004 14978 10008 14976 10012 14974 10015 14977 10018 14980
10022 14982 10017 14982 10013 14983 10009 14985 10005 14986 10002 14990
9998 14991 9996 14987 9992 14986 9988 14985 9983 14985 9979 14986

9975 14986 9971 14989 9969 14993 9969 14996 9966 14994 9963 14990
9960 14993 9960 14997 9956 14997 9953 14994 9949 14996 9946 14994
9939 14993 49 P
9996 14961 10000 14958 10004 14958 10007 14961 10011 14960 10015 14961
10020 14961 10024 14962 10028 14961 10027 14957 10026 14953 10024 14949
10024 14945 10027 14948 10030 14950 10034 14953 10038 14953 10038 14958
10042 14959 10046 14961 10050 14963 10046 14965 10044 14969 10040 14970
10042 14974 10038 14972 10036 14968 10032 14969 10028 14969 10023 14969
10020 14967 10016 14966 10011 14966 10007 14966 10003 14965 9996 14961
36 P
10050 14963 10053 14962 10056 14962 10059 14965 10056 14966 10054 14965
10050 14963 7 P
10056 14960 10060 14959 10062 14956 10064 14959 10067 14960 10070 14959
10073 14961 10071 14963 10068 14962 10064 14961 10061 14962 10056 14960
12:00 PM
10167 15088 10170 15084 10172 15081 10177 15080 10181 15077 10181 15081
10181 15086 10185 15086 10183 15089 10178 15091 10174 15091 10170 15093
10167 15088 13 P
10153 14988 10151 14985 10148 14984 10149 14981 10152 14980 10155 14981
10157 14985 10153 14988 8 P
9939 14957 9942 14955 9943 14952 9947 14953 9949 14952 9953 14953
9956 14953 9959 14953 9961 14956 9963 14958 9960 14960 9957 14959
9954 14959 9950 14960 9947 14960 9944 14960 9939 14957 17 P
9981 14948 9983 14946 9985 14947 9988 14948 9990 14950 9990 14954
9989 14956 9988 14954 9984 14954 9984 14951 9981 14948 11 P
10067 14880 10068 14877 10071 14876 10075 14877 10078 14877 10081 14876
10083 14879 10080 14881 10077 14882 10075 14881 10072 14881 10067 14880
12:00 PM
10097 14876 10099 14874 10101 14872 10102 14869 10105 14867 10106 14870
10108 14873 10106 14875 10102 14875 10097 14876 10 P
10083 14863 10085 14861 10088 14862 10090 14864 10092 14867 10090 14870
10088 14871 10087 14868 10083 14867 10083 14863 10 P
10429 15267 10432 15267 10435 15270 10438 15270 10441 15271 10444 15271
10447 15271 10445 15274 10444 15277 10441 15278 10438 15277 10435 15275
10432 15274 10430 15272 10429 15267 15 P
9908 14951 9911 14951 9914 14949 9916 14950 9920 14951 9921 14954
9919 14958 9916 14958 9913 14956 9911 14954 9908 14951 11 P
9907 14974 9910 14973 9913 14972 9916 14970 9918 14968 9921 14968
9924 14969 9922 14971 9919 14973 9916 14973 9914 14976 9911 14975
9907 14974 13 P
9668 14751 9669 14748 9669 14745 9671 14743 9674 14744 9675 14747
9675 14750 9673 14752 9674 14755 9675 14758 9672 14760 9669 14757
9671 14754 9668 14751 14 P

9196 15599 9199 15596 9203 15598 9205 15601 9209 15603 9214 15604
9218 15604 9219 15609 9218 15613 9218 15617 9215 15620 9211 15622
9209 15618 9204 15618 9204 15614 9205 15609 9201 15609 9198 15606
9196 15599 19 P
8356 14785 8360 14787 8364 14788 8368 14790 8371 14793 8375 14796
8378 14799 8379 14803 8377 14807 8373 14809 8370 14813 8368 14809
8370 14804 8374 14803 8375 14799 8371 14797 8368 14794 8365 14790
8361 14788 8356 14785 20 P
8392 14833 8396 14834 8401 14835 8405 14836 8409 14837 8413 14838
8416 14842 8417 14846 8419 14850 8423 14851 8424 14856 8422 14860
8418 14859 8415 14855 8413 14851 8410 14848 8408 14844 8404 14841
8401 14839 8397 14837 8392 14833 21 P
8475 14886 8471 14884 8467 14882 8463 14880 8459 14878 8455 14876
8455 14872 8452 14869 8448 14867 8444 14864 8441 14862 8437 14860
8437 14856 8434 14853 8430 14850 8426 14849 8430 14847 8434 14849
8438 14850 8442 14851 8446 14853 8449 14855 8452 14859 8455 14862
8458 14866 8461 14869 8464 14871 8467 14875 8470 14877 8473 14881
8475 14886 31 P
8397 14849 8399 14847 8402 14848 8404 14850 8405 14853 8407 14856
8407 14859 8405 14862 8404 14859 8402 14856 8400 14854 8398 14851
8397 14849 13 P
8272 14733 8276 14734 8279 14737 8282 14740 8285 14743 8287 14747
8290 14750 8292 14754 8294 14758 8295 14762 8293 14766 8290 14762
8289 14758 8286 14756 8282 14753 8282 14749 8281 14744 8279 14741
8275 14738 8272 14733 20 P
8293 14780 8292 14777 8295 14776 8297 14777 8299 14780 8301 14783
8301 14786 8299 14784 8295 14783 8293 14780 10 P
8310 14794 8313 14793 8315 14795 8319 14796 8320 14799 8321 14802
8322 14804 8324 14807 8326 14810 8323 14808 8321 14806 8319 14804
8318 14801 8315 14799 8313 14797 8310 14794 16 P
9247 14414 9250 14412 9254 14414 9255 14418 9260 14419 9264 14420
9266 14424 9266 14428 9262 14430 9258 14428 9254 14427 9251 14424
9251 14419 9247 14414 14 P
9224 14344 9228 14341 9231 14343 9236 14342 9240 14342 9243 14345
9240 14348 9235 14349 9231 14349 9231 14354 9230 14358 9228 14362
9225 14359 9225 14354 9224 14350 9224 14344 16 P
9167 14236 9169 14233 9172 14229 9176 14227 9179 14225 9181 14228
9181 14233 9181 14237 9181 14241 9177 14243 9173 14244 9170 14242
9167 14236 13 P
9204 14249 9207 14246 9210 14242 9212 14239 9215 14236 9220 14236
9223 14233 9226 14236 9225 14240 9223 14245 9221 14248 9217 14248
9213 14250 9209 14252 9204 14249 15 P
9221 14208 9224 14206 9229 14206 9231 14202 9234 14199 9238 14196

9240 14200 9238 14205 9238 14209 9240 14213 9238 14216 9235 14220
9233 14224 9231 14228 9228 14230 9224 14228 9223 14223 9223 14219
9225 14215 9225 14211 9221 14208 21 P
9309 14273 9311 14271 9314 14269 9317 14272 9317 14275 9314 14276
9310 14277 9309 14273 8 P
9289 14466 9292 14466 9295 14466 9298 14468 9301 14466 9301 14470
9299 14472 9298 14475 9295 14475 9294 14472 9291 14471 9289 14466
12:00 PM
9287 14456 9289 14453 9291 14450 9294 14448 9295 14446 9296 14448
9295 14452 9297 14453 9294 14454 9294 14457 9291 14459 9287 14456
12:00 PM
9010 13021 9013 13017 9017 13016 9020 13019 9024 13021 9024 13026
9021 13030 9021 13034 9020 13038 9016 13038 9015 13034 9013 13030
9010 13027 9010 13021 14 P
8704 12481 8706 12477 8709 12474 8712 12471 8714 12467 8719 12466
8723 12465 8726 12467 8731 12469 8728 12472 8724 12474 8720 12475
8716 12475 8711 12477 8709 12481 8704 12481 16 P
9538 10853 9542 10847 9547 10843 9552 10838 9557 10833 9561 10828
9566 10823 9571 10818 9577 10821 9584 10817 9590 10815 9595 10810
9600 10806 9606 10801 9611 10797 9615 10792 9620 10787 9626 10783
9632 10779 9638 10776 9645 10773 9648 10768 9654 10764 9661 10764
9668 10761 9673 10757 9679 10754 9686 10752 9692 10748 9699 10747
9705 10744 9711 10740 9716 10737 9723 10733 9728 10729 9735 10727
9741 10725 9747 10721 9753 10718 9760 10716 9766 10715 9773 10716
9780 10714 9787 10712 9793 10711 9800 10708 9806 10705 9812 10702
9819 10699 9825 10697 50 P
9825 10697 9832 10694 9838 10692 9845 10691 9852 10689 9859 10688
9866 10687 9872 10691 9877 10694 9880 10700 9884 10706 9890 10710
9892 10717 9893 10723 9894 10730 9892 10737 9886 10740 9880 10745
9874 10747 9867 10747 9860 10747 9853 10749 9847 10751 9840 10753
9834 10757 9828 10760 9823 10765 9816 10767 9810 10771 9805 10774
9798 10776 9792 10779 9785 10781 9778 10783 9772 10785 9765 10787
9758 10788 9752 10792 9746 10795 9741 10800 9739 10806 9733 10811
9728 10816 9723 10820 9717 10823 9710 10825 9704 10828 9698 10831
9691 10833 9685 10836 50 P
9685 10836 9678 10838 2 P
9678 10838 9672 10841 9668 10847 9665 10853 9661 10859 9658 10865
9654 10871 9650 10876 9644 10880 9639 10884 9632 10888 9626 10890
9619 10891 9612 10893 9605 10892 9598 10892 9592 10890 9585 10888
9578 10886 9572 10884 9567 10879 9562 10874 9557 10870 9551 10866
9545 10862 9538 10853 26 P
9747 10671 9753 10668 9759 10665 9765 10662 9772 10659 9776 10654
9782 10651 9789 10648 9795 10644 9800 10640 9806 10636 9813 10634

9818 10629 9825 10627 9828 10621 9832 10615 9836 10610 9842 10606
9848 10608 9849 10615 9848 10622 9845 10628 9840 10633 9836 10639
9832 10644 9826 10648 9821 10653 9814 10655 9809 10659 9802 10662
9796 10664 9789 10667 9783 10668 9776 10671 9769 10672 9763 10674
9756 10676 9747 10671 38 P
9850 10640 9854 10634 9857 10628 9858 10621 9860 10614 9862 10607
9867 10603 9873 10600 9879 10595 9885 10598 9888 10604 9893 10609
9895 10616 9894 10623 9891 10629 9885 10633 9879 10636 9873 10639
9866 10639 9859 10641 9850 10640 21 P
9983 9961 9977 9965 9971 9966 9971 9973 9966 9978 9960 9981
9958 9974 9951 9972 9955 9966 9960 9961 9966 9957 9969 9951
9971 9944 9976 9939 9977 9932 9978 9925 9977 9918 9975 9912
9978 9906 9983 9901 9988 9896 9993 9891 9996 9885 9998 9878
10004 9875 10010 9879 10015 9883 10021 9886 10028 9885 10035 9886
10042 9883 10045 9877 10052 9877 10059 9878 10065 9879 10072 9876
10078 9873 10085 9871 10091 9869 10098 9867 10105 9865 10111 9861
10117 9857 10123 9854 10130 9855 10137 9856 10143 9859 10140 9865
10138 9872 10141 9878 50 P
10141 9878 10144 9885 10146 9891 10152 9895 10145 9899 10142 9905
10136 9908 10132 9914 10127 9919 10122 9923 10115 9922 10108 9923
10104 9929 10097 9931 10096 9938 10089 9938 10082 9938 10076 9940
10072 9947 10065 9946 10059 9947 10052 9948 10048 9954 10043 9959
10040 9965 10034 9969 10027 9971 10022 9967 10015 9964 10008 9962
10002 9959 9995 9961 9990 9966 9983 9961 34 P
10018 10042 10024 10038 10030 10036 10036 10032 10042 10028 10048 10024
10055 10023 10061 10023 10068 10023 10075 10021 10081 10018 10088 10016
10085 10023 10083 10029 10088 10034 10090 10041 10085 10046 10090 10052
10095 10057 10088 10057 10083 10062 10085 10068 10079 10072 10072 10070
10066 10073 10064 10066 10058 10063 10051 10061 10047 10057 10040 10059
10033 10059 10029 10053 10023 10050 10018 10042 34 P
10109 10149 10111 10143 10110 10136 10115 10131 10113 10124 10119 10121
10125 10118 10132 10117 10131 10124 10138 10125 10143 10129 10149 10125
10156 10125 10163 10124 10170 10124 10176 10120 10183 10120 10180 10126
10174 10130 10179 10134 10175 10140 10169 10142 10162 10142 10155 10143
10153 10149 10146 10149 10149 10143 10143 10144 10136 10146 10129 10147
10123 10145 10116 10148 10109 10149 33 P
10170 10278 10177 10279 10184 10279 10190 10280 10197 10283 10204 10283
10211 10283 10218 10283 10224 10285 10218 10289 10223 10295 10216 10295
10209 10294 10204 10290 10197 10288 10192 10293 10185 10295 10182 10301
10176 10304 10169 10303 10175 10299 10176 10292 10175 10285 10170 10278
24 P
10017 9723 10024 9723 10028 9729 10035 9729 10040 9725 10047 9725
10049 9718 10055 9715 10061 9712 10061 9705 10061 9698 10068 9698

10070 9704 10073 9698 10079 9702 10081 9695 10088 9694 10091 9700
10092 9707 10096 9713 10091 9717 10089 9724 10083 9727 10078 9731
10071 9734 10065 9737 10061 9743 10056 9748 10058 9755 10056 9762
10051 9767 10047 9762 10040 9760 10033 9760 10028 9756 10027 9749
10025 9742 10023 9735 10017 9723 39 P
10098 9792 10104 9788 10110 9784 10114 9779 10119 9774 10125 9770
10130 9765 10136 9764 10142 9760 10149 9763 10144 9769 10141 9775
10141 9782 10136 9786 10142 9789 10137 9795 10131 9797 10124 9800
10117 9802 10111 9800 10104 9799 10098 9792 22 P
10146 9852 10152 9848 10156 9842 10161 9838 10167 9834 10170 9827
10175 9822 10169 9817 10170 9811 10172 9804 10177 9799 10184 9797
10190 9802 10185 9807 10183 9814 10186 9820 10192 9824 10198 9827
10198 9834 10196 9841 10190 9841 10183 9845 10190 9845 10197 9846
10192 9852 10192 9859 10186 9862 10181 9859 10173 9857 10167 9859
10160 9858 10156 9863 10151 9859 10146 9852 34 P
9909 9874 9914 9870 9916 9863 9922 9859 9928 9860 9933 9865
9939 9868 9945 9872 9950 9876 9953 9883 9950 9889 9948 9896
9947 9903 9943 9909 9937 9912 9933 9918 9930 9924 9923 9924
9916 9922 9915 9915 9912 9909 9909 9902 9909 9895 9912 9889
9913 9882 9909 9874 26 P
10051 9976 10055 9970 10059 9965 10066 9967 10070 9972 10071 9979
10078 9982 10083 9987 10076 9991 10070 9993 10063 9995 10057 9992
10053 9987 10051 9976 14 P
10112 10019 10117 10015 10122 10020 10129 10021 10136 10020 10142 10021
10149 10024 10153 10030 10160 10032 10165 10036 10163 10030 10165 10023
10170 10019 10176 10022 10181 10028 10178 10034 10175 10040 10168 10043
10161 10042 10155 10041 10148 10040 10141 10039 10134 10039 10132 10032
10128 10027 10122 10025 10112 10019 27 P
10102 10027 10103 10020 10110 10024 10116 10027 10123 10028 10129 10029
10125 10035 10119 10037 10112 10037 10105 10038 10102 10027 11 P
10327 10294 10329 10290 10331 10294 10336 10294 10338 10290 10343 10289
10342 10293 10341 10297 10343 10302 10344 10306 10340 10306 10336 10304
10332 10302 10330 10298 10327 10294 15 P
10350 10303 10352 10299 10357 10299 10361 10299 10363 10295 10366 10292
10366 10296 10365 10301 10364 10305 10359 10306 10356 10309 10350 10303
12:00 PM
10229 10256 10234 10256 10238 10254 10242 10253 10240 10257 10244 10260
10247 10263 10244 10265 10240 10269 10236 10270 10234 10267 10230 10264
10229 10256 13 P
10168 9888 10171 9884 10168 9881 10169 9876 10172 9874 10177 9874
10177 9878 10176 9882 10176 9887 10179 9890 10184 9889 10188 9891
10186 9895 10182 9896 10180 9900 10182 9904 10181 9908 10178 9905
10173 9903 10169 9903 10171 9899 10170 9894 10168 9888 23 P

9956 9826 9959 9822 9962 9819 9967 9819 9971 9820 9975 9821
9980 9822 9984 9823 9988 9826 9988 9830 9988 9834 9985 9838
9981 9839 9978 9842 9977 9847 9974 9849 9972 9853 9968 9852
9966 9847 9962 9846 9959 9843 9958 9839 9958 9834 9956 9826
24 P
9993 9777 9989 9774 9985 9772 9982 9769 9978 9766 9977 9761
9979 9757 9978 9753 9982 9750 9986 9750 9990 9749 9995 9750
9997 9754 10000 9757 10001 9762 10004 9765 10006 9769 10009 9773
10009 9777 10007 9781 10003 9781 9998 9780 9993 9777 23 P
10097 9757 10101 9756 10103 9753 10106 9749 10109 9746 10112 9743
10116 9741 10119 9737 10123 9739 10128 9737 10131 9736 10135 9739
10139 9739 10144 9739 10144 9743 10140 9742 10139 9747 10135 9748
10132 9745 10128 9746 10123 9746 10119 9748 10116 9750 10112 9753
10115 9757 10117 9761 10113 9762 10110 9765 10105 9765 10102 9763
10097 9757 31 P
10128 9809 10131 9807 10136 9806 10140 9806 10144 9807 10148 9809
10152 9811 10152 9815 10156 9818 10154 9822 10150 9821 10145 9820
10141 9821 10137 9821 10132 9820 10131 9815 10128 9809 17 P
9940 9928 9943 9925 9948 9925 9951 9922 9950 9918 9954 9916
9958 9916 9959 9921 9961 9925 9963 9929 9962 9934 9964 9937
9961 9940 9957 9943 9953 9942 9949 9940 9946 9938 9943 9934
9940 9928 19 P
9999 9738 10000 9735 10000 9731 10001 9728 10000 9725 10003 9727
10005 9729 10008 9731 10009 9735 10011 9737 10014 9739 10014 9742
10013 9745 10009 9746 10007 9745 10003 9744 10002 9741 9999 9738
18 P
9987 9982 9990 9983 9993 9982 9995 9979 9997 9977 9995 9975
9993 9972 9996 9972 9999 9974 10002 9975 10004 9978 10007 9980
10008 9983 10011 9985 10009 9987 10008 9991 10007 9994 10003 9993
10000 9991 10000 9988 9998 9991 9995 9992 9993 9990 9994 9987
9990 9986 9987 9982 26 P
9956 10050 9956 10046 9957 10043 9960 10045 9963 10047 9966 10050
9966 10053 9964 10056 9967 10058 9966 10061 9968 10063 9970 10066
9971 10069 9969 10071 9966 10069 9966 10066 9964 10063 9961 10065
9960 10063 9960 10059 9960 10056 9957 10053 9956 10050 23 P
10347 9977 10348 9974 10350 9971 10353 9971 10356 9971 10359 9971
10359 9974 10357 9977 10354 9979 10351 9979 10347 9977 11 P
9949 9390 9953 9390 9957 9387 9959 9384 9962 9380 9966 9380
9969 9377 9970 9372 9970 9367 9968 9364 9965 9360 9969 9361
9974 9361 9978 9361 9982 9360 9981 9356 9980 9351 9984 9350
9988 9348 9991 9351 9996 9352 9994 9356 9994 9360 9995 9365
9999 9366 10003 9369 10000 9372 9995 9373 9998 9376 10002 9379
10005 9382 10003 9386 10002 9390 10002 9395 9999 9398 10002 9402

10001 9406 9997 9410 9994 9412 9990 9414 9987 9418 9984 9421
9980 9422 9976 9419 9976 9415 9972 9413 9968 9410 9964 9407
9961 9405 9957 9403 50 P
9957 9403 9953 9400 9950 9397 9949 9390 4 P
9905 9385 9907 9381 9911 9381 9916 9381 9920 9380 9924 9378
9928 9376 9930 9372 9933 9369 9938 9370 9942 9369 9946 9371
9950 9373 9947 9377 9952 9377 9954 9380 9957 9384 9955 9387
9950 9387 9947 9390 9948 9394 9946 9398 9942 9396 9937 9396
9933 9394 9928 9394 9924 9396 9921 9399 9916 9398 9913 9396
9909 9399 9906 9397 9908 9392 9905 9385 34 P
9908 9359 9913 9358 9917 9358 9916 9354 9921 9353 9923 9350
9927 9347 9931 9350 9931 9354 9933 9358 9930 9361 9933 9365
9930 9368 9927 9371 9922 9372 9918 9372 9915 9369 9915 9364
9908 9359 19 P
10016 9407 10021 9409 10025 9409 10027 9413 10030 9416 10033 9413
10037 9413 10041 9417 10044 9420 10040 9422 10035 9420 10031 9423
10031 9427 10036 9429 10033 9432 10036 9436 10031 9436 10028 9438
10025 9435 10027 9430 10024 9427 10028 9425 10024 9423 10022 9419
10017 9419 10018 9414 10016 9407 27 P
9607 8814 9610 8807 9611 8800 9614 8794 9618 8788 9622 8783
9621 8776 9615 8772 9621 8769 9620 8762 9627 8764 9634 8764
9640 8767 9647 8770 9654 8772 9653 8779 9660 8776 9665 8772
9668 8778 9662 8783 9664 8790 9669 8786 9676 8789 9678 8782
9685 8784 9681 8790 9687 8793 9687 8800 9680 8800 9674 8804
9671 8810 9672 8816 9669 8823 9667 8830 9669 8836 9674 8842
9681 8843 9679 8849 9673 8854 9667 8857 9661 8861 9654 8860
9647 8858 9643 8852 9641 8845 9644 8838 9637 8835 9633 8829
9628 8824 9621 8821 50 P
9621 8821 9618 8816 9607 8814 3 P
9567 8708 9571 8710 9576 8711 9580 8711 9584 8713 9588 8715
9593 8713 9591 8709 9593 8704 9597 8707 9601 8707 9605 8709
9610 8710 9614 8712 9617 8715 9621 8717 9618 8719 9614 8722
9611 8725 9608 8728 9604 8730 9600 8728 9595 8727 9591 8729
9588 8726 9584 8725 9584 8729 9580 8729 9579 8724 9576 8722
9571 8719 9567 8717 9568 8713 9567 8708 34 P
9558 8649 9561 8645 9564 8649 9568 8651 9572 8653 9576 8655
9579 8652 9583 8650 9583 8654 9587 8657 9590 8660 9593 8664
9598 8664 9593 8666 9590 8664 9585 8663 9581 8660 9577 8661
9574 8665 9571 8669 9567 8669 9566 8664 9563 8662 9562 8658
9561 8653 9558 8649 26 P
9593 8637 9598 8636 9602 8634 9606 8636 9611 8634 9614 8637
9619 8637 9621 8640 9626 8638 9630 8637 9634 8634 9638 8636
9639 8640 9639 8644 9639 8649 9635 8651 9639 8653 9640 8649

9644 8652 9646 8656 9644 8659 9645 8664 9640 8665 9638 8661
9634 8659 9630 8660 9626 8658 9629 8654 9627 8650 9622 8650
9618 8649 9616 8652 9613 8656 9611 8651 9607 8649 9604 8645
9600 8644 9596 8641 9593 8637 39 P
9641 8739 9637 8737 9633 8735 9631 8731 9634 8727 9635 8723
9640 8722 9643 8725 9647 8728 9651 8731 9655 8732 9659 8734
9663 8736 9658 8736 9654 8735 9650 8737 9645 8738 9641 8739
18 P
9580 8434 9583 8431 9587 8428 9590 8425 9590 8429 9590 8434
9593 8432 9598 8431 9602 8428 9606 8429 9610 8426 9610 8430
9607 8434 9603 8435 9599 8438 9596 8441 9592 8444 9588 8446
9584 8447 9588 8444 9591 8441 9587 8442 9585 8438 9580 8434
24 P
9904 5701 9908 5697 9910 5691 9913 5686 9919 5684 9922 5679
9927 5680 9926 5685 9928 5691 9929 5697 9929 5703 9924 5704
9918 5705 9913 5703 9904 5701 15 P
9315 5477 9319 5475 9324 5476 9326 5480 9330 5484 9334 5486
9335 5491 9332 5494 9328 5495 9323 5493 9319 5490 9321 5486
9318 5482 9315 5477 14 P
8866 6170 8872 6167 8878 6162 8882 6156 8889 6154 8896 6156
8902 6159 8902 6166 8907 6172 8912 6176 8919 6180 8924 6185
8931 6185 8938 6185 8945 6182 8942 6188 8940 6195 8939 6202
8935 6208 8930 6203 8928 6210 8921 6207 8920 6200 8918 6207
8914 6201 8911 6195 8911 6202 8912 6209 8905 6209 8901 6203
8897 6197 8896 6190 8892 6196 8889 6202 8883 6198 8880 6192
8876 6186 8882 6188 8885 6182 8878 6182 8884 6179 8891 6179
8898 6179 8896 6172 8889 6168 8886 6162 8881 6167 8875 6172
8872 6178 8866 6170 50 P
8870 6241 8875 6237 8880 6242 8886 6238 8891 6232 8893 6225
8897 6219 8899 6226 8898 6232 8902 6238 8899 6244 8896 6251
8889 6251 8882 6249 8875 6248 8870 6241 16 P
8828 6183 8835 6185 8842 6186 8849 6186 8851 6193 8852 6200
8856 6206 8855 6213 8854 6220 8860 6223 8866 6228 8859 6229
8853 6234 8848 6229 8846 6222 8846 6215 8846 6208 8841 6202
8846 6198 8844 6191 8837 6190 8828 6183 22 P
8840 6064 8845 6063 8849 6064 8854 6064 8852 6068 8855 6071
8852 6075 8851 6080 8851 6084 8847 6085 8844 6088 8843 6084
8842 6079 8844 6075 8844 6070 8840 6064 16 P
8801 5982 8805 5978 8807 5974 8807 5970 8811 5967 8812 5972
8816 5975 8819 5978 8820 5983 8818 5987 8813 5987 8809 5985
8801 5982 13 P
8894 6286 8896 6284 8897 6280 8898 6284 8900 6286 8903 6288
8906 6289 8907 6293 8904 6295 8901 6297 8899 6295 8898 6292

8895 6289 8894 6286 14 P
9121 6141 9121 6136 9122 6132 9123 6128 9124 6123 9125 6119
9126 6123 9124 6128 9124 6132 9124 6137 9126 6141 9127 6146
9127 6150 9129 6155 9130 6159 9133 6163 9135 6167 9137 6170
9137 6175 9134 6171 9132 6167 9129 6163 9128 6159 9124 6155
9123 6151 9122 6147 9121 6141 27 P
8918 7285 8924 7288 8931 7291 8938 7290 8943 7295 8942 7288
8942 7280 8935 7282 8932 7275 8937 7271 8933 7264 8938 7259
8943 7255 8950 7252 8955 7247 8960 7242 8967 7240 8971 7247
8967 7253 8961 7257 8963 7264 8969 7267 8976 7271 8981 7276
8988 7277 8993 7282 9000 7283 9005 7278 9012 7281 9012 7288
9005 7289 8999 7290 8992 7294 8987 7299 8985 7306 8985 7313
8987 7319 8993 7324 8999 7328 9006 7330 9010 7324 9016 7326
9021 7332 9025 7338 9018 7337 9010 7337 9003 7339 8999 7345
8992 7342 8995 7348 50 P
8995 7348 9000 7354 8996 7360 8990 7363 8985 7368 8978 7366
8972 7369 8968 7375 8967 7382 8972 7387 8976 7393 8969 7393
8962 7390 8957 7385 8954 7378 8948 7382 8943 7376 8941 7370
8933 7369 8929 7364 8926 7358 8926 7351 8930 7344 8936 7346
8941 7352 8948 7352 8951 7346 8949 7339 8946 7332 8946 7325
8945 7318 8944 7311 8950 7308 8955 7312 8958 7319 8963 7314
8971 7313 8971 7306 8966 7302 8959 7298 8952 7298 8947 7304
8940 7305 8933 7306 8929 7300 8922 7299 8920 7292 8918 7285
48 P
8800 7143 8805 7138 8802 7132 8807 7127 8814 7126 8820 7123
8826 7127 8833 7129 8839 7125 8837 7118 8836 7111 8842 7108
8849 7106 8856 7106 8859 7112 8853 7118 8857 7124 8864 7125
8869 7130 8862 7128 8857 7133 8852 7128 8845 7128 8839 7132
8833 7136 8828 7141 8828 7148 8821 7150 8819 7156 8814 7151
8810 7145 8800 7143 32 P
8200 6667 8205 6662 8209 6657 8216 6654 8223 6651 8229 6655
8231 6661 8235 6667 8241 6672 8247 6675 8254 6677 8261 6678
8268 6677 8274 6673 8280 6669 8286 6665 8293 6669 8297 6675
8297 6682 8302 6687 8298 6693 8297 6701 8294 6707 8292 6714
8289 6720 8282 6722 8279 6728 8274 6733 8269 6728 8263 6724
8265 6717 8260 6711 8254 6708 8254 6701 8249 6695 8242 6696
8235 6698 8229 6702 8224 6707 8219 6711 8213 6708 8207 6703
8208 6696 8215 6700 8212 6693 8214 6687 8216 6679 8219 6672
8212 6671 8200 6667 50 P
8088 6694 8093 6688 8096 6682 8102 6678 8109 6675 8116 6673
8123 6672 8129 6676 8134 6681 8140 6685 8145 6690 8146 6697
8149 6704 8156 6705 8156 6712 8156 6720 8156 6727 8152 6732
8145 6729 8141 6735 8136 6740 8129 6738 8124 6742 8117 6745

8110 6747 8111 6739 8105 6735 8104 6728 8100 6722 8095 6717
8091 6711 8093 6704 8100 6705 8105 6710 8112 6713 8113 6706
8107 6702 8100 6699 8088 6694 39 P
7690 6184 7697 6184 7704 6186 7711 6188 7718 6186 7717 6178
7723 6182 7729 6185 7736 6187 7743 6188 7750 6190 7757 6190
7754 6197 7746 6198 7745 6205 7745 6212 7745 6219 7748 6226
7752 6232 7759 6234 7766 6231 7772 6228 7779 6228 7780 6236
7775 6241 7775 6248 7777 6255 7783 6259 7782 6265 7788 6269
7793 6265 7800 6266 7802 6273 7809 6275 7801 6275 7797 6281
7791 6284 7786 6289 7780 6285 7773 6286 7769 6280 7762 6281
7756 6278 7753 6285 7749 6291 7743 6295 7737 6299 7732 6304
7725 6300 7724 6293 50 P
7724 6293 7726 6287 7726 6279 7722 6274 7717 6268 7715 6262
7710 6256 7705 6251 7708 6243 7714 6240 7718 6245 7725 6247
7731 6243 7738 6243 7745 6242 7740 6237 7738 6230 7733 6225
7728 6221 7722 6216 7717 6210 7713 6205 7714 6198 7710 6203
7703 6200 7698 6195 7690 6184 27 P
7526 6113 7522 6119 7516 6121 7509 6120 7503 6115 7497 6110
7493 6105 7497 6099 7504 6097 7511 6096 7518 6097 7526 6096
7533 6094 7538 6090 7545 6087 7552 6087 7558 6089 7566 6090
7573 6088 7574 6081 7581 6084 7586 6079 7593 6083 7596 6089
7595 6096 7594 6103 7600 6107 7597 6114 7591 6117 7589 6124
7590 6132 7583 6132 7578 6126 7576 6119 7569 6120 7564 6126
7558 6129 7551 6127 7547 6121 7544 6115 7538 6110 7535 6104
7526 6113 43 P
7527 6180 7520 6179 7513 6179 7510 6173 7506 6167 7498 6166
7491 6165 7493 6157 7499 6154 7506 6153 7513 6151 7520 6149
7522 6155 7523 6163 7527 6169 7533 6172 7527 6180 17 P
7483 6175 7488 6170 7495 6169 7502 6168 7508 6173 7505 6180
7506 6187 7511 6192 7514 6199 7507 6200 7501 6196 7500 6189
7496 6183 7490 6180 7483 6175 15 P
7334 6043 7339 6038 7343 6031 7349 6028 7355 6024 7360 6018
7366 6016 7373 6018 7378 6013 7381 6006 7387 6004 7394 6002
7388 5997 7392 5991 7398 5987 7405 5987 7407 5980 7414 5980
7417 5987 7422 5992 7415 5995 7408 5992 7403 5997 7402 6004
7402 6011 7404 6018 7402 6025 7398 6031 7397 6038 7392 6044
7385 6046 7379 6042 7372 6042 7365 6043 7360 6049 7356 6054
7348 6055 7342 6051 7334 6043 39 P
8712 7276 8716 7270 8720 7265 8721 7257 8724 7251 8731 7254
8736 7258 8743 7258 8744 7265 8741 7272 8741 7279 8740 7286
8743 7293 8745 7299 8748 7306 8746 7313 8750 7319 8750 7326
8750 7333 8746 7340 8740 7337 8737 7331 8733 7325 8727 7321
8726 7313 8724 7307 8721 7300 8719 7293 8717 7287 8712 7276

30 P
8841 7629 8848 7628 8855 7628 8862 7626 8869 7625 8875 7621
8881 7618 8889 7617 8895 7620 8895 7627 8893 7634 8890 7641
8888 7647 8882 7650 8875 7653 8869 7658 8864 7654 8857 7652
8852 7646 8847 7640 8841 7629 21 P
8846 7624 8850 7622 8853 7619 8857 7616 8862 7616 8866 7617
8871 7618 8874 7620 8871 7623 8867 7624 8862 7625 8858 7626
8853 7627 8846 7624 14 P
8893 7958 8895 7954 8898 7950 8898 7946 8902 7946 8904 7950
8908 7947 8912 7946 8916 7947 8921 7948 8926 7949 8927 7953
8931 7956 8934 7959 8939 7959 8942 7961 8946 7964 8945 7969
8941 7971 8936 7970 8933 7967 8932 7963 8930 7959 8926 7960
8927 7964 8922 7965 8918 7963 8914 7965 8909 7966 8905 7965
8901 7965 8897 7964 8893 7958 33 P
9225 8131 9227 8128 9230 8124 9234 8122 9238 8119 9242 8117
9246 8117 9250 8119 9254 8121 9258 8122 9263 8122 9259 8124
9255 8127 9254 8131 9252 8135 9250 8139 9245 8139 9242 8143
9238 8143 9234 8143 9230 8139 9227 8137 9225 8131 23 P
10666 6777 10661 6779 10657 6777 10653 6776 10648 6778 10644 6776
10642 6771 10638 6770 10636 6766 10632 6764 10635 6761 10640 6761
10645 6760 10649 6758 10653 6758 10657 6759 10662 6761 10667 6760
10671 6758 10675 6761 10678 6764 10682 6766 10684 6770 10689 6773
10685 6775 10681 6777 10676 6778 10672 6780 10666 6777 29 P
11075 6565 11078 6562 11083 6561 11087 6560 11092 6561 11096 6561
11100 6563 11104 6566 11106 6570 11103 6574 11098 6576 11094 6576
11089 6576 11085 6574 11081 6572 11077 6570 11075 6565 17 P
11528 6945 11530 6941 11534 6938 11538 6938 11543 6937 11547 6938
11551 6940 11555 6943 11557 6946 11558 6951 11556 6955 11553 6958
11548 6959 11543 6960 11539 6960 11535 6958 11531 6955 11528 6952
11528 6945 19 P
11761 7064 11763 7060 11766 7057 11770 7055 11775 7054 11779 7054
11783 7058 11787 7059 11791 7057 11796 7057 11800 7058 11804 7059
11806 7064 11805 7068 11803 7072 11801 7076 11799 7080 11796 7082
11791 7084 11787 7086 11783 7088 11778 7090 11774 7090 11771 7087
11770 7082 11771 7078 11770 7073 11766 7071 11762 7073 11761 7064
30 P
11528 7005 11530 7003 11534 7002 11537 7000 11540 6999 11541 7002
11539 7005 11536 7007 11533 7009 11530 7007 11528 7005 11 P
11195 6689 11193 6687 11192 6684 11192 6680 11194 6677 11196 6675
11198 6672 11201 6670 11204 6670 11207 6672 11210 6674 11212 6676
11213 6679 11213 6683 11211 6686 11207 6686 11204 6686 11200 6687
11195 6689 19 P
11192 6736 11189 6737 11188 6740 11187 6743 11184 6745 11181 6746

11178 6745 11174 6744 11173 6741 11176 6739 11174 6736 11171 6734
11168 6734 11171 6733 11174 6732 11178 6733 11181 6732 11185 6733
11187 6730 11191 6728 11193 6730 11195 6733 11192 6736 23 P
11233 6730 11234 6727 11238 6725 11241 6725 11244 6728 11246 6730
11246 6734 11245 6737 11242 6739 11240 6741 11237 6741 11235 6738
11234 6735 11233 6730 14 P
11156 6664 11158 6662 11161 6661 11165 6662 11164 6665 11162 6668
11158 6668 11156 6664 8 P
11164 6670 11167 6671 11170 6674 11172 6676 11173 6679 11170 6681
11167 6678 11165 6676 11164 6670 9 P
10966 6636 10968 6636 10970 6637 10972 6638 10975 6639 10972 6639
10970 6639 10969 6638 10966 6636 9 P
10880 7536 10881 7534 10884 7534 10886 7534 10888 7535 10889 7537
10892 7536 10894 7537 10896 7537 10898 7537 10898 7540 10896 7541
10895 7543 10893 7543 10891 7544 10889 7543 10887 7543 10885 7542
10883 7541 10881 7539 10880 7536 21 P
10921 7532 10923 7530 10924 7529 10926 7530 10925 7532 10924 7534
10921 7532 7 P
10957 7633 10958 7632 10961 7631 10963 7632 10964 7634 10963 7635
10961 7637 10959 7637 10958 7636 10957 7633 10 P
10145 7416 10147 7415 10149 7414 10150 7416 10152 7417 10152 7419
10150 7420 10148 7420 10145 7419 10145 7416 10 P
10164 7336 10165 7334 10168 7335 10168 7337 10164 7336 5 P
10096 6965 10097 6963 10099 6962 10101 6961 10103 6960 10105 6960
10108 6961 10109 6960 10111 6961 10112 6963 10110 6964 10109 6966
10108 6968 10106 6969 10104 6969 10102 6968 10101 6966 10099 6965
10096 6965 19 P
8935 6010 8937 6008 8936 6006 8938 6004 8939 6003 8940 6005
8940 6007 8942 6009 8942 6011 8942 6013 8940 6014 8938 6013
8935 6010 13 P
8749 6089 8751 6087 8753 6088 8755 6088 8757 6090 8757 6092
8755 6093 8753 6094 8751 6094 8750 6091 8749 6089 11 P
8906 6005 8907 6003 8909 6003 8912 6003 8913 6005 8912 6008
8909 6008 8907 6007 8906 6005 9 P
8857 6069 8859 6067 8861 6067 8862 6069 8864 6071 8866 6071
8868 6071 8869 6073 8868 6075 8866 6075 8864 6075 8861 6075
8860 6073 8859 6071 8857 6069 15 P
8850 6141 8851 6139 8853 6139 8855 6140 8858 6140 8860 6141
8862 6141 8864 6143 8865 6145 8867 6145 8868 6147 8866 6148
8864 6147 8861 6146 8859 6145 8857 6145 8855 6143 8854 6141
8852 6143 8851 6144 8850 6141 21 P
8850 6015 8852 6014 8854 6014 8855 6016 8853 6017 8850 6015

8854 6006 8857 6007 8859 6008 8860 6010 8859 6012 8857 6012
8856 6010 8854 6006 8 P
8165 6666 8167 6664 8171 6664 8174 6663 8176 6665 8179 6667
8182 6665 8185 6668 8185 6671 8188 6672 8191 6672 8193 6675
8193 6678 8194 6682 8193 6684 8190 6687 8187 6689 8184 6690
8181 6688 8178 6688 8174 6687 8174 6683 8174 6679 8173 6676
8169 6675 8168 6671 8168 6668 8165 6666 28 P
7950 6697 7954 6694 7958 6692 7961 6689 7964 6685 7967 6681
7971 6679 7975 6677 7979 6678 7984 6678 7988 6678 7993 6679
7991 6684 7990 6688 7986 6689 7981 6688 7977 6687 7973 6689
7971 6692 7967 6696 7963 6695 7959 6694 7955 6696 7950 6697
24 P
7953 6699 7957 6696 7961 6696 7965 6698 7970 6696 7973 6693
7975 6689 7980 6689 7984 6691 7988 6692 7992 6690 7994 6685
7998 6684 8003 6687 8007 6687 8006 6691 8003 6694 7999 6697
7997 6701 7993 6704 7988 6705 7984 6706 7979 6708 7975 6707
7971 6706 7966 6705 7961 6703 7957 6702 7953 6699 29 P
8022 6663 8026 6661 8031 6663 8034 6660 8039 6663 8042 6665
8039 6667 8034 6669 8030 6668 8022 6663 10 P
7979 6675 7983 6673 7987 6671 7991 6669 7995 6668 7997 6673
7993 6675 7988 6676 7984 6676 7979 6675 10 P
7942 6708 7943 6704 7945 6701 7947 6700 7951 6698 7952 6702
7950 6704 7947 6707 7946 6709 7944 6712 7942 6708 11 P
8622 7035 8627 7034 8631 7033 8633 7029 8635 7025 8639 7023
8638 7027 8640 7032 8643 7034 8646 7038 8645 7042 8647 7046
8644 7049 8644 7054 8647 7057 8651 7058 8655 7061 8655 7066
8650 7064 8646 7061 8642 7061 8638 7059 8635 7055 8631 7054
8631 7049 8633 7046 8636 7042 8636 7038 8632 7035 8627 7037
8622 7035 31 P
9031 7668 9035 7666 9039 7665 9043 7667 9047 7667 9051 7670
9055 7672 9057 7676 9059 7680 9054 7681 9050 7681 9048 7677
9044 7674 9041 7678 9037 7678 9039 7682 9035 7685 9034 7681
9032 7677 9033 7672 9031 7668 21 P
9075 7721 9070 7720 9067 7717 9063 7714 9060 7712 9060 7707
9059 7703 9058 7699 9062 7695 9062 7700 9062 7705 9064 7708
9068 7710 9073 7711 9077 7709 9081 7706 9083 7709 9082 7714
9079 7716 9075 7721 20 P
9042 7573 9043 7570 9046 7567 9049 7568 9052 7570 9055 7573
9053 7575 9049 7575 9046 7575 9042 7573 10 P
9062 7557 9066 7559 9070 7561 9073 7564 9076 7568 9072 7567
9067 7568 9065 7564 9062 7557 9 P
9316 7804 9320 7802 9324 7805 9326 7808 9331 7807 9329 7811
9326 7815 9322 7814 9318 7811 9316 7804 10 P

9309 7812 9312 7813 9313 7810 9316 7812 9318 7814 9316 7817
9313 7819 9311 7816 9309 7812 9 P
9128 7911 9131 7908 9135 7905 9140 7906 9143 7909 9143 7913
9148 7914 9152 7916 9153 7920 9154 7924 9154 7929 9151 7932
9147 7933 9144 7930 9142 7926 9140 7922 9137 7918 9134 7916
9128 7911 19 P
9084 7869 9088 7868 9093 7868 9095 7873 9099 7872 9103 7873
9104 7877 9104 7882 9104 7886 9100 7887 9099 7883 9095 7885
9090 7883 9087 7881 9087 7876 9084 7869 16 P
8948 7937 8949 7933 8951 7931 8954 7931 8956 7929 8960 7930
8963 7931 8965 7934 8968 7934 8972 7933 8974 7936 8976 7939
8973 7941 8970 7942 8967 7944 8964 7943 8961 7942 8958 7941
8954 7940 8951 7939 8948 7937 21 P
9470 8718 9473 8716 9476 8715 9479 8712 9482 8711 9485 8709
9487 8707 9490 8705 9493 8704 9497 8703 9500 8704 9503 8705
9506 8707 9508 8709 9510 8712 9510 8716 9508 8718 9505 8720
9502 8721 9499 8722 9496 8722 9492 8722 9489 8721 9485 8721
9482 8721 9479 8720 9476 8719 9470 8718 28 P
9422 8731 9425 8730 9427 8727 9429 8724 9432 8723 9436 8722
9439 8721 9442 8723 9442 8726 9442 8730 9439 8732 9437 8735
9436 8738 9433 8740 9430 8741 9426 8742 9424 8743 9420 8744
9417 8744 9417 8741 9418 8738 9419 8735 9422 8731 23 P
9586 8614 9589 8613 9591 8612 9594 8612 9596 8614 9598 8617
9597 8619 9595 8621 9593 8623 9591 8621 9590 8618 9588 8616
9586 8614 13 P
9655 8521 9657 8520 9658 8521 9660 8523 9660 8525 9661 8527
9659 8527 9657 8526 9657 8524 9655 8521 10 P
9648 8527 9651 8527 9651 8525 9653 8524 9655 8525 9657 8527
9659 8528 9660 8530 9662 8532 9663 8534 9662 8535 9660 8535
9657 8535 9656 8533 9655 8531 9654 8530 9652 8530 9648 8527
18 P
9829 9037 9831 9035 9834 9034 9836 9032 9840 9030 9842 9033
9841 9036 9842 9038 9846 9039 9846 9042 9842 9043 9843 9047
9841 9050 9840 9053 9837 9054 9834 9053 9833 9049 9832 9046
9830 9043 9829 9037 20 P
9837 9024 9839 9021 9842 9019 9845 9018 9848 9020 9850 9022
9848 9025 9845 9026 9842 9027 9837 9024 10 P
9964 9034 9968 9034 9971 9035 9969 9032 9971 9030 9970 9027
9967 9025 9969 9022 9973 9023 9974 9021 9977 9023 9979 9025
9982 9023 9982 9027 9983 9030 9985 9033 9987 9035 9984 9036
9981 9036 9977 9037 9977 9041 9977 9044 9974 9043 9971 9043
9970 9040 9967 9037 9964 9034 27 P
9965 8996 9968 8996 9971 8997 9973 9001 9975 8999 9978 9000

9981 9002 9983 9004 9986 9006 9988 9009 9985 9011 9982 9010
9979 9010 9976 9008 9973 9007 9971 9005 9971 9001 9968 8999
9965 8996 19 P
9940 9189 9942 9187 9944 9189 9947 9192 9949 9193 9952 9195
9952 9199 9952 9202 9954 9205 9957 9204 9956 9207 9955 9210
9953 9213 9950 9212 9947 9211 9946 9207 9947 9204 9947 9201
9943 9200 9944 9198 9945 9194 9943 9192 9940 9189 23 P
9946 9165 9950 9165 9952 9163 9955 9163 9958 9164 9958 9168
9955 9170 9958 9172 9960 9175 9962 9177 9966 9178 9969 9178
9969 9182 9973 9182 9976 9183 9973 9184 9969 9184 9967 9182
9964 9180 9962 9178 9958 9178 9956 9176 9953 9176 9949 9176
9947 9174 9949 9171 9946 9165 27 P
9906 9143 9909 9142 9912 9141 9914 9139 9916 9136 9920 9136
9922 9138 9923 9141 9923 9145 9926 9147 9928 9149 9924 9150
9923 9147 9920 9145 9917 9143 9914 9142 9912 9145 9913 9148
9910 9149 9907 9150 9908 9147 9906 9143 22 P
10089 9560 10090 9557 10093 9559 10096 9558 10098 9561 10101 9562
10102 9565 10104 9568 10107 9569 10111 9569 10110 9572 10106 9572
10103 9574 10101 9571 10098 9570 10098 9567 10096 9564 10094 9562
10090 9564 10089 9560 20 P
10094 9721 10097 9720 10100 9718 10103 9717 10106 9715 10109 9713
10111 9711 10114 9709 10115 9712 10118 9713 10122 9712 10125 9712
10127 9709 10130 9707 10130 9710 10131 9714 10128 9715 10125 9714
10122 9714 10119 9716 10116 9716 10112 9717 10110 9720 10106 9720
10106 9723 10104 9726 10101 9726 10098 9728 10095 9727 10096 9724
10094 9721 31 P
10051 9858 10053 9855 10056 9855 10060 9855 10063 9854 10066 9855
10068 9858 10067 9861 10065 9864 10062 9865 10058 9863 10056 9862
10051 9858 13 P
10054 9867 10057 9866 10060 9867 10063 9867 10067 9867 10069 9866
10071 9863 10074 9861 10077 9862 10078 9866 10076 9868 10074 9871
10071 9873 10069 9875 10067 9877 10063 9875 10061 9873 10058 9871
10054 9867 19 P
10039 9863 10040 9860 10044 9861 10047 9861 10047 9865 10048 9868
10047 9871 10043 9872 10041 9871 10040 9867 10039 9863 11 P
10021 9880 10020 9877 10021 9874 10020 9871 10022 9868 10025 9868
10028 9871 10030 9872 10029 9875 10027 9878 10024 9880 10021 9880

12:00 PM

10001 9853 10003 9849 10003 9846 10005 9845 10009 9845 10011 9847
10013 9850 10015 9853 10015 9856 10014 9859 10011 9861 10008 9861
10005 9859 10002 9857 10001 9853 15 P
10280 10369 10284 10368 10286 10366 10290 10365 10293 10366 10294 10368
10298 10368 10298 10372 10295 10372 10292 10370 10290 10372 10287 10375

10284 10376 10283 10373 10285 10370 10280 10369 16 P
10252 10351 10256 10352 10259 10352 10262 10350 10265 10350 10266 10353
10263 10354 10260 10355 10257 10355 10252 10351 10 P
10268 10351 10271 10349 10274 10351 10277 10352 10275 10355 10272 10357
10270 10354 10268 10351 8 P
10334 10338 10337 10336 10339 10337 10343 10339 10339 10341 10334 10338

6:00 PM

9995 10209 9998 10207 10001 10207 10003 10204 10004 10201 10008 10202
10010 10204 10010 10208 10011 10210 10015 10211 10011 10213 10009 10214
10005 10213 10004 10210 10001 10211 9995 10209 16 P
9991 10235 9994 10233 9996 10230 9999 10232 10001 10235 10002 10238
10004 10240 10001 10239 9998 10241 9997 10238 9994 10239 9991 10235

12:00 PM

10100 10241 10103 10241 10106 10243 10109 10245 10108 10248 10104 10249
10106 10245 10102 10245 10100 10241 9 P
10095 10173 10098 10172 10101 10173 10102 10176 10103 10179 10101 10180
10098 10179 10095 10177 10095 10173 9 P
10124 10175 10127 10175 10130 10173 10132 10171 10136 10170 10137 10173
10137 10177 10137 10180 10137 10183 10134 10182 10134 10178 10130 10177
10127 10178 10124 10175 14 P
10191 10128 10194 10128 10197 10129 10200 10129 10201 10125 10204 10123
10207 10124 10209 10127 10205 10129 10203 10131 10203 10134 10200 10137
10197 10136 10195 10132 10191 10128 15 P
10207 10018 10210 10017 10213 10015 10216 10015 10219 10014 10221 10017
10220 10020 10217 10021 10213 10022 10210 10022 10207 10018 11 P
10285 10011 10288 10009 10289 10006 10291 10003 10293 10001 10294 10005
10294 10008 10293 10011 10292 10014 10289 10015 10285 10011 11 P
10304 10011 10305 10008 10307 10006 10311 10005 10311 10002 10312 9999
10314 10001 10313 10005 10313 10008 10311 10011 10309 10013 10304 10011

12:00 PM

10317 10011 10318 10007 10320 10005 10323 10003 10323 10006 10322 10010
10323 10013 10321 10015 10317 10011 9 P
10325 9996 10326 9994 10329 9993 10331 9995 10330 9998 10329 10000
10325 9996 7 P
10204 9858 10206 9855 10207 9852 10210 9852 10211 9855 10209 9859
10209 9862 10205 9862 10204 9858 9 P
10065 10010 10066 10007 10069 10006 10071 10004 10074 10004 10076 10005
10077 10007 10076 10010 10074 10012 10072 10013 10069 10013 10065 10010

12:00 PM

9793 9393 9796 9392 9799 9390 9801 9388 9805 9388 9808 9389
9811 9389 9814 9390 9815 9393 9815 9397 9813 9399 9810 9399
9807 9398 9808 9394 9805 9393 9801 9393 9799 9396 9795 9396
9793 9393 19 P

9980 9158 9982 9155 9982 9152 9985 9154 9988 9156 9990 9158
9993 9160 9990 9162 9987 9161 9983 9161 9980 9158 11 P
9790 8789 9794 8789 9797 8791 9798 8795 9800 8797 9803 8798
9806 8799 9807 8803 9805 8805 9802 8805 9800 8802 9798 8800
9795 8798 9792 8796 9790 8793 9790 8789 16 P
8910 7381 8914 7378 8918 7377 8923 7377 8927 7378 8932 7379
8936 7377 8939 7381 8940 7385 8939 7389 8937 7393 8934 7390
8929 7388 8925 7387 8921 7384 8918 7387 8913 7387 8910 7381
18 P
8857 7378 8860 7375 8864 7374 8869 7373 8871 7377 8875 7379
8878 7382 8880 7386 8883 7390 8883 7395 8878 7394 8875 7391
8871 7388 8868 7385 8866 7381 8864 7377 8862 7381 8857 7378
18 P
8902 7388 8905 7388 8907 7388 8910 7390 8913 7390 8913 7393
8911 7395 8909 7397 8906 7396 8904 7395 8903 7392 8902 7388

12:00 PM

8926 7198 8928 7195 8927 7192 8927 7189 8929 7192 8932 7193
8935 7195 8938 7197 8935 7197 8932 7199 8931 7203 8934 7205
8933 7209 8929 7207 8928 7204 8926 7202 8926 7198 17 P
8930 7223 8932 7219 8934 7217 8937 7218 8940 7218 8943 7219
8947 7220 8949 7222 8947 7224 8945 7227 8945 7230 8942 7229
8939 7227 8936 7228 8936 7225 8930 7223 16 P
8209 6751 8212 6750 8215 6749 8218 6747 8221 6744 8223 6742
8227 6742 8230 6742 8230 6745 8232 6749 8234 6752 8232 6755
8229 6755 8226 6756 8222 6756 8219 6756 8215 6754 8212 6754
8209 6751 19 P
5903 5461 5904 5468 5908 5474 5902 5477 5908 5480 5915 5478
5917 5485 5916 5492 5916 5499 5921 5504 5924 5511 5930 5507
5936 5511 5937 5518 5933 5524 5927 5519 5921 5515 5914 5515
5908 5511 5903 5505 5896 5502 5893 5496 5886 5496 5879 5494
5872 5491 5866 5487 5862 5493 5854 5494 5847 5494 5843 5487
5837 5483 5832 5479 5825 5477 5827 5470 5822 5465 5816 5461
5816 5454 5816 5453 38 P
5839 5453 5846 5454 5847 5453 3 P
5861 5453 5864 5455 5871 5458 5879 5459 5885 5461 5890 5456
5890 5453 7 P
5910 5453 5911 5454 2 P
5911 5454 5903 5461 2 P
5967 5480 5971 5474 5974 5468 5981 5464 5988 5463 5992 5456
5999 5459 6005 5455 6006 5453 9 P
6023 5453 6024 5459 6029 5454 6029 5453 4 P
6040 5453 6038 5458 6042 5465 6038 5470 6034 5477 6031 5483
6024 5485 6017 5488 6010 5489 6004 5484 5997 5483 5990 5485

5984 5488 5977 5488 5967 5480 15 P
5969 5453 5969 5459 5963 5459 5962 5453 5961 5453 5 P
6054 5459 6056 5458 6058 5456 6058 5454 6059 5455 6062 5456
6063 5458 6064 5460 6063 5462 6064 5465 6063 5467 6061 5466
6059 5465 6057 5465 6055 5463 6054 5459 16 P
5540 5469 5543 5465 5545 5461 5547 5457 5549 5453 5 P
5575 5453 5574 5458 5572 5462 5571 5466 5569 5470 5567 5475
5563 5478 5559 5480 5556 5483 5552 5486 5549 5482 5545 5480
5542 5476 5540 5469 14 P
5698 5567 5700 5563 5704 5560 5708 5563 5712 5565 5716 5566
5716 5571 5720 5572 5723 5576 5723 5580 5726 5585 5721 5585
5717 5587 5712 5588 5710 5584 5706 5581 5704 5578 5702 5573
5698 5567 19 P
5790 5572 5794 5569 5798 5566 5800 5562 5805 5561 5809 5561
5808 5566 5806 5570 5802 5573 5799 5575 5795 5572 5790 5572

12:00 PM

6251 5559 6253 5560 6254 5559 6257 5560 6258 5562 6258 5565
6260 5566 6259 5568 6257 5567 6254 5566 6253 5564 6252 5562
6251 5559 13 P
6239 5580 6241 5577 6242 5574 6244 5571 6247 5573 6250 5574
6254 5574 6257 5576 6259 5579 6257 5581 6253 5581 6250 5582
6246 5582 6243 5582 6239 5580 15 P
6252 5647 6253 5644 6257 5644 6259 5641 6260 5638 6263 5640
6262 5643 6259 5646 6256 5647 6256 5651 6252 5647 11 P
12523 8159 12525 8159 12525 8158 12526 8159 12526 8160 12526 8160
12525 8161 12524 8161 12523 8160 12523 8159 10 P
12911 11281 12912 11281 12914 11281 12915 11281 12917 11282 12919 11283
12920 11284 12921 11283 12922 11281 12923 11282 12924 11284 12925 11286
12926 11287 12926 11288 12926 11290 12925 11290 12924 11289 12922 11289
12921 11289 12919 11289 12918 11289 12916 11290 12915 11288 12915 11287
12915 11285 12914 11283 12913 11283 12911 11282 12911 11281 29 P
12896 11256 12897 11256 12898 11256 12898 11256 12897 11258 12895 11258
12895 11257 12896 11256 12896 11256 9 P
12899 11256 12898 11255 12898 11254 12898 11252 12898 11250 12897 11249
12899 11248 12899 11250 12900 11251 12901 11252 12903 11252 12902 11253
12901 11254 12901 11256 12901 11257 12902 11259 12903 11260 12902 11262
12901 11262 12900 11261 12898 11261 12897 11261 12896 11259 12898 11258
12899 11256 25 P
12903 11265 12904 11263 12905 11265 12906 11266 12906 11267 12905 11267
12904 11267 12904 11266 12903 11266 12903 11265 10 P
12905 11271 12906 11271 12906 11272 12905 11272 12905 11271 5 P
12906 11272 12907 11272 12907 11272 12907 11272 12908 11272 12908 11274
12909 11274 12908 11275 12907 11275 12907 11274 12906 11273 12906 11272

12:00 PM

12909 11277 12909 11277 12910 11276 12911 11276 12911 11276 12912 11276
12911 11277 12911 11279 12909 11279 12909 11277 10 P
2297 14405 2329 14399 2320 14346 2297 14349 4 P
2297 14294 2325 14289 2373 14266 2380 14212 2412 14167 2459 14139
2490 14094 2522 14050 2508 13997 2489 13946 2507 13894 2525 13843
2543 13791 2555 13738 2563 13684 2530 13640 2486 13608 2467 13556
2480 13503 2507 13456 2547 13419 2601 13411 2629 13363 2652 13314
2670 13262 2722 13244 2773 13225 2823 13202 2836 13149 2869 13105
2912 13137 2941 13184 2927 13237 2935 13292 2948 13345 3001 13358
3052 13340 3107 13343 3146 13357 3135 13425 3179 13458 3186 13512
3188 13553 3151 13593 3104 13621 3057 13649 46 P
3057 13649 3016 13655 3013 13709 3026 13762 2994 13806 2971 13831
3020 13855 3048 13902 3103 13899 3110 13954 3115 13987 3085 14015
3121 14032 3130 14093 3185 14090 3239 14088 3293 14086 3347 14078
3394 14068 19 P
3394 14068 3397 14013 3416 13962 3466 13939 3513 13912 3560 13885
3610 13863 3651 13826 3684 13784 3718 13740 3770 13723 3819 13701
3861 13666 3905 13634 3957 13618 3972 13565 3946 13517 3950 13463
3938 13409 3930 13369 3863 13335 3862 13280 3850 13227 3859 13173
3879 13122 3873 13067 3866 13013 3812 13014 3757 13016 3702 13017
3648 13018 3593 13014 3540 13026 3490 13049 3450 13011 3464 12958
3483 12907 3452 12862 3397 12864 3342 12866 3288 12873 3233 12869
3186 12897 3153 12941 3120 12985 3065 12992 3011 13000 2944 13010
2907 12969 2904 12914 50 P
2904 12914 2918 12861 2877 12823 2834 12791 2801 12766 2792 12712
2847 12701 2895 12676 2930 12634 2930 12558 2869 12557 2869 12503
2838 12457 2808 12411 2787 12360 2792 12305 2814 12254 2820 12199
2813 12166 2773 12174 2756 12212 2702 12217 2648 12206 2599 12232
2545 12243 2502 12208 2472 12163 2423 12137 2439 12084 2481 12049
2507 12000 2486 11949 2460 11901 2407 11885 2352 11880 2299 11896
2297 11897 37 P
2297 10470 2312 10463 2357 10429 2381 10379 2436 10371 2492 10374
2547 10382 2601 10369 2604 10313 2618 10259 2663 10226 2718 10224
2774 10232 2821 10262 2865 10296 2898 10341 2931 10386 2963 10431
3000 10473 3037 10515 3017 10567 3045 10615 3100 10625 3155 10633
3184 10586 3238 10601 3270 10646 3293 10697 3325 10742 3332 10798
3364 10843 3400 10885 3440 10923 3484 10958 3528 10992 3574 11022
3591 11075 3553 11115 3519 11158 3497 11193 3533 11235 3560 11283
3564 11318 3605 11313 3608 11337 45 P
3608 11337 3611 11358 3556 11364 3501 11371 3448 11356 3396 11336
3357 11321 3315 11358 3278 11398 3253 11448 3219 11492 3164 11499
3140 11548 3125 11579 3081 11546 3043 11517 3009 11560 2980 11607

3000 11626 2967 11670 2917 11693 2862 11701 2808 11693 2753 11695
2698 11687 2646 11705 2599 11734 2561 11775 2534 11779 2536 11827
2481 11824 2420 11828 2410 11788 2354 11791 2297 11812 35 P
3394 14068 3432 14082 3418 14135 3409 14188 3427 14240 3449 14289
3477 14337 3517 14374 3560 14407 3614 14415 3619 14456 11 P
3619 14456 3567 14474 3530 14513 3511 14564 3474 14604 3427 14632
3373 14629 3319 14631 3278 14667 3234 14699 3194 14735 3166 14782
3212 14810 3252 14847 3265 14900 3273 14953 3309 14994 3336 15040
3376 15077 3408 15121 3395 15174 3386 15227 3363 15276 3319 15309
3268 15290 3215 15277 3165 15258 3112 15245 3063 15221 3023 15185
2990 15142 2962 15095 2944 15044 2925 14993 2917 14939 2904 14887
2880 14837 2877 14783 2845 14740 2836 14686 2844 14632 2830 14579
2807 14530 2754 14518 2707 14490 2654 14498 2600 14507 2546 14500
2499 14472 2452 14445 50 P
2452 14445 2398 14443 2344 14447 2297 14459 4 P
3619 14456 3668 14434 3718 14412 3755 14372 3758 14331 3805 14305
3859 14298 3914 14302 3968 14301 4022 14294 4083 14287 4094 14340
4095 14395 4107 14448 4135 14466 4165 14420 4222 14458 4212 14511
4182 14557 4204 14606 4230 14654 4266 14696 4304 14734 4347 14768
4389 14802 4424 14843 4466 14877 4511 14907 4546 14949 4585 14987
4627 15021 4672 15052 4722 15072 4772 15093 4761 15146 4757 15193
4802 15164 4837 15121 4874 15106 4878 15140 4931 15135 4945 15068
4998 15079 5023 15127 5061 15166 5106 15197 5159 15207 5186 15205
5167 15169 5155 15138 50 P
5155 15138 5185 15093 5208 15059 5183 15011 5174 14958 5129 14927
5095 14884 5085 14831 5070 14779 5044 14731 5040 14677 5020 14626
4974 14596 4933 14561 4888 14531 4854 14489 4848 14434 4842 14366
4877 14325 4903 14276 4924 14227 4935 14173 4930 14119 4925 14065
4871 14070 4866 14015 4876 13990 4846 13945 4812 13903 4760 13859
4752 13805 4744 13751 4739 13697 4739 13642 4750 13588 4745 13534
4740 13480 4734 13426 4730 13371 4735 13316 4740 13262 4746 13208
4752 13153 4758 13099 4742 13046 4721 12996 4691 12950 4657 12907
4636 12857 4620 12804 50 P
4620 12804 4594 12756 4584 12702 4578 12647 4557 12597 4580 12562
4615 12529 7 P
4615 12529 4604 12475 4634 12429 4624 12375 4654 12330 4693 12291
4689 12236 4705 12184 4667 12126 4718 12105 4757 12067 4768 12013
4814 11982 4840 11934 4841 11879 4836 11824 4813 11766 17 P
4813 11766 4745 11773 4729 11720 4674 11714 4639 11756 4608 11802
4566 11767 4512 11756 4459 11772 4406 11788 4354 11804 4300 11815
4246 11826 4192 11837 4136 11837 4136 11782 4189 11766 4240 11744
4260 11693 4239 11643 4294 11637 4344 11616 4387 11580 4408 11530
4429 11478 4378 11457 4326 11441 4275 11462 4221 11474 4166 11479

4131 11437 4099 11391 4083 11338 4099 11285 4114 11232 4119 11177
4113 11122 4113 11067 4093 11000 39 P
4093 11000 4037 11001 3981 11001 3943 11041 3909 11084 3874 11127
3835 11167 3784 11189 3742 11225 3700 11261 3662 11301 3638 11326
3608 11337 13 P
4093 11000 4147 10989 4141 10934 4138 10899 4193 10893 4241 10867
4280 10828 4329 10801 4335 10746 4329 10691 4324 10636 4318 10580
4313 10525 4270 10490 4219 10511 4163 10517 4112 10496 4069 10461
4026 10426 3976 10400 3937 10361 3895 10365 3899 10400 3847 10379
3792 10369 3799 10424 3743 10430 3700 10396 3653 10365 3600 10383
3581 10420 3536 10367 3480 10368 3448 10322 3483 10278 3513 10231
3501 10177 3483 10124 3477 10068 3473 10033 3403 10028 3423 9976
3462 9935 3469 9991 3506 10010 3531 9960 3557 9911 3605 9883
3621 9829 3589 9783 50 P
3589 9783 3635 9751 3684 9724 3715 9677 3719 9622 3754 9578
3781 9555 7 P
3781 9555 3787 9611 3819 9657 3840 9709 3867 9758 3903 9801
3939 9844 3991 9822 4024 9812 4040 9865 4090 9892 4145 9902
4201 9902 4254 9886 4297 9850 4347 9824 4401 9835 4453 9857
4458 9913 4431 9961 4452 10013 4504 10035 4555 10057 4625 10051
4621 10009 4677 10004 4732 9999 4788 9994 4824 9952 4871 9921
4922 9901 4978 9891 5033 9886 5087 9871 5127 9833 5168 9794
5204 9751 5248 9717 5302 9702 5357 9715 5412 9711 5467 9718
5510 9755 5555 9788 5632 9783 5629 9741 5601 9743 5581 9690
5577 9621 5630 9601 50 P
5630 9601 5686 9597 5719 9642 5758 9683 5795 9724 5837 9761
5885 9790 5882 9734 5927 9700 5971 9667 5942 9619 5980 9577
5938 9540 5904 9495 5882 9444 5902 9391 5945 9355 5988 9318
6043 9328 6103 9346 20 P
6103 9346 6117 9400 6165 9429 6206 9466 6244 9508 6297 9490
6352 9498 6403 9522 6458 9525 6512 9539 6549 9581 6602 9601
6656 9588 6669 9642 6724 9651 6778 9666 6815 9708 6817 9764
6873 9762 6929 9760 6971 9724 7022 9701 7078 9700 7143 9741
24 P
7143 9741 7127 9794 7118 9849 7119 9905 7104 9959 7059 9991
7044 10045 7045 10101 7046 10143 7008 10183 6979 10213 6981 10269
7022 10308 7062 10345 7106 10380 7076 10427 7034 10463 6986 10491
6933 10509 6883 10532 6841 10568 6819 10603 6804 10642 23 P
6804 10642 6735 10638 6731 10693 6712 10745 6675 10704 6628 10674
6574 10660 6519 10663 6471 10691 6442 10738 6434 10793 6427 10834
6373 10842 6304 10845 6266 10886 6233 10911 6196 10870 6158 10852
6135 10903 6090 10936 6057 10980 6044 11034 6058 11088 6061 11143
6069 11197 6028 11235 5991 11194 5943 11166 5888 11169 5856 11214

5812 11247 5758 11235 5703 11227 5648 11226 5593 11229 5538 11238
5483 11242 5429 11235 5373 11228 5324 11253 5281 11287 5237 11321
5190 11351 5144 11380 5095 11406 5052 11440 5016 11482 4980 11524
4940 11563 4929 11617 50 P
4929 11617 4902 11665 4871 11710 4841 11739 4813 11766 5 P
6804 10642 6817 10697 6840 10747 6880 10785 6924 10819 6977 10833
7060 10839 7067 10895 7069 10950 7070 11005 7071 11061 7067 11116
7069 11171 7076 11226 7082 11274 7054 11272 7053 11230 6998 11232
6963 11233 6923 11195 6878 11227 6844 11271 6790 11279 6742 11306
6744 11361 6746 11417 6708 11457 6658 11480 6610 11508 6613 11563
6615 11611 6656 11610 6666 11570 6721 11578 6796 11583 6804 11638
6822 11690 6818 11745 6841 11795 6888 11824 6941 11839 6948 11894
6949 11948 6940 12003 6942 12058 6982 12095 7037 12099 7084 12072
7132 12045 7147 11992 50 P
7147 11992 7161 11939 7209 11911 7264 11916 7315 11936 7359 11970
7405 12000 7400 12054 7380 12106 7381 12161 7382 12209 7328 12220
7278 12243 7240 12283 7220 12333 7221 12375 16 P
7221 12375 7170 12392 7116 12383 7060 12379 7006 12380 6951 12382
6906 12414 6887 12465 6868 12517 6827 12552 6789 12593 6737 12611
6682 12613 6635 12641 6637 12695 6669 12740 6706 12781 6724 12832
6775 12852 6827 12871 6867 12908 6894 12955 6933 12993 6980 13022
7023 13056 7072 13080 7108 13121 7154 13150 7207 13138 7247 13129
7238 13183 7210 13213 7167 13179 7114 13165 7062 13150 7012 13126
6960 13112 6917 13078 6868 13054 6869 13109 6897 13156 6924 13203
6930 13251 43 P
6930 13251 6887 13218 6859 13216 6836 13265 6782 13272 6738 13239
6695 13206 6654 13242 6599 13244 6545 13247 6498 13275 6451 13303
6399 13321 6350 13344 6300 13368 6246 13376 6206 13382 6165 13388
18 P
6165 13388 6157 13334 6149 13280 6141 13226 6127 13173 6104 13124
6090 13071 6082 13017 6068 12964 6049 12912 6021 12865 5984 12825
5936 12797 5882 12790 5828 12793 5774 12802 5719 12806 5665 12809
5610 12807 5555 12811 5503 12826 5449 12835 5394 12839 5384 12785
5380 12730 5328 12745 5273 12739 5228 12707 5187 12671 5142 12640
5089 12623 5035 12628 4981 12616 4931 12595 4877 12584 4832 12552
4778 12547 4729 12521 4674 12526 4634 12534 4615 12529 41 P
6165 13388 6133 13432 6110 13481 6087 13531 6064 13581 6109 13613
6149 13649 6193 13681 6201 13736 6204 13790 6232 13837 6265 13880
6293 13927 6321 13974 6349 14021 6367 14071 6349 14123 6331 14174
6312 14226 6290 14275 6271 14326 6243 14373 6197 14401 6151 14429
6169 14480 6172 14534 6186 14587 6194 14641 6197 14695 6241 14728
6277 14768 6285 14821 6322 14861 6363 14897 6399 14857 6453 14860
6504 14879 6540 14919 6542 14973 6570 15020 6588 15071 6606 15108

6569 15148 6555 15200 6558 15254 6514 15287 6463 15305 6414 15328
6374 15364 6402 15411 50 P
6402 15411 6434 15455 6487 15468 6539 15481 6588 15504 6620 15548
6566 15550 6513 15558 6520 15612 6524 15680 6483 15681 6486 15735
6440 15776 13 P
6440 15776 6433 15723 6419 15670 6376 15638 6323 15630 6270 15617
6230 15581 6183 15554 6129 15557 6086 15589 6032 15593 5978 15586
5940 15573 5914 15503 5860 15496 5817 15529 5772 15557 5718 15550
5677 15515 5644 15472 5614 15427 5590 15378 5561 15332 5537 15284
5517 15233 5473 15202 5419 15206 5373 15236 5309 15229 5299 15175
5269 15126 5215 15131 5155 15138 33 P
6440 15776 6433 15830 6414 15881 6382 15925 6346 15965 6390 15997
6417 16027 6389 16073 6395 16113 6371 16146 6407 16172 11 P
8148 16172 8163 16144 8184 16095 8200 16043 8252 16058 8304 16048
8358 16053 8413 16053 8460 16027 8514 16028 8545 16003 11 P
8545 16003 8570 16051 8595 16099 8605 16152 8598 16172 5 P
11247 15960 11193 15955 11139 15955 11160 16005 11180 16055 11186 16109
11160 16157 11148 16172 8 P
8545 16003 8508 15964 8474 15921 8427 15895 8465 15858 8414 15841
8384 15766 8426 15732 8468 15698 8520 15683 8551 15639 8599 15614
8653 15615 8693 15615 8705 15562 8712 15509 8729 15457 8755 15410
8786 15365 8835 15328 20 P
8060 14892 8055 14946 8030 14994 7995 15036 8003 15062 8024 15062
8020 15102 7966 15098 7926 15089 7892 15112 7908 15150 7860 15176
7855 15230 7856 15284 7906 15304 7936 15349 7894 15384 7869 15447
7821 15447 7788 15447 20 P
7788 15447 7750 15486 7729 15536 7730 15590 7769 15628 7822 15633
7817 15687 7763 15687 7710 15693 7656 15694 7606 15715 7581 15764
7547 15806 7518 15851 7489 15897 7443 15867 7398 15838 7366 15826
7375 15773 7380 15719 7353 15672 7301 15657 7247 15653 7195 15639
7152 15605 7121 15561 7086 15520 7059 15473 7057 15419 7026 15375
6986 15338 7010 15289 7009 15235 6996 15182 7025 15136 7072 15109
7116 15078 7165 15102 7219 15106 7273 15105 7314 15069 7366 15052
7411 15081 7446 15123 7504 15104 45 P
7504 15104 7555 15124 7594 15162 3 P
7594 15162 7643 15140 7693 15119 7688 15173 7719 15218 7714 15272
7709 15326 7730 15376 8 P
7730 15376 7770 15402 7788 15447 3 P
7504 15104 7514 15051 7558 15020 7568 14967 7573 14913 7593 14862
7567 14815 7531 14773 7505 14725 7494 14672 7495 14618 7507 14565
7534 14518 7583 14494 7637 14490 7691 14497 7744 14510 7796 14524
7832 14543 7870 14565 20 P
6930 13251 6932 13305 6895 13346 6862 13389 6863 13443 6881 13495

6917 13536 6960 13569 7006 13598 7050 13632 7077 13679 7104 13726
7126 13776 7143 13828 7170 13875 7222 13890 7276 13899 7325 13924
7364 13962 7403 14000 7438 14041 7474 14082 7513 14120 7555 14154
7609 14153 7664 14153 7718 14152 7772 14152 7815 14186 7845 14231
7876 14276 7926 14296 7974 14322 8006 14348 8033 14361 35 P
7221 12375 7276 12379 7314 12423 3 P
7314 12423 7320 12478 7332 12531 7381 12556 7434 12571 7488 12576
7538 12554 7577 12515 7615 12475 7630 12423 7651 12372 7676 12323
7725 12349 7746 12365 7788 12330 7822 12307 7875 12323 7926 12344
7960 12386 8009 12412 8064 12406 8099 12364 8114 12311 8114 12270
8169 12270 8220 12292 8236 12344 8235 12385 8274 12424 8308 12448
8356 12422 8382 12471 8403 12522 8423 12573 8438 12625 8493 12621
8548 12627 8585 12666 8626 12667 8634 12656 40 P
7314 12423 7356 12387 7390 12344 7435 12313 7485 12291 7537 12274
7578 12270 7577 12215 7593 12162 7635 12127 7680 12096 7735 12090
7789 12079 7842 12067 7888 12037 7931 11983 7935 11942 7909 11910
7963 11900 8018 11905 8071 11916 8117 11885 8096 11834 8091 11779
8065 11731 8039 11682 8013 11633 8044 11587 8098 11582 8141 11547
8157 11494 8157 11439 8183 11391 8219 11348 8274 11343 8328 11354
8382 11365 8412 11411 8442 11458 8495 11474 8530 11432 8573 11398
8609 11355 8610 11300 8589 11249 8551 11209 8498 11224 8458 11263
8404 11273 8349 11278 50 P
8349 11278 8319 11232 8354 11189 8365 11135 8344 11084 8299 11053
8268 11006 8226 10970 8212 10917 8218 10862 8241 10811 8281 10773
8328 10744 8377 10719 8329 10691 8314 10638 8321 10582 8344 10532
8391 10502 8444 10488 8499 10484 8622 10484 8678 10485 8733 10481
8774 10477 8830 10473 26 P
8830 10473 8881 10495 8936 10491 8973 10449 9009 10407 9056 10377
9108 10357 9162 10370 9209 10340 9216 10285 9261 10317 9314 10335
9338 10385 9391 10403 9446 10405 15 P
9446 10405 9479 10360 9524 10326 9564 10289 9600 10266 9615 10252
6:00 PM
8830 10473 8820 10418 8800 10367 8790 10311 8760 10265 8726 10221
8680 10189 8638 10153 8678 10114 8700 10063 8711 10038 8657 10026
8603 10009 8548 10008 8496 10029 8481 9975 8470 9920 8471 9865
8471 9809 8441 9762 8410 9715 8384 9665 8345 9626 8302 9590
8256 9558 8209 9528 8148 9494 27 P
8148 9494 8108 9533 8065 9569 8010 9580 7954 9585 7899 9591
7844 9603 7795 9629 7779 9683 7758 9735 7705 9751 7678 9784
7629 9758 7612 9705 7585 9656 7534 9635 7484 9609 7428 9605
7393 9649 7416 9700 7463 9730 7499 9773 7503 9815 7457 9846
7402 9853 7358 9818 7314 9783 7265 9758 7218 9728 7176 9733
7143 9741 31 P

8148 9494 8127 9442 8075 9421 8048 9371 8048 9315 8080 9268
8069 9213 8095 9164 8142 9133 8197 9122 8252 9112 8253 9055
8258 8999 8269 8944 8275 8888 8275 8866 16 P
8275 8866 8275 8846 8331 8852 8385 8869 8425 8829 8425 8773
8410 8719 8442 8672 8458 8618 8475 8564 8497 8512 8492 8456
8497 8414 8553 8420 8600 8389 8657 8385 8706 8412 8756 8440
8781 8490 8828 8522 8868 8483 8886 8429 8908 8378 8941 8331
8997 8327 9043 8359 9099 8372 9150 8395 9203 8375 9248 8340
9245 8301 31 P
8275 8866 8219 8866 8219 8810 8180 8770 8133 8738 8084 8711
8030 8695 7978 8673 7938 8634 7894 8598 7854 8638 7819 8682
7765 8666 7711 8650 7684 8600 7648 8557 7603 8522 7547 8522
7547 8466 7588 8457 7587 8401 7597 8345 7608 8289 7581 8240
7549 8193 7501 8162 7447 8147 7391 8137 7347 8101 7310 8058
7304 8002 7292 7946 7312 7893 7338 7843 7291 7812 7249 7773
7227 7721 7226 7678 7268 7677 7251 7623 7214 7580 7169 7544
7124 7509 7096 7460 7064 7413 7040 7361 7023 7306 7010 7251
7003 7194 6966 7151 50 P
6966 7151 6943 7099 6963 7045 6938 7001 6937 6959 5 P
6103 9346 6101 9290 6124 9239 6148 9188 6140 9133 6132 9077
6161 9029 6190 8981 6171 8928 6137 8882 6092 8849 6074 8796
6022 8771 5971 8748 5934 8706 5925 8650 5891 8605 5853 8563
5834 8510 5830 8454 5849 8400 5906 8403 5962 8394 5958 8338
5934 8287 5931 8230 5965 8184 5968 8128 5943 8077 5951 8020
5997 7986 6039 7949 6063 7897 6034 7848 6020 7793 5969 7725
6026 7727 6103 7739 6095 7682 6042 7663 6014 7656 6011 7600
5997 7544 5963 7498 5955 7442 5968 7387 5956 7323 47 P
3781 9555 3775 9499 3753 9448 3746 9392 3757 9337 3721 9294
3776 9282 3742 9213 8 P
3742 9213 3736 9157 3762 9107 3762 9050 3750 8995 3738 8940
3732 8884 3725 8821 3769 8785 3779 8729 3778 8687 3736 8687
3692 8652 3640 8631 3592 8600 3552 8560 3504 8530 3460 8494
3416 8459 3460 8404 3504 8439 3561 8433 3617 8438 3647 8467
3718 8467 3749 8419 3789 8379 3766 8327 3755 8271 3795 8231
3738 8231 3685 8210 3645 8198 3691 8127 3664 8077 3632 8030
3600 7983 3656 7977 3699 7972 3698 7916 3714 7876 3686 7810
42 P
3686 7810 3650 7766 3614 7723 3596 7668 3541 7680 3488 7702
3461 7652 3420 7665 3409 7691 3374 7667 3414 7627 3454 7577
3397 7577 3340 7583 3312 7586 3308 7629 3266 7633 3249 7579
3208 7504 3164 7540 3117 7573 3073 7609 3029 7574 2984 7538
2966 7484 2930 7440 2875 7424 2804 7412 2757 7444 2718 7486
2699 7524 2638 7485 2613 7537 2587 7588 2566 7641 2536 7689

2482 7708 2439 7713 2406 7666 2383 7614 2365 7560 2327 7517
2297 7515 43 P
3686 7810 3743 7810 3800 7804 3792 7733 3849 7727 3857 7798
3904 7830 3944 7870 4001 7870 4006 7813 4051 7777 4106 7766
4150 7802 4190 7842 4237 7874 4277 7914 4333 7926 4377 7890
4429 7912 4482 7934 4532 7908 4580 7877 4612 7830 4602 7774
4580 7721 4581 7665 4636 7649 4686 7645 4681 7588 4626 7570
4611 7515 4600 7460 4590 7403 4546 7366 4507 7325 4467 7284
4431 7240 4395 7195 4407 7139 4421 7114 4471 7141 4502 7190
4542 7231 4595 7210 4632 7166 4644 7110 4656 7054 4640 6999
4626 6950 4684 6946 50 P
4684 6946 4736 6968 4786 6996 4842 6986 4907 6976 5 P
4907 6976 4954 6994 5021 7017 5025 7060 5001 7112 5022 7165
5053 7214 5106 7193 5163 7190 5216 7169 5271 7154 5300 7105
5329 7055 5380 7029 5437 7037 5457 7091 5460 7148 5470 7204
5427 7243 5471 7281 5510 7323 5552 7361 5609 7357 5651 7319
5696 7284 5748 7259 5805 7256 5862 7259 5916 7278 5956 7323
30 P
5956 7323 5985 7273 5999 7218 6056 7221 6112 7230 6169 7238
6224 7252 6280 7261 6315 7215 6353 7173 6325 7141 6356 7111
6308 7079 6253 7062 6210 7025 6165 6989 6138 6938 6098 6897
6058 6857 6104 6823 6150 6789 6196 6755 6250 6774 6307 6777
6359 6803 6411 6778 6444 6834 6501 6838 6558 6836 6612 6856
6666 6876 6719 6896 6765 6863 6786 6809 6841 6791 6853 6757
6909 6773 6899 6829 6889 6886 6901 6935 6937 6959 41 P
4907 6976 4886 6922 4907 6863 4933 6813 4948 6757 4920 6707
4867 6686 4840 6675 4839 6618 4848 6561 4815 6514 4798 6459
4719 6453 4701 6398 4677 6346 4612 6347 4605 6290 4560 6254
4552 6197 4530 6176 4545 6140 4541 6082 4553 6025 4538 5970
4502 5924 4462 5882 4409 5859 4366 5854 4357 5804 4299 5809
4246 5785 4190 5767 4140 5739 4146 5682 4166 5635 4126 5593
4099 5541 4042 5552 3981 5577 3940 5535 3930 5478 3932 5453
42 P
6937 6959 6994 6957 7049 6939 7104 6927 7157 6903 7212 6908
7242 6837 7278 6793 7335 6797 7416 6811 7415 6754 7436 6701
7467 6652 7494 6602 7515 6548 7492 6495 7491 6438 7507 6383
7529 6329 7555 6278 7587 6229 7617 6207 22 P
2297 9444 2313 9434 2364 9410 2418 9397 2474 9389 2529 9397
2582 9417 2627 9451 2650 9502 2683 9547 2740 9545 2794 9532
2850 9541 2894 9575 2931 9617 2959 9666 3009 9691 3063 9673
3072 9617 3097 9567 3147 9592 3197 9617 3250 9600 3306 9593
3315 9538 3325 9483 3323 9426 3333 9371 3380 9339 3419 9298
3412 9242 3432 9190 3442 9163 3479 9207 3500 9244 3499 9187

3492 9131 3483 9090 3538 9078 3590 9056 3645 9066 3672 9115
3684 9171 3714 9200 3742 9213 45 P
5955 5622 5983 5626 6012 5633 6035 5637 4 P
9446 10405 9439 10460 9481 10497 9510 10545 9524 10598 9548 10648
9593 10681 9627 10702 8 P
7594 15162 7599 15188 7602 15216 7587 15238 7565 15253 7544 15271
7542 15298 7528 15321 7528 15346 7554 15343 7581 15343 7602 15342
7627 15355 7652 15362 7679 15362 7705 15369 7730 15376 17 P
3308 4878 3330 4878 3330 4605 3296 4605 3296 4800 3236 4800
3236 4824 3260 4827 3271 4829 3281 4833 3290 4839 3294 4843
3297 4848 3303 4861 3308 4878 15 N F
3523 4878 3545 4878 3545 4605 3511 4605 3511 4800 3450 4800
3450 4824 3475 4827 3486 4829 3496 4833 3504 4839 3508 4843
3512 4848 3518 4861 3523 4878 15 N F
3731 4878 3741 4877 3751 4875 3760 4871 3768 4866 3776 4859
3784 4852 3791 4843 3797 4833 3802 4823 3807 4812 3812 4800
3815 4788 3818 4776 3820 4763 3821 4750 3822 4738 3821 4725
3820 4712 3815 4686 3812 4674 3807 4662 3802 4651 3797 4641
3791 4631 3784 4622 3776 4614 3768 4607 3760 4602 3751 4598
3741 4596 3731 4595 3721 4596 3712 4598 3703 4602 3694 4607
3686 4614 3679 4622 3672 4631 3666 4641 3660 4651 3655 4662
3651 4674 3648 4686 3643 4712 3641 4725 3641 4738 3677 4738
3678 4713 3682 4691 3687 4672 3695 4656 3703 4643 3712 4633
3717 4629 3722 4627 3726 4625 3731 4625 3736 4625 3741 4627
3746 4629 3751 4633 3760 4643 3768 4656 3775 4672 3781 4691
3784 4713 3786 4738 3784 4762 3781 4784 3775 4803 3768 4818
3760 4831 3751 4840 3746 4844 3741 4846 3736 4848 3731 4848
3726 4848 3722 4846 3717 4844 3712 4840 3703 4831 3695 4818
3687 4803 3682 4784 3678 4762 3677 4738 3641 4738 3641 4750
3643 4763 3645 4776 3648 4788 3651 4800 3655 4812 3660 4823
3666 4833 3672 4843 3679 4852 3686 4859 3694 4866 3703 4871
3712 4875 3721 4877 3731 4878 105 N F
3989 4826 3988 4814 3984 4802 3978 4791 3971 4781 3961 4773
3950 4767 3938 4764 3925 4762 3925 4787 3917 4788 3910 4790
3903 4794 3898 4799 3893 4804 3889 4811 3887 4818 3886 4826
3887 4834 3889 4842 3893 4848 3898 4854 3903 4859 3910 4863
3917 4865 3925 4866 3933 4865 3941 4863 3947 4859 3953 4854
3958 4848 3962 4842 3964 4834 3965 4826 3964 4818 3962 4811
3958 4804 3953 4799 3947 4794 3941 4790 3933 4788 3925 4787
3925 4762 3913 4764 3901 4767 3890 4773 3880 4781 3872 4791
3866 4802 3863 4814 3861 4826 3863 4839 3866 4851 3872 4862
3880 4872 3890 4879 3901 4885 3913 4889 3925 4890 3938 4889
3950 4885 3961 4879 3971 4872 3978 4862 3984 4851 3988 4839

3989 4826 67 N F
4042 4886 4237 4886 4237 4854 4079 4854 4079 4764 4231 4764
4231 4731 4079 4731 4079 4637 4243 4637 4243 4605 4042 4605
4042 4886 13 N F
5472 4878 5494 4878 5494 4605 5460 4605 5460 4800 5399 4800
5399 4824 5424 4827 5435 4829 5445 4833 5453 4839 5457 4843
5461 4848 5467 4861 5472 4878 15 N F
5686 4878 5708 4878 5708 4605 5674 4605 5674 4800 5614 4800
5614 4824 5638 4827 5649 4829 5659 4833 5668 4839 5672 4843
5676 4848 5682 4861 5686 4878 15 N F
5925 4878 5949 4878 5949 4701 5991 4701 5991 4669 5949 4669
5949 4605 5915 4605 5915 4669 5801 4669 5801 4701 5831 4701
5915 4701 5915 4820 5831 4701 5801 4701 5801 4705 5925 4878
18 N F
6153 4826 6152 4814 6148 4802 6142 4791 6134 4781 6125 4773
6114 4767 6102 4764 6089 4762 6089 4787 6081 4788 6074 4790
6067 4794 6061 4799 6056 4804 6053 4811 6051 4818 6050 4826
6051 4834 6053 4842 6056 4848 6061 4854 6067 4859 6074 4863
6081 4865 6089 4866 6097 4865 6104 4863 6111 4859 6117 4854
6122 4848 6125 4842 6128 4834 6128 4826 6128 4818 6125 4811
6122 4804 6117 4799 6111 4794 6104 4790 6097 4788 6089 4787
6089 4762 6076 4764 6064 4767 6053 4773 6044 4781 6036 4791
6030 4802 6026 4814 6025 4826 6026 4839 6030 4851 6036 4862
6044 4872 6053 4879 6064 4885 6076 4889 6089 4890 6102 4889
6114 4885 6125 4879 6134 4872 6142 4862 6148 4851 6152 4839
6153 4826 67 N F
6206 4886 6401 4886 6401 4854 6242 4854 6242 4764 6395 4764
6395 4731 6242 4731 6242 4637 6407 4637 6407 4605 6206 4605
6206 4886 13 N F
7620 4878 7642 4878 7642 4605 7608 4605 7608 4800 7548 4800
7548 4824 7572 4827 7583 4829 7593 4833 7602 4839 7606 4843
7610 4848 7616 4861 7620 4878 15 N F
7835 4878 7857 4878 7857 4605 7823 4605 7823 4800 7763 4800
7763 4824 7787 4827 7798 4829 7808 4833 7817 4839 7821 4843
7824 4848 7831 4861 7835 4878 15 N F
8044 4878 8061 4877 8076 4873 8090 4867 8102 4859 8112 4849
8116 4843 8120 4837 8123 4830 8125 4822 8126 4814 8126 4806
8125 4794 8123 4784 8119 4775 8114 4768 8109 4761 8102 4756
8090 4748 8100 4742 8109 4736 8117 4729 8124 4720 8129 4711
8133 4701 8135 4690 8136 4679 8135 4666 8130 4652 8123 4637
8112 4624 8106 4618 8099 4613 8091 4607 8083 4603 8074 4600
8064 4597 8053 4595 8042 4595 8031 4595 8020 4597 8010 4600
8002 4603 7993 4607 7986 4613 7979 4618 7973 4624 7964 4637

7957 4652 7953 4666 7952 4679 7988 4679 7989 4668 7992 4658
7998 4649 8005 4641 8013 4634 8022 4629 8033 4626 8044 4625
8055 4626 8065 4629 8075 4634 8083 4641 8090 4649 8096 4658
8099 4668 8100 4679 8099 4690 8096 4700 8090 4710 8083 4718
8075 4724 8065 4729 8055 4732 8044 4733 8033 4732 8022 4729
8013 4724 8005 4718 7998 4710 7992 4700 7989 4690 7988 4679
7952 4679 7952 4690 7955 4701 7959 4710 7964 4719 7971 4728
7979 4735 7988 4742 7998 4748 7985 4756 7979 4761 7974 4768
7969 4775 7965 4784 7963 4794 7962 4806 7996 4806 7997 4797
8000 4788 8004 4781 8010 4775 8017 4770 8025 4767 8034 4764
8044 4764 8053 4764 8062 4767 8071 4770 8078 4775 8084 4781
8088 4788 8091 4797 8092 4806 8091 4815 8088 4823 8084 4831
8078 4837 8071 4842 8062 4845 8053 4847 8044 4848 8034 4847
8025 4845 8017 4842 8010 4837 8004 4831 8000 4823 7997 4815
7996 4806 7962 4806 7962 4814 7963 4822 7965 4830 7968 4837
7971 4843 7975 4849 7985 4859 7998 4867 8012 4873 8027 4877
8044 4878 145 N F
8302 4826 8300 4814 8297 4802 8291 4791 8283 4781 8274 4773
8263 4767 8251 4764 8238 4762 8238 4787 8230 4788 8223 4790
8216 4794 8210 4799 8205 4804 8202 4811 8199 4818 8199 4826
8199 4834 8202 4842 8205 4848 8210 4854 8216 4859 8223 4863
8230 4865 8238 4866 8246 4865 8253 4863 8260 4859 8266 4854
8271 4848 8274 4842 8276 4834 8277 4826 8276 4818 8274 4811
8271 4804 8266 4799 8260 4794 8253 4790 8246 4788 8238 4787
8238 4762 8225 4764 8213 4767 8202 4773 8193 4781 8185 4791
8179 4802 8175 4814 8174 4826 8175 4839 8179 4851 8185 4862
8193 4872 8202 4879 8213 4885 8225 4889 8238 4890 8251 4889
8263 4885 8274 4879 8283 4872 8291 4862 8297 4851 8300 4839
8302 4826 67 N F
8355 4886 8550 4886 8550 4854 8391 4854 8391 4764 8544 4764
8544 4731 8391 4731 8391 4637 8556 4637 8556 4605 8355 4605
8355 4886 13 N F
9783 4878 9805 4878 9805 4605 9771 4605 9771 4800 9710 4800
9710 4824 9735 4827 9746 4829 9756 4833 9764 4839 9768 4843
9772 4848 9778 4861 9783 4878 15 N F
9905 4784 9939 4784 9940 4797 9943 4810 9947 4821 9953 4830
9961 4838 9971 4843 9982 4847 9995 4848 10006 4847 10015 4844
10024 4840 10032 4834 10039 4826 10043 4818 10047 4807 10048 4796
10047 4784 10044 4774 10039 4766 10032 4759 10024 4752 10013 4744
9985 4727 9962 4714 9943 4700 9928 4686 9917 4671 9908 4656
9903 4640 9900 4623 9899 4605 10082 4605 10082 4637 9937 4637
9941 4649 9946 4660 9952 4668 9960 4676 9970 4684 9982 4691
10014 4709 10032 4718 10047 4728 10059 4739 10068 4751 10075 4763

10080 4775 10083 4788 10084 4800 10083 4808 10082 4816 10080 4824
10077 4832 10073 4838 10069 4845 10063 4851 10058 4856 10045 4866
10030 4872 10013 4877 9995 4878 9977 4877 9968 4875 9959 4872
9951 4869 9944 4865 9937 4861 9931 4855 9925 4849 9920 4842
9916 4834 9912 4826 9909 4816 9907 4806 9906 4795 9905 4784
78 N F
10120 4784 10154 4784 10155 4797 10157 4810 10162 4821 10168 4830
10176 4838 10185 4843 10197 4847 10210 4848 10221 4847 10230 4844
10239 4840 10247 4834 10253 4826 10258 4818 10261 4807 10262 4796
10261 4784 10259 4774 10254 4766 10247 4759 10238 4752 10228 4744
10200 4727 10177 4714 10158 4700 10143 4686 10131 4671 10123 4656
10118 4640 10115 4623 10114 4605 10297 4605 10297 4637 10152 4637
10156 4649 10161 4660 10167 4668 10175 4676 10185 4684 10197 4691
10228 4709 10246 4718 10261 4728 10273 4739 10283 4751 10290 4763
10295 4775 10298 4788 10299 4800 10298 4808 10297 4816 10295 4824
10292 4832 10288 4838 10283 4845 10278 4851 10272 4856 10259 4866
10244 4872 10228 4877 10210 4878 10191 4877 10183 4875 10174 4872
10166 4869 10159 4865 10152 4861 10145 4855 10140 4849 10135 4842
10130 4834 10127 4826 10124 4816 10122 4806 10120 4795 10120 4784
78 N F
10464 4826 10463 4814 10459 4802 10453 4791 10445 4781 10436 4773
10425 4767 10413 4764 10400 4762 10400 4787 10392 4788 10385 4790
10378 4794 10372 4799 10368 4804 10364 4811 10362 4818 10361 4826
10362 4834 10364 4842 10368 4848 10372 4854 10378 4859 10385 4863
10392 4865 10400 4866 10408 4865 10416 4863 10422 4859 10428 4854
10433 4848 10436 4842 10439 4834 10440 4826 10439 4818 10436 4811
10433 4804 10428 4799 10422 4794 10416 4790 10408 4788 10400 4787
10400 4762 10387 4764 10375 4767 10365 4773 10355 4781 10347 4791
10341 4802 10338 4814 10336 4826 10338 4839 10341 4851 10347 4862
10355 4872 10365 4879 10375 4885 10387 4889 10400 4890 10413 4889
10425 4885 10436 4879 10445 4872 10453 4862 10459 4851 10463 4839
10464 4826 67 N F
10517 4886 10712 4886 10712 4854 10553 4854 10553 4764 10706 4764
10706 4731 10553 4731 10553 4637 10718 4637 10718 4605 10517 4605
10517 4886 13 N F
11945 4878 11967 4878 11967 4605 11933 4605 11933 4800 11873 4800
11873 4824 11897 4827 11908 4829 11918 4833 11927 4839 11931 4843
11934 4848 11940 4861 11945 4878 15 N F
12067 4784 12102 4784 12102 4797 12105 4810 12109 4821 12115 4830
12123 4838 12133 4843 12144 4847 12158 4848 12168 4847 12178 4844
12187 4840 12194 4834 12201 4826 12206 4818 12209 4807 12210 4796
12209 4784 12206 4774 12201 4766 12195 4759 12186 4752 12175 4744
12148 4727 12124 4714 12105 4700 12090 4686 12079 4671 12071 4656

12065 4640 12062 4623 12061 4605 12244 4605 12244 4637 12100 4637
12103 4649 12108 4660 12115 4668 12123 4676 12133 4684 12145 4691
12176 4709 12194 4718 12209 4728 12221 4739 12231 4751 12238 4763
12242 4775 12245 4788 12246 4800 12246 4808 12244 4816 12242 4824
12239 4832 12235 4838 12231 4845 12226 4851 12220 4856 12207 4866
12192 4872 12175 4877 12158 4878 12139 4877 12130 4875 12122 4872
12114 4869 12106 4865 12099 4861 12093 4855 12087 4849 12082 4842
12078 4834 12074 4826 12071 4816 12069 4806 12068 4795 12067 4784
78 N F
12455 4808 12421 4808 12418 4817 12415 4825 12410 4832 12405 4838
12398 4842 12391 4845 12384 4847 12377 4848 12369 4848 12362 4846
12356 4844 12350 4841 12344 4837 12339 4833 12335 4827 12330 4821
12323 4806 12318 4789 12315 4768 12314 4746 12323 4754 12331 4761
12339 4767 12347 4770 12355 4773 12363 4775 12381 4776 12394 4774
12408 4770 12421 4764 12434 4754 12440 4748 12445 4742 12450 4734
12454 4727 12457 4718 12459 4708 12461 4698 12461 4687 12427 4685
12426 4696 12422 4707 12417 4718 12410 4727 12402 4734 12392 4740
12382 4744 12371 4746 12359 4744 12349 4740 12340 4734 12331 4727
12324 4718 12319 4707 12315 4696 12314 4685 12315 4674 12319 4663
12324 4653 12331 4644 12340 4636 12349 4630 12359 4626 12371 4625
12382 4626 12392 4630 12402 4636 12410 4644 12417 4653 12422 4663
12426 4674 12427 4685 12461 4687 12460 4671 12455 4655 12449 4639
12444 4632 12439 4625 12433 4619 12426 4613 12419 4608 12411 4603
12402 4600 12392 4597 12382 4595 12371 4595 12358 4595 12347 4598
12337 4601 12327 4605 12319 4611 12311 4618 12305 4626 12299 4634
12294 4644 12289 4655 12286 4666 12283 4678 12279 4704 12278 4731
12279 4748 12280 4764 12282 4779 12285 4793 12288 4806 12293 4818
12298 4829 12304 4839 12311 4848 12318 4856 12326 4862 12335 4868
12344 4872 12354 4876 12365 4878 12377 4878 12392 4877 12407 4873
12420 4866 12431 4857 12440 4847 12447 4835 12452 4822 12455 4808
126 N F
12626 4826 12625 4814 12621 4802 12615 4791 12608 4781 12598 4773
12587 4767 12575 4764 12562 4762 12562 4787 12555 4788 12547 4790
12540 4794 12535 4799 12530 4804 12526 4811 12524 4818 12523 4826
12524 4834 12526 4842 12530 4848 12535 4854 12540 4859 12547 4863
12555 4865 12562 4866 12570 4865 12578 4863 12585 4859 12590 4854
12595 4848 12599 4842 12601 4834 12602 4826 12601 4818 12599 4811
12595 4804 12590 4799 12585 4794 12578 4790 12570 4788 12562 4787
12562 4762 12550 4764 12538 4767 12527 4773 12517 4781 12510 4791
12504 4802 12500 4814 12499 4826 12500 4839 12504 4851 12510 4862
12517 4872 12527 4879 12538 4885 12550 4889 12562 4890 12575 4889
12587 4885 12598 4879 12608 4872 12615 4862 12621 4851 12625 4839
12626 4826 67 N F

12679 4886 12874 4886 12874 4854 12716 4854 12716 4764 12868 4764
12868 4731 12716 4731 12716 4637 12880 4637 12880 4605 12679 4605
12679 4886 13 N F
2316 17019 2338 17019 2338 16745 2304 16745 2304 16940 2244 16940
2244 16964 2268 16967 2279 16970 2289 16973 2298 16980 2301 16984
2305 16989 2311 17002 2316 17019 15 N F
2525 17019 2535 17018 2544 17015 2553 17012 2562 17006 2570 17000
2577 16992 2584 16984 2590 16974 2596 16964 2601 16953 2605 16941
2608 16929 2611 16916 2613 16904 2614 16891 2615 16878 2614 16865
2613 16852 2608 16827 2605 16815 2601 16803 2596 16792 2590 16781
2584 16771 2577 16762 2570 16755 2562 16748 2553 16743 2544 16739
2535 16736 2525 16735 2515 16736 2505 16739 2496 16743 2488 16748
2480 16755 2472 16762 2465 16771 2459 16781 2453 16792 2449 16803
2444 16815 2441 16827 2436 16852 2435 16865 2434 16878 2470 16878
2472 16854 2475 16832 2481 16812 2488 16796 2496 16783 2505 16773
2510 16770 2515 16767 2520 16766 2525 16765 2529 16766 2534 16767
2539 16770 2544 16773 2553 16783 2561 16796 2568 16812 2574 16832
2577 16854 2579 16878 2577 16902 2574 16924 2568 16943 2561 16959
2553 16972 2544 16981 2539 16984 2534 16987 2529 16988 2525 16989
2520 16988 2515 16987 2510 16984 2505 16981 2496 16972 2488 16959
2481 16943 2475 16924 2472 16902 2470 16878 2434 16878 2435 16891
2436 16904 2438 16916 2441 16929 2444 16941 2449 16953 2453 16964
2459 16974 2465 16984 2472 16992 2480 17000 2488 17006 2496 17012
2505 17015 2515 17018 2525 17019 105 N F
2675 17019 2818 17019 2818 16987 2703 16987 2693 16908 2703 16915
2715 16921 2729 16925 2745 16926 2759 16925 2774 16921 2789 16915
2795 16911 2802 16906 2808 16900 2814 16893 2819 16886 2823 16877
2827 16868 2829 16857 2831 16846 2832 16834 2830 16815 2828 16805
2826 16796 2822 16788 2818 16780 2813 16772 2808 16765 2802 16758
2794 16753 2787 16748 2778 16743 2769 16740 2759 16737 2749 16736
2737 16735 2727 16736 2717 16737 2708 16739 2700 16741 2692 16745
2685 16748 2679 16753 2673 16758 2664 16769 2656 16782 2651 16796
2647 16812 2681 16812 2685 16802 2689 16793 2694 16785 2701 16778
2708 16773 2716 16769 2725 16766 2735 16765 2749 16766 2761 16770
2772 16775 2781 16782 2788 16791 2793 16803 2796 16816 2798 16832
2797 16843 2794 16855 2790 16865 2783 16875 2775 16884 2764 16890
2752 16895 2745 16896 2737 16896 2730 16896 2723 16895 2716 16893
2710 16890 2699 16882 2685 16870 2655 16870 2675 17019 83 N F
2997 16967 2996 16954 2992 16942 2986 16931 2978 16922 2969 16914
2958 16908 2946 16904 2933 16903 2933 16928 2925 16928 2918 16931
2911 16934 2905 16939 2901 16945 2897 16952 2895 16959 2894 16967
2895 16975 2897 16982 2901 16989 2905 16995 2911 17000 2918 17003
2925 17006 2933 17006 2941 17006 2949 17003 2955 17000 2961 16995

2966 16989 2970 16982 2972 16975 2973 16967 2972 16959 2970 16952
2966 16945 2961 16939 2955 16934 2949 16931 2941 16928 2933 16928
2933 16903 2920 16904 2908 16908 2898 16914 2888 16922 2880 16931
2874 16942 2871 16954 2869 16967 2871 16980 2874 16992 2880 17003
2888 17012 2898 17020 2908 17026 2920 17030 2933 17031 2946 17030
2958 17026 2969 17020 2978 17012 2986 17003 2992 16992 2996 16980
2997 16967 67 N F
3050 17027 3245 17027 3245 16995 3086 16995 3086 16904 3239 16904
3239 16872 3086 16872 3086 16778 3251 16778 3251 16745 3050 16745
3050 17027 13 N F
4381 17019 4403 17019 4403 16745 4369 16745 4369 16940 4309 16940
4309 16964 4333 16967 4344 16970 4354 16973 4363 16980 4367 16984
4370 16989 4376 17002 4381 17019 15 N F
4596 17019 4618 17019 4618 16745 4584 16745 4584 16940 4523 16940
4523 16964 4548 16967 4559 16970 4569 16973 4577 16980 4581 16984
4585 16989 4591 17002 4596 17019 15 N F
4804 17019 4814 17018 4824 17015 4833 17012 4841 17006 4849 17000
4857 16992 4864 16984 4870 16974 4875 16964 4880 16953 4885 16941
4888 16929 4891 16916 4893 16904 4894 16891 4895 16878 4894 16865
4893 16852 4888 16827 4885 16815 4880 16803 4875 16792 4870 16781
4864 16771 4857 16762 4849 16755 4841 16748 4833 16743 4824 16739
4814 16736 4804 16735 4794 16736 4785 16739 4776 16743 4767 16748
4759 16755 4752 16762 4745 16771 4739 16781 4733 16792 4728 16803
4724 16815 4721 16827 4716 16852 4715 16865 4714 16878 4750 16878
4751 16854 4755 16832 4760 16812 4768 16796 4776 16783 4785 16773
4790 16770 4795 16767 4799 16766 4804 16765 4809 16766 4814 16767
4819 16770 4824 16773 4833 16783 4841 16796 4848 16812 4854 16832
4857 16854 4859 16878 4857 16902 4854 16924 4848 16943 4841 16959
4833 16972 4824 16981 4819 16984 4814 16987 4809 16988 4804 16989
4799 16988 4795 16987 4790 16984 4785 16981 4776 16972 4768 16959
4760 16943 4755 16924 4751 16902 4750 16878 4714 16878 4715 16891
4716 16904 4718 16916 4721 16929 4724 16941 4728 16953 4733 16964
4739 16974 4745 16984 4752 16992 4759 17000 4767 17006 4776 17012
4785 17015 4794 17018 4804 17019 105 N F
5062 16967 5061 16954 5057 16942 5051 16931 5044 16922 5034 16914
5023 16908 5011 16904 4998 16903 4998 16928 4990 16928 4983 16931
4976 16934 4971 16939 4966 16945 4962 16952 4960 16959 4959 16967
4960 16975 4962 16982 4966 16989 4971 16995 4976 17000 4983 17003
4990 17006 4998 17006 5006 17006 5014 17003 5020 17000 5026 16995
5031 16989 5035 16982 5037 16975 5038 16967 5037 16959 5035 16952
5031 16945 5026 16939 5020 16934 5014 16931 5006 16928 4998 16928
4998 16903 4986 16904 4974 16908 4963 16914 4953 16922 4945 16931
4940 16942 4936 16954 4934 16967 4936 16980 4940 16992 4945 17003

4953 17012 4963 17020 4974 17026 4986 17030 4998 17031 5011 17030
5023 17026 5034 17020 5044 17012 5051 17003 5057 16992 5061 16980
5062 16967 67 N F
5115 17027 5310 17027 5310 16995 5152 16995 5152 16904 5304 16904
5304 16872 5152 16872 5152 16778 5316 16778 5316 16745 5115 16745
5115 17027 13 N F
6412 17019 6434 17019 6434 16745 6400 16745 6400 16940 6340 16940
6340 16964 6364 16967 6375 16970 6385 16973 6394 16980 6397 16984
6401 16989 6407 17002 6412 17019 15 N F
6627 17019 6649 17019 6649 16745 6614 16745 6614 16940 6554 16940
6554 16964 6579 16967 6589 16970 6599 16973 6608 16980 6612 16984
6616 16989 6622 17002 6627 17019 15 N F
6771 17019 6914 17019 6914 16987 6799 16987 6789 16908 6799 16915
6811 16921 6825 16925 6841 16926 6855 16925 6870 16921 6884 16915
6891 16911 6898 16906 6904 16900 6910 16893 6915 16886 6919 16877
6923 16868 6925 16857 6927 16846 6928 16834 6926 16815 6924 16805
6922 16796 6918 16788 6914 16780 6909 16772 6904 16765 6897 16758
6890 16753 6883 16748 6874 16743 6865 16740 6855 16737 6845 16736
6833 16735 6823 16736 6813 16737 6804 16739 6796 16741 6788 16745
6781 16748 6775 16753 6769 16758 6760 16769 6752 16782 6747 16796
6743 16812 6777 16812 6781 16802 6785 16793 6790 16785 6797 16778
6804 16773 6812 16769 6821 16766 6831 16765 6845 16766 6857 16770
6868 16775 6877 16782 6884 16791 6889 16803 6892 16816 6893 16832
6893 16843 6890 16855 6886 16865 6879 16875 6871 16884 6860 16890
6848 16895 6841 16896 6833 16896 6825 16896 6819 16895 6812 16893
6806 16890 6795 16882 6781 16870 6751 16870 6771 17019 83 N F
7093 16967 7092 16954 7088 16942 7082 16931 7074 16922 7065 16914
7054 16908 7042 16904 7029 16903 7029 16928 7021 16928 7014 16931
7007 16934 7001 16939 6997 16945 6993 16952 6991 16959 6990 16967
6991 16975 6993 16982 6997 16989 7001 16995 7007 17000 7014 17003
7021 17006 7029 17006 7037 17006 7045 17003 7051 17000 7057 16995
7062 16989 7065 16982 7068 16975 7069 16967 7068 16959 7065 16952
7062 16945 7057 16939 7051 16934 7045 16931 7037 16928 7029 16928
7029 16903 7016 16904 7004 16908 6994 16914 6984 16922 6976 16931
6970 16942 6967 16954 6965 16967 6967 16980 6970 16992 6976 17003
6984 17012 6994 17020 7004 17026 7016 17030 7029 17031 7042 17030
7054 17026 7065 17020 7074 17012 7082 17003 7088 16992 7092 16980
7093 16967 67 N F
7146 17027 7341 17027 7341 16995 7182 16995 7182 16904 7335 16904
7335 16872 7182 16872 7182 16778 7347 16778 7347 16745 7146 16745
7146 17027 13 N F
8433 17019 8455 17019 8455 16745 8421 16745 8421 16940 8361 16940
8361 16964 8385 16967 8396 16970 8406 16973 8415 16980 8419 16984

8422 16989 8428 17002 8433 17019 15 N F
8555 16924 8589 16924 8590 16938 8593 16950 8597 16961 8603 16971
8611 16978 8621 16984 8632 16987 8646 16989 8656 16988 8666 16985
8675 16981 8682 16974 8689 16967 8694 16958 8697 16948 8698 16936
8697 16925 8694 16915 8689 16907 8683 16899 8674 16892 8663 16885
8636 16868 8612 16854 8593 16841 8578 16827 8567 16812 8559 16797
8553 16781 8550 16764 8549 16745 8732 16745 8732 16778 8587 16778
8591 16790 8596 16800 8602 16809 8611 16817 8621 16824 8633 16832
8664 16850 8682 16859 8697 16869 8709 16880 8718 16891 8726 16903
8730 16916 8733 16928 8734 16940 8734 16949 8732 16957 8730 16965
8727 16972 8723 16979 8719 16985 8714 16991 8708 16997 8695 17006
8680 17013 8663 17017 8646 17019 8627 17017 8618 17016 8610 17013
8602 17010 8594 17006 8587 17001 8581 16996 8575 16989 8570 16982
8566 16975 8562 16966 8559 16957 8557 16947 8556 16936 8555 16924
78 N F
8856 17019 8866 17018 8876 17015 8885 17012 8893 17006 8901 17000
8909 16992 8916 16984 8922 16974 8928 16964 8932 16953 8937 16941
8940 16929 8943 16916 8945 16904 8946 16891 8947 16878 8946 16865
8945 16852 8940 16827 8937 16815 8932 16803 8928 16792 8922 16781
8916 16771 8909 16762 8901 16755 8893 16748 8885 16743 8876 16739
8866 16736 8856 16735 8846 16736 8837 16739 8828 16743 8819 16748
8811 16755 8804 16762 8797 16771 8791 16781 8785 16792 8780 16803
8776 16815 8773 16827 8768 16852 8767 16865 8766 16878 8802 16878
8803 16854 8807 16832 8812 16812 8820 16796 8828 16783 8837 16773
8842 16770 8847 16767 8852 16766 8856 16765 8861 16766 8866 16767
8871 16770 8876 16773 8885 16783 8893 16796 8900 16812 8906 16832
8909 16854 8911 16878 8909 16902 8906 16924 8900 16943 8893 16959
8885 16972 8876 16981 8871 16984 8866 16987 8861 16988 8856 16989
8852 16988 8847 16987 8842 16984 8837 16981 8828 16972 8820 16959
8812 16943 8807 16924 8803 16902 8802 16878 8766 16878 8767 16891
8768 16904 8770 16916 8773 16929 8776 16941 8780 16953 8785 16964
8791 16974 8797 16984 8804 16992 8811 17000 8819 17006 8828 17012
8837 17015 8846 17018 8856 17019 105 N F
9114 16967 9113 16954 9109 16942 9103 16931 9096 16922 9086 16914
9075 16908 9063 16904 9050 16903 9050 16928 9042 16928 9035 16931
9028 16934 9023 16939 9018 16945 9014 16952 9012 16959 9011 16967
9012 16975 9014 16982 9018 16989 9023 16995 9028 17000 9035 17003
9042 17006 9050 17006 9058 17006 9066 17003 9072 17000 9078 16995
9083 16989 9087 16982 9089 16975 9090 16967 9089 16959 9087 16952
9083 16945 9078 16939 9072 16934 9066 16931 9058 16928 9050 16928
9050 16903 9038 16904 9026 16908 9015 16914 9005 16922 8997 16931
8992 16942 8988 16954 8986 16967 8988 16980 8992 16992 8997 17003
9005 17012 9015 17020 9026 17026 9038 17030 9050 17031 9063 17030

9075 17026 9086 17020 9096 17012 9103 17003 9109 16992 9113 16980
9114 16967 67 N F
9167 17027 9362 17027 9362 16995 9204 16995 9204 16904 9356 16904
9356 16872 9204 16872 9204 16778 9368 16778 9368 16745 9167 16745
9167 17027 13 N F
10460 17019 10482 17019 10482 16745 10448 16745 10448 16940 10388 16940
10388 16964 10412 16967 10423 16970 10433 16973 10442 16980 10446 16984
10450 16989 10456 17002 10460 17019 15 N F
10583 16924 10617 16924 10618 16938 10620 16950 10625 16961 10631 16971
10639 16978 10648 16984 10660 16987 10673 16989 10683 16988 10693 16985
10702 16981 10710 16974 10716 16967 10721 16958 10724 16948 10725 16936
10724 16925 10721 16915 10717 16907 10710 16899 10701 16892 10691 16885
10663 16868 10640 16854 10621 16841 10606 16827 10594 16812 10586 16797
10581 16781 10578 16764 10577 16745 10759 16745 10759 16778 10615 16778
10618 16790 10623 16800 10630 16809 10638 16817 10648 16824 10660 16832
10691 16850 10709 16859 10724 16869 10736 16880 10746 16891 10753 16903
10758 16916 10761 16928 10761 16940 10761 16949 10760 16957 10757 16965
10754 16972 10751 16979 10746 16985 10741 16991 10735 16997 10722 17006
10707 17013 10691 17017 10673 17019 10654 17017 10645 17016 10637 17013
10629 17010 10622 17006 10615 17001 10608 16996 10603 16989 10598 16982
10593 16975 10590 16966 10587 16957 10585 16947 10583 16936 10583 16924
78 N F
10820 17019 10962 17019 10962 16987 10848 16987 10838 16908 10848 16915
10859 16921 10873 16925 10890 16926 10904 16925 10919 16921 10933 16915
10940 16911 10947 16906 10953 16900 10958 16893 10963 16886 10968 16877
10971 16868 10974 16857 10976 16846 10976 16834 10975 16815 10973 16805
10970 16796 10967 16788 10963 16780 10958 16772 10952 16765 10946 16758
10939 16753 10931 16748 10923 16743 10914 16740 10904 16737 10893 16736
10882 16735 10871 16736 10862 16737 10853 16739 10844 16741 10837 16745
10830 16748 10824 16753 10818 16758 10808 16769 10801 16782 10795 16796
10792 16812 10826 16812 10829 16802 10834 16793 10839 16785 10845 16778
10852 16773 10861 16769 10870 16766 10880 16765 10893 16766 10906 16770
10916 16775 10925 16782 10932 16791 10938 16803 10941 16816 10942 16832
10941 16843 10939 16855 10934 16865 10928 16875 10919 16884 10909 16890
10897 16895 10889 16896 10882 16896 10874 16896 10867 16895 10861 16893
10855 16890 10843 16882 10830 16870 10800 16870 10820 17019 83 N F
11142 16967 11140 16954 11137 16942 11131 16931 11123 16922 11113 16914
11103 16908 11091 16904 11078 16903 11078 16928 11070 16928 11062 16931
11056 16934 11050 16939 11045 16945 11042 16952 11039 16959 11038 16967
11039 16975 11042 16982 11045 16989 11050 16995 11056 17000 11062 17003
11070 17006 11078 17006 11086 17006 11093 17003 11100 17000 11106 16995
11110 16989 11114 16982 11116 16975 11117 16967 11116 16959 11114 16952
11110 16945 11106 16939 11100 16934 11093 16931 11086 16928 11078 16928

11078 16903 11065 16904 11053 16908 11042 16914 11033 16922 11025 16931
11019 16942 11015 16954 11014 16967 11015 16980 11019 16992 11025 17003
11033 17012 11042 17020 11053 17026 11065 17030 11078 17031 11091 17030
11103 17026 11113 17020 11123 17012 11131 17003 11137 16992 11140 16980
11142 16967 67 N F
11195 17027 11390 17027 11390 16995 11231 16995 11231 16904 11383 16904
11383 16872 11231 16872 11231 16778 11396 16778 11396 16745 11195 16745
11195 17027 13 N F
12511 17019 12533 17019 12533 16745 12499 16745 12499 16940 12439 16940
12439 16964 12463 16967 12474 16970 12484 16973 12493 16980 12497 16984
12500 16989 12506 17002 12511 17019 15 N F
12666 16930 12631 16930 12634 16951 12636 16960 12638 16969 12642 16977
12646 16984 12651 16990 12656 16996 12662 17002 12668 17006 12675 17010
12683 17013 12691 17016 12699 17017 12718 17019 12735 17017 12751 17014
12766 17008 12778 17000 12783 16995 12788 16989 12792 16983 12796 16976
12798 16969 12800 16961 12802 16953 12802 16944 12802 16934 12800 16925
12797 16917 12793 16909 12788 16902 12781 16896 12772 16891 12762 16886
12774 16881 12784 16875 12792 16869 12799 16861 12804 16853 12807 16844
12809 16833 12810 16822 12809 16807 12805 16792 12799 16777 12794 16771
12789 16764 12783 16759 12776 16753 12769 16749 12760 16745 12751 16742
12740 16739 12728 16738 12716 16737 12705 16738 12695 16739 12685 16741
12677 16744 12669 16747 12661 16751 12655 16756 12649 16762 12644 16768
12639 16774 12636 16781 12633 16789 12629 16806 12627 16824 12662 16824
12662 16814 12664 16804 12667 16794 12672 16786 12679 16778 12689 16773
12701 16769 12718 16767 12732 16768 12743 16771 12753 16776 12761 16783
12767 16791 12771 16800 12773 16811 12774 16822 12773 16836 12771 16842
12768 16847 12766 16852 12762 16856 12753 16863 12742 16867 12729 16870
12714 16872 12698 16872 12698 16900 12714 16900 12728 16902 12740 16904
12749 16908 12756 16913 12762 16920 12765 16930 12766 16942 12765 16952
12763 16961 12760 16968 12755 16975 12748 16981 12740 16985 12730 16988
12718 16989 12707 16988 12697 16986 12689 16982 12681 16977 12675 16969
12670 16959 12667 16946 12666 16930 123 N F
12934 17019 12944 17018 12954 17015 12963 17012 12971 17006 12979 17000
12987 16992 12994 16984 13000 16974 13006 16964 13011 16953 13015 16941
13018 16929 13021 16916 13023 16904 13024 16891 13025 16878 13024 16865
13023 16852 13018 16827 13015 16815 13011 16803 13006 16792 13000 16781
12994 16771 12987 16762 12979 16755 12971 16748 12963 16743 12954 16739
12944 16736 12934 16735 12925 16736 12915 16739 12906 16743 12898 16748
12890 16755 12882 16762 12875 16771 12869 16781 12863 16792 12858 16803
12854 16815 12851 16827 12846 16852 12845 16865 12844 16878 12880 16878
12882 16854 12885 16832 12891 16812 12898 16796 12906 16783 12915 16773
12920 16770 12925 16767 12930 16766 12934 16765 12939 16766 12944 16767
12949 16770 12954 16773 12963 16783 12971 16796 12978 16812 12984 16832

12987 16854 12989 16878 12987 16902 12984 16924 12978 16943 12971 16959
12963 16972 12954 16981 12949 16984 12944 16987 12939 16988 12934 16989
12930 16988 12925 16987 12920 16984 12915 16981 12906 16972 12898 16959
12891 16943 12885 16924 12882 16902 12880 16878 12844 16878 12845 16891
12846 16904 12848 16916 12851 16929 12854 16941 12858 16953 12863 16964
12869 16974 12875 16984 12882 16992 12890 17000 12898 17006 12906 17012
12915 17015 12925 17018 12934 17019 105 N F
13192 16967 13191 16954 13187 16942 13181 16931 13174 16922 13164 16914
13153 16908 13141 16904 13128 16903 13128 16928 13121 16928 13113 16931
13106 16934 13101 16939 13096 16945 13092 16952 13090 16959 13089 16967
13090 16975 13092 16982 13096 16989 13101 16995 13106 17000 13113 17003
13121 17006 13128 17006 13136 17006 13144 17003 13150 17000 13156 16995
13161 16989 13165 16982 13167 16975 13168 16967 13167 16959 13165 16952
13161 16945 13156 16939 13150 16934 13144 16931 13136 16928 13128 16928
13128 16903 13116 16904 13104 16908 13093 16914 13083 16922 13076 16931
13070 16942 13066 16954 13065 16967 13066 16980 13070 16992 13076 17003
13083 17012 13093 17020 13104 17026 13116 17030 13128 17031 13141 17030
13153 17026 13164 17020 13174 17012 13181 17003 13187 16992 13191 16980
13192 16967 67 N F
13245 17027 13440 17027 13440 16995 13282 16995 13282 16904 13434 16904
13434 16872 13282 16872 13282 16778 13446 16778 13446 16745 13245 16745
13245 17027 13 N F
911 5683 945 5683 946 5696 948 5709 952 5720 959 5729
966 5737 976 5742 988 5746 1001 5747 1011 5746 1021 5743
1030 5739 1038 5733 1044 5725 1049 5717 1052 5706 1053 5695
1052 5683 1049 5673 1045 5665 1038 5658 1029 5651 1018 5643
991 5626 968 5613 949 5599 934 5585 922 5570 914 5555
909 5539 906 5522 905 5504 1087 5504 1087 5536 943 5536
946 5548 951 5559 958 5567 966 5575 976 5583 988 5590
1019 5608 1037 5617 1052 5627 1064 5638 1074 5650 1081 5662
1086 5674 1088 5687 1089 5699 1089 5707 1087 5715 1085 5723
1082 5731 1078 5737 1074 5744 1069 5750 1063 5755 1050 5765
1035 5771 1019 5776 1001 5777 982 5776 973 5774 965 5771
957 5768 949 5764 943 5760 936 5754 931 5748 925 5741
921 5733 917 5725 914 5715 912 5705 911 5694 911 5683
78 N F
1125 5683 1160 5683 1160 5696 1163 5709 1167 5720 1173 5729
1181 5737 1191 5742 1202 5746 1216 5747 1226 5746 1236 5743
1245 5739 1252 5733 1259 5725 1264 5717 1267 5706 1268 5695
1267 5683 1264 5673 1259 5665 1253 5658 1244 5651 1233 5643
1206 5626 1182 5613 1163 5599 1148 5585 1137 5570 1129 5555
1123 5539 1120 5522 1119 5504 1302 5504 1302 5536 1157 5536
1161 5548 1166 5559 1172 5567 1181 5575 1191 5583 1203 5590

1234 5608 1252 5617 1267 5627 1279 5638 1288 5650 1296 5662
1300 5674 1303 5687 1304 5699 1304 5707 1302 5715 1300 5723
1297 5731 1293 5737 1289 5744 1284 5750 1278 5755 1265 5765
1250 5771 1233 5776 1216 5777 1197 5776 1188 5774 1180 5771
1172 5768 1164 5764 1157 5760 1151 5754 1145 5748 1140 5741
1136 5733 1132 5725 1129 5715 1127 5705 1126 5694 1125 5683
78 N F
1470 5725 1468 5713 1465 5701 1459 5690 1451 5680 1441 5672
1430 5666 1419 5663 1406 5661 1406 5686 1398 5687 1390 5689
1384 5693 1378 5698 1373 5703 1369 5710 1367 5717 1366 5725
1367 5733 1369 5741 1373 5747 1378 5753 1384 5758 1390 5762
1398 5764 1406 5765 1414 5764 1421 5762 1428 5758 1434 5753
1438 5747 1442 5741 1444 5733 1445 5725 1444 5717 1442 5710
1438 5703 1434 5698 1428 5693 1421 5689 1414 5687 1406 5686
1406 5661 1393 5663 1381 5666 1370 5672 1361 5680 1353 5690
1347 5701 1343 5713 1342 5725 1343 5738 1347 5750 1353 5761
1361 5771 1370 5778 1381 5784 1393 5788 1406 5789 1419 5788
1430 5784 1441 5778 1451 5771 1459 5761 1465 5750 1468 5738
1470 5725 67 N F
1517 5785 1557 5785 1701 5556 1701 5785 1735 5785 1735 5504
1695 5504 1551 5733 1551 5504 1517 5504 1517 5785 11 N F
911 6859 945 6859 946 6873 948 6886 952 6897 959 6906
966 6913 976 6919 988 6923 1001 6924 1011 6923 1021 6920
1030 6916 1038 6910 1044 6902 1049 6893 1052 6883 1053 6872
1052 6860 1049 6850 1045 6842 1038 6834 1029 6827 1018 6820
991 6803 968 6790 949 6776 934 6762 922 6747 914 6732
909 6716 906 6699 905 6681 1087 6681 1087 6713 943 6713
946 6725 951 6735 958 6744 966 6752 976 6759 988 6767
1019 6785 1037 6794 1052 6804 1064 6815 1074 6827 1081 6839
1086 6851 1088 6863 1089 6876 1089 6884 1087 6892 1085 6900
1082 6907 1078 6914 1074 6921 1069 6927 1063 6932 1050 6941
1035 6948 1019 6952 1001 6954 982 6952 973 6951 965 6948
957 6945 949 6941 943 6936 936 6931 931 6925 925 6918
921 6910 917 6901 914 6892 912 6882 911 6871 911 6859
78 N F
1242 6954 1266 6954 1266 6777 1308 6777 1308 6745 1266 6745
1266 6681 1232 6681 1232 6745 1117 6745 1117 6777 1147 6777
1232 6777 1232 6896 1147 6777 1117 6777 1117 6781 1242 6954
18 N F
1470 6902 1468 6889 1465 6877 1459 6866 1451 6857 1441 6849
1430 6843 1419 6839 1406 6838 1406 6863 1398 6864 1390 6866
1384 6869 1378 6874 1373 6880 1369 6887 1367 6894 1366 6902
1367 6910 1369 6918 1373 6924 1378 6930 1384 6935 1390 6938

1398 6941 1406 6942 1414 6941 1421 6938 1428 6935 1434 6930
1438 6924 1442 6918 1444 6910 1445 6902 1444 6894 1442 6887
1438 6880 1434 6874 1428 6869 1421 6866 1414 6864 1406 6863
1406 6838 1393 6839 1381 6843 1370 6849 1361 6857 1353 6866
1347 6877 1343 6889 1342 6902 1343 6915 1347 6927 1353 6938
1361 6947 1370 6955 1381 6961 1393 6965 1406 6966 1419 6965
1430 6961 1441 6955 1451 6947 1459 6938 1465 6927 1468 6915
1470 6902 67 N F
1517 6962 1557 6962 1701 6733 1701 6962 1735 6962 1735 6681
1695 6681 1551 6910 1551 6681 1517 6681 1517 6962 11 N F
911 8021 945 8021 946 8035 948 8047 952 8058 959 8067
966 8075 976 8081 988 8084 1001 8085 1011 8084 1021 8082
1030 8077 1038 8071 1044 8064 1049 8055 1052 8045 1053 8033
1052 8021 1049 8012 1045 8003 1038 7996 1029 7989 1018 7982
991 7965 968 7951 949 7937 934 7923 922 7909 914 7893
909 7877 906 7860 905 7842 1087 7842 1087 7874 943 7874
946 7887 951 7897 958 7906 966 7913 976 7921 988 7929
1019 7947 1037 7956 1052 7966 1064 7976 1074 7988 1081 8000
1086 8012 1088 8025 1089 8037 1089 8046 1087 8054 1085 8061
1082 8069 1078 8076 1074 8082 1069 8088 1063 8094 1050 8103
1035 8110 1019 8114 1001 8115 982 8114 973 8112 965 8110
957 8106 949 8103 943 8098 936 8092 931 8086 925 8079
921 8071 917 8063 914 8054 912 8044 911 8033 911 8021
78 N F
1298 8045 1264 8045 1262 8054 1258 8062 1253 8069 1248 8075
1241 8079 1235 8083 1227 8085 1220 8085 1212 8085 1206 8084
1199 8081 1193 8078 1188 8074 1182 8070 1178 8064 1174 8058
1167 8043 1162 8026 1159 8006 1157 7983 1166 7992 1174 7999
1182 8004 1190 8008 1198 8010 1206 8012 1224 8013 1237 8012
1251 8008 1265 8001 1277 7991 1283 7986 1288 7979 1293 7972
1297 7964 1300 7955 1303 7946 1304 7935 1304 7925 1270 7923
1269 7934 1265 7945 1260 7955 1253 7964 1245 7972 1235 7978
1225 7981 1214 7983 1203 7981 1192 7978 1183 7972 1174 7964
1167 7955 1162 7945 1159 7934 1157 7923 1159 7911 1162 7900
1167 7890 1174 7881 1183 7873 1192 7867 1203 7864 1214 7862
1225 7864 1235 7867 1245 7873 1253 7881 1260 7890 1265 7900
1269 7911 1270 7923 1304 7925 1303 7908 1299 7892 1292 7877
1287 7869 1282 7862 1276 7856 1270 7850 1262 7845 1254 7841
1245 7837 1235 7834 1225 7833 1214 7832 1201 7833 1190 7835
1180 7838 1170 7843 1162 7848 1154 7855 1148 7863 1142 7872
1137 7881 1133 7892 1129 7903 1126 7915 1123 7941 1121 7969
1122 7985 1123 8001 1125 8016 1128 8030 1132 8043 1136 8055
1141 8066 1147 8076 1154 8085 1161 8093 1169 8100 1178 8105

1188 8110 1198 8113 1208 8115 1220 8115 1236 8114 1250 8110
1263 8103 1274 8095 1283 8084 1290 8072 1295 8059 1298 8045
126 N F
1470 8064 1468 8051 1465 8039 1459 8028 1451 8019 1441 8011
1430 8005 1419 8001 1406 8000 1406 8024 1398 8025 1390 8027
1384 8031 1378 8036 1373 8042 1369 8048 1367 8056 1366 8064
1367 8072 1369 8079 1373 8086 1378 8092 1384 8096 1390 8100
1398 8102 1406 8103 1414 8102 1421 8100 1428 8096 1434 8092
1438 8086 1442 8079 1444 8072 1445 8064 1444 8056 1442 8048
1438 8042 1434 8036 1428 8031 1421 8027 1414 8025 1406 8024
1406 8000 1393 8001 1381 8005 1370 8011 1361 8019 1353 8028
1347 8039 1343 8051 1342 8064 1343 8077 1347 8089 1353 8099
1361 8109 1370 8117 1381 8123 1393 8126 1406 8128 1419 8126
1430 8123 1441 8117 1451 8109 1459 8099 1465 8089 1468 8077
1470 8064 67 N F
1517 8123 1557 8123 1701 7894 1701 8123 1735 8123 1735 7842
1695 7842 1551 8071 1551 7842 1517 7842 1517 8123 11 N F
911 9166 945 9166 946 9180 948 9192 952 9203 959 9213
966 9220 976 9226 988 9229 1001 9231 1011 9230 1021 9227
1030 9223 1038 9217 1044 9209 1049 9200 1052 9190 1053 9178
1052 9167 1049 9157 1045 9149 1038 9141 1029 9134 1018 9127
991 9110 968 9096 949 9083 934 9069 922 9054 914 9039
909 9023 906 9006 905 8987 1087 8987 1087 9020 943 9020
946 9032 951 9042 958 9051 966 9059 976 9066 988 9074
1019 9092 1037 9101 1052 9111 1064 9122 1074 9133 1081 9145
1086 9158 1088 9170 1089 9182 1089 9191 1087 9199 1085 9207
1082 9214 1078 9221 1074 9227 1069 9233 1063 9239 1050 9248
1035 9255 1019 9259 1001 9261 982 9259 973 9258 965 9255
957 9252 949 9248 943 9243 936 9238 931 9231 925 9224
921 9217 917 9208 914 9199 912 9189 911 9178 911 9166
78 N F
1212 9261 1228 9260 1244 9256 1258 9250 1270 9242 1280 9232
1284 9226 1288 9219 1290 9212 1292 9205 1294 9197 1294 9188
1293 9177 1291 9166 1287 9158 1282 9150 1276 9144 1270 9139
1258 9130 1268 9125 1277 9119 1285 9111 1291 9103 1297 9093
1301 9083 1303 9073 1304 9062 1303 9048 1298 9034 1291 9020
1280 9007 1274 9001 1267 8995 1259 8990 1251 8986 1241 8982
1232 8980 1221 8978 1210 8977 1198 8978 1188 8980 1178 8982
1169 8986 1161 8990 1154 8995 1147 9001 1141 9007 1132 9020
1125 9034 1121 9048 1119 9062 1155 9062 1157 9051 1160 9041
1165 9031 1172 9023 1181 9017 1190 9012 1201 9009 1212 9008
1223 9009 1233 9012 1243 9017 1251 9023 1258 9031 1263 9041
1267 9051 1268 9062 1267 9073 1263 9083 1258 9092 1251 9100

1243 9107 1233 9112 1223 9115 1212 9116 1201 9115 1190 9112
1181 9107 1172 9100 1165 9092 1160 9083 1157 9073 1155 9062
1119 9062 1120 9073 1123 9083 1127 9093 1132 9102 1139 9110
1146 9118 1155 9124 1166 9130 1153 9139 1147 9144 1141 9150
1137 9158 1133 9166 1130 9177 1129 9188 1164 9188 1165 9179
1167 9171 1172 9164 1178 9158 1185 9153 1193 9149 1202 9147
1212 9146 1221 9147 1230 9149 1238 9153 1246 9158 1251 9164
1256 9171 1259 9179 1260 9188 1259 9198 1256 9206 1251 9213
1246 9219 1238 9224 1230 9228 1221 9230 1212 9231 1202 9230
1193 9228 1185 9224 1178 9219 1172 9213 1167 9206 1165 9198
1164 9188 1129 9188 1130 9197 1131 9205 1133 9212 1136 9219
1139 9226 1143 9232 1153 9242 1165 9250 1179 9256 1195 9260
1212 9261 145 N F
1470 9209 1468 9196 1465 9184 1459 9173 1451 9164 1441 9156
1430 9150 1419 9146 1406 9145 1406 9170 1398 9170 1390 9173
1384 9176 1378 9181 1373 9187 1369 9194 1367 9201 1366 9209
1367 9217 1369 9224 1373 9231 1378 9237 1384 9242 1390 9245
1398 9248 1406 9248 1414 9248 1421 9245 1428 9242 1434 9237
1438 9231 1442 9224 1444 9217 1445 9209 1444 9201 1442 9194
1438 9187 1434 9181 1428 9176 1421 9173 1414 9170 1406 9170
1406 9145 1393 9146 1381 9150 1370 9156 1361 9164 1353 9173
1347 9184 1343 9196 1342 9209 1343 9222 1347 9234 1353 9245
1361 9254 1370 9262 1381 9268 1393 9272 1406 9273 1419 9272
1430 9268 1441 9262 1451 9254 1459 9245 1465 9234 1468 9222
1470 9209 67 N F
1517 9269 1557 9269 1701 9040 1701 9269 1735 9269 1735 8987
1695 8987 1551 9217 1551 8987 1517 8987 1517 9269 11 N F
943 10308 909 10308 911 10328 913 10337 916 10346 919 10354
923 10361 928 10368 933 10374 939 10379 945 10383 952 10387
960 10390 968 10393 976 10395 995 10396 1012 10395 1028 10391
1043 10385 1055 10377 1060 10372 1065 10366 1069 10360 1073 10354
1076 10346 1078 10339 1079 10330 1079 10322 1079 10311 1077 10302
1074 10294 1070 10286 1065 10280 1058 10274 1049 10268 1039 10263
1051 10258 1061 10253 1069 10246 1076 10239 1081 10230 1085 10221
1087 10210 1087 10199 1086 10184 1082 10169 1076 10155 1072 10148
1066 10142 1060 10136 1054 10131 1046 10126 1037 10122 1028 10119
1017 10117 1006 10115 993 10115 982 10115 972 10116 962 10118
954 10121 946 10124 939 10129 932 10133 926 10139 921 10145
917 10152 913 10159 910 10166 906 10183 905 10201 939 10201
939 10191 941 10181 944 10172 949 10163 956 10156 966 10150
979 10146 995 10145 1009 10145 1021 10148 1030 10153 1038 10160
1044 10168 1048 10177 1050 10188 1051 10199 1050 10213 1048 10219
1046 10225 1043 10229 1039 10233 1030 10240 1019 10244 1006 10247

991 10249 975 10249 975 10277 991 10278 1005 10279 1017 10281
1026 10285 1034 10290 1039 10298 1042 10307 1043 10320 1042 10329
1040 10338 1037 10346 1032 10352 1025 10358 1017 10362 1007 10365
995 10366 984 10365 974 10363 966 10360 958 10354 952 10346
948 10336 944 10323 943 10308 123 N F
1212 10396 1222 10395 1231 10393 1240 10389 1249 10384 1257 10377
1264 10369 1271 10361 1277 10351 1283 10341 1288 10330 1292 10318
1296 10306 1298 10294 1300 10281 1302 10268 1302 10255 1302 10242
1300 10230 1296 10204 1292 10192 1288 10180 1283 10169 1277 10158
1271 10149 1264 10140 1257 10132 1249 10125 1240 10120 1231 10116
1222 10113 1212 10113 1202 10113 1192 10116 1183 10120 1175 10125
1167 10132 1159 10140 1152 10149 1146 10158 1141 10169 1136 10180
1131 10192 1128 10204 1123 10230 1122 10242 1121 10255 1157 10255
1159 10231 1162 10209 1168 10190 1175 10173 1183 10160 1192 10151
1197 10147 1202 10145 1207 10143 1212 10143 1217 10143 1222 10145
1226 10147 1231 10151 1240 10160 1249 10173 1256 10190 1261 10209
1265 10231 1266 10255 1265 10280 1261 10301 1256 10320 1249 10336
1240 10349 1231 10358 1226 10361 1222 10364 1217 10365 1212 10366
1207 10365 1202 10364 1197 10361 1192 10358 1183 10349 1175 10336
1168 10320 1162 10301 1159 10280 1157 10255 1121 10255 1122 10268
1123 10281 1125 10294 1128 10306 1131 10318 1136 10330 1141 10341
1146 10351 1152 10361 1159 10369 1167 10377 1175 10384 1183 10389
1192 10393 1202 10395 1212 10396 105 N F
1470 10344 1468 10331 1465 10319 1459 10308 1451 10299 1441 10291
1430 10285 1419 10281 1406 10280 1406 10305 1398 10306 1390 10308
1384 10312 1378 10316 1373 10322 1369 10329 1367 10336 1366 10344
1367 10352 1369 10360 1373 10366 1378 10372 1384 10377 1390 10380
1398 10383 1406 10384 1414 10383 1421 10380 1428 10377 1434 10372
1438 10366 1442 10360 1444 10352 1445 10344 1444 10336 1442 10329
1438 10322 1434 10316 1428 10312 1421 10308 1414 10306 1406 10305
1406 10280 1393 10281 1381 10285 1370 10291 1361 10299 1353 10308
1347 10319 1343 10331 1342 10344 1343 10357 1347 10369 1353 10380
1361 10389 1370 10397 1381 10403 1393 10407 1406 10408 1419 10407
1430 10403 1441 10397 1451 10389 1459 10380 1465 10369 1468 10357
1470 10344 67 N F
1517 10404 1557 10404 1701 10175 1701 10404 1735 10404 1735 10123
1695 10123 1551 10352 1551 10123 1517 10123 1517 10404 11 N F
943 11433 909 11433 911 11453 913 11462 916 11471 919 11479
923 11486 928 11493 933 11499 939 11504 945 11509 952 11512
960 11516 968 11518 976 11520 995 11521 1012 11520 1028 11516
1043 11510 1055 11502 1060 11497 1065 11491 1069 11485 1073 11479
1076 11472 1078 11464 1079 11456 1079 11447 1079 11437 1077 11427
1074 11419 1070 11412 1065 11405 1058 11399 1049 11393 1039 11388

1051 11384 1061 11378 1069 11371 1076 11364 1081 11355 1085 11346
1087 11336 1087 11324 1086 11309 1082 11294 1076 11280 1072 11273
1066 11267 1060 11261 1054 11256 1046 11251 1037 11247 1028 11244
1017 11242 1006 11240 993 11240 982 11240 972 11241 962 11243
954 11246 946 11250 939 11254 932 11259 926 11264 921 11270
917 11277 913 11284 910 11291 906 11308 905 11326 939 11326
939 11316 941 11306 944 11297 949 11288 956 11281 966 11275
979 11271 995 11270 1009 11270 1021 11273 1030 11278 1038 11285
1044 11293 1048 11303 1050 11313 1051 11324 1050 11338 1048 11344
1046 11350 1043 11355 1039 11359 1030 11365 1019 11370 1006 11372
991 11374 975 11374 975 11403 991 11403 1005 11404 1017 11406
1026 11410 1034 11415 1039 11423 1042 11433 1043 11445 1042 11454
1040 11463 1037 11471 1032 11478 1025 11483 1017 11487 1007 11490
995 11491 984 11490 974 11488 966 11485 958 11479 952 11471
948 11461 944 11448 943 11433 123 N F
1125 11427 1160 11427 1160 11440 1163 11453 1167 11464 1173 11473
1181 11481 1191 11486 1202 11490 1216 11491 1226 11490 1236 11487
1245 11483 1252 11477 1259 11469 1264 11460 1267 11450 1268 11439
1267 11427 1264 11417 1259 11409 1253 11402 1244 11394 1233 11387
1206 11370 1182 11357 1163 11343 1148 11329 1137 11314 1129 11299
1123 11283 1120 11266 1119 11248 1302 11248 1302 11280 1157 11280
1161 11292 1166 11303 1172 11311 1181 11319 1191 11327 1203 11334
1234 11352 1252 11361 1267 11371 1279 11382 1288 11394 1296 11406
1300 11418 1303 11431 1304 11443 1304 11451 1302 11459 1300 11467
1297 11474 1293 11481 1289 11488 1284 11494 1278 11499 1265 11509
1250 11515 1233 11520 1216 11521 1197 11520 1188 11518 1180 11515
1172 11512 1164 11508 1157 11503 1151 11498 1145 11492 1140 11485
1136 11477 1132 11469 1129 11459 1127 11449 1126 11438 1125 11427
78 N F
1470 11469 1468 11457 1465 11445 1459 11434 1451 11424 1441 11416
1430 11410 1419 11407 1406 11405 1406 11430 1398 11431 1390 11433
1384 11437 1378 11442 1373 11447 1369 11454 1367 11461 1366 11469
1367 11477 1369 11485 1373 11491 1378 11497 1384 11502 1390 11506
1398 11508 1406 11509 1414 11508 1421 11506 1428 11502 1434 11497
1438 11491 1442 11485 1444 11477 1445 11469 1444 11461 1442 11454
1438 11447 1434 11442 1428 11437 1421 11433 1414 11431 1406 11430
1406 11405 1393 11407 1381 11410 1370 11416 1361 11424 1353 11434
1347 11445 1343 11457 1342 11469 1343 11482 1347 11494 1353 11505
1361 11515 1370 11522 1381 11528 1393 11532 1406 11533 1419 11532
1430 11528 1441 11522 1451 11515 1459 11505 1465 11494 1468 11482
1470 11469 67 N F
1517 11529 1557 11529 1701 11300 1701 11529 1735 11529 1735 11248
1695 11248 1551 11477 1551 11248 1517 11248 1517 11529 11 N F

943 12551 909 12551 911 12572 913 12581 916 12589 919 12597
923 12605 928 12611 933 12617 939 12622 945 12627 952 12631
960 12634 968 12636 976 12638 995 12640 1012 12638 1028 12635
1043 12629 1055 12620 1060 12615 1065 12610 1069 12604 1073 12597
1076 12590 1078 12582 1079 12574 1079 12565 1079 12555 1077 12546
1074 12538 1070 12530 1065 12523 1058 12517 1049 12512 1039 12507
1051 12502 1061 12496 1069 12490 1076 12482 1081 12474 1085 12464
1087 12454 1087 12443 1086 12427 1082 12412 1076 12398 1072 12392
1066 12385 1060 12379 1054 12374 1046 12370 1037 12366 1028 12362
1017 12360 1006 12359 993 12358 982 12359 972 12360 962 12362
954 12365 946 12368 939 12372 932 12377 926 12382 921 12389
917 12395 913 12402 910 12410 906 12426 905 12445 939 12445
939 12435 941 12425 944 12415 949 12407 956 12399 966 12393
979 12390 995 12388 1009 12389 1021 12392 1030 12397 1038 12403
1044 12412 1048 12421 1050 12431 1051 12443 1050 12457 1048 12463
1046 12468 1043 12473 1039 12477 1030 12483 1019 12488 1006 12491
991 12492 975 12493 975 12521 991 12521 1005 12522 1017 12525
1026 12528 1034 12534 1039 12541 1042 12551 1043 12563 1042 12573
1040 12581 1037 12589 1032 12596 1025 12602 1017 12606 1007 12608
995 12609 984 12609 974 12607 966 12603 958 12598 952 12590
948 12580 944 12567 943 12551 123 N F
1242 12640 1266 12640 1266 12463 1308 12463 1308 12430 1266 12430
1266 12366 1232 12366 1232 12430 1117 12430 1117 12463 1147 12463
1232 12463 1232 12581 1147 12463 1117 12463 1117 12467 1242 12640
18 N F
1470 12588 1468 12575 1465 12563 1459 12552 1451 12543 1441 12535
1430 12529 1419 12525 1406 12524 1406 12548 1398 12549 1390 12551
1384 12555 1378 12560 1373 12566 1369 12572 1367 12580 1366 12588
1367 12596 1369 12603 1373 12610 1378 12616 1384 12620 1390 12624
1398 12626 1406 12627 1414 12626 1421 12624 1428 12620 1434 12616
1438 12610 1442 12603 1444 12596 1445 12588 1444 12580 1442 12572
1438 12566 1434 12560 1428 12555 1421 12551 1414 12549 1406 12548
1406 12524 1393 12525 1381 12529 1370 12535 1361 12543 1353 12552
1347 12563 1343 12575 1342 12588 1343 12601 1347 12613 1353 12623
1361 12633 1370 12641 1381 12647 1393 12650 1406 12652 1419 12650
1430 12647 1441 12641 1451 12633 1459 12623 1465 12613 1468 12601
1470 12588 67 N F
1517 12648 1557 12648 1701 12418 1701 12648 1735 12648 1735 12366
1695 12366 1551 12595 1551 12366 1517 12366 1517 12648 11 N F
943 13664 909 13664 911 13684 913 13694 916 13702 919 13710
923 13717 928 13724 933 13730 939 13735 945 13740 952 13744
960 13747 968 13749 976 13751 995 13752 1012 13751 1028 13747
1043 13741 1055 13733 1060 13728 1065 13723 1069 13717 1073 13710

1076 13703 1078 13695 1079 13687 1079 13678 1079 13668 1077 13659
1074 13650 1070 13643 1065 13636 1058 13630 1049 13624 1039 13620
1051 13615 1061 13609 1069 13602 1076 13595 1081 13587 1085 13577
1087 13567 1087 13555 1086 13540 1082 13525 1076 13511 1072 13504
1066 13498 1060 13492 1054 13487 1046 13482 1037 13478 1028 13475
1017 13473 1006 13471 993 13471 982 13471 972 13473 962 13475
954 13477 946 13481 939 13485 932 13490 926 13495 921 13501
917 13508 913 13515 910 13523 906 13539 905 13557 939 13557
939 13547 941 13538 944 13528 949 13519 956 13512 966 13506
979 13502 995 13501 1009 13502 1021 13504 1030 13509 1038 13516
1044 13524 1048 13534 1050 13544 1051 13555 1050 13570 1048 13576
1046 13581 1043 13586 1039 13590 1030 13596 1019 13601 1006 13604
991 13605 975 13606 975 13634 991 13634 1005 13635 1017 13638
1026 13641 1034 13647 1039 13654 1042 13664 1043 13676 1042 13685
1040 13694 1037 13702 1032 13709 1025 13714 1017 13719 1007 13721
995 13722 984 13722 974 13720 966 13716 958 13710 952 13703
948 13692 944 13680 943 13664 123 N F
1298 13682 1264 13682 1262 13691 1258 13699 1253 13706 1248 13712
1241 13716 1235 13720 1227 13721 1220 13722 1212 13722 1206 13720
1199 13718 1193 13715 1188 13711 1182 13707 1178 13701 1174 13695
1167 13680 1162 13663 1159 13643 1157 13620 1166 13628 1174 13635
1182 13641 1190 13645 1198 13647 1206 13649 1224 13650 1237 13648
1251 13644 1265 13638 1277 13628 1283 13622 1288 13616 1293 13609
1297 13601 1300 13592 1303 13582 1304 13572 1304 13561 1270 13559
1269 13571 1265 13581 1260 13592 1253 13601 1245 13609 1235 13614
1225 13618 1214 13620 1203 13618 1192 13614 1183 13609 1174 13601
1167 13592 1162 13581 1159 13571 1157 13559 1159 13548 1162 13537
1167 13527 1174 13518 1183 13510 1192 13504 1203 13500 1214 13499
1225 13500 1235 13504 1245 13510 1253 13518 1260 13527 1265 13537
1269 13548 1270 13559 1304 13561 1303 13545 1299 13529 1292 13514
1287 13506 1282 13499 1276 13493 1270 13487 1262 13482 1254 13477
1245 13474 1235 13471 1225 13469 1214 13469 1201 13470 1190 13472
1180 13475 1170 13480 1162 13485 1154 13492 1148 13500 1142 13509
1137 13518 1133 13529 1129 13540 1126 13552 1123 13578 1121 13606
1122 13622 1123 13638 1125 13653 1128 13667 1132 13680 1136 13692
1141 13703 1147 13713 1154 13722 1161 13730 1169 13736 1178 13742
1188 13746 1198 13750 1208 13752 1220 13752 1236 13751 1250 13747
1263 13740 1274 13731 1283 13721 1290 13709 1295 13696 1298 13682
126 N F
1470 13701 1468 13688 1465 13676 1459 13665 1451 13655 1441 13647
1430 13642 1419 13638 1406 13637 1406 13661 1398 13662 1390 13664
1384 13668 1378 13673 1373 13678 1369 13685 1367 13693 1366 13701
1367 13708 1369 13716 1373 13723 1378 13728 1384 13733 1390 13737

1398 13739 1406 13740 1414 13739 1421 13737 1428 13733 1434 13728
1438 13723 1442 13716 1444 13708 1445 13701 1444 13693 1442 13685
1438 13678 1434 13673 1428 13668 1421 13664 1414 13662 1406 13661
1406 13637 1393 13638 1381 13642 1370 13647 1361 13655 1353 13665
1347 13676 1343 13688 1342 13701 1343 13713 1347 13725 1353 13736
1361 13746 1370 13754 1381 13759 1393 13763 1406 13765 1419 13763
1430 13759 1441 13754 1451 13746 1459 13736 1465 13725 1468 13713
1470 13701 67 N F
1517 13760 1557 13760 1701 13531 1701 13760 1735 13760 1735 13479
1695 13479 1551 13708 1551 13479 1517 13479 1517 13760 11 N F
943 14770 909 14770 911 14791 913 14800 916 14809 919 14817
923 14824 928 14830 933 14836 939 14842 945 14846 952 14850
960 14853 968 14856 976 14857 995 14859 1012 14858 1028 14854
1043 14848 1055 14840 1060 14835 1065 14829 1069 14823 1073 14816
1076 14809 1078 14801 1079 14793 1079 14784 1079 14774 1077 14765
1074 14757 1070 14749 1065 14742 1058 14736 1049 14731 1039 14726
1051 14721 1061 14715 1069 14709 1076 14701 1081 14693 1085 14684
1087 14673 1087 14662 1086 14647 1082 14632 1076 14617 1072 14611
1066 14604 1060 14599 1054 14593 1046 14589 1037 14585 1028 14582
1017 14579 1006 14578 993 14577 982 14578 972 14579 962 14581
954 14584 946 14587 939 14591 932 14596 926 14602 921 14608
917 14614 913 14621 910 14629 906 14646 905 14664 939 14664
939 14654 941 14644 944 14634 949 14626 956 14618 966 14613
979 14609 995 14608 1009 14608 1021 14611 1030 14616 1038 14623
1044 14631 1048 14640 1050 14651 1051 14662 1050 14676 1048 14682
1046 14687 1043 14692 1039 14696 1030 14703 1019 14707 1006 14710
991 14712 975 14712 975 14740 991 14740 1005 14742 1017 14744
1026 14748 1034 14753 1039 14761 1042 14770 1043 14782 1042 14792
1040 14801 1037 14808 1032 14815 1025 14821 1017 14825 1007 14828
995 14829 984 14828 974 14826 966 14822 958 14817 952 14809
948 14799 944 14786 943 14770 123 N F
1212 14859 1228 14858 1244 14854 1258 14848 1270 14840 1280 14830
1284 14824 1288 14817 1290 14810 1292 14803 1294 14795 1294 14786
1293 14774 1291 14764 1287 14756 1282 14748 1276 14742 1270 14737
1258 14728 1268 14723 1277 14717 1285 14709 1291 14701 1297 14691
1301 14681 1303 14671 1304 14660 1303 14646 1298 14632 1291 14618
1280 14605 1274 14599 1267 14593 1259 14588 1251 14584 1241 14580
1232 14578 1221 14576 1210 14575 1198 14576 1188 14578 1178 14580
1169 14584 1161 14588 1154 14593 1147 14599 1141 14605 1132 14618
1125 14632 1121 14646 1119 14660 1155 14660 1157 14649 1160 14639
1165 14629 1172 14621 1181 14615 1190 14610 1201 14607 1212 14606
1223 14607 1233 14610 1243 14615 1251 14621 1258 14629 1263 14639
1267 14649 1268 14660 1267 14671 1263 14681 1258 14690 1251 14698

1243 14705 1233 14710 1223 14713 1212 14714 1201 14713 1190 14710
1181 14705 1172 14698 1165 14690 1160 14681 1157 14671 1155 14660
1119 14660 1120 14671 1123 14681 1127 14691 1132 14700 1139 14708
1146 14716 1155 14722 1166 14728 1153 14737 1147 14742 1141 14748
1137 14756 1133 14764 1130 14774 1129 14786 1164 14786 1165 14777
1167 14769 1172 14762 1178 14755 1185 14751 1193 14747 1202 14745
1212 14744 1221 14745 1230 14747 1238 14751 1246 14755 1251 14762
1256 14769 1259 14777 1260 14786 1259 14796 1256 14804 1251 14811
1246 14817 1238 14822 1230 14826 1221 14828 1212 14829 1202 14828
1193 14826 1185 14822 1178 14817 1172 14811 1167 14804 1165 14796
1164 14786 1129 14786 1130 14795 1131 14803 1133 14810 1136 14817
1139 14824 1143 14830 1153 14840 1165 14848 1179 14854 1195 14858
1212 14859 145 N F
1470 14807 1468 14794 1465 14782 1459 14771 1451 14762 1441 14754
1430 14748 1419 14744 1406 14743 1406 14768 1398 14768 1390 14771
1384 14774 1378 14779 1373 14785 1369 14792 1367 14799 1366 14807
1367 14815 1369 14822 1373 14829 1378 14835 1384 14840 1390 14843
1398 14846 1406 14846 1414 14846 1421 14843 1428 14840 1434 14835
1438 14829 1442 14822 1444 14815 1445 14807 1444 14799 1442 14792
1438 14785 1434 14779 1428 14774 1421 14771 1414 14768 1406 14768
1406 14743 1393 14744 1381 14748 1370 14754 1361 14762 1353 14771
1347 14782 1343 14794 1342 14807 1343 14820 1347 14832 1353 14843
1361 14852 1370 14860 1381 14866 1393 14870 1406 14871 1419 14870
1430 14866 1441 14860 1451 14852 1459 14843 1465 14832 1468 14820
1470 14807 67 N F
1517 14867 1557 14867 1701 14638 1701 14867 1735 14867 1735 14585
1695 14585 1551 14815 1551 14585 1517 14585 1517 14867 11 N F
1027 15952 1051 15952 1051 15775 1093 15775 1093 15743 1051 15743
1051 15679 1017 15679 1017 15743 903 15743 903 15775 933 15775
1017 15775 1017 15894 933 15775 903 15775 903 15779 1027 15952
18 N F
1212 15952 1222 15951 1231 15949 1240 15945 1249 15940 1257 15933
1264 15925 1271 15917 1277 15907 1283 15897 1288 15886 1292 15874
1296 15862 1298 15850 1300 15837 1302 15824 1302 15811 1302 15798
1300 15785 1296 15760 1292 15748 1288 15736 1283 15725 1277 15714
1271 15705 1264 15696 1257 15688 1249 15681 1240 15676 1231 15672
1222 15669 1212 15669 1202 15669 1192 15672 1183 15676 1175 15681
1167 15688 1159 15696 1152 15705 1146 15714 1141 15725 1136 15736
1131 15748 1128 15760 1123 15785 1122 15798 1121 15811 1157 15811
1159 15787 1162 15765 1168 15746 1175 15729 1183 15716 1192 15707
1197 15703 1202 15701 1207 15699 1212 15699 1217 15699 1222 15701
1226 15703 1231 15707 1240 15716 1249 15729 1256 15746 1261 15765
1265 15787 1266 15811 1265 15836 1261 15857 1256 15876 1249 15892

1240 15905 1231 15914 1226 15917 1222 15920 1217 15921 1212 15922
1207 15921 1202 15920 1197 15917 1192 15914 1183 15905 1175 15892
1168 15876 1162 15857 1159 15836 1157 15811 1121 15811 1122 15824
1123 15837 1125 15850 1128 15862 1131 15874 1136 15886 1141 15897
1146 15907 1152 15917 1159 15925 1167 15933 1175 15940 1183 15945
1192 15949 1202 15951 1212 15952 105 N F
1470 15900 1468 15887 1465 15875 1459 15864 1451 15855 1441 15847
1430 15841 1419 15837 1406 15836 1406 15861 1398 15862 1390 15864
1384 15868 1378 15872 1373 15878 1369 15885 1367 15892 1366 15900
1367 15908 1369 15916 1373 15922 1378 15928 1384 15933 1390 15936
1398 15939 1406 15940 1414 15939 1421 15936 1428 15933 1434 15928
1438 15922 1442 15916 1444 15908 1445 15900 1444 15892 1442 15885
1438 15878 1434 15872 1428 15868 1421 15864 1414 15862 1406 15861
1406 15836 1393 15837 1381 15841 1370 15847 1361 15855 1353 15864
1347 15875 1343 15887 1342 15900 1343 15913 1347 15925 1353 15936
1361 15945 1370 15953 1381 15959 1393 15963 1406 15964 1419 15963
1430 15959 1441 15953 1451 15945 1459 15936 1465 15925 1468 15913
1470 15900 67 N F
1517 15960 1557 15960 1701 15731 1701 15960 1735 15960 1735 15679
1695 15679 1551 15908 1551 15679 1517 15679 1517 15960 11 N F
13570 5585 13604 5585 13605 5599 13607 5611 13611 5622 13618 5632
13625 5639 13635 5645 13647 5648 13660 5650 13670 5649 13680 5646
13689 5642 13697 5636 13703 5628 13708 5619 13711 5609 13712 5597
13711 5586 13708 5576 13704 5568 13697 5560 13688 5553 13677 5546
13650 5529 13627 5515 13608 5502 13593 5488 13581 5473 13573 5458
13567 5442 13564 5425 13564 5406 13746 5406 13746 5439 13602 5439
13605 5451 13610 5461 13617 5470 13625 5478 13635 5485 13647 5493
13678 5511 13696 5520 13711 5530 13723 5541 13733 5552 13740 5564
13745 5577 13747 5589 13748 5601 13748 5610 13746 5618 13744 5626
13741 5633 13737 5640 13733 5646 13728 5652 13722 5658 13709 5667
13694 5674 13678 5678 13660 5680 13641 5678 13632 5677 13624 5674
13616 5671 13608 5667 13602 5662 13595 5657 13589 5650 13584 5643
13580 5636 13576 5627 13573 5618 13571 5608 13570 5597 13570 5585
78 N F
13784 5585 13818 5585 13819 5599 13822 5611 13826 5622 13832 5632
13840 5639 13850 5645 13861 5648 13875 5650 13885 5649 13895 5646
13904 5642 13911 5636 13918 5628 13923 5619 13926 5609 13927 5597
13926 5586 13923 5576 13918 5568 13912 5560 13903 5553 13892 5546
13865 5529 13841 5515 13822 5502 13807 5488 13796 5473 13788 5458
13782 5442 13779 5425 13778 5406 13961 5406 13961 5439 13816 5439
13820 5451 13825 5461 13831 5470 13840 5478 13850 5485 13862 5493
13893 5511 13911 5520 13926 5530 13938 5541 13947 5552 13955 5564
13959 5577 13962 5589 13963 5601 13963 5610 13961 5618 13959 5626

13956 5633 13952 5640 13948 5646 13943 5652 13937 5658 13924 5667
13909 5674 13892 5678 13875 5680 13856 5678 13847 5677 13839 5674
13831 5671 13823 5667 13816 5662 13810 5657 13804 5650 13799 5643
13795 5636 13791 5627 13788 5618 13786 5608 13785 5597 13784 5585
78 N F
14129 5628 14127 5615 14124 5603 14118 5592 14110 5583 14100 5575
14089 5569 14077 5565 14065 5564 14065 5589 14057 5589 14049 5592
14043 5595 14037 5600 14032 5606 14028 5613 14026 5620 14025 5628
14026 5636 14028 5643 14032 5650 14037 5656 14043 5661 14049 5664
14057 5667 14065 5667 14073 5667 14080 5664 14087 5661 14092 5656
14097 5650 14101 5643 14103 5636 14104 5628 14103 5620 14101 5613
14097 5606 14092 5600 14087 5595 14080 5592 14073 5589 14065 5589
14065 5564 14052 5565 14040 5569 14029 5575 14019 5583 14012 5592
14006 5603 14002 5615 14001 5628 14002 5641 14006 5653 14012 5664
14019 5673 14029 5681 14040 5687 14052 5691 14065 5692 14077 5691
14089 5687 14100 5681 14110 5673 14118 5664 14124 5653 14127 5641
14129 5628 67 N F
14176 5688 14216 5688 14360 5459 14360 5688 14394 5688 14394 5406
14354 5406 14210 5636 14210 5406 14176 5406 14176 5688 11 N F
13570 6755 13604 6755 13605 6769 13607 6781 13611 6792 13618 6802
13625 6809 13635 6815 13647 6818 13660 6819 13670 6818 13680 6816
13689 6811 13697 6805 13703 6798 13708 6789 13711 6779 13712 6767
13711 6755 13708 6746 13704 6737 13697 6730 13688 6723 13677 6716
13650 6699 13627 6685 13608 6671 13593 6657 13581 6643 13573 6628
13567 6611 13564 6594 13564 6576 13746 6576 13746 6608 13602 6608
13605 6621 13610 6631 13617 6640 13625 6648 13635 6655 13647 6663
13678 6681 13696 6690 13711 6700 13723 6711 13733 6722 13740 6734
13745 6747 13747 6759 13748 6771 13748 6780 13746 6788 13744 6796
13741 6803 13737 6810 13733 6816 13728 6822 13722 6828 13709 6837
13694 6844 13678 6848 13660 6850 13641 6848 13632 6846 13624 6844
13616 6841 13608 6837 13602 6832 13595 6826 13589 6820 13584 6813
13580 6806 13576 6797 13573 6788 13571 6778 13570 6767 13570 6755
78 N F
13901 6850 13925 6850 13925 6673 13967 6673 13967 6641 13925 6641
13925 6576 13891 6576 13891 6641 13776 6641 13776 6673 13806 6673
13891 6673 13891 6791 13806 6673 13776 6673 13776 6677 13901 6850
18 N F
14129 6798 14127 6785 14124 6773 14118 6762 14110 6753 14100 6745
14089 6739 14077 6735 14065 6734 14065 6758 14057 6759 14049 6762
14043 6765 14037 6770 14032 6776 14028 6782 14026 6790 14025 6798
14026 6806 14028 6813 14032 6820 14037 6826 14043 6830 14049 6834
14057 6836 14065 6837 14073 6836 14080 6834 14087 6830 14092 6826
14097 6820 14101 6813 14103 6806 14104 6798 14103 6790 14101 6782

14097 6776 14092 6770 14087 6765 14080 6762 14073 6759 14065 6758
14065 6734 14052 6735 14040 6739 14029 6745 14019 6753 14012 6762
14006 6773 14002 6785 14001 6798 14002 6811 14006 6823 14012 6834
14019 6843 14029 6851 14040 6857 14052 6861 14065 6862 14077 6861
14089 6857 14100 6851 14110 6843 14118 6834 14124 6823 14127 6811
14129 6798 67 N F
14176 6858 14216 6858 14360 6628 14360 6858 14394 6858 14394 6576
14354 6576 14210 6805 14210 6576 14176 6576 14176 6858 11 N F
13570 7914 13604 7914 13605 7927 13607 7940 13611 7951 13618 7960
13625 7968 13635 7973 13647 7977 13660 7978 13670 7977 13680 7974
13689 7970 13697 7964 13703 7956 13708 7947 13711 7937 13712 7926
13711 7914 13708 7904 13704 7896 13697 7888 13688 7881 13677 7874
13650 7857 13627 7844 13608 7830 13593 7816 13581 7801 13573 7786
13567 7770 13564 7753 13564 7735 13746 7735 13746 7767 13602 7767
13605 7779 13610 7789 13617 7798 13625 7806 13635 7814 13647 7821
13678 7839 13696 7848 13711 7858 13723 7869 13733 7881 13740 7893
13745 7905 13747 7917 13748 7930 13748 7938 13746 7946 13744 7954
13741 7961 13737 7968 13733 7975 13728 7981 13722 7986 13709 7995
13694 8002 13678 8007 13660 8008 13641 8007 13632 8005 13624 8002
13616 7999 13608 7995 13602 7990 13595 7985 13589 7979 13584 7972
13580 7964 13576 7955 13573 7946 13571 7936 13570 7925 13570 7914
78 N F
13957 7938 13923 7938 13921 7947 13917 7955 13912 7962 13907 7968
13900 7972 13893 7975 13886 7977 13879 7978 13871 7977 13864 7976
13858 7974 13852 7971 13846 7967 13841 7962 13837 7957 13833 7951
13826 7936 13821 7919 13817 7898 13816 7875 13825 7884 13833 7891
13841 7896 13849 7900 13857 7903 13865 7905 13883 7906 13896 7904
13910 7900 13924 7893 13936 7884 13942 7878 13947 7872 13952 7864
13956 7856 13959 7848 13962 7838 13963 7828 13963 7817 13929 7815
13928 7826 13924 7837 13919 7847 13912 7857 13904 7864 13894 7870
13884 7874 13873 7875 13862 7874 13851 7870 13842 7864 13833 7857
13826 7847 13821 7837 13818 7826 13816 7815 13818 7804 13821 7793
13826 7783 13833 7774 13842 7766 13851 7760 13862 7756 13873 7755
13884 7756 13894 7760 13904 7766 13912 7774 13919 7783 13924 7793
13928 7804 13929 7815 13963 7817 13962 7801 13958 7785 13951 7769
13946 7762 13941 7755 13935 7749 13929 7743 13921 7738 13913 7733
13904 7730 13894 7727 13884 7725 13873 7725 13860 7725 13849 7727
13839 7731 13829 7735 13821 7741 13813 7748 13807 7756 13801 7764
13796 7774 13792 7785 13788 7796 13785 7808 13782 7834 13780 7861
13781 7878 13782 7894 13784 7908 13787 7922 13791 7935 13795 7948
13800 7959 13806 7969 13813 7978 13820 7985 13828 7992 13837 7998
13846 8002 13857 8005 13867 8007 13879 8008 13895 8007 13909 8002
13922 7996 13933 7987 13942 7977 13949 7965 13954 7952 13957 7938

126 N F
14129 7956 14127 7943 14124 7931 14118 7921 14110 7911 14100 7903
14089 7897 14077 7894 14065 7892 14065 7917 14057 7918 14049 7920
14043 7924 14037 7928 14032 7934 14028 7941 14026 7948 14025 7956
14026 7964 14028 7972 14032 7978 14037 7984 14043 7989 14049 7993
14057 7995 14065 7996 14073 7995 14080 7993 14087 7989 14092 7984
14097 7978 14101 7972 14103 7964 14104 7956 14103 7948 14101 7941
14097 7934 14092 7928 14087 7924 14080 7920 14073 7918 14065 7917
14065 7892 14052 7894 14040 7897 14029 7903 14019 7911 14012 7921
14006 7931 14002 7943 14001 7956 14002 7969 14006 7981 14012 7992
14019 8002 14029 8009 14040 8015 14052 8019 14065 8020 14077 8019
14089 8015 14100 8009 14110 8002 14118 7992 14124 7981 14127 7969
14129 7956 67 N F
14176 8016 14216 8016 14360 7787 14360 8016 14394 8016 14394 7735
14354 7735 14210 7964 14210 7735 14176 7735 14176 8016 11 N F
13570 9056 13604 9056 13605 9069 13607 9082 13611 9093 13618 9102
13625 9110 13635 9115 13647 9119 13660 9120 13670 9119 13680 9116
13689 9112 13697 9106 13703 9098 13708 9090 13711 9079 13712 9068
13711 9056 13708 9046 13704 9038 13697 9031 13688 9024 13677 9016
13650 8999 13627 8986 13608 8972 13593 8958 13581 8943 13573 8928
13567 8912 13564 8895 13564 8877 13746 8877 13746 8909 13602 8909
13605 8921 13610 8932 13617 8940 13625 8948 13635 8956 13647 8963
13678 8981 13696 8990 13711 9000 13723 9011 13733 9023 13740 9035
13745 9047 13747 9060 13748 9072 13748 9080 13746 9088 13744 9096
13741 9104 13737 9110 13733 9117 13728 9123 13722 9128 13709 9138
13694 9144 13678 9149 13660 9150 13641 9149 13632 9147 13624 9145
13616 9141 13608 9137 13602 9133 13595 9127 13589 9121 13584 9114
13580 9106 13576 9098 13573 9088 13571 9078 13570 9067 13570 9056
78 N F
13871 9150 13887 9149 13903 9145 13917 9140 13929 9131 13939 9121
13943 9115 13947 9109 13949 9102 13951 9094 13953 9086 13953 9078
13952 9066 13950 9056 13946 9047 13941 9040 13935 9033 13929 9028
13917 9020 13927 9014 13936 9008 13944 9001 13950 8992 13956 8983
13960 8973 13962 8962 13963 8951 13961 8938 13957 8924 13950 8910
13939 8896 13933 8890 13926 8885 13918 8880 13910 8875 13900 8872
13891 8869 13880 8867 13869 8867 13857 8867 13847 8869 13837 8872
13828 8875 13820 8880 13813 8885 13806 8890 13800 8896 13790 8910
13784 8924 13780 8938 13778 8951 13814 8951 13816 8940 13819 8930
13824 8921 13831 8913 13840 8906 13849 8901 13860 8898 13871 8897
13882 8898 13892 8901 13902 8906 13910 8913 13917 8921 13922 8930
13926 8940 13927 8951 13926 8962 13922 8972 13917 8982 13910 8990
13902 8996 13892 9001 13882 9004 13871 9006 13860 9004 13849 9001
13840 8996 13831 8990 13824 8982 13819 8972 13816 8962 13814 8951

13778 8951 13779 8962 13782 8973 13786 8982 13791 8991 13798 9000
13805 9007 13814 9014 13824 9020 13812 9028 13806 9033 13800 9040
13796 9047 13792 9056 13789 9066 13788 9078 13822 9078 13823 9069
13826 9060 13831 9053 13837 9047 13844 9042 13852 9039 13861 9036
13871 9036 13880 9036 13889 9039 13897 9042 13905 9047 13910 9053
13915 9060 13918 9069 13919 9078 13918 9087 13915 9095 13910 9103
13905 9109 13897 9114 13889 9117 13880 9119 13871 9120 13861 9119
13852 9117 13844 9114 13837 9109 13831 9103 13826 9095 13823 9087
13822 9078 13788 9078 13789 9086 13790 9094 13792 9102 13795 9109
13798 9115 13802 9121 13812 9131 13824 9140 13838 9145 13854 9149
13871 9150 145 N F
14129 9098 14127 9086 14124 9074 14118 9063 14110 9053 14100 9045
14089 9040 14077 9036 14065 9034 14065 9059 14057 9060 14049 9062
14043 9066 14037 9071 14032 9076 14028 9083 14026 9091 14025 9098
14026 9106 14028 9114 14032 9121 14037 9126 14043 9131 14049 9135
14057 9137 14065 9138 14073 9137 14080 9135 14087 9131 14092 9126
14097 9121 14101 9114 14103 9106 14104 9098 14103 9091 14101 9083
14097 9076 14092 9071 14087 9066 14080 9062 14073 9060 14065 9059
14065 9034 14052 9036 14040 9040 14029 9045 14019 9053 14012 9063
14006 9074 14002 9086 14001 9098 14002 9111 14006 9123 14012 9134
14019 9144 14029 9151 14040 9157 14052 9161 14065 9162 14077 9161
14089 9157 14100 9151 14110 9144 14118 9134 14124 9123 14127 9111
14129 9098 67 N F
14176 9158 14216 9158 14360 8929 14360 9158 14394 9158 14394 8877
14354 8877 14210 9106 14210 8877 14176 8877 14176 9158 11 N F
13602 10194 13568 10194 13570 10214 13572 10223 13575 10232 13578 10240
13582 10247 13587 10254 13592 10260 13598 10265 13604 10270 13611 10273
13619 10277 13627 10279 13635 10281 13654 10282 13671 10281 13687 10277
13702 10271 13714 10263 13719 10258 13724 10252 13728 10246 13732 10240
13735 10233 13737 10225 13738 10217 13738 10208 13738 10198 13736 10188
13733 10180 13729 10173 13724 10166 13717 10160 13708 10154 13698 10149
13710 10145 13720 10139 13728 10132 13735 10125 13740 10116 13743 10107
13746 10097 13746 10085 13745 10070 13741 10055 13735 10041 13731 10034
13725 10028 13719 10022 13713 10017 13705 10012 13696 10008 13687 10005
13676 10003 13665 10001 13652 10001 13641 10001 13631 10002 13621 10004
13613 10007 13605 10011 13597 10015 13591 10020 13585 10025 13580 10031
13576 10038 13572 10045 13569 10052 13565 10069 13564 10087 13598 10087
13598 10077 13600 10067 13603 10058 13608 10049 13615 10042 13625 10036
13638 10032 13654 10031 13668 10031 13680 10034 13689 10039 13697 10046
13703 10054 13707 10064 13709 10074 13710 10085 13709 10099 13707 10105
13705 10111 13702 10116 13698 10120 13689 10126 13678 10131 13665 10133
13650 10135 13634 10135 13634 10164 13650 10164 13664 10165 13676 10167
13685 10171 13693 10176 13698 10184 13701 10194 13702 10206 13701 10215

13699 10224 13696 10232 13691 10239 13684 10244 13676 10248 13666 10251
13654 10252 13643 10251 13633 10249 13625 10246 13617 10240 13611 10232
13606 10222 13603 10209 13602 10194 123 N F
13871 10282 13881 10281 13890 10279 13899 10275 13908 10270 13916 10263
13923 10256 13930 10247 13936 10237 13942 10227 13947 10216 13951 10204
13954 10192 13957 10180 13959 10167 13961 10154 13961 10141 13961 10129
13959 10116 13954 10090 13951 10078 13947 10066 13942 10055 13936 10044
13930 10035 13923 10026 13916 10018 13908 10011 13899 10006 13890 10002
13881 10000 13871 9999 13861 10000 13851 10002 13842 10006 13834 10011
13826 10018 13818 10026 13811 10035 13805 10044 13800 10055 13795 10066
13790 10078 13787 10090 13782 10116 13781 10129 13780 10141 13816 10141
13818 10117 13821 10095 13827 10076 13834 10060 13842 10046 13851 10037
13856 10033 13861 10031 13866 10029 13871 10029 13876 10029 13880 10031
13885 10033 13890 10037 13899 10046 13908 10060 13915 10076 13920 10095
13924 10117 13925 10141 13924 10166 13920 10188 13915 10206 13908 10222
13899 10235 13890 10244 13885 10248 13880 10250 13876 10251 13871 10252
13866 10251 13861 10250 13856 10248 13851 10244 13842 10235 13834 10222
13827 10206 13821 10188 13818 10166 13816 10141 13780 10141 13781 10154
13782 10167 13784 10180 13787 10192 13790 10204 13795 10216 13800 10227
13805 10237 13811 10247 13818 10256 13826 10263 13834 10270 13842 10275
13851 10279 13861 10281 13871 10282 105 N F
14129 10230 14127 10218 14124 10206 14118 10195 14110 10185 14100 10177
14089 10171 14077 10168 14065 10166 14065 10191 14057 10192 14049 10194
14043 10198 14037 10202 14032 10208 14028 10215 14026 10222 14025 10230
14026 10238 14028 10246 14032 10252 14037 10258 14043 10263 14049 10267
14057 10269 14065 10270 14073 10269 14080 10267 14087 10263 14092 10258
14097 10252 14101 10246 14103 10238 14104 10230 14103 10222 14101 10215
14097 10208 14092 10202 14087 10198 14080 10194 14073 10192 14065 10191
14065 10166 14052 10168 14040 10171 14029 10177 14019 10185 14012 10195
14006 10206 14002 10218 14001 10230 14002 10243 14006 10255 14012 10266
14019 10276 14029 10283 14040 10289 14052 10293 14065 10294 14077 10293
14089 10289 14100 10283 14110 10276 14118 10266 14124 10255 14127 10243
14129 10230 67 N F
14176 10290 14216 10290 14360 10061 14360 10290 14394 10290 14394 10009
14354 10009 14210 10238 14210 10009 14176 10009 14176 10290 11 N F
13602 11315 13568 11315 13570 11336 13572 11345 13575 11354 13578 11362
13582 11369 13587 11376 13592 11382 13598 11387 13604 11391 13611 11395
13619 11398 13627 11401 13635 11402 13654 11404 13671 11403 13687 11399
13702 11393 13714 11385 13719 11380 13724 11374 13728 11368 13732 11361
13735 11354 13737 11347 13738 11338 13738 11330 13738 11319 13736 11310
13733 11302 13729 11294 13724 11288 13717 11281 13708 11276 13698 11271
13710 11266 13720 11261 13728 11254 13735 11247 13740 11238 13743 11229
13746 11218 13746 11207 13745 11192 13741 11177 13735 11163 13731 11156

13725 11150 13719 11144 13713 11139 13705 11134 13696 11130 13687 11127
13676 11124 13665 11123 13652 11123 13641 11123 13631 11124 13621 11126
13613 11129 13605 11132 13597 11137 13591 11141 13585 11147 13580 11153
13576 11160 13572 11167 13569 11174 13565 11191 13564 11209 13598 11209
13598 11199 13600 11189 13603 11180 13608 11171 13615 11164 13625 11158
13638 11154 13654 11153 13668 11153 13680 11156 13689 11161 13697 11168
13703 11176 13707 11185 13709 11196 13710 11207 13709 11221 13707 11227
13705 11233 13702 11237 13698 11241 13689 11248 13678 11252 13665 11255
13650 11257 13634 11257 13634 11285 13650 11286 13664 11287 13676 11289
13685 11293 13693 11298 13698 11306 13701 11315 13702 11328 13701 11337
13699 11346 13696 11354 13691 11360 13684 11366 13676 11370 13666 11373
13654 11374 13643 11373 13633 11371 13625 11367 13617 11362 13611 11354
13606 11344 13603 11331 13602 11315 123 N F
13784 11309 13818 11309 13819 11323 13822 11336 13826 11347 13832 11356
13840 11363 13850 11369 13861 11373 13875 11374 13885 11373 13895 11370
13904 11366 13911 11360 13918 11352 13923 11343 13926 11333 13927 11321
13926 11310 13923 11300 13918 11292 13912 11284 13903 11277 13892 11270
13865 11253 13841 11240 13822 11226 13807 11212 13796 11197 13788 11182
13782 11166 13779 11149 13778 11131 13961 11131 13961 11163 13816 11163
13820 11175 13825 11185 13831 11194 13840 11202 13850 11209 13862 11217
13893 11235 13911 11244 13926 11254 13938 11265 13947 11277 13955 11289
13959 11301 13962 11313 13963 11326 13963 11334 13961 11342 13959 11350
13956 11357 13952 11364 13948 11371 13943 11377 13937 11382 13924 11391
13909 11398 13892 11402 13875 11404 13856 11402 13847 11401 13839 11398
13831 11395 13823 11391 13816 11386 13810 11381 13804 11375 13799 11368
13795 11360 13791 11351 13788 11342 13786 11332 13785 11321 13784 11309
78 N F
14129 11352 14127 11339 14124 11327 14118 11316 14110 11307 14100 11299
14089 11293 14077 11289 14065 11288 14065 11313 14057 11314 14049 11316
14043 11319 14037 11324 14032 11330 14028 11337 14026 11344 14025 11352
14026 11360 14028 11367 14032 11374 14037 11380 14043 11385 14049 11388
14057 11391 14065 11392 14073 11391 14080 11388 14087 11385 14092 11380
14097 11374 14101 11367 14103 11360 14104 11352 14103 11344 14101 11337
14097 11330 14092 11324 14087 11319 14080 11316 14073 11314 14065 11313
14065 11288 14052 11289 14040 11293 14029 11299 14019 11307 14012 11316
14006 11327 14002 11339 14001 11352 14002 11365 14006 11377 14012 11388
14019 11397 14029 11405 14040 11411 14052 11415 14065 11416 14077 11415
14089 11411 14100 11405 14110 11397 14118 11388 14124 11377 14127 11365
14129 11352 67 N F
14176 11412 14216 11412 14360 11183 14360 11412 14394 11412 14394 11131
14354 11131 14210 11360 14210 11131 14176 11131 14176 11412 11 N F
13602 12430 13568 12430 13570 12451 13572 12460 13575 12469 13578 12477
13582 12484 13587 12490 13592 12496 13598 12502 13604 12506 13611 12510

13619 12513 13627 12515 13635 12517 13654 12519 13671 12517 13687 12514
13702 12508 13714 12500 13719 12495 13724 12489 13728 12483 13732 12476
13735 12469 13737 12461 13738 12453 13738 12444 13738 12434 13736 12425
13733 12417 13729 12409 13724 12402 13717 12396 13708 12391 13698 12386
13710 12381 13720 12375 13728 12369 13735 12361 13740 12353 13743 12344
13746 12333 13746 12322 13745 12307 13741 12292 13735 12277 13731 12271
13725 12264 13719 12259 13713 12253 13705 12249 13696 12245 13687 12242
13676 12239 13665 12238 13652 12237 13641 12238 13631 12239 13621 12241
13613 12244 13605 12247 13597 12251 13591 12256 13585 12262 13580 12268
13576 12274 13572 12281 13569 12289 13565 12306 13564 12324 13598 12324
13598 12314 13600 12304 13603 12294 13608 12286 13615 12278 13625 12273
13638 12269 13654 12267 13668 12268 13680 12271 13689 12276 13697 12282
13703 12291 13707 12300 13709 12311 13710 12322 13709 12336 13707 12342
13705 12347 13702 12352 13698 12356 13689 12363 13678 12367 13665 12370
13650 12372 13634 12372 13634 12400 13650 12400 13664 12402 13676 12404
13685 12408 13693 12413 13698 12420 13701 12430 13702 12442 13701 12452
13699 12461 13696 12468 13691 12475 13684 12481 13676 12485 13666 12488
13654 12489 13643 12488 13633 12486 13625 12482 13617 12477 13611 12469
13606 12459 13603 12446 13602 12430 123 N F
13901 12519 13925 12519 13925 12342 13967 12342 13967 12310 13925 12310
13925 12245 13891 12245 13891 12310 13776 12310 13776 12342 13806 12342
13891 12342 13891 12460 13806 12342 13776 12342 13776 12346 13901 12519
18 N F
14129 12467 14127 12454 14124 12442 14118 12431 14110 12422 14100 12414
14089 12408 14077 12404 14065 12403 14065 12428 14057 12428 14049 12431
14043 12434 14037 12439 14032 12445 14028 12452 14026 12459 14025 12467
14026 12475 14028 12482 14032 12489 14037 12495 14043 12500 14049 12503
14057 12505 14065 12506 14073 12505 14080 12503 14087 12500 14092 12495
14097 12489 14101 12482 14103 12475 14104 12467 14103 12459 14101 12452
14097 12445 14092 12439 14087 12434 14080 12431 14073 12428 14065 12428
14065 12403 14052 12404 14040 12408 14029 12414 14019 12422 14012 12431
14006 12442 14002 12454 14001 12467 14002 12480 14006 12492 14012 12503
14019 12512 14029 12520 14040 12526 14052 12530 14065 12531 14077 12530
14089 12526 14100 12520 14110 12512 14118 12503 14124 12492 14127 12480
14129 12467 67 N F
14176 12527 14216 12527 14360 12298 14360 12527 14394 12527 14394 12245
14354 12245 14210 12474 14210 12245 14176 12245 14176 12527 11 N F
13602 13539 13568 13539 13570 13560 13572 13569 13575 13578 13578 13586
13582 13593 13587 13599 13592 13605 13598 13610 13604 13615 13611 13619
13619 13622 13627 13624 13635 13626 13654 13628 13671 13626 13687 13623
13702 13617 13714 13609 13719 13604 13724 13598 13728 13592 13732 13585
13735 13578 13737 13570 13738 13562 13738 13553 13738 13543 13736 13534
13733 13526 13729 13518 13724 13511 13717 13505 13708 13500 13698 13495

13710 13490 13720 13484 13728 13478 13735 13470 13740 13462 13743 13453
13746 13442 13746 13431 13745 13416 13741 13401 13735 13386 13731 13380
13725 13373 13719 13368 13713 13362 13705 13358 13696 13354 13687 13351
13676 13348 13665 13347 13652 13346 13641 13347 13631 13348 13621 13350
13613 13353 13605 13356 13597 13360 13591 13365 13585 13371 13580 13377
13576 13383 13572 13390 13569 13398 13565 13415 13564 13433 13598 13433
13598 13423 13600 13413 13603 13403 13608 13395 13615 13387 13625 13382
13638 13378 13654 13376 13668 13377 13680 13380 13689 13385 13697 13391
13703 13400 13707 13409 13709 13420 13710 13431 13709 13445 13707 13451
13705 13456 13702 13461 13698 13465 13689 13472 13678 13476 13665 13479
13650 13480 13634 13481 13634 13509 13650 13509 13664 13511 13676 13513
13685 13517 13693 13522 13698 13529 13701 13539 13702 13551 13701 13561
13699 13570 13696 13577 13691 13584 13684 13590 13676 13594 13666 13597
13654 13597 13643 13597 13633 13595 13625 13591 13617 13586 13611 13578
13606 13568 13603 13555 13602 13539 123 N F
13957 13557 13923 13557 13921 13567 13917 13575 13912 13581 13907 13587
13900 13592 13893 13595 13886 13597 13879 13597 13871 13597 13864 13596
13858 13594 13852 13591 13846 13587 13841 13582 13837 13577 13833 13570
13826 13556 13821 13538 13817 13518 13816 13495 13825 13504 13833 13511
13841 13516 13849 13520 13857 13522 13865 13524 13883 13525 13896 13524
13910 13520 13924 13513 13936 13504 13942 13498 13947 13491 13952 13484
13956 13476 13959 13467 13962 13458 13963 13448 13963 13437 13929 13435
13928 13446 13924 13457 13919 13467 13912 13476 13904 13484 13894 13490
13884 13494 13873 13495 13862 13494 13851 13490 13842 13484 13833 13476
13826 13467 13821 13457 13818 13446 13816 13435 13818 13423 13821 13413
13826 13402 13833 13393 13842 13386 13851 13380 13862 13376 13873 13374
13884 13376 13894 13380 13904 13386 13912 13393 13919 13402 13924 13413
13928 13423 13929 13435 13963 13437 13962 13421 13958 13404 13951 13389
13946 13382 13941 13375 13935 13368 13929 13362 13921 13357 13913 13353
13904 13349 13894 13346 13884 13345 13873 13344 13860 13345 13849 13347
13839 13350 13829 13355 13821 13361 13813 13367 13807 13375 13801 13384
13796 13394 13792 13404 13788 13415 13785 13427 13782 13453 13780 13481
13781 13497 13782 13513 13784 13528 13787 13542 13791 13555 13795 13567
13800 13578 13806 13588 13813 13597 13820 13605 13828 13612 13837 13617
13846 13622 13857 13625 13867 13627 13879 13628 13895 13626 13909 13622
13922 13615 13933 13607 13942 13596 13949 13584 13954 13571 13957 13557
126 N F
14129 13576 14127 13563 14124 13551 14118 13540 14110 13531 14100 13523
14089 13517 14077 13513 14065 13512 14065 13536 14057 13537 14049 13540
14043 13543 14037 13548 14032 13554 14028 13561 14026 13568 14025 13576
14026 13584 14028 13591 14032 13598 14037 13604 14043 13609 14049 13612
14057 13614 14065 13615 14073 13614 14080 13612 14087 13609 14092 13604
14097 13598 14101 13591 14103 13584 14104 13576 14103 13568 14101 13561

14097 13554 14092 13548 14087 13543 14080 13540 14073 13537 14065 13536
14065 13512 14052 13513 14040 13517 14029 13523 14019 13531 14012 13540
14006 13551 14002 13563 14001 13576 14002 13589 14006 13601 14012 13612
14019 13621 14029 13629 14040 13635 14052 13639 14065 13640 14077 13639
14089 13635 14100 13629 14110 13621 14118 13612 14124 13601 14127 13589
14129 13576 67 N F
14176 13636 14216 13636 14360 13407 14360 13636 14394 13636 14394 13354
14354 13354 14210 13583 14210 13354 14176 13354 14176 13636 11 N F
13602 14642 13568 14642 13570 14662 13572 14671 13575 14680 13578 14688
13582 14695 13587 14702 13592 14708 13598 14713 13604 14717 13611 14721
13619 14724 13627 14727 13635 14729 13654 14730 13671 14729 13687 14725
13702 14719 13714 14711 13719 14706 13724 14700 13728 14694 13732 14688
13735 14680 13737 14673 13738 14664 13738 14656 13738 14646 13736 14636
13733 14628 13729 14621 13724 14614 13717 14608 13708 14602 13698 14597
13710 14593 13720 14587 13728 14580 13735 14573 13740 14564 13743 14555
13746 14545 13746 14533 13745 14518 13741 14503 13735 14489 13731 14482
13725 14476 13719 14470 13713 14465 13705 14460 13696 14456 13687 14453
13676 14451 13665 14449 13652 14449 13641 14449 13631 14450 13621 14452
13613 14455 13605 14459 13597 14463 13591 14468 13585 14473 13580 14479
13576 14486 13572 14493 13569 14500 13565 14517 13564 14535 13598 14535
13598 14525 13600 14515 13603 14506 13608 14497 13615 14490 13625 14484
13638 14480 13654 14479 13668 14479 13680 14482 13689 14487 13697 14494
13703 14502 13707 14512 13709 14522 13710 14533 13709 14547 13707 14553
13705 14559 13702 14563 13698 14568 13689 14574 13678 14579 13665 14581
13650 14583 13634 14583 13634 14611 13650 14612 13664 14613 13676 14615
13685 14619 13693 14624 13698 14632 13701 14641 13702 14654 13701 14663
13699 14672 13696 14680 13691 14687 13684 14692 13676 14696 13666 14699
13654 14700 13643 14699 13633 14697 13625 14694 13617 14688 13611 14680
13606 14670 13603 14657 13602 14642 123 N F
13871 14730 13887 14729 13903 14725 13917 14719 13929 14711 13939 14701
13943 14695 13947 14689 13949 14682 13951 14674 13953 14666 13953 14658
13952 14646 13950 14636 13946 14627 13941 14620 13935 14613 13929 14608
13917 14599 13927 14594 13936 14588 13944 14580 13950 14572 13956 14563
13960 14553 13962 14542 13963 14531 13961 14518 13957 14503 13950 14489
13939 14476 13933 14470 13926 14464 13918 14459 13910 14455 13900 14452
13891 14449 13880 14447 13869 14447 13857 14447 13847 14449 13837 14452
13828 14455 13820 14459 13813 14464 13806 14470 13800 14476 13790 14489
13784 14503 13780 14518 13778 14531 13814 14531 13816 14520 13819 14510
13824 14501 13831 14493 13840 14486 13849 14481 13860 14478 13871 14477
13882 14478 13892 14481 13902 14486 13910 14493 13917 14501 13922 14510
13926 14520 13927 14531 13926 14542 13922 14552 13917 14562 13910 14570
13902 14576 13892 14581 13882 14584 13871 14585 13860 14584 13849 14581
13840 14576 13831 14570 13824 14562 13819 14552 13816 14542 13814 14531

13778 14531 13779 14542 13782 14553 13786 14562 13791 14571 13798 14580
13805 14587 13814 14594 13824 14599 13812 14608 13806 14613 13800 14620
13796 14627 13792 14636 13789 14646 13788 14658 13822 14658 13823 14648
13826 14640 13831 14633 13837 14627 13844 14622 13852 14618 13861 14616
13871 14616 13880 14616 13889 14618 13897 14622 13905 14627 13910 14633
13915 14640 13918 14648 13919 14658 13918 14667 13915 14675 13910 14683
13905 14689 13897 14693 13889 14697 13880 14699 13871 14700 13861 14699
13852 14697 13844 14693 13837 14689 13831 14683 13826 14675 13823 14667
13822 14658 13788 14658 13789 14666 13790 14674 13792 14682 13795 14689
13798 14695 13802 14701 13812 14711 13824 14719 13838 14725 13854 14729
13871 14730 145 N F
14129 14678 14127 14665 14124 14653 14118 14643 14110 14633 14100 14625
14089 14619 14077 14616 14065 14614 14065 14639 14057 14640 14049 14642
14043 14646 14037 14650 14032 14656 14028 14663 14026 14670 14025 14678
14026 14686 14028 14694 14032 14700 14037 14706 14043 14711 14049 14715
14057 14717 14065 14718 14073 14717 14080 14715 14087 14711 14092 14706
14097 14700 14101 14694 14103 14686 14104 14678 14103 14670 14101 14663
14097 14656 14092 14650 14087 14646 14080 14642 14073 14640 14065 14639
14065 14614 14052 14616 14040 14619 14029 14625 14019 14633 14012 14643
14006 14653 14002 14665 14001 14678 14002 14691 14006 14703 14012 14714
14019 14724 14029 14731 14040 14737 14052 14741 14065 14742 14077 14741
14089 14737 14100 14731 14110 14724 14118 14714 14124 14703 14127 14691
14129 14678 67 N F
14176 14738 14216 14738 14360 14509 14360 14738 14394 14738 14394 14457
14354 14457 14210 14686 14210 14457 14176 14457 14176 14738 11 N F
13688 15828 13712 15828 13712 15651 13754 15651 13754 15619 13712 15619
13712 15555 13678 15555 13678 15619 13564 15619 13564 15651 13594 15651
13678 15651 13678 15770 13594 15651 13564 15651 13564 15655 13688 15828
18 N F
13873 15828 13883 15827 13892 15825 13901 15821 13910 15816 13918 15809
13925 15802 13932 15793 13938 15784 13944 15773 13949 15762 13953 15750
13956 15738 13959 15726 13961 15713 13963 15700 13963 15688 13963 15675
13961 15662 13956 15636 13953 15624 13949 15612 13944 15601 13938 15591
13932 15581 13925 15572 13918 15564 13910 15558 13901 15552 13892 15548
13883 15546 13873 15545 13863 15546 13853 15548 13844 15552 13836 15558
13828 15564 13820 15572 13813 15581 13807 15591 13802 15601 13797 15612
13792 15624 13789 15636 13784 15662 13783 15675 13782 15688 13818 15688
13820 15663 13823 15641 13829 15622 13836 15606 13844 15593 13853 15583
13858 15580 13863 15577 13868 15576 13873 15575 13878 15576 13882 15577
13887 15580 13892 15583 13901 15593 13910 15606 13917 15622 13922 15641
13926 15663 13927 15688 13926 15712 13922 15734 13917 15753 13910 15768
13901 15781 13892 15790 13887 15794 13882 15796 13878 15798 13873 15798
13868 15798 13863 15796 13858 15794 13853 15790 13844 15781 13836 15768

13829 15753 13823 15734 13820 15712 13818 15688 13782 15688 13783 15700
13784 15713 13786 15726 13789 15738 13792 15750 13797 15762 13802 15773
13807 15784 13813 15793 13820 15802 13828 15809 13836 15816 13844 15821
13853 15825 13863 15827 13873 15828 105 N F
14131 15776 14129 15764 14126 15752 14120 15741 14112 15731 14102 15723
14091 15718 14079 15714 14067 15712 14067 15737 14059 15738 14051 15740
14045 15744 14039 15749 14034 15754 14030 15761 14028 15769 14027 15776
14028 15784 14030 15792 14034 15799 14039 15804 14045 15809 14051 15813
14059 15815 14067 15816 14075 15815 14082 15813 14089 15809 14094 15804
14099 15799 14103 15792 14105 15784 14106 15776 14105 15769 14103 15761
14099 15754 14094 15749 14089 15744 14082 15740 14075 15738 14067 15737
14067 15712 14054 15714 14042 15718 14031 15723 14022 15731 14014 15741
14008 15752 14004 15764 14003 15776 14004 15789 14008 15801 14014 15812
14022 15822 14031 15830 14042 15835 14054 15839 14067 15840 14079 15839
14091 15835 14102 15830 14112 15822 14120 15812 14126 15801 14129 15789
14131 15776 67 N F
14178 15836 14218 15836 14362 15607 14362 15836 14396 15836 14396 15555
14356 15555 14212 15784 14212 15555 14178 15555 14178 15836 11 N F
25 W
2646 5172 2646 5453 2 P
3739 5172 3739 5453 2 P
4823 5172 4823 5453 2 P
5903 5172 5903 5453 2 P
6971 5172 6971 5453 2 P
8052 5172 8052 5453 2 P
9133 5172 9133 5453 2 P
10214 5172 10214 5453 2 P
11295 5172 11295 5453 2 P
12376 5172 12376 5453 2 P
2747 16454 2747 16173 2 P
4812 16454 4812 16173 2 P
6843 16454 6843 16173 2 P
8864 16454 8864 16173 2 P
10892 16454 10892 16173 2 P
12942 16454 12942 16173 2 P
2016 5647 2297 5647 2 P
2016 6823 2297 6823 2 P
2016 7980 2297 7980 2 P
2016 9125 2297 9125 2 P
2016 10260 2297 10260 2 P
2016 11387 2297 11387 2 P
2016 12505 2297 12505 2 P
2016 13617 2297 13617 2 P

2016 14723 2297 14723 2 P
2016 15816 2297 15816 2 P
13283 5549 13002 5549 2 P
13283 6719 13002 6719 2 P
13283 7872 13002 7872 2 P
13283 9015 13002 9015 2 P
13283 10147 13002 10147 2 P
13283 11269 13002 11269 2 P
13283 12384 13002 12384 2 P
13283 13492 13002 13492 2 P
13283 14594 13002 14594 2 P
13283 15693 13002 15693 2 P
13002 5453 2297 5453 2297 16173 13002 16173 13002 5453 2297 5453
6:00 PM
7309 17656 7368 17656 7493 17299 7442 17299 7406 17404 7266 17404
7230 17299 7179 17299 7291 17605 7337 17605 7279 17444 7393 17444
7337 17605 7291 17605 7309 17656 15 N F
7526 17557 7564 17557 7564 17518 7575 17531 7586 17542 7597 17550
7608 17556 7618 17560 7628 17563 7637 17564 7646 17564 7661 17563
7678 17559 7687 17555 7696 17551 7705 17545 7714 17538 7722 17530
7730 17520 7737 17509 7743 17495 7748 17481 7752 17464 7754 17445
7755 17424 7755 17406 7753 17389 7750 17373 7745 17359 7740 17347
7734 17336 7727 17326 7720 17317 7711 17309 7703 17303 7694 17298
7684 17293 7675 17290 7665 17288 7655 17287 7646 17286 7635 17287
7624 17288 7614 17290 7604 17294 7595 17299 7585 17306 7576 17314
7567 17324 7567 17192 7526 17192 7526 17427 7567 17427 7567 17416
7568 17405 7573 17386 7580 17368 7589 17354 7600 17341 7606 17336
7613 17332 7620 17329 7626 17326 7633 17325 7641 17324 7652 17326
7665 17330 7677 17337 7682 17342 7688 17348 7693 17354 7697 17362
7702 17370 7705 17379 7708 17390 7710 17401 7711 17413 7712 17427
7711 17439 7710 17451 7708 17462 7704 17472 7701 17481 7696 17489
7691 17497 7686 17503 7674 17513 7662 17521 7651 17525 7641 17526
7634 17526 7627 17524 7621 17522 7614 17519 7602 17510 7590 17498
7581 17484 7573 17467 7570 17458 7568 17448 7567 17437 7567 17427
7526 17427 7526 17557 104 N F
7806 17557 7842 17557 7842 17508 7857 17529 7872 17544 7884 17554
7896 17561 7906 17563 7915 17564 7922 17562 7929 17559 7929 17521
7908 17519 7891 17514 7884 17511 7877 17508 7871 17503 7866 17499
7861 17493 7857 17488 7852 17474 7848 17459 7847 17442 7847 17299
7806 17299 7806 17557 26 N F
7967 17656 8008 17656 8008 17605 7967 17605 7967 17656 5 N F
7967 17557 8008 17557 8008 17299 7967 17299 7967 17557 5 N F
8079 17656 8120 17656 8120 17299 8079 17299 8079 17656 5 N F

2297 17549 2445 17549 2455 17549 2465 17547 2476 17544 2487 17540
2498 17535 2509 17528 2520 17520 2531 17510 2541 17499 2550 17486
2559 17472 2566 17455 2572 17437 2576 17417 2579 17395 2580 17370
2579 17346 2577 17324 2573 17304 2567 17285 2560 17269 2552 17254
2543 17242 2534 17230 2523 17221 2513 17213 2501 17206 2490 17201
2479 17197 2467 17194 2456 17192 2445 17192 2297 17192 2297 17508
2343 17508 2343 17233 2438 17233 2449 17233 2459 17235 2469 17238
2479 17242 2487 17248 2495 17254 2502 17261 2509 17270 2515 17279
2520 17290 2524 17301 2528 17313 2531 17326 2533 17340 2534 17355
2534 17370 2534 17387 2532 17403 2530 17417 2526 17431 2522 17443
2517 17454 2511 17465 2505 17474 2498 17482 2490 17489 2482 17495
2473 17500 2464 17503 2455 17506 2445 17508 2435 17508 2343 17508
2297 17508 2297 17549 74 N F
2644 17549 2708 17549 2812 17238 2917 17549 2980 17549 2980 17192
2937 17192 2937 17490 2835 17192 2787 17192 2687 17490 2687 17192
2644 17192 2644 17549 14 N F
2 O
2458 2789 3323 2789 3323 3744 2458 3744 2458 2789 5 N F
1 O
12 W
2458 2789 3323 2789 3323 3744 2458 3744 2458 2789 5 P
20 O
3323 2789 4188 2789 4188 3744 3323 3744 3323 2789 5 N F
1 O
3323 2789 4188 2789 4188 3744 3323 3744 3323 2789 5 P
38 O
4188 2789 5054 2789 5054 3744 4188 3744 4188 2789 5 N F
1 O
4188 2789 5054 2789 5054 3744 4188 3744 4188 2789 5 P
56 O
5054 2789 5919 2789 5919 3744 5054 3744 5054 2789 5 N F
1 O
5054 2789 5919 2789 5919 3744 5054 3744 5054 2789 5 P
74 O
5919 2789 6784 2789 6784 3744 5919 3744 5919 2789 5 N F
1 O
5919 2789 6784 2789 6784 3744 5919 3744 5919 2789 5 P
92 O
6784 2789 7650 2789 7650 3744 6784 3744 6784 2789 5 N F
1 O
6784 2789 7650 2789 7650 3744 6784 3744 6784 2789 5 P
111 O
7650 2789 8515 2789 8515 3744 7650 3744 7650 2789 5 N F

1 O
7650 2789 8515 2789 8515 3744 7650 3744 7650 2789 5 P
129 O
8515 2789 9380 2789 9380 3744 8515 3744 8515 2789 5 N F
1 O
8515 2789 9380 2789 9380 3744 8515 3744 8515 2789 5 P
147 O
9380 2789 10245 2789 10245 3744 9380 3744 9380 2789 5 N F
1 O
9380 2789 10245 2789 10245 3744 9380 3744 9380 2789 5 P
165 O
10245 2789 11111 2789 11111 3744 10245 3744 10245 2789 5 N F
1 O
10245 2789 11111 2789 11111 3744 10245 3744 10245 2789 5 P
183 O
11111 2789 11976 2789 11976 3744 11111 3744 11111 2789 5 N F
1 O
11111 2789 11976 2789 11976 3744 11111 3744 11111 2789 5 P
201 O
11976 2789 12841 2789 12841 3744 11976 3744 11976 2789 5 N F
1 O
11976 2789 12841 2789 12841 3744 11976 3744 11976 2789 5 P
3169 2500 3179 2499 3188 2497 3196 2493 3205 2488 3212 2482
3219 2474 3226 2466 3232 2457 3237 2447 3242 2437 3249 2414
3254 2390 3255 2366 3254 2341 3249 2317 3242 2294 3237 2283
3232 2273 3226 2264 3219 2256 3212 2248 3205 2242 3196 2237
3188 2233 3179 2231 3169 2230 3160 2231 3151 2233 3142 2237
3134 2242 3127 2248 3119 2256 3113 2264 3107 2273 3102 2283
3097 2294 3090 2317 3085 2341 3083 2366 3118 2366 3119 2342
3122 2321 3128 2303 3134 2288 3142 2275 3151 2266 3155 2263
3160 2260 3165 2259 3169 2258 3174 2259 3179 2260 3183 2263
3188 2266 3197 2275 3204 2288 3211 2303 3216 2321 3220 2342
3221 2366 3220 2389 3216 2410 3211 2428 3204 2443 3197 2455
3188 2464 3179 2469 3174 2470 3169 2471 3165 2470 3160 2469
3151 2464 3142 2455 3134 2443 3128 2428 3122 2410 3119 2389
3118 2366 3083 2366 3085 2390 3090 2414 3097 2437 3102 2447
3107 2457 3113 2466 3119 2474 3127 2482 3134 2488 3142 2493
3151 2497 3160 2499 3169 2500 93 N F
3305 2278 3343 2278 3343 2239 3305 2239 3305 2278 5 N F
3414 2500 3550 2500 3550 2469 3441 2469 3431 2394 3441 2401
3452 2407 3465 2410 3481 2412 3494 2410 3508 2407 3522 2401
3529 2397 3535 2392 3541 2386 3546 2380 3551 2373 3555 2365
3558 2356 3561 2346 3563 2335 3563 2323 3562 2305 3557 2288

3554 2280 3550 2272 3546 2265 3540 2258 3534 2252 3528 2246
3520 2241 3512 2237 3504 2234 3494 2232 3484 2230 3473 2230
3454 2231 3445 2233 3438 2235 3430 2238 3424 2242 3412 2251
3403 2262 3396 2274 3391 2288 3387 2302 3420 2302 3423 2293
3427 2284 3432 2277 3438 2270 3445 2265 3453 2262 3462 2259
3471 2258 3484 2259 3496 2262 3506 2267 3515 2274 3521 2283
3526 2294 3530 2307 3531 2322 3530 2332 3527 2343 3523 2354
3517 2363 3509 2371 3499 2377 3487 2381 3473 2383 3466 2382
3459 2381 3453 2379 3448 2377 3436 2369 3424 2358 3395 2358
3414 2500 79 N F
3932 2500 3942 2499 3951 2497 3959 2493 3968 2488 3975 2482
3982 2474 3989 2466 3995 2457 4000 2447 4005 2437 4012 2414
4017 2390 4018 2366 4017 2341 4012 2317 4005 2294 4000 2283
3995 2273 3989 2264 3982 2256 3975 2248 3968 2242 3959 2237
3951 2233 3942 2231 3932 2230 3923 2231 3914 2233 3905 2237
3897 2242 3890 2248 3882 2256 3876 2264 3870 2273 3865 2283
3860 2294 3853 2317 3848 2341 3846 2366 3881 2366 3882 2342
3885 2321 3891 2303 3897 2288 3905 2275 3914 2266 3918 2263
3923 2260 3928 2259 3932 2258 3937 2259 3942 2260 3946 2263
3951 2266 3960 2275 3967 2288 3974 2303 3979 2321 3983 2342
3984 2366 3983 2389 3979 2410 3974 2428 3967 2443 3960 2455
3951 2464 3942 2469 3937 2470 3932 2471 3928 2470 3923 2469
3914 2464 3905 2455 3897 2443 3891 2428 3885 2410 3882 2389
3881 2366 3846 2366 3848 2390 3853 2414 3860 2437 3865 2447
3870 2457 3876 2466 3882 2474 3890 2482 3897 2488 3905 2493
3914 2497 3923 2499 3932 2500 93 N F
4068 2278 4106 2278 4106 2239 4068 2239 4068 2278 5 N F
4177 2500 4313 2500 4313 2469 4204 2469 4194 2394 4204 2401
4215 2407 4228 2410 4244 2412 4257 2410 4271 2407 4285 2401
4292 2397 4298 2392 4304 2386 4309 2380 4314 2373 4318 2365
4321 2356 4324 2346 4326 2335 4326 2323 4325 2305 4320 2288
4317 2280 4313 2272 4309 2265 4303 2258 4297 2252 4291 2246
4283 2241 4275 2237 4267 2234 4257 2232 4247 2230 4236 2230
4217 2231 4208 2233 4201 2235 4193 2238 4187 2242 4175 2251
4166 2262 4159 2274 4154 2288 4150 2302 4183 2302 4186 2293
4190 2284 4195 2277 4201 2270 4208 2265 4216 2262 4225 2259
4234 2258 4247 2259 4259 2262 4269 2267 4278 2274 4284 2283
4289 2294 4293 2307 4294 2322 4293 2332 4290 2343 4286 2354
4280 2363 4272 2371 4262 2377 4250 2381 4236 2383 4229 2382
4222 2381 4216 2379 4211 2377 4199 2369 4187 2358 4158 2358
4177 2500 79 N F
4382 2500 4517 2500 4517 2469 4408 2469 4399 2394 4408 2401
4419 2407 4433 2410 4448 2412 4462 2410 4476 2407 4490 2401

4496 2397 4502 2392 4508 2386 4514 2380 4518 2373 4523 2365
4526 2356 4529 2346 4530 2335 4531 2323 4529 2305 4525 2288
4522 2280 4518 2272 4513 2265 4508 2258 4502 2252 4495 2246
4488 2241 4480 2237 4471 2234 4462 2232 4452 2230 4441 2230
4421 2231 4413 2233 4405 2235 4398 2238 4391 2242 4380 2251
4371 2262 4364 2274 4358 2288 4355 2302 4387 2302 4391 2293
4395 2284 4400 2277 4406 2270 4413 2265 4421 2262 4429 2259
4439 2258 4452 2259 4463 2262 4474 2267 4482 2274 4489 2283
4494 2294 4497 2307 4498 2322 4497 2332 4495 2343 4491 2354
4485 2363 4477 2371 4467 2377 4455 2381 4441 2383 4433 2382
4427 2381 4421 2379 4415 2377 4404 2369 4391 2358 4362 2358
4382 2500 79 N F
4900 2500 4909 2499 4918 2497 4927 2493 4935 2488 4943 2482
4950 2474 4956 2466 4962 2457 4968 2447 4972 2437 4980 2414
4984 2390 4986 2366 4984 2341 4980 2317 4972 2294 4968 2283
4962 2273 4956 2264 4950 2256 4943 2248 4935 2242 4927 2237
4918 2233 4909 2231 4900 2230 4890 2231 4881 2233 4873 2237
4865 2242 4857 2248 4850 2256 4843 2264 4837 2273 4832 2283
4827 2294 4820 2317 4815 2341 4814 2366 4848 2366 4849 2342
4853 2321 4858 2303 4865 2288 4873 2275 4881 2266 4886 2263
4891 2260 4895 2259 4900 2258 4905 2259 4909 2260 4914 2263
4918 2266 4927 2275 4935 2288 4942 2303 4947 2321 4950 2342
4951 2366 4950 2389 4947 2410 4942 2428 4935 2443 4927 2455
4918 2464 4909 2469 4905 2470 4900 2471 4895 2470 4891 2469
4881 2464 4873 2455 4865 2443 4858 2428 4853 2410 4849 2389
4848 2366 4814 2366 4815 2390 4820 2414 4827 2437 4832 2447
4837 2457 4843 2466 4850 2474 4857 2482 4865 2488 4873 2493
4881 2497 4890 2499 4900 2500 93 N F
5036 2278 5074 2278 5074 2239 5036 2239 5036 2278 5 N F
5288 2433 5255 2433 5253 2441 5250 2449 5245 2456 5240 2461
5234 2465 5227 2468 5220 2470 5213 2471 5206 2470 5200 2469
5194 2467 5188 2464 5183 2461 5178 2456 5173 2451 5169 2445
5163 2431 5158 2414 5155 2395 5154 2373 5162 2382 5170 2388
5177 2393 5185 2397 5193 2399 5201 2401 5217 2402 5230 2401
5243 2397 5256 2390 5268 2381 5274 2376 5279 2370 5283 2363
5287 2355 5290 2347 5292 2338 5293 2328 5294 2318 5261 2316
5260 2327 5257 2337 5252 2347 5245 2355 5237 2363 5228 2368
5218 2372 5208 2373 5197 2372 5187 2368 5178 2363 5170 2355
5163 2347 5158 2337 5155 2327 5154 2316 5155 2305 5158 2295
5163 2285 5170 2276 5178 2269 5187 2263 5197 2260 5208 2258
5218 2260 5228 2263 5237 2269 5245 2276 5252 2285 5257 2295
5260 2305 5261 2316 5294 2318 5292 2302 5289 2287 5282 2272
5278 2265 5273 2259 5267 2253 5261 2247 5254 2242 5246 2238

5238 2234 5228 2232 5218 2230 5208 2230 5196 2230 5185 2232
5175 2235 5166 2240 5158 2245 5151 2252 5145 2259 5139 2268
5134 2277 5130 2287 5127 2297 5124 2309 5121 2333 5120 2360
5120 2376 5121 2391 5123 2405 5126 2418 5129 2430 5134 2442
5139 2452 5144 2462 5151 2471 5158 2478 5165 2485 5174 2490
5183 2494 5192 2497 5203 2499 5213 2500 5228 2498 5242 2494
5254 2488 5265 2480 5274 2470 5281 2458 5285 2446 5288 2433
126 N F
5663 2500 5672 2499 5681 2497 5690 2493 5698 2488 5706 2482
5713 2474 5719 2466 5725 2457 5731 2447 5735 2437 5743 2414
5747 2390 5749 2366 5747 2341 5743 2317 5735 2294 5731 2283
5725 2273 5719 2264 5713 2256 5706 2248 5698 2242 5690 2237
5681 2233 5672 2231 5663 2230 5653 2231 5644 2233 5636 2237
5628 2242 5620 2248 5613 2256 5606 2264 5600 2273 5595 2283
5590 2294 5583 2317 5578 2341 5577 2366 5611 2366 5612 2342
5616 2321 5621 2303 5628 2288 5636 2275 5644 2266 5649 2263
5653 2260 5658 2259 5663 2258 5668 2259 5672 2260 5677 2263
5681 2266 5690 2275 5698 2288 5705 2303 5710 2321 5713 2342
5714 2366 5713 2389 5710 2410 5705 2428 5698 2443 5690 2455
5681 2464 5672 2469 5668 2470 5663 2471 5658 2470 5653 2469
5644 2464 5636 2455 5628 2443 5621 2428 5616 2410 5612 2389
5611 2366 5577 2366 5578 2390 5583 2414 5590 2437 5595 2447
5600 2457 5606 2466 5613 2474 5620 2482 5628 2488 5636 2493
5644 2497 5653 2499 5663 2500 93 N F
5799 2278 5837 2278 5837 2239 5799 2239 5799 2278 5 N F
6051 2433 6018 2433 6016 2441 6013 2449 6008 2456 6003 2461
5997 2465 5990 2468 5983 2470 5976 2471 5969 2470 5963 2469
5957 2467 5951 2464 5946 2461 5941 2456 5936 2451 5932 2445
5926 2431 5921 2414 5918 2395 5917 2373 5925 2382 5933 2388
5940 2393 5948 2397 5956 2399 5964 2401 5980 2402 5993 2401
6006 2397 6019 2390 6031 2381 6037 2376 6042 2370 6046 2363
6050 2355 6053 2347 6055 2338 6056 2328 6057 2318 6024 2316
6023 2327 6020 2337 6015 2347 6008 2355 6000 2363 5991 2368
5981 2372 5971 2373 5960 2372 5950 2368 5941 2363 5933 2355
5926 2347 5921 2337 5918 2327 5917 2316 5918 2305 5921 2295
5926 2285 5933 2276 5941 2269 5950 2263 5960 2260 5971 2258
5981 2260 5991 2263 6000 2269 6008 2276 6015 2285 6020 2295
6023 2305 6024 2316 6057 2318 6055 2302 6052 2287 6045 2272
6041 2265 6036 2259 6030 2253 6024 2247 6017 2242 6009 2238
6001 2234 5991 2232 5981 2230 5971 2230 5959 2230 5948 2232
5938 2235 5929 2240 5921 2245 5914 2252 5908 2259 5902 2268
5897 2277 5893 2287 5890 2297 5887 2309 5884 2333 5883 2360
5883 2376 5884 2391 5886 2405 5889 2418 5892 2430 5897 2442

5902 2452 5907 2462 5914 2471 5921 2478 5928 2485 5937 2490
5946 2494 5955 2497 5966 2499 5976 2500 5991 2498 6005 2494
6017 2488 6028 2480 6037 2470 6044 2458 6048 2446 6051 2433
126 N F
6112 2500 6248 2500 6248 2469 6139 2469 6129 2394 6139 2401
6150 2407 6163 2410 6179 2412 6192 2410 6206 2407 6220 2401
6227 2397 6233 2392 6239 2386 6244 2380 6249 2373 6253 2365
6257 2356 6259 2346 6261 2335 6261 2323 6260 2305 6255 2288
6252 2280 6248 2272 6244 2265 6238 2258 6232 2252 6226 2246
6218 2241 6210 2237 6202 2234 6192 2232 6182 2230 6171 2230
6152 2231 6143 2233 6136 2235 6128 2238 6122 2242 6110 2251
6101 2262 6094 2274 6089 2288 6085 2302 6118 2302 6121 2293
6125 2284 6130 2277 6136 2270 6143 2265 6151 2262 6160 2259
6169 2258 6182 2259 6194 2262 6204 2267 6213 2274 6219 2283
6224 2294 6228 2307 6229 2322 6228 2332 6225 2343 6221 2354
6215 2363 6207 2371 6197 2377 6185 2381 6171 2383 6164 2382
6157 2381 6151 2379 6146 2377 6134 2369 6122 2358 6093 2358
6112 2500 79 N F
6629 2500 6639 2499 6648 2497 6657 2493 6665 2488 6672 2482
6679 2474 6686 2466 6692 2457 6697 2447 6702 2437 6709 2414
6714 2390 6715 2366 6714 2341 6709 2317 6702 2294 6697 2283
6692 2273 6686 2264 6679 2256 6672 2248 6665 2242 6657 2237
6648 2233 6639 2231 6629 2230 6620 2231 6611 2233 6602 2237
6594 2242 6587 2248 6580 2256 6573 2264 6567 2273 6562 2283
6557 2294 6550 2317 6545 2341 6543 2366 6578 2366 6579 2342
6582 2321 6588 2303 6594 2288 6602 2275 6611 2266 6615 2263
6620 2260 6625 2259 6629 2258 6634 2259 6639 2260 6643 2263
6648 2266 6657 2275 6665 2288 6671 2303 6676 2321 6680 2342
6681 2366 6680 2389 6676 2410 6671 2428 6665 2443 6657 2455
6648 2464 6639 2469 6634 2470 6629 2471 6625 2470 6620 2469
6611 2464 6602 2455 6594 2443 6588 2428 6582 2410 6579 2389
6578 2366 6543 2366 6545 2390 6550 2414 6557 2437 6562 2447
6567 2457 6573 2466 6580 2474 6587 2482 6594 2488 6602 2493
6611 2497 6620 2499 6629 2500 93 N F
6765 2278 6803 2278 6803 2239 6765 2239 6765 2278 5 N F
6851 2500 7025 2500 7025 2473 7000 2436 6980 2404 6964 2376
6952 2350 6942 2324 6933 2299 6926 2271 6920 2239 6886 2239
6895 2275 6905 2306 6915 2332 6925 2357 6938 2381 6953 2407
6993 2469 6851 2469 6851 2500 21 N F
7393 2500 7403 2499 7412 2497 7420 2493 7429 2488 7436 2482
7443 2474 7450 2466 7456 2457 7461 2447 7466 2437 7473 2414
7478 2390 7479 2366 7478 2341 7473 2317 7466 2294 7461 2283
7456 2273 7450 2264 7443 2256 7436 2248 7429 2242 7420 2237

7412 2233 7403 2231 7393 2230 7384 2231 7375 2233 7366 2237
7358 2242 7351 2248 7343 2256 7337 2264 7331 2273 7326 2283
7321 2294 7314 2317 7309 2341 7307 2366 7342 2366 7343 2342
7346 2321 7352 2303 7358 2288 7366 2275 7375 2266 7379 2263
7384 2260 7389 2259 7393 2258 7398 2259 7403 2260 7407 2263
7412 2266 7421 2275 7428 2288 7435 2303 7440 2321 7444 2342
7445 2366 7444 2389 7440 2410 7435 2428 7428 2443 7421 2455
7412 2464 7403 2469 7398 2470 7393 2471 7389 2470 7384 2469
7375 2464 7366 2455 7358 2443 7352 2428 7346 2410 7343 2389
7342 2366 7307 2366 7309 2390 7314 2414 7321 2437 7326 2447
7331 2457 7337 2466 7343 2474 7351 2482 7358 2488 7366 2493
7375 2497 7384 2499 7393 2500 93 N F
7529 2278 7567 2278 7567 2239 7529 2239 7529 2278 5 N F
7615 2500 7789 2500 7789 2473 7764 2436 7744 2404 7728 2376
7716 2350 7706 2324 7697 2299 7690 2271 7684 2239 7649 2239
7659 2275 7669 2306 7678 2332 7689 2357 7702 2381 7717 2407
7757 2469 7615 2469 7615 2500 21 N F
7843 2500 7978 2500 7978 2469 7869 2469 7860 2394 7869 2401
7880 2407 7894 2410 7909 2412 7923 2410 7937 2407 7951 2401
7957 2397 7963 2392 7969 2386 7975 2380 7980 2373 7984 2365
7987 2356 7990 2346 7991 2335 7992 2323 7990 2305 7986 2288
7983 2280 7979 2272 7974 2265 7969 2258 7963 2252 7956 2246
7949 2241 7941 2237 7932 2234 7923 2232 7913 2230 7902 2230
7883 2231 7874 2233 7866 2235 7859 2238 7852 2242 7841 2251
7832 2262 7825 2274 7819 2288 7816 2302 7848 2302 7852 2293
7856 2284 7861 2277 7867 2270 7874 2265 7882 2262 7890 2259
7900 2258 7913 2259 7924 2262 7935 2267 7943 2274 7950 2283
7955 2294 7958 2307 7959 2322 7958 2332 7956 2343 7952 2354
7946 2363 7938 2371 7928 2377 7916 2381 7902 2383 7894 2382
7888 2381 7882 2379 7876 2377 7865 2369 7852 2358 7823 2358
7843 2500 79 N F
8361 2500 8370 2499 8379 2497 8388 2493 8396 2488 8404 2482
8411 2474 8417 2466 8423 2457 8429 2447 8433 2437 8441 2414
8445 2390 8447 2366 8445 2341 8441 2317 8433 2294 8429 2283
8423 2273 8417 2264 8411 2256 8404 2248 8396 2242 8388 2237
8379 2233 8370 2231 8361 2230 8351 2231 8342 2233 8334 2237
8326 2242 8318 2248 8311 2256 8304 2264 8299 2273 8293 2283
8288 2294 8281 2317 8276 2341 8275 2366 8309 2366 8310 2342
8314 2321 8319 2303 8326 2288 8334 2275 8342 2266 8347 2263
8352 2260 8356 2259 8361 2258 8366 2259 8370 2260 8375 2263
8379 2266 8388 2275 8396 2288 8403 2303 8408 2321 8411 2342
8412 2366 8411 2389 8408 2410 8403 2428 8396 2443 8388 2455
8379 2464 8370 2469 8366 2470 8361 2471 8356 2470 8352 2469

8342 2464 8334 2455 8326 2443 8319 2428 8314 2410 8310 2389
8309 2366 8275 2366 8276 2390 8281 2414 8288 2437 8293 2447
8299 2457 8304 2466 8311 2474 8318 2482 8326 2488 8334 2493
8342 2497 8351 2499 8361 2500 93 N F
8497 2278 8535 2278 8535 2239 8497 2239 8497 2278 5 N F
8667 2500 8683 2498 8698 2495 8711 2489 8722 2482 8732 2472
8736 2466 8739 2460 8742 2453 8744 2446 8745 2439 8745 2431
8744 2419 8742 2410 8738 2401 8734 2394 8728 2388 8723 2383
8711 2375 8720 2370 8729 2364 8736 2357 8743 2349 8748 2340
8752 2331 8754 2321 8755 2310 8753 2297 8749 2284 8742 2270
8732 2258 8726 2252 8719 2247 8712 2242 8704 2238 8695 2234
8686 2232 8676 2230 8665 2230 8654 2230 8644 2232 8635 2234
8626 2238 8619 2242 8612 2247 8605 2252 8600 2258 8590 2270
8584 2284 8580 2297 8579 2310 8613 2310 8614 2300 8618 2290
8623 2281 8629 2273 8637 2267 8646 2262 8656 2259 8667 2258
8677 2259 8687 2262 8696 2267 8704 2273 8711 2281 8716 2290
8719 2300 8720 2310 8719 2321 8716 2330 8711 2339 8704 2347
8696 2353 8687 2358 8677 2361 8667 2362 8656 2361 8646 2358
8637 2353 8629 2347 8623 2339 8618 2330 8614 2321 8613 2310
8579 2310 8580 2321 8582 2330 8586 2340 8591 2348 8597 2356
8605 2363 8613 2370 8623 2375 8611 2383 8605 2388 8600 2394
8595 2401 8592 2410 8589 2419 8588 2431 8621 2431 8622 2422
8625 2414 8629 2407 8634 2401 8641 2397 8649 2393 8658 2391
8667 2390 8676 2391 8684 2393 8692 2397 8699 2401 8705 2407
8709 2414 8712 2422 8713 2431 8712 2440 8709 2447 8705 2454
8699 2460 8692 2465 8684 2468 8676 2470 8667 2471 8658 2470
8649 2468 8641 2465 8634 2460 8629 2454 8625 2447 8622 2440
8621 2431 8588 2431 8589 2439 8590 2446 8592 2453 8594 2460
8598 2466 8602 2472 8611 2482 8623 2489 8636 2495 8651 2498
8667 2500 145 N F
9124 2500 9133 2499 9142 2497 9151 2493 9159 2488 9167 2482
9174 2474 9180 2466 9186 2457 9192 2447 9196 2437 9204 2414
9208 2390 9210 2366 9208 2341 9204 2317 9196 2294 9192 2283
9186 2273 9180 2264 9174 2256 9167 2248 9159 2242 9151 2237
9142 2233 9133 2231 9124 2230 9114 2231 9105 2233 9097 2237
9089 2242 9081 2248 9074 2256 9067 2264 9062 2273 9056 2283
9051 2294 9044 2317 9039 2341 9038 2366 9072 2366 9073 2342
9077 2321 9082 2303 9089 2288 9097 2275 9105 2266 9110 2263
9115 2260 9119 2259 9124 2258 9129 2259 9133 2260 9138 2263
9142 2266 9151 2275 9159 2288 9166 2303 9171 2321 9174 2342
9175 2366 9174 2389 9171 2410 9166 2428 9159 2443 9151 2455
9142 2464 9133 2469 9129 2470 9124 2471 9119 2470 9115 2469
9105 2464 9097 2455 9089 2443 9082 2428 9077 2410 9073 2389

9072 2366 9038 2366 9039 2390 9044 2414 9051 2437 9056 2447
9062 2457 9067 2466 9074 2474 9081 2482 9089 2488 9097 2493
9105 2497 9114 2499 9124 2500 93 N F
9260 2278 9298 2278 9298 2239 9260 2239 9260 2278 5 N F
9430 2500 9446 2498 9461 2495 9474 2489 9485 2482 9495 2472
9499 2466 9502 2460 9505 2453 9507 2446 9508 2439 9508 2431
9507 2419 9505 2410 9501 2401 9497 2394 9491 2388 9486 2383
9474 2375 9483 2370 9492 2364 9499 2357 9506 2349 9511 2340
9515 2331 9517 2321 9518 2310 9516 2297 9512 2284 9505 2270
9495 2258 9489 2252 9482 2247 9475 2242 9467 2238 9458 2234
9449 2232 9439 2230 9428 2230 9417 2230 9407 2232 9398 2234
9389 2238 9382 2242 9375 2247 9368 2252 9363 2258 9353 2270
9347 2284 9343 2297 9342 2310 9376 2310 9377 2300 9381 2290
9386 2281 9392 2273 9400 2267 9409 2262 9419 2259 9430 2258
9440 2259 9450 2262 9459 2267 9467 2273 9474 2281 9479 2290
9482 2300 9483 2310 9482 2321 9479 2330 9474 2339 9467 2347
9459 2353 9450 2358 9440 2361 9430 2362 9419 2361 9409 2358
9400 2353 9392 2347 9386 2339 9381 2330 9377 2321 9376 2310
9342 2310 9343 2321 9345 2330 9349 2340 9354 2348 9360 2356
9368 2363 9376 2370 9386 2375 9374 2383 9368 2388 9363 2394
9358 2401 9355 2410 9352 2419 9351 2431 9384 2431 9385 2422
9388 2414 9392 2407 9397 2401 9404 2397 9412 2393 9421 2391
9430 2390 9439 2391 9447 2393 9455 2397 9462 2401 9468 2407
9472 2414 9475 2422 9476 2431 9475 2440 9472 2447 9468 2454
9462 2460 9455 2465 9447 2468 9439 2470 9430 2471 9421 2470
9412 2468 9404 2465 9397 2460 9392 2454 9388 2447 9385 2440
9384 2431 9351 2431 9352 2439 9353 2446 9355 2453 9357 2460
9361 2466 9365 2472 9374 2482 9386 2489 9399 2495 9414 2498
9430 2500 145 N F
9573 2500 9709 2500 9709 2469 9600 2469 9590 2394 9600 2401
9611 2407 9624 2410 9640 2412 9653 2410 9667 2407 9681 2401
9688 2397 9694 2392 9700 2386 9705 2380 9710 2373 9714 2365
9718 2356 9720 2346 9722 2335 9722 2323 9721 2305 9716 2288
9713 2280 9709 2272 9705 2265 9699 2258 9693 2252 9687 2246
9679 2241 9671 2237 9663 2234 9653 2232 9643 2230 9632 2230
9613 2231 9604 2233 9597 2235 9589 2238 9583 2242 9571 2251
9562 2262 9555 2274 9550 2288 9546 2302 9579 2302 9582 2293
9586 2284 9591 2277 9597 2270 9604 2265 9612 2262 9621 2259
9630 2258 9643 2259 9655 2262 9665 2267 9674 2274 9680 2283
9686 2294 9689 2307 9690 2322 9689 2332 9686 2343 9682 2354
9676 2363 9668 2371 9658 2377 9646 2381 9632 2383 9625 2382
9618 2381 9612 2379 9607 2377 9596 2369 9583 2358 9554 2358
9573 2500 79 N F

10091 2500 10101 2499 10110 2497 10119 2493 10127 2488 10134 2482
10141 2474 10148 2466 10154 2457 10159 2447 10164 2437 10171 2414
10176 2390 10177 2366 10176 2341 10171 2317 10164 2294 10159 2283
10154 2273 10148 2264 10141 2256 10134 2248 10127 2242 10119 2237
10110 2233 10101 2231 10091 2230 10082 2231 10073 2233 10064 2237
10056 2242 10049 2248 10042 2256 10035 2264 10029 2273 10024 2283
10019 2294 10012 2317 10007 2341 10005 2366 10040 2366 10041 2342
10044 2321 10050 2303 10056 2288 10064 2275 10073 2266 10077 2263
10082 2260 10087 2259 10091 2258 10096 2259 10101 2260 10105 2263
10110 2266 10119 2275 10127 2288 10133 2303 10138 2321 10142 2342
10143 2366 10142 2389 10138 2410 10133 2428 10127 2443 10119 2455
10110 2464 10101 2469 10096 2470 10091 2471 10087 2470 10082 2469
10073 2464 10064 2455 10056 2443 10050 2428 10044 2410 10041 2389
10040 2366 10005 2366 10007 2390 10012 2414 10019 2437 10024 2447
10029 2457 10035 2466 10042 2474 10049 2482 10056 2488 10064 2493
10073 2497 10082 2499 10091 2500 93 N F
10227 2278 10265 2278 10265 2239 10227 2239 10227 2278 5 N F
10397 2500 10407 2499 10416 2498 10424 2496 10432 2493 10440 2489
10447 2484 10454 2478 10460 2471 10465 2463 10470 2454 10474 2443
10478 2432 10481 2419 10483 2405 10484 2390 10485 2373 10485 2353
10483 2334 10480 2317 10477 2302 10472 2289 10467 2277 10461 2267
10455 2258 10448 2251 10440 2245 10432 2240 10424 2236 10415 2233
10407 2231 10390 2230 10376 2231 10364 2234 10353 2239 10342 2246
10333 2256 10326 2268 10319 2282 10315 2299 10348 2299 10351 2289
10354 2281 10359 2274 10364 2268 10370 2264 10376 2261 10383 2259
10391 2258 10399 2259 10406 2260 10412 2263 10418 2266 10423 2270
10428 2275 10436 2287 10443 2302 10447 2318 10450 2336 10451 2356
10442 2347 10433 2340 10425 2335 10418 2332 10410 2329 10404 2328
10391 2327 10381 2328 10372 2330 10363 2332 10355 2335 10348 2339
10341 2344 10335 2349 10330 2355 10325 2361 10321 2367 10315 2382
10312 2397 10311 2413 10344 2413 10345 2403 10348 2393 10352 2384
10358 2375 10366 2367 10375 2361 10386 2357 10397 2356 10409 2357
10419 2361 10428 2367 10436 2375 10442 2384 10447 2393 10450 2403
10451 2413 10450 2424 10447 2434 10442 2443 10436 2452 10428 2460
10419 2466 10409 2470 10397 2471 10386 2470 10375 2466 10366 2460
10358 2452 10352 2443 10348 2434 10345 2424 10344 2413 10311 2413
10311 2422 10312 2430 10314 2438 10316 2446 10320 2454 10323 2461
10328 2468 10333 2474 10339 2479 10346 2484 10353 2489 10361 2493
10369 2496 10378 2498 10387 2499 10397 2500 130 N F
10854 2500 10864 2499 10873 2497 10881 2493 10890 2488 10897 2482
10904 2474 10911 2466 10917 2457 10922 2447 10927 2437 10934 2414
10939 2390 10940 2366 10939 2341 10934 2317 10927 2294 10922 2283
10917 2273 10911 2264 10904 2256 10897 2248 10890 2242 10881 2237

10873 2233 10864 2231 10854 2230 10845 2231 10836 2233 10827 2237
10819 2242 10812 2248 10804 2256 10798 2264 10792 2273 10787 2283
10782 2294 10775 2317 10770 2341 10768 2366 10803 2366 10804 2342
10807 2321 10813 2303 10819 2288 10827 2275 10836 2266 10840 2263
10845 2260 10850 2259 10854 2258 10859 2259 10864 2260 10868 2263
10873 2266 10882 2275 10890 2288 10896 2303 10901 2321 10905 2342
10906 2366 10905 2389 10901 2410 10896 2428 10890 2443 10882 2455
10873 2464 10864 2469 10859 2470 10854 2471 10850 2470 10845 2469
10836 2464 10827 2455 10819 2443 10813 2428 10807 2410 10804 2389
10803 2366 10768 2366 10770 2390 10775 2414 10782 2437 10787 2447
10792 2457 10798 2466 10804 2474 10812 2482 10819 2488 10827 2493
10836 2497 10845 2499 10854 2500 93 N F
10990 2278 11028 2278 11028 2239 10990 2239 10990 2278 5 N F
11160 2500 11170 2499 11179 2498 11187 2496 11195 2493 11203 2489
11210 2484 11217 2478 11223 2471 11228 2463 11233 2454 11237 2443
11241 2432 11244 2419 11246 2405 11247 2390 11248 2373 11248 2353
11246 2334 11243 2317 11240 2302 11235 2289 11230 2277 11224 2267
11218 2258 11211 2251 11203 2245 11195 2240 11187 2236 11178 2233
11170 2231 11153 2230 11139 2231 11127 2234 11116 2239 11105 2246
11096 2256 11089 2268 11082 2282 11078 2299 11111 2299 11114 2289
11117 2281 11122 2274 11127 2268 11133 2264 11139 2261 11146 2259
11154 2258 11162 2259 11169 2260 11175 2263 11181 2266 11186 2270
11191 2275 11199 2287 11206 2302 11210 2318 11213 2336 11214 2356
11205 2347 11196 2340 11188 2335 11181 2332 11173 2329 11167 2328
11154 2327 11144 2328 11135 2330 11126 2332 11118 2335 11111 2339
11104 2344 11098 2349 11093 2355 11088 2361 11084 2367 11078 2382
11075 2397 11074 2413 11107 2413 11108 2403 11110 2393 11115 2384
11121 2375 11129 2367 11138 2361 11149 2357 11160 2356 11172 2357
11182 2361 11191 2367 11199 2375 11205 2384 11210 2393 11213 2403
11214 2413 11213 2424 11210 2434 11205 2443 11199 2452 11191 2460
11182 2466 11172 2470 11160 2471 11149 2470 11138 2466 11129 2460
11121 2452 11115 2443 11110 2434 11108 2424 11107 2413 11074 2413
11074 2422 11075 2430 11077 2438 11079 2446 11083 2454 11086 2461
11091 2468 11096 2474 11102 2479 11109 2484 11116 2489 11124 2493
11132 2496 11141 2498 11150 2499 11160 2500 130 N F
11304 2500 11439 2500 11439 2469 11330 2469 11321 2394 11330 2401
11341 2407 11355 2410 11370 2412 11384 2410 11398 2407 11412 2401
11418 2397 11424 2392 11430 2386 11436 2380 11441 2373 11445 2365
11448 2356 11451 2346 11452 2335 11453 2323 11451 2305 11447 2288
11444 2280 11440 2272 11435 2265 11430 2258 11424 2252 11417 2246
11410 2241 11402 2237 11393 2234 11384 2232 11374 2230 11363 2230
11344 2231 11335 2233 11327 2235 11320 2238 11313 2242 11302 2251
11293 2262 11286 2274 11280 2288 11277 2302 11309 2302 11313 2293

```
11317 2284 11322 2277 11328 2270 11335 2265 11343 2262 11351 2259
11361 2258 11374 2259 11386 2262 11396 2267 11404 2274 11411 2283
11416 2294 11419 2307 11420 2322 11419 2332 11417 2343 11413 2354
11407 2363 11399 2371 11389 2377 11377 2381 11363 2383 11355 2382
11349 2381 11343 2379 11337 2377 11326 2369 11313 2358 11284 2358
11304 2500 79 N F
12000 2495 12021 2495 12021 2234 11988 2234 11988 2420 11931 2420
11931 2443 11954 2446 11964 2448 11974 2452 11982 2458 11989 2466
11995 2478 12000 2495 14 N F
Gr
S
%%Trailer
%%DocumentFonts:
%%Pages: 1
%%EOF
```

Regional loss of dry matter in T1 (May)

```
%%Pages: (atend)
%%PageOrder: Ascend
%%Creator: NCAR GKS
%%DocumentFonts: (atend)
%%EndComments
%%BeginProlog

/M3
{
  2 copy lt {pop} {exch pop} ifelse
  2 copy lt {pop} {exch pop} ifelse
} bind def
/Sc
{
  1. exch sub /ylo exch def
  1. exch sub /mag exch def
  1. exch sub /cyn exch def
  ylo mag cyn M3 /b_cmy exch def
  cyn b_cmy sub /c_cmy exch def
  mag b_cmy sub /m_cmy exch def
  ylo b_cmy sub /y_cmy exch def
  c_cmy m_cmy y_cmy b_cmy setcmykcolor
} bind def
/P
{/n exch def
  newpath
```

```
  moveto
  2 1 n {pop lineto} for
  stroke
 } bind def

/F
{/n exch def
  newpath
  moveto
  2 1 n {pop lineto} for
  closepath
  eofill
} bind def

/B
{ /s exch def
  /n exch def
  1 1 n {newpath pop s 0 360 arc stroke} for
} bind def

/Q
{ /s exch def
  /n exch def
  /ms 0 s sub def
  /ts s s add def
  1 1 n {gsave newpath pop translate
  0 0 moveto ms 0 rmoveto ts 0 rlineto
  0 0 moveto 90 rotate ms  0 rmoveto ts 0 rlineto
  stroke grestore} for
} bind def

/Fs
{ /s exch def
  findfont
  s scalefont
  setfont
} bind def

/V
{ /s exch def
  /n exch def
  /ms 0 s sub def
  /ts s s add def
```

```
  1 1 n {gsave newpath pop translate
  0 0 moveto 30 rotate ms 0 rmoveto  ts 0 rlineto
  0 0 moveto 60 rotate ms 0 rmoveto  ts 0 rlineto
  0 0 moveto 60 rotate ms 0 rmoveto  ts 0 rlineto
  stroke grestore} for
} bind def

/X
{ /s exch def
  /n exch def
  /s s 1.25 mul def
  /ms 0 s sub def
  /ts s s add def
  1 1 n {gsave newpath pop translate
  0 0 moveto 45 rotate ms 0 rmoveto  ts 0 rlineto
  0 0 moveto 90 rotate ms 0 rmoveto  ts 0 rlineto
  stroke grestore} for
} bind def

/Ls
{matrix currentmatrix
 currentrgbcolor
 currentlinewidth
 currentlinecap
 currentlinejoin
 currentdash
} bind def

/Lr
{setdash
 setlinejoin
 setlinecap
 setlinewidth
 setrgbcolor
 setmatrix
} bind def

/M {stroke newpath moveto} bind def
/L {lineto} bind def
/E {eofill} def
/J {setlinejoin} def
/C {setlinecap} def
/W {setlinewidth} def
```

```
/S {stroke showpage} def
/A {scale} def
/U {put} def
/D {setdash} def
/I {currentdash} def
/H {currentlinewidth} def
/G {currentrgbcolor} def
/R {setrgbcolor} def
/N {closepath} def
/K {stroke} def
/Z {rotate} def
/As {ashow} def
/Ch {clippath} def
/Ci {colorimage} def
/Co {concat} def
/Cm {currentmatrix} def
/Cp {clip} def
/Ct {currentpoint} def
/Cu {currentfile} def
/Ex {exch} def
/Im {image} def
/In {initgraphics} def
/Ml {setmiterlimit} def
/Mx {matrix} def
/Np {newpath} def
/Ng {neg} def
/Rh {readhexstring} def
/Rm {rmoveto} def
/Sm {setmatrix} def
/Tr {translate} def
/Gs {gsave} def
/Gr {grestore} def

/Ov
{/strn exch def
 /ispc exch def
 /str1 1 string def
  strn
  {/code exch def
   /chr str1 0 code put str1 def
   0 ispc neg rmoveto
   gsave
   chr stringwidth pop 2 div neg
```

```
   0 rmoveto
   chr false charpath stroke
   grestore
  } forall
} bind def

/Oh
{/strn exch def
 /ispc exch def
 /tstr 1 string def
 strn
   {
    /code exch def
    /chr tstr 0 code put tstr def
    chr false charpath
    currentpoint
    /yt exch def /xt exch def
    stroke
    xt ispc add yt moveto
   } forall
 } bind def

/T 256 array def
/O
{ T
  exch
  get
  aload
  pop
  setrgbcolor
} bind def

/Y
{/strn exch def
 /ispc exch def
 /str1 1 string def
  strn
  {/code exch def
   /chr str1 0 code put str1 def
    0 ispc neg rmoveto
    gsave
      chr stringwidth pop 2 div neg 0 rmoveto
      chr show
```

```
    grestore
  } forall
} def

/Bg
{/ury Ex def /urx Ex def /lly Ex def /llx Ex def
 Np Ls Gr Gs 0 O Ch E Lr
 llx lly M urx lly L urx ury L
 llx ury L llx lly L
 Cp Np
} def

/Oc
{/ury exch def
 /urx exch def
 /lly exch def
 /llx exch def
 Np
 llx lly M urx lly L urx ury L  llx ury L llx lly L
 Cp Np
} def

/Cc
{/CellWidth exch def
 /strn CellWidth 1 add string def
 /strn1 CellWidth 1 add 3 mul string def
 systemdict /colorimage known
 { { Cu strn1 Rh pop } false 3 Ci}
 { { Cu strn1 Rh pop
     /ColorStr exch def
     0 1 CellWidth {
       /YIndx exch def
       /GrayVal YIndx 3 mul def
       strn YIndx
         ColorStr GrayVal       get 0.30 mul
         ColorStr GrayVal 1 add get 0.59 mul
         ColorStr GrayVal 2 add get 0.11 mul
         add add cvi
       put
     } for
     strn
   } Im
 } ifelse
```

```
} def

/Ld
{/ury exch def /urx exch def  /lly exch def /llx exch def
 -90 rotate
 lly urx add neg llx lly sub translate
} bind def
/Pt
{/ury exch def /urx exch def  /lly exch def /llx exch def
 90 rotate
 lly urx add lly llx sub translate
} bind def
%%EndProlog

%%BeginSetup
<</PageSize [00612 00792]>> setpagedevice
%%EndSetup

[1. 1. 1.] [0. 0. 0.] [1. 0. 0.] [0. 1. 0.] [0. 0. 1.] [0. 1. 1.] [1. 0. .8]
[1. 1. 0.] [1. .5 0.] [.6 .8 0.] [0. 1. .6] [0. .5 1.] [.5 0. .8] [1. 0. .6]
[.3 .3 .3] [.7 .7 .7] [1. 1. .3] [.7 1. .5] [.5 1. .6] [.2 1. .7] [.3 .8 .8]
[.5 .7 .8] [.7 .5 .8] [1. .3 .9] [.7 .9 .5] [.4 .9 .5] [.2 .9 .7] [.2 .7 .9]
[.2 .5 1.] [.5 .3 1.] [.7 .2 1.] [.9 .1 1.] [.9 1. .2] [.7 1. .3] [.5 1. .3]
[.2 1. .5] [.2 .8 .6] [.2 .7 .7] [.2 .5 .8] [.2 .4 .9] [.4 .3 .9] [.7 .2 .9]
[.8 .2 .8] [.9 .3 .7] [.8 .4 .6] [.8 .6 .5] [.9 .7 .4] [.9 .7 .3] [1. .9 .9]
[.8 1. .1] [.6 1. .2] [.5 1. .2] [.2 .9 .5] [.2 .8 .5] [.2 .7 .7] [.2 .5 .7]
[.2 .4 .9] [.4 .3 .9] [.5 .2 .9] [.8 .2 .7] [1. .2 .7] [1. .3 .6] [1. .4 .5]
[1. .5 .4] [1. .8 .1] [.7 1. .0] [.6 1. .1] [.4 1. .2] [.1 1. .3] [.1 .8 .4]
[.2 .7 .5] [.1 .6 .7] [.1 .5 .7] [.1 .4 .8] [.2 .3 .9] [.2 .2 1.] [.3 .1 1.]
[.5 .1 .9] [.7 .0 .8] [.9 .0 .7] [.9 .7 .1] [.7 .9 .1] [.5 .9 .1] [.3 .9 .2]
[.1 .9 .3] [.2 .7 .4] [.1 .7 .5] [.1 .5 .6] [.1 .4 .7] [.2 .3 .7] [.2 .2 .8]
[.3 .2 .8] [.5 .1 .7] [.7 .1 .7] [.9 .2 .5] [.8 .2 .5] [.8 .7 .0] [.6 .8 .1]
[.4 .9 .1] [.3 .9 .1] [.1 .9 .2] [.2 .8 .2] [.2 .7 .3] [.2 .6 .5] [.1 .5 .5]
[.1 .4 .6] [.2 .3 .7] [.3 .2 .7] [.4 .2 .6] [.7 .2 .5] [.8 .2 .4] [1. .2 .3]
[.7 .7 .0] [.5 .7 .1] [.4 .8 .1] [.2 .9 .1] [.2 .9 .1] [.2 .8 .2] [.1 .7 .2]
[.1 .7 .3] [.1 .6 .4] [.1 .4 .5] [.1 .4 .5] [.2 .3 .5] [.4 .3 .4] [.5 .3 .4]
[.7 .3 .3] [.8 .3 .2] [.7 .6 .0] [.5 .7 .1] [.4 .7 .1] [.2 .7 .2] [.1 .7 .2]
[.1 .6 .3] [.1 .5 .4] [.1 .4 .4] [.1 .3 .5] [.1 .2 .7] [.1 .1 .7] [.4 .1 .5]
[.5 .1 .5] [.6 .1 .4] [.7 .1 .4] [.9 .1 .3] [.5 .5 .1] [.4 .6 .1] [.3 .6 .1]
[.2 .6 .2] [.1 .5 .2] [.1 .4 .3] [.1 .4 .4] [.2 .3 .4] [.2 .2 .4] [.2 .2 .5]
[.2 .1 .5] [.3 .1 .5] [.4 .1 .4] [.5 .1 .4] [.7 .1 .3] [.8 .1 .2] [.5 .4 .1]
[.4 .5 .1] [.3 .6 .0] [.2 .6 .1] [.1 .7 .1] [.1 .6 .1] [.1 .5 .2] [.1 .4 .2]
[.1 .4 .2] [.1 .3 .3] [.1 .2 .4] [.1 .2 .5] [.1 .1 .5] [.1 .0 .6] [.3 .0 .5]
```

```
[.5 .0 .4] [.7 .3 .0] [.7 .4 .0] [.5 .4 .1] [.3 .4 .2] [.2 .3 .2] [.2 .2 .3]
[.2 .2 .4] [.3 .1 .4] [.5 .1 .3] [.6 .1 .2] [.7 .1 .2] [.8 .0 .2] [1. .0 .2]
[1. .1 .1] [.9 .2 .1] [.8 .2 .1] [.6 .2 .0] [.4 .4 .0] [.2 .4 .1] [.2 .3 .2]
[.2 .2 .2] [.2 .2 .3] [.2 .1 .3] [.3 .1 .3] [.5 .1 .2] [.7 .1 .2] [.8 .0 .2]
[.9 .0 .1] [1. .0 .1] [1. .1 .1] [.8 .2 .0] [.7 .2 .0] [.5 .2 .0] [.4 .3 .0]
[.1 .4 .1] [.1 .4 .1] [.1 .3 .2] [.1 .2 .2] [.1 .2 .3] [.2 .1 .4] [.2 .0 .3]
[.4 .0 .2] [.6 .0 .2] [.7 .0 .1] [.8 .0 .1] [.7 .1 .1] [.7 .1 .1] [.6 .2 .0]
[.4 .2 .0] [.2 .3 .0] [.1 .3 .0] [.1 .2 .1] [.1 .2 .1] [.1 .1 .2] [.1 .1 .3]
[.1 .0 .3] [.1 .0 .2] [.2 .0 .2] [.3 .0 .2] [.3 .0 .1] [.4 .0 .1] [.4 .1 .1]
[.4 .1 .0] [.2 .1 .0] [.0 .0 .0] [.0 .0 .0] [.0 .0 .0] [.0 .0 .0] [.0 .0 .0]
[.0 .0 .0] [.0 .0 .0] [.0 .0 .0] [.0 .0 .0] [.0 .0 .0] [.0 .0 .0] [.0 .0 .0]
[.0 .0 .0] [.0 .0 .0] [.0 .0 .0] [.0 .0 .0]  T astore pop
%%Page: 1 1
0.04000 0.04000 A 1 J 1 C 12 W [] 0 D
T   0 [1.000 1.000 1.000] U
T   1 [0.000 0.000 0.000] U
T   2 [0.000 0.000 1.000] U
T   3 [0.000 0.031 1.000] U
T   4 [0.000 0.059 1.000] U
T   5 [0.000 0.090 1.000] U
T   6 [0.000 0.122 1.000] U
T   7 [0.000 0.153 1.000] U
T   8 [0.000 0.180 1.000] U
T   9 [0.000 0.212 1.000] U
T  10 [0.000 0.243 1.000] U
T  11 [0.000 0.275 1.000] U
T  12 [0.000 0.302 1.000] U
T  13 [0.000 0.333 1.000] U
T  14 [0.000 0.365 1.000] U
T  15 [0.000 0.392 1.000] U
T  16 [0.000 0.424 1.000] U
T  17 [0.000 0.455 1.000] U
T  18 [0.000 0.486 1.000] U
T  19 [0.000 0.514 1.000] U
T  20 [0.000 0.545 1.000] U
T  21 [0.000 0.576 1.000] U
T  22 [0.000 0.608 1.000] U
T  23 [0.000 0.635 1.000] U
T  24 [0.000 0.667 1.000] U
T  25 [0.000 0.698 1.000] U
T  26 [0.000 0.725 1.000] U
T  27 [0.000 0.757 1.000] U
T  28 [0.000 0.788 1.000] U
```

T 29 [0.000 0.820 1.000] U
T 30 [0.000 0.847 1.000] U
T 31 [0.000 0.878 1.000] U
T 32 [0.000 0.910 1.000] U
T 33 [0.000 0.941 1.000] U
T 34 [0.000 0.969 1.000] U
T 35 [0.000 1.000 1.000] U
T 36 [0.000 0.992 0.973] U
T 37 [0.000 0.980 0.941] U
T 38 [0.004 0.973 0.914] U
T 39 [0.004 0.965 0.886] U
T 40 [0.004 0.953 0.859] U
T 41 [0.004 0.945 0.827] U
T 42 [0.008 0.937 0.800] U
T 43 [0.008 0.929 0.773] U
T 44 [0.008 0.918 0.745] U
T 45 [0.008 0.910 0.714] U
T 46 [0.012 0.902 0.686] U
T 47 [0.012 0.890 0.659] U
T 48 [0.012 0.882 0.627] U
T 49 [0.012 0.875 0.600] U
T 50 [0.016 0.863 0.573] U
T 51 [0.016 0.855 0.545] U
T 52 [0.016 0.847 0.514] U
T 53 [0.016 0.839 0.486] U
T 54 [0.020 0.827 0.459] U
T 55 [0.020 0.820 0.431] U
T 56 [0.020 0.812 0.400] U
T 57 [0.020 0.800 0.373] U
T 58 [0.024 0.792 0.345] U
T 59 [0.024 0.784 0.314] U
T 60 [0.024 0.773 0.286] U
T 61 [0.024 0.765 0.259] U
T 62 [0.027 0.757 0.231] U
T 63 [0.027 0.749 0.200] U
T 64 [0.027 0.737 0.173] U
T 65 [0.027 0.729 0.145] U
T 66 [0.031 0.722 0.118] U
T 67 [0.031 0.710 0.086] U
T 68 [0.031 0.702 0.059] U
T 69 [0.059 0.710 0.059] U
T 70 [0.090 0.718 0.055] U
T 71 [0.118 0.729 0.055] U

T 72 [0.145 0.737 0.051] U
T 73 [0.173 0.745 0.051] U
T 74 [0.204 0.753 0.047] U
T 75 [0.231 0.765 0.047] U
T 76 [0.259 0.773 0.043] U
T 77 [0.286 0.780 0.043] U
T 78 [0.318 0.788 0.043] U
T 79 [0.345 0.800 0.039] U
T 80 [0.373 0.808 0.039] U
T 81 [0.400 0.816 0.035] U
T 82 [0.431 0.824 0.035] U
T 83 [0.459 0.835 0.031] U
T 84 [0.486 0.843 0.031] U
T 85 [0.518 0.851 0.031] U
T 86 [0.545 0.859 0.027] U
T 87 [0.573 0.867 0.027] U
T 88 [0.600 0.878 0.024] U
T 89 [0.631 0.886 0.024] U
T 90 [0.659 0.894 0.020] U
T 91 [0.686 0.902 0.020] U
T 92 [0.714 0.914 0.016] U
T 93 [0.745 0.922 0.016] U
T 94 [0.773 0.929 0.016] U
T 95 [0.800 0.937 0.012] U
T 96 [0.827 0.949 0.012] U
T 97 [0.859 0.957 0.008] U
T 98 [0.886 0.965 0.008] U
T 99 [0.914 0.973 0.004] U
T 100 [0.941 0.984 0.004] U
T 101 [0.973 0.992 0.000] U
T 102 [1.000 1.000 0.000] U
T 103 [1.000 0.988 0.000] U
T 104 [1.000 0.980 0.000] U
T 105 [1.000 0.969 0.000] U
T 106 [1.000 0.957 0.000] U
T 107 [1.000 0.945 0.000] U
T 108 [1.000 0.937 0.000] U
T 109 [1.000 0.925 0.000] U
T 110 [1.000 0.914 0.000] U
T 111 [1.000 0.902 0.000] U
T 112 [1.000 0.894 0.000] U
T 113 [1.000 0.882 0.000] U
T 114 [1.000 0.871 0.000] U

T 115 [1.000 0.863 0.000] U
T 116 [1.000 0.851 0.000] U
T 117 [1.000 0.839 0.000] U
T 118 [1.000 0.827 0.000] U
T 119 [1.000 0.820 0.000] U
T 120 [1.000 0.808 0.000] U
T 121 [1.000 0.796 0.000] U
T 122 [1.000 0.784 0.000] U
T 123 [1.000 0.776 0.000] U
T 124 [1.000 0.765 0.000] U
T 125 [1.000 0.753 0.000] U
T 126 [1.000 0.745 0.000] U
T 127 [1.000 0.733 0.000] U
T 128 [1.000 0.722 0.000] U
T 129 [1.000 0.710 0.000] U
T 130 [1.000 0.702 0.000] U
T 131 [1.000 0.690 0.000] U
T 132 [1.000 0.678 0.000] U
T 133 [1.000 0.667 0.000] U
T 134 [1.000 0.659 0.000] U
T 135 [1.000 0.647 0.000] U
T 136 [1.000 0.627 0.000] U
T 137 [1.000 0.608 0.000] U
T 138 [1.000 0.588 0.000] U
T 139 [1.000 0.569 0.000] U
T 140 [1.000 0.549 0.000] U
T 141 [1.000 0.529 0.000] U
T 142 [1.000 0.510 0.000] U
T 143 [1.000 0.490 0.000] U
T 144 [1.000 0.471 0.000] U
T 145 [1.000 0.451 0.000] U
T 146 [1.000 0.431 0.000] U
T 147 [1.000 0.412 0.000] U
T 148 [1.000 0.392 0.000] U
T 149 [1.000 0.373 0.000] U
T 150 [1.000 0.353 0.000] U
T 151 [1.000 0.333 0.000] U
T 152 [1.000 0.314 0.000] U
T 153 [1.000 0.294 0.000] U
T 154 [1.000 0.275 0.000] U
T 155 [1.000 0.255 0.000] U
T 156 [1.000 0.235 0.000] U
T 157 [1.000 0.216 0.000] U

T 158 [1.000 0.196 0.000] U
T 159 [1.000 0.176 0.000] U
T 160 [1.000 0.157 0.000] U
T 161 [1.000 0.137 0.000] U
T 162 [1.000 0.118 0.000] U
T 163 [1.000 0.098 0.000] U
T 164 [1.000 0.078 0.000] U
T 165 [1.000 0.059 0.000] U
T 166 [1.000 0.039 0.000] U
T 167 [1.000 0.020 0.000] U
T 168 [1.000 0.000 0.000] U
T 169 [0.980 0.000 0.016] U
T 170 [0.961 0.000 0.031] U
T 171 [0.941 0.000 0.047] U
T 172 [0.922 0.000 0.063] U
T 173 [0.902 0.000 0.082] U
T 174 [0.878 0.000 0.098] U
T 175 [0.859 0.000 0.114] U
T 176 [0.839 0.000 0.129] U
T 177 [0.820 0.000 0.145] U
T 178 [0.800 0.000 0.161] U
T 179 [0.780 0.000 0.176] U
T 180 [0.761 0.000 0.192] U
T 181 [0.741 0.000 0.212] U
T 182 [0.722 0.000 0.227] U
T 183 [0.702 0.000 0.243] U
T 184 [0.682 0.000 0.259] U
T 185 [0.659 0.000 0.275] U
T 186 [0.639 0.000 0.290] U
T 187 [0.620 0.000 0.306] U
T 188 [0.600 0.000 0.322] U
T 189 [0.580 0.000 0.341] U
T 190 [0.561 0.000 0.357] U
T 191 [0.541 0.000 0.373] U
T 192 [0.522 0.000 0.388] U
T 193 [0.502 0.000 0.404] U
T 194 [0.482 0.000 0.420] U
T 195 [0.463 0.000 0.435] U
T 196 [0.439 0.000 0.451] U
T 197 [0.420 0.000 0.471] U
T 198 [0.400 0.000 0.486] U
T 199 [0.380 0.000 0.502] U
T 200 [0.361 0.000 0.518] U

T 201 [0.341 0.000 0.533] U
-1200 1025 16500 18750 Oc
Gs
1 O
0 O
2300 16170 M 2300 5455 L 2333 5455 L 2366 5455 L 2399 5455 L
2432 5455 L 2466 5455 L 2499 5455 L 2532 5455 L
2565 5455 L 2599 5455 L 2632 5455 L 2665 5455 L
2698 5455 L 2732 5455 L 2765 5455 L 2798 5455 L
2831 5455 L 2864 5455 L 2898 5455 L 2927 5455 L
2926 5459 L 2917 5486 L 2938 5506 L 2967 5505 L
2985 5482 L 2967 5459 L 2967 5455 L 2997 5455 L
3031 5455 L 3064 5455 L 3097 5455 L 3130 5455 L
3164 5455 L 3197 5455 L 3230 5455 L 3263 5455 L
3296 5455 L 3330 5455 L 3363 5455 L 3396 5455 L
3429 5455 L 3463 5455 L 3496 5455 L 3529 5455 L
3562 5455 L 3595 5455 L 3629 5455 L 3662 5455 L
3695 5455 L 3728 5455 L 3762 5455 L 3795 5455 L
3828 5455 L 3861 5455 L 3895 5455 L 3928 5455 L
3961 5455 L 3994 5455 L 4027 5455 L 4061 5455 L
4094 5455 L 4127 5455 L 4160 5455 L 4194 5455 L
4227 5455 L 4260 5455 L 4293 5455 L 4327 5455 L
4360 5455 L 4393 5455 L 4426 5455 L 4459 5455 L
4493 5455 L 4526 5455 L 4559 5455 L 4592 5455 L
4626 5455 L 4659 5455 L 4692 5455 L 4725 5455 L
4759 5455 L 4792 5455 L 4825 5455 L 4858 5455 L
4891 5455 L 4925 5455 L 4958 5455 L 4991 5455 L
5024 5455 L 5058 5455 L 5091 5455 L 5124 5455 L
5157 5455 L 5191 5455 L 5224 5455 L 5257 5455 L
5290 5455 L 5323 5455 L 5357 5455 L 5390 5455 L
5395 5455 L 5407 5466 L 5406 5495 L 5402 5524 L
5404 5553 L 5404 5582 L 5393 5609 L 5369 5625 L
5352 5648 L 5381 5644 L 5387 5616 L 5416 5614 L
5416 5585 L 5411 5557 L 5410 5528 L 5412 5499 L
5430 5476 L 5430 5455 L 5456 5455 L 5490 5455 L
5523 5455 L 5532 5455 L 5539 5469 L 5540 5475 L
5544 5482 L 5551 5487 L 5537 5525 L 5529 5553 L
5547 5530 L 5556 5503 L 5573 5479 L 5592 5457 L
5594 5455 L 5622 5455 L 5644 5455 L 5645 5472 L
5649 5455 L 5656 5455 L 5657 5455 L 5665 5470 L
5675 5497 L 5689 5523 L 5683 5551 L 5659 5567 L
5667 5595 L 5681 5621 L 5681 5650 L 5666 5674 L
5689 5691 L 5674 5715 L 5654 5737 L 5676 5717 L

5703 5726 L 5686 5750 L 5670 5774 L 5650 5795 L
5674 5779 L 5702 5786 L 5688 5811 L 5664 5827 L
5654 5855 L 5627 5866 L 5598 5867 L 5627 5872 L
5655 5876 L 5661 5905 L 5662 5934 L 5645 5957 L
5617 5961 L 5600 5985 L 5627 5975 L 5654 5965 L
5679 5950 L 5677 5921 L 5684 5893 L 5705 5873 L
5723 5896 L 5711 5870 L 5706 5841 L 5712 5830 L
5721 5816 L 5739 5794 L 5745 5765 L 5772 5776 L
5790 5754 L 5797 5725 L 5799 5722 L 5811 5700 L
5823 5674 L 5842 5652 L 5859 5628 L 5853 5600 L
5871 5622 L 5898 5632 L 5926 5625 L 5955 5622 L
5928 5612 L 5902 5598 L 5880 5580 L 5860 5559 L
5880 5538 L 5909 5534 L 5938 5535 L 5967 5536 L
5986 5514 L 6014 5506 L 6043 5507 L 6069 5495 L
6050 5517 L 6058 5545 L 6085 5534 L 6113 5542 L
6119 5571 L 6090 5565 L 6074 5588 L 6051 5570 L
6023 5561 L 6008 5586 L 6036 5590 L 6065 5592 L
6087 5611 L 6061 5624 L 6035 5637 L 6057 5656 L
6085 5663 L 6113 5670 L 6141 5663 L 6159 5640 L
6168 5612 L 6172 5584 L 6200 5590 L 6228 5597 L
6234 5626 L 6207 5636 L 6181 5650 L 6208 5660 L
6209 5689 L 6180 5689 L 6202 5708 L 6216 5734 L
6242 5746 L 6246 5748 L 6256 5751 L 6269 5755 L
6296 5768 L 6324 5762 L 6345 5782 L 6337 5754 L
6314 5736 L 6317 5707 L 6314 5678 L 6325 5651 L
6354 5652 L 6382 5648 L 6403 5628 L 6399 5656 L
6415 5680 L 6431 5705 L 6408 5723 L 6392 5747 L
6417 5732 L 6430 5706 L 6458 5710 L 6472 5735 L
6487 5760 L 6515 5767 L 6544 5767 L 6569 5782 L
6599 5771 L 6577 5763 L 6575 5753 L 6595 5754 L
6614 5755 L 6640 5742 L 6665 5728 L 6637 5727 L
6648 5700 L 6677 5693 L 6705 5699 L 6731 5685 L
6757 5673 L 6754 5702 L 6768 5727 L 6747 5707 L
6730 5731 L 6747 5754 L 6764 5777 L 6785 5797 L
6814 5799 L 6838 5783 L 6866 5775 L 6882 5750 L
6899 5727 L 6921 5747 L 6949 5754 L 6976 5764 L
7003 5775 L 7027 5791 L 7029 5820 L 7046 5796 L
7031 5772 L 7060 5771 L 7082 5790 L 7102 5811 L
7125 5828 L 7149 5844 L 7172 5826 L 7201 5821 L
7229 5828 L 7258 5827 L 7266 5854 L 7287 5874 L
7284 5903 L 7277 5931 L 7291 5957 L 7296 5985 L
7271 5971 L 7268 5999 L 7297 6002 L 7320 5984 L
7341 5964 L 7358 5988 L 7379 5999 L 7361 6015 L

7343 6030 L 7315 6037 L 7292 6055 L 7279 6080 L
7273 6109 L 7245 6113 L 7274 6113 L 7294 6092 L
7317 6074 L 7343 6062 L 7349 6062 L 7372 6062 L
7401 6062 L 7422 6085 L 7434 6099 L 7455 6119 L
7454 6130 L 7455 6156 L 7469 6182 L 7461 6205 L
7487 6216 L 7507 6221 L 7515 6224 L 7527 6217 L
7541 6210 L 7532 6238 L 7559 6227 L 7564 6199 L
7572 6171 L 7590 6186 L 7594 6190 L 7617 6207 L
7641 6223 L 7641 6252 L 7659 6229 L 7651 6201 L
7669 6224 L 7671 6253 L 7676 6281 L 7687 6308 L
7711 6291 L 7724 6307 L 7732 6307 L 7736 6307 L
7759 6291 L 7776 6313 L 7786 6341 L 7785 6369 L
7757 6376 L 7733 6393 L 7762 6390 L 7790 6384 L
7818 6376 L 7840 6357 L 7849 6330 L 7840 6302 L
7838 6273 L 7855 6297 L 7857 6325 L 7860 6354 L
7871 6380 L 7895 6397 L 7891 6426 L 7909 6448 L
7937 6457 L 7946 6430 L 7930 6407 L 7925 6378 L
7937 6404 L 7954 6428 L 7977 6445 L 8001 6460 L
8006 6488 L 8024 6511 L 8013 6537 L 8034 6558 L
8054 6538 L 8077 6556 L 8098 6575 L 8126 6581 L
8120 6609 L 8106 6634 L 8077 6634 L 8074 6663 L
8046 6660 L 8017 6657 L 8000 6661 L 7974 6674 L
7952 6692 L 7941 6704 L 7942 6720 L 7944 6717 L
7954 6704 L 7968 6707 L 7980 6708 L 7997 6709 L
8024 6699 L 8035 6696 L 8051 6691 L 8074 6708 L
8079 6736 L 8054 6749 L 8082 6750 L 8092 6777 L
8109 6754 L 8110 6757 L 8123 6779 L 8125 6808 L
8154 6808 L 8165 6782 L 8157 6754 L 8184 6747 L
8185 6747 L 8198 6773 L 8225 6765 L 8226 6765 L
8253 6770 L 8274 6782 L 8279 6784 L 8273 6812 L
8295 6830 L 8285 6803 L 8310 6790 L 8336 6777 L
8361 6763 L 8360 6734 L 8385 6748 L 8405 6768 L
8394 6794 L 8395 6823 L 8424 6820 L 8437 6845 L
8441 6874 L 8425 6898 L 8403 6880 L 8382 6900 L
8378 6929 L 8355 6945 L 8381 6934 L 8409 6940 L
8402 6968 L 8395 6995 L 8416 6975 L 8417 6947 L
8429 6972 L 8442 6947 L 8437 6919 L 8464 6927 L
8483 6948 L 8511 6944 L 8538 6934 L 8562 6948 L
8534 6955 L 8530 6983 L 8558 6985 L 8587 6984 L
8574 7009 L 8575 7038 L 8562 7012 L 8534 7020 L
8510 7035 L 8501 7062 L 8529 7069 L 8545 7093 L
8562 7116 L 8541 7136 L 8522 7157 L 8548 7168 L
8577 7170 L 8605 7164 L 8588 7140 L 8614 7152 L

8611 7124 L 8601 7097 L 8629 7093 L 8655 7081 L
8655 7081 L 8641 7106 L 8632 7133 L 8648 7157 L
8649 7129 L 8673 7112 L 8701 7114 L 8730 7118 L
8736 7146 L 8753 7151 L 8764 7153 L 8744 7174 L
8727 7197 L 8704 7181 L 8677 7171 L 8687 7198 L
8663 7213 L 8640 7230 L 8619 7249 L 8646 7258 L
8669 7275 L 8694 7289 L 8713 7310 L 8729 7334 L
8741 7360 L 8747 7356 L 8764 7344 L 8754 7317 L
8780 7305 L 8808 7301 L 8813 7273 L 8819 7265 L
8830 7250 L 8827 7221 L 8842 7246 L 8852 7273 L
8879 7264 L 8880 7236 L 8881 7207 L 8889 7214 L
8902 7226 L 8902 7226 L 8908 7254 L 8901 7282 L
8874 7292 L 8868 7320 L 8854 7345 L 8829 7332 L
8821 7360 L 8807 7385 L 8824 7407 L 8850 7418 L
8878 7423 L 8883 7432 L 8892 7448 L 8884 7476 L
8882 7504 L 8891 7531 L 8909 7532 L 8919 7533 L
8928 7560 L 8935 7588 L 8906 7591 L 8881 7603 L
8854 7612 L 8834 7633 L 8850 7656 L 8870 7675 L
8870 7676 L 8887 7699 L 8898 7725 L 8870 7721 L
8893 7737 L 8921 7743 L 8937 7747 L 8949 7750 L
8973 7765 L 8976 7766 L 9001 7771 L 9029 7775 L
9036 7784 L 9046 7797 L 9020 7786 L 8996 7801 L
8979 7824 L 8961 7801 L 8953 7774 L 8927 7762 L
8905 7780 L 8899 7808 L 8886 7833 L 8859 7825 L
8872 7850 L 8895 7867 L 8916 7848 L 8944 7841 L
8967 7824 L 8982 7848 L 8970 7874 L 8966 7902 L
8947 7923 L 8919 7928 L 8891 7928 L 8865 7917 L
8843 7936 L 8841 7964 L 8867 7974 L 8844 7990 L
8849 8017 L 8845 8046 L 8872 8035 L 8894 8017 L
8919 8004 L 8909 8030 L 8901 8058 L 8900 8086 L
8910 8060 L 8927 8037 L 8941 8024 L 8948 8018 L
8953 8046 L 8979 8058 L 8962 8034 L 8990 8027 L
8965 8014 L 8962 7986 L 8968 7984 L 8990 7979 L
9012 7961 L 9017 7989 L 9034 8012 L 9043 8039 L
9071 8036 L 9094 8020 L 9082 7995 L 9057 7982 L
9058 7954 L 9038 7935 L 9009 7933 L 8993 7910 L
8986 7882 L 9014 7882 L 9035 7900 L 9044 7927 L
9072 7930 L 9075 7932 L 9096 7945 L 9100 7947 L
9122 7956 L 9121 7984 L 9142 8004 L 9116 8016 L
9127 8042 L 9102 8056 L 9078 8071 L 9090 8096 L
9118 8091 L 9125 8118 L 9147 8117 L 9153 8116 L
9181 8111 L 9179 8139 L 9152 8146 L 9180 8146 L
9199 8167 L 9204 8195 L 9195 8222 L 9223 8218 L

9238 8222 L 9250 8226 L 9265 8249 L 9251 8274 L
9246 8302 L 9256 8275 L 9280 8261 L 9291 8287 L
9307 8264 L 9313 8274 L 9322 8287 L 9323 8316 L
9326 8321 L 9339 8339 L 9326 8364 L 9332 8391 L
9357 8379 L 9385 8383 L 9384 8411 L 9398 8436 L
9372 8446 L 9359 8471 L 9340 8493 L 9368 8500 L
9387 8520 L 9387 8549 L 9390 8577 L 9418 8580 L
9430 8605 L 9445 8629 L 9463 8651 L 9479 8674 L
9477 8702 L 9452 8716 L 9425 8721 L 9405 8741 L
9377 8746 L 9349 8742 L 9347 8770 L 9369 8752 L
9397 8755 L 9417 8749 L 9424 8748 L 9429 8748 L
9446 8730 L 9474 8731 L 9496 8730 L 9502 8730 L
9529 8727 L 9529 8740 L 9542 8765 L 9540 8793 L
9553 8818 L 9567 8825 L 9578 8831 L 9593 8854 L
9603 8881 L 9581 8898 L 9605 8908 L 9607 8909 L
9616 8936 L 9642 8946 L 9669 8938 L 9652 8916 L
9661 8901 L 9667 8891 L 9676 8865 L 9693 8842 L
9713 8823 L 9734 8842 L 9728 8869 L 9750 8887 L
9762 8912 L 9777 8936 L 9805 8932 L 9806 8931 L
9829 8917 L 9847 8895 L 9859 8920 L 9845 8945 L
9837 8972 L 9823 8996 L 9824 9024 L 9808 9047 L
9796 9073 L 9788 9100 L 9771 9122 L 9744 9129 L
9717 9137 L 9745 9137 L 9773 9137 L 9801 9138 L
9825 9153 L 9837 9166 L 9844 9173 L 9865 9193 L
9857 9220 L 9844 9244 L 9816 9250 L 9801 9274 L
9828 9264 L 9856 9261 L 9874 9282 L 9853 9301 L
9864 9327 L 9839 9339 L 9811 9333 L 9786 9322 L
9807 9340 L 9834 9343 L 9831 9371 L 9804 9377 L
9777 9369 L 9749 9366 L 9750 9394 L 9775 9408 L
9782 9435 L 9787 9462 L 9810 9459 L 9814 9458 L
9832 9436 L 9833 9464 L 9844 9490 L 9836 9463 L
9837 9435 L 9847 9409 L 9874 9416 L 9894 9435 L
9901 9462 L 9899 9490 L 9907 9517 L 9927 9499 L
9918 9472 L 9921 9456 L 9923 9445 L 9925 9417 L
9952 9411 L 9976 9427 L 9980 9432 L 9994 9448 L
9989 9476 L 9964 9488 L 9966 9516 L 9963 9544 L
9989 9554 L 9990 9582 L 9974 9605 L 9977 9633 L
9995 9655 L 9976 9675 L 9949 9668 L 9922 9662 L
9897 9649 L 9896 9621 L 9912 9598 L 9888 9584 L
9881 9611 L 9853 9611 L 9834 9590 L 9808 9582 L
9784 9566 L 9766 9545 L 9775 9572 L 9748 9564 L
9733 9541 L 9724 9568 L 9735 9593 L 9753 9615 L
9780 9609 L 9808 9615 L 9835 9621 L 9857 9639 L

9875 9660 L 9892 9683 L 9915 9698 L 9932 9720 L
9954 9738 L 9968 9761 L 9990 9779 L 10003 9784 L
10010 9787 L 10016 9789 L 10039 9805 L 10046 9832 L
10022 9818 L 9994 9818 L 9966 9817 L 9962 9818 L
9955 9824 L 9944 9834 L 9917 9841 L 9891 9852 L
9864 9857 L 9836 9861 L 9812 9875 L 9798 9899 L
9782 9922 L 9763 9943 L 9746 9965 L 9727 9986 L
9708 10006 L 9685 10022 L 9661 10036 L 9634 10040 L
9606 10039 L 9578 10035 L 9554 10022 L 9531 10005 L
9512 9985 L 9491 9967 L 9467 9953 L 9445 9935 L
9419 9925 L 9391 9925 L 9365 9935 L 9346 9915 L
9371 9902 L 9344 9910 L 9335 9936 L 9341 9964 L
9324 9986 L 9314 10012 L 9326 10037 L 9302 10052 L
9274 10054 L 9246 10051 L 9224 10035 L 9213 10009 L
9197 9987 L 9169 9985 L 9147 10001 L 9128 9981 L
9104 9966 L 9124 9986 L 9141 10008 L 9169 10009 L
9195 10000 L 9194 10028 L 9202 10054 L 9227 10067 L
9254 10073 L 9282 10074 L 9309 10076 L 9337 10076 L
9364 10068 L 9383 10047 L 9411 10050 L 9436 10062 L
9452 10085 L 9468 10107 L 9469 10135 L 9484 10159 L
9504 10178 L 9528 10192 L 9556 10196 L 9571 10219 L
9593 10236 L 9616 10252 L 9643 10256 L 9667 10271 L
9682 10294 L 9702 10313 L 9727 10326 L 9753 10334 L
9780 10341 L 9808 10346 L 9835 10349 L 9863 10352 L
9890 10356 L 9914 10372 L 9920 10399 L 9911 10425 L
9897 10449 L 9884 10474 L 9871 10498 L 9858 10523 L
9844 10547 L 9829 10570 L 9812 10592 L 9789 10608 L
9766 10623 L 9742 10637 L 9714 10637 L 9699 10660 L
9677 10677 L 9651 10689 L 9627 10702 L 9609 10723 L
9595 10747 L 9577 10769 L 9555 10786 L 9531 10799 L
9510 10817 L 9484 10827 L 9458 10836 L 9430 10835 L
9403 10837 L 9378 10849 L 9359 10869 L 9381 10886 L
9368 10911 L 9355 10935 L 9340 10958 L 9312 10961 L
9285 10958 L 9257 10961 L 9231 10970 L 9204 10968 L
9192 10943 L 9165 10937 L 9138 10931 L 9113 10918 L
9087 10910 L 9059 10910 L 9045 10934 L 9072 10928 L
9100 10927 L 9126 10936 L 9147 10954 L 9170 10970 L
9191 10988 L 9215 11002 L 9240 11012 L 9266 11001 L
9293 11001 L 9320 10993 L 9346 11003 L 9361 10980 L
9384 10964 L 9401 10942 L 9420 10922 L 9430 10896 L
9436 10869 L 9462 10861 L 9490 10864 L 9517 10861 L
9532 10884 L 9559 10890 L 9584 10901 L 9610 10910 L
9613 10909 L 9635 10898 L 9662 10892 L 9689 10884 L

9707 10863 L 9732 10852 L 9757 10840 L 9783 10830 L
9810 10826 L 9837 10818 L 9864 10811 L 9889 10821 L
9896 10848 L 9884 10873 L 9870 10897 L 9858 10922 L
9849 10948 L 9829 10968 L 9811 10988 L 9790 11006 L
9765 11019 L 9739 11028 L 9713 11038 L 9686 11038 L
9660 11048 L 9649 11073 L 9629 11092 L 9644 11116 L
9638 11143 L 9638 11171 L 9625 11195 L 9603 11212 L
9578 11222 L 9552 11233 L 9527 11245 L 9502 11255 L
9476 11264 L 9448 11265 L 9429 11285 L 9411 11306 L
9383 11309 L 9399 11332 L 9417 11353 L 9404 11377 L
9378 11387 L 9399 11405 L 9407 11431 L 9406 11459 L
9395 11484 L 9389 11511 L 9379 11537 L 9352 11542 L
9337 11565 L 9332 11592 L 9316 11615 L 9297 11635 L
9286 11660 L 9276 11686 L 9257 11706 L 9251 11732 L
9253 11760 L 9262 11786 L 9240 11802 L 9223 11824 L
9216 11851 L 9220 11878 L 9209 11903 L 9201 11930 L
9175 11939 L 9180 11966 L 9155 11977 L 9155 12004 L
9146 12031 L 9145 12058 L 9136 12084 L 9126 12110 L
9118 12136 L 9110 12162 L 9097 12186 L 9092 12213 L
9078 12237 L 9053 12248 L 9028 12260 L 9002 12266 L
8983 12286 L 8958 12298 L 8932 12307 L 8905 12311 L
8878 12317 L 8865 12293 L 8865 12320 L 8847 12341 L
8820 12347 L 8798 12364 L 8774 12377 L 8751 12391 L
8730 12409 L 8716 12432 L 8712 12459 L 8684 12462 L
8660 12474 L 8635 12461 L 8613 12477 L 8594 12457 L
8602 12484 L 8588 12508 L 8587 12535 L 8596 12561 L
8595 12588 L 8599 12616 L 8614 12639 L 8634 12657 L
8661 12652 L 8673 12677 L 8676 12704 L 8683 12730 L
8692 12757 L 8704 12773 L 8708 12778 L 8733 12791 L
8752 12810 L 8756 12837 L 8770 12861 L 8778 12887 L
8787 12913 L 8781 12939 L 8808 12937 L 8830 12952 L
8840 12927 L 8853 12951 L 8874 12969 L 8890 12947 L
8901 12972 L 8916 12996 L 8927 13020 L 8950 13005 L
8957 13031 L 8955 13059 L 8965 13084 L 8985 13103 L
9010 13113 L 9016 13117 L 9033 13128 L 9026 13102 L
9040 13125 L 9062 13141 L 9074 13166 L 9047 13162 L
9020 13166 L 9036 13189 L 9010 13198 L 8986 13211 L
8978 13237 L 8988 13263 L 9013 13273 L 9039 13264 L
9060 13281 L 9064 13308 L 9082 13288 L 9098 13266 L
9093 13239 L 9083 13213 L 9071 13189 L 9098 13183 L
9124 13192 L 9145 13210 L 9172 13215 L 9199 13219 L
9223 13231 L 9245 13248 L 9240 13275 L 9236 13302 L
9220 13324 L 9226 13351 L 9252 13359 L 9243 13385 L

9258 13408 L 9282 13420 L 9304 13403 L 9328 13390 L
9329 13417 L 9347 13438 L 9346 13465 L 9321 13476 L
9305 13498 L 9279 13492 L 9296 13513 L 9319 13527 L
9345 13520 L 9359 13496 L 9386 13497 L 9386 13525 L
9411 13514 L 9430 13533 L 9453 13548 L 9479 13554 L
9505 13564 L 9530 13574 L 9553 13589 L 9570 13611 L
9566 13637 L 9579 13614 L 9599 13633 L 9626 13636 L
9605 13618 L 9590 13595 L 9617 13602 L 9643 13595 L
9642 13622 L 9668 13632 L 9692 13645 L 9704 13669 L
9729 13680 L 9754 13692 L 9779 13704 L 9768 13729 L
9795 13734 L 9808 13710 L 9818 13685 L 9837 13703 L
9860 13718 L 9859 13745 L 9876 13766 L 9884 13740 L
9867 13719 L 9883 13696 L 9908 13686 L 9881 13682 L
9885 13655 L 9913 13654 L 9930 13651 L 9939 13650 L
9962 13665 L 9969 13671 L 9983 13682 L 9997 13706 L
9998 13706 L 10009 13709 L 10023 13712 L 10012 13687 L
10025 13711 L 10030 13738 L 10029 13765 L 10002 13762 L
9975 13764 L 9995 13782 L 9995 13810 L 10011 13832 L
10034 13846 L 10056 13861 L 10040 13884 L 10033 13910 L
10037 13936 L 10055 13957 L 10066 13968 L 10075 13976 L
10048 13978 L 10024 13965 L 9999 13953 L 9981 13974 L
9955 13966 L 9928 13965 L 9902 13973 L 9878 13987 L
9854 13974 L 9840 13997 L 9849 14023 L 9832 14044 L
9810 14027 L 9787 14012 L 9768 13993 L 9769 13966 L
9754 13989 L 9727 13984 L 9700 13980 L 9684 13958 L
9664 13976 L 9639 13966 L 9612 13961 L 9585 13963 L
9561 13975 L 9542 13995 L 9525 14016 L 9507 14037 L
9532 14048 L 9505 14045 L 9480 14034 L 9453 14031 L
9426 14037 L 9406 14056 L 9402 14083 L 9386 14105 L
9359 14103 L 9342 14125 L 9318 14137 L 9306 14161 L
9279 14158 L 9252 14161 L 9225 14160 L 9204 14142 L
9179 14133 L 9155 14120 L 9128 14115 L 9102 14108 L
9082 14090 L 9057 14081 L 9031 14073 L 9055 14062 L
9051 14035 L 9034 14013 L 9015 13994 L 8996 13974 L
8974 13958 L 8951 13943 L 8925 13936 L 8900 13926 L
8879 13908 L 8866 13884 L 8883 13863 L 8876 13836 L
8856 13817 L 8836 13800 L 8817 13780 L 8790 13773 L
8763 13774 L 8736 13771 L 8711 13782 L 8684 13779 L
8656 13777 L 8629 13778 L 8603 13785 L 8576 13788 L
8555 13806 L 8532 13821 L 8505 13820 L 8502 13847 L
8485 13868 L 8467 13888 L 8473 13915 L 8469 13942 L
8468 13969 L 8470 13996 L 8477 14023 L 8494 14044 L
8511 14065 L 8523 14090 L 8505 14110 L 8480 14121 L

8492 14146 L 8498 14172 L 8491 14198 L 8483 14224 L
8464 14244 L 8459 14271 L 8432 14271 L 8415 14250 L
8424 14276 L 8419 14303 L 8422 14330 L 8408 14353 L
8402 14326 L 8397 14353 L 8373 14340 L 8351 14324 L
8326 14313 L 8302 14301 L 8280 14285 L 8259 14302 L
8232 14306 L 8206 14314 L 8179 14316 L 8152 14313 L
8125 14317 L 8098 14313 L 8075 14327 L 8052 14342 L
8033 14361 L 8010 14376 L 7984 14383 L 7964 14402 L
7945 14422 L 7928 14443 L 7915 14466 L 7903 14491 L
7890 14515 L 7880 14540 L 7870 14565 L 7867 14592 L
7867 14619 L 7872 14646 L 7881 14672 L 7893 14696 L
7906 14720 L 7924 14741 L 7939 14763 L 7964 14774 L
7958 14800 L 7948 14825 L 7967 14845 L 7986 14864 L
8008 14879 L 8034 14888 L 8061 14893 L 8088 14888 L
8111 14875 L 8126 14852 L 8133 14826 L 8151 14806 L
8174 14792 L 8201 14787 L 8223 14803 L 8243 14821 L
8264 14838 L 8284 14856 L 8301 14864 L 8309 14867 L
8326 14867 L 8336 14867 L 8357 14850 L 8371 14850 L
8384 14851 L 8406 14867 L 8406 14867 L 8422 14869 L
8433 14870 L 8458 14880 L 8475 14889 L 8482 14893 L
8504 14909 L 8522 14929 L 8536 14953 L 8553 14974 L
8575 14989 L 8600 15000 L 8583 15021 L 8579 15047 L
8583 15074 L 8575 15100 L 8598 15114 L 8605 15140 L
8613 15166 L 8625 15190 L 8645 15209 L 8667 15225 L
8692 15236 L 8690 15263 L 8710 15281 L 8736 15290 L
8762 15297 L 8788 15305 L 8810 15321 L 8836 15328 L
8851 15350 L 8874 15365 L 8899 15375 L 8925 15383 L
8950 15393 L 8974 15406 L 8998 15418 L 9019 15435 L
9045 15442 L 9071 15449 L 9087 15471 L 9086 15498 L
9099 15521 L 9122 15535 L 9100 15551 L 9122 15567 L
9127 15594 L 9150 15607 L 9164 15630 L 9174 15637 L
9186 15647 L 9204 15667 L 9211 15681 L 9216 15691 L
9228 15705 L 9234 15711 L 9260 15717 L 9262 15720 L
9278 15738 L 9256 15753 L 9267 15777 L 9286 15796 L
9295 15795 L 9311 15793 L 9313 15792 L 9323 15817 L
9342 15836 L 9358 15858 L 9385 15856 L 9407 15840 L
9430 15853 L 9454 15840 L 9481 15841 L 9495 15832 L
9503 15826 L 9523 15807 L 9550 15809 L 9577 15812 L
9598 15828 L 9608 15853 L 9609 15880 L 9629 15898 L
9639 15923 L 9635 15896 L 9622 15872 L 9633 15847 L
9625 15822 L 9639 15798 L 9666 15800 L 9682 15779 L
9705 15764 L 9727 15748 L 9742 15733 L 9754 15732 L
9754 15746 L 9759 15750 L 9769 15746 L 9784 15739 L

9803 15744 L 9801 15751 L 9779 15745 L 9772 15755 L
9766 15762 L 9766 15768 L 9754 15775 L 9732 15791 L
9732 15793 L 9757 15778 L 9777 15766 L 9779 15753 L
9801 15755 L 9806 15746 L 9802 15740 L 9784 15736 L
9760 15748 L 9758 15742 L 9763 15715 L 9754 15698 L
9760 15688 L 9783 15673 L 9803 15655 L 9816 15631 L
9804 15607 L 9786 15586 L 9784 15559 L 9766 15539 L
9752 15516 L 9748 15489 L 9728 15471 L 9715 15447 L
9700 15425 L 9683 15404 L 9667 15382 L 9644 15368 L
9670 15361 L 9651 15342 L 9632 15322 L 9608 15309 L
9581 15310 L 9564 15289 L 9540 15275 L 9518 15261 L
9506 15236 L 9496 15211 L 9517 15195 L 9534 15173 L
9536 15146 L 9534 15125 L 9529 15113 L 9517 15096 L
9494 15087 L 9508 15055 L 9518 15025 L 9541 15011 L
9568 15014 L 9595 15016 L 9619 15028 L 9646 15028 L
9673 15035 L 9700 15033 L 9676 15020 L 9650 15013 L
9627 14998 L 9613 14976 L 9589 14962 L 9563 14955 L
9582 14935 L 9565 14914 L 9587 14898 L 9608 14881 L
9594 14858 L 9568 14849 L 9543 14838 L 9521 14822 L
9495 14819 L 9475 14837 L 9465 14812 L 9451 14789 L
9426 14779 L 9399 14779 L 9372 14772 L 9365 14746 L
9391 14739 L 9369 14722 L 9382 14698 L 9381 14671 L
9402 14654 L 9420 14675 L 9431 14699 L 9458 14699 L
9483 14710 L 9510 14710 L 9536 14719 L 9554 14738 L
9582 14739 L 9609 14741 L 9621 14765 L 9597 14779 L
9571 14785 L 9595 14797 L 9607 14821 L 9629 14838 L
9653 14828 L 9665 14803 L 9672 14799 L 9689 14790 L
9690 14818 L 9679 14842 L 9705 14834 L 9696 14859 L
9716 14841 L 9737 14859 L 9764 14865 L 9756 14891 L
9782 14896 L 9806 14908 L 9782 14920 L 9792 14945 L
9811 14964 L 9834 14978 L 9846 15003 L 9856 15028 L
9879 15042 L 9898 15059 L 9900 15060 L 9915 15059 L
9927 15057 L 9946 15076 L 9950 15103 L 9956 15099 L
9973 15089 L 9991 15110 L 10015 15122 L 10035 15140 L
10042 15146 L 10056 15157 L 10057 15157 L 10071 15163 L
10078 15166 L 10081 15168 L 10088 15170 L 10097 15174 L
10107 15177 L 10131 15189 L 10143 15201 L 10150 15208 L
10170 15216 L 10175 15218 L 10201 15209 L 10226 15219 L
10235 15226 L 10248 15235 L 10223 15246 L 10246 15260 L
10255 15286 L 10257 15285 L 10272 15283 L 10281 15281 L
10284 15281 L 10308 15276 L 10309 15275 L 10321 15268 L
10331 15262 L 10340 15280 L 10343 15286 L 10356 15283 L
10369 15281 L 10383 15304 L 10393 15329 L 10401 15355 L

10403 15328 L 10426 15313 L 10441 15314 L 10453 15314 L
10473 15332 L 10497 15320 L 10524 15318 L 10551 15317 L
10578 15323 L 10600 15338 L 10627 15344 L 10639 15368 L
10644 15341 L 10651 15315 L 10656 15342 L 10661 15369 L
10666 15395 L 10672 15395 L 10693 15397 L 10687 15370 L
10709 15354 L 10733 15342 L 10759 15335 L 10786 15333 L
10796 15307 L 10805 15282 L 10824 15262 L 10813 15237 L
10826 15213 L 10840 15231 L 10843 15234 L 10854 15250 L
10859 15256 L 10856 15283 L 10867 15308 L 10888 15291 L
10914 15283 L 10929 15263 L 10930 15262 L 10954 15275 L
10971 15254 L 10998 15255 L 11015 15243 L 11021 15239 L
11044 15226 L 11070 15234 L 11089 15215 L 11116 15216 L
11139 15230 L 11160 15247 L 11172 15271 L 11177 15283 L
11189 15280 L 11197 15283 L 11202 15289 L 11203 15288 L
11198 15280 L 11189 15277 L 11177 15282 L 11174 15269 L
11163 15241 L 11184 15242 L 11196 15252 L 11225 15269 L
11226 15267 L 11221 15263 L 11197 15251 L 11187 15237 L
11174 15236 L 11150 15223 L 11146 15196 L 11127 15177 L
11128 15150 L 11141 15126 L 11167 15133 L 11153 15109 L
11157 15082 L 11151 15055 L 11144 15029 L 11121 15015 L
11116 14988 L 11125 14962 L 11104 14944 L 11091 14920 L
11082 14895 L 11073 14869 L 11062 14844 L 11070 14818 L
11085 14795 L 11110 14787 L 11103 14813 L 11118 14790 L
11121 14763 L 11147 14772 L 11174 14769 L 11190 14791 L
11214 14779 L 11241 14778 L 11257 14756 L 11230 14752 L
11208 14767 L 11184 14754 L 11157 14751 L 11135 14735 L
11108 14729 L 11085 14715 L 11068 14736 L 11075 14710 L
11071 14683 L 11047 14671 L 11020 14670 L 11031 14645 L
11021 14619 L 11001 14601 L 10991 14576 L 10977 14553 L
10983 14526 L 10984 14499 L 11003 14480 L 11024 14491 L
11034 14487 L 11005 14467 L 11002 14471 L 11004 14478 L
10982 14492 L 10963 14466 L 10943 14449 L 10924 14430 L
10898 14423 L 10924 14416 L 10928 14415 L 10951 14411 L
10960 14414 L 10973 14418 L 10977 14419 L 11002 14429 L
11026 14415 L 11052 14408 L 11079 14411 L 11104 14422 L
11131 14424 L 11156 14414 L 11154 14387 L 11133 14404 L
11106 14399 L 11099 14373 L 11123 14360 L 11098 14350 L
11071 14354 L 11048 14341 L 11063 14318 L 11079 14312 L
11088 14308 L 11114 14300 L 11140 14308 L 11141 14308 L
11160 14322 L 11162 14324 L 11186 14310 L 11181 14283 L
11207 14284 L 11209 14284 L 11217 14310 L 11244 14315 L
11221 14329 L 11248 14331 L 11275 14328 L 11253 14313 L
11268 14290 L 11241 14288 L 11239 14261 L 11212 14254 L

11201 14230 L 11218 14208 L 11229 14233 L 11255 14237 L
11272 14259 L 11286 14268 L 11295 14273 L 11298 14273 L
11322 14271 L 11330 14297 L 11326 14323 L 11323 14351 L
11344 14350 L 11349 14350 L 11350 14350 L 11357 14376 L
11330 14376 L 11304 14386 L 11300 14413 L 11325 14403 L
11352 14404 L 11369 14400 L 11376 14398 L 11379 14397 L
11390 14375 L 11391 14373 L 11400 14366 L 11405 14363 L
11412 14358 L 11413 14357 L 11423 14348 L 11432 14338 L
11449 14316 L 11456 14316 L 11476 14315 L 11479 14342 L
11505 14336 L 11505 14336 L 11509 14309 L 11516 14303 L
11521 14298 L 11529 14290 L 11549 14275 L 11551 14274 L
11557 14300 L 11556 14327 L 11560 14329 L 11582 14336 L
11599 14338 L 11608 14339 L 11629 14357 L 11657 14359 L
11667 14352 L 11679 14344 L 11702 14329 L 11726 14317 L
11753 14322 L 11770 14344 L 11796 14357 L 11778 14334 L
11784 14307 L 11786 14280 L 11798 14256 L 11822 14244 L
11845 14228 L 11867 14214 L 11888 14203 L 11888 14201 L
11865 14211 L 11843 14227 L 11819 14238 L 11795 14251 L
11771 14264 L 11778 14290 L 11765 14314 L 11741 14301 L
11717 14289 L 11722 14262 L 11728 14236 L 11748 14217 L
11774 14210 L 11785 14184 L 11787 14157 L 11763 14145 L
11764 14144 L 11781 14125 L 11803 14108 L 11828 14099 L
11812 14077 L 11837 14065 L 11861 14053 L 11840 14036 L
11813 14032 L 11820 14006 L 11807 13982 L 11823 13960 L
11842 13981 L 11863 13997 L 11868 13970 L 11857 13946 L
11850 13919 L 11877 13913 L 11895 13893 L 11914 13874 L
11941 13868 L 11960 13887 L 11950 13861 L 11928 13845 L
11909 13826 L 11909 13799 L 11895 13822 L 11890 13849 L
11874 13871 L 11847 13876 L 11827 13895 L 11804 13879 L
11780 13892 L 11756 13904 L 11766 13879 L 11741 13891 L
11760 13871 L 11771 13846 L 11750 13863 L 11746 13836 L
11740 13810 L 11739 13837 L 11732 13863 L 11723 13889 L
11697 13880 L 11677 13862 L 11699 13847 L 11717 13827 L
11702 13804 L 11678 13791 L 11693 13769 L 11671 13785 L
11673 13812 L 11666 13838 L 11660 13865 L 11661 13838 L
11636 13826 L 11630 13799 L 11631 13772 L 11604 13766 L
11595 13740 L 11600 13717 L 11601 13714 L 11611 13739 L
11612 13738 L 11621 13734 L 11631 13730 L 11635 13728 L
11634 13700 L 11639 13702 L 11642 13703 L 11652 13706 L
11656 13707 L 11661 13708 L 11679 13688 L 11678 13661 L
11684 13653 L 11689 13647 L 11694 13641 L 11695 13639 L
11697 13630 L 11702 13613 L 11706 13586 L 11720 13566 L
11721 13564 L 11743 13581 L 11732 13606 L 11720 13630 L

11716 13657 L 11691 13668 L 11697 13695 L 11683 13719 L
11702 13738 L 11711 13712 L 11715 13685 L 11733 13706 L
11750 13717 L 11755 13721 L 11761 13694 L 11759 13667 L
11758 13640 L 11782 13627 L 11768 13604 L 11773 13596 L
11779 13585 L 11782 13580 L 11807 13608 L 11806 13593 L
11783 13578 L 11784 13551 L 11788 13548 L 11792 13545 L
11806 13534 L 11793 13510 L 11807 13487 L 11809 13461 L
11810 13459 L 11832 13443 L 11810 13427 L 11826 13421 L
11831 13419 L 11836 13418 L 11847 13408 L 11857 13400 L
11869 13376 L 11889 13357 L 11913 13370 L 11938 13381 L
11960 13397 L 11966 13424 L 11964 13396 L 11944 13379 L
11919 13368 L 11898 13350 L 11873 13338 L 11858 13315 L
11867 13289 L 11877 13291 L 11893 13295 L 11921 13297 L
11947 13303 L 11929 13283 L 11909 13264 L 11925 13241 L
11900 13230 L 11894 13203 L 11867 13202 L 11849 13181 L
11829 13163 L 11810 13143 L 11824 13119 L 11851 13121 L
11878 13126 L 11905 13126 L 11907 13099 L 11880 13101 L
11854 13092 L 11828 13083 L 11816 13059 L 11812 13032 L
11799 13008 L 11825 13003 L 11817 12977 L 11795 12993 L
11789 12966 L 11783 12940 L 11774 12914 L 11800 12907 L
11796 12879 L 11819 12864 L 11845 12857 L 11835 12832 L
11818 12811 L 11799 12831 L 11781 12851 L 11783 12879 L
11770 12854 L 11743 12860 L 11752 12834 L 11764 12822 L
11772 12815 L 11769 12843 L 11789 12824 L 11812 12808 L
11799 12784 L 11774 12774 L 11777 12771 L 11794 12755 L
11811 12777 L 11838 12782 L 11822 12759 L 11833 12734 L
11826 12707 L 11836 12682 L 11860 12695 L 11887 12695 L
11887 12722 L 11878 12748 L 11879 12775 L 11881 12748 L
11905 12735 L 11909 12707 L 11937 12705 L 11912 12694 L
11885 12689 L 11878 12663 L 11851 12667 L 11830 12649 L
11857 12650 L 11884 12647 L 11905 12630 L 11915 12604 L
11888 12602 L 11861 12609 L 11855 12636 L 11828 12634 L
11827 12607 L 11853 12598 L 11873 12580 L 11846 12577 L
11821 12588 L 11806 12612 L 11804 12639 L 11781 12653 L
11772 12627 L 11772 12624 L 11774 12600 L 11787 12575 L
11814 12570 L 11836 12554 L 11848 12549 L 11861 12543 L
11882 12560 L 11910 12559 L 11890 12540 L 11872 12519 L
11879 12493 L 11904 12480 L 11887 12459 L 11910 12444 L
11911 12421 L 11912 12416 L 11934 12433 L 11934 12433 L
11951 12454 L 11955 12481 L 11958 12482 L 11978 12497 L
11981 12510 L 11984 12523 L 12009 12537 L 12009 12564 L
12011 12591 L 12021 12566 L 12024 12539 L 12038 12515 L
12046 12517 L 12064 12522 L 12092 12520 L 12098 12529 L

12108 12542 L 12097 12567 L 12106 12593 L 12109 12621 L
12133 12634 L 12140 12640 L 12153 12653 L 12178 12664 L
12196 12685 L 12222 12693 L 12248 12703 L 12252 12676 L
12234 12655 L 12214 12673 L 12214 12646 L 12215 12619 L
12192 12604 L 12187 12631 L 12176 12606 L 12168 12580 L
12194 12572 L 12209 12595 L 12217 12587 L 12228 12576 L
12245 12554 L 12250 12549 L 12265 12536 L 12277 12561 L
12278 12561 L 12288 12569 L 12299 12577 L 12300 12605 L
12274 12600 L 12277 12627 L 12304 12621 L 12319 12621 L
12331 12621 L 12331 12648 L 12310 12665 L 12287 12681 L
12269 12702 L 12262 12728 L 12285 12743 L 12259 12753 L
12287 12754 L 12313 12761 L 12325 12786 L 12337 12761 L
12346 12748 L 12352 12738 L 12363 12713 L 12354 12688 L
12366 12663 L 12390 12677 L 12389 12704 L 12408 12709 L
12415 12711 L 12442 12705 L 12436 12678 L 12438 12651 L
12447 12654 L 12464 12659 L 12463 12687 L 12454 12713 L
12438 12735 L 12442 12762 L 12437 12789 L 12410 12783 L
12389 12765 L 12365 12778 L 12351 12802 L 12358 12828 L
12375 12807 L 12398 12820 L 12415 12842 L 12442 12848 L
12468 12839 L 12487 12853 L 12490 12855 L 12514 12870 L
12539 12880 L 12546 12906 L 12549 12879 L 12550 12851 L
12551 12851 L 12576 12840 L 12602 12832 L 12629 12832 L
12631 12838 L 12638 12858 L 12646 12854 L 12663 12846 L
12664 12847 L 12688 12856 L 12685 12829 L 12712 12829 L
12719 12803 L 12692 12809 L 12700 12798 L 12708 12787 L
12727 12773 L 12730 12770 L 12741 12795 L 12744 12823 L
12725 12843 L 12726 12871 L 12729 12898 L 12748 12918 L
12730 12939 L 12743 12963 L 12767 12961 L 12771 12961 L
12797 12954 L 12789 12980 L 12783 13006 L 12807 12993 L
12829 12977 L 12833 12977 L 12856 12973 L 12883 12973 L
12895 12976 L 12909 12980 L 12910 12980 L 12911 12980 L
12925 12981 L 12937 12982 L 12952 13005 L 12951 12978 L
12959 12952 L 12980 12969 L 12999 12978 L 12999 13001 L
12998 13007 L 12999 13007 L 12999 14079 L 12999 14085 L
12980 14105 L 12968 14129 L 12961 14156 L 12951 14181 L
12934 14202 L 12915 14222 L 12900 14245 L 12878 14261 L
12864 14284 L 12844 14302 L 12838 14329 L 12819 14348 L
12798 14366 L 12793 14393 L 12772 14411 L 12752 14428 L
12731 14445 L 12712 14465 L 12692 14483 L 12675 14505 L
12656 14524 L 12640 14546 L 12622 14566 L 12606 14588 L
12586 14607 L 12575 14632 L 12563 14657 L 12547 14678 L
12534 14702 L 12519 14725 L 12503 14747 L 12494 14772 L
12476 14793 L 12463 14817 L 12448 14839 L 12438 14865 L

12433 14891 L 12409 14905 L 12384 14913 L 12358 14904 L
12347 14929 L 12328 14948 L 12306 14964 L 12279 14967 L
12256 14981 L 12235 14998 L 12213 15014 L 12196 15036 L
12178 15056 L 12163 15078 L 12147 15100 L 12124 15086 L
12099 15096 L 12072 15102 L 12045 15103 L 12018 15104 L
11995 15119 L 11980 15141 L 11984 15168 L 12007 15183 L
12012 15209 L 11987 15219 L 12013 15226 L 12040 15230 L
12053 15206 L 12045 15232 L 12033 15257 L 12026 15283 L
12029 15310 L 12041 15334 L 12035 15360 L 12009 15368 L
12003 15394 L 12010 15421 L 12022 15445 L 12041 15464 L
12068 15463 L 12081 15487 L 12107 15494 L 12128 15511 L
12149 15529 L 12146 15556 L 12169 15571 L 12182 15594 L
12204 15610 L 12231 15605 L 12258 15601 L 12283 15611 L
12298 15633 L 12324 15624 L 12334 15649 L 12359 15657 L
12377 15678 L 12402 15690 L 12419 15710 L 12445 15720 L
12449 15746 L 12426 15760 L 12434 15786 L 12458 15799 L
12482 15811 L 12506 15823 L 12528 15839 L 12545 15861 L
12561 15882 L 12584 15897 L 12609 15908 L 12621 15932 L
12626 15959 L 12633 15985 L 12638 16012 L 12656 16032 L
12682 16039 L 12698 16061 L 12719 16079 L 12743 16090 L
12767 16102 L 12785 16122 L 12812 16127 L 12836 16139 L
12842 16166 L 12841 16170 L 12833 16170 L 12800 16170 L
12767 16170 L 12734 16170 L 12700 16170 L 12667 16170 L
12634 16170 L 12601 16170 L 12567 16170 L 12534 16170 L
12501 16170 L 12468 16170 L 12435 16170 L 12401 16170 L
12368 16170 L 12335 16170 L 12302 16170 L 12268 16170 L
12235 16170 L 12202 16170 L 12169 16170 L 12135 16170 L
12102 16170 L 12069 16170 L 12036 16170 L 12003 16170 L
11969 16170 L 11936 16170 L 11903 16170 L 11870 16170 L
11836 16170 L 11803 16170 L 11770 16170 L 11737 16170 L
11704 16170 L 11670 16170 L 11637 16170 L 11604 16170 L
11571 16170 L 11537 16170 L 11504 16170 L 11471 16170 L
11438 16170 L 11404 16170 L 11371 16170 L 11338 16170 L
11305 16170 L 11272 16170 L 11238 16170 L 11205 16170 L
11172 16170 L 11139 16170 L 11105 16170 L 11072 16170 L
11039 16170 L 11006 16170 L 10972 16170 L 10939 16170 L
10906 16170 L 10873 16170 L 10840 16170 L 10806 16170 L
10773 16170 L 10740 16170 L 10707 16170 L 10673 16170 L
10640 16170 L 10607 16170 L 10574 16170 L 10540 16170 L
10507 16170 L 10474 16170 L 10441 16170 L 10408 16170 L
10374 16170 L 10341 16170 L 10308 16170 L 10275 16170 L
10241 16170 L 10208 16170 L 10175 16170 L 10142 16170 L
10108 16170 L 10075 16170 L 10042 16170 L 10009 16170 L

9976 16170 L 9942 16170 L 9909 16170 L 9876 16170 L
9843 16170 L 9809 16170 L 9776 16170 L 9743 16170 L
9710 16170 L 9677 16170 L 9643 16170 L 9610 16170 L
9577 16170 L 9544 16170 L 9510 16170 L 9477 16170 L
9444 16170 L 9411 16170 L 9377 16170 L 9344 16170 L
9311 16170 L 9278 16170 L 9245 16170 L 9211 16170 L
9178 16170 L 9145 16170 L 9112 16170 L 9078 16170 L
9045 16170 L 9012 16170 L 8979 16170 L 8945 16170 L
8912 16170 L 8879 16170 L 8846 16170 L 8813 16170 L
8779 16170 L 8746 16170 L 8713 16170 L 8680 16170 L
8646 16170 L 8613 16170 L 8580 16170 L 8547 16170 L
8513 16170 L 8480 16170 L 8447 16170 L 8414 16170 L
8381 16170 L 8347 16170 L 8314 16170 L 8281 16170 L
8248 16170 L 8214 16170 L 8181 16170 L 8148 16170 L
8115 16170 L 8081 16170 L 8048 16170 L 8015 16170 L
7982 16170 L 7949 16170 L 7915 16170 L 7882 16170 L
7849 16170 L 7816 16170 L 7782 16170 L 7749 16170 L
7716 16170 L 7683 16170 L 7650 16170 L 7616 16170 L
7583 16170 L 7550 16170 L 7517 16170 L 7483 16170 L
7450 16170 L 7417 16170 L 7384 16170 L 7350 16170 L
7317 16170 L 7284 16170 L 7251 16170 L 7218 16170 L
7184 16170 L 7151 16170 L 7118 16170 L 7085 16170 L
7051 16170 L 7018 16170 L 6985 16170 L 6952 16170 L
6918 16170 L 6885 16170 L 6852 16170 L 6819 16170 L
6786 16170 L 6752 16170 L 6719 16170 L 6686 16170 L
6653 16170 L 6619 16170 L 6586 16170 L 6553 16170 L
6520 16170 L 6486 16170 L 6453 16170 L 6420 16170 L
6387 16170 L 6354 16170 L 6320 16170 L 6287 16170 L
6254 16170 L 6221 16170 L 6187 16170 L 6154 16170 L
6121 16170 L 6088 16170 L 6054 16170 L 6021 16170 L
5988 16170 L 5955 16170 L 5922 16170 L 5888 16170 L
5855 16170 L 5822 16170 L 5789 16170 L 5755 16170 L
5722 16170 L 5689 16170 L 5656 16170 L 5622 16170 L
5589 16170 L 5556 16170 L 5523 16170 L 5490 16170 L
5456 16170 L 5423 16170 L 5390 16170 L 5357 16170 L
5323 16170 L 5290 16170 L 5257 16170 L 5224 16170 L
5191 16170 L 5157 16170 L 5124 16170 L 5091 16170 L
5058 16170 L 5024 16170 L 4991 16170 L 4958 16170 L
4925 16170 L 4891 16170 L 4858 16170 L 4825 16170 L
4792 16170 L 4759 16170 L 4725 16170 L 4692 16170 L
4659 16170 L 4626 16170 L 4592 16170 L 4559 16170 L
4526 16170 L 4493 16170 L 4459 16170 L 4426 16170 L
4393 16170 L 4360 16170 L 4327 16170 L 4293 16170 L

4260 16170 L 4227 16170 L 4194 16170 L 4160 16170 L
4127 16170 L 4094 16170 L 4061 16170 L 4027 16170 L
3994 16170 L 3961 16170 L 3928 16170 L 3895 16170 L
3861 16170 L 3828 16170 L 3795 16170 L 3762 16170 L
3728 16170 L 3695 16170 L 3662 16170 L 3629 16170 L
3595 16170 L 3562 16170 L 3529 16170 L 3496 16170 L
3463 16170 L 3429 16170 L 3396 16170 L 3363 16170 L
3330 16170 L 3296 16170 L 3263 16170 L 3230 16170 L
3197 16170 L 3164 16170 L 3130 16170 L 3097 16170 L
3064 16170 L 3031 16170 L 2997 16170 L 2964 16170 L
2931 16170 L 2898 16170 L 2864 16170 L 2831 16170 L
2798 16170 L 2765 16170 L 2732 16170 L 2698 16170 L
2665 16170 L 2632 16170 L 2599 16170 L 2565 16170 L
2532 16170 L 2499 16170 L 2466 16170 L 2432 16170 L
2399 16170 L 2366 16170 L 2333 16170 L 2300 16170 L
N E
5542 5477 5541 5470 5543 5466 5545 5461 5547 5457 5548 5455
5556 5455 5575 5455 5575 5458 5573 5462 5571 5466 5569 5471
5567 5475 5563 5478 5559 5480 5556 5483 5552 5486 5549 5482
5545 5480 5542 5477 20 N F
5702 5573 5698 5567 5700 5563 5704 5561 5708 5563 5712 5565
5717 5566 5716 5571 5721 5573 5724 5576 5724 5581 5726 5585
5721 5585 5717 5588 5712 5588 5711 5584 5707 5581 5704 5578
5702 5573 19 N F
5796 5572 5790 5572 5794 5569 5798 5567 5801 5563 5805 5561
5810 5562 5808 5566 5806 5570 5803 5573 5799 5576 5796 5572
12 N F
5817 5461 5816 5455 5822 5455 5855 5455 5865 5455 5871 5458
5879 5459 5885 5461 5890 5456 5890 5455 5910 5455 5903 5461
5904 5468 5908 5474 5902 5477 5909 5480 5915 5478 5917 5485
5916 5492 5916 5499 5921 5504 5924 5511 5930 5507 5936 5511
5937 5519 5933 5524 5927 5520 5921 5516 5914 5515 5908 5511
5903 5506 5897 5502 5893 5496 5886 5497 5879 5495 5872 5491
5866 5488 5862 5494 5855 5495 5847 5494 5843 5488 5838 5483
5832 5479 5825 5477 5827 5471 5823 5465 5817 5461 47 N F
5964 5459 5962 5455 5969 5455 5970 5459 5964 5459 5 N F
5977 5488 5967 5480 5972 5475 5975 5468 5981 5464 5988 5463
5992 5457 5999 5459 6006 5456 6006 5455 6021 5455 6024 5455
6024 5459 6028 5455 6039 5455 6038 5459 6042 5465 6038 5471
6034 5477 6031 5484 6024 5485 6018 5488 6011 5489 6005 5485
5998 5483 5991 5485 5984 5488 5977 5488 28 N F
6055 5463 6054 5459 6056 5458 6058 5457 6058 5455 6059 5455
6060 5456 6062 5456 6063 5458 6064 5460 6064 5462 6064 5465

6063 5467 6061 5466 6059 5465 6057 5465 6055 5463 17 N F
6243 5583 6240 5580 6241 5577 6242 5574 6244 5571 6247 5573
6251 5574 6254 5574 6257 5576 6259 5578 6259 5579 6257 5581
6253 5582 6250 5582 6246 5583 6243 5583 16 N F
6252 5562 6251 5559 6253 5560 6255 5559 6257 5561 6258 5563
6258 5565 6260 5566 6259 5568 6257 5568 6255 5566 6253 5564
6252 5562 13 N F
6256 5651 6252 5647 6253 5644 6257 5644 6259 5641 6260 5638
6263 5640 6262 5643 6260 5646 6257 5647 6256 5651 11 N F
7342 6052 7335 6044 7339 6038 7343 6032 7349 6028 7356 6025
7360 6019 7367 6017 7374 6019 7378 6013 7381 6006 7388 6004
7395 6002 7388 5998 7392 5992 7398 5987 7405 5987 7408 5980
7415 5980 7417 5987 7422 5992 7415 5995 7409 5992 7404 5997
7402 6004 7403 6012 7404 6019 7402 6026 7398 6032 7397 6039
7392 6044 7385 6046 7380 6042 7372 6042 7365 6044 7360 6049
7356 6055 7349 6055 7342 6052 39 N F
7490 6180 7483 6175 7488 6171 7495 6170 7503 6169 7508 6173
7505 6180 7506 6187 7511 6192 7514 6199 7507 6201 7501 6197
7500 6189 7497 6183 7490 6180 15 N F
7498 6166 7491 6165 7493 6158 7499 6154 7506 6153 7513 6151
7520 6149 7522 6156 7523 6163 7527 6169 7533 6172 7527 6180
7520 6179 7513 6179 7510 6173 7506 6167 7498 6166 17 N F
7498 6111 7493 6105 7497 6099 7504 6098 7511 6096 7519 6097
7526 6096 7533 6094 7538 6090 7545 6087 7552 6087 7559 6090
7566 6091 7573 6089 7574 6082 7581 6084 7586 6080 7593 6083
7597 6089 7595 6096 7594 6103 7600 6107 7598 6114 7592 6118
7589 6125 7590 6132 7583 6132 7579 6126 7576 6119 7569 6120
7564 6126 7558 6130 7552 6127 7547 6121 7544 6115 7539 6111
7535 6105 7526 6113 7523 6119 7516 6122 7509 6120 7503 6115
7498 6111 43 N F
7698 6195 7691 6184 7698 6184 7705 6186 7712 6188 7718 6186
7717 6179 7723 6183 7729 6185 7736 6188 7743 6189 7750 6190
7758 6191 7754 6197 7747 6198 7745 6205 7745 6212 7746 6219
7749 6226 7753 6232 7759 6234 7766 6231 7773 6228 7780 6229
7781 6236 7776 6241 7775 6248 7777 6255 7784 6259 7782 6266
7788 6270 7794 6266 7801 6266 7802 6273 7809 6276 7802 6275
7797 6281 7791 6285 7786 6290 7781 6285 7774 6287 7770 6281
7762 6281 7756 6278 7753 6285 7749 6291 7744 6295 7737 6299
7732 6304 7726 6301 7724 6294 7727 6287 7727 6280 7722 6274
7717 6269 7715 6262 7711 6256 7706 6251 7708 6244 7714 6240
7719 6245 7726 6248 7732 6243 7739 6244 7746 6242 7740 6237
7739 6230 7734 6225 7728 6221 7722 6217 7718 6211 7713 6205
7714 6198 7710 6204 7704 6200 7698 6195 76 N F

7944 6713 7942 6708 7943 6705 7945 6702 7948 6700 7950 6699
7951 6699 7953 6702 7950 6704 7948 6707 7946 6710 7944 6713
12 N F
7955 6697 7950 6697 7954 6695 7958 6692 7961 6689 7964 6685
7967 6682 7971 6679 7975 6678 7980 6678 7984 6679 7989 6678
7989 6678 7993 6680 7991 6684 7990 6688 7986 6690 7982 6688
7977 6687 7973 6689 7971 6693 7968 6696 7963 6696 7959 6694
7955 6697 25 N F
7957 6703 7953 6699 7957 6697 7961 6696 7966 6698 7970 6696
7973 6693 7976 6689 7980 6689 7984 6691 7988 6693 7992 6690
7994 6686 7999 6684 8003 6687 8007 6687 8006 6691 8003 6695
7999 6698 7997 6701 7993 6704 7989 6706 7984 6706 7980 6708
7975 6707 7971 6706 7966 6705 7962 6704 7957 6703 29 N F
7984 6676 7979 6675 7983 6673 7987 6671 7991 6669 7996 6669
7997 6673 7993 6675 7989 6677 7984 6676 10 N F
8030 6668 8023 6664 8027 6661 8031 6663 8035 6660 8039 6663
8043 6665 8039 6668 8035 6669 8030 6668 10 N F
8101 6699 8089 6694 8093 6688 8097 6682 8103 6678 8109 6675
8116 6673 8123 6672 8129 6676 8134 6681 8140 6686 8145 6691
8147 6698 8150 6704 8157 6706 8157 6713 8156 6720 8156 6727
8152 6732 8145 6729 8141 6735 8136 6740 8130 6738 8124 6742
8117 6745 8110 6747 8111 6740 8105 6735 8104 6728 8100 6722
8095 6717 8091 6711 8093 6704 8100 6705 8105 6710 8112 6713
8113 6706 8107 6702 8101 6699 39 N F
8169 6668 8165 6666 8167 6664 8171 6664 8174 6663 8176 6666
8179 6667 8182 6666 8185 6668 8185 6671 8188 6673 8191 6672
8193 6675 8194 6679 8195 6682 8193 6685 8190 6687 8187 6689
8184 6690 8181 6688 8178 6688 8175 6687 8174 6683 8174 6680
8173 6677 8170 6675 8169 6672 8169 6668 28 N F
8212 6671 8200 6667 8205 6663 8210 6657 8216 6654 8223 6651
8229 6655 8231 6662 8235 6668 8241 6672 8247 6675 8254 6678
8261 6679 8268 6677 8274 6673 8280 6670 8287 6666 8293 6669
8297 6675 8297 6682 8302 6688 8298 6694 8298 6701 8294 6707
8292 6714 8290 6721 8283 6722 8279 6728 8274 6733 8269 6728
8263 6724 8266 6717 8261 6712 8255 6708 8254 6701 8250 6695
8243 6696 8236 6699 8230 6702 8224 6707 8219 6712 8213 6708
8207 6704 8209 6697 8215 6700 8212 6694 8214 6687 8216 6680
8219 6673 8212 6671 50 N F
8213 6754 8209 6751 8212 6750 8216 6749 8218 6747 8221 6744
8223 6742 8227 6742 8230 6743 8231 6746 8232 6749 8234 6752
8232 6755 8229 6756 8226 6757 8222 6756 8219 6756 8216 6755
8213 6754 19 N F
8275 14738 8272 14733 8276 14734 8279 14737 8283 14740 8285 14743

8288 14747 8290 14751 8293 14754 8294 14758 8295 14762 8293 14766
8291 14763 8289 14759 8286 14756 8283 14753 8282 14749 8281 14745
8279 14741 8275 14738 20 N F
8293 14780 8292 14777 8293 14776 8295 14776 8298 14778 8299 14780
8301 14783 8301 14786 8299 14784 8296 14783 8293 14780 11 N F
8313 14797 8311 14795 8313 14793 8316 14795 8319 14796 8320 14799
8321 14802 8323 14805 8325 14807 8326 14810 8323 14809 8321 14806
8319 14804 8318 14801 8316 14799 8313 14797 16 N F
8362 14788 8356 14786 8361 14787 8365 14788 8369 14790 8372 14793
8375 14796 8378 14799 8379 14803 8377 14807 8373 14810 8371 14813
8369 14809 8370 14805 8374 14804 8375 14799 8371 14797 8368 14794
8365 14791 8362 14788 20 N F
8397 14838 8392 14833 8397 14834 8401 14835 8405 14836 8409 14837
8413 14838 8416 14842 8417 14846 8419 14850 8423 14852 8424 14856
8422 14860 8418 14859 8415 14855 8413 14852 8411 14848 8408 14844
8405 14842 8401 14840 8397 14838 21 N F
8398 14852 8397 14849 8399 14847 8402 14848 8405 14850 8406 14853
8407 14856 8407 14859 8406 14862 8404 14859 8403 14857 8401 14854
8398 14852 13 N F
8431 14850 8426 14849 8430 14847 8434 14849 8438 14850 8442 14851
8446 14853 8450 14856 8453 14859 8456 14862 8458 14866 8461 14869
8464 14872 8467 14875 8470 14878 8473 14881 8475 14886 8471 14884
8467 14882 8463 14881 8460 14878 8456 14877 8455 14873 8452 14870
8448 14868 8445 14865 8441 14862 8437 14860 8437 14856 8434 14853
8431 14850 31 N F
8628 7037 8623 7035 8627 7034 8632 7033 8634 7029 8635 7025
8639 7023 8638 7028 8640 7032 8644 7035 8647 7038 8645 7042
8647 7046 8644 7049 8644 7054 8647 7057 8652 7059 8655 7062
8655 7066 8651 7064 8647 7062 8642 7061 8638 7059 8635 7055
8631 7055 8631 7050 8634 7046 8636 7042 8636 7038 8632 7035
8628 7037 31 N F
8709 12481 8704 12481 8706 12477 8709 12474 8713 12471 8715 12468
8719 12467 8723 12465 8727 12467 8731 12469 8728 12473 8724 12474
8720 12475 8716 12476 8712 12477 8709 12481 16 N F
8717 7287 8712 7276 8717 7270 8721 7265 8721 7258 8724 7251
8731 7254 8736 7258 8743 7258 8744 7266 8741 7272 8742 7279
8741 7286 8743 7293 8746 7300 8748 7306 8747 7313 8750 7320
8750 7327 8750 7334 8747 7340 8740 7337 8737 7331 8733 7325
8727 7321 8727 7314 8725 7307 8721 7301 8720 7294 8717 7287
30 N F
8750 6092 8749 6089 8751 6087 8753 6088 8755 6089 8757 6090
8757 6093 8755 6093 8753 6094 8751 6094 8750 6092 11 N F
8810 7146 8800 7143 8805 7139 8802 7132 8807 7127 8814 7126

8818 7125 8821 7123 8827 7127 8834 7129 8839 7125 8837 7119
8836 7112 8843 7108 8849 7106 8854 7106 8856 7106 8859 7113
8854 7118 8857 7124 8864 7125 8869 7130 8862 7129 8857 7134
8853 7128 8846 7129 8839 7132 8833 7136 8828 7141 8829 7148
8822 7150 8819 7156 8814 7151 8810 7146 34 N F
8809 5985 8802 5982 8805 5978 8807 5974 8808 5970 8812 5968
8812 5972 8816 5975 8819 5978 8820 5983 8818 5987 8813 5987
8809 5985 13 N F
8837 6191 8828 6183 8835 6185 8842 6186 8844 6186 8849 6186
8851 6193 8852 6200 8856 6206 8855 6213 8855 6220 8861 6224
8866 6228 8866 6228 8859 6229 8854 6234 8848 6229 8846 6222
8846 6215 8846 6208 8841 6203 8846 6198 8844 6191 8837 6191
24 N F
8844 6071 8840 6065 8845 6064 8849 6064 8853 6064 8854 6064
8852 6068 8855 6072 8852 6075 8851 6080 8852 6084 8847 6085
8844 6089 8843 6084 8842 6080 8844 6075 8844 6071 17 N F
8848 7641 8842 7629 8849 7629 8854 7628 8856 7628 8863 7626
8870 7625 8876 7621 8882 7618 8889 7618 8896 7620 8895 7628
8894 7634 8891 7641 8888 7648 8882 7651 8875 7654 8870 7658
8864 7654 8857 7652 8852 7646 8848 7641 22 N F
8854 7627 8847 7624 8850 7622 8854 7619 8857 7616 8862 7616
8866 7618 8871 7618 8875 7620 8871 7623 8867 7624 8863 7625
8858 7626 8854 7627 14 N F
8851 6144 8850 6141 8851 6139 8853 6139 8855 6140 8858 6141
8860 6141 8862 6142 8864 6143 8866 6145 8866 6145 8868 6145
8868 6147 8866 6148 8864 6147 8861 6147 8860 6145 8857 6145
8856 6143 8854 6141 8853 6143 8851 6144 22 N F
8853 6017 8850 6015 8852 6014 8854 6014 8855 6016 8853 6017
6 N F
8856 6010 8855 6006 8857 6007 8859 6009 8860 6010 8860 6012
8857 6012 8856 6010 8 N F
8862 7381 8857 7378 8860 7375 8864 7374 8869 7373 8871 7377
8875 7379 8878 7382 8881 7386 8883 7390 8883 7395 8878 7394
8875 7391 8872 7388 8868 7385 8867 7381 8864 7377 8862 7381
18 N F
8859 6071 8857 6069 8859 6068 8860 6068 8861 6068 8862 6070
8864 6071 8866 6071 8869 6071 8869 6073 8868 6075 8866 6076
8864 6075 8861 6075 8861 6073 8859 6071 16 N F
8872 6178 8867 6171 8873 6167 8879 6163 8882 6157 8889 6155
8896 6156 8903 6159 8902 6167 8907 6172 8912 6176 8913 6176
8919 6180 8924 6185 8931 6185 8938 6185 8940 6184 8945 6182
8942 6189 8940 6196 8939 6203 8935 6209 8930 6204 8928 6211
8922 6208 8920 6201 8918 6208 8915 6201 8911 6195 8911 6202

8912 6210 8905 6210 8901 6203 8897 6197 8896 6190 8892 6196
8889 6203 8883 6198 8880 6192 8876 6186 8883 6189 8885 6182
8878 6182 8884 6179 8891 6179 8898 6179 8896 6172 8890 6168
8886 6162 8882 6168 8876 6172 8872 6178 52 N F
8875 6248 8870 6242 8876 6237 8881 6243 8886 6238 8891 6232
8893 6225 8897 6219 8900 6226 8898 6233 8903 6238 8900 6244
8896 6251 8889 6251 8882 6249 8875 6248 16 N F
8897 7964 8893 7958 8896 7954 8898 7950 8898 7946 8903 7946
8905 7950 8908 7948 8913 7946 8917 7947 8921 7948 8926 7949
8927 7953 8931 7956 8934 7959 8939 7960 8943 7961 8946 7965
8945 7969 8941 7971 8937 7970 8933 7967 8933 7963 8930 7959
8926 7961 8927 7965 8923 7965 8919 7964 8914 7965 8910 7966
8906 7965 8901 7965 8897 7964 33 N F
8895 6290 8894 6287 8896 6284 8897 6281 8898 6284 8900 6287
8903 6288 8906 6290 8907 6293 8904 6295 8902 6298 8899 6295
8898 6292 8895 6290 14 N F
8903 7392 8902 7388 8905 7388 8908 7389 8910 7390 8913 7391
8913 7393 8911 7395 8909 7398 8906 7397 8904 7395 8903 7392
12 N F
8908 6007 8906 6005 8908 6004 8910 6003 8912 6003 8913 6006
8912 6008 8910 6008 8908 6007 9 N F
8913 7387 8910 7381 8914 7378 8919 7377 8923 7377 8928 7379
8932 7380 8936 7378 8940 7381 8941 7385 8939 7389 8937 7393
8934 7390 8930 7389 8925 7387 8922 7385 8918 7387 8913 7387
18 N F
8921 7292 8918 7285 8924 7288 8931 7291 8938 7290 8943 7295
8942 7288 8942 7281 8935 7282 8932 7276 8937 7271 8933 7265
8938 7259 8943 7255 8946 7254 8950 7252 8955 7247 8961 7242
8967 7241 8971 7247 8967 7253 8961 7257 8964 7264 8970 7267
8976 7271 8981 7276 8988 7277 8994 7282 9001 7283 9005 7278
9012 7282 9013 7289 9005 7289 8999 7291 8993 7294 8987 7299
8985 7306 8985 7313 8987 7320 8993 7324 8999 7328 9006 7330
9010 7324 9017 7326 9021 7332 9025 7338 9018 7338 9011 7338
9004 7340 8999 7345 8993 7342 8995 7348 9000 7354 8997 7360
8990 7364 8985 7368 8978 7366 8972 7369 8968 7375 8968 7382
8972 7387 8976 7394 8969 7393 8963 7390 8957 7385 8955 7378
8948 7382 8944 7376 8941 7370 8934 7370 8930 7364 8926 7358
8927 7351 8930 7345 8937 7347 8941 7352 8949 7353 8951 7346
8950 7339 8946 7333 8946 7326 8946 7319 8944 7312 8950 7308
8956 7312 8958 7319 8964 7314 8971 7313 8971 7306 8966 7302
8960 7298 8953 7299 8948 7304 8941 7305 8934 7306 8929 7301
8922 7299 8921 7292 98 N F
8928 7204 8926 7202 8926 7198 8929 7196 8927 7192 8927 7189

8928 7190 8929 7192 8933 7193 8935 7195 8938 7197 8935 7197
8933 7200 8931 7203 8934 7205 8933 7209 8930 7207 8928 7204
18 N F
8937 7225 8930 7223 8932 7219 8933 7218 8934 7217 8937 7218
8941 7218 8944 7219 8947 7221 8950 7223 8947 7225 8945 7227
8946 7230 8942 7229 8940 7227 8936 7228 8937 7225 17 N F
8938 6013 8936 6010 8937 6009 8936 6006 8938 6005 8939 6003
8940 6005 8940 6007 8942 6009 8943 6011 8942 6013 8940 6014
8938 6013 13 N F
8952 7939 8948 7937 8949 7934 8951 7931 8954 7931 8957 7929
8960 7930 8963 7932 8965 7934 8969 7934 8972 7934 8974 7936
8976 7939 8974 7941 8970 7942 8968 7944 8964 7943 8961 7942
8958 7941 8955 7941 8952 7939 21 N F
9011 13027 9010 13021 9013 13018 9017 13016 9021 13019 9024 13022
9024 13026 9022 13030 9021 13034 9021 13038 9016 13039 9016 13034
9013 13031 9011 13027 14 N F
9033 7673 9031 7668 9035 7667 9040 7665 9043 7668 9048 7668
9051 7671 9053 7671 9055 7672 9057 7676 9059 7681 9055 7682
9050 7681 9048 7677 9044 7675 9042 7679 9037 7678 9039 7682
9036 7685 9035 7681 9032 7677 9033 7673 22 N F
9046 7575 9042 7573 9044 7570 9046 7568 9050 7569 9052 7571
9055 7573 9053 7576 9050 7576 9046 7575 10 N F
9059 7703 9059 7699 9062 7696 9062 7700 9062 7705 9065 7709
9067 7710 9069 7711 9073 7711 9078 7709 9081 7706 9084 7710
9082 7714 9079 7717 9075 7721 9071 7720 9068 7717 9064 7715
9060 7712 9060 7708 9059 7703 21 N F
9065 7564 9062 7557 9066 7559 9070 7561 9073 7565 9076 7568
9072 7567 9067 7568 9065 7564 9 N F
9088 7876 9084 7869 9089 7868 9093 7869 9095 7873 9099 7872
9104 7873 9104 7878 9104 7882 9105 7887 9100 7887 9099 7883
9095 7885 9091 7884 9087 7881 9088 7876 16 N F
9122 6147 9121 6141 9122 6137 9122 6132 9123 6128 9124 6123
9126 6119 9126 6123 9125 6128 9124 6133 9124 6137 9126 6141
9127 6146 9128 6150 9129 6155 9130 6159 9133 6163 9135 6167
9138 6171 9137 6175 9135 6171 9132 6167 9130 6163 9128 6159
9125 6156 9123 6151 9122 6147 27 N F
9134 7916 9129 7911 9132 7908 9135 7905 9137 7906 9140 7907
9144 7909 9143 7913 9148 7915 9152 7916 9153 7920 9154 7924
9154 7929 9152 7933 9147 7934 9145 7930 9142 7926 9140 7923
9138 7918 9134 7916 20 N F
9140 5927 M 9136 5913 L 9146 5904 L 9161 5907 L 9153 5895 L
9164 5886 L 9176 5877 L 9190 5881 L 9203 5885 L
9217 5890 L 9220 5876 L 9207 5869 L 9193 5867 L

9200 5854 L 9207 5841 L 9211 5827 L 9211 5813 L
9216 5799 L 9221 5786 L 9235 5782 L 9226 5771 L
9232 5757 L 9237 5744 L 9243 5731 L 9249 5718 L
9255 5704 L 9259 5690 L 9259 5676 L 9255 5662 L
9263 5649 L 9276 5643 L 9280 5629 L 9291 5620 L
9302 5610 L 9289 5616 L 9279 5627 L 9272 5639 L
9277 5626 L 9286 5614 L 9296 5603 L 9306 5593 L
9316 5583 L 9328 5575 L 9328 5575 L 9341 5567 L
9347 5580 L 9353 5567 L 9363 5555 L 9373 5546 L
9386 5539 L 9399 5532 L 9412 5525 L 9424 5518 L
9437 5510 L 9447 5500 L 9454 5488 L 9465 5478 L
9472 5465 L 9475 5455 L 9477 5455 L 9510 5455 L
9544 5455 L 9577 5455 L 9608 5455 L 9607 5465 L
9607 5479 L 9609 5494 L 9614 5507 L 9617 5521 L
9622 5535 L 9628 5548 L 9632 5562 L 9635 5576 L
9638 5591 L 9643 5604 L 9646 5619 L 9652 5632 L
9662 5642 L 9665 5657 L 9667 5671 L 9673 5684 L
9685 5693 L 9695 5703 L 9707 5711 L 9720 5718 L
9730 5728 L 9741 5738 L 9751 5749 L 9754 5763 L
9758 5776 L 9753 5790 L 9760 5803 L 9773 5808 L
9784 5818 L 9791 5831 L 9798 5844 L 9802 5858 L
9812 5868 L 9819 5880 L 9824 5894 L 9830 5907 L
9833 5921 L 9838 5935 L 9848 5945 L 9858 5956 L
9859 5970 L 9863 5984 L 9857 5998 L 9859 6012 L
9865 6025 L 9868 6039 L 9872 6053 L 9878 6066 L
9889 6075 L 9891 6089 L 9893 6104 L 9895 6118 L
9899 6132 L 9904 6145 L 9906 6160 L 9911 6173 L
9906 6187 L 9907 6201 L 9910 6215 L 9916 6228 L
9917 6243 L 9917 6257 L 9918 6260 L 9923 6270 L
9924 6285 L 9925 6299 L 9928 6313 L 9934 6327 L
9934 6341 L 9934 6355 L 9940 6369 L 9944 6382 L
9947 6397 L 9949 6411 L 9949 6425 L 9950 6440 L
9951 6454 L 9960 6465 L 9968 6477 L 9960 6489 L
9953 6502 L 9950 6516 L 9955 6530 L 9965 6539 L
9974 6551 L 9973 6565 L 9970 6579 L 9977 6592 L
9989 6601 L 9997 6612 L 10009 6621 L 10014 6634 L
10021 6647 L 10027 6660 L 10026 6674 L 10030 6688 L
10032 6702 L 10034 6716 L 10040 6729 L 10047 6742 L
10058 6751 L 10056 6765 L 10061 6779 L 10073 6787 L
10070 6801 L 10071 6815 L 10068 6829 L 10059 6840 L
10054 6853 L 10050 6867 L 10048 6881 L 10043 6895 L
10039 6909 L 10038 6923 L 10038 6937 L 10039 6951 L
10042 6965 L 10046 6979 L 10054 6991 L 10064 7001 L

10069 7015 L 10080 7024 L 10089 7035 L 10101 7042 L
10104 7044 L 10114 7049 L 10125 7058 L 10117 7070 L
10103 7068 L 10090 7075 L 10089 7090 L 10081 7101 L
10084 7116 L 10073 7125 L 10059 7124 L 10045 7126 L
10031 7122 L 10020 7131 L 10007 7137 L 9994 7131 L
9984 7141 L 9971 7147 L 9960 7156 L 9964 7169 L
9951 7165 L 9940 7174 L 9931 7185 L 9928 7199 L
9916 7207 L 9903 7214 L 9889 7213 L 9875 7209 L
9862 7205 L 9849 7197 L 9840 7186 L 9832 7175 L
9825 7162 L 9817 7150 L 9828 7141 L 9815 7136 L
9806 7126 L 9793 7119 L 9780 7113 L 9766 7111 L
9752 7107 L 9738 7109 L 9724 7103 L 9711 7098 L
9699 7090 L 9687 7083 L 9673 7079 L 9660 7074 L
9649 7065 L 9636 7058 L 9626 7048 L 9618 7033 L
9616 7018 L 9607 7007 L 9599 6995 L 9592 6983 L
9586 6970 L 9582 6956 L 9573 6945 L 9565 6934 L
9571 6921 L 9567 6907 L 9563 6893 L 9554 6882 L
9543 6873 L 9540 6859 L 9529 6849 L 9540 6839 L
9525 6840 L 9511 6838 L 9498 6832 L 9490 6820 L
9496 6807 L 9482 6805 L 9469 6799 L 9464 6785 L
9459 6772 L 9456 6758 L 9452 6744 L 9445 6731 L
9438 6719 L 9427 6709 L 9419 6698 L 9409 6687 L
9404 6674 L 9397 6661 L 9392 6648 L 9386 6634 L
9384 6620 L 9375 6608 L 9371 6595 L 9364 6582 L
9356 6570 L 9368 6562 L 9354 6562 L 9347 6550 L
9340 6537 L 9332 6525 L 9329 6511 L 9325 6497 L
9316 6486 L 9306 6476 L 9296 6465 L 9289 6452 L
9280 6441 L 9274 6429 L 9274 6414 L 9267 6401 L
9262 6388 L 9257 6374 L 9247 6364 L 9256 6353 L
9271 6351 L 9285 6349 L 9299 6352 L 9288 6343 L
9274 6340 L 9259 6339 L 9245 6342 L 9231 6341 L
9222 6329 L 9214 6318 L 9212 6304 L 9210 6289 L
9207 6275 L 9195 6267 L 9189 6254 L 9183 6241 L
9181 6226 L 9180 6212 L 9187 6199 L 9187 6185 L
9193 6172 L 9181 6164 L 9191 6153 L 9188 6139 L
9199 6130 L 9188 6120 L 9192 6106 L 9196 6092 L
9195 6078 L 9192 6064 L 9199 6051 L 9185 6053 L
9183 6039 L 9175 6027 L 9177 6013 L 9166 6004 L
9179 5999 L 9165 5996 L 9164 5982 L 9164 5968 L
9155 5958 L 9169 5954 L 9174 5940 L 9169 5926 L
9158 5917 L 9144 5914 L 9140 5927 L N E
9170 14242 9167 14237 9169 14233 9173 14230 9176 14227 9179 14225
9182 14228 9182 14233 9181 14237 9182 14241 9178 14244 9174 14244

9170 14242 13 N F
9198 15606 9196 15599 9199 15596 9203 15598 9206 15601 9209 15604
9210 15604 9214 15604 9218 15605 9219 15609 9219 15613 9219 15618
9215 15620 9211 15622 9209 15618 9205 15618 9204 15614 9205 15610
9201 15610 9198 15606 20 N F
9209 14252 9205 14249 9207 14246 9210 14243 9212 14239 9216 14236
9220 14236 9223 14233 9226 14237 9225 14241 9224 14245 9221 14249
9217 14248 9214 14251 9209 14252 15 N F
9225 14211 9221 14208 9225 14206 9229 14206 9232 14203 9235 14199
9238 14197 9240 14201 9239 14205 9238 14209 9240 14213 9238 14217
9236 14220 9233 14224 9232 14228 9228 14231 9225 14228 9224 14224
9224 14220 9225 14216 9225 14211 21 N F
9225 14355 9224 14350 9225 14344 9228 14341 9228 14341 9232 14343
9236 14342 9240 14342 9243 14346 9240 14349 9235 14350 9231 14350
9232 14354 9230 14358 9228 14362 9225 14359 9225 14355 17 N F
9227 8137 9225 8132 9227 8128 9230 8124 9234 8122 9238 8119
9242 8117 9246 8117 9250 8119 9254 8121 9259 8122 9263 8123
9259 8125 9255 8127 9254 8131 9252 8135 9250 8139 9246 8140
9243 8143 9238 8144 9234 8143 9230 8140 9227 8137 23 N F
9251 14419 9247 14415 9251 14412 9254 14414 9256 14418 9260 14419
9264 14420 9266 14424 9266 14428 9262 14430 9258 14429 9254 14427
9252 14424 9251 14419 14 N F
9291 14459 9287 14456 9289 14453 9291 14450 9294 14449 9295 14446
9296 14449 9295 14452 9297 14454 9294 14454 9294 14457 9291 14459
12 N F
9291 14471 9289 14466 9291 14467 9292 14467 9295 14466 9298 14468
9301 14467 9301 14470 9299 14472 9298 14475 9295 14475 9294 14472
9291 14471 13 N F
9312 7816 9309 7812 9312 7813 9313 7810 9316 7812 9318 7815
9316 7817 9313 7819 9312 7816 9 N F
9311 14277 9309 14273 9311 14271 9315 14270 9317 14272 9317 14275
9314 14277 9311 14277 8 N F
9318 5482 9316 5477 9320 5475 9324 5477 9327 5481 9330 5484
9334 5487 9335 5491 9332 5494 9328 5495 9324 5493 9320 5490
9321 5486 9318 5482 14 N F
9318 7811 9316 7804 9320 7803 9324 7805 9327 7809 9331 7808
9329 7812 9326 7815 9322 7814 9318 7811 10 N F
9413 15102 9403 15101 9405 15094 9404 15088 9410 15085 9417 15083
9423 15085 9430 15084 9434 15079 9436 15073 9440 15079 9445 15082
9449 15088 9454 15092 9457 15092 9461 15093 9468 15093 9473 15089
9479 15086 9486 15085 9493 15088 9497 15093 9502 15098 9508 15099
9511 15105 9515 15111 9522 15109 9527 15113 9530 15119 9532 15126
9531 15132 9531 15139 9527 15145 9526 15151 9519 15153 9513 15156

9507 15159 9501 15162 9495 15162 9499 15157 9499 15150 9496 15145
9489 15142 9483 15142 9478 15137 9472 15134 9465 15134 9458 15135
9452 15133 9446 15131 9439 15130 9433 15134 9431 15128 9431 15121
9426 15117 9420 15113 9417 15107 9413 15102 58 N F
9421 15022 9416 15017 9422 15014 9429 15012 9433 15017 9435 15024
9440 15028 9445 15033 9449 15039 9450 15045 9456 15049 9462 15049
9469 15049 9471 15042 9467 15038 9462 15032 9458 15027 9455 15021
9452 15015 9459 15013 9465 15011 9472 15010 9477 15006 9484 15007
9490 15010 9490 15017 9494 15022 9497 15029 9497 15035 9494 15042
9488 15046 9482 15049 9489 15048 9496 15048 9502 15050 9500 15059
9497 15065 9491 15067 9485 15069 9478 15071 9472 15070 9466 15066
9462 15071 9457 15075 9450 15074 9446 15068 9443 15062 9443 15055
9439 15050 9432 15049 9425 15049 9423 15042 9425 15036 9419 15035
9422 15029 9421 15022 56 N F
9417 8745 9417 8741 9418 8738 9420 8735 9422 8731 9426 8730
9428 8728 9430 8725 9433 8723 9436 8722 9439 8721 9442 8723
9442 8727 9442 8730 9439 8732 9438 8735 9436 8738 9433 8740
9430 8741 9427 8742 9424 8743 9420 8744 9417 8745 23 N F
9476 8719 9470 8718 9474 8717 9476 8715 9479 8713 9482 8711
9485 8709 9487 8707 9490 8706 9494 8704 9497 8703 9500 8704
9503 8705 9506 8707 9508 8710 9510 8712 9510 8716 9509 8719
9506 8720 9502 8721 9499 8722 9496 8722 9492 8722 9489 8721
9486 8721 9482 8721 9479 8721 9476 8719 28 N F
9546 10862 9538 10853 9542 10848 9548 10843 9553 10839 9558 10834
9562 10828 9566 10823 9571 10818 9578 10821 9584 10818 9590 10815
9595 10810 9600 10806 9606 10802 9611 10797 9616 10792 9621 10787
9626 10783 9632 10780 9638 10776 9645 10774 9649 10768 9655 10765
9662 10764 9668 10762 9673 10757 9680 10755 9686 10752 9692 10749
9699 10747 9705 10745 9711 10740 9717 10737 9723 10734 9728 10729
9735 10728 9742 10726 9747 10722 9753 10718 9756 10717 9760 10716
9767 10715 9774 10716 9780 10715 9787 10713 9794 10711 9800 10708
9806 10706 9813 10702 9819 10699 9825 10697 9832 10695 9838 10692
9845 10691 9852 10689 9859 10688 9860 10688 9866 10688 9872 10691
9878 10694 9881 10700 9885 10706 9891 10710 9892 10717 9893 10723
9894 10730 9892 10737 9886 10740 9880 10745 9874 10747 9867 10748
9860 10748 9853 10749 9847 10752 9840 10753 9834 10757 9828 10760
9823 10765 9817 10768 9811 10771 9805 10775 9798 10777 9792 10779
9785 10781 9779 10783 9772 10785 9765 10787 9758 10789 9753 10792
9746 10795 9742 10801 9739 10807 9734 10812 9728 10816 9723 10821
9717 10823 9710 10826 9704 10828 9698 10832 9691 10834 9685 10836
9678 10838 9672 10842 9668 10847 9665 10853 9662 10860 9658 10865
9655 10871 9650 10876 9645 10881 9639 10885 9633 10888 9626 10890
9619 10891 9613 10893 9606 10893 9599 10892 9592 10891 9585 10889

9579 10887 9572 10884 9567 10879 9562 10875 9557 10870 9551 10866
9546 10862 127 N F
9561 8653 9559 8649 9561 8646 9564 8649 9568 8651 9572 8653
9576 8655 9579 8652 9583 8650 9583 8655 9585 8656 9587 8657
9590 8661 9593 8664 9598 8664 9594 8666 9590 8664 9586 8663
9582 8661 9577 8662 9575 8665 9571 8669 9567 8669 9567 8665
9563 8662 9562 8658 9561 8653 27 N F
9569 8713 9567 8708 9572 8710 9576 8711 9581 8711 9585 8713
9589 8715 9593 8713 9592 8709 9593 8705 9597 8707 9601 8708
9606 8709 9610 8711 9614 8712 9617 8715 9622 8717 9618 8719
9614 8722 9611 8725 9608 8728 9605 8730 9601 8728 9596 8728
9592 8729 9588 8726 9584 8725 9585 8729 9580 8729 9579 8724
9576 8722 9572 8720 9568 8717 9569 8713 34 N F
9585 8438 9580 8435 9583 8431 9587 8429 9590 8426 9591 8430
9590 8434 9594 8432 9598 8431 9602 8429 9607 8429 9610 8426
9610 8430 9607 8434 9603 8435 9600 8439 9597 8442 9592 8444
9589 8447 9585 8447 9588 8444 9592 8442 9587 8442 9585 8438
24 N F
9589 8616 9586 8614 9589 8613 9592 8612 9594 8613 9597 8615
9598 8617 9597 8619 9596 8621 9593 8623 9591 8621 9590 8619
9589 8616 13 N F
9596 8642 9593 8637 9598 8636 9602 8635 9607 8636 9611 8635
9615 8637 9619 8637 9622 8641 9626 8638 9630 8637 9634 8635
9638 8636 9640 8640 9639 8644 9639 8649 9635 8651 9639 8654
9641 8649 9644 8653 9647 8656 9644 8660 9645 8664 9641 8665
9639 8661 9634 8659 9630 8660 9626 8658 9629 8654 9627 8650
9623 8650 9619 8649 9616 8653 9613 8656 9611 8652 9607 8649
9604 8646 9600 8644 9596 8642 39 N F
9618 8816 9608 8814 9611 8807 9611 8800 9614 8794 9618 8788
9623 8783 9622 8776 9616 8773 9622 8769 9621 8762 9627 8764
9634 8764 9641 8767 9641 8768 9647 8770 9654 8772 9653 8779
9660 8777 9665 8772 9668 8779 9662 8783 9664 8790 9670 8786
9676 8789 9678 8783 9685 8784 9681 8790 9687 8793 9688 8800
9681 8800 9675 8804 9671 8810 9672 8817 9670 8823 9667 8830
9670 8837 9674 8842 9681 8843 9679 8849 9673 8854 9667 8857
9661 8861 9654 8860 9648 8858 9643 8852 9642 8845 9644 8839
9638 8835 9633 8829 9628 8825 9622 8821 9618 8816 53 N F
9633 8735 9631 8731 9634 8728 9636 8723 9640 8722 9641 8723
9643 8726 9647 8729 9651 8731 9655 8732 9658 8733 9659 8734
9663 8736 9659 8736 9654 8736 9650 8738 9646 8738 9641 8739
9637 8737 9633 8735 20 N F
9652 8530 9649 8527 9651 8527 9652 8525 9654 8525 9656 8526
9658 8527 9659 8529 9661 8530 9661 8531 9662 8532 9663 8534

9662 8536 9660 8535 9658 8536 9656 8534 9656 8531 9654 8530
9652 8530 19 N F
9657 8524 9655 8522 9657 8520 9659 8522 9660 8523 9661 8525
9661 8528 9659 8528 9657 8526 9657 8524 10 N F
9672 14755 9668 14752 9669 14749 9669 14745 9671 14743 9674 14744
9676 14747 9676 14750 9674 14753 9675 14756 9675 14759 9672 14760
9670 14757 9672 14755 14 N F
9756 10676 9747 10671 9753 10668 9759 10666 9765 10662 9772 10659
9777 10654 9783 10651 9789 10648 9795 10645 9800 10640 9806 10637
9813 10634 9818 10630 9825 10627 9828 10621 9832 10616 9836 10610
9842 10606 9849 10608 9849 10615 9848 10622 9845 10628 9841 10634
9836 10639 9832 10644 9826 10648 9821 10653 9815 10655 9809 10659
9803 10662 9796 10664 9790 10667 9783 10668 9777 10671 9770 10672
9763 10674 9756 10676 38 N F
9792 8796 9791 8793 9791 8789 9794 8790 9797 8792 9798 8795
9800 8798 9803 8798 9806 8800 9807 8803 9806 8806 9802 8805
9800 8802 9798 8800 9795 8798 9792 8796 16 N F
9795 9396 9793 9394 9796 9392 9799 9390 9801 9388 9805 9388
9808 9389 9811 9389 9815 9390 9816 9393 9815 9397 9814 9399
9810 9400 9808 9398 9808 9395 9805 9393 9801 9394 9799 9396
9795 9396 19 N F
9831 9043 9829 9038 9831 9035 9834 9034 9837 9032 9840 9030
9842 9033 9841 9036 9842 9038 9843 9039 9846 9039 9846 9043
9843 9044 9843 9047 9842 9050 9840 9053 9837 9054 9834 9053
9833 9050 9832 9047 9831 9043 21 N F
9842 9027 9838 9024 9839 9021 9842 9019 9845 9018 9848 9020
9850 9022 9849 9025 9845 9026 9842 9027 10 N F
9860 10641 9851 10640 9854 10634 9857 10628 9859 10621 9860 10614
9862 10608 9867 10603 9873 10600 9879 10596 9885 10598 9889 10604
9893 10610 9896 10616 9894 10623 9891 10629 9885 10633 9879 10636
9873 10639 9866 10639 9860 10641 21 N F
9877 14932 9866 14927 9870 14922 9869 14915 9874 14910 9879 14906
9885 14910 9888 14916 9890 14922 9895 14918 9901 14917 9901 14923
9899 14930 9896 14936 9898 14942 9905 14945 9898 14945 9893 14940
9887 14938 9880 14938 9877 14932 21 N F
9913 5703 9905 5701 9908 5697 9910 5691 9913 5686 9919 5684
9922 5679 9928 5680 9927 5686 9928 5691 9930 5697 9929 5703
9924 5705 9918 5705 9913 5703 15 N F
9908 9393 9905 9386 9907 9382 9907 9382 9912 9382 9916 9382
9920 9380 9923 9379 9924 9379 9928 9376 9930 9372 9934 9369
9938 9370 9942 9369 9946 9371 9950 9373 9948 9377 9952 9377
9953 9379 9955 9381 9958 9384 9955 9387 9950 9388 9947 9390
9948 9395 9946 9398 9942 9396 9937 9396 9933 9395 9929 9395

9924 9396 9921 9399 9917 9398 9913 9396 9910 9399 9906 9397
9908 9393 37 N F
9908 9147 9906 9143 9909 9142 9912 9141 9915 9139 9917 9136
9920 9136 9923 9138 9923 9142 9924 9145 9926 9147 9928 9150
9925 9150 9923 9147 9920 9145 9918 9143 9914 9142 9912 9145
9913 9148 9910 9149 9907 9150 9908 9147 22 N F
9911 14976 9907 14974 9911 14974 9914 14972 9916 14971 9916 14971
9918 14968 9921 14969 9925 14969 9922 14972 9920 14973 9916 14973
9915 14976 9911 14976 14 N F
9911 14954 9908 14951 9912 14951 9914 14949 9917 14951 9920 14952
9921 14955 9919 14958 9916 14958 9914 14956 9911 14954 11 N F
9915 9365 9909 9359 9913 9358 9918 9358 9917 9354 9921 9353
9924 9350 9928 9347 9931 9350 9931 9354 9933 9358 9930 9362
9933 9365 9931 9368 9927 9371 9923 9372 9918 9372 9915 9369
9915 9365 19 N F
9913 9882 9909 9875 9914 9870 9916 9863 9922 9859 9929 9860
9934 9865 9940 9869 9945 9873 9951 9877 9953 9883 9951 9890
9949 9897 9948 9903 9944 9909 9937 9912 9934 9918 9930 9924
9923 9924 9916 9922 9915 9916 9912 9909 9910 9903 9910 9896
9912 9889 9913 9882 26 N F
9944 14960 9939 14957 9942 14955 9944 14953 9947 14953 9950 14952
9953 14953 9956 14953 9959 14953 9961 14956 9963 14958 9960 14960
9957 14959 9954 14959 9951 14960 9948 14960 9944 14960 17 N F
9946 14994 9940 14993 9943 14990 9944 14989 9946 14988 9951 14987
9955 14987 9958 14984 9962 14982 9966 14981 9971 14980 9975 14979
9974 14974 9977 14971 9979 14975 9979 14979 9983 14980 9987 14980
9989 14980 9992 14979 9996 14979 10000 14979 10005 14978 10009 14977
10012 14974 10015 14977 10018 14980 10022 14983 10017 14982 10013 14984
10009 14985 10005 14987 10003 14990 9999 14991 9996 14988 9992 14986
9988 14985 9984 14985 9979 14986 9975 14987 9972 14990 9969 14993
9969 14997 9966 14994 9964 14990 9960 14993 9960 14997 9956 14997
9953 14994 9950 14997 9946 14994 51 N F
9943 9192 9940 9190 9942 9187 9944 9190 9947 9192 9950 9194
9952 9196 9952 9199 9952 9203 9954 9205 9958 9204 9956 9207
9956 9210 9953 9213 9950 9213 9947 9211 9946 9208 9947 9204
9947 9201 9943 9201 9945 9198 9945 9194 9943 9192 23 N F
9943 9934 9940 9928 9944 9926 9948 9925 9952 9923 9950 9918
9955 9917 9959 9917 9959 9921 9961 9926 9963 9929 9962 9934
9965 9937 9961 9940 9958 9943 9954 9942 9950 9940 9946 9938
9943 9934 19 N F
9949 9171 9947 9166 9950 9166 9952 9163 9956 9163 9959 9165
9958 9168 9956 9170 9958 9172 9961 9175 9963 9177 9966 9178
9969 9179 9970 9182 9973 9183 9976 9183 9973 9184 9970 9185

9967 9183 9965 9180 9962 9178 9959 9179 9956 9177 9953 9176
9949 9176 9947 9174 9949 9171 27 N F
9951 9397 9949 9390 9953 9390 9957 9388 9959 9384 9962 9380
9967 9380 9970 9377 9970 9372 9970 9368 9968 9364 9965 9360
9970 9362 9970 9362 9974 9362 9978 9362 9979 9362 9983 9360
9982 9356 9980 9351 9985 9350 9988 9348 9990 9350 9992 9351
9996 9352 9994 9356 9994 9361 9995 9365 9999 9367 10003 9369
10000 9373 9996 9373 9998 9376 10002 9379 10005 9382 10004 9387
10002 9391 10002 9395 9999 9399 10002 9402 10001 9407 9998 9410
9994 9412 9990 9414 9988 9418 9985 9422 9980 9422 9977 9420
9976 9415 9972 9413 9968 9410 9965 9408 9961 9405 9957 9403
9953 9401 9951 9397 56 N F
9958 9975 9952 9972 9955 9966 9958 9963 9960 9961 9966 9957
9969 9951 9972 9944 9972 9943 9976 9939 9977 9932 9979 9925
9977 9918 9975 9912 9978 9906 9984 9902 9988 9896 9993 9891
9996 9885 9998 9878 10004 9875 10008 9878 10010 9879 10015 9884
10021 9886 10022 9886 10029 9886 10035 9886 10042 9883 10044 9880
10045 9877 10052 9877 10059 9878 10066 9879 10067 9879 10073 9877
10079 9874 10085 9871 10092 9869 10099 9867 10105 9865 10111 9861
10117 9857 10118 9857 10123 9854 10130 9855 10137 9856 10143 9859
10140 9865 10139 9872 10141 9879 10144 9885 10147 9891 10152 9896
10146 9899 10142 9905 10137 9909 10132 9914 10127 9919 10122 9923
10115 9922 10108 9923 10104 9929 10097 9932 10096 9938 10089 9938
10082 9938 10076 9941 10073 9947 10066 9946 10059 9947 10052 9948
10048 9954 10044 9959 10040 9965 10034 9969 10027 9971 10022 9967
10015 9964 10009 9962 10002 9960 9996 9962 9991 9967 9984 9961
9978 9965 9971 9966 9971 9973 9966 9978 9960 9981 9958 9975
90 N F
9958 10053 9956 10050 9957 10047 9958 10043 9960 10046 9960 10046
9963 10048 9966 10050 9966 10053 9965 10056 9967 10058 9966 10061
9968 10063 9970 10066 9971 10069 9969 10071 9966 10069 9966 10066
9964 10064 9962 10066 9960 10063 9961 10059 9960 10056 9958 10053
24 N F
9958 9834 9956 9826 9959 9823 9962 9820 9967 9819 9971 9820
9975 9821 9980 9822 9984 9824 9988 9826 9989 9830 9988 9835
9986 9838 9981 9839 9979 9843 9978 9847 9974 9849 9972 9853
9968 9852 9967 9848 9962 9846 9959 9843 9958 9839 9958 9834
24 N F
9968 9038 9965 9034 9968 9035 9971 9035 9969 9032 9972 9030
9970 9028 9968 9025 9969 9022 9973 9024 9975 9021 9977 9023
9979 9025 9982 9024 9982 9027 9983 9030 9985 9033 9986 9034
9987 9035 9984 9036 9981 9036 9978 9038 9977 9041 9978 9044
9975 9044 9971 9043 9970 9040 9968 9038 28 N F

9969 8999 9965 8996 9968 8997 9971 8998 9973 9001 9976 8999
9978 9001 9981 9003 9984 9004 9986 9006 9988 9009 9986 9012
9983 9011 9979 9011 9977 9009 9973 9008 9971 9005 9971 9002
9969 8999 19 N F
9979 9766 9978 9762 9979 9758 9979 9753 9982 9750 9986 9750
9991 9750 9995 9750 9997 9754 10000 9758 10001 9762 10005 9765
10006 9769 10009 9773 10010 9777 10007 9781 10003 9782 9999 9780
9993 9777 9989 9774 9986 9772 9982 9769 9979 9766 23 N F
9984 9162 9980 9158 9982 9156 9983 9153 9986 9154 9988 9157
9991 9158 9993 9161 9990 9163 9987 9162 9984 9162 11 N F
9984 14951 9981 14948 9983 14946 9986 14948 9989 14948 9991 14951
9991 14954 9989 14957 9988 14954 9984 14955 9984 14951 11 N F
9991 9986 9987 9982 9990 9983 9994 9982 9995 9979 9998 9977
9995 9975 9993 9972 9997 9972 9999 9974 10002 9976 10004 9978
10007 9980 10009 9983 10011 9985 10009 9988 10009 9991 10007 9994
10004 9993 10001 9991 10001 9988 9999 9991 9995 9992 9993 9990
9994 9987 9991 9986 26 N F
9995 10240 9991 10235 9994 10233 9996 10231 9999 10232 10001 10235
10002 10238 10004 10240 10001 10239 9998 10241 9997 10238 9995 10240
12 N F
10001 10212 9996 10209 9998 10207 10001 10207 10003 10204 10005 10201
10007 10202 10008 10202 10010 10205 10010 10208 10012 10211 10015 10212
10012 10213 10009 10214 10006 10214 10004 10211 10001 10212 17 N F
10003 14965 9997 14961 10000 14958 10004 14958 10007 14961 10011 14960
10016 14962 10020 14962 10024 14962 10028 14961 10028 14957 10026 14953
10024 14949 10024 14945 10027 14948 10031 14951 10034 14954 10038 14954
10038 14958 10042 14959 10045 14960 10046 14961 10050 14963 10046 14965
10044 14969 10040 14971 10042 14975 10039 14972 10036 14968 10032 14970
10028 14970 10024 14969 10020 14968 10016 14966 10011 14966 10007 14966
10003 14965 37 N F
10002 9741 9999 9738 10001 9735 10000 9732 10001 9729 10001 9725
10004 9727 10006 9730 10008 9732 10009 9735 10011 9738 10014 9739
10014 9742 10013 9746 10010 9747 10007 9745 10004 9744 10002 9741
18 N F
10003 9857 10002 9853 10003 9850 10003 9846 10006 9845 10009 9845
10012 9847 10013 9850 10015 9853 10015 9856 10014 9859 10011 9861
10008 9861 10006 9859 10003 9857 15 N F
10018 9415 10017 9408 10021 9409 10025 9409 10027 9413 10030 9417
10033 9414 10038 9414 10041 9417 10044 9420 10040 9422 10035 9421
10031 9423 10032 9427 10036 9429 10033 9432 10036 9436 10031 9436
10028 9439 10026 9435 10027 9431 10025 9427 10028 9425 10024 9423
10022 9419 10017 9419 10018 9415 27 N F
10023 9736 10018 9724 10025 9724 10028 9729 10028 9730 10035 9730

10041 9725 10048 9725 10049 9719 10055 9715 10061 9712 10061 9705
10061 9698 10068 9698 10070 9705 10073 9699 10079 9702 10081 9695
10088 9694 10091 9700 10092 9707 10096 9713 10092 9718 10089 9725
10083 9727 10078 9732 10071 9734 10065 9738 10061 9743 10057 9748
10058 9755 10056 9762 10052 9767 10047 9763 10040 9760 10033 9760
10028 9756 10027 9749 10026 9742 10023 9736 40 N F
10023 10051 10018 10042 10024 10039 10031 10036 10036 10032 10042 10029
10048 10025 10055 10023 10062 10023 10064 10023 10069 10023 10072 10022
10075 10021 10082 10018 10088 10016 10086 10023 10083 10029 10088 10034
10090 10041 10086 10047 10090 10052 10095 10057 10088 10057 10084 10062
10085 10069 10079 10072 10072 10070 10066 10073 10065 10066 10059 10063
10052 10062 10047 10057 10040 10059 10033 10059 10029 10054 10023 10051
36 N F
10021 9880 10020 9877 10021 9874 10020 9871 10022 9868 10025 9869
10028 9871 10031 9872 10029 9875 10027 9878 10025 9880 10021 9880
12 N F
10041 14863 10036 14860 10039 14857 10043 14854 10039 14852 10042 14849
10046 14847 10050 14846 10054 14845 10058 14843 10062 14846 10061 14850
10057 14851 10056 14855 10053 14858 10049 14856 10046 14859 10045 14864
10041 14863 19 N F
10040 9868 10039 9864 10041 9860 10044 9861 10047 9862 10047 9865
10048 9868 10047 9871 10044 9872 10041 9871 10040 9868 11 N F
10054 14965 10051 14964 10054 14963 10057 14963 10059 14965 10057 14967
10054 14965 7 N F
10056 9862 10051 9858 10052 9857 10053 9855 10057 9855 10060 9855
10063 9854 10066 9856 10068 9858 10067 9861 10065 9864 10062 9865
10059 9864 10056 9862 14 N F
10053 9987 10052 9977 10055 9970 10059 9965 10066 9967 10071 9973
10072 9980 10078 9982 10083 9987 10077 9991 10070 9994 10064 9996
10058 9992 10053 9987 14 N F
10059 9871 10054 9867 10057 9866 10060 9868 10062 9867 10063 9867
10067 9868 10070 9866 10072 9863 10074 9861 10077 9862 10078 9866
10077 9869 10074 9871 10071 9873 10069 9876 10067 9877 10064 9876
10062 9873 10059 9871 20 N F
10062 14963 10057 14960 10060 14959 10062 14957 10064 14959 10067 14960
10071 14959 10073 14961 10071 14963 10068 14963 10065 14962 10062 14963
12 N F
10069 10014 10065 10010 10066 10008 10069 10007 10071 10005 10074 10004
10076 10005 10077 10008 10077 10011 10075 10012 10072 10014 10069 10014
12 N F
10072 14881 10067 14880 10069 14878 10072 14876 10075 14878 10078 14878
10081 14876 10083 14879 10080 14881 10078 14883 10075 14881 10072 14881
12 N F

10084 14868 10083 14863 10085 14861 10088 14863 10091 14864 10093 14867
10091 14870 10088 14871 10087 14868 10084 14868 10 N F
10091 9564 10089 9560 10090 9557 10093 9559 10097 9558 10099 9561
10101 9563 10102 9566 10105 9568 10108 9569 10111 9570 10110 9573
10107 9572 10104 9574 10101 9571 10098 9570 10098 9567 10097 9564
10094 9562 10091 9564 20 N F
10096 9724 10094 9721 10097 9720 10100 9718 10103 9717 10104 9717
10106 9715 10109 9713 10112 9711 10114 9709 10115 9712 10119 9713
10122 9712 10125 9712 10127 9710 10130 9707 10130 9711 10132 9714
10129 9715 10125 9715 10122 9714 10119 9716 10116 9717 10113 9718
10110 9720 10107 9720 10106 9724 10104 9726 10101 9726 10099 9728
10095 9728 10096 9724 32 N F
10096 10177 10095 10173 10098 10172 10101 10173 10102 10176 10104 10179
10101 10181 10098 10179 10096 10177 9 N F
10100 6965 10096 6965 10098 6964 10099 6962 10101 6961 10103 6960
10105 6961 10108 6961 10110 6960 10112 6961 10112 6963 10111 6964
10109 6966 10108 6968 10106 6969 10104 6970 10102 6969 10101 6967
10100 6965 19 N F
10102 9763 10097 9758 10099 9757 10101 9757 10104 9753 10106 9749
10109 9746 10112 9743 10116 9741 10120 9738 10124 9739 10128 9738
10132 9736 10135 9739 10140 9739 10144 9739 10145 9743 10140 9742
10140 9747 10136 9748 10132 9745 10128 9746 10123 9746 10119 9748
10116 9751 10112 9754 10115 9757 10117 9761 10113 9763 10110 9766
10105 9766 10102 9763 32 N F
10103 14875 10097 14877 10099 14875 10101 14872 10102 14869 10105 14868
10105 14868 10107 14870 10108 14873 10106 14875 10103 14875 11 N F
10104 9800 10099 9792 10105 9789 10110 9785 10110 9785 10114 9779
10119 9774 10125 9770 10130 9765 10137 9764 10142 9760 10149 9763
10145 9769 10141 9775 10142 9782 10136 9786 10142 9790 10137 9795
10131 9798 10124 9800 10118 9802 10111 9800 10104 9800 23 N F
10103 10245 10100 10242 10101 10241 10103 10241 10105 10242 10106 10243
10109 10245 10108 10248 10105 10249 10106 10246 10103 10245 11 N F
10105 10038 10102 10028 10104 10021 10110 10024 10116 10027 10123 10028
10130 10029 10126 10035 10119 10037 10112 10037 10105 10038 11 N F
10116 10148 10109 10149 10111 10143 10110 10136 10115 10131 10113 10124
10120 10121 10126 10118 10133 10117 10131 10124 10138 10125 10143 10129
10149 10125 10156 10126 10163 10124 10169 10124 10170 10124 10176 10120
10181 10120 10183 10120 10180 10127 10174 10130 10180 10134 10175 10140
10169 10142 10162 10142 10155 10143 10153 10150 10146 10149 10150 10143
10143 10144 10136 10146 10129 10148 10123 10146 10116 10148 35 N F
10122 10025 10112 10020 10118 10015 10122 10020 10129 10022 10136 10020
10142 10022 10149 10024 10153 10030 10156 10031 10160 10032 10165 10036
10163 10030 10165 10023 10171 10019 10177 10022 10181 10028 10178 10035

10175 10041 10169 10043 10162 10042 10155 10042 10148 10040 10141 10040
10134 10039 10133 10032 10128 10027 10122 10025 28 N F
10121 15129 10120 15125 10124 15124 10128 15124 10130 15120 10133 15117
10135 15113 10137 15113 10139 15113 10143 15114 10145 15117 10149 15120
10151 15124 10153 15127 10153 15128 10156 15131 10156 15136 10153 15139
10150 15142 10143 15145 10140 15142 10143 15139 10142 15134 10139 15138
10135 15138 10135 15142 10131 15143 10128 15140 10127 15136 10125 15132
10121 15129 31 N F
10127 10178 10124 10175 10127 10175 10130 10173 10133 10171 10136 10171
10137 10174 10137 10177 10137 10180 10137 10184 10134 10182 10134 10178
10130 10177 10127 10178 14 N F
10131 9815 10128 9810 10131 9808 10136 9806 10140 9806 10145 9807
10148 9810 10150 9810 10153 9811 10152 9815 10156 9818 10154 9822
10150 9821 10145 9820 10141 9822 10137 9821 10133 9820 10131 9815
18 N F
10146 7419 10145 7416 10147 7415 10149 7414 10151 7416 10152 7417
10152 7420 10150 7420 10148 7420 10146 7419 10 N F
10151 9859 10147 9852 10152 9848 10154 9845 10156 9842 10161 9838
10167 9834 10168 9832 10170 9828 10175 9822 10169 9818 10171 9811
10172 9804 10177 9799 10184 9798 10190 9802 10186 9807 10183 9814
10186 9821 10192 9824 10198 9827 10198 9834 10197 9841 10190 9842
10184 9845 10191 9845 10197 9847 10193 9852 10192 9859 10187 9863
10181 9859 10174 9857 10167 9859 10160 9858 10156 9864 10151 9859
36 N F
10151 14985 10148 14984 10149 14981 10152 14980 10153 14981 10155 14982
10157 14985 10153 14988 10151 14985 9 N F
10168 7337 10164 7336 10166 7334 10168 7335 10168 7337 5 N F
10170 15093 10168 15088 10170 15084 10173 15081 10176 15080 10177 15080
10181 15078 10181 15082 10181 15086 10186 15087 10183 15090 10179 15091
10174 15091 10170 15093 14 N F
10171 9884 10168 9881 10169 9877 10173 9874 10177 9874 10178 9878
10176 9883 10177 9887 10179 9890 10184 9890 10188 9891 10186 9895
10182 9897 10180 9901 10182 9904 10181 9909 10178 9905 10174 9904
10170 9903 10171 9899 10170 9895 10168 9888 10171 9884 23 N F
10176 10305 10169 10303 10175 10299 10176 10293 10175 10286 10170 10278
10177 10279 10184 10279 10191 10280 10197 10283 10200 10283 10204 10283
10211 10284 10214 10283 10218 10283 10224 10285 10219 10290 10223 10295
10216 10295 10209 10294 10204 10290 10197 10288 10192 10293 10185 10295
10182 10301 10176 10305 26 N F
10195 10133 10191 10128 10195 10128 10198 10130 10201 10129 10201 10126
10204 10123 10205 10124 10207 10124 10209 10127 10206 10129 10203 10131
10203 10135 10200 10137 10197 10136 10195 10133 16 N F
10205 9862 10204 9858 10206 9855 10207 9852 10211 9852 10211 9856

10209 9859 10209 9862 10205 9862 9 N F
10210 10022 10207 10018 10211 10017 10213 10016 10217 10015 10220 10014
10221 10017 10220 10020 10217 10022 10214 10022 10210 10022 11 N F
10223 14895 10218 14889 10223 14890 10228 14888 10230 14883 10226 14885
10222 14881 10222 14875 10222 14870 10223 14865 10228 14862 10232 14867
10235 14870 10237 14871 10240 14873 10244 14877 10244 14882 10241 14886
10238 14891 10235 14896 10235 14901 10233 14896 10228 14893 10223 14895
24 N F
10231 10264 10230 10257 10234 10256 10238 10254 10243 10254 10240 10258
10244 10260 10247 10263 10244 10266 10241 10269 10237 10271 10234 10267
10231 10264 13 N F
10257 10356 10253 10352 10256 10352 10260 10352 10262 10351 10265 10350
10266 10353 10263 10355 10260 10355 10257 10356 10 N F
10270 10354 10269 10351 10272 10349 10274 10351 10277 10353 10275 10355
10272 10357 10270 10354 8 N F
10285 10371 10281 10369 10284 10368 10286 10366 10290 10366 10290 10366
10293 10366 10295 10369 10298 10369 10299 10372 10295 10373 10292 10371
10290 10373 10287 10375 10284 10377 10284 10373 10285 10371 17 N F
10290 10015 10286 10011 10288 10009 10289 10006 10291 10003 10294 10002
10295 10005 10294 10008 10293 10011 10293 10014 10290 10015 11 N F
10309 10013 10304 10011 10306 10009 10308 10006 10311 10005 10311 10002
10312 9999 10314 10002 10314 10005 10313 10009 10311 10011 10309 10013
12 N F
10321 10015 10317 10011 10319 10008 10321 10005 10324 10003 10324 10007
10322 10010 10323 10013 10321 10015 9 N F
10329 10001 10325 9996 10327 9994 10329 9994 10331 9996 10330 9998
10329 10001 7 N F
10330 10298 10327 10294 10329 10291 10329 10290 10332 10294 10336 10294
10339 10291 10343 10289 10342 10293 10342 10298 10343 10302 10344 10306
10340 10306 10336 10304 10332 10302 10330 10298 16 N F
10340 10341 10334 10339 10337 10336 10340 10338 10340 10338 10343 10340
10340 10341 7 N F
10351 9979 10347 9978 10348 9974 10350 9971 10353 9971 10356 9971
10360 9971 10359 9974 10357 9977 10354 9979 10351 9979 11 N F
10356 10309 10350 10303 10351 10302 10353 10299 10357 10299 10361 10300
10363 10296 10366 10292 10367 10297 10366 10301 10364 10305 10359 10306
10356 10309 13 N F
10430 15272 10429 15268 10433 15268 10435 15270 10438 15270 10441 15272
10445 15271 10448 15272 10446 15274 10444 15277 10441 15278 10438 15277
10436 15275 10433 15274 10430 15272 15 N F
10637 6766 10632 6764 10636 6761 10640 6761 10645 6760 10649 6758
10654 6758 10658 6759 10662 6761 10667 6760 10671 6759 10675 6761
10679 6764 10682 6766 10685 6770 10689 6773 10685 6775 10681 6777

10676 6778 10672 6780 10666 6778 10661 6779 10657 6777 10653 6776
10649 6778 10644 6776 10643 6772 10638 6770 10637 6766 29 N F
10811 15190 10808 15184 10812 15184 10814 15188 10817 15191 10821 15190
10822 15189 10824 15187 10827 15190 10827 15195 10831 15193 10832 15189
10836 15187 10840 15186 10844 15188 10847 15192 10848 15196 10851 15199
10852 15203 10848 15205 10844 15207 10840 15208 10839 15204 10835 15204
10830 15204 10827 15201 10824 15203 10820 15201 10820 15197 10818 15194
10813 15193 10811 15190 32 N F
10812 15136 10811 15131 10814 15131 10813 15128 10816 15125 10819 15126
10822 15126 10824 15129 10824 15133 10821 15135 10820 15138 10820 15141
10817 15139 10815 15137 10812 15136 15 N F
10820 15162 10820 15158 10820 15158 10822 15157 10823 15158 10824 15159
10826 15161 10824 15163 10822 15164 10820 15162 10 N F
10821 15151 10820 15146 10823 15145 10826 15145 10825 15148 10826 15151
10823 15153 10821 15151 8 N F
10848 15183 10846 15179 10848 15175 10846 15170 10850 15167 10853 15170
10857 15171 10857 15176 10859 15179 10856 15182 10858 15186 10854 15188
10850 15186 10848 15183 14 N F
10882 7539 10880 7536 10882 7534 10884 7534 10886 7535 10888 7536
10890 7537 10892 7537 10894 7537 10896 7538 10899 7538 10898 7540
10897 7542 10896 7543 10893 7543 10891 7544 10889 7544 10887 7544
10885 7542 10883 7541 10882 7539 21 N F
10892 14340 10886 14336 10888 14332 10891 14330 10892 14330 10895 14327
10895 14322 10897 14318 10899 14315 10902 14311 10906 14312 10911 14312
10915 14311 10919 14312 10922 14309 10926 14311 10927 14315 10929 14319
10927 14323 10923 14323 10921 14327 10923 14331 10927 14333 10931 14331
10929 14327 10932 14328 10933 14328 10936 14331 10941 14330 10938 14334
10943 14334 10940 14337 10938 14341 10937 14345 10933 14347 10928 14347
10924 14347 10920 14345 10916 14344 10911 14343 10908 14341 10904 14339
10900 14337 10896 14339 10892 14340 45 N F
10908 15175 10903 15169 10908 15169 10911 15173 10914 15175 10918 15175
10922 15177 10925 15180 10927 15184 10931 15186 10934 15189 10935 15194
10939 15194 10938 15198 10942 15199 10946 15198 10948 15200 10949 15201
10952 15204 10951 15209 10946 15208 10942 15207 10945 15211 10941 15212
10944 15215 10940 15216 10942 15220 10939 15222 10938 15226 10941 15230
10945 15229 10945 15233 10941 15235 10938 15238 10933 15238 10929 15239
10926 15236 10922 15234 10926 15231 10925 15227 10921 15225 10924 15222
10925 15218 10921 15215 10917 15213 10916 15209 10911 15210 10908 15207
10905 15203 10904 15199 10908 15201 10912 15202 10916 15203 10920 15202
10918 15198 10913 15197 10915 15193 10919 15192 10920 15187 10918 15183
10914 15184 10909 15184 10909 15179 10908 15175 64 N F
10920 14258 10915 14258 10918 14256 10921 14255 10924 14254 10924 14251
10924 14248 10924 14248 10926 14249 10929 14251 10932 14250 10936 14251

10938 14253 10940 14256 10940 14259 10938 14262 10938 14265 10940 14268
10942 14269 10941 14272 10938 14274 10935 14273 10932 14275 10932 14272
10929 14269 10926 14268 10925 14265 10923 14262 10923 14259 10920 14258
30 N F
10924 7534 10922 7532 10923 7531 10925 7529 10926 7530 10925 7532
10924 7534 7 N F
10934 14642 10928 14640 10931 14637 10935 14636 10939 14637 10937 14641
10938 14645 10942 14642 10946 14642 10949 14645 10952 14642 10956 14640
10959 14638 10963 14636 10967 14634 10972 14634 10972 14638 10975 14635
10979 14635 10982 14638 10979 14642 10977 14646 10975 14649 10971 14651
10971 14656 10967 14656 10963 14656 10959 14658 10955 14660 10951 14661
10948 14664 10947 14660 10944 14657 10942 14653 10938 14652 10933 14651
10933 14647 10934 14642 38 N F
10947 14232 10945 14227 10948 14227 10950 14230 10953 14232 10956 14233
10959 14232 10959 14232 10962 14232 10962 14235 10964 14238 10960 14238
10957 14238 10954 14237 10951 14237 10950 14234 10947 14232 17 N F
10958 7636 10957 7633 10959 7632 10961 7631 10963 7632 10964 7634
10963 7636 10962 7637 10959 7638 10958 7636 10 N F
10969 6638 10966 6636 10968 6636 10971 6637 10973 6638 10975 6639
10973 6640 10970 6640 10969 6638 9 N F
11011 14711 11011 14706 11014 14703 11016 14707 11020 14709 11024 14711
11025 14715 11026 14719 11028 14723 11028 14727 11026 14723 11019 14724
11018 14728 11015 14732 11014 14727 11015 14723 11012 14719 11015 14716
11019 14716 11018 14712 11015 14714 11011 14711 22 N F
11030 6710 11018 6707 11022 6702 11026 6696 11033 6693 11040 6693
11047 6692 11049 6699 11055 6703 11061 6698 11067 6694 11074 6693
11081 6693 11088 6692 11094 6691 11095 6691 11102 6692 11109 6693
11116 6691 11122 6687 11129 6685 11135 6688 11135 6695 11131 6701
11124 6705 11131 6702 11138 6700 11144 6696 11145 6704 11142 6710
11145 6717 11148 6723 11155 6721 11157 6728 11155 6735 11159 6741
11161 6748 11154 6750 11148 6755 11143 6759 11136 6761 11129 6762
11127 6769 11121 6765 11114 6769 11107 6768 11101 6765 11096 6760
11091 6766 11089 6772 11085 6778 11079 6782 11072 6785 11066 6781
11072 6776 11074 6769 11077 6763 11071 6766 11072 6773 11065 6771
11061 6765 11056 6759 11059 6752 11058 6745 11062 6739 11067 6734
11068 6727 11065 6733 11063 6727 11062 6720 11065 6713 11064 6706
11063 6713 11058 6718 11052 6722 11051 6729 11045 6725 11039 6728
11040 6721 11043 6715 11037 6719 11031 6722 11027 6716 11030 6710
84 N F
11059 14266 11058 14263 11061 14261 11064 14262 11067 14261 11070 14261
11071 14264 11072 14265 11073 14267 11074 14270 11077 14268 11078 14271
11078 14274 11079 14277 11079 14281 11076 14280 11073 14280 11069 14280
11066 14279 11069 14277 11072 14276 11071 14273 11071 14269 11068 14270

11065 14270 11062 14272 11062 14269 11059 14266 28 N F
11077 6571 11076 6566 11078 6562 11083 6562 11088 6561 11092 6562
11097 6561 11101 6563 11104 6567 11106 6571 11103 6574 11099 6576
11094 6576 11090 6576 11085 6574 11081 6572 11077 6571 17 N F
11122 14265 11117 14262 11121 14261 11124 14261 11124 14257 11123 14254
11126 14256 11127 14259 11130 14261 11133 14263 11135 14265 11138 14267
11137 14270 11140 14269 11143 14271 11141 14273 11141 14276 11138 14275
11134 14275 11131 14273 11129 14272 11127 14269 11124 14268 11122 14265
24 N F
11157 14243 11152 14241 11156 14241 11159 14239 11161 14237 11163 14235
11166 14236 11163 14238 11165 14241 11163 14243 11160 14244 11157 14243
12 N F
11157 14216 11155 14213 11157 14211 11160 14209 11162 14210 11163 14210
11165 14212 11169 14211 11171 14213 11174 14215 11176 14217 11178 14220
11180 14223 11181 14224 11182 14225 11184 14227 11188 14229 11190 14230
11193 14232 11194 14235 11196 14238 11196 14238 11198 14240 11200 14243
11202 14246 11203 14249 11205 14251 11207 14254 11204 14253 11201 14253
11198 14252 11195 14250 11193 14247 11190 14246 11187 14246 11185 14244
11182 14243 11180 14241 11181 14237 11182 14235 11183 14231 11183 14228
11180 14230 11180 14233 11178 14235 11175 14237 11172 14236 11169 14235
11171 14232 11171 14229 11168 14227 11167 14224 11165 14222 11163 14219
11160 14218 11157 14216 56 N F
11159 6668 11156 6665 11159 6662 11162 6661 11165 6662 11165 6666
11162 6668 11159 6668 8 N F
11166 6676 11164 6670 11168 6671 11170 6674 11172 6676 11173 6680
11170 6681 11168 6679 11166 6676 9 N F
11172 6734 11168 6734 11170 6733 11172 6733 11175 6732 11178 6733
11182 6732 11185 6733 11188 6730 11191 6729 11194 6730 11196 6733
11192 6736 11189 6737 11188 6740 11188 6744 11185 6746 11181 6746
11178 6746 11175 6744 11174 6741 11176 6739 11175 6736 11172 6734
24 N F
11193 6687 11192 6684 11192 6681 11194 6678 11196 6675 11198 6672
11201 6670 11204 6670 11207 6672 11210 6674 11212 6677 11213 6680
11213 6683 11211 6686 11207 6686 11204 6686 11201 6687 11196 6689
11193 6687 19 N F
11229 6801 11225 6794 11231 6789 11237 6791 11237 6791 11243 6796
11250 6797 11255 6792 11259 6786 11264 6781 11263 6773 11260 6767
11253 6765 11249 6759 11254 6754 11261 6752 11268 6750 11273 6745
11280 6741 11287 6740 11293 6743 11298 6748 11299 6755 11306 6754
11312 6750 11319 6753 11322 6759 11323 6766 11323 6773 11323 6780
11324 6787 11323 6795 11319 6801 11323 6807 11319 6813 11319 6820
11323 6826 11328 6831 11333 6837 11333 6844 11338 6849 11342 6855
11345 6861 11343 6868 11347 6874 11352 6879 11357 6884 11358 6891

11357 6898 11358 6905 11351 6905 11344 6906 11343 6899 11342 6892
11338 6886 11334 6880 11331 6873 11336 6869 11333 6862 11332 6855
11330 6848 11323 6850 11316 6848 11313 6841 11306 6839 11306 6832
11303 6825 11305 6818 11299 6814 11292 6814 11285 6813 11279 6809
11272 6809 11265 6807 11263 6814 11259 6820 11252 6823 11252 6816
11250 6809 11246 6803 11241 6807 11233 6806 11229 6801 83 N F
11235 6735 11234 6731 11235 6728 11238 6726 11241 6725 11244 6728
11246 6730 11246 6734 11245 6737 11243 6739 11240 6741 11237 6742
11235 6739 11235 6735 14 N F
11279 12233 11273 12233 11277 12230 11282 12230 11286 12228 11289 12225
11290 12221 11293 12218 11297 12219 11301 12220 11303 12224 11300 12226
11296 12229 11293 12233 11292 12237 11293 12241 11288 12241 11286 12245
11282 12245 11279 12241 11279 12237 11279 12233 22 N F
11291 12571 11288 12567 11289 12562 11292 12559 11293 12563 11295 12567
11300 12567 11301 12571 11303 12575 11304 12580 11302 12584 11298 12586
11298 12581 11296 12577 11294 12573 11291 12571 16 N F
11349 12434 11345 12433 11347 12431 11348 12429 11349 12428 11351 12431
11349 12434 7 N F
11348 12442 11345 12437 11349 12437 11349 12438 11351 12439 11353 12442
11353 12445 11349 12445 11348 12442 9 N F
11353 12417 11348 12420 11350 12417 11352 12414 11354 12411 11355 12408
11358 12410 11361 12409 11364 12411 11363 12414 11360 12415 11357 12417
11355 12414 11353 12417 14 N F
11356 14220 11355 14217 11358 14215 11359 14212 11359 14209 11360 14206
11363 14204 11365 14206 11367 14209 11369 14212 11371 14214 11372 14217
11372 14220 11369 14221 11366 14221 11363 14219 11359 14219 11356 14220
18 N F
11373 12574 11371 12570 11374 12568 11377 12569 11379 12572 11379 12575
11376 12576 11373 12574 8 N F
11383 12581 11379 12576 11382 12576 11384 12579 11386 12581 11383 12581
6 N F
11386 12559 11383 12555 11385 12552 11388 12548 11391 12545 11395 12546
11394 12550 11395 12554 11399 12557 11401 12560 11400 12564 11404 12565
11407 12568 11409 12572 11409 12577 11412 12580 11416 12581 11414 12586
11410 12584 11407 12580 11403 12580 11402 12584 11406 12586 11402 12587
11397 12586 11393 12585 11391 12582 11391 12577 11388 12574 11387 12570
11386 12565 11383 12562 11386 12559 33 N F
11386 14292 11383 14288 11386 14287 11386 14287 11390 14288 11391 14290
11390 14293 11390 14297 11387 14295 11386 14292 10 N F
11400 12617 11396 12617 11399 12615 11402 12614 11402 12614 11400 12617
6 N F
11405 12612 11402 12611 11402 12607 11405 12606 11407 12609 11405 12612
6 N F

11410 12606 11408 12603 11410 12600 11413 12599 11414 12596 11417 12598
11416 12601 11418 12604 11421 12604 11421 12607 11418 12607 11415 12610
11412 12611 11413 12608 11410 12606 15 N F
11415 12560 11414 12557 11417 12556 11421 12557 11423 12560 11425 12562
11426 12565 11423 12566 11419 12565 11418 12562 11415 12560 11 N F
11442 12323 11439 12323 11441 12320 11443 12318 11445 12320 11448 12318
11451 12321 11447 12321 11445 12324 11442 12323 10 N F
11443 14281 11440 14279 11441 14276 11444 14277 11445 14277 11448 14277
11451 14278 11453 14281 11454 14284 11456 14286 11453 14285 11450 14284
11448 14281 11446 14283 11443 14281 15 N F
11502 13908 11501 13904 11502 13901 11505 13899 11505 13902 11506 13905
11506 13909 11509 13907 11512 13908 11511 13911 11508 13912 11505 13913
11502 13911 11502 13908 14 N F
11506 13964 11505 13961 11508 13960 11511 13962 11513 13965 11516 13964
11514 13967 11517 13969 11519 13971 11516 13972 11514 13969 11511 13968
11509 13965 11506 13964 14 N F
11521 13886 11519 13883 11521 13881 11523 13879 11526 13877 11528 13874
11531 13875 11533 13877 11533 13877 11536 13879 11533 13879 11529 13879
11526 13880 11526 13883 11524 13886 11521 13886 16 N F
11530 7007 11528 7005 11531 7003 11534 7002 11537 7001 11539 7000
11540 6999 11541 7003 11539 7006 11536 7007 11533 7009 11530 7007
12 N F
11531 6956 11528 6952 11528 6945 11530 6941 11534 6938 11538 6938
11543 6937 11547 6938 11551 6940 11555 6943 11558 6946 11558 6951
11557 6955 11553 6958 11548 6959 11544 6960 11539 6960 11535 6958
11531 6956 19 N F
11549 14246 11546 14242 11548 14240 11550 14240 11553 14240 11555 14241
11554 14243 11551 14244 11549 14246 9 N F
11553 13923 11551 13919 11552 13920 11554 13920 11557 13919 11554 13918
11553 13915 11556 13913 11559 13914 11561 13917 11563 13919 11564 13922
11563 13925 11560 13927 11557 13926 11554 13926 11553 13923 17 N F
11554 13449 11552 13451 11553 13448 11554 13446 11556 13444 11559 13443
11559 13446 11560 13448 11558 13450 11556 13448 11554 13449 11 N F
11557 13993 11556 13988 11559 13991 11560 13995 11562 13992 11563 13991
11567 13990 11568 13985 11572 13985 11576 13986 11580 13987 11582 13991
11582 13992 11585 13993 11588 13996 11586 14000 11582 14001 11578 14003
11574 14005 11570 14004 11568 14008 11564 14011 11564 14015 11560 14018
11560 14013 11558 14010 11558 14005 11556 14001 11557 13997 11557 13993
30 N F
11559 13969 11558 13965 11560 13962 11560 13961 11562 13959 11565 13959
11567 13959 11568 13959 11570 13961 11569 13964 11570 13967 11567 13967
11565 13969 11562 13971 11559 13969 15 N F
11564 14240 11560 14237 11563 14236 11566 14233 11569 14234 11572 14236

11574 14234 11577 14231 11579 14230 11582 14229 11584 14232 11585 14235
11582 14237 11580 14239 11579 14242 11578 14245 11574 14245 11573 14242
11570 14240 11567 14240 11564 14240 21 N F
11567 13389 11564 13388 11565 13386 11566 13383 11567 13381 11570 13380
11572 13381 11572 13384 11575 13384 11575 13381 11577 13383 11577 13386
11575 13387 11572 13388 11570 13389 11567 13389 16 N F
11573 13691 11570 13689 11571 13686 11573 13688 11575 13689 11578 13690
11581 13689 11583 13690 11583 13692 11584 13693 11586 13695 11585 13697
11585 13700 11582 13701 11583 13698 11581 13696 11580 13694 11577 13694
11574 13693 11573 13691 20 N F
11577 12560 11571 12560 11575 12559 11578 12555 11582 12556 11580 12552
11576 12550 11578 12547 11582 12548 11586 12551 11590 12550 11594 12553
11598 12554 11598 12558 11596 12562 11596 12566 11592 12568 11589 12564
11585 12562 11582 12559 11577 12560 21 N F
11581 14296 11576 14291 11578 14290 11580 14288 11584 14286 11588 14285
11588 14285 11592 14285 11593 14289 11597 14290 11602 14291 11603 14292
11605 14294 11609 14294 11613 14291 11616 14294 11620 14296 11624 14298
11626 14302 11628 14306 11629 14309 11630 14310 11629 14314 11626 14317
11623 14315 11618 14313 11614 14313 11610 14313 11606 14315 11603 14318
11599 14320 11595 14318 11590 14318 11587 14315 11583 14312 11583 14308
11581 14304 11583 14300 11581 14296 39 N F
11588 13442 11583 13442 11585 13440 11587 13438 11589 13437 11589 13440
11588 13442 7 N F
11588 14267 11585 14265 11588 14264 11590 14263 11593 14263 11595 14264
11598 14264 11596 14266 11593 14267 11591 14266 11588 14267 11 N F
11592 13995 11588 13992 11588 13990 11589 13987 11591 13986 11592 13986
11594 13986 11597 13985 11597 13983 11598 13980 11600 13981 11601 13984
11603 13986 11601 13988 11598 13987 11597 13989 11595 13992 11594 13994
11592 13995 19 N F
11596 14229 11591 14228 11592 14227 11593 14225 11595 14223 11597 14220
11600 14219 11603 14217 11606 14217 11609 14215 11607 14218 11605 14220
11604 14223 11605 14227 11605 14230 11602 14230 11599 14229 11596 14229
18 N F
11603 12557 11600 12561 11601 12558 11601 12555 11603 12557 5 N F
11604 13448 11602 13444 11603 13444 11604 13444 11607 13445 11609 13447
11612 13447 11614 13448 11614 13451 11612 13452 11610 13452 11607 13452
11605 13451 11604 13448 14 N F
11603 14276 11602 14274 11602 14270 11604 14269 11605 14270 11606 14271
11605 14274 11603 14276 8 N F
11607 12559 11603 12561 11604 12558 11607 12556 11610 12554 11612 12552
11611 12555 11610 12558 11607 12559 9 N F
11604 12633 11603 12628 11606 12625 11609 12622 11612 12619 11616 12617
11621 12618 11625 12620 11628 12623 11627 12627 11631 12628 11628 12631

11627 12635 11623 12637 11623 12642 11627 12643 11631 12645 11635 12645
11638 12647 11639 12647 11640 12643 11645 12643 11648 12645 11652 12646
11652 12653 11648 12652 11649 12656 11651 12660 11652 12664 11651 12668
11647 12670 11643 12668 11639 12667 11638 12663 11635 12659 11631 12659
11630 12655 11626 12653 11622 12651 11618 12650 11614 12650 11611 12654
11606 12653 11606 12648 11604 12645 11605 12641 11604 12637 11604 12633
48 N F
11616 14262 11607 14262 11610 14259 11614 14257 11617 14254 11621 14252
11625 14251 11625 14251 11629 14249 11633 14246 11633 14246 11633 14246
11636 14243 11636 14239 11635 14235 11638 14232 11641 14229 11643 14232
11647 14235 11649 14239 11653 14241 11655 14245 11655 14249 11654 14253
11651 14256 11647 14258 11643 14258 11638 14259 11635 14262 11631 14264
11628 14267 11628 14271 11628 14276 11629 14280 11632 14283 11629 14287
11625 14286 11622 14284 11617 14282 11614 14280 11615 14275 11615 14271
11615 14267 11616 14262 44 N F
11618 13975 11613 13975 11615 13973 11618 13971 11622 13972 11625 13971
11626 13974 11625 13977 11621 13976 11618 13975 10 N F
11619 12342 11613 12342 11616 12340 11618 12338 11622 12337 11621 12340
11619 12342 7 N F
11619 12615 11617 12611 11618 12606 11618 12602 11619 12599 11619 12598
11622 12594 11625 12597 11627 12593 11630 12590 11634 12592 11637 12588
11641 12587 11642 12587 11645 12588 11649 12591 11649 12595 11652 12598
11655 12601 11656 12605 11660 12605 11660 12604 11661 12609 11663 12613
11656 12614 11652 12617 11649 12620 11644 12620 11642 12623 11641 12627
11638 12631 11636 12627 11635 12623 11631 12620 11634 12617 11632 12613
11631 12617 11627 12618 11623 12617 11619 12615 40 N F
11619 12955 11619 12952 11621 12954 11624 12956 11621 12958 11619 12955
6 N F
11627 12965 11623 12964 11626 12962 11627 12959 11630 12960 11634 12960
11636 12962 11638 12965 11637 12968 11636 12971 11633 12971 11631 12974
11628 12972 11625 12973 11625 12969 11628 12969 11630 12966 11627 12965
18 N F
11630 13972 11626 13970 11628 13968 11631 13968 11635 13967 11638 13966
11639 13963 11642 13962 11644 13965 11641 13966 11639 13969 11638 13972
11635 13973 11633 13975 11630 13972 15 N F
11638 14180 11633 14181 11634 14178 11637 14176 11639 14174 11640 14174
11642 14172 11643 14169 11643 14166 11646 14165 11649 14165 11652 14166
11655 14167 11658 14168 11660 14168 11662 14168 11665 14167 11667 14170
11666 14173 11663 14173 11660 14171 11657 14171 11654 14171 11651 14172
11648 14174 11647 14177 11644 14178 11641 14179 11638 14180 29 N F
11637 12707 11633 12707 11637 12705 11635 12701 11639 12700 11644 12700
11647 12703 11647 12703 11651 12700 11654 12703 11657 12707 11657 12711
11658 12706 11657 12702 11661 12701 11663 12697 11665 12695 11667 12694

11670 12697 11672 12699 11673 12700 11677 12702 11679 12706 11683 12706
11683 12710 11682 12715 11686 12713 11690 12715 11691 12719 11688 12722
11684 12723 11685 12727 11688 12730 11684 12732 11685 12737 11681 12737
11679 12741 11676 12744 11671 12745 11670 12740 11672 12736 11670 12732
11667 12730 11663 12733 11660 12730 11656 12728 11653 12730 11651 12726
11650 12722 11646 12715 11644 12711 11642 12708 11637 12707 53 N F
11635 12364 11635 12360 11638 12360 11641 12358 11640 12361 11643 12364
11645 12366 11646 12364 11646 12360 11648 12362 11648 12365 11647 12369
11643 12369 11640 12368 11637 12366 11635 12364 16 N F
11636 12577 11635 12574 11638 12571 11640 12569 11643 12571 11646 12569
11648 12567 11651 12568 11650 12572 11649 12575 11646 12576 11645 12579
11642 12580 11639 12578 11636 12577 15 N F
11639 12983 11637 12980 11640 12978 11642 12981 11639 12983 5 N F
11640 14024 11637 14019 11641 14017 11644 14015 11649 14014 11651 14014
11653 14013 11657 14013 11662 14014 11665 14016 11667 14020 11663 14022
11661 14018 11656 14019 11652 14021 11648 14021 11644 14023 11640 14024
18 N F
11643 14313 11638 14308 11641 14302 11643 14296 11643 14289 11642 14282
11643 14275 11642 14269 11645 14263 11652 14261 11659 14261 11661 14254
11666 14250 11668 14243 11671 14237 11667 14231 11661 14229 11655 14226
11657 14220 11656 14213 11663 14211 11669 14208 11676 14209 11679 14209
11683 14208 11686 14207 11689 14206 11696 14205 11696 14212 11696 14218
11700 14217 11702 14216 11707 14212 11708 14211 11715 14211 11719 14216
11721 14217 11724 14220 11726 14227 11723 14234 11721 14240 11723 14246
11718 14251 11717 14257 11717 14264 11716 14271 11712 14277 11713 14284
11715 14290 11712 14296 11709 14302 11707 14308 11702 14313 11696 14315
11690 14319 11684 14323 11679 14327 11674 14331 11667 14333 11662 14329
11655 14329 11653 14323 11649 14317 11643 14313 64 N F
11640 12831 11639 12826 11642 12824 11643 12821 11646 12820 11648 12822
11648 12825 11646 12828 11644 12831 11642 12833 11640 12831 11 N F
11644 12555 11642 12553 11643 12553 11646 12553 11646 12553 11649 12554
11650 12550 11648 12547 11649 12544 11652 12545 11652 12548 11652 12551
11652 12554 11654 12557 11654 12557 11651 12557 11648 12556 11644 12555
18 N F
11645 12432 11644 12428 11647 12429 11648 12432 11649 12435 11646 12435
11645 12432 7 N F
11651 13972 11646 13971 11648 13968 11651 13967 11654 13966 11657 13965
11660 13965 11659 13968 11657 13971 11654 13971 11651 13972 11 N F
11649 13144 11647 13141 11650 13140 11651 13142 11652 13145 11649 13144
6 N F
11651 12360 11648 12356 11649 12353 11652 12353 11656 12354 11658 12356
11659 12357 11660 12358 11661 12361 11658 12363 11656 12360 11653 12362
11651 12360 13 N F

11652 12419 11650 12415 11654 12416 11657 12416 11656 12419 11654 12421
11652 12419 7 N F
11658 12816 11652 12813 11656 12813 11661 12813 11661 12809 11664 12812
11667 12815 11669 12811 11671 12810 11673 12810 11677 12809 11681 12806
11683 12810 11686 12813 11685 12817 11687 12820 11689 12824 11685 12826
11681 12827 11681 12832 11681 12836 11685 12838 11686 12842 11684 12846
11686 12849 11686 12854 11690 12855 11694 12856 11697 12859 11701 12861
11697 12863 11695 12860 11690 12860 11686 12859 11682 12859 11681 12854
11678 12851 11676 12848 11674 12844 11671 12840 11667 12838 11663 12837
11659 12836 11657 12832 11656 12828 11653 12824 11657 12822 11661 12820
11658 12816 49 N F
11654 13134 11653 13131 11655 13130 11658 13130 11659 13128 11661 13129
11661 13131 11659 13133 11656 13134 11654 13134 10 N F
11656 12567 11655 12564 11657 12562 11659 12564 11659 12564 11660 12561
11662 12563 11665 12564 11665 12568 11664 12571 11662 12573 11660 12575
11657 12574 11657 12571 11659 12568 11656 12567 16 N F
11657 12400 11655 12396 11658 12396 11659 12396 11661 12398 11660 12401
11657 12400 7 N F
11657 12435 11656 12428 11659 12428 11660 12430 11661 12431 11661 12435
11659 12437 11657 12435 8 N F
11659 12317 11657 12314 11658 12311 11659 12308 11660 12305 11662 12308
11660 12311 11660 12314 11659 12317 9 N F
11659 14135 11658 14131 11661 14129 11664 14128 11665 14125 11665 14122
11668 14120 11670 14118 11673 14119 11674 14122 11677 14123 11679 14121
11679 14118 11682 14119 11683 14119 11685 14118 11687 14115 11688 14112
11690 14115 11692 14118 11693 14121 11692 14124 11691 14127 11688 14128
11685 14130 11683 14133 11680 14133 11677 14134 11676 14131 11673 14129
11670 14130 11668 14132 11666 14135 11664 14137 11660 14137 11659 14135
36 N F
11665 12639 11659 12638 11660 12635 11661 12632 11662 12629 11664 12627
11667 12626 11668 12629 11667 12633 11666 12635 11665 12639 11 N F
11666 12404 11662 12403 11663 12400 11664 12397 11666 12397 11667 12398
11668 12401 11666 12404 8 N F
11666 12396 11662 12394 11664 12391 11668 12391 11671 12391 11669 12394
11666 12396 7 N F
11666 12577 11665 12574 11667 12571 11668 12568 11667 12565 11666 12562
11666 12561 11667 12559 11670 12558 11673 12556 11672 12554 11669 12551
11670 12548 11671 12545 11671 12542 11673 12539 11676 12540 11677 12543
11679 12546 11681 12544 11684 12544 11688 12543 11690 12545 11691 12548
11692 12552 11690 12555 11687 12556 11687 12559 11687 12562 11684 12562
11680 12561 11679 12564 11677 12567 11679 12570 11679 12573 11678 12576
11675 12577 11672 12578 11669 12578 11666 12577 40 N F
11672 12685 11668 12683 11670 12680 11671 12677 11674 12677 11677 12675

11678 12675 11680 12676 11680 12679 11677 12681 11674 12683 11672 12685
12 N F
11670 12381 11669 12378 11670 12375 11674 12375 11675 12373 11677 12370
11680 12369 11680 12373 11682 12375 11682 12379 11680 12376 11678 12379
11676 12381 11673 12382 11670 12381 15 N F
11674 12649 11670 12645 11672 12646 11674 12646 11677 12647 11679 12650
11678 12653 11676 12652 11674 12649 9 N F
11675 12417 11673 12416 11673 12412 11673 12412 11673 12409 11676 12408
11680 12407 11682 12407 11683 12407 11686 12408 11687 12411 11690 12411
11693 12412 11693 12412 11696 12410 11699 12410 11702 12408 11703 12411
11701 12414 11698 12414 11695 12416 11692 12417 11689 12419 11687 12417
11683 12418 11682 12415 11679 12414 11675 12417 28 N F
11677 14174 11674 14170 11676 14168 11677 14171 11679 14173 11679 14176
11677 14174 7 N F
11681 12699 11674 12697 11677 12694 11680 12690 11682 12687 11684 12683
11684 12682 11687 12680 11691 12678 11693 12675 11692 12670 11697 12670
11701 12672 11704 12675 11705 12680 11702 12683 11702 12688 11703 12692
11708 12690 11711 12688 11711 12693 11715 12695 11716 12699 11712 12702
11708 12704 11712 12706 11716 12707 11716 12707 11717 12711 11712 12711
11712 12715 11709 12718 11706 12715 11702 12717 11698 12718 11695 12715
11696 12711 11698 12707 11701 12704 11699 12700 11695 12700 11691 12702
11687 12705 11684 12703 11681 12699 45 N F
11679 12538 11675 12535 11679 12533 11682 12537 11686 12536 11691 12536
11689 12532 11685 12531 11683 12527 11679 12529 11676 12523 11681 12523
11685 12522 11689 12523 11692 12524 11694 12524 11696 12528 11700 12529
11703 12532 11702 12536 11707 12537 11709 12541 11707 12545 11709 12549
11709 12553 11712 12556 11709 12559 11705 12561 11708 12565 11704 12567
11700 12568 11696 12570 11695 12574 11692 12577 11687 12576 11683 12577
11682 12572 11686 12569 11690 12568 11692 12564 11694 12560 11695 12556
11698 12553 11702 12552 11699 12551 11697 12547 11698 12542 11695 12539
11690 12539 11687 12541 11682 12541 11679 12538 52 N F
11682 12405 11677 12404 11678 12401 11680 12399 11682 12401 11685 12401
11685 12404 11682 12405 8 N F
11680 12597 11678 12595 11678 12591 11679 12588 11680 12584 11682 12587
11682 12591 11681 12594 11680 12597 9 N F
11681 13493 11678 13493 11679 13490 11681 13488 11683 13490 11684 13492
11684 13495 11686 13497 11684 13498 11681 13498 11680 13496 11681 13493
12 N F
11684 12675 11680 12674 11680 12673 11681 12671 11683 12668 11687 12669
11685 12672 11684 12675 8 N F
11681 14099 11680 14095 11683 14094 11685 14092 11685 14088 11686 14085
11688 14083 11691 14081 11693 14083 11694 14083 11697 14084 11698 14087
11697 14090 11700 14092 11698 14094 11695 14096 11693 14098 11690 14101

11688 14102 11686 14105 11683 14105 11682 14102 11681 14099 23 N F
11683 12428 11681 12425 11684 12424 11681 12421 11684 12419 11687 12419
11686 12422 11686 12426 11683 12428 9 N F
11688 12654 11681 12652 11685 12651 11688 12648 11689 12646 11690 12644
11692 12640 11693 12640 11697 12640 11701 12638 11700 12634 11700 12629
11704 12629 11704 12629 11707 12627 11711 12630 11713 12633 11718 12634
11719 12630 11722 12627 11721 12623 11725 12621 11728 12621 11730 12621
11734 12621 11736 12625 11735 12629 11735 12634 11733 12637 11731 12641
11733 12644 11729 12646 11727 12650 11723 12652 11720 12649 11715 12649
11712 12652 11710 12656 11705 12657 11702 12654 11698 12656 11693 12657
11692 12653 11688 12654 44 N F
11682 14173 11681 14167 11684 14167 11686 14169 11686 14173 11686 14176
11689 14178 11686 14180 11683 14179 11683 14176 11682 14173 11 N F
11684 12943 11682 12938 11684 12935 11685 12934 11686 12933 11687 12930
11690 12931 11692 12933 11692 12937 11691 12940 11689 12943 11687 12945
11684 12943 13 N F
11686 12495 11682 12495 11683 12493 11684 12492 11686 12490 11686 12486
11688 12484 11689 12485 11690 12485 11692 12488 11693 12491 11696 12492
11698 12495 11698 12498 11695 12496 11694 12499 11697 12501 11694 12502
11692 12505 11689 12504 11688 12501 11687 12498 11686 12495 23 N F
11685 12892 11682 12891 11682 12887 11682 12884 11685 12886 11686 12889
11685 12892 7 N F
11690 12786 11683 12781 11686 12778 11690 12780 11695 12781 11699 12781
11703 12780 11707 12782 11707 12777 11702 12778 11702 12774 11702 12770
11703 12765 11701 12761 11699 12757 11698 12753 11702 12755 11706 12756
11707 12760 11706 12764 11709 12765 11710 12765 11714 12768 11718 12766
11717 12770 11721 12771 11720 12776 11720 12780 11718 12783 11713 12784
11711 12788 11710 12792 11707 12795 11703 12793 11700 12795 11695 12796
11695 12791 11691 12790 11690 12786 39 N F
11684 13483 11683 13481 11684 13478 11687 13478 11687 13478 11687 13480
11688 13483 11689 13485 11686 13485 11684 13483 10 N F
11687 12608 11685 12603 11687 12601 11690 12602 11690 12606 11692 12607
11693 12608 11693 12611 11690 12610 11687 12608 10 N F
11687 12397 11685 12391 11689 12393 11689 12388 11693 12387 11698 12387
11702 12387 11704 12385 11705 12383 11704 12388 11706 12391 11707 12387
11710 12383 11712 12387 11716 12388 11720 12386 11720 12391 11718 12395
11717 12399 11718 12404 11713 12403 11709 12402 11705 12400 11701 12401
11697 12404 11693 12406 11692 12402 11687 12403 11691 12400 11687 12397
30 N F
11687 12640 11685 12635 11689 12634 11691 12637 11691 12640 11689 12643
11687 12640 7 N F
11689 12894 11687 12892 11690 12892 11693 12892 11695 12895 11697 12897
11694 12898 11692 12895 11689 12894 9 N F

11688 14169 11687 14165 11690 14164 11693 14165 11696 14166 11699 14165
11702 14163 11705 14163 11705 14166 11704 14169 11705 14172 11705 14176
11702 14178 11700 14180 11697 14178 11696 14175 11693 14173 11691 14172
11688 14169 19 N F
11695 12667 11687 12665 11691 12664 11693 12663 11696 12663 11699 12660
11703 12662 11707 12662 11711 12659 11713 12656 11716 12652 11720 12654
11724 12655 11726 12659 11725 12664 11723 12668 11726 12670 11724 12674
11720 12674 11719 12679 11716 12681 11714 12677 11716 12673 11712 12673
11708 12671 11704 12669 11700 12668 11695 12667 28 N F
11690 13944 11689 13939 11692 13938 11694 13936 11696 13939 11695 13942
11693 13945 11690 13944 8 N F
11692 12363 11692 12358 11695 12358 11698 12356 11700 12358 11703 12358
11707 12357 11708 12360 11709 12363 11712 12364 11710 12366 11709 12369
11706 12371 11704 12372 11704 12369 11704 12366 11702 12364 11699 12363
11696 12362 11692 12363 20 N F
11698 14154 11693 14153 11696 14150 11700 14149 11702 14145 11701 14140
11706 14140 11705 14144 11707 14148 11709 14151 11707 14155 11702 14154
11698 14154 13 N F
11699 12813 11693 12808 11695 12808 11697 12808 11698 12804 11702 12802
11706 12804 11709 12801 11712 12797 11714 12801 11714 12806 11715 12802
11716 12797 11719 12801 11719 12805 11718 12809 11716 12813 11712 12814
11708 12813 11703 12814 11699 12813 21 N F
11701 12624 11698 12619 11700 12617 11704 12617 11706 12619 11707 12622
11704 12624 11701 12624 8 N F
11703 14032 11701 14026 11704 14022 11705 14018 11706 14014 11706 14010
11710 14012 11711 14013 11713 14015 11715 14012 11715 14007 11719 14009
11721 14013 11724 14016 11725 14020 11727 14024 11731 14025 11731 14030
11730 14034 11729 14038 11725 14039 11720 14039 11716 14039 11712 14039
11711 14035 11707 14033 11703 14032 27 N F
11706 12837 11704 12832 11708 12831 11711 12828 11710 12824 11712 12823
11714 12823 11716 12819 11720 12818 11724 12820 11727 12816 11731 12817
11735 12818 11738 12816 11739 12815 11743 12817 11744 12813 11740 12811
11741 12807 11744 12806 11745 12806 11747 12802 11748 12800 11750 12799
11750 12803 11754 12801 11755 12805 11758 12808 11755 12811 11751 12813
11752 12817 11750 12821 11747 12824 11744 12820 11739 12821 11737 12825
11733 12827 11733 12831 11736 12835 11739 12832 11742 12836 11741 12840
11737 12840 11735 12836 11733 12841 11730 12844 11727 12841 11726 12837
11721 12837 11718 12840 11716 12844 11712 12842 11708 12841 11706 12837
54 N F
11711 13919 11708 13916 11710 13914 11710 13911 11711 13907 11714 13906
11717 13907 11720 13909 11722 13910 11721 13913 11720 13916 11718 13918
11716 13916 11713 13916 11711 13919 15 N F
11711 14135 11709 14130 11712 14128 11715 14127 11716 14130 11716 14134

11714 14136 11711 14135 8 N F
11711 12593 11711 12588 11715 12588 11718 12584 11716 12580 11718 12576
11716 12572 11720 12572 11724 12574 11728 12575 11730 12579 11734 12580
11738 12578 11741 12580 11744 12584 11748 12584 11750 12588 11746 12589
11742 12590 11743 12594 11741 12598 11737 12601 11734 12598 11732 12594
11727 12594 11730 12598 11731 12602 11728 12605 11724 12603 11721 12599
11717 12598 11716 12594 11711 12593 33 N F
11714 12470 11711 12463 11715 12457 11720 12453 11724 12447 11729 12443
11728 12450 11726 12456 11729 12462 11730 12456 11735 12451 11742 12450
11739 12444 11735 12438 11738 12432 11742 12437 11746 12432 11753 12431
11759 12431 11763 12434 11765 12435 11772 12438 11766 12442 11773 12441
11774 12442 11780 12443 11784 12449 11791 12450 11791 12457 11797 12460
11804 12460 11804 12467 11809 12467 11811 12467 11812 12474 11818 12470
11819 12463 11824 12467 11822 12474 11826 12476 11828 12478 11830 12484
11834 12490 11831 12496 11835 12501 11830 12506 11834 12511 11835 12518
11837 12525 11831 12529 11827 12534 11822 12538 11816 12543 11810 12540
11809 12533 11803 12537 11801 12544 11802 12550 11799 12556 11792 12559
11786 12561 11779 12562 11772 12563 11771 12556 11774 12550 11780 12549
11787 12551 11785 12545 11787 12538 11783 12533 11779 12528 11776 12521
11769 12523 11769 12530 11766 12536 11762 12531 11765 12524 11759 12521
11762 12515 11763 12508 11768 12503 11774 12506 11771 12499 11765 12501
11768 12495 11766 12488 11765 12481 11764 12475 11761 12469 11761 12476
11760 12482 11760 12489 11763 12495 11760 12501 11760 12508 11753 12510
11752 12516 11746 12513 11745 12506 11749 12500 11746 12494 11741 12499
11734 12498 11731 12492 11724 12490 11723 12483 11727 12478 11721 12481
11716 12476 11714 12470 110 N F
11713 14150 11712 14145 11713 14141 11714 14141 11717 14139 11721 14139
11723 14143 11725 14143 11727 14143 11730 14147 11734 14148 11739 14147
11743 14148 11747 14150 11748 14154 11750 14158 11750 14162 11747 14165
11745 14169 11741 14171 11737 14172 11733 14172 11729 14173 11725 14175
11721 14177 11717 14175 11715 14171 11716 14167 11715 14163 11713 14159
11715 14154 11713 14150 32 N F
11716 13521 11715 13519 11715 13515 11716 13515 11718 13515 11720 13517
11723 13515 11725 13517 11727 13519 11727 13521 11725 13523 11724 13526
11724 13528 11723 13530 11720 13531 11720 13528 11719 13525 11718 13523
11716 13521 19 N F
11722 13109 11717 13107 11720 13104 11722 13100 11725 13102 11729 13104
11732 13107 11735 13111 11737 13114 11741 13116 11743 13120 11745 13124
11743 13127 11739 13130 11739 13126 11736 13123 11732 13123 11728 13125
11730 13121 11726 13121 11726 13116 11723 13114 11722 13109 23 N F
11730 12615 11729 12610 11732 12609 11735 12609 11738 12610 11740 12613
11742 12610 11744 12613 11745 12616 11743 12618 11745 12620 11747 12623
11744 12624 11740 12623 11738 12621 11736 12618 11736 12615 11733 12616

11730 12615 19 N F
11731 12771 11730 12768 11732 12765 11734 12762 11736 12761 11738 12764
11738 12767 11738 12770 11737 12773 11738 12777 11735 12776 11732 12774
11731 12771 13 N F
11733 13546 11732 13541 11733 13538 11735 13537 11737 13539 11739 13539
11740 13539 11743 13538 11745 13538 11746 13538 11748 13537 11751 13538
11753 13537 11756 13537 11758 13535 11759 13535 11761 13535 11764 13535
11766 13537 11765 13539 11762 13539 11760 13538 11758 13540 11755 13541
11756 13543 11755 13546 11753 13548 11753 13550 11750 13550 11747 13550
11745 13549 11744 13546 11745 13544 11743 13544 11740 13545 11738 13546
11735 13547 11733 13546 38 N F
11746 13257 11742 13255 11745 13254 11748 13254 11746 13257 5 N F
11743 12748 11742 12742 11744 12741 11746 12739 11750 12738 11752 12741
11757 12742 11756 12746 11757 12750 11753 12752 11749 12752 11744 12752
11743 12748 13 N F
11752 14043 11748 14044 11750 14041 11753 14038 11757 14035 11758 14030
11759 14026 11760 14022 11760 14018 11756 14016 11753 14014 11754 14009
11758 14010 11756 14006 11754 14002 11751 13999 11749 13994 11753 13997
11755 14000 11759 13999 11762 13995 11765 13998 11766 14002 11767 14006
11768 14010 11772 14009 11777 14009 11777 14013 11780 14016 11784 14017
11786 14021 11785 14025 11782 14029 11781 14033 11777 14035 11773 14034
11769 14036 11766 14039 11762 14038 11761 14042 11764 14046 11764 14050
11762 14046 11759 14042 11755 14040 11752 14043 46 N F
11751 12791 11748 12792 11750 12788 11752 12785 11752 12781 11752 12778
11750 12775 11753 12774 11756 12775 11760 12775 11762 12777 11761 12780
11761 12783 11759 12786 11757 12788 11755 12791 11751 12791 17 N F
11750 12709 11749 12704 11754 12704 11758 12703 11762 12701 11766 12701
11769 12705 11770 12709 11775 12710 11779 12709 11783 12710 11782 12714
11786 12713 11788 12709 11792 12709 11797 12709 11798 12705 11803 12704
11800 12700 11797 12697 11799 12693 11803 12692 11806 12696 11810 12694
11812 12698 11812 12702 11813 12706 11814 12710 11813 12715 11809 12716
11805 12719 11801 12718 11797 12718 11792 12717 11788 12718 11791 12722
11787 12724 11790 12726 11791 12731 11795 12729 11797 12733 11801 12733
11802 12737 11798 12738 11796 12741 11792 12742 11787 12741 11783 12742
11781 12746 11777 12746 11773 12745 11770 12741 11773 12738 11776 12735
11780 12733 11781 12729 11777 12726 11777 12721 11773 12722 11774 12718
11770 12715 11768 12719 11766 12715 11764 12711 11760 12711 11756 12711
11752 12713 11750 12709 68 N F
11754 13255 11751 13254 11754 13254 11757 13254 11758 13254 11760 13255
11762 13257 11759 13259 11756 13258 11754 13255 10 N F
11758 13238 11756 13234 11758 13232 11760 13229 11763 13231 11763 13234
11761 13237 11758 13238 8 N F
11759 12374 11758 12370 11760 12367 11763 12368 11761 12370 11762 12373

11764 12376 11765 12379 11763 12382 11761 12380 11759 12377 11759 12374
12 N F
11764 12772 11759 12770 11762 12767 11764 12763 11767 12760 11768 12756
11772 12754 11776 12755 11776 12759 11774 12763 11771 12767 11768 12770
11764 12772 13 N F
11762 7073 11761 7064 11763 7061 11766 7057 11771 7055 11775 7054
11779 7055 11783 7058 11787 7059 11791 7057 11796 7057 11800 7058
11804 7060 11806 7064 11805 7068 11804 7073 11801 7076 11800 7081
11796 7083 11791 7084 11787 7086 11783 7088 11779 7090 11774 7090
11771 7087 11771 7083 11771 7078 11771 7074 11766 7072 11762 7073
30 N F
11770 13240 11765 13239 11769 13238 11769 13235 11772 13236 11771 13233
11774 13234 11777 13234 11779 13237 11777 13239 11779 13241 11778 13244
11777 13247 11774 13248 11771 13247 11770 13244 11774 13243 11776 13241
11774 13238 11771 13237 11770 13240 21 N F
11770 13388 11768 13386 11769 13384 11772 13382 11774 13382 11777 13381
11776 13384 11775 13386 11773 13388 11770 13388 10 N F
11776 13263 11771 13262 11774 13261 11774 13261 11777 13261 11781 13260
11779 13263 11776 13263 8 N F
11780 13233 11778 13230 11781 13228 11784 13228 11786 13230 11789 13232
11790 13235 11788 13238 11786 13235 11785 13232 11782 13232 11783 13234
11781 13236 11780 13233 14 N F
11792 13250 11789 13247 11791 13245 11792 13242 11793 13239 11796 13238
11795 13241 11797 13242 11798 13243 11800 13240 11803 13240 11807 13240
11809 13242 11807 13244 11804 13246 11801 13247 11797 13247 11795 13250
11792 13250 19 N F
11797 13204 11793 13201 11796 13200 11797 13196 11799 13194 11801 13196
11801 13200 11800 13203 11797 13204 9 N F
11802 12451 11800 12446 11803 12444 11806 12443 11805 12447 11808 12446
11811 12448 11813 12450 11813 12453 11811 12455 11808 12454 11810 12456
11809 12459 11806 12458 11804 12456 11801 12455 11802 12451 17 N F
11812 11881 11806 11877 11807 11870 11809 11864 11807 11857 11807 11848
11811 11843 11815 11838 11820 11832 11825 11828 11826 11828 11832 11825
11838 11824 11844 11820 11848 11815 11853 11810 11858 11805 11864 11802
11871 11802 11871 11809 11872 11816 11876 11822 11881 11827 11887 11830
11894 11828 11900 11828 11907 11828 11914 11830 11920 11834 11926 11835
11932 11831 11939 11833 11946 11835 11950 11830 11957 11829 11963 11833
11970 11833 11974 11838 11981 11839 11988 11840 11995 11840 12001 11843
12007 11840 12014 11842 12019 11847 12024 11851 12028 11857 12034 11860
12040 11862 12047 11862 12054 11864 12060 11867 12067 11869 12073 11870
12078 11875 12083 11880 12087 11886 12094 11886 12101 11887 12107 11889
12114 11892 12117 11897 12116 11904 12121 11909 12125 11914 12126 11921
12128 11928 12131 11934 12137 11937 12141 11943 12141 11950 12143 11956

12144 11963 12149 11959 12147 11965 12146 11972 12149 11978 12156 11977
12151 11982 12146 11977 12145 11984 12139 11987 12135 11992 12138 11998
12136 12005 12133 12011 12127 12014 12120 12015 12114 12012 12108 12015
12101 12018 12096 12022 12092 12028 12086 12027 12079 12028 12072 12028
12065 12027 12059 12024 12052 12024 12046 12022 12040 12019 12033 12017
12026 12016 12020 12012 12014 12014 12008 12010 12002 12007 11995 12004
11989 12002 11983 11999 11976 11998 11970 11994 11964 11991 11958 11987
11952 11989 11945 11990 11941 11985 11934 11984 11929 11979 11923 11975
11917 11974 11911 11970 11905 11966 11899 11969 11894 11964 11887 11962
11882 11957 11876 11954 11869 11954 11865 11948 11862 11942 11855 11939
11851 11935 11846 11930 11845 11923 11842 11917 11837 11912 11832 11907
11827 11903 11823 11897 11819 11892 11813 11888 11812 11881 143 N F
11809 13260 11807 13259 11809 13257 11811 13258 11812 13260 11809 13260
6 N F
11820 13319 11817 13316 11819 13315 11822 13314 11824 13316 11822 13319
11820 13319 7 N F
11822 13369 11819 13366 11821 13365 11822 13365 11824 13365 11827 13364
11827 13366 11827 13369 11826 13371 11824 13371 11822 13369 11 N F
11826 7122 11819 7120 11823 7114 11827 7109 11830 7102 11835 7096
11839 7091 11842 7084 11835 7081 11835 7074 11837 7067 11834 7061
11828 7058 11825 7051 11832 7048 11837 7043 11840 7037 11836 7031
11825 7031 11829 7025 11834 7020 11840 7015 11847 7013 11848 7021
11850 7014 11855 7009 11862 7012 11869 7014 11876 7016 11882 7019
11889 7021 11896 7022 11903 7023 11910 7024 11917 7023 11924 7025
11931 7027 11937 7022 11933 7028 11928 7033 11924 7039 11918 7044
11911 7044 11904 7046 11898 7048 11894 7055 11887 7057 11880 7056
11874 7058 11867 7060 11863 7066 11865 7073 11860 7078 11853 7081
11849 7087 11848 7094 11844 7100 11839 7104 11834 7110 11829 7115
11826 7122 61 N F
11826 12226 11825 12222 11828 12220 11832 12217 11833 12221 11831 12225
11826 12226 7 N F
11831 13330 11828 13328 11830 13327 11833 13326 11835 13324 11837 13325
11840 13326 11841 13328 11839 13329 11836 13330 11833 13330 11831 13330
12 N F
11840 12220 11834 12216 11838 12215 11841 12211 11843 12207 11847 12207
11845 12211 11847 12215 11851 12216 11852 12221 11848 12222 11844 12219
11840 12220 13 N F
11847 13324 11843 13322 11845 13322 11848 13321 11849 13319 11852 13319
11850 13320 11849 13323 11847 13324 9 N F
11872 13239 11870 13234 11872 13231 11875 13231 11878 13233 11878 13236
11879 13239 11877 13242 11874 13241 11872 13239 10 N F
11906 12354 11904 12351 11907 12350 11908 12353 11910 12356 11914 12356
11914 12359 11916 12362 11914 12364 11911 12366 11910 12363 11908 12360

11907 12357 11906 12354 14 N F
11917 12345 11913 12342 11916 12338 11914 12331 11917 12327 11918 12323
11923 12322 11927 12322 11931 12324 11935 12326 11938 12329 11942 12332
11942 12336 11946 12337 11948 12341 11951 12343 11952 12347 11955 12345
11955 12345 11957 12341 11958 12345 11962 12343 11966 12344 11966 12348
11962 12348 11957 12349 11953 12351 11949 12351 11945 12349 11940 12350
11936 12349 11935 12353 11931 12355 11926 12354 11923 12351 11920 12348
11917 12345 37 N F
11933 12365 11930 12361 11931 12361 11935 12360 11937 12357 11940 12354
11945 12353 11949 12353 11954 12353 11950 12355 11949 12360 11952 12362
11954 12358 11959 12358 11961 12362 11958 12365 11962 12368 11965 12370
11962 12374 11960 12377 11957 12380 11953 12382 11949 12381 11944 12382
11940 12380 11937 12383 11934 12386 11934 12381 11937 12378 11933 12376
11933 12372 11937 12373 11935 12370 11933 12365 34 N F
11949 12392 11949 12386 11952 12386 11955 12388 11958 12386 11960 12389
11963 12389 11962 12392 11961 12395 11958 12396 11955 12396 11952 12394
11949 12392 13 N F
11955 12322 11952 12320 11955 12317 11952 12312 11956 12313 11959 12313
11962 12312 11962 12315 11959 12317 11958 12320 11955 12322 11 N F
11963 12473 11961 12468 11963 12464 11963 12460 11964 12456 11965 12452
11964 12447 11968 12444 11967 12440 11971 12442 11974 12439 11978 12437
11978 12437 11982 12435 11985 12438 11988 12440 11991 12437 11995 12434
11992 12431 11987 12431 11987 12427 11991 12424 11995 12425 11999 12426
12003 12426 12007 12423 12011 12422 12010 12426 12008 12430 12013 12429
12015 12425 12020 12425 12024 12425 12026 12425 12028 12424 12026 12428
12022 12430 12023 12434 12025 12438 12021 12440 12017 12439 12013 12439
12014 12443 12012 12447 12008 12449 12007 12453 12006 12457 12005 12461
12004 12466 12003 12470 12001 12474 11997 12476 11993 12478 11990 12480
11985 12482 11981 12483 11977 12482 11974 12478 11970 12479 11966 12477
11963 12473 61 N F
11974 12344 11969 12341 11972 12337 11974 12333 11975 12329 11978 12332
11981 12328 11983 12324 11987 12323 11989 12326 11989 12331 11991 12334
11990 12339 11994 12341 11993 12345 11990 12343 11989 12347 11987 12351
11982 12352 11983 12356 11987 12358 11991 12359 11993 12363 11990 12365
11986 12367 11982 12368 11978 12371 11974 12368 11974 12364 11970 12364
11971 12359 11975 12360 11977 12356 11977 12352 11974 12349 11974 12344
36 N F
12013 12478 12011 12473 12014 12476 12019 12475 12023 12476 12021 12472
12020 12468 12019 12463 12023 12461 12026 12464 12026 12469 12029 12472
12030 12476 12034 12478 12039 12478 12043 12480 12043 12484 12045 12487
12050 12486 12050 12486 12054 12484 12058 12485 12062 12484 12062 12489
12059 12492 12055 12494 12055 12498 12050 12496 12046 12497 12050 12500
12051 12504 12047 12507 12046 12511 12041 12510 12037 12508 12034 12506

12029 12504 12025 12504 12023 12501 12019 12498 12018 12493 12016 12490
12017 12486 12015 12482 12013 12478 45 N F
12018 12451 12014 12449 12019 12448 12023 12446 12027 12446 12031 12444
12034 12441 12037 12444 12040 12447 12044 12447 12049 12448 12052 12445
12054 12442 12054 12437 12057 12434 12061 12436 12063 12440 12066 12443
12065 12447 12069 12446 12070 12441 12074 12442 12075 12446 12074 12450
12075 12454 12079 12454 12080 12458 12079 12462 12075 12459 12071 12460
12068 12463 12064 12462 12059 12462 12056 12460 12054 12463 12050 12466
12047 12463 12044 12460 12044 12455 12041 12453 12037 12451 12034 12454
12030 12457 12028 12453 12026 12449 12022 12451 12018 12451 47 N F
12023 12380 12022 12377 12023 12374 12022 12368 12025 12366 12027 12364
12029 12366 12029 12370 12031 12372 12032 12375 12030 12378 12029 12381
12026 12382 12023 12380 14 N F
12065 12494 12060 12493 12063 12491 12066 12487 12063 12483 12062 12479
12063 12475 12067 12478 12068 12482 12072 12484 12072 12479 12072 12475
12073 12471 12078 12470 12081 12468 12082 12468 12085 12468 12090 12470
12091 12474 12095 12476 12099 12476 12101 12480 12098 12483 12097 12488
12098 12492 12100 12496 12098 12500 12096 12496 12094 12492 12092 12488
12088 12490 12084 12488 12081 12492 12077 12491 12073 12492 12069 12495
12068 12491 12065 12494 38 N F
12070 12376 12067 12365 12071 12360 12077 12362 12083 12358 12090 12357
12092 12357 12096 12356 12101 12361 12101 12368 12101 12375 12096 12379
12094 12386 12089 12390 12082 12392 12076 12388 12073 12382 12070 12376
18 N F
12110 12268 12107 12262 12109 12258 12113 12258 12117 12259 12120 12263
12118 12267 12114 12269 12110 12268 9 N F
12110 12459 12108 12456 12108 12452 12109 12447 12113 12446 12114 12443
12114 12442 12118 12444 12122 12446 12125 12449 12130 12450 12131 12454
12135 12456 12133 12460 12126 12464 12122 12463 12118 12463 12113 12462
12110 12459 19 N F
12125 12474 12121 12476 12123 12472 12126 12470 12127 12470 12131 12471
12135 12470 12139 12468 12135 12465 12140 12465 12142 12461 12143 12465
12148 12466 12151 12463 12147 12460 12144 12458 12145 12454 12146 12449
12148 12453 12150 12457 12153 12460 12156 12463 12160 12463 12161 12467
12157 12467 12156 12471 12160 12469 12164 12470 12167 12466 12172 12467
12169 12471 12166 12474 12165 12478 12161 12478 12157 12479 12154 12477
12150 12474 12146 12473 12147 12477 12145 12481 12141 12483 12139 12486
12136 12490 12137 12486 12133 12485 12129 12483 12132 12480 12129 12476
12125 12474 49 N F
12136 12530 12129 12528 12133 12525 12136 12522 12136 12521 12137 12518
12137 12513 12140 12510 12143 12507 12145 12511 12144 12515 12148 12514
12147 12509 12149 12505 12150 12501 12152 12505 12154 12509 12155 12513
12154 12517 12152 12521 12150 12525 12148 12529 12144 12531 12140 12532

12136 12530 25 N F
12165 12540 12160 12539 12163 12536 12167 12537 12170 12534 12166 12531
12165 12527 12169 12528 12172 12524 12176 12522 12179 12520 12183 12518
12187 12520 12191 12520 12194 12523 12198 12524 12203 12523 12207 12523
12211 12523 12215 12524 12219 12528 12219 12532 12219 12536 12221 12540
12222 12545 12224 12549 12223 12553 12220 12556 12217 12559 12212 12558
12209 12556 12204 12555 12200 12557 12196 12554 12193 12551 12191 12548
12186 12548 12184 12545 12180 12546 12175 12547 12174 12551 12169 12552
12170 12548 12168 12544 12165 12540 45 N F
12236 12411 12234 12405 12237 12407 12242 12406 12246 12408 12250 12407
12250 12412 12252 12415 12251 12419 12250 12424 12250 12428 12245 12427
12241 12425 12242 12421 12238 12420 12237 12416 12236 12411 17 N F
12274 12325 12269 12321 12271 12317 12272 12313 12276 12311 12277 12306
12279 12303 12283 12300 12287 12300 12291 12299 12294 12303 12290 12302
12286 12304 12284 12308 12281 12311 12279 12315 12277 12319 12279 12323
12278 12328 12274 12325 20 N F
12288 12326 12281 12324 12285 12321 12286 12317 12290 12315 12294 12314
12295 12318 12292 12320 12292 12324 12288 12326 10 N F
12313 12589 12310 12584 12314 12582 12318 12582 12319 12578 12322 12575
12318 12574 12315 12571 12318 12567 12320 12564 12325 12563 12328 12567
12332 12567 12336 12569 12340 12568 12342 12572 12342 12576 12339 12579
12335 12581 12330 12581 12326 12581 12330 12583 12327 12587 12323 12588
12326 12591 12329 12595 12328 12599 12323 12599 12319 12600 12316 12597
12314 12593 12313 12589 32 N F
12323 12552 12321 12547 12326 12547 12330 12548 12334 12549 12337 12545
12332 12545 12328 12546 12329 12542 12333 12540 12338 12541 12342 12539
12342 12535 12340 12531 12344 12528 12347 12532 12350 12534 12354 12536
12358 12537 12362 12540 12363 12544 12361 12548 12360 12552 12355 12553
12353 12557 12349 12559 12346 12563 12345 12558 12341 12557 12337 12554
12334 12557 12329 12558 12326 12560 12321 12559 12325 12556 12323 12552
36 N F
12400 12629 12398 12625 12398 12619 12400 12623 12403 12619 12405 12616
12410 12616 12413 12619 12417 12620 12418 12625 12421 12621 12425 12622
12429 12625 12425 12627 12422 12630 12419 12627 12414 12627 12412 12631
12408 12632 12404 12630 12400 12629 21 N F
12433 12610 12426 12609 12429 12606 12434 12604 12437 12602 12441 12604
12441 12599 12444 12597 12445 12593 12450 12591 12454 12593 12457 12591
12461 12589 12463 12585 12467 12585 12471 12586 12474 12589 12472 12593
12470 12597 12466 12597 12461 12597 12465 12599 12465 12603 12462 12606
12458 12609 12457 12613 12455 12617 12451 12619 12447 12620 12442 12619
12439 12616 12436 12613 12433 12610 33 N F
12458 12794 12457 12787 12458 12780 12461 12774 12465 12769 12470 12764
12472 12758 12477 12753 12480 12747 12477 12740 12475 12734 12477 12733

12482 12732 12484 12725 12489 12721 12496 12720 12503 12721 12509 12724
12509 12726 12511 12731 12507 12737 12505 12743 12511 12747 12516 12751
12523 12752 12524 12746 12524 12739 12525 12732 12526 12731 12528 12726
12530 12719 12536 12718 12539 12718 12543 12718 12545 12720 12549 12722
12556 12723 12560 12717 12566 12720 12571 12721 12573 12721 12569 12726
12566 12732 12570 12737 12574 12743 12572 12750 12571 12757 12570 12763
12569 12770 12572 12776 12572 12783 12566 12787 12560 12785 12555 12789
12542 12787 12536 12784 12533 12778 12527 12775 12522 12770 12517 12774
12515 12781 12509 12783 12502 12783 12503 12790 12498 12795 12495 12801
12496 12808 12501 12812 12504 12818 12505 12825 12503 12831 12500 12837
12494 12841 12487 12842 12481 12839 12476 12835 12472 12829 12472 12822
12476 12817 12470 12815 12463 12813 12463 12806 12463 12799 12458 12794
84 N F
12477 12559 12473 12555 12478 12555 12475 12552 12475 12547 12479 12547
12483 12544 12485 12548 12485 12552 12484 12557 12485 12561 12485 12565
12481 12567 12482 12572 12477 12572 12476 12568 12477 12564 12477 12559
18 N F
12485 8029 12477 8021 12482 8016 12488 8014 12496 8013 12503 8013
12508 8008 12511 8001 12515 7996 12521 7992 12527 7989 12534 7988
12532 7995 12529 8001 12529 8009 12533 8014 12535 8021 12529 8024
12526 8031 12519 8033 12515 8039 12509 8042 12502 8041 12496 8038
12492 8032 12485 8029 26 N F
12525 12806 12523 12802 12524 12798 12526 12794 12528 12790 12532 12789
12536 12788 12541 12788 12545 12789 12549 12790 12550 12794 12554 12796
12558 12794 12562 12792 12566 12793 12570 12791 12574 12792 12578 12789
12582 12789 12581 12793 12577 12796 12573 12797 12570 12800 12569 12804
12571 12808 12570 12813 12566 12815 12562 12817 12558 12819 12560 12823
12556 12824 12552 12821 12551 12817 12550 12813 12550 12808 12548 12804
12545 12807 12545 12811 12545 12816 12546 12820 12549 12824 12550 12828
12551 12832 12551 12836 12547 12835 12542 12833 12537 12829 12535 12825
12534 12821 12532 12817 12529 12814 12528 12810 12525 12806 53 N F
12524 8161 12524 8160 12524 8159 12524 8159 12525 8159 12526 8159
12526 8159 12526 8159 12526 8160 12526 8160 12526 8161 12524 8161
12 N F
12536 8016 12534 8013 12536 8013 12538 8014 12540 8015 12540 8017
12539 8019 12537 8018 12536 8016 9 N F
12543 8019 12542 8015 12545 8016 12546 8018 12545 8020 12543 8019
6 N F
12548 8016 12547 8013 12549 8015 12551 8016 12553 8017 12555 8017
12557 8019 12559 8020 12560 8022 12562 8023 12564 8023 12566 8023
12568 8024 12569 8026 12571 8027 12574 8028 12571 8028 12569 8028
12568 8026 12566 8025 12564 8025 12561 8024 12559 8024 12558 8022
12556 8021 12554 8019 12552 8018 12550 8018 12548 8016 29 N F

12619 12808 12616 12801 12621 12799 12625 12798 12624 12793 12628 12793
12631 12796 12632 12800 12635 12803 12640 12804 12644 12804 12643 12808
12639 12810 12635 12810 12631 12813 12627 12812 12623 12810 12619 12808
18 N F
12639 12792 12633 12789 12637 12788 12638 12784 12642 12784 12645 12781
12649 12781 12653 12779 12654 12783 12651 12787 12656 12787 12660 12788
12663 12791 12660 12794 12656 12796 12653 12799 12650 12802 12646 12804
12642 12802 12639 12799 12636 12796 12639 12792 22 N F
12659 12697 12656 12691 12659 12688 12663 12687 12663 12682 12665 12678
12668 12682 12672 12683 12674 12687 12678 12686 12683 12686 12687 12686
12686 12691 12689 12694 12687 12698 12683 12696 12681 12693 12680 12688
12676 12691 12679 12694 12676 12697 12672 12698 12668 12700 12664 12702
12660 12701 12659 12697 26 N F
12696 8054 12693 8048 12696 8045 12698 8041 12702 8043 12706 8045
12702 8047 12699 8051 12700 8055 12696 8054 10 N F
12721 8183 12715 8170 12722 8173 12729 8174 12735 8177 12733 8184
12727 8187 12721 8183 8 N F
12753 12843 12750 12836 12752 12830 12755 12823 12758 12817 12760 12816
12764 12815 12767 12809 12771 12803 12777 12806 12776 12812 12783 12814
12789 12817 12793 12823 12796 12829 12803 12827 12804 12821 12806 12814
12805 12808 12805 12801 12799 12797 12793 12795 12787 12793 12788 12786
12789 12786 12793 12790 12793 12784 12800 12785 12806 12788 12812 12785
12807 12781 12806 12774 12808 12768 12815 12766 12812 12760 12809 12754
12816 12752 12817 12751 12822 12749 12828 12753 12834 12755 12832 12761
12827 12766 12833 12768 12840 12769 12847 12772 12853 12773 12848 12776
12841 12775 12837 12780 12832 12785 12834 12792 12841 12791 12845 12796
12843 12803 12843 12810 12849 12813 12852 12807 12856 12812 12863 12811
12864 12810 12867 12806 12873 12803 12874 12810 12869 12815 12865 12820
12866 12827 12860 12826 12857 12832 12863 12835 12870 12833 12867 12840
12863 12845 12869 12846 12869 12853 12872 12859 12865 12860 12860 12855
12859 12848 12852 12850 12848 12855 12853 12861 12856 12867 12859 12873
12857 12879 12855 12886 12854 12892 12852 12899 12846 12903 12843 12909
12841 12915 12844 12921 12850 12924 12851 12931 12847 12936 12840 12938
12833 12939 12829 12933 12829 12926 12833 12916 12826 12914 12823 12908
12822 12901 12822 12894 12815 12896 12809 12893 12803 12895 12801 12889
12796 12884 12800 12878 12804 12872 12808 12867 12813 12862 12818 12857
12811 12858 12804 12857 12798 12860 12793 12855 12787 12856 12781 12859
12775 12864 12769 12862 12766 12856 12760 12855 12756 12848 12753 12843
126 N F
12752 12793 12751 12787 12754 12785 12757 12782 12756 12778 12756 12773
12757 12773 12761 12773 12765 12772 12769 12775 12772 12777 12775 12780
12775 12780 12775 12785 12772 12788 12768 12787 12765 12791 12765 12795
12762 12798 12762 12803 12759 12800 12760 12804 12756 12804 12752 12801

12754 12797 12758 12798 12761 12795 12758 12797 12757 12793 12754 12789
12754 12794 12751 12797 12752 12793 33 N F
12757 7950 12754 7945 12756 7941 12760 7939 12760 7943 12761 7948
12757 7950 7 N F
12757 7972 12756 7967 12757 7967 12760 7967 12761 7971 12757 7972
6 N F
12763 12892 12762 12887 12764 12884 12765 12879 12763 12875 12766 12872
12768 12868 12773 12869 12770 12872 12773 12875 12770 12878 12768 12882
12771 12885 12773 12888 12771 12892 12767 12893 12763 12892 17 N F
12772 7966 12769 7962 12773 7959 12775 7955 12780 7957 12783 7959
12781 7963 12779 7967 12775 7969 12772 7966 10 N F
12782 7984 12776 7979 12780 7978 12785 7978 12786 7978 12787 7976
12788 7975 12791 7978 12793 7982 12793 7987 12796 7984 12800 7983
12800 7987 12798 7991 12793 7992 12789 7992 12788 7988 12784 7988
12782 7984 19 N F
12786 7955 12779 7953 12782 7949 12786 7950 12786 7955 5 N F
12787 7949 12783 7944 12786 7940 12787 7945 12787 7949 5 N F
12812 7958 12808 7956 12810 7951 12813 7948 12812 7944 12812 7939
12815 7936 12816 7940 12820 7942 12821 7946 12820 7951 12819 7955
12822 7959 12825 7962 12824 7967 12824 7971 12823 7976 12821 7980
12817 7982 12813 7980 12813 7976 12812 7971 12810 7967 12811 7963
12812 7958 25 N F
12859 7979 12857 7973 12861 7974 12862 7978 12864 7982 12864 7987
12860 7983 12859 7979 8 N F
12885 12955 12882 12951 12886 12950 12889 12947 12889 12943 12892 12939
12888 12937 12892 12935 12896 12934 12897 12930 12896 12925 12899 12922
12901 12923 12904 12923 12905 12927 12903 12931 12903 12936 12904 12940
12906 12944 12907 12948 12906 12953 12905 12957 12900 12956 12898 12960
12895 12962 12892 12959 12888 12958 12885 12955 28 N F
12896 11258 12895 11258 12896 11257 12896 11257 12896 11257 12896 11257
12897 11256 12898 11256 12898 11256 12898 11257 12898 11257 12897 11258
12896 11258 13 N F
12897 11261 12896 11259 12897 11259 12898 11258 12899 11257 12899 11255
12898 11254 12898 11252 12898 11250 12897 11249 12899 11248 12899 11250
12900 11251 12901 11252 12903 11252 12903 11253 12901 11255 12901 11256
12901 11258 12902 11259 12903 11260 12903 11262 12901 11263 12900 11262
12899 11261 12897 11261 26 N F
12904 11267 12903 11266 12903 11265 12904 11263 12905 11264 12906 11265
12906 11265 12906 11266 12906 11268 12906 11268 12905 11268 12904 11267
12904 11267 13 N F
12905 11272 12905 11272 12905 11271 12905 11271 12906 11271 12906 11271
12906 11271 12906 11272 12906 11272 12905 11272 10 N F
12907 11274 12906 11274 12906 11272 12907 11272 12907 11272 12907 11272

12908 11272 12909 11272 12909 11272 12909 11274 12909 11275 12909 11275
12909 11275 12907 11275 12907 11275 12907 11274 16 N F
12909 11279 12909 11279 12909 11278 12910 11277 12910 11277 12910 11277
12910 11276 12911 11276 12912 11276 12912 11277 12912 11277 12911 11278
12911 11279 12911 11279 12909 11279 15 N F
12911 11283 12911 11281 12913 11281 12914 11281 12916 11282 12917 11282
12919 11283 12920 11284 12921 11283 12922 11282 12923 11283 12924 11284
12925 11286 12926 11287 12926 11289 12926 11290 12925 11291 12924 11289
12923 11290 12921 11289 12919 11290 12918 11290 12916 11290 12915 11289
12915 11287 12915 11285 12914 11284 12913 11283 12911 11283 29 N F
12963 7992 12960 7986 12961 7979 12966 7974 12969 7967 12974 7963
12979 7958 12980 7950 12979 7943 12979 7936 12978 7929 12977 7922
12982 7918 12989 7915 12993 7921 12999 7925 12999 7925 12999 8033
12995 8030 12988 8028 12984 8022 12982 8015 12978 8009 12979 8002
12973 7997 12977 7991 12982 7986 12976 7990 12970 7993 12963 7992
30 N F
12974 11621 12970 11609 12977 11607 12984 11607 12988 11602 12995 11601
12999 11606 12999 11607 12999 11637 12993 11637 12998 11632 12991 11634
12994 11627 12988 11623 12987 11616 12994 11619 12999 11623 12995 11617
12995 11610 12988 11612 12983 11616 12980 11622 12976 11628 12974 11621
24 N F
12982 8295 12979 8284 12983 8279 12990 8279 12997 8281 12999 8281
12999 8296 12996 8295 12989 8295 12982 8295 10 N F
12984 11702 12982 11695 12982 11688 12987 11683 12984 11676 12986 11670
12991 11665 12987 11659 12990 11653 12993 11647 12999 11644 12999 11638
12999 11700 12995 11705 12989 11707 12984 11702 16 N F
12983 8119 12983 8112 12985 8105 12988 8099 12992 8093 12996 8086
12999 8084 12999 8119 12996 8116 12991 8121 12986 8126 12983 8119
12 N F
1 O
13000 16171 2298 16171 2298 5454 13000 5454 13000 16171 5 P
Ls Gr Gs Lr
2297 5453 13002 16173 Oc
38 O
2297 6403 2297 6314 2297 6314 2297 7863 2297 7863 2297 7760
2297 7624 2297 7489 2297 7353 2297 7217 2297 7081 2297 6946
2297 6810 2297 6674 2297 6539 2297 6403 16 N F
56 O
2297 7896 2297 7863 2297 7863 2297 9560 2297 9560 2297 9524
2297 9388 2297 9252 2297 9117 2297 8981 2297 8845 2297 8710
2297 8574 2297 8438 2297 8303 2297 8167 2297 8031 2297 7896
18 N F
38 O

2297 9660 2297 9560 2297 9560 2297 11723 2297 11723 2297 11695
2297 11559 2297 11423 2297 11288 2297 11152 2297 11016 2297 10881
2297 10745 2297 10609 2297 10474 2297 10338 2297 10202 2297 10067
2297 9931 2297 9795 2297 9660 21 N F
56 O
2297 11831 2297 11723 2297 11723 2297 11865 2297 11865 2297 11831
6 N F
74 O
2297 11959 2297 11865 2297 11865 2297 11959 2297 11959 5 N F
92 O
2297 11966 2297 11959 2297 11959 2297 12061 2297 12061 2297 11966
6 N F
111 O
2297 12102 2297 12061 2297 12061 2297 12170 2297 12170 2297 12102
6 N F
92 O
2297 12238 2297 12170 2297 12170 2297 12562 2297 12562 2297 12509
2297 12373 2297 12238 8 N F
111 O
2297 12645 2297 12562 2297 12562 2297 12865 2297 12865 2297 12780
2297 12645 7 N F
92 O
2297 12916 2297 12865 2297 12865 2297 13055 2297 13055 2297 13052
2297 12916 7 N F
111 O
2297 13138 2297 13055 2297 13055 2297 13138 2297 13138 5 N F
129 O
2297 13187 2297 13138 2297 13138 2297 13550 2297 13550 2297 13459
2297 13323 2297 13187 8 N F
111 O
2297 13594 2297 13550 2297 13550 2297 14133 2297 14133 2297 14002
2297 13866 2297 13730 2297 13594 9 N F
129 O
2297 14137 2297 14133 2297 14133 2297 14174 2297 14174 2297 14137
6 N F
111 O
2297 14273 2297 14174 2297 14174 2297 14772 2297 14772 2297 14680
2297 14544 2297 14409 2297 14273 9 N F
92 O
2297 14816 2297 14772 2297 14772 2297 15068 2297 15068 2297 14951
2297 14816 7 N F
111 O
2297 15087 2297 15068 2297 15068 2297 15331 2297 15331 2297 15223

2297 15087 7 N F
129 O
2297 15358 2297 15331 2297 15331 2297 15386 2297 15386 2297 15358
6 N F
111 O
2297 15494 2297 15386 2297 15386 2297 15577 2297 15577 2297 15494
6 N F
129 O
2297 15630 2297 15577 2297 15577 2297 15751 2297 15751 2297 15630
6 N F
147 O
2297 15765 2297 15751 2297 15751 2297 15911 2297 15911 2297 15901
2297 15765 7 N F
56 O
2297 6314 2297 5453 2297 5453 2297 5453 2297 6313 2297 6314
6 N F
38 O
2297 7863 2297 6314 2297 6313 2297 7863 2297 7863 5 N F
56 O
2297 9560 2297 7863 2297 7863 2297 9560 2297 9560 5 N F
38 O
2297 11723 2297 9560 2297 9560 2297 11723 2297 11723 5 N F
56 O
2297 11865 2297 11723 2297 11723 2297 11865 2297 11865 5 N F
74 O
2297 11959 2297 11865 2297 11865 2297 11959 2297 11959 5 N F
92 O
2297 12061 2297 11959 2297 11959 2297 12062 2297 12061 5 N F
111 O
2297 12170 2297 12061 2297 12062 2297 12170 2297 12170 5 N F
92 O
2297 12562 2297 12170 2297 12170 2297 12562 2297 12562 5 N F
111 O
2297 12865 2297 12562 2297 12562 2297 12865 2297 12865 5 N F
92 O
2297 13055 2297 12865 2297 12865 2297 13055 2297 13055 5 N F
111 O
2297 13138 2297 13055 2297 13055 2297 13138 2297 13138 5 N F
129 O
2297 13550 2297 13138 2297 13138 2297 13551 2297 13550 5 N F
111 O
2297 14133 2297 13550 2297 13551 2297 14133 2297 14133 5 N F
129 O

2297 14174 2297 14133 2297 14133 2297 14175 2297 14174 5 N F
111 O
2297 14772 2297 14174 2297 14175 2297 14772 2297 14772 5 N F
92 O
2297 15068 2297 14772 2297 14772 2297 15068 2297 15068 5 N F
111 O
2297 15331 2297 15068 2297 15068 2297 15331 2297 15331 5 N F
129 O
2297 15386 2297 15331 2297 15331 2297 15386 2297 15386 5 N F
111 O
2297 15577 2297 15386 2297 15386 2297 15577 2297 15577 5 N F
129 O
2297 15751 2297 15577 2297 15577 2297 15751 2297 15751 5 N F
147 O
2297 15911 2297 15751 2297 15751 2297 15911 2297 15911 5 N F
165 O
2297 16173 2297 15911 2297 15911 2297 16172 2297 16173 2297 16173
6 N F
56 O
2470 5453 M 2297 5453 L 2297 6313 L 2324 6267 L 2433 6185 L
2480 6267 L 2568 6370 L 2662 6403 L 2704 6420 L
2839 6405 L 2842 6403 L 2975 6327 L 3060 6403 L
3110 6521 L 3145 6539 L 3242 6674 L 3213 6810 L
3246 6826 L 3381 6842 L 3466 6946 L 3447 7081 L
3381 7151 L 3246 7156 L 3207 7217 L 3153 7353 L
3116 7489 L 3110 7502 L 2975 7585 L 2839 7561 L
2704 7613 L 2697 7624 L 2641 7760 L 2568 7856 L
2433 7879 L 2297 7863 L 2297 9560 L 2341 9524 L
2433 9461 L 2568 9449 L 2568 8611 L 2559 8574 L
2568 8569 L 2577 8574 L 2568 8611 L 2568 9449 L
2625 9524 L 2704 9624 L 2724 9660 L 2806 9795 L
2839 9821 L 2975 9896 L 3019 9931 L 3110 9998 L
3217 10067 L 3246 10091 L 3369 10202 L 3381 10215 L
3381 9307 L 3246 9300 L 3226 9252 L 3217 9117 L
3110 9020 L 3075 8981 L 2975 8889 L 2847 8845 L
2839 8842 L 2704 8784 L 2600 8710 L 2704 8658 L
2839 8619 L 2975 8622 L 2975 8210 L 2934 8167 L
2975 8140 L 2988 8167 L 2975 8210 L 2975 8622 L
3110 8702 L 3110 8450 L 3079 8438 L 3026 8303 L
3110 8266 L 3110 7802 L 2975 7775 L 2967 7760 L
2975 7741 L 3110 7715 L 3246 7756 L 3251 7760 L
3246 7763 L 3110 7802 L 3110 8266 L 3192 8303 L
3121 8438 L 3110 8450 L 3110 8702 L 3128 8710 L

3246 8759 L 3305 8845 L 3296 8981 L 3378 9117 L
3381 9119 L 3437 9252 L 3381 9307 L 3381 10215 L
3517 10333 L 3517 10206 L 3513 10202 L 3479 10067 L
3517 10052 L 3537 10067 L 3550 10202 L 3517 10206 L
3517 10333 L 3532 10338 L 3652 10388 L 3652 7359 L
3648 7353 L 3517 7239 L 3461 7217 L 3517 7165 L
3652 7119 L 3718 7217 L 3666 7353 L 3652 7359 L
3652 10388 L 3788 10418 L 3923 10455 L 3955 10474 L
4059 10522 L 4194 10542 L 4194 10364 L 4165 10338 L
4059 10254 L 3923 10223 L 3890 10202 L 3830 10067 L
3923 9979 L 3972 10067 L 4059 10160 L 4194 10199 L
4213 10202 L 4244 10338 L 4194 10364 L 4194 10542 L
4330 10594 L 4444 10474 L 4465 10459 L 4601 10397 L
4626 10338 L 4617 10202 L 4667 10067 L 4601 9983 L
4525 9931 L 4465 9874 L 4330 9859 L 4194 9857 L
4104 9795 L 4059 9717 L 3923 9766 L 3885 9660 L
3788 9544 L 3779 9524 L 3746 9388 L 3773 9252 L
3788 9236 L 3788 7524 L 3770 7489 L 3788 7453 L
3788 6686 L 3762 6674 L 3727 6539 L 3755 6403 L
3788 6387 L 3801 6403 L 3835 6539 L 3807 6674 L
3788 6686 L 3788 7453 L 3795 7489 L 3788 7524 L
3788 9236 L 3864 9117 L 3872 8981 L 3854 8845 L
3923 8771 L 3923 8208 L 3885 8167 L 3923 8118 L
3923 6097 L 3865 5996 L 3923 5965 L 4002 5996 L
3923 6097 L 3923 8118 L 4035 8167 L 3923 8208 L
3923 8771 L 4039 8710 L 4050 8574 L 3993 8438 L
4059 8357 L 4059 8073 L 4052 8031 L 4059 8030 L
4065 8031 L 4059 8073 L 4059 8357 L 4124 8438 L
4194 8470 L 4194 7817 L 4078 7760 L 4194 7681 L
4273 7760 L 4194 7817 L 4194 8470 L 4312 8574 L
4330 8586 L 4432 8710 L 4465 8763 L 4574 8710 L
4601 8656 L 4652 8574 L 4601 8528 L 4477 8438 L
4465 8422 L 4330 8321 L 4304 8303 L 4330 8182 L
4330 5936 L 4194 5900 L 4170 5860 L 4194 5835 L
4222 5725 L 4295 5589 L 4330 5555 L 4409 5589 L
4465 5658 L 4514 5725 L 4465 5797 L 4403 5860 L
4330 5936 L 4330 8182 L 4338 8167 L 4465 8133 L
4559 8167 L 4601 8204 L 4680 8167 L 4736 8072 L
4757 8031 L 4736 8013 L 4620 7896 L 4692 7760 L
4736 7750 L 4872 7748 L 4890 7760 L 5007 7837 L
5143 7823 L 5229 7760 L 5151 7624 L 5143 7619 L
5007 7548 L 4872 7503 L 4841 7489 L 4784 7353 L
4872 7317 L 4872 6347 L 4736 6296 L 4710 6267 L

4736 6228 L 4872 6190 L 4965 6267 L 4872 6347 L
4872 7317 L 5007 7331 L 5007 6410 L 4966 6403 L
5007 6348 L 5007 6170 L 4981 6132 L 5007 6127 L
5016 6132 L 5007 6170 L 5007 6348 L 5127 6403 L
5007 6410 L 5007 7331 L 5143 7330 L 5143 6990 L
5106 6946 L 5143 6909 L 5208 6946 L 5143 6990 L
5143 7330 L 5186 7353 L 5278 7415 L 5278 7195 L
5239 7081 L 5278 7021 L 5331 7081 L 5278 7195 L
5278 7415 L 5414 7398 L 5549 7369 L 5549 7338 L
5414 7333 L 5286 7217 L 5414 7128 L 5549 7180 L
5621 7217 L 5549 7338 L 5549 7369 L 5685 7487 L
5686 7489 L 5713 7624 L 5750 7760 L 5820 7875 L
5840 7896 L 5846 8031 L 5893 8167 L 5941 8303 L
5956 8322 L 6091 8355 L 6137 8303 L 6227 8233 L
6264 8303 L 6361 8438 L 6362 8439 L 6363 8438 L
6460 8303 L 6498 8223 L 6633 8176 L 6752 8303 L
6769 8311 L 6904 8323 L 6943 8303 L 6904 8283 L
6769 8284 L 6643 8167 L 6633 8162 L 6498 8097 L
6362 8067 L 6227 8115 L 6162 8031 L 6189 7896 L
6208 7760 L 6227 7717 L 6296 7760 L 6362 7896 L
6362 7896 L 6363 7896 L 6498 7795 L 6576 7760 L
6633 7686 L 6769 7638 L 6843 7760 L 6904 7812 L
6904 7714 L 6777 7624 L 6904 7519 L 7040 7509 L
7040 7477 L 6916 7353 L 7040 7246 L 7078 7353 L
7040 7477 L 7040 7509 L 7170 7624 L 7040 7667 L
6904 7714 L 6904 7812 L 6961 7896 L 6963 8031 L
7040 8127 L 7141 8167 L 7144 8303 L 7175 8313 L
7267 8438 L 7265 8574 L 7311 8584 L 7326 8574 L
7417 8438 L 7407 8303 L 7433 8167 L 7446 8156 L
7460 8167 L 7517 8303 L 7491 8438 L 7469 8574 L
7582 8670 L 7621 8710 L 7717 8833 L 7727 8845 L
7853 8961 L 7976 8845 L 7988 8834 L 8124 8831 L
8151 8845 L 8259 8895 L 8259 8702 L 8124 8590 L
8111 8574 L 7988 8463 L 7927 8438 L 7853 8390 L
7790 8303 L 7853 8255 L 7853 7342 L 7717 7289 L
7673 7217 L 7717 7094 L 7746 7081 L 7766 6946 L
7853 6867 L 7913 6810 L 7988 6770 L 8109 6810 L
8026 6946 L 7988 6967 L 7867 7081 L 7988 7217 L
7991 7217 L 7988 7218 L 7853 7342 L 7853 8255 L
7988 8234 L 8109 8167 L 8124 8154 L 8259 8068 L
8259 7243 L 8179 7217 L 8259 7129 L 8301 7217 L
8259 7243 L 8259 8068 L 8385 8167 L 8259 8290 L
8184 8303 L 8167 8438 L 8259 8511 L 8383 8438 L

8395 8436 L 8396 8438 L 8415 8574 L 8395 8608 L
8259 8702 L 8259 8895 L 8336 8981 L 8259 9047 L
8216 9117 L 8259 9174 L 8372 9252 L 8307 9388 L
8395 9468 L 8485 9388 L 8426 9252 L 8530 9228 L
8666 9169 L 8666 8576 L 8664 8574 L 8666 8570 L
8668 8574 L 8666 8576 L 8666 9169 L 8801 9183 L
8937 9159 L 8937 9079 L 8881 8981 L 8937 8949 L
8970 8981 L 8937 9079 L 8937 9159 L 8998 9252 L
8942 9388 L 9060 9524 L 9072 9553 L 9072 9164 L
8992 9117 L 9072 9095 L 9107 9117 L 9072 9164 L
9072 9553 L 9131 9660 L 9072 9698 L 8937 9740 L
8906 9795 L 8867 9931 L 8801 9987 L 8697 10067 L
8801 10168 L 8832 10202 L 8803 10338 L 8815 10474 L
8801 10498 L 8669 10609 L 8666 10615 L 8583 10745 L
8530 10825 L 8441 10881 L 8419 11016 L 8530 11115 L
8552 11152 L 8604 11288 L 8544 11423 L 8666 11555 L
8692 11559 L 8686 11695 L 8666 11747 L 8653 11831 L
8666 11873 L 8768 11966 L 8801 12032 L 8818 12102 L
8801 12124 L 8666 12134 L 8560 12238 L 8554 12373 L
8530 12382 L 8426 12509 L 8441 12645 L 8414 12780 L
8395 12818 L 8261 12916 L 8259 12917 L 8124 12977 L
7988 12996 L 7853 13031 L 7833 13052 L 7717 13117 L
7582 13119 L 7446 13174 L 7432 13187 L 7429 13323 L
7408 13459 L 7358 13594 L 7311 13618 L 7206 13730 L
7175 13747 L 7040 13752 L 6922 13730 L 6904 13704 L
6890 13594 L 6815 13459 L 6769 13377 L 6633 13353 L
6498 13345 L 6362 13434 L 6313 13323 L 6227 13241 L
6221 13187 L 6212 13052 L 6131 12916 L 6227 12845 L
6362 12861 L 6489 12916 L 6498 12921 L 6633 12988 L
6633 12793 L 6614 12780 L 6532 12645 L 6633 12580 L
6708 12645 L 6660 12780 L 6633 12793 L 6633 12988 L
6769 13014 L 6769 12557 L 6667 12509 L 6769 12482 L
6854 12509 L 6769 12557 L 6769 13014 L 6904 13015 L
7040 12958 L 7175 12998 L 7175 12037 L 7150 11966 L
7175 11882 L 7218 11966 L 7175 12037 L 7175 12998 L
7311 13015 L 7446 13012 L 7446 12748 L 7311 12692 L
7279 12645 L 7311 12586 L 7393 12509 L 7446 12481 L
7507 12509 L 7502 12645 L 7446 12748 L 7446 13012 L
7582 12969 L 7622 12916 L 7717 12814 L 7741 12780 L
7775 12645 L 7717 12532 L 7710 12509 L 7717 12501 L
7817 12373 L 7717 12265 L 7582 12244 L 7526 12238 L
7446 12171 L 7409 12102 L 7446 12090 L 7523 11966 L
7509 11831 L 7512 11695 L 7446 11636 L 7335 11559 L

7311 11539 L 7175 11558 L 7167 11559 L 7175 11591 L
7180 11695 L 7175 11731 L 7040 11718 L 6904 11790 L
6894 11695 L 6903 11559 L 6769 11465 L 6633 11514 L
6538 11423 L 6498 11372 L 6448 11423 L 6498 11516 L
6537 11559 L 6617 11695 L 6606 11831 L 6633 11867 L
6701 11966 L 6666 12102 L 6633 12236 L 6590 12102 L
6498 12010 L 6454 11966 L 6362 11847 L 6328 11831 L
6227 11797 L 6162 11831 L 6143 11966 L 6122 12102 L
6156 12238 L 6120 12373 L 6192 12509 L 6091 12602 L
5956 12515 L 5880 12645 L 5822 12780 L 5820 12781 L
5685 12825 L 5549 12837 L 5414 12794 L 5278 12815 L
5210 12780 L 5143 12712 L 5037 12780 L 5049 12916 L
5143 13030 L 5154 13052 L 5169 13187 L 5143 13215 L
5119 13187 L 5007 13095 L 4872 13066 L 4736 13078 L
4714 13052 L 4601 12956 L 4465 12933 L 4330 12925 L
4316 12916 L 4194 12828 L 4059 12794 L 4023 12780 L
3923 12718 L 3788 12659 L 3652 12649 L 3636 12645 L
3546 12509 L 3652 12455 L 3788 12426 L 3923 12404 L
4059 12399 L 4194 12462 L 4313 12509 L 4330 12521 L
4465 12545 L 4528 12509 L 4601 12476 L 4736 12431 L
4872 12448 L 5006 12509 L 5007 12510 L 5008 12509 L
5007 12497 L 4998 12373 L 5007 12359 L 5140 12238 L
5143 12227 L 5157 12102 L 5143 12056 L 5007 12012 L
4875 12102 L 4872 12128 L 4857 12102 L 4736 12074 L
4601 12079 L 4521 11966 L 4465 11945 L 4330 11922 L
4194 11840 L 4138 11831 L 4059 11812 L 4032 11831 L
3923 11881 L 3788 11839 L 3652 11870 L 3533 11966 L
3517 11972 L 3381 11994 L 3246 11997 L 3110 12009 L
2975 11967 L 2973 11966 L 2912 11831 L 2975 11811 L
3110 11784 L 3110 11572 L 3099 11559 L 3110 11555 L
3246 11553 L 3255 11559 L 3246 11567 L 3110 11572 L
3110 11784 L 3246 11759 L 3358 11695 L 3381 11678 L
3498 11559 L 3517 11507 L 3652 11455 L 3717 11423 L
3788 11406 L 3923 11380 L 3923 11268 L 3788 11268 L
3666 11152 L 3688 11016 L 3788 10923 L 3923 10917 L
4059 10973 L 4118 11016 L 4093 11152 L 4059 11194 L
3923 11268 L 3923 11380 L 4059 11307 L 4091 11288 L
4177 11152 L 4194 11110 L 4330 11058 L 4465 11092 L
4465 11002 L 4330 10951 L 4242 10881 L 4330 10793 L
4465 10747 L 4601 10818 L 4666 10881 L 4601 10960 L
4465 11002 L 4465 11092 L 4601 11090 L 4675 11016 L
4736 10922 L 4785 10881 L 4833 10745 L 4736 10618 L
4601 10713 L 4465 10640 L 4330 10614 L 4221 10745 L

4194 10772 L 4059 10850 L 3923 10834 L 3788 10827 L
3723 10881 L 3652 10940 L 3568 11016 L 3521 11152 L
3517 11217 L 3501 11288 L 3381 11355 L 3246 11388 L
3114 11423 L 3110 11424 L 2975 11456 L 2839 11485 L
2704 11486 L 2568 11532 L 2535 11559 L 2492 11695 L
2433 11739 L 2297 11723 L 2297 11865 L 2428 11966 L
2433 11975 L 2524 12102 L 2521 12238 L 2568 12280 L
2704 12263 L 2714 12238 L 2716 12102 L 2839 12025 L
2975 12068 L 3040 12102 L 3110 12128 L 3246 12102 L
3248 12102 L 3381 12061 L 3517 12025 L 3652 11972 L
3788 11975 L 3923 12050 L 4059 12071 L 4116 12102 L
4194 12175 L 4316 12238 L 4330 12362 L 4335 12373 L
4330 12374 L 4329 12373 L 4194 12285 L 4059 12278 L
3923 12292 L 3788 12305 L 3652 12322 L 3517 12369 L
3511 12373 L 3381 12478 L 3250 12509 L 3246 12515 L
3110 12614 L 3068 12645 L 3072 12780 L 3110 12875 L
3160 12916 L 3234 13052 L 3229 13187 L 3246 13242 L
3381 13308 L 3381 12896 L 3294 12780 L 3381 12739 L
3501 12780 L 3381 12896 L 3381 13308 L 3517 13309 L
3652 13220 L 3663 13187 L 3702 13052 L 3738 12916 L
3788 12886 L 3923 12863 L 4010 12916 L 4048 13052 L
4026 13187 L 4018 13323 L 4059 13418 L 4193 13459 L
4194 13459 L 4195 13459 L 4236 13323 L 4330 13200 L
4336 13187 L 4465 13071 L 4601 13129 L 4655 13187 L
4620 13323 L 4601 13357 L 4552 13459 L 4465 13574 L
4429 13594 L 4330 13726 L 4323 13730 L 4330 13733 L
4465 13817 L 4502 13866 L 4601 13970 L 4656 14002 L
4736 14118 L 4872 14099 L 4918 14002 L 4872 13873 L
4868 13866 L 4801 13730 L 4872 13634 L 4895 13594 L
4872 13517 L 4852 13459 L 4838 13323 L 4872 13279 L
5007 13276 L 5053 13323 L 5143 13423 L 5182 13459 L
5246 13594 L 5244 13730 L 5269 13866 L 5278 13876 L
5414 13997 L 5420 14002 L 5549 14090 L 5644 14137 L
5685 14163 L 5731 14137 L 5750 14002 L 5685 13957 L
5582 13866 L 5549 13850 L 5414 13799 L 5313 13730 L
5295 13594 L 5374 13459 L 5414 13395 L 5481 13323 L
5414 13255 L 5374 13187 L 5347 13052 L 5344 12916 L
5414 12888 L 5455 12916 L 5481 13052 L 5490 13187 L
5549 13245 L 5620 13187 L 5685 13142 L 5820 13152 L
5854 13052 L 5956 12971 L 6091 13025 L 6114 13052 L
6164 13187 L 6153 13323 L 6091 13405 L 5956 13424 L
5854 13323 L 5820 13238 L 5796 13323 L 5702 13459 L
5820 13544 L 5956 13478 L 6081 13594 L 6091 13599 L

6227 13675 L 6281 13730 L 6327 13866 L 6362 13940 L
6429 14002 L 6362 14131 L 6354 14137 L 6311 14273 L
6362 14385 L 6494 14273 L 6498 14272 L 6506 14273 L
6633 14308 L 6712 14409 L 6633 14504 L 6498 14479 L
6362 14424 L 6339 14544 L 6362 14562 L 6492 14680 L
6498 14686 L 6633 14714 L 6726 14680 L 6769 14598 L
6876 14680 L 6904 14693 L 7040 14782 L 7166 14816 L
7175 14817 L 7182 14816 L 7311 14761 L 7446 14740 L
7527 14680 L 7582 14641 L 7717 14588 L 7775 14544 L
7853 14431 L 7853 13771 L 7717 13752 L 7651 13730 L
7717 13645 L 7746 13594 L 7853 13563 L 7928 13594 L
7967 13730 L 7853 13771 L 7853 14431 L 7854 14409 L
7953 14273 L 7988 14245 L 8124 14168 L 8259 14148 L
8297 14137 L 8352 14002 L 8360 13866 L 8395 13829 L
8477 13730 L 8530 13659 L 8666 13600 L 8701 13594 L
8801 13522 L 8879 13459 L 8857 13323 L 8823 13187 L
8826 13052 L 8801 13032 L 8694 12916 L 8666 12807 L
8649 12780 L 8570 12645 L 8582 12509 L 8666 12432 L
8746 12373 L 8801 12317 L 8937 12280 L 9024 12238 L
9072 12151 L 9072 11111 L 8937 11045 L 8909 11016 L
8937 10984 L 9072 10909 L 9198 11016 L 9072 11111 L
9072 12151 L 9083 12102 L 9093 11966 L 9118 11831 L
9208 11726 L 9235 11695 L 9251 11559 L 9341 11423 L
9343 11419 L 9359 11288 L 9343 11153 L 9343 11152 L
9249 11016 L 9229 10881 L 9208 10831 L 9133 10745 L
9072 10736 L 9057 10745 L 8937 10825 L 8801 10773 L
8779 10745 L 8801 10720 L 8937 10654 L 8966 10609 L
8998 10474 L 9072 10385 L 9208 10356 L 9343 10339 L
9344 10338 L 9424 10202 L 9343 10122 L 9280 10067 L
9315 9931 L 9343 9914 L 9370 9931 L 9479 9993 L
9479 8722 L 9462 8710 L 9479 8688 L 9497 8710 L
9479 8722 L 9479 9993 L 9614 9970 L 9614 9453 L
9572 9388 L 9614 9336 L 9668 9388 L 9614 9453 L
9614 9970 L 9735 9931 L 9750 9919 L 9885 9823 L
9939 9795 L 9920 9660 L 9963 9524 L 9926 9388 L
9885 9296 L 9867 9252 L 9768 9117 L 9885 8996 L
10021 9014 L 10156 9016 L 10263 8981 L 10292 8968 L
10427 8917 L 10563 8954 L 10622 8981 L 10698 9009 L
10834 9003 L 10959 9117 L 10969 9153 L 11105 9144 L
11122 9117 L 11105 9094 L 10994 8981 L 10969 8952 L
10874 8845 L 10834 8775 L 10698 8743 L 10659 8710 L
10698 8625 L 10834 8683 L 10969 8691 L 11105 8682 L
11240 8661 L 11240 8169 L 11191 8167 L 11240 8164 L

11242 8167 L 11240 8169 L 11240 8661 L 11353 8710 L
11376 8721 L 11511 8731 L 11647 8760 L 11782 8827 L
11809 8845 L 11918 8901 L 12001 8981 L 12053 9058 L
12061 9117 L 12076 9252 L 12097 9388 L 12116 9524 L
12135 9660 L 12152 9795 L 12169 9931 L 12186 10067 L
12189 10096 L 12238 10202 L 12295 10338 L 12324 10405 L
12372 10474 L 12460 10597 L 12469 10609 L 12560 10745 L
12595 10795 L 12654 10881 L 12731 10966 L 12781 11016 L
12866 11087 L 12972 11152 L 13002 11171 L 13002 10028 L
12866 9999 L 12758 9931 L 12731 9911 L 12626 9795 L
12595 9769 L 12491 9660 L 12460 9610 L 12374 9524 L
12324 9483 L 12267 9388 L 12189 9303 L 12184 9252 L
12175 9117 L 12166 8981 L 12153 8845 L 12140 8710 L
12124 8574 L 12116 8438 L 12101 8303 L 12088 8167 L
12087 8031 L 12084 7896 L 12067 7760 L 12053 7711 L
11918 7632 L 11899 7624 L 11782 7581 L 11647 7526 L
11511 7496 L 11476 7489 L 11376 7480 L 11240 7472 L
11177 7489 L 11124 7624 L 11127 7760 L 11142 7896 L
11105 7958 L 10984 8031 L 10969 8053 L 10888 8167 L
10834 8293 L 10733 8167 L 10757 8031 L 10718 7896 L
10698 7877 L 10670 7896 L 10672 8031 L 10632 8167 L
10563 8234 L 10427 8239 L 10292 8205 L 10156 8274 L
10021 8213 L 9983 8167 L 9885 8055 L 9871 8031 L
9750 7935 L 9652 8031 L 9614 8082 L 9479 8070 L
9345 8167 L 9343 8168 L 9208 8266 L 9131 8303 L
9072 8324 L 8990 8438 L 9072 8541 L 9114 8574 L
9072 8624 L 8937 8602 L 8918 8574 L 8883 8438 L
8801 8363 L 8732 8303 L 8695 8167 L 8751 8031 L
8755 7896 L 8800 7760 L 8801 7759 L 8894 7624 L
8918 7489 L 8838 7353 L 8801 7311 L 8723 7217 L
8666 7165 L 8545 7081 L 8530 7067 L 8395 7047 L
8385 6946 L 8268 6810 L 8259 6804 L 8124 6802 L
8063 6674 L 7988 6575 L 7951 6539 L 7853 6447 L
7717 6440 L 7582 6518 L 7564 6539 L 7577 6674 L
7552 6810 L 7546 6946 L 7446 7044 L 7361 7081 L
7327 7217 L 7311 7235 L 7276 7217 L 7179 7081 L
7175 7080 L 7040 7035 L 6951 7081 L 6904 7106 L
6846 7217 L 6896 7353 L 6769 7474 L 6633 7467 L
6503 7489 L 6498 7491 L 6362 7534 L 6302 7489 L
6227 7453 L 6091 7360 L 6083 7353 L 6091 7345 L
6223 7217 L 6095 7081 L 6227 7001 L 6362 7046 L
6494 7081 L 6498 7083 L 6499 7081 L 6633 6997 L
6675 6946 L 6633 6912 L 6498 6897 L 6439 6810 L

6388 6674 L 6362 6608 L 6227 6547 L 6132 6674 L
6091 6763 L 6078 6810 L 6091 6887 L 6102 6946 L
6091 7037 L 6087 7081 L 5956 7205 L 5836 7081 L
5820 7031 L 5685 7054 L 5641 6946 L 5683 6810 L
5549 6688 L 5510 6674 L 5414 6543 L 5404 6539 L
5278 6478 L 5148 6403 L 5278 6354 L 5414 6389 L
5471 6403 L 5549 6453 L 5571 6403 L 5685 6280 L
5694 6267 L 5806 6132 L 5820 6124 L 5956 6026 L
6091 6002 L 6099 5996 L 6203 5860 L 6227 5798 L
6247 5725 L 6227 5631 L 6226 5589 L 6185 5453 L
6096 5453 L 6055 5453 L 6091 5512 L 6136 5589 L
6091 5656 L 5956 5689 L 5820 5710 L 5810 5725 L
5685 5858 L 5556 5725 L 5549 5713 L 5445 5589 L
5490 5453 L 5405 5453 L 5232 5453 L 5060 5453 L
4887 5453 L 4715 5453 L 4542 5453 L 4369 5453 L
4197 5453 L 4024 5453 L 3851 5453 L 3679 5453 L
3506 5453 L 3333 5453 L 3161 5453 L 2988 5453 L
2815 5453 L 2643 5453 L 2470 5453 L N E
38 O
2297 6313 2297 7863 2433 7879 2568 7856 2641 7760 2697 7624
2704 7613 2839 7561 2975 7585 3110 7502 3116 7489 3153 7353
3207 7217 3246 7156 3381 7151 3447 7081 3466 6946 3381 6842
3246 6826 3213 6810 3242 6674 3145 6539 3110 6521 3060 6403
2975 6327 2842 6403 2839 6405 2704 6420 2662 6403 2568 6370
2480 6267 2433 6185 2324 6267 2297 6313 34 N F
2297 9560 M 2297 11723 L 2433 11739 L 2492 11695 L 2535 11559 L
2568 11532 L 2704 11486 L 2839 11485 L 2975 11456 L
3110 11424 L 3114 11423 L 3246 11388 L 3381 11355 L
3501 11288 L 3517 11217 L 3521 11152 L 3568 11016 L
3652 10940 L 3723 10881 L 3788 10827 L 3923 10834 L
4059 10850 L 4194 10772 L 4221 10745 L 4330 10614 L
4465 10640 L 4601 10713 L 4736 10618 L 4833 10745 L
4785 10881 L 4736 10922 L 4675 11016 L 4601 11090 L
4465 11092 L 4330 11058 L 4194 11110 L 4177 11152 L
4091 11288 L 4059 11307 L 3923 11380 L 3788 11406 L
3717 11423 L 3652 11455 L 3517 11507 L 3498 11559 L
3381 11678 L 3358 11695 L 3246 11759 L 3110 11784 L
2975 11811 L 2912 11831 L 2973 11966 L 2975 11967 L
3110 12009 L 3246 11997 L 3381 11994 L 3517 11972 L
3533 11966 L 3652 11870 L 3788 11839 L 3923 11881 L
4032 11831 L 4059 11812 L 4138 11831 L 4194 11840 L
4330 11922 L 4465 11945 L 4521 11966 L 4601 12079 L
4736 12074 L 4857 12102 L 4872 12128 L 4875 12102 L

5007 12012 L 5143 12056 L 5157 12102 L 5143 12227 L
5140 12238 L 5007 12359 L 4998 12373 L 5007 12497 L
5008 12509 L 5007 12510 L 5006 12509 L 4872 12448 L
4736 12431 L 4601 12476 L 4528 12509 L 4465 12545 L
4330 12521 L 4313 12509 L 4194 12462 L 4059 12399 L
3923 12404 L 3788 12426 L 3652 12455 L 3546 12509 L
3636 12645 L 3652 12649 L 3788 12659 L 3923 12718 L
4023 12780 L 4059 12794 L 4194 12828 L 4316 12916 L
4330 12925 L 4465 12933 L 4601 12956 L 4714 13052 L
4736 13078 L 4872 13066 L 5007 13095 L 5119 13187 L
5143 13215 L 5169 13187 L 5154 13052 L 5143 13030 L
5049 12916 L 5037 12780 L 5143 12712 L 5210 12780 L
5278 12815 L 5414 12794 L 5414 7932 L 5378 7896 L
5400 7760 L 5414 7754 L 5416 7760 L 5426 7896 L
5414 7932 L 5414 12794 L 5549 12837 L 5685 12825 L
5820 12781 L 5822 12780 L 5880 12645 L 5956 12515 L
5956 12480 L 5934 12373 L 5956 12289 L 5956 12221 L
5877 12102 L 5820 12075 L 5756 11966 L 5820 11865 L
5858 11831 L 5956 11706 L 5956 9712 L 5907 9660 L
5820 9541 L 5809 9524 L 5820 9482 L 5956 9391 L
5956 9093 L 5855 8981 L 5932 8845 L 5874 8710 L
5956 8639 L 6021 8710 L 5994 8845 L 6035 8981 L
5956 9093 L 5956 9391 L 5958 9388 L 6041 9252 L
6091 9233 L 6121 9252 L 6227 9373 L 6241 9388 L
6227 9403 L 6105 9524 L 6091 9538 L 6021 9660 L
5956 9712 L 5956 11706 L 6051 11831 L 6041 11966 L
6052 12102 L 5956 12221 L 5956 12289 L 5996 12373 L
5956 12480 L 5956 12515 L 6091 12602 L 6091 10353 L
6044 10338 L 6091 10309 L 6204 10202 L 6145 10067 L
6227 9953 L 6362 10017 L 6412 10067 L 6362 10140 L
6249 10202 L 6227 10220 L 6115 10338 L 6091 10353 L
6091 12602 L 6192 12509 L 6120 12373 L 6156 12238 L
6122 12102 L 6143 11966 L 6162 11831 L 6227 11797 L
6328 11831 L 6362 11847 L 6454 11966 L 6498 12010 L
6590 12102 L 6633 12236 L 6666 12102 L 6701 11966 L
6633 11867 L 6606 11831 L 6617 11695 L 6537 11559 L
6498 11516 L 6448 11423 L 6498 11372 L 6498 10895 L
6475 10881 L 6362 10773 L 6329 10745 L 6362 10717 L
6393 10745 L 6498 10856 L 6518 10881 L 6498 10895 L
6498 11372 L 6538 11423 L 6633 11514 L 6769 11465 L
6903 11559 L 6894 11695 L 6904 11790 L 7040 11718 L
7175 11731 L 7180 11695 L 7175 11591 L 7167 11559 L
7175 11558 L 7175 10617 L 7167 10609 L 7175 10593 L

7185 10609 L 7175 10617 L 7175 11558 L 7311 11539 L
7335 11559 L 7446 11636 L 7512 11695 L 7509 11831 L
7523 11966 L 7446 12090 L 7409 12102 L 7446 12171 L
7526 12238 L 7582 12244 L 7717 12265 L 7717 10771 L
7691 10745 L 7717 10722 L 7748 10745 L 7717 10771 L
7717 12265 L 7817 12373 L 7717 12501 L 7710 12509 L
7717 12532 L 7775 12645 L 7741 12780 L 7717 12814 L
7622 12916 L 7582 12969 L 7446 13012 L 7311 13015 L
7175 12998 L 7040 12958 L 6904 13015 L 6769 13014 L
6633 12988 L 6498 12921 L 6489 12916 L 6362 12861 L
6227 12845 L 6131 12916 L 6212 13052 L 6221 13187 L
6227 13241 L 6313 13323 L 6362 13434 L 6498 13345 L
6633 13353 L 6769 13377 L 6815 13459 L 6890 13594 L
6904 13704 L 6922 13730 L 7040 13752 L 7175 13747 L
7206 13730 L 7311 13618 L 7358 13594 L 7408 13459 L
7429 13323 L 7432 13187 L 7446 13174 L 7582 13119 L
7717 13117 L 7833 13052 L 7853 13031 L 7853 9541 L
7837 9524 L 7853 9503 L 7872 9524 L 7853 9541 L
7853 13031 L 7988 12996 L 7988 11339 L 7939 11288 L
7988 11237 L 8041 11288 L 7988 11339 L 7988 12996 L
8124 12977 L 8124 12180 L 8051 12102 L 8124 12024 L
8205 12102 L 8124 12180 L 8124 12977 L 8259 12917 L
8259 11788 L 8197 11695 L 8259 11614 L 8259 10818 L
8205 10745 L 8259 10680 L 8336 10745 L 8259 10818 L
8259 11614 L 8395 11601 L 8495 11695 L 8395 11787 L
8259 11788 L 8259 12917 L 8261 12916 L 8395 12818 L
8414 12780 L 8441 12645 L 8426 12509 L 8530 12382 L
8554 12373 L 8560 12238 L 8666 12134 L 8801 12124 L
8818 12102 L 8801 12032 L 8768 11966 L 8666 11873 L
8653 11831 L 8666 11747 L 8686 11695 L 8692 11559 L
8666 11555 L 8544 11423 L 8604 11288 L 8552 11152 L
8530 11115 L 8419 11016 L 8441 10881 L 8530 10825 L
8583 10745 L 8666 10615 L 8666 9882 L 8578 9795 L
8666 9714 L 8765 9795 L 8666 9882 L 8666 10615 L
8669 10609 L 8801 10498 L 8815 10474 L 8803 10338 L
8832 10202 L 8801 10168 L 8697 10067 L 8801 9987 L
8867 9931 L 8906 9795 L 8937 9740 L 9072 9698 L
9131 9660 L 9072 9553 L 9060 9524 L 8942 9388 L
8998 9252 L 8937 9159 L 8801 9183 L 8666 9169 L
8530 9228 L 8426 9252 L 8485 9388 L 8395 9468 L
8307 9388 L 8372 9252 L 8259 9174 L 8216 9117 L
8259 9047 L 8336 8981 L 8259 8895 L 8151 8845 L
8124 8831 L 7988 8834 L 7976 8845 L 7853 8961 L

7727 8845 L 7717 8833 L 7621 8710 L 7582 8670 L
7469 8574 L 7491 8438 L 7517 8303 L 7460 8167 L
7446 8156 L 7433 8167 L 7407 8303 L 7417 8438 L
7326 8574 L 7311 8584 L 7265 8574 L 7267 8438 L
7175 8313 L 7144 8303 L 7141 8167 L 7040 8127 L
6963 8031 L 6961 7896 L 6904 7812 L 6843 7760 L
6769 7638 L 6633 7686 L 6576 7760 L 6498 7795 L
6363 7896 L 6362 7896 L 6362 7896 L 6296 7760 L
6227 7717 L 6208 7760 L 6189 7896 L 6162 8031 L
6227 8115 L 6362 8067 L 6498 8097 L 6633 8162 L
6643 8167 L 6769 8284 L 6904 8283 L 6943 8303 L
6904 8323 L 6769 8311 L 6752 8303 L 6633 8176 L
6498 8223 L 6460 8303 L 6363 8438 L 6362 8439 L
6361 8438 L 6264 8303 L 6227 8233 L 6137 8303 L
6091 8355 L 5956 8322 L 5941 8303 L 5893 8167 L
5846 8031 L 5840 7896 L 5820 7875 L 5750 7760 L
5713 7624 L 5686 7489 L 5685 7487 L 5549 7369 L
5414 7398 L 5278 7415 L 5186 7353 L 5143 7330 L
5007 7331 L 4872 7317 L 4784 7353 L 4841 7489 L
4872 7503 L 5007 7548 L 5143 7619 L 5151 7624 L
5229 7760 L 5143 7823 L 5007 7837 L 4890 7760 L
4872 7748 L 4736 7750 L 4692 7760 L 4620 7896 L
4736 8013 L 4757 8031 L 4736 8072 L 4680 8167 L
4601 8204 L 4559 8167 L 4465 8133 L 4338 8167 L
4330 8182 L 4304 8303 L 4330 8321 L 4465 8422 L
4477 8438 L 4601 8528 L 4652 8574 L 4601 8656 L
4574 8710 L 4465 8763 L 4432 8710 L 4330 8586 L
4312 8574 L 4194 8470 L 4124 8438 L 4059 8357 L
3993 8438 L 4050 8574 L 4039 8710 L 3923 8771 L
3854 8845 L 3872 8981 L 3864 9117 L 3788 9236 L
3773 9252 L 3746 9388 L 3779 9524 L 3788 9544 L
3885 9660 L 3923 9766 L 4059 9717 L 4104 9795 L
4194 9857 L 4330 9859 L 4465 9874 L 4525 9931 L
4601 9983 L 4667 10067 L 4617 10202 L 4626 10338 L
4601 10397 L 4465 10459 L 4444 10474 L 4330 10594 L
4194 10542 L 4059 10522 L 3955 10474 L 3923 10455 L
3788 10418 L 3652 10388 L 3532 10338 L 3517 10333 L
3381 10215 L 3369 10202 L 3246 10091 L 3217 10067 L
3110 9998 L 3019 9931 L 2975 9896 L 2839 9821 L
2806 9795 L 2724 9660 L 2704 9624 L 2625 9524 L
2568 9449 L 2433 9461 L 2341 9524 L 2297 9560 L
N E
74 O

2428 11966 M 2297 11865 L 2297 11959 L 2307 11966 L 2403 12102 L
2362 12238 L 2362 12373 L 2433 12407 L 2568 12477 L
2704 12484 L 2829 12373 L 2828 12238 L 2839 12177 L
2975 12173 L 3110 12199 L 3246 12137 L 3381 12113 L
3444 12102 L 3517 12078 L 3652 12023 L 3788 12060 L
3853 12102 L 3788 12209 L 3747 12238 L 3652 12257 L
3517 12324 L 3452 12373 L 3381 12430 L 3246 12475 L
3110 12504 L 3094 12509 L 2975 12582 L 2935 12645 L
2954 12780 L 2975 12842 L 3002 12916 L 3048 13052 L
3071 13187 L 3068 13323 L 3098 13459 L 3110 13482 L
3204 13594 L 3242 13730 L 3246 13754 L 3302 13866 L
3381 13897 L 3458 13866 L 3517 13822 L 3652 13752 L
3697 13730 L 3788 13667 L 3788 13489 L 3780 13459 L
3788 13451 L 3879 13459 L 3788 13489 L 3788 13667 L
3880 13730 L 3923 13771 L 3985 13866 L 4059 13902 L
4170 14002 L 4194 14025 L 4279 14137 L 4330 14241 L
4349 14273 L 4465 14408 L 4466 14409 L 4590 14544 L
4601 14549 L 4621 14544 L 4736 14510 L 4777 14544 L
4848 14680 L 4872 14700 L 5007 14783 L 5051 14680 L
5031 14544 L 5011 14409 L 5046 14273 L 5074 14137 L
5055 14002 L 5007 13903 L 4982 13866 L 4959 13730 L
5007 13666 L 5092 13730 L 5143 13756 L 5205 13866 L
5278 13948 L 5337 14002 L 5414 14080 L 5484 14137 L
5547 14273 L 5549 14278 L 5685 14366 L 5795 14273 L
5820 14258 L 5940 14137 L 5859 14002 L 5833 13866 L
5924 13730 L 5956 13670 L 6091 13681 L 6167 13730 L
6227 13793 L 6263 13866 L 6256 14002 L 6227 14026 L
6152 14137 L 6091 14257 L 6067 14273 L 6079 14409 L
6091 14448 L 6136 14544 L 6227 14558 L 6362 14674 L
6369 14680 L 6493 14816 L 6498 14819 L 6633 14851 L
6769 14903 L 6817 14951 L 6904 15074 L 6925 15087 L
7040 15132 L 7175 15102 L 7311 15130 L 7418 15223 L
7446 15326 L 7582 15352 L 7717 15241 L 7853 15237 L
7902 15223 L 7988 15171 L 8037 15087 L 7988 15020 L
7897 14951 L 7853 14891 L 7717 14918 L 7695 14816 L
7717 14803 L 7853 14786 L 7865 14680 L 7874 14544 L
7886 14409 L 7988 14301 L 8087 14273 L 8124 14248 L
8259 14225 L 8395 14224 L 8412 14137 L 8417 14002 L
8420 13866 L 8530 13750 L 8612 13730 L 8666 13696 L
8797 13730 L 8801 13730 L 8937 13825 L 9003 13866 L
9072 13882 L 9155 13866 L 9208 13844 L 9343 13731 L
9344 13730 L 9343 13729 L 9265 13594 L 9208 13489 L
9186 13459 L 9072 13374 L 9024 13323 L 8941 13187 L

8937 13136 L 8908 13052 L 8801 12967 L 8755 12916 L
8720 12780 L 8666 12701 L 8629 12645 L 8652 12509 L
8666 12497 L 8801 12420 L 8863 12373 L 8937 12345 L
9072 12292 L 9134 12238 L 9134 12102 L 9156 11966 L
9201 11831 L 9208 11822 L 9318 11695 L 9341 11559 L
9343 11556 L 9426 11423 L 9451 11288 L 9479 11253 L
9568 11152 L 9614 11089 L 9750 11031 L 9763 11016 L
9804 10881 L 9750 10801 L 9614 10866 L 9479 10868 L
9379 10745 L 9367 10609 L 9358 10474 L 9479 10348 L
9493 10338 L 9529 10202 L 9614 10099 L 9750 10090 L
9822 10067 L 9885 10016 L 10021 9963 L 10126 9931 L
10144 9795 L 10156 9755 L 10167 9660 L 10292 9526 L
10324 9524 L 10292 9521 L 10156 9495 L 10071 9388 L
10156 9304 L 10292 9265 L 10307 9252 L 10356 9117 L
10427 9064 L 10563 9073 L 10614 9117 L 10563 9206 L
10541 9252 L 10427 9384 L 10423 9388 L 10427 9430 L
10563 9469 L 10698 9455 L 10834 9486 L 10879 9388 L
10969 9320 L 11079 9388 L 10969 9509 L 10917 9524 L
10969 9570 L 11105 9565 L 11133 9524 L 11143 9388 L
11204 9252 L 11240 9191 L 11376 9242 L 11402 9252 L
11511 9306 L 11635 9388 L 11647 9396 L 11782 9487 L
11899 9524 L 11918 9530 L 12034 9660 L 12053 9673 L
12076 9795 L 12097 9931 L 12114 10067 L 12126 10202 L
12139 10338 L 12157 10474 L 12170 10609 L 12184 10745 L
12189 10782 L 12233 10881 L 12294 11016 L 12324 11081 L
12381 11152 L 12460 11263 L 12478 11288 L 12585 11423 L
12595 11439 L 12699 11559 L 12731 11600 L 12845 11695 L
12866 11713 L 13002 11752 L 13002 11171 L 12972 11152 L
12866 11087 L 12781 11016 L 12731 10966 L 12654 10881 L
12595 10795 L 12560 10745 L 12469 10609 L 12460 10597 L
12372 10474 L 12324 10405 L 12295 10338 L 12238 10202 L
12189 10096 L 12186 10067 L 12169 9931 L 12152 9795 L
12135 9660 L 12116 9524 L 12097 9388 L 12076 9252 L
12061 9117 L 12053 9058 L 12001 8981 L 11918 8901 L
11809 8845 L 11782 8827 L 11647 8760 L 11511 8731 L
11376 8721 L 11353 8710 L 11240 8661 L 11105 8682 L
10969 8691 L 10834 8683 L 10698 8625 L 10659 8710 L
10698 8743 L 10834 8775 L 10874 8845 L 10969 8952 L
10994 8981 L 11105 9094 L 11122 9117 L 11105 9144 L
10969 9153 L 10959 9117 L 10834 9003 L 10698 9009 L
10622 8981 L 10563 8954 L 10427 8917 L 10292 8968 L
10263 8981 L 10156 9016 L 10021 9014 L 9885 8996 L
9768 9117 L 9867 9252 L 9885 9296 L 9926 9388 L

9963 9524 L 9920 9660 L 9939 9795 L 9885 9823 L
9750 9919 L 9735 9931 L 9614 9970 L 9479 9993 L
9370 9931 L 9343 9914 L 9315 9931 L 9280 10067 L
9343 10122 L 9424 10202 L 9344 10338 L 9343 10339 L
9208 10356 L 9072 10385 L 8998 10474 L 8966 10609 L
8937 10654 L 8801 10720 L 8779 10745 L 8801 10773 L
8937 10825 L 9057 10745 L 9072 10736 L 9072 10629 L
9056 10609 L 9072 10562 L 9153 10474 L 9208 10452 L
9251 10474 L 9208 10520 L 9112 10609 L 9072 10629 L
9072 10736 L 9133 10745 L 9208 10831 L 9229 10881 L
9249 11016 L 9343 11152 L 9343 11153 L 9359 11288 L
9343 11419 L 9341 11423 L 9251 11559 L 9235 11695 L
9208 11726 L 9118 11831 L 9093 11966 L 9083 12102 L
9072 12151 L 9024 12238 L 8937 12280 L 8801 12317 L
8746 12373 L 8666 12432 L 8582 12509 L 8570 12645 L
8649 12780 L 8666 12807 L 8694 12916 L 8801 13032 L
8826 13052 L 8823 13187 L 8857 13323 L 8879 13459 L
8801 13522 L 8701 13594 L 8666 13600 L 8530 13659 L
8477 13730 L 8395 13829 L 8360 13866 L 8352 14002 L
8297 14137 L 8259 14148 L 8124 14168 L 7988 14245 L
7953 14273 L 7854 14409 L 7853 14431 L 7775 14544 L
7717 14588 L 7582 14641 L 7527 14680 L 7446 14740 L
7311 14761 L 7182 14816 L 7175 14817 L 7166 14816 L
7040 14782 L 6904 14693 L 6876 14680 L 6769 14598 L
6726 14680 L 6633 14714 L 6498 14686 L 6492 14680 L
6362 14562 L 6339 14544 L 6362 14424 L 6498 14479 L
6633 14504 L 6712 14409 L 6633 14308 L 6506 14273 L
6498 14272 L 6494 14273 L 6362 14385 L 6311 14273 L
6354 14137 L 6362 14131 L 6429 14002 L 6362 13940 L
6327 13866 L 6281 13730 L 6227 13675 L 6091 13599 L
6081 13594 L 5956 13478 L 5820 13544 L 5702 13459 L
5796 13323 L 5820 13238 L 5854 13323 L 5956 13424 L
6091 13405 L 6091 13253 L 5976 13187 L 6091 13152 L
6107 13187 L 6091 13253 L 6091 13405 L 6153 13323 L
6164 13187 L 6114 13052 L 6091 13025 L 5956 12971 L
5854 13052 L 5820 13152 L 5685 13142 L 5620 13187 L
5549 13245 L 5490 13187 L 5481 13052 L 5455 12916 L
5414 12888 L 5344 12916 L 5347 13052 L 5374 13187 L
5414 13255 L 5481 13323 L 5414 13395 L 5374 13459 L
5295 13594 L 5313 13730 L 5414 13799 L 5549 13850 L
5582 13866 L 5685 13957 L 5685 13858 L 5549 13800 L
5434 13730 L 5414 13692 L 5369 13594 L 5414 13532 L
5549 13565 L 5573 13594 L 5685 13709 L 5710 13730 L

5685 13858 L 5685 13957 L 5750 14002 L 5731 14137 L
5685 14163 L 5644 14137 L 5549 14090 L 5420 14002 L
5414 13997 L 5278 13876 L 5269 13866 L 5244 13730 L
5246 13594 L 5182 13459 L 5143 13423 L 5053 13323 L
5007 13276 L 4872 13279 L 4838 13323 L 4852 13459 L
4872 13517 L 4895 13594 L 4872 13634 L 4801 13730 L
4868 13866 L 4872 13873 L 4918 14002 L 4872 14099 L
4736 14118 L 4656 14002 L 4601 13970 L 4502 13866 L
4465 13817 L 4330 13733 L 4323 13730 L 4330 13726 L
4429 13594 L 4465 13574 L 4552 13459 L 4601 13357 L
4620 13323 L 4655 13187 L 4601 13129 L 4465 13071 L
4336 13187 L 4330 13200 L 4236 13323 L 4195 13459 L
4194 13459 L 4193 13459 L 4059 13418 L 4018 13323 L
4026 13187 L 4048 13052 L 4010 12916 L 3923 12863 L
3788 12886 L 3738 12916 L 3702 13052 L 3663 13187 L
3652 13220 L 3517 13309 L 3381 13308 L 3246 13242 L
3229 13187 L 3234 13052 L 3160 12916 L 3110 12875 L
3072 12780 L 3068 12645 L 3110 12614 L 3246 12515 L
3250 12509 L 3381 12478 L 3511 12373 L 3517 12369 L
3652 12322 L 3788 12305 L 3923 12292 L 4059 12278 L
4194 12285 L 4329 12373 L 4330 12374 L 4335 12373 L
4330 12362 L 4316 12238 L 4194 12175 L 4116 12102 L
4059 12071 L 3923 12050 L 3788 11975 L 3652 11972 L
3517 12025 L 3381 12061 L 3248 12102 L 3246 12102 L
3110 12128 L 3040 12102 L 2975 12068 L 2839 12025 L
2716 12102 L 2714 12238 L 2704 12263 L 2568 12280 L
2521 12238 L 2524 12102 L 2433 11975 L 2428 11966 L
N E
92 O
2307 11966 M 2297 11959 L 2297 12062 L 2327 12102 L 2297 12170 L
2297 12562 L 2368 12645 L 2349 12780 L 2297 12865 L
2297 13055 L 2433 13079 L 2565 13187 L 2568 13194 L
2568 13006 L 2433 12944 L 2402 12916 L 2433 12790 L
2437 12780 L 2568 12719 L 2670 12780 L 2630 12916 L
2568 13006 L 2568 13194 L 2572 13187 L 2660 13052 L
2704 12992 L 2749 12916 L 2839 12782 L 2839 12682 L
2828 12645 L 2839 12643 L 2840 12645 L 2839 12682 L
2839 12782 L 2914 12916 L 2968 13052 L 2975 13074 L
3009 13187 L 2997 13323 L 2982 13459 L 3001 13594 L
3110 13722 L 3114 13730 L 3148 13866 L 3240 14002 L
3154 14137 L 3110 14172 L 2975 14195 L 2924 14137 L
2917 14002 L 2931 13866 L 2975 13762 L 3072 13730 L
2975 13675 L 2839 13691 L 2815 13730 L 2773 13866 L

2704 13942 L 2674 14002 L 2651 14137 L 2592 14273 L
2568 14301 L 2433 14376 L 2385 14409 L 2350 14544 L
2341 14680 L 2297 14772 L 2297 15068 L 2320 15087 L
2433 15140 L 2513 15223 L 2527 15358 L 2568 15414 L
2673 15494 L 2704 15500 L 2737 15494 L 2839 15455 L
2953 15358 L 2839 15305 L 2756 15223 L 2725 15087 L
2719 14951 L 2704 14875 L 2664 14816 L 2655 14680 L
2704 14654 L 2730 14680 L 2839 14764 L 2867 14816 L
2975 14898 L 3023 14951 L 3110 15055 L 3143 15087 L
3246 15194 L 3246 15122 L 3212 15087 L 3110 14987 L
3080 14951 L 2975 14835 L 2950 14816 L 2892 14680 L
2839 14549 L 2835 14544 L 2704 14438 L 2662 14409 L
2704 14328 L 2839 14311 L 2975 14386 L 3078 14409 L
3110 14449 L 3125 14544 L 3164 14680 L 3242 14816 L
3246 14820 L 3335 14951 L 3328 15087 L 3246 15122 L
3246 15194 L 3365 15223 L 3381 15233 L 3381 14299 L
3367 14273 L 3381 14253 L 3450 14137 L 3517 14076 L
3652 14085 L 3702 14137 L 3652 14200 L 3517 14224 L
3414 14273 L 3381 14299 L 3381 15233 L 3397 15223 L
3517 15140 L 3652 15130 L 3704 15087 L 3729 14951 L
3759 14816 L 3788 14787 L 3923 14727 L 3923 14462 L
3884 14409 L 3916 14273 L 3923 14271 L 4059 14250 L
4104 14273 L 4059 14348 L 4013 14409 L 3923 14462 L
3923 14727 L 4059 14790 L 4194 14765 L 4330 14694 L
4330 14452 L 4219 14409 L 4330 14315 L 4464 14409 L
4330 14452 L 4330 14694 L 4465 14693 L 4601 14698 L
4736 14788 L 4778 14816 L 4872 14879 L 4916 14951 L
4993 15087 L 5007 15126 L 5132 15223 L 5143 15227 L
5148 15223 L 5266 15087 L 5250 14951 L 5185 14816 L
5163 14680 L 5143 14568 L 5134 14544 L 5092 14409 L
5130 14273 L 5143 14229 L 5158 14137 L 5143 14043 L
5132 14002 L 5143 13893 L 5190 14002 L 5278 14014 L
5400 14137 L 5414 14166 L 5483 14273 L 5549 14401 L
5559 14409 L 5658 14544 L 5685 14575 L 5756 14680 L
5814 14816 L 5820 14844 L 5820 14712 L 5806 14680 L
5750 14544 L 5800 14409 L 5820 14374 L 5828 14409 L
5956 14521 L 6018 14544 L 5960 14680 L 5956 14685 L
5820 14712 L 5820 14844 L 5847 14816 L 5956 14774 L
6032 14680 L 6091 14614 L 6227 14608 L 6309 14680 L
6362 14800 L 6376 14816 L 6498 14892 L 6633 14921 L
6696 14951 L 6769 14985 L 6854 15087 L 6904 15140 L
7040 15221 L 7054 15223 L 7175 15270 L 7235 15358 L
7291 15494 L 7311 15511 L 7446 15545 L 7582 15604 L

7717 15528 L 7740 15494 L 7853 15408 L 7988 15434 L
8124 15461 L 8259 15412 L 8370 15358 L 8395 15281 L
8401 15223 L 8395 15196 L 8353 15087 L 8302 14951 L
8259 14943 L 8124 14936 L 7988 14930 L 7875 14816 L
7891 14680 L 7901 14544 L 7917 14409 L 7988 14335 L
8124 14299 L 8259 14286 L 8395 14292 L 8413 14273 L
8439 14137 L 8447 14002 L 8454 13866 L 8530 13786 L
8666 13750 L 8801 13751 L 8918 13866 L 8937 13884 L
9068 14002 L 9072 14006 L 9208 14058 L 9343 14013 L
9398 14002 L 9479 13907 L 9545 13866 L 9548 13730 L
9479 13697 L 9381 13594 L 9343 13584 L 9227 13459 L
9214 13323 L 9208 13317 L 9072 13309 L 8961 13187 L
8965 13052 L 8937 12987 L 8834 12916 L 8801 12860 L
8781 12780 L 8768 12645 L 8801 12606 L 8898 12509 L
8937 12485 L 9072 12385 L 9102 12373 L 9208 12320 L
9311 12238 L 9208 12191 L 9185 12102 L 9208 12005 L
9231 11966 L 9323 11831 L 9343 11805 L 9438 11695 L
9479 11640 L 9565 11559 L 9581 11423 L 9614 11304 L
9620 11288 L 9720 11152 L 9750 11136 L 9862 11016 L
9885 10955 L 9941 10881 L 9976 10745 L 9885 10673 L
9750 10647 L 9699 10745 L 9614 10809 L 9479 10803 L
9432 10745 L 9443 10609 L 9389 10474 L 9479 10380 L
9537 10338 L 9597 10202 L 9614 10181 L 9684 10202 L
9750 10210 L 9882 10338 L 9790 10474 L 9885 10553 L
9946 10609 L 10021 10703 L 10021 10155 L 9946 10067 L
10021 10013 L 10104 10067 L 10021 10155 L 10021 10703 L
10156 10625 L 10177 10609 L 10268 10474 L 10292 10441 L
10292 9736 L 10233 9660 L 10292 9596 L 10363 9660 L
10292 9736 L 10292 10441 L 10414 10338 L 10427 10327 L
10552 10202 L 10563 10192 L 10609 10067 L 10698 9972 L
10716 9931 L 10834 9834 L 10886 9795 L 10969 9721 L
11105 9711 L 11191 9660 L 11240 9573 L 11376 9624 L
11441 9660 L 11511 9704 L 11638 9795 L 11647 9803 L
11782 9891 L 11840 9931 L 11918 9996 L 11982 10067 L
12053 10178 L 12055 10202 L 12065 10338 L 12084 10474 L
12097 10609 L 12110 10745 L 12126 10881 L 12147 11016 L
12175 11152 L 12189 11216 L 12227 11288 L 12294 11423 L
12324 11495 L 12363 11559 L 12443 11695 L 12460 11731 L
12517 11831 L 12595 11962 L 12598 11966 L 12712 12102 L
12731 12128 L 12866 12234 L 12901 12238 L 13002 12245 L
13002 11752 L 12866 11713 L 12845 11695 L 12731 11600 L
12699 11559 L 12595 11439 L 12585 11423 L 12478 11288 L
12460 11263 L 12381 11152 L 12324 11081 L 12294 11016 L

12233 10881 L 12189 10782 L 12184 10745 L 12170 10609 L
12157 10474 L 12139 10338 L 12126 10202 L 12114 10067 L
12097 9931 L 12076 9795 L 12053 9673 L 12034 9660 L
11918 9530 L 11899 9524 L 11782 9487 L 11647 9396 L
11635 9388 L 11511 9306 L 11402 9252 L 11376 9242 L
11240 9191 L 11204 9252 L 11143 9388 L 11133 9524 L
11105 9565 L 10969 9570 L 10917 9524 L 10969 9509 L
11079 9388 L 10969 9320 L 10879 9388 L 10834 9486 L
10698 9455 L 10563 9469 L 10427 9430 L 10423 9388 L
10427 9384 L 10541 9252 L 10563 9206 L 10614 9117 L
10563 9073 L 10427 9064 L 10356 9117 L 10307 9252 L
10292 9265 L 10156 9304 L 10071 9388 L 10156 9495 L
10292 9521 L 10292 9429 L 10156 9395 L 10151 9388 L
10156 9383 L 10292 9350 L 10407 9252 L 10427 9213 L
10446 9252 L 10427 9274 L 10332 9388 L 10292 9429 L
10292 9521 L 10324 9524 L 10292 9526 L 10167 9660 L
10156 9755 L 10144 9795 L 10126 9931 L 10021 9963 L
9885 10016 L 9822 10067 L 9750 10090 L 9614 10099 L
9529 10202 L 9493 10338 L 9479 10348 L 9358 10474 L
9367 10609 L 9379 10745 L 9479 10868 L 9614 10866 L
9750 10801 L 9804 10881 L 9763 11016 L 9750 11031 L
9614 11089 L 9568 11152 L 9479 11253 L 9451 11288 L
9426 11423 L 9343 11556 L 9341 11559 L 9318 11695 L
9208 11822 L 9201 11831 L 9156 11966 L 9134 12102 L
9134 12238 L 9072 12292 L 8937 12345 L 8863 12373 L
8801 12420 L 8666 12497 L 8652 12509 L 8629 12645 L
8666 12701 L 8720 12780 L 8755 12916 L 8801 12967 L
8908 13052 L 8937 13136 L 8941 13187 L 9024 13323 L
9072 13374 L 9186 13459 L 9208 13489 L 9265 13594 L
9343 13729 L 9344 13730 L 9343 13731 L 9208 13844 L
9155 13866 L 9072 13882 L 9003 13866 L 8937 13825 L
8801 13730 L 8797 13730 L 8666 13696 L 8612 13730 L
8530 13750 L 8420 13866 L 8417 14002 L 8412 14137 L
8395 14224 L 8259 14225 L 8124 14248 L 8087 14273 L
7988 14301 L 7886 14409 L 7874 14544 L 7865 14680 L
7853 14786 L 7717 14803 L 7695 14816 L 7717 14918 L
7853 14891 L 7897 14951 L 7988 15020 L 8037 15087 L
7988 15171 L 7902 15223 L 7853 15237 L 7717 15241 L
7582 15352 L 7446 15326 L 7418 15223 L 7311 15130 L
7175 15102 L 7040 15132 L 6925 15087 L 6904 15074 L
6817 14951 L 6769 14903 L 6633 14851 L 6498 14819 L
6493 14816 L 6369 14680 L 6362 14674 L 6227 14558 L
6136 14544 L 6091 14448 L 6079 14409 L 6067 14273 L

6091 14257 L 6091 14079 L 5956 14024 L 5941 14002 L
5956 13947 L 5993 13866 L 6091 13778 L 6176 13866 L
6148 14002 L 6091 14079 L 6091 14257 L 6152 14137 L
6227 14026 L 6256 14002 L 6263 13866 L 6227 13793 L
6167 13730 L 6091 13681 L 5956 13670 L 5924 13730 L
5833 13866 L 5859 14002 L 5940 14137 L 5820 14258 L
5795 14273 L 5685 14366 L 5549 14278 L 5547 14273 L
5484 14137 L 5414 14080 L 5337 14002 L 5278 13948 L
5205 13866 L 5143 13756 L 5092 13730 L 5007 13666 L
4959 13730 L 4982 13866 L 5007 13903 L 5055 14002 L
5074 14137 L 5046 14273 L 5011 14409 L 5031 14544 L
5051 14680 L 5007 14783 L 4872 14700 L 4848 14680 L
4777 14544 L 4736 14510 L 4621 14544 L 4601 14549 L
4590 14544 L 4466 14409 L 4465 14408 L 4349 14273 L
4330 14241 L 4279 14137 L 4194 14025 L 4170 14002 L
4059 13902 L 3985 13866 L 3923 13771 L 3880 13730 L
3788 13667 L 3697 13730 L 3652 13752 L 3517 13822 L
3458 13866 L 3381 13897 L 3302 13866 L 3246 13754 L
3242 13730 L 3204 13594 L 3110 13482 L 3098 13459 L
3068 13323 L 3071 13187 L 3048 13052 L 3002 12916 L
2975 12842 L 2954 12780 L 2935 12645 L 2975 12582 L
3094 12509 L 3110 12504 L 3246 12475 L 3246 12440 L
3110 12437 L 2999 12373 L 3110 12282 L 3137 12238 L
3246 12172 L 3381 12142 L 3517 12152 L 3584 12102 L
3652 12073 L 3724 12102 L 3652 12163 L 3594 12238 L
3517 12279 L 3393 12373 L 3381 12382 L 3246 12440 L
3246 12475 L 3381 12430 L 3452 12373 L 3517 12324 L
3652 12257 L 3747 12238 L 3788 12209 L 3853 12102 L
3788 12060 L 3652 12023 L 3517 12078 L 3444 12102 L
3381 12113 L 3246 12137 L 3110 12199 L 2975 12173 L
2839 12177 L 2828 12238 L 2829 12373 L 2704 12484 L
2568 12477 L 2433 12407 L 2362 12373 L 2362 12238 L
2403 12102 L 2307 11966 L N E
111 O
2327 12102 2297 12062 2297 12170 2327 12102 4 N F
2368 12645 2297 12562 2297 12865 2349 12780 2368 12645 5 N F
2433 13079 2297 13055 2297 13138 2433 13180 2442 13187 2495 13323
2568 13422 2630 13323 2704 13188 2704 13187 2750 13052 2839 12920
2910 13052 2953 13187 2941 13323 2909 13459 2839 13573 2822 13594
2741 13730 2704 13801 2662 13866 2603 14002 2568 14128 2563 14137
2433 14247 2297 14175 2297 14772 2341 14680 2350 14544 2385 14409
2433 14376 2568 14301 2592 14273 2651 14137 2674 14002 2704 13942
2773 13866 2815 13730 2839 13691 2975 13675 3072 13730 2975 13762

2931 13866 2917 14002 2924 14137 2975 14195 3110 14172 3154 14137
3240 14002 3148 13866 3114 13730 3110 13722 3001 13594 2982 13459
2997 13323 3009 13187 2975 13074 2968 13052 2914 12916 2839 12782
2749 12916 2704 12992 2660 13052 2572 13187 2568 13194 2565 13187
2433 13079 67 N F
129 O
2433 13180 2297 13138 2297 13551 2381 13594 2373 13730 2387 13866
2344 14002 2297 14133 2297 14175 2433 14247 2433 14056 2421 14002
2433 13974 2433 13512 2411 13459 2433 13438 2473 13459 2433 13512
2433 13974 2559 13866 2505 13730 2558 13594 2568 13583 2703 13459
2704 13457 2746 13323 2763 13187 2825 13052 2839 13030 2851 13052
2904 13187 2892 13323 2839 13456 2737 13459 2728 13594 2704 13641
2643 13730 2572 13866 2568 13877 2464 14002 2433 14056 2433 14247
2563 14137 2568 14128 2603 14002 2662 13866 2704 13801 2741 13730
2822 13594 2839 13573 2909 13459 2941 13323 2953 13187 2910 13052
2839 12920 2750 13052 2704 13187 2704 13188 2630 13323 2568 13422
2495 13323 2442 13187 2433 13180 63 N F
111 O
2297 13551 2297 14133 2344 14002 2387 13866 2373 13730 2381 13594
2297 13551 7 N F
2320 15087 M 2297 15068 L 2297 15331 L 2323 15358 L 2297 15386 L
2297 15577 L 2433 15559 L 2568 15591 L 2704 15615 L
2747 15630 L 2839 15692 L 2951 15765 L 2975 15772 L
3110 15882 L 3110 15366 L 3106 15358 L 2975 15313 L
2863 15223 L 2839 15191 L 2775 15087 L 2787 14951 L
2839 14879 L 2952 14951 L 2975 14958 L 3091 15087 L
3110 15125 L 3204 15223 L 3128 15358 L 3110 15366 L
3110 15882 L 3138 15901 L 3246 15922 L 3246 15775 L
3231 15765 L 3246 15749 L 3265 15765 L 3246 15775 L
3246 15922 L 3381 15978 L 3432 16037 L 3517 16073 L
3517 15980 L 3476 15901 L 3517 15880 L 3543 15901 L
3517 15980 L 3517 16073 L 3652 16169 L 3659 16172 L
3679 16172 L 3851 16172 L 4024 16172 L 4197 16172 L
4228 16172 L 4330 16089 L 4354 16037 L 4357 15901 L
4330 15849 L 4194 15787 L 4059 15847 L 4039 15901 L
3923 15954 L 3820 15901 L 3923 15780 L 3966 15765 L
3952 15630 L 4059 15551 L 4078 15494 L 4059 15378 L
3975 15358 L 3923 15325 L 3905 15358 L 3852 15494 L
3788 15605 L 3775 15630 L 3652 15737 L 3517 15726 L
3381 15643 L 3367 15630 L 3381 15610 L 3412 15494 L
3417 15358 L 3517 15274 L 3652 15278 L 3788 15282 L
3872 15223 L 3923 15193 L 3997 15087 L 4059 15026 L
4194 15070 L 4330 15031 L 4379 15087 L 4465 15137 L

4601 15141 L 4736 15099 L 4872 15135 L 4912 15223 L
5007 15330 L 5058 15358 L 5143 15452 L 5278 15413 L
5289 15358 L 5308 15223 L 5338 15087 L 5334 14951 L
5278 14850 L 5258 14816 L 5213 14680 L 5210 14544 L
5192 14409 L 5179 14273 L 5204 14137 L 5278 14061 L
5354 14137 L 5414 14265 L 5419 14273 L 5414 14301 L
5373 14409 L 5414 14439 L 5549 14489 L 5592 14544 L
5685 14650 L 5705 14680 L 5760 14816 L 5755 14951 L
5774 15087 L 5820 15157 L 5873 15223 L 5956 15306 L
5956 15104 L 5947 15087 L 5956 15083 L 5960 15087 L
5956 15104 L 5956 15306 L 6016 15358 L 6091 15424 L
6225 15358 L 6144 15223 L 6091 15095 L 6084 15087 L
5956 14964 L 5935 14951 L 5956 14929 L 5979 14816 L
6091 14689 L 6131 14680 L 6227 14658 L 6252 14680 L
6328 14816 L 6362 14906 L 6457 14951 L 6498 14969 L
6633 14982 L 6769 15051 L 6799 15087 L 6904 15198 L
6946 15223 L 7040 15320 L 7091 15358 L 7175 15471 L
7189 15494 L 7311 15595 L 7392 15630 L 7446 15667 L
7580 15765 L 7582 15767 L 7587 15765 L 7717 15720 L
7768 15630 L 7853 15526 L 7988 15564 L 8108 15630 L
8124 15659 L 8144 15630 L 8259 15568 L 8395 15510 L
8429 15494 L 8530 15412 L 8551 15358 L 8530 15240 L
8526 15223 L 8462 15087 L 8401 14951 L 8395 14945 L
8259 14919 L 8124 14909 L 7988 14904 L 7901 14816 L
7917 14680 L 7927 14544 L 7949 14409 L 7988 14368 L
8124 14338 L 8259 14323 L 8395 14321 L 8441 14273 L
8466 14137 L 8477 14002 L 8488 13866 L 8530 13822 L
8666 13782 L 8801 13771 L 8898 13866 L 8937 13904 L
9045 14002 L 9072 14027 L 9208 14130 L 9343 14034 L
9479 14007 L 9484 14002 L 9614 13880 L 9732 13866 L
9659 13730 L 9614 13715 L 9479 13611 L 9463 13594 L
9343 13560 L 9249 13459 L 9237 13323 L 9208 13294 L
9072 13288 L 8981 13187 L 9009 13052 L 8989 12916 L
9046 12780 L 9072 12736 L 9178 12645 L 9208 12623 L
9331 12509 L 9343 12502 L 9479 12429 L 9556 12373 L
9614 12324 L 9693 12238 L 9616 12102 L 9614 12092 L
9611 12102 L 9479 12137 L 9433 12102 L 9366 11966 L
9479 11873 L 9614 11887 L 9634 11831 L 9750 11724 L
9781 11695 L 9811 11559 L 9859 11423 L 9876 11288 L
9885 11264 L 9896 11152 L 10021 11026 L 10030 11016 L
10156 10892 L 10211 10881 L 10292 10821 L 10359 10745 L
10427 10651 L 10438 10609 L 10459 10474 L 10563 10357 L
10586 10338 L 10622 10202 L 10698 10104 L 10747 10067 L

10794 9931 L 10834 9898 L 10969 9806 L 11105 9884 L
11153 9931 L 11240 10024 L 11304 10067 L 11376 10166 L
11399 10202 L 11511 10310 L 11540 10338 L 11647 10424 L
11697 10474 L 11782 10556 L 11839 10609 L 11918 10701 L
11950 10745 L 12034 10881 L 12053 10909 L 12068 11016 L
12096 11152 L 12121 11288 L 12153 11423 L 12187 11559 L
12189 11564 L 12245 11695 L 12294 11831 L 12324 11938 L
12338 11966 L 12408 12102 L 12451 12238 L 12460 12280 L
12483 12373 L 12508 12509 L 12575 12645 L 12595 12652 L
12731 12668 L 12866 12677 L 12936 12645 L 13002 12604 L
13002 12245 L 12901 12238 L 12866 12234 L 12731 12128 L
12712 12102 L 12598 11966 L 12595 11962 L 12517 11831 L
12460 11731 L 12443 11695 L 12363 11559 L 12324 11495 L
12294 11423 L 12227 11288 L 12189 11216 L 12175 11152 L
12147 11016 L 12126 10881 L 12110 10745 L 12097 10609 L
12084 10474 L 12065 10338 L 12055 10202 L 12053 10178 L
11982 10067 L 11918 9996 L 11840 9931 L 11782 9891 L
11647 9803 L 11638 9795 L 11511 9704 L 11441 9660 L
11376 9624 L 11240 9573 L 11191 9660 L 11105 9711 L
10969 9721 L 10886 9795 L 10834 9834 L 10716 9931 L
10698 9972 L 10609 10067 L 10563 10192 L 10552 10202 L
10427 10327 L 10414 10338 L 10292 10441 L 10268 10474 L
10177 10609 L 10156 10625 L 10021 10703 L 9946 10609 L
9885 10553 L 9790 10474 L 9882 10338 L 9750 10210 L
9684 10202 L 9614 10181 L 9597 10202 L 9537 10338 L
9479 10380 L 9389 10474 L 9443 10609 L 9432 10745 L
9479 10803 L 9614 10809 L 9614 10752 L 9546 10745 L
9528 10609 L 9479 10580 L 9420 10474 L 9479 10412 L
9581 10338 L 9614 10270 L 9750 10244 L 9847 10338 L
9750 10440 L 9692 10474 L 9689 10609 L 9623 10745 L
9614 10752 L 9614 10809 L 9699 10745 L 9750 10647 L
9885 10673 L 9976 10745 L 9941 10881 L 9885 10955 L
9862 11016 L 9750 11136 L 9720 11152 L 9620 11288 L
9614 11304 L 9581 11423 L 9565 11559 L 9479 11640 L
9438 11695 L 9343 11805 L 9323 11831 L 9231 11966 L
9208 12005 L 9185 12102 L 9208 12191 L 9311 12238 L
9208 12320 L 9102 12373 L 9072 12385 L 8937 12485 L
8898 12509 L 8801 12606 L 8768 12645 L 8781 12780 L
8801 12860 L 8834 12916 L 8937 12987 L 8965 13052 L
8961 13187 L 9072 13309 L 9208 13317 L 9214 13323 L
9227 13459 L 9343 13584 L 9381 13594 L 9479 13697 L
9548 13730 L 9545 13866 L 9479 13907 L 9398 14002 L
9343 14013 L 9208 14058 L 9072 14006 L 9068 14002 L

8937 13884 L 8918 13866 L 8801 13751 L 8666 13750 L
8530 13786 L 8454 13866 L 8447 14002 L 8439 14137 L
8413 14273 L 8395 14292 L 8259 14286 L 8124 14299 L
7988 14335 L 7917 14409 L 7901 14544 L 7891 14680 L
7875 14816 L 7988 14930 L 8124 14936 L 8259 14943 L
8302 14951 L 8353 15087 L 8395 15196 L 8401 15223 L
8395 15281 L 8370 15358 L 8259 15412 L 8124 15461 L
7988 15434 L 7853 15408 L 7740 15494 L 7717 15528 L
7582 15604 L 7446 15545 L 7311 15511 L 7291 15494 L
7235 15358 L 7175 15270 L 7054 15223 L 7040 15221 L
6904 15140 L 6854 15087 L 6769 14985 L 6696 14951 L
6633 14921 L 6498 14892 L 6376 14816 L 6362 14800 L
6309 14680 L 6227 14608 L 6091 14614 L 6032 14680 L
5956 14774 L 5847 14816 L 5820 14844 L 5814 14816 L
5756 14680 L 5685 14575 L 5658 14544 L 5559 14409 L
5549 14401 L 5483 14273 L 5414 14166 L 5400 14137 L
5278 14014 L 5190 14002 L 5143 13893 L 5132 14002 L
5143 14043 L 5158 14137 L 5143 14229 L 5130 14273 L
5092 14409 L 5134 14544 L 5143 14568 L 5163 14680 L
5185 14816 L 5250 14951 L 5266 15087 L 5148 15223 L
5143 15227 L 5132 15223 L 5007 15126 L 4993 15087 L
4916 14951 L 4872 14879 L 4778 14816 L 4736 14788 L
4601 14698 L 4465 14693 L 4330 14694 L 4194 14765 L
4059 14790 L 3923 14727 L 3788 14787 L 3759 14816 L
3729 14951 L 3704 15087 L 3652 15130 L 3517 15140 L
3397 15223 L 3381 15233 L 3365 15223 L 3246 15194 L
3143 15087 L 3110 15055 L 3023 14951 L 2975 14898 L
2867 14816 L 2839 14764 L 2730 14680 L 2704 14654 L
2655 14680 L 2664 14816 L 2704 14875 L 2719 14951 L
2725 15087 L 2756 15223 L 2839 15305 L 2953 15358 L
2839 15455 L 2737 15494 L 2704 15500 L 2673 15494 L
2568 15414 L 2527 15358 L 2513 15223 L 2433 15140 L
2320 15087 L N E
129 O
2323 15358 2297 15331 2297 15386 2323 15358 4 N F
2433 15559 2297 15577 2297 15751 2433 15686 2568 15704 2704 15714
2764 15765 2839 15810 2975 15866 3014 15901 3110 15943 3246 15978
3340 16037 3381 16053 3517 16114 3598 16172 3659 16172 3652 16169
3517 16073 3432 16037 3381 15978 3246 15922 3138 15901 3110 15882
2975 15772 2951 15765 2839 15692 2747 15630 2704 15615 2568 15591
2433 15559 31 N F
147 O
2433 15686 2297 15751 2297 15911 2392 16037 2433 16108 2516 16172

2568 16172 2568 16050 2554 16037 2568 16005 2672 16037 2568 16050
2568 16172 2643 16172 2815 16172 2988 16172 2990 16172 2975 16150
2847 16037 2839 16024 2752 15901 2839 15874 2893 15901 2975 15964
3110 15989 3246 16034 3250 16037 3381 16088 3517 16154 3543 16172
3598 16172 3517 16114 3381 16053 3340 16037 3246 15978 3110 15943
3014 15901 2975 15866 2839 15810 2764 15765 2704 15714 2568 15704
2433 15686 43 N F
165 O
2392 16037 2297 15911 2297 16172 2470 16172 2516 16172 2433 16108
2392 16037 7 N F
74 O
2433 12944 2402 12916 2433 12790 2437 12780 2568 12719 2670 12780
2630 12916 2568 13006 2433 12944 9 N F
111 O
2433 13512 2411 13459 2433 13438 2473 13459 2433 13512 5 N F
147 O
2433 13974 2421 14002 2433 14056 2464 14002 2568 13877 2572 13866
2643 13730 2704 13641 2728 13594 2737 13459 2839 13456 2839 13333
2833 13323 2821 13187 2839 13150 2855 13187 2843 13323 2839 13333
2839 13456 2892 13323 2904 13187 2851 13052 2839 13030 2825 13052
2763 13187 2746 13323 2704 13457 2703 13459 2568 13583 2558 13594
2505 13730 2559 13866 2433 13974 33 N F
129 O
2568 16050 2554 16037 2568 16005 2672 16037 2568 16050 5 N F
38 O
2568 8611 2559 8574 2568 8569 2577 8574 2568 8611 5 N F
2704 8784 2600 8710 2704 8658 2839 8619 2975 8622 3110 8702
3128 8710 3246 8759 3305 8845 3296 8981 3378 9117 3381 9119
3437 9252 3381 9307 3246 9300 3226 9252 3217 9117 3110 9020
3075 8981 2975 8889 2847 8845 2839 8842 2704 8784 23 N F
74 O
2704 14438 2662 14409 2704 14328 2839 14311 2975 14386 3078 14409
3110 14449 3125 14544 3164 14680 3242 14816 3246 14820 3335 14951
3328 15087 3246 15122 3212 15087 3110 14987 3080 14951 2975 14835
2950 14816 2892 14680 2839 14549 2835 14544 2704 14438 23 N F
165 O
2839 15874 2752 15901 2839 16024 2847 16037 2975 16150 2990 16172
3100 16172 3096 16037 3110 16035 3115 16037 3246 16089 3381 16123
3480 16172 3506 16172 3543 16172 3517 16154 3381 16088 3250 16037
3246 16034 3110 15989 2975 15964 2893 15901 2839 15874 23 N F
129 O
2787 14951 2775 15087 2839 15191 2863 15223 2975 15313 2975 15263
2925 15223 2839 15110 2825 15087 2839 15014 2975 14998 3055 15087

3110 15198 3134 15223 3110 15258 2975 15263 2975 15313 3106 15358
3110 15366 3128 15358 3204 15223 3110 15125 3091 15087 2975 14958
2952 14951 2839 14879 2787 14951 27 N F
165 O
2839 13150 2821 13187 2833 13323 2839 13333 2843 13323 2855 13187
2839 13150 7 N F
147 O
2839 15014 2825 15087 2839 15110 2925 15223 2975 15263 2975 15205
2890 15087 2975 15039 3018 15087 2975 15205 2975 15263 3110 15258
3134 15223 3110 15198 3055 15087 2975 14998 2839 15014 17 N F
111 O
2839 12643 2828 12645 2839 12682 2840 12645 2839 12643 5 N F
165 O
2975 15039 2890 15087 2975 15205 2975 15106 2961 15087 2975 15079
2982 15087 2975 15106 2975 15205 3018 15087 2975 15039 11 N F
38 O
2975 8210 2934 8167 2975 8140 2988 8167 2975 8210 5 N F
183 O
2975 15079 2961 15087 2975 15106 2982 15087 2975 15079 5 N F
38 O
2975 7775 2967 7760 2975 7741 3110 7715 3246 7756 3251 7760
3246 7763 3110 7802 2975 7775 9 N F
165 O
2990 16172 2990 16173 3100 16173 3100 16172 2990 16172 5 N F
3100 16173 2990 16173 2990 16173 3100 16173 3100 16173 5 N F
111 O
3110 12282 2999 12373 3110 12437 3246 12440 3246 12406 3116 12373
3195 12238 3246 12207 3381 12171 3513 12238 3381 12338 3318 12373
3246 12406 3246 12440 3381 12382 3393 12373 3517 12279 3594 12238
3652 12163 3724 12102 3652 12073 3584 12102 3517 12152 3381 12142
3246 12172 3137 12238 3110 12282 27 N F
38 O
3079 8438 3026 8303 3110 8266 3192 8303 3121 8438 3110 8450
3079 8438 7 N F
183 O
3110 16035 3096 16037 3100 16172 3161 16172 3197 16172 3246 16144
3381 16157 3411 16172 3480 16172 3381 16123 3246 16089 3115 16037
3110 16035 13 N F
74 O
3110 11555 3099 11559 3110 11572 3246 11567 3255 11559 3246 11553
3110 11555 7 N F
183 O
3100 16172 3100 16173 3161 16173 3197 16173 3197 16172 3161 16172

3100 16172 7 N F
129 O
3195 12238 3116 12373 3246 12406 3318 12373 3381 12338 3381 12295
3257 12238 3381 12200 3456 12238 3381 12295 3381 12338 3513 12238
3381 12171 3246 12207 3195 12238 15 N F
201 O
3333 16173 3197 16173 3197 16172 3333 16172 3411 16172 3412 16173
3333 16173 7 N F
3246 16144 3197 16172 3333 16172 3411 16172 3381 16157 3246 16144
6 N F
92 O
3246 15775 3231 15765 3246 15749 3265 15765 3246 15775 5 N F
147 O
3381 12200 3257 12238 3381 12295 3381 12251 3352 12238 3381 12229
3399 12238 3381 12251 3381 12295 3456 12238 3381 12200 11 N F
74 O
3381 12739 3294 12780 3381 12896 3501 12780 3381 12739 5 N F
165 O
3381 12229 3352 12238 3381 12251 3399 12238 3381 12229 5 N F
129 O
3381 15610 M 3367 15630 L 3381 15643 L 3517 15726 L 3517 15596 L
3479 15494 L 3517 15376 L 3624 15494 L 3517 15596 L
3517 15726 L 3652 15737 L 3775 15630 L 3788 15605 L
3852 15494 L 3905 15358 L 3923 15325 L 3975 15358 L
4059 15378 L 4078 15494 L 4059 15551 L 3952 15630 L
3966 15765 L 3923 15780 L 3820 15901 L 3923 15954 L
4039 15901 L 4059 15847 L 4194 15787 L 4330 15849 L
4330 15642 L 4306 15630 L 4194 15550 L 4175 15494 L
4194 15441 L 4229 15358 L 4330 15238 L 4465 15343 L
4601 15294 L 4722 15358 L 4711 15494 L 4601 15529 L
4465 15574 L 4344 15630 L 4330 15642 L 4330 15849 L
4357 15901 L 4354 16037 L 4330 16089 L 4228 16172 L
4361 16172 L 4440 16037 L 4465 15944 L 4465 15789 L
4421 15765 L 4465 15728 L 4601 15684 L 4720 15765 L
4601 15775 L 4465 15789 L 4465 15944 L 4500 15901 L
4601 15864 L 4736 15856 L 4872 15805 L 5007 15792 L
5143 15803 L 5143 15766 L 5133 15765 L 5007 15725 L
4893 15630 L 5007 15503 L 5143 15515 L 5278 15512 L
5414 15556 L 5431 15630 L 5414 15663 L 5278 15746 L
5165 15765 L 5143 15766 L 5143 15803 L 5278 15805 L
5414 15793 L 5487 15765 L 5530 15630 L 5515 15494 L
5424 15358 L 5414 15338 L 5378 15223 L 5401 15087 L
5400 14951 L 5314 14816 L 5278 14722 L 5262 14680 L

5278 14565 L 5278 14382 L 5221 14273 L 5250 14137 L
5278 14108 L 5307 14137 L 5359 14273 L 5278 14382 L
5278 14565 L 5414 14546 L 5549 14562 L 5658 14680 L
5685 14761 L 5706 14816 L 5685 14883 L 5655 14951 L
5681 15087 L 5685 15092 L 5775 15223 L 5820 15268 L
5910 15358 L 5956 15468 L 5990 15494 L 6091 15532 L
6181 15494 L 6227 15472 L 6362 15438 L 6484 15494 L
6498 15505 L 6518 15630 L 6573 15765 L 6633 15788 L
6769 15790 L 6769 15699 L 6693 15630 L 6769 15507 L
6769 15396 L 6732 15358 L 6633 15256 L 6582 15223 L
6498 15128 L 6464 15223 L 6362 15348 L 6227 15256 L
6207 15223 L 6148 15087 L 6091 15016 L 6025 14951 L
6022 14816 L 6091 14737 L 6227 14715 L 6289 14816 L
6315 14951 L 6362 14997 L 6498 15065 L 6633 15036 L
6726 15087 L 6769 15117 L 6868 15223 L 6904 15332 L
6929 15358 L 6904 15372 L 6769 15396 L 6769 15507 L
6789 15494 L 6904 15468 L 7040 15477 L 7081 15494 L
7040 15501 L 6942 15630 L 6904 15652 L 6769 15699 L
6769 15790 L 6814 15765 L 6904 15705 L 7036 15630 L
7040 15625 L 7175 15572 L 7246 15630 L 7311 15717 L
7385 15765 L 7446 15801 L 7582 15872 L 7717 15862 L
7819 15901 L 7853 15916 L 7988 15960 L 8124 15908 L
8136 15901 L 8249 15765 L 8259 15753 L 8390 15630 L
8395 15627 L 8530 15613 L 8629 15494 L 8635 15358 L
8552 15223 L 8536 15087 L 8530 15081 L 8423 14951 L
8395 14919 L 8259 14896 L 8124 14882 L 7988 14878 L
7927 14816 L 7944 14680 L 7954 14544 L 7981 14409 L
7988 14401 L 8124 14376 L 8259 14359 L 8395 14350 L
8469 14273 L 8494 14137 L 8506 14002 L 8522 13866 L
8530 13858 L 8666 13814 L 8801 13791 L 8877 13866 L
8937 13924 L 9023 14002 L 9072 14048 L 9184 14137 L
9208 14159 L 9234 14137 L 9343 14056 L 9479 14031 L
9509 14002 L 9614 13904 L 9750 13889 L 9885 13870 L
9892 13866 L 9885 13850 L 9750 13761 L 9731 13730 L
9614 13691 L 9501 13594 L 9479 13571 L 9343 13537 L
9270 13459 L 9261 13323 L 9208 13271 L 9072 13266 L
9000 13187 L 9053 13052 L 9072 12990 L 9208 12925 L
9222 12916 L 9343 12818 L 9402 12780 L 9479 12716 L
9579 12645 L 9614 12624 L 9670 12509 L 9750 12430 L
9807 12373 L 9885 12277 L 9941 12238 L 9976 12102 L
10021 12033 L 10076 11966 L 10081 11831 L 10061 11695 L
10026 11559 L 10021 11531 L 9974 11423 L 9932 11288 L
9946 11152 L 10021 11076 L 10079 11016 L 10156 10941 L

10292 10914 L 10346 10881 L 10427 10793 L 10459 10745 L
10489 10609 L 10512 10474 L 10563 10417 L 10658 10338 L
10682 10202 L 10698 10182 L 10834 10153 L 10885 10202 L
10969 10266 L 11046 10338 L 11105 10377 L 11213 10474 L
11240 10495 L 11376 10606 L 11379 10609 L 11511 10733 L
11522 10745 L 11647 10866 L 11660 10881 L 11760 11016 L
11782 11043 L 11883 11152 L 11918 11198 L 11989 11288 L
12053 11358 L 12068 11423 L 12100 11559 L 12131 11695 L
12170 11831 L 12081 11966 L 12189 12045 L 12207 12102 L
12235 12238 L 12259 12373 L 12279 12509 L 12189 12581 L
12122 12645 L 12103 12780 L 12104 12916 L 12189 13008 L
12247 12916 L 12324 12881 L 12423 12780 L 12460 12744 L
12565 12780 L 12460 12821 L 12363 12916 L 12423 13052 L
12452 13187 L 12416 13323 L 12324 13376 L 12260 13323 L
12229 13187 L 12189 13080 L 12151 13052 L 12053 12961 L
11974 13052 L 11918 13095 L 11908 13187 L 11901 13323 L
11905 13459 L 11865 13594 L 11815 13730 L 11844 13866 L
11918 13927 L 12053 13978 L 12079 14002 L 12189 14079 L
12324 14042 L 12372 14002 L 12428 13866 L 12460 13848 L
12563 13866 L 12595 13870 L 12595 13772 L 12503 13730 L
12460 13682 L 12324 13635 L 12189 13673 L 12068 13594 L
12053 13578 L 12009 13459 L 12017 13323 L 12043 13187 L
12053 13178 L 12063 13187 L 12085 13323 L 12189 13414 L
12254 13459 L 12324 13538 L 12460 13506 L 12526 13459 L
12595 13392 L 12595 13241 L 12578 13187 L 12571 13052 L
12595 12942 L 12729 13052 L 12688 13187 L 12595 13241 L
12595 13392 L 12731 13435 L 12741 13459 L 12754 13594 L
12731 13730 L 12730 13730 L 12595 13772 L 12595 13870 L
12731 13870 L 12746 13866 L 12857 13730 L 12866 13672 L
12866 12960 L 12844 12916 L 12866 12887 L 12949 12916 L
12866 12960 L 12866 13672 L 12948 13730 L 13002 13858 L
13002 12604 L 12936 12645 L 12866 12677 L 12731 12668 L
12595 12652 L 12575 12645 L 12508 12509 L 12483 12373 L
12460 12280 L 12451 12238 L 12408 12102 L 12338 11966 L
12324 11938 L 12294 11831 L 12245 11695 L 12189 11564 L
12187 11559 L 12153 11423 L 12121 11288 L 12096 11152 L
12068 11016 L 12053 10909 L 12034 10881 L 11950 10745 L
11918 10701 L 11839 10609 L 11782 10556 L 11697 10474 L
11647 10424 L 11540 10338 L 11511 10310 L 11399 10202 L
11376 10166 L 11304 10067 L 11240 10024 L 11153 9931 L
11105 9884 L 10969 9806 L 10834 9898 L 10794 9931 L
10747 10067 L 10698 10104 L 10622 10202 L 10586 10338 L
10563 10357 L 10459 10474 L 10438 10609 L 10427 10651 L

10359 10745 L 10292 10821 L 10211 10881 L 10156 10892 L
10030 11016 L 10021 11026 L 9896 11152 L 9885 11264 L
9876 11288 L 9859 11423 L 9811 11559 L 9781 11695 L
9750 11724 L 9634 11831 L 9614 11887 L 9479 11873 L
9366 11966 L 9433 12102 L 9479 12137 L 9611 12102 L
9614 12092 L 9616 12102 L 9693 12238 L 9614 12324 L
9556 12373 L 9479 12429 L 9343 12502 L 9331 12509 L
9208 12623 L 9178 12645 L 9072 12736 L 9046 12780 L
8989 12916 L 9009 13052 L 8981 13187 L 9072 13288 L
9208 13294 L 9237 13323 L 9249 13459 L 9343 13560 L
9463 13594 L 9479 13611 L 9614 13715 L 9659 13730 L
9732 13866 L 9614 13880 L 9484 14002 L 9479 14007 L
9343 14034 L 9208 14130 L 9072 14027 L 9045 14002 L
8937 13904 L 8898 13866 L 8801 13771 L 8666 13782 L
8530 13822 L 8488 13866 L 8477 14002 L 8466 14137 L
8441 14273 L 8395 14321 L 8259 14323 L 8124 14338 L
7988 14368 L 7949 14409 L 7927 14544 L 7917 14680 L
7901 14816 L 7988 14904 L 8124 14909 L 8259 14919 L
8395 14945 L 8401 14951 L 8462 15087 L 8526 15223 L
8530 15240 L 8551 15358 L 8530 15412 L 8429 15494 L
8395 15510 L 8259 15568 L 8144 15630 L 8124 15659 L
8108 15630 L 7988 15564 L 7853 15526 L 7768 15630 L
7717 15720 L 7587 15765 L 7582 15767 L 7580 15765 L
7446 15667 L 7392 15630 L 7311 15595 L 7189 15494 L
7175 15471 L 7091 15358 L 7040 15320 L 6946 15223 L
6904 15198 L 6799 15087 L 6769 15051 L 6633 14982 L
6498 14969 L 6457 14951 L 6362 14906 L 6328 14816 L
6252 14680 L 6227 14658 L 6131 14680 L 6091 14689 L
5979 14816 L 5956 14929 L 5935 14951 L 5956 14964 L
6084 15087 L 6091 15095 L 6144 15223 L 6225 15358 L
6091 15424 L 6016 15358 L 5956 15306 L 5873 15223 L
5820 15157 L 5774 15087 L 5755 14951 L 5760 14816 L
5705 14680 L 5685 14650 L 5592 14544 L 5549 14489 L
5414 14439 L 5373 14409 L 5414 14301 L 5419 14273 L
5414 14265 L 5354 14137 L 5278 14061 L 5204 14137 L
5179 14273 L 5192 14409 L 5210 14544 L 5213 14680 L
5258 14816 L 5278 14850 L 5334 14951 L 5338 15087 L
5308 15223 L 5289 15358 L 5278 15413 L 5143 15452 L
5058 15358 L 5007 15330 L 4912 15223 L 4872 15135 L
4736 15099 L 4601 15141 L 4465 15137 L 4379 15087 L
4330 15031 L 4194 15070 L 4059 15026 L 3997 15087 L
3923 15193 L 3872 15223 L 3788 15282 L 3652 15278 L
3517 15274 L 3417 15358 L 3412 15494 L 3381 15610 L

N E
111 O
3381 14253 3367 14273 3381 14299 3414 14273 3517 14224 3652 14200
3702 14137 3652 14085 3517 14076 3450 14137 3381 14253 11 N F
183 O
3412 16173 3411 16172 3480 16172 3480 16173 3412 16173 5 N F
3412 16173 3412 16173 3480 16173 3480 16173 3412 16173 5 N F
38 O
3517 7239 3461 7217 3517 7165 3652 7119 3718 7217 3666 7353
3652 7359 3648 7353 3517 7239 9 N F
92 O
3517 15980 3476 15901 3517 15880 3543 15901 3517 15980 5 N F
147 O
3517 15376 3479 15494 3517 15596 3624 15494 3517 15376 5 N F
74 O
3517 10052 3479 10067 3513 10202 3517 10206 3550 10202 3537 10067
3517 10052 7 N F
165 O
3480 16173 3480 16172 3506 16172 3543 16172 3543 16173 3506 16173
3480 16173 7 N F
147 O
3543 16173 3543 16172 3598 16172 3598 16173 3543 16173 5 N F
3543 16173 3543 16173 3598 16173 3598 16173 3543 16173 5 N F
129 O
3598 16173 3598 16172 3659 16172 3659 16173 3598 16173 5 N F
3598 16173 3598 16173 3659 16173 3659 16173 3652 16173 3598 16173
6 N F
74 O
3688 11016 3666 11152 3788 11268 3923 11268 3923 11218 3788 11170
3769 11152 3758 11016 3788 10988 3923 10965 4011 11016 4017 11152
3923 11218 3923 11268 4059 11194 4093 11152 4118 11016 4059 10973
3923 10917 3788 10923 3688 11016 21 N F
38 O
3762 6674 3727 6539 3755 6403 3788 6387 3801 6403 3835 6539
3807 6674 3788 6686 3762 6674 9 N F
92 O
3788 10988 3758 11016 3769 11152 3788 11170 3923 11218 3923 11168
3885 11152 3907 11016 3923 11013 3930 11016 3946 11152 3923 11168
3923 11218 4017 11152 4011 11016 3923 10965 3788 10988 17 N F
38 O
3788 7524 3770 7489 3788 7453 3795 7489 3788 7524 5 N F
92 O
3788 13451 3780 13459 3788 13489 3879 13459 3788 13451 5 N F

38 O
3890 10202 3830 10067 3923 9979 3972 10067 4059 10160 4194 10199
4213 10202 4244 10338 4194 10364 4165 10338 4059 10254 3923 10223
3890 10202 13 N F
3923 6097 3865 5996 3923 5965 4002 5996 3923 6097 5 N F
74 O
3923 14462 3884 14409 3916 14273 3923 14271 4059 14250 4104 14273
4059 14348 4013 14409 3923 14462 9 N F
38 O
3923 8208 3885 8167 3923 8118 4035 8167 3923 8208 5 N F
111 O
3907 11016 3885 11152 3923 11168 3946 11152 3930 11016 3923 11013
3907 11016 7 N F
38 O
4059 8073 4052 8031 4059 8030 4065 8031 4059 8073 5 N F
74 O
4194 7681 4078 7760 4194 7817 4273 7760 4194 7681 5 N F
38 O
4194 5900 4170 5860 4194 5835 4222 5725 4295 5589 4330 5555
4409 5589 4465 5658 4514 5725 4465 5797 4403 5860 4330 5936
4194 5900 13 N F
147 O
4194 15441 4175 15494 4194 15550 4306 15630 4330 15642 4330 15510
4312 15494 4330 15409 4376 15494 4330 15510 4330 15642 4344 15630
4465 15574 4601 15529 4711 15494 4722 15358 4601 15294 4465 15343
4330 15238 4229 15358 4194 15441 21 N F
74 O
4330 14452 4219 14409 4330 14315 4464 14409 4330 14452 5 N F
129 O
4330 16173 4227 16173 4227 16173 4361 16173 4361 16173 4330 16173
6 N F
4361 16173 4227 16173 4228 16172 4361 16172 4361 16173 5 N F
74 O
4330 10793 4242 10881 4330 10951 4465 11002 4465 10958 4342 10881
4465 10796 4584 10881 4465 10958 4465 11002 4601 10960 4666 10881
4601 10818 4465 10747 4330 10793 15 N F
165 O
4330 15409 4312 15494 4330 15510 4376 15494 4330 15409 5 N F
92 O
4465 10796 4342 10881 4465 10958 4465 10913 4413 10881 4465 10845
4516 10881 4465 10913 4465 10958 4584 10881 4465 10796 11 N F
147 O
4369 16173 4361 16173 4361 16172 4369 16172 4418 16172 4418 16173

4369 16173 7 N F
4440 16037 M 4361 16172 L 4369 16172 L 4418 16172 L 4465 16099 L
4525 16037 L 4601 15955 L 4736 15940 L 4803 15901 L
4872 15868 L 5007 15830 L 5143 15840 L 5278 15850 L
5414 15853 L 5549 15820 L 5599 15765 L 5627 15630 L
5650 15494 L 5549 15377 L 5536 15358 L 5463 15223 L
5510 15087 L 5500 14951 L 5414 14893 L 5363 14816 L
5331 14680 L 5414 14611 L 5549 14612 L 5612 14680 L
5656 14816 L 5582 14951 L 5582 15087 L 5685 15220 L
5687 15223 L 5820 15354 L 5824 15358 L 5874 15494 L
5951 15630 L 5956 15633 L 6091 15633 L 6094 15630 L
6227 15554 L 6362 15589 L 6404 15630 L 6448 15765 L
6498 15801 L 6633 15840 L 6769 15835 L 6895 15765 L
6904 15759 L 7040 15681 L 7175 15686 L 7233 15765 L
7311 15815 L 7424 15901 L 7446 15921 L 7582 15992 L
7717 16008 L 7786 16037 L 7853 16053 L 7988 16053 L
8012 16037 L 8124 15974 L 8247 15901 L 8259 15887 L
8395 15870 L 8419 15901 L 8495 16037 L 8519 16172 L
8558 16172 L 8666 16116 L 8666 15938 L 8630 15901 L
8666 15777 L 8778 15901 L 8666 15938 L 8666 16116 L
8801 16099 L 8801 15888 L 8671 15765 L 8744 15630 L
8751 15494 L 8801 15472 L 8910 15494 L 8937 15501 L
9072 15607 L 9075 15630 L 9095 15765 L 9072 15850 L
8937 15838 L 8801 15888 L 8801 16099 L 8911 16172 L
9031 16172 L 9203 16172 L 9333 16172 L 9343 16146 L
9383 16172 L 9549 16172 L 9721 16172 L 9885 16172 L
9885 15996 L 9848 15901 L 9867 15765 L 9885 15742 L
9906 15765 L 9920 15901 L 9885 15996 L 9885 16172 L
9894 16172 L 10067 16172 L 10239 16172 L 10275 16172 L
10195 16037 L 10156 15976 L 10106 15901 L 10043 15765 L
10021 15720 L 9943 15630 L 9885 15576 L 9878 15630 L
9750 15764 L 9749 15765 L 9657 15901 L 9614 15964 L
9479 15971 L 9343 15974 L 9310 15901 L 9208 15791 L
9192 15765 L 9097 15630 L 9079 15494 L 9072 15488 L
8937 15471 L 8810 15358 L 8801 15349 L 8666 15340 L
8575 15223 L 8562 15087 L 8530 15054 L 8446 14951 L
8395 14893 L 8259 14872 L 8124 14855 L 7988 14852 L
7953 14816 L 7970 14680 L 7980 14544 L 7988 14507 L
8124 14440 L 8163 14409 L 8259 14395 L 8395 14379 L
8497 14273 L 8521 14137 L 8530 14051 L 8546 14002 L
8604 13866 L 8666 13845 L 8801 13811 L 8857 13866 L
8937 13944 L 9000 14002 L 9072 14069 L 9158 14137 L
9208 14183 L 9263 14137 L 9343 14078 L 9479 14055 L

9534 14002 L 9614 13927 L 9750 13915 L 9885 13899 L
9947 13866 L 9885 13731 L 9884 13730 L 9750 13706 L
9614 13667 L 9530 13594 L 9479 13543 L 9343 13514 L
9292 13459 L 9284 13323 L 9208 13247 L 9072 13244 L
9020 13187 L 9072 13076 L 9208 13063 L 9220 13052 L
9343 12942 L 9406 12916 L 9479 12854 L 9614 12819 L
9750 12781 L 9750 12780 L 9758 12645 L 9766 12509 L
9885 12390 L 9954 12373 L 10021 12325 L 10156 12292 L
10203 12238 L 10292 12117 L 10301 12102 L 10292 12050 L
10280 11966 L 10234 11831 L 10182 11695 L 10156 11674 L
10074 11559 L 10054 11423 L 10021 11371 L 9980 11288 L
9995 11152 L 10021 11126 L 10128 11016 L 10156 10989 L
10292 10965 L 10427 10884 L 10431 10881 L 10519 10745 L
10540 10609 L 10563 10488 L 10698 10493 L 10834 10606 L
10840 10609 L 10969 10678 L 11057 10745 L 11105 10777 L
11225 10881 L 11240 10894 L 11376 11000 L 11391 11016 L
11511 11108 L 11555 11152 L 11647 11246 L 11683 11288 L
11782 11406 L 11797 11423 L 11913 11559 L 11918 11564 L
12021 11695 L 12053 11720 L 12095 11831 L 12053 11880 L
11985 11831 L 11918 11753 L 11875 11831 L 11874 11966 L
11918 12021 L 12053 12060 L 12097 12102 L 12103 12238 L
12123 12373 L 12136 12509 L 12053 12589 L 11960 12509 L
11918 12483 L 11903 12509 L 11899 12645 L 11896 12780 L
11900 12916 L 11892 13052 L 11880 13187 L 11874 13323 L
11878 13459 L 11782 13582 L 11770 13594 L 11703 13730 L
11771 13866 L 11782 13876 L 11916 14002 L 11918 14006 L
12053 14096 L 12104 14137 L 12189 14222 L 12324 14176 L
12365 14137 L 12460 14054 L 12536 14002 L 12595 13967 L
12731 13932 L 12866 13919 L 12927 14002 L 13002 14047 L
13002 13858 L 12948 13730 L 12866 13672 L 12857 13730 L
12746 13866 L 12731 13870 L 12595 13870 L 12563 13866 L
12460 13848 L 12428 13866 L 12372 14002 L 12324 14042 L
12189 14079 L 12079 14002 L 12053 13978 L 11918 13927 L
11844 13866 L 11815 13730 L 11865 13594 L 11905 13459 L
11901 13323 L 11908 13187 L 11918 13095 L 11974 13052 L
12053 12961 L 12151 13052 L 12189 13080 L 12229 13187 L
12260 13323 L 12324 13376 L 12416 13323 L 12452 13187 L
12423 13052 L 12363 12916 L 12460 12821 L 12565 12780 L
12460 12744 L 12423 12780 L 12324 12881 L 12247 12916 L
12189 13008 L 12104 12916 L 12103 12780 L 12122 12645 L
12189 12581 L 12279 12509 L 12259 12373 L 12235 12238 L
12207 12102 L 12189 12045 L 12081 11966 L 12170 11831 L
12131 11695 L 12100 11559 L 12068 11423 L 12053 11358 L

11989 11288 L 11918 11198 L 11883 11152 L 11782 11043 L
11760 11016 L 11660 10881 L 11647 10866 L 11522 10745 L
11511 10733 L 11379 10609 L 11376 10606 L 11240 10495 L
11213 10474 L 11105 10377 L 11046 10338 L 10969 10266 L
10885 10202 L 10834 10153 L 10698 10182 L 10682 10202 L
10658 10338 L 10563 10417 L 10512 10474 L 10489 10609 L
10459 10745 L 10427 10793 L 10346 10881 L 10292 10914 L
10156 10941 L 10079 11016 L 10021 11076 L 9946 11152 L
9932 11288 L 9974 11423 L 10021 11531 L 10026 11559 L
10061 11695 L 10081 11831 L 10076 11966 L 10021 12033 L
9976 12102 L 9941 12238 L 9885 12277 L 9807 12373 L
9750 12430 L 9670 12509 L 9614 12624 L 9579 12645 L
9479 12716 L 9402 12780 L 9343 12818 L 9222 12916 L
9208 12925 L 9072 12990 L 9053 13052 L 9000 13187 L
9072 13266 L 9208 13271 L 9261 13323 L 9270 13459 L
9343 13537 L 9479 13571 L 9501 13594 L 9614 13691 L
9731 13730 L 9750 13761 L 9885 13850 L 9892 13866 L
9885 13870 L 9750 13889 L 9614 13904 L 9509 14002 L
9479 14031 L 9343 14056 L 9234 14137 L 9208 14159 L
9184 14137 L 9072 14048 L 9023 14002 L 8937 13924 L
8877 13866 L 8801 13791 L 8666 13814 L 8530 13858 L
8522 13866 L 8506 14002 L 8494 14137 L 8469 14273 L
8395 14350 L 8259 14359 L 8124 14376 L 7988 14401 L
7981 14409 L 7954 14544 L 7944 14680 L 7927 14816 L
7988 14878 L 8124 14882 L 8259 14896 L 8395 14919 L
8423 14951 L 8530 15081 L 8536 15087 L 8552 15223 L
8635 15358 L 8629 15494 L 8530 15613 L 8395 15627 L
8390 15630 L 8259 15753 L 8249 15765 L 8136 15901 L
8124 15908 L 7988 15960 L 7853 15916 L 7819 15901 L
7717 15862 L 7582 15872 L 7446 15801 L 7385 15765 L
7311 15717 L 7246 15630 L 7175 15572 L 7040 15625 L
7036 15630 L 6904 15705 L 6814 15765 L 6769 15790 L
6633 15788 L 6573 15765 L 6518 15630 L 6498 15505 L
6484 15494 L 6362 15438 L 6227 15472 L 6181 15494 L
6091 15532 L 5990 15494 L 5956 15468 L 5910 15358 L
5820 15268 L 5775 15223 L 5685 15092 L 5681 15087 L
5655 14951 L 5685 14883 L 5706 14816 L 5685 14761 L
5658 14680 L 5549 14562 L 5414 14546 L 5278 14565 L
5262 14680 L 5278 14722 L 5314 14816 L 5400 14951 L
5401 15087 L 5378 15223 L 5414 15338 L 5424 15358 L
5515 15494 L 5530 15630 L 5487 15765 L 5414 15793 L
5278 15805 L 5143 15803 L 5007 15792 L 4872 15805 L
4736 15856 L 4601 15864 L 4500 15901 L 4465 15944 L

4440 16037 L N E
111 O
4465 10845 4413 10881 4465 10913 4516 10881 4465 10845 5 N F
165 O
4465 16173 4418 16173 4418 16173 4490 16173 4490 16173 4465 16173
6 N F
4490 16173 4418 16173 4418 16172 4490 16172 4490 16173 5 N F
4465 16099 4418 16172 4490 16172 4601 16052 4642 16037 4736 16018
4872 15936 4921 15901 5007 15868 5143 15876 5278 15895 5334 15901
5414 15913 5549 15905 5560 15901 5681 15765 5685 15752 5820 15644
5956 15729 6076 15765 6091 15777 6144 15765 6225 15630 6227 15629
6230 15630 6265 15765 6362 15817 6498 15878 6633 15892 6769 15879
6904 15808 6986 15765 7040 15734 7113 15765 7175 15809 7311 15889
7326 15901 7446 16007 7494 16037 7582 16084 7717 16106 7853 16141
7988 16130 8124 16038 8128 16037 8259 15969 8373 16037 8395 16086
8424 16172 8513 16172 8519 16172 8495 16037 8419 15901 8395 15870
8259 15887 8247 15901 8124 15974 8012 16037 7988 16053 7853 16053
7786 16037 7717 16008 7582 15992 7446 15921 7424 15901 7311 15815
7233 15765 7175 15686 7040 15681 6904 15759 6895 15765 6769 15835
6633 15840 6498 15801 6448 15765 6404 15630 6362 15589 6227 15554
6094 15630 6091 15633 5956 15633 5951 15630 5874 15494 5824 15358
5820 15354 5687 15223 5685 15220 5582 15087 5582 14951 5656 14816
5612 14680 5549 14612 5414 14611 5331 14680 5363 14816 5414 14893
5500 14951 5510 15087 5463 15223 5536 15358 5549 15377 5549 14908
5414 14817 5413 14816 5409 14680 5414 14676 5549 14662 5566 14680
5607 14816 5549 14908 5549 15377 5650 15494 5627 15630 5599 15765
5549 15820 5414 15853 5278 15850 5143 15840 5007 15830 4872 15868
4803 15901 4736 15940 4601 15955 4525 16037 4465 16099 125 N F
111 O
4465 15789 4421 15765 4465 15728 4601 15684 4720 15765 4601 15775
4465 15789 7 N F
183 O
4542 16173 4490 16173 4490 16172 4542 16172 4672 16172 4672 16173
4542 16173 7 N F
4601 16052 4490 16172 4542 16172 4672 16172 4736 16148 4824 16037
4872 16007 5007 15910 5143 15918 5278 15951 5414 15971 5549 15951
5672 15901 5685 15887 5820 15771 5956 15828 6039 15901 6091 15972
6200 16037 6227 16065 6362 16104 6431 16037 6498 16011 6633 15983
6769 15940 6816 15901 6904 15857 7040 15793 7175 15897 7183 15901
7311 15947 7408 16037 7446 16088 7582 16158 7645 16172 7650 16172
7822 16172 7995 16172 8023 16172 8124 16097 8259 16044 8356 16172
8424 16172 8395 16086 8373 16037 8259 15969 8128 16037 8124 16038
7988 16130 7853 16141 7717 16106 7582 16084 7494 16037 7446 16007

7326 15901 7311 15889 7175 15809 7113 15765 7040 15734 6986 15765
6904 15808 6769 15879 6633 15892 6498 15878 6362 15817 6265 15765
6230 15630 6227 15629 6225 15630 6144 15765 6091 15777 6076 15765
5956 15729 5820 15644 5685 15752 5681 15765 5560 15901 5549 15905
5414 15913 5334 15901 5278 15895 5143 15876 5007 15868 4921 15901
4872 15936 4736 16018 4642 16037 4601 16052 88 N F
201 O
4736 16148 4672 16172 4715 16172 4887 16172 5060 16172 5232 16172
5405 16172 5578 16172 5750 16172 5923 16172 6096 16172 6268 16172
6441 16172 6614 16172 6786 16172 6959 16172 7132 16172 7304 16172
7477 16172 7645 16172 7582 16158 7446 16088 7408 16037 7311 15947
7183 15901 7175 15897 7040 15793 6904 15857 6816 15901 6769 15940
6633 15983 6498 16011 6431 16037 6362 16104 6227 16065 6200 16037
6091 15972 6039 15901 5956 15828 5820 15771 5685 15887 5672 15901
5549 15951 5414 15971 5278 15951 5143 15918 5007 15910 4872 16007
4824 16037 4736 16148 50 N F
38 O
4736 6296 4710 6267 4736 6228 4872 6190 4965 6267 4872 6347
4736 6296 7 N F
111 O
5007 15725 4893 15630 5007 15503 5143 15515 5278 15512 5414 15556
5431 15630 5414 15663 5278 15746 5165 15765 5143 15766 5133 15765
5007 15725 13 N F
38 O
5007 6410 4966 6403 5007 6348 5127 6403 5007 6410 5 N F
5007 6170 4981 6132 5007 6127 5016 6132 5007 6170 5 N F
5143 6990 5106 6946 5143 6909 5208 6946 5143 6990 5 N F
5278 6478 M 5148 6403 L 5278 6354 L 5414 6389 L 5471 6403 L
5549 6453 L 5571 6403 L 5685 6280 L 5694 6267 L
5806 6132 L 5820 6124 L 5956 6026 L 6091 6002 L
6099 5996 L 6203 5860 L 6227 5798 L 6247 5725 L
6227 5631 L 6226 5589 L 6185 5453 L 6268 5453 L
6441 5453 L 6614 5453 L 6786 5453 L 6959 5453 L
7132 5453 L 7304 5453 L 7477 5453 L 7650 5453 L
7822 5453 L 7995 5453 L 8167 5453 L 8340 5453 L
8513 5453 L 8685 5453 L 8858 5453 L 9031 5453 L
9203 5453 L 9376 5453 L 9549 5453 L 9721 5453 L
9894 5453 L 10067 5453 L 10239 5453 L 10412 5453 L
10584 5453 L 10757 5453 L 10930 5453 L 11102 5453 L
11275 5453 L 11448 5453 L 11620 5453 L 11793 5453 L
11966 5453 L 12138 5453 L 12311 5453 L 12484 5453 L
12656 5453 L 12829 5453 L 12955 5453 L 12995 5589 L
13002 5599 L 13002 10028 L 12866 9999 L 12758 9931 L

12731 9911 L 12626 9795 L 12595 9769 L 12491 9660 L
12460 9610 L 12374 9524 L 12324 9483 L 12267 9388 L
12189 9303 L 12184 9252 L 12175 9117 L 12166 8981 L
12153 8845 L 12140 8710 L 12124 8574 L 12116 8438 L
12101 8303 L 12088 8167 L 12087 8031 L 12084 7896 L
12067 7760 L 12053 7711 L 11918 7632 L 11899 7624 L
11782 7581 L 11647 7526 L 11511 7496 L 11476 7489 L
11376 7480 L 11240 7472 L 11177 7489 L 11124 7624 L
11127 7760 L 11142 7896 L 11105 7958 L 10984 8031 L
10969 8053 L 10888 8167 L 10834 8293 L 10733 8167 L
10757 8031 L 10718 7896 L 10698 7877 L 10670 7896 L
10672 8031 L 10632 8167 L 10563 8234 L 10427 8239 L
10292 8205 L 10156 8274 L 10156 7808 L 10292 7773 L
10307 7760 L 10292 7748 L 10156 7714 L 10045 7760 L
10156 7808 L 10156 8274 L 10021 8213 L 9983 8167 L
9885 8055 L 9885 7810 L 9994 7760 L 9885 7716 L
9885 7143 L 10021 7102 L 10039 7081 L 10085 6946 L
10110 6810 L 10065 6674 L 10021 6635 L 9997 6539 L
9970 6403 L 9927 6267 L 9885 6180 L 9878 6132 L
9839 5996 L 9756 5860 L 9750 5855 L 9614 5739 L
9520 5860 L 9494 5996 L 9490 6132 L 9534 6267 L
9614 6379 L 9624 6403 L 9614 6539 L 9614 6551 L
9596 6674 L 9479 6765 L 9442 6810 L 9479 6944 L
9496 6946 L 9614 7032 L 9627 7081 L 9750 7122 L
9885 7143 L 9885 7716 L 9823 7760 L 9885 7810 L
9885 8055 L 9871 8031 L 9750 7935 L 9652 8031 L
9614 8082 L 9479 8070 L 9479 7916 L 9491 7896 L
9479 7880 L 9472 7896 L 9479 7916 L 9479 8070 L
9345 8167 L 9343 8168 L 9343 6667 L 9446 6539 L
9358 6403 L 9343 6377 L 9331 6403 L 9281 6539 L
9343 6667 L 9343 8168 L 9208 8266 L 9208 5831 L
9343 5745 L 9358 5725 L 9343 5674 L 9279 5589 L
9208 5560 L 9188 5589 L 9134 5725 L 9208 5831 L
9208 8266 L 9131 8303 L 9072 8324 L 8990 8438 L
9072 8541 L 9114 8574 L 9072 8624 L 8937 8602 L
8918 8574 L 8883 8438 L 8801 8363 L 8732 8303 L
8695 8167 L 8751 8031 L 8755 7896 L 8800 7760 L
8801 7759 L 8894 7624 L 8918 7489 L 8838 7353 L
8801 7311 L 8723 7217 L 8666 7165 L 8545 7081 L
8530 7067 L 8395 7047 L 8385 6946 L 8268 6810 L
8259 6804 L 8124 6802 L 8063 6674 L 7988 6575 L
7951 6539 L 7853 6447 L 7717 6440 L 7582 6518 L
7564 6539 L 7577 6674 L 7552 6810 L 7546 6946 L

7446 7044 L 7361 7081 L 7327 7217 L 7311 7235 L
7276 7217 L 7179 7081 L 7175 7080 L 7175 6309 L
7285 6267 L 7311 6255 L 7366 6132 L 7311 6011 L
7303 5996 L 7175 5892 L 7069 5860 L 7040 5848 L
6904 5842 L 6863 5860 L 6874 5996 L 6845 6132 L
6904 6187 L 7040 6200 L 7143 6267 L 7175 6309 L
7175 7080 L 7040 7035 L 6951 7081 L 6904 7106 L
6846 7217 L 6896 7353 L 6769 7474 L 6633 7467 L
6503 7489 L 6498 7491 L 6362 7534 L 6302 7489 L
6227 7453 L 6091 7360 L 6083 7353 L 6091 7345 L
6223 7217 L 6095 7081 L 6227 7001 L 6362 7046 L
6494 7081 L 6498 7083 L 6499 7081 L 6633 6997 L
6675 6946 L 6633 6912 L 6498 6897 L 6439 6810 L
6388 6674 L 6362 6608 L 6227 6547 L 6132 6674 L
6091 6763 L 6078 6810 L 6091 6887 L 6102 6946 L
6091 7037 L 6087 7081 L 5956 7205 L 5836 7081 L
5820 7031 L 5685 7054 L 5641 6946 L 5683 6810 L
5549 6688 L 5510 6674 L 5414 6543 L 5404 6539 L
5278 6478 L N E
147 O
5250 14137 5221 14273 5278 14382 5278 14301 5264 14273 5278 14209
5299 14273 5278 14301 5278 14382 5359 14273 5307 14137 5278 14108
5250 14137 13 N F
38 O
5278 7195 5239 7081 5278 7021 5331 7081 5278 7195 5 N F
165 O
5278 14209 5264 14273 5278 14301 5299 14273 5278 14209 5 N F
38 O
5414 7333 5286 7217 5414 7128 5549 7180 5621 7217 5549 7338
5414 7333 7 N F
92 O
5414 13532 5369 13594 5414 13692 5434 13730 5549 13800 5549 13751
5516 13730 5549 13684 5594 13730 5549 13751 5549 13800 5685 13858
5710 13730 5685 13709 5573 13594 5549 13565 5414 13532 17 N F
56 O
5400 7760 5378 7896 5414 7932 5426 7896 5416 7760 5414 7754
5400 7760 7 N F
183 O
5414 14676 5409 14680 5413 14816 5414 14817 5549 14908 5549 14830
5528 14816 5549 14785 5558 14816 5549 14830 5549 14908 5607 14816
5566 14680 5549 14662 5414 14676 15 N F
74 O
5490 5453 5445 5589 5549 5713 5556 5725 5685 5858 5685 5709

5609 5589 5685 5527 5766 5589 5685 5709 5685 5858 5810 5725
5820 5710 5956 5689 6091 5656 6136 5589 6091 5512 6055 5453
5961 5453 5956 5484 5950 5453 5923 5453 5845 5453 5820 5492
5755 5453 5750 5453 5578 5453 5490 5453 28 N F
111 O
5549 13684 5516 13730 5549 13751 5594 13730 5549 13684 5 N F
201 O
5549 14785 5528 14816 5549 14830 5558 14816 5549 14785 5 N F
92 O
5685 5527 5609 5589 5685 5709 5766 5589 5685 5527 5 N F
74 O
5806 14680 5750 14544 5800 14409 5820 14374 5828 14409 5956 14521
6018 14544 5960 14680 5956 14685 5820 14712 5806 14680 11 N F
56 O
5755 5453 5755 5453 5820 5453 5845 5453 5845 5453 5755 5453
6 N F
5755 5453 5755 5453 5845 5453 5845 5453 5755 5453 5 N F
5820 5492 5755 5453 5845 5453 5820 5492 4 N F
5820 11865 5756 11966 5820 12075 5877 12102 5956 12221 6052 12102
6041 11966 6051 11831 5956 11706 5858 11831 5820 11865 11 N F
5820 9482 5809 9524 5820 9541 5907 9660 5956 9712 6021 9660
6091 9538 6105 9524 6227 9403 6241 9388 6227 9373 6121 9252
6091 9233 6041 9252 5958 9388 5956 9391 5820 9482 17 N F
74 O
5923 5453 5845 5453 5845 5453 5923 5453 5949 5453 5950 5453
5923 5453 7 N F
56 O
5932 8845 5855 8981 5956 9093 6035 8981 5994 8845 6021 8710
5956 8639 5874 8710 5932 8845 9 N F
5956 12289 5934 12373 5956 12480 5996 12373 5956 12289 5 N F
111 O
5956 13947 5941 14002 5956 14024 6091 14079 6148 14002 6176 13866
6091 13778 5993 13866 5956 13947 9 N F
92 O
5956 15104 5947 15087 5956 15083 5960 15087 5956 15104 5 N F
5949 5453 5949 5453 5961 5453 5961 5453 5956 5453 5949 5453
6 N F
5949 5453 5950 5453 5961 5453 5961 5453 5949 5453 5 N F
5961 5453 5950 5453 5956 5484 5961 5453 4 N F
74 O
6055 5453 5961 5453 5961 5453 6055 5453 6055 5453 5 N F
5961 5453 5961 5453 6055 5453 6055 5453 5961 5453 5 N F
92 O

6091 13152 5976 13187 6091 13253 6107 13187 6091 13152 5 N F
147 O
6091 14737 6022 14816 6025 14951 6091 15016 6148 15087 6207 15223
6227 15256 6362 15348 6362 15268 6301 15223 6227 15130 6208 15087
6194 14951 6091 14926 6065 14816 6091 14786 6227 14779 6250 14816
6230 14951 6362 15077 6381 15087 6399 15223 6362 15268 6362 15348
6464 15223 6498 15128 6582 15223 6633 15256 6732 15358 6769 15396
6769 15285 6709 15223 6769 15186 6804 15223 6769 15285 6769 15396
6904 15372 6929 15358 6904 15332 6868 15223 6769 15117 6726 15087
6633 15036 6498 15065 6362 14997 6315 14951 6289 14816 6227 14715
6091 14737 49 N F
56 O
6091 10309 6044 10338 6091 10353 6115 10338 6227 10220 6249 10202
6362 10140 6412 10067 6362 10017 6227 9953 6145 10067 6204 10202
6091 10309 13 N F
6055 5453 6055 5453 6096 5453 6185 5453 6185 5453 6096 5453
6055 5453 7 N F
165 O
6091 14786 6065 14816 6091 14926 6194 14951 6208 15087 6227 15130
6301 15223 6362 15268 6399 15223 6381 15087 6362 15077 6230 14951
6250 14816 6227 14779 6091 14786 15 N F
56 O
6362 10717 6329 10745 6362 10773 6475 10881 6498 10895 6518 10881
6498 10856 6393 10745 6362 10717 9 N F
74 O
6633 12580 6532 12645 6614 12780 6633 12793 6660 12780 6708 12645
6633 12580 7 N F
38 O
6769 12557 6667 12509 6769 12482 6854 12509 6769 12557 5 N F
111 O
6769 15699 6693 15630 6769 15507 6789 15494 6904 15468 7040 15477
7081 15494 7040 15501 6942 15630 6904 15652 6769 15699 11 N F
165 O
6769 15186 6709 15223 6769 15285 6804 15223 6769 15186 5 N F
38 O
6904 7714 6777 7624 6904 7519 7040 7509 7170 7624 7040 7667
6904 7714 7 N F
56 O
6874 5996 6845 6132 6904 6187 7040 6200 7143 6267 7175 6309
7285 6267 7311 6255 7366 6132 7311 6011 7303 5996 7175 5892
7069 5860 7040 5848 6904 5842 6863 5860 6874 5996 17 N F
38 O
7040 7477 6916 7353 7040 7246 7078 7353 7040 7477 5 N F

7175 12037 7150 11966 7175 11882 7218 11966 7175 12037 5 N F
56 O
7175 10593 7167 10609 7175 10617 7185 10609 7175 10593 5 N F
74 O
7311 12586 7279 12645 7311 12692 7446 12748 7502 12645 7507 12509
7446 12481 7393 12509 7311 12586 9 N F
38 O
7717 13752 7651 13730 7717 13645 7746 13594 7853 13563 7928 13594
7967 13730 7853 13771 7717 13752 9 N F
7717 7289 7673 7217 7717 7094 7746 7081 7766 6946 7853 6867
7913 6810 7988 6770 8109 6810 8026 6946 7988 6967 7867 7081
7988 7217 7991 7217 7988 7218 7853 7342 7717 7289 17 N F
56 O
7717 10722 7691 10745 7717 10771 7748 10745 7717 10722 5 N F
38 O
7853 8390 7790 8303 7853 8255 7988 8234 8109 8167 8124 8154
8259 8068 8385 8167 8259 8290 8184 8303 8167 8438 8259 8511
8383 8438 8395 8436 8396 8438 8415 8574 8395 8608 8259 8702
8124 8590 8111 8574 7988 8463 7927 8438 7853 8390 23 N F
56 O
7853 9503 7837 9524 7853 9541 7872 9524 7853 9503 5 N F
7988 11237 7939 11288 7988 11339 8041 11288 7988 11237 5 N F
165 O
7970 14680 M 7953 14816 L 7988 14852 L 8124 14855 L 8259 14872 L
8395 14893 L 8446 14951 L 8530 15054 L 8562 15087 L
8575 15223 L 8666 15340 L 8801 15349 L 8810 15358 L
8937 15471 L 9072 15488 L 9079 15494 L 9097 15630 L
9192 15765 L 9208 15791 L 9310 15901 L 9343 15974 L
9479 15971 L 9614 15964 L 9657 15901 L 9749 15765 L
9750 15764 L 9878 15630 L 9885 15576 L 9943 15630 L
10021 15720 L 10043 15765 L 10106 15901 L 10156 15976 L
10195 16037 L 10275 16172 L 10395 16172 L 10313 16037 L
10292 16009 L 10214 15901 L 10164 15765 L 10156 15690 L
10147 15630 L 10021 15505 L 10009 15494 L 10021 15482 L
10059 15494 L 10156 15597 L 10292 15590 L 10427 15606 L
10504 15630 L 10563 15663 L 10698 15711 L 10834 15679 L
10864 15630 L 10845 15494 L 10834 15475 L 10698 15394 L
10563 15415 L 10512 15358 L 10427 15353 L 10292 15352 L
10166 15223 L 10156 15213 L 10021 15193 L 9907 15087 L
9885 15064 L 9772 14951 L 9750 14930 L 9614 14940 L
9603 14951 L 9586 15087 L 9590 15223 L 9614 15248 L
9719 15358 L 9714 15494 L 9750 15532 L 9750 15505 L
9739 15494 L 9746 15358 L 9660 15223 L 9624 15087 L

9750 15024 L 9815 15087 L 9885 15160 L 10021 15221 L
10029 15223 L 10021 15235 L 9885 15283 L 9792 15358 L
9817 15494 L 9750 15505 L 9750 15532 L 9850 15630 L
9750 15735 L 9722 15765 L 9614 15882 L 9479 15880 L
9343 15880 L 9230 15765 L 9208 15743 L 9119 15630 L
9104 15494 L 9072 15462 L 8937 15448 L 8836 15358 L
8801 15321 L 8666 15311 L 8598 15223 L 8587 15087 L
8530 15026 L 8469 14951 L 8395 14867 L 8259 14848 L
8124 14828 L 7988 14825 L 7979 14816 L 7988 14742 L
8124 14717 L 8149 14680 L 8216 14544 L 8259 14507 L
8395 14409 L 8395 14409 L 8525 14273 L 8530 14241 L
8582 14137 L 8624 14002 L 8666 13919 L 8689 13866 L
8801 13832 L 8836 13866 L 8937 13965 L 8978 14002 L
9072 14090 L 9131 14137 L 9208 14206 L 9292 14137 L
9343 14099 L 9479 14078 L 9558 14002 L 9614 13950 L
9750 13941 L 9885 13928 L 10002 13866 L 9946 13730 L
9885 13693 L 9750 13673 L 9614 13643 L 9558 13594 L
9479 13514 L 9343 13490 L 9314 13459 L 9307 13323 L
9208 13224 L 9072 13223 L 9040 13187 L 9072 13118 L
9208 13112 L 9271 13052 L 9343 12988 L 9479 12938 L
9599 12916 L 9614 12912 L 9632 12916 L 9750 12932 L
9765 12916 L 9794 12780 L 9803 12645 L 9813 12509 L
9885 12437 L 10021 12407 L 10156 12407 L 10275 12509 L
10292 12524 L 10410 12509 L 10427 12485 L 10561 12373 L
10563 12372 L 10593 12238 L 10563 12139 L 10542 12102 L
10427 12041 L 10380 11966 L 10324 11831 L 10292 11747 L
10264 11695 L 10156 11608 L 10122 11559 L 10116 11423 L
10066 11288 L 10146 11152 L 10156 11141 L 10287 11016 L
10292 11016 L 10427 10990 L 10553 10881 L 10563 10850 L
10698 10869 L 10717 10881 L 10834 10961 L 10929 11016 L
10969 11039 L 11105 11139 L 11121 11152 L 11240 11253 L
11280 11288 L 11376 11363 L 11438 11423 L 11511 11500 L
11562 11559 L 11647 11687 L 11655 11695 L 11782 11768 L
11796 11831 L 11807 11966 L 11914 12102 L 11916 12238 L
11918 12242 L 11984 12373 L 11918 12402 L 11857 12509 L
11861 12645 L 11863 12780 L 11867 12916 L 11861 13052 L
11852 13187 L 11848 13323 L 11850 13459 L 11782 13547 L
11738 13594 L 11647 13714 L 11632 13730 L 11647 13745 L
11742 13866 L 11782 13902 L 11888 14002 L 11918 14067 L
12006 14137 L 11918 14241 L 11879 14273 L 11782 14352 L
11647 14392 L 11511 14399 L 11376 14391 L 11240 14390 L
11122 14409 L 11105 14436 L 11093 14544 L 11088 14680 L
11085 14816 L 11105 14835 L 11231 14951 L 11240 15069 L

11240 14838 L 11204 14816 L 11238 14680 L 11239 14544 L
11240 14542 L 11242 14544 L 11245 14680 L 11311 14816 L
11240 14838 L 11240 15069 L 11252 15087 L 11240 15097 L
11144 15223 L 11105 15289 L 11060 15358 L 11105 15389 L
11240 15447 L 11376 15406 L 11400 15358 L 11451 15223 L
11511 15134 L 11525 15087 L 11549 14951 L 11584 14816 L
11647 14755 L 11782 14694 L 11801 14680 L 11911 14544 L
11918 14533 L 12006 14409 L 12053 14335 L 12132 14409 L
12189 14443 L 12227 14409 L 12324 14311 L 12337 14273 L
12460 14154 L 12488 14137 L 12595 14055 L 12707 14002 L
12731 13993 L 12783 14002 L 12852 14137 L 12866 14151 L
12893 14137 L 13002 14096 L 13002 14047 L 12927 14002 L
12866 13919 L 12731 13932 L 12595 13967 L 12536 14002 L
12460 14054 L 12365 14137 L 12324 14176 L 12189 14222 L
12104 14137 L 12053 14096 L 11918 14006 L 11916 14002 L
11782 13876 L 11771 13866 L 11703 13730 L 11770 13594 L
11782 13582 L 11878 13459 L 11874 13323 L 11880 13187 L
11892 13052 L 11900 12916 L 11896 12780 L 11899 12645 L
11903 12509 L 11918 12483 L 11960 12509 L 12053 12589 L
12136 12509 L 12123 12373 L 12103 12238 L 12097 12102 L
12053 12060 L 11918 12021 L 11874 11966 L 11875 11831 L
11918 11753 L 11985 11831 L 12053 11880 L 12095 11831 L
12053 11720 L 12021 11695 L 11918 11564 L 11913 11559 L
11797 11423 L 11782 11406 L 11683 11288 L 11647 11246 L
11555 11152 L 11511 11108 L 11391 11016 L 11376 11000 L
11240 10894 L 11225 10881 L 11105 10777 L 11057 10745 L
10969 10678 L 10840 10609 L 10834 10606 L 10698 10493 L
10563 10488 L 10540 10609 L 10519 10745 L 10431 10881 L
10427 10884 L 10292 10965 L 10156 10989 L 10128 11016 L
10021 11126 L 9995 11152 L 9980 11288 L 10021 11371 L
10054 11423 L 10074 11559 L 10156 11674 L 10182 11695 L
10234 11831 L 10280 11966 L 10292 12050 L 10301 12102 L
10292 12117 L 10203 12238 L 10156 12292 L 10021 12325 L
9954 12373 L 9885 12390 L 9766 12509 L 9758 12645 L
9750 12780 L 9750 12781 L 9614 12819 L 9479 12854 L
9406 12916 L 9343 12942 L 9220 13052 L 9208 13063 L
9072 13076 L 9020 13187 L 9072 13244 L 9208 13247 L
9284 13323 L 9292 13459 L 9343 13514 L 9479 13543 L
9530 13594 L 9614 13667 L 9750 13706 L 9884 13730 L
9885 13731 L 9947 13866 L 9885 13899 L 9750 13915 L
9614 13927 L 9534 14002 L 9479 14055 L 9343 14078 L
9263 14137 L 9208 14183 L 9158 14137 L 9072 14069 L
9000 14002 L 8937 13944 L 8857 13866 L 8801 13811 L

8666 13845 L 8604 13866 L 8546 14002 L 8530 14051 L
8521 14137 L 8497 14273 L 8395 14379 L 8259 14395 L
8163 14409 L 8124 14440 L 7988 14507 L 7980 14544 L
7970 14680 L N E
183 O
7988 14742 M 7979 14816 L 7988 14825 L 8124 14828 L 8259 14848 L
8395 14867 L 8469 14951 L 8530 15026 L 8587 15087 L
8598 15223 L 8666 15311 L 8801 15321 L 8836 15358 L
8937 15448 L 9072 15462 L 9104 15494 L 9119 15630 L
9208 15743 L 9230 15765 L 9343 15880 L 9479 15880 L
9479 15853 L 9343 15853 L 9257 15765 L 9208 15715 L
9140 15630 L 9130 15494 L 9072 15436 L 8937 15425 L
8862 15358 L 8801 15292 L 8666 15281 L 8621 15223 L
8613 15087 L 8530 14998 L 8492 14951 L 8395 14842 L
8259 14824 L 8205 14816 L 8259 14738 L 8320 14680 L
8395 14598 L 8436 14544 L 8530 14419 L 8535 14409 L
8610 14273 L 8659 14137 L 8666 14117 L 8717 14002 L
8756 13866 L 8801 13852 L 8815 13866 L 8937 13985 L
8955 14002 L 9072 14111 L 9105 14137 L 9208 14230 L
9321 14137 L 9343 14121 L 9479 14102 L 9583 14002 L
9614 13973 L 9750 13967 L 9885 13958 L 10021 13902 L
10053 13866 L 10021 13763 L 10008 13730 L 9885 13656 L
9750 13640 L 9614 13619 L 9586 13594 L 9479 13486 L
9343 13467 L 9336 13459 L 9331 13323 L 9208 13201 L
9072 13201 L 9060 13187 L 9072 13161 L 9208 13160 L
9323 13052 L 9343 13033 L 9479 13001 L 9614 12980 L
9750 12985 L 9818 12916 L 9838 12780 L 9848 12645 L
9860 12509 L 9885 12484 L 10021 12460 L 10156 12454 L
10220 12509 L 10292 12575 L 10390 12645 L 10427 12679 L
10563 12767 L 10698 12687 L 10834 12674 L 10865 12645 L
10969 12520 L 11020 12509 L 10969 12435 L 10939 12373 L
10834 12319 L 10753 12238 L 10698 12201 L 10630 12102 L
10608 11966 L 10563 11919 L 10427 11912 L 10383 11831 L
10364 11695 L 10292 11581 L 10249 11559 L 10292 11483 L
10360 11423 L 10427 11379 L 10563 11333 L 10698 11329 L
10834 11384 L 10914 11423 L 10969 11456 L 11105 11542 L
11125 11559 L 11240 11675 L 11257 11695 L 11372 11831 L
11376 11837 L 11469 11966 L 11511 12013 L 11585 12102 L
11647 12169 L 11695 12238 L 11782 12320 L 11811 12373 L
11811 12509 L 11824 12645 L 11830 12780 L 11834 12916 L
11830 13052 L 11823 13187 L 11821 13323 L 11823 13459 L
11782 13512 L 11705 13594 L 11647 13671 L 11595 13730 L
11647 13782 L 11713 13866 L 11782 13929 L 11859 14002 L

11918 14129 L 11928 14137 L 11918 14150 L 11782 14269 L
11776 14273 L 11647 14358 L 11511 14362 L 11376 14355 L
11240 14354 L 11105 14370 L 11068 14409 L 11057 14544 L
11054 14680 L 11053 14816 L 11105 14867 L 11196 14951 L
11205 15087 L 11105 15193 L 11075 15223 L 10969 15338 L
10834 15338 L 10698 15328 L 10563 15327 L 10427 15320 L
10292 15318 L 10199 15223 L 10156 15179 L 10021 15164 L
9938 15087 L 9885 15032 L 9804 14951 L 9750 14898 L
9614 14907 L 9570 14951 L 9558 15087 L 9562 15223 L
9614 15276 L 9692 15358 L 9690 15494 L 9750 15559 L
9822 15630 L 9750 15706 L 9695 15765 L 9614 15853 L
9479 15853 L 9479 15880 L 9614 15882 L 9722 15765 L
9750 15735 L 9850 15630 L 9750 15532 L 9714 15494 L
9719 15358 L 9614 15248 L 9590 15223 L 9586 15087 L
9603 14951 L 9614 14940 L 9750 14930 L 9772 14951 L
9885 15064 L 9907 15087 L 10021 15193 L 10156 15213 L
10166 15223 L 10292 15352 L 10427 15353 L 10512 15358 L
10563 15415 L 10698 15394 L 10834 15475 L 10845 15494 L
10864 15630 L 10834 15679 L 10698 15711 L 10563 15663 L
10504 15630 L 10427 15606 L 10292 15590 L 10156 15597 L
10059 15494 L 10021 15482 L 10009 15494 L 10021 15505 L
10147 15630 L 10156 15690 L 10164 15765 L 10214 15901 L
10292 16009 L 10313 16037 L 10395 16172 L 10412 16172 L
10505 16172 L 10427 16063 L 10411 16037 L 10332 15901 L
10427 15821 L 10563 15872 L 10698 15854 L 10834 15835 L
10969 15868 L 11072 15901 L 11105 15916 L 11127 15901 L
11121 15765 L 11105 15735 L 11007 15630 L 11105 15520 L
11240 15546 L 11376 15545 L 11446 15494 L 11510 15358 L
11511 15357 L 11569 15223 L 11602 15087 L 11645 14951 L
11647 14948 L 11759 14816 L 11782 14801 L 11918 14701 L
11975 14680 L 12053 14622 L 12189 14586 L 12259 14544 L
12324 14485 L 12360 14409 L 12405 14273 L 12460 14220 L
12595 14139 L 12598 14137 L 12731 14069 L 12787 14137 L
12866 14216 L 13002 14163 L 13002 14096 L 12893 14137 L
12866 14151 L 12852 14137 L 12783 14002 L 12731 13993 L
12707 14002 L 12595 14055 L 12488 14137 L 12460 14154 L
12337 14273 L 12324 14311 L 12227 14409 L 12189 14443 L
12132 14409 L 12053 14335 L 12006 14409 L 11918 14533 L
11911 14544 L 11801 14680 L 11782 14694 L 11647 14755 L
11584 14816 L 11549 14951 L 11525 15087 L 11511 15134 L
11451 15223 L 11400 15358 L 11376 15406 L 11240 15447 L
11105 15389 L 11060 15358 L 11105 15289 L 11144 15223 L
11240 15097 L 11252 15087 L 11240 15069 L 11231 14951 L

11105 14835 L 11085 14816 L 11088 14680 L 11093 14544 L
11105 14436 L 11122 14409 L 11240 14390 L 11376 14391 L
11511 14399 L 11647 14392 L 11782 14352 L 11879 14273 L
11918 14241 L 12006 14137 L 11918 14067 L 11888 14002 L
11782 13902 L 11742 13866 L 11647 13745 L 11632 13730 L
11647 13714 L 11738 13594 L 11782 13547 L 11850 13459 L
11848 13323 L 11852 13187 L 11861 13052 L 11867 12916 L
11863 12780 L 11861 12645 L 11857 12509 L 11918 12402 L
11984 12373 L 11918 12242 L 11916 12238 L 11914 12102 L
11807 11966 L 11796 11831 L 11782 11768 L 11655 11695 L
11647 11687 L 11562 11559 L 11511 11500 L 11438 11423 L
11376 11363 L 11280 11288 L 11240 11253 L 11121 11152 L
11105 11139 L 10969 11039 L 10929 11016 L 10834 10961 L
10717 10881 L 10698 10869 L 10563 10850 L 10553 10881 L
10427 10990 L 10292 11016 L 10287 11016 L 10156 11141 L
10146 11152 L 10066 11288 L 10116 11423 L 10122 11559 L
10156 11608 L 10264 11695 L 10292 11747 L 10324 11831 L
10380 11966 L 10427 12041 L 10542 12102 L 10563 12139 L
10593 12238 L 10563 12372 L 10561 12373 L 10427 12485 L
10410 12509 L 10292 12524 L 10275 12509 L 10156 12407 L
10021 12407 L 9885 12437 L 9813 12509 L 9803 12645 L
9794 12780 L 9765 12916 L 9750 12932 L 9632 12916 L
9614 12912 L 9599 12916 L 9479 12938 L 9343 12988 L
9271 13052 L 9208 13112 L 9072 13118 L 9040 13187 L
9072 13223 L 9208 13224 L 9307 13323 L 9314 13459 L
9343 13490 L 9479 13514 L 9558 13594 L 9614 13643 L
9750 13673 L 9885 13693 L 9946 13730 L 10002 13866 L
9885 13928 L 9750 13941 L 9614 13950 L 9558 14002 L
9479 14078 L 9343 14099 L 9292 14137 L 9208 14206 L
9131 14137 L 9072 14090 L 8978 14002 L 8937 13965 L
8836 13866 L 8801 13832 L 8689 13866 L 8666 13919 L
8624 14002 L 8582 14137 L 8530 14241 L 8525 14273 L
8395 14409 L 8395 14409 L 8259 14507 L 8216 14544 L
8149 14680 L 8124 14717 L 7988 14742 L N E
201 O
8124 16097 8023 16172 8167 16172 8340 16172 8356 16172 8259 16044
8124 16097 7 N F
56 O
8124 12024 8051 12102 8124 12180 8205 12102 8124 12024 5 N F
38 O
8259 7243 8179 7217 8259 7129 8301 7217 8259 7243 5 N F
56 O
8259 11614 8197 11695 8259 11788 8395 11787 8495 11695 8395 11601

8259 11614 7 N F
8259 10680 8205 10745 8259 10818 8336 10745 8259 10680 5 N F
201 O
8259 14738 8205 14816 8259 14824 8395 14842 8492 14951 8530 14998
8613 15087 8621 15223 8666 15281 8801 15292 8862 15358 8937 15425
9072 15436 9130 15494 9140 15630 9208 15715 9257 15765 9343 15853
9479 15853 9614 15853 9695 15765 9750 15706 9822 15630 9750 15559
9690 15494 9692 15358 9614 15276 9562 15223 9558 15087 9570 14951
9614 14907 9750 14898 9804 14951 9885 15032 9938 15087 10021 15164
10156 15179 10199 15223 10292 15318 10427 15320 10563 15327 10698 15328
10834 15338 10969 15338 11075 15223 11105 15193 11205 15087 11196 14951
11105 14867 11053 14816 11054 14680 11057 14544 11068 14409 11105 14370
11240 14354 11376 14355 11511 14362 11647 14358 11776 14273 11782 14269
11918 14150 11928 14137 11918 14129 11859 14002 11782 13929 11713 13866
11647 13782 11595 13730 11647 13671 11705 13594 11782 13512 11823 13459
11821 13323 11823 13187 11830 13052 11834 12916 11830 12780 11824 12645
11811 12509 11811 12373 11782 12320 11695 12238 11647 12169 11585 12102
11511 12013 11469 11966 11376 11837 11372 11831 11257 11695 11240 11675
11125 11559 11105 11542 10969 11456 10914 11423 10834 11384 10698 11329
10563 11333 10427 11379 10360 11423 10292 11483 10249 11559 10292 11581
10364 11695 10383 11831 10427 11912 10563 11919 10608 11966 10630 12102
10698 12201 10753 12238 10834 12319 10939 12373 10969 12435 11020 12509
10969 12520 10865 12645 10834 12674 10698 12687 10563 12767 10427 12679
10390 12645 10292 12575 10220 12509 10156 12454 10021 12460 9885 12484
9860 12509 9848 12645 9838 12780 9818 12916 9750 12985 9614 12980
9479 13001 9343 13033 9323 13052 9208 13160 9072 13161 9060 13187
9072 13201 9208 13201 9331 13323 9336 13459 9343 13467 9479 13486
9586 13594 9614 13619 9750 13640 9885 13656 10008 13730 10021 13763
10053 13866 10021 13902 9885 13958 9750 13967 9614 13973 9583 14002
9479 14102 9343 14121 9321 14137 9208 14230 9105 14137 9072 14111
8955 14002 8937 13985 8815 13866 8801 13852 8756 13866 8717 14002
8666 14117 8659 14137 8610 14273 8535 14409 8530 14419 8436 14544
8395 14598 8320 14680 8259 14738 177 N F
183 O
8356 16173 8356 16172 8424 16172 8424 16173 8356 16173 5 N F
8356 16173 8356 16173 8424 16173 8424 16173 8395 16173 8356 16173
6 N F
165 O
8424 16173 8424 16172 8513 16172 8519 16172 8519 16173 8513 16173
8424 16173 7 N F
147 O
8519 16173 8519 16172 8558 16172 8558 16173 8519 16173 5 N F
8519 16173 8519 16173 8558 16173 8558 16173 8530 16173 8519 16173

6 N F
165 O
8666 16116 8558 16172 8685 16172 8858 16172 8911 16172 8801 16099
8666 16116 7 N F
56 O
8666 9714 8578 9795 8666 9882 8765 9795 8666 9714 5 N F
129 O
8666 15938 8630 15901 8666 15777 8778 15901 8666 15938 5 N F
74 O
8666 8570 8664 8574 8666 8576 8668 8574 8666 8570 5 N F
129 O
8801 15888 8671 15765 8744 15630 8751 15494 8801 15472 8910 15494
8937 15501 9072 15607 9075 15630 9095 15765 9072 15850 8937 15838
8801 15888 13 N F
38 O
8937 9079 8881 8981 8937 8949 8970 8981 8937 9079 5 N F
74 O
8937 10984 8909 11016 8937 11045 9072 11111 9198 11016 9072 10909
8937 10984 7 N F
38 O
9072 9164 8992 9117 9072 9095 9107 9117 9072 9164 5 N F
92 O
9072 10562 9056 10609 9072 10629 9112 10609 9208 10520 9251 10474
9208 10452 9153 10474 9072 10562 9 N F
56 O
9188 5589 9134 5725 9208 5831 9343 5745 9358 5725 9343 5674
9279 5589 9208 5560 9188 5589 9 N F
9331 6403 9281 6539 9343 6667 9446 6539 9358 6403 9343 6377
9331 6403 7 N F
165 O
9376 16173 9333 16173 9333 16172 9376 16172 9383 16172 9383 16173
9376 16173 7 N F
9343 16146 9333 16172 9376 16172 9383 16172 9343 16146 5 N F
129 O
9479 10412 9420 10474 9479 10580 9528 10609 9546 10745 9614 10752
9614 10576 9479 10524 9450 10474 9479 10444 9614 10413 9632 10338
9750 10278 9812 10338 9750 10403 9628 10474 9614 10576 9614 10752
9623 10745 9689 10609 9692 10474 9750 10440 9847 10338 9750 10244
9614 10270 9581 10338 9479 10412 27 N F
56 O
9479 6765 9442 6810 9479 6944 9496 6946 9614 7032 9614 6939
9524 6810 9614 6692 9651 6810 9614 6939 9614 7032 9627 7081
9750 7122 9750 6999 9644 6946 9750 6937 9767 6946 9750 6999

9750 7122 9885 7143 9885 6875 9789 6810 9750 6801 9643 6674
9642 6539 9668 6403 9615 6267 9614 6267 9520 6132 9530 5996
9594 5860 9614 5835 9643 5860 9750 5979 9761 5996 9824 6132
9840 6267 9885 6398 9888 6403 9945 6539 9975 6674 9967 6810
9885 6875 9885 7143 10021 7102 10039 7081 10085 6946 10110 6810
10065 6674 10021 6635 9997 6539 9970 6403 9927 6267 9885 6180
9878 6132 9839 5996 9756 5860 9750 5855 9614 5739 9520 5860
9494 5996 9490 6132 9534 6267 9614 6379 9624 6403 9614 6539
9614 6551 9596 6674 9479 6765 69 N F
147 O
9479 10444 9450 10474 9479 10524 9614 10576 9628 10474 9750 10403
9750 10366 9699 10338 9750 10312 9777 10338 9750 10366 9750 10403
9812 10338 9750 10278 9632 10338 9614 10413 9479 10444 17 N F
74 O
9479 8688 9462 8710 9479 8722 9497 8710 9479 8688 5 N F
56 O
9479 7880 9472 7896 9479 7916 9491 7896 9479 7880 5 N F
74 O
9530 5996 9520 6132 9614 6267 9615 6267 9668 6403 9642 6539
9643 6674 9750 6801 9750 6765 9673 6674 9670 6539 9713 6403
9706 6267 9614 6224 9550 6132 9566 5996 9614 5908 9696 5996
9750 6089 9771 6132 9779 6267 9812 6403 9885 6522 9892 6539
9919 6674 9885 6756 9750 6765 9750 6801 9789 6810 9885 6875
9967 6810 9975 6674 9945 6539 9888 6403 9885 6398 9840 6267
9824 6132 9761 5996 9750 5979 9643 5860 9614 5835 9594 5860
9530 5996 43 N F
38 O
9614 6939 9524 6810 9614 6692 9651 6810 9614 6939 5 N F
92 O
9566 5996 9550 6132 9614 6224 9614 6181 9580 6132 9602 5996
9614 5974 9635 5996 9703 6132 9614 6181 9614 6224 9706 6267
9713 6403 9670 6539 9673 6674 9750 6765 9750 6729 9703 6674
9698 6539 9750 6415 9842 6539 9857 6674 9750 6729 9750 6765
9885 6756 9919 6674 9892 6539 9885 6522 9812 6403 9779 6267
9771 6132 9750 6089 9696 5996 9614 5908 9566 5996 35 N F
74 O
9614 9336 9572 9388 9614 9453 9668 9388 9614 9336 5 N F
111 O
9602 5996 9580 6132 9614 6181 9614 6139 9609 6132 9614 6105
9627 6132 9614 6139 9614 6181 9703 6132 9635 5996 9614 5974
9602 5996 13 N F
129 O
9614 6105 9609 6132 9614 6139 9627 6132 9614 6105 5 N F

147 O
9660 15223 9624 15087 9750 15024 9815 15087 9885 15160 10021 15221
10029 15223 10021 15235 9885 15283 9792 15358 9817 15494 9750 15505
9739 15494 9746 15358 9660 15223 15 N F
38 O
9750 6999 9644 6946 9750 6937 9767 6946 9750 6999 5 N F
111 O
9750 6415 9698 6539 9703 6674 9750 6729 9750 6694 9734 6674
9726 6539 9750 6482 9792 6539 9787 6674 9750 6694 9750 6729
9857 6674 9842 6539 9750 6415 15 N F
165 O
9750 10312 9699 10338 9750 10366 9777 10338 9750 10312 5 N F
129 O
9750 6482 9726 6539 9734 6674 9750 6694 9787 6674 9792 6539
9750 6482 7 N F
56 O
9885 7716 9823 7760 9885 7810 9994 7760 9885 7716 5 N F
129 O
9885 15996 9848 15901 9867 15765 9885 15742 9906 15765 9920 15901
9885 15996 7 N F
111 O
10021 10013 9946 10067 10021 10155 10021 10071 10017 10067 10021 10064
10025 10067 10021 10071 10021 10155 10104 10067 10021 10013 11 N F
129 O
10021 10064 10017 10067 10021 10071 10025 10067 10021 10064 5 N F
56 O
10156 7714 10045 7760 10156 7808 10292 7773 10307 7760 10292 7748
10156 7714 7 N F
111 O
10156 9383 10151 9388 10156 9395 10292 9429 10332 9388 10427 9274
10446 9252 10427 9213 10407 9252 10292 9350 10156 9383 11 N F
10292 9596 10233 9660 10292 9736 10363 9660 10292 9596 5 N F
165 O
10275 16172 10275 16173 10395 16173 10395 16172 10275 16172 5 N F
10395 16173 10275 16173 10275 16173 10292 16173 10395 16173 10395 16173
6 N F
201 O
10427 15821 10332 15901 10411 16037 10427 16063 10505 16172 10584 16172
10757 16172 10930 16172 11102 16172 11275 16172 11448 16172 11620 16172
11793 16172 11966 16172 12138 16172 12311 16172 12324 16172 12324 15778
12307 15765 12189 15686 12053 15653 12018 15630 11918 15526 11892 15494
11849 15358 11836 15223 11918 15102 11974 15223 11971 15358 12053 15429
12053 15149 11930 15087 12053 14983 12115 15087 12053 15149 12053 15429

12116 15494 12189 15586 12189 14988 12108 14951 12189 14906 12223 14951
12189 14988 12189 15586 12324 15580 12378 15630 12337 15765 12324 15778
12324 16172 12460 16172 12460 14712 12426 14680 12460 14625 12501 14680
12460 14712 12460 16172 12484 16172 12595 16172 12595 14629 12498 14544
12595 14449 12683 14544 12595 14629 12595 16172 12656 16172 12731 16172
12731 14495 12636 14409 12704 14273 12731 14229 12774 14273 12822 14409
12731 14495 12731 16172 12829 16172 13002 16172 13002 14163 12866 14216
12787 14137 12731 14069 12598 14137 12595 14139 12460 14220 12405 14273
12360 14409 12324 14485 12259 14544 12189 14586 12053 14622 11975 14680
11918 14701 11782 14801 11759 14816 11647 14948 11645 14951 11602 15087
11569 15223 11511 15357 11510 15358 11446 15494 11376 15545 11240 15546
11105 15520 11007 15630 11105 15735 11121 15765 11127 15901 11105 15916
11072 15901 10969 15868 10834 15835 10698 15854 10563 15872 10427 15821
114 N F
183 O
10395 16172 10395 16173 10412 16173 10505 16173 10505 16172 10412 16172
10395 16172 7 N F
38 O
11240 8169 11191 8167 11240 8164 11242 8167 11240 8169 5 N F
147 O
11240 14838 11204 14816 11238 14680 11239 14544 11240 14542 11242 14544
11245 14680 11311 14816 11240 14838 9 N F
183 O
11849 15358 11836 15223 11918 15102 11974 15223 11971 15358 12053 15429
12116 15494 12189 15586 12324 15580 12378 15630 12337 15765 12324 15778
12324 15673 12339 15630 12324 15616 12189 15627 12164 15630 12189 15633
12324 15673 12324 15778 12307 15765 12189 15686 12053 15653 12053 15598
12084 15494 12053 15462 11957 15494 12053 15598 12053 15653 12018 15630
11918 15526 11918 15425 11932 15358 11928 15223 11918 15199 11902 15223
11899 15358 11918 15425 11918 15526 11892 15494 11849 15358 41 N F
165 O
11918 15425 11899 15358 11902 15223 11918 15199 11928 15223 11932 15358
11918 15425 7 N F
183 O
12053 15149 11930 15087 12053 14983 12115 15087 12053 15149 12053 15107
12073 15087 12053 15054 12014 15087 12053 15107 12053 15149 11 N F
165 O
12053 15598 11957 15494 12053 15462 12084 15494 12053 15598 5 N F
111 O
12053 13578 12009 13459 12017 13323 12043 13187 12053 13178 12063 13187
12085 13323 12189 13414 12254 13459 12324 13538 12460 13506 12526 13459
12595 13392 12731 13435 12741 13459 12754 13594 12731 13730 12730 13730
12595 13772 12503 13730 12460 13682 12324 13635 12189 13673 12068 13594

12053 13578 25 N F
165 O
12053 15107 12014 15087 12053 15054 12073 15087 12053 15107 5 N F
183 O
12189 14988 12108 14951 12189 14906 12223 14951 12189 14988 5 N F
165 O
12189 15633 12164 15630 12189 15627 12324 15616 12339 15630 12324 15673
12189 15633 7 N F
183 O
12460 14712 12426 14680 12460 14625 12501 14680 12460 14712 5 N F
12595 14629 12498 14544 12595 14449 12683 14544 12595 14629 12595 14585
12637 14544 12595 14498 12549 14544 12595 14585 12595 14629 11 N F
165 O
12595 14585 12549 14544 12595 14498 12637 14544 12595 14585 5 N F
111 O
12578 13187 12571 13052 12595 12942 12729 13052 12688 13187 12595 13241
12578 13187 7 N F
183 O
12731 14495 12636 14409 12704 14273 12731 14229 12774 14273 12822 14409
12731 14495 12731 14448 12772 14409 12731 14329 12688 14409 12731 14448
12731 14495 13 N F
165 O
12731 14448 12688 14409 12731 14329 12772 14409 12731 14448 5 N F
147 O
12866 12887 12844 12916 12866 12960 12949 12916 12866 12887 5 N F
56 O
12955 5453 12955 5453 13002 5453 13002 5453 12955 5453 5 N F
13002 5453 12955 5453 12995 5589 13002 5599 13002 5453 5 N F
13002 5453 13002 5453 13002 5599 13002 5600 13002 5453 13002 5453
6 N F
38 O
13002 10028 13002 5599 13002 5600 13002 10028 13002 10028 5 N F
56 O
13002 11171 13002 10028 13002 10028 13002 11171 13002 11171 5 N F
74 O
13002 11752 13002 11171 13002 11171 13002 11752 13002 11752 5 N F
92 O
13002 12245 13002 11752 13002 11752 13002 12245 13002 12245 5 N F
111 O
13002 12604 13002 12245 13002 12245 13002 12604 13002 12604 5 N F
129 O
13002 13858 13002 12604 13002 12604 13002 13859 13002 13858 5 N F
147 O

13002 14047 13002 13858 13002 13859 13002 14047 13002 14047 5 N F
165 O
13002 14096 13002 14047 13002 14047 13002 14096 13002 14096 5 N F
183 O
13002 14163 13002 14096 13002 14096 13002 14163 13002 14163 5 N F
201 O
13002 16172 13002 14163 13002 14163 13002 16173 13002 16173 13002 16172
6 N F
38 O
13002 5600 13002 10028 13002 10028 13002 9931 13002 9795 13002 9660
13002 9524 13002 9388 13002 9252 13002 9117 13002 8981 13002 8845
13002 8710 13002 8574 13002 8438 13002 8303 13002 8167 13002 8031
13002 7896 13002 7760 13002 7624 13002 7489 13002 7353 13002 7217
13002 7081 13002 6946 13002 6810 13002 6674 13002 6539 13002 6403
13002 6267 13002 6132 13002 5996 13002 5860 13002 5725 13002 5600
13002 5600 37 N F
56 O
13002 10028 13002 10028 13002 11171 13002 11171 13002 11152 13002 11016
13002 10881 13002 10745 13002 10609 13002 10474 13002 10338 13002 10202
13002 10067 13002 10028 14 N F
74 O
13002 11171 13002 11171 13002 11752 13002 11752 13002 11695 13002 11559
13002 11423 13002 11288 13002 11171 9 N F
92 O
13002 11752 13002 11752 13002 12245 13002 12245 13002 12238 13002 12102
13002 11966 13002 11831 13002 11752 9 N F
111 O
13002 12245 13002 12245 13002 12604 13002 12604 13002 12509 13002 12373
13002 12245 7 N F
129 O
13002 12604 13002 12604 13002 13859 13002 13859 13002 13730 13002 13594
13002 13459 13002 13323 13002 13187 13002 13052 13002 12916 13002 12780
13002 12645 13002 12604 14 N F
147 O
13002 13859 13002 13859 13002 14047 13002 14047 13002 14002 13002 13866
13002 13859 7 N F
165 O
13002 14047 13002 14047 13002 14096 13002 14096 13002 14047 5 N F
183 O
13002 14096 13002 14096 13002 14163 13002 14163 13002 14137 13002 14096
6 N F
Ls Gr Gs Lr
-1200 1025 16500 18750 Oc

1 O
10639 15368 10640 15409 10679 15446 10675 15487 10710 15528 10753 15562
10792 15599 10827 15641 10872 15670 10920 15695 10946 15742 10985 15780
11030 15810 11080 15831 11122 15865 11160 15903 11196 15934 11247 15960
18 P
11247 15960 11300 15971 11330 16016 11355 16064 11389 16106 11419 16151
11428 16172 7 P
12840 16172 12841 16165 12836 16139 12812 16127 12785 16122 12767 16102
12743 16090 12718 16078 12698 16061 12682 16039 12656 16031 12637 16011
12633 15985 12626 15958 12621 15932 12608 15908 12584 15897 12561 15882
12544 15860 12528 15839 12506 15823 12482 15811 12458 15799 12434 15786
12425 15760 12449 15746 12444 15720 12419 15710 12402 15689 12377 15677
12359 15657 12333 15649 12323 15624 12298 15633 12283 15610 12257 15601
12230 15604 12204 15610 12182 15594 12168 15570 12146 15555 12149 15528
12128 15511 12107 15494 12081 15487 12067 15463 12040 15464 12022 15444
12010 15421 12003 15394 50 P
12003 15394 12008 15368 12034 15360 12041 15333 12028 15309 12026 15283
12033 15256 12045 15232 12053 15206 12039 15230 12013 15225 11 P
12013 15225 11986 15218 12012 15209 12006 15183 11984 15168 11980 15141
11994 15118 12018 15104 12045 15102 12072 15102 12098 15095 12124 15086
12147 15100 12162 15078 12178 15056 12196 15035 12213 15014 12235 14998
12256 14981 12279 14966 12306 14963 12328 14948 12347 14928 12358 14904
12383 14913 12409 14904 12433 14891 12438 14864 28 P
12438 14864 12448 14839 12463 14816 12476 14793 12494 14772 12503 14747
12519 14725 12534 14702 12546 14678 12563 14656 12575 14632 12586 14607
12605 14588 12622 14566 12640 14546 12656 14524 12675 14505 12691 14483
12712 14465 12731 14445 12752 14428 12772 14410 12793 14393 12798 14366
12818 14348 12838 14328 12844 14302 12864 14284 12878 14261 12900 14245
12915 14222 12934 14202 12951 14181 12960 14155 12967 14129 12980 14105
12999 14085 13001 14061 38 P
13001 13007 12998 13007 13001 12992 3 P
13001 12979 12980 12968 12959 12951 12951 12978 12952 13005 12936 12982
12909 12980 12883 12972 12855 12973 9 P
12855 12973 12828 12977 12806 12993 12783 13006 12789 12980 12797 12953

6:00 PM

12797 12953 12771 12961 12743 12963 12730 12939 12747 12918 12729 12898
12726 12871 12725 12843 12743 12822 12741 12795 12730 12770 12708 12787
12692 12809 12718 12802 12712 12829 12685 12829 12688 12856 12663 12846
12638 12858 12629 12832 12601 12832 12575 12840 12550 12851 12549 12879
12546 12906 12539 12879 12514 12869 12490 12855 12468 12839 12442 12847
12415 12841 12398 12820 12374 12806 12357 12828 12351 12801 12364 12778
12389 12765 12410 12783 12436 12789 12442 12762 12438 12735 12454 12712
12463 12687 12464 12659 12437 12650 12436 12678 12442 12705 12415 12711

12388 12704 12389 12677 50 P
12389 12677 12366 12663 12354 12687 12363 12713 12352 12738 12337 12761
12324 12786 12313 12761 12286 12754 12259 12753 12284 12743 12262 12728
12269 12701 12287 12680 12309 12664 12331 12648 12331 12621 12303 12621
12276 12627 12273 12600 12300 12604 12299 12577 12276 12561 12265 12536
12245 12554 12228 12575 12208 12595 12194 12571 12167 12580 12176 12606
12187 12631 12192 12604 12215 12618 12214 12646 12214 12673 12234 12655
12252 12676 12247 12703 12222 12693 12196 12684 12178 12663 12153 12653
12133 12634 12109 12620 12106 12593 12097 12567 12107 12542 12091 12519
12064 12521 12037 12515 50 P
12037 12515 12024 12538 2 P
12024 12538 12021 12565 12011 12591 12008 12564 12008 12536 11984 12523
11978 12496 11955 12481 11951 12453 11933 12432 11911 12416 11910 12443
11886 12458 11903 12480 11879 12492 11871 12519 11890 12540 11909 12559
11882 12560 11860 12542 11836 12554 11814 12570 11787 12575 11774 12600
11772 12627 11781 12653 11804 12638 11806 12611 11820 12588 11845 12577
11873 12580 11852 12598 11826 12607 11828 12634 11855 12635 11861 12609
11887 12602 11915 12604 11905 12630 11884 12647 11857 12650 11829 12649
11850 12666 11877 12662 11884 12689 11911 12693 11936 12705 11909 12707
11905 12734 11881 12748 50 P
11881 12748 11879 12775 11878 12748 11887 12722 11887 12694 11859 12695
11836 12681 11826 12707 11832 12734 11822 12759 11838 12782 11811 12777
11794 12755 11773 12773 11799 12784 11811 12808 11789 12823 11769 12842
11771 12815 11752 12834 11743 12860 11770 12854 11782 12878 11781 12851
11799 12831 11817 12811 11835 12832 11845 12857 11818 12864 11796 12879
11800 12906 11773 12913 11783 12939 11789 12966 11795 12993 11816 12977
11825 13003 11798 13007 11812 13031 11816 13058 11828 13083 11853 13092
11879 13101 11906 13098 11905 13126 11878 13126 11851 13121 11824 13119
11810 13143 11829 13163 50 P
11829 13163 11849 13181 2 P
11849 13181 11867 13202 11894 13203 11899 13229 11924 13241 11909 13263
11929 13283 11947 13303 11920 13297 11893 13294 11866 13289 11858 13315
11873 13337 11897 13350 11918 13367 11944 13379 11964 13396 11966 13423
11959 13397 11937 13381 11912 13370 11889 13357 11869 13376 11856 13400
11835 13417 11810 13427 11831 13443 11809 13459 11807 13487 11793 13510
11806 13534 11784 13551 11783 13578 11806 13592 11806 13607 11782 13580
11768 13604 11782 13627 11757 13640 11758 13667 11761 13694 11755 13721
11733 13705 11715 13685 11711 13712 11702 13738 11683 13718 11696 13695
11691 13668 11716 13657 50 P
11716 13657 11719 13630 11732 13606 11743 13581 11721 13564 11705 13586
11701 13613 11695 13639 11678 13660 11678 13688 11660 13708 11634 13700
11635 13727 11610 13739 11601 13713 11595 13740 11604 13766 11630 13772
11629 13799 11636 13825 11661 13837 11660 13865 11666 13838 11672 13812

11671 13784 11693 13769 11677 13791 11702 13804 11717 13826 11699 13847
11676 13862 11697 13880 11723 13889 11732 13863 11739 13837 11740 13809
11746 13836 11750 13863 11771 13846 11760 13871 11741 13890 11766 13879
11756 13904 11779 13891 11804 13879 11826 13895 11847 13876 11873 13871
11890 13849 11894 13822 50 P
11894 13822 11909 13799 2 P
11909 13799 11909 13826 11928 13845 11950 13861 11959 13887 11940 13868
11913 13873 11895 13893 11876 13913 11850 13919 11857 13945 11868 13970
11863 13997 11842 13980 11823 13960 11806 13982 11819 14005 11812 14032
11839 14036 11861 14053 11837 14065 11812 14077 11828 14099 11802 14108
11781 14125 11762 14145 11786 14157 11784 14184 11774 14209 11748 14217
11728 14235 11721 14262 11716 14288 11741 14301 11765 14314 11778 14290
11770 14263 11795 14250 11818 14238 11843 14226 11865 14210 11887 14201
11887 14202 11867 14214 11844 14228 11822 14243 11797 14255 11785 14280
11784 14307 11778 14333 50 P
11778 14333 11796 14357 11770 14344 3 P
11770 14344 11753 14322 11726 14317 11702 14329 11679 14344 11656 14359
11629 14357 11608 14339 11581 14335 11555 14327 11557 14300 11550 14273
11528 14289 11509 14309 11505 14335 11479 14342 11476 14314 11449 14316
11432 14338 11413 14357 11391 14373 11379 14397 11352 14404 11325 14403
11300 14413 11304 14386 11329 14375 11356 14376 11350 14349 11323 14350
11326 14323 11330 14296 11322 14271 11294 14273 11271 14259 11255 14237
11228 14233 11217 14208 11201 14229 11212 14254 11239 14261 11241 14288
11268 14290 11253 14312 11275 14328 11248 14331 11221 14329 11244 14314
11217 14309 11208 14284 50 P
11208 14284 11181 14283 11185 14310 11162 14324 11140 14308 11114 14300
11088 14307 11063 14318 11047 14340 11071 14354 11098 14350 11123 14360
11099 14372 11106 14399 11132 14404 11154 14387 11156 14414 11131 14424
11103 14421 11078 14411 11051 14407 11025 14415 11002 14428 10977 14419
10950 14411 10924 14415 10897 14423 10923 14430 10943 14449 10963 14465
10982 14492 11004 14478 11002 14471 11004 14467 11033 14486 11023 14491
11003 14480 10983 14499 10983 14526 10977 14552 10991 14576 11001 14601
11021 14619 11031 14644 11020 14669 11047 14670 11071 14683 11075 14710
11067 14736 11085 14715 50 P
11085 14715 11108 14729 2 P
11108 14729 11134 14735 11157 14750 11184 14754 11207 14767 11230 14752
11257 14756 11241 14778 11214 14778 11190 14791 11174 14769 11147 14771
11121 14763 11118 14790 11103 14813 11110 14786 11084 14795 11070 14818
11062 14844 11073 14869 11082 14895 11091 14920 11104 14944 11125 14962
11116 14988 11121 15014 11144 15029 11151 15055 11157 15081 11153 15108
11167 15133 11140 15126 11127 15150 11126 15177 11146 15196 11150 15223
11174 15236 11187 15237 11197 15251 11220 15263 11226 15267 11225 15269
11195 15252 11184 15242 11163 15240 11173 15268 11177 15282 11188 15277

11198 15279 11203 15287 50 P
11203 15287 11201 15289 11197 15283 11189 15279 11177 15283 11172 15271
11160 15247 11139 15230 11116 15216 11089 15215 11070 15234 11044 15225
11020 15239 10998 15254 10971 15254 10953 15274 10930 15262 10914 15283
10888 15291 10867 15307 10856 15283 10859 15256 10843 15234 10826 15213
10813 15237 10823 15262 10804 15281 10796 15307 10786 15332 10759 15335
10733 15342 10709 15354 10687 15370 10693 15396 10666 15395 10661 15368
10656 15342 10650 15315 10643 15341 10639 15368 40 P
10639 15368 10627 15344 10600 15338 10578 15323 10551 15317 10524 15318
10497 15320 10473 15332 10453 15313 10426 15313 10403 15328 10401 15355
10393 15329 10383 15304 10369 15280 10343 15286 10331 15262 10307 15276
10281 15281 10255 15285 10246 15260 10223 15246 10247 15235 10226 15218
10200 15209 10175 15218 10150 15208 10131 15188 10106 15177 10081 15167
10056 15157 10035 15140 10015 15121 9991 15110 9973 15089 9949 15102
9946 15075 9926 15057 9900 15060 9879 15042 9856 15027 9846 15002
9834 14978 9811 14964 9792 14945 9781 14920 9806 14908 9782 14896
9755 14890 9763 14864 50 P
9763 14864 9737 14859 9716 14841 9696 14859 9704 14833 9679 14842
9690 14817 9688 14790 9665 14803 9653 14828 9628 14838 9607 14821
9595 14797 9571 14785 9597 14779 9620 14765 9608 14741 9581 14738
9554 14738 9535 14718 9510 14710 9483 14710 9458 14698 9431 14699
9419 14675 9402 14653 9381 14670 9382 14697 9369 14722 9391 14738
9364 14745 9372 14771 9398 14779 9425 14779 9451 14789 9464 14812
9474 14837 9494 14818 9521 14822 9543 14838 9568 14848 9594 14857
9607 14881 9587 14898 9564 14914 9581 14935 9563 14954 9589 14961
9612 14975 9627 14998 50 P
9627 14998 9650 15013 2 P
9650 15013 9675 15020 9699 15033 9672 15034 9646 15028 9619 15027
9594 15015 9567 15014 9541 15011 9517 15025 9507 15055 9494 15087
9517 15096 9529 15113 9533 15125 9536 15146 9533 15173 9517 15194
9496 15211 9506 15236 9517 15260 9540 15275 9564 15289 9581 15310
9608 15309 9632 15322 9650 15342 9670 15361 9644 15368 9667 15382
9683 15403 9700 15424 9714 15447 9728 15470 9748 15489 9752 15516
9766 15538 9783 15559 9786 15586 9803 15607 9816 15631 9803 15655
9782 15673 9760 15688 9754 15698 9762 15715 9758 15741 9760 15748
9784 15735 9802 15740 50 P
9802 15740 9806 15746 9801 15754 9778 15753 9777 15766 9756 15778
9731 15793 9732 15791 9754 15775 9766 15768 9766 15762 9772 15754
9779 15745 9801 15751 9802 15743 9784 15738 9768 15746 9759 15750
9754 15746 9754 15732 9742 15733 9727 15748 9705 15764 9682 15779
9665 15800 9638 15798 9625 15821 9633 15847 9622 15872 9634 15896
9639 15923 9629 15898 9608 15880 9608 15853 9598 15827 9576 15811
9549 15809 9523 15806 9503 15826 9481 15841 9454 15840 9430 15853

9406 15840 9384 15855 9357 15858 9342 15836 9323 15817 9313 15792
9286 15796 9267 15776 50 P
9267 15776 9255 15752 2 P
9255 15752 9278 15738 9260 15717 9234 15711 9216 15690 9204 15666
9185 15647 9164 15630 9150 15607 9127 15593 9122 15567 9100 15550
9122 15535 9099 15521 9086 15497 9087 15470 9071 15449 9045 15442
9019 15435 8998 15417 8974 15406 8950 15392 8925 15383 8899 15375
8874 15364 8851 15350 8835 15328 27 P
8835 15328 8810 15320 8788 15304 8762 15297 8736 15290 8710 15280
8690 15263 8691 15236 8666 15225 8645 15209 8625 15190 8612 15166
8605 15140 8597 15114 8575 15100 8583 15074 8578 15047 8583 15020
8600 15000 8575 14989 8553 14973 8536 14952 8522 14929 8503 14909
8482 14893 8458 14880 8433 14870 8406 14867 8384 14851 8357 14849
8336 14866 8309 14867 8284 14856 8264 14837 8242 14821 8222 14803
8201 14787 8174 14791 8151 14806 8132 14826 8126 14852 8111 14874
8087 14888 8060 14892 44 P
8060 14892 8034 14888 8008 14879 7986 14864 7966 14844 7947 14825
7958 14800 7964 14773 7939 14763 7924 14741 7906 14719 7893 14696
7881 14671 7872 14645 7867 14619 7866 14592 7870 14565 17 P
7870 14565 7879 14539 7890 14514 7903 14490 7914 14466 7928 14443
7945 14421 7964 14402 7984 14383 8010 14376 8033 14361 11 P
8033 14361 8052 14342 8074 14327 8098 14313 8125 14316 8152 14313
8179 14316 8206 14314 8232 14306 8259 14302 8280 14285 8301 14301
8326 14313 8351 14324 8373 14340 8397 14352 8402 14326 8408 14352
8422 14329 8418 14302 8424 14275 8415 14250 8432 14271 8459 14271
8464 14244 8482 14224 8490 14198 8498 14172 8492 14146 8480 14121
8505 14110 8523 14089 8510 14065 8494 14043 8476 14023 8470 13996
8468 13969 8469 13942 8472 13915 8467 13888 8485 13868 8502 13846
8505 13819 8532 13820 8555 13806 8576 13787 8603 13785 8629 13778
8656 13777 8683 13779 50 P
8683 13779 8710 13782 8736 13771 8763 13773 8790 13773 8817 13780
8835 13799 8856 13817 8875 13836 8882 13863 8866 13884 8879 13908
8900 13925 8925 13936 8951 13943 8974 13958 8996 13974 9015 13994
9034 14013 9051 14035 9055 14061 9030 14073 9056 14081 9082 14089
9102 14108 9128 14115 9155 14119 9178 14133 9204 14142 9224 14160
9252 14161 9279 14158 9306 14161 9318 14137 9342 14124 9359 14103
9386 14105 9402 14082 9406 14055 9426 14036 9452 14031 9479 14034
9504 14044 9531 14048 9507 14036 9525 14016 9542 13995 9560 13975
9585 13963 9612 13961 50 P
9612 13961 9639 13966 2 P
9639 13966 9664 13976 9684 13957 9700 13979 9727 13984 9754 13988
9769 13966 9767 13993 9787 14012 9810 14027 9831 14043 9849 14023
9840 13997 9854 13974 9878 13987 9901 13972 9928 13965 9955 13966

9981 13974 9999 13953 10023 13964 10047 13977 10075 13976 10055 13957
10037 13936 10032 13909 10040 13883 10056 13861 10034 13846 10010 13832
9995 13809 9995 13782 9974 13764 10002 13761 10029 13765 10029 13738
10025 13711 10012 13687 10023 13712 9996 13706 9983 13682 9961 13665
9939 13650 9913 13654 9885 13655 9881 13682 9908 13685 9883 13696
9867 13718 9883 13740 50 P
9883 13740 9876 13766 9859 13745 9860 13718 9837 13703 9818 13684
9808 13710 9794 13733 9767 13728 9778 13704 9754 13692 9729 13680
9704 13669 9691 13645 9667 13632 9642 13622 9642 13595 9617 13602
9590 13595 9605 13618 9626 13635 9598 13633 9579 13614 9565 13637
9570 13610 9553 13588 9530 13574 9505 13564 9479 13554 9453 13547
9430 13533 9410 13514 9385 13524 9386 13497 9359 13496 9345 13519
9319 13527 9296 13513 9279 13492 9305 13498 9321 13476 9346 13465
9346 13438 9329 13417 9328 13389 9303 13402 9282 13420 9258 13408
9243 13384 9251 13358 50 P
9251 13358 9225 13350 2 P
9225 13350 9220 13323 9235 13301 9240 13274 9244 13248 9223 13231
9198 13218 9171 13215 9144 13210 9124 13192 9098 13183 9071 13188
9082 13213 9093 13238 9098 13265 9082 13288 9064 13308 9060 13281
9038 13264 9013 13273 8987 13263 8978 13237 8986 13211 9009 13197
9035 13189 9020 13166 9047 13162 9074 13165 9062 13141 9040 13125
9026 13101 9033 13128 9010 13113 8985 13103 8965 13084 8954 13058
8957 13031 8949 13005 8927 13020 8915 12996 8901 12972 8890 12947
8873 12969 8853 12951 8840 12927 8830 12952 8808 12936 8781 12939
8787 12913 8778 12887 50 P
8778 12887 8770 12860 8756 12837 8752 12810 8732 12790 8708 12778
8691 12756 8683 12730 8676 12704 8673 12676 8661 12651 8634 12656
12:00 PM
8634 12656 8613 12638 8599 12615 8595 12588 8595 12561 8586 12535
8588 12508 8601 12484 8594 12457 8613 12477 8635 12461 8659 12474
8684 12462 8711 12459 8716 12432 8730 12409 8751 12391 8774 12377
8798 12364 8820 12347 8847 12340 8865 12320 8865 12292 8878 12317
8905 12311 8932 12306 8958 12297 8982 12286 9001 12266 9028 12259
9053 12248 9078 12237 9092 12213 9096 12186 9109 12162 9117 12135
9126 12109 9136 12084 9145 12058 9146 12030 9154 12004 9155 11976
9180 11966 9175 11939 9201 11929 9209 11903 9220 11878 9216 11851
9223 11824 9240 11802 50 P
9240 11802 9262 11785 9252 11759 9250 11732 9257 11705 9276 11686
9285 11660 9297 11635 9316 11614 9331 11592 9337 11565 9351 11541
9378 11537 9389 11511 9395 11484 9405 11458 9407 11431 9398 11405
9378 11386 9404 11377 9417 11353 9398 11332 9383 11309 9411 11306
9429 11285 9448 11265 9476 11264 9501 11255 9527 11245 9552 11233
9577 11222 9603 11211 9625 11195 9638 11170 9638 11143 9643 11116

9628 11092 9648 11073 9660 11048 9686 11038 9713 11038 9739 11028
9765 11019 9789 11006 9810 10988 9829 10968 9848 10948 9857 10922
9870 10897 9884 10873 50 P
9884 10873 9895 10847 2 P
9895 10847 9889 10820 9863 10810 9836 10818 9810 10826 9782 10829
9757 10839 9732 10852 9706 10863 9688 10884 9662 10892 9635 10898
9610 10910 9584 10900 9558 10890 9531 10884 9517 10860 9490 10864
9462 10861 9436 10869 9430 10896 9420 10922 9401 10942 9384 10964
9361 10979 9345 11002 9319 10992 9293 11001 9265 11001 9240 11012
9215 11002 9190 10988 9169 10970 9147 10953 9126 10936 9100 10926
9072 10928 9045 10934 9059 10910 9087 10910 9113 10918 9138 10931
9164 10937 9192 10942 9203 10968 9231 10970 9257 10961 9284 10958
9312 10961 9339 10958 50 P
9339 10958 9355 10935 9368 10910 9380 10886 9359 10869 9377 10849
9403 10837 9430 10835 9458 10836 9484 10826 9510 10817 9531 10799
9555 10785 9577 10768 9595 10747 9609 10723 9627 10702 17 P
9627 10702 9651 10688 9677 10677 9698 10660 9714 10637 9741 10636
9766 10623 9789 10608 9811 10592 9829 10570 9843 10547 9857 10522
9870 10498 9883 10473 9896 10449 9910 10424 9920 10398 9913 10371
9890 10356 9863 10351 9835 10349 9807 10346 9780 10341 9753 10334
9727 10326 9702 10313 9682 10294 9667 10270 9643 10256 9615 10252
30 P
9615 10252 9593 10236 9571 10219 9556 10196 9528 10191 9503 10178
9484 10159 9469 10135 9468 10107 9452 10084 9436 10062 9411 10050
9383 10047 9364 10067 9337 10076 9309 10076 9281 10073 9254 10073
9227 10067 9202 10054 9194 10027 9195 9999 9169 10009 9141 10007
9124 9985 9104 9966 9128 9981 9147 10001 9169 9984 9196 9986
9213 10009 9223 10035 9246 10051 9274 10053 9302 10052 9325 10037
9314 10012 9323 9986 9341 9964 9335 9936 9344 9909 9371 9901
9346 9915 9365 9935 9391 9925 9419 9925 9445 9935 9466 9953
9490 9967 9512 9985 50 P
9512 9985 9531 10005 9553 10022 9578 10034 9606 10039 9633 10039
9661 10036 9685 10021 9708 10005 9727 9986 9746 9965 9763 9942
9782 9922 9798 9899 9812 9875 9836 9861 9863 9856 9891 9852
9917 9841 9944 9834 9955 9824 9962 9818 9966 9817 9994 9817
10022 9818 10046 9832 10039 9805 10016 9789 9990 9779 9968 9761
9954 9737 9931 9720 9914 9698 9892 9682 9874 9660 9856 9638
9834 9621 9807 9614 9780 9609 9753 9615 9735 9593 9724 9567
9732 9541 9747 9564 9774 9572 9766 9545 9784 9566 9807 9582
9834 9590 9853 9610 50 P
9853 9610 9881 9611 2 P
9881 9611 9888 9584 9912 9597 9896 9621 9897 9648 9921 9662
9949 9668 9976 9675 9995 9655 9977 9633 9974 9605 9990 9582

9989 9554 9963 9543 9966 9516 9963 9488 9989 9476 9994 9448
9975 9426 9952 9411 9924 9417 9923 9444 9917 9472 9927 9498
9906 9517 9899 9490 9901 9462 9894 9435 9874 9416 9847 9409
9837 9435 9835 9463 9844 9490 9833 9464 9832 9436 9814 9458
9786 9462 9782 9435 9774 9407 9750 9393 9749 9365 9777 9369
9803 9377 9831 9371 9834 9343 9806 9340 9785 9321 9811 9332
9839 9338 9864 9327 50 P
9864 9327 9853 9301 9874 9282 9855 9261 9827 9264 9801 9274
9816 9250 9843 9244 9856 9219 9864 9192 9844 9173 9825 9153
9801 9138 9773 9137 9745 9136 9717 9137 9743 9129 9771 9122
9787 9100 9795 9073 9808 9047 9823 9024 9822 8996 9836 8971
9845 8945 9859 8920 9847 8895 9828 8916 9805 8931 9777 8936
9762 8912 9749 8887 9728 8869 9734 8842 9713 8823 9693 8842
9675 8864 9667 8891 9652 8915 9669 8938 9642 8945 9616 8936
9606 8909 9581 8898 9603 8881 9593 8854 9578 8830 9553 8817
9540 8792 9541 8764 50 P
9541 8764 9529 8739 2 P
9529 8739 9529 8727 9502 8730 9474 8731 9446 8730 9429 8748
9424 8748 9396 8755 9369 8751 9347 8770 9349 8742 9377 8745
9404 8740 9424 8720 9452 8716 9477 8702 9479 8674 9463 8650
9445 8629 9430 8605 9418 8579 9390 8577 9386 8548 9387 8520
9368 8500 9340 8493 9358 8471 9371 8446 9398 8436 9384 8411
9385 8383 9357 8378 9332 8391 9325 8363 9339 8339 9322 8315
9322 8287 9306 8263 9291 8287 9280 8261 9256 8275 9245 8301
42 P
9245 8301 9251 8274 9265 8249 9250 8226 9222 8218 9195 8222
9204 8195 9199 8167 9180 8146 9151 8146 9179 8139 9181 8110
9153 8116 9125 8118 9118 8090 9090 8096 9078 8070 9102 8056
9127 8042 9116 8015 9142 8004 9121 7984 9122 7956 9096 7945
9072 7930 9043 7927 9035 7900 9014 7881 8985 7882 8993 7910
9009 7933 9037 7934 9058 7953 9056 7982 9082 7995 9094 8020
9070 8036 9042 8039 9033 8012 9017 7989 9012 7961 8990 7979
8962 7985 8965 8013 8989 8027 8962 8034 8978 8057 8952 8046
8948 8018 8927 8037 50 P
8927 8037 8910 8059 8900 8086 8901 8058 8908 8030 8918 8004
8893 8017 8871 8035 8845 8045 8849 8017 8844 7989 8867 7973
8840 7964 8843 7935 8865 7917 8891 7928 8919 7927 8947 7923
8966 7901 8970 7873 8982 7847 8966 7824 8944 7841 8916 7848
8895 7866 8872 7850 8859 7825 8886 7833 8899 7807 8905 7780
8927 7762 8952 7774 8961 7801 8979 7824 8996 7801 9020 7786
9046 7797 9028 7774 9000 7771 8973 7765 8948 7750 8921 7742
8893 7736 8869 7720 8898 7725 8887 7699 8870 7676 8850 7656
8834 7632 8853 7612 50 P

8853 7612 8880 7603 2 P
8880 7603 8906 7591 8934 7588 8927 7560 8919 7533 8891 7531
8882 7504 8884 7475 8892 7448 8878 7423 8850 7418 8824 7407
8807 7384 8821 7359 8828 7332 8854 7344 8868 7319 8874 7291
8901 7282 8907 7254 8902 7226 8881 7207 8880 7236 8879 7264
8852 7272 8841 7246 8827 7221 8830 7250 8813 7273 8808 7301
8779 7304 8754 7317 8764 7344 8740 7359 8729 7333 8713 7310
8693 7289 8669 7275 8646 7257 8619 7249 8640 7230 8663 7213
8687 7198 8677 7171 8704 7180 8726 7197 8744 7174 8763 7153
8736 7146 8730 7118 50 P
8730 7118 8701 7114 8673 7112 8649 7128 8647 7157 8632 7133
8641 7106 8655 7081 8629 7093 8601 7097 8611 7124 8613 7152
8588 7140 8604 7163 8577 7170 8548 7167 8522 7157 8541 7136
8561 7116 8545 7093 8528 7069 8501 7062 8510 7034 8534 7020
8562 7012 8575 7038 8573 7009 8587 6984 8558 6985 8530 6983
8534 6954 8562 6948 8537 6933 8511 6944 8482 6948 8464 6926
8436 6919 8442 6947 8429 6972 8416 6947 8416 6975 8395 6995
8402 6967 8409 6940 8381 6933 8355 6945 8378 6928 8382 6900
8403 6880 8425 6897 50 P
8425 6897 8441 6874 2 P
8441 6874 8437 6845 8424 6820 8395 6822 8394 6794 8405 6768
8384 6747 8359 6734 8360 6762 8336 6777 8310 6789 8284 6803
8295 6830 8273 6812 8278 6784 8253 6770 8225 6765 8198 6773
8185 6747 8157 6754 8165 6781 8154 6808 8125 6808 8123 6779
8109 6754 8092 6777 8082 6750 8053 6749 8079 6736 8074 6708
8051 6691 8023 6698 7997 6708 7968 6707 7953 6704 7941 6720
7940 6703 7951 6692 7974 6674 7999 6661 8017 6656 8045 6659
8074 6663 8077 6634 8106 6634 8120 6609 8126 6581 8098 6575
8077 6556 8054 6538 50 P
8054 6538 8033 6558 8013 6537 8024 6510 8005 6488 8001 6459
7977 6445 7953 6427 7937 6404 7925 6378 7929 6406 7946 6430
7936 6457 7909 6448 7891 6426 7894 6397 7871 6380 7859 6354
7857 6325 7855 6297 7838 6273 7839 6302 7848 6330 7839 6357
7818 6375 7790 6383 7762 6390 7733 6393 7756 6376 7784 6369
7786 6340 7776 6313 7758 6291 7735 6307 7723 6307 7711 6291
7687 6307 7676 6281 7671 6252 7669 6223 7651 6201 7659 6229
7641 6252 7641 6222 7617 6207 45 P
7617 6207 7594 6189 7571 6171 7564 6199 7558 6227 7532 6238
7540 6210 7515 6223 7487 6215 7460 6205 7469 6181 7455 6155
7454 6129 7455 6119 7434 6099 7422 6085 7401 6062 7372 6062
7343 6062 7316 6074 7294 6092 7273 6113 7245 6113 7273 6108
7279 6080 7292 6054 7315 6037 7343 6030 7379 5998 7358 5987
7341 5964 7319 5984 7296 6002 7268 5999 7271 5970 7295 5985

7291 5956 7277 5931 7284 5903 7287 5874 7266 5854 7258 5826
7228 5828 7200 5820 7172 5826 7149 5844 7125 5828 7101 5811
7082 5790 7059 5771 50 P
7059 5771 7030 5771 7046 5796 7029 5819 7027 5791 7003 5774
6976 5764 6949 5754 6921 5746 6899 5727 6882 5750 6866 5774
6838 5783 6814 5799 6785 5797 6764 5777 6747 5753 6729 5730
6747 5707 6768 5727 6754 5701 6757 5672 6730 5685 6705 5698
6676 5693 6648 5700 6636 5727 6665 5728 6640 5742 6614 5755
6575 5753 6576 5763 6599 5771 6568 5782 6543 5766 6515 5767
6487 5760 6472 5735 6458 5710 6430 5706 6417 5732 6392 5747
6408 5723 6431 5705 6415 5680 6399 5656 6403 5627 6382 5648
6353 5652 6324 5651 50 P
6324 5651 6314 5678 2 P
6314 5678 6317 5707 6314 5736 6337 5753 6345 5781 6324 5762
6295 5767 6269 5755 6242 5746 6216 5733 6202 5708 6179 5689
6209 5689 6208 5660 6181 5650 6206 5635 6234 5625 6228 5597
6200 5590 6172 5584 6168 5612 6159 5640 6141 5663 6113 5670
6084 5663 6056 5656 6035 5637 27 P
6035 5637 6061 5624 6086 5611 6065 5592 6036 5590 6008 5586
6023 5561 6051 5569 6074 5588 6090 5564 6118 5570 6112 5542
6084 5534 6057 5545 6049 5516 6069 5495 6042 5507 6013 5506
5985 5513 5967 5535 5937 5535 5908 5534 5880 5538 5859 5559
5880 5579 5902 5598 5928 5612 5955 5622 28 P
5955 5622 5926 5625 5898 5632 5871 5621 5852 5599 5859 5628
5842 5651 5823 5673 5811 5700 5796 5725 5790 5753 5772 5776
5745 5765 5739 5794 5720 5816 5706 5840 5711 5869 5722 5896
5705 5873 5684 5893 5677 5921 5679 5950 5654 5964 5627 5975
5600 5984 5617 5961 5645 5957 5662 5934 5661 5904 5655 5876
5626 5871 5598 5866 5627 5865 5654 5855 5664 5827 5688 5811
5702 5785 5674 5778 5649 5795 5669 5773 5686 5750 5703 5726
5675 5717 5654 5736 5673 5715 5689 5691 5665 5674 5681 5650
5680 5621 5667 5595 50 P
5667 5595 5659 5567 5682 5551 5689 5522 5674 5497 5665 5469
5656 5453 7 P
5649 5453 5644 5472 5643 5453 3 P
5597 5453 5592 5457 5573 5479 5556 5502 5547 5530 5528 5553
5537 5525 5551 5487 5544 5482 5539 5475 5539 5468 5531 5453

12:00 PM

5429 5453 5430 5476 5412 5498 5410 5527 5410 5557 5415 5585
5415 5614 5386 5615 5380 5644 5351 5648 5369 5625 5393 5608
5404 5582 5403 5553 5402 5524 5405 5494 5407 5466 5392 5453
18 P
2966 5453 2967 5459 2985 5482 2967 5505 2938 5506 2916 5486

2926 5459 2928 5453 8 P
13001 8121 12996 8116 12991 8121 12985 8125 12983 8118 12983 8111
12985 8105 12988 8098 12992 8092 12995 8086 13001 8082 13001 8081

12:00 PM

13001 8033 13001 8033 12994 8030 12988 8028 12984 8022 12981 8015
12978 8009 12978 8002 12973 7997 12977 7991 12982 7985 12976 7990
12969 7993 12962 7992 12960 7986 12961 7979 12966 7973 12968 7967
12974 7963 12979 7957 12979 7950 12979 7943 12979 7936 12977 7929
12976 7922 12982 7918 12988 7915 12993 7920 12999 7925 13001 7925
30 P
11825 7031 11829 7025 11833 7019 11839 7015 11846 7013 11847 7020
11850 7013 11855 7009 11862 7012 11869 7014 11876 7015 11882 7019
11889 7021 11896 7021 11903 7023 11910 7024 11917 7023 11924 7025
11931 7026 11936 7021 11932 7027 11927 7033 11923 7039 11918 7044
11911 7044 11904 7046 11897 7048 11894 7054 11887 7057 11880 7055
11873 7058 11866 7060 11863 7066 11865 7073 11859 7078 11853 7080
11849 7086 11847 7093 11844 7100 11838 7104 11833 7110 11829 7115
11826 7121 11819 7120 11823 7114 11827 7108 11830 7102 11835 7096
11839 7091 11842 7084 50 P
11842 7084 11835 7081 11835 7074 11837 7067 11834 7060 11828 7057
11825 7051 11831 7047 11837 7042 11840 7037 11836 7031 11825 7031

12:00 PM

11225 6793 11231 6789 11237 6791 11242 6796 11250 6797 11255 6792
11259 6786 11264 6780 11263 6773 11259 6767 11253 6764 11249 6758
11254 6754 11261 6752 11268 6749 11273 6744 11279 6741 11286 6740
11293 6742 11298 6748 11299 6755 11306 6753 11312 6750 11319 6752
11322 6758 11323 6766 11322 6773 11322 6780 11324 6787 11323 6794
11319 6800 11322 6807 11319 6813 11319 6820 11322 6826 11328 6831
11332 6836 11333 6843 11338 6849 11342 6854 11345 6861 11342 6868
11346 6874 11352 6879 11356 6883 11358 6890 11357 6897 11358 6905
11351 6905 11344 6906 50 P
11344 6906 11343 6899 11341 6892 11338 6886 11334 6880 11331 6873
11336 6868 11333 6862 11331 6855 11329 6848 11322 6850 11316 6847
11313 6841 11306 6839 11306 6831 11302 6825 11305 6818 11299 6813
11292 6814 11285 6812 11279 6808 11272 6809 11265 6807 11262 6814
11259 6820 11252 6822 11252 6815 11250 6809 11246 6802 11240 6807
11233 6806 11228 6801 11225 6793 33 P
11017 6707 11022 6701 11026 6696 11032 6692 11040 6693 11047 6691
11049 6698 11055 6703 11060 6698 11066 6694 11073 6693 11080 6693
11088 6692 11095 6691 11102 6691 11109 6692 11116 6690 11122 6687
11129 6684 11135 6688 11134 6695 11131 6701 11124 6704 11131 6702
11138 6700 11144 6696 11144 6703 11142 6710 11144 6717 11148 6723
11155 6721 11157 6728 11154 6735 11158 6741 11160 6748 11154 6750

11148 6754 11143 6759 11136 6761 11129 6762 11126 6769 11120 6765
11114 6769 11107 6768 11100 6765 11096 6760 11091 6766 11089 6772
11084 6778 11078 6782 50 P
11078 6782 11072 6784 11066 6781 11071 6776 11073 6769 11077 6763
11070 6766 11071 6773 11065 6770 11060 6764 11056 6758 11059 6752
11058 6745 11062 6739 11067 6734 11068 6727 11065 6733 11062 6727
11062 6720 11064 6713 11064 6706 11063 6713 11058 6718 11052 6722
11051 6729 11045 6724 11039 6728 11039 6721 11043 6715 11037 6719
11031 6722 11026 6716 11030 6710 11017 6707 34 P
9618 7032 9615 7018 9606 7007 9598 6995 9592 6982 9585 6969
9582 6955 9572 6945 9564 6933 9571 6920 9567 6907 9563 6893
9553 6882 9543 6873 9539 6859 9529 6848 9539 6839 9525 6839
9511 6837 9498 6831 9490 6820 9496 6807 9482 6804 9469 6799
9464 6785 9459 6771 9456 6757 9451 6744 9445 6731 9438 6719
9427 6709 9419 6697 9409 6687 9403 6674 9397 6661 9391 6647
9386 6634 9384 6620 9375 6608 9371 6595 9363 6582 9356 6570
9368 6562 9354 6562 9346 6550 9339 6537 9332 6525 9329 6511
9324 6497 9315 6486 50 P
9315 6486 9305 6476 9296 6465 9289 6452 9280 6441 9274 6428
9274 6414 9267 6401 9261 6388 9256 6374 9247 6364 9256 6353
9270 6351 9285 6349 9299 6351 9288 6342 9274 6340 9259 6339
9245 6341 9231 6341 9222 6329 9213 6318 9211 6304 9210 6289
9207 6275 9195 6267 9188 6254 9182 6241 9181 6226 9180 6212
9187 6199 9187 6185 9193 6172 9181 6163 9190 6153 9188 6139
9198 6129 9188 6120 9192 6106 9196 6092 9194 6077 9192 6063
9199 6051 9185 6053 9183 6039 9175 6027 9177 6013 9166 6004
9179 5998 9164 5996 50 P
9164 5996 9163 5982 2 P
9163 5982 9164 5967 9155 5958 9169 5954 9173 5940 9169 5926
9158 5917 9144 5914 9140 5927 9136 5913 9146 5903 9160 5906
9153 5895 9163 5885 9175 5877 9189 5881 9203 5885 9217 5890
9220 5876 9207 5869 9193 5866 9200 5853 9207 5841 9211 5827
9211 5812 9216 5799 9221 5785 9235 5782 9226 5771 9231 5757
9237 5744 9243 5730 9249 5717 9255 5704 9258 5690 9258 5676
9255 5661 9263 5649 9276 5643 9280 5629 9291 5620 9302 5610
9289 5615 9279 5627 9271 5639 9277 5625 9286 5614 9296 5603
9306 5593 9316 5582 50 P
9316 5582 9328 5574 9340 5566 9347 5580 9353 5566 9362 5555
9373 5545 9386 5539 9398 5531 9411 5525 9424 5518 9436 5510
9447 5500 9454 5487 9465 5478 9472 5465 9476 5453 17 P
9607 5453 9607 5465 9606 5479 9608 5494 9614 5507 9617 5521
9622 5535 9627 5548 9632 5562 9635 5576 9638 5590 9642 5604
9646 5618 9652 5632 9662 5642 9664 5657 9667 5671 9673 5684

9684 5693 9695 5703 9707 5711 9720 5717 9730 5727 9740 5738
9751 5748 9754 5763 9758 5776 9753 5790 9760 5803 9773 5808
9784 5818 9790 5831 9798 5843 9801 5857 9812 5868 9819 5880
9824 5894 9830 5907 9833 5921 9837 5935 9848 5944 9857 5956
9859 5970 9862 5984 9857 5997 9859 6012 9865 6025 9868 6039
9872 6053 9877 6066 50 P
9877 6066 9889 6075 9891 6089 9893 6103 9894 6118 9899 6132
9903 6145 9906 6160 9911 6173 9906 6186 9907 6201 9910 6215
9916 6228 9917 6242 9917 6257 9922 6270 9923 6285 9925 6299
9928 6313 9934 6326 9934 6341 9934 6355 9940 6368 9944 6382
9947 6397 9949 6411 9949 6425 9949 6439 9951 6454 9960 6465
9968 6477 9960 6489 9953 6502 9950 6516 9955 6529 9965 6539
9974 6551 9973 6565 9970 6579 9977 6592 9988 6601 9997 6612
10008 6621 10014 6634 10021 6646 10027 6659 10025 6674 10030 6688
10032 6702 49 P
10032 6702 10034 6716 10040 6729 10047 6742 10058 6751 10055 6765
10061 6778 10072 6786 10070 6800 10071 6815 10068 6829 10058 6840
10053 6853 10050 6867 10048 6881 10043 6895 10039 6908 10037 6922
10037 6937 10038 6951 10042 6965 10046 6979 10054 6991 10064 7001
10069 7014 10080 7024 10088 7035 10101 7042 10114 7049 10125 7058
10117 7070 10103 7068 10090 7075 10089 7090 10081 7101 10083 7115
10073 7125 10058 7124 10044 7125 10030 7122 10020 7131 10007 7137
9994 7131 9984 7141 9970 7146 9960 7156 9964 7169 9950 7164
9940 7174 9931 7185 50 P
9931 7185 9927 7199 9916 7207 9903 7214 9889 7213 9875 7209
9861 7204 9849 7197 9840 7186 9832 7174 9825 7162 9817 7150
9828 7141 9815 7136 9805 7126 9793 7119 9780 7113 9766 7111
9752 7107 9738 7108 9724 7103 9711 7098 9699 7090 9687 7082
9673 7079 9660 7074 9648 7065 9636 7058 9626 7048 9618 7032
30 P
12970 11609 12977 11607 12983 11607 12988 11602 12995 11600 12999 11606
13001 11607 7 P
13001 11699 13000 11699 12995 11704 12988 11706 12983 11702 12981 11695
12982 11688 12986 11683 12984 11676 12986 11670 12991 11665 12987 11659
12990 11653 12992 11646 12999 11643 12999 11637 12993 11636 12998 11632
12991 11633 12993 11627 12988 11623 12987 11616 12994 11618 12999 11623
12995 11617 12995 11610 12988 11612 12982 11616 12979 11622 12975 11628
12973 11621 12970 11609 32 P
11806 11848 11811 11843 11815 11837 11819 11832 11825 11828 11831 11825
11838 11824 11844 11820 11848 11815 11853 11810 11858 11805 11864 11802
11871 11802 11871 11809 11872 11815 11876 11821 11880 11827 11886 11829
11893 11828 11900 11828 11907 11827 11913 11830 11919 11834 11926 11834
11932 11831 11939 11833 11945 11835 11950 11829 11957 11829 11963 11832

11970 11832 11974 11838 11981 11839 11987 11840 11994 11840 12001 11842
12007 11840 12013 11841 12018 11847 12024 11851 12028 11856 12033 11860
12040 11862 12047 11862 12054 11863 12060 11867 12066 11869 12073 11870
12078 11875 12082 11880 50 P
12082 11880 12087 11886 12093 11886 12100 11887 12107 11889 12113 11891
12117 11897 12116 11904 12121 11908 12125 11914 12126 11921 12127 11928
12131 11934 12136 11937 12141 11942 12141 11949 12142 11956 12143 11963
12148 11959 12147 11965 12146 11972 12149 11978 12155 11977 12151 11982
12146 11977 12145 11984 12138 11986 12134 11992 12138 11998 12136 12005
12132 12011 12126 12014 12119 12014 12113 12012 12107 12015 12101 12017
12096 12022 12092 12028 12085 12026 12079 12027 12072 12028 12065 12027
12059 12023 12052 12023 12046 12021 12039 12019 12033 12016 12026 12015
12020 12012 12013 12014 50 P
12013 12014 12007 12010 2 P
12007 12010 12001 12007 11995 12004 11989 12001 11983 11999 11976 11997
11970 11994 11964 11991 11958 11987 11952 11989 11945 11990 11941 11984
11934 11983 11929 11979 11923 11975 11917 11973 11911 11969 11905 11966
11899 11968 11893 11964 11887 11961 11882 11957 11876 11954 11869 11954
11865 11948 11862 11942 11855 11939 11850 11934 11845 11929 11845 11923
11842 11917 11837 11911 11832 11907 11826 11902 11823 11897 11818 11891
11813 11888 11812 11881 11806 11877 11806 11870 11809 11864 11807 11857
11806 11848 43 P
12979 8284 12983 8279 12990 8279 12997 8281 13001 8281 5 P
13001 8297 12996 8294 12989 8295 12982 8295 12979 8284 5 P
12715 8170 12721 8173 12729 8174 12735 8177 12733 8184 12726 8187
12721 8183 12715 8170 8 P
12477 8021 12481 8016 12488 8014 12495 8013 12502 8012 12508 8008
12510 8001 12515 7995 12521 7992 12527 7989 12534 7988 12532 7995
12529 8001 12529 8008 12533 8014 12535 8020 12529 8024 12526 8030
12519 8032 12515 8038 12509 8042 12502 8041 12496 8037 12491 8032
12485 8029 12477 8021 26 P
12693 8048 12696 8044 12698 8040 12702 8043 12706 8045 12702 8047
12699 8051 12699 8055 12695 8054 12693 8048 10 P
12534 8012 12536 8013 12538 8014 12540 8015 12540 8017 12538 8019
12537 8017 12535 8016 12534 8012 9 P
12542 8015 12544 8016 12546 8018 12545 8020 12543 8019 12542 8015

6:00 PM

12547 8013 12549 8015 12550 8016 12552 8017 12555 8017 12557 8018
12558 8020 12559 8022 12562 8023 12564 8023 12566 8023 12568 8024
12569 8026 12571 8026 12573 8028 12571 8028 12569 8028 12568 8026
12566 8025 12563 8024 12561 8024 12559 8024 12557 8022 12556 8020
12554 8019 12552 8018 12550 8017 12548 8016 12547 8013 29 P
12807 7955 12810 7951 12813 7948 12812 7944 12812 7939 12814 7936

12816 7940 12820 7942 12821 7946 12820 7951 12819 7955 12821 7959
12825 7962 12824 7966 12824 7971 12823 7975 12820 7979 12817 7982
12812 7980 12812 7976 12812 7971 12810 7967 12810 7963 12812 7958
12807 7955 25 P
12776 7979 12780 7978 12785 7978 12788 7975 12791 7978 12792 7982
12792 7987 12796 7984 12800 7982 12800 7987 12797 7991 12793 7991
12788 7992 12788 7987 12784 7987 12781 7984 12776 7979 17 P
12768 7962 12772 7959 12775 7955 12779 7957 12783 7959 12781 7963
12779 7967 12775 7969 12772 7966 12768 7962 10 P
12779 7952 12781 7949 12786 7950 12785 7954 12779 7952 5 P
12783 7944 12785 7940 12787 7944 12786 7949 12783 7944 5 P
12754 7945 12756 7940 12759 7938 12760 7943 12760 7947 12757 7950
12754 7945 7 P
12756 7967 12760 7966 12761 7971 12757 7972 12756 7967 5 P
12857 7972 12861 7973 12861 7978 12864 7982 12864 7986 12860 7983
12859 7979 12857 7972 8 P
12832 12916 12826 12914 12823 12908 12822 12901 12821 12894 12815 12895
12808 12892 12803 12895 12801 12888 12796 12884 12800 12878 12804 12872
12807 12866 12812 12861 12818 12857 12811 12858 12804 12857 12798 12860
12793 12855 12786 12856 12780 12859 12775 12864 12768 12862 12766 12856
12759 12854 12756 12848 12753 12842 12750 12836 12752 12829 12754 12823
12758 12816 12764 12814 12767 12808 12771 12802 12777 12806 12776 12812
12783 12814 12788 12817 12793 12822 12796 12829 12803 12827 12803 12820
12806 12814 12804 12807 12805 12801 12799 12796 12793 12795 12786 12793
12788 12786 12793 12790 50 P
12793 12790 12793 12783 12800 12785 12806 12788 12812 12785 12806 12781
12805 12774 12808 12768 12814 12766 12811 12760 12809 12754 12815 12752
12822 12749 12827 12753 12834 12755 12832 12761 12826 12766 12833 12768
12840 12769 12846 12772 12853 12772 12847 12775 12840 12774 12836 12780
12832 12785 12834 12792 12841 12791 12845 12796 12842 12802 12842 12809
12848 12812 12852 12806 12856 12812 12862 12811 12867 12806 12873 12803
12873 12810 12868 12815 12864 12820 12866 12827 12859 12826 12857 12832
12863 12835 12870 12833 12867 12839 12862 12845 12869 12846 12869 12853
12872 12859 12865 12859 50 P
12865 12859 12860 12855 2 P
12860 12855 12859 12848 12852 12849 12848 12855 12853 12860 12855 12866
12859 12872 12857 12879 12855 12885 12854 12892 12852 12899 12846 12902
12843 12908 12840 12915 12844 12921 12850 12924 12851 12931 12846 12936
12840 12938 12833 12938 12829 12933 12828 12926 12832 12916 23 P
12542 12787 12536 12784 12533 12778 12527 12774 12522 12769 12517 12774
12515 12781 12509 12782 12502 12783 12503 12790 12498 12795 12495 12801
12495 12808 12501 12812 12503 12818 12505 12825 12503 12831 12500 12837
12494 12841 12487 12841 12481 12839 12476 12834 12471 12829 12471 12822

12476 12817 12469 12814 12463 12813 12463 12806 12462 12799 12458 12793
12456 12787 12458 12780 12461 12774 12465 12768 12470 12764 12471 12758
12476 12753 12479 12746 12477 12740 12475 12733 12482 12732 12484 12725
12489 12721 12496 12720 12503 12721 12509 12724 12510 12730 12507 12736
12505 12743 12511 12747 50 P
12511 12747 12516 12750 12523 12752 12524 12745 12523 12739 12525 12732
12528 12725 12529 12719 12536 12717 12543 12718 12549 12722 12556 12722
12560 12717 12566 12720 12573 12721 12569 12726 12565 12732 12570 12737
12573 12743 12572 12749 12570 12756 12569 12763 12569 12770 12572 12776
12571 12782 12566 12787 12559 12785 12555 12789 12542 12787 29 P
12067 12365 12071 12359 12077 12362 12082 12357 12089 12357 12096 12356
12100 12361 12101 12368 12100 12375 12095 12379 12094 12386 12088 12389
12082 12391 12076 12387 12073 12382 12070 12375 12067 12365 17 P
12233 12404 12237 12406 12241 12406 12246 12408 12250 12407 12250 12411
12252 12415 12250 12419 12249 12423 12249 12428 12245 12427 12241 12425
12242 12421 12237 12420 12237 12416 12235 12411 12233 12404 17 P
12160 12539 12162 12536 12167 12537 12169 12534 12166 12531 12165 12527
12169 12528 12172 12524 12176 12522 12179 12519 12183 12517 12187 12520
12191 12519 12194 12523 12198 12523 12202 12523 12207 12523 12211 12523
12215 12524 12218 12528 12219 12532 12219 12536 12221 12540 12222 12544
12223 12548 12222 12552 12220 12556 12216 12559 12212 12558 12208 12556
12204 12555 12200 12556 12196 12554 12193 12551 12191 12548 12186 12548
12183 12544 12179 12546 12175 12547 12173 12551 12169 12551 12170 12547
12168 12543 12165 12540 12160 12539 45 P
12321 12547 12326 12547 12329 12548 12334 12549 12336 12545 12332 12545
12328 12545 12329 12542 12333 12540 12337 12541 12341 12539 12341 12535
12340 12530 12343 12528 12347 12531 12350 12534 12354 12536 12358 12537
12362 12540 12363 12544 12361 12548 12360 12552 12355 12553 12353 12556
12349 12559 12346 12562 12344 12558 12340 12557 12337 12554 12333 12557
12329 12558 12325 12560 12321 12558 12324 12556 12323 12552 12321 12547
36 P
12426 12608 12429 12605 12434 12604 12437 12602 12441 12603 12441 12599
12444 12596 12445 12593 12449 12591 12453 12593 12457 12590 12461 12589
12462 12585 12467 12585 12471 12585 12474 12589 12472 12593 12470 12596
12465 12596 12461 12597 12465 12598 12465 12603 12462 12606 12458 12609
12456 12613 12455 12617 12451 12618 12447 12620 12442 12618 12438 12616
12436 12613 12433 12609 12426 12608 33 P
12473 12555 12477 12555 12475 12551 12475 12547 12479 12547 12483 12544
12485 12548 12484 12552 12484 12556 12485 12561 12484 12565 12481 12567
12482 12571 12477 12571 12476 12568 12477 12563 12476 12559 12473 12555
18 P
11961 12468 11963 12464 11963 12459 11964 12456 11965 12451 11964 12447
11967 12444 11966 12439 11970 12442 11974 12439 11978 12437 11981 12435

11985 12438 11988 12440 11991 12437 11994 12434 11992 12431 11987 12431
11987 12426 11990 12424 11994 12425 11999 12426 12003 12425 12006 12423
12011 12422 12010 12426 12008 12430 12012 12429 12015 12425 12019 12425
12024 12425 12028 12424 12026 12428 12021 12430 12023 12433 12025 12438
12021 12439 12017 12439 12012 12438 12013 12443 12012 12447 12008 12449
12007 12453 12005 12457 12004 12461 12004 12465 12003 12470 12000 12474
11997 12476 11993 12478 50 P
11993 12478 11990 12480 11985 12482 11981 12483 11977 12481 11974 12478
11970 12478 11966 12477 11963 12473 11961 12468 10 P
11711 12463 11715 12457 11720 12452 11723 12446 11729 12443 11728 12450
11725 12456 11729 12462 11730 12456 11735 12451 11742 12450 11738 12444
11735 12438 11737 12432 11742 12437 11745 12431 11752 12431 11759 12431
11765 12435 11771 12437 11766 12442 11773 12441 11779 12443 11784 12449
11790 12450 11790 12456 11797 12459 11803 12459 11804 12466 11811 12467
11812 12474 11817 12469 11819 12463 11824 12467 11822 12474 11828 12477
11830 12484 11834 12489 11831 12495 11835 12501 11830 12505 11834 12511
11835 12518 11836 12524 11831 12528 11827 12534 11822 12538 11816 12542
11810 12540 11809 12533 50 P
11809 12533 11803 12537 11800 12543 11802 12550 11798 12556 11792 12559
11785 12561 11779 12562 11772 12563 11770 12556 11773 12550 11780 12549
11786 12551 11784 12544 11786 12538 11783 12532 11778 12528 11776 12521
11769 12523 11769 12529 11766 12536 11762 12530 11765 12524 11759 12521
11762 12515 11763 12508 11768 12503 11773 12505 11771 12499 11764 12501
11768 12495 11766 12488 11765 12481 11764 12475 11760 12469 11761 12475
11759 12482 11760 12489 11763 12495 11759 12501 11759 12508 11753 12509
11752 12516 11746 12512 11745 12505 11749 12499 11746 12494 11741 12498
11734 12498 11731 12492 50 P
11731 12492 11724 12490 2 P
11724 12490 11723 12483 11727 12478 11721 12481 11715 12476 11714 12470
11711 12463 7 P
11913 12330 11917 12327 11918 12323 11923 12322 11927 12322 11931 12324
11934 12326 11938 12329 11941 12332 11942 12336 11946 12337 11947 12340
11951 12343 11951 12347 11955 12344 11957 12340 11958 12345 11961 12343
11966 12344 11966 12348 11961 12348 11957 12349 11953 12351 11949 12351
11945 12349 11940 12350 11936 12349 11934 12352 11931 12355 11926 12354
11923 12351 11920 12348 11917 12345 11913 12342 11916 12338 11913 12330
36 P
11930 12361 11934 12360 11937 12357 11940 12353 11945 12353 11949 12352
11953 12353 11950 12355 11949 12359 11952 12362 11954 12358 11959 12358
11961 12362 11958 12365 11961 12368 11965 12370 11962 12373 11960 12377
11957 12380 11952 12382 11949 12381 11944 12382 11940 12380 11937 12383
11933 12385 11934 12381 11937 12378 11933 12376 11932 12372 11937 12373
11934 12369 11933 12365 11930 12361 33 P

11969 12340 11972 12337 11974 12333 11974 12329 11978 12331 11981 12328
11983 12324 11986 12323 11989 12326 11989 12330 11991 12334 11990 12338
11994 12340 11993 12345 11989 12343 11988 12347 11986 12351 11982 12352
11983 12356 11987 12357 11991 12358 11993 12362 11990 12365 11986 12367
11981 12368 11978 12370 11974 12368 11974 12364 11970 12363 11971 12359
11975 12360 11977 12356 11977 12352 11974 12349 11973 12344 11969 12340
36 P
12014 12448 12019 12448 12023 12446 12027 12446 12031 12444 12033 12441
12037 12444 12040 12446 12044 12447 12048 12448 12052 12445 12054 12442
12054 12437 12057 12433 12061 12436 12062 12439 12066 12442 12065 12446
12069 12445 12070 12441 12074 12441 12075 12445 12074 12450 12074 12454
12079 12453 12080 12457 12078 12462 12075 12458 12071 12459 12068 12463
12064 12462 12059 12462 12056 12459 12053 12463 12050 12465 12046 12463
12044 12459 12044 12455 12041 12452 12037 12451 12034 12454 12030 12457
12028 12453 12026 12449 12022 12450 12018 12451 12014 12448 47 P
12010 12473 12014 12476 12018 12475 12023 12476 12021 12472 12020 12468
12019 12463 12023 12461 12026 12464 12026 12469 12028 12472 12030 12476
12034 12478 12038 12478 12042 12479 12042 12484 12045 12487 12050 12486
12053 12484 12058 12484 12062 12484 12062 12489 12059 12491 12054 12494
12054 12498 12050 12496 12046 12497 12050 12500 12051 12504 12047 12507
12045 12510 12041 12510 12037 12508 12033 12505 12029 12504 12025 12504
12023 12501 12019 12497 12018 12493 12015 12490 12017 12486 12015 12482
12013 12478 12010 12473 44 P
12059 12493 12063 12490 12065 12486 12063 12483 12062 12479 12063 12475
12066 12477 12068 12482 12072 12483 12072 12479 12072 12475 12073 12470
12077 12470 12081 12468 12085 12468 12089 12470 12091 12474 12094 12475
12099 12475 12100 12479 12098 12483 12097 12488 12098 12492 12099 12496
12098 12500 12096 12496 12094 12492 12092 12488 12088 12489 12084 12488
12081 12491 12077 12491 12072 12492 12069 12495 12067 12490 12065 12494
12059 12493 37 P
12125 12474 12120 12475 12122 12471 12127 12470 12131 12471 12135 12470
12138 12468 12135 12465 12139 12464 12142 12461 12143 12465 12147 12466
12151 12463 12147 12460 12144 12458 12145 12453 12145 12449 12147 12453
12150 12457 12152 12460 12156 12463 12160 12462 12161 12466 12156 12466
12156 12470 12160 12469 12164 12469 12167 12466 12172 12466 12169 12470
12166 12474 12165 12478 12161 12478 12156 12479 12153 12476 12149 12474
12146 12472 12147 12477 12145 12481 12141 12482 12138 12486 12135 12490
12137 12485 12133 12485 12128 12483 12132 12479 12129 12476 12125 12474
48 P
12129 12528 12133 12525 12135 12522 12137 12517 12137 12513 12140 12510
12143 12507 12145 12511 12144 12515 12148 12514 12147 12509 12149 12505
12150 12501 12152 12505 12153 12509 12155 12513 12153 12517 12152 12521
12150 12525 12147 12529 12144 12531 12139 12531 12135 12530 12129 12528

24 P
12896 12925 12899 12922 12904 12922 12905 12927 12903 12931 12903 12935
12904 12940 12905 12944 12907 12948 12906 12952 12905 12957 12900 12956
12898 12960 12894 12962 12892 12958 12887 12957 12884 12954 12882 12951
12886 12950 12889 12947 12888 12943 12892 12939 12888 12937 12892 12935
12896 12934 12897 12930 12896 12925 27 P
12750 12787 12754 12785 12757 12781 12756 12778 12756 12773 12760 12773
12765 12772 12768 12774 12772 12777 12775 12780 12774 12785 12772 12788
12767 12787 12765 12790 12764 12795 12761 12798 12762 12802 12759 12800
12760 12804 12756 12804 12752 12801 12753 12797 12758 12798 12761 12795
12757 12797 12757 12793 12754 12789 12754 12793 12751 12797 12751 12793
12750 12787 31 P
12763 12875 12765 12871 12768 12868 12772 12868 12770 12872 12773 12875
12770 12878 12767 12881 12770 12885 12773 12888 12771 12892 12767 12893
12763 12892 12761 12887 12764 12884 12765 12879 12763 12875 17 P
12616 12800 12620 12799 12624 12798 12624 12793 12628 12793 12631 12796
12632 12800 12635 12803 12639 12803 12644 12803 12643 12808 12639 12809
12635 12810 12631 12812 12626 12811 12623 12809 12618 12808 12616 12800
18 P
12633 12789 12637 12788 12638 12784 12642 12784 12645 12781 12649 12780
12653 12779 12654 12783 12651 12786 12655 12787 12659 12788 12663 12791
12659 12794 12656 12795 12653 12799 12650 12802 12645 12803 12642 12802
12638 12799 12636 12795 12638 12792 12633 12789 22 P
12656 12691 12659 12688 12663 12687 12663 12682 12665 12678 12667 12682
12672 12683 12673 12687 12678 12686 12682 12686 12686 12686 12686 12690
12689 12694 12687 12697 12683 12696 12680 12692 12679 12688 12676 12691
12679 12694 12676 12697 12671 12697 12668 12700 12664 12702 12659 12701
12658 12696 12656 12691 26 P
12537 12829 12535 12825 12534 12821 12532 12817 12529 12813 12528 12809
12524 12806 12523 12802 12524 12798 12525 12794 12528 12790 12532 12789
12536 12787 12541 12787 12545 12788 12549 12790 12550 12794 12554 12795
12558 12794 12562 12792 12566 12792 12570 12790 12574 12791 12577 12788
12582 12789 12581 12793 12577 12795 12572 12797 12569 12800 12569 12804
12570 12808 12569 12813 12566 12815 12562 12816 12558 12819 12559 12823
12555 12823 12552 12821 12551 12816 12550 12812 12550 12808 12548 12804
12545 12807 12544 12811 12545 12815 12546 12820 12548 12823 12550 12827
12551 12832 12550 12836 50 P
12550 12836 12546 12835 12542 12833 12537 12829 4 P
12398 12618 12400 12622 12402 12619 12405 12616 12409 12615 12413 12618
12417 12620 12417 12624 12421 12621 12425 12622 12428 12624 12425 12627
12422 12630 12418 12627 12414 12627 12412 12631 12408 12632 12404 12630
12400 12628 12398 12624 12398 12618 21 P
12310 12584 12314 12582 12318 12582 12319 12578 12322 12575 12318 12574

12315 12571 12317 12567 12320 12563 12324 12563 12327 12567 12331 12567
12336 12569 12340 12568 12342 12572 12342 12576 12339 12579 12334 12581
12330 12581 12326 12581 12329 12583 12327 12587 12323 12588 12326 12591
12329 12595 12327 12598 12323 12599 12319 12600 12316 12596 12314 12593
12313 12589 12310 12584 32 P
12126 12463 12122 12462 12118 12463 12113 12462 12110 12459 12107 12456
12108 12451 12108 12447 12113 12446 12114 12442 12118 12444 12122 12445
12125 12449 12129 12449 12131 12453 12135 12456 12133 12459 12126 12463
18 P
12268 12321 12270 12317 12272 12313 12275 12310 12276 12306 12279 12303
12282 12300 12287 12300 12291 12299 12294 12303 12289 12302 12286 12304
12284 12308 12280 12311 12279 12315 12277 12319 12279 12323 12277 12327
12274 12325 12268 12321 20 P
12281 12324 12284 12321 12286 12317 12290 12315 12294 12313 12295 12318
12292 12320 12292 12324 12288 12325 12281 12324 10 P
12107 12261 12108 12258 12113 12258 12117 12259 12119 12263 12118 12266
12114 12268 12109 12268 12107 12261 9 P
11834 12216 11838 12214 11840 12211 11843 12207 11847 12207 11845 12211
11846 12215 11851 12216 11851 12220 11847 12222 11844 12219 11839 12220
11834 12216 13 P
11824 12221 11828 12219 11832 12217 11833 12221 11830 12225 11826 12226
11824 12221 7 P
11903 12351 11906 12350 11908 12353 11910 12356 11913 12356 11914 12359
11916 12362 11913 12364 11911 12366 11910 12363 11907 12360 11907 12357
11906 12354 11903 12351 14 P
11949 12386 11952 12385 11954 12387 11957 12386 11959 12389 11963 12389
11962 12392 11960 12395 11958 12396 11954 12395 11952 12393 11949 12392
11949 12386 13 P
11952 12312 11956 12312 11959 12312 11962 12311 11961 12314 11959 12317
11958 12320 11955 12322 11952 12319 11954 12317 11952 12312 11 P
12022 12367 12025 12366 12027 12363 12028 12366 12029 12370 12031 12372
12032 12375 12030 12378 12028 12381 12025 12382 12023 12380 12021 12377
12023 12373 12022 12367 14 P
11273 12232 11277 12230 11281 12230 11285 12228 11288 12225 11290 12221
11293 12217 11297 12219 11301 12219 11303 12224 11299 12226 11296 12229
11293 12232 11292 12237 11293 12240 11288 12241 11286 12245 11282 12244
11279 12241 11279 12237 11279 12232 11273 12232 22 P
11439 12323 11441 12320 11442 12317 11445 12319 11448 12318 11450 12320
11447 12321 11445 12324 11442 12323 11439 12323 10 P
11685 12391 11689 12392 11689 12388 11693 12387 11697 12387 11702 12386
11704 12383 11704 12387 11706 12391 11707 12387 11710 12383 11712 12387
11716 12388 11720 12386 11719 12391 11718 12395 11717 12399 11717 12403
11713 12402 11709 12402 11705 12400 11701 12401 11697 12404 11693 12406

11691 12402 11687 12402 11690 12399 11687 12397 11685 12391 29 P
11692 12358 11695 12358 11697 12356 11700 12358 11703 12358 11707 12357
11708 12359 11709 12363 11712 12363 11710 12366 11709 12369 11706 12370
11703 12372 11704 12369 11704 12366 11702 12364 11698 12363 11695 12362
11692 12363 11692 12358 20 P
11648 12356 11649 12353 11652 12353 11655 12353 11658 12356 11660 12358
11661 12361 11658 12362 11655 12360 11653 12362 11650 12360 11648 12356
12:00 PM
11634 12360 11637 12360 11640 12358 11640 12361 11643 12364 11644 12366
11646 12363 11645 12360 11648 12362 11648 12365 11647 12368 11643 12369
11640 12368 11637 12366 11635 12364 11634 12360 16 P
11675 12417 11672 12415 11673 12412 11673 12409 11676 12408 11679 12407
11683 12407 11686 12408 11687 12411 11690 12410 11693 12412 11696 12410
11699 12410 11702 12408 11703 12411 11701 12413 11698 12414 11695 12416
11692 12417 11689 12419 11687 12417 11683 12417 11682 12414 11679 12414
11675 12417 25 P
11679 12376 11678 12378 11676 12381 11673 12382 11670 12380 11669 12377
11670 12375 11674 12375 11675 12372 11677 12370 11680 12369 11680 12372
11682 12375 11682 12378 11679 12376 15 P
11758 12370 11760 12367 11763 12367 11761 12370 11762 12373 11763 12376
11765 12379 11763 12382 11761 12379 11759 12377 11759 12373 11758 12370
12:00 PM
11382 12555 11385 12551 11387 12548 11391 12545 11395 12545 11394 12550
11395 12554 11398 12556 11401 12560 11400 12564 11404 12565 11407 12568
11409 12572 11408 12576 11412 12580 11415 12581 11414 12585 11410 12583
11407 12580 11403 12580 11402 12584 11406 12586 11401 12587 11397 12586
11393 12585 11391 12581 11391 12577 11388 12574 11387 12569 11386 12565
11383 12562 11386 12559 11382 12555 33 P
11408 12602 11410 12600 11413 12599 11414 12596 11416 12598 11416 12601
11418 12604 11421 12603 11421 12607 11418 12607 11415 12609 11412 12611
11413 12608 11410 12605 11408 12602 15 P
11288 12562 11291 12558 11293 12563 11295 12567 11299 12567 11301 12571
11302 12575 11304 12579 11302 12583 11298 12585 11297 12581 11295 12577
11294 12573 11290 12570 11288 12567 11288 12562 16 P
11345 12433 11347 12430 11348 12428 11351 12430 11349 12433 11345 12433
6:00 PM
11345 12437 11348 12437 11351 12439 11353 12441 11352 12444 11349 12444
11348 12442 11345 12437 8 P
11348 12419 11350 12417 11352 12414 11354 12411 11355 12408 11358 12410
11361 12409 11363 12410 11362 12413 11359 12415 11356 12416 11354 12414
11353 12417 11348 12419 14 P
11371 12570 11373 12568 11376 12569 11379 12571 11379 12575 11375 12575
11373 12574 11371 12570 8 P

11379 12576 11382 12576 11383 12579 11386 12581 11383 12581 11379 12576
6:00 PM
11402 12607 11405 12605 11407 12608 11405 12611 11402 12610 11402 12607
6:00 PM
11571 12560 11575 12558 11577 12555 11582 12555 11580 12552 11575 12550
11578 12547 11582 12548 11586 12550 11590 12549 11593 12552 11597 12553
11598 12558 11596 12562 11596 12566 11591 12567 11589 12564 11585 12562
11582 12559 11577 12559 11571 12560 21 P
11655 12614 11651 12616 11649 12620 11644 12620 11642 12623 11640 12627
11638 12630 11635 12627 11634 12623 11631 12620 11634 12617 11632 12613
11631 12617 11627 12618 11623 12616 11618 12615 11617 12610 11617 12606
11617 12602 11618 12597 11621 12594 11625 12597 11627 12593 11630 12590
11634 12591 11637 12588 11641 12587 11645 12588 11649 12591 11648 12595
11652 12597 11655 12601 11656 12605 11660 12604 11661 12608 11663 12613
11655 12614 37 P
11651 12653 11647 12651 11649 12655 11651 12660 11652 12664 11650 12668
11647 12670 11643 12668 11638 12667 11637 12662 11635 12659 11631 12659
11630 12655 11626 12653 11622 12651 11618 12650 11613 12650 11610 12653
11606 12653 11606 12648 11603 12645 11605 12641 11604 12637 11604 12633
11603 12628 11606 12625 11609 12622 11612 12618 11616 12617 11621 12618
11624 12620 11628 12622 11627 12627 11631 12627 11628 12630 11627 12635
11623 12637 11622 12641 11627 12643 11630 12644 11635 12645 11638 12647
11640 12643 11644 12643 11648 12645 11652 12646 11651 12653 47 P
11414 12556 11417 12556 11420 12557 11422 12560 11425 12562 11426 12565
11422 12565 11419 12565 11418 12562 11415 12560 11414 12556 11 P
11396 12616 11399 12614 11402 12614 11400 12617 11396 12616 5 P
11602 12561 11604 12557 11607 12556 11609 12554 11611 12551 11611 12555
11610 12558 11607 12558 11602 12561 9 P
11600 12561 11601 12557 11601 12554 11603 12556 11600 12561 5 P
11635 12573 11637 12571 11640 12569 11643 12571 11645 12569 11648 12567
11650 12568 11650 12571 11649 12574 11646 12576 11644 12579 11642 12580
11639 12578 11636 12577 11635 12573 15 P
11660 12575 11656 12574 11657 12570 11659 12568 11656 12567 11655 12563
11657 12562 11659 12564 11660 12561 11662 12563 11665 12564 11665 12568
11664 12571 11662 12573 11660 12575 15 P
11666 12562 11667 12558 11670 12558 11673 12556 11671 12553 11669 12551
11669 12548 11671 12545 11671 12542 11673 12539 11676 12540 11677 12543
11678 12545 11681 12544 11684 12543 11688 12543 11690 12545 11691 12548
11691 12551 11690 12554 11687 12555 11687 12558 11687 12562 11683 12561
11680 12561 11678 12564 11677 12567 11678 12569 11678 12573 11678 12576
11675 12577 11672 12578 11669 12578 11665 12577 11664 12574 11667 12571
11668 12568 11667 12565 11666 12562 39 P
11676 12523 11681 12522 11685 12522 11689 12522 11693 12524 11696 12528

11700 12529 11703 12532 11702 12536 11707 12537 11709 12541 11707 12545
11709 12549 11709 12553 11712 12556 11709 12559 11705 12561 11708 12564
11703 12567 11699 12568 11695 12569 11695 12574 11691 12576 11687 12576
11683 12576 11682 12572 11685 12569 11689 12568 11691 12564 11694 12560
11694 12556 11698 12553 11702 12552 11698 12550 11696 12547 11697 12542
11695 12539 11690 12538 11687 12541 11682 12541 11679 12537 11675 12535
11679 12533 11682 12536 11686 12535 11690 12536 11689 12531 11685 12530
11683 12527 11679 12528 50 P
11679 12528 11676 12523 2 P
11642 12552 11645 12553 11649 12553 11649 12550 11648 12547 11649 12544
11651 12544 11651 12548 11652 12551 11651 12554 11654 12556 11650 12557
11648 12556 11644 12555 11642 12552 15 P
11710 12588 11715 12588 11717 12584 11716 12580 11717 12576 11716 12572
11720 12571 11723 12574 11728 12575 11730 12579 11734 12580 11737 12577
11741 12580 11744 12584 11748 12584 11750 12588 11746 12589 11742 12590
11743 12594 11741 12598 11737 12601 11734 12597 11731 12594 11727 12594
11730 12597 11731 12602 11728 12604 11723 12603 11721 12599 11717 12597
11716 12593 11711 12592 11710 12588 33 P
11681 12652 11684 12650 11688 12647 11690 12643 11692 12640 11697 12640
11701 12638 11699 12634 11699 12629 11704 12629 11707 12627 11710 12629
11713 12633 11717 12634 11718 12629 11722 12627 11721 12622 11725 12621
11730 12620 11734 12621 11736 12625 11735 12629 11735 12634 11733 12637
11730 12641 11733 12644 11729 12646 11726 12650 11723 12651 11719 12649
11715 12649 11711 12651 11710 12656 11705 12656 11702 12654 11697 12656
11693 12657 11691 12653 11687 12654 11681 12652 40 P
11687 12665 11691 12663 11695 12662 11699 12660 11703 12662 11707 12662
11710 12659 11713 12655 11716 12652 11719 12654 11724 12655 11725 12659
11725 12663 11723 12667 11726 12670 11724 12674 11719 12674 11719 12679
11716 12681 11714 12677 11716 12673 11711 12673 11708 12671 11704 12669
11699 12668 11695 12667 11687 12665 27 P
11674 12697 11677 12693 11679 12690 11682 12686 11683 12682 11687 12680
11691 12678 11693 12674 11692 12670 11697 12670 11701 12672 11703 12675
11705 12680 11702 12683 11701 12687 11703 12692 11707 12690 11711 12688
11711 12692 11715 12695 11715 12699 11711 12701 11708 12704 11712 12706
11716 12707 11716 12711 11712 12710 11712 12715 11709 12718 11706 12715
11702 12717 11698 12718 11695 12715 11696 12711 11698 12707 11701 12703
11699 12700 11695 12700 11690 12701 11687 12704 11683 12702 11681 12699
11674 12697 43 P
11645 12715 11644 12711 11642 12707 11637 12707 11632 12707 11637 12705
11635 12701 11639 12700 11643 12700 11647 12702 11650 12700 11654 12703
11657 12706 11657 12710 11657 12706 11657 12702 11661 12701 11663 12697
11667 12694 11670 12697 11672 12700 11677 12701 11678 12705 11683 12706
11683 12710 11682 12714 11686 12713 11690 12714 11691 12719 11688 12721

11684 12722 11684 12727 11688 12729 11684 12732 11685 12736 11681 12736
11678 12741 11675 12743 11671 12745 11670 12740 11671 12736 11670 12732
11667 12730 11663 12733 11660 12730 11656 12728 11652 12730 11650 12726
11649 12722 11645 12715 50 P
11679 12674 11681 12670 11683 12668 11687 12669 11684 12672 11683 12675
11679 12674 7 P
11668 12683 11669 12680 11671 12677 11674 12677 11676 12674 11679 12675
11679 12679 11677 12681 11674 12682 11671 12684 11668 12683 11 P
11659 12638 11660 12635 11661 12632 11662 12629 11664 12626 11667 12626
11668 12629 11667 12632 11665 12635 11665 12638 11659 12638 11 P
11729 12609 11732 12609 11735 12608 11738 12610 11739 12613 11742 12610
11744 12613 11745 12616 11742 12618 11745 12620 11746 12623 11743 12624
11740 12623 11737 12621 11736 12618 11736 12615 11733 12615 11730 12615
11729 12609 19 P
11698 12618 11700 12616 11703 12616 11705 12619 11707 12622 11704 12624
11701 12623 11698 12618 8 P
11685 12635 11688 12634 11690 12637 11691 12640 11689 12642 11687 12640
11685 12635 7 P
11670 12645 11674 12646 11676 12647 11678 12650 11678 12653 11675 12651
11674 12649 11670 12645 8 P
11685 12603 11687 12600 11689 12602 11690 12605 11692 12607 11693 12610
11690 12610 11687 12608 11685 12603 9 P
11678 12590 11678 12587 11679 12584 11681 12587 11682 12590 11681 12594
11680 12597 11677 12595 11678 12590 9 P
11682 12494 11684 12492 11686 12489 11685 12486 11687 12483 11690 12485
11692 12488 11692 12491 11696 12492 11698 12495 11698 12498 11695 12496
11694 12499 11697 12501 11694 12502 11692 12505 11689 12503 11687 12501
11687 12497 11685 12495 11682 12494 21 P
11799 12445 11802 12444 11805 12443 11804 12446 11808 12446 11811 12448
11813 12450 11813 12453 11811 12455 11808 12453 11810 12456 11809 12459
11806 12458 11803 12456 11800 12454 11802 12451 11799 12445 17 P
11643 12428 11647 12429 11648 12431 11649 12435 11645 12435 11644 12431
11643 12428 7 P
11656 12428 11659 12428 11661 12431 11661 12434 11659 12437 11657 12435
11656 12428 7 P
11655 12396 11658 12396 11661 12398 11660 12400 11657 12399 11655 12396
6:00 PM
11662 12393 11664 12391 11667 12391 11671 12391 11669 12393 11666 12395
11662 12393 7 P
11662 12403 11663 12399 11664 12396 11667 12397 11668 12400 11666 12403
11662 12403 7 P
11650 12415 11654 12416 11657 12416 11656 12419 11654 12421 11651 12418
11650 12415 7 P

11680 12425 11683 12423 11681 12421 11683 12419 11687 12419 11686 12422
11685 12425 11683 12428 11680 12425 9 P
11676 12404 11677 12401 11679 12398 11682 12400 11685 12401 11685 12404
11682 12404 11676 12404 8 P
11613 12342 11616 12339 11618 12338 11621 12337 11621 12340 11618 12342
11613 12342 7 P
11656 12314 11658 12311 11659 12308 11660 12305 11662 12307 11660 12310
11660 12313 11658 12317 11656 12314 9 P
11749 12703 11754 12704 11758 12702 11762 12701 11766 12701 11769 12704
11770 12709 11775 12710 11779 12709 11783 12710 11782 12714 11786 12713
11788 12709 11792 12709 11796 12708 11798 12704 11802 12703 11800 12700
11797 12696 11799 12693 11803 12692 11805 12696 11809 12694 11812 12697
11811 12702 11813 12706 11813 12710 11812 12714 11809 12716 11805 12719
11800 12718 11796 12717 11792 12716 11788 12717 11790 12721 11786 12723
11790 12726 11791 12730 11795 12729 11796 12733 11800 12733 11802 12737
11798 12737 11796 12741 11791 12742 11787 12741 11783 12741 11781 12746
11777 12746 11772 12745 50 P
11772 12745 11770 12741 11772 12737 11776 12735 11779 12733 11781 12728
11777 12726 11777 12721 11773 12722 11773 12717 11770 12715 11768 12719
11765 12715 11764 12711 11759 12711 11755 12710 11752 12713 11749 12709
11749 12703 19 P
11758 12770 11762 12767 11764 12763 11766 12759 11768 12755 11772 12753
11776 12755 11775 12759 11773 12763 11771 12767 11768 12769 11764 12772
11758 12770 13 P
11742 12741 11745 12739 11750 12737 11752 12741 11756 12741 11756 12746
11757 12750 11753 12752 11749 12752 11744 12752 11742 12748 11742 12741
12:00 PM
11652 12813 11656 12813 11661 12813 11661 12808 11664 12812 11667 12815
11669 12811 11672 12809 11677 12808 11681 12806 11683 12810 11686 12813
11684 12816 11687 12820 11689 12824 11685 12825 11681 12827 11681 12832
11681 12836 11685 12838 11686 12842 11683 12846 11686 12849 11685 12853
11689 12855 11694 12856 11697 12859 11701 12861 11697 12863 11695 12859
11690 12859 11686 12859 11682 12859 11681 12854 11678 12851 11675 12848
11674 12844 11671 12840 11667 12838 11663 12837 11658 12835 11656 12832
11655 12828 11653 12824 11657 12822 11661 12820 11658 12816 11652 12813
48 P
11704 12832 11708 12831 11711 12828 11709 12823 11714 12823 11715 12819
11719 12818 11724 12819 11726 12816 11731 12816 11735 12818 11738 12815
11742 12817 11744 12813 11740 12811 11741 12806 11745 12806 11746 12802
11749 12799 11749 12803 11754 12801 11755 12805 11758 12808 11755 12811
11750 12812 11751 12816 11750 12821 11746 12823 11743 12820 11739 12821
11737 12825 11733 12827 11732 12831 11736 12834 11739 12832 11742 12835
11741 12840 11737 12840 11734 12836 11733 12840 11730 12844 11726 12841

11725 12836 11721 12837 11717 12840 11716 12844 11712 12842 11708 12841
11705 12837 11704 12832 50 P
11693 12808 11697 12808 11698 12803 11702 12802 11706 12803 11709 12801
11712 12797 11714 12801 11714 12806 11715 12801 11716 12797 11719 12800
11719 12805 11718 12809 11716 12813 11711 12814 11707 12813 11703 12813
11699 12812 11693 12808 20 P
11683 12780 11686 12778 11690 12780 11695 12781 11699 12781 11703 12779
11707 12781 11707 12777 11702 12778 11701 12774 11702 12769 11703 12765
11701 12761 11699 12757 11698 12753 11702 12754 11706 12756 11707 12760
11705 12764 11710 12765 11714 12768 11717 12766 11717 12770 11721 12771
11720 12775 11720 12780 11717 12783 11713 12784 11711 12788 11710 12792
11707 12795 11703 12793 11699 12795 11695 12795 11695 12791 11691 12789
11689 12785 11683 12780 38 P
11750 12787 11752 12785 11752 12781 11752 12778 11750 12775 11753 12774
11756 12775 11759 12775 11762 12777 11761 12780 11761 12783 11758 12785
11756 12788 11754 12790 11751 12790 11748 12792 11750 12787 17 P
11730 12768 11732 12765 11733 12762 11736 12760 11738 12763 11737 12767
11737 12770 11737 12773 11738 12776 11735 12775 11731 12774 11731 12771
11730 12768 13 P
11717 13106 11719 13103 11722 13099 11725 13102 11729 13104 11732 13107
11735 13111 11737 13114 11741 13116 11743 13119 11744 13123 11742 13127
11739 13130 11739 13125 11736 13123 11731 13123 11727 13125 11730 13121
11725 13120 11726 13116 11723 13113 11722 13109 11717 13106 23 P
11682 12938 11683 12935 11686 12933 11687 12930 11690 12931 11692 12933
11691 12937 11691 12940 11689 12943 11687 12944 11684 12943 11682 12938
12:00 PM
11623 12964 11626 12962 11627 12959 11630 12960 11633 12960 11636 12962
11638 12964 11637 12967 11635 12971 11632 12971 11630 12974 11628 12972
11624 12972 11624 12969 11628 12968 11630 12966 11627 12965 11623 12964
18 P
11621 12958 11619 12955 11618 12952 11621 12954 11624 12955 11621 12958
6:00 PM
11636 12979 11640 12978 11641 12980 11639 12983 11636 12979 5 P
11687 12892 11690 12892 11693 12892 11695 12895 11697 12897 11694 12898
11692 12895 11689 12894 11687 12892 9 P
11682 12884 11685 12885 11686 12888 11685 12892 11682 12891 11682 12887
11682 12884 7 P
11638 12826 11641 12823 11643 12821 11645 12820 11648 12822 11648 12825
11646 12828 11644 12831 11642 12833 11640 12831 11638 12826 11 P
11652 13130 11655 13129 11658 13130 11658 13128 11661 13129 11661 13131
11658 13133 11656 13134 11654 13133 11652 13130 10 P
11647 13140 11650 13139 11651 13142 11651 13144 11649 13144 11647 13140
6:00 PM

11793 13201 11796 13199 11797 13196 11799 13194 11800 13196 11801 13199
11799 13202 11797 13204 11793 13201 9 P
11870 13234 11872 13231 11875 13230 11877 13232 11878 13236 11879 13239
11877 13242 11873 13241 11871 13238 11870 13234 10 P
11789 13247 11790 13244 11792 13242 11792 13238 11796 13237 11795 13241
11798 13242 11800 13239 11803 13239 11806 13240 11809 13242 11806 13244
11804 13245 11800 13247 11797 13247 11795 13249 11791 13250 11789 13247
18 P
11765 13239 11768 13238 11768 13235 11771 13236 11771 13232 11774 13234
11777 13234 11778 13237 11776 13239 11779 13241 11778 13244 11777 13247
11773 13248 11770 13247 11770 13244 11773 13243 11776 13241 11774 13238
11771 13237 11770 13240 11765 13239 21 P
11778 13229 11781 13228 11784 13228 11786 13230 11789 13232 11790 13235
11788 13237 11786 13235 11785 13232 11782 13231 11783 13234 11781 13236
11779 13232 11778 13229 14 P
11756 13234 11758 13231 11760 13229 11763 13230 11763 13234 11761 13236
11758 13238 11756 13234 8 P
11750 13254 11754 13253 11757 13254 11759 13255 11762 13257 11759 13258
11756 13258 11754 13255 11750 13254 9 P
11771 13261 11774 13261 11777 13261 11781 13260 11778 13263 11775 13262
11771 13261 7 P
11741 13255 11744 13254 11748 13254 11746 13257 11741 13255 5 P
11806 13258 11808 13256 11811 13257 11812 13260 11809 13260 11806 13258
6:00 PM
11817 13316 11819 13315 11822 13314 11823 13316 11822 13318 11819 13318
11817 13316 7 P
11843 13322 11845 13322 11848 13321 11849 13318 11852 13318 11850 13320
11849 13323 11846 13324 11843 13322 9 P
11828 13328 11830 13327 11832 13326 11835 13324 11837 13325 11839 13325
11841 13328 11838 13329 11836 13330 11833 13330 11830 13330 11828 13328
12:00 PM
11818 13366 11821 13365 11824 13364 11826 13363 11827 13366 11827 13369
11826 13371 11823 13371 11822 13369 11818 13366 10 P
11768 13386 11769 13383 11771 13382 11774 13382 11777 13381 11776 13384
11775 13386 11772 13388 11770 13387 11768 13386 10 P
11563 13388 11564 13386 11566 13383 11567 13381 11570 13380 11572 13381
11571 13383 11575 13383 11575 13381 11577 13383 11577 13386 11575 13387
11572 13388 11570 13389 11567 13389 11563 13388 16 P
11583 13442 11584 13440 11587 13438 11589 13436 11589 13439 11588 13442
11583 13442 7 P
11601 13444 11604 13444 11607 13445 11609 13446 11611 13447 11614 13448
11614 13450 11612 13452 11609 13452 11607 13452 11604 13450 11603 13448
11601 13444 13 P

11552 13450 11553 13448 11554 13446 11556 13443 11558 13443 11558 13446
11560 13448 11558 13450 11556 13448 11554 13449 11552 13450 11 P
11715 13515 11718 13515 11720 13516 11722 13515 11724 13516 11726 13519
11726 13521 11724 13523 11723 13525 11724 13528 11723 13530 11720 13531
11719 13528 11719 13525 11717 13523 11715 13521 11715 13519 11715 13515
18 P
11731 13541 11732 13538 11735 13537 11737 13539 11740 13539 11742 13538
11745 13538 11748 13537 11750 13538 11753 13537 11756 13536 11758 13535
11761 13535 11763 13535 11765 13536 11765 13539 11762 13539 11759 13538
11757 13540 11755 13540 11756 13543 11755 13546 11753 13547 11752 13550
11750 13550 11747 13550 11745 13548 11744 13546 11745 13543 11742 13543
11740 13545 11737 13546 11735 13546 11732 13546 11731 13541 35 P
11678 13493 11679 13490 11681 13488 11682 13490 11684 13492 11684 13495
11686 13497 11684 13498 11681 13498 11680 13495 11681 13493 11678 13493

12:00 PM

11683 13480 11684 13477 11687 13477 11687 13480 11688 13482 11688 13485
11685 13485 11684 13483 11683 13480 9 P
11569 13688 11571 13686 11573 13687 11575 13689 11577 13690 11580 13689
11583 13690 11583 13693 11586 13694 11584 13697 11584 13700 11582 13701
11582 13698 11581 13695 11579 13694 11576 13694 11574 13693 11572 13691
11569 13688 19 P
11555 13988 11559 13990 11560 13995 11562 13991 11567 13989 11567 13985
11571 13984 11576 13985 11580 13987 11581 13991 11585 13993 11588 13996
11586 14000 11582 14001 11577 14003 11574 14005 11569 14004 11568 14008
11564 14010 11563 14015 11560 14017 11560 14013 11558 14009 11557 14005
11556 14001 11557 13997 11557 13992 11555 13988 28 P
11587 13992 11588 13990 11589 13987 11591 13986 11594 13986 11596 13985
11597 13982 11597 13979 11600 13981 11601 13984 11602 13985 11601 13987
11598 13987 11597 13989 11595 13991 11594 13994 11591 13995 11587 13992
18 P
11701 14026 11703 14022 11704 14018 11705 14014 11706 14009 11710 14012
11713 14015 11715 14011 11715 14007 11718 14009 11721 14012 11723 14016
11725 14020 11726 14024 11731 14025 11731 14029 11729 14034 11729 14038
11724 14039 11720 14039 11716 14039 11711 14038 11711 14034 11707 14032
11703 14032 11701 14026 26 P
11749 13994 11752 13996 11755 14000 11759 13999 11762 13995 11765 13997
11765 14002 11767 14005 11768 14010 11772 14009 11776 14009 11777 14013
11780 14016 11784 14017 11785 14021 11784 14025 11782 14029 11781 14033
11777 14034 11773 14034 11769 14035 11766 14038 11762 14037 11761 14042
11763 14045 11763 14050 11761 14046 11759 14042 11755 14040 11752 14043
11748 14044 11750 14041 11753 14037 11756 14034 11757 14030 11759 14026
11759 14022 11760 14017 11756 14016 11752 14014 11754 14009 11758 14010
11756 14006 11754 14002 11751 13999 11749 13994 46 P

11636 14018 11641 14017 11644 14015 11649 14014 11652 14013 11657 14012
11661 14013 11665 14016 11667 14020 11663 14022 11661 14018 11656 14018
11652 14021 11648 14021 11644 14022 11640 14024 11636 14018 17 P
11646 13971 11648 13968 11650 13967 11654 13965 11657 13965 11660 13965
11659 13968 11657 13970 11654 13971 11651 13972 11646 13971 11 P
11626 13970 11628 13968 11631 13968 11634 13967 11637 13966 11639 13963
11642 13962 11643 13965 11641 13966 11638 13969 11638 13972 11635 13973
11632 13975 11630 13972 11626 13970 15 P
11613 13975 11615 13972 11618 13971 11621 13972 11624 13971 11626 13974
11624 13977 11621 13976 11618 13975 11613 13975 10 P
11557 13964 11560 13962 11561 13959 11564 13958 11568 13959 11570 13961
11568 13964 11570 13967 11567 13967 11565 13969 11562 13971 11559 13969
11557 13964 13 P
11551 13919 11554 13920 11557 13918 11554 13918 11553 13915 11556 13913
11559 13913 11561 13916 11563 13919 11564 13922 11563 13925 11560 13927
11557 13925 11554 13925 11553 13922 11551 13919 16 P
11500 13903 11502 13901 11504 13898 11505 13902 11506 13905 11506 13908
11509 13907 11512 13908 11510 13911 11507 13912 11504 13913 11502 13911
11502 13908 11500 13903 14 P
11529 13879 11526 13879 11526 13883 11523 13885 11520 13886 11519 13883
11520 13880 11523 13878 11526 13876 11527 13873 11530 13875 11533 13877
11536 13879 11533 13878 11529 13879 15 P
11504 13961 11508 13960 11510 13962 11513 13964 11516 13964 11514 13967
11517 13968 11519 13971 11515 13971 11514 13969 11510 13968 11509 13965
11506 13964 11504 13961 14 P
11689 13939 11691 13937 11694 13935 11695 13938 11695 13942 11693 13944
11690 13944 11689 13939 8 P
11707 13916 11709 13913 11710 13910 11710 13907 11714 13906 11716 13907
11719 13908 11722 13910 11721 13913 11720 13916 11717 13918 11716 13916
11712 13916 11711 13919 11707 13916 15 P
11679 14095 11683 14094 11685 14091 11685 14088 11685 14085 11688 14082
11690 14081 11694 14082 11697 14083 11697 14087 11697 14090 11699 14091
11697 14094 11695 14095 11692 14098 11690 14100 11687 14102 11685 14104
11682 14105 11682 14102 11681 14099 11679 14095 22 P
11658 14131 11660 14128 11663 14128 11664 14124 11665 14121 11668 14120
11670 14117 11673 14119 11674 14122 11677 14123 11679 14121 11679 14117
11682 14119 11684 14117 11687 14115 11688 14112 11690 14115 11692 14117
11692 14121 11692 14123 11690 14127 11688 14128 11685 14130 11683 14132
11679 14133 11676 14134 11676 14130 11673 14129 11670 14130 11668 14132
11665 14135 11663 14137 11660 14137 11658 14134 11658 14131 35 P
11711 14145 11713 14141 11716 14139 11721 14139 11723 14143 11727 14143
11730 14146 11734 14148 11738 14147 11743 14148 11746 14150 11748 14154
11750 14157 11750 14162 11747 14165 11745 14169 11741 14170 11737 14172

11732 14172 11728 14173 11724 14175 11721 14177 11717 14175 11715 14171
11716 14167 11715 14162 11713 14159 11714 14154 11713 14150 11711 14145
30 P
11693 14153 11696 14150 11700 14149 11702 14144 11701 14140 11705 14140
11704 14144 11707 14148 11709 14151 11707 14155 11702 14154 11698 14154
11693 14153 13 P
11687 14164 11690 14163 11692 14165 11696 14166 11699 14165 11701 14163
11704 14162 11704 14166 11704 14169 11705 14172 11704 14175 11702 14177
11699 14180 11696 14178 11696 14175 11693 14173 11690 14172 11688 14169
11687 14164 19 P
11709 14130 11711 14128 11715 14127 11716 14130 11716 14134 11714 14136
11711 14134 11709 14130 8 P
11632 14181 11634 14178 11637 14176 11639 14174 11642 14172 11643 14169
11642 14166 11645 14164 11649 14165 11652 14166 11655 14167 11658 14167
11661 14168 11664 14167 11667 14170 11666 14173 11663 14172 11660 14171
11657 14170 11654 14170 11650 14172 11648 14174 11647 14176 11643 14177
11641 14179 11637 14180 11632 14181 27 P
11681 14167 11684 14166 11685 14169 11686 14173 11686 14176 11689 14178
11686 14180 11683 14179 11683 14175 11682 14172 11681 14167 11 P
11674 14170 11676 14168 11677 14170 11679 14173 11679 14175 11677 14174
11674 14170 7 P
11637 14308 11640 14302 11642 14295 11642 14289 11642 14282 11643 14275
11642 14268 11645 14262 11652 14261 11658 14260 11661 14254 11665 14249
11668 14243 11670 14236 11667 14230 11661 14228 11654 14226 11656 14220
11656 14213 11662 14210 11669 14208 11676 14209 11682 14208 11689 14206
11696 14205 11696 14211 11696 14218 11702 14215 11708 14211 11714 14210
11719 14215 11724 14220 11725 14227 11723 14233 11721 14240 11723 14246
11717 14250 11717 14257 11717 14264 11716 14271 11712 14276 11712 14283
11715 14289 11712 14296 11709 14301 11707 14308 11702 14313 11696 14315
11690 14319 11684 14322 50 P
11684 14322 11678 14326 11674 14331 11667 14333 11661 14329 11655 14329
11652 14322 11649 14317 11643 14313 11637 14308 10 P
11607 14262 11610 14259 11614 14256 11617 14254 11621 14252 11625 14250
11629 14248 11632 14246 11636 14243 11636 14239 11634 14235 11637 14232
11641 14229 11643 14232 11647 14235 11649 14238 11652 14241 11655 14245
11655 14249 11654 14253 11650 14256 11647 14258 11642 14258 11638 14259
11635 14261 11631 14263 11628 14267 11628 14271 11628 14275 11629 14280
11631 14283 11629 14287 11625 14286 11621 14283 11617 14282 11614 14279
11614 14275 11615 14271 11615 14266 11615 14262 11607 14262 41 P
11576 14291 11580 14288 11583 14286 11587 14284 11591 14285 11593 14289
11597 14290 11601 14291 11605 14294 11609 14294 11613 14291 11616 14293
11620 14295 11624 14298 11625 14302 11628 14306 11629 14309 11628 14314
11626 14317 11622 14314 11618 14313 11614 14313 11610 14313 11606 14315

11602 14318 11598 14319 11594 14318 11590 14318 11587 14315 11583 14312
11583 14308 11581 14304 11583 14300 11581 14296 11576 14291 35 P
11591 14228 11593 14225 11595 14222 11597 14220 11600 14219 11602 14216
11605 14216 11609 14215 11607 14218 11604 14220 11604 14223 11604 14226
11605 14229 11602 14229 11598 14229 11595 14229 11591 14228 17 P
11560 14237 11563 14235 11566 14233 11568 14234 11571 14235 11574 14233
11576 14231 11579 14229 11582 14229 11583 14232 11584 14235 11582 14236
11580 14239 11578 14242 11577 14245 11574 14245 11573 14241 11570 14240
11567 14240 11563 14240 11560 14237 21 P
11546 14242 11547 14240 11550 14240 11553 14240 11555 14241 11554 14243
11551 14244 11549 14246 11546 14242 9 P
11602 14269 11604 14269 11605 14271 11605 14274 11603 14275 11602 14273
11602 14269 7 P
11585 14265 11587 14263 11590 14263 11593 14263 11595 14264 11598 14264
11596 14266 11593 14266 11590 14266 11588 14267 11585 14265 11 P
10886 14335 10888 14332 10891 14329 10895 14326 10895 14322 10897 14318
10899 14314 10902 14311 10906 14312 10910 14312 10915 14311 10919 14312
10922 14309 10926 14311 10926 14315 10929 14319 10926 14322 10922 14323
10921 14327 10922 14331 10926 14333 10930 14331 10929 14327 10933 14327
10936 14331 10941 14330 10938 14333 10942 14333 10940 14337 10938 14341
10936 14345 10932 14347 10928 14347 10924 14346 10919 14345 10915 14344
10911 14343 10908 14341 10903 14339 10900 14337 10896 14338 10892 14340
10886 14335 43 P
10915 14258 10918 14256 10921 14254 10924 14254 10924 14251 10923 14248
10926 14249 10929 14250 10932 14250 10935 14250 10937 14253 10939 14256
10939 14259 10937 14262 10937 14265 10939 14267 10942 14269 10941 14272
10938 14273 10935 14273 10932 14275 10931 14272 10929 14269 10926 14268
10925 14265 10923 14262 10922 14259 10919 14258 10915 14258 29 P
10945 14226 10948 14227 10950 14230 10952 14232 10956 14233 10958 14232
10962 14232 10962 14235 10963 14238 10960 14238 10957 14238 10953 14236
10951 14237 10950 14234 10947 14232 10945 14226 16 P
11058 14266 11057 14262 11060 14261 11064 14262 11066 14261 11070 14261
11071 14263 11073 14266 11073 14269 11077 14268 11078 14271 11077 14274
11078 14277 11079 14280 11076 14280 11072 14280 11069 14280 11066 14279
11069 14276 11072 14275 11071 14272 11071 14269 11067 14269 11064 14270
11062 14272 11061 14268 11058 14266 27 P
11117 14261 11120 14261 11124 14260 11124 14257 11123 14254 11125 14256
11127 14259 11130 14261 11132 14262 11135 14265 11137 14267 11137 14270
11140 14269 11143 14271 11141 14273 11140 14276 11137 14275 11134 14274
11131 14273 11129 14272 11127 14268 11124 14267 11122 14265 11117 14261
24 P
11154 14212 11157 14210 11159 14208 11163 14209 11165 14212 11168 14211
11171 14213 11174 14215 11176 14217 11177 14220 11179 14222 11182 14225

11184 14227 11187 14228 11190 14230 11192 14232 11194 14235 11195 14238
11198 14240 11200 14242 11201 14246 11203 14248 11205 14251 11207 14254
11204 14253 11200 14253 11198 14252 11195 14249 11193 14247 11190 14245
11187 14246 11185 14243 11182 14242 11179 14240 11180 14237 11182 14234
11183 14231 11183 14228 11180 14230 11180 14233 11178 14235 11175 14237
11172 14236 11169 14235 11171 14232 11171 14229 11168 14227 11166 14224
11165 14221 11163 14219 50 P
11163 14219 11159 14218 11157 14216 11154 14212 4 P
11355 14216 11358 14214 11359 14212 11358 14208 11359 14205 11362 14204
11365 14206 11367 14209 11369 14212 11371 14214 11372 14217 11372 14220
11369 14221 11366 14220 11362 14219 11359 14219 11356 14220 11355 14216
18 P
11439 14279 11441 14276 11444 14277 11447 14277 11450 14278 11453 14280
11454 14283 11456 14286 11453 14285 11450 14283 11448 14281 11446 14282
11443 14281 11439 14279 14 P
11383 14288 11386 14287 11389 14287 11391 14290 11389 14293 11389 14296
11387 14294 11386 14291 11383 14288 9 P
11152 14241 11156 14240 11158 14239 11161 14237 11163 14234 11165 14236
11163 14238 11165 14241 11163 14243 11159 14244 11156 14243 11152 14241
12:00 PM
10928 14640 10931 14637 10935 14636 10939 14637 10936 14641 10938 14644
10942 14642 10946 14642 10949 14645 10951 14642 10955 14639 10959 14637
10963 14635 10967 14634 10971 14633 10971 14638 10974 14635 10978 14634
10981 14638 10979 14641 10977 14645 10975 14649 10971 14651 10971 14655
10967 14655 10963 14655 10959 14658 10955 14659 10951 14661 10948 14664
10946 14659 10943 14656 10942 14652 10937 14651 10933 14651 10933 14646
10934 14642 10928 14640 38 P
11018 14724 11017 14728 11015 14731 11014 14727 11015 14723 11012 14719
11014 14716 11018 14716 11018 14711 11015 14713 11011 14710 11010 14706
11013 14703 11016 14707 11019 14709 11023 14710 11025 14715 11025 14719
11028 14723 11028 14727 11025 14723 11018 14724 22 P
10903 15169 10907 15169 10910 15172 10914 15175 10918 15174 10922 15177
10925 15180 10927 15184 10931 15186 10934 15189 10935 15193 10939 15194
10938 15198 10942 15199 10946 15197 10949 15200 10951 15204 10950 15209
10946 15208 10942 15207 10944 15210 10941 15212 10944 15215 10939 15216
10942 15219 10938 15222 10937 15226 10941 15229 10945 15229 10944 15233
10941 15234 10937 15238 10933 15237 10929 15238 10925 15236 10922 15233
10925 15231 10924 15227 10921 15225 10924 15221 10924 15217 10921 15215
10917 15213 10916 15209 10911 15210 10908 15206 10905 15203 10904 15199
10908 15201 10912 15202 50 P
10912 15202 10916 15203 10920 15201 10917 15198 10913 15197 10915 15192
10919 15191 10919 15187 10918 15183 10914 15183 10909 15184 10909 15179
10908 15175 10903 15169 14 P

10808 15183 10812 15184 10814 15188 10817 15191 10821 15190 10824 15186
10827 15190 10827 15194 10831 15193 10832 15188 10836 15187 10840 15186
10844 15188 10847 15191 10848 15196 10851 15198 10852 15203 10848 15204
10844 15206 10840 15207 10838 15204 10834 15204 10830 15204 10827 15201
10823 15203 10820 15201 10820 15197 10817 15193 10813 15193 10811 15190
10808 15183 31 P
10846 15170 10849 15167 10852 15170 10857 15171 10856 15176 10859 15179
10856 15182 10858 15186 10854 15188 10850 15186 10848 15183 10845 15179
10848 15175 10846 15170 14 P
10810 15131 10814 15131 10813 15127 10816 15125 10819 15126 10822 15126
10823 15129 10824 15132 10821 15134 10820 15137 10820 15140 10817 15139
10815 15137 10811 15135 10810 15131 15 P
10820 15146 10823 15145 10826 15144 10825 15147 10826 15151 10823 15152
10821 15151 10820 15146 8 P
10819 15158 10822 15157 10824 15159 10825 15161 10824 15163 10822 15164
10820 15162 10819 15158 8 P
9403 15100 9404 15094 9404 15087 9410 15084 9416 15082 9423 15085
9429 15084 9433 15079 9436 15073 9439 15078 9445 15082 9449 15088
9454 15092 9460 15092 9467 15093 9473 15089 9479 15086 9486 15085
9492 15087 9496 15093 9501 15098 9508 15099 9511 15105 9515 15111
9521 15108 9527 15113 9530 15119 9531 15125 9531 15132 9531 15139
9527 15145 9526 15151 9519 15153 9513 15155 9507 15159 9501 15162
9494 15162 9498 15157 9499 15150 9495 15145 9489 15141 9483 15141
9478 15137 9472 15134 9465 15134 9458 15134 9452 15133 9445 15131
9438 15130 9433 15134 50 P
9433 15134 9431 15127 9431 15120 9426 15117 9420 15113 9417 15107
9412 15102 9403 15100 8 P
9500 15059 9497 15065 9491 15067 9484 15069 9478 15071 9471 15069
9466 15065 9462 15071 9457 15075 9450 15073 9446 15068 9443 15062
9443 15055 9438 15050 9432 15049 9425 15048 9423 15042 9425 15035
9418 15034 9422 15028 9420 15022 9416 15016 9422 15014 9429 15012
9433 15017 9435 15024 9440 15028 9444 15033 9449 15038 9450 15045
9456 15048 9462 15049 9469 15048 9471 15042 9466 15038 9462 15032
9458 15027 9455 15020 9452 15014 9458 15013 9465 15011 9472 15010
9477 15006 9484 15006 9489 15010 9490 15016 9494 15022 9497 15028
9496 15035 9493 15041 50 P
9493 15041 9488 15046 9482 15048 9489 15047 9496 15047 9501 15050
9500 15059 7 P
9866 14927 9870 14921 9869 14915 9874 14910 9879 14906 9884 14909
9888 14915 9889 14922 9895 14917 9901 14916 9900 14923 9899 14929
9896 14936 9898 14942 9904 14944 9897 14945 9893 14940 9887 14938
9880 14937 9877 14932 9866 14927 21 P
10217 14889 10223 14889 10228 14887 10230 14882 10225 14885 10222 14880

10222 14875 10222 14870 10223 14864 10228 14862 10231 14866 10235 14870
10240 14873 10244 14876 10244 14882 10240 14886 10238 14890 10235 14895
10235 14901 10233 14896 10228 14893 10223 14894 10217 14889 23 P
10036 14860 10039 14856 10042 14854 10038 14851 10042 14849 10045 14846
10050 14846 10054 14844 10058 14843 10061 14845 10061 14849 10056 14850
10055 14855 10052 14858 10049 14856 10045 14859 10045 14863 10041 14863
10036 14860 19 P
10143 15145 10140 15142 10142 15138 10142 15134 10139 15138 10135 15138
10135 15142 10130 15143 10128 15140 10126 15135 10124 15132 10121 15129
10119 15125 10124 15124 10128 15124 10130 15120 10132 15117 10135 15113
10139 15113 10143 15113 10145 15117 10148 15120 10151 15124 10153 15127
10155 15131 10156 15135 10153 15139 10150 15141 10143 15145 29 P
9939 14993 9942 14990 9946 14988 9950 14987 9955 14986 9958 14984
9962 14982 9966 14981 9970 14980 9974 14978 9974 14974 9977 14971
9979 14975 9978 14979 9983 14980 9987 14980 9991 14979 9996 14978
10000 14979 10004 14978 10008 14976 10012 14974 10015 14977 10018 14980
10022 14982 10017 14982 10013 14983 10009 14985 10005 14986 10002 14990
9998 14991 9996 14987 9992 14986 9988 14985 9983 14985 9979 14986
9975 14986 9971 14989 9969 14993 9969 14996 9966 14994 9963 14990
9960 14993 9960 14997 9956 14997 9953 14994 9949 14996 9946 14994
9939 14993 49 P
9996 14961 10000 14958 10004 14958 10007 14961 10011 14960 10015 14961
10020 14961 10024 14962 10028 14961 10027 14957 10026 14953 10024 14949
10024 14945 10027 14948 10030 14950 10034 14953 10038 14953 10038 14958
10042 14959 10046 14961 10050 14963 10046 14965 10044 14969 10040 14970
10042 14974 10038 14972 10036 14968 10032 14969 10028 14969 10023 14969
10020 14967 10016 14966 10011 14966 10007 14966 10003 14965 9996 14961
36 P
10050 14963 10053 14962 10056 14962 10059 14965 10056 14966 10054 14965
10050 14963 7 P
10056 14960 10060 14959 10062 14956 10064 14959 10067 14960 10070 14959
10073 14961 10071 14963 10068 14962 10064 14961 10061 14962 10056 14960
12:00 PM
10167 15088 10170 15084 10172 15081 10177 15080 10181 15077 10181 15081
10181 15086 10185 15086 10183 15089 10178 15091 10174 15091 10170 15093
10167 15088 13 P
10153 14988 10151 14985 10148 14984 10149 14981 10152 14980 10155 14981
10157 14985 10153 14988 8 P
9939 14957 9942 14955 9943 14952 9947 14953 9949 14952 9953 14953
9956 14953 9959 14953 9961 14956 9963 14958 9960 14960 9957 14959
9954 14959 9950 14960 9947 14960 9944 14960 9939 14957 17 P
9981 14948 9983 14946 9985 14947 9988 14948 9990 14950 9990 14954
9989 14956 9988 14954 9984 14954 9984 14951 9981 14948 11 P

10067 14880 10068 14877 10071 14876 10075 14877 10078 14877 10081 14876
10083 14879 10080 14881 10077 14882 10075 14881 10072 14881 10067 14880

12:00 PM

10097 14876 10099 14874 10101 14872 10102 14869 10105 14867 10106 14870
10108 14873 10106 14875 10102 14875 10097 14876 10 P
10083 14863 10085 14861 10088 14862 10090 14864 10092 14867 10090 14870
10088 14871 10087 14868 10083 14867 10083 14863 10 P
10429 15267 10432 15267 10435 15270 10438 15270 10441 15271 10444 15271
10447 15271 10445 15274 10444 15277 10441 15278 10438 15277 10435 15275
10432 15274 10430 15272 10429 15267 15 P
9908 14951 9911 14951 9914 14949 9916 14950 9920 14951 9921 14954
9919 14958 9916 14958 9913 14956 9911 14954 9908 14951 11 P
9907 14974 9910 14973 9913 14972 9916 14970 9918 14968 9921 14968
9924 14969 9922 14971 9919 14973 9916 14973 9914 14976 9911 14975
9907 14974 13 P
9668 14751 9669 14748 9669 14745 9671 14743 9674 14744 9675 14747
9675 14750 9673 14752 9674 14755 9675 14758 9672 14760 9669 14757
9671 14754 9668 14751 14 P
9196 15599 9199 15596 9203 15598 9205 15601 9209 15603 9214 15604
9218 15604 9219 15609 9218 15613 9218 15617 9215 15620 9211 15622
9209 15618 9204 15618 9204 15614 9205 15609 9201 15609 9198 15606
9196 15599 19 P
8356 14785 8360 14787 8364 14788 8368 14790 8371 14793 8375 14796
8378 14799 8379 14803 8377 14807 8373 14809 8370 14813 8368 14809
8370 14804 8374 14803 8375 14799 8371 14797 8368 14794 8365 14790
8361 14788 8356 14785 20 P
8392 14833 8396 14834 8401 14835 8405 14836 8409 14837 8413 14838
8416 14842 8417 14846 8419 14850 8423 14851 8424 14856 8422 14860
8418 14859 8415 14855 8413 14851 8410 14848 8408 14844 8404 14841
8401 14839 8397 14837 8392 14833 21 P
8475 14886 8471 14884 8467 14882 8463 14880 8459 14878 8455 14876
8455 14872 8452 14869 8448 14867 8444 14864 8441 14862 8437 14860
8437 14856 8434 14853 8430 14850 8426 14849 8430 14847 8434 14849
8438 14850 8442 14851 8446 14853 8449 14855 8452 14859 8455 14862
8458 14866 8461 14869 8464 14871 8467 14875 8470 14877 8473 14881
8475 14886 31 P
8397 14849 8399 14847 8402 14848 8404 14850 8405 14853 8407 14856
8407 14859 8405 14862 8404 14859 8402 14856 8400 14854 8398 14851
8397 14849 13 P
8272 14733 8276 14734 8279 14737 8282 14740 8285 14743 8287 14747
8290 14750 8292 14754 8294 14758 8295 14762 8293 14766 8290 14762
8289 14758 8286 14756 8282 14753 8282 14749 8281 14744 8279 14741
8275 14738 8272 14733 20 P

8293 14780 8292 14777 8295 14776 8297 14777 8299 14780 8301 14783
8301 14786 8299 14784 8295 14783 8293 14780 10 P
8310 14794 8313 14793 8315 14795 8319 14796 8320 14799 8321 14802
8322 14804 8324 14807 8326 14810 8323 14808 8321 14806 8319 14804
8318 14801 8315 14799 8313 14797 8310 14794 16 P
9247 14414 9250 14412 9254 14414 9255 14418 9260 14419 9264 14420
9266 14424 9266 14428 9262 14430 9258 14428 9254 14427 9251 14424
9251 14419 9247 14414 14 P
9224 14344 9228 14341 9231 14343 9236 14342 9240 14342 9243 14345
9240 14348 9235 14349 9231 14349 9231 14354 9230 14358 9228 14362
9225 14359 9225 14354 9224 14350 9224 14344 16 P
9167 14236 9169 14233 9172 14229 9176 14227 9179 14225 9181 14228
9181 14233 9181 14237 9181 14241 9177 14243 9173 14244 9170 14242
9167 14236 13 P
9204 14249 9207 14246 9210 14242 9212 14239 9215 14236 9220 14236
9223 14233 9226 14236 9225 14240 9223 14245 9221 14248 9217 14248
9213 14250 9209 14252 9204 14249 15 P
9221 14208 9224 14206 9229 14206 9231 14202 9234 14199 9238 14196
9240 14200 9238 14205 9238 14209 9240 14213 9238 14216 9235 14220
9233 14224 9231 14228 9228 14230 9224 14228 9223 14223 9223 14219
9225 14215 9225 14211 9221 14208 21 P
9309 14273 9311 14271 9314 14269 9317 14272 9317 14275 9314 14276
9310 14277 9309 14273 8 P
9289 14466 9292 14466 9295 14466 9298 14468 9301 14466 9301 14470
9299 14472 9298 14475 9295 14475 9294 14472 9291 14471 9289 14466
12:00 PM
9287 14456 9289 14453 9291 14450 9294 14448 9295 14446 9296 14448
9295 14452 9297 14453 9294 14454 9294 14457 9291 14459 9287 14456
12:00 PM
9010 13021 9013 13017 9017 13016 9020 13019 9024 13021 9024 13026
9021 13030 9021 13034 9020 13038 9016 13038 9015 13034 9013 13030
9010 13027 9010 13021 14 P
8704 12481 8706 12477 8709 12474 8712 12471 8714 12467 8719 12466
8723 12465 8726 12467 8731 12469 8728 12472 8724 12474 8720 12475
8716 12475 8711 12477 8709 12481 8704 12481 16 P
9538 10853 9542 10847 9547 10843 9552 10838 9557 10833 9561 10828
9566 10823 9571 10818 9577 10821 9584 10817 9590 10815 9595 10810
9600 10806 9606 10801 9611 10797 9615 10792 9620 10787 9626 10783
9632 10779 9638 10776 9645 10773 9648 10768 9654 10764 9661 10764
9668 10761 9673 10757 9679 10754 9686 10752 9692 10748 9699 10747
9705 10744 9711 10740 9716 10737 9723 10733 9728 10729 9735 10727
9741 10725 9747 10721 9753 10718 9760 10716 9766 10715 9773 10716
9780 10714 9787 10712 9793 10711 9800 10708 9806 10705 9812 10702

9819 10699 9825 10697 50 P
9825 10697 9832 10694 9838 10692 9845 10691 9852 10689 9859 10688
9866 10687 9872 10691 9877 10694 9880 10700 9884 10706 9890 10710
9892 10717 9893 10723 9894 10730 9892 10737 9886 10740 9880 10745
9874 10747 9867 10747 9860 10747 9853 10749 9847 10751 9840 10753
9834 10757 9828 10760 9823 10765 9816 10767 9810 10771 9805 10774
9798 10776 9792 10779 9785 10781 9778 10783 9772 10785 9765 10787
9758 10788 9752 10792 9746 10795 9741 10800 9739 10806 9733 10811
9728 10816 9723 10820 9717 10823 9710 10825 9704 10828 9698 10831
9691 10833 9685 10836 50 P
9685 10836 9678 10838 2 P
9678 10838 9672 10841 9668 10847 9665 10853 9661 10859 9658 10865
9654 10871 9650 10876 9644 10880 9639 10884 9632 10888 9626 10890
9619 10891 9612 10893 9605 10892 9598 10892 9592 10890 9585 10888
9578 10886 9572 10884 9567 10879 9562 10874 9557 10870 9551 10866
9545 10862 9538 10853 26 P
9747 10671 9753 10668 9759 10665 9765 10662 9772 10659 9776 10654
9782 10651 9789 10648 9795 10644 9800 10640 9806 10636 9813 10634
9818 10629 9825 10627 9828 10621 9832 10615 9836 10610 9842 10606
9848 10608 9849 10615 9848 10622 9845 10628 9840 10633 9836 10639
9832 10644 9826 10648 9821 10653 9814 10655 9809 10659 9802 10662
9796 10664 9789 10667 9783 10668 9776 10671 9769 10672 9763 10674
9756 10676 9747 10671 38 P
9850 10640 9854 10634 9857 10628 9858 10621 9860 10614 9862 10607
9867 10603 9873 10600 9879 10595 9885 10598 9888 10604 9893 10609
9895 10616 9894 10623 9891 10629 9885 10633 9879 10636 9873 10639
9866 10639 9859 10641 9850 10640 21 P
9983 9961 9977 9965 9971 9966 9971 9973 9966 9978 9960 9981
9958 9974 9951 9972 9955 9966 9960 9961 9966 9957 9969 9951
9971 9944 9976 9939 9977 9932 9978 9925 9977 9918 9975 9912
9978 9906 9983 9901 9988 9896 9993 9891 9996 9885 9998 9878
10004 9875 10010 9879 10015 9883 10021 9886 10028 9885 10035 9886
10042 9883 10045 9877 10052 9877 10059 9878 10065 9879 10072 9876
10078 9873 10085 9871 10091 9869 10098 9867 10105 9865 10111 9861
10117 9857 10123 9854 10130 9855 10137 9856 10143 9859 10140 9865
10138 9872 10141 9878 50 P
10141 9878 10144 9885 10146 9891 10152 9895 10145 9899 10142 9905
10136 9908 10132 9914 10127 9919 10122 9923 10115 9922 10108 9923
10104 9929 10097 9931 10096 9938 10089 9938 10082 9938 10076 9940
10072 9947 10065 9946 10059 9947 10052 9948 10048 9954 10043 9959
10040 9965 10034 9969 10027 9971 10022 9967 10015 9964 10008 9962
10002 9959 9995 9961 9990 9966 9983 9961 34 P
10018 10042 10024 10038 10030 10036 10036 10032 10042 10028 10048 10024

10055 10023 10061 10023 10068 10023 10075 10021 10081 10018 10088 10016
10085 10023 10083 10029 10088 10034 10090 10041 10085 10046 10090 10052
10095 10057 10088 10057 10083 10062 10085 10068 10079 10072 10072 10070
10066 10073 10064 10066 10058 10063 10051 10061 10047 10057 10040 10059
10033 10059 10029 10053 10023 10050 10018 10042 34 P
10109 10149 10111 10143 10110 10136 10115 10131 10113 10124 10119 10121
10125 10118 10132 10117 10131 10124 10138 10125 10143 10129 10149 10125
10156 10125 10163 10124 10170 10124 10176 10120 10183 10120 10180 10126
10174 10130 10179 10134 10175 10140 10169 10142 10162 10142 10155 10143
10153 10149 10146 10149 10149 10143 10143 10144 10136 10146 10129 10147
10123 10145 10116 10148 10109 10149 33 P
10170 10278 10177 10279 10184 10279 10190 10280 10197 10283 10204 10283
10211 10283 10218 10283 10224 10285 10218 10289 10223 10295 10216 10295
10209 10294 10204 10290 10197 10288 10192 10293 10185 10295 10182 10301
10176 10304 10169 10303 10175 10299 10176 10292 10175 10285 10170 10278
24 P
10017 9723 10024 9723 10028 9729 10035 9729 10040 9725 10047 9725
10049 9718 10055 9715 10061 9712 10061 9705 10061 9698 10068 9698
10070 9704 10073 9698 10079 9702 10081 9695 10088 9694 10091 9700
10092 9707 10096 9713 10091 9717 10089 9724 10083 9727 10078 9731
10071 9734 10065 9737 10061 9743 10056 9748 10058 9755 10056 9762
10051 9767 10047 9762 10040 9760 10033 9760 10028 9756 10027 9749
10025 9742 10023 9735 10017 9723 39 P
10098 9792 10104 9788 10110 9784 10114 9779 10119 9774 10125 9770
10130 9765 10136 9764 10142 9760 10149 9763 10144 9769 10141 9775
10141 9782 10136 9786 10142 9789 10137 9795 10131 9797 10124 9800
10117 9802 10111 9800 10104 9799 10098 9792 22 P
10146 9852 10152 9848 10156 9842 10161 9838 10167 9834 10170 9827
10175 9822 10169 9817 10170 9811 10172 9804 10177 9799 10184 9797
10190 9802 10185 9807 10183 9814 10186 9820 10192 9824 10198 9827
10198 9834 10196 9841 10190 9841 10183 9845 10190 9845 10197 9846
10192 9852 10192 9859 10186 9862 10181 9859 10173 9857 10167 9859
10160 9858 10156 9863 10151 9859 10146 9852 34 P
9909 9874 9914 9870 9916 9863 9922 9859 9928 9860 9933 9865
9939 9868 9945 9872 9950 9876 9953 9883 9950 9889 9948 9896
9947 9903 9943 9909 9937 9912 9933 9918 9930 9924 9923 9924
9916 9922 9915 9915 9912 9909 9909 9902 9909 9895 9912 9889
9913 9882 9909 9874 26 P
10051 9976 10055 9970 10059 9965 10066 9967 10070 9972 10071 9979
10078 9982 10083 9987 10076 9991 10070 9993 10063 9995 10057 9992
10053 9987 10051 9976 14 P
10112 10019 10117 10015 10122 10020 10129 10021 10136 10020 10142 10021
10149 10024 10153 10030 10160 10032 10165 10036 10163 10030 10165 10023

10170 10019 10176 10022 10181 10028 10178 10034 10175 10040 10168 10043
10161 10042 10155 10041 10148 10040 10141 10039 10134 10039 10132 10032
10128 10027 10122 10025 10112 10019 27 P
10102 10027 10103 10020 10110 10024 10116 10027 10123 10028 10129 10029
10125 10035 10119 10037 10112 10037 10105 10038 10102 10027 11 P
10327 10294 10329 10290 10331 10294 10336 10294 10338 10290 10343 10289
10342 10293 10341 10297 10343 10302 10344 10306 10340 10306 10336 10304
10332 10302 10330 10298 10327 10294 15 P
10350 10303 10352 10299 10357 10299 10361 10299 10363 10295 10366 10292
10366 10296 10365 10301 10364 10305 10359 10306 10356 10309 10350 10303
12:00 PM
10229 10256 10234 10256 10238 10254 10242 10253 10240 10257 10244 10260
10247 10263 10244 10265 10240 10269 10236 10270 10234 10267 10230 10264
10229 10256 13 P
10168 9888 10171 9884 10168 9881 10169 9876 10172 9874 10177 9874
10177 9878 10176 9882 10176 9887 10179 9890 10184 9889 10188 9891
10186 9895 10182 9896 10180 9900 10182 9904 10181 9908 10178 9905
10173 9903 10169 9903 10171 9899 10170 9894 10168 9888 23 P
9956 9826 9959 9822 9962 9819 9967 9819 9971 9820 9975 9821
9980 9822 9984 9823 9988 9826 9988 9830 9988 9834 9985 9838
9981 9839 9978 9842 9977 9847 9974 9849 9972 9853 9968 9852
9966 9847 9962 9846 9959 9843 9958 9839 9958 9834 9956 9826
24 P
9993 9777 9989 9774 9985 9772 9982 9769 9978 9766 9977 9761
9979 9757 9978 9753 9982 9750 9986 9750 9990 9749 9995 9750
9997 9754 10000 9757 10001 9762 10004 9765 10006 9769 10009 9773
10009 9777 10007 9781 10003 9781 9998 9780 9993 9777 23 P
10097 9757 10101 9756 10103 9753 10106 9749 10109 9746 10112 9743
10116 9741 10119 9737 10123 9739 10128 9737 10131 9736 10135 9739
10139 9739 10144 9739 10144 9743 10140 9742 10139 9747 10135 9748
10132 9745 10128 9746 10123 9746 10119 9748 10116 9750 10112 9753
10115 9757 10117 9761 10113 9762 10110 9765 10105 9765 10102 9763
10097 9757 31 P
10128 9809 10131 9807 10136 9806 10140 9806 10144 9807 10148 9809
10152 9811 10152 9815 10156 9818 10154 9822 10150 9821 10145 9820
10141 9821 10137 9821 10132 9820 10131 9815 10128 9809 17 P
9940 9928 9943 9925 9948 9925 9951 9922 9950 9918 9954 9916
9958 9916 9959 9921 9961 9925 9963 9929 9962 9934 9964 9937
9961 9940 9957 9943 9953 9942 9949 9940 9946 9938 9943 9934
9940 9928 19 P
9999 9738 10000 9735 10000 9731 10001 9728 10000 9725 10003 9727
10005 9729 10008 9731 10009 9735 10011 9737 10014 9739 10014 9742
10013 9745 10009 9746 10007 9745 10003 9744 10002 9741 9999 9738

18 P
9987 9982 9990 9983 9993 9982 9995 9979 9997 9977 9995 9975
9993 9972 9996 9972 9999 9974 10002 9975 10004 9978 10007 9980
10008 9983 10011 9985 10009 9987 10008 9991 10007 9994 10003 9993
10000 9991 10000 9988 9998 9991 9995 9992 9993 9990 9994 9987
9990 9986 9987 9982 26 P
9956 10050 9956 10046 9957 10043 9960 10045 9963 10047 9966 10050
9966 10053 9964 10056 9967 10058 9966 10061 9968 10063 9970 10066
9971 10069 9969 10071 9966 10069 9966 10066 9964 10063 9961 10065
9960 10063 9960 10059 9960 10056 9957 10053 9956 10050 23 P
10347 9977 10348 9974 10350 9971 10353 9971 10356 9971 10359 9971
10359 9974 10357 9977 10354 9979 10351 9979 10347 9977 11 P
9949 9390 9953 9390 9957 9387 9959 9384 9962 9380 9966 9380
9969 9377 9970 9372 9970 9367 9968 9364 9965 9360 9969 9361
9974 9361 9978 9361 9982 9360 9981 9356 9980 9351 9984 9350
9988 9348 9991 9351 9996 9352 9994 9356 9994 9360 9995 9365
9999 9366 10003 9369 10000 9372 9995 9373 9998 9376 10002 9379
10005 9382 10003 9386 10002 9390 10002 9395 9999 9398 10002 9402
10001 9406 9997 9410 9994 9412 9990 9414 9987 9418 9984 9421
9980 9422 9976 9419 9976 9415 9972 9413 9968 9410 9964 9407
9961 9405 9957 9403 50 P
9957 9403 9953 9400 9950 9397 9949 9390 4 P
9905 9385 9907 9381 9911 9381 9916 9381 9920 9380 9924 9378
9928 9376 9930 9372 9933 9369 9938 9370 9942 9369 9946 9371
9950 9373 9947 9377 9952 9377 9954 9380 9957 9384 9955 9387
9950 9387 9947 9390 9948 9394 9946 9398 9942 9396 9937 9396
9933 9394 9928 9394 9924 9396 9921 9399 9916 9398 9913 9396
9909 9399 9906 9397 9908 9392 9905 9385 34 P
9908 9359 9913 9358 9917 9358 9916 9354 9921 9353 9923 9350
9927 9347 9931 9350 9931 9354 9933 9358 9930 9361 9933 9365
9930 9368 9927 9371 9922 9372 9918 9372 9915 9369 9915 9364
9908 9359 19 P
10016 9407 10021 9409 10025 9409 10027 9413 10030 9416 10033 9413
10037 9413 10041 9417 10044 9420 10040 9422 10035 9420 10031 9423
10031 9427 10036 9429 10033 9432 10036 9436 10031 9436 10028 9438
10025 9435 10027 9430 10024 9427 10028 9425 10024 9423 10022 9419
10017 9419 10018 9414 10016 9407 27 P
9607 8814 9610 8807 9611 8800 9614 8794 9618 8788 9622 8783
9621 8776 9615 8772 9621 8769 9620 8762 9627 8764 9634 8764
9640 8767 9647 8770 9654 8772 9653 8779 9660 8776 9665 8772
9668 8778 9662 8783 9664 8790 9669 8786 9676 8789 9678 8782
9685 8784 9681 8790 9687 8793 9687 8800 9680 8800 9674 8804
9671 8810 9672 8816 9669 8823 9667 8830 9669 8836 9674 8842

9681 8843 9679 8849 9673 8854 9667 8857 9661 8861 9654 8860
9647 8858 9643 8852 9641 8845 9644 8838 9637 8835 9633 8829
9628 8824 9621 8821 50 P
9621 8821 9618 8816 9607 8814 3 P
9567 8708 9571 8710 9576 8711 9580 8711 9584 8713 9588 8715
9593 8713 9591 8709 9593 8704 9597 8707 9601 8707 9605 8709
9610 8710 9614 8712 9617 8715 9621 8717 9618 8719 9614 8722
9611 8725 9608 8728 9604 8730 9600 8728 9595 8727 9591 8729
9588 8726 9584 8725 9584 8729 9580 8729 9579 8724 9576 8722
9571 8719 9567 8717 9568 8713 9567 8708 34 P
9558 8649 9561 8645 9564 8649 9568 8651 9572 8653 9576 8655
9579 8652 9583 8650 9583 8654 9587 8657 9590 8660 9593 8664
9598 8664 9593 8666 9590 8664 9585 8663 9581 8660 9577 8661
9574 8665 9571 8669 9567 8669 9566 8664 9563 8662 9562 8658
9561 8653 9558 8649 26 P
9593 8637 9598 8636 9602 8634 9606 8636 9611 8634 9614 8637
9619 8637 9621 8640 9626 8638 9630 8637 9634 8634 9638 8636
9639 8640 9639 8644 9639 8649 9635 8651 9639 8653 9640 8649
9644 8652 9646 8656 9644 8659 9645 8664 9640 8665 9638 8661
9634 8659 9630 8660 9626 8658 9629 8654 9627 8650 9622 8650
9618 8649 9616 8652 9613 8656 9611 8651 9607 8649 9604 8645
9600 8644 9596 8641 9593 8637 39 P
9641 8739 9637 8737 9633 8735 9631 8731 9634 8727 9635 8723
9640 8722 9643 8725 9647 8728 9651 8731 9655 8732 9659 8734
9663 8736 9658 8736 9654 8735 9650 8737 9645 8738 9641 8739
18 P
9580 8434 9583 8431 9587 8428 9590 8425 9590 8429 9590 8434
9593 8432 9598 8431 9602 8428 9606 8429 9610 8426 9610 8430
9607 8434 9603 8435 9599 8438 9596 8441 9592 8444 9588 8446
9584 8447 9588 8444 9591 8441 9587 8442 9585 8438 9580 8434
24 P
9904 5701 9908 5697 9910 5691 9913 5686 9919 5684 9922 5679
9927 5680 9926 5685 9928 5691 9929 5697 9929 5703 9924 5704
9918 5705 9913 5703 9904 5701 15 P
9315 5477 9319 5475 9324 5476 9326 5480 9330 5484 9334 5486
9335 5491 9332 5494 9328 5495 9323 5493 9319 5490 9321 5486
9318 5482 9315 5477 14 P
8866 6170 8872 6167 8878 6162 8882 6156 8889 6154 8896 6156
8902 6159 8902 6166 8907 6172 8912 6176 8919 6180 8924 6185
8931 6185 8938 6185 8945 6182 8942 6188 8940 6195 8939 6202
8935 6208 8930 6203 8928 6210 8921 6207 8920 6200 8918 6207
8914 6201 8911 6195 8911 6202 8912 6209 8905 6209 8901 6203
8897 6197 8896 6190 8892 6196 8889 6202 8883 6198 8880 6192

8876 6186 8882 6188 8885 6182 8878 6182 8884 6179 8891 6179
8898 6179 8896 6172 8889 6168 8886 6162 8881 6167 8875 6172
8872 6178 8866 6170 50 P
8870 6241 8875 6237 8880 6242 8886 6238 8891 6232 8893 6225
8897 6219 8899 6226 8898 6232 8902 6238 8899 6244 8896 6251
8889 6251 8882 6249 8875 6248 8870 6241 16 P
8828 6183 8835 6185 8842 6186 8849 6186 8851 6193 8852 6200
8856 6206 8855 6213 8854 6220 8860 6223 8866 6228 8859 6229
8853 6234 8848 6229 8846 6222 8846 6215 8846 6208 8841 6202
8846 6198 8844 6191 8837 6190 8828 6183 22 P
8840 6064 8845 6063 8849 6064 8854 6064 8852 6068 8855 6071
8852 6075 8851 6080 8851 6084 8847 6085 8844 6088 8843 6084
8842 6079 8844 6075 8844 6070 8840 6064 16 P
8801 5982 8805 5978 8807 5974 8807 5970 8811 5967 8812 5972
8816 5975 8819 5978 8820 5983 8818 5987 8813 5987 8809 5985
8801 5982 13 P
8894 6286 8896 6284 8897 6280 8898 6284 8900 6286 8903 6288
8906 6289 8907 6293 8904 6295 8901 6297 8899 6295 8898 6292
8895 6289 8894 6286 14 P
9121 6141 9121 6136 9122 6132 9123 6128 9124 6123 9125 6119
9126 6123 9124 6128 9124 6132 9124 6137 9126 6141 9127 6146
9127 6150 9129 6155 9130 6159 9133 6163 9135 6167 9137 6170
9137 6175 9134 6171 9132 6167 9129 6163 9128 6159 9124 6155
9123 6151 9122 6147 9121 6141 27 P
8918 7285 8924 7288 8931 7291 8938 7290 8943 7295 8942 7288
8942 7280 8935 7282 8932 7275 8937 7271 8933 7264 8938 7259
8943 7255 8950 7252 8955 7247 8960 7242 8967 7240 8971 7247
8967 7253 8961 7257 8963 7264 8969 7267 8976 7271 8981 7276
8988 7277 8993 7282 9000 7283 9005 7278 9012 7281 9012 7288
9005 7289 8999 7290 8992 7294 8987 7299 8985 7306 8985 7313
8987 7319 8993 7324 8999 7328 9006 7330 9010 7324 9016 7326
9021 7332 9025 7338 9018 7337 9010 7337 9003 7339 8999 7345
8992 7342 8995 7348 50 P
8995 7348 9000 7354 8996 7360 8990 7363 8985 7368 8978 7366
8972 7369 8968 7375 8967 7382 8972 7387 8976 7393 8969 7393
8962 7390 8957 7385 8954 7378 8948 7382 8943 7376 8941 7370
8933 7369 8929 7364 8926 7358 8926 7351 8930 7344 8936 7346
8941 7352 8948 7352 8951 7346 8949 7339 8946 7332 8946 7325
8945 7318 8944 7311 8950 7308 8955 7312 8958 7319 8963 7314
8971 7313 8971 7306 8966 7302 8959 7298 8952 7298 8947 7304
8940 7305 8933 7306 8929 7300 8922 7299 8920 7292 8918 7285
48 P
8800 7143 8805 7138 8802 7132 8807 7127 8814 7126 8820 7123

8826 7127 8833 7129 8839 7125 8837 7118 8836 7111 8842 7108
8849 7106 8856 7106 8859 7112 8853 7118 8857 7124 8864 7125
8869 7130 8862 7128 8857 7133 8852 7128 8845 7128 8839 7132
8833 7136 8828 7141 8828 7148 8821 7150 8819 7156 8814 7151
8810 7145 8800 7143 32 P
8200 6667 8205 6662 8209 6657 8216 6654 8223 6651 8229 6655
8231 6661 8235 6667 8241 6672 8247 6675 8254 6677 8261 6678
8268 6677 8274 6673 8280 6669 8286 6665 8293 6669 8297 6675
8297 6682 8302 6687 8298 6693 8297 6701 8294 6707 8292 6714
8289 6720 8282 6722 8279 6728 8274 6733 8269 6728 8263 6724
8265 6717 8260 6711 8254 6708 8254 6701 8249 6695 8242 6696
8235 6698 8229 6702 8224 6707 8219 6711 8213 6708 8207 6703
8208 6696 8215 6700 8212 6693 8214 6687 8216 6679 8219 6672
8212 6671 8200 6667 50 P
8088 6694 8093 6688 8096 6682 8102 6678 8109 6675 8116 6673
8123 6672 8129 6676 8134 6681 8140 6685 8145 6690 8146 6697
8149 6704 8156 6705 8156 6712 8156 6720 8156 6727 8152 6732
8145 6729 8141 6735 8136 6740 8129 6738 8124 6742 8117 6745
8110 6747 8111 6739 8105 6735 8104 6728 8100 6722 8095 6717
8091 6711 8093 6704 8100 6705 8105 6710 8112 6713 8113 6706
8107 6702 8100 6699 8088 6694 39 P
7690 6184 7697 6184 7704 6186 7711 6188 7718 6186 7717 6178
7723 6182 7729 6185 7736 6187 7743 6188 7750 6190 7757 6190
7754 6197 7746 6198 7745 6205 7745 6212 7745 6219 7748 6226
7752 6232 7759 6234 7766 6231 7772 6228 7779 6228 7780 6236
7775 6241 7775 6248 7777 6255 7783 6259 7782 6265 7788 6269
7793 6265 7800 6266 7802 6273 7809 6275 7801 6275 7797 6281
7791 6284 7786 6289 7780 6285 7773 6286 7769 6280 7762 6281
7756 6278 7753 6285 7749 6291 7743 6295 7737 6299 7732 6304
7725 6300 7724 6293 50 P
7724 6293 7726 6287 7726 6279 7722 6274 7717 6268 7715 6262
7710 6256 7705 6251 7708 6243 7714 6240 7718 6245 7725 6247
7731 6243 7738 6243 7745 6242 7740 6237 7738 6230 7733 6225
7728 6221 7722 6216 7717 6210 7713 6205 7714 6198 7710 6203
7703 6200 7698 6195 7690 6184 27 P
7526 6113 7522 6119 7516 6121 7509 6120 7503 6115 7497 6110
7493 6105 7497 6099 7504 6097 7511 6096 7518 6097 7526 6096
7533 6094 7538 6090 7545 6087 7552 6087 7558 6089 7566 6090
7573 6088 7574 6081 7581 6084 7586 6079 7593 6083 7596 6089
7595 6096 7594 6103 7600 6107 7597 6114 7591 6117 7589 6124
7590 6132 7583 6132 7578 6126 7576 6119 7569 6120 7564 6126
7558 6129 7551 6127 7547 6121 7544 6115 7538 6110 7535 6104
7526 6113 43 P

7527 6180 7520 6179 7513 6179 7510 6173 7506 6167 7498 6166
7491 6165 7493 6157 7499 6154 7506 6153 7513 6151 7520 6149
7522 6155 7523 6163 7527 6169 7533 6172 7527 6180 17 P
7483 6175 7488 6170 7495 6169 7502 6168 7508 6173 7505 6180
7506 6187 7511 6192 7514 6199 7507 6200 7501 6196 7500 6189
7496 6183 7490 6180 7483 6175 15 P
7334 6043 7339 6038 7343 6031 7349 6028 7355 6024 7360 6018
7366 6016 7373 6018 7378 6013 7381 6006 7387 6004 7394 6002
7388 5997 7392 5991 7398 5987 7405 5987 7407 5980 7414 5980
7417 5987 7422 5992 7415 5995 7408 5992 7403 5997 7402 6004
7402 6011 7404 6018 7402 6025 7398 6031 7397 6038 7392 6044
7385 6046 7379 6042 7372 6042 7365 6043 7360 6049 7356 6054
7348 6055 7342 6051 7334 6043 39 P
8712 7276 8716 7270 8720 7265 8721 7257 8724 7251 8731 7254
8736 7258 8743 7258 8744 7265 8741 7272 8741 7279 8740 7286
8743 7293 8745 7299 8748 7306 8746 7313 8750 7319 8750 7326
8750 7333 8746 7340 8740 7337 8737 7331 8733 7325 8727 7321
8726 7313 8724 7307 8721 7300 8719 7293 8717 7287 8712 7276
30 P
8841 7629 8848 7628 8855 7628 8862 7626 8869 7625 8875 7621
8881 7618 8889 7617 8895 7620 8895 7627 8893 7634 8890 7641
8888 7647 8882 7650 8875 7653 8869 7658 8864 7654 8857 7652
8852 7646 8847 7640 8841 7629 21 P
8846 7624 8850 7622 8853 7619 8857 7616 8862 7616 8866 7617
8871 7618 8874 7620 8871 7623 8867 7624 8862 7625 8858 7626
8853 7627 8846 7624 14 P
8893 7958 8895 7954 8898 7950 8898 7946 8902 7946 8904 7950
8908 7947 8912 7946 8916 7947 8921 7948 8926 7949 8927 7953
8931 7956 8934 7959 8939 7959 8942 7961 8946 7964 8945 7969
8941 7971 8936 7970 8933 7967 8932 7963 8930 7959 8926 7960
8927 7964 8922 7965 8918 7963 8914 7965 8909 7966 8905 7965
8901 7965 8897 7964 8893 7958 33 P
9225 8131 9227 8128 9230 8124 9234 8122 9238 8119 9242 8117
9246 8117 9250 8119 9254 8121 9258 8122 9263 8122 9259 8124
9255 8127 9254 8131 9252 8135 9250 8139 9245 8139 9242 8143
9238 8143 9234 8143 9230 8139 9227 8137 9225 8131 23 P
10666 6777 10661 6779 10657 6777 10653 6776 10648 6778 10644 6776
10642 6771 10638 6770 10636 6766 10632 6764 10635 6761 10640 6761
10645 6760 10649 6758 10653 6758 10657 6759 10662 6761 10667 6760
10671 6758 10675 6761 10678 6764 10682 6766 10684 6770 10689 6773
10685 6775 10681 6777 10676 6778 10672 6780 10666 6777 29 P
11075 6565 11078 6562 11083 6561 11087 6560 11092 6561 11096 6561
11100 6563 11104 6566 11106 6570 11103 6574 11098 6576 11094 6576

11089 6576 11085 6574 11081 6572 11077 6570 11075 6565 17 P
11528 6945 11530 6941 11534 6938 11538 6938 11543 6937 11547 6938
11551 6940 11555 6943 11557 6946 11558 6951 11556 6955 11553 6958
11548 6959 11543 6960 11539 6960 11535 6958 11531 6955 11528 6952
11528 6945 19 P
11761 7064 11763 7060 11766 7057 11770 7055 11775 7054 11779 7054
11783 7058 11787 7059 11791 7057 11796 7057 11800 7058 11804 7059
11806 7064 11805 7068 11803 7072 11801 7076 11799 7080 11796 7082
11791 7084 11787 7086 11783 7088 11778 7090 11774 7090 11771 7087
11770 7082 11771 7078 11770 7073 11766 7071 11762 7073 11761 7064
30 P
11528 7005 11530 7003 11534 7002 11537 7000 11540 6999 11541 7002
11539 7005 11536 7007 11533 7009 11530 7007 11528 7005 11 P
11195 6689 11193 6687 11192 6684 11192 6680 11194 6677 11196 6675
11198 6672 11201 6670 11204 6670 11207 6672 11210 6674 11212 6676
11213 6679 11213 6683 11211 6686 11207 6686 11204 6686 11200 6687
11195 6689 19 P
11192 6736 11189 6737 11188 6740 11187 6743 11184 6745 11181 6746
11178 6745 11174 6744 11173 6741 11176 6739 11174 6736 11171 6734
11168 6734 11171 6733 11174 6732 11178 6733 11181 6732 11185 6733
11187 6730 11191 6728 11193 6730 11195 6733 11192 6736 23 P
11233 6730 11234 6727 11238 6725 11241 6725 11244 6728 11246 6730
11246 6734 11245 6737 11242 6739 11240 6741 11237 6741 11235 6738
11234 6735 11233 6730 14 P
11156 6664 11158 6662 11161 6661 11165 6662 11164 6665 11162 6668
11158 6668 11156 6664 8 P
11164 6670 11167 6671 11170 6674 11172 6676 11173 6679 11170 6681
11167 6678 11165 6676 11164 6670 9 P
10966 6636 10968 6636 10970 6637 10972 6638 10975 6639 10972 6639
10970 6639 10969 6638 10966 6636 9 P
10880 7536 10881 7534 10884 7534 10886 7534 10888 7535 10889 7537
10892 7536 10894 7537 10896 7537 10898 7537 10898 7540 10896 7541
10895 7543 10893 7543 10891 7544 10889 7543 10887 7543 10885 7542
10883 7541 10881 7539 10880 7536 21 P
10921 7532 10923 7530 10924 7529 10926 7530 10925 7532 10924 7534
10921 7532 7 P
10957 7633 10958 7632 10961 7631 10963 7632 10964 7634 10963 7635
10961 7637 10959 7637 10958 7636 10957 7633 10 P
10145 7416 10147 7415 10149 7414 10150 7416 10152 7417 10152 7419
10150 7420 10148 7420 10145 7419 10145 7416 10 P
10164 7336 10165 7334 10168 7335 10168 7337 10164 7336 5 P
10096 6965 10097 6963 10099 6962 10101 6961 10103 6960 10105 6960
10108 6961 10109 6960 10111 6961 10112 6963 10110 6964 10109 6966

10108 6968 10106 6969 10104 6969 10102 6968 10101 6966 10099 6965
10096 6965 19 P
8935 6010 8937 6008 8936 6006 8938 6004 8939 6003 8940 6005
8940 6007 8942 6009 8942 6011 8942 6013 8940 6014 8938 6013
8935 6010 13 P
8749 6089 8751 6087 8753 6088 8755 6088 8757 6090 8757 6092
8755 6093 8753 6094 8751 6094 8750 6091 8749 6089 11 P
8906 6005 8907 6003 8909 6003 8912 6003 8913 6005 8912 6008
8909 6008 8907 6007 8906 6005 9 P
8857 6069 8859 6067 8861 6067 8862 6069 8864 6071 8866 6071
8868 6071 8869 6073 8868 6075 8866 6075 8864 6075 8861 6075
8860 6073 8859 6071 8857 6069 15 P
8850 6141 8851 6139 8853 6139 8855 6140 8858 6140 8860 6141
8862 6141 8864 6143 8865 6145 8867 6145 8868 6147 8866 6148
8864 6147 8861 6146 8859 6145 8857 6145 8855 6143 8854 6141
8852 6143 8851 6144 8850 6141 21 P
8850 6015 8852 6014 8854 6014 8855 6016 8853 6017 8850 6015

6:00 PM

8854 6006 8857 6007 8859 6008 8860 6010 8859 6012 8857 6012
8856 6010 8854 6006 8 P
8165 6666 8167 6664 8171 6664 8174 6663 8176 6665 8179 6667
8182 6665 8185 6668 8185 6671 8188 6672 8191 6672 8193 6675
8193 6678 8194 6682 8193 6684 8190 6687 8187 6689 8184 6690
8181 6688 8178 6688 8174 6687 8174 6683 8174 6679 8173 6676
8169 6675 8168 6671 8168 6668 8165 6666 28 P
7950 6697 7954 6694 7958 6692 7961 6689 7964 6685 7967 6681
7971 6679 7975 6677 7979 6678 7984 6678 7988 6678 7993 6679
7991 6684 7990 6688 7986 6689 7981 6688 7977 6687 7973 6689
7971 6692 7967 6696 7963 6695 7959 6694 7955 6696 7950 6697
24 P
7953 6699 7957 6696 7961 6696 7965 6698 7970 6696 7973 6693
7975 6689 7980 6689 7984 6691 7988 6692 7992 6690 7994 6685
7998 6684 8003 6687 8007 6687 8006 6691 8003 6694 7999 6697
7997 6701 7993 6704 7988 6705 7984 6706 7979 6708 7975 6707
7971 6706 7966 6705 7961 6703 7957 6702 7953 6699 29 P
8022 6663 8026 6661 8031 6663 8034 6660 8039 6663 8042 6665
8039 6667 8034 6669 8030 6668 8022 6663 10 P
7979 6675 7983 6673 7987 6671 7991 6669 7995 6668 7997 6673
7993 6675 7988 6676 7984 6676 7979 6675 10 P
7942 6708 7943 6704 7945 6701 7947 6700 7951 6698 7952 6702
7950 6704 7947 6707 7946 6709 7944 6712 7942 6708 11 P
8622 7035 8627 7034 8631 7033 8633 7029 8635 7025 8639 7023
8638 7027 8640 7032 8643 7034 8646 7038 8645 7042 8647 7046

8644 7049 8644 7054 8647 7057 8651 7058 8655 7061 8655 7066
8650 7064 8646 7061 8642 7061 8638 7059 8635 7055 8631 7054
8631 7049 8633 7046 8636 7042 8636 7038 8632 7035 8627 7037
8622 7035 31 P
9031 7668 9035 7666 9039 7665 9043 7667 9047 7667 9051 7670
9055 7672 9057 7676 9059 7680 9054 7681 9050 7681 9048 7677
9044 7674 9041 7678 9037 7678 9039 7682 9035 7685 9034 7681
9032 7677 9033 7672 9031 7668 21 P
9075 7721 9070 7720 9067 7717 9063 7714 9060 7712 9060 7707
9059 7703 9058 7699 9062 7695 9062 7700 9062 7705 9064 7708
9068 7710 9073 7711 9077 7709 9081 7706 9083 7709 9082 7714
9079 7716 9075 7721 20 P
9042 7573 9043 7570 9046 7567 9049 7568 9052 7570 9055 7573
9053 7575 9049 7575 9046 7575 9042 7573 10 P
9062 7557 9066 7559 9070 7561 9073 7564 9076 7568 9072 7567
9067 7568 9065 7564 9062 7557 9 P
9316 7804 9320 7802 9324 7805 9326 7808 9331 7807 9329 7811
9326 7815 9322 7814 9318 7811 9316 7804 10 P
9309 7812 9312 7813 9313 7810 9316 7812 9318 7814 9316 7817
9313 7819 9311 7816 9309 7812 9 P
9128 7911 9131 7908 9135 7905 9140 7906 9143 7909 9143 7913
9148 7914 9152 7916 9153 7920 9154 7924 9154 7929 9151 7932
9147 7933 9144 7930 9142 7926 9140 7922 9137 7918 9134 7916
9128 7911 19 P
9084 7869 9088 7868 9093 7868 9095 7873 9099 7872 9103 7873
9104 7877 9104 7882 9104 7886 9100 7887 9099 7883 9095 7885
9090 7883 9087 7881 9087 7876 9084 7869 16 P
8948 7937 8949 7933 8951 7931 8954 7931 8956 7929 8960 7930
8963 7931 8965 7934 8968 7934 8972 7933 8974 7936 8976 7939
8973 7941 8970 7942 8967 7944 8964 7943 8961 7942 8958 7941
8954 7940 8951 7939 8948 7937 21 P
9470 8718 9473 8716 9476 8715 9479 8712 9482 8711 9485 8709
9487 8707 9490 8705 9493 8704 9497 8703 9500 8704 9503 8705
9506 8707 9508 8709 9510 8712 9510 8716 9508 8718 9505 8720
9502 8721 9499 8722 9496 8722 9492 8722 9489 8721 9485 8721
9482 8721 9479 8720 9476 8719 9470 8718 28 P
9422 8731 9425 8730 9427 8727 9429 8724 9432 8723 9436 8722
9439 8721 9442 8723 9442 8726 9442 8730 9439 8732 9437 8735
9436 8738 9433 8740 9430 8741 9426 8742 9424 8743 9420 8744
9417 8744 9417 8741 9418 8738 9419 8735 9422 8731 23 P
9586 8614 9589 8613 9591 8612 9594 8612 9596 8614 9598 8617
9597 8619 9595 8621 9593 8623 9591 8621 9590 8618 9588 8616
9586 8614 13 P

9655 8521 9657 8520 9658 8521 9660 8523 9660 8525 9661 8527
9659 8527 9657 8526 9657 8524 9655 8521 10 P
9648 8527 9651 8527 9651 8525 9653 8524 9655 8525 9657 8527
9659 8528 9660 8530 9662 8532 9663 8534 9662 8535 9660 8535
9657 8535 9656 8533 9655 8531 9654 8530 9652 8530 9648 8527
18 P
9829 9037 9831 9035 9834 9034 9836 9032 9840 9030 9842 9033
9841 9036 9842 9038 9846 9039 9846 9042 9842 9043 9843 9047
9841 9050 9840 9053 9837 9054 9834 9053 9833 9049 9832 9046
9830 9043 9829 9037 20 P
9837 9024 9839 9021 9842 9019 9845 9018 9848 9020 9850 9022
9848 9025 9845 9026 9842 9027 9837 9024 10 P
9964 9034 9968 9034 9971 9035 9969 9032 9971 9030 9970 9027
9967 9025 9969 9022 9973 9023 9974 9021 9977 9023 9979 9025
9982 9023 9982 9027 9983 9030 9985 9033 9987 9035 9984 9036
9981 9036 9977 9037 9977 9041 9977 9044 9974 9043 9971 9043
9970 9040 9967 9037 9964 9034 27 P
9965 8996 9968 8996 9971 8997 9973 9001 9975 8999 9978 9000
9981 9002 9983 9004 9986 9006 9988 9009 9985 9011 9982 9010
9979 9010 9976 9008 9973 9007 9971 9005 9971 9001 9968 8999
9965 8996 19 P
9940 9189 9942 9187 9944 9189 9947 9192 9949 9193 9952 9195
9952 9199 9952 9202 9954 9205 9957 9204 9956 9207 9955 9210
9953 9213 9950 9212 9947 9211 9946 9207 9947 9204 9947 9201
9943 9200 9944 9198 9945 9194 9943 9192 9940 9189 23 P
9946 9165 9950 9165 9952 9163 9955 9163 9958 9164 9958 9168
9955 9170 9958 9172 9960 9175 9962 9177 9966 9178 9969 9178
9969 9182 9973 9182 9976 9183 9973 9184 9969 9184 9967 9182
9964 9180 9962 9178 9958 9178 9956 9176 9953 9176 9949 9176
9947 9174 9949 9171 9946 9165 27 P
9906 9143 9909 9142 9912 9141 9914 9139 9916 9136 9920 9136
9922 9138 9923 9141 9923 9145 9926 9147 9928 9149 9924 9150
9923 9147 9920 9145 9917 9143 9914 9142 9912 9145 9913 9148
9910 9149 9907 9150 9908 9147 9906 9143 22 P
10089 9560 10090 9557 10093 9559 10096 9558 10098 9561 10101 9562
10102 9565 10104 9568 10107 9569 10111 9569 10110 9572 10106 9572
10103 9574 10101 9571 10098 9570 10098 9567 10096 9564 10094 9562
10090 9564 10089 9560 20 P
10094 9721 10097 9720 10100 9718 10103 9717 10106 9715 10109 9713
10111 9711 10114 9709 10115 9712 10118 9713 10122 9712 10125 9712
10127 9709 10130 9707 10130 9710 10131 9714 10128 9715 10125 9714
10122 9714 10119 9716 10116 9716 10112 9717 10110 9720 10106 9720
10106 9723 10104 9726 10101 9726 10098 9728 10095 9727 10096 9724

10094 9721 31 P
10051 9858 10053 9855 10056 9855 10060 9855 10063 9854 10066 9855
10068 9858 10067 9861 10065 9864 10062 9865 10058 9863 10056 9862
10051 9858 13 P
10054 9867 10057 9866 10060 9867 10063 9867 10067 9867 10069 9866
10071 9863 10074 9861 10077 9862 10078 9866 10076 9868 10074 9871
10071 9873 10069 9875 10067 9877 10063 9875 10061 9873 10058 9871
10054 9867 19 P
10039 9863 10040 9860 10044 9861 10047 9861 10047 9865 10048 9868
10047 9871 10043 9872 10041 9871 10040 9867 10039 9863 11 P
10021 9880 10020 9877 10021 9874 10020 9871 10022 9868 10025 9868
10028 9871 10030 9872 10029 9875 10027 9878 10024 9880 10021 9880
12:00 PM
10001 9853 10003 9849 10003 9846 10005 9845 10009 9845 10011 9847
10013 9850 10015 9853 10015 9856 10014 9859 10011 9861 10008 9861
10005 9859 10002 9857 10001 9853 15 P
10280 10369 10284 10368 10286 10366 10290 10365 10293 10366 10294 10368
10298 10368 10298 10372 10295 10372 10292 10370 10290 10372 10287 10375
10284 10376 10283 10373 10285 10370 10280 10369 16 P
10252 10351 10256 10352 10259 10352 10262 10350 10265 10350 10266 10353
10263 10354 10260 10355 10257 10355 10252 10351 10 P
10268 10351 10271 10349 10274 10351 10277 10352 10275 10355 10272 10357
10270 10354 10268 10351 8 P
10334 10338 10337 10336 10339 10337 10343 10339 10339 10341 10334 10338
6:00 PM
9995 10209 9998 10207 10001 10207 10003 10204 10004 10201 10008 10202
10010 10204 10010 10208 10011 10210 10015 10211 10011 10213 10009 10214
10005 10213 10004 10210 10001 10211 9995 10209 16 P
9991 10235 9994 10233 9996 10230 9999 10232 10001 10235 10002 10238
10004 10240 10001 10239 9998 10241 9997 10238 9994 10239 9991 10235
12:00 PM
10100 10241 10103 10241 10106 10243 10109 10245 10108 10248 10104 10249
10106 10245 10102 10245 10100 10241 9 P
10095 10173 10098 10172 10101 10173 10102 10176 10103 10179 10101 10180
10098 10179 10095 10177 10095 10173 9 P
10124 10175 10127 10175 10130 10173 10132 10171 10136 10170 10137 10173
10137 10177 10137 10180 10137 10183 10134 10182 10134 10178 10130 10177
10127 10178 10124 10175 14 P
10191 10128 10194 10128 10197 10129 10200 10129 10201 10125 10204 10123
10207 10124 10209 10127 10205 10129 10203 10131 10203 10134 10200 10137
10197 10136 10195 10132 10191 10128 15 P
10207 10018 10210 10017 10213 10015 10216 10015 10219 10014 10221 10017
10220 10020 10217 10021 10213 10022 10210 10022 10207 10018 11 P

10285 10011 10288 10009 10289 10006 10291 10003 10293 10001 10294 10005
10294 10008 10293 10011 10292 10014 10289 10015 10285 10011 11 P
10304 10011 10305 10008 10307 10006 10311 10005 10311 10002 10312 9999
10314 10001 10313 10005 10313 10008 10311 10011 10309 10013 10304 10011

12:00 PM

10317 10011 10318 10007 10320 10005 10323 10003 10323 10006 10322 10010
10323 10013 10321 10015 10317 10011 9 P
10325 9996 10326 9994 10329 9993 10331 9995 10330 9998 10329 10000
10325 9996 7 P
10204 9858 10206 9855 10207 9852 10210 9852 10211 9855 10209 9859
10209 9862 10205 9862 10204 9858 9 P
10065 10010 10066 10007 10069 10006 10071 10004 10074 10004 10076 10005
10077 10007 10076 10010 10074 10012 10072 10013 10069 10013 10065 10010

12:00 PM

9793 9393 9796 9392 9799 9390 9801 9388 9805 9388 9808 9389
9811 9389 9814 9390 9815 9393 9815 9397 9813 9399 9810 9399
9807 9398 9808 9394 9805 9393 9801 9393 9799 9396 9795 9396
9793 9393 19 P
9980 9158 9982 9155 9982 9152 9985 9154 9988 9156 9990 9158
9993 9160 9990 9162 9987 9161 9983 9161 9980 9158 11 P
9790 8789 9794 8789 9797 8791 9798 8795 9800 8797 9803 8798
9806 8799 9807 8803 9805 8805 9802 8805 9800 8802 9798 8800
9795 8798 9792 8796 9790 8793 9790 8789 16 P
8910 7381 8914 7378 8918 7377 8923 7377 8927 7378 8932 7379
8936 7377 8939 7381 8940 7385 8939 7389 8937 7393 8934 7390
8929 7388 8925 7387 8921 7384 8918 7387 8913 7387 8910 7381
18 P
8857 7378 8860 7375 8864 7374 8869 7373 8871 7377 8875 7379
8878 7382 8880 7386 8883 7390 8883 7395 8878 7394 8875 7391
8871 7388 8868 7385 8866 7381 8864 7377 8862 7381 8857 7378
18 P
8902 7388 8905 7388 8907 7388 8910 7390 8913 7390 8913 7393
8911 7395 8909 7397 8906 7396 8904 7395 8903 7392 8902 7388

12:00 PM

8926 7198 8928 7195 8927 7192 8927 7189 8929 7192 8932 7193
8935 7195 8938 7197 8935 7197 8932 7199 8931 7203 8934 7205
8933 7209 8929 7207 8928 7204 8926 7202 8926 7198 17 P
8930 7223 8932 7219 8934 7217 8937 7218 8940 7218 8943 7219
8947 7220 8949 7222 8947 7224 8945 7227 8945 7230 8942 7229
8939 7227 8936 7228 8936 7225 8930 7223 16 P
8209 6751 8212 6750 8215 6749 8218 6747 8221 6744 8223 6742
8227 6742 8230 6742 8230 6745 8232 6749 8234 6752 8232 6755
8229 6755 8226 6756 8222 6756 8219 6756 8215 6754 8212 6754

8209 6751 19 P
5903 5461 5904 5468 5908 5474 5902 5477 5908 5480 5915 5478
5917 5485 5916 5492 5916 5499 5921 5504 5924 5511 5930 5507
5936 5511 5937 5518 5933 5524 5927 5519 5921 5515 5914 5515
5908 5511 5903 5505 5896 5502 5893 5496 5886 5496 5879 5494
5872 5491 5866 5487 5862 5493 5854 5494 5847 5494 5843 5487
5837 5483 5832 5479 5825 5477 5827 5470 5822 5465 5816 5461
5816 5454 5816 5453 38 P
5839 5453 5846 5454 5847 5453 3 P
5861 5453 5864 5455 5871 5458 5879 5459 5885 5461 5890 5456
5890 5453 7 P
5910 5453 5911 5454 2 P
5911 5454 5903 5461 2 P
5967 5480 5971 5474 5974 5468 5981 5464 5988 5463 5992 5456
5999 5459 6005 5455 6006 5453 9 P
6023 5453 6024 5459 6029 5454 6029 5453 4 P
6040 5453 6038 5458 6042 5465 6038 5470 6034 5477 6031 5483
6024 5485 6017 5488 6010 5489 6004 5484 5997 5483 5990 5485
5984 5488 5977 5488 5967 5480 15 P
5969 5453 5969 5459 5963 5459 5962 5453 5961 5453 5 P
6054 5459 6056 5458 6058 5456 6058 5454 6059 5455 6062 5456
6063 5458 6064 5460 6063 5462 6064 5465 6063 5467 6061 5466
6059 5465 6057 5465 6055 5463 6054 5459 16 P
5540 5469 5543 5465 5545 5461 5547 5457 5549 5453 5 P
5575 5453 5574 5458 5572 5462 5571 5466 5569 5470 5567 5475
5563 5478 5559 5480 5556 5483 5552 5486 5549 5482 5545 5480
5542 5476 5540 5469 14 P
5698 5567 5700 5563 5704 5560 5708 5563 5712 5565 5716 5566
5716 5571 5720 5572 5723 5576 5723 5580 5726 5585 5721 5585
5717 5587 5712 5588 5710 5584 5706 5581 5704 5578 5702 5573
5698 5567 19 P
5790 5572 5794 5569 5798 5566 5800 5562 5805 5561 5809 5561
5808 5566 5806 5570 5802 5573 5799 5575 5795 5572 5790 5572
12:00 PM
6251 5559 6253 5560 6254 5559 6257 5560 6258 5562 6258 5565
6260 5566 6259 5568 6257 5567 6254 5566 6253 5564 6252 5562
6251 5559 13 P
6239 5580 6241 5577 6242 5574 6244 5571 6247 5573 6250 5574
6254 5574 6257 5576 6259 5579 6257 5581 6253 5581 6250 5582
6246 5582 6243 5582 6239 5580 15 P
6252 5647 6253 5644 6257 5644 6259 5641 6260 5638 6263 5640
6262 5643 6259 5646 6256 5647 6256 5651 6252 5647 11 P
12523 8159 12525 8159 12525 8158 12526 8159 12526 8160 12526 8160

12525 8161 12524 8161 12523 8160 12523 8159 10 P
12911 11281 12912 11281 12914 11281 12915 11281 12917 11282 12919 11283
12920 11284 12921 11283 12922 11281 12923 11282 12924 11284 12925 11286
12926 11287 12926 11288 12926 11290 12925 11290 12924 11289 12922 11289
12921 11289 12919 11289 12918 11289 12916 11290 12915 11288 12915 11287
12915 11285 12914 11283 12913 11283 12911 11282 12911 11281 29 P
12896 11256 12897 11256 12898 11256 12898 11256 12897 11258 12895 11258
12895 11257 12896 11256 12896 11256 9 P
12899 11256 12898 11255 12898 11254 12898 11252 12898 11250 12897 11249
12899 11248 12899 11250 12900 11251 12901 11252 12903 11252 12902 11253
12901 11254 12901 11256 12901 11257 12902 11259 12903 11260 12902 11262
12901 11262 12900 11261 12898 11261 12897 11261 12896 11259 12898 11258
12899 11256 25 P
12903 11265 12904 11263 12905 11265 12906 11266 12906 11267 12905 11267
12904 11267 12904 11266 12903 11266 12903 11265 10 P
12905 11271 12906 11271 12906 11272 12905 11272 12905 11271 5 P
12906 11272 12907 11272 12907 11272 12907 11272 12908 11272 12908 11274
12909 11274 12908 11275 12907 11275 12907 11274 12906 11273 12906 11272
12:00 PM
12909 11277 12909 11277 12910 11276 12911 11276 12911 11276 12912 11276
12911 11277 12911 11279 12909 11279 12909 11277 10 P
2297 14405 2329 14399 2320 14346 2297 14349 4 P
2297 14294 2325 14289 2373 14266 2380 14212 2412 14167 2459 14139
2490 14094 2522 14050 2508 13997 2489 13946 2507 13894 2525 13843
2543 13791 2555 13738 2563 13684 2530 13640 2486 13608 2467 13556
2480 13503 2507 13456 2547 13419 2601 13411 2629 13363 2652 13314
2670 13262 2722 13244 2773 13225 2823 13202 2836 13149 2869 13105
2912 13137 2941 13184 2927 13237 2935 13292 2948 13345 3001 13358
3052 13340 3107 13343 3146 13357 3135 13425 3179 13458 3186 13512
3188 13553 3151 13593 3104 13621 3057 13649 46 P
3057 13649 3016 13655 3013 13709 3026 13762 2994 13806 2971 13831
3020 13855 3048 13902 3103 13899 3110 13954 3115 13987 3085 14015
3121 14032 3130 14093 3185 14090 3239 14088 3293 14086 3347 14078
3394 14068 19 P
3394 14068 3397 14013 3416 13962 3466 13939 3513 13912 3560 13885
3610 13863 3651 13826 3684 13784 3718 13740 3770 13723 3819 13701
3861 13666 3905 13634 3957 13618 3972 13565 3946 13517 3950 13463
3938 13409 3930 13369 3863 13335 3862 13280 3850 13227 3859 13173
3879 13122 3873 13067 3866 13013 3812 13014 3757 13016 3702 13017
3648 13018 3593 13014 3540 13026 3490 13049 3450 13011 3464 12958
3483 12907 3452 12862 3397 12864 3342 12866 3288 12873 3233 12869
3186 12897 3153 12941 3120 12985 3065 12992 3011 13000 2944 13010
2907 12969 2904 12914 50 P

2904 12914 2918 12861 2877 12823 2834 12791 2801 12766 2792 12712
2847 12701 2895 12676 2930 12634 2930 12558 2869 12557 2869 12503
2838 12457 2808 12411 2787 12360 2792 12305 2814 12254 2820 12199
2813 12166 2773 12174 2756 12212 2702 12217 2648 12206 2599 12232
2545 12243 2502 12208 2472 12163 2423 12137 2439 12084 2481 12049
2507 12000 2486 11949 2460 11901 2407 11885 2352 11880 2299 11896
2297 11897 37 P
2297 10470 2312 10463 2357 10429 2381 10379 2436 10371 2492 10374
2547 10382 2601 10369 2604 10313 2618 10259 2663 10226 2718 10224
2774 10232 2821 10262 2865 10296 2898 10341 2931 10386 2963 10431
3000 10473 3037 10515 3017 10567 3045 10615 3100 10625 3155 10633
3184 10586 3238 10601 3270 10646 3293 10697 3325 10742 3332 10798
3364 10843 3400 10885 3440 10923 3484 10958 3528 10992 3574 11022
3591 11075 3553 11115 3519 11158 3497 11193 3533 11235 3560 11283
3564 11318 3605 11313 3608 11337 45 P
3608 11337 3611 11358 3556 11364 3501 11371 3448 11356 3396 11336
3357 11321 3315 11358 3278 11398 3253 11448 3219 11492 3164 11499
3140 11548 3125 11579 3081 11546 3043 11517 3009 11560 2980 11607
3000 11626 2967 11670 2917 11693 2862 11701 2808 11693 2753 11695
2698 11687 2646 11705 2599 11734 2561 11775 2534 11779 2536 11827
2481 11824 2420 11828 2410 11788 2354 11791 2297 11812 35 P
3394 14068 3432 14082 3418 14135 3409 14188 3427 14240 3449 14289
3477 14337 3517 14374 3560 14407 3614 14415 3619 14456 11 P
3619 14456 3567 14474 3530 14513 3511 14564 3474 14604 3427 14632
3373 14629 3319 14631 3278 14667 3234 14699 3194 14735 3166 14782
3212 14810 3252 14847 3265 14900 3273 14953 3309 14994 3336 15040
3376 15077 3408 15121 3395 15174 3386 15227 3363 15276 3319 15309
3268 15290 3215 15277 3165 15258 3112 15245 3063 15221 3023 15185
2990 15142 2962 15095 2944 15044 2925 14993 2917 14939 2904 14887
2880 14837 2877 14783 2845 14740 2836 14686 2844 14632 2830 14579
2807 14530 2754 14518 2707 14490 2654 14498 2600 14507 2546 14500
2499 14472 2452 14445 50 P
2452 14445 2398 14443 2344 14447 2297 14459 4 P
3619 14456 3668 14434 3718 14412 3755 14372 3758 14331 3805 14305
3859 14298 3914 14302 3968 14301 4022 14294 4083 14287 4094 14340
4095 14395 4107 14448 4135 14466 4165 14420 4222 14458 4212 14511
4182 14557 4204 14606 4230 14654 4266 14696 4304 14734 4347 14768
4389 14802 4424 14843 4466 14877 4511 14907 4546 14949 4585 14987
4627 15021 4672 15052 4722 15072 4772 15093 4761 15146 4757 15193
4802 15164 4837 15121 4874 15106 4878 15140 4931 15135 4945 15068
4998 15079 5023 15127 5061 15166 5106 15197 5159 15207 5186 15205
5167 15169 5155 15138 50 P
5155 15138 5185 15093 5208 15059 5183 15011 5174 14958 5129 14927

5095 14884 5085 14831 5070 14779 5044 14731 5040 14677 5020 14626
4974 14596 4933 14561 4888 14531 4854 14489 4848 14434 4842 14366
4877 14325 4903 14276 4924 14227 4935 14173 4930 14119 4925 14065
4871 14070 4866 14015 4876 13990 4846 13945 4812 13903 4760 13859
4752 13805 4744 13751 4739 13697 4739 13642 4750 13588 4745 13534
4740 13480 4734 13426 4730 13371 4735 13316 4740 13262 4746 13208
4752 13153 4758 13099 4742 13046 4721 12996 4691 12950 4657 12907
4636 12857 4620 12804 50 P
4620 12804 4594 12756 4584 12702 4578 12647 4557 12597 4580 12562
4615 12529 7 P
4615 12529 4604 12475 4634 12429 4624 12375 4654 12330 4693 12291
4689 12236 4705 12184 4667 12126 4718 12105 4757 12067 4768 12013
4814 11982 4840 11934 4841 11879 4836 11824 4813 11766 17 P
4813 11766 4745 11773 4729 11720 4674 11714 4639 11756 4608 11802
4566 11767 4512 11756 4459 11772 4406 11788 4354 11804 4300 11815
4246 11826 4192 11837 4136 11837 4136 11782 4189 11766 4240 11744
4260 11693 4239 11643 4294 11637 4344 11616 4387 11580 4408 11530
4429 11478 4378 11457 4326 11441 4275 11462 4221 11474 4166 11479
4131 11437 4099 11391 4083 11338 4099 11285 4114 11232 4119 11177
4113 11122 4113 11067 4093 11000 39 P
4093 11000 4037 11001 3981 11001 3943 11041 3909 11084 3874 11127
3835 11167 3784 11189 3742 11225 3700 11261 3662 11301 3638 11326
3608 11337 13 P
4093 11000 4147 10989 4141 10934 4138 10899 4193 10893 4241 10867
4280 10828 4329 10801 4335 10746 4329 10691 4324 10636 4318 10580
4313 10525 4270 10490 4219 10511 4163 10517 4112 10496 4069 10461
4026 10426 3976 10400 3937 10361 3895 10365 3899 10400 3847 10379
3792 10369 3799 10424 3743 10430 3700 10396 3653 10365 3600 10383
3581 10420 3536 10367 3480 10368 3448 10322 3483 10278 3513 10231
3501 10177 3483 10124 3477 10068 3473 10033 3403 10028 3423 9976
3462 9935 3469 9991 3506 10010 3531 9960 3557 9911 3605 9883
3621 9829 3589 9783 50 P
3589 9783 3635 9751 3684 9724 3715 9677 3719 9622 3754 9578
3781 9555 7 P
3781 9555 3787 9611 3819 9657 3840 9709 3867 9758 3903 9801
3939 9844 3991 9822 4024 9812 4040 9865 4090 9892 4145 9902
4201 9902 4254 9886 4297 9850 4347 9824 4401 9835 4453 9857
4458 9913 4431 9961 4452 10013 4504 10035 4555 10057 4625 10051
4621 10009 4677 10004 4732 9999 4788 9994 4824 9952 4871 9921
4922 9901 4978 9891 5033 9886 5087 9871 5127 9833 5168 9794
5204 9751 5248 9717 5302 9702 5357 9715 5412 9711 5467 9718
5510 9755 5555 9788 5632 9783 5629 9741 5601 9743 5581 9690
5577 9621 5630 9601 50 P

5630 9601 5686 9597 5719 9642 5758 9683 5795 9724 5837 9761
5885 9790 5882 9734 5927 9700 5971 9667 5942 9619 5980 9577
5938 9540 5904 9495 5882 9444 5902 9391 5945 9355 5988 9318
6043 9328 6103 9346 20 P
6103 9346 6117 9400 6165 9429 6206 9466 6244 9508 6297 9490
6352 9498 6403 9522 6458 9525 6512 9539 6549 9581 6602 9601
6656 9588 6669 9642 6724 9651 6778 9666 6815 9708 6817 9764
6873 9762 6929 9760 6971 9724 7022 9701 7078 9700 7143 9741
24 P
7143 9741 7127 9794 7118 9849 7119 9905 7104 9959 7059 9991
7044 10045 7045 10101 7046 10143 7008 10183 6979 10213 6981 10269
7022 10308 7062 10345 7106 10380 7076 10427 7034 10463 6986 10491
6933 10509 6883 10532 6841 10568 6819 10603 6804 10642 23 P
6804 10642 6735 10638 6731 10693 6712 10745 6675 10704 6628 10674
6574 10660 6519 10663 6471 10691 6442 10738 6434 10793 6427 10834
6373 10842 6304 10845 6266 10886 6233 10911 6196 10870 6158 10852
6135 10903 6090 10936 6057 10980 6044 11034 6058 11088 6061 11143
6069 11197 6028 11235 5991 11194 5943 11166 5888 11169 5856 11214
5812 11247 5758 11235 5703 11227 5648 11226 5593 11229 5538 11238
5483 11242 5429 11235 5373 11228 5324 11253 5281 11287 5237 11321
5190 11351 5144 11380 5095 11406 5052 11440 5016 11482 4980 11524
4940 11563 4929 11617 50 P
4929 11617 4902 11665 4871 11710 4841 11739 4813 11766 5 P
6804 10642 6817 10697 6840 10747 6880 10785 6924 10819 6977 10833
7060 10839 7067 10895 7069 10950 7070 11005 7071 11061 7067 11116
7069 11171 7076 11226 7082 11274 7054 11272 7053 11230 6998 11232
6963 11233 6923 11195 6878 11227 6844 11271 6790 11279 6742 11306
6744 11361 6746 11417 6708 11457 6658 11480 6610 11508 6613 11563
6615 11611 6656 11610 6666 11570 6721 11578 6796 11583 6804 11638
6822 11690 6818 11745 6841 11795 6888 11824 6941 11839 6948 11894
6949 11948 6940 12003 6942 12058 6982 12095 7037 12099 7084 12072
7132 12045 7147 11992 50 P
7147 11992 7161 11939 7209 11911 7264 11916 7315 11936 7359 11970
7405 12000 7400 12054 7380 12106 7381 12161 7382 12209 7328 12220
7278 12243 7240 12283 7220 12333 7221 12375 16 P
7221 12375 7170 12392 7116 12383 7060 12379 7006 12380 6951 12382
6906 12414 6887 12465 6868 12517 6827 12552 6789 12593 6737 12611
6682 12613 6635 12641 6637 12695 6669 12740 6706 12781 6724 12832
6775 12852 6827 12871 6867 12908 6894 12955 6933 12993 6980 13022
7023 13056 7072 13080 7108 13121 7154 13150 7207 13138 7247 13129
7238 13183 7210 13213 7167 13179 7114 13165 7062 13150 7012 13126
6960 13112 6917 13078 6868 13054 6869 13109 6897 13156 6924 13203
6930 13251 43 P

6930 13251 6887 13218 6859 13216 6836 13265 6782 13272 6738 13239
6695 13206 6654 13242 6599 13244 6545 13247 6498 13275 6451 13303
6399 13321 6350 13344 6300 13368 6246 13376 6206 13382 6165 13388
18 P
6165 13388 6157 13334 6149 13280 6141 13226 6127 13173 6104 13124
6090 13071 6082 13017 6068 12964 6049 12912 6021 12865 5984 12825
5936 12797 5882 12790 5828 12793 5774 12802 5719 12806 5665 12809
5610 12807 5555 12811 5503 12826 5449 12835 5394 12839 5384 12785
5380 12730 5328 12745 5273 12739 5228 12707 5187 12671 5142 12640
5089 12623 5035 12628 4981 12616 4931 12595 4877 12584 4832 12552
4778 12547 4729 12521 4674 12526 4634 12534 4615 12529 41 P
6165 13388 6133 13432 6110 13481 6087 13531 6064 13581 6109 13613
6149 13649 6193 13681 6201 13736 6204 13790 6232 13837 6265 13880
6293 13927 6321 13974 6349 14021 6367 14071 6349 14123 6331 14174
6312 14226 6290 14275 6271 14326 6243 14373 6197 14401 6151 14429
6169 14480 6172 14534 6186 14587 6194 14641 6197 14695 6241 14728
6277 14768 6285 14821 6322 14861 6363 14897 6399 14857 6453 14860
6504 14879 6540 14919 6542 14973 6570 15020 6588 15071 6606 15108
6569 15148 6555 15200 6558 15254 6514 15287 6463 15305 6414 15328
6374 15364 6402 15411 50 P
6402 15411 6434 15455 6487 15468 6539 15481 6588 15504 6620 15548
6566 15550 6513 15558 6520 15612 6524 15680 6483 15681 6486 15735
6440 15776 13 P
6440 15776 6433 15723 6419 15670 6376 15638 6323 15630 6270 15617
6230 15581 6183 15554 6129 15557 6086 15589 6032 15593 5978 15586
5940 15573 5914 15503 5860 15496 5817 15529 5772 15557 5718 15550
5677 15515 5644 15472 5614 15427 5590 15378 5561 15332 5537 15284
5517 15233 5473 15202 5419 15206 5373 15236 5309 15229 5299 15175
5269 15126 5215 15131 5155 15138 33 P
6440 15776 6433 15830 6414 15881 6382 15925 6346 15965 6390 15997
6417 16027 6389 16073 6395 16113 6371 16146 6407 16172 11 P
8148 16172 8163 16144 8184 16095 8200 16043 8252 16058 8304 16048
8358 16053 8413 16053 8460 16027 8514 16028 8545 16003 11 P
8545 16003 8570 16051 8595 16099 8605 16152 8598 16172 5 P
11247 15960 11193 15955 11139 15955 11160 16005 11180 16055 11186 16109
11160 16157 11148 16172 8 P
8545 16003 8508 15964 8474 15921 8427 15895 8465 15858 8414 15841
8384 15766 8426 15732 8468 15698 8520 15683 8551 15639 8599 15614
8653 15615 8693 15615 8705 15562 8712 15509 8729 15457 8755 15410
8786 15365 8835 15328 20 P
8060 14892 8055 14946 8030 14994 7995 15036 8003 15062 8024 15062
8020 15102 7966 15098 7926 15089 7892 15112 7908 15150 7860 15176
7855 15230 7856 15284 7906 15304 7936 15349 7894 15384 7869 15447

7821 15447 7788 15447 20 P
7788 15447 7750 15486 7729 15536 7730 15590 7769 15628 7822 15633
7817 15687 7763 15687 7710 15693 7656 15694 7606 15715 7581 15764
7547 15806 7518 15851 7489 15897 7443 15867 7398 15838 7366 15826
7375 15773 7380 15719 7353 15672 7301 15657 7247 15653 7195 15639
7152 15605 7121 15561 7086 15520 7059 15473 7057 15419 7026 15375
6986 15338 7010 15289 7009 15235 6996 15182 7025 15136 7072 15109
7116 15078 7165 15102 7219 15106 7273 15105 7314 15069 7366 15052
7411 15081 7446 15123 7504 15104 45 P
7504 15104 7555 15124 7594 15162 3 P
7594 15162 7643 15140 7693 15119 7688 15173 7719 15218 7714 15272
7709 15326 7730 15376 8 P
7730 15376 7770 15402 7788 15447 3 P
7504 15104 7514 15051 7558 15020 7568 14967 7573 14913 7593 14862
7567 14815 7531 14773 7505 14725 7494 14672 7495 14618 7507 14565
7534 14518 7583 14494 7637 14490 7691 14497 7744 14510 7796 14524
7832 14543 7870 14565 20 P
6930 13251 6932 13305 6895 13346 6862 13389 6863 13443 6881 13495
6917 13536 6960 13569 7006 13598 7050 13632 7077 13679 7104 13726
7126 13776 7143 13828 7170 13875 7222 13890 7276 13899 7325 13924
7364 13962 7403 14000 7438 14041 7474 14082 7513 14120 7555 14154
7609 14153 7664 14153 7718 14152 7772 14152 7815 14186 7845 14231
7876 14276 7926 14296 7974 14322 8006 14348 8033 14361 35 P
7221 12375 7276 12379 7314 12423 3 P
7314 12423 7320 12478 7332 12531 7381 12556 7434 12571 7488 12576
7538 12554 7577 12515 7615 12475 7630 12423 7651 12372 7676 12323
7725 12349 7746 12365 7788 12330 7822 12307 7875 12323 7926 12344
7960 12386 8009 12412 8064 12406 8099 12364 8114 12311 8114 12270
8169 12270 8220 12292 8236 12344 8235 12385 8274 12424 8308 12448
8356 12422 8382 12471 8403 12522 8423 12573 8438 12625 8493 12621
8548 12627 8585 12666 8626 12667 8634 12656 40 P
7314 12423 7356 12387 7390 12344 7435 12313 7485 12291 7537 12274
7578 12270 7577 12215 7593 12162 7635 12127 7680 12096 7735 12090
7789 12079 7842 12067 7888 12037 7931 11983 7935 11942 7909 11910
7963 11900 8018 11905 8071 11916 8117 11885 8096 11834 8091 11779
8065 11731 8039 11682 8013 11633 8044 11587 8098 11582 8141 11547
8157 11494 8157 11439 8183 11391 8219 11348 8274 11343 8328 11354
8382 11365 8412 11411 8442 11458 8495 11474 8530 11432 8573 11398
8609 11355 8610 11300 8589 11249 8551 11209 8498 11224 8458 11263
8404 11273 8349 11278 50 P
8349 11278 8319 11232 8354 11189 8365 11135 8344 11084 8299 11053
8268 11006 8226 10970 8212 10917 8218 10862 8241 10811 8281 10773
8328 10744 8377 10719 8329 10691 8314 10638 8321 10582 8344 10532

8391 10502 8444 10488 8499 10484 8622 10484 8678 10485 8733 10481
8774 10477 8830 10473 26 P
8830 10473 8881 10495 8936 10491 8973 10449 9009 10407 9056 10377
9108 10357 9162 10370 9209 10340 9216 10285 9261 10317 9314 10335
9338 10385 9391 10403 9446 10405 15 P
9446 10405 9479 10360 9524 10326 9564 10289 9600 10266 9615 10252
6:00 PM
8830 10473 8820 10418 8800 10367 8790 10311 8760 10265 8726 10221
8680 10189 8638 10153 8678 10114 8700 10063 8711 10038 8657 10026
8603 10009 8548 10008 8496 10029 8481 9975 8470 9920 8471 9865
8471 9809 8441 9762 8410 9715 8384 9665 8345 9626 8302 9590
8256 9558 8209 9528 8148 9494 27 P
8148 9494 8108 9533 8065 9569 8010 9580 7954 9585 7899 9591
7844 9603 7795 9629 7779 9683 7758 9735 7705 9751 7678 9784
7629 9758 7612 9705 7585 9656 7534 9635 7484 9609 7428 9605
7393 9649 7416 9700 7463 9730 7499 9773 7503 9815 7457 9846
7402 9853 7358 9818 7314 9783 7265 9758 7218 9728 7176 9733
7143 9741 31 P
8148 9494 8127 9442 8075 9421 8048 9371 8048 9315 8080 9268
8069 9213 8095 9164 8142 9133 8197 9122 8252 9112 8253 9055
8258 8999 8269 8944 8275 8888 8275 8866 16 P
8275 8866 8275 8846 8331 8852 8385 8869 8425 8829 8425 8773
8410 8719 8442 8672 8458 8618 8475 8564 8497 8512 8492 8456
8497 8414 8553 8420 8600 8389 8657 8385 8706 8412 8756 8440
8781 8490 8828 8522 8868 8483 8886 8429 8908 8378 8941 8331
8997 8327 9043 8359 9099 8372 9150 8395 9203 8375 9248 8340
9245 8301 31 P
8275 8866 8219 8866 8219 8810 8180 8770 8133 8738 8084 8711
8030 8695 7978 8673 7938 8634 7894 8598 7854 8638 7819 8682
7765 8666 7711 8650 7684 8600 7648 8557 7603 8522 7547 8522
7547 8466 7588 8457 7587 8401 7597 8345 7608 8289 7581 8240
7549 8193 7501 8162 7447 8147 7391 8137 7347 8101 7310 8058
7304 8002 7292 7946 7312 7893 7338 7843 7291 7812 7249 7773
7227 7721 7226 7678 7268 7677 7251 7623 7214 7580 7169 7544
7124 7509 7096 7460 7064 7413 7040 7361 7023 7306 7010 7251
7003 7194 6966 7151 50 P
6966 7151 6943 7099 6963 7045 6938 7001 6937 6959 5 P
6103 9346 6101 9290 6124 9239 6148 9188 6140 9133 6132 9077
6161 9029 6190 8981 6171 8928 6137 8882 6092 8849 6074 8796
6022 8771 5971 8748 5934 8706 5925 8650 5891 8605 5853 8563
5834 8510 5830 8454 5849 8400 5906 8403 5962 8394 5958 8338
5934 8287 5931 8230 5965 8184 5968 8128 5943 8077 5951 8020
5997 7986 6039 7949 6063 7897 6034 7848 6020 7793 5969 7725

6026 7727 6103 7739 6095 7682 6042 7663 6014 7656 6011 7600
5997 7544 5963 7498 5955 7442 5968 7387 5956 7323 47 P
3781 9555 3775 9499 3753 9448 3746 9392 3757 9337 3721 9294
3776 9282 3742 9213 8 P
3742 9213 3736 9157 3762 9107 3762 9050 3750 8995 3738 8940
3732 8884 3725 8821 3769 8785 3779 8729 3778 8687 3736 8687
3692 8652 3640 8631 3592 8600 3552 8560 3504 8530 3460 8494
3416 8459 3460 8404 3504 8439 3561 8433 3617 8438 3647 8467
3718 8467 3749 8419 3789 8379 3766 8327 3755 8271 3795 8231
3738 8231 3685 8210 3645 8198 3691 8127 3664 8077 3632 8030
3600 7983 3656 7977 3699 7972 3698 7916 3714 7876 3686 7810
42 P
3686 7810 3650 7766 3614 7723 3596 7668 3541 7680 3488 7702
3461 7652 3420 7665 3409 7691 3374 7667 3414 7627 3454 7577
3397 7577 3340 7583 3312 7586 3308 7629 3266 7633 3249 7579
3208 7504 3164 7540 3117 7573 3073 7609 3029 7574 2984 7538
2966 7484 2930 7440 2875 7424 2804 7412 2757 7444 2718 7486
2699 7524 2638 7485 2613 7537 2587 7588 2566 7641 2536 7689
2482 7708 2439 7713 2406 7666 2383 7614 2365 7560 2327 7517
2297 7515 43 P
3686 7810 3743 7810 3800 7804 3792 7733 3849 7727 3857 7798
3904 7830 3944 7870 4001 7870 4006 7813 4051 7777 4106 7766
4150 7802 4190 7842 4237 7874 4277 7914 4333 7926 4377 7890
4429 7912 4482 7934 4532 7908 4580 7877 4612 7830 4602 7774
4580 7721 4581 7665 4636 7649 4686 7645 4681 7588 4626 7570
4611 7515 4600 7460 4590 7403 4546 7366 4507 7325 4467 7284
4431 7240 4395 7195 4407 7139 4421 7114 4471 7141 4502 7190
4542 7231 4595 7210 4632 7166 4644 7110 4656 7054 4640 6999
4626 6950 4684 6946 50 P
4684 6946 4736 6968 4786 6996 4842 6986 4907 6976 5 P
4907 6976 4954 6994 5021 7017 5025 7060 5001 7112 5022 7165
5053 7214 5106 7193 5163 7190 5216 7169 5271 7154 5300 7105
5329 7055 5380 7029 5437 7037 5457 7091 5460 7148 5470 7204
5427 7243 5471 7281 5510 7323 5552 7361 5609 7357 5651 7319
5696 7284 5748 7259 5805 7256 5862 7259 5916 7278 5956 7323
30 P
5956 7323 5985 7273 5999 7218 6056 7221 6112 7230 6169 7238
6224 7252 6280 7261 6315 7215 6353 7173 6325 7141 6356 7111
6308 7079 6253 7062 6210 7025 6165 6989 6138 6938 6098 6897
6058 6857 6104 6823 6150 6789 6196 6755 6250 6774 6307 6777
6359 6803 6411 6778 6444 6834 6501 6838 6558 6836 6612 6856
6666 6876 6719 6896 6765 6863 6786 6809 6841 6791 6853 6757
6909 6773 6899 6829 6889 6886 6901 6935 6937 6959 41 P

4907 6976 4886 6922 4907 6863 4933 6813 4948 6757 4920 6707
4867 6686 4840 6675 4839 6618 4848 6561 4815 6514 4798 6459
4719 6453 4701 6398 4677 6346 4612 6347 4605 6290 4560 6254
4552 6197 4530 6176 4545 6140 4541 6082 4553 6025 4538 5970
4502 5924 4462 5882 4409 5859 4366 5854 4357 5804 4299 5809
4246 5785 4190 5767 4140 5739 4146 5682 4166 5635 4126 5593
4099 5541 4042 5552 3981 5577 3940 5535 3930 5478 3932 5453
42 P
6937 6959 6994 6957 7049 6939 7104 6927 7157 6903 7212 6908
7242 6837 7278 6793 7335 6797 7416 6811 7415 6754 7436 6701
7467 6652 7494 6602 7515 6548 7492 6495 7491 6438 7507 6383
7529 6329 7555 6278 7587 6229 7617 6207 22 P
2297 9444 2313 9434 2364 9410 2418 9397 2474 9389 2529 9397
2582 9417 2627 9451 2650 9502 2683 9547 2740 9545 2794 9532
2850 9541 2894 9575 2931 9617 2959 9666 3009 9691 3063 9673
3072 9617 3097 9567 3147 9592 3197 9617 3250 9600 3306 9593
3315 9538 3325 9483 3323 9426 3333 9371 3380 9339 3419 9298
3412 9242 3432 9190 3442 9163 3479 9207 3500 9244 3499 9187
3492 9131 3483 9090 3538 9078 3590 9056 3645 9066 3672 9115
3684 9171 3714 9200 3742 9213 45 P
5955 5622 5983 5626 6012 5633 6035 5637 4 P
9446 10405 9439 10460 9481 10497 9510 10545 9524 10598 9548 10648
9593 10681 9627 10702 8 P
7594 15162 7599 15188 7602 15216 7587 15238 7565 15253 7544 15271
7542 15298 7528 15321 7528 15346 7554 15343 7581 15343 7602 15342
7627 15355 7652 15362 7679 15362 7705 15369 7730 15376 17 P
3308 4878 3330 4878 3330 4605 3296 4605 3296 4800 3236 4800
3236 4824 3260 4827 3271 4829 3281 4833 3290 4839 3294 4843
3297 4848 3303 4861 3308 4878 15 N F
3523 4878 3545 4878 3545 4605 3511 4605 3511 4800 3450 4800
3450 4824 3475 4827 3486 4829 3496 4833 3504 4839 3508 4843
3512 4848 3518 4861 3523 4878 15 N F
3731 4878 3741 4877 3751 4875 3760 4871 3768 4866 3776 4859
3784 4852 3791 4843 3797 4833 3802 4823 3807 4812 3812 4800
3815 4788 3818 4776 3820 4763 3821 4750 3822 4738 3821 4725
3820 4712 3815 4686 3812 4674 3807 4662 3802 4651 3797 4641
3791 4631 3784 4622 3776 4614 3768 4607 3760 4602 3751 4598
3741 4596 3731 4595 3721 4596 3712 4598 3703 4602 3694 4607
3686 4614 3679 4622 3672 4631 3666 4641 3660 4651 3655 4662
3651 4674 3648 4686 3643 4712 3641 4725 3641 4738 3677 4738
3678 4713 3682 4691 3687 4672 3695 4656 3703 4643 3712 4633
3717 4629 3722 4627 3726 4625 3731 4625 3736 4625 3741 4627
3746 4629 3751 4633 3760 4643 3768 4656 3775 4672 3781 4691

3784 4713 3786 4738 3784 4762 3781 4784 3775 4803 3768 4818
3760 4831 3751 4840 3746 4844 3741 4846 3736 4848 3731 4848
3726 4848 3722 4846 3717 4844 3712 4840 3703 4831 3695 4818
3687 4803 3682 4784 3678 4762 3677 4738 3641 4738 3641 4750
3643 4763 3645 4776 3648 4788 3651 4800 3655 4812 3660 4823
3666 4833 3672 4843 3679 4852 3686 4859 3694 4866 3703 4871
3712 4875 3721 4877 3731 4878 105 N F
3989 4826 3988 4814 3984 4802 3978 4791 3971 4781 3961 4773
3950 4767 3938 4764 3925 4762 3925 4787 3917 4788 3910 4790
3903 4794 3898 4799 3893 4804 3889 4811 3887 4818 3886 4826
3887 4834 3889 4842 3893 4848 3898 4854 3903 4859 3910 4863
3917 4865 3925 4866 3933 4865 3941 4863 3947 4859 3953 4854
3958 4848 3962 4842 3964 4834 3965 4826 3964 4818 3962 4811
3958 4804 3953 4799 3947 4794 3941 4790 3933 4788 3925 4787
3925 4762 3913 4764 3901 4767 3890 4773 3880 4781 3872 4791
3866 4802 3863 4814 3861 4826 3863 4839 3866 4851 3872 4862
3880 4872 3890 4879 3901 4885 3913 4889 3925 4890 3938 4889
3950 4885 3961 4879 3971 4872 3978 4862 3984 4851 3988 4839
3989 4826 67 N F
4042 4886 4237 4886 4237 4854 4079 4854 4079 4764 4231 4764
4231 4731 4079 4731 4079 4637 4243 4637 4243 4605 4042 4605
4042 4886 13 N F
5472 4878 5494 4878 5494 4605 5460 4605 5460 4800 5399 4800
5399 4824 5424 4827 5435 4829 5445 4833 5453 4839 5457 4843
5461 4848 5467 4861 5472 4878 15 N F
5686 4878 5708 4878 5708 4605 5674 4605 5674 4800 5614 4800
5614 4824 5638 4827 5649 4829 5659 4833 5668 4839 5672 4843
5676 4848 5682 4861 5686 4878 15 N F
5925 4878 5949 4878 5949 4701 5991 4701 5991 4669 5949 4669
5949 4605 5915 4605 5915 4669 5801 4669 5801 4701 5831 4701
5915 4701 5915 4820 5831 4701 5801 4701 5801 4705 5925 4878
18 N F
6153 4826 6152 4814 6148 4802 6142 4791 6134 4781 6125 4773
6114 4767 6102 4764 6089 4762 6089 4787 6081 4788 6074 4790
6067 4794 6061 4799 6056 4804 6053 4811 6051 4818 6050 4826
6051 4834 6053 4842 6056 4848 6061 4854 6067 4859 6074 4863
6081 4865 6089 4866 6097 4865 6104 4863 6111 4859 6117 4854
6122 4848 6125 4842 6128 4834 6128 4826 6128 4818 6125 4811
6122 4804 6117 4799 6111 4794 6104 4790 6097 4788 6089 4787
6089 4762 6076 4764 6064 4767 6053 4773 6044 4781 6036 4791
6030 4802 6026 4814 6025 4826 6026 4839 6030 4851 6036 4862
6044 4872 6053 4879 6064 4885 6076 4889 6089 4890 6102 4889
6114 4885 6125 4879 6134 4872 6142 4862 6148 4851 6152 4839

6153 4826 67 N F
6206 4886 6401 4886 6401 4854 6242 4854 6242 4764 6395 4764
6395 4731 6242 4731 6242 4637 6407 4637 6407 4605 6206 4605
6206 4886 13 N F
7620 4878 7642 4878 7642 4605 7608 4605 7608 4800 7548 4800
7548 4824 7572 4827 7583 4829 7593 4833 7602 4839 7606 4843
7610 4848 7616 4861 7620 4878 15 N F
7835 4878 7857 4878 7857 4605 7823 4605 7823 4800 7763 4800
7763 4824 7787 4827 7798 4829 7808 4833 7817 4839 7821 4843
7824 4848 7831 4861 7835 4878 15 N F
8044 4878 8061 4877 8076 4873 8090 4867 8102 4859 8112 4849
8116 4843 8120 4837 8123 4830 8125 4822 8126 4814 8126 4806
8125 4794 8123 4784 8119 4775 8114 4768 8109 4761 8102 4756
8090 4748 8100 4742 8109 4736 8117 4729 8124 4720 8129 4711
8133 4701 8135 4690 8136 4679 8135 4666 8130 4652 8123 4637
8112 4624 8106 4618 8099 4613 8091 4607 8083 4603 8074 4600
8064 4597 8053 4595 8042 4595 8031 4595 8020 4597 8010 4600
8002 4603 7993 4607 7986 4613 7979 4618 7973 4624 7964 4637
7957 4652 7953 4666 7952 4679 7988 4679 7989 4668 7992 4658
7998 4649 8005 4641 8013 4634 8022 4629 8033 4626 8044 4625
8055 4626 8065 4629 8075 4634 8083 4641 8090 4649 8096 4658
8099 4668 8100 4679 8099 4690 8096 4700 8090 4710 8083 4718
8075 4724 8065 4729 8055 4732 8044 4733 8033 4732 8022 4729
8013 4724 8005 4718 7998 4710 7992 4700 7989 4690 7988 4679
7952 4679 7952 4690 7955 4701 7959 4710 7964 4719 7971 4728
7979 4735 7988 4742 7998 4748 7985 4756 7979 4761 7974 4768
7969 4775 7965 4784 7963 4794 7962 4806 7996 4806 7997 4797
8000 4788 8004 4781 8010 4775 8017 4770 8025 4767 8034 4764
8044 4764 8053 4764 8062 4767 8071 4770 8078 4775 8084 4781
8088 4788 8091 4797 8092 4806 8091 4815 8088 4823 8084 4831
8078 4837 8071 4842 8062 4845 8053 4847 8044 4848 8034 4847
8025 4845 8017 4842 8010 4837 8004 4831 8000 4823 7997 4815
7996 4806 7962 4806 7962 4814 7963 4822 7965 4830 7968 4837
7971 4843 7975 4849 7985 4859 7998 4867 8012 4873 8027 4877
8044 4878 145 N F
8302 4826 8300 4814 8297 4802 8291 4791 8283 4781 8274 4773
8263 4767 8251 4764 8238 4762 8238 4787 8230 4788 8223 4790
8216 4794 8210 4799 8205 4804 8202 4811 8199 4818 8199 4826
8199 4834 8202 4842 8205 4848 8210 4854 8216 4859 8223 4863
8230 4865 8238 4866 8246 4865 8253 4863 8260 4859 8266 4854
8271 4848 8274 4842 8276 4834 8277 4826 8276 4818 8274 4811
8271 4804 8266 4799 8260 4794 8253 4790 8246 4788 8238 4787
8238 4762 8225 4764 8213 4767 8202 4773 8193 4781 8185 4791

8179 4802 8175 4814 8174 4826 8175 4839 8179 4851 8185 4862
8193 4872 8202 4879 8213 4885 8225 4889 8238 4890 8251 4889
8263 4885 8274 4879 8283 4872 8291 4862 8297 4851 8300 4839
8302 4826 67 N F
8355 4886 8550 4886 8550 4854 8391 4854 8391 4764 8544 4764
8544 4731 8391 4731 8391 4637 8556 4637 8556 4605 8355 4605
8355 4886 13 N F
9783 4878 9805 4878 9805 4605 9771 4605 9771 4800 9710 4800
9710 4824 9735 4827 9746 4829 9756 4833 9764 4839 9768 4843
9772 4848 9778 4861 9783 4878 15 N F
9905 4784 9939 4784 9940 4797 9943 4810 9947 4821 9953 4830
9961 4838 9971 4843 9982 4847 9995 4848 10006 4847 10015 4844
10024 4840 10032 4834 10039 4826 10043 4818 10047 4807 10048 4796
10047 4784 10044 4774 10039 4766 10032 4759 10024 4752 10013 4744
9985 4727 9962 4714 9943 4700 9928 4686 9917 4671 9908 4656
9903 4640 9900 4623 9899 4605 10082 4605 10082 4637 9937 4637
9941 4649 9946 4660 9952 4668 9960 4676 9970 4684 9982 4691
10014 4709 10032 4718 10047 4728 10059 4739 10068 4751 10075 4763
10080 4775 10083 4788 10084 4800 10083 4808 10082 4816 10080 4824
10077 4832 10073 4838 10069 4845 10063 4851 10058 4856 10045 4866
10030 4872 10013 4877 9995 4878 9977 4877 9968 4875 9959 4872
9951 4869 9944 4865 9937 4861 9931 4855 9925 4849 9920 4842
9916 4834 9912 4826 9909 4816 9907 4806 9906 4795 9905 4784
78 N F
10120 4784 10154 4784 10155 4797 10157 4810 10162 4821 10168 4830
10176 4838 10185 4843 10197 4847 10210 4848 10221 4847 10230 4844
10239 4840 10247 4834 10253 4826 10258 4818 10261 4807 10262 4796
10261 4784 10259 4774 10254 4766 10247 4759 10238 4752 10228 4744
10200 4727 10177 4714 10158 4700 10143 4686 10131 4671 10123 4656
10118 4640 10115 4623 10114 4605 10297 4605 10297 4637 10152 4637
10156 4649 10161 4660 10167 4668 10175 4676 10185 4684 10197 4691
10228 4709 10246 4718 10261 4728 10273 4739 10283 4751 10290 4763
10295 4775 10298 4788 10299 4800 10298 4808 10297 4816 10295 4824
10292 4832 10288 4838 10283 4845 10278 4851 10272 4856 10259 4866
10244 4872 10228 4877 10210 4878 10191 4877 10183 4875 10174 4872
10166 4869 10159 4865 10152 4861 10145 4855 10140 4849 10135 4842
10130 4834 10127 4826 10124 4816 10122 4806 10120 4795 10120 4784
78 N F
10464 4826 10463 4814 10459 4802 10453 4791 10445 4781 10436 4773
10425 4767 10413 4764 10400 4762 10400 4787 10392 4788 10385 4790
10378 4794 10372 4799 10368 4804 10364 4811 10362 4818 10361 4826
10362 4834 10364 4842 10368 4848 10372 4854 10378 4859 10385 4863
10392 4865 10400 4866 10408 4865 10416 4863 10422 4859 10428 4854

10433 4848 10436 4842 10439 4834 10440 4826 10439 4818 10436 4811
10433 4804 10428 4799 10422 4794 10416 4790 10408 4788 10400 4787
10400 4762 10387 4764 10375 4767 10365 4773 10355 4781 10347 4791
10341 4802 10338 4814 10336 4826 10338 4839 10341 4851 10347 4862
10355 4872 10365 4879 10375 4885 10387 4889 10400 4890 10413 4889
10425 4885 10436 4879 10445 4872 10453 4862 10459 4851 10463 4839
10464 4826 67 N F
10517 4886 10712 4886 10712 4854 10553 4854 10553 4764 10706 4764
10706 4731 10553 4731 10553 4637 10718 4637 10718 4605 10517 4605
10517 4886 13 N F
11945 4878 11967 4878 11967 4605 11933 4605 11933 4800 11873 4800
11873 4824 11897 4827 11908 4829 11918 4833 11927 4839 11931 4843
11934 4848 11940 4861 11945 4878 15 N F
12067 4784 12102 4784 12102 4797 12105 4810 12109 4821 12115 4830
12123 4838 12133 4843 12144 4847 12158 4848 12168 4847 12178 4844
12187 4840 12194 4834 12201 4826 12206 4818 12209 4807 12210 4796
12209 4784 12206 4774 12201 4766 12195 4759 12186 4752 12175 4744
12148 4727 12124 4714 12105 4700 12090 4686 12079 4671 12071 4656
12065 4640 12062 4623 12061 4605 12244 4605 12244 4637 12100 4637
12103 4649 12108 4660 12115 4668 12123 4676 12133 4684 12145 4691
12176 4709 12194 4718 12209 4728 12221 4739 12231 4751 12238 4763
12242 4775 12245 4788 12246 4800 12246 4808 12244 4816 12242 4824
12239 4832 12235 4838 12231 4845 12226 4851 12220 4856 12207 4866
12192 4872 12175 4877 12158 4878 12139 4877 12130 4875 12122 4872
12114 4869 12106 4865 12099 4861 12093 4855 12087 4849 12082 4842
12078 4834 12074 4826 12071 4816 12069 4806 12068 4795 12067 4784
78 N F
12455 4808 12421 4808 12418 4817 12415 4825 12410 4832 12405 4838
12398 4842 12391 4845 12384 4847 12377 4848 12369 4848 12362 4846
12356 4844 12350 4841 12344 4837 12339 4833 12335 4827 12330 4821
12323 4806 12318 4789 12315 4768 12314 4746 12323 4754 12331 4761
12339 4767 12347 4770 12355 4773 12363 4775 12381 4776 12394 4774
12408 4770 12421 4764 12434 4754 12440 4748 12445 4742 12450 4734
12454 4727 12457 4718 12459 4708 12461 4698 12461 4687 12427 4685
12426 4696 12422 4707 12417 4718 12410 4727 12402 4734 12392 4740
12382 4744 12371 4746 12359 4744 12349 4740 12340 4734 12331 4727
12324 4718 12319 4707 12315 4696 12314 4685 12315 4674 12319 4663
12324 4653 12331 4644 12340 4636 12349 4630 12359 4626 12371 4625
12382 4626 12392 4630 12402 4636 12410 4644 12417 4653 12422 4663
12426 4674 12427 4685 12461 4687 12460 4671 12455 4655 12449 4639
12444 4632 12439 4625 12433 4619 12426 4613 12419 4608 12411 4603
12402 4600 12392 4597 12382 4595 12371 4595 12358 4595 12347 4598
12337 4601 12327 4605 12319 4611 12311 4618 12305 4626 12299 4634

12294 4644 12289 4655 12286 4666 12283 4678 12279 4704 12278 4731
12279 4748 12280 4764 12282 4779 12285 4793 12288 4806 12293 4818
12298 4829 12304 4839 12311 4848 12318 4856 12326 4862 12335 4868
12344 4872 12354 4876 12365 4878 12377 4878 12392 4877 12407 4873
12420 4866 12431 4857 12440 4847 12447 4835 12452 4822 12455 4808
126 N F
12626 4826 12625 4814 12621 4802 12615 4791 12608 4781 12598 4773
12587 4767 12575 4764 12562 4762 12562 4787 12555 4788 12547 4790
12540 4794 12535 4799 12530 4804 12526 4811 12524 4818 12523 4826
12524 4834 12526 4842 12530 4848 12535 4854 12540 4859 12547 4863
12555 4865 12562 4866 12570 4865 12578 4863 12585 4859 12590 4854
12595 4848 12599 4842 12601 4834 12602 4826 12601 4818 12599 4811
12595 4804 12590 4799 12585 4794 12578 4790 12570 4788 12562 4787
12562 4762 12550 4764 12538 4767 12527 4773 12517 4781 12510 4791
12504 4802 12500 4814 12499 4826 12500 4839 12504 4851 12510 4862
12517 4872 12527 4879 12538 4885 12550 4889 12562 4890 12575 4889
12587 4885 12598 4879 12608 4872 12615 4862 12621 4851 12625 4839
12626 4826 67 N F
12679 4886 12874 4886 12874 4854 12716 4854 12716 4764 12868 4764
12868 4731 12716 4731 12716 4637 12880 4637 12880 4605 12679 4605
12679 4886 13 N F
2316 17019 2338 17019 2338 16745 2304 16745 2304 16940 2244 16940
2244 16964 2268 16967 2279 16970 2289 16973 2298 16980 2301 16984
2305 16989 2311 17002 2316 17019 15 N F
2525 17019 2535 17018 2544 17015 2553 17012 2562 17006 2570 17000
2577 16992 2584 16984 2590 16974 2596 16964 2601 16953 2605 16941
2608 16929 2611 16916 2613 16904 2614 16891 2615 16878 2614 16865
2613 16852 2608 16827 2605 16815 2601 16803 2596 16792 2590 16781
2584 16771 2577 16762 2570 16755 2562 16748 2553 16743 2544 16739
2535 16736 2525 16735 2515 16736 2505 16739 2496 16743 2488 16748
2480 16755 2472 16762 2465 16771 2459 16781 2453 16792 2449 16803
2444 16815 2441 16827 2436 16852 2435 16865 2434 16878 2470 16878
2472 16854 2475 16832 2481 16812 2488 16796 2496 16783 2505 16773
2510 16770 2515 16767 2520 16766 2525 16765 2529 16766 2534 16767
2539 16770 2544 16773 2553 16783 2561 16796 2568 16812 2574 16832
2577 16854 2579 16878 2577 16902 2574 16924 2568 16943 2561 16959
2553 16972 2544 16981 2539 16984 2534 16987 2529 16988 2525 16989
2520 16988 2515 16987 2510 16984 2505 16981 2496 16972 2488 16959
2481 16943 2475 16924 2472 16902 2470 16878 2434 16878 2435 16891
2436 16904 2438 16916 2441 16929 2444 16941 2449 16953 2453 16964
2459 16974 2465 16984 2472 16992 2480 17000 2488 17006 2496 17012
2505 17015 2515 17018 2525 17019 105 N F
2675 17019 2818 17019 2818 16987 2703 16987 2693 16908 2703 16915

2715 16921 2729 16925 2745 16926 2759 16925 2774 16921 2789 16915
2795 16911 2802 16906 2808 16900 2814 16893 2819 16886 2823 16877
2827 16868 2829 16857 2831 16846 2832 16834 2830 16815 2828 16805
2826 16796 2822 16788 2818 16780 2813 16772 2808 16765 2802 16758
2794 16753 2787 16748 2778 16743 2769 16740 2759 16737 2749 16736
2737 16735 2727 16736 2717 16737 2708 16739 2700 16741 2692 16745
2685 16748 2679 16753 2673 16758 2664 16769 2656 16782 2651 16796
2647 16812 2681 16812 2685 16802 2689 16793 2694 16785 2701 16778
2708 16773 2716 16769 2725 16766 2735 16765 2749 16766 2761 16770
2772 16775 2781 16782 2788 16791 2793 16803 2796 16816 2798 16832
2797 16843 2794 16855 2790 16865 2783 16875 2775 16884 2764 16890
2752 16895 2745 16896 2737 16896 2730 16896 2723 16895 2716 16893
2710 16890 2699 16882 2685 16870 2655 16870 2675 17019 83 N F
2997 16967 2996 16954 2992 16942 2986 16931 2978 16922 2969 16914
2958 16908 2946 16904 2933 16903 2933 16928 2925 16928 2918 16931
2911 16934 2905 16939 2901 16945 2897 16952 2895 16959 2894 16967
2895 16975 2897 16982 2901 16989 2905 16995 2911 17000 2918 17003
2925 17006 2933 17006 2941 17006 2949 17003 2955 17000 2961 16995
2966 16989 2970 16982 2972 16975 2973 16967 2972 16959 2970 16952
2966 16945 2961 16939 2955 16934 2949 16931 2941 16928 2933 16928
2933 16903 2920 16904 2908 16908 2898 16914 2888 16922 2880 16931
2874 16942 2871 16954 2869 16967 2871 16980 2874 16992 2880 17003
2888 17012 2898 17020 2908 17026 2920 17030 2933 17031 2946 17030
2958 17026 2969 17020 2978 17012 2986 17003 2992 16992 2996 16980
2997 16967 67 N F
3050 17027 3245 17027 3245 16995 3086 16995 3086 16904 3239 16904
3239 16872 3086 16872 3086 16778 3251 16778 3251 16745 3050 16745
3050 17027 13 N F
4381 17019 4403 17019 4403 16745 4369 16745 4369 16940 4309 16940
4309 16964 4333 16967 4344 16970 4354 16973 4363 16980 4367 16984
4370 16989 4376 17002 4381 17019 15 N F
4596 17019 4618 17019 4618 16745 4584 16745 4584 16940 4523 16940
4523 16964 4548 16967 4559 16970 4569 16973 4577 16980 4581 16984
4585 16989 4591 17002 4596 17019 15 N F
4804 17019 4814 17018 4824 17015 4833 17012 4841 17006 4849 17000
4857 16992 4864 16984 4870 16974 4875 16964 4880 16953 4885 16941
4888 16929 4891 16916 4893 16904 4894 16891 4895 16878 4894 16865
4893 16852 4888 16827 4885 16815 4880 16803 4875 16792 4870 16781
4864 16771 4857 16762 4849 16755 4841 16748 4833 16743 4824 16739
4814 16736 4804 16735 4794 16736 4785 16739 4776 16743 4767 16748
4759 16755 4752 16762 4745 16771 4739 16781 4733 16792 4728 16803
4724 16815 4721 16827 4716 16852 4715 16865 4714 16878 4750 16878
4751 16854 4755 16832 4760 16812 4768 16796 4776 16783 4785 16773

4790 16770 4795 16767 4799 16766 4804 16765 4809 16766 4814 16767
4819 16770 4824 16773 4833 16783 4841 16796 4848 16812 4854 16832
4857 16854 4859 16878 4857 16902 4854 16924 4848 16943 4841 16959
4833 16972 4824 16981 4819 16984 4814 16987 4809 16988 4804 16989
4799 16988 4795 16987 4790 16984 4785 16981 4776 16972 4768 16959
4760 16943 4755 16924 4751 16902 4750 16878 4714 16878 4715 16891
4716 16904 4718 16916 4721 16929 4724 16941 4728 16953 4733 16964
4739 16974 4745 16984 4752 16992 4759 17000 4767 17006 4776 17012
4785 17015 4794 17018 4804 17019 105 N F
5062 16967 5061 16954 5057 16942 5051 16931 5044 16922 5034 16914
5023 16908 5011 16904 4998 16903 4998 16928 4990 16928 4983 16931
4976 16934 4971 16939 4966 16945 4962 16952 4960 16959 4959 16967
4960 16975 4962 16982 4966 16989 4971 16995 4976 17000 4983 17003
4990 17006 4998 17006 5006 17006 5014 17003 5020 17000 5026 16995
5031 16989 5035 16982 5037 16975 5038 16967 5037 16959 5035 16952
5031 16945 5026 16939 5020 16934 5014 16931 5006 16928 4998 16928
4998 16903 4986 16904 4974 16908 4963 16914 4953 16922 4945 16931
4940 16942 4936 16954 4934 16967 4936 16980 4940 16992 4945 17003
4953 17012 4963 17020 4974 17026 4986 17030 4998 17031 5011 17030
5023 17026 5034 17020 5044 17012 5051 17003 5057 16992 5061 16980
5062 16967 67 N F
5115 17027 5310 17027 5310 16995 5152 16995 5152 16904 5304 16904
5304 16872 5152 16872 5152 16778 5316 16778 5316 16745 5115 16745
5115 17027 13 N F
6412 17019 6434 17019 6434 16745 6400 16745 6400 16940 6340 16940
6340 16964 6364 16967 6375 16970 6385 16973 6394 16980 6397 16984
6401 16989 6407 17002 6412 17019 15 N F
6627 17019 6649 17019 6649 16745 6614 16745 6614 16940 6554 16940
6554 16964 6579 16967 6589 16970 6599 16973 6608 16980 6612 16984
6616 16989 6622 17002 6627 17019 15 N F
6771 17019 6914 17019 6914 16987 6799 16987 6789 16908 6799 16915
6811 16921 6825 16925 6841 16926 6855 16925 6870 16921 6884 16915
6891 16911 6898 16906 6904 16900 6910 16893 6915 16886 6919 16877
6923 16868 6925 16857 6927 16846 6928 16834 6926 16815 6924 16805
6922 16796 6918 16788 6914 16780 6909 16772 6904 16765 6897 16758
6890 16753 6883 16748 6874 16743 6865 16740 6855 16737 6845 16736
6833 16735 6823 16736 6813 16737 6804 16739 6796 16741 6788 16745
6781 16748 6775 16753 6769 16758 6760 16769 6752 16782 6747 16796
6743 16812 6777 16812 6781 16802 6785 16793 6790 16785 6797 16778
6804 16773 6812 16769 6821 16766 6831 16765 6845 16766 6857 16770
6868 16775 6877 16782 6884 16791 6889 16803 6892 16816 6893 16832
6893 16843 6890 16855 6886 16865 6879 16875 6871 16884 6860 16890
6848 16895 6841 16896 6833 16896 6825 16896 6819 16895 6812 16893

6806 16890 6795 16882 6781 16870 6751 16870 6771 17019 83 N F
7093 16967 7092 16954 7088 16942 7082 16931 7074 16922 7065 16914
7054 16908 7042 16904 7029 16903 7029 16928 7021 16928 7014 16931
7007 16934 7001 16939 6997 16945 6993 16952 6991 16959 6990 16967
6991 16975 6993 16982 6997 16989 7001 16995 7007 17000 7014 17003
7021 17006 7029 17006 7037 17006 7045 17003 7051 17000 7057 16995
7062 16989 7065 16982 7068 16975 7069 16967 7068 16959 7065 16952
7062 16945 7057 16939 7051 16934 7045 16931 7037 16928 7029 16928
7029 16903 7016 16904 7004 16908 6994 16914 6984 16922 6976 16931
6970 16942 6967 16954 6965 16967 6967 16980 6970 16992 6976 17003
6984 17012 6994 17020 7004 17026 7016 17030 7029 17031 7042 17030
7054 17026 7065 17020 7074 17012 7082 17003 7088 16992 7092 16980
7093 16967 67 N F
7146 17027 7341 17027 7341 16995 7182 16995 7182 16904 7335 16904
7335 16872 7182 16872 7182 16778 7347 16778 7347 16745 7146 16745
7146 17027 13 N F
8433 17019 8455 17019 8455 16745 8421 16745 8421 16940 8361 16940
8361 16964 8385 16967 8396 16970 8406 16973 8415 16980 8419 16984
8422 16989 8428 17002 8433 17019 15 N F
8555 16924 8589 16924 8590 16938 8593 16950 8597 16961 8603 16971
8611 16978 8621 16984 8632 16987 8646 16989 8656 16988 8666 16985
8675 16981 8682 16974 8689 16967 8694 16958 8697 16948 8698 16936
8697 16925 8694 16915 8689 16907 8683 16899 8674 16892 8663 16885
8636 16868 8612 16854 8593 16841 8578 16827 8567 16812 8559 16797
8553 16781 8550 16764 8549 16745 8732 16745 8732 16778 8587 16778
8591 16790 8596 16800 8602 16809 8611 16817 8621 16824 8633 16832
8664 16850 8682 16859 8697 16869 8709 16880 8718 16891 8726 16903
8730 16916 8733 16928 8734 16940 8734 16949 8732 16957 8730 16965
8727 16972 8723 16979 8719 16985 8714 16991 8708 16997 8695 17006
8680 17013 8663 17017 8646 17019 8627 17017 8618 17016 8610 17013
8602 17010 8594 17006 8587 17001 8581 16996 8575 16989 8570 16982
8566 16975 8562 16966 8559 16957 8557 16947 8556 16936 8555 16924
78 N F
8856 17019 8866 17018 8876 17015 8885 17012 8893 17006 8901 17000
8909 16992 8916 16984 8922 16974 8928 16964 8932 16953 8937 16941
8940 16929 8943 16916 8945 16904 8946 16891 8947 16878 8946 16865
8945 16852 8940 16827 8937 16815 8932 16803 8928 16792 8922 16781
8916 16771 8909 16762 8901 16755 8893 16748 8885 16743 8876 16739
8866 16736 8856 16735 8846 16736 8837 16739 8828 16743 8819 16748
8811 16755 8804 16762 8797 16771 8791 16781 8785 16792 8780 16803
8776 16815 8773 16827 8768 16852 8767 16865 8766 16878 8802 16878
8803 16854 8807 16832 8812 16812 8820 16796 8828 16783 8837 16773
8842 16770 8847 16767 8852 16766 8856 16765 8861 16766 8866 16767

8871 16770 8876 16773 8885 16783 8893 16796 8900 16812 8906 16832
8909 16854 8911 16878 8909 16902 8906 16924 8900 16943 8893 16959
8885 16972 8876 16981 8871 16984 8866 16987 8861 16988 8856 16989
8852 16988 8847 16987 8842 16984 8837 16981 8828 16972 8820 16959
8812 16943 8807 16924 8803 16902 8802 16878 8766 16878 8767 16891
8768 16904 8770 16916 8773 16929 8776 16941 8780 16953 8785 16964
8791 16974 8797 16984 8804 16992 8811 17000 8819 17006 8828 17012
8837 17015 8846 17018 8856 17019 105 N F
9114 16967 9113 16954 9109 16942 9103 16931 9096 16922 9086 16914
9075 16908 9063 16904 9050 16903 9050 16928 9042 16928 9035 16931
9028 16934 9023 16939 9018 16945 9014 16952 9012 16959 9011 16967
9012 16975 9014 16982 9018 16989 9023 16995 9028 17000 9035 17003
9042 17006 9050 17006 9058 17006 9066 17003 9072 17000 9078 16995
9083 16989 9087 16982 9089 16975 9090 16967 9089 16959 9087 16952
9083 16945 9078 16939 9072 16934 9066 16931 9058 16928 9050 16928
9050 16903 9038 16904 9026 16908 9015 16914 9005 16922 8997 16931
8992 16942 8988 16954 8986 16967 8988 16980 8992 16992 8997 17003
9005 17012 9015 17020 9026 17026 9038 17030 9050 17031 9063 17030
9075 17026 9086 17020 9096 17012 9103 17003 9109 16992 9113 16980
9114 16967 67 N F
9167 17027 9362 17027 9362 16995 9204 16995 9204 16904 9356 16904
9356 16872 9204 16872 9204 16778 9368 16778 9368 16745 9167 16745
9167 17027 13 N F
10460 17019 10482 17019 10482 16745 10448 16745 10448 16940 10388 16940
10388 16964 10412 16967 10423 16970 10433 16973 10442 16980 10446 16984
10450 16989 10456 17002 10460 17019 15 N F
10583 16924 10617 16924 10618 16938 10620 16950 10625 16961 10631 16971
10639 16978 10648 16984 10660 16987 10673 16989 10683 16988 10693 16985
10702 16981 10710 16974 10716 16967 10721 16958 10724 16948 10725 16936
10724 16925 10721 16915 10717 16907 10710 16899 10701 16892 10691 16885
10663 16868 10640 16854 10621 16841 10606 16827 10594 16812 10586 16797
10581 16781 10578 16764 10577 16745 10759 16745 10759 16778 10615 16778
10618 16790 10623 16800 10630 16809 10638 16817 10648 16824 10660 16832
10691 16850 10709 16859 10724 16869 10736 16880 10746 16891 10753 16903
10758 16916 10761 16928 10761 16940 10761 16949 10760 16957 10757 16965
10754 16972 10751 16979 10746 16985 10741 16991 10735 16997 10722 17006
10707 17013 10691 17017 10673 17019 10654 17017 10645 17016 10637 17013
10629 17010 10622 17006 10615 17001 10608 16996 10603 16989 10598 16982
10593 16975 10590 16966 10587 16957 10585 16947 10583 16936 10583 16924
78 N F
10820 17019 10962 17019 10962 16987 10848 16987 10838 16908 10848 16915
10859 16921 10873 16925 10890 16926 10904 16925 10919 16921 10933 16915
10940 16911 10947 16906 10953 16900 10958 16893 10963 16886 10968 16877

10971 16868 10974 16857 10976 16846 10976 16834 10975 16815 10973 16805
10970 16796 10967 16788 10963 16780 10958 16772 10952 16765 10946 16758
10939 16753 10931 16748 10923 16743 10914 16740 10904 16737 10893 16736
10882 16735 10871 16736 10862 16737 10853 16739 10844 16741 10837 16745
10830 16748 10824 16753 10818 16758 10808 16769 10801 16782 10795 16796
10792 16812 10826 16812 10829 16802 10834 16793 10839 16785 10845 16778
10852 16773 10861 16769 10870 16766 10880 16765 10893 16766 10906 16770
10916 16775 10925 16782 10932 16791 10938 16803 10941 16816 10942 16832
10941 16843 10939 16855 10934 16865 10928 16875 10919 16884 10909 16890
10897 16895 10889 16896 10882 16896 10874 16896 10867 16895 10861 16893
10855 16890 10843 16882 10830 16870 10800 16870 10820 17019 83 N F
11142 16967 11140 16954 11137 16942 11131 16931 11123 16922 11113 16914
11103 16908 11091 16904 11078 16903 11078 16928 11070 16928 11062 16931
11056 16934 11050 16939 11045 16945 11042 16952 11039 16959 11038 16967
11039 16975 11042 16982 11045 16989 11050 16995 11056 17000 11062 17003
11070 17006 11078 17006 11086 17006 11093 17003 11100 17000 11106 16995
11110 16989 11114 16982 11116 16975 11117 16967 11116 16959 11114 16952
11110 16945 11106 16939 11100 16934 11093 16931 11086 16928 11078 16928
11078 16903 11065 16904 11053 16908 11042 16914 11033 16922 11025 16931
11019 16942 11015 16954 11014 16967 11015 16980 11019 16992 11025 17003
11033 17012 11042 17020 11053 17026 11065 17030 11078 17031 11091 17030
11103 17026 11113 17020 11123 17012 11131 17003 11137 16992 11140 16980
11142 16967 67 N F
11195 17027 11390 17027 11390 16995 11231 16995 11231 16904 11383 16904
11383 16872 11231 16872 11231 16778 11396 16778 11396 16745 11195 16745
11195 17027 13 N F
12511 17019 12533 17019 12533 16745 12499 16745 12499 16940 12439 16940
12439 16964 12463 16967 12474 16970 12484 16973 12493 16980 12497 16984
12500 16989 12506 17002 12511 17019 15 N F
12666 16930 12631 16930 12634 16951 12636 16960 12638 16969 12642 16977
12646 16984 12651 16990 12656 16996 12662 17002 12668 17006 12675 17010
12683 17013 12691 17016 12699 17017 12718 17019 12735 17017 12751 17014
12766 17008 12778 17000 12783 16995 12788 16989 12792 16983 12796 16976
12798 16969 12800 16961 12802 16953 12802 16944 12802 16934 12800 16925
12797 16917 12793 16909 12788 16902 12781 16896 12772 16891 12762 16886
12774 16881 12784 16875 12792 16869 12799 16861 12804 16853 12807 16844
12809 16833 12810 16822 12809 16807 12805 16792 12799 16777 12794 16771
12789 16764 12783 16759 12776 16753 12769 16749 12760 16745 12751 16742
12740 16739 12728 16738 12716 16737 12705 16738 12695 16739 12685 16741
12677 16744 12669 16747 12661 16751 12655 16756 12649 16762 12644 16768
12639 16774 12636 16781 12633 16789 12629 16806 12627 16824 12662 16824
12662 16814 12664 16804 12667 16794 12672 16786 12679 16778 12689 16773
12701 16769 12718 16767 12732 16768 12743 16771 12753 16776 12761 16783

12767 16791 12771 16800 12773 16811 12774 16822 12773 16836 12771 16842
12768 16847 12766 16852 12762 16856 12753 16863 12742 16867 12729 16870
12714 16872 12698 16872 12698 16900 12714 16900 12728 16902 12740 16904
12749 16908 12756 16913 12762 16920 12765 16930 12766 16942 12765 16952
12763 16961 12760 16968 12755 16975 12748 16981 12740 16985 12730 16988
12718 16989 12707 16988 12697 16986 12689 16982 12681 16977 12675 16969
12670 16959 12667 16946 12666 16930 123 N F
12934 17019 12944 17018 12954 17015 12963 17012 12971 17006 12979 17000
12987 16992 12994 16984 13000 16974 13006 16964 13011 16953 13015 16941
13018 16929 13021 16916 13023 16904 13024 16891 13025 16878 13024 16865
13023 16852 13018 16827 13015 16815 13011 16803 13006 16792 13000 16781
12994 16771 12987 16762 12979 16755 12971 16748 12963 16743 12954 16739
12944 16736 12934 16735 12925 16736 12915 16739 12906 16743 12898 16748
12890 16755 12882 16762 12875 16771 12869 16781 12863 16792 12858 16803
12854 16815 12851 16827 12846 16852 12845 16865 12844 16878 12880 16878
12882 16854 12885 16832 12891 16812 12898 16796 12906 16783 12915 16773
12920 16770 12925 16767 12930 16766 12934 16765 12939 16766 12944 16767
12949 16770 12954 16773 12963 16783 12971 16796 12978 16812 12984 16832
12987 16854 12989 16878 12987 16902 12984 16924 12978 16943 12971 16959
12963 16972 12954 16981 12949 16984 12944 16987 12939 16988 12934 16989
12930 16988 12925 16987 12920 16984 12915 16981 12906 16972 12898 16959
12891 16943 12885 16924 12882 16902 12880 16878 12844 16878 12845 16891
12846 16904 12848 16916 12851 16929 12854 16941 12858 16953 12863 16964
12869 16974 12875 16984 12882 16992 12890 17000 12898 17006 12906 17012
12915 17015 12925 17018 12934 17019 105 N F
13192 16967 13191 16954 13187 16942 13181 16931 13174 16922 13164 16914
13153 16908 13141 16904 13128 16903 13128 16928 13121 16928 13113 16931
13106 16934 13101 16939 13096 16945 13092 16952 13090 16959 13089 16967
13090 16975 13092 16982 13096 16989 13101 16995 13106 17000 13113 17003
13121 17006 13128 17006 13136 17006 13144 17003 13150 17000 13156 16995
13161 16989 13165 16982 13167 16975 13168 16967 13167 16959 13165 16952
13161 16945 13156 16939 13150 16934 13144 16931 13136 16928 13128 16928
13128 16903 13116 16904 13104 16908 13093 16914 13083 16922 13076 16931
13070 16942 13066 16954 13065 16967 13066 16980 13070 16992 13076 17003
13083 17012 13093 17020 13104 17026 13116 17030 13128 17031 13141 17030
13153 17026 13164 17020 13174 17012 13181 17003 13187 16992 13191 16980
13192 16967 67 N F
13245 17027 13440 17027 13440 16995 13282 16995 13282 16904 13434 16904
13434 16872 13282 16872 13282 16778 13446 16778 13446 16745 13245 16745
13245 17027 13 N F
911 5683 945 5683 946 5696 948 5709 952 5720 959 5729
966 5737 976 5742 988 5746 1001 5747 1011 5746 1021 5743
1030 5739 1038 5733 1044 5725 1049 5717 1052 5706 1053 5695

1052 5683 1049 5673 1045 5665 1038 5658 1029 5651 1018 5643
991 5626 968 5613 949 5599 934 5585 922 5570 914 5555
909 5539 906 5522 905 5504 1087 5504 1087 5536 943 5536
946 5548 951 5559 958 5567 966 5575 976 5583 988 5590
1019 5608 1037 5617 1052 5627 1064 5638 1074 5650 1081 5662
1086 5674 1088 5687 1089 5699 1089 5707 1087 5715 1085 5723
1082 5731 1078 5737 1074 5744 1069 5750 1063 5755 1050 5765
1035 5771 1019 5776 1001 5777 982 5776 973 5774 965 5771
957 5768 949 5764 943 5760 936 5754 931 5748 925 5741
921 5733 917 5725 914 5715 912 5705 911 5694 911 5683
78 N F
1125 5683 1160 5683 1160 5696 1163 5709 1167 5720 1173 5729
1181 5737 1191 5742 1202 5746 1216 5747 1226 5746 1236 5743
1245 5739 1252 5733 1259 5725 1264 5717 1267 5706 1268 5695
1267 5683 1264 5673 1259 5665 1253 5658 1244 5651 1233 5643
1206 5626 1182 5613 1163 5599 1148 5585 1137 5570 1129 5555
1123 5539 1120 5522 1119 5504 1302 5504 1302 5536 1157 5536
1161 5548 1166 5559 1172 5567 1181 5575 1191 5583 1203 5590
1234 5608 1252 5617 1267 5627 1279 5638 1288 5650 1296 5662
1300 5674 1303 5687 1304 5699 1304 5707 1302 5715 1300 5723
1297 5731 1293 5737 1289 5744 1284 5750 1278 5755 1265 5765
1250 5771 1233 5776 1216 5777 1197 5776 1188 5774 1180 5771
1172 5768 1164 5764 1157 5760 1151 5754 1145 5748 1140 5741
1136 5733 1132 5725 1129 5715 1127 5705 1126 5694 1125 5683
78 N F
1470 5725 1468 5713 1465 5701 1459 5690 1451 5680 1441 5672
1430 5666 1419 5663 1406 5661 1406 5686 1398 5687 1390 5689
1384 5693 1378 5698 1373 5703 1369 5710 1367 5717 1366 5725
1367 5733 1369 5741 1373 5747 1378 5753 1384 5758 1390 5762
1398 5764 1406 5765 1414 5764 1421 5762 1428 5758 1434 5753
1438 5747 1442 5741 1444 5733 1445 5725 1444 5717 1442 5710
1438 5703 1434 5698 1428 5693 1421 5689 1414 5687 1406 5686
1406 5661 1393 5663 1381 5666 1370 5672 1361 5680 1353 5690
1347 5701 1343 5713 1342 5725 1343 5738 1347 5750 1353 5761
1361 5771 1370 5778 1381 5784 1393 5788 1406 5789 1419 5788
1430 5784 1441 5778 1451 5771 1459 5761 1465 5750 1468 5738
1470 5725 67 N F
1517 5785 1557 5785 1701 5556 1701 5785 1735 5785 1735 5504
1695 5504 1551 5733 1551 5504 1517 5504 1517 5785 11 N F
911 6859 945 6859 946 6873 948 6886 952 6897 959 6906
966 6913 976 6919 988 6923 1001 6924 1011 6923 1021 6920
1030 6916 1038 6910 1044 6902 1049 6893 1052 6883 1053 6872
1052 6860 1049 6850 1045 6842 1038 6834 1029 6827 1018 6820

991 6803 968 6790 949 6776 934 6762 922 6747 914 6732
909 6716 906 6699 905 6681 1087 6681 1087 6713 943 6713
946 6725 951 6735 958 6744 966 6752 976 6759 988 6767
1019 6785 1037 6794 1052 6804 1064 6815 1074 6827 1081 6839
1086 6851 1088 6863 1089 6876 1089 6884 1087 6892 1085 6900
1082 6907 1078 6914 1074 6921 1069 6927 1063 6932 1050 6941
1035 6948 1019 6952 1001 6954 982 6952 973 6951 965 6948
957 6945 949 6941 943 6936 936 6931 931 6925 925 6918
921 6910 917 6901 914 6892 912 6882 911 6871 911 6859
78 N F
1242 6954 1266 6954 1266 6777 1308 6777 1308 6745 1266 6745
1266 6681 1232 6681 1232 6745 1117 6745 1117 6777 1147 6777
1232 6777 1232 6896 1147 6777 1117 6777 1117 6781 1242 6954
18 N F
1470 6902 1468 6889 1465 6877 1459 6866 1451 6857 1441 6849
1430 6843 1419 6839 1406 6838 1406 6863 1398 6864 1390 6866
1384 6869 1378 6874 1373 6880 1369 6887 1367 6894 1366 6902
1367 6910 1369 6918 1373 6924 1378 6930 1384 6935 1390 6938
1398 6941 1406 6942 1414 6941 1421 6938 1428 6935 1434 6930
1438 6924 1442 6918 1444 6910 1445 6902 1444 6894 1442 6887
1438 6880 1434 6874 1428 6869 1421 6866 1414 6864 1406 6863
1406 6838 1393 6839 1381 6843 1370 6849 1361 6857 1353 6866
1347 6877 1343 6889 1342 6902 1343 6915 1347 6927 1353 6938
1361 6947 1370 6955 1381 6961 1393 6965 1406 6966 1419 6965
1430 6961 1441 6955 1451 6947 1459 6938 1465 6927 1468 6915
1470 6902 67 N F
1517 6962 1557 6962 1701 6733 1701 6962 1735 6962 1735 6681
1695 6681 1551 6910 1551 6681 1517 6681 1517 6962 11 N F
911 8021 945 8021 946 8035 948 8047 952 8058 959 8067
966 8075 976 8081 988 8084 1001 8085 1011 8084 1021 8082
1030 8077 1038 8071 1044 8064 1049 8055 1052 8045 1053 8033
1052 8021 1049 8012 1045 8003 1038 7996 1029 7989 1018 7982
991 7965 968 7951 949 7937 934 7923 922 7909 914 7893
909 7877 906 7860 905 7842 1087 7842 1087 7874 943 7874
946 7887 951 7897 958 7906 966 7913 976 7921 988 7929
1019 7947 1037 7956 1052 7966 1064 7976 1074 7988 1081 8000
1086 8012 1088 8025 1089 8037 1089 8046 1087 8054 1085 8061
1082 8069 1078 8076 1074 8082 1069 8088 1063 8094 1050 8103
1035 8110 1019 8114 1001 8115 982 8114 973 8112 965 8110
957 8106 949 8103 943 8098 936 8092 931 8086 925 8079
921 8071 917 8063 914 8054 912 8044 911 8033 911 8021
78 N F
1298 8045 1264 8045 1262 8054 1258 8062 1253 8069 1248 8075

1241 8079 1235 8083 1227 8085 1220 8085 1212 8085 1206 8084
1199 8081 1193 8078 1188 8074 1182 8070 1178 8064 1174 8058
1167 8043 1162 8026 1159 8006 1157 7983 1166 7992 1174 7999
1182 8004 1190 8008 1198 8010 1206 8012 1224 8013 1237 8012
1251 8008 1265 8001 1277 7991 1283 7986 1288 7979 1293 7972
1297 7964 1300 7955 1303 7946 1304 7935 1304 7925 1270 7923
1269 7934 1265 7945 1260 7955 1253 7964 1245 7972 1235 7978
1225 7981 1214 7983 1203 7981 1192 7978 1183 7972 1174 7964
1167 7955 1162 7945 1159 7934 1157 7923 1159 7911 1162 7900
1167 7890 1174 7881 1183 7873 1192 7867 1203 7864 1214 7862
1225 7864 1235 7867 1245 7873 1253 7881 1260 7890 1265 7900
1269 7911 1270 7923 1304 7925 1303 7908 1299 7892 1292 7877
1287 7869 1282 7862 1276 7856 1270 7850 1262 7845 1254 7841
1245 7837 1235 7834 1225 7833 1214 7832 1201 7833 1190 7835
1180 7838 1170 7843 1162 7848 1154 7855 1148 7863 1142 7872
1137 7881 1133 7892 1129 7903 1126 7915 1123 7941 1121 7969
1122 7985 1123 8001 1125 8016 1128 8030 1132 8043 1136 8055
1141 8066 1147 8076 1154 8085 1161 8093 1169 8100 1178 8105
1188 8110 1198 8113 1208 8115 1220 8115 1236 8114 1250 8110
1263 8103 1274 8095 1283 8084 1290 8072 1295 8059 1298 8045
126 N F
1470 8064 1468 8051 1465 8039 1459 8028 1451 8019 1441 8011
1430 8005 1419 8001 1406 8000 1406 8024 1398 8025 1390 8027
1384 8031 1378 8036 1373 8042 1369 8048 1367 8056 1366 8064
1367 8072 1369 8079 1373 8086 1378 8092 1384 8096 1390 8100
1398 8102 1406 8103 1414 8102 1421 8100 1428 8096 1434 8092
1438 8086 1442 8079 1444 8072 1445 8064 1444 8056 1442 8048
1438 8042 1434 8036 1428 8031 1421 8027 1414 8025 1406 8024
1406 8000 1393 8001 1381 8005 1370 8011 1361 8019 1353 8028
1347 8039 1343 8051 1342 8064 1343 8077 1347 8089 1353 8099
1361 8109 1370 8117 1381 8123 1393 8126 1406 8128 1419 8126
1430 8123 1441 8117 1451 8109 1459 8099 1465 8089 1468 8077
1470 8064 67 N F
1517 8123 1557 8123 1701 7894 1701 8123 1735 8123 1735 7842
1695 7842 1551 8071 1551 7842 1517 7842 1517 8123 11 N F
911 9166 945 9166 946 9180 948 9192 952 9203 959 9213
966 9220 976 9226 988 9229 1001 9231 1011 9230 1021 9227
1030 9223 1038 9217 1044 9209 1049 9200 1052 9190 1053 9178
1052 9167 1049 9157 1045 9149 1038 9141 1029 9134 1018 9127
991 9110 968 9096 949 9083 934 9069 922 9054 914 9039
909 9023 906 9006 905 8987 1087 8987 1087 9020 943 9020
946 9032 951 9042 958 9051 966 9059 976 9066 988 9074
1019 9092 1037 9101 1052 9111 1064 9122 1074 9133 1081 9145

1086 9158 1088 9170 1089 9182 1089 9191 1087 9199 1085 9207
1082 9214 1078 9221 1074 9227 1069 9233 1063 9239 1050 9248
1035 9255 1019 9259 1001 9261 982 9259 973 9258 965 9255
957 9252 949 9248 943 9243 936 9238 931 9231 925 9224
921 9217 917 9208 914 9199 912 9189 911 9178 911 9166
78 N F
1212 9261 1228 9260 1244 9256 1258 9250 1270 9242 1280 9232
1284 9226 1288 9219 1290 9212 1292 9205 1294 9197 1294 9188
1293 9177 1291 9166 1287 9158 1282 9150 1276 9144 1270 9139
1258 9130 1268 9125 1277 9119 1285 9111 1291 9103 1297 9093
1301 9083 1303 9073 1304 9062 1303 9048 1298 9034 1291 9020
1280 9007 1274 9001 1267 8995 1259 8990 1251 8986 1241 8982
1232 8980 1221 8978 1210 8977 1198 8978 1188 8980 1178 8982
1169 8986 1161 8990 1154 8995 1147 9001 1141 9007 1132 9020
1125 9034 1121 9048 1119 9062 1155 9062 1157 9051 1160 9041
1165 9031 1172 9023 1181 9017 1190 9012 1201 9009 1212 9008
1223 9009 1233 9012 1243 9017 1251 9023 1258 9031 1263 9041
1267 9051 1268 9062 1267 9073 1263 9083 1258 9092 1251 9100
1243 9107 1233 9112 1223 9115 1212 9116 1201 9115 1190 9112
1181 9107 1172 9100 1165 9092 1160 9083 1157 9073 1155 9062
1119 9062 1120 9073 1123 9083 1127 9093 1132 9102 1139 9110
1146 9118 1155 9124 1166 9130 1153 9139 1147 9144 1141 9150
1137 9158 1133 9166 1130 9177 1129 9188 1164 9188 1165 9179
1167 9171 1172 9164 1178 9158 1185 9153 1193 9149 1202 9147
1212 9146 1221 9147 1230 9149 1238 9153 1246 9158 1251 9164
1256 9171 1259 9179 1260 9188 1259 9198 1256 9206 1251 9213
1246 9219 1238 9224 1230 9228 1221 9230 1212 9231 1202 9230
1193 9228 1185 9224 1178 9219 1172 9213 1167 9206 1165 9198
1164 9188 1129 9188 1130 9197 1131 9205 1133 9212 1136 9219
1139 9226 1143 9232 1153 9242 1165 9250 1179 9256 1195 9260
1212 9261 145 N F
1470 9209 1468 9196 1465 9184 1459 9173 1451 9164 1441 9156
1430 9150 1419 9146 1406 9145 1406 9170 1398 9170 1390 9173
1384 9176 1378 9181 1373 9187 1369 9194 1367 9201 1366 9209
1367 9217 1369 9224 1373 9231 1378 9237 1384 9242 1390 9245
1398 9248 1406 9248 1414 9248 1421 9245 1428 9242 1434 9237
1438 9231 1442 9224 1444 9217 1445 9209 1444 9201 1442 9194
1438 9187 1434 9181 1428 9176 1421 9173 1414 9170 1406 9170
1406 9145 1393 9146 1381 9150 1370 9156 1361 9164 1353 9173
1347 9184 1343 9196 1342 9209 1343 9222 1347 9234 1353 9245
1361 9254 1370 9262 1381 9268 1393 9272 1406 9273 1419 9272
1430 9268 1441 9262 1451 9254 1459 9245 1465 9234 1468 9222
1470 9209 67 N F

1517 9269 1557 9269 1701 9040 1701 9269 1735 9269 1735 8987
1695 8987 1551 9217 1551 8987 1517 8987 1517 9269 11 N F
943 10308 909 10308 911 10328 913 10337 916 10346 919 10354
923 10361 928 10368 933 10374 939 10379 945 10383 952 10387
960 10390 968 10393 976 10395 995 10396 1012 10395 1028 10391
1043 10385 1055 10377 1060 10372 1065 10366 1069 10360 1073 10354
1076 10346 1078 10339 1079 10330 1079 10322 1079 10311 1077 10302
1074 10294 1070 10286 1065 10280 1058 10274 1049 10268 1039 10263
1051 10258 1061 10253 1069 10246 1076 10239 1081 10230 1085 10221
1087 10210 1087 10199 1086 10184 1082 10169 1076 10155 1072 10148
1066 10142 1060 10136 1054 10131 1046 10126 1037 10122 1028 10119
1017 10117 1006 10115 993 10115 982 10115 972 10116 962 10118
954 10121 946 10124 939 10129 932 10133 926 10139 921 10145
917 10152 913 10159 910 10166 906 10183 905 10201 939 10201
939 10191 941 10181 944 10172 949 10163 956 10156 966 10150
979 10146 995 10145 1009 10145 1021 10148 1030 10153 1038 10160
1044 10168 1048 10177 1050 10188 1051 10199 1050 10213 1048 10219
1046 10225 1043 10229 1039 10233 1030 10240 1019 10244 1006 10247
991 10249 975 10249 975 10277 991 10278 1005 10279 1017 10281
1026 10285 1034 10290 1039 10298 1042 10307 1043 10320 1042 10329
1040 10338 1037 10346 1032 10352 1025 10358 1017 10362 1007 10365
995 10366 984 10365 974 10363 966 10360 958 10354 952 10346
948 10336 944 10323 943 10308 123 N F
1212 10396 1222 10395 1231 10393 1240 10389 1249 10384 1257 10377
1264 10369 1271 10361 1277 10351 1283 10341 1288 10330 1292 10318
1296 10306 1298 10294 1300 10281 1302 10268 1302 10255 1302 10242
1300 10230 1296 10204 1292 10192 1288 10180 1283 10169 1277 10158
1271 10149 1264 10140 1257 10132 1249 10125 1240 10120 1231 10116
1222 10113 1212 10113 1202 10113 1192 10116 1183 10120 1175 10125
1167 10132 1159 10140 1152 10149 1146 10158 1141 10169 1136 10180
1131 10192 1128 10204 1123 10230 1122 10242 1121 10255 1157 10255
1159 10231 1162 10209 1168 10190 1175 10173 1183 10160 1192 10151
1197 10147 1202 10145 1207 10143 1212 10143 1217 10143 1222 10145
1226 10147 1231 10151 1240 10160 1249 10173 1256 10190 1261 10209
1265 10231 1266 10255 1265 10280 1261 10301 1256 10320 1249 10336
1240 10349 1231 10358 1226 10361 1222 10364 1217 10365 1212 10366
1207 10365 1202 10364 1197 10361 1192 10358 1183 10349 1175 10336
1168 10320 1162 10301 1159 10280 1157 10255 1121 10255 1122 10268
1123 10281 1125 10294 1128 10306 1131 10318 1136 10330 1141 10341
1146 10351 1152 10361 1159 10369 1167 10377 1175 10384 1183 10389
1192 10393 1202 10395 1212 10396 105 N F
1470 10344 1468 10331 1465 10319 1459 10308 1451 10299 1441 10291
1430 10285 1419 10281 1406 10280 1406 10305 1398 10306 1390 10308

1384 10312 1378 10316 1373 10322 1369 10329 1367 10336 1366 10344
1367 10352 1369 10360 1373 10366 1378 10372 1384 10377 1390 10380
1398 10383 1406 10384 1414 10383 1421 10380 1428 10377 1434 10372
1438 10366 1442 10360 1444 10352 1445 10344 1444 10336 1442 10329
1438 10322 1434 10316 1428 10312 1421 10308 1414 10306 1406 10305
1406 10280 1393 10281 1381 10285 1370 10291 1361 10299 1353 10308
1347 10319 1343 10331 1342 10344 1343 10357 1347 10369 1353 10380
1361 10389 1370 10397 1381 10403 1393 10407 1406 10408 1419 10407
1430 10403 1441 10397 1451 10389 1459 10380 1465 10369 1468 10357
1470 10344 67 N F
1517 10404 1557 10404 1701 10175 1701 10404 1735 10404 1735 10123
1695 10123 1551 10352 1551 10123 1517 10123 1517 10404 11 N F
943 11433 909 11433 911 11453 913 11462 916 11471 919 11479
923 11486 928 11493 933 11499 939 11504 945 11509 952 11512
960 11516 968 11518 976 11520 995 11521 1012 11520 1028 11516
1043 11510 1055 11502 1060 11497 1065 11491 1069 11485 1073 11479
1076 11472 1078 11464 1079 11456 1079 11447 1079 11437 1077 11427
1074 11419 1070 11412 1065 11405 1058 11399 1049 11393 1039 11388
1051 11384 1061 11378 1069 11371 1076 11364 1081 11355 1085 11346
1087 11336 1087 11324 1086 11309 1082 11294 1076 11280 1072 11273
1066 11267 1060 11261 1054 11256 1046 11251 1037 11247 1028 11244
1017 11242 1006 11240 993 11240 982 11240 972 11241 962 11243
954 11246 946 11250 939 11254 932 11259 926 11264 921 11270
917 11277 913 11284 910 11291 906 11308 905 11326 939 11326
939 11316 941 11306 944 11297 949 11288 956 11281 966 11275
979 11271 995 11270 1009 11270 1021 11273 1030 11278 1038 11285
1044 11293 1048 11303 1050 11313 1051 11324 1050 11338 1048 11344
1046 11350 1043 11355 1039 11359 1030 11365 1019 11370 1006 11372
991 11374 975 11374 975 11403 991 11403 1005 11404 1017 11406
1026 11410 1034 11415 1039 11423 1042 11433 1043 11445 1042 11454
1040 11463 1037 11471 1032 11478 1025 11483 1017 11487 1007 11490
995 11491 984 11490 974 11488 966 11485 958 11479 952 11471
948 11461 944 11448 943 11433 123 N F
1125 11427 1160 11427 1160 11440 1163 11453 1167 11464 1173 11473
1181 11481 1191 11486 1202 11490 1216 11491 1226 11490 1236 11487
1245 11483 1252 11477 1259 11469 1264 11460 1267 11450 1268 11439
1267 11427 1264 11417 1259 11409 1253 11402 1244 11394 1233 11387
1206 11370 1182 11357 1163 11343 1148 11329 1137 11314 1129 11299
1123 11283 1120 11266 1119 11248 1302 11248 1302 11280 1157 11280
1161 11292 1166 11303 1172 11311 1181 11319 1191 11327 1203 11334
1234 11352 1252 11361 1267 11371 1279 11382 1288 11394 1296 11406
1300 11418 1303 11431 1304 11443 1304 11451 1302 11459 1300 11467
1297 11474 1293 11481 1289 11488 1284 11494 1278 11499 1265 11509

1250 11515 1233 11520 1216 11521 1197 11520 1188 11518 1180 11515
1172 11512 1164 11508 1157 11503 1151 11498 1145 11492 1140 11485
1136 11477 1132 11469 1129 11459 1127 11449 1126 11438 1125 11427
78 N F
1470 11469 1468 11457 1465 11445 1459 11434 1451 11424 1441 11416
1430 11410 1419 11407 1406 11405 1406 11430 1398 11431 1390 11433
1384 11437 1378 11442 1373 11447 1369 11454 1367 11461 1366 11469
1367 11477 1369 11485 1373 11491 1378 11497 1384 11502 1390 11506
1398 11508 1406 11509 1414 11508 1421 11506 1428 11502 1434 11497
1438 11491 1442 11485 1444 11477 1445 11469 1444 11461 1442 11454
1438 11447 1434 11442 1428 11437 1421 11433 1414 11431 1406 11430
1406 11405 1393 11407 1381 11410 1370 11416 1361 11424 1353 11434
1347 11445 1343 11457 1342 11469 1343 11482 1347 11494 1353 11505
1361 11515 1370 11522 1381 11528 1393 11532 1406 11533 1419 11532
1430 11528 1441 11522 1451 11515 1459 11505 1465 11494 1468 11482
1470 11469 67 N F
1517 11529 1557 11529 1701 11300 1701 11529 1735 11529 1735 11248
1695 11248 1551 11477 1551 11248 1517 11248 1517 11529 11 N F
943 12551 909 12551 911 12572 913 12581 916 12589 919 12597
923 12605 928 12611 933 12617 939 12622 945 12627 952 12631
960 12634 968 12636 976 12638 995 12640 1012 12638 1028 12635
1043 12629 1055 12620 1060 12615 1065 12610 1069 12604 1073 12597
1076 12590 1078 12582 1079 12574 1079 12565 1079 12555 1077 12546
1074 12538 1070 12530 1065 12523 1058 12517 1049 12512 1039 12507
1051 12502 1061 12496 1069 12490 1076 12482 1081 12474 1085 12464
1087 12454 1087 12443 1086 12427 1082 12412 1076 12398 1072 12392
1066 12385 1060 12379 1054 12374 1046 12370 1037 12366 1028 12362
1017 12360 1006 12359 993 12358 982 12359 972 12360 962 12362
954 12365 946 12368 939 12372 932 12377 926 12382 921 12389
917 12395 913 12402 910 12410 906 12426 905 12445 939 12445
939 12435 941 12425 944 12415 949 12407 956 12399 966 12393
979 12390 995 12388 1009 12389 1021 12392 1030 12397 1038 12403
1044 12412 1048 12421 1050 12431 1051 12443 1050 12457 1048 12463
1046 12468 1043 12473 1039 12477 1030 12483 1019 12488 1006 12491
991 12492 975 12493 975 12521 991 12521 1005 12522 1017 12525
1026 12528 1034 12534 1039 12541 1042 12551 1043 12563 1042 12573
1040 12581 1037 12589 1032 12596 1025 12602 1017 12606 1007 12608
995 12609 984 12609 974 12607 966 12603 958 12598 952 12590
948 12580 944 12567 943 12551 123 N F
1242 12640 1266 12640 1266 12463 1308 12463 1308 12430 1266 12430
1266 12366 1232 12366 1232 12430 1117 12430 1117 12463 1147 12463
1232 12463 1232 12581 1147 12463 1117 12463 1117 12467 1242 12640
18 N F

1470 12588 1468 12575 1465 12563 1459 12552 1451 12543 1441 12535
1430 12529 1419 12525 1406 12524 1406 12548 1398 12549 1390 12551
1384 12555 1378 12560 1373 12566 1369 12572 1367 12580 1366 12588
1367 12596 1369 12603 1373 12610 1378 12616 1384 12620 1390 12624
1398 12626 1406 12627 1414 12626 1421 12624 1428 12620 1434 12616
1438 12610 1442 12603 1444 12596 1445 12588 1444 12580 1442 12572
1438 12566 1434 12560 1428 12555 1421 12551 1414 12549 1406 12548
1406 12524 1393 12525 1381 12529 1370 12535 1361 12543 1353 12552
1347 12563 1343 12575 1342 12588 1343 12601 1347 12613 1353 12623
1361 12633 1370 12641 1381 12647 1393 12650 1406 12652 1419 12650
1430 12647 1441 12641 1451 12633 1459 12623 1465 12613 1468 12601
1470 12588 67 N F
1517 12648 1557 12648 1701 12418 1701 12648 1735 12648 1735 12366
1695 12366 1551 12595 1551 12366 1517 12366 1517 12648 11 N F
943 13664 909 13664 911 13684 913 13694 916 13702 919 13710
923 13717 928 13724 933 13730 939 13735 945 13740 952 13744
960 13747 968 13749 976 13751 995 13752 1012 13751 1028 13747
1043 13741 1055 13733 1060 13728 1065 13723 1069 13717 1073 13710
1076 13703 1078 13695 1079 13687 1079 13678 1079 13668 1077 13659
1074 13650 1070 13643 1065 13636 1058 13630 1049 13624 1039 13620
1051 13615 1061 13609 1069 13602 1076 13595 1081 13587 1085 13577
1087 13567 1087 13555 1086 13540 1082 13525 1076 13511 1072 13504
1066 13498 1060 13492 1054 13487 1046 13482 1037 13478 1028 13475
1017 13473 1006 13471 993 13471 982 13471 972 13473 962 13475
954 13477 946 13481 939 13485 932 13490 926 13495 921 13501
917 13508 913 13515 910 13523 906 13539 905 13557 939 13557
939 13547 941 13538 944 13528 949 13519 956 13512 966 13506
979 13502 995 13501 1009 13502 1021 13504 1030 13509 1038 13516
1044 13524 1048 13534 1050 13544 1051 13555 1050 13570 1048 13576
1046 13581 1043 13586 1039 13590 1030 13596 1019 13601 1006 13604
991 13605 975 13606 975 13634 991 13634 1005 13635 1017 13638
1026 13641 1034 13647 1039 13654 1042 13664 1043 13676 1042 13685
1040 13694 1037 13702 1032 13709 1025 13714 1017 13719 1007 13721
995 13722 984 13722 974 13720 966 13716 958 13710 952 13703
948 13692 944 13680 943 13664 123 N F
1298 13682 1264 13682 1262 13691 1258 13699 1253 13706 1248 13712
1241 13716 1235 13720 1227 13721 1220 13722 1212 13722 1206 13720
1199 13718 1193 13715 1188 13711 1182 13707 1178 13701 1174 13695
1167 13680 1162 13663 1159 13643 1157 13620 1166 13628 1174 13635
1182 13641 1190 13645 1198 13647 1206 13649 1224 13650 1237 13648
1251 13644 1265 13638 1277 13628 1283 13622 1288 13616 1293 13609
1297 13601 1300 13592 1303 13582 1304 13572 1304 13561 1270 13559
1269 13571 1265 13581 1260 13592 1253 13601 1245 13609 1235 13614

1225 13618 1214 13620 1203 13618 1192 13614 1183 13609 1174 13601
1167 13592 1162 13581 1159 13571 1157 13559 1159 13548 1162 13537
1167 13527 1174 13518 1183 13510 1192 13504 1203 13500 1214 13499
1225 13500 1235 13504 1245 13510 1253 13518 1260 13527 1265 13537
1269 13548 1270 13559 1304 13561 1303 13545 1299 13529 1292 13514
1287 13506 1282 13499 1276 13493 1270 13487 1262 13482 1254 13477
1245 13474 1235 13471 1225 13469 1214 13469 1201 13470 1190 13472
1180 13475 1170 13480 1162 13485 1154 13492 1148 13500 1142 13509
1137 13518 1133 13529 1129 13540 1126 13552 1123 13578 1121 13606
1122 13622 1123 13638 1125 13653 1128 13667 1132 13680 1136 13692
1141 13703 1147 13713 1154 13722 1161 13730 1169 13736 1178 13742
1188 13746 1198 13750 1208 13752 1220 13752 1236 13751 1250 13747
1263 13740 1274 13731 1283 13721 1290 13709 1295 13696 1298 13682
126 N F
1470 13701 1468 13688 1465 13676 1459 13665 1451 13655 1441 13647
1430 13642 1419 13638 1406 13637 1406 13661 1398 13662 1390 13664
1384 13668 1378 13673 1373 13678 1369 13685 1367 13693 1366 13701
1367 13708 1369 13716 1373 13723 1378 13728 1384 13733 1390 13737
1398 13739 1406 13740 1414 13739 1421 13737 1428 13733 1434 13728
1438 13723 1442 13716 1444 13708 1445 13701 1444 13693 1442 13685
1438 13678 1434 13673 1428 13668 1421 13664 1414 13662 1406 13661
1406 13637 1393 13638 1381 13642 1370 13647 1361 13655 1353 13665
1347 13676 1343 13688 1342 13701 1343 13713 1347 13725 1353 13736
1361 13746 1370 13754 1381 13759 1393 13763 1406 13765 1419 13763
1430 13759 1441 13754 1451 13746 1459 13736 1465 13725 1468 13713
1470 13701 67 N F
1517 13760 1557 13760 1701 13531 1701 13760 1735 13760 1735 13479
1695 13479 1551 13708 1551 13479 1517 13479 1517 13760 11 N F
943 14770 909 14770 911 14791 913 14800 916 14809 919 14817
923 14824 928 14830 933 14836 939 14842 945 14846 952 14850
960 14853 968 14856 976 14857 995 14859 1012 14858 1028 14854
1043 14848 1055 14840 1060 14835 1065 14829 1069 14823 1073 14816
1076 14809 1078 14801 1079 14793 1079 14784 1079 14774 1077 14765
1074 14757 1070 14749 1065 14742 1058 14736 1049 14731 1039 14726
1051 14721 1061 14715 1069 14709 1076 14701 1081 14693 1085 14684
1087 14673 1087 14662 1086 14647 1082 14632 1076 14617 1072 14611
1066 14604 1060 14599 1054 14593 1046 14589 1037 14585 1028 14582
1017 14579 1006 14578 993 14577 982 14578 972 14579 962 14581
954 14584 946 14587 939 14591 932 14596 926 14602 921 14608
917 14614 913 14621 910 14629 906 14646 905 14664 939 14664
939 14654 941 14644 944 14634 949 14626 956 14618 966 14613
979 14609 995 14608 1009 14608 1021 14611 1030 14616 1038 14623
1044 14631 1048 14640 1050 14651 1051 14662 1050 14676 1048 14682

1046 14687 1043 14692 1039 14696 1030 14703 1019 14707 1006 14710
991 14712 975 14712 975 14740 991 14740 1005 14742 1017 14744
1026 14748 1034 14753 1039 14761 1042 14770 1043 14782 1042 14792
1040 14801 1037 14808 1032 14815 1025 14821 1017 14825 1007 14828
995 14829 984 14828 974 14826 966 14822 958 14817 952 14809
948 14799 944 14786 943 14770 123 N F
1212 14859 1228 14858 1244 14854 1258 14848 1270 14840 1280 14830
1284 14824 1288 14817 1290 14810 1292 14803 1294 14795 1294 14786
1293 14774 1291 14764 1287 14756 1282 14748 1276 14742 1270 14737
1258 14728 1268 14723 1277 14717 1285 14709 1291 14701 1297 14691
1301 14681 1303 14671 1304 14660 1303 14646 1298 14632 1291 14618
1280 14605 1274 14599 1267 14593 1259 14588 1251 14584 1241 14580
1232 14578 1221 14576 1210 14575 1198 14576 1188 14578 1178 14580
1169 14584 1161 14588 1154 14593 1147 14599 1141 14605 1132 14618
1125 14632 1121 14646 1119 14660 1155 14660 1157 14649 1160 14639
1165 14629 1172 14621 1181 14615 1190 14610 1201 14607 1212 14606
1223 14607 1233 14610 1243 14615 1251 14621 1258 14629 1263 14639
1267 14649 1268 14660 1267 14671 1263 14681 1258 14690 1251 14698
1243 14705 1233 14710 1223 14713 1212 14714 1201 14713 1190 14710
1181 14705 1172 14698 1165 14690 1160 14681 1157 14671 1155 14660
1119 14660 1120 14671 1123 14681 1127 14691 1132 14700 1139 14708
1146 14716 1155 14722 1166 14728 1153 14737 1147 14742 1141 14748
1137 14756 1133 14764 1130 14774 1129 14786 1164 14786 1165 14777
1167 14769 1172 14762 1178 14755 1185 14751 1193 14747 1202 14745
1212 14744 1221 14745 1230 14747 1238 14751 1246 14755 1251 14762
1256 14769 1259 14777 1260 14786 1259 14796 1256 14804 1251 14811
1246 14817 1238 14822 1230 14826 1221 14828 1212 14829 1202 14828
1193 14826 1185 14822 1178 14817 1172 14811 1167 14804 1165 14796
1164 14786 1129 14786 1130 14795 1131 14803 1133 14810 1136 14817
1139 14824 1143 14830 1153 14840 1165 14848 1179 14854 1195 14858
1212 14859 145 N F
1470 14807 1468 14794 1465 14782 1459 14771 1451 14762 1441 14754
1430 14748 1419 14744 1406 14743 1406 14768 1398 14768 1390 14771
1384 14774 1378 14779 1373 14785 1369 14792 1367 14799 1366 14807
1367 14815 1369 14822 1373 14829 1378 14835 1384 14840 1390 14843
1398 14846 1406 14846 1414 14846 1421 14843 1428 14840 1434 14835
1438 14829 1442 14822 1444 14815 1445 14807 1444 14799 1442 14792
1438 14785 1434 14779 1428 14774 1421 14771 1414 14768 1406 14768
1406 14743 1393 14744 1381 14748 1370 14754 1361 14762 1353 14771
1347 14782 1343 14794 1342 14807 1343 14820 1347 14832 1353 14843
1361 14852 1370 14860 1381 14866 1393 14870 1406 14871 1419 14870
1430 14866 1441 14860 1451 14852 1459 14843 1465 14832 1468 14820
1470 14807 67 N F

1517 14867 1557 14867 1701 14638 1701 14867 1735 14867 1735 14585
1695 14585 1551 14815 1551 14585 1517 14585 1517 14867 11 N F
1027 15952 1051 15952 1051 15775 1093 15775 1093 15743 1051 15743
1051 15679 1017 15679 1017 15743 903 15743 903 15775 933 15775
1017 15775 1017 15894 933 15775 903 15775 903 15779 1027 15952
18 N F
1212 15952 1222 15951 1231 15949 1240 15945 1249 15940 1257 15933
1264 15925 1271 15917 1277 15907 1283 15897 1288 15886 1292 15874
1296 15862 1298 15850 1300 15837 1302 15824 1302 15811 1302 15798
1300 15785 1296 15760 1292 15748 1288 15736 1283 15725 1277 15714
1271 15705 1264 15696 1257 15688 1249 15681 1240 15676 1231 15672
1222 15669 1212 15669 1202 15669 1192 15672 1183 15676 1175 15681
1167 15688 1159 15696 1152 15705 1146 15714 1141 15725 1136 15736
1131 15748 1128 15760 1123 15785 1122 15798 1121 15811 1157 15811
1159 15787 1162 15765 1168 15746 1175 15729 1183 15716 1192 15707
1197 15703 1202 15701 1207 15699 1212 15699 1217 15699 1222 15701
1226 15703 1231 15707 1240 15716 1249 15729 1256 15746 1261 15765
1265 15787 1266 15811 1265 15836 1261 15857 1256 15876 1249 15892
1240 15905 1231 15914 1226 15917 1222 15920 1217 15921 1212 15922
1207 15921 1202 15920 1197 15917 1192 15914 1183 15905 1175 15892
1168 15876 1162 15857 1159 15836 1157 15811 1121 15811 1122 15824
1123 15837 1125 15850 1128 15862 1131 15874 1136 15886 1141 15897
1146 15907 1152 15917 1159 15925 1167 15933 1175 15940 1183 15945
1192 15949 1202 15951 1212 15952 105 N F
1470 15900 1468 15887 1465 15875 1459 15864 1451 15855 1441 15847
1430 15841 1419 15837 1406 15836 1406 15861 1398 15862 1390 15864
1384 15868 1378 15872 1373 15878 1369 15885 1367 15892 1366 15900
1367 15908 1369 15916 1373 15922 1378 15928 1384 15933 1390 15936
1398 15939 1406 15940 1414 15939 1421 15936 1428 15933 1434 15928
1438 15922 1442 15916 1444 15908 1445 15900 1444 15892 1442 15885
1438 15878 1434 15872 1428 15868 1421 15864 1414 15862 1406 15861
1406 15836 1393 15837 1381 15841 1370 15847 1361 15855 1353 15864
1347 15875 1343 15887 1342 15900 1343 15913 1347 15925 1353 15936
1361 15945 1370 15953 1381 15959 1393 15963 1406 15964 1419 15963
1430 15959 1441 15953 1451 15945 1459 15936 1465 15925 1468 15913
1470 15900 67 N F
1517 15960 1557 15960 1701 15731 1701 15960 1735 15960 1735 15679
1695 15679 1551 15908 1551 15679 1517 15679 1517 15960 11 N F
13570 5585 13604 5585 13605 5599 13607 5611 13611 5622 13618 5632
13625 5639 13635 5645 13647 5648 13660 5650 13670 5649 13680 5646
13689 5642 13697 5636 13703 5628 13708 5619 13711 5609 13712 5597
13711 5586 13708 5576 13704 5568 13697 5560 13688 5553 13677 5546
13650 5529 13627 5515 13608 5502 13593 5488 13581 5473 13573 5458

13567 5442 13564 5425 13564 5406 13746 5406 13746 5439 13602 5439
13605 5451 13610 5461 13617 5470 13625 5478 13635 5485 13647 5493
13678 5511 13696 5520 13711 5530 13723 5541 13733 5552 13740 5564
13745 5577 13747 5589 13748 5601 13748 5610 13746 5618 13744 5626
13741 5633 13737 5640 13733 5646 13728 5652 13722 5658 13709 5667
13694 5674 13678 5678 13660 5680 13641 5678 13632 5677 13624 5674
13616 5671 13608 5667 13602 5662 13595 5657 13589 5650 13584 5643
13580 5636 13576 5627 13573 5618 13571 5608 13570 5597 13570 5585
78 N F
13784 5585 13818 5585 13819 5599 13822 5611 13826 5622 13832 5632
13840 5639 13850 5645 13861 5648 13875 5650 13885 5649 13895 5646
13904 5642 13911 5636 13918 5628 13923 5619 13926 5609 13927 5597
13926 5586 13923 5576 13918 5568 13912 5560 13903 5553 13892 5546
13865 5529 13841 5515 13822 5502 13807 5488 13796 5473 13788 5458
13782 5442 13779 5425 13778 5406 13961 5406 13961 5439 13816 5439
13820 5451 13825 5461 13831 5470 13840 5478 13850 5485 13862 5493
13893 5511 13911 5520 13926 5530 13938 5541 13947 5552 13955 5564
13959 5577 13962 5589 13963 5601 13963 5610 13961 5618 13959 5626
13956 5633 13952 5640 13948 5646 13943 5652 13937 5658 13924 5667
13909 5674 13892 5678 13875 5680 13856 5678 13847 5677 13839 5674
13831 5671 13823 5667 13816 5662 13810 5657 13804 5650 13799 5643
13795 5636 13791 5627 13788 5618 13786 5608 13785 5597 13784 5585
78 N F
14129 5628 14127 5615 14124 5603 14118 5592 14110 5583 14100 5575
14089 5569 14077 5565 14065 5564 14065 5589 14057 5589 14049 5592
14043 5595 14037 5600 14032 5606 14028 5613 14026 5620 14025 5628
14026 5636 14028 5643 14032 5650 14037 5656 14043 5661 14049 5664
14057 5667 14065 5667 14073 5667 14080 5664 14087 5661 14092 5656
14097 5650 14101 5643 14103 5636 14104 5628 14103 5620 14101 5613
14097 5606 14092 5600 14087 5595 14080 5592 14073 5589 14065 5589
14065 5564 14052 5565 14040 5569 14029 5575 14019 5583 14012 5592
14006 5603 14002 5615 14001 5628 14002 5641 14006 5653 14012 5664
14019 5673 14029 5681 14040 5687 14052 5691 14065 5692 14077 5691
14089 5687 14100 5681 14110 5673 14118 5664 14124 5653 14127 5641
14129 5628 67 N F
14176 5688 14216 5688 14360 5459 14360 5688 14394 5688 14394 5406
14354 5406 14210 5636 14210 5406 14176 5406 14176 5688 11 N F
13570 6755 13604 6755 13605 6769 13607 6781 13611 6792 13618 6802
13625 6809 13635 6815 13647 6818 13660 6819 13670 6818 13680 6816
13689 6811 13697 6805 13703 6798 13708 6789 13711 6779 13712 6767
13711 6755 13708 6746 13704 6737 13697 6730 13688 6723 13677 6716
13650 6699 13627 6685 13608 6671 13593 6657 13581 6643 13573 6628
13567 6611 13564 6594 13564 6576 13746 6576 13746 6608 13602 6608

13605 6621 13610 6631 13617 6640 13625 6648 13635 6655 13647 6663
13678 6681 13696 6690 13711 6700 13723 6711 13733 6722 13740 6734
13745 6747 13747 6759 13748 6771 13748 6780 13746 6788 13744 6796
13741 6803 13737 6810 13733 6816 13728 6822 13722 6828 13709 6837
13694 6844 13678 6848 13660 6850 13641 6848 13632 6846 13624 6844
13616 6841 13608 6837 13602 6832 13595 6826 13589 6820 13584 6813
13580 6806 13576 6797 13573 6788 13571 6778 13570 6767 13570 6755
78 N F
13901 6850 13925 6850 13925 6673 13967 6673 13967 6641 13925 6641
13925 6576 13891 6576 13891 6641 13776 6641 13776 6673 13806 6673
13891 6673 13891 6791 13806 6673 13776 6673 13776 6677 13901 6850
18 N F
14129 6798 14127 6785 14124 6773 14118 6762 14110 6753 14100 6745
14089 6739 14077 6735 14065 6734 14065 6758 14057 6759 14049 6762
14043 6765 14037 6770 14032 6776 14028 6782 14026 6790 14025 6798
14026 6806 14028 6813 14032 6820 14037 6826 14043 6830 14049 6834
14057 6836 14065 6837 14073 6836 14080 6834 14087 6830 14092 6826
14097 6820 14101 6813 14103 6806 14104 6798 14103 6790 14101 6782
14097 6776 14092 6770 14087 6765 14080 6762 14073 6759 14065 6758
14065 6734 14052 6735 14040 6739 14029 6745 14019 6753 14012 6762
14006 6773 14002 6785 14001 6798 14002 6811 14006 6823 14012 6834
14019 6843 14029 6851 14040 6857 14052 6861 14065 6862 14077 6861
14089 6857 14100 6851 14110 6843 14118 6834 14124 6823 14127 6811
14129 6798 67 N F
14176 6858 14216 6858 14360 6628 14360 6858 14394 6858 14394 6576
14354 6576 14210 6805 14210 6576 14176 6576 14176 6858 11 N F
13570 7914 13604 7914 13605 7927 13607 7940 13611 7951 13618 7960
13625 7968 13635 7973 13647 7977 13660 7978 13670 7977 13680 7974
13689 7970 13697 7964 13703 7956 13708 7947 13711 7937 13712 7926
13711 7914 13708 7904 13704 7896 13697 7888 13688 7881 13677 7874
13650 7857 13627 7844 13608 7830 13593 7816 13581 7801 13573 7786
13567 7770 13564 7753 13564 7735 13746 7735 13746 7767 13602 7767
13605 7779 13610 7789 13617 7798 13625 7806 13635 7814 13647 7821
13678 7839 13696 7848 13711 7858 13723 7869 13733 7881 13740 7893
13745 7905 13747 7917 13748 7930 13748 7938 13746 7946 13744 7954
13741 7961 13737 7968 13733 7975 13728 7981 13722 7986 13709 7995
13694 8002 13678 8007 13660 8008 13641 8007 13632 8005 13624 8002
13616 7999 13608 7995 13602 7990 13595 7985 13589 7979 13584 7972
13580 7964 13576 7955 13573 7946 13571 7936 13570 7925 13570 7914
78 N F
13957 7938 13923 7938 13921 7947 13917 7955 13912 7962 13907 7968
13900 7972 13893 7975 13886 7977 13879 7978 13871 7977 13864 7976
13858 7974 13852 7971 13846 7967 13841 7962 13837 7957 13833 7951

13826 7936 13821 7919 13817 7898 13816 7875 13825 7884 13833 7891
13841 7896 13849 7900 13857 7903 13865 7905 13883 7906 13896 7904
13910 7900 13924 7893 13936 7884 13942 7878 13947 7872 13952 7864
13956 7856 13959 7848 13962 7838 13963 7828 13963 7817 13929 7815
13928 7826 13924 7837 13919 7847 13912 7857 13904 7864 13894 7870
13884 7874 13873 7875 13862 7874 13851 7870 13842 7864 13833 7857
13826 7847 13821 7837 13818 7826 13816 7815 13818 7804 13821 7793
13826 7783 13833 7774 13842 7766 13851 7760 13862 7756 13873 7755
13884 7756 13894 7760 13904 7766 13912 7774 13919 7783 13924 7793
13928 7804 13929 7815 13963 7817 13962 7801 13958 7785 13951 7769
13946 7762 13941 7755 13935 7749 13929 7743 13921 7738 13913 7733
13904 7730 13894 7727 13884 7725 13873 7725 13860 7725 13849 7727
13839 7731 13829 7735 13821 7741 13813 7748 13807 7756 13801 7764
13796 7774 13792 7785 13788 7796 13785 7808 13782 7834 13780 7861
13781 7878 13782 7894 13784 7908 13787 7922 13791 7935 13795 7948
13800 7959 13806 7969 13813 7978 13820 7985 13828 7992 13837 7998
13846 8002 13857 8005 13867 8007 13879 8008 13895 8007 13909 8002
13922 7996 13933 7987 13942 7977 13949 7965 13954 7952 13957 7938
126 N F
14129 7956 14127 7943 14124 7931 14118 7921 14110 7911 14100 7903
14089 7897 14077 7894 14065 7892 14065 7917 14057 7918 14049 7920
14043 7924 14037 7928 14032 7934 14028 7941 14026 7948 14025 7956
14026 7964 14028 7972 14032 7978 14037 7984 14043 7989 14049 7993
14057 7995 14065 7996 14073 7995 14080 7993 14087 7989 14092 7984
14097 7978 14101 7972 14103 7964 14104 7956 14103 7948 14101 7941
14097 7934 14092 7928 14087 7924 14080 7920 14073 7918 14065 7917
14065 7892 14052 7894 14040 7897 14029 7903 14019 7911 14012 7921
14006 7931 14002 7943 14001 7956 14002 7969 14006 7981 14012 7992
14019 8002 14029 8009 14040 8015 14052 8019 14065 8020 14077 8019
14089 8015 14100 8009 14110 8002 14118 7992 14124 7981 14127 7969
14129 7956 67 N F
14176 8016 14216 8016 14360 7787 14360 8016 14394 8016 14394 7735
14354 7735 14210 7964 14210 7735 14176 7735 14176 8016 11 N F
13570 9056 13604 9056 13605 9069 13607 9082 13611 9093 13618 9102
13625 9110 13635 9115 13647 9119 13660 9120 13670 9119 13680 9116
13689 9112 13697 9106 13703 9098 13708 9090 13711 9079 13712 9068
13711 9056 13708 9046 13704 9038 13697 9031 13688 9024 13677 9016
13650 8999 13627 8986 13608 8972 13593 8958 13581 8943 13573 8928
13567 8912 13564 8895 13564 8877 13746 8877 13746 8909 13602 8909
13605 8921 13610 8932 13617 8940 13625 8948 13635 8956 13647 8963
13678 8981 13696 8990 13711 9000 13723 9011 13733 9023 13740 9035
13745 9047 13747 9060 13748 9072 13748 9080 13746 9088 13744 9096
13741 9104 13737 9110 13733 9117 13728 9123 13722 9128 13709 9138

13694 9144 13678 9149 13660 9150 13641 9149 13632 9147 13624 9145
13616 9141 13608 9137 13602 9133 13595 9127 13589 9121 13584 9114
13580 9106 13576 9098 13573 9088 13571 9078 13570 9067 13570 9056
78 N F
13871 9150 13887 9149 13903 9145 13917 9140 13929 9131 13939 9121
13943 9115 13947 9109 13949 9102 13951 9094 13953 9086 13953 9078
13952 9066 13950 9056 13946 9047 13941 9040 13935 9033 13929 9028
13917 9020 13927 9014 13936 9008 13944 9001 13950 8992 13956 8983
13960 8973 13962 8962 13963 8951 13961 8938 13957 8924 13950 8910
13939 8896 13933 8890 13926 8885 13918 8880 13910 8875 13900 8872
13891 8869 13880 8867 13869 8867 13857 8867 13847 8869 13837 8872
13828 8875 13820 8880 13813 8885 13806 8890 13800 8896 13790 8910
13784 8924 13780 8938 13778 8951 13814 8951 13816 8940 13819 8930
13824 8921 13831 8913 13840 8906 13849 8901 13860 8898 13871 8897
13882 8898 13892 8901 13902 8906 13910 8913 13917 8921 13922 8930
13926 8940 13927 8951 13926 8962 13922 8972 13917 8982 13910 8990
13902 8996 13892 9001 13882 9004 13871 9006 13860 9004 13849 9001
13840 8996 13831 8990 13824 8982 13819 8972 13816 8962 13814 8951
13778 8951 13779 8962 13782 8973 13786 8982 13791 8991 13798 9000
13805 9007 13814 9014 13824 9020 13812 9028 13806 9033 13800 9040
13796 9047 13792 9056 13789 9066 13788 9078 13822 9078 13823 9069
13826 9060 13831 9053 13837 9047 13844 9042 13852 9039 13861 9036
13871 9036 13880 9036 13889 9039 13897 9042 13905 9047 13910 9053
13915 9060 13918 9069 13919 9078 13918 9087 13915 9095 13910 9103
13905 9109 13897 9114 13889 9117 13880 9119 13871 9120 13861 9119
13852 9117 13844 9114 13837 9109 13831 9103 13826 9095 13823 9087
13822 9078 13788 9078 13789 9086 13790 9094 13792 9102 13795 9109
13798 9115 13802 9121 13812 9131 13824 9140 13838 9145 13854 9149
13871 9150 145 N F
14129 9098 14127 9086 14124 9074 14118 9063 14110 9053 14100 9045
14089 9040 14077 9036 14065 9034 14065 9059 14057 9060 14049 9062
14043 9066 14037 9071 14032 9076 14028 9083 14026 9091 14025 9098
14026 9106 14028 9114 14032 9121 14037 9126 14043 9131 14049 9135
14057 9137 14065 9138 14073 9137 14080 9135 14087 9131 14092 9126
14097 9121 14101 9114 14103 9106 14104 9098 14103 9091 14101 9083
14097 9076 14092 9071 14087 9066 14080 9062 14073 9060 14065 9059
14065 9034 14052 9036 14040 9040 14029 9045 14019 9053 14012 9063
14006 9074 14002 9086 14001 9098 14002 9111 14006 9123 14012 9134
14019 9144 14029 9151 14040 9157 14052 9161 14065 9162 14077 9161
14089 9157 14100 9151 14110 9144 14118 9134 14124 9123 14127 9111
14129 9098 67 N F
14176 9158 14216 9158 14360 8929 14360 9158 14394 9158 14394 8877
14354 8877 14210 9106 14210 8877 14176 8877 14176 9158 11 N F

13602 10194 13568 10194 13570 10214 13572 10223 13575 10232 13578 10240
13582 10247 13587 10254 13592 10260 13598 10265 13604 10270 13611 10273
13619 10277 13627 10279 13635 10281 13654 10282 13671 10281 13687 10277
13702 10271 13714 10263 13719 10258 13724 10252 13728 10246 13732 10240
13735 10233 13737 10225 13738 10217 13738 10208 13738 10198 13736 10188
13733 10180 13729 10173 13724 10166 13717 10160 13708 10154 13698 10149
13710 10145 13720 10139 13728 10132 13735 10125 13740 10116 13743 10107
13746 10097 13746 10085 13745 10070 13741 10055 13735 10041 13731 10034
13725 10028 13719 10022 13713 10017 13705 10012 13696 10008 13687 10005
13676 10003 13665 10001 13652 10001 13641 10001 13631 10002 13621 10004
13613 10007 13605 10011 13597 10015 13591 10020 13585 10025 13580 10031
13576 10038 13572 10045 13569 10052 13565 10069 13564 10087 13598 10087
13598 10077 13600 10067 13603 10058 13608 10049 13615 10042 13625 10036
13638 10032 13654 10031 13668 10031 13680 10034 13689 10039 13697 10046
13703 10054 13707 10064 13709 10074 13710 10085 13709 10099 13707 10105
13705 10111 13702 10116 13698 10120 13689 10126 13678 10131 13665 10133
13650 10135 13634 10135 13634 10164 13650 10164 13664 10165 13676 10167
13685 10171 13693 10176 13698 10184 13701 10194 13702 10206 13701 10215
13699 10224 13696 10232 13691 10239 13684 10244 13676 10248 13666 10251
13654 10252 13643 10251 13633 10249 13625 10246 13617 10240 13611 10232
13606 10222 13603 10209 13602 10194 123 N F
13871 10282 13881 10281 13890 10279 13899 10275 13908 10270 13916 10263
13923 10256 13930 10247 13936 10237 13942 10227 13947 10216 13951 10204
13954 10192 13957 10180 13959 10167 13961 10154 13961 10141 13961 10129
13959 10116 13954 10090 13951 10078 13947 10066 13942 10055 13936 10044
13930 10035 13923 10026 13916 10018 13908 10011 13899 10006 13890 10002
13881 10000 13871 9999 13861 10000 13851 10002 13842 10006 13834 10011
13826 10018 13818 10026 13811 10035 13805 10044 13800 10055 13795 10066
13790 10078 13787 10090 13782 10116 13781 10129 13780 10141 13816 10141
13818 10117 13821 10095 13827 10076 13834 10060 13842 10046 13851 10037
13856 10033 13861 10031 13866 10029 13871 10029 13876 10029 13880 10031
13885 10033 13890 10037 13899 10046 13908 10060 13915 10076 13920 10095
13924 10117 13925 10141 13924 10166 13920 10188 13915 10206 13908 10222
13899 10235 13890 10244 13885 10248 13880 10250 13876 10251 13871 10252
13866 10251 13861 10250 13856 10248 13851 10244 13842 10235 13834 10222
13827 10206 13821 10188 13818 10166 13816 10141 13780 10141 13781 10154
13782 10167 13784 10180 13787 10192 13790 10204 13795 10216 13800 10227
13805 10237 13811 10247 13818 10256 13826 10263 13834 10270 13842 10275
13851 10279 13861 10281 13871 10282 105 N F
14129 10230 14127 10218 14124 10206 14118 10195 14110 10185 14100 10177
14089 10171 14077 10168 14065 10166 14065 10191 14057 10192 14049 10194
14043 10198 14037 10202 14032 10208 14028 10215 14026 10222 14025 10230
14026 10238 14028 10246 14032 10252 14037 10258 14043 10263 14049 10267

14057 10269 14065 10270 14073 10269 14080 10267 14087 10263 14092 10258
14097 10252 14101 10246 14103 10238 14104 10230 14103 10222 14101 10215
14097 10208 14092 10202 14087 10198 14080 10194 14073 10192 14065 10191
14065 10166 14052 10168 14040 10171 14029 10177 14019 10185 14012 10195
14006 10206 14002 10218 14001 10230 14002 10243 14006 10255 14012 10266
14019 10276 14029 10283 14040 10289 14052 10293 14065 10294 14077 10293
14089 10289 14100 10283 14110 10276 14118 10266 14124 10255 14127 10243
14129 10230 67 N F
14176 10290 14216 10290 14360 10061 14360 10290 14394 10290 14394 10009
14354 10009 14210 10238 14210 10009 14176 10009 14176 10290 11 N F
13602 11315 13568 11315 13570 11336 13572 11345 13575 11354 13578 11362
13582 11369 13587 11376 13592 11382 13598 11387 13604 11391 13611 11395
13619 11398 13627 11401 13635 11402 13654 11404 13671 11403 13687 11399
13702 11393 13714 11385 13719 11380 13724 11374 13728 11368 13732 11361
13735 11354 13737 11347 13738 11338 13738 11330 13738 11319 13736 11310
13733 11302 13729 11294 13724 11288 13717 11281 13708 11276 13698 11271
13710 11266 13720 11261 13728 11254 13735 11247 13740 11238 13743 11229
13746 11218 13746 11207 13745 11192 13741 11177 13735 11163 13731 11156
13725 11150 13719 11144 13713 11139 13705 11134 13696 11130 13687 11127
13676 11124 13665 11123 13652 11123 13641 11123 13631 11124 13621 11126
13613 11129 13605 11132 13597 11137 13591 11141 13585 11147 13580 11153
13576 11160 13572 11167 13569 11174 13565 11191 13564 11209 13598 11209
13598 11199 13600 11189 13603 11180 13608 11171 13615 11164 13625 11158
13638 11154 13654 11153 13668 11153 13680 11156 13689 11161 13697 11168
13703 11176 13707 11185 13709 11196 13710 11207 13709 11221 13707 11227
13705 11233 13702 11237 13698 11241 13689 11248 13678 11252 13665 11255
13650 11257 13634 11257 13634 11285 13650 11286 13664 11287 13676 11289
13685 11293 13693 11298 13698 11306 13701 11315 13702 11328 13701 11337
13699 11346 13696 11354 13691 11360 13684 11366 13676 11370 13666 11373
13654 11374 13643 11373 13633 11371 13625 11367 13617 11362 13611 11354
13606 11344 13603 11331 13602 11315 123 N F
13784 11309 13818 11309 13819 11323 13822 11336 13826 11347 13832 11356
13840 11363 13850 11369 13861 11373 13875 11374 13885 11373 13895 11370
13904 11366 13911 11360 13918 11352 13923 11343 13926 11333 13927 11321
13926 11310 13923 11300 13918 11292 13912 11284 13903 11277 13892 11270
13865 11253 13841 11240 13822 11226 13807 11212 13796 11197 13788 11182
13782 11166 13779 11149 13778 11131 13961 11131 13961 11163 13816 11163
13820 11175 13825 11185 13831 11194 13840 11202 13850 11209 13862 11217
13893 11235 13911 11244 13926 11254 13938 11265 13947 11277 13955 11289
13959 11301 13962 11313 13963 11326 13963 11334 13961 11342 13959 11350
13956 11357 13952 11364 13948 11371 13943 11377 13937 11382 13924 11391
13909 11398 13892 11402 13875 11404 13856 11402 13847 11401 13839 11398
13831 11395 13823 11391 13816 11386 13810 11381 13804 11375 13799 11368

13795 11360 13791 11351 13788 11342 13786 11332 13785 11321 13784 11309
78 N F
14129 11352 14127 11339 14124 11327 14118 11316 14110 11307 14100 11299
14089 11293 14077 11289 14065 11288 14065 11313 14057 11314 14049 11316
14043 11319 14037 11324 14032 11330 14028 11337 14026 11344 14025 11352
14026 11360 14028 11367 14032 11374 14037 11380 14043 11385 14049 11388
14057 11391 14065 11392 14073 11391 14080 11388 14087 11385 14092 11380
14097 11374 14101 11367 14103 11360 14104 11352 14103 11344 14101 11337
14097 11330 14092 11324 14087 11319 14080 11316 14073 11314 14065 11313
14065 11288 14052 11289 14040 11293 14029 11299 14019 11307 14012 11316
14006 11327 14002 11339 14001 11352 14002 11365 14006 11377 14012 11388
14019 11397 14029 11405 14040 11411 14052 11415 14065 11416 14077 11415
14089 11411 14100 11405 14110 11397 14118 11388 14124 11377 14127 11365
14129 11352 67 N F
14176 11412 14216 11412 14360 11183 14360 11412 14394 11412 14394 11131
14354 11131 14210 11360 14210 11131 14176 11131 14176 11412 11 N F
13602 12430 13568 12430 13570 12451 13572 12460 13575 12469 13578 12477
13582 12484 13587 12490 13592 12496 13598 12502 13604 12506 13611 12510
13619 12513 13627 12515 13635 12517 13654 12519 13671 12517 13687 12514
13702 12508 13714 12500 13719 12495 13724 12489 13728 12483 13732 12476
13735 12469 13737 12461 13738 12453 13738 12444 13738 12434 13736 12425
13733 12417 13729 12409 13724 12402 13717 12396 13708 12391 13698 12386
13710 12381 13720 12375 13728 12369 13735 12361 13740 12353 13743 12344
13746 12333 13746 12322 13745 12307 13741 12292 13735 12277 13731 12271
13725 12264 13719 12259 13713 12253 13705 12249 13696 12245 13687 12242
13676 12239 13665 12238 13652 12237 13641 12238 13631 12239 13621 12241
13613 12244 13605 12247 13597 12251 13591 12256 13585 12262 13580 12268
13576 12274 13572 12281 13569 12289 13565 12306 13564 12324 13598 12324
13598 12314 13600 12304 13603 12294 13608 12286 13615 12278 13625 12273
13638 12269 13654 12267 13668 12268 13680 12271 13689 12276 13697 12282
13703 12291 13707 12300 13709 12311 13710 12322 13709 12336 13707 12342
13705 12347 13702 12352 13698 12356 13689 12363 13678 12367 13665 12370
13650 12372 13634 12372 13634 12400 13650 12400 13664 12402 13676 12404
13685 12408 13693 12413 13698 12420 13701 12430 13702 12442 13701 12452
13699 12461 13696 12468 13691 12475 13684 12481 13676 12485 13666 12488
13654 12489 13643 12488 13633 12486 13625 12482 13617 12477 13611 12469
13606 12459 13603 12446 13602 12430 123 N F
13901 12519 13925 12519 13925 12342 13967 12342 13967 12310 13925 12310
13925 12245 13891 12245 13891 12310 13776 12310 13776 12342 13806 12342
13891 12342 13891 12460 13806 12342 13776 12342 13776 12346 13901 12519
18 N F
14129 12467 14127 12454 14124 12442 14118 12431 14110 12422 14100 12414
14089 12408 14077 12404 14065 12403 14065 12428 14057 12428 14049 12431

14043 12434 14037 12439 14032 12445 14028 12452 14026 12459 14025 12467
14026 12475 14028 12482 14032 12489 14037 12495 14043 12500 14049 12503
14057 12505 14065 12506 14073 12505 14080 12503 14087 12500 14092 12495
14097 12489 14101 12482 14103 12475 14104 12467 14103 12459 14101 12452
14097 12445 14092 12439 14087 12434 14080 12431 14073 12428 14065 12428
14065 12403 14052 12404 14040 12408 14029 12414 14019 12422 14012 12431
14006 12442 14002 12454 14001 12467 14002 12480 14006 12492 14012 12503
14019 12512 14029 12520 14040 12526 14052 12530 14065 12531 14077 12530
14089 12526 14100 12520 14110 12512 14118 12503 14124 12492 14127 12480
14129 12467 67 N F
14176 12527 14216 12527 14360 12298 14360 12527 14394 12527 14394 12245
14354 12245 14210 12474 14210 12245 14176 12245 14176 12527 11 N F
13602 13539 13568 13539 13570 13560 13572 13569 13575 13578 13578 13586
13582 13593 13587 13599 13592 13605 13598 13610 13604 13615 13611 13619
13619 13622 13627 13624 13635 13626 13654 13628 13671 13626 13687 13623
13702 13617 13714 13609 13719 13604 13724 13598 13728 13592 13732 13585
13735 13578 13737 13570 13738 13562 13738 13553 13738 13543 13736 13534
13733 13526 13729 13518 13724 13511 13717 13505 13708 13500 13698 13495
13710 13490 13720 13484 13728 13478 13735 13470 13740 13462 13743 13453
13746 13442 13746 13431 13745 13416 13741 13401 13735 13386 13731 13380
13725 13373 13719 13368 13713 13362 13705 13358 13696 13354 13687 13351
13676 13348 13665 13347 13652 13346 13641 13347 13631 13348 13621 13350
13613 13353 13605 13356 13597 13360 13591 13365 13585 13371 13580 13377
13576 13383 13572 13390 13569 13398 13565 13415 13564 13433 13598 13433
13598 13423 13600 13413 13603 13403 13608 13395 13615 13387 13625 13382
13638 13378 13654 13376 13668 13377 13680 13380 13689 13385 13697 13391
13703 13400 13707 13409 13709 13420 13710 13431 13709 13445 13707 13451
13705 13456 13702 13461 13698 13465 13689 13472 13678 13476 13665 13479
13650 13480 13634 13481 13634 13509 13650 13509 13664 13511 13676 13513
13685 13517 13693 13522 13698 13529 13701 13539 13702 13551 13701 13561
13699 13570 13696 13577 13691 13584 13684 13590 13676 13594 13666 13597
13654 13597 13643 13597 13633 13595 13625 13591 13617 13586 13611 13578
13606 13568 13603 13555 13602 13539 123 N F
13957 13557 13923 13557 13921 13567 13917 13575 13912 13581 13907 13587
13900 13592 13893 13595 13886 13597 13879 13597 13871 13597 13864 13596
13858 13594 13852 13591 13846 13587 13841 13582 13837 13577 13833 13570
13826 13556 13821 13538 13817 13518 13816 13495 13825 13504 13833 13511
13841 13516 13849 13520 13857 13522 13865 13524 13883 13525 13896 13524
13910 13520 13924 13513 13936 13504 13942 13498 13947 13491 13952 13484
13956 13476 13959 13467 13962 13458 13963 13448 13963 13437 13929 13435
13928 13446 13924 13457 13919 13467 13912 13476 13904 13484 13894 13490
13884 13494 13873 13495 13862 13494 13851 13490 13842 13484 13833 13476
13826 13467 13821 13457 13818 13446 13816 13435 13818 13423 13821 13413

13826 13402 13833 13393 13842 13386 13851 13380 13862 13376 13873 13374
13884 13376 13894 13380 13904 13386 13912 13393 13919 13402 13924 13413
13928 13423 13929 13435 13963 13437 13962 13421 13958 13404 13951 13389
13946 13382 13941 13375 13935 13368 13929 13362 13921 13357 13913 13353
13904 13349 13894 13346 13884 13345 13873 13344 13860 13345 13849 13347
13839 13350 13829 13355 13821 13361 13813 13367 13807 13375 13801 13384
13796 13394 13792 13404 13788 13415 13785 13427 13782 13453 13780 13481
13781 13497 13782 13513 13784 13528 13787 13542 13791 13555 13795 13567
13800 13578 13806 13588 13813 13597 13820 13605 13828 13612 13837 13617
13846 13622 13857 13625 13867 13627 13879 13628 13895 13626 13909 13622
13922 13615 13933 13607 13942 13596 13949 13584 13954 13571 13957 13557
126 N F
14129 13576 14127 13563 14124 13551 14118 13540 14110 13531 14100 13523
14089 13517 14077 13513 14065 13512 14065 13536 14057 13537 14049 13540
14043 13543 14037 13548 14032 13554 14028 13561 14026 13568 14025 13576
14026 13584 14028 13591 14032 13598 14037 13604 14043 13609 14049 13612
14057 13614 14065 13615 14073 13614 14080 13612 14087 13609 14092 13604
14097 13598 14101 13591 14103 13584 14104 13576 14103 13568 14101 13561
14097 13554 14092 13548 14087 13543 14080 13540 14073 13537 14065 13536
14065 13512 14052 13513 14040 13517 14029 13523 14019 13531 14012 13540
14006 13551 14002 13563 14001 13576 14002 13589 14006 13601 14012 13612
14019 13621 14029 13629 14040 13635 14052 13639 14065 13640 14077 13639
14089 13635 14100 13629 14110 13621 14118 13612 14124 13601 14127 13589
14129 13576 67 N F
14176 13636 14216 13636 14360 13407 14360 13636 14394 13636 14394 13354
14354 13354 14210 13583 14210 13354 14176 13354 14176 13636 11 N F
13602 14642 13568 14642 13570 14662 13572 14671 13575 14680 13578 14688
13582 14695 13587 14702 13592 14708 13598 14713 13604 14717 13611 14721
13619 14724 13627 14727 13635 14729 13654 14730 13671 14729 13687 14725
13702 14719 13714 14711 13719 14706 13724 14700 13728 14694 13732 14688
13735 14680 13737 14673 13738 14664 13738 14656 13738 14646 13736 14636
13733 14628 13729 14621 13724 14614 13717 14608 13708 14602 13698 14597
13710 14593 13720 14587 13728 14580 13735 14573 13740 14564 13743 14555
13746 14545 13746 14533 13745 14518 13741 14503 13735 14489 13731 14482
13725 14476 13719 14470 13713 14465 13705 14460 13696 14456 13687 14453
13676 14451 13665 14449 13652 14449 13641 14449 13631 14450 13621 14452
13613 14455 13605 14459 13597 14463 13591 14468 13585 14473 13580 14479
13576 14486 13572 14493 13569 14500 13565 14517 13564 14535 13598 14535
13598 14525 13600 14515 13603 14506 13608 14497 13615 14490 13625 14484
13638 14480 13654 14479 13668 14479 13680 14482 13689 14487 13697 14494
13703 14502 13707 14512 13709 14522 13710 14533 13709 14547 13707 14553
13705 14559 13702 14563 13698 14568 13689 14574 13678 14579 13665 14581
13650 14583 13634 14583 13634 14611 13650 14612 13664 14613 13676 14615

13685 14619 13693 14624 13698 14632 13701 14641 13702 14654 13701 14663
13699 14672 13696 14680 13691 14687 13684 14692 13676 14696 13666 14699
13654 14700 13643 14699 13633 14697 13625 14694 13617 14688 13611 14680
13606 14670 13603 14657 13602 14642 123 N F
13871 14730 13887 14729 13903 14725 13917 14719 13929 14711 13939 14701
13943 14695 13947 14689 13949 14682 13951 14674 13953 14666 13953 14658
13952 14646 13950 14636 13946 14627 13941 14620 13935 14613 13929 14608
13917 14599 13927 14594 13936 14588 13944 14580 13950 14572 13956 14563
13960 14553 13962 14542 13963 14531 13961 14518 13957 14503 13950 14489
13939 14476 13933 14470 13926 14464 13918 14459 13910 14455 13900 14452
13891 14449 13880 14447 13869 14447 13857 14447 13847 14449 13837 14452
13828 14455 13820 14459 13813 14464 13806 14470 13800 14476 13790 14489
13784 14503 13780 14518 13778 14531 13814 14531 13816 14520 13819 14510
13824 14501 13831 14493 13840 14486 13849 14481 13860 14478 13871 14477
13882 14478 13892 14481 13902 14486 13910 14493 13917 14501 13922 14510
13926 14520 13927 14531 13926 14542 13922 14552 13917 14562 13910 14570
13902 14576 13892 14581 13882 14584 13871 14585 13860 14584 13849 14581
13840 14576 13831 14570 13824 14562 13819 14552 13816 14542 13814 14531
13778 14531 13779 14542 13782 14553 13786 14562 13791 14571 13798 14580
13805 14587 13814 14594 13824 14599 13812 14608 13806 14613 13800 14620
13796 14627 13792 14636 13789 14646 13788 14658 13822 14658 13823 14648
13826 14640 13831 14633 13837 14627 13844 14622 13852 14618 13861 14616
13871 14616 13880 14616 13889 14618 13897 14622 13905 14627 13910 14633
13915 14640 13918 14648 13919 14658 13918 14667 13915 14675 13910 14683
13905 14689 13897 14693 13889 14697 13880 14699 13871 14700 13861 14699
13852 14697 13844 14693 13837 14689 13831 14683 13826 14675 13823 14667
13822 14658 13788 14658 13789 14666 13790 14674 13792 14682 13795 14689
13798 14695 13802 14701 13812 14711 13824 14719 13838 14725 13854 14729
13871 14730 145 N F
14129 14678 14127 14665 14124 14653 14118 14643 14110 14633 14100 14625
14089 14619 14077 14616 14065 14614 14065 14639 14057 14640 14049 14642
14043 14646 14037 14650 14032 14656 14028 14663 14026 14670 14025 14678
14026 14686 14028 14694 14032 14700 14037 14706 14043 14711 14049 14715
14057 14717 14065 14718 14073 14717 14080 14715 14087 14711 14092 14706
14097 14700 14101 14694 14103 14686 14104 14678 14103 14670 14101 14663
14097 14656 14092 14650 14087 14646 14080 14642 14073 14640 14065 14639
14065 14614 14052 14616 14040 14619 14029 14625 14019 14633 14012 14643
14006 14653 14002 14665 14001 14678 14002 14691 14006 14703 14012 14714
14019 14724 14029 14731 14040 14737 14052 14741 14065 14742 14077 14741
14089 14737 14100 14731 14110 14724 14118 14714 14124 14703 14127 14691
14129 14678 67 N F
14176 14738 14216 14738 14360 14509 14360 14738 14394 14738 14394 14457
14354 14457 14210 14686 14210 14457 14176 14457 14176 14738 11 N F

13688 15828 13712 15828 13712 15651 13754 15651 13754 15619 13712 15619
13712 15555 13678 15555 13678 15619 13564 15619 13564 15651 13594 15651
13678 15651 13678 15770 13594 15651 13564 15651 13564 15655 13688 15828
18 N F
13873 15828 13883 15827 13892 15825 13901 15821 13910 15816 13918 15809
13925 15802 13932 15793 13938 15784 13944 15773 13949 15762 13953 15750
13956 15738 13959 15726 13961 15713 13963 15700 13963 15688 13963 15675
13961 15662 13956 15636 13953 15624 13949 15612 13944 15601 13938 15591
13932 15581 13925 15572 13918 15564 13910 15558 13901 15552 13892 15548
13883 15546 13873 15545 13863 15546 13853 15548 13844 15552 13836 15558
13828 15564 13820 15572 13813 15581 13807 15591 13802 15601 13797 15612
13792 15624 13789 15636 13784 15662 13783 15675 13782 15688 13818 15688
13820 15663 13823 15641 13829 15622 13836 15606 13844 15593 13853 15583
13858 15580 13863 15577 13868 15576 13873 15575 13878 15576 13882 15577
13887 15580 13892 15583 13901 15593 13910 15606 13917 15622 13922 15641
13926 15663 13927 15688 13926 15712 13922 15734 13917 15753 13910 15768
13901 15781 13892 15790 13887 15794 13882 15796 13878 15798 13873 15798
13868 15798 13863 15796 13858 15794 13853 15790 13844 15781 13836 15768
13829 15753 13823 15734 13820 15712 13818 15688 13782 15688 13783 15700
13784 15713 13786 15726 13789 15738 13792 15750 13797 15762 13802 15773
13807 15784 13813 15793 13820 15802 13828 15809 13836 15816 13844 15821
13853 15825 13863 15827 13873 15828 105 N F
14131 15776 14129 15764 14126 15752 14120 15741 14112 15731 14102 15723
14091 15718 14079 15714 14067 15712 14067 15737 14059 15738 14051 15740
14045 15744 14039 15749 14034 15754 14030 15761 14028 15769 14027 15776
14028 15784 14030 15792 14034 15799 14039 15804 14045 15809 14051 15813
14059 15815 14067 15816 14075 15815 14082 15813 14089 15809 14094 15804
14099 15799 14103 15792 14105 15784 14106 15776 14105 15769 14103 15761
14099 15754 14094 15749 14089 15744 14082 15740 14075 15738 14067 15737
14067 15712 14054 15714 14042 15718 14031 15723 14022 15731 14014 15741
14008 15752 14004 15764 14003 15776 14004 15789 14008 15801 14014 15812
14022 15822 14031 15830 14042 15835 14054 15839 14067 15840 14079 15839
14091 15835 14102 15830 14112 15822 14120 15812 14126 15801 14129 15789
14131 15776 67 N F
14178 15836 14218 15836 14362 15607 14362 15836 14396 15836 14396 15555
14356 15555 14212 15784 14212 15555 14178 15555 14178 15836 11 N F
25 W
2646 5172 2646 5453 2 P
3739 5172 3739 5453 2 P
4823 5172 4823 5453 2 P
5903 5172 5903 5453 2 P
6971 5172 6971 5453 2 P
8052 5172 8052 5453 2 P

9133 5172 9133 5453 2 P
10214 5172 10214 5453 2 P
11295 5172 11295 5453 2 P
12376 5172 12376 5453 2 P
2747 16454 2747 16173 2 P
4812 16454 4812 16173 2 P
6843 16454 6843 16173 2 P
8864 16454 8864 16173 2 P
10892 16454 10892 16173 2 P
12942 16454 12942 16173 2 P
2016 5647 2297 5647 2 P
2016 6823 2297 6823 2 P
2016 7980 2297 7980 2 P
2016 9125 2297 9125 2 P
2016 10260 2297 10260 2 P
2016 11387 2297 11387 2 P
2016 12505 2297 12505 2 P
2016 13617 2297 13617 2 P
2016 14723 2297 14723 2 P
2016 15816 2297 15816 2 P
13283 5549 13002 5549 2 P
13283 6719 13002 6719 2 P
13283 7872 13002 7872 2 P
13283 9015 13002 9015 2 P
13283 10147 13002 10147 2 P
13283 11269 13002 11269 2 P
13283 12384 13002 12384 2 P
13283 13492 13002 13492 2 P
13283 14594 13002 14594 2 P
13283 15693 13002 15693 2 P
13002 5453 2297 5453 2297 16173 13002 16173 13002 5453 2297 5453
6:00 PM
7210 17656 7274 17656 7378 17345 7483 17656 7546 17656 7546 17299
7503 17299 7503 17598 7401 17299 7353 17299 7253 17598 7253 17299
7210 17299 7210 17656 14 N F
7613 17480 7614 17496 7618 17511 7621 17518 7625 17525 7630 17531
7636 17538 7642 17543 7650 17549 7658 17553 7667 17557 7678 17560
7689 17562 7701 17564 7714 17564 7729 17564 7745 17562 7761 17558
7776 17552 7783 17548 7790 17543 7796 17538 7801 17532 7805 17525
7809 17518 7811 17510 7811 17501 7811 17337 7812 17333 7815 17329
7818 17325 7823 17323 7828 17321 7833 17320 7838 17321 7842 17322
7842 17291 7832 17289 7816 17289 7805 17289 7797 17290 7790 17292
7785 17295 7781 17299 7777 17305 7771 17322 7749 17306 7739 17300

7730 17296 7721 17293 7711 17290 7700 17289 7686 17289 7675 17289
7665 17290 7655 17292 7647 17295 7639 17298 7633 17302 7627 17306
7622 17311 7617 17316 7614 17322 7609 17334 7606 17347 7605 17360
7643 17360 7644 17353 7647 17346 7651 17339 7657 17333 7665 17329
7674 17325 7685 17323 7697 17322 7714 17324 7729 17329 7741 17335
7752 17342 7760 17350 7766 17359 7769 17367 7771 17376 7771 17427
7751 17419 7736 17416 7689 17409 7675 17405 7665 17400 7657 17394
7651 17388 7647 17382 7645 17375 7643 17368 7643 17360 7605 17360
7606 17379 7609 17395 7614 17407 7622 17417 7631 17425 7643 17431
7656 17435 7671 17439 7737 17450 7753 17454 7764 17459 7767 17461
7769 17464 7770 17467 7771 17470 7771 17493 7770 17497 7768 17502
7764 17508 7758 17513 7750 17518 7740 17522 7729 17525 7714 17526
7699 17525 7687 17522 7676 17517 7668 17511 7661 17504 7657 17496
7654 17488 7653 17480 7613 17480 129 N F
7862 17557 7908 17557 7972 17358 8043 17557 8089 17557 7969 17233
7961 17219 7953 17209 7944 17202 7936 17197 7928 17194 7920 17192
7903 17192 7894 17192 7880 17197 7880 17233 7896 17230 7903 17229
7911 17230 7918 17233 7924 17237 7929 17244 7934 17253 7949 17296
7862 17557 25 N F
2297 17549 2445 17549 2455 17549 2465 17547 2476 17544 2487 17540
2498 17535 2509 17528 2520 17520 2531 17510 2541 17499 2550 17486
2559 17472 2566 17455 2572 17437 2576 17417 2579 17395 2580 17370
2579 17346 2577 17324 2573 17304 2567 17285 2560 17269 2552 17254
2543 17242 2534 17230 2523 17221 2513 17213 2501 17206 2490 17201
2479 17197 2467 17194 2456 17192 2445 17192 2297 17192 2297 17508
2343 17508 2343 17233 2438 17233 2449 17233 2459 17235 2469 17238
2479 17242 2487 17248 2495 17254 2502 17261 2509 17270 2515 17279
2520 17290 2524 17301 2528 17313 2531 17326 2533 17340 2534 17355
2534 17370 2534 17387 2532 17403 2530 17417 2526 17431 2522 17443
2517 17454 2511 17465 2505 17474 2498 17482 2490 17489 2482 17495
2473 17500 2464 17503 2455 17506 2445 17508 2435 17508 2343 17508
2297 17508 2297 17549 74 N F
2644 17549 2708 17549 2812 17238 2917 17549 2980 17549 2980 17192
2937 17192 2937 17490 2835 17192 2787 17192 2687 17490 2687 17192
2644 17192 2644 17549 14 N F
2 O
2458 2789 3323 2789 3323 3744 2458 3744 2458 2789 5 N F
1 O
12 W
2458 2789 3323 2789 3323 3744 2458 3744 2458 2789 5 P
20 O
3323 2789 4188 2789 4188 3744 3323 3744 3323 2789 5 N F
1 O

3323 2789 4188 2789 4188 3744 3323 3744 3323 2789 5 P
38 O
4188 2789 5054 2789 5054 3744 4188 3744 4188 2789 5 N F
1 O
4188 2789 5054 2789 5054 3744 4188 3744 4188 2789 5 P
56 O
5054 2789 5919 2789 5919 3744 5054 3744 5054 2789 5 N F
1 O
5054 2789 5919 2789 5919 3744 5054 3744 5054 2789 5 P
74 O
5919 2789 6784 2789 6784 3744 5919 3744 5919 2789 5 N F
1 O
5919 2789 6784 2789 6784 3744 5919 3744 5919 2789 5 P
92 O
6784 2789 7650 2789 7650 3744 6784 3744 6784 2789 5 N F
1 O
6784 2789 7650 2789 7650 3744 6784 3744 6784 2789 5 P
111 O
7650 2789 8515 2789 8515 3744 7650 3744 7650 2789 5 N F
1 O
7650 2789 8515 2789 8515 3744 7650 3744 7650 2789 5 P
129 O
8515 2789 9380 2789 9380 3744 8515 3744 8515 2789 5 N F
1 O
8515 2789 9380 2789 9380 3744 8515 3744 8515 2789 5 P
147 O
9380 2789 10245 2789 10245 3744 9380 3744 9380 2789 5 N F
1 O
9380 2789 10245 2789 10245 3744 9380 3744 9380 2789 5 P
165 O
10245 2789 11111 2789 11111 3744 10245 3744 10245 2789 5 N F
1 O
10245 2789 11111 2789 11111 3744 10245 3744 10245 2789 5 P
183 O
11111 2789 11976 2789 11976 3744 11111 3744 11111 2789 5 N F
1 O
11111 2789 11976 2789 11976 3744 11111 3744 11111 2789 5 P
201 O
11976 2789 12841 2789 12841 3744 11976 3744 11976 2789 5 N F
1 O
11976 2789 12841 2789 12841 3744 11976 3744 11976 2789 5 P
3167 2500 3177 2499 3186 2497 3195 2493 3203 2488 3210 2482
3217 2474 3224 2466 3230 2457 3235 2447 3240 2437 3247 2414

3252 2390 3253 2366 3252 2341 3247 2317 3240 2294 3235 2283
3230 2273 3224 2264 3217 2256 3210 2248 3203 2242 3195 2237
3186 2233 3177 2231 3167 2230 3158 2231 3149 2233 3140 2237
3132 2242 3125 2248 3118 2256 3111 2264 3105 2273 3100 2283
3095 2294 3088 2317 3083 2341 3081 2366 3116 2366 3117 2342
3120 2321 3126 2303 3132 2288 3140 2275 3149 2266 3153 2263
3158 2260 3163 2259 3167 2258 3172 2259 3177 2260 3181 2263
3186 2266 3195 2275 3203 2288 3209 2303 3214 2321 3218 2342
3219 2366 3218 2389 3214 2410 3209 2428 3203 2443 3195 2455
3186 2464 3177 2469 3172 2470 3167 2471 3163 2470 3158 2469
3149 2464 3140 2455 3132 2443 3126 2428 3120 2410 3117 2389
3116 2366 3081 2366 3083 2390 3088 2414 3095 2437 3100 2447
3105 2457 3111 2466 3118 2474 3125 2482 3132 2488 3140 2493
3149 2497 3158 2499 3167 2500 93 N F
3303 2278 3341 2278 3341 2239 3303 2239 3303 2278 5 N F
3502 2500 3525 2500 3525 2331 3565 2331 3565 2301 3525 2301
3525 2239 3492 2239 3492 2301 3383 2301 3383 2331 3412 2331
3492 2331 3492 2444 3412 2331 3383 2331 3383 2335 3502 2500
18 N F
3930 2500 3940 2499 3949 2497 3958 2493 3966 2488 3973 2482
3980 2474 3987 2466 3993 2457 3998 2447 4003 2437 4010 2414
4015 2390 4016 2366 4015 2341 4010 2317 4003 2294 3998 2283
3993 2273 3987 2264 3980 2256 3973 2248 3966 2242 3958 2237
3949 2233 3940 2231 3930 2230 3921 2231 3912 2233 3903 2237
3895 2242 3888 2248 3881 2256 3874 2264 3868 2273 3863 2283
3858 2294 3851 2317 3846 2341 3844 2366 3879 2366 3880 2342
3883 2321 3889 2303 3895 2288 3903 2275 3912 2266 3916 2263
3921 2260 3926 2259 3930 2258 3935 2259 3940 2260 3944 2263
3949 2266 3958 2275 3966 2288 3972 2303 3977 2321 3981 2342
3982 2366 3981 2389 3977 2410 3972 2428 3966 2443 3958 2455
3949 2464 3940 2469 3935 2470 3930 2471 3926 2470 3921 2469
3912 2464 3903 2455 3895 2443 3889 2428 3883 2410 3880 2389
3879 2366 3844 2366 3846 2390 3851 2414 3858 2437 3863 2447
3868 2457 3874 2466 3881 2474 3888 2482 3895 2488 3903 2493
3912 2497 3921 2499 3930 2500 93 N F
4066 2278 4104 2278 4104 2239 4066 2239 4066 2278 5 N F
4265 2500 4288 2500 4288 2331 4328 2331 4328 2301 4288 2301
4288 2239 4255 2239 4255 2301 4146 2301 4146 2331 4175 2331
4255 2331 4255 2444 4175 2331 4146 2331 4146 2335 4265 2500
18 N F
4469 2500 4492 2500 4492 2331 4533 2331 4533 2301 4492 2301
4492 2239 4460 2239 4460 2301 4351 2301 4351 2331 4380 2331
4460 2331 4460 2444 4380 2331 4351 2331 4351 2335 4469 2500

18 N F
4798 2500 4807 2499 4816 2497 4825 2493 4833 2488 4840 2482
4847 2474 4854 2466 4860 2457 4865 2447 4870 2437 4877 2414
4882 2390 4884 2366 4882 2341 4877 2317 4870 2294 4865 2283
4860 2273 4854 2264 4847 2256 4840 2248 4833 2242 4825 2237
4816 2233 4807 2231 4798 2230 4788 2231 4779 2233 4770 2237
4762 2242 4755 2248 4748 2256 4741 2264 4735 2273 4730 2283
4725 2294 4718 2317 4713 2341 4712 2366 4746 2366 4747 2342
4751 2321 4756 2303 4762 2288 4770 2275 4779 2266 4784 2263
4788 2260 4793 2259 4798 2258 4802 2259 4807 2260 4812 2263
4816 2266 4825 2275 4833 2288 4839 2303 4845 2321 4848 2342
4849 2366 4848 2389 4845 2410 4839 2428 4833 2443 4825 2455
4816 2464 4807 2469 4802 2470 4798 2471 4793 2470 4788 2469
4779 2464 4770 2455 4762 2443 4756 2428 4751 2410 4747 2389
4746 2366 4712 2366 4713 2390 4718 2414 4725 2437 4730 2447
4735 2457 4741 2466 4748 2474 4755 2482 4762 2488 4770 2493
4779 2497 4788 2499 4798 2500 93 N F
4933 2278 4972 2278 4972 2239 4933 2239 4933 2278 5 N F
5132 2500 5155 2500 5155 2331 5195 2331 5195 2301 5155 2301
5155 2239 5123 2239 5123 2301 5014 2301 5014 2331 5042 2331
5123 2331 5123 2444 5042 2331 5014 2331 5014 2335 5132 2500
18 N F
5308 2500 5324 2498 5339 2495 5352 2489 5364 2482 5373 2472
5377 2466 5380 2460 5383 2453 5385 2446 5386 2439 5386 2431
5385 2419 5383 2410 5379 2401 5375 2394 5370 2388 5364 2383
5352 2375 5361 2370 5370 2364 5378 2357 5384 2349 5389 2340
5393 2331 5395 2321 5396 2310 5394 2297 5390 2284 5383 2270
5373 2258 5367 2252 5361 2247 5353 2242 5345 2238 5336 2234
5327 2232 5317 2230 5306 2230 5295 2230 5285 2232 5276 2234
5268 2238 5260 2242 5253 2247 5246 2252 5241 2258 5232 2270
5225 2284 5221 2297 5220 2310 5254 2310 5256 2300 5259 2290
5264 2281 5270 2273 5278 2267 5287 2262 5297 2259 5308 2258
5318 2259 5328 2262 5337 2267 5345 2273 5352 2281 5357 2290
5360 2300 5361 2310 5360 2321 5357 2330 5352 2339 5345 2347
5337 2353 5328 2358 5318 2361 5308 2362 5297 2361 5287 2358
5278 2353 5270 2347 5264 2339 5259 2330 5256 2321 5254 2310
5220 2310 5221 2321 5223 2330 5227 2340 5232 2348 5238 2356
5246 2363 5254 2370 5264 2375 5252 2383 5246 2388 5241 2394
5236 2401 5233 2410 5230 2419 5230 2431 5262 2431 5263 2422
5266 2414 5270 2407 5276 2401 5283 2397 5290 2393 5299 2391
5308 2390 5317 2391 5326 2393 5333 2397 5340 2401 5346 2407
5350 2414 5353 2422 5354 2431 5353 2440 5350 2447 5346 2454
5340 2460 5333 2465 5326 2468 5317 2470 5308 2471 5299 2470

5290 2468 5283 2465 5276 2460 5270 2454 5266 2447 5263 2440
5262 2431 5230 2431 5230 2439 5231 2446 5233 2453 5236 2460
5239 2466 5243 2472 5252 2482 5264 2489 5277 2495 5292 2498
5308 2500 145 N F
5663 2500 5672 2499 5681 2497 5690 2493 5698 2488 5706 2482
5713 2474 5719 2466 5725 2457 5731 2447 5735 2437 5743 2414
5747 2390 5749 2366 5747 2341 5743 2317 5735 2294 5731 2283
5725 2273 5719 2264 5713 2256 5706 2248 5698 2242 5690 2237
5681 2233 5672 2231 5663 2230 5653 2231 5644 2233 5636 2237
5628 2242 5620 2248 5613 2256 5606 2264 5600 2273 5595 2283
5590 2294 5583 2317 5578 2341 5577 2366 5611 2366 5612 2342
5616 2321 5621 2303 5628 2288 5636 2275 5644 2266 5649 2263
5653 2260 5658 2259 5663 2258 5668 2259 5672 2260 5677 2263
5681 2266 5690 2275 5698 2288 5705 2303 5710 2321 5713 2342
5714 2366 5713 2389 5710 2410 5705 2428 5698 2443 5690 2455
5681 2464 5672 2469 5668 2470 5663 2471 5658 2470 5653 2469
5644 2464 5636 2455 5628 2443 5621 2428 5616 2410 5612 2389
5611 2366 5577 2366 5578 2390 5583 2414 5590 2437 5595 2447
5600 2457 5606 2466 5613 2474 5620 2482 5628 2488 5636 2493
5644 2497 5653 2499 5663 2500 93 N F
5799 2278 5837 2278 5837 2239 5799 2239 5799 2278 5 N F
5908 2500 6043 2500 6043 2469 5934 2469 5925 2394 5934 2401
5945 2407 5959 2410 5974 2412 5988 2410 6002 2407 6016 2401
6022 2397 6028 2392 6034 2386 6040 2380 6044 2373 6049 2365
6052 2356 6054 2346 6056 2335 6057 2323 6055 2305 6051 2288
6048 2280 6044 2272 6039 2265 6034 2258 6028 2252 6021 2246
6014 2241 6006 2237 5997 2234 5988 2232 5978 2230 5967 2230
5947 2231 5939 2233 5931 2235 5924 2238 5917 2242 5906 2251
5897 2262 5890 2274 5884 2288 5881 2302 5913 2302 5917 2293
5921 2284 5926 2277 5932 2270 5939 2265 5947 2262 5955 2259
5965 2258 5978 2259 5989 2262 6000 2267 6008 2274 6015 2283
6020 2294 6023 2307 6024 2322 6023 2332 6021 2343 6017 2354
6010 2363 6003 2371 5993 2377 5981 2381 5967 2383 5959 2382
5953 2381 5947 2379 5941 2377 5930 2369 5917 2358 5888 2358
5908 2500 79 N F
6091 2410 6124 2410 6124 2423 6127 2435 6131 2445 6137 2454
6144 2461 6153 2466 6164 2470 6177 2471 6187 2470 6196 2467
6205 2463 6212 2457 6218 2450 6223 2442 6226 2432 6227 2421
6226 2410 6223 2401 6219 2393 6212 2386 6204 2379 6194 2372
6167 2356 6145 2343 6127 2330 6113 2317 6102 2303 6094 2288
6089 2273 6086 2257 6085 2239 6259 2239 6259 2270 6122 2270
6125 2282 6130 2291 6136 2300 6144 2307 6153 2314 6165 2322
6194 2339 6211 2347 6226 2357 6237 2367 6246 2378 6253 2390

6258 2402 6260 2413 6261 2425 6261 2433 6259 2441 6257 2448
6254 2455 6251 2462 6247 2468 6236 2479 6224 2488 6210 2494
6194 2498 6177 2500 6159 2498 6143 2494 6135 2491 6128 2487
6121 2483 6115 2478 6110 2472 6105 2465 6101 2458 6097 2450
6095 2441 6093 2431 6091 2421 6091 2410 76 N F
6528 2500 6538 2499 6547 2497 6555 2493 6563 2488 6571 2482
6578 2474 6585 2466 6590 2457 6596 2447 6601 2437 6608 2414
6613 2390 6614 2366 6613 2341 6608 2317 6601 2294 6596 2283
6590 2273 6585 2264 6578 2256 6571 2248 6563 2242 6555 2237
6547 2233 6538 2231 6528 2230 6519 2231 6510 2233 6501 2237
6493 2242 6485 2248 6478 2256 6472 2264 6466 2273 6460 2283
6456 2294 6448 2317 6444 2341 6442 2366 6476 2366 6478 2342
6481 2321 6486 2303 6493 2288 6501 2275 6510 2266 6514 2263
6519 2260 6523 2259 6528 2258 6533 2259 6537 2260 6542 2263
6547 2266 6555 2275 6563 2288 6570 2303 6575 2321 6579 2342
6580 2366 6579 2389 6575 2410 6570 2428 6563 2443 6555 2455
6547 2464 6537 2469 6533 2470 6528 2471 6523 2470 6519 2469
6510 2464 6501 2455 6493 2443 6486 2428 6481 2410 6478 2389
6476 2366 6442 2366 6444 2390 6448 2414 6456 2437 6460 2447
6466 2457 6472 2466 6478 2474 6485 2482 6493 2488 6501 2493
6510 2497 6519 2499 6528 2500 93 N F
6664 2278 6702 2278 6702 2239 6664 2239 6664 2278 5 N F
6773 2500 6908 2500 6908 2469 6800 2469 6790 2394 6799 2401
6811 2407 6824 2410 6840 2412 6853 2410 6867 2407 6881 2401
6887 2397 6894 2392 6900 2386 6905 2380 6910 2373 6914 2365
6917 2356 6920 2346 6921 2335 6922 2323 6920 2305 6916 2288
6913 2280 6909 2272 6904 2265 6899 2258 6893 2252 6886 2246
6879 2241 6871 2237 6862 2234 6853 2232 6843 2230 6832 2230
6813 2231 6804 2233 6796 2235 6789 2238 6782 2242 6771 2251
6762 2262 6755 2274 6750 2288 6746 2302 6779 2302 6782 2293
6786 2284 6791 2277 6797 2270 6804 2265 6812 2262 6821 2259
6830 2258 6843 2259 6855 2262 6865 2267 6873 2274 6880 2283
6885 2294 6888 2307 6889 2322 6889 2332 6886 2343 6882 2354
6876 2363 6868 2371 6858 2377 6846 2381 6832 2383 6825 2382
6818 2381 6812 2379 6806 2377 6795 2369 6782 2358 6754 2358
6773 2500 79 N F
7121 2433 7088 2433 7086 2441 7083 2449 7078 2456 7073 2461
7067 2465 7060 2468 7053 2470 7046 2471 7039 2470 7033 2469
7026 2467 7021 2464 7015 2461 7011 2456 7006 2451 7002 2445
6996 2431 6991 2414 6988 2395 6987 2373 6995 2382 7002 2388
7010 2393 7018 2397 7025 2399 7033 2401 7050 2402 7063 2401
7076 2397 7089 2390 7101 2381 7107 2376 7112 2370 7116 2363
7120 2355 7123 2347 7125 2338 7126 2328 7126 2318 7094 2316

7093 2327 7090 2337 7085 2347 7078 2355 7070 2363 7061 2368
7051 2372 7040 2373 7030 2372 7020 2368 7011 2363 7003 2355
6996 2347 6991 2337 6988 2327 6987 2316 6988 2305 6991 2295
6996 2285 7003 2276 7011 2269 7020 2263 7030 2260 7040 2258
7051 2260 7061 2263 7070 2269 7078 2276 7085 2285 7090 2295
7093 2305 7094 2316 7126 2318 7125 2302 7121 2287 7115 2272
7111 2265 7106 2259 7100 2253 7094 2247 7087 2242 7079 2238
7070 2234 7061 2232 7051 2230 7040 2230 7029 2230 7018 2232
7008 2235 6999 2240 6991 2245 6984 2252 6978 2259 6972 2268
6967 2277 6963 2287 6960 2297 6957 2309 6954 2333 6952 2360
6953 2376 6954 2391 6956 2405 6959 2418 6962 2430 6966 2442
6971 2452 6977 2462 6983 2471 6990 2478 6998 2485 7006 2490
7015 2494 7025 2497 7035 2499 7046 2500 7061 2498 7075 2494
7087 2488 7098 2480 7106 2470 7113 2458 7118 2446 7121 2433
126 N F
7496 2500 7505 2499 7514 2497 7523 2493 7531 2488 7538 2482
7546 2474 7552 2466 7558 2457 7563 2447 7568 2437 7575 2414
7580 2390 7582 2366 7580 2341 7575 2317 7568 2294 7563 2283
7558 2273 7552 2264 7546 2256 7538 2248 7531 2242 7523 2237
7514 2233 7505 2231 7496 2230 7486 2231 7477 2233 7469 2237
7460 2242 7453 2248 7446 2256 7439 2264 7433 2273 7428 2283
7423 2294 7416 2317 7411 2341 7410 2366 7444 2366 7445 2342
7449 2321 7454 2303 7461 2288 7468 2275 7477 2266 7482 2263
7486 2260 7491 2259 7496 2258 7500 2259 7505 2260 7510 2263
7514 2266 7523 2275 7531 2288 7537 2303 7543 2321 7546 2342
7547 2366 7546 2389 7543 2410 7537 2428 7531 2443 7523 2455
7514 2464 7505 2469 7500 2470 7496 2471 7491 2470 7486 2469
7477 2464 7468 2455 7461 2443 7454 2428 7449 2410 7445 2389
7444 2366 7410 2366 7411 2390 7416 2414 7423 2437 7428 2447
7433 2457 7439 2466 7446 2474 7453 2482 7460 2488 7469 2493
7477 2497 7486 2499 7496 2500 93 N F
7631 2278 7670 2278 7670 2239 7631 2239 7631 2278 5 N F
7884 2433 7851 2433 7849 2441 7846 2449 7841 2456 7836 2461
7830 2465 7823 2468 7816 2470 7809 2471 7802 2470 7796 2469
7789 2467 7784 2464 7778 2461 7774 2456 7769 2451 7765 2445
7759 2431 7754 2414 7751 2395 7750 2373 7758 2382 7765 2388
7773 2393 7781 2397 7788 2399 7796 2401 7813 2402 7826 2401
7839 2397 7852 2390 7864 2381 7870 2376 7875 2370 7879 2363
7883 2355 7886 2347 7888 2338 7889 2328 7889 2318 7857 2316
7856 2327 7853 2337 7848 2347 7841 2355 7833 2363 7824 2368
7814 2372 7803 2373 7793 2372 7783 2368 7774 2363 7766 2355
7759 2347 7754 2337 7751 2327 7750 2316 7751 2305 7754 2295
7759 2285 7766 2276 7774 2269 7783 2263 7793 2260 7803 2258

7814 2260 7824 2263 7833 2269 7841 2276 7848 2285 7853 2295
7856 2305 7857 2316 7889 2318 7888 2302 7884 2287 7878 2272
7874 2265 7869 2259 7863 2253 7857 2247 7850 2242 7842 2238
7833 2234 7824 2232 7814 2230 7803 2230 7792 2230 7781 2232
7771 2235 7762 2240 7754 2245 7747 2252 7741 2259 7735 2268
7730 2277 7726 2287 7723 2297 7720 2309 7717 2333 7715 2360
7716 2376 7717 2391 7719 2405 7722 2418 7725 2430 7729 2442
7734 2452 7740 2462 7746 2471 7753 2478 7761 2485 7769 2490
7778 2494 7788 2497 7798 2499 7809 2500 7824 2498 7838 2494
7850 2488 7861 2480 7869 2470 7876 2458 7881 2446 7884 2433
126 N F
8257 2500 8266 2499 8275 2497 8284 2493 8292 2488 8300 2482
8307 2474 8313 2466 8319 2457 8324 2447 8329 2437 8337 2414
8341 2390 8343 2366 8341 2341 8337 2317 8329 2294 8324 2283
8319 2273 8313 2264 8307 2256 8300 2248 8292 2242 8284 2237
8275 2233 8266 2231 8257 2230 8247 2231 8238 2233 8230 2237
8221 2242 8214 2248 8207 2256 8200 2264 8194 2273 8189 2283
8184 2294 8177 2317 8172 2341 8171 2366 8205 2366 8206 2342
8210 2321 8215 2303 8222 2288 8229 2275 8238 2266 8243 2263
8247 2260 8252 2259 8257 2258 8261 2259 8266 2260 8271 2263
8275 2266 8284 2275 8292 2288 8299 2303 8304 2321 8307 2342
8308 2366 8307 2389 8304 2410 8299 2428 8292 2443 8284 2455
8275 2464 8266 2469 8261 2470 8257 2471 8252 2470 8247 2469
8238 2464 8229 2455 8222 2443 8215 2428 8210 2410 8206 2389
8205 2366 8171 2366 8172 2390 8177 2414 8184 2437 8189 2447
8194 2457 8200 2466 8207 2474 8214 2482 8221 2488 8230 2493
8238 2497 8247 2499 8257 2500 93 N F
8392 2278 8431 2278 8431 2239 8392 2239 8392 2278 5 N F
8645 2433 8612 2433 8610 2441 8607 2449 8602 2456 8597 2461
8591 2465 8584 2468 8577 2470 8570 2471 8563 2470 8557 2469
8551 2467 8545 2464 8540 2461 8535 2456 8530 2451 8526 2445
8520 2431 8515 2414 8512 2395 8511 2373 8519 2382 8526 2388
8534 2393 8542 2397 8550 2399 8557 2401 8574 2402 8587 2401
8600 2397 8613 2390 8625 2381 8631 2376 8636 2370 8640 2363
8644 2355 8647 2347 8649 2338 8650 2328 8650 2318 8618 2316
8617 2327 8614 2337 8609 2347 8602 2355 8594 2363 8585 2368
8575 2372 8564 2373 8554 2372 8544 2368 8535 2363 8527 2355
8520 2347 8515 2337 8512 2327 8511 2316 8512 2305 8515 2295
8520 2285 8527 2276 8535 2269 8544 2263 8554 2260 8564 2258
8575 2260 8585 2263 8594 2269 8602 2276 8609 2285 8614 2295
8617 2305 8618 2316 8650 2318 8649 2302 8645 2287 8639 2272
8635 2265 8630 2259 8624 2253 8618 2247 8611 2242 8603 2238
8594 2234 8585 2232 8575 2230 8564 2230 8553 2230 8542 2232

8532 2235 8523 2240 8515 2245 8508 2252 8502 2259 8496 2268
8491 2277 8487 2287 8484 2297 8481 2309 8478 2333 8477 2360
8477 2376 8478 2391 8480 2405 8483 2418 8486 2430 8491 2442
8495 2452 8501 2462 8507 2471 8515 2478 8522 2485 8531 2490
8540 2494 8549 2497 8559 2499 8570 2500 8585 2498 8599 2494
8611 2488 8622 2480 8631 2470 8637 2458 8642 2446 8645 2433
126 N F
8796 2500 8819 2500 8819 2331 8859 2331 8859 2301 8819 2301
8819 2239 8786 2239 8786 2301 8677 2301 8677 2331 8706 2331
8786 2331 8786 2444 8706 2331 8677 2331 8677 2335 8796 2500
18 N F
9124 2500 9133 2499 9142 2497 9151 2493 9159 2488 9167 2482
9174 2474 9180 2466 9186 2457 9192 2447 9196 2437 9204 2414
9208 2390 9210 2366 9208 2341 9204 2317 9196 2294 9192 2283
9186 2273 9180 2264 9174 2256 9167 2248 9159 2242 9151 2237
9142 2233 9133 2231 9124 2230 9114 2231 9105 2233 9097 2237
9089 2242 9081 2248 9074 2256 9067 2264 9062 2273 9056 2283
9051 2294 9044 2317 9039 2341 9038 2366 9072 2366 9073 2342
9077 2321 9082 2303 9089 2288 9097 2275 9105 2266 9110 2263
9115 2260 9119 2259 9124 2258 9129 2259 9133 2260 9138 2263
9142 2266 9151 2275 9159 2288 9166 2303 9171 2321 9174 2342
9175 2366 9174 2389 9171 2410 9166 2428 9159 2443 9151 2455
9142 2464 9133 2469 9129 2470 9124 2471 9119 2470 9115 2469
9105 2464 9097 2455 9089 2443 9082 2428 9077 2410 9073 2389
9072 2366 9038 2366 9039 2390 9044 2414 9051 2437 9056 2447
9062 2457 9067 2466 9074 2474 9081 2482 9089 2488 9097 2493
9105 2497 9114 2499 9124 2500 93 N F
9260 2278 9298 2278 9298 2239 9260 2239 9260 2278 5 N F
9512 2433 9479 2433 9477 2441 9474 2449 9469 2456 9464 2461
9458 2465 9451 2468 9444 2470 9437 2471 9430 2470 9424 2469
9418 2467 9412 2464 9407 2461 9402 2456 9397 2451 9393 2445
9387 2431 9382 2414 9379 2395 9378 2373 9386 2382 9394 2388
9401 2393 9409 2397 9417 2399 9425 2401 9441 2402 9454 2401
9467 2397 9480 2390 9492 2381 9498 2376 9503 2370 9507 2363
9511 2355 9514 2347 9516 2338 9517 2328 9518 2318 9485 2316
9484 2327 9481 2337 9476 2347 9469 2355 9461 2363 9452 2368
9442 2372 9432 2373 9421 2372 9411 2368 9402 2363 9394 2355
9387 2347 9382 2337 9379 2327 9378 2316 9379 2305 9382 2295
9387 2285 9394 2276 9402 2269 9411 2263 9421 2260 9432 2258
9442 2260 9452 2263 9461 2269 9469 2276 9476 2285 9481 2295
9484 2305 9485 2316 9518 2318 9516 2302 9513 2287 9506 2272
9502 2265 9497 2259 9491 2253 9485 2247 9478 2242 9470 2238
9462 2234 9452 2232 9442 2230 9432 2230 9420 2230 9409 2232

9399 2235 9390 2240 9382 2245 9375 2252 9369 2259 9363 2268
9358 2277 9354 2287 9351 2297 9348 2309 9345 2333 9344 2360
9344 2376 9345 2391 9347 2405 9350 2418 9353 2430 9358 2442
9363 2452 9368 2462 9375 2471 9382 2478 9389 2485 9398 2490
9407 2494 9416 2497 9427 2499 9437 2500 9452 2498 9466 2494
9478 2488 9489 2480 9498 2470 9505 2458 9509 2446 9512 2433
126 N F
9634 2500 9650 2498 9665 2495 9678 2489 9690 2482 9699 2472
9703 2466 9707 2460 9709 2453 9711 2446 9712 2439 9713 2431
9712 2419 9709 2410 9706 2401 9701 2394 9696 2388 9690 2383
9678 2375 9688 2370 9696 2364 9704 2357 9710 2349 9715 2340
9719 2331 9721 2321 9722 2310 9721 2297 9716 2284 9709 2270
9699 2258 9693 2252 9687 2247 9679 2242 9671 2238 9663 2234
9653 2232 9643 2230 9632 2230 9622 2230 9612 2232 9602 2234
9594 2238 9586 2242 9579 2247 9573 2252 9567 2258 9558 2270
9551 2284 9548 2297 9546 2310 9581 2310 9582 2300 9585 2290
9590 2281 9597 2273 9605 2267 9614 2262 9624 2259 9634 2258
9645 2259 9655 2262 9664 2267 9672 2273 9678 2281 9683 2290
9687 2300 9688 2310 9687 2321 9683 2330 9678 2339 9672 2347
9664 2353 9655 2358 9645 2361 9634 2362 9624 2361 9614 2358
9605 2353 9597 2347 9590 2339 9585 2330 9582 2321 9581 2310
9546 2310 9547 2321 9549 2330 9553 2340 9558 2348 9565 2356
9572 2363 9581 2370 9590 2375 9578 2383 9573 2388 9567 2394
9563 2401 9559 2410 9557 2419 9556 2431 9588 2431 9589 2422
9592 2414 9596 2407 9602 2401 9609 2397 9617 2393 9625 2391
9634 2390 9643 2391 9652 2393 9660 2397 9666 2401 9672 2407
9676 2414 9679 2422 9680 2431 9679 2440 9676 2447 9672 2454
9666 2460 9660 2465 9652 2468 9643 2470 9634 2471 9625 2470
9617 2468 9609 2465 9602 2460 9596 2454 9592 2447 9589 2440
9588 2431 9556 2431 9556 2439 9557 2446 9559 2453 9562 2460
9565 2466 9569 2472 9579 2482 9590 2489 9603 2495 9618 2498
9634 2500 145 N F
9989 2500 9999 2499 10008 2497 10016 2493 10024 2488 10032 2482
10039 2474 10046 2466 10051 2457 10057 2447 10062 2437 10069 2414
10074 2390 10075 2366 10074 2341 10069 2317 10062 2294 10057 2283
10051 2273 10046 2264 10039 2256 10032 2248 10024 2242 10016 2237
10008 2233 9999 2231 9989 2230 9980 2231 9971 2233 9962 2237
9954 2242 9946 2248 9939 2256 9933 2264 9927 2273 9921 2283
9917 2294 9909 2317 9905 2341 9903 2366 9938 2366 9939 2342
9942 2321 9947 2303 9954 2288 9962 2275 9971 2266 9975 2263
9980 2260 9984 2259 9989 2258 9994 2259 9998 2260 10003 2263
10008 2266 10016 2275 10024 2288 10031 2303 10036 2321 10040 2342
10041 2366 10040 2389 10036 2410 10031 2428 10024 2443 10016 2455

10008 2464 9998 2469 9994 2470 9989 2471 9984 2470 9980 2469
9971 2464 9962 2455 9954 2443 9947 2428 9942 2410 9939 2389
9938 2366 9903 2366 9905 2390 9909 2414 9917 2437 9921 2447
9927 2457 9933 2466 9939 2474 9946 2482 9954 2488 9962 2493
9971 2497 9980 2499 9989 2500 93 N F
10125 2278 10163 2278 10163 2239 10125 2239 10125 2278 5 N F
10211 2500 10385 2500 10385 2473 10360 2436 10340 2404 10324 2376
10311 2350 10301 2324 10293 2299 10286 2271 10280 2239 10245 2239
10255 2275 10264 2306 10274 2332 10285 2357 10298 2381 10313 2407
10352 2469 10211 2469 10211 2500 21 N F
10417 2410 10450 2410 10451 2423 10453 2435 10457 2445 10463 2454
10470 2461 10480 2466 10491 2470 10503 2471 10513 2470 10522 2467
10531 2463 10538 2457 10544 2450 10549 2442 10552 2432 10553 2421
10552 2410 10549 2401 10545 2393 10538 2386 10530 2379 10520 2372
10494 2356 10472 2343 10454 2330 10439 2317 10428 2303 10420 2288
10415 2273 10412 2257 10412 2239 10586 2239 10586 2270 10448 2270
10451 2282 10456 2291 10462 2300 10470 2307 10479 2314 10491 2322
10521 2339 10538 2347 10552 2357 10564 2367 10573 2378 10579 2390
10584 2402 10587 2413 10587 2425 10587 2433 10586 2441 10584 2448
10581 2455 10577 2462 10573 2468 10563 2479 10550 2488 10536 2494
10520 2498 10503 2500 10485 2498 10469 2494 10461 2491 10454 2487
10448 2483 10442 2478 10436 2472 10431 2465 10427 2458 10424 2450
10421 2441 10419 2431 10418 2421 10417 2410 76 N F
10854 2500 10864 2499 10873 2497 10881 2493 10890 2488 10897 2482
10904 2474 10911 2466 10917 2457 10922 2447 10927 2437 10934 2414
10939 2390 10940 2366 10939 2341 10934 2317 10927 2294 10922 2283
10917 2273 10911 2264 10904 2256 10897 2248 10890 2242 10881 2237
10873 2233 10864 2231 10854 2230 10845 2231 10836 2233 10827 2237
10819 2242 10812 2248 10804 2256 10798 2264 10792 2273 10787 2283
10782 2294 10775 2317 10770 2341 10768 2366 10803 2366 10804 2342
10807 2321 10813 2303 10819 2288 10827 2275 10836 2266 10840 2263
10845 2260 10850 2259 10854 2258 10859 2259 10864 2260 10868 2263
10873 2266 10882 2275 10890 2288 10896 2303 10901 2321 10905 2342
10906 2366 10905 2389 10901 2410 10896 2428 10890 2443 10882 2455
10873 2464 10864 2469 10859 2470 10854 2471 10850 2470 10845 2469
10836 2464 10827 2455 10819 2443 10813 2428 10807 2410 10804 2389
10803 2366 10768 2366 10770 2390 10775 2414 10782 2437 10787 2447
10792 2457 10798 2466 10804 2474 10812 2482 10819 2488 10827 2493
10836 2497 10845 2499 10854 2500 93 N F
10990 2278 11028 2278 11028 2239 10990 2239 10990 2278 5 N F
11076 2500 11250 2500 11250 2473 11225 2436 11205 2404 11189 2376
11177 2350 11167 2324 11158 2299 11151 2271 11145 2239 11111 2239
11120 2275 11130 2306 11140 2332 11150 2357 11163 2381 11178 2407

11218 2469 11076 2469 11076 2500 21 N F
11447 2433 11414 2433 11412 2441 11409 2449 11405 2456 11399 2461
11393 2465 11386 2468 11380 2470 11372 2471 11365 2470 11359 2469
11353 2467 11347 2464 11342 2461 11337 2456 11332 2451 11328 2445
11322 2431 11317 2414 11314 2395 11313 2373 11321 2382 11329 2388
11336 2393 11344 2397 11352 2399 11360 2401 11376 2402 11389 2401
11402 2397 11415 2390 11427 2381 11433 2376 11438 2370 11442 2363
11446 2355 11449 2347 11451 2338 11452 2328 11453 2318 11420 2316
11419 2327 11416 2337 11411 2347 11404 2355 11396 2363 11387 2368
11377 2372 11367 2373 11356 2372 11346 2368 11337 2363 11329 2355
11323 2347 11317 2337 11314 2327 11313 2316 11314 2305 11317 2295
11323 2285 11329 2276 11337 2269 11346 2263 11356 2260 11367 2258
11377 2260 11387 2263 11396 2269 11404 2276 11411 2285 11416 2295
11419 2305 11420 2316 11453 2318 11451 2302 11448 2287 11441 2272
11437 2265 11432 2259 11426 2253 11420 2247 11413 2242 11405 2238
11397 2234 11387 2232 11377 2230 11367 2230 11355 2230 11344 2232
11334 2235 11326 2240 11317 2245 11310 2252 11304 2259 11298 2268
11294 2277 11289 2287 11286 2297 11283 2309 11280 2333 11279 2360
11279 2376 11280 2391 11282 2405 11285 2418 11289 2430 11293 2442
11298 2452 11303 2462 11310 2471 11317 2478 11324 2485 11333 2490
11342 2494 11351 2497 11362 2499 11372 2500 11388 2498 11401 2494
11413 2488 11424 2480 11433 2470 11440 2458 11444 2446 11447 2433
126 N F
11822 2500 11831 2499 11840 2497 11849 2493 11857 2488 11865 2482
11872 2474 11878 2466 11884 2457 11890 2447 11894 2437 11902 2414
11906 2390 11908 2366 11906 2341 11902 2317 11894 2294 11890 2283
11884 2273 11878 2264 11872 2256 11865 2248 11857 2242 11849 2237
11840 2233 11831 2231 11822 2230 11812 2231 11803 2233 11795 2237
11787 2242 11779 2248 11772 2256 11765 2264 11760 2273 11754 2283
11750 2294 11742 2317 11737 2341 11736 2366 11770 2366 11771 2342
11775 2321 11780 2303 11787 2288 11795 2275 11803 2266 11808 2263
11813 2260 11817 2259 11822 2258 11827 2259 11831 2260 11836 2263
11840 2266 11849 2275 11857 2288 11864 2303 11869 2321 11872 2342
11874 2366 11872 2389 11869 2410 11864 2428 11857 2443 11849 2455
11840 2464 11831 2469 11827 2470 11822 2471 11817 2470 11813 2469
11803 2464 11795 2455 11787 2443 11780 2428 11775 2410 11771 2389
11770 2366 11736 2366 11737 2390 11742 2414 11750 2437 11754 2447
11760 2457 11765 2466 11772 2474 11779 2482 11787 2488 11795 2493
11803 2497 11812 2499 11822 2500 93 N F
11958 2278 11996 2278 11996 2239 11958 2239 11958 2278 5 N F
12128 2500 12144 2498 12159 2495 12172 2489 12183 2482 12193 2472
12197 2466 12200 2460 12203 2453 12205 2446 12206 2439 12206 2431
12205 2419 12203 2410 12199 2401 12195 2394 12189 2388 12184 2383

```
12172 2375 12181 2370 12190 2364 12197 2357 12204 2349 12209 2340
12213 2331 12215 2321 12216 2310 12214 2297 12210 2284 12203 2270
12193 2258 12187 2252 12180 2247 12173 2242 12165 2238 12156 2234
12147 2232 12137 2230 12126 2230 12115 2230 12105 2232 12096 2234
12087 2238 12080 2242 12073 2247 12066 2252 12061 2258 12051 2270
12045 2284 12041 2297 12040 2310 12074 2310 12075 2300 12079 2290
12084 2281 12090 2273 12098 2267 12107 2262 12117 2259 12128 2258
12138 2259 12148 2262 12157 2267 12165 2273 12172 2281 12177 2290
12180 2300 12181 2310 12180 2321 12177 2330 12172 2339 12165 2347
12157 2353 12148 2358 12138 2361 12128 2362 12117 2361 12107 2358
12098 2353 12090 2347 12084 2339 12079 2330 12075 2321 12074 2310
12040 2310 12041 2321 12043 2330 12047 2340 12052 2348 12058 2356
12066 2363 12074 2370 12084 2375 12072 2383 12066 2388 12061 2394
12056 2401 12053 2410 12050 2419 12049 2431 12082 2431 12083 2422
12086 2414 12090 2407 12095 2401 12102 2397 12110 2393 12119 2391
12128 2390 12137 2391 12145 2393 12153 2397 12160 2401 12166 2407
12170 2414 12173 2422 12174 2431 12173 2440 12170 2447 12166 2454
12160 2460 12153 2465 12145 2468 12137 2470 12128 2471 12119 2470
12110 2468 12102 2465 12095 2460 12090 2454 12086 2447 12083 2440
12082 2431 12049 2431 12050 2439 12051 2446 12053 2453 12055 2460
12059 2466 12063 2472 12072 2482 12084 2489 12097 2495 12112 2498
12128 2500 145 N F
Gr
S
%%Trailer
%%DocumentFonts:
%%Pages: 1
%%EOF
```

Regional loss of dry matter in T2 (March)

```
%%Pages: (atend)
%%PageOrder: Ascend
%%Creator: NCAR GKS
%%DocumentFonts: (atend)
%%EndComments
%%BeginProlog

/M3
{
  2 copy lt {pop} {exch pop} ifelse
  2 copy lt {pop} {exch pop} ifelse
} bind def
```

```
/Sc
{
  1. exch sub /ylo exch def
  1. exch sub /mag exch def
  1. exch sub /cyn exch def
  ylo mag cyn M3 /b_cmy exch def
  cyn b_cmy sub /c_cmy exch def
  mag b_cmy sub /m_cmy exch def
  ylo b_cmy sub /y_cmy exch def
  c_cmy m_cmy y_cmy b_cmy setcmykcolor
} bind def
/P
{/n exch def
  newpath
  moveto
  2 1 n {pop lineto} for
  stroke
 } bind def

/F
{/n exch def
  newpath
  moveto
  2 1 n {pop lineto} for
  closepath
  eofill
} bind def

/B
{ /s exch def
  /n exch def
  1 1 n {newpath pop s 0 360 arc stroke} for
} bind def

/Q
{ /s exch def
  /n exch def
  /ms 0 s sub def
  /ts s s add def
  1 1 n {gsave newpath pop translate
  0 0 moveto ms 0 rmoveto ts 0 rlineto
  0 0 moveto 90 rotate ms  0 rmoveto ts 0 rlineto
  stroke grestore} for
```

```
} bind def

/Fs
{ /s exch def
  findfont
  s scalefont
  setfont
} bind def

/V
{ /s exch def
  /n exch def
  /ms 0 s sub def
  /ts s s add def
  1 1 n {gsave newpath pop translate
  0 0 moveto 30 rotate ms 0 rmoveto  ts 0 rlineto
  0 0 moveto 60 rotate ms 0 rmoveto  ts 0 rlineto
  0 0 moveto 60 rotate ms 0 rmoveto  ts 0 rlineto
  stroke grestore} for
} bind def

/X
{ /s exch def
  /n exch def
  /s s 1.25 mul def
  /ms 0 s sub def
  /ts s s add def
  1 1 n {gsave newpath pop translate
  0 0 moveto 45 rotate ms 0 rmoveto  ts 0 rlineto
  0 0 moveto 90 rotate ms 0 rmoveto  ts 0 rlineto
  stroke grestore} for
} bind def

/Ls
{matrix currentmatrix
 currentrgbcolor
 currentlinewidth
 currentlinecap
 currentlinejoin
 currentdash
} bind def

/Lr
```

```
{setdash
 setlinejoin
 setlinecap
 setlinewidth
 setrgbcolor
 setmatrix
} bind def

/M {stroke newpath moveto} bind def
/L {lineto} bind def
/E {eofill} def
/J {setlinejoin} def
/C {setlinecap} def
/W {setlinewidth} def
/S {stroke showpage} def
/A {scale} def
/U {put} def
/D {setdash} def
/I {currentdash} def
/H {currentlinewidth} def
/G {currentrgbcolor} def
/R {setrgbcolor} def
/N {closepath} def
/K {stroke} def
/Z {rotate} def
/As {ashow} def
/Ch {clippath} def
/Ci {colorimage} def
/Co {concat} def
/Cm {currentmatrix} def
/Cp {clip} def
/Ct {currentpoint} def
/Cu {currentfile} def
/Ex {exch} def
/Im {image} def
/In {initgraphics} def
/Ml {setmiterlimit} def
/Mx {matrix} def
/Np {newpath} def
/Ng {neg} def
/Rh {readhexstring} def
/Rm {rmoveto} def
/Sm {setmatrix} def
```

```
/Tr {translate} def
/Gs {gsave} def
/Gr {grestore} def

/Ov
{/strn exch def
 /ispc exch def
 /str1 1 string def
  strn
  {/code exch def
   /chr str1 0 code put str1 def
   0 ispc neg rmoveto
   gsave
   chr stringwidth pop 2 div neg
   0 rmoveto
   chr false charpath stroke
   grestore
  } forall
} bind def

/Oh
{/strn exch def
 /ispc exch def
 /tstr 1 string def
 strn
   {
    /code exch def
    /chr tstr 0 code put tstr def
    chr false charpath
    currentpoint
    /yt exch def /xt exch def
    stroke
    xt ispc add yt moveto
   } forall
 } bind def

/T 256 array def
/O
{ T
  exch
  get
  aload
  pop
```

```
  setrgbcolor
} bind def

/Y
{/strn exch def
 /ispc exch def
 /str1 1 string def
  strn
  {/code exch def
   /chr str1 0 code put str1 def
    0 ispc neg rmoveto
    gsave
      chr stringwidth pop 2 div neg 0 rmoveto
      chr show
    grestore
  } forall
} def

/Bg
{/ury Ex def /urx Ex def /lly Ex def /llx Ex def
 Np Ls Gr Gs 0 O Ch E Lr
 llx lly M urx lly L urx ury L
 llx ury L llx lly L
 Cp Np
} def

/Oc
{/ury exch def
 /urx exch def
 /lly exch def
 /llx exch def
 Np
 llx lly M urx lly L urx ury L  llx ury L llx lly L
 Cp Np
} def

/Cc
{/CellWidth exch def
 /strn CellWidth 1 add string def
 /strn1 CellWidth 1 add 3 mul string def
 systemdict /colorimage known
 { { Cu strn1 Rh pop } false 3 Ci}
 { { Cu strn1 Rh pop
```

```
     /ColorStr exch def
     0 1 CellWidth {
       /YIndx exch def
       /GrayVal YIndx 3 mul def
       strn YIndx
         ColorStr GrayVal       get 0.30 mul
         ColorStr GrayVal 1 add get 0.59 mul
         ColorStr GrayVal 2 add get 0.11 mul
         add add cvi
       put
     } for
     strn
   } Im
 } ifelse
} def

/Ld
{/ury exch def /urx exch def  /lly exch def /llx exch def
 -90 rotate
 lly urx add neg llx lly sub translate
} bind def
/Pt
{/ury exch def /urx exch def  /lly exch def /llx exch def
 90 rotate
 lly urx add lly llx sub translate
} bind def
%%EndProlog

%%BeginSetup
<</PageSize [00612 00792]>> setpagedevice
%%EndSetup

[1. 1. 1.] [0. 0. 0.] [1. 0. 0.] [0. 1. 0.] [0. 0. 1.] [0. 1. 1.] [1. 0. .8]
[1. 1. 0.] [1. .5 0.] [.6 .8 0.] [0. 1. .6] [0. .5 1.] [.5 0. .8] [1. 0. .6]
[.3 .3 .3] [.7 .7 .7] [1. 1. .3] [.7 1. .5] [.5 1. .6] [.2 1. .7] [.3 .8 .8]
[.5 .7 .8] [.7 .5 .8] [1. .3 .9] [.7 .9 .5] [.4 .9 .5] [.2 .9 .7] [.2 .7 .9]
[.2 .5 1.] [.5 .3 1.] [.7 .2 1.] [.9 .1 1.] [.9 1. .2] [.7 1. .3] [.5 1. .3]
[.2 1. .5] [.2 .8 .6] [.2 .7 .7] [.2 .5 .8] [.2 .4 .9] [.4 .3 .9] [.7 .2 .9]
[.8 .2 .8] [.9 .3 .7] [.8 .4 .6] [.8 .6 .5] [.9 .7 .4] [.9 .7 .3] [1. .9 .9]
[.8 1. .1] [.6 1. .2] [.5 1. .2] [.2 .9 .5] [.2 .8 .5] [.2 .7 .7] [.2 .5 .7]
[.2 .4 .9] [.4 .3 .9] [.5 .2 .9] [.8 .2 .7] [1. .2 .7] [1. .3 .6] [1. .4 .5]
[1. .5 .4] [1. .8 .1] [.7 1. .0] [.6 1. .1] [.4 1. .2] [.1 1. .3] [.1 .8 .4]
[.2 .7 .5] [.1 .6 .7] [.1 .5 .7] [.1 .4 .8] [.2 .3 .9] [.2 .2 1.] [.3 .1 1.]
```

```
[.5 .1 .9] [.7 .0 .8] [.9 .0 .7] [.9 .7 .1] [.7 .9 .1] [.5 .9 .1] [.3 .9 .2]
[.1 .9 .3] [.2 .7 .4] [.1 .7 .5] [.1 .5 .6] [.1 .4 .7] [.2 .3 .7] [.2 .2 .8]
[.3 .2 .8] [.5 .1 .7] [.7 .1 .7] [.9 .2 .5] [.8 .2 .5] [.8 .7 .0] [.6 .8 .1]
[.4 .9 .1] [.3 .9 .1] [.1 .9 .2] [.2 .8 .2] [.2 .7 .3] [.2 .6 .5] [.1 .5 .5]
[.1 .4 .6] [.2 .3 .7] [.3 .2 .7] [.4 .2 .6] [.7 .2 .5] [.8 .2 .4] [1. .2 .3]
[.7 .7 .0] [.5 .7 .1] [.4 .8 .1] [.2 .9 .1] [.2 .9 .1] [.2 .8 .2] [.1 .7 .2]
[.1 .7 .3] [.1 .6 .4] [.1 .4 .5] [.1 .4 .5] [.2 .3 .5] [.4 .3 .4] [.5 .3 .4]
[.7 .3 .3] [.8 .3 .2] [.7 .6 .0] [.5 .7 .1] [.4 .7 .1] [.2 .7 .2] [.1 .7 .2]
[.1 .6 .3] [.1 .5 .4] [.1 .4 .4] [.1 .3 .5] [.1 .2 .7] [.1 .1 .7] [.4 .1 .5]
[.5 .1 .5] [.6 .1 .4] [.7 .1 .4] [.9 .1 .3] [.5 .5 .1] [.4 .6 .1] [.3 .6 .1]
[.2 .6 .2] [.1 .5 .2] [.1 .4 .3] [.1 .4 .4] [.2 .3 .4] [.2 .2 .4] [.2 .2 .5]
[.2 .1 .5] [.3 .1 .5] [.4 .1 .4] [.5 .1 .4] [.7 .1 .3] [.8 .1 .2] [.5 .4 .1]
[.4 .5 .1] [.3 .6 .0] [.2 .6 .1] [.1 .7 .1] [.1 .6 .1] [.1 .5 .2] [.1 .4 .2]
[.1 .4 .2] [.1 .3 .3] [.1 .2 .4] [.1 .2 .5] [.1 .1 .5] [.1 .0 .6] [.3 .0 .5]
[.5 .0 .4] [.7 .3 .0] [.7 .4 .0] [.5 .4 .1] [.3 .4 .2] [.2 .3 .2] [.2 .2 .3]
[.2 .2 .4] [.3 .1 .4] [.5 .1 .3] [.6 .1 .2] [.7 .1 .2] [.8 .0 .2] [1. .0 .2]
[1. .1 .1] [.9 .2 .1] [.8 .2 .1] [.6 .2 .0] [.4 .4 .0] [.2 .4 .1] [.2 .3 .2]
[.2 .2 .2] [.2 .2 .3] [.2 .1 .3] [.3 .1 .3] [.5 .1 .2] [.7 .1 .2] [.8 .0 .2]
[.9 .0 .1] [1. .0 .1] [1. .1 .1] [.8 .2 .0] [.7 .2 .0] [.5 .2 .0] [.4 .3 .0]
[.1 .4 .1] [.1 .4 .1] [.1 .3 .2] [.1 .2 .2] [.1 .2 .3] [.2 .1 .4] [.2 .0 .3]
[.4 .0 .2] [.6 .0 .2] [.7 .0 .1] [.8 .0 .1] [.7 .1 .1] [.7 .1 .1] [.6 .2 .0]
[.4 .2 .0] [.2 .3 .0] [.1 .3 .0] [.1 .2 .1] [.1 .2 .1] [.1 .1 .2] [.1 .1 .3]
[.1 .0 .3] [.1 .0 .2] [.2 .0 .2] [.3 .0 .2] [.3 .0 .1] [.4 .0 .1] [.4 .1 .1]
[.4 .1 .0] [.2 .1 .0] [.0 .0 .0] [.0 .0 .0] [.0 .0 .0] [.0 .0 .0] [.0 .0 .0]
[.0 .0 .0] [.0 .0 .0] [.0 .0 .0] [.0 .0 .0] [.0 .0 .0] [.0 .0 .0] [.0 .0 .0]
[.0 .0 .0] [.0 .0 .0] [.0 .0 .0] [.0 .0 .0]  T astore pop
%%Page: 1 1
0.04000 0.04000 A 1 J 1 C 12 W [] 0 D
T   0 [1.000 1.000 1.000] U
T   1 [0.000 0.000 0.000] U
T   2 [0.000 0.000 1.000] U
T   3 [0.000 0.031 1.000] U
T   4 [0.000 0.059 1.000] U
T   5 [0.000 0.090 1.000] U
T   6 [0.000 0.122 1.000] U
T   7 [0.000 0.153 1.000] U
T   8 [0.000 0.180 1.000] U
T   9 [0.000 0.212 1.000] U
T  10 [0.000 0.243 1.000] U
T  11 [0.000 0.275 1.000] U
T  12 [0.000 0.302 1.000] U
T  13 [0.000 0.333 1.000] U
T  14 [0.000 0.365 1.000] U
```

T 15 [0.000 0.392 1.000] U
T 16 [0.000 0.424 1.000] U
T 17 [0.000 0.455 1.000] U
T 18 [0.000 0.486 1.000] U
T 19 [0.000 0.514 1.000] U
T 20 [0.000 0.545 1.000] U
T 21 [0.000 0.576 1.000] U
T 22 [0.000 0.608 1.000] U
T 23 [0.000 0.635 1.000] U
T 24 [0.000 0.667 1.000] U
T 25 [0.000 0.698 1.000] U
T 26 [0.000 0.725 1.000] U
T 27 [0.000 0.757 1.000] U
T 28 [0.000 0.788 1.000] U
T 29 [0.000 0.820 1.000] U
T 30 [0.000 0.847 1.000] U
T 31 [0.000 0.878 1.000] U
T 32 [0.000 0.910 1.000] U
T 33 [0.000 0.941 1.000] U
T 34 [0.000 0.969 1.000] U
T 35 [0.000 1.000 1.000] U
T 36 [0.000 0.992 0.973] U
T 37 [0.000 0.980 0.941] U
T 38 [0.004 0.973 0.914] U
T 39 [0.004 0.965 0.886] U
T 40 [0.004 0.953 0.859] U
T 41 [0.004 0.945 0.827] U
T 42 [0.008 0.937 0.800] U
T 43 [0.008 0.929 0.773] U
T 44 [0.008 0.918 0.745] U
T 45 [0.008 0.910 0.714] U
T 46 [0.012 0.902 0.686] U
T 47 [0.012 0.890 0.659] U
T 48 [0.012 0.882 0.627] U
T 49 [0.012 0.875 0.600] U
T 50 [0.016 0.863 0.573] U
T 51 [0.016 0.855 0.545] U
T 52 [0.016 0.847 0.514] U
T 53 [0.016 0.839 0.486] U
T 54 [0.020 0.827 0.459] U
T 55 [0.020 0.820 0.431] U
T 56 [0.020 0.812 0.400] U
T 57 [0.020 0.800 0.373] U

T 58 [0.024 0.792 0.345] U
T 59 [0.024 0.784 0.314] U
T 60 [0.024 0.773 0.286] U
T 61 [0.024 0.765 0.259] U
T 62 [0.027 0.757 0.231] U
T 63 [0.027 0.749 0.200] U
T 64 [0.027 0.737 0.173] U
T 65 [0.027 0.729 0.145] U
T 66 [0.031 0.722 0.118] U
T 67 [0.031 0.710 0.086] U
T 68 [0.031 0.702 0.059] U
T 69 [0.059 0.710 0.059] U
T 70 [0.090 0.718 0.055] U
T 71 [0.118 0.729 0.055] U
T 72 [0.145 0.737 0.051] U
T 73 [0.173 0.745 0.051] U
T 74 [0.204 0.753 0.047] U
T 75 [0.231 0.765 0.047] U
T 76 [0.259 0.773 0.043] U
T 77 [0.286 0.780 0.043] U
T 78 [0.318 0.788 0.043] U
T 79 [0.345 0.800 0.039] U
T 80 [0.373 0.808 0.039] U
T 81 [0.400 0.816 0.035] U
T 82 [0.431 0.824 0.035] U
T 83 [0.459 0.835 0.031] U
T 84 [0.486 0.843 0.031] U
T 85 [0.518 0.851 0.031] U
T 86 [0.545 0.859 0.027] U
T 87 [0.573 0.867 0.027] U
T 88 [0.600 0.878 0.024] U
T 89 [0.631 0.886 0.024] U
T 90 [0.659 0.894 0.020] U
T 91 [0.686 0.902 0.020] U
T 92 [0.714 0.914 0.016] U
T 93 [0.745 0.922 0.016] U
T 94 [0.773 0.929 0.016] U
T 95 [0.800 0.937 0.012] U
T 96 [0.827 0.949 0.012] U
T 97 [0.859 0.957 0.008] U
T 98 [0.886 0.965 0.008] U
T 99 [0.914 0.973 0.004] U
T 100 [0.941 0.984 0.004] U

T 101 [0.973 0.992 0.000] U
T 102 [1.000 1.000 0.000] U
T 103 [1.000 0.988 0.000] U
T 104 [1.000 0.980 0.000] U
T 105 [1.000 0.969 0.000] U
T 106 [1.000 0.957 0.000] U
T 107 [1.000 0.945 0.000] U
T 108 [1.000 0.937 0.000] U
T 109 [1.000 0.925 0.000] U
T 110 [1.000 0.914 0.000] U
T 111 [1.000 0.902 0.000] U
T 112 [1.000 0.894 0.000] U
T 113 [1.000 0.882 0.000] U
T 114 [1.000 0.871 0.000] U
T 115 [1.000 0.863 0.000] U
T 116 [1.000 0.851 0.000] U
T 117 [1.000 0.839 0.000] U
T 118 [1.000 0.827 0.000] U
T 119 [1.000 0.820 0.000] U
T 120 [1.000 0.808 0.000] U
T 121 [1.000 0.796 0.000] U
T 122 [1.000 0.784 0.000] U
T 123 [1.000 0.776 0.000] U
T 124 [1.000 0.765 0.000] U
T 125 [1.000 0.753 0.000] U
T 126 [1.000 0.745 0.000] U
T 127 [1.000 0.733 0.000] U
T 128 [1.000 0.722 0.000] U
T 129 [1.000 0.710 0.000] U
T 130 [1.000 0.702 0.000] U
T 131 [1.000 0.690 0.000] U
T 132 [1.000 0.678 0.000] U
T 133 [1.000 0.667 0.000] U
T 134 [1.000 0.659 0.000] U
T 135 [1.000 0.647 0.000] U
T 136 [1.000 0.627 0.000] U
T 137 [1.000 0.608 0.000] U
T 138 [1.000 0.588 0.000] U
T 139 [1.000 0.569 0.000] U
T 140 [1.000 0.549 0.000] U
T 141 [1.000 0.529 0.000] U
T 142 [1.000 0.510 0.000] U
T 143 [1.000 0.490 0.000] U

T 144 [1.000 0.471 0.000] U
T 145 [1.000 0.451 0.000] U
T 146 [1.000 0.431 0.000] U
T 147 [1.000 0.412 0.000] U
T 148 [1.000 0.392 0.000] U
T 149 [1.000 0.373 0.000] U
T 150 [1.000 0.353 0.000] U
T 151 [1.000 0.333 0.000] U
T 152 [1.000 0.314 0.000] U
T 153 [1.000 0.294 0.000] U
T 154 [1.000 0.275 0.000] U
T 155 [1.000 0.255 0.000] U
T 156 [1.000 0.235 0.000] U
T 157 [1.000 0.216 0.000] U
T 158 [1.000 0.196 0.000] U
T 159 [1.000 0.176 0.000] U
T 160 [1.000 0.157 0.000] U
T 161 [1.000 0.137 0.000] U
T 162 [1.000 0.118 0.000] U
T 163 [1.000 0.098 0.000] U
T 164 [1.000 0.078 0.000] U
T 165 [1.000 0.059 0.000] U
T 166 [1.000 0.039 0.000] U
T 167 [1.000 0.020 0.000] U
T 168 [1.000 0.000 0.000] U
T 169 [0.980 0.000 0.016] U
T 170 [0.961 0.000 0.031] U
T 171 [0.941 0.000 0.047] U
T 172 [0.922 0.000 0.063] U
T 173 [0.902 0.000 0.082] U
T 174 [0.878 0.000 0.098] U
T 175 [0.859 0.000 0.114] U
T 176 [0.839 0.000 0.129] U
T 177 [0.820 0.000 0.145] U
T 178 [0.800 0.000 0.161] U
T 179 [0.780 0.000 0.176] U
T 180 [0.761 0.000 0.192] U
T 181 [0.741 0.000 0.212] U
T 182 [0.722 0.000 0.227] U
T 183 [0.702 0.000 0.243] U
T 184 [0.682 0.000 0.259] U
T 185 [0.659 0.000 0.275] U
T 186 [0.639 0.000 0.290] U

T 187 [0.620 0.000 0.306] U
T 188 [0.600 0.000 0.322] U
T 189 [0.580 0.000 0.341] U
T 190 [0.561 0.000 0.357] U
T 191 [0.541 0.000 0.373] U
T 192 [0.522 0.000 0.388] U
T 193 [0.502 0.000 0.404] U
T 194 [0.482 0.000 0.420] U
T 195 [0.463 0.000 0.435] U
T 196 [0.439 0.000 0.451] U
T 197 [0.420 0.000 0.471] U
T 198 [0.400 0.000 0.486] U
T 199 [0.380 0.000 0.502] U
T 200 [0.361 0.000 0.518] U
T 201 [0.341 0.000 0.533] U
-1150 1075 16450 18700 Oc
Gs
1 O
0 O
2300 16217 M 2300 5503 L 2333 5503 L 2367 5503 L 2400 5503 L
2433 5503 L 2466 5503 L 2499 5503 L 2533 5503 L
2566 5503 L 2599 5503 L 2632 5503 L 2666 5503 L
2699 5503 L 2732 5503 L 2765 5503 L 2798 5503 L
2832 5503 L 2865 5503 L 2898 5503 L 2928 5503 L
2927 5506 L 2917 5534 L 2939 5553 L 2968 5552 L
2986 5530 L 2968 5507 L 2967 5503 L 2998 5503 L
3031 5503 L 3064 5503 L 3098 5503 L 3131 5503 L
3164 5503 L 3197 5503 L 3230 5503 L 3264 5503 L
3297 5503 L 3330 5503 L 3363 5503 L 3397 5503 L
3430 5503 L 3463 5503 L 3496 5503 L 3529 5503 L
3563 5503 L 3596 5503 L 3629 5503 L 3662 5503 L
3696 5503 L 3729 5503 L 3762 5503 L 3795 5503 L
3828 5503 L 3862 5503 L 3895 5503 L 3928 5503 L
3961 5503 L 3995 5503 L 4028 5503 L 4061 5503 L
4094 5503 L 4128 5503 L 4161 5503 L 4194 5503 L
4227 5503 L 4260 5503 L 4294 5503 L 4327 5503 L
4360 5503 L 4393 5503 L 4427 5503 L 4460 5503 L
4493 5503 L 4526 5503 L 4559 5503 L 4593 5503 L
4626 5503 L 4659 5503 L 4692 5503 L 4726 5503 L
4759 5503 L 4792 5503 L 4825 5503 L 4859 5503 L
4892 5503 L 4925 5503 L 4958 5503 L 4991 5503 L
5025 5503 L 5058 5503 L 5091 5503 L 5124 5503 L
5158 5503 L 5191 5503 L 5224 5503 L 5257 5503 L

5290 5503 L 5324 5503 L 5357 5503 L 5390 5503 L
5395 5503 L 5408 5513 L 5406 5542 L 5403 5571 L
5404 5600 L 5404 5630 L 5394 5657 L 5369 5673 L
5352 5696 L 5381 5692 L 5387 5663 L 5416 5662 L
5416 5633 L 5411 5604 L 5410 5575 L 5412 5546 L
5430 5524 L 5430 5503 L 5457 5503 L 5490 5503 L
5523 5503 L 5532 5503 L 5539 5516 L 5540 5523 L
5544 5530 L 5551 5535 L 5537 5572 L 5529 5600 L
5547 5578 L 5556 5550 L 5573 5527 L 5592 5505 L
5594 5503 L 5623 5503 L 5644 5503 L 5645 5520 L
5649 5503 L 5656 5503 L 5658 5503 L 5665 5518 L
5675 5545 L 5689 5570 L 5683 5599 L 5659 5615 L
5668 5643 L 5681 5668 L 5682 5698 L 5666 5722 L
5690 5738 L 5674 5763 L 5654 5784 L 5676 5765 L
5703 5774 L 5686 5797 L 5670 5821 L 5650 5842 L
5674 5826 L 5702 5833 L 5688 5859 L 5664 5875 L
5654 5902 L 5627 5913 L 5598 5914 L 5627 5919 L
5655 5924 L 5661 5952 L 5663 5981 L 5645 6005 L
5617 6009 L 5600 6032 L 5628 6023 L 5654 6012 L
5680 5998 L 5678 5969 L 5684 5941 L 5705 5921 L
5723 5944 L 5711 5917 L 5706 5889 L 5713 5877 L
5721 5864 L 5740 5842 L 5745 5813 L 5772 5824 L
5790 5801 L 5797 5773 L 5799 5769 L 5811 5748 L
5823 5721 L 5842 5699 L 5859 5676 L 5853 5647 L
5872 5669 L 5898 5680 L 5926 5673 L 5955 5670 L
5928 5660 L 5902 5646 L 5880 5627 L 5860 5607 L
5880 5586 L 5909 5582 L 5938 5582 L 5967 5584 L
5986 5561 L 6014 5553 L 6043 5555 L 6069 5543 L
6050 5564 L 6058 5592 L 6085 5582 L 6113 5590 L
6119 5618 L 6090 5612 L 6074 5636 L 6051 5617 L
6024 5609 L 6008 5633 L 6037 5638 L 6066 5639 L
6087 5659 L 6061 5672 L 6035 5685 L 6057 5704 L
6085 5711 L 6113 5717 L 6141 5711 L 6159 5688 L
6169 5660 L 6172 5631 L 6200 5638 L 6229 5645 L
6234 5673 L 6207 5683 L 6182 5697 L 6209 5708 L
6209 5737 L 6180 5737 L 6202 5756 L 6216 5782 L
6242 5794 L 6247 5795 L 6256 5799 L 6270 5803 L
6296 5815 L 6324 5810 L 6345 5829 L 6338 5801 L
6315 5784 L 6317 5755 L 6314 5726 L 6325 5699 L
6354 5700 L 6382 5696 L 6403 5675 L 6399 5704 L
6415 5728 L 6431 5752 L 6408 5771 L 6392 5795 L
6417 5780 L 6430 5754 L 6458 5757 L 6472 5783 L
6487 5808 L 6515 5815 L 6544 5814 L 6569 5830 L

6599 5819 L 6577 5810 L 6575 5801 L 6595 5802 L
6614 5803 L 6640 5790 L 6666 5776 L 6637 5775 L
6649 5748 L 6677 5741 L 6705 5746 L 6731 5733 L
6757 5720 L 6754 5749 L 6768 5774 L 6747 5755 L
6730 5778 L 6747 5801 L 6764 5825 L 6785 5845 L
6814 5847 L 6838 5830 L 6866 5823 L 6882 5798 L
6899 5775 L 6921 5794 L 6949 5802 L 6976 5811 L
7003 5823 L 7027 5839 L 7029 5867 L 7046 5844 L
7031 5819 L 7060 5819 L 7082 5837 L 7102 5858 L
7125 5876 L 7149 5891 L 7173 5874 L 7201 5868 L
7229 5876 L 7258 5874 L 7266 5902 L 7287 5922 L
7284 5951 L 7277 5979 L 7291 6004 L 7296 6033 L
7271 6018 L 7268 6047 L 7297 6049 L 7320 6032 L
7341 6012 L 7358 6035 L 7379 6046 L 7361 6062 L
7343 6078 L 7315 6085 L 7292 6102 L 7279 6128 L
7273 6156 L 7245 6161 L 7274 6160 L 7294 6140 L
7317 6122 L 7343 6110 L 7349 6110 L 7372 6110 L
7401 6110 L 7422 6133 L 7434 6147 L 7455 6167 L
7454 6177 L 7455 6203 L 7469 6229 L 7461 6252 L
7487 6263 L 7507 6269 L 7515 6271 L 7527 6265 L
7541 6258 L 7532 6285 L 7559 6275 L 7564 6246 L
7572 6219 L 7590 6234 L 7594 6237 L 7617 6255 L
7641 6270 L 7641 6299 L 7659 6277 L 7651 6249 L
7669 6271 L 7671 6300 L 7676 6329 L 7687 6355 L
7711 6339 L 7724 6355 L 7732 6355 L 7736 6354 L
7759 6338 L 7776 6361 L 7786 6388 L 7785 6417 L
7757 6424 L 7733 6441 L 7762 6438 L 7790 6431 L
7818 6423 L 7840 6405 L 7849 6377 L 7840 6350 L
7838 6321 L 7855 6344 L 7857 6373 L 7860 6402 L
7871 6428 L 7894 6445 L 7891 6473 L 7909 6496 L
7937 6504 L 7946 6477 L 7930 6454 L 7925 6426 L
7937 6452 L 7954 6475 L 7977 6493 L 8001 6507 L
8006 6536 L 8024 6558 L 8013 6585 L 8034 6605 L
8054 6586 L 8077 6604 L 8098 6623 L 8126 6629 L
8120 6657 L 8106 6682 L 8077 6682 L 8074 6710 L
8046 6708 L 8017 6704 L 8000 6709 L 7974 6722 L
7952 6740 L 7940 6751 L 7942 6767 L 7944 6764 L
7954 6752 L 7968 6755 L 7980 6755 L 7997 6756 L
8023 6746 L 8035 6743 L 8051 6739 L 8074 6755 L
8079 6784 L 8054 6797 L 8082 6798 L 8092 6825 L
8109 6802 L 8110 6804 L 8123 6827 L 8125 6855 L
8154 6856 L 8165 6829 L 8157 6801 L 8184 6795 L
8185 6795 L 8198 6821 L 8225 6813 L 8226 6813 L

8253 6818 L 8274 6829 L 8278 6832 L 8273 6860 L
8295 6878 L 8285 6851 L 8310 6837 L 8336 6825 L
8361 6810 L 8359 6781 L 8385 6795 L 8405 6815 L
8394 6842 L 8395 6870 L 8424 6867 L 8437 6893 L
8441 6921 L 8425 6945 L 8403 6928 L 8382 6948 L
8378 6976 L 8355 6993 L 8381 6981 L 8409 6987 L
8402 7015 L 8395 7043 L 8416 7023 L 8416 6994 L
8429 7020 L 8442 6994 L 8437 6966 L 8464 6974 L
8483 6996 L 8511 6992 L 8537 6981 L 8562 6996 L
8534 7002 L 8530 7031 L 8558 7033 L 8587 7031 L
8573 7056 L 8575 7085 L 8562 7060 L 8534 7067 L
8510 7082 L 8501 7110 L 8529 7117 L 8545 7140 L
8561 7164 L 8541 7184 L 8522 7205 L 8548 7215 L
8577 7218 L 8604 7211 L 8588 7188 L 8614 7200 L
8611 7171 L 8601 7145 L 8629 7141 L 8654 7129 L
8655 7129 L 8641 7154 L 8632 7181 L 8648 7205 L
8649 7176 L 8673 7160 L 8701 7162 L 8730 7166 L
8736 7194 L 8753 7198 L 8763 7201 L 8744 7221 L
8727 7245 L 8704 7228 L 8677 7219 L 8687 7245 L
8663 7261 L 8640 7278 L 8619 7297 L 8646 7305 L
8669 7322 L 8693 7337 L 8713 7357 L 8729 7381 L
8740 7407 L 8747 7403 L 8764 7391 L 8754 7365 L
8780 7352 L 8808 7349 L 8813 7320 L 8819 7312 L
8830 7297 L 8827 7269 L 8842 7294 L 8852 7320 L
8879 7312 L 8880 7283 L 8881 7255 L 8889 7262 L
8902 7273 L 8902 7274 L 8907 7302 L 8901 7330 L
8874 7339 L 8868 7367 L 8854 7392 L 8828 7380 L
8821 7407 L 8807 7432 L 8824 7455 L 8850 7466 L
8878 7471 L 8883 7479 L 8892 7496 L 8884 7523 L
8882 7552 L 8891 7579 L 8909 7580 L 8919 7581 L
8927 7608 L 8935 7635 L 8906 7638 L 8880 7651 L
8853 7659 L 8834 7680 L 8850 7704 L 8869 7723 L
8870 7723 L 8887 7746 L 8898 7773 L 8870 7768 L
8893 7784 L 8921 7790 L 8937 7795 L 8948 7798 L
8973 7813 L 8976 7813 L 9000 7818 L 9028 7822 L
9036 7831 L 9046 7845 L 9020 7834 L 8996 7849 L
8979 7871 L 8961 7849 L 8953 7822 L 8927 7809 L
8905 7827 L 8899 7855 L 8886 7880 L 8859 7872 L
8872 7897 L 8895 7914 L 8916 7895 L 8944 7889 L
8966 7872 L 8982 7895 L 8970 7921 L 8966 7949 L
8947 7970 L 8919 7975 L 8891 7976 L 8865 7965 L
8843 7983 L 8841 8011 L 8867 8021 L 8844 8037 L
8849 8065 L 8845 8093 L 8872 8083 L 8893 8065 L

8918 8051 L 8909 8078 L 8901 8105 L 8900 8134 L
8910 8107 L 8927 8084 L 8941 8072 L 8948 8066 L
8953 8094 L 8978 8105 L 8962 8082 L 8990 8075 L
8965 8061 L 8962 8033 L 8967 8032 L 8990 8026 L
9012 8008 L 9017 8036 L 9033 8060 L 9043 8086 L
9071 8083 L 9094 8068 L 9082 8042 L 9057 8030 L
9058 8001 L 9037 7982 L 9009 7981 L 8993 7957 L
8985 7930 L 9014 7929 L 9035 7948 L 9044 7975 L
9072 7977 L 9075 7979 L 9096 7993 L 9100 7994 L
9122 8004 L 9121 8032 L 9142 8051 L 9116 8063 L
9127 8089 L 9102 8104 L 9078 8118 L 9090 8143 L
9118 8138 L 9125 8166 L 9147 8164 L 9153 8164 L
9181 8158 L 9179 8186 L 9152 8194 L 9180 8194 L
9199 8215 L 9204 8243 L 9195 8269 L 9223 8266 L
9238 8270 L 9250 8273 L 9265 8297 L 9251 8321 L
9246 8349 L 9256 8323 L 9280 8308 L 9291 8335 L
9307 8311 L 9313 8321 L 9322 8335 L 9323 8363 L
9326 8368 L 9339 8386 L 9326 8411 L 9332 8439 L
9357 8426 L 9385 8430 L 9384 8459 L 9398 8483 L
9371 8494 L 9358 8519 L 9340 8540 L 9368 8548 L
9387 8568 L 9387 8596 L 9390 8624 L 9418 8627 L
9430 8653 L 9445 8677 L 9463 8698 L 9479 8721 L
9477 8750 L 9452 8763 L 9424 8768 L 9405 8788 L
9377 8793 L 9349 8789 L 9347 8817 L 9368 8799 L
9397 8802 L 9417 8797 L 9424 8795 L 9429 8796 L
9446 8778 L 9474 8778 L 9496 8777 L 9502 8777 L
9529 8775 L 9528 8787 L 9542 8812 L 9540 8840 L
9553 8865 L 9567 8872 L 9578 8878 L 9593 8902 L
9603 8928 L 9581 8945 L 9604 8956 L 9607 8957 L
9616 8983 L 9642 8993 L 9669 8985 L 9652 8963 L
9661 8948 L 9667 8939 L 9675 8912 L 9693 8890 L
9713 8870 L 9734 8889 L 9728 8916 L 9749 8935 L
9762 8960 L 9777 8983 L 9805 8979 L 9805 8979 L
9829 8964 L 9846 8942 L 9859 8968 L 9845 8992 L
9836 9019 L 9823 9044 L 9824 9072 L 9808 9095 L
9795 9120 L 9788 9147 L 9771 9170 L 9744 9177 L
9717 9184 L 9745 9184 L 9773 9185 L 9801 9186 L
9825 9200 L 9837 9213 L 9844 9221 L 9865 9240 L
9856 9267 L 9843 9292 L 9816 9297 L 9801 9321 L
9827 9312 L 9855 9308 L 9874 9330 L 9853 9349 L
9864 9375 L 9838 9386 L 9811 9380 L 9785 9369 L
9806 9388 L 9834 9391 L 9831 9418 L 9804 9424 L
9777 9417 L 9749 9413 L 9750 9441 L 9774 9455 L

9782 9482 L 9786 9510 L 9810 9506 L 9814 9505 L
9832 9484 L 9833 9512 L 9844 9537 L 9836 9511 L
9837 9483 L 9847 9456 L 9873 9464 L 9894 9482 L
9901 9510 L 9899 9538 L 9906 9565 L 9927 9546 L
9918 9519 L 9921 9503 L 9923 9492 L 9924 9464 L
9952 9459 L 9975 9474 L 9980 9479 L 9993 9495 L
9989 9523 L 9963 9535 L 9966 9563 L 9963 9591 L
9989 9601 L 9990 9629 L 9974 9652 L 9977 9680 L
9995 9702 L 9976 9723 L 9949 9715 L 9921 9709 L
9897 9696 L 9896 9668 L 9912 9645 L 9888 9631 L
9881 9658 L 9853 9658 L 9834 9637 L 9807 9629 L
9784 9614 L 9766 9593 L 9774 9619 L 9747 9612 L
9732 9588 L 9724 9615 L 9735 9641 L 9753 9662 L
9780 9656 L 9807 9662 L 9835 9669 L 9856 9686 L
9874 9708 L 9891 9730 L 9914 9746 L 9932 9768 L
9954 9785 L 9968 9809 L 9990 9826 L 10003 9831 L
10010 9834 L 10016 9837 L 10039 9852 L 10046 9879 L
10022 9865 L 9994 9865 L 9966 9864 L 9962 9865 L
9955 9871 L 9944 9882 L 9917 9889 L 9891 9899 L
9864 9904 L 9836 9909 L 9812 9923 L 9798 9947 L
9782 9969 L 9763 9990 L 9746 10012 L 9727 10033 L
9708 10053 L 9685 10069 L 9661 10084 L 9634 10087 L
9606 10086 L 9578 10082 L 9553 10069 L 9531 10053 L
9512 10032 L 9490 10015 L 9467 10000 L 9445 9983 L
9419 9973 L 9391 9972 L 9365 9983 L 9346 9962 L
9371 9949 L 9344 9957 L 9335 9984 L 9341 10011 L
9323 10033 L 9314 10059 L 9326 10085 L 9302 10099 L
9274 10101 L 9246 10099 L 9223 10082 L 9213 10057 L
9197 10034 L 9169 10032 L 9147 10049 L 9128 10028 L
9104 10013 L 9124 10033 L 9141 10055 L 9169 10056 L
9195 10047 L 9194 10075 L 9202 10102 L 9226 10114 L
9254 10121 L 9281 10121 L 9309 10123 L 9337 10123 L
9364 10115 L 9383 10095 L 9411 10097 L 9436 10110 L
9452 10132 L 9468 10155 L 9469 10183 L 9484 10206 L
9503 10226 L 9528 10239 L 9555 10243 L 9571 10267 L
9593 10283 L 9616 10299 L 9643 10303 L 9667 10318 L
9682 10341 L 9702 10360 L 9727 10373 L 9753 10381 L
9780 10388 L 9807 10393 L 9835 10396 L 9863 10399 L
9890 10403 L 9913 10419 L 9920 10446 L 9910 10472 L
9896 10496 L 9884 10521 L 9870 10545 L 9857 10570 L
9844 10594 L 9829 10618 L 9811 10639 L 9789 10656 L
9766 10670 L 9741 10684 L 9714 10684 L 9698 10708 L
9676 10725 L 9651 10736 L 9627 10749 L 9609 10770 L

9595 10794 L 9577 10816 L 9555 10833 L 9531 10846 L
9510 10864 L 9484 10874 L 9458 10884 L 9430 10882 L
9403 10884 L 9378 10896 L 9359 10916 L 9381 10933 L
9367 10958 L 9355 10982 L 9340 11006 L 9312 11008 L
9285 11006 L 9257 11008 L 9231 11018 L 9203 11015 L
9192 10990 L 9165 10984 L 9138 10978 L 9113 10965 L
9087 10957 L 9059 10957 L 9045 10981 L 9072 10976 L
9100 10974 L 9126 10984 L 9147 11001 L 9169 11017 L
9190 11035 L 9215 11049 L 9240 11059 L 9265 11048 L
9293 11048 L 9320 11040 L 9345 11050 L 9361 11027 L
9384 11011 L 9401 10989 L 9420 10969 L 9430 10943 L
9436 10916 L 9462 10908 L 9490 10911 L 9517 10908 L
9531 10931 L 9558 10937 L 9584 10948 L 9610 10957 L
9612 10956 L 9635 10945 L 9662 10939 L 9689 10931 L
9706 10910 L 9732 10899 L 9757 10887 L 9783 10877 L
9810 10873 L 9837 10865 L 9863 10858 L 9889 10868 L
9895 10895 L 9884 10920 L 9870 10944 L 9858 10969 L
9848 10995 L 9829 11015 L 9811 11035 L 9790 11053 L
9765 11066 L 9739 11075 L 9713 11085 L 9686 11085 L
9660 11095 L 9648 11120 L 9629 11139 L 9643 11163 L
9637 11190 L 9638 11218 L 9625 11242 L 9603 11259 L
9577 11269 L 9552 11280 L 9527 11292 L 9502 11302 L
9475 11311 L 9448 11312 L 9429 11332 L 9411 11353 L
9383 11357 L 9399 11380 L 9417 11400 L 9404 11424 L
9378 11434 L 9399 11452 L 9407 11478 L 9405 11506 L
9395 11531 L 9389 11558 L 9379 11584 L 9352 11589 L
9337 11612 L 9331 11639 L 9316 11662 L 9297 11682 L
9286 11707 L 9276 11733 L 9257 11753 L 9250 11779 L
9252 11807 L 9262 11833 L 9240 11849 L 9223 11871 L
9216 11898 L 9220 11925 L 9209 11951 L 9201 11977 L
9175 11986 L 9180 12013 L 9155 12024 L 9154 12051 L
9146 12078 L 9145 12105 L 9136 12131 L 9126 12157 L
9118 12183 L 9109 12209 L 9096 12233 L 9092 12260 L
9078 12284 L 9053 12295 L 9028 12307 L 9001 12313 L
8983 12333 L 8958 12345 L 8932 12354 L 8905 12358 L
8878 12364 L 8865 12340 L 8865 12367 L 8847 12388 L
8820 12394 L 8798 12411 L 8774 12424 L 8751 12438 L
8730 12456 L 8716 12479 L 8712 12506 L 8684 12509 L
8660 12521 L 8635 12508 L 8613 12524 L 8594 12504 L
8601 12531 L 8588 12555 L 8587 12582 L 8595 12608 L
8595 12635 L 8599 12663 L 8614 12686 L 8634 12704 L
8661 12699 L 8673 12724 L 8676 12751 L 8683 12777 L
8692 12803 L 8704 12820 L 8708 12825 L 8733 12838 L

8752 12857 L 8755 12884 L 8770 12908 L 8778 12934 L
8787 12960 L 8781 12986 L 8808 12983 L 8830 12999 L
8840 12974 L 8853 12998 L 8873 13016 L 8890 12994 L
8901 13019 L 8916 13042 L 8927 13067 L 8950 13052 L
8957 13078 L 8955 13106 L 8965 13131 L 8985 13150 L
9010 13160 L 9016 13164 L 9033 13175 L 9026 13148 L
9040 13172 L 9062 13188 L 9074 13213 L 9047 13209 L
9020 13213 L 9035 13236 L 9010 13244 L 8986 13258 L
8978 13284 L 8987 13310 L 9013 13320 L 9038 13311 L
9060 13328 L 9064 13355 L 9082 13335 L 9098 13312 L
9093 13286 L 9083 13260 L 9071 13235 L 9098 13230 L
9124 13239 L 9145 13257 L 9171 13262 L 9198 13266 L
9223 13278 L 9244 13295 L 9240 13322 L 9236 13349 L
9220 13371 L 9225 13397 L 9251 13406 L 9243 13432 L
9258 13455 L 9282 13467 L 9304 13450 L 9328 13437 L
9329 13464 L 9347 13485 L 9346 13512 L 9321 13523 L
9305 13545 L 9279 13539 L 9296 13560 L 9319 13574 L
9345 13566 L 9359 13543 L 9386 13544 L 9385 13571 L
9411 13561 L 9430 13580 L 9453 13595 L 9479 13601 L
9505 13611 L 9530 13621 L 9553 13636 L 9570 13657 L
9566 13684 L 9579 13661 L 9599 13680 L 9626 13682 L
9605 13665 L 9590 13642 L 9616 13649 L 9643 13642 L
9642 13669 L 9668 13679 L 9691 13692 L 9704 13716 L
9729 13727 L 9754 13739 L 9778 13751 L 9768 13776 L
9794 13781 L 9808 13757 L 9817 13731 L 9837 13750 L
9860 13765 L 9859 13792 L 9876 13813 L 9884 13787 L
9867 13765 L 9883 13743 L 9908 13733 L 9881 13729 L
9885 13702 L 9912 13701 L 9930 13698 L 9939 13696 L
9961 13712 L 9969 13718 L 9983 13729 L 9996 13753 L
9998 13753 L 10009 13756 L 10023 13759 L 10012 13734 L
10025 13758 L 10029 13785 L 10028 13812 L 10002 13808 L
9974 13811 L 9995 13829 L 9995 13856 L 10010 13879 L
10034 13893 L 10056 13908 L 10040 13930 L 10032 13956 L
10037 13983 L 10055 14004 L 10066 14015 L 10074 14023 L
10047 14025 L 10023 14011 L 9999 14000 L 9981 14021 L
9955 14013 L 9928 14012 L 9902 14020 L 9878 14033 L
9854 14021 L 9840 14044 L 9849 14070 L 9831 14091 L
9810 14074 L 9787 14059 L 9767 14040 L 9769 14013 L
9754 14035 L 9727 14031 L 9700 14026 L 9684 14005 L
9664 14023 L 9639 14013 L 9612 14008 L 9585 14010 L
9561 14022 L 9542 14042 L 9525 14063 L 9507 14083 L
9532 14095 L 9505 14092 L 9479 14081 L 9452 14078 L
9426 14084 L 9406 14103 L 9402 14129 L 9386 14152 L

9359 14150 L 9342 14171 L 9318 14184 L 9306 14208 L
9279 14205 L 9252 14208 L 9225 14207 L 9204 14189 L
9179 14180 L 9155 14166 L 9128 14162 L 9102 14155 L
9082 14136 L 9056 14128 L 9030 14120 L 9055 14108 L
9051 14082 L 9034 14060 L 9015 14041 L 8996 14021 L
8974 14005 L 8951 13990 L 8925 13983 L 8900 13973 L
8879 13955 L 8866 13931 L 8883 13910 L 8876 13883 L
8856 13864 L 8835 13847 L 8816 13827 L 8790 13820 L
8763 13820 L 8736 13818 L 8711 13828 L 8683 13826 L
8656 13824 L 8629 13825 L 8603 13832 L 8576 13835 L
8555 13853 L 8532 13867 L 8505 13866 L 8502 13893 L
8485 13914 L 8467 13935 L 8472 13962 L 8469 13989 L
8468 14016 L 8470 14043 L 8477 14070 L 8494 14091 L
8511 14112 L 8523 14137 L 8505 14157 L 8480 14168 L
8492 14193 L 8498 14219 L 8491 14245 L 8483 14271 L
8464 14291 L 8459 14318 L 8432 14318 L 8415 14297 L
8424 14323 L 8419 14349 L 8422 14376 L 8408 14399 L
8402 14373 L 8397 14399 L 8373 14387 L 8351 14371 L
8326 14360 L 8302 14348 L 8280 14332 L 8259 14349 L
8232 14353 L 8206 14361 L 8179 14363 L 8152 14360 L
8125 14364 L 8098 14360 L 8074 14374 L 8052 14389 L
8033 14408 L 8010 14423 L 7984 14430 L 7964 14449 L
7945 14468 L 7928 14489 L 7915 14513 L 7903 14537 L
7890 14561 L 7880 14587 L 7870 14612 L 7867 14639 L
7867 14666 L 7872 14693 L 7881 14718 L 7893 14743 L
7906 14767 L 7924 14787 L 7939 14810 L 7964 14820 L
7958 14847 L 7948 14872 L 7967 14892 L 7986 14911 L
8008 14926 L 8034 14935 L 8061 14939 L 8087 14935 L
8111 14921 L 8126 14899 L 8133 14873 L 8151 14853 L
8174 14839 L 8201 14834 L 8223 14850 L 8243 14868 L
8264 14884 L 8284 14903 L 8301 14911 L 8309 14914 L
8326 14914 L 8336 14913 L 8357 14896 L 8370 14897 L
8384 14898 L 8406 14914 L 8406 14914 L 8422 14916 L
8433 14917 L 8458 14927 L 8475 14936 L 8482 14940 L
8504 14956 L 8522 14976 L 8536 14999 L 8553 15020 L
8575 15036 L 8600 15047 L 8583 15067 L 8579 15094 L
8583 15121 L 8574 15147 L 8598 15161 L 8605 15187 L
8613 15213 L 8625 15237 L 8645 15256 L 8666 15272 L
8691 15283 L 8690 15310 L 8710 15327 L 8736 15337 L
8762 15344 L 8788 15351 L 8810 15368 L 8836 15375 L
8851 15397 L 8874 15411 L 8899 15422 L 8925 15430 L
8950 15439 L 8974 15453 L 8998 15464 L 9019 15482 L
9045 15489 L 9071 15496 L 9087 15517 L 9086 15544 L

9099 15568 L 9122 15582 L 9100 15597 L 9122 15614 L
9127 15640 L 9150 15653 L 9164 15677 L 9173 15684 L
9186 15693 L 9204 15713 L 9211 15728 L 9216 15738 L
9228 15752 L 9234 15758 L 9260 15764 L 9262 15767 L
9278 15784 L 9255 15799 L 9267 15824 L 9286 15843 L
9295 15842 L 9311 15839 L 9313 15839 L 9323 15864 L
9342 15883 L 9358 15905 L 9384 15903 L 9406 15887 L
9430 15900 L 9454 15887 L 9481 15888 L 9494 15879 L
9503 15872 L 9522 15853 L 9549 15856 L 9576 15858 L
9598 15874 L 9608 15900 L 9609 15927 L 9629 15945 L
9639 15970 L 9635 15943 L 9622 15919 L 9633 15894 L
9625 15868 L 9638 15845 L 9665 15847 L 9682 15825 L
9705 15811 L 9727 15795 L 9742 15780 L 9754 15779 L
9754 15792 L 9759 15797 L 9769 15793 L 9784 15785 L
9803 15791 L 9801 15797 L 9778 15791 L 9772 15801 L
9766 15809 L 9766 15815 L 9754 15822 L 9731 15838 L
9731 15840 L 9756 15825 L 9777 15813 L 9778 15800 L
9801 15801 L 9806 15793 L 9802 15787 L 9784 15782 L
9760 15795 L 9758 15788 L 9763 15762 L 9754 15745 L
9760 15735 L 9783 15719 L 9803 15701 L 9816 15678 L
9804 15653 L 9786 15633 L 9784 15606 L 9766 15585 L
9751 15563 L 9748 15536 L 9728 15517 L 9714 15494 L
9700 15471 L 9683 15450 L 9667 15429 L 9644 15414 L
9670 15407 L 9650 15389 L 9632 15369 L 9608 15356 L
9581 15356 L 9563 15336 L 9540 15322 L 9517 15307 L
9506 15283 L 9495 15258 L 9517 15241 L 9533 15220 L
9536 15193 L 9533 15171 L 9529 15160 L 9517 15143 L
9494 15134 L 9507 15102 L 9517 15071 L 9541 15058 L
9568 15061 L 9595 15063 L 9619 15074 L 9646 15075 L
9672 15081 L 9699 15080 L 9676 15067 L 9650 15060 L
9627 15045 L 9612 15022 L 9589 15008 L 9563 15001 L
9581 14982 L 9564 14960 L 9587 14945 L 9608 14928 L
9594 14904 L 9568 14895 L 9543 14885 L 9521 14869 L
9494 14865 L 9475 14884 L 9465 14859 L 9451 14836 L
9425 14826 L 9398 14826 L 9372 14818 L 9365 14792 L
9391 14785 L 9369 14769 L 9381 14745 L 9381 14717 L
9402 14700 L 9419 14721 L 9431 14746 L 9458 14745 L
9483 14757 L 9510 14757 L 9535 14765 L 9554 14785 L
9581 14786 L 9608 14788 L 9620 14812 L 9597 14826 L
9571 14832 L 9595 14844 L 9607 14868 L 9628 14885 L
9653 14874 L 9665 14850 L 9672 14846 L 9689 14837 L
9690 14864 L 9679 14889 L 9704 14880 L 9696 14906 L
9716 14888 L 9737 14906 L 9763 14912 L 9755 14937 L

9782 14943 L 9806 14955 L 9782 14966 L 9792 14991 L
9811 15011 L 9834 15025 L 9846 15050 L 9856 15075 L
9879 15089 L 9898 15105 L 9899 15107 L 9914 15105 L
9926 15104 L 9946 15123 L 9949 15149 L 9956 15146 L
9973 15136 L 9991 15157 L 10015 15168 L 10035 15187 L
10042 15193 L 10056 15204 L 10056 15204 L 10071 15210 L
10078 15213 L 10081 15214 L 10088 15217 L 10097 15220 L
10106 15224 L 10131 15235 L 10143 15248 L 10150 15255 L
10170 15263 L 10175 15265 L 10201 15256 L 10226 15265 L
10235 15272 L 10247 15282 L 10223 15293 L 10246 15307 L
10254 15332 L 10256 15332 L 10272 15330 L 10281 15328 L
10284 15327 L 10308 15323 L 10308 15322 L 10321 15314 L
10331 15308 L 10340 15326 L 10343 15333 L 10356 15330 L
10369 15327 L 10383 15351 L 10393 15376 L 10401 15402 L
10403 15375 L 10426 15360 L 10441 15360 L 10453 15360 L
10473 15379 L 10497 15367 L 10524 15365 L 10551 15364 L
10578 15370 L 10600 15385 L 10626 15391 L 10639 15415 L
10644 15388 L 10650 15362 L 10656 15388 L 10660 15415 L
10666 15442 L 10672 15442 L 10693 15443 L 10687 15417 L
10709 15401 L 10733 15388 L 10759 15382 L 10786 15379 L
10796 15354 L 10804 15328 L 10823 15309 L 10813 15284 L
10826 15260 L 10840 15278 L 10843 15281 L 10854 15297 L
10859 15303 L 10855 15330 L 10867 15355 L 10887 15337 L
10914 15330 L 10929 15310 L 10930 15308 L 10953 15321 L
10971 15301 L 10998 15302 L 11015 15290 L 11020 15286 L
11044 15272 L 11069 15281 L 11089 15262 L 11116 15262 L
11139 15277 L 11160 15294 L 11172 15318 L 11176 15330 L
11189 15326 L 11196 15330 L 11202 15336 L 11203 15334 L
11198 15326 L 11188 15324 L 11176 15329 L 11173 15315 L
11163 15287 L 11184 15288 L 11196 15299 L 11225 15316 L
11226 15314 L 11220 15310 L 11196 15298 L 11187 15284 L
11174 15283 L 11150 15269 L 11146 15242 L 11126 15224 L
11127 15197 L 11140 15173 L 11167 15180 L 11153 15155 L
11156 15128 L 11151 15102 L 11144 15076 L 11121 15061 L
11116 15035 L 11124 15009 L 11104 14991 L 11091 14967 L
11082 14942 L 11073 14916 L 11062 14891 L 11070 14865 L
11084 14842 L 11110 14833 L 11103 14860 L 11118 14837 L
11121 14810 L 11147 14819 L 11174 14816 L 11190 14838 L
11214 14825 L 11241 14825 L 11257 14803 L 11230 14799 L
11207 14814 L 11183 14801 L 11156 14797 L 11134 14782 L
11108 14776 L 11084 14762 L 11067 14783 L 11075 14757 L
11071 14730 L 11047 14717 L 11020 14716 L 11031 14691 L
11021 14666 L 11001 14648 L 10991 14623 L 10977 14599 L

10983 14573 L 10983 14546 L 11003 14527 L 11023 14538 L
11033 14533 L 11004 14514 L 11002 14517 L 11003 14525 L
10981 14539 L 10963 14512 L 10943 14496 L 10923 14477 L
10897 14470 L 10923 14462 L 10928 14462 L 10950 14458 L
10960 14461 L 10972 14464 L 10976 14466 L 11002 14476 L
11025 14462 L 11051 14454 L 11078 14458 L 11103 14469 L
11130 14471 L 11156 14461 L 11154 14434 L 11132 14451 L
11106 14446 L 11099 14419 L 11123 14407 L 11098 14397 L
11071 14401 L 11047 14388 L 11063 14365 L 11079 14358 L
11088 14355 L 11114 14347 L 11140 14355 L 11140 14355 L
11159 14369 L 11162 14371 L 11185 14357 L 11181 14330 L
11206 14331 L 11208 14331 L 11217 14357 L 11244 14361 L
11221 14376 L 11248 14378 L 11275 14375 L 11253 14359 L
11268 14337 L 11241 14335 L 11238 14308 L 11212 14301 L
11201 14276 L 11217 14255 L 11228 14280 L 11255 14284 L
11271 14306 L 11286 14315 L 11294 14320 L 11298 14320 L
11322 14318 L 11330 14343 L 11326 14370 L 11323 14397 L
11343 14397 L 11349 14397 L 11350 14397 L 11356 14423 L
11329 14423 L 11304 14433 L 11300 14460 L 11325 14449 L
11352 14451 L 11369 14446 L 11375 14445 L 11378 14444 L
11389 14422 L 11391 14420 L 11400 14413 L 11405 14410 L
11412 14404 L 11413 14404 L 11422 14394 L 11432 14384 L
11449 14363 L 11456 14363 L 11476 14362 L 11478 14389 L
11504 14383 L 11505 14383 L 11509 14356 L 11515 14350 L
11520 14345 L 11528 14337 L 11549 14321 L 11550 14320 L
11557 14347 L 11555 14374 L 11560 14375 L 11581 14382 L
11598 14385 L 11608 14386 L 11629 14403 L 11656 14406 L
11667 14398 L 11679 14391 L 11702 14376 L 11726 14364 L
11753 14369 L 11770 14390 L 11796 14404 L 11778 14381 L
11783 14354 L 11785 14327 L 11797 14302 L 11822 14291 L
11844 14275 L 11867 14261 L 11887 14249 L 11887 14248 L
11865 14258 L 11843 14273 L 11818 14285 L 11794 14298 L
11770 14311 L 11778 14337 L 11765 14361 L 11741 14348 L
11716 14336 L 11721 14309 L 11728 14282 L 11748 14264 L
11774 14256 L 11784 14231 L 11787 14204 L 11762 14192 L
11763 14191 L 11781 14172 L 11802 14155 L 11828 14146 L
11812 14124 L 11836 14112 L 11861 14100 L 11839 14083 L
11812 14079 L 11819 14053 L 11806 14029 L 11823 14007 L
11841 14027 L 11863 14044 L 11868 14017 L 11857 13992 L
11850 13966 L 11876 13960 L 11895 13940 L 11913 13920 L
11940 13915 L 11959 13934 L 11950 13908 L 11928 13892 L
11908 13873 L 11909 13846 L 11894 13869 L 11889 13896 L
11873 13918 L 11847 13923 L 11826 13941 L 11804 13926 L

11779 13938 L 11755 13951 L 11766 13926 L 11741 13938 L
11760 13918 L 11771 13893 L 11750 13910 L 11746 13883 L
11740 13856 L 11739 13884 L 11732 13910 L 11723 13936 L
11697 13927 L 11676 13909 L 11699 13894 L 11717 13873 L
11701 13851 L 11677 13838 L 11693 13816 L 11671 13831 L
11672 13859 L 11666 13885 L 11660 13912 L 11660 13885 L
11636 13872 L 11629 13846 L 11630 13819 L 11604 13813 L
11594 13787 L 11600 13764 L 11600 13761 L 11610 13786 L
11612 13785 L 11621 13781 L 11630 13777 L 11635 13775 L
11634 13747 L 11639 13749 L 11642 13750 L 11651 13753 L
11656 13754 L 11660 13755 L 11678 13735 L 11678 13708 L
11683 13700 L 11688 13694 L 11694 13688 L 11695 13686 L
11697 13676 L 11701 13660 L 11705 13633 L 11720 13613 L
11721 13611 L 11742 13628 L 11732 13653 L 11720 13677 L
11716 13704 L 11691 13715 L 11696 13742 L 11682 13766 L
11702 13785 L 11710 13759 L 11715 13732 L 11733 13752 L
11749 13764 L 11755 13768 L 11761 13741 L 11758 13714 L
11757 13687 L 11781 13674 L 11768 13651 L 11772 13643 L
11778 13632 L 11781 13627 L 11807 13655 L 11806 13639 L
11783 13625 L 11784 13598 L 11787 13595 L 11791 13592 L
11806 13581 L 11793 13557 L 11807 13534 L 11809 13508 L
11809 13506 L 11831 13490 L 11809 13474 L 11826 13468 L
11830 13466 L 11835 13464 L 11846 13455 L 11856 13447 L
11869 13423 L 11888 13404 L 11912 13417 L 11937 13428 L
11959 13444 L 11966 13471 L 11964 13443 L 11943 13425 L
11918 13414 L 11897 13397 L 11873 13385 L 11858 13362 L
11866 13336 L 11877 13338 L 11893 13342 L 11920 13344 L
11947 13350 L 11928 13330 L 11909 13311 L 11924 13288 L
11899 13277 L 11894 13250 L 11867 13249 L 11849 13228 L
11828 13210 L 11810 13190 L 11823 13166 L 11851 13168 L
11878 13173 L 11905 13173 L 11906 13146 L 11879 13148 L
11853 13139 L 11827 13130 L 11816 13106 L 11811 13079 L
11798 13055 L 11825 13050 L 11816 13024 L 11794 13040 L
11788 13013 L 11783 12987 L 11773 12961 L 11800 12953 L
11796 12926 L 11818 12911 L 11845 12904 L 11835 12879 L
11817 12858 L 11799 12878 L 11780 12898 L 11782 12925 L
11770 12901 L 11743 12907 L 11752 12881 L 11764 12869 L
11771 12862 L 11769 12889 L 11789 12870 L 11811 12855 L
11799 12831 L 11773 12821 L 11776 12818 L 11794 12802 L
11810 12824 L 11837 12829 L 11822 12806 L 11832 12781 L
11826 12754 L 11835 12729 L 11859 12742 L 11887 12742 L
11887 12769 L 11878 12795 L 11879 12822 L 11881 12795 L
11905 12782 L 11909 12754 L 11936 12752 L 11911 12740 L

11884 12736 L 11877 12710 L 11850 12713 L 11829 12696 L
11856 12697 L 11884 12694 L 11905 12677 L 11915 12651 L
11887 12649 L 11861 12656 L 11855 12683 L 11827 12681 L
11826 12654 L 11852 12645 L 11873 12627 L 11845 12624 L
11820 12635 L 11806 12659 L 11804 12686 L 11780 12700 L
11772 12674 L 11772 12671 L 11774 12647 L 11787 12622 L
11814 12617 L 11835 12601 L 11847 12596 L 11861 12590 L
11882 12607 L 11909 12606 L 11890 12587 L 11871 12566 L
11879 12540 L 11903 12527 L 11886 12506 L 11909 12491 L
11911 12468 L 11911 12463 L 11933 12480 L 11933 12480 L
11951 12501 L 11955 12528 L 11957 12529 L 11977 12544 L
11981 12557 L 11984 12570 L 12008 12583 L 12008 12611 L
12011 12638 L 12021 12613 L 12023 12586 L 12037 12562 L
12045 12564 L 12064 12568 L 12091 12567 L 12098 12576 L
12107 12589 L 12096 12614 L 12105 12640 L 12109 12668 L
12133 12681 L 12139 12687 L 12153 12700 L 12178 12711 L
12196 12731 L 12222 12740 L 12247 12750 L 12251 12723 L
12234 12702 L 12213 12720 L 12214 12693 L 12215 12666 L
12191 12651 L 12187 12678 L 12176 12653 L 12167 12627 L
12193 12619 L 12208 12642 L 12216 12634 L 12228 12623 L
12244 12601 L 12249 12596 L 12265 12583 L 12276 12608 L
12277 12608 L 12288 12616 L 12298 12624 L 12300 12652 L
12273 12647 L 12276 12674 L 12303 12668 L 12319 12668 L
12331 12668 L 12331 12695 L 12309 12712 L 12287 12727 L
12269 12749 L 12261 12775 L 12284 12790 L 12259 12800 L
12286 12801 L 12313 12808 L 12324 12833 L 12337 12808 L
12346 12795 L 12352 12785 L 12363 12760 L 12353 12735 L
12366 12710 L 12389 12724 L 12388 12751 L 12407 12756 L
12415 12758 L 12441 12752 L 12436 12725 L 12437 12698 L
12446 12701 L 12464 12706 L 12463 12734 L 12453 12760 L
12438 12782 L 12442 12809 L 12436 12836 L 12409 12830 L
12389 12812 L 12364 12825 L 12351 12849 L 12357 12875 L
12374 12854 L 12398 12867 L 12415 12889 L 12441 12895 L
12468 12886 L 12487 12900 L 12490 12902 L 12513 12917 L
12539 12926 L 12546 12953 L 12549 12926 L 12550 12898 L
12550 12898 L 12575 12887 L 12601 12879 L 12628 12879 L
12631 12885 L 12638 12905 L 12645 12901 L 12662 12893 L
12664 12894 L 12688 12903 L 12684 12876 L 12712 12876 L
12718 12850 L 12692 12856 L 12699 12845 L 12708 12834 L
12726 12820 L 12730 12817 L 12741 12842 L 12743 12870 L
12725 12890 L 12726 12918 L 12728 12945 L 12747 12965 L
12730 12986 L 12743 13010 L 12766 13008 L 12770 13008 L
12797 13001 L 12789 13027 L 12782 13053 L 12806 13040 L

12828 13024 L 12833 13024 L 12855 13020 L 12883 13020 L
12894 13023 L 12908 13027 L 12909 13027 L 12911 13027 L
12924 13028 L 12936 13029 L 12951 13052 L 12951 13025 L
12958 12999 L 12979 13016 L 12999 13025 L 12999 13048 L
12997 13054 L 12999 13054 L 12999 14126 L 12998 14132 L
12980 14152 L 12967 14176 L 12960 14202 L 12950 14228 L
12933 14249 L 12914 14269 L 12899 14291 L 12878 14308 L
12864 14331 L 12843 14349 L 12837 14376 L 12818 14395 L
12798 14413 L 12793 14440 L 12772 14457 L 12751 14475 L
12730 14492 L 12711 14512 L 12691 14530 L 12675 14552 L
12656 14571 L 12640 14593 L 12621 14613 L 12605 14635 L
12586 14654 L 12574 14679 L 12563 14703 L 12546 14725 L
12534 14749 L 12519 14772 L 12503 14794 L 12494 14819 L
12476 14839 L 12463 14863 L 12448 14886 L 12438 14911 L
12432 14938 L 12409 14952 L 12383 14960 L 12358 14951 L
12347 14976 L 12328 14995 L 12305 15011 L 12278 15013 L
12256 15028 L 12235 15045 L 12213 15061 L 12196 15082 L
12178 15103 L 12162 15125 L 12146 15147 L 12123 15133 L
12098 15142 L 12072 15149 L 12045 15149 L 12018 15151 L
11994 15165 L 11980 15188 L 11983 15215 L 12006 15230 L
12012 15256 L 11986 15265 L 12012 15273 L 12039 15276 L
12053 15253 L 12044 15279 L 12033 15303 L 12026 15329 L
12028 15356 L 12041 15381 L 12034 15407 L 12008 15415 L
12002 15441 L 12009 15467 L 12022 15491 L 12040 15511 L
12067 15510 L 12081 15533 L 12107 15541 L 12128 15558 L
12149 15575 L 12146 15602 L 12168 15618 L 12182 15641 L
12204 15657 L 12230 15651 L 12257 15648 L 12282 15658 L
12298 15680 L 12323 15671 L 12333 15696 L 12359 15704 L
12377 15724 L 12401 15736 L 12419 15757 L 12444 15766 L
12448 15793 L 12425 15807 L 12434 15833 L 12457 15846 L
12482 15858 L 12506 15870 L 12528 15886 L 12544 15907 L
12561 15929 L 12584 15943 L 12608 15955 L 12621 15979 L
12626 16005 L 12633 16032 L 12637 16058 L 12655 16078 L
12681 16086 L 12697 16108 L 12718 16125 L 12743 16137 L
12767 16149 L 12785 16169 L 12811 16174 L 12836 16186 L
12841 16213 L 12840 16217 L 12833 16217 L 12800 16217 L
12766 16217 L 12733 16217 L 12700 16217 L 12667 16217 L
12633 16217 L 12600 16217 L 12567 16217 L 12534 16217 L
12501 16217 L 12467 16217 L 12434 16217 L 12401 16217 L
12368 16217 L 12334 16217 L 12301 16217 L 12268 16217 L
12235 16217 L 12201 16217 L 12168 16217 L 12135 16217 L
12102 16217 L 12069 16217 L 12035 16217 L 12002 16217 L
11969 16217 L 11936 16217 L 11902 16217 L 11869 16217 L

11836 16217 L 11803 16217 L 11770 16217 L 11736 16217 L
11703 16217 L 11670 16217 L 11637 16217 L 11603 16217 L
11570 16217 L 11537 16217 L 11504 16217 L 11471 16217 L
11437 16217 L 11404 16217 L 11371 16217 L 11338 16217 L
11304 16217 L 11271 16217 L 11238 16217 L 11205 16217 L
11171 16217 L 11138 16217 L 11105 16217 L 11072 16217 L
11039 16217 L 11005 16217 L 10972 16217 L 10939 16217 L
10906 16217 L 10872 16217 L 10839 16217 L 10806 16217 L
10773 16217 L 10740 16217 L 10706 16217 L 10673 16217 L
10640 16217 L 10607 16217 L 10573 16217 L 10540 16217 L
10507 16217 L 10474 16217 L 10440 16217 L 10407 16217 L
10374 16217 L 10341 16217 L 10308 16217 L 10274 16217 L
10241 16217 L 10208 16217 L 10175 16217 L 10141 16217 L
10108 16217 L 10075 16217 L 10042 16217 L 10009 16217 L
9975 16217 L 9942 16217 L 9909 16217 L 9876 16217 L
9842 16217 L 9809 16217 L 9776 16217 L 9743 16217 L
9710 16217 L 9676 16217 L 9643 16217 L 9610 16217 L
9577 16217 L 9543 16217 L 9510 16217 L 9477 16217 L
9444 16217 L 9410 16217 L 9377 16217 L 9344 16217 L
9311 16217 L 9278 16217 L 9244 16217 L 9211 16217 L
9178 16217 L 9145 16217 L 9111 16217 L 9078 16217 L
9045 16217 L 9012 16217 L 8979 16217 L 8945 16217 L
8912 16217 L 8879 16217 L 8846 16217 L 8812 16217 L
8779 16217 L 8746 16217 L 8713 16217 L 8680 16217 L
8646 16217 L 8613 16217 L 8580 16217 L 8547 16217 L
8513 16217 L 8480 16217 L 8447 16217 L 8414 16217 L
8380 16217 L 8347 16217 L 8314 16217 L 8281 16217 L
8248 16217 L 8214 16217 L 8181 16217 L 8148 16217 L
8115 16217 L 8081 16217 L 8048 16217 L 8015 16217 L
7982 16217 L 7949 16217 L 7915 16217 L 7882 16217 L
7849 16217 L 7816 16217 L 7782 16217 L 7749 16217 L
7716 16217 L 7683 16217 L 7650 16217 L 7616 16217 L
7583 16217 L 7550 16217 L 7517 16217 L 7483 16217 L
7450 16217 L 7417 16217 L 7384 16217 L 7350 16217 L
7317 16217 L 7284 16217 L 7251 16217 L 7218 16217 L
7184 16217 L 7151 16217 L 7118 16217 L 7085 16217 L
7051 16217 L 7018 16217 L 6985 16217 L 6952 16217 L
6919 16217 L 6885 16217 L 6852 16217 L 6819 16217 L
6786 16217 L 6752 16217 L 6719 16217 L 6686 16217 L
6653 16217 L 6619 16217 L 6586 16217 L 6553 16217 L
6520 16217 L 6487 16217 L 6453 16217 L 6420 16217 L
6387 16217 L 6354 16217 L 6320 16217 L 6287 16217 L
6254 16217 L 6221 16217 L 6188 16217 L 6154 16217 L

6121 16217 L 6088 16217 L 6055 16217 L 6021 16217 L
5988 16217 L 5955 16217 L 5922 16217 L 5889 16217 L
5855 16217 L 5822 16217 L 5789 16217 L 5756 16217 L
5722 16217 L 5689 16217 L 5656 16217 L 5623 16217 L
5589 16217 L 5556 16217 L 5523 16217 L 5490 16217 L
5457 16217 L 5423 16217 L 5390 16217 L 5357 16217 L
5324 16217 L 5290 16217 L 5257 16217 L 5224 16217 L
5191 16217 L 5158 16217 L 5124 16217 L 5091 16217 L
5058 16217 L 5025 16217 L 4991 16217 L 4958 16217 L
4925 16217 L 4892 16217 L 4859 16217 L 4825 16217 L
4792 16217 L 4759 16217 L 4726 16217 L 4692 16217 L
4659 16217 L 4626 16217 L 4593 16217 L 4559 16217 L
4526 16217 L 4493 16217 L 4460 16217 L 4427 16217 L
4393 16217 L 4360 16217 L 4327 16217 L 4294 16217 L
4260 16217 L 4227 16217 L 4194 16217 L 4161 16217 L
4128 16217 L 4094 16217 L 4061 16217 L 4028 16217 L
3995 16217 L 3961 16217 L 3928 16217 L 3895 16217 L
3862 16217 L 3828 16217 L 3795 16217 L 3762 16217 L
3729 16217 L 3696 16217 L 3662 16217 L 3629 16217 L
3596 16217 L 3563 16217 L 3529 16217 L 3496 16217 L
3463 16217 L 3430 16217 L 3397 16217 L 3363 16217 L
3330 16217 L 3297 16217 L 3264 16217 L 3230 16217 L
3197 16217 L 3164 16217 L 3131 16217 L 3098 16217 L
3064 16217 L 3031 16217 L 2998 16217 L 2965 16217 L
2931 16217 L 2898 16217 L 2865 16217 L 2832 16217 L
2798 16217 L 2765 16217 L 2732 16217 L 2699 16217 L
2666 16217 L 2632 16217 L 2599 16217 L 2566 16217 L
2533 16217 L 2499 16217 L 2466 16217 L 2433 16217 L
2400 16217 L 2367 16217 L 2333 16217 L 2300 16217 L
N E
5543 5524 5541 5517 5543 5513 5545 5509 5547 5505 5548 5503
5556 5503 5575 5503 5575 5505 5573 5510 5571 5514 5569 5518
5567 5522 5563 5525 5560 5528 5556 5531 5552 5534 5550 5530
5545 5528 5543 5524 20 N F
5702 5621 5699 5615 5700 5611 5704 5608 5708 5611 5712 5612
5717 5614 5717 5618 5721 5620 5724 5624 5724 5628 5726 5633
5721 5633 5717 5635 5713 5636 5711 5631 5707 5629 5704 5625
5702 5621 19 N F
5796 5620 5790 5619 5794 5617 5798 5614 5801 5611 5805 5609
5810 5609 5808 5614 5806 5618 5803 5621 5799 5623 5796 5620
12 N F
5817 5509 5816 5503 5822 5503 5855 5503 5865 5503 5872 5506
5879 5506 5885 5509 5890 5504 5890 5503 5910 5503 5904 5508

5904 5516 5908 5522 5902 5525 5909 5528 5916 5525 5918 5532
5916 5539 5916 5547 5921 5552 5924 5559 5930 5555 5936 5559
5937 5566 5933 5572 5927 5567 5921 5563 5914 5563 5908 5558
5903 5553 5897 5550 5893 5544 5886 5544 5879 5542 5872 5539
5866 5535 5862 5541 5855 5542 5848 5542 5844 5535 5838 5531
5832 5527 5825 5525 5828 5518 5823 5513 5817 5509 47 N F
5964 5506 5963 5503 5970 5503 5970 5507 5964 5506 5 N F
5977 5535 5967 5528 5972 5522 5975 5516 5981 5512 5988 5511
5992 5505 5999 5506 6006 5503 6006 5503 6021 5503 6024 5503
6024 5507 6028 5503 6039 5503 6038 5506 6042 5512 6038 5519
6034 5525 6031 5531 6024 5532 6018 5536 6011 5537 6005 5532
5998 5531 5991 5533 5984 5536 5977 5535 28 N F
6055 5511 6054 5507 6056 5505 6058 5504 6058 5503 6060 5503
6060 5503 6062 5504 6064 5506 6065 5508 6064 5510 6064 5512
6063 5514 6061 5514 6059 5512 6057 5512 6055 5511 17 N F
6243 5630 6240 5628 6241 5625 6242 5622 6245 5619 6248 5621
6251 5622 6254 5622 6257 5624 6259 5626 6259 5626 6257 5629
6253 5629 6250 5630 6247 5630 6243 5630 16 N F
6252 5610 6251 5607 6253 5608 6255 5607 6257 5608 6258 5610
6259 5612 6260 5614 6259 5616 6257 5615 6255 5614 6254 5612
6252 5610 13 N F
6256 5699 6252 5695 6253 5692 6257 5691 6259 5689 6260 5685
6263 5688 6262 5691 6260 5693 6257 5695 6256 5699 11 N F
7342 6099 7335 6091 7339 6086 7343 6079 7349 6076 7356 6072
7360 6066 7367 6064 7374 6066 7378 6060 7381 6054 7388 6051
7395 6049 7388 6045 7392 6039 7398 6035 7405 6034 7408 6027
7415 6027 7417 6034 7422 6040 7415 6042 7409 6040 7404 6045
7402 6052 7403 6059 7404 6066 7402 6073 7398 6079 7397 6086
7392 6092 7385 6094 7380 6090 7372 6090 7365 6091 7361 6097
7356 6102 7349 6103 7342 6099 39 N F
7490 6228 7483 6223 7488 6218 7496 6217 7503 6216 7508 6221
7505 6228 7506 6235 7511 6240 7514 6247 7507 6248 7501 6244
7500 6237 7497 6231 7490 6228 15 N F
7498 6214 7491 6212 7493 6205 7499 6201 7506 6200 7513 6199
7520 6197 7522 6203 7523 6211 7527 6217 7533 6220 7527 6228
7520 6226 7513 6227 7510 6220 7506 6215 7498 6214 17 N F
7498 6158 7493 6153 7497 6147 7504 6145 7511 6144 7519 6145
7526 6144 7533 6142 7538 6138 7545 6135 7552 6135 7559 6137
7566 6138 7573 6136 7574 6129 7581 6132 7586 6127 7593 6131
7597 6137 7595 6144 7594 6151 7600 6155 7598 6162 7592 6165
7589 6172 7590 6179 7583 6179 7579 6173 7576 6167 7569 6168
7564 6173 7558 6177 7552 6175 7548 6169 7544 6163 7539 6158
7535 6152 7526 6161 7523 6167 7516 6169 7509 6167 7503 6163

7498 6158 43 N F
7698 6243 7691 6232 7698 6232 7705 6233 7712 6236 7718 6233
7717 6226 7723 6230 7729 6233 7736 6235 7743 6236 7750 6238
7758 6238 7754 6245 7747 6245 7745 6252 7745 6260 7746 6267
7749 6273 7753 6279 7759 6282 7766 6279 7773 6276 7780 6276
7781 6283 7775 6289 7775 6296 7777 6303 7784 6306 7782 6313
7788 6317 7794 6313 7801 6314 7802 6321 7809 6323 7802 6323
7797 6329 7791 6332 7786 6337 7781 6333 7774 6334 7770 6328
7762 6329 7756 6326 7753 6333 7749 6339 7744 6343 7737 6346
7732 6351 7725 6348 7724 6341 7727 6334 7727 6327 7722 6322
7717 6316 7715 6309 7711 6304 7706 6298 7708 6291 7714 6288
7719 6293 7726 6295 7732 6291 7739 6291 7746 6290 7740 6285
7739 6278 7734 6273 7728 6268 7722 6264 7718 6258 7713 6253
7714 6246 7710 6251 7704 6248 7698 6243 76 N F
7944 6760 7942 6755 7943 6752 7945 6749 7948 6747 7950 6747
7951 6746 7953 6749 7950 6752 7948 6754 7946 6757 7944 6760
12 N F
7955 6744 7950 6745 7954 6742 7958 6740 7961 6736 7964 6733
7967 6729 7971 6727 7975 6725 7980 6726 7984 6726 7989 6726
7989 6726 7993 6727 7991 6731 7990 6736 7986 6737 7982 6735
7977 6734 7973 6737 7971 6740 7968 6744 7963 6743 7959 6741
7955 6744 25 N F
7957 6750 7953 6747 7957 6744 7961 6743 7966 6745 7970 6744
7973 6741 7975 6737 7980 6736 7984 6739 7988 6740 7992 6737
7994 6733 7999 6732 8002 6734 8007 6734 8006 6739 8003 6742
7999 6745 7997 6749 7993 6752 7989 6753 7984 6754 7980 6755
7975 6755 7971 6754 7966 6753 7962 6751 7957 6750 29 N F
7984 6724 7979 6723 7983 6721 7987 6719 7991 6716 7996 6716
7997 6721 7993 6723 7989 6724 7984 6724 10 N F
8030 6716 8023 6711 8027 6709 8031 6710 8035 6708 8039 6710
8043 6713 8039 6715 8035 6717 8030 6716 10 N F
8100 6747 8089 6741 8093 6735 8097 6729 8103 6726 8109 6723
8116 6721 8123 6719 8129 6724 8134 6729 8140 6733 8145 6738
8147 6745 8150 6752 8157 6753 8157 6760 8156 6767 8156 6775
8152 6780 8145 6777 8141 6782 8136 6788 8130 6786 8124 6790
8117 6793 8110 6794 8111 6787 8105 6783 8104 6776 8100 6769
8095 6764 8091 6758 8093 6751 8100 6753 8105 6758 8112 6761
8113 6754 8107 6750 8100 6747 39 N F
8169 6716 8165 6714 8167 6712 8171 6712 8174 6710 8176 6713
8179 6715 8182 6713 8185 6715 8185 6718 8188 6720 8191 6720
8193 6723 8194 6726 8195 6729 8193 6732 8190 6734 8187 6736
8184 6737 8181 6735 8178 6735 8175 6734 8174 6731 8174 6727
8173 6724 8170 6723 8169 6719 8169 6716 28 N F

8212 6719 8200 6715 8205 6710 8210 6704 8216 6701 8223 6699
8229 6702 8231 6709 8235 6715 8241 6719 8247 6723 8254 6725
8261 6726 8268 6725 8274 6721 8280 6717 8286 6713 8293 6717
8297 6723 8297 6730 8302 6735 8298 6741 8298 6748 8294 6754
8292 6761 8290 6768 8283 6770 8279 6776 8274 6781 8269 6775
8263 6771 8266 6765 8261 6759 8255 6755 8254 6748 8250 6742
8243 6744 8236 6746 8230 6750 8224 6755 8219 6759 8213 6756
8207 6751 8209 6744 8215 6748 8212 6741 8214 6734 8216 6727
8219 6720 8212 6719 50 N F
8212 6801 8209 6799 8212 6798 8215 6796 8218 6794 8221 6792
8223 6790 8227 6789 8230 6790 8231 6793 8232 6797 8234 6799
8232 6803 8229 6803 8226 6804 8222 6804 8219 6804 8216 6802
8212 6801 19 N F
8275 14785 8272 14780 8276 14781 8279 14784 8283 14787 8285 14790
8287 14794 8290 14797 8293 14801 8294 14805 8295 14809 8293 14813
8291 14809 8289 14805 8286 14803 8282 14800 8282 14796 8281 14791
8279 14788 8275 14785 20 N F
8293 14827 8292 14824 8293 14823 8295 14823 8298 14824 8299 14827
8301 14830 8301 14833 8299 14831 8296 14830 8293 14827 11 N F
8313 14844 8311 14841 8313 14839 8316 14841 8318 14843 8320 14846
8321 14849 8323 14852 8325 14854 8326 14857 8323 14855 8321 14853
8319 14851 8318 14848 8316 14846 8313 14844 16 N F
8362 14835 8356 14833 8361 14834 8365 14835 8369 14837 8372 14840
8375 14843 8378 14846 8379 14850 8377 14854 8373 14856 8370 14860
8369 14856 8370 14852 8374 14850 8375 14846 8371 14844 8368 14841
8365 14837 8362 14835 20 N F
8397 14885 8392 14880 8397 14881 8401 14882 8405 14883 8409 14884
8413 14885 8416 14889 8417 14893 8419 14897 8423 14899 8424 14903
8422 14906 8418 14905 8415 14902 8413 14898 8410 14895 8408 14891
8405 14888 8401 14886 8397 14885 21 N F
8398 14898 8397 14896 8399 14894 8402 14895 8405 14897 8406 14900
8407 14903 8407 14906 8406 14909 8404 14906 8402 14904 8401 14901
8398 14898 13 N F
8431 14897 8426 14896 8430 14894 8434 14896 8438 14897 8442 14898
8446 14900 8450 14903 8452 14906 8456 14909 8458 14913 8461 14916
8464 14919 8467 14922 8470 14925 8473 14928 8475 14933 8471 14931
8467 14929 8463 14927 8459 14925 8455 14924 8455 14919 8452 14916
8448 14914 8445 14912 8441 14909 8437 14907 8437 14903 8434 14899
8431 14897 31 N F
8628 7084 8623 7083 8627 7081 8631 7081 8634 7077 8635 7072
8639 7071 8638 7075 8640 7079 8643 7082 8646 7086 8645 7090
8647 7094 8644 7097 8644 7101 8647 7105 8652 7106 8655 7109
8654 7114 8651 7111 8646 7109 8642 7109 8638 7106 8635 7103

8631 7102 8631 7097 8633 7093 8636 7090 8636 7085 8632 7083
8628 7084 31 N F
8709 12528 8704 12528 8706 12524 8709 12521 8712 12518 8715 12515
8719 12514 8723 12512 8727 12514 8731 12516 8728 12519 8724 12521
8720 12522 8716 12522 8711 12524 8709 12528 16 N F
8717 7335 8712 7324 8716 7318 8721 7312 8721 7305 8724 7299
8731 7302 8736 7306 8743 7306 8744 7313 8741 7320 8742 7327
8741 7334 8743 7341 8746 7347 8748 7354 8747 7361 8750 7367
8750 7374 8750 7381 8747 7388 8740 7385 8737 7379 8733 7372
8727 7368 8727 7361 8724 7355 8721 7348 8719 7341 8717 7335
30 N F
8750 6139 8749 6136 8751 6135 8753 6136 8755 6136 8757 6138
8757 6140 8755 6141 8753 6142 8751 6141 8750 6139 11 N F
8810 7193 8800 7191 8805 7186 8802 7180 8807 7174 8814 7174
8818 7172 8821 7171 8827 7175 8833 7177 8839 7173 8837 7166
8836 7159 8842 7156 8849 7153 8853 7153 8856 7153 8859 7160
8854 7165 8857 7171 8864 7172 8869 7178 8862 7176 8857 7181
8853 7175 8845 7176 8839 7180 8833 7183 8828 7189 8828 7196
8822 7197 8819 7204 8814 7199 8810 7193 34 N F
8809 6033 8802 6029 8805 6026 8807 6022 8808 6017 8812 6015
8812 6020 8816 6022 8819 6026 8820 6030 8818 6035 8813 6034
8809 6033 13 N F
8837 6238 8828 6231 8835 6232 8842 6234 8844 6234 8849 6233
8851 6240 8852 6247 8856 6253 8855 6260 8854 6268 8860 6272
8866 6276 8866 6276 8859 6277 8853 6282 8848 6277 8846 6270
8846 6263 8846 6255 8841 6250 8846 6246 8844 6239 8837 6238
24 N F
8844 6118 8840 6113 8845 6111 8849 6112 8853 6112 8854 6112
8852 6116 8855 6119 8852 6123 8851 6127 8851 6132 8847 6133
8844 6136 8843 6132 8842 6127 8844 6123 8844 6118 17 N F
8848 7688 8842 7677 8849 7676 8854 7676 8856 7675 8863 7673
8870 7673 8875 7669 8882 7665 8889 7665 8895 7668 8895 7675
8894 7682 8890 7688 8888 7695 8882 7698 8875 7701 8869 7705
8863 7701 8857 7699 8852 7694 8848 7688 22 N F
8854 7674 8846 7672 8850 7669 8853 7666 8857 7664 8862 7664
8866 7665 8871 7666 8875 7668 8871 7671 8867 7672 8862 7673
8858 7673 8854 7674 14 N F
8851 6192 8850 6188 8851 6186 8853 6187 8855 6187 8858 6188
8860 6188 8862 6189 8864 6191 8865 6192 8866 6192 8868 6192
8868 6195 8866 6195 8864 6195 8861 6194 8860 6193 8857 6192
8856 6191 8854 6189 8853 6191 8851 6192 22 N F
8853 6065 8850 6063 8852 6061 8854 6061 8855 6064 8853 6065
6 N F

8856 6058 8855 6054 8857 6055 8859 6056 8860 6058 8859 6060
8857 6060 8856 6058 8 N F
8862 7429 8857 7426 8860 7422 8864 7421 8869 7421 8871 7425
8875 7426 8878 7430 8880 7434 8883 7437 8883 7442 8878 7442
8875 7438 8872 7436 8868 7433 8867 7429 8864 7425 8862 7429
18 N F
8859 6119 8857 6117 8859 6115 8859 6115 8861 6115 8862 6117
8864 6119 8866 6119 8869 6119 8869 6121 8868 6123 8866 6123
8864 6123 8861 6122 8861 6120 8859 6119 16 N F
8872 6226 8867 6219 8873 6215 8878 6210 8882 6204 8889 6202
8896 6204 8903 6207 8902 6214 8907 6220 8912 6224 8913 6224
8919 6228 8924 6233 8931 6233 8938 6232 8940 6231 8945 6229
8942 6236 8940 6243 8939 6250 8935 6256 8930 6251 8928 6258
8921 6255 8920 6248 8918 6255 8915 6249 8911 6243 8910 6250
8912 6257 8905 6257 8901 6251 8897 6245 8896 6238 8892 6244
8889 6250 8883 6246 8880 6240 8876 6234 8883 6236 8885 6229
8878 6230 8884 6226 8891 6227 8898 6227 8896 6220 8890 6216
8886 6210 8881 6215 8875 6219 8872 6226 52 N F
8875 6296 8870 6289 8876 6285 8881 6290 8886 6285 8891 6280
8893 6273 8897 6267 8900 6273 8898 6280 8903 6285 8900 6292
8896 6298 8889 6299 8882 6297 8875 6296 16 N F
8897 8011 8893 8006 8895 8002 8898 7998 8898 7993 8903 7993
8904 7998 8908 7995 8912 7994 8917 7995 8921 7996 8926 7996
8927 8001 8930 8004 8934 8006 8939 8007 8943 8009 8946 8012
8945 8017 8941 8018 8937 8018 8933 8015 8932 8010 8930 8007
8926 8008 8927 8012 8923 8012 8918 8011 8914 8013 8910 8013
8905 8012 8901 8012 8897 8011 33 N F
8895 6337 8894 6334 8896 6332 8897 6328 8898 6331 8900 6334
8903 6336 8906 6337 8907 6341 8904 6343 8902 6345 8899 6343
8898 6340 8895 6337 14 N F
8903 7440 8902 7436 8905 7436 8908 7436 8910 7437 8913 7438
8913 7441 8910 7442 8909 7445 8906 7444 8904 7443 8903 7440
12 N F
8908 6054 8906 6053 8908 6051 8910 6050 8912 6051 8913 6053
8912 6055 8910 6055 8908 6054 9 N F
8913 7434 8910 7428 8914 7426 8918 7425 8923 7425 8928 7426
8932 7427 8936 7425 8939 7428 8941 7433 8939 7437 8937 7441
8934 7438 8930 7436 8925 7434 8921 7432 8918 7435 8913 7434
18 N F
8921 7339 8918 7332 8924 7335 8931 7338 8938 7338 8943 7342
8942 7335 8942 7328 8935 7330 8932 7323 8937 7318 8933 7312
8938 7307 8943 7302 8945 7301 8950 7300 8955 7295 8960 7290
8967 7288 8971 7294 8967 7301 8961 7304 8964 7311 8970 7315

8976 7318 8981 7323 8988 7325 8993 7329 9000 7330 9005 7325
9011 7329 9012 7336 9005 7336 8998 7338 8992 7342 8987 7347
8985 7353 8985 7360 8987 7367 8993 7372 8999 7375 9006 7377
9010 7372 9017 7374 9021 7379 9025 7385 9018 7385 9011 7385
9004 7387 8999 7392 8992 7389 8995 7396 9000 7401 8996 7408
8990 7411 8985 7416 8978 7413 8972 7416 8968 7422 8968 7429
8972 7435 8976 7441 8969 7441 8963 7437 8957 7432 8954 7426
8948 7429 8943 7424 8941 7417 8934 7417 8930 7411 8926 7406
8926 7398 8930 7392 8937 7394 8941 7400 8948 7400 8951 7394
8950 7387 8946 7380 8946 7373 8945 7366 8944 7359 8950 7355
8955 7360 8958 7366 8964 7362 8971 7361 8971 7354 8966 7350
8960 7346 8952 7346 8948 7351 8941 7352 8934 7354 8929 7348
8922 7346 8921 7339 98 N F
8928 7252 8926 7249 8926 7245 8929 7243 8927 7240 8927 7237
8928 7237 8929 7239 8932 7241 8935 7243 8938 7244 8935 7245
8933 7247 8931 7250 8934 7253 8933 7256 8930 7255 8928 7252
18 N F
8936 7272 8930 7270 8932 7267 8933 7266 8934 7264 8937 7266
8940 7265 8944 7267 8947 7268 8950 7270 8947 7272 8944 7274
8945 7278 8942 7277 8940 7275 8936 7276 8936 7272 17 N F
8938 6061 8936 6058 8937 6056 8936 6054 8937 6052 8939 6051
8940 6053 8940 6055 8942 6056 8943 6059 8942 6061 8940 6061
8938 6061 13 N F
8951 7987 8948 7984 8949 7981 8951 7978 8954 7978 8957 7976
8960 7978 8963 7979 8965 7982 8968 7982 8972 7981 8974 7983
8976 7986 8973 7989 8970 7990 8967 7992 8964 7990 8961 7989
8958 7988 8955 7988 8951 7987 21 N F
9011 13074 9010 13068 9013 13064 9017 13063 9020 13066 9024 13069
9024 13073 9022 13077 9021 13081 9021 13085 9016 13086 9015 13081
9013 13078 9011 13074 14 N F
9033 7720 9031 7716 9035 7714 9039 7712 9043 7715 9047 7715
9051 7718 9053 7719 9055 7720 9057 7724 9059 7728 9054 7729
9050 7729 9048 7725 9044 7722 9041 7726 9037 7725 9039 7730
9036 7733 9035 7728 9032 7725 9033 7720 22 N F
9046 7623 9042 7620 9044 7617 9046 7615 9049 7616 9052 7618
9055 7620 9053 7623 9049 7623 9046 7623 10 N F
9059 7751 9058 7746 9062 7743 9062 7748 9062 7752 9064 7756
9067 7757 9069 7758 9073 7759 9077 7757 9081 7754 9083 7757
9082 7762 9079 7764 9075 7768 9071 7768 9067 7764 9064 7762
9060 7760 9060 7755 9059 7751 21 N F
9065 7611 9062 7605 9066 7607 9070 7608 9073 7612 9076 7615
9071 7615 9067 7616 9065 7611 9 N F
9087 7924 9084 7917 9089 7916 9093 7916 9095 7920 9099 7919

9103 7920 9104 7925 9104 7929 9104 7934 9100 7934 9098 7930
9095 7933 9091 7931 9087 7928 9087 7924 16 N F
9122 6194 9121 6189 9121 6184 9122 6180 9123 6175 9124 6171
9125 6166 9126 6171 9125 6175 9124 6180 9124 6185 9126 6189
9127 6193 9127 6198 9129 6202 9130 6207 9132 6211 9135 6214
9138 6218 9137 6223 9134 6219 9132 6215 9130 6211 9128 6207
9125 6203 9123 6199 9122 6194 27 N F
9134 7964 9128 7959 9132 7956 9135 7953 9137 7953 9140 7954
9144 7956 9143 7961 9148 7962 9152 7963 9152 7968 9154 7972
9154 7976 9152 7980 9147 7981 9145 7977 9142 7974 9140 7970
9138 7966 9134 7964 20 N F
9140 5975 M 9136 5961 L 9146 5951 L 9160 5955 L 9152 5942 L
9164 5933 L 9175 5925 L 9189 5929 L 9203 5933 L
9217 5938 L 9220 5923 L 9207 5917 L 9193 5914 L
9200 5901 L 9207 5889 L 9211 5875 L 9211 5860 L
9216 5847 L 9221 5833 L 9235 5830 L 9226 5818 L
9232 5805 L 9237 5792 L 9243 5778 L 9249 5765 L
9255 5752 L 9259 5738 L 9258 5723 L 9255 5709 L
9263 5697 L 9276 5691 L 9280 5677 L 9291 5667 L
9302 5658 L 9289 5663 L 9279 5674 L 9271 5687 L
9277 5673 L 9286 5662 L 9296 5651 L 9306 5641 L
9316 5630 L 9328 5623 L 9328 5622 L 9340 5614 L
9347 5627 L 9353 5614 L 9362 5603 L 9373 5593 L
9386 5587 L 9398 5579 L 9412 5573 L 9424 5566 L
9436 5558 L 9447 5548 L 9454 5535 L 9465 5526 L
9472 5513 L 9475 5503 L 9477 5503 L 9510 5503 L
9543 5503 L 9577 5503 L 9607 5503 L 9607 5512 L
9606 5527 L 9608 5541 L 9614 5555 L 9616 5569 L
9622 5583 L 9628 5596 L 9632 5610 L 9635 5624 L
9638 5638 L 9643 5652 L 9646 5666 L 9652 5679 L
9662 5690 L 9664 5704 L 9667 5718 L 9673 5732 L
9684 5740 L 9695 5750 L 9707 5758 L 9720 5765 L
9730 5775 L 9740 5786 L 9750 5796 L 9754 5810 L
9758 5824 L 9753 5838 L 9760 5851 L 9773 5856 L
9784 5865 L 9790 5878 L 9797 5891 L 9801 5905 L
9812 5915 L 9819 5928 L 9824 5941 L 9830 5955 L
9832 5969 L 9838 5982 L 9848 5992 L 9857 6003 L
9859 6018 L 9863 6032 L 9857 6045 L 9859 6060 L
9865 6073 L 9868 6087 L 9872 6101 L 9878 6114 L
9889 6123 L 9891 6137 L 9892 6151 L 9894 6166 L
9899 6179 L 9903 6193 L 9906 6207 L 9911 6221 L
9906 6234 L 9907 6249 L 9910 6263 L 9916 6276 L
9917 6290 L 9917 6305 L 9918 6307 L 9923 6318 L

9924 6332 L 9925 6347 L 9928 6361 L 9934 6374 L
9934 6388 L 9934 6403 L 9939 6416 L 9944 6430 L
9947 6444 L 9949 6458 L 9949 6473 L 9949 6487 L
9951 6502 L 9960 6513 L 9968 6525 L 9960 6537 L
9953 6549 L 9950 6563 L 9955 6577 L 9965 6587 L
9974 6598 L 9972 6613 L 9970 6627 L 9977 6640 L
9988 6648 L 9997 6660 L 10008 6668 L 10014 6682 L
10021 6694 L 10026 6707 L 10026 6722 L 10030 6735 L
10032 6749 L 10034 6764 L 10040 6777 L 10047 6789 L
10058 6799 L 10055 6813 L 10060 6826 L 10073 6834 L
10070 6848 L 10071 6862 L 10068 6877 L 10058 6887 L
10053 6901 L 10050 6915 L 10048 6929 L 10043 6942 L
10039 6956 L 10037 6970 L 10038 6985 L 10039 6999 L
10041 7013 L 10046 7027 L 10054 7038 L 10064 7049 L
10069 7062 L 10080 7071 L 10088 7083 L 10101 7090 L
10104 7091 L 10113 7097 L 10125 7105 L 10117 7117 L
10103 7116 L 10090 7123 L 10089 7137 L 10081 7149 L
10083 7163 L 10073 7173 L 10059 7172 L 10044 7173 L
10030 7170 L 10020 7179 L 10006 7185 L 9994 7178 L
9984 7189 L 9971 7194 L 9959 7203 L 9964 7217 L
9950 7212 L 9940 7221 L 9931 7233 L 9927 7247 L
9916 7255 L 9903 7262 L 9889 7260 L 9875 7257 L
9862 7252 L 9849 7245 L 9840 7234 L 9832 7222 L
9824 7210 L 9817 7198 L 9828 7189 L 9815 7184 L
9805 7173 L 9793 7167 L 9780 7161 L 9766 7158 L
9752 7155 L 9738 7156 L 9724 7151 L 9711 7145 L
9699 7138 L 9687 7130 L 9673 7127 L 9660 7121 L
9649 7112 L 9636 7106 L 9626 7096 L 9618 7080 L
9615 7066 L 9607 7055 L 9598 7043 L 9592 7030 L
9586 7017 L 9582 7003 L 9573 6993 L 9564 6981 L
9571 6968 L 9567 6954 L 9562 6941 L 9553 6930 L
9543 6920 L 9540 6906 L 9529 6896 L 9540 6886 L
9525 6887 L 9511 6885 L 9498 6879 L 9490 6868 L
9496 6855 L 9482 6852 L 9469 6846 L 9464 6833 L
9459 6819 L 9455 6805 L 9452 6792 L 9445 6779 L
9438 6767 L 9427 6757 L 9419 6745 L 9409 6734 L
9403 6721 L 9397 6708 L 9392 6695 L 9386 6682 L
9384 6668 L 9375 6656 L 9371 6642 L 9363 6630 L
9356 6618 L 9368 6610 L 9354 6610 L 9347 6597 L
9340 6585 L 9332 6573 L 9329 6559 L 9324 6545 L
9315 6534 L 9305 6523 L 9296 6513 L 9289 6500 L
9280 6489 L 9274 6476 L 9274 6462 L 9267 6449 L
9261 6436 L 9257 6422 L 9247 6411 L 9256 6400 L

9270 6398 L 9285 6397 L 9299 6399 L 9288 6390 L
9274 6388 L 9259 6387 L 9245 6389 L 9231 6389 L
9222 6377 L 9213 6366 L 9212 6351 L 9210 6337 L
9207 6323 L 9195 6314 L 9188 6302 L 9183 6288 L
9181 6274 L 9180 6260 L 9187 6247 L 9187 6232 L
9192 6219 L 9181 6211 L 9190 6201 L 9188 6187 L
9199 6177 L 9188 6168 L 9192 6154 L 9196 6140 L
9194 6126 L 9192 6111 L 9199 6099 L 9185 6101 L
9183 6087 L 9175 6075 L 9177 6060 L 9166 6052 L
9179 6046 L 9165 6044 L 9163 6030 L 9164 6015 L
9155 6006 L 9169 6001 L 9173 5988 L 9169 5974 L
9158 5965 L 9144 5961 L 9140 5975 L N E
9170 14289 9167 14284 9169 14280 9172 14277 9176 14274 9179 14272
9181 14275 9181 14280 9181 14284 9181 14288 9178 14291 9173 14291
9170 14289 13 N F
9198 15653 9196 15646 9199 15643 9203 15645 9206 15648 9209 15650
9209 15650 9214 15651 9218 15652 9219 15656 9219 15660 9219 15664
9215 15667 9211 15669 9209 15665 9205 15665 9204 15661 9205 15657
9201 15656 9198 15653 20 N F
9209 14298 9204 14296 9207 14293 9210 14290 9212 14286 9215 14283
9220 14283 9223 14280 9226 14283 9225 14288 9223 14292 9221 14295
9217 14295 9213 14298 9209 14298 15 N F
9225 14258 9221 14255 9225 14253 9229 14253 9232 14250 9234 14246
9238 14244 9240 14247 9239 14252 9238 14256 9240 14260 9238 14264
9235 14267 9233 14271 9232 14275 9228 14278 9225 14275 9223 14271
9224 14266 9225 14262 9225 14258 21 N F
9225 14401 9224 14397 9224 14391 9228 14388 9228 14388 9232 14390
9236 14389 9240 14389 9243 14392 9240 14395 9235 14396 9231 14396
9231 14401 9230 14405 9228 14409 9225 14406 9225 14401 17 N F
9227 8184 9225 8179 9227 8175 9230 8172 9234 8169 9238 8167
9242 8164 9246 8165 9250 8167 9254 8169 9259 8170 9263 8170
9259 8172 9255 8174 9253 8178 9252 8183 9250 8187 9246 8187
9242 8191 9238 8191 9233 8190 9230 8187 9227 8184 23 N F
9251 14466 9247 14461 9250 14459 9254 14461 9256 14465 9260 14466
9264 14467 9266 14471 9266 14475 9262 14477 9258 14476 9254 14474
9251 14471 9251 14466 14 N F
9291 14506 9287 14503 9289 14500 9291 14497 9293 14495 9295 14493
9296 14496 9295 14499 9297 14501 9294 14501 9294 14504 9291 14506
12 N F
9291 14518 9289 14513 9291 14513 9292 14514 9295 14513 9298 14515
9301 14513 9301 14517 9299 14519 9298 14522 9295 14522 9294 14519
9291 14518 13 N F
9311 7864 9309 7859 9312 7861 9313 7857 9316 7859 9318 7862

9316 7865 9313 7867 9311 7864 9 N F
9311 14324 9309 14320 9311 14318 9314 14317 9317 14319 9317 14322
9314 14323 9311 14324 8 N F
9318 5530 9315 5525 9320 5523 9324 5524 9326 5528 9330 5532
9333 5534 9335 5538 9332 5542 9328 5543 9323 5541 9320 5538
9320 5533 9318 5530 14 N F
9318 7859 9316 7852 9320 7850 9324 7853 9326 7856 9331 7855
9329 7859 9326 7862 9322 7861 9318 7859 10 N F
9412 15149 9403 15148 9405 15141 9404 15134 9410 15131 9416 15129
9423 15132 9429 15131 9433 15126 9436 15119 9439 15125 9445 15129
9449 15135 9454 15139 9457 15139 9461 15139 9468 15140 9473 15136
9479 15133 9486 15132 9492 15134 9496 15140 9501 15144 9508 15146
9511 15152 9515 15157 9521 15156 9527 15160 9530 15166 9532 15172
9531 15179 9531 15186 9527 15192 9526 15198 9519 15200 9513 15203
9507 15206 9501 15209 9494 15209 9499 15204 9499 15197 9495 15192
9489 15189 9482 15189 9478 15183 9472 15181 9465 15181 9458 15181
9452 15180 9445 15177 9439 15177 9433 15181 9431 15174 9431 15168
9426 15164 9420 15160 9417 15154 9412 15149 58 N F
9420 15069 9416 15064 9422 15061 9429 15059 9433 15064 9435 15070
9440 15075 9445 15080 9449 15085 9450 15092 9456 15096 9462 15096
9469 15095 9471 15089 9466 15084 9462 15079 9458 15074 9455 15067
9452 15062 9458 15060 9465 15058 9472 15057 9477 15053 9484 15053
9489 15057 9490 15064 9494 15069 9497 15075 9496 15082 9493 15088
9488 15093 9482 15095 9489 15094 9495 15094 9502 15097 9500 15106
9497 15112 9491 15114 9484 15116 9478 15118 9471 15116 9466 15112
9462 15118 9457 15122 9450 15120 9446 15115 9443 15109 9443 15102
9439 15097 9432 15096 9425 15095 9423 15089 9425 15082 9418 15081
9422 15075 9420 15069 56 N F
9417 8792 9417 8788 9418 8785 9419 8782 9422 8779 9425 8778
9427 8775 9429 8772 9432 8771 9436 8769 9439 8769 9441 8771
9441 8774 9442 8777 9439 8779 9437 8782 9436 8785 9433 8787
9430 8789 9427 8790 9424 8791 9420 8791 9417 8792 23 N F
9476 8767 9470 8765 9473 8764 9476 8762 9479 8760 9482 8758
9485 8757 9487 8754 9490 8753 9493 8752 9496 8750 9500 8751
9503 8753 9506 8754 9508 8757 9510 8760 9510 8763 9508 8766
9505 8768 9502 8769 9499 8769 9496 8770 9492 8769 9489 8769
9486 8769 9482 8769 9479 8768 9476 8767 28 N F
9545 10909 9538 10900 9542 10895 9547 10890 9552 10886 9557 10881
9562 10875 9566 10870 9571 10865 9577 10868 9583 10865 9590 10862
9595 10858 9600 10853 9606 10849 9611 10844 9616 10839 9620 10834
9626 10830 9632 10827 9638 10824 9645 10821 9648 10815 9654 10812
9661 10811 9668 10809 9673 10804 9679 10802 9686 10799 9692 10796
9699 10794 9705 10792 9711 10787 9717 10784 9723 10781 9728 10776

9735 10775 9741 10773 9747 10769 9753 10765 9756 10764 9760 10763
9766 10762 9773 10763 9780 10762 9787 10760 9793 10758 9800 10756
9806 10753 9812 10749 9819 10746 9825 10744 9832 10742 9838 10739
9845 10738 9852 10736 9858 10735 9859 10735 9865 10735 9872 10738
9878 10741 9880 10748 9885 10753 9890 10757 9892 10764 9893 10771
9894 10777 9891 10784 9886 10787 9880 10792 9874 10794 9867 10795
9860 10795 9853 10796 9847 10799 9840 10800 9834 10804 9828 10807
9823 10812 9817 10815 9811 10818 9805 10822 9798 10824 9791 10826
9785 10829 9778 10831 9772 10832 9765 10834 9758 10836 9752 10839
9746 10842 9742 10848 9739 10854 9734 10859 9728 10863 9723 10868
9717 10870 9710 10873 9704 10876 9698 10879 9691 10881 9685 10883
9678 10885 9672 10889 9668 10894 9665 10900 9662 10907 9658 10912
9654 10918 9650 10924 9644 10928 9639 10932 9632 10935 9626 10937
9619 10938 9612 10940 9605 10940 9599 10939 9592 10938 9585 10936
9579 10934 9572 10931 9567 10927 9562 10922 9557 10917 9551 10913
9545 10909 127 N F
9561 8701 9558 8697 9561 8693 9564 8696 9568 8698 9572 8701
9576 8703 9579 8700 9583 8698 9583 8702 9584 8703 9587 8705
9590 8708 9593 8711 9593 8711 9598 8711 9594 8714 9590 8711
9585 8710 9581 8708 9577 8709 9574 8713 9571 8716 9567 8716
9567 8712 9563 8710 9562 8705 9561 8701 28 N F
9568 8760 9567 8756 9571 8757 9576 8758 9580 8759 9584 8761
9589 8762 9593 8760 9591 8756 9593 8752 9597 8755 9601 8755
9606 8756 9610 8758 9614 8760 9617 8763 9621 8764 9618 8767
9614 8769 9611 8773 9608 8776 9604 8778 9600 8775 9596 8775
9591 8776 9588 8774 9584 8772 9584 8777 9580 8776 9579 8772
9575 8769 9571 8767 9568 8765 9568 8760 34 N F
9585 8486 9580 8482 9583 8479 9587 8476 9590 8473 9590 8477
9590 8482 9594 8480 9598 8478 9602 8476 9606 8476 9610 8473
9610 8478 9607 8481 9603 8483 9599 8486 9596 8489 9592 8491
9589 8494 9584 8495 9588 8492 9591 8489 9587 8490 9585 8486
24 N F
9588 8664 9586 8661 9589 8661 9591 8659 9594 8660 9596 8662
9598 8664 9597 8667 9595 8669 9593 8670 9591 8669 9590 8666
9588 8664 13 N F
9596 8689 9593 8684 9598 8684 9602 8682 9606 8683 9611 8682
9614 8685 9619 8684 9622 8688 9626 8686 9630 8684 9634 8682
9638 8683 9639 8687 9639 8692 9639 8696 9635 8699 9639 8701
9640 8697 9643 8700 9647 8703 9644 8707 9645 8711 9641 8712
9638 8708 9634 8706 9630 8708 9626 8705 9629 8702 9627 8698
9623 8698 9618 8696 9616 8700 9613 8703 9611 8699 9607 8697
9604 8693 9600 8691 9596 8689 39 N F
9617 8863 9608 8861 9610 8855 9611 8848 9614 8841 9618 8836

9622 8830 9622 8823 9615 8820 9621 8816 9620 8809 9627 8811
9634 8812 9640 8815 9641 8815 9647 8817 9654 8819 9653 8826
9660 8824 9665 8820 9668 8826 9662 8830 9664 8837 9670 8833
9676 8836 9678 8830 9685 8832 9681 8837 9687 8840 9688 8848
9681 8848 9675 8851 9671 8857 9672 8864 9670 8871 9667 8877
9670 8884 9674 8889 9681 8890 9679 8897 9673 8901 9667 8904
9661 8908 9654 8907 9647 8905 9643 8900 9642 8893 9643 8886
9637 8882 9633 8877 9628 8872 9622 8869 9617 8863 53 N F
9633 8782 9631 8778 9634 8775 9636 8771 9640 8770 9641 8770
9643 8773 9647 8776 9651 8778 9655 8779 9657 8781 9659 8781
9663 8783 9658 8784 9654 8783 9650 8785 9645 8785 9641 8787
9637 8784 9633 8782 20 N F
9652 8577 9648 8575 9651 8574 9651 8572 9654 8572 9656 8573
9657 8574 9659 8576 9660 8578 9661 8579 9662 8579 9663 8581
9662 8583 9660 8582 9657 8583 9656 8581 9656 8579 9654 8578
9652 8577 19 N F
9656 8571 9655 8569 9657 8567 9658 8569 9660 8571 9661 8573
9661 8575 9659 8575 9657 8573 9656 8571 10 N F
9671 14801 9668 14798 9669 14795 9669 14792 9671 14790 9674 14791
9676 14794 9675 14797 9674 14799 9674 14802 9675 14806 9672 14806
9670 14804 9671 14801 14 N F
9756 10723 9747 10718 9753 10715 9759 10713 9765 10709 9771 10706
9776 10701 9783 10698 9789 10695 9795 10692 9800 10687 9806 10684
9813 10681 9818 10677 9824 10674 9828 10668 9832 10663 9836 10657
9842 10653 9848 10656 9849 10663 9848 10669 9845 10676 9840 10681
9836 10686 9832 10691 9826 10695 9821 10700 9814 10702 9809 10707
9803 10709 9796 10712 9790 10714 9783 10716 9776 10718 9770 10720
9763 10721 9756 10723 38 N F
9792 8843 9791 8841 9791 8837 9794 8837 9797 8839 9798 8842
9800 8845 9803 8845 9806 8847 9807 8850 9805 8853 9802 8853
9800 8850 9798 8847 9795 8846 9792 8843 16 N F
9795 9444 9793 9441 9796 9439 9799 9438 9801 9435 9805 9436
9808 9436 9811 9437 9814 9438 9815 9441 9815 9444 9813 9447
9810 9447 9807 9445 9808 9442 9805 9441 9801 9441 9799 9443
9795 9444 19 N F
9831 9091 9829 9085 9831 9083 9834 9081 9837 9079 9840 9078
9842 9080 9841 9083 9842 9085 9843 9086 9846 9086 9846 9090
9843 9091 9843 9094 9841 9097 9840 9100 9837 9101 9834 9100
9832 9097 9832 9094 9831 9091 21 N F
9842 9074 9838 9072 9839 9069 9842 9066 9845 9065 9848 9067
9850 9069 9848 9072 9845 9073 9842 9074 10 N F
9859 10689 9851 10687 9854 10681 9857 10675 9858 10668 9860 10662
9862 10655 9867 10650 9873 10647 9879 10643 9885 10645 9889 10651

9893 10657 9895 10663 9894 10670 9891 10677 9885 10680 9879 10683
9873 10686 9866 10687 9859 10689 21 N F
9877 14978 9866 14974 9870 14968 9869 14962 9874 14957 9879 14953
9885 14956 9888 14962 9889 14969 9895 14965 9901 14963 9900 14970
9898 14976 9896 14983 9898 14989 9904 14991 9898 14992 9893 14987
9886 14985 9880 14985 9877 14978 21 N F
9913 5751 9904 5749 9908 5744 9910 5739 9913 5734 9918 5732
9922 5727 9927 5728 9926 5733 9928 5739 9929 5744 9929 5750
9924 5752 9918 5753 9913 5751 15 N F
9908 9440 9905 9433 9907 9429 9907 9429 9911 9429 9916 9429
9920 9428 9923 9427 9924 9426 9928 9424 9930 9420 9933 9417
9938 9417 9942 9416 9946 9419 9950 9420 9947 9424 9952 9425
9953 9426 9954 9428 9957 9431 9955 9435 9950 9435 9947 9438
9948 9442 9946 9445 9942 9444 9937 9443 9933 9442 9928 9442
9924 9443 9921 9447 9917 9445 9913 9443 9910 9446 9906 9444
9908 9440 37 N F
9908 9195 9905 9190 9909 9189 9912 9189 9914 9186 9916 9184
9920 9184 9923 9185 9923 9189 9923 9192 9926 9194 9928 9197
9924 9198 9923 9195 9920 9193 9917 9190 9914 9190 9912 9192
9913 9195 9910 9196 9907 9198 9908 9195 22 N F
9911 15022 9907 15021 9910 15020 9913 15019 9916 15017 9916 15017
9918 15015 9921 15016 9924 15016 9922 15018 9919 15020 9916 15020
9914 15023 9911 15022 14 N F
9911 15001 9908 14998 9911 14998 9914 14996 9917 14998 9920 14998
9920 15001 9919 15004 9916 15005 9913 15003 9911 15001 11 N F
9915 9412 9908 9406 9913 9405 9917 9406 9916 9401 9921 9401
9924 9397 9927 9395 9931 9397 9931 9401 9933 9405 9930 9409
9933 9412 9930 9415 9927 9418 9923 9420 9918 9419 9915 9416
9915 9412 19 N F
9913 9929 9909 9922 9914 9917 9916 9911 9922 9906 9928 9907
9933 9912 9939 9916 9945 9920 9950 9924 9953 9931 9951 9937
9948 9944 9947 9951 9943 9956 9937 9959 9933 9965 9930 9971
9923 9971 9916 9970 9915 9963 9912 9956 9909 9950 9910 9943
9912 9936 9913 9929 26 N F
9944 15007 9939 15004 9942 15002 9943 14999 9946 15000 9950 14999
9953 15000 9956 15000 9959 15000 9961 15003 9963 15005 9960 15006
9957 15006 9954 15006 9951 15007 9947 15007 9944 15007 17 N F
9946 15041 9939 15040 9942 15037 9944 15036 9946 15035 9950 15034
9955 15033 9958 15031 9962 15029 9966 15028 9971 15027 9974 15025
9974 15021 9977 15018 9979 15022 9979 15026 9983 15027 9987 15027
9989 15027 9991 15026 9996 15025 10000 15026 10004 15025 10008 15023
10012 15021 10015 15024 10018 15027 10022 15029 10017 15029 10013 15030
10009 15032 10005 15033 10002 15037 9998 15038 9996 15034 9992 15033

9988 15032 9983 15032 9979 15033 9975 15033 9972 15036 9969 15039
9969 15044 9966 15041 9964 15037 9960 15040 9960 15044 9956 15044
9953 15041 9950 15044 9946 15041 51 N F
9943 9239 9939 9237 9942 9234 9944 9237 9947 9239 9949 9241
9952 9243 9952 9247 9952 9250 9954 9252 9957 9252 9956 9254
9955 9258 9953 9260 9950 9260 9947 9258 9946 9255 9947 9252
9946 9248 9943 9248 9944 9245 9945 9242 9943 9239 23 N F
9943 9982 9940 9976 9943 9973 9948 9973 9951 9970 9950 9966
9954 9964 9959 9964 9959 9969 9961 9973 9963 9977 9962 9981
9964 9985 9961 9987 9958 9990 9953 9989 9950 9987 9946 9985
9943 9982 19 N F
9949 9218 9946 9213 9950 9213 9952 9211 9955 9210 9958 9212
9958 9215 9955 9218 9958 9220 9960 9222 9963 9225 9966 9226
9969 9226 9969 9229 9973 9230 9976 9230 9973 9232 9970 9232
9967 9230 9964 9228 9962 9226 9958 9226 9956 9224 9952 9223
9949 9224 9947 9221 9949 9218 27 N F
9951 9444 9949 9437 9953 9437 9957 9435 9959 9431 9962 9428
9966 9427 9969 9424 9970 9420 9970 9415 9968 9411 9965 9408
9969 9409 9970 9409 9974 9409 9978 9409 9978 9409 9982 9407
9981 9403 9980 9399 9984 9398 9988 9395 9990 9397 9991 9399
9996 9400 9994 9403 9994 9408 9995 9412 9999 9414 10003 9416
10000 9420 9995 9420 9998 9424 10002 9426 10005 9429 10004 9434
10002 9438 10002 9442 9999 9446 10002 9450 10001 9454 9997 9457
9994 9459 9990 9462 9987 9465 9984 9469 9980 9469 9977 9467
9976 9463 9972 9460 9968 9458 9964 9455 9961 9453 9957 9451
9953 9448 9951 9444 56 N F
9958 10022 9951 10019 9955 10013 9958 10011 9960 10008 9966 10004
9969 9998 9971 9992 9972 9991 9976 9986 9977 9979 9978 9973
9977 9966 9975 9959 9978 9953 9984 9949 9988 9944 9992 9938
9996 9932 9998 9925 10004 9922 10008 9925 10010 9926 10015 9931
10021 9933 10021 9933 10028 9933 10035 9934 10042 9931 10044 9927
10045 9924 10052 9925 10059 9926 10066 9926 10066 9926 10072 9924
10079 9921 10085 9918 10092 9916 10098 9915 10105 9912 10111 9908
10117 9904 10117 9904 10123 9901 10130 9902 10137 9904 10143 9906
10140 9912 10139 9919 10141 9926 10144 9932 10146 9939 10152 9943
10145 9946 10142 9952 10136 9956 10132 9961 10127 9967 10121 9970
10114 9969 10108 9970 10104 9976 10097 9979 10096 9986 10089 9985
10082 9985 10076 9988 10073 9994 10066 9994 10059 9994 10052 9996
10048 10001 10043 10006 10039 10012 10034 10017 10027 10018 10021 10014
10015 10011 10008 10010 10002 10007 9995 10009 9990 10014 9984 10008
9978 10012 9971 10013 9971 10020 9966 10025 9960 10029 9958 10022
90 N F
9957 10101 9956 10097 9957 10094 9958 10091 9960 10093 9960 10093

9963 10095 9965 10097 9966 10100 9964 10103 9967 10106 9966 10109
9968 10110 9970 10113 9971 10116 9969 10119 9966 10117 9966 10113
9964 10111 9961 10113 9960 10110 9960 10107 9959 10103 9957 10101
24 N F
9958 9881 9956 9873 9959 9870 9962 9867 9967 9866 9971 9867
9975 9869 9980 9869 9984 9871 9988 9873 9988 9877 9988 9882
9985 9885 9981 9887 9978 9890 9977 9894 9974 9896 9972 9901
9968 9899 9966 9895 9962 9893 9959 9890 9958 9886 9958 9881
24 N F
9967 9085 9965 9081 9968 9082 9971 9083 9969 9080 9972 9078
9970 9075 9967 9072 9969 9070 9972 9071 9974 9068 9977 9070
9979 9073 9982 9071 9982 9074 9983 9077 9985 9080 9986 9081
9987 9083 9984 9083 9980 9083 9978 9085 9977 9088 9978 9092
9974 9091 9971 9090 9970 9087 9967 9085 28 N F
9968 9047 9965 9043 9968 9044 9971 9045 9973 9048 9975 9046
9978 9048 9981 9050 9983 9052 9986 9054 9988 9056 9986 9059
9983 9058 9979 9058 9976 9056 9973 9055 9971 9052 9971 9049
9968 9047 19 N F
9979 9813 9978 9809 9979 9805 9978 9800 9982 9798 9986 9797
9990 9797 9995 9798 9997 9801 10000 9805 10001 9809 10004 9812
10006 9816 10009 9820 10010 9824 10007 9828 10003 9829 9999 9827
9993 9824 9989 9822 9985 9819 9982 9816 9979 9813 23 N F
9983 9209 9979 9206 9982 9203 9982 9200 9985 9202 9987 9204
9991 9205 9992 9208 9990 9210 9987 9209 9983 9209 11 N F
9984 14998 9980 14995 9983 14992 9985 14994 9988 14995 9990 14998
9991 15001 9989 15004 9987 15001 9984 15001 9984 14998 11 N F
9990 10033 9987 10029 9990 10031 9993 10029 9995 10026 9997 10024
9995 10022 9993 10019 9996 10019 9999 10021 10002 10023 10004 10025
10007 10028 10008 10030 10011 10033 10009 10035 10008 10038 10006 10041
10003 10040 10000 10039 10000 10035 9998 10038 9995 10039 9992 10037
9994 10034 9990 10033 26 N F
9994 10287 9991 10283 9994 10280 9996 10278 9999 10279 10000 10282
10002 10285 10004 10287 10001 10287 9998 10288 9997 10285 9994 10287
12 N F
10001 10259 9995 10256 9998 10254 10001 10255 10003 10252 10004 10249
10006 10249 10008 10249 10010 10252 10010 10255 10012 10258 10015 10259
10012 10260 10009 10261 10005 10261 10004 10258 10001 10259 17 N F
10003 15012 9996 15008 9999 15005 10004 15005 10007 15008 10011 15007
10015 15008 10020 15009 10024 15009 10028 15008 10027 15004 10026 15000
10024 14996 10024 14992 10027 14995 10030 14997 10034 15000 10038 15000
10038 15005 10042 15006 10045 15007 10046 15008 10050 15010 10046 15012
10044 15016 10040 15018 10042 15021 10039 15019 10036 15015 10032 15017
10028 15016 10024 15016 10020 15014 10016 15013 10011 15013 10007 15013

10003 15012 37 N F
10002 9788 9999 9786 10000 9783 10000 9779 10001 9776 10000 9773
10003 9774 10006 9777 10008 9779 10009 9782 10011 9785 10014 9786
10014 9790 10013 9793 10010 9794 10006 9793 10003 9791 10002 9788
18 N F
10002 9904 10001 9900 10003 9897 10003 9894 10006 9892 10009 9892
10011 9894 10013 9897 10015 9900 10015 9903 10014 9907 10011 9908
10008 9908 10005 9906 10002 9904 15 N F
10018 9462 10016 9455 10021 9456 10025 9456 10027 9460 10030 9464
10033 9461 10038 9461 10041 9464 10044 9468 10040 9469 10035 9468
10031 9470 10032 9475 10036 9476 10033 9479 10036 9483 10031 9483
10027 9486 10025 9482 10027 9478 10024 9474 10028 9472 10024 9470
10022 9466 10017 9466 10018 9462 27 N F
10023 9783 10017 9771 10024 9771 10027 9776 10028 9777 10035 9777
10040 9772 10047 9773 10049 9766 10055 9763 10061 9759 10061 9752
10061 9745 10068 9745 10070 9752 10073 9746 10079 9749 10081 9742
10088 9741 10091 9747 10092 9754 10096 9760 10091 9765 10089 9772
10083 9774 10078 9779 10071 9781 10065 9785 10061 9790 10056 9796
10058 9803 10056 9809 10052 9815 10047 9810 10040 9808 10033 9808
10028 9803 10027 9796 10025 9789 10023 9783 40 N F
10023 10098 10018 10090 10024 10086 10030 10083 10036 10080 10042 10076
10048 10072 10054 10071 10061 10070 10064 10070 10068 10070 10072 10069
10075 10068 10081 10066 10088 10064 10085 10070 10083 10077 10088 10082
10090 10088 10085 10094 10090 10099 10095 10104 10088 10104 10083 10109
10085 10116 10079 10119 10072 10117 10066 10120 10065 10114 10058 10110
10052 10109 10047 10104 10040 10107 10033 10106 10029 10101 10023 10098
36 N F
10021 9928 10019 9925 10021 9922 10020 9918 10021 9915 10025 9916
10027 9918 10030 9919 10029 9923 10027 9925 10025 9927 10021 9928
12 N F
10040 14910 10036 14907 10039 14904 10042 14901 10039 14899 10042 14896
10045 14893 10050 14892 10054 14891 10058 14890 10061 14892 10061 14897
10056 14898 10055 14902 10052 14905 10048 14903 10046 14906 10045 14910
10040 14910 19 N F
10040 9915 10039 9911 10040 9908 10044 9908 10047 9909 10047 9912
10048 9915 10047 9918 10044 9919 10040 9918 10040 9915 11 N F
10054 15012 10050 15011 10053 15010 10057 15009 10059 15011 10056 15013
10054 15012 7 N F
10056 9909 10051 9905 10052 9905 10053 9903 10056 9902 10060 9902
10063 9902 10066 9903 10068 9906 10067 9908 10065 9911 10062 9912
10059 9911 10056 9909 14 N F
10053 10034 10051 10024 10054 10017 10059 10012 10066 10015 10070 10020
10071 10027 10078 10029 10083 10035 10076 10038 10070 10041 10064 10043

10058 10040 10053 10034 14 N F
10059 9918 10054 9914 10057 9913 10060 9915 10062 9914 10063 9914
10066 9915 10069 9913 10072 9911 10074 9908 10077 9910 10078 9913
10076 9916 10074 9918 10071 9920 10069 9923 10066 9925 10063 9923
10061 9920 10059 9918 20 N F
10061 15010 10056 15007 10059 15005 10062 15003 10064 15006 10067 15007
10070 15006 10073 15008 10071 15010 10067 15010 10064 15008 10061 15010
12 N F
10069 10061 10065 10057 10066 10055 10069 10054 10071 10052 10074 10052
10076 10052 10077 10055 10076 10058 10074 10060 10072 10061 10069 10061
12 N F
10072 14928 10067 14927 10068 14925 10071 14923 10074 14924 10078 14925
10081 14923 10083 14926 10080 14928 10078 14929 10075 14928 10072 14928
12 N F
10083 14914 10083 14910 10085 14908 10088 14909 10090 14911 10092 14914
10091 14917 10088 14918 10087 14915 10083 14914 10 N F
10090 9611 10089 9608 10090 9605 10093 9606 10096 9605 10098 9608
10101 9610 10102 9613 10104 9615 10107 9617 10111 9617 10110 9620
10106 9619 10103 9621 10101 9619 10098 9618 10098 9614 10096 9611
10093 9610 10090 9611 20 N F
10096 9772 10094 9769 10097 9767 10100 9765 10103 9764 10103 9764
10106 9763 10109 9761 10111 9758 10114 9756 10115 9759 10118 9760
10122 9759 10125 9759 10127 9757 10130 9755 10130 9758 10131 9761
10128 9762 10125 9762 10122 9761 10119 9763 10116 9764 10112 9765
10110 9767 10107 9768 10106 9771 10104 9773 10100 9773 10098 9776
10095 9775 10096 9772 32 N F
10095 10224 10094 10220 10098 10220 10101 10220 10102 10223 10103 10226
10101 10228 10098 10226 10095 10224 9 N F
10099 7012 10096 7013 10097 7011 10099 7010 10101 7009 10103 7008
10105 7008 10107 7008 10109 7008 10112 7008 10112 7011 10110 7012
10109 7014 10108 7016 10106 7017 10104 7017 10102 7016 10101 7014
10099 7012 19 N F
10102 14922 10097 14924 10099 14921 10101 14919 10102 14916 10104 14915
10105 14914 10106 14917 10108 14920 10106 14922 10102 14922 11 N F
10101 9810 10097 9805 10098 9805 10101 9804 10103 9800 10106 9796
10109 9793 10112 9790 10116 9788 10119 9785 10123 9787 10127 9785
10132 9783 10135 9786 10139 9787 10144 9786 10144 9790 10140 9790
10140 9794 10135 9795 10132 9792 10128 9794 10123 9794 10119 9795
10115 9798 10112 9801 10115 9804 10117 9808 10113 9810 10110 9813
10105 9813 10101 9810 32 N F
10104 9847 10098 9840 10104 9836 10110 9832 10110 9832 10113 9826
10119 9821 10125 9818 10129 9813 10136 9811 10142 9808 10149 9810
10145 9816 10141 9822 10141 9829 10136 9833 10141 9837 10137 9843

10131 9845 10124 9847 10117 9849 10111 9847 10104 9847 23 N F
10102 10292 10100 10289 10101 10289 10103 10288 10105 10289 10106 10290
10109 10292 10107 10295 10104 10296 10106 10293 10102 10292 11 N F
10105 10085 10102 10075 10103 10068 10110 10071 10116 10074 10122 10076
10129 10077 10125 10082 10119 10084 10112 10084 10105 10085 11 N F
10116 10195 10108 10197 10111 10190 10110 10183 10114 10178 10113 10171
10119 10169 10126 10165 10133 10165 10131 10171 10138 10172 10143 10176
10149 10173 10156 10173 10163 10171 10168 10171 10170 10171 10175 10168
10181 10167 10182 10167 10180 10174 10174 10177 10179 10182 10175 10187
10169 10190 10162 10189 10155 10190 10153 10197 10146 10196 10149 10190
10142 10191 10136 10193 10129 10195 10123 10193 10116 10195 35 N F
10122 10072 10112 10067 10117 10062 10122 10068 10129 10069 10135 10067
10142 10069 10149 10072 10153 10077 10156 10078 10160 10079 10165 10084
10163 10077 10165 10071 10170 10066 10176 10070 10180 10075 10178 10082
10175 10088 10168 10090 10161 10089 10154 10089 10148 10087 10141 10087
10134 10087 10133 10080 10128 10075 10122 10072 28 N F
10121 15176 10120 15172 10124 15171 10128 15171 10130 15167 10132 15163
10134 15160 10137 15160 10139 15160 10143 15160 10145 15164 10148 15167
10151 15171 10153 15174 10153 15174 10155 15178 10156 15182 10153 15185
10150 15188 10143 15192 10140 15189 10142 15185 10141 15181 10139 15184
10134 15184 10135 15189 10130 15190 10128 15187 10126 15183 10124 15179
10121 15176 31 N F
10127 10225 10124 10223 10127 10223 10130 10220 10133 10219 10136 10218
10137 10221 10137 10224 10137 10228 10137 10231 10134 10229 10133 10225
10130 10224 10127 10225 14 N F
10131 9863 10127 9857 10131 9855 10136 9854 10140 9853 10144 9855
10148 9857 10150 9857 10152 9858 10152 9863 10156 9865 10154 9869
10149 9868 10145 9867 10141 9869 10137 9869 10132 9867 10131 9863
18 N F
10145 7467 10145 7464 10147 7463 10149 7461 10150 7463 10152 7465
10152 7467 10150 7468 10147 7468 10145 7467 10 N F
10151 9907 10147 9900 10152 9895 10154 9892 10156 9889 10161 9885
10167 9881 10168 9880 10170 9875 10175 9870 10169 9865 10170 9858
10172 9852 10177 9847 10184 9845 10190 9849 10185 9855 10183 9861
10186 9868 10192 9872 10198 9875 10198 9882 10196 9888 10189 9889
10183 9893 10190 9892 10197 9894 10193 9899 10192 9906 10186 9910
10180 9906 10174 9905 10167 9907 10160 9905 10156 9911 10151 9907
36 N F
10151 15032 10148 15031 10149 15028 10152 15027 10153 15027 10155 15029
10156 15031 10153 15035 10151 15032 9 N F
10168 7385 10164 7383 10166 7382 10168 7382 10168 7385 5 N F
10170 15140 10167 15135 10170 15131 10173 15128 10176 15127 10177 15127
10180 15124 10181 15129 10181 15133 10185 15133 10183 15137 10178 15137

10174 15138 10170 15140 14 N F
10171 9932 10168 9928 10169 9924 10172 9922 10177 9921 10177 9926
10176 9930 10176 9934 10179 9938 10184 9937 10188 9938 10186 9942
10182 9944 10180 9948 10182 9952 10181 9956 10178 9952 10174 9951
10169 9951 10171 9946 10170 9942 10168 9935 10171 9932 23 N F
10176 10352 10169 10350 10175 10347 10176 10340 10175 10333 10170 10326
10177 10326 10184 10326 10190 10328 10197 10330 10200 10330 10204 10330
10211 10331 10213 10330 10218 10330 10224 10333 10219 10337 10223 10342
10216 10342 10209 10341 10204 10337 10197 10336 10192 10340 10185 10342
10182 10349 10176 10352 26 N F
10195 10180 10191 10176 10194 10176 10197 10177 10201 10176 10201 10173
10204 10171 10205 10171 10207 10172 10208 10174 10206 10176 10203 10179
10203 10182 10200 10184 10197 10183 10195 10180 16 N F
10205 9909 10204 9905 10206 9902 10207 9899 10210 9900 10211 9903
10209 9906 10209 9909 10205 9909 9 N F
10210 10069 10207 10066 10210 10065 10213 10063 10216 10062 10220 10061
10221 10064 10220 10068 10217 10069 10213 10069 10210 10069 11 N F
10223 14941 10217 14936 10223 14936 10228 14934 10230 14929 10225 14932
10222 14927 10222 14922 10221 14917 10223 14911 10228 14909 10231 14913
10235 14917 10236 14918 10240 14920 10244 14924 10244 14929 10241 14933
10238 14938 10235 14942 10235 14948 10233 14943 10228 14940 10223 14941
24 N F
10230 10312 10229 10304 10234 10303 10238 10302 10242 10301 10240 10305
10243 10307 10247 10310 10244 10313 10240 10316 10236 10318 10234 10314
10230 10312 13 N F
10256 10403 10253 10399 10256 10399 10259 10400 10262 10398 10265 10397
10266 10400 10263 10402 10260 10402 10256 10403 10 N F
10270 10401 10268 10398 10271 10397 10274 10399 10277 10400 10275 10402
10272 10404 10270 10401 8 N F
10285 10418 10280 10416 10284 10415 10286 10413 10289 10413 10289 10413
10293 10413 10295 10416 10298 10416 10298 10419 10295 10420 10292 10418
10290 10420 10287 10422 10284 10424 10283 10421 10285 10418 17 N F
10289 10062 10285 10058 10288 10057 10289 10053 10291 10050 10294 10049
10295 10052 10294 10055 10293 10058 10293 10062 10289 10062 11 N F
10308 10061 10304 10059 10305 10056 10307 10053 10311 10053 10311 10049
10312 10046 10314 10049 10314 10052 10313 10056 10310 10058 10308 10061
12 N F
10321 10063 10317 10058 10318 10055 10320 10052 10323 10051 10323 10054
10322 10057 10323 10060 10321 10063 9 N F
10328 10048 10325 10044 10326 10041 10329 10041 10331 10043 10330 10045
10328 10048 7 N F
10330 10345 10327 10341 10328 10338 10329 10337 10331 10341 10336 10342
10338 10338 10342 10336 10342 10341 10342 10345 10343 10349 10344 10353

10340 10353 10336 10351 10332 10349 10330 10345 16 N F
10340 10389 10334 10386 10336 10383 10340 10385 10340 10385 10342 10387
10340 10389 7 N F
10351 10027 10347 10025 10348 10022 10350 10019 10353 10018 10356 10018
10359 10018 10359 10022 10357 10024 10354 10026 10351 10027 11 N F
10356 10357 10350 10350 10351 10349 10352 10347 10357 10346 10361 10347
10363 10343 10366 10339 10366 10344 10365 10348 10363 10352 10359 10353
10356 10357 13 N F
10430 15319 10429 15314 10432 15314 10435 15316 10438 15317 10441 15318
10444 15318 10448 15318 10445 15321 10444 15324 10441 15325 10438 15324
10435 15322 10432 15321 10430 15319 15 N F
10636 6813 10632 6812 10635 6809 10640 6809 10644 6807 10649 6806
10653 6806 10658 6807 10662 6808 10666 6808 10671 6806 10675 6808
10678 6811 10682 6814 10685 6818 10689 6820 10685 6823 10680 6825
10676 6826 10672 6827 10665 6825 10661 6827 10657 6824 10653 6824
10648 6825 10644 6824 10642 6819 10638 6818 10636 6813 29 N F
10811 15236 10808 15230 10812 15231 10814 15235 10817 15238 10821 15236
10822 15236 10824 15234 10827 15237 10827 15241 10831 15240 10832 15235
10836 15234 10840 15233 10844 15235 10847 15238 10848 15242 10851 15245
10852 15250 10848 15251 10844 15253 10840 15255 10839 15250 10834 15250
10830 15251 10827 15248 10823 15250 10820 15248 10820 15244 10817 15240
10813 15240 10811 15236 32 N F
10812 15182 10810 15178 10813 15177 10813 15174 10816 15172 10819 15173
10822 15173 10823 15176 10824 15179 10821 15181 10820 15184 10820 15188
10817 15186 10815 15184 10812 15182 15 N F
10820 15209 10819 15205 10820 15205 10822 15204 10823 15205 10824 15206
10826 15208 10824 15210 10822 15211 10820 15209 10 N F
10820 15197 10820 15193 10823 15191 10826 15191 10825 15194 10826 15197
10823 15199 10820 15197 8 N F
10848 15229 10845 15226 10848 15222 10846 15217 10849 15214 10852 15217
10857 15218 10856 15222 10859 15226 10856 15229 10858 15233 10854 15235
10850 15233 10848 15229 14 N F
10881 7587 10880 7583 10881 7581 10884 7581 10886 7582 10888 7583
10889 7585 10892 7584 10894 7584 10896 7585 10898 7585 10898 7587
10896 7589 10895 7591 10893 7591 10891 7591 10889 7591 10886 7591
10885 7590 10883 7588 10881 7587 21 N F
10892 14387 10886 14383 10888 14379 10891 14377 10891 14376 10894 14373
10895 14369 10897 14365 10899 14361 10902 14358 10906 14359 10910 14359
10915 14358 10919 14359 10922 14356 10926 14358 10927 14362 10929 14366
10926 14369 10922 14370 10920 14374 10922 14378 10926 14380 10930 14378
10929 14374 10932 14374 10933 14375 10936 14378 10940 14377 10938 14381
10942 14380 10940 14384 10938 14388 10936 14392 10932 14394 10928 14394
10924 14393 10920 14392 10915 14391 10911 14390 10907 14388 10903 14386

10900 14384 10896 14385 10892 14387 45 N F
10908 15222 10903 15216 10907 15216 10910 15219 10914 15222 10918 15222
10922 15224 10925 15227 10927 15231 10931 15233 10933 15236 10935 15240
10939 15241 10938 15245 10942 15246 10946 15244 10948 15246 10949 15247
10951 15251 10950 15255 10946 15255 10942 15254 10944 15257 10941 15259
10944 15262 10939 15263 10942 15266 10938 15269 10937 15273 10940 15276
10945 15275 10944 15280 10941 15282 10937 15285 10933 15284 10929 15285
10925 15282 10922 15281 10925 15278 10924 15274 10921 15272 10924 15269
10924 15264 10921 15262 10917 15260 10915 15256 10911 15256 10908 15254
10905 15250 10904 15246 10908 15248 10912 15249 10916 15250 10920 15248
10917 15245 10913 15243 10915 15239 10919 15238 10919 15234 10918 15230
10913 15230 10909 15230 10909 15226 10908 15222 64 N F
10919 14305 10915 14305 10918 14303 10921 14301 10924 14301 10924 14298
10923 14295 10924 14295 10926 14296 10929 14298 10932 14297 10935 14298
10937 14300 10939 14303 10939 14306 10937 14309 10937 14312 10939 14315
10942 14316 10940 14319 10938 14320 10934 14320 10932 14322 10931 14318
10929 14316 10926 14315 10925 14312 10923 14309 10922 14306 10919 14305
30 N F
10924 7582 10921 7580 10923 7578 10924 7577 10926 7578 10925 7580
10924 7582 7 N F
10933 14689 10928 14687 10931 14684 10935 14683 10939 14684 10936 14687
10938 14691 10941 14689 10946 14689 10949 14692 10951 14688 10955 14687
10959 14685 10963 14682 10967 14681 10971 14680 10971 14685 10974 14681
10979 14681 10981 14685 10979 14688 10977 14692 10974 14696 10971 14698
10971 14702 10967 14702 10962 14702 10959 14705 10955 14707 10951 14708
10948 14711 10946 14707 10943 14703 10941 14700 10937 14698 10933 14698
10933 14694 10933 14689 38 N F
10947 14279 10945 14273 10948 14274 10950 14277 10953 14279 10955 14280
10958 14279 10959 14279 10962 14278 10962 14282 10963 14285 10960 14285
10957 14284 10954 14283 10950 14284 10949 14281 10947 14279 17 N F
10958 7684 10957 7681 10958 7679 10961 7679 10963 7679 10964 7681
10963 7683 10961 7685 10959 7685 10958 7684 10 N F
10968 6686 10966 6684 10968 6683 10970 6684 10972 6686 10974 6687
10972 6687 10970 6687 10968 6686 9 N F
11011 14757 11010 14753 11013 14750 11016 14753 11019 14756 11023 14758
11025 14762 11025 14766 11027 14770 11027 14774 11025 14770 11018 14771
11017 14775 11015 14778 11014 14774 11014 14770 11012 14766 11014 14762
11019 14763 11018 14758 11014 14760 11011 14757 22 N F
11030 6758 11017 6755 11022 6749 11026 6743 11033 6740 11040 6741
11047 6739 11049 6746 11055 6750 11060 6746 11066 6742 11073 6741
11081 6741 11088 6740 11094 6739 11095 6738 11102 6739 11109 6740
11116 6738 11122 6734 11129 6732 11135 6736 11135 6743 11130 6749
11124 6752 11131 6750 11138 6748 11144 6744 11144 6751 11142 6758

11144 6765 11148 6771 11155 6769 11157 6776 11154 6782 11158 6788
11160 6795 11154 6798 11148 6802 11143 6807 11136 6809 11129 6810
11126 6816 11120 6813 11114 6816 11107 6816 11100 6813 11095 6807
11091 6813 11089 6820 11084 6826 11078 6830 11072 6832 11066 6828
11071 6824 11073 6817 11077 6811 11070 6813 11071 6820 11064 6818
11060 6812 11056 6806 11059 6800 11058 6793 11062 6787 11067 6782
11068 6774 11065 6781 11062 6774 11061 6767 11064 6761 11063 6754
11063 6761 11058 6766 11052 6770 11050 6777 11045 6772 11039 6776
11040 6769 11043 6762 11037 6767 11031 6770 11026 6764 11030 6758
84 N F
11058 14312 11057 14309 11060 14308 11063 14309 11066 14308 11070 14308
11071 14311 11072 14312 11073 14313 11074 14317 11076 14315 11078 14318
11077 14321 11078 14324 11079 14327 11076 14327 11072 14327 11069 14327
11066 14326 11069 14324 11072 14323 11071 14319 11071 14316 11068 14316
11064 14317 11061 14319 11061 14315 11058 14312 28 N F
11077 6618 11075 6613 11078 6610 11083 6609 11087 6608 11092 6609
11096 6609 11100 6611 11104 6614 11106 6618 11103 6622 11098 6623
11094 6624 11089 6624 11085 6622 11081 6620 11077 6618 17 N F
11122 14312 11117 14308 11120 14308 11124 14307 11124 14304 11123 14301
11125 14303 11127 14306 11129 14308 11132 14310 11135 14311 11137 14314
11137 14317 11140 14316 11143 14317 11141 14320 11140 14323 11137 14322
11134 14321 11131 14320 11128 14319 11127 14315 11124 14314 11122 14312
24 N F
11156 14290 11152 14288 11155 14287 11158 14286 11161 14284 11162 14281
11165 14283 11163 14285 11165 14288 11162 14290 11159 14291 11156 14290
12 N F
11157 14263 11154 14259 11157 14257 11159 14255 11162 14256 11162 14257
11165 14258 11168 14258 11171 14260 11174 14262 11176 14264 11177 14267
11179 14270 11181 14271 11182 14272 11184 14274 11187 14275 11190 14277
11192 14279 11194 14282 11195 14285 11196 14285 11198 14287 11200 14290
11201 14293 11203 14296 11205 14298 11206 14301 11203 14300 11200 14300
11197 14299 11195 14297 11193 14294 11190 14292 11187 14293 11185 14291
11182 14289 11179 14287 11180 14284 11182 14281 11182 14278 11182 14275
11180 14277 11180 14280 11177 14282 11175 14284 11172 14283 11169 14282
11171 14279 11170 14276 11168 14274 11166 14271 11165 14269 11162 14266
11159 14265 11157 14263 56 N F
11158 6715 11156 6712 11158 6710 11161 6708 11164 6710 11164 6713
11162 6715 11158 6715 8 N F
11165 6723 11164 6718 11167 6719 11170 6721 11172 6724 11173 6727
11170 6729 11167 6726 11165 6723 9 N F
11171 6782 11168 6781 11170 6781 11171 6781 11175 6780 11178 6780
11181 6780 11185 6780 11187 6778 11190 6776 11193 6778 11196 6781
11192 6784 11189 6785 11188 6788 11187 6791 11184 6793 11181 6794

11177 6793 11174 6792 11173 6789 11176 6787 11174 6784 11171 6782
24 N F
11193 6735 11192 6732 11192 6728 11194 6725 11196 6722 11198 6720
11201 6717 11204 6718 11207 6720 11209 6722 11212 6724 11213 6727
11212 6731 11211 6734 11207 6734 11204 6734 11200 6735 11196 6737
11193 6735 19 N F
11228 6848 11225 6841 11230 6836 11236 6839 11237 6839 11242 6844
11250 6845 11255 6840 11259 6834 11263 6828 11263 6821 11259 6815
11253 6812 11249 6806 11254 6801 11261 6800 11268 6797 11273 6792
11279 6789 11286 6787 11293 6790 11298 6796 11299 6803 11306 6801
11312 6798 11319 6800 11322 6806 11323 6813 11322 6821 11322 6828
11323 6835 11323 6842 11319 6848 11322 6854 11319 6861 11319 6868
11323 6874 11328 6879 11332 6884 11333 6891 11338 6897 11342 6902
11345 6909 11342 6915 11346 6921 11351 6926 11356 6931 11358 6938
11357 6945 11357 6953 11350 6952 11343 6954 11343 6947 11341 6939
11338 6933 11333 6928 11331 6921 11336 6916 11333 6910 11331 6903
11329 6896 11322 6898 11316 6895 11312 6889 11306 6886 11305 6879
11302 6873 11304 6866 11299 6861 11292 6861 11285 6860 11279 6856
11272 6857 11265 6855 11262 6862 11259 6868 11252 6870 11252 6863
11249 6856 11246 6850 11240 6855 11233 6854 11228 6848 83 N F
11234 6783 11233 6778 11235 6775 11237 6773 11241 6773 11243 6775
11246 6778 11246 6781 11245 6784 11243 6787 11240 6789 11236 6789
11235 6786 11234 6783 14 N F
11279 12280 11273 12280 11277 12277 11281 12277 11285 12275 11288 12272
11290 12268 11293 12265 11297 12266 11301 12267 11303 12271 11299 12273
11296 12276 11293 12280 11291 12284 11292 12288 11288 12288 11286 12292
11282 12292 11279 12288 11279 12284 11279 12280 22 N F
11290 12618 11288 12614 11288 12609 11291 12606 11293 12610 11295 12614
11299 12614 11301 12618 11302 12622 11304 12626 11302 12631 11298 12633
11297 12628 11295 12624 11294 12620 11290 12618 16 N F
11349 12481 11345 12480 11347 12478 11348 12476 11349 12475 11350 12478
11349 12481 7 N F
11348 12489 11345 12484 11348 12484 11349 12485 11351 12486 11353 12488
11352 12492 11349 12492 11348 12489 9 N F
11353 12464 11348 12467 11350 12464 11352 12461 11353 12458 11355 12455
11358 12457 11360 12456 11363 12458 11362 12461 11359 12462 11356 12464
11354 12461 11353 12464 14 N F
11356 14267 11355 14263 11357 14262 11359 14259 11358 14255 11359 14252
11362 14251 11365 14253 11366 14256 11369 14258 11370 14261 11372 14264
11372 14267 11369 14268 11365 14267 11362 14266 11359 14266 11356 14267
18 N F
11372 12621 11371 12617 11373 12615 11376 12616 11378 12619 11379 12622
11375 12622 11372 12621 8 N F

11383 12628 11379 12623 11382 12623 11383 12626 11386 12628 11383 12628
6 N F
11386 12606 11382 12602 11385 12599 11387 12595 11391 12592 11395 12593
11394 12597 11394 12601 11398 12603 11401 12607 11399 12611 11404 12612
11407 12615 11409 12619 11408 12624 11411 12627 11415 12628 11414 12632
11410 12631 11407 12627 11403 12627 11402 12631 11405 12633 11401 12634
11397 12633 11393 12632 11390 12628 11391 12624 11388 12621 11387 12617
11386 12612 11383 12609 11386 12606 33 N F
11386 14338 11383 14335 11386 14334 11386 14334 11389 14334 11391 14337
11390 14340 11389 14344 11387 14342 11386 14338 10 N F
11400 12664 11396 12664 11398 12662 11401 12661 11402 12661 11400 12664
6 N F
11405 12659 11402 12658 11402 12654 11405 12653 11406 12656 11405 12659
6 N F
11410 12653 11408 12650 11410 12647 11413 12646 11414 12643 11416 12645
11416 12648 11417 12651 11420 12651 11421 12654 11417 12654 11415 12657
11412 12658 11413 12655 11410 12653 15 N F
11415 12607 11414 12604 11417 12603 11420 12604 11422 12607 11425 12609
11425 12612 11422 12613 11419 12612 11417 12609 11415 12607 11 N F
11441 12370 11438 12370 11441 12367 11442 12365 11445 12367 11448 12365
11450 12368 11447 12368 11445 12371 11441 12370 10 N F
11443 14328 11439 14325 11441 14323 11444 14324 11445 14324 11447 14324
11450 14325 11453 14327 11454 14330 11456 14333 11453 14332 11450 14330
11448 14328 11445 14329 11443 14328 15 N F
11502 13955 11500 13951 11502 13948 11504 13946 11505 13949 11505 13952
11505 13955 11508 13954 11512 13955 11510 13958 11507 13959 11504 13960
11502 13958 11502 13955 14 N F
11506 14011 11504 14008 11507 14007 11510 14009 11512 14011 11516 14011
11514 14014 11517 14015 11518 14018 11515 14019 11513 14016 11510 14015
11509 14012 11506 14011 14 N F
11520 13933 11518 13930 11520 13927 11523 13925 11525 13923 11527 13921
11530 13922 11533 13924 11533 13924 11535 13926 11532 13926 11529 13926
11525 13927 11526 13930 11523 13932 11520 13933 16 N F
11530 7055 11527 7052 11530 7051 11534 7050 11537 7048 11539 7047
11540 7047 11541 7050 11539 7053 11536 7055 11533 7056 11530 7055
12 N F
11531 7003 11528 7000 11528 6993 11530 6989 11533 6986 11538 6985
11542 6985 11547 6985 11551 6987 11554 6991 11557 6994 11558 6999
11556 7003 11552 7005 11548 7007 11544 7007 11539 7008 11535 7006
11531 7003 19 N F
11549 14293 11546 14289 11547 14287 11550 14287 11553 14287 11555 14288
11554 14290 11551 14291 11549 14293 9 N F
11553 13969 11551 13966 11552 13966 11554 13967 11557 13965 11554 13965

11553 13962 11556 13960 11559 13961 11561 13963 11563 13966 11564 13969
11562 13972 11560 13974 11557 13973 11554 13973 11553 13969 17 N F
11554 13496 11552 13498 11553 13495 11554 13493 11556 13491 11558 13490
11558 13492 11559 13495 11558 13497 11556 13495 11554 13496 11 N F
11557 14039 11555 14035 11559 14038 11560 14042 11562 14039 11562 14038
11566 14036 11567 14032 11571 14031 11576 14032 11580 14034 11581 14038
11582 14038 11585 14040 11588 14043 11585 14047 11581 14048 11577 14050
11574 14052 11569 14051 11568 14055 11564 14058 11563 14062 11560 14065
11560 14060 11558 14056 11557 14052 11556 14048 11557 14044 11557 14039
30 N F
11559 14016 11557 14012 11559 14009 11560 14008 11561 14006 11564 14006
11567 14006 11568 14006 11570 14008 11568 14011 11570 14014 11567 14014
11565 14016 11562 14018 11559 14016 15 N F
11563 14287 11560 14284 11563 14282 11565 14280 11568 14281 11571 14282
11574 14280 11576 14278 11579 14276 11582 14276 11583 14279 11584 14282
11582 14283 11580 14286 11578 14289 11577 14292 11574 14291 11573 14289
11570 14287 11567 14287 11563 14287 21 N F
11567 13436 11563 13435 11565 13433 11566 13430 11567 13428 11570 13427
11572 13428 11572 13431 11574 13431 11575 13428 11577 13430 11577 13433
11575 13434 11572 13435 11569 13436 11567 13436 16 N F
11572 13738 11569 13735 11571 13733 11573 13734 11575 13736 11577 13736
11580 13736 11583 13737 11583 13738 11583 13740 11585 13742 11584 13744
11584 13747 11582 13748 11582 13745 11581 13743 11579 13741 11576 13741
11574 13740 11572 13738 20 N F
11577 12606 11571 12607 11575 12606 11577 12602 11582 12603 11579 12599
11575 12597 11578 12594 11582 12595 11586 12598 11590 12597 11593 12600
11597 12600 11598 12605 11596 12609 11595 12613 11591 12615 11589 12611
11585 12609 11581 12606 11577 12606 21 N F
11580 14343 11576 14337 11577 14337 11580 14335 11583 14333 11587 14331
11588 14331 11591 14332 11593 14336 11597 14337 11601 14338 11603 14339
11605 14341 11609 14341 11612 14338 11616 14340 11620 14343 11624 14345
11626 14349 11628 14353 11629 14356 11629 14357 11628 14361 11626 14364
11622 14362 11618 14360 11614 14360 11609 14360 11606 14362 11602 14365
11598 14366 11594 14365 11590 14365 11586 14362 11583 14359 11583 14355
11581 14351 11582 14347 11580 14343 39 N F
11587 13489 11583 13489 11584 13487 11586 13485 11589 13484 11589 13486
11587 13489 7 N F
11588 14314 11585 14311 11587 14311 11590 14310 11592 14310 11595 14311
11598 14311 11596 14313 11593 14313 11590 14313 11588 14314 11 N F
11591 14042 11587 14039 11588 14037 11588 14034 11591 14033 11591 14033
11594 14033 11596 14032 11597 14029 11597 14027 11600 14028 11601 14031
11602 14033 11600 14034 11598 14034 11597 14036 11595 14038 11593 14040
11591 14042 19 N F

11595 14276 11591 14275 11591 14274 11593 14272 11594 14269 11597 14267
11600 14265 11602 14264 11606 14264 11608 14262 11607 14265 11604 14267
11604 14270 11604 14273 11605 14277 11602 14276 11598 14276 11595 14276
18 N F
11603 12604 11600 12608 11601 12605 11601 12602 11603 12604 5 N F
11603 13495 11601 13491 11602 13491 11604 13491 11606 13492 11609 13493
11611 13494 11614 13495 11614 13497 11612 13499 11609 13499 11607 13499
11604 13498 11603 13495 14 N F
11603 14323 11601 14320 11601 14316 11604 14316 11605 14317 11606 14318
11605 14321 11603 14323 8 N F
11606 12606 11602 12608 11604 12605 11607 12603 11609 12601 11612 12599
11611 12602 11610 12605 11606 12606 9 N F
11604 12680 11603 12675 11606 12672 11609 12669 11612 12666 11616 12664
11620 12665 11624 12667 11628 12669 11626 12674 11631 12675 11628 12678
11627 12682 11623 12684 11622 12689 11626 12690 11630 12692 11635 12692
11638 12694 11639 12694 11640 12690 11644 12690 11648 12692 11652 12693
11651 12700 11647 12699 11649 12703 11651 12707 11652 12711 11650 12715
11646 12717 11642 12715 11638 12714 11637 12710 11635 12706 11631 12706
11630 12702 11626 12700 11622 12698 11618 12697 11613 12697 11610 12700
11606 12700 11606 12695 11603 12692 11605 12688 11604 12684 11604 12680
48 N F
11615 14309 11607 14309 11610 14306 11614 14304 11617 14301 11621 14299
11624 14298 11625 14298 11629 14295 11632 14293 11632 14293 11632 14293
11636 14290 11636 14286 11634 14282 11637 14279 11640 14276 11643 14279
11646 14282 11649 14285 11653 14288 11655 14292 11655 14296 11654 14300
11650 14303 11646 14304 11642 14305 11638 14306 11635 14308 11631 14311
11628 14314 11628 14318 11628 14322 11629 14327 11631 14330 11629 14334
11625 14333 11621 14330 11617 14329 11614 14326 11614 14322 11615 14318
11614 14313 11615 14309 44 N F
11618 14022 11613 14022 11615 14019 11618 14018 11621 14019 11624 14018
11626 14021 11624 14024 11621 14023 11618 14022 10 N F
11618 12389 11613 12389 11615 12387 11618 12385 11621 12384 11621 12387
11618 12389 7 N F
11618 12662 11617 12658 11617 12653 11617 12649 11618 12646 11619 12645
11621 12641 11625 12644 11627 12640 11630 12637 11634 12639 11637 12635
11641 12634 11641 12634 11645 12635 11648 12638 11648 12642 11652 12644
11655 12648 11656 12652 11659 12651 11660 12651 11661 12656 11663 12660
11655 12661 11651 12664 11648 12667 11644 12667 11642 12670 11640 12674
11638 12678 11635 12674 11634 12670 11631 12667 11634 12664 11632 12660
11631 12664 11627 12665 11622 12664 11618 12662 40 N F
11619 13002 11618 12999 11621 13001 11624 13003 11621 13005 11619 13002
6 N F
11627 13012 11623 13011 11626 13009 11627 13006 11630 13007 11633 13007

11636 13009 11638 13012 11637 13015 11635 13018 11632 13018 11630 13021
11628 13019 11624 13020 11624 13016 11628 13016 11630 13013 11627 13012
18 N F
11630 14019 11626 14017 11628 14015 11631 14015 11634 14014 11637 14013
11639 14010 11642 14009 11643 14012 11640 14013 11638 14016 11638 14019
11635 14020 11632 14022 11630 14019 15 N F
11637 14227 11632 14228 11634 14225 11636 14223 11639 14221 11640 14221
11641 14219 11643 14216 11642 14212 11645 14212 11649 14212 11652 14213
11655 14214 11658 14214 11660 14215 11661 14215 11664 14214 11666 14217
11666 14220 11663 14219 11660 14218 11657 14217 11653 14217 11650 14218
11648 14221 11646 14224 11643 14225 11640 14226 11637 14227 29 N F
11637 12754 11633 12754 11636 12752 11635 12748 11639 12747 11643 12747
11646 12749 11647 12750 11650 12747 11654 12750 11657 12753 11657 12758
11657 12753 11657 12749 11661 12748 11663 12744 11665 12742 11666 12741
11669 12744 11671 12746 11672 12747 11677 12749 11678 12752 11683 12753
11682 12757 11682 12762 11686 12760 11690 12761 11691 12766 11688 12769
11683 12770 11684 12774 11688 12777 11684 12779 11685 12784 11680 12784
11679 12788 11675 12791 11671 12791 11670 12787 11671 12783 11670 12779
11666 12777 11663 12780 11660 12777 11656 12775 11652 12777 11650 12773
11649 12769 11645 12762 11643 12758 11641 12755 11637 12754 53 N F
11635 12411 11634 12407 11637 12407 11640 12405 11640 12408 11642 12411
11644 12413 11646 12411 11645 12407 11648 12409 11647 12412 11646 12415
11643 12416 11640 12415 11637 12413 11635 12411 16 N F
11636 12624 11635 12621 11637 12618 11640 12616 11643 12618 11645 12616
11647 12614 11650 12615 11650 12619 11648 12622 11646 12623 11644 12626
11641 12627 11639 12625 11636 12624 15 N F
11639 13029 11636 13027 11639 13025 11641 13027 11639 13029 5 N F
11640 14071 11636 14066 11640 14064 11644 14062 11648 14061 11651 14061
11653 14060 11657 14060 11661 14060 11665 14063 11666 14067 11662 14069
11660 14065 11656 14066 11652 14068 11648 14068 11644 14069 11640 14071
18 N F
11643 14360 11637 14355 11640 14349 11642 14343 11642 14336 11642 14329
11643 14322 11642 14316 11645 14310 11652 14308 11658 14307 11660 14301
11665 14296 11668 14290 11670 14284 11667 14278 11661 14275 11654 14273
11656 14267 11656 14260 11662 14258 11669 14255 11675 14256 11679 14255
11682 14255 11686 14254 11689 14253 11695 14252 11695 14258 11696 14265
11699 14264 11702 14262 11706 14259 11707 14258 11714 14258 11719 14263
11721 14264 11724 14267 11725 14274 11723 14280 11721 14287 11723 14293
11717 14298 11717 14304 11717 14311 11715 14318 11712 14324 11712 14330
11715 14337 11712 14343 11708 14348 11707 14355 11702 14360 11695 14362
11690 14366 11684 14369 11679 14373 11674 14378 11667 14380 11661 14376
11654 14376 11653 14369 11649 14364 11643 14360 64 N F
11639 12878 11638 12873 11641 12871 11643 12868 11646 12867 11648 12869

11648 12872 11646 12875 11644 12878 11642 12880 11639 12878 11 N F
11644 12602 11642 12600 11643 12600 11645 12600 11645 12600 11648 12600
11649 12597 11648 12594 11648 12591 11652 12592 11652 12595 11652 12598
11651 12601 11653 12604 11654 12604 11650 12604 11647 12603 11644 12602
18 N F
11644 12479 11643 12475 11647 12476 11648 12479 11649 12482 11645 12482
11644 12479 7 N F
11651 14019 11646 14018 11648 14015 11650 14014 11654 14013 11657 14012
11660 14012 11659 14015 11657 14017 11654 14018 11651 14019 11 N F
11649 13191 11647 13187 11650 13186 11651 13189 11651 13192 11649 13191
6 N F
11650 12407 11647 12403 11649 12400 11652 12400 11655 12401 11658 12403
11658 12404 11660 12405 11661 12408 11658 12410 11655 12407 11653 12409
11650 12407 13 N F
11652 12466 11650 12462 11653 12463 11656 12463 11656 12466 11653 12468
11652 12466 7 N F
11658 12863 11652 12860 11656 12860 11660 12860 11661 12856 11664 12859
11667 12862 11668 12858 11671 12857 11673 12857 11677 12856 11681 12853
11682 12857 11686 12860 11684 12864 11687 12867 11689 12871 11685 12873
11681 12874 11681 12879 11681 12883 11685 12885 11686 12889 11683 12893
11686 12896 11685 12901 11689 12902 11694 12903 11697 12906 11701 12908
11697 12910 11694 12907 11690 12907 11686 12906 11681 12906 11681 12901
11678 12898 11675 12895 11673 12891 11671 12887 11667 12885 11663 12884
11658 12883 11656 12879 11655 12875 11653 12871 11657 12869 11660 12867
11658 12863 49 N F
11653 13180 11653 13177 11655 13176 11658 13177 11658 13175 11661 13176
11660 13178 11658 13180 11656 13181 11653 13180 10 N F
11656 12614 11654 12611 11657 12609 11659 12611 11659 12611 11659 12608
11662 12610 11665 12611 11665 12615 11664 12618 11662 12620 11659 12622
11656 12621 11656 12618 11659 12615 11656 12614 16 N F
11657 12447 11655 12443 11658 12443 11658 12443 11661 12445 11660 12448
11657 12447 7 N F
11656 12482 11655 12475 11659 12475 11660 12477 11661 12478 11661 12482
11659 12484 11656 12482 8 N F
11658 12364 11656 12361 11658 12358 11659 12355 11660 12352 11662 12355
11660 12358 11659 12361 11658 12364 9 N F
11658 14181 11658 14178 11660 14176 11663 14175 11664 14172 11665 14169
11667 14166 11670 14164 11673 14166 11674 14169 11677 14170 11679 14168
11679 14164 11681 14166 11682 14166 11684 14165 11686 14162 11688 14159
11689 14162 11692 14164 11692 14168 11692 14171 11690 14174 11687 14175
11685 14177 11683 14179 11679 14180 11676 14181 11676 14177 11673 14176
11670 14177 11667 14179 11666 14182 11663 14184 11660 14184 11658 14181
36 N F

11665 12686 11659 12685 11660 12682 11660 12679 11662 12676 11664 12673
11667 12673 11668 12676 11667 12679 11665 12682 11665 12686 11 N F
11666 12451 11661 12450 11662 12447 11664 12444 11666 12444 11667 12444
11667 12448 11666 12451 8 N F
11666 12443 11662 12441 11664 12438 11667 12438 11670 12438 11669 12441
11666 12443 7 N F
11665 12624 11664 12621 11666 12618 11668 12615 11667 12612 11666 12609
11666 12608 11667 12606 11670 12605 11673 12603 11671 12601 11669 12598
11669 12595 11671 12592 11671 12589 11673 12586 11676 12587 11677 12590
11678 12593 11681 12591 11684 12591 11687 12590 11690 12592 11690 12595
11691 12599 11690 12602 11687 12603 11686 12606 11686 12609 11683 12609
11680 12608 11678 12611 11676 12614 11678 12617 11678 12620 11678 12623
11675 12624 11672 12625 11668 12625 11665 12624 40 N F
11671 12732 11668 12730 11669 12727 11671 12724 11674 12724 11676 12722
11678 12722 11679 12723 11679 12726 11677 12728 11674 12729 11671 12732
12 N F
11670 12428 11669 12425 11670 12422 11673 12422 11675 12420 11676 12417
11680 12416 11680 12420 11682 12422 11682 12426 11679 12423 11678 12426
11676 12428 11673 12429 11670 12428 15 N F
11673 12696 11670 12692 11672 12693 11673 12693 11676 12694 11678 12697
11678 12700 11675 12699 11673 12696 9 N F
11675 12464 11672 12463 11673 12459 11673 12456 11676 12455 11679 12454
11681 12454 11683 12454 11686 12455 11687 12458 11690 12458 11693 12459
11693 12459 11696 12457 11699 12457 11701 12455 11703 12458 11701 12460
11698 12461 11695 12463 11692 12464 11689 12466 11686 12464 11683 12465
11682 12462 11679 12461 11675 12464 27 N F
11677 14221 11674 14217 11676 14215 11677 14218 11679 14220 11679 14223
11677 14221 7 N F
11681 12746 11674 12744 11677 12740 11679 12737 11682 12734 11683 12730
11683 12729 11687 12727 11691 12725 11693 12721 11692 12717 11696 12717
11700 12719 11703 12722 11705 12727 11702 12730 11701 12735 11703 12739
11707 12737 11711 12735 11711 12740 11715 12742 11715 12746 11711 12749
11708 12751 11712 12753 11715 12754 11716 12754 11716 12758 11712 12758
11712 12762 11709 12765 11706 12762 11702 12764 11698 12765 11694 12762
11696 12758 11698 12754 11701 12751 11699 12747 11694 12747 11690 12749
11687 12752 11683 12750 11681 12746 45 N F
11679 12585 11675 12582 11679 12580 11682 12584 11686 12583 11690 12583
11689 12579 11685 12577 11683 12574 11679 12575 11676 12570 11680 12570
11685 12569 11689 12570 11692 12571 11693 12571 11696 12575 11700 12576
11703 12579 11702 12583 11706 12584 11708 12588 11706 12592 11708 12596
11709 12600 11712 12603 11709 12606 11705 12608 11707 12612 11703 12614
11699 12615 11695 12617 11695 12621 11691 12624 11687 12623 11682 12624
11682 12619 11685 12616 11689 12615 11691 12611 11694 12607 11694 12603

11698 12600 11702 12599 11698 12598 11696 12594 11697 12589 11694 12586
11690 12586 11686 12588 11682 12588 11679 12585 52 N F
11681 12451 11676 12451 11677 12448 11679 12446 11682 12448 11685 12448
11685 12451 11681 12451 8 N F
11680 12644 11677 12642 11678 12638 11679 12634 11680 12631 11681 12634
11681 12638 11680 12641 11680 12644 9 N F
11680 13540 11678 13540 11679 13537 11681 13535 11682 13537 11684 13539
11684 13542 11686 13544 11683 13545 11681 13545 11680 13542 11680 13540
12 N F
11683 12722 11679 12721 11680 12720 11681 12718 11683 12715 11686 12716
11684 12719 11683 12722 8 N F
11681 14146 11680 14142 11683 14141 11685 14139 11685 14135 11685 14132
11687 14130 11690 14128 11693 14129 11693 14130 11697 14130 11697 14134
11697 14137 11699 14139 11697 14141 11694 14142 11692 14145 11690 14147
11687 14149 11685 14152 11682 14152 11682 14149 11681 14146 23 N F
11683 12475 11680 12472 11683 12471 11681 12468 11683 12466 11686 12466
11686 12469 11685 12473 11683 12475 9 N F
11687 12701 11680 12699 11684 12697 11688 12695 11688 12693 11690 12691
11692 12687 11693 12687 11696 12687 11700 12685 11699 12681 11699 12676
11704 12676 11704 12676 11707 12674 11710 12677 11713 12680 11717 12681
11718 12677 11722 12674 11721 12670 11725 12668 11727 12668 11729 12668
11734 12668 11735 12672 11735 12676 11735 12681 11733 12684 11730 12688
11733 12691 11729 12693 11727 12697 11723 12699 11719 12696 11715 12696
11711 12699 11709 12703 11705 12704 11701 12701 11697 12703 11693 12704
11691 12700 11687 12701 44 N F
11682 14219 11681 14214 11684 14213 11686 14216 11686 14219 11686 14223
11688 14225 11686 14227 11683 14226 11683 14222 11682 14219 11 N F
11684 12990 11681 12985 11683 12982 11685 12981 11686 12980 11686 12977
11690 12978 11692 12980 11691 12984 11691 12987 11689 12990 11686 12991
11684 12990 13 N F
11685 12542 11681 12541 11683 12540 11684 12539 11686 12537 11685 12533
11687 12530 11689 12532 11690 12532 11692 12535 11692 12538 11695 12539
11698 12542 11698 12545 11695 12543 11694 12546 11697 12548 11694 12549
11692 12552 11689 12551 11687 12548 11686 12545 11685 12542 23 N F
11685 12939 11682 12938 11682 12934 11682 12931 11685 12933 11686 12936
11685 12939 7 N F
11689 12832 11682 12828 11686 12825 11690 12827 11694 12828 11699 12828
11703 12826 11707 12828 11706 12824 11702 12825 11701 12821 11702 12816
11702 12812 11701 12808 11699 12804 11698 12800 11702 12802 11706 12803
11707 12807 11705 12811 11709 12812 11710 12812 11713 12815 11717 12813
11716 12817 11721 12818 11720 12823 11720 12827 11717 12830 11713 12831
11711 12835 11710 12839 11707 12842 11703 12840 11699 12842 11695 12843
11695 12838 11691 12836 11689 12832 39 N F

11683 13530 11683 13527 11684 13525 11686 13524 11686 13524 11687 13527
11688 13530 11688 13532 11685 13532 11683 13530 10 N F
11687 12655 11685 12650 11686 12648 11689 12649 11690 12652 11691 12654
11692 12655 11693 12658 11689 12657 11687 12655 10 N F
11687 12444 11685 12438 11689 12440 11689 12435 11693 12434 11697 12434
11702 12434 11703 12432 11704 12430 11704 12435 11706 12438 11707 12434
11710 12430 11712 12434 11716 12435 11720 12433 11719 12438 11718 12442
11717 12446 11717 12451 11713 12450 11709 12449 11704 12447 11700 12448
11697 12451 11693 12453 11691 12449 11687 12450 11690 12447 11687 12444
30 N F
11687 12687 11685 12682 11688 12681 11690 12684 11691 12687 11688 12690
11687 12687 7 N F
11689 12941 11686 12939 11690 12939 11693 12939 11695 12942 11697 12944
11694 12945 11692 12942 11689 12941 9 N F
11688 14216 11687 14212 11690 14210 11692 14212 11696 14213 11699 14212
11701 14210 11704 14210 11704 14213 11704 14216 11705 14219 11704 14222
11702 14225 11699 14227 11696 14225 11696 14222 11693 14220 11690 14219
11688 14216 19 N F
11695 12714 11687 12712 11691 12711 11693 12710 11695 12710 11699 12707
11703 12709 11707 12709 11710 12706 11713 12703 11715 12699 11719 12701
11723 12702 11725 12706 11725 12711 11723 12715 11726 12717 11724 12721
11719 12721 11719 12726 11715 12728 11713 12724 11716 12720 11711 12720
11707 12718 11704 12716 11699 12715 11695 12714 28 N F
11690 13991 11688 13986 11691 13984 11694 13983 11695 13985 11695 13989
11693 13991 11690 13991 8 N F
11692 12410 11692 12405 11695 12405 11697 12403 11700 12405 11703 12405
11706 12404 11708 12407 11709 12410 11712 12410 11710 12413 11709 12416
11706 12418 11703 12419 11703 12416 11704 12413 11702 12411 11698 12410
11695 12409 11692 12410 20 N F
11698 14201 11693 14200 11696 14197 11700 14196 11701 14192 11701 14187
11705 14186 11704 14191 11707 14195 11709 14198 11706 14202 11702 14201
11698 14201 13 N F
11699 12859 11693 12855 11695 12855 11697 12855 11698 12851 11702 12849
11706 12851 11709 12848 11711 12844 11713 12848 11713 12853 11714 12849
11716 12844 11719 12848 11719 12852 11718 12856 11716 12860 11711 12861
11707 12860 11703 12861 11699 12859 21 N F
11701 12671 11698 12666 11700 12664 11703 12664 11705 12666 11706 12669
11704 12671 11701 12671 8 N F
11703 14079 11701 14073 11703 14069 11704 14065 11705 14061 11706 14057
11710 14059 11711 14060 11713 14062 11715 14058 11714 14054 11718 14056
11721 14060 11723 14063 11725 14067 11727 14071 11731 14072 11730 14076
11729 14081 11728 14085 11724 14086 11720 14086 11716 14086 11711 14086
11711 14081 11707 14079 11703 14079 27 N F

11705 12884 11703 12879 11708 12878 11711 12875 11709 12871 11711 12870
11714 12870 11715 12866 11719 12865 11724 12866 11727 12863 11731 12864
11735 12865 11738 12863 11738 12862 11742 12864 11744 12860 11740 12858
11740 12854 11744 12853 11745 12853 11746 12849 11748 12847 11749 12846
11749 12850 11753 12848 11755 12852 11758 12855 11754 12858 11750 12860
11751 12864 11750 12868 11746 12871 11743 12867 11739 12868 11737 12872
11733 12874 11732 12878 11735 12881 11739 12879 11742 12883 11741 12887
11737 12887 11734 12883 11733 12888 11730 12891 11727 12888 11725 12884
11721 12884 11717 12887 11716 12891 11712 12889 11708 12888 11705 12884
54 N F
11711 13966 11707 13963 11709 13961 11710 13957 11710 13954 11713 13953
11716 13954 11719 13955 11722 13957 11721 13960 11720 13963 11717 13965
11715 13963 11712 13963 11711 13966 15 N F
11710 14182 11709 14177 11711 14175 11714 14174 11716 14177 11716 14181
11713 14183 11710 14182 8 N F
11711 12639 11710 12635 11715 12635 11717 12631 11716 12627 11717 12623
11716 12619 11720 12619 11723 12621 11728 12622 11729 12626 11734 12627
11737 12625 11741 12627 11744 12631 11748 12631 11750 12635 11746 12636
11741 12637 11742 12641 11740 12645 11737 12648 11734 12645 11731 12641
11727 12641 11730 12645 11731 12649 11727 12652 11723 12650 11721 12646
11717 12645 11715 12641 11711 12639 33 N F
11713 12517 11711 12510 11715 12504 11720 12500 11723 12494 11729 12490
11728 12497 11726 12503 11728 12509 11730 12503 11735 12498 11742 12497
11738 12491 11735 12485 11737 12479 11741 12484 11745 12479 11752 12478
11759 12478 11763 12481 11765 12482 11771 12485 11766 12489 11773 12488
11774 12489 11779 12490 11783 12496 11790 12497 11790 12504 11796 12507
11803 12507 11804 12514 11809 12514 11811 12514 11812 12521 11817 12517
11819 12510 11824 12514 11822 12521 11826 12523 11828 12524 11830 12531
11834 12537 11831 12543 11835 12548 11830 12553 11834 12558 11835 12565
11836 12572 11831 12576 11827 12581 11821 12585 11816 12590 11810 12587
11808 12580 11803 12584 11800 12591 11802 12597 11798 12603 11792 12606
11785 12608 11779 12609 11772 12610 11770 12603 11773 12597 11780 12596
11787 12598 11784 12592 11786 12585 11783 12580 11778 12575 11775 12568
11769 12570 11768 12577 11766 12583 11762 12577 11765 12571 11759 12568
11762 12562 11763 12555 11767 12550 11774 12552 11771 12546 11764 12548
11768 12542 11766 12535 11765 12528 11764 12522 11760 12516 11761 12523
11759 12529 11760 12536 11763 12542 11759 12548 11759 12555 11753 12557
11752 12563 11746 12560 11745 12553 11748 12547 11746 12541 11741 12546
11734 12545 11730 12539 11724 12537 11722 12530 11727 12525 11721 12528
11715 12523 11713 12517 110 N F
11713 14197 11711 14192 11713 14188 11713 14187 11716 14185 11721 14186
11723 14190 11724 14190 11727 14190 11730 14193 11734 14195 11738 14194
11742 14195 11746 14197 11747 14201 11750 14205 11750 14209 11747 14212

11745 14216 11741 14218 11737 14219 11732 14219 11728 14220 11724 14222
11721 14224 11717 14222 11715 14218 11715 14214 11715 14209 11713 14205
11714 14201 11713 14197 32 N F
11715 13568 11715 13566 11715 13562 11716 13562 11717 13562 11720 13564
11722 13562 11724 13564 11726 13566 11726 13568 11724 13570 11723 13572
11724 13575 11722 13577 11720 13578 11719 13575 11719 13572 11717 13570
11715 13568 19 N F
11722 13156 11716 13154 11719 13150 11721 13147 11725 13149 11729 13151
11732 13154 11734 13158 11737 13161 11741 13163 11743 13167 11744 13171
11742 13174 11739 13177 11739 13173 11735 13170 11731 13170 11727 13172
11730 13168 11725 13168 11726 13163 11722 13161 11722 13156 23 N F
11730 12662 11728 12657 11732 12656 11735 12656 11738 12657 11739 12660
11741 12657 11744 12660 11745 12663 11742 12665 11744 12667 11746 12670
11743 12671 11740 12670 11737 12668 11736 12665 11736 12662 11733 12663
11730 12662 19 N F
11731 12818 11730 12815 11732 12812 11733 12809 11736 12808 11738 12810
11737 12814 11737 12817 11737 12820 11738 12823 11735 12822 11731 12821
11731 12818 13 N F
11732 13593 11731 13588 11732 13585 11735 13584 11737 13586 11739 13586
11740 13586 11742 13585 11745 13585 11746 13585 11747 13584 11750 13585
11753 13584 11756 13584 11758 13582 11759 13582 11760 13582 11763 13582
11765 13584 11765 13586 11762 13586 11759 13585 11757 13587 11755 13588
11756 13590 11755 13593 11753 13595 11752 13597 11749 13597 11747 13597
11745 13595 11744 13593 11745 13591 11742 13590 11740 13592 11737 13593
11735 13593 11732 13593 38 N F
11746 13304 11741 13302 11744 13301 11748 13301 11746 13304 5 N F
11742 12795 11742 12789 11743 12788 11745 12786 11749 12785 11752 12788
11756 12788 11756 12793 11757 12797 11753 12799 11748 12799 11744 12799
11742 12795 13 N F
11752 14090 11747 14091 11750 14088 11753 14085 11756 14081 11757 14077
11759 14073 11759 14069 11760 14064 11756 14063 11752 14060 11754 14056
11758 14057 11756 14053 11754 14049 11751 14046 11749 14041 11752 14044
11755 14047 11759 14046 11761 14042 11765 14044 11765 14049 11767 14053
11768 14057 11772 14056 11776 14056 11776 14060 11780 14063 11784 14064
11785 14068 11784 14072 11782 14076 11781 14080 11777 14081 11773 14081
11769 14082 11766 14086 11761 14084 11761 14089 11763 14092 11763 14097
11761 14093 11759 14089 11755 14087 11752 14090 46 N F
11751 12838 11748 12839 11750 12834 11752 12832 11752 12828 11752 12825
11750 12822 11753 12821 11756 12822 11759 12822 11762 12824 11761 12827
11761 12830 11758 12833 11756 12835 11754 12838 11751 12838 17 N F
11749 12756 11749 12751 11753 12751 11757 12750 11762 12748 11766 12748
11768 12752 11770 12756 11774 12757 11778 12756 11783 12757 11781 12761
11786 12760 11788 12756 11792 12756 11796 12756 11798 12752 11802 12750

11800 12747 11797 12744 11799 12740 11803 12739 11805 12743 11809 12741
11812 12745 11811 12749 11813 12753 11813 12757 11812 12762 11808 12763
11805 12766 11801 12765 11796 12765 11792 12764 11788 12765 11790 12769
11786 12771 11790 12773 11791 12777 11795 12776 11796 12780 11800 12780
11802 12784 11798 12785 11795 12788 11791 12789 11787 12788 11783 12789
11781 12793 11776 12793 11772 12792 11769 12788 11772 12785 11776 12782
11779 12780 11780 12776 11777 12773 11777 12768 11773 12769 11773 12765
11770 12762 11767 12766 11765 12762 11764 12758 11759 12758 11755 12758
11751 12760 11749 12756 68 N F
11754 13302 11750 13301 11753 13300 11757 13301 11758 13301 11759 13302
11762 13304 11759 13306 11756 13305 11754 13302 10 N F
11758 13285 11756 13281 11758 13279 11760 13276 11763 13278 11762 13281
11760 13284 11758 13285 8 N F
11759 12421 11757 12417 11760 12414 11763 12415 11761 12417 11762 12420
11763 12423 11764 12426 11763 12429 11760 12427 11759 12424 11759 12421
12 N F
11764 12819 11758 12817 11761 12814 11764 12810 11766 12807 11768 12803
11772 12801 11776 12802 11775 12806 11773 12810 11771 12814 11767 12817
11764 12819 13 N F
11762 7120 11761 7112 11763 7108 11766 7105 11770 7103 11775 7101
11779 7102 11782 7105 11787 7107 11791 7105 11795 7104 11800 7105
11804 7107 11806 7111 11805 7116 11803 7120 11801 7124 11799 7128
11795 7131 11791 7132 11787 7133 11783 7136 11778 7137 11774 7138
11770 7135 11770 7130 11771 7126 11770 7121 11766 7119 11762 7120
30 N F
11769 13287 11765 13286 11768 13285 11768 13282 11771 13283 11771 13280
11774 13281 11777 13281 11778 13284 11776 13286 11779 13288 11778 13291
11776 13294 11773 13295 11770 13294 11770 13291 11773 13290 11775 13288
11774 13285 11771 13284 11769 13287 21 N F
11770 13435 11767 13433 11769 13430 11771 13429 11774 13429 11777 13428
11776 13431 11774 13433 11772 13435 11770 13435 10 N F
11775 13310 11771 13308 11773 13308 11774 13308 11777 13307 11780 13307
11778 13310 11775 13310 8 N F
11779 13280 11778 13277 11780 13275 11784 13275 11786 13277 11789 13279
11789 13282 11787 13285 11786 13282 11785 13279 11781 13278 11783 13281
11780 13283 11779 13280 14 N F
11791 13297 11788 13294 11790 13292 11792 13289 11792 13285 11795 13285
11795 13288 11797 13289 11798 13289 11800 13287 11803 13286 11806 13287
11809 13289 11806 13291 11803 13293 11800 13294 11797 13294 11795 13296
11791 13297 19 N F
11797 13251 11793 13248 11796 13246 11797 13243 11799 13241 11800 13243
11801 13247 11799 13250 11797 13251 9 N F
11801 12498 11799 12493 11802 12491 11805 12490 11804 12494 11807 12493

11810 12495 11813 12497 11813 12500 11810 12502 11808 12501 11810 12503
11809 12506 11806 12505 11803 12503 11800 12502 11801 12498 17 N F
11812 11928 11806 11924 11806 11918 11808 11911 11807 11904 11807 11895
11811 11890 11815 11885 11819 11879 11825 11875 11826 11875 11831 11872
11838 11871 11844 11867 11848 11862 11853 11857 11858 11852 11864 11849
11870 11849 11871 11856 11872 11863 11876 11869 11880 11874 11886 11877
11893 11875 11900 11875 11907 11875 11913 11877 11919 11881 11926 11882
11932 11878 11939 11880 11945 11882 11950 11877 11957 11876 11963 11880
11970 11880 11974 11885 11980 11886 11987 11887 11994 11887 12001 11890
12007 11887 12013 11889 12018 11894 12023 11898 12027 11904 12033 11907
12040 11909 12047 11909 12054 11911 12060 11914 12066 11916 12073 11917
12077 11923 12082 11927 12086 11933 12093 11933 12100 11934 12107 11936
12113 11939 12117 11944 12116 11951 12121 11956 12124 11961 12126 11968
12127 11975 12130 11981 12136 11985 12141 11990 12141 11997 12142 12003
12143 12010 12148 12006 12147 12012 12145 12019 12149 12025 12155 12024
12151 12029 12146 12024 12145 12031 12138 12034 12134 12039 12138 12045
12136 12052 12132 12058 12126 12061 12119 12062 12113 12059 12107 12062
12101 12065 12096 12069 12092 12075 12085 12074 12078 12075 12072 12075
12065 12074 12059 12071 12052 12071 12045 12069 12039 12066 12033 12064
12026 12063 12020 12059 12013 12061 12007 12057 12001 12054 11995 12051
11989 12049 11982 12046 11976 12045 11970 12041 11963 12038 11958 12034
11951 12036 11945 12038 11941 12032 11934 12031 11929 12026 11923 12022
11916 12021 11911 12017 11905 12013 11898 12016 11893 12011 11887 12009
11881 12005 11875 12001 11868 12001 11865 11995 11861 11989 11855 11987
11850 11982 11845 11977 11845 11970 11841 11964 11837 11959 11832 11954
11826 11950 11823 11944 11818 11939 11812 11935 11812 11928 143 N F
11809 13307 11806 13306 11808 13304 11811 13305 11812 13307 11809 13307
6 N F
11819 13366 11816 13363 11819 13362 11822 13361 11823 13363 11822 13366
11819 13366 7 N F
11821 13416 11818 13413 11821 13412 11822 13412 11824 13411 11826 13410
11827 13413 11827 13416 11826 13418 11823 13418 11821 13416 11 N F
11826 7169 11819 7168 11823 7162 11827 7156 11830 7150 11834 7144
11839 7138 11841 7132 11835 7129 11834 7122 11836 7115 11834 7108
11827 7105 11825 7098 11831 7095 11836 7090 11840 7084 11836 7078
11825 7078 11828 7072 11833 7067 11839 7063 11846 7061 11847 7068
11849 7061 11855 7057 11862 7059 11868 7062 11875 7063 11882 7066
11889 7069 11896 7069 11903 7071 11910 7071 11917 7071 11924 7073
11931 7074 11936 7069 11932 7075 11927 7080 11923 7086 11918 7091
11911 7091 11904 7093 11897 7096 11894 7102 11887 7105 11880 7103
11873 7106 11866 7107 11863 7114 11865 7121 11859 7125 11853 7128
11849 7134 11847 7141 11844 7147 11838 7152 11833 7157 11829 7162
11826 7169 61 N F

11826 12273 11824 12269 11828 12267 11832 12264 11833 12268 11830 12272
11826 12273 7 N F
11830 13377 11827 13375 11830 13374 11832 13373 11834 13371 11837 13372
11839 13373 11841 13375 11838 13376 11836 13377 11833 13377 11830 13377
12 N F
11839 12267 11834 12263 11838 12262 11841 12258 11842 12254 11847 12254
11845 12258 11846 12262 11850 12263 11851 12268 11847 12269 11844 12266
11839 12267 13 N F
11846 13371 11842 13369 11845 13369 11848 13368 11849 13366 11852 13366
11849 13367 11849 13370 11846 13371 9 N F
11871 13286 11869 13280 11871 13278 11875 13278 11877 13280 11878 13283
11879 13286 11877 13289 11873 13288 11871 13286 10 N F
11905 12401 11903 12398 11906 12397 11908 12400 11910 12403 11913 12403
11914 12406 11916 12409 11913 12411 11911 12413 11909 12410 11908 12407
11907 12404 11905 12401 14 N F
11916 12392 11913 12389 11915 12385 11913 12378 11916 12374 11918 12370
11922 12369 11927 12369 11930 12371 11934 12373 11938 12376 11941 12379
11942 12383 11946 12384 11947 12388 11951 12390 11951 12394 11955 12392
11955 12392 11957 12388 11958 12392 11961 12390 11966 12391 11966 12395
11961 12395 11957 12396 11953 12398 11949 12398 11944 12396 11940 12397
11936 12396 11934 12400 11930 12402 11926 12401 11923 12398 11919 12395
11916 12392 37 N F
11933 12412 11930 12408 11930 12408 11934 12407 11937 12404 11940 12401
11944 12400 11949 12400 11953 12400 11949 12402 11948 12407 11952 12409
11954 12405 11958 12405 11961 12409 11958 12412 11961 12415 11965 12417
11962 12421 11959 12424 11956 12427 11952 12429 11948 12428 11944 12429
11940 12427 11937 12430 11933 12433 11934 12428 11937 12425 11933 12423
11932 12419 11936 12420 11934 12417 11933 12412 34 N F
11949 12439 11949 12433 11952 12433 11954 12435 11957 12433 11959 12436
11962 12436 11962 12439 11960 12442 11957 12443 11954 12443 11952 12441
11949 12439 13 N F
11955 12368 11952 12367 11954 12364 11952 12359 11955 12360 11958 12360
11962 12359 11961 12362 11959 12364 11958 12367 11955 12368 11 N F
11963 12520 11961 12515 11962 12511 11962 12507 11963 12503 11965 12498
11964 12494 11967 12491 11966 12487 11970 12489 11974 12486 11977 12484
11978 12484 11981 12482 11984 12485 11988 12487 11991 12484 11994 12481
11991 12478 11987 12478 11987 12474 11990 12471 11994 12472 11998 12473
12003 12473 12006 12470 12010 12469 12010 12473 12008 12477 12012 12476
12015 12472 12019 12472 12023 12472 12025 12472 12028 12471 12025 12475
12021 12477 12023 12481 12025 12485 12021 12487 12017 12486 12012 12486
12013 12490 12012 12494 12008 12496 12007 12500 12005 12504 12004 12508
12004 12513 12003 12517 12000 12521 11997 12523 11993 12525 11989 12527
11985 12529 11981 12530 11977 12528 11974 12525 11969 12526 11965 12524

11963 12520 61 N F
11973 12391 11969 12388 11972 12384 11974 12380 11974 12376 11978 12379
11981 12375 11982 12371 11986 12370 11989 12373 11989 12378 11991 12381
11990 12386 11994 12388 11993 12392 11989 12390 11988 12394 11986 12398
11982 12399 11982 12403 11987 12405 11991 12406 11993 12409 11989 12412
11986 12414 11981 12415 11977 12418 11974 12415 11974 12411 11969 12411
11970 12406 11975 12407 11977 12403 11977 12399 11974 12396 11973 12391
36 N F
12012 12525 12010 12520 12014 12523 12018 12522 12022 12523 12021 12519
12020 12515 12019 12510 12023 12508 12025 12511 12026 12516 12028 12519
12030 12523 12034 12525 12038 12525 12042 12527 12042 12531 12045 12534
12049 12533 12050 12533 12053 12531 12057 12532 12062 12531 12062 12536
12058 12539 12054 12541 12054 12545 12050 12543 12046 12544 12049 12547
12051 12551 12047 12554 12045 12558 12041 12557 12037 12555 12033 12553
12029 12551 12025 12551 12022 12548 12019 12545 12018 12540 12015 12537
12017 12533 12015 12529 12012 12525 45 N F
12017 12498 12014 12495 12018 12495 12022 12493 12027 12493 12030 12491
12033 12488 12036 12491 12039 12494 12044 12494 12048 12495 12052 12492
12054 12489 12054 12484 12057 12481 12061 12483 12063 12487 12066 12490
12065 12494 12069 12493 12070 12488 12074 12489 12075 12493 12074 12497
12074 12501 12079 12501 12080 12505 12078 12509 12075 12506 12070 12507
12068 12510 12063 12509 12059 12509 12056 12507 12053 12510 12050 12513
12046 12510 12044 12507 12044 12502 12041 12500 12036 12498 12033 12501
12030 12504 12028 12500 12026 12496 12022 12497 12017 12498 47 N F
12022 12427 12021 12424 12022 12421 12022 12415 12024 12413 12027 12411
12028 12413 12029 12417 12031 12419 12031 12422 12030 12425 12029 12428
12025 12429 12022 12427 14 N F
12065 12541 12059 12540 12063 12538 12065 12534 12063 12530 12062 12526
12063 12522 12066 12525 12068 12529 12072 12531 12071 12526 12072 12522
12073 12518 12077 12517 12081 12515 12082 12515 12085 12515 12089 12517
12090 12521 12094 12523 12099 12523 12100 12527 12098 12530 12097 12535
12097 12539 12099 12543 12098 12547 12096 12543 12093 12539 12092 12535
12088 12537 12084 12535 12081 12539 12076 12538 12072 12539 12069 12542
12067 12538 12065 12541 38 N F
12070 12422 12067 12412 12070 12406 12077 12409 12082 12405 12089 12404
12092 12404 12096 12403 12100 12408 12101 12415 12100 12422 12095 12426
12094 12433 12088 12437 12082 12439 12076 12435 12072 12429 12070 12422
18 N F
12109 12315 12107 12309 12108 12305 12113 12305 12117 12306 12119 12310
12117 12314 12114 12316 12109 12315 9 N F
12110 12506 12107 12503 12108 12498 12108 12494 12113 12493 12114 12490
12114 12489 12118 12491 12122 12493 12125 12496 12129 12497 12131 12501
12134 12503 12133 12507 12126 12510 12122 12510 12117 12510 12113 12509

12110 12506 19 N F
12125 12521 12120 12523 12122 12519 12126 12517 12126 12517 12130 12518
12135 12517 12138 12515 12135 12512 12139 12512 12142 12508 12143 12512
12147 12513 12150 12510 12147 12507 12143 12505 12144 12501 12145 12496
12147 12500 12150 12504 12152 12507 12156 12510 12160 12509 12161 12514
12157 12513 12156 12518 12160 12516 12164 12517 12167 12513 12171 12514
12169 12517 12166 12521 12165 12525 12161 12525 12156 12526 12153 12524
12149 12521 12145 12520 12146 12524 12145 12528 12141 12530 12138 12533
12135 12537 12137 12533 12132 12532 12128 12530 12131 12527 12129 12523
12125 12521 49 N F
12135 12577 12129 12575 12132 12572 12135 12569 12135 12568 12137 12565
12137 12560 12139 12557 12143 12554 12144 12558 12143 12562 12148 12561
12147 12556 12149 12552 12150 12548 12152 12552 12153 12556 12155 12560
12153 12564 12151 12568 12150 12572 12147 12576 12144 12578 12139 12579
12135 12577 25 N F
12165 12587 12159 12586 12162 12583 12167 12584 12169 12581 12166 12578
12165 12574 12169 12575 12171 12571 12175 12569 12179 12567 12183 12565
12186 12567 12191 12567 12193 12570 12198 12571 12202 12570 12206 12570
12211 12570 12215 12571 12218 12575 12219 12579 12219 12583 12220 12587
12222 12592 12223 12596 12222 12600 12219 12603 12216 12606 12212 12605
12208 12603 12204 12602 12200 12603 12196 12601 12193 12598 12190 12595
12186 12595 12183 12592 12179 12593 12175 12594 12173 12598 12169 12599
12170 12594 12167 12591 12165 12587 45 N F
12235 12458 12233 12452 12237 12454 12241 12453 12245 12455 12250 12454
12250 12459 12252 12462 12250 12466 12249 12471 12249 12475 12245 12474
12241 12472 12241 12468 12237 12467 12237 12463 12235 12458 17 N F
12274 12372 12268 12368 12270 12364 12272 12360 12275 12358 12276 12353
12279 12350 12282 12347 12287 12347 12291 12346 12293 12350 12289 12349
12285 12351 12284 12355 12280 12358 12278 12362 12277 12366 12278 12370
12277 12374 12274 12372 20 N F
12288 12373 12281 12371 12284 12368 12286 12364 12290 12362 12294 12361
12295 12365 12291 12367 12292 12371 12288 12373 10 N F
12313 12636 12309 12631 12313 12629 12318 12629 12318 12625 12322 12622
12318 12621 12315 12618 12317 12614 12320 12611 12324 12610 12327 12614
12331 12614 12335 12616 12340 12615 12342 12619 12341 12623 12338 12626
12334 12628 12330 12628 12325 12628 12329 12630 12327 12634 12323 12635
12326 12638 12328 12642 12327 12646 12323 12646 12319 12647 12316 12644
12313 12640 12313 12636 32 N F
12323 12599 12321 12594 12325 12594 12329 12595 12334 12596 12336 12592
12332 12592 12327 12593 12329 12589 12333 12587 12337 12588 12341 12586
12341 12582 12340 12578 12343 12575 12346 12579 12350 12581 12354 12583
12358 12584 12361 12587 12362 12591 12361 12595 12359 12599 12355 12600
12352 12604 12348 12606 12346 12610 12344 12605 12340 12604 12337 12601

12333 12604 12329 12605 12325 12607 12321 12606 12324 12603 12323 12599
36 N F
12400 12676 12397 12672 12398 12666 12400 12670 12402 12666 12405 12663
12409 12663 12412 12666 12416 12667 12417 12672 12420 12668 12425 12669
12428 12672 12425 12674 12421 12677 12418 12674 12414 12674 12412 12678
12407 12679 12404 12677 12400 12676 21 N F
12432 12657 12426 12656 12429 12653 12433 12651 12437 12649 12441 12651
12440 12646 12444 12644 12445 12640 12449 12638 12453 12640 12457 12638
12461 12636 12462 12632 12467 12632 12471 12633 12474 12636 12472 12640
12470 12644 12465 12644 12461 12644 12465 12646 12465 12650 12461 12653
12458 12656 12456 12660 12455 12664 12450 12666 12446 12667 12442 12666
12438 12663 12435 12660 12432 12657 33 N F
12458 12841 12456 12834 12458 12827 12461 12821 12465 12816 12470 12811
12471 12804 12476 12800 12479 12794 12477 12787 12475 12781 12477 12780
12481 12779 12484 12772 12489 12768 12496 12767 12502 12768 12508 12771
12509 12772 12510 12778 12507 12784 12504 12790 12510 12794 12516 12798
12523 12799 12524 12792 12523 12786 12525 12779 12525 12778 12527 12772
12529 12766 12536 12765 12538 12765 12543 12765 12544 12766 12548 12769
12555 12770 12560 12764 12566 12767 12571 12767 12573 12768 12569 12773
12565 12779 12570 12784 12573 12790 12572 12797 12570 12804 12569 12810
12568 12817 12572 12823 12571 12830 12566 12834 12559 12832 12554 12836
12542 12834 12536 12831 12533 12825 12527 12822 12522 12817 12517 12821
12515 12828 12508 12830 12502 12830 12502 12837 12498 12842 12494 12848
12495 12855 12501 12859 12503 12865 12505 12872 12503 12878 12500 12884
12494 12888 12487 12889 12481 12886 12475 12882 12471 12876 12471 12869
12476 12864 12469 12862 12463 12860 12463 12853 12462 12846 12458 12841
84 N F
12476 12606 12473 12602 12477 12602 12475 12599 12475 12594 12479 12594
12482 12591 12485 12595 12484 12599 12484 12604 12485 12608 12484 12612
12480 12614 12481 12619 12477 12619 12475 12615 12477 12611 12476 12606
18 N F
12485 8076 12477 8069 12481 8063 12488 8061 12495 8061 12502 8060
12507 8055 12510 8049 12514 8043 12520 8039 12527 8036 12534 8035
12531 8042 12529 8049 12528 8056 12532 8062 12535 8068 12529 8072
12525 8078 12519 8080 12515 8086 12509 8090 12502 8089 12495 8085
12491 8080 12485 8076 26 N F
12524 12853 12523 12849 12524 12845 12525 12841 12527 12837 12532 12836
12536 12835 12540 12835 12544 12836 12549 12837 12549 12841 12554 12843
12557 12841 12561 12839 12566 12840 12570 12838 12574 12838 12577 12836
12582 12836 12580 12840 12577 12843 12572 12844 12569 12847 12568 12851
12570 12855 12569 12860 12566 12862 12561 12864 12558 12866 12559 12870
12555 12871 12552 12868 12550 12864 12549 12859 12550 12855 12548 12851
12545 12854 12544 12858 12545 12863 12546 12867 12548 12871 12550 12875

12551 12879 12550 12883 12546 12882 12542 12880 12537 12876 12535 12872
12534 12868 12532 12864 12529 12861 12527 12856 12524 12853 53 N F
12524 8208 12523 8207 12523 8207 12523 8206 12525 8206 12525 8206
12525 8206 12526 8206 12526 8207 12526 8208 12525 8208 12524 8208
12 N F
12535 8063 12533 8060 12535 8061 12538 8062 12540 8062 12540 8065
12538 8067 12537 8065 12535 8063 9 N F
12542 8067 12542 8063 12544 8064 12546 8065 12544 8067 12542 8067
6 N F
12548 8064 12547 8061 12548 8062 12550 8064 12552 8064 12555 8065
12556 8066 12558 8068 12559 8070 12561 8070 12563 8071 12566 8071
12568 8072 12569 8074 12571 8074 12573 8075 12571 8076 12569 8075
12567 8074 12565 8072 12563 8072 12561 8072 12559 8071 12557 8070
12556 8068 12554 8066 12552 8066 12550 8065 12548 8064 29 N F
12618 12855 12616 12848 12620 12846 12624 12845 12623 12840 12628 12840
12631 12843 12632 12847 12635 12850 12639 12851 12643 12851 12643 12855
12639 12857 12634 12857 12631 12859 12626 12858 12622 12857 12618 12855
18 N F
12638 12839 12633 12836 12637 12835 12637 12831 12642 12831 12645 12828
12649 12828 12653 12826 12654 12830 12651 12833 12655 12834 12659 12835
12663 12838 12659 12841 12655 12843 12653 12846 12649 12849 12645 12851
12641 12849 12638 12846 12635 12843 12638 12839 22 N F
12658 12744 12656 12738 12659 12735 12663 12734 12662 12729 12664 12725
12667 12729 12671 12730 12673 12734 12678 12733 12682 12733 12686 12733
12686 12738 12689 12741 12687 12745 12683 12743 12680 12739 12679 12735
12676 12738 12679 12741 12676 12744 12671 12745 12668 12747 12664 12749
12659 12748 12658 12744 26 N F
12695 8101 12693 8096 12696 8092 12698 8088 12702 8090 12706 8093
12701 8094 12699 8098 12699 8103 12695 8101 10 N F
12721 8230 12715 8218 12721 8221 12728 8221 12735 8225 12733 8231
12726 8234 12721 8230 8 N F
12752 12890 12750 12883 12752 12876 12754 12870 12757 12864 12759 12863
12764 12862 12767 12856 12771 12850 12777 12853 12776 12859 12782 12861
12788 12864 12793 12870 12795 12876 12802 12874 12803 12868 12806 12861
12804 12855 12805 12848 12799 12844 12792 12842 12786 12840 12788 12833
12788 12833 12793 12837 12793 12831 12800 12832 12806 12835 12812 12832
12806 12828 12805 12821 12808 12815 12814 12813 12811 12807 12809 12801
12815 12799 12816 12798 12822 12796 12827 12800 12834 12802 12831 12808
12826 12813 12833 12815 12840 12816 12846 12819 12853 12819 12847 12823
12840 12821 12836 12827 12832 12832 12834 12839 12840 12838 12845 12843
12842 12850 12842 12857 12848 12859 12852 12853 12856 12859 12862 12858
12863 12857 12866 12853 12873 12850 12873 12857 12868 12862 12864 12867
12866 12874 12859 12873 12857 12879 12863 12882 12869 12880 12866 12886

12862 12892 12869 12893 12869 12900 12872 12906 12865 12907 12860 12902
12858 12895 12852 12897 12848 12902 12852 12908 12855 12914 12859 12920
12856 12926 12854 12933 12854 12939 12851 12946 12846 12950 12843 12956
12840 12962 12843 12968 12849 12971 12850 12978 12846 12983 12840 12985
12833 12986 12829 12980 12828 12973 12832 12963 12826 12961 12823 12955
12822 12948 12821 12941 12814 12943 12808 12939 12802 12942 12800 12936
12796 12931 12800 12925 12803 12919 12807 12914 12812 12909 12817 12904
12810 12905 12804 12904 12797 12907 12793 12902 12786 12903 12780 12906
12775 12911 12768 12909 12766 12903 12759 12902 12756 12895 12752 12890
126 N F
12751 12840 12750 12834 12754 12832 12757 12829 12755 12825 12756 12820
12756 12820 12760 12820 12765 12819 12768 12822 12772 12824 12775 12827
12775 12827 12774 12832 12771 12835 12767 12834 12765 12838 12764 12842
12761 12845 12762 12850 12758 12847 12759 12851 12755 12851 12752 12848
12753 12844 12758 12845 12761 12842 12757 12844 12756 12840 12754 12836
12754 12841 12751 12844 12751 12840 33 N F
12756 7997 12754 7992 12755 7988 12759 7986 12759 7991 12760 7995
12756 7997 7 N F
12756 8020 12755 8015 12756 8014 12759 8014 12761 8019 12756 8020
6 N F
12762 12939 12761 12934 12764 12931 12764 12926 12763 12922 12765 12919
12768 12915 12772 12916 12769 12919 12773 12922 12769 12925 12767 12929
12770 12932 12773 12935 12771 12939 12766 12940 12762 12939 17 N F
12771 8013 12768 8009 12772 8006 12775 8003 12779 8004 12783 8007
12781 8011 12779 8015 12775 8016 12771 8013 10 N F
12781 8031 12775 8026 12780 8026 12784 8026 12785 8025 12786 8024
12788 8022 12790 8026 12792 8030 12792 8034 12795 8031 12800 8030
12800 8034 12797 8038 12793 8039 12788 8039 12788 8035 12783 8035
12781 8031 19 N F
12785 8002 12779 8000 12781 7996 12786 7998 12785 8002 5 N F
12786 7996 12783 7991 12785 7988 12787 7992 12786 7996 5 N F
12811 8006 12807 8003 12809 7999 12812 7996 12812 7991 12812 7987
12814 7983 12816 7987 12820 7989 12821 7994 12820 7998 12819 8003
12821 8007 12824 8010 12823 8014 12824 8019 12823 8023 12820 8027
12816 8029 12812 8028 12812 8023 12811 8019 12809 8015 12810 8010
12811 8006 25 N F
12859 8026 12856 8020 12861 8021 12861 8026 12863 8029 12863 8034
12860 8031 12859 8026 8 N F
12884 13002 12881 12998 12886 12997 12889 12994 12888 12989 12891 12986
12888 12984 12891 12982 12896 12981 12896 12976 12896 12972 12899 12969
12901 12969 12903 12970 12904 12974 12903 12978 12903 12983 12904 12987
12905 12991 12906 12995 12906 13000 12904 13004 12900 13003 12898 13007
12894 13009 12892 13006 12887 13004 12884 13002 28 N F

12895 11305 12895 11305 12895 11304 12895 11304 12896 11304 12896 11304
12897 11303 12897 11303 12898 11303 12897 11304 12897 11304 12896 11305
12895 11305 13 N F
12896 11308 12896 11307 12896 11306 12897 11305 12898 11304 12898 11302
12898 11301 12898 11299 12897 11298 12897 11296 12898 11295 12899 11297
12899 11299 12901 11299 12903 11299 12902 11301 12901 11302 12900 11303
12901 11305 12902 11306 12903 11308 12902 11309 12901 11310 12900 11309
12898 11308 12896 11308 26 N F
12903 11314 12903 11313 12903 11312 12904 11311 12904 11311 12905 11312
12905 11312 12906 11313 12906 11315 12905 11315 12905 11315 12904 11314
12903 11314 13 N F
12904 11319 12904 11319 12904 11318 12905 11318 12905 11318 12905 11318
12905 11318 12905 11319 12905 11320 12904 11319 10 N F
12906 11322 12906 11321 12906 11319 12906 11319 12906 11319 12907 11319
12907 11319 12908 11319 12908 11319 12908 11321 12909 11322 12909 11322
12908 11322 12907 11322 12906 11322 12906 11322 16 N F
12909 11326 12909 11326 12909 11325 12909 11325 12909 11324 12910 11324
12910 11323 12910 11323 12911 11323 12911 11324 12911 11324 12911 11325
12911 11326 12911 11326 12909 11326 15 N F
12911 11330 12911 11328 12912 11328 12914 11329 12915 11329 12917 11329
12918 11330 12919 11331 12921 11330 12922 11329 12923 11330 12923 11331
12924 11333 12925 11334 12926 11336 12926 11337 12924 11338 12924 11336
12922 11337 12920 11336 12919 11337 12917 11337 12915 11337 12915 11336
12915 11334 12915 11332 12914 11331 12912 11330 12911 11330 29 N F
12962 8040 12959 8033 12961 8026 12965 8021 12968 8014 12974 8010
12979 8005 12979 7998 12979 7991 12979 7984 12977 7977 12976 7970
12982 7965 12988 7963 12993 7968 12999 7972 12999 7972 12999 8080
12994 8078 12988 8075 12984 8069 12981 8063 12978 8056 12978 8049
12973 8044 12977 8039 12981 8033 12976 8038 12969 8040 12962 8040
30 N F
12973 11668 12970 11656 12977 11654 12983 11654 12988 11649 12994 11648
12998 11653 12999 11654 12999 11684 12992 11684 12997 11679 12991 11681
12993 11674 12988 11670 12987 11663 12993 11666 12999 11670 12995 11664
12994 11657 12988 11659 12982 11663 12979 11669 12975 11675 12973 11668
24 N F
12981 8342 12978 8332 12983 8326 12990 8326 12996 8328 12999 8328
12999 8343 12996 8342 12988 8343 12981 8342 10 N F
12983 11749 12981 11742 12982 11736 12986 11730 12984 11724 12985 11717
12990 11712 12987 11706 12990 11700 12992 11694 12998 11691 12999 11685
12999 11747 12995 11752 12988 11754 12983 11749 16 N F
12983 8166 12983 8159 12985 8152 12988 8146 12992 8140 12995 8134
12999 8131 12999 8167 12996 8164 12991 8169 12985 8173 12983 8166
12 N F

1 O
13000 16218 2299 16218 2299 5502 13000 5502 13000 16218 5 P
Ls Gr Gs Lr
2298 5501 13001 16219 Oc
129 O
2298 5637 2298 5527 2298 5527 2298 7055 2298 7055 2298 6993
2298 6858 2298 6722 2298 6586 2298 6451 2298 6315 2298 6179
2298 6044 2298 5908 2298 5772 2298 5637 16 N F
147 O
2298 7129 2298 7055 2298 7055 2298 7270 2298 7270 2298 7265
2298 7129 7 N F
129 O
2298 7400 2298 7270 2298 7270 2298 7767 2298 7767 2298 7672
2298 7536 2298 7400 8 N F
147 O
2298 7807 2298 7767 2298 7767 2298 8282 2298 8282 2298 8214
2298 8079 2298 7943 2298 7807 9 N F
165 O
2298 8350 2298 8282 2298 8282 2298 8747 2298 8747 2298 8621
2298 8486 2298 8350 8 N F
147 O
2298 8757 2298 8747 2298 8747 2298 9351 2298 9351 2298 9300
2298 9164 2298 9028 2298 8893 2298 8757 10 N F
165 O
2298 9435 2298 9351 2298 9351 2298 9483 2298 9483 2298 9435
6 N F
147 O
2298 9571 2298 9483 2298 9483 2298 10136 2298 10136 2298 10114
2298 9978 2298 9842 2298 9707 2298 9571 10 N F
129 O
2298 10249 2298 10136 2298 10136 2298 11418 2298 11418 2298 11335
2298 11199 2298 11064 2298 10928 2298 10792 2298 10656 2298 10521
2298 10385 2298 10249 14 N F
147 O
2298 11471 2298 11418 2298 11418 2298 11816 2298 11816 2298 11742
2298 11606 2298 11471 8 N F
165 O
2298 11878 2298 11816 2298 11816 2298 11992 2298 11992 2298 11878
6 N F
147 O
2298 5527 2298 5501 2298 5501 2298 5501 2298 5527 2298 5527
6 N F
129 O

2298 7055 2298 5527 2298 5527 2298 7055 2298 7055 5 N F
147 O
2298 7270 2298 7055 2298 7055 2298 7270 2298 7270 5 N F
129 O
2298 7767 2298 7270 2298 7270 2298 7767 2298 7767 5 N F
147 O
2298 8282 2298 7767 2298 7767 2298 8282 2298 8282 5 N F
165 O
2298 8747 2298 8282 2298 8282 2298 8747 2298 8747 5 N F
147 O
2298 9351 2298 8747 2298 8747 2298 9351 2298 9351 5 N F
165 O
2298 9483 2298 9351 2298 9351 2298 9483 2298 9483 5 N F
147 O
2298 10136 2298 9483 2298 9483 2298 10136 2298 10136 5 N F
129 O
2298 11418 2298 10136 2298 10136 2298 11418 2298 11418 5 N F
147 O
2298 11816 2298 11418 2298 11418 2298 11816 2298 11816 5 N F
165 O
2298 11992 2298 11816 2298 11816 2298 11992 2298 11992 5 N F
183 O
2298 16219 2298 11992 2298 11992 2298 16219 2298 16219 2298 16219
6 N F
147 O
2504 5501 2298 5501 2298 5527 2433 5581 2536 5637 2569 5666
2607 5637 2598 5501 2504 5501 9 N F
129 O
2298 5527 M 2298 7055 L 2433 7074 L 2498 7129 L 2569 7223 L
2704 7185 L 2837 7129 L 2704 7064 L 2651 6993 L
2674 6858 L 2704 6792 L 2829 6722 L 2840 6720 L
2975 6658 L 3024 6586 L 3111 6463 L 3176 6586 L
3246 6644 L 3382 6685 L 3414 6722 L 3496 6858 L
3517 6978 L 3551 6993 L 3653 7003 L 3676 6993 L
3788 6948 L 3924 6886 L 3949 6858 L 3924 6780 L
3913 6722 L 3922 6586 L 3924 6582 L 3969 6586 L
4059 6592 L 4187 6722 L 4195 6735 L 4330 6793 L
4330 5735 L 4304 5637 L 4330 5618 L 4339 5637 L
4330 5735 L 4330 6793 L 4426 6858 L 4466 6865 L
4601 6916 L 4737 6946 L 4858 6858 L 4872 6835 L
4952 6722 L 5008 6681 L 5143 6707 L 5160 6722 L
5167 6858 L 5143 6876 L 5047 6993 L 5143 7127 L
5146 7129 L 5258 7265 L 5279 7298 L 5414 7385 L

5550 7368 L 5550 7223 L 5414 7159 L 5380 7129 L
5325 6993 L 5414 6894 L 5550 6896 L 5576 6993 L
5598 7129 L 5550 7223 L 5550 7368 L 5685 7268 L
5700 7265 L 5820 7244 L 5865 7265 L 5956 7319 L
6034 7265 L 6091 7192 L 6158 7265 L 6189 7400 L
6227 7425 L 6362 7494 L 6498 7500 L 6633 7512 L
6702 7536 L 6769 7632 L 6801 7672 L 6769 7742 L
6754 7807 L 6691 7943 L 6769 8067 L 6904 8027 L
6962 8079 L 7040 8123 L 7175 8118 L 7236 8079 L
7270 7943 L 7311 7907 L 7367 7943 L 7446 7983 L
7525 7943 L 7476 7807 L 7446 7767 L 7311 7725 L
7239 7672 L 7291 7536 L 7311 7528 L 7446 7491 L
7505 7400 L 7582 7297 L 7666 7400 L 7717 7460 L
7853 7469 L 7988 7425 L 8124 7414 L 8259 7435 L
8395 7442 L 8530 7449 L 8666 7452 L 8793 7536 L
8801 7550 L 8850 7672 L 8882 7807 L 8801 7868 L
8781 7943 L 8790 8079 L 8801 8199 L 8937 8176 L
9010 8214 L 9072 8234 L 9108 8214 L 9208 8176 L
9343 8160 L 9410 8079 L 9382 7943 L 9343 7839 L
9331 7807 L 9343 7790 L 9395 7807 L 9479 7830 L
9614 7857 L 9642 7807 L 9749 7752 L 9885 7684 L
9913 7672 L 10020 7610 L 10156 7669 L 10168 7672 L
10291 7686 L 10427 7726 L 10473 7672 L 10562 7643 L
10698 7670 L 10833 7629 L 10915 7536 L 10969 7504 L
11104 7486 L 11240 7470 L 11375 7480 L 11511 7490 L
11646 7502 L 11782 7516 L 11912 7536 L 11917 7539 L
12048 7672 L 12053 7677 L 12075 7807 L 12090 7943 L
12100 8079 L 12110 8214 L 12121 8350 L 12128 8486 L
12136 8621 L 12142 8757 L 12149 8893 L 12156 9028 L
12165 9164 L 12177 9300 L 12185 9435 L 12188 9481 L
12224 9571 L 12277 9707 L 12319 9842 L 12324 9853 L
12427 9978 L 12459 10015 L 12574 10114 L 12595 10138 L
12715 10249 L 12730 10264 L 12866 10381 L 12873 10385 L
13001 10505 L 13001 8060 L 12996 8079 L 12967 8214 L
12943 8350 L 12930 8486 L 12906 8621 L 12892 8757 L
12866 8842 L 12730 8864 L 12595 8808 L 12459 8800 L
12365 8757 L 12324 8735 L 12254 8621 L 12188 8520 L
12187 8486 L 12181 8350 L 12175 8214 L 12169 8079 L
12165 7943 L 12162 7807 L 12154 7672 L 12140 7536 L
12053 7445 L 11917 7430 L 11782 7424 L 11646 7415 L
11511 7409 L 11375 7402 L 11343 7400 L 11240 7368 L
11104 7373 L 11040 7400 L 10969 7406 L 10833 7443 L
10698 7473 L 10562 7479 L 10427 7454 L 10291 7429 L

10156 7407 L 10020 7415 L 9885 7424 L 9749 7441 L
9614 7477 L 9479 7469 L 9343 7458 L 9208 7449 L
9072 7421 L 9023 7400 L 8937 7354 L 8819 7265 L
8801 7252 L 8709 7129 L 8666 7080 L 8588 6993 L
8530 6939 L 8465 6993 L 8395 7097 L 8350 7129 L
8259 7259 L 8197 7129 L 8124 7045 L 7988 7032 L
7853 7069 L 7741 6993 L 7717 6970 L 7614 6858 L
7582 6815 L 7564 6858 L 7511 6993 L 7446 7044 L
7311 7072 L 7175 7006 L 7151 6993 L 7040 6932 L
6970 6858 L 6904 6780 L 6769 6795 L 6633 6755 L
6498 6736 L 6490 6722 L 6488 6586 L 6362 6498 L
6227 6491 L 6091 6451 L 6090 6451 L 5956 6374 L
5825 6451 L 5851 6586 L 5820 6599 L 5794 6586 L
5685 6464 L 5677 6451 L 5664 6315 L 5651 6179 L
5685 6087 L 5685 5723 L 5578 5637 L 5685 5580 L
5742 5637 L 5685 5723 L 5685 6087 L 5727 6044 L
5820 5947 L 5836 5908 L 5949 5772 L 5956 5769 L
6091 5703 L 6126 5637 L 6091 5531 L 6077 5501 L
6003 5501 L 5797 5501 L 5591 5501 L 5385 5501 L
5180 5501 L 4974 5501 L 4768 5501 L 4562 5501 L
4356 5501 L 4150 5501 L 3945 5501 L 3739 5501 L
3533 5501 L 3327 5501 L 3121 5501 L 2916 5501 L
2710 5501 L 2598 5501 L 2607 5637 L 2569 5666 L
2536 5637 L 2433 5581 L 2298 5527 L N E
147 O
2433 7074 M 2298 7055 L 2298 7270 L 2409 7400 L 2433 7427 L
2525 7536 L 2473 7672 L 2433 7702 L 2298 7767 L
2298 8282 L 2366 8350 L 2433 8424 L 2496 8350 L
2489 8214 L 2569 8111 L 2704 8109 L 2745 8214 L
2840 8339 L 2850 8350 L 2946 8486 L 2975 8558 L
3080 8486 L 3111 8481 L 3206 8350 L 3111 8291 L
3027 8214 L 2975 8105 L 2935 8079 L 2975 8048 L
2975 7169 L 2844 7129 L 2973 6993 L 2975 6992 L
2979 6993 L 3031 7129 L 2975 7169 L 2975 8048 L
3077 7943 L 3111 7925 L 3246 7886 L 3343 7807 L
3246 7716 L 3142 7672 L 3182 7536 L 3232 7400 L
3246 7369 L 3382 7393 L 3394 7400 L 3512 7536 L
3496 7672 L 3517 7677 L 3653 7781 L 3673 7807 L
3740 7943 L 3768 8079 L 3788 8130 L 3814 8214 L
3867 8350 L 3817 8486 L 3919 8621 L 3924 8652 L
3935 8757 L 3940 8893 L 3993 9028 L 4034 9164 L
4059 9220 L 4176 9300 L 4195 9312 L 4330 9427 L
4337 9435 L 4330 9492 L 4195 9538 L 4155 9571 L

4164 9707 L 4195 9808 L 4330 9837 L 4353 9842 L
4466 9851 L 4466 8808 L 4424 8757 L 4330 8653 L
4300 8621 L 4195 8512 L 4154 8486 L 4080 8350 L
4195 8217 L 4330 8248 L 4466 8334 L 4466 8075 L
4330 8045 L 4195 8024 L 4059 7964 L 4014 7943 L
3986 7807 L 3946 7672 L 3924 7616 L 3896 7536 L
3924 7480 L 4059 7528 L 4075 7536 L 4195 7593 L
4195 7044 L 4110 6993 L 4195 6911 L 4330 6925 L
4347 6993 L 4330 7009 L 4195 7044 L 4195 7593 L
4330 7650 L 4352 7672 L 4433 7807 L 4466 7845 L
4540 7943 L 4466 8075 L 4466 8334 L 4492 8350 L
4601 8447 L 4737 8479 L 4751 8486 L 4764 8621 L
4737 8632 L 4636 8757 L 4601 8801 L 4466 8808 L
4466 9851 L 4599 9978 L 4601 9980 L 4669 10114 L
4649 10249 L 4612 10385 L 4601 10416 L 4466 10515 L
4454 10521 L 4330 10636 L 4195 10592 L 4059 10568 L
3924 10528 L 3909 10521 L 3788 10467 L 3653 10437 L
3517 10389 L 3512 10385 L 3382 10269 L 3364 10249 L
3246 10134 L 3219 10114 L 3111 10034 L 3015 9978 L
2975 9943 L 2840 9849 L 2831 9842 L 2726 9707 L
2704 9668 L 2643 9571 L 2569 9466 L 2433 9463 L
2298 9483 L 2298 10136 L 2433 10122 L 2446 10114 L
2550 9978 L 2569 9952 L 2672 9978 L 2704 9986 L
2840 10041 L 2975 10093 L 3020 10114 L 3111 10163 L
3246 10248 L 3247 10249 L 3346 10385 L 3382 10427 L
3517 10516 L 3550 10521 L 3653 10543 L 3788 10617 L
3869 10656 L 3788 10730 L 3741 10792 L 3653 10850 L
3537 10928 L 3517 10945 L 3382 10961 L 3280 11064 L
3246 11084 L 3143 11199 L 3111 11264 L 3048 11335 L
2975 11362 L 2840 11397 L 2704 11425 L 2569 11429 L
2433 11453 L 2298 11418 L 2298 11816 L 2433 11842 L
2518 11742 L 2569 11619 L 2578 11606 L 2704 11561 L
2840 11558 L 2975 11529 L 3111 11494 L 3204 11471 L
3246 11455 L 3382 11427 L 3496 11335 L 3506 11199 L
3517 11185 L 3569 11064 L 3653 10989 L 3717 10928 L
3788 10876 L 3924 10871 L 4059 10894 L 4195 10816 L
4217 10792 L 4330 10664 L 4466 10700 L 4601 10735 L
4737 10663 L 4839 10792 L 4778 10928 L 4737 10958 L
4659 11064 L 4601 11104 L 4466 11113 L 4330 11091 L
4195 11153 L 4169 11199 L 4087 11335 L 4059 11353 L
3924 11407 L 3788 11432 L 3653 11460 L 3517 11411 L
3486 11471 L 3455 11606 L 3382 11704 L 3334 11742 L
3246 11773 L 3111 11795 L 2975 11815 L 2840 11863 L

2786 11878 L 2704 11947 L 2613 12013 L 2704 12039 L
2840 12027 L 2975 12060 L 3111 12101 L 3246 12082 L
3246 11900 L 3201 11878 L 3246 11873 L 3346 11878 L
3246 11900 L 3246 12082 L 3382 12066 L 3510 12013 L
3517 12007 L 3653 11915 L 3754 11878 L 3788 11861 L
3865 11878 L 3924 11905 L 3924 11682 L 3788 11669 L
3699 11606 L 3788 11581 L 3924 11564 L 4007 11606 L
3924 11682 L 3924 11905 L 3985 11878 L 4059 11848 L
4195 11865 L 4208 11878 L 4330 11952 L 4466 12012 L
4473 12013 L 4601 12083 L 4737 12117 L 4872 12098 L
5008 12028 L 5052 12013 L 5143 11999 L 5279 11977 L
5355 12013 L 5279 12094 L 5249 12149 L 5143 12264 L
5122 12285 L 5008 12388 L 4934 12420 L 4872 12448 L
4761 12420 L 4737 12416 L 4729 12420 L 4601 12502 L
4466 12553 L 4330 12533 L 4195 12450 L 4140 12420 L
4059 12385 L 3924 12388 L 3788 12400 L 3653 12417 L
3646 12420 L 3517 12505 L 3406 12556 L 3517 12675 L
3559 12692 L 3653 12717 L 3788 12738 L 3924 12780 L
4045 12827 L 4059 12834 L 4059 12634 L 3924 12599 L
3839 12556 L 3924 12530 L 4059 12530 L 4111 12556 L
4059 12634 L 4059 12834 L 4195 12882 L 4330 12955 L
4330 12696 L 4279 12692 L 4330 12690 L 4384 12692 L
4330 12696 L 4330 12955 L 4431 12963 L 4466 12965 L
4601 12982 L 4737 13036 L 4872 13091 L 4888 13099 L
5008 13124 L 5096 13099 L 5038 12963 L 5062 12827 L
5143 12698 L 5279 12733 L 5414 12765 L 5550 12788 L
5550 12467 L 5532 12420 L 5550 12394 L 5563 12420 L
5550 12467 L 5550 12788 L 5685 12795 L 5685 11972 L
5550 11951 L 5515 11878 L 5414 11794 L 5279 11782 L
5143 11857 L 5112 11878 L 5008 11910 L 4895 11878 L
4872 11856 L 4801 11742 L 4737 11691 L 4601 11728 L
4498 11606 L 4543 11471 L 4466 11417 L 4330 11367 L
4316 11335 L 4328 11199 L 4330 11198 L 4466 11199 L
4466 11199 L 4601 11297 L 4737 11259 L 4783 11199 L
4872 11146 L 5008 11090 L 5045 11064 L 5105 10928 L
5135 10792 L 5008 10684 L 4998 10656 L 5008 10596 L
5008 10447 L 4872 10391 L 4864 10385 L 4863 10249 L
4872 10236 L 4872 7431 L 4843 7400 L 4872 7371 L
4980 7400 L 4872 7431 L 4872 10236 L 5008 10232 L
5048 10249 L 5049 10385 L 5008 10447 L 5008 10596 L
5143 10633 L 5207 10656 L 5279 10752 L 5279 7566 L
5143 7562 L 5054 7536 L 5143 7455 L 5279 7507 L
5363 7536 L 5279 7566 L 5279 10752 L 5414 10709 L

5503 10656 L 5550 10559 L 5550 7817 L 5540 7807 L
5485 7672 L 5550 7536 L 5685 7660 L 5689 7672 L
5685 7715 L 5598 7807 L 5550 7817 L 5550 10559 L
5584 10656 L 5685 10766 L 5685 10151 L 5620 10114 L
5685 10031 L 5703 10114 L 5685 10151 L 5685 10766 L
5690 10792 L 5757 10928 L 5745 11064 L 5788 11199 L
5820 11228 L 5956 11311 L 5969 11335 L 6091 11438 L
6227 11414 L 6227 11212 L 6091 11201 L 6089 11199 L
6091 11199 L 6227 11195 L 6232 11199 L 6227 11212 L
6227 11414 L 6290 11471 L 6227 11526 L 6091 11584 L
5956 11526 L 5842 11606 L 5820 11616 L 5738 11742 L
5708 11878 L 5685 11972 L 5685 12795 L 5803 12692 L
5820 12556 L 5802 12420 L 5792 12285 L 5803 12149 L
5820 12100 L 5848 12013 L 5820 11904 L 5815 11878 L
5820 11838 L 5851 11742 L 5956 11687 L 5995 11742 L
6083 11878 L 6091 11978 L 6107 11878 L 6227 11831 L
6362 11859 L 6362 11751 L 6227 11750 L 6091 11749 L
6086 11742 L 6091 11670 L 6227 11714 L 6362 11711 L
6362 11202 L 6361 11199 L 6227 11104 L 6091 11113 L
5956 11143 L 5853 11064 L 5903 10928 L 5870 10792 L
5883 10656 L 5869 10521 L 5895 10385 L 5956 10252 L
6003 10249 L 6052 10114 L 5964 9978 L 6074 9842 L
5956 9817 L 5844 9707 L 5820 9674 L 5765 9571 L
5797 9435 L 5820 9405 L 5956 9319 L 5965 9300 L
5956 9247 L 5933 9164 L 5840 9028 L 5883 8893 L
5820 8765 L 5816 8757 L 5820 8732 L 5956 8628 L
5956 8578 L 5899 8486 L 5915 8350 L 5910 8214 L
5956 8081 L 6020 8214 L 6017 8350 L 6003 8486 L
5956 8578 L 5956 8628 L 6091 8664 L 6116 8757 L
6091 8860 L 6086 8893 L 6056 9028 L 5981 9164 L
6091 9210 L 6227 9263 L 6241 9300 L 6271 9435 L
6362 9509 L 6399 9571 L 6407 9707 L 6362 9777 L
6258 9842 L 6255 9978 L 6355 10114 L 6362 10189 L
6381 10249 L 6362 10309 L 6291 10385 L 6227 10490 L
6217 10521 L 6227 10528 L 6362 10573 L 6498 10545 L
6568 10521 L 6633 10500 L 6652 10521 L 6769 10627 L
6808 10656 L 6769 10770 L 6633 10736 L 6559 10792 L
6574 10928 L 6498 10975 L 6370 11064 L 6367 11199 L
6362 11202 L 6362 11711 L 6443 11742 L 6362 11751 L
6362 11859 L 6384 11878 L 6472 12013 L 6498 12040 L
6610 12149 L 6633 12205 L 6656 12149 L 6713 12013 L
6633 11892 L 6622 11878 L 6625 11742 L 6513 11606 L
6498 11590 L 6437 11471 L 6498 11400 L 6544 11471 L

6633 11557 L 6633 11477 L 6626 11471 L 6633 11456 L
6655 11471 L 6633 11477 L 6633 11557 L 6769 11517 L
6904 11593 L 6970 11606 L 7040 11642 L 7099 11606 L
7175 11600 L 7311 11584 L 7342 11606 L 7446 11683 L
7518 11742 L 7504 11878 L 7522 12013 L 7471 12149 L
7528 12285 L 7582 12310 L 7669 12420 L 7697 12556 L
7717 12589 L 7795 12692 L 7800 12827 L 7717 12925 L
7582 12958 L 7562 12963 L 7446 12987 L 7311 13001 L
7175 13000 L 7067 12963 L 7040 12948 L 7015 12963 L
6904 13005 L 6769 13024 L 6633 13028 L 6498 12982 L
6426 12963 L 6362 12946 L 6258 12963 L 6227 12983 L
6202 13099 L 6221 13234 L 6227 13286 L 6262 13370 L
6272 13506 L 6358 13641 L 6362 13655 L 6498 13756 L
6583 13777 L 6633 13801 L 6769 13793 L 6904 13823 L
6955 13777 L 7040 13738 L 7175 13727 L 7311 13681 L
7445 13641 L 7446 13641 L 7505 13506 L 7494 13370 L
7582 13244 L 7594 13234 L 7717 13164 L 7771 13099 L
7853 12976 L 7853 12214 L 7803 12149 L 7717 12112 L
7657 12013 L 7582 11933 L 7567 11878 L 7582 11825 L
7678 11742 L 7582 11606 L 7581 11606 L 7446 11580 L
7311 11504 L 7175 11496 L 7091 11471 L 7040 11436 L
6904 11379 L 6769 11356 L 6754 11335 L 6746 11199 L
6769 11159 L 6833 11064 L 6769 10971 L 6761 10928 L
6769 10923 L 6904 10916 L 6904 10541 L 6769 10522 L
6768 10521 L 6769 10516 L 6904 10411 L 6928 10521 L
6904 10541 L 6904 10916 L 6993 10928 L 6931 11064 L
7040 11145 L 7040 10911 L 7004 10792 L 6986 10656 L
7040 10596 L 7052 10656 L 7175 10787 L 7175 10726 L
7109 10656 L 7175 10581 L 7175 10176 L 7130 10114 L
7175 10068 L 7270 10114 L 7175 10176 L 7175 10581 L
7249 10656 L 7175 10726 L 7175 10787 L 7291 10792 L
7175 10818 L 7040 10911 L 7040 11145 L 7163 11199 L
7175 11207 L 7188 11199 L 7311 11093 L 7390 11064 L
7446 10983 L 7582 11024 L 7717 11007 L 7761 10928 L
7717 10847 L 7644 10792 L 7582 10742 L 7446 10770 L
7345 10656 L 7446 10526 L 7452 10521 L 7446 10518 L
7347 10385 L 7346 10249 L 7446 10141 L 7500 10114 L
7515 9978 L 7446 9877 L 7441 9842 L 7446 9839 L
7446 8638 L 7391 8621 L 7311 8604 L 7175 8584 L
7073 8486 L 7175 8365 L 7311 8383 L 7411 8350 L
7373 8214 L 7446 8167 L 7501 8214 L 7459 8350 L
7564 8486 L 7447 8621 L 7446 8638 L 7446 9839 L
7582 9757 L 7628 9707 L 7717 9626 L 7853 9696 L

7853 9627 L 7790 9571 L 7853 9499 L 7908 9571 L
7853 9627 L 7853 9696 L 7975 9571 L 7988 9479 L
7988 9335 L 7853 9312 L 7836 9300 L 7853 9230 L
7853 8955 L 7782 8893 L 7717 8827 L 7650 8757 L
7582 8672 L 7541 8621 L 7582 8580 L 7624 8621 L
7717 8699 L 7788 8757 L 7853 8822 L 7927 8757 L
7988 8697 L 8043 8757 L 7988 8817 L 7921 8893 L
7853 8955 L 7853 9230 L 7890 9164 L 7988 9135 L
8046 9164 L 8003 9300 L 7988 9335 L 7988 9479 L
8035 9435 L 8124 9383 L 8203 9435 L 8259 9506 L
8395 9558 L 8395 9492 L 8328 9435 L 8395 9348 L
8456 9435 L 8395 9492 L 8395 9558 L 8528 9435 L
8530 9411 L 8530 9169 L 8482 9164 L 8510 9028 L
8530 8989 L 8666 8997 L 8666 8727 L 8558 8621 L
8530 8595 L 8432 8486 L 8395 8435 L 8325 8350 L
8395 8272 L 8530 8271 L 8601 8350 L 8666 8481 L
8676 8486 L 8784 8621 L 8666 8727 L 8666 8997 L
8695 9028 L 8666 9054 L 8539 9164 L 8530 9169 L
8530 9411 L 8558 9435 L 8642 9571 L 8530 9688 L
8496 9707 L 8417 9842 L 8491 9978 L 8395 10077 L
8375 10114 L 8395 10147 L 8520 10249 L 8530 10253 L
8666 10269 L 8666 9909 L 8600 9842 L 8666 9777 L
8742 9842 L 8666 9909 L 8666 10269 L 8740 10385 L
8685 10521 L 8666 10535 L 8530 10611 L 8510 10656 L
8395 10762 L 8337 10792 L 8302 10928 L 8345 11064 L
8395 11139 L 8461 11199 L 8530 11255 L 8612 11335 L
8666 11429 L 8699 11471 L 8718 11606 L 8666 11739 L
8664 11742 L 8530 11841 L 8395 11876 L 8259 11866 L
8228 11878 L 8124 11945 L 8063 12013 L 7988 12092 L
7921 12149 L 7853 12214 L 7853 12976 L 7889 12963 L
7988 12937 L 8124 12937 L 8259 12943 L 8395 12938 L
8530 12952 L 8633 12963 L 8666 12973 L 8795 13099 L
8801 13101 L 8937 13107 L 9029 13099 L 9072 13095 L
9072 11168 L 8977 11064 L 9072 10960 L 9072 9540 L
8978 9435 L 9072 9329 L 9208 9317 L 9270 9435 L
9208 9484 L 9072 9540 L 9072 10960 L 9180 11064 L
9072 11168 L 9072 13095 L 9208 13073 L 9343 12973 L
9376 12963 L 9479 12885 L 9479 12055 L 9404 12013 L
9479 11965 L 9479 10919 L 9357 10792 L 9385 10656 L
9415 10521 L 9479 10443 L 9492 10385 L 9564 10249 L
9614 10223 L 9614 9684 L 9526 9571 L 9492 9435 L
9479 9342 L 9433 9300 L 9374 9164 L 9343 9105 L
9279 9028 L 9208 8964 L 9127 8893 L 9204 8757 L

9208 8755 L 9343 8643 L 9378 8621 L 9479 8581 L
9614 8601 L 9674 8621 L 9614 8683 L 9479 8725 L
9459 8757 L 9479 8814 L 9520 8893 L 9614 8985 L
9720 9028 L 9749 9066 L 9885 9055 L 9996 9164 L
9885 9290 L 9820 9300 L 9749 9365 L 9742 9435 L
9701 9571 L 9614 9684 L 9614 10223 L 9690 10249 L
9749 10258 L 9870 10385 L 9837 10521 L 9884 10656 L
9873 10792 L 9749 10885 L 9614 10916 L 9479 10919 L
9479 11965 L 9510 12013 L 9479 12055 L 9479 12885 L
9614 12869 L 9749 12869 L 9758 12827 L 9761 12692 L
9765 12556 L 9885 12442 L 10011 12420 L 10020 12413 L
10075 12420 L 10156 12423 L 10170 12420 L 10241 12285 L
10289 12149 L 10234 12013 L 10170 11878 L 10156 11857 L
10076 11742 L 10020 11629 L 9989 11606 L 9906 11471 L
9885 11383 L 9855 11335 L 9815 11199 L 9885 11101 L
9935 11064 L 10020 10950 L 10020 10219 L 9915 10114 L
10020 10011 L 10120 10114 L 10020 10219 L 10020 10950 L
10156 10932 L 10199 10928 L 10291 10883 L 10291 9819 L
10183 9707 L 10291 9584 L 10409 9707 L 10291 9819 L
10291 10883 L 10372 10792 L 10427 10722 L 10439 10656 L
10438 10521 L 10477 10385 L 10562 10279 L 10569 10249 L
10600 10114 L 10680 9978 L 10698 9917 L 10754 9842 L
10698 9798 L 10562 9750 L 10524 9707 L 10562 9649 L
10629 9571 L 10577 9435 L 10562 9434 L 10557 9435 L
10427 9512 L 10291 9560 L 10156 9555 L 10040 9435 L
10156 9315 L 10194 9300 L 10184 9164 L 10291 9067 L
10352 9028 L 10291 8907 L 10286 8893 L 10156 8835 L
10020 8791 L 9955 8757 L 9885 8728 L 9783 8621 L
9866 8486 L 9885 8460 L 9952 8486 L 9992 8621 L
10020 8692 L 10156 8653 L 10276 8757 L 10291 8865 L
10337 8893 L 10427 8915 L 10560 9028 L 10562 9029 L
10695 9164 L 10698 9194 L 10701 9164 L 10833 9035 L
10869 9028 L 10833 9011 L 10728 8893 L 10698 8881 L
10562 8865 L 10433 8757 L 10562 8640 L 10567 8621 L
10698 8515 L 10828 8621 L 10833 8626 L 10969 8640 L
11019 8621 L 11104 8530 L 11204 8486 L 11134 8350 L
11240 8242 L 11265 8214 L 11240 8172 L 11104 8147 L
11038 8079 L 11104 8027 L 11163 7943 L 11168 7807 L
11191 7672 L 11240 7580 L 11375 7611 L 11511 7665 L
11525 7672 L 11646 7748 L 11743 7807 L 11782 7835 L
11893 7943 L 11917 7974 L 11964 8079 L 12025 8214 L
12053 8277 L 12060 8350 L 12070 8486 L 12079 8621 L
12088 8757 L 12096 8893 L 12104 9028 L 12112 9164 L

12121 9300 L 12128 9435 L 12135 9571 L 12142 9707 L
12148 9842 L 12158 9978 L 12169 10114 L 12182 10249 L
12188 10314 L 12212 10385 L 12245 10521 L 12275 10656 L
12312 10792 L 12324 10840 L 12356 10928 L 12423 11064 L
12459 11133 L 12505 11199 L 12595 11327 L 12602 11335 L
12710 11471 L 12730 11493 L 12866 11596 L 12889 11606 L
13001 11655 L 13001 10505 L 12873 10385 L 12866 10381 L
12730 10264 L 12715 10249 L 12595 10138 L 12574 10114 L
12459 10015 L 12427 9978 L 12324 9853 L 12319 9842 L
12277 9707 L 12224 9571 L 12188 9481 L 12185 9435 L
12177 9300 L 12165 9164 L 12156 9028 L 12149 8893 L
12142 8757 L 12136 8621 L 12128 8486 L 12121 8350 L
12110 8214 L 12100 8079 L 12090 7943 L 12075 7807 L
12053 7677 L 12048 7672 L 11917 7539 L 11912 7536 L
11782 7516 L 11646 7502 L 11511 7490 L 11375 7480 L
11240 7470 L 11104 7486 L 10969 7504 L 10915 7536 L
10833 7629 L 10698 7670 L 10562 7643 L 10473 7672 L
10427 7726 L 10291 7686 L 10168 7672 L 10156 7669 L
10020 7610 L 9913 7672 L 9885 7684 L 9749 7752 L
9642 7807 L 9614 7857 L 9479 7830 L 9395 7807 L
9343 7790 L 9331 7807 L 9343 7839 L 9382 7943 L
9410 8079 L 9343 8160 L 9208 8176 L 9108 8214 L
9072 8234 L 9010 8214 L 8937 8176 L 8801 8199 L
8790 8079 L 8781 7943 L 8801 7868 L 8882 7807 L
8850 7672 L 8801 7550 L 8793 7536 L 8666 7452 L
8530 7449 L 8395 7442 L 8259 7435 L 8124 7414 L
7988 7425 L 7853 7469 L 7717 7460 L 7666 7400 L
7582 7297 L 7505 7400 L 7446 7491 L 7311 7528 L
7291 7536 L 7239 7672 L 7311 7725 L 7446 7767 L
7476 7807 L 7525 7943 L 7446 7983 L 7367 7943 L
7311 7907 L 7270 7943 L 7236 8079 L 7175 8118 L
7040 8123 L 6962 8079 L 6904 8027 L 6769 8067 L
6691 7943 L 6754 7807 L 6769 7742 L 6801 7672 L
6769 7632 L 6702 7536 L 6633 7512 L 6498 7500 L
6362 7494 L 6227 7425 L 6189 7400 L 6158 7265 L
6091 7192 L 6034 7265 L 5956 7319 L 5865 7265 L
5820 7244 L 5700 7265 L 5685 7268 L 5550 7368 L
5414 7385 L 5279 7298 L 5258 7265 L 5146 7129 L
5143 7127 L 5047 6993 L 5143 6876 L 5167 6858 L
5160 6722 L 5143 6707 L 5008 6681 L 4952 6722 L
4872 6835 L 4858 6858 L 4737 6946 L 4601 6916 L
4466 6865 L 4426 6858 L 4330 6793 L 4195 6735 L
4187 6722 L 4059 6592 L 3969 6586 L 3924 6582 L

3922 6586 L 3913 6722 L 3924 6780 L 3949 6858 L
3924 6886 L 3788 6948 L 3676 6993 L 3653 7003 L
3551 6993 L 3517 6978 L 3496 6858 L 3414 6722 L
3382 6685 L 3246 6644 L 3176 6586 L 3111 6463 L
3024 6586 L 2975 6658 L 2840 6720 L 2829 6722 L
2704 6792 L 2674 6858 L 2651 6993 L 2704 7064 L
2837 7129 L 2704 7185 L 2569 7223 L 2498 7129 L
2433 7074 L N E
129 O
2298 7270 2298 7767 2433 7702 2473 7672 2525 7536 2433 7427
2409 7400 2298 7270 8 N F
165 O
2366 8350 2298 8282 2298 8747 2425 8621 2433 8586 2524 8486
2569 8450 2587 8486 2704 8603 2768 8621 2840 8637 2975 8652
3109 8757 3111 8763 3136 8893 3111 8905 2975 8897 2840 8900
2704 8949 2569 8946 2433 8974 2370 9028 2394 9164 2340 9300
2298 9351 2298 9483 2433 9463 2569 9466 2643 9571 2704 9668
2726 9707 2831 9842 2840 9849 2975 9943 3015 9978 3111 10034
3219 10114 3246 10134 3364 10249 3382 10269 3512 10385 3517 10389
3653 10437 3653 10262 3517 10254 3512 10249 3463 10114 3517 10098
3547 10114 3653 10224 3666 10249 3653 10262 3653 10437 3788 10467
3909 10521 3924 10528 4059 10568 4059 9474 4022 9435 4059 9392
4091 9435 4059 9474 4059 10568 4195 10592 4330 10636 4330 10119
4232 10114 4330 10105 4351 10114 4330 10119 4330 10636 4454 10521
4466 10515 4601 10416 4612 10385 4649 10249 4669 10114 4601 9980
4599 9978 4466 9851 4353 9842 4330 9837 4195 9808 4164 9707
4155 9571 4195 9538 4330 9492 4337 9435 4330 9427 4195 9312
4176 9300 4059 9220 4034 9164 3993 9028 3940 8893 3935 8757
3924 8652 3919 8621 3817 8486 3867 8350 3814 8214 3788 8130
3768 8079 3740 7943 3673 7807 3653 7781 3517 7677 3496 7672
3512 7536 3394 7400 3382 7393 3246 7369 3232 7400 3182 7536
3142 7672 3246 7716 3343 7807 3246 7886 3111 7925 3077 7943
2975 8048 2935 8079 2975 8105 3027 8214 3111 8291 3206 8350
3111 8481 3080 8486 2975 8558 2946 8486 2850 8350 2840 8339
2745 8214 2704 8109 2569 8111 2489 8214 2496 8350 2433 8424
2366 8350 139 N F
147 O
2298 8747 2298 9351 2340 9300 2394 9164 2370 9028 2433 8974
2569 8946 2704 8949 2840 8900 2975 8897 3111 8905 3136 8893
3111 8763 3109 8757 2975 8652 2840 8637 2768 8621 2704 8603
2587 8486 2569 8450 2524 8486 2433 8586 2425 8621 2298 8747
24 N F
129 O

2298 10136 2298 11418 2433 11453 2569 11429 2704 11425 2840 11397
2975 11362 2975 10545 2918 10521 2975 10490 3015 10521 2975 10545
2975 11362 3048 11335 3111 11264 3143 11199 3246 11084 3280 11064
3382 10961 3517 10945 3537 10928 3653 10850 3741 10792 3788 10730
3869 10656 3788 10617 3653 10543 3550 10521 3517 10516 3382 10427
3346 10385 3247 10249 3246 10248 3111 10163 3020 10114 2975 10093
2840 10041 2704 9986 2672 9978 2569 9952 2550 9978 2446 10114
2433 10122 2298 10136 44 N F
165 O
2433 11842 M 2298 11816 L 2298 11992 L 2331 12013 L 2406 12149 L
2370 12285 L 2416 12420 L 2433 12430 L 2569 12514 L
2704 12527 L 2821 12420 L 2822 12285 L 2833 12149 L
2840 12146 L 2857 12149 L 2975 12218 L 3111 12249 L
3246 12197 L 3354 12149 L 3382 12144 L 3517 12121 L
3653 12046 L 3788 12062 L 3924 12146 L 3951 12149 L
3924 12178 L 3798 12285 L 3788 12285 L 3653 12307 L
3517 12355 L 3442 12420 L 3382 12451 L 3246 12496 L
3121 12556 L 3111 12561 L 2975 12653 L 2947 12692 L
2975 12816 L 2977 12827 L 3034 12963 L 3080 13099 L
3104 13234 L 3104 13370 L 3111 13393 L 3246 13505 L
3247 13506 L 3332 13641 L 3382 13770 L 3517 13742 L
3613 13641 L 3653 13547 L 3664 13506 L 3719 13370 L
3716 13234 L 3734 13099 L 3781 12963 L 3788 12959 L
3924 12942 L 3980 12963 L 3999 13099 L 3963 13234 L
3924 13363 L 3908 13370 L 3924 13377 L 3971 13506 L
3954 13641 L 4004 13777 L 4059 13850 L 4117 13913 L
4195 13972 L 4330 14028 L 4330 13528 L 4313 13506 L
4300 13370 L 4330 13290 L 4350 13234 L 4466 13142 L
4599 13234 L 4516 13370 L 4466 13480 L 4400 13506 L
4330 13528 L 4330 14028 L 4357 14048 L 4415 14184 L
4466 14310 L 4473 14320 L 4601 14383 L 4737 14407 L
4872 14393 L 4912 14320 L 5008 14193 L 5011 14184 L
5008 14170 L 4977 14048 L 4932 13913 L 4916 13777 L
4943 13641 L 4935 13506 L 4872 13409 L 4854 13370 L
4872 13352 L 5008 13363 L 5017 13370 L 5078 13506 L
5124 13641 L 5143 13670 L 5202 13641 L 5279 13625 L
5399 13506 L 5414 13483 L 5414 13179 L 5356 13099 L
5396 12963 L 5414 12959 L 5430 12963 L 5494 13099 L
5414 13179 L 5414 13483 L 5475 13370 L 5550 13242 L
5563 13234 L 5685 13136 L 5709 13099 L 5770 12963 L
5820 12940 L 5866 12963 L 5956 13018 L 5956 11915 L
5947 11878 L 5956 11863 L 5964 11878 L 5956 11915 L
5956 13018 L 6037 13099 L 6091 13157 L 6111 13234 L

6099 13370 L 6091 13388 L 5956 13447 L 5820 13438 L
5685 13499 L 5681 13506 L 5685 13561 L 5779 13641 L
5820 13648 L 5833 13641 L 5956 13594 L 5994 13641 L
6091 13680 L 6196 13777 L 6227 13815 L 6265 13913 L
6293 14048 L 6243 14184 L 6227 14231 L 6193 14320 L
6212 14455 L 6227 14480 L 6289 14591 L 6362 14648 L
6439 14727 L 6498 14786 L 6633 14837 L 6688 14862 L
6769 14899 L 6863 14998 L 6904 15047 L 7017 15134 L
7040 15149 L 7175 15138 L 7216 15134 L 7311 15111 L
7446 15126 L 7534 14998 L 7582 14908 L 7599 14862 L
7717 14800 L 7853 14772 L 7863 14727 L 7882 14591 L
7896 14455 L 7988 14338 L 8110 14320 L 8124 14316 L
8259 14279 L 8395 14242 L 8423 14184 L 8439 14048 L
8448 13913 L 8530 13825 L 8666 13808 L 8801 13808 L
8937 13850 L 9072 13890 L 9208 13898 L 9343 13910 L
9354 13913 L 9407 14048 L 9479 14053 L 9483 14048 L
9550 13913 L 9543 13777 L 9493 13641 L 9479 13626 L
9343 13596 L 9273 13506 L 9275 13370 L 9208 13304 L
9072 13302 L 9019 13234 L 9072 13187 L 9208 13188 L
9303 13099 L 9343 13063 L 9479 13044 L 9614 13036 L
9749 13049 L 9835 12963 L 9846 12827 L 9848 12692 L
9852 12556 L 9885 12525 L 10020 12514 L 10156 12515 L
10201 12556 L 10291 12646 L 10355 12692 L 10427 12763 L
10513 12827 L 10562 12876 L 10611 12827 L 10698 12771 L
10833 12760 L 10901 12692 L 10969 12600 L 11104 12557 L
11107 12556 L 11104 12555 L 10969 12446 L 10944 12420 L
10833 12361 L 10729 12285 L 10698 12262 L 10618 12149 L
10564 12013 L 10562 12011 L 10427 12000 L 10355 11878 L
10291 11784 L 10259 11742 L 10156 11664 L 10110 11606 L
10073 11471 L 10020 11415 L 9975 11335 L 9973 11199 L
10020 11154 L 10114 11064 L 10156 11026 L 10291 11017 L
10427 10961 L 10460 10928 L 10514 10792 L 10522 10656 L
10523 10521 L 10562 10469 L 10643 10385 L 10655 10249 L
10698 10184 L 10766 10114 L 10789 9978 L 10833 9933 L
10833 9403 L 10761 9300 L 10833 9289 L 10833 9170 L
10827 9164 L 10833 9158 L 10839 9164 L 10833 9170 L
10833 9289 L 10952 9164 L 10969 9096 L 11104 9160 L
11108 9164 L 11104 9176 L 10989 9300 L 10969 9307 L
10833 9403 L 10833 9933 L 10925 9842 L 10969 9789 L
10969 9473 L 10937 9435 L 10969 9406 L 11024 9435 L
10969 9473 L 10969 9789 L 11104 9754 L 11151 9707 L
11180 9571 L 11167 9435 L 11182 9300 L 11207 9164 L
11104 9054 L 11074 9028 L 10969 8934 L 10918 8893 L

10833 8794 L 10698 8770 L 10680 8757 L 10694 8621 L
10698 8618 L 10702 8621 L 10833 8732 L 10969 8735 L
11104 8709 L 11205 8621 L 11240 8594 L 11279 8621 L
11375 8705 L 11461 8621 L 11457 8486 L 11375 8385 L
11289 8350 L 11373 8214 L 11375 8196 L 11511 8197 L
11533 8214 L 11646 8310 L 11682 8350 L 11782 8481 L
11784 8486 L 11849 8621 L 11917 8735 L 11926 8757 L
11976 8893 L 12039 9028 L 12053 9059 L 12059 9164 L
12066 9300 L 12072 9435 L 12076 9571 L 12079 9707 L
12082 9842 L 12085 9978 L 12089 10114 L 12094 10249 L
12098 10385 L 12102 10521 L 12106 10656 L 12110 10792 L
12115 10928 L 12123 11064 L 12132 11199 L 12143 11335 L
12153 11471 L 12168 11606 L 12188 11730 L 12191 11742 L
12202 11878 L 12216 12013 L 12219 12149 L 12254 12285 L
12295 12420 L 12324 12492 L 12364 12556 L 12408 12692 L
12459 12762 L 12595 12798 L 12730 12809 L 12788 12827 L
12866 12864 L 13001 12887 L 13001 11655 L 12889 11606 L
12866 11596 L 12730 11493 L 12710 11471 L 12602 11335 L
12595 11327 L 12505 11199 L 12459 11133 L 12423 11064 L
12356 10928 L 12324 10840 L 12312 10792 L 12275 10656 L
12245 10521 L 12212 10385 L 12188 10314 L 12182 10249 L
12169 10114 L 12158 9978 L 12148 9842 L 12142 9707 L
12135 9571 L 12128 9435 L 12121 9300 L 12112 9164 L
12104 9028 L 12096 8893 L 12088 8757 L 12079 8621 L
12070 8486 L 12060 8350 L 12053 8277 L 12025 8214 L
11964 8079 L 11917 7974 L 11893 7943 L 11782 7835 L
11743 7807 L 11646 7748 L 11525 7672 L 11511 7665 L
11375 7611 L 11240 7580 L 11191 7672 L 11168 7807 L
11163 7943 L 11104 8027 L 11038 8079 L 11104 8147 L
11240 8172 L 11265 8214 L 11240 8242 L 11134 8350 L
11204 8486 L 11104 8530 L 11019 8621 L 10969 8640 L
10833 8626 L 10828 8621 L 10698 8515 L 10567 8621 L
10562 8640 L 10433 8757 L 10562 8865 L 10698 8881 L
10728 8893 L 10833 9011 L 10869 9028 L 10833 9035 L
10701 9164 L 10698 9194 L 10695 9164 L 10562 9029 L
10560 9028 L 10427 8915 L 10337 8893 L 10291 8865 L
10276 8757 L 10156 8653 L 10020 8692 L 9992 8621 L
9952 8486 L 9885 8460 L 9866 8486 L 9783 8621 L
9885 8728 L 9955 8757 L 10020 8791 L 10156 8835 L
10286 8893 L 10291 8907 L 10352 9028 L 10291 9067 L
10184 9164 L 10194 9300 L 10156 9315 L 10040 9435 L
10156 9555 L 10291 9560 L 10291 9464 L 10156 9457 L
10135 9435 L 10156 9414 L 10291 9390 L 10366 9300 L

10388 9164 L 10427 9131 L 10562 9140 L 10586 9164 L
10562 9195 L 10482 9300 L 10427 9347 L 10326 9435 L
10291 9464 L 10291 9560 L 10427 9512 L 10557 9435 L
10562 9434 L 10577 9435 L 10629 9571 L 10562 9649 L
10524 9707 L 10562 9750 L 10698 9798 L 10754 9842 L
10698 9917 L 10680 9978 L 10600 10114 L 10569 10249 L
10562 10279 L 10477 10385 L 10438 10521 L 10439 10656 L
10427 10722 L 10372 10792 L 10291 10883 L 10199 10928 L
10156 10932 L 10020 10950 L 9935 11064 L 9885 11101 L
9815 11199 L 9855 11335 L 9885 11383 L 9906 11471 L
9989 11606 L 10020 11629 L 10076 11742 L 10156 11857 L
10170 11878 L 10234 12013 L 10289 12149 L 10241 12285 L
10170 12420 L 10156 12423 L 10075 12420 L 10020 12413 L
10011 12420 L 9885 12442 L 9765 12556 L 9761 12692 L
9758 12827 L 9749 12869 L 9614 12869 L 9479 12885 L
9376 12963 L 9343 12973 L 9208 13073 L 9072 13095 L
9029 13099 L 8937 13107 L 8801 13101 L 8795 13099 L
8666 12973 L 8633 12963 L 8530 12952 L 8395 12938 L
8259 12943 L 8124 12937 L 7988 12937 L 7889 12963 L
7853 12976 L 7771 13099 L 7717 13164 L 7594 13234 L
7582 13244 L 7494 13370 L 7505 13506 L 7446 13641 L
7445 13641 L 7311 13681 L 7175 13727 L 7040 13738 L
6955 13777 L 6904 13823 L 6769 13793 L 6633 13801 L
6583 13777 L 6498 13756 L 6362 13655 L 6358 13641 L
6272 13506 L 6262 13370 L 6227 13286 L 6221 13234 L
6202 13099 L 6227 12983 L 6258 12963 L 6362 12946 L
6426 12963 L 6498 12982 L 6633 13028 L 6633 12900 L
6498 12847 L 6442 12827 L 6402 12692 L 6362 12641 L
6291 12556 L 6227 12444 L 6218 12420 L 6227 12406 L
6362 12308 L 6396 12285 L 6362 12239 L 6319 12149 L
6362 12014 L 6465 12149 L 6498 12173 L 6521 12285 L
6506 12420 L 6541 12556 L 6633 12653 L 6683 12692 L
6769 12801 L 6769 12604 L 6706 12556 L 6769 12527 L
6853 12556 L 6769 12604 L 6769 12801 L 6865 12827 L
6769 12848 L 6633 12900 L 6633 13028 L 6769 13024 L
6904 13005 L 6904 12701 L 6889 12692 L 6904 12655 L
6943 12692 L 6904 12701 L 6904 13005 L 7015 12963 L
7040 12948 L 7067 12963 L 7175 13000 L 7311 13001 L
7446 12987 L 7446 12832 L 7386 12827 L 7311 12813 L
7214 12692 L 7175 12601 L 7040 12575 L 7020 12556 L
6904 12459 L 6769 12435 L 6738 12420 L 6726 12285 L
6767 12149 L 6769 12146 L 6769 11628 L 6754 11606 L
6769 11602 L 6775 11606 L 6769 11628 L 6769 12146 L

6775 12149 L 6904 12274 L 6912 12285 L 7040 12400 L
7063 12420 L 7175 12533 L 7273 12420 L 7309 12285 L
7311 12283 L 7312 12285 L 7446 12412 L 7466 12420 L
7582 12521 L 7592 12556 L 7582 12626 L 7553 12692 L
7454 12827 L 7446 12832 L 7446 12987 L 7562 12963 L
7582 12958 L 7717 12925 L 7800 12827 L 7795 12692 L
7717 12589 L 7697 12556 L 7669 12420 L 7582 12310 L
7528 12285 L 7471 12149 L 7522 12013 L 7504 11878 L
7518 11742 L 7446 11683 L 7342 11606 L 7311 11584 L
7175 11600 L 7099 11606 L 7040 11642 L 6970 11606 L
6904 11593 L 6769 11517 L 6633 11557 L 6544 11471 L
6498 11400 L 6437 11471 L 6498 11590 L 6513 11606 L
6625 11742 L 6622 11878 L 6633 11892 L 6713 12013 L
6656 12149 L 6633 12205 L 6610 12149 L 6498 12040 L
6472 12013 L 6384 11878 L 6362 11859 L 6227 11831 L
6107 11878 L 6091 11978 L 6083 11878 L 5995 11742 L
5956 11687 L 5851 11742 L 5820 11838 L 5815 11878 L
5820 11904 L 5848 12013 L 5820 12100 L 5803 12149 L
5792 12285 L 5802 12420 L 5820 12556 L 5803 12692 L
5685 12795 L 5550 12788 L 5414 12765 L 5279 12733 L
5143 12698 L 5062 12827 L 5038 12963 L 5096 13099 L
5008 13124 L 4888 13099 L 4872 13091 L 4737 13036 L
4601 12982 L 4466 12965 L 4431 12963 L 4330 12955 L
4195 12882 L 4059 12834 L 4045 12827 L 3924 12780 L
3788 12738 L 3653 12717 L 3559 12692 L 3517 12675 L
3406 12556 L 3517 12505 L 3646 12420 L 3653 12417 L
3788 12400 L 3924 12388 L 4059 12385 L 4140 12420 L
4195 12450 L 4330 12533 L 4466 12553 L 4601 12502 L
4729 12420 L 4737 12416 L 4761 12420 L 4872 12448 L
4934 12420 L 5008 12388 L 5122 12285 L 5143 12264 L
5249 12149 L 5279 12094 L 5355 12013 L 5279 11977 L
5143 11999 L 5052 12013 L 5008 12028 L 4872 12098 L
4737 12117 L 4601 12083 L 4473 12013 L 4466 12012 L
4330 11952 L 4208 11878 L 4195 11865 L 4059 11848 L
3985 11878 L 3924 11905 L 3865 11878 L 3788 11861 L
3754 11878 L 3653 11915 L 3517 12007 L 3510 12013 L
3382 12066 L 3246 12082 L 3111 12101 L 2975 12060 L
2840 12027 L 2704 12039 L 2613 12013 L 2704 11947 L
2786 11878 L 2840 11863 L 2975 11815 L 3111 11795 L
3246 11773 L 3334 11742 L 3382 11704 L 3455 11606 L
3486 11471 L 3517 11411 L 3653 11460 L 3788 11432 L
3924 11407 L 3924 11271 L 3788 11259 L 3723 11199 L
3739 11064 L 3788 11018 L 3924 11012 L 4059 11059 L

4067 11064 L 4070 11199 L 4059 11214 L 3924 11271 L
3924 11407 L 4059 11353 L 4087 11335 L 4169 11199 L
4195 11153 L 4330 11091 L 4466 11113 L 4466 10991 L
4330 10953 L 4293 10928 L 4330 10889 L 4466 10854 L
4601 10911 L 4615 10928 L 4601 10946 L 4466 10991 L
4466 11113 L 4601 11104 L 4659 11064 L 4737 10958 L
4778 10928 L 4839 10792 L 4737 10663 L 4601 10735 L
4466 10700 L 4330 10664 L 4217 10792 L 4195 10816 L
4059 10894 L 3924 10871 L 3788 10876 L 3717 10928 L
3653 10989 L 3569 11064 L 3517 11185 L 3506 11199 L
3496 11335 L 3382 11427 L 3246 11455 L 3204 11471 L
3111 11494 L 2975 11529 L 2840 11558 L 2704 11561 L
2578 11606 L 2569 11619 L 2518 11742 L 2433 11842 L
N E
183 O
2331 12013 M 2298 11992 L 2298 16219 L 2504 16219 L 2569 16219 L
2569 12975 L 2546 12963 L 2548 12827 L 2569 12812 L
2592 12827 L 2579 12963 L 2569 12975 L 2569 16219 L
2710 16219 L 2916 16219 L 3111 16219 L 3111 14870 L
3105 14862 L 3111 14855 L 3116 14862 L 3111 14870 L
3111 16219 L 3121 16219 L 3327 16219 L 3533 16219 L
3739 16219 L 3945 16219 L 4150 16219 L 4356 16219 L
4562 16219 L 4768 16219 L 4974 16219 L 5180 16219 L
5385 16219 L 5591 16219 L 5685 16219 L 5685 14291 L
5550 14250 L 5508 14184 L 5414 14096 L 5357 14048 L
5279 13956 L 5237 13913 L 5186 13777 L 5279 13660 L
5384 13777 L 5414 13789 L 5550 13850 L 5651 13913 L
5685 13937 L 5802 14048 L 5810 14184 L 5685 14291 L
5685 16219 L 5797 16219 L 6003 16219 L 6209 16219 L
6415 16219 L 6620 16219 L 6826 16219 L 7032 16219 L
7238 16219 L 7444 16219 L 7650 16219 L 7855 16219 L
8061 16219 L 8267 16219 L 8473 16219 L 8679 16219 L
8884 16219 L 9090 16219 L 9296 16219 L 9502 16219 L
9708 16219 L 9914 16219 L 10119 16219 L 10325 16219 L
10531 16219 L 10737 16219 L 10943 16219 L 11149 16219 L
11354 16219 L 11560 16219 L 11766 16219 L 11972 16219 L
12178 16219 L 12324 16219 L 12324 12835 L 12316 12827 L
12324 12808 L 12354 12827 L 12324 12835 L 12324 16219 L
12383 16219 L 12589 16219 L 12595 16219 L 12595 13656 L
12573 13641 L 12595 13552 L 12595 13302 L 12570 13234 L
12558 13099 L 12575 12963 L 12595 12933 L 12730 12900 L
12756 12963 L 12822 13099 L 12730 13234 L 12730 13234 L
12595 13302 L 12595 13552 L 12623 13641 L 12595 13656 L

12595 16219 L 12795 16219 L 13001 16219 L 13001 13180 L
12881 13099 L 13001 13038 L 13001 12887 L 12866 12864 L
12788 12827 L 12730 12809 L 12595 12798 L 12459 12762 L
12408 12692 L 12364 12556 L 12324 12492 L 12295 12420 L
12254 12285 L 12219 12149 L 12216 12013 L 12202 11878 L
12191 11742 L 12188 11730 L 12168 11606 L 12153 11471 L
12143 11335 L 12132 11199 L 12123 11064 L 12115 10928 L
12110 10792 L 12106 10656 L 12102 10521 L 12098 10385 L
12094 10249 L 12089 10114 L 12085 9978 L 12082 9842 L
12079 9707 L 12076 9571 L 12072 9435 L 12066 9300 L
12059 9164 L 12053 9059 L 12039 9028 L 11976 8893 L
11926 8757 L 11917 8735 L 11849 8621 L 11784 8486 L
11782 8481 L 11682 8350 L 11646 8310 L 11533 8214 L
11511 8197 L 11375 8196 L 11373 8214 L 11289 8350 L
11375 8385 L 11457 8486 L 11461 8621 L 11375 8705 L
11279 8621 L 11240 8594 L 11205 8621 L 11104 8709 L
10969 8735 L 10833 8732 L 10702 8621 L 10698 8618 L
10694 8621 L 10680 8757 L 10698 8770 L 10833 8794 L
10918 8893 L 10969 8934 L 11074 9028 L 11104 9054 L
11207 9164 L 11182 9300 L 11167 9435 L 11180 9571 L
11151 9707 L 11104 9754 L 10969 9789 L 10925 9842 L
10833 9933 L 10789 9978 L 10766 10114 L 10698 10184 L
10655 10249 L 10643 10385 L 10562 10469 L 10523 10521 L
10522 10656 L 10514 10792 L 10460 10928 L 10427 10961 L
10291 11017 L 10156 11026 L 10114 11064 L 10020 11154 L
9973 11199 L 9975 11335 L 10020 11415 L 10073 11471 L
10110 11606 L 10156 11664 L 10259 11742 L 10291 11784 L
10355 11878 L 10427 12000 L 10562 12011 L 10564 12013 L
10618 12149 L 10698 12262 L 10729 12285 L 10833 12361 L
10944 12420 L 10969 12446 L 11104 12555 L 11107 12556 L
11104 12557 L 10969 12600 L 10901 12692 L 10833 12760 L
10698 12771 L 10611 12827 L 10562 12876 L 10513 12827 L
10427 12763 L 10355 12692 L 10291 12646 L 10201 12556 L
10156 12515 L 10020 12514 L 9885 12525 L 9852 12556 L
9848 12692 L 9846 12827 L 9835 12963 L 9749 13049 L
9614 13036 L 9479 13044 L 9343 13063 L 9303 13099 L
9208 13188 L 9072 13187 L 9019 13234 L 9072 13302 L
9208 13304 L 9275 13370 L 9273 13506 L 9343 13596 L
9479 13626 L 9493 13641 L 9543 13777 L 9550 13913 L
9483 14048 L 9479 14053 L 9407 14048 L 9354 13913 L
9343 13910 L 9208 13898 L 9072 13890 L 8937 13850 L
8801 13808 L 8666 13808 L 8530 13825 L 8448 13913 L
8439 14048 L 8423 14184 L 8395 14242 L 8259 14279 L

8124 14316 L 8110 14320 L 7988 14338 L 7896 14455 L
7882 14591 L 7863 14727 L 7853 14772 L 7717 14800 L
7599 14862 L 7582 14908 L 7534 14998 L 7446 15126 L
7311 15111 L 7216 15134 L 7175 15138 L 7040 15149 L
7017 15134 L 6904 15047 L 6863 14998 L 6769 14899 L
6688 14862 L 6633 14837 L 6498 14786 L 6439 14727 L
6362 14648 L 6289 14591 L 6227 14480 L 6212 14455 L
6193 14320 L 6227 14231 L 6243 14184 L 6293 14048 L
6265 13913 L 6227 13815 L 6196 13777 L 6091 13680 L
5994 13641 L 5956 13594 L 5833 13641 L 5820 13648 L
5779 13641 L 5685 13561 L 5681 13506 L 5685 13499 L
5820 13438 L 5956 13447 L 6091 13388 L 6099 13370 L
6111 13234 L 6091 13157 L 6037 13099 L 5956 13018 L
5866 12963 L 5820 12940 L 5770 12963 L 5709 13099 L
5685 13136 L 5563 13234 L 5550 13242 L 5475 13370 L
5414 13483 L 5399 13506 L 5279 13625 L 5202 13641 L
5143 13670 L 5124 13641 L 5078 13506 L 5017 13370 L
5008 13363 L 4872 13352 L 4854 13370 L 4872 13409 L
4935 13506 L 4943 13641 L 4916 13777 L 4932 13913 L
4977 14048 L 5008 14170 L 5011 14184 L 5008 14193 L
4912 14320 L 4872 14393 L 4737 14407 L 4601 14383 L
4473 14320 L 4466 14310 L 4415 14184 L 4357 14048 L
4330 14028 L 4195 13972 L 4117 13913 L 4059 13850 L
4004 13777 L 3954 13641 L 3971 13506 L 3924 13377 L
3908 13370 L 3924 13363 L 3963 13234 L 3999 13099 L
3980 12963 L 3924 12942 L 3788 12959 L 3781 12963 L
3734 13099 L 3716 13234 L 3719 13370 L 3664 13506 L
3653 13547 L 3613 13641 L 3517 13742 L 3382 13770 L
3332 13641 L 3247 13506 L 3246 13505 L 3111 13393 L
3104 13370 L 3104 13234 L 3080 13099 L 3034 12963 L
2977 12827 L 2975 12816 L 2947 12692 L 2975 12653 L
3111 12561 L 3121 12556 L 3246 12496 L 3382 12451 L
3442 12420 L 3517 12355 L 3653 12307 L 3788 12285 L
3798 12285 L 3924 12178 L 3951 12149 L 3924 12146 L
3788 12062 L 3653 12046 L 3517 12121 L 3382 12144 L
3354 12149 L 3246 12197 L 3111 12249 L 2975 12218 L
2857 12149 L 2840 12146 L 2833 12149 L 2822 12285 L
2821 12420 L 2704 12527 L 2569 12514 L 2433 12430 L
2416 12420 L 2370 12285 L 2406 12149 L 2331 12013 L
N E
165 O
2569 12975 2546 12963 2548 12827 2569 12812 2592 12827 2579 12963
2569 12975 7 N F

129 O
2975 7169 2844 7129 2973 6993 2975 6992 2979 6993 3031 7129
2975 7169 7 N F
147 O
2975 10490 2918 10521 2975 10545 3015 10521 2975 10490 5 N F
165 O
3111 14870 3105 14862 3111 14855 3116 14862 3111 14870 5 N F
129 O
3246 11900 3201 11878 3246 11873 3346 11878 3246 11900 5 N F
183 O
3517 10098 3463 10114 3512 10249 3517 10254 3653 10262 3666 10249
3653 10224 3547 10114 3517 10098 9 N F
129 O
3788 11669 3699 11606 3788 11581 3924 11564 4007 11606 3924 11682
3788 11669 7 N F
183 O
3739 11064 3723 11199 3788 11259 3924 11271 4059 11214 4070 11199
4067 11064 4059 11059 3924 11012 3788 11018 3739 11064 11 N F
129 O
3924 12599 3839 12556 3924 12530 4059 12530 4111 12556 4059 12634
3924 12599 7 N F
165 O
3924 7480 3896 7536 3924 7616 3946 7672 3986 7807 4014 7943
4059 7964 4195 8024 4330 8045 4466 8075 4540 7943 4466 7845
4433 7807 4352 7672 4330 7650 4195 7593 4075 7536 4059 7528
3924 7480 19 N F
147 O
4059 9474 4022 9435 4059 9392 4091 9435 4059 9474 5 N F
165 O
4195 8217 4080 8350 4154 8486 4195 8512 4300 8621 4330 8653
4424 8757 4466 8808 4601 8801 4636 8757 4737 8632 4764 8621
4751 8486 4737 8479 4601 8447 4492 8350 4466 8334 4330 8248
4195 8217 19 N F
129 O
4195 7044 4110 6993 4195 6911 4330 6925 4347 6993 4330 7009
4195 7044 7 N F
183 O
4330 10105 4232 10114 4330 10119 4351 10114 4330 10105 5 N F
129 O
4330 12696 4279 12692 4330 12690 4384 12692 4330 12696 5 N F
183 O
4330 10889 4293 10928 4330 10953 4466 10991 4601 10946 4615 10928
4601 10911 4466 10854 4330 10889 9 N F

4330 13290 4300 13370 4313 13506 4330 13528 4400 13506 4466 13480
4516 13370 4599 13234 4466 13142 4350 13234 4330 13290 11 N F
111 O
4330 5735 4304 5637 4330 5618 4339 5637 4330 5735 5 N F
129 O
4330 11367 4316 11335 4328 11199 4330 11198 4466 11199 4466 11199
4601 11297 4737 11259 4783 11199 4872 11146 5008 11090 5045 11064
5105 10928 5135 10792 5008 10684 4998 10656 5008 10596 5143 10633
5207 10656 5279 10752 5414 10709 5503 10656 5550 10559 5584 10656
5685 10766 5690 10792 5757 10928 5745 11064 5788 11199 5820 11228
5956 11311 5969 11335 6091 11438 6227 11414 6290 11471 6227 11526
6091 11584 5956 11526 5842 11606 5820 11616 5738 11742 5708 11878
5685 11972 5550 11951 5515 11878 5414 11794 5279 11782 5143 11857
5112 11878 5008 11910 5008 11373 5109 11335 5008 11292 4981 11335
5008 11373 5008 11910 4895 11878 4872 11856 4801 11742 4737 11691
4601 11728 4498 11606 4543 11471 4466 11417 4330 11367 65 N F
4872 7431 4843 7400 4872 7371 4980 7400 4872 7431 5 N F
4864 10385 4863 10249 4872 10236 5008 10232 5048 10249 5049 10385
5008 10447 4872 10391 4864 10385 9 N F
111 O
5008 11373 4981 11335 5008 11292 5109 11335 5008 11373 5 N F
129 O
5143 7562 5054 7536 5143 7455 5279 7507 5363 7536 5279 7566
5143 7562 7 N F
165 O
5237 13913 5186 13777 5279 13660 5384 13777 5414 13789 5550 13850
5651 13913 5685 13937 5802 14048 5810 14184 5685 14291 5550 14250
5508 14184 5414 14096 5357 14048 5279 13956 5237 13913 17 N F
147 O
5414 6894 5325 6993 5380 7129 5414 7159 5550 7223 5598 7129
5576 6993 5550 6896 5414 6894 9 N F
183 O
5396 12963 5356 13099 5414 13179 5494 13099 5430 12963 5414 12959
5396 12963 7 N F
129 O
5540 7807 5485 7672 5550 7536 5685 7660 5689 7672 5685 7715
5598 7807 5550 7817 5540 7807 9 N F
5550 12467 5532 12420 5550 12394 5563 12420 5550 12467 5 N F
147 O
5685 5580 5578 5637 5685 5723 5742 5637 5685 5580 5 N F
129 O
5685 10151 5620 10114 5685 10031 5703 10114 5685 10151 5 N F
111 O

5664 6315 M 5651 6179 L 5685 6087 L 5727 6044 L 5820 5947 L
5836 5908 L 5949 5772 L 5956 5769 L 6091 5703 L
6126 5637 L 6091 5531 L 6077 5501 L 6209 5501 L
6240 5501 L 6227 5544 L 6225 5637 L 6227 5652 L
6339 5772 L 6362 5812 L 6415 5772 L 6498 5751 L
6633 5742 L 6707 5772 L 6769 5804 L 6904 5812 L
7040 5831 L 7175 5907 L 7176 5908 L 7311 6023 L
7333 6044 L 7414 6179 L 7446 6263 L 7582 6291 L
7717 6285 L 7853 6267 L 7988 6282 L 8049 6315 L
8124 6363 L 8241 6451 L 8259 6471 L 8395 6561 L
8430 6586 L 8530 6646 L 8642 6722 L 8666 6735 L
8801 6836 L 8855 6858 L 8937 6894 L 9072 6922 L
9208 6917 L 9279 6858 L 9268 6722 L 9243 6586 L
9300 6451 L 9343 6419 L 9367 6451 L 9449 6586 L
9445 6722 L 9479 6739 L 9492 6722 L 9512 6586 L
9544 6451 L 9505 6315 L 9479 6220 L 9450 6179 L
9457 6044 L 9466 5908 L 9479 5833 L 9486 5772 L
9590 5637 L 9614 5624 L 9654 5637 L 9749 5682 L
9858 5772 L 9885 5890 L 9911 5772 L 9904 5637 L
9885 5611 L 9836 5501 L 9914 5501 L 10119 5501 L
10325 5501 L 10531 5501 L 10737 5501 L 10943 5501 L
11149 5501 L 11354 5501 L 11560 5501 L 11766 5501 L
11972 5501 L 12178 5501 L 12383 5501 L 12589 5501 L
12795 5501 L 13001 5501 L 13001 6753 L 12998 6858 L
13001 6886 L 13001 7222 L 12997 7265 L 12980 7400 L
12980 7536 L 12997 7672 L 13001 7686 L 13001 8060 L
12996 8079 L 12967 8214 L 12943 8350 L 12930 8486 L
12906 8621 L 12892 8757 L 12866 8842 L 12730 8864 L
12595 8808 L 12459 8800 L 12365 8757 L 12324 8735 L
12254 8621 L 12188 8520 L 12187 8486 L 12181 8350 L
12175 8214 L 12169 8079 L 12165 7943 L 12162 7807 L
12154 7672 L 12140 7536 L 12053 7445 L 11917 7430 L
11782 7424 L 11646 7415 L 11511 7409 L 11375 7402 L
11343 7400 L 11240 7368 L 11104 7373 L 11040 7400 L
10969 7406 L 10833 7443 L 10698 7473 L 10698 5941 L
10833 5934 L 10868 5908 L 10899 5772 L 10833 5685 L
10698 5697 L 10562 5711 L 10496 5772 L 10562 5866 L
10671 5908 L 10698 5941 L 10698 7473 L 10562 7479 L
10562 6861 L 10607 6858 L 10599 6722 L 10562 6651 L
10521 6722 L 10539 6858 L 10562 6861 L 10562 7479 L
10427 7454 L 10427 6459 L 10428 6451 L 10427 6449 L
10426 6451 L 10427 6459 L 10427 7454 L 10291 7429 L
10291 5918 L 10325 5908 L 10291 5899 L 10278 5908 L

10291 5918 L 10291 7429 L 10156 7407 L 10020 7415 L
10020 7178 L 10053 7129 L 10085 6993 L 10115 6858 L
10098 6722 L 10020 6624 L 10005 6586 L 9971 6451 L
9947 6315 L 9897 6179 L 9885 6167 L 9845 6044 L
9798 5908 L 9749 5851 L 9652 5772 L 9614 5740 L
9585 5772 L 9523 5908 L 9507 6044 L 9506 6179 L
9549 6315 L 9614 6426 L 9628 6451 L 9620 6586 L
9614 6670 L 9604 6722 L 9614 6737 L 9664 6858 L
9749 6952 L 9836 6993 L 9885 7074 L 9973 7129 L
10020 7178 L 10020 7415 L 9885 7424 L 9749 7441 L
9614 7477 L 9479 7469 L 9343 7458 L 9208 7449 L
9072 7421 L 9023 7400 L 8937 7354 L 8819 7265 L
8801 7252 L 8709 7129 L 8666 7080 L 8588 6993 L
8530 6939 L 8465 6993 L 8395 7097 L 8350 7129 L
8259 7259 L 8197 7129 L 8124 7045 L 7988 7032 L
7853 7069 L 7741 6993 L 7717 6970 L 7614 6858 L
7582 6815 L 7564 6858 L 7511 6993 L 7446 7044 L
7311 7072 L 7175 7006 L 7151 6993 L 7040 6932 L
6970 6858 L 6904 6780 L 6769 6795 L 6633 6755 L
6498 6736 L 6490 6722 L 6488 6586 L 6362 6498 L
6227 6491 L 6091 6451 L 6090 6451 L 5956 6374 L
5825 6451 L 5851 6586 L 5820 6599 L 5794 6586 L
5685 6464 L 5677 6451 L 5664 6315 L N E
165 O
5797 9435 5765 9571 5820 9674 5844 9707 5956 9817 5956 9614
5933 9571 5956 9556 5987 9571 5956 9614 5956 9817 6074 9842
5964 9978 6052 10114 6003 10249 5956 10252 5895 10385 5869 10521
5883 10656 5870 10792 5903 10928 5853 11064 5956 11143 6091 11113
6227 11104 6361 11199 6362 11202 6362 10822 6323 10792 6362 10764
6389 10792 6362 10822 6362 11202 6367 11199 6370 11064 6498 10975
6574 10928 6559 10792 6633 10736 6769 10770 6808 10656 6769 10627
6652 10521 6633 10500 6568 10521 6498 10545 6362 10573 6227 10528
6217 10521 6227 10490 6227 10163 6188 10114 6227 10073 6227 9755
6207 9707 6227 9689 6245 9707 6227 9755 6227 10073 6264 10114
6227 10163 6227 10490 6291 10385 6362 10309 6381 10249 6362 10189
6355 10114 6255 9978 6258 9842 6362 9777 6407 9707 6399 9571
6362 9509 6271 9435 6241 9300 6227 9263 6091 9210 5981 9164
6056 9028 6086 8893 6091 8860 6116 8757 6091 8664 5956 8628
5820 8732 5816 8757 5820 8765 5883 8893 5840 9028 5933 9164
5956 9247 5965 9300 5956 9319 5820 9405 5797 9435 95 N F
5915 8350 5899 8486 5956 8578 6003 8486 6017 8350 6020 8214
5956 8081 5910 8214 5915 8350 9 N F
183 O

5956 9556 5933 9571 5956 9614 5987 9571 5956 9556 5 N F
5956 11863 5947 11878 5956 11915 5964 11878 5956 11863 5 N F
111 O
6077 5501 6077 5501 6209 5501 6240 5501 6240 5501 6209 5501
6077 5501 7 N F
129 O
6091 11749 6086 11742 6091 11670 6227 11714 6362 11711 6443 11742
6362 11751 6227 11750 6091 11749 9 N F
6091 11201 6089 11199 6091 11199 6227 11195 6232 11199 6227 11212
6091 11201 7 N F
183 O
6227 10073 6188 10114 6227 10163 6264 10114 6227 10073 5 N F
147 O
6227 9755 6207 9707 6227 9689 6245 9707 6227 9755 5 N F
183 O
6227 12406 6218 12420 6227 12444 6291 12556 6362 12641 6402 12692
6442 12827 6498 12847 6633 12900 6769 12848 6865 12827 6769 12801
6683 12692 6633 12653 6541 12556 6506 12420 6521 12285 6498 12173
6465 12149 6362 12014 6319 12149 6362 12239 6396 12285 6362 12308
6227 12406 25 N F
92 O
6227 5652 6225 5637 6227 5544 6240 5501 6415 5501 6620 5501
6826 5501 7032 5501 7238 5501 7444 5501 7650 5501 7658 5501
7717 5510 7853 5508 7988 5505 8124 5507 8157 5501 8267 5501
8473 5501 8679 5501 8884 5501 9090 5501 9296 5501 9502 5501
9708 5501 9836 5501 9885 5611 9904 5637 9911 5772 9885 5890
9858 5772 9749 5682 9654 5637 9614 5624 9590 5637 9486 5772
9479 5833 9466 5908 9457 6044 9450 6179 9479 6220 9505 6315
9544 6451 9512 6586 9492 6722 9479 6739 9445 6722 9449 6586
9367 6451 9343 6419 9300 6451 9243 6586 9268 6722 9279 6858
9208 6917 9072 6922 8937 6894 8937 6368 9072 6337 9082 6315
9193 6179 9208 6162 9343 6163 9374 6044 9344 5908 9343 5907
9339 5908 9208 5955 9208 5836 9322 5772 9220 5637 9208 5631
9205 5637 9156 5772 9208 5836 9208 5955 9184 5908 9089 5772
9087 5637 9072 5553 8937 5563 8801 5601 8666 5628 8629 5637
8530 5696 8395 5755 8363 5772 8342 5908 8380 6044 8395 6052
8530 6164 8552 6179 8666 6257 8757 6315 8801 6340 8937 6368
8937 6894 8855 6858 8801 6836 8666 6735 8642 6722 8530 6646
8430 6586 8395 6561 8259 6471 8241 6451 8124 6363 8049 6315
7988 6282 7853 6267 7717 6285 7582 6291 7446 6263 7414 6179
7333 6044 7311 6023 7176 5908 7175 5907 7040 5831 6904 5812
6769 5804 6707 5772 6633 5742 6498 5751 6415 5772 6362 5812
6339 5772 6227 5652 128 N F

183 O
6362 10764 6323 10792 6362 10822 6389 10792 6362 10764 5 N F
129 O
6633 11477 6626 11471 6633 11456 6655 11471 6633 11477 5 N F
147 O
6769 12604 6706 12556 6769 12527 6853 12556 6769 12604 5 N F
183 O
6767 12149 6726 12285 6738 12420 6769 12435 6904 12459 7020 12556
7040 12575 7175 12601 7214 12692 7311 12813 7386 12827 7446 12832
7454 12827 7553 12692 7582 12626 7592 12556 7582 12521 7466 12420
7446 12412 7312 12285 7311 12283 7309 12285 7273 12420 7175 12533
7063 12420 7040 12400 6912 12285 6904 12274 6775 12149 6769 12146
6767 12149 31 N F
129 O
6754 11335 6746 11199 6769 11159 6833 11064 6769 10971 6761 10928
6769 10923 6904 10916 6993 10928 6931 11064 7040 11145 7163 11199
7175 11207 7188 11199 7311 11093 7390 11064 7446 10983 7582 11024
7717 11007 7761 10928 7717 10847 7717 10359 7853 10356 7914 10249
7988 10175 8078 10114 8124 10072 8198 9978 8124 9902 7988 9953
7890 9978 7853 9993 7836 9978 7717 9920 7629 9978 7611 10114
7648 10249 7717 10359 7717 10847 7644 10792 7582 10742 7446 10770
7345 10656 7446 10526 7452 10521 7446 10518 7347 10385 7346 10249
7446 10141 7500 10114 7515 9978 7446 9877 7441 9842 7446 9839
7582 9757 7628 9707 7717 9626 7853 9696 7975 9571 7988 9479
8035 9435 8124 9383 8203 9435 8259 9506 8395 9558 8528 9435
8530 9411 8558 9435 8642 9571 8530 9688 8496 9707 8417 9842
8491 9978 8395 10077 8375 10114 8395 10147 8520 10249 8530 10253
8666 10269 8740 10385 8685 10521 8666 10535 8530 10611 8510 10656
8395 10762 8337 10792 8302 10928 8345 11064 8395 11139 8461 11199
8530 11255 8612 11335 8666 11429 8699 11471 8718 11606 8666 11739
8664 11742 8530 11841 8395 11876 8259 11866 8228 11878 8124 11945
8063 12013 7988 12092 7988 11452 8102 11335 7988 11215 7869 11335
7988 11452 7988 12092 7921 12149 7853 12214 7803 12149 7717 12112
7657 12013 7582 11933 7567 11878 7582 11825 7678 11742 7582 11606
7581 11606 7446 11580 7311 11504 7175 11496 7091 11471 7040 11436
6904 11379 6769 11356 6754 11335 129 N F
183 O
6769 11602 6754 11606 6769 11628 6775 11606 6769 11602 5 N F
129 O
6769 10522 6768 10521 6769 10516 6904 10411 6928 10521 6904 10541
6769 10522 7 N F
147 O
6904 12701 6889 12692 6904 12655 6943 12692 6904 12701 5 N F

129 O
7004 10792 6986 10656 7040 10596 7052 10656 7175 10787 7291 10792
7175 10818 7040 10911 7004 10792 9 N F
7175 8584 7073 8486 7175 8365 7311 8383 7411 8350 7373 8214
7446 8167 7501 8214 7459 8350 7564 8486 7447 8621 7446 8638
7391 8621 7311 8604 7175 8584 15 N F
165 O
7175 10581 7109 10656 7175 10726 7175 10666 7166 10656 7175 10646
7185 10656 7175 10666 7175 10726 7249 10656 7175 10581 11 N F
129 O
7175 10176 7130 10114 7175 10068 7270 10114 7175 10176 5 N F
183 O
7175 10646 7166 10656 7175 10666 7185 10656 7175 10646 5 N F
165 O
7582 8580 7541 8621 7582 8672 7650 8757 7717 8827 7782 8893
7853 8955 7921 8893 7988 8817 8043 8757 7988 8697 7927 8757
7853 8822 7788 8757 7717 8699 7624 8621 7582 8580 17 N F
147 O
7629 9978 7611 10114 7648 10249 7717 10359 7853 10356 7914 10249
7988 10175 8078 10114 8124 10072 8198 9978 8124 9902 7988 9953
7890 9978 7853 9993 7836 9978 7717 9920 7629 9978 17 N F
111 O
7855 5501 7658 5501 7717 5510 7853 5508 7988 5505 8124 5507
8157 5501 8061 5501 7855 5501 9 N F
165 O
7853 9499 7790 9571 7853 9627 7908 9571 7853 9499 5 N F
129 O
7853 9312 7836 9300 7853 9230 7890 9164 7988 9135 8046 9164
8003 9300 7988 9335 7853 9312 9 N F
147 O
7988 11215 7869 11335 7988 11452 7988 11394 7928 11335 7988 11274
8046 11335 7988 11394 7988 11452 8102 11335 7988 11215 11 N F
165 O
7988 11274 7928 11335 7988 11394 7988 11337 7986 11335 7988 11333
7990 11335 7988 11337 7988 11394 8046 11335 7988 11274 11 N F
183 O
7988 11333 7986 11335 7988 11337 7990 11335 7988 11333 5 N F
165 O
8395 8272 8325 8350 8395 8435 8432 8486 8530 8595 8558 8621
8666 8727 8784 8621 8676 8486 8666 8481 8601 8350 8530 8271
8395 8272 13 N F
8395 9348 8328 9435 8395 9492 8456 9435 8395 9348 5 N F
74 O

8380 6044 8342 5908 8363 5772 8395 5755 8530 5696 8629 5637
8666 5628 8801 5601 8937 5563 9072 5553 9087 5637 9089 5772
9184 5908 9208 5955 9339 5908 9343 5907 9344 5908 9374 6044
9343 6163 9208 6162 9193 6179 9082 6315 9072 6337 8937 6368
8801 6340 8757 6315 8666 6257 8552 6179 8530 6164 8395 6052
8380 6044 31 N F
165 O
8510 9028 8482 9164 8530 9169 8539 9164 8666 9054 8695 9028
8666 8997 8530 8989 8510 9028 9 N F
8666 9777 8600 9842 8666 9909 8742 9842 8666 9777 5 N F
9072 10960 8977 11064 9072 11168 9072 11092 9046 11064 9072 11035
9102 11064 9072 11092 9072 11168 9180 11064 9072 10960 11 N F
9072 9329 8978 9435 9072 9540 9072 9459 9051 9435 9072 9411
9109 9435 9072 9459 9072 9540 9208 9484 9270 9435 9208 9317
9072 9329 13 N F
183 O
9072 11035 9046 11064 9072 11092 9102 11064 9072 11035 5 N F
9072 9411 9051 9435 9072 9459 9109 9435 9072 9411 5 N F
165 O
9204 8757 9127 8893 9208 8964 9279 9028 9343 9105 9374 9164
9433 9300 9479 9342 9479 9060 9419 9028 9479 8985 9519 9028
9479 9060 9479 9342 9492 9435 9526 9571 9614 9684 9614 9485
9578 9435 9614 9377 9652 9435 9614 9485 9614 9684 9701 9571
9742 9435 9749 9365 9820 9300 9885 9290 9996 9164 9885 9055
9749 9066 9720 9028 9614 8985 9520 8893 9479 8814 9459 8757
9479 8725 9614 8683 9674 8621 9614 8601 9479 8581 9378 8621
9343 8643 9208 8755 9204 8757 45 N F
111 O
9205 5637 9156 5772 9208 5836 9322 5772 9220 5637 9208 5631
9205 5637 7 N F
165 O
9385 10656 9357 10792 9479 10919 9479 10835 9437 10792 9479 10731
9534 10792 9479 10835 9479 10919 9614 10916 9614 10494 9569 10385
9614 10331 9749 10338 9794 10385 9749 10456 9614 10494 9614 10916
9749 10885 9873 10792 9884 10656 9837 10521 9870 10385 9749 10258
9690 10249 9614 10223 9564 10249 9492 10385 9479 10443 9415 10521
9385 10656 31 N F
9479 11965 9404 12013 9479 12055 9510 12013 9479 11965 5 N F
147 O
9479 9060 9419 9028 9479 8985 9519 9028 9479 9060 5 N F
183 O
9479 10731 9437 10792 9479 10835 9534 10792 9479 10731 5 N F
129 O

9507 6044 9506 6179 9549 6315 9614 6426 9628 6451 9620 6586
9614 6670 9604 6722 9614 6737 9664 6858 9749 6952 9836 6993
9885 7074 9885 6933 9749 6871 9737 6858 9658 6722 9664 6586
9674 6451 9614 6350 9594 6315 9544 6179 9547 6044 9573 5908
9614 5835 9701 5908 9749 5954 9794 6044 9834 6179 9866 6315
9885 6350 9921 6451 9957 6586 10008 6722 10017 6858 9885 6933
9885 7074 9973 7129 10020 7178 10053 7129 10085 6993 10115 6858
10098 6722 10020 6624 10005 6586 9971 6451 9947 6315 9897 6179
9885 6167 9845 6044 9798 5908 9749 5851 9652 5772 9614 5740
9585 5772 9523 5908 9507 6044 57 N F
147 O
9547 6044 9544 6179 9594 6315 9614 6350 9674 6451 9664 6586
9658 6722 9737 6858 9749 6871 9885 6933 9885 6839 9749 6792
9706 6722 9707 6586 9719 6451 9677 6315 9614 6263 9581 6179
9586 6044 9614 5936 9730 6044 9749 6068 9779 6179 9794 6315
9846 6451 9885 6497 9910 6586 9936 6722 9885 6839 9885 6933
10017 6858 10008 6722 9957 6586 9921 6451 9885 6350 9866 6315
9834 6179 9794 6044 9749 5954 9701 5908 9614 5835 9573 5908
9547 6044 43 N F
183 O
9614 10331 9569 10385 9614 10494 9749 10456 9794 10385 9749 10338
9614 10331 7 N F
9614 9377 9578 9435 9614 9485 9652 9435 9614 9377 5 N F
165 O
9586 6044 9581 6179 9614 6263 9677 6315 9719 6451 9707 6586
9706 6722 9749 6792 9885 6839 9936 6722 9910 6586 9885 6497
9846 6451 9794 6315 9779 6179 9749 6068 9730 6044 9614 5936
9586 6044 19 N F
10020 10011 9915 10114 10020 10219 10020 10128 10006 10114 10020 10100
10034 10114 10020 10128 10020 10219 10120 10114 10020 10011 11 N F
183 O
10020 10100 10006 10114 10020 10128 10034 10114 10020 10100 5 N F
10156 9414 10135 9435 10156 9457 10291 9464 10326 9435 10427 9347
10482 9300 10562 9195 10586 9164 10562 9140 10427 9131 10388 9164
10366 9300 10291 9390 10156 9414 15 N F
165 O
10291 9584 10183 9707 10291 9819 10291 9722 10277 9707 10291 9690
10307 9707 10291 9722 10291 9819 10409 9707 10291 9584 11 N F
183 O
10291 9690 10277 9707 10291 9722 10307 9707 10291 9690 5 N F
92 O
10291 5918 10278 5908 10291 5899 10325 5908 10291 5918 5 N F
10427 6459 10426 6451 10427 6449 10428 6451 10427 6459 5 N F

10562 5866 10496 5772 10562 5711 10698 5697 10833 5685 10899 5772
10868 5908 10833 5934 10698 5941 10671 5908 10562 5866 11 N F
10539 6858 10521 6722 10562 6651 10599 6722 10607 6858 10562 6861
10539 6858 7 N F
147 O
10833 9403 10761 9300 10833 9289 10952 9164 10969 9096 11104 9160
11108 9164 11104 9176 10989 9300 10969 9307 10833 9403 11 N F
183 O
10833 9158 10827 9164 10833 9170 10839 9164 10833 9158 5 N F
10969 9406 10937 9435 10969 9473 11024 9435 10969 9406 5 N F
165 O
12324 12835 12316 12827 12324 12808 12354 12827 12324 12835 5 N F
12570 13234 12558 13099 12575 12963 12595 12933 12730 12900 12756 12963
12822 13099 12730 13234 12730 13234 12595 13302 12570 13234 11 N F
12595 13656 12573 13641 12595 13552 12623 13641 12595 13656 5 N F
13001 13180 12881 13099 13001 13038 13001 13180 4 N F
129 O
12997 7265 12980 7400 12980 7536 12997 7672 13001 7686 13001 7222
12997 7265 7 N F
13001 6753 12998 6858 13001 6886 13001 6753 4 N F
111 O
13001 5501 13001 5501 13001 6753 13001 6751 13001 5501 13001 5501
6 N F
129 O
13001 6886 13001 6753 13001 6751 13001 6887 13001 6886 5 N F
111 O
13001 7222 13001 6886 13001 6887 13001 7221 13001 7222 5 N F
129 O
13001 7686 13001 7222 13001 7221 13001 7686 13001 7686 5 N F
111 O
13001 8060 13001 7686 13001 7686 13001 8060 13001 8060 5 N F
129 O
13001 10505 13001 8060 13001 8060 13001 10505 13001 10505 5 N F
147 O
13001 11655 13001 10505 13001 10505 13001 11655 13001 11655 5 N F
165 O
13001 12887 13001 11655 13001 11655 13001 12887 13001 12887 5 N F
183 O
13001 13038 13001 12887 13001 12887 13001 13038 13001 13038 5 N F
165 O
13001 13180 13001 13038 13001 13038 13001 13180 13001 13180 5 N F
183 O
13001 16219 13001 13180 13001 13180 13001 16219 13001 16219 13001 16219

6 N F
129 O
13001 6750 13001 6751 13001 6887 13001 6887 13001 6858 13001 6750
6 N F
111 O
13001 6887 13001 7221 13001 7221 13001 7129 13001 6993 13001 6887
13001 6887 7 N F
129 O
13001 7221 13001 7221 13001 7686 13001 7686 13001 7672 13001 7536
13001 7400 13001 7265 13001 7221 9 N F
111 O
13001 7686 13001 8060 13001 8060 13001 7943 13001 7807 13001 7686
13001 7686 7 N F
129 O
13001 8060 13001 8060 13001 10505 13001 10505 13001 10385 13001 10249
13001 10114 13001 9978 13001 9842 13001 9707 13001 9571 13001 9435
13001 9300 13001 9164 13001 9028 13001 8893 13001 8757 13001 8621
13001 8486 13001 8350 13001 8214 13001 8079 13001 8060 23 N F
147 O
13001 10505 13001 10505 13001 11655 13001 11655 13001 11606 13001 11471
13001 11335 13001 11199 13001 11064 13001 10928 13001 10792 13001 10656
13001 10521 13001 10505 14 N F
165 O
13001 11655 13001 11655 13001 12887 13001 12887 13001 12827 13001 12692
13001 12556 13001 12420 13001 12285 13001 12149 13001 12013 13001 11878
13001 11742 13001 11655 14 N F
183 O
13001 12887 13001 12887 13001 13038 13001 13038 13001 12963 13001 12887
6 N F
165 O
13001 13038 13001 13180 13001 13180 13001 13099 13001 13038 13001 13038
6 N F
Ls Gr Gs Lr
-1150 1075 16450 18700 Oc
1 O
10638 15415 10639 15455 10678 15493 10675 15533 10710 15574 10753 15608
10791 15646 10826 15687 10872 15717 10920 15742 10946 15789 10984 15827
11030 15857 11080 15877 11122 15912 11160 15949 11196 15980 11246 16007
18 P
11246 16007 11300 16018 11330 16063 11355 16110 11389 16152 11419 16197
11427 16219 7 P
12839 16219 12841 16212 12835 16186 12811 16174 12785 16169 12766 16149
12742 16136 12718 16125 12697 16107 12681 16086 12655 16078 12637 16058

12633 16031 12625 16005 12620 15978 12608 15955 12583 15943 12561 15928
12544 15907 12527 15885 12505 15870 12481 15858 12457 15845 12433 15833
12425 15807 12448 15793 12444 15766 12418 15757 12401 15736 12376 15724
12359 15704 12333 15696 12323 15671 12298 15680 12282 15657 12257 15648
12230 15651 12204 15657 12181 15641 12168 15617 12146 15602 12148 15575
12127 15558 12107 15540 12081 15533 12067 15510 12040 15511 12022 15491
12009 15467 12002 15441 50 P
12002 15441 12008 15415 12034 15407 12040 15380 12028 15356 12025 15329
12033 15303 12044 15279 12053 15253 12039 15276 12012 15272 11 P
12012 15272 11986 15265 12012 15256 12006 15230 11983 15215 11980 15188
11994 15165 12017 15151 12045 15149 12071 15148 12098 15142 12123 15132
12146 15147 12162 15125 12178 15103 12195 15082 12212 15061 12234 15045
12255 15027 12278 15013 12305 15010 12328 14995 12346 14975 12357 14950
12383 14960 12409 14951 12432 14938 12437 14911 28 P
12437 14911 12447 14886 12462 14863 12475 14839 12493 14819 12502 14793
12518 14771 12533 14749 12546 14725 12562 14703 12574 14678 12586 14654
12605 14635 12621 14613 12640 14593 12655 14571 12675 14552 12691 14530
12711 14511 12730 14492 12751 14475 12772 14457 12792 14439 12798 14413
12817 14395 12837 14375 12843 14349 12863 14331 12878 14308 12899 14291
12914 14268 12933 14249 12950 14227 12960 14202 12967 14176 12979 14152
12998 14132 13001 14107 38 P
13001 13054 12997 13054 13001 13039 3 P
13001 13026 12979 13016 12958 12998 12950 13025 12951 13052 12936 13029
12909 13027 12882 13019 12855 13020 9 P
12855 13020 12828 13024 12806 13040 12782 13053 12788 13026 12797 13000
6:00 PM
12797 13000 12770 13008 12743 13010 12729 12986 12747 12965 12728 12945
12726 12917 12725 12890 12743 12869 12741 12842 12729 12817 12707 12833
12691 12856 12718 12849 12712 12876 12684 12876 12687 12903 12662 12893
12637 12905 12628 12879 12601 12878 12575 12887 12549 12898 12548 12925
12546 12953 12539 12926 12513 12917 12490 12902 12467 12886 12441 12894
12415 12888 12398 12867 12374 12853 12357 12875 12350 12848 12364 12825
12388 12812 12409 12830 12436 12836 12441 12809 12438 12782 12453 12759
12462 12733 12463 12706 12437 12698 12436 12725 12441 12752 12415 12757
12388 12751 12389 12724 50 P
12389 12724 12365 12710 12353 12734 12363 12760 12351 12785 12337 12808
12324 12833 12312 12808 12286 12800 12258 12799 12284 12790 12261 12775
12269 12748 12286 12727 12309 12712 12331 12695 12330 12668 12303 12668
12276 12674 12273 12647 12300 12651 12298 12624 12276 12607 12265 12583
12244 12600 12228 12622 12208 12642 12193 12618 12167 12627 12176 12653
12187 12678 12191 12651 12215 12665 12214 12693 12213 12720 12234 12702
12251 12723 12247 12750 12222 12740 12195 12731 12178 12711 12152 12699
12132 12681 12108 12667 12105 12640 12096 12614 12107 12589 12091 12566

12063 12568 12037 12562 50 P
12037 12562 12023 12585 2 P
12023 12585 12021 12613 12010 12638 12008 12611 12008 12583 11984 12570
11977 12543 11955 12528 11951 12500 11933 12479 11911 12463 11909 12490
11886 12506 11903 12527 11879 12540 11871 12566 11889 12586 11909 12606
11881 12607 11860 12590 11835 12600 11814 12617 11786 12622 11774 12647
11771 12674 11780 12700 11803 12686 11806 12658 11820 12635 11845 12624
11872 12627 11852 12645 11826 12654 11827 12681 11854 12683 11860 12656
11887 12649 11915 12651 11905 12677 11883 12694 11856 12697 11829 12696
11850 12713 11877 12710 11884 12736 11911 12740 11936 12752 11909 12754
11905 12781 11881 12795 50 P
11881 12795 11879 12822 11877 12795 11887 12769 11886 12741 11859 12742
11835 12728 11825 12754 11832 12781 11822 12806 11837 12829 11810 12824
11794 12802 11773 12820 11799 12831 11811 12855 11788 12870 11768 12889
11771 12862 11751 12881 11743 12907 11770 12901 11782 12925 11780 12898
11799 12878 11817 12857 11835 12878 11845 12904 11818 12910 11796 12926
11800 12953 11773 12960 11782 12986 11788 13013 11794 13040 11816 13023
11825 13050 11798 13054 11811 13079 11815 13105 11827 13130 11853 13139
11879 13148 11906 13145 11904 13173 11877 13173 11851 13168 11823 13166
11809 13190 11828 13209 50 P
11828 13209 11848 13228 2 P
11848 13228 11867 13249 11894 13250 11899 13277 11924 13288 11909 13310
11928 13330 11947 13350 11920 13344 11893 13341 11866 13336 11858 13361
11873 13385 11897 13396 11918 13414 11943 13425 11963 13443 11965 13470
11959 13444 11937 13428 11912 13417 11888 13403 11868 13422 11856 13447
11835 13464 11809 13473 11831 13490 11809 13506 11807 13533 11793 13557
11806 13581 11783 13598 11782 13625 11806 13639 11806 13655 11781 13627
11767 13650 11781 13674 11757 13686 11758 13714 11760 13741 11755 13768
11732 13752 11715 13732 11710 13758 11701 13784 11682 13765 11696 13742
11691 13715 11716 13704 50 P
11716 13704 11720 13677 11731 13652 11742 13627 11721 13611 11705 13633
11701 13659 11694 13686 11677 13707 11678 13735 11660 13755 11634 13747
11635 13774 11610 13786 11600 13760 11594 13787 11604 13812 11630 13818
11629 13846 11636 13872 11660 13884 11659 13912 11665 13885 11672 13859
11670 13831 11693 13815 11677 13838 11701 13850 11716 13873 11699 13893
11676 13909 11696 13927 11722 13935 11731 13910 11738 13883 11739 13856
11745 13883 11749 13910 11771 13892 11760 13918 11741 13937 11766 13926
11755 13951 11779 13938 11803 13926 11826 13941 11846 13923 11873 13918
11889 13896 11894 13869 50 P
11894 13869 11909 13846 2 P
11909 13846 11908 13873 11927 13892 11949 13908 11959 13934 11940 13914
11913 13920 11895 13940 11876 13960 11850 13966 11856 13992 11868 14017
11863 14044 11841 14027 11823 14007 11806 14028 11819 14052 11812 14078

11839 14083 11860 14099 11836 14112 11812 14124 11828 14146 11802 14155
11781 14172 11762 14191 11786 14204 11784 14231 11774 14256 11748 14264
11728 14282 11721 14309 11716 14335 11741 14347 11764 14360 11778 14337
11770 14310 11794 14297 11818 14285 11843 14273 11865 14257 11887 14248
11887 14249 11867 14260 11844 14275 11822 14290 11797 14302 11785 14326
11783 14354 11777 14380 50 P
11777 14380 11795 14404 11770 14390 3 P
11770 14390 11752 14369 11725 14364 11701 14376 11678 14390 11656 14406
11629 14403 11608 14386 11581 14382 11555 14374 11556 14346 11550 14320
11528 14336 11509 14356 11504 14382 11478 14388 11476 14361 11448 14363
11432 14384 11412 14403 11390 14420 11378 14444 11352 14450 11325 14449
11300 14459 11304 14432 11329 14422 11356 14423 11349 14396 11323 14397
11325 14370 11330 14343 11322 14317 11294 14319 11271 14305 11255 14283
11228 14279 11217 14254 11200 14276 11212 14301 11238 14308 11240 14335
11268 14337 11252 14359 11274 14375 11247 14377 11221 14375 11243 14361
11217 14357 11208 14331 50 P
11208 14331 11181 14330 11185 14357 11161 14371 11139 14355 11114 14347
11087 14354 11063 14365 11047 14387 11071 14401 11098 14397 11123 14407
11099 14419 11105 14445 11132 14450 11153 14434 11156 14461 11130 14471
11103 14468 11078 14458 11051 14454 11025 14462 11001 14475 10976 14465
10950 14458 10923 14462 10897 14469 10923 14477 10942 14496 10962 14512
10981 14538 11003 14525 11001 14517 11004 14514 11033 14533 11023 14538
11003 14527 10983 14545 10983 14573 10976 14599 10991 14622 11000 14648
11021 14666 11030 14691 11020 14716 11047 14717 11071 14730 11074 14757
11067 14783 11084 14762 50 P
11084 14762 11108 14775 2 P
11108 14775 11134 14782 11156 14797 11183 14801 11207 14814 11230 14799
11257 14802 11240 14825 11214 14825 11189 14837 11173 14815 11146 14819
11121 14809 11118 14837 11102 14860 11109 14833 11084 14842 11070 14865
11062 14891 11072 14916 11081 14941 11091 14967 11104 14991 11124 15009
11116 15034 11121 15061 11144 15075 11151 15102 11156 15128 11152 15155
11166 15180 11140 15173 11127 15197 11126 15224 11146 15242 11150 15269
11173 15282 11186 15284 11196 15297 11220 15310 11226 15313 11224 15316
11195 15299 11184 15288 11163 15287 11173 15315 11176 15329 11188 15324
11197 15326 11203 15334 50 P
11203 15334 11201 15336 11196 15330 11188 15326 11176 15330 11172 15318
11159 15294 11138 15276 11116 15262 11088 15261 11069 15281 11043 15272
11020 15286 10998 15301 10971 15301 10953 15321 10929 15308 10913 15330
10887 15337 10866 15354 10855 15330 10859 15303 10842 15281 10825 15260
10812 15284 10823 15309 10804 15328 10796 15354 10786 15379 10759 15382
10732 15388 10709 15401 10687 15417 10693 15443 10666 15442 10660 15415
10656 15388 10650 15362 10643 15388 10638 15415 40 P
10638 15415 10626 15390 10600 15385 10577 15369 10551 15364 10524 15365

10497 15367 10472 15379 10452 15360 10425 15360 10403 15374 10400 15402
10393 15375 10383 15351 10369 15327 10342 15332 10331 15308 10307 15322
10281 15328 10254 15332 10246 15306 10222 15293 10247 15282 10226 15265
10201 15255 10175 15265 10150 15254 10131 15235 10106 15224 10081 15214
10056 15203 10034 15187 10015 15168 9991 15156 9973 15136 9949 15149
9946 15122 9926 15104 9899 15106 9879 15089 9856 15074 9845 15049
9834 15025 9811 15011 9792 14991 9781 14966 9806 14955 9782 14942
9755 14937 9763 14911 50 P
9763 14911 9737 14905 9716 14888 9696 14906 9704 14880 9678 14889
9690 14864 9688 14837 9665 14850 9653 14874 9628 14885 9607 14868
9595 14843 9571 14832 9597 14826 9620 14812 9608 14787 9581 14785
9554 14785 9535 14765 9509 14756 9482 14757 9458 14745 9431 14745
9419 14721 9402 14700 9381 14717 9381 14744 9369 14769 9391 14785
9364 14792 9372 14818 9398 14826 9425 14826 9451 14835 9464 14859
9474 14884 9494 14865 9521 14869 9543 14885 9568 14895 9594 14904
9607 14927 9587 14945 9564 14960 9581 14981 9562 15001 9589 15008
9612 15022 9627 15045 50 P
9627 15045 9649 15060 2 P
9649 15060 9675 15067 9699 15080 9672 15081 9646 15075 9619 15074
9595 15062 9567 15061 9540 15057 9517 15071 9507 15102 9494 15134
9517 15143 9529 15160 9533 15171 9536 15192 9533 15219 9517 15241
9495 15258 9506 15282 9517 15307 9540 15322 9563 15336 9581 15356
9608 15355 9632 15368 9650 15388 9670 15407 9644 15414 9667 15428
9683 15450 9699 15471 9714 15494 9728 15517 9748 15536 9751 15562
9766 15585 9783 15606 9786 15632 9804 15653 9815 15678 9803 15701
9782 15719 9760 15734 9754 15744 9762 15762 9757 15788 9760 15794
9784 15782 9801 15786 50 P
9801 15786 9806 15792 9800 15801 9778 15800 9777 15813 9756 15824
9731 15840 9731 15837 9754 15821 9766 15815 9766 15808 9772 15801
9778 15791 9801 15797 9803 15791 9784 15785 9768 15793 9759 15797
9754 15792 9754 15779 9742 15779 9726 15794 9705 15811 9682 15825
9665 15847 9638 15845 9624 15868 9633 15894 9622 15919 9634 15943
9639 15969 9629 15944 9609 15926 9608 15899 9598 15874 9576 15858
9549 15856 9522 15853 9503 15872 9481 15888 9454 15887 9430 15899
9406 15886 9384 15902 9357 15905 9342 15883 9322 15864 9313 15838
9286 15843 9267 15823 50 P
9267 15823 9255 15799 2 P
9255 15799 9278 15784 9260 15764 9233 15758 9215 15737 9204 15713
9185 15693 9164 15677 9150 15653 9126 15640 9121 15614 9100 15597
9122 15582 9099 15568 9086 15544 9087 15517 9071 15495 9045 15489
9018 15482 8998 15464 8974 15453 8950 15439 8924 15430 8899 15422
8874 15411 8851 15396 8835 15374 27 P
8835 15374 8809 15367 8787 15351 8762 15344 8735 15337 8710 15327

8690 15309 8691 15282 8666 15272 8645 15255 8625 15237 8612 15213
8605 15187 8597 15161 8574 15147 8583 15121 8578 15094 8582 15067
8600 15046 8575 15036 8553 15020 8535 14999 8522 14976 8503 14956
8482 14940 8458 14927 8433 14917 8406 14914 8384 14898 8357 14896
8336 14913 8309 14914 8284 14903 8264 14884 8242 14868 8222 14849
8200 14833 8174 14839 8151 14853 8132 14872 8126 14899 8111 14921
8087 14934 8061 14939 44 P
8061 14939 8034 14934 8008 14926 7985 14911 7967 14891 7948 14872
7958 14847 7964 14820 7939 14809 7923 14787 7906 14766 7893 14742
7881 14718 7872 14692 7867 14666 7867 14638 7869 14612 17 P
7869 14612 7879 14586 7890 14561 7902 14537 7915 14513 7928 14489
7945 14468 7964 14449 7984 14430 8010 14423 8033 14408 11 P
8033 14408 8052 14389 8074 14373 8098 14360 8125 14364 8151 14360
8179 14363 8206 14361 8231 14353 8258 14349 8279 14331 8301 14347
8325 14360 8351 14371 8372 14387 8396 14399 8402 14373 8407 14399
8422 14376 8418 14349 8424 14322 8414 14297 8432 14318 8459 14317
8464 14290 8482 14271 8490 14245 8498 14219 8492 14193 8480 14168
8505 14157 8523 14136 8510 14112 8494 14090 8476 14069 8470 14043
8468 14016 8469 13989 8472 13962 8467 13935 8484 13914 8502 13893
8505 13866 8532 13867 8555 13853 8575 13834 8603 13832 8629 13825
8656 13824 8683 13826 50 P
8683 13826 8710 13828 8735 13818 8763 13820 8790 13820 8816 13827
8835 13846 8856 13864 8875 13883 8882 13909 8866 13931 8879 13955
8900 13972 8925 13982 8951 13990 8974 14005 8996 14021 9015 14041
9034 14060 9051 14081 9055 14108 9030 14120 9056 14128 9082 14136
9102 14155 9128 14162 9155 14166 9178 14180 9204 14189 9225 14207
9251 14208 9279 14205 9306 14208 9318 14184 9342 14171 9359 14150
9386 14152 9401 14129 9406 14102 9426 14083 9452 14078 9479 14081
9504 14091 9531 14095 9507 14083 9525 14063 9542 14041 9560 14021
9584 14010 9612 14008 50 P
9612 14008 9639 14013 2 P
9639 14013 9664 14023 9683 14004 9700 14026 9727 14031 9754 14035
9769 14013 9767 14040 9787 14059 9810 14073 9831 14090 9849 14070
9840 14044 9854 14020 9878 14033 9901 14019 9927 14012 9955 14013
9981 14020 9999 14000 10023 14011 10047 14024 10074 14023 10055 14004
10037 13983 10032 13956 10040 13930 10056 13908 10034 13893 10010 13878
9994 13856 9994 13829 9974 13811 10001 13808 10028 13812 10029 13785
10025 13758 10012 13734 10023 13759 9996 13753 9983 13729 9961 13712
9939 13696 9912 13701 9885 13702 9880 13729 9908 13732 9883 13743
9867 13765 9883 13787 50 P
9883 13787 9876 13813 9858 13792 9860 13764 9837 13750 9817 13731
9808 13757 9794 13781 9767 13776 9778 13750 9753 13739 9729 13727
9704 13716 9691 13692 9667 13679 9642 13669 9643 13642 9616 13649

9590 13642 9604 13665 9625 13682 9598 13680 9579 13661 9565 13684
9569 13657 9553 13635 9530 13621 9504 13611 9479 13601 9452 13594
9429 13580 9410 13561 9385 13571 9386 13544 9358 13543 9345 13566
9319 13574 9296 13560 9279 13538 9305 13545 9320 13522 9345 13512
9347 13485 9329 13464 9327 13436 9303 13449 9282 13466 9257 13455
9243 13431 9251 13406 50 P
9251 13406 9225 13397 2 P
9225 13397 9219 13371 9235 13348 9240 13321 9244 13294 9222 13278
9198 13265 9171 13261 9144 13257 9124 13238 9098 13230 9071 13235
9082 13260 9092 13286 9098 13312 9082 13335 9063 13355 9060 13328
9038 13311 9012 13320 8987 13309 8978 13284 8986 13258 9009 13244
9035 13236 9020 13213 9047 13209 9074 13212 9061 13188 9040 13172
9025 13148 9033 13174 9010 13160 8985 13150 8965 13131 8954 13105
8957 13078 8950 13052 8927 13067 8915 13042 8901 13019 8890 12994
8873 13016 8852 12998 8840 12974 8830 12999 8808 12983 8780 12986
8787 12960 8778 12933 50 P
8778 12933 8770 12908 8755 12884 8751 12857 8732 12838 8708 12825
8691 12803 8683 12777 8676 12751 8672 12724 8661 12699 8634 12704

12:00 PM

8634 12704 8613 12685 8599 12662 8595 12635 8595 12608 8587 12582
8588 12554 8601 12530 8594 12504 8613 12524 8635 12508 8660 12520
8684 12508 8711 12506 8715 12479 8730 12456 8750 12438 8774 12423
8798 12411 8820 12394 8846 12387 8865 12367 8865 12339 8878 12364
8904 12358 8932 12353 8958 12344 8982 12333 9001 12313 9028 12306
9053 12295 9078 12284 9092 12260 9096 12233 9109 12209 9117 12182
9126 12157 9136 12131 9145 12105 9146 12078 9154 12051 9155 12024
9180 12013 9175 11986 9200 11976 9209 11950 9220 11925 9215 11898
9222 11871 9239 11849 50 P
9239 11849 9261 11833 9252 11806 9250 11779 9257 11752 9276 11733
9285 11707 9297 11682 9316 11662 9331 11639 9336 11612 9351 11589
9378 11584 9388 11558 9394 11531 9405 11505 9407 11478 9398 11451
9378 11433 9403 11424 9417 11400 9398 11379 9383 11356 9410 11353
9428 11332 9448 11312 9475 11311 9501 11302 9527 11292 9552 11280
9577 11269 9603 11258 9625 11242 9638 11217 9637 11189 9643 11163
9629 11139 9648 11120 9660 11095 9685 11085 9713 11085 9739 11075
9765 11066 9789 11053 9811 11035 9829 11015 9848 10995 9857 10968
9870 10944 9884 10920 50 P
9884 10920 9895 10895 2 P
9895 10895 9889 10867 9863 10858 9836 10865 9810 10873 9782 10876
9756 10887 9732 10898 9706 10909 9688 10931 9662 10939 9635 10945
9610 10957 9584 10948 9558 10937 9531 10931 9517 10908 9489 10911
9462 10908 9435 10916 9430 10943 9420 10969 9401 10989 9384 11011
9361 11027 9345 11050 9319 11039 9293 11047 9265 11048 9240 11059

9214 11049 9190 11035 9169 11017 9147 11001 9125 10983 9099 10974
9072 10975 9045 10981 9059 10957 9087 10957 9113 10965 9138 10978
9164 10984 9192 10989 9203 11015 9231 11017 9257 11008 9284 11005
9312 11008 9340 11005 50 P
9340 11005 9354 10982 9367 10957 9380 10933 9358 10916 9377 10896
9402 10884 9430 10882 9458 10883 9484 10874 9509 10864 9531 10846
9555 10832 9577 10815 9595 10794 9609 10770 9626 10749 17 P
9626 10749 9651 10736 9676 10724 9698 10708 9713 10684 9741 10684
9766 10670 9789 10655 9811 10639 9829 10617 9843 10594 9857 10570
9870 10545 9883 10520 9896 10496 9910 10472 9920 10446 9913 10419
9890 10403 9863 10399 9835 10396 9807 10393 9780 10388 9753 10381
9726 10373 9702 10360 9682 10341 9667 10318 9643 10303 9616 10299
30 P
9616 10299 9593 10283 9571 10266 9555 10243 9528 10239 9503 10226
9484 10206 9468 10182 9468 10155 9452 10131 9435 10109 9410 10097
9383 10094 9363 10115 9337 10123 9309 10123 9281 10121 9253 10120
9226 10114 9202 10101 9194 10074 9195 10046 9168 10056 9140 10055
9124 10032 9104 10013 9127 10028 9146 10049 9169 10031 9196 10034
9212 10056 9223 10082 9246 10098 9273 10101 9301 10099 9325 10085
9314 10059 9323 10032 9340 10011 9335 9984 9344 9957 9370 9949
9346 9962 9365 9982 9391 9972 9419 9972 9444 9982 9466 10000
9490 10014 9511 10032 50 P
9511 10032 9531 10052 9553 10069 9578 10082 9605 10086 9633 10087
9661 10084 9684 10069 9707 10052 9727 10032 9746 10012 9762 9989
9782 9969 9797 9946 9812 9922 9836 9908 9863 9903 9891 9899
9916 9888 9944 9881 9955 9871 9962 9865 9966 9864 9994 9865
10022 9865 10046 9879 10039 9852 10015 9836 9989 9826 9968 9808
9954 9785 9931 9767 9914 9745 9891 9730 9874 9707 9856 9686
9834 9668 9807 9661 9780 9656 9753 9662 9735 9640 9724 9615
9732 9588 9747 9611 9774 9619 9766 9593 9784 9613 9807 9629
9834 9637 9853 9658 50 P
9853 9658 9880 9658 2 P
9880 9658 9887 9631 9912 9645 9896 9668 9897 9696 9921 9709
9948 9715 9976 9722 9994 9702 9977 9680 9974 9652 9989 9629
9988 9601 9963 9591 9965 9563 9963 9535 9988 9523 9993 9495
9975 9474 9951 9459 9924 9463 9923 9491 9918 9519 9927 9546
9906 9565 9899 9537 9901 9509 9894 9482 9873 9463 9846 9456
9837 9482 9835 9510 9843 9537 9832 9511 9832 9483 9814 9505
9786 9510 9782 9482 9774 9455 9750 9441 9749 9413 9777 9416
9804 9424 9831 9418 9834 9390 9806 9388 9785 9369 9811 9380
9838 9386 9864 9374 50 P
9864 9374 9853 9348 9873 9329 9855 9308 9827 9311 9801 9321
9815 9297 9843 9291 9856 9267 9864 9240 9844 9220 9825 9200

9801 9185 9772 9184 9745 9184 9717 9184 9743 9176 9771 9169
9787 9147 9795 9120 9807 9094 9824 9071 9822 9043 9836 9019
9845 8992 9858 8967 9846 8942 9828 8964 9805 8979 9777 8983
9762 8959 9749 8934 9727 8916 9734 8889 9713 8870 9692 8889
9675 8912 9667 8938 9652 8963 9669 8985 9642 8993 9616 8983
9607 8956 9581 8945 9603 8928 9593 8901 9578 8878 9553 8865
9539 8840 9542 8812 50 P
9542 8812 9528 8787 2 P
9528 8787 9529 8774 9502 8777 9474 8778 9445 8777 9429 8795
9423 8795 9397 8802 9368 8799 9347 8817 9349 8789 9377 8793
9404 8788 9424 8768 9452 8763 9477 8749 9479 8721 9463 8697
9444 8676 9430 8652 9418 8627 9390 8624 9386 8596 9387 8567
9367 8547 9340 8540 9358 8518 9371 8494 9398 8483 9384 8459
9385 8430 9357 8426 9332 8439 9326 8411 9338 8386 9322 8363
9321 8334 9306 8311 9291 8334 9280 8308 9255 8323 9245 8349
42 P
9245 8349 9250 8321 9265 8297 9250 8273 9222 8265 9195 8269
9204 8242 9199 8214 9180 8193 9152 8193 9179 8186 9181 8158
9153 8163 9125 8165 9118 8138 9090 8143 9077 8118 9102 8103
9126 8089 9116 8063 9141 8051 9121 8032 9122 8003 9096 7992
9072 7977 9044 7975 9035 7948 9014 7929 8985 7929 8993 7957
9009 7980 9037 7982 9058 8001 9057 8029 9082 8042 9094 8068
9070 8083 9043 8086 9033 8060 9017 8036 9011 8008 8989 8026
8962 8033 8965 8061 8989 8075 8962 8082 8978 8105 8952 8093
8948 8065 8927 8084 50 P
8927 8084 8910 8107 8900 8133 8901 8105 8908 8078 8918 8051
8893 8064 8871 8082 8845 8093 8849 8064 8843 8037 8867 8021
8841 8011 8843 7983 8864 7964 8891 7976 8919 7975 8947 7970
8966 7949 8970 7921 8982 7895 8966 7871 8944 7888 8916 7895
8895 7914 8872 7897 8858 7872 8886 7880 8899 7855 8904 7827
8927 7809 8952 7822 8961 7849 8979 7871 8996 7848 9019 7833
9046 7844 9028 7822 9000 7818 8972 7813 8948 7798 8921 7790
8893 7784 8870 7768 8898 7772 8887 7746 8870 7723 8850 7703
8834 7680 8853 7659 50 P
8853 7659 8880 7650 2 P
8880 7650 8906 7638 8934 7635 8927 7608 8919 7580 8891 7579
8882 7551 8884 7523 8892 7495 8878 7471 8850 7466 8824 7454
8807 7432 8821 7407 8828 7379 8854 7392 8867 7367 8874 7339
8900 7329 8907 7302 8902 7274 8881 7254 8879 7283 8879 7311
8852 7320 8842 7293 8827 7269 8829 7297 8813 7320 8808 7348
8779 7352 8754 7365 8764 7391 8740 7407 8729 7381 8713 7357
8693 7337 8669 7322 8646 7305 8619 7297 8640 7277 8663 7261
8686 7245 8676 7218 8703 7228 8727 7245 8743 7221 8763 7201

8735 7194 8729 7166 50 P
8729 7166 8701 7161 8672 7160 8649 7176 8647 7204 8632 7180
8641 7153 8655 7128 8629 7140 8600 7144 8611 7171 8613 7199
8588 7188 8604 7211 8576 7217 8548 7215 8522 7204 8541 7183
8561 7163 8545 7140 8529 7117 8501 7109 8510 7082 8534 7067
8561 7060 8575 7085 8573 7056 8587 7031 8558 7033 8530 7031
8534 7002 8562 6996 8537 6981 8511 6991 8482 6996 8464 6974
8436 6966 8441 6994 8429 7020 8416 6994 8416 7022 8395 7042
8402 7015 8409 6987 8381 6981 8354 6992 8378 6976 8382 6948
8402 6928 8425 6945 50 P
8425 6945 8440 6921 2 P
8440 6921 8437 6892 8424 6867 8395 6870 8393 6841 8405 6815
8385 6795 8359 6781 8360 6810 8336 6825 8310 6837 8285 6851
8295 6877 8272 6859 8278 6832 8253 6818 8225 6812 8197 6820
8185 6794 8157 6801 8164 6829 8154 6856 8125 6855 8123 6827
8108 6801 8091 6825 8082 6798 8053 6796 8079 6784 8074 6755
8051 6738 8023 6746 7996 6756 7968 6755 7953 6751 7942 6767
7940 6751 7952 6740 7974 6722 7999 6709 8017 6704 8045 6707
8074 6710 8077 6681 8105 6681 8120 6657 8126 6629 8098 6622
8077 6603 8054 6585 50 P
8054 6585 8033 6605 8013 6585 8024 6558 8005 6536 8001 6507
7976 6493 7953 6475 7937 6452 7925 6425 7929 6454 7946 6477
7936 6504 7909 6495 7891 6473 7894 6445 7871 6428 7859 6401
7857 6373 7855 6344 7838 6321 7839 6350 7849 6377 7839 6404
7817 6423 7790 6431 7762 6437 7733 6440 7757 6423 7785 6417
7786 6388 7776 6361 7758 6338 7735 6354 7723 6355 7711 6339
7687 6355 7676 6329 7671 6300 7669 6271 7651 6248 7659 6276
7641 6299 7641 6270 7616 6254 45 P
7616 6254 7594 6237 7571 6218 7564 6246 7558 6275 7532 6285
7540 6258 7515 6271 7487 6263 7461 6252 7469 6229 7455 6203
7454 6177 7455 6166 7434 6146 7422 6133 7401 6109 7372 6110
7343 6110 7317 6122 7294 6139 7273 6160 7245 6161 7273 6156
7279 6127 7292 6102 7315 6084 7343 6077 7379 6046 7358 6035
7340 6012 7319 6031 7297 6049 7268 6047 7271 6018 7296 6032
7291 6004 7277 5978 7284 5950 7287 5921 7266 5902 7258 5874
7229 5876 7201 5868 7172 5874 7149 5891 7124 5876 7101 5858
7081 5837 7059 5818 50 P
7059 5818 7030 5819 7046 5843 7029 5867 7027 5838 7003 5822
6976 5811 6949 5802 6921 5794 6899 5775 6882 5798 6866 5822
6838 5830 6814 5846 6785 5844 6764 5825 6747 5801 6730 5778
6746 5754 6768 5774 6753 5749 6757 5720 6731 5733 6705 5746
6676 5741 6648 5748 6636 5774 6665 5776 6640 5789 6614 5803
6575 5801 6577 5810 6599 5818 6569 5829 6544 5814 6515 5814

6487 5808 6472 5783 6458 5757 6429 5753 6417 5779 6392 5794
6408 5770 6430 5752 6415 5728 6399 5704 6403 5675 6382 5696
6353 5699 6324 5698 50 P
6324 5698 6314 5726 2 P
6314 5726 6317 5754 6314 5783 6338 5801 6345 5829 6324 5810
6295 5815 6269 5803 6242 5793 6216 5781 6202 5756 6180 5737
6209 5736 6208 5707 6181 5697 6207 5683 6234 5673 6229 5644
6200 5637 6172 5631 6168 5660 6159 5687 6141 5710 6113 5717
6085 5711 6057 5704 6035 5684 27 P
6035 5684 6061 5671 6087 5659 6065 5639 6036 5638 6008 5633
6023 5609 6051 5617 6073 5636 6090 5612 6118 5618 6113 5590
6085 5582 6058 5592 6050 5564 6069 5543 6043 5555 6014 5553
5985 5561 5967 5583 5938 5582 5908 5581 5880 5586 5860 5606
5880 5627 5902 5645 5928 5659 5955 5669 28 P
5955 5669 5926 5672 5898 5680 5871 5669 5853 5647 5859 5676
5842 5699 5823 5721 5811 5748 5797 5772 5790 5801 5771 5824
5745 5813 5739 5841 5720 5863 5706 5888 5711 5917 5723 5943
5705 5920 5684 5940 5677 5969 5680 5997 5654 6012 5627 6023
5600 6032 5617 6009 5645 6004 5662 5981 5661 5952 5655 5924
5626 5919 5598 5914 5627 5913 5654 5902 5664 5875 5688 5858
5702 5833 5674 5826 5650 5842 5670 5821 5686 5797 5703 5773
5675 5764 5654 5784 5674 5762 5689 5738 5666 5722 5681 5697
5681 5668 5667 5642 50 P
5667 5642 5659 5615 5683 5598 5689 5570 5675 5544 5665 5517
5656 5501 7 P
5649 5501 5645 5520 5644 5501 3 P
5596 5501 5592 5505 5573 5527 5556 5550 5547 5578 5529 5600
5537 5572 5551 5535 5544 5529 5540 5522 5539 5516 5531 5501

12:00 PM

5429 5501 5430 5523 5412 5546 5410 5575 5410 5604 5415 5633
5415 5662 5386 5663 5380 5691 5352 5696 5369 5672 5393 5656
5404 5629 5404 5600 5402 5571 5406 5542 5407 5513 5392 5501
18 P
2967 5501 2968 5507 2986 5529 2968 5552 2939 5553 2917 5534
2926 5506 2928 5501 8 P
13001 8169 12995 8163 12990 8169 12985 8172 12982 8166 12982 8159
12985 8152 12988 8146 12991 8140 12995 8134 13001 8129 13001 8128

12:00 PM

13001 8081 13000 8081 12994 8078 12987 8075 12983 8069 12981 8063
12978 8056 12978 8049 12973 8044 12976 8038 12981 8033 12976 8037
12969 8040 12962 8040 12959 8033 12960 8026 12965 8021 12968 8014
12973 8010 12978 8005 12979 7998 12979 7991 12978 7983 12976 7977
12976 7969 12981 7965 12988 7963 12993 7968 12998 7972 13001 7972

30 P
11825 7078 11828 7072 11833 7067 11839 7063 11846 7061 11847 7068
11849 7061 11855 7056 11861 7059 11868 7062 11875 7063 11882 7066
11888 7068 11895 7069 11902 7070 11910 7071 11917 7070 11924 7072
11931 7074 11936 7069 11932 7075 11927 7080 11923 7086 11918 7091
11910 7091 11904 7093 11897 7096 11894 7102 11887 7104 11880 7103
11873 7105 11866 7107 11863 7113 11864 7120 11859 7125 11852 7128
11848 7134 11847 7141 11844 7147 11838 7152 11833 7157 11829 7162
11825 7169 11818 7168 11822 7161 11826 7156 11830 7149 11834 7143
11838 7138 11841 7131 50 P
11841 7131 11835 7128 11834 7121 11836 7114 11833 7108 11827 7105
11824 7098 11831 7095 11836 7090 11840 7084 11836 7078 11825 7078
12:00 PM
11224 6841 11230 6836 11237 6839 11242 6843 11249 6844 11254 6840
11259 6834 11263 6828 11262 6821 11259 6814 11252 6812 11249 6806
11254 6801 11261 6800 11267 6797 11273 6792 11279 6788 11286 6787
11293 6790 11297 6795 11298 6802 11305 6801 11311 6797 11318 6800
11322 6806 11323 6813 11322 6820 11322 6828 11323 6835 11323 6842
11319 6848 11322 6854 11318 6861 11318 6868 11322 6873 11327 6879
11332 6884 11332 6891 11337 6896 11342 6902 11345 6908 11342 6915
11346 6921 11351 6926 11356 6931 11358 6938 11357 6945 11357 6953
11350 6952 11343 6954 50 P
11343 6954 11343 6946 11341 6939 11338 6933 11333 6927 11331 6921
11336 6916 11332 6910 11331 6903 11329 6896 11322 6897 11316 6895
11312 6889 11305 6886 11305 6879 11302 6872 11304 6865 11299 6861
11291 6861 11284 6860 11279 6856 11272 6857 11265 6855 11262 6861
11259 6868 11252 6870 11251 6863 11249 6856 11245 6850 11240 6855
11233 6854 11228 6848 11224 6841 33 P
11017 6755 11021 6749 11026 6743 11032 6740 11040 6740 11047 6739
11049 6746 11055 6750 11060 6745 11066 6742 11073 6741 11080 6740
11087 6740 11094 6738 11101 6739 11109 6740 11115 6738 11122 6734
11128 6732 11135 6735 11134 6743 11130 6749 11124 6752 11130 6749
11137 6747 11144 6744 11144 6751 11142 6758 11144 6764 11148 6770
11155 6768 11156 6775 11154 6782 11158 6788 11160 6795 11153 6798
11148 6802 11142 6806 11135 6808 11128 6809 11126 6816 11120 6813
11114 6816 11107 6815 11100 6813 11095 6807 11091 6813 11088 6820
11084 6825 11078 6829 50 P
11078 6829 11071 6832 11065 6828 11071 6823 11073 6816 11077 6811
11070 6813 11071 6820 11064 6818 11060 6812 11056 6806 11059 6800
11058 6793 11062 6787 11066 6781 11067 6774 11064 6780 11062 6774
11061 6767 11064 6761 11063 6753 11062 6761 11057 6765 11051 6770
11050 6777 11045 6772 11038 6776 11039 6769 11042 6762 11037 6766
11030 6770 11026 6764 11029 6757 11017 6755 34 P

9618 7079 9615 7066 9607 7054 9598 7042 9592 7030 9585 7017
9582 7003 9572 6992 9564 6981 9571 6968 9567 6954 9562 6940
9553 6929 9542 6920 9539 6906 9529 6896 9539 6886 9525 6887
9511 6885 9497 6879 9489 6868 9496 6855 9481 6852 9468 6846
9464 6833 9459 6819 9455 6805 9451 6791 9444 6779 9437 6766
9427 6756 9419 6745 9409 6734 9403 6721 9397 6708 9391 6695
9386 6681 9384 6667 9375 6656 9371 6642 9363 6630 9356 6617
9368 6609 9354 6609 9347 6597 9339 6585 9332 6572 9329 6558
9324 6545 9315 6534 50 P
9315 6534 9305 6523 9296 6513 9289 6500 9280 6489 9273 6476
9273 6461 9267 6449 9261 6435 9256 6422 9247 6411 9256 6400
9270 6398 9284 6396 9299 6399 9287 6390 9273 6387 9259 6387
9245 6389 9231 6388 9222 6376 9213 6365 9212 6351 9210 6337
9206 6323 9195 6314 9188 6301 9182 6288 9181 6274 9180 6259
9186 6247 9186 6232 9192 6219 9181 6211 9190 6201 9187 6186
9198 6177 9188 6167 9192 6153 9196 6140 9194 6125 9192 6111
9199 6098 9185 6101 9183 6087 9175 6074 9177 6060 9166 6051
9179 6046 9164 6044 50 P
9164 6044 9163 6030 2 P
9163 6030 9164 6015 9155 6005 9169 6001 9173 5988 9169 5974
9157 5964 9144 5961 9139 5975 9135 5961 9146 5951 9160 5954
9152 5942 9163 5933 9175 5925 9189 5929 9203 5933 9217 5938
9220 5923 9207 5917 9193 5914 9199 5901 9207 5889 9211 5875
9211 5860 9216 5847 9221 5833 9235 5829 9226 5818 9231 5805
9237 5791 9242 5778 9249 5765 9255 5751 9258 5737 9258 5723
9255 5709 9262 5697 9276 5690 9279 5676 9291 5667 9302 5658
9289 5663 9279 5674 9271 5686 9277 5673 9286 5662 9296 5651
9306 5641 9316 5630 50 P
9316 5630 9328 5622 9340 5614 9347 5627 9352 5614 9362 5603
9373 5593 9386 5586 9398 5579 9411 5573 9424 5566 9436 5558
9446 5548 9454 5535 9465 5526 9472 5513 9475 5501 17 P
9608 5501 9607 5512 9606 5527 9608 5541 9614 5555 9616 5569
9622 5583 9627 5595 9632 5609 9634 5624 9638 5638 9643 5652
9646 5666 9652 5679 9662 5690 9664 5704 9667 5718 9673 5732
9684 5740 9695 5750 9707 5758 9720 5765 9730 5775 9740 5785
9750 5796 9754 5810 9758 5824 9753 5838 9759 5850 9772 5856
9784 5865 9790 5878 9797 5891 9801 5905 9811 5915 9819 5927
9824 5941 9829 5954 9832 5969 9837 5982 9848 5992 9857 6003
9859 6018 9862 6032 9857 6045 9858 6059 9865 6073 9868 6087
9872 6101 9877 6114 50 P
9877 6114 9889 6122 9891 6137 9892 6151 9894 6165 9898 6179
9903 6193 9906 6207 9911 6220 9905 6234 9906 6248 9910 6262
9915 6276 9917 6290 9917 6304 9922 6318 9923 6332 9924 6346

9927 6361 9933 6374 9934 6388 9934 6403 9939 6416 9944 6430
9947 6444 9949 6458 9949 6473 9949 6487 9951 6501 9959 6513
9967 6524 9960 6537 9952 6549 9950 6563 9955 6577 9965 6587
9973 6598 9972 6613 9970 6627 9977 6639 9988 6648 9996 6659
10008 6668 10013 6681 10021 6694 10026 6707 10025 6721 10030 6735
10032 6749 49 P
10032 6749 10034 6763 10039 6777 10046 6789 10057 6799 10055 6813
10060 6826 10072 6834 10070 6848 10071 6862 10068 6876 10058 6887
10053 6900 10050 6914 10048 6928 10043 6942 10039 6956 10037 6970
10037 6984 10038 6999 10041 7013 10046 7026 10054 7038 10064 7048
10068 7062 10080 7071 10088 7083 10101 7090 10113 7096 10124 7105
10116 7117 10102 7116 10090 7123 10088 7137 10080 7149 10083 7163
10073 7172 10058 7171 10044 7173 10030 7169 10019 7178 10006 7184
9993 7178 9984 7188 9970 7194 9959 7203 9964 7217 9950 7212
9940 7221 9931 7232 50 P
9931 7232 9927 7246 9915 7255 9903 7261 9889 7260 9875 7256
9861 7252 9849 7245 9840 7234 9832 7222 9824 7210 9817 7197
9828 7188 9815 7184 9805 7173 9792 7167 9779 7160 9766 7158
9752 7154 9737 7156 9724 7150 9711 7145 9698 7138 9687 7130
9673 7126 9659 7121 9648 7112 9636 7106 9625 7096 9618 7079
30 P
12969 11655 12976 11654 12983 11654 12987 11648 12994 11647 12998 11653
13001 11654 7 P
13001 11746 12999 11747 12994 11752 12988 11754 12983 11749 12981 11742
12982 11735 12986 11730 12983 11723 12985 11717 12990 11712 12986 11706
12989 11700 12992 11693 12998 11690 12999 11683 12992 11683 12997 11678
12990 11681 12993 11674 12987 11670 12987 11663 12993 11666 12998 11669
12994 11664 12994 11657 12988 11659 12982 11663 12979 11669 12975 11675
12973 11668 12969 11655 32 P
11806 11895 11811 11890 11815 11884 11819 11879 11825 11875 11831 11872
11838 11871 11844 11867 11847 11861 11852 11857 11858 11852 11864 11848
11870 11849 11871 11855 11872 11862 11875 11868 11880 11873 11886 11876
11893 11875 11900 11875 11906 11874 11913 11877 11919 11880 11926 11881
11932 11878 11938 11880 11945 11882 11949 11876 11956 11876 11962 11879
11969 11880 11974 11885 11980 11886 11987 11887 11994 11887 12000 11889
12006 11887 12013 11888 12018 11894 12023 11898 12027 11903 12033 11907
12040 11909 12047 11909 12053 11910 12060 11913 12066 11916 12072 11917
12077 11922 12082 11927 50 P
12082 11927 12086 11933 12093 11933 12100 11934 12106 11936 12113 11938
12117 11944 12115 11951 12121 11955 12124 11961 12126 11968 12127 11975
12130 11981 12136 11984 12141 11989 12140 11996 12142 12003 12143 12010
12148 12005 12147 12012 12145 12019 12148 12025 12155 12024 12150 12029
12145 12024 12144 12031 12138 12033 12134 12039 12137 12045 12135 12052

12132 12058 12126 12061 12119 12061 12113 12058 12107 12062 12100 12065
12096 12069 12092 12075 12085 12073 12078 12074 12071 12074 12064 12074
12058 12071 12052 12071 12045 12068 12039 12066 12032 12063 12026 12062
12019 12059 12013 12061 50 P
12013 12061 12007 12057 2 P
12007 12057 12001 12053 11995 12051 11988 12049 11982 12045 11975 12044
11969 12041 11963 12038 11958 12034 11951 12036 11944 12037 11940 12032
11933 12030 11929 12026 11923 12022 11916 12021 11910 12017 11904 12013
11898 12015 11893 12011 11887 12009 11881 12004 11875 12001 11868 12001
11865 11995 11861 11989 11855 11986 11850 11981 11845 11976 11845 11970
11841 11964 11837 11958 11831 11954 11826 11950 11823 11944 11818 11939
11812 11935 11811 11928 11806 11924 11806 11917 11808 11911 11807 11904
11806 11895 43 P
12978 8331 12982 8326 12989 8326 12996 8328 13001 8328 5 P
13001 8344 12995 8342 12988 8342 12981 8342 12978 8331 5 P
12715 8218 12721 8220 12728 8221 12735 8224 12733 8231 12726 8234
12720 8230 12715 8218 8 P
12476 8068 12481 8063 12488 8061 12495 8061 12502 8060 12507 8055
12510 8049 12514 8043 12520 8039 12526 8036 12534 8035 12531 8042
12528 8049 12528 8056 12532 8061 12535 8068 12528 8071 12525 8078
12518 8080 12515 8086 12509 8090 12501 8089 12495 8085 12491 8079
12484 8076 12476 8068 26 P
12692 8096 12696 8092 12698 8088 12701 8090 12705 8093 12701 8094
12699 8098 12699 8103 12695 8101 12692 8096 10 P
12533 8060 12535 8061 12537 8061 12539 8062 12539 8064 12538 8067
12537 8065 12535 8063 12533 8060 9 P
12542 8063 12544 8063 12545 8065 12544 8067 12542 8067 12542 8063
6:00 PM
12547 8060 12548 8062 12550 8063 12552 8064 12554 8064 12556 8066
12558 8067 12559 8069 12561 8070 12563 8070 12566 8071 12568 8071
12569 8074 12571 8074 12573 8075 12571 8076 12569 8075 12567 8074
12565 8072 12563 8072 12561 8071 12559 8071 12557 8069 12555 8068
12554 8066 12552 8065 12549 8065 12548 8063 12547 8060 29 P
12807 8003 12809 7999 12812 7996 12812 7991 12812 7986 12814 7983
12815 7987 12819 7989 12820 7993 12820 7998 12819 8003 12821 8006
12824 8009 12823 8014 12823 8018 12822 8023 12820 8027 12816 8029
12812 8027 12812 8023 12811 8019 12809 8014 12810 8010 12811 8006
12807 8003 25 P
12775 8026 12780 8026 12784 8025 12787 8022 12790 8026 12792 8029
12792 8034 12795 8031 12799 8029 12799 8034 12797 8038 12792 8039
12788 8039 12787 8035 12783 8035 12781 8031 12775 8026 17 P
12768 8008 12772 8006 12774 8003 12779 8004 12783 8006 12780 8011
12778 8014 12774 8016 12771 8013 12768 8008 10 P

12778 8000 12781 7996 12785 7998 12785 8002 12778 8000 5 P
12782 7991 12785 7987 12786 7992 12786 7996 12782 7991 5 P
12754 7992 12755 7988 12759 7986 12759 7991 12760 7995 12756 7997
12754 7992 7 P
12755 8014 12759 8014 12760 8018 12756 8019 12755 8014 5 P
12856 8020 12860 8021 12861 8025 12863 8029 12863 8034 12859 8030
12858 8026 12856 8020 8 P
12832 12963 12826 12961 12822 12955 12822 12948 12821 12941 12814 12942
12808 12939 12802 12942 12800 12935 12795 12931 12799 12925 12803 12919
12807 12913 12812 12909 12817 12904 12810 12905 12803 12904 12797 12907
12793 12902 12786 12903 12780 12906 12774 12911 12768 12909 12765 12903
12759 12901 12756 12895 12752 12889 12750 12883 12751 12876 12754 12870
12757 12863 12763 12861 12766 12855 12770 12849 12777 12852 12776 12859
12782 12861 12788 12864 12792 12869 12795 12876 12802 12874 12803 12867
12806 12861 12804 12854 12804 12848 12799 12844 12792 12842 12786 12839
12787 12833 12793 12837 50 P
12793 12837 12793 12830 12799 12831 12805 12834 12812 12832 12806 12828
12805 12821 12807 12814 12814 12813 12810 12807 12809 12800 12815 12798
12821 12796 12827 12800 12834 12802 12831 12808 12826 12813 12832 12815
12839 12816 12846 12818 12852 12819 12846 12823 12840 12821 12836 12826
12831 12832 12834 12838 12840 12838 12845 12843 12842 12849 12842 12856
12848 12859 12851 12853 12856 12859 12862 12858 12866 12853 12873 12850
12873 12857 12868 12862 12864 12867 12865 12874 12859 12873 12856 12878
12863 12882 12869 12880 12866 12886 12862 12892 12868 12893 12868 12899
12871 12906 12865 12906 50 P
12865 12906 12859 12902 2 P
12859 12902 12858 12895 12851 12896 12848 12902 12852 12908 12855 12913
12859 12919 12856 12926 12854 12932 12853 12939 12851 12946 12845 12949
12842 12955 12840 12962 12843 12968 12849 12970 12850 12977 12846 12983
12839 12985 12832 12986 12829 12980 12828 12973 12832 12963 23 P
12542 12834 12535 12831 12532 12825 12526 12821 12522 12817 12517 12821
12515 12827 12508 12830 12501 12830 12502 12837 12497 12842 12494 12848
12495 12854 12501 12859 12503 12865 12504 12871 12503 12878 12499 12884
12494 12888 12487 12889 12480 12886 12475 12881 12471 12876 12471 12869
12475 12864 12469 12861 12462 12860 12462 12853 12462 12846 12458 12840
12456 12834 12457 12827 12460 12821 12465 12816 12469 12811 12471 12804
12476 12799 12479 12793 12476 12787 12474 12781 12481 12778 12483 12772
12489 12768 12495 12767 12502 12768 12508 12771 12510 12778 12506 12783
12504 12790 12510 12793 50 P
12510 12793 12516 12798 12522 12799 12524 12792 12523 12785 12525 12778
12527 12772 12529 12766 12535 12764 12542 12765 12548 12769 12555 12769
12559 12764 12566 12767 12573 12768 12568 12773 12565 12779 12569 12784
12573 12790 12571 12797 12570 12803 12569 12810 12568 12817 12571 12823

12571 12830 12566 12833 12559 12831 12554 12836 12542 12834 29 P
12066 12412 12070 12406 12076 12408 12082 12405 12089 12404 12096 12402
12100 12408 12101 12415 12100 12422 12095 12426 12093 12433 12088 12436
12081 12438 12076 12435 12072 12429 12070 12422 12066 12412 17 P
12233 12451 12237 12454 12241 12453 12245 12454 12250 12454 12250 12458
12252 12462 12250 12466 12249 12470 12249 12475 12244 12474 12241 12472
12241 12468 12237 12467 12236 12462 12235 12458 12233 12451 17 P
12159 12586 12162 12583 12166 12584 12169 12580 12166 12578 12164 12573
12169 12575 12171 12571 12175 12569 12179 12566 12183 12564 12186 12567
12191 12566 12193 12570 12198 12570 12202 12570 12206 12570 12211 12570
12215 12571 12218 12575 12219 12579 12219 12583 12220 12587 12221 12591
12223 12596 12222 12599 12219 12603 12216 12606 12212 12605 12208 12603
12204 12602 12199 12603 12195 12601 12192 12598 12190 12594 12185 12595
12183 12591 12179 12593 12175 12594 12173 12598 12169 12599 12169 12594
12167 12591 12164 12587 12159 12586 45 P
12321 12593 12325 12594 12329 12595 12334 12596 12336 12592 12331 12592
12327 12592 12329 12589 12332 12586 12337 12588 12341 12586 12341 12582
12339 12577 12343 12575 12346 12578 12350 12580 12353 12583 12358 12584
12361 12587 12362 12591 12361 12595 12359 12599 12354 12600 12352 12604
12348 12606 12345 12609 12344 12605 12340 12604 12336 12601 12333 12604
12329 12605 12325 12607 12321 12606 12324 12603 12322 12599 12321 12593
36 P
12426 12655 12429 12653 12433 12651 12437 12649 12440 12650 12440 12646
12444 12643 12445 12639 12449 12637 12453 12640 12457 12637 12461 12636
12462 12632 12466 12632 12470 12632 12474 12636 12472 12640 12469 12643
12465 12643 12460 12644 12465 12646 12465 12650 12461 12653 12458 12656
12456 12660 12454 12664 12450 12665 12446 12667 12441 12665 12438 12663
12435 12660 12432 12656 12426 12655 33 P
12473 12602 12477 12602 12474 12598 12474 12594 12479 12594 12482 12591
12484 12595 12484 12599 12483 12604 12484 12608 12484 12612 12480 12614
12481 12618 12477 12619 12475 12614 12476 12610 12476 12606 12473 12602
18 P
11960 12515 11962 12511 11962 12507 11963 12502 11965 12498 11964 12494
11967 12491 11966 12486 11970 12488 11974 12486 11977 12484 11981 12482
11984 12485 11988 12487 11991 12484 11994 12481 11991 12478 11987 12478
11987 12473 11990 12471 11994 12472 11998 12473 12003 12472 12006 12470
12010 12469 12010 12473 12008 12477 12012 12476 12014 12472 12019 12472
12023 12472 12027 12471 12025 12475 12021 12477 12023 12480 12025 12485
12020 12486 12016 12486 12012 12486 12013 12490 12011 12494 12007 12495
12006 12500 12005 12504 12004 12508 12004 12512 12003 12517 12000 12520
11996 12522 11992 12525 50 P
11992 12525 11989 12527 11985 12529 11981 12530 11976 12528 11974 12525
11969 12526 11965 12523 11963 12520 11960 12515 10 P

11710 12509 11715 12504 11720 12499 11723 12493 11729 12490 11728 12497
11725 12503 11728 12509 11729 12502 11735 12498 11742 12497 11738 12491
11734 12485 11737 12479 11741 12484 11745 12478 11752 12478 11759 12478
11765 12482 11771 12484 11766 12489 11773 12488 11779 12490 11783 12495
11790 12497 11790 12503 11796 12506 11803 12507 11803 12513 11810 12514
11811 12521 11817 12516 11818 12509 11823 12514 11822 12521 11828 12524
11830 12530 11833 12536 11830 12542 11835 12548 11830 12553 11834 12558
11835 12565 11836 12571 11830 12575 11826 12581 11821 12585 11816 12589
11809 12586 11808 12580 50 P
11808 12580 11802 12584 11800 12590 11801 12597 11798 12603 11792 12606
11785 12608 11778 12608 11772 12610 11770 12603 11773 12597 11780 12596
11786 12598 11784 12592 11786 12585 11782 12579 11778 12575 11775 12568
11768 12570 11768 12576 11766 12583 11761 12577 11765 12571 11758 12568
11761 12562 11763 12555 11767 12550 11773 12552 11771 12546 11764 12547
11767 12541 11766 12535 11765 12528 11763 12521 11760 12515 11760 12522
11759 12529 11760 12536 11763 12542 11759 12548 11759 12555 11752 12556
11751 12563 11746 12560 11745 12553 11748 12547 11745 12540 11741 12546
11734 12545 11730 12539 50 P
11730 12539 11724 12537 2 P
11724 12537 11722 12530 11727 12525 11720 12527 11715 12523 11713 12516
11710 12509 7 P
11913 12377 11916 12374 11918 12370 11922 12369 11926 12369 11930 12371
11934 12373 11938 12376 11941 12379 11941 12383 11946 12384 11947 12388
11951 12390 11951 12394 11954 12391 11956 12387 11958 12392 11961 12390
11966 12391 11965 12395 11961 12395 11956 12395 11953 12398 11948 12398
11944 12396 11940 12397 11935 12395 11934 12400 11930 12401 11926 12401
11922 12398 11919 12395 11916 12392 11913 12389 11915 12385 11913 12377
36 P
11930 12408 11934 12407 11937 12404 11940 12400 11944 12400 11948 12399
11953 12400 11949 12402 11948 12407 11952 12409 11954 12405 11958 12405
11960 12409 11958 12412 11961 12415 11965 12417 11961 12420 11959 12424
11956 12427 11952 12429 11948 12428 11944 12429 11940 12427 11937 12430
11933 12433 11933 12428 11937 12424 11932 12423 11932 12419 11936 12420
11934 12416 11933 12412 11930 12408 33 P
11969 12387 11971 12384 11974 12380 11974 12376 11978 12378 11981 12375
11982 12371 11986 12369 11989 12373 11989 12377 11990 12381 11989 12385
11994 12387 11993 12392 11989 12390 11988 12394 11986 12398 11982 12399
11982 12403 11987 12405 11991 12405 11992 12409 11989 12412 11985 12414
11981 12415 11977 12417 11974 12415 11974 12411 11969 12411 11970 12406
11975 12407 11976 12403 11977 12399 11974 12395 11973 12391 11969 12387
36 P
12014 12495 12018 12495 12022 12493 12026 12493 12030 12491 12033 12487
12036 12491 12039 12494 12043 12494 12048 12494 12052 12492 12054 12488

12053 12484 12056 12480 12060 12483 12062 12486 12065 12489 12064 12493
12069 12492 12069 12488 12074 12488 12075 12492 12073 12497 12074 12501
12078 12500 12079 12505 12078 12508 12075 12506 12070 12506 12068 12509
12063 12509 12058 12509 12055 12506 12053 12510 12049 12513 12046 12510
12043 12506 12044 12502 12040 12499 12036 12498 12033 12501 12029 12504
12027 12500 12026 12496 12021 12497 12017 12498 12014 12495 47 P
12010 12520 12013 12522 12018 12522 12022 12522 12020 12519 12019 12514
12019 12510 12023 12508 12025 12511 12026 12515 12028 12519 12029 12523
12034 12525 12038 12525 12042 12526 12042 12530 12045 12534 12049 12533
12053 12531 12057 12532 12062 12531 12061 12535 12058 12539 12054 12541
12054 12545 12050 12543 12046 12544 12049 12547 12050 12551 12047 12554
12045 12557 12040 12557 12036 12555 12033 12553 12029 12551 12024 12551
12022 12547 12019 12544 12018 12540 12015 12537 12017 12533 12014 12529
12012 12525 12010 12520 44 P
12059 12540 12063 12537 12065 12534 12062 12530 12062 12526 12063 12521
12066 12525 12068 12529 12071 12530 12071 12526 12071 12522 12072 12518
12077 12517 12081 12514 12085 12515 12089 12517 12090 12521 12094 12522
12098 12522 12100 12527 12097 12530 12097 12534 12097 12539 12099 12543
12097 12547 12096 12543 12093 12539 12092 12535 12088 12536 12084 12535
12081 12539 12076 12538 12072 12539 12068 12542 12067 12537 12064 12541
12059 12540 37 P
12124 12521 12120 12522 12122 12519 12126 12516 12130 12518 12134 12517
12138 12514 12135 12512 12139 12511 12142 12508 12143 12512 12147 12513
12150 12510 12147 12507 12143 12505 12144 12500 12145 12496 12147 12500
12149 12504 12152 12507 12155 12510 12159 12509 12161 12513 12156 12513
12155 12518 12159 12516 12164 12516 12166 12513 12171 12514 12169 12517
12165 12520 12165 12525 12161 12525 12156 12526 12153 12523 12149 12521
12145 12519 12146 12523 12144 12528 12140 12529 12138 12533 12135 12536
12136 12532 12132 12532 12128 12530 12131 12527 12128 12523 12124 12521
48 P
12129 12575 12132 12572 12135 12569 12136 12564 12137 12560 12139 12556
12143 12554 12144 12558 12143 12562 12147 12561 12147 12556 12148 12552
12150 12548 12151 12551 12153 12556 12154 12560 12153 12564 12151 12568
12149 12572 12147 12576 12143 12578 12139 12578 12135 12577 12129 12575
24 P
12895 12972 12899 12969 12903 12969 12904 12974 12903 12978 12902 12982
12903 12987 12905 12991 12906 12995 12906 12999 12904 13003 12900 13003
12897 13006 12894 13009 12891 13005 12887 13004 12884 13001 12881 12998
12885 12997 12888 12994 12888 12989 12891 12986 12887 12984 12891 12982
12895 12981 12896 12976 12895 12972 27 P
12750 12834 12754 12831 12756 12828 12755 12824 12756 12820 12760 12820
12764 12819 12768 12821 12771 12824 12774 12827 12774 12831 12771 12834
12767 12834 12764 12837 12764 12842 12761 12845 12762 12849 12758 12847

12759 12851 12755 12851 12752 12848 12753 12844 12757 12845 12761 12842
12757 12844 12756 12839 12754 12836 12754 12840 12751 12844 12751 12839
12750 12834 31 P
12762 12922 12765 12918 12768 12915 12772 12916 12769 12919 12772 12921
12769 12925 12767 12928 12770 12932 12773 12935 12770 12939 12766 12940
12762 12938 12761 12934 12764 12931 12764 12926 12762 12922 17 P
12615 12847 12620 12846 12624 12845 12623 12840 12627 12839 12631 12842
12632 12847 12634 12850 12639 12850 12643 12850 12642 12855 12638 12856
12634 12857 12631 12859 12626 12858 12622 12856 12618 12855 12615 12847
18 P
12632 12835 12636 12835 12637 12831 12641 12831 12645 12828 12649 12827
12653 12826 12654 12830 12650 12833 12655 12834 12659 12835 12662 12838
12659 12841 12655 12842 12653 12846 12649 12849 12645 12850 12641 12849
12638 12846 12635 12842 12638 12839 12632 12835 22 P
12655 12738 12658 12735 12663 12733 12662 12729 12664 12725 12667 12728
12671 12730 12673 12734 12677 12733 12682 12733 12686 12733 12685 12738
12689 12741 12686 12745 12683 12743 12680 12739 12679 12735 12676 12738
12678 12741 12675 12744 12671 12745 12668 12747 12663 12749 12659 12748
12658 12743 12655 12738 26 P
12537 12876 12534 12872 12533 12868 12531 12864 12528 12860 12527 12856
12524 12853 12522 12849 12524 12845 12525 12841 12527 12837 12531 12835
12535 12834 12540 12834 12544 12835 12548 12837 12549 12841 12553 12842
12557 12840 12561 12838 12566 12839 12569 12838 12574 12838 12577 12835
12581 12835 12580 12840 12576 12842 12572 12844 12569 12847 12568 12851
12570 12855 12569 12860 12565 12862 12561 12863 12557 12866 12559 12870
12555 12871 12552 12868 12550 12863 12549 12859 12549 12855 12547 12851
12544 12854 12544 12858 12545 12862 12545 12867 12548 12870 12549 12874
12551 12878 12550 12883 50 P
12550 12883 12546 12882 12542 12880 12537 12876 4 P
12397 12665 12400 12669 12402 12666 12405 12663 12409 12662 12412 12665
12416 12667 12417 12671 12420 12668 12424 12669 12428 12671 12425 12674
12421 12677 12418 12674 12414 12674 12411 12678 12407 12679 12404 12677
12400 12675 12397 12671 12397 12665 21 P
12309 12631 12313 12629 12317 12629 12318 12625 12322 12622 12317 12621
12315 12618 12317 12614 12320 12611 12324 12610 12327 12613 12331 12614
12335 12616 12339 12615 12342 12619 12341 12623 12338 12626 12334 12628
12330 12628 12325 12628 12329 12630 12327 12634 12322 12635 12325 12638
12328 12642 12327 12646 12323 12646 12318 12647 12316 12643 12313 12640
12312 12635 12309 12631 32 P
12126 12510 12121 12509 12117 12510 12113 12509 12110 12506 12107 12502
12107 12498 12108 12494 12112 12493 12114 12489 12118 12491 12122 12492
12125 12495 12129 12497 12130 12500 12134 12502 12132 12506 12126 12510
18 P

12268 12367 12270 12364 12271 12359 12275 12357 12276 12353 12279 12349
12282 12346 12286 12347 12291 12346 12293 12349 12289 12349 12285 12351
12284 12355 12280 12358 12278 12362 12277 12366 12278 12370 12277 12374
12273 12371 12268 12367 20 P
12281 12371 12284 12368 12286 12364 12289 12362 12294 12360 12295 12365
12291 12367 12292 12371 12287 12372 12281 12371 10 P
12106 12309 12108 12305 12112 12305 12117 12306 12119 12309 12117 12313
12113 12315 12109 12315 12106 12309 9 P
11833 12263 11838 12262 11840 12258 11842 12254 11846 12254 11844 12258
11846 12262 11850 12263 11851 12267 11847 12269 11843 12266 11839 12267
11833 12263 13 P
11824 12269 11828 12266 11831 12264 11833 12268 11830 12272 11825 12273
11824 12269 7 P
11903 12398 11906 12397 11908 12400 11910 12402 11913 12402 11913 12406
11915 12408 11913 12411 11911 12413 11909 12410 11907 12407 11906 12404
11905 12401 11903 12398 14 P
11948 12433 11952 12433 11954 12435 11957 12433 11959 12436 11962 12436
11962 12439 11960 12442 11957 12443 11954 12442 11951 12440 11948 12439
11948 12433 13 P
11952 12359 11955 12359 11958 12359 11961 12358 11961 12362 11959 12364
11958 12367 11954 12368 11952 12366 11954 12364 11952 12359 11 P
12021 12414 12024 12413 12026 12411 12028 12413 12028 12416 12031 12419
12031 12422 12029 12424 12028 12428 12025 12428 12022 12427 12021 12423
12022 12420 12021 12414 14 P
11273 12279 11276 12277 11281 12277 11285 12275 11288 12272 11290 12268
11292 12264 11296 12266 11301 12266 11302 12271 11299 12273 11296 12276
11293 12279 11291 12284 11292 12288 11288 12288 11286 12292 11281 12292
11279 12288 11279 12284 11279 12279 11273 12279 22 P
11438 12370 11440 12367 11442 12364 11445 12366 11448 12365 11450 12367
11447 12368 11445 12371 11441 12370 11438 12370 10 P
11685 12438 11689 12440 11688 12435 11693 12434 11697 12434 11701 12434
11704 12430 11703 12434 11706 12438 11707 12434 11709 12430 11712 12434
11716 12435 11720 12433 11719 12437 11717 12442 11716 12446 11717 12450
11713 12449 11708 12449 11704 12447 11700 12448 11696 12451 11693 12453
11691 12449 11687 12449 11690 12447 11687 12443 11685 12438 29 P
11692 12405 11695 12405 11697 12403 11700 12405 11703 12405 11706 12404
11707 12406 11708 12409 11712 12410 11710 12413 11709 12416 11706 12417
11703 12419 11703 12416 11703 12413 11701 12411 11698 12410 11695 12409
11692 12409 11692 12405 20 P
11647 12403 11649 12400 11652 12400 11655 12400 11657 12403 11660 12405
11660 12408 11657 12409 11655 12407 11652 12409 11650 12407 11647 12403

12:00 PM

11634 12407 11637 12407 11640 12405 11640 12408 11642 12411 11644 12413

11646 12411 11645 12407 11648 12409 11647 12412 11646 12415 11643 12416
11640 12415 11637 12413 11634 12411 11634 12407 16 P
11675 12464 11672 12462 11672 12459 11673 12456 11676 12455 11679 12454
11682 12454 11686 12455 11687 12457 11689 12457 11693 12459 11695 12457
11699 12456 11701 12455 11702 12458 11700 12460 11697 12461 11694 12463
11691 12464 11688 12466 11686 12464 11683 12464 11681 12461 11678 12461
11675 12464 25 P
11679 12422 11677 12426 11676 12428 11672 12429 11670 12428 11669 12424
11670 12421 11673 12422 11674 12419 11676 12416 11679 12416 11680 12419
11682 12422 11681 12425 11679 12422 15 P
11757 12416 11759 12414 11763 12414 11761 12417 11762 12420 11763 12423
11764 12426 11763 12429 11760 12427 11759 12423 11758 12420 11757 12416
12:00 PM
11382 12602 11384 12598 11387 12595 11390 12592 11395 12592 11394 12597
11394 12601 11398 12603 11401 12607 11399 12611 11404 12612 11406 12615
11408 12619 11408 12624 11411 12627 11415 12628 11413 12632 11410 12631
11407 12627 11403 12627 11402 12631 11405 12633 11401 12634 11397 12633
11392 12632 11390 12628 11390 12624 11388 12621 11387 12617 11386 12612
11383 12609 11385 12606 11382 12602 33 P
11408 12649 11410 12647 11412 12646 11413 12643 11416 12645 11416 12648
11417 12651 11420 12650 11420 12654 11417 12654 11414 12656 11412 12658
11412 12655 11410 12653 11408 12649 15 P
11288 12609 11291 12606 11293 12610 11295 12613 11299 12614 11301 12618
11302 12622 11303 12626 11302 12631 11298 12632 11297 12628 11295 12624
11294 12620 11290 12618 11288 12614 11288 12609 16 P
11345 12480 11347 12477 11348 12475 11350 12477 11349 12480 11345 12480
6:00 PM
11345 12484 11348 12484 11351 12486 11353 12488 11352 12491 11349 12492
11347 12488 11345 12484 8 P
11348 12466 11349 12464 11352 12461 11353 12458 11354 12455 11357 12457
11360 12456 11363 12457 11362 12461 11359 12462 11356 12463 11354 12461
11353 12464 11348 12466 14 P
11370 12617 11373 12615 11376 12616 11378 12618 11378 12621 11375 12622
11372 12621 11370 12617 8 P
11378 12623 11382 12623 11383 12626 11386 12628 11383 12627 11378 12623
6:00 PM
11402 12654 11405 12653 11406 12655 11405 12658 11402 12657 11402 12654
6:00 PM
11571 12607 11575 12605 11577 12602 11582 12603 11579 12599 11575 12597
11578 12593 11582 12595 11585 12597 11590 12596 11593 12599 11597 12600
11597 12605 11595 12608 11595 12613 11591 12614 11589 12611 11584 12609
11581 12606 11577 12606 11571 12607 21 P
11655 12661 11651 12663 11648 12667 11644 12666 11641 12670 11640 12674

11637 12678 11635 12674 11634 12670 11630 12667 11634 12664 11631 12660
11631 12664 11627 12665 11622 12663 11618 12662 11616 12657 11617 12653
11617 12649 11619 12644 11621 12641 11625 12644 11626 12640 11629 12637
11634 12639 11636 12635 11641 12634 11645 12635 11648 12637 11648 12642
11651 12644 11655 12647 11656 12651 11660 12651 11660 12655 11663 12660
11655 12661 37 P
11651 12699 11647 12698 11649 12703 11650 12706 11652 12711 11650 12715
11646 12717 11642 12715 11638 12714 11637 12710 11635 12706 11630 12706
11629 12702 11626 12699 11622 12698 11618 12697 11613 12697 11610 12700
11606 12699 11606 12695 11603 12692 11605 12688 11604 12684 11604 12679
11602 12675 11606 12672 11608 12669 11612 12665 11616 12664 11620 12665
11624 12667 11628 12669 11626 12674 11630 12674 11628 12678 11627 12682
11622 12684 11622 12689 11626 12690 11630 12691 11635 12692 11638 12694
11640 12690 11644 12690 11648 12692 11652 12693 11651 12699 47 P
11413 12604 11417 12603 11420 12604 11422 12606 11424 12608 11425 12612
11422 12612 11419 12612 11417 12609 11414 12607 11413 12604 11 P
11396 12663 11398 12661 11401 12661 11400 12664 11396 12663 5 P
11602 12607 11604 12605 11606 12603 11609 12601 11611 12598 11610 12601
11609 12605 11606 12605 11602 12607 9 P
11599 12607 11601 12605 11600 12601 11602 12604 11599 12607 5 P
11635 12620 11637 12618 11640 12616 11642 12618 11645 12616 11647 12613
11650 12615 11650 12618 11648 12621 11645 12623 11644 12626 11641 12627
11639 12625 11636 12624 11635 12620 15 P
11659 12622 11656 12621 11656 12618 11658 12615 11656 12613 11654 12611
11657 12608 11659 12611 11659 12608 11662 12610 11665 12611 11665 12614
11664 12618 11662 12620 11659 12622 15 P
11665 12608 11666 12606 11670 12605 11672 12603 11671 12600 11669 12598
11669 12595 11671 12592 11671 12589 11672 12586 11676 12586 11676 12590
11678 12593 11680 12591 11684 12591 11687 12590 11689 12592 11690 12595
11691 12598 11689 12601 11686 12603 11686 12606 11686 12609 11683 12608
11680 12608 11678 12611 11676 12614 11678 12617 11678 12620 11678 12623
11674 12624 11671 12625 11668 12625 11665 12624 11664 12621 11666 12618
11667 12615 11666 12612 11665 12608 39 P
11676 12570 11680 12569 11684 12569 11689 12569 11693 12571 11695 12575
11699 12576 11703 12579 11702 12583 11706 12584 11708 12588 11706 12592
11708 12596 11708 12600 11712 12603 11708 12606 11705 12608 11707 12611
11703 12613 11699 12615 11695 12617 11694 12621 11691 12624 11686 12623
11682 12624 11681 12619 11685 12616 11689 12614 11691 12611 11693 12607
11694 12603 11698 12600 11702 12599 11698 12597 11696 12593 11697 12589
11694 12586 11689 12585 11686 12588 11681 12587 11678 12584 11675 12582
11679 12580 11681 12583 11686 12583 11690 12583 11688 12579 11685 12577
11683 12573 11679 12575 50 P
11679 12575 11676 12570 2 P

11642 12599 11645 12600 11648 12600 11649 12597 11648 12594 11648 12591
11651 12591 11651 12594 11651 12598 11651 12601 11654 12604 11650 12604
11647 12603 11644 12602 11642 12599 15 P
11710 12635 11714 12634 11717 12631 11716 12627 11717 12623 11715 12619
11720 12618 11723 12621 11728 12622 11729 12626 11734 12627 11737 12625
11741 12627 11743 12631 11748 12631 11750 12635 11745 12636 11741 12637
11742 12641 11740 12644 11737 12648 11734 12644 11731 12641 11727 12641
11730 12644 11730 12649 11727 12651 11723 12650 11721 12646 11716 12644
11715 12640 11711 12639 11710 12635 33 P
11680 12699 11684 12697 11687 12695 11689 12691 11692 12687 11696 12687
11700 12685 11699 12681 11699 12676 11703 12676 11707 12674 11710 12676
11713 12680 11717 12681 11718 12676 11721 12674 11721 12669 11725 12668
11729 12667 11734 12668 11735 12672 11735 12676 11735 12681 11732 12684
11730 12688 11732 12691 11729 12693 11727 12697 11722 12698 11719 12696
11715 12696 11711 12698 11709 12703 11705 12704 11701 12701 11697 12703
11693 12704 11691 12700 11687 12700 11680 12699 40 P
11687 12712 11691 12710 11695 12710 11699 12707 11702 12708 11707 12709
11710 12706 11712 12703 11715 12699 11719 12701 11723 12702 11725 12706
11724 12711 11722 12714 11725 12717 11723 12721 11719 12721 11719 12725
11715 12727 11713 12724 11715 12720 11711 12720 11707 12718 11703 12716
11699 12715 11695 12714 11687 12712 27 P
11674 12743 11676 12740 11679 12736 11681 12733 11683 12729 11686 12726
11691 12725 11693 12721 11692 12717 11696 12717 11700 12719 11703 12722
11704 12726 11702 12730 11701 12734 11703 12738 11707 12737 11711 12735
11710 12739 11714 12742 11715 12746 11711 12748 11708 12751 11712 12753
11716 12754 11716 12758 11712 12757 11712 12762 11709 12765 11706 12762
11702 12764 11698 12765 11694 12762 11695 12758 11698 12754 11700 12750
11699 12747 11694 12747 11690 12748 11687 12752 11683 12749 11681 12746
11674 12743 43 P
11645 12762 11643 12758 11641 12754 11637 12754 11632 12754 11636 12752
11634 12748 11638 12747 11643 12747 11647 12749 11650 12747 11654 12750
11656 12753 11657 12757 11657 12753 11657 12749 11661 12748 11663 12743
11666 12741 11669 12743 11672 12747 11677 12748 11678 12752 11683 12753
11682 12757 11681 12761 11685 12760 11689 12761 11691 12766 11687 12768
11683 12769 11684 12774 11687 12776 11684 12779 11684 12783 11680 12784
11678 12788 11675 12791 11671 12791 11670 12787 11671 12783 11670 12779
11666 12777 11663 12780 11659 12777 11656 12775 11652 12777 11650 12773
11649 12769 11645 12762 50 P
11679 12720 11681 12718 11683 12715 11686 12716 11684 12719 11683 12722
11679 12720 7 P
11667 12730 11669 12727 11671 12724 11674 12724 11676 12721 11679 12722
11679 12726 11676 12728 11673 12729 11671 12731 11667 12730 11 P
11658 12685 11659 12682 11660 12679 11662 12676 11663 12673 11667 12673

11667 12676 11666 12679 11665 12682 11664 12685 11658 12685 11 P
11728 12656 11731 12656 11735 12655 11738 12657 11739 12660 11741 12657
11743 12660 11744 12663 11742 12665 11744 12667 11746 12670 11743 12671
11740 12670 11737 12668 11736 12665 11736 12662 11732 12662 11729 12662
11728 12656 19 P
11698 12665 11700 12663 11703 12663 11705 12666 11706 12669 11703 12671
11700 12670 11698 12665 8 P
11685 12682 11688 12681 11690 12684 11691 12687 11688 12690 11687 12686
11685 12682 7 P
11670 12692 11673 12693 11676 12694 11678 12697 11678 12700 11675 12698
11673 12696 11670 12692 8 P
11685 12650 11686 12647 11689 12649 11689 12652 11692 12654 11692 12657
11689 12657 11686 12655 11685 12650 9 P
11678 12637 11678 12634 11679 12631 11681 12634 11681 12637 11680 12641
11679 12644 11677 12641 11678 12637 9 P
11681 12541 11684 12539 11685 12536 11685 12533 11687 12530 11689 12532
11692 12535 11692 12538 11695 12539 11698 12542 11698 12545 11695 12543
11694 12546 11696 12548 11694 12549 11692 12552 11689 12550 11687 12548
11686 12544 11685 12541 11681 12541 21 P
11799 12493 11802 12491 11805 12490 11804 12493 11807 12493 11810 12494
11812 12497 11812 12500 11810 12502 11807 12500 11809 12503 11809 12506
11806 12505 11803 12503 11800 12501 11801 12498 11799 12493 17 P
11643 12475 11646 12476 11648 12478 11648 12482 11645 12482 11644 12478
11643 12475 7 P
11655 12475 11658 12475 11660 12478 11660 12481 11658 12484 11656 12482
11655 12475 7 P
11655 12443 11658 12442 11660 12444 11659 12448 11656 12447 11655 12443

6:00 PM

11662 12440 11664 12438 11667 12437 11670 12438 11669 12441 11665 12442
11662 12440 7 P
11661 12449 11662 12447 11663 12443 11666 12444 11667 12448 11666 12450
11661 12449 7 P
11650 12462 11653 12463 11656 12463 11656 12466 11653 12468 11651 12465
11650 12462 7 P
11680 12472 11683 12470 11680 12468 11683 12466 11686 12466 11686 12469
11685 12472 11683 12475 11680 12472 9 P
11676 12451 11677 12448 11679 12445 11681 12448 11685 12448 11685 12451
11681 12451 11676 12451 8 P
11613 12388 11615 12387 11618 12385 11621 12384 11620 12387 11618 12389
11613 12388 7 P
11656 12361 11657 12358 11658 12355 11659 12352 11662 12355 11660 12357
11659 12360 11658 12364 11656 12361 9 P
11749 12750 11753 12750 11757 12749 11761 12748 11766 12748 11768 12752

11770 12755 11774 12757 11778 12756 11782 12757 11781 12761 11785 12760
11787 12756 11792 12756 11796 12755 11797 12752 11802 12750 11800 12747
11796 12743 11799 12739 11803 12739 11805 12743 11809 12741 11811 12745
11811 12749 11812 12753 11813 12757 11812 12761 11808 12763 11804 12766
11800 12765 11796 12764 11792 12763 11787 12764 11790 12768 11786 12770
11790 12773 11790 12777 11795 12776 11796 12780 11800 12780 11802 12784
11797 12784 11795 12788 11791 12789 11787 12788 11782 12788 11780 12792
11776 12792 11772 12791 50 P
11772 12791 11769 12788 11772 12785 11775 12782 11779 12779 11780 12775
11777 12773 11777 12768 11772 12769 11773 12764 11770 12762 11767 12766
11765 12762 11764 12758 11759 12758 11755 12757 11751 12760 11749 12756
11749 12750 19 P
11758 12817 11761 12813 11764 12810 11766 12806 11767 12802 11772 12800
11776 12802 11775 12806 11773 12810 11771 12813 11767 12817 11764 12819
11758 12817 13 P
11742 12788 11745 12786 11749 12784 11751 12788 11756 12788 11756 12792
11757 12797 11752 12799 11748 12798 11744 12799 11742 12795 11742 12788

12:00 PM

11651 12860 11656 12860 11660 12860 11661 12855 11663 12859 11666 12862
11668 12858 11672 12856 11677 12855 11680 12853 11682 12857 11686 12860
11684 12863 11687 12867 11688 12871 11685 12872 11680 12874 11681 12878
11680 12883 11685 12885 11686 12889 11683 12892 11686 12896 11685 12901
11689 12902 11693 12903 11696 12906 11701 12908 11697 12910 11694 12906
11690 12906 11686 12905 11681 12905 11681 12901 11678 12898 11675 12895
11673 12890 11670 12887 11666 12885 11662 12884 11658 12882 11656 12879
11655 12875 11652 12871 11656 12869 11660 12867 11658 12863 11651 12860
48 P
11703 12878 11708 12878 11710 12875 11709 12870 11713 12870 11715 12866
11719 12865 11723 12866 11727 12863 11731 12863 11735 12865 11738 12862
11742 12864 11743 12860 11740 12857 11740 12853 11744 12853 11746 12849
11749 12845 11749 12850 11753 12848 11754 12852 11758 12855 11754 12857
11750 12859 11751 12863 11749 12868 11746 12870 11743 12867 11739 12868
11736 12871 11732 12874 11732 12878 11735 12881 11739 12879 11742 12882
11741 12887 11736 12887 11734 12883 11732 12887 11730 12891 11726 12888
11725 12884 11721 12884 11717 12887 11715 12891 11712 12889 11707 12888
11705 12884 11703 12878 50 P
11692 12855 11697 12855 11698 12850 11701 12849 11706 12850 11709 12848
11711 12844 11713 12848 11713 12853 11714 12848 11716 12844 11718 12847
11718 12852 11717 12856 11715 12860 11711 12861 11707 12860 11702 12860
11699 12859 11692 12855 20 P
11682 12827 11686 12825 11689 12827 11694 12828 11698 12828 11702 12826
11706 12828 11706 12824 11702 12825 11701 12820 11701 12816 11702 12812
11701 12808 11699 12804 11698 12800 11701 12802 11706 12803 11707 12807

11705 12811 11709 12812 11713 12814 11717 12813 11716 12817 11721 12818
11720 12823 11720 12827 11717 12830 11713 12831 11710 12835 11710 12839
11706 12842 11702 12840 11699 12842 11694 12842 11694 12838 11691 12836
11689 12832 11682 12827 38 P
11750 12834 11751 12831 11751 12828 11752 12825 11750 12822 11752 12821
11756 12822 11759 12821 11761 12824 11760 12827 11760 12830 11758 12832
11756 12835 11754 12838 11751 12837 11748 12838 11750 12834 17 P
11730 12814 11732 12812 11733 12809 11736 12807 11737 12810 11737 12813
11737 12817 11737 12820 11737 12823 11734 12822 11731 12821 11731 12818
11730 12814 13 P
11716 13153 11719 13150 11721 13146 11724 13149 11729 13151 11731 13154
11734 13158 11737 13161 11741 13162 11742 13166 11744 13171 11742 13174
11738 13176 11739 13172 11735 13169 11731 13170 11727 13172 11729 13168
11725 13167 11725 13163 11722 13160 11722 13156 11716 13153 23 P
11681 12984 11683 12982 11686 12980 11686 12977 11689 12977 11692 12980
11691 12983 11691 12987 11689 12989 11686 12991 11683 12990 11681 12984

12:00 PM

11623 13011 11626 13009 11627 13006 11630 13007 11633 13006 11635 13009
11637 13011 11636 13015 11635 13017 11632 13018 11630 13021 11627 13019
11624 13019 11624 13016 11627 13015 11630 13013 11627 13012 11623 13011
18 P
11621 13004 11619 13002 11618 12998 11621 13001 11623 13003 11621 13004

6:00 PM

11636 13026 11639 13025 11641 13027 11638 13029 11636 13026 5 P
11686 12939 11689 12939 11693 12939 11694 12942 11696 12944 11693 12945
11692 12942 11688 12941 11686 12939 9 P
11682 12931 11685 12932 11685 12935 11685 12939 11681 12937 11682 12934
11682 12931 7 P
11638 12873 11641 12870 11642 12868 11645 12867 11648 12869 11648 12872
11646 12875 11644 12877 11641 12880 11639 12878 11638 12873 11 P
11652 13177 11655 13176 11657 13177 11658 13174 11660 13175 11660 13178
11658 13180 11656 13181 11653 13180 11652 13177 10 P
11647 13187 11649 13186 11651 13188 11651 13191 11649 13191 11647 13187

6:00 PM

11793 13247 11796 13246 11797 13243 11799 13240 11800 13243 11801 13246
11799 13249 11796 13251 11793 13247 9 P
11869 13280 11871 13278 11874 13278 11877 13279 11877 13282 11879 13286
11876 13288 11873 13288 11871 13286 11869 13280 10 P
11788 13294 11790 13292 11792 13288 11792 13285 11795 13284 11795 13287
11797 13289 11800 13287 11803 13286 11806 13287 11809 13289 11806 13291
11803 13293 11800 13294 11797 13294 11794 13296 11791 13296 11788 13294
18 P
11765 13286 11768 13285 11768 13282 11771 13282 11771 13279 11773 13281

11777 13281 11778 13283 11776 13286 11779 13288 11778 13291 11776 13294
11773 13295 11770 13294 11770 13290 11773 13290 11775 13287 11773 13285
11770 13284 11769 13287 11765 13286 21 P
11778 13277 11780 13274 11783 13274 11786 13277 11788 13279 11789 13282
11787 13285 11785 13282 11785 13279 11781 13278 11783 13281 11780 13283
11779 13280 11778 13277 14 P
11756 13281 11758 13279 11760 13276 11763 13277 11762 13281 11760 13283
11757 13285 11756 13281 8 P
11750 13301 11753 13300 11756 13300 11759 13302 11761 13304 11759 13306
11756 13304 11753 13302 11750 13301 9 P
11770 13308 11773 13308 11777 13307 11780 13307 11778 13310 11775 13309
11770 13308 7 P
11741 13301 11744 13301 11748 13301 11745 13303 11741 13301 5 P
11806 13306 11808 13303 11810 13304 11811 13307 11809 13307 11806 13306
6:00 PM
11816 13363 11818 13361 11821 13361 11823 13363 11822 13365 11819 13365
11816 13363 7 P
11842 13368 11845 13368 11847 13368 11848 13365 11851 13366 11849 13367
11848 13370 11846 13371 11842 13368 9 P
11827 13375 11830 13374 11832 13373 11834 13371 11837 13372 11839 13373
11840 13375 11838 13375 11836 13377 11833 13377 11830 13377 11827 13375
12:00 PM
11818 13413 11821 13412 11823 13411 11826 13410 11826 13413 11826 13415
11825 13418 11823 13418 11821 13416 11818 13413 10 P
11767 13432 11768 13430 11771 13429 11774 13429 11776 13428 11775 13431
11774 13433 11772 13435 11770 13434 11767 13432 10 P
11563 13435 11564 13432 11565 13430 11567 13428 11569 13427 11572 13428
11571 13430 11574 13430 11575 13428 11577 13430 11577 13432 11575 13434
11572 13435 11569 13436 11566 13436 11563 13435 16 P
11583 13489 11584 13487 11586 13485 11589 13484 11589 13486 11587 13488
11583 13489 7 P
11601 13491 11604 13491 11606 13492 11608 13493 11611 13494 11614 13494
11614 13497 11612 13499 11609 13498 11606 13499 11604 13498 11603 13495
11601 13491 13 P
11551 13498 11553 13495 11554 13492 11555 13491 11558 13490 11558 13492
11559 13495 11557 13497 11556 13494 11554 13496 11551 13498 11 P
11715 13562 11717 13562 11720 13563 11722 13562 11724 13563 11726 13565
11726 13568 11724 13570 11723 13572 11724 13575 11722 13577 11720 13577
11719 13574 11718 13572 11717 13570 11715 13568 11714 13565 11715 13562
18 P
11731 13587 11732 13585 11735 13584 11737 13586 11739 13585 11742 13584
11745 13585 11747 13584 11750 13584 11752 13584 11755 13584 11758 13582
11760 13581 11763 13582 11765 13584 11764 13586 11761 13585 11759 13585

11757 13586 11754 13587 11756 13590 11754 13592 11753 13594 11752 13597
11749 13597 11746 13597 11744 13595 11743 13593 11745 13590 11742 13590
11739 13592 11737 13593 11735 13593 11732 13592 11731 13587 35 P
11677 13540 11679 13537 11680 13535 11682 13537 11684 13539 11684 13542
11686 13544 11683 13545 11680 13545 11680 13542 11680 13540 11677 13540

12:00 PM

11683 13527 11684 13524 11686 13524 11686 13527 11687 13529 11688 13532
11685 13532 11683 13530 11683 13527 9 P
11569 13735 11570 13733 11573 13734 11575 13736 11577 13736 11580 13736
11582 13737 11583 13740 11585 13741 11584 13744 11584 13747 11582 13748
11582 13745 11581 13742 11579 13741 11576 13741 11573 13740 11572 13738
11569 13735 19 P
11555 14035 11558 14037 11560 14041 11562 14038 11566 14036 11567 14032
11571 14031 11576 14032 11579 14033 11581 14038 11585 14040 11588 14043
11585 14046 11581 14048 11577 14049 11573 14052 11569 14051 11567 14055
11564 14058 11563 14062 11560 14064 11560 14060 11557 14056 11557 14052
11556 14048 11557 14044 11557 14039 11555 14035 28 P
11587 14039 11587 14037 11588 14034 11591 14033 11593 14033 11596 14032
11597 14029 11597 14026 11599 14028 11600 14031 11602 14032 11600 14034
11598 14033 11597 14036 11595 14038 11593 14040 11591 14042 11587 14039
18 P
11701 14073 11703 14069 11704 14065 11705 14061 11706 14056 11709 14059
11713 14062 11715 14058 11714 14054 11718 14056 11720 14060 11723 14063
11724 14067 11727 14071 11730 14072 11730 14076 11729 14080 11728 14084
11724 14086 11720 14085 11715 14086 11711 14085 11710 14081 11707 14079
11702 14078 11701 14073 26 P
11749 14040 11752 14043 11754 14047 11759 14046 11761 14042 11765 14044
11765 14048 11766 14053 11767 14057 11772 14055 11776 14055 11776 14060
11779 14063 11783 14063 11785 14068 11784 14072 11781 14075 11781 14080
11777 14081 11772 14081 11768 14082 11765 14085 11761 14084 11760 14088
11763 14092 11763 14096 11761 14092 11759 14089 11755 14087 11751 14090
11747 14091 11750 14087 11753 14084 11756 14081 11757 14077 11758 14073
11759 14068 11759 14064 11756 14063 11752 14060 11753 14056 11758 14056
11756 14053 11754 14049 11751 14046 11749 14040 46 P
11636 14066 11640 14064 11644 14061 11648 14061 11652 14060 11657 14060
11661 14060 11664 14063 11666 14067 11662 14069 11660 14065 11656 14066
11652 14067 11648 14068 11643 14069 11640 14071 11636 14066 17 P
11646 14018 11648 14015 11650 14013 11654 14012 11656 14012 11660 14012
11659 14014 11657 14017 11654 14018 11650 14019 11646 14018 11 P
11626 14017 11628 14014 11631 14015 11634 14014 11637 14013 11638 14010
11642 14009 11643 14012 11640 14013 11638 14016 11638 14019 11635 14020
11632 14021 11630 14019 11626 14017 15 P
11612 14021 11615 14019 11618 14018 11621 14018 11624 14018 11626 14020

11624 14024 11621 14023 11618 14022 11612 14021 10 P
11557 14011 11559 14009 11561 14006 11564 14005 11568 14005 11570 14008
11568 14011 11570 14014 11566 14013 11564 14016 11562 14017 11558 14016
11557 14011 13 P
11551 13966 11554 13967 11557 13965 11554 13964 11553 13961 11556 13960
11558 13961 11561 13963 11562 13966 11563 13969 11562 13972 11560 13974
11557 13973 11554 13973 11553 13969 11551 13966 16 P
11500 13950 11501 13948 11504 13946 11505 13949 11505 13952 11505 13955
11508 13954 11511 13955 11510 13958 11507 13959 11504 13960 11501 13958
11501 13955 11500 13950 14 P
11528 13926 11525 13926 11525 13930 11523 13932 11520 13933 11518 13930
11520 13927 11522 13925 11525 13923 11527 13920 11530 13921 11533 13924
11535 13926 11532 13925 11528 13926 15 P
11504 14007 11507 14007 11510 14009 11512 14011 11515 14011 11514 14013
11517 14015 11518 14018 11515 14018 11513 14016 11510 14014 11508 14012
11506 14011 11504 14007 14 P
11688 13985 11691 13984 11694 13982 11695 13985 11694 13988 11693 13991
11689 13991 11688 13985 8 P
11707 13963 11709 13960 11709 13957 11710 13954 11713 13953 11716 13954
11719 13955 11722 13957 11720 13960 11720 13963 11717 13965 11715 13962
11712 13963 11710 13966 11707 13963 15 P
11679 14142 11683 14141 11684 14138 11685 14135 11685 14132 11687 14129
11690 14128 11693 14129 11696 14130 11697 14133 11696 14137 11699 14138
11697 14141 11694 14142 11692 14145 11690 14147 11687 14148 11685 14151
11682 14152 11681 14148 11680 14146 11679 14142 22 P
11658 14178 11660 14175 11663 14174 11664 14172 11665 14168 11667 14166
11670 14164 11672 14166 11673 14169 11677 14170 11679 14167 11678 14164
11681 14166 11684 14165 11686 14162 11687 14159 11689 14162 11692 14164
11692 14167 11692 14170 11690 14173 11687 14175 11685 14177 11683 14179
11679 14180 11676 14180 11676 14177 11672 14176 11669 14176 11667 14179
11665 14182 11663 14184 11659 14184 11658 14181 11658 14178 35 P
11711 14191 11713 14188 11716 14185 11720 14186 11722 14190 11727 14190
11730 14193 11734 14195 11738 14194 11742 14195 11746 14196 11747 14201
11750 14204 11750 14209 11746 14212 11744 14216 11741 14217 11736 14219
11732 14218 11728 14219 11724 14222 11721 14224 11717 14222 11715 14218
11715 14214 11714 14209 11713 14205 11714 14201 11713 14197 11711 14191
30 P
11692 14200 11696 14197 11700 14195 11701 14191 11701 14187 11705 14186
11704 14190 11706 14194 11709 14198 11706 14201 11702 14201 11698 14201
11692 14200 13 P
11686 14211 11689 14210 11692 14212 11695 14212 11699 14211 11701 14210
11704 14209 11704 14212 11703 14216 11705 14219 11704 14222 11702 14224
11699 14226 11696 14225 11695 14222 11693 14220 11690 14218 11688 14216

11686 14211 19 P
11708 14177 11711 14175 11714 14174 11715 14177 11715 14180 11713 14182
11710 14181 11708 14177 8 P
11632 14227 11634 14225 11636 14223 11639 14221 11641 14218 11642 14216
11642 14212 11645 14211 11648 14212 11651 14212 11655 14214 11658 14214
11661 14215 11664 14214 11666 14217 11666 14220 11663 14219 11659 14218
11656 14217 11653 14217 11650 14218 11648 14221 11646 14223 11643 14224
11640 14225 11637 14226 11632 14227 27 P
11680 14214 11684 14213 11685 14216 11686 14219 11686 14223 11688 14225
11685 14226 11683 14225 11683 14222 11681 14219 11680 14214 11 P
11673 14217 11676 14215 11677 14217 11679 14219 11678 14222 11676 14221
11673 14217 7 P
11637 14355 11640 14349 11642 14343 11642 14336 11642 14329 11643 14322
11642 14315 11645 14309 11651 14308 11658 14307 11660 14301 11665 14296
11667 14290 11670 14283 11667 14278 11660 14275 11654 14273 11656 14267
11656 14260 11662 14258 11669 14255 11675 14255 11682 14254 11688 14253
11695 14251 11695 14258 11695 14265 11702 14262 11707 14258 11714 14258
11718 14262 11723 14267 11725 14274 11723 14280 11720 14286 11722 14293
11717 14297 11716 14304 11717 14311 11715 14317 11712 14323 11712 14330
11715 14336 11712 14343 11708 14348 11706 14355 11702 14360 11695 14362
11689 14365 11684 14369 50 P
11684 14369 11678 14373 11673 14378 11667 14379 11661 14376 11654 14375
11652 14369 11648 14364 11643 14360 11637 14355 10 P
11607 14308 11610 14305 11614 14303 11617 14301 11621 14298 11625 14297
11629 14295 11632 14293 11635 14290 11636 14286 11634 14282 11637 14279
11640 14275 11643 14279 11646 14282 11649 14285 11652 14288 11655 14292
11655 14296 11654 14300 11650 14302 11646 14304 11642 14304 11637 14305
11634 14308 11630 14310 11628 14314 11627 14318 11628 14322 11629 14326
11631 14330 11629 14333 11625 14333 11621 14330 11617 14329 11613 14326
11614 14322 11614 14317 11614 14313 11615 14309 11607 14308 41 P
11576 14337 11579 14335 11583 14332 11587 14331 11591 14332 11592 14336
11597 14337 11601 14337 11604 14340 11608 14340 11612 14338 11616 14340
11620 14343 11623 14345 11626 14349 11627 14352 11629 14356 11628 14360
11626 14364 11622 14361 11618 14360 11614 14360 11609 14360 11605 14362
11602 14365 11598 14366 11594 14365 11590 14364 11586 14361 11583 14359
11582 14354 11580 14351 11582 14346 11580 14343 11576 14337 35 P
11590 14274 11592 14272 11594 14269 11597 14267 11599 14265 11602 14264
11605 14264 11608 14262 11607 14265 11604 14267 11604 14270 11604 14273
11605 14276 11601 14276 11598 14275 11595 14276 11590 14274 17 P
11560 14283 11563 14282 11565 14280 11568 14281 11571 14282 11573 14280
11576 14278 11578 14276 11582 14275 11583 14279 11584 14281 11582 14283
11579 14286 11578 14289 11577 14292 11574 14291 11572 14288 11570 14287
11566 14287 11563 14287 11560 14283 21 P

11546 14289 11547 14287 11550 14287 11553 14286 11555 14288 11554 14290
11551 14290 11549 14292 11546 14289 9 P
11601 14316 11604 14316 11605 14318 11605 14321 11602 14322 11601 14320
11601 14316 7 P
11584 14311 11587 14310 11590 14310 11592 14310 11595 14311 11598 14311
11595 14312 11593 14313 11590 14313 11587 14314 11584 14311 11 P
10886 14382 10887 14378 10891 14376 10894 14373 10895 14369 10897 14365
10899 14361 10902 14358 10906 14359 10910 14358 10914 14357 10919 14359
10922 14356 10926 14357 10926 14361 10928 14365 10926 14369 10922 14370
10920 14374 10922 14378 10926 14380 10930 14378 10928 14374 10933 14374
10936 14377 10940 14376 10938 14380 10942 14380 10940 14383 10938 14388
10936 14392 10932 14393 10928 14393 10924 14393 10919 14392 10915 14391
10911 14390 10907 14388 10903 14386 10899 14383 10896 14385 10892 14387
10886 14382 43 P
10914 14304 10917 14302 10920 14301 10924 14301 10924 14297 10923 14295
10926 14296 10928 14297 10932 14297 10935 14297 10937 14300 10939 14303
10939 14306 10937 14309 10937 14312 10939 14314 10942 14316 10940 14318
10938 14320 10934 14319 10932 14322 10931 14318 10929 14316 10926 14315
10925 14311 10922 14309 10922 14305 10919 14304 10914 14304 29 P
10944 14273 10948 14274 10949 14276 10953 14279 10955 14280 10958 14279
10961 14278 10962 14281 10963 14285 10960 14285 10956 14284 10954 14283
10950 14284 10949 14281 10947 14279 10944 14273 16 P
11058 14312 11057 14309 11060 14308 11063 14309 11066 14307 11070 14308
11071 14310 11072 14313 11073 14316 11076 14315 11078 14318 11077 14321
11078 14324 11079 14327 11076 14326 11072 14326 11069 14327 11066 14325
11068 14324 11071 14322 11071 14319 11071 14316 11067 14316 11064 14317
11061 14318 11061 14315 11058 14312 27 P
11117 14308 11120 14308 11123 14307 11124 14304 11122 14301 11125 14303
11127 14305 11129 14308 11132 14309 11135 14311 11137 14314 11136 14317
11139 14316 11143 14317 11141 14319 11140 14323 11137 14322 11134 14321
11131 14320 11128 14318 11127 14315 11124 14314 11122 14312 11117 14308
24 P
11154 14259 11157 14257 11159 14255 11162 14257 11165 14258 11168 14258
11171 14260 11173 14261 11175 14264 11177 14267 11179 14269 11181 14272
11184 14274 11187 14275 11190 14276 11192 14279 11194 14282 11195 14285
11197 14287 11200 14289 11201 14293 11202 14295 11204 14298 11206 14301
11203 14300 11200 14300 11197 14298 11195 14296 11193 14294 11190 14292
11187 14293 11185 14290 11181 14289 11179 14287 11180 14284 11181 14281
11182 14278 11182 14275 11179 14277 11180 14280 11177 14282 11174 14284
11172 14283 11168 14281 11171 14279 11170 14276 11167 14274 11166 14271
11164 14268 11162 14266 50 P
11162 14266 11159 14265 11156 14262 11154 14259 4 P
11354 14263 11357 14261 11359 14258 11358 14255 11359 14252 11362 14251

11365 14253 11366 14255 11368 14258 11370 14261 11372 14264 11372 14267
11368 14267 11365 14267 11362 14266 11359 14266 11356 14267 11354 14263
18 P
11439 14325 11441 14323 11444 14324 11447 14324 11450 14325 11453 14327
11454 14330 11455 14333 11453 14331 11449 14330 11448 14328 11445 14329
11442 14328 11439 14325 14 P
11383 14335 11385 14334 11389 14334 11391 14337 11389 14340 11389 14343
11387 14342 11386 14338 11383 14335 9 P
11152 14288 11155 14287 11158 14286 11160 14284 11162 14281 11165 14283
11163 14285 11164 14287 11162 14290 11159 14290 11156 14289 11152 14288
12:00 PM
10927 14687 10931 14684 10934 14683 10939 14684 10936 14687 10938 14691
10941 14688 10946 14689 10949 14692 10951 14688 10955 14686 10959 14684
10963 14682 10967 14680 10971 14680 10971 14684 10974 14681 10978 14681
10981 14685 10978 14688 10977 14692 10974 14695 10970 14698 10971 14702
10967 14702 10962 14702 10959 14705 10955 14706 10950 14707 10948 14711
10946 14706 10943 14703 10941 14699 10937 14698 10933 14698 10933 14693
10933 14689 10927 14687 38 P
11018 14771 11017 14775 11014 14778 11014 14774 11014 14770 11012 14766
11014 14762 11018 14762 11018 14758 11014 14760 11011 14757 11010 14753
11013 14750 11015 14753 11019 14755 11023 14757 11025 14762 11025 14766
11027 14770 11027 14774 11025 14770 11018 14771 22 P
10903 15216 10907 15216 10910 15219 10913 15222 10918 15222 10921 15224
10925 15227 10927 15231 10931 15232 10933 15236 10934 15240 10939 15241
10938 15245 10942 15246 10946 15244 10949 15247 10951 15251 10950 15255
10946 15255 10942 15253 10944 15257 10940 15259 10943 15262 10939 15263
10942 15266 10938 15268 10937 15273 10940 15276 10944 15275 10944 15279
10940 15281 10937 15284 10933 15284 10928 15285 10925 15282 10921 15280
10925 15278 10924 15274 10920 15272 10923 15268 10924 15264 10920 15261
10917 15260 10915 15256 10911 15256 10907 15253 10905 15250 10904 15246
10907 15248 10912 15249 50 P
10912 15249 10915 15250 10919 15248 10917 15245 10913 15243 10914 15239
10919 15238 10919 15234 10918 15230 10913 15230 10909 15230 10909 15226
10907 15222 10903 15216 14 P
10808 15230 10812 15231 10813 15235 10817 15238 10820 15236 10824 15233
10826 15237 10827 15241 10831 15239 10831 15235 10835 15233 10840 15233
10844 15234 10846 15238 10847 15242 10851 15245 10852 15249 10847 15251
10844 15253 10840 15254 10838 15250 10834 15250 10830 15251 10827 15247
10823 15249 10819 15247 10820 15243 10817 15240 10813 15240 10810 15236
10808 15230 31 P
10846 15217 10849 15213 10852 15217 10856 15218 10856 15222 10859 15226
10855 15229 10857 15233 10854 15234 10850 15233 10848 15229 10845 15225
10848 15222 10846 15217 14 P

10810 15177 10813 15177 10813 15174 10815 15172 10818 15173 10821 15173
10823 15176 10824 15179 10821 15181 10819 15184 10819 15187 10817 15185
10815 15183 10811 15182 10810 15177 15 P
10819 15192 10823 15191 10826 15191 10825 15194 10825 15197 10823 15199
10820 15197 10819 15192 8 P
10819 15205 10821 15204 10824 15205 10825 15208 10824 15210 10821 15211
10820 15209 10819 15205 8 P
9402 15147 9404 15141 9403 15134 9409 15131 9416 15129 9422 15132
9429 15131 9433 15126 9436 15119 9439 15125 9445 15129 9449 15134
9454 15139 9460 15139 9467 15139 9473 15135 9479 15132 9486 15132
9492 15134 9496 15140 9501 15144 9508 15146 9511 15152 9515 15157
9521 15155 9526 15159 9529 15166 9531 15172 9530 15178 9530 15185
9527 15191 9525 15198 9519 15199 9513 15202 9507 15206 9501 15209
9494 15209 9499 15204 9499 15197 9495 15191 9489 15188 9482 15188
9478 15183 9472 15181 9465 15181 9458 15181 9451 15180 9445 15177
9438 15177 9432 15181 50 P
9432 15181 9430 15174 9431 15167 9426 15163 9420 15160 9417 15154
9412 15149 9402 15147 8 P
9500 15106 9497 15112 9490 15113 9484 15116 9478 15118 9471 15116
9466 15112 9462 15118 9456 15121 9450 15120 9446 15114 9442 15109
9442 15102 9438 15097 9431 15096 9425 15095 9423 15089 9425 15082
9418 15081 9421 15075 9420 15069 9416 15063 9422 15060 9429 15059
9433 15064 9435 15070 9440 15075 9444 15080 9449 15085 9450 15092
9455 15095 9462 15096 9469 15095 9471 15089 9466 15084 9462 15078
9458 15073 9455 15067 9452 15061 9458 15060 9465 15058 9471 15057
9477 15053 9484 15053 9489 15056 9490 15063 9494 15069 9496 15075
9496 15082 9493 15088 50 P
9493 15088 9488 15092 9482 15095 9488 15094 9495 15094 9501 15097
9500 15106 7 P
9866 14974 9870 14968 9869 14961 9874 14957 9879 14952 9885 14956
9887 14962 9889 14969 9894 14964 9901 14963 9900 14970 9898 14976
9895 14983 9898 14989 9904 14991 9898 14991 9893 14986 9886 14985
9879 14984 9877 14978 9866 14974 21 P
10217 14936 10223 14936 10227 14934 10230 14929 10225 14932 10222 14927
10222 14922 10221 14917 10223 14911 10228 14908 10231 14913 10235 14917
10240 14919 10244 14924 10244 14929 10240 14933 10238 14938 10235 14942
10234 14948 10233 14942 10228 14940 10223 14941 10217 14936 23 P
10036 14906 10039 14903 10042 14900 10038 14898 10042 14896 10045 14893
10049 14892 10053 14891 10058 14890 10061 14892 10060 14896 10056 14897
10055 14901 10052 14905 10048 14903 10045 14906 10044 14910 10040 14910
10036 14906 19 P
10143 15191 10139 15189 10142 15185 10141 15181 10138 15184 10134 15184
10135 15189 10130 15190 10128 15187 10126 15182 10124 15178 10121 15176

10119 15171 10124 15170 10128 15170 10130 15167 10132 15163 10134 15159
10138 15160 10143 15160 10145 15164 10148 15167 10151 15170 10153 15174
10155 15178 10155 15182 10153 15185 10150 15188 10143 15191 29 P
9939 15040 9942 15036 9946 15034 9950 15034 9955 15033 9958 15031
9962 15028 9966 15027 9970 15027 9974 15025 9974 15021 9977 15018
9979 15021 9978 15026 9983 15026 9987 15027 9991 15026 9995 15025
10000 15025 10004 15025 10008 15023 10012 15020 10015 15024 10017 15027
10021 15029 10017 15029 10013 15030 10009 15031 10005 15033 10002 15036
9998 15038 9995 15034 9992 15033 9987 15032 9983 15032 9979 15033
9974 15033 9971 15036 9969 15039 9969 15043 9966 15041 9963 15037
9960 15039 9960 15043 9956 15043 9952 15041 9949 15043 9946 15041
9939 15040 49 P
9996 15007 9999 15004 10003 15005 10007 15007 10011 15007 10015 15008
10020 15009 10024 15009 10028 15008 10027 15004 10025 14999 10024 14996
10024 14991 10027 14995 10030 14997 10034 15000 10038 15000 10038 15004
10042 15005 10046 15007 10049 15010 10046 15012 10044 15016 10039 15017
10042 15021 10038 15018 10036 15014 10032 15016 10028 15016 10023 15016
10020 15014 10015 15012 10011 15013 10007 15013 10002 15012 9996 15007
36 P
10050 15010 10053 15010 10056 15009 10059 15011 10056 15013 10053 15012
10050 15010 7 P
10056 15006 10059 15005 10061 15003 10064 15005 10067 15006 10070 15006
10073 15007 10071 15010 10067 15009 10064 15008 10061 15010 10056 15006
12:00 PM
10167 15134 10169 15131 10172 15127 10176 15126 10180 15124 10181 15128
10181 15133 10185 15133 10182 15137 10178 15137 10174 15138 10170 15139
10167 15134 13 P
10152 15034 10151 15032 10147 15031 10148 15027 10152 15027 10154 15028
10156 15031 10152 15034 8 P
9939 15004 9942 15002 9943 14999 9946 15000 9949 14999 9952 15000
9956 14999 9959 15000 9961 15003 9963 15005 9960 15006 9957 15005
9954 15006 9950 15006 9947 15006 9944 15006 9939 15004 17 P
9980 14995 9983 14992 9985 14994 9988 14995 9990 14997 9990 15000
9988 15003 9987 15000 9984 15001 9984 14998 9980 14995 11 P
10066 14927 10068 14925 10071 14923 10074 14924 10078 14925 10080 14923
10082 14925 10080 14928 10077 14929 10075 14927 10072 14928 10066 14927
12:00 PM
10096 14924 10099 14921 10101 14919 10102 14915 10104 14914 10106 14917
10108 14920 10106 14922 10102 14922 10096 14924 10 P
10082 14910 10085 14907 10088 14909 10090 14911 10092 14914 10090 14917
10087 14918 10086 14914 10083 14914 10082 14910 10 P
10429 15314 10432 15314 10435 15316 10438 15317 10441 15318 10444 15318
10447 15318 10445 15320 10444 15323 10441 15325 10438 15324 10435 15322

10432 15320 10429 15319 10429 15314 15 P
9908 14998 9911 14998 9914 14996 9916 14997 9920 14998 9920 15001
9919 15004 9916 15005 9913 15003 9911 15000 9908 14998 11 P
9907 15021 9910 15020 9913 15019 9916 15017 9918 15014 9921 15016
9924 15016 9922 15018 9919 15019 9916 15020 9914 15022 9911 15022
9907 15021 13 P
9668 14798 9669 14795 9669 14792 9670 14789 9674 14791 9675 14793
9675 14797 9673 14799 9674 14802 9675 14805 9672 14806 9669 14804
9671 14801 9668 14798 14 P
9196 15646 9199 15643 9203 15644 9205 15648 9209 15650 9213 15651
9218 15651 9219 15656 9219 15660 9218 15664 9214 15666 9211 15668
9209 15665 9204 15665 9204 15660 9205 15656 9200 15656 9198 15653
9196 15646 19 P
8356 14833 8360 14833 8365 14835 8368 14836 8371 14840 8374 14843
8378 14846 8379 14850 8377 14854 8373 14856 8370 14860 8368 14855
8370 14851 8374 14850 8374 14846 8371 14844 8368 14841 8365 14837
8361 14835 8356 14833 20 P
8392 14880 8396 14880 8401 14882 8404 14883 8409 14884 8413 14885
8415 14889 8417 14893 8419 14897 8423 14898 8424 14903 8422 14906
8417 14905 8415 14901 8412 14898 8410 14894 8408 14891 8404 14888
8401 14886 8396 14884 8392 14880 21 P
8475 14933 8470 14931 8467 14929 8463 14927 8459 14925 8455 14924
8455 14919 8452 14916 8448 14914 8444 14911 8441 14908 8437 14907
8437 14903 8434 14899 8430 14897 8426 14896 8430 14893 8433 14896
8438 14897 8442 14898 8446 14899 8450 14902 8452 14906 8455 14908
8458 14912 8460 14915 8464 14919 8467 14921 8470 14925 8473 14928
8475 14933 31 P
8396 14895 8399 14893 8402 14894 8404 14897 8406 14899 8407 14903
8407 14906 8406 14909 8404 14906 8402 14903 8400 14901 8398 14898
8396 14895 13 P
8272 14780 8276 14780 8279 14783 8283 14786 8285 14790 8287 14793
8289 14797 8292 14800 8294 14805 8295 14809 8293 14813 8291 14809
8289 14805 8285 14802 8282 14800 8282 14796 8281 14791 8278 14787
8275 14785 8272 14780 20 P
8293 14827 8292 14823 8294 14822 8297 14824 8299 14827 8301 14829
8301 14833 8298 14831 8295 14829 8293 14827 10 P
8310 14841 8313 14839 8315 14841 8318 14843 8320 14846 8321 14849
8322 14851 8324 14854 8326 14857 8323 14855 8321 14853 8318 14851
8318 14848 8315 14846 8313 14843 8310 14841 16 P
9247 14461 9250 14458 9254 14461 9255 14465 9260 14466 9264 14466
9266 14470 9266 14475 9262 14477 9257 14475 9254 14474 9251 14470
9250 14466 9247 14461 14 P
9224 14390 9227 14388 9232 14389 9236 14389 9240 14388 9242 14392

9239 14395 9235 14396 9231 14396 9231 14401 9230 14404 9228 14408
9225 14406 9225 14401 9224 14397 9224 14390 16 P
9167 14283 9169 14280 9172 14276 9176 14274 9179 14271 9181 14275
9181 14279 9181 14283 9181 14288 9177 14290 9173 14291 9169 14289
9167 14283 13 P
9204 14296 9207 14293 9210 14289 9212 14286 9215 14283 9219 14282
9223 14280 9226 14283 9225 14287 9223 14292 9221 14295 9217 14295
9213 14297 9209 14298 9204 14296 15 P
9221 14255 9225 14252 9229 14253 9232 14249 9234 14246 9238 14243
9240 14247 9239 14251 9238 14255 9240 14259 9238 14264 9235 14267
9233 14271 9231 14275 9228 14278 9225 14275 9223 14271 9224 14266
9225 14262 9225 14258 9221 14255 21 P
9309 14320 9311 14317 9314 14316 9316 14318 9316 14322 9314 14323
9311 14324 9309 14320 8 P
9289 14513 9292 14513 9295 14513 9298 14515 9300 14513 9301 14516
9299 14518 9298 14522 9294 14522 9293 14519 9291 14517 9289 14513
12:00 PM
9287 14502 9289 14500 9291 14497 9293 14495 9295 14493 9296 14495
9294 14499 9297 14500 9294 14501 9293 14504 9291 14506 9287 14502
12:00 PM
9010 13068 9012 13064 9017 13063 9020 13066 9024 13068 9024 13073
9021 13076 9021 13081 9021 13085 9016 13085 9015 13081 9013 13077
9010 13074 9010 13068 14 P
8704 12528 8706 12523 8708 12520 8712 12518 8714 12514 8719 12514
8723 12512 8726 12514 8730 12515 8728 12519 8724 12521 8720 12521
8715 12522 8711 12524 8709 12528 8704 12528 16 P
9538 10900 9542 10895 9547 10890 9552 10886 9557 10880 9561 10875
9566 10869 9571 10865 9577 10868 9583 10865 9589 10862 9595 10857
9600 10853 9605 10848 9611 10844 9615 10839 9620 10834 9626 10830
9632 10826 9638 10823 9644 10821 9648 10815 9654 10811 9661 10811
9668 10809 9673 10804 9679 10802 9685 10799 9691 10796 9698 10794
9705 10791 9710 10787 9717 10784 9723 10781 9728 10776 9734 10775
9741 10773 9747 10768 9753 10765 9759 10763 9766 10762 9773 10763
9780 10761 9786 10759 9793 10758 9800 10755 9806 10753 9812 10749
9818 10746 9825 10744 50 P
9825 10744 9832 10741 9838 10739 9844 10738 9851 10736 9858 10735
9865 10734 9871 10738 9877 10741 9880 10747 9884 10753 9890 10756
9891 10763 9893 10770 9894 10777 9891 10783 9885 10787 9880 10791
9873 10794 9866 10795 9860 10794 9853 10796 9847 10798 9840 10800
9834 10804 9828 10807 9822 10812 9817 10815 9811 10818 9804 10822
9798 10823 9791 10826 9785 10829 9778 10830 9771 10832 9765 10834
9758 10836 9752 10839 9746 10842 9741 10847 9738 10854 9733 10858
9728 10863 9723 10868 9717 10870 9710 10872 9704 10875 9698 10879

9691 10881 9684 10883 50 P
9684 10883 9678 10885 2 P
9678 10885 9672 10889 9668 10894 9665 10900 9661 10907 9658 10912
9654 10918 9649 10923 9644 10928 9638 10931 9632 10935 9626 10937
9619 10938 9612 10940 9605 10939 9598 10939 9591 10937 9585 10936
9578 10933 9572 10931 9567 10926 9562 10922 9557 10917 9551 10913
9545 10909 9538 10900 26 P
9746 10718 9753 10715 9759 10712 9765 10709 9771 10706 9776 10701
9782 10698 9789 10695 9795 10691 9800 10687 9806 10683 9812 10681
9818 10677 9824 10674 9827 10668 9832 10662 9836 10657 9842 10653
9848 10655 9848 10662 9848 10669 9844 10675 9840 10681 9836 10686
9832 10691 9826 10695 9821 10700 9814 10702 9808 10706 9803 10709
9796 10711 9789 10714 9783 10715 9776 10718 9769 10719 9763 10721
9756 10723 9746 10718 38 P
9850 10687 9854 10681 9856 10675 9858 10668 9860 10661 9862 10655
9867 10650 9873 10647 9878 10642 9885 10645 9889 10651 9892 10657
9895 10663 9894 10670 9891 10676 9885 10680 9879 10683 9872 10686
9865 10686 9859 10688 9850 10687 21 P
9983 10008 9977 10012 9971 10013 9970 10020 9966 10025 9960 10028
9957 10022 9951 10019 9955 10013 9960 10008 9965 10004 9969 9998
9971 9991 9976 9986 9977 9979 9978 9972 9977 9965 9974 9959
9978 9953 9983 9949 9988 9943 9992 9938 9996 9932 9998 9925
10004 9922 10009 9926 10015 9931 10021 9933 10028 9932 10035 9934
10041 9930 10045 9924 10052 9924 10058 9925 10065 9926 10072 9924
10078 9921 10085 9918 10092 9916 10098 9914 10104 9912 10110 9908
10116 9904 10123 9901 10130 9902 10136 9903 10143 9906 10139 9912
10138 9919 10141 9925 50 P
10141 9925 10144 9932 10146 9938 10152 9943 10145 9946 10142 9952
10136 9956 10131 9961 10127 9966 10121 9970 10114 9969 10107 9970
10103 9976 10097 9979 10095 9985 10088 9985 10081 9985 10075 9988
10072 9994 10065 9993 10058 9994 10052 9995 10048 10001 10043 10006
10039 10012 10034 10016 10027 10017 10021 10014 10015 10010 10008 10009
10002 10007 9995 10009 9990 10014 9983 10008 34 P
10017 10090 10023 10086 10030 10083 10036 10079 10042 10076 10047 10071
10054 10070 10061 10070 10068 10070 10075 10068 10081 10065 10088 10063
10085 10070 10083 10077 10088 10081 10089 10088 10085 10094 10090 10099
10095 10104 10088 10104 10083 10109 10085 10116 10079 10119 10072 10116
10066 10120 10064 10113 10058 10110 10051 10108 10046 10104 10040 10106
10033 10106 10029 10101 10022 10098 10017 10090 34 P
10108 10197 10111 10190 10109 10183 10114 10178 10113 10171 10119 10168
10125 10165 10132 10164 10130 10171 10137 10172 10143 10176 10148 10172
10155 10173 10162 10171 10169 10171 10175 10168 10182 10167 10180 10174
10174 10177 10179 10181 10175 10187 10168 10189 10161 10189 10154 10190

10153 10197 10146 10196 10149 10190 10142 10191 10136 10193 10129 10195
10122 10193 10116 10195 10108 10197 33 P
10169 10325 10176 10326 10183 10326 10190 10327 10197 10330 10203 10330
10210 10330 10217 10330 10224 10333 10218 10336 10223 10342 10216 10342
10209 10341 10203 10336 10196 10335 10191 10340 10185 10342 10181 10348
10175 10351 10168 10350 10175 10346 10176 10340 10175 10333 10169 10325
24 P
10017 9771 10024 9771 10028 9776 10035 9777 10040 9772 10047 9772
10048 9766 10054 9762 10061 9759 10060 9752 10060 9745 10067 9745
10070 9752 10073 9745 10079 9749 10081 9742 10087 9740 10090 9747
10092 9754 10096 9760 10091 9765 10089 9772 10082 9774 10078 9779
10071 9781 10065 9785 10060 9790 10056 9795 10058 9802 10056 9809
10051 9814 10046 9809 10039 9808 10032 9807 10027 9803 10027 9796
10025 9789 10022 9783 10017 9771 39 P
10098 9839 10104 9836 10110 9832 10113 9825 10118 9821 10124 9817
10129 9813 10136 9811 10142 9807 10148 9810 10144 9816 10141 9822
10141 9829 10136 9833 10141 9837 10137 9842 10130 9845 10124 9846
10117 9849 10110 9847 10103 9846 10098 9839 22 P
10146 9899 10152 9895 10155 9889 10161 9885 10167 9881 10169 9874
10174 9870 10169 9865 10170 9858 10172 9851 10177 9846 10183 9845
10189 9849 10185 9854 10183 9861 10186 9867 10191 9871 10198 9874
10198 9881 10196 9888 10189 9889 10183 9892 10190 9892 10197 9894
10193 9899 10192 9906 10186 9909 10180 9906 10173 9904 10167 9906
10160 9905 10156 9911 10151 9906 10146 9899 34 P
9909 9922 9914 9917 9916 9910 9921 9906 9928 9907 9933 9912
9939 9916 9945 9920 9950 9924 9953 9930 9950 9937 9948 9944
9947 9950 9943 9956 9937 9959 9933 9965 9930 9971 9923 9971
9916 9970 9914 9963 9912 9956 9909 9950 9909 9943 9912 9936
9913 9929 9909 9922 26 P
10051 10023 10054 10017 10059 10012 10066 10014 10070 10020 10071 10027
10078 10029 10082 10034 10076 10037 10070 10041 10063 10043 10057 10039
10053 10034 10051 10023 14 P
10112 10067 10117 10062 10122 10067 10129 10069 10135 10067 10142 10069
10148 10071 10153 10077 10159 10079 10165 10083 10162 10077 10165 10070
10170 10066 10176 10069 10180 10075 10177 10081 10175 10088 10168 10090
10161 10089 10154 10088 10147 10087 10140 10087 10133 10086 10132 10079
10128 10074 10121 10072 10112 10067 27 P
10101 10074 10103 10067 10109 10071 10115 10074 10122 10076 10129 10076
10125 10082 10118 10084 10111 10084 10104 10085 10101 10074 11 P
10326 10341 10328 10337 10331 10341 10335 10341 10338 10337 10342 10336
10342 10340 10341 10344 10343 10349 10344 10353 10340 10353 10336 10351
10332 10349 10329 10345 10326 10341 15 P
10350 10350 10352 10346 10356 10346 10361 10347 10363 10342 10365 10339

10366 10343 10365 10348 10363 10352 10359 10353 10356 10356 10350 10350

12:00 PM

10229 10304 10233 10303 10238 10301 10242 10301 10240 10305 10243 10307
10247 10310 10244 10313 10240 10315 10236 10318 10234 10314 10230 10311
10229 10304 13 P
10168 9935 10170 9931 10167 9928 10168 9924 10172 9921 10176 9921
10177 9925 10176 9929 10176 9934 10179 9937 10183 9937 10188 9938
10186 9942 10182 9944 10180 9948 10182 9951 10180 9956 10177 9952
10173 9951 10169 9950 10170 9946 10170 9942 10168 9935 23 P
9956 9873 9959 9870 9962 9866 9966 9866 9971 9867 9975 9868
9979 9869 9984 9871 9987 9873 9988 9877 9988 9881 9985 9885
9981 9886 9978 9890 9977 9894 9973 9896 9972 9900 9968 9899
9966 9895 9962 9893 9958 9890 9958 9886 9958 9881 9956 9873
24 P
9993 9824 9989 9822 9985 9819 9981 9816 9978 9813 9977 9809
9979 9804 9978 9800 9981 9797 9986 9797 9990 9797 9994 9797
9997 9801 9999 9805 10001 9809 10004 9812 10006 9816 10008 9819
10009 9824 10007 9828 10002 9829 9999 9827 9993 9824 23 P
10096 9805 10101 9803 10103 9800 10106 9796 10108 9793 10112 9790
10116 9788 10119 9785 10123 9786 10127 9785 10131 9783 10135 9786
10139 9786 10144 9786 10144 9790 10140 9789 10139 9794 10135 9795
10132 9792 10128 9793 10123 9793 10119 9795 10115 9797 10112 9801
10115 9804 10117 9808 10112 9810 10109 9813 10105 9813 10101 9810
10096 9805 31 P
10127 9857 10131 9854 10135 9853 10140 9853 10144 9854 10148 9857
10152 9858 10152 9863 10155 9865 10153 9869 10149 9868 10145 9867
10141 9868 10136 9868 10132 9867 10131 9863 10127 9857 17 P
9940 9975 9943 9973 9948 9972 9951 9970 9950 9965 9954 9964
9958 9964 9959 9968 9960 9972 9963 9977 9962 9981 9964 9985
9960 9987 9957 9990 9953 9989 9949 9987 9945 9985 9942 9981
9940 9975 19 P
9999 9785 10000 9782 10000 9779 10001 9776 10000 9772 10003 9774
10005 9776 10008 9779 10009 9782 10010 9785 10014 9786 10014 9789
10013 9793 10009 9794 10006 9793 10003 9791 10002 9788 9999 9785
18 P
9987 10029 9990 10030 9993 10029 9995 10026 9997 10024 9994 10022
9993 10019 9996 10019 9999 10021 10002 10023 10004 10025 10006 10027
10008 10030 10010 10032 10008 10035 10008 10038 10006 10041 10003 10040
10000 10038 10000 10035 9998 10038 9995 10038 9992 10037 9993 10034
9990 10033 9987 10029 26 P
9955 10097 9956 10094 9957 10091 9960 10093 9963 10094 9965 10097
9966 10100 9964 10103 9966 10105 9965 10108 9968 10110 9970 10113
9971 10116 9969 10119 9966 10116 9966 10113 9964 10110 9961 10113

9960 10109 9960 10106 9959 10103 9957 10100 9955 10097 23 P
10347 10024 10348 10021 10349 10019 10353 10017 10356 10018 10359 10018
10358 10021 10357 10024 10354 10026 10350 10027 10347 10024 11 P
9948 9437 9953 9437 9957 9435 9959 9431 9962 9427 9966 9427
9969 9424 9970 9419 9970 9415 9967 9411 9965 9407 9969 9409
9973 9409 9978 9409 9982 9407 9981 9403 9980 9398 9984 9397
9988 9395 9991 9398 9995 9399 9993 9403 9994 9407 9994 9412
9999 9413 10002 9416 10000 9420 9995 9420 9998 9423 10002 9426
10005 9429 10003 9433 10001 9438 10001 9442 9999 9446 10001 9449
10001 9454 9997 9456 9993 9459 9989 9461 9987 9465 9984 9468
9980 9469 9976 9467 9976 9462 9972 9460 9968 9457 9964 9455
9960 9452 9956 9451 50 P
9956 9451 9952 9448 9950 9444 9948 9437 4 P
9905 9433 9907 9428 9911 9428 9915 9429 9920 9427 9924 9426
9928 9424 9930 9419 9933 9417 9937 9417 9942 9416 9945 9418
9950 9420 9947 9424 9951 9424 9954 9428 9957 9431 9954 9434
9950 9434 9947 9438 9948 9441 9945 9445 9941 9444 9937 9443
9933 9441 9928 9442 9924 9443 9921 9446 9916 9445 9913 9443
9909 9446 9906 9444 9907 9440 9905 9433 34 P
9908 9406 9913 9405 9917 9405 9916 9401 9921 9400 9923 9397
9927 9395 9931 9397 9930 9401 9933 9405 9929 9409 9933 9412
9930 9415 9927 9418 9922 9419 9918 9419 9914 9416 9915 9412
9908 9406 19 P
10016 9455 10020 9456 10025 9456 10027 9460 10029 9463 10033 9461
10037 9461 10041 9464 10044 9467 10039 9469 10035 9468 10031 9470
10031 9474 10036 9476 10033 9479 10035 9483 10031 9483 10027 9485
10025 9482 10027 9477 10024 9474 10028 9472 10024 9470 10021 9466
10017 9466 10017 9462 10016 9455 27 P
9607 8861 9610 8855 9610 8848 9614 8841 9617 8835 9622 8830
9622 8823 9615 8820 9621 8816 9620 8809 9627 8811 9634 8811
9640 8814 9647 8817 9653 8819 9653 8826 9659 8824 9665 8820
9668 8826 9662 8830 9663 8837 9669 8833 9676 8836 9678 8830
9685 8831 9681 8837 9687 8840 9687 8848 9680 8848 9674 8851
9670 8857 9672 8864 9669 8871 9667 8877 9669 8884 9674 8889
9681 8890 9678 8896 9673 8901 9667 8904 9661 8908 9654 8907
9647 8905 9643 8899 9641 8893 9643 8886 9637 8882 9633 8877
9627 8872 9622 8869 50 P
9622 8869 9617 8863 9607 8861 3 P
9567 8756 9571 8757 9575 8758 9580 8758 9584 8760 9588 8761
9593 8760 9591 8756 9593 8752 9596 8754 9601 8754 9605 8756
9609 8758 9614 8759 9617 8763 9621 8764 9617 8766 9614 8768
9611 8772 9608 8775 9604 8777 9600 8775 9596 8775 9591 8776
9588 8773 9583 8772 9584 8777 9580 8776 9579 8771 9575 8769

9571 8767 9567 8765 9568 8760 9567 8756 34 P
9558 8696 9561 8693 9564 8696 9567 8698 9572 8700 9575 8702
9579 8700 9583 8697 9583 8702 9587 8704 9589 8708 9593 8711
9597 8711 9594 8714 9589 8711 9585 8710 9581 8708 9577 8709
9574 8713 9571 8716 9566 8716 9566 8711 9562 8709 9562 8705
9561 8701 9558 8696 26 P
9593 8684 9597 8683 9602 8682 9606 8683 9610 8682 9614 8685
9619 8684 9622 8688 9625 8686 9630 8684 9633 8682 9638 8683
9639 8687 9639 8692 9638 8696 9634 8699 9639 8701 9640 8696
9643 8700 9646 8703 9644 8707 9645 8711 9640 8712 9638 8708
9634 8706 9630 8707 9626 8705 9629 8702 9627 8697 9623 8697
9618 8696 9616 8700 9612 8703 9610 8699 9607 8696 9604 8693
9600 8691 9596 8689 9593 8684 39 P
9640 8786 9637 8784 9633 8781 9631 8778 9634 8775 9636 8771
9640 8770 9643 8773 9646 8775 9651 8778 9655 8779 9659 8781
9662 8783 9658 8784 9654 8782 9649 8785 9645 8785 9640 8786
18 P
9580 8482 9583 8479 9587 8476 9590 8473 9590 8477 9589 8481
9593 8480 9597 8478 9602 8476 9606 8476 9610 8473 9609 8477
9607 8481 9602 8482 9599 8486 9596 8489 9592 8490 9588 8494
9584 8494 9588 8491 9591 8489 9587 8489 9585 8486 9580 8482
24 P
9904 5748 9908 5744 9910 5739 9913 5734 9918 5732 9921 5727
9927 5727 9926 5733 9928 5739 9929 5744 9929 5750 9923 5752
9918 5753 9913 5750 9904 5748 15 P
9315 5524 9319 5522 9323 5524 9326 5528 9329 5531 9333 5534
9335 5538 9332 5542 9327 5543 9323 5541 9319 5538 9320 5533
9318 5529 9315 5524 14 P
8866 6218 8872 6214 8878 6210 8882 6204 8889 6202 8896 6203
8902 6206 8902 6214 8907 6219 8912 6224 8918 6227 8923 6232
8931 6232 8938 6232 8945 6229 8942 6236 8940 6243 8938 6250
8935 6256 8930 6251 8928 6258 8921 6255 8920 6248 8918 6255
8914 6249 8910 6243 8910 6250 8911 6257 8904 6257 8901 6251
8896 6245 8896 6237 8892 6244 8888 6250 8882 6246 8880 6239
8875 6233 8882 6236 8885 6229 8878 6230 8884 6226 8891 6226
8898 6226 8895 6219 8889 6216 8886 6209 8881 6215 8875 6219
8872 6225 8866 6218 50 P
8870 6289 8875 6284 8880 6290 8886 6285 8891 6279 8893 6273
8896 6266 8899 6273 8898 6280 8902 6285 8899 6291 8896 6298
8889 6299 8882 6296 8875 6295 8870 6289 16 P
8828 6231 8835 6232 8842 6234 8849 6233 8851 6240 8852 6247
8856 6253 8855 6260 8854 6267 8860 6271 8866 6276 8859 6277
8853 6281 8848 6276 8845 6269 8846 6262 8845 6255 8841 6250

8846 6245 8844 6239 8837 6238 8828 6231 22 P
8840 6112 8844 6111 8849 6112 8853 6111 8851 6116 8855 6119
8852 6123 8851 6127 8851 6132 8846 6132 8844 6136 8843 6131
8842 6127 8844 6123 8843 6118 8840 6112 16 P
8801 6029 8805 6026 8807 6022 8807 6017 8811 6015 8812 6019
8816 6022 8819 6026 8820 6030 8817 6034 8813 6034 8809 6032
8801 6029 13 P
8894 6334 8896 6331 8896 6328 8898 6331 8900 6334 8902 6336
8906 6337 8907 6340 8904 6343 8901 6345 8899 6343 8898 6339
8895 6337 8894 6334 14 P
9120 6189 9121 6184 9121 6180 9123 6175 9124 6170 9125 6166
9126 6171 9124 6175 9124 6180 9124 6184 9125 6189 9127 6193
9127 6198 9128 6202 9130 6206 9132 6210 9135 6214 9138 6218
9137 6223 9134 6219 9132 6215 9130 6211 9127 6206 9124 6203
9123 6198 9121 6194 9120 6189 27 P
8917 7332 8924 7335 8930 7338 8937 7338 8943 7342 8942 7335
8942 7328 8935 7329 8931 7323 8937 7318 8933 7312 8938 7306
8943 7302 8950 7299 8955 7294 8960 7290 8967 7288 8971 7294
8967 7301 8961 7304 8964 7311 8969 7315 8976 7318 8981 7323
8988 7324 8993 7329 9000 7330 9005 7325 9011 7329 9012 7336
9005 7336 8998 7338 8992 7341 8987 7346 8985 7353 8985 7360
8987 7367 8993 7372 8998 7375 9005 7377 9010 7372 9016 7373
9021 7379 9025 7385 9017 7385 9010 7384 9003 7387 8998 7392
8992 7389 8995 7396 50 P
8995 7396 9000 7401 8996 7408 8990 7411 8985 7416 8978 7413
8972 7416 8967 7422 8967 7429 8972 7434 8975 7440 8968 7440
8963 7437 8957 7432 8954 7426 8948 7429 8943 7424 8940 7417
8933 7417 8930 7411 8925 7405 8926 7398 8930 7392 8937 7394
8941 7400 8948 7400 8951 7393 8949 7386 8946 7380 8946 7373
8945 7366 8944 7359 8950 7355 8955 7360 8958 7366 8964 7361
8971 7361 8971 7353 8965 7350 8959 7346 8952 7346 8947 7351
8940 7352 8933 7353 8929 7348 8922 7346 8921 7339 8917 7332
48 P
8799 7191 8805 7186 8802 7179 8807 7174 8814 7174 8821 7170
8826 7175 8833 7177 8839 7173 8836 7166 8836 7159 8842 7155
8849 7153 8856 7153 8858 7160 8853 7165 8857 7171 8864 7172
8869 7177 8862 7176 8857 7181 8852 7175 8845 7176 8839 7180
8833 7183 8828 7188 8828 7196 8821 7197 8819 7204 8814 7198
8809 7193 8799 7191 32 P
8200 6714 8205 6710 8209 6704 8216 6701 8222 6698 8229 6702
8231 6709 8235 6715 8241 6719 8247 6723 8254 6725 8261 6726
8268 6724 8274 6721 8280 6717 8286 6713 8293 6716 8297 6722
8297 6729 8302 6735 8298 6741 8298 6748 8294 6754 8292 6761

8289 6768 8283 6770 8279 6776 8273 6780 8269 6775 8263 6771
8265 6764 8260 6759 8255 6755 8253 6748 8249 6742 8242 6744
8235 6746 8229 6750 8224 6754 8219 6759 8212 6756 8207 6751
8208 6744 8215 6748 8212 6741 8214 6734 8216 6727 8218 6720
8211 6719 8200 6714 50 P
8089 6741 8092 6735 8097 6729 8103 6726 8109 6722 8116 6721
8123 6719 8129 6723 8134 6728 8140 6733 8144 6738 8147 6745
8149 6751 8156 6753 8156 6760 8156 6767 8156 6775 8151 6780
8145 6777 8141 6782 8136 6788 8129 6785 8124 6790 8117 6792
8110 6794 8111 6787 8105 6783 8104 6776 8100 6769 8095 6764
8091 6758 8093 6751 8100 6752 8105 6757 8112 6761 8113 6753
8107 6750 8100 6747 8089 6741 39 P
7690 6231 7698 6231 7705 6233 7712 6235 7718 6233 7716 6226
7722 6230 7729 6233 7736 6235 7743 6236 7750 6238 7757 6238
7754 6244 7746 6245 7745 6252 7745 6259 7745 6266 7748 6273
7752 6279 7759 6282 7766 6279 7772 6276 7779 6276 7780 6283
7775 6288 7775 6295 7777 6302 7784 6306 7781 6313 7787 6317
7793 6313 7801 6314 7802 6321 7808 6323 7801 6323 7797 6329
7790 6332 7786 6337 7780 6332 7773 6334 7770 6328 7762 6329
7756 6325 7753 6332 7749 6338 7743 6343 7737 6346 7732 6351
7726 6348 7724 6341 50 P
7724 6341 7727 6334 7727 6327 7722 6322 7717 6316 7714 6309
7710 6303 7705 6298 7707 6291 7714 6287 7719 6293 7726 6295
7731 6290 7738 6291 7745 6289 7740 6285 7738 6277 7734 6273
7728 6268 7722 6264 7717 6258 7713 6253 7714 6245 7710 6251
7703 6247 7698 6243 7690 6231 27 P
7526 6160 7522 6166 7515 6169 7509 6167 7503 6163 7498 6158
7493 6153 7497 6146 7504 6145 7511 6144 7518 6144 7525 6144
7532 6142 7538 6138 7544 6134 7552 6134 7558 6137 7566 6138
7572 6136 7574 6129 7581 6132 7586 6127 7592 6131 7596 6137
7595 6144 7593 6151 7599 6154 7598 6161 7591 6165 7589 6172
7590 6179 7583 6179 7578 6173 7576 6166 7569 6168 7564 6173
7558 6177 7551 6175 7547 6169 7544 6162 7539 6158 7534 6152
7526 6160 43 P
7527 6227 7520 6226 7513 6227 7510 6220 7505 6215 7498 6213
7491 6212 7492 6205 7499 6201 7506 6200 7513 6198 7520 6196
7522 6203 7523 6210 7526 6217 7533 6219 7527 6227 17 P
7483 6223 7488 6218 7495 6217 7503 6216 7508 6220 7505 6227
7506 6234 7511 6240 7513 6246 7506 6248 7500 6244 7500 6237
7497 6230 7490 6227 7483 6223 15 P
7334 6091 7339 6085 7343 6079 7349 6075 7355 6072 7360 6066
7366 6064 7373 6066 7378 6060 7381 6054 7388 6051 7395 6049
7388 6045 7392 6039 7398 6035 7405 6034 7407 6027 7415 6027

7417 6034 7421 6039 7415 6042 7409 6039 7404 6045 7402 6052
7403 6059 7404 6066 7402 6073 7398 6079 7397 6086 7392 6091
7385 6094 7379 6089 7372 6089 7365 6091 7360 6096 7356 6102
7348 6102 7342 6099 7334 6091 39 P
8712 7324 8716 7318 8720 7312 8721 7305 8724 7298 8730 7302
8736 7305 8743 7305 8743 7313 8741 7319 8741 7326 8740 7333
8742 7340 8746 7347 8748 7353 8747 7360 8750 7367 8750 7374
8749 7381 8747 7388 8740 7384 8736 7379 8733 7372 8727 7368
8726 7361 8724 7354 8721 7348 8719 7341 8717 7334 8712 7324
30 P
8842 7677 8849 7676 8856 7675 8862 7673 8870 7673 8875 7668
8881 7665 8888 7665 8895 7667 8895 7675 8893 7681 8890 7688
8888 7695 8881 7698 8875 7701 8869 7705 8863 7701 8857 7699
8852 7693 8848 7688 8842 7677 21 P
8846 7671 8850 7669 8853 7666 8857 7663 8862 7664 8866 7665
8870 7665 8874 7667 8871 7670 8866 7672 8862 7672 8858 7673
8853 7674 8846 7671 14 P
8893 8005 8895 8001 8898 7998 8898 7993 8902 7993 8904 7997
8908 7995 8912 7993 8917 7994 8921 7996 8925 7996 8927 8000
8930 8004 8934 8006 8938 8007 8943 8008 8945 8012 8945 8016
8941 8018 8936 8018 8933 8014 8932 8010 8930 8006 8925 8008
8927 8012 8923 8012 8918 8011 8914 8012 8909 8013 8905 8012
8901 8012 8896 8011 8893 8005 33 P
9225 8179 9227 8175 9230 8171 9234 8169 9238 8167 9241 8164
9246 8164 9250 8167 9254 8169 9258 8170 9263 8170 9259 8172
9255 8174 9253 8178 9252 8182 9250 8186 9245 8187 9242 8190
9238 8191 9233 8190 9230 8187 9226 8184 9225 8179 23 P
10665 6825 10661 6826 10657 6824 10652 6823 10648 6825 10644 6823
10642 6819 10638 6818 10636 6813 10632 6812 10635 6808 10640 6808
10644 6807 10649 6806 10653 6806 10658 6806 10661 6808 10666 6807
10671 6806 10675 6808 10678 6811 10682 6814 10685 6818 10688 6820
10685 6822 10680 6825 10676 6826 10672 6827 10665 6825 29 P
11075 6613 11078 6609 11083 6609 11087 6608 11092 6609 11096 6608
11100 6610 11103 6614 11105 6618 11102 6622 11098 6623 11093 6623
11089 6623 11085 6622 11081 6619 11076 6618 11075 6613 17 P
11528 6993 11529 6989 11533 6986 11537 6985 11542 6984 11547 6985
11551 6987 11554 6990 11557 6993 11557 6998 11556 7003 11552 7005
11548 7006 11543 7007 11539 7007 11534 7005 11531 7003 11528 6999
11528 6993 19 P
11760 7112 11763 7108 11766 7104 11770 7103 11774 7101 11779 7102
11782 7105 11787 7106 11790 7105 11795 7104 11800 7105 11804 7107
11806 7111 11805 7116 11803 7120 11801 7124 11799 7128 11795 7130
11790 7131 11786 7133 11782 7135 11778 7137 11773 7138 11770 7134

11770 7130 11770 7125 11770 7121 11766 7119 11761 7120 11760 7112
30 P
11527 7052 11530 7050 11533 7049 11536 7048 11540 7047 11541 7050
11539 7053 11536 7055 11533 7056 11530 7055 11527 7052 11 P
11195 6736 11193 6735 11192 6731 11192 6728 11194 6725 11196 6722
11197 6719 11200 6717 11204 6717 11207 6719 11209 6722 11212 6724
11213 6727 11212 6730 11210 6734 11207 6734 11203 6734 11200 6735
11195 6736 19 P
11192 6783 11189 6785 11187 6787 11187 6791 11184 6793 11180 6793
11177 6793 11174 6792 11173 6788 11175 6786 11174 6783 11171 6781
11167 6781 11171 6780 11174 6780 11178 6780 11181 6779 11185 6780
11187 6778 11190 6776 11193 6778 11195 6780 11192 6783 23 P
11233 6778 11234 6775 11237 6773 11240 6772 11243 6775 11245 6777
11246 6781 11245 6784 11242 6787 11239 6788 11236 6789 11235 6786
11234 6783 11233 6778 14 P
11156 6712 11158 6709 11161 6708 11164 6709 11164 6713 11161 6715
11158 6715 11156 6712 8 P
11164 6717 11167 6719 11169 6721 11172 6723 11173 6727 11170 6728
11167 6726 11165 6723 11164 6717 9 P
10965 6684 10968 6683 10970 6684 10972 6685 10974 6686 10972 6687
10970 6687 10968 6686 10965 6684 9 P
10880 7583 10881 7581 10883 7581 10885 7582 10888 7583 10889 7585
10891 7584 10893 7584 10896 7585 10898 7585 10898 7587 10896 7589
10895 7590 10893 7590 10891 7591 10889 7591 10886 7591 10884 7589
10883 7588 10881 7586 10880 7583 21 P
10921 7580 10922 7578 10924 7576 10926 7578 10925 7579 10924 7581
10921 7580 7 P
10956 7680 10958 7679 10960 7678 10962 7679 10963 7681 10962 7683
10961 7685 10959 7685 10957 7684 10956 7680 10 P
10145 7464 10147 7462 10148 7461 10150 7463 10152 7465 10152 7467
10150 7467 10147 7467 10145 7466 10145 7464 10 P
10164 7383 10165 7381 10167 7382 10167 7384 10164 7383 5 P
10095 7013 10097 7011 10099 7010 10101 7008 10103 7007 10105 7008
10107 7008 10109 7007 10111 7008 10112 7011 10110 7012 10109 7013
10108 7015 10106 7017 10103 7017 10101 7016 10101 7014 10099 7012
10095 7013 19 P
8935 6058 8937 6056 8936 6054 8937 6052 8939 6051 8939 6053
8940 6055 8942 6056 8943 6058 8942 6061 8940 6061 8938 6060
8935 6058 13 P
8749 6136 8750 6135 8752 6135 8755 6136 8756 6138 8757 6140
8755 6140 8753 6141 8751 6141 8750 6139 8749 6136 11 P
8906 6052 8907 6051 8909 6050 8911 6051 8913 6053 8911 6055
8909 6055 8907 6054 8906 6052 9 P

8857 6117 8859 6115 8861 6115 8862 6117 8864 6118 8866 6118
8868 6118 8869 6120 8868 6123 8865 6123 8863 6123 8861 6122
8860 6120 8858 6119 8857 6117 15 P
8849 6188 8851 6186 8853 6186 8855 6187 8857 6188 8859 6188
8862 6189 8863 6190 8865 6192 8867 6192 8867 6194 8865 6195
8863 6194 8861 6194 8859 6193 8857 6192 8856 6190 8853 6189
8852 6190 8850 6191 8849 6188 21 P
8850 6062 8852 6061 8854 6061 8855 6063 8853 6064 8850 6062

6:00 PM

8855 6054 8857 6054 8858 6056 8860 6058 8859 6060 8857 6059
8856 6058 8855 6054 8 P
8164 6713 8167 6712 8171 6711 8173 6710 8176 6713 8179 6715
8182 6713 8184 6715 8185 6718 8187 6720 8191 6720 8192 6722
8193 6726 8194 6729 8193 6732 8190 6734 8187 6736 8184 6737
8181 6735 8177 6735 8175 6734 8174 6730 8173 6727 8172 6724
8170 6722 8168 6719 8169 6715 8164 6713 28 P
7950 6744 7954 6742 7958 6740 7961 6736 7964 6733 7967 6729
7970 6727 7975 6725 7980 6726 7984 6726 7989 6726 7993 6727
7990 6731 7990 6736 7985 6737 7982 6735 7977 6734 7973 6736
7970 6740 7968 6743 7963 6743 7959 6741 7955 6744 7950 6744
24 P
7953 6747 7956 6744 7961 6743 7965 6745 7969 6744 7973 6740
7975 6736 7980 6736 7984 6738 7988 6740 7991 6737 7994 6733
7998 6731 8002 6734 8007 6734 8005 6738 8003 6742 7999 6745
7996 6749 7992 6751 7988 6753 7984 6753 7980 6755 7975 6755
7970 6754 7966 6752 7962 6751 7957 6750 7953 6747 29 P
8023 6711 8026 6709 8031 6710 8034 6708 8039 6710 8042 6712
8039 6715 8034 6716 8030 6715 8023 6711 10 P
7979 6722 7983 6721 7987 6718 7991 6716 7996 6716 7997 6720
7992 6722 7988 6724 7984 6724 7979 6722 10 P
7942 6755 7943 6752 7945 6749 7947 6747 7951 6746 7952 6749
7950 6751 7947 6754 7946 6757 7944 6760 7942 6755 11 P
8623 7082 8627 7081 8631 7080 8633 7076 8634 7072 8639 7070
8638 7075 8640 7079 8643 7082 8646 7085 8645 7090 8647 7093
8644 7097 8644 7101 8647 7104 8652 7106 8655 7109 8654 7113
8650 7111 8646 7109 8642 7109 8638 7106 8635 7103 8631 7102
8631 7097 8633 7093 8636 7090 8635 7085 8632 7083 8627 7084
8623 7082 31 P
9031 7715 9035 7714 9039 7712 9043 7715 9047 7715 9051 7717
9055 7720 9057 7723 9059 7728 9054 7729 9050 7729 9047 7724
9044 7722 9041 7725 9037 7725 9039 7729 9036 7732 9034 7728
9032 7724 9032 7720 9031 7715 21 P
9075 7768 9070 7767 9067 7764 9063 7762 9059 7759 9060 7755

9059 7750 9058 7746 9061 7743 9062 7748 9062 7752 9064 7756
9068 7758 9073 7758 9077 7757 9081 7753 9083 7757 9082 7762
9079 7764 9075 7768 20 P
9042 7620 9044 7617 9046 7615 9049 7616 9052 7618 9054 7620
9053 7623 9049 7623 9046 7622 9042 7620 10 P
9061 7604 9066 7607 9070 7608 9073 7611 9076 7615 9071 7614
9067 7615 9065 7611 9061 7604 9 P
9315 7851 9320 7850 9323 7852 9326 7856 9330 7855 9329 7859
9326 7862 9321 7861 9318 7858 9315 7851 10 P
9309 7859 9312 7861 9313 7857 9315 7859 9318 7862 9316 7865
9313 7866 9311 7863 9309 7859 9 P
9128 7958 9132 7956 9135 7952 9139 7954 9144 7956 9143 7961
9147 7962 9152 7963 9152 7968 9154 7971 9154 7976 9151 7980
9147 7981 9145 7977 9142 7973 9139 7970 9138 7965 9133 7963
9128 7958 19 P
9084 7916 9088 7915 9093 7916 9095 7920 9099 7919 9103 7920
9104 7925 9104 7929 9104 7934 9099 7934 9098 7930 9095 7933
9090 7931 9087 7928 9087 7923 9084 7916 16 P
8947 7984 8949 7981 8951 7978 8954 7978 8957 7976 8960 7977
8963 7979 8965 7982 8968 7982 8972 7980 8974 7983 8976 7986
8973 7988 8970 7990 8967 7991 8964 7990 8961 7989 8958 7988
8954 7988 8951 7986 8947 7984 21 P
9470 8765 9473 8764 9476 8761 9479 8760 9482 8758 9485 8756
9487 8754 9490 8752 9493 8751 9496 8750 9500 8751 9503 8752
9506 8754 9508 8757 9510 8759 9510 8763 9508 8766 9505 8767
9502 8768 9499 8768 9495 8770 9492 8769 9489 8768 9485 8768
9482 8768 9479 8768 9475 8766 9470 8765 28 P
9422 8778 9425 8777 9427 8774 9429 8772 9432 8770 9435 8769
9438 8768 9441 8771 9441 8774 9442 8777 9439 8779 9437 8782
9436 8785 9433 8787 9430 8788 9427 8789 9423 8791 9420 8791
9416 8792 9416 8788 9417 8785 9419 8782 9422 8778 23 P
9586 8661 9589 8660 9591 8659 9594 8660 9596 8661 9598 8664
9597 8666 9595 8668 9593 8670 9590 8668 9589 8666 9588 8664
9586 8661 13 P
9655 8569 9656 8567 9658 8569 9660 8571 9660 8573 9661 8575
9659 8575 9657 8573 9656 8571 9655 8569 10 P
9648 8574 9650 8574 9651 8572 9653 8572 9655 8573 9657 8574
9659 8576 9660 8578 9661 8579 9663 8581 9661 8582 9659 8582
9657 8583 9656 8581 9655 8579 9654 8578 9651 8577 9648 8574
18 P
9829 9085 9830 9082 9834 9081 9836 9079 9839 9077 9842 9080
9840 9083 9842 9085 9846 9086 9846 9090 9842 9091 9843 9094
9841 9097 9840 9100 9836 9101 9833 9100 9832 9097 9832 9093

9830 9090 9829 9085 20 P
9837 9071 9839 9069 9841 9066 9844 9065 9848 9066 9850 9069
9848 9072 9845 9073 9842 9074 9837 9071 10 P
9964 9081 9967 9082 9971 9082 9969 9079 9971 9077 9970 9075
9967 9072 9969 9070 9972 9071 9974 9068 9977 9070 9979 9072
9982 9071 9981 9074 9983 9077 9985 9080 9987 9083 9984 9083
9980 9083 9977 9085 9977 9088 9977 9092 9974 9091 9971 9090
9970 9087 9967 9085 9964 9081 27 P
9964 9043 9968 9044 9971 9045 9972 9048 9975 9045 9978 9048
9980 9050 9983 9051 9986 9054 9988 9056 9986 9058 9982 9058
9979 9058 9976 9056 9973 9055 9971 9052 9971 9049 9968 9047
9964 9043 19 P
9939 9236 9942 9234 9944 9236 9947 9239 9949 9241 9951 9243
9951 9246 9951 9250 9954 9252 9957 9251 9956 9254 9955 9257
9953 9260 9949 9260 9947 9258 9946 9255 9947 9251 9946 9248
9943 9248 9944 9244 9944 9241 9942 9239 9939 9236 23 P
9946 9213 9949 9213 9952 9210 9955 9210 9958 9212 9958 9215
9955 9217 9958 9220 9960 9222 9962 9225 9965 9226 9969 9226
9969 9229 9972 9229 9976 9230 9973 9232 9969 9232 9966 9230
9964 9227 9962 9225 9958 9226 9956 9224 9952 9223 9949 9224
9947 9221 9948 9218 9946 9213 27 P
9905 9190 9908 9189 9912 9188 9914 9186 9916 9183 9920 9183
9922 9185 9923 9189 9923 9192 9926 9194 9928 9197 9924 9197
9922 9194 9920 9192 9917 9190 9914 9190 9912 9192 9913 9195
9909 9196 9906 9197 9908 9194 9905 9190 22 P
10088 9607 10090 9604 10093 9606 10096 9605 10098 9608 10101 9610
10101 9613 10104 9615 10107 9616 10110 9617 10109 9620 10106 9619
10103 9621 10101 9618 10097 9617 10098 9614 10096 9611 10093 9609
10090 9611 10088 9607 20 P
10094 9768 10097 9767 10100 9765 10103 9764 10106 9762 10108 9760
10111 9758 10114 9756 10115 9759 10118 9760 10121 9759 10125 9759
10127 9757 10129 9754 10130 9758 10131 9761 10128 9762 10125 9761
10122 9761 10118 9763 10115 9764 10112 9765 10110 9767 10107 9767
10106 9771 10103 9773 10100 9773 10098 9775 10095 9775 10095 9771
10094 9768 31 P
10051 9905 10053 9902 10056 9902 10059 9902 10063 9901 10066 9903
10068 9905 10066 9908 10065 9911 10061 9912 10058 9911 10056 9909
10051 9905 13 P
10053 9914 10057 9913 10060 9915 10063 9914 10066 9915 10069 9913
10072 9910 10074 9908 10077 9909 10078 9913 10076 9916 10073 9917
10071 9920 10069 9923 10066 9924 10063 9923 10061 9920 10058 9918
10053 9914 19 P
10039 9910 10040 9907 10043 9908 10046 9908 10047 9912 10048 9915

10046 9918 10043 9919 10040 9918 10039 9915 10039 9910 11 P
10021 9927 10019 9924 10021 9921 10020 9918 10021 9915 10024 9915
10027 9917 10030 9919 10029 9922 10027 9925 10024 9927 10021 9927
12:00 PM
10001 9900 10002 9897 10002 9893 10006 9892 10009 9892 10011 9894
10013 9897 10015 9900 10015 9903 10014 9906 10011 9908 10008 9908
10005 9906 10002 9904 10001 9900 15 P
10280 10416 10283 10415 10286 10413 10289 10413 10292 10413 10295 10415
10298 10415 10298 10419 10295 10420 10292 10418 10289 10420 10287 10422
10284 10424 10283 10420 10285 10418 10280 10416 16 P
10252 10399 10256 10399 10259 10399 10262 10397 10265 10397 10266 10400
10263 10401 10260 10401 10256 10403 10252 10399 10 P
10268 10398 10271 10396 10274 10398 10277 10399 10275 10402 10272 10404
10270 10401 10268 10398 8 P
10334 10385 10336 10383 10339 10385 10342 10386 10339 10389 10334 10385
6:00 PM
9995 10256 9998 10254 10001 10254 10002 10251 10004 10248 10007 10249
10009 10251 10009 10255 10012 10257 10015 10259 10012 10260 10008 10261
10005 10261 10003 10257 10000 10258 9995 10256 16 P
9991 10283 9993 10280 9995 10278 9999 10279 10000 10282 10001 10285
10004 10287 10001 10286 9998 10288 9997 10285 9994 10286 9991 10283
12:00 PM
10100 10289 10103 10288 10106 10290 10108 10292 10107 10295 10104 10296
10106 10293 10102 10292 10100 10289 9 P
10094 10220 10097 10219 10101 10220 10102 10223 10103 10226 10100 10228
10097 10226 10095 10224 10094 10220 9 P
10124 10222 10127 10222 10130 10220 10132 10219 10136 10218 10137 10221
10136 10224 10137 10227 10136 10230 10133 10228 10133 10225 10130 10224
10127 10225 10124 10222 14 P
10191 10175 10194 10175 10197 10177 10200 10176 10201 10172 10203 10170
10206 10171 10208 10174 10205 10176 10203 10178 10202 10181 10200 10184
10197 10183 10195 10180 10191 10175 15 P
10207 10065 10210 10064 10213 10063 10216 10062 10219 10061 10221 10064
10219 10067 10217 10069 10213 10069 10210 10069 10207 10065 11 P
10285 10058 10288 10056 10289 10053 10290 10050 10294 10049 10295 10052
10294 10055 10293 10058 10292 10062 10289 10062 10285 10058 11 P
10303 10058 10305 10056 10307 10053 10310 10052 10311 10049 10312 10046
10313 10049 10313 10052 10313 10056 10310 10058 10308 10060 10303 10058
12:00 PM
10317 10058 10318 10055 10320 10052 10323 10050 10323 10053 10321 10057
10323 10060 10321 10063 10317 10058 9 P
10325 10043 10326 10041 10329 10041 10331 10043 10329 10045 10328 10048
10325 10043 7 P

10204 9905 10205 9902 10207 9899 10210 9900 10210 9903 10209 9906
10208 9909 10205 9909 10204 9905 9 P
10065 10057 10066 10055 10068 10053 10071 10051 10073 10051 10076 10052
10077 10055 10076 10057 10074 10059 10072 10060 10069 10060 10065 10057

12:00 PM

9793 9441 9796 9439 9799 9438 9801 9435 9804 9435 9807 9436
9811 9437 9814 9438 9815 9440 9814 9444 9813 9446 9810 9447
9807 9445 9807 9442 9804 9440 9801 9441 9798 9443 9795 9443
9793 9441 19 P
9979 9205 9981 9203 9982 9200 9985 9201 9987 9204 9991 9205
9992 9207 9990 9210 9987 9208 9983 9208 9979 9205 11 P
9790 8836 9794 8837 9797 8838 9797 8842 9799 8845 9803 8845
9805 8846 9806 8850 9805 8853 9801 8852 9800 8850 9797 8847
9795 8845 9792 8843 9790 8840 9790 8836 16 P
8910 7428 8914 7425 8918 7425 8923 7425 8927 7426 8932 7427
8936 7425 8939 7428 8940 7432 8938 7437 8937 7440 8933 7438
8929 7436 8925 7434 8921 7432 8917 7434 8913 7434 8910 7428
18 P
8857 7425 8859 7422 8864 7421 8869 7421 8871 7425 8875 7426
8878 7430 8880 7433 8882 7437 8882 7442 8878 7441 8875 7438
8871 7436 8868 7432 8866 7428 8864 7424 8862 7429 8857 7425
18 P
8902 7436 8904 7436 8907 7436 8910 7437 8913 7438 8913 7440
8910 7442 8909 7445 8906 7444 8903 7443 8903 7440 8902 7436

12:00 PM

8926 7245 8928 7242 8927 7240 8927 7236 8929 7239 8932 7240
8935 7242 8938 7244 8935 7245 8932 7247 8931 7250 8933 7253
8932 7256 8929 7254 8928 7252 8926 7249 8926 7245 17 P
8930 7270 8931 7267 8933 7264 8937 7266 8940 7265 8943 7267
8946 7268 8949 7270 8947 7272 8944 7274 8945 7277 8942 7276
8939 7275 8936 7275 8936 7272 8930 7270 16 P
8209 6799 8212 6797 8215 6796 8218 6794 8221 6792 8223 6790
8227 6789 8230 6790 8230 6793 8231 6797 8234 6799 8232 6802
8229 6803 8226 6804 8222 6804 8219 6804 8215 6802 8212 6801
8209 6799 19 P
5904 5508 5904 5515 5908 5521 5901 5524 5908 5527 5915 5525
5918 5532 5916 5539 5916 5547 5921 5552 5924 5558 5930 5554
5936 5559 5937 5566 5933 5572 5927 5567 5921 5563 5914 5563
5908 5558 5903 5553 5897 5550 5893 5543 5885 5544 5878 5542
5872 5539 5866 5535 5862 5541 5855 5542 5847 5541 5843 5535
5838 5530 5832 5527 5825 5525 5827 5518 5822 5513 5817 5508
5816 5501 5816 5501 38 P
5839 5501 5846 5502 5847 5501 3 P

5861 5501 5864 5502 5871 5505 5878 5506 5885 5509 5890 5503
5891 5501 7 P
5911 5501 5912 5501 2 P
5912 5501 5904 5508 2 P
5967 5528 5972 5522 5974 5515 5981 5512 5988 5510 5992 5505
5999 5506 6006 5503 6006 5501 9 P
6023 5501 6024 5506 6029 5502 6030 5501 4 P
6039 5501 6038 5506 6042 5512 6038 5519 6034 5524 6031 5531
6024 5532 6017 5536 6010 5536 6005 5532 5998 5531 5991 5533
5984 5536 5977 5535 5967 5528 15 P
5969 5501 5970 5507 5964 5506 5962 5501 5962 5501 5 P
6054 5507 6056 5505 6058 5504 6058 5502 6060 5503 6062 5503
6063 5506 6064 5508 6064 5510 6064 5512 6063 5514 6061 5514
6059 5512 6057 5512 6055 5510 6054 5507 16 P
5540 5517 5543 5513 5545 5509 5547 5505 5549 5501 5 P
5575 5501 5574 5505 5573 5509 5571 5514 5569 5518 5567 5522
5563 5525 5559 5528 5556 5530 5552 5534 5549 5530 5545 5528
5542 5524 5540 5517 14 P
5698 5615 5700 5611 5704 5608 5708 5611 5712 5612 5717 5614
5717 5618 5720 5620 5724 5623 5724 5628 5726 5632 5721 5633
5717 5635 5712 5635 5710 5631 5706 5629 5704 5625 5702 5621
5698 5615 19 P
5790 5619 5794 5616 5798 5614 5800 5611 5805 5609 5810 5609
5808 5613 5806 5618 5803 5621 5798 5623 5796 5620 5790 5619
12:00 PM
6251 5606 6253 5608 6255 5607 6256 5608 6258 5610 6258 5612
6260 5614 6259 5616 6256 5615 6255 5614 6253 5612 6252 5610
6251 5606 13 P
6240 5628 6241 5625 6242 5621 6244 5619 6247 5620 6251 5622
6254 5622 6257 5623 6259 5626 6256 5628 6253 5629 6249 5629
6246 5630 6243 5630 6240 5628 15 P
6252 5694 6253 5692 6256 5691 6259 5689 6260 5685 6263 5687
6262 5691 6260 5693 6256 5695 6256 5698 6252 5694 11 P
12523 8206 12524 8206 12525 8206 12525 8206 12525 8207 12526 8207
12525 8208 12524 8208 12523 8207 12523 8206 10 P
12910 11328 12912 11328 12914 11328 12915 11329 12917 11329 12918 11330
12919 11331 12921 11330 12921 11328 12923 11330 12923 11331 12924 11333
12925 11334 12925 11335 12925 11337 12924 11337 12923 11336 12922 11336
12920 11336 12918 11336 12917 11337 12915 11337 12915 11335 12915 11334
12915 11332 12914 11331 12912 11330 12910 11329 12910 11328 29 P
12896 11304 12896 11303 12897 11303 12897 11304 12896 11305 12895 11305
12895 11304 12895 11304 12896 11304 9 P
12898 11304 12898 11302 12897 11300 12897 11299 12897 11297 12896 11295

12898 11295 12899 11297 12899 11298 12901 11299 12902 11299 12902 11300
12901 11301 12900 11303 12900 11305 12901 11306 12902 11307 12902 11309
12901 11309 12899 11308 12898 11308 12896 11308 12896 11306 12897 11305
12898 11304 25 P
12902 11312 12903 11311 12905 11312 12906 11313 12906 11314 12904 11314
12903 11314 12903 11313 12902 11313 12902 11312 10 P
12904 11318 12905 11318 12905 11319 12904 11319 12904 11318 5 P
12906 11319 12906 11319 12907 11319 12907 11319 12908 11319 12908 11321
12908 11321 12908 11322 12907 11322 12906 11321 12906 11321 12906 11319
12:00 PM
12909 11325 12909 11325 12910 11323 12910 11323 12911 11323 12911 11323
12911 11325 12910 11326 12909 11326 12909 11325 10 P
2298 14452 2330 14446 2321 14393 2298 14396 4 P
2298 14341 2325 14336 2374 14312 2381 14258 2413 14214 2459 14185
2491 14141 2523 14097 2509 14044 2490 13993 2507 13941 2526 13890
2543 13838 2556 13785 2563 13730 2530 13687 2486 13655 2468 13604
2480 13550 2508 13503 2548 13466 2602 13458 2630 13410 2653 13361
2671 13309 2722 13290 2774 13272 2823 13249 2837 13195 2869 13152
2913 13184 2941 13231 2927 13285 2935 13338 2948 13392 3001 13405
3053 13387 3108 13390 3146 13404 3135 13472 3179 13505 3187 13559
3188 13600 3151 13640 3104 13668 3057 13696 46 P
3057 13696 3017 13701 3014 13756 3027 13809 2994 13853 2971 13878
3021 13902 3048 13948 3103 13946 3111 14000 3115 14034 3085 14061
3122 14079 3130 14140 3185 14137 3239 14135 3294 14133 3348 14125
3394 14115 19 P
3394 14115 3397 14060 3417 14009 3466 13986 3513 13959 3561 13932
3610 13909 3651 13874 3685 13831 3718 13788 3770 13770 3820 13748
3861 13712 3906 13681 3958 13664 3973 13612 3946 13564 3950 13509
3939 13456 3930 13416 3863 13382 3862 13327 3850 13274 3860 13220
3880 13169 3873 13115 3867 13060 3812 13061 3757 13062 3702 13064
3648 13065 3593 13061 3540 13073 3490 13096 3451 13058 3464 13005
3484 12954 3452 12909 3397 12911 3343 12913 3288 12920 3234 12917
3186 12944 3153 12988 3120 13032 3066 13039 3012 13047 2944 13057
2907 13016 2905 12961 50 P
2905 12961 2918 12908 2878 12871 2834 12838 2801 12813 2793 12759
2847 12748 2895 12722 2931 12681 2931 12605 2869 12605 2869 12549
2839 12504 2809 12458 2788 12407 2793 12352 2815 12301 2820 12246
2813 12213 2773 12221 2757 12259 2702 12264 2648 12253 2600 12279
2546 12290 2503 12256 2472 12210 2424 12184 2439 12131 2482 12096
2507 12047 2486 11996 2460 11948 2407 11932 2352 11927 2299 11944
2298 11944 37 P
2298 10517 2313 10510 2357 10477 2382 10426 2436 10418 2492 10421
2547 10429 2601 10416 2605 10360 2619 10306 2663 10273 2719 10271

2774 10279 2822 10309 2866 10343 2899 10388 2931 10433 2964 10478
3000 10520 3037 10562 3018 10614 3046 10662 3100 10672 3155 10681
3185 10633 3238 10648 3271 10694 3293 10744 3325 10790 3332 10845
3364 10890 3401 10932 3441 10971 3484 11005 3528 11039 3575 11069
3592 11122 3554 11162 3519 11205 3497 11240 3533 11283 3560 11330
3564 11365 3606 11360 3608 11384 45 P
3608 11384 3611 11405 3556 11411 3501 11418 3448 11403 3396 11383
3358 11369 3316 11405 3278 11445 3253 11494 3220 11539 3165 11546
3141 11596 3125 11626 3082 11593 3043 11563 3010 11607 2981 11655
3001 11674 2968 11718 2917 11741 2862 11748 2808 11740 2753 11742
2699 11734 2646 11753 2599 11781 2562 11822 2535 11826 2537 11874
2482 11872 2420 11875 2410 11835 2355 11838 2298 11859 35 P
3394 14115 3432 14129 3418 14181 3410 14235 3427 14287 3450 14336
3477 14383 3517 14421 3560 14453 3614 14463 3619 14503 11 P
3619 14503 3568 14521 3531 14560 3512 14611 3475 14651 3427 14678
3373 14675 3319 14677 3279 14714 3235 14745 3194 14782 3166 14828
3213 14857 3253 14893 3266 14946 3273 15000 3309 15040 3337 15087
3377 15124 3409 15168 3395 15220 3387 15274 3363 15323 3319 15355
3268 15337 3216 15323 3165 15305 3113 15291 3063 15268 3023 15232
2991 15189 2963 15142 2944 15091 2926 15040 2918 14986 2904 14933
2881 14884 2878 14830 2845 14786 2837 14733 2845 14679 2831 14626
2808 14577 2755 14564 2708 14537 2654 14545 2600 14553 2547 14546
2500 14519 2453 14492 50 P
2453 14492 2398 14490 2344 14494 2298 14506 4 P
3619 14503 3669 14481 3719 14458 3756 14419 3759 14378 3806 14351
3860 14345 3914 14349 3968 14347 4023 14341 4083 14334 4095 14387
4096 14442 4107 14495 4136 14513 4165 14467 4222 14505 4213 14558
4183 14603 4205 14654 4231 14701 4266 14743 4305 14780 4347 14814
4389 14849 4424 14890 4466 14924 4512 14954 4546 14996 4585 15034
4627 15068 4672 15098 4722 15119 4772 15140 4762 15193 4757 15240
4802 15210 4837 15168 4874 15153 4878 15187 4931 15181 4945 15115
4998 15126 5023 15174 5061 15212 5106 15243 5159 15254 5186 15252
5168 15216 5155 15184 50 P
5155 15184 5185 15140 5209 15106 5184 15058 5174 15005 5129 14974
5095 14932 5085 14878 5070 14826 5045 14778 5040 14723 5019 14673
4975 14643 4933 14608 4888 14577 4854 14535 4849 14481 4842 14413
4877 14372 4903 14324 4924 14273 4935 14220 4930 14166 4925 14111
4871 14117 4866 14062 4877 14037 4847 13991 4813 13949 4760 13906
4753 13852 4744 13798 4739 13743 4740 13689 4750 13635 4745 13581
4740 13527 4735 13472 4730 13418 4735 13364 4741 13309 4747 13254
4752 13200 4758 13146 4742 13093 4721 13043 4691 12997 4657 12954
4636 12904 4620 12851 50 P
4620 12851 4595 12803 4584 12749 4578 12695 4558 12643 4581 12610

4614 12576 7 P
4614 12576 4604 12522 4635 12476 4624 12422 4655 12377 4694 12338
4689 12283 4705 12230 4667 12173 4718 12152 4757 12114 4768 12060
4814 12030 4841 11981 4841 11926 4836 11871 4813 11813 17 P
4813 11813 4745 11820 4729 11767 4675 11761 4639 11803 4609 11849
4566 11814 4512 11803 4459 11819 4407 11835 4354 11851 4300 11862
4246 11873 4192 11884 4137 11884 4136 11830 4189 11813 4240 11791
4261 11740 4239 11690 4294 11684 4345 11663 4388 11627 4409 11577
4430 11526 4379 11504 4326 11489 4275 11510 4221 11521 4166 11526
4131 11484 4100 11438 4083 11385 4099 11332 4114 11279 4120 11224
4114 11169 4113 11114 4093 11047 39 P
4093 11047 4038 11047 3982 11048 3943 11088 3909 11131 3874 11174
3836 11214 3785 11236 3743 11272 3700 11308 3662 11348 3638 11372
3608 11384 13 P
4093 11047 4147 11036 4141 10981 4138 10946 4193 10940 4242 10914
4281 10875 4330 10848 4335 10794 4330 10738 4324 10683 4319 10627
4313 10572 4270 10537 4219 10558 4164 10564 4112 10543 4069 10507
4026 10472 3976 10447 3937 10408 3895 10412 3899 10447 3847 10426
3793 10416 3799 10471 3744 10477 3700 10443 3654 10413 3600 10429
3582 10467 3536 10414 3481 10415 3449 10369 3483 10326 3513 10278
3501 10224 3484 10171 3477 10115 3473 10081 3404 10076 3424 10023
3462 9983 3469 10038 3506 10057 3532 10008 3557 9958 3606 9931
3621 9877 3590 9831 50 P
3590 9831 3635 9799 3684 9772 3715 9725 3720 9669 3755 9625
3781 9603 7 P
3781 9603 3787 9658 3819 9704 3841 9756 3868 9806 3904 9848
3939 9891 3991 9870 4024 9859 4041 9913 4090 9939 4145 9949
4201 9949 4255 9933 4298 9897 4347 9871 4402 9882 4453 9904
4458 9960 4432 10009 4453 10060 4504 10082 4556 10104 4625 10098
4621 10056 4677 10051 4733 10046 4788 10042 4824 9999 4871 9968
4923 9948 4978 9938 5033 9934 5087 9918 5127 9880 5168 9841
5204 9799 5248 9765 5302 9750 5357 9762 5413 9758 5468 9766
5510 9802 5556 9835 5632 9830 5629 9788 5602 9790 5582 9738
5578 9668 5630 9648 50 P
5630 9648 5686 9645 5719 9689 5757 9730 5796 9772 5837 9809
5885 9837 5882 9781 5927 9747 5971 9714 5942 9666 5980 9624
5938 9587 5905 9542 5882 9491 5902 9439 5945 9402 5987 9366
6043 9376 6103 9393 20 P
6103 9393 6117 9447 6165 9476 6207 9514 6244 9555 6297 9537
6352 9545 6403 9569 6458 9573 6513 9587 6550 9629 6602 9648
6657 9635 6670 9690 6724 9698 6779 9713 6815 9755 6817 9811
6873 9809 6928 9808 6971 9771 7022 9748 7078 9747 7143 9788
24 P

7143 9788 7128 9842 7118 9897 7119 9952 7105 10006 7059 10038
7044 10092 7046 10148 7047 10190 7008 10230 6979 10261 6981 10317
7022 10355 7062 10393 7106 10427 7076 10474 7034 10511 6986 10538
6933 10556 6883 10578 6841 10615 6819 10651 6804 10689 23 P
6804 10689 6735 10685 6731 10740 6712 10793 6675 10751 6628 10722
6574 10708 6519 10710 6471 10738 6442 10786 6434 10840 6428 10881
6373 10889 6304 10893 6267 10933 6233 10958 6196 10917 6158 10900
6135 10950 6090 10982 6058 11027 6044 11081 6058 11135 6061 11190
6070 11245 6029 11282 5991 11241 5943 11213 5889 11216 5856 11261
5812 11294 5758 11281 5703 11274 5648 11272 5593 11276 5538 11285
5484 11289 5429 11282 5374 11275 5325 11300 5281 11334 5238 11368
5191 11398 5144 11427 5095 11453 5052 11487 5016 11529 4980 11571
4941 11610 4929 11663 50 P
4929 11663 4902 11712 4871 11758 4842 11787 4813 11813 5 P
6804 10689 6817 10744 6840 10794 6880 10832 6924 10866 6977 10881
7061 10886 7067 10942 7069 10997 7070 11052 7071 11108 7068 11163
7069 11218 7076 11273 7082 11321 7054 11319 7053 11278 6998 11279
6963 11280 6923 11242 6878 11274 6845 11318 6790 11326 6742 11353
6744 11408 6746 11464 6708 11504 6658 11527 6610 11555 6613 11610
6615 11659 6656 11657 6666 11617 6721 11625 6796 11630 6804 11685
6822 11737 6818 11792 6841 11842 6888 11872 6941 11886 6948 11940
6949 11995 6940 12050 6942 12105 6982 12143 7037 12146 7084 12119
7132 12092 7147 12039 50 P
7147 12039 7162 11986 7209 11959 7264 11963 7315 11983 7359 12017
7405 12047 7401 12102 7380 12153 7381 12208 7382 12256 7329 12267
7278 12289 7240 12329 7221 12380 7222 12422 16 P
7222 12422 7170 12439 7115 12429 7061 12426 7006 12427 6951 12429
6906 12461 6887 12512 6868 12563 6826 12600 6789 12640 6737 12657
6682 12660 6635 12688 6637 12742 6670 12786 6706 12827 6724 12880
6775 12898 6826 12918 6866 12955 6893 13002 6934 13040 6980 13069
7023 13102 7072 13126 7108 13168 7154 13197 7208 13185 7247 13176
7238 13230 7210 13260 7167 13226 7114 13211 7062 13197 7013 13173
6960 13159 6917 13125 6868 13101 6869 13155 6897 13203 6924 13250
6930 13298 43 P
6930 13298 6887 13265 6860 13263 6835 13312 6782 13319 6738 13286
6695 13253 6654 13289 6599 13292 6545 13294 6498 13322 6451 13350
6399 13368 6350 13391 6301 13414 6247 13422 6206 13429 6166 13435
18 P
6166 13435 6158 13381 6149 13327 6141 13273 6128 13220 6104 13171
6090 13117 6082 13064 6068 13010 6050 12959 6021 12912 5984 12872
5937 12844 5883 12837 5828 12840 5774 12849 5719 12853 5665 12856
5610 12854 5556 12858 5502 12873 5449 12882 5394 12886 5385 12832
5380 12777 5328 12792 5274 12785 5228 12754 5187 12719 5142 12687

5090 12670 5035 12675 4981 12663 4931 12642 4878 12631 4832 12600
4778 12594 4729 12568 4675 12573 4635 12580 4614 12576 41 P
6166 13435 6133 13479 6110 13528 6087 13578 6065 13627 6109 13659
6149 13696 6193 13728 6201 13782 6204 13837 6232 13883 6265 13927
6293 13974 6321 14020 6349 14067 6368 14118 6349 14170 6331 14221
6313 14272 6290 14322 6272 14373 6244 14420 6197 14447 6151 14476
6169 14527 6172 14581 6186 14634 6194 14688 6197 14742 6241 14774
6277 14814 6285 14868 6322 14908 6362 14944 6399 14904 6453 14907
6504 14925 6540 14965 6543 15020 6570 15067 6588 15118 6606 15155
6569 15195 6556 15247 6558 15301 6514 15333 6463 15352 6414 15375
6374 15411 6402 15458 50 P
6402 15458 6434 15501 6487 15515 6540 15528 6588 15551 6620 15595
6566 15597 6513 15605 6521 15658 6523 15726 6483 15728 6486 15782
6441 15823 13 P
6441 15823 6433 15770 6420 15717 6376 15685 6323 15677 6270 15664
6230 15628 6183 15600 6129 15603 6086 15636 6032 15639 5978 15632
5940 15619 5914 15549 5860 15542 5818 15575 5771 15604 5718 15597
5677 15561 5644 15519 5615 15473 5590 15425 5561 15379 5537 15331
5517 15280 5473 15249 5419 15253 5373 15282 5310 15275 5300 15222
5269 15173 5215 15178 5155 15184 33 P
6441 15823 6433 15877 6414 15928 6382 15971 6346 16011 6390 16043
6417 16074 6389 16120 6395 16160 6371 16192 6407 16219 11 P
8148 16219 8163 16191 8184 16141 8200 16090 8252 16105 8305 16095
8358 16100 8412 16100 8460 16075 8514 16075 8546 16049 11 P
8546 16049 8570 16097 8595 16145 8605 16198 8598 16219 5 P
11246 16007 11193 16001 11138 16002 11159 16052 11180 16101 11186 16155
11160 16203 11148 16219 8 P
8546 16049 8508 16010 8474 15968 8427 15942 8466 15904 8414 15888
8384 15813 8426 15778 8468 15745 8520 15730 8551 15685 8599 15660
8653 15661 8693 15662 8705 15609 8712 15555 8728 15504 8755 15457
8786 15412 8835 15374 20 P
8061 14939 8055 14993 8030 15041 7995 15083 8003 15109 8024 15109
8019 15149 7966 15144 7926 15137 7892 15159 7908 15197 7860 15223
7855 15276 7855 15331 7905 15351 7936 15396 7894 15431 7868 15493
7821 15494 7787 15494 20 P
7787 15494 7750 15532 7729 15583 7730 15637 7768 15675 7822 15680
7817 15734 7763 15734 7709 15740 7656 15741 7606 15762 7581 15810
7547 15852 7518 15898 7489 15943 7443 15914 7398 15885 7366 15872
7376 15819 7380 15765 7353 15718 7301 15703 7247 15700 7195 15685
7152 15652 7121 15608 7086 15567 7059 15520 7057 15466 7026 15422
6986 15384 7011 15336 7008 15282 6997 15229 7025 15183 7072 15156
7116 15124 7165 15149 7218 15153 7273 15151 7314 15116 7366 15099
7411 15128 7446 15169 7504 15151 45 P

7504 15151 7555 15171 7593 15209 3 P
7593 15209 7643 15187 7693 15166 7688 15220 7719 15265 7714 15319
7709 15373 7730 15423 8 P
7730 15423 7770 15449 7787 15494 3 P
7504 15151 7514 15097 7558 15067 7568 15013 7573 14959 7593 14908
7567 14861 7532 14820 7505 14772 7494 14719 7495 14665 7507 14612
7534 14565 7583 14541 7637 14537 7691 14544 7744 14557 7796 14571
7832 14589 7869 14612 20 P
6930 13298 6932 13352 6895 13392 6861 13436 6863 13490 6881 13542
6917 13583 6960 13616 7006 13645 7049 13678 7077 13726 7104 13774
7126 13823 7143 13875 7170 13922 7222 13936 7276 13946 7324 13971
7364 14009 7403 14046 7438 14088 7474 14129 7513 14167 7555 14201
7609 14200 7664 14200 7718 14199 7773 14198 7815 14233 7845 14278
7876 14323 7926 14343 7974 14369 8006 14395 8033 14408 35 P
7222 12422 7276 12426 7314 12470 3 P
7314 12470 7321 12525 7332 12578 7381 12603 7434 12618 7489 12622
7539 12601 7577 12562 7615 12522 7630 12470 7651 12419 7676 12370
7725 12395 7746 12413 7788 12377 7822 12354 7875 12370 7926 12391
7961 12433 8009 12459 8063 12454 8098 12411 8114 12358 8114 12317
8169 12317 8220 12338 8236 12391 8235 12433 8274 12471 8308 12494
8357 12469 8382 12518 8402 12569 8423 12619 8438 12672 8493 12668
8547 12674 8585 12713 8626 12713 8634 12704 40 P
7314 12470 7356 12435 7390 12392 7435 12360 7485 12338 7538 12322
7578 12317 7577 12262 7593 12209 7635 12174 7680 12143 7735 12137
7788 12125 7842 12115 7888 12083 7931 12030 7935 11989 7909 11958
7962 11946 8017 11952 8071 11962 8117 11932 8096 11881 8091 11826
8065 11777 8039 11729 8013 11681 8043 11634 8098 11629 8141 11594
8157 11541 8157 11486 8183 11437 8219 11395 8273 11390 8328 11401
8381 11412 8412 11458 8442 11505 8495 11521 8530 11479 8573 11444
8609 11403 8610 11347 8589 11295 8551 11256 8497 11271 8458 11310
8404 11320 8349 11325 50 P
8349 11325 8318 11279 8354 11236 8365 11183 8344 11131 8299 11100
8268 11054 8226 11017 8212 10964 8218 10909 8241 10859 8281 10820
8328 10790 8377 10766 8329 10738 8314 10685 8321 10630 8344 10579
8390 10549 8444 10535 8500 10531 8622 10532 8678 10532 8733 10528
8775 10525 8830 10520 26 P
8830 10520 8881 10542 8936 10538 8972 10496 9009 10454 9055 10424
9108 10404 9162 10417 9209 10387 9216 10332 9261 10364 9314 10382
9338 10432 9391 10450 9446 10452 15 P
9446 10452 9479 10407 9523 10374 9564 10335 9600 10314 9616 10299

6:00 PM

8830 10520 8820 10466 8800 10413 8790 10359 8760 10312 8726 10268
8680 10236 8638 10200 8677 10162 8700 10110 8711 10085 8656 10073

8603 10056 8547 10056 8496 10076 8480 10023 8470 9968 8470 9912
8471 9856 8440 9809 8410 9762 8384 9713 8345 9673 8302 9637
8256 9606 8209 9575 8148 9541 27 P
8148 9541 8108 9581 8065 9616 8010 9627 7954 9633 7899 9639
7844 9650 7795 9676 7779 9730 7758 9782 7705 9799 7679 9831
7629 9806 7612 9752 7585 9703 7534 9682 7484 9657 7428 9652
7394 9696 7416 9747 7463 9778 7499 9820 7503 9862 7457 9894
7402 9900 7358 9865 7315 9831 7265 9805 7218 9775 7176 9780
7143 9788 31 P
8148 9541 8127 9490 8075 9468 8048 9419 8048 9362 8079 9316
8069 9261 8095 9211 8142 9180 8197 9169 8252 9158 8252 9102
8258 9047 8270 8991 8275 8935 8276 8913 16 P
8276 8913 8276 8893 8331 8899 8385 8916 8425 8877 8425 8820
8410 8766 8441 8720 8458 8666 8475 8611 8497 8560 8493 8503
8497 8461 8553 8467 8600 8437 8657 8432 8706 8459 8756 8487
8781 8537 8828 8569 8868 8530 8886 8476 8908 8425 8940 8378
8997 8374 9043 8406 9098 8419 9150 8442 9203 8422 9247 8388
9245 8349 31 P
8276 8913 8219 8913 8219 8857 8179 8817 8133 8785 8083 8759
8030 8743 7977 8721 7938 8681 7894 8645 7854 8686 7818 8729
7765 8713 7710 8697 7684 8648 7648 8604 7604 8569 7547 8570
7547 8514 7588 8505 7587 8448 7598 8392 7608 8337 7580 8287
7549 8241 7502 8210 7447 8194 7391 8184 7347 8149 7310 8106
7304 8049 7292 7994 7312 7941 7338 7890 7290 7859 7250 7820
7227 7768 7226 7725 7268 7724 7251 7671 7214 7627 7169 7592
7124 7557 7097 7507 7064 7460 7041 7408 7022 7354 7010 7298
7003 7241 6966 7198 50 P
6966 7198 6942 7146 6963 7092 6938 7049 6937 7006 5 P
6103 9393 6101 9337 6124 9286 6149 9235 6140 9180 6132 9124
6161 9076 6190 9028 6171 8974 6138 8929 6093 8896 6073 8843
6023 8819 5972 8795 5934 8753 5925 8697 5891 8652 5853 8610
5834 8558 5831 8501 5849 8448 5906 8450 5962 8441 5959 8385
5935 8334 5932 8277 5965 8232 5968 8175 5943 8124 5951 8068
5997 8034 6039 7996 6063 7944 6034 7895 6020 7841 5969 7772
6026 7775 6103 7786 6095 7730 6042 7711 6014 7704 6011 7647
5997 7592 5963 7546 5955 7489 5969 7434 5956 7370 47 P
3781 9603 3775 9547 3753 9495 3747 9439 3757 9384 3721 9341
3776 9329 3743 9260 8 P
3743 9260 3736 9204 3762 9154 3762 9098 3750 9043 3738 8987
3733 8931 3726 8869 3769 8832 3779 8777 3779 8734 3737 8735
3693 8699 3640 8678 3593 8647 3553 8608 3505 8577 3461 8542
3417 8507 3460 8451 3505 8487 3561 8480 3617 8485 3648 8515
3719 8514 3749 8467 3789 8426 3767 8374 3756 8318 3795 8278

3738 8278 3686 8257 3645 8245 3692 8174 3664 8124 3633 8077
3600 8030 3657 8024 3699 8020 3699 7963 3715 7923 3687 7858
42 P
3687 7858 3650 7814 3614 7770 3597 7716 3541 7728 3489 7750
3461 7699 3420 7712 3410 7738 3374 7715 3414 7674 3455 7624
3397 7624 3341 7631 3312 7634 3309 7676 3266 7681 3249 7627
3208 7551 3164 7588 3118 7620 3073 7657 3029 7621 2984 7586
2967 7531 2930 7488 2875 7472 2805 7459 2758 7492 2718 7533
2699 7572 2639 7533 2613 7584 2587 7635 2567 7688 2536 7737
2482 7755 2440 7760 2407 7714 2383 7661 2365 7608 2328 7565
2298 7563 43 P
3687 7858 3744 7857 3800 7851 3793 7780 3850 7774 3857 7845
3904 7877 3945 7917 4002 7917 4007 7860 4051 7824 4107 7813
4150 7849 4191 7890 4238 7921 4278 7962 4334 7973 4378 7937
4430 7959 4482 7982 4532 7956 4580 7925 4612 7878 4602 7822
4581 7769 4582 7712 4636 7696 4686 7692 4681 7635 4627 7618
4611 7563 4601 7507 4590 7451 4547 7414 4507 7373 4467 7332
4431 7287 4396 7242 4407 7187 4421 7161 4471 7189 4502 7237
4542 7278 4596 7257 4632 7213 4645 7157 4656 7101 4641 7046
4627 6998 4684 6993 50 P
4684 6993 4736 7016 4786 7044 4843 7034 4907 7024 5 P
4907 7024 4954 7042 5022 7064 5025 7107 5002 7160 5022 7213
5053 7261 5106 7241 5163 7237 5217 7217 5272 7202 5300 7152
5329 7102 5380 7077 5437 7084 5457 7138 5460 7195 5470 7252
5428 7291 5471 7329 5510 7370 5552 7408 5609 7405 5651 7366
5697 7332 5748 7307 5805 7304 5862 7306 5916 7325 5956 7370
30 P
5956 7370 5985 7321 5999 7266 6056 7268 6113 7277 6169 7286
6224 7299 6281 7308 6315 7262 6354 7220 6325 7189 6356 7159
6308 7126 6254 7109 6210 7072 6166 7036 6139 6985 6099 6945
6058 6904 6104 6870 6150 6836 6196 6802 6251 6822 6307 6825
6359 6850 6411 6826 6444 6882 6501 6885 6558 6883 6612 6904
6666 6924 6719 6944 6766 6911 6786 6857 6840 6839 6853 6805
6909 6820 6899 6877 6889 6933 6900 6982 6937 7006 41 P
4907 7024 4886 6970 4907 6911 4933 6860 4949 6805 4921 6754
4867 6734 4840 6723 4839 6665 4849 6609 4816 6562 4798 6507
4719 6501 4701 6446 4678 6393 4613 6395 4606 6338 4560 6302
4553 6245 4531 6224 4546 6187 4541 6130 4554 6073 4538 6017
4503 5971 4462 5929 4409 5906 4366 5902 4357 5851 4299 5856
4246 5833 4191 5815 4140 5787 4147 5729 4167 5683 4126 5641
4099 5589 4042 5600 3981 5625 3940 5583 3930 5526 3932 5501
42 P
6937 7006 6994 7005 7049 6986 7105 6974 7157 6951 7211 6956

7242 6885 7278 6840 7335 6844 7416 6859 7415 6802 7436 6748
7467 6700 7493 6649 7515 6595 7492 6543 7491 6485 7507 6430
7529 6376 7555 6325 7587 6277 7616 6254 22 P
2298 9491 2313 9481 2364 9457 2419 9444 2475 9436 2530 9445
2583 9464 2628 9498 2651 9549 2684 9594 2740 9592 2795 9579
2850 9588 2895 9622 2932 9665 2960 9713 3010 9738 3063 9720
3072 9665 3097 9615 3147 9639 3198 9665 3251 9647 3306 9640
3316 9585 3325 9530 3324 9474 3334 9418 3380 9386 3419 9346
3412 9290 3433 9237 3443 9211 3480 9254 3500 9291 3499 9234
3492 9178 3483 9137 3539 9125 3590 9103 3645 9113 3672 9163
3684 9218 3714 9247 3743 9260 45 P
5955 5669 5984 5673 6012 5681 6035 5684 4 P
9446 10452 9439 10507 9481 10544 9510 10591 9524 10645 9548 10695
9593 10728 9626 10749 8 P
7593 15209 7599 15235 7602 15262 7587 15285 7565 15300 7544 15318
7542 15345 7527 15367 7528 15393 7554 15389 7582 15389 7603 15389
7626 15401 7652 15409 7679 15409 7706 15416 7730 15423 17 P
3308 4926 3330 4926 3330 4653 3296 4653 3296 4847 3236 4847
3236 4872 3260 4874 3271 4877 3281 4881 3290 4887 3294 4891
3298 4896 3304 4909 3308 4926 15 N F
3523 4926 3545 4926 3545 4653 3511 4653 3511 4847 3451 4847
3451 4872 3475 4874 3486 4877 3496 4881 3505 4887 3509 4891
3512 4896 3518 4909 3523 4926 15 N F
3732 4926 3742 4925 3751 4923 3760 4919 3769 4913 3777 4907
3784 4899 3791 4891 3797 4881 3803 4871 3808 4860 3812 4848
3816 4836 3818 4824 3820 4811 3822 4798 3822 4785 3822 4772
3820 4759 3818 4747 3816 4734 3812 4722 3808 4710 3803 4699
3797 4688 3791 4678 3784 4670 3777 4662 3769 4655 3760 4650
3751 4646 3742 4643 3732 4643 3722 4643 3712 4646 3703 4650
3695 4655 3687 4662 3679 4670 3673 4678 3666 4688 3661 4699
3656 4710 3652 4722 3648 4734 3645 4747 3643 4759 3642 4772
3641 4785 3678 4785 3679 4761 3682 4739 3688 4720 3695 4703
3703 4690 3712 4681 3717 4677 3722 4675 3727 4673 3732 4673
3737 4673 3742 4675 3746 4677 3751 4681 3760 4690 3769 4703
3776 4720 3781 4739 3785 4761 3786 4785 3785 4810 3781 4831
3776 4850 3769 4866 3760 4879 3751 4888 3746 4891 3742 4894
3737 4895 3732 4896 3727 4895 3722 4894 3717 4891 3712 4888
3703 4879 3695 4866 3688 4850 3682 4831 3679 4810 3678 4785
3641 4785 3642 4798 3643 4811 3645 4824 3648 4836 3652 4848
3656 4860 3661 4871 3666 4881 3673 4891 3679 4899 3687 4907
3695 4913 3703 4919 3712 4923 3722 4925 3732 4926 107 N F
3990 4874 3988 4861 3985 4849 3979 4838 3971 4829 3961 4821
3951 4815 3939 4811 3926 4810 3926 4835 3918 4836 3910 4838

3904 4841 3898 4846 3893 4852 3889 4859 3887 4866 3886 4874
3887 4882 3889 4889 3893 4896 3898 4902 3904 4907 3910 4910
3918 4913 3926 4914 3934 4913 3941 4910 3948 4907 3954 4902
3958 4896 3962 4889 3964 4882 3965 4874 3964 4866 3962 4859
3958 4852 3954 4846 3948 4841 3941 4838 3934 4836 3926 4835
3926 4810 3913 4811 3901 4815 3890 4821 3881 4829 3873 4838
3867 4849 3863 4861 3862 4874 3863 4887 3867 4899 3873 4910
3881 4919 3890 4927 3901 4933 3913 4937 3926 4938 3939 4937
3951 4933 3961 4927 3971 4919 3979 4910 3985 4899 3988 4887
3990 4874 67 N F
4043 4934 4237 4934 4237 4902 4079 4902 4079 4811 4231 4811
4231 4779 4079 4779 4079 4685 4243 4685 4243 4653 4043 4653
4043 4934 13 N F
5472 4926 5494 4926 5494 4653 5460 4653 5460 4847 5400 4847
5400 4872 5424 4874 5435 4877 5445 4881 5454 4887 5458 4891
5461 4896 5467 4909 5472 4926 15 N F
5687 4926 5709 4926 5709 4653 5675 4653 5675 4847 5614 4847
5614 4872 5639 4874 5650 4877 5660 4881 5668 4887 5672 4891
5676 4896 5682 4909 5687 4926 15 N F
5925 4926 5949 4926 5949 4749 5992 4749 5992 4717 5949 4717
5949 4653 5915 4653 5915 4717 5801 4717 5801 4749 5831 4749
5915 4749 5915 4868 5831 4749 5801 4749 5801 4753 5925 4926
18 N F
6153 4874 6152 4861 6148 4849 6142 4838 6134 4829 6125 4821
6114 4815 6102 4811 6089 4810 6089 4835 6081 4836 6074 4838
6067 4841 6061 4846 6057 4852 6053 4859 6051 4866 6050 4874
6051 4882 6053 4889 6057 4896 6061 4902 6067 4907 6074 4910
6081 4913 6089 4914 6097 4913 6105 4910 6111 4907 6117 4902
6122 4896 6126 4889 6128 4882 6129 4874 6128 4866 6126 4859
6122 4852 6117 4846 6111 4841 6105 4838 6097 4836 6089 4835
6089 4810 6076 4811 6064 4815 6054 4821 6044 4829 6036 4838
6030 4849 6027 4861 6025 4874 6027 4887 6030 4899 6036 4910
6044 4919 6054 4927 6064 4933 6076 4937 6089 4938 6102 4937
6114 4933 6125 4927 6134 4919 6142 4910 6148 4899 6152 4887
6153 4874 67 N F
6206 4934 6401 4934 6401 4902 6242 4902 6242 4811 6395 4811
6395 4779 6242 4779 6242 4685 6407 4685 6407 4653 6206 4653
6206 4934 13 N F
7620 4926 7642 4926 7642 4653 7608 4653 7608 4847 7548 4847
7548 4872 7572 4874 7583 4877 7593 4881 7602 4887 7606 4891
7610 4896 7616 4909 7620 4926 15 N F
7835 4926 7857 4926 7857 4653 7823 4653 7823 4847 7763 4847
7763 4872 7787 4874 7798 4877 7808 4881 7817 4887 7821 4891

7824 4896 7831 4909 7835 4926 15 N F
8044 4926 8061 4925 8076 4921 8090 4915 8102 4907 8108 4902
8112 4897 8116 4891 8120 4884 8122 4877 8124 4870 8126 4862
8126 4854 8125 4842 8123 4831 8119 4823 8114 4815 8109 4809
8102 4804 8090 4795 8100 4790 8109 4784 8117 4776 8124 4768
8129 4759 8133 4749 8135 4738 8136 4727 8135 4713 8130 4699
8123 4685 8112 4672 8106 4666 8099 4660 8091 4655 8083 4651
8074 4647 8064 4645 8053 4643 8042 4643 8031 4643 8020 4645
8010 4647 8001 4651 7993 4655 7986 4660 7979 4666 7973 4672
7964 4685 7957 4699 7953 4713 7952 4727 7988 4727 7989 4716
7992 4706 7998 4696 8004 4688 8013 4682 8022 4677 8033 4674
8044 4673 8055 4674 8065 4677 8075 4682 8083 4688 8090 4696
8096 4706 8099 4716 8100 4727 8099 4738 8096 4748 8090 4757
8083 4765 8075 4772 8065 4777 8055 4780 8044 4781 8033 4780
8022 4777 8013 4772 8004 4765 7998 4757 7992 4748 7989 4738
7988 4727 7952 4727 7952 4738 7955 4748 7959 4758 7964 4767
7971 4775 7979 4783 7988 4790 7998 4795 7985 4804 7979 4809
7974 4815 7969 4823 7965 4831 7962 4842 7962 4854 7996 4854
7997 4844 8000 4836 8004 4829 8010 4823 8017 4818 8025 4814
8034 4812 8044 4811 8053 4812 8062 4814 8071 4818 8078 4823
8084 4829 8088 4836 8091 4844 8092 4854 8091 4863 8088 4871
8084 4878 8078 4884 8071 4889 8062 4893 8053 4895 8044 4896
8034 4895 8025 4893 8017 4889 8010 4884 8004 4878 8000 4871
7997 4863 7996 4854 7962 4854 7962 4862 7963 4870 7965 4877
7968 4884 7971 4891 7975 4897 7980 4902 7985 4907 7998 4915
8012 4921 8027 4925 8044 4926 147 N F
8302 4874 8300 4861 8297 4849 8291 4838 8283 4829 8273 4821
8263 4815 8251 4811 8238 4810 8238 4835 8230 4836 8222 4838
8216 4841 8210 4846 8205 4852 8202 4859 8199 4866 8198 4874
8199 4882 8202 4889 8205 4896 8210 4902 8216 4907 8222 4910
8230 4913 8238 4914 8246 4913 8253 4910 8260 4907 8266 4902
8270 4896 8274 4889 8276 4882 8277 4874 8276 4866 8274 4859
8270 4852 8266 4846 8260 4841 8253 4838 8246 4836 8238 4835
8238 4810 8225 4811 8213 4815 8202 4821 8193 4829 8185 4838
8179 4849 8175 4861 8174 4874 8175 4887 8179 4899 8185 4910
8193 4919 8202 4927 8213 4933 8225 4937 8238 4938 8251 4937
8263 4933 8273 4927 8283 4919 8291 4910 8297 4899 8300 4887
8302 4874 67 N F
8355 4934 8549 4934 8549 4902 8391 4902 8391 4811 8543 4811
8543 4779 8391 4779 8391 4685 8556 4685 8556 4653 8355 4653
8355 4934 13 N F
9783 4926 9805 4926 9805 4653 9770 4653 9770 4847 9710 4847
9710 4872 9735 4874 9745 4877 9755 4881 9764 4887 9768 4891

9772 4896 9778 4909 9783 4926 15 N F
9905 4831 9939 4831 9940 4845 9942 4858 9947 4869 9953 4878
9961 4885 9970 4891 9982 4895 9995 4896 10006 4895 10015 4892
10024 4888 10032 4882 10038 4874 10043 4865 10046 4855 10047 4843
10046 4832 10044 4822 10039 4814 10032 4806 10023 4799 10013 4792
9985 4775 9962 4762 9943 4748 9928 4734 9916 4719 9908 4704
9903 4688 9900 4671 9899 4653 10082 4653 10082 4685 9937 4685
9941 4697 9946 4707 9952 4716 9960 4724 9970 4731 9982 4739
10013 4757 10031 4766 10046 4776 10058 4787 10068 4799 10075 4811
10080 4823 10083 4835 10084 4847 10083 4856 10082 4864 10080 4872
10077 4879 10073 4886 10068 4893 10063 4899 10057 4904 10044 4913
10029 4920 10013 4924 9995 4926 9976 4924 9968 4923 9959 4920
9951 4917 9944 4913 9937 4908 9931 4903 9925 4897 9920 4890
9915 4882 9912 4873 9909 4864 9907 4854 9905 4843 9905 4831
78 N F
10120 4831 10154 4831 10155 4845 10157 4858 10161 4869 10168 4878
10175 4885 10185 4891 10197 4895 10210 4896 10220 4895 10230 4892
10239 4888 10247 4882 10253 4874 10258 4865 10261 4855 10262 4843
10261 4832 10258 4822 10254 4814 10247 4806 10238 4799 10227 4792
10200 4775 10177 4762 10158 4748 10143 4734 10131 4719 10123 4704
10118 4688 10115 4671 10114 4653 10296 4653 10296 4685 10152 4685
10155 4697 10160 4707 10167 4716 10175 4724 10185 4731 10197 4739
10228 4757 10246 4766 10261 4776 10273 4787 10283 4799 10290 4811
10295 4823 10297 4835 10298 4847 10298 4856 10296 4864 10294 4872
10291 4879 10287 4886 10283 4893 10278 4899 10272 4904 10259 4913
10244 4920 10228 4924 10210 4926 10191 4924 10182 4923 10174 4920
10166 4917 10158 4913 10152 4908 10145 4903 10140 4897 10134 4890
10130 4882 10126 4873 10124 4864 10121 4854 10120 4843 10120 4831
78 N F
10464 4874 10463 4861 10459 4849 10453 4838 10445 4829 10436 4821
10425 4815 10413 4811 10400 4810 10400 4835 10392 4836 10385 4838
10378 4841 10372 4846 10367 4852 10364 4859 10361 4866 10361 4874
10361 4882 10364 4889 10367 4896 10372 4902 10378 4907 10385 4910
10392 4913 10400 4914 10408 4913 10415 4910 10422 4907 10428 4902
10433 4896 10436 4889 10438 4882 10439 4874 10438 4866 10436 4859
10433 4852 10428 4846 10422 4841 10415 4838 10408 4836 10400 4835
10400 4810 10387 4811 10375 4815 10364 4821 10355 4829 10347 4838
10341 4849 10337 4861 10336 4874 10337 4887 10341 4899 10347 4910
10355 4919 10364 4927 10375 4933 10387 4937 10400 4938 10413 4937
10425 4933 10436 4927 10445 4919 10453 4910 10459 4899 10463 4887
10464 4874 67 N F
10517 4934 10712 4934 10712 4902 10553 4902 10553 4811 10706 4811
10706 4779 10553 4779 10553 4685 10718 4685 10718 4653 10517 4653

10517 4934 13 N F
11945 4926 11967 4926 11967 4653 11933 4653 11933 4847 11872 4847
11872 4872 11897 4874 11908 4877 11917 4881 11926 4887 11930 4891
11934 4896 11940 4909 11945 4926 15 N F
12067 4831 12101 4831 12102 4845 12105 4858 12109 4869 12115 4878
12123 4885 12132 4891 12144 4895 12157 4896 12168 4895 12177 4892
12186 4888 12194 4882 12200 4874 12205 4865 12208 4855 12209 4843
12209 4832 12206 4822 12201 4814 12194 4806 12185 4799 12175 4792
12147 4775 12124 4762 12105 4748 12090 4734 12079 4719 12070 4704
12065 4688 12062 4671 12061 4653 12244 4653 12244 4685 12099 4685
12103 4697 12108 4707 12114 4716 12122 4724 12132 4731 12144 4739
12175 4757 12193 4766 12208 4776 12221 4787 12230 4799 12237 4811
12242 4823 12245 4835 12246 4847 12245 4856 12244 4864 12242 4872
12239 4879 12235 4886 12230 4893 12225 4899 12220 4904 12206 4913
12191 4920 12175 4924 12157 4926 12138 4924 12130 4923 12121 4920
12113 4917 12106 4913 12099 4908 12093 4903 12087 4897 12082 4890
12077 4882 12074 4873 12071 4864 12069 4854 12067 4843 12067 4831
78 N F
12454 4856 12420 4856 12418 4865 12414 4873 12410 4880 12404 4885
12398 4890 12391 4893 12384 4895 12376 4896 12369 4895 12362 4894
12355 4892 12349 4889 12344 4885 12339 4880 12334 4875 12330 4869
12323 4854 12318 4836 12315 4816 12314 4793 12322 4802 12330 4809
12338 4814 12346 4818 12354 4821 12363 4822 12380 4823 12393 4822
12407 4818 12421 4811 12434 4802 12440 4796 12445 4789 12449 4782
12453 4774 12457 4765 12459 4756 12460 4746 12460 4735 12426 4733
12425 4744 12422 4755 12416 4765 12409 4774 12401 4782 12392 4788
12381 4792 12370 4793 12359 4792 12349 4788 12339 4782 12331 4774
12324 4765 12318 4755 12315 4744 12314 4733 12315 4722 12318 4711
12324 4701 12331 4691 12339 4684 12349 4678 12359 4674 12370 4673
12381 4674 12392 4678 12401 4684 12409 4691 12416 4701 12422 4711
12425 4722 12426 4733 12460 4735 12459 4719 12455 4703 12448 4687
12444 4680 12439 4673 12433 4666 12426 4661 12419 4655 12410 4651
12401 4647 12392 4645 12381 4643 12370 4643 12358 4643 12346 4645
12336 4649 12327 4653 12318 4659 12311 4666 12304 4673 12298 4682
12293 4692 12289 4702 12285 4714 12283 4726 12279 4751 12278 4779
12278 4796 12279 4811 12281 4826 12284 4840 12288 4853 12292 4865
12298 4876 12304 4886 12310 4895 12318 4903 12326 4910 12334 4916
12344 4920 12354 4923 12365 4925 12376 4926 12392 4924 12406 4920
12419 4914 12430 4905 12439 4895 12447 4883 12452 4870 12454 4856
126 N F
12626 4874 12625 4861 12621 4849 12615 4838 12607 4829 12598 4821
12587 4815 12575 4811 12562 4810 12562 4835 12554 4836 12547 4838
12540 4841 12534 4846 12529 4852 12526 4859 12523 4866 12523 4874

12523 4882 12526 4889 12529 4896 12534 4902 12540 4907 12547 4910
12554 4913 12562 4914 12570 4913 12577 4910 12584 4907 12590 4902
12595 4896 12598 4889 12601 4882 12601 4874 12601 4866 12598 4859
12595 4852 12590 4846 12584 4841 12577 4838 12570 4836 12562 4835
12562 4810 12549 4811 12537 4815 12526 4821 12517 4829 12509 4838
12503 4849 12499 4861 12498 4874 12499 4887 12503 4899 12509 4910
12517 4919 12526 4927 12537 4933 12549 4937 12562 4938 12575 4937
12587 4933 12598 4927 12607 4919 12615 4910 12621 4899 12625 4887
12626 4874 67 N F
12679 4934 12874 4934 12874 4902 12715 4902 12715 4811 12868 4811
12868 4779 12715 4779 12715 4685 12880 4685 12880 4653 12679 4653
12679 4934 13 N F
2316 17065 2338 17065 2338 16792 2304 16792 2304 16987 2244 16987
2244 17011 2268 17014 2279 17016 2289 17020 2298 17026 2302 17030
2306 17035 2312 17048 2316 17065 15 N F
2525 17065 2535 17064 2545 17062 2554 17058 2562 17053 2570 17046
2577 17039 2584 17030 2591 17021 2596 17010 2601 16999 2605 16987
2609 16975 2612 16963 2614 16950 2615 16937 2615 16925 2615 16912
2614 16899 2612 16886 2609 16873 2605 16861 2601 16849 2596 16838
2591 16828 2584 16818 2577 16809 2570 16801 2562 16795 2554 16789
2545 16785 2535 16783 2525 16782 2515 16783 2506 16785 2497 16789
2488 16795 2480 16801 2473 16809 2466 16818 2460 16828 2454 16838
2449 16849 2445 16861 2441 16873 2438 16886 2436 16899 2435 16912
2435 16925 2471 16925 2472 16900 2476 16878 2481 16859 2488 16843
2496 16830 2506 16820 2510 16817 2515 16814 2520 16813 2525 16812
2530 16813 2535 16814 2540 16817 2545 16820 2554 16830 2562 16843
2569 16859 2574 16878 2578 16900 2579 16925 2578 16949 2574 16971
2569 16990 2562 17005 2554 17018 2545 17027 2540 17031 2535 17033
2530 17035 2525 17035 2520 17035 2515 17033 2510 17031 2506 17027
2496 17018 2488 17005 2481 16990 2476 16971 2472 16949 2471 16925
2435 16925 2435 16937 2436 16950 2438 16963 2441 16975 2445 16987
2449 16999 2454 17010 2460 17021 2466 17030 2473 17039 2480 17046
2488 17053 2497 17058 2506 17062 2515 17064 2525 17065 107 N F
2676 17065 2818 17065 2818 17033 2704 17033 2694 16955 2703 16962
2715 16968 2729 16971 2746 16973 2760 16972 2775 16968 2789 16962
2796 16957 2803 16952 2809 16946 2814 16940 2819 16932 2824 16924
2827 16914 2830 16904 2832 16893 2832 16880 2831 16861 2829 16852
2826 16843 2823 16834 2819 16826 2814 16819 2808 16812 2802 16805
2795 16799 2787 16794 2779 16790 2770 16787 2760 16784 2749 16782
2738 16782 2727 16782 2718 16783 2709 16785 2700 16788 2693 16791
2686 16795 2679 16799 2674 16804 2664 16816 2657 16829 2651 16843
2647 16858 2682 16858 2685 16848 2690 16839 2695 16831 2701 16825
2708 16819 2717 16815 2726 16813 2736 16812 2749 16813 2762 16816

2772 16821 2781 16829 2788 16838 2794 16849 2797 16863 2798 16878
2797 16890 2795 16901 2790 16912 2784 16922 2775 16930 2765 16937
2752 16941 2745 16942 2738 16943 2730 16942 2723 16941 2717 16939
2711 16936 2699 16928 2686 16917 2656 16917 2676 17065 83 N F
2998 17014 2996 17001 2993 16989 2987 16978 2979 16968 2969 16960
2959 16955 2947 16951 2934 16949 2934 16974 2926 16975 2918 16977
2912 16981 2906 16986 2901 16991 2898 16998 2895 17006 2894 17014
2895 17021 2898 17029 2901 17036 2906 17041 2912 17046 2918 17050
2926 17052 2934 17053 2942 17052 2949 17050 2956 17046 2962 17041
2966 17036 2970 17029 2972 17021 2973 17014 2972 17006 2970 16998
2966 16991 2962 16986 2956 16981 2949 16977 2942 16975 2934 16974
2934 16949 2921 16951 2909 16955 2898 16960 2889 16968 2881 16978
2875 16989 2871 17001 2870 17014 2871 17026 2875 17038 2881 17049
2889 17059 2898 17067 2909 17072 2921 17076 2934 17078 2947 17076
2959 17072 2969 17067 2979 17059 2987 17049 2993 17038 2996 17026
2998 17014 67 N F
3051 17073 3245 17073 3245 17041 3087 17041 3087 16951 3239 16951
3239 16919 3087 16919 3087 16824 3251 16824 3251 16792 3051 16792
3051 17073 13 N F
4381 17065 4403 17065 4403 16792 4369 16792 4369 16987 4309 16987
4309 17011 4333 17014 4344 17016 4354 17020 4363 17026 4367 17030
4371 17035 4377 17048 4381 17065 15 N F
4596 17065 4618 17065 4618 16792 4584 16792 4584 16987 4524 16987
4524 17011 4548 17014 4559 17016 4569 17020 4578 17026 4582 17030
4585 17035 4591 17048 4596 17065 15 N F
4805 17065 4815 17064 4824 17062 4833 17058 4842 17053 4850 17046
4857 17039 4864 17030 4870 17021 4876 17010 4881 16999 4885 16987
4889 16975 4891 16963 4893 16950 4895 16937 4895 16925 4895 16912
4893 16899 4891 16886 4889 16873 4885 16861 4881 16849 4876 16838
4870 16828 4864 16818 4857 16809 4850 16801 4842 16795 4833 16789
4824 16785 4815 16783 4805 16782 4795 16783 4785 16785 4776 16789
4768 16795 4760 16801 4752 16809 4745 16818 4739 16828 4734 16838
4729 16849 4724 16861 4721 16873 4718 16886 4716 16899 4715 16912
4714 16925 4751 16925 4752 16900 4755 16878 4761 16859 4768 16843
4776 16830 4785 16820 4790 16817 4795 16814 4800 16813 4805 16812
4810 16813 4815 16814 4819 16817 4824 16820 4833 16830 4842 16843
4849 16859 4854 16878 4858 16900 4859 16925 4858 16949 4854 16971
4849 16990 4842 17005 4833 17018 4824 17027 4819 17031 4815 17033
4810 17035 4805 17035 4800 17035 4795 17033 4790 17031 4785 17027
4776 17018 4768 17005 4761 16990 4755 16971 4752 16949 4751 16925
4714 16925 4715 16937 4716 16950 4718 16963 4721 16975 4724 16987
4729 16999 4734 17010 4739 17021 4745 17030 4752 17039 4760 17046
4768 17053 4776 17058 4785 17062 4795 17064 4805 17065 107 N F

5063 17014 5061 17001 5058 16989 5052 16978 5044 16968 5034 16960
5023 16955 5011 16951 4999 16949 4999 16974 4991 16975 4983 16977
[truncated: 341,472 more chars]
